# Supplementary material for: An ancestral genomic sequence that serves as a nucleation site for de novo gene birth
Source: PLoS One. 2022 May 12;17(5):e0267864. doi: 10.1371/journal.pone.0267864 (PMC9097989; doi:10.1371/journal.pone.0267864)
Supplement: S1 Fig — a. Sequence alignment of the (GGT1), RefSeqGene with the GGT1-spacer sequences from mouse and primate species. b. Alignment of sequence from the Microcebus murinus (gray mouse) lemur with part of the 3’ end sequence the (GGT1), RefSeqGene sequence. (PDF) [file pone.0267864.s001.pdf]

Detection of an ancestral genomic sequence that serves as a nucleation site for de novo gene birth

Nicholas Delihas
Department of Microbiology and Immunology, Renaissance School of Medicine, Stony Brook University, Stony Brook, N.Y., United States of America

S1 Fig. a. Sequence alignment of the (GGT1), RefSeqGene (that extends 2010 bp beyond the *GGT1* gene at its 3′ end) with the GGT1-spacer sequences from mouse and primate species. (from Homo sapiens gamma-glutamyltransferase 1 (GGT1), RefSeqGene on chromosome 22, NCBI Reference Sequence: NG_008111.1. Purple highlight represents close similarities in sequences. **b.** Alignment of sequence from the *Microcebus murinus* (gray mouse) lemur with part of the 3′ end sequence the (GGT1), RefSeqGene sequence.

```
Percent Identity  Matrix - created by Clustal2.1          %ientity

     1: GGT1.END-GGT5.end.75422027-75453034.mouse          54.10
     2: GGT1.END-GGT5.START.75422027-75425161.mouse        54.10
     3: GGT1.end-GGT5.beginining.Philippine.tarsier.ref    72.36
     4: GGT1.end-FAM247.start.Rhesus.ref.                  90.48

     5: GGT1.new.NCBI.RefSeqGene                          100.00
     6: GGT1.end-start.BCRP3..human.ref                   100.00
     7: GGT1end-LOC749026.end.7456450-7520130.chimp        98.65
     8: GGT1.end-FAM247.start.chimp.ref                    98.65
```

```
 CLUSTAL O(1.2.4) multiple sequence alignment

GGT1.END-GGT5.end.75422027-75453034.mouse            ---------atcatggactttagtgtcttgtgtatgaaatggagtcatctcacttggaaa  51
GGT1.END-GGT5.START.75422027-75425161.mouse          ---------atcatggactttagtgtcttgtgtatgaaatggagtcatctcacttggaaa  51
GGT1.end-GGT5.beginining.Philippine.tarsier.ref      ggctcccactctctgggcctcggtgtcctgtgtgtgaattggagacatcgggct-ggcag  59
GGT1.end-FAM247.start.Rhesus.ref.                    ----------ctctgggcctcagtgtattgtgtgtgaaatggaaccatctggctggggag  50
GGT1.new.NCBI.RefSeqGene                             -ctccccactctctgggcctcagtgtattgtgtgtgaaatggagccatctggctggggag  59
GGT1.end-start.BCRP3..human.ref                      -ctccccactctctgggcctcagtgtattgtgtgtgaaatggagccatctggctggggag  59
GGT1end-LOC749026.end.7456450-7520130.chimp          ----------ctctgggcctcagtgtattctgtgtgaaatggagccatctggctggggag  50
GGT1.end-FAM247.start.chimp.ref                      ----------ctctgggcctcagtgtattctgtgtgaaatggagccatctggctggggag  50
                                                                  *** * *  ****  * *** **** ****  ****   ** ** *

GGT1.END-GGT5.end.75422027-75453034.mouse            atgacaaggcaggatcctaagacttaccgtgatgttaagtcatgaagctaacaccaacct  111
GGT1.END-GGT5.START.75422027-75425161.mouse          atgacaaggcaggatcctaagacttaccgtgatgttaagtcatgaagctaacaccaacct  111
GGT1.end-GGT5.beginining.Philippine.tarsier.ref      caatggtgtgg-ggggttctgaggtcttcaca---------ctggagctccctcagcatt  109
GGT1.end-FAM247.start.Rhesus.ref.                    gaatggagaggtgggattcggagatcttcacactgcggtcgctggaactagcctcagtat  110
GGT1.new.NCBI.RefSeqGene                             gaacagagaggtgggattcggagatcttcacaatgcgggcactggaactagcctcagcat  119
GGT1.end-start.BCRP3..human.ref                      gaacagagaggtgggattcggagatcttcacaatgcgggcactggaactagcctcagcat  119
GGT1end-LOC749026.end.7456450-7520130.chimp          gaacagagaggtgggattcggagatcttcacaatgtgggcactggaactagcctcaacat  110
GGT1.end-FAM247.start.chimp.ref                      gaacagagaggtgggattcggagatcttcacaatgtgggcactggaactagcctcaacat  110
                                                            *    *    *  **  *      *          ** * **  *       *

GGT1.END-GGT5.end.75422027-75453034.mouse            ttaagtggggggaatcccaccgtgcctg--------------------------------  139
GGT1.END-GGT5.START.75422027-75425161.mouse          ttaagtggggggaatcccaccgtgcctg--------------------------------  139
GGT1.end-GGT5.beginining.Philippine.tarsier.ref      tttcagtagggcgagagcctgggggctggtggcgagggacaggggcaggccagtgctaag  169
GGT1.end-FAM247.start.Rhesus.ref.                    cttcagcgtggggagagccaggtgcgtg--gctagggaccaggggaaggtccatgccaac  168
GGT1.new.NCBI.RefSeqGene                             cttcagcatggggagagccaggcacatg--gctgggggccaggggaaggttcacaccaag  177
GGT1.end-start.BCRP3..human.ref                      cttcagcatggggagagccaggcacatg--gctgggggccaggggaaggttcacaccaag  177
GGT1end-LOC749026.end.7456450-7520130.chimp          cttcagcatggggagagccaggcacatg--gctgggggccaggggaaggttcacaccaag  168
GGT1.end-FAM247.start.chimp.ref                      cttcagcatggggagagccaggcacatg--gctgggggccaggggaaggttcacaccaag  168
                                                      *       **  *   *   *    **

GGT1.END-GGT5.end.75422027-75453034.mouse            -------------------agagttagggggatggggtaaacagaacacctaaattgtcc  180
GGT1.END-GGT5.START.75422027-75425161.mouse          -------------------agagttagggggatggggtaaacagaacacctaaattgtcc  180
GGT1.end-GGT5.beginining.Philippine.tarsier.ref      cacttgctcctcctctcccagagctcctggataggactccgggtcctcccttcttggagt  229
GGT1.end-FAM247.start.Rhesus.ref.                    ccctgccccttcccaccctgatccattggactttggggccaggtgctcccttattggggc  228
GGT1.new.NCBI.RefSeqGene                             ctctgccccttcccaccctgatccctcggactttggggccaggccctcccttactggggc  237
GGT1.end-start.BCRP3..human.ref                      ctctgccccttcccaccctgatccctcggactttggggccaggccctcccttactggggc  237
GGT1end-LOC749026.end.7456450-7520130.chimp          ctctgccccttcccaccctgatccctcggactttggggccaggccctcccttactggggc  228
GGT1.end-FAM247.start.chimp.ref                      ctctgccccttcccaccctgatccctcggactttggggccaggccctcccttactggggc  228
                                                                                 *     *             ***   * *

GGT1.END-GGT5.end.75422027-75453034.mouse            taagcagtgacacttgc-aatatcagccactagagggcgccccgaccttgccattaactg  239
GGT1.END-GGT5.START.75422027-75425161.mouse          taagcagtgacacttgc-aatatcagccactagagggcgccccgaccttgccattaactg  239
GGT1.end-GGT5.beginining.Philippine.tarsier.ref      agggcagcgacactacctagtacctgccaccaggggcaccagggcactgacctttactt  289
GGT1.end-FAM247.start.Rhesus.ref.                    tgcacagtgac---acctaggactagccaccagggggtgccgcgcccctggtgctttctt  285
GGT1.new.NCBI.RefSeqGene                             tgggcagtgacactacctaggatcagccaccagggggtaccacgaccctggcactttctt  297
GGT1.end-start.BCRP3..human.ref                      tgggcagtgacactacctaggatcagccaccagggggtaccacgaccctggcactttctt  297
```

```
GGT1end-LOC749026.end.7456450-7520130.chimp          tgggcagtgacactacctaggatcagccaccggggggtgccacgaccctggcactttctt  288
GGT1.end-FAM247.start.chimp.ref                      tgggcagtgacactacctaggatcagccaccggggggtgccacgaccctggcactttctt  288
                                                         *** ***     * *  *   *****  * ***  **  * *  **     *  **

GGT1.END-GGT5.end.75422027-75453034.mouse            gaacaaagcaatgaca-ctgtagctgtgggtggagaat---gcttctaggacagatgtg-  294
GGT1.END-GGT5.START.75422027-75425161.mouse          gaacaaagcaatgaca-ctgtagctgtgggtggagaat---gcttctaggacagatgtg-  294
GGT1.end-GGT5.beginining.Philippine.tarsier.ref      ggacagacggtagcgggcagatgccaggaactcgggtgtctcctcctcagacccgcgggt  349
GGT1.end-FAM247.start.Rhesus.ref.                    aggcagtgggtggccagctgatgctgggaacctgggca---ccttctcagacccatgggc  342
GGT1.new.NCBI.RefSeqGene                             aggcagagggtggccagctgatgctgggaacccgggtg---ccttcttagacccgtaggc  354
GGT1.end-start.BCRP3..human.ref                      aggcagagggtggccagctgatgctgggaacccgggtg---ccttcttagacccgtaggc  354
GGT1end-LOC749026.end.7456450-7520130.chimp          aggcagagggtggccagctgatgctgggaacccggcg---ccttctcagacccgtaggc  345
GGT1.end-FAM247.start.chimp.ref                      aggcagagggtggccagctgatgctgggaacccggcg---ccttctcagacccgtaggc  345
                                                        **        *    * *  **    *       *       ** **  ***       *

GGT1.END-GGT5.end.75422027-75453034.mouse            -----ggctctcattggcccgcactta-gggggtaaaactgaa-----------ggagag  337
GGT1.END-GGT5.START.75422027-75425161.mouse          -----ggctctcattggcccgcactta-gggggtaaaactgaa-----------ggagag  337
GGT1.end-GGT5.beginining.Philippine.tarsier.ref      gtcccggctcgccctgctgaggacaaacagaggtgaagctgaggtctgaggagtgggagt  409
GGT1.end-FAM247.start.Rhesus.ref.                    atcca-actcatcctgctaatgacacg-ggaggtgaagctgagttccaaggaatgggaat  400
GGT1.new.NCBI.RefSeqGene                             gtcca-gctcaccctgccgatgacact-ggaggtgaagctgaggtccgaggaatggggac  412
GGT1.end-start.BCRP3..human.ref                      gtcca-gctcaccctgccgatgacact-ggaggtgaagctgaggtccgaggaatggggac  412
GGT1end-LOC749026.end.7456450-7520130.chimp          gtcca-gctcaccctgccgatgacact-ggaggtgaagctgaggtccgaggaatggggac  403
GGT1.end-FAM247.start.chimp.ref                      gtcca-gctcaccctgccgatgacact-ggaggtgaagctgaggtccgaggaatggggac  403
                                                           ***    **       **      * *** ** ****             **

GGT1.END-GGT5.end.75422027-75453034.mouse            tagactagagacaaaaacaaggcaatttggctaaggccatacttgaggtcactttccaag  397
GGT1.END-GGT5.START.75422027-75425161.mouse          tagactagagacaaaaacaaggcaatttggctaaggccatacttgaggtcactttccaag  397
GGT1.end-GGT5.beginining.Philippine.tarsier.ref      tgggcaagaggctggaacaaaacatctcagtcagaccctggg----gggggctttccaag  465
GGT1.end-FAM247.start.Rhesus.ref.                    tgggcatcacgctagaggaaaacatcttagtcagagccaagcccctgggggggtttccaag  460
GGT1.new.NCBI.RefSeqGene                             tgggcaacaggctggaggaaaacatctcggtcagagccacgcccctgggggggtttccaag  472
GGT1.end-start.BCRP3..human.ref                      tgggcaacaggctggaggaaaacatctcggtcagagccacgcccctgggggggtttccaag  472
GGT1end-LOC749026.end.7456450-7520130.chimp          tgggcaacaggctggaggaaaacatctcggtcagagccacgcccctgggggggtttccaag  463
GGT1.end-FAM247.start.chimp.ref                      tgggcaacaggctggaggaaaacatctcggtcagagccacgcccctgggggggtttccaag  463
                                                     * * *   *  *   *  **  **  *  *  *   **         **    ********

GGT1.END-GGT5.end.75422027-75453034.mouse            cacaaacccagagtga---aagccaagcttg--------tcttgcaagaggcccagttct  446
GGT1.END-GGT5.START.75422027-75425161.mouse          cacaaacccagagtga---aagccaagcttg--------tcttgcaagaggcccagttct  446
GGT1.end-GGT5.beginining.Philippine.tarsier.ref      cacaaactctgagtgaaagaaacccagcttgtgatcagctccagagggaggcccgattct  525
GGT1.end-FAM247.start.Rhesus.ref.                    tataagcccagagtga---aacccaagcttgtgaccctctccagagggaggcctggtttt  517
GGT1.new.NCBI.RefSeqGene                             tttaagcccagagtga---aacccaagcttgtgatcctctccagagggaggcctggttct  529
GGT1.end-start.BCRP3..human.ref                      tttaagcccagagtga---aacccaagcttgtgatcctctccagagggaggcctggttct  529
GGT1end-LOC749026.end.7456450-7520130.chimp          tttaagcccagagtga---aacccaagcttgtgatcctctccagagggaggcctggttct  520
GGT1.end-FAM247.start.chimp.ref                      tttaagcccagagtga---aacccaagcttgtgatcctctccagagggaggcctggttct  520
                                                        ** * * ******   ** ** ******        **  *   ******   ** *

GGT1.END-GGT5.end.75422027-75453034.mouse            caaacaaacagacagtcccagcaggtccgccagctatagaaaacccacagatccacaagg  506
GGT1.END-GGT5.START.75422027-75425161.mouse          caaacaaacagacagtcccagcaggtccgccagctatagaaaacccacagatccacaagg  506
GGT1.end-GGT5.beginining.Philippine.tarsier.ref      caggaa-------------------cagcagatcgcagtga------cttggaccagatg  560
GGT1.end-FAM247.start.Rhesus.ref.                    cagggaacagcaaatgggaagaggtccccagattccagggatcagggcttggaccagctg  577
GGT1.new.NCBI.RefSeqGene                             cagggaacagcaaacgggaagatgtccccagatcccagggatcagggcttggaccagccg  589
GGT1.end-start.BCRP3..human.ref                      cagggaacagcaaacgggaagatgtccccagatcccagggatcagggcttggaccagccg  589
GGT1end-LOC749026.end.7456450-7520130.chimp          cagggaacagcaaacgggaagatgtccccagatcccagggatcagggcttggaccagccg  580
GGT1.end-FAM247.start.chimp.ref                      cagggaacagcaaacgggaagatgtccccagatcccagggatcagggcttggaccagccg  580
                                                     **   *                         *        *      *     *     *

GGT1.END-GGT5.end.75422027-75453034.mouse            ggaacacagctca---caatggatgagcccagaagagcaccattagatacatccctcctt  563
GGT1.END-GGT5.START.75422027-75425161.mouse          ggaacacagctca---caatggatgagcccagaagagcaccattagatacatccctcctt  563
GGT1.end-GGT5.beginining.Philippine.tarsier.ref      ggaatgtggccccaggaaggggaggcatctggaagggaattgttggttacagcgcccctt-  619
GGT1.end-FAM247.start.Rhesus.ref.                    gggacgcagccca---gagggagtgggtctggaagggaacagctagacacagcagcctt-  633
GGT1.new.NCBI.RefSeqGene                             gggacgcagccca---gagggagtgggtccagaaggaaacagctagacacagcagcctt-  645
GGT1.end-start.BCRP3..human.ref                      gggacgcagccca---gagggagtgggtccagaaggaaacagctagacacagcagcctt-  645
GGT1end-LOC749026.end.7456450-7520130.chimp          gggacgcagccca---gagggagtgggtccggaaggaaacagctcgacacagcagcctt-  636
GGT1.end-FAM247.start.chimp.ref                      gggacgcagccca---gagggagtgggtccggaaggaaacagctcgacacagcagcctt-  636
                                                     ** *    ** *      *  *    *   * ****   *    * *  *** *   * *

GGT1.END-GGT5.end.75422027-75453034.mouse            gtgttagtcagggtttctattgctgcaatgaaagaccatgaccaaagcagcttggagagg  623
GGT1.END-GGT5.START.75422027-75425161.mouse          gtgttagtcagggtttctattgctgcaatgaaagaccatgaccaaagcagcttggagagg  623
GGT1.end-GGT5.beginining.Philippine.tarsier.ref      ------------------------------------------------------------  619
GGT1.end-FAM247.start.Rhesus.ref.                    ------------------------------------------------------------  633
GGT1.new.NCBI.RefSeqGene                             ------------------------------------------------------------  645
GGT1.end-start.BCRP3..human.ref                      ------------------------------------------------------------  645
GGT1end-LOC749026.end.7456450-7520130.chimp          ------------------------------------------------------------  636
GGT1.end-FAM247.start.chimp.ref                      ------------------------------------------------------------  636

GGT1.END-GGT5.end.75422027-75453034.mouse            aaagggtttatttggcttacatttccacatcacagttgaccatgaaaggtaaggacaggc  683
GGT1.END-GGT5.START.75422027-75425161.mouse          aaagggtttatttggcttacatttccacatcacagttgaccatgaaaggtaaggacaggc  683
GGT1.end-GGT5.beginining.Philippine.tarsier.ref      ------------------------------------------------------------  619
GGT1.end-FAM247.start.Rhesus.ref.                    ------------------------------------------------------------  633
GGT1.new.NCBI.RefSeqGene                             ------------------------------------------------------------  645
GGT1.end-start.BCRP3..human.ref                      ------------------------------------------------------------  645
GGT1end-LOC749026.end.7456450-7520130.chimp          ------------------------------------------------------------  636
GGT1.end-FAM247.start.chimp.ref                      ------------------------------------------------------------  636

GGT1.END-GGT5.end.75422027-75453034.mouse            actcagacgggccaggaacctggagacaggagccaatacaggggccatggaagagtgcga  743
GGT1.END-GGT5.START.75422027-75425161.mouse          actcagacgggccaggaacctggagacaggagccaatacaggggccatggaagagtgcga  743
GGT1.end-GGT5.beginining.Philippine.tarsier.ref      ------------------------------------------------------------  619
GGT1.end-FAM247.start.Rhesus.ref.                    ------------------------------------------------------------  633
GGT1.new.NCBI.RefSeqGene                             ------------------------------------------------------------  645
GGT1.end-start.BCRP3..human.ref                      ------------------------------------------------------------  645
GGT1end-LOC749026.end.7456450-7520130.chimp          ------------------------------------------------------------  636
GGT1.end-FAM247.start.chimp.ref                      ------------------------------------------------------------  636
```

```
GGT1.END-GGT5.end.75422027-75453034.mouse          cttgctcctcatggcttgttcagccttctttcttatagaacccaggaccttcagcccagg  803
GGT1.END-GGT5.START.75422027-75425161.mouse        cttgctcctcatggcttgttcagccttctttcttatagaacccaggaccttcagcccagg  803
GGT1.end-GGT5.beginining.Philippine.tarsier.ref    ------------------------------------------------------------  619
GGT1.end-FAM247.start.Rhesus.ref.                  ------------------------------------------------------------  633
GGT1.new.NCBI.RefSeqGene                           ------------------------------------------------------------  645
GGT1.end-start.BCRP3..human.ref                    ------------------------------------------------------------  645
GGT1end-LOC749026.end.7456450-7520130.chimp        ------------------------------------------------------------  636
GGT1.end-FAM247.start.chimp.ref                    ------------------------------------------------------------  636

GGT1.END-GGT5.end.75422027-75453034.mouse          gatggcgccacccacaataggctaaaccctcctccatccatcactaatttaaaaactgtc  863
GGT1.END-GGT5.START.75422027-75425161.mouse        gatggcgccacccacaataggctaaaccctcctccatccatcactaatttaaaaactgtc  863
GGT1.end-GGT5.beginining.Philippine.tarsier.ref    ------------------------------------------------------------  619
GGT1.end-FAM247.start.Rhesus.ref.                  ------------------------------------------------------------  633
GGT1.new.NCBI.RefSeqGene                           ------------------------------------------------------------  645
GGT1.end-start.BCRP3..human.ref                    ------------------------------------------------------------  645
GGT1end-LOC749026.end.7456450-7520130.chimp        ------------------------------------------------------------  636
GGT1.end-FAM247.start.chimp.ref                    ------------------------------------------------------------  636

GGT1.END-GGT5.end.75422027-75453034.mouse          ctgcaggcttgcccacagctagatcttatggaggtattttctcagttgaggctccctctc  923
GGT1.END-GGT5.START.75422027-75425161.mouse        ctgcaggcttgcccacagctagatcttatggaggtattttctcagttgaggctccctctc  923
GGT1.end-GGT5.beginining.Philippine.tarsier.ref    ------------------------------------------------------------  619
GGT1.end-FAM247.start.Rhesus.ref.                  ------------------------------------------------------------  633
GGT1.new.NCBI.RefSeqGene                           ------------------------------------------------------------  645
GGT1.end-start.BCRP3..human.ref                    ------------------------------------------------------------  645
GGT1end-LOC749026.end.7456450-7520130.chimp        ------------------------------------------------------------  636
GGT1.end-FAM247.start.chimp.ref                    ------------------------------------------------------------  636

GGT1.END-GGT5.end.75422027-75453034.mouse          tgatgactctagtttgtgtcaagttgatataaaactagccaacacactctttaagggcag  983
GGT1.END-GGT5.START.75422027-75425161.mouse        tgatgactctagtttgtgtcaagttgatataaaactagccaacacactctttaagggcag  983
GGT1.end-GGT5.beginining.Philippine.tarsier.ref    ----------------------------------------------tgccaaggggcccg  633
GGT1.end-FAM247.start.Rhesus.ref.                  ----------------------------------------------caccactgg--cag  645
GGT1.new.NCBI.RefSeqGene                           ----------------------------------------------caccatcgg--cag  657
GGT1.end-start.BCRP3..human.ref                    ----------------------------------------------caccatcgg--cag  657
GGT1end-LOC749026.end.7456450-7520130.chimp        ----------------------------------------------caccatcgg--cag  648
GGT1.end-FAM247.start.chimp.ref                    ----------------------------------------------caccatcgg--cag  648
                                                                                                   *     *  * *

GGT1.END-GGT5.end.75422027-75453034.mouse          cccctcatgctgtgtcaaggtctatagaactcacagctgtg---------accacgaggt  1034
GGT1.END-GGT5.START.75422027-75425161.mouse        cccctcatgctgtgtcaaggtctatagaactcacagctgtg---------accacgaggt  1034
GGT1.end-GGT5.beginining.Philippine.tarsier.ref    tccctcaggccctccttcagggtccgcttacctgttctccacacagttgtaacacctggg  693
GGT1.end-FAM247.start.Rhesus.ref.                  cccctcccggcctccctcggggcctgc-tccctcctccaagcaccgttccaacacctggg  704
GGT1.new.NCBI.RefSeqGene                           cccctccaggcctccctcagggcctgc-tccctcctctgtgcacagttccaacacctggg  716
GGT1.end-start.BCRP3..human.ref                    cccctccaggcctccctcagggcctgc-tccctcctctgtgcacagttccaacacctggg  716
GGT1end-LOC749026.end.7456450-7520130.chimp        cccctccaggcctccctcggggcctgc-tccctcctctgtgcacagttccaacacctggg  707
GGT1.end-FAM247.start.chimp.ref                    cccctccaggcctccctcggggcctgc-tccctcctctgtgcacagttccaacacctggg  707
                                                    *****  *   *      *           *    *             * ***  **

GGT1.END-GGT5.end.75422027-75453034.mouse          aaagagacacttggagacaatgtagaagtgagccaccgg-----ggtagtcacagcctag  1089
GGT1.END-GGT5.START.75422027-75425161.mouse        aaagagacacttggagacaatgtagaagtgagccaccgg-----ggtagtcacagcctag  1089
GGT1.end-GGT5.beginining.Philippine.tarsier.ref    atg---------------------------------------------------------  696
GGT1.end-FAM247.start.Rhesus.ref.                  gcagggttctgggaaaggctggtggaggtgggctggtggggggcggtgatcacagcccag  764
GGT1.new.NCBI.RefSeqGene                           gcagggttctgggaagggctggtggaggtgggctggtgggaggcggtgatcacagcccag  776
GGT1.end-start.BCRP3..human.ref                    gcagggttctgggaagggctggtggaggtgggctggtgggaggcggtgatcacagcccag  776
GGT1end-LOC749026.end.7456450-7520130.chimp        gcagggttctgggaagggctggtggaggtgggctggtgggaggcggtgatcacagcccag  767
GGT1.end-FAM247.start.chimp.ref                    gcagggttctgggaagggctggtggaggtgggctggtgggaggcggtgatcacagcccag  767

GGT1.END-GGT5.end.75422027-75453034.mouse          gagctaaatattgccccttaaagtgagcacaagttgggggtccctgagggaagggaccca  1149
GGT1.END-GGT5.START.75422027-75425161.mouse        gagctaaatattgccccttaaagtgagcacaagttgggggtccctgagggaagggaccca  1149
GGT1.end-GGT5.beginining.Philippine.tarsier.ref    ------------------------------------------------------------  696
GGT1.end-FAM247.start.Rhesus.ref.                  catctgggtatcacca---------------------ggggcactggggccagggcccag  803
GGT1.new.NCBI.RefSeqGene                           cacctggatatcacca---------------------ggggcactggggccaggggccag  815
GGT1.end-start.BCRP3..human.ref                    cacctggatatcacca---------------------ggggcactggggccaggggccag  815
GGT1end-LOC749026.end.7456450-7520130.chimp        cacctggatatcacca---------------------ggggcactggggccaggggccag  806
GGT1.end-FAM247.start.chimp.ref                    cacctggatatcacca---------------------ggggcactggggccaggggccag  806

GGT1.END-GGT5.end.75422027-75453034.mouse          gtggctggaggagggtcc-------caggaggctagaagtcctggtgtttccaacctgcc  1202
GGT1.END-GGT5.START.75422027-75425161.mouse        gtggctggaggagggtcc-------caggaggctagaagtcctggtgtttccaacctgcc  1202
GGT1.end-GGT5.beginining.Philippine.tarsier.ref    -----------------------------------gaaaatttggtgcttccaaagtgcc  721
GGT1.end-FAM247.start.Rhesus.ref.                  gtgaagccaggtcggggctctcctttagaagccccgaaaacctggtgataccaaagggcc  863
GGT1.new.NCBI.RefSeqGene                           gtgaggccaggtcggggctatccttcaggatccccgaagacctggtgattccaaagggcc  875
GGT1.end-start.BCRP3..human.ref                    gtgaggccaggtcggggctatccttcaggatccccgaagacctggtgattccaaagggcc  875
GGT1end-LOC749026.end.7456450-7520130.chimp        gtgaggccaggtcggggctatccttcaggagccccgaaaacctggtgattccaaagggcc  866
GGT1.end-FAM247.start.chimp.ref                    gtgaggccaggtcggggctatccttcaggagccccgaaaacctggtgattccaaagggcc  866
                                                                                      ***    ***** * ****   ***

GGT1.END-GGT5.end.75422027-75453034.mouse          catgggaaagcagggttttcctgtaggtgtg--------ctccatgtctgagctctaaag  1254
GGT1.END-GGT5.START.75422027-75425161.mouse        catgggaaagcagggttttcctgtaggtgtg--------ctccatgtctgagctctaaag  1254
GGT1.end-GGT5.beginining.Philippine.tarsier.ref    caca-aggaaatgggttttgagcctgcagagtac----------------accccaggag  764
GGT1.end-FAM247.start.Rhesus.ref.                  cacagacaaacagggtttgtgcctgcggagttgagtaccaccgggtctaagccctggag  923
GGT1.new.NCBI.RefSeqGene                           catagacaaacagggttttctgcctgtggagtcaagtcccactgggtctgagctctggag  935
GGT1.end-start.BCRP3..human.ref                    catagacaaacagggttttctgcctgtggagtcaagtcccactgggtctgagctctggag  935
GGT1end-LOC749026.end.7456450-7520130.chimp        catagacaaacagggttttctgcctgtggagtcaagtcccactgggtctgagctctggag  926
GGT1.end-FAM247.start.chimp.ref                    catagacaaacagggttttctgcctgtggagtcaagtcccactgggtctgagctctggag  926
                                                   **      *   *******      *  *  *                   * * *   **

GGT1.END-GGT5.end.75422027-75453034.mouse          ctctctatctctggggctc--cttggggaacacaga--agctgggctgaagggggggggtg  1310
GGT1.END-GGT5.START.75422027-75425161.mouse        ctctctatctctggggctc--cttggggaacacaga--agctgggctgaagggggggggtg  1310
```

```
GGT1.end-GGT5.beginining.Philippine.tarsier.ref   ggctttgtctctggagttccccaggggtgagatgactgggtgagatgacaggggtgagtg  824
GGT1.end-FAM247.start.Rhesus.ref.                 ggctgtgtccctggggctccccaggggtgagatgga--ggtgggctcaactg---gtgta  978
GGT1.new.NCBI.RefSeqGene                          ggctgtgtctctggggctctgcaggggtgagatgga--ggtgggctcaactg---gtgta  990
GGT1.end-start.BCRP3..human.ref                   ggctgtgtctctggggctctgcaggggtgagatgga--ggtgggctcaactg---gtgta  990
GGT1end-LOC749026.end.7456450-7520130.chimp       ggctgtgtctctggggctctgcaagggtgagatgga--ggtgggctcaaatg---gtgta  981
GGT1.end-FAM247.start.chimp.ref                   ggctgtgtctctggggctctgcaagggtgagatgga--ggtgggctcaaatg---gtgta  981
                                                    ** * ** **** * **  *  ***  * *       *   *       *   * **

GGT1.END-GGT5.end.75422027-75453034.mouse         ccagccattcctcaatccatatttga----------------------------------  1336
GGT1.END-GGT5.START.75422027-75425161.mouse       ccagccattcctcaatccatatttga----------------------------------  1336
GGT1.end-GGT5.beginining.Philippine.tarsier.ref   caagtcactactcaatcctaaattta----------------------------------  850
GGT1.end-FAM247.start.Rhesus.ref.                 cccgtcactcctcaatccttattttatgtatttaatttttaaaaaaatttttatttgaac  1038
GGT1.new.NCBI.RefSeqGene                          caagtcactcttcaatccttattttatttatttaatttttttaaaaa---aaatttaaac  1047
GGT1.end-start.BCRP3..human.ref                   caagtcactcttcaatccttattttatttatttaatttttttaaaaa---aaatttaaac  1047
GGT1end-LOC749026.end.7456450-7520130.chimp       caagtcactcctcaatccttattttatttatttaattttttt-aaaa---aaatttaaac  1037
GGT1.end-FAM247.start.chimp.ref                   caagtcactcctcaatccttattttatttatttaattttttt-aaaa---aaatttaaac  1037
                                                  *  * ** *  *******  * ** *

GGT1.END-GGT5.end.75422027-75453034.mouse         ------------------------------------------------------------  1336
GGT1.END-GGT5.START.75422027-75425161.mouse       ------------------------------------------------------------  1336
GGT1.end-GGT5.beginining.Philippine.tarsier.ref   ------------------------------------------------------------  850
GGT1.end-FAM247.start.Rhesus.ref.                 aaatagagatggggtctcactatgttgaccaggctggtcttaaactcttgacttcaagca  1098
GGT1.new.NCBI.RefSeqGene                          caatagagatggggtctcactatgttgatcaggctggtctt-aactcctgacttcaagca  1106
GGT1.end-start.BCRP3..human.ref                   caatagagatggggtctcactatgttgatcaggctggtctt-aactcctgacttcaagca  1106
GGT1end-LOC749026.end.7456450-7520130.chimp       caatagagatggggtctcactatgttgatcaggctggtctt-aactcctgacttcaagca  1096
GGT1.end-FAM247.start.chimp.ref                   caatagagatggggtctcactatgttgatcaggctggtctt-aactcctgacttcaagca  1096


GGT1.END-GGT5.end.75422027-75453034.mouse         ------------------------------------------------------------  1336
GGT1.END-GGT5.START.75422027-75425161.mouse       ------------------------------------------------------------  1336
GGT1.end-GGT5.beginining.Philippine.tarsier.ref   ------------------------------------------------------------  850
GGT1.end-FAM247.start.Rhesus.ref.                 gtcctcctagcttggcct-ccaaagtgctaggattactttggggattactttagggatga  1157
GGT1.new.NCBI.RefSeqGene                          gtcccccatctcagtctcccaaagtgctaggattac----------------aggggtga  1151
GGT1.end-start.BCRP3..human.ref                   gtcccccatctcagtctcccaaagtgctaggattac----------------aggggtga  1151
GGT1end-LOC749026.end.7456450-7520130.chimp       gtcccccatgtcagtctcccaaagtgctaggattac----------------aggggtga  1141
GGT1.end-FAM247.start.chimp.ref                   gtcccccatgtcagtctcccaaagtgctaggattac----------------aggggtga  1141


GGT1.END-GGT5.end.75422027-75453034.mouse         ------------------------------------------------------------  1336
GGT1.END-GGT5.START.75422027-75425161.mouse       ------------------------------------------------------------  1336
GGT1.end-GGT5.beginining.Philippine.tarsier.ref   -------------------------------gcttgagagaaaaggtcatggccccattt  879
GGT1.end-FAM247.start.Rhesus.ref.                 gtcactgcacgcggcctcaatccttattttggcctgaaaggaaaggctgtggccccgttt  1217
GGT1.new.NCBI.RefSeqGene                          gacactgcacccggcctcaatccttattttggcctgagaggaaaggccgtggccccattt  1211
GGT1.end-start.BCRP3..human.ref                   gacactgcacccggcctcaatccttattttggcctgagaggaaaggccgtggccccattt  1211
GGT1end-LOC749026.end.7456450-7520130.chimp       gccactgcacccggcctcaatccttattttggcctgagaggaaaggccgtggccccattt  1201
GGT1.end-FAM247.start.chimp.ref                   gccactgcacccggcctcaatccttattttggcctgagaggaaaggccgtggccccattt  1201


GGT1.END-GGT5.end.75422027-75453034.mouse         --aggggagaaggcaggattccctaggccaatatattaagatatggtatatcagtgtttc  1394
GGT1.END-GGT5.START.75422027-75425161.mouse       --aggggagaaggcaggattccctaggccaatatattaagatatggtatatcagtgtttc  1394
GGT1.end-GGT5.beginining.Philippine.tarsier.ref   acaggggagaagactgaggccagaagggagggcctt---cctctgagtcgcacagcactg  936
GGT1.end-FAM247.start.Rhesus.ref.                 gcaggggagaagactgaggctggagggcaggcctt---gctctgggttgcacagcagca  1274
GGT1.new.NCBI.RefSeqGene                          gcaggggagaagactgaagctggaggggcaggcctt---gctctgggttgcacagcagca  1268
GGT1.end-start.BCRP3..human.ref                   gcaggggagaagactgaagctggaggggcaggcctt---gctctgggttgcacagcagca  1268
GGT1end-LOC749026.end.7456450-7520130.chimp       gcaggggagaagactgaagctggaggggcaggcctt---gctctgggttgcacagcagca  1258
GGT1.end-FAM247.start.chimp.ref                   gcaggggagaagactgaagctggaggggcaggcctt---gctctgggttgcacagcagca  1258
                                                    ********** * *         **        *     * **

GGT1.END-GGT5.end.75422027-75453034.mouse         atgccccactggactggccggagatttccatggacccagggtgcttgaagtgtaccgc--  1452
GGT1.END-GGT5.START.75422027-75425161.mouse       atgccccactggactggccggagatttccatggacccagggtgcttgaagtgtaccgc--  1452
GGT1.end-GGT5.beginining.Philippine.tarsier.ref   agggaagtgggggctggccatgaggctccctggccccaacatgctga-caggtgtacctc  995
GGT1.end-FAM247.start.Rhesus.ref.                 agagaagtgggagctggccatgaggcttcctggacccgaagcactggtggggttcaccct  1334
GGT1.new.NCBI.RefSeqGene                          acagaagtgggagctggccacgaggcttcctcgactcgacacactggtggggtacaccct  1328
GGT1.end-start.BCRP3..human.ref                   acagaagtgggagctggccacgaggcttcctcgactcgacacactggtggggtacaccct  1328
GGT1end-LOC749026.end.7456450-7520130.chimp       agagaagtgggagctggccacgaggcttcctcgactcgacacactggtggggtacaccct  1318
GGT1.end-FAM247.start.chimp.ref                   agagaagtgggagctggccacgaggcttcctcgactcgacacactggtggggtacaccct  1318
                                                  *         *  ******       * * * * * *      **      **    *

GGT1.END-GGT5.end.75422027-75453034.mouse         -gctctcccagacccactgcgtatagcccagagcttctttg--gagacttggggacttga  1509
GGT1.END-GGT5.START.75422027-75425161.mouse       -gctctcccagacccactgcgtatagcccagagcttctttg--gagacttggggacttga  1509
GGT1.end-GGT5.beginining.Philippine.tarsier.ref   agtgtccctggccctatggggcttagtccaaggctacctca----ggggctggtacttga  1051
GGT1.end-FAM247.start.Rhesus.ref.                 ggttcttcaggtcccatggggctcagcccaggactaccttggtgggggtgggagacttaa  1394
GGT1.new.NCBI.RefSeqGene                          ggttctccaggtcccatggggctcagcccaggactacctcg--gggggtgagggacttaa  1386
GGT1.end-start.BCRP3..human.ref                   ggttctccaggtcccatggggctcagcccaggactacctcg--gggggtgagggacttaa  1386
GGT1end-LOC749026.end.7456450-7520130.chimp       ggttctccaggtcccatggggctcagcccaggactacctcg--gggggtgagggacttaa  1376
GGT1.end-FAM247.start.chimp.ref                   ggttctccaggtcccatggggctcagcccaggactacctcg--gggggtgagggacttaa  1376
                                                   *     *  * ** *  * *   ** ***   ** * *      *     *  **** *

GGT1.END-GGT5.end.75422027-75453034.mouse         gtgaccatcccctttcta---gtgcctttccatgttacttcctggataagggcctttgga  1566
GGT1.END-GGT5.START.75422027-75425161.mouse       gtgaccatcccctttcta---gtgcctttccatgttacttcctggataagggcctttgga  1566
GGT1.end-GGT5.beginining.Philippine.tarsier.ref   attctctcttttcttccaactgtcccctcccccatcacctcctggataaggacactcagg  1111
GGT1.end-FAM247.start.Rhesus.ref.                 atcctctccttcattctcattgtcccttcccccatcatttcctgaggaagcacattcagg  1454
GGT1.new.NCBI.RefSeqGene                          atcctctccttcattctcatcgccccttcccccatcatttcctgaggaaggacattcagg  1446
GGT1.end-start.BCRP3..human.ref                   atcctctccttcattctcatcgccccttcccccatcatttcctgaggaaggacattcagg  1446
GGT1end-LOC749026.end.7456450-7520130.chimp       atcctctccttcattctcatcaccccttcccccatcatttcctgaggaaggacattcagg  1436
GGT1.end-FAM247.start.chimp.ref                   atcctctccttcattctcatcaccccttcccccatcatttcctgaggaaggacattcagg  1436
                                                   *   *       ***        ** * **    * *  *****   ***  *  *  *

GGT1.END-GGT5.end.75422027-75453034.mouse         gccaccagacctggcctt------------------------------------------  1584
GGT1.END-GGT5.START.75422027-75425161.mouse       gccaccagacctggcctt------------------------------------------  1584
GGT1.end-GGT5.beginining.Philippine.tarsier.ref   cccttcccagcggggcct------------------------------------------  1129
GGT1.end-FAM247.start.Rhesus.ref.                 gacctc------------------------------------------------------  1460
GGT1.new.NCBI.RefSeqGene                          gacctgaaggagcggcctgcccctccacatctgtgggtgtttctcatcaggtgggacaag  1506
```

```
GGT1.end-start.BCRP3..human.ref                  gacctgaaggagcggcctgcccctccacatctgtgggtgtttctcatcaggtgggacaag  1506
GGT1end-LOC749026.end.7456450-7520130.chimp      gacctgaaggagcggcctgcccctccacatctgtgggtgtttctcatcaggtgggacaag  1496
GGT1.end-FAM247.start.chimp.ref                  gacctgaaggagcggcctgcccctccacatctgtgggtgtttctcatcaggtgggacaag  1496
                                                   *

GGT1.END-GGT5.end.75422027-75453034.mouse        ------------------------------------------------------------  1584
GGT1.END-GGT5.START.75422027-75425161.mouse      ------------------------------------------------------------  1584
GGT1.end-GGT5.begininig.Philippine.tarsier.ref   ------------------------------------------------------------  1129
GGT1.end-FAM247.start.Rhesus.ref.                ------------------------------------------------------------  1460
GGT1.new.NCBI.RefSeqGene                         agactgagaaaagaaagagacacagagacaaagtatagagaaagaaaagtgggcccaggg  1566
GGT1.end-start.BCRP3..human.ref                  agactgagaaaagaaagagacacagagacaaagtatagagaaagaaaagtgggcccaggg  1566
GGT1end-LOC749026.end.7456450-7520130.chimp      agactgagaaaagaaagagacacagagacaaagtatagagaaagaaaagtgggcccaggg  1556
GGT1.end-FAM247.start.chimp.ref                  agactgagaaaagaaagagacacagagacaaagtatagagaaagaaaagtgggcccaggg  1556


GGT1.END-GGT5.end.75422027-75453034.mouse        ------------------------------------------------------------  1584
GGT1.END-GGT5.START.75422027-75425161.mouse      ------------------------------------------------------------  1584
GGT1.end-GGT5.begininig.Philippine.tarsier.ref   ------------------------------------------------------------  1129
GGT1.end-FAM247.start.Rhesus.ref.                ------------------------------------------------------------  1460
GGT1.new.NCBI.RefSeqGene                         gacctgcgctcagcatacagaggccccacgctggcatcagtctctgagttccctagtatt  1626
GGT1.end-start.BCRP3..human.ref                  gacctgcgctcagcatacagaggccccacgctggcatcagtctctgagttccctagtatt  1626
GGT1end-LOC749026.end.7456450-7520130.chimp      gacctgcgctcagcatatggaggacccacgctggcaccagtctctgagttccctagtatt  1616
GGT1.end-FAM247.start.chimp.ref                  gacctgcgctcagcatatggaggacccacgctggcaccagtctctgagttccctagtatt  1616


GGT1.END-GGT5.end.75422027-75453034.mouse        ------------------------------------------------------------  1584
GGT1.END-GGT5.START.75422027-75425161.mouse      ------------------------------------------------------------  1584
GGT1.end-GGT5.begininig.Philippine.tarsier.ref   ------------------------------------------------------------  1129
GGT1.end-FAM247.start.Rhesus.ref.                ------------------------------------------------------------  1460
GGT1.new.NCBI.RefSeqGene                         tattgatcattatctctaccatctcagagaggggggatgtagcaggacaatatggtaatag  1686
GGT1.end-start.BCRP3..human.ref                  tattgatcattatctctaccatctcagagaggggggatgtagcaggacaatatggtaatag  1686
GGT1end-LOC749026.end.7456450-7520130.chimp      tattgatcattatctctaccatctcagagaggggggatgtggcaggacaacatggtaatag  1676
GGT1.end-FAM247.start.chimp.ref                  tattgatcattatctctaccatctcagagaggggggatgtggcaggacaacatggtaatag  1676


GGT1.END-GGT5.end.75422027-75453034.mouse        ------------------------------------------------------------  1584
GGT1.END-GGT5.START.75422027-75425161.mouse      ------------------------------------------------------------  1584
GGT1.end-GGT5.begininig.Philippine.tarsier.ref   ------------------------------------------------------------  1129
GGT1.end-FAM247.start.Rhesus.ref.                ------------------------------------------------------------  1460
GGT1.new.NCBI.RefSeqGene                         tggggagagggtcagcaggaaaacacgtgaacaaatgtctctgtgtcataaacaaggtta  1746
GGT1.end-start.BCRP3..human.ref                  tggggagagggtcagcaggaaaacacgtgaacaaatgtctctgtgtcataaacaaggtta  1746
GGT1end-LOC749026.end.7456450-7520130.chimp      tggggcgagggtcagcaggaaaacacgtgaacaaatgtctctgtgtcataaacaaggtta  1736
GGT1.end-FAM247.start.chimp.ref                  tggggcgagggtcagcaggaaaacacgtgaacaaatgtctctgtgtcataaacaaggtta  1736


GGT1.END-GGT5.end.75422027-75453034.mouse        ------------------------------------------------------------  1584
GGT1.END-GGT5.START.75422027-75425161.mouse      ------------------------------------------------------------  1584
GGT1.end-GGT5.begininig.Philippine.tarsier.ref   ------------------------------------------------------------  1129
GGT1.end-FAM247.start.Rhesus.ref.                ------------------------------------------------------------  1460
GGT1.new.NCBI.RefSeqGene                         agaaaaaggtgctgtgctttgatgtgcatatacataaacatctcaatgcattaaagagca  1806
GGT1.end-start.BCRP3..human.ref                  agaaaaaggtgctgtgctttgatgtgcatatacataaacatctcaatgcattaaagagca  1806
GGT1end-LOC749026.end.7456450-7520130.chimp      agaaaaaggtgctgtgctttgatgtgcatatacataaacatctcaatgcattaaagagca  1796
GGT1.end-FAM247.start.chimp.ref                  agaaaaaggtgctgtgctttgatgtgcatatacataaacatctcaatgcattaaagagca  1796


GGT1.END-GGT5.end.75422027-75453034.mouse        ------------------------------------------------------------  1584
GGT1.END-GGT5.START.75422027-75425161.mouse      ------------------------------------------------------------  1584
GGT1.end-GGT5.begininig.Philippine.tarsier.ref   ------------------------------------------------------------  1129
GGT1.end-FAM247.start.Rhesus.ref.                ------------------------------------------------------------  1460
GGT1.new.NCBI.RefSeqGene                         gtattgccaccagcatgtcccacctccagccctaaggcagttttctcctatctcagtaga  1866
GGT1.end-start.BCRP3..human.ref                  gtattgccaccagcatgtcccacctccagccctaaggcagttttctcctatctcagtaga  1866
GGT1end-LOC749026.end.7456450-7520130.chimp      gtattgccaccagcatgtcccacctccagccctaaggcagttttctcctatctcagtaga  1856
GGT1.end-FAM247.start.chimp.ref                  gtattgccaccagcatgtcccacctccagccctaaggcagttttctcctatctcagtaga  1856


GGT1.END-GGT5.end.75422027-75453034.mouse        ------------------------------------------------------------  1584
GGT1.END-GGT5.START.75422027-75425161.mouse      ------------------------------------------------------------  1584
GGT1.end-GGT5.begininig.Philippine.tarsier.ref   ------------------------------------------------------------  1129
GGT1.end-FAM247.start.Rhesus.ref.                ------------------------------------------------------------  1460
GGT1.new.NCBI.RefSeqGene                         tggaatatacaattgggttttac------acattcctttgcccagggacgatcaggagac  1920
GGT1.end-start.BCRP3..human.ref                  tggaatatacaattgggttttac------acattcctttgcccagggacgatcaggagac  1920
GGT1end-LOC749026.end.7456450-7520130.chimp      tggaatatacaattgggttttacaccgagacattcctttgcccagggacgatcaggagac  1916
GGT1.end-FAM247.start.chimp.ref                  tggaatatacaattgggttttacaccgagacattcctttgcccagggacgatcaggagac  1916


GGT1.END-GGT5.end.75422027-75453034.mouse        ------------------------------------------------------------  1584
GGT1.END-GGT5.START.75422027-75425161.mouse      ------------------------------------------------------------  1584
GGT1.end-GGT5.begininig.Philippine.tarsier.ref   ------------------------------------------------------------  1129
GGT1.end-FAM247.start.Rhesus.ref.                ------------------------------------------------------------  1460
GGT1.new.NCBI.RefSeqGene                         agatgccttcctcttatctcaactgcaaagaggccttccttcctcttatactaatcctcc  1980
GGT1.end-start.BCRP3..human.ref                  agatgccttcctcttatctcaactgcaaagaggccttccttcctcttatactaatcctcc  1980
GGT1end-LOC749026.end.7456450-7520130.chimp      agatgccttcctcttgtctcaactgcaaagaggccttccttcctcttatactaatcctcc  1976
GGT1.end-FAM247.start.chimp.ref                  agatgccttcctcttgtctcaactgcaaagaggccttccttcctcttatactaatcctcc  1976


GGT1.END-GGT5.end.75422027-75453034.mouse        ------------------------------------------------------------  1584
GGT1.END-GGT5.START.75422027-75425161.mouse      ------------------------------------------------------------  1584
GGT1.end-GGT5.begininig.Philippine.tarsier.ref   ------------------------------------------------------------  1129
GGT1.end-FAM247.start.Rhesus.ref.                ------------------------------------------------------------  1460
GGT1.new.NCBI.RefSeqGene                         tcagcacagaccctttacgggtgtcgggct------------------------------  2010
GGT1.end-start.BCRP3..human.ref                  tcagcacagaccctttacgggtgtcgggctggggaacggtcaggtcttttccttcccaca  2040
GGT1end-LOC749026.end.7456450-7520130.chimp      tcagcacagaccctttacgggtgtcgggctgggggacggtcaggtctttctcttcccacg  2036
GGT1.end-FAM247.start.chimp.ref                  tcagcacagaccctttacgggtgtcgggctgggggacggtcaggtctttctcttcccacg  2036
```

S1 Fig. b. Alignment of sequence from the *Microcebus murinus* (gray mouse) lemur with part of the 3′ end sequence the (GGT1), RefSeqGene sequence.

```
# Aligned_sequences: 2
# 1: lemur.1-1004
# 2: human.1-1031 represents the lAST 1031 bp of the (GGT1), RefSeqGene that extends 2010 bp beyond the GGT1 gene at its 3' end.
# Matrix: EDNAFULL
# Gap_penalty: 16
# Extend_penalty: 4
#
# Length: 1042
# Identity:     870/1042 (83.5%)
# Similarity:   870/1042 (83.5%)
# Gaps:          49/1042 ( 4.7%)
# Score: 3482
#
#
#=======================================

lemur.1-1004       1 --------CTCTGGGCCTC--TGTCCTGTGTGTAAAATGGAGCCATCTGG     40
                             |||||||||||  |||..|||||||.||||||||||||||||
human.1-1031       1 TCCCCACTCTCTGGGCCTCAGTGTATTGTGTGTGAAATGGAGCCATCTGG     50

lemur.1-1004      41 CTGGGGAGGAACGGAGAGGTGGGATTCAGAGATCTTCACACTGCAGGCGC     90
                     ||||||||||||.||||||||||||||.||||||||||||.|||.|||.|
human.1-1031      51 CTGGGGAGGAACAGAGAGGTGGGATTCGGAGATCTTCACAATGCGGGCAC    100

lemur.1-1004      91 TGGAACGAGCTTCAGCATCTTCAGCACGGGGAGAGCCAGGCGCATGGCTG    140
                     ||||||.|||.|||||||||||||||.||||||||||||||.||||||||
human.1-1031     101 TGGAACTAGCCTCAGCATCTTCAGCATGGGGAGAGCCAGGCACATGGCTG    150

lemur.1-1004     141 GGGGCCGGGGGCAGGTCTGCGCCAAGCCCTGCCCCACCCAACCCTGAGCA    190
                     ||||||.||||.||||...|.||||||.|||||||..||.|||||||.|.
human.1-1031     151 GGGGCCAGGGGAAGGTTCACACCAAGCTCTGCCCCTTCCCACCCTGATCC    200

lemur.1-1004     191 CTAAGACTT-GGGGACAGGCCCTCCCTTCCTGGGGCTGGGCAGCGACACT    239
                     ||..||||| ||||.|||||||||||||.||||||||||||||.||||||
human.1-1031     201 CTCGGACTTTGGGGCCAGGCCCTCCCTTACTGGGGCTGGGCAGTGACACT    250

lemur.1-1004     240 ACCTAGGATCAGCCACCAGAGTGCGCCACGACCCTAGCACTT----AGGC    285
                     |||||||||||||||||||.|.|..||||||||||.||||||    ||||
human.1-1031     251 ACCTAGGATCAGCCACCAGGGGGTACCACGACCCTGGCACTTTCTTAGGC    300

lemur.1-1004     286 AGAGGGTGGCCAGCTGTTGCCAGGAACCCCGGGCGCCTTCTCAGACCCGC    335
                     ||||||||||||||||.|||..||||||| |||.|||||||.|||||||.
human.1-1031     301 AGAGGGTGGCCAGCTGATGCTGGGAACCC-GGGTGCCTTCTTAGACCCGT    349

lemur.1-1004     336 GGGCGTCCAGCTAACCCTGCCGAGGACACAGGGAGGTGAAGCTGAGGTCT    385
                     .|||||||||||.||||||||||.|||||.|| ||||||||||||||||.
human.1-1031     350 AGGCGTCCAGCTCACCCTGCCGATGACACTGG-AGGTGAAGCTGAGGTCC    398

lemur.1-1004     386 GAGGAGTGGGGATTGGGCAAGAGGCTGAAGCAAAACATCCCAGTCAGAGC    435
                     |||||.||||||.|||||||.||||||.||.||||||||.|.||||||||
human.1-1031     399 GAGGAATGGGGACTGGGCAACAGGCTGGAGGAAAACATCTCGGTCAGAGC    448

lemur.1-1004     436 CAAGCCCTTGGAGGGGGGTGCTTTCCAAGTGCAAGCCCAGAATGAAACCC    485
                     ||.||||.|||.|||      |||||||||..|||||||||.||||||||
human.1-1031     449 CACGCCCCTGGGGGG------TTTCCAAGTTTAAGCCCAGAGTGAAACCC    492

lemur.1-1004     486 AAGCTTGTGATTCTCTCCAGAGGGAGGCCTGTTTCTCAGGGAACAGCAAA    535
                     |||||||||||.|||||||||||||||||||.||||||||||||||||||
human.1-1031     493 AAGCTTGTGATCCTCTCCAGAGGGAGGCCTGGTTCTCAGGGAACAGCAAA    542

lemur.1-1004     536 CTGGAAGAGGTCCCCAGATCCCAGGGATCAGGGCTTGGCCCAGCTGGGGA    585
                     |.||||||.|||||||||||||||||||||||||||||.|||||.|||||
human.1-1031     543 CGGGAAGATGTCCCCAGATCCCAGGGATCAGGGCTTGGACCAGCCGGGGA    592

lemur.1-1004     586 TACAGCCCAGAGGGAGTGGGTCTTGAAGGGATCAGCTAGACACGGTGGCC    635
                     ..||||||||||||||||||||..|||||.|.|||||||||||.|..|||
human.1-1031     593 CGCAGCCCAGAGGGAGTGGGTCCAGAAGGAAACAGCTAGACACAGCAGCC    642

lemur.1-1004     636 TTCACCATCGGCAGCCCCGCCAGGCCTTCCTC----CCTTGTCC----TC    677
                     ||||||||||||||||||.||||||||.||||    |||..|||    ||
human.1-1031     643 TTCACCATCGGCAGCCCCTCCAGGCCTCCCTCAGGGCCTGCTCCCTCCTC    692

lemur.1-1004     678 TGCACACACGTTTTCCAACAC-TGGGGAGGGGTTCTGGGCAAGGCTGGTG    726
                     ||..||||.   ||||||||| |||||..||||||||||.|.||||||||
human.1-1031     693 TGTGCACAG---TTCCAACACCTGGGGCAGGGTTCTGGGAAGGGCTGGTG    739

lemur.1-1004     727 GAGGTGGGCTGGTGAGGGGTGGTGGTCACAGCCCAGCACCTGGATATCGC    776
                     ||||||||||||||.|.||.||||.|||||||||||||||||||||||.|
human.1-1031     740 GAGGTGGGCTGGTGGGAGGCGGTGATCACAGCCCAGCACCTGGATATCAC    789

lemur.1-1004     777 CAGGGGCACTGGGGCCAGGGACCA--------CA--TCATGGCTCTTGTT    816
                     ||||||||||||||||||||.|||        ||  ||..||||.|..||
```

```
human.1-1031     790 CAGGGGCACTGGGGCCAGGGGCCAGGTGAGGCCAGGTCGGGGCTATCCTT    839

lemur.1-1004     817 CAGGAGCCT-GAAAACCTGGTGACTGCAAAGGGCCCACAGGCAAACAGGG    865
                     |||||.||. |||.|||||||||.|.|||||||||||.||.|||||||||
human.1-1031     840 CAGGATCCCCGAAGACCTGGTGATTCCAAAGGGCCCATAGACAAACAGGG    889

lemur.1-1004     866 TTTTGTGCCTGCCAAGGAGAGTTCCACTGGGTCTGAGCCCTGGAGGGTTC    915
                     ||||.||||||...||...|||.|||||||||||||||.||||||||.|.
human.1-1031     890 TTTTCTGCCTGTGGAGTCAAGTCCCACTGGGTCTGAGCTCTGGAGGGCTG    939

lemur.1-1004     916 TGCCTCTGGGACTCCCCAGGGGTGAGATGGAGGTGGGCTTAACTGGTGTA    965
                     ||.|||||||.|||..|||||||||||||||||||||||.||||||||||
human.1-1031     940 TGTCTCTGGGGCTCTGCAGGGGTGAGATGGAGGTGGGCTCAACTGGTGTA    989

lemur.1-1004     966 CAAGTGACTCCTCAATCCTTATTTACTTAATTT---TTTTTT   1004
                     |||||.||||.|||||||||||||||..||.||||   ||||||
human.1-1031     990 CAAGTCACTCTTCAATCCTTATTTTATTTATTTAATTTTTTT   1031
```
