# Supplementary material for: An ancestral genomic sequence that serves as a nucleation site for de novo gene birth
Source: PLoS One. 2022 May 12;17(5):e0267864. doi: 10.1371/journal.pone.0267864 (PMC9097989; doi:10.1371/journal.pone.0267864)
Supplement: S2 Fig — (PDF) [file pone.0267864.s002.pdf]

Detection of an ancestral genomic sequence that serves as a nucleation site for de novo gene birth

Nicholas Delihas
Department of Microbiology and Immunology, Renaissance School of Medicine, Stony Brook University, Stony Brook, N.Y., United States of America

S2 Fig. Complete spacer nucleotide sequences aligned. Several invariant sequences are highlighted (light blue), the FAM247 start sequence (green), and the mouse *GGT5* sequence (red). The sequences that have been used can be obtained from the author Email: Nicholas.delihas@stonybrook.edu. The file size was too large when included.

```
CLUSTAL O(1.2.4) multiple sequence alignment


GGT5.total.&minus3200bp.mouse                   gaagcctttgcgggctgaactttccatatcatggactttagtgtcttgtgtatgaaatgg    60
GGT5.nt.mouse                                   ------------------------------------------------------------    0
FAM247.LOC105372935.ref.human                   ------------------------------------------------------------    0
GGT1.end-GGT5.beginining.Philippine.tarsier.ref ------------------------------------------------------------    0
GGT5.end-GGT1.beginning.Rhesus.28419652-28635852 ----------------------------ctctgggcctcagtgtattgtgtgtgaaatgg    32
spacer.GGT1.end-start.BCRP3..human.ref          -------------------ctccccactctctgggcctcagtgtattgtgtgtgaaatgg    41
GGT1end-LOC749026.end.7456450-7520130.chimp     ----------------------------ctctgggcctcagtgtattctgtgtgaaatgg    32


GGT5.total.&minus3200bp.mouse                   agtcatctcacttggaa--aatgacaaggcaggatcctaagacttaccgtgatgttaagt    118
GGT5.nt.mouse                                   ------------------------------------------------------------    0
FAM247.LOC105372935.ref.human                   ------------------------------------------------------------    0
GGT1.end-GGT5.beginining.Philippine.tarsier.ref ------------------------------------------------------------    0
GGT5.end-GGT1.beginning.Rhesus.28419652-28635852 aaccatctggctggggaggaatggagaggtgggattcggagatcttcac-actgcggtcg    91
spacer.GGT1.end-start.BCRP3..human.ref          agccatctggctggggaggaacagagaggtgggattcggagatcttcac-aatgcgggca    100
GGT1end-LOC749026.end.7456450-7520130.chimp     agccatctggctggggaggaacagagaggtgggattcggagatcttcac-aatgtgggca    91


GGT5.total.&minus3200bp.mouse                   catgaagctaacaccaacctttaagtgggggaatccca---ccgtgcctgagagttagg    175
GGT5.nt.mouse                                   ------------------------------------------------------------    0
FAM247.LOC105372935.ref.human                   ------------------------------------------------------------    0
GGT1.end-GGT5.beginining.Philippine.tarsier.ref ------------------------------------------------------------    0
GGT5.end-GGT1.beginning.Rhesus.28419652-28635852 ctggaactagcctcagtatcttcagcgtggggagagccaggtgcgtggctagggaccagg    151
spacer.GGT1.end-start.BCRP3..human.ref          ctggaactagcctcagcatcttcagcatggggagagccaggcacatggctgggggccagg    160
GGT1end-LOC749026.end.7456450-7520130.chimp     ctggaactagcctcaacatcttcagcatggggagagccaggcacatggctgggggccagg    151


GGT5.total.&minus3200bp.mouse                   ggga---------------------------------------------tggggtaaaca    190
GGT5.nt.mouse                                   ------------------------------------------------------------    0
FAM247.LOC105372935.ref.human                   ------------------------------------------------------------    0
GGT1.end-GGT5.beginining.Philippine.tarsier.ref ------------------------------------------------------------    0
GGT5.end-GGT1.beginning.Rhesus.28419652-28635852 ggaaggtccatgccaacccctgccccttcccaccctgatccattggactttggggccagg    211
spacer.GGT1.end-start.BCRP3..human.ref          ggaaggttcacaccaagctctgccccttcccaccctgatccctcggactttggggccagg    220
GGT1end-LOC749026.end.7456450-7520130.chimp     ggaaggttcacaccaagctctgccccttcccaccctgatccctcggactttggggccagg    211


GGT5.total.&minus3200bp.mouse                   gaacacctaaattgtcctaagcagtgacacttgc-aatatcagccactagagggcgcccc    249
GGT5.nt.mouse                                   ------------------------------------------------------------    0
FAM247.LOC105372935.ref.human                   ------------------------------------------------------------    0
GGT1.end-GGT5.beginining.Philippine.tarsier.ref ------------------------------------------------------------    0
GGT5.end-GGT1.beginning.Rhesus.28419652-28635852 tgctcccttattggggctgcacagtgac---acctaggactagccaccagggggtgccgc    268
spacer.GGT1.end-start.BCRP3..human.ref          ccctcccttactggggctgggcagtgacactacctaggatcagccaccagggggtaccac    280
GGT1end-LOC749026.end.7456450-7520130.chimp     ccctcccttactggggctgggcagtgacactacctaggatcagccaccgggggtgccac    271


GGT5.total.&minus3200bp.mouse                   gaccttgccattaactggaacaaagcaatgaca-ctgtagctgtgggtggagaatgcttc    308
GGT5.nt.mouse                                   ------------------------------------------------------------    0
FAM247.LOC105372935.ref.human                   ------------------------------------------------------------    0
GGT1.end-GGT5.beginining.Philippine.tarsier.ref ------------------------------------------------------------    0
GGT5.end-GGT1.beginning.Rhesus.28419652-28635852 gcccctggtgctttcttaggcagtgggtggccagctgatgctgggaacctgggcaccttc    328
spacer.GGT1.end-start.BCRP3..human.ref          gaccctggcactttcttaggcagagggtggccagctgatgctgggaacccgggtgccttc    340
GGT1end-LOC749026.end.7456450-7520130.chimp     gaccctggcactttcttaggcagagggtggccagctgatgctgggaacccgggcgccttc    331


GGT5.total.&minus3200bp.mouse                   taggacagatgtg-----ggctctcattggcccgcacttaggggtaaaactgaa-----    358
GGT5.nt.mouse                                   ------------------------------------------------------------    0
FAM247.LOC105372935.ref.human                   ------------------------------------------------------------    0
GGT1.end-GGT5.beginining.Philippine.tarsier.ref ------------------------------------------------------------    0
GGT5.end-GGT1.beginning.Rhesus.28419652-28635852 tcagacccatgggcatccaactcatcctgctaatgacacgggaggtgaagctgagttcca    388
spacer.GGT1.end-start.BCRP3..human.ref          ttagacccgtaggcgtccagctcaccctgccgatgacactggaggtgaagctgaggtccg    400
GGT1end-LOC749026.end.7456450-7520130.chimp     tcagacccgtaggcgtccagctcaccctgccgatgacactggaggtgaagctgaggtccg    391


GGT5.total.&minus3200bp.mouse                   ------ggagagtagactagagacaaaaacaaggcaatttggctaaggccatacttgagg    412
GGT5.nt.mouse                                   ------------------------------------------------------------    0
FAM247.LOC105372935.ref.human                   ------------------------------------------------------------    0
GGT1.end-GGT5.beginining.Philippine.tarsier.ref ------------------------------------------------------------    0
```

```
GGT5.end-GGT1.beginning.Rhesus.28419652-28635852      aggaatgggaattgggcatcacgctagaggaaaacatcttagtcagagccaagcccctgg    448
spacer.GGT1.end-start.BCRP3..human.ref                aggaatggggactgggcaacaggctggaggaaaacatctcggtcagagccacgcccctgg    460
GGT1end-LOC749026.end.7456450-7520130.chimp           aggaatggggactgggcaacaggctggaggaaaacatctcggtcagagccacgcccctgg    451


GGT5.total.&minus3200bp.mouse                         tcactttccaagcacaaacccagagtgaaagccaagcttg--------tcttgcaagagg    464
GGT5.nt.mouse                                         ------------------------------------------------------------    0
FAM247.LOC105372935.ref.human                         ------------------------------------------------------------    0
GGT1.end-GGT5.begining.Philippine.tarsier.ref         ------------------------------------------------------------    0
GGT5.end-GGT1.beginning.Rhesus.28419652-28635852      ggggtttccaagtataagcccagagtgaaacccaagcttgtgaccctctccagagggagg    508
spacer.GGT1.end-start.BCRP3..human.ref                ggggtttccaagtttaagcccagagtgaaacccaagcttgtgatcctctccagagggagg    520
GGT1end-LOC749026.end.7456450-7520130.chimp           ggggtttccaagtttaagcccagagtgaaacccaagcttgtgatcctctccagagggagg    511


GGT5.total.&minus3200bp.mouse                         cccagttctcaaacaaacagacagtcccagcaggtccgccagctatagaaaacccacaga    524
GGT5.nt.mouse                                         ------------------------------------------------------------    0
FAM247.LOC105372935.ref.human                         ------------------------------------------------------------    0
GGT1.end-GGT5.begining.Philippine.tarsier.ref         ------------------------------------------------------------    0
GGT5.end-GGT1.beginning.Rhesus.28419652-28635852      cctggttttcagggaacagcaaatgggaagaggtccccagattccagggatcagggcttg    568
spacer.GGT1.end-start.BCRP3..human.ref                cctggttctcagggaacagcaaacgggaagatgtccccagatcccagggatcagggcttg    580
GGT1end-LOC749026.end.7456450-7520130.chimp           cctggttctcagggaacagcaaacgggaagatgtccccagatcccagggatcagggcttg    571


GGT5.total.&minus3200bp.mouse                         tccacaaggggaacacagctcacaatggatgagcccagaagagcaccattagatacatcc    584
GGT5.nt.mouse                                         ------------------------------------------------------------    0
FAM247.LOC105372935.ref.human                         ------------------------------------------------------------    0
GGT1.end-GGT5.begining.Philippine.tarsier.ref         ------------------------------------------------------------    0
GGT5.end-GGT1.beginning.Rhesus.28419652-28635852      gaccagctggggacgcagcccagagggagtgggtctggaagggaacagctagacacagca    628
spacer.GGT1.end-start.BCRP3..human.ref                gaccagccggggacgcagcccagagggagtgggtccagaaggaaacagctagacacagca    640
GGT1end-LOC749026.end.7456450-7520130.chimp           gaccagccggggacgcagcccagagggagtgggtccggaaggaaacagctcgacacagca    631


GGT5.total.&minus3200bp.mouse                         ctccttgtgttagtcagggtttctattgctgcaatgaaagaccatgaccaaagcagcttg    644
GGT5.nt.mouse                                         ------------------------------------------------------------    0
FAM247.LOC105372935.ref.human                         ------------------------------------------------------------    0
GGT1.end-GGT5.begining.Philippine.tarsier.ref         ------------------------------------------------------------    0
GGT5.end-GGT1.beginning.Rhesus.28419652-28635852      gccttcaccactggcagcccctccc-----------------------------------    653
spacer.GGT1.end-start.BCRP3..human.ref                gccttcaccatcggcagcccctcca-----------------------------------    665
GGT1end-LOC749026.end.7456450-7520130.chimp           gccttcaccatcggcagcccctcca-----------------------------------    656


GGT5.total.&minus3200bp.mouse                         gagaggaaagggtttatttggcttacatttccacatcacagttgaccatgaaaggtaagg    704
GGT5.nt.mouse                                         ------------------------------------------------------------    0
FAM247.LOC105372935.ref.human                         ------------------------------------------------------------    0
GGT1.end-GGT5.begining.Philippine.tarsier.ref         ------------------------------------------------------------    0
GGT5.end-GGT1.beginning.Rhesus.28419652-28635852      ------------------------------------------------------------    653
spacer.GGT1.end-start.BCRP3..human.ref                ------------------------------------------------------------    665
GGT1end-LOC749026.end.7456450-7520130.chimp           ------------------------------------------------------------    656


GGT5.total.&minus3200bp.mouse                         acaggcactcagacgggccaggaacctggagacaggagccaatacaggggccatggaaga    764
GGT5.nt.mouse                                         ------------------------------------------------------------    0
FAM247.LOC105372935.ref.human                         ------------------------------------------------------------    0
GGT1.end-GGT5.begining.Philippine.tarsier.ref         ------------------------------------------------------------    0
GGT5.end-GGT1.beginning.Rhesus.28419652-28635852      ------------------------------------------------------------    653
spacer.GGT1.end-start.BCRP3..human.ref                ------------------------------------------------------------    665
GGT1end-LOC749026.end.7456450-7520130.chimp           ------------------------------------------------------------    656


GGT5.total.&minus3200bp.mouse                         gtgcgacttgctcctcatggcttgttcagccttctttcttatagaacccaggaccttcag    824
GGT5.nt.mouse                                         ------------------------------------------------------------    0
FAM247.LOC105372935.ref.human                         ------------------------------------------------------------    0
GGT1.end-GGT5.begining.Philippine.tarsier.ref         ------------------------------------------------------------    0
GGT5.end-GGT1.beginning.Rhesus.28419652-28635852      ------------------------------------------------------------    653
spacer.GGT1.end-start.BCRP3..human.ref                ------------------------------------------------------------    665
GGT1end-LOC749026.end.7456450-7520130.chimp           ------------------------------------------------------------    656


GGT5.total.&minus3200bp.mouse                         cccagggatggcgccacccacaataggctaaaccctcctccatccatcactaatttaaaa    884
GGT5.nt.mouse                                         ------------------------------------------------------------    0
FAM247.LOC105372935.ref.human                         ------------------------------------------------------------    0
GGT1.end-GGT5.begining.Philippine.tarsier.ref         ------------------------------------------------------------    0
GGT5.end-GGT1.beginning.Rhesus.28419652-28635852      ------------ggcctccctcggggcctgctccctcctccaagcaccgttcc-------    694
spacer.GGT1.end-start.BCRP3..human.ref                ------------ggcctccctcagggcctgctccctcctctgtgcacagttcc-------    706
GGT1end-LOC749026.end.7456450-7520130.chimp           ------------ggcctccctcggggcctgctccctcctctgtgcacagttcc-------    697


GGT5.total.&minus3200bp.mouse                         actgtcctgcaggcttgcccacagctagatcttatggaggtattttctcagttgaggctc    944
GGT5.nt.mouse                                         ------------------------------------------------------------    0
FAM247.LOC105372935.ref.human                         ------------------------------------------------------------    0
GGT1.end-GGT5.begining.Philippine.tarsier.ref         ------------------------------------------------------------    0
GGT5.end-GGT1.beginning.Rhesus.28419652-28635852      ------------------------------------------------------------    694
spacer.GGT1.end-start.BCRP3..human.ref                ------------------------------------------------------------    706
GGT1end-LOC749026.end.7456450-7520130.chimp           ------------------------------------------------------------    697


GGT5.total.&minus3200bp.mouse                         cctctctgatgactctagtttgtgtcaagttgatataaaactagccaacacactctttaa    1004
GGT5.nt.mouse                                         ------------------------------------------------------------    0
FAM247.LOC105372935.ref.human                         ------------------------------------------------------------    0
GGT1.end-GGT5.begining.Philippine.tarsier.ref         ------------------------------------------------------------    0
GGT5.end-GGT1.beginning.Rhesus.28419652-28635852      ------------------------------------------------------------    694
spacer.GGT1.end-start.BCRP3..human.ref                ------------------------------------------------------------    706
GGT1end-LOC749026.end.7456450-7520130.chimp           ------------------------------------------------------------    697
```

```
GGT5.total.&minus3200bp.mouse                      gggcagcccctcatgctgtgtcaaggtctatagaactcacagctgtgaccacgaggtaaa    1064
GGT5.nt.mouse                                      ------------------------------------------------------------    0
FAM247.LOC105372935.ref.human                      ------------------------------------------------------------    0
GGT1.end-GGT5.begining.Philippine.tarsier.ref      ------------------------------------------------------------    0
GGT5.end-GGT1.beginning.Rhesus.28419652-28635852   -----------------------------------------------aacacctggggca    707
spacer.GGT1.end-start.BCRP3..human.ref             -----------------------------------------------aacacctggggca    719
GGT1end-LOC749026.end.7456450-7520130.chimp        -----------------------------------------------aacacctggggca    710

GGT5.total.&minus3200bp.mouse                      gagacacttggagacaatgtagaagtgagccaccgg-----ggtagtcacagcctaggag    1119
GGT5.nt.mouse                                      ------------------------------------------------------------    0
FAM247.LOC105372935.ref.human                      ------------------------------------------------------------    0
GGT1.end-GGT5.begining.Philippine.tarsier.ref      ------------------------------------------------------------    0
GGT5.end-GGT1.beginning.Rhesus.28419652-28635852   gggttctgggaaaggctggtggaggtgggctggtgggggggcggtgatcacagcccagcat    767
spacer.GGT1.end-start.BCRP3..human.ref             gggttctgggaagggctggtggaggtgggctggtgggaggcggtgatcacagcccagcac    779
GGT1end-LOC749026.end.7456450-7520130.chimp        gggttctgggaagggctggtggaggtgggctggtgggaggcggtgatcacagcccagcac    770

GGT5.total.&minus3200bp.mouse                      ctaaatattgccccttaaagtgagcacaagttgggggtccctgagggaagggacccagtg    1179
GGT5.nt.mouse                                      ------------------------------------------------------------    0
FAM247.LOC105372935.ref.human                      ------------------------------------------------------------    0
GGT1.end-GGT5.begining.Philippine.tarsier.ref      ------------------------------------------------------------    0
GGT5.end-GGT1.beginning.Rhesus.28419652-28635852   ctgggtatcacca---------------------ggggcactggggccagggcccaggtg    806
spacer.GGT1.end-start.BCRP3..human.ref             ctggatatcacca---------------------ggggcactggggccaggggccaggtg    818
GGT1end-LOC749026.end.7456450-7520130.chimp        ctggatatcacca---------------------ggggcactggggccaggggccaggtg    809

GGT5.total.&minus3200bp.mouse                      gctggaggagggtcc-------caggaggctagaagtcctggtgtttccaacctgcccat    1232
GGT5.nt.mouse                                      ------------------------------------------------------------    0
FAM247.LOC105372935.ref.human                      ------------------------------------------------------------    0
GGT1.end-GGT5.begining.Philippine.tarsier.ref      ------------------------------------------------------------    0
GGT5.end-GGT1.beginning.Rhesus.28419652-28635852   aagccaggtcggggctctcctttagaagccccgaaaacctggtgataccaaagggcccac    866
spacer.GGT1.end-start.BCRP3..human.ref             aggccaggtcggggctatccttcaggatccccgaagacctggtgattccaaagggcccat    878
GGT1end-LOC749026.end.7456450-7520130.chimp        aggccaggtcggggctatccttcaggagccccgaaaacctggtgattccaaagggcccat    869

GGT5.total.&minus3200bp.mouse                      gggaaagcagggttttcctgtaggtgtg--------ctccatgtctgagctctaaagctc    1284
GGT5.nt.mouse                                      ------------------------------------------------------------    0
FAM247.LOC105372935.ref.human                      ------------------------------------------------------------    0
GGT1.end-GGT5.begining.Philippine.tarsier.ref      ------------------------------------------------------------    0
GGT5.end-GGT1.beginning.Rhesus.28419652-28635852   agacaaacagggttttgtgcctgcggagttgagtaccaccgggtctaagccctggagggc    926
spacer.GGT1.end-start.BCRP3..human.ref             agacaaacagggttttctgcctgtggagtcaagtcccactgggtctgagctctggagggc    938
GGT1end-LOC749026.end.7456450-7520130.chimp        agacaaacagggttttctgcctgtggagtcaagtcccactgggtctgagctctggagggc    929

GGT5.total.&minus3200bp.mouse                      tctatctctggggctccttggggaacaca-gaagctgggctgaagggggggtgccagcc    1343
GGT5.nt.mouse                                      ------------------------------------------------------------    0
FAM247.LOC105372935.ref.human                      ------------------------------------------------------------    0
GGT1.end-GGT5.begining.Philippine.tarsier.ref      ------------------------------------------------------------    0
GGT5.end-GGT1.beginning.Rhesus.28419652-28635852   tgtgtccctggggctccccaggggtgagatggaggtgggctcaa--ctggtgtacccgtc    984
spacer.GGT1.end-start.BCRP3..human.ref             tgtgtctctggggctctgcaggggtgagatggaggtgggctcaa--ctggtgtacaagtc    996
GGT1end-LOC749026.end.7456450-7520130.chimp        tgtgtctctggggctctgcaagggtgagatggaggtgggctcaa--atggtgtacaagtc    987

GGT5.total.&minus3200bp.mouse                      attcctcaatccatatttgaa---------------------------------------    1364
GGT5.nt.mouse                                      ------------------------------------------------------------    0
FAM247.LOC105372935.ref.human                      ------------------------------------------------------------    0
GGT1.end-GGT5.begining.Philippine.tarsier.ref      ------------------------------------------------------------    0
GGT5.end-GGT1.beginning.Rhesus.28419652-28635852   actcctcaatccttattttatgtatttaatttttaaaaaaatttttatttgaacaaatag    1044
spacer.GGT1.end-start.BCRP3..human.ref             actcttcaatccttattttatttatttaattttttttaaaaa---aaatttaaaccaatag    1053
GGT1end-LOC749026.end.7456450-7520130.chimp        actcctcaatccttattttatttatttaattttttttaa-aa---aaatttaaaccaatag    1043

GGT5.total.&minus3200bp.mouse                      ------------------------------------------------------------    1364
GGT5.nt.mouse                                      ------------------------------------------------------------    0
FAM247.LOC105372935.ref.human                      ------------------------------------------------------------    0
GGT1.end-GGT5.begining.Philippine.tarsier.ref      -----ggctcccactctctgggcctcggtgtcctg---tgtgtgaattggaga-------    45
GGT5.end-GGT1.beginning.Rhesus.28419652-28635852   agatggggtctcactatgttgaccaggctggtcttaaactcttgacttcaagcagtcctc    1104
spacer.GGT1.end-start.BCRP3..human.ref             agatggggtctcactatgttgatcaggctggtctt-aactcctgacttcaagcagtcccc    1112
GGT1end-LOC749026.end.7456450-7520130.chimp        agatggggtctcactatgttgatcaggctggtctt-aactcctgacttcaagcagtcccc    1102

GGT5.total.&minus3200bp.mouse                      ----------------------------------------------ggggagaaggcagg    1378
GGT5.nt.mouse                                      ------------------------------------------------------------    0
FAM247.LOC105372935.ref.human                      ------------------------------------------------------------    0
GGT1.end-GGT5.begining.Philippine.tarsier.ref      -----------catcgggctggcagcaatggtgtggggggttctgaggtctt-------c    87
GGT5.end-GGT1.beginning.Rhesus.28419652-28635852   ctagcttggcct-ccaaagtgctaggattactttggggattactttagggatgagtcact    1163
spacer.GGT1.end-start.BCRP3..human.ref             ccatctcagtctcccaaagtgctaggattac---------------aggggtgagacact    1157
GGT1end-LOC749026.end.7456450-7520130.chimp        ccatgtcagtctcccaaagtgctaggattac---------------aggggtgagccact    1147

GGT5.total.&minus3200bp.mouse                      attccctaggccaatatattaagatatggtatatcagtgtttcatgccccactggactgg    1438
GGT5.nt.mouse                                      ------------------------------------------------------------    0
FAM247.LOC105372935.ref.human                      ------------------------------------------------------------    0
GGT1.end-GGT5.begining.Philippine.tarsier.ref      acactggagctccctcagcattttcagtagggcgagagcctgggggctggtggcgaggg    147
GGT5.end-GGT1.beginning.Rhesus.28419652-28635852   gcacgcggcctcaatccttattttggcctgaaaggaaaggctgtggccccgtttgcaggg    1223
spacer.GGT1.end-start.BCRP3..human.ref             gcacccggcctcaatccttattttggcctgagaggaaaggccgtggccccatttgcaggg    1217
GGT1end-LOC749026.end.7456450-7520130.chimp        gcacccggcctcaatccttattttggcctgagaggaaaggccgtggccccatttgcaggg    1207

GGT5.total.&minus3200bp.mouse                      ccggagatttccat----------------------------------------------    1452
```

```
GGT5.nt.mouse                                         ------------------------------------------------------------    0
FAM247.LOC105372935.ref.human                         ------------------------------------------------------------    0
GGT1.end-GGT5.begining.Philippine.tarsier.ref         ac--aggggcaggc-cagtgctaagcacttgctcct------------------------    180
GGT5.end-GGT1.beginning.Rhesus.28419652-28635852      gagaagactgaggctggaggggcaggccttgctctgggttgcacagcagcaagagaagtg    1283
spacer.GGT1.end-start.BCRP3..human.ref                gagaagactgaagctggaggggcaggccttgctctgggttgcacagcagcaacagaagtg    1277
GGT1end-LOC749026.end.7456450-7520130.chimp           gagaagactgaagctggaggggcaggccttgctctgggttgcacagcagcaagagaagtg    1267


GGT5.total.&minus3200bp.mouse                         ----------------------ggacccagggtgcttgaagtgtaccgc---gctctccc    1487
GGT5.nt.mouse                                         ------------------------------------------------------------    0
FAM247.LOC105372935.ref.human                         ------------------------------------------------------------    0
GGT1.end-GGT5.begining.Philippine.tarsier.ref         ----------------------cctctcc-----------------------------ca    189
GGT5.end-GGT1.beginning.Rhesus.28419652-28635852      ggagctggccatgaggcttcctggacccgaagcactggtggggttcaccctggttcttca    1343
spacer.GGT1.end-start.BCRP3..human.ref                ggagctggccacgaggcttcctcgactcgacacactggtggggtacaccctggttctcca    1337
GGT1end-LOC749026.end.7456450-7520130.chimp           ggagctggccacgaggcttcctcgactcgacacactggtggggtacaccctggttctcca    1327


GGT5.total.&minus3200bp.mouse                         agacccactgcgtatagcccagagcttctttg--gagacttggggacttgagtgaccatc    1545
GGT5.nt.mouse                                         ------------------------------------------------------------    0
FAM247.LOC105372935.ref.human                         ------------------------------------------------------------    0
GGT1.end-GGT5.begining.Philippine.tarsier.ref         gagctcctggataggactccgggtcctcccttcttggagtagggcagcgac---actacc    246
GGT5.end-GGT1.beginning.Rhesus.28419652-28635852      ggtcccatggggctcagcccaggactaccttggtgggggtgggagacttaa---atcctc    1400
spacer.GGT1.end-start.BCRP3..human.ref                ggtcccatggggctcagcccaggactacctcg--gggggtgagggacttaa---atcctc    1392
GGT1end-LOC749026.end.7456450-7520130.chimp           ggtcccatggggctcagcccaggactacctcg--gggggtgagggacttaa---atcctc    1382


GGT5.total.&minus3200bp.mouse                         ccctttctagt-----------gcctttccatgttacttcctggataagggcct------    1588
GGT5.nt.mouse                                         ------------------------------------------------------------    0
FAM247.LOC105372935.ref.human                         ------------------------------------------------------------    0
GGT1.end-GGT5.begining.Philippine.tarsier.ref         tagtacctgccaccagggggcaccagggcactgacctttacttggacagacggtagcggg    306
GGT5.end-GGT1.beginning.Rhesus.28419652-28635852      tccttcattct----cattgt-cccttcccccatcatttcctgaggaagcacat-tcagg    1454
spacer.GGT1.end-start.BCRP3..human.ref                tccttcattct----catcgc-cccttcccccatcatttcctgaggaaggacat-tcagg    1446
GGT1end-LOC749026.end.7456450-7520130.chimp           tccttcattct----catcac-cccttcccccatcatttcctgaggaaggacat-tcagg    1436


GGT5.total.&minus3200bp.mouse                         ------------------------------------------------------------    1588
GGT5.nt.mouse                                         ------------------------------------------------------------    0
FAM247.LOC105372935.ref.human                         ------------------------------------------------------------    0
GGT1.end-GGT5.begining.Philippine.tarsier.ref         cagatgccaggaactcgggtgtctcctcctcagacccgcgggtgtcccggctcgccctgc    366
GGT5.end-GGT1.beginning.Rhesus.28419652-28635852      gacctc------------------------------------------------------    1460
spacer.GGT1.end-start.BCRP3..human.ref                gacctg-----aaggagcggcctgcccctccacatctgtgggtgtttctcatcaggtggg    1501
GGT1end-LOC749026.end.7456450-7520130.chimp           gacctg-----aaggagcggcctgcccctccacatctgtgggtgtttctcatcaggtggg    1491


GGT5.total.&minus3200bp.mouse                         ------------------------------------------------------------    1588
GGT5.nt.mouse                                         ------------------------------------------------------------    0
FAM247.LOC105372935.ref.human                         ------------------------------------------------------------    0
GGT1.end-GGT5.begining.Philippine.tarsier.ref         t---------gaggacaaa--cagaggtgaagctgaggtctgaggagtgggagttgggca    415
GGT5.end-GGT1.beginning.Rhesus.28419652-28635852      ------------------------------------------------------------    1460
spacer.GGT1.end-start.BCRP3..human.ref                acaagagactgagaaaagaaagagacacagagacaaagtatagagaaagaaaagtgggcc    1561
GGT1end-LOC749026.end.7456450-7520130.chimp           acaagagactgagaaaagaaagagacacagagacaaagtatagagaaagaaaagtgggcc    1551


GGT5.total.&minus3200bp.mouse                         ------------------------------------------------------------    1588
GGT5.nt.mouse                                         ------------------------------------------------------------    0
FAM247.LOC105372935.ref.human                         ------------------------------------------------------------    0
GGT1.end-GGT5.begining.Philippine.tarsier.ref         agaggctggaacaaaacatctcagtcagaccctggggggggctttccaagcacaaactct    475
GGT5.end-GGT1.beginning.Rhesus.28419652-28635852      ------------------------------------------------------------    1460
spacer.GGT1.end-start.BCRP3..human.ref                caggggacctg----------cgctcagcatacagaggccccacgctggcatcagtctct    1611
GGT1end-LOC749026.end.7456450-7520130.chimp           caggggacctg----------cgctcagcatatggaggacccacgctggcaccagtctct    1601


GGT5.total.&minus3200bp.mouse                         ------------------------------------------------------------    1588
GGT5.nt.mouse                                         ------------------------------------------------------------    0
FAM247.LOC105372935.ref.human                         ------------------------------------------------------------    0
GGT1.end-GGT5.begining.Philippine.tarsier.ref         gagtgaaagaaacccagcttgtgatcag------ctccagagggaggcccgattctcagg    529
GGT5.end-GGT1.beginning.Rhesus.28419652-28635852      ------------------------------------------------------------    1460
spacer.GGT1.end-start.BCRP3..human.ref                gagttccctagtatttattgatcattatctctaccatctcagagagggggatgtagcagg    1671
GGT1end-LOC749026.end.7456450-7520130.chimp           gagttccctagtatttattgatcattatctctaccatctcagagagggggatgtggcagg    1661


GGT5.total.&minus3200bp.mouse                         ------------------------------------------------------------    1588
GGT5.nt.mouse                                         ------------------------------------------------------------    0
FAM247.LOC105372935.ref.human                         ------------------------------------------------------------    0
GGT1.end-GGT5.begining.Philippine.tarsier.ref         aacagcagatcgcagtga---------------------cttggaccagatgggaatgtg    568
GGT5.end-GGT1.beginning.Rhesus.28419652-28635852      ------------------------------------------------------------    1460
spacer.GGT1.end-start.BCRP3..human.ref                acaatatggtaatagtggggagagggtcagcaggaaaacacgtgaacaaatgtctctgtg    1731
GGT1end-LOC749026.end.7456450-7520130.chimp           acaacatggtaatagtggggcgagggtcagcaggaaaacacgtgaacaaatgtctctgtg    1721


GGT5.total.&minus3200bp.mouse                         ------------------------------------------------------------    1588
GGT5.nt.mouse                                         ------------------------------------------------------------    0
FAM247.LOC105372935.ref.human                         ------------------------------------------------------------    0
GGT1.end-GGT5.begining.Philippine.tarsier.ref         gccccaggaaggggaggcatctg------------gaagggaa---------------tt    601
GGT5.end-GGT1.beginning.Rhesus.28419652-28635852      ------------------------------------------------------------    1460
spacer.GGT1.end-start.BCRP3..human.ref                tcataaacaaggttaagaaaaaggtgctgtgctttgatgtgcatatacataaacatctca    1791
GGT1end-LOC749026.end.7456450-7520130.chimp           tcataaacaaggttaagaaaaaggtgctgtgctttgatgtgcatatacataaacatctca    1781


GGT5.total.&minus3200bp.mouse                         ------------------------------------------------------------    1588
GGT5.nt.mouse                                         ------------------------------------------------------------    0
FAM247.LOC105372935.ref.human                         ------------------------------------------------------------    0
GGT1.end-GGT5.begining.Philippine.tarsier.ref         gttggttacagcgccctttgccaaggggcccgtccct---caggccctccttcaggtcc     658
```

```
GGT5.end-GGT1.beginning.Rhesus.28419652-28635852       ------------------------------------------------------------   1460
spacer.GGT1.end-start.BCRP3..human.ref                 atgcattaaagagcagtattgccaccagcatgtcccacctccagccctaaggcagttttc   1851
GGT1end-LOC749026.end.7456450-7520130.chimp            atgcattaaagagcagtattgccaccagcatgtcccacctccagccctaaggcagttttc   1841


GGT5.total.&minus3200bp.mouse                          ------------------------------------------------------------   1588
GGT5.nt.mouse                                          ------------------------------------------------------------   0
FAM247.LOC105372935.ref.human                          ------------------------------------------------------------   0
GGT1.end-GGT5.begininig.Philippine.tarsier.ref         gcttacctgttctccacacagttgtaacacctgggatggaaaatttggtgctt-------   711
GGT5.end-GGT1.beginning.Rhesus.28419652-28635852       ------------------------------------------------------------   1460
spacer.GGT1.end-start.BCRP3..human.ref                 tccta-------tctc---------agtagatggaatatacaattgggttttaca-----   1890
GGT1end-LOC749026.end.7456450-7520130.chimp            tccta-------tctc---------agtagatggaatatacaattgggttttacaccgag   1885


GGT5.total.&minus3200bp.mouse                          ------------------------------------------------------------   1588
GGT5.nt.mouse                                          ------------------------------------------------------------   0
FAM247.LOC105372935.ref.human                          ------------------------------------------------------------   0
GGT1.end-GGT5.begininig.Philippine.tarsier.ref         -----c-----caaagtgcccacaaggaaatgggttttgagcctgcagagtacaccc-ca   760
GGT5.end-GGT1.beginning.Rhesus.28419652-28635852       ------------------------------------------------------------   1460
spacer.GGT1.end-start.BCRP3..human.ref                 -cattcctttgcccagggacgatcaggagacagatgcc-ttcctcttatctcaactgcaa   1948
GGT1end-LOC749026.end.7456450-7520130.chimp            acattcctttgcccagggacgatcaggagacagatgcc-ttcctcttgtctcaactgcaa   1944


GGT5.total.&minus3200bp.mouse                          ------------------------------------------------------------   1588
GGT5.nt.mouse                                          ------------------------------------------------------------   0
FAM247.LOC105372935.ref.human                          ------------------------------------------------------------   0
GGT1.end-GGT5.begininig.Philippine.tarsier.ref         ggagggctttgtc--------tctggagttccccaggggtgagatgactgggtgagatga   812
GGT5.end-GGT1.beginning.Rhesus.28419652-28635852       ------------------------------------------------------------   1460
spacer.GGT1.end-start.BCRP3..human.ref                 agaggccttccttcctcttatactaatcctcctcagcacagaccctttacgggtgtcggg   2008
GGT1end-LOC749026.end.7456450-7520130.chimp            agaggccttccttcctcttatactaatcctcctcagcacagaccctttacgggtgtcggg   2004


GGT5.total.&minus3200bp.mouse                          --------------------------------------------------------ttgg   1592
GGT5.nt.mouse                                          ------------------------------------------------------------   0
FAM247.LOC105372935.ref.human                          ------------------------------------------------------------   0
GGT1.end-GGT5.begininig.Philippine.tarsier.ref         caggggtgagtgcaagtca--------------ctactcaatcctaaatttagct-tgag   857
GGT5.end-GGT1.beginning.Rhesus.28419652-28635852       ------------------------------------------------------------   1460
spacer.GGT1.end-start.BCRP3..human.ref                 ctggggaacggtcaggtcttttccttcccacaaggccatatttcagactgtcacatgggg   2068
GGT1end-LOC749026.end.7456450-7520130.chimp            ctgggggacggtcaggtctttctcttcccacgaggccatatttcagactgtcacatgggg   2064


GGT5.total.&minus3200bp.mouse                          agcca-------------------------------------------------------   1597
GGT5.nt.mouse                                          ------------------------------------------------------------   0
FAM247.LOC105372935.ref.human                          ------------------------------------------------------------   0
GGT1.end-GGT5.begininig.Philippine.tarsier.ref         agaaa----aggtcatggccccatttacaggggagaagac--tgaggccaga--------   903
GGT5.end-GGT1.beginning.Rhesus.28419652-28635852       ------------------------------------------------------------   1460
spacer.GGT1.end-start.BCRP3..human.ref                 agaaaccttggacaatacctggctttcctaggcagaggtccctgcggccttctgcagtgt   2128
GGT1end-LOC749026.end.7456450-7520130.chimp            agaaaccttggacaatacctggctttcctaggcagaggtccctgcggccttctgcagtgt   2124


GGT5.total.&minus3200bp.mouse                          ------------------------------------------------------------   1597
GGT5.nt.mouse                                          ------------------------------------------------------------   0
FAM247.LOC105372935.ref.human                          ------------------------------------------------------------   0
GGT1.end-GGT5.begininig.Philippine.tarsier.ref         -----------------------aggg----agggccttcct---ctgagtcgcacagca   933
GGT5.end-GGT1.beginning.Rhesus.28419652-28635852       ------------------------------------------------------------   1460
spacer.GGT1.end-start.BCRP3..human.ref                 tttgtgcccctgcttacttgagattagggagtggtgatgacttttaacaagcatgctgcc   2188
GGT1end-LOC749026.end.7456450-7520130.chimp            tttgtgtccctgcttacttgagattagggagtggtgatgacttttaacaagcatgttgcc   2184


GGT5.total.&minus3200bp.mouse                          ------------------------------------------------------------   1597
GGT5.nt.mouse                                          ------------------------------------------------------------   0
FAM247.LOC105372935.ref.human                          ------------------------------------------------------------   0
GGT1.end-GGT5.begininig.Philippine.tarsier.ref         ctgagggaagtgggggctggccatgaggctccctggccccaaca--------tgctgaca   985
GGT5.end-GGT1.beginning.Rhesus.28419652-28635852       ------------------------------------------------------------   1460
spacer.GGT1.end-start.BCRP3..human.ref                 ttcaagcatttgtttaacaaagcacatcctgcacagccctgaatccattaaaccttgagt   2248
GGT1end-LOC749026.end.7456450-7520130.chimp            ttaaagcatttgtttaacaaagcacatcctgcatagccctaaatccattaaaccttgagt   2244


GGT5.total.&minus3200bp.mouse                          ------------------------------------------------------------   1597
GGT5.nt.mouse                                          ------------------------------------------------------------   0
FAM247.LOC105372935.ref.human                          ------------------------------------------------------------   0
GGT1.end-GGT5.begininig.Philippine.tarsier.ref         ggtgtacctcagtgtccctggccctatggggcttagtc-------------caaggctac   1032
GGT5.end-GGT1.beginning.Rhesus.28419652-28635852       ------------------------------------------------------------   1460
spacer.GGT1.end-start.BCRP3..human.ref                 cgacacagtacttgtttctgtgagcacagggttggggatagggttacagattaacagcat   2308
GGT1end-LOC749026.end.7456450-7520130.chimp            cgacacagtacatgtttctgtgagcacagggttggggatagggttacagattaacagcat   2304


GGT5.total.&minus3200bp.mouse                          ------------------------------------------------------------   1597
GGT5.nt.mouse                                          ------------------------------------------------------------   0
FAM247.LOC105372935.ref.human                          ------------------------------------------------------------   0
GGT1.end-GGT5.begininig.Philippine.tarsier.ref         ctcaggggctggtacttgaattctctcttttcttccaactgtcccctcccccatcacctc   1092
GGT5.end-GGT1.beginning.Rhesus.28419652-28635852       ------------------------------------------------------------   1460
spacer.GGT1.end-start.BCRP3..human.ref                 ctcaaggcaaaaga--atttttcttactacagaacaaaatggagcctcttacgtctactt   2366
GGT1end-LOC749026.end.7456450-7520130.chimp            ctcaaggcaaaaga--atttttcttactacagaacaaaatggagcctcttacgtctactt   2362


GGT5.total.&minus3200bp.mouse                          ----------------------------------------------------------cc   1599
GGT5.nt.mouse                                          ------------------------------------------------------------   0
FAM247.LOC105372935.ref.human                          ------------------------------------------------------------   0
GGT1.end-GGT5.begininig.Philippine.tarsier.ref         ctgg---------------------ataaggacactcaggcccttcccagcggggcctcc   1131
GGT5.end-GGT1.beginning.Rhesus.28419652-28635852       ----------------------------------------------------------cc   1462
spacer.GGT1.end-start.BCRP3..human.ref                 ctttctacatagacacagtaacagtctgatatctctttcttttccccacagggaccttcc   2426
GGT1end-LOC749026.end.7456450-7520130.chimp            ctttctacatagacacagtaacagtctgatatctctttcttttccccacagggaccttcc   2422
```

```
GGT5.total.&minus3200bp.mouse                       agacctggccttcccagccaagtccagtatgatagcaa-------gccttgagaatccgt    1652
GGT5.nt.mouse                                       ------------------------------------------------------------    0
FAM247.LOC105372935.ref.human                       ------------------------------------------------------------    0
GGT1.end-GGT5.beginining.Philippine.tarsier.ref     ccagggcgcct--ctggctgttcccagcatgatacgcattcctttccctgggcctttgct    1189
GGT5.end-GGT1.beginning.Rhesus.28419652-28635852    tggctgtgcct--cagtccaaaaccagaatgacacgcattcctttccctgggcctttgct    1520
spacer.GGT1.end-start.BCRP3..human.ref              tggctgtgcct--cggatcaggaccagaatgacacccattcatttccctgggcctttgct    2484
GGT1end-LOC749026.end.7456450-7520130.chimp         tggctgtgcct--aggatcaggaccagaatgacacccattcatttccctgggcctttgct    2480


GGT5.total.&minus3200bp.mouse                       agaactgcctctgaactctggtctgcgcctgaccaggatgtcccagccagggaccaggag    1712
GGT5.nt.mouse                                       ------------------------------------------------------------    0
FAM247.LOC105372935.ref.human                       ------------------------------------------------------------    0
GGT1.end-GGT5.beginining.Philippine.tarsier.ref     ctggtggtgcttacaccctggcctctgcctaattgggatggcc------agggagaggag    1243
GGT5.end-GGT1.beginning.Rhesus.28419652-28635852    caggcggtccctgcaccctggcctctgcctgaccagggtggtg------gggagaggagg    1574
spacer.GGT1.end-start.BCRP3..human.ref              ccggtggtccctgcaccctggcctctgcctgacgaggatggtg------gggagaggagg    2538
GGT1end-LOC749026.end.7456450-7520130.chimp         cgggcggtccctgcaccctggcctctgcctgaccaggatggtg------gggagaggagg    2534


GGT5.total.&minus3200bp.mouse                       gggaagataa--cctacccttgtgtctgcttgttccccccatactgctttggccttaggg    1770
GGT5.nt.mouse                                       ------------------------------------------------------------    0
FAM247.LOC105372935.ref.human                       ------------------------------------------------------------    0
GGT1.end-GGT5.beginining.Philippine.tarsier.ref     agggaatatccctgactcccactgtctccactgtccc----tgctgttctggcttctggg    1299
GGT5.end-GGT1.beginning.Rhesus.28419652-28635852    ggggacgtcc--cctccgctgctgtctccactgttcc----tgctgccctggcctctggg    1628
spacer.GGT1.end-start.BCRP3..human.ref              ggggacatcc--cccacgctgctgtctccactgt----------------ggcctctgag    2580
GGT1end-LOC749026.end.7456450-7520130.chimp         ggggatgtcc--cccacgctgctgtctccactgttcc----tgctgcccaggcctctgag    2588


GGT5.total.&minus3200bp.mouse                       gacagtggcccg-----------tgagtggactggcctacattcaagaatgtgctttggt    1819
GGT5.nt.mouse                                       ------------------------------------------------------------    0
FAM247.LOC105372935.ref.human                       ------------------------------------------------------------    0
GGT1.end-GGT5.beginining.Philippine.tarsier.ref     tttcaggactac-----agcctgtgggcgggatggcctgggcccaggaatgcacttttgt    1354
GGT5.end-GGT1.beginning.Rhesus.28419652-28635852    cttccaggactgcagtgggtgggtgggtgggctggcctgagcccaggaatgcacttcggc    1688
spacer.GGT1.end-start.BCRP3..human.ref              cttccaggactgcagcgggtgggtgggtggcctggcctaagcccaggaatgcacttcagc    2640
GGT1end-LOC749026.end.7456450-7520130.chimp         cttccaggactgcagcgggtgggtgggtggcctggcctaagcccaggaatgcacttcagc    2648


GGT5.total.&minus3200bp.mouse                       tcctggttgagcaatgacatgacattgtagtgttggggacaactgggatggggaagactc    1879
GGT5.nt.mouse                                       ------------------------------------------------------------    0
FAM247.LOC105372935.ref.human                       ------------------------------------------------------------    0
GGT1.end-GGT5.beginining.Philippine.tarsier.ref     tactggttgtgca-----atgttgctggggttaagggggggcaggaggaggtgcctata--    1407
GGT5.end-GGT1.beginning.Rhesus.28419652-28635852    tcctggttgagca-----aagtcactgagacttgggagtcgggtcgggttgggaggaggc    1743
spacer.GGT1.end-start.BCRP3..human.ref              tcctggttgagca-----atgtcactgaggcttgggagtcgggtggggacgggaggaggc    2695
GGT1end-LOC749026.end.7456450-7520130.chimp         tcctggtagagca-----atgtcactgaggcttgggagtcgggtggggacgggaggaggc    2703


GGT5.total.&minus3200bp.mouse                       ccacagacactcccctcttcct-ctaggaagatgtgggaacaggcttacag---------    1929
GGT5.nt.mouse                                       ------------------------------------------------------------    0
FAM247.LOC105372935.ref.human                       ------------------------------------------------------------    0
GGT1.end-GGT5.beginining.Philippine.tarsier.ref     -----------gcccccactgtgagcagcagctgtgggaacagcctacaggaaggcctgc    1456
GGT5.end-GGT1.beginning.Rhesus.28419652-28635852    gtccacaggcccccactac---gaaaggcagctgtgggaacagtctgcctg---------    1791
spacer.GGT1.end-start.BCRP3..human.ref              gtccgcaggcccccccctaccgtgagaggcagccgtgggaacagcctacctc---------    2746
GGT1end-LOC749026.end.7456450-7520130.chimp         gtccgcaggcc-cccctaccgtgagaggcagccgtgggaacagcctacctc---------    2753


GGT5.total.&minus3200bp.mouse                       --gaaacagcctcccttgtccaggcttagcagggactctgggtgtgaca------cttaa    1981
GGT5.nt.mouse                                       ------------------------------------------------------------    0
FAM247.LOC105372935.ref.human                       ------------------------------------------------------------    0
GGT1.end-GGT5.beginining.Philippine.tarsier.ref     ctcttatggccgctccaggctgagctgaccagaggctctggc-tggacatgggggtctgg    1515
GGT5.end-GGT1.beginning.Rhesus.28419652-28635852    --taaacaaccactccagcccaggctgaccaggggctctggctgggacattgggatctgg    1849
spacer.GGT1.end-start.BCRP3..human.ref              --taaacaatcgctgcagcccaggctgaccaggggctctggc-cggacataggggcctgg    2803
GGT1end-LOC749026.end.7456450-7520130.chimp         --taaacaatcactgtagcccaggctgaccaggggctctggc-cggacagaggggcctgg    2810


GGT5.total.&minus3200bp.mouse                       caggatgggtggccagtgcgg--gcattccctctctgggcctcttttttttttttttttt    2039
GGT5.nt.mouse                                       ------------------------------------------------------------    0
FAM247.LOC105372935.ref.human                       ------------------------------------------------------------    0
GGT1.end-GGT5.beginining.Philippine.tarsier.ref     caggctgtgtgtcctgtgagcatacagtccctctctgggcctcagtgtctctgctgtagt    1575
GGT5.end-GGT1.beginning.Rhesus.28419652-28635852    caggctgtgtggcctgtaaggacacagtctgtctctgtgcctcagtttctctgctgccca    1909
spacer.GGT1.end-start.BCRP3..human.ref              caggctgtgtggcctgtaaggacacagtctgtctctgtgcctcagtttctctgctgccca    2863
GGT1end-LOC749026.end.7456450-7520130.chimp         caggctgtgtggcctgtaaggacacagtctgtctctgtgcctcagtttctctgctgccca    2870


GGT5.total.&minus3200bp.mouse                       tttttttttggcattgccaagccgggggtacgaagatgggggcgggggggcatgctcagt    2099
GGT5.nt.mouse                                       ------------------------------------------------------------    0
FAM247.LOC105372935.ref.human                       ------------------------------------------------------------    0
GGT1.end-GGT5.beginining.Philippine.tarsier.ref     aggag----gtgggttctgactgcaggtgtagtcacctggggcaagcactgctcacctgg    1631
GGT5.end-GGT1.beginning.Rhesus.28419652-28635852    gttgg-----gcgtcccagactccaggtgtagacatctggagcaggcagtgctcagctgg    1964
spacer.GGT1.end-start.BCRP3..human.ref              gatgg----agaggcccagactccaggtgtagacatctggagcaggcagtgttcagctgg    2919
GGT1end-LOC749026.end.7456450-7520130.chimp         gatgg----agaggcccagactccaggtgtagacatctggagcaggcagtgttcagctgg    2926


GGT5.total.&minus3200bp.mouse                       ggaaagagagtgaggaatgatggga----------aggcctgaaaccaagagctcttctg    2149
GGT5.nt.mouse                                       ------------------------------------------------------------    0
FAM247.LOC105372935.ref.human                       ------------------------------------------------------------    0
GGT1.end-GGT5.beginining.Philippine.tarsier.ref     aaagaagtggggaagataggtggggcctctgtggcaagtgtttagcccatagcctctgca    1691
GGT5.end-GGT1.beginning.Rhesus.28419652-28635852    gaaggaagtggggaggactggaggagccatgtgtgaaggattcca------acccacatc    2018
spacer.GGT1.end-start.BCRP3..human.ref              ggagggagcggggaggactatgggggccacgtgggaagaagtcca------gcccacatc    2973
GGT1end-LOC749026.end.7456450-7520130.chimp         ggagggagcggtgaggactatgggggccacgtgggaagaagtcca------gcccacatc    2980


GGT5.total.&minus3200bp.mouse                       ccctgcacatgtattaattctgatcacgagagcacattggtgggtcctcactgctccagc    2209
```

```
GGT5.nt.mouse                                         ------------------------------------------------------------    0
FAM247.LOC105372935.ref.human                         ------------------------------------------------------------    0
GGT1.end-GGT5.beginining.Philippine.tarsier.ref       ctccacacccctgctgagcctggccaagagagaccctcagtgggtactcactcccaggct    1751
GGT5.end-GGT1.beginning.Rhesus.28419652-28635852      acctgcacccctgctgagcctggtcaacagagcccctcagtgggtcctcactcccctggc    2078
spacer.GGT1.end-start.BCRP3..human.ref                acctgcacccctgctgagcctggtcaacagaggccctcagtgggtcctcactctcctggc    3033
GGT1end-LOC749026.end.7456450-7520130.chimp           acctgcacccctgctgagcctggtcaacagaggccctcagtgggtcctcactctcctggc    3040


GGT5.total.&minus3200bp.mouse                         tgcctccc----actggaacatcctgagatcatgg----aaagataggacccaagccagg    2261
GGT5.nt.mouse                                         ------------------------------------------------------------    0
FAM247.LOC105372935.ref.human                         ------------------------------------------------------------    0
GGT1.end-GGT5.beginining.Philippine.tarsier.ref       gcccccccccccgttgggtaccctgtggcctggggcgggcaaatagggcccaggtcagt    1811
GGT5.end-GGT1.beginning.Rhesus.28419652-28635852      tgcctccc----ggttaggcaccctgagggctggggagaacagg-----gccaggccagt    2129
spacer.GGT1.end-start.BCRP3..human.ref                tgcctccc----atttaggcaccctgaggcctggggagaacaga-----gccaggccagt    3084
GGT1end-LOC749026.end.7456450-7520130.chimp           tgcctccc----atttaggcaccctgaggcctggggagaacaga-----gccaggccagt    3091


GGT5.total.&minus3200bp.mouse                         ct-cccagaggaggaggctcaccagcacagaaatagccctgagttcttctgg--------    2312
GGT5.nt.mouse                                         ------------------------------------------------------------    0
FAM247.LOC105372935.ref.human                         ------------------------------------------------------------    0
GGT1.end-GGT5.beginining.Philippine.tarsier.ref       gtccccagaagagttgggatggac-----------------------acagagatgt-gg    1847
GGT5.end-GGT1.beginning.Rhesus.28419652-28635852      gtccccagagaggctgcgctgccagcacagtaatagcggatttggattcagggaagcaga    2189
spacer.GGT1.end-start.BCRP3..human.ref                gtccccagagaggctgcgctgccagcacagtagtagcagatttggattcagggaagtaga    3144
GGT1end-LOC749026.end.7456450-7520130.chimp           gtccccagagaggctgcgctgccagcacagtagtagcagatttggattcagggaagtaga    3151


GGT5.total.&minus3200bp.mouse                         -actcagcaagtgggcctctgagctgcagaaaaagggagggcacttgagcagggcagcct    2371
GGT5.nt.mouse                                         ------------------------------------------------------------    0
FAM247.LOC105372935.ref.human                         ------------------------------------------------------------    0
GGT1.end-GGT5.beginining.Philippine.tarsier.ref       accccagccagggcacagcagagctgcag-gcgagcatggggggctcaggcagggcagcct    1906
GGT5.end-GGT1.beginning.Rhesus.28419652-28635852      cccgcagccagggtggggaagagctgcag-gctgggcgtggcacctaggcggcacagcct    2248
spacer.GGT1.end-start.BCRP3..human.ref                cctgcagccagggtgggaaagagctgcag-gcggggtggagcccccacatggcacagccc    3203
GGT1end-LOC749026.end.7456450-7520130.chimp           cctgcagccagggtgggaaagagctgcag-gcggggtggagcccccacatggcacagccc    3210


GGT5.total.&minus3200bp.mouse                         cgtcctttagaggtctaaggttgagtttatgggtcactatgtcccagcactgtgtgtcag    2431
GGT5.nt.mouse                                         ------------------------------------------------------------    0
FAM247.LOC105372935.ref.human                         ------------------------------------------------------------    0
GGT1.end-GGT5.beginining.Philippine.tarsier.ref       ccttctctggaggtccatggctgcatttccaggacagcaggccccagggatgggta----    1962
GGT5.end-GGT1.beginning.Rhesus.28419652-28635852      ccctccctggag-gcccacgctgcatttccaggacagcaagtcccagggatggatg----    2303
spacer.GGT1.end-start.BCRP3..human.ref                ccctccttggag-gtctatgctgcatttccaggacagcaagtcccagggatggatg----    3258
GGT1end-LOC749026.end.7456450-7520130.chimp           ccctccctggag-gtctgtgctgcatttccaggacagcaagtcccagggatggatg----    3265


GGT5.total.&minus3200bp.mouse                         cagcactgggtgtcagggcactggacatctggcctgcctgcccactcccttcccacccca    2491
GGT5.nt.mouse                                         ------------------------------------------------------------    0
FAM247.LOC105372935.ref.human                         ------------------------------------------------------------    0
GGT1.end-GGT5.beginining.Philippine.tarsier.ref       --a--tccagtgccaagg-gctgaacgcatggcccgtctgcgttctccatgtgggtgtct    2017
GGT5.end-GGT1.beginning.Rhesus.28419652-28635852      --gtcccaggtgccaagg-gctagaggcatggtctgtctgcattccccacatggacgtct    2360
spacer.GGT1.end-start.BCRP3..human.ref                --gtgcctggtgccaagg-gctagaggcatggtctgtctgcattccctacaggggcatct    3315
GGT1end-LOC749026.end.7456450-7520130.chimp           --gtgccaggtgccaagg-gctagaggcatggtctgtctgcattccccacaggggcgtct    3322


GGT5.total.&minus3200bp.mouse                         tgctcagatgtttggatgaggctgtgccaggctgtaataaaacggtcccgacgaggctgg    2551
GGT5.nt.mouse                                         ------------------------------------------------------------    0
FAM247.LOC105372935.ref.human                         ------------------------------------------------------------    0
GGT1.end-GGT5.beginining.Philippine.tarsier.ref       tgcagtcatgggcgtttggtgctct-----------------------------------    2042
GGT5.end-GGT1.beginning.Rhesus.28419652-28635852      tgtagtcaccagcgtttgatgctgt-----------------------------------    2385
spacer.GGT1.end-start.BCRP3..human.ref                tgtagtcaccagcatttgatgctgt-----------------------------------    3340
GGT1end-LOC749026.end.7456450-7520130.chimp           tatagtcaccagcatttgatgctgt-----------------------------------    3347


GGT5.total.&minus3200bp.mouse                         agagctggctcagcagttaaaagcactgactgctcttcctgaggtcctgactctaaatcc    2611
GGT5.nt.mouse                                         ------------------------------------------------------------    0
FAM247.LOC105372935.ref.human                         ------------------------------------------------------------    0
GGT1.end-GGT5.beginining.Philippine.tarsier.ref       -------------------------cgagtctgcctatcccctgtgcagactgggaagct    2077
GGT5.end-GGT1.beginning.Rhesus.28419652-28635852      -------------------------caagtcccccctgtcctctctgcggactgagaagcc    2420
spacer.GGT1.end-start.BCRP3..human.ref                -------------------------caagtcccccctgtcctctgtgcagactgggaagcc    3375
GGT1end-LOC749026.end.7456450-7520130.chimp           -------------------------caagtcccccctgtcctctgtgcagactgggaagcc    3382


GGT5.total.&minus3200bp.mouse                         cagcaaccacatggtggct-----------------------------------------    2630
GGT5.nt.mouse                                         ------------------------------------------------------------    0
FAM247.LOC105372935.ref.human                         ------------------------------------------------------------    0
GGT1.end-GGT5.beginining.Philippine.tarsier.ref       caggcccccctcctggccaccctggggatggggggtggtgtgccagctgcagctgagtagg    2137
GGT5.end-GGT1.beginning.Rhesus.28419652-28635852      cttggtcatccttaggg-g------gttgtggaacccaaaccaggctgcagaagcatagg    2473
spacer.GGT1.end-start.BCRP3..human.ref                cttggtcaccctgggggg------gttggggggacccaggccaggctgcagaaacataag    3429
GGT1end-LOC749026.end.7456450-7520130.chimp           cttggtcaccctggggg-g------gttggggggacccaggccaggctgcagaaacataag    3435


GGT5.total.&minus3200bp.mouse                         ---------cacaaccatctgtaatgagatctgatgccctcttctggtgtgtctgaagac    2681
GGT5.nt.mouse                                         ------------------------------------------------------------    0
FAM247.LOC105372935.ref.human                         ------------------------------------------------------------    0
GGT1.end-GGT5.beginining.Philippine.tarsier.ref       gacttgaacctgggccctgagtgacaccaccttatgtcctctc-----------------    2180
GGT5.end-GGT1.beginning.Rhesus.28419652-28635852      gacttgaacccaagttttaagtgacaccacctttgtcccctc----------------    2517
spacer.GGT1.end-start.BCRP3..human.ref                gacttgaacccgggtcctgagtgacaccaccttgggtcctcctc----------------    3473
GGT1end-LOC749026.end.7456450-7520130.chimp           gacttgaacccgggtcctgagtgacaccaccttgggtcctcctc----------------    3479


GGT5.total.&minus3200bp.mouse                         agctacagtgtacttacatataataaataaataaataaataaataaataaatctttt    2741
GGT5.nt.mouse                                         ------------------------------------------------------------    0
FAM247.LOC105372935.ref.human                         ------------------------------------------------------------    0
GGT1.end-GGT5.beginining.Philippine.tarsier.ref       ------------------------------------------------------------    2180
```

```
GGT5.end-GGT1.beginning.Rhesus.28419652-28635852      ------------------------------------------------------------    2517
spacer.GGT1.end-start.BCRP3..human.ref                ------------------------------------------------------------    3473
GGT1end-LOC749026.end.7456450-7520130.chimp           ------------------------------------------------------------    3479


GGT5.total.&minus3200bp.mouse                         aaaatatttaaaagattcatttctataaaaatttaaaaattaaaacagcacggtcctg    2801
GGT5.nt.mouse                                         ------------------------------------------------------------    0
FAM247.LOC105372935.ref.human                         ------------------------------------------------------------    0
GGT1.end-GGT5.beginining.Philippine.tarsier.ref       ------------------------------------------------------------    2180
GGT5.end-GGT1.beginning.Rhesus.28419652-28635852      ------------------------------------------------------------    2517
spacer.GGT1.end-start.BCRP3..human.ref                ------------------------------------------------------------    3473
GGT1end-LOC749026.end.7456450-7520130.chimp           ------------------------------------------------------------    3479


GGT5.total.&minus3200bp.mouse                         agtggctaccttggtctaccttcagccccatctctgtaccagctgggtgattgcattgag    2861
GGT5.nt.mouse                                         ------------------------------------------------------------    0
FAM247.LOC105372935.ref.human                         ------------------------------------------------------------    0
GGT1.end-GGT5.beginining.Philippine.tarsier.ref       --------tccctctgtctcttcagccccactttgaagctacctgggctgggccatgcag    2232
GGT5.end-GGT1.beginning.Rhesus.28419652-28635852      --------cctcggtctctgttcagttccacttcgatattgcctgtgctgggccatgcag    2569
spacer.GGT1.end-start.BCRP3..human.ref                --------cctctgcctctgttcagctccaccttgatggtgactaggctgggccatgcgg    3525
GGT1end-LOC749026.end.7456450-7520130.chimp           --------cctctgcctctgttcagctccaccttgatgctgactaggctgggccatgcgg    3531


GGT5.total.&minus3200bp.mouse                         aggctgcctgggtggaaccagttgggagtggaagctggggaatggagtt-----------    2910
GGT5.nt.mouse                                         ------------------------------------------------------------    0
FAM247.LOC105372935.ref.human                         ------------------------------------------------------------    0
GGT1.end-GGT5.beginining.Philippine.tarsier.ref       a--------gagggtgagaattagaggt-gggagttggagtgg---gct---ccaccctg    2277
GGT5.end-GGT1.beginning.Rhesus.28419652-28635852      a--------gagggttaggggatagagatgggaactggggagtggggctccactctcaga    2621
spacer.GGT1.end-start.BCRP3..human.ref                a--------gagggttaggggatagagatgggagctggggagcagggctccactctggga    3577
GGT1end-LOC749026.end.7456450-7520130.chimp           a--------gagggttaggggatagagatgggagctggggagcagggctccactctggga    3583


GGT5.total.&minus3200bp.mouse                         ---------------------------------tcaccctgcaggagacaccctgaccct    2937
GGT5.nt.mouse                                         ------------------------------------------------------------    0
FAM247.LOC105372935.ref.human                         ------------------------------------------------------------    0
GGT1.end-GGT5.beginining.Philippine.tarsier.ref       tggggccaggcttacctgatccaggataggaaactgggtggccccagctctgcttttctg    2337
GGT5.end-GGT1.beginning.Rhesus.28419652-28635852      gaggggcagccttgctggatccaggggagatagttgagcagccccagctctgctttcccg    2681
spacer.GGT1.end-start.BCRP3..human.ref                ggggggcagccttgccggatccagggcag--agttaagcggccccagctctgctttccta    3635
GGT1end-LOC749026.end.7456450-7520130.chimp           ggggggcagccttgccggatccagggcag--agttaagcggccccagctctgctttccta    3641


GGT5.total.&minus3200bp.mouse                         gagctgttgggaggcaggaaaacagtgagagaggttccctgagttccatgtcccagttcc    2997
GGT5.nt.mouse                                         ------------------------------------------------------------    0
FAM247.LOC105372935.ref.human                         ------------------------------------------------------------    0
GGT1.end-GGT5.beginining.Philippine.tarsier.ref       gtgttgggttt-------------------------cttgggagccctgcctgggcctcc    2372
GGT5.end-GGT1.beginning.Rhesus.28419652-28635852      gagctgctgggaaccccaggaatggtgtg-gagattcctgggagctctgcccccacttga    2740
spacer.GGT1.end-start.BCRP3..human.ref                gagctgctgagaacccgggaaatggtgtg-gaggttccggggagccctgcccctacctgg    3694
GGT1end-LOC749026.end.7456450-7520130.chimp           gagctgctgagaacccaggaaatggtgtg-gagtttccggggaaccctgcccctacctgg    3700


GGT5.total.&minus3200bp.mouse                         cct------gggtgcatcaagcatcacatttccaatggcactgggactgaggagtctttg    3051
GGT5.nt.mouse                                         ------------------------------------------------------------    0
FAM247.LOC105372935.ref.human                         ------------------------------------------------------------    0
GGT1.end-GGT5.beginining.Philippine.tarsier.ref       cctcccactgggtcccccaggcgccaaggcctcaagcatattcagcggggatggg-----    2427
GGT5.end-GGT1.beginning.Rhesus.28419652-28635852      caa---ccacagtgcagcaggcaccaagttctcctgcacattgggacagtgtgaccctgg    2797
spacer.GGT1.end-start.BCRP3..human.ref                caa---ccgcagtgcagcaggcaccaagttctcctgcacattgcgacagtgtgaccctgg    3751
GGT1end-LOC749026.end.7456450-7520130.chimp           caa---ccgcagtgcagcaggcaccaagttctcctgcacattgcgacagtgtgaccctgg    3757


GGT5.total.&minus3200bp.mouse                         ggtggtgttggggcagcagggcaggccatgggatcaactggcgatggaagagttaacagc    3111
GGT5.nt.mouse                                         ------------------------------------------------------------    0
FAM247.LOC105372935.ref.human                         ------------------------------------------------------------    0
GGT1.end-GGT5.beginining.Philippine.tarsier.ref       ---------------------------------accacggcagcaagggagttaaccg-c    2453
GGT5.end-GGT1.beginning.Rhesus.28419652-28635852      gctctggt--tagtggcaggtggggccttgggtcctaccagcagtgagggagttagca-c    2854
spacer.GGT1.end-start.BCRP3..human.ref                gctctggc--gggcagtaggtggggcctttggacctaccagcagtgagggagttaaca-c    3808
GGT1end-LOC749026.end.7456450-7520130.chimp           gctctggc--gggcggtaggtggggcctttggacctaccagcagtgagggagttaaca-c    3814


GGT5.total.&minus3200bp.mouse                         ggcagctggctcttctcaagaaaaaaaaaactccctg------tagatgcctggcttgcc    3165
GGT5.nt.mouse                                         --------------------------------------------------------tgcc    4
FAM247.LOC105372935.ref.human                         ------------------------------------------------------------    0
GGT1.end-GGT5.beginining.Philippine.tarsier.ref       agcagctggctcctgtagcaaagaa--aactcccccagacgctttgctgcctggccttcc    2511
GGT5.end-GGT1.beginning.Rhesus.28419652-28635852      agcagctggctcctctagggaaggaaaactcccttcagacactttggtgcctggcctcct    2914
spacer.GGT1.end-start.BCRP3..human.ref                agcagctgactcctctaggcaaggaaaactcccctcagacgctttgctgcctggcctcct    3868
GGT1end-LOC749026.end.7456450-7520130.chimp           agcagctgactcctctaggcaaggaaaactcccctcagatgctttgctgcctggcctcct    3874


GGT5.total.&minus3200bp.mouse                         tccagggttgagcctcgggagctgaaaactgcaagttcagacctgtggctagttctgc--    3223
GGT5.nt.mouse                                         tccagggttgagcctcgggagctgaaaactgcaagttcagacctgtggctagttctgc--    62
FAM247.LOC105372935.ref.human                         ----------------------tgaaaactagaagttgaggcatgagtttggccactc--    36
GGT1.end-GGT5.beginining.Philippine.tarsier.ref       gccagggctgaga--acagggctgaaaactggaagttgaggcgtgagcatagcacactct    2569
GGT5.end-GGT1.beginning.Rhesus.28419652-28635852      gccaggaaca-ag--caggagctgaaaactagaagttgaggcataagtttggccactc--    2969
spacer.GGT1.end-start.BCRP3..human.ref                gccagcaaca-ag--caggagctgaaaaccagaagttgaggcgtgagtttggtca-----    3920
GGT1end-LOC749026.end.7456450-7520130.chimp           gccagcaaca-ag--caggagctgaaaactagaagttgaggcgtgagtttggccactc--    3929
                                                                            *******   ***** ** * *  *   * *

GGT5.total.&minus3200bp.mouse                         ---------ctctggaggagagtgtagcagcagcctggccacagcctgccgtctgtctat    3274
GGT5.nt.mouse                                         ---------ctctggaggagagtgtagcagcagcctggccacagcctgccgtctgtctat    113
FAM247.LOC105372935.ref.human                         -----cgtagtgtgcacttggtgagggcagcagctcgccaca--gctgccagccatctgt    89
GGT1.end-GGT5.beginining.Philippine.tarsier.ref       ccctccgaagtgagcgcttgaagagggcagcagctctgtcaccagctgctggctgccag-    2628
GGT5.end-GGT1.beginning.Rhesus.28419652-28635852      -----tgtagtgtgtacctggggagggcagcagctcgccaca--gctgccagctg-----    3017
spacer.GGT1.end-start.BCRP3..human.ref                ------------------------------------------------------------    3920
GGT1end-LOC749026.end.7456450-7520130.chimp           -----cgtagtgtgcacttggtgagggcagcagctcgccaca--gctgccagccgtctgt    3982
```

```
GGT5.total.&minus3200bp.mouse                         ccataaacccaggctggctggcttcctgtttctcctctgt---aac----ctgcctggcc    3327
GGT5.nt.mouse                                         ccataaacccaggctggctggcttcctgtttctcctctgt---aac----ctgcctggcc    166
FAM247.LOC105372935.ref.human                         ccat-----tcacccatctgtccatctggcagcccgctgttcagacctgtctgtctgtcc    144
GGT1.end-GGT5.begininig.Philippine.tarsier.ref        --------------ccgctg-ttcttacgctccccgctgcccagac----ctgtctgtcc    2669
GGT5.end-GGT1.beginning.Rhesus.28419652-28635852      ccag-----ccgtctacccattcacctggcagcccgcttttcagac----ctgcctgtcc    3068
spacer.GGT1.end-start.BCRP3..human.ref                ------------------------------------------------------------    3920
GGT1end-LOC749026.end.7456450-7520130.chimp           ccat-----tcacccatctgtccatctggcagcccgctgttcagacccgtctgtctgtcc    4037

GGT5.total.&minus3200bp.mouse                         acctatctgtaaggctgtccgtc---------------------------------atcc    3354
GGT5.nt.mouse                                         acctatctgtaaggctgtccgtc---------------------------------atcc    193
FAM247.LOC105372935.ref.human                         gcccatctgtaagcccatctctgtcccattgtctatctgaccatctttctcttactgtcc    204
GGT1.end-GGT5.begininig.Philippine.tarsier.ref        acccacctgta----------------agcccatctttgaccatccttctcttactgttc    2713
GGT5.end-GGT1.beginning.Rhesus.28419652-28635852      acccatctataagcccatctctgtcccgttgtctatctgaccatctttctcttactgtcc    3128
spacer.GGT1.end-start.BCRP3..human.ref                ------------------------------------------------------------    3920
GGT1end-LOC749026.end.7456450-7520130.chimp           gcccatctgtaagcccatctctgtcccattgtctatctgaccatctttctcttactgtcc    4097

GGT5.total.&minus3200bp.mouse                         tttctgtccagttgtctggcctgtgtggctgtctgt--------------cttgacccat    3400
GGT5.nt.mouse                                         tttctgtccagttgtctggcctgtgtggctgtctgt--------------cttgacccat    239
FAM247.LOC105372935.ref.human                         tctttgtctagctatctggcctgtctgtcgatccatcttcgtgtctgtcttcagccccca    264
GGT1.end-GGT5.begininig.Philippine.tarsier.ref        tctctgtccagctgtctggcctgtctatccatctgcttgtctgtctctggc------ctc    2767
GGT5.end-GGT1.beginning.Rhesus.28419652-28635852      tctctgtccagcaatctggcctgtctgtcgatccatcttcttgtctaac-tgtggcccca    3187
spacer.GGT1.end-start.BCRP3..human.ref                ------------------------------------------------------------    3920
GGT1end-LOC749026.end.7456450-7520130.chimp           tctttatctagctatctggcctatccgtcgatccatcttcgtgtctgtcttcagccccca    4157

GGT5.total.&minus3200bp.mouse                         cgatctttcccctctgtccaatagcctgaga-tcaccctgtgtagcttcttttcccttca    3459
GGT5.nt.mouse                                         cgatctttcccctctgtccaatagcctgaga-tcaccctgtgtagcttcttttcccttca    298
FAM247.LOC105372935.ref.human                         cctgtttgtccatctgtccaattacctgtgagtctatctatgcatcttcttgtccattca    324
GGT1.end-GGT5.begininig.Philippine.tarsier.ref        cctgttcgtccatctgtcca-gtcattgggagcccatgtgagcctactcttgtccattca    2826
GGT5.end-GGT1.beginning.Rhesus.28419652-28635852      cctatttgtccatctgtccaattacctttgattctatctgtgcatcttcttgtccatcca    3247
spacer.GGT1.end-start.BCRP3..human.ref                ------------------------------------------------------------    3920
GGT1end-LOC749026.end.7456450-7520130.chimp           cctgtttgtccatctgtccaattacctgtgagtctatctatgcatcttcttgtccattca    4217

GGT5.total.&minus3200bp.mouse                         gtttgtcagtcagtttgtctgtctctgtctgtccactgccctgtgtgtccagttgcctcc    3519
GGT5.nt.mouse                                         gtttgtcagtcagtttgtctgtctctgtctgtccactgccctgtgtgtccagttgcctcc    358
FAM247.LOC105372935.ref.human                         tctgcccaccca---tctgtccctccatctgcccaccggcct-----------------c    364
GGT1.end-GGT5.begininig.Philippine.tarsier.ref        tctgctcttcca---tctgtccatctgtctgttcattggcct-----------------a    2866
GGT5.end-GGT1.beginning.Rhesus.28419652-28635852      tctgcccaccca---tctgtccctgtgtctgctcactggcct-----------------c    3287
spacer.GGT1.end-start.BCRP3..human.ref                ------------------------------------------------------------    3920
GGT1end-LOC749026.end.7456450-7520130.chimp           tctgcccaccca---tctgtccctccgtctgcccaccggcct-----------------c    4257

GGT5.total.&minus3200bp.mouse                         ctctctgtccctgggccactgagccatggcttggggtcacagggccacggtctgcctggt    3579
GGT5.nt.mouse                                         ctctctgtccctgggccactgagccatggcttggggtcacagggccacggtctgcctggt    418
FAM247.LOC105372935.ref.human                         ccctctccttctgggccgcagagccatggcccaggactacggagccatgggtgacctggt    424
GGT1.end-GGT5.begininig.Philippine.tarsier.ref        tctgctc-----------------------------------------------------    2873
GGT5.end-GGT1.beginning.Rhesus.28419652-28635852      ccctctcctcctgggccacagagccatggcccagggctgtgggtccttggtcagcctggt    3347
spacer.GGT1.end-start.BCRP3..human.ref                ------------------------------------------------------------    3920
GGT1end-LOC749026.end.7456450-7520130.chimp           ccctctccttctgggccgcagagccatggcccaggactgcagagccatggttggcctggt    4317

GGT5.total.&minus3200bp.mouse                         cct------gctgggtgtaggtctaggtctggttatcgttgtgttggctgcggtcctttc    3633
GGT5.nt.mouse                                         cct------gctgggtgtaggtctaggtctggttatcgttgtgttggctgcggtcctttc    472
FAM247.LOC105372935.ref.human                         cctgctggggctggggctggggctggcgctggctgtcattgtgctggctgtggtcctctc    484
GGT1.end-GGT5.begininig.Philippine.tarsier.ref        ------------------------------------------------------------    2873
GGT5.end-GGT1.beginning.Rhesus.28419652-28635852      gct------gctggggctggggctggggctggctgtcactgtgctggctgtggtcctctc    3401
spacer.GGT1.end-start.BCRP3..human.ref                ------------------------------------------------------------    3920
GGT1end-LOC749026.end.7456450-7520130.chimp           cct------gctggggctggggctggcgctggctgtcattgtgctggctgt---------    4362

GGT5.total.&minus3200bp.mouse                         tcctcgtcaagcctcttgtggtcccggtgccttcacgcgtgctgcggtagcggctgactc    3693
GGT5.nt.mouse                                         tcctcgtcaagcctcttgtggtcccggtgccttcacgcgtgctgcggtagcggctgactc    532
FAM247.LOC105372935.ref.human                         tcgacaccaggccccatttgaccccc-ggcctttgcccacgccgctgttgctgctgactc    543
GGT1.end-GGT5.begininig.Philippine.tarsier.ref        ------------------------------------------------------------    2873
GGT5.end-GGT1.beginning.Rhesus.28419652-28635852      tcgccaccagactccctgtggcccccaggcctttgcccacgctactgttgctgctgactc    3461
spacer.GGT1.end-start.BCRP3..human.ref                ------------------------------------------------------------    3920
GGT1end-LOC749026.end.7456450-7520130.chimp           ------------------------------------------------------------    4362

GGT5.total.&minus3200bp.mouse                         caagatctgctcggatattggacggtgagtaaaaagtgggtgtgagctcagtggcccttg    3753
GGT5.nt.mouse                                         caagatctgctcggatattggacggtgagtaaaaagtgggtgtgagctcagtggcccttg    592
FAM247.LOC105372935.ref.human                         caaggtcttctcaaatattgtacggtgagtgagacgtgggaggaagctgggtggcctttg    603
GGT1.end-GGT5.begininig.Philippine.tarsier.ref        ------------------------------------------------------------    2873
GGT5.end-GGT1.beginning.Rhesus.28419652-28635852      caaggtctgctcaaatattggactgtgagtgagacgtgggaggaagctgggtggcctttg    3521
spacer.GGT1.end-start.BCRP3..human.ref                ------------------------------------------------------------    3920
GGT1end-LOC749026.end.7456450-7520130.chimp           ------------------------------------------------------------    4362

GGT5.total.&minus3200bp.mouse                         gcagctagcgggttgtgcctggagactgtgtaactg------------------------    3789
GGT5.nt.mouse                                         gcagctagcgggttgtgcctggagactgtgtaactg------------------------    628
FAM247.LOC105372935.ref.human                         gcagccagcccctcctggagaaggcgtgtgtgtgtgagcatgtgtgtgtgtgagagatta    663
GGT1.end-GGT5.begininig.Philippine.tarsier.ref        ------------------------------------------------------------    2873
GGT5.end-GGT1.beginning.Rhesus.28419652-28635852      gcagccagcccctcctggagaaggcgtgtgtgtttgagtgtgtgagtgtgtgggcgtgcg    3581
spacer.GGT1.end-start.BCRP3..human.ref                ------------------------------------------------------------    3920
GGT1end-LOC749026.end.7456450-7520130.chimp           ------------------------------------------------------------    4362

GGT5.total.&minus3200bp.mouse                         -----------tggtgggggtgtgaagagggacacgcgagtgccggctgt----------    3828
```

```
GGT5.nt.mouse                                       -----------tggtgggggtgtgaagagggacacgcgagtgccggctgt----------    667
FAM247.LOC105372935.ref.human                       tgtgtga----gtgtgtgtgggtatatgtgtgagtgtgtttgtggggtgtgggtgtgtgt    719
GGT1.end-GGT5.begining.Philippine.tarsier.ref       ------------------------------------------------------------    2873
GGT5.end-GGT1.beginning.Rhesus.28419652-28635852    tgtgtgattgcgtgtgtgagtgtgtatgtatgtgtgtgagtgggggtgtgggggtgtgt     3641
spacer.GGT1.end-start.BCRP3..human.ref              ------------------------------------------------------------    3920
GGT1end-LOC749026.end.7456450-7520130.chimp         ------------------------------------------------------------    4362

GGT5.total.&minus3200bp.mouse                       ------------------------------------------------------------    3828
GGT5.nt.mouse                                       ------------------------------------------------------------    667
FAM247.LOC105372935.ref.human                       gaatgtgtgtgatcgtgtttgggtgtgtgtatgtgtgagtgtgggtgtgtgtgaatgtgt    779
GGT1.end-GGT5.begining.Philippine.tarsier.ref       ------------------------------------------------------------    2873
GGT5.end-GGT1.beginning.Rhesus.28419652-28635852    gaatgtgtgtgattgtgtttgggggt------------------gtatgtgtgggtgtgt    3683
spacer.GGT1.end-start.BCRP3..human.ref              ------------------------------------------------------------    3920
GGT1end-LOC749026.end.7456450-7520130.chimp         ------------------------------------------------------------    4362

GGT5.total.&minus3200bp.mouse                       ------------------------------------------------------------    3828
GGT5.nt.mouse                                       ------------------------------------------------------------    667
FAM247.LOC105372935.ref.human                       gtgattgtgtttgtgtatgtgtgtgtgggtgtgtgtgagtatatgtgagtgtgagtgtgt    839
GGT1.end-GGT5.begining.Philippine.tarsier.ref       ------------------------------------------------------------    2873
GGT5.end-GGT1.beginning.Rhesus.28419652-28635852    gtgattgtgtttgggtatgtgtgggtgtatgtgtgagagtgagtg---------------    3728
spacer.GGT1.end-start.BCRP3..human.ref              ------------------------------------------------------------    3920
GGT1end-LOC749026.end.7456450-7520130.chimp         ------------------------------------------------------------    4362

GGT5.total.&minus3200bp.mouse                       ------------------------------------------------------------    3828
GGT5.nt.mouse                                       ------------------------------------------------------------    667
FAM247.LOC105372935.ref.human                       ggggtgtgggtgggtgtgaatgtgtgtgattgtgtttcgctgtgtgagggtgtgtgtga    899
GGT1.end-GGT5.begining.Philippine.tarsier.ref       ------------------------------------------------------------    2873
GGT5.end-GGT1.beginning.Rhesus.28419652-28635852    caggggtgtgcgtgtgtgaatgtgtgtgattgtgtttgggtgtgtgtgtgggagggtga    3788
spacer.GGT1.end-start.BCRP3..human.ref              ------------------------------------------------------------    3920
GGT1end-LOC749026.end.7456450-7520130.chimp         ------------------------------------------------------------    4362

GGT5.total.&minus3200bp.mouse                       ------------------------------------------------------------    3828
GGT5.nt.mouse                                       ------------------------------------------------------------    667
FAM247.LOC105372935.ref.human                       ctgtgagtgtgtgagtgtgggtgtgtgggtgtgtgtaaatgtgtgagtgtgagtatgggg    959
GGT1.end-GGT5.begining.Philippine.tarsier.ref       ------------------------------------------------------------    2873
GGT5.end-GGT1.beginning.Rhesus.28419652-28635852    gtgtgactgtgtgggag-gggtgtgggtgtgtgtgaatgtgtgtgattgtgggtatgtgt    3847
spacer.GGT1.end-start.BCRP3..human.ref              ------------------------------------------------------------    3920
GGT1end-LOC749026.end.7456450-7520130.chimp         ------------------------------------------------------------    4362

GGT5.total.&minus3200bp.mouse                       ------------------------------------------------------------    3828
GGT5.nt.mouse                                       ------------------------------------------------------------    667
FAM247.LOC105372935.ref.human                       gggtgggtatgtgtgaatgtgtgtgattgtgtgtgggtatatttgtgggggtgtgtgtgt    1019
GGT1.end-GGT5.begining.Philippine.tarsier.ref       ------------------------------------------------------------    2873
GGT5.end-GGT1.beginning.Rhesus.28419652-28635852    atgtgtgggtgtgtaagtgcgtgtgtgtgtgggtgtatgtatgtgtgcgtgtgtgagtgt    3907
spacer.GGT1.end-start.BCRP3..human.ref              ------------------------------------------------------------    3920
GGT1end-LOC749026.end.7456450-7520130.chimp         ------------------------------------------------------------    4362

GGT5.total.&minus3200bp.mouse                       ------------------------------------------------------gcttgc    3834
GGT5.nt.mouse                                       ------------------------------------------------------gcttgc    673
FAM247.LOC105372935.ref.human                       gtgtgcacgtgtgtgtgtgtgcacgtgcactggcccaggaagcaggagcc----gtgtgt    1075
GGT1.end-GGT5.begining.Philippine.tarsier.ref       ------------------------------------------------------------    2873
GGT5.end-GGT1.beginning.Rhesus.28419652-28635852    gtgt----gtgcgtgtgtgtacaagtgcactggcccaggaagcaggagccgtgtgtgtgt    3963
spacer.GGT1.end-start.BCRP3..human.ref              ------------------------------------------------------------    3920
GGT1end-LOC749026.end.7456450-7520130.chimp         ------------------------------------------------------------    4362

GGT5.total.&minus3200bp.mouse                       ggtggggtaaaggccatgtgtggtttggcttcctacaacagtcccagggcccttgcacag    3894
GGT5.nt.mouse                                       ggtggggtaaaggccatgtgtggtttggcttcctacaacagtcccagggcccttgcacag    733
FAM247.LOC105372935.ref.human                       gtgggcttcagcacctgcagggcttgagcgcaaggagacagcctcagggcccttgcacag    1135
GGT1.end-GGT5.begining.Philippine.tarsier.ref       ------------------------------------------------------------    2873
GGT5.end-GGT1.beginning.Rhesus.28419652-28635852    gtgggcttcagcacctgcagggcttgggcacaaggaggcagcctcagggcccttgcacag    4023
spacer.GGT1.end-start.BCRP3..human.ref              ------------------------------------------------------------    3920
GGT1end-LOC749026.end.7456450-7520130.chimp         ------------------------------------------------------------    4362

GGT5.total.&minus3200bp.mouse                       aatg---gtgtatgtgtgtccatagggaagatggggactctggacagcattgcaaagatg    3951
GGT5.nt.mouse                                       aatg---gtgtatgtgtgtccatagggaagatggggactctggacagcattgcaaagatg    790
FAM247.LOC105372935.ref.human                       aacaggcggcagggtgtgcccgtggggcagatggggacttggggacaatggtggt-----    1190
GGT1.end-GGT5.begining.Philippine.tarsier.ref       ------------------------------------------------------------    2873
GGT5.end-GGT1.beginning.Rhesus.28419652-28635852    aacaggtggcagggtgtgcccatggggcagatggtgatttagggaca---gtcat-----    4075
spacer.GGT1.end-start.BCRP3..human.ref              ------------------------------------------------------------    3920
GGT1end-LOC749026.end.7456450-7520130.chimp         ------------------------------------------------------------    4362

GGT5.total.&minus3200bp.mouse                       gtgtgagtctattattgaagatcggctatacagtgctggttggaaaggcagactggatga    4011
GGT5.nt.mouse                                       gtgtgagtctattattgaagatcggctatacagtgctggttggaaaggcagactggatga    850
FAM247.LOC105372935.ref.human                       gtgtgagtccatacctggctccaggattcaggaggcccatttgcatatccca--ggtggg    1248
GGT1.end-GGT5.begining.Philippine.tarsier.ref       ------------------------------------------------------------    2873
GGT5.end-GGT1.beginning.Rhesus.28419652-28635852    gtgtgagtccacacctggctccaggattcaggagacccatttgcacatccca--ggtgg-    4132
spacer.GGT1.end-start.BCRP3..human.ref              ------------------------------------------------------------    3920
GGT1end-LOC749026.end.7456450-7520130.chimp         ------------------------------------------------------------    4362

GGT5.total.&minus3200bp.mouse                       gacctgtgactttgtgcctgctggttctgaccaagactggaccttttt------------    4059
GGT5.nt.mouse                                       gacctgtgactttgtgcctgctggttctgaccaagactggaccttttt------------    898
FAM247.LOC105372935.ref.human                       aacctgtctggccccgcctgaccctgctggccggcgcaggccccttcagtgaggccaatt    1308
GGT1.end-GGT5.begining.Philippine.tarsier.ref       ------------------------------------------------------------    2873
```

```
GGT5.end-GGT1.beginning.Rhesus.28419652-28635852      ----------------------------ggccagtacaggccccttcagtgaggccaatt    4164
spacer.GGT1.end-start.BCRP3..human.ref                ------------------------------------------------------------    3920
GGT1end-LOC749026.end.7456450-7520130.chimp           ------------------------------------------------------------    4362


GGT5.total.&minus3200bp.mouse                         ----------------------cagaaacctgggcaacgggcttcag-ggttccatacat    4096
GGT5.nt.mouse                                         ----------------------cagaaacctgggcaacgggcttcag-ggttccatacat    935
FAM247.LOC105372935.ref.human                         ctccaaggctgcggtcttctcccagggtcatgggtgaaggggtttggaggctccctgcgt    1368
GGT1.end-GGT5.beginining.Philippine.tarsier.ref       ------------------------------------------------------------    2873
GGT5.end-GGT1.beginning.Rhesus.28419652-28635852      ctccaaggctggggtcttctcccagggtcataggtgaagggcttcagaggctccctgtgt    4224
spacer.GGT1.end-start.BCRP3..human.ref                ------------------------------------------------------------    3920
GGT1end-LOC749026.end.7456450-7520130.chimp           ------------------------------------------------------------    4362


GGT5.total.&minus3200bp.mouse                         tggt-ctgccctgccacag-agagagggaggtcccagggccagtataagaaagcac----    4150
GGT5.nt.mouse                                         tggt-ctgccctgccacag-agagagggaggtcccagggccagtataagaaagcac----    989
FAM247.LOC105372935.ref.human                         gggtactggcctgctggggtacacacaatgctgccatagccagtctgcccctacacccag    1428
GGT1.end-GGT5.beginining.Philippine.tarsier.ref       ------------------------------------------------------------    2873
GGT5.end-GGT1.beginning.Rhesus.28419652-28635852      gggtactggcctgctggggtacacacaatgctgccacagccagtctgccccagcttccag    4284
spacer.GGT1.end-start.BCRP3..human.ref                ------------------------------------------------------------    3920
GGT1end-LOC749026.end.7456450-7520130.chimp           ------------------------------------------------------------    4362


GGT5.total.&minus3200bp.mouse                         -------------------------------------------gttacagctgtcaggac    4167
GGT5.nt.mouse                                         -------------------------------------------gttacagctgtcaggac    1006
FAM247.LOC105372935.ref.human                         cccggggccacatctcaggtctctcagtcctgaggagcccggtgccccacccctcacatc    1488
GGT1.end-GGT5.beginining.Philippine.tarsier.ref       ------------------------------------------------------------    2873
GGT5.end-GGT1.beginning.Rhesus.28419652-28635852      ctgggggccacatctcgggtttctctgtcctggggagcctggtgccccacccctcacatc    4344
spacer.GGT1.end-start.BCRP3..human.ref                ------------------------------------------------------------    3920
GGT1end-LOC749026.end.7456450-7520130.chimp           ------------------------------------------------------------    4362


GGT5.total.&minus3200bp.mouse                         ctccctctctgtggcagggtctggccttggtgagtggagtgactgatgtggaggctggga    4227
GGT5.nt.mouse                                         ctccctctctgtggcagggtctggccttggtgagtggagtgactgatgtggaggctggga    1066
FAM247.LOC105372935.ref.human                         ctctctccctgagtcagggcctgggtctcgtgagctgagtgactgatacttggtgt----    1544
GGT1.end-GGT5.beginining.Philippine.tarsier.ref       ------------------------------------------------------------    2873
GGT5.end-GGT1.beginning.Rhesus.28419652-28635852      ctctctccctgagtcagggcctgggtctcctgagctgagtgactgatacttggtgt----    4400
spacer.GGT1.end-start.BCRP3..human.ref                ------------------------------------------------------------    3920
GGT1end-LOC749026.end.7456450-7520130.chimp           ------------------------------------------------------------    4362


GGT5.total.&minus3200bp.mouse                         aggagggagagagacagacagagacagacacagacacagagagaaagagaggcagagaca    4287
GGT5.nt.mouse                                         aggagggagagagacagacagagacagacacagacacagagagaaagagaggcagagaca    1126
FAM247.LOC105372935.ref.human                         ------------------------------------------------------------    1544
GGT1.end-GGT5.beginining.Philippine.tarsier.ref       ------------------------------------------------------------    2873
GGT5.end-GGT1.beginning.Rhesus.28419652-28635852      ------------------------------------------------------------    4400
spacer.GGT1.end-start.BCRP3..human.ref                ------------------------------------------------------------    3920
GGT1end-LOC749026.end.7456450-7520130.chimp           ------------------------------------------------------------    4362


GGT5.total.&minus3200bp.mouse                         gagagacagagacagagagacagagagaggaagagtggggagagagagacagacagagag    4347
GGT5.nt.mouse                                         gagagacagagacagagagacagagagaggaagagtggggagagagagacagacagagag    1186
FAM247.LOC105372935.ref.human                         ------------------------------------------------------------    1544
GGT1.end-GGT5.beginining.Philippine.tarsier.ref       ------------------------------------------------------------    2873
GGT5.end-GGT1.beginning.Rhesus.28419652-28635852      ------------------------------------------------------------    4400
spacer.GGT1.end-start.BCRP3..human.ref                ------------------------------------------------------------    3920
GGT1end-LOC749026.end.7456450-7520130.chimp           ------------------------------------------------------------    4362


GGT5.total.&minus3200bp.mouse                         atagacagagagacagacagagagagaggcagagagagaggcagagagagaggcagagag    4407
GGT5.nt.mouse                                         atagacagagagacagacagagagagaggcagagagagaggcagagagagaggcagagag    1246
FAM247.LOC105372935.ref.human                         ------------------------------------------------------------    1544
GGT1.end-GGT5.beginining.Philippine.tarsier.ref       ------------------------------------------------------------    2873
GGT5.end-GGT1.beginning.Rhesus.28419652-28635852      ------------------------------------------------------------    4400
spacer.GGT1.end-start.BCRP3..human.ref                ------------------------------------------------------------    3920
GGT1end-LOC749026.end.7456450-7520130.chimp           ------------------------------------------------------------    4362


GGT5.total.&minus3200bp.mouse                         acagacagagagagaggcagagagagaggcagagagaagctacagtggttgtttcctgac    4467
GGT5.nt.mouse                                         acagacagagagagaggcagagagagaggcagagagaagctacagtggttgtttcctgac    1306
FAM247.LOC105372935.ref.human                         ------------cctggatgagggcgtggtggagaggggccacagcgggtgtttcctgac    1592
GGT1.end-GGT5.beginining.Philippine.tarsier.ref       ------------------------------------------------------------    2873
GGT5.end-GGT1.beginning.Rhesus.28419652-28635852      ------------cctgaatgagggtgtggtggagaggggccacggcgggtgtttcctgac    4448
spacer.GGT1.end-start.BCRP3..human.ref                ------------------------------------------------------------    3920
GGT1end-LOC749026.end.7456450-7520130.chimp           ------------------------------------------------------------    4362


GGT5.total.&minus3200bp.mouse                         ---cttccaggaaacccagcccatgggaagcctatgctacccaccatgctgccacaggat    4524
GGT5.nt.mouse                                         ---cttccaggaaacccagcccatgggaagcctatgctacccaccatgctgccacaggat    1363
FAM247.LOC105372935.ref.human                         cctcttccaggaagg-------------------tgctgctgccgctgcagggaggaca-    1632
GGT1.end-GGT5.beginining.Philippine.tarsier.ref       ------------------------------------------------------------    2873
GGT5.end-GGT1.beginning.Rhesus.28419652-28635852      cctcttccaggaaacccagcccaagggaggccttcgctgctgccactgcagagaggaca-    4507
spacer.GGT1.end-start.BCRP3..human.ref                ------------------------------------------------------------    3920
GGT1end-LOC749026.end.7456450-7520130.chimp           ------------------------------------------------------------    4362


GGT5.total.&minus3200bp.mouse                         taccctgaggcaagaagtcactcctggtcccctgcctcccactggggccctgaaa-cctg    4583
GGT5.nt.mouse                                         taccctgaggcaagaagtcactcctggtcccctgcctcccactggggccctgaaa-cctg    1422
FAM247.LOC105372935.ref.human                         ------cacacaggatgccccttcttgccccctgcctcccattgggcccacaaaagccag    1686
GGT1.end-GGT5.beginining.Philippine.tarsier.ref       ------------------------------------------------------------    2873
GGT5.end-GGT1.beginning.Rhesus.28419652-28635852      ------catacaggacgccccttcctgccccctgcctgccattgggcccacaaaagccgg    4561
spacer.GGT1.end-start.BCRP3..human.ref                ------------------------------------------------------------    3920
GGT1end-LOC749026.end.7456450-7520130.chimp           ------------------------------------------------------------    4362
```

```
GGT5.total.&minus3200bp.mouse                         gacaagcctctcctttttttctggtcatctgggtctccttcccagaatatctgcctggtag    4643
GGT5.nt.mouse                                         gacaagcctctcctttttttctggtcatctgggtctccttcccagaatatctgcctggtag    1482
FAM247.LOC105372935.ref.human                         ggcaagcctcccctccctgccagccacct-ggtctgcttcccagaaattctgtcttgcag    1745
GGT1.end-GGT5.beginining.Philippine.tarsier.ref       ------------------------------------------------------------    2873
GGT5.end-GGT1.beginning.Rhesus.28419652-28635852      ggcaagcctcccctccctg-cagccacct-ggtctgcttcccagaagctctgtcttgcag    4619
spacer.GGT1.end-start.BCRP3..human.ref                ------------------------------------------------------------    3920
GGT1end-LOC749026.end.7456450-7520130.chimp           ------------------------------------------------------------    4362

GGT5.total.&minus3200bp.mouse                         cccactgtcagggttcaagtgctttgtaaacccaaggctgggtcctgcagcaggcagagt    4703
GGT5.nt.mouse                                         cccactgtcagggttcaagtgctttgtaaacccaaggctgggtcctgcagcaggcagagt    1542
FAM247.LOC105372935.ref.human                         gctgttgggaggatcccagtactttgtaaactaaagcaagggaggagtggccg-------    1798
GGT1.end-GGT5.beginining.Philippine.tarsier.ref       ------------------------------------------------------------    2873
GGT5.end-GGT1.beginning.Rhesus.28419652-28635852      gctgttgggaggatcccagtgctttgtaaactaaagcaagggaggcgtggccg-------    4672
spacer.GGT1.end-start.BCRP3..human.ref                ------------------------------------------------------------    3920
GGT1end-LOC749026.end.7456450-7520130.chimp           ------------------------------------------------------------    4362

GGT5.total.&minus3200bp.mouse                         ggtggtcctggcccaaaataccatgctgttatgctttttttttgactgttattgaatgtac    4763
GGT5.nt.mouse                                         ggtggtcctggcccaaaataccatgctgttatgctttttttttgactgttattgaatgtac    1602
FAM247.LOC105372935.ref.human                         ------------------------------------------------------------    1798
GGT1.end-GGT5.beginining.Philippine.tarsier.ref       ------------------------------------------------------------    2873
GGT5.end-GGT1.beginning.Rhesus.28419652-28635852      ------------------------------------------------------------    4672
spacer.GGT1.end-start.BCRP3..human.ref                ------------------------------------------------------------    3920
GGT1end-LOC749026.end.7456450-7520130.chimp           ------------------------------------------------------------    4362

GGT5.total.&minus3200bp.mouse                         cttctgtctgtctgtccttccttcttccttccttacatcctttcttccttctgtccatcc    4823
GGT5.nt.mouse                                         cttctgtctgtctgtccttccttcttccttccttacatcctttcttccttctgtccatcc    1662
FAM247.LOC105372935.ref.human                         ------------------------------------------------------------    1798
GGT1.end-GGT5.beginining.Philippine.tarsier.ref       ------------------------------------------------------------    2873
GGT5.end-GGT1.beginning.Rhesus.28419652-28635852      ------------------------------------------------------------    4672
spacer.GGT1.end-start.BCRP3..human.ref                ------------------------------------------------------------    3920
GGT1end-LOC749026.end.7456450-7520130.chimp           ------------------------------------------------------------    4362

GGT5.total.&minus3200bp.mouse                         ttccatctgtcctttgtctgtttgtccttcaaaccatctgtccttccttccttccttcct    4883
GGT5.nt.mouse                                         ttccatctgtcctttgtctgtttgtccttcaaaccatctgtccttccttccttccttcct    1722
FAM247.LOC105372935.ref.human                         ------------------------------------------------------------    1798
GGT1.end-GGT5.beginining.Philippine.tarsier.ref       ------------------------------------------------------------    2873
GGT5.end-GGT1.beginning.Rhesus.28419652-28635852      ------------------------------------------------------------    4672
spacer.GGT1.end-start.BCRP3..human.ref                ------------------------------------------------------------    3920
GGT1end-LOC749026.end.7456450-7520130.chimp           ------------------------------------------------------------    4362

GGT5.total.&minus3200bp.mouse                         tccttctgtccatccttctttctgtctgtctgttcatctgtttttccttccatccttcct    4943
GGT5.nt.mouse                                         tccttctgtccatccttctttctgtctgtctgttcatctgtttttccttccatccttcct    1782
FAM247.LOC105372935.ref.human                         ------------------------------------------------------------    1798
GGT1.end-GGT5.beginining.Philippine.tarsier.ref       ------------------------------------------------------------    2873
GGT5.end-GGT1.beginning.Rhesus.28419652-28635852      ------------------------------------------------------------    4672
spacer.GGT1.end-start.BCRP3..human.ref                ------------------------------------------------------------    3920
GGT1end-LOC749026.end.7456450-7520130.chimp           ------------------------------------------------------------    4362

GGT5.total.&minus3200bp.mouse                         tctgtctatccttccttccttctgtccatccttccttccttctgtctgtccttccttctg    5003
GGT5.nt.mouse                                         tctgtctatccttccttccttctgtccatccttccttccttctgtctgtccttccttctg    1842
FAM247.LOC105372935.ref.human                         ------------------------------------------------------------    1798
GGT1.end-GGT5.beginining.Philippine.tarsier.ref       ------------------------------------------------------------    2873
GGT5.end-GGT1.beginning.Rhesus.28419652-28635852      ------------------------------------------------------------    4672
spacer.GGT1.end-start.BCRP3..human.ref                ------------------------------------------------------------    3920
GGT1end-LOC749026.end.7456450-7520130.chimp           ------------------------------------------------------------    4362

GGT5.total.&minus3200bp.mouse                         tctgtctttccttccttctgtccttccttctgtttgtcctgccttccttcccgcctccct    5063
GGT5.nt.mouse                                         tctgtctttccttccttctgtccttccttctgtttgtcctgccttccttcccgcctccct    1902
FAM247.LOC105372935.ref.human                         ------ttctctctctttgttcattcattcaccttttcattcattccttcttccctccat    1852
GGT1.end-GGT5.beginining.Philippine.tarsier.ref       ------------------------------------------------------------    2873
GGT5.end-GGT1.beginning.Rhesus.28419652-28635852      ------ttctctctctttgttcattcattcaccttttgagtcatttcttcctccctccat    4726
spacer.GGT1.end-start.BCRP3..human.ref                ------------------------------------------------------------    3920
GGT1end-LOC749026.end.7456450-7520130.chimp           ------------------------------------------------------------    4362

GGT5.total.&minus3200bp.mouse                         tattccatctgtctacccattctcttccacctccctcgctcacagtagcccgcaaccctg    5123
GGT5.nt.mouse                                         tattccatctgtctacccattctcttccacctccctcgctcacagtagcccgcaaccctg    1962
FAM247.LOC105372935.ref.human                         tcccccatctgtgcatccttccctgccctgattgctcatgccacccccccccagcccctc    1912
GGT1.end-GGT5.beginining.Philippine.tarsier.ref       ------------------------------------------------------------    2873
GGT5.end-GGT1.beginning.Rhesus.28419652-28635852      taccccatctgtccatccttccctgccctgattgctcatgcca--cacccccagcccctc    4784
spacer.GGT1.end-start.BCRP3..human.ref                ------------------------------------------------------------    3920
GGT1end-LOC749026.end.7456450-7520130.chimp           ------------------------------------------------------------    4362

GGT5.total.&minus3200bp.mouse                         ctaacctggtcctctgagttcccagacttcctggaggattcacatctacatttaacttgg    5183
GGT5.nt.mouse                                         ctaacctggtcctctgagttcccagacttcctggaggattcacatctacatttaacttgg    2022
FAM247.LOC105372935.ref.human                         ctgacctggtcctttggtttctcttcagggct--------------ttctgtctcctccc    1958
GGT1.end-GGT5.beginining.Philippine.tarsier.ref       ------------------------------------------------------------    2873
GGT5.end-GGT1.beginning.Rhesus.28419652-28635852      ctgacctggtcctttggtttctcttcatggct--------------ttctgtctcctccc    4830
spacer.GGT1.end-start.BCRP3..human.ref                ------------------------------------------------------------    3920
GGT1end-LOC749026.end.7456450-7520130.chimp           ------------------------------------------------------------    4362

GGT5.total.&minus3200bp.mouse                         aactccatgggaatagcagccc--ggatcagtagggac---agtcctttctacgtcacat    5238
```

```
GGT5.nt.mouse                                       aactccatgggaatagcagccc--ggatcagtagggac---agtcctttctacgtcacat    2077
FAM247.LOC105372935.ref.human                       acagggctgagaatggcagctcagggacaagtaggggctggggactgcttagtctcccca    2018
GGT1.end-GGT5.beginining.Philippine.tarsier.ref     ------------------------------------------------------------    2873
GGT5.end-GGT1.beginning.Rhesus.28419652-28635852    acagggctgagaatggcagctcagggacaagtagagcctggtgactgcttggtctcccgg    4890
spacer.GGT1.end-start.BCRP3..human.ref              ------------------------------------------------------------    3920
GGT1end-LOC749026.end.7456450-7520130.chimp         ------------------------------------------------------------    4362


GGT5.total.&minus3200bp.mouse                       ggcgctcccaagggactcaa------------------------gagttaagaccaacag    5274
GGT5.nt.mouse                                       ggcgctcccaagggactcaa------------------------gagttaagaccaacag    2113
FAM247.LOC105372935.ref.human                       gtggctctcaggggatttgagggtttgacgccagctgccaccccaggctgtgcccctcct    2078
GGT1.end-GGT5.beginining.Philippine.tarsier.ref     ------------------------------------------------------------    2873
GGT5.end-GGT1.beginning.Rhesus.28419652-28635852    gtggctcctaggggatttgagggattgatgcctgctg-------aggctgtgcccctcct    4943
spacer.GGT1.end-start.BCRP3..human.ref              ------------------------------------------------------------    3920
GGT1end-LOC749026.end.7456450-7520130.chimp         ------------------------------------------------------------    4362


GGT5.total.&minus3200bp.mouse                       ttgctct----gtctccatgagatgcagcatctatttaatccttctagaatacaaaattg    5330
GGT5.nt.mouse                                       ttgctct----gtctccatgagatgcagcatctatttaatccttctagaatacaaaattg    2169
FAM247.LOC105372935.ref.human                       ctgctcgggaggacatacagagatgcgacacccacttaaac--tcgaagttgcaaagatg    2136
GGT1.end-GGT5.beginining.Philippine.tarsier.ref     ------------------------------------------------------------    2873
GGT5.end-GGT1.beginning.Rhesus.28419652-28635852    ctgctcaggaggacatacagagatgtggcacccacttaaac--tcaaagttgcacagatg    5001
spacer.GGT1.end-start.BCRP3..human.ref              ------------------------------------------------------------    3920
GGT1end-LOC749026.end.7456450-7520130.chimp         ------------------------------------------------------------    4362


GGT5.total.&minus3200bp.mouse                       taactgaggtggggtatgtgtgcacgctcaggccccacagaaaaccagccagaagagcag    5390
GGT5.nt.mouse                                       taactgaggtggggtatgtgtgcacgctcaggccccacagaaaaccagccagaagagcag    2229
FAM247.LOC105372935.ref.human                       caaatgagactggg----------gtctcaggcaccagagaccacccgtgggcacgtggc    2186
GGT1.end-GGT5.beginining.Philippine.tarsier.ref     ------------------------------------------------------------    2873
GGT5.end-GGT1.beginning.Rhesus.28419652-28635852    caaatgagactggg----------gtctcaggcaccagagacca-ccgtgggcacgtgac    5050
spacer.GGT1.end-start.BCRP3..human.ref              ------------------------------------------------------------    3920
GGT1end-LOC749026.end.7456450-7520130.chimp         ------------------------------------------------------------    4362


GGT5.total.&minus3200bp.mouse                       cctctgtgaacaaaggggcgtgcttctactatctgtgagaaggct---------------    5435
GGT5.nt.mouse                                       cctctgtgaacaaaggggcgtgcttctactatctgtgagaaggct---------------    2274
FAM247.LOC105372935.ref.human                       ttttgggagtggggacctgctgccacagatctctga--agagtctggacctgctgggtct    2244
GGT1.end-GGT5.beginining.Philippine.tarsier.ref     ------------------------------------------------------------    2873
GGT5.end-GGT1.beginning.Rhesus.28419652-28635852    ctttgggagtggggacctgctgccacagatctctgagtggagtctggacctactgggtct    5110
spacer.GGT1.end-start.BCRP3..human.ref              ------------------------------------------------------------    3920
GGT1end-LOC749026.end.7456450-7520130.chimp         ------------------------------------------------------------    4362


GGT5.total.&minus3200bp.mouse                       --------------------ctctgaacagggagcattggtctgcgaggcccaggctgtg    5475
GGT5.nt.mouse                                       --------------------ctctgaacagggagcattggtctgcgaggcccaggctgtg    2314
FAM247.LOC105372935.ref.human                       ccccgagtgactgtctggggggtctccatagcatgccctgctgtgtgcgtgacggtcactg    2304
GGT1.end-GGT5.beginining.Philippine.tarsier.ref     ------------------------------------------------------------    2873
GGT5.end-GGT1.beginning.Rhesus.28419652-28635852    ccccaagtgactgtctgggggtctctgtagcatgccctgctgtgtacgtgagggtcagtg    5170
spacer.GGT1.end-start.BCRP3..human.ref              ------------------------------------------------------------    3920
GGT1end-LOC749026.end.7456450-7520130.chimp         ------------------------------------------------------------    4362


GGT5.total.&minus3200bp.mouse                       gttggtgctaagagcctgcactctaaactctcaagct---------------tcccctgg    5520
GGT5.nt.mouse                                       gttggtgctaagagcctgcactctaaactctcaagct---------------tcccctgg    2359
FAM247.LOC105372935.ref.human                       gttgggtaggggtctctactctaaagctccctctgccggcatcccctccaactctccctt    2364
GGT1.end-GGT5.beginining.Philippine.tarsier.ref     ------------------------------------------------------------    2873
GGT5.end-GGT1.beginning.Rhesus.28419652-28635852    gttggggaggggtctctgctctaatgcttcctacactggcactccctcaaactcccttgg    5230
spacer.GGT1.end-start.BCRP3..human.ref              ------------------------------------------------------------    3920
GGT1end-LOC749026.end.7456450-7520130.chimp         ------------------------------------------------------------    4362


GGT5.total.&minus3200bp.mouse                       tggaaagcggatgtgggttcccccacagtgttttgttaaactcttcttgcttcctgttct    5580
GGT5.nt.mouse                                       tggaaagcggatgtgggttcccccacagtgttttgttaaactcttcttgcttcctgttct    2419
FAM247.LOC105372935.ref.human                       ggtgaagagaggatgtggtttgccccagtgttttatcaaacaactct---ctccacttcc    2421
GGT1.end-GGT5.beginining.Philippine.tarsier.ref     ------------------------------------------------------------    2873
GGT5.end-GGT1.beginning.Rhesus.28419652-28635852    tgaagagagaggatgtggtttgccgcagtgttttatcgaacaactct---ctccacttcc    5287
spacer.GGT1.end-start.BCRP3..human.ref              ------------------------------------------------------------    3920
GGT1end-LOC749026.end.7456450-7520130.chimp         ------------------------------------------------------------    4362


GGT5.total.&minus3200bp.mouse                       tgttttaagaagtcaagggtgggagggaacct----atggacccagctgcttaccgtaag    5636
GGT5.nt.mouse                                       tgttttaagaagtcaagggtgggagggaacct----atggacccagctgcttaccgtaag    2475
FAM247.LOC105372935.ref.human                       agttttaagaagctgggagtggaagagagcctggggctggccccagctgc-tgctgtgaa    2480
GGT1.end-GGT5.beginining.Philippine.tarsier.ref     ------------------------------------------------------------    2873
GGT5.end-GGT1.beginning.Rhesus.28419652-28635852    tgttttcagaagccgggagtggaagagagcctggggctggccccagctgc-tgctgcgga    5346
spacer.GGT1.end-start.BCRP3..human.ref              ------------------------------------------------------------    3920
GGT1end-LOC749026.end.7456450-7520130.chimp         ------------------------------------------------------------    4362


GGT5.total.&minus3200bp.mouse                       agagtgcttggtgaccatagccagactaggccttcaggaaggtgctgttgtgtgtctcct    5696
GGT5.nt.mouse                                       agagtgcttggtgaccatagccagactaggccttcaggaaggtgctgttgtgtgtctcct    2535
FAM247.LOC105372935.ref.human                       acaggggtcactggacgctgggaccctggccgggctggctggagg------------cct    2528
GGT1.end-GGT5.beginining.Philippine.tarsier.ref     ------------------------------------------------------------    2873
GGT5.end-GGT1.beginning.Rhesus.28419652-28635852    acaggggtcactggacgctgggaccctggccgggctggctggggg------------cct    5394
spacer.GGT1.end-start.BCRP3..human.ref              ------------------------------------------------------------    3920
GGT1end-LOC749026.end.7456450-7520130.chimp         ------------------------------------------------------------    4362


GGT5.total.&minus3200bp.mouse                       ggccaagagcttttcattcgacttaggctgtctg--------cccgggggaagaacctgg    5748
GGT5.nt.mouse                                       ggccaagagcttttcattcgacttaggctgtctg--------cccgggggaagaacctgg    2587
FAM247.LOC105372935.ref.human                       caggaagaggcctgctacagcgtcatcctggccaagattcctccctgcagaggaccctgg    2588
GGT1.end-GGT5.beginining.Philippine.tarsier.ref     ------------------------------------------------------------    2873
```

```
GGT5.end-GGT1.beginning.Rhesus.28419652-28635852      ccggaagaggcctgctgcagcgtcatcctggccgagatccctccctgcaggggcccctgg    5454
spacer.GGT1.end-start.BCRP3..human.ref                ------------------------------------------------------------    3920
GGT1end-LOC749026.end.7456450-7520130.chimp           ------------------------------------------------------------    4362


GGT5.total.&minus3200bp.mouse                         ccagtgtcactgtgggaagtcttggtgctactggtattcccccctactgttgctgctgct    5808
GGT5.nt.mouse                                         ccagtgtcactgtgggaagtcttggtgctactggtattcccccctactgttgctgctgct    2647
FAM247.LOC105372935.ref.human                         cca-cgctgccacagggtctgctggggccaccagaagcccatgctcctg-----------    2636
GGT1.end-GGT5.begining.Philippine.tarsier.ref         ------------------------------------------------------------    2873
GGT5.end-GGT1.beginning.Rhesus.28419652-28635852      cca-tgctgccgcagggtctgctggggccaccagaagcccacgctcctg-----------    5502
spacer.GGT1.end-start.BCRP3..human.ref                ------------------------------------------------------------    3920
GGT1end-LOC749026.end.7456450-7520130.chimp           ------------------------------------------------------------    4362


GGT5.total.&minus3200bp.mouse                         gttcttgccaggatgggaacgctgtggaagggtcagtctttctaaagtccctcacctctc    5868
GGT5.nt.mouse                                         gttcttgccaggatgggaacgctgtggaagggtcagtctttctaaagtccctcacctctc    2707
FAM247.LOC105372935.ref.human                         ------------------------------------------------cctccatctctc    2648
GGT1.end-GGT5.begining.Philippine.tarsier.ref         ------------------------------------------------------------    2873
GGT5.end-GGT1.beginning.Rhesus.28419652-28635852      ------------------------------------------------cctccatctctg    5514
spacer.GGT1.end-start.BCRP3..human.ref                ------------------------------------------------------------    3920
GGT1end-LOC749026.end.7456450-7520130.chimp           ------------------------------------------------------------    4362


GGT5.total.&minus3200bp.mouse                         ccctgggacaccagtgtgcccaaccctaggtacttaatggccacacccttatttaccaaa    5928
GGT5.nt.mouse                                         ccctgggacaccagtgtgcccaaccctaggtacttaatggccacacccttatttaccaaa    2767
FAM247.LOC105372935.ref.human                         ccctctgtgctcacctctcaccaggaggccctcccagagttcagtgtcc-----------    2697
GGT1.end-GGT5.begining.Philippine.tarsier.ref         ------------------------------------------------------------    2873
GGT5.end-GGT1.beginning.Rhesus.28419652-28635852      ccctgtgtgctcacctctcaccagcaggccctcccagagtccagtctct-----------    5563
spacer.GGT1.end-start.BCRP3..human.ref                ------------------------------------------------------------    3920
GGT1end-LOC749026.end.7456450-7520130.chimp           ------------------------------------------------------------    4362


GGT5.total.&minus3200bp.mouse                         atcatagaaggtaaggctgctttggggttctggcgagtaggtttctggagggctgtcatc    5988
GGT5.nt.mouse                                         atcatagaaggtaaggctgctttggggttctggcgagtaggtttctggagggctgtcatc    2827
FAM247.LOC105372935.ref.human                         ------------------------------------------------------------    2697
GGT1.end-GGT5.begining.Philippine.tarsier.ref         ------------------------------------------------------------    2873
GGT5.end-GGT1.beginning.Rhesus.28419652-28635852      ------------------------------------------------------------    5563
spacer.GGT1.end-start.BCRP3..human.ref                ------------------------------------------------------------    3920
GGT1end-LOC749026.end.7456450-7520130.chimp           ------------------------------------------------------------    4362


GGT5.total.&minus3200bp.mouse                         tggactgacactgggctacaggttggggtgacaactgaaccccaaaggctgagtgtctcc    6048
GGT5.nt.mouse                                         tggactgacactgggctacaggttggggtgacaactgaaccccaaaggctgagtgtctcc    2887
FAM247.LOC105372935.ref.human                         -------------------------------------------------------tgctt    2702
GGT1.end-GGT5.begining.Philippine.tarsier.ref         ------------------------------------------------------------    2873
GGT5.end-GGT1.beginning.Rhesus.28419652-28635852      -------------------------------------------------------tctgc    5568
spacer.GGT1.end-start.BCRP3..human.ref                ------------------------------------------------------------    3920
GGT1end-LOC749026.end.7456450-7520130.chimp           ------------------------------------------------------------    4362


GGT5.total.&minus3200bp.mouse                         tgctgcttcatgtccacctggcatctgagggttgcactcttcgttcaga----ggagagc    6104
GGT5.nt.mouse                                         tgctgcttcatgtccacctggcatctgagggttgcactcttcgttcaga----ggagagc    2943
FAM247.LOC105372935.ref.human                         ttttttttt---------ttttttgtgacggtgtctcactctgtcaccaggctggagtgc    2753
GGT1.end-GGT5.begining.Philippine.tarsier.ref         ------------------------------------------------------------    2873
GGT5.end-GGT1.beginning.Rhesus.28419652-28635852      tctttttttgtttgtttgttttttgagatggtgtctcactctgtcaccaggctggagtgc    5628
spacer.GGT1.end-start.BCRP3..human.ref                ------------------------------------------------------------    3920
GGT1end-LOC749026.end.7456450-7520130.chimp           ------------------------------------------------------------    4362


GGT5.total.&minus3200bp.mouse                         agtcacacaagccaagctctatggcaccatgtgggcctcagt--------ttcctctctg    6156
GGT5.nt.mouse                                         agtcacacaagccaagctctatggcaccatgtgggcctcagt--------ttcctctctg    2995
FAM247.LOC105372935.ref.human                         agtggcgcgatctcagcttactgcaacctctgcttcctcggttcaaatgattctcctgcc    2813
GGT1.end-GGT5.begining.Philippine.tarsier.ref         ------------------------------------------------------------    2873
GGT5.end-GGT1.beginning.Rhesus.28419652-28635852      agtggcgcaatctcggctcactgaaatttc------------------------------    5658
spacer.GGT1.end-start.BCRP3..human.ref                ------------------------------------------------------------    3920
GGT1end-LOC749026.end.7456450-7520130.chimp           ------------------------------------------------------------    4362


GGT5.total.&minus3200bp.mouse                         tgatatggcttttaggctgctacctccttcatgggacgccagggaacacacaggtgaatt    6216
GGT5.nt.mouse                                         tgatatggcttttaggctgctacctccttcatgggacgccagggaacacacaggtgaatt    3055
FAM247.LOC105372935.ref.human                         tcagcctcctgagtagctgggactacaggtgccagccaccacgccccgctaatttctgta    2873
GGT1.end-GGT5.begining.Philippine.tarsier.ref         ------------------------------------------------------------    2873
GGT5.end-GGT1.beginning.Rhesus.28419652-28635852      ------------------------------------------------------------    5658
spacer.GGT1.end-start.BCRP3..human.ref                ------------------------------------------------------------    3920
GGT1end-LOC749026.end.7456450-7520130.chimp           ------------------------------------------------------------    4362


GGT5.total.&minus3200bp.mouse                         cacctcacacagtggacgtgttccttcagtagctagacagtcatttgcctggattcatct    6276
GGT5.nt.mouse                                         cacctcacacagtggacgtgttccttcagtagctagacagtcatttgcctggattcatct    3115
FAM247.LOC105372935.ref.human                         tttttagtagagacggggtttcaccgtgttggccaggatggtctctatctcttgattcac    2933
GGT1.end-GGT5.begining.Philippine.tarsier.ref         ------------------------------------------------------------    2873
GGT5.end-GGT1.beginning.Rhesus.28419652-28635852      ------------------------------------------------------------    5658
spacer.GGT1.end-start.BCRP3..human.ref                ------------------------------------------------------------    3920
GGT1end-LOC749026.end.7456450-7520130.chimp           ------------------------------------------------------------    4362


GGT5.total.&minus3200bp.mouse                         ggacataggccaagatctcaggacccaaggggagattagggtgtggctcctggttctcat    6336
GGT5.nt.mouse                                         ggacataggccaagatctcaggacccaaggggagattagggtgtggctcctggttctcat    3175
FAM247.LOC105372935.ref.human                         ccgccttggccacccaaagtgctggcattacaggagtgagtcatggcacctgg-cctcat    2992
GGT1.end-GGT5.begining.Philippine.tarsier.ref         ------------------------------------------------------------    2873
GGT5.end-GGT1.beginning.Rhesus.28419652-28635852      ---------------------------------------------------------cgt    5661
spacer.GGT1.end-start.BCRP3..human.ref                ------------------------------------------------------------    3920
GGT1end-LOC749026.end.7456450-7520130.chimp           ------------------------------------------------------------    4362
```

```
GGT5.total.&minus3200bp.mouse                         cttccaggacttgggcttcagtttctcctctttcttcctctcaccctgaaacttgttctg    6396
GGT5.nt.mouse                                         cttccaggacttgggcttcagtttctcctctttcttcctctcaccctgaaacttgttctg    3235
FAM247.LOC105372935.ref.human                         ctcctactctttcagcaccaggtttt----------------------------------    3018
GGT1.end-GGT5.begining.Philippine.tarsier.ref         ------------------------------------------------------------    2873
GGT5.end-GGT1.beginning.Rhesus.28419652-28635852      ctcctactctttcagcatcaggtttt----------------------------------    5687
spacer.GGT1.end-start.BCRP3..human.ref                ------------------------------------------------------------    3920
GGT1end-LOC749026.end.7456450-7520130.chimp           ------------------------------------------------------------    4362

GGT5.total.&minus3200bp.mouse                         agaccttccttgttctccatattcccggccagctcaccaggaagtgactgccttccccaa    6456
GGT5.nt.mouse                                         agaccttccttgttctccatattcccggccagctcaccaggaagtgactgccttccccaa    3295
FAM247.LOC105372935.ref.human                         ------------------------------------------------------------    3018
GGT1.end-GGT5.begining.Philippine.tarsier.ref         ------------------------------------------------------------    2873
GGT5.end-GGT1.beginning.Rhesus.28419652-28635852      ------------------------------------------------------------    5687
spacer.GGT1.end-start.BCRP3..human.ref                ------------------------------------------------------------    3920
GGT1end-LOC749026.end.7456450-7520130.chimp           ------------------------------------------------------------    4362

GGT5.total.&minus3200bp.mouse                         ccactggggctcaggatgaaaagctctttgcacctagggatggcatacgtatacgcaaac    6516
GGT5.nt.mouse                                         ccactggggctcaggatgaaaagctctttgcacctagggatggcatacgtatacgcaaac    3355
FAM247.LOC105372935.ref.human                         ------------------------------------------------------------    3018
GGT1.end-GGT5.begining.Philippine.tarsier.ref         ------------------------------------------------------------    2873
GGT5.end-GGT1.beginning.Rhesus.28419652-28635852      ------------------------------------------------------------    5687
spacer.GGT1.end-start.BCRP3..human.ref                ------------------------------------------------------------    3920
GGT1end-LOC749026.end.7456450-7520130.chimp           ------------------------------------------------------------    4362

GGT5.total.&minus3200bp.mouse                         gcctgtgtgaacacacacggctgagtgagcctgtccaggctgtagtggtgacatcagaga    6576
GGT5.nt.mouse                                         gcctgtgtgaacacacacggctgagtgagcctgtccaggctgtagtggtgacatcagaga    3415
FAM247.LOC105372935.ref.human                         ------------------------------------------------------------    3018
GGT1.end-GGT5.begining.Philippine.tarsier.ref         ------------------------------------------------------------    2873
GGT5.end-GGT1.beginning.Rhesus.28419652-28635852      ------------------------------------------------------------    5687
spacer.GGT1.end-start.BCRP3..human.ref                ------------------------------------------------------------    3920
GGT1end-LOC749026.end.7456450-7520130.chimp           ------------------------------------------------------------    4362

GGT5.total.&minus3200bp.mouse                         agcctgtcgcttccttgggaacaagtacatgggaaatgtttcagccttttactgacccag    6636
GGT5.nt.mouse                                         agcctgtcgcttccttgggaacaagtacatgggaaatgtttcagccttttactgacccag    3475
FAM247.LOC105372935.ref.human                         -----------------------attcttgggattctgctacagccggagcccctgggtg    3055
GGT1.end-GGT5.begining.Philippine.tarsier.ref         ------------------------------------------------------------    2873
GGT5.end-GGT1.beginning.Rhesus.28419652-28635852      -----------------------attactgggattctgctacagccagagaccctgggag    5724
spacer.GGT1.end-start.BCRP3..human.ref                ------------------------------------------------------------    3920
GGT1end-LOC749026.end.7456450-7520130.chimp           ------------------------------------------------------------    4362

GGT5.total.&minus3200bp.mouse                         actgatcacatttcccaagggagggaagccaggccccagaagctgcctcacctcttgcaa    6696
GGT5.nt.mouse                                         actgatcacatttcccaagggagggaagccaggccccagaagctgcctcacctcttgcaa    3535
FAM247.LOC105372935.ref.human                         cgagttcctaaggtttctgtgagtgtggacccagcaccgtgcctagtagacatacaaaag    3115
GGT1.end-GGT5.begining.Philippine.tarsier.ref         ------------------------------------------------------------    2873
GGT5.end-GGT1.beginning.Rhesus.28419652-28635852      cagattcctaaggcttatgtgagtgtggacccagcaccgtgcctagcagacatacaaaag    5784
spacer.GGT1.end-start.BCRP3..human.ref                ------------------------------------------------------------    3920
GGT1end-LOC749026.end.7456450-7520130.chimp           ------------------------------------------------------------    4362

GGT5.total.&minus3200bp.mouse                         ctacctagtaggaatatagccctactgtagcagggctactatagacccaggctcctggct    6756
GGT5.nt.mouse                                         ctacctagtaggaatatagccctactgtagcagggctactatagacccaggctcctggct    3595
FAM247.LOC105372935.ref.human                         gagcatggtgacagtg-aggtctgtcatctccagcataatgactgttttgatccttgtaa    3174
GGT1.end-GGT5.begining.Philippine.tarsier.ref         ------------------------------------------------------------    2873
GGT5.end-GGT1.beginning.Rhesus.28419652-28635852      gagcatggtgacagtg-aggtctgtcatctccagcttaatgactgttttgatccttgtca    5843
spacer.GGT1.end-start.BCRP3..human.ref                ------------------------------------------------------------    3920
GGT1end-LOC749026.end.7456450-7520130.chimp           ------------------------------------------------------------    4362

GGT5.total.&minus3200bp.mouse                         tatccctgtggggttggggatacatgtgggtaagtcatg--ggccctgcaacatctaata    6814
GGT5.nt.mouse                                         tatccctgtggggttggggatacatgtgggtaagtcatg--ggccctgcaacatctaata    3653
FAM247.LOC105372935.ref.human                         aaaaggtgatttttggctgggtgtggtggctcacacctgtaatcccagcactttgggagg    3234
GGT1.end-GGT5.begining.Philippine.tarsier.ref         ------------------------------------------------------------    2873
GGT5.end-GGT1.beginning.Rhesus.28419652-28635852      aaaaggtgatttttggctgagcatggtggctcacacctgtaatcccagcactttgggagg    5903
spacer.GGT1.end-start.BCRP3..human.ref                ------------------------------------------------------------    3920
GGT1end-LOC749026.end.7456450-7520130.chimp           ------------------------------------------------------------    4362

GGT5.total.&minus3200bp.mouse                         cgtttgtcatagattaaaaatgggagggggctggagagatggctaaacaggtaagagca    6874
GGT5.nt.mouse                                         cgtttgtcatagattaaaaatgggagggggctggagagatggctaaacaggtaagagca    3713
FAM247.LOC105372935.ref.human                         ccgagggggtggctcacttgaggtcaggagttggagaccagcctgggcaacatggtgaa    3294
GGT1.end-GGT5.begining.Philippine.tarsier.ref         ------------------------------------------------------------    2873
GGT5.end-GGT1.beginning.Rhesus.28419652-28635852      ccgaggcgggtggatcacttgaggtcaggagttggagaccagcctgggcaacatggtgaa    5963
spacer.GGT1.end-start.BCRP3..human.ref                ------------------------------------------------------------    3920
GGT1end-LOC749026.end.7456450-7520130.chimp           ------------------------------------------------------------    4362

GGT5.total.&minus3200bp.mouse                         ccaactgctcttctgaaggtcctgagctca-------------------------aatcc    6909
GGT5.nt.mouse                                         ccaactgctcttctgaaggtcctgagctca-------------------------aatcc    3748
FAM247.LOC105372935.ref.human                         accatgtctctactaaaaatacaaaaattagctgggcatggtagcaggtgcctgtaatcc    3354
GGT1.end-GGT5.begining.Philippine.tarsier.ref         ------------------------------------------------------------    2873
GGT5.end-GGT1.beginning.Rhesus.28419652-28635852      accccgtctctactaaaaatacaaaaattagctgggcatggtagcgggtgcctgtaatcc    6023
spacer.GGT1.end-start.BCRP3..human.ref                ------------------------------------------------------------    3920
GGT1end-LOC749026.end.7456450-7520130.chimp           ------------------------------------------------------------    4362

GGT5.total.&minus3200bp.mouse                         cagcaaccacatagtggctcacaaccaccagtaatgacatctgacgccctcttctggtgg    6969
```

```
GGT5.nt.mouse                                      cagcaaccacatagtggctcacaaccaccagtaatgacatctgacgccctcttctggtgg    3808
FAM247.LOC105372935.ref.human                      cagatac-----------------------------------------------------    3361
GGT1.end-GGT5.beginining.Philippine.tarsier.ref    ------------------------------------------------------------    2873
GGT5.end-GGT1.beginning.Rhesus.28419652-28635852   cagctac-----------------------------------------------------    6030
spacer.GGT1.end-start.BCRP3..human.ref             ------------------------------------------------------------    3920
GGT1end-LOC749026.end.7456450-7520130.chimp        ------------------------------------------------------------    4362

GGT5.total.&minus3200bp.mouse                      gtctgaagacagctacagtgtacttatttgtaataataaataaatctttgggcaagagca    7029
GGT5.nt.mouse                                      gtctgaagacagctacagtgtacttatttgtaataataaataaatctttgggcaagagca    3868
FAM247.LOC105372935.ref.human                      -ttgggaggctgagacaggagaatcacttgaacccaggaggcaaaggtttcagtaagcca    3420
GGT1.end-GGT5.beginining.Philippine.tarsier.ref    ------------------------------------------------------------    2873
GGT5.end-GGT1.beginning.Rhesus.28419652-28635852   -ttgggagtctgagacaggagaatcacttgaacccaagaggcaaatgttgcagtgagcca    6089
spacer.GGT1.end-start.BCRP3..human.ref             ------------------------------------------------------------    3920
GGT1end-LOC749026.end.7456450-7520130.chimp        ------------------------------------------------------------    4362

GGT5.total.&minus3200bp.mouse                      agcagggccaaccagagcaaatggggttggccagagcgagcagagttctaaattcaattc    7089
GGT5.nt.mouse                                      agcagggccaaccagagcaaatggggttggccagagcgagcagagttctaaattcaattc    3928
FAM247.LOC105372935.ref.human                      agattgcaccactgcactccagcctgggtgacagagcaagacttggtctcaaaaaaaaaa    3480
GGT1.end-GGT5.beginining.Philippine.tarsier.ref    ------------------------------------------------------------    2873
GGT5.end-GGT1.beginning.Rhesus.28419652-28635852   agatagcaccactgcactacagcctgggtgacagagcaagacttggtctcaaaaaaaaaa    6149
spacer.GGT1.end-start.BCRP3..human.ref             ------------------------------------------------------------    3920
GGT1end-LOC749026.end.7456450-7520130.chimp        ------------------------------------------------------------    4362

GGT5.total.&minus3200bp.mouse                      ccaacaaccagatgaaggctcaccaccatctgtacagctacagtgtgtactcatatatat    7149
GGT5.nt.mouse                                      ccaacaaccagatgaaggctcaccaccatctgtacagctacagtgtgtactcatatatat    3988
FAM247.LOC105372935.ref.human                      aaaagaaagaaagaaaagtttatatttttgt-----tctaatggttatcttaatatcgtc    3535
GGT1.end-GGT5.beginining.Philippine.tarsier.ref    ------------------------------------------------------------    2873
GGT5.end-GGT1.beginning.Rhesus.28419652-28635852   aaaa-----aaagaaaagtttatatttttgt-----tctaaaacttatcttaatgtcttc    6199
spacer.GGT1.end-start.BCRP3..human.ref             ------------------------------------------------------------    3920
GGT1end-LOC749026.end.7456450-7520130.chimp        ------------------------------------------------------------    4362

GGT5.total.&minus3200bp.mouse                      ataataaataaataaacccttttttaaaaaaatgggaggggtaggcgcatgctcactggt    7209
GGT5.nt.mouse                                      ataataaataaataaacccttttttaaaaaaatgggaggggtaggcgcatgctcactggt    4048
FAM247.LOC105372935.ref.human                      attctataattatatgttttatataattataatagctatataagatataatacccctagt    3595
GGT1.end-GGT5.beginining.Philippine.tarsier.ref    ------------------------------------------------------------    2873
GGT5.end-GGT1.beginning.Rhesus.28419652-28635852   attctata--------ttttatataattataagagctatataagatatactacccctagt    6251
spacer.GGT1.end-start.BCRP3..human.ref             ------------------------------------------------------------    3920
GGT1end-LOC749026.end.7456450-7520130.chimp        ------------------------------------------------------------    4362

GGT5.total.&minus3200bp.mouse                      acagtacttacctaacatacatgaggttcccggttacatttccatcagggtaaaatgagg    7269
GGT5.nt.mouse                                      acagtacttacctaacatacatgaggttcccggttacatttccatcagggtaaaatgagg    4108
FAM247.LOC105372935.ref.human                      atgttgtttttggatattctacttgctcctgatggttaatttatgtgtcaacttggcta    3655
GGT1.end-GGT5.beginining.Philippine.tarsier.ref    ------------------------------------------------------------    2873
GGT5.end-GGT1.beginning.Rhesus.28419652-28635852   actttgtttttggatattctattcactcctgatggttaatttatgtgtcaactttgcta    6311
spacer.GGT1.end-start.BCRP3..human.ref             ------------------------------------------------------------    3920
GGT1end-LOC749026.end.7456450-7520130.chimp        ------------------------------------------------------------    4362

GGT5.total.&minus3200bp.mouse                      aacaactacactctgtcat-------------------------------aacctaggga    7298
GGT5.nt.mouse                                      aacaactacactctgtcat-------------------------------aacctaggga    4137
FAM247.LOC105372935.ref.human                      agctatggtgtcctgttgtttggtcaaatacttgtcaatatcttgctgggaggttatttc    3715
GGT1.end-GGT5.beginining.Philippine.tarsier.ref    ------------------------------------------------------------    2873
GGT5.end-GGT1.beginning.Rhesus.28419652-28635852   agctatgatgccctgttgtttggtcaaatacttctcaatatcttgctgggaggttatctc    6371
spacer.GGT1.end-start.BCRP3..human.ref             ------------------------------------------------------------    3920
GGT1end-LOC749026.end.7456450-7520130.chimp        ------------------------------------------------------------    4362

GGT5.total.&minus3200bp.mouse                      cacaatgtgaccaagagggacacagccctgactttgtgggaggaa---------------    7343
GGT5.nt.mouse                                      cacaatgtgaccaagagggacacagccctgactttgtgggaggaa---------------    4182
FAM247.LOC105372935.ref.human                      atagatgtgattaacactgacagtcaattgactttaagtaaaacagattacccaccataa    3775
GGT1.end-GGT5.beginining.Philippine.tarsier.ref    ------------------------------------------------------------    2873
GGT5.end-GGT1.beginning.Rhesus.28419652-28635852   atagatgtgattaacattgacagtcagctgactttaggtaaaaca---------------    6416
spacer.GGT1.end-start.BCRP3..human.ref             ------------------------------------------------------------    3920
GGT1end-LOC749026.end.7456450-7520130.chimp        ------------------------------------------------------------    4362

GGT5.total.&minus3200bp.mouse                      -atacaaacagatctgtaactctgttgca-------------------------------    7371
GGT5.nt.mouse                                      -atacaaacagatctgtaactctgttgca-------------------------------    4210
FAM247.LOC105372935.ref.human                      tatgggtgggccacctccaatcagttgaaggccttaagaacaaaaactgaggtttcccag    3835
GGT1.end-GGT5.beginining.Philippine.tarsier.ref    ------------------------------------------------------------    2873
GGT5.end-GGT1.beginning.Rhesus.28419652-28635852   -atgtgattaacgctgacagtcagttgac------tttaagtaaaactgaggtttcccag    6469
spacer.GGT1.end-start.BCRP3..human.ref             ------------------------------------------------------------    3920
GGT1end-LOC749026.end.7456450-7520130.chimp        ------------------------------------------------------------    4362

GGT5.total.&minus3200bp.mouse                      ------------------------------------------------------------    7371
GGT5.nt.mouse                                      ------------------------------------------------------------    4210
FAM247.LOC105372935.ref.human                      agaagcaggaattctgcttcaagactgtaacacacaaaccctgcctgagtttctggcctg    3895
GGT1.end-GGT5.beginining.Philippine.tarsier.ref    ------------------------------------------------------------    2873
GGT5.end-GGT1.beginning.Rhesus.28419652-28635852   agaagcaggaattctgctttaacactataacatgtaaatcctgcctgagtttctggcctg    6529
spacer.GGT1.end-start.BCRP3..human.ref             ------------------------------------------------------------    3920
GGT1end-LOC749026.end.7456450-7520130.chimp        ------------------------------------------------------------    4362

GGT5.total.&minus3200bp.mouse                      ------------------------------------------------------------    7371
GGT5.nt.mouse                                      ------------------------------------------------------------    4210
FAM247.LOC105372935.ref.human                      ctgactgctctacagattttaggttccagacttcgagatcaactcttacctgaatttata    3955
GGT1.end-GGT5.beginining.Philippine.tarsier.ref    ------------------------------------------------------------    2873
```

```
GGT5.end-GGT1.beginning.Rhesus.28419652-28635852      ctgactgctctccaggttttaggttccagacttcgagatcaactcttacctgaatttata    6589
spacer.GGT1.end-start.BCRP3..human.ref                ------------------------------------------------------------    3920
GGT1end-LOC749026.end.7456450-7520130.chimp           ------------------------------------------------------------    4362


GGT5.total.&minus3200bp.mouse                         ---------------------------tggttaggtaggatcactgtttatgtgaggttt    7404
GGT5.nt.mouse                                         ---------------------------tggttaggtaggatcactgtttatgtgaggttt    4243
FAM247.LOC105372935.ref.human                         gcctgctggcttgccctacagattttaaaacttgctagtccccacaatcatgtgagccaa    4015
GGT1.end-GGT5.beginining.Philippine.tarsier.ref       ------------------------------------------------------------    2873
GGT5.end-GGT1.beginning.Rhesus.28419652-28635852      agctgctggttcgccatacagatttt-aaacttgctagtccccacaaccgtgtgagccaa    6648
spacer.GGT1.end-start.BCRP3..human.ref                ------------------------------------------------------------    3920
GGT1end-LOC749026.end.7456450-7520130.chimp           ------------------------------------------------------------    4362


GGT5.total.&minus3200bp.mouse                         ctgttctgtgaccctctctccctcccggagagagtttttgtgttatcaggacaattctct    7464
GGT5.nt.mouse                                         ctgttctgtgaccctctctccctcccggagagagtttttgtgttatcaggacaattctct    4303
FAM247.LOC105372935.ref.human                         ttcctaaataaatctctctctatgtataatctattggtttagtttctctgaaaaactttc    4075
GGT1.end-GGT5.beginining.Philippine.tarsier.ref       ------------------------------------------------------------    2873
GGT5.end-GGT1.beginning.Rhesus.28419652-28635852      ttcctaaataaatctctctctatgtataacctattggtttagtttctctaaaaaccttttt    6708
spacer.GGT1.end-start.BCRP3..human.ref                ------------------------------------------------------------    3920
GGT1end-LOC749026.end.7456450-7520130.chimp           ------------------------------------------------------------    4362


GGT5.total.&minus3200bp.mouse                         tggcttagtcatccccacggtgattacagttgggagccatcatgcctagcagcatggagc    7524
GGT5.nt.mouse                                         tggcttagtcatccccacggtgattacagttgggagccatcatgcctagcagcatggagc    4363
FAM247.LOC105372935.ref.human                         acatccagtttcctggttgttaagaa----------------------------------    4101
GGT1.end-GGT5.beginining.Philippine.tarsier.ref       ------------------------------------------------------------    2873
GGT5.end-GGT1.beginning.Rhesus.28419652-28635852      acatctagtttcctggatgttaagta----------------------------------    6734
spacer.GGT1.end-start.BCRP3..human.ref                ------------------------------------------------------------    3920
GGT1end-LOC749026.end.7456450-7520130.chimp           ------------------------------------------------------------    4362


GGT5.total.&minus3200bp.mouse                         tattccctaccctcttgctgccatttttattaggttgtccattagatgtgttttctgtc    7584
GGT5.nt.mouse                                         tattccctaccctcttgctgccatttttattaggttgtccattagatgtgttttctgtc    4423
FAM247.LOC105372935.ref.human                         --------------ttaccgaaactagctagtaacttctttttttttttttttttttgag    4147
GGT1.end-GGT5.beginining.Philippine.tarsier.ref       ------------------------------------------------------------    2873
GGT5.end-GGT1.beginning.Rhesus.28419652-28635852      --------------atactgaaactagctagtaacttc--ttttcttttttttttttgag    6778
spacer.GGT1.end-start.BCRP3..human.ref                ------------------------------------------------------------    3920
GGT1end-LOC749026.end.7456450-7520130.chimp           ------------------------------------------------------------    4362


GGT5.total.&minus3200bp.mouse                         ttgttatttctctccctttaatatgcagcaagtcttaagcctcctttgtattcctagcat    7644
GGT5.nt.mouse                                         ttgttatttctctccctttaatatgcagcaagtcttaagcctcctttgtattcctagcat    4483
FAM247.LOC105372935.ref.human                         acagagttttgctcttgttgcccaggctggaatgcaatggcacaatctca-gctcaccgc    4206
GGT1.end-GGT5.beginining.Philippine.tarsier.ref       ------------------------------------------------------------    2873
GGT5.end-GGT1.beginning.Rhesus.28419652-28635852      atggaattttgctcttgttgcccaggctggagtgcagtgccgcgatcttg-gctcaccgc    6837
spacer.GGT1.end-start.BCRP3..human.ref                ------------------------------------------------------------    3920
GGT1end-LOC749026.end.7456450-7520130.chimp           ------------------------------------------------------------    4362


GGT5.total.&minus3200bp.mouse                         gaactccgcttaatagggattcaatatgactttttatatcatagatgatcacaattggac    7704
GGT5.nt.mouse                                         gaactccgcttaatagggattcaatatgactttttatatcatagatgatcacaattggac    4543
FAM247.LOC105372935.ref.human                         aacctccacttcct--gggtccaagcaattctcctccctcagcctcctgagtagctggga    4264
GGT1.end-GGT5.beginining.Philippine.tarsier.ref       ------------------------------------------------------------    2873
GGT5.end-GGT1.beginning.Rhesus.28419652-28635852      aacctccacttcct--gggtccaagcgattctcctccctcagcctcccgagtagctggga    6895
spacer.GGT1.end-start.BCRP3..human.ref                ------------------------------------------------------------    3920
GGT1end-LOC749026.end.7456450-7520130.chimp           ------------------------------------------------------------    4362


GGT5.total.&minus3200bp.mouse                         ttactaatttttgtcttgatgatcagcaatctctgtaattttcctttgttatgatgcctc    7764
GGT5.nt.mouse                                         ttactaatttttgtcttgatgatcagcaatctctgtaattttcctttgttatgatgcctc    4603
FAM247.LOC105372935.ref.human                         ttacaggcatgtgccaccatgcttggctaatttttgtatttttagtagagacagggcttc    4324
GGT1.end-GGT5.beginining.Philippine.tarsier.ref       ------------------------------------------------------------    2873
GGT5.end-GGT1.beginning.Rhesus.28419652-28635852      ttacaggcatgtgccaccatgctcggctaatttttgtatttttagtagagatggggcttc    6955
spacer.GGT1.end-start.BCRP3..human.ref                ------------------------------------------------------------    3920
GGT1end-LOC749026.end.7456450-7520130.chimp           ------------------------------------------------------------    4362
```
