# Supplementary material for: An ancestral genomic sequence that serves as a nucleation site for de novo gene birth
Source: PLoS One. 2022 May 12;17(5):e0267864. doi: 10.1371/journal.pone.0267864 (PMC9097989; doi:10.1371/journal.pone.0267864)
Supplement: S3 Fig — (PDF) [file pone.0267864.s003.pdf]

Detection of an ancestral genomic sequence that serves as a nucleation site for de novo gene birth

Nicholas Delihas
Department of Microbiology and Immunology, Renaissance School of Medicine, Stony Brook University, Stony Brook, N.Y., United States of America

S3 Fig. Amino Acid sequence alignment of *GGT5* from various species.

```
#
#
#  Percent Identity  Matrix - created by Clustal2.1
#
#

     1: GGT5B.aa.zebafish                                       100.00  100.00   46.08   43.98   47.90   47.52
     2: GGT5b.aa./protein_id="XP_002667883.3.zebrafish          100.00  100.00   46.08   43.98   47.90   47.52
     3: GGT5.aa.oposssum                                         46.08   46.08  100.00   57.48   59.06   60.75
     4: GGT5.aa./protein_id="XP_006513710.1.isoform1.mouse       43.98   43.98   57.48  100.00   78.79   79.86
     5: GGT5.aa.protein_id="XP_008067295.1.isoform1.tarsier      47.90   47.90   59.06   78.79  100.00   84.84
     6: GGT5.aa.protein_id="XP_011528435.1.isoform.X1.human      47.52   47.52   60.75   79.86   84.84  100.00
```

```
CLUSTAL O(1.2.4) multiple sequence alignment


GGT5B.aa.zebgafish                                      MAKSQSRRCCFCLLALVCT--AAIICICILFSK-----QKCDFTRAAVSADSLMCSDIGR 53
GGT5b.aa./protein_id="XP_002667883.3.zebrafish          MAKSQSRRCCFCLLALVCT--AAIICICILFSK-----QKCDFTRAAVSADSLMCSDIGR 53
GGT5.aa.oposssum                                        MARPGGRAVCLILLAAG--LLAAIIAAACTLGRAAATCPAASYRTAAVAADTPRCSAIGR 58
GGT5.aa./protein_id="XP_006513710.1.isoform1.mouse      MAWGHRATVCLVLLGVGLGL--VIVVLAAVLSPRQASCGPGAFTRAAVAADSKICSDIGR 58
GGT5.aa.protein_id="XP_008067295.1.isoform1.tarsier     MAWGCRAIISLVLLGLGLGLALVIIVLAVVLPRHQAPCGPQAFAHAAIAADSKVCSDIGR 60
GGT5.aa.protein_id="XP_011528435.1.isoform.X1.human     MARGYGATVSLVLLGLG--LALAVIVLAVVLSRHQAPCGPQAFAHAAVAADSKVCSDIGR 58
                                                        **       .: **.       .::  .  :           :  **::**:  ** ***

GGT5B.aa.zebgafish                                      DILRQGGSAVDAAIAALLCTGLVNPQSMGLGGGSIFTIMD-NTGKVKIISSRETVPKGVR 112
GGT5b.aa./protein_id="XP_002667883.3.zebrafish          DILRQGGSAVDAAIAALLCTGLVNPQSMGLGGGSIFTIMD-NTGKVKIISSRETVPKGVR 112
GGT5.aa.oposssum                                        DTLRRGGSPVDAAIAALLCTTVLNPQSMGLGGGVIXXXXX-------------------- 98
GGT5.aa./protein_id="XP_006513710.1.isoform1.mouse      ------------------------------------------------------------ 58
GGT5.aa.protein_id="XP_008067295.1.isoform1.tarsier     AILQQRGSPVDATIAALVCTSVINPQSMGLGGGVIFTIYNAKTGKVEVINARETVPVSHD 120
GGT5.aa.protein_id="XP_011528435.1.isoform.X1.human     AILQQQGSPVDATIAALVCTSVVNPQSMGLGGGVIFTIYNVTTGKVEVINARETVPASHA 118


GGT5B.aa.zebgafish                                      RDLLKDCPRVLAIVTGSQWIGVPGEIRGYEEAHRLYGKLPWYKLFEPSIKLAREGFPMPE 172
GGT5b.aa./protein_id="XP_002667883.3.zebrafish          RDLLKDCPRVLAIVTGSQWIGVPGEIRGYEEAHRLYGKLPWYKLFEPSIKLAREGFPMPE 172
GGT5.aa.oposssum                                        ------------XTPGAYWIGVPGELRGYQLAHQRHGRLPWAQLFQPTIRLLREGLRVPR 146
GGT5.aa./protein_id="XP_006513710.1.isoform1.mouse      ------------------------------------------------------------ 58
GGT5.aa.protein_id="XP_008067295.1.isoform1.tarsier     PALLDQCEKALPLGTGAQWIGVPGELRGYAKAHHRHGRLPWAQLFQPTIALLRRGHRVPP 180
GGT5.aa.protein_id="XP_011528435.1.isoform.X1.human     PSLLDQCAQALPLGTGAQWIGVPGELRGYAEAHRRHGRLPWAQLFQPTIALLRGGHVVAP 178


GGT5B.aa.zebgafish                                      YLSQFIQHAYIKQILFSGTGLCELFCKNNNTEVLGPGDTLKFPQLAKTLETIAKEGADAF 232
GGT5b.aa./protein_id="XP_002667883.3.zebrafish          YLSQFIQHAYIKQILFSGTGLCELFCKNNNTEVLGPGDTLKFPQLAKTLETIAKEGADAF 232
GGT5.aa.oposssum                                        VLSLFL-NSFLSPAIRG-SSLGRLFF--NGSRPLAEGEPLPWPELARTLETVAEKGADEL 202
GGT5.aa./protein_id="XP_006513710.1.isoform1.mouse      ----------------------QLFF--NGTETLRSQDPFPWPALANTLETVAKEGAEVL 94
GGT5.aa.protein_id="XP_008067295.1.isoform1.tarsier     VLGQFLNNSFLRPVLHR-STLRKLFF--NGTEPLRPQDLLQWPELATTLETVATEGAESF 237
GGT5.aa.protein_id="XP_011528435.1.isoform.X1.human     VLSRFLHNSILRPSLQA-STLRQLFF--NGTEPLRPQDPLPWPALATTLETVATEGVEVF 235
                                                                              .**   *.:. *   : : :* ** ****:* :*.: :

GGT5B.aa.zebgafish                                      YSGKIAKDLIQDIQARNGALSLDDLKNFKVRVSDPWTVDLGEYKMHFPPPPAGGAILSFI 292
GGT5b.aa./protein_id="XP_002667883.3.zebrafish          YSGKIAKDLIQDIQARNGALSLDDLKNFKVRVSDPWTVDLGEYKMHFPPPPAGGAILSFI 292
GGT5.aa.oposssum                                        YTGALAESLLADLGGEGGTLTREDLAGFQAEVAEPLTVSLGNYTLYTPPPPAGGALLGFV 262
GGT5.aa./protein_id="XP_006513710.1.isoform1.mouse      YTGRLGRMLVEDIAKQGSLLTVQDLAAFQPEVVEPLEMPLGNYTLYSPPPPAGGAILSFI 154
GGT5.aa.protein_id="XP_008067295.1.isoform1.tarsier     YTGRLGQMLVEDIAKEGSQLTLQDLEAFQPEVVDALEVALGDYTLYSPPPPAGGAILSFI 297
GGT5.aa.protein_id="XP_011528435.1.isoform.X1.human     YTGRLGQMLVEDIAKEGSQLTLQDLAKFQPEVVDALEVPLGDYTLYSPPPPAGGAILSFI 295
                                                        *:* :.. *: *:  ... *: :**  *: .* :   : **:*.:: ********:*.*:

GGT5B.aa.zebgafish                                      LKLMHEFGLSPASIQGD-QKNLTLHRYIEAVKFANGQRRFLKDPEFNSGDM---KSVTDE 348
GGT5b.aa./protein_id="XP_002667883.3.zebrafish          LKLMHEFGLSPASIQGD-QKNLTLHRYIEAVKFANGQRRFLKDPEFNSGDM---KSVTDE 348
GGT5.aa.oposssum                                        LNVLRGFRFS------PDGKVDTYHRVVETLKFASGLKWALRDPRSHPEPLGGRLDLLSE 316
GGT5.aa./protein_id="XP_006513710.1.isoform1.mouse      LNVLKGFNFSAETVARPGGEVNMYHHLVETLKFAVGQRWRLWDPSSHPGIQNISRDLLRE 214
GGT5.aa.protein_id="XP_008067295.1.isoform1.tarsier     LKVLRGFNFSAESVTRPEERVNVYHHLVETLKFAEGQRWRLWDPHSHLEVQNASQDLLGE 357
GGT5.aa.protein_id="XP_011528435.1.isoform.X1.human     LNVLRGFNFSTESMARPEGRVNVYHHLVETLKFAKGQRWRLGDPRSHPKLQNASRDLLGE 355
                                                        *:::: * :*          .     *: :*::*** * :  * **  :        .:  *

GGT5B.aa.zebgafish                                      KFIKQIRALITDSGTHE-DSYYNIT---PAVDRFGTTHVSVLAADGSAVSVTSTINHMLG 404
GGT5b.aa./protein_id="XP_002667883.3.zebrafish          KFIKQIRALITDSGTHE-DSYYNIT---PAVDRFGTTHVSVLAADGSAVSVTSTINHMLG 404
GGT5.aa.oposssum                                        ELAQRVRALIGPRGDRP-AAHYNVSLPGAGGQETGTSHVAVLGPDGSAVSATSTINTPFG 375
GGT5.aa./protein_id="XP_006513710.1.isoform1.mouse      DLAQRIRQQIDGRGDHHQLSHY--NLTGVRGNRMGTSHVSVLGEDGSAVAATSTINTPFG 272
GGT5.aa.protein_id="XP_008067295.1.isoform1.tarsier     ALAQHIRQQIDGRGDH-QLSHY--HLTRAWDHRTGTAHVSVLGEDGSAVAATSTINTPFG 414
GGT5.aa.protein_id="XP_011528435.1.isoform.X1.human     TLAQLIRQQIDGRGDH-QLSHY--SLAEAWGHGTGTSHVSVLGEDGSAVAATSTINTPFG 412
                                                         : : :*  *   * :   ::*         .  **:**:**. *****:.*****  :*
```

```
GGT5B.aa.zebgafish                              SAVYSPKTGIILNNELVDFCNRAD-----------------------------SVTPGEQ 435
GGT5b.aa./protein_id="XP_002667883.3.zebrafish  SAVYSPKTGIILNNELVDFCNRAD-----------------------------SVTPGEQ 435
GGT5.aa.oposssum                                SMVYSPRTGLILNNQLLDLCWRTRPDSGELL----------------------DPAPGER 413
GGT5.aa./protein_id="XP_006513710.1.isoform1.mouse  AMVYSPRTGILLNNELLDLCWRHMPTSPITP----------------------PPVPGER 310
GGT5.aa.protein_id="XP_008067295.1.isoform1.tarsier  SMVYSPRTGILLNNELLDLCWRRPPGSGITPSPE-----VSGDRVGGAARGCWPPVPGER 469
GGT5.aa.protein_id="XP_011528435.1.isoform.X1.human  AMVYSPRTGIILNNELLDLCERCPRGSGTTPSPDAVCSAVSGDRVGGAPGRCWPPVPGER 472
                                                : ****:**::***:*:*:* *                                .***:

GGT5B.aa.zebgafish                              PPSSMAPAILQS--QQKTLVIGGSGGSMITSAMALSIMNHLWFGMNLNDSIAAKIVFVGS 493
GGT5b.aa./protein_id="XP_002667883.3.zebrafish  PPSSMAPAILQS--QQKTLVIGGSGGSMITSAMALSIMNHLWFGMNLNDSIAAKIVFVGS 493
GGT5.aa.oposssum                                PPSLMVPSILLSQDRKAKLVIGGSGGEMILPATALAIMNNLWFGLDLHEAIKAKILHVRP 473
GGT5.aa./protein_id="XP_006513710.1.isoform1.mouse  PPSSMVPSILVNKGQGSKLVIGGAGGELIISAVAQTIMNKLWLGFDLTEAIASPILHVNS 370
GGT5.aa.protein_id="XP_008067295.1.isoform1.tarsier  PPSSMAPSILINKAQGSKLVIGGAGGELIISAMAQVIINKLWLGFNLTAAIEAPILHVNR 529
GGT5.aa.protein_id="XP_011528435.1.isoform.X1.human  SPSSMVPSILINKAQGSKLVIGGAGGELIISAVAQAIMSKLWLGFDLRAAIAAPILHVNS 532
                                                 ** *.*:** .  :  .*****:**.:*  * *  *:.:**:*::*  :* : *:.*

GGT5B.aa.zebgafish                              NNMVNFEKGYDS-------------------------------SAIKAMEKLGHN--VMY 520
GGT5b.aa./protein_id="XP_002667883.3.zebrafish  NNMVNFEKGYDS-------------------------------SAIKAMEKLGHN--VMY 520
GGT5.aa.oposssum                                TMEVLFEPDFAQ-------------------------------EVQDGLVSRGHQ--RQE 500
GGT5.aa./protein_id="XP_006513710.1.isoform1.mouse  KGHVEYEPKFNQ-------------------------------EVQKGLQDRGQIQSQSQ 399
GGT5.aa.protein_id="XP_008067295.1.isoform1.tarsier  KGHVEYEPHFNQ-------------------------------EVQKGLEDRGQN--QSR 556
GGT5.aa.protein_id="XP_011528435.1.isoform.X1.human  KGCVEYEPNFSQKQDLAALPRLVSNSWPQVILLPQPPKLLALQEVQRGLQDRGQN--QTQ 590
                                                .  * :*  : .                                ..  .: . *:

GGT5B.aa.zebgafish                              NPYFFNVVNGISKE-GQCISAISDARKQGQSAGY 553
GGT5b.aa./protein_id="XP_002667883.3.zebrafish  NPYFFNVVNGISKE-GQCISAISDARKQGQSAGY 553
GGT5.aa.oposssum                                TKFWLNVVQAVAQDARGCIYAESDERKLGEAAGY 534
GGT5.aa./protein_id="XP_006513710.1.isoform1.mouse  RPVFLNAVQAVFQE-GPCVYAASDLRKAGKASGY 432
GGT5.aa.protein_id="XP_008067295.1.isoform1.tarsier  RPFFLNVVQAVSQE-GACVYAASDPRKGGEAAGY 589
GGT5.aa.protein_id="XP_011528435.1.isoform.X1.human  RPFFLNVVQAVSQE-GACVYAVSDLRKSGEAAGY 623
                                                   ::*.*:.: ::   *: * ** ** *:::**
```

```
>GGT5.aa.oposssum
MARPGGRAVCLILLAAGLLAAIIAAACTLGRAAATCPAASYRTA
                    AVAADTPRCSAIGRDTLRRGGSPVDAAIAALLCTTVLNPQSMGLGGGVIXXXXXXTPG
                    AYWIGVPGELRGYQLAHQRHGRLPWAQLFQPTIRLLREGLRVPRVLSLFLNSFLSPAI
                    RGSSLGRLFFNGSRPLAEGEPLPWPELARTLETVAEKGADELYTGALAESLLADLGGE
                    GGTLTREDLAGFQAEVAEPLTVSLGNYTLYTPPPPAGGALLGFVLNVLRGFRFSPDGK
                    VDTYHRVVETLKFASGLKWALRDPRSHPEPLGGRLDLLSEELAQRVRALIGPRGDRPA
                    AHYNVSLPGAGGQETGTSHVAVLGPDGSAVSATSTINTPFGSMVYSPRTGLILNNQLL
                    DLCWRTRPDSGELLDPAPGERPPSLMVPSILLSQDRKAKLVIGGSGGEMILPATALAI
                    MNNLWFGLDLHEAIKAKILHVRPTMEVLFEPDFAQEVQDGLVSRGHQRQETKFWLNVV
                    QAVAQDARGCIYAESDERKLGEAAGY

>GGT5B.aa.zebgafish
MAKSQSRRCCFCLLALVCTAAIICICILFSKQKCDFTRAAVSAD
                    SLMCSDIGRDILRQGGSAVDAAIAALLCTGLVNPQSMGLGGGSIFTIMDNTGKVKIIS
                    SRETVPKGVRRDLLKDCPRVLAIVTGSQWIGVPGEIRGYEEAHRLYGKLPWYKLFEPS
                    IKLAREGFPMPEYLSQFIQHAYIKQILFSGTGLCELFCKNNNTEVLGPGDTLKFPQLA
                    KTLETIAKEGADAFYSGKIAKDLIQDIQARNGALSLDDLKNFKVRVSDPWTVDLGEYK
                    MHFPPPPAGGAILSFILKLMHEFGLSPASIQGDQKNLTLHRYIEAVKFANGQRRFLKD
                    PEFNSGDMKSVTDEKFIKQIRALITDSGTHEDSYYNITPAVDRFGTTHVSVLAADGSA
                    VSVTSTINHMLGSAVYSPKTGIILNNELVDFCNRADSVTPGEQPPSSMAPAILQSQQK
                    TLVIGGSGGSMITSAMALSIMNHLWFGMNLNDSIAAKIVFVGSNNMVNFEKGYDSSAI
                    KAMEKLGHNVMYNPYFFNVVNGISKEGQCISAISDARKQGQSAGY

>GGT5b.aa./protein_id="XP_002667883.3.zebrafish
MAKSQSRRCCFCLLALVCTAAIICICILFSKQKCDFTRAAVSAD
                    SLMCSDIGRDILRQGGSAVDAAIAALLCTGLVNPQSMGLGGGSIFTIMDNTGKVKIIS
                    SRETVPKGVRRDLLKDCPRVLAIVTGSQWIGVPGEIRGYEEAHRLYGKLPWYKLFEPS
                    IKLAREGFPMPEYLSQFIQHAYIKQILFSGTGLCELFCKNNNTEVLGPGDTLKFPQLA
                    KTLETIAKEGADAFYSGKIAKDLIQDIQARNGALSLDDLKNFKVRVSDPWTVDLGEYK
                    MHFPPPPAGGAILSFILKLMHEFGLSPASIQGDQKNLTLHRYIEAVKFANGQRRFLKD
                    PEFNSGDMKSVTDEKFIKQIRALITDSGTHEDSYYNITPAVDRFGTTHVSVLAADGSA
                    VSVTSTINHMLGSAVYSPKTGIILNNELVDFCNRADSVTPGEQPPSSMAPAILQSQQK
                    TLVIGGSGGSMITSAMALSIMNHLWFGMNLNDSIAAKIVFVGSNNMVNFEKGYDSSAI
                    KAMEKLGHNVMYNPYFFNVVNGISKEGQCISAISDARKQGQSAGY

>GGT5.aa./protein_id="XP_006513710.1.isoform1.mouse
MAWGHRATVCLVLLGVGLGLVIVVLAAVLSPRQASCGPGAFTRA
                    AVAADSKICSDIGRQLFFNGTETLRSQDPFPWPALANTLETVAKEGAEVLYTGRLGRM
                    LVEDIAKQGSLLTVQDLAAFQPEVVEPLEMPLGNYTLYSPPPPAGGAILSFILNVLKG
                    FNFSAETVARPGGEVNMYHHLVETLKFAVGQRWRLWDPSSHPGIQNISRDLLREDLAQ
                    RIRQQIDGRGDHHQLSHYNLTGVRGNRMGTSHVSVLGEDGSAVAATSTINTPFGAMVY
                    SPRTGILLNNELLDLCWRHMPTSPITPPPVPGERPPSSMVPSILVNKGQGSKLVIGGA
                    GGELIISAVAQTIMNKLWLGFDLTEAIASPILHVNSKGHVEYEPKFNQEVQKGLQDRG
                    QIQSQSQRPVFLNAVQAVFQEGPCVYAASDLRKAGKASGY

>GGT5.aa.protein_id="XP_008067295.1.isoform1.tarsier
"MAWGCRAIISLVLLGLGLGLALVIIVLAVVLPRHQAPCGPQAFA
                    HAAIAADSKVCSDIGRAILQQRGSPVDATIAALVCTSVINPQSMGLGGGVIFTIYNAK
                    TGKVEVINARETVPVSHDPALLDQCEKALPLGTGAQWIGVPGELRGYAKAHHRHGRLP
                    WAQLFQPTIALLRRGHRVPPVLGQFLNNSFLRPVLHRSTLRKLFFNGTEPLRPQDLLQ
```

WPELATTLETVATEGAESFYTGRLGQMLVEDIAKEGSQLTLQDLEAFQPEVVDALEVA
LGDYTLYSPPPPAGGAILSFILKVLRGFNFSAESVTRPEERVNVYHHLVETLKFAEGQ
RWRLWDPHSHLEVQNASQDLLGEALAQHIRQQIDGRGDHQLSHYHLTRAWDHRTGTAH
VSVLGEDGSAVAATSTINTPFGSMVYSPRTGILLNNELLDLCWRRPPGSGITPSPEVS
GDRVGGAARGCWPPVPGERPPSSMAPSILINKAQGSKLVIGGAGGELIISAMAQVIIN
KLWLGFNLTAAIEAPILHVNRKGHVEYEPHFNQEVQKGLEDRGQNQSRRPFFLNVVQA
VSQEGACVYAASDPRKGGEAAGY"

>GGT5.aa.protein_id="XP_011528435.1.isoform.X1.human

MARGYGATVSLVLLGLGLALAVIVLAVVLSRHQAPCGPQAFAHA
AVAADSKVCSDIGRAILQQQGSPVDATIAALVCTSVVNPQSMGLGGGVIFTIYNVTTG
KVEVINARETVPASHAPSLLDQCAQALPLGTGAQWIGVPGELRGYAEAHRRHGRLPWA
QLFQPTIALLRGGHVVAPVLSRFLHNSILRPSLQASTLRQLFFNGTEPLRPQDPLPWP
ALATTLETVATEGVEVFYTGRLGQMLVEDIAKEGSQLTLQDLAKFQPEVVDALEVPLG
DYTLYSPPPPAGGAILSFILNVLRGFNFSTESMARPEGRVNVYHHLVETLKFAKGQRW
RLGDPRSHPKLQNASRDLLGETLAQLIRQQIDGRGDHQLSHYSLAEAWGHGTGTSHVS
VLGEDGSAVAATSTINTPFGAMVYSPRTGIILNNELLDLCERCPRGSGTTPSPDAVCS
AVSGDRVGGAPGRCWPPVPGERSPSSMVPSILINKAQGSKLVIGGAGGELIISAVAQA
IMSKLWLGFDLRAAIAAPILHVNSKGCVEYEPNFSQKQDLAALPRLVSNSWPQVILLP
QPPKLLALQEVQRGLQDRGQNQTQRPFFLNVVQAVSQEGACVYAVSDLRKSGEAAGY
