# Supplementary material for: An ancestral genomic sequence that serves as a nucleation site for de novo gene birth
Source: PLoS One. 2022 May 12;17(5):e0267864. doi: 10.1371/journal.pone.0267864 (PMC9097989; doi:10.1371/journal.pone.0267864)
Supplement: S4 Fig — (PDF) [file pone.0267864.s004.pdf]

Detection of an ancestral genomic sequence that serves as a nucleation site for de novo gene birth

Nicholas Delihas
Department of Microbiology and Immunology, Renaissance School of Medicine, Stony Brook University, Stony Brook, N.Y., United States of America

S4 Fig. The alignment of the BCRP3 sequence present in the Rhesus locus that contains the sequence between *LOC106996293* and *GGT1*. The sequences used are at the end of the file.

```
CLUSTAL O(1.2.4) multiple sequence alignment


LOC106996293.end.GGT1.start.28595839-28635852.Rhesus      ctctgggcctcagtgtattgtgtgtgaaatggaaccatctggctggggaggaatggagag    60
BCRP3.HUMAN.NCBI.REF                                      ------------------------------------------------------------    0

LOC106996293.end.GGT1.start.28595839-28635852.Rhesus      gtgggattcggagatcttcacactgcggtcgctggaactagcctcagtatcttcagcgtg    120
BCRP3.HUMAN.NCBI.REF                                      ------------------------------------------------------------    0

LOC106996293.end.GGT1.start.28595839-28635852.Rhesus      gggagagccaggtgcgtggctagggaccaggggaaggtccatgccaacccctgccccttc    180
BCRP3.HUMAN.NCBI.REF                                      ------------------------------------------------------------    0

LOC106996293.end.GGT1.start.28595839-28635852.Rhesus      ccaccctgatccattggactttggggccaggtgctcccttattggggctgcacagtgaca    240
BCRP3.HUMAN.NCBI.REF                                      ------------------------------------------------------------    0

LOC106996293.end.GGT1.start.28595839-28635852.Rhesus      cctaggactagccaccaggggggtgccgcgcccctggtgctttcttaggcagtgggtggcc    300
BCRP3.HUMAN.NCBI.REF                                      ------------------------------------------------------------    0

LOC106996293.end.GGT1.start.28595839-28635852.Rhesus      agctgatgctgggaacctgggcaccttctcagacccatgggcatccaactcatcctgcta    360
BCRP3.HUMAN.NCBI.REF                                      ------------------------------------------------------------    0

LOC106996293.end.GGT1.start.28595839-28635852.Rhesus      atgacacgggaggtgaagctgagttccaaggaatgggaattgggcatcacgctagaggaa    420
BCRP3.HUMAN.NCBI.REF                                      ------------------------------------------------------------    0

LOC106996293.end.GGT1.start.28595839-28635852.Rhesus      aacatcttagtcagagccaagcccctgggggtttccaagtataagcccagagtgaaacc    480
BCRP3.HUMAN.NCBI.REF                                      ------------------------------------------------------------    0

LOC106996293.end.GGT1.start.28595839-28635852.Rhesus      caagcttgtgaccctctccagagggaggcctggttttcagggaacagcaaatgggaagag    540
BCRP3.HUMAN.NCBI.REF                                      ------------------------------------------------------------    0

LOC106996293.end.GGT1.start.28595839-28635852.Rhesus      gtccccagattccagggatcagggcttggaccagctggggacgcagcccagagggagtgg    600
BCRP3.HUMAN.NCBI.REF                                      ------------------------------------------------------------    0

LOC106996293.end.GGT1.start.28595839-28635852.Rhesus      gtctggaagggaacagctagacacagcagccttcaccactggcagcccctcccggcctcc    660
BCRP3.HUMAN.NCBI.REF                                      ------------------------------------------------------------    0

LOC106996293.end.GGT1.start.28595839-28635852.Rhesus      ctcggggcctgctccctcctccaagcaccgttccaacacctggggcagggttctgggaaa    720
BCRP3.HUMAN.NCBI.REF                                      ------------------------------------------------------------    0

LOC106996293.end.GGT1.start.28595839-28635852.Rhesus      ggctggtggaggtgggctggtgggggggcggtgatcacagcccagcatctgggtatcacca    780
BCRP3.HUMAN.NCBI.REF                                      ------------------------------------------------------------    0

LOC106996293.end.GGT1.start.28595839-28635852.Rhesus      ggggcactggggccagggcccaggtgaagccaggtcggggctctcctttagaagccccga    840
BCRP3.HUMAN.NCBI.REF                                      ------------------------------------------------------------    0

LOC106996293.end.GGT1.start.28595839-28635852.Rhesus      aaacctggtgataccaaagggcccacagacaaacagggttttgtgcctgcggagttgagt    900
BCRP3.HUMAN.NCBI.REF                                      ------------------------------------------------------------    0

LOC106996293.end.GGT1.start.28595839-28635852.Rhesus      accaccgggtctaagccctggagggctgtgtccctggggctccccaggggtgagatggag    960
BCRP3.HUMAN.NCBI.REF                                      ------------------------------------------------------------    0

LOC106996293.end.GGT1.start.28595839-28635852.Rhesus      gtgggctcaactggtgtacccgtcactcctcaatccttattttatgtatttaatttttaa    1020
```

```
BCRP3.HUMAN.NCBI.REF                                ------------------------------------------------------------   0

LOC106996293.end.GGT1.start.28595839-28635852.Rhesus  aaaaattttatttgaacaaatagagatggggtctcactatgttgaccaggctggtctta   1080
BCRP3.HUMAN.NCBI.REF                                ------------------------------------------------------------   0

LOC106996293.end.GGT1.start.28595839-28635852.Rhesus  aactcttgacttcaagcagtcctcctagcttggcctccaaagtgctaggattactttggg   1140
BCRP3.HUMAN.NCBI.REF                                ------------------------------------------------------------   0

LOC106996293.end.GGT1.start.28595839-28635852.Rhesus  gattactttagggatgagtcactgcacgcggcctcaatccttattttggcctgaaaggaa   1200
BCRP3.HUMAN.NCBI.REF                                ------------------------------------------------------------   0

LOC106996293.end.GGT1.start.28595839-28635852.Rhesus  aggctgtggccccgtttgcaggggagaagactgaggctggagggggcaggccttgctctgg   1260
BCRP3.HUMAN.NCBI.REF                                ------------------------------------------------------------   0

LOC106996293.end.GGT1.start.28595839-28635852.Rhesus  gttgcacagcagcaagagaagtgggagctggccatgaggcttcctggacccgaagcactg   1320
BCRP3.HUMAN.NCBI.REF                                ------------------------------------------------------------   0

LOC106996293.end.GGT1.start.28595839-28635852.Rhesus  gtggggttcaccctggttcttcaggtcccatggggctcagcccaggactaccttggtggg   1380
BCRP3.HUMAN.NCBI.REF                                ------------------------------------------------------------   0

LOC106996293.end.GGT1.start.28595839-28635852.Rhesus  ggtgggagacttaaatcctctccttcattctcattgtcccttcccccatcatttcctgag   1440
BCRP3.HUMAN.NCBI.REF                                ------------------------------------------------------------   0

LOC106996293.end.GGT1.start.28595839-28635852.Rhesus  gaagcacattcagggacctccctggctgtgcctcagtccaaaaccagaatgacacgcatt   1500
BCRP3.HUMAN.NCBI.REF                                ------------------------------------------------------------   0

LOC106996293.end.GGT1.start.28595839-28635852.Rhesus  cctttccctgggcctttgctcaggcggtccctgcaccctggcctctgcctgaccagggtg   1560
BCRP3.HUMAN.NCBI.REF                                ------------------------------------------------------------   0

LOC106996293.end.GGT1.start.28595839-28635852.Rhesus  gtggggagaggagggggacgtcccctccgctgctgtctccactgttcctgctgccctgg   1620
BCRP3.HUMAN.NCBI.REF                                ------------------------------------------------------------   0

LOC106996293.end.GGT1.start.28595839-28635852.Rhesus  cctctgggcttccaggactgcagtgggtgggtgggtgggctggcctgagcccaggaatgc   1680
BCRP3.HUMAN.NCBI.REF                                ------------------------------------------------------------   0

LOC106996293.end.GGT1.start.28595839-28635852.Rhesus  acttcggctcctggttgagcaaagtcactgagacttgggagtcgggtcgggttgggagga   1740
BCRP3.HUMAN.NCBI.REF                                ------------------------------------------------------------   0

LOC106996293.end.GGT1.start.28595839-28635852.Rhesus  ggcgtccacaggcccccactacgaaaggcagctgtgggaacagtctgcctgtaaacaacc   1800
BCRP3.HUMAN.NCBI.REF                                ------------------------------------------------------------   0

LOC106996293.end.GGT1.start.28595839-28635852.Rhesus  actccagcccaggctgaccaggggctctggctgggacattgggatctggcaggctgtgtg   1860
BCRP3.HUMAN.NCBI.REF                                ------------------------------------------------------------   0

LOC106996293.end.GGT1.start.28595839-28635852.Rhesus  gcctgtaaggacacagtctgtctctgtgcctcagtttctctgctgcccagttgggcgtcc   1920
BCRP3.HUMAN.NCBI.REF                                ------------------------------------------------------------   0

LOC106996293.end.GGT1.start.28595839-28635852.Rhesus  cagactccaggtgtagacatctggagcaggcagtgctcagctgggaaggaagtggggagg   1980
BCRP3.HUMAN.NCBI.REF                                ------------------------------------------------------------   0

LOC106996293.end.GGT1.start.28595839-28635852.Rhesus  actggaggagccatgtgtgaaggattccaacccacatcacctgcacccctgctgagcctg   2040
BCRP3.HUMAN.NCBI.REF                                ------------------------------------------------------------   0

LOC106996293.end.GGT1.start.28595839-28635852.Rhesus  gtcaacagagcccctcagtgggtcctcactcccctggctgcctcccggttaggcaccctg   2100
BCRP3.HUMAN.NCBI.REF                                ------------------------------------------------------------   0

LOC106996293.end.GGT1.start.28595839-28635852.Rhesus  agggctggggagaacagggccaggccagtgtccccagagaggctgcgctgccagcacagt   2160
BCRP3.HUMAN.NCBI.REF                                ------------------------------------------------------------   0

LOC106996293.end.GGT1.start.28595839-28635852.Rhesus  aatagcggatttggattcagggaagcagacccgcagccagggtggggaagagctgcaggc   2220
BCRP3.HUMAN.NCBI.REF                                ------------------------------------------------------------   0

LOC106996293.end.GGT1.start.28595839-28635852.Rhesus  tgggcgtggcacctaggcggcacagcctccctccctggaggcccacgctgcatttccagg   2280
BCRP3.HUMAN.NCBI.REF                                ------------------------------------------------------------   0

LOC106996293.end.GGT1.start.28595839-28635852.Rhesus  acagcaagtcccagggatggatggtcccaggtgccaagggctagaggcatggtctgtctg   2340
BCRP3.HUMAN.NCBI.REF                                ------------------------------------------------------------   0

LOC106996293.end.GGT1.start.28595839-28635852.Rhesus  cattccccacatggacgtcttgtagtcaccagcgtttgatgctgtcaagtccccctgtcc   2400
BCRP3.HUMAN.NCBI.REF                                ------------------------------------------------------------   0
```

```
LOC106996293.end.GGT1.start.28595839-28635852.Rhesus      tctctgcggactgagaagcccttggtcatccttagggggttgtggaacccaaaccaggct      2460
BCRP3.HUMAN.NCBI.REF                                      ------------------------------------------------------------      0

LOC106996293.end.GGT1.start.28595839-28635852.Rhesus      gcagaagcatagggacttgaacccaagttttaagtgacaccaccttttgtccccctccct      2520
BCRP3.HUMAN.NCBI.REF                                      ------------------------------------------------------------      0

LOC106996293.end.GGT1.start.28595839-28635852.Rhesus      cggtctctgttcagttccacttcgatattgcctgtgctgggccatgcagagagggttagg      2580
BCRP3.HUMAN.NCBI.REF                                      ------------------------------------------------------------      0

LOC106996293.end.GGT1.start.28595839-28635852.Rhesus      ggatagagatgggaactggggagtggggctccactctcagagaggggcagccttgctgga      2640
BCRP3.HUMAN.NCBI.REF                                      ------------------------------------------------------------      0

LOC106996293.end.GGT1.start.28595839-28635852.Rhesus      tccaggggagatagttgagcagccccagctctgctttcccggagctgctgggaaccccag      2700
BCRP3.HUMAN.NCBI.REF                                      ------------------------------------------------------------      0

LOC106996293.end.GGT1.start.28595839-28635852.Rhesus      gaatggtgtggagattcctgggagctctgcccccacttgacaaccacagtgcagcaggca      2760
BCRP3.HUMAN.NCBI.REF                                      ------------------------------------------------------------      0

LOC106996293.end.GGT1.start.28595839-28635852.Rhesus      ccaagttctcctgcacattgggacagtgtgaccctgggctctggttagtggcaggtgggg      2820
BCRP3.HUMAN.NCBI.REF                                      ------------------------------------------------------------      0

LOC106996293.end.GGT1.start.28595839-28635852.Rhesus      ccttgggtcctaccagcagtgagggagttagcacagcagctggctcctctagggaaggaa      2880
BCRP3.HUMAN.NCBI.REF                                      ------------------------------------------------------------      0

LOC106996293.end.GGT1.start.28595839-28635852.Rhesus      aactcccttcagacactttggtgcctggcctcctgccaggaacaagcaggagctgaaaac      2940
BCRP3.HUMAN.NCBI.REF                                      ------------------------------------------------------------      0

LOC106996293.end.GGT1.start.28595839-28635852.Rhesus      tagaagttgaggcataagtttggccactctgtagtgtgtacctggggagggcagcagctc      3000
BCRP3.HUMAN.NCBI.REF                                      -------------------------actccgtagtgtgcacttggtgagggcagcagctc      35
                                                                                   **** ******** ** *** **************

LOC106996293.end.GGT1.start.28595839-28635852.Rhesus      gccacagctgccagctg-----ccagccgtctacccattcacctggcagcccgcttttca      3055
BCRP3.HUMAN.NCBI.REF                                      gccacagctgccagccgtctgtccattcacccatctgtccatctggcagcccgctgttca      95
                                                          *************** *      ***  *  * * *  * ** ************* ****

LOC106996293.end.GGT1.start.28595839-28635852.Rhesus      gac----ctgcctgtccacccatctataagcccatctctgtcccgttgtctatctgacca      3111
BCRP3.HUMAN.NCBI.REF                                      gacccgtctgtctgtccgcccatctgtaagcccatctctgtcccattgtctatctgacca      155
                                                          ***    *** ****** ******* ****************** ***************

LOC106996293.end.GGT1.start.28595839-28635852.Rhesus      tctttctcttactgtcctctctgtccagcaatctggcctgtctgtcgatccatcttcttg      3171
BCRP3.HUMAN.NCBI.REF                                      tctttctcttactgtcctctttgtctagctatctggcctatctgtcgatccatcttcgtg      215
                                                          ******************* **** *** ********* ***************** **

LOC106996293.end.GGT1.start.28595839-28635852.Rhesus      tctaac-tgtggccccacctatttgtccatctgtccaattacctttgattctatctgtgc      3230
BCRP3.HUMAN.NCBI.REF                                      tctgtcttcagccccccacctgtttgtccatctgtccaattacctgtgagtctatctatgc      275
                                                          ***   * *   * ******** ********************** *** ******* ***

LOC106996293.end.GGT1.start.28595839-28635852.Rhesus      atcttcttgtccatccatctgcccacccatctgtccctgtgtctgctcactggcctcccc      3290
BCRP3.HUMAN.NCBI.REF                                      accttcttgtccattcatctgcccacccatctgtccctccgtctgcccaccggcctcccc      335
                                                          * ************ ***********************  ****** *** *********

LOC106996293.end.GGT1.start.28595839-28635852.Rhesus      tctcctcctgggccacagagccatggcccagggctgtgggtccttggtcagcctggtgct      3350
BCRP3.HUMAN.NCBI.REF                                      tctccttctgggccgcagagccatggcccaggactgcagagccatggttggcctggtcct      395
                                                          ****** ******* ***************** ***   *  ** ****  ******* **

LOC106996293.end.GGT1.start.28595839-28635852.Rhesus      gctggggctggggctggggctggctgtcactgtgctggctgtggtcctctctcgccacca      3410
BCRP3.HUMAN.NCBI.REF                                      gctggggctggggcttgtgctggctgtcattgtgctggctgtggtcctctctcgacacca      455
                                                          *************** * *********** ************************ *****

LOC106996293.end.GGT1.start.28595839-28635852.Rhesus      gactccctgtggcccccaggcctttgcccacgctactgttgctgctgactccaaggtctg      3470
BCRP3.HUMAN.NCBI.REF                                      ggccccatttgaccccc-ggcctttgcccacgccgctgttgctgctgactccaaggtctg      514
                                                          * * ** * ** ***** ***************  *************************

LOC106996293.end.GGT1.start.28595839-28635852.Rhesus      ctcaaatattggactgtgagtgagacgtgggaggaagctgggtggcctttggcagccagc      3530
BCRP3.HUMAN.NCBI.REF                                      ctcggatattggacggtgagtgagacgtgggaggaagctgggtggccctggcagccagc      574
                                                          ***  ********* ******************************** ************

LOC106996293.end.GGT1.start.28595839-28635852.Rhesus      ccctcctggagaaggcgtgtgtgtgtttgagtgtgtgagtgtgtgggcgtgcgtgtgtgatt      3590
BCRP3.HUMAN.NCBI.REF                                      ccctcctggagaaggcgtgtgtgtgagagtgtgtgtgtgtgagcatgtgtgtgtgtgaga      634
                                                          ***********************  ********* ***** *   *** ********

LOC106996293.end.GGT1.start.28595839-28635852.Rhesus      gcgtgtgtgagtgtgtatg------tatgtgtgtgagtggggggtgtgggggtgtgtgaa      3644
BCRP3.HUMAN.NCBI.REF                                      gagtatgtgtcagtgtgtgtgggtatatgagtgtgagtgtggggtgtgggtgtgtgtgaa      694
                                                          * ** ****   **** **      **** ********* ********** *********

LOC106996293.end.GGT1.start.28595839-28635852.Rhesus      tgtgtgtgattgtgtttgggggtgtat--------gtgtgggtgtgtgtgattgtgtttg      3696
BCRP3.HUMAN.NCBI.REF                                      tgtgtgtgatcgtgtttgggtgtgtgtatgtgtgagtgtgggtgtgtgtgaatgtgtgtg      754
                                                          ********** ********* **** *        **************** ***** **

LOC106996293.end.GGT1.start.28595839-28635852.Rhesus      ggtatgtgtgggtgtatgtgtgagagtgagtg---------------caggggtgtgcg      3741
BCRP3.HUMAN.NCBI.REF                                      agtgtgtttgtgtgtatgtgtgagtgtgggtgggggtatatgagtgtgagtgtgtgggtg      814
                                                           ** *** ** ************* *** ***                ** * *** * *
```

```
LOC106996293.end.GGT1.start.28595839-28635852.Rhesus      tgtgtgaatgtgtgtgattgtgtttgggtgtgtgtgtgggagggtgagtgtgactgtgtg     3801
BCRP3.HUMAN.NCBI.REF                                      ggtgtgaacgtgtgtgattgtgttttgctgtgtgagggtgtgtgtgactatgagtgtgt-     873
                                                           ******* **************** * ****** * * * * **** * *** *****

LOC106996293.end.GGT1.start.28595839-28635852.Rhesus      ggaggggtgtgggtgtgtgtgaatgtgtgtgattgtgggtatgtgtatgt----------     3851
BCRP3.HUMAN.NCBI.REF                                      ----gagtgtgggtgtgtgtaaatgtgtgtgattgtgtgagtgtatgtgtgggtgtgagt     929
                                                              * ************** **************** *  *** * ***

LOC106996293.end.GGT1.start.28595839-28635852.Rhesus      --------------------------gtgggtgtgtaagtgcgtgtgtgtgtgggtgtat     3885
BCRP3.HUMAN.NCBI.REF                                      gtgtgagtgtgagtatgggggtgtgggtgtgtgtgaatgtgcgtgattgtgtgtgggtat     989
                                                                                    *** ***** * *******  ****** * ****

LOC106996293.end.GGT1.start.28595839-28635852.Rhesus      gtatgtgtgcgtgtgtgagtgtgtgtgtgcgtgtgtgtacaagtgcactggcccaggaag     3945
BCRP3.HUMAN.NCBI.REF                                      gtgtgtgtgtgtgtgagtgtgtgtgtgcgtgtgtgtgcacgtgcactggcccaggcag     1049
                                                          ** ****** ***** * ******************** ** *************** **

LOC106996293.end.GGT1.start.28595839-28635852.Rhesus      caggagccgtgtgtgtgtgtgggcttcagcacctgcagggcttgggcacaaggaggcagc     4005
BCRP3.HUMAN.NCBI.REF                                      caggagcc----atgtgtgtgggcttcagcacctgcagggcttgagcgcaaggagacagc     1105
                                                          ********     ******************************* ** ******* ****

LOC106996293.end.GGT1.start.28595839-28635852.Rhesus      ctcagggcccttgcacagaacaggtggcagggtgtgcccatggggcagatggtgatttag     4065
BCRP3.HUMAN.NCBI.REF                                      ctcagggcccttgcacagaacaggcggcagggtgtgcccgtggggcagatggggacttgg     1165
                                                          ************************ ************** ************ ** ** *

LOC106996293.end.GGT1.start.28595839-28635852.Rhesus      ggaca---gtcatgtgtgagtccacacctggctccaggattcaggagacccatttgcaca     4122
BCRP3.HUMAN.NCBI.REF                                      ggacaatggtggtgtgtgagtccatacctggctccaggattcaggaggcccatttgcaca     1225
                                                          *****   **  ************ ********************** ************

LOC106996293.end.GGT1.start.28595839-28635852.Rhesus      tcccaggtgg-----------------------------ggccagtacaggccccttcag     4153
BCRP3.HUMAN.NCBI.REF                                      tcccaggtgggaacctgtctggccccggctgaccctgctggccggtgcaggccccttcag     1285
                                                          **********                             **** ** *************

LOC106996293.end.GGT1.start.28595839-28635852.Rhesus      tgaggccaattctccaaggctggggtcttctcccagggtcataggtgaagggcttcagag     4213
BCRP3.HUMAN.NCBI.REF                                      tgaggccaattctccaaggctgcggtcttctcccagggtcatgggtgaaggggtttggag     1345
                                                          ********************** ******************* ********* **  ***

LOC106996293.end.GGT1.start.28595839-28635852.Rhesus      gctccctgtgtgggtactggcctgctggggtacacacaatgctgccacagccagtctgcc     4273
BCRP3.HUMAN.NCBI.REF                                      gctccctgcgtgggtactggcctgctgggttacacacaatgctgccatagccagtctgcc     1405
                                                          ******** ******************** ***************** ************

LOC106996293.end.GGT1.start.28595839-28635852.Rhesus      ccagcttccagctgggggccacatctcgggtttctctgtcctggggagcctggtgcccca     4333
BCRP3.HUMAN.NCBI.REF                                      cctacacccagcctggggccacatctcaggtctctcagtcctgaggagcccggtgcccca     1465
                                                          **  *  *****  ************* *** **** ****** ****** *********

LOC106996293.end.GGT1.start.28595839-28635852.Rhesus      cccctcacatcctctctccctgagtcagggcctgggtctcctgagctgagtgactgatac     4393
BCRP3.HUMAN.NCBI.REF                                      cccctcacatcctctctccctgagtcagggcctgggtctcgtgagctgagtgactgatac     1525
                                                          **************************************** *******************

LOC106996293.end.GGT1.start.28595839-28635852.Rhesus      ttggtgtcctgaatgagggtgtggtggagaggggccacggcgggtgtttcctgaccctct     4453
BCRP3.HUMAN.NCBI.REF                                      ttggtgtcctggatgagggcgtgatggagaggggccacagcgggtgtttcctgaccctct     1585
                                                          *********** ******* *** ************** *********************

LOC106996293.end.GGT1.start.28595839-28635852.Rhesus      tccaggaaacccagcccaagggaggccttcgctgctgccactgcagagaggacacataca     4513
BCRP3.HUMAN.NCBI.REF                                      tccaggaagg-------------------tgctgctgccgctgcagggaggacacataca     1626
                                                          ********                      ********* ****** *************

LOC106996293.end.GGT1.start.28595839-28635852.Rhesus      ggacgccccttcctgcccccctgcctgccattgggcccacaaaagccggggcaagcctccc     4573
BCRP3.HUMAN.NCBI.REF                                      ggatgccccttcctgcccccctgcctcccattgggcccacaaaagccagggcaagcctccc     1686
                                                          *** ********************* ******************** *************

LOC106996293.end.GGT1.start.28595839-28635852.Rhesus      ctccctg-cagccacctggtctgcttcccagaagctctgtcttgcaggctgttgggagga     4632
BCRP3.HUMAN.NCBI.REF                                      ctccctgccagccacctggtctgcttcccagaaattctgtcttgcaggctgttgggagga     1746
                                                          ******* *************************  *************************

LOC106996293.end.GGT1.start.28595839-28635852.Rhesus      tcccagtgctttgtaaactaaagcaagggaggcgtggccgttctctctctttgttcattc     4692
BCRP3.HUMAN.NCBI.REF                                      tcccagtactttgtaaactaaagcaagggaggagtggccgttctctc----tgttcattc     1802
                                                          ******* ************************ **************    *********

LOC106996293.end.GGT1.start.28595839-28635852.Rhesus      attcaccttttgagtcatttcttcctccctccattaccccatctgtccatccttccctgc     4752
BCRP3.HUMAN.NCBI.REF                                      attcaccttttcattcattccttcttccctccattcccccatctgtccatccttccctgc     1862
                                                          *********** * ***** **** ********** ************************

LOC106996293.end.GGT1.start.28595839-28635852.Rhesus      cctgattgctcatgcca----cacccccagcccctcctgacctggtcctttggtttctct     4808
BCRP3.HUMAN.NCBI.REF                                      cctgattgctcatgccaccgccccccgcagcccctcctgacctggtcctttggtttctct     1922
                                                          *****************    * *** *********************************

LOC106996293.end.GGT1.start.28595839-28635852.Rhesus      tcatggctttctgtctcctcccacagggctgagaatggcagctcagggacaagtagagcc     4868
BCRP3.HUMAN.NCBI.REF                                      tcagggatttctgtctcctcccacagggctgagaatggcagctcagggacaagtaggggc     1982
                                                          *** ** ************************************************* * *

LOC106996293.end.GGT1.start.28595839-28635852.Rhesus      tggtgactgcttggtctcccgggtggctcctaggggatttgagggattgatgcctgctg-     4927
BCRP3.HUMAN.NCBI.REF                                      tggggactgcttagtctccccagtggctctcaggggatttgagggtttgacgccagctgc     2042
                                                          *** ******** *******  *******  ************** **** *** ****

LOC106996293.end.GGT1.start.28595839-28635852.Rhesus      ------aggctgtgcccctcctctgctcaggaggacatacagagatgtggcacccactta     4981
BCRP3.HUMAN.NCBI.REF                                      caccccaggctgtgcccctcctctgctcaggaggacatacag-gatgcaacacccactta     2101
                                                                ************************************ ****   **********

LOC106996293.end.GGT1.start.28595839-28635852.Rhesus      aactcaaagttgcacagatgcaaatgagactggggtctcaggcaccagagacca-ccgtg     5040
BCRP3.HUMAN.NCBI.REF                                      aactcgaagttgcaaagatgcaaatgagactggggtctcaggcaccagagaccacccgtg     2161
                                                          ***** ******** *************************************** *****
```

```
LOC106996293.end.GGT1.start.28595839-28635852.Rhesus    ggcacgtgacctttgggagtggggacctgctgccacagatctctgagtggagtctggacc    5100
BCRP3.HUMAN.NCBI.REF                                    ggcacgtggcttttgggattggagacctgctgccacagatctctga--agagtctggacc    2219
                                                        ******** * ******* *** ***********************   ***********

LOC106996293.end.GGT1.start.28595839-28635852.Rhesus    tactgggtctccccaagtgactgtctggggtctctgtagcatgccctgctgtgtacgtg    5160
BCRP3.HUMAN.NCBI.REF                                    tgctgggtctccccaagtgactctctggggtctccatagcatgccctgctgtgtgcatg    2279
                                                        * ******************** ************  ****************** * **

LOC106996293.end.GGT1.start.28595839-28635852.Rhesus    agggtcagtggttggggaggggtctctgctctaatgcttcctacactggcactccctcaa    5220
BCRP3.HUMAN.NCBI.REF                                    acggtcactggttgggtaggggtctctactctaaagctccctctgccggcatcccctcga    2339
                                                        * ***** ******** ********** ****** *** ***   * ****  ***** *

LOC106996293.end.GGT1.start.28595839-28635852.Rhesus    a--ctcccttggtgaagagagaggatgtggtttgccgcagtgttttatcgaacaactctc    5278
BCRP3.HUMAN.NCBI.REF                                    actctcccttggtgaagagagaggatgtggtttgccccagtgttttatcaaacaactctc    2399
                                                        *  ******************************** ************ **********

LOC106996293.end.GGT1.start.28595839-28635852.Rhesus    tccacttcctgttttcagaagccgggagtggaagagagcctggggctggccccagctgct    5338
BCRP3.HUMAN.NCBI.REF                                    tccacttcctgttttaagaagctgggagtggaagagagcctggggctggccccagctgct    2459
                                                        *************** ****** *************************************

LOC106996293.end.GGT1.start.28595839-28635852.Rhesus    gctgcggaacaggggtcactggacgctgggaccctggccgggctggctgggggcctccgg    5398
BCRP3.HUMAN.NCBI.REF                                    gctgcgaaacaggggtcactggacgctgggaccctggccgggctggctggaggcctcagg    2519
                                                        ****** ******************************************* ****** **

LOC106996293.end.GGT1.start.28595839-28635852.Rhesus    aagaggcctgctgcagcgtcatcctggccgagatccctccctgcaggggcccctggccat    5458
BCRP3.HUMAN.NCBI.REF                                    aagaggcctgctacagtgtcatcctggccaagattcctccctgcagaggaccctggccac    2579
                                                        ************ *** ************ **** *********** ** *********

LOC106996293.end.GGT1.start.28595839-28635852.Rhesus    gctgccgcagggtctgctggggccaccagaagcccacgctcctgcctccatctctgccct    5518
BCRP3.HUMAN.NCBI.REF                                    gctgccacagggtctgctggggccaccagaagcccatgctcctgcctccatctctcccct    2639
                                                        ****** **************************** ****************** ****

LOC106996293.end.GGT1.start.28595839-28635852.Rhesus    gtgtgctcacctctcaccagcaggccctcccagagtccagtctcttctgctctttttttg    5578
BCRP3.HUMAN.NCBI.REF                                    ctgtgctcacctctcaccaggaggccctcccagagttcagtgtcctgcttttttttt--    2697
                                                         ****************** *************** **** ** *    * ******

LOC106996293.end.GGT1.start.28595839-28635852.Rhesus    tttgtttgtttttttgagatggtgtctcactctgtcaccaggctggagtgcagtggcgcaa    5638
BCRP3.HUMAN.NCBI.REF                                    --------tttttttagatggtgtctcgttctgtcaccaggctggagtgcagtggcgcga    2749
                                                                ****** ************  ***************************** *

LOC106996293.end.GGT1.start.28595839-28635852.Rhesus    tctcggctcactgaaatttc----------------------------------------    5658
BCRP3.HUMAN.NCBI.REF                                    tctcagctcactgcaacctctgcttccttggttcaaatgattctcctgcctcagcctcct    2809
                                                        **** ******** **  **

LOC106996293.end.GGT1.start.28595839-28635852.Rhesus    ------------------------------------------------------------    5658
BCRP3.HUMAN.NCBI.REF                                    gagtagctgggactacaggtgccagccaccacgcccaggtaatttttgtatttttagtag    2869


LOC106996293.end.GGT1.start.28595839-28635852.Rhesus    ------------------------------------------------------------    5658
BCRP3.HUMAN.NCBI.REF                                    agacggggtttcaccatgttggccaggatggtctctatctcttgattcgcccgccttggc    2929


LOC106996293.end.GGT1.start.28595839-28635852.Rhesus    ----------------------------------------------cgtctcctactctt    5672
BCRP3.HUMAN.NCBI.REF                                    ctcccaaagtgctggaattacaggagtgagtcatggcacccggcctcatctcctactctt    2989
                                                                                                      * ************

LOC106996293.end.GGT1.start.28595839-28635852.Rhesus    tcagcatcaggttttattactgggattctgctacagccagagaccctgggagcagattcc    5732
BCRP3.HUMAN.NCBI.REF                                    tcagcaccaggttttactcttgggattctgctacagccgcagcccctgggtgcgagttcc    3049
                                                        ****** ********* *  ******************  ** ******* **   ****

LOC106996293.end.GGT1.start.28595839-28635852.Rhesus    taaggcttatgtgagtgtggacccagcaccgtgcctagcagacatacaaaaggagcatgg    5792
BCRP3.HUMAN.NCBI.REF                                    taagctttctgtgagtgtggacccagcaccgtgcctagtagacatacaaaaggagcatgg    3109
                                                        ****  ** ***************************** *********************

LOC106996293.end.GGT1.start.28595839-28635852.Rhesus    tgacagtgaggtctgtcatctccagcttaatgactgttttgatccttgtcaaaaaggtga    5852
BCRP3.HUMAN.NCBI.REF                                    tgacagtgaggtctgtcatctccagcataatgactgttttgatccttgtaaaaaaggtga    3169
                                                        ************************** ********************** **********

LOC106996293.end.GGT1.start.28595839-28635852.Rhesus    tttttggctgagcatggtggctcacacctgtaatcccagcactttgggaggccgaggcgg    5912
BCRP3.HUMAN.NCBI.REF                                    tttttggctgggtgtggtggctcacacctgtaatcccagcactttgggaggccgatgggg    3229
                                                        ********** *  ***************************************** * **

LOC106996293.end.GGT1.start.28595839-28635852.Rhesus    gtggatcacttgaggtcaggagttggagaccagcctgggcaacatggtgaaaccccgtct    5972
BCRP3.HUMAN.NCBI.REF                                    gtggctcacttgaggtcaggagttggagcccagcctgggcaacatggtgaaaccacgtct    3289
                                                        **** *********************** ************************* *****

LOC106996293.end.GGT1.start.28595839-28635852.Rhesus    ctactaaaaatacaaaaattagctgggcatggtagcgggtgcctgtaatcccagctactt    6032
BCRP3.HUMAN.NCBI.REF                                    ctactaaaaatacaaaaattagctgggcatggtaacggatgcctgtaatcccagctactt    3349
                                                        ********************************** *** *********************

LOC106996293.end.GGT1.start.28595839-28635852.Rhesus    gggagtctgagacaggagaatcacttgaacccaagaggcaaatgttgcagtgagccaaga    6092
BCRP3.HUMAN.NCBI.REF                                    gggaggctgagacaggagaatcacttgaacccaggaggcaaaggttgcggtaagccaaga    3409
                                                        ***** *************************** ******** ***** ** ********

LOC106996293.end.GGT1.start.28595839-28635852.Rhesus    tagcaccactgcactacagcctgggtgacagagcaagacttggtctcaaaaaaaaaaaaa    6152
BCRP3.HUMAN.NCBI.REF                                    ttgtaccactgcactccagcctgggtgacagagcaagacttggtctcaaaaaaaaaaaaa    3469
                                                        * * *********** ********************************************

LOC106996293.end.GGT1.start.28595839-28635852.Rhesus    a-------aaagaaaagtttatatttttgttctaaaacttatcttaatgtcttcattcta    6205
BCRP3.HUMAN.NCBI.REF                                    aaagaaagaaagaaaagtttatatttttgttctaatggttatcttaatatcgtcattcta    3529
                                                        *       ***************************   ********** ** ********

LOC106996293.end.GGT1.start.28595839-28635852.Rhesus    ta--------ttttatataattataagagctatataagatatactacccctagtactttg    6257
```

```
BCRP3.HUMAN.NCBI.REF                                    taattgtatgttttatataattataatagctatataagatataatacccctagtatgttg    3589
                                                        **        **************** **************** ***********  ***

LOC106996293.end.GGT1.start.28595839-28635852.Rhesus    ttttttggatattctattcactcctgatggttaatttatgtgtcaactttgctaagctat    6317
BCRP3.HUMAN.NCBI.REF                                    ttttttggatattctacttgctcctgatggttaatttatatgtcaacttggctaagctat    3649
                                                        **************** *  ******************* ********* **********

LOC106996293.end.GGT1.start.28595839-28635852.Rhesus    gatgccctgttgtttggtcaaatacttctcaatatcttgctgggaggttatctcatagat    6377
BCRP3.HUMAN.NCBI.REF                                    ggtgccccgttgtttggtcaaatacttgtcaatatcttgctgggaggttatttcatagat    3709
                                                        * ***** ******************* *********************** ********

LOC106996293.end.GGT1.start.28595839-28635852.Rhesus    gtgattaacattgacagtcagctgactttaggtaaaaca----------------atgtg    6421
BCRP3.HUMAN.NCBI.REF                                    gtgattaacactgacagtcagttgactttaagtaaaacagattacccaccataatatggg    3769
                                                        ********** ********** ******** ********                *** *

LOC106996293.end.GGT1.start.28595839-28635852.Rhesus    attaacgctgacagtcagttgac------tttaagtaaaactgaggtttcccagagaagc    6475
BCRP3.HUMAN.NCBI.REF                                    tgggccacctccaatcagttgaaggccgtaagaacaaaaactgaggtttcccagagaagc    3829
                                                             * *   ** ********          **  ************************

LOC106996293.end.GGT1.start.28595839-28635852.Rhesus    aggaattctgctttaacactataacatgtaaatcctgcctgagtttctggcctgctgact    6535
BCRP3.HUMAN.NCBI.REF                                    aggaattctgcctcaagactgtaacacacaaaccctgcctgagtttctggcctgctgact    3889
                                                        *********** * ** *** *****   *** ***************************

LOC106996293.end.GGT1.start.28595839-28635852.Rhesus    gctctccaggttttaggttccagacttcgagatcaactcttacctgaatttataagctgc    6595
BCRP3.HUMAN.NCBI.REF                                    gctctacagagtttaggttccagacttcgagatcaactcttacctgaatttatagcctgc    3949
                                                        ***** ***  *******************************************  ****

LOC106996293.end.GGT1.start.28595839-28635852.Rhesus    tggttcgccatacagattttaaacttgctagtccccacaaccgtgtgagccaattcctaa    6655
BCRP3.HUMAN.NCBI.REF                                    tggcttgccctacagattttaaacttgctagtccccacaatcatgtgagccaattcctca    4009
                                                        *** * *** ****************************** * *************** *

LOC106996293.end.GGT1.start.28595839-28635852.Rhesus    ataaatctctctctatgtataacctattggtttagtttctctaaaaaccttttacatcta    6715
BCRP3.HUMAN.NCBI.REF                                    ataaatctctctctatgtataatctattggtttagtttctctgaaaagctttcacatcca    4069
                                                        ********************** ******************* **** **** ***** *

LOC106996293.end.GGT1.start.28595839-28635852.Rhesus    gtttcctggatgttaagtaatactgaaactagctagtaacttc---------ttttcttt    6766
BCRP3.HUMAN.NCBI.REF                                    gtttcctggatgttaagaattactgaaactagctagtaacttcttttttttttttttttt    4129
                                                        ***************** * ***********************         **** ***

LOC106996293.end.GGT1.start.28595839-28635852.Rhesus    tttttttttgagatggaattttgctcttgttgcccaggctggagtgcagtgccgcgatct    6826
BCRP3.HUMAN.NCBI.REF                                    tttttttttgagacagagttttgctcttgttgcccaggctggaatgcaatggcacaatct    4189
                                                        *************  ** ************************* **** ** * * ****

LOC106996293.end.GGT1.start.28595839-28635852.Rhesus    tggctcaccgcaacctccacttcctgggtccaagcgattctcctccctcagcctcccgag    6886
BCRP3.HUMAN.NCBI.REF                                    cagctcaccgcaacctccacttcctgggtccaagcaattctcctccctcagcctcctgag    4249
                                                          ********************************* ******************** ***

LOC106996293.end.GGT1.start.28595839-28635852.Rhesus    tagctgggattacaggcatgtgccaccatgctcggctaatttttgtatttttagtagaga    6946
BCRP3.HUMAN.NCBI.REF                                    tagctgggattacaggcatgtgccaccatgcttggctaatttttgtatttttagtagaga    4309
                                                        ******************************** ***************************

LOC106996293.end.GGT1.start.28595839-28635852.Rhesus    tggggcttcttcatgttggtcaggctggtctcgaactcccaacctcaggtgatccacctg    7006
BCRP3.HUMAN.NCBI.REF                                    cagggcttctccatgttggtcaggctggtcttgaactcccaacctcaggtgat-cagccg    4368
                                                          ******** ******************** ********************* ** * *

LOC106996293.end.GGT1.start.28595839-28635852.Rhesus    ccttggcctcacaaagtgctgggattacaggcatgagccaccgctcccggctcctcgtaa    7066
BCRP3.HUMAN.NCBI.REF                                    ccttggcctcacaaagtgctggaattacaggcatgagccaccgcacctggctcctagtaa    4428
                                                        ********************** ********************* ** ******* ****

LOC106996293.end.GGT1.start.28595839-28635852.Rhesus    cttcttcttttctgtgatatgtctcttatctctaata-----------------------    7103
BCRP3.HUMAN.NCBI.REF                                    attcttcttttccgtgatgtgtctcttacctctaataatacttttcttcttttttttttt    4488
                                                         *********** ***** ********* ********

LOC106996293.end.GGT1.start.28595839-28635852.Rhesus    ------------------------------------------------------------    7103
BCRP3.HUMAN.NCBI.REF                                    ttgagacggagtctcgttctgtcgcccaggcgggagtgctgtggcgcgatctccgctcac    4548


LOC106996293.end.GGT1.start.28595839-28635852.Rhesus    ------------------------------------------------------------    7103
BCRP3.HUMAN.NCBI.REF                                    tgcaagctccgccttccgggttcacgccattctcctgcctcaacctcccgagtagctggg    4608


LOC106996293.end.GGT1.start.28595839-28635852.Rhesus    ------------------------------------------------------------    7103
BCRP3.HUMAN.NCBI.REF                                    actacaggcgcccgccactgcgcccggctaatttttttgtatttttagtagagacggggtt    4668


LOC106996293.end.GGT1.start.28595839-28635852.Rhesus    ------------------------------------------------------------    7103
BCRP3.HUMAN.NCBI.REF                                    tcaccgtggtctcgatctcctgacctcgtgatccgcccgcctcggcctcccaaagtgctg    4728


LOC106996293.end.GGT1.start.28595839-28635852.Rhesus    -------------------------------atacttttcttcttaaagtctacttcatt    7132
BCRP3.HUMAN.NCBI.REF                                    ggattacaggcgtgagccaccgcgtccggccatacttttcttcttaaagtctacttcatt    4788
                                                                                       *****************************

LOC106996293.end.GGT1.start.28595839-28635852.Rhesus    aaaaatagtaatgctgggcatggtggctcatgcctgtaatctcggcactttgttggaggt    7192
BCRP3.HUMAN.NCBI.REF                                    aaaaatagttatgctgggcatggtggctcatggctgtaatctcggcactttgttggaggt    4848
                                                        ********* ********************** ***************************

LOC106996293.end.GGT1.start.28595839-28635852.Rhesus    tgaggtgggtggatcactgaagcccaggagttcaagaccagcctgggcaacatggcaaga    7252
BCRP3.HUMAN.NCBI.REF                                    cgaggtgggtggatcactgaagcccaggagttcaagaccaacctgggcaacgtggcgaga    4908
                                                         *************************************** ********** **** ***

LOC106996293.end.GGT1.start.28595839-28635852.Rhesus    ccctggctctacagaaaaatacaaaaattagccgggtgtggctaatataattctaagtta    7312
BCRP3.HUMAN.NCBI.REF                                    ccctgcctctaca-aaaaatacaaaaattagctgggtgtggctaatat------------    4955
```

```
                                                          ***** ******* ****************** ***************

LOC106996293.end.GGT1.start.28595839-28635852.Rhesus      gcacacctgtagtcccagctacttgggatgctgaggtgggagaatcgcttgaacctagaa     7372
BCRP3.HUMAN.NCBI.REF                                      --acacttgtagtcccagctacttgggatgctgaggtgggagaatcgcttgagcctagaa     5013
                                                            **** ********************************************** *******

LOC106996293.end.GGT1.start.28595839-28635852.Rhesus      gggggagattgctgtgagccaagatcatgtcactgcactccagcctgggagacagagtga     7432
BCRP3.HUMAN.NCBI.REF                                      gggagagattgctgtaagccaagatcacatcactgcactccagcctgggagacagagtga     5073
                                                          *** *********** ***********  *******************************

LOC106996293.end.GGT1.start.28595839-28635852.Rhesus      ggctctatctcaaaaaaaaaaaaaaaaaagaagttatacagctttcttggttaatgcatg     7492
BCRP3.HUMAN.NCBI.REF                                      ggctctatctccaaaaaaaaaaaaaaaaa-aagttatacagctttcttggttagtgcatg     5132
                                                          *********** ***************** *********************** ******

LOC106996293.end.GGT1.start.28595839-28635852.Rhesus      -----catattttcattattttccacctttctgtatccttatataaaaggcattagttg     7547
BCRP3.HUMAN.NCBI.REF                                      catgccatattttcattattttccacctctctgtatccttatataaaaggcattagttg     5192
                                                               *********************** *******************************

LOC106996293.end.GGT1.start.28595839-28635852.Rhesus      ggtt----tttatttccaattagtttaattttattatccttttaaatgtaactaattat     7603
BCRP3.HUMAN.NCBI.REF                                      ggttttactttattttcaattattttaattttattgtccttttaaatgtaactaatgat     5252
                                                          ****    ******* ****** ************* ******************** **

LOC106996293.end.GGT1.start.28595839-28635852.Rhesus      ttatttgggttgaaagccaccaccaatttgttttccatgcctactctctttcttcttatc     7663
BCRP3.HUMAN.NCBI.REF                                      ttatttgggttgaaacccaccaccaatttgttttccatgcctattctatttcttcttatc     5312
                                                          *************** *************************** *** ************

LOC106996293.end.GGT1.start.28595839-28635852.Rhesus      tcctcccacatcttgttttgcatttattatttttattatttaatttcctc---ctctatt     7720
BCRP3.HUMAN.NCBI.REF                                      tcctctcacatcttgttttggatttattatttttattatttaatttcctccttctctatt     5372
                                                          ***** ************** *****************************   *******

LOC106996293.end.GGT1.start.28595839-28635852.Rhesus      agttttgtaactgtgcagtcttggagttattttaaaaga-gacagtagattattttagag     7779
BCRP3.HUMAN.NCBI.REF                                      agtttcatagctctgcagtcttagagttattttaaaagatgacagtggattattttagag     5432
                                                          *****  ** ** ********* **************** ****** *************

LOC106996293.end.GGT1.start.28595839-28635852.Rhesus      cttacaacatgcatccttcacttaccaaagtctaacatgagctagtactttttttgttgtt     7839
BCRP3.HUMAN.NCBI.REF                                      cttacaacatgcatccttcacttatcaaagtctaacatgagctagtacttt-------tt     5485
                                                          ************************ **************************       **

LOC106996293.end.GGT1.start.28595839-28635852.Rhesus      gtcgtcattgagacagagggagtctcgctctgctgtccaggctggagtgcagtggagcaa     7899
BCRP3.HUMAN.NCBI.REF                                      gttgttgttgagatagagagagtcttcctctgctgcccaggctggagtgcagtggagcaa     5545
                                                          ** **  ****** **** ******  ******** ************************

LOC106996293.end.GGT1.start.28595839-28635852.Rhesus      tcttggttcactgcaacctctgcctcttgggttcaagcaattctcctgcctcagtctcct     7959
BCRP3.HUMAN.NCBI.REF                                      tcttggttcactgcaacctccacttcttgggttcaagcagttctcctgcctcagtcacct     5605
                                                          ********************  * *************** **************** ***

LOC106996293.end.GGT1.start.28595839-28635852.Rhesus      gagtagctgggatcacaggcgtgcaccactatgcccagctaatttttgtattcttttttt     8019
BCRP3.HUMAN.NCBI.REF                                      gagtagctgggaccacaggtgtgcaccactatgcccggccaatttttgtattc-tttttt     5664
                                                          ************ ****** **************** ** ************* ******

LOC106996293.end.GGT1.start.28595839-28635852.Rhesus      agtagagacagggtttcaccatgttggccaggctggtcttgaactcctgaccttaagaga     8079
BCRP3.HUMAN.NCBI.REF                                      agtagagacagggtttcaccatgttggccaggctggtcttgaactcctgaccttaagaga     5724
                                                          ************************************************************

LOC106996293.end.GGT1.start.28595839-28635852.Rhesus      tccgcctgcctcggcgtcccaaaatgttgggattacaggcataaaccactgcgcctagcc     8139
BCRP3.HUMAN.NCBI.REF                                      tctgcctacctcggcgtcctaaagtgttgggattacaggcatgagccaccgcgcccagcc     5784
                                                          ** **** *********** *** ****************** * **** ***** ****

LOC106996293.end.GGT1.start.28595839-28635852.Rhesus      tatgagttagtacttctatccccttcctagtcagtacaagaaccttggaacaggaatgaa     8199
BCRP3.HUMAN.NCBI.REF                                      tatgagttagtacttctatgctcttcctagtcagtacaagaaccttggaacaggaactaa     5844
                                                          ******************* * **********************************  **

LOC106996293.end.GGT1.start.28595839-28635852.Rhesus      atttacccccaatgacttatatgctaatatttttgtgtttttaaatatatgtatatgtg     8259
BCRP3.HUMAN.NCBI.REF                                      atttaccccagtgacttatatgctaatatttttgtgtattttaaatatatatgt-----     5899
                                                          *********** ************************** ************ * *

LOC106996293.end.GGT1.start.28595839-28635852.Rhesus      cagctgggtgcggtggctcatgcctgtaatcccagcactttgggaggccgaggcgggcag     8319
BCRP3.HUMAN.NCBI.REF                                      ------------------------------------------------------------     5899


LOC106996293.end.GGT1.start.28595839-28635852.Rhesus      atcacaaggtcaggaaatcgagaccatcctggctaacacggtgaaactctgtttctacta     8379
BCRP3.HUMAN.NCBI.REF                                      ------------------------------------------------------------     5899


LOC106996293.end.GGT1.start.28595839-28635852.Rhesus      aaaatacaaaaaactggctgggcgcaatggctcacacctgtaatcccagcactttaggag     8439
BCRP3.HUMAN.NCBI.REF                                      ------------------------------------------------------------     5899


LOC106996293.end.GGT1.start.28595839-28635852.Rhesus      gccgaggcggaaggacaacctgaggtcaggagtttgagaccagcctgaccaacatgcaga     8499
BCRP3.HUMAN.NCBI.REF                                      ------------------------------------------------------------     5899


LOC106996293.end.GGT1.start.28595839-28635852.Rhesus      aaccccatctctactaaaaatacaaaattagtcgggcatgatggcgcatgcctgtaatcc     8559
BCRP3.HUMAN.NCBI.REF                                      ------------------------------------------------------------     5899


LOC106996293.end.GGT1.start.28595839-28635852.Rhesus      cagctactcgggaggctgaggcaggagaattgcttgaacctgggaggcagaggttgcagt     8619
BCRP3.HUMAN.NCBI.REF                                      ------------------------------------------------------------     5899


LOC106996293.end.GGT1.start.28595839-28635852.Rhesus      gagctgagatcgcacctttgcactccagcctgggcaacaagagcaaaactccatctcaaa     8679
BCRP3.HUMAN.NCBI.REF                                      ------------------------------------------------------------     5899
```

```
LOC106996293.end.GGT1.start.28595839-28635852.Rhesus      aaaaaaagataaaataaaaaactaactaactaaataatacaaattagtcgggcgtggtgg     8739
BCRP3.HUMAN.NCBI.REF                                      ------------------------------------------------------------     5899

LOC106996293.end.GGT1.start.28595839-28635852.Rhesus      cgcatgcctgtaatcccagctactctagaggctgaggcaggagaatggtgtgaacctggg     8799
BCRP3.HUMAN.NCBI.REF                                      ------------------------------------------------------------     5899

LOC106996293.end.GGT1.start.28595839-28635852.Rhesus      agacggagattgcagtgagccgagatcgcaccactgcactccagcctgggcgacagagtg     8859
BCRP3.HUMAN.NCBI.REF                                      ------------------------------------------------------------     5899

LOC106996293.end.GGT1.start.28595839-28635852.Rhesus      agactccgtctcaaaaaaaaaaaaaaaaatatatatatatatatatttatattttgtgtg     8919
BCRP3.HUMAN.NCBI.REF                                      ------------------------------------------------------------     5899

LOC106996293.end.GGT1.start.28595839-28635852.Rhesus      tgtgcgtgcatagatgtatctgtgtgtttttttgtgtttttattcttatttatgttgaga     8979
BCRP3.HUMAN.NCBI.REF                                      -----gtgcatagatgtatctgtgtg-ttttttgtgtttttattcttatttatgttgaga     5953
                                                               ********************* *********************************

LOC106996293.end.GGT1.start.28595839-28635852.Rhesus      gtgtagagctatgtaaaaataaacagaattgtataatgaagccccatgtatccattcaat     9039
BCRP3.HUMAN.NCBI.REF                                      gtgtagagctatgtaagagtaaagagaattgtgtaatgaagccccgagtatccattcaat     6013
                                                          **************** * **** ******** ************  *************

LOC106996293.end.GGT1.start.28595839-28635852.Rhesus      ttcaacaacaatcttatggccaagctaatttcatgtatactctttctctttcctgcttcc     9099
BCRP3.HUMAN.NCBI.REF                                      ttcaacaacaatctcatggccaagctaatttcatgtata------ctctttcctgcttcc     6067
                                                          ************** ************************      ***************

LOC106996293.end.GGT1.start.28595839-28635852.Rhesus      ttctaccccacattatttcagtgcaaatcccagatatataactttacccatacatatttc     9159
BCRP3.HUMAN.NCBI.REF                                      ctctaccccacattatttcagtgcaaatcccagatatataactgtacccatacatatttc     6127
                                                           ****************************************** ****************

LOC106996293.end.GGT1.start.28595839-28635852.Rhesus      agtatgctttatttattttaaaccccacaagatatcattttctatactactataattta     9219
BCRP3.HUMAN.NCBI.REF                                      agtatgttttatttattttaaaccccacaatatatcattttctatactactgtaatttca     6187
                                                          ****** *********************** ******************** ****** *

LOC106996293.end.GGT1.start.28595839-28635852.Rhesus      taacaataacattcatttagatttactcaaacatttacttcttctgttaccctttatttt     9279
BCRP3.HUMAN.NCBI.REF                                      taccaataacattcatttagatttacccacacgtttacctcttctgttaccctttatttt     6247
                                                          ** *********************** ** ** ***** *********************

LOC106996293.end.GGT1.start.28595839-28635852.Rhesus      tatttataaaaatatatttgggaaaaaatatatttgcacacatagtcaaggatctcctga     9339
BCRP3.HUMAN.NCBI.REF                                      tatttataaaaatatctttgggaagaaatatctttcagcacatggtcaaggatctcctga     6307
                                                          *************** ******** ****** ***   ***** ****************

LOC106996293.end.GGT1.start.28595839-28635852.Rhesus      gggctatgtcatggccaaaatatacatatatattccatatttatatctatctatatatac     9399
BCRP3.HUMAN.NCBI.REF                                      gggctatgtcatgggcaaaatatacatatatattccatatatatacacacatatacacac     6367
                                                          ************** ************************* ****   *  **** * **

LOC106996293.end.GGT1.start.28595839-28635852.Rhesus      acacacatacacacacacacatatacacacacaaacatatatgtattccactttcacttt     9459
BCRP3.HUMAN.NCBI.REF                                      acacacatatatacac------atatacacacacacatatatatattccactttcacttt     6421
                                                          ********* * ****      *** ******* ******** *****************

LOC106996293.end.GGT1.start.28595839-28635852.Rhesus      ----tttatttgttttttgagaccgagtctcgctctctcgctctgttgcccaggctggag     9515
BCRP3.HUMAN.NCBI.REF                                      tttgtttgtttgttttttgagaccgag--------tctcgctctgtggcccaggctggag     6473
                                                              *** *******************        *********** *************

LOC106996293.end.GGT1.start.28595839-28635852.Rhesus      tgcagtggtgcgatctcagctcactgcaacttctgcctcctgggttcaagtgattctcct     9575
BCRP3.HUMAN.NCBI.REF                                      tgcggtggtgggatctcagctcactgcaacttctgcctcctgggttcaggtgattctcct     6533
                                                          *** ****** ************************************* ***********

LOC106996293.end.GGT1.start.28595839-28635852.Rhesus      gtctcagcctcc-aaatatctgggattacaggcatgagccaccacgcctggctaattttt     9634
BCRP3.HUMAN.NCBI.REF                                      gtctcagcctcctgagtagctgggattacaggtgttagccatcacgtctggctaa-----     6588
                                                          ************  * ** *************  * ***** **** ********

LOC106996293.end.GGT1.start.28595839-28635852.Rhesus      ttttttttttttttttttttgagacgaagtctccctctgttgcccaggctggagtgcagt     9694
BCRP3.HUMAN.NCBI.REF                                      ------------------------------------------------------------     6588

LOC106996293.end.GGT1.start.28595839-28635852.Rhesus      ggcacgatcttggcgcactgcaacctctactttcctggttcaagcaattcccctgcctca     9754
BCRP3.HUMAN.NCBI.REF                                      ------------------------------------------------------------     6588

LOC106996293.end.GGT1.start.28595839-28635852.Rhesus      gcctcccgagtagctgggattacaggtgcacaccaccatgcccagataatttttttgtat     9814
BCRP3.HUMAN.NCBI.REF                                      ------------------------------------------------------------     6588

LOC106996293.end.GGT1.start.28595839-28635852.Rhesus      ttgtagtagagacagggtttgcctatgatggccagactgttctcaaacttccgacctctg     9874
BCRP3.HUMAN.NCBI.REF                                      ------------------------------------------------------------     6588

LOC106996293.end.GGT1.start.28595839-28635852.Rhesus      gcaatccactcaccttggcctcccaaagtgctggggttataggcatgagccaccatgcct     9934
BCRP3.HUMAN.NCBI.REF                                      ------------------------------------------------------------     6588

LOC106996293.end.GGT1.start.28595839-28635852.Rhesus      ggcctgttttttttttttttttttttttgagatagagtctcgttctgtcacccaggctg     9994
BCRP3.HUMAN.NCBI.REF                                      -------tttttgttgtttttttttttttgagacggagtatcgctctgtcacccaggctg     6641
                                                                 ***** ** *****************  **** *** ****************

LOC106996293.end.GGT1.start.28595839-28635852.Rhesus      gagtgcagtggcaagatcttggctcactgcaacctccgtctctggggttcaagcaattct     10054
BCRP3.HUMAN.NCBI.REF                                      gagtgcagtggcaagatcttgtctcactgcaacctccacctctcaggttcaagcaattct     6701
                                                          ********************* ***************  ****  ***************
```

```
LOC106996293.end.GGT1.start.28595839-28635852.Rhesus    tgtgcctcaaga-attgagtagctgggattacaggcgcccaccaccacatctggctaatt    10113
BCRP3.HUMAN.NCBI.REF                                    tgtgcctcagcctcctgagtagctgggattacaggcatccaccaccacatctggctaatt    6761
                                                        *********      *********************  **********************

LOC106996293.end.GGT1.start.28595839-28635852.Rhesus    tgtgtatttttggtagagatggagcttcaccatgttggccaggctggtcttgaactcctg    10173
BCRP3.HUMAN.NCBI.REF                                    tgtgtatttttggtagagatggggtttcaccatgttggccaggctggtctcgaacttctg    6821
                                                        ********************** * ************************* ***** ***

LOC106996293.end.GGT1.start.28595839-28635852.Rhesus    acctcaagtgatccacctatctcggccttccaaagtgctgggattacagacatgagccac    10233
BCRP3.HUMAN.NCBI.REF                                    acctcaggtgatccacctgcctcggccttccaaagtgctgggattacaggcatgagccac    6881
                                                        ****** ***********  ***************************** **********

LOC106996293.end.GGT1.start.28595839-28635852.Rhesus    catgcccggccattttcacttttgaaggatattgttagtgcacataaaattctaggttgg    10293
BCRP3.HUMAN.NCBI.REF                                    catgcccagccattttcacttttgaaggatattgttaatgagcatagaattctaggttgg    6941
                                                        ******* ***************************** **  **** *************

LOC106996293.end.GGT1.start.28595839-28635852.Rhesus    cagatattttctttcctcagtttgaaaacatgattcccttgtatttgatttctcctgttt    10353
BCRP3.HUMAN.NCBI.REF                                    cagatattttctttcctcagtttgaaaacatgattcccttgtatctgatttctcctgctt    7001
                                                        ******************************************** ************ **

LOC106996293.end.GGT1.start.28595839-28635852.Rhesus    ttattgagaagccaattctcaatctaattttgctcatttgaaggcaatgtcttttttttgt    10413
BCRP3.HUMAN.NCBI.REF                                    ttattgggaagccaattctcaatctaattttgctcatttgaaggcaatggctttttctgt    7061
                                                        ****** ****************************************** ****** ***

LOC106996293.end.GGT1.start.28595839-28635852.Rhesus    tgttgtgtttttgagatggagtctcactctgtcgcccaggctggagtgcagtggcgtga    10473
BCRP3.HUMAN.NCBI.REF                                    tgttgtgttttctgaggtggagtctcactctgtcacccaggctggactgcagtggtgcaa    7121
                                                        *********** **** ***************** *********** ******** *  *

LOC106996293.end.GGT1.start.28595839-28635852.Rhesus    tctcagttcactgcaagttctgcctcctgggttcacgccattcttctgcctcaggctccc    10533
BCRP3.HUMAN.NCBI.REF                                    tctcagctcactgcaacctctgcctcctgggttcaagtgattcttctgcctcagcctccc    7181
                                                        ****** *********  ***************** *  *************** *****

LOC106996293.end.GGT1.start.28595839-28635852.Rhesus    gagtagctgggactacaggtgcccacaaccactcccagctaatttttgtatttttagta    10593
BCRP3.HUMAN.NCBI.REF                                    aagtagctgggattacaggtgtccaccatcacacctggc---------------------    7220
                                                         *********** ******** **** * *** **  **

LOC106996293.end.GGT1.start.28595839-28635852.Rhesus    gagatgggatttcaccatgttagccaggatggtctcgatctcctgaacttgtgatccacc    10653
BCRP3.HUMAN.NCBI.REF                                    ------------------------------------------------------------    7220


LOC106996293.end.GGT1.start.28595839-28635852.Rhesus    tgtcttggcctcccaaagtgctgggattacaggtgtgagccaccgcacctggccccttaa    10713
BCRP3.HUMAN.NCBI.REF                                    ---------------------------------------------------------taa    7223
                                                                                                                 ***

LOC106996293.end.GGT1.start.28595839-28635852.Rhesus    ttgttgtatttttaatagagatgaattttgccgtgttggtcaggctgatcccgaactct    10773
BCRP3.HUMAN.NCBI.REF                                    ttgttgtatttttaatagagatgaacttttgccatgttggtcaggctgatcccaaactcc    7283
                                                        ************************* ******* ******************* *****

LOC106996293.end.GGT1.start.28595839-28635852.Rhesus    tcgtttcaggtaatccaccctcctcagcctcccaaagtgctgggattacaggtgtgagca    10833
BCRP3.HUMAN.NCBI.REF                                    tcatttcaggtgatccgcccgcctcagcctcccaaa-tgctgggattacaggcatgagac    7342
                                                        ** ******** **** *** *************** ***************  ****

LOC106996293.end.GGT1.start.28595839-28635852.Rhesus    acccctacaccccggcctgaaggcagtatctttttttcctctggctgctttaaaaaggttt    10893
BCRP3.HUMAN.NCBI.REF                                    agcccacaaccctggcctgcaggcagtatctttttttcctctggctgctttgaaaagtttt    7402
                                                        * ***   **** ****** ****************************** ***** ***

LOC106996293.end.GGT1.start.28595839-28635852.Rhesus    gtccttggctttgagcagttttacaccgatgcatttaggtggttcttcattctatgactt    10953
BCRP3.HUMAN.NCBI.REF                                    gtcttt-gttttgagcag-tttacactgatgcatttaggtggctcctcattccatgactt    7460
                                                        *** ** * ********* ******* *************** ** ****** *******

LOC106996293.end.GGT1.start.28595839-28635852.Rhesus    cattctttttttgtccattttagaaaattctcagctttatctcttcaagtattacgtcttt    11013
BCRP3.HUMAN.NCBI.REF                                    gattctttttttgtccattttagaaaactgccagctttatctcttcaagtattatgtcttc    7520
                                                         ************************* *  *********************** *****

LOC106996293.end.GGT1.start.28595839-28635852.Rhesus    cccatcctctctcttctctccttatgagactccaatttcacatgacttataccttgttaa    11073
BCRP3.HUMAN.NCBI.REF                                    cccatcctctctctactctccttatgagactccaatttcacatgacttatgccttgttaa    7580
                                                        ************** *********************************** *********

LOC106996293.end.GGT1.start.28595839-28635852.Rhesus    agtatctcccatgtctgttaatccatttcctgtgtgttctgtctatttttctctttgtac    11133
BCRP3.HUMAN.NCBI.REF                                    agtatccccccatgtctcttaatccatttcctgtatgttctatctgtttttctctttgtac    7640
                                                        ****** ********* **************** ****** *** ***************

LOC106996293.end.GGT1.start.28595839-28635852.Rhesus    ttcaatttgtacattttgtatcagactatctcccaattagccggacatggtggtggggca    11193
BCRP3.HUMAN.NCBI.REF                                    ttcaatttgtatagtttgtatcaaactatctcccaattagccgggcgtggtggt-gggtg    7699
                                                        *********** * ********* ******************** * ******* ***

LOC106996293.end.GGT1.start.28595839-28635852.Rhesus    cctgtaaccccagctacttgggaggctgaggcaggagaatggcttgaacccgggaggtga    11253
BCRP3.HUMAN.NCBI.REF                                    cctgtaatcccagctacttgggagcctgaggcaggagaattgcttgaacccgagaggtgg    7759
                                                        ******* **************** *************** *********** ******

LOC106996293.end.GGT1.start.28595839-28635852.Rhesus    aggttgcagtgagctgagatcatgccactgtcctccagcctgggcaacagagtaaggctc    11313
BCRP3.HUMAN.NCBI.REF                                    aagttgtagtgagccgagatcatgccactgcactccagcctgggcaacagagtgagaccc    7819
                                                        * **** ******* ***************  ********************* ** * *

LOC106996293.end.GGT1.start.28595839-28635852.Rhesus    tgtctcaaataaataaaaaataaataaataccagttcattatttatttatttattttta    11373
BCRP3.HUMAN.NCBI.REF                                    tgtctcaataaataaataaataaataaataccagttcactattt---------tttttta    7870
                                                        ********  **  ** ********************* *****         *******

LOC106996293.end.GGT1.start.28595839-28635852.Rhesus    tgtttgtgtctactgtgctgttcaaattgagttcctaattccgtttttttttt----ttt    11429
BCRP3.HUMAN.NCBI.REF                                    tgtttgtgtctagtgtgctgttcaaattgagttcctaattccattttttttttagacttt    7930
                                                        ************ ***************************** **********    ***

LOC106996293.end.GGT1.start.28595839-28635852.Rhesus    tttttttgagacacagtctctatctgttgctcaggctggagttcagtggtacgatctcaa    11489
```

```
BCRP3.HUMAN.NCBI.REF                                      tttttttg------agtctctatctgttgcccaggctggagttcagtggtgcaatctcaa     7984
                                                          ********      **************** ******************* * *******

LOC106996293.end.GGT1.start.28595839-28635852.Rhesus      ctcactgcagcctctgcctcccaggttcaagcgattctcgtgcctcagcctcctgagtaa     11549
BCRP3.HUMAN.NCBI.REF                                      ctcactgtagcctccacctcccaggttcaagcgattctcatgcctcagcctctcgagtaa     8044
                                                          ******* ******  *********************** ************  ******

LOC106996293.end.GGT1.start.28595839-28635852.Rhesus      ctgggattaccaccatgcctaactcagttttgtgtttttttttgttttttttttttttga     11609
BCRP3.HUMAN.NCBI.REF                                      ctgggattaccaccacgcctaact------------------------------------     8068
                                                          *************** ********

LOC106996293.end.GGT1.start.28595839-28635852.Rhesus      gacggagtctcgctctgtcgcccagcccaggctggagtgcagtggcgcgatctcggctca     11669
BCRP3.HUMAN.NCBI.REF                                      ------------------------------------------------------------     8068


LOC106996293.end.GGT1.start.28595839-28635852.Rhesus      ctgcaagctccgcctcccgggttcacgccattctcctgcctcagcctcccgagtagctgg     11729
BCRP3.HUMAN.NCBI.REF                                      ------------------------------------------------------------     8068


LOC106996293.end.GGT1.start.28595839-28635852.Rhesus      gactacaggcgcccacaaccgcgcccggctaatttttttgtatttttagtagagacgggt     11789
BCRP3.HUMAN.NCBI.REF                                      ------------------------------------------------------------     8068


LOC106996293.end.GGT1.start.28595839-28635852.Rhesus      ttcaccgtggtctcgatctcctgaccttgtgatccgcccgtctcggcctcccaaagtgct     11849
BCRP3.HUMAN.NCBI.REF                                      ------------------------------------------------------------     8068


LOC106996293.end.GGT1.start.28595839-28635852.Rhesus      gggattacaggcgtgagccaccgcgcccggccagttttgtgtttttagtagagacaggtt     11909
BCRP3.HUMAN.NCBI.REF                                      -------------------------------catttttgtatttttagtagagatggggt     8097
                                                                                         ** ****** *************  ** *

LOC106996293.end.GGT1.start.28595839-28635852.Rhesus      tc-gccatgttggccaggctggtcttgaactcctggcctcatgtgattcacc--ctgctg     11966
BCRP3.HUMAN.NCBI.REF                                      tctgccatgttggccaggctggtcttgaactcttggccttatgtgattggcctacctctg     8157
                                                          ** ***************************** ****** ********  **  *  ***

LOC106996293.end.GGT1.start.28595839-28635852.Rhesus      tctcccaacgtgctgggattataggcgtgagccaccactcccagcctcc---------at     12017
BCRP3.HUMAN.NCBI.REF                                      tctcccaaagtgctgggattataggcctaaaccaccactcccagcctccttttttttttt     8217
                                                          ******** ***************** * * ******************          *

LOC106996293.end.GGT1.start.28595839-28635852.Rhesus      tttttttttttgagacggagtctcgctctgttgcccaggctggagtagagtggcacgatc     12077
BCRP3.HUMAN.NCBI.REF                                      tttttttttttgagacggagtctcgctctgtcgcccaggctggagtagagtggtgcgatc     8277
                                                          ******************************* *********************  *****

LOC106996293.end.GGT1.start.28595839-28635852.Rhesus      tcggctcactggaacctccacctcccggttcaagagattctcctgtctcaatctcccgag     12137
BCRP3.HUMAN.NCBI.REF                                      tcggctcactgcaacctccccctcccagttcaagtgattctcctgcctcagcctcccgag     8337
                                                          *********** ******* ****** ******* ********** ****  ********

LOC106996293.end.GGT1.start.28595839-28635852.Rhesus      tagctgggactacaggcacatgccaccatgcctggctaatttttgtaattttagtagaga     12197
BCRP3.HUMAN.NCBI.REF                                      tagctaggactataggagcatgccaccatgcctggctaatttttgtaattttagtagaga     8397
                                                          ***** ****** ***  ******************************************

LOC106996293.end.GGT1.start.28595839-28635852.Rhesus      tggggtttcaccatattggtcaggctggtcttgaactcctgacctcaggtgatccaccca     12257
BCRP3.HUMAN.NCBI.REF                                      tggggattcaccatattggtcaggctggtcttgaacttctgacctcaggtgatctaccca     8457
                                                          ***** ******************************* **************** *****

LOC106996293.end.GGT1.start.28595839-28635852.Rhesus      cctcagcctcccaaagtgctgggattacaggagtgagtcaccatgcccagtgcgtctatt     12317
BCRP3.HUMAN.NCBI.REF                                      cctcagcctcccaaagtgctgggattacaggcgtgagtcaccacgcctagtgcatcca-t     8516
                                                          ******************************* *********** *** ***** ** * *

LOC106996293.end.GGT1.start.28595839-28635852.Rhesus      tttttatagtggccagttttctgattaaattc----ttgtttctttatatccttgaatat     12373
BCRP3.HUMAN.NCBI.REF                                      tttttgtagttgccagttttctgatgaaattcttaattgtttctttatatccttg-atat     8575
                                                          ***** **** ************** ******    ******************* ****

LOC106996293.end.GGT1.start.28595839-28635852.Rhesus      agatataaagtacttattttaaagttcatggtctgacaatttcataatctagagatccta     12433
BCRP3.HUMAN.NCBI.REF                                      acatgtaaagaacttattttaaagtacatggtctgatgattttataatctggagatccta     8635
                                                          * ** ***** ************** **********  **** ******* *********

LOC106996293.end.GGT1.start.28595839-28635852.Rhesus      tgggccttttaaaaaattgtctctgctttctcttgagctttgttcctgctgtcttatttc     12493
BCRP3.HUMAN.NCBI.REF                                      tgggcctttttaaaagttgtctgtgctttctcttgagcttttttcctgctgtcttatttc     8695
                                                          ********** **** ****** ****************** ******************

LOC106996293.end.GGT1.start.28595839-28635852.Rhesus      cttgtttgcttggttgtttttaatttggcaatggagggtgtgtataaaaattgttagaaa     12553
BCRP3.HUMAN.NCBI.REF                                      cttgtttgcttagttgtttttaatttggcaatggaagttgtgtataaaaatcgttacaaa     8755
                                                          *********** *********************** * ************* **** ***

LOC106996293.end.GGT1.start.28595839-28635852.Rhesus      taa-----ttttttttttgagatggagtctcgctctgtt-cccaggctggagtgcaatga     12607
BCRP3.HUMAN.NCBI.REF                                      taattttttttttttttttgagatggagtctcgctttgttgcccaagctggagtgcaatga     8815
                                                          ***     ************************** **** **** ***************

LOC106996293.end.GGT1.start.28595839-28635852.Rhesus      catgatctcggctcaccgcaacctccacatcccagggttcaactctcctacctccgcctc     12667
BCRP3.HUMAN.NCBI.REF                                      cgtgatctcggctcactgcaacctctgcatcccagggttcaattctcctacctcagcctc     8875
                                                          * ************** ********  *************** *********** *****

LOC106996293.end.GGT1.start.28595839-28635852.Rhesus      ccgactagctgggattacaggcaggtgccagcatgcctggctaatttttgtatttttagt     12727
BCRP3.HUMAN.NCBI.REF                                      ccaagtagctgggattgcaggcaggtgccagcacgcctggctaatttttgtatttttagt     8935
                                                          ** * *********** **************** **************************

LOC106996293.end.GGT1.start.28595839-28635852.Rhesus      agagatggggtttcaccatgttggtcaggctggtctcgaactcctgacctcatgatctgc     12787
BCRP3.HUMAN.NCBI.REF                                      agagatgggtttttaccatgttggtcaggctggtctcagactcctgacctcgtgatctgc     8995
                                                          ********* *** ***********************  ************ ********

LOC106996293.end.GGT1.start.28595839-28635852.Rhesus      ctgcctcagcctcccaaagtgctgggattacaggcgtgagccactgtacccagcaagaaa     12847
BCRP3.HUMAN.NCBI.REF                                      ccacctcagcctcccaaagtgctgggattacaggcgtgagccactgcgcccagccagaaa     9055
```

```
                                                      *  *******************************************  ****** *****

LOC106996293.end.GGT1.start.28595839-28635852.Rhesus  taatttttaaaaataatttccagccccagcacgatggctcatgcttgtaatctcatcact     12907
BCRP3.HUMAN.NCBI.REF                                  taatttttaaaaataattttgagccccagcatgatggctcatgcttgtaatcccatcact     9115
                                                      *******************  ********** ******************** *******

LOC106996293.end.GGT1.start.28595839-28635852.Rhesus  ttgggaggctgaggcaggcagattgcttgagcctagaagttcaaaatcagcctgcgcaac     12967
BCRP3.HUMAN.NCBI.REF                                  ttgggaggctgaggcgggcagattgcttgagcctaggagttcaagatcagcctgtacaac     9175
                                                      *************** ******************** ******* *********  ****

LOC106996293.end.GGT1.start.28595839-28635852.Rhesus  atggtgaaaccccatctctacaaaaaataaaaaactagcta---ggcgtggtagtgtgtg     13024
BCRP3.HUMAN.NCBI.REF                                  atggtgaaaccccatctctacaaaaaataaaaaattagctgtgtgtggtggtggtgtgtg     9235
                                                      ********************************** *****    *  ***** *******

LOC106996293.end.GGT1.start.28595839-28635852.Rhesus  cctgtagtcccaggtgtttgggatgctgaggtgggagtctcacttgaaccaaggtgatcg     13084
BCRP3.HUMAN.NCBI.REF                                  cctgtagtcccagctgtttgggacgctgaggtgggaggctcacttgagcctgggtgatcg     9295
                                                      ************* ********* ************* ********* **  ********

LOC106996293.end.GGT1.start.28595839-28635852.Rhesus  acgctgcagtgagccatgatcctgagactgcactccaacctgggcaacagagtgagatgc     13144
BCRP3.HUMAN.NCBI.REF                                  aggctgcagtgagccatgatcctgagactgcactccagcctgggcaacagagtgagatgc     9355
                                                      * *********************************** **********************

LOC106996293.end.GGT1.start.28595839-28635852.Rhesus  tgtctc----aaataaataaataaaaataaaataacatgaggcctagaagtctgaaattc     13200
BCRP3.HUMAN.NCBI.REF                                  tgtctcaaataaataaataaataaaaataaaatactttgaggcctaggggtctaaaattc     9415
                                                      ******    ************************   **********  **** ******

LOC106996293.end.GGT1.start.28595839-28635852.Rhesus  tgggatctcccttatgcatttgagcggctgagatgatctgaagctggatccagtgctcct     13260
BCRP3.HUMAN.NCBI.REF                                  tgagatctcctttatgcatttgagtgactgagatgatctgaagctggatccagtgctcct     9475
                                                      ** ******* ************* * *********************************

LOC106996293.end.GGT1.start.28595839-28635852.Rhesus  gagggctgctctatttctggttgactgtcactcctagagtaagaaacctgcaccccacgt     13320
BCRP3.HUMAN.NCBI.REF                                  gagggctgctttatttctggttgactgtgactcctagagtaagaaacctgcaccccacat     9535
                                                      ********** ***************** ***************************** *

LOC106996293.end.GGT1.start.28595839-28635852.Rhesus  gtggggcattatggcattgcctccctcagccacgtgaataggtcaacagcactgctctag     13380
BCRP3.HUMAN.NCBI.REF                                  gtggggcattatggcatcccctccctcagccacatgagtaagtcaacagcactgctctag     9595
                                                      *****************  ************** *** ** *******************

LOC106996293.end.GGT1.start.28595839-28635852.Rhesus  accaggtgtggtggctcacgcctatagtcccagctactcaggagactgaagtaggaggat     13440
BCRP3.HUMAN.NCBI.REF                                  accaggtgtggtggctcacacctatagtcccagctactcgggagactgaggcaggaggat     9655
                                                      ******************* ******************* ********* * ********

LOC106996293.end.GGT1.start.28595839-28635852.Rhesus  tacttcaggccaggaatttgagaccaccctgagcaata---tattaggttggtacaaaag     13497
BCRP3.HUMAN.NCBI.REF                                  tgcttcaggccaggaatttgagaccagccagagcaatatattattaggttggtacaaaag     9715
                                                      * ************************ ** ********   *******************

LOC106996293.end.GGT1.start.28595839-28635852.Rhesus  taagtgtggtttttgccattaaaagtaatagcgaccctgtctcagcaaaaaaaa----aa     13553
BCRP3.HUMAN.NCBI.REF                                  taattgcagtgtttgcctttaaaagtaatggcaaccctgtttcagcaaaataaaaagcaa     9775
                                                      *** **  ** ****** *********** ** ******* ********* ***    **

LOC106996293.end.GGT1.start.28595839-28635852.Rhesus  aaaaaaaaaaaaaagggaagaagagaaaaagaatcagctgggcatggtggctcacacctg     13613
BCRP3.HUMAN.NCBI.REF                                  aaaaaaaaaaaaaaaaaaaaaagaaaggaagaatcagctgggcgtggtggctcacgcctc     9835
                                                      **************   ** **** *  *************** *********** ***

LOC106996293.end.GGT1.start.28595839-28635852.Rhesus  taatcccagtactttgggaggccgagttgggtggatcacaaggtcaggagattgagacca     13673
BCRP3.HUMAN.NCBI.REF                                  taatcccagcactttgggaggccaaggcgggcagatcatgagatcaggagatcgagacca     9895
                                                      ********* ************* **  ***  *****  ** ********* *******

LOC106996293.end.GGT1.start.28595839-28635852.Rhesus  tc----ctaacatggtgaaaccccgtctctactgaaaatatgacaaattagctgggcatg     13729
BCRP3.HUMAN.NCBI.REF                                  tcctggctaacacggtgaaaccccatttctactaaaaatacaaaaaattagccgggcatg     9955
                                                      **    ****** *********** * ****** ******  * ******** *******

LOC106996293.end.GGT1.start.28595839-28635852.Rhesus  gtagtgggcacctgtagtcccagctattcgggaggctgaggcaggagaatggcgtgaacc     13789
BCRP3.HUMAN.NCBI.REF                                  gtggcaggcgcctgtagtcccagctactcaggaggcggaggcaggagaatggcatgaacc     10015
                                                      ** *  *** **************** ** ****** **************** ******

LOC106996293.end.GGT1.start.28595839-28635852.Rhesus  caggaggcggaacttgcagtgagccgagatcgcaccactgcactccagcctgggtgacag     13849
BCRP3.HUMAN.NCBI.REF                                  caggaggtggaggttgcagtgagccgagatcatgccactgcactccagcctgggtgacag     10075
                                                      ******* ***  ******************   **************************

LOC106996293.end.GGT1.start.28595839-28635852.Rhesus  agtgagactccgtctcaaaaaaaaaaaaagaaagaaagaaagaaaaaaaccccactgctc     13909
BCRP3.HUMAN.NCBI.REF                                  agtgagactccgtctcaaaaaaaaaaaaaaaagaat----------------cactgctc     10119
                                                      ***************************** **  *                 ********

LOC106996293.end.GGT1.start.28595839-28635852.Rhesus  tgtctctcagcctcctcttccaggattggctgtcatcttgaggggaatgctggccttgcc     13969
BCRP3.HUMAN.NCBI.REF                                  tgtctctcagcctcctcttccaagattggccgtcgccttgaggggaatgctggccttgcc     10179
                                                      ********************** ******* ***  ************************

LOC106996293.end.GGT1.start.28595839-28635852.Rhesus  tgtctccagccttgttcttctctgcctcttatgcctttaagcacatgttttctatttgct     14029
BCRP3.HUMAN.NCBI.REF                                  tgtctccagccctgtacctctctgcctcctatgcctttaagcacatgttttctatttgct     10239
                                                      *********** *** * ********** *******************************

LOC106996293.end.GGT1.start.28595839-28635852.Rhesus  gggctgtgaaatctgcacttcatctgatggggtttgctttataggtgactagatcctttt     14089
BCRP3.HUMAN.NCBI.REF                                  gggctgtgaaatctgctcttcatctgatggggtttgctttataggtgactagatcctttt     10299
                                                      **************** *******************************************

LOC106996293.end.GGT1.start.28595839-28635852.Rhesus  ctcttggtggttttagaatttgcatcttcacattgactttaaatagtctgattacagttt     14149
BCRP3.HUMAN.NCBI.REF                                  ctcttggtggttttagaattcgcattttcacattgaccttaaatagtctgattatagttt     10359
                                                      ******************** **** *********** **************** *****

LOC106996293.end.GGT1.start.28595839-28635852.Rhesus  gccatggcaaagaccctttgcattgcattgtttggggatatttgaccctcttttatctgg     14209
BCRP3.HUMAN.NCBI.REF                                  gccacggcaaagaccctttgcattgcattgtttggggatatttgagcctcctctatctgg     10419
                                                      **** **************************************** **** * *******
```

```
LOC106996293.end.GGT1.start.28595839-28635852.Rhesus      atgtctaatctcctgttagatgtgagtagttttc---attatttcattaatgggctggca     14266
BCRP3.HUMAN.NCBI.REF                                      atgtctaatctcttgttagatgtgagtagttttcattattattttattaatgggctggca     10479
                                                          ************ *********************   ******* ***************

LOC106996293.end.GGT1.start.28595839-28635852.Rhesus      tgtggaccgtggttccaggggggctcagagaggcagccgcctgatgtct-gaccgcttct     14325
BCRP3.HUMAN.NCBI.REF                                      tgtgggccgtggttccaggcaggctcagaggggcagctgcctgatgtctggacagcttct     10539
                                                          ***** *************  ********* ****** *********** *** ******

LOC106996293.end.GGT1.start.28595839-28635852.Rhesus      ctttctgtctcttttcttacctggactctgggttgcttgttagctgcttctgccagttct     14385
BCRP3.HUMAN.NCBI.REF                                      ctttctg--tcttttcttacctggactctgggttgcttgttagctgcttctgccagttct     10597
                                                          *******  ***************************************************

LOC106996293.end.GGT1.start.28595839-28635852.Rhesus      gagttttcaaggggagaggggcccagtgatggctgttctttgaaggaaagggaagaatgt     14445
BCRP3.HUMAN.NCBI.REF                                      gagttttcaaggggagaggggcccagtgatggctgttctttgaaggaaagggaagaatgt     10657
                                                          ************************************************************

LOC106996293.end.GGT1.start.28595839-28635852.Rhesus      ctcctgtttaacacgtttctatgtttccagttagttagttaggttagttagttagttaga     14505
BCRP3.HUMAN.NCBI.REF                                      ctcctgtttaacatgtttctatgtttccagtta---------cttggttagttagttaga     10708
                                                          ************* *******************          ** **************

LOC106996293.end.GGT1.start.28595839-28635852.Rhesus      gccagggtatctcactctgttgcacaggctggagtgcaatggcatgatcttggctcacag     14565
BCRP3.HUMAN.NCBI.REF                                      gccagggtctctcgctctgttgcgcaggctggagtgcaatggcatgatcgtggctcacag     10768
                                                          ******** **** ********* ************************* **********

LOC106996293.end.GGT1.start.28595839-28635852.Rhesus      tagcctccacctaccaggctcaagcagtgctcccaccccagcctcccaagtagctgggac     14625
BCRP3.HUMAN.NCBI.REF                                      cagcctccaccttccaggctcgagcaatgctcccacctcagcctctcaagcagctgggac     10828
                                                           *********** ******** **** ********** ******* **** *********

LOC106996293.end.GGT1.start.28595839-28635852.Rhesus      tgcaggcatgtgccaccatgcttggctaccttaaaaaaatttttttttttttttttgata     14685
BCRP3.HUMAN.NCBI.REF                                      tgcaggtatgtgccaccatgcttggctgcctttttaaa-----tttttttttttttaata     10883
                                                          ****** ******************** ****   ***     ************* ***

LOC106996293.end.GGT1.start.28595839-28635852.Rhesus      cagacgaggtctcactatattgcccaggttggtattaaactcatgggctcaagtgatcct     14745
BCRP3.HUMAN.NCBI.REF                                      cagacaaggtctcactatattgcccaggctggtcttaaactcatgggctcaagtgatact     10943
                                                          ***** ********************** **** *********************** **

LOC106996293.end.GGT1.start.28595839-28635852.Rhesus      cctgccttggcctttctaagtgctgatattacaggcagggcttcaattttttaaagctcc     14805
BCRP3.HUMAN.NCBI.REF                                      cctgcctcggcctttcaaagtgctgatatcacaggcagggtttccattttttaaagctcc     11003
                                                          ******* ******** ************ ********** *** ***************

LOC106996293.end.GGT1.start.28595839-28635852.Rhesus      cagcaggggtattaaagtctccctttccagagaaagcgcactctgtccccatccctcatg     14865
BCRP3.HUMAN.NCBI.REF                                      cagcagtggta-taaactcctcctttccagagaaagcgcactctgtccgcatccctcatg     11062
                                                          ****** **** **** **  *************************** ***********

LOC106996293.end.GGT1.start.28595839-28635852.Rhesus      ttatcctctcctgcctctgcttagggttcactctgggggaat-tgccacttgagagattc     14924
BCRP3.HUMAN.NCBI.REF                                      ttgtcctctcctgcctctgcttagggttcactccgggggaaagtgccacttgagagtttc     11122
                                                          ** ****************************** *******  ************* ***

LOC106996293.end.GGT1.start.28595839-28635852.Rhesus      ctttttgtgtgtggcttctgactgaccggtccctgctcacagctgctgcttctcagggtg     14984
BCRP3.HUMAN.NCBI.REF                                      ctttttgtgtgtgg-ttctgactgactgctccctgctcacagatgctgattctcagggtg     11181
                                                          ************** *********** * ************* ***** ***********

LOC106996293.end.GGT1.start.28595839-28635852.Rhesus      gggtccctgaggcctggagtgtggcctctgaaaaccttcagggccagaagcagaatgaga     15044
BCRP3.HUMAN.NCBI.REF                                      gggtccctgaggcctggagtgtggcctctgacgaccttcagggccaggtgtggaatgaga     11241
                                                          *******************************  **************  *  ********

LOC106996293.end.GGT1.start.28595839-28635852.Rhesus      gcctgtggccacatagcccctggtgggagacgtcctgccaccctttgcttctctgtgtca     15104
BCRP3.HUMAN.NCBI.REF                                      gcctgtggccacatggcccccggtgggagacgtcccgccgccctttgcttctctgtgcca     11301
                                                          ************** ***** ************** *** ***************** **

LOC106996293.end.GGT1.start.28595839-28635852.Rhesus      ctctggctgtacatttcagatccctgggaaacgttaactggtaggacctagaaggggagg     15164
BCRP3.HUMAN.NCBI.REF                                      ctctggctgcacagttcagagccttgggaaatgttaaccagtaggacctagacggggagg     11361
                                                          ********* *** ****** ** ******* ******  ************ *******

LOC106996293.end.GGT1.start.28595839-28635852.Rhesus      tgaggaggggtcacgccccaggtgtgcctgtggtgagcctttgtgctgagcaggtgcagg     15224
BCRP3.HUMAN.NCBI.REF                                      tgagaaggggtcacccccccaggtgtgcctgtggtgagccttcgtgctgagcaggtgcagg     11421
                                                          **** ********* ************************** ******************

LOC106996293.end.GGT1.start.28595839-28635852.Rhesus      gagggaggcccaggtgcacacatctgtgaagtaggggcagctggttgggctccttgacct     15284
BCRP3.HUMAN.NCBI.REF                                      gagggaggcccaggtgcacacacctgtgaagtaggggcagctggctgggctccttgacct     11481
                                                          ********************* ********************** ***************

LOC106996293.end.GGT1.start.28595839-28635852.Rhesus      gctccagaacttcttattttctagccacttcacctgcagaaggcccaggtggctgtggtc     15344
BCRP3.HUMAN.NCBI.REF                                      gctccagagcttcttattttctggccacttcacctgcagaaggcccaggtggctgtggcc     11541
                                                          ******** ************* *********************************** *

LOC106996293.end.GGT1.start.28595839-28635852.Rhesus      tctagggtcccttgcagtgaacataccgcctgtcctcagctcctctctaggcctggtgct     15404
BCRP3.HUMAN.NCBI.REF                                      tctagggtcccttgcc--------------------------------------------     11557
                                                          ***************

LOC106996293.end.GGT1.start.28595839-28635852.Rhesus      gtgctcagcaccgtcgtatgtgtgtatgtgtgcacgcacgtgaatgtgtgcaggcatcat     15464
BCRP3.HUMAN.NCBI.REF                                      ------------------------------------------------------------     11557


LOC106996293.end.GGT1.start.28595839-28635852.Rhesus      gaggtgtggtccctgctcttaggcagcttgccttgtggctgaaatgaaccatcacctgca     15524
BCRP3.HUMAN.NCBI.REF                                      ------------------------------------------------------------     11557


LOC106996293.end.GGT1.start.28595839-28635852.Rhesus      tcaaggaaacacaaggccagatgcagtggccttgtctggagtcagatggtggctccgtga     15584
BCRP3.HUMAN.NCBI.REF                                      ------------------------------------------------------------     11557
```

```
LOC106996293.end.GGT1.start.28595839-28635852.Rhesus      cctggcttcactccagaactacctggggtttctagttgtgcaaatcccaggtcccaccca     15644
BCRP3.HUMAN.NCBI.REF                                      ------------------------------------------------------------     11557


LOC106996293.end.GGT1.start.28595839-28635852.Rhesus      gattatgagtccagttttctacaggtggagcccaggaaggtgtatttttaacacgatggt     15704
BCRP3.HUMAN.NCBI.REF                                      ------------------------------------------------------------     11557


LOC106996293.end.GGT1.start.28595839-28635852.Rhesus      actgctcattcacccagcccagccagtggtctctgtgtacccaggaactgctgacacaga     15764
BCRP3.HUMAN.NCBI.REF                                      ------------------------------------------------------------     11557


LOC106996293.end.GGT1.start.28595839-28635852.Rhesus      gctggtgctccccataaaacagaagacagagccttcacggacgggggacgtgtggcttgg     15824
BCRP3.HUMAN.NCBI.REF                                      ------------------------------------------------------------     11557


LOC106996293.end.GGT1.start.28595839-28635852.Rhesus      tcatgacagggtatttcgtaccagcacagactgtgttcaggtgacattaaggacaagttc     15884
BCRP3.HUMAN.NCBI.REF                                      ------------------------------------------------------------     11557


LOC106996293.end.GGT1.start.28595839-28635852.Rhesus      ctgcaggctagctgcctggacagggctgggtgggggtagaagaggggtcacagaggggct     15944
BCRP3.HUMAN.NCBI.REF                                      ------------------------------------------------------------     11557


LOC106996293.end.GGT1.start.28595839-28635852.Rhesus      tcctcccggccgcctcaccagctgcttgatttagggcttggctctgggtcatcctgggcc     16004
BCRP3.HUMAN.NCBI.REF                                      ------------------------------------------------------------     11557


LOC106996293.end.GGT1.start.28595839-28635852.Rhesus      tgattctgaaccacgggactggtgtggcctgcaggctcctgccacaagctgttcatggtg     16064
BCRP3.HUMAN.NCBI.REF                                      ------------------------------------------------------------     11557


LOC106996293.end.GGT1.start.28595839-28635852.Rhesus      caggggagaacagcgtccacagttccccagacagcagcagtggataccaggcccccagg     16124
BCRP3.HUMAN.NCBI.REF                                      ------------------------------------------------------------     11557


LOC106996293.end.GGT1.start.28595839-28635852.Rhesus      agttgttactgaagttgctgctggacaactcgcccttcactgagctccacatagcacccg     16184
BCRP3.HUMAN.NCBI.REF                                      ------------------------------------------------------------     11557


LOC106996293.end.GGT1.start.28595839-28635852.Rhesus      tggtgatgggagccgggtggagagagtctgccagcctgtgcatcagctcccaactgggag     16244
BCRP3.HUMAN.NCBI.REF                                      -------------------------------------tgtcctcagctcccaactgggag     11580
                                                                                                  * ******************

LOC106996293.end.GGT1.start.28595839-28635852.Rhesus      gggcagagggaggagggggtgggagccccaggcagcagggctctgggagcagtggggccc     16304
BCRP3.HUMAN.NCBI.REF                                      gggcagagggaggagggggtggagaccccaggcagcagggctctgggagcagtggggccc     11640
                                                          **********************   ***********************************

LOC106996293.end.GGT1.start.28595839-28635852.Rhesus      tgggttccaggggtgtctggcaggcccctccttactctacctcttttggcctctgggtgg     16364
BCRP3.HUMAN.NCBI.REF                                      tgggtcccaggggtgtctggcaggcccctccttactctacgtc--tcggcctctggatgg     11698
                                                          ***** ********************************** **  * ********* ***

LOC106996293.end.GGT1.start.28595839-28635852.Rhesus      agatgctggccacagtcaggctctgcctctgactaaggactggagaagtggcgggtgtgg     16424
BCRP3.HUMAN.NCBI.REF                                      aggtgctggctgcagtcgggctctgcctctgactaagggttggggaagtggcgggtgtgg     11758
                                                          ** *******  ***** ********************  *** ****************

LOC106996293.end.GGT1.start.28595839-28635852.Rhesus      gctgctgccccgtgcagcctctgaacagaccccagggcctctgccaatcatgactccttg     16484
BCRP3.HUMAN.NCBI.REF                                      gctgctgccccgtggggcctctgaacagaccccagggcctctgccaatcatgactccttc     11818
                                                          **************  *******************************************

LOC106996293.end.GGT1.start.28595839-28635852.Rhesus      ctttcagctggacccacaggccctgcaggacagagactgacagtatgctgtcatcaccat     16544
BCRP3.HUMAN.NCBI.REF                                      ctttcagctggacccgcaggccctgcaggacagagactggcagcgcgccgtcatcgccat     11878
                                                          *************** *********************** ***   ** ****** ****

LOC106996293.end.GGT1.start.28595839-28635852.Rhesus      gaatggggtatgtgtccctgggacttttctggtgcccacatcccccagaagggtagggtg     16604
BCRP3.HUMAN.NCBI.REF                                      gaatggggtacgtgtccgtgggactctcctggcgcccacttcccccagaaggatagggtg     11938
                                                          ********** ****** ******* * **** ****** ************ *******

LOC106996293.end.GGT1.start.28595839-28635852.Rhesus      gcctctattcatttcaaatcggtcagaggtggctgagcctgagccagcgtctgacacgga     16664
BCRP3.HUMAN.NCBI.REF                                      gcctctgttcatttcaaatcagtcagaggtggctgagcctgaggcagcatctgagaggga     11998
                                                          ****** ************* ********************** **** ***** * ***

LOC106996293.end.GGT1.start.28595839-28635852.Rhesus      gcctggttggaggagggaggttccccgaagagcagaatcgccgtgcccgggaatcgtca-     16723
BCRP3.HUMAN.NCBI.REF                                      gcctggttggagaagggagg-gcccccaagagcagaatcaccatgcacgggaatcgtcat     12057
                                                          ************ *******  **** ************ ** *** ************ 

LOC106996293.end.GGT1.start.28595839-28635852.Rhesus      -cactgactgggatgcagttgccagccaggccctgagcatccctcctcaaacaaaggtct     16782
BCRP3.HUMAN.NCBI.REF                                      tcattggctggaatgcagttgccagccaggccctgagcatccctcctcaaacaaaggtct     12117
                                                           ** ** **** ************************************************

LOC106996293.end.GGT1.start.28595839-28635852.Rhesus      catggcaccaccaggacaggtggggcctccactcggtgacctggggactgcatgtagaaa     16842
BCRP3.HUMAN.NCBI.REF                                      catggcaccaccaggacaggtggggcctccactcagggacctgggggctgcccatagaaa     12177
                                                          ********************************** * ********* ****   ******

LOC106996293.end.GGT1.start.28595839-28635852.Rhesus      tggagacctctgatttgtctttaggtaccccagaaaggtttacaccttaaaagcaatgac     16902
BCRP3.HUMAN.NCBI.REF                                      tggagaccccctgatttgtctttaggtaccccagaaaggtttagaccttaaaagcaatgac     12237
                                                          ******** ********************************** ****************

LOC106996293.end.GGT1.start.28595839-28635852.Rhesus      acacccaaaaaggcctgggcatatatggtaaaatgttaatatttgatgattcttggcttt     16962
BCRP3.HUMAN.NCBI.REF                                      acacccaaaaaggcccgggtataaatggtaaaatgttaatatttga-gattcttggcttt     12296
                                                          *************** *** *** ********************** *************

LOC106996293.end.GGT1.start.28595839-28635852.Rhesus      ttcttatactattctgtctttcctacttaatttttaattgttattaagaaagagagagtg     17022
```

```
BCRP3.HUMAN.NCBI.REF                                    ttcttacattattctgtctttccttcttaatttttaattgttactaagagaaagc---tg     12353
                                                        ****** * *************** ***************** ***** * **    **

LOC106996293.end.GGT1.start.28595839-28635852.Rhesus    ggcacagtacacctatggtctgagctactctagtggctgagccaggagaatcactggagc     17082
BCRP3.HUMAN.NCBI.REF                                    gtcacagtaca-ctataatctcagctactctggaggctgagccaggagaatcactggagc     12412
                                                        * ********* ****  *** ********* * **************************

LOC106996293.end.GGT1.start.28595839-28635852.Rhesus    ccaagagttcgagtacagcctgggcaacattgcaagatcccatat-tttaaaaaaagtaa     17141
BCRP3.HUMAN.NCBI.REF                                    ccaagagtttgattacagcctgggcaacattgcaagatcccatatctaaaaaaaaagcaa     12472
                                                        ********* ** ******************************** *  ******** **

LOC106996293.end.GGT1.start.28595839-28635852.Rhesus    gcaaccaagagaagcagcggggattttaggaggtggttctgcagaagccagtcctttaca     17201
BCRP3.HUMAN.NCBI.REF                                    gcaagcaagagaagcagcggggattttaggaggtgcttctgcagaaaccagtcgtttata     12532
                                                        **** ****************************** ********** ****** **** *

LOC106996293.end.GGT1.start.28595839-28635852.Rhesus    ccatcttcaacaatcctggctcttgctgaggtagactaggggattccctgaggggcagcc     17261
BCRP3.HUMAN.NCBI.REF                                    tcatcttcaacaatcctggctcttgctgaagtagactaggggcttccccgaggggcggct     12592
                                                         **************************** ************ ***** ******* **

LOC106996293.end.GGT1.start.28595839-28635852.Rhesus    ctacctcatgctgagacctctgcatgccttcagagtggaaatatttgatgagactccaag     17321
BCRP3.HUMAN.NCBI.REF                                    ccacctcatgctgagacctctgcatgccttggggggtggaaatatttgatgagactcccag     12652
                                                        * ****************************  * *********************** **

LOC106996293.end.GGT1.start.28595839-28635852.Rhesus    gggcccttgagaccttgggctatgagggccagaaagattagtggactatgccctttctcc     17381
BCRP3.HUMAN.NCBI.REF                                    gggtccttgggaccttgggctgtgaggaccagaaggattagaggactgtgccccttctcc     12712
                                                        *** ***** *********** ***** ****** ****** ***** ***** ******

LOC106996293.end.GGT1.start.28595839-28635852.Rhesus    cctctatagattgaagtaaagctcttggtcaagttcaacagcagagagttcaacttgaag     17441
BCRP3.HUMAN.NCBI.REF                                    ccactgtagatcgaagtaaagctctcggtcaagttcaacagcagggagttcagcttgaag     12772
                                                        ** ** ***** ************* ****************** ******* *******

LOC106996293.end.GGT1.start.28595839-28635852.Rhesus    aggatgccatcatgaaa----acaggggtcttcggagctgagattgctgtggtcaccaag     17497
BCRP3.HUMAN.NCBI.REF                                    aggatgccgtcccgaaaacagacaggggtcttcggagtcaagattgctgtggtcaccaag     12832
                                                        ******** **  ****    ****************   ********************

LOC106996293.end.GGT1.start.28595839-28635852.Rhesus    tgagtggggaggggcttgggctcatgcactgagggtgcatgtcccttcagctgtttctgc     17557
BCRP3.HUMAN.NCBI.REF                                    tgagtggggaggggcttgggctcacgcactgagggtgcctgtcccttcagctgtttctgc     12892
                                                        ************************ ************* *********************

LOC106996293.end.GGT1.start.28595839-28635852.Rhesus    agagaagagcatgtgtgggtctctcctctctctgtgtggccacttcgtggtgaggtcagg     17617
BCRP3.HUMAN.NCBI.REF                                    agaaaagagcatgtgtgggtctctcctctctgtgcatggccactgcacggtgaggtcagg     12952
                                                        *** *************************** **  ******** *  ************

LOC106996293.end.GGT1.start.28595839-28635852.Rhesus    ccccagggaacacttggtgtgtttagctacctcctgtgtttactcaacaccagctcagga     17677
BCRP3.HUMAN.NCBI.REF                                    ccccagggaacac--ggcgtcttcagctacctcctgtgtttcctgcaaaccagctcagga     13010
                                                        *************  ** ** ** ***************** **  * ************

LOC106996293.end.GGT1.start.28595839-28635852.Rhesus    atgtccttgccaccttgcttggaagcagtagggctggctccaggaactgcccgagtgca-     17736
BCRP3.HUMAN.NCBI.REF                                    atgtccttgccaccttgcttggaagcagta-ggctggctccaggaactgcccaagtgcag     13069
                                                        ****************************** ******************** ******

LOC106996293.end.GGT1.start.28595839-28635852.Rhesus    -gttttctgccctcgcttggaatttgtcatggtcccaggttcctgttgaatggccataac     17795
BCRP3.HUMAN.NCBI.REF                                    ggttttctgcccttgcttggaattagtcacggtcccagattcctgttgaatggccataac     13129
                                                         ************ ********** **** ******** *********************

LOC106996293.end.GGT1.start.28595839-28635852.Rhesus    ccctgcccctttgtcacaagtcagttgccaagagaagcctgttgggtttgagagcagttc     17855
BCRP3.HUMAN.NCBI.REF                                    ccctgcccctttgtcacgagtcagttgccaagagaagcctgtttggtttgagagcagttc     13189
                                                        ***************** ************************* ****************

LOC106996293.end.GGT1.start.28595839-28635852.Rhesus    ttgcagacacagaccacttcctctgagaattcatttgcttccccaggttggaatccggct     17915
BCRP3.HUMAN.NCBI.REF                                    atgcagacatagaccacttcctctgagaattcatttgcttccccaggatggaatctggct     13249
                                                         ******** ************************************* ******* ****

LOC106996293.end.GGT1.start.28595839-28635852.Rhesus    gggcccctgaacttgctggtcatgtgggccggggcctccatcaatcataccccggactcc     17975
BCRP3.HUMAN.NCBI.REF                                    gggcctctgaccttgctggtcacgtgggccggggcctccatcagtcataccctggactcc     13309
                                                        ***** **** *********** ********************* ********* *******

LOC106996293.end.GGT1.start.28595839-28635852.Rhesus    tttctgtgtctaaacagcac-actcacccccaactgcacggcagccacttgcgtagcact     18034
BCRP3.HUMAN.NCBI.REF                                    tatctgtgtctaaacaccacgccccacccccaactgcacggcagccactcgcatagcact     13369
                                                        * ************** ***  * ************************* ** *******

LOC106996293.end.GGT1.start.28595839-28635852.Rhesus    ctgggagggctctgggcatgagcagcgaggactccagcagcagccccccaaataaccac     18094
BCRP3.HUMAN.NCBI.REF                                    ctgggagggctgtgggcatgagcagcgaggactccatgagcagctcccccagataagccc     13429
                                                        *********** ************************  ****** ****** **** * *

LOC106996293.end.GGT1.start.28595839-28635852.Rhesus    tgctaatgagggtggcttgagaagcggctttgatgtgctcctaaacccagttgcaaaaca     18154
BCRP3.HUMAN.NCBI.REF                                    tgctaatgaggg-ggcttgcgaagcagctttgatgtgctggtaaatccaggtgcaaaaca     13488
                                                        ************ ****** ***** **************  **** **** *********

LOC106996293.end.GGT1.start.28595839-28635852.Rhesus    aagctaaagttaaggcctcagcacagcgctctgttctaactttgaagtattcttactcta     18214
BCRP3.HUMAN.NCBI.REF                                    gaactcaagttagggcctccgcacagcactgcgttctaactgtgaaggattcttactcta     13548
                                                         * ** ****** ****** ******* **  ********* ***** ************

LOC106996293.end.GGT1.start.28595839-28635852.Rhesus    gtgtcctgtgtggcggtattggaattgttcagtgctaggactcagaggagtaaagcactt     18274
BCRP3.HUMAN.NCBI.REF                                    gtgtcctgtgtggaggtattggaattgtccattgctaagactcagaggagaaaagcactt     13608
                                                        ************* ************** ** ***** ************ *********

LOC106996293.end.GGT1.start.28595839-28635852.Rhesus    agcagcgcaggacttagagcgctggtgctgaggcaacccttcattcattcattggatgtg     18334
BCRP3.HUMAN.NCBI.REF                                    agcatcgcaggacttggagcaccggtgctgaggcaacccttcattcattcgtcggatgtg     13668
                                                        **** ********** **** * *************************** * *******

LOC106996293.end.GGT1.start.28595839-28635852.Rhesus    tgttaaggcccaggcccagggcaggggtcagggattctcttctcacacagcacgcgggtg     18394
BCRP3.HUMAN.NCBI.REF                                    tgtta------aggcccagggcaggggtcagggattctcctctcacacagcacgtgggtg     13722
```

```
                                                             *****      **************************** ************** *****

LOC106996293.end.GGT1.start.28595839-28635852.Rhesus         gcaggaccaacaccgggtctgatctcccagctggggacacaggctgctaaccccaggcct     18454
BCRP3.HUMAN.NCBI.REF                                         gcaggaccaacaccgggtctgacctcccagccgggggcacaggctgctaaccccaggcct     13782
                                                             ********************** ******** **** ***********************

LOC106996293.end.GGT1.start.28595839-28635852.Rhesus         ggaatctgtcagatgccctttctgtgctgactttacttagacaggcctcctgaccttccc     18514
BCRP3.HUMAN.NCBI.REF                                         ggaatctgtcagatgcccttcctgtgctgacttgacttagacaggcctcctgaccttccc     13842
                                                             ******************** ************ **************************

LOC106996293.end.GGT1.start.28595839-28635852.Rhesus         acaaagatcatgtgtgacttgcaggggttctggctgcttgaaagg-tcctgagacagtac     18573
BCRP3.HUMAN.NCBI.REF                                         gcaaaggtcatgtgtgattcgcaggggttctggccgcttgaaaggttcctgagaaagcac     13902
                                                              ***** ********** * ************** ********** ******** ** **

LOC106996293.end.GGT1.start.28595839-28635852.Rhesus         atgcaatgaggactgagcttgcagagggagcacaggcatgcagaaggttctctgtgcagc     18633
BCRP3.HUMAN.NCBI.REF                                         atgccatgaggacagagcttgcagagggaggacaggcatgcagaaggctctgtgtgcagc     13962
                                                             **** ******** **************** **************** *** ********

LOC106996293.end.GGT1.start.28595839-28635852.Rhesus         cccacacctagtcaccttaaccatcatcctcactccacctcc------------atagag     18681
BCRP3.HUMAN.NCBI.REF                                         cccagacctgggtaccttcgtcaccgtcctcaccccacctccgggtgtgcagatagggag     14022
                                                             **** **** *  *****   ** * ******* ********            *  ***

LOC106996293.end.GGT1.start.28595839-28635852.Rhesus         caggtctcctgtg-tgtggcccacgcagggctgccaggacactgagaacatttcctcctc     18740
BCRP3.HUMAN.NCBI.REF                                         caggcctcctgtgttatggcccaagcggggctgttaggacactgagaacattccctcctc     14082
                                                             **** ******** * ******* ** ******  ***************** *******

LOC106996293.end.GGT1.start.28595839-28635852.Rhesus         ccgcaggagacagaggtccgaggtgccctacatcgtacgccagtgcgtggaggagctcaa     18800
BCRP3.HUMAN.NCBI.REF                                         ccgcaggagagagaggtccaaggtgccctacatcatgcgccagtgcgtggaggagatcga     14142
                                                             ********** ******** ************** * ****************** ** *

LOC106996293.end.GGT1.start.28595839-28635852.Rhesus         gcgccgaggcatggaggaggtgggcatctaccgcgtgtccggagtggccacggacatcca     18860
BCRP3.HUMAN.NCBI.REF                                         gcgccgaggcatggaggaggtgggcatctaccgcgtgtccggtgtggccacggacatcca     14202
                                                             ****************************************** *****************

LOC106996293.end.GGT1.start.28595839-28635852.Rhesus         ggcactgaaggcagccttcgacgtcagtcagtggtggcctggggaggacaggatggaggt     18920
BCRP3.HUMAN.NCBI.REF                                         ggcactgaaggcaggcttcaacgtcagtgagtgtcggcctgcgcaggacgggatggaggt     14262
                                                             ************** **** ******** ****  ****** * ***** **********

LOC106996293.end.GGT1.start.28595839-28635852.Rhesus         gtgggaggcggtgtccgcgatgagatctcagagtgctccatggtccgggcatgtcacatt     18980
BCRP3.HUMAN.NCBI.REF                                         gtgggcagtggtgtccgcgatgagatctcagagtgctccatggcccaggcatgtcacatc     14322
                                                             *****  * ********************************** ** ************

LOC106996293.end.GGT1.start.28595839-28635852.Rhesus         cttctctgtgtcttttcttcatttacagtgttactatttttaaaaaagagaagacaagaa     19040
BCRP3.HUMAN.NCBI.REF                                         cttctctgtgtcttttcttcatttactgttttattattttaaaaaaagagaaaacaagag     14382
                                                             ************************** ** *** ****** *********** ******

LOC106996293.end.GGT1.start.28595839-28635852.Rhesus         ttatagaaatagcttctgtagaagccagtttttaaaccatcctagccatgcatgccactt     19100
BCRP3.HUMAN.NCBI.REF                                         ttgtacaaacagcttctatagaagccagtttttacaccatcgtacccactcatgccactt     14442
                                                             ** ** *** ******* **************** ****** ** ***  **********

LOC106996293.end.GGT1.start.28595839-28635852.Rhesus         gctggggtgaaccaggggcttctgtggggccttggccttcctgccttgggggtggacagg     19160
BCRP3.HUMAN.NCBI.REF                                         ggtggagtggaccaggggcttctgtggggacttggccttcctgccttgggggtggacagg     14502
                                                             * *** *** ******************* ******************************

LOC106996293.end.GGT1.start.28595839-28635852.Rhesus         aggtggaagcccaggactcagtgcagtctgtccactgccctgtgtgacgatgcggtgggc     19220
BCRP3.HUMAN.NCBI.REF                                         aggtggaagcccaggactcagtgcggtctgtccactgccctgtatgaggatgtggtgggc     14562
                                                             ************************ ****************** *** **** *******

LOC106996293.end.GGT1.start.28595839-28635852.Rhesus         agaggacactgatgggacccagctcaggctggggctgcagcatctctgcctccatttcac     19280
BCRP3.HUMAN.NCBI.REF                                         agagggcactgatgaaattcagcgcaggccggggctgcagcatctccgcctccatctcac     14622
                                                             ***** ********  *  **** ***** **************** ******** ****

LOC106996293.end.GGT1.start.28595839-28635852.Rhesus         caactctctcaggctatgaaagacatggacctgcctcaagtgccagaggaggacacagag     19340
BCRP3.HUMAN.NCBI.REF                                         caaccctcacaggccttgaaggacccagactggcctcaaatgccaggggagggcactgag     14682
                                                             **** *** *****  **** ***   ***  ******* ****** ***** *** ***

LOC106996293.end.GGT1.start.28595839-28635852.Rhesus         gccccagagggtcctttccagcatcttcaaagcaacaggattttgtgcctgcagaccctt     19400
BCRP3.HUMAN.NCBI.REF                                         accccagagggtccttcccagcatcttcaaagcaacaggattttgtgcctgcagaccctt     14742
                                                              *************** *******************************************

LOC106996293.end.GGT1.start.28595839-28635852.Rhesus         ctttggggcacacaccactgaccctgaccaggacccctagaatgcctatcacccctgggt     19460
BCRP3.HUMAN.NCBI.REF                                         ctttgcagcacacaccacccaccctgaccaggacccctagaatgcccagcatccctgga     14802
                                                             *****  ***********  ************************** * ** *******

LOC106996293.end.GGT1.start.28595839-28635852.Rhesus         gggccctgtggtaatt-----tccctctgggggcccagaatagacctggcctgcggtgag     19515
BCRP3.HUMAN.NCBI.REF                                         gggccctgtggtagtttcagctccctctgggggcccagaatgaacctggcctgtggtgag     14862
                                                             ************* **     ********************  ********** ******

LOC106996293.end.GGT1.start.28595839-28635852.Rhesus         gacgcaagcaccagtggcccattgggtccaaaggaagacattgattcaaacactgaaacc     19575
BCRP3.HUMAN.NCBI.REF                                         gatgtaagcaccaatggccaattgggtccaaaggaagacaccggttcaaacactgaaacc     14922
                                                             ** * ******** ***** ********************  * ****************

LOC106996293.end.GGT1.start.28595839-28635852.Rhesus         aatcagattctcccacagccttcctgccatcagaagacactggtgcagggttggttgcta     19635
BCRP3.HUMAN.NCBI.REF                                         aatcagattctcccacggccttcctgctatcagacgacactggtgcaggggtggttgcta     14982
                                                             **************** ********** ****** *************** *********

LOC106996293.end.GGT1.start.28595839-28635852.Rhesus         tgtacagggcagagtcacccgattcccacgcaggcactgtgtcctgctgtgctggcctcc     19695
BCRP3.HUMAN.NCBI.REF                                         tgtacagggcagagccacccaatccccacgcaggcgctgtgtcctgccacgttggcctcc     15042
                                                             ************** ***** ** *********** ***********   * ********

LOC106996293.end.GGT1.start.28595839-28635852.Rhesus         tcctggccatcacatcgggccaagcaggggagaggaatgggaatgcccatgcaccccccat     19755
BCRP3.HUMAN.NCBI.REF                                         tcctggccatcacatcaggccaagcaggggagaggaatgggaatgcccacgcacccctat     15102
                                                             **************** ******************************** ******* **
```

```
LOC106996293.end.GGT1.start.28595839-28635852.Rhesus      caactctgcagacacagaaccacacacagctcttgggaggggtcagatgagctgcttaaa     19815
BCRP3.HUMAN.NCBI.REF                                      caactctgcagacacagaaccatgcacagctcttgggaggagtcagatgagctgctcaaa     15162
                                                          **********************  **************** *************** ***

LOC106996293.end.GGT1.start.28595839-28635852.Rhesus      gccggggagggacccgcacagtggtcaacatggcagggacggtgctttagccaagcctgg     19875
BCRP3.HUMAN.NCBI.REF                                      gcccaggagggacccgcacagtggtcagtgtggcagggacggtgctttagccaaggcagg     15222
                                                          ***  **********************   ************************* * **

LOC106996293.end.GGT1.start.28595839-28635852.Rhesus      gatggtgggagactcacttgggatcctgaaggaggccgctgcatttccatgctctttcca     19935
BCRP3.HUMAN.NCBI.REF                                      gatggtgggtgactcactcaggatcttcaaggaggccgctgcatttccgtgctctttcca     15282
                                                          ********* ********  ***** * ******************** ***********

LOC106996293.end.GGT1.start.28595839-28635852.Rhesus      gataacaaggacgtgtcggtgatgatgagcgagatggacgtgaacgccatcgcagggacg     19995
BCRP3.HUMAN.NCBI.REF                                      gataacaaggacgtgtcggtgatgatgagcgagatggacgtgaacgccatcgcaggcacg     15342
                                                          ******************************************************** ***

LOC106996293.end.GGT1.start.28595839-28635852.Rhesus      ctgaagctgtacttccgtgagctgcccgagcccctcttcaccgacgagttctaccccaac     20055
BCRP3.HUMAN.NCBI.REF                                      ctgaagctgtacttccgtgagctgcccgagcccctcttcactgacgagttctaccccaac     15402
                                                          ***************************************** ******************

LOC106996293.end.GGT1.start.28595839-28635852.Rhesus      ttcgccgagggcatcggtgagcactggaggccttggcctcatgggagacgtctcctccac     20115
BCRP3.HUMAN.NCBI.REF                                      ttcgcagagggcatcggtgagcactggaggccttggcctcatgggagacgtctcctccac     15462
                                                          ***** ******************************************************

LOC106996293.end.GGT1.start.28595839-28635852.Rhesus      atgcactgctgcccttggaggctgtgaaaagtgatgtgggaacctgagctgtgccccc     20175
BCRP3.HUMAN.NCBI.REF                                      gtgcactgctgccctcggaggctgtgaaaagcgaggtgggaacctgagctgtaacccc     15522
                                                           ************** *************** ** ******************  ****

LOC106996293.end.GGT1.start.28595839-28635852.Rhesus      tctgccatggtcggtgttttaacccaacctcagaaaaca--ggaccaaaatcaagcctgt     20233
BCRP3.HUMAN.NCBI.REF                                      tctgccgtggtcggcattttaacccaacctcaaaaagcaggggaccagaaccgagcctgt     15582
                                                          ****** *******  **************** *** **  ****** ** * *******

LOC106996293.end.GGT1.start.28595839-28635852.Rhesus      cctggaagacctcgcccatccccagaggctccccgtccctattcctcaaggagaccaag     20293
BCRP3.HUMAN.NCBI.REF                                      cctggaaggccttgcccatccccagaggctccccatccctactcctcaaggagaccaag     15642
                                                          ******** *** ********************* ****** ******************

LOC106996293.end.GGT1.start.28595839-28635852.Rhesus      agggtgaaatggtcagcactgccgtgctgtggggtcctaaagtctgctgtcctccttcct     20353
BCRP3.HUMAN.NCBI.REF                                      aggctgaaatagtcagcactgctgtgctatggggtcctaaagtctgctgtcctccttcct     15702
                                                          *** ****** *********** ***** *******************************

LOC106996293.end.GGT1.start.28595839-28635852.Rhesus      gcagaccaggactgaaggagc--gcccaggtgctctagccacgggtcctggcccagtcaa     20411
BCRP3.HUMAN.NCBI.REF                                      gcagaccagggctgaaggagggtgcctgggtgctcttgccatgggtcctggtccagccaa     15762
                                                          ********** *********   ***  ******** **** ********* **** ***

LOC106996293.end.GGT1.start.28595839-28635852.Rhesus      gcatgggttcaaacctggcctgacccttagtcaacctgcaggctgatggctagagtgggt     20471
BCRP3.HUMAN.NCBI.REF                                      gcatggtttcaaacatgacctgacccttagtcaacctggaggctgatgtctagagcgggt     15822
                                                          ****** ******* ** ******************** ********* ****** ****

LOC106996293.end.GGT1.start.28595839-28635852.Rhesus      gctggttcatgtggcacctgtagtctccacatcacccttagggcaggtctgcctcccagg     20531
BCRP3.HUMAN.NCBI.REF                                      gctggtgcgtgcagcacctgtggcctctgcatcacccttagggcaggtctgcctcccggg     15882
                                                          ****** * **  ******** * ***  **************************** **

LOC106996293.end.GGT1.start.28595839-28635852.Rhesus      cccatgcacagagga-------------cctgcaggtggccctgtggtgtccaggacaat     20578
BCRP3.HUMAN.NCBI.REF                                      cccatgcacagaggacctggtctcccagcctgcaggtgcccctgtggtgtccaggacgac     15942
                                                          ***************             ********** ****************** *

LOC106996293.end.GGT1.start.28595839-28635852.Rhesus      gagggagtctctgcatacttggtggggctggagccctcccacttcccacctctttgtgtc     20638
BCRP3.HUMAN.NCBI.REF                                      gaggggtctctgtgtacttggtggggctgggaccctcccacttcccacctccttgtgtc     16002
                                                          ***** *******  ****************  ******************* *******

LOC106996293.end.GGT1.start.28595839-28635852.Rhesus      cctcactccc--gtttcattccacgccaaacctcccctaccttgggctcccctggggagg     20696
BCRP3.HUMAN.NCBI.REF                                      cctcactcccctgtttcattccatgctgagcctcccctgccttgggct-ccctggggagg     16061
                                                          **********  *********** **  * ******** ********* ***********

LOC106996293.end.GGT1.start.28595839-28635852.Rhesus      gggtggtggcaggagatgcccaagtgcagctctgtccatgagtgactgctctcgcagctc     20756
BCRP3.HUMAN.NCBI.REF                                      gggtggtggcaggagttgcccgagggcagctctgcccatgagcagctgctctagcggctc     16121
                                                          *************** ***** ** ********* *******   ******* ** ****

LOC106996293.end.GGT1.start.28595839-28635852.Rhesus      ctcctgctgctgttcgccgggtgctgctgacccctgtgaggtggagaaaaggcgttcagg     20816
BCRP3.HUMAN.NCBI.REF                                      ctcctgctgctgttcgccgggtgctgctgacccctgcgaggtagagaaaaggcgttcagg     16181
                                                          ************************************ ***** *****************

LOC106996293.end.GGT1.start.28595839-28635852.Rhesus      tggctcataccccacaccagcaccccttgcagagtcctcactgggggccagagctgtggg     20876
BCRP3.HUMAN.NCBI.REF                                      tggttcacaccccacacaggtgcccctcacagggtcctcactggcggccagcgctgtggg     16241
                                                          *** *** *********  *  *****  *** *********** ****** ********

LOC106996293.end.GGT1.start.28595839-28635852.Rhesus      actgaggatgatgacaaccctgggctatgcagggacacgagccccaggcactccacgtaa     20936
BCRP3.HUMAN.NCBI.REF                                      tgtgacgatgatgacaagcctaaactgcgcaaggactcgtgtcccgggcgctccatgtga     16301
                                                            *** *********** ***   **  *** **** ** * *** *** ***** ** *

LOC106996293.end.GGT1.start.28595839-28635852.Rhesus      ccacctcaggagaggtttctcaggagagcaaaaattacacggggcaggctgggcatggtg     20996
BCRP3.HUMAN.NCBI.REF                                      ccacctcgggagaggtctccggcttgtcgtaacc--------------------------     16335
16320 bp

                                                          ******* ******** **      *    **

LOC106996293.end.GGT1.start.28595839-28635852.Rhesus      gctcacacctataatcccagcactttgggaggcccaggcgggtggattacctgaggtcag     21056
BCRP3.HUMAN.NCBI.REF                                      ------------------------------------------------------------     16335


LOC106996293.end.GGT1.start.28595839-28635852.Rhesus      gagttcaagaccagcctggccaacatggtgaaaccctgtctctactaaaaatacaaaaat     21116
BCRP3.HUMAN.NCBI.REF                                      ------------------------------------------------------------     16335
```

```
LOC106996293.end.GGT1.start.28595839-28635852.Rhesus      tagctggacctggtggcacatgcctgtaatcccagctactcaggaggctgaggcagggga     21176
BCRP3.HUMAN.NCBI.REF                                      ------------------------------------------------------------     16335

LOC106996293.end.GGT1.start.28595839-28635852.Rhesus      attgcttgagcccggaggcagaggttgcagtgagctgagatcatgtctctgcactccagc     21236
BCRP3.HUMAN.NCBI.REF                                      ------------------------------------------------------------     16335

LOC106996293.end.GGT1.start.28595839-28635852.Rhesus      ctggctgagagagcaagactctgtcttaaaaaaaaaaaaaaaaaaattacgcggggcagaa     21296
BCRP3.HUMAN.NCBI.REF                                      ------------------------------------------------------------     16335

LOC106996293.end.GGT1.start.28595839-28635852.Rhesus      gaaagaaggcatggaggcacgggtgatgggcggagggcccggctgcctgcggcccatcgt     21356
BCRP3.HUMAN.NCBI.REF                                      ------------------------------------------------------------     16335

LOC106996293.end.GGT1.start.28595839-28635852.Rhesus      gcttgcctgcgcagcctgggttgggggtgcggagagtgggcgaccatggggtggtgtggc     21416
BCRP3.HUMAN.NCBI.REF                                      ------------------------------------------------------------     16335

LOC106996293.end.GGT1.start.28595839-28635852.Rhesus      ctggccccagctccagcatcattgtctccacagggacgtgcaggcgctctggaccaatga     21476
BCRP3.HUMAN.NCBI.REF                                      ------------------------------------------------------------     16335

LOC106996293.end.GGT1.start.28595839-28635852.Rhesus      ccacgcgctggcctggcacctgagcaatgacttccgagaggaccctgtggcctgggcacg     21536
BCRP3.HUMAN.NCBI.REF                                      ------------------------------------------------------------     16335

LOC106996293.end.GGT1.start.28595839-28635852.Rhesus      cacttagtgccaggcctgggaggagctggaggatcagctgcccagtttcctggaggagct     21596
BCRP3.HUMAN.NCBI.REF                                      ------------------------------------------------------------     16335

LOC106996293.end.GGT1.start.28595839-28635852.Rhesus      gccggactgcccctgcaccctgacccaggcccgggcggactccggccgcttcttcgtgag     21656
BCRP3.HUMAN.NCBI.REF                                      ------------------------------------------------------------     16335

LOC106996293.end.GGT1.start.28595839-28635852.Rhesus      cctccaatcagggcccaggagaggggatgagggtgtcgcctccccactgaggacagcacc     21716
BCRP3.HUMAN.NCBI.REF                                      ------------------------------------------------------------     16335

LOC106996293.end.GGT1.start.28595839-28635852.Rhesus      aggggaggcagatagaggtgtcctggagggtggggcgggggtctcagggcacctgcagag     21776
BCRP3.HUMAN.NCBI.REF                                      ------------------------------------------------------------     16335

LOC106996293.end.GGT1.start.28595839-28635852.Rhesus      ttggcctcgggaaggggatgacagaaccggaggccactgggtgacagccacctgctgctc     21836
BCRP3.HUMAN.NCBI.REF                                      ------------------------------------------------------------     16335

LOC106996293.end.GGT1.start.28595839-28635852.Rhesus      tgcagacggactacggctgtgacatggagcagggcagtgtgtgcacctaccaccctgggg     21896
BCRP3.HUMAN.NCBI.REF                                      ------------------------------------------------------------     16335

LOC106996293.end.GGT1.start.28595839-28635852.Rhesus      ccgtgcactgtgtgcgctctgtgcaggccaggtgagcccccaggctggggccggggtggg     21956
BCRP3.HUMAN.NCBI.REF                                      ------------------------------------------------------------     16335

LOC106996293.end.GGT1.start.28595839-28635852.Rhesus      gactggggacaggggtgggctcccaacagtggcctggccgtgacccactggctcccgcag     22016
BCRP3.HUMAN.NCBI.REF                                      --------------------------------caggggagtgacccactgcctcctgcag     16363
                                                                                          *  **  *********** **** ****

LOC106996293.end.GGT1.start.28595839-28635852.Rhesus      ccctcggtacagctcgggtcagcagtgctgctacacagcggacgggacgcagctcctgat     22076
BCRP3.HUMAN.NCBI.REF                                      ctctttc-----------------------------------------------------     16370
                                                          * **

LOC106996293.end.GGT1.start.28595839-28635852.Rhesus      ggctgactccagcagcggcagcactcccgaccgcggccatgactggggcgcaccccgtt     22136
BCRP3.HUMAN.NCBI.REF                                      ------------------------------------------------------------     16370

LOC106996293.end.GGT1.start.28595839-28635852.Rhesus      ccgcacgccaccccgagtgcccggcatgtcccactggctctacgatgtcctcagcttcta     22196
BCRP3.HUMAN.NCBI.REF                                      ------------------------------------------------------------     16370

LOC106996293.end.GGT1.start.28595839-28635852.Rhesus      ttattgctgcctctgggcacccgactgcgcccgctacatgcaacggcggccctccaatga     22256
BCRP3.HUMAN.NCBI.REF                                      ------------------------------------------------------------     16370

LOC106996293.end.GGT1.start.28595839-28635852.Rhesus      ctgccgcaactaccagcccccgcgactaggtgggtgccatcctgtgccccggaccctggg     22316
BCRP3.HUMAN.NCBI.REF                                      ------------------------------------------------------------     16370

LOC106996293.end.GGT1.start.28595839-28635852.Rhesus      aaagatcgggctgggctggggtgcaccccacctgaccctccactctcaccccagcctccg     22376
BCRP3.HUMAN.NCBI.REF                                      ------------------------------------------------------------     16370

LOC106996293.end.GGT1.start.28595839-28635852.Rhesus      ccttcggagacccacactttgtgacctttgacggcaccaacttcacattcaatgggcgcg     22436
BCRP3.HUMAN.NCBI.REF                                      ------------------------------------------------------------     16370

LOC106996293.end.GGT1.start.28595839-28635852.Rhesus      gagagtacgtgctgctagaggcagtgctgactgatctgagggtgcaggcgcgggcccagc     22496
BCRP3.HUMAN.NCBI.REF                                      ------------------------------------------------------------     16370
```

```
LOC106996293.end.GGT1.start.28595839-28635852.Rhesus      cagggaggatgtccaatggtgaggccagggctaggggctgctctgggtggcacagggtag      22556
BCRP3.HUMAN.NCBI.REF                                      ------------------------------------------------------------      16370

LOC106996293.end.GGT1.start.28595839-28635852.Rhesus      atccaaggtgggaggctggagccaagtggcgcccgttccgctcccaccaccacaggcaca      22616
BCRP3.HUMAN.NCBI.REF                                      ------------------------------------------------------------      16370

LOC106996293.end.GGT1.start.28595839-28635852.Rhesus      cagacccgtggcacagggctgactgcaatggccgtccaggagggcaactcagacgtggtg      22676
BCRP3.HUMAN.NCBI.REF                                      ------------------------------------------------------------      16370

LOC106996293.end.GGT1.start.28595839-28635852.Rhesus      gaggtcaggctggccaacgggaccagaggtctggaggtgctgctgaaccaggaggtgctg      22736
BCRP3.HUMAN.NCBI.REF                                      ------------------------------------------------------------      16370

LOC106996293.end.GGT1.start.28595839-28635852.Rhesus      agcttcgccgagcagagctggatggacctgaagggtgagtagtccagccacgtgaggctt      22796
BCRP3.HUMAN.NCBI.REF                                      ------------------------------------------------------------      16370

LOC106996293.end.GGT1.start.28595839-28635852.Rhesus      cgggctgccctcacctcctccccattcctgcggggagactgaggggaagccctgggcctt      22856
BCRP3.HUMAN.NCBI.REF                                      ------------------------------------------------------------      16370

LOC106996293.end.GGT1.start.28595839-28635852.Rhesus      cacgcctctcccagccctggctagaggcctgggcggtccgacctcaggccttcacaccca      22916
BCRP3.HUMAN.NCBI.REF                                      ------------------------------------------------------------      16370

LOC106996293.end.GGT1.start.28595839-28635852.Rhesus      ccaaggtgcccacatcatacccacctggtcaaaagccaagaggccaggatgggggacat      22976
BCRP3.HUMAN.NCBI.REF                                      ------------------------------------------------------------      16370

LOC106996293.end.GGT1.start.28595839-28635852.Rhesus      gtcctccctacagagcatcccgggagccatctggagggaactcacccggtaacgttcacc      23036
BCRP3.HUMAN.NCBI.REF                                      ------------------------------------------------------------      16370

LOC106996293.end.GGT1.start.28595839-28635852.Rhesus      gctggcctgcacagagccactcttgtgctcctgtcactccctcagtcctcaaaagccact      23096
BCRP3.HUMAN.NCBI.REF                                      ------------------------------------------------------------      16370

LOC106996293.end.GGT1.start.28595839-28635852.Rhesus      gcaaggtcgccagccctgcacggttaaggatgtccctggcaagcggtagcgccagcatcc      23156
BCRP3.HUMAN.NCBI.REF                                      ------------------------------------------------------------      16370

LOC106996293.end.GGT1.start.28595839-28635852.Rhesus      gaaccccttgcttcagggaactgagcgaatgaaaagattcctggttgggatgggtgggga      23216
BCRP3.HUMAN.NCBI.REF                                      ------------------------------------------------------------      16370

LOC106996293.end.GGT1.start.28595839-28635852.Rhesus      cctgggaggggcctgtcagcactgagggtacaaggaccctcgggacgtgcccaggcaggt      23276
BCRP3.HUMAN.NCBI.REF                                      ------------------------------------------------------------      16370

LOC106996293.end.GGT1.start.28595839-28635852.Rhesus      gtggctgctgcagccaaggccagagggacccacacgtgcatctcagcatgggggggttcac      23336
BCRP3.HUMAN.NCBI.REF                                      ------------------------------------------------------------      16370

LOC106996293.end.GGT1.start.28595839-28635852.Rhesus      cgcaggggctgcacctcaggcgccgctgggggttccaccacaagcaaggcaggggtggtg      23396
BCRP3.HUMAN.NCBI.REF                                      ------------------------------------------------------------      16370

LOC106996293.end.GGT1.start.28595839-28635852.Rhesus      gagggtcatggtcaacagaattggccctggggacgccctcccttcccctgcaggtgccct      23456
BCRP3.HUMAN.NCBI.REF                                      ------------------------------------------------------------      16370

LOC106996293.end.GGT1.start.28595839-28635852.Rhesus      cacccacagcccctgggtgcaaccgccgccaccaccgcaaggcctgcaaagatgatgccc      23516
BCRP3.HUMAN.NCBI.REF                                      ------------------------------------------------------------      16370

LOC106996293.end.GGT1.start.28595839-28635852.Rhesus      aaaaacaccgtgtagctgcgaactggtggggaagcgagtgtcagcgaggggccctctggg      23576
BCRP3.HUMAN.NCBI.REF                                      ------------------------------------------------------------      16370

LOC106996293.end.GGT1.start.28595839-28635852.Rhesus      gtggtgagtggggacaggtgcaggcaggtatatgagcagggtgtcctcacctggctggca      23636
BCRP3.HUMAN.NCBI.REF                                      ------------------------------------------------------------      16370

LOC106996293.end.GGT1.start.28595839-28635852.Rhesus      ctccggggtgggtgaggaccaggtgccatcagcctggcaggtgctggtctctgccctggc      23696
BCRP3.HUMAN.NCBI.REF                                      ------------------------------------------------------------      16370

LOC106996293.end.GGT1.start.28595839-28635852.Rhesus      caggctgtagccgttgtcacagtggaagtagatggtggaacctgccaggtacctgttgcc      23756
BCRP3.HUMAN.NCBI.REF                                      ------------------------------------------------------------      16370

LOC106996293.end.GGT1.start.28595839-28635852.Rhesus      cttcttttgtccgttgggaggtggggccagccagccacaggacactactgggatggcggc      23816
BCRP3.HUMAN.NCBI.REF                                      ------------------------------------------------------------      16370

LOC106996293.end.GGT1.start.28595839-28635852.Rhesus      agtgtgtgctgctgcgacacaggaagggcaacatgtgagaccccccagcccctgtcccga      23876
BCRP3.HUMAN.NCBI.REF                                      ------------------------------------------------------------      16370

LOC106996293.end.GGT1.start.28595839-28635852.Rhesus      gaccccagggcagccggtgggaccgccgagcctacttctaactgcccagcctggaaacag      23936
```

```
BCRP3.HUMAN.NCBI.REF                                        ------------------------------------------------------------  16370

LOC106996293.end.GGT1.start.28595839-28635852.Rhesus        gaacctaaaaagtattgatgacagagatagagagagggctggggccctgctcattttgtct  23996
BCRP3.HUMAN.NCBI.REF                                        ------------------------------------------------------------  16370

LOC106996293.end.GGT1.start.28595839-28635852.Rhesus        ggagcttaactcacttgaggtaaaagtttgtggctggcagagaggttggtaaatgaggga  24056
BCRP3.HUMAN.NCBI.REF                                        ------------------------------------------------------------  16370

LOC106996293.end.GGT1.start.28595839-28635852.Rhesus        gaaattgcaaaaaggagagagtcactgattgtaggaatcccaaattctaaatacaactga  24116
BCRP3.HUMAN.NCBI.REF                                        ------------------------------------------------------------  16370

LOC106996293.end.GGT1.start.28595839-28635852.Rhesus        ggacaaagccctggctgcccctgcactctgtgtgtgggggaagatgtacggagcccagct  24176
BCRP3.HUMAN.NCBI.REF                                        ------------------------------------------------------------  16370

LOC106996293.end.GGT1.start.28595839-28635852.Rhesus        tccaactcacttggtcaaatgaggaaactctggggaaaacgcaaagtctctacagtcagc  24236
BCRP3.HUMAN.NCBI.REF                                        ------------------------------------------------------------  16370

LOC106996293.end.GGT1.start.28595839-28635852.Rhesus        actgcttggggaggagagtggctgtgggtgctcaaggaggtggaacttcattccccaggc  24296
BCRP3.HUMAN.NCBI.REF                                        ------------------------------------------------------------  16370

LOC106996293.end.GGT1.start.28595839-28635852.Rhesus        ctgggtcccagaggaggacggcctgtgtgctgtgctggacggtgatgttcggcttctccc  24356
BCRP3.HUMAN.NCBI.REF                                        ------------------------------------------------------------  16370

LOC106996293.end.GGT1.start.28595839-28635852.Rhesus        caggtgggagccagcagctgctgaggaagggtgatgtggaacaaaggagagagcctgggt  24416
BCRP3.HUMAN.NCBI.REF                                        ------------------------------------------------------------  16370

LOC106996293.end.GGT1.start.28595839-28635852.Rhesus        ctgaggtgtgtggtggttttaatggatctgggtttctaatgagacacccccggcgctgat  24476
BCRP3.HUMAN.NCBI.REF                                        ------------------------------------------------------------  16370

LOC106996293.end.GGT1.start.28595839-28635852.Rhesus        gtgacaaaatcacaccattgtgtctagtgttgtatcttccggggcaggagatggtggttt  24536
BCRP3.HUMAN.NCBI.REF                                        ------------------------------------------------------------  16370

LOC106996293.end.GGT1.start.28595839-28635852.Rhesus        ctgtcctgggggctgagcccctgcaggaggctggaccagcacgggtagatgcagagacgc  24596
BCRP3.HUMAN.NCBI.REF                                        ------------------------------------------------------------  16370

LOC106996293.end.GGT1.start.28595839-28635852.Rhesus        gtgagcacagggcagggtccaccctggggtgggcagagctgggagggtaggcctgcaagg  24656
BCRP3.HUMAN.NCBI.REF                                        ------------------------------------------------------------  16370

LOC106996293.end.GGT1.start.28595839-28635852.Rhesus        gcctcaggggcctgtgcttgggtcatgtgcgaggacagtgtggaagaggggacccaggca  24716
BCRP3.HUMAN.NCBI.REF                                        ------------------------------------------------------------  16370

LOC106996293.end.GGT1.start.28595839-28635852.Rhesus        ggatggggacacccataaccctcctcctgaggacccaaagagaaggcagagctgcctcaa  24776
BCRP3.HUMAN.NCBI.REF                                        ------------------------------------------------------------  16370

LOC106996293.end.GGT1.start.28595839-28635852.Rhesus        atctttccatatgacagttcattttgtgtgtcaaattaggtaggccacagaacccagtca  24836
BCRP3.HUMAN.NCBI.REF                                        ------------------------------------------------------------  16370

LOC106996293.end.GGT1.start.28595839-28635852.Rhesus        tacactcaaacagaaatctagatgtgtccatgacggtatttttaaagatgggattcacat  24896
BCRP3.HUMAN.NCBI.REF                                        ------------------------------------------------------------  16370

LOC106996293.end.GGT1.start.28595839-28635852.Rhesus        ttaaagcagcagactttgattagccttcatctgtttttttgcgtttttgttgttgttgttg  24956
BCRP3.HUMAN.NCBI.REF                                        ------------------------------------------------------------  16370

LOC106996293.end.GGT1.start.28595839-28635852.Rhesus        tttttgtttttgctttgagacagggtcttgctctgttactcaggctggagtgtgggagt  25016
BCRP3.HUMAN.NCBI.REF                                        ------------------------------------------------------------  16370

LOC106996293.end.GGT1.start.28595839-28635852.Rhesus        gcaaccttggctcactgcagcctcaacctcctaggctcaaacaatccacccacctccgcc  25076
BCRP3.HUMAN.NCBI.REF                                        ------------------------------------------------------------  16370

LOC106996293.end.GGT1.start.28595839-28635852.Rhesus        tcctgagtagctgggaccacaggtgtacagccccacatgtggctaattattttattttat  25136
BCRP3.HUMAN.NCBI.REF                                        ------------------------------------------------------------  16370

LOC106996293.end.GGT1.start.28595839-28635852.Rhesus        tttttgtagagactgggttttgccatgttgcccaagctggtctccaattcctgagctcaa  25196
BCRP3.HUMAN.NCBI.REF                                        ------------------------------------------------------------  16370

LOC106996293.end.GGT1.start.28595839-28635852.Rhesus        gtgatccacccacctcagcctcccaaattgctgggattacaggtgtgagccaccgcacct  25256
BCRP3.HUMAN.NCBI.REF                                        ------------------------------------------------------------  16370

LOC106996293.end.GGT1.start.28595839-28635852.Rhesus        ggccaagttcatcatttacaatgtgagtcatgtcctttacccaatcagttgacagccttg  25316
BCRP3.HUMAN.NCBI.REF                                        ------------------------------------------------------------  16370
```

```
LOC106996293.end.GGT1.start.28595839-28635852.Rhesus      atagaaataagatgacgactgagctgttacctctgagatgacaataagaaacacatcacc     25376
BCRP3.HUMAN.NCBI.REF                                      ------------------------------------------------------------     16370

LOC106996293.end.GGT1.start.28595839-28635852.Rhesus      caggtggagatgacagagactttgcttgggtaagagcctatcagatccatgtcaccccaa     25436
BCRP3.HUMAN.NCBI.REF                                      ------------------------------------------------------------     16370

LOC106996293.end.GGT1.start.28595839-28635852.Rhesus      gtccatgttgtaggtgacagcacagttttagctcagcaacgctcagccccgggccggctg     25496
BCRP3.HUMAN.NCBI.REF                                      ------------------------------------------------------------     16370

LOC106996293.end.GGT1.start.28595839-28635852.Rhesus      cacccacactctgcccatcagccccagagccgtgtgtcttgcacgggtctgtgcctcttc     25556
BCRP3.HUMAN.NCBI.REF                                      ------------------------------------------------------------     16370

LOC106996293.end.GGT1.start.28595839-28635852.Rhesus      cctgactgcagctgtgcttgctccttggctcaccctttctggttcctctaactttccacc     25616
BCRP3.HUMAN.NCBI.REF                                      ------------------------------------------------------------     16370

LOC106996293.end.GGT1.start.28595839-28635852.Rhesus      agcatccttcacctccccttcctcagatacaagcaggccccatggacccagaacaggatt     25676
BCRP3.HUMAN.NCBI.REF                                      ------------------------------------------------------------     16370

LOC106996293.end.GGT1.start.28595839-28635852.Rhesus      ctttcagtacttaagttttacttccatttttttatacttttcaaactaagcttggcttga     25736
BCRP3.HUMAN.NCBI.REF                                      ------------------------------------------------------------     16370

LOC106996293.end.GGT1.start.28595839-28635852.Rhesus      cccaaatcaacatctttcgacctccaccaaagtctccaacgagatatttttatcttaaac     25796
BCRP3.HUMAN.NCBI.REF                                      ------------------------------------------------------------     16370

LOC106996293.end.GGT1.start.28595839-28635852.Rhesus      cacaaatttctccttgaaaaaacataattagggtgaggcaatatgaacaatggatagagc     25856
BCRP3.HUMAN.NCBI.REF                                      ------------------------------------------------------------     16370

LOC106996293.end.GGT1.start.28595839-28635852.Rhesus      taaaggttaaactaaatataagcatatggccccgtcagatctagttgtttgaggaaaatc     25916
BCRP3.HUMAN.NCBI.REF                                      ------------------------------------------------------------     16370

LOC106996293.end.GGT1.start.28595839-28635852.Rhesus      aggtgttgtttgaagaactgacaacagaaggcatgcctgagctagacttgggaaaagtca     25976
BCRP3.HUMAN.NCBI.REF                                      ------------------------------------------------------------     16370

LOC106996293.end.GGT1.start.28595839-28635852.Rhesus      ccagccaagatgactgtgcaatcccggagaacagaagggcggttttgcttacttctctgt     26036
BCRP3.HUMAN.NCBI.REF                                      ------------------------------------------------------------     16370

LOC106996293.end.GGT1.start.28595839-28635852.Rhesus      aggtcttgggcactaagtcttagagttgctatgccgtacattacagtaatagcctcatcc     26096
BCRP3.HUMAN.NCBI.REF                                      ------------------------------------------------------------     16370

LOC106996293.end.GGT1.start.28595839-28635852.Rhesus      tcaggctgcagcccagaggtgtgcagaatccctgcattggctgaggcatctttacttcca     26156
BCRP3.HUMAN.NCBI.REF                                      ------------------------------------------------------------     16370

LOC106996293.end.GGT1.start.28595839-28635852.Rhesus      gagaagcagctggggactccagagccctggtgtttatttgagtctgaatagtattctcag     26216
BCRP3.HUMAN.NCBI.REF                                      ------------------------------------------------------------     16370

LOC106996293.end.GGT1.start.28595839-28635852.Rhesus      gagaaactggggagggatccctggcttttgctaggaccagagtattgagaagtcatcaac     26276
BCRP3.HUMAN.NCBI.REF                                      ------------------------------------------------------------     16370

LOC106996293.end.GGT1.start.28595839-28635852.Rhesus      acggaagcacagaagccactgctcagggtgcaggtgagtctggcagatgctcccagggat     26336
BCRP3.HUMAN.NCBI.REF                                      ------------------------------------------------------------     16370

LOC106996293.end.GGT1.start.28595839-28635852.Rhesus      gcagagagggagggcggctgagtcagcatgggctgggagagggaacctgcagacagatag     26396
BCRP3.HUMAN.NCBI.REF                                      ----------------------------------------------------------ag     16372
                                                                                                                    **

LOC106996293.end.GGT1.start.28595839-28635852.Rhesus      tcctaggtgctaggacagtgaccagggttaagctgtttggggtataagccagaggagga     26456
BCRP3.HUMAN.NCBI.REF                                      acccagttgcaaggaagagctgcatgctcaacctgttgttgtccct--------------     16418
                                                           ** ** *** ****       ** * * ** *****   *    *

LOC106996293.end.GGT1.start.28595839-28635852.Rhesus      tgacacaggctcaggacctgaccctggacatcaaaagactgttcctatctgtgcagtgag     26516
BCRP3.HUMAN.NCBI.REF                                      ------------------------------------------------------------     16418

LOC106996293.end.GGT1.start.28595839-28635852.Rhesus      agaggaacatgaggaggaggggatccaggccctggtggacacagccctcctgaaacctga     26576
BCRP3.HUMAN.NCBI.REF                                      ---------------------gccggaggccaacctgctcaccttccttttccttctaga     16457
                                                                               *    *****    **  ***   ***  *    *  **

LOC106996293.end.GGT1.start.28595839-28635852.Rhesus      gtgccgtgctgggctgcacaggcctttttttatcttctccacttgcctggtggggaggggc     26636
BCRP3.HUMAN.NCBI.REF                                      ccacctggaaaggtagcccagctctcttgtggctgcccaggactccaggtctccaggccg     16517
                                                             **  *   **  ** ***  ** ** *  ** * *      ** ***    ***

LOC106996293.end.GGT1.start.28595839-28635852.Rhesus      tgttcatgtcaatttgtttgatccactggtgatctctgcctcaccctgagaaccagctga     26696
BCRP3.HUMAN.NCBI.REF                                      ttggggtgcccctctgctcccacca-----------------------------------     16542
                                                          *     ** *  * ** *    ***
```

```
LOC106996293.end.GGT1.start.28595839-28635852.Rhesus      ggtctgagcttggttctttagacagaggttctgaggggtgagactcagagtttgccccat      26756
BCRP3.HUMAN.NCBI.REF                                      ------------------------------------------------------------      16542

LOC106996293.end.GGT1.start.28595839-28635852.Rhesus      tggagggaccaaggaggaagcagcttctgggaccttccacagatctgtgagcacgagact      26816
BCRP3.HUMAN.NCBI.REF                                      ------------------------------------------------------------      16542

LOC106996293.end.GGT1.start.28595839-28635852.Rhesus      ctgcccatcttggaaggacacagctgctgactcctcagcagcaaacacaggctttgctgc      26876
BCRP3.HUMAN.NCBI.REF                                      ------------------------------------------------------------      16542

LOC106996293.end.GGT1.start.28595839-28635852.Rhesus      cctagccctggggagatagggggcggcacagctgcgggtgctcctgtgcagaccctgag      26936
BCRP3.HUMAN.NCBI.REF                                      ------------------------------------------------------------      16542

LOC106996293.end.GGT1.start.28595839-28635852.Rhesus      acagacccacagtgccctctgctgctcgctggccaccctgcagtctctcagcggaaactg      26996
BCRP3.HUMAN.NCBI.REF                                      ------------------------------------------------------------      16542

LOC106996293.end.GGT1.start.28595839-28635852.Rhesus      tgagtggagtcacctgagagccaggccactgtcaggccacagaatctgtgagtcaagact      27056
BCRP3.HUMAN.NCBI.REF                                      ------------------------------------------------------------      16542

LOC106996293.end.GGT1.start.28595839-28635852.Rhesus      ctggctctgccccgtgttactgacaggaataagaatcctttactcatgaggtttttctgg      27116
BCRP3.HUMAN.NCBI.REF                                      ------------------------------------------------------------      16542

LOC106996293.end.GGT1.start.28595839-28635852.Rhesus      atttccaagagaagcccacgagggaaatctgatgaccatccaatgtgaggaggctgcagg      27176
BCRP3.HUMAN.NCBI.REF                                      ------------------------------------------------------------      16542

LOC106996293.end.GGT1.start.28595839-28635852.Rhesus      gagcggggaatgcagagggctctggaagcgaagacctgaacagctccctccacaggggcc      27236
BCRP3.HUMAN.NCBI.REF                                      ------------------------------------------------------------      16542

LOC106996293.end.GGT1.start.28595839-28635852.Rhesus      agggaaggaggcaactgatagttggatattgtaaatgggaaaatgtctcacatgtctggc      27296
BCRP3.HUMAN.NCBI.REF                                      ------------------------------------------------------------      16542

LOC106996293.end.GGT1.start.28595839-28635852.Rhesus      ctccatcctggatgattttactcttaggccacatacgttgctgcctctcctgccacagga      27356
BCRP3.HUMAN.NCBI.REF                                      ------------------------------------------------------------      16542

LOC106996293.end.GGT1.start.28595839-28635852.Rhesus      tgcctctcctgagacccaataccatggatgggtggctcagcacagcctgggccctggacc      27416
BCRP3.HUMAN.NCBI.REF                                      ------------------------------------------------------------      16542

LOC106996293.end.GGT1.start.28595839-28635852.Rhesus      ctgagagatgagtgtggtgcaaaggaacatgctttagtccactccggaaacactagcaaa      27476
BCRP3.HUMAN.NCBI.REF                                      ------------------------------------------------------------      16542

LOC106996293.end.GGT1.start.28595839-28635852.Rhesus      agaccaggacaaagcagcagtgagacaggcgtgaggacgtcaaagtgaaaccatgaaaga      27536
BCRP3.HUMAN.NCBI.REF                                      ------------------------------------------------------------      16542

LOC106996293.end.GGT1.start.28595839-28635852.Rhesus      ccactgtggtgaagcagtggacagagaacaggagtggtggaggcgtccccaggggtaaca      27596
BCRP3.HUMAN.NCBI.REF                                      ------------------------------------------------------------      16542

LOC106996293.end.GGT1.start.28595839-28635852.Rhesus      ccaggttttgtccacggaggtacaaatttacttgttcaggtcatctcagtgccctggatc      27656
BCRP3.HUMAN.NCBI.REF                                      ------------------------------------------------------------      16542

LOC106996293.end.GGT1.start.28595839-28635852.Rhesus      ttcttgaagtgaaaaatcactgaagtctttgtgctttctgacattctgggccctctcttc      27716
BCRP3.HUMAN.NCBI.REF                                      ------------------------------------------------------------      16542

LOC106996293.end.GGT1.start.28595839-28635852.Rhesus      cttcatcttcctttgctgccccagcttgaagagggtctgcattggcacattcagtgaggg      27776
BCRP3.HUMAN.NCBI.REF                                      ------------------------------------------------------------      16542

LOC106996293.end.GGT1.start.28595839-28635852.Rhesus      gctctgaggcggtggtgtcctccctgcgtggacattttccaccagaggctgagaggtaga      27836
BCRP3.HUMAN.NCBI.REF                                      ------------------------------------------------------------      16542

LOC106996293.end.GGT1.start.28595839-28635852.Rhesus      tcaagtgctgagttcctcttctcaaatgcagctcactcccccgctctctgaatgagaacc      27896
BCRP3.HUMAN.NCBI.REF                                      ------------------------------------------------------------      16542

LOC106996293.end.GGT1.start.28595839-28635852.Rhesus      tgttatctatgtgtgtttctcttatttccacccagggacgcttttgttaaaactgtgtat      27956
BCRP3.HUMAN.NCBI.REF                                      ------------------------------------------------------------      16542

LOC106996293.end.GGT1.start.28595839-28635852.Rhesus      atctcctctgtttaaggggcagcggtggccgcaggtgctcctcatgcttgaaggaatggg      28016
BCRP3.HUMAN.NCBI.REF                                      ------------------------------------------------------------      16542

LOC106996293.end.GGT1.start.28595839-28635852.Rhesus      aggggagtgtccacagtggctcggctccttggctgggcgcaatcactggcagggtcagct      28076
BCRP3.HUMAN.NCBI.REF                                      ------------------------------------------------------------      16542
```

```
LOC106996293.end.GGT1.start.28595839-28635852.Rhesus      ccaagtcctcattagaccagcgcccaggctccttagcccaggaatgtccagaatctgggc     28136
BCRP3.HUMAN.NCBI.REF                                      ------------------------------------------------------------     16542

LOC106996293.end.GGT1.start.28595839-28635852.Rhesus      acagagcccaggcctcaggccaccatggcccacctgagcagctcctcctctgcacttcct     28196
BCRP3.HUMAN.NCBI.REF                                      -----------gacccccagcaccaaggaccttttcccccgacccctgtctgcagtaac-     16590
                                                                     * * *    ***** ** **   *   * *  **   ****** *  *

LOC106996293.end.GGT1.start.28595839-28635852.Rhesus      ggggcccggcatggaggcacagcccccaaggctgctggaggatgaccagggcctatcaac     28256
BCRP3.HUMAN.NCBI.REF                                      ------------------------------------------------------------     16590

LOC106996293.end.GGT1.start.28595839-28635852.Rhesus      atttgtggagagggtcctgaatgggggctcaggcagggtagcattggtcagatttgcccc     28316
BCRP3.HUMAN.NCBI.REF                                      ------------------------------------------------------------     16590

LOC106996293.end.GGT1.start.28595839-28635852.Rhesus      ttaggtctcagagaaagtgctggagagggtgaggctggaggttgggggctgggtggagaa     28376
BCRP3.HUMAN.NCBI.REF                                      ------------------------------------------------------------     16590

LOC106996293.end.GGT1.start.28595839-28635852.Rhesus      cggggttgggaggagaggaggggggggagttgagcccccctcagtgtggcctgcagcgctgg     28436
BCRP3.HUMAN.NCBI.REF                                      ------------------------------------------------------------     16590

LOC106996293.end.GGT1.start.28595839-28635852.Rhesus      gagtggccacatgggggcagcagagtgtaggaacagcgagtctgggtgccgggaggttgg     28496
BCRP3.HUMAN.NCBI.REF                                      ------------------------------------------------------------     16590

LOC106996293.end.GGT1.start.28595839-28635852.Rhesus      gagggcagtcagccccgccctgcagctggatcaacagggttggtcacaactgcagccaag     28556
BCRP3.HUMAN.NCBI.REF                                      -------------------------------------------tcactgcttctaaggac     16607
                                                                                                     ****  ** *     *

LOC106996293.end.GGT1.start.28595839-28635852.Rhesus      gtccgccacagcccacccctctgccgcccctcacctgggccatccccccatctcaggagt     28616
BCRP3.HUMAN.NCBI.REF                                      tagcaccactgccaccccacccctgcctctcctctttgccaccctcctccctctgcact     16667
                                                             * **** ***  **** *  * *** ***  **  **** ** **   *** * * *

LOC106996293.end.GGT1.start.28595839-28635852.Rhesus      gttgaagaggttgcacaggttgccccaggtcattcagcaggcactgaccatagggtggga     28676
BCRP3.HUMAN.NCBI.REF                                      gt----------------------------------------------------------     16669
                                                          **

LOC106996293.end.GGT1.start.28595839-28635852.Rhesus      gtccaggcctcccacctacttggccttgcccttggcgaatgggagagctggggaggcaga     28736
BCRP3.HUMAN.NCBI.REF                                      ------------------------------------------------------------     16669

LOC106996293.end.GGT1.start.28595839-28635852.Rhesus      ggcaggtctcatctgaggccttcctggaggaggggaatgtgcatgatggggcctgtgtgc     28796
BCRP3.HUMAN.NCBI.REF                                      ------------------------------------------------------------     16669

LOC106996293.end.GGT1.start.28595839-28635852.Rhesus      gggaacagagctgagagggttgcagggcagggtggaaggaggctagcagatgggaatggg     28856
BCRP3.HUMAN.NCBI.REF                                      ------------------------------------------------------------     16669

LOC106996293.end.GGT1.start.28595839-28635852.Rhesus      gtctggttcctgtctttctagacctcactctgttgcttccccattccctgggttttcacc     28916
BCRP3.HUMAN.NCBI.REF                                      ------------------------------------------------------------     16669

LOC106996293.end.GGT1.start.28595839-28635852.Rhesus      tccaggcacggtggggctgagggccagccgcttggccagctgggcaggcactttcctccc     28976
BCRP3.HUMAN.NCBI.REF                                      ------------------------------------------------------------     16669

LOC106996293.end.GGT1.start.28595839-28635852.Rhesus      tacagatgaggtaaggcagggctcccctctcactgtctgaatccccagcgcaccctgtgc     29036
BCRP3.HUMAN.NCBI.REF                                      ------------------------------------------------------------     16669

LOC106996293.end.GGT1.start.28595839-28635852.Rhesus      catgccaggcctgggccaccacgtgtcagtgggtgagaggtcagtttcctggtcaaggcc     29096
BCRP3.HUMAN.NCBI.REF                                      ------------------------------------------------------------     16669

LOC106996293.end.GGT1.start.28595839-28635852.Rhesus      tggacatgagggagcgggcacaggtttacaggggggtccaagaggggcaggggcagcttgg     29156
BCRP3.HUMAN.NCBI.REF                                      ------------------------------------------------------------     16669

LOC106996293.end.GGT1.start.28595839-28635852.Rhesus      cagctcatcaggtccactagagggtgtcccagacaagaacagcccccgaactgaagtcac     29216
BCRP3.HUMAN.NCBI.REF                                      ------------------------------------------------------------     16669

LOC106996293.end.GGT1.start.28595839-28635852.Rhesus      caaacaggttggattctcagagctctgattggttggacaaggtgttctgggtagaggaac     29276
BCRP3.HUMAN.NCBI.REF                                      ------------------------------------------------------------     16669

LOC106996293.end.GGT1.start.28595839-28635852.Rhesus      agtctatgcaaaggctcagaggaagtgcagtcgtatctgcggaatggcaggggtgggggc     29336
BCRP3.HUMAN.NCBI.REF                                      ------------------------------------------------------------     16669

LOC106996293.end.GGT1.start.28595839-28635852.Rhesus      cagccaaggccacccctaccaggccaaggcagttagacttcatcctaagagcaaagtgtg     29396
BCRP3.HUMAN.NCBI.REF                                      ------------------------------------------------------------     16669

LOC106996293.end.GGT1.start.28595839-28635852.Rhesus      gcctgcctcgggctcccccacccccaccaatctccctccaggatgggcccttggtctgcc     29456
BCRP3.HUMAN.NCBI.REF                                      ------------------------------------------------------------     16669

LOC106996293.end.GGT1.start.28595839-28635852.Rhesus      cccgcacctcccctttccagcaggagaaagggtgccctgtctgtggccctagtcccagac     29516
```

```
BCRP3.HUMAN.NCBI.REF                                      ------------------------------------------------------------     16669

LOC106996293.end.GGT1.start.28595839-28635852.Rhesus      atcagatgttcccagtgacaggggcgggccagcctgggaagccccaccacctgccagggg     29576
BCRP3.HUMAN.NCBI.REF                                      ------------------------------------------------------------     16669

LOC106996293.end.GGT1.start.28595839-28635852.Rhesus      cttttcctggggcctgaaagagacagaggcagggcctgtgcaaaggcggaaggcctggag     29636
BCRP3.HUMAN.NCBI.REF                                      --------------------------------ggccttaacaaagagctcagagctttgg     16697
                                                                                          *****   *****     **  **   *

LOC106996293.end.GGT1.start.28595839-28635852.Rhesus      ccggtgcagactctctagagacatcaaaggcggtgacctccgcctcagggggatggtctc     29696
BCRP3.HUMAN.NCBI.REF                                      ccgtggccagcagtgcacttggacccccctcttccctcccaagcacatcatgaagacctc     16757
                                                          ***  **   *  *  *     * *     *      * *   * **    ** *  ***

LOC106996293.end.GGT1.start.28595839-28635852.Rhesus      ccaaacagcagcccagggcttgcaggacgggcagccatgggagctttgcgggtggttcac     29756
BCRP3.HUMAN.NCBI.REF                                      cc---catcagcccagagctggcc------------------------------------     16778
                                                          **   ** ******** *** **

LOC106996293.end.GGT1.start.28595839-28635852.Rhesus      ggtggggttgaaatgacagagtatcagagtcacccagtggcctgtaccgtcgtggtggta     29816
BCRP3.HUMAN.NCBI.REF                                      ------------------------------------------------------------     16778

LOC106996293.end.GGT1.start.28595839-28635852.Rhesus      ccaatcagagtggggcagatggtaacccagggctttggaaacacaggtgatgagagagct     29876
BCRP3.HUMAN.NCBI.REF                                      ------------------------------------------------------------     16778

LOC106996293.end.GGT1.start.28595839-28635852.Rhesus      ggggggtgtaacaggagctgatgaggtccctgcaggatcccaagacccaccttgggcaga     29936
BCRP3.HUMAN.NCBI.REF                                      ------------------------------------------------------------     16778

LOC106996293.end.GGT1.start.28595839-28635852.Rhesus      agggcaaagggaggaaggagtgtgctatgggagccaggtccaggtccccatggaagtggg     29996
BCRP3.HUMAN.NCBI.REF                                      ------------------------------------------------------------     16778

LOC106996293.end.GGT1.start.28595839-28635852.Rhesus      tgtcacacaggcctgtgggccgaggggagtcacagaggtgacagcccagctgcaggtccc     30056
BCRP3.HUMAN.NCBI.REF                                      ---------------------------------------------------ccttgtcct     16787
                                                                                                              *  ****

LOC106996293.end.GGT1.start.28595839-28635852.Rhesus      cgttgactagagcagggaagccccacttctctgtgccccctgccctccagcctcccacta     30116
BCRP3.HUMAN.NCBI.REF                                      gggccactgagacccagaagtaccaaggctggagtcagcttgcagcacagccagggtcga     16847
                                                           *   ***    *   ****  ***   **     *  * ***    *****     * *

LOC106996293.end.GGT1.start.28595839-28635852.Rhesus      ggtctcttgttggctga-------------gcacagtgggtgcggctgacctgggggcca     30163
BCRP3.HUMAN.NCBI.REF                                      ggtcactccctccctgaggactctagcacggcacagcccctctgcctctctcctggtggt     16907
                                                          **** **   *  ****             ******    *  * **  *    **

LOC106996293.end.GGT1.start.28595839-28635852.Rhesus      ggatctgactcagtacagtacagtacagccacagctggggagcgagggaccccctcctct     30223
BCRP3.HUMAN.NCBI.REF                                      ggcgttgaaacagcaccctctgcttcggtcctctacagggtggcagagaaggaggcggtc     16967
                                                          **   ***  *** **  *    * * * *       *** *  ** **      *

LOC106996293.end.GGT1.start.28595839-28635852.Rhesus      gccacgctgcttccttctgcaagctccagggtggccgagtgcctgctgtgggccagagct     30283
BCRP3.HUMAN.NCBI.REF                                      aataaggtg--tccctgcacaacctcgccactgtctttggccccacgctgctccggccct     17025
                                                             * * **  *** *   *** ***     ** *   *  **  *  **  ** *  **

LOC106996293.end.GGT1.start.28595839-28635852.Rhesus      ccactaggcccatcaggacccccctgtcctcagggtccagtgctgccaaggaggagggag     30343
BCRP3.HUMAN.NCBI.REF                                      ccgagaaggagagcaagctccctgccaaccccagccagcctgtcaccatgactgacagca     17085
                                                          **   * *   * ** *  ***      * *  *      **   *** *   **  *

LOC106996293.end.GGT1.start.28595839-28635852.Rhesus      gggtcggtgcagcgcattgtaggagtgggaagtcagggaggcgtcctggaggagtccggc     30403
BCRP3.HUMAN.NCBI.REF                                      gg----------------------------------------------------------     17087
                                                          **

LOC106996293.end.GGT1.start.28595839-28635852.Rhesus      ttcccacaggccccctccaggctttcgccagctccctttgttgcaaggatgtaaggatga     30463
BCRP3.HUMAN.NCBI.REF                                      ------------------------------------------------------------     17087

LOC106996293.end.GGT1.start.28595839-28635852.Rhesus      ctgactttggaagccatgccaaccaagtccctgctgacctgatctcccaccactactgtt     30523
BCRP3.HUMAN.NCBI.REF                                      ------------------------------------------------------------     17087

LOC106996293.end.GGT1.start.28595839-28635852.Rhesus      ccaggaccaggaaataaacttcttttctgattaagccttaatatgtccaattaagcctta     30583
BCRP3.HUMAN.NCBI.REF                                      ------------------------------------------------------------     17087

LOC106996293.end.GGT1.start.28595839-28635852.Rhesus      atatggaccatttgttcaagcagttggttggtcctgagaaaggaaagaaacttctctttg     30643
BCRP3.HUMAN.NCBI.REF                                      ------------------------------------------------------------     17087

LOC106996293.end.GGT1.start.28595839-28635852.Rhesus      gtgagacagggtctcactctgccacccaggctggagtgcagtggtgcgatcatagctcat     30703
BCRP3.HUMAN.NCBI.REF                                      ------------------------------------------------------------     17087

LOC106996293.end.GGT1.start.28595839-28635852.Rhesus      ggccgcctcaacctcccaggctcaggtgatcctcctgcctcagcctcacaagtagctggg     30763
BCRP3.HUMAN.NCBI.REF                                      ------------------------------------------------------------     17087

LOC106996293.end.GGT1.start.28595839-28635852.Rhesus      gccacaggcacgcccaggctggtcttgaactcctgggatcaagcaatcctcctgcctcgg     30823
BCRP3.HUMAN.NCBI.REF                                      -------------------------------------------------tccttggaggt     17098
                                                                                                           ****     *

LOC106996293.end.GGT1.start.28595839-28635852.Rhesus      cctcccaaaatgctgggattacaggcacgatccactgcacccggccaaaaataaactttt     30883
BCRP3.HUMAN.NCBI.REF                                      catgtctcaggtatgggaagacagtctccagcccatgca---------------------     17137
```

```
                                                          * *  *  *    *****  **** * * * **  ****

LOC106996293.end.GGT1.start.28595839-28635852.Rhesus      tacctgaggaatgagtgccccctttaattatcaggcccagagaggcattaaaatgtgaca     30943
BCRP3.HUMAN.NCBI.REF                                      ------------------------------------------------------------     17137


LOC106996293.end.GGT1.start.28595839-28635852.Rhesus      gggtgtcacagcagtcgtgcctcactcccaccttgatgccacttgctaggtgggttctag     31003
BCRP3.HUMAN.NCBI.REF                                      ------------------------------------------------------------     17137


LOC106996293.end.GGT1.start.28595839-28635852.Rhesus      actaaccgatgtcaagtagccataaaatgccatgtgctggacaccatgatgtaggggcca     31063
BCRP3.HUMAN.NCBI.REF                                      ------------------------------------------------------------     17137


LOC106996293.end.GGT1.start.28595839-28635852.Rhesus      caggaaaacttccccttcaccctctgaaggttcaaggaaaaatcagctcacaaaaggcag     31123
BCRP3.HUMAN.NCBI.REF                                      ------------------------------------------------------------     17137


LOC106996293.end.GGT1.start.28595839-28635852.Rhesus      attaattggaaaacaagccatgcaaatttattagcatgcattggggagaatcatagagtc     31183
BCRP3.HUMAN.NCBI.REF                                      ------------------------------------------------------------     17137


LOC106996293.end.GGT1.start.28595839-28635852.Rhesus      attgccgaatatcccagataaagatgcttatataccccgcttcttaggggaaagggagat     31243
BCRP3.HUMAN.NCBI.REF                                      ------------------------------------------------------------     17137


LOC106996293.end.GGT1.start.28595839-28635852.Rhesus      gggaatctctcctttgatactcagctgcagccagactcctgcaggaagacccccaacctc     31303
BCRP3.HUMAN.NCBI.REF                                      ------------------------------------------------------------     17137


LOC106996293.end.GGT1.start.28595839-28635852.Rhesus      ccactcctacaacgcactacactggccactccctcctcctcggcagcactggaccctgag     31363
BCRP3.HUMAN.NCBI.REF                                      -----------------------------------------------accccagcctgac     17150
                                                                                                         **   * *****

LOC106996293.end.GGT1.start.28595839-28635852.Rhesus      gggaggggtctcctgatggcccacagcgggcacttgatcaccccgggctggcaggagatg     31423
BCRP3.HUMAN.NCBI.REF                                      agaggtggcctctgcctgccccacccccagtcctgcccatcttccgacttgcattgtatg     17210
                                                           *  * ** ***    ** *****  *  *  **      *  * * ** ***    ***

LOC106996293.end.GGT1.start.28595839-28635852.Rhesus      ggagtgaggatga---tttcagaaggacaagtacatgatttgggctggggaggatttttg     31480
BCRP3.HUMAN.NCBI.REF                                      tggtggtggctgagattcagagagagggacttgcctaggtttgcatggatgggagtgat-     17269
                                                           *   * ** ***   *   ***  *  *  * * *   ** *  ***   *** *  *

LOC106996293.end.GGT1.start.28595839-28635852.Rhesus      tttgtttgttgtaagagggagtctacctctgtcaccagcctggaccacccagctacttgg     31540
BCRP3.HUMAN.NCBI.REF                                      -------------agggggtgcccaggccacctcctggtcctgctggtgcaccttgctgg     17316
                                                                       ** *** * * *   *     *  * *  *      ** **   ***

LOC106996293.end.GGT1.start.28595839-28635852.Rhesus      gagattgagccactgcccctggcctgggtggggagaatttaattggtttaaagaacgtac     31600
BCRP3.HUMAN.NCBI.REF                                      gggctt------------------------------------------------------     17322
                                                          * * **

LOC106996293.end.GGT1.start.28595839-28635852.Rhesus      agggccgggcgcggtggctcatgcctgtaatcccagcactttgcgaagctgaggcgggcg     31660
BCRP3.HUMAN.NCBI.REF                                      ------------------------------------------------------------     17322


LOC106996293.end.GGT1.start.28595839-28635852.Rhesus      gatcaggagatcaggagatcgagatcattttggctaacatggtgaaaccccgtctctact     31720
BCRP3.HUMAN.NCBI.REF                                      ------------------------------------------------------------     17322


LOC106996293.end.GGT1.start.28595839-28635852.Rhesus      aaaactacaaaaaattagccaggtgtggtggggggcatctgtagtcacagctactcagga     31780
BCRP3.HUMAN.NCBI.REF                                      ------------------------------------------------------------     17322


LOC106996293.end.GGT1.start.28595839-28635852.Rhesus      ggctgaggcaggagactcgcatgaactcaggaggtggagcttgcagtgagccgagattgc     31840
BCRP3.HUMAN.NCBI.REF                                      ------------------------------------------------------------     17322


LOC106996293.end.GGT1.start.28595839-28635852.Rhesus      accactgcacaccagcctgggcgacagagcgagaccccgtctcaaaaaacaaaaacaaaa     31900
BCRP3.HUMAN.NCBI.REF                                      ------------------------------------------------------------     17322


LOC106996293.end.GGT1.start.28595839-28635852.Rhesus      caaacaaaaaaaacatacaatggggctgggcacagtggttcatgcttgtaatcccagcac     31960
BCRP3.HUMAN.NCBI.REF                                      --------aaaaccaccccaagtgttcgggtgtggtggctcatgcctgtaatcccagcac     17374
                                                                  **** **  * * * *   ***    **** ****** **************

LOC106996293.end.GGT1.start.28595839-28635852.Rhesus      tttgggaggccgaggcagggggattgcgaggtcaggagtttcagaccagcctggccaaca     32020
BCRP3.HUMAN.NCBI.REF                                      tttgggaggccgaggcaggacaact--gaacccaggtgtttgagaccagtctgggcaatg     17432
                                                          *******************   * *  **   **** **** ******* **** ***

LOC106996293.end.GGT1.start.28595839-28635852.Rhesus      ttgtgaaatcccgcctctactaaaaatataaaaaattagccgggtgtggtggtgtgcacc     32080
BCRP3.HUMAN.NCBI.REF                                      tag---------------------------------------------------------     17435
                                                          * *

LOC106996293.end.GGT1.start.28595839-28635852.Rhesus      tgtaatcccagctacttgggaggctgaggcaggagaatcacctgaatctgggaggcacag     32140
BCRP3.HUMAN.NCBI.REF                                      ------------------------------------------------------------     17435


LOC106996293.end.GGT1.start.28595839-28635852.Rhesus      gttgcagtgagccaagatcatgccactgcacgccagcctgggcaacagtgtgagactcgg     32200
BCRP3.HUMAN.NCBI.REF                                      ------------------------------------------------------------     17435


LOC106996293.end.GGT1.start.28595839-28635852.Rhesus      tctcaaaaaaaaaaaaagaacatacaaacatataatgggcctggctcggtggctcatgcc     32260
BCRP3.HUMAN.NCBI.REF                                      ------------------------------------------------------------     17435
```

```
LOC106996293.end.GGT1.start.28595839-28635852.Rhesus      tgtaatcccagcactccagagggtgaagcaggcagattatgagatcaatagatcaagacc     32320
BCRP3.HUMAN.NCBI.REF                                      ------------------------------------------------------------     17435

LOC106996293.end.GGT1.start.28595839-28635852.Rhesus      atcctggccaacatggtgaaaccccatctttatgaaaaatac----aaaaattagccagg     32376
BCRP3.HUMAN.NCBI.REF                                      -----------------caaaccccatctctagaaaaaatacaaagaaaaattagtcagg     17478
                                                                            *********** **  ********    ********* ****

LOC106996293.end.GGT1.start.28595839-28635852.Rhesus      cgtggtggtgcacacctgtaatcccagctacttggaaggctgaggcaggagaatggcttg     32436
BCRP3.HUMAN.NCBI.REF                                      cattgtggcacacatctgtaatcctaggtatctgggaggctgacacaggaggattgcttg     17538
                                                          * * ****  **** ********* ** **  *** *******  ****** ** *****

LOC106996293.end.GGT1.start.28595839-28635852.Rhesus      cacctgggaggcggagattgcagtgagccaagatcgcaccactgcacgccagcct-ggtg     32495
BCRP3.HUMAN.NCBI.REF                                      agcccaggagttagaggctgcagtgatccatgatggagccactgtactccagcctggggg     17598
                                                            **  ****   ***  ******** *** *** *  ****** ** ******* ** *

LOC106996293.end.GGT1.start.28595839-28635852.Rhesus      acagagcaagac----tccatctcaaaaacaaacaaacaaaaacaaacaaaaccatacag     32551
BCRP3.HUMAN.NCBI.REF                                      acagagcaaggccctgtgcatctctaaaataaataatca---------------------     17637
                                                          ********** *    * ****** **** *** ** **

LOC106996293.end.GGT1.start.28595839-28635852.Rhesus      cgccctgggacaaagtctgttgggcctgaagagcaaacaatagattgtgacgagtctgtc     32611
BCRP3.HUMAN.NCBI.REF                                      ------------------------------------------------------------     17637

LOC106996293.end.GGT1.start.28595839-28635852.Rhesus      caggtgtgttgacagttttcagtctttcttcctgtgacaggagttcagttaaggaagact     32671
BCRP3.HUMAN.NCBI.REF                                      ------------------------------------------------------------     17637

LOC106996293.end.GGT1.start.28595839-28635852.Rhesus      cacaggaggggccagagctcatggttttcttctttggcagatccagacttcaggcagata     32731
BCRP3.HUMAN.NCBI.REF                                      ------------------------------------------------------------     17637

LOC106996293.end.GGT1.start.28595839-28635852.Rhesus      aggaacttcagagaacaacttcatcctgagctctggctgacagactgagaccgaggcggg     32791
BCRP3.HUMAN.NCBI.REF                                      ------------------------------------------------------------     17637

LOC106996293.end.GGT1.start.28595839-28635852.Rhesus      gagaggtcagagaccttgtggcttcttcttcagttcagtgccatattttggggtatcagt     32851
BCRP3.HUMAN.NCBI.REF                                      ------------------------------------------------------------     17637

LOC106996293.end.GGT1.start.28595839-28635852.Rhesus      ttctgagccccagcaataactcatactctatagttcaacagtaaccagccaggcaaggag     32911
BCRP3.HUMAN.NCBI.REF                                      ------------------------------------------------------------     17637

LOC106996293.end.GGT1.start.28595839-28635852.Rhesus      cagtggctcacatctgtaatcctagtgctctgggagatggaggcaagaggatcgcttgag     32971
BCRP3.HUMAN.NCBI.REF                                      ------------------------------------------------------------     17637

LOC106996293.end.GGT1.start.28595839-28635852.Rhesus      gccaggagttcaagaccagtatgggcaacatagtggagcctgcctacaaacacacacaca     33031
BCRP3.HUMAN.NCBI.REF                                      ---------------------------------------------ccccccacccaacaa     17652
                                                                                                        *   *** **   *

LOC106996293.end.GGT1.start.28595839-28635852.Rhesus      cacacatttagccatgacagcatgcacctgtaatcgcagctactcaggagactgaggcaa     33091
BCRP3.HUMAN.NCBI.REF                                      gtcatgccttgtcaggaccccaccccacccccgtctcactgtaaggggttcatgacacca     17712
                                                            **    * * ** ***  **  *  *     ** **        **    ***  * *

LOC106996293.end.GGT1.start.28595839-28635852.Rhesus      gaggatcatttgagccctggaggatgag--gctgcaatgagccatgatgacaccactgcc     33149
BCRP3.HUMAN.NCBI.REF                                      gcaggggtttctagcacctgaggtggacttgggggcttgggccccaaagacctccccacc     17772
                                                          *  *    **  *** *  ****  **   *  *   ** ***   * ***  * *  **

LOC106996293.end.GGT1.start.28595839-28635852.Rhesus      aacaga-gcgagatctcatctcaaaaaataaaataaaataaagccaatcactaacgcgtg     33208
BCRP3.HUMAN.NCBI.REF                                      agcagctgtgagcccccctctgagccact-------------------------------     17801
                                                          * ***  * ***  * * *** *   * *

LOC106996293.end.GGT1.start.28595839-28635852.Rhesus      ttattgaaactgcctttgcaaaaaagcataattgaggaaattatgacagtgaaagaaatc     33268
BCRP3.HUMAN.NCBI.REF                                      ------------------------------------------------------------     17801

LOC106996293.end.GGT1.start.28595839-28635852.Rhesus      agaggtgaccaactctccaagagtctgaacctcctcaaattgctgctggggataacatca     33328
BCRP3.HUMAN.NCBI.REF                                      ------------------------------------------------------------     17801

LOC106996293.end.GGT1.start.28595839-28635852.Rhesus      ctattgtaaaacttgagatcagggcttgagatattttgcagaccctgcactccatggatc     33388
BCRP3.HUMAN.NCBI.REF                                      ------------------------------------------------------------     17801

LOC106996293.end.GGT1.start.28595839-28635852.Rhesus      agctgacgccacccagactgctattctggctcaaccagttctgccatcacagccaggaac     33448
BCRP3.HUMAN.NCBI.REF                                      ------------------------------------------------------------     17801

LOC106996293.end.GGT1.start.28595839-28635852.Rhesus      agaagacagcaagaaaaactcatttccacccgctgtgattccatcttcaacctgaccaat     33508
BCRP3.HUMAN.NCBI.REF                                      ------------------------------------------------------------     17801

LOC106996293.end.GGT1.start.28595839-28635852.Rhesus      caacactccccatttcccaagcccctacctgccaaattatctttaaaaactttggtcagg     33568
BCRP3.HUMAN.NCBI.REF                                      ------------------------------------------------------------     17801

LOC106996293.end.GGT1.start.28595839-28635852.Rhesus      cgcctagactcatgtcagtaatcccagtaatccctgcagtttgggagactgaggcaggcg     33628
BCRP3.HUMAN.NCBI.REF                                      ------------------------------------------------------------     17801
```

```
LOC106996293.end.GGT1.start.28595839-28635852.Rhesus      gatcacttgaggtcaggagttcaagatcagcctggccaacacggtgaaacctcgtctcta      33688
BCRP3.HUMAN.NCBI.REF                                      ------------------------------------------------------------      17801

LOC106996293.end.GGT1.start.28595839-28635852.Rhesus      ctaaaatcgcaataattagctggatgtggtggcacatgcctgtagtcccagctgctcagg      33748
BCRP3.HUMAN.NCBI.REF                                      ------------------------------------------------------------      17801

LOC106996293.end.GGT1.start.28595839-28635852.Rhesus      aggctgaggcatgagaatcacttgaacccagcaggtggaggttgtagtgagcgtctcaaa      33808
BCRP3.HUMAN.NCBI.REF                                      ------------------------------------------------------------      17801

LOC106996293.end.GGT1.start.28595839-28635852.Rhesus      aaaacaaaaagcaaaaaacaaaaaactctgatccccgaatccttggggagactgatttga      33868
BCRP3.HUMAN.NCBI.REF                                      ------------------------------------------------------------      17801

LOC106996293.end.GGT1.start.28595839-28635852.Rhesus      gcgataataaaactccgattgactctgcatgaattactctttgtccactgcgactcccct      33928
BCRP3.HUMAN.NCBI.REF                                      ------------------------------------------------------------      17801

LOC106996293.end.GGT1.start.28595839-28635852.Rhesus      gtcttgaaaaataggctctgtctaggcagctggcaaggtgaacccattgcagttacatta      33988
BCRP3.HUMAN.NCBI.REF                                      ------------------------------------------------------------      17801

LOC106996293.end.GGT1.start.28595839-28635852.Rhesus      cctctctaaaccactgagaattcctgctaaacaattttatatgaaaccactccctatcct      34048
BCRP3.HUMAN.NCBI.REF                                      ------------------------------------------------------------      17801

LOC106996293.end.GGT1.start.28595839-28635852.Rhesus      cttttactatttatttatttatttttttgagccagaatctcgctttgtcacccaggctgc      34108
BCRP3.HUMAN.NCBI.REF                                      ------------------------------------------------------------      17801

LOC106996293.end.GGT1.start.28595839-28635852.Rhesus      agtgcagtggcgcgatcttggctcattgcagcctccacctcctgggttcaagggattctt      34168
BCRP3.HUMAN.NCBI.REF                                      ------------------------------------------------------------      17801

LOC106996293.end.GGT1.start.28595839-28635852.Rhesus      ctgcctcagcctcccaagtagctgggaccaccagtggtgtgcgccaccactcccagctaa      34228
BCRP3.HUMAN.NCBI.REF                                      ------------------------------------------------------------      17801

LOC106996293.end.GGT1.start.28595839-28635852.Rhesus      tttttgtttgtttgtttgttttgagacgtagttttgctcttgttgcccagctggactgca      34288
BCRP3.HUMAN.NCBI.REF                                      ------------------------------------------------------------      17801

LOC106996293.end.GGT1.start.28595839-28635852.Rhesus      atggcacaatcttggctcaccaaaacctctgcctcccaggttcaagcgattctcctgcct      34348
BCRP3.HUMAN.NCBI.REF                                      ------------------------------------------------------------      17801

LOC106996293.end.GGT1.start.28595839-28635852.Rhesus      cagcctcccaagtagctgggattgcaggcatgtgccaccacacccaactaatctaatttt      34408
BCRP3.HUMAN.NCBI.REF                                      ------------------------------------------------------------      17801

LOC106996293.end.GGT1.start.28595839-28635852.Rhesus      gtatcgttagtagagatggggtttcactctgttggccaggctggtcttaaactcccaacc      34468
BCRP3.HUMAN.NCBI.REF                                      ------------------------------------------------------------      17801

LOC106996293.end.GGT1.start.28595839-28635852.Rhesus      tcaggtgatctgcccaccttggcctcccaaagtgctgagattacaggtgtgtgccaccat      34528
BCRP3.HUMAN.NCBI.REF                                      ------------------------------------------------------------      17801

LOC106996293.end.GGT1.start.28595839-28635852.Rhesus      gcccagtctccttatcctcttttgtctttaaagaaactgcttttgggccgggcgcggtgg      34588
BCRP3.HUMAN.NCBI.REF                                      ------------------------------------ctcctcttccccactctgcgaggg      17825
                                                                                              ** ** **   *     ***  **

LOC106996293.end.GGT1.start.28595839-28635852.Rhesus      ctcaagcctgtaatcccagcactttgggaggccgaggcgggcggatcacaaggtcacgag      34648
BCRP3.HUMAN.NCBI.REF                                      caggacgaggtgctgctgtacttcttgcggctggaggccatccctgccctgaacagcaag      17885
                                                          *   *    **  * *      * * *  *   *****   *    * *       * **

LOC106996293.end.GGT1.start.28595839-28635852.Rhesus      atcgagaccacagtgaaaccccgtctctactaaaaatacaaaaaattagccgggtgcggt      34708
BCRP3.HUMAN.NCBI.REF                                      agacagagcatcctgttctccaccgatgtctaaaggtcccagtccatctcctggaggcgg      17945
                                                          *   *** **   **    **        *****  * * *     *  ** ** *  *

LOC106996293.end.GGT1.start.28595839-28635852.Rhesus      ggcgggcgcctg------------tagtcccagctactcaggaggctgaggcaggagaat      34756
BCRP3.HUMAN.NCBI.REF                                      acagatggcctggaaacctctggctaatcgggccatctgtagagtgggaatcaagatttt      18005
                                                             *   *****            ** **    *  **   ***   **  ** **   *

LOC106996293.end.GGT1.start.28595839-28635852.Rhesus      ggtgggaacccgggaggcggagcttgcagtgagccgagatcgcgccactgcactccagcc      34816
BCRP3.HUMAN.NCBI.REF                                      ctgaggcatccttgggccacccccaggtgtcaggcca-----------------------      18042
                                                              ** * **  * * *    *  *  ** ** * *

LOC106996293.end.GGT1.start.28595839-28635852.Rhesus      tgggcaacagcgtgagactccgtctcaaaaaaaaaaaaaaaaagaaactgcttttgtggcc      34876
BCRP3.HUMAN.NCBI.REF                                      ------------------tctgccaagagacagcggcccaaagcagaaggacaggtggcc      18084
                                                                            ** * *   * * *       **** *   *     ******

LOC106996293.end.GGT1.start.28595839-28635852.Rhesus      aggcatggtggctcacgtctgtaatctcaccactttgggaggccaaggcaggtggatcat      34936
BCRP3.HUMAN.NCBI.REF                                      tgggcagatcccgcccaggtctgaaagccccaggctggcctcagactgtgggtttttat      18144
                                                           **   * *  * * *   * * *   * ***   ***      *  *  ***   * **

LOC106996293.end.GGT1.start.28595839-28635852.Rhesus      gaggtcaggagttcaagatcagcctggccgagatggtgaaacctcatctctactaaaaat      34996
BCRP3.HUMAN.NCBI.REF                                      gtggcca-----------------------------------------------------      18151
                                                          * ** **

LOC106996293.end.GGT1.start.28595839-28635852.Rhesus      acaaaacgttagctgggcatggtggtgggcgcctgtaagctactcgtgaggctgaggcag      35056
```

```
BCRP3.HUMAN.NCBI.REF                                      ------------------------------------------------------------      18151

LOC106996293.end.GGT1.start.28595839-28635852.Rhesus      agaactgctcgaacccggaaggcagaagttgcagtgagccaagatcatgccactgcactc      35116
BCRP3.HUMAN.NCBI.REF                                      ------------------------------------------------------------      18151

LOC106996293.end.GGT1.start.28595839-28635852.Rhesus      cagcctgggtgacagagtgaaactgtctcaaaaagcaacaacaacaacaataaaaac      35176
BCRP3.HUMAN.NCBI.REF                                      ------------------------------------------------------------      18151

LOC106996293.end.GGT1.start.28595839-28635852.Rhesus      aaaacaaaactgcttttgggccaggcatggtggttcatgcctgtaatcatagcactttgg      35236
BCRP3.HUMAN.NCBI.REF                                      ------------------------------------------------------------      18151

LOC106996293.end.GGT1.start.28595839-28635852.Rhesus      gaggctgagttgggaggatcacctgagttcagaaattggaggccagactgggcaacatag      35296
BCRP3.HUMAN.NCBI.REF                                      ---cccgagggcgccccaagccagttcatctcggagtccaggcctggccctgggagacag      18208
                                                              * ***   *    *   *      **    * *  ***** * *   *  * * **

LOC106996293.end.GGT1.start.28595839-28635852.Rhesus      tgagacacca-tctctttaaaaacaaaaacaaacaaacaaacaaaaactgcttgtaacaa      35355
BCRP3.HUMAN.NCBI.REF                                      ggtgaaagcagtggtttttatgaacttaacttatagagtccaaaagatttctactgaatc      18268
                                                           * ** * ** *   *** *  *    ***  * * *     *** * * **  * *

LOC106996293.end.GGT1.start.28595839-28635852.Rhesus      aggtcgaatgaagcactccccaaggcaacttggaagtgtgtctggggccactgttctcaa      35415
BCRP3.HUMAN.NCBI.REF                                      acttgtcaagaagcgccctctctggggagaagggaacgtgactggattccc---tcactg      18325
                                                          *  *   * ***** * * *   **  *   ** *  *** ****   * *   ** *

LOC106996293.end.GGT1.start.28595839-28635852.Rhesus      ccttggcccaaataaactatactaattttgcctcagtttcttcctttaggtcaatagtcc      35475
BCRP3.HUMAN.NCBI.REF                                      ttgtatcttgaataaacgctgct------------gcttcatcctgtgggggccgtggcc      18373
                                                             *  *   *******  * **            * *** **** * **      * **

LOC106996293.end.GGT1.start.28595839-28635852.Rhesus      ctgtaacaatacaccaaaggcataggtataaaaagatggccttacctttttcattagtgt      35535
BCRP3.HUMAN.NCBI.REF                                      ctgtccctgtgtgggtggggcctc------------------ttccatttccctgactta      18415
                                                          ****  *  *        *** *                   * ** *** * * * *

LOC106996293.end.GGT1.start.28595839-28635852.Rhesus      ggaaaataaatactgtgtgtgcaaggtgaaaaaaatctaaacagaacaattcagggaact      35595
BCRP3.HUMAN.NCBI.REF                                      gaaaccacactccacttctaacagggtttgagaggcttggtcagcactgggtagcg----      18471
                                                          * **    * * *  *     ** ***   * *    *   *** **      * *

LOC106996293.end.GGT1.start.28595839-28635852.Rhesus      agcccctggctaggggacattgaagctgtgctcagccttttgcttctttaaacactatgg      35655
BCRP3.HUMAN.NCBI.REF                                      ------------------------------------------------------------      18471

LOC106996293.end.GGT1.start.28595839-28635852.Rhesus      tactgtgtaaataagttcaggttaacctggaggagaaattcacacgagtagatcagctgg      35715
BCRP3.HUMAN.NCBI.REF                                      ------------------------------------------------------------      18471

LOC106996293.end.GGT1.start.28595839-28635852.Rhesus      ttaaatggcacaagcattttctgtctcaagttatactggtgtaatctcagctcactgaaa      35775
BCRP3.HUMAN.NCBI.REF                                      ------------------------------------------------------------      18471

LOC106996293.end.GGT1.start.28595839-28635852.Rhesus      cctctgctgagttcaagccattctgcaaatattcacccattgtgagttgtcttttcactg      35835
BCRP3.HUMAN.NCBI.REF                                      ----------------------------ttttgactccattcttggctttcttctttttc      18503
                                                                                       * *    ***** *  * * **** *   *

LOC106996293.end.GGT1.start.28595839-28635852.Rhesus      tttcagtgatgtcttttgttgaacagaatttcttaattttcatatagtccaatttatcat      35895
BCRP3.HUMAN.NCBI.REF                                      tttccagaaggatttttgtgcagaa-----------------------------------      18528
                                                          ****    * *  ******  *  *

LOC106996293.end.GGT1.start.28595839-28635852.Rhesus      tttttcttctatactagcttttgtgtcctgtttaagaaaaaatttggtatgcaacaaa      35955
BCRP3.HUMAN.NCBI.REF                                      ------------------------------------------------------------      18528

LOC106996293.end.GGT1.start.28595839-28635852.Rhesus      ataaaaaaacagaatttaaaaaagagaagcatttacctactctaaggtcacaaagatact      36015
BCRP3.HUMAN.NCBI.REF                                      ------------------------------------------------------------      18528

LOC106996293.end.GGT1.start.28595839-28635852.Rhesus      ctctttctttctgtaaatactacttaatattttatttgattatttatttatttatttatg      36075
BCRP3.HUMAN.NCBI.REF                                      ------------------------------------------------------------      18528

LOC106996293.end.GGT1.start.28595839-28635852.Rhesus      tatttttggagatgggggtctcactacgttgcccaggctggtttcgaactccttggctca      36135
BCRP3.HUMAN.NCBI.REF                                      ------------------------------------------------------------      18528

LOC106996293.end.GGT1.start.28595839-28635852.Rhesus      agtgatcactcggttgctgtatttttagtacagacaggatttcaccatgctgtcctggct      36195
BCRP3.HUMAN.NCBI.REF                                      ----atgggtcttttgttgccgtgttagtcctccttgga-------aggcagctcagaag      18577
                                                              **   **  *** **   * ***** *     ***       * ** *  * *

LOC106996293.end.GGT1.start.28595839-28635852.Rhesus      gatctcgaactcttgacctcgtgatctgcccgcctggggctcctaaagtgctaggattac      36255
BCRP3.HUMAN.NCBI.REF                                      gcctgtgaaatgtcgggggacaggacccccagggagggaatcccaggctacgcaccttag      18637
                                                          *     *** * * *       *  *  ** *   ***  *** *   * *     ***

LOC106996293.end.GGT1.start.28595839-28635852.Rhesus      agatgtgagccaccgcgcctggcttgtttgtttttgagatggagtcttgctctgtcaccc      36315
BCRP3.HUMAN.NCBI.REF                                      ------------------------------------------------------------      18637

LOC106996293.end.GGT1.start.28595839-28635852.Rhesus      aggctggagtgcagtggtgcgatctccactcaatgcaacatcttccttccaggttcaagc      36375
BCRP3.HUMAN.NCBI.REF                                      ---------------ggttcgttctccagggagagcgacctcgtcccccgatcctgaccg      18682
                                                                         *** ** ******   *  ** ** ***  * *   * *

LOC106996293.end.GGT1.start.28595839-28635852.Rhesus      aattctcctgcctcagtctcccaatcagctgagatcacagatgcctgcaaccacaactgg      36435
BCRP3.HUMAN.NCBI.REF                                      cccttccggcccacgctctcc---------------------------------------      18703
```

```
                                                             *  *   ** *  *****

LOC106996293.end.GGT1.start.28595839-28635852.Rhesus      ctaatttttttgtatttttagtagagacaaggtttcaccatgttggccaggatgctctta     36495
BCRP3.HUMAN.NCBI.REF                                      --------------tgtttggcttccacaggcctggacttctctggcttctctgcccaca     18749
                                                                        * *** *     *** *  *  **     ****     *** *  *

LOC106996293.end.GGT1.start.28595839-28635852.Rhesus      aactcctgacctcaagtaatccacctacctcagccttccaaagtgctgggattgcaggca     36555
BCRP3.HUMAN.NCBI.REF                                      cactccctgcccccagt-gtccctgcccctgccccagcacaggtgacttcatttctgtcc     18808
                                                           *****   ** * ***  ***     ***   **  *  * ***     *** * * *

LOC106996293.end.GGT1.start.28595839-28635852.Rhesus      gtgagccacagtggctggcctatttattttgatactt-----------------------     36592
BCRP3.HUMAN.NCBI.REF                                      tctcagctcagtggactcgctcatcttttgtataagtctccacttggtggcagcagcttg     18868
                                                                * ******     **  *  ***  ***  *

LOC106996293.end.GGT1.start.28595839-28635852.Rhesus      ------------------aatttttcttcttcttttcttttcctttttttttttttt     36634
BCRP3.HUMAN.NCBI.REF                                      ctgatgacttgttttaaaactttcatcctaaataaccttttgatacttgaatatttttaa     18928
                                                                            * ***  ** *   *    ** *  * ***   * *****

LOC106996293.end.GGT1.start.28595839-28635852.Rhesus      ttttgagatggagtttcactcttgttgcccgacctggagtgcaatggtgccatctcggct     36694
BCRP3.HUMAN.NCBI.REF                                      gttttatacatagtttctaattttttttccgaaca-------------------------     18962
                                                           *** * *   ******    ** ** **  **

LOC106996293.end.GGT1.start.28595839-28635852.Rhesus      caccacaacttctgcctcgcaggttcaagcaattctcctgcctcagcctcctgagtagct     36754
BCRP3.HUMAN.NCBI.REF                                      ------------------------------------------------------------     18962

LOC106996293.end.GGT1.start.28595839-28635852.Rhesus      gggattacaggcacacaccaccacatccagttaattttgtatttttagtagagatgggtt     36814
BCRP3.HUMAN.NCBI.REF                                      ------------------------------------------------------------     18962

LOC106996293.end.GGT1.start.28595839-28635852.Rhesus      ttctccatgttgatcaggctggtcttgaactcccgacctcaggtgatccgcccgcctcca     36874
BCRP3.HUMAN.NCBI.REF                                      ------------------------------------------------------------     18962

LOC106996293.end.GGT1.start.28595839-28635852.Rhesus      cctctcaaactgttgggattacaggcatgagccaccatgctcggccttgattcttatttt     36934
BCRP3.HUMAN.NCBI.REF                                      -----------------gatccagatacctaataagatgctggaatgtaatccctggaca     19005
                                                                             * ***  *      *  ***** *    * ** * *

LOC106996293.end.GGT1.start.28595839-28635852.Rhesus      ctacgtaactgtttaagcgatgaattttcctctgagc-cctgcattagctgtaccctata     36993
BCRP3.HUMAN.NCBI.REF                                      atccgtgtc-ctggcagcatttggtcttcctctaagcgcctggctccgctgttctcagga     19064
                                                           * ***  *  *   ***  *   * ******* *** ****  *  ***** * *   *

LOC106996293.end.GGT1.start.28595839-28635852.Rhesus      gggtctgatctgtgctgattatagtgcaacttgaagatgctgcccttttggtgttgattt     37053
BCRP3.HUMAN.NCBI.REF                                      gtg---------------------------------------------------------     19067
                                                          * *

LOC106996293.end.GGT1.start.28595839-28635852.Rhesus      cctctgtgtaacaggatgtttcaagagccagctgtaagagtctctggctgggctgggaat     37113
BCRP3.HUMAN.NCBI.REF                                      ------------------------------------------------------------     19067

LOC106996293.end.GGT1.start.28595839-28635852.Rhesus      gcacattggctggcactgctggtggtgcagcattgatgtgagggcagggcctgggcaggt     37173
BCRP3.HUMAN.NCBI.REF                                      ------------------------------------------------------------     19067

LOC106996293.end.GGT1.start.28595839-28635852.Rhesus      tccccagggtgggatgagcagggagggagaggaatgggcttcctccagggcctcaggaac     37233
BCRP3.HUMAN.NCBI.REF                                      ------------------------------------------------------------     19067

LOC106996293.end.GGT1.start.28595839-28635852.Rhesus      caaggaagtctccgaactacctccaggcagggtcagatgtgcattttccaaagcttattg     37293
BCRP3.HUMAN.NCBI.REF                                      ------------------------------------------------------------     19067

LOC106996293.end.GGT1.start.28595839-28635852.Rhesus      cattaagagtgaggagtagactgtggtggtgccagcagcataaagactgaaaagaaataa     37353
BCRP3.HUMAN.NCBI.REF                                      ------------------------------------------------------------     19067

LOC106996293.end.GGT1.start.28595839-28635852.Rhesus      ggaggtggagccagggctgaaccacacagctggacgggatgggcgggggcagtggggggg     37413
BCRP3.HUMAN.NCBI.REF                                      ggttctgaagtctctggagaacaggatacgtggagggttaggaagggggcca---------     19118
                                                          **   ** ** *   *  ****   * *  **** **   **  **** **

LOC106996293.end.GGT1.start.28595839-28635852.Rhesus      ggattctacaagtagcacaggagggaggcgaggcgggcaccacagagctttcagctgcct     37473
BCRP3.HUMAN.NCBI.REF                                      ------------------------------------------------------------     19118

LOC106996293.end.GGT1.start.28595839-28635852.Rhesus      cacggctgacttctatttggcttgttgagtccaaggtgcctgtgcaccttgtccagatgc     37533
BCRP3.HUMAN.NCBI.REF                                      ------------------------------------------------------------     19118

LOC106996293.end.GGT1.start.28595839-28635852.Rhesus      tctggaggtggttggggaattgctcgtgtatgtgagtctgagactgcacacagctcggcc     37593
BCRP3.HUMAN.NCBI.REF                                      ------------------------------------------------------------     19118

LOC106996293.end.GGT1.start.28595839-28635852.Rhesus      aatgccaacatggagatccatcagttcacaagtggtgaacgagccttctctgggtgggtg     37653
BCRP3.HUMAN.NCBI.REF                                      ------------------------------------------------------------     19118

LOC106996293.end.GGT1.start.28595839-28635852.Rhesus      gcagtcccaggttggccaagagtccagggagtgcaccctcagtctgcaacagagttagaa     37713
BCRP3.HUMAN.NCBI.REF                                      -------------ggcctagagacgggagactccctcccggagcaggtggaggcacagga     19165
                                                                       **** **** *  * ** * *  **     * *    **    ** *

LOC106996293.end.GGT1.start.28595839-28635852.Rhesus      tccggcaccacggagccagggttgcacccctgctgcggggaacaggagccccttttcctc     37773
BCRP3.HUMAN.NCBI.REF                                      ccattcgctac---cccatctgccggcacctgcgggggagcccaggcattctttgtaagc     19222
                                                           *   * * **    ***        * ***** * ** *  ****    * ** *   *
```

```
LOC106996293.end.GGT1.start.28595839-28635852.Rhesus      tc-cctgacatgcagggacaaagttatcatgctctgtgggtgccgctgggaggcactgat     37832
BCRP3.HUMAN.NCBI.REF                                      cctcctgaccacctggctcaaagaaaaca-------------------------------     19251
                                                           * ******   * **  *****  * **

LOC106996293.end.GGT1.start.28595839-28635852.Rhesus      aggaaagactggccaggccttcccaagcaagctccaggcaacctcccagcaccccggccc     37892
BCRP3.HUMAN.NCBI.REF                                      ------------------------------------------------------------     19251


LOC106996293.end.GGT1.start.28595839-28635852.Rhesus      agatgtaggtgtggtctgggagcacagagccaggttcccaccgatggggcacctgaggtt     37952
BCRP3.HUMAN.NCBI.REF                                      ------------------------------------------------------------     19251


LOC106996293.end.GGT1.start.28595839-28635852.Rhesus      tgtgtctagcagccttttcttcctttggttccctttttcctgtttggcccacagggtttgg     38012
BCRP3.HUMAN.NCBI.REF                                      ------------------------------------------------------------     19251


LOC106996293.end.GGT1.start.28595839-28635852.Rhesus      gacaggtggctgctaccaacaggtgtcaaggtggccccacagacctgagccagccacttc     38072
BCRP3.HUMAN.NCBI.REF                                      gaagcatggaggccgccaagtattttcaagaaataatcccatgaacatggcatcactttt     19311
                                                          **    ***  **  ****    * *****       * **       * ** *   **

LOC106996293.end.GGT1.start.28595839-28635852.Rhesus      tccatagccctcccctgcccatgaaggactgctcagaggggcccccattgagagctggat     38132
BCRP3.HUMAN.NCBI.REF                                      ttagaaagaggggcttggggcaggcagaggagagaagggagatcaaactgagagccaagt     19371
                                                          *    *       * **     *   **      *  ** *  *  * *******    *

LOC106996293.end.GGT1.start.28595839-28635852.Rhesus      ctggacttggttaaactgcaaggggagaagctctgtcaccctgcagggcgtgagttcagc     38192
BCRP3.HUMAN.NCBI.REF                                      ttccagacggt---cctgcaggaggaggatgcagctgcccagagggaa----------     19418
                                                           *  *   ***    ***** * ***** * *    *  **   ****

LOC106996293.end.GGT1.start.28595839-28635852.Rhesus      tgaagcattgggaccatggcgctggggagccaactaggggggaggctaccacacagagcgc     38252
BCRP3.HUMAN.NCBI.REF                                      -gcaggatcacatttaaggaagtgtgtggggtccctggatgacaccagcacccagtgcgg     19477
                                                           * ** **       * **   ** *  *    *  **  **  * * *** *** ***

LOC106996293.end.GGT1.start.28595839-28635852.Rhesus      ctgagttctgggcacggcccacagagagatcagtgatggcggcaggtctgcccgagtggg     38312
BCRP3.HUMAN.NCBI.REF                                      ctctg-tctggcaaccgctcccaaggtggcaggagtgggtgtcccctgtgtgtcagtggg     19536
                                                          **  * *****  ** ** * **  * *    * *  ** * *   * **    ******

LOC106996293.end.GGT1.start.28595839-28635852.Rhesus      tgggccggcttggccccctatctccaaagtgagccagcttttgctccctgacctcctcct     38372
BCRP3.HUMAN.NCBI.REF                                      cagctcctgctgaacccacagctcactggggag---------------------------     19569
                                                            *  *    **  ***  * ***    * ***

LOC106996293.end.GGT1.start.28595839-28635852.Rhesus      agtctgccagacccccccttccagccacgcagtcaagcccacattaggaattagccccatc     38432
BCRP3.HUMAN.NCBI.REF                                      ------------------------------------------------------------     19569


LOC106996293.end.GGT1.start.28595839-28635852.Rhesus      cagcccctctcccacatacgacctccaaatatgtccatgtcccaatccctacaacccaag     38492
BCRP3.HUMAN.NCBI.REF                                      ---------------------cctgacagtggggccatgtgcctgacactcctctctgct     19608
                                                                               ***   * *  * ****** **   * ** *   *

LOC106996293.end.GGT1.start.28595839-28635852.Rhesus      attagaccttatgtggcaagagtgactttgaagatatgattcaa---------ttaagga     38543
BCRP3.HUMAN.NCBI.REF                                      tgtggacctggcaaggcagggagcagaaaacagagctacttgaaggctttctgtctgcgt     19668
                                                            * *****     **** *    *      ***  *  ** **         *    *

LOC106996293.end.GGT1.start.28595839-28635852.Rhesus      ttttgagatggggagattatcctggaccatccaggtgggcccactatgcccccaaaattc     38603
BCRP3.HUMAN.NCBI.REF                                      ctgtgtgcagtgtggatttagttgtgcttttttacttgctgggagagcacagccaccattt     19728
                                                           * ** *  * *  ****    **  *  *  *  **     *     *  ***  ***

LOC106996293.end.GGT1.start.28595839-28635852.Rhesus      acatgttgaaagctaatcaccaatgtgatagtatcagcaggtggggcctttgggatgtgg     38663
BCRP3.HUMAN.NCBI.REF                                      acaagcagtg--------------------------------------------------     19738
                                                          *** *  *

LOC106996293.end.GGT1.start.28595839-28635852.Rhesus      ttaggtcatgagggtagagccctcatgaatgggattagcgccctataaaagagacccccag     38723
BCRP3.HUMAN.NCBI.REF                                      ------------------------------------------------------------     19738


LOC106996293.end.GGT1.start.28595839-28635852.Rhesus      aagccgggcatagtgggctgcacctacagaaccagctactgaggaggctgaggcaggaag     38783
BCRP3.HUMAN.NCBI.REF                                      ------------------------------------------------------------     19738


LOC106996293.end.GGT1.start.28595839-28635852.Rhesus      atctcttgggtacagcagagagctctatgatcacgccactgcactccagccttggtgaca     38843
BCRP3.HUMAN.NCBI.REF                                      ------------------------------------------tcaccctcgtgggtggcg     19756
                                                                                                    * **  * * **** *

LOC106996293.end.GGT1.start.28595839-28635852.Rhesus      gagtgagaccctgtctctaaagtaattaaataaaagggaccccagagagctagctagctt     38903
BCRP3.HUMAN.NCBI.REF                                      aggacagaacaggagcctctgctctctgtacctatctgggcccggtgggctcccttgtcc     19816
                                                            *  *** *  *   **     *   *  *   *   *  *** * * ***  ** *

LOC106996293.end.GGT1.start.28595839-28635852.Rhesus      cttccactatgtcagttagaaggcgccatataggccgggcgcggtggctcaagcctgtaa     38963
BCRP3.HUMAN.NCBI.REF                                      tggcttccatctctgtctca----------------------------------------     19836
                                                             *  * ** ** **    *

LOC106996293.end.GGT1.start.28595839-28635852.Rhesus      tcccagcactttgggaggccgagacaggcggatcacgaggtcaggagatcgagaccatcc     39023
BCRP3.HUMAN.NCBI.REF                                      --------------------------------------------------gcgaccattc     19846
                                                                                                            * ****** *

LOC106996293.end.GGT1.start.28595839-28635852.Rhesus      tggctaacacggtgaaaccccgtctctactaaaaatacaagaaaattagccgggcgaggt     39083
BCRP3.HUMAN.NCBI.REF                                      agccctgcacaggaacacatgttgcttagaaaagccaaat---------ccagcccttgt     19897
                                                           * *   *** *  * **    *   **  ***   * *          ** * *   **

LOC106996293.end.GGT1.start.28595839-28635852.Rhesus      ggcaggcgcctgtagtcccagctactcgggaggctgaggcaggagaatggcgtgaacccg     39143
BCRP3.HUMAN.NCBI.REF                                      ctctgcctcctctggtctc-----------------------------------------     19916
                                                            * * * *** * *** *
```

```
LOC106996293.end.GGT1.start.28595839-28635852.Rhesus      ggaggcggagcttgcagtgagccgagatcgcgctactgcactccagcctggggcacagag     39203
BCRP3.HUMAN.NCBI.REF                                      ------------------------------------------------------------     19916


LOC106996293.end.GGT1.start.28595839-28635852.Rhesus      caagactccgtctcaaaaaaaaaaaaaaaaaaaagaaggccatatatgaggacgtgggcc     39263
BCRP3.HUMAN.NCBI.REF                                      --------------------------------atgatgtgcatctgttaccttgaaactg     19944
                                                                                          * ** *  *** * * *    *

LOC106996293.end.GGT1.start.28595839-28635852.Rhesus      ctcacaggacatcaaatctgccagcaccttgatcttggacttcccagcctccagcgctgt     39323
BCRP3.HUMAN.NCBI.REF                                      gaaaccagtctatcaatgtctgtgccaattttttattccctccccaacctccttccccat     20004
                                                             **  * *    *** *    **   **  *  *   ** **** *****  * *  *

LOC106996293.end.GGT1.start.28595839-28635852.Rhesus      gagcaataaattt---------------ctgtaaaatgctataagccacccactttccag     39368
BCRP3.HUMAN.NCBI.REF                                      acgactttttatttatgtaggatgtgtgctgtctaatgatgggatgaccacacttttcca     20064
                                                            *   *    **               ****  **** *   *    * ****** *

LOC106996293.end.GGT1.start.28595839-28635852.Rhesus      tatttttgttatggcagcccaggcagactaaaacaacctctgtggcacatttggg-----     39423
BCRP3.HUMAN.NCBI.REF                                      tgttctaaaagt-gctcctctcccacagggtcccagggctggtggttgctttgggtctac     20123
                                                          * ** *     * **  * *   ** *      **      ****    ******

LOC106996293.end.GGT1.start.28595839-28635852.Rhesus      ------------------------------------------------------------     39423
BCRP3.HUMAN.NCBI.REF                                      agctacgtcttacccgcctcctgcctcaacagcctgtgtggtggcaaagccggtgtgggg     20183


LOC106996293.end.GGT1.start.28595839-28635852.Rhesus      --------------gtgatgtaggaacagccgatgttcccaggaaccagtgaggggcagg     39469
BCRP3.HUMAN.NCBI.REF                                      ctggggaacgcagcgttctccaggaggggacccggctctccttctgcagtgcaggcga--     20241
                                                                        **  *  ****   * *   * ** *      *****  **  *

LOC106996293.end.GGT1.start.28595839-28635852.Rhesus      agtcctagatccctctagcgagatccccagagagcgccatgtacccgccctgcagctggc     39529
BCRP3.HUMAN.NCBI.REF                                      aggcctagatgccagtgtgacctcccacaaggcgtggcttccagactccc-----cggcc     20296
                                                          ** ******* **  *        ** **  * * * * *  *  * ***     * * *

LOC106996293.end.GGT1.start.28595839-28635852.Rhesus      aaagctgtttatggaagttaggggcctgttggtttagagcggccattctcaaccaggggg     39589
BCRP3.HUMAN.NCBI.REF                                      ggaagtgatgcttttttgccgcgggccctgggtttgaagcagcctggctttctcttggta     20356
                                                            *  ** *  *        * ** *  * *****  *** ***   **    *  **

LOC106996293.end.GGT1.start.28595839-28635852.Rhesus      atgtggcactgtcttgagacagttttggttgtcatgagtgggtgtgtgtggtggggagtg     39649
BCRP3.HUMAN.NCBI.REF                                      agtggctggtgtctt---------------------------------------------     20371
                                                          *   *    ******

LOC106996293.end.GGT1.start.28595839-28635852.Rhesus      ctaccgcatcacacaggctgaggcacgggatgctgctcaacatcctacagtgtggggacg     39709
BCRP3.HUMAN.NCBI.REF                                      ---------------agcagctgcaatctgagctcagccacctacacaccaccgtggccg     20416
                                                                          ** *  ***      ***   * ** * *        * ** **

LOC106996293.end.GGT1.start.28595839-28635852.Rhesus      gccctcacaacaaagaatcacttcattcaaaacatggatgagggccaggcgcagtgactc     39769
BCRP3.HUMAN.NCBI.REF                                      acactttcattaaaaagtttcctgagacga------------------------------     20446
                                                           * **  **  *** * *  * * *  * *

LOC106996293.end.GGT1.start.28595839-28635852.Rhesus      acacctgtaatcccagcattttaggaggccgaagcaggaggattgtttgaagccaggagt     39829
BCRP3.HUMAN.NCBI.REF                                      ------------------------------------------------------------     20446


LOC106996293.end.GGT1.start.28595839-28635852.Rhesus      ttgagaccagcctgggcaacatggtgaaaccccatctttacaaaaaaagcaaaaaaccag     39889
BCRP3.HUMAN.NCBI.REF                                      ------------------------------------------------------------     20446


LOC106996293.end.GGT1.start.28595839-28635852.Rhesus      ctgggcgtggcgatgcacacctgtactcccagctacttggaagcctgaggtgggaggatg     39949
BCRP3.HUMAN.NCBI.REF                                      ------------------------------------------------------------     20446


LOC106996293.end.GGT1.start.28595839-28635852.Rhesus      gcttgagcccaggaagtcaaggctggagtgagctgtgattgtaacagtacactccaacct     40009
BCRP3.HUMAN.NCBI.REF                                      ------------------------------------------------------------     20446


LOC106996293.end.GGT1.start.28595839-28635852.Rhesus      gggca   40014
BCRP3.HUMAN.NCBI.REF                                      -----   20446
 ========================================================================================
```

LOC106996293.***end.GGT1.has.human.BCRP3.to.position.16320 bp.Rhesus

```
>LOC106996293.end.GGT1.start.28595839-28635852.Rhesus
ctctgggcctcagtgtattgtgtgtgaaatggaaccatctggctggggaggaatggagag
gtgggattcggagatcttcacactgcggtcgctggaactagcctcagtatcttcagcgtg
gggagagccaggtgcgtggctagggaccaggggaaggtccatgccaacccctgcccttc
ccaccctgatccattggactttggggccaggtgctcccttattggggctgcacagtgaca
cctaggactagccaccagggggtgccgcgcccctggtgctttcttaggcagtgggtggcc
agctgatgctgggaacctgggcaccttctcagacccatgggcatccaactcatcctgcta
atgacacgggaggtgaagctgagttccaaggaatgggaattgggcatcacgctagaggaa
aacatcttagtcagagccaagcccctggggggtttccaagtataagcccagagtgaaacc
caagcttgtgaccctctccagagggaggcctggttttcagggaacagcaaatgggaagag
gtccccagattccagggatcagggcttggaccagctggggacgcagcccagagggagtgg
gtctggaagggaacagctagacacagcagccttcaccactggcagcccctcccggcctcc
ctcggggcctgctccctcctccaagcaccgttccaacacctggggcagggttctgggaaa
ggctggtggaggtgggctggtgggggcggtgatcacagcccagcatctgggtatcacca
ggggcactggggccagggcccaggtgaagccaggtcggggctctcctttagaagccccga
aaacctggtgataccaaagggcccacagacaaacagggttttgtgcctgcggagttgagt
accaccgggtctaagccctggagggctgtgtccctggggctccccaggggtgagatggag
gtgggctcaactggtgtacccgtcactcctcaatccttattttatgtatttaatttttaa
aaaaatttttatttgaacaaatagagatggggtctcactatgttgaccaggctggtctta
aactcttgacttcaagcagtcctcctagcttggcctccaaagtgctaggattactttggg
```

gattactttagggatgagtcactgcacgcggcctcaatccttattttggcctgaaaggaa
aggctgtggccccgtttgcaggggagaagactgaggctggaggggcaggccttgctctgg
gttgcacagcagcaagagaagtgggagctggccatgaggcttcctggacccgaagcactg
gtggggttcaccctggttcttcaggtcccatggggctcagcccaggactaccttggtggg
ggtgggagacttaaatcctctccttcattctcattgtcccttcccccatcatttcctgag
gaagcacattcagggacctccctggctgtgcctcagtccaaaaccagaatgacacgcatt
cctttccctgggcctttgctcaggcggtccctgcaccctggcctctgcctgaccagggtg
gtggggagaggagggggggacgtcccctccgctgctgtctccactgttcctgctgccctgg
cctctgggcttccaggactgcagtgggtgggtgggtgggctggcctgagcccaggaatgc
acttcggctcctggttgagcaaagtcactgagacttgggagtcgggtcgggttgggagga
ggcgtccacaggcccccactacgaaaggcagctgtgggaacagtctgcctgtaaacaacc
actccagcccaggctgaccaggggctctggctgggacattgggatctggcaggctgtgtg
gcctgtaaggacacagtctgtctctgtgcctcagtttctctgctgcccagttgggcgtcc
cagactccaggtgtagacatctggagcaggcagtgctcagctgggaaggaagtggggagg
actggaggagccatgtgtgaaggattccaacccacatcacctgcacccctgctgagcctg
gtcaacagagcccctcagtgggtcctcactcccctggctgcctcccggttaggcaccctg
agggctggggagaacagggccaggccagtgtccccagagaggctgcgctgccagcacagt
aatagcggatttggattcagggaagcagacccgcagccagggtggggaagagctgcaggc
tgggcgtggcacctaggcggcacagcctccctccctggaggcccacgctgcatttccagg
acagcaagtcccagggatggatggtcccaggtgccaagggctagaggcatggtctgtctg
cattccccacatggacgtcttgtagtcaccagcgtttgatgctgtcaagtccccctgtcc
tctctgcggactgagaagcccttggtcatccttaggggggttgtggaacccaaaccaggct
gcagaagcatagggacttgaacccaagttttaagtgacaccaccttttgtccccctccct
cggtctctgttcagttccacttcgatattgcctgtgctgggccatgcagagagggttagg
ggatagagatgggaactggggagtggggctccactctcagagaggggcagccttgctgga
tccaggggagatagttgagcagccccagctctgctttcccggagctgctgggaaccccag
gaatggtgtggagattcctgggagctctgcccccacttgacaaccacagtgcagcaggca
ccaagttctcctgcacattgggacagtgtgaccctgggctctggttagtggcaggtgggg
ccttgggtcctaccagcagtgagggagttagcacagcagctggctcctctagggaaggaa
aactcccttcagacactttggtgcctggcctcctgccaggaacaagcaggagctgaaaac
tagaagttgaggcataagtttggccactctgtagtgtgtacctggggagggcagcagctc
gccacagctgccagctgccagccgtctacccattcacctggcagcccgcttttcagacct
gcctgtccacccatctataagcccatctctgtcccgttgtctatctgaccatctttctct
tactgtcctctctgtccagcaatctggcctgtctgtcgatccatcttcttgtctaactgt
ggccccacctatttgtccatctgtccaattacctttgattctatctgtgcatcttcttgt
ccatccatctgcccacccatctgtccctgtgtctgctcactggcctcccctctcctcctg
ggccacagagccatggcccagggctgtgggtccttggtcagcctggtgctgctggggctg
gggctggggctggctgtcactgtgctggctgtggtcctctctcgccaccagactccctgt
ggcccccaggcctttgcccacgctactgttgctgctgactccaaggtctgctcaaatatt
ggactgtgagtgagacgtgggaggaagctgggtggcctttggcagccagcccctcctgga
gaaggcgtgtgtgtttgagtgtgtgagtgtgtgggcgtgcgtgtgtgattgcgtgtgtga
gtgtgtatgtatgtgtgtgagtggggggtgtgggggtgtgtgaatgtgtgtgattgtgtt
tgggggtgtatgtgtgggtgtgtgtgattgtgtttgggtatgtgtgggtgtatgtgtgag
agtgagtgcagggggtgtgcgtgtgtgaatgtgtgtgattgtgtttgggtgtgtgtgtgg
gagggtgagtgtgactgtgtgggaggggtgtgggtgtgtgtgaatgtgtgtgattgtggg
tatgtgtatgtgtgggtgtgtaagtgcgtgtgtgtgtgggtgtatgtatgtgtgcgtgtg
tgagtgtgtgtgtgcgtgtgtgtacaagtgcactggcccaggaagcaggagccgtgtgtg
tgtgtgggcttcagcacctgcagggcttgggcacaaggaggcagcctcagggcccttgca
cagaacaggtggcagggtgtgcccatggggcagatggtgatttagggacagtcatgtgtg
agtccacacctggctccaggattcaggagacccatttgcacatcccaggtggggccagta
caggccccttcagtgaggccaattctccaaggctggggtcttctcccagggtcataggtg
aagggcttcagaggctccctgtgtgggtactggcctgctggggtacacacaatgctgcca
cagccagtctgccccagcttccagctgggggccacatctcgggtttctctgtcctgggga
gcctggtgccccacccctcacatcctctctccctgagtcagggcctgggtctcctgagct
gagtgactgatacttggtgtcctgaatgagggtgtggtggagaggggccacggcgggtgt
ttcctgaccctcttccaggaaacccagcccaagggaggccttcgctgctgccactgcaga
gaggacacatacaggacgccccttcctgccccctgcctgccattgggcccacaaaagccg
gggcaagcctcccctccctgcagccacctggtctgcttcccagaagctctgtcttgcagg
ctgttgggaggatcccagtgctttgtaaactaaagcaagggaggcgtggccgttctctct
ctttgttcattcattcaccttttgagtcatttcttcctccctccattaccccatctgtcc
atccttccctgccctgattgctcatgccacacccccagcccctcctgacctggtcctttg
gtttctcttcatggctttctgtctcctcccacagggctgagaatggcagctcagggacaa
gtagagcctggtgactgcttggtctcccgggtggctcctaggggatttgagggattgatg
cctgctgaggctgtgcccctcctctgctcaggaggacatacagagatgtggcacccactt
aaactcaaagttgcacagatgcaaatgagactggggtctcaggcaccagagaccaccgtg
ggcacgtgacctttgggagtggggacctgctgccacagatctctgagtggagtctggacc
tactgggtctccccaagtgactgtctgggggtctctgtagcatgccctgctgtgtacgtg
agggtcagtggttggggaggggtctctgctctaatgcttcctacactggcactccctcaa
actcccttggtgaagagagaggatgtggtttgccgcagtgttttatcgaacaactctctc
cacttcctgttttcagaagccgggagtggaagagagcctggggctggccccagctgctgc
tgcggaacaggggtcactggacgctgggaccctggccgggctggctgggggcctccggaa
gaggcctgctgcagcgtcatcctggccgagatccctccctgcaggggcccctggccatgc
tgccgcagggtctgctggggccaccagaagcccacgctcctgcctccatctctgccctgt
gtgctcacctctcaccagcaggccctcccagagtccagtctcttctgctcttttttttgtt
tgtttgtttttttgagatggtgtctcactctgtcaccaggctggagtgcagtggcgcaatc
tcggctcactgaaatttccgtctcctactctttcagcatcaggttttattactgggattc
tgctacagccagagaccctgggagcagattcctaaggcttatgtgagtgtggacccagca
ccgtgcctagcagacatacaaaaggagcatggtgacagtgaggtctgtcatctccagctt
aatgactgttttgatccttgtcaaaaaggtgatttttggctgagcatggtggctcacacc
tgtaatcccagcactttgggaggccgaggcgggtggatcacttgaggtcaggagttggag
accagcctgggcaacatggtgaaaccccgtctctactaaaaatacaaaaattagctgggc
atggtagcgggtgcctgtaatcccagctacttgggagtctgagacaggagaatcacttga
acccaagaggcaaatgttgcagtgagccaagatagcaccactgcactacagcctgggtga
cagagcaagacttggtctcaaaaaaaaaaaaaaaaaaagaaaagtttatatttttgttctaa
aacttatcttaatgtcttcattctatattttatataattataagagctatataagatata
ctacccctagtactttgtttttggatattctattcactcctgatggttaatttatgtgt
caactttgctaagctatgatgccctgttgtttggtcaaatacttctcaatatcttgctgg
gaggttatctcatagatgtgattaacattgacagtcagctgactttaggtaaaacaatgt
gattaacgctgacagtcagttgactttaagtaaaactgaggtttcccagagaagcaggaa
ttctgctttaacactataacatgtaaatcctgcctgagtttctggcctgctgactgctct
ccaggttttaggttccagacttcgagatcaactcttacctgaatttataagctgctggtt
cgccatacagattttaaacttgctagtccccacaaccgtgtgagccaattcctaaataaa
tctctctctatgtataacctattggtttagtttctctaaaaaccttttacatctagtttc

ctggatgttaagtaatactgaaactagctagtaacttcttttcttttttttttttgagat
ggaattttgctcttgttgcccaggctggagtgcagtgccgcgatcttggctcaccgcaac
ctccacttcctgggtccaagcgattctcctccctcagcctcccgagtagctgggattaca
ggcatgtgccaccatgctcggctaattttgtatttttagtagagatggggcttcttcat
gttggtcaggctggtctcgaactcccaacctcaggtgatccacctgccttggcctcacaa
agtgctgggattacaggcatgagccaccgctcccggctcctcgtaacttcttcttttctg
tgatatgtctcttatctctaataatacttttcttcttaaagtctacttcattaaaaatag
taatgctgggcatggtggctcatgcctgtaatctcggcactttgttggaggttgaggtgg
gtggatcactgaagcccaggagttcaagaccagcctgggcaacatggcaagaccctggct
ctacagaaaaatacaaaaattagccgggtgtggctaatataattctaagttagcacacct
gtagtcccagctacttgggatgctgaggtgggagaatcgcttgaacctagaagggggaga
ttgctgtgagccaagatcatgtcactgcactccagcctgggagacagagtgaggctctat
ctcaaaaaaaaaaaaaaaaaaagaagttatacagctttcttggttaatgcatgcatatttt
tcattattttccacctttctgtatccttatataaaaggcattagttgggtttttatttcc
aattagtttaatttttattatccttttaaatgtaactaattatttatttgggttgaaagc
caccaccaatttgttttccatgcctactctctttcttcttatctcctcccacatcttgtt
ttgcatttattatttttattatttaatttcctcctctattagttttgtaactgtgcagtc
ttggagttattttaaaagagacagtagattattttagagcttacaacatgcatccttcac
ttaccaaagtctaacatgagctagtactttttttgttgttgtcgtcattgagacagaggga
gtctcgctctgctgtccaggctggagtgcagtggagcaatcttggttcactgcaacctct
gcctcttgggttcaagcaattctcctgcctcagtctcctgagtagctgggatcacaggcg
tgcaccactatgcccagctaattttttgtattcttttttttagtagagacagggtttcacca
tgttggccaggctggtcttgaactcctgaccttaagagatccgcctgcctcggcgtccca
aaatgttgggattacaggcataaaccactgcgcctagcctatgagttagtacttctatcc
ccttcctagtcagtacaagaaccttggaacaggaatgaaatttacccccaatgacttata
tgctaatatttttgtgtttttttaaatatatgtatatgtgcagctgggtgcggtggctcat
gcctgtaatcccagcactttgggaggccgaggcgggcagatcacaaggtcaggaaatcga
gaccatcctggctaacacggtgaaactctgtttctactaaaaatacaaaaaactggctgg
gcgcaatggctcacacctgtaatcccagcactttaggaggccgaggcggaaggacaacct
gaggtcaggagtttgagaccagcctgaccaacatgcagaaaccccatctctactaaaaat
acaaaattagtcgggcatgatggcgcatgcctgtaatcccagctactcgggaggctgagg
caggagaattgcttgaacctgggaggcagaggttgcagtgagctgagatcgcacctttgc
actccagcctgggcaacaagagcaaaactccatctcaaaaaaaaaaagataaaataaaaaa
ctaactaactaaataatacaaattagtcgggcgtggtggcgcatgcctgtaatcccagct
actctagaggctgaggcaggagaatggtgtgaacctgggagacggagattgcagtgagcc
gagatcgcaccactgcactccagcctgggcgacagagtgagactccgtctcaaaaaaaaa
aaaaaatatatatatatatatatatttatattttgtgtgtgtgcgtgcatagatgtatct
gtgtgtttttttgtgtttttattcttatttatgttgagagtgtagagctatgtaaaaata
aacagaattgtataatgaagccccatgtatccattcaatttcaacaacaatcttatggcc
aagctaatttcatgtatactctttctctttcctgcttccttctaccccacattatttcag
tgcaaatcccagatatataactttacccatacatatttcagtatgctttatttattttaa
accccacaagatatcattttctatactactataattttataacaataacattcatttaga
tttactcaaacatttacttcttctgttaccctttatttttatttataaaaatatatttgg
gaaaaaatatatttgcacacatagtcaaggatctcctgagggctatgtcatggccaaaat
atacatatatattccatatttatatctatctatatatacacacacatacacacacacaca
tatacacacacaaacatatatgtattccactttcactttttttatttgttttttgagaccg
agtctcgctctctcgctctgttgcccaggctggagtgcagtggtgcgatctcagctcact
gcaacttctgcctcctgggttcaagtgattctcctgtctcagcctccaaatatctgggat
tacaggcatgagccaccacgcctggctaatttttttttttttttttttttttttgagacg
aagtctccctctgttgcccaggctggagtgcagtggcacgatcttggcgcactgcaacct
ctactttcctggttcaagcaattcccctgcctcagcctcccgagtagctgggattacagg
tgcacaccaccatgcccagataattttttttgtatttgtagtagagacagggtttgcctat
gatggccagactgttctcaaacttccgacctctggcaatccactcaccttggcctcccaa
agtgctggggttataggcatgagccaccatgcctggcctgttttttttttttttttttttt
tttgagatagagtctcgttctgtcacccaggctggagtgcagtggcaagatcttggctca
ctgcaacctccgtctctggggttcaagcaattcttgtgcctcaagaattgagtagctggg
attacaggcgcccaccaccacatctggctaatttgtgtatttttggtagagatggagctt
caccatgttggccaggctggtcttgaactcctgacctcaagtgatccacctatctcggcc
ttccaaagtgctgggattacagacatgagccaccatgcccggccattttcacttttgaag
gatattgttagtgcacataaaattctaggttggcagatattttctttcctcagtttgaaa
acatgattcccttgtatttgatttctcctgtttttattgagaagccaattctcaatctaa
ttttgctcatttgaaggcaatgtcttttttttgttgttgtgtttttttgagatggagtctca
ctctgtcgcccaggctggagtgcagtggcgtgatctcagttcactgcaagttctgcctcc
tgggttcacgccattcttctgcctcaggctcccgagtagctgggactacaggtgcccaca
accactcccagctaatttttttgtatttttagtagagatgggatttcaccatgttagccag
gatggtctcgatctcctgaacttgtgatccacctgtcttggcctcccaaagtgctgggat
tacaggtgtgagccaccgcacctggccccttaattgttgtatttttaatagagatgaatt
tttgccgtgttggtcaggctgatcccgaactcttcgtttcaggtaatccaccctcctcag
cctcccaaagtgctgggattacaggtgtgagcaacccctacaccccggcctgaaggcagt
atcttttttcctctggctgctttaaaaaggtttgtccttggctttgagcagttttacacc
gatgcatttaggtggttcttcattctatgacttcattcttttttttgtccattttagaaaat
tctcagctttatctcttcaagtattacgtctttcccatcctctctcttctctccttatga
gactccaatttcacatgacttataccttgttaaagtatctcccatgtctgttaatccatt
tcctgtgtgttctgtctatttttctctttgtacttcaatttgtacattttgtatcagact
atctcccaattagccggacatggtggtggggcacctgtaaccccagctacttgggaggct
gaggcaggagaatggcttgaacccgggaggtgaaggttgcagtgagctgagatcatgcca
ctgtcctccagcctgggcaacagagtaaggctctgtctcaaataaataaaaaaataaataa
ataccagttcattatttatttatttatttttatgtttgtgtctactgtgctgttcaaat
tgagttcctaattccgttttttttttttttttttttttgagacacagtctctatctgttgct
caggctggagttcagtggtacgatctcaactcactgcagcctctgcctcccaggttcaag
cgattctcgtgcctcagcctcctgagtaactgggattaccaccatgcctaactcagtttt
gtgttttttttgtttttttttttttttgagacggagtctcgctctgtcgcccagcccagg
ctggagtgcagtggcgcgatctcggctcactgcaagctccgcctcccgggttcacgccat
tctcctgcctcagcctcccgagtagctgggactacaggcgcccacaaccgcgcccggcta
atttttttgtatttttagtagagacggggtttcaccgtggtctcgatctcctgaccttgtg
atccgcccgtctcggcctcccaaagtgctgggattacaggcgtgagccaccgcgcccggc
cagttttgtgtttttagtagagacaggtttcgccatgttggccaggctggtcttgaactc
ctggcctcatgtgattcaccctgctgtctcccaacgtgctgggattataggcgtgagcca
ccactcccagcctccatttttttttttttgagacggagtctcgctctgttgcccaggctgg
agtagagtggcacgatctcggctcactggaacctccacctcccggttcaagagattctcc
tgtctcaatctcccgagtagctgggactacaggcacatgccaccatgcctggctaatttt
tgtaattttagtagagatggggtttcaccatattggtcaggctggtcttgaactcctgac
ctcaggtgatccacccacctcagcctcccaaagtgctgggattacaggagtgagtcacca

tgcccagtgcgtctattttttatagtggccagttttctgattaaattcttgtttctta
tatccttgaatatagatataaagtacttattttaaagttcatggtctgacaatttcataa
tctagagatcctatgggccttttaaaaaattgtctctgctttctcttgagctttgttcct
gctgtcttatttccttgtttgcttggttgtttttaatttggcaatggagggtgtgtataa
aaattgttagaaataattttttttttgagatggagtctcgctctgttcccaggctggagt
gcaatgacatgatctcggctcaccgcaacctccacatcccagggttcaactctcctacct
ccgcctcccgactagctgggattacaggcaggtgccagcatgcctggctaatttttgtat
ttttagtagagatggggtttcaccatgttggtcaggctggtctcgaactcctgacctcat
gatctgcctgcctcagcctcccaaagtgctgggattacaggcgtgagccactgtacccag
caagaaataatttttaaaaataatttccagccccagcacgatggctcatgcttgtaatct
catcactttgggaggctgaggcaggcagattgcttgagcctagaagttcaaaatcagcct
gcgcaacatggtgaaaccccatctctacaaaaaataaaaaactagctaggcgtggtagtg
tgtgcctgtagtcccaggtgtttgggatgctgaggtgggagtctcacttgaaccaaggtg
atcgacgctgcagtgagccatgatcctgagactgcactccaacctgggcaacagagtgag
atgctgtctcaaataaataaataaaaataaaataacatgaggcctagaagtctgaaattc
tgggatctcccttatgcatttgagcggctgagatgatctgaagctggatccagtgctcct
gagggctgctctatttctggttgactgtcactcctagagtaagaaacctgcaccccacgt
gtggggcattatggcattgcctccctcagccacgtgaataggtcaacagcactgctctag
accaggtgtggtggctcacgcctatagtcccagctactcaggagactgaagtaggaggat
tacttcaggccaggaatttgagaccaccctgagcaatatattaggttggtacaaaagtaa
gtgtggtttttgccattaaaagtaatagcgaccctgtctcagcaaaaaaaaaaaaaaaaa
aaaaaaagggaagaagagaaaaagaatcagctgggcatggtggctcacacctgtaatccc
agtactttgggaggccgagttgggtggatcacaaggtcaggagattgagaccatcctaac
atggtgaaaccccgtctctactgaaaatatgacaaattagctgggcatggtagtgggcac
ctgtagtcccagctattcgggaggctgaggcaggagaatggcgtgaacccaggaggcgga
acttgcagtgagccgagatcgcaccactgcactccagcctgggtgacagagtgagactcc
gtctcaaaaaaaaaaaaagaaagaaagaaagaaaaaaaccccactgctctgtctctcagc
ctcctcttccaggattggctgtcatcttgaggggaatgctggccttgcctgtctccagcc
ttgttcttctctgcctcttatgcctttaagcacatgttttctatttgctgggctgtgaaa
tctgcacttcatctgatggggtttgctttataggtgactagatccttttctcttggtggt
tttagaatttgcatcttcacattgactttaaatagtctgattacagtttgccatggcaaa
gaccctttgcattgcattgtttggggatatttgaccctcttttatctggatgtctaatct
cctgttagatgtgagtagttttcattatttcattaatgggctggcatgtggaccgtggtt
ccaggggggctcagagaggcagccgcctgatgtctgaccgcttctctttctgtctctttt
cttacctggactctgggttgcttgttagctgcttctgccagttctgagttttcaaggga
gaggggcccagtgatggctgttctttgaaggaaagggaagaatgtctcctgtttaacacg
tttctatgtttccagttagttagttaggttagttagttagttagagccagggtatctcac
tctgttgcacaggctggagtgcaatggcatgatcttggctcacagtagcctccacctacc
aggctcaagcagtgctcccaccccagcctcccaagtagctgggactgcaggcatgtgcca
ccatgcttggctaccttaaaaaaaatttttttttttttttgatacagacgaggtctcac
tatattgcccaggttggtattaaactcatgggctcaagtgatcctcctgccttggccttt
ctaagtgctgatattacaggcagggcttcaatttttaaagctcccagcaggggtattaa
agtctccctttccagagaaagcgcactctgtccccatccctcatgttatcctctcctgcc
tctgcttagggttcactctgggggaattgccacttgagagattcctttttgtgtgtggct
tctgactgaccggtccctgctcacagctgctgcttctcagggtggggtccctgaggcctg
gagtgtggcctctgaaaaccttcagggccagaagcagaatgagagcctgtggccacatag
cccctggtgggagacgtcctgccaccctttgcttctctgtgtcactctggctgtacattt
cagatccctgggaaacgttaactggtaggacctagaaggggaggtgaggaggggtcacgc
cccaggtgtgcctgtggtgagcctttgtgctgagcaggtgcagggagggaggcccaggtg
cacacatctgtgaagtaggggcagctggttgggctccttgacctgctccagaacttctta
ttttctagccacttcacctgcagaaggcccaggtggctgtggtctctagggtcccttgca
gtgaacataccgcctgtcctcagctcctctctaggcctggtgctgtgctcagcaccgtcg
tatgtgtgtatgtgtgcacgcacgtgaatgtgtgcaggcatcatgaggtgtggtccctgc
tcttaggcagcttgccttgtggctgaaatgaaccatcacctgcatcaaggaaacacaagg
ccagatgcagtggccttgtctggagtcagatggtggctccgtgacctggcttcactccag
aactacctggggtttctagttgtgcaaatcccaggtcccacccagattatgagtccagtt
ttctacaggtggagcccaggaaggtgtatttttaacacgatggtactgctcattcaccca
gcccagccagtggtctctgtgtacccaggaactgctgacacagagctggtgctccccata
aaacagaagacagagccttcacggacgggggacgtgtggcttggtcatgacagggtattt
cgtaccagcacagactgtgttcaggtgacattaaggacaagttcctgcaggctagctgcc
tggacagggctgggtgggggtagaagaggggtcacagaggggcttcctcccggccgcctc
accagctgcttgatttagggcttggctctgggtcatcctgggcctgattctgaaccacgg
gactggtgtggcctgcaggctcctgccacaagctgttcatggtgcagggggagaacagcg
tccacagttccccagacagcagcagtggataccaggcccccaggagttgttactgaagtt
gctgctggacaactcgcccttcactgagctccacatagcacccgtggtgatgggagccgg
gtggagagagtctgccagcctgtgcatcagctcccaactgggaggggcagagggaggagg
gggtgggagccccaggcagcagggctctgggagcagtggggccctgggttccaggggtgt
ctggcaggcccctccttactctacctcttttggcctctgggtggagatgctggccacagt
caggctctgcctctgactaaggactggagaagtggcgggtgtgggctgctgccccgtgca
gcctctgaacagaccccagggcctctgccaatcatgactccttgctttcagctggaccca
caggccctgcaggacagagactgacagtatgctgtcatcaccatgaatggggtatgtgtc
cctgggacttttctggtgcccacatcccccagaagggtagggtggcctctattcatttca
aatcggtcagaggtggctgagcctgagccagcgtctgacacggagcctggttggaggagg
gaggttccccgaagagcagaatcgccgtgcccgggaatcgtcacactgactgggatgcag
ttgccagccaggccctgagcatccctcctcaaacaaaggtctcatggcaccaccaggaca
ggtggggcctccactcggtgacctggggactgcatgtagaaatggagacctctgatttgt
ctttaggtaccccagaaaggtttacaccttaaaagcaatgacacacccaaaaaggcctgg
gcatatatggtaaaatgttaatatttgatgattcttggctttttcttatactattctgtc
tttcctacttaatttttaattgttattaagaaagagagagtgggcacagtacacctatgg
tctgagctactctagtggctgagccaggagaatcactggagcccaagagttcgagtacag
cctgggcaacattgcaagatcccatattttaaaaaaagtaagcaaccaagagaagcagcg
gggattttaggaggtggttctgcagaagccagtcctttacaccatcttcaacaatcctgg
ctcttgctgaggtagactaggggattccctgaggggcagccctacctcatgctgagacct
ctgcatgccttcagagtggaaatatttgatgagactccaaggggcccttgagaccttggg
ctatgagggccagaaagattagtggactatgccctttctcccctctatagattgaagtaa
agctcttggtcaagttcaacagcagagagttcaacttgaagaggatgccatcatgaaaac
aggggtcttcggagctgagattgctgtggtcaccaagtgagtggggaggggcttgggctc
atgcactgagggtgcatgtcccttcagctgtttctgcagagaagagcatgtgtgggtctc
tcctctctctgtgtggccacttcgtggtgaggtcaggccccagggaacacttggtgtgtt
tagctacctcctgtgtttactcaacaccagctcaggaatgtccttgccaccttgcttgga
agcagtagggctggctccaggaactgcccgagtgcagttttctgccctcgcttggaattt
gtcatggtcccaggttcctgttgaatggccataacccctgcccctttgtcacaagtcagt
tgccaagagaagcctgttgggtttgagagcagttcttgcagacacagaccacttcctctg

agaattcatttgcttccccaggttggaatccggctgggcccctgaacttgctggtcatgt
gggccggggcctccatcaatcataccccggactcctttctgtgtctaaacagcacactca
cccccaactgcacggcagccacttgcgtagcactctgggagggctctgggcatgagcagc
gaggactccagcagcagcccccccaaataaccactgctaatgagggtggcttgagaagcg
gctttgatgtgctcctaaacccagttgcaaaacaaagctaaagttaaggcctcagcacag
cgctctgttctaactttgaagtattcttactctagtgtcctgtgtggcggtattggaatt
gttcagtgctaggactcagaggagtaaagcacttagcagcgcaggacttagagcgctggt
gctgaggcaacccttcattcattcattggatgtgtgttaaggcccaggcccagggcaggg
gtcagggattctcttctcacacagcacgcgggtggcaggaccaacaccgggtctgatctc
ccagctggggacacaggctgctaaccccaggcctggaatctgtcagatgccctttctgtg
ctgactttacttagacaggcctcctgaccttcccacaaagatcatgtgtgacttgcaggg
gttctggctgcttgaaaggtcctgagacagtacatgcaatgaggactgagcttgcagagg
gagcacaggcatgcagaaggttctctgtgcagccccacacctagtcaccttaaccatcat
cctcactccacctccatagagcaggtctcctgtgtgtggcccacgcagggctgccaggac
actgagaacatttcctcctcccgcaggagacagaggtccgaggtgccctacatcgtacgc
cagtgcgtggaggagctcaagcgccgaggcatggaggaggtgggcatctaccgcgtgtcc
ggagtggccacggacatccaggcactgaaggcagccttcgacgtcagtcagtggtggcct
ggggaggacaggatggaggtgtgggaggcggtgtccgcgatgagatctcagagtgctcca
tggtccgggcatgtcacattcttctctgtgtcttttcttcatttacagtgttactatttt
taaaaaagagaagacaagaattatagaaatagcttctgtagaagccagtttttaaaccat
cctagccatgcatgccacttgctggggtgaaccaggggcttctgtggggccttggccttc
ctgccttgggggtggacaggaggtggaagcccaggactcagtgcagtctgtccactgccc
tgtgtgacgatgcggtgggcagaggacactgatgggacccagctcaggctggggctgcag
catctctgcctccatttcaccaactctctcaggctatgaaagacatggacctgcctcaag
tgccagaggaggacacagaggccccagagggtcctttccagcatcttcaaagcaacagga
ttttgtgcctgcagacccttctttggggcacacaccactgaccctgaccaggacccctag
aatgcctatcacccctgggtgggccctgtggtaatttccctctgggggcccagaatagac
ctggcctgcggtgaggacgcaagcaccagtggcccattgggtccaaaggaagacattgat
tcaaacactgaaaccaatcagattctcccacagccttcctgccatcagaagacactggtg
cagggttggttgctatgtacagggcagagtcacccgattcccacgcaggcactgtgtcct
gctgtgctggcctcctcctggccatcacatcgggccaagcaggggagaggaatgggaatg
cccatgcacccccatcaactctgcagacacagaaccacacacagctcttgggaggggtca
gatgagctgcttaaagccggggagggacccgcacagtggtcaacatggcagggacggtgc
tttagccaagcctgggatggtgggagactcacttgggatcctgaaggaggccgctgcatt
tccatgctctttccagataacaaggacgtgtcggtgatgatgagcgagatggacgtgaac
gccatcgcagggacgctgaagctgtacttccgtgagctgcccgagcccctcttcaccgac
gagttctaccccaacttcgccgagggcatcggtgagcactggaggccttggcctcatggg
agacgtctcctccacatgcactgctgcccttggaggctgtgaaaagtgatgtgtgggaac
ctgagctgtgccccctctgccatggtcggtgttttaacccaacctcagaaaacaggacca
aaatcaagcctgtcctggaagacctcgcccatccccagagggctccccgtccctattcct
caaggagaccaagagggtgaaatggtcagcactgccgtgctgtggggtcctaaagtctgc
tgtcctccttcctgcagaccaggactgaaggagcgcccaggtgctctagccacgggtcct
ggcccagtcaagcatgggttcaaacctggcctgacccttagtcaacctgcaggctgatgg
ctagagtgggtgctggttcatgtggcacctgtagtctccacatcacccttagggcaggtc
tgcctcccaggcccatgcacagaggacctgcaggtggccctgtggtgtccaggacaatga
gggagtctctgcatacttggtggggctggagccctcccacttcccacctctttgtgtccc
tcactcccgtttcattccacgccaaacctcccctaccttgggctcccctggggaggggt
ggtggcaggagatgcccaagtgcagctctgtccatgagtgactgctctcgcagctcctcc
tgctgctgttcgccgggtgctgctgacccctgtgaggtggagaaaaggcgttcaggtggc
tcataccccacaccagcaccccttgcagagtcctcactgggggccagagctgtgggactg
aggatgatgacaaccctgggctatgcagggacacgagccccaggcactccacgtaaccac
ctcaggagaggtttctcaggagagcaaaaattacacggggcaggctgggcatggtggctc
acacctataatcccagcactttgggaggcccaggcgggtggattacctgaggtcaggagt
tcaagaccagcctggccaacatggtgaaaccctgtctctactaaaaatacaaaaattagc
tggacctggtggcacatgcctgtaatcccagctactcaggaggctgaggcaggggaattg
cttgagcccggaggcagaggttgcagtgagctgagatcatgtctctgcactccagcctgg
ctgagagagcaagactctgtcttaaaaaaaaaaaaaaaaaaaattacgcggggcagaagaaa
gaaggcatggaggcacgggtgatgggcggagggcccggctgcctgcggcccatcgtgctt
gcctgcgcagcctgggttgggggtgcggagagtgggcgaccatggggtggtgtggcctgg
ccccagctccagcatcattgtctccacagggacgtgcaggcgctctggaccaatgaccac
gcgctggcctggcacctgagcaatgacttccgagaggaccctgtggcctgggcacgcact
tagtgccaggcctgggaggagctggaggatcagctgcccagtttcctggaggagctgccg
gactgcccctgcaccctgacccaggcccgggcggactccggccgcttcttcgtgagcctc
caatcagggcccaggagaggggatgagggtgtcgcctccccactgaggacagcaccaggg
gaggcagatagaggtgtcctggagggtggggcgggggtctcagggcacctgcagagttgg
cctcgggaaggggatgacagaaccggaggccactgggtgacagccacctgctgctctgca
gacggactacggctgtgacatggagcagggcagtgtgtgcacctaccaccctggggccgt
gcactgtgtgcgctctgtgcaggccaggtgagcccccaggctggggccggggtggggact
ggggacaggggtgggctcccaacagtggcctggccgtgacccactggctcccgcagccct
cggtacagctcgggtcagcagtgctgctacacagcggacgggacgcagctcctgatggct
gactccagcagcggcagcactcccgaccgcggccatgactggggcgcacccccgttccgc
acgccaccccgagtgcccggcatgtcccactggctctacgatgtcctcagcttctattat
tgctgcctctgggcacccgactgcgcccgctacatgcaacggcggccctccaatgactgc
cgcaactaccagcccccgcgactaggtgggtgccatcctgtgccccggaccctgggaaag
atcgggctgggctggggtgcaccccacctgaccctccactctcaccccagcctccgcctt
cggagacccacactttgtgacctttgacggcaccaacttcacattcaatgggcgcggaga
gtacgtgctgctagaggcagtgctgactgatctgagggtgcaggcgcgggcccagccagg
gaggatgtccaatggtgaggccagggctaggggctgctctgggtggcacagggtagatcc
aaggtgggaggctggagccaagtggcgcccgttccgctcccaccaccacaggcacacaga
cccgtggcacagggctgactgcaatggccgtccaggagggcaactcagacgtggtggagg
tcaggctggccaacggaccagaggtctggaggtgctgctgaaccaggaggtgctgagct
tcgccgagcagagctggatggacctgaagggtgagtagtccagccacgtgaggcttcggg
ctgccctcacctcctccccattcctgcggggagactgaggggaagccctgggccttcacg
cctctcccagccctggctagaggcctgggcggtccgacctcaggccttcacacccaccaa
ggtgcccacatcatacccacctggtcaaaagccaagaggccaggatgggggacatgtcc
tccctacagagcatcccgggagccatctggagggaactcacccggtaacgttcaccgctg
gcctgcacagagccactcttgtgctcctgtcactccctcagtcctcaaaagccactgcaa
ggtcgccagccctgcacggttaaggatgtccctggcaagcggtagcgccagcatccgaac
cccttgcttcagggaactgagcgaatgaaaagattcctggttgggatgggtggggacctg
ggaggggcctgtcagcactgagggtacaaggaccctcgggacgtgcccaggcaggtgtgg
ctgctgcagccaaggccagagggacccacacgtgcatctcagcatgggggttcaccgca
ggggctgcacctcaggcgccgctgggggttccaccacaagcaaggcaggggtggtggagg
gtcatggtcaacagaattggccctggggacgccctcccttcccctgcaggtgccctcacc

cacagcccctgggtgcaaccgccgccaccaccgcaaggcctgcaaagatgatgcccaaaa
acaccgtgtagctgcgaactggtggggaagcgagtgtcagcgaggggccctctggggtgg
tgagtggggacaggtgcaggcaggtatatgagcagggtgtcctcacctggctggcactcc
ggggtgggtgaggaccaggtgccatcagcctggcaggtgctggtctctgccctggccagg
ctgtagccgttgtcacagtggaagtagatggtggaacctgccaggtacctgttgcccttc
ttttgtccgttgggaggtggggccagccagccacaggacactactgggatggcggcagtg
tgtgctgctgcgacacaggaagggcaacatgtgagaccccccagcccctgtcccgagacc
ccagggcagccggtggaccgccgagcctacttctaactgcccagcctggaaacaggaac
ctaaaaagtattgatgacagagataggagagggctggggccctgctcattttgtctggag
cttaactcacttgaggtaaaagtttgtggctggcagagaggttggtaaatgagggagaaa
ttgcaaaaaggagagagtcactgattgtaggaatcccaaattctaaatacaactgaggac
aaagccctggctgcccctgcactctgtgtgtgggggaagatgtacggagcccagcttcca
actcacttggtcaaatgaggaaactctggggaaaacgcaaagtctctacagtcagcactg
cttggggaggagagtggctgtgggtgctcaaggaggtggaacttcattccccaggcctgg
gtcccagaggaggacggcctgtgtgctgtgctggacggtgatgttcggcttctccccagg
tgggagccagcagctgctgaggaagggtgatgtggaacaaaggagagagcctgggtctga
ggtgtgtggtggttttaatggatctgggtttctaatgagacacccccggcgctgatgtga
caaaatcacaccattgtgtctagtgttgtatcttccggggcaggagatggtggtttctgt
cctgggggctgagcccctgcaggaggctggaccagcacgggtagatgcagagacgcgtga
gcacagggcagggtccaccctggggtggcagagctgggagggtaggcctgcaagggcct
caggggcctgtgcttgggtcatgtgcgaggacagtgtggaagaggggacccaggcaggat
ggggacacccataaccctcctcctgaggacccaaagagaaggcagagctgcctcaaatct
ttccatatgacagttcattttgtgtgtcaaattaggtaggccacagaacccagtcataca
ctcaaacagaaatctagatgtgtccatgacggtatttttaaagatgggattcacatttaa
agcagcagactttgattagccttcatctgtttttgcgtttttgttgttgttgttgtttt
ttgtttttgctttgagacagggtcttgctctgttactcaggctggagtgtgggagtgcaa
ccttggctcactgcagcctcaacctcctaggctcaaacaatccacccacctccgcctcct
gagtagctgggaccacaggtgtacagccccacatgtggctaattattttattttattttt
tgtagagactgggttttgccatgttgcccaagctggtctccaattcctgagctcaagtga
tccacccacctcagcctcccaaattgctgggattacaggtgtgagccaccgcacctggcc
aagttcatcatttacaatgtgagtcatgtcctttacccaatcagttgacagccttgatag
aaataagatgacgactgagctgttacctctgagatgacaataagaaacacatcacccagg
tggagatgacagagactttgcttgggtaagagcctatcagatccatgtcaccccaagtcc
atgttgtaggtgacagcacagttttagctcagcaacgctcagccccgggccggctgcacc
cacactctgcccatcagcccagagccgtgtgtcttgcacgggtctgtgcctcttccctg
actgcagctgtgcttgctccttggctcaccctttctggttcctctaactttccaccagca
tccttcacctccccttcctcagatacaagcaggccccatggacccagaacaggattcttt
cagtacttaagttttacttccatttttttatacttttcaaactaagcttggcttgaccca
aatcaacatctttcgacctccaccaaagtctccaacgagatatttttatcttaaaccaca
aatttctccttgaaaaaacataattagggtgaggcaatatgaacaatggatagagctaaa
ggttaaactaaatataagcatatggccccgtcagatctagttgtttgaggaaaatcaggt
gttgtttgaagaactgacaacagaaggcatgcctgagctagacttgggaaaagtcaccag
ccaagatgactgtgcaatcccggagaacagaagggcggttttgcttacttctctgtaggt
cttgggcactaagtcttagagttgctatgccgtacattacagtaatagcctcatcctcag
gctgcagcccagaggtgtgcagaatccctgcattggctgaggcatctttacttccagaga
agcagctggggactccagagccctggtgtttatttgagtctgaatagtattctcaggaga
aactggggagggatccctggcttttgctaggaccagagtattgagaagtcatcaacacgg
aagcacagaagccactgctcagggtgcaggtgagtctggcagatgctcccagggatgcag
agagggagggcggctgagtcagcatgggctgggagagggaacctgcagacagatagtcct
aggtgctaggacagtgaccagggttaagctgtttgggggtataagccagaggaggatgac
acaggctcaggacctgaccctggacatcaaaagactgttcctatctgtgcagtgagagag
gaacatgaggaggaggggatccaggccctggtggacacagccctcctgaaacctgagtgc
cgtgctgggctgcacaggcctttttttatcttctccacttgcctggtggggaggggctgtt
catgtcaatttgtttgatccactggtgatctctgcctcaccctgagaaccagctgaggtc
tgagcttggttctttagacagaggttctgaggggtgagactcagagtttgccccattgga
gggaccaaggaggaagcagcttctgggaccttccacagatctgtgagcacgagactctgc
ccatcttggaaggacacagctgctgactcctcagcagcaaacacaggctttgctgcccta
gccctggggagatagggggggcggcacagctgcgggtgctcctgtgcagaccctgagacag
acccacagtgccctctgctgctcgctggccaccctgcagtctctcagcggaaactgtgag
tggagtcacctgagagccaggccactgtcaggccacagaatctgtgagtcaagactctgg
ctctgccccgtgttactgacaggaataagaatcctttactcatgaggtttttctggattt
ccaagagaagcccacgagggaaatctgatgaccatccaatgtgaggaggctgcagggagc
ggggaatgcagagggctctggaagcgaagacctgaacagctccctccacaggggccaggg
aaggaggcaactgatagttggatattgtaaatgggaaaatgtctcacatgtctggcctcc
atcctggatgattttactcttaggccacatacgttgctgcctctcctgccacaggatgcc
tctcctgagacccaataccatggatgggtggctcagcacagcctgggccctggaccctga
gagatgagtgtggtgcaaaggaacatgctttagtccactccggaaacactagcaaaagac
caggacaaagcagcagtgagacaggcgtgaggacgtcaaagtgaaaccatgaaagaccac
tgtggtgaagcagtggacagagaacaggagtggtggaggcgtccccaggggtaacaccag
gttttgtccacggaggtacaaatttacttgttcaggtcatctcagtgccctggatcttct
tgaagtgaaaaatcactgaagtctttgtgctttctgacattctgggccctctcttccttc
atcttcctttgctgccccagcttgaagagggtctgcattggcacattcagtgaggggctc
tgaggcggtggtgtcctccctgcgtggacattttccaccagaggctgagaggtagatcaa
gtgctgagttcctcttctcaaatgcagctcactcccccgctctctgaatgagaacctgtt
atctatgtgtgtttctcttatttccacccagggacgcttttgttaaaactgtgtatatct
cctctgtttaaggggcagcggtggccgcaggtgctcctcatgcttgaaggaatgggaggg
gagtgtccacagtggctcggctccttggctgggcgcaatcactggcagggtcagctccaa
gtcctcattagaccagcgcccaggctccttagcccaggaatgtccagaatctgggcacag
agcccaggcctcaggccaccatggcccacctgagcagctcctcctctgcacttcctgggg
cccggcatggaggcacagcccccaaggctgctggaggatgaccagggcctatcaacattt
gtggagagggtcctgaatgggggctcaggcagggtagcattggtcagatttgccccttag
gtctcagagaaagtgctggagagggtgaggctggaggttgggggctgggtggagaacggg
gttgggaggagaggaggggggggagttgagccccctcagtgtggcctgcagcgctgggagt
ggccacatgggggcagcagagtgtaggaacagcgagtctgggtgccgggaggttgggagg
gcagtcagccccgccctgcagctggatcaacagggttggtcacaactgcagccaaggtcc
gccacagcccacccctctgccgcccctcacctgggccatcccccccatctcaggagtgttg
aagaggttgcacaggttgccccaggtcattcagcaggcactgaccatagggtgggagtcc
aggcctcccacctacttggccttgcccttggcgaatgggagagctggggaggcagaggca
ggtctcatctgaggccttcctggaggaggggaatgtgcatgatggggcctgtgtgcggga
acagagctgagagggttgcagggcagggtggaaggaggctagcagatgggaatggggtct
ggttcctgtctttctagacctcactctgttgcttccccattccctgggttttcacctcca
ggcacggtggggctgagggccagccgcttggccagctgggcaggcactttcctccctaca
gatgaggtaaggcagggctcccctctcactgtctgaatccccagcgcaccctgtgccatg

ccaggcctgggccaccacgtgtcagtgggtgagaggtcagtttcctggtcaaggcctgga
catgagggagcgggcacaggtttacagggggtccaagaggggcaggggcagcttggcagc
tcatcaggtccactagagggtgtcccagacaagaacagcccccgaactgaagtcaccaaa
caggttggattctcagagctctgattggttggacaaggtgttctgggtagaggaacagtc
tatgcaaaggctcagaggaagtgcagtcgtatctgcggaatggcaggggtgggggccagc
caaggccacccctaccaggccaaggcagttagacttcatcctaagagcaaagtgtggcct
gcctcgggctcccccacccccaccaatctccctccaggatgggcccttggtctgcccccg
cacctcccctttccagcaggagaaagggtgccctgtctgtggccctagtcccagacatca
gatgttcccagtgacaggggcgggccagcctgggaagccccaccacctgccaggggcttt
tcctggggcctgaaagagacagaggcagggcctgtgcaaaggcggaaggcctggagccgg
tgcagactctctagagacatcaaaggcggtgacctccgcctcaggggggatggtctcccaa
acagcagcccagggcttgcaggacgggcagccatgggagctttgcgggtggttcacggtg
gggttgaaatgacagagtatcagagtcacccagtggcctgtaccgtcgtggtggtaccaa
tcagagtggggcagatggtaacccagggctttggaaacacaggtgatgagagagctgggg
ggtgtaacaggagctgatgaggtccctgcaggatcccaagacccaccttgggcagaaggg
caaagggaggaaggagtgtgctatgggagccaggtccaggtccccatggaagtgggtgtc
acacaggcctgtgggccgaggggagtcacagaggtgacagcccagctgcaggtccccgtt
gactagagcagggaagccccacttctctgtgccccctgccctccagcctcccactaggtc
tcttgttggctgagcacagtgggtgcggctgacctgggggccaggatctgactcagtaca
gtacagtacagccacagctggggagcgagggacccccctcctctgccacgctgcttccttc
tgcaagctccagggtggccgagtgcctgctgtgggccagagctccactaggcccatcagg
acccccctgtcctcagggtccagtgctgccaaggaggagggaggggtcggtgcagcgcat
tgtaggagtgggaagtcagggaggcgtcctggaggagtccggcttcccacaggccccctc
caggctttcgccagctccctttgttgcaaggatgtaaggatgactgactttggaagccat
gccaaccaagtccctgctgacctgatctcccaccactactgttccaggaccaggaaataa
acttcttttctgattaagccttaatatgtccaattaagccttaatatggaccatttgttc
aagcagttggttggtcctgagaaaggaaagaaacttctctttggtgagacagggtctcac
tctgccacccaggctggagtgcagtggtgcgatcatagctcatggccgcctcaacctccc
aggctcaggtgatcctcctgcctcagcctcacaagtagctggggccacaggcacgcccag
gctggtcttgaactcctgggatcaagcaatcctcctgcctcggcctcccaaaatgctggg
attacaggcacgatccactgcacccggccaaaaataaactttttacctgaggaatgagtg
ccccctttaattatcaggcccagagaggcattaaaatgtgacagggtgtcacagcagtcg
tgcctcactcccaccttgatgccacttgctaggtgggttctagactaaccgatgtcaagt
agccataaaatgccatgtgctggacaccatgatgtaggggccacaggaaaacttcccctt
caccctctgaaggttcaaggaaaaatcagctcacaaaaggcagattaattggaaaacaag
ccatgcaaatttattagcatgcattggggagaatcatagagtcattgccgaatatcccag
ataaagatgcttatataccccgcttcttaggggaaagggagatgggaatctctcctttga
tactcagctgcagccagactcctgcaggaagacccccaacctcccactcctacaacgcac
tacactggccactccctcctcctcggcagcactggaccctgaggggaggggtctcctgat
ggcccacagcgggcacttgatcaccccgggctggcaggagatgggagtgaggatgatttc
agaaggacaagtacatgatttgggctggggaggatttttgtttgtttgttgtaagaggga
gtctacctctgtcaccagcctggaccacccagctacttgggagattgagccactgcccct
ggcctgggtggggagaatttaattggtttaaagaacgtacagggccgggcgcggtggctc
atgcctgtaatcccagcactttgcgaagctgaggcgggcggatcaggagatcaggagatc
gagatcattttggctaacatggtgaaaccccgtctctactaaaactacaaaaaattagcc
aggtgtggtgggggcatctgtagtcacagctactcaggaggctgaggcaggagactcgc
atgaactcaggaggtggagcttgcagtgagccgagattgcaccactgcacaccagcctgg
gcgacagagcgagaccccgtctcaaaaaacaaaaacaaaacaaacaaaaaaaaacatacaa
tggggctgggcacagtggttcatgcttgtaatcccagcactttgggaggccgaggcaggg
ggattgcgaggtcaggagtttcagaccagcctggccaacattgtgaaatcccgcctctac
taaaaatataaaaaattagccgggtgtggtggtgtgcacctgtaatcccagctacttggg
aggctgaggcaggagaatcacctgaatctgggaggcacaggttgcagtgagccaagatca
tgccactgcacgccagcctgggcaacagtgtgagactcggtctcaaaaaaaaaaaaaagaa
catacaaacatataatgggcctggctcggtggctcatgcctgtaatcccagcactccaga
gggtgaagcaggcagattatgagatcaatagatcaagaccatcctggccaacatggtgaa
accccatctttatgaaaaatacaaaaattagccaggcgtggtggtgcacacctgtaatcc
cagctacttggaaggctgaggcaggagaatggcttgcacctgggaggcggagattgcagt
gagccaagatcgcaccactgcacgccagcctggtgacagagcaagactccatctcaaaaa
caaacaaacaaaaacaaacaaaaccatacagcgccctgggacaaagtctgttgggcctga
agagcaaacaatagattgtgacgagtctgtccaggtgtgttgacagttttcagtctttct
tcctgtgacaggagttcagttaaggaagactcacaggaggggccagagctcatggttttc
ttctttggcagatccagacttcaggcagataaggaacttcagagaacaacttcatcctga
gctctggctgacagactgagaccgaggcggggagaggtcagagaccttgtggcttcttct
tcagttcagtgccatattttggggtatcagtttctgagccccagcaataactcatactct
atagttcaacagtaaccagccaggcaaggagcagtggctcacatctgtaatcctagtgct
ctgggagatggaggcaagaggatcgcttgaggccaggagttcaagaccagtatgggcaac
atagtggagcctgcctacaaacacacacacacacacatttagccatgacagcatgcacct
gtaatcgcagctactcaggagactgaggcaagaggatcatttgagccctggaggatgagg
ctgcaatgagccatgatgacaccactgccaacagagcgagatctcatctcaaaaaataaa
ataaaataaagccaatcactaacgcgtgttattgaaactgcctttgcaaaaaagcataat
tgaggaaattatgacagtgaaagaaatcagaggtgaccaactctccaagagtctgaacct
cctcaaattgctgctggggataacatcactattgtaaaacttgagatcagggcttgagat
attttgcagaccctgcactccatggatcagctgacgccacccagactgctattctggctc
aaccagttctgccatcacagccaggaacagaagacagcaagaaaaactcatttccacccg
ctgtgattccatcttcaacctgaccaatcaacactccccatttcccaagcccctacctgc
caaattatctttaaaaactttggtcaggcgcctagactcatgtcagtaatcccagtaatc
cctgcagtttgggagactgaggcaggcggatcacttgaggtcaggagttcaagatcagcc
tggccaacacggtgaaacctcgtctctactaaaatcgcaataattagctggatgtggtgg
cacatgcctgtagtcccagctgctcaggaggctgaggcatgagaatcacttgaacccagc
aggtggaggttgtagtgagcgtctcaaaaaaacaaaaagcaaaaaacaaaaaactctgat
ccccgaatccttggggagactgatttgagcgataataaaactccgattgactctgcatga
attactctttgtccactgcgactcccctgtcttgaaaaataggctctgtctaggcagctg
gcaaggtgaacccattgcagttacattacctctctaaaccactgagaattcctgctaaac
aattttatatgaaaccactccctatcctcttttactatttatttatttatttttttgagc
cagaatctcgctttgtcacccaggctgcagtgcagtggcgcgatcttggctcattgcagc
ctccacctcctgggttcaagggattcttctgcctcagcctcccaagtagctgggaccacc
agtggtgtgcgccaccactcccagctaatttttgtttgtttgtttgttttgagacgtagt
tttgctcttgttgcccagctggactgcaatggcacaatcttggctcaccaaaacctctgc
ctcccaggttcaagcgattctcctgcctcagcctcccaagtagctgggattgcaggcatg
tgccaccacacccaactaatctaattttgtatcgttagtagagatggggtttcactctgt
tggccaggctggtcttaaactcccaacctcaggtgatctgcccaccttggcctcccaaag
tgctgagattacaggtgtgtgccaccatgcccagtctccttatcctcttttgtctttaaa
gaaactgcttttgggccgggcgcggtggctcaagcctgtaatcccagcactttgggaggc

cgaggcgggcggatcacaaggtcacgagatcgagaccacagtgaaaccccgtctctacta
aaaatacaaaaaattagccgggtgcggtggcgggcgcctgtagtcccagctactcaggag
gctgaggcaggagaatggtgggaacccgggaggcggagcttgcagtgagccgagatcgcg
ccactgcactccagcctgggcaacagcgtgagactccgtctcaaaaaaaaaaaaaaaaga
aactgcttttgtggccaggcatggtggctcacgtctgtaatctcaccactttgggaggcc
aaggcaggtggatcatgaggtcaggagttcaagatcagcctggccgagatggtgaaacct
catctctactaaaaatacaaaacgttagctgggcatggtggtgggcgcctgtaagctact
cgtgaggctgaggcagagaactgctcgaacccggaaggcagaagttgcagtgagccaaga
tcatgccactgcactccagcctgggtgacagagtgaaactgtctcaaaaagcaacaacaa
caacaacaataaaaacaaaacaaaactgcttttgggccaggcatggtggttcatgcctgt
aatcatagcactttgggaggctgagttgggaggatcacctgagttcagaaattggaggcc
agactgggcaacatagtgagacaccatctctttaaaaacaaaaacaaacaaacaaacaaa
aactgcttgtaacaaaggtcgaatgaagcactccccaaggcaacttggaagtgtgtctgg
ggccactgttctcaaccttggcccaaataaactatactaattttgcctcagtttcttcct
ttaggtcaatagtccctgtaacaatacaccaaaggcataggtataaaaagatggccttac
ctttttcattagtgtggaaaataaatactgtgtgtgcaaggtgaaaaaaatctaaacaga
acaattcagggaactagcccctggctaggggacattgaagctgtgctcagccttttgctt
ctttaaacactatggtactgtgtaaataagttcaggttaacctggaggagaaattcacac
gagtagatcagctggttaaatggcacaagcattttctgtctcaagttatactggtgtaat
ctcagctcactgaaacctctgctgagttcaagccattctgcaaatattcacccattgtga
gttgtcttttcactgtttcagtgatgtcttttgttgaacagaatttcttaattttcatat
agtccaatttatcatttttttcttctatactagcttttgtgtcctgtttaagaaaaaatt
ttggtatgcaacaaaataaaaaaacagaatttaaaaaagagaagcatttacctactctaa
ggtcacaaagatactctctttctttctgtaaatactacttaatattttatttgattattt
atttatttatttatgtatttttggagatgggggtctcactacgttgcccaggctggtttc
gaactccttggctcaagtgatcactcggttgctgtatttttagtacagacaggatttcac
catgctgtcctggctgatctcgaactcttgacctcgtgatctgcccgcctggggctccta
aagtgctaggattacagatgtgagccaccgcgcctggcttgtttgtttttgagatggagt
cttgctctgtcacccaggctggagtgcagtggtgcgatctccactcaatgcaacatcttc
cttccaggttcaagcaattctcctgcctcagtctcccaatcagctgagatcacagatgcc
tgcaaccacaactggctaatttttttgtatttttagtagagacaaggtttcaccatgttg
gccaggatgctcttaaactcctgacctcaagtaatccacctacctcagccttccaaagtg
ctgggattgcaggcagtgagccacagtggctggcctatttattttgatacttaatttttt
ctttctttcttttcttttcctttttttttttttttttgagatggagtttcactcttgtt
gcccgacctggagtgcaatggtgccatctcggctcaccacaacttctgcctcgcaggttc
aagcaattctcctgcctcagcctcctgagtagctgggattacaggcacacaccaccacat
ccagttaattttgtatttttagtagagatgggttttctccatgttgatcaggctggtctt
gaactcccgacctcaggtgatccgcccgcctccacctctcaaactgttgggattacaggc
atgagccaccatgctcggccttgattcttattttctacgtaactgtttaagcgatgaatt
ttcctctgagccctgcattagctgtaccctatagggtctgatctgtgctgattatagtgc
aacttgaagatgctgcccttttggtgttgatttcctctgtgtaacaggatgtttcaagag
ccagctgtaagagtctctggctgggctgggaatgcacattggctggcactgctggtggtg
cagcattgatgtgagggcagggcctgggcaggttccccagggtgggatgagcagggaggg
agaggaatgggcttcctccagggcctcaggaaccaaggaagtctccgaactacctccagg
cagggtcagatgtgcattttccaaagcttattgcattaagagtgaggagtagactgtggt
ggtgccagcagcataaagactgaaaagaaataaggaggtggagccagggctgaaccacac
agctggacgggatgggcgggggcagtgggggggggattctacaagtagcacaggagggag
gcgaggcgggcaccacagagctttcagctgcctcacggctgacttctatttggcttgttg
agtccaaggtgcctgtgcaccttgtccagatgctctggaggtggttggggaattgctcgt
gtatgtgagtctgagactgcacacagctcggccaatgccaacatggagatccatcagttc
acaagtggtgaacgagccttctctgggtgggtggcagtcccaggttggccaagagtccag
ggagtgcaccctcagtctgcaacagagttagaatccggcaccacggagccagggttgcac
ccctgctgcggggaacaggagccccttttcctctccctgacatgcagggacaaagttatc
atgctctgtgggtgccgctgggaggcactgataggaaagactggccaggccttcccaagc
aagctccaggcaacctcccagcaccccggcccagatgtaggtgtggtctgggagcacaga
gccaggttcccaccgatggggcacctgaggtttgtgtctagcagccttttcttcctttgg
ttccctttttcctgtttggcccacagggtttgggacaggtggctgctaccaacaggtgtca
aggtggccccacagacctgagccagccacttctccatagccctcccctgcccatgaagga
ctgctcagaggggcccccattgagagctggatctggacttggttaaactgcaaggggaga
agctctgtcaccctgcagggcgtgagttcagctgaagcattgggaccatggcgctgggga
gccaactagggggaggctaccacacagagcgcctgagttctgggcacggcccacagagag
atcagtgatggcggcaggtctgcccgagtgggtgggccggcttggccccctatctccaaa
gtgagccagcttttgctccctgacctcctcctagtctgccagacccccccttccagccacg
cagtcaagcccacattaggaattagccccatccagcccctctcccacatacgacctccaa
atatgtccatgtcccaatccctacaacccaagattagaccttatgtggcaagagtgactt
tgaagatatgattcaattaaggattttgagatggggagattatcctggaccatccaggtg
ggcccactatgcccccaaaattcacatgttgaaagctaatcaccaatgtgatagtatcag
caggtggggcctttgggatgtggttaggtcatgagggtagagccctcatgaatgggatta
gcgccctataaaagagaccccagaagccgggcatagtgggctgcacctacagaaccagct
actgaggaggctgaggcaggaagatctcttgggtacagcagagagctctatgatcacgcc
actgcactccagccttggtgacagagtgagaccctgtctctaaagtaattaaataaaagg
gaccccagagagctagctagcttcttccactatgtcagttagaaggcgccatataggccg
ggcgcggtggctcaagcctgtaatcccagcactttgggaggccgagacaggcggatcacg
aggtcaggagatcgagaccatcctggctaacacggtgaaaccccgtctctactaaaaata
caagaaaattagccgggcgaggtggcaggcgcctgtagtcccagctactcgggaggctga
ggcaggagaatggcgtgaacccgggaggcggagcttgcagtgagccgagatcgcgctact
gcactccagcctggggcacagagcaagactccgtctcaaaaaaaaaaaaaaaaaaaaagaa
ggccatatatgaggacgtgggccctcacaggacatcaaatctgccagcaccttgatcttg
gacttcccagcctccagcgctgtgagcaataaatttctgtaaaatgctataagccaccca
ctttccagtatttttgttatggcagcccaggcagactaaaacaacctctgtggcacattt
ggggtgatgtaggaacagccgatgttcccaggaaccagtgaggggcaggagtcctagatc
cctctagcgagatccccagagagcgccatgtacccgccctgcagctggcaaagctgttta
tggaagttaggggcctgttggtttagagcggccattctcaaccaggggggatgtggcactg
tcttgagacagttttggttgtcatgagtgggtgtgtgtggtggggagtgctaccgcatca
cacaggctgaggcacgggatgctgctcaacatcctacagtgtggggacggccctcacaac
aaagaatcacttcattcaaaacatggatgagggccaggcgcagtgactcacacctgtaat
cccagcattttaggaggccgaagcaggaggattgtttgaagccaggagtttgagaccagc
ctgggcaacatggtgaaaccccatctttacaaaaaaagcaaaaaaccagctgggcgtggc
gatgcacacctgtactcccagctacttggaagcctgaggtgggaggatggcttgagccca
ggaagtcaaggctggagtgagctgtgattgtaacagtacactccaacctgggca

>BCRP3.HUMAN.NCBI.REF
actccgtagtgtgcacttggtgagggcagcagctcgccacagctgccagccgtctgtcca
ttcacccatctgtccatctggcagcccgctgttcagacccgtctgtctgtccgcccatct
gtaagcccatctctgtcccattgtctatctgaccatctttctcttactgtcctctttgtc
tagctatctggcctatctgtcgatccatcttcgtgtctgtcttcagccccccacctgtttg
tccatctgtccaattacctgtgagtctatctatgcaccttcttgtccattcatctgccca
cccatctgtccctccgtctgcccaccggcctcccctctccttctgggccgcagagccatg
gcccaggactgcagagccatggttggcctggtcctgctggggctggggcttgtgctggct
gtcattgtgctggctgtggtcctctctcgacaccaggccccatttgacccccggcctttg
cccacgccgctgttgctgctgactccaaggtctgctcggatattggacggtgagtgagac
gtgggaggaagctgggtggcccttggcagccagcccctcctggagaaggcgtgtgtgtga
gagtgtgtgtgtgtgagcatgtgtgtgtgtgagagagtatgtgtcagtgtgtgtgggtat
atgagtgtgagtgtggggtgtgggtgtgtgtgaatgtgtgtgatcgtgtttgggtgtgtg
tatgtgtgagtgtgggtgtgtgtgaatgtgtgtgagtgtgtttgtgtgtatgtgtgagtg
tgggtgggggtatatgagtgtgagtgtgtgggtgggtgtgaacgtgtgtgattgtgtttt
gctgtgtgagggtgtgtgtgactatgagtgtgtgagtgtgggtgtgtgtaaatgtgtgtg
attgtgtgagtgtatgtgtgggtgtgagtgtgtgagtgtgagtatgggggtgtgggtgtg
tgtgaatgtgcgtgattgtgtgtgggtatgtgtgtgtgtgtgagtgtgtgtgtgtgcg
tgtgtgtgcacgtgcactggcccaggcagcaggagccatgtgtgtgggcttcagcacctg
cagggcttgagcgcaaggagacagcctcagggcccttgcacagaacaggcggcagggtgt
gcccgtggggcagatggggacttggggacaatggtggtgtgtgagtccatacctggctcc
aggattcaggaggcccatttgcacatcccaggtgggaacctgtctggccccggctgaccc
tgctggccggtgcaggccccttcagtgaggccaattctccaaggctgcggtcttctccca
gggtcatgggtgaagggggtttggaggctccctgcgtgggtactggcctgctgggttacac
acaatgctgccatagccagtctgcccctacacccagcctggggccacatctcaggtctct
cagtcctgaggagcccggtgccccacccctcacatcctctctccctgagtcagggcctgg
gtctcgtgagctgagtgactgatacttggtgtcctggatgagggcgtgatggagaggggc
cacagcgggtgtttcctgaccctcttccaggaaggtgctgctgccgctgcagggaggaca
catacaggatgccccttcctgccccctgcctcccattgggcccacaaaagccagggcaag
cctcccctccctgccagccacctggtctgcttcccagaaattctgtcttgcaggctgttg
ggaggatcccagtactttgtaaactaaagcaagggaggagtggccgttctctctgttcat
tcattcaccttttcattcattccttcttccctccattcccccatctgtccatccttccct
gccctgattgctcatgccaccgccccccgcagcccctcctgacctggtcctttggtttct
cttcagggatttctgtctcctcccacagggctgagaatggcagctcagggacaagtaggg
gctggggactgcttagtctccccagtggctctcaggggatttgagggtttgacgccagct
gccaccccaggctgtgcccctcctctgctcaggaggacatacaggatgcaacacccactt
aaactcgaagttgcaaagatgcaaatgagactggggtctcaggcaccagagaccacccgt
gggcacgtggcttttgggattggagacctgctgccacagatctctgaagagtctggacct
gctgggtctccccaagtgactctctgggggtctccatagcatgccctgctgtgtgcatga
cggtcactggttgggtaggggtctctactctaaagctccctctgccggcatcccctcgaa
ctctcccttggtgaagagagaggatgtggtttgccccagtgttttatcaaacaactctct
ccacttcctgttttaagaagctgggagtggaagagagcctggggctggccccagctgctg
ctgcgaaacaggggtcactggacgctgggaccctggccgggctggctggaggcctcagga
agaggcctgctacagtgtcatcctggccaagattcctccctgcagaggaccctggccacg
ctgccacagggtctgctggggccaccagaagcccatgctcctgcctccatctctcccctc
tgtgctcacctctcaccaggaggccctcccagagttcagtgtcctgctttttttttttt
ttttagatggtgtctcgttctgtcaccaggctggagtgcagtggcgcgatctcagctcac
tgcaacctctgcttccttggttcaaatgattctcctgcctcagcctcctgagtagctggg
actacaggtgccagccaccacgcccaggtaatttttgtatttttagtagagacggggttt
caccatgttggccaggatggtctctatctcttgattcgcccgccttggcctcccaaagtg
ctggaattacaggagtgagtcatggcacccggcctcatctcctactctttcagcaccagg
ttttactcttgggattctgctacagccgcagcccctgggtgcgagttcctaagctttctg
tgagtgtggacccagcaccgtgcctagtagacatacaaaaggagcatggtgacagtgagg
tctgtcatctccagcataatgactgttttgatccttgtaaaaaaggtgatttttggctgg
gtgtggtggctcacacctgtaatcccagcactttgggaggccgatgggggtggctcactt
gaggtcaggagttggagcccagcctgggcaacatggtgaaaccacgtctctactaaaaat
acaaaaattagctgggcatggtaacggatgcctgtaatcccagctacttgggaggctgag
acaggagaatcacttgaacccaggaggcaaaggttgcggtaagccaagattgtaccactg
cactccagcctgggtgacagagcaagacttggtctcaaaaaaaaaaaaaaaaagaaagaaa
gaaaagtttatatttttgttctaatggttatcttaatatcgtcattctataattgtatgt
tttatataattataatagctatataagatataatacccctagtatgttgtttttggata
ttctacttgctcctgatggttaatttatatgtcaacttggctaagctatggtgccccgtt
gtttggtcaaatacttgtcaatatcttgctgggaggttatttcatagatgtgattaacac
tgacagtcagttgactttaagtaaaacagattacccaccataatatgggtgggccacctc
caatcagttgaaggccgtaagaacaaaaactgaggtttcccagagaagcaggaattctgc
ctcaagactgtaacacacaaaccctgcctgagtttctggcctgctgactgctctacagag
tttaggttccagacttcgagatcaactcttacctgaatttatagcctgctggcttgccct
acagattttaaacttgctagtccccacaatcatgtgagccaattcctcaataaatctctc
tctatgtataatctattggtttagtttctctgaaaagctttcacatccagtttcctggat
gttaagaattactgaaactagctagtaacttcttttttttttttttttttttttttga
gacagagttttgctcttgttgcccaggctggaatgcaatggcacaatctcagctcaccgc
aacctccacttcctgggtccaagcaattctcctccctcagcctcctgagtagctgggatt
acaggcatgtgccaccatgcttggctaatttttgtatttttagtagagacagggcttctc
catgttggtcaggctggtcttgaactcccaacctcaggtgatcagccgccttggcctcac
aaagtgctggaattacaggcatgagccaccgcacctggctcctagtaaattcttcttttc
cgtgatgtgtctcttacctctaataatacttttcttcttttttttttttgagacggagt
ctcgttctgtcgcccaggcgggagtgctgtggcgcgatctccgctcactgcaagctccgc
cttccgggttcacgccattctcctgcctcaacctcccgagtagctgggactacaggcgcc
cgccactgcgcccggctaatttttttgtatttttagtagagacggggtttcaccgtggtct
cgatctcctgacctcgtgatccgcccgcctcggcctcccaaagtgctgggattacaggcg
tgagccaccgcgtccggccatacttttcttcttaaagtctacttcattaaaaatagttat
gctgggcatggtggctcatggctgtaatctcggcactttgttggaggtcgaggtgggtgg
atcactgaagcccaggagttcaagaccaacctgggcaacgtggcgagaccctgcctctac
aaaaaatacaaaaattagctgggtgtggctaatatacacttgtagtcccagctacttggg
atgctgaggtgggagaatcgcttgagcctagaagggagagattgctgtaagccaagatca
catcactgcactccagcctgggagacagagtgaggctctatctccaaaaaaaaaaaaaaaa
aaaagttatacagctttcttggttagtgcatgcatgccatatttttcattattttccacc
tctctgtatccttatataaaaggcattagttgggttttactttattttcaattattttaa
tttttattgtccttttaaatgtaactaatgatttatttgggttgaaacccaccaccaatt
tgttttccatgcctattctatttcttcttatctcctctcacatcttgttttggatttatt

atttttattatttaatttcctccttctctattagtttcatagctctgcagtcttagagtt
attttaaaagatgacagtggattattttagagcttacaacatgcatccttcacttatcaa
agtctaacatgagctagtactttttgttgttgttgagatagagagagtcttcctctgctg
cccaggctggagtgcagtggagcaatcttggttcactgcaacctccacttcttgggttca
agcagttctcctgcctcagtcacctgagtagctgggaccacaggtgtgcaccactatgcc
cggccaatttttgtattctttttagtagagacagggtttcaccatgttggccaggctgg
tcttgaactcctgaccttaagagatctgcctacctcggcgtcctaaagtgttgggattac
aggcatgagccaccgcgcccagcctatgagttagtacttctatgctcttcctagtcagta
caagaaccttggaacaggaactaaatttacccccagtgacttatatgctaatatttttgt
gtattttaaatatatatgtgtgcatagatgtatctgtgtgtttttttgtgtttttattctt
atttatgttgagagtgtagagctatgtaagagtaaagagaattgtgtaatgaagccccga
gtatccattcaatttcaacaacaatctcatggccaagctaatttcatgtatactctttcc
tgcttccctctaccccacattatttcagtgcaaatcccagatatataactgtacccatac
atatttcagtatgttttatttattttaaaccccacaatatatcattttctatactactgt
aatttcataccaataacattcatttagatttacccacacgtttacctcttctgttaccct
ttatttttatttataaaaaatatctttgggaagaaatatctttcagcacatggtcaaggat
ctcctgagggctatgtcatgggcaaaatatacatatatattccatatatatacacacata
tacacacacacacatatatacacatatacacacacacatatatatattccactttcactt
ttttgtttgtttgtttttttgagaccgagtctcgctctgtggcccaggctggagtgcggtg
gtgggatctcagctcactgcaacttctgcctcctgggttcaggtgattctcctgtctcag
cctcctgagtagctgggattacaggtgttagccatcacgtctggctaatttttgttgttt
tttttttttgagacggagtatcgctctgtcacccaggctggagtgcagtggcaagatct
tgtctcactgcaacctccacctctcaggttcaagcaattcttgtgcctcagcctcctgag
tagctgggattacaggcatccaccaccacatctggctaatttgtgtatttttggtagaga
tggggtttcaccatgttggccaggctggtctcgaacttctgacctcaggtgatccacctg
cctcggccttccaaagtgctgggattacaggcatgagccaccatgcccagccattttcac
ttttgaaggatattgttaatgagcatagaattctaggttggcagatattttctttcctca
gtttgaaaacatgattcccttgtatctgatttctcctgcttttattgggaagccaattct
caatctaattttgctcatttgaaggcaatggctttttctgttgttgtgttttctgaggtg
gagtctcactctgtcacccaggctggactgcagtggtgcaatctcagctcactgcaacct
ctgcctcctgggttcaagtgattcttctgcctcagcctcccaagtagctgggattacagg
tgtccaccatcacacctggctaattgttgtatttttaatagagatgaacttttgccatgt
tggtcaggctgatcccaaactcctcatttcaggtgatccgcccgcctcagcctcccaaat
gctgggattacaggcatgagacagcccacaaccctggcctgcaggcagtatcttttttcc
tctggctgctttgaaaagttttgtctttgttttgagcagtttacactgatgcatttaggt
ggctcctcattccatgacttgattctttttgtccattttagaaaactgccagctttatc
tcttcaagtattatgtcttccccatcctctctctactctccttatgagactccaatttca
catgacttatgccttgttaaagtatcccccatgtctcttaatccatttcctgtatgttct
atctgtttttctctttgtacttcaatttgtatagtttgtatcaaactatctcccaattag
ccgggcgtggtggtgggtgcctgtaatcccagctacttgggagcctgaggcaggagaatt
gcttgaacccgagaggtggaagttgtagtgagccgagatcatgccactgcactccagcct
gggcaacagagtgagaccctgtctcaataaataaataaataaataaataccagttcacta
tttttttttatgtttgtgtctagtgtgctgttcaaattgagttcctaattccattttttt
tttagactttttttttttgagtctctatctgttgcccaggctggagttcagtggtgcaatc
tcaactcactgtagcctccacctcccaggttcaagcgattctcatgcctcagcctctcga
gtaactgggattaccaccacgcctaactcatttttgtatttttagtagagatggggttct
gccatgttggccaggctggtcttgaactcttggccttatgtgattggcctacctctgtct
cccaaagtgctgggattataggcctaaaccaccactcccagcctccttttttttttttttt
tttttttgagacggagtctcgctctgtcgcccaggctggagtagagtggtgcgatctcg
gctcactgcaacctccccctcccagttcaagtgattctcctgcctcagcctcccgagtag
ctaggactataggagcatgccaccatgcctggctaatttttgtaattttagtagagatgg
ggattcaccatattggtcaggctggtcttgaacttctgacctcaggtgatctacccacct
cagcctcccaaagtgctgggattacaggcgtgagtcaccacgcctagtgcatccattttt
tgtagttgccagttttctgatgaaattcttaattgtttctttatatccttgatatacatg
taaagaacttattttaaagtacatggtctgatgattttataatctggagatcctatgggc
ctttttaaaagttgtctgtgctttctcttgagctttttttcctgctgtcttatttccttgt
ttgcttagttgtttttaatttggcaatggaagttgtgtataaaaaatcgttacaaataatt
tttttttttttttgagatggagtctcgctttgttgcccaagctggagtgcaatgacgtga
tctcggctcactgcaacctctgcatcccagggttcaattctcctacctcagcctcccaag
tagctgggattgcaggcaggtgccagcacgcctggctaatttttgtatttttagtagaga
tgggtttttaccatgttggtcaggctggtctcagactcctgacctcgtgatctgcccacc
tcagcctcccaaagtgctgggattacaggcgtgagccactgcgcccagccagaaataatt
tttaaaaataattttgagccccagcatgatggctcatgcttgtaatcccatcactttggg
aggctgaggcgggcagattgcttgagcctaggagttcaagatcagcctgtacaacatggt
gaaaccccatctctacaaaaaataaaaaaattagctgtgtgtggtggtggtgtgtgcctgt
agtcccagctgtttgggacgctgaggtgggaggctcacttgagcctgggtgatcgaggct
gcagtgagccatgatcctgagactgcactccagcctgggcaacagagtgagatgctgtct
caaataaataaataaataaaaaataaaatactttgaggcctaggggtctaaaattctgaga
tctcctttatgcatttgagtgactgagatgatctgaagctggatccagtgctcctgaggg
ctgctttatttctggttgactgtgactcctagagtaagaaacctgcaccccacatgtggg
gcattatggcatcccctccctcagccacatgagtaagtcaacagcactgctctagaccag
gtgtggtggctcacacctatagtcccagctactcgggagactgaggcaggaggattgctt
caggccaggaatttgagaccagccagagcaatatattattaggttggtacaaaagtaatt
gcagtgtttgcctttaaaagtaatggcaaccctgtttcagcaaaataaaaagcaaaaaaa
aaaaaaaaaaaaaaaaaagaaaggaagaatcagctgggcgtggtggctcacgcctctaatc
ccagcactttgggaggccaaggcgggcagatcatgagatcaggagatcgagaccatcctg
gctaacacggtgaaaccccatttctactaaaaatacaaaaaaattagccgggcatggtggc
aggcgcctgtagtcccagctactcaggaggcggaggcaggagaatggcatgaacccagga
ggtggaggttgcagtgagccgagatcatgccactgcactccagcctgggtgacagagtga
gactccgtctcaaaaaaaaaaaaaaaaagaatcactgctctgtctctcagcctcctcttcc
aagattggccgtcgccttgaggggaatgctggccttgcctgtctccagccctgtacctct
ctgcctcctatgcctttaagcacatgttttctatttgctgggctgtgaaatctgctcttc
atctgatggggtttgctttataggtgactagatccttttctcttggtggttttagaattc
gcattttcacattgaccttaaatagtctgattatagtttgccacggcaaagaccctttgc
attgcattgtttggggatatttgagcctcctctatctggatgtctaatctcttgttagat
gtgagtagttttcattattattttattaatgggctggcatgtgggccgtggttccaggca
ggctcagaggggcagctgcctgatgtctggacagcttctctttctgtcttttcttacctg
gactctgggttgcttgttagctgcttctgccagttctgagttttcaaggggagaggggcc
cagtgatggctgttctttgaaggaaagggaagaatgtctcctgtttaacatgtttctatg
tttccagttacttggttagttagttagagccagggtctctcgctctgttgcgcaggctgg
agtgcaatggcatgatcgtggctcacagcagcctccaccttccaggctcgagcaatgctc
ccacctcagcctctcaagcagctgggactgcaggtatgtgccaccatgcttggctgcctt
tttaaatttttttttttttaatacagacaaggtctcactatattgcccaggctggtctta

aactcatgggctcaagtgatactcctgcctcggcctttcaaagtgctgatatcacaggca
gggtttccatttttaaagctcccagcagtggtataaactcctcctttccagagaaagcg
cactctgtccgcatccctcatgttgtcctctcctgcctctgcttagggttcactccgggg
gaaagtgccacttgagagtttccttttgtgtgtggttctgactgactgctccctgctca
cagatgctgattctcagggtggggtccctgaggcctggagtgtggcctctgacgaccttc
agggccaggtgtggaatgagagcctgtggccacatggcccccggtgggagacgtcccgcc
gccctttgcttctctgtgccactctggctgcacagttcagagccttgggaaatgttaacc
agtaggacctagacggggaggtgagaaggggtcaccccccaggtgtgcctgtggtgagcc
ttcgtgctgagcaggtgcagggagggaggcccaggtgcacacacctgtgaagtaggggca
gctggctgggctccttgacctgctccagagcttcttattttctggccacttcacctgcag
aaggcccaggtggctgtggcctctagggtcccttgcctgtcctcagctcccaactgggag
gggcagagggaggagggggtggagaccccaggcagcagggctctgggagcagtggggccc
tgggtcccaggggtgtctggcaggcccctccttactctacgtctcggcctctggatggag
gtgctggctgcagtcgggctctgcctctgactaagggttggggaagtggcgggtgtgggc
tgctgccccgtggggcctctgaacagaccccagggcctctgccaatcatgactccttcct
ttcagctggacccgcaggccctgcaggacagagactggcagcgcgccgtcatcgccatga
atggggtacgtgtccgtgggactctcctggcgcccacttcccccagaaggatagggtggc
ctctgttcatttcaaatcagtcagaggtggctgagcctgaggcagcatctgagagggagc
ctggttggagaagggagggcccccaagagcagaatcaccatgcacgggaatcgtcattca
ttggctggaatgcagttgccagccaggccctgagcatccctcctcaaacaaaggtctcat
ggcaccaccaggacaggtggggcctccactcagggacctgggggctgcccatagaaatgg
agacccctgatttgtctttaggtaccccagaaaggtttagaccttaaaagcaatgacaca
cccaaaaaggcccgggtataaatggtaaaatgttaatatttgagattcttggctttttct
tacattattctgtctttccttcttaatttttaattgttactaagagaaagctggtcacag
tacactataatctcagctactctggaggctgagccaggagaatcactggagcccaagagt
ttgattacagcctgggcaacattgcaagatcccatatctaaaaaaaaaagcaagcaagcaa
gagaagcagcggggattttaggaggtgcttctgcagaaaccagtcgtttatatcatcttc
aacaatcctggctcttgctgaagtagactaggggcttccccgaggggcggctccacctca
tgctgagacctctgcatgccttgggggtggaaatatttgatgagactcccaggggtcctt
gggaccttgggctgtgaggaccagaaggattagaggactgtgccccttctccccactgta
gatcgaagtaaagctctcggtcaagttcaacagcagggagttcagcttgaagaggatgcc
gtcccgaaaacagacaggggtcttcggagtcaagattgctgtggtcaccaagtgagtggg
gaggggcttgggctcacgcactgagggtgcctgtcccttcagctgtttctgcagaaaaga
gcatgtgtgggtctctcctctctgtgcatggccactgcacggtgaggtcaggccccaggg
aacacggcgtcttcagctacctcctgtgtttcctgcaaaccagctcaggaatgtccttgc
caccttgcttggaagcagtaggctggctccaggaactgcccaagtgcagggttttctgcc
cttgcttggaattagtcacggtcccagattcctgttgaatggccataacccctgcccctt
tgtcacgagtcagttgccaagagaagcctgtttggtttgagagcagttcatgcagacata
gaccacttcctctgagaattcatttgcttccccaggatggaatctggctgggcctctgac
cttgctggtcacgtgggccggggcctccatcagtcataccctggactcctatctgtgtct
aaacaccacgccccacccccaactgcacggcagccactcgcatagcactctgggagggct
gtgggcatgagcagcgaggactccatgagcagctcccccagataagccctgctaatgagg
gggcttgcgaagcagctttgatgtgctggtaaatccaggtgcaaaacagaactcaagtta
gggcctccgcacagcactgcgttctaactgtgaaggattcttactctagtgtcctgtgtg
gaggtattggaattgtccattgctaagactcagaggagaaaagcacttagcatcgcagga
cttggagcaccggtgctgaggcaacccttcattcattcgtcggatgtgtgttaaggccca
gggcaggggtcagggattctcctctcacacagcacgtgggtggcaggaccaacaccgggt
ctgacctcccagccgggggcacaggctgctaaccccaggcctggaatctgtcagatgccc
ttcctgtgctgacttgacttagacaggcctcctgaccttcccgcaaaggtcatgtgtgat
tcgcaggggttctggccgcttgaaaggttcctgagaaagcacatgccatgaggacagagc
ttgcagagggaggacaggcatgcagaaggctctgtgtgcagccccagacctgggtacctt
cgtcaccgtcctcaccccacctccgggtgtgcagatagggagcaggcctcctgtgttatg
gcccaagcggggctgttaggacactgagaacattccctcctcccgcaggagagagaggtc
caaggtgccctacatcatgcgccagtgcgtggaggagatcgagcgccgaggcatggagga
ggtgggcatctaccgcgtgtccggtgtggccacggacatccaggcactgaaggcaggctt
caacgtcagtgagtgtcggcctgcgcaggacgggatggaggtgtgggcagtggtgtccgc
gatgagatctcagagtgctccatggcccaggcatgtcacatccttctctgtgtcttttct
tcatttactgttttattattttaaaaaaagagaaaacaagagttgtacaaacagcttcta
tagaagccagtttttacaccatcgtacccactcatgccacttggtggagtggaccagggg
cttctgtggggacttggccttcctgccttgggggtggacaggaggtggaagcccaggact
cagtgcggtctgtccactgccctgtatgaggatgtggtgggcagagggcactgatgaaat
tcagcgcaggccggggctgcagcatctccgcctccatctcaccaaccctcacaggccttg
aaggacccagactggcctcaaatgccaggggagggcactgagaccccagagggtccttcc
cagcatcttcaaagcaacaggattttgtgcctgcagacccttctttgcagcacacaccac
ccaccctgaccaggacccctagaatgcccagcatccctgggagggccctgtggtagtttc
agctccctctgggggcccagaatgaacctggcctgtggtgaggatgtaagcaccaatggc
caattgggtccaaaggaagacaccggttcaaacactgaaaccaatcagattctcccacgg
ccttcctgctatcagacgacactggtgcaggggtggttgctatgtacagggcagagccac
ccaatccccacgcaggcgctgtgtcctgccacgttggcctcctcctggccatcacatcag
gccaagcaggggagaggaatgggaatgcccacgcacccctatcaactctgcagacacaga
accatgcacagctcttgggaggagtcagatgagctgctcaaagcccaggagggacccgca
cagtggtcagtgtggcagggacggtgctttagccaaggcagggatggtgggtgactcact
caggatcttcaaggaggccgctgcatttccgtgctctttccagataacaaggacgtgtcg
gtgatgatgagcgagatggacgtgaacgccatcgcaggcacgctgaagctgtacttccgt
gagctgcccgagcccctcttcactgacgagttctaccccaacttcgcagagggcatcggt
gagcactggaggccttggcctcatgggagacgtctcctccacgtgcactgctgccctcgg
aggctgtgaaaagcgaggtgtgggaacctgagctgtaacccctctgccgtggtcggcatt
ttaacccaacctcaaaaagcaggggaccagaaccgagcctgtcctggaaggccttgccca
tccccagagggctccccatccctactcctcaaggagaccaagaggctgaaatagtcagca
ctgctgtgctatggggtcctaaagtctgctgtcctccttcctgcagaccagggctgaagg
agggtgcctgggtgctcttgccatgggtcctggtccagccaagcatggtttcaaacatga
cctgacccttagtcaacctggaggctgatgtctagagcgggtgctggtgcgtgcagcacc
tgtggcctctgcatcacccttagggcaggtctgcctcccgggcccatgcacagaggacct
ggtctcccagcctgcaggtgcccctgtggtgtccaggacgacgaggggggtctctgtgtac
ttggtggggctgggaccctcccacttcccacctccttgtgtccctcactcccctgtttca
ttccatgctgagcctcccctgccttgggctccctggggagggggtggtggcaggagttgc
ccgagggcagctctgcccatgagcagctgctctagcggctcctcctgctgctgttcgccg
ggtgctgctgacccctgcgaggtagagaaaaggcgttcaggtggttcacaccccacacag
gtgcccctcacagggtcctcactggcggccagcgctgtgggtgtgacgatgatgacaagc
ctaaactgcgcaaggactcgtgtcccgggcgctccatgtgaccacctcgggagaggtctc
cggcttgtcgtaacccaggggagtgacccactgcctcctgcagctctttcagacccagtt
gcaaggaagagctgcatgctcaacctgttgttgtccctgccggaggccaacctgctcacc
ttccttttccttctagaccacctggaaaggtagcccagctctcttgtggctgcccaggac

tccaggtctccaggccgttggggtgcccctctgctcccaccagacccccagcaccaagga
ccttttcccccgacccctgtctgcagtaactcactgcttctaaggactagcaccactgcc
acccccacccctgcctctcctctttgccaccctcctccctctgcactgtggccttaacaa
agagctcagagctttggccgtggccagcagtgcacttggaccccccctcttccctcccaag
cacatcatgaagacctccccatcagcccagagctggccccttgtcctgggccactgagac
ccagaagtaccaaggctggagtcagcttgcagcacagccagggtcgaggtcactccctcc
ctgaggactctagcacggcacagcccctctgcctctctcctggtggtggcgttgaaacag
caccctctgcttcggtcctctacagggtggcagagaaggaggcggtcaataaggtgtccc
tgcacaacctcgccactgtctttggccccacgctgctccggccctccgagaaggagagca
agctccctgccaaccccagccagcctgtcaccatgactgacagcaggtccttggaggtca
tgtctcaggtatgggaagacagtctccagcccatgcaaccccagcctgacagaggtggcc
tctgcctgccccacccccagtcctgcccatcttccgacttgcattgtatgtggtggtggc
tgagattcagagagagggacttgcctaggtttgcatggatgggagtgatagggggtgccc
aggccacctcctggtcctgctggtgcaccttgctgggggcttaaaaccaccccaagtgtt
cgggtgtggtggctcatgcctgtaatcccagcactttgggaggccgaggcaggacaactg
aacccaggtgtttgagaccagtctgggcaatgtagcaaaccccatctctagaaaaaatac
aaagaaaaattagtcaggcattgtggcacacatctgtaatcctaggtatctgggaggctg
acacaggaggattgcttgagcccaggagttagaggctgcagtgatccatgatggagccac
tgtactccagcctgggggacagagcaaggccctgtgcatctctaaaataaataatcaccc
cccacccaacaagtcatgccttgtcaggaccccaccccacccccgtctcactgtaagggg
ttcatgacaccagcaggggtttctagcacctgaggtggacttgggggcttgggccccaaa
gacctccccaccagcagctgtgagcccccctctgagccactctcctcttccccactctgc
gagggcaggacgaggtgctgctgtacttcttgcggctggaggccatccctgccctgaaca
gcaagagacagagcatcctgttctccaccgatgtctaaaggtcccagtccatctcctgga
ggcggacagatggcctggaaacctctggctaatcgggccatctgtagagtgggaatcaag
attttctgaggcatccttgggccacccccaggtgtcaggccatctgccaagagacagcgg
cccaaagcagaaggacaggtggcctgggcagatcccgcccaggtctgaaagccccaggct
ggcctcagactgtgggttttttatgtggccacccgagggcgccccaagccagttcatctc
ggagtccaggcctggccctgggagacagggtgaaagcagtggtttttatgaacttaactt
atagagtccaaaagatttctactgaatcacttgtcaagaagcgccctctctggggagaag
ggaacgtgactggattccctcactgttgtatcttgaataaacgctgctgcttcatcctgt
gggggccgtggccctgtccctgtgtgggtggggcctcttccatttccctgacttagaaac
cacactccacttctaacagggtttgagaggcttggtcagcactgggtagcgttttgactc
cattcttggctttcttctttttctttccagaaggatttttgtgcagaaatgggtcttttg
ttgccgtgttagtcctccttggaaggcagctcagaaggcctgtgaaatgtcgggggacag
gacccccagggagggaatcccaggctacgcaccttagggttcgttctccagggagagcga
cctcgtcccccgatcctgaccgcccttccggcccacgctctcctgtttggcttccacagg
cctggacttctctggcttctctgcccacacactccctgcccccagtgtccctgcccctgc
cccagcacaggtgacttcatttctgtcctctcagctcagtggactcgctcatcttttgta
taagtctccacttggtggcagcagcttgctgatgacttgttttaaaactttcatcctaaa
taaccttttgatacttgaatatttttaagttttatacatagtttctaatttttttccgaa
cagatccagatacctaataagatgctggaatgtaatccctggacaatccgtgtcctggca
gcatttggtcttcctctaagcgcctggctccgctgttctcaggagtgggttctgaagtct
ctggagaacaggatacgtggagggttaggaaggggccaggcctagagacgggagactccc
tcccggagcaggtggaggcacaggaccattcgctaccccatctgccggcacctgcggggg
agcccaggcattctttgtaagccctcctgaccacctggctcaaagaaaacagaagcatgg
aggccgccaagtattttcaagaaataatcccatgaacatggcatcactttttttagaaaga
ggggcttggggcaggcagaggagagaagggagatcaaactgagagccaagtttccagacg
gtcctgcaggaggagaggatgcagctgcccagagggaagcaggatcacatttaaggaagt
gtgtggggtccctggatgacaccagcacccagtgcggctctgtctggcaaccgctcccaa
ggtggcaggagtgggtgtcccctgtgtgtcagtgggcagctcctgctgaacccacagctc
actggggagcctgacagtggggccatgtgcctgacactcctctctgcttgtggacctggc
aaggcagggagcagaaaacagagctacttgaaggctttctgtctgcgtctgtgtgcagtg
tggatttagttgtgctttttacttgctgggagagcacagccaccatttacaagcagtgtc
accctcgtgggtggcgaggacagaacaggagcctctgctctctgtacctatctgggcccg
gtgggctcccttgtcctggcttccatctctgtctcagcgaccattcagccctgcacagga
acacatgttgcttagaaaagccaaatccagcccttgtctctgcctcctctggtctcatga
tgtgcatctgttaccttgaaactggaaaccagtctatcaatgtctgtgccaattttttat
tccctccccaacctccttccccatacgactttttatttatgtaggatgtgtgctgtctaa
tgatgggatgaccacacttttccatgttctaaaagtgctcctctcccacagggtcccagg
gctggtggttgctttgggtctacagctacgtcttacccgcctcctgcctcaacagcctgt
gtggtggcaaagccggtgtggggctggggaacgcagcgttctccaggaggggacccggct
ctccttctgcagtgcaggcgaaggcctagatgccagtgtgacctcccacaaggcgtggct
tccagactccccggccggaagtgatgctttttgccgcgggccctgggtttgaagcagcc
tggctttctcttggtaagtggctggtgtcttagcagctgcaatctgagctcagccaccta
cacaccaccgtggccgacactttcattaaaaagtttcctgagacga
