# Supplementary material for: An ancestral genomic sequence that serves as a nucleation site for de novo gene birth
Source: PLoS One. 2022 May 12;17(5):e0267864. doi: 10.1371/journal.pone.0267864 (PMC9097989; doi:10.1371/journal.pone.0267864)
Supplement: S5 Fig — (PDF) [file pone.0267864.s005.pdf]

Detection of an ancestral genomic sequence that serves as a nucleation site for de novo gene birth

Nicholas Delihas
Department of Microbiology and Immunology, Renaissance School of Medicine, Stony Brook University, Stony Brook, N.Y., United States of America

S5 Fig. a. Pink highlight, perfect tandem repeats (except for AluSg4-LIMEg) in the human *BCRP3* gene Alu/LINE TE tandem repeat sequence. Compare with the Rhesus tandem repeats in S5 Fig. b., below, which shows imperfect repeats. Data kindly provided by Dr. Jessica Storer and obtained with an updated RepeatMasker program.

					bp position	bp position			TE	
574	23.7	7.3	3.3	BCRP3.NCBIref	5930	6134	-14312	C	L1MC5	LINE/L1
302	22.6	0	9.5	BCRP3.NCBIref	6122	6248	-14198	C	L1MEg	LINE/L1
245	17.8	2.2	0	BCRP3.NCBIref	6279	6323	-14123	+	MER4E1	LTR/ERV1
16	16.9	2.9	0	BCRP3.NCBIref	6327	6347	-14099	+	(AT)n	Simple_repeat
29	2.2	8.5	0	BCRP3.NCBIref	6348	6394	-14052	+	(ATATACACAC) n	Simple_repeat
16	16.9	2.9	0	BCRP3.NCBIref	6395	6407	-14039	+	(AT)n	Simple_repeat
1241	14.8	0	0	BCRP3.NCBIref	6419	6588	-13858	C	AluSg	SINE/Alu
2365	10.5	0	0	BCRP3.NCBIref	6589	6892	-13554	C	AluSx1	SINE/Alu
1241	14.8	0	0	BCRP3.NCBIref	6893	6904	-13542	C	AluSg	SINE/Alu
1059	27.7	5.4	1.6	BCRP3.NCBIref	6905	7052	-13394	C	L1MEg	LINE/L1
2037	14.8	0.3	0	BCRP3.NCBIref	7053	7343	-13103	C	AluSx1	SINE/Alu
1059	27.7	5.4	1.6	BCRP3.NCBIref	7344	7674	-12772	C	L1MEg	LINE/L1
1282	12.1	0	0	BCRP3.NCBIref	7675	7847	-12599	+	AluSg4	SINE/Alu
696	25.1	12.3	3.2	BCRP3.NCBIref	7850	7913	-12533	C	L1MEg	LINE/L1
1661	14.3	5.5	1.3	BCRP3.NCBIref	7914	8203	-12243	C	AluSx	SINE/Alu
2366	9.1	0.3	0	BCRP3.NCBIref	8207	8504	-11942	C	AluSx	SINE/Alu
696	25.1	12.3	3.2	BCRP3.NCBIref	8505	8758	-11688	C	L1MEg	LINE/L1
2090	9.6	2	0.3	BCRP3.NCBIref	8759	9050	-11396	C	AluSx	SINE/Alu
696	25.1	12.3	3.2	BCRP3.NCBIref	9051	9083	-11363	C	L1MEg	LINE/L1
1847	15.3	0.3	1	BCRP3.NCBIref	9084	9387	-11059	+	AluJb	SINE/Alu
696	25.1	12.3	3.2	BCRP3.NCBIref	9388	9515	-10931	C	L1MEg	LINE/L1
656	17.2	0	0	BCRP3.NCBIref	9597	9695	-10751	+	FLAM_A	SINE/Alu
352	7.8	0	0	BCRP3.NCBIref	9699	9749	-10697	C	MADE1	DNA/TcMar-Mariner
28	10.8	0	0	BCRP3.NCBIref	9762	9801	-10645	+	(A)n	Simple_repeat
2491	7.4	0	0	BCRP3.NCBIref	9812	10110	-10336	+	AluY	SINE/Alu
942	20.2	1.8	0	BCRP3.NCBIref	10242	10469	-9977	C	L1M2	LINE/L1

S5 Fig. b.

					bp position	bp position			TE	
510	23.5	5.7	4.7	GGT1_RHESUS .REF.rev.compl	8956	9165	-25275	C	L1MC5	LINE/L1
259	20.2	0.8	10.3	GGT1_RHESUS .REF.rev.compl	9154	9280	-25160	C	L1MEg	LINE/L1
24	34.9	1.1	0	GGT1_RHESUS .REF.rev.compl	9359	9445	-24995	+	(AT)n	Simple_repeat
1111	10.8	1.6	4.4	GGT1_RHESUS .REF.rev.compl	9457	9629	-24811	C	AluSx	SINE/Alu
1928	15.1	0	0.7	GGT1_RHESUS .REF.rev.compl	9630	9938	-24502	C	AluSx	SINE/Alu
2124	12.2	0.7	0.3	GGT1_RHESUS .REF.rev.compl	9941	10244	-24196	C	AluSx	SINE/Alu
544	21.5	1.3	0.7	GGT1_RHESUS .REF.rev.compl	10253	10402	-24038	C	L1MEg	LINE/L1
2369	10.5	0	0	GGT1_RHESUS .REF.rev.compl	10403	10707	-23733	C	AluY	SINE/Alu
841	17.5	0	0	GGT1_RHESUS .REF.rev.compl	10711	10836	-23604	C	AluSz6	SINE/Alu
596	24.4	14.5	0.3	GGT1_RHESUS .REF.rev.compl	10851	11167	-23273	C	L1MEg	LINE/L1
1217	13.8	0	0.6	GGT1_RHESUS .REF.rev.compl	11168	11341	-23099	+	AluSg4	SINE/Alu
16	0	4.3	0	GGT1_RHESUS .REF.rev.compl	11352	11374	-23066	+	(TTAT)n	Simple_repeat
1699	17.1	1	0	GGT1_RHESUS .REF.rev.compl	11417	11583	-22857	C	AluSx	SINE/Alu
2580	0.7	0	1.7	GGT1_RHESUS .REF.rev.compl	11584	11881	-22559	C	AluYRb3	SINE/Alu
1699	17.1	1	0	GGT1_RHESUS .REF.rev.compl	11882	12012	-22428	C	AluSx	SINE/Alu
2270	10	0.3	0	GGT1_RHESUS .REF.rev.compl	12017	12307	-22133	C	AluSx	SINE/Alu

552	27.7	7	2.9	GGT1_RHESUS .REF.rev.compl	12317	12556	-21884	C	L1MEg	LINE/L1
2013	9.8	2.5	0.3	GGT1_RHESUS .REF.rev.compl	12557	12841	-21599	C	AluSx	SINE/Alu
552	27.7	7	2.9	GGT1_RHESUS .REF.rev.compl	12842	12875	-21565	C	L1MEg	LINE/L1
1743	18.3	0.3	0	GGT1_RHESUS .REF.rev.compl	12876	13175	-21265	+	AluJb	SINE/Alu
552	27.7	7	2.9	GGT1_RHESUS .REF.rev.compl	13176	13300	-21140	C	L1MEg	LINE/L1
555	27.5	0	8.1	GGT1_RHESUS .REF.rev.compl	13382	13567	-20873	+	AluJb	SINE/Alu
2309	9.8	1.3	0	GGT1_RHESUS .REF.rev.compl	13590	13895	-20545	+	AluY	SINE/Alu
759	22.7	3.1	0	GGT1_RHESUS .REF.rev.compl	14032	14256	-20184	C	*L1M2*	LINE/L1
