# Supplementary material for: An ancestral genomic sequence that serves as a nucleation site for de novo gene birth
Source: PLoS One. 2022 May 12;17(5):e0267864. doi: 10.1371/journal.pone.0267864 (PMC9097989; doi:10.1371/journal.pone.0267864)
Supplement: S6 Fig — (PDF) [file pone.0267864.s006.pdf]

Detection of an ancestral genomic sequence that serves as a nucleation site for de novo gene birth

Nicholas Delihas
Department of Microbiology and Immunology, Renaissance School of Medicine, Stony Brook University, Stony Brook, N.Y., United States of America

S6 Fig. Alignment of the BCRP3 sequence from the baboon with the BCRP3 and Rhesus BCRP3 sequences. The highlighted area represents the identity of the baboon sequence with that of the human *BCRP3.* The sequences used are at the end of the file.

```
#
#  Percent Identity  Matrix - created by Clustal2.1
#
#

     1: Papio.anubis.clone.rp41-133b2.8034-40080.revcompl.Baboon         100.00   76.92   71.11
     2: BCRP3.HUMAN.NCBI.REF                                              76.92  100.00   83.07
     3: LOC106996293.glutathione.hydrolase5.proenzyme-like-GGT1.rhesus.   71.11   83.07  100.00
```

```
CLUSTAL O(1.2.4) multiple sequence alignment

Papio.anubis.clone.rp41-133b2.8034-40080.revcompl.Baboon            ------------------------------------------------------------    0
BCRP3.HUMAN.NCBI.REF                                                ------------------------------------------------------------    0
LOC106996293.glutathione.hydrolase5.proenzyme-like-GGT1.rhesus.     gatcacaaggtcaggagttcaagaccagcctggccaatatggtgaaaccccgtctctacc    60

Papio.anubis.clone.rp41-133b2.8034-40080.revcompl.Baboon            ------------------------------------------------------------    0
BCRP3.HUMAN.NCBI.REF                                                ------------------------------------------------------------    0
LOC106996293.glutathione.hydrolase5.proenzyme-like-GGT1.rhesus.     aaaaatacaaaaattagccaggcatggtggcgggcttctgtagtcccagctactcaggcg    120

Papio.anubis.clone.rp41-133b2.8034-40080.revcompl.Baboon            ------------------------------------------------------------    0
BCRP3.HUMAN.NCBI.REF                                                ------------------------------------------------------------    0
LOC106996293.glutathione.hydrolase5.proenzyme-like-GGT1.rhesus.     cctaagacaggagaatgccttgaacctggcggggcggaggttgcagtgagcggagatcat    180

Papio.anubis.clone.rp41-133b2.8034-40080.revcompl.Baboon            ------------------------------------------------------------    0
BCRP3.HUMAN.NCBI.REF                                                ------------------------------------------------------------    0
LOC106996293.glutathione.hydrolase5.proenzyme-like-GGT1.rhesus.     gccagcctgggcaacagagtaagagtgtcaaaaaaaaaaaattatatatatatgtgtgta    240

Papio.anubis.clone.rp41-133b2.8034-40080.revcompl.Baboon            ------------------------------------------------------------    0
BCRP3.HUMAN.NCBI.REF                                                ------------------------------------------------------------    0
LOC106996293.glutathione.hydrolase5.proenzyme-like-GGT1.rhesus.     tgtgtatagtgcatagttggaaaagaaaatactggttttcacgggtgattggcagctagc    300

Papio.anubis.clone.rp41-133b2.8034-40080.revcompl.Baboon            ------------------------------------------------------------    0
BCRP3.HUMAN.NCBI.REF                                                ------------------------------------------------------------    0
LOC106996293.glutathione.hydrolase5.proenzyme-like-GGT1.rhesus.     tgtaagtggaagaagtgcattttcctttctttccaggtcccagagaaaaacctccatgct    360

Papio.anubis.clone.rp41-133b2.8034-40080.revcompl.Baboon            ------------------------------------------------------------    0
BCRP3.HUMAN.NCBI.REF                                                ------------------------------------------------------------    0
LOC106996293.glutathione.hydrolase5.proenzyme-like-GGT1.rhesus.     gggttgatagcagcagctaggatcagttgcccaatactctggattctcgagatgaagacc    420

Papio.anubis.clone.rp41-133b2.8034-40080.revcompl.Baboon            ------------------------------------------------------------    0
BCRP3.HUMAN.NCBI.REF                                                ------------------------------------------------------------    0
LOC106996293.glutathione.hydrolase5.proenzyme-like-GGT1.rhesus.     gtctgcagaggctgacctttcctgcaggctcatgtcctaaagcaaattatttgcactcaa    480

Papio.anubis.clone.rp41-133b2.8034-40080.revcompl.Baboon            ------------------------------------------------------------    0
BCRP3.HUMAN.NCBI.REF                                                ------------------------------------------------------------    0
LOC106996293.glutathione.hydrolase5.proenzyme-like-GGT1.rhesus.     atatcctcccttgtaggacagaaaagaagttcccaagtcttggtcacattgggcctgtgg    540

Papio.anubis.clone.rp41-133b2.8034-40080.revcompl.Baboon            ------------------------------------------------------------    0
BCRP3.HUMAN.NCBI.REF                                                ------------------------------------------------------------    0
LOC106996293.glutathione.hydrolase5.proenzyme-like-GGT1.rhesus.     agtacagtatcttttctacatatttgcttttgatgttctcagcatccctgcaaggcaggt    600

Papio.anubis.clone.rp41-133b2.8034-40080.revcompl.Baboon            ------------------------------------------------------------    0
BCRP3.HUMAN.NCBI.REF                                                ------------------------------------------------------------    0
LOC106996293.glutathione.hydrolase5.proenzyme-like-GGT1.rhesus.     tgaggtgtgaggagtctcagtcccagggagctgaaggcagactgcaagagctgggtatgg    660

Papio.anubis.clone.rp41-133b2.8034-40080.revcompl.Baboon            ------------------------------------------------------------    0
BCRP3.HUMAN.NCBI.REF                                                ------------------------------------------------------------    0
LOC106996293.glutathione.hydrolase5.proenzyme-like-GGT1.rhesus.     atgccgcactcctgggcctgggccgcctgggttttcttcagcctgggaagtacttccaaa    720

Papio.anubis.clone.rp41-133b2.8034-40080.revcompl.Baboon            ------------------------------------------------------------    0
BCRP3.HUMAN.NCBI.REF                                                ------------------------------------------------------------    0
LOC106996293.glutathione.hydrolase5.proenzyme-like-GGT1.rhesus.     cccactcctcgctagtctcttacaatcctgtgggagggcaaagcctaaatcatcatggct    780

Papio.anubis.clone.rp41-133b2.8034-40080.revcompl.Baboon            ------------------------------------------------------------    0
BCRP3.HUMAN.NCBI.REF                                                ------------------------------------------------------------    0
LOC106996293.glutathione.hydrolase5.proenzyme-like-GGT1.rhesus.     ccttccaccaactagctgcagatcaggaaagtgaggtccagggaggtcctgactggcttt    840

Papio.anubis.clone.rp41-133b2.8034-40080.revcompl.Baboon            ------------------------------------------------------------    0
BCRP3.HUMAN.NCBI.REF                                                ------------------------------------------------------------    0
LOC106996293.glutathione.hydrolase5.proenzyme-like-GGT1.rhesus.     gctccagggacaatgaggctagaggtctgttccccacaggaggaccatgatggttctcct    900

Papio.anubis.clone.rp41-133b2.8034-40080.revcompl.Baboon            ------------------------------------------------------------    0
BCRP3.HUMAN.NCBI.REF                                                ------------------------------------------------------------    0
LOC106996293.glutathione.hydrolase5.proenzyme-like-GGT1.rhesus.     gcttcaaatggcctctttggaaggggcttgctcatgtaggtgaatcctggggctgagcag    960

Papio.anubis.clone.rp41-133b2.8034-40080.revcompl.Baboon            ------------------------------------------------------------    0
BCRP3.HUMAN.NCBI.REF                                                ------------------------------------------------------------    0
LOC106996293.glutathione.hydrolase5.proenzyme-like-GGT1.rhesus.     cctcagtgacagtccctgaaatggctgcaggtgagggagcagcatgcaggcattaacgtc    1020

Papio.anubis.clone.rp41-133b2.8034-40080.revcompl.Baboon            ------------------------------------------------------------    0
BCRP3.HUMAN.NCBI.REF                                                ------------------------------------------------------------    0
LOC106996293.glutathione.hydrolase5.proenzyme-like-GGT1.rhesus.     acagactgacatcaggccacctagacttacatcctggctcctctggtttctggctctttg    1080

Papio.anubis.clone.rp41-133b2.8034-40080.revcompl.Baboon            ------------------------------------------------------------    0
BCRP3.HUMAN.NCBI.REF                                                ------------------------------------------------------------    0
LOC106996293.glutathione.hydrolase5.proenzyme-like-GGT1.rhesus.     aagttggacaagaaatcccctttttttttcttttgagagacagagtcttgctccatcaccc    1140

Papio.anubis.clone.rp41-133b2.8034-40080.revcompl.Baboon            ------------------------------------------------------------    0
BCRP3.HUMAN.NCBI.REF                                                ------------------------------------------------------------    0
LOC106996293.glutathione.hydrolase5.proenzyme-like-GGT1.rhesus.     aggctgtggtgcaatggcgtgatctcagctcactgccacttctaccctccaggttcaagc    1200

Papio.anubis.clone.rp41-133b2.8034-40080.revcompl.Baboon            ------------------------------------------------------------    0
BCRP3.HUMAN.NCBI.REF                                                ------------------------------------------------------------    0
LOC106996293.glutathione.hydrolase5.proenzyme-like-GGT1.rhesus.     aattctcctgcctcagcctcccaagtagctgggattacaggcgctcaccaacatgcctgg    1260
```

```
Papio.anubis.clone.rp41-133b2.8034-40080.revcompl.Baboon             ------------------------------------------------------------    0
BCRP3.HUMAN.NCBI.REF                                                 ------------------------------------------------------------    0
LOC106996293.glutathione.hydrolase5.proenzyme-like-GGT1.rhesus.      ttaatttttttttccttctatttttagtagagtggggtttcaccatgttggccagcctgg    1320

Papio.anubis.clone.rp41-133b2.8034-40080.revcompl.Baboon             ------------------------------------------------------------    0
BCRP3.HUMAN.NCBI.REF                                                 ------------------------------------------------------------    0
LOC106996293.glutathione.hydrolase5.proenzyme-like-GGT1.rhesus.      tcttgaacttctgacctcaggtgatccagcgccttggcctcccaaagtgctggtattaca    1380

Papio.anubis.clone.rp41-133b2.8034-40080.revcompl.Baboon             ------------------------------------------------------------    0
BCRP3.HUMAN.NCBI.REF                                                 ------------------------------------------------------------    0
LOC106996293.glutathione.hydrolase5.proenzyme-like-GGT1.rhesus.      ggcgtgagccatgccacccgactgacaagtcccctttcttaacctccttctgcctcagtc    1440

Papio.anubis.clone.rp41-133b2.8034-40080.revcompl.Baboon             ------------------------------------------------------------    0
BCRP3.HUMAN.NCBI.REF                                                 ------------------------------------------------------------    0
LOC106996293.glutathione.hydrolase5.proenzyme-like-GGT1.rhesus.      gacttacctggaaaacagagcacctaccttcttcaaatgagaagagaattaaaggagtat    1500

Papio.anubis.clone.rp41-133b2.8034-40080.revcompl.Baboon             ------------------------------------------------------------    0
BCRP3.HUMAN.NCBI.REF                                                 ------------------------------------------------------------    0
LOC106996293.glutathione.hydrolase5.proenzyme-like-GGT1.rhesus.      aaggccatcagaacagtacctagcccaagggaggctatggaagtgcttgttgaattaaaa    1560

Papio.anubis.clone.rp41-133b2.8034-40080.revcompl.Baboon             ------------------------------------------------------------    0
BCRP3.HUMAN.NCBI.REF                                                 ------------------------------------------------------------    0
LOC106996293.glutathione.hydrolase5.proenzyme-like-GGT1.rhesus.      aatggcttaatctctggcctagtcccaaaggtttgggttcctgccattgccacccagaga    1620

Papio.anubis.clone.rp41-133b2.8034-40080.revcompl.Baboon             ------------------------------------------------------------    0
BCRP3.HUMAN.NCBI.REF                                                 ------------------------------------------------------------    0
LOC106996293.glutathione.hydrolase5.proenzyme-like-GGT1.rhesus.      gccctgggtggaggccctgctgacattgaagagtgcctgccctcctcccctcaatgcggt    1680

Papio.anubis.clone.rp41-133b2.8034-40080.revcompl.Baboon             ------------------------------------------------------------    0
BCRP3.HUMAN.NCBI.REF                                                 ------------------------------------------------------------    0
LOC106996293.glutathione.hydrolase5.proenzyme-like-GGT1.rhesus.      agacactttccaaaacttacgtcatcttcactccagtgagataatgggaggcttggtgta    1740

Papio.anubis.clone.rp41-133b2.8034-40080.revcompl.Baboon             ------------------------------------------------------------    0
BCRP3.HUMAN.NCBI.REF                                                 ------------------------------------------------------------    0
LOC106996293.glutathione.hydrolase5.proenzyme-like-GGT1.rhesus.      cctcgttcctaagatgagggaacaggctgtgagaggaccacatcaccagcaagtgacagg    1800

Papio.anubis.clone.rp41-133b2.8034-40080.revcompl.Baboon             ------------------------------------------------------------    0
BCRP3.HUMAN.NCBI.REF                                                 ------------------------------------------------------------    0
LOC106996293.glutathione.hydrolase5.proenzyme-like-GGT1.rhesus.      ctgggaccaaggccaggcctgtctgcctcctagactaggcctgtgtgaggaccggcctta    1860

Papio.anubis.clone.rp41-133b2.8034-40080.revcompl.Baboon             ------------------------------------------------------------    0
BCRP3.HUMAN.NCBI.REF                                                 ------------------------------------------------------------    0
LOC106996293.glutathione.hydrolase5.proenzyme-like-GGT1.rhesus.      ctacagcccacttgcttcctcctctgtttagagcttgggctggagctgtcctggccagag    1920

Papio.anubis.clone.rp41-133b2.8034-40080.revcompl.Baboon             ------------------------------------------------------------    0
BCRP3.HUMAN.NCBI.REF                                                 ------------------------------------------------------------    0
LOC106996293.glutathione.hydrolase5.proenzyme-like-GGT1.rhesus.      ctctctgcctcaccaggctgcatgagccacaagctgccacttcctggaacttggtggccc    1980

Papio.anubis.clone.rp41-133b2.8034-40080.revcompl.Baboon             ------------------------------------------------------------    0
BCRP3.HUMAN.NCBI.REF                                                 ------------------------------------------------------------    0
LOC106996293.glutathione.hydrolase5.proenzyme-like-GGT1.rhesus.      tgccctggacggccttgctggtggtcatgaaggctccgtgaaagctgtgaacacctgcat    2040

Papio.anubis.clone.rp41-133b2.8034-40080.revcompl.Baboon             ------------------------------------------------------------    0
BCRP3.HUMAN.NCBI.REF                                                 ------------------------------------------------------------    0
LOC106996293.glutathione.hydrolase5.proenzyme-like-GGT1.rhesus.      ggcagtgaggccagccttccccacttgatccgtttcaggctctggctccgctactacgtc    2100

Papio.anubis.clone.rp41-133b2.8034-40080.revcompl.Baboon             ------------------------------------------------------------    0
BCRP3.HUMAN.NCBI.REF                                                 ------------------------------------------------------------    0
LOC106996293.glutathione.hydrolase5.proenzyme-like-GGT1.rhesus.      gtgtgcttctgtccaagaggcagagactgaaaccatctgccgccgcctggattcctgcag    2160

Papio.anubis.clone.rp41-133b2.8034-40080.revcompl.Baboon             ------------------------------------------------------------    0
BCRP3.HUMAN.NCBI.REF                                                 ------------------------------------------------------------    0
LOC106996293.glutathione.hydrolase5.proenzyme-like-GGT1.rhesus.      agcttgcctaggacaccagctcaggtccttgtgcaccatcctggtaggaatctctcagtg    2220

Papio.anubis.clone.rp41-133b2.8034-40080.revcompl.Baboon             ------------------------------------------------------------    0
BCRP3.HUMAN.NCBI.REF                                                 ------------------------------------------------------------    0
LOC106996293.glutathione.hydrolase5.proenzyme-like-GGT1.rhesus.      acggtggagggtttgtgggctggcttagggccaggggtgggcagaaagcagggcacaagg    2280

Papio.anubis.clone.rp41-133b2.8034-40080.revcompl.Baboon             ------------------------------------------------------------    0
BCRP3.HUMAN.NCBI.REF                                                 ------------------------------------------------------------    0
LOC106996293.glutathione.hydrolase5.proenzyme-like-GGT1.rhesus.      gaccggagagcttggcatttctccagcaggagtttcagggagagctcagatgtctgctta    2340

Papio.anubis.clone.rp41-133b2.8034-40080.revcompl.Baboon             ------------------------------------------------------------    0
BCRP3.HUMAN.NCBI.REF                                                 ------------------------------------------------------------    0
LOC106996293.glutathione.hydrolase5.proenzyme-like-GGT1.rhesus.      tgtgacaggtttatcagcctgcctggcccagggtgggctctcatagttttgatttaaata    2400

Papio.anubis.clone.rp41-133b2.8034-40080.revcompl.Baboon             ------------------------------------------------------------    0
BCRP3.HUMAN.NCBI.REF                                                 ------------------------------------------------------------    0
LOC106996293.glutathione.hydrolase5.proenzyme-like-GGT1.rhesus.      atagcttgctcacagaataagtgcatgttggtttgagctttcattttctcataatcctct    2460

Papio.anubis.clone.rp41-133b2.8034-40080.revcompl.Baboon             ------------------------------------------------------------    0
BCRP3.HUMAN.NCBI.REF                                                 ------------------------------------------------------------    0
LOC106996293.glutathione.hydrolase5.proenzyme-like-GGT1.rhesus.      ctgctggcccctcccaccatctaccccattttgcctcactctggggagcctgaacccccct    2520

Papio.anubis.clone.rp41-133b2.8034-40080.revcompl.Baboon             ------------------------------------------------------------    0
BCRP3.HUMAN.NCBI.REF                                                 ------------------------------------------------------------    0
LOC106996293.glutathione.hydrolase5.proenzyme-like-GGT1.rhesus.      gttccagagtaggggcatagggaaatgcctctatggcttctgagctgctgggcagggcca    2580

Papio.anubis.clone.rp41-133b2.8034-40080.revcompl.Baboon             ------------------------------------------------------------    0
BCRP3.HUMAN.NCBI.REF                                                 ------------------------------------------------------------    0
LOC106996293.glutathione.hydrolase5.proenzyme-like-GGT1.rhesus.      gtccctaaaacaaggagagctgggagggggctggaccccgatggagtgctgagagctgaa    2640

Papio.anubis.clone.rp41-133b2.8034-40080.revcompl.Baboon             ------------------------------------------------------------    0
BCRP3.HUMAN.NCBI.REF                                                 ------------------------------------------------------------    0
LOC106996293.glutathione.hydrolase5.proenzyme-like-GGT1.rhesus.      agtcctatttaacaatggggagggagcagaaaccccgaaaagggaaaggcctggccaggt    2700

Papio.anubis.clone.rp41-133b2.8034-40080.revcompl.Baboon             ------------------------------------------------------------    0
BCRP3.HUMAN.NCBI.REF                                                 ------------------------------------------------------------    0
LOC106996293.glutathione.hydrolase5.proenzyme-like-GGT1.rhesus.      cccacgcaagtcaggagcagagcagggctgaagcgtgacatctccagtctctccaaagcc    2760

Papio.anubis.clone.rp41-133b2.8034-40080.revcompl.Baboon             ------------------------------------------------------------    0
BCRP3.HUMAN.NCBI.REF                                                 ------------------------------------------------------------    0
LOC106996293.glutathione.hydrolase5.proenzyme-like-GGT1.rhesus.      tttgggtgaaaagtccacctaaagcagtgagatcagtggggcctaaggaagatcctttttc    2820

Papio.anubis.clone.rp41-133b2.8034-40080.revcompl.Baboon             ------------------------------------------------------------    0
BCRP3.HUMAN.NCBI.REF                                                 ------------------------------------------------------------    0
LOC106996293.glutathione.hydrolase5.proenzyme-like-GGT1.rhesus.      ctcactgcctccatcccagtcctgggacccaacaccgtcctgccctcaccctccttgacc    2880

Papio.anubis.clone.rp41-133b2.8034-40080.revcompl.Baboon             ------------------------------------------------------------    0
BCRP3.HUMAN.NCBI.REF                                                 ------------------------------------------------------------    0
LOC106996293.glutathione.hydrolase5.proenzyme-like-GGT1.rhesus.      atctccttctgccctgagaggctgttatgcatccagccagcctgggaatgtgctggaatc    2940

Papio.anubis.clone.rp41-133b2.8034-40080.revcompl.Baboon             ------------------------------------------------------------    0
BCRP3.HUMAN.NCBI.REF                                                 ------------------------------------------------------------    0
LOC106996293.glutathione.hydrolase5.proenzyme-like-GGT1.rhesus.      tgtgcaccacggcggatggatacgccgctcacagtcctctgtgggccgtggctgtgagga    3000
```

```
Papio.anubis.clone.rp41-133b2.8034-40080.revcompl.Baboon            ------------------------------------------------------------    0
BCRP3.HUMAN.NCBI.REF                                                ------------------------------------------------------------    0
LOC106996293.glutathione.hydrolase5.proenzyme-like-GGT1.rhesus.     aaagtcagagctggtgggttggggtgtgtaggcccaggaggacccctgcctcctccttag    3060

Papio.anubis.clone.rp41-133b2.8034-40080.revcompl.Baboon            ------------------------------------------------------------    0
BCRP3.HUMAN.NCBI.REF                                                ------------------------------------------------------------    0
LOC106996293.glutathione.hydrolase5.proenzyme-like-GGT1.rhesus.     ccttggagcctccctctgctgctgagtttctcctcctctagcaggggcccactgcccca    3120

Papio.anubis.clone.rp41-133b2.8034-40080.revcompl.Baboon            ------------------------------------------------------------    0
BCRP3.HUMAN.NCBI.REF                                                ------------------------------------------------------------    0
LOC106996293.glutathione.hydrolase5.proenzyme-like-GGT1.rhesus.     gaccttacctcctggctcagcacatgatttccctactctgggcctcagtatcccccgga    3180

Papio.anubis.clone.rp41-133b2.8034-40080.revcompl.Baboon            ------------------------------------------------------------    0
BCRP3.HUMAN.NCBI.REF                                                ------------------------------------------------------------    0
LOC106996293.glutathione.hydrolase5.proenzyme-like-GGT1.rhesus.     catctctaaattcctttcccgcctgccctgcctgtctcctggttgggctgaactgtaact    3240

Papio.anubis.clone.rp41-133b2.8034-40080.revcompl.Baboon            ------------------------------------------------------------    0
BCRP3.HUMAN.NCBI.REF                                                ------------------------------------------------------------    0
LOC106996293.glutathione.hydrolase5.proenzyme-like-GGT1.rhesus.     cctcacacagggctgagggtgactgtggcatcaggcttctggttgggagcccaccctgat    3300

Papio.anubis.clone.rp41-133b2.8034-40080.revcompl.Baboon            ------------------------------------------------------------    0
BCRP3.HUMAN.NCBI.REF                                                ------------------------------------------------------------    0
LOC106996293.glutathione.hydrolase5.proenzyme-like-GGT1.rhesus.     ggtgcttggggaggaatggacatggttcccccctgccagggacagccctaggactaaggcc    3360

Papio.anubis.clone.rp41-133b2.8034-40080.revcompl.Baboon            ------------------------------------------------------------    0
BCRP3.HUMAN.NCBI.REF                                                ------------------------------------------------------------    0
LOC106996293.glutathione.hydrolase5.proenzyme-like-GGT1.rhesus.     ccctgctgctggctatgcccacctccacccccgcatgctctcccacatgggggacccc    3420

Papio.anubis.clone.rp41-133b2.8034-40080.revcompl.Baboon            ------------------------------------------------------------    0
BCRP3.HUMAN.NCBI.REF                                                ------------------------------------------------------------    0
LOC106996293.glutathione.hydrolase5.proenzyme-like-GGT1.rhesus.     aaggcctagagggaacacacaggcactttgtggcctgctgtgtctcctaaccccacagag    3480

Papio.anubis.clone.rp41-133b2.8034-40080.revcompl.Baboon            ------------------------------------------------------------    0
BCRP3.HUMAN.NCBI.REF                                                ------------------------------------------------------------    0
LOC106996293.glutathione.hydrolase5.proenzyme-like-GGT1.rhesus.     ctgcccttcgccaagtacaggaggcctgggactgggtactgggggtggcaggcagcactg    3540

Papio.anubis.clone.rp41-133b2.8034-40080.revcompl.Baboon            ------------------------------------------------------------    0
BCRP3.HUMAN.NCBI.REF                                                ------------------------------------------------------------    0
LOC106996293.glutathione.hydrolase5.proenzyme-like-GGT1.rhesus.     taccctgtgggaggcaggcaccaaatcctcccacttagtcactgtggcccctgcctctgg    3600

Papio.anubis.clone.rp41-133b2.8034-40080.revcompl.Baboon            ------------------------------------------------------------    0
BCRP3.HUMAN.NCBI.REF                                                ------------------------------------------------------------    0
LOC106996293.glutathione.hydrolase5.proenzyme-like-GGT1.rhesus.     tctgtgcagccttgtcccccgcccagagggctctggtggcaaggctggcagcccgggctc    3660

Papio.anubis.clone.rp41-133b2.8034-40080.revcompl.Baboon            ------------------------------------------------------------    0
BCRP3.HUMAN.NCBI.REF                                                ------------------------------------------------------------    0
LOC106996293.glutathione.hydrolase5.proenzyme-like-GGT1.rhesus.     gacgtgagcttgctgtggggctccatgtaaagctttgctcctcctggggctcccaacctg    3720

Papio.anubis.clone.rp41-133b2.8034-40080.revcompl.Baboon            ------------------------------------------------------------    0
BCRP3.HUMAN.NCBI.REF                                                ------------------------------------------------------------    0
LOC106996293.glutathione.hydrolase5.proenzyme-like-GGT1.rhesus.     ctccttcgcagctccccttctcaaggagcccaagcgctgcccagctgtgagggtctgctc    3780

Papio.anubis.clone.rp41-133b2.8034-40080.revcompl.Baboon            ------------------------------------------------------------    0
BCRP3.HUMAN.NCBI.REF                                                ------------------------------------------------------------    0
LOC106996293.glutathione.hydrolase5.proenzyme-like-GGT1.rhesus.     cctggaggcctcctttaactccccagcccacagggatggacccaggacccccatatctta    3840

Papio.anubis.clone.rp41-133b2.8034-40080.revcompl.Baboon            ------------------------------------------------------------    0
BCRP3.HUMAN.NCBI.REF                                                ------------------------------------------------------------    0
LOC106996293.glutathione.hydrolase5.proenzyme-like-GGT1.rhesus.     gactccatgggggatttccccaaggaacacctgggggaaggtcagtgtgacctttgtccc    3900

Papio.anubis.clone.rp41-133b2.8034-40080.revcompl.Baboon            ------------------------------------------------------------    0
BCRP3.HUMAN.NCBI.REF                                                ------------------------------------------------------------    0
LOC106996293.glutathione.hydrolase5.proenzyme-like-GGT1.rhesus.     tcccttttggatagacacgccaccctttctccccaccccagcagcagggctgttgctcag    3960

Papio.anubis.clone.rp41-133b2.8034-40080.revcompl.Baboon            ------------------------------------------------------------    0
BCRP3.HUMAN.NCBI.REF                                                ------------------------------------------------------------    0
LOC106996293.glutathione.hydrolase5.proenzyme-like-GGT1.rhesus.     tgaccctgagatcggtgatgcagaaaagcagaggcgggtgtcactctttattgcggggtc    4020

Papio.anubis.clone.rp41-133b2.8034-40080.revcompl.Baboon            ------------------------------------------------------------    0
BCRP3.HUMAN.NCBI.REF                                                ------------------------------------------------------------    0
LOC106996293.glutathione.hydrolase5.proenzyme-like-GGT1.rhesus.     cacactatgggtgttagggctcctcccactgagggaagcctgagcctctagccggggctg    4080

Papio.anubis.clone.rp41-133b2.8034-40080.revcompl.Baboon            ------------------------------------------------------------    0
BCRP3.HUMAN.NCBI.REF                                                ------------------------------------------------------------    0
LOC106996293.glutathione.hydrolase5.proenzyme-like-GGT1.rhesus.     gcctcctggcctcctatgagtccattctccttctgtccccttaatctcaggctgagcatt    4140

Papio.anubis.clone.rp41-133b2.8034-40080.revcompl.Baboon            ------------------------------------------------------------    0
BCRP3.HUMAN.NCBI.REF                                                ------------------------------------------------------------    0
LOC106996293.glutathione.hydrolase5.proenzyme-like-GGT1.rhesus.     tgcactggatctctgggggcctgtgagtcctcctcgaccttcatggcccactaccatgtg    4200

Papio.anubis.clone.rp41-133b2.8034-40080.revcompl.Baboon            ------------------------------------------------------------    0
BCRP3.HUMAN.NCBI.REF                                                ------------------------------------------------------------    0
LOC106996293.glutathione.hydrolase5.proenzyme-like-GGT1.rhesus.     cttgagggcatcacaagtcagtagcaatgagccaggtgggtggtgggtcacctggtgcag    4260

Papio.anubis.clone.rp41-133b2.8034-40080.revcompl.Baboon            ------------------------------------------------------------    0
BCRP3.HUMAN.NCBI.REF                                                ------------------------------------------------------------    0
LOC106996293.glutathione.hydrolase5.proenzyme-like-GGT1.rhesus.     caggcccggggctcgggtcccagcccagcacctgtggagtcccaggtggaggcaggggtg    4320

Papio.anubis.clone.rp41-133b2.8034-40080.revcompl.Baboon            ------------------------------------------------------------    0
BCRP3.HUMAN.NCBI.REF                                                ------------------------------------------------------------    0
LOC106996293.glutathione.hydrolase5.proenzyme-like-GGT1.rhesus.     gtggttccggctgcaccaccctcaggctcaaggtccaggccctcgtagatggtggggagt    4380

Papio.anubis.clone.rp41-133b2.8034-40080.revcompl.Baboon            ------------------------------------------------------------    0
BCRP3.HUMAN.NCBI.REF                                                ------------------------------------------------------------    0
LOC106996293.glutathione.hydrolase5.proenzyme-like-GGT1.rhesus.     gaggtgcgctggctcagccgccggcgcaggccaaccagcaggggctggggcagggaagcc    4440

Papio.anubis.clone.rp41-133b2.8034-40080.revcompl.Baboon            ------------------------------------------------------------    0
BCRP3.HUMAN.NCBI.REF                                                ------------------------------------------------------------    0
LOC106996293.glutathione.hydrolase5.proenzyme-like-GGT1.rhesus.     acatccacgttgttgcccaggtctacccaggccagtacagggaacttggtggtgtccttg    4500

Papio.anubis.clone.rp41-133b2.8034-40080.revcompl.Baboon            ------------------------------------------------------------    0
BCRP3.HUMAN.NCBI.REF                                                ------------------------------------------------------------    0
LOC106996293.glutathione.hydrolase5.proenzyme-like-GGT1.rhesus.     atggcgtcggtgagcttgcgggcagcagcccgcgtcagccggttgccgttgagcaggagc    4560

Papio.anubis.clone.rp41-133b2.8034-40080.revcompl.Baboon            ------------------------------------------------------------    0
BCRP3.HUMAN.NCBI.REF                                                ------------------------------------------------------------    0
LOC106996293.glutathione.hydrolase5.proenzyme-like-GGT1.rhesus.     tgggtgaggcggggcagcgcccacaggctgggcagcagcaggtgcagcagctcatcactc    4620

Papio.anubis.clone.rp41-133b2.8034-40080.revcompl.Baboon            ------------------------------------------------------------    0
BCRP3.HUMAN.NCBI.REF                                                ------------------------------------------------------------    0
LOC106996293.glutathione.hydrolase5.proenzyme-like-GGT1.rhesus.     agccctgtgaagctcaggtccagcaccgccagcacagcaccgtggctgctcagatagtgt    4680

Papio.anubis.clone.rp41-133b2.8034-40080.revcompl.Baboon            ------------------------------------------------------------    0
BCRP3.HUMAN.NCBI.REF                                                ------------------------------------------------------------    0
LOC106996293.glutathione.hydrolase5.proenzyme-like-GGT1.rhesus.     gtgatgtgctgcacgtcccgtaccgacagtgggatgcctgagaggtccacagtctcctct    4740
```

```
Papio.anubis.clone.rp41-133b2.8034-40080.revcompl.Baboon            ------------------------------------------------------------    0
BCRP3.HUMAN.NCBI.REF                                                ------------------------------------------------------------    0
LOC106996293.glutathione.hydrolase5.proenzyme-like-GGT1.rhesus.     gccagtggagtcttctggaggctgcccttgaggctgtgggagggtggcaggcggggatgg    4800

Papio.anubis.clone.rp41-133b2.8034-40080.revcompl.Baboon            ------------------------------------------------------------    0
BCRP3.HUMAN.NCBI.REF                                                ------------------------------------------------------------    0
LOC106996293.glutathione.hydrolase5.proenzyme-like-GGT1.rhesus.     actaggcaaccaggcagtccccccactgaccagagacagtaacctgtcaggaaaagccca    4860

Papio.anubis.clone.rp41-133b2.8034-40080.revcompl.Baboon            ------------------------------------------------------------    0
BCRP3.HUMAN.NCBI.REF                                                ------------------------------------------------------------    0
LOC106996293.glutathione.hydrolase5.proenzyme-like-GGT1.rhesus.     ggcctatggtctggcaaagccaggttcaaactgtcactttctttcttttacttttttttt    4920

Papio.anubis.clone.rp41-133b2.8034-40080.revcompl.Baboon            ------------------------------------------------------------    0
BCRP3.HUMAN.NCBI.REF                                                ------------------------------------------------------------    0
LOC106996293.glutathione.hydrolase5.proenzyme-like-GGT1.rhesus.     tttttttttgagacagttttactcttgttgcccaggctgaagcgcaatggcacgatctcg    4980

Papio.anubis.clone.rp41-133b2.8034-40080.revcompl.Baboon            ------------------------------------------------------------    0
BCRP3.HUMAN.NCBI.REF                                                ------------------------------------------------------------    0
LOC106996293.glutathione.hydrolase5.proenzyme-like-GGT1.rhesus.     gttcactgcaacctctgcctcctgggttgaaacgattctcctgcctcagcctcctgagta    5040

Papio.anubis.clone.rp41-133b2.8034-40080.revcompl.Baboon            ------------------------------------------------------------    0
BCRP3.HUMAN.NCBI.REF                                                ------------------------------------------------------------    0
LOC106996293.glutathione.hydrolase5.proenzyme-like-GGT1.rhesus.     gctgggattacaggcgtgcaccaccatgcccggctaattttatatttttagtagagacag    5100

Papio.anubis.clone.rp41-133b2.8034-40080.revcompl.Baboon            ------------------------------------------------------------    0
BCRP3.HUMAN.NCBI.REF                                                ------------------------------------------------------------    0
LOC106996293.glutathione.hydrolase5.proenzyme-like-GGT1.rhesus.     ggtttctccatgtttgtcaggctggtctcgaactcctcacctcaggtgatccgaccgcct    5160

Papio.anubis.clone.rp41-133b2.8034-40080.revcompl.Baboon            ------------------------------------------------------------    0
BCRP3.HUMAN.NCBI.REF                                                ------------------------------------------------------------    0
LOC106996293.glutathione.hydrolase5.proenzyme-like-GGT1.rhesus.     cagcctcccaaagtggtgggattacaggcgtgagacaccaagcctggcccaaactgtcac    5220

Papio.anubis.clone.rp41-133b2.8034-40080.revcompl.Baboon            ------------------------------------------------------------    0
BCRP3.HUMAN.NCBI.REF                                                ------------------------------------------------------------    0
LOC106996293.glutathione.hydrolase5.proenzyme-like-GGT1.rhesus.     tttctaagtgggtggccatgggcaatgctcagagcctctgtttcccctttagtgaaaatg    5280

Papio.anubis.clone.rp41-133b2.8034-40080.revcompl.Baboon            ------------------------------------------------------------    0
BCRP3.HUMAN.NCBI.REF                                                ------------------------------------------------------------    0
LOC106996293.glutathione.hydrolase5.proenzyme-like-GGT1.rhesus.     ggacgtggttgttgtgagaattgaatacacacacagtaaggctctatcttcatgaactat    5340

Papio.anubis.clone.rp41-133b2.8034-40080.revcompl.Baboon            ------------------------------------------------------------    0
BCRP3.HUMAN.NCBI.REF                                                ------------------------------------------------------------    0
LOC106996293.glutathione.hydrolase5.proenzyme-like-GGT1.rhesus.     tatttgaaatctcactgtgtgcccagcatctccacacttgttacattgacatctcactta    5400

Papio.anubis.clone.rp41-133b2.8034-40080.revcompl.Baboon            ------------------------------------------------------------    0
BCRP3.HUMAN.NCBI.REF                                                ------------------------------------------------------------    0
LOC106996293.glutathione.hydrolase5.proenzyme-like-GGT1.rhesus.     tttttcagataggtgagataactcttccatggccacacaccactaagcggggaaccagat    5460

Papio.anubis.clone.rp41-133b2.8034-40080.revcompl.Baboon            ------------------------------------------------------------    0
BCRP3.HUMAN.NCBI.REF                                                ------------------------------------------------------------    0
LOC106996293.glutathione.hydrolase5.proenzyme-like-GGT1.rhesus.     ccccaaactgagatgctctgactcttgagcccacatgtttttttccctgtgccacttctg    5520

Papio.anubis.clone.rp41-133b2.8034-40080.revcompl.Baboon            ------------------------------------------------------------    0
BCRP3.HUMAN.NCBI.REF                                                ------------------------------------------------------------    0
LOC106996293.glutathione.hydrolase5.proenzyme-like-GGT1.rhesus.     aggtcgattcttttagagtagggagaaattcttctacaaagggatggctattaggctata    5580

Papio.anubis.clone.rp41-133b2.8034-40080.revcompl.Baboon            ------------------------------------------------------------    0
BCRP3.HUMAN.NCBI.REF                                                ------------------------------------------------------------    0
LOC106996293.glutathione.hydrolase5.proenzyme-like-GGT1.rhesus.     cagggcaccagggctagagagggacaggcccagggtagtgggaggggaggtcctgggtgt    5640

Papio.anubis.clone.rp41-133b2.8034-40080.revcompl.Baboon            ------------------------------------------------------------    0
BCRP3.HUMAN.NCBI.REF                                                ------------------------------------------------------------    0
LOC106996293.glutathione.hydrolase5.proenzyme-like-GGT1.rhesus.     gcaggctggggacctgccctgggggagggcttcctgtgttttctcggaagggaagttcag    5700

Papio.anubis.clone.rp41-133b2.8034-40080.revcompl.Baboon            ------------------------------------------------------------    0
BCRP3.HUMAN.NCBI.REF                                                ------------------------------------------------------------    0
LOC106996293.glutathione.hydrolase5.proenzyme-like-GGT1.rhesus.     agactgagaaaggacccccccctctggccccaccaaccctctaggtctctccccagttac    5760

Papio.anubis.clone.rp41-133b2.8034-40080.revcompl.Baboon            ------------------------------------------------------------    0
BCRP3.HUMAN.NCBI.REF                                                ------------------------------------------------------------    0
LOC106996293.glutathione.hydrolase5.proenzyme-like-GGT1.rhesus.     ctgaacagagccaactaggcagacaggccttttgggggattgacagcagtgagcacaccc    5820

Papio.anubis.clone.rp41-133b2.8034-40080.revcompl.Baboon            ------------------------------------------------------------    0
BCRP3.HUMAN.NCBI.REF                                                ------------------------------------------------------------    0
LOC106996293.glutathione.hydrolase5.proenzyme-like-GGT1.rhesus.     tcaggacccccacctgccatggcagggaacaaccttgtgcggtccaagctcagcgtggcc    5880

Papio.anubis.clone.rp41-133b2.8034-40080.revcompl.Baboon            ------------------------------------------------------------    0
BCRP3.HUMAN.NCBI.REF                                                ------------------------------------------------------------    0
LOC106996293.glutathione.hydrolase5.proenzyme-like-GGT1.rhesus.     tgttaggcccaaggtgggagggcagacaggggaggcctgctaaggtaagctcccggcagc    5940

Papio.anubis.clone.rp41-133b2.8034-40080.revcompl.Baboon            ------------------------------------------------------------    0
BCRP3.HUMAN.NCBI.REF                                                ------------------------------------------------------------    0
LOC106996293.glutathione.hydrolase5.proenzyme-like-GGT1.rhesus.     agttcaggcccctgggcctgggtgcctgtggccagctctgggctgagcctgtgtgtgaag    6000

Papio.anubis.clone.rp41-133b2.8034-40080.revcompl.Baboon            ------------------------------------------------------------    0
BCRP3.HUMAN.NCBI.REF                                                ------------------------------------------------------------    0
LOC106996293.glutathione.hydrolase5.proenzyme-like-GGT1.rhesus.     ctccatggatagagtcactccaggatggagtgtctgagagggacccatggcagagggagg    6060

Papio.anubis.clone.rp41-133b2.8034-40080.revcompl.Baboon            ------------------------------------------------------------    0
BCRP3.HUMAN.NCBI.REF                                                ------------------------------------------------------------    0
LOC106996293.glutathione.hydrolase5.proenzyme-like-GGT1.rhesus.     tactgggcagccacggaggcctcagctttaggcagcctggcagtggcaaagaattcccaa    6120

Papio.anubis.clone.rp41-133b2.8034-40080.revcompl.Baboon            ------------------------------------------------------------    0
BCRP3.HUMAN.NCBI.REF                                                ------------------------------------------------------------    0
LOC106996293.glutathione.hydrolase5.proenzyme-like-GGT1.rhesus.     caaggtacaggaaaatcagggtagagtgtggccttaggtatgccacttctccgaacctca    6180

Papio.anubis.clone.rp41-133b2.8034-40080.revcompl.Baboon            ------------------------------------------------------------    0
BCRP3.HUMAN.NCBI.REF                                                ------------------------------------------------------------    0
LOC106996293.glutathione.hydrolase5.proenzyme-like-GGT1.rhesus.     gtttccttccctctaaaatgagaatcactatccagaatctgcctcccccacaacctgttg    6240

Papio.anubis.clone.rp41-133b2.8034-40080.revcompl.Baboon            ------------------------------------------------------------    0
BCRP3.HUMAN.NCBI.REF                                                ------------------------------------------------------------    0
LOC106996293.glutathione.hydrolase5.proenzyme-like-GGT1.rhesus.     ggaagtcttagctgaaaatgccttctgaggtcacctcagctgttgggtggagggctgggg    6300

Papio.anubis.clone.rp41-133b2.8034-40080.revcompl.Baboon            ------------------------------------------------------------    0
BCRP3.HUMAN.NCBI.REF                                                ------------------------------------------------------------    0
LOC106996293.glutathione.hydrolase5.proenzyme-like-GGT1.rhesus.     tgtccaggtcttgtaagaggccagggattgagggacgtgtgaatggcaggaggcaggacc    6360

Papio.anubis.clone.rp41-133b2.8034-40080.revcompl.Baboon            ------------------------------------------------------------    0
BCRP3.HUMAN.NCBI.REF                                                ------------------------------------------------------------    0
LOC106996293.glutathione.hydrolase5.proenzyme-like-GGT1.rhesus.     gtcccatgggaaatggcaaacgggtcactcagagggctcagcttggcttgggaagcatct    6420

Papio.anubis.clone.rp41-133b2.8034-40080.revcompl.Baboon            ------------------------------------------------------------    0
BCRP3.HUMAN.NCBI.REF                                                ------------------------------------------------------------    0
LOC106996293.glutathione.hydrolase5.proenzyme-like-GGT1.rhesus.     gctgccacaggtcaggctgacttccagggtagggccctcatgtggaccaggtaagggcct    6480
```

```
Papio.anubis.clone.rp41-133b2.8034-40080.revcompl.Baboon             ------------------------------------------------------------    0
BCRP3.HUMAN.NCBI.REF                                                 ------------------------------------------------------------    0
LOC106996293.glutathione.hydrolase5.proenzyme-like-GGT1.rhesus.      caccaggtgggatttcagcaccagcctcttggtgagagtgcagcctgagaagggaccttg    6540

Papio.anubis.clone.rp41-133b2.8034-40080.revcompl.Baboon             ------------------------------------------------------------    0
BCRP3.HUMAN.NCBI.REF                                                 ------------------------------------------------------------    0
LOC106996293.glutathione.hydrolase5.proenzyme-like-GGT1.rhesus.      gaacagcagtgaggaggggcagtggctaggctccccttaccagctctgggtcttcctctg    6600

Papio.anubis.clone.rp41-133b2.8034-40080.revcompl.Baboon             ------------------------------------------------------------    0
BCRP3.HUMAN.NCBI.REF                                                 ------------------------------------------------------------    0
LOC106996293.glutathione.hydrolase5.proenzyme-like-GGT1.rhesus.      gcacaggctggacccttgctgctgcttcgagtgaggggtgaggtggtagatgagctgtcg    6660

Papio.anubis.clone.rp41-133b2.8034-40080.revcompl.Baboon             ------------------------------------------------------------    0
BCRP3.HUMAN.NCBI.REF                                                 ------------------------------------------------------------    0
LOC106996293.glutathione.hydrolase5.proenzyme-like-GGT1.rhesus.      gcagatcttctccgaggacttccagagctcgtagtcctgtgggagggtagcgtcaccatg    6720

Papio.anubis.clone.rp41-133b2.8034-40080.revcompl.Baboon             ------------------------------------------------------------    0
BCRP3.HUMAN.NCBI.REF                                                 ------------------------------------------------------------    0
LOC106996293.glutathione.hydrolase5.proenzyme-like-GGT1.rhesus.      gtgaacctgagcccctcctgcccacctcagggccttggggagaggggcccgggcagccaa    6780

Papio.anubis.clone.rp41-133b2.8034-40080.revcompl.Baboon             ------------------------------------------------------------    0
BCRP3.HUMAN.NCBI.REF                                                 ------------------------------------------------------------    0
LOC106996293.glutathione.hydrolase5.proenzyme-like-GGT1.rhesus.      gtgtgtgtgtcagcacagccgtgcaccatgcatcactgagctgcccaccagggcctcccc    6840

Papio.anubis.clone.rp41-133b2.8034-40080.revcompl.Baboon             ------------------------------------------------------------    0
BCRP3.HUMAN.NCBI.REF                                                 ------------------------------------------------------------    0
LOC106996293.glutathione.hydrolase5.proenzyme-like-GGT1.rhesus.      ctcccaccccccaacccggctgtagcctggcacagagccaggggaggctgccctctggga    6900

Papio.anubis.clone.rp41-133b2.8034-40080.revcompl.Baboon             ------------------------------------------------------------    0
BCRP3.HUMAN.NCBI.REF                                                 ------------------------------------------------------------    0
LOC106996293.glutathione.hydrolase5.proenzyme-like-GGT1.rhesus.      gctggacaggctggtccagtcccgcagtgcccagtgggaggacgcccagacaatgggccc    6960

Papio.anubis.clone.rp41-133b2.8034-40080.revcompl.Baboon             ------------------------------------------------------------    0
BCRP3.HUMAN.NCBI.REF                                                 ------------------------------------------------------------    0
LOC106996293.glutathione.hydrolase5.proenzyme-like-GGT1.rhesus.      ttgttccttgtcctcaggcccagggccagcgtcttgggctatcagccggctgcctgccca    7020

Papio.anubis.clone.rp41-133b2.8034-40080.revcompl.Baboon             ------------------------------------------------------------    0
BCRP3.HUMAN.NCBI.REF                                                 ------------------------------------------------------------    0
LOC106996293.glutathione.hydrolase5.proenzyme-like-GGT1.rhesus.      ccctcagcacccctcggggacttgccacagggggaggaggccagaccaggcccctcgagg    7080

Papio.anubis.clone.rp41-133b2.8034-40080.revcompl.Baboon             ------------------------------------------------------------    0
BCRP3.HUMAN.NCBI.REF                                                 ------------------------------------------------------------    0
LOC106996293.glutathione.hydrolase5.proenzyme-like-GGT1.rhesus.      gagccctgttccattgacgccaatcccagggagtaggaacaagccaggtcagcatgttct    7140

Papio.anubis.clone.rp41-133b2.8034-40080.revcompl.Baboon             ------------------------------------------------------------    0
BCRP3.HUMAN.NCBI.REF                                                 ------------------------------------------------------------    0
LOC106996293.glutathione.hydrolase5.proenzyme-like-GGT1.rhesus.      tcttcctttcactctccaagcgagactgccccgtggggtctgggtctgggctgacagggc    7200

Papio.anubis.clone.rp41-133b2.8034-40080.revcompl.Baboon             ------------------------------------------------------------    0
BCRP3.HUMAN.NCBI.REF                                                 ------------------------------------------------------------    0
LOC106996293.glutathione.hydrolase5.proenzyme-like-GGT1.rhesus.      tgggggatgtaatgttctacagcaccaccgcctggctctctcttagtcctaactgggctc    7260

Papio.anubis.clone.rp41-133b2.8034-40080.revcompl.Baboon             ------------------------------------------------------------    0
BCRP3.HUMAN.NCBI.REF                                                 ------------------------------------------------------------    0
LOC106996293.glutathione.hydrolase5.proenzyme-like-GGT1.rhesus.      ctgcagaggcccagctcagggcagaatccagggtgcgcggcccacaaagctggcactgag    7320

Papio.anubis.clone.rp41-133b2.8034-40080.revcompl.Baboon             ------------------------------------------------------------    0
BCRP3.HUMAN.NCBI.REF                                                 ------------------------------------------------------------    0
LOC106996293.glutathione.hydrolase5.proenzyme-like-GGT1.rhesus.      catgctccactgcatcctttctccagtccctctgtgaacagccccaggagcaggcggcac    7380

Papio.anubis.clone.rp41-133b2.8034-40080.revcompl.Baboon             ------------------------------------------------------------    0
BCRP3.HUMAN.NCBI.REF                                                 ------------------------------------------------------------    0
LOC106996293.glutathione.hydrolase5.proenzyme-like-GGT1.rhesus.      acacagcagctgcaagccctggccattcctctgatggctgtggggtaggctcagagtcct    7440

Papio.anubis.clone.rp41-133b2.8034-40080.revcompl.Baboon             ------------------------------------------------------------    0
BCRP3.HUMAN.NCBI.REF                                                 ------------------------------------------------------------    0
LOC106996293.glutathione.hydrolase5.proenzyme-like-GGT1.rhesus.      aggggttgactatgcagagacctgggcattcagagctggcagggcccatagcggataccc    7500

Papio.anubis.clone.rp41-133b2.8034-40080.revcompl.Baboon             ------------------------------------------------------------    0
BCRP3.HUMAN.NCBI.REF                                                 ------------------------------------------------------------    0
LOC106996293.glutathione.hydrolase5.proenzyme-like-GGT1.rhesus.      ttgttttggtgcgggactcactgtgactaggagtcgggggacagtggctggtcacatggc    7560

Papio.anubis.clone.rp41-133b2.8034-40080.revcompl.Baboon             ------------------------------------------------------------    0
BCRP3.HUMAN.NCBI.REF                                                 ------------------------------------------------------------    0
LOC106996293.glutathione.hydrolase5.proenzyme-like-GGT1.rhesus.      agcctcatggcggctcgatgctcatggcaggacatctgcaggtgctgtcagagtggggca    7620

Papio.anubis.clone.rp41-133b2.8034-40080.revcompl.Baboon             ------------------------------------------------------------    0
BCRP3.HUMAN.NCBI.REF                                                 ------------------------------------------------------------    0
LOC106996293.glutathione.hydrolase5.proenzyme-like-GGT1.rhesus.      gccgtggggtagaggctgcaggtggcaaactccaccccagcaatgaggagggccttgctt    7680

Papio.anubis.clone.rp41-133b2.8034-40080.revcompl.Baboon             ------------------------------------------------------------    0
BCRP3.HUMAN.NCBI.REF                                                 ------------------------------------------------------------    0
LOC106996293.glutathione.hydrolase5.proenzyme-like-GGT1.rhesus.      atgaccccagctgttgtgggtggaagcagtgagcacctgtccccaagggtgtctgtacag    7740

Papio.anubis.clone.rp41-133b2.8034-40080.revcompl.Baboon             ------------------------------------------------------------    0
BCRP3.HUMAN.NCBI.REF                                                 ------------------------------------------------------------    0
LOC106996293.glutathione.hydrolase5.proenzyme-like-GGT1.rhesus.      gatggagactggtggacagaagggagctctagccaagggcggctctcttctggcccagga    7800

Papio.anubis.clone.rp41-133b2.8034-40080.revcompl.Baboon             ------------------------------------------------------------    0
BCRP3.HUMAN.NCBI.REF                                                 ------------------------------------------------------------    0
LOC106996293.glutathione.hydrolase5.proenzyme-like-GGT1.rhesus.      actcagaggctgggggtctggttccgttagtggatttctcagaacagtttgtgtccctca    7860

Papio.anubis.clone.rp41-133b2.8034-40080.revcompl.Baboon             ------------------------------------------------------------    0
BCRP3.HUMAN.NCBI.REF                                                 ------------------------------------------------------------    0
LOC106996293.glutathione.hydrolase5.proenzyme-like-GGT1.rhesus.      gggtccccatatgacttctgcccagctagtctcccctgcctgggtccccaggcctggtgc    7920

Papio.anubis.clone.rp41-133b2.8034-40080.revcompl.Baboon             ------------------------------------------------------------    0
BCRP3.HUMAN.NCBI.REF                                                 ------------------------------------------------------------    0
LOC106996293.glutathione.hydrolase5.proenzyme-like-GGT1.rhesus.      acagtgctgccacacagaaaacgggtctgcccaggactctgagggtccgctcacacttgc    7980

Papio.anubis.clone.rp41-133b2.8034-40080.revcompl.Baboon             ------------------------------------------------------------    0
BCRP3.HUMAN.NCBI.REF                                                 ------------------------------------------------------------    0
LOC106996293.glutathione.hydrolase5.proenzyme-like-GGT1.rhesus.      tgtagctggtggccagcctaatatactgatgctggcagcaggtctcagacagggactgtg    8040

Papio.anubis.clone.rp41-133b2.8034-40080.revcompl.Baboon             ------------------------------------------------------------    0
BCRP3.HUMAN.NCBI.REF                                                 ------------------------------------------------------------    0
LOC106996293.glutathione.hydrolase5.proenzyme-like-GGT1.rhesus.      acttgcctaaggccatacagcaagccacagaaggacctagcttcccagacctcagccctg    8100

Papio.anubis.clone.rp41-133b2.8034-40080.revcompl.Baboon             ------------------------------------------------------------    0
BCRP3.HUMAN.NCBI.REF                                                 ------------------------------------------------------------    0
LOC106996293.glutathione.hydrolase5.proenzyme-like-GGT1.rhesus.      ggctctgctggcccctgtccaccacagtgcccagcccagcgcccatgcctgccagcctca    8160

Papio.anubis.clone.rp41-133b2.8034-40080.revcompl.Baboon             ------------------------------------------------------------    0
BCRP3.HUMAN.NCBI.REF                                                 ------------------------------------------------------------    0
LOC106996293.glutathione.hydrolase5.proenzyme-like-GGT1.rhesus.      ccttcttggggcactgcagttcccgggccaggttcataagcaggtcatgggagatggggt    8220
```

```
Papio.anubis.clone.rp41-133b2.8034-40080.revcompl.Baboon           ------------------------------------------------------------    0
BCRP3.HUMAN.NCBI.REF                                               ------------------------------------------------------------    0
LOC106996293.glutathione.hydrolase5.proenzyme-like-GGT1.rhesus.    cgaccaggttgaggaaggccacatctctgtagaggatgtcagtaaggagggacccctcaa    8280

Papio.anubis.clone.rp41-133b2.8034-40080.revcompl.Baboon           ------------------------------------------------------------    0
BCRP3.HUMAN.NCBI.REF                                               ------------------------------------------------------------    0
LOC106996293.glutathione.hydrolase5.proenzyme-like-GGT1.rhesus.    ggcccaggtcctgtgagtgagtggtgaagagaaagttgagtgagccagtcgggtctccca    8340

Papio.anubis.clone.rp41-133b2.8034-40080.revcompl.Baboon           ------------------------------------------------------------    0
BCRP3.HUMAN.NCBI.REF                                               ------------------------------------------------------------    0
LOC106996293.glutathione.hydrolase5.proenzyme-like-GGT1.rhesus.    tctccaggtcccaccccagccagccagatgctggttatgccccttcctgtctccatctcc    8400

Papio.anubis.clone.rp41-133b2.8034-40080.revcompl.Baboon           ------------------------------------------------------------    0
BCRP3.HUMAN.NCBI.REF                                               ------------------------------------------------------------    0
LOC106996293.glutathione.hydrolase5.proenzyme-like-GGT1.rhesus.    tccctaaggctggctgtattctccccacaggcgaccatgggccaactgtccagtggtggg    8460

Papio.anubis.clone.rp41-133b2.8034-40080.revcompl.Baboon           ------------------------------------------------------------    0
BCRP3.HUMAN.NCBI.REF                                               ------------------------------------------------------------    0
LOC106996293.glutathione.hydrolase5.proenzyme-like-GGT1.rhesus.    caaaccctttcatttttccttatttttgagacagggtcttgctctgtcacccaggctag    8520

Papio.anubis.clone.rp41-133b2.8034-40080.revcompl.Baboon           ------------------------------------------------------------    0
BCRP3.HUMAN.NCBI.REF                                               ------------------------------------------------------------    0
LOC106996293.glutathione.hydrolase5.proenzyme-like-GGT1.rhesus.    agtccagtggtgtgatctcggctcattgtaacctctgcctcctcaagggatcctcccacc    8580

Papio.anubis.clone.rp41-133b2.8034-40080.revcompl.Baboon           ------------------------------------------------------------    0
BCRP3.HUMAN.NCBI.REF                                               ------------------------------------------------------------    0
LOC106996293.glutathione.hydrolase5.proenzyme-like-GGT1.rhesus.    tcagcctcctgaggagctggggctacagataggcactactatgcttagctaattaaaaaa    8640

Papio.anubis.clone.rp41-133b2.8034-40080.revcompl.Baboon           ------------------------------------------------------------    0
BCRP3.HUMAN.NCBI.REF                                               ------------------------------------------------------------    0
LOC106996293.glutathione.hydrolase5.proenzyme-like-GGT1.rhesus.    aatttttgtagagatggggtctcactatgttgcccaggctggtctcaaactcctgggct    8700

Papio.anubis.clone.rp41-133b2.8034-40080.revcompl.Baboon           ------------------------------------------------------------    0
BCRP3.HUMAN.NCBI.REF                                               ------------------------------------------------------------    0
LOC106996293.glutathione.hydrolase5.proenzyme-like-GGT1.rhesus.    caagcgatcctcccaacgtgctgggattacaggtgtaagccacttggccttcgtggaccc    8760

Papio.anubis.clone.rp41-133b2.8034-40080.revcompl.Baboon           ------------------------------------------------------------    0
BCRP3.HUMAN.NCBI.REF                                               ------------------------------------------------------------    0
LOC106996293.glutathione.hydrolase5.proenzyme-like-GGT1.rhesus.    tttggataagtagaggggagggacatgggagccatcagggaagtgataagtgaattccag    8820

Papio.anubis.clone.rp41-133b2.8034-40080.revcompl.Baboon           ------------------------------------------------------------    0
BCRP3.HUMAN.NCBI.REF                                               ------------------------------------------------------------    0
LOC106996293.glutathione.hydrolase5.proenzyme-like-GGT1.rhesus.    aacccttagaccagttccctcgaggtaaagtgttaggctatgacactgccttctctgggc    8880

Papio.anubis.clone.rp41-133b2.8034-40080.revcompl.Baboon           ------------------------------------------------------------    0
BCRP3.HUMAN.NCBI.REF                                               ------------------------------------------------------------    0
LOC106996293.glutathione.hydrolase5.proenzyme-like-GGT1.rhesus.    ttagggcttatcagtgggattctttggggtctctcctcttatggacgggggtttgctctt    8940

Papio.anubis.clone.rp41-133b2.8034-40080.revcompl.Baboon           ------------------------------------------------------------    0
BCRP3.HUMAN.NCBI.REF                                               ------------------------------------------------------------    0
LOC106996293.glutathione.hydrolase5.proenzyme-like-GGT1.rhesus.    cttatgacccctcaaattcccctggccccagcattcccttcagaccctttgccttcccac    9000

Papio.anubis.clone.rp41-133b2.8034-40080.revcompl.Baboon           ------------------------------------------------------------    0
BCRP3.HUMAN.NCBI.REF                                               ------------------------------------------------------------    0
LOC106996293.glutathione.hydrolase5.proenzyme-like-GGT1.rhesus.    ctatcccttggttttcctgagctgctgccagcatctgtttgcggacaggtctggatcctt    9060

Papio.anubis.clone.rp41-133b2.8034-40080.revcompl.Baboon           ------------------------------------------------------------    0
BCRP3.HUMAN.NCBI.REF                                               ------------------------------------------------------------    0
LOC106996293.glutathione.hydrolase5.proenzyme-like-GGT1.rhesus.    cccttgggggcggacctctctctcagatgtctcaaaggcatgctaccctgaagccggcct    9120

Papio.anubis.clone.rp41-133b2.8034-40080.revcompl.Baboon           ------------------------------------------------------------    0
BCRP3.HUMAN.NCBI.REF                                               ------------------------------------------------------------    0
LOC106996293.glutathione.hydrolase5.proenzyme-like-GGT1.rhesus.    caccacccatgccccatgccccagttcagtgtagggcgcctccatgcccatcttcccagg    9180

Papio.anubis.clone.rp41-133b2.8034-40080.revcompl.Baboon           ------------------------------------------------------------    0
BCRP3.HUMAN.NCBI.REF                                               ------------------------------------------------------------    0
LOC106996293.glutathione.hydrolase5.proenzyme-like-GGT1.rhesus.    cactggggccagaagccctggaaccccccaacactctgccctcctcctctctcccccagc    9240

Papio.anubis.clone.rp41-133b2.8034-40080.revcompl.Baboon           ------------------------------------------------------------    0
BCRP3.HUMAN.NCBI.REF                                               ------------------------------------------------------------    0
LOC106996293.glutathione.hydrolase5.proenzyme-like-GGT1.rhesus.    tcacctagtcatcccttctctccccaccactcctgccaggattcaggcctccagcctctt    9300

Papio.anubis.clone.rp41-133b2.8034-40080.revcompl.Baboon           ------------------------------------------------------------    0
BCRP3.HUMAN.NCBI.REF                                               ------------------------------------------------------------    0
LOC106996293.glutathione.hydrolase5.proenzyme-like-GGT1.rhesus.    ctcctacccatccctcaccccaccgcctgcacagacctgccctggccttcctgcctcact    9360

Papio.anubis.clone.rp41-133b2.8034-40080.revcompl.Baboon           ------------------------------------------------------------    0
BCRP3.HUMAN.NCBI.REF                                               ------------------------------------------------------------    0
LOC106996293.glutathione.hydrolase5.proenzyme-like-GGT1.rhesus.    cctccactttctcctcctgtctactcccacaggactggtgtggggctgaacactttcaga    9420

Papio.anubis.clone.rp41-133b2.8034-40080.revcompl.Baboon           ------------------------------------------------------------    0
BCRP3.HUMAN.NCBI.REF                                               ------------------------------------------------------------    0
LOC106996293.glutathione.hydrolase5.proenzyme-like-GGT1.rhesus.    gggtctctctctctctcttttttttttgagaccgagttttgctcctgttgcccaagctgg    9480

Papio.anubis.clone.rp41-133b2.8034-40080.revcompl.Baboon           ------------------------------------------------------------    0
BCRP3.HUMAN.NCBI.REF                                               ------------------------------------------------------------    0
LOC106996293.glutathione.hydrolase5.proenzyme-like-GGT1.rhesus.    agtgcaatggtgtgatgtcagctcactgcaacctctgtctcccaggttcaagcaactctc    9540

Papio.anubis.clone.rp41-133b2.8034-40080.revcompl.Baboon           ------------------------------------------------------------    0
BCRP3.HUMAN.NCBI.REF                                               ------------------------------------------------------------    0
LOC106996293.glutathione.hydrolase5.proenzyme-like-GGT1.rhesus.    ctgcctcagcctcccgagtaggtgggattacaggcatgcgccaccttgcccagctaattt    9600

Papio.anubis.clone.rp41-133b2.8034-40080.revcompl.Baboon           ------------------------------------------------------------    0
BCRP3.HUMAN.NCBI.REF                                               ------------------------------------------------------------    0
LOC106996293.glutathione.hydrolase5.proenzyme-like-GGT1.rhesus.    tgtatttttagtagagatgcggtttctccatgttgatcaggctggtctcgaactcccgac    9660

Papio.anubis.clone.rp41-133b2.8034-40080.revcompl.Baboon           ------------------------------------------------------------    0
BCRP3.HUMAN.NCBI.REF                                               ------------------------------------------------------------    0
LOC106996293.glutathione.hydrolase5.proenzyme-like-GGT1.rhesus.    ttcaggtgatctgcccaccttggcctcccaaagtgttgggattacaggtgtgagccactg    9720

Papio.anubis.clone.rp41-133b2.8034-40080.revcompl.Baboon           ------------------------------------------------------------    0
BCRP3.HUMAN.NCBI.REF                                               ------------------------------------------------------------    0
LOC106996293.glutathione.hydrolase5.proenzyme-like-GGT1.rhesus.    cgtctggcccagagggtctctttagaggagagattgagagctgatatggcagccacagcc    9780

Papio.anubis.clone.rp41-133b2.8034-40080.revcompl.Baboon           ------------------------------------------------------------    0
BCRP3.HUMAN.NCBI.REF                                               ------------------------------------------------------------    0
LOC106996293.glutathione.hydrolase5.proenzyme-like-GGT1.rhesus.    aggaggggcctctagaccccatgtgggccttctccaatccataggtgatatgctgtgtgc    9840

Papio.anubis.clone.rp41-133b2.8034-40080.revcompl.Baboon           ------------------------------------------------------------    0
BCRP3.HUMAN.NCBI.REF                                               ------------------------------------------------------------    0
LOC106996293.glutathione.hydrolase5.proenzyme-like-GGT1.rhesus.    gtgggtcccaagcacaggaagttgggccaggcagggaagtccgtgggtcacagctgcagg    9900

Papio.anubis.clone.rp41-133b2.8034-40080.revcompl.Baboon           ------------------------------------------------------------    0
BCRP3.HUMAN.NCBI.REF                                               ------------------------------------------------------------    0
LOC106996293.glutathione.hydrolase5.proenzyme-like-GGT1.rhesus.    gtccttgcaggttatggagcaggataggcccctggctggacctcaaatcccaacctctga    9960
```

```
Papio.anubis.clone.rp41-133b2.8034-40080.revcompl.Baboon          ------------------------------------------------------------    0
BCRP3.HUMAN.NCBI.REF                                              ------------------------------------------------------------    0
LOC106996293.glutathione.hydrolase5.proenzyme-like-GGT1.rhesus.   gacagaaagaggtgacctagctggtctctgaggacccttctttcctccagtttcccaact    10020

Papio.anubis.clone.rp41-133b2.8034-40080.revcompl.Baboon          ------------------------------------------------------------    0
BCRP3.HUMAN.NCBI.REF                                              ------------------------------------------------------------    0
LOC106996293.glutathione.hydrolase5.proenzyme-like-GGT1.rhesus.   aaaatgcccaactggtgtcttgggctccagacggcatcccagactgagatccctgcctct    10080

Papio.anubis.clone.rp41-133b2.8034-40080.revcompl.Baboon          ------------------------------------------------------------    0
BCRP3.HUMAN.NCBI.REF                                              ------------------------------------------------------------    0
LOC106996293.glutathione.hydrolase5.proenzyme-like-GGT1.rhesus.   gggtgcctggctgattcagtgctgctctgctgtttgccacagatgccagctagggaagat    10140

Papio.anubis.clone.rp41-133b2.8034-40080.revcompl.Baboon          ------------------------------------------------------------    0
BCRP3.HUMAN.NCBI.REF                                              ------------------------------------------------------------    0
LOC106996293.glutathione.hydrolase5.proenzyme-like-GGT1.rhesus.   ccagaaggaaggctggctgatgagcctggcagacccaaacagggccccagatcgcaacct    10200

Papio.anubis.clone.rp41-133b2.8034-40080.revcompl.Baboon          ------------------------------------------------------------    0
BCRP3.HUMAN.NCBI.REF                                              ------------------------------------------------------------    0
LOC106996293.glutathione.hydrolase5.proenzyme-like-GGT1.rhesus.   gccagcctgccttccgctggtctagacagctcctcttaaaggggcagctccatttttcca    10260

Papio.anubis.clone.rp41-133b2.8034-40080.revcompl.Baboon          ------------------------------------------------------------    0
BCRP3.HUMAN.NCBI.REF                                              ------------------------------------------------------------    0
LOC106996293.glutathione.hydrolase5.proenzyme-like-GGT1.rhesus.   gcctgctgaacccagagttggaggaggtggagaagggtagagaggaagaagggctctggc    10320

Papio.anubis.clone.rp41-133b2.8034-40080.revcompl.Baboon          ------------------------------------------------------------    0
BCRP3.HUMAN.NCBI.REF                                              ------------------------------------------------------------    0
LOC106996293.glutathione.hydrolase5.proenzyme-like-GGT1.rhesus.   cctggcttgccccagtttttctctgtggaccctgggatgaccacgtctccccatgcctca    10380

Papio.anubis.clone.rp41-133b2.8034-40080.revcompl.Baboon          ------------------------------------------------------------    0
BCRP3.HUMAN.NCBI.REF                                              ------------------------------------------------------------    0
LOC106996293.glutathione.hydrolase5.proenzyme-like-GGT1.rhesus.   gtttccccatttctagtataccttctgcaggccaaacacctgcatggctgactccctgct    10440

Papio.anubis.clone.rp41-133b2.8034-40080.revcompl.Baboon          ------------------------------------------------------------    0
BCRP3.HUMAN.NCBI.REF                                              ------------------------------------------------------------    0
LOC106996293.glutathione.hydrolase5.proenzyme-like-GGT1.rhesus.   tacaggatggggcaggagtggtggcagctgctgcaggggatggggacggggtggtgagga    10500

Papio.anubis.clone.rp41-133b2.8034-40080.revcompl.Baboon          ------------------------------------------------------------    0
BCRP3.HUMAN.NCBI.REF                                              ------------------------------------------------------------    0
LOC106996293.glutathione.hydrolase5.proenzyme-like-GGT1.rhesus.   aagtgacttgcccaggtgatggtgggaccagcatcctgggacgacccagggcttgggatc    10560

Papio.anubis.clone.rp41-133b2.8034-40080.revcompl.Baboon          ------------------------------------------------------------    0
BCRP3.HUMAN.NCBI.REF                                              ------------------------------------------------------------    0
LOC106996293.glutathione.hydrolase5.proenzyme-like-GGT1.rhesus.   tgggtttagtgtacctggtgtgtgtaggagcagggccaccactggaccaagtgttgtgat    10620

Papio.anubis.clone.rp41-133b2.8034-40080.revcompl.Baboon          ------------------------------------------------------------    0
BCRP3.HUMAN.NCBI.REF                                              ------------------------------------------------------------    0
LOC106996293.glutathione.hydrolase5.proenzyme-like-GGT1.rhesus.   gcacagatactaggggtgtgtactctaagggcatgagctgcctgctggagaactggtgat    10680

Papio.anubis.clone.rp41-133b2.8034-40080.revcompl.Baboon          ------------------------------------------------------------    0
BCRP3.HUMAN.NCBI.REF                                              ------------------------------------------------------------    0
LOC106996293.glutathione.hydrolase5.proenzyme-like-GGT1.rhesus.   gcatgcggtggctctgtgtgggctgccatggtgtcttgggatgaggctgcaccggcagac    10740

Papio.anubis.clone.rp41-133b2.8034-40080.revcompl.Baboon          ------------------------------------------------------------    0
BCRP3.HUMAN.NCBI.REF                                              ------------------------------------------------------------    0
LOC106996293.glutathione.hydrolase5.proenzyme-like-GGT1.rhesus.   cagccctgggtcctctctccagggtccagtgcctccccaatcctggaggaaggggaagct    10800

Papio.anubis.clone.rp41-133b2.8034-40080.revcompl.Baboon          ------------------------------------------------------------    0
BCRP3.HUMAN.NCBI.REF                                              ------------------------------------------------------------    0
LOC106996293.glutathione.hydrolase5.proenzyme-like-GGT1.rhesus.   accttcttccattgtctctcaatacaagatgattcgacctcacttgccactcctcccggg    10860

Papio.anubis.clone.rp41-133b2.8034-40080.revcompl.Baboon          ------------------------------------------------------------    0
BCRP3.HUMAN.NCBI.REF                                              ------------------------------------------------------------    0
LOC106996293.glutathione.hydrolase5.proenzyme-like-GGT1.rhesus.   gcccttcccatcttgccagggcctgaatctgagcttttagctccaggctggggccaccct    10920

Papio.anubis.clone.rp41-133b2.8034-40080.revcompl.Baboon          ------------------------------------------------------------    0
BCRP3.HUMAN.NCBI.REF                                              ------------------------------------------------------------    0
LOC106996293.glutathione.hydrolase5.proenzyme-like-GGT1.rhesus.   gaacacacacaacccctctcaaaccgcagagacccggacacactcagcggccccttctc    10980

Papio.anubis.clone.rp41-133b2.8034-40080.revcompl.Baboon          ------------------------------------------------------------    0
BCRP3.HUMAN.NCBI.REF                                              ------------------------------------------------------------    0
LOC106996293.glutathione.hydrolase5.proenzyme-like-GGT1.rhesus.   cacacacggtccgccggcctcacccagccctcgctccagccggccgccctctagaccact    11040

Papio.anubis.clone.rp41-133b2.8034-40080.revcompl.Baboon          ------------------------------------------------------------    0
BCRP3.HUMAN.NCBI.REF                                              ------------------------------------------------------------    0
LOC106996293.glutathione.hydrolase5.proenzyme-like-GGT1.rhesus.   caagaccccgcgcccagcagcccgtcgcgccctcggaacccgtccgtgcgcgcccaagac    11100

Papio.anubis.clone.rp41-133b2.8034-40080.revcompl.Baboon          ------------------------------------------------------------    0
BCRP3.HUMAN.NCBI.REF                                              ------------------------------------------------------------    0
LOC106996293.glutathione.hydrolase5.proenzyme-like-GGT1.rhesus.   cagctccgcctcgcctcccgacccatacatgcccacaacccataacctcgcgcctcctag    11160

Papio.anubis.clone.rp41-133b2.8034-40080.revcompl.Baboon          ------------------------------------------------------------    0
BCRP3.HUMAN.NCBI.REF                                              ------------------------------------------------------------    0
LOC106996293.glutathione.hydrolase5.proenzyme-like-GGT1.rhesus.   acccccagaccctccctggccgccagccccggggcggccggctccttctctcgcctggcg    11220

Papio.anubis.clone.rp41-133b2.8034-40080.revcompl.Baboon          ------------------------------------------------------------    0
BCRP3.HUMAN.NCBI.REF                                              ------------------------------------------------------------    0
LOC106996293.glutathione.hydrolase5.proenzyme-like-GGT1.rhesus.   caggaggcgcagcagctgccgggcgcgctccggccgccgctcgcggagcgtggactggat    11280

Papio.anubis.clone.rp41-133b2.8034-40080.revcompl.Baboon          ------------------------------------------------------------    0
BCRP3.HUMAN.NCBI.REF                                              ------------------------------------------------------------    0
LOC106996293.glutathione.hydrolase5.proenzyme-like-GGT1.rhesus.   ctcgcgaagccaccgcacccggcgctcgtagggcgcgggcctgcagccggccaccgcccc    11340

Papio.anubis.clone.rp41-133b2.8034-40080.revcompl.Baboon          ------------------------------------------------------------    0
BCRP3.HUMAN.NCBI.REF                                              ------------------------------------------------------------    0
LOC106996293.glutathione.hydrolase5.proenzyme-like-GGT1.rhesus.   ggcctcggagcctgcctcaggcccggcccgccggcccagccgcgcccccatggccaccgc    11400

Papio.anubis.clone.rp41-133b2.8034-40080.revcompl.Baboon          ------------------------------------------------------------    0
BCRP3.HUMAN.NCBI.REF                                              ------------------------------------------------------------    0
LOC106996293.glutathione.hydrolase5.proenzyme-like-GGT1.rhesus.   gcggcgggcgccgaactcacagaaggctggacggtctgcaccccgcctccggggccgccc    11460

Papio.anubis.clone.rp41-133b2.8034-40080.revcompl.Baboon          ------------------------------------------------------------    0
BCRP3.HUMAN.NCBI.REF                                              ------------------------------------------------------------    0
LOC106996293.glutathione.hydrolase5.proenzyme-like-GGT1.rhesus.   atagtcgccccgcccccacccaccccggccccgcccccacccaccccggccccgccccgt    11520

Papio.anubis.clone.rp41-133b2.8034-40080.revcompl.Baboon          ------------------------------------------------------------    0
BCRP3.HUMAN.NCBI.REF                                              ------------------------------------------------------------    0
LOC106996293.glutathione.hydrolase5.proenzyme-like-GGT1.rhesus.   gcgggtatgaaggctgagcgcccgccccaccccgcgccggagcgaggccagtgggttcct    11580

Papio.anubis.clone.rp41-133b2.8034-40080.revcompl.Baboon          ------------------------------------------------------------    0
BCRP3.HUMAN.NCBI.REF                                              ------------------------------------------------------------    0
LOC106996293.glutathione.hydrolase5.proenzyme-like-GGT1.rhesus.   cgatttggcctggtcttactggaggaggccctgcctgtgtcagtcttggcgctagagtcc    11640

Papio.anubis.clone.rp41-133b2.8034-40080.revcompl.Baboon          ------------------------------------------------------------    0
BCRP3.HUMAN.NCBI.REF                                              ------------------------------------------------------------    0
LOC106996293.glutathione.hydrolase5.proenzyme-like-GGT1.rhesus.   aaggacctacagttgccaggggagacgcggagctcggcccagatgaggtggcttttggca    11700
```

```
Papio.anubis.clone.rp41-133b2.8034-40080.revcompl.Baboon           ------------------------------------------------------------    0
BCRP3.HUMAN.NCBI.REF                                               ------------------------------------------------------------    0
LOC106996293.glutathione.hydrolase5.proenzyme-like-GGT1.rhesus.    cctctctaccttctggcccaagtgctgactggggaatagtcgctttgacggtgcagagca    11760

Papio.anubis.clone.rp41-133b2.8034-40080.revcompl.Baboon           ------------------------------------------------------------    0
BCRP3.HUMAN.NCBI.REF                                               ------------------------------------------------------------    0
LOC106996293.glutathione.hydrolase5.proenzyme-like-GGT1.rhesus.    ttccaggcgggtgtgggggttccactgcgtgtgccagcattaggtattgcccccacctgc    11820

Papio.anubis.clone.rp41-133b2.8034-40080.revcompl.Baboon           ------------------------------------------------------------    0
BCRP3.HUMAN.NCBI.REF                                               ------------------------------------------------------------    0
LOC106996293.glutathione.hydrolase5.proenzyme-like-GGT1.rhesus.    ttttagaaatctgccctgcatggtgaatctccgtctctactaaaatacaaaaattaggcc    11880

Papio.anubis.clone.rp41-133b2.8034-40080.revcompl.Baboon           ------------------------------------------------------------    0
BCRP3.HUMAN.NCBI.REF                                               ------------------------------------------------------------    0
LOC106996293.glutathione.hydrolase5.proenzyme-like-GGT1.rhesus.    gggcgcgatggctcattcctgtaatcccagcactttgggaggccgaggcagacggaatct    11940

Papio.anubis.clone.rp41-133b2.8034-40080.revcompl.Baboon           ------------------------------------------------------------    0
BCRP3.HUMAN.NCBI.REF                                               ------------------------------------------------------------    0
LOC106996293.glutathione.hydrolase5.proenzyme-like-GGT1.rhesus.    gaggcccggagttcgagaccagcctggctaatatggtgaaaccccatttttactgaaaat    12000

Papio.anubis.clone.rp41-133b2.8034-40080.revcompl.Baboon           ------------------------------------------------------------    0
BCRP3.HUMAN.NCBI.REF                                               ------------------------------------------------------------    0
LOC106996293.glutathione.hydrolase5.proenzyme-like-GGT1.rhesus.    acaaaaaattagcccggcgtggtggtgggcgcctgtaatcccagctacttgggaggctga    12060

Papio.anubis.clone.rp41-133b2.8034-40080.revcompl.Baboon           ------------------------------------------------------------    0
BCRP3.HUMAN.NCBI.REF                                               ------------------------------------------------------------    0
LOC106996293.glutathione.hydrolase5.proenzyme-like-GGT1.rhesus.    ggtaggagaatcaattgaaccggggaggcggaggttgcagtgagccgagatagatggtgc    12120

Papio.anubis.clone.rp41-133b2.8034-40080.revcompl.Baboon           ------------------------------------------------------------    0
BCRP3.HUMAN.NCBI.REF                                               ------------------------------------------------------------    0
LOC106996293.glutathione.hydrolase5.proenzyme-like-GGT1.rhesus.    cactgcactccaccctgggtgacagcaaaactatgtctcagaaaagaaaagaaaagaaga    12180

Papio.anubis.clone.rp41-133b2.8034-40080.revcompl.Baboon           ------------------------------------------------------------    0
BCRP3.HUMAN.NCBI.REF                                               ------------------------------------------------------------    0
LOC106996293.glutathione.hydrolase5.proenzyme-like-GGT1.rhesus.    aaaggaaaaaaaagaaaagagaaaagaaaaggaatctttcctgcagctggcagttgcggt    12240

Papio.anubis.clone.rp41-133b2.8034-40080.revcompl.Baboon           ------------------------------------------------------------    0
BCRP3.HUMAN.NCBI.REF                                               ------------------------------------------------------------    0
LOC106996293.glutathione.hydrolase5.proenzyme-like-GGT1.rhesus.    gtaggacctgctcacactgcatggacactcctgcactggcaaatatttactgcctcctgc    12300

Papio.anubis.clone.rp41-133b2.8034-40080.revcompl.Baboon           ------------------------------------------------------------    0
BCRP3.HUMAN.NCBI.REF                                               ------------------------------------------------------------    0
LOC106996293.glutathione.hydrolase5.proenzyme-like-GGT1.rhesus.    tttttggtttggggcaggcccccacctctgactccagcctggggaataaaccggatgaag    12360

Papio.anubis.clone.rp41-133b2.8034-40080.revcompl.Baboon           ------------------------------------------------------------    0
BCRP3.HUMAN.NCBI.REF                                               ------------------------------------------------------------    0
LOC106996293.glutathione.hydrolase5.proenzyme-like-GGT1.rhesus.    agtgaggttaaccgttaagcccagctctaggcaggctggcagcaggcgggctgttatctg    12420

Papio.anubis.clone.rp41-133b2.8034-40080.revcompl.Baboon           ------------------------------------------------------------    0
BCRP3.HUMAN.NCBI.REF                                               ------------------------------------------------------------    0
LOC106996293.glutathione.hydrolase5.proenzyme-like-GGT1.rhesus.    ccacttcagtcttcctccctgataaggggttggggggttggggcaggcagggtgagatg    12480

Papio.anubis.clone.rp41-133b2.8034-40080.revcompl.Baboon           ------------------------------------------------------------    0
BCRP3.HUMAN.NCBI.REF                                               ------------------------------------------------------------    0
LOC106996293.glutathione.hydrolase5.proenzyme-like-GGT1.rhesus.    ggggccctggctcttctgaggaccagggtgaagtacggacccaccctgctcaaggagtat    12540

Papio.anubis.clone.rp41-133b2.8034-40080.revcompl.Baboon           ------------------------------------------------------------    0
BCRP3.HUMAN.NCBI.REF                                               ------------------------------------------------------------    0
LOC106996293.glutathione.hydrolase5.proenzyme-like-GGT1.rhesus.    cccacttcccagggccccttcacagaagtctaggtctctgttggaggcctgctgggccc    12600

Papio.anubis.clone.rp41-133b2.8034-40080.revcompl.Baboon           ------------------------------------------------------------    0
BCRP3.HUMAN.NCBI.REF                                               ------------------------------------------------------------    0
LOC106996293.glutathione.hydrolase5.proenzyme-like-GGT1.rhesus.    ctgcagccccaatcccagctctgggaccctcacatctgggtgggggatgtttgaggcact    12660

Papio.anubis.clone.rp41-133b2.8034-40080.revcompl.Baboon           ------------------------------------------------------------    0
BCRP3.HUMAN.NCBI.REF                                               ------------------------------------------------------------    0
LOC106996293.glutathione.hydrolase5.proenzyme-like-GGT1.rhesus.    tatctgcagtccccacttccccaagcaaggagcatgtgtctagcctgtgaggatgggtac    12720

Papio.anubis.clone.rp41-133b2.8034-40080.revcompl.Baboon           ------------------------------------------------------------    0
BCRP3.HUMAN.NCBI.REF                                               ------------------------------------------------------------    0
LOC106996293.glutathione.hydrolase5.proenzyme-like-GGT1.rhesus.    ctgttgtgcaggaaggactgagcaccgtccgagggcatatagtgagggacaacagccaat    12780

Papio.anubis.clone.rp41-133b2.8034-40080.revcompl.Baboon           ------------------------------------------------------------    0
BCRP3.HUMAN.NCBI.REF                                               ------------------------------------------------------------    0
LOC106996293.glutathione.hydrolase5.proenzyme-like-GGT1.rhesus.    cacccgtgtgtgtgtgtgtgtgtgtgtgtgtgtgtgtgtgtcttttaattaccctatgaaga    12840

Papio.anubis.clone.rp41-133b2.8034-40080.revcompl.Baboon           ------------------------------------------------------------    0
BCRP3.HUMAN.NCBI.REF                                               ------------------------------------------------------------    0
LOC106996293.glutathione.hydrolase5.proenzyme-like-GGT1.rhesus.    ttcaaggttccttcctttgggttaagcactgcctgcccagagatggtagtagggagaggg    12900

Papio.anubis.clone.rp41-133b2.8034-40080.revcompl.Baboon           ------------------------------------------------------------    0
BCRP3.HUMAN.NCBI.REF                                               ------------------------------------------------------------    0
LOC106996293.glutathione.hydrolase5.proenzyme-like-GGT1.rhesus.    cggggtgctaggctctgtcttcctgtggggctgggtccctgagacaagacagaggacagc    12960

Papio.anubis.clone.rp41-133b2.8034-40080.revcompl.Baboon           ------------------------------------------------------------    0
BCRP3.HUMAN.NCBI.REF                                               ------------------------------------------------------------    0
LOC106996293.glutathione.hydrolase5.proenzyme-like-GGT1.rhesus.    aggatgtcttagccccttgaccatccacagacccccaacccctgctggagtggtacttgc    13020

Papio.anubis.clone.rp41-133b2.8034-40080.revcompl.Baboon           ------------------------------------------------------------    0
BCRP3.HUMAN.NCBI.REF                                               ------------------------------------------------------------    0
LOC106996293.glutathione.hydrolase5.proenzyme-like-GGT1.rhesus.    atccccttctcccctggtacctgccagctggacccaacaacctctcctcccgctacagtg    13080

Papio.anubis.clone.rp41-133b2.8034-40080.revcompl.Baboon           ------------------------------------------------------------    0
BCRP3.HUMAN.NCBI.REF                                               ------------------------------------------------------------    0
LOC106996293.glutathione.hydrolase5.proenzyme-like-GGT1.rhesus.    cagccagtattgtcctgaggccaccttggcaccataggtttcctccctggcaaacctgca    13140

Papio.anubis.clone.rp41-133b2.8034-40080.revcompl.Baboon           ------------------------------------------------------------    0
BCRP3.HUMAN.NCBI.REF                                               ------------------------------------------------------------    0
LOC106996293.glutathione.hydrolase5.proenzyme-like-GGT1.rhesus.    ttacggccttgcgtgccttcccccagagccaaggcgctgttgctgaactgaagtggcgac    13200

Papio.anubis.clone.rp41-133b2.8034-40080.revcompl.Baboon           ------------------------------------------------------------    0
BCRP3.HUMAN.NCBI.REF                                               ------------------------------------------------------------    0
LOC106996293.glutathione.hydrolase5.proenzyme-like-GGT1.rhesus.    agaccgggtcagagctttaagcagcagccccagtgccctagggacctctcagaccccctg    13260

Papio.anubis.clone.rp41-133b2.8034-40080.revcompl.Baboon           ------------------------------------------------------------    0
BCRP3.HUMAN.NCBI.REF                                               ------------------------------------------------------------    0
LOC106996293.glutathione.hydrolase5.proenzyme-like-GGT1.rhesus.    cccagagcaggcagcaggtaagccctcctccccttcccctgcagcctcagggctgaagcc    13320

Papio.anubis.clone.rp41-133b2.8034-40080.revcompl.Baboon           ------------------------------------------------------------    0
BCRP3.HUMAN.NCBI.REF                                               ------------------------------------------------------------    0
LOC106996293.glutathione.hydrolase5.proenzyme-like-GGT1.rhesus.    agagctgggagggctcccagatcctgaccatgtcctcccagaggctgagccaggcctgga    13380

Papio.anubis.clone.rp41-133b2.8034-40080.revcompl.Baboon           ------------------------------------------------------------    0
BCRP3.HUMAN.NCBI.REF                                               ------------------------------------------------------------    0
LOC106996293.glutathione.hydrolase5.proenzyme-like-GGT1.rhesus.    cactgtcccctcgaatgtgagaagactgggcagggggaggggagggaggaggggatggag    13440
```

```
Papio.anubis.clone.rp41-133b2.8034-40080.revcompl.Baboon            ------------------------------------------------------------    0
BCRP3.HUMAN.NCBI.REF                                                ------------------------------------------------------------    0
LOC106996293.glutathione.hydrolase5.proenzyme-like-GGT1.rhesus.     ggctgagccttgaggcatgaaagaggcaacctaggcagtggtatgggggagtgcccagag    13500

Papio.anubis.clone.rp41-133b2.8034-40080.revcompl.Baboon            ------------------------------------------------------------    0
BCRP3.HUMAN.NCBI.REF                                                ------------------------------------------------------------    0
LOC106996293.glutathione.hydrolase5.proenzyme-like-GGT1.rhesus.     tttagagtcctgtggaaggcagagaactgccccagcccctgcctctcatcctccaccctc    13560

Papio.anubis.clone.rp41-133b2.8034-40080.revcompl.Baboon            ------------------------------------------------------------    0
BCRP3.HUMAN.NCBI.REF                                                ------------------------------------------------------------    0
LOC106996293.glutathione.hydrolase5.proenzyme-like-GGT1.rhesus.     tgtgtccctggctgagaggttcctagaggacgtttttccagcaaaagagggagggtggtg    13620

Papio.anubis.clone.rp41-133b2.8034-40080.revcompl.Baboon            ------------------------------------------------------------    0
BCRP3.HUMAN.NCBI.REF                                                ------------------------------------------------------------    0
LOC106996293.glutathione.hydrolase5.proenzyme-like-GGT1.rhesus.     gctgggcctagatccttgaatgggcttttctctgcctgcttgcagggcgcctagggtaaa    13680

Papio.anubis.clone.rp41-133b2.8034-40080.revcompl.Baboon            ------------------------------------------------------------    0
BCRP3.HUMAN.NCBI.REF                                                ------------------------------------------------------------    0
LOC106996293.glutathione.hydrolase5.proenzyme-like-GGT1.rhesus.     tgggctcttggagacaatttctgacctaacctgacttgaacaggaacccccagaggcaga    13740

Papio.anubis.clone.rp41-133b2.8034-40080.revcompl.Baboon            ------------------------------------------------------------    0
BCRP3.HUMAN.NCBI.REF                                                ------------------------------------------------------------    0
LOC106996293.glutathione.hydrolase5.proenzyme-like-GGT1.rhesus.     gctatcccctcaggcgtggtggcgtgattccacctcgccagcccatgtgttccacatgga    13800

Papio.anubis.clone.rp41-133b2.8034-40080.revcompl.Baboon            ------------------------------------------------------------    0
BCRP3.HUMAN.NCBI.REF                                                ------------------------------------------------------------    0
LOC106996293.glutathione.hydrolase5.proenzyme-like-GGT1.rhesus.     aggctgctggcccctctccctggggttaccctgagcagcagagctgtgcttgttgagacc    13860

Papio.anubis.clone.rp41-133b2.8034-40080.revcompl.Baboon            ------------------------------------------------------------    0
BCRP3.HUMAN.NCBI.REF                                                ------------------------------------------------------------    0
LOC106996293.glutathione.hydrolase5.proenzyme-like-GGT1.rhesus.     caggggggtggagggattgcgatattgtcctctcagctcccagggctgatgtgggagctca    13920

Papio.anubis.clone.rp41-133b2.8034-40080.revcompl.Baboon            ------------------------------------------------------------    0
BCRP3.HUMAN.NCBI.REF                                                ------------------------------------------------------------    0
LOC106996293.glutathione.hydrolase5.proenzyme-like-GGT1.rhesus.     agggcttaccctggatccttttgggtagccctgccatccctagggagtcagtgaggtcag    13980

Papio.anubis.clone.rp41-133b2.8034-40080.revcompl.Baboon            ------------------------------------------------------------    0
BCRP3.HUMAN.NCBI.REF                                                ------------------------------------------------------------    0
LOC106996293.glutathione.hydrolase5.proenzyme-like-GGT1.rhesus.     aggttgggagctggagaatcaggtagggaggacacagctagacctcaggctctaatggga    14040

Papio.anubis.clone.rp41-133b2.8034-40080.revcompl.Baboon            ------------------------------------------------------------    0
BCRP3.HUMAN.NCBI.REF                                                ------------------------------------------------------------    0
LOC106996293.glutathione.hydrolase5.proenzyme-like-GGT1.rhesus.     acatactaggctccaagtgggacctgccaggagacaggaattgatgacagccacaccttt    14100

Papio.anubis.clone.rp41-133b2.8034-40080.revcompl.Baboon            ------------------------------------------------------------    0
BCRP3.HUMAN.NCBI.REF                                                ------------------------------------------------------------    0
LOC106996293.glutathione.hydrolase5.proenzyme-like-GGT1.rhesus.     ggcgtctcgagccctctgccatccatgcagtggcccagttcaaatgacaggaacacaagg    14160

Papio.anubis.clone.rp41-133b2.8034-40080.revcompl.Baboon            ------------------------------------------------------------    0
BCRP3.HUMAN.NCBI.REF                                                ------------------------------------------------------------    0
LOC106996293.glutathione.hydrolase5.proenzyme-like-GGT1.rhesus.     tccagatggctcagtgggctggtagagggctgtagccagggctggaactggccttgcact    14220

Papio.anubis.clone.rp41-133b2.8034-40080.revcompl.Baboon            ------------------------------------------------------------    0
BCRP3.HUMAN.NCBI.REF                                                ------------------------------------------------------------    0
LOC106996293.glutathione.hydrolase5.proenzyme-like-GGT1.rhesus.     gcctctgggcccttctctacccacctggtggtattcttcaagcctaggaggaaaaaaggc    14280

Papio.anubis.clone.rp41-133b2.8034-40080.revcompl.Baboon            ------------------------------------------------------------    0
BCRP3.HUMAN.NCBI.REF                                                ------------------------------------------------------------    0
LOC106996293.glutathione.hydrolase5.proenzyme-like-GGT1.rhesus.     cagctcagggttaatgtaacctcagcatgtagctgggtggcactgaacctcctggtagcc    14340

Papio.anubis.clone.rp41-133b2.8034-40080.revcompl.Baboon            ------------------------------------------------------------    0
BCRP3.HUMAN.NCBI.REF                                                ------------------------------------------------------------    0
LOC106996293.glutathione.hydrolase5.proenzyme-like-GGT1.rhesus.     actgcaaagaaggatacagaatgatttgggggcattcactaggaggaaggaagcagatgg    14400

Papio.anubis.clone.rp41-133b2.8034-40080.revcompl.Baboon            ------------------------------------------------------------    0
BCRP3.HUMAN.NCBI.REF                                                ------------------------------------------------------------    0
LOC106996293.glutathione.hydrolase5.proenzyme-like-GGT1.rhesus.     atactctgtggagacaggatgtcccctgcgcttgctctacagttacgggttgtcaacctt    14460

Papio.anubis.clone.rp41-133b2.8034-40080.revcompl.Baboon            ------------------------------------------------------------    0
BCRP3.HUMAN.NCBI.REF                                                ------------------------------------------------------------    0
LOC106996293.glutathione.hydrolase5.proenzyme-like-GGT1.rhesus.     ctgcagggccaggaattgtcatgaggctgtagtaggcagaagaacttggcggtggggagg    14520

Papio.anubis.clone.rp41-133b2.8034-40080.revcompl.Baboon            ------------------------------------------------------------    0
BCRP3.HUMAN.NCBI.REF                                                ------------------------------------------------------------    0
LOC106996293.glutathione.hydrolase5.proenzyme-like-GGT1.rhesus.     gtgagatcacaggtataggctcagggcctggattcaaatcccctgggctgccacttgttg    14580

Papio.anubis.clone.rp41-133b2.8034-40080.revcompl.Baboon            ------------------------------------------------------------    0
BCRP3.HUMAN.NCBI.REF                                                ------------------------------------------------------------    0
LOC106996293.glutathione.hydrolase5.proenzyme-like-GGT1.rhesus.     gatgtgtaaccacagacagagtaaggacctgccatagcaaattacacaacccacttccgt    14640

Papio.anubis.clone.rp41-133b2.8034-40080.revcompl.Baboon            ------------------------------------------------------------    0
BCRP3.HUMAN.NCBI.REF                                                ------------------------------------------------------------    0
LOC106996293.glutathione.hydrolase5.proenzyme-like-GGT1.rhesus.     ggcttaaagcaacagaatagttctggaggctagtagtctgaaatccaagtataggacaag    14700

Papio.anubis.clone.rp41-133b2.8034-40080.revcompl.Baboon            ------------------------------------------------------------    0
BCRP3.HUMAN.NCBI.REF                                                ------------------------------------------------------------    0
LOC106996293.glutathione.hydrolase5.proenzyme-like-GGT1.rhesus.     gcacgctccctctgaggactccaggggaagatccttccttgcttcttcagcttctcatgg    14760

Papio.anubis.clone.rp41-133b2.8034-40080.revcompl.Baboon            ------------------------------------------------------------    0
BCRP3.HUMAN.NCBI.REF                                                ------------------------------------------------------------    0
LOC106996293.glutathione.hydrolase5.proenzyme-like-GGT1.rhesus.     ctcctggcaatccttggccttccttggctgcaactgcatcattccagtctctgaaaccat    14820

Papio.anubis.clone.rp41-133b2.8034-40080.revcompl.Baboon            ------------------------------------------------------------    0
BCRP3.HUMAN.NCBI.REF                                                ------------------------------------------------------------    0
LOC106996293.glutathione.hydrolase5.proenzyme-like-GGT1.rhesus.     ctacctgtgtgtctgtgtggccttcacatagccttccttatttctgcaatggggtctcac    14880

Papio.anubis.clone.rp41-133b2.8034-40080.revcompl.Baboon            ------------------------------------------------------------    0
BCRP3.HUMAN.NCBI.REF                                                ------------------------------------------------------------    0
LOC106996293.glutathione.hydrolase5.proenzyme-like-GGT1.rhesus.     tatgttgcccaggctggtcttgaactcctgggtccaagcagtcctcctgcctcagcctcc    14940

Papio.anubis.clone.rp41-133b2.8034-40080.revcompl.Baboon            ------------------------------------------------------------    0
BCRP3.HUMAN.NCBI.REF                                                ------------------------------------------------------------    0
LOC106996293.glutathione.hydrolase5.proenzyme-like-GGT1.rhesus.     caagtagctgggattatgggtgagtgccaccatgccagctcacatggcctccttgtaaga    15000

Papio.anubis.clone.rp41-133b2.8034-40080.revcompl.Baboon            ------------------------------------------------------------    0
BCRP3.HUMAN.NCBI.REF                                                ------------------------------------------------------------    0
LOC106996293.glutathione.hydrolase5.proenzyme-like-GGT1.rhesus.     actctagtggctgggtgctagatcacgcctgtaatccagcacttttggaggctgatgcag    15060

Papio.anubis.clone.rp41-133b2.8034-40080.revcompl.Baboon            ------------------------------------------------------------    0
BCRP3.HUMAN.NCBI.REF                                                ------------------------------------------------------------    0
LOC106996293.glutathione.hydrolase5.proenzyme-like-GGT1.rhesus.     acagatcatgaggtcaggagattgagaccatcctggccaacatggtgaaaccctgtctct    15120

Papio.anubis.clone.rp41-133b2.8034-40080.revcompl.Baboon            ------------------------------------------------------------    0
BCRP3.HUMAN.NCBI.REF                                                ------------------------------------------------------------    0
LOC106996293.glutathione.hydrolase5.proenzyme-like-GGT1.rhesus.     actaaaatacggaaaaaaaaaaaaaaaattagccaggtgcggtgatgcgtgcttgcagtc    15180
```

```
Papio.anubis.clone.rp41-133b2.8034-40080.revcompl.Baboon          ------------------------------------------------------------    0
BCRP3.HUMAN.NCBI.REF                                              ------------------------------------------------------------    0
LOC106996293.glutathione.hydrolase5.proenzyme-like-GGT1.rhesus.   ctagctacttgggaggctgaggcaggggaatcgcttgaacccgggaggcataagttgcag    15240

Papio.anubis.clone.rp41-133b2.8034-40080.revcompl.Baboon          ------------------------------------------------------------    0
BCRP3.HUMAN.NCBI.REF                                              ------------------------------------------------------------    0
LOC106996293.glutathione.hydrolase5.proenzyme-like-GGT1.rhesus.   tgagctgagattgtgcgactctactccagcctggcgacagagcaaggctctatcaaaaaa    15300

Papio.anubis.clone.rp41-133b2.8034-40080.revcompl.Baboon          ------------------------------------------------------------    0
BCRP3.HUMAN.NCBI.REF                                              ------------------------------------------------------------    0
LOC106996293.glutathione.hydrolase5.proenzyme-like-GGT1.rhesus.   acaaaacaaaacaaaacaaaaaaactcaagctactggatttaggactcattcattctttt    15360

Papio.anubis.clone.rp41-133b2.8034-40080.revcompl.Baboon          ------------------------------------------------------------    0
BCRP3.HUMAN.NCBI.REF                                              ------------------------------------------------------------    0
LOC106996293.glutathione.hydrolase5.proenzyme-like-GGT1.rhesus.   attaattttaattttttttttgagacagggtcttactctgttgcccaggctggagtgca    15420

Papio.anubis.clone.rp41-133b2.8034-40080.revcompl.Baboon          ------------------------------------------------------------    0
BCRP3.HUMAN.NCBI.REF                                              ------------------------------------------------------------    0
LOC106996293.glutathione.hydrolase5.proenzyme-like-GGT1.rhesus.   atggcacgatcttgactcactgaagcctccacttcccaggttcaagcaattctcctgcct    15480

Papio.anubis.clone.rp41-133b2.8034-40080.revcompl.Baboon          ------------------------------------------------------------    0
BCRP3.HUMAN.NCBI.REF                                              ------------------------------------------------------------    0
LOC106996293.glutathione.hydrolase5.proenzyme-like-GGT1.rhesus.   cagccttctaagtagctgggcttacaggcgcccggcaccacacctagataatttttgtat    15540

Papio.anubis.clone.rp41-133b2.8034-40080.revcompl.Baboon          ------------------------------------------------------------    0
BCRP3.HUMAN.NCBI.REF                                              ------------------------------------------------------------    0
LOC106996293.glutathione.hydrolase5.proenzyme-like-GGT1.rhesus.   ttttagtagagacaggatttcaccatattggtcaggctggtcttgaactcctgacctcag    15600

Papio.anubis.clone.rp41-133b2.8034-40080.revcompl.Baboon          ------------------------------------------------------------    0
BCRP3.HUMAN.NCBI.REF                                              ------------------------------------------------------------    0
LOC106996293.glutathione.hydrolase5.proenzyme-like-GGT1.rhesus.   gttatccacccccatttacctcccaaagtcctgggattacaggcgtgagccactgtgccc    15660

Papio.anubis.clone.rp41-133b2.8034-40080.revcompl.Baboon          ------------------------------------------------------------    0
BCRP3.HUMAN.NCBI.REF                                              ------------------------------------------------------------    0
LOC106996293.glutathione.hydrolase5.proenzyme-like-GGT1.rhesus.   tgcagggctcattcattctaatcccgtatggtctcatcttaactaattacttatgtagag    15720

Papio.anubis.clone.rp41-133b2.8034-40080.revcompl.Baboon          ------------------------------------------------------------    0
BCRP3.HUMAN.NCBI.REF                                              ------------------------------------------------------------    0
LOC106996293.glutathione.hydrolase5.proenzyme-like-GGT1.rhesus.   accctatttccaaataaggtcatgtgaggaaaaaccaactcaaaccgtgttttttggctg    15780

Papio.anubis.clone.rp41-133b2.8034-40080.revcompl.Baboon          ------------------------------------------------------------    0
BCRP3.HUMAN.NCBI.REF                                              ------------------------------------------------------------    0
LOC106996293.glutathione.hydrolase5.proenzyme-like-GGT1.rhesus.   aatacggtggctcacgcctatcatcttagcactttgggaggccaaggtgggcggatcact    15840

Papio.anubis.clone.rp41-133b2.8034-40080.revcompl.Baboon          ------------------------------------------------------------    0
BCRP3.HUMAN.NCBI.REF                                              ------------------------------------------------------------    0
LOC106996293.glutathione.hydrolase5.proenzyme-like-GGT1.rhesus.   tgaggctgggagtttgagaccagcctggccaacatagcaaaaccccgtctttactaaaaa    15900

Papio.anubis.clone.rp41-133b2.8034-40080.revcompl.Baboon          ------------------------------------------------------------    0
BCRP3.HUMAN.NCBI.REF                                              ------------------------------------------------------------    0
LOC106996293.glutathione.hydrolase5.proenzyme-like-GGT1.rhesus.   atacaaaaaaatacaaaaattagcctgagtagcaggcgcctgtagtcccagctactcagg    15960

Papio.anubis.clone.rp41-133b2.8034-40080.revcompl.Baboon          ------------------------------------------------------------    0
BCRP3.HUMAN.NCBI.REF                                              ------------------------------------------------------------    0
LOC106996293.glutathione.hydrolase5.proenzyme-like-GGT1.rhesus.   aggctgagggaggagaatcgcttgaacccaggaggtggaggtttcagtgagctgagatag    16020

Papio.anubis.clone.rp41-133b2.8034-40080.revcompl.Baboon          ------------------------------------------------------------    0
BCRP3.HUMAN.NCBI.REF                                              ------------------------------------------------------------    0
LOC106996293.glutathione.hydrolase5.proenzyme-like-GGT1.rhesus.   cacaaccacattccagcctgggtgacagagtgagactccatctcaaaacacacacacaca    16080

Papio.anubis.clone.rp41-133b2.8034-40080.revcompl.Baboon          ------------------------------------------------------------    0
BCRP3.HUMAN.NCBI.REF                                              ------------------------------------------------------------    0
LOC106996293.glutathione.hydrolase5.proenzyme-like-GGT1.rhesus.   cacacacacacacacacacacacacacacacacaactactgtgttttccttctgttgtca    16140

Papio.anubis.clone.rp41-133b2.8034-40080.revcompl.Baboon          ------------------------------------------------------------    0
BCRP3.HUMAN.NCBI.REF                                              ------------------------------------------------------------    0
LOC106996293.glutathione.hydrolase5.proenzyme-like-GGT1.rhesus.   caagacaacaatcatcacagaagacttctgtgaccaaatgtgtggattctccccacacac    16200

Papio.anubis.clone.rp41-133b2.8034-40080.revcompl.Baboon          ------------------------------------------------------------    0
BCRP3.HUMAN.NCBI.REF                                              ------------------------------------------------------------    0
LOC106996293.glutathione.hydrolase5.proenzyme-like-GGT1.rhesus.   ccagcaagaatcaattctgcagccgacgccagctggtgtcttccaattcaactcaattct    16260

Papio.anubis.clone.rp41-133b2.8034-40080.revcompl.Baboon          ------------------------------------------------------------    0
BCRP3.HUMAN.NCBI.REF                                              ------------------------------------------------------------    0
LOC106996293.glutathione.hydrolase5.proenzyme-like-GGT1.rhesus.   catgctgtctacctggagatggcctcagaaaccacaggttgagggcttggtcccacaaga    16320

Papio.anubis.clone.rp41-133b2.8034-40080.revcompl.Baboon          ------------------------------------------------------------    0
BCRP3.HUMAN.NCBI.REF                                              ------------------------------------------------------------    0
LOC106996293.glutathione.hydrolase5.proenzyme-like-GGT1.rhesus.   ccacccctcatcccaccagtcacaagtctgggctttgggaacatctgactaatcggcttc    16380

Papio.anubis.clone.rp41-133b2.8034-40080.revcompl.Baboon          ------------------------------------------------------------    0
BCRP3.HUMAN.NCBI.REF                                              ------------------------------------------------------------    0
LOC106996293.glutathione.hydrolase5.proenzyme-like-GGT1.rhesus.   tagttggggttcctacagcaccctctttgggttcaattaatttgctagagtagctcacac    16440

Papio.anubis.clone.rp41-133b2.8034-40080.revcompl.Baboon          ------------------------------------------------------------    0
BCRP3.HUMAN.NCBI.REF                                              ------------------------------------------------------------    0
LOC106996293.glutathione.hydrolase5.proenzyme-like-GGT1.rhesus.   aactcagggagacctgtttgtcggtttatttcaaaggatgttttattttattttctcctc    16500

Papio.anubis.clone.rp41-133b2.8034-40080.revcompl.Baboon          ------------------------------------------------------------    0
BCRP3.HUMAN.NCBI.REF                                              ------------------------------------------------------------    0
LOC106996293.glutathione.hydrolase5.proenzyme-like-GGT1.rhesus.   agacatactttatcatcacttaaagtcagtagctcagaggaaactttaaaagctacaaac    16560

Papio.anubis.clone.rp41-133b2.8034-40080.revcompl.Baboon          ------------------------------------------------------------    0
BCRP3.HUMAN.NCBI.REF                                              ------------------------------------------------------------    0
LOC106996293.glutathione.hydrolase5.proenzyme-like-GGT1.rhesus.   aggcggggcacagtggctcatgcctgtaatcccagcactttgggcggtcgaggcaggtgg    16620

Papio.anubis.clone.rp41-133b2.8034-40080.revcompl.Baboon          ------------------------------------------------------------    0
BCRP3.HUMAN.NCBI.REF                                              ------------------------------------------------------------    0
LOC106996293.glutathione.hydrolase5.proenzyme-like-GGT1.rhesus.   atcacttgaggtcaggagctggagaccagtctggccaacatggtgaaatcccgtctctac    16680

Papio.anubis.clone.rp41-133b2.8034-40080.revcompl.Baboon          ------------------------------------------------------------    0
BCRP3.HUMAN.NCBI.REF                                              ------------------------------------------------------------    0
LOC106996293.glutathione.hydrolase5.proenzyme-like-GGT1.rhesus.   taaaaatacaaaattagctggctgtggtggcaggagcctgtaatcccagctacttaggat    16740

Papio.anubis.clone.rp41-133b2.8034-40080.revcompl.Baboon          ------------------------------------------------------------    0
BCRP3.HUMAN.NCBI.REF                                              ------------------------------------------------------------    0
LOC106996293.glutathione.hydrolase5.proenzyme-like-GGT1.rhesus.   gctgaggcaggataatcatcagctgaacccgagaggcagaagttgcagtgagctgagatc    16800

Papio.anubis.clone.rp41-133b2.8034-40080.revcompl.Baboon          ------------------------------------------------------------    0
BCRP3.HUMAN.NCBI.REF                                              ------------------------------------------------------------    0
LOC106996293.glutathione.hydrolase5.proenzyme-like-GGT1.rhesus.   atgcctctgcgctccagcctgggcaacagagagagagactgtctcaaaaaaaaaaaaaaa    16860

Papio.anubis.clone.rp41-133b2.8034-40080.revcompl.Baboon          ------------------------------------------------------------    0
BCRP3.HUMAN.NCBI.REF                                              ------------------------------------------------------------    0
LOC106996293.glutathione.hydrolase5.proenzyme-like-GGT1.rhesus.   aaagataaaggctacaaatagacagtcagatgaagagatacatagggtaggactggaaga    16920
```

```
Papio.anubis.clone.rp41-133b2.8034-40080.revcompl.Baboon        ------------------------------------------------------------    0
BCRP3.HUMAN.NCBI.REF                                            ------------------------------------------------------------    0
LOC106996293.glutathione.hydrolase5.proenzyme-like-GGT1.rhesus.  ctcccaagtacagaagtttctgtcctggtggagttggacacgtggatgagtggggtttt    16980

Papio.anubis.clone.rp41-133b2.8034-40080.revcompl.Baboon        ------------------------------------------------------------    0
BCRP3.HUMAN.NCBI.REF                                            ------------------------------------------------------------    0
LOC106996293.glutathione.hydrolase5.proenzyme-like-GGT1.rhesus.  tattgttgcccaggctggagtacagtggcactaactcggctcactacaacctctgcctcc    17040

Papio.anubis.clone.rp41-133b2.8034-40080.revcompl.Baboon        ------------------------------------------------------------    0
BCRP3.HUMAN.NCBI.REF                                            ------------------------------------------------------------    0
LOC106996293.glutathione.hydrolase5.proenzyme-like-GGT1.rhesus.  tgtgctcaagtgatcctcccctctcagcctcccaagtagctgggaccacaggcatgtgcc    17100

Papio.anubis.clone.rp41-133b2.8034-40080.revcompl.Baboon        ------------------------------------------------------------    0
BCRP3.HUMAN.NCBI.REF                                            ------------------------------------------------------------    0
LOC106996293.glutathione.hydrolase5.proenzyme-like-GGT1.rhesus.  accatgtccagctaatttttttttttttttagttttggtagagatggggtctgtctatat    17160

Papio.anubis.clone.rp41-133b2.8034-40080.revcompl.Baboon        ------------------------------------------------------------    0
BCRP3.HUMAN.NCBI.REF                                            ------------------------------------------------------------    0
LOC106996293.glutathione.hydrolase5.proenzyme-like-GGT1.rhesus.  tgctcaggctgactcctggtctcaagccatccacccacctcgccctcccaaagtgctctg    17220

Papio.anubis.clone.rp41-133b2.8034-40080.revcompl.Baboon        ------------------------------------------------------------    0
BCRP3.HUMAN.NCBI.REF                                            ------------------------------------------------------------    0
LOC106996293.glutathione.hydrolase5.proenzyme-like-GGT1.rhesus.  attataggcgtgagccactgcacctggccgtggatgagttcttattcaccttcctgtcag    17280

Papio.anubis.clone.rp41-133b2.8034-40080.revcompl.Baboon        ------------------------------------------------------------    0
BCRP3.HUMAN.NCBI.REF                                            ------------------------------------------------------------    0
LOC106996293.glutathione.hydrolase5.proenzyme-like-GGT1.rhesus.  cctccacatgaagttccccaaaccccgtcctcttgggccttttttaaaaataaattaatta    17340

Papio.anubis.clone.rp41-133b2.8034-40080.revcompl.Baboon        ------------------------------------------------------------    0
BCRP3.HUMAN.NCBI.REF                                            ------------------------------------------------------------    0
LOC106996293.glutathione.hydrolase5.proenzyme-like-GGT1.rhesus.  aaaaattaaggacagggtctcactatgttgcccaggctggtcttgaactcctggtctcaa    17400

Papio.anubis.clone.rp41-133b2.8034-40080.revcompl.Baboon        ------------------------------------------------------------    0
BCRP3.HUMAN.NCBI.REF                                            ------------------------------------------------------------    0
LOC106996293.glutathione.hydrolase5.proenzyme-like-GGT1.rhesus.  gtgatccacccaccttgtcctcccgtagtgctgggattacaagattagagatgccagctg    17460

Papio.anubis.clone.rp41-133b2.8034-40080.revcompl.Baboon        ------------------------------------------------------------    0
BCRP3.HUMAN.NCBI.REF                                            ------------------------------------------------------------    0
LOC106996293.glutathione.hydrolase5.proenzyme-like-GGT1.rhesus.  tcaggtggctaggtggagtcagtgtaaagtcctagaagatgcattttagtaaaaacagcc    17520

Papio.anubis.clone.rp41-133b2.8034-40080.revcompl.Baboon        ------------------------------------------------------------    0
BCRP3.HUMAN.NCBI.REF                                            ------------------------------------------------------------    0
LOC106996293.glutathione.hydrolase5.proenzyme-like-GGT1.rhesus.  acctgacagctggcatccatccattcagtgaagattgttgggcactgtggatactgtgga    17580

Papio.anubis.clone.rp41-133b2.8034-40080.revcompl.Baboon        ------------------------------------------------------------    0
BCRP3.HUMAN.NCBI.REF                                            ------------------------------------------------------------    0
LOC106996293.glutathione.hydrolase5.proenzyme-like-GGT1.rhesus.  ggaggcagcagtagcagctggccctgcagagtgaggtgcagtgctggagggcttcaggag    17640

Papio.anubis.clone.rp41-133b2.8034-40080.revcompl.Baboon        ------------------------------------------------------------    0
BCRP3.HUMAN.NCBI.REF                                            ------------------------------------------------------------    0
LOC106996293.glutathione.hydrolase5.proenzyme-like-GGT1.rhesus.  gaggtggcattgtgatcggtgtctgaagaatgcctcaggccatgacaatggagccagtgt    17700

Papio.anubis.clone.rp41-133b2.8034-40080.revcompl.Baboon        ------------------------------------------------------------    0
BCRP3.HUMAN.NCBI.REF                                            ------------------------------------------------------------    0
LOC106996293.glutathione.hydrolase5.proenzyme-like-GGT1.rhesus.  aaagtcccaaagatcaggaaggagcaagaggaaaggatagttcatatccaggcacacccc    17760

Papio.anubis.clone.rp41-133b2.8034-40080.revcompl.Baboon        ------------------------------------------------------------    0
BCRP3.HUMAN.NCBI.REF                                            ------------------------------------------------------------    0
LOC106996293.glutathione.hydrolase5.proenzyme-like-GGT1.rhesus.  tccagaaaaaaaaaaaagaggaaaggatgggcgccagcaaggtaggacaacaggggcctc    17820

Papio.anubis.clone.rp41-133b2.8034-40080.revcompl.Baboon        ------------------------------------------------------------    0
BCRP3.HUMAN.NCBI.REF                                            ------------------------------------------------------------    0
LOC106996293.glutathione.hydrolase5.proenzyme-like-GGT1.rhesus.  tagagtgttttcttcccaggaggggaggggtgtgctcagatttacatgctgagtgcctgg    17880

Papio.anubis.clone.rp41-133b2.8034-40080.revcompl.Baboon        ------------------------------------------------------------    0
BCRP3.HUMAN.NCBI.REF                                            ------------------------------------------------------------    0
LOC106996293.glutathione.hydrolase5.proenzyme-like-GGT1.rhesus.  actgcaatagggaaatagcagaggggctggggcctgtgactgggagtctccaggtgagag    17940

Papio.anubis.clone.rp41-133b2.8034-40080.revcompl.Baboon        ------------------------------------------------------------    0
BCRP3.HUMAN.NCBI.REF                                            ------------------------------------------------------------    0
LOC106996293.glutathione.hydrolase5.proenzyme-like-GGT1.rhesus.  gattggcccgtggggactggcggtcaggtgaggctgggagtgaggtgtagaggtgggagc    18000

Papio.anubis.clone.rp41-133b2.8034-40080.revcompl.Baboon        ------------------------------------------------------------    0
BCRP3.HUMAN.NCBI.REF                                            ------------------------------------------------------------    0
LOC106996293.glutathione.hydrolase5.proenzyme-like-GGT1.rhesus.  agctggacaggactagtgaggatgcaggggcggctgccccgggctagactctggctgctc    18060

Papio.anubis.clone.rp41-133b2.8034-40080.revcompl.Baboon        ------------------------------------------------------------    0
BCRP3.HUMAN.NCBI.REF                                            ------------------------------------------------------------    0
LOC106996293.glutathione.hydrolase5.proenzyme-like-GGT1.rhesus.  tggccgttggaggtctggggaggcagctgcttagctcaggctcgggcacgtccacccggc    18120

Papio.anubis.clone.rp41-133b2.8034-40080.revcompl.Baboon        ------------------------------------------------------------    0
BCRP3.HUMAN.NCBI.REF                                            ------------------------------------------------------------    0
LOC106996293.glutathione.hydrolase5.proenzyme-like-GGT1.rhesus.  acgtctgaaacattagccagtggggatgcagactcgtcagcgagagcattcctgagccca    18180

Papio.anubis.clone.rp41-133b2.8034-40080.revcompl.Baboon        ------------------------------------------------------------    0
BCRP3.HUMAN.NCBI.REF                                            ------------------------------------------------------------    0
LOC106996293.glutathione.hydrolase5.proenzyme-like-GGT1.rhesus.  gcatccaagaggcaaaggaagttgggaaggtgctgctaagaggtgggggaggtccccaaa    18240

Papio.anubis.clone.rp41-133b2.8034-40080.revcompl.Baboon        ------------------------------------------------------------    0
BCRP3.HUMAN.NCBI.REF                                            ------------------------------------------------------------    0
LOC106996293.glutathione.hydrolase5.proenzyme-like-GGT1.rhesus.  agtccaggtgagagcatggcatgtgtggaaagtgctgggcagttttggcatcagtgaaga    18300

Papio.anubis.clone.rp41-133b2.8034-40080.revcompl.Baboon        ------------------------------------------------------------    0
BCRP3.HUMAN.NCBI.REF                                            ------------------------------------------------------------    0
LOC106996293.glutathione.hydrolase5.proenzyme-like-GGT1.rhesus.  cacggggggctgtgtgtgaacaggtggggttgggcgtcaccagggagtctttagacagga    18360

Papio.anubis.clone.rp41-133b2.8034-40080.revcompl.Baboon        ------------------------------------------------------------    0
BCRP3.HUMAN.NCBI.REF                                            ------------------------------------------------------------    0
LOC106996293.glutathione.hydrolase5.proenzyme-like-GGT1.rhesus.  gcgggttggctctcctggagatcaggccatggcttggtgtcgggactggggcatggacag    18420

Papio.anubis.clone.rp41-133b2.8034-40080.revcompl.Baboon        ------------------------------------------------------------    0
BCRP3.HUMAN.NCBI.REF                                            ------------------------------------------------------------    0
LOC106996293.glutathione.hydrolase5.proenzyme-like-GGT1.rhesus.  tcttcctccacccccaacctctccttggagctttgagacactgcagataattagcaggtg    18480

Papio.anubis.clone.rp41-133b2.8034-40080.revcompl.Baboon        ------------------------------------------------------------    0
BCRP3.HUMAN.NCBI.REF                                            ------------------------------------------------------------    0
LOC106996293.glutathione.hydrolase5.proenzyme-like-GGT1.rhesus.  atgtgataaatactacctgtggagggtgccttgggcagggaagacagctctgctctgtcc    18540

Papio.anubis.clone.rp41-133b2.8034-40080.revcompl.Baboon        ------------------------------------------------------------    0
BCRP3.HUMAN.NCBI.REF                                            ------------------------------------------------------------    0
LOC106996293.glutathione.hydrolase5.proenzyme-like-GGT1.rhesus.  tgctctgaggtgctcatcaggaaaggagagaaactactacagacccagtgttggagcctg    18600

Papio.anubis.clone.rp41-133b2.8034-40080.revcompl.Baboon        ------------------------------------------------------------    0
BCRP3.HUMAN.NCBI.REF                                            ------------------------------------------------------------    0
LOC106996293.glutathione.hydrolase5.proenzyme-like-GGT1.rhesus.  gccagaccctgtgaggcctcgaaagtagcctaccaagggaagggaaagcctaccaagata    18660
```

```
Papio.anubis.clone.rp41-133b2.8034-40080.revcompl.Baboon            ------------------------------------------------------------    0
BCRP3.HUMAN.NCBI.REF                                                ------------------------------------------------------------    0
LOC106996293.glutathione.hydrolase5.proenzyme-like-GGT1.rhesus.     cccacactttcagtggctggggttgtttgaacccagggactctagggcccatgcctctgc    18720

Papio.anubis.clone.rp41-133b2.8034-40080.revcompl.Baboon            ------------------------------------------------------------    0
BCRP3.HUMAN.NCBI.REF                                                ------------------------------------------------------------    0
LOC106996293.glutathione.hydrolase5.proenzyme-like-GGT1.rhesus.     catcctgcctctgagtttgggagttggacttggtccatcaagggccgggccagccctgct    18780

Papio.anubis.clone.rp41-133b2.8034-40080.revcompl.Baboon            ------------------------------------------------------------    0
BCRP3.HUMAN.NCBI.REF                                                ------------------------------------------------------------    0
LOC106996293.glutathione.hydrolase5.proenzyme-like-GGT1.rhesus.     gctgagtgctcccaggcatgcacaagctgacagttctgaagggcccacgcacttggccat    18840

Papio.anubis.clone.rp41-133b2.8034-40080.revcompl.Baboon            ------------------------------------------------------------    0
BCRP3.HUMAN.NCBI.REF                                                ------------------------------------------------------------    0
LOC106996293.glutathione.hydrolase5.proenzyme-like-GGT1.rhesus.     gctccagcctatgtcctcacatcaaaggtaggtgctggaatagcaaatgctgcagatggc    18900

Papio.anubis.clone.rp41-133b2.8034-40080.revcompl.Baboon            ------------------------------------------------------------    0
BCRP3.HUMAN.NCBI.REF                                                ------------------------------------------------------------    0
LOC106996293.glutathione.hydrolase5.proenzyme-like-GGT1.rhesus.     acagggggtgttggccaggctctgggggcacaggagctctgcccatgcccacagttggcc    18960

Papio.anubis.clone.rp41-133b2.8034-40080.revcompl.Baboon            ------------------------------------------------------------    0
BCRP3.HUMAN.NCBI.REF                                                ------------------------------------------------------------    0
LOC106996293.glutathione.hydrolase5.proenzyme-like-GGT1.rhesus.     tgtgctgacctccctccatcacaactgagtggcacccgccctttcattgagctgaagtct    19020

Papio.anubis.clone.rp41-133b2.8034-40080.revcompl.Baboon            ------------------------------------------------------------    0
BCRP3.HUMAN.NCBI.REF                                                ------------------------------------------------------------    0
LOC106996293.glutathione.hydrolase5.proenzyme-like-GGT1.rhesus.     tccttccaaaatcccttcagtgtggtctttgtggctcagagaggctgtggccaggtacaa    19080

Papio.anubis.clone.rp41-133b2.8034-40080.revcompl.Baboon            ------------------------------------------------------------    0
BCRP3.HUMAN.NCBI.REF                                                ------------------------------------------------------------    0
LOC106996293.glutathione.hydrolase5.proenzyme-like-GGT1.rhesus.     gatctggggtggcccctgcttctctgctctgtggcctttgacagtgggcaggatctgagg    19140

Papio.anubis.clone.rp41-133b2.8034-40080.revcompl.Baboon            ------------------------------------------------------------    0
BCRP3.HUMAN.NCBI.REF                                                ------------------------------------------------------------    0
LOC106996293.glutathione.hydrolase5.proenzyme-like-GGT1.rhesus.     tcaggcccacttgcagcaggtgttacaaagaggcaaattggggcttaatgttggaaagaa    19200

Papio.anubis.clone.rp41-133b2.8034-40080.revcompl.Baboon            ------------------------------------------------------------    0
BCRP3.HUMAN.NCBI.REF                                                ------------------------------------------------------------    0
LOC106996293.glutathione.hydrolase5.proenzyme-like-GGT1.rhesus.     cttgccaaccaactatagtcccgttgtctttggaggtagtgagctccccattggggaagg    19260

Papio.anubis.clone.rp41-133b2.8034-40080.revcompl.Baboon            ------------------------------------------------------------    0
BCRP3.HUMAN.NCBI.REF                                                ------------------------------------------------------------    0
LOC106996293.glutathione.hydrolase5.proenzyme-like-GGT1.rhesus.     tatgtaagccaacctttctgggatgttggaagatcagagaccaaacacttgagattctgt    19320

Papio.anubis.clone.rp41-133b2.8034-40080.revcompl.Baboon            ------------------------------------------------------------    0
BCRP3.HUMAN.NCBI.REF                                                ------------------------------------------------------------    0
LOC106996293.glutathione.hydrolase5.proenzyme-like-GGT1.rhesus.     gatcctagtattaactcaaatgctatgatgcttggatttgaagtcagttctatttagcca    19380

Papio.anubis.clone.rp41-133b2.8034-40080.revcompl.Baboon            ------------------------------------------------------------    0
BCRP3.HUMAN.NCBI.REF                                                ------------------------------------------------------------    0
LOC106996293.glutathione.hydrolase5.proenzyme-like-GGT1.rhesus.     gtttaccaagtgtctccttggtgcaggacaccagggaacacaaaatgtcctggataatac    19440

Papio.anubis.clone.rp41-133b2.8034-40080.revcompl.Baboon            ------------------------------------------------------------    0
BCRP3.HUMAN.NCBI.REF                                                ------------------------------------------------------------    0
LOC106996293.glutathione.hydrolase5.proenzyme-like-GGT1.rhesus.     tgggtttctggctcccagggttccctcagatgttgttccagtggttctagaatataggcc    19500

Papio.anubis.clone.rp41-133b2.8034-40080.revcompl.Baboon            ------------------------------------------------------------    0
BCRP3.HUMAN.NCBI.REF                                                ------------------------------------------------------------    0
LOC106996293.glutathione.hydrolase5.proenzyme-like-GGT1.rhesus.     aggcatggcttgcatctggacccatcagcattgctcgctcaatgtgagcttcatgactgc    19560

Papio.anubis.clone.rp41-133b2.8034-40080.revcompl.Baboon            ------------------------------------------------------------    0
BCRP3.HUMAN.NCBI.REF                                                ------------------------------------------------------------    0
LOC106996293.glutathione.hydrolase5.proenzyme-like-GGT1.rhesus.     agttctcacgcccccacgtgcccccctaccccgccccgccatgacccttaccatggccca    19620

Papio.anubis.clone.rp41-133b2.8034-40080.revcompl.Baboon            ------------------------------------------------------------    0
BCRP3.HUMAN.NCBI.REF                                                ------------------------------------------------------------    0
LOC106996293.glutathione.hydrolase5.proenzyme-like-GGT1.rhesus.     ctcagggcacaaggctttctggatgattcctgatactcagtgtggtcagcttcctggacc    19680

Papio.anubis.clone.rp41-133b2.8034-40080.revcompl.Baboon            ------------------------------------------------------------    0
BCRP3.HUMAN.NCBI.REF                                                ------------------------------------------------------------    0
LOC106996293.glutathione.hydrolase5.proenzyme-like-GGT1.rhesus.     tggcagtggggtggggctgcaatgagacgccagtcgcaggtagtggtttgcacagccttc    19740

Papio.anubis.clone.rp41-133b2.8034-40080.revcompl.Baboon            ------------------------------------------------------------    0
BCRP3.HUMAN.NCBI.REF                                                ------------------------------------------------------------    0
LOC106996293.glutathione.hydrolase5.proenzyme-like-GGT1.rhesus.     ctggtgtcgtgggtcactgtcttgccaagtggctttgggcaggtcccttcccctctcagg    19800

Papio.anubis.clone.rp41-133b2.8034-40080.revcompl.Baboon            ------------------------------------------------------------    0
BCRP3.HUMAN.NCBI.REF                                                ------------------------------------------------------------    0
LOC106996293.glutathione.hydrolase5.proenzyme-like-GGT1.rhesus.     ccgcaagttcctatgccaggagcttgttttctcagatacagagctggggcagatcccagc    19860

Papio.anubis.clone.rp41-133b2.8034-40080.revcompl.Baboon            ------------------------------------------------------------    0
BCRP3.HUMAN.NCBI.REF                                                ------------------------------------------------------------    0
LOC106996293.glutathione.hydrolase5.proenzyme-like-GGT1.rhesus.     ccgctgaaacagacagggctttggaggccatggtgggcccatttctcagaggggccgtgt    19920

Papio.anubis.clone.rp41-133b2.8034-40080.revcompl.Baboon            ------------------------------------------------------------    0
BCRP3.HUMAN.NCBI.REF                                                ------------------------------------------------------------    0
LOC106996293.glutathione.hydrolase5.proenzyme-like-GGT1.rhesus.     ggctggatgctgagccaggcactggactggccccttgcgcctcctgtcaggctgaggccc    19980

Papio.anubis.clone.rp41-133b2.8034-40080.revcompl.Baboon            ------------------------------------------------------------    0
BCRP3.HUMAN.NCBI.REF                                                ------------------------------------------------------------    0
LOC106996293.glutathione.hydrolase5.proenzyme-like-GGT1.rhesus.     cggaggcaccgtgggctgctggtgccagcacacaggggtgataaggagatgaaagtcact    20040

Papio.anubis.clone.rp41-133b2.8034-40080.revcompl.Baboon            ------------------------------------------------------------    0
BCRP3.HUMAN.NCBI.REF                                                ------------------------------------------------------------    0
LOC106996293.glutathione.hydrolase5.proenzyme-like-GGT1.rhesus.     agaatggtggggcggcacccgccccgggctgtgttggcagctgtccctggctggaggcca    20100

Papio.anubis.clone.rp41-133b2.8034-40080.revcompl.Baboon            ------------------------------------------------------------    0
BCRP3.HUMAN.NCBI.REF                                                ------------------------------------------------------------    0
LOC106996293.glutathione.hydrolase5.proenzyme-like-GGT1.rhesus.     gcttggccctggaacactcctcaaggaacaaggacacagagagccagctcagccagcctc    20160

Papio.anubis.clone.rp41-133b2.8034-40080.revcompl.Baboon            ------------------------------------------------------------    0
BCRP3.HUMAN.NCBI.REF                                                ------------------------------------------------------------    0
LOC106996293.glutathione.hydrolase5.proenzyme-like-GGT1.rhesus.     cacctagcgccccaatcagcactcccttcttcccatgacccctgccctttgcccggctcc    20220

Papio.anubis.clone.rp41-133b2.8034-40080.revcompl.Baboon            ------------------------------------------------------------    0
BCRP3.HUMAN.NCBI.REF                                                ------------------------------------------------------------    0
LOC106996293.glutathione.hydrolase5.proenzyme-like-GGT1.rhesus.     tggaactggtcaaggagctaatggacacccaccagggtcttaatgagtgagccagggtta    20280

Papio.anubis.clone.rp41-133b2.8034-40080.revcompl.Baboon            ------------------------------------------------------------    0
BCRP3.HUMAN.NCBI.REF                                                ------------------------------------------------------------    0
LOC106996293.glutathione.hydrolase5.proenzyme-like-GGT1.rhesus.     atggttattgggcctggtgacacattcaccagcctcccagggatctcaaagttcagactg    20340

Papio.anubis.clone.rp41-133b2.8034-40080.revcompl.Baboon            ------------------------------------------------------------    0
BCRP3.HUMAN.NCBI.REF                                                ------------------------------------------------------------    0
LOC106996293.glutathione.hydrolase5.proenzyme-like-GGT1.rhesus.     gcctggccttgaaggtcttagctcggccacctccttttccagccttgacacctcggggct    20400
```

```
Papio.anubis.clone.rp41-133b2.8034-40080.revcompl.Baboon           ------------------------------------------------------------    0
BCRP3.HUMAN.NCBI.REF                                               ------------------------------------------------------------    0
LOC106996293.glutathione.hydrolase5.proenzyme-like-GGT1.rhesus.    caggaagggcctagggtggtcaagaggtcccctgcagccctcacttcctggaaaaggctc    20460

Papio.anubis.clone.rp41-133b2.8034-40080.revcompl.Baboon           ------------------------------------------------------------    0
BCRP3.HUMAN.NCBI.REF                                               ------------------------------------------------------------    0
LOC106996293.glutathione.hydrolase5.proenzyme-like-GGT1.rhesus.    catttcctggccttcagggttaggtggttccttctgcctggcaggtcctccccaaatcca    20520

Papio.anubis.clone.rp41-133b2.8034-40080.revcompl.Baboon           ------------------------------------------------------------    0
BCRP3.HUMAN.NCBI.REF                                               ------------------------------------------------------------    0
LOC106996293.glutathione.hydrolase5.proenzyme-like-GGT1.rhesus.    atcccagcttgggctggattcccaaggcccctattctggttggtgtctgccccttcccta    20580

Papio.anubis.clone.rp41-133b2.8034-40080.revcompl.Baboon           ------------------------------------------------------------    0
BCRP3.HUMAN.NCBI.REF                                               ------------------------------------------------------------    0
LOC106996293.glutathione.hydrolase5.proenzyme-like-GGT1.rhesus.    gggctcttcccccctggggtgaccatactgtctcatgcccagccaggttggggcatgagac    20640

Papio.anubis.clone.rp41-133b2.8034-40080.revcompl.Baboon           ------------------------------------------------------------    0
BCRP3.HUMAN.NCBI.REF                                               ------------------------------------------------------------    0
LOC106996293.glutathione.hydrolase5.proenzyme-like-GGT1.rhesus.    ctggagagaagaatctggaggggccaagatttgatgggagaatgaatgatggaatgaaag    20700

Papio.anubis.clone.rp41-133b2.8034-40080.revcompl.Baboon           ------------------------------------------------------------    0
BCRP3.HUMAN.NCBI.REF                                               ------------------------------------------------------------    0
LOC106996293.glutathione.hydrolase5.proenzyme-like-GGT1.rhesus.    aaggaatttaagcatgtgccttgctaagagtgacaattctggcaagctgcagaccatgtg    20760

Papio.anubis.clone.rp41-133b2.8034-40080.revcompl.Baboon           ------------------------------------------------------------    0
BCRP3.HUMAN.NCBI.REF                                               ------------------------------------------------------------    0
LOC106996293.glutathione.hydrolase5.proenzyme-like-GGT1.rhesus.    tggacctcgatctgagggctgagaggcttgggcctgccagccctgtgggggcacccaccc    20820

Papio.anubis.clone.rp41-133b2.8034-40080.revcompl.Baboon           ------------------------------------------------------------    0
BCRP3.HUMAN.NCBI.REF                                               ------------------------------------------------------------    0
LOC106996293.glutathione.hydrolase5.proenzyme-like-GGT1.rhesus.    catctcatctcattgaatagtgcagagcaggtgagggcatgatggacaaatgggcctcat    20880

Papio.anubis.clone.rp41-133b2.8034-40080.revcompl.Baboon           ------------------------------------------------------------    0
BCRP3.HUMAN.NCBI.REF                                               ------------------------------------------------------------    0
LOC106996293.glutathione.hydrolase5.proenzyme-like-GGT1.rhesus.    gcccctgcttcaactgcctggggtcctccatcagcctgtctgctcagaaccctgaaattg    20940

Papio.anubis.clone.rp41-133b2.8034-40080.revcompl.Baboon           ------------------------------------------------------------    0
BCRP3.HUMAN.NCBI.REF                                               ------------------------------------------------------------    0
LOC106996293.glutathione.hydrolase5.proenzyme-like-GGT1.rhesus.    ggggttgaggaaggaccttcctggatcatgtgactcccttcagcccaggtgaccagggtc    21000

Papio.anubis.clone.rp41-133b2.8034-40080.revcompl.Baboon           ------------------------------------------------------------    0
BCRP3.HUMAN.NCBI.REF                                               ------------------------------------------------------------    0
LOC106996293.glutathione.hydrolase5.proenzyme-like-GGT1.rhesus.    cttggagtgacagttcctagagccctgcctgtagccctgtaagttggggagacggcctgt    21060

Papio.anubis.clone.rp41-133b2.8034-40080.revcompl.Baboon           ------------------------------------------------------------    0
BCRP3.HUMAN.NCBI.REF                                               ------------------------------------------------------------    0
LOC106996293.glutathione.hydrolase5.proenzyme-like-GGT1.rhesus.    tgggagaggcatccctgccctggggtcgtagaggtgatctaggctcctcagaccttgtgg    21120

Papio.anubis.clone.rp41-133b2.8034-40080.revcompl.Baboon           ------------------------------------------------------------    0
BCRP3.HUMAN.NCBI.REF                                               ------------------------------------------------------------    0
LOC106996293.glutathione.hydrolase5.proenzyme-like-GGT1.rhesus.    ggcctcagatgcttacatctccagctcctcctggtgtgggcatctggctgcaccccagct    21180

Papio.anubis.clone.rp41-133b2.8034-40080.revcompl.Baboon           ------------------------------------------------------------    0
BCRP3.HUMAN.NCBI.REF                                               ------------------------------------------------------------    0
LOC106996293.glutathione.hydrolase5.proenzyme-like-GGT1.rhesus.    ctgtggactcatctcccagggaacctttggtctaatctgcctcccactgaaacctggcca    21240

Papio.anubis.clone.rp41-133b2.8034-40080.revcompl.Baboon           ------------------------------------------------------------    0
BCRP3.HUMAN.NCBI.REF                                               ------------------------------------------------------------    0
LOC106996293.glutathione.hydrolase5.proenzyme-like-GGT1.rhesus.    cattcagctccaccaaggccttcagagctcaggtctctcattcagggtttgaggccccgc    21300

Papio.anubis.clone.rp41-133b2.8034-40080.revcompl.Baboon           ------------------------------------------------------------    0
BCRP3.HUMAN.NCBI.REF                                               ------------------------------------------------------------    0
LOC106996293.glutathione.hydrolase5.proenzyme-like-GGT1.rhesus.    cagacagacctgcagccgtcccagatcacacagccccagggatgggaccaggagccagca    21360

Papio.anubis.clone.rp41-133b2.8034-40080.revcompl.Baboon           ------------------------------------------------------------    0
BCRP3.HUMAN.NCBI.REF                                               ------------------------------------------------------------    0
LOC106996293.glutathione.hydrolase5.proenzyme-like-GGT1.rhesus.    agtgtcccacctggagcagttcctgtgcctttaaagccttccctcccccaccccgcccc    21420

Papio.anubis.clone.rp41-133b2.8034-40080.revcompl.Baboon           ------------------------------------------------------------    0
BCRP3.HUMAN.NCBI.REF                                               ------------------------------------------------------------    0
LOC106996293.glutathione.hydrolase5.proenzyme-like-GGT1.rhesus.    acccacccccgggccactaggggagggaaggaggagctgggtcacagcagggaatcttac    21480

Papio.anubis.clone.rp41-133b2.8034-40080.revcompl.Baboon           ------------------------------------------------------------    0
BCRP3.HUMAN.NCBI.REF                                               ------------------------------------------------------------    0
LOC106996293.glutathione.hydrolase5.proenzyme-like-GGT1.rhesus.    cttggtttgctggatgaccggaccaggagtcgggtgagccaggaagtgagggtagctggc    21540

Papio.anubis.clone.rp41-133b2.8034-40080.revcompl.Baboon           ------------------------------------------------------------    0
BCRP3.HUMAN.NCBI.REF                                               ------------------------------------------------------------    0
LOC106996293.glutathione.hydrolase5.proenzyme-like-GGT1.rhesus.    tgtgccccagtgctgtgtgacccagaggtgccactcaccctctctgaactggtgaacatc    21600

Papio.anubis.clone.rp41-133b2.8034-40080.revcompl.Baboon           ------------------------------------------------------------    0
BCRP3.HUMAN.NCBI.REF                                               ------------------------------------------------------------    0
LOC106996293.glutathione.hydrolase5.proenzyme-like-GGT1.rhesus.    ataggtggggaggctcaggccagggcactcccaggagtgtctggaggcctgagtttcatt    21660

Papio.anubis.clone.rp41-133b2.8034-40080.revcompl.Baboon           ------------------------------------------------------------    0
BCRP3.HUMAN.NCBI.REF                                               ------------------------------------------------------------    0
LOC106996293.glutathione.hydrolase5.proenzyme-like-GGT1.rhesus.    ctcagctctgccatatgcttgctgcactctagaggagctcctcttcctctccgagcctcg    21720

Papio.anubis.clone.rp41-133b2.8034-40080.revcompl.Baboon           ------------------------------------------------------------    0
BCRP3.HUMAN.NCBI.REF                                               ------------------------------------------------------------    0
LOC106996293.glutathione.hydrolase5.proenzyme-like-GGT1.rhesus.    gtttatgcgcctgtgtggtgggagtgagttgcacttcagggtgaagggggtgagactggt    21780

Papio.anubis.clone.rp41-133b2.8034-40080.revcompl.Baboon           ------------------------------------------------------------    0
BCRP3.HUMAN.NCBI.REF                                               ------------------------------------------------------------    0
LOC106996293.glutathione.hydrolase5.proenzyme-like-GGT1.rhesus.    gtgggcgcaccctgcagaaggatcccacaggtgggcagagccctgggtttttttatccgac    21840

Papio.anubis.clone.rp41-133b2.8034-40080.revcompl.Baboon           ------------------------------------------------------------    0
BCRP3.HUMAN.NCBI.REF                                               ------------------------------------------------------------    0
LOC106996293.glutathione.hydrolase5.proenzyme-like-GGT1.rhesus.    tgagtctgggctggggggtggcctccgtttcttgactgatgagtgggtttgagtgaggta    21900

Papio.anubis.clone.rp41-133b2.8034-40080.revcompl.Baboon           ------------------------------------------------------------    0
BCRP3.HUMAN.NCBI.REF                                               ------------------------------------------------------------    0
LOC106996293.glutathione.hydrolase5.proenzyme-like-GGT1.rhesus.    tgcgggtgggtgaaggagggctggggggagtcacctgacttacgtgaagaaacccttgag    21960

Papio.anubis.clone.rp41-133b2.8034-40080.revcompl.Baboon           ------------------------------------------------------------    0
BCRP3.HUMAN.NCBI.REF                                               ------------------------------------------------------------    0
LOC106996293.glutathione.hydrolase5.proenzyme-like-GGT1.rhesus.    acagctgtggcttcttggaattaagagggaagagtgcagcatggagaaactgaggcccgg    22020

Papio.anubis.clone.rp41-133b2.8034-40080.revcompl.Baboon           ------------------------------------------------------------    0
BCRP3.HUMAN.NCBI.REF                                               ------------------------------------------------------------    0
LOC106996293.glutathione.hydrolase5.proenzyme-like-GGT1.rhesus.    gaggatttggcagccctgagtgggggtggggggatcagctctggaatagagccagagctg    22080

Papio.anubis.clone.rp41-133b2.8034-40080.revcompl.Baboon           ------------------------------------------------------------    0
BCRP3.HUMAN.NCBI.REF                                               ------------------------------------------------------------    0
LOC106996293.glutathione.hydrolase5.proenzyme-like-GGT1.rhesus.    tgatggggttgggggaccctgtgtccttgggccgtgcttgtcccactcctgggctctgtg    22140
```

```
Papio.anubis.clone.rp41-133b2.8034-40080.revcompl.Baboon          ------------------------------------------------------------    0
BCRP3.HUMAN.NCBI.REF                                               ------------------------------------------------------------    0
LOC106996293.glutathione.hydrolase5.proenzyme-like-GGT1.rhesus.    tgtggctgtggattggggtgggacagggctggttgtgcgaggggtcattgcccaactcca    22200

Papio.anubis.clone.rp41-133b2.8034-40080.revcompl.Baboon          ------------------------------------------------------------    0
BCRP3.HUMAN.NCBI.REF                                               ------------------------------------------------------------    0
LOC106996293.glutathione.hydrolase5.proenzyme-like-GGT1.rhesus.    gagggcgcctgccacctctcagctctctgtagggatacatatagttccttcaacaggttt    22260

Papio.anubis.clone.rp41-133b2.8034-40080.revcompl.Baboon          ------------------------------------------------------------    0
BCRP3.HUMAN.NCBI.REF                                               ------------------------------------------------------------    0
LOC106996293.glutathione.hydrolase5.proenzyme-like-GGT1.rhesus.    ccagcaggtagaggttattaaatcttactgaaggggtgtttttctaattgtcaactctg    22320

Papio.anubis.clone.rp41-133b2.8034-40080.revcompl.Baboon          ------------------------------------------------------------    0
BCRP3.HUMAN.NCBI.REF                                               ------------------------------------------------------------    0
LOC106996293.glutathione.hydrolase5.proenzyme-like-GGT1.rhesus.    cgccgcaagggtggaaactgtgagagactgattccaactccaggtctgggtggtaagcat    22380

Papio.anubis.clone.rp41-133b2.8034-40080.revcompl.Baboon          ------------------------------------------------------------    0
BCRP3.HUMAN.NCBI.REF                                               ------------------------------------------------------------    0
LOC106996293.glutathione.hydrolase5.proenzyme-like-GGT1.rhesus.    ccagtccaggggtgtagacaggcctggggaagcctcagacatctgtgcgttctcataccc    22440

Papio.anubis.clone.rp41-133b2.8034-40080.revcompl.Baboon          ------------------------------------------------------------    0
BCRP3.HUMAN.NCBI.REF                                               ------------------------------------------------------------    0
LOC106996293.glutathione.hydrolase5.proenzyme-like-GGT1.rhesus.    agggatagtgactccatgctgggggctgtcagcgtaaggggtgggtgggtcctgggctta    22500

Papio.anubis.clone.rp41-133b2.8034-40080.revcompl.Baboon          ------------------------------------------------------------    0
BCRP3.HUMAN.NCBI.REF                                               ------------------------------------------------------------    0
LOC106996293.glutathione.hydrolase5.proenzyme-like-GGT1.rhesus.    cctgcaggtctgagacctcctggggccagctgacctcggtaaatccctttttgtctaagct    22560

Papio.anubis.clone.rp41-133b2.8034-40080.revcompl.Baboon          ------------------------------------------------------------    0
BCRP3.HUMAN.NCBI.REF                                               ------------------------------------------------------------    0
LOC106996293.glutathione.hydrolase5.proenzyme-like-GGT1.rhesus.    tcagtttcctgcctgtgaatggggttgcggctgtgctccagtttcacccttgtggctctg    22620

Papio.anubis.clone.rp41-133b2.8034-40080.revcompl.Baboon          ------------------------------------------------------------    0
BCRP3.HUMAN.NCBI.REF                                               ------------------------------------------------------------    0
LOC106996293.glutathione.hydrolase5.proenzyme-like-GGT1.rhesus.    gggttgtggtgaaaaagccatcaagctgggttggagactggctgccgtgtctacctcttc    22680

Papio.anubis.clone.rp41-133b2.8034-40080.revcompl.Baboon          ------------------------------------------------------------    0
BCRP3.HUMAN.NCBI.REF                                               ------------------------------------------------------------    0
LOC106996293.glutathione.hydrolase5.proenzyme-like-GGT1.rhesus.    ctcatactcctctctctgctgcatcctgggaagctgctcagctcagcctagattaggctc    22740

Papio.anubis.clone.rp41-133b2.8034-40080.revcompl.Baboon          ------------------------------------------------------------    0
BCRP3.HUMAN.NCBI.REF                                               ------------------------------------------------------------    0
LOC106996293.glutathione.hydrolase5.proenzyme-like-GGT1.rhesus.    agttgtgtctgtgtgtgcacgtgattgcacctgttggaagtgtgtggtactgacaccaga    22800

Papio.anubis.clone.rp41-133b2.8034-40080.revcompl.Baboon          ------------------------------------------------------------    0
BCRP3.HUMAN.NCBI.REF                                               ------------------------------------------------------------    0
LOC106996293.glutathione.hydrolase5.proenzyme-like-GGT1.rhesus.    gtcagtgtctctgggtgagtgaggcttgcacatttctggggacagggaactcactacctt    22860

Papio.anubis.clone.rp41-133b2.8034-40080.revcompl.Baboon          ------------------------------------------------------------    0
BCRP3.HUMAN.NCBI.REF                                               ------------------------------------------------------------    0
LOC106996293.glutathione.hydrolase5.proenzyme-like-GGT1.rhesus.    atgtgcccagaacaagagctttggggtctggagaagacttctaggccaacccctgcagtc    22920

Papio.anubis.clone.rp41-133b2.8034-40080.revcompl.Baboon          ------------------------------------------------------------    0
BCRP3.HUMAN.NCBI.REF                                               ------------------------------------------------------------    0
LOC106996293.glutathione.hydrolase5.proenzyme-like-GGT1.rhesus.    tttcctcaggtgacatggcttccccagacccacttccctggaggtgccctgtctgcattc    22980

Papio.anubis.clone.rp41-133b2.8034-40080.revcompl.Baboon          ------------------------------------------------------------    0
BCRP3.HUMAN.NCBI.REF                                               ------------------------------------------------------------    0
LOC106996293.glutathione.hydrolase5.proenzyme-like-GGT1.rhesus.    aaggggtagaaggctgactgggacagaacgcgacacactcagcatgtgaggaaaagcctc    23040

Papio.anubis.clone.rp41-133b2.8034-40080.revcompl.Baboon          ------------------------------------------------------------    0
BCRP3.HUMAN.NCBI.REF                                               ------------------------------------------------------------    0
LOC106996293.glutathione.hydrolase5.proenzyme-like-GGT1.rhesus.    cttcattctgtaggccctacctctgttgacatgacctttgataaagtgcctcccttcctg    23100

Papio.anubis.clone.rp41-133b2.8034-40080.revcompl.Baboon          ------------------------------------------------------------    0
BCRP3.HUMAN.NCBI.REF                                               ------------------------------------------------------------    0
LOC106996293.glutathione.hydrolase5.proenzyme-like-GGT1.rhesus.    tctcccctctctggaaccctcagctgcttccacgcttcagctgtgccccatcgcaggcag    23160

Papio.anubis.clone.rp41-133b2.8034-40080.revcompl.Baboon          ------------------------------------------------------------    0
BCRP3.HUMAN.NCBI.REF                                               ------------------------------------------------------------    0
LOC106996293.glutathione.hydrolase5.proenzyme-like-GGT1.rhesus.    ctctctgtctccctactttccccagcccagggttttccttttggggtcagctgcgggggt    23220

Papio.anubis.clone.rp41-133b2.8034-40080.revcompl.Baboon          ------------------------------------------------------------    0
BCRP3.HUMAN.NCBI.REF                                               ------------------------------------------------------------    0
LOC106996293.glutathione.hydrolase5.proenzyme-like-GGT1.rhesus.    ctggcccatcctttgcccactcagacattcttcctgcccacctgctgctgagaatcctgc    23280

Papio.anubis.clone.rp41-133b2.8034-40080.revcompl.Baboon          ------------------------------------------------------------    0
BCRP3.HUMAN.NCBI.REF                                               ------------------------------------------------------------    0
LOC106996293.glutathione.hydrolase5.proenzyme-like-GGT1.rhesus.    atgtctcatatgctctcaggcagagaggaaactgctgagcaggccagggcaggggacgga    23340

Papio.anubis.clone.rp41-133b2.8034-40080.revcompl.Baboon          ------------------------------------------------------------    0
BCRP3.HUMAN.NCBI.REF                                               ------------------------------------------------------------    0
LOC106996293.glutathione.hydrolase5.proenzyme-like-GGT1.rhesus.    gccctgtgactctccactttagacccctcccagctgactgctgtggaatgcagccactta    23400

Papio.anubis.clone.rp41-133b2.8034-40080.revcompl.Baboon          ------------------------------------------------------------    0
BCRP3.HUMAN.NCBI.REF                                               ------------------------------------------------------------    0
LOC106996293.glutathione.hydrolase5.proenzyme-like-GGT1.rhesus.    gcagagccaaatcccctgaggttcctgtctgctgtctagccttcagggacaggtcatggg    23460

Papio.anubis.clone.rp41-133b2.8034-40080.revcompl.Baboon          ------------------------------------------------------------    0
BCRP3.HUMAN.NCBI.REF                                               ------------------------------------------------------------    0
LOC106996293.glutathione.hydrolase5.proenzyme-like-GGT1.rhesus.    gccttggttcctgccccgtcattttcccagtcaccctgatcttcaggggaggaacagcc    23520

Papio.anubis.clone.rp41-133b2.8034-40080.revcompl.Baboon          ------------------------------------------------------------    0
BCRP3.HUMAN.NCBI.REF                                               ------------------------------------------------------------    0
LOC106996293.glutathione.hydrolase5.proenzyme-like-GGT1.rhesus.    agggaaagggtttggtcatagcacctccaccctagggctattgaggatctcacagttgtg    23580

Papio.anubis.clone.rp41-133b2.8034-40080.revcompl.Baboon          ------------------------------------------------------------    0
BCRP3.HUMAN.NCBI.REF                                               ------------------------------------------------------------    0
LOC106996293.glutathione.hydrolase5.proenzyme-like-GGT1.rhesus.    tgtctggtgggttctgtccagagcccatttgagaacagtgggtgacaggacaggcctatg    23640

Papio.anubis.clone.rp41-133b2.8034-40080.revcompl.Baboon          ------------------------------------------------------------    0
BCRP3.HUMAN.NCBI.REF                                               ------------------------------------------------------------    0
LOC106996293.glutathione.hydrolase5.proenzyme-like-GGT1.rhesus.    tgacccaggcaggcagcaatattgggtcagccttcgtgtccccttctgtcagctggggca    23700

Papio.anubis.clone.rp41-133b2.8034-40080.revcompl.Baboon          ------------------------------------------------------------    0
BCRP3.HUMAN.NCBI.REF                                               ------------------------------------------------------------    0
LOC106996293.glutathione.hydrolase5.proenzyme-like-GGT1.rhesus.    gcctggaaggatgattgtggggtaggtgttatgggaatagacctcaggtggaggctgcag    23760

Papio.anubis.clone.rp41-133b2.8034-40080.revcompl.Baboon          ------------------------------------------------------------    0
BCRP3.HUMAN.NCBI.REF                                               ------------------------------------------------------------    0
LOC106996293.glutathione.hydrolase5.proenzyme-like-GGT1.rhesus.    gggctctccggcactgtaggcacagcaggcccaggagcaggggaggcagccatgactcgt    23820

Papio.anubis.clone.rp41-133b2.8034-40080.revcompl.Baboon          ------------------------------------------------------------    0
BCRP3.HUMAN.NCBI.REF                                               ------------------------------------------------------------    0
LOC106996293.glutathione.hydrolase5.proenzyme-like-GGT1.rhesus.    ggagcctctggcccatatccttggcagatgagggccacaggggaatgggcagcagtgtcc    23880
```

```
Papio.anubis.clone.rp41-133b2.8034-40080.revcompl.Baboon          ------------------------------------------------------------    0
BCRP3.HUMAN.NCBI.REF                                               ------------------------------------------------------------    0
LOC106996293.glutathione.hydrolase5.proenzyme-like-GGT1.rhesus.    aaggtcccctgggctgggcccacggctgttcctggctcagacttcttggtgggctggtca    23940

Papio.anubis.clone.rp41-133b2.8034-40080.revcompl.Baboon          ------------------------------------------------------------    0
BCRP3.HUMAN.NCBI.REF                                               ------------------------------------------------------------    0
LOC106996293.glutathione.hydrolase5.proenzyme-like-GGT1.rhesus.    ggaacatgcagtaacttggggcagttaccaagtggccaaggctgcgctattgggctgtgt    24000

Papio.anubis.clone.rp41-133b2.8034-40080.revcompl.Baboon          ------------------------------------------------------------    0
BCRP3.HUMAN.NCBI.REF                                               ------------------------------------------------------------    0
LOC106996293.glutathione.hydrolase5.proenzyme-like-GGT1.rhesus.    gagcttgggccagctcaggccctctctggaccctgcctttctgggctgttcaggtggttc    24060

Papio.anubis.clone.rp41-133b2.8034-40080.revcompl.Baboon          ------------------------------------------------------------    0
BCRP3.HUMAN.NCBI.REF                                               ------------------------------------------------------------    0
LOC106996293.glutathione.hydrolase5.proenzyme-like-GGT1.rhesus.    cttgggcctggagtgctaatgtttctggatgggcagcagaaccggtctgcactcagggcc    24120

Papio.anubis.clone.rp41-133b2.8034-40080.revcompl.Baboon          ------------------------------------------------------------    0
BCRP3.HUMAN.NCBI.REF                                               ------------------------------------------------------------    0
LOC106996293.glutathione.hydrolase5.proenzyme-like-GGT1.rhesus.    ccaggccatgttcccggaacacacctttagcattgacatcagggtgtggtgaggcccctt    24180

Papio.anubis.clone.rp41-133b2.8034-40080.revcompl.Baboon          ------------------------------------------------------------    0
BCRP3.HUMAN.NCBI.REF                                               ------------------------------------------------------------    0
LOC106996293.glutathione.hydrolase5.proenzyme-like-GGT1.rhesus.    aggctgggttctggtctggtgccaggggcaccactactccactgcctccagagccatctc    24240

Papio.anubis.clone.rp41-133b2.8034-40080.revcompl.Baboon          ------------------------------------------------------------    0
BCRP3.HUMAN.NCBI.REF                                               ------------------------------------------------------------    0
LOC106996293.glutathione.hydrolase5.proenzyme-like-GGT1.rhesus.    tgggacactggctgtgagttcagatgttctgaacagggacagggaaagccagaggaggac    24300

Papio.anubis.clone.rp41-133b2.8034-40080.revcompl.Baboon          ------------------------------------------------------------    0
BCRP3.HUMAN.NCBI.REF                                               ------------------------------------------------------------    0
LOC106996293.glutathione.hydrolase5.proenzyme-like-GGT1.rhesus.    ccagcctggggctagctggaggggctcgtgggcagacagcgccctttggagggaactgag    24360

Papio.anubis.clone.rp41-133b2.8034-40080.revcompl.Baboon          ------------------------------------------------------------    0
BCRP3.HUMAN.NCBI.REF                                               ------------------------------------------------------------    0
LOC106996293.glutathione.hydrolase5.proenzyme-like-GGT1.rhesus.    tctggaaggaaaccctttccccgggtcacagcacctggcacccagggccctcccagggct    24420

Papio.anubis.clone.rp41-133b2.8034-40080.revcompl.Baboon          ------------------------------------------------------------    0
BCRP3.HUMAN.NCBI.REF                                               ------------------------------------------------------------    0
LOC106996293.glutathione.hydrolase5.proenzyme-like-GGT1.rhesus.    gcgtgaactttagtcacatggtgacaggccgagtcaccgtgccaagtcgctgtgcgcctc    24480

Papio.anubis.clone.rp41-133b2.8034-40080.revcompl.Baboon          ------------------------------------------------------------    0
BCRP3.HUMAN.NCBI.REF                                               ------------------------------------------------------------    0
LOC106996293.glutathione.hydrolase5.proenzyme-like-GGT1.rhesus.    cttgctgctgtgacgtcagcctccccttcctcccagccaggctggacctctgtgagaggc    24540

Papio.anubis.clone.rp41-133b2.8034-40080.revcompl.Baboon          ------------------------------------------------------------    0
BCRP3.HUMAN.NCBI.REF                                               ------------------------------------------------------------    0
LOC106996293.glutathione.hydrolase5.proenzyme-like-GGT1.rhesus.    ctgcctgtcctgcaccctgtgcagatgcctcccactgtctgctggggctgatgggcacct    24600

Papio.anubis.clone.rp41-133b2.8034-40080.revcompl.Baboon          ------------------------------------------------------------    0
BCRP3.HUMAN.NCBI.REF                                               ------------------------------------------------------------    0
LOC106996293.glutathione.hydrolase5.proenzyme-like-GGT1.rhesus.    cctggctggtctcttgtactaggtaagctcatgggtcctccggctgctcctgctccttcc    24660

Papio.anubis.clone.rp41-133b2.8034-40080.revcompl.Baboon          ------------------------------------------------------------    0
BCRP3.HUMAN.NCBI.REF                                               ------------------------------------------------------------    0
LOC106996293.glutathione.hydrolase5.proenzyme-like-GGT1.rhesus.    ctgcctctgctcctcctcggaggtggccacccccagatcccagtcccgattcagaggccc    24720

Papio.anubis.clone.rp41-133b2.8034-40080.revcompl.Baboon          ------------------------------------------------------------    0
BCRP3.HUMAN.NCBI.REF                                               ------------------------------------------------------------    0
LOC106996293.glutathione.hydrolase5.proenzyme-like-GGT1.rhesus.    cctgaggagcactgcagggggccgcaggcgtggctctgagccactctggagagtgggga    24780

Papio.anubis.clone.rp41-133b2.8034-40080.revcompl.Baboon          ------------------------------------------------------------    0
BCRP3.HUMAN.NCBI.REF                                               ------------------------------------------------------------    0
LOC106996293.glutathione.hydrolase5.proenzyme-like-GGT1.rhesus.    gcccagccagttctgtggccgggactttcccaggcagacaagtctgtctcttcctcccca    24840

Papio.anubis.clone.rp41-133b2.8034-40080.revcompl.Baboon          ------------------------------------------------------------    0
BCRP3.HUMAN.NCBI.REF                                               ------------------------------------------------------------    0
LOC106996293.glutathione.hydrolase5.proenzyme-like-GGT1.rhesus.    gcgggtgcagcccagaactgtcttctgaggaagaggtgctctcctgggcccccactgtcc    24900

Papio.anubis.clone.rp41-133b2.8034-40080.revcompl.Baboon          ------------------------------------------------------------    0
BCRP3.HUMAN.NCBI.REF                                               ------------------------------------------------------------    0
LOC106996293.glutathione.hydrolase5.proenzyme-like-GGT1.rhesus.    ccaagcctcaggtaagcccatcagggtcacaaggaaggggatctgggtttgaggccaacc    24960

Papio.anubis.clone.rp41-133b2.8034-40080.revcompl.Baboon          ------------------------------------------------------------    0
BCRP3.HUMAN.NCBI.REF                                               ------------------------------------------------------------    0
LOC106996293.glutathione.hydrolase5.proenzyme-like-GGT1.rhesus.    atggcaactgacttactttctagcctcagtttcccctagggtgtgcagctctcacactgt    25020

Papio.anubis.clone.rp41-133b2.8034-40080.revcompl.Baboon          ------------------------------------------------------------    0
BCRP3.HUMAN.NCBI.REF                                               ------------------------------------------------------------    0
LOC106996293.glutathione.hydrolase5.proenzyme-like-GGT1.rhesus.    ttgggtgacagccagccctctgggcttagacattctttcagtgagtcttgagggtggagg    25080

Papio.anubis.clone.rp41-133b2.8034-40080.revcompl.Baboon          ------------------------------------------------------------    0
BCRP3.HUMAN.NCBI.REF                                               ------------------------------------------------------------    0
LOC106996293.glutathione.hydrolase5.proenzyme-like-GGT1.rhesus.    gatgggaaatggaagcccagggcctcagcagggtgtcttcctccgagctggggacatctg    25140

Papio.anubis.clone.rp41-133b2.8034-40080.revcompl.Baboon          ------------------------------------------------------------    0
BCRP3.HUMAN.NCBI.REF                                               ------------------------------------------------------------    0
LOC106996293.glutathione.hydrolase5.proenzyme-like-GGT1.rhesus.    cctgtgggaggctgggcccaacctcccttgctaacctgccctgggaggaagtgacagggc    25200

Papio.anubis.clone.rp41-133b2.8034-40080.revcompl.Baboon          ------------------------------------------------------------    0
BCRP3.HUMAN.NCBI.REF                                               ------------------------------------------------------------    0
LOC106996293.glutathione.hydrolase5.proenzyme-like-GGT1.rhesus.    ccagcactgcctactcccctccccatgcttcctggcagtctgagcctggctgggtgtccc    25260

Papio.anubis.clone.rp41-133b2.8034-40080.revcompl.Baboon          ------------------------------------------------------------    0
BCRP3.HUMAN.NCBI.REF                                               ------------------------------------------------------------    0
LOC106996293.glutathione.hydrolase5.proenzyme-like-GGT1.rhesus.    ctgctcctccaggagcctacttgggcctttgggtagacagattaacagacaggggaggct    25320

Papio.anubis.clone.rp41-133b2.8034-40080.revcompl.Baboon          ------------------------------------------------------------    0
BCRP3.HUMAN.NCBI.REF                                               ------------------------------------------------------------    0
LOC106996293.glutathione.hydrolase5.proenzyme-like-GGT1.rhesus.    gggtcatggttgggccaccccaggaccctgatggggggctcagctcatgaccctgagcc    25380

Papio.anubis.clone.rp41-133b2.8034-40080.revcompl.Baboon          ------------------------------------------------------------    0
BCRP3.HUMAN.NCBI.REF                                               ------------------------------------------------------------    0
LOC106996293.glutathione.hydrolase5.proenzyme-like-GGT1.rhesus.    tgggagagatgaggccatgccttccagggcactcagcatgacccggcccagtggacggga    25440

Papio.anubis.clone.rp41-133b2.8034-40080.revcompl.Baboon          ------------------------------------------------------------    0
BCRP3.HUMAN.NCBI.REF                                               ------------------------------------------------------------    0
LOC106996293.glutathione.hydrolase5.proenzyme-like-GGT1.rhesus.    ctgggtagcttccttggtgcaggggggttgtcatgctaggatgggggcaccgaccaggcca    25500

Papio.anubis.clone.rp41-133b2.8034-40080.revcompl.Baboon          ------------------------------------------------------------    0
BCRP3.HUMAN.NCBI.REF                                               ------------------------------------------------------------    0
LOC106996293.glutathione.hydrolase5.proenzyme-like-GGT1.rhesus.    ggcccccgccccatgacatgtggtggaaatgtccgtttgttttgttttgtttttttgcttt    25560

Papio.anubis.clone.rp41-133b2.8034-40080.revcompl.Baboon          ------------------------------------------------------------    0
BCRP3.HUMAN.NCBI.REF                                               ------------------------------------------------------------    0
LOC106996293.glutathione.hydrolase5.proenzyme-like-GGT1.rhesus.    tttttttgagatggagtttcactgttgttgcccaagctggagtgcagtggtgcaatcttg    25620
```

```
Papio.anubis.clone.rp41-133b2.8034-40080.revcompl.Baboon          ------------------------------------------------------------    0
BCRP3.HUMAN.NCBI.REF                                              ------------------------------------------------------------    0
LOC106996293.glutathione.hydrolase5.proenzyme-like-GGT1.rhesus.   cctcactgcaacttctgccccccaggttcaagcaattctcctgcctcagctcccgaatag    25680

Papio.anubis.clone.rp41-133b2.8034-40080.revcompl.Baboon          ------------------------------------------------------------    0
BCRP3.HUMAN.NCBI.REF                                              ------------------------------------------------------------    0
LOC106996293.glutathione.hydrolase5.proenzyme-like-GGT1.rhesus.   ctggtattacaggcacctgccaccatacccagctaatttttttgtatttttagtggagacg    25740

Papio.anubis.clone.rp41-133b2.8034-40080.revcompl.Baboon          ------------------------------------------------------------    0
BCRP3.HUMAN.NCBI.REF                                              ------------------------------------------------------------    0
LOC106996293.glutathione.hydrolase5.proenzyme-like-GGT1.rhesus.   ggggtttcaccatgttggccaggctggtctcaaactcttcacctcaggtgatccacctgc    25800

Papio.anubis.clone.rp41-133b2.8034-40080.revcompl.Baboon          ------------------------------------------------------------    0
BCRP3.HUMAN.NCBI.REF                                              ------------------------------------------------------------    0
LOC106996293.glutathione.hydrolase5.proenzyme-like-GGT1.rhesus.   ttctgcctcccaaagtgctgggattataggcacaagcccctgcatctggccatgtgatag    25860

Papio.anubis.clone.rp41-133b2.8034-40080.revcompl.Baboon          ------------------------------------------------------------    0
BCRP3.HUMAN.NCBI.REF                                              ------------------------------------------------------------    0
LOC106996293.glutathione.hydrolase5.proenzyme-like-GGT1.rhesus.   gaatgttctgtgtccataatggatactgcactttgctggtccagctcctgagtctccttg    25920

Papio.anubis.clone.rp41-133b2.8034-40080.revcompl.Baboon          ------------------------------------------------------------    0
BCRP3.HUMAN.NCBI.REF                                              ------------------------------------------------------------    0
LOC106996293.glutathione.hydrolase5.proenzyme-like-GGT1.rhesus.   gacctccaggaatcggtgtttctatcaggaacccttaaccctgtcccagactccctgctg    25980

Papio.anubis.clone.rp41-133b2.8034-40080.revcompl.Baboon          ------------------------------------------------------------    0
BCRP3.HUMAN.NCBI.REF                                              ------------------------------------------------------------    0
LOC106996293.glutathione.hydrolase5.proenzyme-like-GGT1.rhesus.   ggacccagggtgttggagtggcaagagggctgtcaggcctggtgaagggtgtgagctgtc    26040

Papio.anubis.clone.rp41-133b2.8034-40080.revcompl.Baboon          ------------------------------------------------------------    0
BCRP3.HUMAN.NCBI.REF                                              ------------------------------------------------------------    0
LOC106996293.glutathione.hydrolase5.proenzyme-like-GGT1.rhesus.   ccatggggcagggaggaggcaggacctgttctgtagctggacagacagagccctctagct    26100

Papio.anubis.clone.rp41-133b2.8034-40080.revcompl.Baboon          ------------------------------------------------------------    0
BCRP3.HUMAN.NCBI.REF                                              ------------------------------------------------------------    0
LOC106996293.glutathione.hydrolase5.proenzyme-like-GGT1.rhesus.   gctttctggaagactgaagggcaggtgatgttggagggagggagtgcaggcagggtctct    26160

Papio.anubis.clone.rp41-133b2.8034-40080.revcompl.Baboon          ------------------------------------------------------------    0
BCRP3.HUMAN.NCBI.REF                                              ------------------------------------------------------------    0
LOC106996293.glutathione.hydrolase5.proenzyme-like-GGT1.rhesus.   gagggagttcaggtcagaaacaagtggcacctggattcaggctgtggtggtcacggtggg    26220

Papio.anubis.clone.rp41-133b2.8034-40080.revcompl.Baboon          ------------------------------------------------------------    0
BCRP3.HUMAN.NCBI.REF                                              ------------------------------------------------------------    0
LOC106996293.glutathione.hydrolase5.proenzyme-like-GGT1.rhesus.   gatgaggggctgcttcggattctgctggggatgtggggtggtgcgctgctgcttgactac    26280

Papio.anubis.clone.rp41-133b2.8034-40080.revcompl.Baboon          ------------------------------------------------------------    0
BCRP3.HUMAN.NCBI.REF                                              ------------------------------------------------------------    0
LOC106996293.glutathione.hydrolase5.proenzyme-like-GGT1.rhesus.   tcccagtcccctctgctcttggtgtctgcatccagggctgggaggcggtcaaatgtttca    26340

Papio.anubis.clone.rp41-133b2.8034-40080.revcompl.Baboon          ------------------------------------------------------------    0
BCRP3.HUMAN.NCBI.REF                                              ------------------------------------------------------------    0
LOC106996293.glutathione.hydrolase5.proenzyme-like-GGT1.rhesus.   cactattggccccaggcctgccaagcctggggaggtggccacccttccacgatggcattt    26400

Papio.anubis.clone.rp41-133b2.8034-40080.revcompl.Baboon          ------------------------------------------------------------    0
BCRP3.HUMAN.NCBI.REF                                              ------------------------------------------------------------    0
LOC106996293.glutathione.hydrolase5.proenzyme-like-GGT1.rhesus.   ggatgttccctgtatgtggggagggcacaggggctccattcctagaccacctctgggaca    26460

Papio.anubis.clone.rp41-133b2.8034-40080.revcompl.Baboon          ------------------------------------------------------------    0
BCRP3.HUMAN.NCBI.REF                                              ------------------------------------------------------------    0
LOC106996293.glutathione.hydrolase5.proenzyme-like-GGT1.rhesus.   gtgtgtctggctctgaggtcagatgctctgccctgggacagggtggagtggaggggaacc    26520

Papio.anubis.clone.rp41-133b2.8034-40080.revcompl.Baboon          ------------------------------------------------------------    0
BCRP3.HUMAN.NCBI.REF                                              ------------------------------------------------------------    0
LOC106996293.glutathione.hydrolase5.proenzyme-like-GGT1.rhesus.   cagcttggggctcattggaggggcttgctggcagacactgcactttgtgggaaactgagt    26580

Papio.anubis.clone.rp41-133b2.8034-40080.revcompl.Baboon          ------------------------------------------------------------    0
BCRP3.HUMAN.NCBI.REF                                              ------------------------------------------------------------    0
LOC106996293.glutathione.hydrolase5.proenzyme-like-GGT1.rhesus.   gtgggaggggggaacccccctacctctgtcaccacatccctcttcgctgttggggtcatacc    26640

Papio.anubis.clone.rp41-133b2.8034-40080.revcompl.Baboon          ------------------------------------------------------------    0
BCRP3.HUMAN.NCBI.REF                                              ------------------------------------------------------------    0
LOC106996293.glutathione.hydrolase5.proenzyme-like-GGT1.rhesus.   tgtcacctactgccatagccatgagacttcccaagggtcactgctgcacctcactgcaca    26700

Papio.anubis.clone.rp41-133b2.8034-40080.revcompl.Baboon          ------------------------------------------------------------    0
BCRP3.HUMAN.NCBI.REF                                              ------------------------------------------------------------    0
LOC106996293.glutathione.hydrolase5.proenzyme-like-GGT1.rhesus.   gcccagaagggagtccacaggggacatacagtgagcaagagacctgtgcccctcaggcct    26760

Papio.anubis.clone.rp41-133b2.8034-40080.revcompl.Baboon          ------------------------------------------------------------    0
BCRP3.HUMAN.NCBI.REF                                              ------------------------------------------------------------    0
LOC106996293.glutathione.hydrolase5.proenzyme-like-GGT1.rhesus.   cctgggggtgtccccaatgcagccatgataataatcacagccaccattcaccaagccctt    26820

Papio.anubis.clone.rp41-133b2.8034-40080.revcompl.Baboon          ------------------------------------------------------------    0
BCRP3.HUMAN.NCBI.REF                                              ------------------------------------------------------------    0
LOC106996293.glutathione.hydrolase5.proenzyme-like-GGT1.rhesus.   cccacagtctaacctactctattcacaacactctcagcagcaaggcaagtgaggtgctgc    26880

Papio.anubis.clone.rp41-133b2.8034-40080.revcompl.Baboon          ------------------------------------------------------------    0
BCRP3.HUMAN.NCBI.REF                                              ------------------------------------------------------------    0
LOC106996293.glutathione.hydrolase5.proenzyme-like-GGT1.rhesus.   cgtcatccagactggatggttcattgatttgcctgaggccccacggcaggtgagtggcaa    26940

Papio.anubis.clone.rp41-133b2.8034-40080.revcompl.Baboon          ------------------------------------------------------------    0
BCRP3.HUMAN.NCBI.REF                                              ------------------------------------------------------------    0
LOC106996293.glutathione.hydrolase5.proenzyme-like-GGT1.rhesus.   gtccagcctcagagcagggcaggctagctatgctccctgagccccctttgccatgcttac    27000

Papio.anubis.clone.rp41-133b2.8034-40080.revcompl.Baboon          ------------------------------------------------------------    0
BCRP3.HUMAN.NCBI.REF                                              ------------------------------------------------------------    0
LOC106996293.glutathione.hydrolase5.proenzyme-like-GGT1.rhesus.   cacatgcacatcctgggcttgtgcaggaatgccctgtctcctgcgtgccctgctctgtgc    27060

Papio.anubis.clone.rp41-133b2.8034-40080.revcompl.Baboon          ------------------------------------------------------------    0
BCRP3.HUMAN.NCBI.REF                                              ------------------------------------------------------------    0
LOC106996293.glutathione.hydrolase5.proenzyme-like-GGT1.rhesus.   aaaaccctctttgagctgtgcctgggagatatgctgagagaattcatggaaacaaatgtg    27120

Papio.anubis.clone.rp41-133b2.8034-40080.revcompl.Baboon          ------------------------------------------------------------    0
BCRP3.HUMAN.NCBI.REF                                              ------------------------------------------------------------    0
LOC106996293.glutathione.hydrolase5.proenzyme-like-GGT1.rhesus.   ttactgacagcctctttgcctccagagttcaactggagaccagagaaatcagctagaggc    27180

Papio.anubis.clone.rp41-133b2.8034-40080.revcompl.Baboon          ------------------------------------------------------------    0
BCRP3.HUMAN.NCBI.REF                                              ------------------------------------------------------------    0
LOC106996293.glutathione.hydrolase5.proenzyme-like-GGT1.rhesus.   agagggaggtcacacggagtcccccagaaaggactgggctacgcgcgcttcaggtaacct    27240

Papio.anubis.clone.rp41-133b2.8034-40080.revcompl.Baboon          ------------------------------------------------------------    0
BCRP3.HUMAN.NCBI.REF                                              ------------------------------------------------------------    0
LOC106996293.glutathione.hydrolase5.proenzyme-like-GGT1.rhesus.   tcttgacctttaggagaatgagaaggctgcctgaccagagagtctctgaagaagattctg    27300

Papio.anubis.clone.rp41-133b2.8034-40080.revcompl.Baboon          ------------------------------------------------------------    0
BCRP3.HUMAN.NCBI.REF                                              ------------------------------------------------------------    0
LOC106996293.glutathione.hydrolase5.proenzyme-like-GGT1.rhesus.   tggctacatgctcctgcagagtgtgagggagaccctggttatttcctcagctgtttccac    27360
```

```
Papio.anubis.clone.rp41-133b2.8034-40080.revcompl.Baboon           ------------------------------------------------------------    0
BCRP3.HUMAN.NCBI.REF                                               ------------------------------------------------------------    0
LOC106996293.glutathione.hydrolase5.proenzyme-like-GGT1.rhesus.    caaatcctcccatctttcgcggccaacaccccaggcaaggcttggtgcccccgtctgctg    27420

Papio.anubis.clone.rp41-133b2.8034-40080.revcompl.Baboon           ------------------------------------------------------------    0
BCRP3.HUMAN.NCBI.REF                                               ------------------------------------------------------------    0
LOC106996293.glutathione.hydrolase5.proenzyme-like-GGT1.rhesus.    ctggacagtaagtcctggctccgtggcagtgaatctgtggggtactctgattgtgggtac    27480

Papio.anubis.clone.rp41-133b2.8034-40080.revcompl.Baboon           ------------------------------------------------------------    0
BCRP3.HUMAN.NCBI.REF                                               ------------------------------------------------------------    0
LOC106996293.glutathione.hydrolase5.proenzyme-like-GGT1.rhesus.    tatggaagctaaaccccatgctccaggtggggtggagggtcctcaggagactcctggaca    27540

Papio.anubis.clone.rp41-133b2.8034-40080.revcompl.Baboon           ------------------------------------------------------------    0
BCRP3.HUMAN.NCBI.REF                                               ------------------------------------------------------------    0
LOC106996293.glutathione.hydrolase5.proenzyme-like-GGT1.rhesus.    gtgccagactctgggctgaggtgggggacacaagagaaaccaggccaggcccgtccctac    27600

Papio.anubis.clone.rp41-133b2.8034-40080.revcompl.Baboon           ------------------------------------------------------------    0
BCRP3.HUMAN.NCBI.REF                                               ------------------------------------------------------------    0
LOC106996293.glutathione.hydrolase5.proenzyme-like-GGT1.rhesus.    cagagcttctccctaagcagtggaggctcagccactgtgagaaggtaggcaaggccctgc    27660

Papio.anubis.clone.rp41-133b2.8034-40080.revcompl.Baboon           ------------------------------------------------------------    0
BCRP3.HUMAN.NCBI.REF                                               ------------------------------------------------------------    0
LOC106996293.glutathione.hydrolase5.proenzyme-like-GGT1.rhesus.    agaaagaggggtgtggaaatatgggggctcccaggaagggcccctgctggagatggggtt    27720

Papio.anubis.clone.rp41-133b2.8034-40080.revcompl.Baboon           ------------------------------------------------------------    0
BCRP3.HUMAN.NCBI.REF                                               ------------------------------------------------------------    0
LOC106996293.glutathione.hydrolase5.proenzyme-like-GGT1.rhesus.    cttaccaggatggactctgaagataagcagggaggatttgggagggcagagatgaggccc    27780

Papio.anubis.clone.rp41-133b2.8034-40080.revcompl.Baboon           ------------------------------------------------------------    0
BCRP3.HUMAN.NCBI.REF                                               ------------------------------------------------------------    0
LOC106996293.glutathione.hydrolase5.proenzyme-like-GGT1.rhesus.    agagcttctgacagagggcatggcctgggcaaaggtctaggggccggacagcctgcacga    27840

Papio.anubis.clone.rp41-133b2.8034-40080.revcompl.Baboon           ------------------------------------------------------------    0
BCRP3.HUMAN.NCBI.REF                                               ------------------------------------------------------------    0
LOC106996293.glutathione.hydrolase5.proenzyme-like-GGT1.rhesus.    gttctgggaagtgggaaggagacactgggcctgtgtttctgaggcctgactttcgactgt    27900

Papio.anubis.clone.rp41-133b2.8034-40080.revcompl.Baboon           ------------------------------------------------------------    0
BCRP3.HUMAN.NCBI.REF                                               ------------------------------------------------------------    0
LOC106996293.glutathione.hydrolase5.proenzyme-like-GGT1.rhesus.    gccctgttggggagggaccagggaatgtatgaggctggacctgaccctgctccttaccct    27960

Papio.anubis.clone.rp41-133b2.8034-40080.revcompl.Baboon           ------------------------------------------------------------    0
BCRP3.HUMAN.NCBI.REF                                               ------------------------------------------------------------    0
LOC106996293.glutathione.hydrolase5.proenzyme-like-GGT1.rhesus.    gtgggtgcagcagagccatgaagaagaggttagtggtgctgggcctgctggccgtggtcc    28020

Papio.anubis.clone.rp41-133b2.8034-40080.revcompl.Baboon           ------------------------------------------------------------    0
BCRP3.HUMAN.NCBI.REF                                               ------------------------------------------------------------    0
LOC106996293.glutathione.hydrolase5.proenzyme-like-GGT1.rhesus.    tggtgctggtcattgttggcctctgtctctggctgccctcggcctccaaggaacctgaca    28080

Papio.anubis.clone.rp41-133b2.8034-40080.revcompl.Baboon           ------------------------------------------------------------    0
BCRP3.HUMAN.NCBI.REF                                               ------------------------------------------------------------    0
LOC106996293.glutathione.hydrolase5.proenzyme-like-GGT1.rhesus.    accacgtgtacactagggctgccgtggctgcagatgccaagcagtgctcggagattggga    28140

Papio.anubis.clone.rp41-133b2.8034-40080.revcompl.Baboon           ------------------------------------------------------------    0
BCRP3.HUMAN.NCBI.REF                                               ------------------------------------------------------------    0
LOC106996293.glutathione.hydrolase5.proenzyme-like-GGT1.rhesus.    ggtgagtggggcagggcatgggggcatgggccatgaaaactgggcaagtggacctgagca    28200

Papio.anubis.clone.rp41-133b2.8034-40080.revcompl.Baboon           ------------------------------------------------------------    0
BCRP3.HUMAN.NCBI.REF                                               ------------------------------------------------------------    0
LOC106996293.glutathione.hydrolase5.proenzyme-like-GGT1.rhesus.    ataccttcacccctctgagacttagtttccccacatgtaagctttgcttggactctctca    28260

Papio.anubis.clone.rp41-133b2.8034-40080.revcompl.Baboon           ------------------------------------------------------------    0
BCRP3.HUMAN.NCBI.REF                                               ------------------------------------------------------------    0
LOC106996293.glutathione.hydrolase5.proenzyme-like-GGT1.rhesus.    gtagcctctgggaaggggacggtgactccgagagcagggtttggatgtcctagagccaaa    28320

Papio.anubis.clone.rp41-133b2.8034-40080.revcompl.Baboon           ------------------------------------------------------------    0
BCRP3.HUMAN.NCBI.REF                                               ------------------------------------------------------------    0
LOC106996293.glutathione.hydrolase5.proenzyme-like-GGT1.rhesus.    cagcgccccttttctcagttctaagagtctctttctctttggataaactctactgttgta    28380

Papio.anubis.clone.rp41-133b2.8034-40080.revcompl.Baboon           ------------------------------------------------------------    0
BCRP3.HUMAN.NCBI.REF                                               ------------------------------------------------------------    0
LOC106996293.glutathione.hydrolase5.proenzyme-like-GGT1.rhesus.    ttgtttggttgttatttttacttatttcttcatatatcaatttatttagagatggacagt    28440

Papio.anubis.clone.rp41-133b2.8034-40080.revcompl.Baboon           ------------------------------------------------------------    0
BCRP3.HUMAN.NCBI.REF                                               ------------------------------------------------------------    0
LOC106996293.glutathione.hydrolase5.proenzyme-like-GGT1.rhesus.    ctcgctctgtcaccaggctggagtgcagtggtgcaatctcagttaactgcaacctctgcc    28500

Papio.anubis.clone.rp41-133b2.8034-40080.revcompl.Baboon           ------------------------------------------------------------    0
BCRP3.HUMAN.NCBI.REF                                               ------------------------------------------------------------    0
LOC106996293.glutathione.hydrolase5.proenzyme-like-GGT1.rhesus.    tcccaagttcaagtgattctcatgtctaagccttccaagtagctgggattacaggcatgt    28560

Papio.anubis.clone.rp41-133b2.8034-40080.revcompl.Baboon           ------------------------------------------------------------    0
BCRP3.HUMAN.NCBI.REF                                               ------------------------------------------------------------    0
LOC106996293.glutathione.hydrolase5.proenzyme-like-GGT1.rhesus.    gccaccacacctagctaatttgtgtgtgtgtgtgtgtgtgtgtgtgtgtgtgtgtgtgtg    28620

Papio.anubis.clone.rp41-133b2.8034-40080.revcompl.Baboon           ------------------------------------------------------------    0
BCRP3.HUMAN.NCBI.REF                                               ------------------------------------------------------------    0
LOC106996293.glutathione.hydrolase5.proenzyme-like-GGT1.rhesus.    tgtgtttctttttctgagacagagtctcactctgtcacccaggctggagggcagtggtgc    28680

Papio.anubis.clone.rp41-133b2.8034-40080.revcompl.Baboon           ------------------------------------------------------------    0
BCRP3.HUMAN.NCBI.REF                                               ------------------------------------------------------------    0
LOC106996293.glutathione.hydrolase5.proenzyme-like-GGT1.rhesus.    gatcttggcttactgcaacctccacctcccgggttcaagtgattctcctgcctcagcctc    28740

Papio.anubis.clone.rp41-133b2.8034-40080.revcompl.Baboon           ------------------------------------------------------------    0
BCRP3.HUMAN.NCBI.REF                                               ------------------------------------------------------------    0
LOC106996293.glutathione.hydrolase5.proenzyme-like-GGT1.rhesus.    cacagtagctgggactacaggcttatgccaccatgcccggctaatttttgtatttttagt    28800

Papio.anubis.clone.rp41-133b2.8034-40080.revcompl.Baboon           ------------------------------------------------------------    0
BCRP3.HUMAN.NCBI.REF                                               ------------------------------------------------------------    0
LOC106996293.glutathione.hydrolase5.proenzyme-like-GGT1.rhesus.    agagacaggatttcactatgttggccaggctggtcttgaactcctgacctcatgatccac    28860

Papio.anubis.clone.rp41-133b2.8034-40080.revcompl.Baboon           ------------------------------------------------------------    0
BCRP3.HUMAN.NCBI.REF                                               ------------------------------------------------------------    0
LOC106996293.glutathione.hydrolase5.proenzyme-like-GGT1.rhesus.    ctgcctctgcctctcaaagtgctgggattacaggcatgagccaccacacctggcttaatt    28920

Papio.anubis.clone.rp41-133b2.8034-40080.revcompl.Baboon           ------------------------------------------------------------    0
BCRP3.HUMAN.NCBI.REF                                               ------------------------------------------------------------    0
LOC106996293.glutathione.hydrolase5.proenzyme-like-GGT1.rhesus.    atggtgtttttagtagagacagggtttcaccatgttggtcaggctggtctcgaactcctg    28980

Papio.anubis.clone.rp41-133b2.8034-40080.revcompl.Baboon           ------------------------------------------------------------    0
BCRP3.HUMAN.NCBI.REF                                               ------------------------------------------------------------    0
LOC106996293.glutathione.hydrolase5.proenzyme-like-GGT1.rhesus.    acctcaggggatccacccacttgggcctcccaaagtgttgggattacaggtgtgagccac    29040

Papio.anubis.clone.rp41-133b2.8034-40080.revcompl.Baboon           ------------------------------------------------------------    0
BCRP3.HUMAN.NCBI.REF                                               ------------------------------------------------------------    0
LOC106996293.glutathione.hydrolase5.proenzyme-like-GGT1.rhesus.    cacgcctggcttattttttttttttttaaggtggagtcttactctgtcacccaggctggag    29100
```

```
Papio.anubis.clone.rp41-133b2.8034-40080.revcompl.Baboon            ------------------------------------------------------------    0
BCRP3.HUMAN.NCBI.REF                                                ------------------------------------------------------------    0
LOC106996293.glutathione.hydrolase5.proenzyme-like-GGT1.rhesus.     tgtaggggagcgatcattgcccactgcagttcaaactcttgggtttaagtgatccccctg    29160

Papio.anubis.clone.rp41-133b2.8034-40080.revcompl.Baboon            ------------------------------------------------------------    0
BCRP3.HUMAN.NCBI.REF                                                ------------------------------------------------------------    0
LOC106996293.glutathione.hydrolase5.proenzyme-like-GGT1.rhesus.     cctcagattcctaaagtactgggattatagctgtgagccatggtgcctggcctctactgt    29220

Papio.anubis.clone.rp41-133b2.8034-40080.revcompl.Baboon            ------------------------------------------------------------    0
BCRP3.HUMAN.NCBI.REF                                                ------------------------------------------------------------    0
LOC106996293.glutathione.hydrolase5.proenzyme-like-GGT1.rhesus.     tttattctattttagactcctatctcatgttatatacaaagacttaaacagaaaatatct    29280

Papio.anubis.clone.rp41-133b2.8034-40080.revcompl.Baboon            ------------------------------------------------------------    0
BCRP3.HUMAN.NCBI.REF                                                ------------------------------------------------------------    0
LOC106996293.glutathione.hydrolase5.proenzyme-like-GGT1.rhesus.     tatgtttttgatattttgagatagggtcttgttctgtcacccagactggagtgcagcggc    29340

Papio.anubis.clone.rp41-133b2.8034-40080.revcompl.Baboon            ------------------------------------------------------------    0
BCRP3.HUMAN.NCBI.REF                                                ------------------------------------------------------------    0
LOC106996293.glutathione.hydrolase5.proenzyme-like-GGT1.rhesus.     atgatcatagctcactgcagccttgagctcttgggcttaagccatctttccacctcagcc    29400

Papio.anubis.clone.rp41-133b2.8034-40080.revcompl.Baboon            ------------------------------------------------------------    0
BCRP3.HUMAN.NCBI.REF                                                ------------------------------------------------------------    0
LOC106996293.glutathione.hydrolase5.proenzyme-like-GGT1.rhesus.     tcctgagtcactaggactacaggtgtgcatcacacctgactaattaaaaaagactgtttt    29460

Papio.anubis.clone.rp41-133b2.8034-40080.revcompl.Baboon            ------------------------------------------------------------    0
BCRP3.HUMAN.NCBI.REF                                                ------------------------------------------------------------    0
LOC106996293.glutathione.hydrolase5.proenzyme-like-GGT1.rhesus.     gtagagatgaggtctcactatattgcccaggctggtcttgaactcctggcctcaagtgat    29520

Papio.anubis.clone.rp41-133b2.8034-40080.revcompl.Baboon            ------------------------------------------------------------    0
BCRP3.HUMAN.NCBI.REF                                                ------------------------------------------------------------    0
LOC106996293.glutathione.hydrolase5.proenzyme-like-GGT1.rhesus.     cctccaccttggcctcccaaagtgttgagattataggtgtaagccaccatctctagccgg    29580

Papio.anubis.clone.rp41-133b2.8034-40080.revcompl.Baboon            ------------------------------------------------------------    0
BCRP3.HUMAN.NCBI.REF                                                ------------------------------------------------------------    0
LOC106996293.glutathione.hydrolase5.proenzyme-like-GGT1.rhesus.     aaaaaaaaaaaaaaaaaaaaaaagttattaataaaatatagcaatttccctttttgtacc    29640

Papio.anubis.clone.rp41-133b2.8034-40080.revcompl.Baboon            ------------------------------------------------------------    0
BCRP3.HUMAN.NCBI.REF                                                ------------------------------------------------------------    0
LOC106996293.glutathione.hydrolase5.proenzyme-like-GGT1.rhesus.     aattataaaagtcatgtacatttgtttgcttgaaaaagaggaaactatctgggcgaggtg    29700

Papio.anubis.clone.rp41-133b2.8034-40080.revcompl.Baboon            ------------------------------------------------------------    0
BCRP3.HUMAN.NCBI.REF                                                ------------------------------------------------------------    0
LOC106996293.glutathione.hydrolase5.proenzyme-like-GGT1.rhesus.     gttcacacctgtaaccccagcattttgggaggctgaggcgggcagaccacccaaggtcac    29760

Papio.anubis.clone.rp41-133b2.8034-40080.revcompl.Baboon            ------------------------------------------------------------    0
BCRP3.HUMAN.NCBI.REF                                                ------------------------------------------------------------    0
LOC106996293.glutathione.hydrolase5.proenzyme-like-GGT1.rhesus.     gagttcgagaccagcctggccaacatggtgaaactccgtccctattaaaattacaaaata    29820

Papio.anubis.clone.rp41-133b2.8034-40080.revcompl.Baboon            ------------------------------------------------------------    0
BCRP3.HUMAN.NCBI.REF                                                ------------------------------------------------------------    0
LOC106996293.glutathione.hydrolase5.proenzyme-like-GGT1.rhesus.     gttagctgggcgtggtggaatgcacctgtaatcccagctacgcaggaggctgaggcagga    29880

Papio.anubis.clone.rp41-133b2.8034-40080.revcompl.Baboon            ------------------------------------------------------------    0
BCRP3.HUMAN.NCBI.REF                                                ------------------------------------------------------------    0
LOC106996293.glutathione.hydrolase5.proenzyme-like-GGT1.rhesus.     gaattgcttgaacccaggaggcggaggttgcagtgagctaagatcacgccactgcactcc    29940

Papio.anubis.clone.rp41-133b2.8034-40080.revcompl.Baboon            ------------------------------------------------------------    0
BCRP3.HUMAN.NCBI.REF                                                ------------------------------------------------------------    0
LOC106996293.glutathione.hydrolase5.proenzyme-like-GGT1.rhesus.     agcctgggtgactgagtgaaactctatcttagaaaaaaaaagaaaaaagaaaaagaggaa    30000

Papio.anubis.clone.rp41-133b2.8034-40080.revcompl.Baboon            ------------------------------------------------------------    0
BCRP3.HUMAN.NCBI.REF                                                ------------------------------------------------------------    0
LOC106996293.glutathione.hydrolase5.proenzyme-like-GGT1.rhesus.     actgtaatcccagcacttcgggaggttgaggcgataggattgctttagaccatgagttca    30060

Papio.anubis.clone.rp41-133b2.8034-40080.revcompl.Baboon            ------------------------------------------------------------    0
BCRP3.HUMAN.NCBI.REF                                                ------------------------------------------------------------    0
LOC106996293.glutathione.hydrolase5.proenzyme-like-GGT1.rhesus.     agaccagcctgggcaacatagaaagaccctatctctacaaaaagacaaaaaattgccagc    30120

Papio.anubis.clone.rp41-133b2.8034-40080.revcompl.Baboon            ------------------------------------------------------------    0
BCRP3.HUMAN.NCBI.REF                                                ------------------------------------------------------------    0
LOC106996293.glutathione.hydrolase5.proenzyme-like-GGT1.rhesus.     catggtggttcttacctgtagtcccagctactcaggagactgaagtgggaggattacttg    30180

Papio.anubis.clone.rp41-133b2.8034-40080.revcompl.Baboon            ------------------------------------------------------------    0
BCRP3.HUMAN.NCBI.REF                                                ------------------------------------------------------------    0
LOC106996293.glutathione.hydrolase5.proenzyme-like-GGT1.rhesus.     agcccaggaggtcaaggctgcactgagccatgacaatgccactgcacttcatcctggtga    30240

Papio.anubis.clone.rp41-133b2.8034-40080.revcompl.Baboon            ------------------------------------------------------------    0
BCRP3.HUMAN.NCBI.REF                                                ------------------------------------------------------------    0
LOC106996293.glutathione.hydrolase5.proenzyme-like-GGT1.rhesus.     caaggtgagactctgtcttataaaacaaaggccaggcacagtggctcacacctgtaatac    30300

Papio.anubis.clone.rp41-133b2.8034-40080.revcompl.Baboon            ------------------------------------------------------------    0
BCRP3.HUMAN.NCBI.REF                                                ------------------------------------------------------------    0
LOC106996293.glutathione.hydrolase5.proenzyme-like-GGT1.rhesus.     cagcactttgggaggccaaggtgggtggattacttgaacccaggagttcttgaccagcct    30360

Papio.anubis.clone.rp41-133b2.8034-40080.revcompl.Baboon            ------------------------------------------------------------    0
BCRP3.HUMAN.NCBI.REF                                                ------------------------------------------------------------    0
LOC106996293.glutathione.hydrolase5.proenzyme-like-GGT1.rhesus.     gggcaacatggcaaaatccgatctttacaaaacacacaaacaaacaaaattggctgggca    30420

Papio.anubis.clone.rp41-133b2.8034-40080.revcompl.Baboon            ------------------------------------------------------------    0
BCRP3.HUMAN.NCBI.REF                                                ------------------------------------------------------------    0
LOC106996293.glutathione.hydrolase5.proenzyme-like-GGT1.rhesus.     tggtggcatgtgcctatagtcccagctacttgggaggctgaggtgggagggtcaattgag    30480

Papio.anubis.clone.rp41-133b2.8034-40080.revcompl.Baboon            ------------------------------------------------------------    0
BCRP3.HUMAN.NCBI.REF                                                ------------------------------------------------------------    0
LOC106996293.glutathione.hydrolase5.proenzyme-like-GGT1.rhesus.     cccaggagattgaggctgctgtgatctgagatcacaccactgcactccagcctgagcaac    30540

Papio.anubis.clone.rp41-133b2.8034-40080.revcompl.Baboon            ------------------------------------------------------------    0
BCRP3.HUMAN.NCBI.REF                                                ------------------------------------------------------------    0
LOC106996293.glutathione.hydrolase5.proenzyme-like-GGT1.rhesus.     aaagagagacaaaaaaaaaaaaaaaaaaggcgacaaggtgggtggatcatgagctcaaga    30600

Papio.anubis.clone.rp41-133b2.8034-40080.revcompl.Baboon            ------------------------------------------------------------    0
BCRP3.HUMAN.NCBI.REF                                                ------------------------------------------------------------    0
LOC106996293.glutathione.hydrolase5.proenzyme-like-GGT1.rhesus.     gatggagaccatcctggccaacatagtgaagccccatctctcctaaaaatataaaaatta    30660

Papio.anubis.clone.rp41-133b2.8034-40080.revcompl.Baboon            ------------------------------------------------------------    0
BCRP3.HUMAN.NCBI.REF                                                ------------------------------------------------------------    0
LOC106996293.glutathione.hydrolase5.proenzyme-like-GGT1.rhesus.     gccaggggtggtggcgtgtgcctgtagtcccagctactcgggaggctgaggcaggagaat    30720

Papio.anubis.clone.rp41-133b2.8034-40080.revcompl.Baboon            ------------------------------------------------------------    0
BCRP3.HUMAN.NCBI.REF                                                ------------------------------------------------------------    0
LOC106996293.glutathione.hydrolase5.proenzyme-like-GGT1.rhesus.     ggcgtgaacccaggaggcagaggttgcagtgagctgagatagtgccactgcactcaggcc    30780

Papio.anubis.clone.rp41-133b2.8034-40080.revcompl.Baboon            ------------------------------------------------------------    0
BCRP3.HUMAN.NCBI.REF                                                ------------------------------------------------------------    0
LOC106996293.glutathione.hydrolase5.proenzyme-like-GGT1.rhesus.     tggcaacagagcaagattctgcctcaaaaaaaaaaagaaaaagaaaaactaaaaacaaaa    30840
```

```
Papio.anubis.clone.rp41-133b2.8034-40080.revcompl.Baboon            ------------------------------------------------------------    0
BCRP3.HUMAN.NCBI.REF                                                ------------------------------------------------------------    0
LOC106996293.glutathione.hydrolase5.proenzyme-like-GGT1.rhesus.     accccaaaactcgaatggacttctcttccatcctccgttaggcaggtgggcagcagggtg    30900

Papio.anubis.clone.rp41-133b2.8034-40080.revcompl.Baboon            ------------------------------------------------------------    0
BCRP3.HUMAN.NCBI.REF                                                ------------------------------------------------------------    0
LOC106996293.glutathione.hydrolase5.proenzyme-like-GGT1.rhesus.     tgaatgtggggccaggatggaagcctgcaggttctcacgtctttatgtgccacatggcag    30960

Papio.anubis.clone.rp41-133b2.8034-40080.revcompl.Baboon            ------------------------------------------------------------    0
BCRP3.HUMAN.NCBI.REF                                                ------------------------------------------------------------    0
LOC106996293.glutathione.hydrolase5.proenzyme-like-GGT1.rhesus.     ggacgcactgcaggacggtggctctgcggtggatgcagccattgcagccctgttgtgtat    31020

Papio.anubis.clone.rp41-133b2.8034-40080.revcompl.Baboon            ------------------------------------------------------------    0
BCRP3.HUMAN.NCBI.REF                                                ------------------------------------------------------------    0
LOC106996293.glutathione.hydrolase5.proenzyme-like-GGT1.rhesus.     ggggctcatgaatgcccacagcatgggcatcgggggcggcctgttcctcaccatctacaa    31080

Papio.anubis.clone.rp41-133b2.8034-40080.revcompl.Baboon            ------------------------------------------------------------    0
BCRP3.HUMAN.NCBI.REF                                                ------------------------------------------------------------    0
LOC106996293.glutathione.hydrolase5.proenzyme-like-GGT1.rhesus.     cagcaccacgcgtgagtgccttgggagaggcgagggagaggggcagggggtgtgggttgg    31140

Papio.anubis.clone.rp41-133b2.8034-40080.revcompl.Baboon            ------------------------------------------------------------    0
BCRP3.HUMAN.NCBI.REF                                                ------------------------------------------------------------    0
LOC106996293.glutathione.hydrolase5.proenzyme-like-GGT1.rhesus.     gccgcggcacagctgggtggtccctgggctcacgcagcataaagggtttgggtgggtctg    31200

Papio.anubis.clone.rp41-133b2.8034-40080.revcompl.Baboon            ------------------------------------------------------------    0
BCRP3.HUMAN.NCBI.REF                                                ------------------------------------------------------------    0
LOC106996293.glutathione.hydrolase5.proenzyme-like-GGT1.rhesus.     cctgcctacctgcttctccttctaggaaaagctgaggtcatcaacgcccgcgaggtggcc    31260

Papio.anubis.clone.rp41-133b2.8034-40080.revcompl.Baboon            ------------------------------------------------------------    0
BCRP3.HUMAN.NCBI.REF                                                ------------------------------------------------------------    0
LOC106996293.glutathione.hydrolase5.proenzyme-like-GGT1.rhesus.     cccaggctggcctttgccagcatgttcaacagctcacagcagtcccagaatggtaagctg    31320

Papio.anubis.clone.rp41-133b2.8034-40080.revcompl.Baboon            ------------------------------------------------------------    0
BCRP3.HUMAN.NCBI.REF                                                ------------------------------------------------------------    0
LOC106996293.glutathione.hydrolase5.proenzyme-like-GGT1.rhesus.     tcctgcaagcttggggtgtgggtgcagagccagctgggccactgggaaggggccttgccc    31380

Papio.anubis.clone.rp41-133b2.8034-40080.revcompl.Baboon            ------------------------------------------------------------    0
BCRP3.HUMAN.NCBI.REF                                                ------------------------------------------------------------    0
LOC106996293.glutathione.hydrolase5.proenzyme-like-GGT1.rhesus.     acaggatcctggccctgtcagggtccaggggcagttctagcacctccatcccttcctggc    31440

Papio.anubis.clone.rp41-133b2.8034-40080.revcompl.Baboon            ------------------------------------------------------------    0
BCRP3.HUMAN.NCBI.REF                                                ------------------------------------------------------------    0
LOC106996293.glutathione.hydrolase5.proenzyme-like-GGT1.rhesus.     cccatagcaccctcccacaatgagtggtcaggaccatcctcaccagggtaaagggctggg    31500

Papio.anubis.clone.rp41-133b2.8034-40080.revcompl.Baboon            ------------------------------------------------------------    0
BCRP3.HUMAN.NCBI.REF                                                ------------------------------------------------------------    0
LOC106996293.glutathione.hydrolase5.proenzyme-like-GGT1.rhesus.     agcttctgttatttctgctaaggcctccggggccaccctgtgcagcacacagagaataat    31560

Papio.anubis.clone.rp41-133b2.8034-40080.revcompl.Baboon            ------------------------------------------------------------    0
BCRP3.HUMAN.NCBI.REF                                                ------------------------------------------------------------    0
LOC106996293.glutathione.hydrolase5.proenzyme-like-GGT1.rhesus.     tattacactagcagaccttatggaccagggctcactggggcccacgctctgctctgtgct    31620

Papio.anubis.clone.rp41-133b2.8034-40080.revcompl.Baboon            ------------------------------------------------------------    0
BCRP3.HUMAN.NCBI.REF                                                ------------------------------------------------------------    0
LOC106996293.glutathione.hydrolase5.proenzyme-like-GGT1.rhesus.     tttcatccatgagtccctcacacccctccctgctcctttggggtaggggatgctatgtgg    31680

Papio.anubis.clone.rp41-133b2.8034-40080.revcompl.Baboon            ------------------------------------------------------------    0
BCRP3.HUMAN.NCBI.REF                                                ------------------------------------------------------------    0
LOC106996293.glutathione.hydrolase5.proenzyme-like-GGT1.rhesus.     gttcccattttacagggtgcagatgctgaggcccagagaggtcatgaaaatcagctgaag    31740

Papio.anubis.clone.rp41-133b2.8034-40080.revcompl.Baboon            ------------------------------------------------------------    0
BCRP3.HUMAN.NCBI.REF                                                ------------------------------------------------------------    0
LOC106996293.glutathione.hydrolase5.proenzyme-like-GGT1.rhesus.     tcatacagctgggaggtggtgaagttaaaattgaacccaagctgtctataccctgccttt    31800

Papio.anubis.clone.rp41-133b2.8034-40080.revcompl.Baboon            ------------------------------------------------------------    0
BCRP3.HUMAN.NCBI.REF                                                ------------------------------------------------------------    0
LOC106996293.glutathione.hydrolase5.proenzyme-like-GGT1.rhesus.     tcaacaggcatcccattcacttatttgttcatttgtggggatggggctctagagtgtgag    31860

Papio.anubis.clone.rp41-133b2.8034-40080.revcompl.Baboon            ------------------------------------------------------------    0
BCRP3.HUMAN.NCBI.REF                                                ------------------------------------------------------------    0
LOC106996293.glutathione.hydrolase5.proenzyme-like-GGT1.rhesus.     gtggagtctctcttttctaatctggtcttcagtggagaggaggcccccaaattccccagg    31920

Papio.anubis.clone.rp41-133b2.8034-40080.revcompl.Baboon            ------------------------------------------------------------    0
BCRP3.HUMAN.NCBI.REF                                                ------------------------------------------------------------    0
LOC106996293.glutathione.hydrolase5.proenzyme-like-GGT1.rhesus.     tacctgaggggaagccactgtccatccaggaagccactgtccatcctcaaaggaggcaca    31980

Papio.anubis.clone.rp41-133b2.8034-40080.revcompl.Baboon            ------------------------------------------------------------    0
BCRP3.HUMAN.NCBI.REF                                                ------------------------------------------------------------    0
LOC106996293.glutathione.hydrolase5.proenzyme-like-GGT1.rhesus.     atagattatgagattaaaaattggaagattgggtgggtctcaacagctcacgcctataat    32040

Papio.anubis.clone.rp41-133b2.8034-40080.revcompl.Baboon            ------------------------------------------------------------    0
BCRP3.HUMAN.NCBI.REF                                                ------------------------------------------------------------    0
LOC106996293.glutathione.hydrolase5.proenzyme-like-GGT1.rhesus.     cctagtacttaaggaggccaagtcaggaggactgcatcagcccaggagtttgagaccagc    32100

Papio.anubis.clone.rp41-133b2.8034-40080.revcompl.Baboon            ------------------------------------------------------------    0
BCRP3.HUMAN.NCBI.REF                                                ------------------------------------------------------------    0
LOC106996293.glutathione.hydrolase5.proenzyme-like-GGT1.rhesus.     ctgggcaacgtagcaagactccgtctctacaaaaaatgaaaaaaaaaaaaattagccagg    32160

Papio.anubis.clone.rp41-133b2.8034-40080.revcompl.Baboon            ------------------------------------------------------------    0
BCRP3.HUMAN.NCBI.REF                                                ------------------------------------------------------------    0
LOC106996293.glutathione.hydrolase5.proenzyme-like-GGT1.rhesus.     tgtggtggtatgtgtctatggtcccagctcctcgggaggctgaggtgggaggatcacttg    32220

Papio.anubis.clone.rp41-133b2.8034-40080.revcompl.Baboon            ------------------------------------------------------------    0
BCRP3.HUMAN.NCBI.REF                                                ------------------------------------------------------------    0
LOC106996293.glutathione.hydrolase5.proenzyme-like-GGT1.rhesus.     agcccatgaggttgaggctgcagtgagccatgattgtaccactgcactctagcttgggca    32280

Papio.anubis.clone.rp41-133b2.8034-40080.revcompl.Baboon            ------------------------------------------------------------    0
BCRP3.HUMAN.NCBI.REF                                                ------------------------------------------------------------    0
LOC106996293.glutathione.hydrolase5.proenzyme-like-GGT1.rhesus.     acaaagcaagaccctgtcaaaaaaaaaaaaaaaatgctggatgtggtggctcacgcttgt    32340

Papio.anubis.clone.rp41-133b2.8034-40080.revcompl.Baboon            ------------------------------------------------------------    0
BCRP3.HUMAN.NCBI.REF                                                ------------------------------------------------------------    0
LOC106996293.glutathione.hydrolase5.proenzyme-like-GGT1.rhesus.     aatcccagcactctgggaggctgaggcgggtggatcacctgggtcaggagttcaagacca    32400

Papio.anubis.clone.rp41-133b2.8034-40080.revcompl.Baboon            ------------------------------------------------------------    0
BCRP3.HUMAN.NCBI.REF                                                ------------------------------------------------------------    0
LOC106996293.glutathione.hydrolase5.proenzyme-like-GGT1.rhesus.     tcctggccaacatagtgaaaccctgtctctactaaaagcataataattagccaggcatgt    32460

Papio.anubis.clone.rp41-133b2.8034-40080.revcompl.Baboon            ------------------------------------------------------------    0
BCRP3.HUMAN.NCBI.REF                                                ------------------------------------------------------------    0
LOC106996293.glutathione.hydrolase5.proenzyme-like-GGT1.rhesus.     tggcatgggcctgtaatcccagctactcaggagtctaaggcaggagaatcgcttgaaccc    32520

Papio.anubis.clone.rp41-133b2.8034-40080.revcompl.Baboon            ------------------------------------------------------------    0
BCRP3.HUMAN.NCBI.REF                                                ------------------------------------------------------------    0
LOC106996293.glutathione.hydrolase5.proenzyme-like-GGT1.rhesus.     gggaggtggaggttacagtgagccgagatcatgccactgtactccagcctgggcaacaag    32580
```

```
Papio.anubis.clone.rp41-133b2.8034-40080.revcompl.Baboon              ------------------------------------------------------------    0
BCRP3.HUMAN.NCBI.REF                                                  ------------------------------------------------------------    0
LOC106996293.glutathione.hydrolase5.proenzyme-like-GGT1.rhesus.       agcgaagctcggtctcaaaaaaaaaaaaaagttggaggatggaggggcagggcacactca    32640

Papio.anubis.clone.rp41-133b2.8034-40080.revcompl.Baboon              ------------------------------------------------------------    0
BCRP3.HUMAN.NCBI.REF                                                  ------------------------------------------------------------    0
LOC106996293.glutathione.hydrolase5.proenzyme-like-GGT1.rhesus.       ccatagcaagtcttagacctcaggtgaggatcctgggtggtgccctttggagtcttctac    32700

Papio.anubis.clone.rp41-133b2.8034-40080.revcompl.Baboon              ------------------------------------------------------------    0
BCRP3.HUMAN.NCBI.REF                                                  ------------------------------------------------------------    0
LOC106996293.glutathione.hydrolase5.proenzyme-like-GGT1.rhesus.       aacatactcaatctttgattttttttttttttttttttttgagatggagtctcactcctg    32760

Papio.anubis.clone.rp41-133b2.8034-40080.revcompl.Baboon              ------------------------------------------------------------    0
BCRP3.HUMAN.NCBI.REF                                                  ------------------------------------------------------------    0
LOC106996293.glutathione.hydrolase5.proenzyme-like-GGT1.rhesus.       ttgcccagactagagtgcagtggtgcaatctctgctcactgcaacctccgcctcccagtt    32820

Papio.anubis.clone.rp41-133b2.8034-40080.revcompl.Baboon              ------------------------------------------------------------    0
BCRP3.HUMAN.NCBI.REF                                                  ------------------------------------------------------------    0
LOC106996293.glutathione.hydrolase5.proenzyme-like-GGT1.rhesus.       tcaagtgattctcctgcctcagcctcccatagctgggattacaggttcccggggcccagc    32880

Papio.anubis.clone.rp41-133b2.8034-40080.revcompl.Baboon              ------------------------------------------------------------    0
BCRP3.HUMAN.NCBI.REF                                                  ------------------------------------------------------------    0
LOC106996293.glutathione.hydrolase5.proenzyme-like-GGT1.rhesus.       caattttttctatttttagtagagagggggtttcactatgttgcccaggttggtctcgaa    32940

Papio.anubis.clone.rp41-133b2.8034-40080.revcompl.Baboon              ------------------------------------------------------------    0
BCRP3.HUMAN.NCBI.REF                                                  ------------------------------------------------------------    0
LOC106996293.glutathione.hydrolase5.proenzyme-like-GGT1.rhesus.       ctcctgacctcatgatctgcacccccccccgccctcggccttccaaagtgctggaattac    33000

Papio.anubis.clone.rp41-133b2.8034-40080.revcompl.Baboon              ------------------------------------------------------------    0
BCRP3.HUMAN.NCBI.REF                                                  ------------------------------------------------------------    0
LOC106996293.glutathione.hydrolase5.proenzyme-like-GGT1.rhesus.       aggtgtgagctactgcaccgaccaatctttcatttttttaatactcattaagaaactcag    33060

Papio.anubis.clone.rp41-133b2.8034-40080.revcompl.Baboon              ------------------------------------------------------------    0
BCRP3.HUMAN.NCBI.REF                                                  ------------------------------------------------------------    0
LOC106996293.glutathione.hydrolase5.proenzyme-like-GGT1.rhesus.       catctgtagacatgaagttgctcagggtaagagaatgcaggaatcataggcttggtacct    33120

Papio.anubis.clone.rp41-133b2.8034-40080.revcompl.Baboon              ------------------------------------------------------------    0
BCRP3.HUMAN.NCBI.REF                                                  ------------------------------------------------------------    0
LOC106996293.glutathione.hydrolase5.proenzyme-like-GGT1.rhesus.       tgtggatgcttggaatcatttctttttttttttttttttttttgagacggagtctcgctc    33180

Papio.anubis.clone.rp41-133b2.8034-40080.revcompl.Baboon              ------------------------------------------------------------    0
BCRP3.HUMAN.NCBI.REF                                                  ------------------------------------------------------------    0
LOC106996293.glutathione.hydrolase5.proenzyme-like-GGT1.rhesus.       tgtcacccaggctggagtgcagtggccggatctcagctcactgcaagctccgccccccgg    33240

Papio.anubis.clone.rp41-133b2.8034-40080.revcompl.Baboon              ------------------------------------------------------------    0
BCRP3.HUMAN.NCBI.REF                                                  ------------------------------------------------------------    0
LOC106996293.glutathione.hydrolase5.proenzyme-like-GGT1.rhesus.       gtttacgccattctcctgcctcagcctcccgagtagctgggactacaggcgcccaccatc    33300

Papio.anubis.clone.rp41-133b2.8034-40080.revcompl.Baboon              ------------------------------------------------------------    0
BCRP3.HUMAN.NCBI.REF                                                  ------------------------------------------------------------    0
LOC106996293.glutathione.hydrolase5.proenzyme-like-GGT1.rhesus.       tcgcccggctagtttttttgtatttttttagtagagacaaggtttcaccgggttagccatg    33360

Papio.anubis.clone.rp41-133b2.8034-40080.revcompl.Baboon              ------------------------------------------------------------    0
BCRP3.HUMAN.NCBI.REF                                                  ------------------------------------------------------------    0
LOC106996293.glutathione.hydrolase5.proenzyme-like-GGT1.rhesus.       atggtctcgatctcctaacctcgtgatccgcccgtctcggcctcctaaagtgctgggatt    33420

Papio.anubis.clone.rp41-133b2.8034-40080.revcompl.Baboon              ------------------------------------------------------------    0
BCRP3.HUMAN.NCBI.REF                                                  ------------------------------------------------------------    0
LOC106996293.glutathione.hydrolase5.proenzyme-like-GGT1.rhesus.       acaggcttgagccaccgcgcccggcctgaaatcgtttctttaactagaatgtattgagca    33480

Papio.anubis.clone.rp41-133b2.8034-40080.revcompl.Baboon              ------------------------------------------------------------    0
BCRP3.HUMAN.NCBI.REF                                                  ------------------------------------------------------------    0
LOC106996293.glutathione.hydrolase5.proenzyme-like-GGT1.rhesus.       tcatttaaagaatcaggtgttgttcctgaagctggggtgaaaaacaaagatggcagatga    33540

Papio.anubis.clone.rp41-133b2.8034-40080.revcompl.Baboon              ------------------------------------------------------------    0
BCRP3.HUMAN.NCBI.REF                                                  ------------------------------------------------------------    0
LOC106996293.glutathione.hydrolase5.proenzyme-like-GGT1.rhesus.       aatctgtgacattccaggtgggaaggagaaactaggcaggtgtgggtgtgttacgggctg    33600

Papio.anubis.clone.rp41-133b2.8034-40080.revcompl.Baboon              ------------------------------------------------------------    0
BCRP3.HUMAN.NCBI.REF                                                  ------------------------------------------------------------    0
LOC106996293.glutathione.hydrolase5.proenzyme-like-GGT1.rhesus.       tggaaaaacagacctgggggcctcaaaatctgggattctatggagggtgacctagtcag    33660

Papio.anubis.clone.rp41-133b2.8034-40080.revcompl.Baboon              ------------------------------------------------------------    0
BCRP3.HUMAN.NCBI.REF                                                  ------------------------------------------------------------    0
LOC106996293.glutathione.hydrolase5.proenzyme-like-GGT1.rhesus.       ggaggggatatttgagcaaagaccgagaggcaggggtggggtcaggggagctttgtggtg    33720

Papio.anubis.clone.rp41-133b2.8034-40080.revcompl.Baboon              ------------------------------------------------------------    0
BCRP3.HUMAN.NCBI.REF                                                  ------------------------------------------------------------    0
LOC106996293.glutathione.hydrolase5.proenzyme-like-GGT1.rhesus.       tctgggggaagaccaagtgatccaggtgtgatgggcagggacagagccctgtggggagca    33780

Papio.anubis.clone.rp41-133b2.8034-40080.revcompl.Baboon              ------------------------------------------------------------    0
BCRP3.HUMAN.NCBI.REF                                                  ------------------------------------------------------------    0
LOC106996293.glutathione.hydrolase5.proenzyme-like-GGT1.rhesus.       ggggaggctgcagcaattgacccgaggggaacggcccaggttgtgtgggatctcttaggt    33840

Papio.anubis.clone.rp41-133b2.8034-40080.revcompl.Baboon              ------------------------------------------------------------    0
BCRP3.HUMAN.NCBI.REF                                                  ------------------------------------------------------------    0
LOC106996293.glutathione.hydrolase5.proenzyme-like-GGT1.rhesus.       catggtgaagcctctgcctcttgtctcaaagaagtgggggcctgtggagtgtgtgagtgg    33900

Papio.anubis.clone.rp41-133b2.8034-40080.revcompl.Baboon              ------------------------------------------------------------    0
BCRP3.HUMAN.NCBI.REF                                                  ------------------------------------------------------------    0
LOC106996293.glutathione.hydrolase5.proenzyme-like-GGT1.rhesus.       agggggatgtatctgacctgtggacccccttgggttgctgtgtaagggacagggagcctgt    33960

Papio.anubis.clone.rp41-133b2.8034-40080.revcompl.Baboon              ------------------------------------------------------------    0
BCRP3.HUMAN.NCBI.REF                                                  ------------------------------------------------------------    0
LOC106996293.glutathione.hydrolase5.proenzyme-like-GGT1.rhesus.       ggggacctgtccggaggtcactgcagtaattcatggagagggtggtgggaagtggctgac    34020

Papio.anubis.clone.rp41-133b2.8034-40080.revcompl.Baboon              ------------------------------------------------------------    0
BCRP3.HUMAN.NCBI.REF                                                  ------------------------------------------------------------    0
LOC106996293.glutathione.hydrolase5.proenzyme-like-GGT1.rhesus.       actggacagacacacttggaagtggagcctgtggatttgaggatgggttgggtgtgatgc    34080

Papio.anubis.clone.rp41-133b2.8034-40080.revcompl.Baboon              ------------------------------------------------------------    0
BCRP3.HUMAN.NCBI.REF                                                  ------------------------------------------------------------    0
LOC106996293.glutathione.hydrolase5.proenzyme-like-GGT1.rhesus.       tggtgtagggagtccccgaggaccccagatcctttgtctgtacctggaaggatggggtga    34140

Papio.anubis.clone.rp41-133b2.8034-40080.revcompl.Baboon              ------------------------------------------------------------    0
BCRP3.HUMAN.NCBI.REF                                                  ------------------------------------------------------------    0
LOC106996293.glutathione.hydrolase5.proenzyme-like-GGT1.rhesus.       ccctaacggagacaggcagggctctcaggaagcaggtctgcgtggacactcaggagctcg    34200

Papio.anubis.clone.rp41-133b2.8034-40080.revcompl.Baboon              ------------------------------------------------------------    0
BCRP3.HUMAN.NCBI.REF                                                  ------------------------------------------------------------    0
LOC106996293.glutathione.hydrolase5.proenzyme-like-GGT1.rhesus.       gttttgggcacgtttaagtttgagatgctttcttcgagcaggcaggtagatacttgcgtc    34260

Papio.anubis.clone.rp41-133b2.8034-40080.revcompl.Baboon              ------------------------------------------------------------    0
BCRP3.HUMAN.NCBI.REF                                                  ------------------------------------------------------------    0
LOC106996293.glutathione.hydrolase5.proenzyme-like-GGT1.rhesus.       tggcgatttgaggagcagtccaggccagcaggtcaatttggcagttgtcagtgcttaaat    34320
```

```
Papio.anubis.clone.rp41-133b2.8034-40080.revcompl.Baboon          ------------------------------------------------------------    0
BCRP3.HUMAN.NCBI.REF                                              ------------------------------------------------------------    0
LOC106996293.glutathione.hydrolase5.proenzyme-like-GGT1.rhesus.   ggaggctgaagctcagatgtcagacagaccaccaggagagagagcaaagacagagggcag    34380

Papio.anubis.clone.rp41-133b2.8034-40080.revcompl.Baboon          ------------------------------------------------------------    0
BCRP3.HUMAN.NCBI.REF                                              ------------------------------------------------------------    0
LOC106996293.glutathione.hydrolase5.proenzyme-like-GGT1.rhesus.   agcaggagctaggatggcaggcgggggacacacgggtggagccaggtgctgggatgcagg    34440

Papio.anubis.clone.rp41-133b2.8034-40080.revcompl.Baboon          ------------------------------------------------------------    0
BCRP3.HUMAN.NCBI.REF                                              ------------------------------------------------------------    0
LOC106996293.glutathione.hydrolase5.proenzyme-like-GGT1.rhesus.   ggtggctctcagggagagtgatgagcctagtaaagctgagagggggcactgggtctggca    34500

Papio.anubis.clone.rp41-133b2.8034-40080.revcompl.Baboon          ------------------------------------------------------------    0
BCRP3.HUMAN.NCBI.REF                                              ------------------------------------------------------------    0
LOC106996293.glutathione.hydrolase5.proenzyme-like-GGT1.rhesus.   gtgtgggggtcgccggagagcttggcaagtatagtggcatgacagcctgattggagtgag    34560

Papio.anubis.clone.rp41-133b2.8034-40080.revcompl.Baboon          ------------------------------------------------------------    0
BCRP3.HUMAN.NCBI.REF                                              ------------------------------------------------------------    0
LOC106996293.glutathione.hydrolase5.proenzyme-like-GGT1.rhesus.   gtcaggagaagatttttttctgatattgatacatgatattttctatacttatgggtacat    34620

Papio.anubis.clone.rp41-133b2.8034-40080.revcompl.Baboon          ------------------------------------------------------------    0
BCRP3.HUMAN.NCBI.REF                                              ------------------------------------------------------------    0
LOC106996293.glutathione.hydrolase5.proenzyme-like-GGT1.rhesus.   gtgagtgcttgttacatgcatagtgtatggtgatcaaggcagggtatttggggtctccat    34680

Papio.anubis.clone.rp41-133b2.8034-40080.revcompl.Baboon          ------------------------------------------------------------    0
BCRP3.HUMAN.NCBI.REF                                              ------------------------------------------------------------    0
LOC106996293.glutathione.hydrolase5.proenzyme-like-GGT1.rhesus.   caccttgagtatttttcattcctgtttgttagcaccatagtcttctcttccagttactct    34740

Papio.anubis.clone.rp41-133b2.8034-40080.revcompl.Baboon          ------------------------------------------------------------    0
BCRP3.HUMAN.NCBI.REF                                              ------------------------------------------------------------    0
LOC106996293.glutathione.hydrolase5.proenzyme-like-GGT1.rhesus.   gaaatatacaaaatactgttgttaagcattgtcaccccgctctgctgtcaaacattagaa    34800

Papio.anubis.clone.rp41-133b2.8034-40080.revcompl.Baboon          ------------------------------------------------------------    0
BCRP3.HUMAN.NCBI.REF                                              ------------------------------------------------------------    0
LOC106996293.glutathione.hydrolase5.proenzyme-like-GGT1.rhesus.   ctgctgtcttgactgggcctgcggtgcggtggctcatgcctgtcatcccaacaccttgga    34860

Papio.anubis.clone.rp41-133b2.8034-40080.revcompl.Baboon          ------------------------------------------------------------    0
BCRP3.HUMAN.NCBI.REF                                              ------------------------------------------------------------    0
LOC106996293.glutathione.hydrolase5.proenzyme-like-GGT1.rhesus.   aagctgaggtgggtggattacctgaggtcaggagtttgaaaccagccttgccaacatggc    34920

Papio.anubis.clone.rp41-133b2.8034-40080.revcompl.Baboon          ------------------------------------------------------------    0
BCRP3.HUMAN.NCBI.REF                                              ------------------------------------------------------------    0
LOC106996293.glutathione.hydrolase5.proenzyme-like-GGT1.rhesus.   gaaaccccatctctactaaaaatacaaaaattagcccggcgttctggcatgtgcctgtaa    34980

Papio.anubis.clone.rp41-133b2.8034-40080.revcompl.Baboon          ------------------------------------------------------------    0
BCRP3.HUMAN.NCBI.REF                                              ------------------------------------------------------------    0
LOC106996293.glutathione.hydrolase5.proenzyme-like-GGT1.rhesus.   tcccagctacttgggaggctgaggcaggagagttacttgaacccaggaggcggaggttgc    35040

Papio.anubis.clone.rp41-133b2.8034-40080.revcompl.Baboon          ------------------------------------------------------------    0
BCRP3.HUMAN.NCBI.REF                                              ------------------------------------------------------------    0
LOC106996293.glutathione.hydrolase5.proenzyme-like-GGT1.rhesus.   agtgagctgagatcatgccactgaactccagtctgggacacacagcaagactgtctcaaa    35100

Papio.anubis.clone.rp41-133b2.8034-40080.revcompl.Baboon          ------------------------------------------------------------    0
BCRP3.HUMAN.NCBI.REF                                              ------------------------------------------------------------    0
LOC106996293.glutathione.hydrolase5.proenzyme-like-GGT1.rhesus.   aaaaaaaaaaaagaaagaaagaaaaaagaaaaaaaaagaagttctgtctaactgcaaggg    35160

Papio.anubis.clone.rp41-133b2.8034-40080.revcompl.Baboon          ------------------------------------------------------------    0
BCRP3.HUMAN.NCBI.REF                                              ------------------------------------------------------------    0
LOC106996293.glutathione.hydrolase5.proenzyme-like-GGT1.rhesus.   ggaaggaatcatgtgctgggtgtcagaccttccaggatgtatgtgtgcagcttctaagaa    35220

Papio.anubis.clone.rp41-133b2.8034-40080.revcompl.Baboon          ------------------------------------------------------------    0
BCRP3.HUMAN.NCBI.REF                                              ------------------------------------------------------------    0
LOC106996293.glutathione.hydrolase5.proenzyme-like-GGT1.rhesus.   ttgaaaccactggctcttggaaacttgtgccaggcttcagggtgggagaggtagttctag    35280

Papio.anubis.clone.rp41-133b2.8034-40080.revcompl.Baboon          ------------------------------------------------------------    0
BCRP3.HUMAN.NCBI.REF                                              ------------------------------------------------------------    0
LOC106996293.glutathione.hydrolase5.proenzyme-like-GGT1.rhesus.   agccacagccaccaagcctgggagtagccaaatggcccccgtcatctctggcgagagcag    35340

Papio.anubis.clone.rp41-133b2.8034-40080.revcompl.Baboon          ------------------------------------------------------------    0
BCRP3.HUMAN.NCBI.REF                                              ------------------------------------------------------------    0
LOC106996293.glutathione.hydrolase5.proenzyme-like-GGT1.rhesus.   gaaaatacctgggcgcagaggccacggttgtatctttctgggcaaagggtcagtgtctat    35400

Papio.anubis.clone.rp41-133b2.8034-40080.revcompl.Baboon          ------------------------------------------------------------    0
BCRP3.HUMAN.NCBI.REF                                              ------------------------------------------------------------    0
LOC106996293.glutathione.hydrolase5.proenzyme-like-GGT1.rhesus.   agtgtacatatggtcagtgaggctcgggggggaagcaggcccaggggtctgtttccagtga    35460

Papio.anubis.clone.rp41-133b2.8034-40080.revcompl.Baboon          ------------------------------------------------------------    0
BCRP3.HUMAN.NCBI.REF                                              ------------------------------------------------------------    0
LOC106996293.glutathione.hydrolase5.proenzyme-like-GGT1.rhesus.   cctcctcgaaagtcagaactggaaggcaaaaccccttataggctgggtgcacctgtagtc    35520

Papio.anubis.clone.rp41-133b2.8034-40080.revcompl.Baboon          ------------------------------------------------------------    0
BCRP3.HUMAN.NCBI.REF                                              ------------------------------------------------------------    0
LOC106996293.glutathione.hydrolase5.proenzyme-like-GGT1.rhesus.   ccagtatgtcgggatgctgaggtgggaggatcacttgaggccaggagtttgagaccagtc    35580

Papio.anubis.clone.rp41-133b2.8034-40080.revcompl.Baboon          ------------------------------------------------------------    0
BCRP3.HUMAN.NCBI.REF                                              ------------------------------------------------------------    0
LOC106996293.glutathione.hydrolase5.proenzyme-like-GGT1.rhesus.   ttggcaacatagcaagaccccgtctctacaaaaaaataaacaataaaaaattaggctcgg    35640

Papio.anubis.clone.rp41-133b2.8034-40080.revcompl.Baboon          ------------------------------------------------------------    0
BCRP3.HUMAN.NCBI.REF                                              ------------------------------------------------------------    0
LOC106996293.glutathione.hydrolase5.proenzyme-like-GGT1.rhesus.   tgtggtggctcacacctgtaatcacagaactttgagaggctgagcggggtggatcacctg    35700

Papio.anubis.clone.rp41-133b2.8034-40080.revcompl.Baboon          ------------------------------------------------------------    0
BCRP3.HUMAN.NCBI.REF                                              ------------------------------------------------------------    0
LOC106996293.glutathione.hydrolase5.proenzyme-like-GGT1.rhesus.   atgttagaaattcaggaccagcctgacgaacctggtgaaacctgtctctactaaaattac    35760

Papio.anubis.clone.rp41-133b2.8034-40080.revcompl.Baboon          ------------------------------------------------------------    0
BCRP3.HUMAN.NCBI.REF                                              ------------------------------------------------------------    0
LOC106996293.glutathione.hydrolase5.proenzyme-like-GGT1.rhesus.   aaaaattagcaaggcgtggtggtgcatgcctgtaatcccagctacttgggaggctgaggc    35820

Papio.anubis.clone.rp41-133b2.8034-40080.revcompl.Baboon          ------------------------------------------------------------    0
BCRP3.HUMAN.NCBI.REF                                              ------------------------------------------------------------    0
LOC106996293.glutathione.hydrolase5.proenzyme-like-GGT1.rhesus.   aggagaattatttgaactcgggaggtggaggttggagtgagccaagattgtgccattgca    35880

Papio.anubis.clone.rp41-133b2.8034-40080.revcompl.Baboon          ------------------------------------------------------------    0
BCRP3.HUMAN.NCBI.REF                                              ------------------------------------------------------------    0
LOC106996293.glutathione.hydrolase5.proenzyme-like-GGT1.rhesus.   ctccagcctgggcaacaagagtgaaactctatctccccccccaaaaaaagccacatgtgg    35940

Papio.anubis.clone.rp41-133b2.8034-40080.revcompl.Baboon          ------------------------------------------------------------    0
BCRP3.HUMAN.NCBI.REF                                              ------------------------------------------------------------    0
LOC106996293.glutathione.hydrolase5.proenzyme-like-GGT1.rhesus.   tggggtgtgtctatagtctcaggtacttctgaggctgaggtgggaggatcgcttgagcct    36000

Papio.anubis.clone.rp41-133b2.8034-40080.revcompl.Baboon          ------------------------------------------------------------    0
BCRP3.HUMAN.NCBI.REF                                              ------------------------------------------------------------    0
LOC106996293.glutathione.hydrolase5.proenzyme-like-GGT1.rhesus.   ggaaaaccaaggctgcagtgagccgtgattgcaccactccactccagctggggcaacaga    36060
```

```
Papio.anubis.clone.rp41-133b2.8034-40080.revcompl.Baboon            ------------------------------------------------------------    0
BCRP3.HUMAN.NCBI.REF                                                ------------------------------------------------------------    0
LOC106996293.glutathione.hydrolase5.proenzyme-like-GGT1.rhesus.     gtgagactctgtctcaaaacaaacaaaaaacccttatagttggatggggaaactgaggct    36120

Papio.anubis.clone.rp41-133b2.8034-40080.revcompl.Baboon            ------------------------------------------------------------    0
BCRP3.HUMAN.NCBI.REF                                                ------------------------------------------------------------    0
LOC106996293.glutathione.hydrolase5.proenzyme-like-GGT1.rhesus.     gggagaggggacaggacggaggttaaggctcaggtcttgcctctggagcagttgaaaaga    36180

Papio.anubis.clone.rp41-133b2.8034-40080.revcompl.Baboon            ------------------------------------------------------------    0
BCRP3.HUMAN.NCBI.REF                                                ------------------------------------------------------------    0
LOC106996293.glutathione.hydrolase5.proenzyme-like-GGT1.rhesus.     ggaagagagccctctcttggggctggctgattcttgagggtggcctgtgcttgacctggg    36240

Papio.anubis.clone.rp41-133b2.8034-40080.revcompl.Baboon            ------------------------------------------------------------    0
BCRP3.HUMAN.NCBI.REF                                                ------------------------------------------------------------    0
LOC106996293.glutathione.hydrolase5.proenzyme-like-GGT1.rhesus.     ttgggcaggacctgctcttgctttggcacattctggtggagcccatgagtgttacaggat    36300

Papio.anubis.clone.rp41-133b2.8034-40080.revcompl.Baboon            ------------------------------------------------------------    0
BCRP3.HUMAN.NCBI.REF                                                ------------------------------------------------------------    0
LOC106996293.glutathione.hydrolase5.proenzyme-like-GGT1.rhesus.     aagggcttgtggtcagtgagatgggaggggatctggcctggcacaggattttagacatgc    36360

Papio.anubis.clone.rp41-133b2.8034-40080.revcompl.Baboon            ------------------------------------------------------------    0
BCRP3.HUMAN.NCBI.REF                                                ------------------------------------------------------------    0
LOC106996293.glutathione.hydrolase5.proenzyme-like-GGT1.rhesus.     aagcacctgcacagacacctcatcctgggacagcaaaacccagttgcatgctactgcttc    36420

Papio.anubis.clone.rp41-133b2.8034-40080.revcompl.Baboon            ------------------------------------------------------------    0
BCRP3.HUMAN.NCBI.REF                                                ------------------------------------------------------------    0
LOC106996293.glutathione.hydrolase5.proenzyme-like-GGT1.rhesus.     ccctcctgtgccctcctcagacatccctggtccatgtacactcctgcctgccgagcccag    36480

Papio.anubis.clone.rp41-133b2.8034-40080.revcompl.Baboon            ------------------------------------------------------------    0
BCRP3.HUMAN.NCBI.REF                                                ------------------------------------------------------------    0
LOC106996293.glutathione.hydrolase5.proenzyme-like-GGT1.rhesus.     atatgaccttaaacatccccttctgctgggtgtggtggttcactcctggaatctcagctg    36540

Papio.anubis.clone.rp41-133b2.8034-40080.revcompl.Baboon            ------------------------------------------------------------    0
BCRP3.HUMAN.NCBI.REF                                                ------------------------------------------------------------    0
LOC106996293.glutathione.hydrolase5.proenzyme-like-GGT1.rhesus.     cttgggaggctgaggtgggagaattacttgagtccaggagtttgagaccaggctgggaaa    36600

Papio.anubis.clone.rp41-133b2.8034-40080.revcompl.Baboon            ------------------------------------------------------------    0
BCRP3.HUMAN.NCBI.REF                                                ------------------------------------------------------------    0
LOC106996293.glutathione.hydrolase5.proenzyme-like-GGT1.rhesus.     gatggcaagaccccatctcaaaaaagaaaaaaaaaatatttttcgtcctaagcctcattg    36660

Papio.anubis.clone.rp41-133b2.8034-40080.revcompl.Baboon            ------------------------------------------------------------    0
BCRP3.HUMAN.NCBI.REF                                                ------------------------------------------------------------    0
LOC106996293.glutathione.hydrolase5.proenzyme-like-GGT1.rhesus.     acccatctgtaaaatgagtcagggaatgtgcctgggatgctgcctgtgagagattctgat    36720

Papio.anubis.clone.rp41-133b2.8034-40080.revcompl.Baboon            ------------------------------------------------------------    0
BCRP3.HUMAN.NCBI.REF                                                ------------------------------------------------------------    0
LOC106996293.glutathione.hydrolase5.proenzyme-like-GGT1.rhesus.     gttccctactcagggccactaactgtagctctctctccccagggggggctgtcagtggcgg    36780

Papio.anubis.clone.rp41-133b2.8034-40080.revcompl.Baboon            ------------------------------------------------------------    0
BCRP3.HUMAN.NCBI.REF                                                ------------------------------------------------------------    0
LOC106996293.glutathione.hydrolase5.proenzyme-like-GGT1.rhesus.     tgcccggggagatccgaggctatgagctggcgcaccagcggcatgggcggctgccctggg    36840

Papio.anubis.clone.rp41-133b2.8034-40080.revcompl.Baboon            ------------------------------------------------------------    0
BCRP3.HUMAN.NCBI.REF                                                ------------------------------------------------------------    0
LOC106996293.glutathione.hydrolase5.proenzyme-like-GGT1.rhesus.     ctcgcctcttccggcccagcatccagctggcccgccagggcttccccgtgggcaagggct    36900

Papio.anubis.clone.rp41-133b2.8034-40080.revcompl.Baboon            ------------------------------------------------------------    0
BCRP3.HUMAN.NCBI.REF                                                ------------------------------------------------------------    0
LOC106996293.glutathione.hydrolase5.proenzyme-like-GGT1.rhesus.     tggcagcagccctggaaaacaagcggaccgtcatcgagcagcagcctgtcttgtggtatg    36960

Papio.anubis.clone.rp41-133b2.8034-40080.revcompl.Baboon            ------------------------------------------------------------    0
BCRP3.HUMAN.NCBI.REF                                                ------------------------------------------------------------    0
LOC106996293.glutathione.hydrolase5.proenzyme-like-GGT1.rhesus.     tctgtgggtgcggccccctgacacaggcagtgcaggcacagcccaaggaccctgcaggcc    37020

Papio.anubis.clone.rp41-133b2.8034-40080.revcompl.Baboon            ------------------------------------------------------------    0
BCRP3.HUMAN.NCBI.REF                                                ------------------------------------------------------------    0
LOC106996293.glutathione.hydrolase5.proenzyme-like-GGT1.rhesus.     cctagcagcagtggagcggccctctgccttcaggaccccgcactgataatgggctgagga    37080

Papio.anubis.clone.rp41-133b2.8034-40080.revcompl.Baboon            ------------------------------------------------------------    0
BCRP3.HUMAN.NCBI.REF                                                ------------------------------------------------------------    0
LOC106996293.glutathione.hydrolase5.proenzyme-like-GGT1.rhesus.     gatgcagacccttcccaccacgtgtggggacacattctgaggtggggtcccagtggccac    37140

Papio.anubis.clone.rp41-133b2.8034-40080.revcompl.Baboon            ------------------------------------------------------------    0
BCRP3.HUMAN.NCBI.REF                                                ------------------------------------------------------------    0
LOC106996293.glutathione.hydrolase5.proenzyme-like-GGT1.rhesus.     tgtggctggccgtgtgtcctgagtggcaagggacactaggaggctcccggaagggacatt    37200

Papio.anubis.clone.rp41-133b2.8034-40080.revcompl.Baboon            ------------------------------------------------------------    0
BCRP3.HUMAN.NCBI.REF                                                ------------------------------------------------------------    0
LOC106996293.glutathione.hydrolase5.proenzyme-like-GGT1.rhesus.     gggaggatgagtgctgagtgacagagccaccaacctgtgacaggtgctgccccagctttg    37260

Papio.anubis.clone.rp41-133b2.8034-40080.revcompl.Baboon            ------------------------------------------------------------    0
BCRP3.HUMAN.NCBI.REF                                                ------------------------------------------------------------    0
LOC106996293.glutathione.hydrolase5.proenzyme-like-GGT1.rhesus.     ttccgttctcctgtgtgggtgggtgtggggggtggtctagctgagtccatcccacctgct    37320

Papio.anubis.clone.rp41-133b2.8034-40080.revcompl.Baboon            ------------------------------------------------------------    0
BCRP3.HUMAN.NCBI.REF                                                ------------------------------------------------------------    0
LOC106996293.glutathione.hydrolase5.proenzyme-like-GGT1.rhesus.     tcctcacgtgagccccctctgccccagtgaggtgttctgccgggatggaaaggtgcttcg    37380

Papio.anubis.clone.rp41-133b2.8034-40080.revcompl.Baboon            ------------------------------------------------------------    0
BCRP3.HUMAN.NCBI.REF                                                ------------------------------------------------------------    0
LOC106996293.glutathione.hydrolase5.proenzyme-like-GGT1.rhesus.     ggagggggagagactgaccctgccgcggctggctgacacctacgagacgctggccatcga    37440

Papio.anubis.clone.rp41-133b2.8034-40080.revcompl.Baboon            ------------------------------------------------------------    0
BCRP3.HUMAN.NCBI.REF                                                ------------------------------------------------------------    0
LOC106996293.glutathione.hydrolase5.proenzyme-like-GGT1.rhesus.     gggtgcccaggccttctacaacggcagcctcacggcccagattgtgaaggacatccaggc    37500

Papio.anubis.clone.rp41-133b2.8034-40080.revcompl.Baboon            ------------------------------------------------------------    0
BCRP3.HUMAN.NCBI.REF                                                ------------------------------------------------------------    0
LOC106996293.glutathione.hydrolase5.proenzyme-like-GGT1.rhesus.     agccggtgagtgggtaacctcaggggcctgggtgaggaaccctgcagtggaaaccctgag    37560

Papio.anubis.clone.rp41-133b2.8034-40080.revcompl.Baboon            ------------------------------------------------------------    0
BCRP3.HUMAN.NCBI.REF                                                ------------------------------------------------------------    0
LOC106996293.glutathione.hydrolase5.proenzyme-like-GGT1.rhesus.     ctgtggcccagagccctggggtcttcctgtcctgcctgagcctgcaggaagttcctcgtg    37620

Papio.anubis.clone.rp41-133b2.8034-40080.revcompl.Baboon            ------------------------------------------------------------    0
BCRP3.HUMAN.NCBI.REF                                                ------------------------------------------------------------    0
LOC106996293.glutathione.hydrolase5.proenzyme-like-GGT1.rhesus.     gaggagtgtcagtggccggccatgtgggttcacagctcctgggtatgtcaaagccaagag    37680

Papio.anubis.clone.rp41-133b2.8034-40080.revcompl.Baboon            ------------------------------------------------------------    0
BCRP3.HUMAN.NCBI.REF                                                ------------------------------------------------------------    0
LOC106996293.glutathione.hydrolase5.proenzyme-like-GGT1.rhesus.     aggccttgcggtccaggagagcaagcccctggtggggtaaatgcaggtgtaggcaagagc    37740

Papio.anubis.clone.rp41-133b2.8034-40080.revcompl.Baboon            ------------------------------------------------------------    0
BCRP3.HUMAN.NCBI.REF                                                ------------------------------------------------------------    0
LOC106996293.glutathione.hydrolase5.proenzyme-like-GGT1.rhesus.     cagggctagggaagcactagaatatggcctgaagatccaggaggacttggaggaggtggt    37800
```

```
Papio.anubis.clone.rp41-133b2.8034-40080.revcompl.Baboon           ------------------------------------------------------------    0
BCRP3.HUMAN.NCBI.REF                                                ------------------------------------------------------------    0
LOC106996293.glutathione.hydrolase5.proenzyme-like-GGT1.rhesus.     ggctgggctgcagataactttgttaggcagagaaaggaagggattcctagcagaggagca    37860

Papio.anubis.clone.rp41-133b2.8034-40080.revcompl.Baboon           ------------------------------------------------------------    0
BCRP3.HUMAN.NCBI.REF                                                ------------------------------------------------------------    0
LOC106996293.glutathione.hydrolase5.proenzyme-like-GGT1.rhesus.     gctgggctaaggcccagggagagggggctttgattcaccaagagggttacaagggatgag    37920

Papio.anubis.clone.rp41-133b2.8034-40080.revcompl.Baboon           ------------------------------------------------------------    0
BCRP3.HUMAN.NCBI.REF                                                ------------------------------------------------------------    0
LOC106996293.glutathione.hydrolase5.proenzyme-like-GGT1.rhesus.     ggtcaccttgagagaggcgtggggaaggggctttgtggggcaggggcctggagcttggct    37980

Papio.anubis.clone.rp41-133b2.8034-40080.revcompl.Baboon           ------------------------------------------------------------    0
BCRP3.HUMAN.NCBI.REF                                                ------------------------------------------------------------    0
LOC106996293.glutathione.hydrolase5.proenzyme-like-GGT1.rhesus.     gtgactttcttcaggtaatttttgtcactgtttcatggagaaggatgactagtgtgtcga    38040

Papio.anubis.clone.rp41-133b2.8034-40080.revcompl.Baboon           ------------------------------------------------------------    0
BCRP3.HUMAN.NCBI.REF                                                ------------------------------------------------------------    0
LOC106996293.glutathione.hydrolase5.proenzyme-like-GGT1.rhesus.     cctttaccactgaggctggaaattagcatgccaataccctgtctgtctggagctgactcc    38100

Papio.anubis.clone.rp41-133b2.8034-40080.revcompl.Baboon           ------------------------------------------------------------    0
BCRP3.HUMAN.NCBI.REF                                                ------------------------------------------------------------    0
LOC106996293.glutathione.hydrolase5.proenzyme-like-GGT1.rhesus.     aggagaattaagagcctccctccctccctctatccattcatcatgaggagaagaggccaa    38160

Papio.anubis.clone.rp41-133b2.8034-40080.revcompl.Baboon           ------------------------------------------------------------    0
BCRP3.HUMAN.NCBI.REF                                                ------------------------------------------------------------    0
LOC106996293.glutathione.hydrolase5.proenzyme-like-GGT1.rhesus.     gcagcagggacaccggcaggaattctccacttagaaaaggccctctgagctgggcgcggt    38220

Papio.anubis.clone.rp41-133b2.8034-40080.revcompl.Baboon           ------------------------------------------------------------    0
BCRP3.HUMAN.NCBI.REF                                                ------------------------------------------------------------    0
LOC106996293.glutathione.hydrolase5.proenzyme-like-GGT1.rhesus.     ggctcacgtctgtaatcccagcactttgggaggccgaggcaggtggatcacctgacgtca    38280

Papio.anubis.clone.rp41-133b2.8034-40080.revcompl.Baboon           ------------------------------------------------------------    0
BCRP3.HUMAN.NCBI.REF                                                ------------------------------------------------------------    0
LOC106996293.glutathione.hydrolase5.proenzyme-like-GGT1.rhesus.     ggagttcaagaccagtctggccaacatggtgaaaccctgtctctactaaaaatacaaaat    38340

Papio.anubis.clone.rp41-133b2.8034-40080.revcompl.Baboon           ------------------------------------------------------------    0
BCRP3.HUMAN.NCBI.REF                                                ------------------------------------------------------------    0
LOC106996293.glutathione.hydrolase5.proenzyme-like-GGT1.rhesus.     tagtcgggcatggtgggttgtgcctttaatcccagctacttgggaggccacggcaggaga    38400

Papio.anubis.clone.rp41-133b2.8034-40080.revcompl.Baboon           ------------------------------------------------------------    0
BCRP3.HUMAN.NCBI.REF                                                ------------------------------------------------------------    0
LOC106996293.glutathione.hydrolase5.proenzyme-like-GGT1.rhesus.     attgctagaacccgggaggctgaggttgtagtgagccaagattgcaccactgcactccac    38460

Papio.anubis.clone.rp41-133b2.8034-40080.revcompl.Baboon           ------------------------------------------------------------    0
BCRP3.HUMAN.NCBI.REF                                                ------------------------------------------------------------    0
LOC106996293.glutathione.hydrolase5.proenzyme-like-GGT1.rhesus.     agatactccgtctcaaaaaaaaaaaaaaaaaaaaaaaaaggaaagaaaaggccctctgag    38520

Papio.anubis.clone.rp41-133b2.8034-40080.revcompl.Baboon           ------------------------------------------------------------    0
BCRP3.HUMAN.NCBI.REF                                                ------------------------------------------------------------    0
LOC106996293.glutathione.hydrolase5.proenzyme-like-GGT1.rhesus.     gccaagcttggtgtctcacacctgtaatcccaacactttgggaggctgaggtagaagttg    38580

Papio.anubis.clone.rp41-133b2.8034-40080.revcompl.Baboon           ------------------------------------------------------------    0
BCRP3.HUMAN.NCBI.REF                                                ------------------------------------------------------------    0
LOC106996293.glutathione.hydrolase5.proenzyme-like-GGT1.rhesus.     aggccagaagtccatgacaagcctgggcaacatagtgagactacaaaaaacatattgaca    38640

Papio.anubis.clone.rp41-133b2.8034-40080.revcompl.Baboon           ------------------------------------------------------------    0
BCRP3.HUMAN.NCBI.REF                                                ------------------------------------------------------------    0
LOC106996293.glutathione.hydrolase5.proenzyme-like-GGT1.rhesus.     ctctacataaatatagtgggggtggggctggggaaccagaaaacaaaaaattcaaagcat    38700

Papio.anubis.clone.rp41-133b2.8034-40080.revcompl.Baboon           ------------------------------------------------------------    0
BCRP3.HUMAN.NCBI.REF                                                ------------------------------------------------------------    0
LOC106996293.glutathione.hydrolase5.proenzyme-like-GGT1.rhesus.     actgaaaagaaatacagcaagaccctacaacaaaattaaaatttgctgagtgtggtggtg    38760

Papio.anubis.clone.rp41-133b2.8034-40080.revcompl.Baboon           ------------------------------------------------------------    0
BCRP3.HUMAN.NCBI.REF                                                ------------------------------------------------------------    0
LOC106996293.glutathione.hydrolase5.proenzyme-like-GGT1.rhesus.     ccacctttagcctcagctactggggaggatctgctggaggatcacttgagcccaggagtt    38820

Papio.anubis.clone.rp41-133b2.8034-40080.revcompl.Baboon           ------------------------------------------------------------    0
BCRP3.HUMAN.NCBI.REF                                                ------------------------------------------------------------    0
LOC106996293.glutathione.hydrolase5.proenzyme-like-GGT1.rhesus.     caaggctgtgattaagccactgcactccagcctgggtgacagagcaagatcctatctcta    38880

Papio.anubis.clone.rp41-133b2.8034-40080.revcompl.Baboon           ------------------------------------------------------------    0
BCRP3.HUMAN.NCBI.REF                                                ------------------------------------------------------------    0
LOC106996293.glutathione.hydrolase5.proenzyme-like-GGT1.rhesus.     aaaaagaaaaaggaaaacactgtttggccatagaggagagaaggtagacaagaagccagt    38940

Papio.anubis.clone.rp41-133b2.8034-40080.revcompl.Baboon           ------------------------------------------------------------    0
BCRP3.HUMAN.NCBI.REF                                                ------------------------------------------------------------    0
LOC106996293.glutathione.hydrolase5.proenzyme-like-GGT1.rhesus.     aagccaagcaggcagagggcaggtggccccagcccagctgtggggtggtgagcagtgtca    39000

Papio.anubis.clone.rp41-133b2.8034-40080.revcompl.Baboon           ------------------------------------------------------------    0
BCRP3.HUMAN.NCBI.REF                                                ------------------------------------------------------------    0
LOC106996293.glutathione.hydrolase5.proenzyme-like-GGT1.rhesus.     gggagacaggagttcaagagcgagtgaggttcccaagggggcaaggttgcctgtggcccc    39060

Papio.anubis.clone.rp41-133b2.8034-40080.revcompl.Baboon           ------------------------------------------------------------    0
BCRP3.HUMAN.NCBI.REF                                                ------------------------------------------------------------    0
LOC106996293.glutathione.hydrolase5.proenzyme-like-GGT1.rhesus.     ctcccagggcacagtcccacgcctccagtagtgcactctgtcctccctggtaggttcagg    39120

Papio.anubis.clone.rp41-133b2.8034-40080.revcompl.Baboon           ------------------------------------------------------------    0
BCRP3.HUMAN.NCBI.REF                                                ------------------------------------------------------------    0
LOC106996293.glutathione.hydrolase5.proenzyme-like-GGT1.rhesus.     cttttccccaccactatggtgcagccatgcttgcccccaacaccactggtgcagctccat    39180

Papio.anubis.clone.rp41-133b2.8034-40080.revcompl.Baboon           ------------------------------------------------------------    0
BCRP3.HUMAN.NCBI.REF                                                ------------------------------------------------------------    0
LOC106996293.glutathione.hydrolase5.proenzyme-like-GGT1.rhesus.     cctccacacaagggtgcagccccttcccagcgcccattcgagctgctgtcccattgcagg    39240

Papio.anubis.clone.rp41-133b2.8034-40080.revcompl.Baboon           ------------------------------------------------------------    0
BCRP3.HUMAN.NCBI.REF                                                ------------------------------------------------------------    0
LOC106996293.glutathione.hydrolase5.proenzyme-like-GGT1.rhesus.     gggcattgtgacagccgaggacctgaacaactaccgcgctgagctgattgagcatccgct    39300

Papio.anubis.clone.rp41-133b2.8034-40080.revcompl.Baboon           ------------------------------------------------------------    0
BCRP3.HUMAN.NCBI.REF                                                ------------------------------------------------------------    0
LOC106996293.glutathione.hydrolase5.proenzyme-like-GGT1.rhesus.     gaacatcagcctgggagatgcggtgctgtacatgcccagcgcgccgctcagcgggcccgt    39360

Papio.anubis.clone.rp41-133b2.8034-40080.revcompl.Baboon           ------------------------------------------------------------    0
BCRP3.HUMAN.NCBI.REF                                                ------------------------------------------------------------    0
LOC106996293.glutathione.hydrolase5.proenzyme-like-GGT1.rhesus.     gctggccctcatcctcaacatccttaaaggtgagtggtcgtgccacagccctgtggtagg    39420

Papio.anubis.clone.rp41-133b2.8034-40080.revcompl.Baboon           ------------------------------------------------------------    0
BCRP3.HUMAN.NCBI.REF                                                ------------------------------------------------------------    0
LOC106996293.glutathione.hydrolase5.proenzyme-like-GGT1.rhesus.     acccatgacacgccccacccctgctgcagctctgctggcccccatgccacgtctttgcat    39480

Papio.anubis.clone.rp41-133b2.8034-40080.revcompl.Baboon           ------------------------------------------------------------    0
BCRP3.HUMAN.NCBI.REF                                                ------------------------------------------------------------    0
LOC106996293.glutathione.hydrolase5.proenzyme-like-GGT1.rhesus.     cactgagctcccagggagtgctcctgtgtcacagctcaccatgtcctgaaggaggcagtg    39540
```

```
Papio.anubis.clone.rp41-133b2.8034-40080.revcompl.Baboon             ------------------------------------------------------------    0
BCRP3.HUMAN.NCBI.REF                                                  ------------------------------------------------------------    0
LOC106996293.glutathione.hydrolase5.proenzyme-like-GGT1.rhesus.       cagagccacagggctgaagtgggcaatgctcgagggttggaggaggaacaggagtcatca    39600

Papio.anubis.clone.rp41-133b2.8034-40080.revcompl.Baboon             ------------------------------------------------------------    0
BCRP3.HUMAN.NCBI.REF                                                  ------------------------------------------------------------    0
LOC106996293.glutathione.hydrolase5.proenzyme-like-GGT1.rhesus.       ggacggagagaggtacaggagctcagggctgcagggcttggtccagagggcatccctggt    39660

Papio.anubis.clone.rp41-133b2.8034-40080.revcompl.Baboon             ------------------------------------------------------------    0
BCRP3.HUMAN.NCBI.REF                                                  ------------------------------------------------------------    0
LOC106996293.glutathione.hydrolase5.proenzyme-like-GGT1.rhesus.       ccagtgggtggccctgccacttggtcattgcatgaccagagctgatgcgtgacgtgagat    39720

Papio.anubis.clone.rp41-133b2.8034-40080.revcompl.Baboon             ------------------------------------------------------------    0
BCRP3.HUMAN.NCBI.REF                                                  ------------------------------------------------------------    0
LOC106996293.glutathione.hydrolase5.proenzyme-like-GGT1.rhesus.       ccaggttggggagccttcaccttacttcactctcaccacagccttctgaagcagctgctg    39780

Papio.anubis.clone.rp41-133b2.8034-40080.revcompl.Baboon             ------------------------------------------------------------    0
BCRP3.HUMAN.NCBI.REF                                                  ------------------------------------------------------------    0
LOC106996293.glutathione.hydrolase5.proenzyme-like-GGT1.rhesus.       ctattggttattaaatgctccttgaggtgggcaaagtggctgaggaaggtgttcaccctc    39840

Papio.anubis.clone.rp41-133b2.8034-40080.revcompl.Baboon             ------------------------------------------------------------    0
BCRP3.HUMAN.NCBI.REF                                                  ------------------------------------------------------------    0
LOC106996293.glutathione.hydrolase5.proenzyme-like-GGT1.rhesus.       tgagtccctcagatcctctggggctcagcaacgtgcacctggctctgatcaaccagggta    39900

Papio.anubis.clone.rp41-133b2.8034-40080.revcompl.Baboon             ------------------------------------------------------------    0
BCRP3.HUMAN.NCBI.REF                                                  ------------------------------------------------------------    0
LOC106996293.glutathione.hydrolase5.proenzyme-like-GGT1.rhesus.       caacttctcccgggcgagcgtggagaccccggagcagaagggcctaacgtaccaccgcat    39960

Papio.anubis.clone.rp41-133b2.8034-40080.revcompl.Baboon             ------------------------------------------------------------    0
BCRP3.HUMAN.NCBI.REF                                                  ------------------------------------------------------------    0
LOC106996293.glutathione.hydrolase5.proenzyme-like-GGT1.rhesus.       cgtagaggcttttcggtttgcctatgccaagaggaccctgcttggggaccccaagtttgt    40020

Papio.anubis.clone.rp41-133b2.8034-40080.revcompl.Baboon             ------------------------------------------------------------    0
BCRP3.HUMAN.NCBI.REF                                                  ------------------------------------------------------------    0
LOC106996293.glutathione.hydrolase5.proenzyme-like-GGT1.rhesus.       ggatgtgactgaggtaaggggcagaggctggcccactctaggcgtggggcctgctgtaga    40080

Papio.anubis.clone.rp41-133b2.8034-40080.revcompl.Baboon             ------------------------------------------------------------    0
BCRP3.HUMAN.NCBI.REF                                                  ------------------------------------------------------------    0
LOC106996293.glutathione.hydrolase5.proenzyme-like-GGT1.rhesus.       ggcatcaggtgggctccccagggcggctgcagcctcacatatgctttatgaatccattcc    40140

Papio.anubis.clone.rp41-133b2.8034-40080.revcompl.Baboon             ------------------------------------------------------------    0
BCRP3.HUMAN.NCBI.REF                                                  ------------------------------------------------------------    0
LOC106996293.glutathione.hydrolase5.proenzyme-like-GGT1.rhesus.       tgccacaaactttgattgcgggcctactgtgtgctcagatggactggtcggtgaccccgg    40200

Papio.anubis.clone.rp41-133b2.8034-40080.revcompl.Baboon             ------------------------------------------------------------    0
BCRP3.HUMAN.NCBI.REF                                                  ------------------------------------------------------------    0
LOC106996293.glutathione.hydrolase5.proenzyme-like-GGT1.rhesus.       gtcttggcctctgccccacagaactgacagtgtggggaattagtggccacactcctacct    40260

Papio.anubis.clone.rp41-133b2.8034-40080.revcompl.Baboon             ------------------------------------------------------------    0
BCRP3.HUMAN.NCBI.REF                                                  ------------------------------------------------------------    0
LOC106996293.glutathione.hydrolase5.proenzyme-like-GGT1.rhesus.       caagtgctttgcacatgctgtggttctcgagctcagtgctgagatgaggaacgcatgggg    40320

Papio.anubis.clone.rp41-133b2.8034-40080.revcompl.Baboon             ------------------------------------------------------------    0
BCRP3.HUMAN.NCBI.REF                                                  ------------------------------------------------------------    0
LOC106996293.glutathione.hydrolase5.proenzyme-like-GGT1.rhesus.       gcattgcagccctcgggcatgaggagaaggacaggtgaaagggagagctccatgctgggt    40380

Papio.anubis.clone.rp41-133b2.8034-40080.revcompl.Baboon             ------------------------------------------------------------    0
BCRP3.HUMAN.NCBI.REF                                                  ------------------------------------------------------------    0
LOC106996293.glutathione.hydrolase5.proenzyme-like-GGT1.rhesus.       ccctgtgagaccctgtcatatcccttcccgctgaggatcctcacatccctccttacctac    40440

Papio.anubis.clone.rp41-133b2.8034-40080.revcompl.Baboon             ------------------------------------------------------------    0
BCRP3.HUMAN.NCBI.REF                                                  ------------------------------------------------------------    0
LOC106996293.glutathione.hydrolase5.proenzyme-like-GGT1.rhesus.       tcgggtccttggcattcccaggcagacctgcagacccctccacactgaccagggacctcc    40500

Papio.anubis.clone.rp41-133b2.8034-40080.revcompl.Baboon             ------------------------------------------------------------    0
BCRP3.HUMAN.NCBI.REF                                                  ------------------------------------------------------------    0
LOC106996293.glutathione.hydrolase5.proenzyme-like-GGT1.rhesus.       ctggaggggcctcagctgcccgggtggtgtcttctctttccccatgagcattctgcacct    40560

Papio.anubis.clone.rp41-133b2.8034-40080.revcompl.Baboon             ------------------------------------------------------------    0
BCRP3.HUMAN.NCBI.REF                                                  ------------------------------------------------------------    0
LOC106996293.glutathione.hydrolase5.proenzyme-like-GGT1.rhesus.       ctgactcctgctgtagccagtgacctggtgtcttgtctctttgaggggacagggccacta    40620

Papio.anubis.clone.rp41-133b2.8034-40080.revcompl.Baboon             ------------------------------------------------------------    0
BCRP3.HUMAN.NCBI.REF                                                  ------------------------------------------------------------    0
LOC106996293.glutathione.hydrolase5.proenzyme-like-GGT1.rhesus.       cagtgtgtacctctgccctcccttttagctaaggccagctcttccgtccacacgctggct    40680

Papio.anubis.clone.rp41-133b2.8034-40080.revcompl.Baboon             ------------------------------------------------------------    0
BCRP3.HUMAN.NCBI.REF                                                  ------------------------------------------------------------    0
LOC106996293.glutathione.hydrolase5.proenzyme-like-GGT1.rhesus.       cagggcactgttcctacaatgctcctctctgcctctgaccatttccttctttataacgga    40740

Papio.anubis.clone.rp41-133b2.8034-40080.revcompl.Baboon             ------------------------------------------------------------    0
BCRP3.HUMAN.NCBI.REF                                                  ------------------------------------------------------------    0
LOC106996293.glutathione.hydrolase5.proenzyme-like-GGT1.rhesus.       ttattcccgaaagaaaggcagctcttgtagctctagaaacttccatctgctgcctccttc    40800

Papio.anubis.clone.rp41-133b2.8034-40080.revcompl.Baboon             ------------------------------------------------------------    0
BCRP3.HUMAN.NCBI.REF                                                  ------------------------------------------------------------    0
LOC106996293.glutathione.hydrolase5.proenzyme-like-GGT1.rhesus.       tgtcctctgctccccttacagcaaacatggctgggctatgtcctctctcccttcagggtg    40860

Papio.anubis.clone.rp41-133b2.8034-40080.revcompl.Baboon             ------------------------------------------------------------    0
BCRP3.HUMAN.NCBI.REF                                                  ------------------------------------------------------------    0
LOC106996293.glutathione.hydrolase5.proenzyme-like-GGT1.rhesus.       tcccctcccctgctctatccccatgccaccagattgccacatccagcctcagtttcccca    40920

Papio.anubis.clone.rp41-133b2.8034-40080.revcompl.Baboon             ------------------------------------------------------------    0
BCRP3.HUMAN.NCBI.REF                                                  ------------------------------------------------------------    0
LOC106996293.glutathione.hydrolase5.proenzyme-like-GGT1.rhesus.       tcaggccccactcaacagcatctcacacagctcaccacactctacttgagttttcatttt    40980

Papio.anubis.clone.rp41-133b2.8034-40080.revcompl.Baboon             ------------------------------------------------------------    0
BCRP3.HUMAN.NCBI.REF                                                  ------------------------------------------------------------    0
LOC106996293.glutathione.hydrolase5.proenzyme-like-GGT1.rhesus.       gcaaattttcacacctacgaaaatgtaaaaaaaaacccaaaccgtccaagtgtcaatgtt    41040

Papio.anubis.clone.rp41-133b2.8034-40080.revcompl.Baboon             ------------------------------------------------------------    0
BCRP3.HUMAN.NCBI.REF                                                  ------------------------------------------------------------    0
LOC106996293.glutathione.hydrolase5.proenzyme-like-GGT1.rhesus.       ccctttccctagagtccacagtgtggacgtttctgctggatttcctctctctccgtctcc    41100

Papio.anubis.clone.rp41-133b2.8034-40080.revcompl.Baboon             ------------------------------------------------------------    0
BCRP3.HUMAN.NCBI.REF                                                  ------------------------------------------------------------    0
LOC106996293.glutathione.hydrolase5.proenzyme-like-GGT1.rhesus.       acccacgtctattgggattcgtgtttttctgagggatttgacagtaggttgctgatgtca    41160

Papio.anubis.clone.rp41-133b2.8034-40080.revcompl.Baboon             ------------------------------------------------------------    0
BCRP3.HUMAN.NCBI.REF                                                  ------------------------------------------------------------    0
LOC106996293.glutathione.hydrolase5.proenzyme-like-GGT1.rhesus.       cacctcagggtgtgtctcaagagtgagacttgaacataccacagcaggatgctggggtga    41220

Papio.anubis.clone.rp41-133b2.8034-40080.revcompl.Baboon             ------------------------------------------------------------    0
BCRP3.HUMAN.NCBI.REF                                                  ------------------------------------------------------------    0
LOC106996293.glutathione.hydrolase5.proenzyme-like-GGT1.rhesus.       cacaacctactgtcccttctctttacctacagtaggctcccttggctgttttgtttctgt    41280
```

```
Papio.anubis.clone.rp41-133b2.8034-40080.revcompl.Baboon          ------------------------------------------------------------    0
BCRP3.HUMAN.NCBI.REF                                              ------------------------------------------------------------    0
LOC106996293.glutathione.hydrolase5.proenzyme-like-GGT1.rhesus.   tgtgtttttgtttggacacagagtctctgtcatccaggttggagtgcagtggtgtgatct    41340

Papio.anubis.clone.rp41-133b2.8034-40080.revcompl.Baboon          ------------------------------------------------------------    0
BCRP3.HUMAN.NCBI.REF                                              ------------------------------------------------------------    0
LOC106996293.glutathione.hydrolase5.proenzyme-like-GGT1.rhesus.   cggctcactgcagcctcaacctccagggctcaagcaatcctcccacttcagcccctacaa    41400

Papio.anubis.clone.rp41-133b2.8034-40080.revcompl.Baboon          ------------------------------------------------------------    0
BCRP3.HUMAN.NCBI.REF                                              ------------------------------------------------------------    0
LOC106996293.glutathione.hydrolase5.proenzyme-like-GGT1.rhesus.   gcagctgggaccacaggcacatgccaccaaacctggctaattttgtatttttttttaga    41460

Papio.anubis.clone.rp41-133b2.8034-40080.revcompl.Baboon          ------------------------------------------------------------    0
BCRP3.HUMAN.NCBI.REF                                              ------------------------------------------------------------    0
LOC106996293.glutathione.hydrolase5.proenzyme-like-GGT1.rhesus.   gatagggtcttgccacattgcccagactggtctcaaactcctgggctcaaacgatcctcc    41520

Papio.anubis.clone.rp41-133b2.8034-40080.revcompl.Baboon          ------------------------------------------------------------    0
BCRP3.HUMAN.NCBI.REF                                              ------------------------------------------------------------    0
LOC106996293.glutathione.hydrolase5.proenzyme-like-GGT1.rhesus.   cacctcagcctcccaaagcgctgggcttacaggtgtgaaccacagtgctcgacctgtttt    41580

Papio.anubis.clone.rp41-133b2.8034-40080.revcompl.Baboon          ------------------------------------------------------------    0
BCRP3.HUMAN.NCBI.REF                                              ------------------------------------------------------------    0
LOC106996293.glutathione.hydrolase5.proenzyme-like-GGT1.rhesus.   gttgattttaaagcccagggcagtggtctggaagatgtcccacatcctatttgcctttc    41640

Papio.anubis.clone.rp41-133b2.8034-40080.revcompl.Baboon          ------------------------------------------------------------    0
BCRP3.HUMAN.NCBI.REF                                              ------------------------------------------------------------    0
LOC106996293.glutathione.hydrolase5.proenzyme-like-GGT1.rhesus.   tgtttccttaggggcagattcagacagagcaccttgccctgcgattgctaatctgtgggg    41700

Papio.anubis.clone.rp41-133b2.8034-40080.revcompl.Baboon          ------------------------------------------------------------    0
BCRP3.HUMAN.NCBI.REF                                              ------------------------------------------------------------    0
LOC106996293.glutathione.hydrolase5.proenzyme-like-GGT1.rhesus.   gtgttgctgaaactctcctgaggcattttcttcagccttcagggcccatcctctctgtct    41760

Papio.anubis.clone.rp41-133b2.8034-40080.revcompl.Baboon          ------------------------------------------------------------    0
BCRP3.HUMAN.NCBI.REF                                              ------------------------------------------------------------    0
LOC106996293.glutathione.hydrolase5.proenzyme-like-GGT1.rhesus.   cctccttcacctcctcatctcctcctcgccacggccttggggaccttggccaggctcgtg    41820

Papio.anubis.clone.rp41-133b2.8034-40080.revcompl.Baboon          ------------------------------------------------------------    0
BCRP3.HUMAN.NCBI.REF                                              ------------------------------------------------------------    0
LOC106996293.glutathione.hydrolase5.proenzyme-like-GGT1.rhesus.   gcatccagcagcctctcaatgtcaatacctccatgttcatccctcagcccgcccttgccg    41880

Papio.anubis.clone.rp41-133b2.8034-40080.revcompl.Baboon          ------------------------------------------------------------    0
BCRP3.HUMAN.NCBI.REF                                              ------------------------------------------------------------    0
LOC106996293.glutathione.hydrolase5.proenzyme-like-GGT1.rhesus.   gtgcacccatgcttatacatgcgcccatgctcttcaggtctgatgaagcatccaaaggca    41940

Papio.anubis.clone.rp41-133b2.8034-40080.revcompl.Baboon          ------------------------------------------------------------    0
BCRP3.HUMAN.NCBI.REF                                              ------------------------------------------------------------    0
LOC106996293.glutathione.hydrolase5.proenzyme-like-GGT1.rhesus.   agctcctgaccgtcgccaaacctgccccttctgcagggtttacctccctagttggcgcca    42000

Papio.anubis.clone.rp41-133b2.8034-40080.revcompl.Baboon          ------------------------------------------------------------    0
BCRP3.HUMAN.NCBI.REF                                              ------------------------------------------------------------    0
LOC106996293.glutathione.hydrolase5.proenzyme-like-GGT1.rhesus.   tcctcaattcttctctttctcccacccaggccatcatctcttgcctggttgatacccaca    42060

Papio.anubis.clone.rp41-133b2.8034-40080.revcompl.Baboon          ------------------------------------------------------------    0
BCRP3.HUMAN.NCBI.REF                                              ------------------------------------------------------------    0
LOC106996293.glutathione.hydrolase5.proenzyme-like-GGT1.rhesus.   gcctcccctttgggctttatctttatccccctcacaactgccagagggaccctgtgaaaa    42120

Papio.anubis.clone.rp41-133b2.8034-40080.revcompl.Baboon          ------------------------------------------------------------    0
BCRP3.HUMAN.NCBI.REF                                              ------------------------------------------------------------    0
LOC106996293.glutathione.hydrolase5.proenzyme-like-GGT1.rhesus.   cactccccagcctgctcattcctctgcccaaagcctgcgtggcacagagcaaaagccagc    42180

Papio.anubis.clone.rp41-133b2.8034-40080.revcompl.Baboon          ------------------------------------------------------------    0
BCRP3.HUMAN.NCBI.REF                                              ------------------------------------------------------------    0
LOC106996293.glutathione.hydrolase5.proenzyme-like-GGT1.rhesus.   cattatgggacctaggaggtcctgtgggatgggccccagcctgcatcttcatattcttct    42240

Papio.anubis.clone.rp41-133b2.8034-40080.revcompl.Baboon          ------------------------------------------------------------    0
BCRP3.HUMAN.NCBI.REF                                              ------------------------------------------------------------    0
LOC106996293.glutathione.hydrolase5.proenzyme-like-GGT1.rhesus.   cctccctaacccattcactctctgcctatcgcttaccagcctataccacctgcctcaggg    42300

Papio.anubis.clone.rp41-133b2.8034-40080.revcompl.Baboon          ------------------------------------------------------------    0
BCRP3.HUMAN.NCBI.REF                                              ------------------------------------------------------------    0
LOC106996293.glutathione.hydrolase5.proenzyme-like-GGT1.rhesus.   cctttgcactgaccattaaggccgcattccaggctcttttcacatgttgcctcctccgag    42360

Papio.anubis.clone.rp41-133b2.8034-40080.revcompl.Baboon          ------------------------------------------------------------    0
BCRP3.HUMAN.NCBI.REF                                              ------------------------------------------------------------    0
LOC106996293.glutathione.hydrolase5.proenzyme-like-GGT1.rhesus.   aagccctccctgaccactctacccatacctcatgcctctcaattccccttacctggtttg    42420

Papio.anubis.clone.rp41-133b2.8034-40080.revcompl.Baboon          ------------------------------------------------------------    0
BCRP3.HUMAN.NCBI.REF                                              ------------------------------------------------------------    0
LOC106996293.glutathione.hydrolase5.proenzyme-like-GGT1.rhesus.   tggtttcagcactttcctcatgtgcgtttgtttttcttggcgtgagggcagggacctaag    42480

Papio.anubis.clone.rp41-133b2.8034-40080.revcompl.Baboon          ------------------------------------------------------------    0
BCRP3.HUMAN.NCBI.REF                                              ------------------------------------------------------------    0
LOC106996293.glutathione.hydrolase5.proenzyme-like-GGT1.rhesus.   tgtctgttccctgttgattttccagtgccaggcatgcagtgcaaactctagaaatctatt    42540

Papio.anubis.clone.rp41-133b2.8034-40080.revcompl.Baboon          ------------------------------------------------------------    0
BCRP3.HUMAN.NCBI.REF                                              ------------------------------------------------------------    0
LOC106996293.glutathione.hydrolase5.proenzyme-like-GGT1.rhesus.   tttgcatgagtgaatgagtgattgaatgcggcaagggtctggaggctgagggccagacag    42600

Papio.anubis.clone.rp41-133b2.8034-40080.revcompl.Baboon          ------------------------------------------------------------    0
BCRP3.HUMAN.NCBI.REF                                              ------------------------------------------------------------    0
LOC106996293.glutathione.hydrolase5.proenzyme-like-GGT1.rhesus.   acattcagagttgctggaaggcgacagagacagagagtcagactggtcatgcaaggtgct    42660

Papio.anubis.clone.rp41-133b2.8034-40080.revcompl.Baboon          ------------------------------------------------------------    0
BCRP3.HUMAN.NCBI.REF                                              ------------------------------------------------------------    0
LOC106996293.glutathione.hydrolase5.proenzyme-like-GGT1.rhesus.   gggcctgcccttgggccctggggagccatggaaggctgtgggtgccagagggttgtggtc    42720

Papio.anubis.clone.rp41-133b2.8034-40080.revcompl.Baboon          ------------------------------------------------------------    0
BCRP3.HUMAN.NCBI.REF                                              ------------------------------------------------------------    0
LOC106996293.glutathione.hydrolase5.proenzyme-like-GGT1.rhesus.   agagccacagtcgggggccttctgagacctgtgccccctccccaccctccctccccacct    42780

Papio.anubis.clone.rp41-133b2.8034-40080.revcompl.Baboon          ------------------------------------------------------------    0
BCRP3.HUMAN.NCBI.REF                                              ------------------------------------------------------------    0
LOC106996293.glutathione.hydrolase5.proenzyme-like-GGT1.rhesus.   cctcaggccagctctggggtctcggcaggtggtccgcaacatgacctccgagttcttcgc    42840

Papio.anubis.clone.rp41-133b2.8034-40080.revcompl.Baboon          ------------------------------------------------------------    0
BCRP3.HUMAN.NCBI.REF                                              ------------------------------------------------------------    0
LOC106996293.glutathione.hydrolase5.proenzyme-like-GGT1.rhesus.   tgcccagctccgggcccagatctctgatgataccactcacccgatctcctactacaaacc    42900

Papio.anubis.clone.rp41-133b2.8034-40080.revcompl.Baboon          ------------------------------------------------------------    0
BCRP3.HUMAN.NCBI.REF                                              ------------------------------------------------------------    0
LOC106996293.glutathione.hydrolase5.proenzyme-like-GGT1.rhesus.   cgagttctacacaccggatgatgggggcactgcccacctgtctgtcgtcgcagaggacgg    42960

Papio.anubis.clone.rp41-133b2.8034-40080.revcompl.Baboon          ------------------------------------------------------------    0
BCRP3.HUMAN.NCBI.REF                                              ------------------------------------------------------------    0
LOC106996293.glutathione.hydrolase5.proenzyme-like-GGT1.rhesus.   cagtgctgtgtccgccaccagcaccatcaacctctagtaggggctgctgggccgcctggg    43020
```

```
Papio.anubis.clone.rp41-133b2.8034-40080.revcompl.Baboon            ------------------------------------------------------------    0
BCRP3.HUMAN.NCBI.REF                                                ------------------------------------------------------------    0
LOC106996293.glutathione.hydrolase5.proenzyme-like-GGT1.rhesus.     tgggacagggccaggggcatgtggtccagggactgcccacttatccagtaaggtggctcc    43080

Papio.anubis.clone.rp41-133b2.8034-40080.revcompl.Baboon            ------------------------------------------------------------    0
BCRP3.HUMAN.NCBI.REF                                                ------------------------------------------------------------    0
LOC106996293.glutathione.hydrolase5.proenzyme-like-GGT1.rhesus.     atcaccccttttcctggtgggaaactgaggcccgaccttggtagcttatcctgggcctct    43140

Papio.anubis.clone.rp41-133b2.8034-40080.revcompl.Baboon            ------------------------------------------------------------    0
BCRP3.HUMAN.NCBI.REF                                                ------------------------------------------------------------    0
LOC106996293.glutathione.hydrolase5.proenzyme-like-GGT1.rhesus.     cagggagtaagtttgagcccaggttgggtcgggcgaggtcaggcgctgtctgacctggct    43200

Papio.anubis.clone.rp41-133b2.8034-40080.revcompl.Baboon            ------------------------------------------------------------    0
BCRP3.HUMAN.NCBI.REF                                                ------------------------------------------------------------    0
LOC106996293.glutathione.hydrolase5.proenzyme-like-GGT1.rhesus.     gggcggtagctttggctccaaggtccgctccccagtcagcgggatcctgttcaatgatga    43260

Papio.anubis.clone.rp41-133b2.8034-40080.revcompl.Baboon            ------------------------------------------------------------    0
BCRP3.HUMAN.NCBI.REF                                                ------------------------------------------------------------    0
LOC106996293.glutathione.hydrolase5.proenzyme-like-GGT1.rhesus.     aatggatgacttcagctctcccagcatcaccaatcagtttggggtgccccctcacctgc    43320

Papio.anubis.clone.rp41-133b2.8034-40080.revcompl.Baboon            ------------------------------------------------------------    0
BCRP3.HUMAN.NCBI.REF                                                ------------------------------------------------------------    0
LOC106996293.glutathione.hydrolase5.proenzyme-like-GGT1.rhesus.     caatttcatccagccaggtatggggtggaggtctgggggtggagggctggggtggagagg    43380

Papio.anubis.clone.rp41-133b2.8034-40080.revcompl.Baboon            ------------------------------------------------------------    0
BCRP3.HUMAN.NCBI.REF                                                ------------------------------------------------------------    0
LOC106996293.glutathione.hydrolase5.proenzyme-like-GGT1.rhesus.     gggggtgtcctgggcaggcagctaaccagcatccccgccttctcccatcggccacaggga    43440

Papio.anubis.clone.rp41-133b2.8034-40080.revcompl.Baboon            ------------------------------------------------------------    0
BCRP3.HUMAN.NCBI.REF                                                ------------------------------------------------------------    0
LOC106996293.glutathione.hydrolase5.proenzyme-like-GGT1.rhesus.     agcagccgctctcgtccatgtgtccaacgatcatggtgggccaggacggccaggtccgga    43500

Papio.anubis.clone.rp41-133b2.8034-40080.revcompl.Baboon            ------------------------------------------------------------    0
BCRP3.HUMAN.NCBI.REF                                                ------------------------------------------------------------    0
LOC106996293.glutathione.hydrolase5.proenzyme-like-GGT1.rhesus.     tggtggtgggagcttctgggggcacgcagatcaccacggccactgcactggtatgtgtca    43560

Papio.anubis.clone.rp41-133b2.8034-40080.revcompl.Baboon            ------------------------------------------------------------    0
BCRP3.HUMAN.NCBI.REF                                                ------------------------------------------------------------    0
LOC106996293.glutathione.hydrolase5.proenzyme-like-GGT1.rhesus.     ccccttttctccctggcgctgcccaccctgcacagcccccaggccatgctgatcacactc    43620

Papio.anubis.clone.rp41-133b2.8034-40080.revcompl.Baboon            ------------------------------------------------------------    0
BCRP3.HUMAN.NCBI.REF                                                ------------------------------------------------------------    0
LOC106996293.glutathione.hydrolase5.proenzyme-like-GGT1.rhesus.     ccatgccccaggcaatcatctacaacctctggttcggctatgacgtgaagcgggccgtgg    43680

Papio.anubis.clone.rp41-133b2.8034-40080.revcompl.Baboon            ------------------------------------------------------------    0
BCRP3.HUMAN.NCBI.REF                                                ------------------------------------------------------------    0
LOC106996293.glutathione.hydrolase5.proenzyme-like-GGT1.rhesus.     aggagccccggctgcacaaccagcttctacccaacgtcacaacagtggagagaaacattg    43740

Papio.anubis.clone.rp41-133b2.8034-40080.revcompl.Baboon            ------------------------------------------------------------    0
BCRP3.HUMAN.NCBI.REF                                                ------------------------------------------------------------    0
LOC106996293.glutathione.hydrolase5.proenzyme-like-GGT1.rhesus.     accaggtgcgctgggggttggagaaactgagtcacggtgtggggtcccagggcatcctgg    43800

Papio.anubis.clone.rp41-133b2.8034-40080.revcompl.Baboon            ------------------------------------------------------------    0
BCRP3.HUMAN.NCBI.REF                                                ------------------------------------------------------------    0
LOC106996293.glutathione.hydrolase5.proenzyme-like-GGT1.rhesus.     actggaggcctggatcatcatggagtggacaatggttggtgtcctctctctggtgcctgg    43860

Papio.anubis.clone.rp41-133b2.8034-40080.revcompl.Baboon            ------------------------------------------------------------    0
BCRP3.HUMAN.NCBI.REF                                                ------------------------------------------------------------    0
LOC106996293.glutathione.hydrolase5.proenzyme-like-GGT1.rhesus.     gccatctggagcccctgtgccatgagggccaagccacctgctccagtgagacccagcagg    43920

Papio.anubis.clone.rp41-133b2.8034-40080.revcompl.Baboon            ------------------------------------------------------------    0
BCRP3.HUMAN.NCBI.REF                                                ------------------------------------------------------------    0
LOC106996293.glutathione.hydrolase5.proenzyme-like-GGT1.rhesus.     ccccaacctgctcttcctgatgacctggcccaaaatggcaccacctgggctgaggcctgt    43980

Papio.anubis.clone.rp41-133b2.8034-40080.revcompl.Baboon            ------------------------------------------------------------    0
BCRP3.HUMAN.NCBI.REF                                                ------------------------------------------------------------    0
LOC106996293.glutathione.hydrolase5.proenzyme-like-GGT1.rhesus.     gaccacacaggcatggttcaggtggcatctggaaccctgctcaggcttcctgtctcctcc    44040

Papio.anubis.clone.rp41-133b2.8034-40080.revcompl.Baboon            ------------------------------------------------------------    0
BCRP3.HUMAN.NCBI.REF                                                ------------------------------------------------------------    0
LOC106996293.glutathione.hydrolase5.proenzyme-like-GGT1.rhesus.     cacccccaggcagtgactgcagccctggaaacccggcaccatgacactgagatcgcatcc    44100

Papio.anubis.clone.rp41-133b2.8034-40080.revcompl.Baboon            ------------------------------------------------------------    0
BCRP3.HUMAN.NCBI.REF                                                ------------------------------------------------------------    0
LOC106996293.glutathione.hydrolase5.proenzyme-like-GGT1.rhesus.     accttcatcgctgtggtgcaagctgtcgtccacacacctggtggctgggcagctgcctcg    44160

Papio.anubis.clone.rp41-133b2.8034-40080.revcompl.Baboon            ------------------------------------------------------------    0
BCRP3.HUMAN.NCBI.REF                                                ------------------------------------------------------------    0
LOC106996293.glutathione.hydrolase5.proenzyme-like-GGT1.rhesus.     gactccaggaaaggcggggagcctgctggttactgagtgcttggggcggacaaagctgac    44220

Papio.anubis.clone.rp41-133b2.8034-40080.revcompl.Baboon            ------------------------------------------------------------    0
BCRP3.HUMAN.NCBI.REF                                                ------------------------------------------------------------    0
LOC106996293.glutathione.hydrolase5.proenzyme-like-GGT1.rhesus.     cagcaatccagggacaagatactcaccaggaccaggaaggggactttggtgtcccctgtg    44280

Papio.anubis.clone.rp41-133b2.8034-40080.revcompl.Baboon            ------------------------------------------------------------    0
BCRP3.HUMAN.NCBI.REF                                                ------------------------------------------------------------    0
LOC106996293.glutathione.hydrolase5.proenzyme-like-GGT1.rhesus.     agtggtagagcatcacaataaatgaggccactgtgtcaggctccaggcagcctccctggc    44340

Papio.anubis.clone.rp41-133b2.8034-40080.revcompl.Baboon            ------------------------------------------------------------    0
BCRP3.HUMAN.NCBI.REF                                                ------------------------------------------------------------    0
LOC106996293.glutathione.hydrolase5.proenzyme-like-GGT1.rhesus.     ctggctccccactctctgggcctcagtgtattgtgtgtgaaatggaaccatctggctggg    44400

Papio.anubis.clone.rp41-133b2.8034-40080.revcompl.Baboon            ------------------------------------------------------------    0
BCRP3.HUMAN.NCBI.REF                                                ------------------------------------------------------------    0
LOC106996293.glutathione.hydrolase5.proenzyme-like-GGT1.rhesus.     gaggaatggagaggtgggattcggagatcttcacactgcggtcgctggaactagcctcag    44460

Papio.anubis.clone.rp41-133b2.8034-40080.revcompl.Baboon            ------------------------------------------------------------    0
BCRP3.HUMAN.NCBI.REF                                                ------------------------------------------------------------    0
LOC106996293.glutathione.hydrolase5.proenzyme-like-GGT1.rhesus.     tatcttcagcgtggggagagccaggtgcgtggctagggaccaggggaaggtccatgccaa    44520

Papio.anubis.clone.rp41-133b2.8034-40080.revcompl.Baboon            ------------------------------------------------------------    0
BCRP3.HUMAN.NCBI.REF                                                ------------------------------------------------------------    0
LOC106996293.glutathione.hydrolase5.proenzyme-like-GGT1.rhesus.     cccctgccccttcccaccctgatccattggactttggggccaggtgctcccttattgggg    44580

Papio.anubis.clone.rp41-133b2.8034-40080.revcompl.Baboon            ------------------------------------------------------------    0
BCRP3.HUMAN.NCBI.REF                                                ------------------------------------------------------------    0
LOC106996293.glutathione.hydrolase5.proenzyme-like-GGT1.rhesus.     ctgcacagtgacacctaggactagccaccagggggtgccgcgcccctggtgctttcttag    44640

Papio.anubis.clone.rp41-133b2.8034-40080.revcompl.Baboon            ------------------------------------------------------------    0
BCRP3.HUMAN.NCBI.REF                                                ------------------------------------------------------------    0
LOC106996293.glutathione.hydrolase5.proenzyme-like-GGT1.rhesus.     gcagtgggtggccagctgatgctgggaacctgggcaccttctcagacccatgggcatcca    44700

Papio.anubis.clone.rp41-133b2.8034-40080.revcompl.Baboon            ------------------------------------------------------------    0
BCRP3.HUMAN.NCBI.REF                                                ------------------------------------------------------------    0
LOC106996293.glutathione.hydrolase5.proenzyme-like-GGT1.rhesus.     actcatcctgctaatgacacgggaggtgaagctgagttccaaggaatgggaattgggcat    44760
```

```
Papio.anubis.clone.rp41-133b2.8034-40080.revcompl.Baboon          ------------------------------------------------------------    0
BCRP3.HUMAN.NCBI.REF                                              ------------------------------------------------------------    0
LOC106996293.glutathione.hydrolase5.proenzyme-like-GGT1.rhesus.   cacgctagaggaaaacatcttagtcagagccaagcccctggggggtttccaagtataagc    44820

Papio.anubis.clone.rp41-133b2.8034-40080.revcompl.Baboon          -----------------------atcctctccagagggaggcctggttctcagggaacag    37
BCRP3.HUMAN.NCBI.REF                                              ------------------------------------------------------------    0
LOC106996293.glutathione.hydrolase5.proenzyme-like-GGT1.rhesus.   ccagagtgaaacccaagcttgtgaccctctccagagggaggcctggttttcagggaacag    44880

Papio.anubis.clone.rp41-133b2.8034-40080.revcompl.Baboon          caaatgggaagaggtccccagatcccagggatcagggcttggaccagctggggacacagc    97
BCRP3.HUMAN.NCBI.REF                                              ------------------------------------------------------------    0
LOC106996293.glutathione.hydrolase5.proenzyme-like-GGT1.rhesus.   caaatgggaagaggtccccagattccagggatcagggcttggaccagctggggacgcagc    44940

Papio.anubis.clone.rp41-133b2.8034-40080.revcompl.Baboon          ccagagggagtgggtctggaagggaacagctagacacagcagccttcaccatcggcagcc    157
BCRP3.HUMAN.NCBI.REF                                              ------------------------------------------------------------    0
LOC106996293.glutathione.hydrolase5.proenzyme-like-GGT1.rhesus.   ccagagggagtgggtctggaagggaacagctagacacagcagccttcaccactggcagcc    45000

Papio.anubis.clone.rp41-133b2.8034-40080.revcompl.Baboon          cctcccggcctccctcggggtctgctccctcctccgggcacagttccaacacctggggca    217
BCRP3.HUMAN.NCBI.REF                                              ------------------------------------------------------------    0
LOC106996293.glutathione.hydrolase5.proenzyme-like-GGT1.rhesus.   cctcccggcctccctcggggcctgctccctcctccaagcaccgttccaacacctggggca    45060

Papio.anubis.clone.rp41-133b2.8034-40080.revcompl.Baboon          gggttctgggaaa--------------ggctggtgggaggctgttatcacagcccagcac    263
BCRP3.HUMAN.NCBI.REF                                              ------------------------------------------------------------    0
LOC106996293.glutathione.hydrolase5.proenzyme-like-GGT1.rhesus.   gggttctgggaaaggctggtggaggtgggctggtggggggcggtgatcacagcccagcat    45120

Papio.anubis.clone.rp41-133b2.8034-40080.revcompl.Baboon          ctgagtatcaccagggtcactggggccagggcccaggtgaagccaggtcggggctctcct    323
BCRP3.HUMAN.NCBI.REF                                              ------------------------------------------------------------    0
LOC106996293.glutathione.hydrolase5.proenzyme-like-GGT1.rhesus.   ctgggtatcaccaggggcactggggccagggcccaggtgaagccaggtcggggctctcct    45180

Papio.anubis.clone.rp41-133b2.8034-40080.revcompl.Baboon          ttagaagccctgaaaacctggtgataccaaagggcccacagacaaacagggttttgtgcc    383
BCRP3.HUMAN.NCBI.REF                                              ------------------------------------------------------------    0
LOC106996293.glutathione.hydrolase5.proenzyme-like-GGT1.rhesus.   ttagaagccccgaaaacctggtgataccaaagggcccacagacaaacagggttttgtgcc    45240

Papio.anubis.clone.rp41-133b2.8034-40080.revcompl.Baboon          tgcggagttgagtaccagcgggtctaagccctggagggctgtgtccctggggctccccag    443
BCRP3.HUMAN.NCBI.REF                                              ------------------------------------------------------------    0
LOC106996293.glutathione.hydrolase5.proenzyme-like-GGT1.rhesus.   tgcggagttgagtaccaccgggtctaagccctggagggctgtgtccctggggctccccag    45300

Papio.anubis.clone.rp41-133b2.8034-40080.revcompl.Baboon          gggtgagatggaggtgggctcaactggtgtacacgtcactcctcaatccttattttattt    503
BCRP3.HUMAN.NCBI.REF                                              ------------------------------------------------------------    0
LOC106996293.glutathione.hydrolase5.proenzyme-like-GGT1.rhesus.   gggtgagatggaggtgggctcaactggtgtacccgtcactcctcaatccttattttatgt    45360

Papio.anubis.clone.rp41-133b2.8034-40080.revcompl.Baboon          atttaatttaaaaaac------tatttaaacaaatagagatggggtctcactatgttgac    557
BCRP3.HUMAN.NCBI.REF                                              ------------------------------------------------------------    0
LOC106996293.glutathione.hydrolase5.proenzyme-like-GGT1.rhesus.   atttaatttttaaaaaaattttttatttgaacaaatagagatggggtctcactatgttgac    45420

Papio.anubis.clone.rp41-133b2.8034-40080.revcompl.Baboon          caggctggtcttaaactcctgacttcaagcaatcctcccatctcggcctcaaaagtgcta    617
BCRP3.HUMAN.NCBI.REF                                              ------------------------------------------------------------    0
LOC106996293.glutathione.hydrolase5.proenzyme-like-GGT1.rhesus.   caggctggtcttaaactcttgacttcaagcagtcctcctagcttggcctccaaagtgcta    45480

Papio.anubis.clone.rp41-133b2.8034-40080.revcompl.Baboon          ggattac---------------agggatgagccactgcacccagcctcaatccttatttt    662
BCRP3.HUMAN.NCBI.REF                                              ------------------------------------------------------------    0
LOC106996293.glutathione.hydrolase5.proenzyme-like-GGT1.rhesus.   ggattactttggggattactttagggatgagtcactgcacgcggcctcaatccttatttt    45540

Papio.anubis.clone.rp41-133b2.8034-40080.revcompl.Baboon          ggcctgagaggaaaggccgtggcccagtttgcaggggagaagactgaggctggaggggca    722
BCRP3.HUMAN.NCBI.REF                                              ------------------------------------------------------------    0
LOC106996293.glutathione.hydrolase5.proenzyme-like-GGT1.rhesus.   ggcctgaaaggaaaggctgtggccccgtttgcaggggagaagactgaggctggaggggca    45600

Papio.anubis.clone.rp41-133b2.8034-40080.revcompl.Baboon          ggccttgctctgggtggcacagcagcaagagaagtgggagctggccacgaggcttcctgg    782
BCRP3.HUMAN.NCBI.REF                                              ------------------------------------------------------------    0
LOC106996293.glutathione.hydrolase5.proenzyme-like-GGT1.rhesus.   ggccttgctctgggttgcacagcagcaagagaagtgggagctggccatgaggcttcctgg    45660

Papio.anubis.clone.rp41-133b2.8034-40080.revcompl.Baboon          acctgatacgctgctggggtacaccctggttctccaggtcccatggggctcagcccagga    842
BCRP3.HUMAN.NCBI.REF                                              ------------------------------------------------------------    0
LOC106996293.glutathione.hydrolase5.proenzyme-like-GGT1.rhesus.   acccgaagcactggtggggttcaccctggttcttcaggtcccatggggctcagcccagga    45720

Papio.anubis.clone.rp41-133b2.8034-40080.revcompl.Baboon          ctacttcg--ggaggtgggagacttaaaccccctctttccttctcatagtcccttctccc    900
BCRP3.HUMAN.NCBI.REF                                              ------------------------------------------------------------    0
LOC106996293.glutathione.hydrolase5.proenzyme-like-GGT1.rhesus.   ctaccttggtgggggtgggagacttaaatcctctccttcattctcattgtcccttccccc    45780

Papio.anubis.clone.rp41-133b2.8034-40080.revcompl.Baboon          atcatttcctgaggaaggacattcagggacctccctggctgtgcctcag-----gactag    955
BCRP3.HUMAN.NCBI.REF                                              ------------------------------------------------------------    0
LOC106996293.glutathione.hydrolase5.proenzyme-like-GGT1.rhesus.   atcatttcctgaggaagcacattcagggacctccctggctgtgcctcagtccaaaaccag    45840

Papio.anubis.clone.rp41-133b2.8034-40080.revcompl.Baboon          aatgacacccattcctttccctgggcctttgctcaggcggtccctgcaccctggcctctg    1015
BCRP3.HUMAN.NCBI.REF                                              ------------------------------------------------------------    0
LOC106996293.glutathione.hydrolase5.proenzyme-like-GGT1.rhesus.   aatgacacgcattcctttccctgggcctttgctcaggcggtccctgcaccctggcctctg    45900

Papio.anubis.clone.rp41-133b2.8034-40080.revcompl.Baboon          cctgaccaggatggtggggagaggaggggggacgtcccctccgctgctgtctccactgtt    1075
BCRP3.HUMAN.NCBI.REF                                              ------------------------------------------------------------    0
LOC106996293.glutathione.hydrolase5.proenzyme-like-GGT1.rhesus.   cctgaccagggtggtggggagaggaggggggacgtcccctccgctgctgtctccactgtt    45960

Papio.anubis.clone.rp41-133b2.8034-40080.revcompl.Baboon          cctgctgccctgacctctgggcttccaggactgcagtgggtgggtgggtgggctggcctg    1135
BCRP3.HUMAN.NCBI.REF                                              ------------------------------------------------------------    0
LOC106996293.glutathione.hydrolase5.proenzyme-like-GGT1.rhesus.   cctgctgccctggcctctgggcttccaggactgcagtgggtgggtgggtgggctggcctg    46020

Papio.anubis.clone.rp41-133b2.8034-40080.revcompl.Baboon          agcccaggaatgcacttcggctcctggttgagcaatgtcactgaggcttgggagtcgggt    1195
BCRP3.HUMAN.NCBI.REF                                              ------------------------------------------------------------    0
LOC106996293.glutathione.hydrolase5.proenzyme-like-GGT1.rhesus.   agcccaggaatgcacttcggctcctggttgagcaaagtcactgagacttgggagtcgggt    46080

Papio.anubis.clone.rp41-133b2.8034-40080.revcompl.Baboon          cgggttgggaggaggagtccacaagcccccactgtgaaaggcagccgtgggaacagtctg    1255
BCRP3.HUMAN.NCBI.REF                                              ------------------------------------------------------------    0
LOC106996293.glutathione.hydrolase5.proenzyme-like-GGT1.rhesus.   cgggttgggaggaggcgtccacaggcccccactacgaaaggcagctgtgggaacagtctg    46140

Papio.anubis.clone.rp41-133b2.8034-40080.revcompl.Baboon          cctgtaaacaaccactccagcccaggctgaccaggggctctggct-ggacataggggcct    1314
BCRP3.HUMAN.NCBI.REF                                              ------------------------------------------------------------    0
LOC106996293.glutathione.hydrolase5.proenzyme-like-GGT1.rhesus.   cctgtaaacaaccactccagcccaggctgaccaggggctctggctgggacattgggatct    46200

Papio.anubis.clone.rp41-133b2.8034-40080.revcompl.Baboon          ggcaggctgtgtggcctgtaaggacacagtctgtctctgtgcctcagtttctctgctgcc    1374
BCRP3.HUMAN.NCBI.REF                                              ------------------------------------------------------------    0
LOC106996293.glutathione.hydrolase5.proenzyme-like-GGT1.rhesus.   ggcaggctgtgtggcctgtaaggacacagtctgtctctgtgcctcagtttctctgctgcc    46260

Papio.anubis.clone.rp41-133b2.8034-40080.revcompl.Baboon          cagttgggcgtcccagactccaggtgtagacatctggagcaggcagtgctcagctaggaa    1434
BCRP3.HUMAN.NCBI.REF                                              ------------------------------------------------------------    0
LOC106996293.glutathione.hydrolase5.proenzyme-like-GGT1.rhesus.   cagttgggcgtcccagactccaggtgtagacatctggagcaggcagtgctcagctgggaa    46320

Papio.anubis.clone.rp41-133b2.8034-40080.revcompl.Baboon          ggaagtggggaggactggaggagccacgtgtgaaggattccaacccacatcacctgcacc    1494
BCRP3.HUMAN.NCBI.REF                                              ------------------------------------------------------------    0
LOC106996293.glutathione.hydrolase5.proenzyme-like-GGT1.rhesus.   ggaagtggggaggactggaggagccatgtgtgaaggattccaacccacatcacctgcacc    46380

Papio.anubis.clone.rp41-133b2.8034-40080.revcompl.Baboon          cctgctgagcctggtcaacagagcccctcagtgggtcctcactcccctggctgcctcccg    1554
BCRP3.HUMAN.NCBI.REF                                              ------------------------------------------------------------    0
LOC106996293.glutathione.hydrolase5.proenzyme-like-GGT1.rhesus.   cctgctgagcctggtcaacagagcccctcagtgggtcctcactcccctggctgcctcccg    46440

Papio.anubis.clone.rp41-133b2.8034-40080.revcompl.Baboon          gttaggcaccctgaggcctagggagaacagggccaggccagtgtccccagagaggctgca    1614
BCRP3.HUMAN.NCBI.REF                                              ------------------------------------------------------------    0
LOC106996293.glutathione.hydrolase5.proenzyme-like-GGT1.rhesus.   gttaggcaccctgagggctggggagaacagggccaggccagtgtccccagagaggctgcg    46500
```

```
Papio.anubis.clone.rp41-133b2.8034-40080.revcompl.Baboon           ctgccagcacagtaatagcggatttggattcagggaagcagacccgcagccagggtgggg    1674
BCRP3.HUMAN.NCBI.REF                                                ------------------------------------------------------------    0
LOC106996293.glutathione.hydrolase5.proenzyme-like-GGT1.rhesus.     ctgccagcacagtaatagcggatttggattcagggaagcagacccgcagccagggtgggg    46560


Papio.anubis.clone.rp41-133b2.8034-40080.revcompl.Baboon           aagagctgcaggctgggcgtggcacctaggcggcacagcctccctccctggaggcccacg    1734
BCRP3.HUMAN.NCBI.REF                                                ------------------------------------------------------------    0
LOC106996293.glutathione.hydrolase5.proenzyme-like-GGT1.rhesus.     aagagctgcaggctgggcgtggcacctaggcggcacagcctccctccctggaggcccacg    46620


Papio.anubis.clone.rp41-133b2.8034-40080.revcompl.Baboon           ctgcatttccaggacagcaagtcccagggatggatggtcccaggtgccaagggctagagg    1794
BCRP3.HUMAN.NCBI.REF                                                ------------------------------------------------------------    0
LOC106996293.glutathione.hydrolase5.proenzyme-like-GGT1.rhesus.     ctgcatttccaggacagcaagtcccagggatggatggtcccaggtgccaagggctagagg    46680


Papio.anubis.clone.rp41-133b2.8034-40080.revcompl.Baboon           catggtctgtctgcattccccacatggacgtcttgtagtcaccagcgtttgatgctgtca    1854
BCRP3.HUMAN.NCBI.REF                                                ------------------------------------------------------------    0
LOC106996293.glutathione.hydrolase5.proenzyme-like-GGT1.rhesus.     catggtctgtctgcattccccacatggacgtcttgtagtcaccagcgtttgatgctgtca    46740


Papio.anubis.clone.rp41-133b2.8034-40080.revcompl.Baboon           agtgaccctgtcctctgtgcagaccgggaagcccttggcta-ccctggagggttatggga    1913
BCRP3.HUMAN.NCBI.REF                                                ------------------------------------------------------------    0
LOC106996293.glutathione.hydrolase5.proenzyme-like-GGT1.rhesus.     agtcccccctgtcctctctgcggactgagaagcccttggtcatccttagggggttgtggaa    46800


Papio.anubis.clone.rp41-133b2.8034-40080.revcompl.Baboon           cccaggccaggctgcagaagcatagggacttgaacccaagttttaagtgacaccactttg    1973
BCRP3.HUMAN.NCBI.REF                                                ------------------------------------------------------------    0
LOC106996293.glutathione.hydrolase5.proenzyme-like-GGT1.rhesus.     cccaaaccaggctgcagaagcatagggacttgaacccaagttttaagtgacaccaccttt    46860


Papio.anubis.clone.rp41-133b2.8034-40080.revcompl.Baboon           tgtcccccctccctctgtctctgtttagcgccacctcgatgctgcttgtgctgggccatgc    2033
BCRP3.HUMAN.NCBI.REF                                                ------------------------------------------------------------    0
LOC106996293.glutathione.hydrolase5.proenzyme-like-GGT1.rhesus.     tgtcccccctccctcggtctctgttcagttccacttcgatattgcctgtgctgggccatgc    46920


Papio.anubis.clone.rp41-133b2.8034-40080.revcompl.Baboon           aaagagggttaggggatagagatgggagctggggagtgcggctccactctgggag-gggg    2092
BCRP3.HUMAN.NCBI.REF                                                ------------------------------------------------------------    0
LOC106996293.glutathione.hydrolase5.proenzyme-like-GGT1.rhesus.     agagagggttaggggatagagatgggaactggggagtggggctccactctcagagagggg    46980


Papio.anubis.clone.rp41-133b2.8034-40080.revcompl.Baboon           cagccttgctggatccaggggagatagttgagcagccccagctctgctttcccggagctg    2152
BCRP3.HUMAN.NCBI.REF                                                ------------------------------------------------------------    0
LOC106996293.glutathione.hydrolase5.proenzyme-like-GGT1.rhesus.     cagccttgctggatccaggggagatagttgagcagccccagctctgctttcccggagctg    47040


Papio.anubis.clone.rp41-133b2.8034-40080.revcompl.Baboon           ccgggaaccccgggaatggtgtggagattcctgggagccctgcccccacctg-caaccgc    2211
BCRP3.HUMAN.NCBI.REF                                                ------------------------------------------------------------    0
LOC106996293.glutathione.hydrolase5.proenzyme-like-GGT1.rhesus.     ctgggaaccccgggaatggtgtggagattcctgggagctctgcccccacttgacaaccac    47100


Papio.anubis.clone.rp41-133b2.8034-40080.revcompl.Baboon           agtgcagcaggcgccaagttctccagcacattgggacagtgtgatcctggcctctggtga    2271
BCRP3.HUMAN.NCBI.REF                                                ------------------------------------------------------------    0
LOC106996293.glutathione.hydrolase5.proenzyme-like-GGT1.rhesus.     agtgcagcaggcaccaagttctcctgcacattgggacagtgtgaccctgggctctggtta    47160


Papio.anubis.clone.rp41-133b2.8034-40080.revcompl.Baboon           gtggcaggcaggggcctttggacctaccggcagtgagggagttaacacagcagctggctc    2331
BCRP3.HUMAN.NCBI.REF                                                ------------------------------------------------------------    0
LOC106996293.glutathione.hydrolase5.proenzyme-like-GGT1.rhesus.     gtggcagg-tggggccttgggtcctaccagcagtgagggagttagcacagcagctggctc    47219


Papio.anubis.clone.rp41-133b2.8034-40080.revcompl.Baboon           ctctaggcaaggaaaactcccctcagacgctttgctgcctggcctcctgccaggaacaag    2391
BCRP3.HUMAN.NCBI.REF                                                ------------------------------------------------------------    0
LOC106996293.glutathione.hydrolase5.proenzyme-like-GGT1.rhesus.     ctctagggaaggaaaactcccttcagacactttggtgcctggcctcctgccaggaacaag    47279


Papio.anubis.clone.rp41-133b2.8034-40080.revcompl.Baboon           caggagctgaaaactagaagttgaggcgtgagtttggccactccatagtgtgcatttagg    2451
BCRP3.HUMAN.NCBI.REF                                                ---------------------------------------actccgtagtgtgcacttggt    21
LOC106996293.glutathione.hydrolase5.proenzyme-like-GGT1.rhesus.     caggagctgaaaactagaagttgaggcataagtttggccactctgtagtgtgtacctggg    47339
                                                                                                           ****  ******* *  * *

Papio.anubis.clone.rp41-133b2.8034-40080.revcompl.Baboon           gagggcagcagcacgtcacagccgccggccgcaagccatccgtccattcactcatctgtc    2511
BCRP3.HUMAN.NCBI.REF                                                gagggcagcagctcgccacagctgccagccg-------tctgtccattcacccatctgtc    74
LOC106996293.glutathione.hydrolase5.proenzyme-like-GGT1.rhesus.     gagggcagcagctcgccacagctgccagctg------------ccagccgtctacccatt    47387
                                                                    ************ ** ****** *** ** *            ***  *    * *  *

Papio.anubis.clone.rp41-133b2.8034-40080.revcompl.Baboon           catctggcagcccgctgttcagacctatctgtctgtccacccatctgtaagcctgtctct    2571
BCRP3.HUMAN.NCBI.REF                                                catctggcagcccgctgttcagacccgtctgtctgtccgcccatctgtaagcccatctct    134
LOC106996293.glutathione.hydrolase5.proenzyme-like-GGT1.rhesus.     cacctggcagcccgcttttcagac----ctgcctgtccacccatctataagcccatctct    47443
                                                                    ** ************* *******    *** ****** ******* ******  *****

Papio.anubis.clone.rp41-133b2.8034-40080.revcompl.Baboon           gtcccattgtctatctgaccatctttctcttactgtcctcttcgtccagctatctggcct    2631
BCRP3.HUMAN.NCBI.REF                                                gtcccattgtctatctgaccatctttctcttactgtcctctttgtctagctatctggcct    194
LOC106996293.glutathione.hydrolase5.proenzyme-like-GGT1.rhesus.     gtcccgttgtctatctgaccatctttctcttactgtcctctctgtccagcaatctggcct    47503
                                                                    ***** ***********************************  *** *** *********

Papio.anubis.clone.rp41-133b2.8034-40080.revcompl.Baboon           gtctgttgatccatcttcgtgtctgtc-tgtggccccacctgtttgtccatctgtccaat    2690
BCRP3.HUMAN.NCBI.REF                                                atctgtcgatccatcttcgtgtctgtcttcagcccccacctgtttgtccatctgtccaat    254
LOC106996293.glutathione.hydrolase5.proenzyme-like-GGT1.rhesus.     gtctgtcgatccatcttcttgtctaac-tgtggccccacctatttgtccatctgtccaat    47562
                                                                     ***** *********** *****  * *  * ******** ******************

Papio.anubis.clone.rp41-133b2.8034-40080.revcompl.Baboon           tatctgtgagtctatctgtgcatcttcttgtccatccatctgcccacccatctgtccctg    2750
BCRP3.HUMAN.NCBI.REF                                                tacctgtgagtctatctatgcaccttcttgtccattcatctgcccacccatctgtccctc    314
LOC106996293.glutathione.hydrolase5.proenzyme-like-GGT1.rhesus.     tacctttgattctatctgtgcatcttcttgtccatccatctgcccacccatctgtccctg    47622
                                                                    ** ** *** ******* **** ************ ***********************

Papio.anubis.clone.rp41-133b2.8034-40080.revcompl.Baboon           tgtctgctcaccggcctccccccctctcctcctgggccacagagccatggcccggggctgt    2810
BCRP3.HUMAN.NCBI.REF                                                cgtctgcccaccggcct--cccctctccttctgggccgcagagccatggcccaggactgc    372
LOC106996293.glutathione.hydrolase5.proenzyme-like-GGT1.rhesus.     tgtctgctcactggcct--cccctctcctcctgggccacagagccatggcccagggctgt    47680
                                                                     ****** *** *****  ********** ******* ************** ** ***

Papio.anubis.clone.rp41-133b2.8034-40080.revcompl.Baboon           ggggccacggtcggcctagtcctgctggggctggggctggcgctggctgtcattgtgctg    2870
BCRP3.HUMAN.NCBI.REF                                                agagccatggttggcctggtcctgctggggctggggcttgtgctggctgtcattgtgctg    432
LOC106996293.glutathione.hydrolase5.proenzyme-like-GGT1.rhesus.     gggtccttggtcagcctggtgctgctggggctggggctggggctggctgtcactgtgctg    47740
                                                                     *  **  ***  **** ** ***************** * *********** *******

Papio.anubis.clone.rp41-133b2.8034-40080.revcompl.Baboon           gctgtgatcctctctcgccaccaagccccctgcggcccccaggcctttgcccacgctgct    2930
BCRP3.HUMAN.NCBI.REF                                                gctgtggtcctctctcgacaccaggccccatttgacccc-ggcctttgcccacgccgct    491
LOC106996293.glutathione.hydrolase5.proenzyme-like-GGT1.rhesus.     gctgtggtcctctctcgccaccagactccctgtggcccccaggcctttgcccacgctact    47800
                                                                    ****** ********** *****  * ** *  * ***** ***************  **

Papio.anubis.clone.rp41-133b2.8034-40080.revcompl.Baboon           gttgctgccgactccaaggtttgctcggatattggacggtgagtgagaggtggcggggag    2990
BCRP3.HUMAN.NCBI.REF                                                gttgctgctgactccaaggtctgctcggatattggacggtgagtgagacgtgggaggaag    551
LOC106996293.glutathione.hydrolase5.proenzyme-like-GGT1.rhesus.     gttgctgctgactccaaggtctgctcaaatattggactgtgagtgagacgtgggaggaag    47860
                                                                    ******** *********** *****  ********* ********** ****  ** **

Papio.anubis.clone.rp41-133b2.8034-40080.revcompl.Baboon           ctgggtggcccttggcagccagcccctcctggagaaggtgcgtgtgtgtgagtgtgtgta    3050
BCRP3.HUMAN.NCBI.REF                                                ctgggtggcccttggcagccagcccctcctggagaaggcgtgtgtgtgagagtgtgtgtg    611
LOC106996293.glutathione.hydrolase5.proenzyme-like-GGT1.rhesus.     ctgggtggcctttggcagccagcccctcctggagaaggcgtgtgtgtttgagtgtgtgag    47920
                                                                    ********** *************************** * ******  *********

Papio.anubis.clone.rp41-133b2.8034-40080.revcompl.Baboon           tgggtgtgtg--------------------------------------------------    3060
BCRP3.HUMAN.NCBI.REF                                                tgtgagcatgtgtgtgtgtgagagagtatgtgtcagtgtgtgtgggtatatgagtgtgag    671
LOC106996293.glutathione.hydrolase5.proenzyme-like-GGT1.rhesus.     tgtgtgggcgtgcgtgtgtgattgcgtgt------gtgagtgtgtatgtatgtgtgtgag    47974
                                                                    ** * *   *

Papio.anubis.clone.rp41-133b2.8034-40080.revcompl.Baboon           ----------gggagtgtgtgtgagtgtgtgattgcgtgcgtgtgtgaga----------    3100
BCRP3.HUMAN.NCBI.REF                                                tgtggggtgtgggtgtgtgtgaatgtgtgtgatcgtgtttgggtgtgtgtatgtgtgagt    731
LOC106996293.glutathione.hydrolase5.proenzyme-like-GGT1.rhesus.     tggggggtgtgggggtgtgtgaatgtgtgtgattgtgtttgggggtgtat--------gt    48026
                                                                              *** *******   ********* * **  * * ***

Papio.anubis.clone.rp41-133b2.8034-40080.revcompl.Baboon           ------------------------------------------------------------    3100
BCRP3.HUMAN.NCBI.REF                                                gtgggtgtgtgtgaatgtgtgtgagtgtgtttgtgtgtatgtgtgagtgtgggtgggggt    791
LOC106996293.glutathione.hydrolase5.proenzyme-like-GGT1.rhesus.     gtgggtgtgtgtgattgtgtttgggtatgtgtgggtgtatgtgtgagagtgagtg-----    48081


Papio.anubis.clone.rp41-133b2.8034-40080.revcompl.Baboon           ------------------------------------------------------------    3100
BCRP3.HUMAN.NCBI.REF                                                atatgagtgtgagtgtgtgggtgggtgtgaacgtgtgtgattgtgttttgctgtgtgagg    851
LOC106996293.glutathione.hydrolase5.proenzyme-like-GGT1.rhesus.     ----------tagggggtgtgcgtgtgtgaatgtgtgtgattgtgtttgggtgtgtgtgt    48131


Papio.anubis.clone.rp41-133b2.8034-40080.revcompl.Baboon           ------------------------------gtgtgagtgtgtgtgagtgtgtggggtgtg    3133
BCRP3.HUMAN.NCBI.REF                                                gtgtgtgtgactatgagtgtgt-----gagtgtgggtgtgtgtaaatgtgtgtgattgtg    906
LOC106996293.glutathione.hydrolase5.proenzyme-like-GGT1.rhesus.     gggagggtgagtgtgactgtgtgggaggggtgtgggtgtgtgtgaatgtgtgtgattgtg    48191
                                                                                               * *** * ******** * ****** *  ****
```

```
Papio.anubis.clone.rp41-133b2.8034-40080.revcompl.Baboon              ggaatgtgtgtgggagtgtgtgtgtgtgagtgtgc--atgtgtgtgggtgtgggggtgaa    3191
BCRP3.HUMAN.NCBI.REF                                                  tgagtgtatgtgtgggtgtgagtgtgtgagtgtgagtatgggggtgtgggtgtgtgtgaa    966
LOC106996293.glutathione.hydrolase5.proenzyme-like-GGT1.rhesus.       ggtatgtgtatgt-------------------------------------gtgggtgtgta    48215
                                                                       *  *** * **                                     *** * *** *

Papio.anubis.clone.rp41-133b2.8034-40080.revcompl.Baboon              tgtgtgtgattgtgtgggggtatgtgtatgtgtgtgtgtgtgtgtgtgtgtg--------    3243
BCRP3.HUMAN.NCBI.REF                                                  tgtgcgtgattgtgtgtgggtatgtgtgtgtgtgtgagtgtgtgtgtgcgtgtgtg    1026
LOC106996293.glutathione.hydrolase5.proenzyme-like-GGT1.rhesus.       agtgcgtgtgtgtgtgggtgtatgtatgtgtgcgtgtgtgagtgtgtgtgtgcgtgtgtg    48275
                                                                       *** ***  ****** * ****** * **** ***** * ***********

Papio.anubis.clone.rp41-133b2.8034-40080.revcompl.Baboon              --cacgtgcactggcccaggaagcaggagccgtg--tgtgtgtgggcttcagcacctgca    3299
BCRP3.HUMAN.NCBI.REF                                                  tgcacgtgcactggcccaggcagcaggagcc----atgtgtgtgggcttcagcacctgca    1082
LOC106996293.glutathione.hydrolase5.proenzyme-like-GGT1.rhesus.       tacaagtgcactggcccaggaagcaggagccgtgtgtgtgtgtgggcttcagcacctgca    48335
                                                                        ** *************** **********     ************************

Papio.anubis.clone.rp41-133b2.8034-40080.revcompl.Baboon              gggcttgggcgcaaggaggcagcctcagggcccttgcacagaacaggtggcagggtgtgc    3359
BCRP3.HUMAN.NCBI.REF                                                  gggcttgagcgcaaggagacagcctcagggcccttgcacagaacaggcggcagggtgtgc    1142
LOC106996293.glutathione.hydrolase5.proenzyme-like-GGT1.rhesus.       gggcttgggcacaaggaggcagcctcagggcccttgcacagaacaggtggcagggtgtgc    48395
                                                                      ******* ** ******* **************************** ************

Papio.anubis.clone.rp41-133b2.8034-40080.revcompl.Baboon              ccatggggcagatggtgatttagggaca---gtcgtgtgtgagtccacacctggctccag    3416
BCRP3.HUMAN.NCBI.REF                                                  ccgtggggcagatggggacttggggacaatggtggtgtgtgagtccatacctggctccag    1202
LOC106996293.glutathione.hydrolase5.proenzyme-like-GGT1.rhesus.       ccatggggcagatggtgatttagggaca---gtcatgtgtgagtccacacctggctccag    48452
                                                                      ** ************ ** ** ******   **  ************ ************

Papio.anubis.clone.rp41-133b2.8034-40080.revcompl.Baboon              gattcaggaggctcatttgcatatcctaggtgggaaccggtctggccccgcctgaccctg    3476
BCRP3.HUMAN.NCBI.REF                                                  gattcaggaggcccatttgcacatcccaggtgggaacctgtctggccccggctgaccctg    1262
LOC106996293.glutathione.hydrolase5.proenzyme-like-GGT1.rhesus.       gattcaggagacccatttgcacatcccaggtgg---------------------------    48485
                                                                      ********** * ******** **** ******

Papio.anubis.clone.rp41-133b2.8034-40080.revcompl.Baboon              ctggccagtgcagccccttcagtgaggccaattctccaaggctggggtcttctcccagg    3536
BCRP3.HUMAN.NCBI.REF                                                  ctggccggtgcaggccccttcagtgaggccaattctccaaggctgcggtcttctcccagg    1322
LOC106996293.glutathione.hydrolase5.proenzyme-like-GGT1.rhesus.       --ggccagtacaggccccttcagtgaggccaattctccaaggctggggtcttctcccagg    48543
                                                                        **** ** *** ******************************* **************

Papio.anubis.clone.rp41-133b2.8034-40080.revcompl.Baboon              gtcataggtgaagggcttcagaggctccctgtgtgggtactggcctgctggggtacacac    3596
BCRP3.HUMAN.NCBI.REF                                                  gtcatgggtgaaggggtttggaggctccctgcgtgggtactggcctgctgggttacacac    1382
LOC106996293.glutathione.hydrolase5.proenzyme-like-GGT1.rhesus.       gtcataggtgaagggcttcagaggctccctgtgtgggtactggcctgctggggtacacac    48603
                                                                      ***** ********* **  *********** ******************** *******

Papio.anubis.clone.rp41-133b2.8034-40080.revcompl.Baboon              aatgctgccacagccagtctgccccaactcccagcccatgaccacatctcgggtctctct    3656
BCRP3.HUMAN.NCBI.REF                                                  aatgctgccatagccagtctgcccctacacccagcctggggccacatctcaggtctctca    1442
LOC106996293.glutathione.hydrolase5.proenzyme-like-GGT1.rhesus.       aatgctgccacagccagtctgccccagcttccagctgggggccacatctcgggtttctct    48663
                                                                      ********** **************  *  *****    * ********* *** ****

Papio.anubis.clone.rp41-133b2.8034-40080.revcompl.Baboon              gtcctggggaggctggtgccccacccctcacatcctctctccctgagtcagggcctgggt    3716
BCRP3.HUMAN.NCBI.REF                                                  gtcctgaggagcccggtgccccacccctcacatcctctctccctgagtcagggcctgggt    1502
LOC106996293.glutathione.hydrolase5.proenzyme-like-GGT1.rhesus.       gtcctggggagcctggtgccccacccctcacatcctctctccctgagtcagggcctgggt    48723
                                                                      ****** **** * **********************************************

Papio.anubis.clone.rp41-133b2.8034-40080.revcompl.Baboon              ctcgtgagctgagcgactgatacttggtgtcctggatgagggtgtggtggagagaggcca    3776
BCRP3.HUMAN.NCBI.REF                                                  ctcgtgagctgagtgactgatacttggtgtcctggatgagggcgtgatggagaggggcca    1562
LOC106996293.glutathione.hydrolase5.proenzyme-like-GGT1.rhesus.       ctcctgagctgagtgactgatacttggtgtcctgaatgagggtgtggtggagaggggcca    48783
                                                                      *** ********* ******************** ******* *** ******* *****

Papio.anubis.clone.rp41-133b2.8034-40080.revcompl.Baboon              cggcgggtgtttcctgaccctcttccaggaaacccagcccaagggaggccttcgctgctg    3836
BCRP3.HUMAN.NCBI.REF                                                  cagcgggtgtttcctgaccctcttccaggaagg-------------------tgctgctg    1603
LOC106996293.glutathione.hydrolase5.proenzyme-like-GGT1.rhesus.       cggcgggtgtttcctgaccctcttccaggaaacccagcccaagggaggccttcgctgctg    48843
                                                                      * *****************************                      *******

Papio.anubis.clone.rp41-133b2.8034-40080.revcompl.Baboon              ccactgcagagaggacacatacaggaggccccttcctgccccctgcctcccattgggccc    3896
BCRP3.HUMAN.NCBI.REF                                                  ccgctgcagggaggacacatacaggatgccccttcctgccccctgcctcccattgggccc    1663
LOC106996293.glutathione.hydrolase5.proenzyme-like-GGT1.rhesus.       ccactgcagagaggacacatacaggacgccccttcctgccccctgcctgccattgggccc    48903
                                                                      ** ****** **************** ********************* ***********

Papio.anubis.clone.rp41-133b2.8034-40080.revcompl.Baboon              acaaaagccgggggaagcctcccctccctg-cagccacctggtctgcttcccagaagttc    3955
BCRP3.HUMAN.NCBI.REF                                                  acaaaagccagggcaagcctcccctccctgccagccacctggtctgcttcccagaaattc    1723
LOC106996293.glutathione.hydrolase5.proenzyme-like-GGT1.rhesus.       acaaaagccggggcaagcctcccctccctg-cagccacctggtctgcttcccagaagctc    48962
                                                                      ********* *** **************** *************************  **

Papio.anubis.clone.rp41-133b2.8034-40080.revcompl.Baboon              tgtcttgcagtccgttgggaggatcccagtgctttgtaaactaaagcaagggaggcgtgg    4015
BCRP3.HUMAN.NCBI.REF                                                  tgtcttgcaggctgttgggaggatcccagtactttgtaaactaaagcaagggaggagtgg    1783
LOC106996293.glutathione.hydrolase5.proenzyme-like-GGT1.rhesus.       tgtcttgcaggctgttgggaggatcccagtgctttgtaaactaaagcaagggaggcgtgg    49022
                                                                      ********** * ***************** ************************ ****

Papio.anubis.clone.rp41-133b2.8034-40080.revcompl.Baboon              ccgttctctctctttgttcattcattcaccttttgagtcattccttcctccctccattcc    4075
BCRP3.HUMAN.NCBI.REF                                                  ccgttctctc----tgttcattcattcaccttttcattcattccttcttccctccattcc    1839
LOC106996293.glutathione.hydrolase5.proenzyme-like-GGT1.rhesus.       ccgttctctctctttgttcattcattcaccttttgagtcatttcttcctccctccattac    49082
                                                                      **********    ******************** * ***** **** ********** *

Papio.anubis.clone.rp41-133b2.8034-40080.revcompl.Baboon              cccatctgtccatccttccctgccctgattgctcatgcca----ccccccagcccctcc    4131
BCRP3.HUMAN.NCBI.REF                                                  cccatctgtccatccttccctgccctgattgctcatgccaccgccccccgcagcccctcc    1899
LOC106996293.glutathione.hydrolase5.proenzyme-like-GGT1.rhesus.       cccatctgtccatccttccctgccctgattgctcatgcca----cacccccagcccctcc    49138
                                                                      ****************************************    * *** **********

Papio.anubis.clone.rp41-133b2.8034-40080.revcompl.Baboon              tgacctgatcatttggtttctcttcagggattttgtctcctcccacagggctgagaatg    4191
BCRP3.HUMAN.NCBI.REF                                                  tgacctggtcctttggtttctcttcagggatttctgtctcctcccacagggctgagaatg    1959
LOC106996293.glutathione.hydrolase5.proenzyme-like-GGT1.rhesus.       tgacctggtcctttggtttctctccatggctttctgtctcctcccacagggctgagaatg    49198
                                                                      ******* ** ************ ** ** *** **************************

Papio.anubis.clone.rp41-133b2.8034-40080.revcompl.Baboon              gcagctcagggacaagtgggggctggggactgcttggtctccccagtggctcccagggga    4251
BCRP3.HUMAN.NCBI.REF                                                  gcagctcagggacaagtaggggctggggactgcttagtctccccagtggctctcagggga    2019
LOC106996293.glutathione.hydrolase5.proenzyme-like-GGT1.rhesus.       gcagctcagggacaagtagagcctggtgactgcttggtctcccgggtggctcctagggga    49258
                                                                      ***************** * * **** ******** *******  *******  ******

Papio.anubis.clone.rp41-133b2.8034-40080.revcompl.Baboon              tttgagggattgatgccagctgccaccccaggctgtgcccctcctctgctcaggaggaca    4311
BCRP3.HUMAN.NCBI.REF                                                  tttgagggtttgacgccagctgccaccccaggctgtgcccctcctctgctcaggaggaca    2079
LOC106996293.glutathione.hydrolase5.proenzyme-like-GGT1.rhesus.       tttgagggattgatgcctgctg-------aggctgtgcccctcctctgctcaggaggaca    49311
                                                                      ******** **** *** ****       *******************************

Papio.anubis.clone.rp41-133b2.8034-40080.revcompl.Baboon              tacagagatgtggcacccacttaaactcaaagttgcaaagatgcaaatgagactggggtc    4371
BCRP3.HUMAN.NCBI.REF                                                  tacag-gatgcaacacccacttaaactcgaagttgcaaagatgcaaatgagactggggtc    2138
LOC106996293.glutathione.hydrolase5.proenzyme-like-GGT1.rhesus.       tacagagatgtggcacccacttaaactcaaagttgcacagatgcaaatgagactggggtc    49371
                                                                      ***** ****   *************** ******** **********************

Papio.anubis.clone.rp41-133b2.8034-40080.revcompl.Baboon              tcaggcaccagagacca-ccgtgggcacatggcctttgggagtggggacctgctgccaca    4430
BCRP3.HUMAN.NCBI.REF                                                  tcaggcaccagagaccacccgtgggcacgtggcttttgggattggagacctgctgccaca    2198
LOC106996293.glutathione.hydrolase5.proenzyme-like-GGT1.rhesus.       tcaggcaccagagacca-ccgtgggcacgtgacctttgggagtggggacctgctgccaca    49430
                                                                      ***************** ********** ** * ******* *** **************

Papio.anubis.clone.rp41-133b2.8034-40080.revcompl.Baboon              gatctctgaatggagtctggacctgctaggtctccccgagtgactgtctgggggtctcca    4490
BCRP3.HUMAN.NCBI.REF                                                  gatctctga--agagtctggacctgctgggtctccccaagtgactctctgggggtctcca    2256
LOC106996293.glutathione.hydrolase5.proenzyme-like-GGT1.rhesus.       gatctctgagtggagtctggacctactgggtctccccaagtgactgtctgggggtctctg    49490
                                                                      *********   ************ ** ********* ******* ************

Papio.anubis.clone.rp41-133b2.8034-40080.revcompl.Baboon              tagtgtgccctgctgtgcgcgtgagggtcagtggttggggaggggtctctgctctaatgc    4550
BCRP3.HUMAN.NCBI.REF                                                  tagcatgccctgctgtgtgcatgacggtcactggttgggtaggggtctctactctaaagc    2316
LOC106996293.glutathione.hydrolase5.proenzyme-like-GGT1.rhesus.       tagcatgccctgctgtgtacgtgagggtcagtggttggggaggggtctctgctctaatgc    49550
                                                                      ***  ************  * *** ***** ******** ********** ****** **

Papio.anubis.clone.rp41-133b2.8034-40080.revcompl.Baboon              tccctccgccggcactccctcaaactctcccttggtgaggagagaggatgtggtttgccc    4610
BCRP3.HUMAN.NCBI.REF                                                  tccctctgccggcatcccctcgaactctcccttggtgaagagagaggatgtggtttgccc    2376
LOC106996293.glutathione.hydrolase5.proenzyme-like-GGT1.rhesus.       ttcctacaccggcactccctcaaactctcccttggtgaagagagaggatgtggtttgccc    49610
                                                                      * ***   ******  ***** **************** *********************

Papio.anubis.clone.rp41-133b2.8034-40080.revcompl.Baboon              cagtgttttatcgaacaactctctccacttcctgttttcagaagctgggagtggaagaga    4670
BCRP3.HUMAN.NCBI.REF                                                  cagtgttttatcaaacaactctctccacttcctgttttaagaagctgggagtggaagaga    2436
LOC106996293.glutathione.hydrolase5.proenzyme-like-GGT1.rhesus.       cagtgttttatcgaacaactctctccacttcctgttttcagaagccgggagtggaagaga    49670
                                                                      ************ ************************* ****** **************

Papio.anubis.clone.rp41-133b2.8034-40080.revcompl.Baboon              gcctggggctggccccagctgctgctgcggaacaggggtcactggacgctgggaccctgg    4730
BCRP3.HUMAN.NCBI.REF                                                  gcctggggctggccccagctgctgctgcgaaacaggggtcactggacgctgggaccctgg    2496
LOC106996293.glutathione.hydrolase5.proenzyme-like-GGT1.rhesus.       gcctggggctggccccagctgctgctgcggaacaggggtcactggacgctgggaccctgg    49730
                                                                      ***************************** ******************************

Papio.anubis.clone.rp41-133b2.8034-40080.revcompl.Baboon              ccgggctggctgggggcctcaggaagaggcctgctgcagcgtcatcctggccaagatccc    4790
BCRP3.HUMAN.NCBI.REF                                                  ccgggctggctggaggcctcaggaagaggcctgctacagtgtcatcctggccaagattcc    2556
LOC106996293.glutathione.hydrolase5.proenzyme-like-GGT1.rhesus.       ccgggctggctgggggcctcaggaagaggcctgctgcagcgtcatcctggccgagatccc    49790
                                                                      ************* ********************* *** ************ **** **

Papio.anubis.clone.rp41-133b2.8034-40080.revcompl.Baboon              tccctgcaggggcccctggccatgctgccgcagggtctgctggggccaccagaagcccat    4850
BCRP3.HUMAN.NCBI.REF                                                  tccctgcagaggaccctggccacgctgccacagggtctgctggggccaccagaagcccat    2616
LOC106996293.glutathione.hydrolase5.proenzyme-like-GGT1.rhesus.       tccctgcaggggcccctggccatgctgccgcagggtctgctggggccaccagaagcccac    49850
                                                                      ********* ** ********* ****** *****************************
```

```
Papio.anubis.clone.rp41-133b2.8034-40080.revcompl.Baboon          gctcctgcctccatctctcccctctgtgctcacctctcaccagcaggccctcccagagtc    4910
BCRP3.HUMAN.NCBI.REF                                              gctcctgcctccatctctcccctctgtgctcacctctcaccaggaggccctcccagagtt    2676
LOC106996293.glutathione.hydrolase5.proenzyme-like-GGT1.rhesus.   gctcctgcctccatctctgccctgtgtgctcacctctcaccagcaggccctcccagagtc    49910
                                                                  ****************** **** ******************* ***************

Papio.anubis.clone.rp41-133b2.8034-40080.revcompl.Baboon          cagtctc--ctgctgtttgtttgtttgtttg---tttgagacagtgtcttgctctgtcac    4965
BCRP3.HUMAN.NCBI.REF                                              cagtgtcctg----------cttttttttttttttttagatggtgtctcgttctgtcac    2726
LOC106996293.glutathione.hydrolase5.proenzyme-like-GGT1.rhesus.   cagtctcttctgctctttttttgtttgtttgtttttgagatggtgtctcactctgtcac    49970
                                                                  **** **              * *** ***    *** ***  ******   ********

Papio.anubis.clone.rp41-133b2.8034-40080.revcompl.Baboon          caggctggagtgcagtggcgcgatttcggctcactgcaacctccacctcctcggttcaaa    5025
BCRP3.HUMAN.NCBI.REF                                              caggctggagtgcagtggcgcgatctcagctcactgcaacctctgcttccttggttcaaa    2786
LOC106996293.glutathione.hydrolase5.proenzyme-like-GGT1.rhesus.   caggctggagtgcagtggcgcaatctcggctcactgaaatttc-----------------    50013
                                                                  ********************* ** ** ******** **  **

Papio.anubis.clone.rp41-133b2.8034-40080.revcompl.Baboon          tgattctcctgcctcagcctcctgagtagctgggactacaggcgccagccaccatgccca    5085
BCRP3.HUMAN.NCBI.REF                                              tgattctcctgcctcagcctcctgagtagctgggactacaggtgccagccaccacgccca    2846
LOC106996293.glutathione.hydrolase5.proenzyme-like-GGT1.rhesus.   ------------------------------------------------------------    50013


Papio.anubis.clone.rp41-133b2.8034-40080.revcompl.Baboon          gttaatttttgtatttttggtagagacagggtttcactatgttggccaggatggtctcaa    5145
BCRP3.HUMAN.NCBI.REF                                              ggtaatttttgtatttttagtagagacggggtttcaccatgttggccaggatggtctcta    2906
LOC106996293.glutathione.hydrolase5.proenzyme-like-GGT1.rhesus.   ------------------------------------------------------------    50013


Papio.anubis.clone.rp41-133b2.8034-40080.revcompl.Baboon          tctcttgacctcatgatttgcccacctcggcctcccaaactgctgggattacaagagtga    5205
BCRP3.HUMAN.NCBI.REF                                              tctct--------tgattcgcccgccttggcctcccaaagtgctggaattacaggagtga    2958
LOC106996293.glutathione.hydrolase5.proenzyme-like-GGT1.rhesus.   ------------------------------------------------------------    50013


Papio.anubis.clone.rp41-133b2.8034-40080.revcompl.Baboon          gccacggtgcccggcccagtctcctactctttcgg---caggttttattcttgggattct    5262
BCRP3.HUMAN.NCBI.REF                                              gtcatggcacccggcctcatctcctactctttcagcaccaggttttactcttgggattct    3018
LOC106996293.glutathione.hydrolase5.proenzyme-like-GGT1.rhesus.   -----------------cgtctcctactctttcagcatcaggttttattactgggattct    50056
                                                                                     ************** *   ********* *  *********

Papio.anubis.clone.rp41-133b2.8034-40080.revcompl.Baboon          gctacagccagc-accccgggtgtgagttcctaaggcttatgtgagtgtgaacccagcac    5321
BCRP3.HUMAN.NCBI.REF                                              gctacagccgcagcccctgggtgcgagttcctaagctttctgtgagtgtggacccagcac    3078
LOC106996293.glutathione.hydrolase5.proenzyme-like-GGT1.rhesus.   gctacagccagagaccctgggagcagattcctaaggcttatgtgagtgtggacccagcac    50116
                                                                  *********     *** *** *    ********  ** ********** *********

Papio.anubis.clone.rp41-133b2.8034-40080.revcompl.Baboon          catgcctagtagacatacaaaagaagcatggtga-------------catttccagcata    5368
BCRP3.HUMAN.NCBI.REF                                              cgtgcctagtagacatacaaaaggagcatggtgacagtgaggtctgtcatctccagcata    3138
LOC106996293.glutathione.hydrolase5.proenzyme-like-GGT1.rhesus.   cgtgcctagtagacatacaaaaggagcatggtgacagtgaggtctgtcatctccagctta    50176
                                                                  * ******************** **********             *** ****** **

Papio.anubis.clone.rp41-133b2.8034-40080.revcompl.Baboon          atgactg--gagatccttgtcaaaaaggtgatttttggctgagcatggtggctcacacct    5426
BCRP3.HUMAN.NCBI.REF                                              atgactgttttgatccttgtaaaaaaggtgatttttggctgggtgtggtggctcacacct    3198
LOC106996293.glutathione.hydrolase5.proenzyme-like-GGT1.rhesus.   atgactgttttgatccttgtcaaaaaggtgatttttggctgagcatggtggctcacacct    50236
                                                                  *******    ********* ******************* *  ***************

Papio.anubis.clone.rp41-133b2.8034-40080.revcompl.Baboon          gtaatcccagcactttgggaggccgaggcgggtggatcacttgacgtcaggagttggaga    5486
BCRP3.HUMAN.NCBI.REF                                              gtaatcccagcactttgggaggccgatggggggtggctcacttgaggtcaggagttggagc    3258
LOC106996293.glutathione.hydrolase5.proenzyme-like-GGT1.rhesus.   gtaatcccagcactttgggaggccgaggcgggtggatcacttgaggtcaggagttggaga    50296
                                                                  ************************** * ****** ******** **************

Papio.anubis.clone.rp41-133b2.8034-40080.revcompl.Baboon          ccagcctgggcaacatggtgaaaccccgtctctactgaaaatacaaaaattagccgggca    5546
BCRP3.HUMAN.NCBI.REF                                              ccagcctgggcaacatggtgaaaccacgtctctactaaaaatacaaaaattagctgggca    3318
LOC106996293.glutathione.hydrolase5.proenzyme-like-GGT1.rhesus.   ccagcctgggcaacatggtgaaaccccgtctctactaaaaatacaaaaattagctgggca    50356
                                                                  ************************* ********** ***************** *****

Papio.anubis.clone.rp41-133b2.8034-40080.revcompl.Baboon          tggtagcaggtgcctataatcccagctactcgggaggctgaggcaggagaatcacttgaa    5606
BCRP3.HUMAN.NCBI.REF                                              tggtaacggatgcctgtaatcccagctacttgggaggctgagacaggagaatcacttgaa    3378
LOC106996293.glutathione.hydrolase5.proenzyme-like-GGT1.rhesus.   tggtagcgggtgcctgtaatcccagctacttgggagtctgagacaggagaatcacttgaa    50416
                                                                  ***** * * ***** ************** ***** ***** *****************

Papio.anubis.clone.rp41-133b2.8034-40080.revcompl.Baboon          cctgggaggcaaaggttgcagtaagccaagattgcaccactgcactacagcctgggtgac    5666
BCRP3.HUMAN.NCBI.REF                                              cccaggaggcaaaggttgcggtaagccaagattgtaccactgcactccagcctgggtgac    3438
LOC106996293.glutathione.hydrolase5.proenzyme-like-GGT1.rhesus.   cccaagaggcaaatgttgcagtgagccaagatagcaccactgcactacagcctgggtgac    50476
                                                                  **   ******** ***** ** ********* * *********** *************

Papio.anubis.clone.rp41-133b2.8034-40080.revcompl.Baboon          agagcaagatttggtctcaaaaaacag------------agagaaaagtttatatttttg    5714
BCRP3.HUMAN.NCBI.REF                                              agagcaagacttggtctcaaaaaaaaaaaaaaaagaaagaaagaaaagtttatatttttg    3498
LOC106996293.glutathione.hydrolase5.proenzyme-like-GGT1.rhesus.   agagcaagacttggtctcaaaaaaaaaaaaa--------aaagaaaagtttatatttttg    50528
                                                                  ********* ************** *             * *******************

Papio.anubis.clone.rp41-133b2.8034-40080.revcompl.Baboon          ttctaatggttatcttaatatcttcattctataattatatgttttacataattataatca    5774
BCRP3.HUMAN.NCBI.REF                                              ttctaatggttatcttaatatcgtcattctataattgtatgttttatataattataatag    3558
LOC106996293.glutathione.hydrolase5.proenzyme-like-GGT1.rhesus.   ttctaaaacttatcttaatgtcttcattctata--------ttttatataattataagag    50580
                                                                  ******   ********** ** **********        ***** **********

Papio.anubis.clone.rp41-133b2.8034-40080.revcompl.Baboon          ctacataagatatactacccctagtactttgtttttggatattctgtttgctcctgatg    5834
BCRP3.HUMAN.NCBI.REF                                              ctatataagatataatacccctagtatgttgtttttggatattctacttgctcctgatg    3618
LOC106996293.glutathione.hydrolase5.proenzyme-like-GGT1.rhesus.   ctatataagatatactacccctagtactttgtttttggatattctattcactcctgatg    50640
                                                                  *** ********** ***********  ******************  *  *********

Papio.anubis.clone.rp41-133b2.8034-40080.revcompl.Baboon          gttaatgtatgtgccaacttgccttagttatgatgccccattgtttggtcaaatacttgt    5894
BCRP3.HUMAN.NCBI.REF                                              gttaatttatatgtcaacttggctaagctatggtgccccgttgtttggtcaaatacttgt    3678
LOC106996293.glutathione.hydrolase5.proenzyme-like-GGT1.rhesus.   gttaatttatgtgtcaactttgctaagctatgatgccctgttgtttggtcaaatacttct    50700
                                                                  ****** *** ** ******  ** ** **** *****  ***************** *

Papio.anubis.clone.rp41-133b2.8034-40080.revcompl.Baboon          cagtatcttgctgggaggttatttcatagatgtaattaacattgacaggcagttgacttt    5954
BCRP3.HUMAN.NCBI.REF                                              caatatcttgctgggaggttatttcatagatgtgattaacactgacagtcagttgacttt    3738
LOC106996293.glutathione.hydrolase5.proenzyme-like-GGT1.rhesus.   caatatcttgctgggaggttatctcatagatgtgattaacattgacagtcagctgacttt    50760
                                                                  ** ******************* ********** ******* ****** *** *******

Papio.anubis.clone.rp41-133b2.8034-40080.revcompl.Baboon          aagtgaaacagaatactcaccataatatgggtgggccacctccaatcagttgaaggcctt    6014
BCRP3.HUMAN.NCBI.REF                                              aagtaaaacagattacccaccataatatgggtgggccacctccaatcagttgaaggccgt    3798
LOC106996293.glutathione.hydrolase5.proenzyme-like-GGT1.rhesus.   aggtaaaaca----------------atgtgattaacgctgacagtcagttgac------    50798
                                                                  * ** *****                *** *     * *   ** ********

Papio.anubis.clone.rp41-133b2.8034-40080.revcompl.Baboon          aagaacaaaaactgaggtttcccagagaagcaggaattctgcttcaagactataacatat    6074
BCRP3.HUMAN.NCBI.REF                                              aagaacaaaaactgaggtttcccagagaagcaggaattctgcctcaagactgtaacacac    3858
LOC106996293.glutathione.hydrolase5.proenzyme-like-GGT1.rhesus.   tttaagtaaaactgaggtttcccagagaagcaggaattctgctttaacactataacatgt    50858
                                                                     **  *********************************** * ** *** *****

Papio.anubis.clone.rp41-133b2.8034-40080.revcompl.Baboon          aaatcctgcctgagtttctggcctgctgactgctctacaggttttaggttccagacttcg    6134
BCRP3.HUMAN.NCBI.REF                                              aaaccctgcctgagtttctggcctgctgactgctctacagagtttaggttccagacttcg    3918
LOC106996293.glutathione.hydrolase5.proenzyme-like-GGT1.rhesus.   aaatcctgcctgagtttctggcctgctgactgctctccaggttttaggttccagacttcg    50918
                                                                  *** ******************************** ***  ******************

Papio.anubis.clone.rp41-133b2.8034-40080.revcompl.Baboon          agatcaactcttaccttaatttatagcctgtgggcatgccctacagattttaaacttgct    6194
BCRP3.HUMAN.NCBI.REF                                              agatcaactcttacctgaatttatagcctgctggcttgccctacagattttaaacttgct    3978
LOC106996293.glutathione.hydrolase5.proenzyme-like-GGT1.rhesus.   agatcaactcttacctgaatttataagctgctggttcgccatacagattttaaacttgct    50978
                                                                  **************** ********  ***  **   *** *******************

Papio.anubis.clone.rp41-133b2.8034-40080.revcompl.Baboon          agtccccacagtcatgtgagccaattcctaaataaatctctctctgtctatgtataacct    6254
BCRP3.HUMAN.NCBI.REF                                              agtccccacaatcatgtgagccaattcctcaataaatctctc----tctatgtataatct    4034
LOC106996293.glutathione.hydrolase5.proenzyme-like-GGT1.rhesus.   agtccccacaaccgtgtgagccaattcctaaataaatctctc----tctatgtataacct    51034
                                                                  **********  * *************** ************    *********** **

Papio.anubis.clone.rp41-133b2.8034-40080.revcompl.Baboon          gttggtttagtttctctaaaaaacttttacatccagtttcctggatgttaagtaataccg    6314
BCRP3.HUMAN.NCBI.REF                                              attggtttagtttctctgaaaagctttcacatccagtttcctggatgttaagaattactg    4094
LOC106996293.glutathione.hydrolase5.proenzyme-like-GGT1.rhesus.   attggtttagtttctctaaaaaccttttacatctagtttcctggatgttaagtaatactg    51094
                                                                   **************** **** **** ***** ****************** * *** *

Papio.anubis.clone.rp41-133b2.8034-40080.revcompl.Baboon          aacctagctagtaacttcttcctttttttttttttttttttttttgagccggagtttcg    6374
BCRP3.HUMAN.NCBI.REF                                              aaactagctagtaacttctt--tttttttttttttttttttttttttgagacagagttttg    4152
LOC106996293.glutathione.hydrolase5.proenzyme-like-GGT1.rhesus.   aaactagctagtaacttc-----------ttttcttttttttttttgagatggaattttg    51143
                                                                  ** ***************           **** ***************   ** *** *

Papio.anubis.clone.rp41-133b2.8034-40080.revcompl.Baboon          ctcttgttgcccaggctggagtgcagtggtgagatctcggctcaccgcaacctccgcctc    6434
BCRP3.HUMAN.NCBI.REF                                              ctcttgttgcccaggctggaatgcaatggcacaatctcagctcaccgcaacctccacttc    4212
LOC106996293.glutathione.hydrolase5.proenzyme-like-GGT1.rhesus.   ctcttgttgcccaggctggagtgcagtgccgcgatcttggctcaccgcaacctccacttc    51203
                                                                  ******************** **** **    ****  **************** * **

Papio.anubis.clone.rp41-133b2.8034-40080.revcompl.Baboon          ctgggttcaagcgattctcctgccttagcctcccaagtagctgggattacaggcatgtgc    6494
BCRP3.HUMAN.NCBI.REF                                              ctgggtccaagcaattctcctccctcagcctcctgagtagctgggattacaggcatgtgc    4272
LOC106996293.glutathione.hydrolase5.proenzyme-like-GGT1.rhesus.   ctgggtccaagcgattctcctccctcagcctcccgagtagctgggattacaggcatgtgc    51263
                                                                  ****** ***** ******** *** *******  *************************

Papio.anubis.clone.rp41-133b2.8034-40080.revcompl.Baboon          caccacacctggctaa-ttttgtatttttggtagaggcagggtttctccatgtgggtcag    6553
BCRP3.HUMAN.NCBI.REF                                              caccatgcttggctaatttttgtatttttagtagagacagggcttctccatgttggtcag    4332
LOC106996293.glutathione.hydrolase5.proenzyme-like-GGT1.rhesus.   caccatgctcggctaatttttgtatttttagtagagatggggcttcttcatgttggtcag    51323
                                                                  *****  *  ****** ************ ******   *** **** ***** ******
```

```
Papio.anubis.clone.rp41-133b2.8034-40080.revcompl.Baboon          attggtctcaaactcccgaactcatgtgatctgcctgccttggcctcccaaagtgctggg    6613
BCRP3.HUMAN.NCBI.REF                                               gctggtcttgaactcccaacctcaggtgat-cagccgccttggcctcacaaagtgctgga    4391
LOC106996293.glutathione.hydrolase5.proenzyme-like-GGT1.rhesus.   gctggtctcgaactcccaacctcaggtgatccacctgccttggcctcacaaagtgctggg    51383
                                                                     ******  ******* * **** *****    * *********** ***********

Papio.anubis.clone.rp41-133b2.8034-40080.revcompl.Baboon          attacaggcgtgagccaccgcacccgtctcctagtaatttcttcttttccgcgatgtgtg    6673
BCRP3.HUMAN.NCBI.REF                                               attacaggcatgagccaccgcacctggctcctagtaaattcttcttttccgtgatgtgtc    4451
LOC106996293.glutathione.hydrolase5.proenzyme-like-GGT1.rhesus.   attacaggcatgagccaccgctcccggctcctcgtaacttcttcttttctgtgatatgtc    51443
                                                                   ********* *********** ** * ***** **** *********** * *** ***

Papio.anubis.clone.rp41-133b2.8034-40080.revcompl.Baboon          tcttatctctaata----------------------------------------------    6687
BCRP3.HUMAN.NCBI.REF                                               tcttacctctaataatacttttcttcttttttttttttttgagacggagtctcgttctgtc    4511
LOC106996293.glutathione.hydrolase5.proenzyme-like-GGT1.rhesus.   tcttatctctaata----------------------------------------------    51457
                                                                   ***** ********

Papio.anubis.clone.rp41-133b2.8034-40080.revcompl.Baboon          ------------------------------------------------------------    6687
BCRP3.HUMAN.NCBI.REF                                               gcccaggcgggagtgctgtggcgcgatctccgctcactgcaagctccgccttccgggttc    4571
LOC106996293.glutathione.hydrolase5.proenzyme-like-GGT1.rhesus.   ------------------------------------------------------------    51457


Papio.anubis.clone.rp41-133b2.8034-40080.revcompl.Baboon          ------------------------------------------------------------    6687
BCRP3.HUMAN.NCBI.REF                                               acgccattctcctgcctcaacctcccgagtagctgggactacaggcgcccgccactgcgc    4631
LOC106996293.glutathione.hydrolase5.proenzyme-like-GGT1.rhesus.   ------------------------------------------------------------    51457


Papio.anubis.clone.rp41-133b2.8034-40080.revcompl.Baboon          ------------------------------------------------------------    6687
BCRP3.HUMAN.NCBI.REF                                               ccggctaatttttgtatttttagtagagacggggtttcaccgtggtctcgatctcctga    4691
LOC106996293.glutathione.hydrolase5.proenzyme-like-GGT1.rhesus.   ------------------------------------------------------------    51457


Papio.anubis.clone.rp41-133b2.8034-40080.revcompl.Baboon          ------------------------------------------------------------    6687
BCRP3.HUMAN.NCBI.REF                                               cctcgtgatccgcccgcctcggcctcccaaagtgctgggattacaggcgtgagccaccgc    4751
LOC106996293.glutathione.hydrolase5.proenzyme-like-GGT1.rhesus.   ------------------------------------------------------------    51457


Papio.anubis.clone.rp41-133b2.8034-40080.revcompl.Baboon          --------atacttttcttcttgaagtctacttcattaaaaatagtaatgctgggcatgg    6739
BCRP3.HUMAN.NCBI.REF                                               gtccggccatacttttcttcttaaagtctacttcattaaaaatagttatgctgggcatgg    4811
LOC106996293.glutathione.hydrolase5.proenzyme-like-GGT1.rhesus.   --------atacttttcttcttaaagtctacttcattaaaaatagtaatgctgggcatgg    51509
                                                                           ************** ********************** ************

Papio.anubis.clone.rp41-133b2.8034-40080.revcompl.Baboon          tggctcatgcctgtaatctcagcactttg--ggaggtcaaggtgggtggatcgtcaaagc    6797
BCRP3.HUMAN.NCBI.REF                                               tggctcatggctgtaatctcggcactttgttggaggtcgaggtgggtggatcactgaagc    4871
LOC106996293.glutathione.hydrolase5.proenzyme-like-GGT1.rhesus.   tggctcatgcctgtaatctcggcactttgttggaggttgaggtgggtggatcactgaagc    51569
                                                                   ********* ********** ********  ******  *************    ****

Papio.anubis.clone.rp41-133b2.8034-40080.revcompl.Baboon          ccaggagttcaagaccagcctgggcaacatggtgagactctgcctctac--aaaaataca    6855
BCRP3.HUMAN.NCBI.REF                                               ccaggagttcaagaccaacctgggcaacgtggcgagaccctgcctctaca-aaaaataca    4930
LOC106996293.glutathione.hydrolase5.proenzyme-like-GGT1.rhesus.   ccaggagttcaagaccagcctgggcaacatggcaagaccctggctctacagaaaaataca    51629
                                                                   ***************** ********** ***  **** *** ******  *********

Papio.anubis.clone.rp41-133b2.8034-40080.revcompl.Baboon          aaaattagctgggtgtggttaatataattctaacttggcacacttgtagtcccagctact    6915
BCRP3.HUMAN.NCBI.REF                                               aaaattagctgggtgtggctaatat--------------acacttgtagtcccagctact    4976
LOC106996293.glutathione.hydrolase5.proenzyme-like-GGT1.rhesus.   aaaattagccgggtgtggctaatataattctaagttagcacacctgtagtcccagctact    51689
                                                                   ********* ******** ******              **** ****************

Papio.anubis.clone.rp41-133b2.8034-40080.revcompl.Baboon          tgggaggttgagggggagaatcacatgagcctagaagggagagattgctgtgagccaag    6975
BCRP3.HUMAN.NCBI.REF                                               tgggatgctgaggtgggagaatcgcttgagcctagaagggagagattgctgtaagccaag    5036
LOC106996293.glutathione.hydrolase5.proenzyme-like-GGT1.rhesus.   tgggatgctgaggtgggagaatcgcttgaacctagaaggggagattgctgtgagccaag    51749
                                                                   ***** * ***** ********* * *** ********** *********** *******

Papio.anubis.clone.rp41-133b2.8034-40080.revcompl.Baboon          atcacgtcactgcgctccagcctgggagacagagtgaggctctatctcaaaaaaaaagaa    7035
BCRP3.HUMAN.NCBI.REF                                               atcacatcactgcactccagcctgggagacagagtgaggctctatctccaaaa-----aa    5091
LOC106996293.glutathione.hydrolase5.proenzyme-like-GGT1.rhesus.   atcatgtcactgcactccagcctgggagacagagtgaggctctatctcaaaaa-----aa    51804
                                                                   ****  ******* ********************************** ****     **

Papio.anubis.clone.rp41-133b2.8034-40080.revcompl.Baboon          aaaaaaaaaaaaagttatacagctttctgggttagtgcatgtatgatatattttttcatta    7095
BCRP3.HUMAN.NCBI.REF                                               aaaaaaaaaaaaaagttatacagctttcttggttagtgcatgcatgccatatttttcatta    5151
LOC106996293.glutathione.hydrolase5.proenzyme-like-GGT1.rhesus.   aaaaaaaaaagaagttatacagctttcttggttaatgcatg-----catatttttcatta    51859
                                                                   ********** ***************** ***** ******      *************

Papio.anubis.clone.rp41-133b2.8034-40080.revcompl.Baboon          ttttccacctttctgtatccttatataaaagccattagttgggttttactttatttccaa    7155
BCRP3.HUMAN.NCBI.REF                                               ttttccacctctctgtatccttatataaaaggcattagttgggttttactttattttcaa    5211
LOC106996293.glutathione.hydrolase5.proenzyme-like-GGT1.rhesus.   ttttccacctttctgtatccttatataaaaggcattagttgggtt----tttatttccaa    51915
                                                                   ********** ******************* *************    ******* ***

Papio.anubis.clone.rp41-133b2.8034-40080.revcompl.Baboon          ttactttaatttttattgattgtccttttaaatgtaactaatgatttatttgggttgaaa    7215
BCRP3.HUMAN.NCBI.REF                                               ttattttaattttt----attgtccttttaaatgtaactaatgatttatttgggttgaaa    5267
LOC106996293.glutathione.hydrolase5.proenzyme-like-GGT1.rhesus.   ttagtttaattttt----attatccttttaaatgtaactaattatttatttgggttgaaa    51971
                                                                   *** **********    *** ******************** *****************

Papio.anubis.clone.rp41-133b2.8034-40080.revcompl.Baboon          gccaccaccagtttgttttccctgcctgttctgtttcttcttatctcctctcacatcttg    7275
BCRP3.HUMAN.NCBI.REF                                               cccaccaccaatttgttttccatgcctattctatttcttcttatctcctctcacatcttg    5327
LOC106996293.glutathione.hydrolase5.proenzyme-like-GGT1.rhesus.   gccaccaccaatttgttttccatgcctactctctttcttcttatctcctcccacatcttg    52031
                                                                    ********* ********** *****  *** ***************** *********

Papio.anubis.clone.rp41-133b2.8034-40080.revcompl.Baboon          ttttgcatttattatttttattattcaatttcctgcttctctataagtttcataactgta    7335
BCRP3.HUMAN.NCBI.REF                                               ttttggatttattatttttattatttaatttcctccttctctattagtttcatagctctg    5387
LOC106996293.glutathione.hydrolase5.proenzyme-like-GGT1.rhesus.   ttttgcatttattatttttattatttaatttcctc---ctctattagttttgtaactgtg    52088
                                                                   ***** ****************** ********    ****** *****  ** ** *

Papio.anubis.clone.rp41-133b2.8034-40080.revcompl.Baboon          cagtcttggagttattttaaaagatgacagcagattattttagagcttacaacatgcatc    7395
BCRP3.HUMAN.NCBI.REF                                               cagtcttagagttattttaaaagatgacagtggattattttagagcttacaacatgcatc    5447
LOC106996293.glutathione.hydrolase5.proenzyme-like-GGT1.rhesus.   cagtcttggagttattttaaaaga-gacagtagattattttagagcttacaacatgcatc    52147
                                                                   ******* **************** *****  ****************************

Papio.anubis.clone.rp41-133b2.8034-40080.revcompl.Baboon          cttcacttaccaaagtcttaacatgagttagtaccttttttttgttgatttgtttttttctt    7455
BCRP3.HUMAN.NCBI.REF                                               cttcacttatcaaagtc-taacatgagctagtactttt------------ttgttgttgtt    5494
LOC106996293.glutathione.hydrolase5.proenzyme-like-GGT1.rhesus.   cttcacttaccaaagtc-taacatgagctagtactttttttgt-----tgttgtcgtcatt    52201
                                                                   ********* ******* ********* ****** **            *  *  *  **

Papio.anubis.clone.rp41-133b2.8034-40080.revcompl.Baboon          gagatagagggagtcttgctctgctgtccaggctggagtgcagtggagcaatcttggttc    7515
BCRP3.HUMAN.NCBI.REF                                               gagatagagagagtcttcctctgctgcccaggctggagtgcagtggagcaatcttggttc    5554
LOC106996293.glutathione.hydrolase5.proenzyme-like-GGT1.rhesus.   gagacagagggagtctcgctctgctgtccaggctggagtgcagtggagcaatcttggttc    52261
                                                                   **** **** ******  ******** *********************************

Papio.anubis.clone.rp41-133b2.8034-40080.revcompl.Baboon          actgcaacctccgcctcttaggttcaagcaattctcctgcctcagtctcctgagtagctg    7575
BCRP3.HUMAN.NCBI.REF                                               actgcaacctccacttcttgggttcaagcagttctcctgcctcagtcacctgagtagctg    5614
LOC106996293.glutathione.hydrolase5.proenzyme-like-GGT1.rhesus.   actgcaacctctgcctcttgggttcaagcaattctcctgcctcagtctcctgagtagctg    52321
                                                                   ***********  * **** ********** **************** ************

Papio.anubis.clone.rp41-133b2.8034-40080.revcompl.Baboon          ggaccacaggcgtgcaccactatgcccagctaacttttgttt---ttttctagtagagac    7632
BCRP3.HUMAN.NCBI.REF                                               ggaccacaggtgtgcaccactatgcccggccaatttttgtattc-tttttagtagagac    5673
LOC106996293.glutathione.hydrolase5.proenzyme-like-GGT1.rhesus.   ggatcacaggcgtgcaccactatgcccagctaatttttgtattctttttttagtagagac    52381
                                                                   *** ****** **************** ** ** ****** *   **** **********

Papio.anubis.clone.rp41-133b2.8034-40080.revcompl.Baboon          agggtttcaccatgttggccaggctgaccttgaactcctgaccttaagagatctgcctgc    7692
BCRP3.HUMAN.NCBI.REF                                               agggtttcaccatgttggccaggctggtcttgaactcctgaccttaagagatctgcctac    5733
LOC106996293.glutathione.hydrolase5.proenzyme-like-GGT1.rhesus.   agggtttcaccatgttggccaggctggtcttgaactcctgaccttaagagatccgcctgc    52441
                                                                   **************************  ************************ **** *

Papio.anubis.clone.rp41-133b2.8034-40080.revcompl.Baboon          ctcggcgtcctaaagtgttgggattacaggcatgagccaccacgcccagcctatgagtta    7752
BCRP3.HUMAN.NCBI.REF                                               ctcggcgtcctaaagtgttgggattacaggcatgagccaccgcgcccagcctatgagtta    5793
LOC106996293.glutathione.hydrolase5.proenzyme-like-GGT1.rhesus.   ctcggcgtcccaaaatgttgggattacaggcataaaccactgcgcctagcctatgagtta    52501
                                                                   ********** *** ****************** * ****  **** *************

Papio.anubis.clone.rp41-133b2.8034-40080.revcompl.Baboon          gtatttctatcctcttcctagttagtacaagaaccttggaacaggaacgaaatttgcccc    7812
BCRP3.HUMAN.NCBI.REF                                               gtacttctatgctcttcctagtcagtacaagaaccttggaacaggaactaaatttacccc    5853
LOC106996293.glutathione.hydrolase5.proenzyme-like-GGT1.rhesus.   gtacttctatccccttcctagtcagtacaagaaccttggaacaggaatgaaatttacccc    52561
                                                                   *** ****** * ********* ************************  ****** ****

Papio.anubis.clone.rp41-133b2.8034-40080.revcompl.Baboon          caacgacttatatgttaatacttttgtgtattttaaatatgcgtgt--------------    7858
BCRP3.HUMAN.NCBI.REF                                               cagtgacttatatgctaatatttttgtgtattttaaatatatatgt--------------    5899
LOC106996293.glutathione.hydrolase5.proenzyme-like-GGT1.rhesus.   caatgacttatatgctaatattttgtgtttttaaatatatgtatatgtgcagctgggt    52621
                                                                   **  ********** ***** ******** *********   * *

Papio.anubis.clone.rp41-133b2.8034-40080.revcompl.Baboon          ------------------------------------------------------------    7858
BCRP3.HUMAN.NCBI.REF                                               ------------------------------------------------------------    5899
LOC106996293.glutathione.hydrolase5.proenzyme-like-GGT1.rhesus.   gcggtggctcatgcctgtaatcccagcactttgggaggccgaggcgggcagatcacaagg    52681


Papio.anubis.clone.rp41-133b2.8034-40080.revcompl.Baboon          ------------------------------------------------------------    7858
BCRP3.HUMAN.NCBI.REF                                               ------------------------------------------------------------    5899
LOC106996293.glutathione.hydrolase5.proenzyme-like-GGT1.rhesus.   tcaggaaatcgagaccatcctggctaacacggtgaaactctgtttctactaaaatacaa    52741
```

```
Papio.anubis.clone.rp41-133b2.8034-40080.revcompl.Baboon          ------------------------------------------------------------    7858
BCRP3.HUMAN.NCBI.REF                                              ------------------------------------------------------------    5899
LOC106996293.glutathione.hydrolase5.proenzyme-like-GGT1.rhesus.   aaaactggctgggcgcaatggctcacacctgtaatcccagcactttaggaggccgaggcg    52801


Papio.anubis.clone.rp41-133b2.8034-40080.revcompl.Baboon          ------------------------------------------------------------    7858
BCRP3.HUMAN.NCBI.REF                                              ------------------------------------------------------------    5899
LOC106996293.glutathione.hydrolase5.proenzyme-like-GGT1.rhesus.   gaaggacaacctgaggccaggagtttgagaccagcctgaccaacatgcagaaaccccatc    52861


Papio.anubis.clone.rp41-133b2.8034-40080.revcompl.Baboon          ------------------------------------------------------------    7858
BCRP3.HUMAN.NCBI.REF                                              ------------------------------------------------------------    5899
LOC106996293.glutathione.hydrolase5.proenzyme-like-GGT1.rhesus.   tctactaaaaatacaaaattagtcgggcatgatggcgcatgcctgtaatcccagctactc    52921


Papio.anubis.clone.rp41-133b2.8034-40080.revcompl.Baboon          ------------------------------------------------------------    7858
BCRP3.HUMAN.NCBI.REF                                              ------------------------------------------------------------    5899
LOC106996293.glutathione.hydrolase5.proenzyme-like-GGT1.rhesus.   gggaggctgaggcaggagaattgcttgaacctgggaggcagaggttgcagtgagctgaga    52981


Papio.anubis.clone.rp41-133b2.8034-40080.revcompl.Baboon          ------------------------------------------------------------    7858
BCRP3.HUMAN.NCBI.REF                                              ------------------------------------------------------------    5899
LOC106996293.glutathione.hydrolase5.proenzyme-like-GGT1.rhesus.   tcgcacctttgcactccagcctgggcaacaagagcaaaactccatctcaaaaaaaaaaga    53041


Papio.anubis.clone.rp41-133b2.8034-40080.revcompl.Baboon          ------------------------------------------------------------    7858
BCRP3.HUMAN.NCBI.REF                                              ------------------------------------------------------------    5899
LOC106996293.glutathione.hydrolase5.proenzyme-like-GGT1.rhesus.   taaaataaaaaactaactaactaaataatacaaattagtcgggcgtggtggcgcatgcct    53101


Papio.anubis.clone.rp41-133b2.8034-40080.revcompl.Baboon          ------------------------------------------------------------    7858
BCRP3.HUMAN.NCBI.REF                                              ------------------------------------------------------------    5899
LOC106996293.glutathione.hydrolase5.proenzyme-like-GGT1.rhesus.   gtaatcccagctactctagaggctgaggcaggagaatggtgtgaacctgggagacggaga    53161


Papio.anubis.clone.rp41-133b2.8034-40080.revcompl.Baboon          ------------------------------------------------------------    7858
BCRP3.HUMAN.NCBI.REF                                              ------------------------------------------------------------    5899
LOC106996293.glutathione.hydrolase5.proenzyme-like-GGT1.rhesus.   ttgcagtgagccgagatcgcaccactgcactccagcctgggcgacagagtgagactccgt    53221


Papio.anubis.clone.rp41-133b2.8034-40080.revcompl.Baboon          ------------------------------------------------gtgtgtgtatgt    7870
BCRP3.HUMAN.NCBI.REF                                              ----------------------------------------------------------gt    5901
LOC106996293.glutathione.hydrolase5.proenzyme-like-GGT1.rhesus.   ctcaaaaaaaaaaaaatatatatatatatatatatatatttatattttgtgtgtgtgcgt    53281
                                                                                                                            **

Papio.anubis.clone.rp41-133b2.8034-40080.revcompl.Baboon          gcatagatgtatctgtgtg-ttttctgtgtttttattcttatttatgttgagagtataga    7929
BCRP3.HUMAN.NCBI.REF                                              gcatagatgtatctgtgtg-tttttgtgtttttattcttatttatgttgagagtgtaga    5960
LOC106996293.glutathione.hydrolase5.proenzyme-like-GGT1.rhesus.   gcatagatgtatctgtgtgttttttgtgtttttattcttatttatgttgagagtgtaga    53341
                                                                  ******************* **** ****************************** ****

Papio.anubis.clone.rp41-133b2.8034-40080.revcompl.Baboon          gctatgtaaaagtaaagagaattgtataatgaagccccgtgtatccattcaatttcaaca    7989
BCRP3.HUMAN.NCBI.REF                                              gctatgtaagagtaaagagaattgtgtaatgaagccccgagtatccattcaatttcaaca    6020
LOC106996293.glutathione.hydrolase5.proenzyme-like-GGT1.rhesus.   gctatgtaaaataaacagaattgtataatgaagccccatgtatccattcaatttcaaca    53401
                                                                  ********* * **** ******** ************  ********************

Papio.anubis.clone.rp41-133b2.8034-40080.revcompl.Baboon          acaatcttatggccaagctaatttcatgtatactctttctctttcctgcttccttctacc    8049
BCRP3.HUMAN.NCBI.REF                                              acaatctcatggccaagctaatttcatgtata------ctctttcctgcttccctctacc    6074
LOC106996293.glutathione.hydrolase5.proenzyme-like-GGT1.rhesus.   acaatcttatggccaagctaatttcatgtatactctttctctttcctgcttccttctacc    53461
                                                                  ******* ************************      *************** ******

Papio.anubis.clone.rp41-133b2.8034-40080.revcompl.Baboon          ccacattatttcagtgcaaat-ccagatatataactttacccatacatatttcagtatat    8108
BCRP3.HUMAN.NCBI.REF                                              ccacattatttcagtgcaaatcccagatatataactgtacccatacatatttcagtatgt    6134
LOC106996293.glutathione.hydrolase5.proenzyme-like-GGT1.rhesus.   ccacattatttcagtgcaaatcccagatatataactttacccatacatatttcagtatgc    53521
                                                                  ********************* ************** *********************

Papio.anubis.clone.rp41-133b2.8034-40080.revcompl.Baboon          tttatttattttaaaccccacaagatatcattttctatactactataattttataccaat    8168
BCRP3.HUMAN.NCBI.REF                                              tttatttattttaaaccccacaatatatcattttctatactactgtaatttcataccaat    6194
LOC106996293.glutathione.hydrolase5.proenzyme-like-GGT1.rhesus.   tttatttattttaaaccccacaagatatcattttctatactactataattttataacaat    53581
                                                                  *********************** ******************** ****** *** ****

Papio.anubis.clone.rp41-133b2.8034-40080.revcompl.Baboon          aacattcatttagatttacccacacatttaccacttctgttacccttttttttttttttt    8228
BCRP3.HUMAN.NCBI.REF                                              aacattcatttagatttacccacacgtttacctcttctgtt-------------------    6235
LOC106996293.glutathione.hydrolase5.proenzyme-like-GGT1.rhesus.   aacattcatttagatttactcaaacatttacttcttctgtt-------------------    53622
                                                                  ******************* ** ** *****  ********

Papio.anubis.clone.rp41-133b2.8034-40080.revcompl.Baboon          ttgagacagagtgtcgctctgtcacccaggctggcgcaatctgggctcactgcaagctcc    8288
BCRP3.HUMAN.NCBI.REF                                              ------------------------------------------------------------    6235
LOC106996293.glutathione.hydrolase5.proenzyme-like-GGT1.rhesus.   ------------------------------------------------------------    53622


Papio.anubis.clone.rp41-133b2.8034-40080.revcompl.Baboon          gcctcccaggttcacgccattctcctgcctcagcctcccgagtagctgggactacaggtg    8348
BCRP3.HUMAN.NCBI.REF                                              ------------------------------------------------------------    6235
LOC106996293.glutathione.hydrolase5.proenzyme-like-GGT1.rhesus.   ------------------------------------------------------------    53622


Papio.anubis.clone.rp41-133b2.8034-40080.revcompl.Baboon          cccgccaccatgcccagctaatttttgtatttttagtagagacaggtttcactgtgtt    8408
BCRP3.HUMAN.NCBI.REF                                              ------------------------------------------------------------    6235
LOC106996293.glutathione.hydrolase5.proenzyme-like-GGT1.rhesus.   ------------------------------------------------------------    53622


Papio.anubis.clone.rp41-133b2.8034-40080.revcompl.Baboon          agccaggatggtctcgatctcctaatctcgtgatacacccgcctcggcctcccaaaatgc    8468
BCRP3.HUMAN.NCBI.REF                                              ------------------------------------------------------------    6235
LOC106996293.glutathione.hydrolase5.proenzyme-like-GGT1.rhesus.   ------------------------------------------------------------    53622


Papio.anubis.clone.rp41-133b2.8034-40080.revcompl.Baboon          tgagattacaggcatgagccaccgtgcctggccaccctttgtttttatttataaaaataa    8528
BCRP3.HUMAN.NCBI.REF                                              ---------------------------------accctttatttttatttataaaaatat    6262
LOC106996293.glutathione.hydrolase5.proenzyme-like-GGT1.rhesus.   ---------------------------------accctttatttttatttataaaaatat    53649
                                                                                                   ******* ******************

Papio.anubis.clone.rp41-133b2.8034-40080.revcompl.Baboon          ctttgggaaaaaatatctttgggcacatggtcaaggatctcctgagggctgtgacatggg    8588
BCRP3.HUMAN.NCBI.REF                                              ctttgggaagaaatatctttcagcacatggtcaaggatctcctgagggctatgtcatggg    6322
LOC106996293.glutathione.hydrolase5.proenzyme-like-GGT1.rhesus.   atttgggaaaaaatatatttgcacacatagtcaaggatctcctgagggctatgtcatggc    53709
                                                                   ******** ****** ***   ***** ********************* ** *****

Papio.anubis.clone.rp41-133b2.8034-40080.revcompl.Baboon          taaaatatacatatatattccatatatatat-----------------------------    8619
BCRP3.HUMAN.NCBI.REF                                              caaaatatacatatatattccatatatatacacacatatacacacacacacatatataca    6382
LOC106996293.glutathione.hydrolase5.proenzyme-like-GGT1.rhesus.   caaaatatacatatatattccatatttatatctatctatatatacacacacatacacaca    53769
                                                                   ************************ ****

Papio.anubis.clone.rp41-133b2.8034-40080.revcompl.Baboon          ---------------------atatatatattccactttcactcttttgt-------ttt    8651
BCRP3.HUMAN.NCBI.REF                                              c------atatacacacacacatatatatattccactttcacttttttgtttgtttgttt    6436
LOC106996293.glutathione.hydrolase5.proenzyme-like-GGT1.rhesus.   cacatatatacacacacaaacatatatgtattccactttcacttt----tttatttgttt    53825
                                                                                       ****** *************** *    *       ***

Papio.anubis.clone.rp41-133b2.8034-40080.revcompl.Baboon          tttgagaccaag--------tctcactctgttacccaggctggagtgcagtggtgtgatc    8703
BCRP3.HUMAN.NCBI.REF                                              tttgagaccgag--------tctcgctctgtggcccaggctggagtgcggtggtgggatc    6488
LOC106996293.glutathione.hydrolase5.proenzyme-like-GGT1.rhesus.   tttgagaccgagtctcgctctctcgctctgttgcccaggctggagtgcagtggtgcgatc    53885
                                                                  ********* **        **** ******  *************** ****** ****

Papio.anubis.clone.rp41-133b2.8034-40080.revcompl.Baboon          tcagctcactgcaacttctgcctcccaggttcaagtgattctcctgtctcagcctcctga    8763
BCRP3.HUMAN.NCBI.REF                                              tcagctcactgcaacttctgcctcctgggttcaggtgattctcctgtctcagcctcctga    6548
LOC106996293.glutathione.hydrolase5.proenzyme-like-GGT1.rhesus.   tcagctcactgcaacttctgcctcctgggttcaagtgattctcctgtctcagcctcc-aa    53944
                                                                  *************************  ****** ***********************  *

Papio.anubis.clone.rp41-133b2.8034-40080.revcompl.Baboon          gtagctgggattccaggcgtgagccaccaagcctggctaa--tttttgtacttttttttt    8821
BCRP3.HUMAN.NCBI.REF                                              gtagctgggattacaggtgttagccatcacgtctggctaa--------------------    6588
LOC106996293.glutathione.hydrolase5.proenzyme-like-GGT1.rhesus.   atatctgggattacaggcatgagccaccacgcctggctaatttttttttttttttttttt    54004
                                                                   ** ******** ****  * ***** ** * ********

Papio.anubis.clone.rp41-133b2.8034-40080.revcompl.Baboon          tttttgagatagagtcttgctctgtcgcccaggctggagtgcagtggcacaatctccact    8881
BCRP3.HUMAN.NCBI.REF                                              ------------------------------------------------------------    6588
LOC106996293.glutathione.hydrolase5.proenzyme-like-GGT1.rhesus.   tttttgagacgaagtctccctctgttgcccaggctggagtgcagtggcacgatcttggcg    54064


Papio.anubis.clone.rp41-133b2.8034-40080.revcompl.Baboon          cactgcaacctccacttccctggttcaagcagtttccctgcctcagcctcccgagtagct    8941
BCRP3.HUMAN.NCBI.REF                                              ------------------------------------------------------------    6588
LOC106996293.glutathione.hydrolase5.proenzyme-like-GGT1.rhesus.   cactgcaacctctactttcctggttcaagcaattcccctgcctcagcctcccgagtagct    54124


Papio.anubis.clone.rp41-133b2.8034-40080.revcompl.Baboon          gggattacaacaggtgcatgacaccatgccctgctaatttttttatatttatggtagaga    9001
BCRP3.HUMAN.NCBI.REF                                              ------------------------------------------------------------    6588
LOC106996293.glutathione.hydrolase5.proenzyme-like-GGT1.rhesus.   gggatt---acaggtgcacaccaccatgcccagataatttttttgtatttgtagtagaga    54181
```

```
Papio.anubis.clone.rp41-133b2.8034-40080.revcompl.Baboon              cagagtttccccatgttggccagactggtcttgaactcctgacctcaggcaattcacctg    9061
BCRP3.HUMAN.NCBI.REF                                                  ------------------------------------------------------------    6588
LOC106996293.glutathione.hydrolase5.proenzyme-like-GGT1.rhesus.       cagggtttgcctatgatggccagactgttctcaaacttccgacctctggcaatccactca    54241


Papio.anubis.clone.rp41-133b2.8034-40080.revcompl.Baboon              ccttgacctcccaaagtgcagagattacaggcgtgagtcactgcacc-------------    9108
BCRP3.HUMAN.NCBI.REF                                                  ------------------------------------------------------------    6588
LOC106996293.glutathione.hydrolase5.proenzyme-like-GGT1.rhesus.       ccttggcctcccaaagtgctggggttataggcatgagccgccaccctgcctggcctgttt    54301


Papio.anubis.clone.rp41-133b2.8034-40080.revcompl.Baboon              -----------tggccctttttttgttttttaagacagattctcactctgtcccccag    9157
BCRP3.HUMAN.NCBI.REF                                                  -----------tttttgttgttttttttttttgagacggagtatcgctctgtcacccag    6637
LOC106996293.glutathione.hydrolase5.proenzyme-like-GGT1.rhesus.       tttttttttttttttttttttttttttttttttgagatagagtctcgttctgtcacccag    54361
                                                                                 *     ** ***** ******* ***  ** * **  ****** *****

Papio.anubis.clone.rp41-133b2.8034-40080.revcompl.Baboon              actggagtgcagtggcgagatctcagctccctgcaatctccacctacagggttcaaacga    9217
BCRP3.HUMAN.NCBI.REF                                                  gctggagtgcagtggcaagatcttgtctcactgcaacctccacctctcaggttcaagcaa    6697
LOC106996293.glutathione.hydrolase5.proenzyme-like-GGT1.rhesus.       gctggagtgcagtggcaagatcttggctcactgcaacctccgtctctggggttcaagcaa    54421
                                                                       *************** ******   *** ****** ****  **    ******* * *

Papio.anubis.clone.rp41-133b2.8034-40080.revcompl.Baboon              ctcttgtgcctcagctttgcaagtagctgggattacaggtacccaccaccacacctgtct    9277
BCRP3.HUMAN.NCBI.REF                                                  ttcttgtgcctcagcctcctgagtagctgggattacaggcatccaccaccacatctggct    6757
LOC106996293.glutathione.hydrolase5.proenzyme-like-GGT1.rhesus.       ttcttgtgcctcaaga-attgagtagctgggattacaggcgcccaccaccacatctggct    54480
                                                                       ************        ******************   *********** *** **

Papio.anubis.clone.rp41-133b2.8034-40080.revcompl.Baboon              aatttttgtatttttggcagagatggggttttaccatgttggccaggctggtctcgaact    9337
BCRP3.HUMAN.NCBI.REF                                                  aatttgtgtatttttggtagagatggggtttcaccatgttggccaggctggtctcgaact    6817
LOC106996293.glutathione.hydrolase5.proenzyme-like-GGT1.rhesus.       aatttgtgtatttttggtagagatggagcttcaccatgttggccaggctggtcttgaact    54540
                                                                      ***** *********** ******** * ** ********************* *****

Papio.anubis.clone.rp41-133b2.8034-40080.revcompl.Baboon              cctgacctcaggtgatccactcacctcggccttccaaagtgctgggattacaggcatgag    9397
BCRP3.HUMAN.NCBI.REF                                                  tctgacctcaggtgatccacctgcctcggccttccaaagtgctgggattacaggcatgag    6877
LOC106996293.glutathione.hydrolase5.proenzyme-like-GGT1.rhesus.       cctgacctcaagtgatccacctatctcggccttccaaagtgctgggattacagacatgag    54600
                                                                       ********* *********    ***************************** ******

Papio.anubis.clone.rp41-133b2.8034-40080.revcompl.Baboon              ccaccgtggctggccattttcacttttgaaggatattgttagtgggcgtagaattctagg    9457
BCRP3.HUMAN.NCBI.REF                                                  ccaccatgcccagccattttcacttttgaaggatattgttaatgagcatagaattctagg    6937
LOC106996293.glutathione.hydrolase5.proenzyme-like-GGT1.rhesus.       ccaccatgcccggccattttcacttttgaaggatattgttagtgcacataaaattctagg    54660
                                                                      ***** ** *  **************************** **  * ** *********

Papio.anubis.clone.rp41-133b2.8034-40080.revcompl.Baboon              ttggcagatattttctttcctcagtttggaaacatggttcccttgtatctgatttatctt    9517
BCRP3.HUMAN.NCBI.REF                                                  ttggcagatattttctttcctcagtttgaaaacatgattcccttgtatctgatttctcct    6997
LOC106996293.glutathione.hydrolase5.proenzyme-like-GGT1.rhesus.       ttggcagatattttctttcctcagtttgaaaacatgattcccttgtatttgatttctcct    54720
                                                                      **************************** ******* *********** ****** ** *

Papio.anubis.clone.rp41-133b2.8034-40080.revcompl.Baboon              gtttttattgggaagccaattctcaatctaattttgctcatttgaaagtaatgtcttttt    9577
BCRP3.HUMAN.NCBI.REF                                                  gcttttattgggaagccaattctcaatctaattttgctcatttgaaggcaatggcttttt    7057
LOC106996293.glutathione.hydrolase5.proenzyme-like-GGT1.rhesus.       gtttttattgagaagccaattctcaatctaattttgctcatttgaaggcaatgtcttttt    54780
                                                                      * ******** *********************************** * **** ******

Papio.anubis.clone.rp41-133b2.8034-40080.revcompl.Baboon              atgttgttttgtttttgagatggagtctcactctgtcgcccaagctggagtgcagctgt    9637
BCRP3.HUMAN.NCBI.REF                                                  ctgttgttgtgttttctgaggtggagtctcactctgtcacccaggctggactgcagtggt    7117
LOC106996293.glutathione.hydrolase5.proenzyme-like-GGT1.rhesus.       ttgttgttgtgtttttgagatggagtctcactctgtcgcccaggctggagtgcagtggc    54840
                                                                       ******* ****** **** ***************** **** ****** *****  *

Papio.anubis.clone.rp41-133b2.8034-40080.revcompl.Baboon              gcgatctcagctcactgcaagctctgcctcctgggttcaagcaattc-tctgcctcagcc    9696
BCRP3.HUMAN.NCBI.REF                                                  gcaatctcagctcactgcaacctctgcctcctgggttcaagtgattcttctgcctcagcc    7177
LOC106996293.glutathione.hydrolase5.proenzyme-like-GGT1.rhesus.       gtgatctcagttcactgcaagttctgcctcctgggttcacgccattcttctgcctcaggc    54900
                                                                      *  ******* *********  ***************** *  **** ********** *

Papio.anubis.clone.rp41-133b2.8034-40080.revcompl.Baboon              tccaaagtagctgggattacaggtgtccactaccacgcccagc-----------------    9739
BCRP3.HUMAN.NCBI.REF                                                  tcccaagtagctgggattacaggtgtccaccatcacacctggc-----------------    7220
LOC106996293.glutathione.hydrolase5.proenzyme-like-GGT1.rhesus.       tcccgagtagctgggactacaggtgcccacaaccactcccagctaatttttgtattttt    54960
                                                                      ***  *********** ******** **** * *** **  **

Papio.anubis.clone.rp41-133b2.8034-40080.revcompl.Baboon              ------------------------------------------------------------    9739
BCRP3.HUMAN.NCBI.REF                                                  ------------------------------------------------------------    7220
LOC106996293.glutathione.hydrolase5.proenzyme-like-GGT1.rhesus.       agtagagatgggatttcaccatgttagccaggatggtctcgatctcctgaacttgtgatc    55020


Papio.anubis.clone.rp41-133b2.8034-40080.revcompl.Baboon              ------------------------------------------------------------    9739
BCRP3.HUMAN.NCBI.REF                                                  ------------------------------------------------------------    7220
LOC106996293.glutathione.hydrolase5.proenzyme-like-GGT1.rhesus.       cacctgtcctggcctcccaaagtgctgggattacaggtgtgagccaccgcacctggcccc    55080


Papio.anubis.clone.rp41-133b2.8034-40080.revcompl.Baboon              -taatttttgtatttttagtagagatggattttgccatattggccaggctggtcttgaa    9798
BCRP3.HUMAN.NCBI.REF                                                  -taattgttgtatttttaatagagatgaacttttgccatgttggtcaggctgatcccaaa    7279
LOC106996293.glutathione.hydrolase5.proenzyme-like-GGT1.rhesus.       ttaattgttgtatttttaatagagatgaattttgccgtgttggtcaggctgatcccgaa    55140
                                                                       ***** *********** ******** * ******* * **** ******* **   **

Papio.anubis.clone.rp41-133b2.8034-40080.revcompl.Baboon              ctcctcagttcaggtgatccacctgcctcagcctcccaaa-tgctgggattacaggcgtg    9857
BCRP3.HUMAN.NCBI.REF                                                  ctcctcatttcaggtgatccgcccgcctcagcctcccaaa-tgctgggattacaggcatg    7338
LOC106996293.glutathione.hydrolase5.proenzyme-like-GGT1.rhesus.       ctcttcgtttcaggtaatccaccctcctcagcctcccaaagtgctgggattacaggtgtg    55200
                                                                      *** **  ******* **** **  *************** ***************  **

Papio.anubis.clone.rp41-133b2.8034-40080.revcompl.Baboon              agccgcccccctcaccctggccagaaggcagtatcttttttttcctctggctgctttaaaaa    9917
BCRP3.HUMAN.NCBI.REF                                                  agacagcccacaaccctggcctgcaggcagtatc-ttttttcctctggctgctttgaaaa    7397
LOC106996293.glutathione.hydrolase5.proenzyme-like-GGT1.rhesus.       agcaacccctacaccccggcctgaaggcagtatc-ttttttcctctggctgctttaaaaa    55259
                                                                      **    ***   **** **** * ********** ****************** ****

Papio.anubis.clone.rp41-133b2.8034-40080.revcompl.Baboon              gttt--gcctttactttgagcagttttacactgatacatttaggtggctcttcattccat    9975
BCRP3.HUMAN.NCBI.REF                                                  gttttgt-ctttgttttgagcag-tttacactgatgcatttaggtggctcctcattccat    7455
LOC106996293.glutathione.hydrolase5.proenzyme-like-GGT1.rhesus.       ggtttgtccttggctttgagcagttttacaccgatgcatttaggtggttcttcattctat    55319
                                                                      * **    ***   ********* ******* *** *********** ** ****** **

Papio.anubis.clone.rp41-133b2.8034-40080.revcompl.Baboon              ggcttgattctttttttgtccattttagaaaattctcagttttatctcttcaagtattacg    10035
BCRP3.HUMAN.NCBI.REF                                                  gacttgattctttttttgtccattttagaaaactgccagctttatctcttcaagtattatg    7515
LOC106996293.glutathione.hydrolase5.proenzyme-like-GGT1.rhesus.       gacttcattctttttttgtccattttagaaaattctcagctttatctcttcaagtattacg    55379
                                                                      * *** ************************* *  *** ****************** *

Papio.anubis.clone.rp41-133b2.8034-40080.revcompl.Baboon              tcttccccatcctctctctactctccttatgagactccaatttcacatgacctatgcctt    10095
BCRP3.HUMAN.NCBI.REF                                                  tcttccccatcctctctctactctccttatgagactccaatttcacatgacttatgcctt    7575
LOC106996293.glutathione.hydrolase5.proenzyme-like-GGT1.rhesus.       tctttcccatcctctctcttctctccttatgagactccaatttcacatgacttataccttt    55439
                                                                      **** ************** ****************************** *** ****

Papio.anubis.clone.rp41-133b2.8034-40080.revcompl.Baboon              gttaaagtatccccccatgtctcttaatccatttcctgtatgttttatctatttttctcct    10155
BCRP3.HUMAN.NCBI.REF                                                  gttaaagtatccccccatgtctcttaatccatttcctgtatgttctatctgtttttctctt    7635
LOC106996293.glutathione.hydrolase5.proenzyme-like-GGT1.rhesus.       gttaaagtatctcccatgtctgttaatccatttcctgtgtgttctgtctatttttctctt    55499
                                                                      *********** ********* **************** **** * *** ******** *

Papio.anubis.clone.rp41-133b2.8034-40080.revcompl.Baboon              tgtacttcattttgtatattttgtatcaaactgtctcccaattagccaggcgtggtggcg    10215
BCRP3.HUMAN.NCBI.REF                                                  tgtacttcaatttgtatagtttgtatcaaactatctcccaattagccgggcgtggtggtg    7695
LOC106996293.glutathione.hydrolase5.proenzyme-like-GGT1.rhesus.       tgtacttcaatttgtacattttgtatcagactatctcccaattagccggacatggtggtg    55559
                                                                      ********* ****** * ********* *** ************** * * ****** *

Papio.anubis.clone.rp41-133b2.8034-40080.revcompl.Baboon              ggt-gcctgtaatcccagctactcagaaggccaaggtgggagaattgcttgaacccaggg    10274
BCRP3.HUMAN.NCBI.REF                                                  ggt-gcctgtaatcccagctacttgggagcctgaggcaggagaattgcttgaacccgaga    7754
LOC106996293.glutathione.hydrolase5.proenzyme-like-GGT1.rhesus.       gggcacctgtaaccccagctacttgggaggctgaggcaggagaatggcttgaacccggga    55619
                                                                      **   ******* **********  * ** *  ***  ******* **********  *

Papio.anubis.clone.rp41-133b2.8034-40080.revcompl.Baboon              ag-ggaggttgcagtgagcagagatcatgccagatcatgccattgcactccagcctgggt    10333
BCRP3.HUMAN.NCBI.REF                                                  ggtggaagttgtagtgagccgagatcatgccac----------tgcactccagcctgggc    7804
LOC106996293.glutathione.hydrolase5.proenzyme-like-GGT1.rhesus.       ggtgaaggttgcagtgagctgagatcatgccac----------tgtcctccagcctgggc    55669
                                                                       * * * **** ******* ************           **  ************

Papio.anubis.clone.rp41-133b2.8034-40080.revcompl.Baboon              gacagagtgagactctgtctcaaa-aataaataaataaataaaaataaaaaccctа    10392
BCRP3.HUMAN.NCBI.REF                                                  aacagagtgagaccctgtctcaataaataaataaataaat----------------    7848
LOC106996293.glutathione.hydrolase5.proenzyme-like-GGT1.rhesus.       aacagagtaaggctctgtctcaaataaataaaaaataaataaat----------------    55713
                                                                       ******* ** * *********  **  ** ************

Papio.anubis.clone.rp41-133b2.8034-40080.revcompl.Baboon              tctcccagttcacaatttttttttttttttttttttttgcatgtttgtctctagtctgc    10452
BCRP3.HUMAN.NCBI.REF                                                  -----------accagttcactattt---------ttttttatgtttgtgtctagtgtgc    7888
LOC106996293.glutathione.hydrolase5.proenzyme-like-GGT1.rhesus.       -----------accagttcattatttatttatttatttttatgtttgtgtctactgtgc    55762
                                                                                 ** * **   * ***         ****  ******** **** * ***

Papio.anubis.clone.rp41-133b2.8034-40080.revcompl.Baboon              tgttcaaattcagttcctaattccattttttttttt-ttttgtgagactcagtctctatc    10511
BCRP3.HUMAN.NCBI.REF                                                  tgttcaaattgagttcctaattccatttttttttttagactttttttttttgagtctctatc    7948
LOC106996293.glutathione.hydrolase5.proenzyme-like-GGT1.rhesus.       tgttcaaattgagttcctaattccattttttttttttttttttttgagacacagtctctatc    55822
                                                                      ********** ************************    ** *       **********

Papio.anubis.clone.rp41-133b2.8034-40080.revcompl.Baboon              tgctgcccaggctggagtgcagtggtgagatctcaactcactgtagcctccacctccc-g    10570
BCRP3.HUMAN.NCBI.REF                                                  tgttgcccaggctggagttcagtggtgcaatctcaactcactgtagcctccacctcccag    8008
LOC106996293.glutathione.hydrolase5.proenzyme-like-GGT1.rhesus.       tgttgctcaggctggagttcagtggtacgatctcaactcactgcagcctctgcctcccag    55882
                                                                      ** *** *********** *******   ************** ******  ****** *
```

```
Papio.anubis.clone.rp41-133b2.8034-40080.revcompl.Baboon             gttcaagcgattctcatgcctcagcctcctgaataactgggattaccaccatgcctagct    10630
BCRP3.HUMAN.NCBI.REF                                                 gttcaagcgattctcatgcctcagcctctcgagtaactgggattaccaccacgcctaact    8068
LOC106996293.glutathione.hydrolase5.proenzyme-like-GGT1.rhesus.      gttcaagcgattctcgtgcctcagcctcctgagtaactgggattaccaccatgcctaact    55942
                                                                     *************** ************  ** ****************** ***** **

Papio.anubis.clone.rp41-133b2.8034-40080.revcompl.Baboon             ------------------------------------------------------------    10630
BCRP3.HUMAN.NCBI.REF                                                 ------------------------------------------------------------    8068
LOC106996293.glutathione.hydrolase5.proenzyme-like-GGT1.rhesus.      cagttttgtgtttttttttgttttttttttttttgagacggagtctcgctctgtcgccca    56002


Papio.anubis.clone.rp41-133b2.8034-40080.revcompl.Baboon             ------------------------------------------------------------    10630
BCRP3.HUMAN.NCBI.REF                                                 ------------------------------------------------------------    8068
LOC106996293.glutathione.hydrolase5.proenzyme-like-GGT1.rhesus.      gcccaggctggagtgcagtggcgcgatctcggctcactgcaagctccgcctcccgggttc    56062


Papio.anubis.clone.rp41-133b2.8034-40080.revcompl.Baboon             ------------------------------------------------------------    10630
BCRP3.HUMAN.NCBI.REF                                                 ------------------------------------------------------------    8068
LOC106996293.glutathione.hydrolase5.proenzyme-like-GGT1.rhesus.      acgccattctcctgcctcagcctcccgagtagctgggactacaggcgcccacaaccgcgc    56122


Papio.anubis.clone.rp41-133b2.8034-40080.revcompl.Baboon             ------------------------------------------------------------    10630
BCRP3.HUMAN.NCBI.REF                                                 ------------------------------------------------------------    8068
LOC106996293.glutathione.hydrolase5.proenzyme-like-GGT1.rhesus.      ccggctaatttttgtatttttagtagagacggggtttcaccgtggtctcgatctcctga    56182


Papio.anubis.clone.rp41-133b2.8034-40080.revcompl.Baboon             ------------------------------------------------------------    10630
BCRP3.HUMAN.NCBI.REF                                                 ------------------------------------------------------------    8068
LOC106996293.glutathione.hydrolase5.proenzyme-like-GGT1.rhesus.      ccttgtgatccgcccgtctcggcctcccaaagtgctgggattacaggcgtgagccaccgc    56242


Papio.anubis.clone.rp41-133b2.8034-40080.revcompl.Baboon             -------catttttgtatttttagtagaggtggtgcttcgccatgttagccaggctagtc    10683
BCRP3.HUMAN.NCBI.REF                                                 -------catttttgtatttttagtagagatggggttctgccatgttggccaggctggtc    8121
LOC106996293.glutathione.hydrolase5.proenzyme-like-GGT1.rhesus.      gcccggccagttttgtgtttttagtagagacaggtttc-gccatgttggccaggctggtc    56301
                                                                            ** ****** ************   *   *  ******** ******** ***

Papio.anubis.clone.rp41-133b2.8034-40080.revcompl.Baboon             ttgaactcccagcctcgtgtgattctcctgcctctgcctcccaaagtgctgggattatag    10743
BCRP3.HUMAN.NCBI.REF                                                 ttgaactcttggccttatgtgattggcctacctctgtctcccaaagtgctgggattatag    8181
LOC106996293.glutathione.hydrolase5.proenzyme-like-GGT1.rhesus.      ttgaactcctggcctcatgtgattcacc--ctgctgtctcccaacgtgctgggattatag    56359
                                                                     ********   ****  *******  **  *  *** ******* ***************

Papio.anubis.clone.rp41-133b2.8034-40080.revcompl.Baboon             gcctaagccaccactcccagcctcc-----------------------------------    10768
BCRP3.HUMAN.NCBI.REF                                                 gcctaaaccaccactcccagcctcctttttttttttttttttttttgagacggagtctc    8241
LOC106996293.glutathione.hydrolase5.proenzyme-like-GGT1.rhesus.      gcgtgagccaccactcccagcctcc--------atttttttttttttgagacggagtctc    56411
                                                                     ** * * ******************

Papio.anubis.clone.rp41-133b2.8034-40080.revcompl.Baboon             ------------------------------------------------------------    10768
BCRP3.HUMAN.NCBI.REF                                                 gctctgtcgcccaggctggagtagagtggtgcgatctcggctcactgcaacctcccctc    8301
LOC106996293.glutathione.hydrolase5.proenzyme-like-GGT1.rhesus.      gctctgttgcccaggctggagtagagtggcacgatctcggctcactggaacctccacctc    56471


Papio.anubis.clone.rp41-133b2.8034-40080.revcompl.Baboon             ------------------------------------------------------------    10768
BCRP3.HUMAN.NCBI.REF                                                 ccagttcaagtgattctcctgcctcagcctcccgagtagctaggactataggagcatgcc    8361
LOC106996293.glutathione.hydrolase5.proenzyme-like-GGT1.rhesus.      ccggttcaagagattctcctgtctcaatctcccgagtagctgggactacaggcacatgcc    56531


Papio.anubis.clone.rp41-133b2.8034-40080.revcompl.Baboon             ------------------------------------------------------------    10768
BCRP3.HUMAN.NCBI.REF                                                 accatgcctggctaatttttgtaattttagtagagatggggattcaccatattggtcagg    8421
LOC106996293.glutathione.hydrolase5.proenzyme-like-GGT1.rhesus.      accatgcctggctaatttttgtaattttagtagagatggggtttcaccatattggtcagg    56591


Papio.anubis.clone.rp41-133b2.8034-40080.revcompl.Baboon             ------------------------------------------------------------    10768
BCRP3.HUMAN.NCBI.REF                                                 ctggtcttgaacttctgacctcaggtgatctacccacctcagcctcccaaagtgctggga    8481
LOC106996293.glutathione.hydrolase5.proenzyme-like-GGT1.rhesus.      ctggtcttgaactcctgacctcaggtgatccacccacctcagcctcccaaagtgctggga    56651


Papio.anubis.clone.rp41-133b2.8034-40080.revcompl.Baboon             ----------------------------------atttttaatagttgctagttttttga    10794
BCRP3.HUMAN.NCBI.REF                                                 ttacaggcgtgagtcaccacgcctagtgcatcca-ttttttgtagttgccagttttctga    8540
LOC106996293.glutathione.hydrolase5.proenzyme-like-GGT1.rhesus.      ttacaggagtgagtcaccatgcccagtgcgtctatttttttatagtggccagttttctga    56711
                                                                                                        *****  **** ** ****** ***

Papio.anubis.clone.rp41-133b2.8034-40080.revcompl.Baboon             taaaattc----ttgtttctttatatccttgaatatagatataaagtacttattttaaag    10850
BCRP3.HUMAN.NCBI.REF                                                 tgaaattcttaattgtttctttatatccttg-atatacatgtaaagaacttattttaaag    8599
LOC106996293.glutathione.hydrolase5.proenzyme-like-GGT1.rhesus.      ttaaattc----ttgtttctttatatccttgaatatagatataaagtacttattttaaag    56767
                                                                     * ******    ******************* ***** ** ***** *************

Papio.anubis.clone.rp41-133b2.8034-40080.revcompl.Baboon             tacatggtctgagagttccataatctggagatcctataggcctttttaaaagttgtctgt    10910
BCRP3.HUMAN.NCBI.REF                                                 tacatggtctgatgattttataatctggagatcctatgggcctttttaaaagttgtctgt    8659
LOC106996293.glutathione.hydrolase5.proenzyme-like-GGT1.rhesus.      ttcatggtctgacaatttcataatctagagatcctatgggccttttaaaaaattgtctct    56827
                                                                     * **********   **  ******* ********** ******** **** ****** *

Papio.anubis.clone.rp41-133b2.8034-40080.revcompl.Baboon             gctttctcttgaggtttgttcctgctgtcttatttccttgtttgcttggttgtttttaat    10970
BCRP3.HUMAN.NCBI.REF                                                 gctttctcttgagcttttttcctgctgtcttatttccttgtttgcttagttgtttttaat    8719
LOC106996293.glutathione.hydrolase5.proenzyme-like-GGT1.rhesus.      gctttctcttgagctttgttcctgctgtcttatttccttgtttgcttggttgtttttaat    56887
                                                                     ************* *** ***************************** ************

Papio.anubis.clone.rp41-133b2.8034-40080.revcompl.Baboon             ttagcactggagggtatgtataaaaattgtggccgggcgcggtggctcaagcctgtaatc    11030
BCRP3.HUMAN.NCBI.REF                                                 ttggcaatggaagttgtgtataaaaatcgttacaaataattttttttttttttttgagatg    8779
LOC106996293.glutathione.hydrolase5.proenzyme-like-GGT1.rhesus.      ttggcaatggagggtgtgtataaaaattgttagaaataa-----ttttttttttgagatg    56942
                                                                     ** *** **** * * *********** **                *      **  **

Papio.anubis.clone.rp41-133b2.8034-40080.revcompl.Baboon             ccagcactttgggaggccgagacgggcgaatcacgaggtcaggagatcgagaccatcctg    11090
BCRP3.HUMAN.NCBI.REF                                                 gagtctcgctttgttgcccaagctggagtgcaatgacgtgatctcggctcactgcaacct    8839
LOC106996293.glutathione.hydrolase5.proenzyme-like-GGT1.rhesus.      gagtctcgctctgtt-cccaggctggagtgcaatgacatgatctcggctcaccgcaacct    57001
                                                                         * *  *  *   ** *  * ** *    * **  * *      *         *

Papio.anubis.clone.rp41-133b2.8034-40080.revcompl.Baboon             gctaacacggtgaaaccccgtctctactaaaaaatacaaaaactagccgggcaaggtggc    11150
BCRP3.HUMAN.NCBI.REF                                                 ctgcatcccagggttcaattctcctacctcagcctcc--caagtagctgggattgcaggc    8897
LOC106996293.glutathione.hydrolase5.proenzyme-like-GGT1.rhesus.      ccacatcccagggttcaactctcctacctccgcctcc--cgactagctgggattacaggc    57059
                                                                         *  *   *   *       ****       * *    * **** ***      ***

Papio.anubis.clone.rp41-133b2.8034-40080.revcompl.Baboon             gggcgcctgtagtcccagctactcgggaggctgaggcaggagaatggcgtaaatccggga    11210
BCRP3.HUMAN.NCBI.REF                                                 aggtgccagcacgcctggctaatttttgtatttttagtagagatgggtttttaccatgtt    8957
LOC106996293.glutathione.hydrolase5.proenzyme-like-GGT1.rhesus.      aggtgccagcatgcctggctaatttttgtatttttagtagagatggggtttcaccatgtt    57119
                                                                      ** *** * *  **  **** *       *       ****  **  *  * *  *

Papio.anubis.clone.rp41-133b2.8034-40080.revcompl.Baboon             ggcagagcttg----------------cagtgagctgagatccggccactgcactccagc    11254
BCRP3.HUMAN.NCBI.REF                                                 ggtcaggctggtctcagactcctgacctcgtgatctgcccacctcagcctcccaaagtgc    9017
LOC106996293.glutathione.hydrolase5.proenzyme-like-GGT1.rhesus.      ggtcaggctggtctcgaactcctgacctcatgatctgcctgcctcagcctcccaaagtgc    57179
                                                                     **    *** *                   *** ***    **     ** *      **

Papio.anubis.clone.rp41-133b2.8034-40080.revcompl.Baboon             ctggacgacagagccagactccgtctcaaaaaaaaaaaaaaaaaaaaaaaaaaaaaaatt    11314
BCRP3.HUMAN.NCBI.REF                                                 tgggattacaggcgtgagccactgcgcccagccagaaataatttttaaaaataat-----    9072
LOC106996293.glutathione.hydrolase5.proenzyme-like-GGT1.rhesus.      tgggattacaggcgtgagccactgtacccagcaagaaataa-ttttaaaaataat-----    57233
                                                                       ***  ****       *  *    *  *   * *** **     ***** **

Papio.anubis.clone.rp41-133b2.8034-40080.revcompl.Baboon             gttagaggctgggtgcggtggctcatgcttgtaatcccagcacttcgggaggccgaggtg    11374
BCRP3.HUMAN.NCBI.REF                                                 -tttgagccccagcatgatggctcatgcttgtaatcccatcactttgggaggctgaggcg    9131
LOC106996293.glutathione.hydrolase5.proenzyme-like-GGT1.rhesus.      -ttccagccccagcacgatggctcatgcttgtaatctcatcactttgggaggctgaggca    57292
                                                                      **  ** *   *   * ****************** ** ***** ******* ****

Papio.anubis.clone.rp41-133b2.8034-40080.revcompl.Baboon             ggcagat--catgaggtcaggagttcaagaccagcctggccaacacagcgaaaccctgtc    11432
BCRP3.HUMAN.NCBI.REF                                                 ggcagattgcttgagcctaggagttcaagatcagcctgtacaacatggtgaaaccccatc    9191
LOC106996293.glutathione.hydrolase5.proenzyme-like-GGT1.rhesus.      ggcagattgcttgagcctagaagttcaaaatcagcctgcgcaacatggtgaaaccccatc    57352
                                                                     *******  * ****   ** ******* * *******  *****  * *******  **

Papio.anubis.clone.rp41-133b2.8034-40080.revcompl.Baboon             tctactaaaaagacaaaaattagctg---agcgtggtggcgggtggctgtaatcccagct    11489
BCRP3.HUMAN.NCBI.REF                                                 tctacaaaaaata-aaaaattagctgtgtgtggtggtggtgtgtgcctgtagtcccagct    9250
LOC106996293.glutathione.hydrolase5.proenzyme-like-GGT1.rhesus.      tctacaaaaaata-aaaaactagcta---ggcgtggtagtgtgtgcctgtagtcccaggt    57408
                                                                     ***** ***** * ***** *****       ***** * * *** ***** ****** *

Papio.anubis.clone.rp41-133b2.8034-40080.revcompl.Baboon             acttgggaggctgagacaggagaattgcttgaacccaggagatggaggctgcagtgagct    11549
BCRP3.HUMAN.NCBI.REF                                                 gtttgggacgctgaggtgggaggctcacttgagcctgggtgatcgaggctgcagtgagcc    9310
LOC106996293.glutathione.hydrolase5.proenzyme-like-GGT1.rhesus.      gtttgggatgctgaggtgggagtctcacttgaaccaaggtgatcgacgctgcagtgagcc    57468
                                                                       ****** ******   ****  *  ***** **  ** *** ** ************

Papio.anubis.clone.rp41-133b2.8034-40080.revcompl.Baboon             gagatcgaggcactgcactccagcctgggcaacagagtgaaatgctgtctcaaataaata    11609
BCRP3.HUMAN.NCBI.REF                                                 atgatcctgagactgcactccagcctgggcaacagagtgagatgctgtctcaaataaata    9370
LOC106996293.glutathione.hydrolase5.proenzyme-like-GGT1.rhesus.      atgatcctgagactgcactccaacctgggcaacagagtgagatgctgtctc----aaata    57524
                                                                       ****  *  *********** ***************** **********    *****

Papio.anubis.clone.rp41-133b2.8034-40080.revcompl.Baboon             aataaataaaaataaaataatttgaggcctaggagtctgaaattctgggatgtcctttat    11669
BCRP3.HUMAN.NCBI.REF                                                 aataaataaaaataaaatactttgaggcctaggggtctaaaattctgagatctcctttat    9430
LOC106996293.glutathione.hydrolase5.proenzyme-like-GGT1.rhesus.      aataaataaaaataaaataacatgaggcctagaagtctgaaattctgggatctcccttat    57584
                                                                     *******************   **********  **** ******** *** *** ****
```

```
Papio.anubis.clone.rp41-133b2.8034-40080.revcompl.Baboon             gcaatttgagtggctgagatgatctgaagctggatccagtgctcctgagggctgctctat    11729
BCRP3.HUMAN.NCBI.REF                                                 gc-atttgagtgactgagatgatctgaagctggatccagtgctcctgagggctgctttat    9489
LOC106996293.glutathione.hydrolase5.proenzyme-like-GGT1.rhesus.      gc-atttgagcggctgagatgatctgaagctggatccagtgctcctgagggctgctctat    57643
                                                                     ** ******* * ******************************************* ***

Papio.anubis.clone.rp41-133b2.8034-40080.revcompl.Baboon             ttctggttgactgtcactcctagagtaagaaacctgcaccccacgtgtggggcattatgg    11789
BCRP3.HUMAN.NCBI.REF                                                 ttctggttgactgtgactcctagagtaagaaacctgcaccccacatgtggggcattatgg    9549
LOC106996293.glutathione.hydrolase5.proenzyme-like-GGT1.rhesus.      ttctggttgactgtcactcctagagtaagaaacctgcaccccacgtgtggggcattatgg    57703
                                                                     ************** ***************************** ***************

Papio.anubis.clone.rp41-133b2.8034-40080.revcompl.Baboon             catcccctcccttagccacgtgagtaggtcaacagcactgctctagaccaggtgtggtgg    11849
BCRP3.HUMAN.NCBI.REF                                                 catcccctccctcagccacatgagtaagtcaacagcactgctctagaccaggtgtggtgg    9609
LOC106996293.glutathione.hydrolase5.proenzyme-like-GGT1.rhesus.      cattgcctccctcagccacgtgaataggtcaacagcactgctctagaccaggtgtggtgg    57763
                                                                     ***  ******* ****** *** ** *********************************

Papio.anubis.clone.rp41-133b2.8034-40080.revcompl.Baboon             ctcacgcctatagtcccagctactcgggagactgaggcaggaagattgcttcaggccagg    11909
BCRP3.HUMAN.NCBI.REF                                                 ctcacacctatagtcccagctactcgggagactgaggcaggaggattgcttcaggccagg    9669
LOC106996293.glutathione.hydrolase5.proenzyme-like-GGT1.rhesus.      ctcacgcctatagtcccagctactcaggagactgaagtaggaggattacttcaggccagg    57823
                                                                     ***** ******************* ********* * **** **** ************

Papio.anubis.clone.rp41-133b2.8034-40080.revcompl.Baboon             aatttgagaccagcctgagcaata---tgttaggttggtacaaaggtaattgcagttttt    11966
BCRP3.HUMAN.NCBI.REF                                                 aatttgagaccagccagagcaatatattattaggttggtacaaaagtaattgcagtgttt    9729
LOC106996293.glutathione.hydrolase5.proenzyme-like-GGT1.rhesus.      aatttgagaccaccctgagcaata---tattaggttggtacaaaagtaagtgtggttttt    57880
                                                                     ************ ** ********   * *************** **** **  ** ***

Papio.anubis.clone.rp41-133b2.8034-40080.revcompl.Baboon             accattaaaagtaatggtgaccctgtctcagcaaaataaaaagcaaaaaaaaaaaaaaa-    12025
BCRP3.HUMAN.NCBI.REF                                                 gcctttaaaagtaatggcaaccctgtttcagcaaaataaaaagcaaaaaaaaaaaaaaaa    9789
LOC106996293.glutathione.hydrolase5.proenzyme-like-GGT1.rhesus.      gccattaaaagtaatagcgaccctgtctcagcaaaaaaa----aaaaaaaaaaaaaaaa    57936
                                                                      ** *********** *  ******* ********* ***    ***************
```

End of identity of baboon BCRP3 sequence

```
Papio.anubis.clone.rp41-133b2.8034-40080.revcompl.Baboon             ------------------------------------------------------------    12025
BCRP3.HUMAN.NCBI.REF                                                 aaaaaaaagaaaggaagaatcagctgggcgtggtggctcacgcctctaatcccagcactt    9849
LOC106996293.glutathione.hydrolase5.proenzyme-like-GGT1.rhesus.      gggaagaagagaaaaagaatcagctgggcatggtggctcacacctgtaatcccagtactt    57996


Papio.anubis.clone.rp41-133b2.8034-40080.revcompl.Baboon             ------------------------------------------------------------    12025
BCRP3.HUMAN.NCBI.REF                                                 tgggaggccaaggcgggcagatcatgagatcaggagatcgagaccatcctggctaacacg    9909
LOC106996293.glutathione.hydrolase5.proenzyme-like-GGT1.rhesus.      tgggaggccgagttgggtggatcacaaggtcaggagattgagaccatc----ctaacatg    58052


Papio.anubis.clone.rp41-133b2.8034-40080.revcompl.Baboon             ------------------------------------------------------------    12025
BCRP3.HUMAN.NCBI.REF                                                 gtgaaaccccatttctactaaaaatacaaaaaattagccgggcatggtggcaggcgcctg    9969
LOC106996293.glutathione.hydrolase5.proenzyme-like-GGT1.rhesus.      gtgaaaccccgtctctactgaaaatatgacaaattagctgggcatggtagtgggcacctg    58112


Papio.anubis.clone.rp41-133b2.8034-40080.revcompl.Baboon             ------------------------------------------------------------    12025
BCRP3.HUMAN.NCBI.REF                                                 tagtcccagctactcaggaggcggaggcaggagaatggcatgaacccaggaggtggaggt    10029
LOC106996293.glutathione.hydrolase5.proenzyme-like-GGT1.rhesus.      tagtcccagctattcgggaggctgaggcaggagaatggcgtgaacccaggaggcggaact    58172


Papio.anubis.clone.rp41-133b2.8034-40080.revcompl.Baboon             ------------------------------------------------------------    12025
BCRP3.HUMAN.NCBI.REF                                                 tgcagtgagccgagatcatgccactgcactccagcctgggtgacagagtgagactccgtc    10089
LOC106996293.glutathione.hydrolase5.proenzyme-like-GGT1.rhesus.      tgcagtgagccgagatcgcaccactgcactccagcctgggtgacagagtgagactccgtc    58232


Papio.anubis.clone.rp41-133b2.8034-40080.revcompl.Baboon             --aaaaaaggaagaaagagaaaaaagaaaaaaaaaaatcaccaatctatttctcag----    12079
BCRP3.HUMAN.NCBI.REF                                                 tcaaaaaaaaaaaaaaaagaat----------------cactgctctgtctctcagcctc    10133
LOC106996293.glutathione.hydrolase5.proenzyme-like-GGT1.rhesus.      tcaaaaaaaaaaaaaagaaagaaagaaagaaaaaaacccactgctctgtctctcagcctc    58292
                                                                       ******  ** **  *  *                ***   *** * ******

Papio.anubis.clone.rp41-133b2.8034-40080.revcompl.Baboon             ---------gattggctgtcgccttgaggggaatgctggccttgtttgtctccagccctg    12130
BCRP3.HUMAN.NCBI.REF                                                 ctcttccaagattggccgtcgccttgaggggaatgctggccttgcctgtctccagccctg    10193
LOC106996293.glutathione.hydrolase5.proenzyme-like-GGT1.rhesus.      ctcttccaggattggctgtcatcttgaggggaatgctggccttgcctgtctccagccttg    58352
                                                                              ******* ***  **********************  *********** **

Papio.anubis.clone.rp41-133b2.8034-40080.revcompl.Baboon             tacttctctgcctcctatgcctttaag--catgtttcctatttgctgggcttttcta-gt    12187
BCRP3.HUMAN.NCBI.REF                                                 tacctctctgcctcctatgcctttaagcacatgttttctatttgctgggctgtgaaatct    10253
LOC106996293.glutathione.hydrolase5.proenzyme-like-GGT1.rhesus.      ttcttctctgcctcttatgcctttaagcacatgttttctatttgctgggctgtgaaatct    58412
                                                                     * * ********** ************  ******* ************** *   *  *

Papio.anubis.clone.rp41-133b2.8034-40080.revcompl.Baboon             tctctgcgggaattgtggtctgaattacag------------------------------    12217
BCRP3.HUMAN.NCBI.REF                                                 gctcttcatctgatggggtttgctttataggtgactagatcctttctcttggtggtttt    10313
LOC106996293.glutathione.hydrolase5.proenzyme-like-GGT1.rhesus.      gcacttcatctgatggggtttgctttataggtgactagatcctttctcttggtggtttt    58472
                                                                      * ** *      ** *** **  *** **

Papio.anubis.clone.rp41-133b2.8034-40080.revcompl.Baboon             ------------------------------------------------------------    12217
BCRP3.HUMAN.NCBI.REF                                                 agaattcgcattttcacattgaccttaaatagtctgattatagtttgccacggcaaagac    10373
LOC106996293.glutathione.hydrolase5.proenzyme-like-GGT1.rhesus.      agaatttgcatcttcacattgactttaaatagtctgattacagtttgccatggcaaagac    58532


Papio.anubis.clone.rp41-133b2.8034-40080.revcompl.Baboon             ------------------------------------------------------------    12217
BCRP3.HUMAN.NCBI.REF                                                 cctttgcattgcattgtttggggatatttgagcctcctctatctggatgtctaatctctt    10433
LOC106996293.glutathione.hydrolase5.proenzyme-like-GGT1.rhesus.      cctttgcattgcattgtttggggatatttgaccctcttttatctggatgtctaatctcct    58592


Papio.anubis.clone.rp41-133b2.8034-40080.revcompl.Baboon             ------------------------------------------------------------    12217
BCRP3.HUMAN.NCBI.REF                                                 gttagatgtgagtagttttcattattatttattaatgggctggcatgtgggccgtggtt    10493
LOC106996293.glutathione.hydrolase5.proenzyme-like-GGT1.rhesus.      gttagatgtgagtagttttc---attatttcattaatgggctggcatgtggaccgtggtt    58649


Papio.anubis.clone.rp41-133b2.8034-40080.revcompl.Baboon             -------------------------------------------------------tctcc    12222
BCRP3.HUMAN.NCBI.REF                                                 ccaggcaggctcagaggggcagctgcctgatgtctggacagcttctctttctg--tcttt    10551
LOC106996293.glutathione.hydrolase5.proenzyme-like-GGT1.rhesus.      ccagggggctcagagaggcagccgcctgatgtct-gaccgcttctctttctgtctcttt    58708
                                                                                                                            ***

Papio.anubis.clone.rp41-133b2.8034-40080.revcompl.Baboon             cattaccagga-------------------------------------------------    12233
BCRP3.HUMAN.NCBI.REF                                                 tcttacctggactctgggttgcttgttagctgcttctgccagttctgagttttcaagggg    10611
LOC106996293.glutathione.hydrolase5.proenzyme-like-GGT1.rhesus.      tcttacctggactctgggttgcttgttagctgcttctgccagttctgagttttcaagggg    58768
                                                                       ***** ***

Papio.anubis.clone.rp41-133b2.8034-40080.revcompl.Baboon             ------------------------------------------------------------    12233
BCRP3.HUMAN.NCBI.REF                                                 agaggggcccagtgatggctgttctttgaaggaaagggaagaatgtctcctgtttaacat    10671
LOC106996293.glutathione.hydrolase5.proenzyme-like-GGT1.rhesus.      agaggggcccagtgatggctgttctttgaaggaaagggaagaatgtctcctgtttaacac    58828


Papio.anubis.clone.rp41-133b2.8034-40080.revcompl.Baboon             ------------------------------------------------------------    12233
BCRP3.HUMAN.NCBI.REF                                                 gtttctatgtttccagtta---------cttggttagttagttagagccagggtctctcg    10722
LOC106996293.glutathione.hydrolase5.proenzyme-like-GGT1.rhesus.      atttctatgtttccagttagttagttaggttagttagttagttagagccagggtatctca    58888


Papio.anubis.clone.rp41-133b2.8034-40080.revcompl.Baboon             ------------------------------------------------------------    12233
BCRP3.HUMAN.NCBI.REF                                                 ctctgttgcgcaggctggagtgcaatggcatgatcgtggctcacagcagcctccaccttc    10782
LOC106996293.glutathione.hydrolase5.proenzyme-like-GGT1.rhesus.      ctctgttgcacaggctggagtgcaatggcatgatcttggctcacagtagcctccacctac    58948


Papio.anubis.clone.rp41-133b2.8034-40080.revcompl.Baboon             ------------------------------------------------------------    12233
BCRP3.HUMAN.NCBI.REF                                                 caggctcgagcaatgctcccacctcagcctctcaagcagctgggactgcaggtatgtgcc    10842
LOC106996293.glutathione.hydrolase5.proenzyme-like-GGT1.rhesus.      caggctcaagcagtgctcccaccccagcctcccaagtagctgggactgcaggcatgtgcc    59008


Papio.anubis.clone.rp41-133b2.8034-40080.revcompl.Baboon             ------------------------------------------------------------    12233
BCRP3.HUMAN.NCBI.REF                                                 accatgcttggctgcctttttaaa-----ttttttttttttttaatacagacaaggtctca    10897
LOC106996293.glutathione.hydrolase5.proenzyme-like-GGT1.rhesus.      accatgcttggctaccttaaaaaaatttttttttttttttgatacagacgaggtctca    59068


Papio.anubis.clone.rp41-133b2.8034-40080.revcompl.Baboon             ------------------------------------------------------------    12233
BCRP3.HUMAN.NCBI.REF                                                 ctatattgcccaggctggtcttaaactcatgggctcaagtgatactcctgcctcggcctt    10957
LOC106996293.glutathione.hydrolase5.proenzyme-like-GGT1.rhesus.      ctatattgcccaggttggtattaaactcatgggctcaagtgatcctcctgccttggcctt    59128


Papio.anubis.clone.rp41-133b2.8034-40080.revcompl.Baboon             ------------------agtctgacttacatttaacatatttcttttttttttttttt    12275
BCRP3.HUMAN.NCBI.REF                                                 tcaaagtgctgatatcacaggca-------------------------------------    10980
LOC106996293.glutathione.hydrolase5.proenzyme-like-GGT1.rhesus.      tctaagtgctgatattacaggcagggcttcaattttttttttttttttttttttttttt    59188
                                                                                       ** *

Papio.anubis.clone.rp41-133b2.8034-40080.revcompl.Baboon             tgagacggagtctcactctgtcgcccaggctggagtgcagtggcgcaatcttggctcatt    12335
BCRP3.HUMAN.NCBI.REF                                                 ------------------------------------------------------------    10980
LOC106996293.glutathione.hydrolase5.proenzyme-like-GGT1.rhesus.      tgagacggagtctcgctctgtcgcccaagctggagtgcagtggccggatctcagctcact    59248


Papio.anubis.clone.rp41-133b2.8034-40080.revcompl.Baboon             gcaagctccgcctcccaggtttacgccattctcctgcctcagcctcctgagtagctggga    12395
BCRP3.HUMAN.NCBI.REF                                                 ------------------------------------------------------------    10980
LOC106996293.glutathione.hydrolase5.proenzyme-like-GGT1.rhesus.      gcaagctccgccgcccgagtttacaccattctcctgcctcagcctcccgagtagctggga    59308
```

```
Papio.anubis.clone.rp41-133b2.8034-40080.revcompl.Baboon            ctacaggcgcctgccatcatgtccggctaattttt--gtattttttagtagagacggggt    12453
BCRP3.HUMAN.NCBI.REF                                                ------------------------------------------------------------    10980
LOC106996293.glutathione.hydrolase5.proenzyme-like-GGT1.rhesus.     ctacaggcgcctgccacctcgcccggctagttttttttgtattttttagtagagacggggt    59368


Papio.anubis.clone.rp41-133b2.8034-40080.revcompl.Baboon            ttcaccgtgttagccaggatggtctcaatctcctgacctcgtgatctgcccgcctcggcc    12513
BCRP3.HUMAN.NCBI.REF                                                ------------------------------------------------------------    10980
LOC106996293.glutathione.hydrolase5.proenzyme-like-GGT1.rhesus.     ttcaccgtgttagccaggatggtcttgatctcctgacctcgtgatccgcccgtctcggcc    59428


Papio.anubis.clone.rp41-133b2.8034-40080.revcompl.Baboon            tcccaaagtgctaggactacaggtgtgagccatcgtgcccagccttagcagatttttttt    12573
BCRP3.HUMAN.NCBI.REF                                                ---------------------------------------------gggtttccatttttt    10995
LOC106996293.glutathione.hydrolase5.proenzyme-like-GGT1.rhesus.     tcccaaagtgctgggattacaggcttgagccaccgcgcccggcctgggcttcaatttttt    59488
                                                                                                                   *      ******

Papio.anubis.clone.rp41-133b2.8034-40080.revcompl.Baboon            ttttaatgtaagggagcaa-----------------------------------------    12592
BCRP3.HUMAN.NCBI.REF                                                aaagctcccagcagtggta-taaactcctcctttccagagaaagcgcactctgtccgcat    11054
LOC106996293.glutathione.hydrolase5.proenzyme-like-GGT1.rhesus.     aaagctcccagcaggggtattaaagtctccctttccagagaaagcgcactctgtccccat    59548
                                                                             *   * *  *

Papio.anubis.clone.rp41-133b2.8034-40080.revcompl.Baboon            ------------------------------------------------------------    12592
BCRP3.HUMAN.NCBI.REF                                                ccctcatgttgtcctctcctgcctctgcttagggttcactccgggggaaagtgccacttg    11114
LOC106996293.glutathione.hydrolase5.proenzyme-like-GGT1.rhesus.     ccctcatgttatcctctcctgcctctgcttagggttcactctgggggaat-tgccacttg    59607


Papio.anubis.clone.rp41-133b2.8034-40080.revcompl.Baboon            ------------------------------------------------------------    12592
BCRP3.HUMAN.NCBI.REF                                                agagtttcctttttgtgtgtgg-ttctgactgactgctccctgctcacagatgctgattc    11173
LOC106996293.glutathione.hydrolase5.proenzyme-like-GGT1.rhesus.     agagattcctttttgtgtgtggcttctgactgaccggtccctgctcacagctgctgcttc    59667


Papio.anubis.clone.rp41-133b2.8034-40080.revcompl.Baboon            ------------------ggctgactctggggcctgggtaggaggccattgcaataaccc    12634
BCRP3.HUMAN.NCBI.REF                                                tcagggtggggtccctgaggcctggagtgtggcctctgacgaccttcagggccaggtgtg    11233
LOC106996293.glutathione.hydrolase5.proenzyme-like-GGT1.rhesus.     tcagggtggggtccctgaggcctggagtgtggcctctgaaaccttcagggccagaagca    59727
                                                                                      ***      ** *****  *        **  ** *

Papio.anubis.clone.rp41-133b2.8034-40080.revcompl.Baboon            aggtgagacgggttggtggcttggaccaatgtggtggagatgg-----------------    12677
BCRP3.HUMAN.NCBI.REF                                                gaatgagagcctgtggccacatggcccccggtgggagacgtcccgccgccctttgcttct    11293
LOC106996293.glutathione.hydrolase5.proenzyme-like-GGT1.rhesus.     gaatgagagcctgtggccacatagcccctggtgggagacgtcctgccaccctttgcttct    59787
                                                                       *****     ***   * * * **   ****  **  *

Papio.anubis.clone.rp41-133b2.8034-40080.revcompl.Baboon            ------------------------------------------------------------    12677
BCRP3.HUMAN.NCBI.REF                                                ctgtgccactctggctgcacagttcagagccttgggaaatgttaaccagtaggacctaga    11353
LOC106996293.glutathione.hydrolase5.proenzyme-like-GGT1.rhesus.     ctgtgtcactctggctgtacatttcagatccctgggaaacgttaactggtaggacctaga    59847


Papio.anubis.clone.rp41-133b2.8034-40080.revcompl.Baboon            ------------------------------------------------------------    12677
BCRP3.HUMAN.NCBI.REF                                                cggggaggtgagaaggggtcaccccccaggtgtgcctgtggtgagccttcgtgctgagca    11413
LOC106996293.glutathione.hydrolase5.proenzyme-like-GGT1.rhesus.     aggggaggtgaggaggggtcacgccccaggtgtgcctgtggtgagcctttgtgctgagca    59907


Papio.anubis.clone.rp41-133b2.8034-40080.revcompl.Baboon            ------------------------------------------------------------    12677
BCRP3.HUMAN.NCBI.REF                                                ggtgcagggagggaggcccaggtgcacacacctgtgaagtaggggcagctggctgggctc    11473
LOC106996293.glutathione.hydrolase5.proenzyme-like-GGT1.rhesus.     ggtgcagggagggaggcccaggtgcacacatctgtgaagtaggggcagctggttgggctc    59967


Papio.anubis.clone.rp41-133b2.8034-40080.revcompl.Baboon            ------------------------------------------------------------    12677
BCRP3.HUMAN.NCBI.REF                                                cttgacctgctccagagcttcttatttctggccacttcacctgcagaaggcccaggtgg    11533
LOC106996293.glutathione.hydrolase5.proenzyme-like-GGT1.rhesus.     cttgacctgctccagaacttcttatttctagccacttcacctgcagaaggcccaggtgg    60027


Papio.anubis.clone.rp41-133b2.8034-40080.revcompl.Baboon            ------------------------------------------------------------    12677
BCRP3.HUMAN.NCBI.REF                                                ctgtggcctctagggtcccttgcc------------------------------------    11557
LOC106996293.glutathione.hydrolase5.proenzyme-like-GGT1.rhesus.     ctgtggtctctagggtcccttgcagtgaacataccgcctgtcctcagctcctctctaggc    60087


Papio.anubis.clone.rp41-133b2.8034-40080.revcompl.Baboon            ------------------------------------------------------------    12677
BCRP3.HUMAN.NCBI.REF                                                ------------------------------------------------------------    11557
LOC106996293.glutathione.hydrolase5.proenzyme-like-GGT1.rhesus.     ctggtgctgtgctcagcaccgtcgtatgtgtgtatgtgtgcacgcacgtgaatgtgtgca    60147


Papio.anubis.clone.rp41-133b2.8034-40080.revcompl.Baboon            ------------------------------------------------------------    12677
BCRP3.HUMAN.NCBI.REF                                                ------------------------------------------------------------    11557
LOC106996293.glutathione.hydrolase5.proenzyme-like-GGT1.rhesus.     ggcatcatgaggtgtggtccctgctcttaggcagcttgccttgtggctgaaatgaaccat    60207


Papio.anubis.clone.rp41-133b2.8034-40080.revcompl.Baboon            ------------------------------------------------------------    12677
BCRP3.HUMAN.NCBI.REF                                                ------------------------------------------------------------    11557
LOC106996293.glutathione.hydrolase5.proenzyme-like-GGT1.rhesus.     cacctgcatcaaggaaacacaaggccagatgcagtggccttgtctggagtcagatggtgg    60267


Papio.anubis.clone.rp41-133b2.8034-40080.revcompl.Baboon            ------------------------------------------------------------    12677
BCRP3.HUMAN.NCBI.REF                                                ------------------------------------------------------------    11557
LOC106996293.glutathione.hydrolase5.proenzyme-like-GGT1.rhesus.     ctccgtgacctggcttcactccagaactacctggggtttctagttgtgcaaatcccaggt    60327


Papio.anubis.clone.rp41-133b2.8034-40080.revcompl.Baboon            ------------------------------------------------------------    12677
BCRP3.HUMAN.NCBI.REF                                                ------------------------------------------------------------    11557
LOC106996293.glutathione.hydrolase5.proenzyme-like-GGT1.rhesus.     cccacccagattatgagtccagttttctacaggtggagcccaggaaggtgtatttttaac    60387


Papio.anubis.clone.rp41-133b2.8034-40080.revcompl.Baboon            ------------------------------------------------------------    12677
BCRP3.HUMAN.NCBI.REF                                                ------------------------------------------------------------    11557
LOC106996293.glutathione.hydrolase5.proenzyme-like-GGT1.rhesus.     acgatggtactgctcattcacccagcccagccagtggtctctgtgtacccaggaactgct    60447


Papio.anubis.clone.rp41-133b2.8034-40080.revcompl.Baboon            ------------------------------------------------------------    12677
BCRP3.HUMAN.NCBI.REF                                                ------------------------------------------------------------    11557
LOC106996293.glutathione.hydrolase5.proenzyme-like-GGT1.rhesus.     gacacagagctggtgctccccataaaacagaagacagagccttcacggacgggggacgtg    60507


Papio.anubis.clone.rp41-133b2.8034-40080.revcompl.Baboon            ------------------------------------------------------------    12677
BCRP3.HUMAN.NCBI.REF                                                ------------------------------------------------------------    11557
LOC106996293.glutathione.hydrolase5.proenzyme-like-GGT1.rhesus.     tggcttggtcatgacagggtatttcgtaccagcacagactgtgttcaggtgacattaagg    60567


Papio.anubis.clone.rp41-133b2.8034-40080.revcompl.Baboon            ------------------------------------------------------------    12677
BCRP3.HUMAN.NCBI.REF                                                ------------------------------------------------------------    11557
LOC106996293.glutathione.hydrolase5.proenzyme-like-GGT1.rhesus.     acaagttcctgcaggctagctgcctggacagggctgggtgggggtagaagaggggtcaca    60627


Papio.anubis.clone.rp41-133b2.8034-40080.revcompl.Baboon            ------------------------------------------------------------    12677
BCRP3.HUMAN.NCBI.REF                                                ------------------------------------------------------------    11557
LOC106996293.glutathione.hydrolase5.proenzyme-like-GGT1.rhesus.     gaggggcttcctcccggccgcctcaccagctgcttgatttagggcttggctctgggtcat    60687


Papio.anubis.clone.rp41-133b2.8034-40080.revcompl.Baboon            ------------------------------------------------------------    12677
BCRP3.HUMAN.NCBI.REF                                                ------------------------------------------------------------    11557
LOC106996293.glutathione.hydrolase5.proenzyme-like-GGT1.rhesus.     cctgggcctgattctgaaccacgggactggtgtggcctgcaggctcctgccacaagctgt    60747


Papio.anubis.clone.rp41-133b2.8034-40080.revcompl.Baboon            ------------------------------------------------------------    12677
BCRP3.HUMAN.NCBI.REF                                                ------------------------------------------------------------    11557
LOC106996293.glutathione.hydrolase5.proenzyme-like-GGT1.rhesus.     tcatggtgcagggggagaacagtgtccacagttccccagacagcagcagtggataccagg    60807


Papio.anubis.clone.rp41-133b2.8034-40080.revcompl.Baboon            ------------------------------------------------------------    12677
BCRP3.HUMAN.NCBI.REF                                                ------------------------------------------------------------    11557
LOC106996293.glutathione.hydrolase5.proenzyme-like-GGT1.rhesus.     cccccaggagttgttactgaagttgctgctggacaactcgcccttcactgagctccacat    60867


Papio.anubis.clone.rp41-133b2.8034-40080.revcompl.Baboon            ---------------------------------------------aggtagaacctccaa    12692
BCRP3.HUMAN.NCBI.REF                                                ---------------------------------------------tgtcctcagctccca    11572
LOC106996293.glutathione.hydrolase5.proenzyme-like-GGT1.rhesus.     agcacccgtggtgatgggagccgggtggagagagtctgccagcctgtgcatcagctccca    60927
                                                                                                                        * **** *

Papio.anubis.clone.rp41-133b2.8034-40080.revcompl.Baboon            cctgagtcgctggtgggttggatgtggggaaagaaaaaagtgaccaatccaggacggctc    12752
BCRP3.HUMAN.NCBI.REF                                                actgggaggggcagagggaggagggggtggag---------accccaggcagcagggctc    11623
LOC106996293.glutathione.hydrolase5.proenzyme-like-GGT1.rhesus.     actgggaggggcagagggaggagggggtggga---------gccccaggcagcagggctc    60978
                                                                     *** *  *      **  *** * ** *              ** *  *** * *****

Papio.anubis.clone.rp41-133b2.8034-40080.revcompl.Baboon            aaggg--tgtagggcgctgagcaaaaggggggagtgg-----------------------    12787
BCRP3.HUMAN.NCBI.REF                                                tgggagcagtggggccctgggtcccaggggtgtctggcaggcccctccttactctacgtc    11683
LOC106996293.glutathione.hydrolase5.proenzyme-like-GGT1.rhesus.     tgggagcagtggggccctgggttccaggggtgtctggcaggcccctccttactctacctc    61038
```

```
                                                                    **    ** **** *** *    ***** *  ***
Papio.anubis.clone.rp41-133b2.8034-40080.revcompl.Baboon          ------------------------------------------------------------    12787
BCRP3.HUMAN.NCBI.REF                                              --tcggcctctggatggaggtgctggctgcagtcgggctctgcctctgactaagggttgg    11741
LOC106996293.glutathione.hydrolase5.proenzyme-like-GGT1.rhesus.   ttttggcctctgggtggagatgctggccacagtcaggctctgcctctgactaaggactgg    61098


Papio.anubis.clone.rp41-133b2.8034-40080.revcompl.Baboon          ------------------------------------------------------------    12787
BCRP3.HUMAN.NCBI.REF                                              ggaagtggcgggtgtgggctgctgccccgtggggcctctgaacagaccccagggcctctg    11801
LOC106996293.glutathione.hydrolase5.proenzyme-like-GGT1.rhesus.   agaagtggcgggtgtgggctgctgccccgtgcagcctctgaacagacgccagggcctctg    61158


Papio.anubis.clone.rp41-133b2.8034-40080.revcompl.Baboon          ------------------------------------------------------------    12787
BCRP3.HUMAN.NCBI.REF                                              ccaatcatgactccttcctttcagctggacccgcaggccctgcaggacagagactggcag    11861
LOC106996293.glutathione.hydrolase5.proenzyme-like-GGT1.rhesus.   ccaatcatgactccttgctttcagctggacccacaggccctgcaggacagagactgacag    61218


Papio.anubis.clone.rp41-133b2.8034-40080.revcompl.Baboon          ------------------------------------------------------------    12787
BCRP3.HUMAN.NCBI.REF                                              cgcgccgtcatcgccatgaatggggtacgtgtccgtgggactctcctggcgcccacttcc    11921
LOC106996293.glutathione.hydrolase5.proenzyme-like-GGT1.rhesus.   tatgctgtcatcaccatgaatggggtatgtgtccgtgggacttttctggtgcccacatcc    61278


Papio.anubis.clone.rp41-133b2.8034-40080.revcompl.Baboon          ------------------------------------------------------------    12787
BCRP3.HUMAN.NCBI.REF                                              cccagaaggatagggtggcctctgttcatttcaaatcagtcagaggtggctgagcctgag    11981
LOC106996293.glutathione.hydrolase5.proenzyme-like-GGT1.rhesus.   cccagaagggtagggtggcctctattcatttcaaatcggtcagaggtggctgagcctgag    61338


Papio.anubis.clone.rp41-133b2.8034-40080.revcompl.Baboon          ------------------------------------------------------------    12787
BCRP3.HUMAN.NCBI.REF                                              gcagcatctgagagggagcctggttggagaagggagg-gcccccaagagcagaatcacca    12040
LOC106996293.glutathione.hydrolase5.proenzyme-like-GGT1.rhesus.   ccagcgtctgacacggagcctggttggaggagggaggttccccgaagagcagaatcgccg    61398


Papio.anubis.clone.rp41-133b2.8034-40080.revcompl.Baboon          ------------------------------------------------------------    12787
BCRP3.HUMAN.NCBI.REF                                              tgcacgggaatcgtcattcattggctggaatgcagttgccagccaggccctgagcatccc    12100
LOC106996293.glutathione.hydrolase5.proenzyme-like-GGT1.rhesus.   tgcccgggaatcgtca--cactgactgggatgcagttgccagccaggccctgagcatccc    61456


Papio.anubis.clone.rp41-133b2.8034-40080.revcompl.Baboon          ------------------------------------------------------------    12787
BCRP3.HUMAN.NCBI.REF                                              tcctcaaacaaaggtctcatggcaccaccaggacaggtggggcctccactcagggacctg    12160
LOC106996293.glutathione.hydrolase5.proenzyme-like-GGT1.rhesus.   tcctcaaacaaaggtctcatggcaccaccaggacaggtggggcctccactcggtgacctg    61516


Papio.anubis.clone.rp41-133b2.8034-40080.revcompl.Baboon          ------------------------------ttgtgtctatttctaaaacaacgaggact    12816
BCRP3.HUMAN.NCBI.REF                                              ggggctgcccatagaaatggagacccctgatttgtctttaggtaccccagaaaggtttag    12220
LOC106996293.glutathione.hydrolase5.proenzyme-like-GGT1.rhesus.   gggactgcatgtagaaatggagacctctgatttgtctttaggtaccccagaaaggtttag    61576
                                                                                                 **** * **  *     * ** *

Papio.anubis.clone.rp41-133b2.8034-40080.revcompl.Baboon          aggagatgagccattttgcggggaatgttggaaggaaaatcaaaagtacattttaggtca    12876
BCRP3.HUMAN.NCBI.REF                                              accttaaaagcaatgacacacccaaaaaggcccgggtataaatggtaaaatgttaatatt    12280
LOC106996293.glutathione.hydrolase5.proenzyme-like-GGT1.rhesus.   accttaaaagcaatgacacacccaaaaaggcctgggcatatatggtaaaatgttaatatt    61636
                                                                  *    *  *** **    *    **    *   **  *   *     * ** ***

Papio.anubis.clone.rp41-133b2.8034-40080.revcompl.Baboon          tggtatgtttgaaatgtttcttagac-agccggtgcaggtatcttgtgcatagctgggta    12935
BCRP3.HUMAN.NCBI.REF                                              tga-gattcttggctttttcttacattattctgtctttccttcttaatttttaattgtta    12339
LOC106996293.glutathione.hydrolase5.proenzyme-like-GGT1.rhesus.   tgatgattcttggctttttcttatactattctgtctttcctacttaatttttaattgtta    61696
                                                                  **     * *    * ******* *  *  * **        ***     *   * * **

Papio.anubis.clone.rp41-133b2.8034-40080.revcompl.Baboon          taagtgtctggagtgtagggagaggac-----tggctggagataaaattggggagttacc    12990
BCRP3.HUMAN.NCBI.REF                                              ctaagagaaagc---tggtcacagtaca-ctataatctcagctactctggaggctgagcc    12395
LOC106996293.glutathione.hydrolase5.proenzyme-like-GGT1.rhesus.   ttaagaaagagagagtgggcacagtacacctatggtctgagctactctagtggctgagcc    61756
                                                                    *       *    * *  * ** **     *      ** **   * * **     **

Papio.anubis.clone.rp41-133b2.8034-40080.revcompl.Baboon          agcacacaggctgcatctcatgccat-gagcctagatggagtcacccggggagaacagat    13049
BCRP3.HUMAN.NCBI.REF                                              aggagaatcactggagcccaagagtttgattacagcctgggcaacattgcaagatcccat    12455
LOC106996293.glutathione.hydrolase5.proenzyme-like-GGT1.rhesus.   aggagaatcactggagcccaagagttcgagtacagcctgggcaacattgcaagatcccat    61816
                                                                  ** * *    *** * * ** *   * **    **   * *  **   *  *** *  **

Papio.anubis.clone.rp41-133b2.8034-40080.revcompl.Baboon          aggtgcacaaggaccacggaccaagcctggggcccccaacatcaggagtcgagggaggtg    13109
BCRP3.HUMAN.NCBI.REF                                              atctaaaaaaaaagcaagcaagca------------------------------------    12479
LOC106996293.glutathione.hydrolase5.proenzyme-like-GGT1.rhesus.   at-tttaaaaaaagtaagcaacca------------------------------------    61839
                                                                  *  *  * **  *  * * *   *

Papio.anubis.clone.rp41-133b2.8034-40080.revcompl.Baboon          ggaggaaaaccaaagcctggcagggtaggggaggtgggtgcagggtaccgggccgttccc    13169
BCRP3.HUMAN.NCBI.REF                                              --------------------------agagaagcagcggggattttaggaggtgcttctg    12513
LOC106996293.glutathione.hydrolase5.proenzyme-like-GGT1.rhesus.   --------------------------agagaagcagcggggattttaggaggtggttctg    61873
                                                                                            ** * **  * * * *   **   **   ***

Papio.anubis.clone.rp41-133b2.8034-40080.revcompl.Baboon          tcttggggctttggtttccccatctgtaaccattttccccatt-----------------    13212
BCRP3.HUMAN.NCBI.REF                                              cagaaaccagtcgtttatatcatcttcaacaatcctggctcttgctgaagtagactaggg    12573
LOC106996293.glutathione.hydrolase5.proenzyme-like-GGT1.rhesus.   cagaagccagtcctttacaccatcttcaacaatcctggctcttgctgaggtagactaggg    61933
                                                                            *   **    *****  *** **  *  *  **

Papio.anubis.clone.rp41-133b2.8034-40080.revcompl.Baboon          ------------------------------------------------------------    13212
BCRP3.HUMAN.NCBI.REF                                              gcttccccgaggggcggctccacctcatgctgagacctctgcatgccttgggggtggaaa    12633
LOC106996293.glutathione.hydrolase5.proenzyme-like-GGT1.rhesus.   gattccctgaggggcagccctaccccatgctgagacctctgcatgccttcagagtggaaa    61993


Papio.anubis.clone.rp41-133b2.8034-40080.revcompl.Baboon          ------------------------------------------------------------    13212
BCRP3.HUMAN.NCBI.REF                                              tatttgatgagactcccaggggtccttgggaccttgggctgtgaggaccagaaggattag    12693
LOC106996293.glutathione.hydrolase5.proenzyme-like-GGT1.rhesus.   tatttgatgagactccaaggggcccttgagaccttgggctatgagggccagaaagattag    62053


Papio.anubis.clone.rp41-133b2.8034-40080.revcompl.Baboon          ------------------------------------------------------------    13212
BCRP3.HUMAN.NCBI.REF                                              aggactgtgccccttctccccactgtagatcgaagtaaagctctcggtcaagttcaacag    12753
LOC106996293.glutathione.hydrolase5.proenzyme-like-GGT1.rhesus.   tggactatgccctttctcccctctatagattgaagtaaagctcttggtcaagttcaacag    62113


Papio.anubis.clone.rp41-133b2.8034-40080.revcompl.Baboon          ------------------------------------------------------------    13212
BCRP3.HUMAN.NCBI.REF                                              cagggagttcagcttgaagaggatgccgtcccgaaaacagacaggggtcttcggagtcaa    12813
LOC106996293.glutathione.hydrolase5.proenzyme-like-GGT1.rhesus.   cagagagttcaacttgaagaggatgccatcatgaaa----acaggggtcttcggagctga    62169


Papio.anubis.clone.rp41-133b2.8034-40080.revcompl.Baboon          --ttgccccggaagccaaaacagttgacaaggctggaagcagacaggttggggaagcaag    13270
BCRP3.HUMAN.NCBI.REF                                              gattgctgtggtcaccaagtgagtggggaggggcttgggctcacgcactgagggtgcct-    12872
LOC106996293.glutathione.hydrolase5.proenzyme-like-GGT1.rhesus.   gattgctgtggtcaccaagtgagtggggaggggcttgggctcatgcactgagggtgcat-    62228
                                                                    ****   **   ****   *** *  * **      **  *     ** **  **

Papio.anubis.clone.rp41-133b2.8034-40080.revcompl.Baboon          ggtgcattccatttatttaggaggtaagacctt---------------------------    13303
BCRP3.HUMAN.NCBI.REF                                              -gtcccttcagctgtttctgcagaaaagagcatgtgtgggtctctcctctctgtgcatgg    12931
LOC106996293.glutathione.hydrolase5.proenzyme-like-GGT1.rhesus.   -gtcccttcagctgtttctgcagagaagagcatgtgtgggtctctcctctctctgtgtgg    62287
                                                                   ** * ***   *  **  * **  **** * *

Papio.anubis.clone.rp41-133b2.8034-40080.revcompl.Baboon          ------------------------------------------------------------    13303
BCRP3.HUMAN.NCBI.REF                                              ccactgcacggtgaggtcaggccccagggaacac--ggcgtcttcagctacctcctgtgt    12989
LOC106996293.glutathione.hydrolase5.proenzyme-like-GGT1.rhesus.   ccgctgcgtggtgaggtcaggccccagggaacacttggtgtgttcagctacctcctgtgt    62347


Papio.anubis.clone.rp41-133b2.8034-40080.revcompl.Baboon          ------------------------------------------------------------    13303
BCRP3.HUMAN.NCBI.REF                                              ttcctgcaaaccagctcaggaatgtccttgccaccttgcttggaagcagta-ggctggct    13048
LOC106996293.glutathione.hydrolase5.proenzyme-like-GGT1.rhesus.   ttactcaacaccagctcaggaatgtccttgccaccttgcttggaagcagtagggctggct    62407


Papio.anubis.clone.rp41-133b2.8034-40080.revcompl.Baboon          ------------------------------------------------------------    13303
BCRP3.HUMAN.NCBI.REF                                              ccaggaactgcccaagtgcagggttttctgcccttgcttggaattagtcacggtcccaga    13108
LOC106996293.glutathione.hydrolase5.proenzyme-like-GGT1.rhesus.   ccaggaactgcccgagtgca--gttttctgccctcgcttggaatttgtcatggtcccagg    62465


Papio.anubis.clone.rp41-133b2.8034-40080.revcompl.Baboon          ------------------------------------------------------------    13303
BCRP3.HUMAN.NCBI.REF                                              ttcctgttgaatggccataacccctgcccctttgtcacgagtcagttgccaagagaagcc    13168
LOC106996293.glutathione.hydrolase5.proenzyme-like-GGT1.rhesus.   ttcctgttgaatggccataacccctgcccctttgtcacaagtcagttgccaagagaagcc    62525


Papio.anubis.clone.rp41-133b2.8034-40080.revcompl.Baboon          ------------------------------------------------------------    13303
BCRP3.HUMAN.NCBI.REF                                              tgtttggtttgagagcagttcatgcagacatagaccacttcctctgagaattcatttgct    13228
LOC106996293.glutathione.hydrolase5.proenzyme-like-GGT1.rhesus.   tgttgggtttgagagcagttcttgcagacacagaccacttcctctgagaattcatttgct    62585


Papio.anubis.clone.rp41-133b2.8034-40080.revcompl.Baboon          ------------------------------------------------------------    13303
BCRP3.HUMAN.NCBI.REF                                              tccccaggatggaatctggctgggcctctgaccttgctggtcacgtgggccggggcctcc    13288
LOC106996293.glutathione.hydrolase5.proenzyme-like-GGT1.rhesus.   tccccaggttggaatccggctgggcccctgaacttgctggtcatgtgggccggggcctcc    62645


Papio.anubis.clone.rp41-133b2.8034-40080.revcompl.Baboon          ------------------------------------------------------------    13303
BCRP3.HUMAN.NCBI.REF                                              atcagtcataccctggactcctatctgtgtctaaacaccacgccccacccccaactgcac    13348
LOC106996293.glutathione.hydrolase5.proenzyme-like-GGT1.rhesus.   atcaatcataccccggactcctttctgtgtctaaacagcac-actcacccccaactgcac    62704
```

```
Papio.anubis.clone.rp41-133b2.8034-40080.revcompl.Baboon              ------------------------------------------------------------   13303
BCRP3.HUMAN.NCBI.REF                                                  ggcagccactcgcatagcactctgggagggctgtgggcatgagcagcgaggactccatga   13408
LOC106996293.glutathione.hydrolase5.proenzyme-like-GGT1.rhesus.       ggcagccacttgcgtagcactctgggagggctctgggcatgagcagcgaggactccagca   62764


Papio.anubis.clone.rp41-133b2.8034-40080.revcompl.Baboon              ----------------------------------tgtctgggatgggaatttaaagtgtt   13329
BCRP3.HUMAN.NCBI.REF                                                  gcagctcccccagataagccctgctaatgaggg-ggcttgcgaagcagctttgatgtgct   13467
LOC106996293.glutathione.hydrolase5.proenzyme-like-GGT1.rhesus.       gcagcccccccaaataaccactgctaatgagggtggcttgagaagcggctttgatgtgct   62824
                                                                                                         *  ** ** *    *** * *** *

Papio.anubis.clone.rp41-133b2.8034-40080.revcompl.Baboon              tttccctccagt------------------------------------------ttgtca   13347
BCRP3.HUMAN.NCBI.REF                                                  ggtaaatccaggtgcaaaacagaactcaagttagggcctccgcacagcactgcgttctaa   13527
LOC106996293.glutathione.hydrolase5.proenzyme-like-GGT1.rhesus.       cctaaacccagttgcaaaacaaagctaaagttaaggcctcagcacagcgctctgttctaa   62884
                                                                        *    ****                                           ** * *

Papio.anubis.clone.rp41-133b2.8034-40080.revcompl.Baboon              tttggctttgtttatggtgttttattcttattaaaattagtgcttatg------------   13395
BCRP3.HUMAN.NCBI.REF                                                  ctgtgaaggattcttactctagtgtcctgtgtggaggtattggaattgtccattgctaag   13587
LOC106996293.glutathione.hydrolase5.proenzyme-like-GGT1.rhesus.       ctttgaagtattcttactctagtgtcctgtgtggcggtattggaattgttcagtgctagg   62944
                                                                       *  *     **  *  * *  * * **   *     ** **    **

Papio.anubis.clone.rp41-133b2.8034-40080.revcompl.Baboon              ------------------------------------------------------------   13395
BCRP3.HUMAN.NCBI.REF                                                  actcagaggagaaaagcacttagcatcgcaggacttggagcaccggtgctgaggcaaccc   13647
LOC106996293.glutathione.hydrolase5.proenzyme-like-GGT1.rhesus.       actcagaggagtaaagcacttagcagcgcaggacttagagcgctggtgctgaggcaaccc   63004


Papio.anubis.clone.rp41-133b2.8034-40080.revcompl.Baboon              ------------------------------------------------------------   13395
BCRP3.HUMAN.NCBI.REF                                                  ttcattcattcgtcggatgtgtgtta------aggcccagggcaggggtcaggattctc   13701
LOC106996293.glutathione.hydrolase5.proenzyme-like-GGT1.rhesus.       ttcattcattcattggatgtgtgttaaggcccaggcccagggcaggggtcagggattctc   63064


Papio.anubis.clone.rp41-133b2.8034-40080.revcompl.Baboon              ------------------------------------------------------------   13395
BCRP3.HUMAN.NCBI.REF                                                  ctctcacacagcacgtgggtggcaggaccaacaccgggtctgacctcccagccgggggca   13761
LOC106996293.glutathione.hydrolase5.proenzyme-like-GGT1.rhesus.       ttctcacacagcacgcgggtggcaggaccaacaccgggtctgatctcccagctggggaca   63124


Papio.anubis.clone.rp41-133b2.8034-40080.revcompl.Baboon              --------------cctgacttgtttatttcatcttctggctctgtgctctctttcccat   13441
BCRP3.HUMAN.NCBI.REF                                                  caggctgctaaccccaggcctggaatctgtcagatgcccttcctgtgctgacttgactta   13821
LOC106996293.glutathione.hydrolase5.proenzyme-like-GGT1.rhesus.       caggctgctaaccccaggcctggaatctgtcagatgccctttctgtgctgactttactta   63184
                                                                                    *  * ** *  * * ***  * *     *******  ***  *

Papio.anubis.clone.rp41-133b2.8034-40080.revcompl.Baboon              tccaagactacatgtgtccctatggcgcccatgtgtgccacgttgggcccagggag----   13497
BCRP3.HUMAN.NCBI.REF                                                  gacaggcctcctgaccttcccgcaaaggtcatgtgtgattcgcaggggttctggccgctt   13881
LOC106996293.glutathione.hydrolase5.proenzyme-like-GGT1.rhesus.       gacaggcctcctgaccttcccacaaagatcatgtgtgacttgcaggggttctggctgctt   63244
                                                                        ** * ** *     * **      *  ********    *  ***     **

Papio.anubis.clone.rp41-133b2.8034-40080.revcompl.Baboon              ------------------------------------------------------------   13497
BCRP3.HUMAN.NCBI.REF                                                  gaaaggttcctgagaaagcacatgccatgaggacagagcttgcagagggaggacaggcat   13941
LOC106996293.glutathione.hydrolase5.proenzyme-like-GGT1.rhesus.       gaaagg-tcctgagacagtacatgcaatgaggactgagcttgcagagggagcacaggcat   63303


Papio.anubis.clone.rp41-133b2.8034-40080.revcompl.Baboon              ------------------------------------------------------------   13497
BCRP3.HUMAN.NCBI.REF                                                  gcagaaggctctgtgtgcagccccagacctgggtaccttcgtcaccgtcctcaccccacc   14001
LOC106996293.glutathione.hydrolase5.proenzyme-like-GGT1.rhesus.       gcagaaggttctctgtgcagccccacacctagtcaccttaaccatcatcctcactccacc   63363


Papio.anubis.clone.rp41-133b2.8034-40080.revcompl.Baboon              ------------------------------------------------------------   13497
BCRP3.HUMAN.NCBI.REF                                                  tccgggtgtgcagatagggagcaggcctcctgtgttatggcccaagcggggctgttagga   14061
LOC106996293.glutathione.hydrolase5.proenzyme-like-GGT1.rhesus.       tcc------------atagagcaggtctcctgtg-tgtggcccacgcagggctgccagga   63410


Papio.anubis.clone.rp41-133b2.8034-40080.revcompl.Baboon              ------------------------------------------------------------   13497
BCRP3.HUMAN.NCBI.REF                                                  cactgagaacattccctcctcccgcaggagagagaggtccaaggtgccctacatcatgcg   14121
LOC106996293.glutathione.hydrolase5.proenzyme-like-GGT1.rhesus.       cactgagaacatttcctcctcccgcaggagacagaggtccgaggtgccctacatcgtacg   63470


Papio.anubis.clone.rp41-133b2.8034-40080.revcompl.Baboon              ------------------------------------------------------------   13497
BCRP3.HUMAN.NCBI.REF                                                  ccagtgcgtggaggagatcgagcgccgaggcatggaggaggtgggcatctaccgcgtgtc   14181
LOC106996293.glutathione.hydrolase5.proenzyme-like-GGT1.rhesus.       ccagtgcgtggaggagctcaagcgccgaggcatggaggaggtgggcatctaccgcgtgtc   63530


Papio.anubis.clone.rp41-133b2.8034-40080.revcompl.Baboon              ------------------------------------------------------------   13497
BCRP3.HUMAN.NCBI.REF                                                  cggtgtggccacggacatccaggcactgaaggcaggcttcaacgtcagtgagtgtcggcc   14241
LOC106996293.glutathione.hydrolase5.proenzyme-like-GGT1.rhesus.       cggagtggccacggacatccaggcactgaaggcagccttcgacgtcagtcagtggtggcc   63590


Papio.anubis.clone.rp41-133b2.8034-40080.revcompl.Baboon              ------------------------------------------------------------   13497
BCRP3.HUMAN.NCBI.REF                                                  tgcgcaggacgggatggaggtgtgggcagtggtgtccgcgatgagatctcagagtgctcc   14301
LOC106996293.glutathione.hydrolase5.proenzyme-like-GGT1.rhesus.       tggggaggacaggatggaggtgtgggaggcggtgtccgcgatgagatctcagagtgctcc   63650


Papio.anubis.clone.rp41-133b2.8034-40080.revcompl.Baboon              ------------------------------------------------------------   13497
BCRP3.HUMAN.NCBI.REF                                                  atggcccaggcatgtcacatccttctctgtgtcttttcttcatttactgttttattattt   14361
LOC106996293.glutathione.hydrolase5.proenzyme-like-GGT1.rhesus.       atggtccgggcatgtcacattcttctctgtgtcttttcttcatttacagtgttactattt   63710


Papio.anubis.clone.rp41-133b2.8034-40080.revcompl.Baboon              ------------------------------------------------------------   13497
BCRP3.HUMAN.NCBI.REF                                                  taaaaaaagagaaaacaagagttgtacaaacagcttctatagaagccagtttttacacca   14421
LOC106996293.glutathione.hydrolase5.proenzyme-like-GGT1.rhesus.       ttaaaaaagagaagacaagaattatagaaatagcttctgtagaagccagtttttaaacca   63770


Papio.anubis.clone.rp41-133b2.8034-40080.revcompl.Baboon              ------------------------------------------------------ggccat   13503
BCRP3.HUMAN.NCBI.REF                                                  tcgtacccactcatgccacttggtggagtggaccaggggcttctgtggggacttggcctt   14481
LOC106996293.glutathione.hydrolase5.proenzyme-like-GGT1.rhesus.       tcctagccatgcatgccacttgctggggtgaaccaggggcttctgtggggccttggcctt   63830
                                                                                                                            **** *

Papio.anubis.clone.rp41-133b2.8034-40080.revcompl.Baboon              gtggctcaggagctggggagggtttgtgtccagatggaaccgccaggtccagccacagac   13563
BCRP3.HUMAN.NCBI.REF                                                  cctgccttggggtggacaggaggtggaagcccaggactcagtgcggtctgtccactgcc   14541
LOC106996293.glutathione.hydrolase5.proenzyme-like-GGT1.rhesus.       cctgccttggggtggacaggaggtggaagcccaggactcagtgcagtctgtccactgcc   63890
                                                                         **   ** * ***  ***   **    *  * *   * *    ***   **** * *

Papio.anubis.clone.rp41-133b2.8034-40080.revcompl.Baboon              accttagccgaaggagggtgctagggccagaggtcccatggggtgggtgcagggac----   13619
BCRP3.HUMAN.NCBI.REF                                                  ctgtatgaggatgtggtgggcagagggcactgatgaaattcagcgcaggccggggctgca   14601
LOC106996293.glutathione.hydrolase5.proenzyme-like-GGT1.rhesus.       ctgtgtgacgatgcggtgggcagaggacactgatggacccagctcaggctggggctgca   63950
                                                                         *  *  ** *  * * **   ** **  * *   *    *     ** *** *

Papio.anubis.clone.rp41-133b2.8034-40080.revcompl.Baboon              ------------------------------------------------------------   13619
BCRP3.HUMAN.NCBI.REF                                                  gcatctccgcctccatctcaccaaccctcacaggccttgaaggacccagactggcctcaa   14661
LOC106996293.glutathione.hydrolase5.proenzyme-like-GGT1.rhesus.       gcatctctgcctccatttcaccaactctctcaggctatgaaagacatggacctgcctcaa   64010


Papio.anubis.clone.rp41-133b2.8034-40080.revcompl.Baboon              ------------------------------------------------------------   13619
BCRP3.HUMAN.NCBI.REF                                                  atgccaggggagggcactgagaccccagagggtccttcccagcatcttcaaagcaacagg   14721
LOC106996293.glutathione.hydrolase5.proenzyme-like-GGT1.rhesus.       gtgccagaggaggacacagaggccccagagggtcctttccagcatcttcaaagcaacagg   64070


Papio.anubis.clone.rp41-133b2.8034-40080.revcompl.Baboon              ------------------------------------------------------------   13619
BCRP3.HUMAN.NCBI.REF                                                  attttgtgcctgcagacccttctttgcagcacacaccacccaccctgaccaggacccta   14781
LOC106996293.glutathione.hydrolase5.proenzyme-like-GGT1.rhesus.       attttgtgcctgcagacccttctttggggcacacaccactgaccctgaccaggacccta   64130


Papio.anubis.clone.rp41-133b2.8034-40080.revcompl.Baboon              ------------------------------------------------------------   13619
BCRP3.HUMAN.NCBI.REF                                                  gaatgcccagcatccctgggagggccctgtggtagtttcagctccctctgggggcccaga   14841
LOC106996293.glutathione.hydrolase5.proenzyme-like-GGT1.rhesus.       gaatgcctatcacccctgggtgggccctgtggtaatt-----tccctctgggggcccaga   64185


Papio.anubis.clone.rp41-133b2.8034-40080.revcompl.Baboon              ------------------------------------------------------------   13619
BCRP3.HUMAN.NCBI.REF                                                  atgaacctggcctgtggtgaggatgtaagcaccaatggccaattgggtccaaaggaagac   14901
LOC106996293.glutathione.hydrolase5.proenzyme-like-GGT1.rhesus.       atagacctggcctgcggtgaggacgcaagcaccagtggcccattgggtccaaaggaagac   64245


Papio.anubis.clone.rp41-133b2.8034-40080.revcompl.Baboon              ------------------------------------------------------------   13619
BCRP3.HUMAN.NCBI.REF                                                  accggttcaaacactgaaaccaatcagattctcccacggccttcctgctatcagacgaca   14961
LOC106996293.glutathione.hydrolase5.proenzyme-like-GGT1.rhesus.       attgattcaaacactgaaaccaatcagattctcccacagccttcctgccatcagaagaca   64305


Papio.anubis.clone.rp41-133b2.8034-40080.revcompl.Baboon              ------------------------------------------------------------   13619
BCRP3.HUMAN.NCBI.REF                                                  ctggtgcaggggtggttgctatgtacagggcagagccacccaatccccacgcaggcgctg   15021
LOC106996293.glutathione.hydrolase5.proenzyme-like-GGT1.rhesus.       ctggtgcagggttggttgctatgtacagggcagagtcacccgattcccacgcaggcactg   64365


Papio.anubis.clone.rp41-133b2.8034-40080.revcompl.Baboon              ------------------------------------------------------------   13619
BCRP3.HUMAN.NCBI.REF                                                  tgtcctgccacgttggcctcctcctggccatcacatcaggccaagcaggggagaggaatg   15081
LOC106996293.glutathione.hydrolase5.proenzyme-like-GGT1.rhesus.       tgtcctgctgtgctggcctcctcctggccatcacatcgggccaagcaggggagaggaatg   64425
```

```
Papio.anubis.clone.rp41-133b2.8034-40080.revcompl.Baboon              ------------------------------------------------------------    13619
BCRP3.HUMAN.NCBI.REF                                                  ggaatgcccacgcacccctatcaactctgcagacacagaaccatgcacagctcttgggag    15141
LOC106996293.glutathione.hydrolase5.proenzyme-like-GGT1.rhesus.       ggaatgcccatgcacccccatcaactctgcagacacagaaccacacacagctcttgggag    64485

Papio.anubis.clone.rp41-133b2.8034-40080.revcompl.Baboon              ------------------------------------------------------------    13619
BCRP3.HUMAN.NCBI.REF                                                  gagtcagatgagctgctcaaagcccaggagggacccgcacagtggtcagtgtggcaggga    15201
LOC106996293.glutathione.hydrolase5.proenzyme-like-GGT1.rhesus.       gggtcagatgagctgcttaaagccggggagggacccgcacagtggtcaacatggcaggga    64545

Papio.anubis.clone.rp41-133b2.8034-40080.revcompl.Baboon              ------------------------------------------------------------    13619
BCRP3.HUMAN.NCBI.REF                                                  cggtgctttagccaaggcagggatggtgggtgactcactcaggatcttcaaggaggccgc    15261
LOC106996293.glutathione.hydrolase5.proenzyme-like-GGT1.rhesus.       cggtgctttagccaagcctgggatggtgggagactcacttgggatcctgaaggaggccgc    64605

Papio.anubis.clone.rp41-133b2.8034-40080.revcompl.Baboon              ------------------------------------------------------------    13619
BCRP3.HUMAN.NCBI.REF                                                  tgcatttccgtgctctttccagataacaaggacgtgtcggtgatgatgagcgagatggac    15321
LOC106996293.glutathione.hydrolase5.proenzyme-like-GGT1.rhesus.       tgcatttccatgctctttccagataacaaggacgtgtcggtgatgatgagcgagatggac    64665

Papio.anubis.clone.rp41-133b2.8034-40080.revcompl.Baboon              ------------------------------------------------------------    13619
BCRP3.HUMAN.NCBI.REF                                                  gtgaacgccatcgcaggcacgctgaagctgtacttccgtgagctgcccgagcccctcttc    15381
LOC106996293.glutathione.hydrolase5.proenzyme-like-GGT1.rhesus.       gtgaacgccatcgcagggacgctgaagctgtacttccgtgagctgcccgagcccctcttc    64725

Papio.anubis.clone.rp41-133b2.8034-40080.revcompl.Baboon              ------------------------------------------------------------    13619
BCRP3.HUMAN.NCBI.REF                                                  actgacgagttctaccccaacttcgcagagggcatcggtgagcactggaggccttggcct    15441
LOC106996293.glutathione.hydrolase5.proenzyme-like-GGT1.rhesus.       accgacgagttctaccccaacttcgccgagggcatcggtgagcactggaggccttggcct    64785

Papio.anubis.clone.rp41-133b2.8034-40080.revcompl.Baboon              ------------------------------------------------------------    13619
BCRP3.HUMAN.NCBI.REF                                                  catgggagacgtctcctccacgtgcactgctgccctcggaggctgtgaaaagcgaggtgt    15501
LOC106996293.glutathione.hydrolase5.proenzyme-like-GGT1.rhesus.       catgggagacgtctcctccacatgcactgctgcccttggaggctgtgaaaagtgatgtgt    64845

Papio.anubis.clone.rp41-133b2.8034-40080.revcompl.Baboon              ------------------------------------------------------------    13619
BCRP3.HUMAN.NCBI.REF                                                  gggaacctgagctgtaacccctctgccgtggtcggcattttaacccaacctcaaaaagca    15561
LOC106996293.glutathione.hydrolase5.proenzyme-like-GGT1.rhesus.       gggaacctgagctgtgcccccctctgccatggtcggtgttttaacccaacctcagaaaaca    64905

Papio.anubis.clone.rp41-133b2.8034-40080.revcompl.Baboon              ------------------------------------------------------------    13619
BCRP3.HUMAN.NCBI.REF                                                  ggggaccagaaccgagcctgtcctggaaggccttgcccatccccagagggctccccatcc    15621
LOC106996293.glutathione.hydrolase5.proenzyme-like-GGT1.rhesus.       --ggaccaaaatcaagcctgtcctggaagacctcgcccatccccagagggctccccgtcc    64963

Papio.anubis.clone.rp41-133b2.8034-40080.revcompl.Baboon              ------------------------------------------------------------    13619
BCRP3.HUMAN.NCBI.REF                                                  ctactcctcaaggagaccaagaggctgaaatagtcagcactgctgtgctatggggtccta    15681
LOC106996293.glutathione.hydrolase5.proenzyme-like-GGT1.rhesus.       ctattcctcaaggagaccaagagggtgaaatggtcagcactgctgtgctgtggggtccta    65023

Papio.anubis.clone.rp41-133b2.8034-40080.revcompl.Baboon              ------------------------------------------------------------    13619
BCRP3.HUMAN.NCBI.REF                                                  aagtctgctgtcctccttcctgcagaccagggctgaaggagggtgcctgggtgctcttgc    15741
LOC106996293.glutathione.hydrolase5.proenzyme-like-GGT1.rhesus.       aagtctgctgtcctccttcctgcagaccaggactgaaggagc--gcccaggtgctctagc    65081

Papio.anubis.clone.rp41-133b2.8034-40080.revcompl.Baboon              ------------------------------------------------------------    13619
BCRP3.HUMAN.NCBI.REF                                                  catgggtcctggtccagccaagcatggtttcaaacatgacctgacccttagtcaacctgg    15801
LOC106996293.glutathione.hydrolase5.proenzyme-like-GGT1.rhesus.       cacgggttctggcccagtcaagcatgggttcaaacctggcctgacccttagtcaacctgc    65141

Papio.anubis.clone.rp41-133b2.8034-40080.revcompl.Baboon              ------------------------------------------------------------    13619
BCRP3.HUMAN.NCBI.REF                                                  aggctgatgtctagagcgggtgctggtgcgtgcagcacctgtggcctctgcatcaccctt    15861
LOC106996293.glutathione.hydrolase5.proenzyme-like-GGT1.rhesus.       aggctgatggctagagtgggtgctggttcatgtggcacctgtagtctccacatcaccctt    65201

Papio.anubis.clone.rp41-133b2.8034-40080.revcompl.Baboon              ------------------------------------------------------------    13619
BCRP3.HUMAN.NCBI.REF                                                  agggcaggtctgcctcccgggcccatgcacagaggacctggtctcccagcctgcaggtgc    15921
LOC106996293.glutathione.hydrolase5.proenzyme-like-GGT1.rhesus.       agggcaggtctgcctcccaggcccatgcacagagga-------------cctgcaggtgg    65248

Papio.anubis.clone.rp41-133b2.8034-40080.revcompl.Baboon              -----------------------------------------------ccaggtccactcc    13632
BCRP3.HUMAN.NCBI.REF                                                  ccctgtggtgtccaggacgacgaggggggtctctgtgtacttggtggggctgggaccctcc    15981
LOC106996293.glutathione.hydrolase5.proenzyme-like-GGT1.rhesus.       ccctgtggtgtccaggacaatgagggagtctctgcatacttggtggggctggagccctcc    65308
                                                                                                                  * **  * ****

Papio.anubis.clone.rp41-133b2.8034-40080.revcompl.Baboon              ctctttgggccttggtttccccatctgcaac-----------------------------    13663
BCRP3.HUMAN.NCBI.REF                                                  cacttcccacctccttgtgtccctcactcccctgtttcattccatgctgagcctcccctg    16041
LOC106996293.glutathione.hydrolase5.proenzyme-like-GGT1.rhesus.       cacttcccacctctttgtgtccctcactccc--gtttcattccacgccaaacctcccta    65366
                                                                      * ***    ***   * *  ** **     *

Papio.anubis.clone.rp41-133b2.8034-40080.revcompl.Baboon              ------------------------------------------------------------    13663
BCRP3.HUMAN.NCBI.REF                                                  ccttgggct-ccctggggagggggtggtggcaggagttgcccgagggcagctctgcccat    16100
LOC106996293.glutathione.hydrolase5.proenzyme-like-GGT1.rhesus.       ccttgggctcccctggggagggggtggtggcaggagatgcccaagtgcagctctgtccat    65426

Papio.anubis.clone.rp41-133b2.8034-40080.revcompl.Baboon              ------------------------------------------------------------    13663
BCRP3.HUMAN.NCBI.REF                                                  gagcagctgctctagcggctcctcctgctgctgttcgccgggtgctgctgacccctgcga    16160
LOC106996293.glutathione.hydrolase5.proenzyme-like-GGT1.rhesus.       gagtgactgctctcgcagctcctcctgctgctgttcgccgggtgctgctgacccctgtga    65486

Papio.anubis.clone.rp41-133b2.8034-40080.revcompl.Baboon              ------------------------------------------------------------    13663
BCRP3.HUMAN.NCBI.REF                                                  ggtagagaaaaggcgttcaggtggttcacaccccacacaggtgcccctcacagggtcctc    16220
LOC106996293.glutathione.hydrolase5.proenzyme-like-GGT1.rhesus.       ggtggagaaaaggcgttcaggtggctcataccccacaccagcgccccttgcagagtcctc    65546

Papio.anubis.clone.rp41-133b2.8034-40080.revcompl.Baboon              ------------------------------------------------------------    13663
BCRP3.HUMAN.NCBI.REF                                                  actggcggccagcgctgtgggtgtgacgatgatgacaagcctaaactgcgcaaggactcg    16280
LOC106996293.glutathione.hydrolase5.proenzyme-like-GGT1.rhesus.       actgggggccagagctgtgggactgaggatgatgacaaccctgggctatgcagggacacg    65606

Papio.anubis.clone.rp41-133b2.8034-40080.revcompl.Baboon              ---------------------------------------------tgggatttaaatgct    13678
BCRP3.HUMAN.NCBI.REF                                                  tgtcccgggcgctccatgtgaccacctcgggagaggtctccggcttgtcgtaacc-----    16335
LOC106996293.glutathione.hydrolase5.proenzyme-like-GGT1.rhesus.       agccccaggcactccacgtaaccacctcaggagaggtttctcaggagagcaaaaattaca    65666
```

End of Rhesus ident. with BCRP3

*

```
Papio.anubis.clone.rp41-133b2.8034-40080.revcompl.Baboon              ggcttctgaccgggtgcggtggctcatgtctgtaatcccggcactttgggaggctgaggt    13738
BCRP3.HUMAN.NCBI.REF                                                  ------------------------------------------------------------    16335
LOC106996293.glutathione.hydrolase5.proenzyme-like-GGT1.rhesus.       cggggcaggctgggcatggtggctcacgcctataatcccagcactttgggaggcccaggc    65726

Papio.anubis.clone.rp41-133b2.8034-40080.revcompl.Baboon              gggcagatcacctgatgtcgggagttcgagaccagcttgaccaacatggagaaaccctgt    13798
BCRP3.HUMAN.NCBI.REF                                                  ------------------------------------------------------------    16335
LOC106996293.glutathione.hydrolase5.proenzyme-like-GGT1.rhesus.       gggtggattacctgaggtcaggagttcaagaccagcctggccaacatggtgaaaccctgt    65786

Papio.anubis.clone.rp41-133b2.8034-40080.revcompl.Baboon              ctctactaaaaatac-aaaattagccgggcatggtggcacatgcctgtaatcccagctac    13857
BCRP3.HUMAN.NCBI.REF                                                  ------------------------------------------------------------    16335
LOC106996293.glutathione.hydrolase5.proenzyme-like-GGT1.rhesus.       ctctactaaaaatacaaaaattagctggacctggtggcacatgcctgtaatcccagctac    65846

Papio.anubis.clone.rp41-133b2.8034-40080.revcompl.Baboon              tcgggaggctgaggcaggagaatcacttgaacccgggaggcagaggttgtggtgagctga    13917
BCRP3.HUMAN.NCBI.REF                                                  ------------------------------------------------------------    16335
LOC106996293.glutathione.hydrolase5.proenzyme-like-GGT1.rhesus.       tcaggaggctgaggcaggggaattgcttgagccc-ggaggcagaggttgcagtgagctga    65905

Papio.anubis.clone.rp41-133b2.8034-40080.revcompl.Baboon              aatcgtgccattgccctatagcctgggcaacaagagtgaaactctgtccaaaaaaaaaga    13977
BCRP3.HUMAN.NCBI.REF                                                  ------------------------------------------------------------    16335
LOC106996293.glutathione.hydrolase5.proenzyme-like-GGT1.rhesus.       gatcatgtctctgcactccagcctggctga-gagagcaagactctgtcttaaaaaaaaaa    65964

Papio.anubis.clone.rp41-133b2.8034-40080.revcompl.Baboon              aaaagttgg---------------------------------------------------    13986
BCRP3.HUMAN.NCBI.REF                                                  ------------------------------------------------------------    16335
LOC106996293.glutathione.hydrolase5.proenzyme-like-GGT1.rhesus.       aaaattacacggggcagaagaaagaaggcatggaggcacgggtgatgggcggagggcccg    66024

Papio.anubis.clone.rp41-133b2.8034-40080.revcompl.Baboon              -------------cttcttgaggttctggggagcattcc-tgggtgtaagggaagaggtt    14032
BCRP3.HUMAN.NCBI.REF                                                  ------------------------------------------------------------    16335
LOC106996293.glutathione.hydrolase5.proenzyme-like-GGT1.rhesus.       gctgcctgcggcccatcgtgcttgcctgcgcagcctgggttgggggtgcggagagtgggc    66084

Papio.anubis.clone.rp41-133b2.8034-40080.revcompl.Baboon              gtcctcggtgagctgtgcaggcctgtgccccagggacagggtcacagtgtaggccaggct    14092
```

```
BCRP3.HUMAN.NCBI.REF                                              ------------------------------------------------------------    16335
LOC106996293.glutathione.hydrolase5.proenzyme-like-GGT1.rhesus.   gaccatggggtggtgt----ggcctggccccagctccagcatcattgtctccacagggac    66140

Papio.anubis.clone.rp41-133b2.8034-40080.revcompl.Baboon          gaccagcccctcagcactga----------------------------------------    14112
BCRP3.HUMAN.NCBI.REF                                              ------------------------------------------------------------    16335
LOC106996293.glutathione.hydrolase5.proenzyme-like-GGT1.rhesus.   gtgcaggcgctctggaccaatgaccacgcgctggcctggcacctgagcaatgacttccga    66200

Papio.anubis.clone.rp41-133b2.8034-40080.revcompl.Baboon          ------------------------------------------------------------    14112
BCRP3.HUMAN.NCBI.REF                                              ------------------------------------------------------------    16335
LOC106996293.glutathione.hydrolase5.proenzyme-like-GGT1.rhesus.   gaggaccctgtggcctgggcacgcacttagtgccaggcctgggaggagctggaggatcag    66260

Papio.anubis.clone.rp41-133b2.8034-40080.revcompl.Baboon          ------------------------------------------------------------    14112
BCRP3.HUMAN.NCBI.REF                                              ------------------------------------------------------------    16335
LOC106996293.glutathione.hydrolase5.proenzyme-like-GGT1.rhesus.   ctgcccagtttcctggaggagctgccggactgcccctgcaccctgacccaggcccgggcg    66320

Papio.anubis.clone.rp41-133b2.8034-40080.revcompl.Baboon          ------------------------------------------------------------    14112
BCRP3.HUMAN.NCBI.REF                                              ------------------------------------------------------------    16335
LOC106996293.glutathione.hydrolase5.proenzyme-like-GGT1.rhesus.   gactccggccgcttcttcgtgagcctccaatcagggcccaggagaggggatgagggtgtc    66380

Papio.anubis.clone.rp41-133b2.8034-40080.revcompl.Baboon          ------------------------------------------------------------    14112
BCRP3.HUMAN.NCBI.REF                                              ------------------------------------------------------------    16335
LOC106996293.glutathione.hydrolase5.proenzyme-like-GGT1.rhesus.   gcctccccactgaggacagcaccaggggaggcagatagaggtgtcctggagggtggggcg    66440

Papio.anubis.clone.rp41-133b2.8034-40080.revcompl.Baboon          ------------------------------------------------------------    14112
BCRP3.HUMAN.NCBI.REF                                              ------------------------------------------------------------    16335
LOC106996293.glutathione.hydrolase5.proenzyme-like-GGT1.rhesus.   ggggtctcagggcacctgcagagttggcctcgggaaggggatgacagaaccggaggccac    66500

Papio.anubis.clone.rp41-133b2.8034-40080.revcompl.Baboon          ------------------------------------------------------------    14112
BCRP3.HUMAN.NCBI.REF                                              ------------------------------------------------------------    16335
LOC106996293.glutathione.hydrolase5.proenzyme-like-GGT1.rhesus.   tgggtgacagccacctgctgctctgcagacggactacggctgtgacatggagcagggcag    66560

Papio.anubis.clone.rp41-133b2.8034-40080.revcompl.Baboon          ------------------------------------------------------------    14112
BCRP3.HUMAN.NCBI.REF                                              ------------------------------------------------------------    16335
LOC106996293.glutathione.hydrolase5.proenzyme-like-GGT1.rhesus.   tgtgtgcacctaccaccctggggccgtgcactgtgtgcgctctgtgcaggccaggtgagc    66620

Papio.anubis.clone.rp41-133b2.8034-40080.revcompl.Baboon          ------------------------------------------------------------    14112
BCRP3.HUMAN.NCBI.REF                                              -------------------------------------------------------caggg    16340
LOC106996293.glutathione.hydrolase5.proenzyme-like-GGT1.rhesus.   ccccaggctggggccggggtggggactggggacaggggtgggctcccaacagtggcctgg    66680

Papio.anubis.clone.rp41-133b2.8034-40080.revcompl.Baboon          ------------------------------------------------------------    14112
BCRP3.HUMAN.NCBI.REF                                              gagtgacccactgcctcctgcagctctttc------------------------------    16370
LOC106996293.glutathione.hydrolase5.proenzyme-like-GGT1.rhesus.   ccgtgacccactggctcccgcagccctcggtacagctcgggtcagcagtgctgctacaca    66740

Papio.anubis.clone.rp41-133b2.8034-40080.revcompl.Baboon          ------------------------------------------------------------    14112
BCRP3.HUMAN.NCBI.REF                                              ------------------------------------------------------------    16370
LOC106996293.glutathione.hydrolase5.proenzyme-like-GGT1.rhesus.   gcggacgggacgcagctcctgatggctgactccagcagcggcagcactcccgaccgcggc    66800

Papio.anubis.clone.rp41-133b2.8034-40080.revcompl.Baboon          ------------------------------------------------------------    14112
BCRP3.HUMAN.NCBI.REF                                              ------------------------------------------------------------    16370
LOC106996293.glutathione.hydrolase5.proenzyme-like-GGT1.rhesus.   catgactggggcgcacccccgttccgcacgccaccccgagtgcccggcatgtcccactgg    66860

Papio.anubis.clone.rp41-133b2.8034-40080.revcompl.Baboon          ------------------------------------------------------------    14112
BCRP3.HUMAN.NCBI.REF                                              ------------------------------------------------------------    16370
LOC106996293.glutathione.hydrolase5.proenzyme-like-GGT1.rhesus.   ctctacgatgtcctcagcttctattattgctgcctctgggcacccgactgcgcccgctac    66920

Papio.anubis.clone.rp41-133b2.8034-40080.revcompl.Baboon          ------------------------------------------------------------    14112
BCRP3.HUMAN.NCBI.REF                                              ------------------------------------------------------------    16370
LOC106996293.glutathione.hydrolase5.proenzyme-like-GGT1.rhesus.   atgcaacggcggccctccaatgactgccgcaactaccagcccccgcgactaggtgggtgc    66980

Papio.anubis.clone.rp41-133b2.8034-40080.revcompl.Baboon          ------------------------------------------------------------    14112
BCRP3.HUMAN.NCBI.REF                                              ------------------------------------------------------------    16370
LOC106996293.glutathione.hydrolase5.proenzyme-like-GGT1.rhesus.   catcctgtgccccggaccctgggaaagatcggctgggctggggtgcaccccacctgacc    67040

Papio.anubis.clone.rp41-133b2.8034-40080.revcompl.Baboon          ------------------------------------------------------------    14112
BCRP3.HUMAN.NCBI.REF                                              ------------------------------------------------------------    16370
LOC106996293.glutathione.hydrolase5.proenzyme-like-GGT1.rhesus.   ctccactctcaccccagcctccgccttcggagacccacactttgtgacctttgacggcac    67100

Papio.anubis.clone.rp41-133b2.8034-40080.revcompl.Baboon          ------------------------------------------------------------    14112
BCRP3.HUMAN.NCBI.REF                                              ------------------------------------------------------------    16370
LOC106996293.glutathione.hydrolase5.proenzyme-like-GGT1.rhesus.   caacttcacattcaatgggcgcggagagtacgtgctgctagaggcagtgctgactgatct    67160

Papio.anubis.clone.rp41-133b2.8034-40080.revcompl.Baboon          ------------------------------------------------------------    14112
BCRP3.HUMAN.NCBI.REF                                              ------------------------------------------------------------    16370
LOC106996293.glutathione.hydrolase5.proenzyme-like-GGT1.rhesus.   gagggtgcaggcgcgggcccagccagggaggatgtccaatggtgaggccagggctagggg    67220

Papio.anubis.clone.rp41-133b2.8034-40080.revcompl.Baboon          ------------------------------------------------------------    14112
BCRP3.HUMAN.NCBI.REF                                              ------------------------------------------------------------    16370
LOC106996293.glutathione.hydrolase5.proenzyme-like-GGT1.rhesus.   ctgctctgggtggcacagggtagatccaaggtgggaggctggagccaagtggcgcccgtt    67280

Papio.anubis.clone.rp41-133b2.8034-40080.revcompl.Baboon          ------------------------------------------------------------    14112
BCRP3.HUMAN.NCBI.REF                                              ------------------------------------------------------------    16370
LOC106996293.glutathione.hydrolase5.proenzyme-like-GGT1.rhesus.   ccgctcccaccaccacaggcacacagacccgtggcacagggctgactgcaatggccgtcc    67340

Papio.anubis.clone.rp41-133b2.8034-40080.revcompl.Baboon          ------------------------------------------------------------    14112
BCRP3.HUMAN.NCBI.REF                                              ------------------------------------------------------------    16370
LOC106996293.glutathione.hydrolase5.proenzyme-like-GGT1.rhesus.   aggagggcaactcagacgtggtggaggtcaggctggccaacgggaccagaggtctggagg    67400

Papio.anubis.clone.rp41-133b2.8034-40080.revcompl.Baboon          ------------------------------------------------------------    14112
BCRP3.HUMAN.NCBI.REF                                              ------------------------------------------------------------    16370
LOC106996293.glutathione.hydrolase5.proenzyme-like-GGT1.rhesus.   tgctgctgaaccaggaggtgctgagcttcgccgagcagagctggatggacctgaagggtg    67460

Papio.anubis.clone.rp41-133b2.8034-40080.revcompl.Baboon          ------------------------------------------------------------    14112
BCRP3.HUMAN.NCBI.REF                                              ------------------------------------------------------------    16370
LOC106996293.glutathione.hydrolase5.proenzyme-like-GGT1.rhesus.   agtagtccagccacgtgaggcttcgggctgccctcacctcctccccattcctgcggggag    67520

Papio.anubis.clone.rp41-133b2.8034-40080.revcompl.Baboon          ------------------------------------------------------------    14112
BCRP3.HUMAN.NCBI.REF                                              ------------------------------------------------------------    16370
LOC106996293.glutathione.hydrolase5.proenzyme-like-GGT1.rhesus.   actgaggggaagccctgggccttcacgcctctcccagccctggctagaggcctgggcggt    67580

Papio.anubis.clone.rp41-133b2.8034-40080.revcompl.Baboon          ------------------------------------------------------------    14112
BCRP3.HUMAN.NCBI.REF                                              ------------------------------------------------------------    16370
LOC106996293.glutathione.hydrolase5.proenzyme-like-GGT1.rhesus.   ccgacctcaggccttcacacccaccaaggtgcccacatcatacccacctggtcaaaagcc    67640

Papio.anubis.clone.rp41-133b2.8034-40080.revcompl.Baboon          ------------------------------------------------------------    14112
BCRP3.HUMAN.NCBI.REF                                              ------------------------------------------------------------    16370
LOC106996293.glutathione.hydrolase5.proenzyme-like-GGT1.rhesus.   aagaggccaggatgggggacatgtcctccctacagagcatcccgggagccatctggagg    67700

Papio.anubis.clone.rp41-133b2.8034-40080.revcompl.Baboon          ------------------------------------------------------------    14112
BCRP3.HUMAN.NCBI.REF                                              ------------------------------------------------------------    16370
LOC106996293.glutathione.hydrolase5.proenzyme-like-GGT1.rhesus.   gaactcacccggtaacgttcaccgctggcctgcacagagccactcttgtgctcctgtcac    67760

Papio.anubis.clone.rp41-133b2.8034-40080.revcompl.Baboon          ------------------------------------------------------------    14112
BCRP3.HUMAN.NCBI.REF                                              ------------------------------------------------------------    16370
LOC106996293.glutathione.hydrolase5.proenzyme-like-GGT1.rhesus.   tccctcagtcctcaaaagccactgcaaggtcgccagccctgcacggttaaggatgtccct    67820

Papio.anubis.clone.rp41-133b2.8034-40080.revcompl.Baboon          ------------------------------------------------------------    14112
```

```
BCRP3.HUMAN.NCBI.REF                                          ------------------------------------------------------------    16370
LOC106996293.glutathione.hydrolase5.proenzyme-like-GGT1.rhesus.   ggcaagcggtagcgccagcatccgaaccccttgcttcagggaactgagcgaatgaaaaga    67880

Papio.anubis.clone.rp41-133b2.8034-40080.revcompl.Baboon      ------------------------------------------------------------    14112
BCRP3.HUMAN.NCBI.REF                                          ------------------------------------------------------------    16370
LOC106996293.glutathione.hydrolase5.proenzyme-like-GGT1.rhesus.   ttcctggttgggatgggtggggacctgggaggggcctgtcagcactgagggtacaaggac    67940

Papio.anubis.clone.rp41-133b2.8034-40080.revcompl.Baboon      ------------------------------------------------------------    14112
BCRP3.HUMAN.NCBI.REF                                          ------------------------------------------------------------    16370
LOC106996293.glutathione.hydrolase5.proenzyme-like-GGT1.rhesus.   cctcgggacgtgcccaggcaggtgtggctgctgcagccaaggccagagggacccacacgt    68000

Papio.anubis.clone.rp41-133b2.8034-40080.revcompl.Baboon      ------------------------------------------------------------    14112
BCRP3.HUMAN.NCBI.REF                                          ------------------------------------------------------------    16370
LOC106996293.glutathione.hydrolase5.proenzyme-like-GGT1.rhesus.   gcatctcagcatgggggttcaccgcaggggctgcacctcaggcgccgctgggggttcca    68060

Papio.anubis.clone.rp41-133b2.8034-40080.revcompl.Baboon      ------------------------------------------------------------    14112
BCRP3.HUMAN.NCBI.REF                                          ------------------------------------------------------------    16370
LOC106996293.glutathione.hydrolase5.proenzyme-like-GGT1.rhesus.   ccacaagcaaggcaggggtggtggagggtcatggtcaacagaattggccctggggacgcc    68120

Papio.anubis.clone.rp41-133b2.8034-40080.revcompl.Baboon      ------------------------------------------------------------    14112
BCRP3.HUMAN.NCBI.REF                                          ------------------------------------------------------------    16370
LOC106996293.glutathione.hydrolase5.proenzyme-like-GGT1.rhesus.   ctcccttcccctgcaggtgccctcacccacagcccctgggtgcaaccgccgccaccaccg    68180

Papio.anubis.clone.rp41-133b2.8034-40080.revcompl.Baboon      ------------------------------------------------------------    14112
BCRP3.HUMAN.NCBI.REF                                          ------------------------------------------------------------    16370
LOC106996293.glutathione.hydrolase5.proenzyme-like-GGT1.rhesus.   caaggcctgcaaagatgatgcccaaaaacaccgtgtagctgcgaactggtggggaagcga    68240

Papio.anubis.clone.rp41-133b2.8034-40080.revcompl.Baboon      ------------------------------------------------------------    14112
BCRP3.HUMAN.NCBI.REF                                          ------------------------------------------------------------    16370
LOC106996293.glutathione.hydrolase5.proenzyme-like-GGT1.rhesus.   gtgtcagcgaggggccctctggggtggtgagtggggacaggtgcaggcaggtatatgagc    68300

Papio.anubis.clone.rp41-133b2.8034-40080.revcompl.Baboon      ------------------------------------------------------------    14112
BCRP3.HUMAN.NCBI.REF                                          ------------------------------------------------------------    16370
LOC106996293.glutathione.hydrolase5.proenzyme-like-GGT1.rhesus.   agggtgtcctcacctggctggcactccggggtgggtgaggaccaggtgccatcagcctgg    68360

Papio.anubis.clone.rp41-133b2.8034-40080.revcompl.Baboon      ------------------------------------------------------------    14112
BCRP3.HUMAN.NCBI.REF                                          ------------------------------------------------------------    16370
LOC106996293.glutathione.hydrolase5.proenzyme-like-GGT1.rhesus.   caggtgctggtctctgccctggccaggctgtagccgttgtcacagtggaagtagatggtg    68420

Papio.anubis.clone.rp41-133b2.8034-40080.revcompl.Baboon      ------------------------------------------------------------    14112
BCRP3.HUMAN.NCBI.REF                                          ------------------------------------------------------------    16370
LOC106996293.glutathione.hydrolase5.proenzyme-like-GGT1.rhesus.   gaacctgccaggtacctgttgccctccttttgtccgttgggaggtggggccagccagcca    68480

Papio.anubis.clone.rp41-133b2.8034-40080.revcompl.Baboon      ------------------------------------------------------------    14112
BCRP3.HUMAN.NCBI.REF                                          ------------------------------------------------------------    16370
LOC106996293.glutathione.hydrolase5.proenzyme-like-GGT1.rhesus.   caggacactactgggatggcggcagtgtgtgctgctgcgacacaggaagggcaacatgtg    68540

Papio.anubis.clone.rp41-133b2.8034-40080.revcompl.Baboon      ------------------------------------------------------------    14112
BCRP3.HUMAN.NCBI.REF                                          ------------------------------------------------------------    16370
LOC106996293.glutathione.hydrolase5.proenzyme-like-GGT1.rhesus.   agaccccccagcccctgtcccgagaccccagggcagccggtgggaccgccgagcctactt    68600

Papio.anubis.clone.rp41-133b2.8034-40080.revcompl.Baboon      ------------------------------------------------------------    14112
BCRP3.HUMAN.NCBI.REF                                          ------------------------------------------------------------    16370
LOC106996293.glutathione.hydrolase5.proenzyme-like-GGT1.rhesus.   ctaactgcccagcctggaaacaggaacctaaaaagtattgatgacagagataggagaggg    68660

Papio.anubis.clone.rp41-133b2.8034-40080.revcompl.Baboon      ------------------------------------------------------------    14112
BCRP3.HUMAN.NCBI.REF                                          ------------------------------------------------------------    16370
LOC106996293.glutathione.hydrolase5.proenzyme-like-GGT1.rhesus.   ctggggccctgctcattttgtctggagcttaactcacttgaggtaaaagtttgtggctgg    68720

Papio.anubis.clone.rp41-133b2.8034-40080.revcompl.Baboon      ------------------------------------------------------------    14112
BCRP3.HUMAN.NCBI.REF                                          ------------------------------------------------------------    16370
LOC106996293.glutathione.hydrolase5.proenzyme-like-GGT1.rhesus.   cagagaggttggtaaatgagggagaaattgcaaaaaggagagagtcactgattgtaggaa    68780

Papio.anubis.clone.rp41-133b2.8034-40080.revcompl.Baboon      ------------------------------------------------------------    14112
BCRP3.HUMAN.NCBI.REF                                          ------------------------------------------------------------    16370
LOC106996293.glutathione.hydrolase5.proenzyme-like-GGT1.rhesus.   tcccaaattctaaatacaactgaggacaaagccctggctgcccctgcactctgtgtgtgg    68840

Papio.anubis.clone.rp41-133b2.8034-40080.revcompl.Baboon      ------------------------------------------------------------    14112
BCRP3.HUMAN.NCBI.REF                                          ------------------------------------------------------------    16370
LOC106996293.glutathione.hydrolase5.proenzyme-like-GGT1.rhesus.   gggaagatgtacggagcccagcttccaactcacttggtcaaatgaggaaactctggggaa    68900

Papio.anubis.clone.rp41-133b2.8034-40080.revcompl.Baboon      ------------------------------------------------------------    14112
BCRP3.HUMAN.NCBI.REF                                          ------------------------------------------------------------    16370
LOC106996293.glutathione.hydrolase5.proenzyme-like-GGT1.rhesus.   aacgcaaagtctctacagtcagcactgcttggggaggagagtggctgtgggtgctcaagg    68960

Papio.anubis.clone.rp41-133b2.8034-40080.revcompl.Baboon      ------------------------------------------------------------    14112
BCRP3.HUMAN.NCBI.REF                                          ------------------------------------------------------------    16370
LOC106996293.glutathione.hydrolase5.proenzyme-like-GGT1.rhesus.   aggtggaacttcattccccaggcctgggtcccagaggaggacggcctgtgtgctgtgctg    69020

Papio.anubis.clone.rp41-133b2.8034-40080.revcompl.Baboon      ----------------------------agggcccacctcttgctgcccatgatagatgg    14144
BCRP3.HUMAN.NCBI.REF                                          ------------------------------------------------------------    16370
LOC106996293.glutathione.hydrolase5.proenzyme-like-GGT1.rhesus.   gacggtgatgttcggcttctccccaggtgggagccagcagctgctgaggaagggtgatgt    69080

Papio.anubis.clone.rp41-133b2.8034-40080.revcompl.Baboon      ggaggggtaggccagagcatgcatttctttcttttctttctttctttctctctctctttc    14204
BCRP3.HUMAN.NCBI.REF                                          ------------------------------------------------------------    16370
LOC106996293.glutathione.hydrolase5.proenzyme-like-GGT1.rhesus.   ggaacaa-aggagagagcctgggtctgaggtgtgtggtggttttaatggatctgggtttc    69139

Papio.anubis.clone.rp41-133b2.8034-40080.revcompl.Baboon      t-----------------------------------------------------------    14205
BCRP3.HUMAN.NCBI.REF                                          ------------------------------------------------------------    16370
LOC106996293.glutathione.hydrolase5.proenzyme-like-GGT1.rhesus.   taatgagacacccccggcgctgatgtgacaaaatcacaccattgtgtctagtgttgtatc    69199

Papio.anubis.clone.rp41-133b2.8034-40080.revcompl.Baboon      ------------------------------------------------------------    14205
BCRP3.HUMAN.NCBI.REF                                          ------------------------------------------------------------    16370
LOC106996293.glutathione.hydrolase5.proenzyme-like-GGT1.rhesus.   ttccggggcaggagatggtggtttctgtcctgggggctgagcccctgcaggaggctggac    69259

Papio.anubis.clone.rp41-133b2.8034-40080.revcompl.Baboon      ------------------------------------------------------------    14205
BCRP3.HUMAN.NCBI.REF                                          ------------------------------------------------------------    16370
LOC106996293.glutathione.hydrolase5.proenzyme-like-GGT1.rhesus.   cagcacgggtagatgcagagacgcgtgagcacagggcagggtccaccctggggtgggcag    69319

Papio.anubis.clone.rp41-133b2.8034-40080.revcompl.Baboon      ------------------------------------------------------------    14205
BCRP3.HUMAN.NCBI.REF                                          ------------------------------------------------------------    16370
LOC106996293.glutathione.hydrolase5.proenzyme-like-GGT1.rhesus.   agctgggagggtaggcctgcaagggcctcaggggcctgtgcttgggtcatgtgcgaggac    69379

Papio.anubis.clone.rp41-133b2.8034-40080.revcompl.Baboon      ------------------------------------------------------------    14205
BCRP3.HUMAN.NCBI.REF                                          ------------------------------------------------------------    16370
LOC106996293.glutathione.hydrolase5.proenzyme-like-GGT1.rhesus.   agtgtggaagaggggacccaggcaggatggggacacccataaccctcctcctgaggaccc    69439

Papio.anubis.clone.rp41-133b2.8034-40080.revcompl.Baboon      ------------------------------------------------------------    14205
BCRP3.HUMAN.NCBI.REF                                          ------------------------------------------------------------    16370
LOC106996293.glutathione.hydrolase5.proenzyme-like-GGT1.rhesus.   aaagagaaggcagagctgcctcaaatctttccatatgacagttcattttgtgtgtcaaat    69499

Papio.anubis.clone.rp41-133b2.8034-40080.revcompl.Baboon      ---------------------------------------ttctttcttttttctttcttt    14226
BCRP3.HUMAN.NCBI.REF                                          ------------------------------------------------------------    16370
LOC106996293.glutathione.hydrolase5.proenzyme-like-GGT1.rhesus.   taggtaggccacagaacccagtcatacactcaaacagaaatctagatgtgtccatgacgg    69559

Papio.anubis.clone.rp41-133b2.8034-40080.revcompl.Baboon      ctttcttttctttctttctttcttttctttcatctttctttttctttctttcttttcttt    14286
```

```
BCRP3.HUMAN.NCBI.REF                                             ------------------------------------------------------------    16370
LOC106996293.glutathione.hydrolase5.proenzyme-like-GGT1.rhesus.  tatttttaaagatgggattcacatttaaagcagcagactttgattagccttcatctgttt    69619

Papio.anubis.clone.rp41-133b2.8034-40080.revcompl.Baboon         catctttctttttctttctttcttttctttctccttccttccttccttccttccttcctt    14346
BCRP3.HUMAN.NCBI.REF                                             ------------------------------------------------------------    16370
LOC106996293.glutathione.hydrolase5.proenzyme-like-GGT1.rhesus.  tttgcgtttttgttgttgttgttgttttttgtttttg-----------------------    69656

Papio.anubis.clone.rp41-133b2.8034-40080.revcompl.Baboon         ccttccttccctctctctctctctgtctctctctttctgagatactcttactctgttg    14406
BCRP3.HUMAN.NCBI.REF                                             ------------------------------------------------------------    16370
LOC106996293.glutathione.hydrolase5.proenzyme-like-GGT1.rhesus.  ----------------------------------ctttgagacagggtcttgctctgtta    69682

Papio.anubis.clone.rp41-133b2.8034-40080.revcompl.Baboon         c-caggctggagtgcaatggcgcgattttggctcactgtaacctctgcctgccaggttca    14465
BCRP3.HUMAN.NCBI.REF                                             ------------------------------------------------------------    16370
LOC106996293.glutathione.hydrolase5.proenzyme-like-GGT1.rhesus.  ctcaggctggagtgtgggagtgcaaccttggctcactgcagcctcaacctcctaggctca    69742

Papio.anubis.clone.rp41-133b2.8034-40080.revcompl.Baboon         agcgattctcctgcctcagcctcccgtgtagctgggattacaggtgcccaccatcacata    14525
BCRP3.HUMAN.NCBI.REF                                             ------------------------------------------------------------    16370
LOC106996293.glutathione.hydrolase5.proenzyme-like-GGT1.rhesus.  aacaatccacccacctccgcctcctgagtagctgggaccacaggtgtacagccccacatg    69802

Papio.anubis.clone.rp41-133b2.8034-40080.revcompl.Baboon         cagctaa-----gttttgtatttttagtagagacagggtttcaccatgttggccaggctg    14580
BCRP3.HUMAN.NCBI.REF                                             ------------------------------------------------------------    16370
LOC106996293.glutathione.hydrolase5.proenzyme-like-GGT1.rhesus.  tggctaattattttattttatttttgtagagactgggttttgccatgttgcccaagctg    69862

Papio.anubis.clone.rp41-133b2.8034-40080.revcompl.Baboon         gtctcgaactcctgacctcaagtgatccgcctgcctcggcctcccaaagtgctgggatta    14640
BCRP3.HUMAN.NCBI.REF                                             ------------------------------------------------------------    16370
LOC106996293.glutathione.hydrolase5.proenzyme-like-GGT1.rhesus.  gtctccaattcctgagctcaagtgatccacccacctcagcctcccaaattgctgggatta    69922

Papio.anubis.clone.rp41-133b2.8034-40080.revcompl.Baboon         caggcgtgagccaccatgcttggcctaggccaaagcatttccataggctcagggccccag    14700
BCRP3.HUMAN.NCBI.REF                                             ------------------------------------------------------------    16370
LOC106996293.glutathione.hydrolase5.proenzyme-like-GGT1.rhesus.  caggtgtgagccaccgcacctggccaagttcatcatttacaatgtgagtcatgtccttta    69982

Papio.anubis.clone.rp41-133b2.8034-40080.revcompl.Baboon         gagaatttgtggggcttgtcagctcaagccccatgccctggtggtgggaaggatcctggg    14760
BCRP3.HUMAN.NCBI.REF                                             ------------------------------------------------------------    16370
LOC106996293.glutathione.hydrolase5.proenzyme-like-GGT1.rhesus.  cccaatcagttg------------------------------------------------    69994

Papio.anubis.clone.rp41-133b2.8034-40080.revcompl.Baboon         gccacctgccaaagaccagctttgaagtagttgtggccagag-tgtccctagtgagatgg    14819
BCRP3.HUMAN.NCBI.REF                                             ------------------------------------------------------------    16370
LOC106996293.glutathione.hydrolase5.proenzyme-like-GGT1.rhesus.  ----------acagccttgatagaaataagatgacgactgagctgttacctctgagatga    70044

Papio.anubis.clone.rp41-133b2.8034-40080.revcompl.Baboon         ggagtggagtcccat------ggtgggctccagggccattgtgagggactaagaggtggc    14873
BCRP3.HUMAN.NCBI.REF                                             ------------------------------------------------------------    16370
LOC106996293.glutathione.hydrolase5.proenzyme-like-GGT1.rhesus.  caataagaaacacatcacccaggtggagatgacagagactttgcttgggtaagagcctat    70104

Papio.anubis.clone.rp41-133b2.8034-40080.revcompl.Baboon         ctaggcctggttctgcctatggcatat---------------------------------    14900
BCRP3.HUMAN.NCBI.REF                                             ------------------------------------------------------------    16370
LOC106996293.glutathione.hydrolase5.proenzyme-like-GGT1.rhesus.  cagatccatgtcaccccaagtccatgttgtaggtgacagcacagttttagctcagcaacg    70164

Papio.anubis.clone.rp41-133b2.8034-40080.revcompl.Baboon         ------------------------------------------------------------    14900
BCRP3.HUMAN.NCBI.REF                                             ------------------------------------------------------------    16370
LOC106996293.glutathione.hydrolase5.proenzyme-like-GGT1.rhesus.  ctcagccccgggccggctgcacccacactctgcccatcagccccagagccgtgtgtcttg    70224

Papio.anubis.clone.rp41-133b2.8034-40080.revcompl.Baboon         ------------------------------------------------------------    14900
BCRP3.HUMAN.NCBI.REF                                             ------------------------------------------------------------    16370
LOC106996293.glutathione.hydrolase5.proenzyme-like-GGT1.rhesus.  cacgggtctgtgcctcttccctgactgcagctgtgcttgctccttggctcaccctttctg    70284

Papio.anubis.clone.rp41-133b2.8034-40080.revcompl.Baboon         ------------------------------------------------------------    14900
BCRP3.HUMAN.NCBI.REF                                             ------------------------------------------------------------    16370
LOC106996293.glutathione.hydrolase5.proenzyme-like-GGT1.rhesus.  gttcctctaactttccaccagcatccttcacctccccttcctcagatacaagcaggcccc    70344

Papio.anubis.clone.rp41-133b2.8034-40080.revcompl.Baboon         ------------------------------------------------------------    14900
BCRP3.HUMAN.NCBI.REF                                             ------------------------------------------------------------    16370
LOC106996293.glutathione.hydrolase5.proenzyme-like-GGT1.rhesus.  atggacccagaacaggattctttcagtacttaagttttacttccatttttttatactttt    70404

Papio.anubis.clone.rp41-133b2.8034-40080.revcompl.Baboon         ------------------------------------------------------------    14900
BCRP3.HUMAN.NCBI.REF                                             ------------------------------------------------------------    16370
LOC106996293.glutathione.hydrolase5.proenzyme-like-GGT1.rhesus.  caaactaagcttggcttgacccaaatcaacatctttcgacctccaccaaagtctccaacg    70464

Papio.anubis.clone.rp41-133b2.8034-40080.revcompl.Baboon         ------------------------------------------------------------    14900
BCRP3.HUMAN.NCBI.REF                                             ------------------------------------------------------------    16370
LOC106996293.glutathione.hydrolase5.proenzyme-like-GGT1.rhesus.  agatatttttatcttaaaccacaaatttctccttgaaaaaacataattagggtgaggcaa    70524

Papio.anubis.clone.rp41-133b2.8034-40080.revcompl.Baboon         ------------------------------------------------------------    14900
BCRP3.HUMAN.NCBI.REF                                             ------------------------------------------------------------    16370
LOC106996293.glutathione.hydrolase5.proenzyme-like-GGT1.rhesus.  tatgaacaatggatagagctaaaggttaaactaaatataagcatatggccccgtcagatc    70584

Papio.anubis.clone.rp41-133b2.8034-40080.revcompl.Baboon         ---------------gagctggtgctgctgggagcaatggcaacagaagcactttgcaaa    14945
BCRP3.HUMAN.NCBI.REF                                             ------------------------------------------------------------    16370
LOC106996293.glutathione.hydrolase5.proenzyme-like-GGT1.rhesus.  tagttgtttgaggaaaatcaggtgttgtttgaagaactgacaacagaaggcat-----gc    70639

Papio.anubis.clone.rp41-133b2.8034-40080.revcompl.Baboon         ctgagaagcaccttgtatatggcaaaggtcagtgtaaatggcatcacatttgcagctgta    15005
BCRP3.HUMAN.NCBI.REF                                             ------------------------------------------------------------    16370
LOC106996293.glutathione.hydrolase5.proenzyme-like-GGT1.rhesus.  ctgagctagacttgggaaaagtcaccagccaagatgactgtgcaatcccggagaacagaa    70699

Papio.anubis.clone.rp41-133b2.8034-40080.revcompl.Baboon         gaatgttgtggacctgtgagcagctggatttg----------------------------    15037
BCRP3.HUMAN.NCBI.REF                                             ------------------------------------------------------------    16370
LOC106996293.glutathione.hydrolase5.proenzyme-like-GGT1.rhesus.  gggcggttttgcttacttctctgtaggtcttgggcactaagtcttagagttgctatgccg    70759

Papio.anubis.clone.rp41-133b2.8034-40080.revcompl.Baboon         ------------------------------------------------------------    15037
BCRP3.HUMAN.NCBI.REF                                             ------------------------------------------------------------    16370
LOC106996293.glutathione.hydrolase5.proenzyme-like-GGT1.rhesus.  tacattacagtaatagcctcatcctcaggctgcagcccagaggtgtgcagaatccctgca    70819

Papio.anubis.clone.rp41-133b2.8034-40080.revcompl.Baboon         ----------------------------------------atggggaagcctggttgtgt    15057
BCRP3.HUMAN.NCBI.REF                                             ------------------------------------------------------------    16370
LOC106996293.glutathione.hydrolase5.proenzyme-like-GGT1.rhesus.  ttggctgaggcatctttacttccagagaagcagctggggactccagagccctggtgttta    70879

Papio.anubis.clone.rp41-133b2.8034-40080.revcompl.Baboon         tctgtgacctaatctggggtttggtggcagcagatgggccacctctg-------------    15104
BCRP3.HUMAN.NCBI.REF                                             ------------------------------------------------------------    16370
LOC106996293.glutathione.hydrolase5.proenzyme-like-GGT1.rhesus.  tttgagtctgaatagtattctcaggagaaactggggagggatccctggcttttgctagga    70939

Papio.anubis.clone.rp41-133b2.8034-40080.revcompl.Baboon         ------------------------------------------------------------    15104
BCRP3.HUMAN.NCBI.REF                                             ------------------------------------------------------------    16370
LOC106996293.glutathione.hydrolase5.proenzyme-like-GGT1.rhesus.  ccagagtattgagaagtcatcaacacggaagcacagaagccactgctcagggtgcaggtg    70999

Papio.anubis.clone.rp41-133b2.8034-40080.revcompl.Baboon         ------------------------------------------------------------    15104
BCRP3.HUMAN.NCBI.REF                                             ------------------------------------------------------------    16370
LOC106996293.glutathione.hydrolase5.proenzyme-like-GGT1.rhesus.  agtctggcagatgctcccagggatgcagagagggagggcggctgagtcagcatgggctgg    71059

Papio.anubis.clone.rp41-133b2.8034-40080.revcompl.Baboon         ----------------------tcaccattctccaatgagtgccagctgagcaagcctag    15142
BCRP3.HUMAN.NCBI.REF                                             ----------------------agacccagttgcaaggaagagctgcatgctcaacctgt    16408
LOC106996293.glutathione.hydrolase5.proenzyme-like-GGT1.rhesus.  gagagggaacctgcagacagatagtcctaggtgctaggacagtgaccagggttaagctgt    71119
                                                                                          **    * * * **       *      *  **

Papio.anubis.clone.rp41-133b2.8034-40080.revcompl.Baboon         tgggg-----aggcagggggaggggagccccaccgccgactctgtgccgtgtcccctaga    15197
BCRP3.HUMAN.NCBI.REF                                             tgttgtccct--------------------------------------------------    16418
LOC106996293.glutathione.hydrolase5.proenzyme-like-GGT1.rhesus.  ttgggggtataagccagaggaggatgacacaggctcaggacctgaccctggacatcaaaa    71179
                                                                 *   *

Papio.anubis.clone.rp41-133b2.8034-40080.revcompl.Baboon         gcc-------atcctccagcagcagggctcacccgtggacgccaccatcgcggctctcgt    15250
```

```
BCRP3.HUMAN.NCBI.REF                                                  ---------------------------------------------gccggaggccaacct    16433
LOC106996293.glutathione.hydrolase5.proenzyme-like-GGT1.rhesus.       gactgttcctatctgtgcagtgagagaggaacatgaggaggaggggatccagggccctggt    71239
                                                                                                                          ***    *

Papio.anubis.clone.rp41-133b2.8034-40080.revcompl.Baboon              ctgcaccagcgttgtcaatcctcagagcatggcctgggcggaggggtcatcttcaccat    15310
BCRP3.HUMAN.NCBI.REF                                                  gctcaccttcctttccttctagaccacc-tggaaaggtagcccagctctcttgtggctg    16492
LOC106996293.glutathione.hydrolase5.proenzyme-like-GGT1.rhesus.       ggacacagccctcctgaaacctgagtgcc-gtgctgggctgcacaggcctttttttatctt    71298
                                                                         ***   * *  *    *   *   *    *   **  *    *  *   *    *

Papio.anubis.clone.rp41-133b2.8034-40080.revcompl.Baboon              ctacaatgtgacgacaggtgggc-------------------------------------    15333
BCRP3.HUMAN.NCBI.REF                                                  cccaggactccaggtctccaggccgttggggtgcccctctgctcccacca----------    16542
LOC106996293.glutathione.hydrolase5.proenzyme-like-GGT1.rhesus.       ctccacttgcctggtggggaggggctgttcatgtcaatttgtttgatccactggtgatct    71358
                                                                      *           *       **

Papio.anubis.clone.rp41-133b2.8034-40080.revcompl.Baboon              ------------------------------------------------------------    15333
BCRP3.HUMAN.NCBI.REF                                                  ------------------------------------------------------------    16542
LOC106996293.glutathione.hydrolase5.proenzyme-like-GGT1.rhesus.       ctgcctcaccctgagaaccagctgaggtctgagcttggttctttagacagaggttctgag    71418

Papio.anubis.clone.rp41-133b2.8034-40080.revcompl.Baboon              -------------------ccacatgtgacctgccatggggaagatgggcttgtccacac    15374
BCRP3.HUMAN.NCBI.REF                                                  ------------------------------------------------------------    16542
LOC106996293.glutathione.hydrolase5.proenzyme-like-GGT1.rhesus.       gggtgagactcagagtttgccccattggagggaccaaggaggaagcagcttctgggacct    71478

Papio.anubis.clone.rp41-133b2.8034-40080.revcompl.Baboon              cacagaggaacccccttctccagccagtcccatccactcaagaccgcttctgggtgctctt    15434
BCRP3.HUMAN.NCBI.REF                                                  ------------------------------------------------------------    16542
LOC106996293.glutathione.hydrolase5.proenzyme-like-GGT1.rhesus.       tccacagatctgtgagcacgagactctgcccatcttggaaggacacagctgctgactcct    71538

Papio.anubis.clone.rp41-133b2.8034-40080.revcompl.Baboon              gggcccctgccctgccctcttcacctcagctgtgcaaagcccttgtgtcaactcctgtc    15494
BCRP3.HUMAN.NCBI.REF                                                  ------------------------------------------------------------    16542
LOC106996293.glutathione.hydrolase5.proenzyme-like-GGT1.rhesus.       cagcagcaaacacaggctttgctgccctagccctggggagatagggggggcggcacagctg    71598

Papio.anubis.clone.rp41-133b2.8034-40080.revcompl.Baboon              cccctgttagcccagagtccatctcataatgcagagatggaaactgaggcctg--gagca    15552
BCRP3.HUMAN.NCBI.REF                                                  ------------------------------------------------------------    16542
LOC106996293.glutathione.hydrolase5.proenzyme-like-GGT1.rhesus.       cgggtgctcctgtgcagaccctgagacagacccacagtgccctctgctgctcgctggcca    71658

Papio.anubis.clone.rp41-133b2.8034-40080.revcompl.Baboon              ggccaggggctgtctgcagggtccgtgaccgctcacctgccctgccctactctagggaag    15612
BCRP3.HUMAN.NCBI.REF                                                  ------------------------------------------------------------    16542
LOC106996293.glutathione.hydrolase5.proenzyme-like-GGT1.rhesus.       ccctgcagtctctcagcagaaactgtgagt------------------------------    71688

Papio.anubis.clone.rp41-133b2.8034-40080.revcompl.Baboon              gtggaggtcatcaatgcccgggagacagtgccagcccaccacgccccgagcctgctggac    15672
BCRP3.HUMAN.NCBI.REF                                                  ------------------------------------------------------------    16542
LOC106996293.glutathione.hydrolase5.proenzyme-like-GGT1.rhesus.       --------------------ggagtcacctgagagccaggccactgtcaggccacagaat    71728

Papio.anubis.clone.rp41-133b2.8034-40080.revcompl.Baboon              cggtgtgcacaggctctgccgctgggtagaggtgaggccccatgggggtcccccactccc    15732
BCRP3.HUMAN.NCBI.REF                                                  ------------------------------------------------------------    16542
LOC106996293.glutathione.hydrolase5.proenzyme-like-GGT1.rhesus.       ctgtgagtcaagactctggctctgccccgtgttactg----acaggaataagaatccttt    71784

Papio.anubis.clone.rp41-133b2.8034-40080.revcompl.Baboon              accccccaggccactaggactgaccacaacacccccaaa---------------------    15772
BCRP3.HUMAN.NCBI.REF                                                  ------------------------------------------------------------    16542
LOC106996293.glutathione.hydrolase5.proenzyme-like-GGT1.rhesus.       actcatgaggtttttctggatttccaagagaagcccacgagggaaatctgatgaccatcc    71844

Papio.anubis.clone.rp41-133b2.8034-40080.revcompl.Baboon              ------------------------------------------------------------    15772
BCRP3.HUMAN.NCBI.REF                                                  ------------------------------------------------------------    16542
LOC106996293.glutathione.hydrolase5.proenzyme-like-GGT1.rhesus.       aatgtgaggaggctgcagggagcggggaatgcagagggctctggaagcgaagacctgaac    71904

Papio.anubis.clone.rp41-133b2.8034-40080.revcompl.Baboon              ------------------------------------------------------------    15772
BCRP3.HUMAN.NCBI.REF                                                  ------------------------------------------------------------    16542
LOC106996293.glutathione.hydrolase5.proenzyme-like-GGT1.rhesus.       agctccctccacaggggccagggaaggaggcaactgatagttggatattgtaaatgggaa    71964

Papio.anubis.clone.rp41-133b2.8034-40080.revcompl.Baboon              ------------------------------------------------------------    15772
BCRP3.HUMAN.NCBI.REF                                                  ------------------------------------------------------------    16542
LOC106996293.glutathione.hydrolase5.proenzyme-like-GGT1.rhesus.       aatgtctcacatgtctggcctccatcctggatgattttactcttaggccacatacgttgc    72024

Papio.anubis.clone.rp41-133b2.8034-40080.revcompl.Baboon              ------------------------------------------------------------    15772
BCRP3.HUMAN.NCBI.REF                                                  ------------------------------------------------------------    16542
LOC106996293.glutathione.hydrolase5.proenzyme-like-GGT1.rhesus.       tgcctctcctgccacaggatgcctctcctgagacccaataccatggatgggtggctcagc    72084

Papio.anubis.clone.rp41-133b2.8034-40080.revcompl.Baboon              ------------------------------------------------------------    15772
BCRP3.HUMAN.NCBI.REF                                                  ------------------------------------------------------------    16542
LOC106996293.glutathione.hydrolase5.proenzyme-like-GGT1.rhesus.       acagcctgggccctggaccctgagagatgagtgtggtgcaaaggaacatgctttagtcca    72144

Papio.anubis.clone.rp41-133b2.8034-40080.revcompl.Baboon              ------------------------------------------------------------    15772
BCRP3.HUMAN.NCBI.REF                                                  ------------------------------------------------------------    16542
LOC106996293.glutathione.hydrolase5.proenzyme-like-GGT1.rhesus.       ctccggaaacactagcaaaagaccaggacaaagcagcagtgagacaggcgtgaggacgtc    72204

Papio.anubis.clone.rp41-133b2.8034-40080.revcompl.Baboon              ------------------------------------------------------------    15772
BCRP3.HUMAN.NCBI.REF                                                  ------------------------------------------------------------    16542
LOC106996293.glutathione.hydrolase5.proenzyme-like-GGT1.rhesus.       aaagtgaaaccatgaaagaccactgtggtgaagcagtggacagagaacaggagtggtgga    72264

Papio.anubis.clone.rp41-133b2.8034-40080.revcompl.Baboon              ------------------------------------------------------------    15772
BCRP3.HUMAN.NCBI.REF                                                  ------------------------------------------------------------    16542
LOC106996293.glutathione.hydrolase5.proenzyme-like-GGT1.rhesus.       ggcgtccccaggggtaacaccaggttttgtccacggaggtacaaatttacttgttcaggt    72324

Papio.anubis.clone.rp41-133b2.8034-40080.revcompl.Baboon              ------------------------------------------------------------    15772
BCRP3.HUMAN.NCBI.REF                                                  ------------------------------------------------------------    16542
LOC106996293.glutathione.hydrolase5.proenzyme-like-GGT1.rhesus.       catctcagtgccctggatcttcttgaagtgaaaaatcactgaagtctttgtgctttctga    72384

Papio.anubis.clone.rp41-133b2.8034-40080.revcompl.Baboon              ------------------------------------------------------------    15772
BCRP3.HUMAN.NCBI.REF                                                  ------------------------------------------------------------    16542
LOC106996293.glutathione.hydrolase5.proenzyme-like-GGT1.rhesus.       cattctgggccctctcttccttcatcttcctttgctgccccagcttgaagagggtctgca    72444

Papio.anubis.clone.rp41-133b2.8034-40080.revcompl.Baboon              ------------------------------------------------------------    15772
BCRP3.HUMAN.NCBI.REF                                                  ------------------------------------------------------------    16542
LOC106996293.glutathione.hydrolase5.proenzyme-like-GGT1.rhesus.       ttggcacattcagtgaggggctctgaggcggtggtgtcctccctgcgtggacattttcca    72504

Papio.anubis.clone.rp41-133b2.8034-40080.revcompl.Baboon              ------------------------------------------------------------    15772
BCRP3.HUMAN.NCBI.REF                                                  ------------------------------------------------------------    16542
LOC106996293.glutathione.hydrolase5.proenzyme-like-GGT1.rhesus.       ccagaggctgagaggtagatcaagtgctgagttcctcttctcaaatgcagctcactcccc    72564

Papio.anubis.clone.rp41-133b2.8034-40080.revcompl.Baboon              ------------------------------------------------------------    15772
BCRP3.HUMAN.NCBI.REF                                                  ------------------------------------------------------------    16542
LOC106996293.glutathione.hydrolase5.proenzyme-like-GGT1.rhesus.       cgctctctgaatgagaacctgttatctatgtgtgtttctcttatttccacccagggacgc    72624

Papio.anubis.clone.rp41-133b2.8034-40080.revcompl.Baboon              ------------------------------------------------------------    15772
BCRP3.HUMAN.NCBI.REF                                                  ------------------------------------------------------------    16542
LOC106996293.glutathione.hydrolase5.proenzyme-like-GGT1.rhesus.       ttttgttaaaactgtgtatatctcctctgtttaaggggcagcggtggccgcaggtgctcc    72684

Papio.anubis.clone.rp41-133b2.8034-40080.revcompl.Baboon              ------------------------------------------------------------    15772
BCRP3.HUMAN.NCBI.REF                                                  ------------------------------------------------------------    16542
LOC106996293.glutathione.hydrolase5.proenzyme-like-GGT1.rhesus.       tcatgcttgaaggaatgggaggggagtgtccacagtggctcggctccttggctgggcgca    72744

Papio.anubis.clone.rp41-133b2.8034-40080.revcompl.Baboon              ------------------------------------------------------------    15772
BCRP3.HUMAN.NCBI.REF                                                  ------------------------------------------------------------    16542
LOC106996293.glutathione.hydrolase5.proenzyme-like-GGT1.rhesus.       atcactggcagggtcagctccaagtcctcattagaccagcgcccaggctccttagcccag    72804

Papio.anubis.clone.rp41-133b2.8034-40080.revcompl.Baboon              ------------------------------ggatcagtgcctcatggacaggctgtgact    15802
BCRP3.HUMAN.NCBI.REF                                                  ------------------------------gacccccagcaccaaggacctttttccccg    16572
LOC106996293.glutathione.hydrolase5.proenzyme-like-GGT1.rhesus.       gaatgtccagaatctgggcacagagcccaggcctcaggccaccatggcccacctgagcag    72864
                                                                                                    *   *    *  ** ** *    *

Papio.anubis.clone.rp41-133b2.8034-40080.revcompl.Baboon              ccctggcactgggggacagggagtctatggggaacccccaaccccaacctgcagagattg    15862
```

```
BCRP3.HUMAN.NCBI.REF                                            acccctgtctgca-----------------------------------------------   16585
LOC106996293.glutathione.hydrolase5.proenzyme-like-GGT1.rhesus. ctcctcctctgcacttcctggggcccggcatggaggcacagcccccaaggctgctggagg   72924
                                                                  *     ***

Papio.anubis.clone.rp41-133b2.8034-40080.revcompl.Baboon        agggattgggaaggtccccccttgggaatgggcgagctgaggtgatcctgggagagggcca   15922
BCRP3.HUMAN.NCBI.REF                                            ------------------------------------------------------------   16585
LOC106996293.glutathione.hydrolase5.proenzyme-like-GGT1.rhesus. atgaccagggcctatcaacatttgtggagagggtcctgaatgggggctcaggcagggtag   72984

Papio.anubis.clone.rp41-133b2.8034-40080.revcompl.Baboon        ggctggacaaa---ggcctggaaggttgagtggagatgggttggtgggacagggtggggc   15979
BCRP3.HUMAN.NCBI.REF                                            ------------------------------------------------------------   16585
LOC106996293.glutathione.hydrolase5.proenzyme-like-GGT1.rhesus. cattggtcagatttgccccttaggtctcagagaaagtgctggagagggtgaggctggagg   73044

Papio.anubis.clone.rp41-133b2.8034-40080.revcompl.Baboon        tcctggggccagtttgtgcagggccccttgggcctggccatggactttgtggggagccac   16039
BCRP3.HUMAN.NCBI.REF                                            ------------------------------------------------------------   16585
LOC106996293.glutathione.hydrolase5.proenzyme-like-GGT1.rhesus. t--tgggggctgggtggagaacggggttgggaggagaggagggggggagttgagccccct   73102

Papio.anubis.clone.rp41-133b2.8034-40080.revcompl.Baboon        aggtggtctgagcagggagggacatggtcgggtgtgggctccagaaagctcactctggct   16099
BCRP3.HUMAN.NCBI.REF                                            ------------------------------------------------------------   16585
LOC106996293.glutathione.hydrolase5.proenzyme-like-GGT1.rhesus. cagtgtggcctgcagcgctgggagtggccacatgggggcagcagagtg------------   73150

Papio.anubis.clone.rp41-133b2.8034-40080.revcompl.Baboon        gctgtagctggggggccacacgtaggggtgatggcgaacccctgggaaggcctggaagcc   16159
BCRP3.HUMAN.NCBI.REF                                            ------------------------------------------------------------   16585
LOC106996293.glutathione.hydrolase5.proenzyme-like-GGT1.rhesus. ----------------------------------------------------taggaaca   73158

Papio.anubis.clone.rp41-133b2.8034-40080.revcompl.Baboon        acaggtttgagtgctgggatgt------gccatgcaggggcccagtggatcggggtgccc   16213
BCRP3.HUMAN.NCBI.REF                                            ------------------------------------------------------------   16585
LOC106996293.glutathione.hydrolase5.proenzyme-like-GGT1.rhesus. gcgagtctgggtgccgggaggttgggagggcagtcagccccgccctgcagctggatcaac   73218

Papio.anubis.clone.rp41-133b2.8034-40080.revcompl.Baboon        ggggagctccgcggctac-gccgaggcccaccgccgccatggccgcctgccctgggcgca   16272
BCRP3.HUMAN.NCBI.REF                                            ---gtaactcactgcttctaaggactagcaccactgccaccccacc--cctgcctctcc   16640
LOC106996293.glutathione.hydrolase5.proenzyme-like-GGT1.rhesus. agggttggtcacaactgcagccaaggtccgccacagcccacccctct--gccgcccctca   73276
                                                                   *     * *  ** *     *    * ** * ***    ** *    *     * *

Papio.anubis.clone.rp41-133b2.8034-40080.revcompl.Baboon        gctgttccagcccaccatc---gccctgctccgagggggcatgtggtggcccccgtcct   16329
BCRP3.HUMAN.NCBI.REF                                            tctttgccaccctcctccctctgcactgt-------------------------------   16669
LOC106996293.glutathione.hydrolase5.proenzyme-like-GGT1.rhesus. cctgggccatccccccatctcaggagtgttgaagaggttgcacaggttgccccaggtcat   73336
                                                                 **   *** **  *   *   *   **

Papio.anubis.clone.rp41-133b2.8034-40080.revcompl.Baboon        cagccagttcctgcacaacgg------cttcctgcggccttccttgcaggcgtcaaccct   16383
BCRP3.HUMAN.NCBI.REF                                            ------------------------------------------------------------   16669
LOC106996293.glutathione.hydrolase5.proenzyme-like-GGT1.rhesus. tcagcaggcactgaccatagggtgggagtccaggcctcccacctacttggccttgccctt   73396

Papio.anubis.clone.rp41-133b2.8034-40080.revcompl.Baboon        gcggtgagccccacatggtggccctgggctcctgggttcaaggccacatcctgtctgggc   16443
BCRP3.HUMAN.NCBI.REF                                            ------------------------------------------------------------   16669
LOC106996293.glutathione.hydrolase5.proenzyme-like-GGT1.rhesus. ggcgaatgggagagctggggaggcagaggcaggtctcatctgaggccttcctggaggagg   73456

Papio.anubis.clone.rp41-133b2.8034-40080.revcompl.Baboon        ccagtccacaccccagctctgcctcagtccctcctacactggaggattgacccttggggt   16503
BCRP3.HUMAN.NCBI.REF                                            ------------------------------------------------------------   16669
LOC106996293.glutathione.hydrolase5.proenzyme-like-GGT1.rhesus. ggaatgtgcatgatggggcctgtgtgcgggaacagagctgagagggttgcagggcagggt   73516

Papio.anubis.clone.rp41-133b2.8034-40080.revcompl.Baboon        gctggggaccttaaggcccagacagagagcactttcctgt----------ttcagtttat   16553
BCRP3.HUMAN.NCBI.REF                                            ------------------------------------------------------------   16669
LOC106996293.glutathione.hydrolase5.proenzyme-like-GGT1.rhesus. ggaaggaggctagcagatgggaatggggtctggttcctgtctttctagacctcactctgt   73576

Papio.anubis.clone.rp41-133b2.8034-40080.revcompl.Baboon        ggccttccggtcccctgtggcacagcctaactcgggtgaggctgcggggagtttgtcgcc   16613
BCRP3.HUMAN.NCBI.REF                                            ------------------------------------------------------------   16669
LOC106996293.glutathione.hydrolase5.proenzyme-like-GGT1.rhesus. tgcttccccattccctggg---------ttttcacctccaggcacggtggggctgagggc   73627

Papio.anubis.clone.rp41-133b2.8034-40080.revcompl.Baboon        caagtgctaggactccacggtgtggatttgggctctgccaccagccctggccccggcctc   16673
BCRP3.HUMAN.NCBI.REF                                            ------------------------------------------------------------   16669
LOC106996293.glutathione.hydrolase5.proenzyme-like-GGT1.rhesus. cagccgcttggccagctgggcaggcactttcctccctacagatgaggtaaggcagggctc   73687

Papio.anubis.clone.rp41-133b2.8034-40080.revcompl.Baboon        ctcctccc----------------------------------------------------   16681
BCRP3.HUMAN.NCBI.REF                                            ------------------------------------------------------------   16669
LOC106996293.glutathione.hydrolase5.proenzyme-like-GGT1.rhesus. ccctctcactgtctgaatccccagcgcaccctgtgccatgccaggcctgggccaccacgt   73747

Papio.anubis.clone.rp41-133b2.8034-40080.revcompl.Baboon        ------------------------------------------------------------   16681
BCRP3.HUMAN.NCBI.REF                                            ------------------------------------------------------------   16669
LOC106996293.glutathione.hydrolase5.proenzyme-like-GGT1.rhesus. gtcagtgggtgagaggtcagtttcctggtcaaggcctggacatgagggagcgggcacagg   73807

Papio.anubis.clone.rp41-133b2.8034-40080.revcompl.Baboon        ------------------------------------------------------------   16681
BCRP3.HUMAN.NCBI.REF                                            ------------------------------------------------------------   16669
LOC106996293.glutathione.hydrolase5.proenzyme-like-GGT1.rhesus. tttacagggggcccaagaggggcaggggcagcttggcagctcatcaggtccactagaggg   73867

Papio.anubis.clone.rp41-133b2.8034-40080.revcompl.Baboon        ------------------------------------------------------------   16681
BCRP3.HUMAN.NCBI.REF                                            ------------------------------------------------------------   16669
LOC106996293.glutathione.hydrolase5.proenzyme-like-GGT1.rhesus. tgtcccagacaagaacagcccccgaactgaagtcaccaaacaggttggattctcagagct   73927

Papio.anubis.clone.rp41-133b2.8034-40080.revcompl.Baboon        ------------------------------------------------------------   16681
BCRP3.HUMAN.NCBI.REF                                            ------------------------------------------------------------   16669
LOC106996293.glutathione.hydrolase5.proenzyme-like-GGT1.rhesus. ctgattggttggacaaggtgttctgggtagaggaacagtctatgcaaaggctcagaggaa   73987

Papio.anubis.clone.rp41-133b2.8034-40080.revcompl.Baboon        ------------------------------------------------------------   16681
BCRP3.HUMAN.NCBI.REF                                            ------------------------------------------------------------   16669
LOC106996293.glutathione.hydrolase5.proenzyme-like-GGT1.rhesus. gtgcagtcgtatctgcggaatggcaggggtgggggccagccaaggccacccctaccaggc   74047

Papio.anubis.clone.rp41-133b2.8034-40080.revcompl.Baboon        ------------------------------------------------------------   16681
BCRP3.HUMAN.NCBI.REF                                            ------------------------------------------------------------   16669
LOC106996293.glutathione.hydrolase5.proenzyme-like-GGT1.rhesus. caaggcagttagacttcatcctaagagcaaagtgtggcctgcctcgggctcccccacccc   74107

Papio.anubis.clone.rp41-133b2.8034-40080.revcompl.Baboon        ------------------------------------------------------------   16681
BCRP3.HUMAN.NCBI.REF                                            ------------------------------------------------------------   16669
LOC106996293.glutathione.hydrolase5.proenzyme-like-GGT1.rhesus. caccaatctccctccaggatgggcccttggtctgcccccgcacctcccctttccagcatg   74167

Papio.anubis.clone.rp41-133b2.8034-40080.revcompl.Baboon        ------------------------------------------------------------   16681
BCRP3.HUMAN.NCBI.REF                                            ------------------------------------------------------------   16669
LOC106996293.glutathione.hydrolase5.proenzyme-like-GGT1.rhesus. agaaagggtgccctgtctgtggccctagtcccagacatcagatgttcccagtgacagggg   74227

Papio.anubis.clone.rp41-133b2.8034-40080.revcompl.Baboon        ------------------------------------------------------------   16681
BCRP3.HUMAN.NCBI.REF                                            ------------------------------------------------------------   16669
LOC106996293.glutathione.hydrolase5.proenzyme-like-GGT1.rhesus. cgggccagcctgggaagccccaccacctgccaggggcttttcctggggcctgaaagagac   74287

Papio.anubis.clone.rp41-133b2.8034-40080.revcompl.Baboon        --------tctctctgtcatggcaggagggcttcaactgccattgtcacccgccccaccc   16733
BCRP3.HUMAN.NCBI.REF                                            --------ggccttaacaaagagctcagagctttggccgtggccagcagtgcacttggac   16721
LOC106996293.glutathione.hydrolase5.proenzyme-like-GGT1.rhesus. agaggcagggcctgtgcaaaggcggaaggcctggagccggtgcagactctctagagacat   74347
                                                                           **     * *     **  **    * *       *

Papio.anubis.clone.rp41-133b2.8034-40080.revcompl.Baboon        agcccactggcagcccctcccaggagtgaggagatgacaggccctaggtggacccaggcc   16793
BCRP3.HUMAN.NCBI.REF                                            ccccctcttccctcccaagcacatcatgaagacctccc---catcagcccagagct----   16774
LOC106996293.glutathione.hydrolase5.proenzyme-like-GGT1.rhesus. caaaggcggtgacctccgcctcaggggatggtctcccaaacagcagcccagggcttgca   74407
                                                                      *      * *   *       ** *   *  *   *   **       *

Papio.anubis.clone.rp41-133b2.8034-40080.revcompl.Baboon        ccactgggaaatctgccctccctggaccagggccatgagcggggtgggtggtgtcagtcc   16853
BCRP3.HUMAN.NCBI.REF                                            ------------------------------------------------------------   16774
LOC106996293.glutathione.hydrolase5.proenzyme-like-GGT1.rhesus. ggacgggcagccatgggagctttgcgggtggttcacggtggggttgaaatgacagagtat   74467

Papio.anubis.clone.rp41-133b2.8034-40080.revcompl.Baboon        caccacctcctagggaatctgtcccccggtgtcc--------------------------   16887
BCRP3.HUMAN.NCBI.REF                                            ------------------------------------------------------------   16774
LOC106996293.glutathione.hydrolase5.proenzyme-like-GGT1.rhesus. cagagtcacccagtggcctgtaccgtcgtggtggtaccaatcagagtggggcagatggta   74527

Papio.anubis.clone.rp41-133b2.8034-40080.revcompl.Baboon        ------------------------------------------------------------   16887
```

```
BCRP3.HUMAN.NCBI.REF                                                 ------------------------------------------------------------    16774
LOC106996293.glutathione.hydrolase5.proenzyme-like-GGT1.rhesus.      acccagggctttggaaacacaggtgatgagagagctggggggtgtaacaggagctgatga    74587


Papio.anubis.clone.rp41-133b2.8034-40080.revcompl.Baboon             ------------------------------------------------------------    16887
BCRP3.HUMAN.NCBI.REF                                                 ------------------------------------------------------------    16774
LOC106996293.glutathione.hydrolase5.proenzyme-like-GGT1.rhesus.      ggtccctgcaggatcccaagacccaccttgggcagaagggcaaagggaggaaggagtgtg    74647


Papio.anubis.clone.rp41-133b2.8034-40080.revcompl.Baboon             ------------------------------------------------------------    16887
BCRP3.HUMAN.NCBI.REF                                                 ------------------------------------------------------------    16774
LOC106996293.glutathione.hydrolase5.proenzyme-like-GGT1.rhesus.      ctatgggagccaggtccaggtccccatggaagtgggtgtcacacaggcctgtgggccgag    74707


Papio.anubis.clone.rp41-133b2.8034-40080.revcompl.Baboon             -----------------------accctagcgcctcgggaccctgggtcagggcaccccc    16924
BCRP3.HUMAN.NCBI.REF                                                 -----------------------ggccccttgtcctgggccactgagacccagaagtacc    16811
LOC106996293.glutathione.hydrolase5.proenzyme-like-GGT1.rhesus.      gggagtcacagaggtgacagcccagctgcaggtccccgttgactagagcagggaagcccc    74767
                                                                                              *     * *   *    **    *   * *   **

Papio.anubis.clone.rp41-133b2.8034-40080.revcompl.Baboon             actgtagggtcctgaggactccacccacgagagccccgaaagagtgtggctgc-------    16977
BCRP3.HUMAN.NCBI.REF                                                 aaggctggagtcagcttgcagcacagccagggtcgaggtcactccctccctgaggactct    16871
LOC106996293.glutathione.hydrolase5.proenzyme-like-GGT1.rhesus.      acttctctgtgccccctgccctccagcctcccactaggtctcttgttggctga-------    74820
                                                                     *          *      *    *   *     *   *        *  ***

Papio.anubis.clone.rp41-133b2.8034-40080.revcompl.Baboon             ------ctctaggacctgcccccaccctgaggctccccccatcccccagccagctcttct    17031
BCRP3.HUMAN.NCBI.REF                                                 agcacggcacagcccctctgcctctctcctggtggtggcgttgaaacagcaccctctgct    16931
LOC106996293.glutathione.hydrolase5.proenzyme-like-GGT1.rhesus.      ------gcacagtgggtgcggctgacctgggggccaggatctgactcagtacagtacagt    74874
                                                                               **    *    *   *    **         *    ***     *    *

Papio.anubis.clone.rp41-133b2.8034-40080.revcompl.Baboon             tcaacgggacagaacccctgaggcctcaggacccactcccatggcctgcgctggccacca    17091
BCRP3.HUMAN.NCBI.REF                                                 tcggt------cctctacagggtggcagagaaggaggcggtcaataaggtg--tccctgc    16983
LOC106996293.glutathione.hydrolase5.proenzyme-like-GGT1.rhesus.      acagc------cacagctggggagcgagggaccccctcctctgccacgctgcttccttct    74928
                                                                      *                 * *       **      *         *      **

Papio.anubis.clone.rp41-133b2.8034-40080.revcompl.Baboon             ccctggagaccgtggccacagagggtgcggaggtcttctacacggggaggctgggccaga    17151
BCRP3.HUMAN.NCBI.REF                                                 acaacctcgccactgtctttggccccacgctgctccggccctccgagaaggagagcaagc    17043
LOC106996293.glutathione.hydrolase5.proenzyme-like-GGT1.rhesus.      gcaagctccagggtggccgagtgcctgctgtgggccagagctccactaggcccatcagga    74988
                                                                      *            * *   *      *   *  *     * *    * *     *  *

Papio.anubis.clone.rp41-133b2.8034-40080.revcompl.Baboon             tgctggtggaggacattgccaaggaaggtcagcctccttgagatcccatgccctgtccct    17211
BCRP3.HUMAN.NCBI.REF                                                 tccctgccaaccccagccagcctgtcaccatgactgacagcagg----------------    17087
LOC106996293.glutathione.hydrolase5.proenzyme-like-GGT1.rhesus.      cccccctgtcctcagggtccagtgctgccaaggaggagggaggggtcggtgcagcgcatt    75048
                                                                       *                    *       *       *

Papio.anubis.clone.rp41-133b2.8034-40080.revcompl.Baboon             gctggaggaagagccaagttctgggcactgggggttgagggcaggctccttcccaggccc    17271
BCRP3.HUMAN.NCBI.REF                                                 ------------------------------------------------------------    17087
LOC106996293.glutathione.hydrolase5.proenzyme-like-GGT1.rhesus.      gtaggagtgggaagtcagggaggcgtcctggag---gagtccggcttcccacaggccccc    75105


Papio.anubis.clone.rp41-133b2.8034-40080.revcompl.Baboon             tgagggagtgctggaggtgcctgggaccccgagaggtcatgc-acctagctctgccagcc    17330
BCRP3.HUMAN.NCBI.REF                                                 ------------------------------------------------------------    17087
LOC106996293.glutathione.hydrolase5.proenzyme-like-GGT1.rhesus.      tccaggctttcgccagctccctttgttgcaaggatgtaaggatgactgactttggaagcc    75165


Papio.anubis.clone.rp41-133b2.8034-40080.revcompl.Baboon             caaggacccctgtggtccaggacctcagagtgcagagcctgcgggttagagccagcagag    17390
BCRP3.HUMAN.NCBI.REF                                                 ------------------------------------------------------------    17087
LOC106996293.glutathione.hydrolase5.proenzyme-like-GGT1.rhesus.      atgccaaccaagtccctgctgacctgatctcccaccactactgttccaggaccaggaaat    75225


Papio.anubis.clone.rp41-133b2.8034-40080.revcompl.Baboon             gggcgttactgatggtcacaacaggcctgggtagctgagctgac----------------    17434
BCRP3.HUMAN.NCBI.REF                                                 ------------------------------------------------------------    17087
LOC106996293.glutathione.hydrolase5.proenzyme-like-GGT1.rhesus.      aa-----acttcttttctgattaagccttaatatgtccaattaagccttaatatggacca    75280


Papio.anubis.clone.rp41-133b2.8034-40080.revcompl.Baboon             ------------------------------------------------------------    17434
BCRP3.HUMAN.NCBI.REF                                                 ------------------------------------------------------------    17087
LOC106996293.glutathione.hydrolase5.proenzyme-like-GGT1.rhesus.      tttgttcaagcagttggttggtcctgagaaaggaaagaaacttctctttggtgagacagg    75340


Papio.anubis.clone.rp41-133b2.8034-40080.revcompl.Baboon             ------------------------------------------------------------    17434
BCRP3.HUMAN.NCBI.REF                                                 ------------------------------------------------------------    17087
LOC106996293.glutathione.hydrolase5.proenzyme-like-GGT1.rhesus.      gtctcactctgccacccaggctggagtgcagtggtgcgatcatagctcatggccgcctca    75400


Papio.anubis.clone.rp41-133b2.8034-40080.revcompl.Baboon             ------------------------------------------------------------    17434
BCRP3.HUMAN.NCBI.REF                                                 ------------------------------------------------------------    17087
LOC106996293.glutathione.hydrolase5.proenzyme-like-GGT1.rhesus.      acctcccaggctcaggtgatcctcctgcctcagcctcacaagtagctggggccacaggca    75460


Papio.anubis.clone.rp41-133b2.8034-40080.revcompl.Baboon             ---------------------------------------cgttctcacaggctctcaaag    17455
BCRP3.HUMAN.NCBI.REF                                                 ---------------------------------------tccttggaggtcatgtctcag    17108
LOC106996293.glutathione.hydrolase5.proenzyme-like-GGT1.rhesus.      cgcccaggctggtcttgaactcctgggatcaagcaatcctcctgcctcggcctcccaaaa    75520
                                                                                                               *         *  *  *

Papio.anubis.clone.rp41-133b2.8034-40080.revcompl.Baboon             gccagctccctctcccatcttccccttcacctcccc------agcctccctgcctctggt    17509
BCRP3.HUMAN.NCBI.REF                                                 gtatgggaagacagtctccagcccatgca-------------------------------    17137
LOC106996293.glutathione.hydrolase5.proenzyme-like-GGT1.rhesus.      tgctgggattacaggcacgatccactgcacccggccaaaaataaactttttacctgagga    75580
                                                                         *      *   *     **  * **

Papio.anubis.clone.rp41-133b2.8034-40080.revcompl.Baboon             ctctgtccacccctccatgcttgaccccagggggccggtcctgaggccaccgcctacaga    17569
BCRP3.HUMAN.NCBI.REF                                                 ------------------------------------------------------------    17137
LOC106996293.glutathione.hydrolase5.proenzyme-like-GGT1.rhesus.      atgagtgccccctttaattatcaggcccagagaggcattaaaatgtgacagggtgtcaca    75640


Papio.anubis.clone.rp41-133b2.8034-40080.revcompl.Baboon             ccaaccatgaccctctgtgtccacaaggctagagcacgagtggg----------------    17613
BCRP3.HUMAN.NCBI.REF                                                 ------------------------------------------------------------    17137
LOC106996293.glutathione.hydrolase5.proenzyme-like-GGT1.rhesus.      gcagtcgtgcctcactcccaccttgatgccacttgctaggtgggttctagactaaccgat    75700


Papio.anubis.clone.rp41-133b2.8034-40080.revcompl.Baboon             ------------------------------------------------------------    17613
BCRP3.HUMAN.NCBI.REF                                                 ------------------------------------------------------------    17137
LOC106996293.glutathione.hydrolase5.proenzyme-like-GGT1.rhesus.      gtcaagtagccataaaatgccatgtgctggacaccatgatgtaggggccacaggaaaact    75760


Papio.anubis.clone.rp41-133b2.8034-40080.revcompl.Baboon             ------------------------------------------------------------    17613
BCRP3.HUMAN.NCBI.REF                                                 ------------------------------------------------------------    17137
LOC106996293.glutathione.hydrolase5.proenzyme-like-GGT1.rhesus.      tccccttcaccctctgaaggttcaaggaaaaatcagctcacaaaaggcagattaattgga    75820


Papio.anubis.clone.rp41-133b2.8034-40080.revcompl.Baboon             ------------------------------------------------------------    17613
BCRP3.HUMAN.NCBI.REF                                                 ------------------------------------------------------------    17137
LOC106996293.glutathione.hydrolase5.proenzyme-like-GGT1.rhesus.      aaacaagccatgcaaatttattagcatgcattggggagaatcatagagtcattgccgaat    75880


Papio.anubis.clone.rp41-133b2.8034-40080.revcompl.Baboon             ------------------------------------------------------------    17613
BCRP3.HUMAN.NCBI.REF                                                 ------------------------------------------------------------    17137
LOC106996293.glutathione.hydrolase5.proenzyme-like-GGT1.rhesus.      atcccagataaagatgcttatataccccgcttcttaggggaaagggagatgggaatctct    75940


Papio.anubis.clone.rp41-133b2.8034-40080.revcompl.Baboon             -----------------------------------------------atcccattgtgtg    17626
BCRP3.HUMAN.NCBI.REF                                                 ------------------------------------------------------------    17137
LOC106996293.glutathione.hydrolase5.proenzyme-like-GGT1.rhesus.      cctttgatactcagctgcagccagactcctgcaggaagacccccaacctcccactcctac    76000


Papio.anubis.clone.rp41-133b2.8034-40080.revcompl.Baboon             acctgacaccagtggctattcctccctgcctcagtttccccatctgttcatggagccctc    17686
BCRP3.HUMAN.NCBI.REF                                                 -------------------------------------accccagcctgacagaggtggcc    17160
LOC106996293.glutathione.hydrolase5.proenzyme-like-GGT1.rhesus.      aacgcactacactggccactccctcctcctcggcagcactggaccctgaggggaggggtc    76060
                                                                                                           *       *    *  *    *

Papio.anubis.clone.rp41-133b2.8034-40080.revcompl.Baboon             tccgtctcaccccagggagccagctgacgctgcaggacctg-------------------    17727
BCRP3.HUMAN.NCBI.REF                                                 tctgcctgccccacccccagtcctgcccatcttccgacttgcattgtatgtggtggtggc    17220
LOC106996293.glutathione.hydrolase5.proenzyme-like-GGT1.rhesus.      tcctgatggcccacagcgggcacttgatcaccccgggctggcaggagatgggagtgagga    76120
                                                                     **    *  ***                       * *  *

Papio.anubis.clone.rp41-133b2.8034-40080.revcompl.Baboon             ------------------------------------------------------------    17727
BCRP3.HUMAN.NCBI.REF                                                 tgagattcagagagagggacttgcctaggtttgcatggatgggagtgat-----------    17269
LOC106996293.glutathione.hydrolase5.proenzyme-like-GGT1.rhesus.      tga---tttcagaaggacaagtacatgatttgggctggggaggattttgtttgtttgtt    76177


Papio.anubis.clone.rp41-133b2.8034-40080.revcompl.Baboon             ------------------------------------------------------------    17727
BCRP3.HUMAN.NCBI.REF                                                 ---agggggtgcccaggccacctcctggtcctgctggtgcaccttgctgggggctt----    17322
LOC106996293.glutathione.hydrolase5.proenzyme-like-GGT1.rhesus.      gtaagagggagtctacctctgtcaccagcctggaccacccagctacttgggagattgagc    76237


Papio.anubis.clone.rp41-133b2.8034-40080.revcompl.Baboon             ------------------------------------------------------------    17727
```

```
BCRP3.HUMAN.NCBI.REF                                              ------------------------------------------------------------    17322
LOC106996293.glutathione.hydrolase5.proenzyme-like-GGT1.rhesus.   cactgcccctggcctgggtggggagaatttaattggtttaaagaacgtacagggccgggc    76297

Papio.anubis.clone.rp41-133b2.8034-40080.revcompl.Baboon          ------------------------------------------------------------    17727
BCRP3.HUMAN.NCBI.REF                                              ------------------------------------------------------------    17322
LOC106996293.glutathione.hydrolase5.proenzyme-like-GGT1.rhesus.   gcggtggctcatgcctgtaatcccagcactttgcgaagctgaggcgggcggatcaggaga    76357

Papio.anubis.clone.rp41-133b2.8034-40080.revcompl.Baboon          ------------------------------------------------------------    17727
BCRP3.HUMAN.NCBI.REF                                              ------------------------------------------------------------    17322
LOC106996293.glutathione.hydrolase5.proenzyme-like-GGT1.rhesus.   tcgagatcattttggctaacatggtgaaaccccgtctctactaaaactacaaaaaattag    76417

Papio.anubis.clone.rp41-133b2.8034-40080.revcompl.Baboon          ------------------------------------------------------------    17727
BCRP3.HUMAN.NCBI.REF                                              ------------------------------------------------------------    17322
LOC106996293.glutathione.hydrolase5.proenzyme-like-GGT1.rhesus.   ccaggtgtggtgggggcatctgtagtcacagctactcaggaggctgaggcaggagactc    76477

Papio.anubis.clone.rp41-133b2.8034-40080.revcompl.Baboon          ------------------------------------------------------------    17727
BCRP3.HUMAN.NCBI.REF                                              ------------------------------------------------------------    17322
LOC106996293.glutathione.hydrolase5.proenzyme-like-GGT1.rhesus.   gcatgaactcaggaggtggagcttgcagtgagccgagattgcaccactgcacaccagcct    76537

Papio.anubis.clone.rp41-133b2.8034-40080.revcompl.Baboon          ------------------------------------------------------------    17727
BCRP3.HUMAN.NCBI.REF                                              --------------------------------------------------aaaaccaccc    17332
LOC106996293.glutathione.hydrolase5.proenzyme-like-GGT1.rhesus.   gggcgacagagcgagaccccgtctcaaaaaacaaaaacaaaacaaacaaaaaaaacatac    76597

Papio.anubis.clone.rp41-133b2.8034-40080.revcompl.Baboon          ------------------------------------------------------------    17727
BCRP3.HUMAN.NCBI.REF                                              caagtgttcgggtgtggtggctcatgcctgtaatcccagcactttgggaggccgaggcag    17392
LOC106996293.glutathione.hydrolase5.proenzyme-like-GGT1.rhesus.   aatggggctgggcacagtggttcatgcttgtaatcccagcactttgggaggccgaggcag    76657

Papio.anubis.clone.rp41-133b2.8034-40080.revcompl.Baboon          ------------------------------------------------------------    17727
BCRP3.HUMAN.NCBI.REF                                              gacaact--gaacccaggtgtttgagaccagtctgggcaatgtag---------------    17435
LOC106996293.glutathione.hydrolase5.proenzyme-like-GGT1.rhesus.   ggggattgcgaggtcaggagtttcagaccagcctggccaacattgtgaaatcccgcctct    76717

Papio.anubis.clone.rp41-133b2.8034-40080.revcompl.Baboon          ----------------------------------------------------gccaaatt    17735
BCRP3.HUMAN.NCBI.REF                                              ------------------------------------------------------------    17435
LOC106996293.glutathione.hydrolase5.proenzyme-like-GGT1.rhesus.   actaaaaatataaaaaattagccgggtgtggtggtgtgcacctgtaatcccagctacttg    76777

Papio.anubis.clone.rp41-133b2.8034-40080.revcompl.Baboon          ccagcccgaggtagtggacgctctgga------ggtgcccctgggggactataccctgta    17789
BCRP3.HUMAN.NCBI.REF                                              ------------------------------------------------------------    17435
LOC106996293.glutathione.hydrolase5.proenzyme-like-GGT1.rhesus.   ggaggctgaggcaggagaatcacctgaatctgggaggcacaggttgcagtgagccaagat    76837

Papio.anubis.clone.rp41-133b2.8034-40080.revcompl.Baboon          ctcaccaccgccacctgcagggggtgccattctcagcttcatcctcaatgtgctaagagg    17849
BCRP3.HUMAN.NCBI.REF                                              ------------------------------------------------------------    17435
LOC106996293.glutathione.hydrolase5.proenzyme-like-GGT1.rhesus.   catgccactgcacgccagcctgggcaacagtgtgagactcggtctcaaaaaaaaaaaaag    76897

Papio.anubis.clone.rp41-133b2.8034-40080.revcompl.Baboon          taaagccc----------------------------------------------------    17857
BCRP3.HUMAN.NCBI.REF                                              ------------------------------------------------------------    17435
LOC106996293.glutathione.hydrolase5.proenzyme-like-GGT1.rhesus.   aacatacaaacatataatgggcctggctcggtggctcatgcctgtaatcccagcactcca    76957

Papio.anubis.clone.rp41-133b2.8034-40080.revcompl.Baboon          ------------------------------------------------------------    17857
BCRP3.HUMAN.NCBI.REF                                              -----------------------------------------------------------c    17436
LOC106996293.glutathione.hydrolase5.proenzyme-like-GGT1.rhesus.   gagggtgaagcaggcagattatgagatcaatagatcaagaccatcctggccaacatggtg    77017

Papio.anubis.clone.rp41-133b2.8034-40080.revcompl.Baboon          ------------------------------------------------------------    17857
BCRP3.HUMAN.NCBI.REF                                              aaaccccatctctagaaaaaatacaaagaaaaattagtcaggcattgtggcacacatctg    17496
LOC106996293.glutathione.hydrolase5.proenzyme-like-GGT1.rhesus.   aaaccccatctttatgaaaaatac----aaaaattagccaggcgtggtggtgcacacctg    77073

Papio.anubis.clone.rp41-133b2.8034-40080.revcompl.Baboon          ------------------------------------------------------------    17857
BCRP3.HUMAN.NCBI.REF                                              taatcctaggtatctgggaggctgacacaggaggattgcttgagcccaggagttagaggc    17556
LOC106996293.glutathione.hydrolase5.proenzyme-like-GGT1.rhesus.   taatcccagctacttggaaggctgaggcaggagaatggcttgcacctgggaggcggagat    77133

Papio.anubis.clone.rp41-133b2.8034-40080.revcompl.Baboon          --------------------------------------ctgcccagagctctggcccccc    17879
BCRP3.HUMAN.NCBI.REF                                              tgcagtgatccatgatggagccactgtactccagcctgggggacagagcaaggccctgtg    17616
LOC106996293.glutathione.hydrolase5.proenzyme-like-GGT1.rhesus.   tgcagtgagccaagatcgcaccactgcacgccagcct-ggtgacagagcaagac----tc    77188
                                                                                                             ******

Papio.anubis.clone.rp41-133b2.8034-40080.revcompl.Baboon          acacccgggaactcagactccaagcccagccaaggcccagctcagcctcctcctccataa    17939
BCRP3.HUMAN.NCBI.REF                                              catctctaaaataaataatcacccccccacccaa---------------------------    17649
LOC106996293.glutathione.hydrolase5.proenzyme-like-GGT1.rhesus.   catctcaaaaacaaacaaacaaaaacaaacaaaaccata---cagcgccctgggacaaag    77245
                                                                     * *   **   * *  *     * * * **

Papio.anubis.clone.rp41-133b2.8034-40080.revcompl.Baboon          accgggttcacagatgcacaatgtgaagactgagctgtgtaaaccagtcatgatggtggg    17999
BCRP3.HUMAN.NCBI.REF                                              ------------------caagtcatgccttgtcaggaccccaccccacccccgtctcac    17691
LOC106996293.glutathione.hydrolase5.proenzyme-like-GGT1.rhesus.   tctgttgggcctgaagagcaaacaatagattgtgacgagtctgtccaggtgtgttgacag    77305
                                                                                    ***         **    *       *

Papio.anubis.clone.rp41-133b2.8034-40080.revcompl.Baboon          ttttagagctgacctccggctccacccatacgtgaggctttgctgaagggtgtgacatcg    18059
BCRP3.HUMAN.NCBI.REF                                              tgtaaggggttcatgacaccagcaggggtttct-agcacctgaggtggacttgggggctt    17750
LOC106996293.glutathione.hydrolase5.proenzyme-like-GGT1.rhesus.   ttttcagtctttcttcctgtgacaggagtt------cagttaaggaagactcacaggagg    77359
                                                                  * *      *      *     **    *           *   *  *  *

Papio.anubis.clone.rp41-133b2.8034-40080.revcompl.Baboon          tttgtccagagtcctcctcccactaggtgaccagtcccagactca---------------    18104
BCRP3.HUMAN.NCBI.REF                                              gggccccaaagacctccccaccagcagctgtgagccccccctctgagccactctcctcttc    17810
LOC106996293.glutathione.hydrolase5.proenzyme-like-GGT1.rhesus.   ggcc--------------------------------------------------------    77363

Papio.anubis.clone.rp41-133b2.8034-40080.revcompl.Baboon          --------------------gaagcaggtgggctg-----tgtgggtggtgggctgccca    18139
BCRP3.HUMAN.NCBI.REF                                              cccactctgcgagggcaggacgaggtgctgctgtacttcttgcggctggaggccatccct    17870
LOC106996293.glutathione.hydrolase5.proenzyme-like-GGT1.rhesus.   --------------------agagctcatggttttcttctt-tggcagatccagacttca    77402
                                                                                      **    **   *      *  **  *          *

Papio.anubis.clone.rp41-133b2.8034-40080.revcompl.Baboon          tcacggacatgcacctgggggagtgaaaccagcccagcccaaggcagcctgcaggggggtt    18199
BCRP3.HUMAN.NCBI.REF                                              gccctgaacagcaagagacagagcatcctgttctccaccgatgtctaaaggtcccagtcc    17930
LOC106996293.glutathione.hydrolase5.proenzyme-like-GGT1.rhesus.   ggcagataaggaacttcagagaacaacttcatcctgagctctggctgacagactgagacc    77462
                                                                            * *       **          *     *   * *     *     *

Papio.anubis.clone.rp41-133b2.8034-40080.revcompl.Baboon          ctggcagtgccaaggcctggggctgtctgtgctggcttaatattagtgtcctgtgt----    18255
BCRP3.HUMAN.NCBI.REF                                              atctcctggaggcg-gacagatggcctggaaacctctggctaatcgggccatctgtagag    17989
LOC106996293.glutathione.hydrolase5.proenzyme-like-GGT1.rhesus.   gaggcggggagaggtcagagaccttgtggcttcttcttcagttcagtgccatattt----    77518
                                                                      *   *    *     *        *      **        * * * * * *

Papio.anubis.clone.rp41-133b2.8034-40080.revcompl.Baboon          -------------------------------------ctgccattacaaatcaccacaag    18278
BCRP3.HUMAN.NCBI.REF                                              tgggaatcaagattttctgaggcatccttgggccacccccaggtgtcaggccat------    18043
LOC106996293.glutathione.hydrolase5.proenzyme-like-GGT1.rhesus.   tggggtatcagtttctgagccccagcaataactcatactctatagttcaacagtaaccag    77578
                                                                                                       *

Papio.anubis.clone.rp41-133b2.8034-40080.revcompl.Baboon          cttggtggcttaaaataacagcaatgtgtcctctcacagttctggaagccagaagcccga    18338
BCRP3.HUMAN.NCBI.REF                                              -ctgccaagagacagcggcccaaagcagaaggacaggtggcctgggc-------------    18089
LOC106996293.glutathione.hydrolase5.proenzyme-like-GGT1.rhesus.   ccaggcaaggagcagtggctcacatctgtaatcctagtgctctgggagatggaggcaaga    77638
                                                                     *         *    *    *   *          *  ****

Papio.anubis.clone.rp41-133b2.8034-40080.revcompl.Baboon          tatctaggtgtccacagggctgcattccc-------------------------------    18367
BCRP3.HUMAN.NCBI.REF                                              ----agatcccgcccaggtctgaaagccccaggctggcctcagactgtgggtttttttatg    18145
LOC106996293.glutathione.hydrolase5.proenzyme-like-GGT1.rhesus.   ggatcgcttgaggccaggagttcaagaccagtatgggcaacatagtggagcctgcctaca    77698
                                                                                ****  *  *   **

Papio.anubis.clone.rp41-133b2.8034-40080.revcompl.Baboon          ------------------------------------------------------------    18367
BCRP3.HUMAN.NCBI.REF                                              tggccacccgagggcgccccaagccagttca-----------------------------    18176
LOC106996293.glutathione.hydrolase5.proenzyme-like-GGT1.rhesus.   aacacacacacacacacatttagccatgccagcatgcacctgtaatcgcagctactcagg    77758

Papio.anubis.clone.rp41-133b2.8034-40080.revcompl.Baboon          --------------------ttgggggctccaggggagagtctgttctttgccttgttga    18407
BCRP3.HUMAN.NCBI.REF                                              ------------------------------------------------------------    18176
LOC106996293.glutathione.hydrolase5.proenzyme-like-GGT1.rhesus.   agactgaggcaagaggatcatttgagccctggaggatgaggctgcaatgagccatgatga    77818

Papio.anubis.clone.rp41-133b2.8034-40080.revcompl.Baboon          cctaaataaaaaacagaacaaggctgttgtttctttgaacaacattcagagaaaatgatg    18467
BCRP3.HUMAN.NCBI.REF                                              ------------------------------------------------------------    18176
LOC106996293.glutathione.hydrolase5.proenzyme-like-GGT1.rhesus.   caccactgccaacagagcgagatctcatct--------caaaaaataaaataaaataaag    77870

Papio.anubis.clone.rp41-133b2.8034-40080.revcompl.Baboon          tttattccagagtaggcatcgcggtgggaatacccacgctacggtaaactgtgtgtgtgt    18527
```

```
BCRP3.HUMAN.NCBI.REF                                               ------------------------------------------------------------    18176
LOC106996293.glutathione.hydrolase5.proenzyme-like-GGT1.rhesus.    ccaatcactaacgcgttat-----tgaaactgcctttgcaaaaaagcataattgaggaaa    77925


Papio.anubis.clone.rp41-133b2.8034-40080.revcompl.Baboon           tcagggaggtgaaggaagacaaggttctcaa-----------------------------    18559
BCRP3.HUMAN.NCBI.REF                                               ------------------------------------------------------------    18176
LOC106996293.glutathione.hydrolase5.proenzyme-like-GGT1.rhesus.    ttatgacagtgaaagaaatcagaggtgaccaactctccaagagtctgaacctcctcaaat    77985


Papio.anubis.clone.rp41-133b2.8034-40080.revcompl.Baboon           ------------------------------------------------------------    18559
BCRP3.HUMAN.NCBI.REF                                               ------------------------------------------------------------    18176
LOC106996293.glutathione.hydrolase5.proenzyme-like-GGT1.rhesus.    tgctgctggggataacatcactattgtaaaacttgagatcagggcttgagatattttgca    78045


Papio.anubis.clone.rp41-133b2.8034-40080.revcompl.Baboon           ------------------------------------------------------------    18559
BCRP3.HUMAN.NCBI.REF                                               ------------------------------------------------------------    18176
LOC106996293.glutathione.hydrolase5.proenzyme-like-GGT1.rhesus.    gaccctgcactccatggatcagctgacgccacccagactgctattctggctcaaccagtt    78105


Papio.anubis.clone.rp41-133b2.8034-40080.revcompl.Baboon           ------------------------------------------------------------    18559
BCRP3.HUMAN.NCBI.REF                                               ------------------------------------------------------------    18176
LOC106996293.glutathione.hydrolase5.proenzyme-like-GGT1.rhesus.    ctgccatcacagccaggaacagaagacagcaagaaaaactcatttccacccgctgtgatt    78165


Papio.anubis.clone.rp41-133b2.8034-40080.revcompl.Baboon           ------------------------------------------------------------    18559
BCRP3.HUMAN.NCBI.REF                                               ------------------------------------------------------------    18176
LOC106996293.glutathione.hydrolase5.proenzyme-like-GGT1.rhesus.    ccatcttcaacctgaccaatcaacactccccatttcccaagcccctacctgccaaattat    78225


Papio.anubis.clone.rp41-133b2.8034-40080.revcompl.Baboon           ------------------------------------------------------------    18559
BCRP3.HUMAN.NCBI.REF                                               ------------------------------------------------------------    18176
LOC106996293.glutathione.hydrolase5.proenzyme-like-GGT1.rhesus.    ctttaaaaactttggtcaggcgcctagactcatgtcagtaatcccagtaatccctgcagt    78285


Papio.anubis.clone.rp41-133b2.8034-40080.revcompl.Baboon           ------------------------------------------------------------    18559
BCRP3.HUMAN.NCBI.REF                                               ------------------------------------------------------------    18176
LOC106996293.glutathione.hydrolase5.proenzyme-like-GGT1.rhesus.    ttgggagactgaggcaggcggatcacttgaggtcaggagttcaagatcagcctggccaac    78345


Papio.anubis.clone.rp41-133b2.8034-40080.revcompl.Baboon           ------------------------------------------------------------    18559
BCRP3.HUMAN.NCBI.REF                                               ------------------------------------------------------------    18176
LOC106996293.glutathione.hydrolase5.proenzyme-like-GGT1.rhesus.    acggtgaaacctcgtctctactaaaatcgcaataattagctggatgtggtggcacatgcc    78405


Papio.anubis.clone.rp41-133b2.8034-40080.revcompl.Baboon           ------------------------------------------------------------    18559
BCRP3.HUMAN.NCBI.REF                                               ------------------------------------------------------------    18176
LOC106996293.glutathione.hydrolase5.proenzyme-like-GGT1.rhesus.    tgtagtcccagctgctcaggaggctgaggcatgagaatcacttgaacccagcaggtggag    78465


Papio.anubis.clone.rp41-133b2.8034-40080.revcompl.Baboon           ------------------------------------------------------------    18559
BCRP3.HUMAN.NCBI.REF                                               ------------------------------------------------------------    18176
LOC106996293.glutathione.hydrolase5.proenzyme-like-GGT1.rhesus.    gttgtagtgagcgtctcaaaaaaacaaaaagcaaaaaacaaaaaactctgatccccgaat    78525


Papio.anubis.clone.rp41-133b2.8034-40080.revcompl.Baboon           ------------------------------------------------------------    18559
BCRP3.HUMAN.NCBI.REF                                               ------------------------------------------------------------    18176
LOC106996293.glutathione.hydrolase5.proenzyme-like-GGT1.rhesus.    ccttggggagactgatttgagcgataataaaactccgattgactctgcatgaattactct    78585


Papio.anubis.clone.rp41-133b2.8034-40080.revcompl.Baboon           ------------------------------------------------------------    18559
BCRP3.HUMAN.NCBI.REF                                               ---------------------tctcggagtccaggcctggccctggga---gacagggtg    18212
LOC106996293.glutathione.hydrolase5.proenzyme-like-GGT1.rhesus.    ttgtccactgcgactcccctgtcttgaaaaataggctctgtctaggcagctggcaaggtg    78645


Papio.anubis.clone.rp41-133b2.8034-40080.revcompl.Baboon           aaagaaaatgaggaagagtatataattttttttgcttataatcaatgataatttattgtat    18619
BCRP3.HUMAN.NCBI.REF                                               aaagcagtggtttttatgaacttaacttatagagtccaaaagatttctactgaatcactt    18272
LOC106996293.glutathione.hydrolase5.proenzyme-like-GGT1.rhesus.    aacccattgcagttacattacctctctaaaccactgagaattcctgctaaacaattttat    78705
                                                                   **   *             *  *   *      *   **    *  **    **    *

Papio.anubis.clone.rp41-133b2.8034-40080.revcompl.Baboon           at-----------tttattttttcattgcattgttattattattattgagatggtgtct    18668
BCRP3.HUMAN.NCBI.REF                                               gtcaagaagcgccctctc------------------------------------------    18290
LOC106996293.glutathione.hydrolase5.proenzyme-like-GGT1.rhesus.    atgaaaccactccctatcctcttttactatttatttatttatttttttgagccagaatct    78765
                                                                    *            *

Papio.anubis.clone.rp41-133b2.8034-40080.revcompl.Baboon           tgctctgtcacttaggctagagcacagtggcacaatctcggttcactgcaagctctactt    18728
BCRP3.HUMAN.NCBI.REF                                               ----------------------tggggagaaggaacgtgactggattc--cctcactgt    18326
LOC106996293.glutathione.hydrolase5.proenzyme-like-GGT1.rhesus.    cgctttgtcacccaggctgcagtgcagtggcgcgatcttggctcattgcagcctccacct    78825
                                                                                             * *     * *  *  *   * *   ***    *

Papio.anubis.clone.rp41-133b2.8034-40080.revcompl.Baboon           cctgggttcaagcagttctcctgactcagcctcccaagtagctgggat------------    18776
BCRP3.HUMAN.NCBI.REF                                               tgtatcttgaataaacgctgctgcttcatcctgtgggggccgtggccctgtccctgtgtg    18386
LOC106996293.glutathione.hydrolase5.proenzyme-like-GGT1.rhesus.    cctgggttcaagggattcttctgcctcagcctcccaagtagctgggac------------    78873
                                                                     *   ** **      ** ***  *** ***     *    ***

Papio.anubis.clone.rp41-133b2.8034-40080.revcompl.Baboon           ------------------------------------------------------------    18776
BCRP3.HUMAN.NCBI.REF                                               ggtggggcctcttccatttccctgacttagaaaccacactccacttctaacagggtttga    18446
LOC106996293.glutathione.hydrolase5.proenzyme-like-GGT1.rhesus.    ------------------------------------------------------------    78873


Papio.anubis.clone.rp41-133b2.8034-40080.revcompl.Baboon           -------------tacaggcatgggccaccacatgccgctgatttttatcttttttttt    18823
BCRP3.HUMAN.NCBI.REF                                               gaggcttggtcagcactgggtagcgttttgactccattcttggctt--tcttctttttct    18504
LOC106996293.glutathione.hydrolase5.proenzyme-like-GGT1.rhesus.    ----------caccagtggtgtgcgccaccactcccagctaatttt--tgtttgtttgtt    78921
                                                                                 *  **   * *     **      **     **  * **  ***  *

Papio.anubis.clone.rp41-133b2.8034-40080.revcompl.Baboon           ttcctttttttttttttgagacagagtcttgatctgtcgcccagactagagtgcaatggca    18883
BCRP3.HUMAN.NCBI.REF                                               ttccagaaggatttttgtgcagaaatgggtcttttgttgccgtgttagtc----------    18554
LOC106996293.glutathione.hydrolase5.proenzyme-like-GGT1.rhesus.    t----------gttttgagacgtagttttgctcttgttgcccagctggactgcaatggca    78971
                                                                   *           ***** *    * *     * *     **     * *

Papio.anubis.clone.rp41-133b2.8034-40080.revcompl.Baboon           tgatctcagctcattgcaacctctgcctctcaggtacaagcgattctcctgcctcagcct    18943
BCRP3.HUMAN.NCBI.REF                                               ------------------------------------------------------------    18554
LOC106996293.glutathione.hydrolase5.proenzyme-like-GGT1.rhesus.    caatcttggctcaccaaaacctctgcctcccaggttcaagcgattctcctgcctcagcct    79031


Papio.anubis.clone.rp41-133b2.8034-40080.revcompl.Baboon           ctcaagtagctgggattacaggtgcctgccatcatacccggttaat----ttttgtattt    18999
BCRP3.HUMAN.NCBI.REF                                               ------------------------------------------------------------    18554
LOC106996293.glutathione.hydrolase5.proenzyme-like-GGT1.rhesus.    cccaagtagctgggattgcaggcatgtgccaccacacccaactaatctaattttgtatcg    79091


Papio.anubis.clone.rp41-133b2.8034-40080.revcompl.Baboon           ttggtagagataag--tttgccatgttgatcaggctggtcttgaactcctgatgtcaggt    19057
BCRP3.HUMAN.NCBI.REF                                               ------------------------------------------------------------    18554
LOC106996293.glutathione.hydrolase5.proenzyme-like-GGT1.rhesus.    ttagtagagatggggtttcactctgttggccaggctggtcttaaactcccaacctcaggt    79151


Papio.anubis.clone.rp41-133b2.8034-40080.revcompl.Baboon           gatccgcccacctcggcctcccaaagtgctaggattacaggcttgagccaccatgcccag    19117
BCRP3.HUMAN.NCBI.REF                                               ------------------------------------------------------------    18554
LOC106996293.glutathione.hydrolase5.proenzyme-like-GGT1.rhesus.    gatctgcccaccttggcctcccaaagtgctgagattacaggtgtgtgccaccatgcccag    79211


Papio.anubis.clone.rp41-133b2.8034-40080.revcompl.Baboon           atgatttttttc------------------------------------------------    19129
BCRP3.HUMAN.NCBI.REF                                               ------------------------------------------------------------    18554
LOC106996293.glutathione.hydrolase5.proenzyme-like-GGT1.rhesus.    tctccttatcctcttttgtctttaaagaaactgcttttgggccgggcgcggtggctcaag    79271


Papio.anubis.clone.rp41-133b2.8034-40080.revcompl.Baboon           ------------------------------------------------------------    19129
BCRP3.HUMAN.NCBI.REF                                               ------------------------------------------------------------    18554
LOC106996293.glutathione.hydrolase5.proenzyme-like-GGT1.rhesus.    cctgtaatcccagcactttgggaggccgaggcgggcggatcacaaggtcacgagatcgag    79331


Papio.anubis.clone.rp41-133b2.8034-40080.revcompl.Baboon           ------------------------------------------------------------    19129
BCRP3.HUMAN.NCBI.REF                                               ------------------------------------------------------------    18554
LOC106996293.glutathione.hydrolase5.proenzyme-like-GGT1.rhesus.    accacagtgaaaccccgtctctactaaaaatacaaaaaattagccgggtgcggtggcggg    79391


Papio.anubis.clone.rp41-133b2.8034-40080.revcompl.Baboon           ------------------------------------------------------------    19129
BCRP3.HUMAN.NCBI.REF                                               ------------------------------------------------------------    18554
LOC106996293.glutathione.hydrolase5.proenzyme-like-GGT1.rhesus.    cgcctgtagtcccagctactcaggaggctgaggcaggagaatggtgggaacccgggaggc    79451


Papio.anubis.clone.rp41-133b2.8034-40080.revcompl.Baboon           ------------------------------------------------------------    19129
BCRP3.HUMAN.NCBI.REF                                               ------------------------------------------------------------    18554
LOC106996293.glutathione.hydrolase5.proenzyme-like-GGT1.rhesus.    ggagcttgcagtgagccgagatcgcgccactgcactccagcctgggcaacagcgtgagac    79511


Papio.anubis.clone.rp41-133b2.8034-40080.revcompl.Baboon           ------------------------------------------------------------    19129
```

```
BCRP3.HUMAN.NCBI.REF                                                  ------------------------------------------------------------    18554
LOC106996293.glutathione.hydrolase5.proenzyme-like-GGT1.rhesus.       tccgtctcaaaaaaaaaaaaaaaaaaaaaaaaaaagaaactgcttttgtggccaggcat    79571

Papio.anubis.clone.rp41-133b2.8034-40080.revcompl.Baboon              ------------------------------------------------------------    19129
BCRP3.HUMAN.NCBI.REF                                                  ------------------------------------------------------------    18554
LOC106996293.glutathione.hydrolase5.proenzyme-like-GGT1.rhesus.       ggtggctcacgtctgtaatctcaccactttgggaggccaaggcaggtggatcatgaggtc    79631

Papio.anubis.clone.rp41-133b2.8034-40080.revcompl.Baboon              ------------------------------------------------------------    19129
BCRP3.HUMAN.NCBI.REF                                                  ------------------------------------------------------------    18554
LOC106996293.glutathione.hydrolase5.proenzyme-like-GGT1.rhesus.       aggagttcaagatcagcctggccgagatggtgaaacctcatctctactaaaaatacaaaa    79691

Papio.anubis.clone.rp41-133b2.8034-40080.revcompl.Baboon              ------------------------------------------------------------    19129
BCRP3.HUMAN.NCBI.REF                                                  ---------------------------------ctccttggaaggcagctcagaaggcct    18581
LOC106996293.glutathione.hydrolase5.proenzyme-like-GGT1.rhesus.       cgttagctgggcatggtggtgggcgcctgtaagctactcgtgaggctgaggcagagaact    79751

Papio.anubis.clone.rp41-133b2.8034-40080.revcompl.Baboon              ------------------------------------------------------------    19129
BCRP3.HUMAN.NCBI.REF                                                  gtgaaatgtcgggggacaggacccccagggagggaatcccaggctacgcaccttag----    18637
LOC106996293.glutathione.hydrolase5.proenzyme-like-GGT1.rhesus.       gctcgaacccggaaggcagaagttgcagtgagccaagatcatgccactgcactccagcct    79811

Papio.anubis.clone.rp41-133b2.8034-40080.revcompl.Baboon              ------------------------------------------------------------    19129
BCRP3.HUMAN.NCBI.REF                                                  ------------------------------------------------------------    18637
LOC106996293.glutathione.hydrolase5.proenzyme-like-GGT1.rhesus.       gggtgacagagtgaaactgtctcaaaaagcaacaacaacaacaacaataaaaacaaaaca    79871

Papio.anubis.clone.rp41-133b2.8034-40080.revcompl.Baboon              ------------------------------------------------------------    19129
BCRP3.HUMAN.NCBI.REF                                                  ------------------------------------------------------------    18637
LOC106996293.glutathione.hydrolase5.proenzyme-like-GGT1.rhesus.       aaactgcttttgggccaggcatggtggttcatgcctgtaatcatagcactttgggaggct    79931

Papio.anubis.clone.rp41-133b2.8034-40080.revcompl.Baboon              ------------------------------------------------------------    19129
BCRP3.HUMAN.NCBI.REF                                                  ------------------------------------------------------------    18637
LOC106996293.glutathione.hydrolase5.proenzyme-like-GGT1.rhesus.       gagttgggaggatcacctgagttcagaaattggaggccagactgggcaacatagtgagac    79991

Papio.anubis.clone.rp41-133b2.8034-40080.revcompl.Baboon              ------------------------------------------------------------    19129
BCRP3.HUMAN.NCBI.REF                                                  ------------------------------------------------------------    18637
LOC106996293.glutathione.hydrolase5.proenzyme-like-GGT1.rhesus.       accatctctttaaaaacaaaaacaaacaaacaaacaaaaactgcttgtaacaaaggtcga    80051

Papio.anubis.clone.rp41-133b2.8034-40080.revcompl.Baboon              ------------------------------------------------------------    19129
BCRP3.HUMAN.NCBI.REF                                                  ------------------------------------------------------------    18637
LOC106996293.glutathione.hydrolase5.proenzyme-like-GGT1.rhesus.       atgaagcactccccaaggcaacttggaagtgtgtctggggccactgttctcaaccttggc    80111

Papio.anubis.clone.rp41-133b2.8034-40080.revcompl.Baboon              ------------------------------------------------------------    19129
BCRP3.HUMAN.NCBI.REF                                                  ------------------------------------------------------------    18637
LOC106996293.glutathione.hydrolase5.proenzyme-like-GGT1.rhesus.       ccaaataaactatactaattttgcctcagtttcttcctttaggtcaatagtccctgtaac    80171

Papio.anubis.clone.rp41-133b2.8034-40080.revcompl.Baboon              ------------------------------------------------------------    19129
BCRP3.HUMAN.NCBI.REF                                                  ------------------------------------------------------------    18637
LOC106996293.glutathione.hydrolase5.proenzyme-like-GGT1.rhesus.       aatacaccaaaggcataggtataaaaagatggccttaccttttttcattagtgtggaaaat    80231

Papio.anubis.clone.rp41-133b2.8034-40080.revcompl.Baboon              ------------------------------------------------------------    19129
BCRP3.HUMAN.NCBI.REF                                                  ------------------------------------------------------------    18637
LOC106996293.glutathione.hydrolase5.proenzyme-like-GGT1.rhesus.       aaatactgtgtgtgcaaggtgaaaaaaatctaaacagaacaattcagggaactagcccct    80291

Papio.anubis.clone.rp41-133b2.8034-40080.revcompl.Baboon              ------------------------------------------------------------    19129
BCRP3.HUMAN.NCBI.REF                                                  ------------------------------------------------------------    18637
LOC106996293.glutathione.hydrolase5.proenzyme-like-GGT1.rhesus.       ggctaggggacattgaagctgtgctcagccttttgcttctttaaacactatggtactgtg    80351

Papio.anubis.clone.rp41-133b2.8034-40080.revcompl.Baboon              ------------------------------------------------------------    19129
BCRP3.HUMAN.NCBI.REF                                                  ------------------------------------------------------------    18637
LOC106996293.glutathione.hydrolase5.proenzyme-like-GGT1.rhesus.       taaataagttcaggttaacctggaggagaaattcacacgagtagatcagctggttaaatg    80411

Papio.anubis.clone.rp41-133b2.8034-40080.revcompl.Baboon              ------------------------------------------------------------    19129
BCRP3.HUMAN.NCBI.REF                                                  ------------------------------------------------------------    18637
LOC106996293.glutathione.hydrolase5.proenzyme-like-GGT1.rhesus.       gcacaagcattttctgtctcaagttatactggtgtaatctcagctcactgaaacctctgc    80471

Papio.anubis.clone.rp41-133b2.8034-40080.revcompl.Baboon              ------------------------------------------------------------    19129
BCRP3.HUMAN.NCBI.REF                                                  ------------------------------------------------------------    18637
LOC106996293.glutathione.hydrolase5.proenzyme-like-GGT1.rhesus.       tgagttcaagccattctgcaaatattcacccattgtgagttgtcttttcactgtttcagt    80531

Papio.anubis.clone.rp41-133b2.8034-40080.revcompl.Baboon              ------------------------------------------------------------    19129
BCRP3.HUMAN.NCBI.REF                                                  ------------------------------------------------------------    18637
LOC106996293.glutathione.hydrolase5.proenzyme-like-GGT1.rhesus.       gatgtcttttgttgaacagaatttcttaattttcatatagtccaatttatcattttttttc    80591

Papio.anubis.clone.rp41-133b2.8034-40080.revcompl.Baboon              ------------------------------------------------------------    19129
BCRP3.HUMAN.NCBI.REF                                                  ------------------------------------------------------------    18637
LOC106996293.glutathione.hydrolase5.proenzyme-like-GGT1.rhesus.       ttctatactagcttttgtgtcctgtttaagaaaaaattttggtatgcaacaaaataaaaa    80651

Papio.anubis.clone.rp41-133b2.8034-40080.revcompl.Baboon              ------------------------------------------------------------    19129
BCRP3.HUMAN.NCBI.REF                                                  ------------------------------------------------------------    18637
LOC106996293.glutathione.hydrolase5.proenzyme-like-GGT1.rhesus.       aacagaatttaaaaaagagaagcatttacctactctaaggtcacaaagatactctctttc    80711

Papio.anubis.clone.rp41-133b2.8034-40080.revcompl.Baboon              ------------------------------------------------------------    19129
BCRP3.HUMAN.NCBI.REF                                                  ------------------------------------------------------------    18637
LOC106996293.glutathione.hydrolase5.proenzyme-like-GGT1.rhesus.       tttctgtaaatactacttaatattttatttgattatttatttatttatttatgtattttt    80771

Papio.anubis.clone.rp41-133b2.8034-40080.revcompl.Baboon              ------------------------------------------------------------    19129
BCRP3.HUMAN.NCBI.REF                                                  ------------------------------------------------------------    18637
LOC106996293.glutathione.hydrolase5.proenzyme-like-GGT1.rhesus.       ggagatgggggtctcactacgttgcccaggctggtttcgaactccttggctcaagtgatc    80831

Papio.anubis.clone.rp41-133b2.8034-40080.revcompl.Baboon              ------------------------------------------------------------    19129
BCRP3.HUMAN.NCBI.REF                                                  ------------------------------------------------------------    18637
LOC106996293.glutathione.hydrolase5.proenzyme-like-GGT1.rhesus.       actcggttgctgtattttagtacagacaggatttcaccatgctgtcctggctgatctcg    80891

Papio.anubis.clone.rp41-133b2.8034-40080.revcompl.Baboon              ------------------------------------------------------------    19129
BCRP3.HUMAN.NCBI.REF                                                  ------------------------------------------------------------    18637
LOC106996293.glutathione.hydrolase5.proenzyme-like-GGT1.rhesus.       aactcttgacctcgtgatctgcccgcctggggctcctaaagtgctaggattacagatgtg    80951

Papio.anubis.clone.rp41-133b2.8034-40080.revcompl.Baboon              ------------------------------------------------------------    19129
BCRP3.HUMAN.NCBI.REF                                                  ------------------------------------------------------------    18637
LOC106996293.glutathione.hydrolase5.proenzyme-like-GGT1.rhesus.       agccaccgcgcctggcttgtttgtttttgagatggagtcttgctctgtcacccaggctgg    81011

Papio.anubis.clone.rp41-133b2.8034-40080.revcompl.Baboon              ------------------------------------------------------------    19129
BCRP3.HUMAN.NCBI.REF                                                  --------ggttcgttctccagggagagcgacctcgtcccccgatcctgaccgcccttcc    18689
LOC106996293.glutathione.hydrolase5.proenzyme-like-GGT1.rhesus.       agtgcagtggtgcgatctccactcaatgcaacatcttccttccaggttcaagcaattctc    81071

Papio.anubis.clone.rp41-133b2.8034-40080.revcompl.Baboon              ------------------------------------------------------------    19129
BCRP3.HUMAN.NCBI.REF                                                  ggcccacgctctcc----------------------------------------------    18703
LOC106996293.glutathione.hydrolase5.proenzyme-like-GGT1.rhesus.       ctgcctcagtctcccaatcagctgagatcacagatgcctgcaaccacaactggctaattt    81131

Papio.anubis.clone.rp41-133b2.8034-40080.revcompl.Baboon              -------tttttagtagagacggggtttcaccatgttggcctggctggtcttgaactctt    19182
BCRP3.HUMAN.NCBI.REF                                                  -------tgtttggcttccacaggcctggacttctctggcttctctgcccacacactccc    18756
LOC106996293.glutathione.hydrolase5.proenzyme-like-GGT1.rhesus.       ttttgtatttttagtagagacaaggtttcaccatgttggccaggatgctcttaaactcct    81191
                                                                             * *** *     **  *  *  **     ****     **  *    ****

Papio.anubis.clone.rp41-133b2.8034-40080.revcompl.Baboon              gaccttaactgacccacttgccttagcctcccaaagtgctgggatttcagac-gtgagcc    19241
BCRP3.HUMAN.NCBI.REF                                                  tgcccccagt-gtccctgcccctgccccagcacaggtgacttcatttctgtcctctcagc    18815
LOC106996293.glutathione.hydrolase5.proenzyme-like-GGT1.rhesus.       gacctcaagtaatccacctacctcagccttccaaagtgctgggattgcaggcagtgagcc    81251
                                                                        **   * *   **     ***   **  *  * ***     *** * * *       *

Papio.anubis.clone.rp41-133b2.8034-40080.revcompl.Baboon              acctctgcctggctattttatttttatttcattagttttgggggaacacgtggtgttcgg    19301
```

```
BCRP3.HUMAN.NCBI.REF                                          tcagtggactcgctcatcttttgtataagtctccacttggtggcagcagcttgctgatga   18875
LOC106996293.glutathione.hydrolase5.proenzyme-like-GGT1.rhesus.   acagtggctggcctatttattttgatactt------------------------------   81281
                                                               *    *     **  *   **

Papio.anubis.clone.rp41-133b2.8034-40080.revcompl.Baboon      ctgcatggaaaagtt------------------atttagtggatgacttctttttttgag   19343
BCRP3.HUMAN.NCBI.REF                                          cttgttttaaaactttcatcctaaataacctttttgatacttgaatatttttaagttttat   18935
LOC106996293.glutathione.hydrolase5.proenzyme-like-GGT1.rhesus.   -----------aattttttctttctttcttttcttttcctttttttttttttttttgag   81330
                                                                         * **                     *  *       ** *   *** *

Papio.anubis.clone.rp41-133b2.8034-40080.revcompl.Baboon      atggaatctcactc-tgtggcccaggctggagagcagtggcatgatcttagctcactgca   19402
BCRP3.HUMAN.NCBI.REF                                          acatagtttctaatttttttccgaac----------------------------------   18961
LOC106996293.glutathione.hydrolase5.proenzyme-like-GGT1.rhesus.   atggagtttcactcttgttgcccgacctggagtgcaatggtgccatctcggctcaccaca   81390
                                                              *   * * **     * *   **

Papio.anubis.clone.rp41-133b2.8034-40080.revcompl.Baboon      acctctgcctcccagattcaagcaattctccagtctcagcct------------------   19444
BCRP3.HUMAN.NCBI.REF                                          ------------------------------------------------------------   18961
LOC106996293.glutathione.hydrolase5.proenzyme-like-GGT1.rhesus.   acttctgcctcgcaggttcaagcaattctcctgcctcagcctcctgagtagctgggatta   81450

Papio.anubis.clone.rp41-133b2.8034-40080.revcompl.Baboon      ----------------------------------tttttagtaaaaaaggggtttcacca   19470
BCRP3.HUMAN.NCBI.REF                                          ------------------------------------------------------------   18961
LOC106996293.glutathione.hydrolase5.proenzyme-like-GGT1.rhesus.   caggcacacaccaccacatccagttaattttgtatttttagtagagatgggttttctcca   81510

Papio.anubis.clone.rp41-133b2.8034-40080.revcompl.Baboon      tgttggccaggctggtctcgaactcctgacttcaagtgatctgcctgcctcagcctccca   19530
BCRP3.HUMAN.NCBI.REF                                          ------------------------------------------------------------   18961
LOC106996293.glutathione.hydrolase5.proenzyme-like-GGT1.rhesus.   tgttgatcaggctggtcttgaactcccgacctcaggtgatccgcccgcctccacctctca   81570

Papio.anubis.clone.rp41-133b2.8034-40080.revcompl.Baboon      aagtgctgggattacaggcatgagccactgtgcctgtcctttagtggtgatttctgagac   19590
BCRP3.HUMAN.NCBI.REF                                          ---------agatccagatacctaataagatgctggaatgtaatccctggacaatccgtg   19012
LOC106996293.glutathione.hydrolase5.proenzyme-like-GGT1.rhesus.   aactgttgggattacaggcatgagccaccatgctcggccttgattcttatttctacgta   81630
                                                                          * ***  *      *   ***  *    * *    *      *  *

Papio.anubis.clone.rp41-133b2.8034-40080.revcompl.Baboon      cttggtgcacccattacccaagcagtacac------------------------------   19620
BCRP3.HUMAN.NCBI.REF                                          tc-ctggcagcatttggtcttcctctaagcgcctggctccgctgttctcaggagtg----   19067
LOC106996293.glutathione.hydrolase5.proenzyme-like-GGT1.rhesus.   actgtttaagcgatgaattttcctctgagc-cctgcattagctgtaccctatagggtctg   81689
                                                                      * *  *        *  *   *

Papio.anubis.clone.rp41-133b2.8034-40080.revcompl.Baboon      ------------------------------------------------------------   19620
BCRP3.HUMAN.NCBI.REF                                          ------------------------------------------------------------   19067
LOC106996293.glutathione.hydrolase5.proenzyme-like-GGT1.rhesus.   atctgtgctgattatagtgcaacttgaagatgctgcccttttggtgttgatttcctctgt   81749

Papio.anubis.clone.rp41-133b2.8034-40080.revcompl.Baboon      ------------------------------------------------------------   19620
BCRP3.HUMAN.NCBI.REF                                          ------------------------------------------------------------   19067
LOC106996293.glutathione.hydrolase5.proenzyme-like-GGT1.rhesus.   gtaacaggatgtttcaagagccagctgtaagagtctctggctgggctgggaatgcacatt   81809

Papio.anubis.clone.rp41-133b2.8034-40080.revcompl.Baboon      ------------------------------------------------------------   19620
BCRP3.HUMAN.NCBI.REF                                          ------------------------------------------------------------   19067
LOC106996293.glutathione.hydrolase5.proenzyme-like-GGT1.rhesus.   ggctggcactgctggtggtgcagcattgatgtgagggcagggcctgggcaggttccccag   81869

Papio.anubis.clone.rp41-133b2.8034-40080.revcompl.Baboon      ------------------------------------------------------------   19620
BCRP3.HUMAN.NCBI.REF                                          ------------------------------------------------------------   19067
LOC106996293.glutathione.hydrolase5.proenzyme-like-GGT1.rhesus.   ggtgggatgagcagggagggagaggaatgggcttcctccagggcctcaggaaccaaggaa   81929

Papio.anubis.clone.rp41-133b2.8034-40080.revcompl.Baboon      ------------------------------------------------------------   19620
BCRP3.HUMAN.NCBI.REF                                          ------------------------------------------------------------   19067
LOC106996293.glutathione.hydrolase5.proenzyme-like-GGT1.rhesus.   gtctccgaactacctccaggcagggtcagatgtgcattttccaaagcttattgcattaag   81989

Papio.anubis.clone.rp41-133b2.8034-40080.revcompl.Baboon      ------------------------------------------------------------   19620
BCRP3.HUMAN.NCBI.REF                                          -----------------------------------------------------ggttctg   19074
LOC106996293.glutathione.hydrolase5.proenzyme-like-GGT1.rhesus.   agtgaggagtagactgtggtggtgccagcagcataaagactgaaaagaaataaggaggtg   82049

Papio.anubis.clone.rp41-133b2.8034-40080.revcompl.Baboon      ------------------------------------------------------------   19620
BCRP3.HUMAN.NCBI.REF                                          aagtctctggagaacaggatacgtggagggttaggaaggggcca----------------   19118
LOC106996293.glutathione.hydrolase5.proenzyme-like-GGT1.rhesus.   gagccagggctgaaccacacagctggacgggatgggcgggggcagtgggggggggattct   82109

Papio.anubis.clone.rp41-133b2.8034-40080.revcompl.Baboon      ------------------------------------------------------------   19620
BCRP3.HUMAN.NCBI.REF                                          ------------------------------------------------------------   19118
LOC106996293.glutathione.hydrolase5.proenzyme-like-GGT1.rhesus.   acaagtagcacaggagggaggcgaggcgggcaccacagagctttcagctgcctcacggct   82169

Papio.anubis.clone.rp41-133b2.8034-40080.revcompl.Baboon      ------------------------------------------------------------   19620
BCRP3.HUMAN.NCBI.REF                                          ------------------------------------------------------------   19118
LOC106996293.glutathione.hydrolase5.proenzyme-like-GGT1.rhesus.   gacttctatttggcttgttgagtccaaggtgcctgtgcaccttgtccagatgctctggag   82229

Papio.anubis.clone.rp41-133b2.8034-40080.revcompl.Baboon      ------------------------------------------------------------   19620
BCRP3.HUMAN.NCBI.REF                                          ------------------------------------------------------------   19118
LOC106996293.glutathione.hydrolase5.proenzyme-like-GGT1.rhesus.   gtggttggggaattgctcgtgtatgtgagtctgagactgcacacagctcggccaatgcca   82289

Papio.anubis.clone.rp41-133b2.8034-40080.revcompl.Baboon      ------------------------------------------------------------   19620
BCRP3.HUMAN.NCBI.REF                                          ------------------------------------------------------------   19118
LOC106996293.glutathione.hydrolase5.proenzyme-like-GGT1.rhesus.   acatggagatccatcagttcacaagtggtgaacgagccttctctgggtgggtggcagtcc   82349

Papio.anubis.clone.rp41-133b2.8034-40080.revcompl.Baboon      ------------------------------------------------------------   19620
BCRP3.HUMAN.NCBI.REF                                          ------ggcctagagacgggagactccctcccggagcaggtggaggcacaggaccattcg   19172
LOC106996293.glutathione.hydrolase5.proenzyme-like-GGT1.rhesus.   caggttggccaagagtccagggagtgcaccctcagtctgcaacagagttagaatccggca   82409

Papio.anubis.clone.rp41-133b2.8034-40080.revcompl.Baboon      ------------------------------------------------------------   19620
BCRP3.HUMAN.NCBI.REF                                          ctac---cccatctgccggcacctgcgggggagcccaggcattctttgtaagccctcctg   19229
LOC106996293.glutathione.hydrolase5.proenzyme-like-GGT1.rhesus.   ccacggagccagggttgcacccctgctgcggggaacaggagccccttttcctctc-cctg   82468

Papio.anubis.clone.rp41-133b2.8034-40080.revcompl.Baboon      ------------------------------------------------------------   19620
BCRP3.HUMAN.NCBI.REF                                          accacctggctcaaagaaaacagaagcatgg-----------------------------   19260
LOC106996293.glutathione.hydrolase5.proenzyme-like-GGT1.rhesus.   acatgcagggacaaagttatcatgctctgtgggtgccgctgggaggcactgataggaaag   82528

Papio.anubis.clone.rp41-133b2.8034-40080.revcompl.Baboon      ------------------------------------------------------------   19620
BCRP3.HUMAN.NCBI.REF                                          ------------------------------------------------------------   19260
LOC106996293.glutathione.hydrolase5.proenzyme-like-GGT1.rhesus.   actggccaggccttcccaagcaagctccaggcaacctcccagcaccccggcccagatgta   82588

Papio.anubis.clone.rp41-133b2.8034-40080.revcompl.Baboon      ------------------------------------------------------------   19620
BCRP3.HUMAN.NCBI.REF                                          ------------------------------------------------------------   19260
LOC106996293.glutathione.hydrolase5.proenzyme-like-GGT1.rhesus.   ggtgtggtctgggagcacagagccaggttcccaccgtggggcacctgaggtttgtgtcta   82648

Papio.anubis.clone.rp41-133b2.8034-40080.revcompl.Baboon      ------------------------------------------------------------   19620
BCRP3.HUMAN.NCBI.REF                                          ------------------------------------------------------------   19260
LOC106996293.glutathione.hydrolase5.proenzyme-like-GGT1.rhesus.   gcagccttttcttcctttggttcccttttcctgtttggcccacagggtttgggacaggtg   82708

Papio.anubis.clone.rp41-133b2.8034-40080.revcompl.Baboon      ------------------------------------------------------------   19620
BCRP3.HUMAN.NCBI.REF                                          ------------------------------------------------------------   19260
LOC106996293.glutathione.hydrolase5.proenzyme-like-GGT1.rhesus.   gctgctaccaacaggtctcaaggtggccccacagacctgagccagccacttctccatagc   82768

Papio.anubis.clone.rp41-133b2.8034-40080.revcompl.Baboon      ------------------------------------------------------------   19620
BCRP3.HUMAN.NCBI.REF                                          ------------------------------------------------------------   19260
LOC106996293.glutathione.hydrolase5.proenzyme-like-GGT1.rhesus.   cctcccctgcccatgaagactgctcagaggggccccattgagagctggatctgggacttt   82828

Papio.anubis.clone.rp41-133b2.8034-40080.revcompl.Baboon      ------------------------------------------------------------   19620
BCRP3.HUMAN.NCBI.REF                                          ------------------------------------------------------------   19260
LOC106996293.glutathione.hydrolase5.proenzyme-like-GGT1.rhesus.   ggttaaactgcaaggggagaagctctgtcaccctgcagggcgtgaagttcagctgaagcc   82888

Papio.anubis.clone.rp41-133b2.8034-40080.revcompl.Baboon      ------------------------------------------------------------   19620
BCRP3.HUMAN.NCBI.REF                                          ------------------------------------------------------------   19260
LOC106996293.glutathione.hydrolase5.proenzyme-like-GGT1.rhesus.   attgggaccatggcctgggagcccaactagggggaggctaccacacagagcgcctgagtt   82948

Papio.anubis.clone.rp41-133b2.8034-40080.revcompl.Baboon      ------------------------------------------------------------   19620
```

```
BCRP3.HUMAN.NCBI.REF                                           ------------------------------------------------------------    19260
LOC106996293.glutathione.hydrolase5.proenzyme-like-GGT1.rhesus.    ctggcagcggcccaagagagtcagtgatggcggcaggtctgcccgagggtgggccggctt    83008

Papio.anubis.clone.rp41-133b2.8034-40080.revcompl.Baboon           ------------------------------------------------------------    19620
BCRP3.HUMAN.NCBI.REF                                           ------------------------------------------------------------    19260
LOC106996293.glutathione.hydrolase5.proenzyme-like-GGT1.rhesus.    ggcccctcatctcaaagtgagccagcttttgctccctgaccttcctcctagtctgccaga    83068

Papio.anubis.clone.rp41-133b2.8034-40080.revcompl.Baboon           ------------------------------------------------------------    19620
BCRP3.HUMAN.NCBI.REF                                           ------------------------------------------------------------    19260
LOC106996293.glutathione.hydrolase5.proenzyme-like-GGT1.rhesus.    ccaccccttccagccacgcagtcaagcccacattaggaattagccccatccagcccctct    83128

Papio.anubis.clone.rp41-133b2.8034-40080.revcompl.Baboon           ------------------------------------------------------------    19620
BCRP3.HUMAN.NCBI.REF                                           --------aggccgccaagtattttcaagaaataatcccatgaacatggcatca---ctt    19309
LOC106996293.glutathione.hydrolase5.proenzyme-like-GGT1.rhesus.    cccacatacgacctccaaatatgtccatgtcccaatccctacaacccaagattagacctt    83188

Papio.anubis.clone.rp41-133b2.8034-40080.revcompl.Baboon           ------------------------------------------------------------    19620
BCRP3.HUMAN.NCBI.REF                                           ttttagaaagaggggcttggggcaggcagaggagagaagggagatcaaactgagagccaa    19369
LOC106996293.glutathione.hydrolase5.proenzyme-like-GGT1.rhesus.    atgtggcaagagtgactttgaagatatgattcaattaaggattttgagatggggagatta    83248

Papio.anubis.clone.rp41-133b2.8034-40080.revcompl.Baboon           ------------------------------------------------------------    19620
BCRP3.HUMAN.NCBI.REF                                           gttt--------------------------------------------------------    19373
LOC106996293.glutathione.hydrolase5.proenzyme-like-GGT1.rhesus.    tcctggaccatccaggtgggcccactatgcccccaaaattcacatgttgaaagctaatca    83308

Papio.anubis.clone.rp41-133b2.8034-40080.revcompl.Baboon           ------------------------------------------------------------    19620
BCRP3.HUMAN.NCBI.REF                                           ------------------------------------------------------------    19373
LOC106996293.glutathione.hydrolase5.proenzyme-like-GGT1.rhesus.    ccaatgtgatagtatcagcaggtggggcctttgggatgtggttaggtcatgagggtagag    83368

Papio.anubis.clone.rp41-133b2.8034-40080.revcompl.Baboon           ------------------------------------------------------------    19620
BCRP3.HUMAN.NCBI.REF                                           ------------------------------------------------ccagacggtcct    19385
LOC106996293.glutathione.hydrolase5.proenzyme-like-GGT1.rhesus.    ccctcatgaatgggattagcgccctataaaagagaccccagaagccgggcatagtgggct    83428

Papio.anubis.clone.rp41-133b2.8034-40080.revcompl.Baboon           ------------------------------------------------------------    19620
BCRP3.HUMAN.NCBI.REF                                           gcaggaggagaggatgcagctgcccagagggaagcaggatcacatttaaggaagtgtgtg    19445
LOC106996293.glutathione.hydrolase5.proenzyme-like-GGT1.rhesus.    gcacctacagaaccagctactgaggaggctgaggcaggaagatctcttgggtacagcaga    83488

Papio.anubis.clone.rp41-133b2.8034-40080.revcompl.Baboon           ------------------------------------------------------------    19620
BCRP3.HUMAN.NCBI.REF                                           gggtccctggatgacaccagcaccc-----------------------------------    19470
LOC106996293.glutathione.hydrolase5.proenzyme-like-GGT1.rhesus.    gagctctatgatcacgccactgcactccagccttggtgacagagtgagaccctgtctcta    83548

Papio.anubis.clone.rp41-133b2.8034-40080.revcompl.Baboon           ------------------------------------------------------------    19620
BCRP3.HUMAN.NCBI.REF                                           ------------------------------------------------------------    19470
LOC106996293.glutathione.hydrolase5.proenzyme-like-GGT1.rhesus.    aagtaattaaataaaagggaccccagagagctagctagcttcttccactatgtcagttag    83608

Papio.anubis.clone.rp41-133b2.8034-40080.revcompl.Baboon           ------------------------------------------------------------    19620
BCRP3.HUMAN.NCBI.REF                                           ------------------------------------------------------------    19470
LOC106996293.glutathione.hydrolase5.proenzyme-like-GGT1.rhesus.    aaggcgccatataggccgggcgcggtggctcaagcctgtaatcccagcactttgggaggc    83668

Papio.anubis.clone.rp41-133b2.8034-40080.revcompl.Baboon           ------------------------------------------------------------    19620
BCRP3.HUMAN.NCBI.REF                                           ------------------------------------------------------------    19470
LOC106996293.glutathione.hydrolase5.proenzyme-like-GGT1.rhesus.    cgagacaggcggatcacgaggtcaggagatcgagaccatcctggctaacacggtgaaacc    83728

Papio.anubis.clone.rp41-133b2.8034-40080.revcompl.Baboon           ------------------------------------------------------------    19620
BCRP3.HUMAN.NCBI.REF                                           ------------------------------------------------------------    19470
LOC106996293.glutathione.hydrolase5.proenzyme-like-GGT1.rhesus.    ctgtctctactaaaaatacaagaaaattagccgggcgaggtggcaggcgcctgtagtccc    83788

Papio.anubis.clone.rp41-133b2.8034-40080.revcompl.Baboon           ------------------------------------------------------------    19620
BCRP3.HUMAN.NCBI.REF                                           ------------------------------------------------------------    19470
LOC106996293.glutathione.hydrolase5.proenzyme-like-GGT1.rhesus.    agctactcgggaggctgaggcaggagaatggcgtgaacccgggaggcggagcttgcagtg    83848

Papio.anubis.clone.rp41-133b2.8034-40080.revcompl.Baboon           ------------------------------------------------------------    19620
BCRP3.HUMAN.NCBI.REF                                           ------------------------------------------------------------    19470
LOC106996293.glutathione.hydrolase5.proenzyme-like-GGT1.rhesus.    agccgagatcgcgctactgcactccagcctggggcacagagcaagactccgtctcaaaaa    83908

Papio.anubis.clone.rp41-133b2.8034-40080.revcompl.Baboon           ------------------------------------------------------------    19620
BCRP3.HUMAN.NCBI.REF                                           ------------------------------------------------------------    19470
LOC106996293.glutathione.hydrolase5.proenzyme-like-GGT1.rhesus.    aaaaaaaaaaaaaaagaaggcgccatatatgaggacgtgggccctcacaggacatcaaat    83968

Papio.anubis.clone.rp41-133b2.8034-40080.revcompl.Baboon           ------------------------------------------------------------    19620
BCRP3.HUMAN.NCBI.REF                                           ------------------------------------------------------------    19470
LOC106996293.glutathione.hydrolase5.proenzyme-like-GGT1.rhesus.    ctgccagcaccttgatcttggacttcccagcctccagcgctgtgagcaataaatttctgt    84028

Papio.anubis.clone.rp41-133b2.8034-40080.revcompl.Baboon           ------------------------------------------------------------    19620
BCRP3.HUMAN.NCBI.REF                                           ------------------------------------------------------------    19470
LOC106996293.glutathione.hydrolase5.proenzyme-like-GGT1.rhesus.    aaaatgctataagccacccactttccagtatttttgttatggcagcccaggcagactaaa    84088

Papio.anubis.clone.rp41-133b2.8034-40080.revcompl.Baboon           ------------------------------------------------------------    19620
BCRP3.HUMAN.NCBI.REF                                           ------------------------------------------------------------    19470
LOC106996293.glutathione.hydrolase5.proenzyme-like-GGT1.rhesus.    acaacctctgtggcacatttggggtgatgtaggaacagccgatgttcccaggaaccagtg    84148

Papio.anubis.clone.rp41-133b2.8034-40080.revcompl.Baboon           ------------------------------------------------------------    19620
BCRP3.HUMAN.NCBI.REF                                           ------------------------------------------------------------    19470
LOC106996293.glutathione.hydrolase5.proenzyme-like-GGT1.rhesus.    aggggcaggagtcctagatccctctagcgagatccccagagagcgccatgtacccgccct    84208

Papio.anubis.clone.rp41-133b2.8034-40080.revcompl.Baboon           ------------------------------------------------------------    19620
BCRP3.HUMAN.NCBI.REF                                           ------------------------------------------------------------    19470
LOC106996293.glutathione.hydrolase5.proenzyme-like-GGT1.rhesus.    gcagctggcaaagctgtttatggaagttaggggcctgttggtttagagcggccattctca    84268

Papio.anubis.clone.rp41-133b2.8034-40080.revcompl.Baboon           ------------------------------------------------------------    19620
BCRP3.HUMAN.NCBI.REF                                           --------agtgcggctctgtctggcaaccgctcccaaggtggcaggagtgggtgt----    19518
LOC106996293.glutathione.hydrolase5.proenzyme-like-GGT1.rhesus.    accaggggggatgtggcactgtcttgagacagtt--ttggttgtcatgagtgggtgtgtgt    84326

Papio.anubis.clone.rp41-133b2.8034-40080.revcompl.Baboon           ------------------------------------------------------------    19620
BCRP3.HUMAN.NCBI.REF                                           ------------------------------------------------------------    19518
LOC106996293.glutathione.hydrolase5.proenzyme-like-GGT1.rhesus.    ggtggggagtgctaccgcatcacacaggctgaggcacgggatgctgctcaacatcctaca    84386

Papio.anubis.clone.rp41-133b2.8034-40080.revcompl.Baboon           ------------------------------------------------------------    19620
BCRP3.HUMAN.NCBI.REF                                           ------------------------------------------------------------    19518
LOC106996293.glutathione.hydrolase5.proenzyme-like-GGT1.rhesus.    gtgtggggacggccctcacaacaaagaatcacttcattcaaaacatggatgagggccagg    84446

Papio.anubis.clone.rp41-133b2.8034-40080.revcompl.Baboon           ------------------------------------------------------------    19620
BCRP3.HUMAN.NCBI.REF                                           ------------------------------------------------------------    19518
LOC106996293.glutathione.hydrolase5.proenzyme-like-GGT1.rhesus.    cgcagtgactcacacctgtaatcccagcattttaggaggccgaagcaggaggattgtttg    84506

Papio.anubis.clone.rp41-133b2.8034-40080.revcompl.Baboon           ------------------------------------------------------------    19620
BCRP3.HUMAN.NCBI.REF                                           -------------------cccctgtgtgtcagtgggcagctcctgctgaacccacagct    19559
LOC106996293.glutathione.hydrolase5.proenzyme-like-GGT1.rhesus.    aagccaggagtttgagaccagcctgggcaacatggtgaaaccccatctttacaaaaaaag    84566

Papio.anubis.clone.rp41-133b2.8034-40080.revcompl.Baboon           ------------------------------------------------------------    19620
BCRP3.HUMAN.NCBI.REF                                           cactggggagcctgacagtggggccatgtgcctgacactcctctctgcttgtggacctgg    19619
LOC106996293.glutathione.hydrolase5.proenzyme-like-GGT1.rhesus.    caaaaaaccagctgggcgtggcgatgcacacctgt-actcccagctacttggaagcctga    84625

Papio.anubis.clone.rp41-133b2.8034-40080.revcompl.Baboon           ------------------------------------------------------------    19620
BCRP3.HUMAN.NCBI.REF                                           caaggcagggagcagaaaacagagctacttgaag--------------------------    19653
LOC106996293.glutathione.hydrolase5.proenzyme-like-GGT1.rhesus.    ggtgggaggatggcttgagcccaggaagtcaaggctggagtgagctgtgattgtaacagt    84685

Papio.anubis.clone.rp41-133b2.8034-40080.revcompl.Baboon           ------------------------------------------------------------    19620
```

```
BCRP3.HUMAN.NCBI.REF                                                  ------------------------------------------------------------    19653
LOC106996293.glutathione.hydrolase5.proenzyme-like-GGT1.rhesus.       acactccaacctgggcaacagagcgagatgctgcctcaaaaaaaaaaaaaaaaattgtac    84745

Papio.anubis.clone.rp41-133b2.8034-40080.revcompl.Baboon              ------------------------------------------------------------    19620
BCRP3.HUMAN.NCBI.REF                                                  ------------------------------------------------------------    19653
LOC106996293.glutathione.hydrolase5.proenzyme-like-GGT1.rhesus.       atatagtaaaaattatcaccaatgccctaatgccccagaagaaaatcactttacagactg    84805

Papio.anubis.clone.rp41-133b2.8034-40080.revcompl.Baboon              ------------------------------------------------------------    19620
BCRP3.HUMAN.NCBI.REF                                                  ------------------------------------------------------------    19653
LOC106996293.glutathione.hydrolase5.proenzyme-like-GGT1.rhesus.       ccacaggaaaaacaacctcttttacaaataaagtcacaaatataaattaaaaattacaca    84865

Papio.anubis.clone.rp41-133b2.8034-40080.revcompl.Baboon              ------------------------------------------------------------    19620
BCRP3.HUMAN.NCBI.REF                                                  ------------------------------------------------------------    19653
LOC106996293.glutathione.hydrolase5.proenzyme-like-GGT1.rhesus.       aggaaaaagtcaccatgagggagagtctgagaggccccagccaggaaactaaaactcact    84925

Papio.anubis.clone.rp41-133b2.8034-40080.revcompl.Baboon              ------------------------------------------------------------    19620
BCRP3.HUMAN.NCBI.REF                                                  ---------------------------------------gctttctgtctgcgtctgtgt    19674
LOC106996293.glutathione.hydrolase5.proenzyme-like-GGT1.rhesus.       tctgaaaaatggcatcaaccacagaccagagaccaaggtgctgagattctcagcctggga    84985

Papio.anubis.clone.rp41-133b2.8034-40080.revcompl.Baboon              ------------------------------------------------------------    19620
BCRP3.HUMAN.NCBI.REF                                                  gcagtgtggatttagttgtgctttttacttgctgggagagcaca-----------gccac    19723
LOC106996293.glutathione.hydrolase5.proenzyme-like-GGT1.rhesus.       gcagtgtagacaacaccctgctctctgtcttggaggccacggaactggcctggaggccac    85045

Papio.anubis.clone.rp41-133b2.8034-40080.revcompl.Baboon              ------------------------------------------------------------    19620
BCRP3.HUMAN.NCBI.REF                                                  catttacaagcagtgtcaccctcgtgggtggcgaggacagaacaggagcctctgctctct    19783
LOC106996293.glutathione.hydrolase5.proenzyme-like-GGT1.rhesus.       agtggcctcacaaggtccccaactcctatggtccccaaagctgaagtgtacctggcctaa    85105

Papio.anubis.clone.rp41-133b2.8034-40080.revcompl.Baboon              ------------------------------------------------------------    19620
BCRP3.HUMAN.NCBI.REF                                                  gtacctatctgg------------------------------------------------    19795
LOC106996293.glutathione.hydrolase5.proenzyme-like-GGT1.rhesus.       gaatctcagtgactgagttcctctcaggtggctttgagcagcatggacctggggagtggg    85165

Papio.anubis.clone.rp41-133b2.8034-40080.revcompl.Baboon              ------------------------------------------------------------    19620
BCRP3.HUMAN.NCBI.REF                                                  ------------------------------------------------------------    19795
LOC106996293.glutathione.hydrolase5.proenzyme-like-GGT1.rhesus.       ctctgccctaggcatccagcttggacctcagcctcacctggctgaagctgggctcgtact    85225

Papio.anubis.clone.rp41-133b2.8034-40080.revcompl.Baboon              ------------------------------------------------------------    19620
BCRP3.HUMAN.NCBI.REF                                                  ------------------------------------------------------------    19795
LOC106996293.glutathione.hydrolase5.proenzyme-like-GGT1.rhesus.       ccacacggcccttactgttgacatgcaggatgggggctgcaatggccgctctcaggtcaa    85285

Papio.anubis.clone.rp41-133b2.8034-40080.revcompl.Baboon              ------------------------------------------------------------    19620
BCRP3.HUMAN.NCBI.REF                                                  ------------------------------------------------------------    19795
LOC106996293.glutathione.hydrolase5.proenzyme-like-GGT1.rhesus.       agccaagccacagcttgttcatgatggcctgggggagagatgagggatgcagggagaaag    85345

Papio.anubis.clone.rp41-133b2.8034-40080.revcompl.Baboon              ------------------------------------------------------------    19620
BCRP3.HUMAN.NCBI.REF                                                  ------------------------------------------------------------    19795
LOC106996293.glutathione.hydrolase5.proenzyme-like-GGT1.rhesus.       ggggtaccctctaccctacttcagccctcacatccccactccagggagagtcagacagta    85405

Papio.anubis.clone.rp41-133b2.8034-40080.revcompl.Baboon              ------------------------------------------------------------    19620
BCRP3.HUMAN.NCBI.REF                                                  ------------------------------------------------------------    19795
LOC106996293.glutathione.hydrolase5.proenzyme-like-GGT1.rhesus.       tgctgcccagagaggagacactggagcccagagcccccccagactcacctgggccacggc    85465

Papio.anubis.clone.rp41-133b2.8034-40080.revcompl.Baboon              ------------------------------------------------------------    19620
BCRP3.HUMAN.NCBI.REF                                                  ------------------------------------------------------------    19795
LOC106996293.glutathione.hydrolase5.proenzyme-like-GGT1.rhesus.       agagatgatgagctccccaccagccccgccaatcactagcttcgacccctgggctttgct    85525

Papio.anubis.clone.rp41-133b2.8034-40080.revcompl.Baboon              ------------------------------------------------------------    19620
BCRP3.HUMAN.NCBI.REF                                                  ------------------------------------------------------------    19795
LOC106996293.glutathione.hydrolase5.proenzyme-like-GGT1.rhesus.       gatcaggatggagggcaccatggaggacggggaccgctcgcctggaactgggggccagca    85585

Papio.anubis.clone.rp41-133b2.8034-40080.revcompl.Baboon              --------------------------------------------------------actg    19624
BCRP3.HUMAN.NCBI.REF                                                  -gcccggtgggctcccttgtcctggcttccatctctgtctcagcgaccattca---gccc    19851
LOC106996293.glutathione.hydrolase5.proenzyme-like-GGT1.rhesus.       ccctccgggagctccatccaccctgtctccattcactgctgagcaaacagcagcttagcg    85645

Papio.anubis.clone.rp41-133b2.8034-40080.revcompl.Baboon              tacctagtgtgtagtcttttatccctcatctgcctcccacccttccccttgagttcccac    19684
BCRP3.HUMAN.NCBI.REF                                                  tgcacaggaacacatgttgcttagaaaagccaaatccagcccttgtctctgcctcctctg    19911
LOC106996293.glutathione.hydrolase5.proenzyme-like-GGT1.rhesus.       agtgcagtgacccatccaggggtcagcatgcagccccagcccagccccctctctcctctc    85705
                                                                           **       *            *       **  ***    *  *   * * *

Papio.anubis.clone.rp41-133b2.8034-40080.revcompl.Baboon              g-----------------------------------------------------------    19685
BCRP3.HUMAN.NCBI.REF                                                  gtctcatgatgtgcatctgttaccttgaaactggaaaccagtctatcaatgtctgtgcca    19971
LOC106996293.glutathione.hydrolase5.proenzyme-like-GGT1.rhesus.       ccctggtgggggcccttgtggaccccgggtgggaagccttgttctcaccaggtgagggg    85765

Papio.anubis.clone.rp41-133b2.8034-40080.revcompl.Baboon              ------------------------------------------------------------    19685
BCRP3.HUMAN.NCBI.REF                                                  atttttattccctccccaacctccttccccatacg---acttttatttatgtaggatg    20028
LOC106996293.glutathione.hydrolase5.proenzyme-like-GGT1.rhesus.       gtagtgccggaaccccgggggcatcgctcgcataagtccaggagctcgttgttgaggatg    85825

Papio.anubis.clone.rp41-133b2.8034-40080.revcompl.Baboon              ------------------------------------------------------------    19685
BCRP3.HUMAN.NCBI.REF                                                  tgtgctgtctaatg----------------------------------------------    20042
LOC106996293.glutathione.hydrolase5.proenzyme-like-GGT1.rhesus.       atgcctgtccatggtgaatacaccatcgctccaaagctgcagccgtggaggcagagcctg    85885

Papio.anubis.clone.rp41-133b2.8034-40080.revcompl.Baboon              ------------------------------------------------------------    19685
BCRP3.HUMAN.NCBI.REF                                                  -------------------atgggatgaccacacttttccatgttctaaaagtgctcctc    20083
LOC106996293.glutathione.hydrolase5.proenzyme-like-GGT1.rhesus.       ggctcccaccagacaccgcctgccctgcccaccttgcaggacccccctcactttacagctg    85945

Papio.anubis.clone.rp41-133b2.8034-40080.revcompl.Baboon              ------------------------------------------------------------    19685
BCRP3.HUMAN.NCBI.REF                                                  tcccacagggtcccagggctggtggttgctttgggtctacagctacgtc-----------    20132
LOC106996293.glutathione.hydrolase5.proenzyme-like-GGT1.rhesus.       ccccgaagcatgctggagccactgttctctccctggcatcacccaggaggggtccccaaa    86005

Papio.anubis.clone.rp41-133b2.8034-40080.revcompl.Baboon              ------------------------------------------------------------    19685
BCRP3.HUMAN.NCBI.REF                                                  ---------------------------------------ttacccgcctcctgcctcaac    20153
LOC106996293.glutathione.hydrolase5.proenzyme-like-GGT1.rhesus.       gacccacctgtgcccccatatcagggcacccttctgagtgaggccgcccattcccaggta    86065

Papio.anubis.clone.rp41-133b2.8034-40080.revcompl.Baboon              ------------------------------------------------------------    19685
BCRP3.HUMAN.NCBI.REF                                                  agcctgtgtggtggcaaagccggtgtggggctggggaacgcagcgttctccaggaggga    20213
LOC106996293.glutathione.hydrolase5.proenzyme-like-GGT1.rhesus.       agtcttggctcggggccaccccagcagcagcctttccagccagagatgtcaggggtgggg    86125

Papio.anubis.clone.rp41-133b2.8034-40080.revcompl.Baboon              ------------------------------------------------------------    19685
BCRP3.HUMAN.NCBI.REF                                                  cccggctctccttctgcagtgcaggcgaaggcctagatgccagtgtgacctcccacaagg    20273
LOC106996293.glutathione.hydrolase5.proenzyme-like-GGT1.rhesus.       gcctcttttgaaacagaggaggagacaaaggccaaaagcaaggt----cctgccccatgg    86181

Papio.anubis.clone.rp41-133b2.8034-40080.revcompl.Baboon              ------------------------------------------------------------    19685
BCRP3.HUMAN.NCBI.REF                                                  cgtggcttccagactccccggccggaagtgatgctttttttgccgcgggccctgggtttga    20333
LOC106996293.glutathione.hydrolase5.proenzyme-like-GGT1.rhesus.       ggacaagtgaggggacatggggctgggggagtgcaggtctaggagaggaggagtgaggcc    86241

Papio.anubis.clone.rp41-133b2.8034-40080.revcompl.Baboon              ------------------------------------------------------------    19685
BCRP3.HUMAN.NCBI.REF                                                  agcagcct----------------------------------------------------    20341
LOC106996293.glutathione.hydrolase5.proenzyme-like-GGT1.rhesus.       accagccttcccccaggccccatgcacggtgtgttgatggtgctggtggcagccacggcg    86301

Papio.anubis.clone.rp41-133b2.8034-40080.revcompl.Baboon              ------------------------------------------------------------    19685
BCRP3.HUMAN.NCBI.REF                                                  ------------------------------------------------------------    20341
LOC106996293.glutathione.hydrolase5.proenzyme-like-GGT1.rhesus.       ctgccatcctcccccagcacagacatgggacatgcctgtcccatggccccaggcctcggc    86361

Papio.anubis.clone.rp41-133b2.8034-40080.revcompl.Baboon              ------------------------------------------------------------    19685
BCRP3.HUMAN.NCBI.REF                                                  ------------------------------------------------------------    20341
LOC106996293.glutathione.hydrolase5.proenzyme-like-GGT1.rhesus.       caagctgtagtgactgagctggtggtccccccggccatcgaactgttggcggatgagctg    86421

Papio.anubis.clone.rp41-133b2.8034-40080.revcompl.Baboon              ------------------------------------------------------------    19685
```

```
BCRP3.HUMAN.NCBI.REF                                                  ------------------------------------------------------------    20341
LOC106996293.glutathione.hydrolase5.proenzyme-like-GGT1.rhesus.       ggccagggtctcccccagcaggtcctgggaggcattctggggtgcgtgggcaagtggcag    86481

Papio.anubis.clone.rp41-133b2.8034-40080.revcompl.Baboon              ------------------------------------------------------------    19685
BCRP3.HUMAN.NCBI.REF                                                  ------------------------------------------------------------    20341
LOC106996293.glutathione.hydrolase5.proenzyme-like-GGT1.rhesus.       ggtcagtgaggggcaaggcgggatgtgcagaaccaagggcaggacaagctcagcccccat    86541

Papio.anubis.clone.rp41-133b2.8034-40080.revcompl.Baboon              ------------------------------------------------------------    19685
BCRP3.HUMAN.NCBI.REF                                                  ------------------------------------------------------------    20341
LOC106996293.glutathione.hydrolase5.proenzyme-like-GGT1.rhesus.       gggggccacctccagcctgtcctctaccttgtggccaggcctccctcaagcccaggctag    86601

Papio.anubis.clone.rp41-133b2.8034-40080.revcompl.Baboon              ------------------------------------------------------------    19685
BCRP3.HUMAN.NCBI.REF                                                  ------------------------------------------------------------    20341
LOC106996293.glutathione.hydrolase5.proenzyme-like-GGT1.rhesus.       gtctagactctgcccaccctccaggtcctttgagatatggtgccactcctgcatctgacc    86661

Papio.anubis.clone.rp41-133b2.8034-40080.revcompl.Baboon              ------------------------------------------------------------    19685
BCRP3.HUMAN.NCBI.REF                                                  ------------------------------------------------------------    20341
LOC106996293.glutathione.hydrolase5.proenzyme-like-GGT1.rhesus.       cctgtacctgccaccccgggacccctttcagtcctgccctggcctagaaccacatacatc    86721

Papio.anubis.clone.rp41-133b2.8034-40080.revcompl.Baboon              ------------------------------------------------------------    19685
BCRP3.HUMAN.NCBI.REF                                                  ------------------------------------------------------------    20341
LOC106996293.glutathione.hydrolase5.proenzyme-like-GGT1.rhesus.       tccctccaacctcaccctgctgggcctgacccttctttcatgctaagtctcaagccaggg    86781

Papio.anubis.clone.rp41-133b2.8034-40080.revcompl.Baboon              ------------------------------------------------------------    19685
BCRP3.HUMAN.NCBI.REF                                                  ------------------------------------------------------------    20341
LOC106996293.glutathione.hydrolase5.proenzyme-like-GGT1.rhesus.       aggaggacagcccccagcccagcaacctcagtaacctcacctggagcttcgggtggctg    86841

Papio.anubis.clone.rp41-133b2.8034-40080.revcompl.Baboon              ------------------------------------------------------------    19685
BCRP3.HUMAN.NCBI.REF                                                  ------------------------------------------------------------    20341
LOC106996293.glutathione.hydrolase5.proenzyme-like-GGT1.rhesus.       cgagggtcccccagcctccacctctgccccctggcaaacttgagcttctccacaaggtgg    86901

Papio.anubis.clone.rp41-133b2.8034-40080.revcompl.Baboon              ------------------------------------------------------------    19685
BCRP3.HUMAN.NCBI.REF                                                  ------------------------------------------------------------    20341
LOC106996293.glutathione.hydrolase5.proenzyme-like-GGT1.rhesus.       tggtacatgttcaccctcccctcaggcctggccacagactctgctgagaagctgaaccct    86961

Papio.anubis.clone.rp41-133b2.8034-40080.revcompl.Baboon              ------------------------------------------------------------    19685
BCRP3.HUMAN.NCBI.REF                                                  ------------------------------------------------------------    20341
LOC106996293.glutathione.hydrolase5.proenzyme-like-GGT1.rhesus.       ggagagggttcagggcacaggttgcggtcaccctatgtaatggattgaatgttgcccccc    87021

Papio.anubis.clone.rp41-133b2.8034-40080.revcompl.Baboon              ------------------------------------------------------------    19685
BCRP3.HUMAN.NCBI.REF                                                  ------------------------------------------------------------    20341
LOC106996293.glutathione.hydrolase5.proenzyme-like-GGT1.rhesus.       ccgcagaaggattcacccacatcctaattcccaaaacctgtggatactaccttatacagc    87081

Papio.anubis.clone.rp41-133b2.8034-40080.revcompl.Baboon              ---------------------------------------------gtccattatagcatt    19700
BCRP3.HUMAN.NCBI.REF                                                  ------------------------------------------------------------    20341
LOC106996293.glutathione.hydrolase5.proenzyme-like-GGT1.rhesus.       acaacagtgaccttaagtggaaaagtatgttaaatatatatatataattttttttttt    87141

Papio.anubis.clone.rp41-133b2.8034-40080.revcompl.Baboon              atccccctgagacagagtctcgctctgtcaccaaagctggggtgcaatggtgc-atctcg    19759
BCRP3.HUMAN.NCBI.REF                                                  ----------------ggctttctcttggtaagtggctggtgtcttagcagctgcaatct    20385
LOC106996293.glutathione.hydrolase5.proenzyme-like-GGT1.rhesus.       ttttttttgagacagagtctcactctgttgcccaggctggagtgcaatggccctgtctca    87201
                                                                                      * **  ****         ***** **   *          **

Papio.anubis.clone.rp41-133b2.8034-40080.revcompl.Baboon              gttcactacaacctccgccacctggattcaagtgattttcctgcctcagcctcc-caagt    19818
BCRP3.HUMAN.NCBI.REF                                                  gagctcagccacctacaca-ccaccgtggccgacactttcattaaaagtttcctgagac    20444
LOC106996293.glutathione.hydrolase5.proenzyme-like-GGT1.rhesus.       gctcactgcaacctccgcctcccaggttccagtgattcttgtgcctcagtctcctgaaac    87261
                                                                      *  * *  * **** * *  **    *    *  * * *  *     **  ***  *

Papio.anubis.clone.rp41-133b2.8034-40080.revcompl.Baboon              agcgcctgccaccacatctttttttttttttttttgtattattagtaaagatggggttt    19878
BCRP3.HUMAN.NCBI.REF                                                  ga----------------------------------------------------------    20446
LOC106996293.glutathione.hydrolase5.proenzyme-like-GGT1.rhesus.       tacaggtgttcgccaccatgcccaactaattttttttgtatgtttagtagggacaaggttt    87321

Papio.anubis.clone.rp41-133b2.8034-40080.revcompl.Baboon              caccatgttggccaggctggtcttgaactcctgacctcaggtgatccgcctgccttgtcc    19938
BCRP3.HUMAN.NCBI.REF                                                  ------------------------------------------------------------    20446
LOC106996293.glutathione.hydrolase5.proenzyme-like-GGT1.rhesus.       tgccatgttgttcaggctggtctcgaactcctggcctcaagtgatctgcctgccttggcc    87381

Papio.anubis.clone.rp41-133b2.8034-40080.revcompl.Baboon              tcccaaagtgctgggattataggtgtgagccaccatgctgggc-----------------    19981
BCRP3.HUMAN.NCBI.REF                                                  ------------------------------------------------------------    20446
LOC106996293.glutathione.hydrolase5.proenzyme-like-GGT1.rhesus.       tcccaaagtgctaggatcacaggtgtgaaccaccatgcccggcctttttttgtgtgttttg    87441

Papio.anubis.clone.rp41-133b2.8034-40080.revcompl.Baboon              ------------------------------------------------------------    19981
BCRP3.HUMAN.NCBI.REF                                                  ------------------------------------------------------------    20446
LOC106996293.glutathione.hydrolase5.proenzyme-like-GGT1.rhesus.       ttttgttttttttttttttgagagggagtcttgctctatcacccaggctggagtgtgtggc    87501

Papio.anubis.clone.rp41-133b2.8034-40080.revcompl.Baboon              ------------------------------------------------------------    19981
BCRP3.HUMAN.NCBI.REF                                                  ------------------------------------------------------------    20446
LOC106996293.glutathione.hydrolase5.proenzyme-like-GGT1.rhesus.       acgatcttgacatgactaaaatagtccctatgaagccaatagggccattggtatcatgta    87561

Papio.anubis.clone.rp41-133b2.8034-40080.revcompl.Baboon              ------------------------------------------------------------    19981
BCRP3.HUMAN.NCBI.REF                                                  ------------------------------------------------------------    20446
LOC106996293.glutathione.hydrolase5.proenzyme-like-GGT1.rhesus.       gaaggatacaagacacgaaacactgtgtgccagcagactggtatcatgtctgtgtcttta    87621

Papio.anubis.clone.rp41-133b2.8034-40080.revcompl.Baboon              ------------------------------------------------------------    19981
BCRP3.HUMAN.NCBI.REF                                                  ------------------------------------------------------------    20446
LOC106996293.glutathione.hydrolase5.proenzyme-like-GGT1.rhesus.       gcaggagtcctcacagctgtccacacttcagtccaggggctattctctggattctgctcc    87681

Papio.anubis.clone.rp41-133b2.8034-40080.revcompl.Baboon              ------------------------------------------------------------    19981
BCRP3.HUMAN.NCBI.REF                                                  ------------------------------------------------------------    20446
LOC106996293.glutathione.hydrolase5.proenzyme-like-GGT1.rhesus.       ccaagtgacacctcaggtgcaaaggaacaaggtcacatgggtgggcacagggctacccgg    87741

Papio.anubis.clone.rp41-133b2.8034-40080.revcompl.Baboon              ------------------------------------------------------------    19981
BCRP3.HUMAN.NCBI.REF                                                  ------------------------------------------------------------    20446
LOC106996293.glutathione.hydrolase5.proenzyme-like-GGT1.rhesus.       cttagaagcctgtgtcccaaaaagaaactgagatttcttttaacagtcccacggtcaaag    87801

Papio.anubis.clone.rp41-133b2.8034-40080.revcompl.Baboon              ------------------------------------------------------------    19981
BCRP3.HUMAN.NCBI.REF                                                  ------------------------------------------------------------    20446
LOC106996293.glutathione.hydrolase5.proenzyme-like-GGT1.rhesus.       ttttcaagagtcccagcaatggacatgcctagttacaaacaccactgcactcagggaaac    87861

Papio.anubis.clone.rp41-133b2.8034-40080.revcompl.Baboon              ------------------------------------------------------------    19981
BCRP3.HUMAN.NCBI.REF                                                  ------------------------------------------------------------    20446
LOC106996293.glutathione.hydrolase5.proenzyme-like-GGT1.rhesus.       ccaaagtatggtttacctcttgatttttctccaaagtaaaccaccaaaaagttgaccat    87921

Papio.anubis.clone.rp41-133b2.8034-40080.revcompl.Baboon              ------------------------------------------------------------    19981
BCRP3.HUMAN.NCBI.REF                                                  ------------------------------------------------------------    20446
LOC106996293.glutathione.hydrolase5.proenzyme-like-GGT1.rhesus.       agacttcaaaacatttctaacaatagcaaagttcagttgatggcagacttcaaaaatcgc    87981

Papio.anubis.clone.rp41-133b2.8034-40080.revcompl.Baboon              ------------------------------------------------------------    19981
BCRP3.HUMAN.NCBI.REF                                                  ------------------------------------------------------------    20446
LOC106996293.glutathione.hydrolase5.proenzyme-like-GGT1.rhesus.       aattactcttctctaactctagatgcccccattgtttacaattcattcatagctctccca    88041

Papio.anubis.clone.rp41-133b2.8034-40080.revcompl.Baboon              ------------------------------------------------------------    19981
BCRP3.HUMAN.NCBI.REF                                                  ------------------------------------------------------------    20446
LOC106996293.glutathione.hydrolase5.proenzyme-like-GGT1.rhesus.       attcagatgatgatgaccaactatgggccattttatatgtagtctggtaacccaacaca    88101

Papio.anubis.clone.rp41-133b2.8034-40080.revcompl.Baboon              ------------------------------------------------------------    19981
BCRP3.HUMAN.NCBI.REF                                                  ------------------------------------------------------------    20446
LOC106996293.glutathione.hydrolase5.proenzyme-like-GGT1.rhesus.       gatgacattccgccttttccaggtttccaagggaactgactcagaaagttaacccaagtt    88161

Papio.anubis.clone.rp41-133b2.8034-40080.revcompl.Baboon              ------------------------------------------------------------    19981
```

```
BCRP3.HUMAN.NCBI.REF                                             ------------------------------------------------------------    20446
LOC106996293.glutathione.hydrolase5.proenzyme-like-GGT1.rhesus.  catatttatttctagagaaagataaaaaaaattttctaactttattcatgcatacctaat    88221

Papio.anubis.clone.rp41-133b2.8034-40080.revcompl.Baboon         ------------------------------------------------------------    19981
BCRP3.HUMAN.NCBI.REF                                             ------------------------------------------------------------    20446
LOC106996293.glutathione.hydrolase5.proenzyme-like-GGT1.rhesus.  acaagggtacacgaaatttctgagagaccttcaacctcactgccaaattttcaagagctg    88281

Papio.anubis.clone.rp41-133b2.8034-40080.revcompl.Baboon         ------------------------------------------------------------    19981
BCRP3.HUMAN.NCBI.REF                                             ------------------------------------------------------------    20446
LOC106996293.glutathione.hydrolase5.proenzyme-like-GGT1.rhesus.  tgacaaaccttaatgtcaaaccaagactgattttatagcagaactgccatcacatctgtc    88341

Papio.anubis.clone.rp41-133b2.8034-40080.revcompl.Baboon         ------------------------------------------------------------    19981
BCRP3.HUMAN.NCBI.REF                                             ------------------------------------------------------------    20446
LOC106996293.glutathione.hydrolase5.proenzyme-like-GGT1.rhesus.  aggtggatctgccatggactttttttgtgtcgtcttccccatgtctctgagttgttgaaag    88401

Papio.anubis.clone.rp41-133b2.8034-40080.revcompl.Baboon         --------------------------tccattatatcattccggttttttggagatggag    20015
BCRP3.HUMAN.NCBI.REF                                             ------------------------------------------------------------    20446
LOC106996293.glutathione.hydrolase5.proenzyme-like-GGT1.rhesus.  tatcacagccccaaacacatttgtttttctttcgcttttttttttttttggagatggag    88461

Papio.anubis.clone.rp41-133b2.8034-40080.revcompl.Baboon         tttcactcttgtcacccaggctggagtgcaacgcaacggtgagctctcagctcactgcaa    20075
BCRP3.HUMAN.NCBI.REF                                             ------------------------------------------------------------    20446
LOC106996293.glutathione.hydrolase5.proenzyme-like-GGT1.rhesus.  tctcactc-tatctctcaggctggagagcagtggcac-----aatctcggctcactgcaa    88515

Papio.anubis.clone.rp41-133b2.8034-40080.revcompl.Baboon         cctccacctcccg-gttcaagcgattctcctgcctcagcctccctagtagctaggactac    20134
BCRP3.HUMAN.NCBI.REF                                             ------------------------------------------------------------    20446
LOC106996293.glutathione.hydrolase5.proenzyme-like-GGT1.rhesus.  cctccgcctcctgagttcaagcgattctcctgcctcagcctccagagtagctgggactac    88575

Papio.anubis.clone.rp41-133b2.8034-40080.revcompl.Baboon         ccatgtgtgccaccatgcccagctaatttttatattattagtagagacagggtttcatca    20194
BCRP3.HUMAN.NCBI.REF                                             ------------------------------------------------------------    20446
LOC106996293.glutathione.hydrolase5.proenzyme-like-GGT1.rhesus.  aggcatgtgccaccaagcctggctaatttt-----------tagagatggggtttcacca    88624

Papio.anubis.clone.rp41-133b2.8034-40080.revcompl.Baboon         tgttagccagactggtctcgaactcctgaactcaggtgattcacccgccttggcctccca    20254
BCRP3.HUMAN.NCBI.REF                                             ------------------------------------------------------------    20446
LOC106996293.glutathione.hydrolase5.proenzyme-like-GGT1.rhesus.  tgttggccagactggtctcgaactcctgacctcaggtgatccacccgacttggcctccca    88684

Papio.anubis.clone.rp41-133b2.8034-40080.revcompl.Baboon         aagtgctaggattataggcataaactactgcgcctggcccagtatattgtttttattcct    20314
BCRP3.HUMAN.NCBI.REF                                             ------------------------------------------------------------    20446
LOC106996293.glutathione.hydrolase5.proenzyme-like-GGT1.rhesus.  aagtgctgggattacaggcatgagccactgcgcccggcaggcaaacacatttctagttca    88744

Papio.anubis.clone.rp41-133b2.8034-40080.revcompl.Baboon         ttg---------------------------------------------------------    20317
BCRP3.HUMAN.NCBI.REF                                             ------------------------------------------------------------    20446
LOC106996293.glutathione.hydrolase5.proenzyme-like-GGT1.rhesus.  tgcatacatatgccaggtgggatgaacctgccctggggcccccgatggtgctcatgggta    88804

Papio.anubis.clone.rp41-133b2.8034-40080.revcompl.Baboon         ------------------------------------------------------------    20317
BCRP3.HUMAN.NCBI.REF                                             ------------------------------------------------------------    20446
LOC106996293.glutathione.hydrolase5.proenzyme-like-GGT1.rhesus.  gagcagggtgatcccaggctccaagatggcaggcttggatgccggcagcaggtggtctgg    88864

Papio.anubis.clone.rp41-133b2.8034-40080.revcompl.Baboon         ------------------------------------------------------------    20317
BCRP3.HUMAN.NCBI.REF                                             ------------------------------------------------------------    20446
LOC106996293.glutathione.hydrolase5.proenzyme-like-GGT1.rhesus.  cctgttgttaggccccctgctggtgcaagcacaaaccagaagtggcaggaagggcaggtc    88924

Papio.anubis.clone.rp41-133b2.8034-40080.revcompl.Baboon         ------------------------------------------------------------    20317
BCRP3.HUMAN.NCBI.REF                                             ------------------------------------------------------------    20446
LOC106996293.glutathione.hydrolase5.proenzyme-like-GGT1.rhesus.  tatccccaggtccctggatggagtacttgggcacttgtggggtgggggttctttgggagg    88984

Papio.anubis.clone.rp41-133b2.8034-40080.revcompl.Baboon         ------------------------------------------------------------    20317
BCRP3.HUMAN.NCBI.REF                                             ------------------------------------------------------------    20446
LOC106996293.glutathione.hydrolase5.proenzyme-like-GGT1.rhesus.  ggttagcctgtcctcaggccctgtgggagcctacatggacacaggttgtggtgggtgggc    89044

Papio.anubis.clone.rp41-133b2.8034-40080.revcompl.Baboon         ------------------------------------------------------------    20317
BCRP3.HUMAN.NCBI.REF                                             ------------------------------------------------------------    20446
LOC106996293.glutathione.hydrolase5.proenzyme-like-GGT1.rhesus.  tggggtgatcctcaggtccctgaatgtcatccttgggcaccaggggcagacggcctgggt    89104

Papio.anubis.clone.rp41-133b2.8034-40080.revcompl.Baboon         ------------------------------------------------------------    20317
BCRP3.HUMAN.NCBI.REF                                             ------------------------------------------------------------    20446
LOC106996293.glutathione.hydrolase5.proenzyme-like-GGT1.rhesus.  ctgttgtcagtcctccctgtggtatgtgtctgtgcccatggcaacaggtgggcagagcaa    89164

Papio.anubis.clone.rp41-133b2.8034-40080.revcompl.Baboon         ------------------------------------------------------------    20317
BCRP3.HUMAN.NCBI.REF                                             ------------------------------------------------------------    20446
LOC106996293.glutathione.hydrolase5.proenzyme-like-GGT1.rhesus.  tttccaggctcccgggtggtttgcttgggtggcagtggtgctgactggttgtgagcgggg    89224

Papio.anubis.clone.rp41-133b2.8034-40080.revcompl.Baboon         ------------------------------------------------------------    20317
BCRP3.HUMAN.NCBI.REF                                             ------------------------------------------------------------    20446
LOC106996293.glutathione.hydrolase5.proenzyme-like-GGT1.rhesus.  tgagtctgcattcaatagccaactcagagaccccagatgccattttccatgacaactggc    89284

Papio.anubis.clone.rp41-133b2.8034-40080.revcompl.Baboon         ------------------------------------------------------------    20317
BCRP3.HUMAN.NCBI.REF                                             ------------------------------------------------------------    20446
LOC106996293.glutathione.hydrolase5.proenzyme-like-GGT1.rhesus.  ctgtctggacattgtgccatgtggctggccccacactaggccaggcctccatgagtataa    89344

Papio.anubis.clone.rp41-133b2.8034-40080.revcompl.Baboon         ------------------------------------------------------------    20317
BCRP3.HUMAN.NCBI.REF                                             ------------------------------------------------------------    20446
LOC106996293.glutathione.hydrolase5.proenzyme-like-GGT1.rhesus.  ggaaacatgggaggcaggaggtcggggccctgccctgcggagggagaagaatgggtcata    89404

Papio.anubis.clone.rp41-133b2.8034-40080.revcompl.Baboon         ------------------------------------------------------------    20317
BCRP3.HUMAN.NCBI.REF                                             ------------------------------------------------------------    20446
LOC106996293.glutathione.hydrolase5.proenzyme-like-GGT1.rhesus.  ggcagtggtcattgagcagaagagagaatgagcagaggatgctgcgaggcagtgagtata    89464

Papio.anubis.clone.rp41-133b2.8034-40080.revcompl.Baboon         ------------------------------------------------------------    20317
BCRP3.HUMAN.NCBI.REF                                             ------------------------------------------------------------    20446
LOC106996293.glutathione.hydrolase5.proenzyme-like-GGT1.rhesus.  aggaatcatatagctgaaggtattcatctgacatataccggaaactaatacagagtaaga    89524

Papio.anubis.clone.rp41-133b2.8034-40080.revcompl.Baboon         ------------------------------------------------------------    20317
BCRP3.HUMAN.NCBI.REF                                             ------------------------------------------------------------    20446
LOC106996293.glutathione.hydrolase5.proenzyme-like-GGT1.rhesus.  aaatacaaaaaccaaataatccaacaaaacttgggcaaagaatctatagacattgctgaa    89584

Papio.anubis.clone.rp41-133b2.8034-40080.revcompl.Baboon         ------------------------------------------------------------    20317
BCRP3.HUMAN.NCBI.REF                                             ------------------------------------------------------------    20446
LOC106996293.glutathione.hydrolase5.proenzyme-like-GGT1.rhesus.  aagaaggcaggaaatgaactaacaggtaaaggaaaagcttgtccacatcactaatcatca    89644

Papio.anubis.clone.rp41-133b2.8034-40080.revcompl.Baboon         ------------------------------------------------------------    20317
BCRP3.HUMAN.NCBI.REF                                             ------------------------------------------------------------    20446
LOC106996293.glutathione.hydrolase5.proenzyme-like-GGT1.rhesus.  aaaaaatttaaagcaaagccacactaagagagtgtccccatccacataagatgtatgttg    89704

Papio.anubis.clone.rp41-133b2.8034-40080.revcompl.Baboon         ------------------------------------------------------------    20317
BCRP3.HUMAN.NCBI.REF                                             ------------------------------------------------------------    20446
LOC106996293.glutathione.hydrolase5.proenzyme-like-GGT1.rhesus.  ccaaaaacaaaaacagaattttgaaaaggcaatcaacagtgtagatttgcagagagggaa    89764

Papio.anubis.clone.rp41-133b2.8034-40080.revcompl.Baboon         ------------------------------------------------------------    20317
BCRP3.HUMAN.NCBI.REF                                             ------------------------------------------------------------    20446
LOC106996293.glutathione.hydrolase5.proenzyme-like-GGT1.rhesus.  actctttgacactactgatgggaatgtaaattgatgtacacgctatgagcaatagtcggc    89824

Papio.anubis.clone.rp41-133b2.8034-40080.revcompl.Baboon         -----------------------------------cgtcctcatagcttagctttggtat    20342
BCRP3.HUMAN.NCBI.REF                                             ------------------------------------------------------------    20446
LOC106996293.glutathione.hydrolase5.proenzyme-like-GGT1.rhesus.  agttcctcctcaaatgaaaaatacaactactatgtcatccaggaatctcagcactgggaa    89884

Papio.anubis.clone.rp41-133b2.8034-40080.revcompl.Baboon         ataaagctaagcttagctttgcgtcctcatagcttagctttggtatataactgtttt---    20399
```

```
BCRP3.HUMAN.NCBI.REF                                              ------------------------------------------------------------    20446
LOC106996293.glutathione.hydrolase5.proenzyme-like-GGT1.rhesus.   atacaaagtacatggatgtattatgctccctgattaaaatgacatagaagagctatagt    89944

Papio.anubis.clone.rp41-133b2.8034-40080.revcompl.Baboon          ------------------------------------------------------------    20399
BCRP3.HUMAN.NCBI.REF                                              ------------------------------------------------------------    20446
LOC106996293.glutathione.hydrolase5.proenzyme-like-GGT1.rhesus.   gatccaaacaatatggtattggcattaacacaaaaatatagaacagacacaacgagggag    90004

Papio.anubis.clone.rp41-133b2.8034-40080.revcompl.Baboon          ------------------------------------------------------------    20399
BCRP3.HUMAN.NCBI.REF                                              ------------------------------------------------------------    20446
LOC106996293.glutathione.hydrolase5.proenzyme-like-GGT1.rhesus.   ccaaataccaaacccaaattcatatatgatagatacattaaaaaaaaatacacccaggga    90064

Papio.anubis.clone.rp41-133b2.8034-40080.revcompl.Baboon          ------------------------------------------------------------    20399
BCRP3.HUMAN.NCBI.REF                                              ------------------------------------------------------------    20446
LOC106996293.glutathione.hydrolase5.proenzyme-like-GGT1.rhesus.   atgggagaccccaggctgccttttgccgggatgctgaagtgtcctggggtctacagatgc    90124

Papio.anubis.clone.rp41-133b2.8034-40080.revcompl.Baboon          ------------------------------------------------------------    20399
BCRP3.HUMAN.NCBI.REF                                              ------------------------------------------------------------    20446
LOC106996293.glutathione.hydrolase5.proenzyme-like-GGT1.rhesus.   cgctcacctgggctctggatggtttctactccgtaaggatttccagagtttcccctcagc    90184

Papio.anubis.clone.rp41-133b2.8034-40080.revcompl.Baboon          ------------------------------------------------------------    20399
BCRP3.HUMAN.NCBI.REF                                              ------------------------------------------------------------    20446
LOC106996293.glutathione.hydrolase5.proenzyme-like-GGT1.rhesus.   caccctcagagtcaggggggctcccatgggccagtgaccatctgtcccagtctttcgtgt    90244

Papio.anubis.clone.rp41-133b2.8034-40080.revcompl.Baboon          ------------------------------------------------------------    20399
BCRP3.HUMAN.NCBI.REF                                              ------------------------------------------------------------    20446
LOC106996293.glutathione.hydrolase5.proenzyme-like-GGT1.rhesus.   ctccatgacacactgtgtgacctatgtatactgcaatatgtaggtgcgtgtattgaaagc    90304

Papio.anubis.clone.rp41-133b2.8034-40080.revcompl.Baboon          ------------------------------------------------------------    20399
BCRP3.HUMAN.NCBI.REF                                              ------------------------------------------------------------    20446
LOC106996293.glutathione.hydrolase5.proenzyme-like-GGT1.rhesus.   gtacagttaagatgacctacaaccgatgctactcgaagtcttccagagatttttcagata    90364

Papio.anubis.clone.rp41-133b2.8034-40080.revcompl.Baboon          ------------------------------------------------------------    20399
BCRP3.HUMAN.NCBI.REF                                              ------------------------------------------------------------    20446
LOC106996293.glutathione.hydrolase5.proenzyme-like-GGT1.rhesus.   tccccagaccaaaacaaactagtgacattccatggttttgcaaaccagaaccaacaactg    90424

Papio.anubis.clone.rp41-133b2.8034-40080.revcompl.Baboon          ------------------------------------------------------------    20399
BCRP3.HUMAN.NCBI.REF                                              ------------------------------------------------------------    20446
LOC106996293.glutathione.hydrolase5.proenzyme-like-GGT1.rhesus.   cacagaaacagctccttatgcaccatcagacactctccagacctgccccacattcacaaa    90484

Papio.anubis.clone.rp41-133b2.8034-40080.revcompl.Baboon          ------------------------------------------------------------    20399
BCRP3.HUMAN.NCBI.REF                                              ------------------------------------------------------------    20446
LOC106996293.glutathione.hydrolase5.proenzyme-like-GGT1.rhesus.   cctaacgaacgagcacgaacccaggcagtcttcactcctaagggccatttaacaacctct    90544

Papio.anubis.clone.rp41-133b2.8034-40080.revcompl.Baboon          ------------------------------------------------------------    20399
BCRP3.HUMAN.NCBI.REF                                              ------------------------------------------------------------    20446
LOC106996293.glutathione.hydrolase5.proenzyme-like-GGT1.rhesus.   atcagtcatgcagcctttgatctgggccaggctgccgacacacaggggtgaattgtgcct    90604

Papio.anubis.clone.rp41-133b2.8034-40080.revcompl.Baboon          ------------------------------------------------------------    20399
BCRP3.HUMAN.NCBI.REF                                              ------------------------------------------------------------    20446
LOC106996293.glutathione.hydrolase5.proenzyme-like-GGT1.rhesus.   ctaatgctgagtgttgcttcccctcactgggctaacccccacccttgcaccaagaagggt    90664

Papio.anubis.clone.rp41-133b2.8034-40080.revcompl.Baboon          ------------------------------------------------------------    20399
BCRP3.HUMAN.NCBI.REF                                              ------------------------------------------------------------    20446
LOC106996293.glutathione.hydrolase5.proenzyme-like-GGT1.rhesus.   ccctagggaaactgctgttcgacatccaggagcccatgtggaatgttccagcaacctgga    90724

Papio.anubis.clone.rp41-133b2.8034-40080.revcompl.Baboon          ------------------------------------------------------------    20399
BCRP3.HUMAN.NCBI.REF                                              ------------------------------------------------------------    20446
LOC106996293.glutathione.hydrolase5.proenzyme-like-GGT1.rhesus.   tgacctcggccacctctagtaccagcgtttccctctgtggggctcccttcctggctctta    90784

Papio.anubis.clone.rp41-133b2.8034-40080.revcompl.Baboon          ------------------------------------------------------------    20399
BCRP3.HUMAN.NCBI.REF                                              ------------------------------------------------------------    20446
LOC106996293.glutathione.hydrolase5.proenzyme-like-GGT1.rhesus.   ctcatgaaggctgatctaaagtggctgagatgtcctgctcccttagccacccaggtcacc    90844

Papio.anubis.clone.rp41-133b2.8034-40080.revcompl.Baboon          ------------------------------------------------------------    20399
BCRP3.HUMAN.NCBI.REF                                              ------------------------------------------------------------    20446
LOC106996293.glutathione.hydrolase5.proenzyme-like-GGT1.rhesus.   ctgatggcatcacactggccagaggcagatgtctgcattcaagaatgaggtattcacagt    90904

Papio.anubis.clone.rp41-133b2.8034-40080.revcompl.Baboon          ------------------------------------------------------------    20399
BCRP3.HUMAN.NCBI.REF                                              ------------------------------------------------------------    20446
LOC106996293.glutathione.hydrolase5.proenzyme-like-GGT1.rhesus.   ctgacttccctggtgcaggggacaatgtctggcacctccagacagccagaaccaggtaag    90964

Papio.anubis.clone.rp41-133b2.8034-40080.revcompl.Baboon          ------------------------------------------------------------    20399
BCRP3.HUMAN.NCBI.REF                                              ------------------------------------------------------------    20446
LOC106996293.glutathione.hydrolase5.proenzyme-like-GGT1.rhesus.   gaatccagctctgacacagctcaaagactctgtggcagacctggagagattctccaggcc    91024

Papio.anubis.clone.rp41-133b2.8034-40080.revcompl.Baboon          ------------------------------------------------------------    20399
BCRP3.HUMAN.NCBI.REF                                              ------------------------------------------------------------    20446
LOC106996293.glutathione.hydrolase5.proenzyme-like-GGT1.rhesus.   tgtgtcctctcctggctcactggattcttaggaggattactgagacactgggcttaaagc    91084

Papio.anubis.clone.rp41-133b2.8034-40080.revcompl.Baboon          ------------------------------------------------------------    20399
BCRP3.HUMAN.NCBI.REF                                              ------------------------------------------------------------    20446
LOC106996293.glutathione.hydrolase5.proenzyme-like-GGT1.rhesus.   tctcagcctagggcccagaatgccacagggaatcaaatcagggcgatcaaggactaaaaa    91144

Papio.anubis.clone.rp41-133b2.8034-40080.revcompl.Baboon          ------------------------------------------------------------    20399
BCRP3.HUMAN.NCBI.REF                                              ------------------------------------------------------------    20446
LOC106996293.glutathione.hydrolase5.proenzyme-like-GGT1.rhesus.   cgtctccttggctgggaatggggtatcagggttgcagagacaaggggcacagagtgatgc    91204

Papio.anubis.clone.rp41-133b2.8034-40080.revcompl.Baboon          ------------------------------------------------------------    20399
BCRP3.HUMAN.NCBI.REF                                              ------------------------------------------------------------    20446
LOC106996293.glutathione.hydrolase5.proenzyme-like-GGT1.rhesus.   ctgtaccatcagaggatctggtccaccctgggctcctcaacctgagtgtgaggccttgct    91264

Papio.anubis.clone.rp41-133b2.8034-40080.revcompl.Baboon          ------------------------------------------------------------    20399
BCRP3.HUMAN.NCBI.REF                                              ------------------------------------------------------------    20446
LOC106996293.glutathione.hydrolase5.proenzyme-like-GGT1.rhesus.   gggacctgagctcaccttccccttgggagtccattgtgactacagtccagcgggatcaat    91324

Papio.anubis.clone.rp41-133b2.8034-40080.revcompl.Baboon          ------------------------------------------------------------    20399
BCRP3.HUMAN.NCBI.REF                                              ------------------------------------------------------------    20446
LOC106996293.glutathione.hydrolase5.proenzyme-like-GGT1.rhesus.   gtcctcactgatgccagacagaagtcctgggggatgcggagggcatcctgtagcagcagg    91384

Papio.anubis.clone.rp41-133b2.8034-40080.revcompl.Baboon          ------------------------------------------------------------    20399
BCRP3.HUMAN.NCBI.REF                                              ------------------------------------------------------------    20446
LOC106996293.glutathione.hydrolase5.proenzyme-like-GGT1.rhesus.   cagtctgggtggtccacaggtgtgtgcttcagcatgtcctgggaagggatgagctggtga    91444

Papio.anubis.clone.rp41-133b2.8034-40080.revcompl.Baboon          ------------------------------------------------------------    20399
BCRP3.HUMAN.NCBI.REF                                              ------------------------------------------------------------    20446
LOC106996293.glutathione.hydrolase5.proenzyme-like-GGT1.rhesus.   attgagggatgtgtttccagggatcagtgagaagcccctggggaatgctcagtcctcacc    91504

Papio.anubis.clone.rp41-133b2.8034-40080.revcompl.Baboon          ------------------------------------------------------------    20399
BCRP3.HUMAN.NCBI.REF                                              ------------------------------------------------------------    20446
LOC106996293.glutathione.hydrolase5.proenzyme-like-GGT1.rhesus.   gagagaattcaaagggcaggccctcagggacccacactcatgcctgcaaaatctgaactg    91564

Papio.anubis.clone.rp41-133b2.8034-40080.revcompl.Baboon          ------------------------------------------------------------    20399
BCRP3.HUMAN.NCBI.REF                                              ------------------------------------------------------------    20446
LOC106996293.glutathione.hydrolase5.proenzyme-like-GGT1.rhesus.   aaaattagaatcggagcagcagggttgactctaaaagggccccagctggcttttctccct    91624

Papio.anubis.clone.rp41-133b2.8034-40080.revcompl.Baboon          ------------------------------------------------------------    20399
```

```
BCRP3.HUMAN.NCBI.REF                                          ------------------------------------------------------------    20446
LOC106996293.glutathione.hydrolase5.proenzyme-like-GGT1.rhesus.   gctgcagtctttactcttttctcctggcagaggcagccaggtggaggctcatcaaaccaa    91684

Papio.anubis.clone.rp41-133b2.8034-40080.revcompl.Baboon      ------------------------------------------------------------    20399
BCRP3.HUMAN.NCBI.REF                                          ------------------------------------------------------------    20446
LOC106996293.glutathione.hydrolase5.proenzyme-like-GGT1.rhesus.   gcagctgggagagacacccctctacccaccctgcagctgctccaaggatactggagaccg    91744

Papio.anubis.clone.rp41-133b2.8034-40080.revcompl.Baboon      -------gggcttggcttggtagctcatgcctg---------------------------    20425
BCRP3.HUMAN.NCBI.REF                                          ------------------------------------------------------------    20446
LOC106996293.glutathione.hydrolase5.proenzyme-like-GGT1.rhesus.   aaagacaggggttggggaagcaggacgttcctgcctccactgagagcaattggccaaatg    91804

Papio.anubis.clone.rp41-133b2.8034-40080.revcompl.Baboon      ------------------------------------------------------------    20425
BCRP3.HUMAN.NCBI.REF                                          ------------------------------------------------------------    20446
LOC106996293.glutathione.hydrolase5.proenzyme-like-GGT1.rhesus.   ccaagctcagtagctaagctctattgaggccctggccctgttggcatggttaccacaggc    91864

Papio.anubis.clone.rp41-133b2.8034-40080.revcompl.Baboon      ------------------------------------------------------------    20425
BCRP3.HUMAN.NCBI.REF                                          ------------------------------------------------------------    20446
LOC106996293.glutathione.hydrolase5.proenzyme-like-GGT1.rhesus.   cagggaggcatactcagggcccaggatccctctgcctgacttttcaaatggtctgttctc    91924

Papio.anubis.clone.rp41-133b2.8034-40080.revcompl.Baboon      ------------------------------------------------------------    20425
BCRP3.HUMAN.NCBI.REF                                          ------------------------------------------------------------    20446
LOC106996293.glutathione.hydrolase5.proenzyme-like-GGT1.rhesus.   agtgactctggtgccctcccacaccgggctcagtcttctgagaaactcctaagccctctc    91984

Papio.anubis.clone.rp41-133b2.8034-40080.revcompl.Baboon      ------------------------------------------------------------    20425
BCRP3.HUMAN.NCBI.REF                                          ------------------------------------------------------------    20446
LOC106996293.glutathione.hydrolase5.proenzyme-like-GGT1.rhesus.   tggcccttcaggggccttaacactggtgcataggaccaggatgctctgagtgacttactt    92044

Papio.anubis.clone.rp41-133b2.8034-40080.revcompl.Baboon      ------------------------------------------------------------    20425
BCRP3.HUMAN.NCBI.REF                                          ------------------------------------------------------------    20446
LOC106996293.glutathione.hydrolase5.proenzyme-like-GGT1.rhesus.   ggtcaatgggctggtacagcagagctgttggggacagcagagagaaaaggacaatgaca    92104

Papio.anubis.clone.rp41-133b2.8034-40080.revcompl.Baboon      ------------------------------------------------------------    20425
BCRP3.HUMAN.NCBI.REF                                          ------------------------------------------------------------    20446
LOC106996293.glutathione.hydrolase5.proenzyme-like-GGT1.rhesus.   tacaaaaaaccactcaacacctcacagaccagtttttcccccacccaccttgctccccc    92164

Papio.anubis.clone.rp41-133b2.8034-40080.revcompl.Baboon      ------------------------------------------------------------    20425
BCRP3.HUMAN.NCBI.REF                                          ------------------------------------------------------------    20446
LOC106996293.glutathione.hydrolase5.proenzyme-like-GGT1.rhesus.   cgtgaacacctgcttgcaggaagccaaagagggtccacagcagcctccacctctgttatc    92224

Papio.anubis.clone.rp41-133b2.8034-40080.revcompl.Baboon      ------------------------------------------------------------    20425
BCRP3.HUMAN.NCBI.REF                                          ------------------------------------------------------------    20446
LOC106996293.glutathione.hydrolase5.proenzyme-like-GGT1.rhesus.   actccaatggcagaccaagcagcaagaacaggtgacacacagccctggtgaccgtgtccc    92284

Papio.anubis.clone.rp41-133b2.8034-40080.revcompl.Baboon      ------------------------------------------------------------    20425
BCRP3.HUMAN.NCBI.REF                                          ------------------------------------------------------------    20446
LOC106996293.glutathione.hydrolase5.proenzyme-like-GGT1.rhesus.   ctctctacccatgcctattctcccatcagacttggcttcttcgagggctgccccagtcct    92344

Papio.anubis.clone.rp41-133b2.8034-40080.revcompl.Baboon      ------------------------------------------------------------    20425
BCRP3.HUMAN.NCBI.REF                                          ------------------------------------------------------------    20446
LOC106996293.glutathione.hydrolase5.proenzyme-like-GGT1.rhesus.   gtccaggccttacctggctcagagcagatgatcccccgcacctcccaccctgaatcacac    92404

Papio.anubis.clone.rp41-133b2.8034-40080.revcompl.Baboon      ------------------------------------------------------------    20425
BCRP3.HUMAN.NCBI.REF                                          ------------------------------------------------------------    20446
LOC106996293.glutathione.hydrolase5.proenzyme-like-GGT1.rhesus.   ggctacacccagcacatgctacagaggcccagcacactctgggtcacatccagaaactct    92464

Papio.anubis.clone.rp41-133b2.8034-40080.revcompl.Baboon      ------------------------------------------------------------    20425
BCRP3.HUMAN.NCBI.REF                                          ------------------------------------------------------------    20446
LOC106996293.glutathione.hydrolase5.proenzyme-like-GGT1.rhesus.   acagtaggggaggggacacgctgtttcccaggaagggcagctccccaggcctggtcattc    92524

Papio.anubis.clone.rp41-133b2.8034-40080.revcompl.Baboon      ------------------------------------------------------------    20425
BCRP3.HUMAN.NCBI.REF                                          ------------------------------------------------------------    20446
LOC106996293.glutathione.hydrolase5.proenzyme-like-GGT1.rhesus.   tcagactccttcagtcaaaggccggccacagccagctgggatcgccatgcttcacgctga    92584

Papio.anubis.clone.rp41-133b2.8034-40080.revcompl.Baboon      ------------------------------------------------------------    20425
BCRP3.HUMAN.NCBI.REF                                          ------------------------------------------------------------    20446
LOC106996293.glutathione.hydrolase5.proenzyme-like-GGT1.rhesus.   caaacatgactggccttgcctcccctatgggaagagacccctgcacacagcctcaaaccc    92644

Papio.anubis.clone.rp41-133b2.8034-40080.revcompl.Baboon      ------------------------------------------------------------    20425
BCRP3.HUMAN.NCBI.REF                                          ------------------------------------------------------------    20446
LOC106996293.glutathione.hydrolase5.proenzyme-like-GGT1.rhesus.   agacataacaggacaggacacgctgggcgctagggaggccagccagatctccacacaaag    92704

Papio.anubis.clone.rp41-133b2.8034-40080.revcompl.Baboon      ------------------------------------------------------------    20425
BCRP3.HUMAN.NCBI.REF                                          ------------------------------------------------------------    20446
LOC106996293.glutathione.hydrolase5.proenzyme-like-GGT1.rhesus.   gctctgctcttaagcaggaggcagcctcaggggcaaaagtttcctgaagcaactcaggtc    92764

Papio.anubis.clone.rp41-133b2.8034-40080.revcompl.Baboon      ------------------------------------------------------------    20425
BCRP3.HUMAN.NCBI.REF                                          ------------------------------------------------------------    20446
LOC106996293.glutathione.hydrolase5.proenzyme-like-GGT1.rhesus.   aggcctcacatacaagagccccagaggctgcaaaagcaccagctcagccagccaagaacc    92824

Papio.anubis.clone.rp41-133b2.8034-40080.revcompl.Baboon      ------------------------------------------------------------    20425
BCRP3.HUMAN.NCBI.REF                                          ------------------------------------------------------------    20446
LOC106996293.glutathione.hydrolase5.proenzyme-like-GGT1.rhesus.   cagggccacacccttctgaaacccagacaccaagtgaagatcgctgggattcagcagaac    92884

Papio.anubis.clone.rp41-133b2.8034-40080.revcompl.Baboon      ------------------------------------------------------------    20425
BCRP3.HUMAN.NCBI.REF                                          ------------------------------------------------------------    20446
LOC106996293.glutathione.hydrolase5.proenzyme-like-GGT1.rhesus.   aggaggacgtgacagctggcggaccaacacttcccaacacccttccagcaagcagctgca    92944

Papio.anubis.clone.rp41-133b2.8034-40080.revcompl.Baboon      ------------------------------------------------------------    20425
BCRP3.HUMAN.NCBI.REF                                          ------------------------------------------------------------    20446
LOC106996293.glutathione.hydrolase5.proenzyme-like-GGT1.rhesus.   cccctcctgatgcgagtcaccccacttcggtcctgcacctggatgcctctgtccagggta    93004

Papio.anubis.clone.rp41-133b2.8034-40080.revcompl.Baboon      ------------------------------------------------------------    20425
BCRP3.HUMAN.NCBI.REF                                          ------------------------------------------------------------    20446
LOC106996293.glutathione.hydrolase5.proenzyme-like-GGT1.rhesus.   gcatatttggcctcctgtcataggagcagtgagaggcaggatggtggtctgggaagaagg    93064

Papio.anubis.clone.rp41-133b2.8034-40080.revcompl.Baboon      ------------------------------------------------------------    20425
BCRP3.HUMAN.NCBI.REF                                          ------------------------------------------------------------    20446
LOC106996293.glutathione.hydrolase5.proenzyme-like-GGT1.rhesus.   atcctagactgggtgtcccataggaccaggttgtgacagatcccacagccaacactcatg    93124

Papio.anubis.clone.rp41-133b2.8034-40080.revcompl.Baboon      ------------------------------------------------------------    20425
BCRP3.HUMAN.NCBI.REF                                          ------------------------------------------------------------    20446
LOC106996293.glutathione.hydrolase5.proenzyme-like-GGT1.rhesus.   ccccagccaacccccaaacccaatagctccccaaagcccttgatgtaaatagcctgtgca    93184

Papio.anubis.clone.rp41-133b2.8034-40080.revcompl.Baboon      ------------------------------------------------------------    20425
BCRP3.HUMAN.NCBI.REF                                          ------------------------------------------------------------    20446
LOC106996293.glutathione.hydrolase5.proenzyme-like-GGT1.rhesus.   ctgctaagcagacacctcagggaacaatctgatggctgtgaagaagatgcagcctcaaca    93244

Papio.anubis.clone.rp41-133b2.8034-40080.revcompl.Baboon      ------------------------------------------------------------    20425
BCRP3.HUMAN.NCBI.REF                                          ------------------------------------------------------------    20446
LOC106996293.glutathione.hydrolase5.proenzyme-like-GGT1.rhesus.   gggttccacaagccttctgcaagcaaagccttctttgcttgggtgcctactcaaagcact    93304

Papio.anubis.clone.rp41-133b2.8034-40080.revcompl.Baboon      ------------------taatcccagcgctttgggaggccaaggcgggc--agatcaca    20465
BCRP3.HUMAN.NCBI.REF                                          ------------------------------------------------------------    20446
LOC106996293.glutathione.hydrolase5.proenzyme-like-GGT1.rhesus.   agggaagatcttaaaagataaattcagcattgtgggaggcagaggcaggagtacagcttg    93364

Papio.anubis.clone.rp41-133b2.8034-40080.revcompl.Baboon      aggtcaggagattgagaccatcctggatagcacagtgaaaccctgtctctactaaaaata    20525
```

```
BCRP3.HUMAN.NCBI.REF                                              ------------------------------------------------------------    20446
LOC106996293.glutathione.hydrolase5.proenzyme-like-GGT1.rhesus.   aggttaggactttaagaccagcctgagtgcaatggtgagaccctgtctctacaaaaca--    93422

Papio.anubis.clone.rp41-133b2.8034-40080.revcompl.Baboon          caaaaaattagccaggcattgtggtgggcgcctgtagtcccagctgcttgggaggctgag    20585
BCRP3.HUMAN.NCBI.REF                                              ------------------------------------------------------------    20446
LOC106996293.glutathione.hydrolase5.proenzyme-like-GGT1.rhesus.   -caaaaattagcatggcatggtggtacatgcccatagtcccagccacttgggaggctgag    93481

Papio.anubis.clone.rp41-133b2.8034-40080.revcompl.Baboon          gcaggagaatggtgtgaacccaggagatggagcttgcagtgagccgagatcatgccactg    20645
BCRP3.HUMAN.NCBI.REF                                              ------------------------------------------------------------    20446
LOC106996293.glutathione.hydrolase5.proenzyme-like-GGT1.rhesus.   aagggaggattccttgagcccgggaggtcaaggttgcagtgc-ccatgatcatgccactg    93540

Papio.anubis.clone.rp41-133b2.8034-40080.revcompl.Baboon          ctctccagcctgggggacagagtgagatcc-gtctcaaacaaacaaacaaacaaacaaag    20704
BCRP3.HUMAN.NCBI.REF                                              ------------------------------------------------------------    20446
LOC106996293.glutathione.hydrolase5.proenzyme-like-GGT1.rhesus.   tgttacaacctgggccacagcacaagatcctgttacaatcaaacaaacaaacaaacaacc    93600

Papio.anubis.clone.rp41-133b2.8034-40080.revcompl.Baboon          aaaaaaccgtttt-----------------------------------------------    20717
BCRP3.HUMAN.NCBI.REF                                              ------------------------------------------------------------    20446
LOC106996293.glutathione.hydrolase5.proenzyme-like-GGT1.rhesus.   aaagatgcctgttttctgccccatcaaataaacccaaatctctgcagggtgacacagggt    93660

Papio.anubis.clone.rp41-133b2.8034-40080.revcompl.Baboon          ------------------------------------------------------------    20717
BCRP3.HUMAN.NCBI.REF                                              ------------------------------------------------------------    20446
LOC106996293.glutathione.hydrolase5.proenzyme-like-GGT1.rhesus.   atacacatttttaaatatctcctttcctgaagtaaatgtatagtaatggctgaaaaccac    93720

Papio.anubis.clone.rp41-133b2.8034-40080.revcompl.Baboon          ------------------------------------------------------------    20717
BCRP3.HUMAN.NCBI.REF                                              ------------------------------------------------------------    20446
LOC106996293.glutathione.hydrolase5.proenzyme-like-GGT1.rhesus.   tgagttatgtaaatacacttcctatgacttatttgtagccctttcgtgtctagcaatttc    93780

Papio.anubis.clone.rp41-133b2.8034-40080.revcompl.Baboon          ------------------------------------------------------------    20717
BCRP3.HUMAN.NCBI.REF                                              ------------------------------------------------------------    20446
LOC106996293.glutathione.hydrolase5.proenzyme-like-GGT1.rhesus.   tacagaccctctccgtgggctttggaagccggaatgggcagggccaggtgtgaggtatcc    93840

Papio.anubis.clone.rp41-133b2.8034-40080.revcompl.Baboon          ------------------------------------------------------------    20717
BCRP3.HUMAN.NCBI.REF                                              ------------------------------------------------------------    20446
LOC106996293.glutathione.hydrolase5.proenzyme-like-GGT1.rhesus.   tagcacactgaggctagagcacagctcccagctcaggggggacccatttctgaggctaagg    93900

Papio.anubis.clone.rp41-133b2.8034-40080.revcompl.Baboon          ------------------------------------------------------------    20717
BCRP3.HUMAN.NCBI.REF                                              ------------------------------------------------------------    20446
LOC106996293.glutathione.hydrolase5.proenzyme-like-GGT1.rhesus.   cacccaccagggactcagcctatcttcagagggaaaatcacacctcagaaccccagagac    93960

Papio.anubis.clone.rp41-133b2.8034-40080.revcompl.Baboon          ------------------------------------------------------------    20717
BCRP3.HUMAN.NCBI.REF                                              ------------------------------------------------------------    20446
LOC106996293.glutathione.hydrolase5.proenzyme-like-GGT1.rhesus.   ccaaaagaaagttcgaatttggcacatacctgccagtgggtcctggagtggcagccccag    94020

Papio.anubis.clone.rp41-133b2.8034-40080.revcompl.Baboon          ------------------------------------------------------------    20717
BCRP3.HUMAN.NCBI.REF                                              ------------------------------------------------------------    20446
LOC106996293.glutathione.hydrolase5.proenzyme-like-GGT1.rhesus.   ggaaaaccgccattgcactgtcagctccctccagccttctctcaggtcccagggggtgga    94080

Papio.anubis.clone.rp41-133b2.8034-40080.revcompl.Baboon          ------------------------------------------------------------    20717
BCRP3.HUMAN.NCBI.REF                                              ------------------------------------------------------------    20446
LOC106996293.glutathione.hydrolase5.proenzyme-like-GGT1.rhesus.   ggtctctgactaagctcaaaggtctcaacaaacggcgggttgaacctctgaggagttatt    94140

Papio.anubis.clone.rp41-133b2.8034-40080.revcompl.Baboon          ------------------------------------------------------------    20717
BCRP3.HUMAN.NCBI.REF                                              ------------------------------------------------------------    20446
LOC106996293.glutathione.hydrolase5.proenzyme-like-GGT1.rhesus.   aacaggcctatagttacctcaagactccacctgaccagggccaaaaatgagtcctgggag    94200

Papio.anubis.clone.rp41-133b2.8034-40080.revcompl.Baboon          ------------------------------------------------------------    20717
BCRP3.HUMAN.NCBI.REF                                              ------------------------------------------------------------    20446
LOC106996293.glutathione.hydrolase5.proenzyme-like-GGT1.rhesus.   ggcagccaggaagacagcagggcagagaggaaacacaggaggagggggccctgggaccac    94260

Papio.anubis.clone.rp41-133b2.8034-40080.revcompl.Baboon          ------------------------------------------------------------    20717
BCRP3.HUMAN.NCBI.REF                                              ------------------------------------------------------------    20446
LOC106996293.glutathione.hydrolase5.proenzyme-like-GGT1.rhesus.   atcagatatggggaaaccaggggaacgagggcagccctgatgattgtgctccccataggc    94320

Papio.anubis.clone.rp41-133b2.8034-40080.revcompl.Baboon          ------------------------------------------------------------    20717
BCRP3.HUMAN.NCBI.REF                                              ------------------------------------------------------------    20446
LOC106996293.glutathione.hydrolase5.proenzyme-like-GGT1.rhesus.   cctgcatgtctgtgtcccctgagaattcagggtgaggagctcactcttgcccttcacctg    94380

Papio.anubis.clone.rp41-133b2.8034-40080.revcompl.Baboon          ------------------------------------------------------------    20717
BCRP3.HUMAN.NCBI.REF                                              ------------------------------------------------------------    20446
LOC106996293.glutathione.hydrolase5.proenzyme-like-GGT1.rhesus.   ttcctgggctctggcaggctggcctgcttcctgccccagcctcacccactctccccactc    94440

Papio.anubis.clone.rp41-133b2.8034-40080.revcompl.Baboon          ------------------------------------------------------------    20717
BCRP3.HUMAN.NCBI.REF                                              ------------------------------------------------------------    20446
LOC106996293.glutathione.hydrolase5.proenzyme-like-GGT1.rhesus.   ctcaccactgatatgggcaagttgatctctctggtttgctggcctctcactctctgccca    94500

Papio.anubis.clone.rp41-133b2.8034-40080.revcompl.Baboon          ------------------------------------------------------------    20717
BCRP3.HUMAN.NCBI.REF                                              ------------------------------------------------------------    20446
LOC106996293.glutathione.hydrolase5.proenzyme-like-GGT1.rhesus.   atggcaccgagttaacttctttgcatgctttattttctgtgattaaaaagggaagcaatt    94560

Papio.anubis.clone.rp41-133b2.8034-40080.revcompl.Baboon          ------------------------------------------------------------    20717
BCRP3.HUMAN.NCBI.REF                                              ------------------------------------------------------------    20446
LOC106996293.glutathione.hydrolase5.proenzyme-like-GGT1.rhesus.   ctcactaacgccatttctcagaggagaaaacaggcacagggatgttctatgacttcctca    94620

Papio.anubis.clone.rp41-133b2.8034-40080.revcompl.Baboon          ------------------------------------------------------------    20717
BCRP3.HUMAN.NCBI.REF                                              ------------------------------------------------------------    20446
LOC106996293.glutathione.hydrolase5.proenzyme-like-GGT1.rhesus.   cggtcaccaagctacaagtgggagctgggctttgaatggaggtctacccatgctcctaaa    94680

Papio.anubis.clone.rp41-133b2.8034-40080.revcompl.Baboon          ------------------------------------------------------------    20717
BCRP3.HUMAN.NCBI.REF                                              ------------------------------------------------------------    20446
LOC106996293.glutathione.hydrolase5.proenzyme-like-GGT1.rhesus.   tttgagccgtcctccaaagaaaggacagcattgtaaaagctcagtgaatgtggcaggaga    94740

Papio.anubis.clone.rp41-133b2.8034-40080.revcompl.Baboon          ------------------------------------------------------------    20717
BCRP3.HUMAN.NCBI.REF                                              ------------------------------------------------------------    20446
LOC106996293.glutathione.hydrolase5.proenzyme-like-GGT1.rhesus.   cttcactaaggcctgaccaggctggtaccctgatatccaacttccagcctccagaactgg    94800

Papio.anubis.clone.rp41-133b2.8034-40080.revcompl.Baboon          ------------------------------------------------------------    20717
BCRP3.HUMAN.NCBI.REF                                              ------------------------------------------------------------    20446
LOC106996293.glutathione.hydrolase5.proenzyme-like-GGT1.rhesus.   gagaaacataattctgtttctgatgagctccccaggctatggctctttgttgtaacatcc    94860

Papio.anubis.clone.rp41-133b2.8034-40080.revcompl.Baboon          ------------------------------------------------------------    20717
BCRP3.HUMAN.NCBI.REF                                              ------------------------------------------------------------    20446
LOC106996293.glutathione.hydrolase5.proenzyme-like-GGT1.rhesus.   tgaactagcccagaagtgcgttccatcatgtgtgtcctattccatcattcagaagggagt    94920

Papio.anubis.clone.rp41-133b2.8034-40080.revcompl.Baboon          ------------------------------------------------------------    20717
BCRP3.HUMAN.NCBI.REF                                              ------------------------------------------------------------    20446
LOC106996293.glutathione.hydrolase5.proenzyme-like-GGT1.rhesus.   cactaacccctcagtgctgtctttgtggatgctgcccaccctcggctgagatggggcaca    94980

Papio.anubis.clone.rp41-133b2.8034-40080.revcompl.Baboon          ------------------------------------------------------------    20717
BCRP3.HUMAN.NCBI.REF                                              ------------------------------------------------------------    20446
LOC106996293.glutathione.hydrolase5.proenzyme-like-GGT1.rhesus.   gggtctgggtctaaacaaaattggaaggcactggaaggtgcaatgtcagaggaggaggat    95040

Papio.anubis.clone.rp41-133b2.8034-40080.revcompl.Baboon          ------------------------------------------------------------    20717
BCRP3.HUMAN.NCBI.REF                                              ------------------------------------------------------------    20446
LOC106996293.glutathione.hydrolase5.proenzyme-like-GGT1.rhesus.   gtctgaatttcgtttaatctcaacctctactggtgcatcatgaaagaggaggcccagagc    95100

Papio.anubis.clone.rp41-133b2.8034-40080.revcompl.Baboon          ------------------------------------------------------------    20717
```

```
BCRP3.HUMAN.NCBI.REF                                             ------------------------------------------------------------    20446
LOC106996293.glutathione.hydrolase5.proenzyme-like-GGT1.rhesus.  aaaagtgtcttctcctgagaccacactgtggaccccaggcccactggagttctcttccat    95160

Papio.anubis.clone.rp41-133b2.8034-40080.revcompl.Baboon         ------------------------------------------------------------    20717
BCRP3.HUMAN.NCBI.REF                                             ------------------------------------------------------------    20446
LOC106996293.glutathione.hydrolase5.proenzyme-like-GGT1.rhesus.  gctacctctcaacgcttcttgtatttcttccatctgtaactgtgtgcaatatctacaggg    95220

Papio.anubis.clone.rp41-133b2.8034-40080.revcompl.Baboon         ------------------------------------------------------------    20717
BCRP3.HUMAN.NCBI.REF                                             ------------------------------------------------------------    20446
LOC106996293.glutathione.hydrolase5.proenzyme-like-GGT1.rhesus.  aacatcacatcatgagttttgtaagatctatctcaaaacacacctcatgagttccctagg    95280

Papio.anubis.clone.rp41-133b2.8034-40080.revcompl.Baboon         ------------------------------------------------------------    20717
BCRP3.HUMAN.NCBI.REF                                             ------------------------------------------------------------    20446
LOC106996293.glutathione.hydrolase5.proenzyme-like-GGT1.rhesus.  agggagatgctagcataacccccattgtgcgtgctgggaaggccccaaaacaccaaagga    95340

Papio.anubis.clone.rp41-133b2.8034-40080.revcompl.Baboon         ------------------------------------------------------------    20717
BCRP3.HUMAN.NCBI.REF                                             ------------------------------------------------------------    20446
LOC106996293.glutathione.hydrolase5.proenzyme-like-GGT1.rhesus.  gttctccagggtctcacaactgctaagataaaggttgtttgacccagaactgtctcctta    95400

Papio.anubis.clone.rp41-133b2.8034-40080.revcompl.Baboon         ------------------------------------------------------------    20717
BCRP3.HUMAN.NCBI.REF                                             ------------------------------------------------------------    20446
LOC106996293.glutathione.hydrolase5.proenzyme-like-GGT1.rhesus.  aaactgggaccctggttacatccaggtcttcccaaggtgctgaaggggcacactttggca    95460

Papio.anubis.clone.rp41-133b2.8034-40080.revcompl.Baboon         ------------------------------------------------------------    20717
BCRP3.HUMAN.NCBI.REF                                             ------------------------------------------------------------    20446
LOC106996293.glutathione.hydrolase5.proenzyme-like-GGT1.rhesus.  taatggtgaaaggtaaggagttgatgggggaaggtgagcggccagaagcccaatgaggcc    95520

Papio.anubis.clone.rp41-133b2.8034-40080.revcompl.Baboon         ------------------------------------------------------------    20717
BCRP3.HUMAN.NCBI.REF                                             ------------------------------------------------------------    20446
LOC106996293.glutathione.hydrolase5.proenzyme-like-GGT1.rhesus.  tttgagtaggtcttatggaaaggaggagcttacaggatggaacctacaagatgtgacctc    95580

Papio.anubis.clone.rp41-133b2.8034-40080.revcompl.Baboon         ------------------------------------------------------------    20717
BCRP3.HUMAN.NCBI.REF                                             ------------------------------------------------------------    20446
LOC106996293.glutathione.hydrolase5.proenzyme-like-GGT1.rhesus.  acttccttgctgacagaccccaacagaacttagaattctggtaactaggcacccatatta    95640

Papio.anubis.clone.rp41-133b2.8034-40080.revcompl.Baboon         ------------------------------------------------------------    20717
BCRP3.HUMAN.NCBI.REF                                             ------------------------------------------------------------    20446
LOC106996293.glutathione.hydrolase5.proenzyme-like-GGT1.rhesus.  tacacacagccccatttccagctcagttggtagttgacacccaagggaacaacatgttct    95700

Papio.anubis.clone.rp41-133b2.8034-40080.revcompl.Baboon         ------------------------------------------------------------    20717
BCRP3.HUMAN.NCBI.REF                                             ------------------------------------------------------------    20446
LOC106996293.glutathione.hydrolase5.proenzyme-like-GGT1.rhesus.  cttgctgtgtcccgacgtgctgtcgacctcctcgtcacagaagaggccaaaatgagagtc    95760

Papio.anubis.clone.rp41-133b2.8034-40080.revcompl.Baboon         ------------------------------------------------------------    20717
BCRP3.HUMAN.NCBI.REF                                             ------------------------------------------------------------    20446
LOC106996293.glutathione.hydrolase5.proenzyme-like-GGT1.rhesus.  tttctcgagaatatagacattggttcaatcctcaccctcgacgcctctggccatttgcca    95820

Papio.anubis.clone.rp41-133b2.8034-40080.revcompl.Baboon         ------------------------------------------------------------    20717
BCRP3.HUMAN.NCBI.REF                                             ------------------------------------------------------------    20446
LOC106996293.glutathione.hydrolase5.proenzyme-like-GGT1.rhesus.  gaaggcacccacaggtaacacaggacccagggcaggcccatccaggccaggcctcagctg    95880

Papio.anubis.clone.rp41-133b2.8034-40080.revcompl.Baboon         ------------------------------------------------------------    20717
BCRP3.HUMAN.NCBI.REF                                             ------------------------------------------------------------    20446
LOC106996293.glutathione.hydrolase5.proenzyme-like-GGT1.rhesus.  tgtccatcagggcagcgctgagcccctccccatgtgtttggggaaacaggagaagaggg    95940

Papio.anubis.clone.rp41-133b2.8034-40080.revcompl.Baboon         ------------------------------------------------------------    20717
BCRP3.HUMAN.NCBI.REF                                             ------------------------------------------------------------    20446
LOC106996293.glutathione.hydrolase5.proenzyme-like-GGT1.rhesus.  gccctccctggacaggagcaggtaggttgttgggggttggagcctggtcggtatgtcag    96000

Papio.anubis.clone.rp41-133b2.8034-40080.revcompl.Baboon         ------------------------------------------------------------    20717
BCRP3.HUMAN.NCBI.REF                                             ------------------------------------------------------------    20446
LOC106996293.glutathione.hydrolase5.proenzyme-like-GGT1.rhesus.  gttcacagcccaggccatgccttcaggatgggaaggtgagtgtcgaggacatagtttgtc    96060

Papio.anubis.clone.rp41-133b2.8034-40080.revcompl.Baboon         ------------------------------------------------------------    20717
BCRP3.HUMAN.NCBI.REF                                             ------------------------------------------------------------    20446
LOC106996293.glutathione.hydrolase5.proenzyme-like-GGT1.rhesus.  tgctcagatctgaatcccaagacctcaagcttcatctgccagagacgtcaacatcctctg    96120

Papio.anubis.clone.rp41-133b2.8034-40080.revcompl.Baboon         ------------------------------------------------------------    20717
BCRP3.HUMAN.NCBI.REF                                             ------------------------------------------------------------    20446
LOC106996293.glutathione.hydrolase5.proenzyme-like-GGT1.rhesus.  gctggaggtctatataaatgctcctatgtgccaaatcctgttgcggaagggggcgccagg    96180

Papio.anubis.clone.rp41-133b2.8034-40080.revcompl.Baboon         ------------------------------------------------------------    20717
BCRP3.HUMAN.NCBI.REF                                             ------------------------------------------------------------    20446
LOC106996293.glutathione.hydrolase5.proenzyme-like-GGT1.rhesus.  aaggagttctaatggggtatcccactatggggtcagccaggcaggccactgacacctctt    96240

Papio.anubis.clone.rp41-133b2.8034-40080.revcompl.Baboon         ------------------------------------------------------------    20717
BCRP3.HUMAN.NCBI.REF                                             ------------------------------------------------------------    20446
LOC106996293.glutathione.hydrolase5.proenzyme-like-GGT1.rhesus.  cgcctggctctcgggcaatttctttgcagagctccacacagcagatcaagcaggagctgt    96300

Papio.anubis.clone.rp41-133b2.8034-40080.revcompl.Baboon         ------------------------------------------------------------    20717
BCRP3.HUMAN.NCBI.REF                                             ------------------------------------------------------------    20446
LOC106996293.glutathione.hydrolase5.proenzyme-like-GGT1.rhesus.  tggatggattccatttctccatcttcttcaaagaagggcaggggccgcctgctaccaacc    96360

Papio.anubis.clone.rp41-133b2.8034-40080.revcompl.Baboon         ------------------------------------------------------------    20717
BCRP3.HUMAN.NCBI.REF                                             ------------------------------------------------------------    20446
LOC106996293.glutathione.hydrolase5.proenzyme-like-GGT1.rhesus.  atggccagtgctggtctggggtgagattgggcacagaagggtctccacagacagggactc    96420

Papio.anubis.clone.rp41-133b2.8034-40080.revcompl.Baboon         ------------------------------------------------------------    20717
BCRP3.HUMAN.NCBI.REF                                             ------------------------------------------------------------    20446
LOC106996293.glutathione.hydrolase5.proenzyme-like-GGT1.rhesus.  cagaaaaccagggtggacccaggactcaaatacgaatatggggactcactaggctctgtc    96480

Papio.anubis.clone.rp41-133b2.8034-40080.revcompl.Baboon         ------------------------------------------------------------    20717
BCRP3.HUMAN.NCBI.REF                                             ------------------------------------------------------------    20446
LOC106996293.glutathione.hydrolase5.proenzyme-like-GGT1.rhesus.  ctagggctttcacacttgagtgagccgctgtcctctcccaaaggaatctggctgccatta    96540

Papio.anubis.clone.rp41-133b2.8034-40080.revcompl.Baboon         ------------------------------------------------------------    20717
BCRP3.HUMAN.NCBI.REF                                             ------------------------------------------------------------    20446
LOC106996293.glutathione.hydrolase5.proenzyme-like-GGT1.rhesus.  gtccccagtggcaacttggttacaggcaaagtccctttcacctatcagaggaagatcaga    96600

Papio.anubis.clone.rp41-133b2.8034-40080.revcompl.Baboon         ------------------------------------------------------------    20717
BCRP3.HUMAN.NCBI.REF                                             ------------------------------------------------------------    20446
LOC106996293.glutathione.hydrolase5.proenzyme-like-GGT1.rhesus.  gaaaatccttctgttttcgctttatactatgccaggagtatcccagcatgttgctataag    96660

Papio.anubis.clone.rp41-133b2.8034-40080.revcompl.Baboon         ------------------------------------------------------------    20717
BCRP3.HUMAN.NCBI.REF                                             ------------------------------------------------------------    20446
LOC106996293.glutathione.hydrolase5.proenzyme-like-GGT1.rhesus.  aattgggtacaatggtaacactgtagaaacatactcatactgttgtcattttaactcccc    96720

Papio.anubis.clone.rp41-133b2.8034-40080.revcompl.Baboon         ------------------------------------------------------------    20717
BCRP3.HUMAN.NCBI.REF                                             ------------------------------------------------------------    20446
LOC106996293.glutathione.hydrolase5.proenzyme-like-GGT1.rhesus.  accctaagtgtatctccggcaacaggcagctcctctgccctgccgacctcttccaggact    96780

Papio.anubis.clone.rp41-133b2.8034-40080.revcompl.Baboon         ------------------------------------------------------------    20717
BCRP3.HUMAN.NCBI.REF                                             ------------------------------------------------------------    20446
LOC106996293.glutathione.hydrolase5.proenzyme-like-GGT1.rhesus.  taaaatccaccacttctacacaaacgagttgcgggggtggtgattacaacaaaagaaggt    96840

Papio.anubis.clone.rp41-133b2.8034-40080.revcompl.Baboon         ------------------------------------------------------------    20717
```

```
BCRP3.HUMAN.NCBI.REF                                              ------------------------------------------------------------    20446
LOC106996293.glutathione.hydrolase5.proenzyme-like-GGT1.rhesus.   gaagctcctctatgcatctgtctttaaagtgtcatctcagagaacgcatccctgcagacc    96900

Papio.anubis.clone.rp41-133b2.8034-40080.revcompl.Baboon          ------------------------------------------------------------    20717
BCRP3.HUMAN.NCBI.REF                                              ------------------------------------------------------------    20446
LOC106996293.glutathione.hydrolase5.proenzyme-like-GGT1.rhesus.   cactgggcaggagctgcaccctcgcatgtcactgtaatattccttaacggaggccctgtt    96960

Papio.anubis.clone.rp41-133b2.8034-40080.revcompl.Baboon          ------------------------------------------------------------    20717
BCRP3.HUMAN.NCBI.REF                                              ------------------------------------------------------------    20446
LOC106996293.glutathione.hydrolase5.proenzyme-like-GGT1.rhesus.   ccacagtgggtcagcctctcagtgctggtggagcctgtcatgataaaattccctatctag    97020

Papio.anubis.clone.rp41-133b2.8034-40080.revcompl.Baboon          ------------------------------------------------------------    20717
BCRP3.HUMAN.NCBI.REF                                              ------------------------------------------------------------    20446
LOC106996293.glutathione.hydrolase5.proenzyme-like-GGT1.rhesus.   gcagcctttctataggcagcataatcctaagtgtgtcctaataggctggagcagtttccc    97080

Papio.anubis.clone.rp41-133b2.8034-40080.revcompl.Baboon          ------------------------------------------------------------    20717
BCRP3.HUMAN.NCBI.REF                                              ------------------------------------------------------------    20446
LOC106996293.glutathione.hydrolase5.proenzyme-like-GGT1.rhesus.   agggctgccataacatgccacgctgactgaagggcttaaccacagaaatctggttcctca    97140

Papio.anubis.clone.rp41-133b2.8034-40080.revcompl.Baboon          ------------------------------------------------------------    20717
BCRP3.HUMAN.NCBI.REF                                              ------------------------------------------------------------    20446
LOC106996293.glutathione.hydrolase5.proenzyme-like-GGT1.rhesus.   caattctggagactggaagcccaatgtcaagctgtcagtctggggtggtttctctgggcc    97200

Papio.anubis.clone.rp41-133b2.8034-40080.revcompl.Baboon          ------------------------------------------------------------    20717
BCRP3.HUMAN.NCBI.REF                                              ------------------------------------------------------------    20446
LOC106996293.glutathione.hydrolase5.proenzyme-like-GGT1.rhesus.   tctctccttgccgtggagatggctgacttctatctcggtcagcacagggtcttctcttca    97260

Papio.anubis.clone.rp41-133b2.8034-40080.revcompl.Baboon          ------------------------------------------------------------    20717
BCRP3.HUMAN.NCBI.REF                                              ------------------------------------------------------------    20446
LOC106996293.glutathione.hydrolase5.proenzyme-like-GGT1.rhesus.   acttctctgtgcgctgcccacctatccttgtaagaaccccaggcatattaacttaggggc    97320

Papio.anubis.clone.rp41-133b2.8034-40080.revcompl.Baboon          ------------------------------------------------------------    20717
BCRP3.HUMAN.NCBI.REF                                              ------------------------------------------------------------    20446
LOC106996293.glutathione.hydrolase5.proenzyme-like-GGT1.rhesus.   caccctaatgaactcacttcacctgaattacctcttggagaactctgtgtccaaacacag    97380

Papio.anubis.clone.rp41-133b2.8034-40080.revcompl.Baboon          ------------------------------------------------------------    20717
BCRP3.HUMAN.NCBI.REF                                              ------------------------------------------------------------    20446
LOC106996293.glutathione.hydrolase5.proenzyme-like-GGT1.rhesus.   tcacattctgagtctctggggttaagaatagaaacagttacatttgggggaggggacaca    97440

Papio.anubis.clone.rp41-133b2.8034-40080.revcompl.Baboon          ------------------------------------------------------------    20717
BCRP3.HUMAN.NCBI.REF                                              ------------------------------------------------------------    20446
LOC106996293.glutathione.hydrolase5.proenzyme-like-GGT1.rhesus.   attcaacccataaaatacacaaatcccctgtggaagacacacccgggaaagggaaatctg    97500

Papio.anubis.clone.rp41-133b2.8034-40080.revcompl.Baboon          ------------------------------------------------------------    20717
BCRP3.HUMAN.NCBI.REF                                              ------------------------------------------------------------    20446
LOC106996293.glutathione.hydrolase5.proenzyme-like-GGT1.rhesus.   ggtgaaaggatgtgcagatagcaagtttcaacagctctagccagctgactttccaaaagg    97560

Papio.anubis.clone.rp41-133b2.8034-40080.revcompl.Baboon          ------------------------------------------------------------    20717
BCRP3.HUMAN.NCBI.REF                                              ------------------------------------------------------------    20446
LOC106996293.glutathione.hydrolase5.proenzyme-like-GGT1.rhesus.   gctccagctgctcacacggtcaccagcagcctctgaaggtagctgcttctttgaatgcaa    97620

Papio.anubis.clone.rp41-133b2.8034-40080.revcompl.Baboon          ------------------------------------------------------------    20717
BCRP3.HUMAN.NCBI.REF                                              ------------------------------------------------------------    20446
LOC106996293.glutathione.hydrolase5.proenzyme-like-GGT1.rhesus.   cctcccctgaatactaacatgctcttaactttttgccaatctgctgtctgtgtagcagag    97680

Papio.anubis.clone.rp41-133b2.8034-40080.revcompl.Baboon          ------------------------------------------------------------    20717
BCRP3.HUMAN.NCBI.REF                                              ------------------------------------------------------------    20446
LOC106996293.glutathione.hydrolase5.proenzyme-like-GGT1.rhesus.   aaaattaccttgtttcatttccagtttcaaacatacatttccgtaacctttcccggtttc    97740

Papio.anubis.clone.rp41-133b2.8034-40080.revcompl.Baboon          ------------------------------------------------------------    20717
BCRP3.HUMAN.NCBI.REF                                              ------------------------------------------------------------    20446
LOC106996293.glutathione.hydrolase5.proenzyme-like-GGT1.rhesus.   aatacctgcctgcgttttctcttccacaagtcacctggcagatcctaggcgttgggatgc    97800

Papio.anubis.clone.rp41-133b2.8034-40080.revcompl.Baboon          ------------------------------------------------------------    20717
BCRP3.HUMAN.NCBI.REF                                              ------------------------------------------------------------    20446
LOC106996293.glutathione.hydrolase5.proenzyme-like-GGT1.rhesus.   gcttcctcccaccctggagtcttccggcattttccccttggccagggctgggagggccat    97860

Papio.anubis.clone.rp41-133b2.8034-40080.revcompl.Baboon          ------------------------------------------------------------    20717
BCRP3.HUMAN.NCBI.REF                                              ------------------------------------------------------------    20446
LOC106996293.glutathione.hydrolase5.proenzyme-like-GGT1.rhesus.   agctgtttctccagactcacattcatgttaaagaacaaaaacaatcatatccttttaagt    97920

Papio.anubis.clone.rp41-133b2.8034-40080.revcompl.Baboon          ------------------------------------------------------------    20717
BCRP3.HUMAN.NCBI.REF                                              ------------------------------------------------------------    20446
LOC106996293.glutathione.hydrolase5.proenzyme-like-GGT1.rhesus.   caaaggcttcttctagagcatcttgtctggtgcgctgtcactaaaataaggaggggatga    97980

Papio.anubis.clone.rp41-133b2.8034-40080.revcompl.Baboon          ------------------------------------------------------------    20717
BCRP3.HUMAN.NCBI.REF                                              ------------------------------------------------------------    20446
LOC106996293.glutathione.hydrolase5.proenzyme-like-GGT1.rhesus.   gacctccccaggccgttcccttccatacagaagcggagcctcaggtggcctagagggaat    98040

Papio.anubis.clone.rp41-133b2.8034-40080.revcompl.Baboon          ------------------------------------------------------------    20717
BCRP3.HUMAN.NCBI.REF                                              ------------------------------------------------------------    20446
LOC106996293.glutathione.hydrolase5.proenzyme-like-GGT1.rhesus.   ttcatccagtcctccctgtggaaggtgctctcctctgacccgggtgctgctgagcaagca    98100

Papio.anubis.clone.rp41-133b2.8034-40080.revcompl.Baboon          ------------------------------------------------------------    20717
BCRP3.HUMAN.NCBI.REF                                              ------------------------------------------------------------    20446
LOC106996293.glutathione.hydrolase5.proenzyme-like-GGT1.rhesus.   tctgacatccaaaagcccgcatctcctcttctccaccatgagggtgattttgaggattg    98160

Papio.anubis.clone.rp41-133b2.8034-40080.revcompl.Baboon          ------------------------------------------------------------    20717
BCRP3.HUMAN.NCBI.REF                                              ------------------------------------------------------------    20446
LOC106996293.glutathione.hydrolase5.proenzyme-like-GGT1.rhesus.   cagggatgatgctcttcatgcaggctgttgatacagagggtgctattgccccacagctga    98220

Papio.anubis.clone.rp41-133b2.8034-40080.revcompl.Baboon          ------------------------------------------------------------    20717
BCRP3.HUMAN.NCBI.REF                                              ------------------------------------------------------------    20446
LOC106996293.glutathione.hydrolase5.proenzyme-like-GGT1.rhesus.   cattcaccttgaaggcatgaatcattttatccttctccatcctcacatgcaattttacct    98280

Papio.anubis.clone.rp41-133b2.8034-40080.revcompl.Baboon          ------------------------------------------------------------    20717
BCRP3.HUMAN.NCBI.REF                                              ------------------------------------------------------------    20446
LOC106996293.glutathione.hydrolase5.proenzyme-like-GGT1.rhesus.   tgaaagcctagaacccttttcaccatgggcacccagggtctccacatgcacaagtccctg    98340

Papio.anubis.clone.rp41-133b2.8034-40080.revcompl.Baboon          ------------------------------------------------------------    20717
BCRP3.HUMAN.NCBI.REF                                              ------------------------------------------------------------    20446
LOC106996293.glutathione.hydrolase5.proenzyme-like-GGT1.rhesus.   aagggttgatgcttcttggagtgatttccagagcccggtcctgtggtggttcatggtgtt    98400

Papio.anubis.clone.rp41-133b2.8034-40080.revcompl.Baboon          ------------------------------------------------------------    20717
BCRP3.HUMAN.NCBI.REF                                              ------------------------------------------------------------    20446
LOC106996293.glutathione.hydrolase5.proenzyme-like-GGT1.rhesus.   acactgactcccatgtccctccttgtccccctagtatgaaaatgagatctgcaggatgcc    98460

Papio.anubis.clone.rp41-133b2.8034-40080.revcompl.Baboon          ------------------------------------------------------------    20717
BCRP3.HUMAN.NCBI.REF                                              ------------------------------------------------------------    20446
LOC106996293.glutathione.hydrolase5.proenzyme-like-GGT1.rhesus.   aactttccagcccagcacaggggagaagctattggagtccctggttccagcctttctaac    98520

Papio.anubis.clone.rp41-133b2.8034-40080.revcompl.Baboon          ------------------------------------------------------------    20717
BCRP3.HUMAN.NCBI.REF                                              ------------------------------------------------------------    20446
LOC106996293.glutathione.hydrolase5.proenzyme-like-GGT1.rhesus.   taaacccatctcctcctatgccacctgcctgggtccctcctgggactttatcaccgtgcc    98580

Papio.anubis.clone.rp41-133b2.8034-40080.revcompl.Baboon          ------------------------------------------------------------    20717
```

```
BCRP3.HUMAN.NCBI.REF                                              ------------------------------------------------------------    20446
LOC106996293.glutathione.hydrolase5.proenzyme-like-GGT1.rhesus.   acactttttggaactactggttagaaggtgagtgtccatcccctccaaggcacggaggtg    98640

Papio.anubis.clone.rp41-133b2.8034-40080.revcompl.Baboon          ------------------------------------------------------------    20717
BCRP3.HUMAN.NCBI.REF                                              ------------------------------------------------------------    20446
LOC106996293.glutathione.hydrolase5.proenzyme-like-GGT1.rhesus.   cctctgtcccctactggctgtgtcttgaagatgcacctcttggagcctcagttggctcct    98700

Papio.anubis.clone.rp41-133b2.8034-40080.revcompl.Baboon          ------------------------------------------------------------    20717
BCRP3.HUMAN.NCBI.REF                                              ------------------------------------------------------------    20446
LOC106996293.glutathione.hydrolase5.proenzyme-like-GGT1.rhesus.   gtggaacagggagtaatgagaagatgaagctcacagggctcttgtagggactgaatgatc    98760

Papio.anubis.clone.rp41-133b2.8034-40080.revcompl.Baboon          -----------------------------------------gaaatgatcatctttggtt    20736
BCRP3.HUMAN.NCBI.REF                                              ------------------------------------------------------------    20446
LOC106996293.glutathione.hydrolase5.proenzyme-like-GGT1.rhesus.   taagacacagcaaacaaaggggtccgtagagcctaaaactctggacaatctgctgtgggt    98820

Papio.anubis.clone.rp41-133b2.8034-40080.revcompl.Baboon          actgggatgcatg-----------------------------------------------    20749
BCRP3.HUMAN.NCBI.REF                                              ------------------------------------------------------------    20446
LOC106996293.glutathione.hydrolase5.proenzyme-like-GGT1.rhesus.   gctgtgattcatgtttattacttttctgttcccactcactgaagcagctctcagcattct    98880

Papio.anubis.clone.rp41-133b2.8034-40080.revcompl.Baboon          ------------------------------------------------------------    20749
BCRP3.HUMAN.NCBI.REF                                              ------------------------------------------------------------    20446
LOC106996293.glutathione.hydrolase5.proenzyme-like-GGT1.rhesus.   gccgcaatggcccatctctcttgctgtttggaaaggcacatagaaagcaagtctgcaatg    98940

Papio.anubis.clone.rp41-133b2.8034-40080.revcompl.Baboon          ------------------------------------------------------------    20749
BCRP3.HUMAN.NCBI.REF                                              ------------------------------------------------------------    20446
LOC106996293.glutathione.hydrolase5.proenzyme-like-GGT1.rhesus.   gcattgctaaaggatgtctgagtttccttctggctggactttctccttgacacagacaga    99000

Papio.anubis.clone.rp41-133b2.8034-40080.revcompl.Baboon          ------------------------------------------------------------    20749
BCRP3.HUMAN.NCBI.REF                                              ------------------------------------------------------------    20446
LOC106996293.glutathione.hydrolase5.proenzyme-like-GGT1.rhesus.   agaggggtcccttcatgctgaaaaaggagccacaggcccactcagacatctggagaggct    99060

Papio.anubis.clone.rp41-133b2.8034-40080.revcompl.Baboon          ------------------------------------------------------------    20749
BCRP3.HUMAN.NCBI.REF                                              ------------------------------------------------------------    20446
LOC106996293.glutathione.hydrolase5.proenzyme-like-GGT1.rhesus.   cactgggttctccaatggttggggttcactcattcaacacatacatacaaaacacctcat    99120

Papio.anubis.clone.rp41-133b2.8034-40080.revcompl.Baboon          ------------------------------------------------------------    20749
BCRP3.HUMAN.NCBI.REF                                              ------------------------------------------------------------    20446
LOC106996293.glutathione.hydrolase5.proenzyme-like-GGT1.rhesus.   ttgtgcatcgttcttcttgtgacttggcctaatttcataaacaaggcagatgacaatccc    99180

Papio.anubis.clone.rp41-133b2.8034-40080.revcompl.Baboon          ------------------------------------------------------------    20749
BCRP3.HUMAN.NCBI.REF                                              ------------------------------------------------------------    20446
LOC106996293.glutathione.hydrolase5.proenzyme-like-GGT1.rhesus.   tgggcctcaattctactcagagtcaggtgctcacagtagacagaataaacaaatcatatc    99240

Papio.anubis.clone.rp41-133b2.8034-40080.revcompl.Baboon          ------------------------------------------------------------    20749
BCRP3.HUMAN.NCBI.REF                                              ------------------------------------------------------------    20446
LOC106996293.glutathione.hydrolase5.proenzyme-like-GGT1.rhesus.   tacagaatgttagaggggaaaccctcatggcctcctctacatatggtggcatcctcccag    99300

Papio.anubis.clone.rp41-133b2.8034-40080.revcompl.Baboon          ------------------------------------------------------------    20749
BCRP3.HUMAN.NCBI.REF                                              ------------------------------------------------------------    20446
LOC106996293.glutathione.hydrolase5.proenzyme-like-GGT1.rhesus.   attctgactagaatgacggagcccaacaagtataaactggggggacttagggttctgaaa    99360

Papio.anubis.clone.rp41-133b2.8034-40080.revcompl.Baboon          ------------------------------------------------------------    20749
BCRP3.HUMAN.NCBI.REF                                              ------------------------------------------------------------    20446
LOC106996293.glutathione.hydrolase5.proenzyme-like-GGT1.rhesus.   ggcctcttcacccacaaaacatgggaggaaatatgtggactctggctggggagagaataa    99420

Papio.anubis.clone.rp41-133b2.8034-40080.revcompl.Baboon          ------------------------------------------------------------    20749
BCRP3.HUMAN.NCBI.REF                                              ------------------------------------------------------------    20446
LOC106996293.glutathione.hydrolase5.proenzyme-like-GGT1.rhesus.   aggagccctggggttcatgtcttataattcccacaacaaggctgacatttgggaacttcc    99480

Papio.anubis.clone.rp41-133b2.8034-40080.revcompl.Baboon          ------------------------------------------------------------    20749
BCRP3.HUMAN.NCBI.REF                                              ------------------------------------------------------------    20446
LOC106996293.glutathione.hydrolase5.proenzyme-like-GGT1.rhesus.   cctgcaagaggcaaatagtgagttctgtaaatgaagacttaggtccattgcaaaggagag    99540

Papio.anubis.clone.rp41-133b2.8034-40080.revcompl.Baboon          ------------------------------------------------------------    20749
BCRP3.HUMAN.NCBI.REF                                              ------------------------------------------------------------    20446
LOC106996293.glutathione.hydrolase5.proenzyme-like-GGT1.rhesus.   gtgagggtggggtcacaactggccactgagagacccatccccaccactgtggcttcccag    99600

Papio.anubis.clone.rp41-133b2.8034-40080.revcompl.Baboon          ------------------------------------------------------------    20749
BCRP3.HUMAN.NCBI.REF                                              ------------------------------------------------------------    20446
LOC106996293.glutathione.hydrolase5.proenzyme-like-GGT1.rhesus.   ctctccctgtcctcctccacccaacatctgcccctaccctcctaaccccaggaccagggg    99660

Papio.anubis.clone.rp41-133b2.8034-40080.revcompl.Baboon          ------------------------------------------------------------    20749
BCRP3.HUMAN.NCBI.REF                                              ------------------------------------------------------------    20446
LOC106996293.glutathione.hydrolase5.proenzyme-like-GGT1.rhesus.   aaccaaagctggagctttgatgagcaagctgctcacaaatctgcctggagctgcagtctt    99720

Papio.anubis.clone.rp41-133b2.8034-40080.revcompl.Baboon          ------------------------------------------------------------    20749
BCRP3.HUMAN.NCBI.REF                                              ------------------------------------------------------------    20446
LOC106996293.glutathione.hydrolase5.proenzyme-like-GGT1.rhesus.   gagtgcccaggtgcacagtgctgtgctccagggccattggaaagagaatgtcagtgggac    99780

Papio.anubis.clone.rp41-133b2.8034-40080.revcompl.Baboon          ------------------------------------------------------------    20749
BCRP3.HUMAN.NCBI.REF                                              ------------------------------------------------------------    20446
LOC106996293.glutathione.hydrolase5.proenzyme-like-GGT1.rhesus.   gccggggcacacaagggtctgtagagccctgctgcatggccaggtctgccccttccagga    99840

Papio.anubis.clone.rp41-133b2.8034-40080.revcompl.Baboon          ------------------------------------------------------------    20749
BCRP3.HUMAN.NCBI.REF                                              ------------------------------------------------------------    20446
LOC106996293.glutathione.hydrolase5.proenzyme-like-GGT1.rhesus.   cagcactgatggcttggggtagggtggggctgtcctctacacaggcagcaagaggccagg    99900

Papio.anubis.clone.rp41-133b2.8034-40080.revcompl.Baboon          ------------------------------------------------------------    20749
BCRP3.HUMAN.NCBI.REF                                              ------------------------------------------------------------    20446
LOC106996293.glutathione.hydrolase5.proenzyme-like-GGT1.rhesus.   gacccagaaccacgcaagggtgccctggagggttgtgtgggagaaggccaggcctctgac    99960

Papio.anubis.clone.rp41-133b2.8034-40080.revcompl.Baboon          ------------------------------------------------------------    20749
BCRP3.HUMAN.NCBI.REF                                              ------------------------------------------------------------    20446
LOC106996293.glutathione.hydrolase5.proenzyme-like-GGT1.rhesus.   tcagctgtccactccatcaccagcaccaccacctccattttgttcacctggccccctgaa    100020

Papio.anubis.clone.rp41-133b2.8034-40080.revcompl.Baboon          ------------------------------------------------------------    20749
BCRP3.HUMAN.NCBI.REF                                              ------------------------------------------------------------    20446
LOC106996293.glutathione.hydrolase5.proenzyme-like-GGT1.rhesus.   aacacctcagtttgctgccaggccctgcaacggtcatctttctggataaagctggccttc    100080

Papio.anubis.clone.rp41-133b2.8034-40080.revcompl.Baboon          ------------------------------------------------------------    20749
BCRP3.HUMAN.NCBI.REF                                              ------------------------------------------------------------    20446
LOC106996293.glutathione.hydrolase5.proenzyme-like-GGT1.rhesus.   aggaccttcgcctggcctggactgggcgtggaggaccatcaggaaattgtcctaggccag    100140

Papio.anubis.clone.rp41-133b2.8034-40080.revcompl.Baboon          ------------------------------------------------------------    20749
BCRP3.HUMAN.NCBI.REF                                              ------------------------------------------------------------    20446
LOC106996293.glutathione.hydrolase5.proenzyme-like-GGT1.rhesus.   ttggtgcttccggagcccaaggaggccaagccagatggtgagaagactttccgggggtgg    100200

Papio.anubis.clone.rp41-133b2.8034-40080.revcompl.Baboon          ------------------------------------------------------------    20749
BCRP3.HUMAN.NCBI.REF                                              ------------------------------------------------------------    20446
LOC106996293.glutathione.hydrolase5.proenzyme-like-GGT1.rhesus.   tgttttggggctataattcccttaaattccatccaggcatttctatacttggaaatccca    100260

Papio.anubis.clone.rp41-133b2.8034-40080.revcompl.Baboon          ------------------------------------------------------------    20749
BCRP3.HUMAN.NCBI.REF                                              ------------------------------------------------------------    20446
LOC106996293.glutathione.hydrolase5.proenzyme-like-GGT1.rhesus.   gtgaagagtgactggaatggtgaccttttctccttttccagatcctgctccacctcctgg    100320

Papio.anubis.clone.rp41-133b2.8034-40080.revcompl.Baboon          ------------------------------------------------------------    20749
```

```
BCRP3.HUMAN.NCBI.REF                                               ------------------------------------------------------------    20446
LOC106996293.glutathione.hydrolase5.proenzyme-like-GGT1.rhesus.    gcaacacgcattaacaatgctggccctggagccagcaccaccactgctggcggacctgcg    100380

Papio.anubis.clone.rp41-133b2.8034-40080.revcompl.Baboon           ------------------------------------------------------------    20749
BCRP3.HUMAN.NCBI.REF                                               ------------------------------------------------------------    20446
LOC106996293.glutathione.hydrolase5.proenzyme-like-GGT1.rhesus.    gcctgctctagagccagagtcacctgtagccctggatgcaccaggatatctacattcagc    100440

Papio.anubis.clone.rp41-133b2.8034-40080.revcompl.Baboon           ------------------------------------------------------------    20749
BCRP3.HUMAN.NCBI.REF                                               ------------------------------------------------------------    20446
LOC106996293.glutathione.hydrolase5.proenzyme-like-GGT1.rhesus.    atcagcaccagcaccaggggaagggccccctccaggaacagtgctggagccacagtcagc    100500

Papio.anubis.clone.rp41-133b2.8034-40080.revcompl.Baboon           ------------------------------------------------------------    20749
BCRP3.HUMAN.NCBI.REF                                               ------------------------------------------------------------    20446
LOC106996293.glutathione.hydrolase5.proenzyme-like-GGT1.rhesus.    cccagagtcaccctgtccctgtcccgggactgtccagagccaacacactgaggagctgcc    100560

Papio.anubis.clone.rp41-133b2.8034-40080.revcompl.Baboon           ------------------------------------------------------------    20749
BCRP3.HUMAN.NCBI.REF                                               ------------------------------------------------------------    20446
LOC106996293.glutathione.hydrolase5.proenzyme-like-GGT1.rhesus.    ggacatcacgaccttccctcccaggctgctggctgagcagctgacccttatggatgcggt    100620

Papio.anubis.clone.rp41-133b2.8034-40080.revcompl.Baboon           ------------------------------------------------------------    20749
BCRP3.HUMAN.NCBI.REF                                               ------------------------------------------------------------    20446
LOC106996293.glutathione.hydrolase5.proenzyme-like-GGT1.rhesus.    gagcagctgggctttgcaggctgtgcctctggcaccagctgtctcaggccagcctgtctc    100680

Papio.anubis.clone.rp41-133b2.8034-40080.revcompl.Baboon           ------------------------------------------------------------    20749
BCRP3.HUMAN.NCBI.REF                                               ------------------------------------------------------------    20446
LOC106996293.glutathione.hydrolase5.proenzyme-like-GGT1.rhesus.    tgaggagcggccaatgccctgggtccagtttcagccccacttcttaccaaccatgggatc    100740

Papio.anubis.clone.rp41-133b2.8034-40080.revcompl.Baboon           ------------------------------------------------------------    20749
BCRP3.HUMAN.NCBI.REF                                               ------------------------------------------------------------    20446
LOC106996293.glutathione.hydrolase5.proenzyme-like-GGT1.rhesus.    tggatgagtttcctcacccacaagccttccctgtctgcatgtggacagcagagatgggac    100800

Papio.anubis.clone.rp41-133b2.8034-40080.revcompl.Baboon           ------------------------------------------------------------    20749
BCRP3.HUMAN.NCBI.REF                                               ------------------------------------------------------------    20446
LOC106996293.glutathione.hydrolase5.proenzyme-like-GGT1.rhesus.    atcgcccgtgccagctgcacagagtgactgtgcagactgaatgacaggggatggacagaa    100860

Papio.anubis.clone.rp41-133b2.8034-40080.revcompl.Baboon           ------------------------------------------------------------    20749
BCRP3.HUMAN.NCBI.REF                                               ------------------------------------------------------------    20446
LOC106996293.glutathione.hydrolase5.proenzyme-like-GGT1.rhesus.    agcaggacagggcaggtgatcaccgaggggcaggcagggccatgggtcactcacccagct    100920

Papio.anubis.clone.rp41-133b2.8034-40080.revcompl.Baboon           ------------------------------------------------------------    20749
BCRP3.HUMAN.NCBI.REF                                               ------------------------------------------------------------    20446
LOC106996293.glutathione.hydrolase5.proenzyme-like-GGT1.rhesus.    gctcaggagcctcactaccctcagcacttattaggtacctgatgcatactagattctatg    100980

Papio.anubis.clone.rp41-133b2.8034-40080.revcompl.Baboon           ------------------------------------------------------------    20749
BCRP3.HUMAN.NCBI.REF                                               ------------------------------------------------------------    20446
LOC106996293.glutathione.hydrolase5.proenzyme-like-GGT1.rhesus.    gcagacacccaaacagagcccaaggttgcagctgctgcaaggaaactgcaccagtaggga    101040

Papio.anubis.clone.rp41-133b2.8034-40080.revcompl.Baboon           ------------------------------------------------------------    20749
BCRP3.HUMAN.NCBI.REF                                               ------------------------------------------------------------    20446
LOC106996293.glutathione.hydrolase5.proenzyme-like-GGT1.rhesus.    gagaaggagtagagggctgattgggatgggaggaagatgaggcctcaggatggggaggcc    101100

Papio.anubis.clone.rp41-133b2.8034-40080.revcompl.Baboon           ------------------------------------------------------------    20749
BCRP3.HUMAN.NCBI.REF                                               ------------------------------------------------------------    20446
LOC106996293.glutathione.hydrolase5.proenzyme-like-GGT1.rhesus.    tgagccaccttctagttcttggaatgaggatggcctgggagaaaatgtcactctctcttc    101160

Papio.anubis.clone.rp41-133b2.8034-40080.revcompl.Baboon           ------------------------------------------------------------    20749
BCRP3.HUMAN.NCBI.REF                                               ------------------------------------------------------------    20446
LOC106996293.glutathione.hydrolase5.proenzyme-like-GGT1.rhesus.    ccacccttgttggtttctgggacatgatgcattcagggccctcggtgggcagaaaaccaa    101220

Papio.anubis.clone.rp41-133b2.8034-40080.revcompl.Baboon           ------------------------------------------------------------    20749
BCRP3.HUMAN.NCBI.REF                                               ------------------------------------------------------------    20446
LOC106996293.glutathione.hydrolase5.proenzyme-like-GGT1.rhesus.    acccagggactcccacaagtctggagcatattttaaagctttctgagctcaagctcggtt    101280

Papio.anubis.clone.rp41-133b2.8034-40080.revcompl.Baboon           ------------------------------------------------------------    20749
BCRP3.HUMAN.NCBI.REF                                               ------------------------------------------------------------    20446
LOC106996293.glutathione.hydrolase5.proenzyme-like-GGT1.rhesus.    cctgccagagactgtggataactgtgagctcagtccctgcctgggactgtgggtgactct    101340

Papio.anubis.clone.rp41-133b2.8034-40080.revcompl.Baboon           ------------------------------------------------------------    20749
BCRP3.HUMAN.NCBI.REF                                               ------------------------------------------------------------    20446
LOC106996293.glutathione.hydrolase5.proenzyme-like-GGT1.rhesus.    gagctggggtgtgctgtgtccatgacactctcctccttccccaaaggatctgttcaagaa    101400

Papio.anubis.clone.rp41-133b2.8034-40080.revcompl.Baboon           ------------------------------------------------------------    20749
BCRP3.HUMAN.NCBI.REF                                               ------------------------------------------------------------    20446
LOC106996293.glutathione.hydrolase5.proenzyme-like-GGT1.rhesus.    ggtggagctctacgaatgcttgggctccatctggggccaacgacatcagaaggggagtga    101460

Papio.anubis.clone.rp41-133b2.8034-40080.revcompl.Baboon           ------------------------------------------------------------    20749
BCRP3.HUMAN.NCBI.REF                                               ------------------------------------------------------------    20446
LOC106996293.glutathione.hydrolase5.proenzyme-like-GGT1.rhesus.    gcacgtggcacccacagtttgtgccaccattgcacacttcaacaggctcgccaactgtgt    101520

Papio.anubis.clone.rp41-133b2.8034-40080.revcompl.Baboon           ------------------------------------------------------------    20749
BCRP3.HUMAN.NCBI.REF                                               ------------------------------------------------------------    20446
LOC106996293.glutathione.hydrolase5.proenzyme-like-GGT1.rhesus.    caccacctcctgcctcggggaccacagcatgagggtccaggatagggccagggtggtgga    101580

Papio.anubis.clone.rp41-133b2.8034-40080.revcompl.Baboon           ------------------------------------------------------------    20749
BCRP3.HUMAN.NCBI.REF                                               ------------------------------------------------------------    20446
LOC106996293.glutathione.hydrolase5.proenzyme-like-GGT1.rhesus.    gcactggatcaaggtggccagggtaagccatggttgggccttgggattccctctttaaaa    101640

Papio.anubis.clone.rp41-133b2.8034-40080.revcompl.Baboon           ------------------------------------------------------------    20749
BCRP3.HUMAN.NCBI.REF                                               ------------------------------------------------------------    20446
LOC106996293.glutathione.hydrolase5.proenzyme-like-GGT1.rhesus.    atggggaactgcctcttctcctccatcggctttcaggattggcatctgtatctctagcct    101700

Papio.anubis.clone.rp41-133b2.8034-40080.revcompl.Baboon           ------------------------------------------------------------    20749
BCRP3.HUMAN.NCBI.REF                                               ------------------------------------------------------------    20446
LOC106996293.glutathione.hydrolase5.proenzyme-like-GGT1.rhesus.    gagccctacacatcccctaggcccttcttctctgaacttccctgaccttgacccccatgg    101760

Papio.anubis.clone.rp41-133b2.8034-40080.revcompl.Baboon           ------------------------------------------------------------    20749
BCRP3.HUMAN.NCBI.REF                                               ------------------------------------------------------------    20446
LOC106996293.glutathione.hydrolase5.proenzyme-like-GGT1.rhesus.    cccagtggtggctgctcacgtctgacctgggatcttccttgggttgaactgaaatctttc    101820

Papio.anubis.clone.rp41-133b2.8034-40080.revcompl.Baboon           ------------------------------------------------------------    20749
BCRP3.HUMAN.NCBI.REF                                               ------------------------------------------------------------    20446
LOC106996293.glutathione.hydrolase5.proenzyme-like-GGT1.rhesus.    tagatgagtgacattcactcagccccaggtgtaccctcctgaggctccctgggcctctgc    101880

Papio.anubis.clone.rp41-133b2.8034-40080.revcompl.Baboon           ------------------------------------------------------------    20749
BCRP3.HUMAN.NCBI.REF                                               ------------------------------------------------------------    20446
LOC106996293.glutathione.hydrolase5.proenzyme-like-GGT1.rhesus.    ttcattcaagaagggagatctcagcagagggggctgaggctgaagtgggtctgactccaa    101940

Papio.anubis.clone.rp41-133b2.8034-40080.revcompl.Baboon           ------------------------------------------------------------    20749
BCRP3.HUMAN.NCBI.REF                                               ------------------------------------------------------------    20446
LOC106996293.glutathione.hydrolase5.proenzyme-like-GGT1.rhesus.    ctctggacctcacagctcactcttccctctccaggagtgcctaagcctcaacaacttctc    102000

Papio.anubis.clone.rp41-133b2.8034-40080.revcompl.Baboon           ------------------------------------------------------------    20749
BCRP3.HUMAN.NCBI.REF                                               ------------------------------------------------------------    20446
LOC106996293.glutathione.hydrolase5.proenzyme-like-GGT1.rhesus.    ctcagtgcacgccatcgtctctgctctgcgcagcaacccaatacatcggctacacaagac    102060

Papio.anubis.clone.rp41-133b2.8034-40080.revcompl.Baboon           ------------------------------------------------------------    20749
```

```
BCRP3.HUMAN.NCBI.REF                                               ------------------------------------------------------------    20446
LOC106996293.glutathione.hydrolase5.proenzyme-like-GGT1.rhesus.    gtgggcagcagtgtccaggtgaggagctccctccacgggagcaccagtgttgacttaggg    102120

Papio.anubis.clone.rp41-133b2.8034-40080.revcompl.Baboon           ------------------------------------------------------------    20749
BCRP3.HUMAN.NCBI.REF                                               ------------------------------------------------------------    20446
LOC106996293.glutathione.hydrolase5.proenzyme-like-GGT1.rhesus.    acccataggtctccccatgtgccctcaacgattctgaaaggttcttggagaacagggatg    102180

Papio.anubis.clone.rp41-133b2.8034-40080.revcompl.Baboon           ------------------------------------------------------------    20749
BCRP3.HUMAN.NCBI.REF                                               ------------------------------------------------------------    20446
LOC106996293.glutathione.hydrolase5.proenzyme-like-GGT1.rhesus.    ctggaggcagggatgggctggtaggtgtggtcactaagctgccctggactcctaggcaag    102240

Papio.anubis.clone.rp41-133b2.8034-40080.revcompl.Baboon           ------------------------------------------------------------    20749
BCRP3.HUMAN.NCBI.REF                                               ------------------------------------------------------------    20446
LOC106996293.glutathione.hydrolase5.proenzyme-like-GGT1.rhesus.    gatttctaactcaggagtaaggtttttaaccatcaggaacagactggagccaactggag    102300

Papio.anubis.clone.rp41-133b2.8034-40080.revcompl.Baboon           ------------------------------------------------------------    20749
BCRP3.HUMAN.NCBI.REF                                               ------------------------------------------------------------    20446
LOC106996293.glutathione.hydrolase5.proenzyme-like-GGT1.rhesus.    gctttcaggtgtttgcacccagcagtggaactgtgtccagctggaagctaactgtgaaca    102360

Papio.anubis.clone.rp41-133b2.8034-40080.revcompl.Baboon           ------------------------------------------------------------    20749
BCRP3.HUMAN.NCBI.REF                                               ------------------------------------------------------------    20446
LOC106996293.glutathione.hydrolase5.proenzyme-like-GGT1.rhesus.    cgcaggggctcatgtgaagtggagatgggccaggggaggagcatgacagtcccaccctgg    102420

Papio.anubis.clone.rp41-133b2.8034-40080.revcompl.Baboon           ------------------------------------------------------------    20749
BCRP3.HUMAN.NCBI.REF                                               ------------------------------------------------------------    20446
LOC106996293.glutathione.hydrolase5.proenzyme-like-GGT1.rhesus.    tcctctggagcccttgtcatcagacgaccccactggaaactctcacgcaggaagctgaga    102480

Papio.anubis.clone.rp41-133b2.8034-40080.revcompl.Baboon           ------------------------------------------------------------    20749
BCRP3.HUMAN.NCBI.REF                                               ------------------------------------------------------------    20446
LOC106996293.glutathione.hydrolase5.proenzyme-like-GGT1.rhesus.    ttcactgggttttcaaacaaaagggattggaattcacaaatctcccccgtgattcccaaa    102540

Papio.anubis.clone.rp41-133b2.8034-40080.revcompl.Baboon           ------------------------------------------------------------    20749
BCRP3.HUMAN.NCBI.REF                                               ------------------------------------------------------------    20446
LOC106996293.glutathione.hydrolase5.proenzyme-like-GGT1.rhesus.    tttacccttttttctttcttctatcaatagcaaaagctcaaaatatctaaaagaactctg    102600

Papio.anubis.clone.rp41-133b2.8034-40080.revcompl.Baboon           ------------------------------------------------------------    20749
BCRP3.HUMAN.NCBI.REF                                               ------------------------------------------------------------    20446
LOC106996293.glutathione.hydrolase5.proenzyme-like-GGT1.rhesus.    caaaaaagacactgcagtgaagagggacctgctgatcaaggtacagtggagtctgggaga    102660

Papio.anubis.clone.rp41-133b2.8034-40080.revcompl.Baboon           ------------------------------------------------------------    20749
BCRP3.HUMAN.NCBI.REF                                               ------------------------------------------------------------    20446
LOC106996293.glutathione.hydrolase5.proenzyme-like-GGT1.rhesus.    tgtgggacaagtgtttaagggtcagaggaaagagtgagtttggaagggcattgatcccgg    102720

Papio.anubis.clone.rp41-133b2.8034-40080.revcompl.Baboon           ------------------------------------------------------------    20749
BCRP3.HUMAN.NCBI.REF                                               ------------------------------------------------------------    20446
LOC106996293.glutathione.hydrolase5.proenzyme-like-GGT1.rhesus.    tgtgcagtggttattttgtaatgttttgacttacctactaaaagtggacttgaaaaattc    102780

Papio.anubis.clone.rp41-133b2.8034-40080.revcompl.Baboon           ------------------------------------------------------------    20749
BCRP3.HUMAN.NCBI.REF                                               ------------------------------------------------------------    20446
LOC106996293.glutathione.hydrolase5.proenzyme-like-GGT1.rhesus.    cctccatgcctactttgggcaaacaggaggagaggtgtgtgggtcgatgggcacgtgggg    102840

Papio.anubis.clone.rp41-133b2.8034-40080.revcompl.Baboon           ------------------------------------------------------------    20749
BCRP3.HUMAN.NCBI.REF                                               ------------------------------------------------------------    20446
LOC106996293.glutathione.hydrolase5.proenzyme-like-GGT1.rhesus.    gcacgggggcagcaggccctggaaataggatgtggcaatggctgctgggcttctgagtga    102900

Papio.anubis.clone.rp41-133b2.8034-40080.revcompl.Baboon           ------------------------------------------------------------    20749
BCRP3.HUMAN.NCBI.REF                                               ------------------------------------------------------------    20446
LOC106996293.glutathione.hydrolase5.proenzyme-like-GGT1.rhesus.    gggtgatgagctgcagcattagcaggactctggctcccatgctggtccatgctgctggca    102960

Papio.anubis.clone.rp41-133b2.8034-40080.revcompl.Baboon           ------------------------------------------------------------    20749
BCRP3.HUMAN.NCBI.REF                                               ------------------------------------------------------------    20446
LOC106996293.glutathione.hydrolase5.proenzyme-like-GGT1.rhesus.    tggagcttcctccaggctgggggtggtcatggtaggtgggactttcttccttcctcaaa    103020

Papio.anubis.clone.rp41-133b2.8034-40080.revcompl.Baboon           ------------------------------------------------------------    20749
BCRP3.HUMAN.NCBI.REF                                               ------------------------------------------------------------    20446
LOC106996293.glutathione.hydrolase5.proenzyme-like-GGT1.rhesus.    ctggccgcaattccttaggaagccaggcctccgctgctgcttctgtctgcagcgcacctc    103080

Papio.anubis.clone.rp41-133b2.8034-40080.revcompl.Baboon           ------------------------------------------------------------    20749
BCRP3.HUMAN.NCBI.REF                                               ------------------------------------------------------------    20446
LOC106996293.glutathione.hydrolase5.proenzyme-like-GGT1.rhesus.    catgggcagggactgcagtccacactggggaaagagggaaccacaacagaggaagctca    103140

Papio.anubis.clone.rp41-133b2.8034-40080.revcompl.Baboon           ------------------------------------------------------------    20749
BCRP3.HUMAN.NCBI.REF                                               ------------------------------------------------------------    20446
LOC106996293.glutathione.hydrolase5.proenzyme-like-GGT1.rhesus.    tatgccagggagtccagtagactgcccagctatgggtcccaatgggcaaactcaggacag    103200

Papio.anubis.clone.rp41-133b2.8034-40080.revcompl.Baboon           ------------------------------------------------------------    20749
BCRP3.HUMAN.NCBI.REF                                               ------------------------------------------------------------    20446
LOC106996293.glutathione.hydrolase5.proenzyme-like-GGT1.rhesus.    atgtatgtgtttgctgggactccccactctgccctttgcagacatctgaaaatggtcatg    103260

Papio.anubis.clone.rp41-133b2.8034-40080.revcompl.Baboon           ------------------------------------------------------------    20749
BCRP3.HUMAN.NCBI.REF                                               ------------------------------------------------------------    20446
LOC106996293.glutathione.hydrolase5.proenzyme-like-GGT1.rhesus.    tcacaggattctcaccaattagcagtgacacatgctcatgacaagtatctgggggatcca    103320

Papio.anubis.clone.rp41-133b2.8034-40080.revcompl.Baboon           ------------------------------------------------------------    20749
BCRP3.HUMAN.NCBI.REF                                               ------------------------------------------------------------    20446
LOC106996293.glutathione.hydrolase5.proenzyme-like-GGT1.rhesus.    tgcattcctaggggatcctccctgaccagatctcagaaacctccatgcaaatgagaaggc    103380

Papio.anubis.clone.rp41-133b2.8034-40080.revcompl.Baboon           ------------------------------------------------------------    20749
BCRP3.HUMAN.NCBI.REF                                               ------------------------------------------------------------    20446
LOC106996293.glutathione.hydrolase5.proenzyme-like-GGT1.rhesus.    aacatgtcaccccacccaggattctggaaaccctgccatgtccgtgagagatgtcgcct    103440

Papio.anubis.clone.rp41-133b2.8034-40080.revcompl.Baboon           ------------------------------------------------------------    20749
BCRP3.HUMAN.NCBI.REF                                               ------------------------------------------------------------    20446
LOC106996293.glutathione.hydrolase5.proenzyme-like-GGT1.rhesus.    caggaggccacatcctgagtggaggaagagagttctgtgcacggaactcccctgggggat    103500

Papio.anubis.clone.rp41-133b2.8034-40080.revcompl.Baboon           ------------------------------------------------------------    20749
BCRP3.HUMAN.NCBI.REF                                               ------------------------------------------------------------    20446
LOC106996293.glutathione.hydrolase5.proenzyme-like-GGT1.rhesus.    cactggagaggccaaaccatggatttggcatggcgcaaacccagtttgtgtggcagagac    103560

Papio.anubis.clone.rp41-133b2.8034-40080.revcompl.Baboon           ------------------------------------------------------------    20749
BCRP3.HUMAN.NCBI.REF                                               ------------------------------------------------------------    20446
LOC106996293.glutathione.hydrolase5.proenzyme-like-GGT1.rhesus.    tccagtggggctcagataggcaggtgccacttaaccaggtctcctaaaatgccctgtccc    103620

Papio.anubis.clone.rp41-133b2.8034-40080.revcompl.Baboon           ------------------------------------------------------------    20749
BCRP3.HUMAN.NCBI.REF                                               ------------------------------------------------------------    20446
LOC106996293.glutathione.hydrolase5.proenzyme-like-GGT1.rhesus.    tttcccatcaagactctgcaaggctggagacctagacactcagagactccaaagaacaag    103680

Papio.anubis.clone.rp41-133b2.8034-40080.revcompl.Baboon           ------------------------------------------------------------    20749
BCRP3.HUMAN.NCBI.REF                                               ------------------------------------------------------------    20446
LOC106996293.glutathione.hydrolase5.proenzyme-like-GGT1.rhesus.    accctgatgggtggtggtgctgggatatggggtgagggcagccgagacagagcctccagg    103740

Papio.anubis.clone.rp41-133b2.8034-40080.revcompl.Baboon           ------------------------------------------------------------    20749
BCRP3.HUMAN.NCBI.REF                                               ------------------------------------------------------------    20446
LOC106996293.glutathione.hydrolase5.proenzyme-like-GGT1.rhesus.    atggggaggaggtgccttctcttttggggccctggggagtcactgcccactcttggtctc    103800

Papio.anubis.clone.rp41-133b2.8034-40080.revcompl.Baboon           ------------------------------------------------------------    20749
```

```
BCRP3.HUMAN.NCBI.REF                                            ------------------------------------------------------------    20446
LOC106996293.glutathione.hydrolase5.proenzyme-like-GGT1.rhesus.     tgtttccttatctggaaaatgaagggatgctgagcctgtagtgcaggcctcacagggtgg    103860

Papio.anubis.clone.rp41-133b2.8034-40080.revcompl.Baboon        ------------------------------------------------------------    20749
BCRP3.HUMAN.NCBI.REF                                            ------------------------------------------------------------    20446
LOC106996293.glutathione.hydrolase5.proenzyme-like-GGT1.rhesus.     aaatgaggttcaagaaaagaaagcaattggagggtgctcgtgaatggttcttcctcagag    103920

Papio.anubis.clone.rp41-133b2.8034-40080.revcompl.Baboon        ------------------------------------------------------------    20749
BCRP3.HUMAN.NCBI.REF                                            ------------------------------------------------------------    20446
LOC106996293.glutathione.hydrolase5.proenzyme-like-GGT1.rhesus.     ggatgagggggagaacaatgacaacagctacaggaaacagtactcaggaggtcctgtgag    103980

Papio.anubis.clone.rp41-133b2.8034-40080.revcompl.Baboon        ------------------------------------------------------------    20749
BCRP3.HUMAN.NCBI.REF                                            ------------------------------------------------------------    20446
LOC106996293.glutathione.hydrolase5.proenzyme-like-GGT1.rhesus.     gtagctgtggttttcatggctctttatagaaaggaaacagtctcagggaggcctggctgc    104040

Papio.anubis.clone.rp41-133b2.8034-40080.revcompl.Baboon        ------------------------------------------------------------    20749
BCRP3.HUMAN.NCBI.REF                                            ------------------------------------------------------------    20446
LOC106996293.glutathione.hydrolase5.proenzyme-like-GGT1.rhesus.     atgagtgggtgacacacacagggagtgtggagctggccagtggtatgagcactgtgccag    104100

Papio.anubis.clone.rp41-133b2.8034-40080.revcompl.Baboon        ------------------------------------------------------------    20749
BCRP3.HUMAN.NCBI.REF                                            ------------------------------------------------------------    20446
LOC106996293.glutathione.hydrolase5.proenzyme-like-GGT1.rhesus.     gtgactcacgccagtccctggagatccaagtgtggggtggctggggtgtaactgggggaa    104160

Papio.anubis.clone.rp41-133b2.8034-40080.revcompl.Baboon        ------------------------------------------------------------    20749
BCRP3.HUMAN.NCBI.REF                                            ------------------------------------------------------------    20446
LOC106996293.glutathione.hydrolase5.proenzyme-like-GGT1.rhesus.     gggaggagagcctcactgtccctgtccctgacacctggcaggcggggagctttaaggtgg    104220

Papio.anubis.clone.rp41-133b2.8034-40080.revcompl.Baboon        ------------------------------------------------------------    20749
BCRP3.HUMAN.NCBI.REF                                            ------------------------------------------------------------    20446
LOC106996293.glutathione.hydrolase5.proenzyme-like-GGT1.rhesus.     ccacccaggagaggaacccccagagagcccagatgaggctacagaggcagaagaaggtga    104280

Papio.anubis.clone.rp41-133b2.8034-40080.revcompl.Baboon        ------------------------------------------------------------    20749
BCRP3.HUMAN.NCBI.REF                                            ------------------------------------------------------------    20446
LOC106996293.glutathione.hydrolase5.proenzyme-like-GGT1.rhesus.     gtgagactgtggcatggagggaccgcaggggatcagaggacagggctcttttccccacca    104340

Papio.anubis.clone.rp41-133b2.8034-40080.revcompl.Baboon        ------------------------------------------------------------    20749
BCRP3.HUMAN.NCBI.REF                                            ------------------------------------------------------------    20446
LOC106996293.glutathione.hydrolase5.proenzyme-like-GGT1.rhesus.     gctggaggcctccatatcaacacagcgggggcttcctccccagccctgccctcctatggc    104400

Papio.anubis.clone.rp41-133b2.8034-40080.revcompl.Baboon        --------------------------------------------------gaaaagtgat    20759
BCRP3.HUMAN.NCBI.REF                                            ------------------------------------------------------------    20446
LOC106996293.glutathione.hydrolase5.proenzyme-like-GGT1.rhesus.     cactgggcctggaaaacctccattggagagaccagagcaagggtctgggaaagcagaact    104460

Papio.anubis.clone.rp41-133b2.8034-40080.revcompl.Baboon        aagggtggcactagtctgaggctggacaggccactcctgggcagatgt------------    20807
BCRP3.HUMAN.NCBI.REF                                            ------------------------------------------------------------    20446
LOC106996293.glutathione.hydrolase5.proenzyme-like-GGT1.rhesus.     cagagtgtccctagggtaaggctgggaccacaggcccttgtgacttgttaaaatcccacc    104520

Papio.anubis.clone.rp41-133b2.8034-40080.revcompl.Baboon        ------------------------------------------------------------    20807
BCRP3.HUMAN.NCBI.REF                                            ------------------------------------------------------------    20446
LOC106996293.glutathione.hydrolase5.proenzyme-like-GGT1.rhesus.     atagaggtaactaggagtgattgccagacttggaggtcagctggaatagaggaggcaggg    104580

Papio.anubis.clone.rp41-133b2.8034-40080.revcompl.Baboon        ------------------------------------------------------------    20807
BCRP3.HUMAN.NCBI.REF                                            ------------------------------------------------------------    20446
LOC106996293.glutathione.hydrolase5.proenzyme-like-GGT1.rhesus.     aattgggaaaggcagctgagggtctttggctgctacaactgggagacctggggacggggg    104640

Papio.anubis.clone.rp41-133b2.8034-40080.revcompl.Baboon        ------------------------------------------------------------    20807
BCRP3.HUMAN.NCBI.REF                                            ------------------------------------------------------------    20446
LOC106996293.glutathione.hydrolase5.proenzyme-like-GGT1.rhesus.     ttctgggacacaggggctgatgggatttcatgggacagggccttgggcaagcacctgagg    104700

Papio.anubis.clone.rp41-133b2.8034-40080.revcompl.Baboon        ------------------------------------------------------------    20807
BCRP3.HUMAN.NCBI.REF                                            ------------------------------------------------------------    20446
LOC106996293.glutathione.hydrolase5.proenzyme-like-GGT1.rhesus.     gtcaatactcatcaccactaccctcccacctccccaccctccatggcacagggcgtgg    104760

Papio.anubis.clone.rp41-133b2.8034-40080.revcompl.Baboon        ------------------------------------------------------------    20807
BCRP3.HUMAN.NCBI.REF                                            ------------------------------------------------------------    20446
LOC106996293.glutathione.hydrolase5.proenzyme-like-GGT1.rhesus.     tccccttcctgggggattttctgactgagttacacaggttggattcagccatcccggatg    104820

Papio.anubis.clone.rp41-133b2.8034-40080.revcompl.Baboon        ------------------------------------------------------------    20807
BCRP3.HUMAN.NCBI.REF                                            ------------------------------------------------------------    20446
LOC106996293.glutathione.hydrolase5.proenzyme-like-GGT1.rhesus.     atctggatgtgagtgaccctggggcaggctgcttgggaaccaggatcctgaggcttggga    104880

Papio.anubis.clone.rp41-133b2.8034-40080.revcompl.Baboon        ------------------------------------------------------------    20807
BCRP3.HUMAN.NCBI.REF                                            ------------------------------------------------------------    20446
LOC106996293.glutathione.hydrolase5.proenzyme-like-GGT1.rhesus.     ggacaggggcctggaaggagcccttagatcttagcccttggaaaacctcctctcctgaga    104940

Papio.anubis.clone.rp41-133b2.8034-40080.revcompl.Baboon        ------------------------------------------------------------    20807
BCRP3.HUMAN.NCBI.REF                                            ------------------------------------------------------------    20446
LOC106996293.glutathione.hydrolase5.proenzyme-like-GGT1.rhesus.     gcctcatagctgctcctgtgggtgggagtgtcaggcccatctcattacctctgactgaca    105000

Papio.anubis.clone.rp41-133b2.8034-40080.revcompl.Baboon        ------------------------------------------------------------    20807
BCRP3.HUMAN.NCBI.REF                                            ------------------------------------------------------------    20446
LOC106996293.glutathione.hydrolase5.proenzyme-like-GGT1.rhesus.     gaggctccatggcagtcagcagtccctatccctgagtggtgaagctgcagagctgcctga    105060

Papio.anubis.clone.rp41-133b2.8034-40080.revcompl.Baboon        ------------------------------------------------------------    20807
BCRP3.HUMAN.NCBI.REF                                            ------------------------------------------------------------    20446
LOC106996293.glutathione.hydrolase5.proenzyme-like-GGT1.rhesus.     ctgcagcgctgctgaggtgtgggctgagctggactcagcctctccctagggtagtcccat    105120

Papio.anubis.clone.rp41-133b2.8034-40080.revcompl.Baboon        ------------------------------------------------------------    20807
BCRP3.HUMAN.NCBI.REF                                            ------------------------------------------------------------    20446
LOC106996293.glutathione.hydrolase5.proenzyme-like-GGT1.rhesus.     aataggagggtgaaattgagcctttccaagggcaggcaattccaggataactgagcactt    105180

Papio.anubis.clone.rp41-133b2.8034-40080.revcompl.Baboon        ------------------------------------------------------------    20807
BCRP3.HUMAN.NCBI.REF                                            ------------------------------------------------------------    20446
LOC106996293.glutathione.hydrolase5.proenzyme-like-GGT1.rhesus.     tctttctaagggagacctcagtttctctgtctgtcatctcagagggttggggcagaaggt    105240

Papio.anubis.clone.rp41-133b2.8034-40080.revcompl.Baboon        ------------------------------------------------------------    20807
BCRP3.HUMAN.NCBI.REF                                            ------------------------------------------------------------    20446
LOC106996293.glutathione.hydrolase5.proenzyme-like-GGT1.rhesus.     ccctgagcctcagctcccatgcccactgccctagagcccggagcctgaggcaggttcacg    105300

Papio.anubis.clone.rp41-133b2.8034-40080.revcompl.Baboon        ----------------------------------ctttgcagaagtattttttgtgtaag    20833
BCRP3.HUMAN.NCBI.REF                                            ------------------------------------------------------------    20446
LOC106996293.glutathione.hydrolase5.proenzyme-like-GGT1.rhesus.     ttgtgtcatgtgcacatcccctgaccctggtggccctggcagtagtgcagcatgggaagg    105360

Papio.anubis.clone.rp41-133b2.8034-40080.revcompl.Baboon        gttgtgatggcctttgtgtaaggttgtgag------------------------------    20863
BCRP3.HUMAN.NCBI.REF                                            ------------------------------------------------------------    20446
LOC106996293.glutathione.hydrolase5.proenzyme-like-GGT1.rhesus.     gatggggtggggctgttgtgggccagggaccttttttgatgggctctatctgacttccagg    105420

Papio.anubis.clone.rp41-133b2.8034-40080.revcompl.Baboon        ------------------------------------------------------------    20863
BCRP3.HUMAN.NCBI.REF                                            ------------------------------------------------------------    20446
LOC106996293.glutathione.hydrolase5.proenzyme-like-GGT1.rhesus.     gcaacaccaacaagaggagaaaggtgagcagctgggacattcacgttggatgaggttggg    105480

Papio.anubis.clone.rp41-133b2.8034-40080.revcompl.Baboon        ------------------------------------------------------------    20863
BCRP3.HUMAN.NCBI.REF                                            ------------------------------------------------------------    20446
LOC106996293.glutathione.hydrolase5.proenzyme-like-GGT1.rhesus.     gatgtggacgtcacagtccaccctggacaggacactccctggctccatcctctacatctt    105540

Papio.anubis.clone.rp41-133b2.8034-40080.revcompl.Baboon        ------------------------------------------------------------    20863
```

```
BCRP3.HUMAN.NCBI.REF                                          ------------------------------------------------------------    20446
LOC106996293.glutathione.hydrolase5.proenzyme-like-GGT1.rhesus.    agttttactgggtttgacacacacaggaggaggagacctatctcagtggagaaagtggga    105600

Papio.anubis.clone.rp41-133b2.8034-40080.revcompl.Baboon      ------------------------------------------------------------    20863
BCRP3.HUMAN.NCBI.REF                                          ------------------------------------------------------------    20446
LOC106996293.glutathione.hydrolase5.proenzyme-like-GGT1.rhesus.    aaggcactggaatgaaagaccaattcctgcaggaggggctatttacatccaactctgaga    105660

Papio.anubis.clone.rp41-133b2.8034-40080.revcompl.Baboon      ------------------------------------------------------------    20863
BCRP3.HUMAN.NCBI.REF                                          ------------------------------------------------------------    20446
LOC106996293.glutathione.hydrolase5.proenzyme-like-GGT1.rhesus.    acaggctgggggctgcattgggccccctcagagaacggccccagggaccagccccttctc    105720

Papio.anubis.clone.rp41-133b2.8034-40080.revcompl.Baboon      ------------------------------------------------------------    20863
BCRP3.HUMAN.NCBI.REF                                          ------------------------------------------------------------    20446
LOC106996293.glutathione.hydrolase5.proenzyme-like-GGT1.rhesus.    cctgcctgccaaaggtcccctcagcgtcttccacccaggccctgtcagcatcctgtcctc    105780

Papio.anubis.clone.rp41-133b2.8034-40080.revcompl.Baboon      ------------------------------------------------------------    20863
BCRP3.HUMAN.NCBI.REF                                          ------------------------------------------------------------    20446
LOC106996293.glutathione.hydrolase5.proenzyme-like-GGT1.rhesus.    tgtctctaggaggtccgagttctgcaggaattgcagctactccaagtggctgccatgaat    105840

Papio.anubis.clone.rp41-133b2.8034-40080.revcompl.Baboon      ------------------------------------------------------------    20863
BCRP3.HUMAN.NCBI.REF                                          ------------------------------------------------------------    20446
LOC106996293.glutathione.hydrolase5.proenzyme-like-GGT1.rhesus.    tacaggcttcagcctctggagaaatttgtcacttgtttctcaagaatggagcagctcagt    105900

Papio.anubis.clone.rp41-133b2.8034-40080.revcompl.Baboon      -----------------------------------------------------------t    20864
BCRP3.HUMAN.NCBI.REF                                          ------------------------------------------------------------    20446
LOC106996293.glutathione.hydrolase5.proenzyme-like-GGT1.rhesus.    gacaaggagaggtgagggcctgggagatgggcagagggtgggagaaagctctccattttt    105960

Papio.anubis.clone.rp41-133b2.8034-40080.revcompl.Baboon      tgtttgtttttttgaggcagggtcttgctctgtcacctgggctggagtgcagtggcactgt    20924
BCRP3.HUMAN.NCBI.REF                                          ------------------------------------------------------------    20446
LOC106996293.glutathione.hydrolase5.proenzyme-like-GGT1.rhesus.    tttttttttttttgagacggagtctcgctctgccgcccaggctggagtgcagtggccggat    106020

Papio.anubis.clone.rp41-133b2.8034-40080.revcompl.Baboon      caaggctcactgcagcttcaacctcac---------------------------------    20951
BCRP3.HUMAN.NCBI.REF                                          ------------------------------------------------------------    20446
LOC106996293.glutathione.hydrolase5.proenzyme-like-GGT1.rhesus.    ctcaggtcactgcatgctccaccacccgggttcacgccattctcctgcctcagcctcccg    106080

Papio.anubis.clone.rp41-133b2.8034-40080.revcompl.Baboon      ------------------------------------------------------------    20951
BCRP3.HUMAN.NCBI.REF                                          ------------------------------------------------------------    20446
LOC106996293.glutathione.hydrolase5.proenzyme-like-GGT1.rhesus.    agtagttgggactacaggcgcccgccaccgcgcccggctagtttttttgtatttttttagta    106140

Papio.anubis.clone.rp41-133b2.8034-40080.revcompl.Baboon      ------------------------------------------------------------    20951
BCRP3.HUMAN.NCBI.REF                                          ------------------------------------------------------------    20446
LOC106996293.glutathione.hydrolase5.proenzyme-like-GGT1.rhesus.    gagacggggtttcaccgtgttagccaggatggtctcgatctcctgacctcgtgatccgcc    106200

Papio.anubis.clone.rp41-133b2.8034-40080.revcompl.Baboon      ------------------------------------------------------------    20951
BCRP3.HUMAN.NCBI.REF                                          ------------------------------------------------------------    20446
LOC106996293.glutathione.hydrolase5.proenzyme-like-GGT1.rhesus.    cgtctcggcctcccaaagtgctgggattacaggcttgagccaccgcgcccggcctccatt    106260

Papio.anubis.clone.rp41-133b2.8034-40080.revcompl.Baboon      ------------------------------------------------------------    20951
BCRP3.HUMAN.NCBI.REF                                          ------------------------------------------------------------    20446
LOC106996293.glutathione.hydrolase5.proenzyme-like-GGT1.rhesus.    tttttttaacgtggtctggctctgtcgcccaggctgcactgcagtgtcaccatctctgct    106320

Papio.anubis.clone.rp41-133b2.8034-40080.revcompl.Baboon      ------------------------------------------------------------    20951
BCRP3.HUMAN.NCBI.REF                                          ------------------------------------------------------------    20446
LOC106996293.glutathione.hydrolase5.proenzyme-like-GGT1.rhesus.    cactgcaacatctgcctcctgggctcaagcccttcctcagcctcccaagcagctgggatt    106380

Papio.anubis.clone.rp41-133b2.8034-40080.revcompl.Baboon      ------------------------------------------------------------    20951
BCRP3.HUMAN.NCBI.REF                                          ------------------------------------------------------------    20446
LOC106996293.glutathione.hydrolase5.proenzyme-like-GGT1.rhesus.    actgctatccaccaccaaatccggtcgttcccctgttgttgttctggtataggtggggtt    106440

Papio.anubis.clone.rp41-133b2.8034-40080.revcompl.Baboon      ----------------------------------------ctcaatcctgtcacctcagc    20971
BCRP3.HUMAN.NCBI.REF                                          ------------------------------------------------------------    20446
LOC106996293.glutathione.hydrolase5.proenzyme-like-GGT1.rhesus.    tcacgatgatgtccaggctggtctcaaactcctggcctcaagcaatccacccacctcagc    106500

Papio.anubis.clone.rp41-133b2.8034-40080.revcompl.Baboon      cttccaaagtgctgggattacaggtgtgaaccaccacatgtggct-----gcagctctat    21026
BCRP3.HUMAN.NCBI.REF                                          ------------------------------------------------------------    20446
LOC106996293.glutathione.hydrolase5.proenzyme-like-GGT1.rhesus.    ctcccaaagtactgacagtacaggtgtcagccaccccacctggcctagaggaggctctcc    106560

Papio.anubis.clone.rp41-133b2.8034-40080.revcompl.Baboon      tttgacaattttcaccgcctcttaccacttccagtggctgccagcgt-------------    21073
BCRP3.HUMAN.NCBI.REF                                          ------------------------------------------------------------    20446
LOC106996293.glutathione.hydrolase5.proenzyme-like-GGT1.rhesus.    tgtggacagcagcagagagcctatggccatgactccactgccagcatcaaaccttgttgc    106620

Papio.anubis.clone.rp41-133b2.8034-40080.revcompl.Baboon      ---------------------------------------------------------tcc    21076
BCRP3.HUMAN.NCBI.REF                                          ------------------------------------------------------------    20446
LOC106996293.glutathione.hydrolase5.proenzyme-like-GGT1.rhesus.    atggggactgctggggacccaggattccagctggtcaggcaatgagaggggacctgatgt    106680

Papio.anubis.clone.rp41-133b2.8034-40080.revcompl.Baboon      ttggcttgtggctgcctcactccagtctctgtctcc------------------------    21112
BCRP3.HUMAN.NCBI.REF                                          ------------------------------------------------------------    20446
LOC106996293.glutathione.hydrolase5.proenzyme-like-GGT1.rhesus.    gtagcttatggtggcctcacagctgcttctctgtcctgcagccacaagctgtcctgtcag    106740

Papio.anubis.clone.rp41-133b2.8034-40080.revcompl.Baboon      ------------------------------------------------------------    21112
BCRP3.HUMAN.NCBI.REF                                          ------------------------------------------------------------    20446
LOC106996293.glutathione.hydrolase5.proenzyme-like-GGT1.rhesus.    ctggagcccgaatcccagtaggctggaaacatcctgcagtggctggggccgcactgggat    106800

Papio.anubis.clone.rp41-133b2.8034-40080.revcompl.Baboon      ------------------------------------------------------------    21112
BCRP3.HUMAN.NCBI.REF                                          ------------------------------------------------------------    20446
LOC106996293.glutathione.hydrolase5.proenzyme-like-GGT1.rhesus.    gctggccagaacaccagctctgcatcatccttcacccagaccgtgacaccaggaaaccac    106860

Papio.anubis.clone.rp41-133b2.8034-40080.revcompl.Baboon      ------------------------------------------------------------    21112
BCRP3.HUMAN.NCBI.REF                                          ------------------------------------------------------------    20446
LOC106996293.glutathione.hydrolase5.proenzyme-like-GGT1.rhesus.    atctaggtggcttgcagctcagctacatcttgaccccactcctcaacaccagctgctcct    106920

Papio.anubis.clone.rp41-133b2.8034-40080.revcompl.Baboon      ------------------------------------------------------------    21112
BCRP3.HUMAN.NCBI.REF                                          ------------------------------------------------------------    20446
LOC106996293.glutathione.hydrolase5.proenzyme-like-GGT1.rhesus.    cctagccaggatcaggccatgggacttttgcatgtcaggcgggaaaccattttatgttta    106980

Papio.anubis.clone.rp41-133b2.8034-40080.revcompl.Baboon      ------------------------------------------------------------    21112
BCRP3.HUMAN.NCBI.REF                                          ------------------------------------------------------------    20446
LOC106996293.glutathione.hydrolase5.proenzyme-like-GGT1.rhesus.    ttttctttagtgtataagtaacgggtttttttccttaactttcgttaaaataaaattttaa    107040

Papio.anubis.clone.rp41-133b2.8034-40080.revcompl.Baboon      ------------------------------------------------------------    21112
BCRP3.HUMAN.NCBI.REF                                          ------------------------------------------------------------    20446
LOC106996293.glutathione.hydrolase5.proenzyme-like-GGT1.rhesus.    aacactactcagaatgttctagtgttggaatgagaatagtaactccagtgcagtaaccgt    107100

Papio.anubis.clone.rp41-133b2.8034-40080.revcompl.Baboon      ------------------------------------------------------------    21112
BCRP3.HUMAN.NCBI.REF                                          ------------------------------------------------------------    20446
LOC106996293.glutathione.hydrolase5.proenzyme-like-GGT1.rhesus.    ataccagtctttaggattcatagtctagcatgtcacattgaggctatggctgaacaagtc    107160

Papio.anubis.clone.rp41-133b2.8034-40080.revcompl.Baboon      ------------------------------------------------------------    21112
BCRP3.HUMAN.NCBI.REF                                          ------------------------------------------------------------    20446
LOC106996293.glutathione.hydrolase5.proenzyme-like-GGT1.rhesus.    atggaatagcgtttgcattacgcctgccaactgtttaatgttttccttctttattcaaat    107220

Papio.anubis.clone.rp41-133b2.8034-40080.revcompl.Baboon      ------------------------------------------------------------    21112
BCRP3.HUMAN.NCBI.REF                                          ------------------------------------------------------------    20446
LOC106996293.glutathione.hydrolase5.proenzyme-like-GGT1.rhesus.    taattactgctaactctgtttaaggaaaacaaaaaataaaacaaacaaactaaaaaacac    107280

Papio.anubis.clone.rp41-133b2.8034-40080.revcompl.Baboon      ------------------------------------------------------------    21112
```

```
BCRP3.HUMAN.NCBI.REF                                             ------------------------------------------------------------    20446
LOC106996293.glutathione.hydrolase5.proenzyme-like-GGT1.rhesus.  ttaaaaaccacagtatgtcacccaatctatgtcacttgttctactgaccagttactctca    107340

Papio.anubis.clone.rp41-133b2.8034-40080.revcompl.Baboon         ------------------------------------------------------------    21112
BCRP3.HUMAN.NCBI.REF                                             ------------------------------------------------------------    20446
LOC106996293.glutathione.hydrolase5.proenzyme-like-GGT1.rhesus.  aacttaaattctagttaaattcaagttccaccgggttactctactttttttaagttgtaaa    107400

Papio.anubis.clone.rp41-133b2.8034-40080.revcompl.Baboon         ------------------------------------------------------------    21112
BCRP3.HUMAN.NCBI.REF                                             ------------------------------------------------------------    20446
LOC106996293.glutathione.hydrolase5.proenzyme-like-GGT1.rhesus.  tatttaatgaatcacttaaatatttactgaagggctggagatgggaggtagttatgcaag    107460

Papio.anubis.clone.rp41-133b2.8034-40080.revcompl.Baboon         ------------------------------------------------------------    21112
BCRP3.HUMAN.NCBI.REF                                             ------------------------------------------------------------    20446
LOC106996293.glutathione.hydrolase5.proenzyme-like-GGT1.rhesus.  tggtagggctggccttctccagaagtcctgtgcagaagggagctggccatgagtctccag    107520

Papio.anubis.clone.rp41-133b2.8034-40080.revcompl.Baboon         ------------------------------------------------------------    21112
BCRP3.HUMAN.NCBI.REF                                             ------------------------------------------------------------    20446
LOC106996293.glutathione.hydrolase5.proenzyme-like-GGT1.rhesus.  gaaacacagataaacttttcccactatggatcttcccagactttctggcacctctcccct    107580

Papio.anubis.clone.rp41-133b2.8034-40080.revcompl.Baboon         ------------------------------------------------------------    21112
BCRP3.HUMAN.NCBI.REF                                             ------------------------------------------------------------    20446
LOC106996293.glutathione.hydrolase5.proenzyme-like-GGT1.rhesus.  aagggaacatcaaggaaggagagaattgtttgaacccagtatctaaaggatgtcccacag    107640

Papio.anubis.clone.rp41-133b2.8034-40080.revcompl.Baboon         ------------------------------------------------------------    21112
BCRP3.HUMAN.NCBI.REF                                             ------------------------------------------------------------    20446
LOC106996293.glutathione.hydrolase5.proenzyme-like-GGT1.rhesus.  cttaatttgaacacaagccccactgtggtatatcagacaaaaacaagtcaaagaaatcaa    107700

Papio.anubis.clone.rp41-133b2.8034-40080.revcompl.Baboon         ------------------------------------------------------------    21112
BCRP3.HUMAN.NCBI.REF                                             ------------------------------------------------------------    20446
LOC106996293.glutathione.hydrolase5.proenzyme-like-GGT1.rhesus.  catttcagggtctgaaatataataccccccaaatcagcacaaaataaaacacttaaatcca    107760

Papio.anubis.clone.rp41-133b2.8034-40080.revcompl.Baboon         ------------------------------------------------------------    21112
BCRP3.HUMAN.NCBI.REF                                             ------------------------------------------------------------    20446
LOC106996293.glutathione.hydrolase5.proenzyme-like-GGT1.rhesus.  gtcctggagccacaaatctcctataagaaaagtgggagtttatttcagcaaaacttgtat    107820

Papio.anubis.clone.rp41-133b2.8034-40080.revcompl.Baboon         ------------------------------------------------------------    21112
BCRP3.HUMAN.NCBI.REF                                             ------------------------------------------------------------    20446
LOC106996293.glutathione.hydrolase5.proenzyme-like-GGT1.rhesus.  ctatgcatgcagtttgcaaaaaagaaagaaaacaaaagcaaaaattatctatggcaacaa    107880

Papio.anubis.clone.rp41-133b2.8034-40080.revcompl.Baboon         ------------------------------------------------------------    21112
BCRP3.HUMAN.NCBI.REF                                             ------------------------------------------------------------    20446
LOC106996293.glutathione.hydrolase5.proenzyme-like-GGT1.rhesus.  acccttagaccatgcaattgatttggcaatactttttctttcttattttttgttcttttt    107940

Papio.anubis.clone.rp41-133b2.8034-40080.revcompl.Baboon         ------------------------------------------------------------    21112
BCRP3.HUMAN.NCBI.REF                                             ------------------------------------------------------------    20446
LOC106996293.glutathione.hydrolase5.proenzyme-like-GGT1.rhesus.  ggatgggacacaaaaatcactgctagcaaatgtgaacacaaacacatgggactatacatt    108000

Papio.anubis.clone.rp41-133b2.8034-40080.revcompl.Baboon         ------------------------------------------------------------    21112
BCRP3.HUMAN.NCBI.REF                                             ------------------------------------------------------------    20446
LOC106996293.glutathione.hydrolase5.proenzyme-like-GGT1.rhesus.  atcttagagcttctgcatgggaaagagaaacaacgaaccattaaagaaggcatcctgatg    108060

Papio.anubis.clone.rp41-133b2.8034-40080.revcompl.Baboon         ------------------------------------------------------------    21112
BCRP3.HUMAN.NCBI.REF                                             ------------------------------------------------------------    20446
LOC106996293.glutathione.hydrolase5.proenzyme-like-GGT1.rhesus.  agttcatgtcctttgtagggacatggatgaagctggaaaccatcattctcagcaaactat    108120

Papio.anubis.clone.rp41-133b2.8034-40080.revcompl.Baboon         ------------------------------------------------------------    21112
BCRP3.HUMAN.NCBI.REF                                             ------------------------------------------------------------    20446
LOC106996293.glutathione.hydrolase5.proenzyme-like-GGT1.rhesus.  tgcaagaacagaaaaccaaacaccgcatgttctcactcataggtgggaattgaacagtga    108180

Papio.anubis.clone.rp41-133b2.8034-40080.revcompl.Baboon         ------------------------------------------------------------    21112
BCRP3.HUMAN.NCBI.REF                                             ------------------------------------------------------------    20446
LOC106996293.glutathione.hydrolase5.proenzyme-like-GGT1.rhesus.  gaacacttggacacaggaaggggaatatcgcacactggggcttgtggtgggttcgggggt    108240

Papio.anubis.clone.rp41-133b2.8034-40080.revcompl.Baboon         ------------------------------------------------------------    21112
BCRP3.HUMAN.NCBI.REF                                             ------------------------------------------------------------    20446
LOC106996293.glutathione.hydrolase5.proenzyme-like-GGT1.rhesus.  tgggggagggatagaataggagatatacctaatgtaagtgacgagttaataggtgcagca    108300

Papio.anubis.clone.rp41-133b2.8034-40080.revcompl.Baboon         ------------------------------------------------------------    21112
BCRP3.HUMAN.NCBI.REF                                             ------------------------------------------------------------    20446
LOC106996293.glutathione.hydrolase5.proenzyme-like-GGT1.rhesus.  catcaacatggcacatgtatacatatgcaacaaacctgcacgttgtgcacatgtacccta    108360

Papio.anubis.clone.rp41-133b2.8034-40080.revcompl.Baboon         ------------------------------------------------------------    21112
BCRP3.HUMAN.NCBI.REF                                             ------------------------------------------------------------    20446
LOC106996293.glutathione.hydrolase5.proenzyme-like-GGT1.rhesus.  gaacataaagtataataataataataaaagaaggcatcctacagattgaaagaaagttca    108420

Papio.anubis.clone.rp41-133b2.8034-40080.revcompl.Baboon         ------------------------------------------------------------    21112
BCRP3.HUMAN.NCBI.REF                                             ------------------------------------------------------------    20446
LOC106996293.glutathione.hydrolase5.proenzyme-like-GGT1.rhesus.  gcagaatcgtatctgtgaaaggggttgcaatctaacatgtatgagaaactaacactactc    108480

Papio.anubis.clone.rp41-133b2.8034-40080.revcompl.Baboon         ------------------------------------------------------------    21112
BCRP3.HUMAN.NCBI.REF                                             ------------------------------------------------------------    20446
LOC106996293.glutathione.hydrolase5.proenzyme-like-GGT1.rhesus.  taactcggaaaatgaacacaacctaatacccaagctaaaactgggcaaagaacctgaaca    108540

Papio.anubis.clone.rp41-133b2.8034-40080.revcompl.Baboon         ------------------------------------------------------------    21112
BCRP3.HUMAN.NCBI.REF                                             ------------------------------------------------------------    20446
LOC106996293.glutathione.hydrolase5.proenzyme-like-GGT1.rhesus.  ggcacatctgaaaagtaatcagggaattgactgacaggtcacagagaagttgctcagttt    108600

Papio.anubis.clone.rp41-133b2.8034-40080.revcompl.Baboon         ------------------------------------------------------------    21112
BCRP3.HUMAN.NCBI.REF                                             ------------------------------------------------------------    20446
LOC106996293.glutathione.hydrolase5.proenzyme-like-GGT1.rhesus.  cactaatcctcacacacatgtctaagtgcaaaccacactcagatacctctcactctaatt    108660

Papio.anubis.clone.rp41-133b2.8034-40080.revcompl.Baboon         ------------------------------------------------------------    21112
BCRP3.HUMAN.NCBI.REF                                             ------------------------------------------------------------    20446
LOC106996293.glutathione.hydrolase5.proenzyme-like-GGT1.rhesus.  agaatgaaacttaccaaaaacaacaaaaaacccatacattcacaggcagtggccaatgta    108720

Papio.anubis.clone.rp41-133b2.8034-40080.revcompl.Baboon         ------------------------------------------------------------    21112
BCRP3.HUMAN.NCBI.REF                                             ------------------------------------------------------------    20446
LOC106996293.glutathione.hydrolase5.proenzyme-like-GGT1.rhesus.  gaaatgcagaaaaagagattttatactctattggtgagaatgtacattactatacacacg    108780

Papio.anubis.clone.rp41-133b2.8034-40080.revcompl.Baboon         ------------------------------------------------------------    21112
BCRP3.HUMAN.NCBI.REF                                             ------------------------------------------------------------    20446
LOC106996293.glutathione.hydrolase5.proenzyme-like-GGT1.rhesus.  aagcgtaacagtggaaggtgacttaaatattttacaactacccgttcacccaggaatccc    108840

Papio.anubis.clone.rp41-133b2.8034-40080.revcompl.Baboon         ------------------------------------------------------------    21112
BCRP3.HUMAN.NCBI.REF                                             ------------------------------------------------------------    20446
LOC106996293.glutathione.hydrolase5.proenzyme-like-GGT1.rhesus.  accactggttacacacagaaagcacatggaatctgttatgttgaagagatatcggccttc    108900

Papio.anubis.clone.rp41-133b2.8034-40080.revcompl.Baboon         ------------------------------------------------------------    21112
BCRP3.HUMAN.NCBI.REF                                             ------------------------------------------------------------    20446
LOC106996293.glutathione.hydrolase5.proenzyme-like-GGT1.rhesus.  ttatggcaattgaagcactatgcacaatagcgaaggtatccaatccacctacctgttcgt    108960

Papio.anubis.clone.rp41-133b2.8034-40080.revcompl.Baboon         ------------------------------------------------------------    21112
BCRP3.HUMAN.NCBI.REF                                             ------------------------------------------------------------    20446
LOC106996293.glutathione.hydrolase5.proenzyme-like-GGT1.rhesus.  gcagagataaagggataaagaaactgcagtacacaatggaatcctccttggccatagaaa    109020

Papio.anubis.clone.rp41-133b2.8034-40080.revcompl.Baboon         ------------------------------------------------------------    21112
```

```
BCRP3.HUMAN.NCBI.REF                                              ------------------------------------------------------------    20446
LOC106996293.glutathione.hydrolase5.proenzyme-like-GGT1.rhesus.   tccatgaaaccatgtcatctgcagcaacatacagaaacctggaggacacgaccttcaagg    109080

Papio.anubis.clone.rp41-133b2.8034-40080.revcompl.Baboon          ------------------------------------------------------------    21112
BCRP3.HUMAN.NCBI.REF                                              ------------------------------------------------------------    20446
LOC106996293.glutathione.hydrolase5.proenzyme-like-GGT1.rhesus.   aaatgagtcaggaagagagagacaaacactgcaagatttcatgcatgtgagaatgagatc    109140

Papio.anubis.clone.rp41-133b2.8034-40080.revcompl.Baboon          ------------------------------------------------------------    21112
BCRP3.HUMAN.NCBI.REF                                              ------------------------------------------------------------    20446
LOC106996293.glutathione.hydrolase5.proenzyme-like-GGT1.rhesus.   aactttctctctaaacgactttatctcctagaagtagaacattccacagtcgtgaagaga    109200

Papio.anubis.clone.rp41-133b2.8034-40080.revcompl.Baboon          ------------------------------------------------------------    21112
BCRP3.HUMAN.NCBI.REF                                              ------------------------------------------------------------    20446
LOC106996293.glutathione.hydrolase5.proenzyme-like-GGT1.rhesus.   gcctgggggttggggagttggggcaggaactgggaaatggatacaatattacactcagat    109260

Papio.anubis.clone.rp41-133b2.8034-40080.revcompl.Baboon          ------------------------------------------------------------    21112
BCRP3.HUMAN.NCBI.REF                                              ------------------------------------------------------------    20446
LOC106996293.glutathione.hydrolase5.proenzyme-like-GGT1.rhesus.   gacaggaataaattcagctgttctattccacagtaggatgtctagagttaactgtatcct    109320

Papio.anubis.clone.rp41-133b2.8034-40080.revcompl.Baboon          ------------------------------------------------------------    21112
BCRP3.HUMAN.NCBI.REF                                              ------------------------------------------------------------    20446
LOC106996293.glutathione.hydrolase5.proenzyme-like-GGT1.rhesus.   accgtattttccaaaaaaggctgaaaagaaggattctgaatattgcaaccatagagaact    109380

Papio.anubis.clone.rp41-133b2.8034-40080.revcompl.Baboon          ------------------------------------------------------------    21112
BCRP3.HUMAN.NCBI.REF                                              ------------------------------------------------------------    20446
LOC106996293.glutathione.hydrolase5.proenzyme-like-GGT1.rhesus.   aataaataataactacacaaggtaacagagacaatgccttgctttcattattacacaagg    109440

Papio.anubis.clone.rp41-133b2.8034-40080.revcompl.Baboon          ------------------------------------------------------------    21112
BCRP3.HUMAN.NCBI.REF                                              ------------------------------------------------------------    20446
LOC106996293.glutathione.hydrolase5.proenzyme-like-GGT1.rhesus.   tacatatgcatggattataatgtcccactctactctttaattgtatacctttactagaga    109500

Papio.anubis.clone.rp41-133b2.8034-40080.revcompl.Baboon          ------------------------------------------------------------    21112
BCRP3.HUMAN.NCBI.REF                                              ------------------------------------------------------------    20446
LOC106996293.glutathione.hydrolase5.proenzyme-like-GGT1.rhesus.   gcaaattggttttaaggacatagaaataaacaatgctgaagttcatgtgagaccaggaaa    109560

Papio.anubis.clone.rp41-133b2.8034-40080.revcompl.Baboon          ------------------------------------------------------------    21112
BCRP3.HUMAN.NCBI.REF                                              ------------------------------------------------------------    20446
LOC106996293.glutathione.hydrolase5.proenzyme-like-GGT1.rhesus.   tgccctgcatttccaaagcaattctgagaaatccaaactacattggatgcatcacacccc    109620

Papio.anubis.clone.rp41-133b2.8034-40080.revcompl.Baboon          ------------------------------------------------------------    21112
BCRP3.HUMAN.NCBI.REF                                              ------------------------------------------------------------    20446
LOC106996293.glutathione.hydrolase5.proenzyme-like-GGT1.rhesus.   ctgatgtgaaatttcactcaaactctagggacctgcttccacatggatgagtggaagaga    109680

Papio.anubis.clone.rp41-133b2.8034-40080.revcompl.Baboon          ------------------------------------------------------------    21112
BCRP3.HUMAN.NCBI.REF                                              ------------------------------------------------------------    20446
LOC106996293.glutathione.hydrolase5.proenzyme-like-GGT1.rhesus.   acagagaacctgaagtaaacccacacacctcatactacctgatgctggatgaaatacaca    109740

Papio.anubis.clone.rp41-133b2.8034-40080.revcompl.Baboon          ------------------------------------------------------------    21112
BCRP3.HUMAN.NCBI.REF                                              ------------------------------------------------------------    20446
LOC106996293.glutathione.hydrolase5.proenzyme-like-GGT1.rhesus.   atgataagtaaaagggaaagaactcccttttccatagttttgggataagtggcgagccat    109800

Papio.anubis.clone.rp41-133b2.8034-40080.revcompl.Baboon          ------------------------------------------------------------    21112
BCRP3.HUMAN.NCBI.REF                                              ------------------------------------------------------------    20446
LOC106996293.glutathione.hydrolase5.proenzyme-like-GGT1.rhesus.   atgcagaagagtaagtacaactaggcctctacttctcactatgtaccaaagttcactcag    109860

Papio.anubis.clone.rp41-133b2.8034-40080.revcompl.Baboon          ------------------------------------------------------------    21112
BCRP3.HUMAN.NCBI.REF                                              ------------------------------------------------------------    20446
LOC106996293.glutathione.hydrolase5.proenzyme-like-GGT1.rhesus.   atgaatgaaagatgtaaatggaagacctcaaagtacaaaaatcctacaagagaccctagg    109920

Papio.anubis.clone.rp41-133b2.8034-40080.revcompl.Baboon          ------------------------------------------------------------    21112
BCRP3.HUMAN.NCBI.REF                                              ------------------------------------------------------------    20446
LOC106996293.glutathione.hydrolase5.proenzyme-like-GGT1.rhesus.   aaatacccttcttgacatcagctttgacaaagcatttatatgcctaagatgccacatgac    109980

Papio.anubis.clone.rp41-133b2.8034-40080.revcompl.Baboon          ------------------------------------------------------------    21112
BCRP3.HUMAN.NCBI.REF                                              ------------------------------------------------------------    20446
LOC106996293.glutathione.hydrolase5.proenzyme-like-GGT1.rhesus.   acggtaacaacagcaataatcaacatgtgggacctaatacactaaagagccaccacacaa    110040

Papio.anubis.clone.rp41-133b2.8034-40080.revcompl.Baboon          ------------------------------------------------------------    21112
BCRP3.HUMAN.NCBI.REF                                              ------------------------------------------------------------    20446
LOC106996293.glutathione.hydrolase5.proenzyme-like-GGT1.rhesus.   cacaagaaatcacccacagagtagacagacaacatacaggatgagagaaaatgttcccaa    110100

Papio.anubis.clone.rp41-133b2.8034-40080.revcompl.Baboon          ------------------------------------------------------------    21112
BCRP3.HUMAN.NCBI.REF                                              ------------------------------------------------------------    20446
LOC106996293.glutathione.hydrolase5.proenzyme-like-GGT1.rhesus.   actatgtgcctgaccaaggcctaatatccagaatctaccttaaagaccttacagaaatca    110160

Papio.anubis.clone.rp41-133b2.8034-40080.revcompl.Baboon          ------------------------------------------------------------    21112
BCRP3.HUMAN.NCBI.REF                                              ------------------------------------------------------------    20446
LOC106996293.glutathione.hydrolase5.proenzyme-like-GGT1.rhesus.   attagccaacccgcctaataattagtaggcaatggatataaacacacacttctcgaaaga    110220

Papio.anubis.clone.rp41-133b2.8034-40080.revcompl.Baboon          ------------------------------------------------------------    21112
BCRP3.HUMAN.NCBI.REF                                              ------------------------------------------------------------    20446
LOC106996293.glutathione.hydrolase5.proenzyme-like-GGT1.rhesus.   tgatgtgagagcaaccaacaaattgaaaatatgctcaacctaattgtcagagaaatataa    110280

Papio.anubis.clone.rp41-133b2.8034-40080.revcompl.Baboon          ------------------------------------------------------------    21112
BCRP3.HUMAN.NCBI.REF                                              ------------------------------------------------------------    20446
LOC106996293.glutathione.hydrolase5.proenzyme-like-GGT1.rhesus.   atcaaaagcacaatgagatatatctctcgctggtcagaatgggggttacacagtttaaaa    110340

Papio.anubis.clone.rp41-133b2.8034-40080.revcompl.Baboon          ------------------------------------------------------------    21112
BCRP3.HUMAN.NCBI.REF                                              ------------------------------------------------------------    20446
LOC106996293.glutathione.hydrolase5.proenzyme-like-GGT1.rhesus.   aaaaaaggacactggcgaggcagcagagaaaggggacactggtccacttttggtgaaaat    110400

Papio.anubis.clone.rp41-133b2.8034-40080.revcompl.Baboon          ------------------------------------------------------------    21112
BCRP3.HUMAN.NCBI.REF                                              ------------------------------------------------------------    20446
LOC106996293.glutathione.hydrolase5.proenzyme-like-GGT1.rhesus.   gcaaagtacttcagacaccatggaaagcacttcggagattgctcaaagaacttaaaccag    110460

Papio.anubis.clone.rp41-133b2.8034-40080.revcompl.Baboon          ------------------------------------------------------------    21112
BCRP3.HUMAN.NCBI.REF                                              ------------------------------------------------------------    20446
LOC106996293.glutathione.hydrolase5.proenzyme-like-GGT1.rhesus.   aactaccatctgacccagcaatcacaccactggggtatacacagaggaaaataaatcctt    110520

Papio.anubis.clone.rp41-133b2.8034-40080.revcompl.Baboon          ------------------------------------------------------------    21112
BCRP3.HUMAN.NCBI.REF                                              ------------------------------------------------------------    20446
LOC106996293.glutathione.hydrolase5.proenzyme-like-GGT1.rhesus.   ctgtgcaaaacacgcatgcacacaaatggtcatggcagcagtatttacaatggcaaacat    110580

Papio.anubis.clone.rp41-133b2.8034-40080.revcompl.Baboon          ------------------------------------------------------------    21112
BCRP3.HUMAN.NCBI.REF                                              ------------------------------------------------------------    20446
LOC106996293.glutathione.hydrolase5.proenzyme-like-GGT1.rhesus.   gtgaaatcagcctaggtaccggtcaacagtggatcggaaaaggaaaatgtggtacctata    110640

Papio.anubis.clone.rp41-133b2.8034-40080.revcompl.Baboon          ------------------------------------------------------------    21112
BCRP3.HUMAN.NCBI.REF                                              ------------------------------------------------------------    20446
LOC106996293.glutathione.hydrolase5.proenzyme-like-GGT1.rhesus.   taccacaaaaaactaggcaggaattaaaagaaaaaagaaggaaatcatgtccttggcagc    110700

Papio.anubis.clone.rp41-133b2.8034-40080.revcompl.Baboon          ------------------------------------------------------------    21112
BCRP3.HUMAN.NCBI.REF                                              ------------------------------------------------------------    20446
LOC106996293.glutathione.hydrolase5.proenzyme-like-GGT1.rhesus.   aacatgaaaggagctggaggccattatctaaagagaaataaggaaaacacagaacaccaa    110760

Papio.anubis.clone.rp41-133b2.8034-40080.revcompl.Baboon          ------------------------------------------------------------    21112
```

```
BCRP3.HUMAN.NCBI.REF                                              ------------------------------------------------------------    20446
LOC106996293.glutathione.hydrolase5.proenzyme-like-GGT1.rhesus.   atgccacatgttctcacttctaaggtggagctaacattgaatacaccctaccgtaaaagt    110820

Papio.anubis.clone.rp41-133b2.8034-40080.revcompl.Baboon          ------------------------------------------------------------    21112
BCRP3.HUMAN.NCBI.REF                                              ------------------------------------------------------------    20446
LOC106996293.glutathione.hydrolase5.proenzyme-like-GGT1.rhesus.   gaaaacaacagacactggtgactatcagatgaaaaaggaagaagggatgaggtatgggct    110880

Papio.anubis.clone.rp41-133b2.8034-40080.revcompl.Baboon          ------------------------------------------------------------    21112
BCRP3.HUMAN.NCBI.REF                                              ------------------------------------------------------------    20446
LOC106996293.glutathione.hydrolase5.proenzyme-like-GGT1.rhesus.   gaagacctatctggtgggtccacccactgcctgcatgataaggttgcttggaccccaagt    110940

Papio.anubis.clone.rp41-133b2.8034-40080.revcompl.Baboon          ------------------------------------------------------------    21112
BCRP3.HUMAN.NCBI.REF                                              ------------------------------------------------------------    20446
LOC106996293.glutathione.hydrolase5.proenzyme-like-GGT1.rhesus.   ctcagtgtcatgcaatataccaaagacagtaactaatctgcctttgtacactttagtctg    111000

Papio.anubis.clone.rp41-133b2.8034-40080.revcompl.Baboon          ------------------------------------------------------------    21112
BCRP3.HUMAN.NCBI.REF                                              ------------------------------------------------------------    20446
LOC106996293.glutathione.hydrolase5.proenzyme-like-GGT1.rhesus.   taataaaggtggatattatgtaataattacaaacttagaagttaaaagcatgaatgaaaa    111060

Papio.anubis.clone.rp41-133b2.8034-40080.revcompl.Baboon          ------------------------------------------------------------    21112
BCRP3.HUMAN.NCBI.REF                                              ------------------------------------------------------------    20446
LOC106996293.glutathione.hydrolase5.proenzyme-like-GGT1.rhesus.   acacaaacttctacaattatcaaaacaatcttttatttggcataaactaagaaagtggac    111120

Papio.anubis.clone.rp41-133b2.8034-40080.revcompl.Baboon          ------------------------------------------------------------    21112
BCRP3.HUMAN.NCBI.REF                                              ------------------------------------------------------------    20446
LOC106996293.glutathione.hydrolase5.proenzyme-like-GGT1.rhesus.   agaatgagtaaaccaaatactcaaccccaatttacatatattgaacacgtgtttcaaaag    111180

Papio.anubis.clone.rp41-133b2.8034-40080.revcompl.Baboon          ------------------------------------------------------------    21112
BCRP3.HUMAN.NCBI.REF                                              ------------------------------------------------------------    20446
LOC106996293.glutathione.hydrolase5.proenzyme-like-GGT1.rhesus.   aacaccaaaaacacacaatgggcaaaagagagactgttcaatagatgattttgagcaaac    111240

Papio.anubis.clone.rp41-133b2.8034-40080.revcompl.Baboon          ------------------------------------------------------------    21112
BCRP3.HUMAN.NCBI.REF                                              ------------------------------------------------------------    20446
LOC106996293.glutathione.hydrolase5.proenzyme-like-GGT1.rhesus.   tgaatattcacatagaaaacactgaaataggacccttgtgtcatgcaacacaaaaatcaa    111300

Papio.anubis.clone.rp41-133b2.8034-40080.revcompl.Baboon          ------------------------------------------------------------    21112
BCRP3.HUMAN.NCBI.REF                                              ------------------------------------------------------------    20446
LOC106996293.glutathione.hydrolase5.proenzyme-like-GGT1.rhesus.   tgcaaaatacattaaagacctaaaactcaattctgaaaccacacaactcctacaagaaaa    111360

Papio.anubis.clone.rp41-133b2.8034-40080.revcompl.Baboon          ------------------------------------------------------------    21112
BCRP3.HUMAN.NCBI.REF                                              ------------------------------------------------------------    20446
LOC106996293.glutathione.hydrolase5.proenzyme-like-GGT1.rhesus.   catagggtgtgcatgtattttagaaaagaaatccatgcatgtaggctgctaaaggcatta    111420

Papio.anubis.clone.rp41-133b2.8034-40080.revcompl.Baboon          ------------------------------------------------------------    21112
BCRP3.HUMAN.NCBI.REF                                              ------------------------------------------------------------    20446
LOC106996293.glutathione.hydrolase5.proenzyme-like-GGT1.rhesus.   aaaaaactttaaaacaaggaaacacccatagacaatgtaatggcttggcattttcactca    111480

Papio.anubis.clone.rp41-133b2.8034-40080.revcompl.Baboon          ------------------------------------------------------------    21112
BCRP3.HUMAN.NCBI.REF                                              ------------------------------------------------------------    20446
LOC106996293.glutathione.hydrolase5.proenzyme-like-GGT1.rhesus.   aaagctggggacgtggttccacagaaccgtggaacacattagaagacccagatataaacc    111540

Papio.anubis.clone.rp41-133b2.8034-40080.revcompl.Baboon          ------------------------------------------------------------    21112
BCRP3.HUMAN.NCBI.REF                                              ------------------------------------------------------------    20446
LOC106996293.glutathione.hydrolase5.proenzyme-like-GGT1.rhesus.   tgcacatctgaaaccatctgacacttgaaaaaaatcaacaaaaataagtgatggagaaag    111600

Papio.anubis.clone.rp41-133b2.8034-40080.revcompl.Baboon          ------------------------------------------------------------    21112
BCRP3.HUMAN.NCBI.REF                                              ------------------------------------------------------------    20446
LOC106996293.glutathione.hydrolase5.proenzyme-like-GGT1.rhesus.   gactccctatcagtaagtggtgctgggataagtggctagctagatgtagaataaataaca    111660

Papio.anubis.clone.rp41-133b2.8034-40080.revcompl.Baboon          ------------------------------------------------------------    21112
BCRP3.HUMAN.NCBI.REF                                              ------------------------------------------------------------    20446
LOC106996293.glutathione.hydrolase5.proenzyme-like-GGT1.rhesus.   ctgggcccctgtgtctcaccatgtacagaaaataactcaaaatgaatcaaagtttgaaat    111720

Papio.anubis.clone.rp41-133b2.8034-40080.revcompl.Baboon          ------------------------------------------------------------    21112
BCRP3.HUMAN.NCBI.REF                                              ------------------------------------------------------------    20446
LOC106996293.glutathione.hydrolase5.proenzyme-like-GGT1.rhesus.   gcgaaatccagggtgggcgcagtggctcattcctggaatccatgcactttgggagccaaa    111780

Papio.anubis.clone.rp41-133b2.8034-40080.revcompl.Baboon          ------------------------------------------------------------    21112
BCRP3.HUMAN.NCBI.REF                                              ------------------------------------------------------------    20446
LOC106996293.glutathione.hydrolase5.proenzyme-like-GGT1.rhesus.   gtgggtagatgacttgaagtcaggagttcaagaccagcctggtcgacatagtgaaaccct    111840

Papio.anubis.clone.rp41-133b2.8034-40080.revcompl.Baboon          ------------------------------------------------------------    21112
BCRP3.HUMAN.NCBI.REF                                              ------------------------------------------------------------    20446
LOC106996293.glutathione.hydrolase5.proenzyme-like-GGT1.rhesus.   gcctctaataaaaaaacaaaagttagctgggcgtggtggcgcgtgcctgtactctcagct    111900

Papio.anubis.clone.rp41-133b2.8034-40080.revcompl.Baboon          ------------------------------------------------------------    21112
BCRP3.HUMAN.NCBI.REF                                              ------------------------------------------------------------    20446
LOC106996293.glutathione.hydrolase5.proenzyme-like-GGT1.rhesus.   actcaggagattgaggcaagagaatcacttgaacccagaagctggagggtgcagtgagcc    111960

Papio.anubis.clone.rp41-133b2.8034-40080.revcompl.Baboon          ------------------------------------------------------------    21112
BCRP3.HUMAN.NCBI.REF                                              ------------------------------------------------------------    20446
LOC106996293.glutathione.hydrolase5.proenzyme-like-GGT1.rhesus.   aagatcgctccattgcattccagcctgtacaacagagtgagactcagtctctaaataaat    112020

Papio.anubis.clone.rp41-133b2.8034-40080.revcompl.Baboon          ------------------------------------------------------------    21112
BCRP3.HUMAN.NCBI.REF                                              ------------------------------------------------------------    20446
LOC106996293.glutathione.hydrolase5.proenzyme-like-GGT1.rhesus.   acataaatacataaaaatgcaagacctgaaatgaaaaagtcctgcacgagaatctagcaa    112080

Papio.anubis.clone.rp41-133b2.8034-40080.revcompl.Baboon          ------------------------------------------------------------    21112
BCRP3.HUMAN.NCBI.REF                                              ------------------------------------------------------------    20446
LOC106996293.glutathione.hydrolase5.proenzyme-like-GGT1.rhesus.   atacccttctcaacagaggctttggcaaagcatttatatgtcaagtccccaaaagcaatg    112140

Papio.anubis.clone.rp41-133b2.8034-40080.revcompl.Baboon          ------------------------------------------------------------    21112
BCRP3.HUMAN.NCBI.REF                                              ------------------------------------------------------------    20446
LOC106996293.glutathione.hydrolase5.proenzyme-like-GGT1.rhesus.   gcaacaaaaacaattactgataagtgggacctaatactcaaaagagctgctgcacagaac    112200

Papio.anubis.clone.rp41-133b2.8034-40080.revcompl.Baboon          ------------------------------------------------------------    21112
BCRP3.HUMAN.NCBI.REF                                              ------------------------------------------------------------    20446
LOC106996293.glutathione.hydrolase5.proenzyme-like-GGT1.rhesus.   aagaaacaaccaacagagtcagcacacagcctatagaatgagggaacatactccccaact    112260

Papio.anubis.clone.rp41-133b2.8034-40080.revcompl.Baboon          ------------------------------------------------------------    21112
BCRP3.HUMAN.NCBI.REF                                              ------------------------------------------------------------    20446
LOC106996293.glutathione.hydrolase5.proenzyme-like-GGT1.rhesus.   acgcatctgaaaaacgtctaatatccaggatttatcgtaaagaccttaaacaaataaaat    112320

Papio.anubis.clone.rp41-133b2.8034-40080.revcompl.Baboon          ------------------------------------------------------------    21112
BCRP3.HUMAN.NCBI.REF                                              ------------------------------------------------------------    20446
LOC106996293.glutathione.hydrolase5.proenzyme-like-GGT1.rhesus.   cagaaaaaaaacccaacttataaatgggcatgggacatgaacacacacttaaaagaaggt    112380

Papio.anubis.clone.rp41-133b2.8034-40080.revcompl.Baboon          ------------------------------------------------------------    21112
BCRP3.HUMAN.NCBI.REF                                              ------------------------------------------------------------    20446
LOC106996293.glutathione.hydrolase5.proenzyme-like-GGT1.rhesus.   gtaccagtaaccaatgagcatgaaaacatgttcgatctcactgatcatcagagaaatgca    112440

Papio.anubis.clone.rp41-133b2.8034-40080.revcompl.Baboon          ------------------------------------------------------------    21112
BCRP3.HUMAN.NCBI.REF                                              ------------------------------------------------------------    20446
LOC106996293.glutathione.hydrolase5.proenzyme-like-GGT1.rhesus.   aatcagaaacatactgagataccatctcacactggtcagaatggcaattatgacacacag    112500

Papio.anubis.clone.rp41-133b2.8034-40080.revcompl.Baboon          ------------------------------------------------------------    21112
```

```
BCRP3.HUMAN.NCBI.REF                                              ------------------------------------------------------------    20446
LOC106996293.glutathione.hydrolase5.proenzyme-like-GGT1.rhesus.   tccacaacaacagaggctggtggggcagatgagcaaagaaatgcaggtccactgttgggg    112560

Papio.anubis.clone.rp41-133b2.8034-40080.revcompl.Baboon          ------------------------------------------------------------    21112
BCRP3.HUMAN.NCBI.REF                                              ------------------------------------------------------------    20446
LOC106996293.glutathione.hydrolase5.proenzyme-like-GGT1.rhesus.   gaaatgcaaactagttcagacccccctggagagcagtgtggacatttctcaaagaacctaa   112620

Papio.anubis.clone.rp41-133b2.8034-40080.revcompl.Baboon          ------------------------------------------------------------    21112
BCRP3.HUMAN.NCBI.REF                                              ------------------------------------------------------------    20446
LOC106996293.glutathione.hydrolase5.proenzyme-like-GGT1.rhesus.   aagagaactaccacccaacgctgcaaccccactcctgaggatctacccaaaggaaaatcc    112680

Papio.anubis.clone.rp41-133b2.8034-40080.revcompl.Baboon          ------------------------------------------------------------    21112
BCRP3.HUMAN.NCBI.REF                                              ------------------------------------------------------------    20446
LOC106996293.glutathione.hydrolase5.proenzyme-like-GGT1.rhesus.   ttccatccaaaacatgcacgcactcgtatgttcatggcagtactactcacaatggtaaag    112740

Papio.anubis.clone.rp41-133b2.8034-40080.revcompl.Baboon          ------------------------------------------------------------    21112
BCRP3.HUMAN.NCBI.REF                                              ------------------------------------------------------------    20446
LOC106996293.glutathione.hydrolase5.proenzyme-like-GGT1.rhesus.   acacggaatcagccttggtgcccatcagcagtggatcagagaaaggaaatgtggtacata    112800

Papio.anubis.clone.rp41-133b2.8034-40080.revcompl.Baboon          ------------------------------------------------------------    21112
BCRP3.HUMAN.NCBI.REF                                              ------------------------------------------------------------    20446
LOC106996293.glutathione.hydrolase5.proenzyme-like-GGT1.rhesus.   cacaccacggaacactacacagccataaaaaacaaatccatgcccttaccagaaacctgg    112860

Papio.anubis.clone.rp41-133b2.8034-40080.revcompl.Baboon          ------------------------------------------------------------    21112
BCRP3.HUMAN.NCBI.REF                                              ------------------------------------------------------------    20446
LOC106996293.glutathione.hydrolase5.proenzyme-like-GGT1.rhesus.   acagagctgcaggccattacgaacaaaaggcaagaacagaaaaccacaattttcaaaata    112920

Papio.anubis.clone.rp41-133b2.8034-40080.revcompl.Baboon          ------------------------------------------------------------    21112
BCRP3.HUMAN.NCBI.REF                                              ------------------------------------------------------------    20446
LOC106996293.glutathione.hydrolase5.proenzyme-like-GGT1.rhesus.   gcttcagaggtcagttgtgaatattctctccacagagaaatcataactcaagctcacaga    112980

Papio.anubis.clone.rp41-133b2.8034-40080.revcompl.Baboon          ------------------------------------------------------------    21112
BCRP3.HUMAN.NCBI.REF                                              ------------------------------------------------------------    20446
LOC106996293.glutathione.hydrolase5.proenzyme-like-GGT1.rhesus.   ggtgccagacactgcaacttcataatgatgctacgtttacacagatcaaattgtcccttg    113040

Papio.anubis.clone.rp41-133b2.8034-40080.revcompl.Baboon          ------------------------------------------------------------    21112
BCRP3.HUMAN.NCBI.REF                                              ------------------------------------------------------------    20446
LOC106996293.glutathione.hydrolase5.proenzyme-like-GGT1.rhesus.   cactcactggttatacaggcatactctacggcaatgaaactctgtcccattttggtaaca    113100

Papio.anubis.clone.rp41-133b2.8034-40080.revcompl.Baboon          ------------------------------------------------------------    21112
BCRP3.HUMAN.NCBI.REF                                              ------------------------------------------------------------    20446
LOC106996293.glutathione.hydrolase5.proenzyme-like-GGT1.rhesus.   ttcattctcaccggagtaagatgatatctgagtgtggttttgattgacttctcgtgagga    113160

Papio.anubis.clone.rp41-133b2.8034-40080.revcompl.Baboon          ---------------------atggtcacactggtttctcctctgctctgtgtgtcctct    21151
BCRP3.HUMAN.NCBI.REF                                              ------------------------------------------------------------    20446
LOC106996293.glutathione.hydrolase5.proenzyme-like-GGT1.rhesus.   tcagtaatgttgattcagaatattttacctgtgcatccttctcaggtcttcaggtccttt    113220

Papio.anubis.clone.rp41-133b2.8034-40080.revcompl.Baboon          cctctgtgt---------------------------------------------------    21160
BCRP3.HUMAN.NCBI.REF                                              ------------------------------------------------------------    20446
LOC106996293.glutathione.hydrolase5.proenzyme-like-GGT1.rhesus.   gcccagtcttacacatcaaatagaaacaagttcacgataacctggcaaacatctctaacc    113280

Papio.anubis.clone.rp41-133b2.8034-40080.revcompl.Baboon          ------------------------------------------------------------    21160
BCRP3.HUMAN.NCBI.REF                                              ------------------------------------------------------------    20446
LOC106996293.glutathione.hydrolase5.proenzyme-like-GGT1.rhesus.   acagcaaaatgtaaatcaaaaccacacttagataccatctcattctaattggaatgagtg    113340

Papio.anubis.clone.rp41-133b2.8034-40080.revcompl.Baboon          ------------------------------------------------------------    21160
BCRP3.HUMAN.NCBI.REF                                              ------------------------------------------------------------    20446
LOC106996293.glutathione.hydrolase5.proenzyme-like-GGT1.rhesus.   ttacaaaaaaagacaaaatcattgaaagaaaggcaaatgctggtgtgtttccagagagag    113400

Papio.anubis.clone.rp41-133b2.8034-40080.revcompl.Baboon          ------------------------------------------------------------    21160
BCRP3.HUMAN.NCBI.REF                                              ------------------------------------------------------------    20446
LOC106996293.glutathione.hydrolase5.proenzyme-like-GGT1.rhesus.   agagacagagagagacagtcttctgcacagttcgtgagaaggtaaactagtgcacacgct    113460

Papio.anubis.clone.rp41-133b2.8034-40080.revcompl.Baboon          ------------------------------------------------------------    21160
BCRP3.HUMAN.NCBI.REF                                              ------------------------------------------------------------    20446
LOC106996293.glutathione.hydrolase5.proenzyme-like-GGT1.rhesus.   acagaaaccacttggagattcctccaagccttaaaagactccaattaccatttcaaccag    113520

Papio.anubis.clone.rp41-133b2.8034-40080.revcompl.Baboon          ------------------------------------------------------------    21160
BCRP3.HUMAN.NCBI.REF                                              ------------------------------------------------------------    20446
LOC106996293.glutathione.hydrolase5.proenzyme-like-GGT1.rhesus.   caattctactactggaaatacactcaaagccattgaaatcaggacaccaaagacagacct    113580

Papio.anubis.clone.rp41-133b2.8034-40080.revcompl.Baboon          ------------------------------------------------------------    21160
BCRP3.HUMAN.NCBI.REF                                              ------------------------------------------------------------    20446
LOC106996293.glutathione.hydrolase5.proenzyme-like-GGT1.rhesus.   acccacccatgtggatttcagcactgttcccaagagccagtgtaagcatcaacctacctg    113640

Papio.anubis.clone.rp41-133b2.8034-40080.revcompl.Baboon          ------------------------------------------------------------    21160
BCRP3.HUMAN.NCBI.REF                                              ------------------------------------------------------------    20446
LOC106996293.glutathione.hydrolase5.proenzyme-like-GGT1.rhesus.   cttatccacagacgaaggaagaaagaagctgctccacatgtacacaccgggatactcttc    113700

Papio.anubis.clone.rp41-133b2.8034-40080.revcompl.Baboon          ------------------------------------------------------------    21160
BCRP3.HUMAN.NCBI.REF                                              ------------------------------------------------------------    20446
LOC106996293.glutathione.hydrolase5.proenzyme-like-GGT1.rhesus.   acacagaaaaccacaatgacatcatatcgtggggaaccacacggttgcacctggagggca    113760

Papio.anubis.clone.rp41-133b2.8034-40080.revcompl.Baboon          ------------------------------------------------------------    21160
BCRP3.HUMAN.NCBI.REF                                              ------------------------------------------------------------    20446
LOC106996293.glutathione.hydrolase5.proenzyme-like-GGT1.rhesus.   tgatggtaaattaaatgagcaaaggggggagagaaacaaacctgagtcatctcacacatgc    113820

Papio.anubis.clone.rp41-133b2.8034-40080.revcompl.Baboon          ------------------------------------------------------------    21160
BCRP3.HUMAN.NCBI.REF                                              ------------------------------------------------------------    20446
LOC106996293.glutathione.hydrolase5.proenzyme-like-GGT1.rhesus.   agaatctgagaaactctatctcaaagacctggcgagcacaatattggtttccagagattg    113880

Papio.anubis.clone.rp41-133b2.8034-40080.revcompl.Baboon          ------------------------------------------------------------    21160
BCRP3.HUMAN.NCBI.REF                                              ------------------------------------------------------------    20446
LOC106996293.glutathione.hydrolase5.proenzyme-like-GGT1.rhesus.   ggggaaaacaggggtaatgggcagggattgtaaatgggtacaaagttacacacaaatgag    113940

Papio.anubis.clone.rp41-133b2.8034-40080.revcompl.Baboon          --------------------------------------gtctgtcttacaagtacactgt    21182
BCRP3.HUMAN.NCBI.REF                                              ------------------------------------------------------------    20446
LOC106996293.glutathione.hydrolase5.proenzyme-like-GGT1.rhesus.   aggaagaaaattctgttgttctattgcactgcagggtgaccagggttgacagtatcggca    114000

Papio.anubis.clone.rp41-133b2.8034-40080.revcompl.Baboon          gatcacattcagggcacatctagataacccaggatcatctcctcctctcaaaatgtttta    21242
BCRP3.HUMAN.NCBI.REF                                              ------------------------------------------------------------    20446
LOC106996293.glutathione.hydrolase5.proenzyme-like-GGT1.rhesus.   catcactttcaaggcagcttgatagaatactgaaggttctcagcataaaggaataaacaa    114060

Papio.anubis.clone.rp41-133b2.8034-40080.revcompl.Baboon          ------------------------------------------------------------    21242
BCRP3.HUMAN.NCBI.REF                                              ------------------------------------------------------------    20446
LOC106996293.glutathione.hydrolase5.proenzyme-like-GGT1.rhesus.   tgggaaaaggtaacagaatcactaagtaccctgatttcattgttccacaaggtacgcatg    114120

Papio.anubis.clone.rp41-133b2.8034-40080.revcompl.Baboon          ------------------------------------------------------------    21242
BCRP3.HUMAN.NCBI.REF                                              ------------------------------------------------------------    20446
LOC106996293.glutathione.hydrolase5.proenzyme-like-GGT1.rhesus.   ggccataatgccacactctaccctctcgttgtattgatcctttactatgcaatacatttg    114180

Papio.anubis.clone.rp41-133b2.8034-40080.revcompl.Baboon          ------------------------------------------------------------    21242
BCRP3.HUMAN.NCBI.REF                                              ------------------------------------------------------------    20446
LOC106996293.glutathione.hydrolase5.proenzyme-like-GGT1.rhesus.   ttgaaagaaatagaaacaggcgctgctaatattcatatgagaccatgaagtgccctgagt    114240

Papio.anubis.clone.rp41-133b2.8034-40080.revcompl.Baboon          ------------------------------------------------------------    21242
```

```
BCRP3.HUMAN.NCBI.REF                                               ------------------------------------------------------------    20446
LOC106996293.glutathione.hydrolase5.proenzyme-like-GGT1.rhesus.    ttcatcctcagcaatcctgagacatccagactacgtcagattttaaatttcatggaaaag    114300

Papio.anubis.clone.rp41-133b2.8034-40080.revcompl.Baboon           ------------------------------------------------------------    21242
BCRP3.HUMAN.NCBI.REF                                               ------------------------------------------------------------    20446
LOC106996293.glutathione.hydrolase5.proenzyme-like-GGT1.rhesus.    ccacggacccacttccacacagagcagtggaacacaagagaggaccaagaagttaaacca    114360

Papio.anubis.clone.rp41-133b2.8034-40080.revcompl.Baboon           ------------------------------------------------------------    21242
BCRP3.HUMAN.NCBI.REF                                               ------------------------------------------------------------    20446
LOC106996293.glutathione.hydrolase5.proenzyme-like-GGT1.rhesus.    cacactgaaaaccatctgatctatcatagaatccacaaaaataagcaaagggaaaaggac    114420

Papio.anubis.clone.rp41-133b2.8034-40080.revcompl.Baboon           ------------------------------------------------------------    21242
BCRP3.HUMAN.NCBI.REF                                               ------------------------------------------------------------    20446
LOC106996293.glutathione.hydrolase5.proenzyme-like-GGT1.rhesus.    accctgttcaatcactgatgctgaaataagcggctagccatgtgcagaataacactaggc    114480

Papio.anubis.clone.rp41-133b2.8034-40080.revcompl.Baboon           ------------------------------------------------------------    21242
BCRP3.HUMAN.NCBI.REF                                               ------------------------------------------------------------    20446
LOC106996293.glutathione.hydrolase5.proenzyme-like-GGT1.rhesus.    ccctaactcccaacacacacaaagaggaactcaagatgaatggaagatttcaatgtaaaa    114540

Papio.anubis.clone.rp41-133b2.8034-40080.revcompl.Baboon           ------------------------------------------------------------    21242
BCRP3.HUMAN.NCBI.REF                                               ------------------------------------------------------------    20446
LOC106996293.glutathione.hydrolase5.proenzyme-like-GGT1.rhesus.    actcaaactattaaaatcttaccagaaaactcgtgaaatacccttctccacataggcttt    114600

Papio.anubis.clone.rp41-133b2.8034-40080.revcompl.Baboon           ------------------------------------------------------------    21242
BCRP3.HUMAN.NCBI.REF                                               ------------------------------------------------------------    20446
LOC106996293.glutathione.hydrolase5.proenzyme-like-GGT1.rhesus.    ggcaaagcatttagatggctaagtcctgaagagcaacggcaacaaaaacaaaaattggca    114660

Papio.anubis.clone.rp41-133b2.8034-40080.revcompl.Baboon           ------------------------------------------------------------    21242
BCRP3.HUMAN.NCBI.REF                                               ------------------------------------------------------------    20446
LOC106996293.glutathione.hydrolase5.proenzyme-like-GGT1.rhesus.    agtcagacctaatacctgaaagagctgctgcacagcaagagagactacaacagagtaaac    114720

Papio.anubis.clone.rp41-133b2.8034-40080.revcompl.Baboon           ------------------------------------------------------------    21242
BCRP3.HUMAN.NCBI.REF                                               ------------------------------------------------------------    20446
LOC106996293.glutathione.hydrolase5.proenzyme-like-GGT1.rhesus.    agacagcgtacagaatgggagaacatgttcccaaactgtgcctctgaccaacgtctgata    114780

Papio.anubis.clone.rp41-133b2.8034-40080.revcompl.Baboon           ------------------------------------------------------------    21242
BCRP3.HUMAN.NCBI.REF                                               ------------------------------------------------------------    20446
LOC106996293.glutathione.hydrolase5.proenzyme-like-GGT1.rhesus.    tgcagaatctacaaggcccttcaacaagtcaaccagaggaataaacagaaaaagaatatc    114840

Papio.anubis.clone.rp41-133b2.8034-40080.revcompl.Baboon           ------------------------------------------------------------    21242
BCRP3.HUMAN.NCBI.REF                                               ------------------------------------------------------------    20446
LOC106996293.glutathione.hydrolase5.proenzyme-like-GGT1.rhesus.    ccattaatatacaggccagggatgtgaacacacacttctcaaaagtcgatgtacaagcaa    114900

Papio.anubis.clone.rp41-133b2.8034-40080.revcompl.Baboon           ------------------------------------------------------------    21242
BCRP3.HUMAN.NCBI.REF                                               ------------------------------------------------------------    20446
LOC106996293.glutathione.hydrolase5.proenzyme-like-GGT1.rhesus.    ctaacaaataggaaaacatgctcaagctcactcattaacagagagctgccaatcaaaagc    114960

Papio.anubis.clone.rp41-133b2.8034-40080.revcompl.Baboon           ------------------------------------------------------------    21242
BCRP3.HUMAN.NCBI.REF                                               ------------------------------------------------------------    20446
LOC106996293.glutathione.hydrolase5.proenzyme-like-GGT1.rhesus.    acgacgagatgccatcccacgcaggtcacaatggtgaggaccacacagacaaagaacaca    115020

Papio.anubis.clone.rp41-133b2.8034-40080.revcompl.Baboon           ------------------------------------------------------------    21242
BCRP3.HUMAN.NCBI.REF                                               ------------------------------------------------------------    20446
LOC106996293.glutathione.hydrolase5.proenzyme-like-GGT1.rhesus.    cgctggtgaggcagccaaggaaaggaaacactggtatgccttggggggaatgaaaactag    115080

Papio.anubis.clone.rp41-133b2.8034-40080.revcompl.Baboon           ------------------------------------------------------------    21242
BCRP3.HUMAN.NCBI.REF                                               ------------------------------------------------------------    20446
LOC106996293.glutathione.hydrolase5.proenzyme-like-GGT1.rhesus.    tacagacaccattgaaagcagatcagggatttctcaaagagctgaaaacagaactaccat    115140

Papio.anubis.clone.rp41-133b2.8034-40080.revcompl.Baboon           ------------------------------------------------------------    21242
BCRP3.HUMAN.NCBI.REF                                               ------------------------------------------------------------    20446
LOC106996293.glutathione.hydrolase5.proenzyme-like-GGT1.rhesus.    ctcacccagaaagccctctcctgtgcatccacccaaaggaaaatcaacccttctatccaa    115200

Papio.anubis.clone.rp41-133b2.8034-40080.revcompl.Baboon           ------------------------------------------------------------    21242
BCRP3.HUMAN.NCBI.REF                                               ------------------------------------------------------------    20446
LOC106996293.glutathione.hydrolase5.proenzyme-like-GGT1.rhesus.    aagacacagacacttgtatgttcatggcagtgttattcacaatgtcaaagacatggaatc    115260

Papio.anubis.clone.rp41-133b2.8034-40080.revcompl.Baboon           ------------------------------------------------------------    21242
BCRP3.HUMAN.NCBI.REF                                               ------------------------------------------------------------    20446
LOC106996293.glutathione.hydrolase5.proenzyme-like-GGT1.rhesus.    aacctagccatcaacagtggatgagagaccggcacagtgactcacacctgtaattctagc    115320

Papio.anubis.clone.rp41-133b2.8034-40080.revcompl.Baboon           ------------------------------------------------------------    21242
BCRP3.HUMAN.NCBI.REF                                               ------------------------------------------------------------    20446
LOC106996293.glutathione.hydrolase5.proenzyme-like-GGT1.rhesus.    actttgggaggccgaggcaggtggatcgcttcagttcagaattcaagaccagcctgagca    115380

Papio.anubis.clone.rp41-133b2.8034-40080.revcompl.Baboon           ------------------------------------------------------------    21242
BCRP3.HUMAN.NCBI.REF                                               ------------------------------------------------------------    20446
LOC106996293.glutathione.hydrolase5.proenzyme-like-GGT1.rhesus.    aaatgatgaaatcccatctctacaataatacaaaaattagctggatgtggtggcgggcac    115440

Papio.anubis.clone.rp41-133b2.8034-40080.revcompl.Baboon           ------------------------------------------------------------    21242
BCRP3.HUMAN.NCBI.REF                                               ------------------------------------------------------------    20446
LOC106996293.glutathione.hydrolase5.proenzyme-like-GGT1.rhesus.    ctgtagtcctatctaatctggaggctgaggtgggaggatcacctgagcccagaaggtgga    115500

Papio.anubis.clone.rp41-133b2.8034-40080.revcompl.Baboon           ------------------------------------------------------------    21242
BCRP3.HUMAN.NCBI.REF                                               ------------------------------------------------------------    20446
LOC106996293.glutathione.hydrolase5.proenzyme-like-GGT1.rhesus.    aactgcaggccattgttataagtgaactaaggcccaaatagaaaaccaaacgccacatgt    115560

Papio.anubis.clone.rp41-133b2.8034-40080.revcompl.Baboon           ------------------------------------------------------------    21242
BCRP3.HUMAN.NCBI.REF                                               ------------------------------------------------------------    20446
LOC106996293.glutathione.hydrolase5.proenzyme-like-GGT1.rhesus.    tctcacttgtaagcgggagctaaactttgaatggactcaaacataaagaagagaacaaca    115620

Papio.anubis.clone.rp41-133b2.8034-40080.revcompl.Baboon           ------------------------------------------------------------    21242
BCRP3.HUMAN.NCBI.REF                                               ------------------------------------------------------------    20446
LOC106996293.glutathione.hydrolase5.proenzyme-like-GGT1.rhesus.    gacacctggaatgaccaccacaacaataaaaaaacaggagagagggagcgtgtgctgaag    115680

Papio.anubis.clone.rp41-133b2.8034-40080.revcompl.Baboon           ------------------------------------------------------------    21242
BCRP3.HUMAN.NCBI.REF                                               ------------------------------------------------------------    20446
LOC106996293.glutathione.hydrolase5.proenzyme-like-GGT1.rhesus.    acccaacctgtgggccctctgctcactgcctgggttacggggttaatgggaccccaagtc    115740

Papio.anubis.clone.rp41-133b2.8034-40080.revcompl.Baboon           ------------------------------------------------------------    21242
BCRP3.HUMAN.NCBI.REF                                               ------------------------------------------------------------    20446
LOC106996293.glutathione.hydrolase5.proenzyme-like-GGT1.rhesus.    tcagcgtgatgcaatcaacagatgtaactaacttgcatatgtaccctttaaactataata    115800

Papio.anubis.clone.rp41-133b2.8034-40080.revcompl.Baboon           ------------------------------------------------------------    21242
BCRP3.HUMAN.NCBI.REF                                               ------------------------------------------------------------    20446
LOC106996293.glutathione.hydrolase5.proenzyme-like-GGT1.rhesus.    ataagagtagaatgcattaaaaaaaatccaaggttagaaccaaagaaaacaataaatgaa    115860

Papio.anubis.clone.rp41-133b2.8034-40080.revcompl.Baboon           ------------------------------------------------------------    21242
BCRP3.HUMAN.NCBI.REF                                               ------------------------------------------------------------    20446
LOC106996293.glutathione.hydrolase5.proenzyme-like-GGT1.rhesus.    aaaacaatctacttatccaaacaatccatgactgtcacaaacacagaacgaggacctaag    115920

Papio.anubis.clone.rp41-133b2.8034-40080.revcompl.Baboon           ------------------------------------------------------------    21242
BCRP3.HUMAN.NCBI.REF                                               ------------------------------------------------------------    20446
LOC106996293.glutathione.hydrolase5.proenzyme-like-GGT1.rhesus.    tgacagaaagaggcagccaaatactatattcaacatgatatgtagggaacacatttttca    115980

Papio.anubis.clone.rp41-133b2.8034-40080.revcompl.Baboon           ------------------------------------------------------------    21242
```

```
BCRP3.HUMAN.NCBI.REF                                             ------------------------------------------------------------    20446
LOC106996293.glutathione.hydrolase5.proenzyme-like-GGT1.rhesus.  aaaccccaaaaagacacaatgggaaagggagagtctggttcaattactgattttgagaaa    116040

Papio.anubis.clone.rp41-133b2.8034-40080.revcompl.Baboon         ------------------------------------------------------------    21242
BCRP3.HUMAN.NCBI.REF                                             ------------------------------------------------------------    20446
LOC106996293.glutathione.hydrolase5.proenzyme-like-GGT1.rhesus.  actgaatctccacaggaaaaacactgaaatagaacccttacaatgcacaatacacaaaat    116100

Papio.anubis.clone.rp41-133b2.8034-40080.revcompl.Baboon         ------------------------------------------------------------    21242
BCRP3.HUMAN.NCBI.REF                                             ------------------------------------------------------------    20446
LOC106996293.glutathione.hydrolase5.proenzyme-like-GGT1.rhesus.  caacacaaaatgaattaaagacctaaactcaatcttgaatttgtaaaggtcctataagaa    116160

Papio.anubis.clone.rp41-133b2.8034-40080.revcompl.Baboon         ------------------------------------------------------------    21242
BCRP3.HUMAN.NCBI.REF                                             ------------------------------------------------------------    20446
LOC106996293.glutathione.hydrolase5.proenzyme-like-GGT1.rhesus.  aatatagagtgggtatatattttaggaaacccagtatctatgcaaacaggctgcaaaagg    116220

Papio.anubis.clone.rp41-133b2.8034-40080.revcompl.Baboon         ------------------------------------------------------------    21242
BCRP3.HUMAN.NCBI.REF                                             ------------------------------------------------------------    20446
LOC106996293.glutathione.hydrolase5.proenzyme-like-GGT1.rhesus.  taaaaaagcatcataacactgaaaaaaaaaaaagtagggaaactacacccagagacaacg    116280

Papio.anubis.clone.rp41-133b2.8034-40080.revcompl.Baboon         ------------------------------------------------------------    21242
BCRP3.HUMAN.NCBI.REF                                             ------------------------------------------------------------    20446
LOC106996293.glutathione.hydrolase5.proenzyme-like-GGT1.rhesus.  caaggcttggttctgtgtgtgtgtgcatgtgtgcatgcatgtgcacgcacatgcacacat    116340

Papio.anubis.clone.rp41-133b2.8034-40080.revcompl.Baboon         ------------------------------------------------------------    21242
BCRP3.HUMAN.NCBI.REF                                             ------------------------------------------------------------    20446
LOC106996293.glutathione.hydrolase5.proenzyme-like-GGT1.rhesus.  gtttttgtgtttagtggaagtcgggtgttgaacacaaaaatcactgctcacatacaaaac    116400

Papio.anubis.clone.rp41-133b2.8034-40080.revcompl.Baboon         ------------------------------------------------------------    21242
BCRP3.HUMAN.NCBI.REF                                             ------------------------------------------------------------    20446
LOC106996293.glutathione.hydrolase5.proenzyme-like-GGT1.rhesus.  tataaccatataagactagggacattttgcaacttctacagggaaaagaaaacaatgaaa    116460

Papio.anubis.clone.rp41-133b2.8034-40080.revcompl.Baboon         ------------------------------------------------------------    21242
BCRP3.HUMAN.NCBI.REF                                             ------------------------------------------------------------    20446
LOC106996293.glutathione.hydrolase5.proenzyme-like-GGT1.rhesus.  caaatttaaaaaagcatgctaaagactgggagaaactataaaaaatcctaaccctgaaag    116520

Papio.anubis.clone.rp41-133b2.8034-40080.revcompl.Baboon         ------------------------------------------------------------    21242
BCRP3.HUMAN.NCBI.REF                                             ------------------------------------------------------------    20446
LOC106996293.glutathione.hydrolase5.proenzyme-like-GGT1.rhesus.  aggttgttatctaacacgtacaataaactgatatgactaagtgaaaaacaacaaaaacaa    116580

Papio.anubis.clone.rp41-133b2.8034-40080.revcompl.Baboon         ------------------------------------------------------------    21242
BCRP3.HUMAN.NCBI.REF                                             ------------------------------------------------------------    20446
LOC106996293.glutathione.hydrolase5.proenzyme-like-GGT1.rhesus.  caaaaaatatccaaggtaaaaggatctaaatagacctgagtgaaaagaaagcaggaaact    116640

Papio.anubis.clone.rp41-133b2.8034-40080.revcompl.Baboon         ----------------------------------------------------------ca    21244
BCRP3.HUMAN.NCBI.REF                                             ------------------------------------------------------------    20446
LOC106996293.glutathione.hydrolase5.proenzyme-like-GGT1.rhesus.  gacccacaggtgacagttcctcaatatcactaatcctcacaaaaatgtgaatcaaaacca    116700

Papio.anubis.clone.rp41-133b2.8034-40080.revcompl.Baboon         tactttaggctgggtgtggtagctcatgcctgtaatcccagcactttgggaggctaaggc    21304
BCRP3.HUMAN.NCBI.REF                                             ------------------------------------------------------------    20446
LOC106996293.glutathione.hydrolase5.proenzyme-like-GGT1.rhesus.  tactcggggccgggcgcggtggctcaagcctgtaatcccagcactttgggaggccgagac    116760

Papio.anubis.clone.rp41-133b2.8034-40080.revcompl.Baboon         gggtggatcacttgaggtcaggagttggagaccagcctggctaacacagtgaagccccat    21364
BCRP3.HUMAN.NCBI.REF                                             ------------------------------------------------------------    20446
LOC106996293.glutathione.hydrolase5.proenzyme-like-GGT1.rhesus.  gggtggatcac--gaggtcaggagatcaagaccatcctggctaacacagtgaaaccccgt    116818

Papio.anubis.clone.rp41-133b2.8034-40080.revcompl.Baboon         ctctactaaaaa--tacaaaaattagccaggcatggtggcgtgcgcctgcagtcctagct    21422
BCRP3.HUMAN.NCBI.REF                                             ------------------------------------------------------------    20446
LOC106996293.glutathione.hydrolase5.proenzyme-like-GGT1.rhesus.  ctctactaaaaaatacaaaaaactagccgggcgaggtagcgggcgcctgtagtcccagct    116878

Papio.anubis.clone.rp41-133b2.8034-40080.revcompl.Baboon         actcagaaggctgaggcgaaagaataacttgaatccaggaggtggagattgcagtaagct    21482
BCRP3.HUMAN.NCBI.REF                                             ------------------------------------------------------------    20446
LOC106996293.glutathione.hydrolase5.proenzyme-like-GGT1.rhesus.  acacgggaggctgaggcaggagaatggcgtaaacccgggaggcggagcttgcaatgagct    116938

Papio.anubis.clone.rp41-133b2.8034-40080.revcompl.Baboon         gagatcgcgccac-acactccagcctgggcgacagaggaagactcctccaaaaaacacaa    21541
BCRP3.HUMAN.NCBI.REF                                             ------------------------------------------------------------    20446
LOC106996293.glutathione.hydrolase5.proenzyme-like-GGT1.rhesus.  gagatccagccactgcactccagcctgggcgacagagcgagactccgtctcaaaaaaaaa    116998

Papio.anubis.clone.rp41-133b2.8034-40080.revcompl.Baboon         aaa---------------------------------------------------------    21544
BCRP3.HUMAN.NCBI.REF                                             ------------------------------------------------------------    20446
LOC106996293.glutathione.hydrolase5.proenzyme-like-GGT1.rhesus.  aaaaaaaaaaaaaaaaaaaaccatactcggttaccatctaactccacctagaacgagtatt    117058

Papio.anubis.clone.rp41-133b2.8034-40080.revcompl.Baboon         ------------------------------------------------------------    21544
BCRP3.HUMAN.NCBI.REF                                             ------------------------------------------------------------    20446
LOC106996293.glutathione.hydrolase5.proenzyme-like-GGT1.rhesus.  acaaaacacaaaaatcaaaattgttgaaggcaatgctcagtgctgacttgcaagaaaaaa    117118

Papio.anubis.clone.rp41-133b2.8034-40080.revcompl.Baboon         ------------------------------------------------------------    21544
BCRP3.HUMAN.NCBI.REF                                             ------------------------------------------------------------    20446
LOC106996293.glutathione.hydrolase5.proenzyme-like-GGT1.rhesus.  aaaaaaatcctctacactcttcatggaatgaaaattactatataaactacaaaaaaattt    117178

Papio.anubis.clone.rp41-133b2.8034-40080.revcompl.Baboon         ---------------------------aggcggggcatgatggctcatgcctgtaatccc    21577
BCRP3.HUMAN.NCBI.REF                                             ------------------------------------------------------------    20446
LOC106996293.glutathione.hydrolase5.proenzyme-like-GGT1.rhesus.  tggaagttccttaaaaaagtaaaaggtaggccgggtgcagtggctcacacctgtaatctc    117238

Papio.anubis.clone.rp41-133b2.8034-40080.revcompl.Baboon         agcactttgggaggccaaggcagacagatcacttgaggccaggagtttgagaccagcctg    21637
BCRP3.HUMAN.NCBI.REF                                             ------------------------------------------------------------    20446
LOC106996293.glutathione.hydrolase5.proenzyme-like-GGT1.rhesus.  agcactttgggaggctgagacaggtggatcacctgaggtcaggagttcgagaccagcctc    117298

Papio.anubis.clone.rp41-133b2.8034-40080.revcompl.Baboon         aacaacatggcgaaaccctgtctctactaaaatac-------------------------    21673
BCRP3.HUMAN.NCBI.REF                                             ------------------------------------------------------------    20446
LOC106996293.glutathione.hydrolase5.proenzyme-like-GGT1.rhesus.  attaacatggtgaaatcccatctctactaaaaatacaaaaattagctaggcgtggtggca    117358

Papio.anubis.clone.rp41-133b2.8034-40080.revcompl.Baboon         ------------------------------------------------------------    21673
BCRP3.HUMAN.NCBI.REF                                             ------------------------------------------------------------    20446
LOC106996293.glutathione.hydrolase5.proenzyme-like-GGT1.rhesus.  ggtgcctgtattctcagccacttgggaggctgaggtgggagaatcacttgaatgcaggag    117418

Papio.anubis.clone.rp41-133b2.8034-40080.revcompl.Baboon         ------------------------------------------------------------    21673
BCRP3.HUMAN.NCBI.REF                                             ------------------------------------------------------------    20446
LOC106996293.glutathione.hydrolase5.proenzyme-like-GGT1.rhesus.  gtgaagtttgcattgagccgaaattgccccactgcactccagcctgggagacaagagcaa    117478

Papio.anubis.clone.rp41-133b2.8034-40080.revcompl.Baboon         ------------------------------------------------------------    21673
BCRP3.HUMAN.NCBI.REF                                             ------------------------------------------------------------    20446
LOC106996293.glutathione.hydrolase5.proenzyme-like-GGT1.rhesus.  gactccgtctttaaaaaaaaaagtacaactgccactccagctgacaatcccaccactggg    117538

Papio.anubis.clone.rp41-133b2.8034-40080.revcompl.Baboon         ------------------------------------------------------------    21673
BCRP3.HUMAN.NCBI.REF                                             ------------------------------------------------------------    20446
LOC106996293.glutathione.hydrolase5.proenzyme-like-GGT1.rhesus.  tacatgtttagagaaaacaaaaaccctgtgttatctgccttttcatgtgtcctgaagcac    117598

Papio.anubis.clone.rp41-133b2.8034-40080.revcompl.Baboon         ------------------------------------------------------------    21673
BCRP3.HUMAN.NCBI.REF                                             ------------------------------------------------------------    20446
LOC106996293.glutathione.hydrolase5.proenzyme-like-GGT1.rhesus.  tagtcacaatagccgagatgtggaatcaacctacctgtctatccacacatgaagggataa    117658

Papio.anubis.clone.rp41-133b2.8034-40080.revcompl.Baboon         ------------------------------------------------------------    21673
BCRP3.HUMAN.NCBI.REF                                             ------------------------------------------------------------    20446
LOC106996293.glutathione.hydrolase5.proenzyme-like-GGT1.rhesus.  aagaaccacagtatatatacacaatgaaacacacatcagctctaatacttcaggaaatca    117718

Papio.anubis.clone.rp41-133b2.8034-40080.revcompl.Baboon         ------------------------------------------------------------    21673
```

```
BCRP3.HUMAN.NCBI.REF                                                ------------------------------------------------------------    20446
LOC106996293.glutathione.hydrolase5.proenzyme-like-GGT1.rhesus.     tgtcatctgcaaccacttggagaaacctggaagacaattaggtgaaatgaaacgagtctg    117778

Papio.anubis.clone.rp41-133b2.8034-40080.revcompl.Baboon            ------------------------------------------------------------    21673
BCRP3.HUMAN.NCBI.REF                                                ------------------------------------------------------------    20446
LOC106996293.glutathione.hydrolase5.proenzyme-like-GGT1.rhesus.     ctagggagagacccacgctgcatgacatcaggcatgtggaatccaaaaaccttgtctcc    117838

Papio.anubis.clone.rp41-133b2.8034-40080.revcompl.Baboon            ------------------------------------------------------------    21673
BCRP3.HUMAN.NCBI.REF                                                ------------------------------------------------------------    20446
LOC106996293.glutathione.hydrolase5.proenzyme-like-GGT1.rhesus.     ttgaagcacaacttccaataggggttactggaggctggggagagcagggagcctgggcag    117898

Papio.anubis.clone.rp41-133b2.8034-40080.revcompl.Baboon            ------------------------------------------------------------    21673
BCRP3.HUMAN.NCBI.REF                                                ------------------------------------------------------------    20446
LOC106996293.glutathione.hydrolase5.proenzyme-like-GGT1.rhesus.     ggattggtaacaggtacagagtgatgctcagatacaaggaatccattctggtgttctatt    117958

Papio.anubis.clone.rp41-133b2.8034-40080.revcompl.Baboon            ------------------------------------------------------------    21673
BCRP3.HUMAN.NCBI.REF                                                ------------------------------------------------------------    20446
LOC106996293.glutathione.hydrolase5.proenzyme-like-GGT1.rhesus.     gcacaacagggtgactaggtgattagggtcaacagaatctagtaaatttttcaaaatca    118018

Papio.anubis.clone.rp41-133b2.8034-40080.revcompl.Baboon            ------------------------------------------------------------    21673
BCRP3.HUMAN.NCBI.REF                                                ------------------------------------------------------------    20446
LOC106996293.glutathione.hydrolase5.proenzyme-like-GGT1.rhesus.     gctggaaaatatggttctgaatatcctctccacagagaaataataatgatggaaggtcac    118078

Papio.anubis.clone.rp41-133b2.8034-40080.revcompl.Baboon            ------------------------------------------------------------    21673
BCRP3.HUMAN.NCBI.REF                                                ------------------------------------------------------------    20446
LOC106996293.glutathione.hydrolase5.proenzyme-like-GGT1.rhesus.     acagatgccagatacgatgatttgatcatgatgctacacaaacatatgtcaaaatgtccc    118138

Papio.anubis.clone.rp41-133b2.8034-40080.revcompl.Baboon            ------------------------------------------------------------    21673
BCRP3.HUMAN.NCBI.REF                                                ------------------------------------------------------------    20446
LOC106996293.glutathione.hydrolase5.proenzyme-like-GGT1.rhesus.     ttgcacccttgggttgggggtgtgtgtatactctatagaaataaaactgccatcaaatgt    118198

Papio.anubis.clone.rp41-133b2.8034-40080.revcompl.Baboon            ------------------------------------------------------------    21673
BCRP3.HUMAN.NCBI.REF                                                ------------------------------------------------------------    20446
LOC106996293.glutathione.hydrolase5.proenzyme-like-GGT1.rhesus.     tggtagcattcattttaactggagtgagatgaaatccgagtgtggttttctcacactttt    118258

Papio.anubis.clone.rp41-133b2.8034-40080.revcompl.Baboon            ------------------------------------------------------------    21673
BCRP3.HUMAN.NCBI.REF                                                ------------------------------------------------------------    20446
LOC106996293.glutathione.hydrolase5.proenzyme-like-GGT1.rhesus.     gtgtgaatcagcgatggtgagaatcttctctttgacctgtgcatcaacctcatgtcttca    118318

Papio.anubis.clone.rp41-133b2.8034-40080.revcompl.Baboon            ------------------------------------------------------------    21673
BCRP3.HUMAN.NCBI.REF                                                ------------------------------------------------------------    20446
LOC106996293.glutathione.hydrolase5.proenzyme-like-GGT1.rhesus.     ggtcctccgcccagggctacataacaaattaaaaacaaattcacaataacctagcaagta    118378

Papio.anubis.clone.rp41-133b2.8034-40080.revcompl.Baboon            ------------------------------------------------------------    21673
BCRP3.HUMAN.NCBI.REF                                                ------------------------------------------------------------    20446
LOC106996293.glutathione.hydrolase5.proenzyme-like-GGT1.rhesus.     tcacttatcatacacacacaaaaatcaaaaacacactcaagatttcatctcactctacat    118438

Papio.anubis.clone.rp41-133b2.8034-40080.revcompl.Baboon            ------------------------------------------------------------    21673
BCRP3.HUMAN.NCBI.REF                                                ------------------------------------------------------------    20446
LOC106996293.glutathione.hydrolase5.proenzyme-like-GGT1.rhesus.     tcaatggatgccataaaaaaaaaattgaaacatattaaaacacaaaacaatcaaggtgtg    118498

Papio.anubis.clone.rp41-133b2.8034-40080.revcompl.Baboon            ------------------------------------------------------------    21673
BCRP3.HUMAN.NCBI.REF                                                ------------------------------------------------------------    20446
LOC106996293.glutathione.hydrolase5.proenzyme-like-GGT1.rhesus.     gatttccagaggaggaacttgtctccacatttggtgggaaatggtaaactagtatgcaca    118558

Papio.anubis.clone.rp41-133b2.8034-40080.revcompl.Baboon            ------------------------------------------------------------    21673
BCRP3.HUMAN.NCBI.REF                                                ------------------------------------------------------------    20446
LOC106996293.glutathione.hydrolase5.proenzyme-like-GGT1.rhesus.     ctagaagaaaccacttagatgttccttgaaattgtaaaactacaactaccattgtctcca    118618

Papio.anubis.clone.rp41-133b2.8034-40080.revcompl.Baboon            ------------------------------------------------------------    21673
BCRP3.HUMAN.NCBI.REF                                                ------------------------------------------------------------    20446
LOC106996293.glutathione.hydrolase5.proenzyme-like-GGT1.rhesus.     gcaattctattcctagctatatacacagagtgcacgatcaggacatggaagagattatct    118678

Papio.anubis.clone.rp41-133b2.8034-40080.revcompl.Baboon            ------------------------------------------------------------    21673
BCRP3.HUMAN.NCBI.REF                                                ------------------------------------------------------------    20446
LOC106996293.glutathione.hydrolase5.proenzyme-like-GGT1.rhesus.     gtcatcccaggtggaattcagccctatgcccaaaagccaagataagaaatcagccgacct    118738

Papio.anubis.clone.rp41-133b2.8034-40080.revcompl.Baboon            ------------------------------------------------------------    21673
BCRP3.HUMAN.NCBI.REF                                                ------------------------------------------------------------    20446
LOC106996293.glutathione.hydrolase5.proenzyme-like-GGT1.rhesus.     gtctatacacagatgaagggatgaagaatctctagtatacagacacaatggaatactttt    118798

Papio.anubis.clone.rp41-133b2.8034-40080.revcompl.Baboon            ------------------------------------------------------------    21673
BCRP3.HUMAN.NCBI.REF                                                ------------------------------------------------------------    20446
LOC106996293.glutathione.hydrolase5.proenzyme-like-GGT1.rhesus.     gcaccatcaaagttcatggaatcctaccatttccagcaatatggctgaacccggagatat    118858

Papio.anubis.clone.rp41-133b2.8034-40080.revcompl.Baboon            ------------------------------------------------------------    21673
BCRP3.HUMAN.NCBI.REF                                                ------------------------------------------------------------    20446
LOC106996293.glutathione.hydrolase5.proenzyme-like-GGT1.rhesus.     tctgttcaatcaaatcagaaaggcagacaaagaccaagcctgcatgttctcactcatgtg    118918

Papio.anubis.clone.rp41-133b2.8034-40080.revcompl.Baboon            ------------------------------------------------------------    21673
BCRP3.HUMAN.NCBI.REF                                                ------------------------------------------------------------    20446
LOC106996293.glutathione.hydrolase5.proenzyme-like-GGT1.rhesus.     ggagctgaaaaatttaactcactgaaggtgaaaatacagcagtggtcaccacaggctagg    118978

Papio.anubis.clone.rp41-133b2.8034-40080.revcompl.Baboon            ------------------------------------------------------------    21673
BCRP3.HUMAN.NCBI.REF                                                ------------------------------------------------------------    20446
LOC106996293.glutathione.hydrolase5.proenzyme-like-GGT1.rhesus.     gggaggagaggacatcaaggactggcaataggtacacagagagttacaggagaggaatac    119038

Papio.anubis.clone.rp41-133b2.8034-40080.revcompl.Baboon            ------------------------------------------------------------    21673
BCRP3.HUMAN.NCBI.REF                                                ------------------------------------------------------------    20446
LOC106996293.glutathione.hydrolase5.proenzyme-like-GGT1.rhesus.     attctggtattccactccaaagcagggtgatgatacttaacaatatggtcgtgctgtttt    119098

Papio.anubis.clone.rp41-133b2.8034-40080.revcompl.Baboon            ------------------------------------------------------------    21673
BCRP3.HUMAN.NCBI.REF                                                ------------------------------------------------------------    20446
LOC106996293.glutathione.hydrolase5.proenzyme-like-GGT1.rhesus.     caaaatagctactaaggaggattttgaatgttcacacatcgaagatatcaaacctacacg    119158

Papio.anubis.clone.rp41-133b2.8034-40080.revcompl.Baboon            ------------------------------------------------------------    21673
BCRP3.HUMAN.NCBI.REF                                                ------------------------------------------------------------    20446
LOC106996293.glutathione.hydrolase5.proenzyme-like-GGT1.rhesus.     tgttcacaagcatgggaaactccctgattttatccatactcaaggtatacaaacaagtat    119218

Papio.anubis.clone.rp41-133b2.8034-40080.revcompl.Baboon            ------------------------------------------------------------    21673
BCRP3.HUMAN.NCBI.REF                                                ------------------------------------------------------------    20446
LOC106996293.glutathione.hydrolase5.proenzyme-like-GGT1.rhesus.     cacaatgtcccaatgcacccctaattctggacatgaagtataaaacaaatgttttatgaa    119278

Papio.anubis.clone.rp41-133b2.8034-40080.revcompl.Baboon            ------------------------------------------------------------    21673
BCRP3.HUMAN.NCBI.REF                                                ------------------------------------------------------------    20446
LOC106996293.glutathione.hydrolase5.proenzyme-like-GGT1.rhesus.     atgttaacaatacgcggctaaaaattcacatggaaccactaaagacccaaagaacctgaa    119338

Papio.anubis.clone.rp41-133b2.8034-40080.revcompl.Baboon            ------------------------------------------------------------    21673
BCRP3.HUMAN.NCBI.REF                                                ------------------------------------------------------------    20446
LOC106996293.glutathione.hydrolase5.proenzyme-like-GGT1.rhesus.     tatggaaagcaatcccagggaatacaaactcaatgggaggccgcacactccccaatttca    119398

Papio.anubis.clone.rp41-133b2.8034-40080.revcompl.Baboon            ------------------------------------------------------------    21673
BCRP3.HUMAN.NCBI.REF                                                ------------------------------------------------------------    20446
LOC106996293.glutathione.hydrolase5.proenzyme-like-GGT1.rhesus.     aaatacatgtaaaacctccacttgggccgggcgcggtggctcaagcctgtaatcccagca    119458

Papio.anubis.clone.rp41-133b2.8034-40080.revcompl.Baboon            ------------------------------------------------------------    21673
```

```
BCRP3.HUMAN.NCBI.REF                                              ------------------------------------------------------------    20446
LOC106996293.glutathione.hydrolase5.proenzyme-like-GGT1.rhesus.   ctttgggaggccgagacgggcggatcacgaggtcaggagatcgagaccatcctggctaac    119518

Papio.anubis.clone.rp41-133b2.8034-40080.revcompl.Baboon          -----------------------aaaaaaagaaaaaattagctgggcgtggtggcgaacg    21710
BCRP3.HUMAN.NCBI.REF                                              ------------------------------------------------------------    20446
LOC106996293.glutathione.hydrolase5.proenzyme-like-GGT1.rhesus.   acagtgaaaccccgtctctactaaaaaatacaaaaaactagccgggcgaggtggtgggcg    119578

Papio.anubis.clone.rp41-133b2.8034-40080.revcompl.Baboon          cacgtagtatcagctactcaggagactgaggcacaagaaccccttgaacctgggagatgg    21770
BCRP3.HUMAN.NCBI.REF                                              ------------------------------------------------------------    20446
LOC106996293.glutathione.hydrolase5.proenzyme-like-GGT1.rhesus.   cctgtagtcccagctactcgggaggctgaggcaggagaacggcgtaaacccaggaggcgg    119638

Papio.anubis.clone.rp41-133b2.8034-40080.revcompl.Baboon          aggttgcagtgagctgagactgcaccattgcactccagcctgggcgacagagtgagactc    21830
BCRP3.HUMAN.NCBI.REF                                              ------------------------------------------------------------    20446
LOC106996293.glutathione.hydrolase5.proenzyme-like-GGT1.rhesus.   agcttgcagtgagctgagatccggccactgcactccagcctgggcggcagagtaagactc    119698

Papio.anubis.clone.rp41-133b2.8034-40080.revcompl.Baboon          tgtctcaaaaaaaatcgttaaca-------------------------------------    21853
BCRP3.HUMAN.NCBI.REF                                              ------------------------------------------------------------    20446
LOC106996293.glutathione.hydrolase5.proenzyme-like-GGT1.rhesus.   cgtctcaaaaaaacaaaaaaacaaaaacaaaaacaaaaacaaaaaacctccactcatcca    119758

Papio.anubis.clone.rp41-133b2.8034-40080.revcompl.Baboon          ------------------------------------------------------------    21853
BCRP3.HUMAN.NCBI.REF                                              ------------------------------------------------------------    20446
LOC106996293.glutathione.hydrolase5.proenzyme-like-GGT1.rhesus.   aagactacagtactggcaaaaaaatgaatccatgaacctagggacataagagaacctaaa    119818

Papio.anubis.clone.rp41-133b2.8034-40080.revcompl.Baboon          ------------------------------------------------------------    21853
BCRP3.HUMAN.NCBI.REF                                              ------------------------------------------------------------    20446
LOC106996293.glutathione.hydrolase5.proenzyme-like-GGT1.rhesus.   cgcacacctgtatgcagtcaacacatttttttaaagaatgcctagaagatgcaatgagg    119878

Papio.anubis.clone.rp41-133b2.8034-40080.revcompl.Baboon          ----------------------tacttttccacttaaggtattagtcactcttgtgctgt    21891
BCRP3.HUMAN.NCBI.REF                                              ------------------------------------------------------------    20446
LOC106996293.glutathione.hydrolase5.proenzyme-like-GGT1.rhesus.   ctgggtgctatggcacctgtactcctagtgctttcagagactgagtcaggtgtggtggcg    119938

Papio.anubis.clone.rp41-133b2.8034-40080.revcompl.Baboon          gtaagtagtatccacaggttttgggaattaggatgtgggtggagctctctggggaggggg    21951
BCRP3.HUMAN.NCBI.REF                                              ------------------------------------------------------------    20446
LOC106996293.glutathione.hydrolase5.proenzyme-like-GGT1.rhesus.   cacaccagcaatcccagccacttgggaagatgaggcaggagaatcgctcgaagcaaggag    119998

Papio.anubis.clone.rp41-133b2.8034-40080.revcompl.Baboon          gca---------------------------------------------------------    21954
BCRP3.HUMAN.NCBI.REF                                              ------------------------------------------------------------    20446
LOC106996293.glutathione.hydrolase5.proenzyme-like-GGT1.rhesus.   gcagatgttgcagagagctgagatctcgccactgcactccagtgtggacgacagcatgag    120058

Papio.anubis.clone.rp41-133b2.8034-40080.revcompl.Baboon          ------------------------------------------------------------    21954
BCRP3.HUMAN.NCBI.REF                                              ------------------------------------------------------------    20446
LOC106996293.glutathione.hydrolase5.proenzyme-like-GGT1.rhesus.   tgggactacatcccaaagcggaaaaaagatgagatgaggagtcagtagtctgttcaactg    120118

Papio.anubis.clone.rp41-133b2.8034-40080.revcompl.Baboon          ------------------------------------------------------------    21954
BCRP3.HUMAN.NCBI.REF                                              ------------------------------------------------------------    20446
LOC106996293.glutathione.hydrolase5.proenzyme-like-GGT1.rhesus.   agcagtctgaaacgactggatagccacatacaaaagaatgaaacaagacataagaccttg    120178

Papio.anubis.clone.rp41-133b2.8034-40080.revcompl.Baboon          ---------------------------------acattcaaccctttacatagg------    21975
BCRP3.HUMAN.NCBI.REF                                              ------------------------------------------------------------    20446
LOC106996293.glutathione.hydrolase5.proenzyme-like-GGT1.rhesus.   atgttatacaatacacaaatataaactcaaaatacattaaaccctaaacacaagacctaa    120238

Papio.anubis.clone.rp41-133b2.8034-40080.revcompl.Baboon          ------------------------------------------------------------    21975
BCRP3.HUMAN.NCBI.REF                                              ------------------------------------------------------------    20446
LOC106996293.glutathione.hydrolase5.proenzyme-like-GGT1.rhesus.   aagcataaaactgctacaggaaaacacagagtgagcttaagtttaggaaaacccgaatct    120298

Papio.anubis.clone.rp41-133b2.8034-40080.revcompl.Baboon          ------------------------------------------------------------    21975
BCRP3.HUMAN.NCBI.REF                                              ------------------------------------------------------------    20446
LOC106996293.glutathione.hydrolase5.proenzyme-like-GGT1.rhesus.   aagctcacaggatgtaaaagcaaacaataggaaaaaaaaaaaaagaaaaacagaaaaaaa    120358

Papio.anubis.clone.rp41-133b2.8034-40080.revcompl.Baboon          ------------------------------------------------------------    21975
BCRP3.HUMAN.NCBI.REF                                              ------------------------------------------------------------    20446
LOC106996293.glutathione.hydrolase5.proenzyme-like-GGT1.rhesus.   accacttccaacaacataagtcaaattttctagtttttgttattgactttaccttacaaa    120418

Papio.anubis.clone.rp41-133b2.8034-40080.revcompl.Baboon          -------------------------------------gtgaccccaacctgtgccccgac    21998
BCRP3.HUMAN.NCBI.REF                                              ------------------------------------------------------------    20446
LOC106996293.glutathione.hydrolase5.proenzyme-like-GGT1.rhesus.   aacaatcactgctagccagtcatgctggctcacatctgtaaatccaggacattgggaggc    120478

Papio.anubis.clone.rp41-133b2.8034-40080.revcompl.Baboon          ccctctccagggttcaacttctcagcagagtctgtggccaggcctgaggggagggtgaac    22058
BCRP3.HUMAN.NCBI.REF                                              ------------------------------------------------------------    20446
LOC106996293.glutathione.hydrolase5.proenzyme-like-GGT1.rhesus.   cgaggcaggtggatcacctgaggtcaggaattcgagaccagactggccaacatggtgaaa    120538

Papio.anubis.clone.rp41-133b2.8034-40080.revcompl.Baboon          atgtaccaccaccttgtggagacgctcaagtttgccagggggcagag-------------    22105
BCRP3.HUMAN.NCBI.REF                                              ------------------------------------------------------------    20446
LOC106996293.glutathione.hydrolase5.proenzyme-like-GGT1.rhesus.   ccctgtctctac----taaaaatacaaaaattagccaggagtggtggcgcacctgtag    120594

Papio.anubis.clone.rp41-133b2.8034-40080.revcompl.Baboon          ------------------------------------------------------------    22105
BCRP3.HUMAN.NCBI.REF                                              ------------------------------------------------------------    20446
LOC106996293.glutathione.hydrolase5.proenzyme-like-GGT1.rhesus.   tcccagctacttgggaggctgaggcaagagaatcaatcgaacccgggaagcggaggttgc    120654

Papio.anubis.clone.rp41-133b2.8034-40080.revcompl.Baboon          ------------------------------------------------------------    22105
BCRP3.HUMAN.NCBI.REF                                              ------------------------------------------------------------    20446
LOC106996293.glutathione.hydrolase5.proenzyme-like-GGT1.rhesus.   agtgagccaagatcatgacaccacactctagcctgggcaacacagcaagactccgtctca    120714

Papio.anubis.clone.rp41-133b2.8034-40080.revcompl.Baboon          ------------------------------------------------------------    22105
BCRP3.HUMAN.NCBI.REF                                              ------------------------------------------------------------    20446
LOC106996293.glutathione.hydrolase5.proenzyme-like-GGT1.rhesus.   aaaaaaaaaaaaaaaggaaaaacaaaaataaaaaaatcacaactaaaataatcaagaaac    120774

Papio.anubis.clone.rp41-133b2.8034-40080.revcompl.Baboon          ------------------------------------------------------------    22105
BCRP3.HUMAN.NCBI.REF                                              ------------------------------------------------------------    20446
LOC106996293.glutathione.hydrolase5.proenzyme-like-GGT1.rhesus.   aaaaacacaaaaaatcctgaagaagttctgcacattaaagaaagcaatgcaccaaccaac    120834

Papio.anubis.clone.rp41-133b2.8034-40080.revcompl.Baboon          ------------------------------------------------------------    22105
BCRP3.HUMAN.NCBI.REF                                              ------------------------------------------------------------    20446
LOC106996293.glutathione.hydrolase5.proenzyme-like-GGT1.rhesus.   actccgaaggcattcttcagatagaaaggacattataaggaatcatgcatctgaaactac    120894

Papio.anubis.clone.rp41-133b2.8034-40080.revcompl.Baboon          ------------------------------------------------------------    22105
BCRP3.HUMAN.NCBI.REF                                              ------------------------------------------------------------    20446
LOC106996293.glutathione.hydrolase5.proenzyme-like-GGT1.rhesus.   cacaccactgaggagcaaaacacacaaaaccaactaacccaaccaaaactgggacacaat    120954

Papio.anubis.clone.rp41-133b2.8034-40080.revcompl.Baboon          ------------------------------------------------------------    22105
BCRP3.HUMAN.NCBI.REF                                              ------------------------------------------------------------    20446
LOC106996293.glutathione.hydrolase5.proenzyme-like-GGT1.rhesus.   cctgaacagacatcactaaaaagacaatgcacaattcactaacatgtgaaaagggcgatt    121014

Papio.anubis.clone.rp41-133b2.8034-40080.revcompl.Baboon          ------------------------------------------------------------    22105
BCRP3.HUMAN.NCBI.REF                                              ------------------------------------------------------------    20446
LOC106996293.glutathione.hydrolase5.proenzyme-like-GGT1.rhesus.   ctcaacattactaatcatctctaaactgtaaatgaaaaccacactcagatacacactcac    121074

Papio.anubis.clone.rp41-133b2.8034-40080.revcompl.Baboon          ----------------------------------------------gtggaggctggggg    22119
BCRP3.HUMAN.NCBI.REF                                              ------------------------------------------------------------    20446
LOC106996293.glutathione.hydrolase5.proenzyme-like-GGT1.rhesus.   cctaatttgaacaaataaaagaaaccaattatttcaaacaaattccctggaggaagttgc    121134

Papio.anubis.clone.rp41-133b2.8034-40080.revcompl.Baboon          accctcgcagccacccgaagctccaggtgaggttgctgaggttgctgggatgggggctg    22179
BCRP3.HUMAN.NCBI.REF                                              ------------------------------------------------------------    20446
LOC106996293.glutathione.hydrolase5.proenzyme-like-GGT1.rhesus.   acacacagctactctcacactgttgggagaaatcactgttattacagacactatgaaaac    121194

Papio.anubis.clone.rp41-133b2.8034-40080.revcompl.Baboon          tcctctctggctcaggacttagcatgaaaggagggtcaggcctggcagggggaggttgga    22239
```

```
BCRP3.HUMAN.NCBI.REF                                                ------------------------------------------------------------    20446
LOC106996293.glutathione.hydrolase5.proenzyme-like-GGT1.rhesus.     ctct---tggagcttcccaaacaatcaaaaaggaaactaccatttaatctggcaattcca    121251

Papio.anubis.clone.rp41-133b2.8034-40080.revcompl.Baboon            gggagatgtatgtggttctaggccagggcaggactgaaagggatcccggggtggcaggta    22299
BCRP3.HUMAN.NCBI.REF                                                ------------------------------------------------------------    20446
LOC106996293.glutathione.hydrolase5.proenzyme-like-GGT1.rhesus.     gcagtgggtatatacttagggacagtgaaatcactgcatgaaagccatagctgccttccc    121311

Papio.anubis.clone.rp41-133b2.8034-40080.revcompl.Baboon            cgggggtcagatgcaggagtggcaccatatctcaaaggacctggagggtgggcagagtct    22359
BCRP3.HUMAN.NCBI.REF                                                ------------------------------------------------------------    20446
LOC106996293.glutathione.hydrolase5.proenzyme-like-GGT1.rhesus.     aggttgaccgaagcactatacacaata---------------------------------    121338

Papio.anubis.clone.rp41-133b2.8034-40080.revcompl.Baboon            agacttagcctgcgcttgagggaggcctggccacaaggtagaggacaggctggaggtggc    22419
BCRP3.HUMAN.NCBI.REF                                                ------------------------------------------------------------    20446
LOC106996293.glutathione.hydrolase5.proenzyme-like-GGT1.rhesus.     ------------------------------------------------------------    121338

Papio.anubis.clone.rp41-133b2.8034-40080.revcompl.Baboon            ccccatgggagctgagcttgtcctgcccttggttctatggatcccgcttggcccctcact    22479
BCRP3.HUMAN.NCBI.REF                                                ------------------------------------------------------------    20446
LOC106996293.glutathione.hydrolase5.proenzyme-like-GGT1.rhesus.     ------------------------------------------------------------    121338

Papio.anubis.clone.rp41-133b2.8034-40080.revcompl.Baboon            gatcctgccacctgcccacccaccccagaatgcctcccgggacctgctagaggagaccct    22539
BCRP3.HUMAN.NCBI.REF                                                ------------------------------------------------------------    20446
LOC106996293.glutathione.hydrolase5.proenzyme-like-GGT1.rhesus.     ---------------------------------------------gctaaggtatgcaat    121353

Papio.anubis.clone.rp41-133b2.8034-40080.revcompl.Baboon            ggcccagctcatccgccaacagatcgatggccggggggaccaccagctcag---------    22590
BCRP3.HUMAN.NCBI.REF                                                ------------------------------------------------------------    20446
LOC106996293.glutathione.hydrolase5.proenzyme-like-GGT1.rhesus.     caacctacctatccatcaactgatgaagggatgaaggaacttcagtatctggacacaacg    121413

Papio.anubis.clone.rp41-133b2.8034-40080.revcompl.Baboon            ------------------------------------------------------------    22590
BCRP3.HUMAN.NCBI.REF                                                ------------------------------------------------------------    20446
LOC106996293.glutathione.hydrolase5.proenzyme-like-GGT1.rhesus.     aaatacgccccatcaacaaagattgatgacataatgtcttttgtggcaacacggataaac    121473

Papio.anubis.clone.rp41-133b2.8034-40080.revcompl.Baboon            ------------------------------------------------------------    22590
BCRP3.HUMAN.NCBI.REF                                                ------------------------------------------------------------    20446
LOC106996293.glutathione.hydrolase5.proenzyme-like-GGT1.rhesus.     ctggaagacattacactcagtggaatcagccaggcagggaaacaaaaacactgcatcatc    121533

Papio.anubis.clone.rp41-133b2.8034-40080.revcompl.Baboon            ------------------------------------------------------------    22590
BCRP3.HUMAN.NCBI.REF                                                ------------------------------------------------------------    20446
LOC106996293.glutathione.hydrolase5.proenzyme-like-GGT1.rhesus.     tgacccatgaggagactaaaaaagaattatctaacggaaagtgaaagtacaatagtggtt    121593

Papio.anubis.clone.rp41-133b2.8034-40080.revcompl.Baboon            ------------------------------------------------------------    22590
BCRP3.HUMAN.NCBI.REF                                                ------------------------------------------------------------    20446
LOC106996293.glutathione.hydrolase5.proenzyme-like-GGT1.rhesus.     atcacatgctgggggaggagagggcatgggctggcattggtaatgggtaccaagttgca    121653

Papio.anubis.clone.rp41-133b2.8034-40080.revcompl.Baboon            ------------------------------------------------------------    22590
BCRP3.HUMAN.NCBI.REF                                                ------------------------------------------------------------    20446
LOC106996293.glutathione.hydrolase5.proenzyme-like-GGT1.rhesus.     cttccaggggaggaattaatgctggtgatctactccacaggagggtgactacaagtaaca    121713

Papio.anubis.clone.rp41-133b2.8034-40080.revcompl.Baboon            ------------------------------------------------------------    22590
BCRP3.HUMAN.NCBI.REF                                                ------------------------------------------------------------    20446
LOC106996293.glutathione.hydrolase5.proenzyme-like-GGT1.rhesus.     atacggtaggatctctttcaatagaggtagaaaggagaggaacactgaaggttcacacaa    121773

Papio.anubis.clone.rp41-133b2.8034-40080.revcompl.Baboon            ------------------------------------------------------------    22590
BCRP3.HUMAN.NCBI.REF                                                ------------------------------------------------------------    20446
LOC106996293.glutathione.hydrolase5.proenzyme-like-GGT1.rhesus.     caaacaaataaaaactagacatgggaatagatatgcaaaacaccctgacttcatcattcc    121833

Papio.anubis.clone.rp41-133b2.8034-40080.revcompl.Baboon            ------------------------------------------------------------    22590
BCRP3.HUMAN.NCBI.REF                                                ------------------------------------------------------------    20446
LOC106996293.glutathione.hydrolase5.proenzyme-like-GGT1.rhesus.     tcaaagtatgcaagtaaacaaaatcccactgtacccccctaattttggacatgtatgacaa    121893

Papio.anubis.clone.rp41-133b2.8034-40080.revcompl.Baboon            ------------------------------------------------------------    22590
BCRP3.HUMAN.NCBI.REF                                                ------------------------------------------------------------    20446
LOC106996293.glutathione.hydrolase5.proenzyme-like-GGT1.rhesus.     actcaattttaaaactataaatggacaatggtaaaattcacagagaaccagggaagaccc    121953

Papio.anubis.clone.rp41-133b2.8034-40080.revcompl.Baboon            ------------------------------------------------------------    22590
BCRP3.HUMAN.NCBI.REF                                                ------------------------------------------------------------    20446
LOC106996293.glutathione.hydrolase5.proenzyme-like-GGT1.rhesus.     tgaaaatccaaagcacttcccagaaagagaaaatcacttgattgcctcacgctccgcgat    122013

Papio.anubis.clone.rp41-133b2.8034-40080.revcompl.Baboon            ------------------------------------------------------------    22590
BCRP3.HUMAN.NCBI.REF                                                ------------------------------------------------------------    20446
LOC106996293.glutathione.hydrolase5.proenzyme-like-GGT1.rhesus.     gtcaaattacatcaaaaagctatacttaccgaggcagcaaggaactggcataaaaataga    122073

Papio.anubis.clone.rp41-133b2.8034-40080.revcompl.Baboon            ------------------------------------------------------------    22590
BCRP3.HUMAN.NCBI.REF                                                ------------------------------------------------------------    20446
LOC106996293.glutathione.hydrolase5.proenzyme-like-GGT1.rhesus.     aacatttatcattggacagtattgggtagccaacagacaaatctgaatgtacttacagtc    122133

Papio.anubis.clone.rp41-133b2.8034-40080.revcompl.Baboon            ------------------------------------------------------------    22590
BCRP3.HUMAN.NCBI.REF                                                ------------------------------------------------------------    20446
LOC106996293.glutathione.hydrolase5.proenzyme-like-GGT1.rhesus.     aacagatttctcaaaaggccaggcgcggtggctcacgtctgtaatcccaacattttggga    122193

Papio.anubis.clone.rp41-133b2.8034-40080.revcompl.Baboon            ------------------------------------------------------------    22590
BCRP3.HUMAN.NCBI.REF                                                ------------------------------------------------------------    20446
LOC106996293.glutathione.hydrolase5.proenzyme-like-GGT1.rhesus.     ggcaacggctggcgaaccacttgatgtcaggagtttgggatcagcctaacatggtgataa    122253

Papio.anubis.clone.rp41-133b2.8034-40080.revcompl.Baboon            --ccactacagcttggccgaggcctggggccacgggacaggcacatcccatgtgtctgtg    22648
BCRP3.HUMAN.NCBI.REF                                                ------------------------------------------------------------    20446
LOC106996293.glutathione.hydrolase5.proenzyme-like-GGT1.rhesus.     taaaaatagaaaaattagccagcatggtggcatgtgactgtaatcccagctacttgggg    122313

Papio.anubis.clone.rp41-133b2.8034-40080.revcompl.Baboon            ctgggggaggatg-------------gcagtgccgtggctgccaccagcaccatcaacac    22695
BCRP3.HUMAN.NCBI.REF                                                ------------------------------------------------------------    20446
LOC106996293.glutathione.hydrolase5.proenzyme-like-GGT1.rhesus.     ctgaggcaggagaatcgcttggatctgggaggcagaggttgcagtgagcaaagatcacac    122373

Papio.anubis.clone.rp41-133b2.8034-40080.revcompl.Baboon            accgtgcgtggggcctgggggaag------------------------------------    22719
BCRP3.HUMAN.NCBI.REF                                                ------------------------------------------------------------    20446
LOC106996293.glutathione.hydrolase5.proenzyme-like-GGT1.rhesus.     cacttcactctagcctgggcaaaaaagcaacactctgtatcaaaacaaacaaacaaacaa    122433

Papio.anubis.clone.rp41-133b2.8034-40080.revcompl.Baboon            ------------------------------------------------------------    22719
BCRP3.HUMAN.NCBI.REF                                                ------------------------------------------------------------    20446
LOC106996293.glutathione.hydrolase5.proenzyme-like-GGT1.rhesus.     acaaaaaacccaaacaaaatgggaaacagctagtctgttctgtaaatgactttgagaaaa    122493

Papio.anubis.clone.rp41-133b2.8034-40080.revcompl.Baboon            ------------------------------------------------------------    22719
BCRP3.HUMAN.NCBI.REF                                                ------------------------------------------------------------    20446
LOC106996293.glutathione.hydrolase5.proenzyme-like-GGT1.rhesus.     ctggaaacccacacacaaaacatggaaacctccttgagcttatccttaatccttatgcaa    122553

Papio.anubis.clone.rp41-133b2.8034-40080.revcompl.Baboon            ----------------gctggtggcctcactcctcctctcctagacctgcactcccccag    22763
BCRP3.HUMAN.NCBI.REF                                                ------------------------------------------------------------    20446
LOC106996293.glutathione.hydrolase5.proenzyme-like-GGT1.rhesus.     ggtacacaagtgctgtaatgtccccattgcacccctaattctggacatgcagtatgaacc    122613

Papio.anubis.clone.rp41-133b2.8034-40080.revcompl.Baboon            ccccatgtcccctcacttgtccccatggggcagcaccttgcttttgccctttgtctcctc    22823
BCRP3.HUMAN.NCBI.REF                                                ------------------------------------------------------------    20446
LOC106996293.glutathione.hydrolase5.proenzyme-like-GGT1.rhesus.     aaacttgtcatgaaatgtaactatacacagctaaaaattcacatagaactaccatagact    122673

Papio.anubis.clone.rp41-133b2.8034-40080.revcompl.Baboon            ctctgtttcaaaag----------------------------------------------    22837
BCRP3.HUMAN.NCBI.REF                                                ------------------------------------------------------------    20446
LOC106996293.glutathione.hydrolase5.proenzyme-like-GGT1.rhesus.     ctgaatatgcaaagcaatcctaggaaatataaacttgtttggaggtctcacactgttcaa    122733

Papio.anubis.clone.rp41-133b2.8034-40080.revcompl.Baboon            ------------------------------------------------------------    22837
```

```
BCRP3.HUMAN.NCBI.REF                                               ------------------------------------------------------------   20446
LOC106996293.glutathione.hydrolase5.proenzyme-like-GGT1.rhesus.    tttcaaattacatggaaaagatctactcatccaaagactatggcagtggcctaacaatga   122793

Papio.anubis.clone.rp41-133b2.8034-40080.revcompl.Baboon           ------------------------------------------------------------   22837
BCRP3.HUMAN.NCBI.REF                                               ------------------------------------------------------------   20446
LOC106996293.glutathione.hydrolase5.proenzyme-like-GGT1.rhesus.    aaccatgcagacataacagaacctaaacgtacatctgtatggagtcaacacgctgttttt   122853

Papio.anubis.clone.rp41-133b2.8034-40080.revcompl.Baboon           ------------------------------------------------------------   22837
BCRP3.HUMAN.NCBI.REF                                               ------------------------------------------------------------   20446
LOC106996293.glutathione.hydrolase5.proenzyme-like-GGT1.rhesus.    tcaagagaatgcctggaagacaaaatgaggagtgggaggtctgttcaattaatgaatctg   122913

Papio.anubis.clone.rp41-133b2.8034-40080.revcompl.Baboon           ------------------------------------------------------------   22837
BCRP3.HUMAN.NCBI.REF                                               ------------------------------------------------------------   20446
LOC106996293.glutathione.hydrolase5.proenzyme-like-GGT1.rhesus.    aaaatactggatatccacatgaaaaagaatgaaataggacccttatgttacacaatacac   122973

Papio.anubis.clone.rp41-133b2.8034-40080.revcompl.Baboon           ------------------------------------aggcccccacccctgacacctctg   22861
BCRP3.HUMAN.NCBI.REF                                               ------------------------------------------------------------   20446
LOC106996293.glutathione.hydrolase5.proenzyme-like-GGT1.rhesus.    aaataccaacttgaaataaatgaaaacctaaacacaagacccaaaagcataaaacttcta   123033

Papio.anubis.clone.rp41-133b2.8034-40080.revcompl.Baboon           gctggaagggctgctgctggggtggccccgagctaagacttacctgggaatgggcgg---   22918
BCRP3.HUMAN.NCBI.REF                                               ------------------------------------------------------------   20446
LOC106996293.glutathione.hydrolase5.proenzyme-like-GGT1.rhesus.    caggaaaacacagagtgagtggaagctcagggcaacttcatctatgctcataggttgcaa   123093

Papio.anubis.clone.rp41-133b2.8034-40080.revcompl.Baboon           ------------------------------------------------------------   22918
BCRP3.HUMAN.NCBI.REF                                               ------------------------------------------------------------   20446
LOC106996293.glutathione.hydrolase5.proenzyme-like-GGT1.rhesus.    aaagaaaaaacaggaaatacatggaaaatacattcaaaatcaatgaaatgtatcaaaatt   123153

Papio.anubis.clone.rp41-133b2.8034-40080.revcompl.Baboon           ------------------------------------------------------------   22918
BCRP3.HUMAN.NCBI.REF                                               ------------------------------------------------------------   20446
LOC106996293.glutathione.hydrolase5.proenzyme-like-GGT1.rhesus.    gtgatttttctttctggcctgacccaaaatcgctgctaacaaaggaaaactcaacacgt   123213

Papio.anubis.clone.rp41-133b2.8034-40080.revcompl.Baboon           ---------------------------------------------------cctcactca   22927
BCRP3.HUMAN.NCBI.REF                                               ------------------------------------------------------------   20446
LOC106996293.glutathione.hydrolase5.proenzyme-like-GGT1.rhesus.    gaaaaagaaaactcgagaactacggcatggtaaagggagcaatgaaccaatcaacaatga   123273

Papio.anubis.clone.rp41-133b2.8034-40080.revcompl.Baboon           gaagggtgccctgatatgggggcacaggtgggtctttggggacccctcctgggtgatgcc   22987
BCRP3.HUMAN.NCBI.REF                                               ------------------------------------------------------------   20446
LOC106996293.glutathione.hydrolase5.proenzyme-like-GGT1.rhesus.    gaaggcattcttcatataggg---------------------------------------   123294

Papio.anubis.clone.rp41-133b2.8034-40080.revcompl.Baboon           agggagagaacagtggctccagcatgcttcggggcagctgtaaagtgagggggtcctgca   23047
BCRP3.HUMAN.NCBI.REF                                               ------------------------------------------------------------   20446
LOC106996293.glutathione.hydrolase5.proenzyme-like-GGT1.rhesus.    ------------------agggcatgatgaggaatcctttatctgaaagaggttgttaca   123336

Papio.anubis.clone.rp41-133b2.8034-40080.revcompl.Baboon           aggtgggcagggcaggcggtgtctggtgggagcccaggctctgcctccacggctgcagct   23107
BCRP3.HUMAN.NCBI.REF                                               ------------------------------------------------------------   20446
LOC106996293.glutathione.hydrolase5.proenzyme-like-GGT1.rhesus.    tggtatgcacgagaaactaatgctactaagaagcaaaa----------------------   123374

Papio.anubis.clone.rp41-133b2.8034-40080.revcompl.Baboon           ttggagcgatggtgtattcaccacggacaggcatcatcctcaacaacgagctcctggact   23167
BCRP3.HUMAN.NCBI.REF                                               ------------------------------------------------------------   20446
LOC106996293.glutathione.hydrolase5.proenzyme-like-GGT1.rhesus.    ------------------------------------------------------------   123374

Papio.anubis.clone.rp41-133b2.8034-40080.revcompl.Baboon           tatgcgagcgatgcccccggggttccggcaccacccccctcacctggtgagaacaaggctt   23227
BCRP3.HUMAN.NCBI.REF                                               ------------------------------------------------------------   20446
LOC106996293.glutathione.hydrolase5.proenzyme-like-GGT1.rhesus.    ------------------------catgcaaggccaagtcacctggccaaagttgggcaa   123410

Papio.anubis.clone.rp41-133b2.8034-40080.revcompl.Baboon           cccacctggga------tccacaagggcccccaccaggggagaggagagagggggctgg   23281
BCRP3.HUMAN.NCBI.REF                                               ------------------------------------------------------------   20446
LOC106996293.glutathione.hydrolase5.proenzyme-like-GGT1.rhesus.    tgaacctgaagagatatttctcaaaagtcccagctactcgggaagctgaggtgggagtga   123470

Papio.anubis.clone.rp41-133b2.8034-40080.revcompl.Baboon           gctggggctgcatgc---------------------------------------------   23296
BCRP3.HUMAN.NCBI.REF                                               ------------------------------------------------------------   20446
LOC106996293.glutathione.hydrolase5.proenzyme-like-GGT1.rhesus.    gctgagatcgcacccctacactccagcaaccatagccagacactgtatcaaaaaaaaaaa   123530

Papio.anubis.clone.rp41-133b2.8034-40080.revcompl.Baboon           ------------------------------------------------------------   23296
BCRP3.HUMAN.NCBI.REF                                               ------------------------------------------------------------   20446
LOC106996293.glutathione.hydrolase5.proenzyme-like-GGT1.rhesus.    aaaaaggaaaaaagaaaaaagaaataaactatgcagagaaagataaacatcgcattgtct   123590

Papio.anubis.clone.rp41-133b2.8034-40080.revcompl.Baboon           ------------------------------------------------------------   23296
BCRP3.HUMAN.NCBI.REF                                               ------------------------------------------------------------   20446
LOC106996293.glutathione.hydrolase5.proenzyme-like-GGT1.rhesus.    cactcaggaggaatctacaaaactttatttcctagaactagtaagcataactttctaaat   123650

Papio.anubis.clone.rp41-133b2.8034-40080.revcompl.Baboon           ------------------------------------------------------------   23296
BCRP3.HUMAN.NCBI.REF                                               ------------------------------------------------------------   20446
LOC106996293.glutathione.hydrolase5.proenzyme-like-GGT1.rhesus.    gggttggggttcaaaggtggggcatggtcagagattggaaacaggtataaagttacatat   123710

Papio.anubis.clone.rp41-133b2.8034-40080.revcompl.Baboon           ------------------------------------------------------------   23296
BCRP3.HUMAN.NCBI.REF                                               ------------------------------------------------------------   20446
LOC106996293.glutathione.hydrolase5.proenzyme-like-GGT1.rhesus.    tcccatgagaggaatgaaacctgtagttcttttccacagcagcagggtgactaggtctaa   123770

Papio.anubis.clone.rp41-133b2.8034-40080.revcompl.Baboon           ------------------------------------------------------------   23296
BCRP3.HUMAN.NCBI.REF                                               ------------------------------------------------------------   20446
LOC106996293.glutathione.hydrolase5.proenzyme-like-GGT1.rhesus.    cattatcagagtgtacttttcaagaaagctagaaaagaggattctgaatgtcttcacctc   123830

Papio.anubis.clone.rp41-133b2.8034-40080.revcompl.Baboon           ------------------------------------------------------------   23296
BCRP3.HUMAN.NCBI.REF                                               ------------------------------------------------------------   20446
LOC106996293.glutathione.hydrolase5.proenzyme-like-GGT1.rhesus.    actctatgaggaaagacagatgctaaataccctgatttcatcattacacaatgtatgcat   123890

Papio.anubis.clone.rp41-133b2.8034-40080.revcompl.Baboon           ------------------tgacccctggatgggtcactgcacttgctaagccgctgtttg   23338
BCRP3.HUMAN.NCBI.REF                                               ------------------------------------------------------------   20446
LOC106996293.glutathione.hydrolase5.proenzyme-like-GGT1.rhesus.    ggaccaaagtgtctcattctaccctctggtagtacacctttaccacagggtaaatttttg   123950

Papio.anubis.clone.rp41-133b2.8034-40080.revcompl.Baboon           ctcagcagtgaatggagacagggtgggtggagctcccggagggtgctggcccccagttcc   23398
BCRP3.HUMAN.NCBI.REF                                               ------------------------------------------------------------   20446
LOC106996293.glutathione.hydrolase5.proenzyme-like-GGT1.rhesus.    taaagaaatgtaagggaacaggctgag---------------------------------   123977

Papio.anubis.clone.rp41-133b2.8034-40080.revcompl.Baboon           aggcgagcggtccccatcctccatggtgccctccatcctgatcagcaaagcccaggggtc   23458
BCRP3.HUMAN.NCBI.REF                                               ------------------------------------------------------------   20446
LOC106996293.glutathione.hydrolase5.proenzyme-like-GGT1.rhesus.    ------------------------------------------------------------   123977

Papio.anubis.clone.rp41-133b2.8034-40080.revcompl.Baboon           gaagctagtgattggcggggctggcggggagctcatcatctctgccgtggcccaggtgag   23518
BCRP3.HUMAN.NCBI.REF                                               ------------------------------------------------------------   20446
LOC106996293.glutathione.hydrolase5.proenzyme-like-GGT1.rhesus.    ------------------------------------------------------------   123977

Papio.anubis.clone.rp41-133b2.8034-40080.revcompl.Baboon           tctgggggactctgggctccagtgtctcctctctggcagcatactgtctgactctccc   23578
BCRP3.HUMAN.NCBI.REF                                               ------------------------------------------------------------   20446
LOC106996293.glutathione.hydrolase5.proenzyme-like-GGT1.rhesus.    --catggtggctcatgcctgcaatcccagcactttcagaggctgaggcaggagtattgct   124035

Papio.anubis.clone.rp41-133b2.8034-40080.revcompl.Baboon           tggagtggggatgtgagggctgaagtagggtagagggtacccccctttctccctgcatccc   23638
BCRP3.HUMAN.NCBI.REF                                               ------------------------------------------------------------   20446
LOC106996293.glutathione.hydrolase5.proenzyme-like-GGT1.rhesus.    tgaacccaagaagtgcaggctgcagtg---------------------------------   124062

Papio.anubis.clone.rp41-133b2.8034-40080.revcompl.Baboon           tcatctctcccccaggccatcatgaacaagttgtggcttggctttgacctgagagcggcc   23698
BCRP3.HUMAN.NCBI.REF                                               ------------------------------------------------------------   20446
LOC106996293.glutathione.hydrolase5.proenzyme-like-GGT1.rhesus.    ------------------------------------------------------------   124062

Papio.anubis.clone.rp41-133b2.8034-40080.revcompl.Baboon           attgcagcccccatcctgcatgtcaacagtaagggccgtgtggagtacgagcccagcttc   23758
```

```
BCRP3.HUMAN.NCBI.REF                                            ------------------------------------------------------------    20446
LOC106996293.glutathione.hydrolase5.proenzyme-like-GGT1.rhesus.   ------------------------------------------------------------    124062

Papio.anubis.clone.rp41-133b2.8034-40080.revcompl.Baboon        agccaggtgaggctgaggtccaagctggatgcctagggcagagcccactccccaagtccg    23818
BCRP3.HUMAN.NCBI.REF                                            ------------------------------------------------------------    20446
LOC106996293.glutathione.hydrolase5.proenzyme-like-GGT1.rhesus.   ----------------------------------agccaagattgcaccactgcactcca    124088

Papio.anubis.clone.rp41-133b2.8034-40080.revcompl.Baboon        tgctgctcaaagccacctgagaggaactcagtcactgagattcttaggccaggtacactt    23878
BCRP3.HUMAN.NCBI.REF                                            ------------------------------------------------------------    20446
LOC106996293.glutathione.hydrolase5.proenzyme-like-GGT1.rhesus.   gtctgggcaacagagtgagacactgtctcaaaaaataaaaat------------------    124130

Papio.anubis.clone.rp41-133b2.8034-40080.revcompl.Baboon        cagctttgggggccataggagttggggacctcgatgggtgaggctgtcagtgacctcca    23938
BCRP3.HUMAN.NCBI.REF                                            ------------------------------------------------------------    20446
LOC106996293.glutathione.hydrolase5.proenzyme-like-GGT1.rhesus.   -----------------------------------------------taaaaaaaaaaaa    124143

Papio.anubis.clone.rp41-133b2.8034-40080.revcompl.Baboon        ggccagttctgtggcctccaagacagacagtagggtgttgtctacactgctcccaggctg    23998
BCRP3.HUMAN.NCBI.REF                                            ------------------------------------------------------------    20446
LOC106996293.glutathione.hydrolase5.proenzyme-like-GGT1.rhesus.   gatgacgttcaagtgatcaaccaaatgaaaaaatgtgcaacct-cagcgcaatgcaactc    124202

Papio.anubis.clone.rp41-133b2.8034-40080.revcompl.Baboon        aggatctcagcaacttggtctctctggtctgtcattgatgccatttttcagaagtgagtt    24058
BCRP3.HUMAN.NCBI.REF                                            ------------------------------------------------------------    20446
LOC106996293.glutathione.hydrolase5.proenzyme-like-GGT1.rhesus.   ccaaacacaatgagatatcatctcacgttggtcacaatgggcatttttcaaagtcaaaa    124262

Papio.anubis.clone.rp41-133b2.8034-40080.revcompl.Baboon        ttcctagctggggcctctcagactctccctcatg--------------------------    24092
BCRP3.HUMAN.NCBI.REF                                            ------------------------------------------------------------    20446
LOC106996293.glutathione.hydrolase5.proenzyme-like-GGT1.rhesus.   caacatgctggcgaggctgataatctgtcccaaatcaaggcagcaaaattcctgcaaaga    124322

Papio.anubis.clone.rp41-133b2.8034-40080.revcompl.Baboon        ------------------------------------------------------------    24092
BCRP3.HUMAN.NCBI.REF                                            ------------------------------------------------------------    20446
LOC106996293.glutathione.hydrolase5.proenzyme-like-GGT1.rhesus.   aaaaaaactaaagcatgtctgccagctgctggtctgtgttagagaaggctctttctaggg    124382

Papio.anubis.clone.rp41-133b2.8034-40080.revcompl.Baboon        ------------------------------------------------------------    24092
BCRP3.HUMAN.NCBI.REF                                            ------------------------------------------------------------    20446
LOC106996293.glutathione.hydrolase5.proenzyme-like-GGT1.rhesus.   gcaccagagggaatggagtgtaattcaaactggaagactttcaaagccagagctagctca    124442

Papio.anubis.clone.rp41-133b2.8034-40080.revcompl.Baboon        ------------------------------------------------------------    24092
BCRP3.HUMAN.NCBI.REF                                            ------------------------------------------------------------    20446
LOC106996293.glutathione.hydrolase5.proenzyme-like-GGT1.rhesus.   cctattgaggctataccttttaactaaactcaactgcagtagtcaggactcttgttgcaa    124502

Papio.anubis.clone.rp41-133b2.8034-40080.revcompl.Baboon        --------------------------------------------gtgactatttccttgt    24108
BCRP3.HUMAN.NCBI.REF                                            ------------------------------------------------------------    20446
LOC106996293.glutathione.hydrolase5.proenzyme-like-GGT1.rhesus.   gtgacagaaacccaactcaaactagcttaagcctaaagacagatttatggactatctcat    124562

Papio.anubis.clone.rp41-133b2.8034-40080.revcompl.Baboon        gtgatttgtaacttatatatttgtgactttatttgtaaaagaggttgtttttc-------    24161
BCRP3.HUMAN.NCBI.REF                                            ------------------------------------------------------------    20446
LOC106996293.glutathione.hydrolase5.proenzyme-like-GGT1.rhesus.   ggaattcataaacaagatagttctgactctctctcagacctcattggccttaagaacaat    124622

Papio.anubis.clone.rp41-133b2.8034-40080.revcompl.Baboon        ------------------------------------------------------------    24161
BCRP3.HUMAN.NCBI.REF                                            ------------------------------------------------------------    20446
LOC106996293.glutathione.hydrolase5.proenzyme-like-GGT1.rhesus.   atggagtcagagactcaaacaccactctcttctcttgtcatgtctctgtttctttgagat    124682

Papio.anubis.clone.rp41-133b2.8034-40080.revcompl.Baboon        ------------------------------------------------------------    24161
BCRP3.HUMAN.NCBI.REF                                            ------------------------------------------------------------    20446
LOC106996293.glutathione.hydrolase5.proenzyme-like-GGT1.rhesus.   ttgtccttttttctcagcaattcccccaatcctctagttcacataagatggagtggagtc    124742

Papio.anubis.clone.rp41-133b2.8034-40080.revcompl.Baboon        -------------------------------------------------------ctgtg    24166
BCRP3.HUMAN.NCBI.REF                                            ------------------------------------------------------------    20446
LOC106996293.glutathione.hydrolase5.proenzyme-like-GGT1.rhesus.   tccctctgtcgcccaggcaggagtgcagtggcacagtcttggctcactgcaacctctgtc    124802

Papio.anubis.clone.rp41-133b2.8034-40080.revcompl.Baboon        gcagtctataaagtgatttcttctggggcattggtggtaatttttactacaggtgtgtg    24226
BCRP3.HUMAN.NCBI.REF                                            ------------------------------------------------------------    20446
LOC106996293.glutathione.hydrolase5.proenzyme-like-GGT1.rhesus.   tcctgggttcaagcgattcttgtgcctcagcttccgagtggctgggatgacaggcatctg    124862

Papio.anubis.clone.rp41-133b2.8034-40080.revcompl.Baboon        ccaccacacctggatagtttttatttttttgtttttcgtagagacagggtctctcgccat    24286
BCRP3.HUMAN.NCBI.REF                                            ------------------------------------------------------------    20446
LOC106996293.glutathione.hydrolase5.proenzyme-like-GGT1.rhesus.   ccaccacgtccagctaacttttgtatttttagt--------agagacggattttcgccat    124914

Papio.anubis.clone.rp41-133b2.8034-40080.revcompl.Baboon        gttgcctaggctggtcttgaactcctgagctcacgcaatcttcccaccttggcctcccaa    24346
BCRP3.HUMAN.NCBI.REF                                            ------------------------------------------------------------    20446
LOC106996293.glutathione.hydrolase5.proenzyme-like-GGT1.rhesus.   gttggccgggctggtttcgaactcctga---caggtgatccacctgccttggccttgcaa    124971

Papio.anubis.clone.rp41-133b2.8034-40080.revcompl.Baboon        aatgctggaattataggtgtgagccaccatgtctggc-----------------------    24383
BCRP3.HUMAN.NCBI.REF                                            ------------------------------------------------------------    20446
LOC106996293.glutathione.hydrolase5.proenzyme-like-GGT1.rhesus.   agtgctgggattacatacgtgagccaccgcgcccggctactgatgcaataatatattctt    125031

Papio.anubis.clone.rp41-133b2.8034-40080.revcompl.Baboon        ------------------------------------------------------------    24383
BCRP3.HUMAN.NCBI.REF                                            ------------------------------------------------------------    20446
LOC106996293.glutathione.hydrolase5.proenzyme-like-GGT1.rhesus.   aactctggttcaaagactttttgaagaagcagataatgagcatgacagaattgaaactga    125091

Papio.anubis.clone.rp41-133b2.8034-40080.revcompl.Baboon        ------------------------------------------------------------    24383
BCRP3.HUMAN.NCBI.REF                                            ------------------------------------------------------------    20446
LOC106996293.glutathione.hydrolase5.proenzyme-like-GGT1.rhesus.   tttggggaaacgattaaaatctccatctaggttgggcgcgatggttcacacctataatcc    125151

Papio.anubis.clone.rp41-133b2.8034-40080.revcompl.Baboon        ------------------------------------------------------------    24383
BCRP3.HUMAN.NCBI.REF                                            ------------------------------------------------------------    20446
LOC106996293.glutathione.hydrolase5.proenzyme-like-GGT1.rhesus.   caacactttgggaggccaagacaggcagatcacttgcggtcaggagtttgaaaccagccc    125211

Papio.anubis.clone.rp41-133b2.8034-40080.revcompl.Baboon        ------------------------------------------------------------    24383
BCRP3.HUMAN.NCBI.REF                                            ------------------------------------------------------------    20446
LOC106996293.glutathione.hydrolase5.proenzyme-like-GGT1.rhesus.   agcaaacatgctgaaaccccatctctactaaaaatacaaaaatttgcttggtgttttggt    125271

Papio.anubis.clone.rp41-133b2.8034-40080.revcompl.Baboon        ------------------------------------------------------------    24383
BCRP3.HUMAN.NCBI.REF                                            ------------------------------------------------------------    20446
LOC106996293.glutathione.hydrolase5.proenzyme-like-GGT1.rhesus.   gtgcacctgtaatcccagctactcaggaagctgagccatgaggatcacttgaacccagga    125331

Papio.anubis.clone.rp41-133b2.8034-40080.revcompl.Baboon        ------------------------------------------------------------    24383
BCRP3.HUMAN.NCBI.REF                                            ------------------------------------------------------------    20446
LOC106996293.glutathione.hydrolase5.proenzyme-like-GGT1.rhesus.   ggtggaggttgcagtgagccacgatcacgtcactgcactccagcctgggcaacagagtga    125391

Papio.anubis.clone.rp41-133b2.8034-40080.revcompl.Baboon        ------------------------------------------------------------    24383
BCRP3.HUMAN.NCBI.REF                                            ------------------------------------------------------------    20446
LOC106996293.glutathione.hydrolase5.proenzyme-like-GGT1.rhesus.   gaccctgtctcaaaaaaaaaaaaaaaaaaaaaaatccccattcatacatgatgataccaa    125451

Papio.anubis.clone.rp41-133b2.8034-40080.revcompl.Baboon        --------------ctacaaacatttttttgattccccacaaaagcc-------cagggt    24422
BCRP3.HUMAN.NCBI.REF                                            ------------------------------------------------------------    20446
LOC106996293.glutathione.hydrolase5.proenzyme-like-GGT1.rhesus.   aagatcaaaagaaagcagaacgatttttttggagcaaaacacactgcttgctgtcaataa    125511

Papio.anubis.clone.rp41-133b2.8034-40080.revcompl.Baboon        gcagctccaggggctgaattctcctggttgactcctcctccatccatagacagagacttg    24482
BCRP3.HUMAN.NCBI.REF                                            ------------------------------------------------------------    20446
LOC106996293.glutathione.hydrolase5.proenzyme-like-GGT1.rhesus.   aaagattacagtcctaacttttcccccaagaattctgtcagataatctcaaagttatcaa    125571

Papio.anubis.clone.rp41-133b2.8034-40080.revcompl.Baboon        ccttccttggagaatctttgggttgatggatgccctttgttatgacctatttaaaagatt    24542
BCRP3.HUMAN.NCBI.REF                                            ------------------------------------------------------------    20446
LOC106996293.glutathione.hydrolase5.proenzyme-like-GGT1.rhesus.   aatgaaatgggattatttaaagatgaagga------------------cttaaagaaaaa    125613

Papio.anubis.clone.rp41-133b2.8034-40080.revcompl.Baboon        gataggtccgggcactgtgg-tcatggctgtaatcccagcactttgggaggcagaggcag    24601
```

```
BCRP3.HUMAN.NCBI.REF                                            ------------------------------------------------------------    20446
LOC106996293.glutathione.hydrolase5.proenzyme-like-GGT1.rhesus.   tacttggccaggcacggtgactaatgcttgtaatcccagcactttggaaggccgaggctg    125673

Papio.anubis.clone.rp41-133b2.8034-40080.revcompl.Baboon        gtggataacctgaggtgaggacttccaggccagcctggccaacatggtgaaaccccgtct    24661
BCRP3.HUMAN.NCBI.REF                                            ------------------------------------------------------------    20446
LOC106996293.glutathione.hydrolase5.proenzyme-like-GGT1.rhesus.   gtggatcacctgaggtcaggagttcaagaccagcctggccaacatcatgaaaccccgtct    125733

Papio.anubis.clone.rp41-133b2.8034-40080.revcompl.Baboon        ctagtaaaaatacaaaaattagccaggcatggtggcgcacacctgtaatcccagctactc    24721
BCRP3.HUMAN.NCBI.REF                                            ------------------------------------------------------------    20446
LOC106996293.glutathione.hydrolase5.proenzyme-like-GGT1.rhesus.   ccactaaacatacaaaaattagccgggcatggtggccggtgcctctaatcccagctactc    125793

Papio.anubis.clone.rp41-133b2.8034-40080.revcompl.Baboon        aggaggctgaggcaggagaatcgcttgaacctgggaggc---------------------    24760
BCRP3.HUMAN.NCBI.REF                                            ------------------------------------------------------------    20446
LOC106996293.glutathione.hydrolase5.proenzyme-like-GGT1.rhesus.   gggaggatgaggcaagagaatcgcttaaacccaggaggcagagggtgtgcacctgtaatc    125853

Papio.anubis.clone.rp41-133b2.8034-40080.revcompl.Baboon        -------------------------------------------------ggaggttgcag    24771
BCRP3.HUMAN.NCBI.REF                                            ------------------------------------------------------------    20446
LOC106996293.glutathione.hydrolase5.proenzyme-like-GGT1.rhesus.   ccagttactggggaggctgaggcacgagaattgcttgagttcaggagatggaggttgcag    125913

Papio.anubis.clone.rp41-133b2.8034-40080.revcompl.Baboon        tgagctgagatcgtgccattgcacgatttttgttgcctgggcaacaaacgcaaaattctg    24831
BCRP3.HUMAN.NCBI.REF                                            ------------------------------------------------------------    20446
LOC106996293.glutathione.hydrolase5.proenzyme-like-GGT1.rhesus.   tgaactgagatctcaccactgcac------tccagcctgggtgacagaatgaga---ctg    125964

Papio.anubis.clone.rp41-133b2.8034-40080.revcompl.Baboon        tctcaaaaaaaaaaaaaaaaaagattgatatattttcaagactccaaacttggttcctga    24891
BCRP3.HUMAN.NCBI.REF                                            ------------------------------------------------------------    20446
LOC106996293.glutathione.hydrolase5.proenzyme-like-GGT1.rhesus.   cctcaaaaaaaaaaaaaaaaaaaagtgctatttt--------------------------    125998

Papio.anubis.clone.rp41-133b2.8034-40080.revcompl.Baboon        ggtcagttcagttcagctaactgaggttcaaggtcctcaaagctgcctctcctgttatga    24951
BCRP3.HUMAN.NCBI.REF                                            ------------------------------------------------------------    20446
LOC106996293.glutathione.hydrolase5.proenzyme-like-GGT1.rhesus.   ------------------------------aagtgttttatgattaaacttctgtaattg    126028

Papio.anubis.clone.rp41-133b2.8034-40080.revcompl.Baboon        gatttcttcggtctcaagtccaccttccaagcccttagaacctgagaaccatggactggg    25011
BCRP3.HUMAN.NCBI.REF                                            ------------------------------------------------------------    20446
LOC106996293.glutathione.hydrolase5.proenzyme-like-GGT1.rhesus.   tct---attgcactaagttcattatttttattgtattaaactttagattcatggggcaca    126085

Papio.anubis.clone.rp41-133b2.8034-40080.revcompl.Baboon        accttagtgtagtaacagagtgtagtccccaacagtcttagtcttcacaccttgagacta    25071
BCRP3.HUMAN.NCBI.REF                                            ------------------------------------------------------------    20446
LOC106996293.glutathione.hydrolase5.proenzyme-like-GGT1.rhesus.   tgtgcaggtgtgttcca-tgagtacactgcaaaatgctaaggcttg--------------    126130

Papio.anubis.clone.rp41-133b2.8034-40080.revcompl.Baboon        aggacctcagcctgttaggctcaaattcttggagtttgcaggtctccctccccatgtagg    25131
BCRP3.HUMAN.NCBI.REF                                            ------------------------------------------------------------    20446
LOC106996293.glutathione.hydrolase5.proenzyme-like-GGT1.rhesus.   ------------------------------ggcttctattgaactcatcacccaaacact    126160

Papio.anubis.clone.rp41-133b2.8034-40080.revcompl.Baboon        gtccttcgcatgacaggaagaccctctcagggctctcaggttccagggatcttagatatg    25191
BCRP3.HUMAN.NCBI.REF                                            ------------------------------------------------------------    20446
LOC106996293.glutathione.hydrolase5.proenzyme-like-GGT1.rhesus.   gaccatagtacccaatgactagtttttcaaccctttcccctccttccctccccacatgtg    126220

Papio.anubis.clone.rp41-133b2.8034-40080.revcompl.Baboon        gaggatttcaagatccagggcacatgctttggagccctcctcccttgactctttatttat    25251
BCRP3.HUMAN.NCBI.REF                                            ------------------------------------------------------------    20446
LOC106996293.glutathione.hydrolase5.proenzyme-like-GGT1.rhesus.   gagttcctagtgtctgttatttccatctttatgtccat-ttgaaccaactgtttagctat    126279

Papio.anubis.clone.rp41-133b2.8034-40080.revcompl.Baboon        ttatttatttatttatttatttatttaggcagtctcactctgtcactcaggctggagtgc    25311
BCRP3.HUMAN.NCBI.REF                                            ------------------------------------------------------------    20446
LOC106996293.glutathione.hydrolase5.proenzyme-like-GGT1.rhesus.   ttatttatttatttatttttgag---acagagtcttgctccttcaccctggatggagtgc    126336

Papio.anubis.clone.rp41-133b2.8034-40080.revcompl.Baboon        agtggcacaatcttgcctcaccacaacctccgcttcccaggttcaagcaattcttctgcc    25371
BCRP3.HUMAN.NCBI.REF                                            ------------------------------------------------------------    20446
LOC106996293.glutathione.hydrolase5.proenzyme-like-GGT1.rhesus.   agtggctccatctcagctcactgcaatattcacctcccgggttcaagcgattctccagcc    126396

Papio.anubis.clone.rp41-133b2.8034-40080.revcompl.Baboon        tcagcc-tctgagtagctgggactacaggcgtg-caaaccaagcctggctaatttttgta    25429
BCRP3.HUMAN.NCBI.REF                                            ------------------------------------------------------------    20446
LOC106996293.glutathione.hydrolase5.proenzyme-like-GGT1.rhesus.   tcagccttctgagtagctgggattacaggcaggtgccaccacaccaggcaaacttttgca    126456

Papio.anubis.clone.rp41-133b2.8034-40080.revcompl.Baboon        tttttagtaaagacggggt-----------------------------------------    25448
BCRP3.HUMAN.NCBI.REF                                            ------------------------------------------------------------    20446
LOC106996293.glutathione.hydrolase5.proenzyme-like-GGT1.rhesus.   attttagtagaaatgggattccaccatgttggccaggctggtatcgaactcctgacctca    126516

Papio.anubis.clone.rp41-133b2.8034-40080.revcompl.Baboon        ------------------------------------------------------------    25448
BCRP3.HUMAN.NCBI.REF                                            ------------------------------------------------------------    20446
LOC106996293.glutathione.hydrolase5.proenzyme-like-GGT1.rhesus.   agtaatccacctgctttgccctcccagagtgctgggattataggcatgagccactgtacc    126576

Papio.anubis.clone.rp41-133b2.8034-40080.revcompl.Baboon        ------------------------------------------------------------    25448
BCRP3.HUMAN.NCBI.REF                                            ------------------------------------------------------------    20446
LOC106996293.glutathione.hydrolase5.proenzyme-like-GGT1.rhesus.   ttgcctatttttaaataatacttaattttctttcattttgctaataatttagagtacaat    126636

Papio.anubis.clone.rp41-133b2.8034-40080.revcompl.Baboon        ------------------------------------------------------------    25448
BCRP3.HUMAN.NCBI.REF                                            ------------------------------------------------------------    20446
LOC106996293.glutathione.hydrolase5.proenzyme-like-GGT1.rhesus.   tatataatgatattaactttttttgggggggtgggacagggtctcactctgtcacccagga    126696

Papio.anubis.clone.rp41-133b2.8034-40080.revcompl.Baboon        ------------------------------------------------------------    25448
BCRP3.HUMAN.NCBI.REF                                            ------------------------------------------------------------    20446
LOC106996293.glutathione.hydrolase5.proenzyme-like-GGT1.rhesus.   tggagtgcaatggctcaatcatgcttccatgcagcctcaaattcctgggctcatgtgatc    126756

Papio.anubis.clone.rp41-133b2.8034-40080.revcompl.Baboon        ------------------------------------------------------------    25448
BCRP3.HUMAN.NCBI.REF                                            ------------------------------------------------------------    20446
LOC106996293.glutathione.hydrolase5.proenzyme-like-GGT1.rhesus.   ctcccacctcagcctcccaaacagctgggaccacagccatgcaccaccatgtccagctaa    126816

Papio.anubis.clone.rp41-133b2.8034-40080.revcompl.Baboon        ------------------------------------------------------------    25448
BCRP3.HUMAN.NCBI.REF                                            ------------------------------------------------------------    20446
LOC106996293.glutathione.hydrolase5.proenzyme-like-GGT1.rhesus.   tttttctatttttgtggagacagggtcttgctatgttgcccggggggtgtcaaattcctg    126876

Papio.anubis.clone.rp41-133b2.8034-40080.revcompl.Baboon        ------------------------------------------------------------    25448
BCRP3.HUMAN.NCBI.REF                                            ------------------------------------------------------------    20446
LOC106996293.glutathione.hydrolase5.proenzyme-like-GGT1.rhesus.   agctcaagcaatctgcccgccttggcttcccaaagtgctgggattagaggccgaaaccac    126936

Papio.anubis.clone.rp41-133b2.8034-40080.revcompl.Baboon        ------------------------------------------------------------    25448
BCRP3.HUMAN.NCBI.REF                                            ------------------------------------------------------------    20446
LOC106996293.glutathione.hydrolase5.proenzyme-like-GGT1.rhesus.   cgtccctggcctaataatgtagaatcttacttaatgtttcatagtatgattttcaaagtt    126996

Papio.anubis.clone.rp41-133b2.8034-40080.revcompl.Baboon        ------------------------------------------------------------    25448
BCRP3.HUMAN.NCBI.REF                                            ------------------------------------------------------------    20446
LOC106996293.glutathione.hydrolase5.proenzyme-like-GGT1.rhesus.   tgtatttgaaatgtatttaaatatgtagtttgatcttttgaaaaacatttgaattcctaa    127056

Papio.anubis.clone.rp41-133b2.8034-40080.revcompl.Baboon        ------------------------------------------------------------    25448
BCRP3.HUMAN.NCBI.REF                                            ------------------------------------------------------------    20446
LOC106996293.glutathione.hydrolase5.proenzyme-like-GGT1.rhesus.   cagattataaaatactattgtgcccaggaggtggaggttgcaatgaacagagatcaaact    127116

Papio.anubis.clone.rp41-133b2.8034-40080.revcompl.Baboon        ------------------------------------------------------------    25448
BCRP3.HUMAN.NCBI.REF                                            ------------------------------------------------------------    20446
LOC106996293.glutathione.hydrolase5.proenzyme-like-GGT1.rhesus.   gctgcactccagcctgggtgacagagcaagatgccagctcaaaaaaaaacaaaacaaaac    127176

Papio.anubis.clone.rp41-133b2.8034-40080.revcompl.Baboon        ------------------------------------------------------------    25448
BCRP3.HUMAN.NCBI.REF                                            ------------------------------------------------------------    20446
LOC106996293.glutathione.hydrolase5.proenzyme-like-GGT1.rhesus.   aaaaaaaactagttgtgttagggttcaaaccagagaagcagaaccagtaggaaagataca    127236

Papio.anubis.clone.rp41-133b2.8034-40080.revcompl.Baboon        ------------------------------------------------------------    25448
```

```
BCRP3.HUMAN.NCBI.REF                                              ------------------------------------------------------------    20446
LOC106996293.glutathione.hydrolase5.proenzyme-like-GGT1.rhesus.   cacattcagatatacataaacatatagtgacacacatctgcatagatatagaaacggatg    127296

Papio.anubis.clone.rp41-133b2.8034-40080.revcompl.Baboon          --ttccggccgggcgcggtggctcaagcctgtaatcccagcactttgggaggccgagacg    25506
BCRP3.HUMAN.NCBI.REF                                              ------------------------------------------------------------    20446
LOC106996293.glutathione.hydrolase5.proenzyme-like-GGT1.rhesus.   gcttctagccaggcacagtggctcacacctgtaatcccagcactttgggaggccgaggca    127356

Papio.anubis.clone.rp41-133b2.8034-40080.revcompl.Baboon          ggcggatcac--gaggtcaggagatcgagaccatcctggctaacacggtgaaaccctgtc    25564
BCRP3.HUMAN.NCBI.REF                                              ------------------------------------------------------------    20446
LOC106996293.glutathione.hydrolase5.proenzyme-like-GGT1.rhesus.   ggcagatcacctgaggtcaggagtttgagaccagcctggccaacatgatgaaactctgtc    127416

Papio.anubis.clone.rp41-133b2.8034-40080.revcompl.Baboon          tctactaaaaatacaaaaaactagccgggcgaagtggcgggcgcctgtggtcccagcta    25624
BCRP3.HUMAN.NCBI.REF                                              ------------------------------------------------------------    20446
LOC106996293.glutathione.hydrolase5.proenzyme-like-GGT1.rhesus.   tctaataaaaa--tacaaaaattagccaggcatggtggtgcatacctgtagttccagcaa    127474

Papio.anubis.clone.rp41-133b2.8034-40080.revcompl.Baboon          ctcgggaggctgaggcaggagaatggcgtgaacccgggaggcggagcttgcagtgagctg    25684
BCRP3.HUMAN.NCBI.REF                                              ------------------------------------------------------------    20446
LOC106996293.glutathione.hydrolase5.proenzyme-like-GGT1.rhesus.   atcgggaggctgaggcaggagaattgcttgaacccgggaagcagcagttgcagtgagccg    127534

Papio.anubis.clone.rp41-133b2.8034-40080.revcompl.Baboon          agatccggccaccgcactccagcctgggcgacagagccagactcagtctcaaaaaaaaaa    25744
BCRP3.HUMAN.NCBI.REF                                              ------------------------------------------------------------    20446
LOC106996293.glutathione.hydrolase5.proenzyme-like-GGT1.rhesus.   agatcacgccactgcactccagcctgggtgacaaagctagactccatctctaacaaacaa    127594

Papio.anubis.clone.rp41-133b2.8034-40080.revcompl.Baboon          aaaaaaaaaaaaa-----------------------------------------------    25757
BCRP3.HUMAN.NCBI.REF                                              ------------------------------------------------------------    20446
LOC106996293.glutathione.hydrolase5.proenzyme-like-GGT1.rhesus.   acaaacaaacaaacaaaaaagctatatacagacatacatctagatagatagatatattcg    127654

Papio.anubis.clone.rp41-133b2.8034-40080.revcompl.Baboon          ------------------------------------------------------------    25757
BCRP3.HUMAN.NCBI.REF                                              ------------------------------------------------------------    20446
LOC106996293.glutathione.hydrolase5.proenzyme-like-GGT1.rhesus.   taaaagcttggcttctgcatttgtagggttggctaagcaagcccagagctcgcaggactg    127714

Papio.anubis.clone.rp41-133b2.8034-40080.revcompl.Baboon          ------------------------------------------------------------    25757
BCRP3.HUMAN.NCBI.REF                                              ------------------------------------------------------------    20446
LOC106996293.glutathione.hydrolase5.proenzyme-like-GGT1.rhesus.   gccgtcaggaacgacaatcaccacccaggcagggccaaagcttgcttttcacaggcactc    127774

Papio.anubis.clone.rp41-133b2.8034-40080.revcompl.Baboon          ------------------------------------------------------------    25757
BCRP3.HUMAN.NCBI.REF                                              ------------------------------------------------------------    20446
LOC106996293.glutathione.hydrolase5.proenzyme-like-GGT1.rhesus.   aggaagaaagatccagagggaagggagcacaattgtggactcggctgctatttgaagtct    127834

Papio.anubis.clone.rp41-133b2.8034-40080.revcompl.Baboon          ------------------------------------------------------------    25757
BCRP3.HUMAN.NCBI.REF                                              ------------------------------------------------------------    20446
LOC106996293.glutathione.hydrolase5.proenzyme-like-GGT1.rhesus.   gtgttccaggaagtcctaagccctctcttttctaaaaaaagaaacaaactttttggcttg    127894

Papio.anubis.clone.rp41-133b2.8034-40080.revcompl.Baboon          ------------------------------------------------------------    25757
BCRP3.HUMAN.NCBI.REF                                              ------------------------------------------------------------    20446
LOC106996293.glutathione.hydrolase5.proenzyme-like-GGT1.rhesus.   tggtgccacaagctgtagtctcagctgctcaggaggttgaggcaggagaattgcttgagc    127954

Papio.anubis.clone.rp41-133b2.8034-40080.revcompl.Baboon          ------------------------------------------------------------    25757
BCRP3.HUMAN.NCBI.REF                                              ------------------------------------------------------------    20446
LOC106996293.glutathione.hydrolase5.proenzyme-like-GGT1.rhesus.   acaggagttagaatctagcctgagcagcataacaagactttggttctaaaaagcatttaa    128014

Papio.anubis.clone.rp41-133b2.8034-40080.revcompl.Baboon          ------------------------------------------------------------    25757
BCRP3.HUMAN.NCBI.REF                                              ------------------------------------------------------------    20446
LOC106996293.glutathione.hydrolase5.proenzyme-like-GGT1.rhesus.   aaaatagtccggctgcagtggctcacaaccgtagtcccagcactttgggaggctaaggca    128074

Papio.anubis.clone.rp41-133b2.8034-40080.revcompl.Baboon          ------------------------------------------------------------    25757
BCRP3.HUMAN.NCBI.REF                                              ------------------------------------------------------------    20446
LOC106996293.glutathione.hydrolase5.proenzyme-like-GGT1.rhesus.   ggtgaactgcttgaagtcaggagttcaagaccagtctggccaacatggtgaaaccccgtc    128134

Papio.anubis.clone.rp41-133b2.8034-40080.revcompl.Baboon          ------------------------------------------------------------    25757
BCRP3.HUMAN.NCBI.REF                                              ------------------------------------------------------------    20446
LOC106996293.glutathione.hydrolase5.proenzyme-like-GGT1.rhesus.   tctactaaaaatacaaaaatcagccgcgcgtggtggcgcatgcctgtaatcccagctact    128194

Papio.anubis.clone.rp41-133b2.8034-40080.revcompl.Baboon          ------------------------------------------------------------    25757
BCRP3.HUMAN.NCBI.REF                                              ------------------------------------------------------------    20446
LOC106996293.glutathione.hydrolase5.proenzyme-like-GGT1.rhesus.   tgggaggctgaggtggaagaattgcttgaacctgggaggcagaggttgcagtgagccgag    128254

Papio.anubis.clone.rp41-133b2.8034-40080.revcompl.Baboon          ------------------------------------------------------------    25757
BCRP3.HUMAN.NCBI.REF                                              ------------------------------------------------------------    20446
LOC106996293.glutathione.hydrolase5.proenzyme-like-GGT1.rhesus.   atctcgccattgcactccagcctgggcaacaagagcgaaatcccatctaaaaaaaaaatt    128314

Papio.anubis.clone.rp41-133b2.8034-40080.revcompl.Baboon          ------------------------------------------------------------    25757
BCRP3.HUMAN.NCBI.REF                                              ------------------------------------------------------------    20446
LOC106996293.glutathione.hydrolase5.proenzyme-like-GGT1.rhesus.   tttttgtaatgaaaaaagttttggagataaggggtcttgccctgttgaccaggctggaac    128374

Papio.anubis.clone.rp41-133b2.8034-40080.revcompl.Baboon          ------------------------------------------------------------    25757
BCRP3.HUMAN.NCBI.REF                                              ------------------------------------------------------------    20446
LOC106996293.glutathione.hydrolase5.proenzyme-like-GGT1.rhesus.   acagtggcacagtcacacctcactgcagcctcaaactcttgggctcaagcaattctccct    128434

Papio.anubis.clone.rp41-133b2.8034-40080.revcompl.Baboon          ------------------------------------------------------------    25757
BCRP3.HUMAN.NCBI.REF                                              ------------------------------------------------------------    20446
LOC106996293.glutathione.hydrolase5.proenzyme-like-GGT1.rhesus.   cctcagcccccctagcagctgggactgtaagtgcataccaccatgctgactaactaaaag    128494

Papio.anubis.clone.rp41-133b2.8034-40080.revcompl.Baboon          -----------------agacggggtttcctcatgttggccaggctggtctggaactccc    25800
BCRP3.HUMAN.NCBI.REF                                              ------------------------------------------------------------    20446
LOC106996293.glutathione.hydrolase5.proenzyme-like-GGT1.rhesus.   aaaaaaaaaatttagagagatgtagtctcgctatgttgcccaggctagtctcacactcct    128554

Papio.anubis.clone.rp41-133b2.8034-40080.revcompl.Baboon          aacctcaagtgagctgcccaccttggcctcccaaaatgctgggcttacgagcatgagcca    25860
BCRP3.HUMAN.NCBI.REF                                              ------------------------------------------------------------    20446
LOC106996293.glutathione.hydrolase5.proenzyme-like-GGT1.rhesus.   gacctcaa--gatcttcccgccttagcctcccaaagtgctaggat-------gtgagcca    128605

Papio.anubis.clone.rp41-133b2.8034-40080.revcompl.Baboon          ctgtgcctggccctcccttgactcttgactgaaggacctttgtcctcgtgaacatcaatt    25920
BCRP3.HUMAN.NCBI.REF                                              ------------------------------------------------------------    20446
LOC106996293.glutathione.hydrolase5.proenzyme-like-GGT1.rhesus.   ccattcccggccccagtaaaccaatactgatacgttattattaactaaggttcatcttga    128665

Papio.anubis.clone.rp41-133b2.8034-40080.revcompl.Baboon          ctcaggacctttcactctgaggacctgaaatgctgagaatttgggagatgacagtctggg    25980
BCRP3.HUMAN.NCBI.REF                                              ------------------------------------------------------------    20446
LOC106996293.glutathione.hydrolase5.proenzyme-like-GGT1.rhesus.   ttcagatttctttactatttcccctaatgtccttttcctgttccaagatctcatccagca    128725

Papio.anubis.clone.rp41-133b2.8034-40080.revcompl.Baboon          gactgggattaatggaatccagtggcccacgaacttaaggttctcagttcccttggggag    26040
BCRP3.HUMAN.NCBI.REF                                              ------------------------------------------------------------    20446
LOC106996293.glutathione.hydrolase5.proenzyme-like-GGT1.rhesus.   gaccatactatgtttagttct---------------------------------------    128746

Papio.anubis.clone.rp41-133b2.8034-40080.revcompl.Baboon          ttggaatgtcagctattcaggtctagggctttccatggagtaaatcctaaactctgggtt    26100
BCRP3.HUMAN.NCBI.REF                                              ------------------------------------------------------------    20446
LOC106996293.glutathione.hydrolase5.proenzyme-like-GGT1.rhesus.   ------------------------------------------------------------    128746

Papio.anubis.clone.rp41-133b2.8034-40080.revcompl.Baboon          ggagacttcgagcctccaaggaccttcacagctaaggcccagggactagggtgaggagag    26160
BCRP3.HUMAN.NCBI.REF                                              ------------------------------------------------------------    20446
LOC106996293.glutathione.hydrolase5.proenzyme-like-GGT1.rhesus.   ------------------------------------------------------------    128746

Papio.anubis.clone.rp41-133b2.8034-40080.revcompl.Baboon          tctttgatcctcagagtcttggagtttggccagtggactctgaggaatggagtctctgag    26220
BCRP3.HUMAN.NCBI.REF                                              ------------------------------------------------------------    20446
LOC106996293.glutathione.hydrolase5.proenzyme-like-GGT1.rhesus.   ------------------------------------------------------------    128746

Papio.anubis.clone.rp41-133b2.8034-40080.revcompl.Baboon          cactgtaagtccagctttggcttcagtagtgaggatcttggccttcaagtctaaggacag    26280
```

```
BCRP3.HUMAN.NCBI.REF                                              ------------------------------------------------------------    20446
LOC106996293.glutathione.hydrolase5.proenzyme-like-GGT1.rhesus.   ------------------------------------------------------------    128746

Papio.anubis.clone.rp41-133b2.8034-40080.revcompl.Baboon          tgggcaattccacggattgtcaggcacggggaacttatagccaaatgtgcagggctccaa    26340
BCRP3.HUMAN.NCBI.REF                                              ------------------------------------------------------------    20446
LOC106996293.glutathione.hydrolase5.proenzyme-like-GGT1.rhesus.   -------------------------------taagtctcctcaggattgctggagtgcag    128775

Papio.anubis.clone.rp41-133b2.8034-40080.revcompl.Baboon          agacctcatttgccctgtcagcagctcaggccatgtggcatcacctgaagcacctagat-    26399
BCRP3.HUMAN.NCBI.REF                                              ------------------------------------------------------------    20446
LOC106996293.glutathione.hydrolase5.proenzyme-like-GGT1.rhesus.   tgactccatctcagctcactgcaacctccgaccaccaagttcaagctattctcctgcctc    128835

Papio.anubis.clone.rp41-133b2.8034-40080.revcompl.Baboon          -gtctccggaatctcaggccctgcggggtaaggtccccgggccattagtctttttttttt    26458
BCRP3.HUMAN.NCBI.REF                                              ------------------------------------------------------------    20446
LOC106996293.glutathione.hydrolase5.proenzyme-like-GGT1.rhesus.   agcctcccgagttgctgggattacaggcacctgccaccacgcccagctaattattattat    128895

Papio.anubis.clone.rp41-133b2.8034-40080.revcompl.Baboon          ctttctttttttggcttgttattgttgttgagacacagtttcactctgtcacccaggccg    26518
BCRP3.HUMAN.NCBI.REF                                              ------------------------------------------------------------    20446
LOC106996293.glutathione.hydrolase5.proenzyme-like-GGT1.rhesus.   ttttttt--------------------ttgagagggagcctccctctgctccccaggctg    128935

Papio.anubis.clone.rp41-133b2.8034-40080.revcompl.Baboon          gagtgcagtggcgcgatctcagctcattgcaacctccacctcctgggttcaagtgattct    26578
BCRP3.HUMAN.NCBI.REF                                              ------------------------------------------------------------    20446
LOC106996293.glutathione.hydrolase5.proenzyme-like-GGT1.rhesus.   gagtgcagtggggcaatcttggctcactgcaacctctgcctcctgggttcaaacaattct    128995

Papio.anubis.clone.rp41-133b2.8034-40080.revcompl.Baboon          cctgcctcagcctcccgagtagctgggattacaggcccacaccaccatgcctggctaatt    26638
BCRP3.HUMAN.NCBI.REF                                              ------------------------------------------------------------    20446
LOC106996293.glutathione.hydrolase5.proenzyme-like-GGT1.rhesus.   cctgcctcagcttccctagtagctgggattacatgtgtgtactaccactcactgtctttt    129055

Papio.anubis.clone.rp41-133b2.8034-40080.revcompl.Baboon          tttgtattattagta----------gagatggggttttgccatgttggccaggctggtct    26688
BCRP3.HUMAN.NCBI.REF                                              ------------------------------------------------------------    20446
LOC106996293.glutathione.hydrolase5.proenzyme-like-GGT1.rhesus.   ttttttttttttttggagacagtgtgagatggggtttcaccacattggccaggctggtct    129115

Papio.anubis.clone.rp41-133b2.8034-40080.revcompl.Baboon          ggaactcctgacctcaggt-atccgcctgcctcggcctcccaaattgctgggattacagg    26747
BCRP3.HUMAN.NCBI.REF                                              ------------------------------------------------------------    20446
LOC106996293.glutathione.hydrolase5.proenzyme-like-GGT1.rhesus.   agaactcctggcctcaggtcatccacccacctcagcctcccaaagtgctgggattacagg    129175

Papio.anubis.clone.rp41-133b2.8034-40080.revcompl.Baboon          tgtgagtgactgcacccagccaagttctcaggtcattagtcttaaatggtatctggaaac    26807
BCRP3.HUMAN.NCBI.REF                                              ------------------------------------------------------------    20446
LOC106996293.glutathione.hydrolase5.proenzyme-like-GGT1.rhesus.   tgtcagccactgtgcccaggctattctgcacttcacttgcaaaatatttttccctttaca    129235

Papio.anubis.clone.rp41-133b2.8034-40080.revcompl.Baboon          acggggccctcgtagcacaggtccctgtactcta--------------------------    26841
BCRP3.HUMAN.NCBI.REF                                              ------------------------------------------------------------    20446
LOC106996293.glutathione.hydrolase5.proenzyme-like-GGT1.rhesus.   ataaattgctctatgctgcatcacctttcctgtgtgtttctttttttaaatttttattta    129295

Papio.anubis.clone.rp41-133b2.8034-40080.revcompl.Baboon          ------------------------------------------------------------    26841
BCRP3.HUMAN.NCBI.REF                                              ------------------------------------------------------------    20446
LOC106996293.glutathione.hydrolase5.proenzyme-like-GGT1.rhesus.   ttttaattatttattctttttgaaacagggtctcactatgttgcccaggctggtctctaa    129355

Papio.anubis.clone.rp41-133b2.8034-40080.revcompl.Baboon          ------------------------------------------------------------    26841
BCRP3.HUMAN.NCBI.REF                                              ------------------------------------------------------------    20446
LOC106996293.glutathione.hydrolase5.proenzyme-like-GGT1.rhesus.   tctttgagctcaagcgatccgctcaccatggcctcccaaagtgctgggatgacaggcatt    129415

Papio.anubis.clone.rp41-133b2.8034-40080.revcompl.Baboon          ------------------------------------------------------------    26841
BCRP3.HUMAN.NCBI.REF                                              ------------------------------------------------------------    20446
LOC106996293.glutathione.hydrolase5.proenzyme-like-GGT1.rhesus.   agccaccacacccagcctaaattctttgaaactgacaactgcggccccacaacagctgtc    129475

Papio.anubis.clone.rp41-133b2.8034-40080.revcompl.Baboon          -------gaacctctgcccctttacagactggtagttcgtgaggaccatagcccccaggc    26894
BCRP3.HUMAN.NCBI.REF                                              ------------------------------------------------------------    20446
LOC106996293.glutathione.hydrolase5.proenzyme-like-GGT1.rhesus.   aatcattgtaccactgcca---tccagcctggttgacagtgtaaggcctc-atctcacaa    129531

Papio.anubis.clone.rp41-133b2.8034-40080.revcompl.Baboon          aaggtacttggcatcttaggaacaagcacaaggctagggctactttggtctctgacttcc    26954
BCRP3.HUMAN.NCBI.REF                                              ------------------------------------------------------------    20446
LOC106996293.glutathione.hydrolase5.proenzyme-like-GGT1.rhesus.   aaggaaaaagatatattaaaaataaataaataaataaatatttttttttccttaaaaaca    129591

Papio.anubis.clone.rp41-133b2.8034-40080.revcompl.Baboon          ttgaagtccacaaattcagggtcttgagtctgagcctgtaggcccccatgccctgagtgt    27014
BCRP3.HUMAN.NCBI.REF                                              ------------------------------------------------------------    20446
LOC106996293.glutathione.hydrolase5.proenzyme-like-GGT1.rhesus.   aggtatcccagtcatgcacggta-------------------------------------    129614

Papio.anubis.clone.rp41-133b2.8034-40080.revcompl.Baboon          ctcacactgtgggactgtgtgtgcctttagcttagggatctaatttccttggattcatgg    27074
BCRP3.HUMAN.NCBI.REF                                              ------------------------------------------------------------    20446
LOC106996293.glutathione.hydrolase5.proenzyme-like-GGT1.rhesus.   --------gctcacacctgtaatccttgcactttgggaggccaaggcaggtgagtcatct    129666

Papio.anubis.clone.rp41-133b2.8034-40080.revcompl.Baboon          gtattcacgtcct-----------------------------------------------    27087
BCRP3.HUMAN.NCBI.REF                                              ------------------------------------------------------------    20446
LOC106996293.glutathione.hydrolase5.proenzyme-like-GGT1.rhesus.   gaggtcaggagttcaagaccaacccagacctacatggtgaaaccccgtctctactaaaca    129726

Papio.anubis.clone.rp41-133b2.8034-40080.revcompl.Baboon          ------------------------------------------------------------    27087
BCRP3.HUMAN.NCBI.REF                                              ------------------------------------------------------------    20446
LOC106996293.glutathione.hydrolase5.proenzyme-like-GGT1.rhesus.   aaaaccacaaaaattggctggacatggtggaaggtgcctgtaatccctgctactagggag    129786

Papio.anubis.clone.rp41-133b2.8034-40080.revcompl.Baboon          ------------------------------------------------------------    27087
BCRP3.HUMAN.NCBI.REF                                              ------------------------------------------------------------    20446
LOC106996293.glutathione.hydrolase5.proenzyme-like-GGT1.rhesus.   gctgagggaggagaatcacttgaacttgggaggtggaggttgcagtgagcccaagagtgc    129846

Papio.anubis.clone.rp41-133b2.8034-40080.revcompl.Baboon          ------------------------------------------------------------    27087
BCRP3.HUMAN.NCBI.REF                                              ------------------------------------------------------------    20446
LOC106996293.glutathione.hydrolase5.proenzyme-like-GGT1.rhesus.   accagtgccctccagtctgggctcctacagagtgagactccagctcaaaaacaaatacaa    129906

Papio.anubis.clone.rp41-133b2.8034-40080.revcompl.Baboon          ------------------------------------------------------------    27087
BCRP3.HUMAN.NCBI.REF                                              ------------------------------------------------------------    20446
LOC106996293.glutathione.hydrolase5.proenzyme-like-GGT1.rhesus.   acaaaaaagacagggtctccctctgttgcccagaccagagtgcagtgacgcggtgacagc    129966

Papio.anubis.clone.rp41-133b2.8034-40080.revcompl.Baboon          ---------------------------------------tatcctagctgaattctccac    27108
BCRP3.HUMAN.NCBI.REF                                              ------------------------------------------------------------    20446
LOC106996293.glutathione.hydrolase5.proenzyme-like-GGT1.rhesus.   tcactgcacccttgacctcccaggctcaactgattctcctgcctcagtagctgggactac    130026

Papio.anubis.clone.rp41-133b2.8034-40080.revcompl.Baboon          ccacagacaccagaagggccctaaagttgtgggttcctgggtgtctaaggtcttgacatc    27168
BCRP3.HUMAN.NCBI.REF                                              ------------------------------------------------------------    20446
LOC106996293.glutathione.hydrolase5.proenzyme-like-GGT1.rhesus.   cagcacacatcaccatgactggagaatttttaaatttttttgtgctgatggtcttgctatg    130086

Papio.anubis.clone.rp41-133b2.8034-40080.revcompl.Baboon          gtgtgttga---------------------------------------------------    27177
BCRP3.HUMAN.NCBI.REF                                              ------------------------------------------------------------    20446
LOC106996293.glutathione.hydrolase5.proenzyme-like-GGT1.rhesus.   ttgcccagcctggtcttgaactcttggcctgaagtgatcctcctaccaagtcctccttac    130146

Papio.anubis.clone.rp41-133b2.8034-40080.revcompl.Baboon          ------------------------------------------------------------    27177
BCRP3.HUMAN.NCBI.REF                                              ------------------------------------------------------------    20446
LOC106996293.glutathione.hydrolase5.proenzyme-like-GGT1.rhesus.   aaagagctgggatgacaggcgtgagccaccgcacccggccccctgaagctgcctgttcct    130206

Papio.anubis.clone.rp41-133b2.8034-40080.revcompl.Baboon          ------------------------------------------------------------    27177
BCRP3.HUMAN.NCBI.REF                                              ------------------------------------------------------------    20446
LOC106996293.glutathione.hydrolase5.proenzyme-like-GGT1.rhesus.   tgagcacgatgtatatcaaggggagctagagtgagagcagcaccgtggggaagggacagg    130266

Papio.anubis.clone.rp41-133b2.8034-40080.revcompl.Baboon          ------------------------------------------------------------    27177
BCRP3.HUMAN.NCBI.REF                                              ------------------------------------------------------------    20446
LOC106996293.glutathione.hydrolase5.proenzyme-like-GGT1.rhesus.   gctgccaccctgagctatgtcatgttaggccagacccctcttctccaagcgagatcaacc    130326

Papio.anubis.clone.rp41-133b2.8034-40080.revcompl.Baboon          ------------------------------------------------------------    27177
```

```
BCRP3.HUMAN.NCBI.REF                                                   ------------------------------------------------------------    20446
LOC106996293.glutathione.hydrolase5.proenzyme-like-GGT1.rhesus.        acatgagtgatctcaggagcccgtgcccgctcagacagcctggattttagcttcgtaggt    130386

Papio.anubis.clone.rp41-133b2.8034-40080.revcompl.Baboon               ------------------gatcataggtcttttttgaaaattttttgaggcaaggtcttg    27219
BCRP3.HUMAN.NCBI.REF                                                   ------------------------------------------------------------    20446
LOC106996293.glutathione.hydrolase5.proenzyme-like-GGT1.rhesus.        ggatgggaacctgtgaaggttcagagatgctggatgaaccttttcctgggagagctttc    130446

Papio.anubis.clone.rp41-133b2.8034-40080.revcompl.Baboon               taaccgcggattcatc--------------------------------------------    27235
BCRP3.HUMAN.NCBI.REF                                                   ------------------------------------------------------------    20446
LOC106996293.glutathione.hydrolase5.proenzyme-like-GGT1.rhesus.        caaacagggaacaaacagagtcacccggggccccgctgagggaggatcctaccagaagcc    130506

Papio.anubis.clone.rp41-133b2.8034-40080.revcompl.Baboon               ------------------------------------------------------------    27235
BCRP3.HUMAN.NCBI.REF                                                   ------------------------------------------------------------    20446
LOC106996293.glutathione.hydrolase5.proenzyme-like-GGT1.rhesus.        gtcatcactgtctcactccacaccggtggtgatggtgatgtcatcgatgtaggtagttgg    130566

Papio.anubis.clone.rp41-133b2.8034-40080.revcompl.Baboon               ------------------------------------------------------------    27235
BCRP3.HUMAN.NCBI.REF                                                   ------------------------------------------------------------    20446
LOC106996293.glutathione.hydrolase5.proenzyme-like-GGT1.rhesus.        tgtcgtgtacagaagcacagaccgctgtagtcccgcatagttgaagaagtcaaagtctgt    130626

Papio.anubis.clone.rp41-133b2.8034-40080.revcompl.Baboon               ------------------------------------------------------------    27235
BCRP3.HUMAN.NCBI.REF                                                   ------------------------------------------------------------    20446
LOC106996293.glutathione.hydrolase5.proenzyme-like-GGT1.rhesus.        gttctgaacaaagtaacccttgggatacctagggcaggaggactgtggttcgctgggccc    130686

Papio.anubis.clone.rp41-133b2.8034-40080.revcompl.Baboon               ------------------------------------------------------------    27235
BCRP3.HUMAN.NCBI.REF                                                   ------------------------------------------------------------    20446
LOC106996293.glutathione.hydrolase5.proenzyme-like-GGT1.rhesus.        ttgccatgggtcttttgccctcaggtcacttgcctccccagatcagggcctcctctctgt    130746

Papio.anubis.clone.rp41-133b2.8034-40080.revcompl.Baboon               ------------------------aagacaggggaattgcaatagagaaagagtaattca    27271
BCRP3.HUMAN.NCBI.REF                                                   ------------------------------------------------------------    20446
LOC106996293.glutathione.hydrolase5.proenzyme-like-GGT1.rhesus.        gtaggccaccctgtccctggcaggaagaccacagggtggggcgggaaggtgggtgcgcgc    130806

Papio.anubis.clone.rp41-133b2.8034-40080.revcompl.Baboon               ggcagagcctgctgggtgggagaccggagttttattattactcaaatcagtctccctgaa    27331
BCRP3.HUMAN.NCBI.REF                                                   ------------------------------------------------------------    20446
LOC106996293.glutathione.hydrolase5.proenzyme-like-GGT1.rhesus.        agtggaggcaggatggtactcacttggagttgtcagttgtgtacggatggtccctggtgg    130866

Papio.anubis.clone.rp41-133b2.8034-40080.revcompl.Baboon               cattcggggagtagagttttttcgtgtttgttttgttttgt-------------------    27372
BCRP3.HUMAN.NCBI.REF                                                   ------------------------------------------------------------    20446
LOC106996293.glutathione.hydrolase5.proenzyme-like-GGT1.rhesus.        cagggtggggggcgagcgtgttgttgatggcgacagttgatgacgcagaggcgggagggc    130926

Papio.anubis.clone.rp41-133b2.8034-40080.revcompl.Baboon               ------------------------------------------------------------    27372
BCRP3.HUMAN.NCBI.REF                                                   ------------------------------------------------------------    20446
LOC106996293.glutathione.hydrolase5.proenzyme-like-GGT1.rhesus.        aggggccccacccggaatagactgctgctgtcagcctcgaaggggaggtagcccctttca    130986

Papio.anubis.clone.rp41-133b2.8034-40080.revcompl.Baboon               ------------------------------------------------------------    27372
BCRP3.HUMAN.NCBI.REF                                                   ------------------------------------------------------------    20446
LOC106996293.glutathione.hydrolase5.proenzyme-like-GGT1.rhesus.        tgctctagcttgtggacaccaatcacccactgcagacatagggagatatgagaaggggggt    131046

Papio.anubis.clone.rp41-133b2.8034-40080.revcompl.Baboon               ------------------------------------------------------------    27372
BCRP3.HUMAN.NCBI.REF                                                   ------------------------------------------------------------    20446
LOC106996293.glutathione.hydrolase5.proenzyme-like-GGT1.rhesus.        ggcaggtcggggcatgaggaggggctctgctatggggcacgccctcacataacccgcagc    131106

Papio.anubis.clone.rp41-133b2.8034-40080.revcompl.Baboon               ------------------------------------------------------------    27372
BCRP3.HUMAN.NCBI.REF                                                   ------------------------------------------------------------    20446
LOC106996293.glutathione.hydrolase5.proenzyme-like-GGT1.rhesus.        atggggctcaagggcctgccagccagacgagaaaaaggacatcccagtggggtagatcag    131166

Papio.anubis.clone.rp41-133b2.8034-40080.revcompl.Baboon               ------------------------------------------------------------    27372
BCRP3.HUMAN.NCBI.REF                                                   ------------------------------------------------------------    20446
LOC106996293.glutathione.hydrolase5.proenzyme-like-GGT1.rhesus.        gccacctatccacctacacagggcacagcccccagggcagggtaaggccccccactgtcc    131226

Papio.anubis.clone.rp41-133b2.8034-40080.revcompl.Baboon               ------------------------------------------------------------    27372
BCRP3.HUMAN.NCBI.REF                                                   ------------------------------------------------------------    20446
LOC106996293.glutathione.hydrolase5.proenzyme-like-GGT1.rhesus.        caccccaggagacaggctgtgggtggaatctgagggtcccatgctgttcagcagctgtgc    131286

Papio.anubis.clone.rp41-133b2.8034-40080.revcompl.Baboon               ------------------------------------------------------------    27372
BCRP3.HUMAN.NCBI.REF                                                   ------------------------------------------------------------    20446
LOC106996293.glutathione.hydrolase5.proenzyme-like-GGT1.rhesus.        ccacacacctgccctgcctgctcctggccacactgaccacaatggcatagaagtgggcac    131346

Papio.anubis.clone.rp41-133b2.8034-40080.revcompl.Baboon               ------------------------------------------------------------    27372
BCRP3.HUMAN.NCBI.REF                                                   ------------------------------------------------------------    20446
LOC106996293.glutathione.hydrolase5.proenzyme-like-GGT1.rhesus.        tgccaatcctcagcaccactcttatgtgcaggtcccggatccatcgcttcagcaggggca    131406

Papio.anubis.clone.rp41-133b2.8034-40080.revcompl.Baboon               ------------------------------------------------------------    27372
BCRP3.HUMAN.NCBI.REF                                                   ------------------------------------------------------------    20446
LOC106996293.glutathione.hydrolase5.proenzyme-like-GGT1.rhesus.        cctcccgttcataccacatccagctgacaaaatgctgcagccgccagtcctggctgatgt    131466

Papio.anubis.clone.rp41-133b2.8034-40080.revcompl.Baboon               ------------------------------------------------------------    27372
BCRP3.HUMAN.NCBI.REF                                                   ------------------------------------------------------------    20446
LOC106996293.glutathione.hydrolase5.proenzyme-like-GGT1.rhesus.        cattgaaaatggaaactggtatgtccaaggtggggcccgactgcgaagagaagggccggg    131526

Papio.anubis.clone.rp41-133b2.8034-40080.revcompl.Baboon               ------------------------------------------------------------    27372
BCRP3.HUMAN.NCBI.REF                                                   ------------------------------------------------------------    20446
LOC106996293.glutathione.hydrolase5.proenzyme-like-GGT1.rhesus.        ctcagctcctaggcccacaaagggatccgggaaaacagtgctgcacagctgtgcccccag    131586

Papio.anubis.clone.rp41-133b2.8034-40080.revcompl.Baboon               ------------------------------------------------------------    27372
BCRP3.HUMAN.NCBI.REF                                                   ------------------------------------------------------------    20446
LOC106996293.glutathione.hydrolase5.proenzyme-like-GGT1.rhesus.        gcctagcacaagccctgacacatggtgaggactcctggcagaaaagacagataacttgct    131646

Papio.anubis.clone.rp41-133b2.8034-40080.revcompl.Baboon               ------------------------------------------------------------    27372
BCRP3.HUMAN.NCBI.REF                                                   ------------------------------------------------------------    20446
LOC106996293.glutathione.hydrolase5.proenzyme-like-GGT1.rhesus.        gatgacaggtcaactgccagggcagtttgtgcacctgggccactggggtcagaagttcga    131706

Papio.anubis.clone.rp41-133b2.8034-40080.revcompl.Baboon               ------------------------------------------------------------    27372
BCRP3.HUMAN.NCBI.REF                                                   ------------------------------------------------------------    20446
LOC106996293.glutathione.hydrolase5.proenzyme-like-GGT1.rhesus.        gaccagcctggccaatatggtgaaaccccatctctactaaaaatacaaaaattagctggg    131766

Papio.anubis.clone.rp41-133b2.8034-40080.revcompl.Baboon               ------------------------------------------------------------    27372
BCRP3.HUMAN.NCBI.REF                                                   ------------------------------------------------------------    20446
LOC106996293.glutathione.hydrolase5.proenzyme-like-GGT1.rhesus.        ggtggtggcggcgcctgtagtcccagctactcaggaggctgaggcaggagaatcgcttga    131826

Papio.anubis.clone.rp41-133b2.8034-40080.revcompl.Baboon               ------------------------------------------------------------    27372
BCRP3.HUMAN.NCBI.REF                                                   ------------------------------------------------------------    20446
LOC106996293.glutathione.hydrolase5.proenzyme-like-GGT1.rhesus.        acccagaaggcggaggctgcagtgagccaagaccatgccatcgcactccagcctgggtga    131886

Papio.anubis.clone.rp41-133b2.8034-40080.revcompl.Baboon               ------------------------------------------------------------    27372
BCRP3.HUMAN.NCBI.REF                                                   ------------------------------------------------------------    20446
LOC106996293.glutathione.hydrolase5.proenzyme-like-GGT1.rhesus.        cacggcgagactctgtctcaaaaaaaaagaaaaagaaaaagaggatgtagatcaggcaaa    131946

Papio.anubis.clone.rp41-133b2.8034-40080.revcompl.Baboon               ------------------------------------------------------------    27372
BCRP3.HUMAN.NCBI.REF                                                   ------------------------------------------------------------    20446
LOC106996293.glutathione.hydrolase5.proenzyme-like-GGT1.rhesus.        cgcggcccagacctcaccggatcccacttctttgctgaccacatccaagtcaggagggca    132006

Papio.anubis.clone.rp41-133b2.8034-40080.revcompl.Baboon               ------------------------------------------------------------    27372
BCRP3.HUMAN.NCBI.REF                                                   ------------------------------------------------------------    20446
LOC106996293.glutathione.hydrolase5.proenzyme-like-GGT1.rhesus.        acaggggtggcgtctttggcctgatgtcacagtctgtcctctatctgaaccctgaaaacc    132066

Papio.anubis.clone.rp41-133b2.8034-40080.revcompl.Baboon               ------------------------------------------------------------    27372
```

```
BCRP3.HUMAN.NCBI.REF                                               ------------------------------------------------------------    20446
LOC106996293.glutathione.hydrolase5.proenzyme-like-GGT1.rhesus.    aacgtaccttccccctcagcagagccgacatcagaatgtctaaactgtcccctgctggc    132126

Papio.anubis.clone.rp41-133b2.8034-40080.revcompl.Baboon           ------------------------------------------------------------    27372
BCRP3.HUMAN.NCBI.REF                                               ------------------------------------------------------------    20446
LOC106996293.glutathione.hydrolase5.proenzyme-like-GGT1.rhesus.    gatctctgggcccagctctgtgcagcgcctgctgctccggcctgacgagtgtctggtggc    132186

Papio.anubis.clone.rp41-133b2.8034-40080.revcompl.Baboon           ------------------------------------------------------------    27372
BCRP3.HUMAN.NCBI.REF                                               ------------------------------------------------------------    20446
LOC106996293.glutathione.hydrolase5.proenzyme-like-GGT1.rhesus.    tcatgcctataatcagtgctttgggaagccaacgtgggaagatggcctgaggccagtagt    132246

Papio.anubis.clone.rp41-133b2.8034-40080.revcompl.Baboon           ------------------------------------------------------------    27372
BCRP3.HUMAN.NCBI.REF                                               ------------------------------------------------------------    20446
LOC106996293.glutathione.hydrolase5.proenzyme-like-GGT1.rhesus.    tcgagatcagcctggataacatagtgagaccctgtatcttaaaaaacaaaacaaaacgaa    132306

Papio.anubis.clone.rp41-133b2.8034-40080.revcompl.Baboon           ----tttgttttgtttttgagacagagtcttgctctgtcgcccaggctggagtgcagtgg    27428
BCRP3.HUMAN.NCBI.REF                                               ------------------------------------------------------------    20446
LOC106996293.glutathione.hydrolase5.proenzyme-like-GGT1.rhesus.    aaagttggtttttgttttgagacagagtttcagtctgtcgcacaggctggagtgcagtgg    132366

Papio.anubis.clone.rp41-133b2.8034-40080.revcompl.Baboon           catgat---------ctgtaacctctgcctcctggggtcaagcaattctcatgtctcagc    27479
BCRP3.HUMAN.NCBI.REF                                               ------------------------------------------------------------    20446
LOC106996293.glutathione.hydrolase5.proenzyme-like-GGT1.rhesus.    cttgatctcggctcactgcaacctccgactccctggttcaaacaattctcctgcctctgc    132426

Papio.anubis.clone.rp41-133b2.8034-40080.revcompl.Baboon           ctcccaagtagctgggatttcagatgtgtgccaccaagcctggctaatttttgtattttt    27539
BCRP3.HUMAN.NCBI.REF                                               ------------------------------------------------------------    20446
LOC106996293.glutathione.hydrolase5.proenzyme-like-GGT1.rhesus.    ctcccaagtagctgggattacaggtgctcatcgccacgcccggctaatttttgtgttttt    132486

Papio.anubis.clone.rp41-133b2.8034-40080.revcompl.Baboon           agcagaaacagggtttctccatgttggccaggatggtctcgaactcctgacctcaggtga    27599
BCRP3.HUMAN.NCBI.REF                                               ------------------------------------------------------------    20446
LOC106996293.glutathione.hydrolase5.proenzyme-like-GGT1.rhesus.    agtagggacggggtttcaccacgttggccggggtggtcttgaactcctggcctccggtaa    132546

Papio.anubis.clone.rp41-133b2.8034-40080.revcompl.Baboon           tctgcccacctcggcctcccaaagtgctgggattacaggcatgaccaccgcacctggcta    27659
BCRP3.HUMAN.NCBI.REF                                               ------------------------------------------------------------    20446
LOC106996293.glutathione.hydrolase5.proenzyme-like-GGT1.rhesus.    tccgcccgcctcagcctcccaaggtgctgggattacaggtgtgagccaccaagcccgacc    132606

Papio.anubis.clone.rp41-133b2.8034-40080.revcompl.Baboon           gggtgcagtgttttt---------------------------------------------    27674
BCRP3.HUMAN.NCBI.REF                                               ------------------------------------------------------------    20446
LOC106996293.glutathione.hydrolase5.proenzyme-like-GGT1.rhesus.    aagtttcttttctttgttttttttttttttttttttttaattaggcatggcggccccagct    132666

Papio.anubis.clone.rp41-133b2.8034-40080.revcompl.Baboon           ------------------------------------------------------------    27674
BCRP3.HUMAN.NCBI.REF                                               ------------------------------------------------------------    20446
LOC106996293.glutathione.hydrolase5.proenzyme-like-GGT1.rhesus.    aagttgcaaggctggggcgggagaatcgcttgagctcagaagctaggattgtgccactcc    132726

Papio.anubis.clone.rp41-133b2.8034-40080.revcompl.Baboon           ------------------------------------------------------------    27674
BCRP3.HUMAN.NCBI.REF                                               ------------------------------------------------------------    20446
LOC106996293.glutathione.hydrolase5.proenzyme-like-GGT1.rhesus.    gctgcaacctgggcggcaggaccaaacacagtctcaaaaacatttcaaaaattaaaaata    132786

Papio.anubis.clone.rp41-133b2.8034-40080.revcompl.Baboon           ------------------------------------------------------------    27674
BCRP3.HUMAN.NCBI.REF                                               ------------------------------------------------------------    20446
LOC106996293.glutathione.hydrolase5.proenzyme-like-GGT1.rhesus.    aatattaggagactgcccgggctcagccttcgcctccggaggcggctggacaggagcgct    132846

Papio.anubis.clone.rp41-133b2.8034-40080.revcompl.Baboon           ------------------------------------------------------------    27674
BCRP3.HUMAN.NCBI.REF                                               ------------------------------------------------------------    20446
LOC106996293.glutathione.hydrolase5.proenzyme-like-GGT1.rhesus.    tcctcctcctcccgcctcctccgccctggccctcctgggtccccctagtcaccccagtac    132906

Papio.anubis.clone.rp41-133b2.8034-40080.revcompl.Baboon           ------------------------------------------------------------    27674
BCRP3.HUMAN.NCBI.REF                                               ------------------------------------------------------------    20446
LOC106996293.glutathione.hydrolase5.proenzyme-like-GGT1.rhesus.    cccagtcctccaaccccgcgcccccaagcccattgacctttaacctcctccgggcccccca    132966

Papio.anubis.clone.rp41-133b2.8034-40080.revcompl.Baboon           ------------------------------------------------------------    27674
BCRP3.HUMAN.NCBI.REF                                               ------------------------------------------------------------    20446
LOC106996293.glutathione.hydrolase5.proenzyme-like-GGT1.rhesus.    gctcccgggcactcaggtgaaggaggccgcagggcgccggggcttaggccattcccaacg    133026

Papio.anubis.clone.rp41-133b2.8034-40080.revcompl.Baboon           ------------------------------------------------------------    27674
BCRP3.HUMAN.NCBI.REF                                               ------------------------------------------------------------    20446
LOC106996293.glutathione.hydrolase5.proenzyme-like-GGT1.rhesus.    ccaggcttccgggggcctctcgcaacggtcgcgcctacctctgctcctagaagcccggga    133086

Papio.anubis.clone.rp41-133b2.8034-40080.revcompl.Baboon           ------------------------------------------------------------    27674
BCRP3.HUMAN.NCBI.REF                                               ------------------------------------------------------------    20446
LOC106996293.glutathione.hydrolase5.proenzyme-like-GGT1.rhesus.    gtcggttgttggagaagtcggcttggaagcttcagaggccgtccagctccttgcgctccc    133146

Papio.anubis.clone.rp41-133b2.8034-40080.revcompl.Baboon           ------------------------------------------------------------    27674
BCRP3.HUMAN.NCBI.REF                                               ------------------------------------------------------------    20446
LOC106996293.glutathione.hydrolase5.proenzyme-like-GGT1.rhesus.    gcgatgcgctctgccggatgtacagcatcccgcccagcagcgcctgcgggcagccacaca    133206

Papio.anubis.clone.rp41-133b2.8034-40080.revcompl.Baboon           ------------------------------------------------------------    27674
BCRP3.HUMAN.NCBI.REF                                               ------------------------------------------------------------    20446
LOC106996293.glutathione.hydrolase5.proenzyme-like-GGT1.rhesus.    acagccgccggagagccgcccaggacaccgccaccagccaggtcatacttcccgctcctc    133266

Papio.anubis.clone.rp41-133b2.8034-40080.revcompl.Baboon           ------------------------------------------------------------    27674
BCRP3.HUMAN.NCBI.REF                                               ------------------------------------------------------------    20446
LOC106996293.glutathione.hydrolase5.proenzyme-like-GGT1.rhesus.    ctcttgtcagccagtctgaggggatgagagcggcgcaggggaagccctgcgggcttgtcg    133326

Papio.anubis.clone.rp41-133b2.8034-40080.revcompl.Baboon           ------------------------------------------------------------    27674
BCRP3.HUMAN.NCBI.REF                                               ------------------------------------------------------------    20446
LOC106996293.glutathione.hydrolase5.proenzyme-like-GGT1.rhesus.    cctgaggcgcgtgcggcatggcgagcgtgaggccttgggcgccatgtttgttgagggcga    133386

Papio.anubis.clone.rp41-133b2.8034-40080.revcompl.Baboon           ------------------------------------------------------------    27674
BCRP3.HUMAN.NCBI.REF                                               ------------------------------------------------------------    20446
LOC106996293.glutathione.hydrolase5.proenzyme-like-GGT1.rhesus.    gtgccaggcctggaggggcggggcagtgccaggcgctgctcagtagaacctgtggctgga    133446

Papio.anubis.clone.rp41-133b2.8034-40080.revcompl.Baboon           ------------------------------------------------------------    27674
BCRP3.HUMAN.NCBI.REF                                               ------------------------------------------------------------    20446
LOC106996293.glutathione.hydrolase5.proenzyme-like-GGT1.rhesus.    atccagggctaggcctttggggaagcgggacccccaccctcagctccagccaagaagggc    133506

Papio.anubis.clone.rp41-133b2.8034-40080.revcompl.Baboon           ------------------------------------------------------------    27674
BCRP3.HUMAN.NCBI.REF                                               ------------------------------------------------------------    20446
LOC106996293.glutathione.hydrolase5.proenzyme-like-GGT1.rhesus.    gtcgtctgctgccatcccagcagaggctggatgatccagggccgcaggtcaggacagaac    133566

Papio.anubis.clone.rp41-133b2.8034-40080.revcompl.Baboon           ------------------------------------------------------------    27674
BCRP3.HUMAN.NCBI.REF                                               ------------------------------------------------------------    20446
LOC106996293.glutathione.hydrolase5.proenzyme-like-GGT1.rhesus.    ccccgagattggtggccttactgtttcttcaaggggcacctccacttgctttccccagca    133626

Papio.anubis.clone.rp41-133b2.8034-40080.revcompl.Baboon           ------------------------------------------------------------    27674
BCRP3.HUMAN.NCBI.REF                                               ------------------------------------------------------------    20446
LOC106996293.glutathione.hydrolase5.proenzyme-like-GGT1.rhesus.    ggaattggagcaggccccagcggagcctcttccatgactgctggatgcagcctcttgcag    133686

Papio.anubis.clone.rp41-133b2.8034-40080.revcompl.Baboon           ------------------------------------------------------------    27674
BCRP3.HUMAN.NCBI.REF                                               ------------------------------------------------------------    20446
LOC106996293.glutathione.hydrolase5.proenzyme-like-GGT1.rhesus.    ggatgcatgtccttgggaggcggctgtagccttgaccttggatctctgggaccctgaccc    133746

Papio.anubis.clone.rp41-133b2.8034-40080.revcompl.Baboon           ------------aaggacagcttggtgggttgggggaagccaatgagccaggagtgctga    27722
BCRP3.HUMAN.NCBI.REF                                               ------------------------------------------------------------    20446
LOC106996293.glutathione.hydrolase5.proenzyme-like-GGT1.rhesus.    taaccaaggggacagaagttccttcttggctttgaagatacagggagagaggccaggtgc    133806

Papio.anubis.clone.rp41-133b2.8034-40080.revcompl.Baboon           c--tggtcagggatgaaatcacagggagttggctg-------------------------    27755
```

```
BCRP3.HUMAN.NCBI.REF                                             ------------------------------------------------------------    20446
LOC106996293.glutathione.hydrolase5.proenzyme-like-GGT1.rhesus.  agtgggtcaggcctgtaatcccagcactttggggggctgaggtggtcggatcacctgag    133866

Papio.anubis.clone.rp41-133b2.8034-40080.revcompl.Baboon         ------------------------------------------------------------    27755
BCRP3.HUMAN.NCBI.REF                                             ------------------------------------------------------------    20446
LOC106996293.glutathione.hydrolase5.proenzyme-like-GGT1.rhesus.  gccagtagttcgagaccagcctggccaacacagcgaagccccctctcaaatactaaaaac    133926

Papio.anubis.clone.rp41-133b2.8034-40080.revcompl.Baboon         ------------------------------------------------------------    27755
BCRP3.HUMAN.NCBI.REF                                             ------------------------------------------------------------    20446
LOC106996293.glutathione.hydrolase5.proenzyme-like-GGT1.rhesus.  agaaaaaaaaaaaaaaaaaaaaaaggaggagccaggcatgatagcaggcgcctgtaatccc    133986

Papio.anubis.clone.rp41-133b2.8034-40080.revcompl.Baboon         ------------------------------------------------------------    27755
BCRP3.HUMAN.NCBI.REF                                             ------------------------------------------------------------    20446
LOC106996293.glutathione.hydrolase5.proenzyme-like-GGT1.rhesus.  agctactcaggaggctgaagctgaagaatcacttgaacccaggaggcgaaggttgcagtg    134046

Papio.anubis.clone.rp41-133b2.8034-40080.revcompl.Baboon         ------------------------------------------------------------    27755
BCRP3.HUMAN.NCBI.REF                                             ------------------------------------------------------------    20446
LOC106996293.glutathione.hydrolase5.proenzyme-like-GGT1.rhesus.  agccgaggtcacaccactgcagtccaacctgggtgacagagcaagaatctatctcaaaaa    134106

Papio.anubis.clone.rp41-133b2.8034-40080.revcompl.Baboon         ------------------------------------------------------------    27755
BCRP3.HUMAN.NCBI.REF                                             ------------------------------------------------------------    20446
LOC106996293.glutathione.hydrolase5.proenzyme-like-GGT1.rhesus.  aaaaaaaaaaaaaaattgcagggagagggtgccctgctggaccccactttactccagtagg    134166

Papio.anubis.clone.rp41-133b2.8034-40080.revcompl.Baboon         ------------------------------------------------------------    27755
BCRP3.HUMAN.NCBI.REF                                             ------------------------------------------------------------    20446
LOC106996293.glutathione.hydrolase5.proenzyme-like-GGT1.rhesus.  gaaggcaccgtgtggaagggtagaggaagagatctggagacagtaaaagagacataggtt    134226

Papio.anubis.clone.rp41-133b2.8034-40080.revcompl.Baboon         ------------------------------------------------------------    27755
BCRP3.HUMAN.NCBI.REF                                             ------------------------------------------------------------    20446
LOC106996293.glutathione.hydrolase5.proenzyme-like-GGT1.rhesus.  tattaaggggactttcacacaggtgcagtggctgtggctggacatgagaaccactacatt    134286

Papio.anubis.clone.rp41-133b2.8034-40080.revcompl.Baboon         ------------------------------------------------------------    27755
BCRP3.HUMAN.NCBI.REF                                             ------------------------------------------------------------    20446
LOC106996293.glutathione.hydrolase5.proenzyme-like-GGT1.rhesus.  tgtaaaaagcaagcagcttccataacattttcacctagcatcctcgacctgttaccggtg    134346

Papio.anubis.clone.rp41-133b2.8034-40080.revcompl.Baboon         ------------------------------------------------------------    27755
BCRP3.HUMAN.NCBI.REF                                             ------------------------------------------------------------    20446
LOC106996293.glutathione.hydrolase5.proenzyme-like-GGT1.rhesus.  gaaggtatccgagttcctggtggtgaatccatacacaactgaaacaacctcaattcttgc    134406

Papio.anubis.clone.rp41-133b2.8034-40080.revcompl.Baboon         ------------------------------------------------------------    27755
BCRP3.HUMAN.NCBI.REF                                             ------------------------------------------------------------    20446
LOC106996293.glutathione.hydrolase5.proenzyme-like-GGT1.rhesus.  ctcctcagaagaaagaatttaaccgagggtcataaagtagaaaccagatagagataagtt    134466

Papio.anubis.clone.rp41-133b2.8034-40080.revcompl.Baboon         -------------------------------------------ggcccggtggctcacgc    27772
BCRP3.HUMAN.NCBI.REF                                             ------------------------------------------------------------    20446
LOC106996293.glutathione.hydrolase5.proenzyme-like-GGT1.rhesus.  gcagagcaggagtaaaattttattaaaaagcaggccaggaacaggcgtggtggctcatgc    134526

Papio.anubis.clone.rp41-133b2.8034-40080.revcompl.Baboon         ctgtaatcccagcactttgggaggctgaggtgggtggatcacctgaggtcg------gga    27826
BCRP3.HUMAN.NCBI.REF                                             ------------------------------------------------------------    20446
LOC106996293.glutathione.hydrolase5.proenzyme-like-GGT1.rhesus.  ctgtaatcccagttctttgggaggctgagcccagtggatcacctgagatcaggagttgga    134586

Papio.anubis.clone.rp41-133b2.8034-40080.revcompl.Baboon         gaccagcctggccaacatggcgaaaacctgtctctactaaaaatacaaaaattagctggg    27886
BCRP3.HUMAN.NCBI.REF                                             ------------------------------------------------------------    20446
LOC106996293.glutathione.hydrolase5.proenzyme-like-GGT1.rhesus.  gaccagcctggccaacatggtgaaaccatgtctctactaaaaatacaaaaattagctgga    134646

Papio.anubis.clone.rp41-133b2.8034-40080.revcompl.Baboon         catagtggcaggcatctgtaatcccagctactggggaggctgaggcaagagaatcacttg    27946
BCRP3.HUMAN.NCBI.REF                                             ------------------------------------------------------------    20446
LOC106996293.glutathione.hydrolase5.proenzyme-like-GGT1.rhesus.  cgtgatggtgagtgcctgtaatcccagctacttgggaggctgaggcaggagaatctcttc    134706

Papio.anubis.clone.rp41-133b2.8034-40080.revcompl.Baboon         aatctggaagg-ggaagttgcagtgagccaagatcatgccattgcatgccagcatgggtg    28005
BCRP3.HUMAN.NCBI.REF                                             ------------------------------------------------------------    20446
LOC106996293.glutathione.hydrolase5.proenzyme-like-GGT1.rhesus.  aacctgggaggtggaggttgtagtgagctgagattgagccactgcactccagcctgggt-    134765

Papio.anubis.clone.rp41-133b2.8034-40080.revcompl.Baboon         acagagcaggactccatctcaaaaacagacacacacacacacacaaaccgggaaatcgta    28065
BCRP3.HUMAN.NCBI.REF                                             ------------------------------------------------------------    20446
LOC106996293.glutathione.hydrolase5.proenzyme-like-GGT1.rhesus.  atagagtgagatagtatttaaaaaatggttactaaaaaaactttagaataggaagtaaag    134825

Papio.anubis.clone.rp41-133b2.8034-40080.revcompl.Baboon         gaatcttagggagttgaaactgtc------------------------------------    28089
BCRP3.HUMAN.NCBI.REF                                             ------------------------------------------------------------    20446
LOC106996293.glutathione.hydrolase5.proenzyme-like-GGT1.rhesus.  gaaagaaggaaagtacaacttggaagagggccaagcggtcgacctgagaaaccaagtgcc    134885

Papio.anubis.clone.rp41-133b2.8034-40080.revcompl.Baboon         ------------------------------------------------ttcttgagctga    28101
BCRP3.HUMAN.NCBI.REF                                             ------------------------------------------------------------    20446
LOC106996293.glutathione.hydrolase5.proenzyme-like-GGT1.rhesus.  cagcttgacctcttgactcagagtttcacaggttggcatcctttcaggatcttgtcactc    134945

Papio.anubis.clone.rp41-133b2.8034-40080.revcompl.Baboon         gtcagttcctgggtgggggccacaaga------tcagatgagccagttaatccatctgga    28155
BCRP3.HUMAN.NCBI.REF                                             ------------------------------------------------------------    20446
LOC106996293.glutathione.hydrolase5.proenzyme-like-GGT1.rhesus.  ctgattccttagcttggggctccgaacggactttcttctttgaccgtaaattcattctga    135005

Papio.anubis.clone.rp41-133b2.8034-40080.revcompl.Baboon         tggtgccagctgat----------------------------------------------    28169
BCRP3.HUMAN.NCBI.REF                                             ------------------------------------------------------------    20446
LOC106996293.glutathione.hydrolase5.proenzyme-like-GGT1.rhesus.  gggtacacatcaattatggctgtcgggtgcctctgccatacagaggggtccctctggtcc    135065

Papio.anubis.clone.rp41-133b2.8034-40080.revcompl.Baboon         ------------------------------------------------------------    28169
BCRP3.HUMAN.NCBI.REF                                             ------------------------------------------------------------    20446
LOC106996293.glutathione.hydrolase5.proenzyme-like-GGT1.rhesus.  taggaacctggactggacacagttgtttgtacgattactcctccacccctttttctcatgc    135125

Papio.anubis.clone.rp41-133b2.8034-40080.revcompl.Baboon         ccatcaagtgcagggtctgcaaaatatctccaacactgatcttagg---agcagtttagg    28226
BCRP3.HUMAN.NCBI.REF                                             ------------------------------------------------------------    20446
LOC106996293.glutathione.hydrolase5.proenzyme-like-GGT1.rhesus.  tcgccaaatccaccttctgagaatggaccttaatactggaaatggagtccccagcatagt    135185

Papio.anubis.clone.rp41-133b2.8034-40080.revcompl.Baboon         gagggtcagaatcttgtagccttcagctgcatgactcctaaaccgtaatttctaatcttg    28286
BCRP3.HUMAN.NCBI.REF                                             ------------------------------------------------------------    20446
LOC106996293.glutathione.hydrolase5.proenzyme-like-GGT1.rhesus.  gaaaataaatacattgtag-----aaactgacaaggcctaaaggctttttcccaaatgtt    135240

Papio.anubis.clone.rp41-133b2.8034-40080.revcompl.Baboon         tggctaatgtgagtcctatgaaggcaatctagtgctcaggcaagaaggaggtctgctttg    28346
BCRP3.HUMAN.NCBI.REF                                             ------------------------------------------------------------    20446
LOC106996293.glutathione.hydrolase5.proenzyme-like-GGT1.rhesus.  tgtgcttttttttcttattttatttctttaaaatttacttgtatat-------tccttta    135293

Papio.anubis.clone.rp41-133b2.8034-40080.revcompl.Baboon         ggaaagcgctgttactgtctttgttttaaactatcaactaagtttctctcaaagttagtt    28406
BCRP3.HUMAN.NCBI.REF                                             ------------------------------------------------------------    20446
LOC106996293.glutathione.hydrolase5.proenzyme-like-GGT1.rhesus.  gtgaaatgcttacatgcttaaattaggatatagttcactgtttatgaggcagctttaggc    135353

Papio.anubis.clone.rp41-133b2.8034-40080.revcompl.Baboon         cagcctacacccaggaatgaacaaggacaactgggaggtt--------------------    28446
BCRP3.HUMAN.NCBI.REF                                             ------------------------------------------------------------    20446
LOC106996293.glutathione.hydrolase5.proenzyme-like-GGT1.rhesus.  cacatttaacaaaagaaagaaaaagtaggatgcaaaagtttatacagtatgtataaatca    135413

Papio.anubis.clone.rp41-133b2.8034-40080.revcompl.Baboon         ------------------------------------------------------------    28446
BCRP3.HUMAN.NCBI.REF                                             ------------------------------------------------------------    20446
LOC106996293.glutathione.hydrolase5.proenzyme-like-GGT1.rhesus.  gtaattcaggacgggcttagtggctcacacctgtaatcccagaactttgggagggggagg    135473

Papio.anubis.clone.rp41-133b2.8034-40080.revcompl.Baboon         ------------------------------------------------------------    28446
BCRP3.HUMAN.NCBI.REF                                             ------------------------------------------------------------    20446
LOC106996293.glutathione.hydrolase5.proenzyme-like-GGT1.rhesus.  caggaggatgacttgagcccaggagtttgtgaccagcctggacaacataatgagaccact    135533

Papio.anubis.clone.rp41-133b2.8034-40080.revcompl.Baboon         ------------------------------------------------------------    28446
```

```
BCRP3.HUMAN.NCBI.REF                                                  ------------------------------------------------------------    20446
LOC106996293.glutathione.hydrolase5.proenzyme-like-GGT1.rhesus.       gtctctacaagaaatacaaaaaaacaaaaaaaaaattgcccaggcggggtggcacacacc    135593

Papio.anubis.clone.rp41-133b2.8034-40080.revcompl.Baboon              ------------------------------------------------------------    28446
BCRP3.HUMAN.NCBI.REF                                                  ------------------------------------------------------------    20446
LOC106996293.glutathione.hydrolase5.proenzyme-like-GGT1.rhesus.       tatcatcccaggtacatgagaggccgaggcaggagcattgatgaaccccgggaagcagag    135653

Papio.anubis.clone.rp41-133b2.8034-40080.revcompl.Baboon              ------------------------------------------------------------    28446
BCRP3.HUMAN.NCBI.REF                                                  ------------------------------------------------------------    20446
LOC106996293.glutathione.hydrolase5.proenzyme-like-GGT1.rhesus.       gttgcagtgagccaagattgtgccactgcactccagcctaggtgacagagcgagacccca    135713

Papio.anubis.clone.rp41-133b2.8034-40080.revcompl.Baboon              ------------------------agaagcaagatggagttgattaagttagatctctca    28482
BCRP3.HUMAN.NCBI.REF                                                  ------------------------------------------------------------    20446
LOC106996293.glutathione.hydrolase5.proenzyme-like-GGT1.rhesus.       tctcaaacaaaaaaaaaaaagaaaagaataagactaaagggtgccagaaaagatgccttc    135773

Papio.anubis.clone.rp41-133b2.8034-40080.revcompl.Baboon              ctgtctcagtcataattttgcaaaggtggtttcagtcttgctcttttgcccaggctggag    28542
BCRP3.HUMAN.NCBI.REF                                                  ------------------------------------------------------------    20446
LOC106996293.glutathione.hydrolase5.proenzyme-like-GGT1.rhesus.       ctttttct-ttttcttttttttttttttttttgagtctcaccctgtcacccagactggag    135832

Papio.anubis.clone.rp41-133b2.8034-40080.revcompl.Baboon              tgcagtgtcacgatcaaggctcactgtagcctcaaactcctggcctcaagggatcctcct    28602
BCRP3.HUMAN.NCBI.REF                                                  ------------------------------------------------------------    20446
LOC106996293.glutathione.hydrolase5.proenzyme-like-GGT1.rhesus.       ggcaatggcccgatctcagctcactgcaacctccacctccctggttcaagtgattctcct    135892

Papio.anubis.clone.rp41-133b2.8034-40080.revcompl.Baboon              gcttcagcctcctgagtagctgggactacaagcacagggccaccacacccgactaatttt    28662
BCRP3.HUMAN.NCBI.REF                                                  ------------------------------------------------------------    20446
LOC106996293.glutathione.hydrolase5.proenzyme-like-GGT1.rhesus.       gcctcagcctcctgagtagctgg--attacagatgctcaccaccacacccagctagtttt    135950

Papio.anubis.clone.rp41-133b2.8034-40080.revcompl.Baboon              tttttcttttcttttctttttttaataaagacgtgatctcgctatgttgcccaggctggtc    28722
BCRP3.HUMAN.NCBI.REF                                                  ------------------------------------------------------------    20446
LOC106996293.glutathione.hydrolase5.proenzyme-like-GGT1.rhesus.       tgt-------------atttttaacaacgacagggtttcactatgttaaccaggctggcc    135997

Papio.anubis.clone.rp41-133b2.8034-40080.revcompl.Baboon              ttgaactcctgagctcaaaagatcctcctgcctcagcctccaaaagtgctgggattacag    28782
BCRP3.HUMAN.NCBI.REF                                                  ------------------------------------------------------------    20446
LOC106996293.glutathione.hydrolase5.proenzyme-like-GGT1.rhesus.       tcaaactcctgacctcaggtgatccacccacctcggcctcccaaagtcctggaattacag    136057

Papio.anubis.clone.rp41-133b2.8034-40080.revcompl.Baboon              gtgtgagccaccgggcccagcccaat----------------------------------    28808
BCRP3.HUMAN.NCBI.REF                                                  ------------------------------------------------------------    20446
LOC106996293.glutathione.hydrolase5.proenzyme-like-GGT1.rhesus.       gcgtgagccaccacgcctggccaattcaagtatttcttcagtttacagttggctaagaaa    136117

Papio.anubis.clone.rp41-133b2.8034-40080.revcompl.Baboon              ------------------------------------------------------------    28808
BCRP3.HUMAN.NCBI.REF                                                  ------------------------------------------------------------    20446
LOC106996293.glutathione.hydrolase5.proenzyme-like-GGT1.rhesus.       gcaaagctttatctaaaaccttggggtcagcagaaggggtgttaagtttctgcctgtgag    136177

Papio.anubis.clone.rp41-133b2.8034-40080.revcompl.Baboon              ------------------------------------------------------------    28808
BCRP3.HUMAN.NCBI.REF                                                  ------------------------------------------------------------    20446
LOC106996293.glutathione.hydrolase5.proenzyme-like-GGT1.rhesus.       tgtgattccctccaggcccctcaggaagaaatttggaacaaagaacgccagtcagagttc    136237

Papio.anubis.clone.rp41-133b2.8034-40080.revcompl.Baboon              ------------------------------------------------------------    28808
BCRP3.HUMAN.NCBI.REF                                                  ------------------------------------------------------------    20446
LOC106996293.glutathione.hydrolase5.proenzyme-like-GGT1.rhesus.       agtcctcggttcacccttatctgaggtcgaagggaaagctgtcaccatttttcatccggt    136297

Papio.anubis.clone.rp41-133b2.8034-40080.revcompl.Baboon              ------------------------------------------------------------    28808
BCRP3.HUMAN.NCBI.REF                                                  ------------------------------------------------------------    20446
LOC106996293.glutathione.hydrolase5.proenzyme-like-GGT1.rhesus.       ggagatctgcgcttccgaaaacaacttagagacatttgtcaagatgtcatctttactttc    136357

Papio.anubis.clone.rp41-133b2.8034-40080.revcompl.Baboon              ------------------------------------------------------------    28808
BCRP3.HUMAN.NCBI.REF                                                  ------------------------------------------------------------    20446
LOC106996293.glutathione.hydrolase5.proenzyme-like-GGT1.rhesus.       tataggggatcagatatcttatggctgtaaccaacttggatggctattgttctgagctct    136417

Papio.anubis.clone.rp41-133b2.8034-40080.revcompl.Baboon              -------------------ttttgatttcttgtagagacggtgtcttgctttg------    28843
BCRP3.HUMAN.NCBI.REF                                                  ------------------------------------------------------------    20446
LOC106996293.glutathione.hydrolase5.proenzyme-like-GGT1.rhesus.       tattaccttttcttttcttttcttttttttttgagacggagtctcactctgtctccc    136477

Papio.anubis.clone.rp41-133b2.8034-40080.revcompl.Baboon              ----------------------ttgttgccctggctgctctcaaactcctggcctcaagt    28881
BCRP3.HUMAN.NCBI.REF                                                  ------------------------------------------------------------    20446
LOC106996293.glutathione.hydrolase5.proenzyme-like-GGT1.rhesus.       aggctggagagcagtggtccaatctaagcccactgcaccgtccacctcctgggttcaagc    136537

Papio.anubis.clone.rp41-133b2.8034-40080.revcompl.Baboon              gatcctcccaccttgg-ctcccaagtggctaacactgtaggtatgtgccacca----tgc    28936
BCRP3.HUMAN.NCBI.REF                                                  ------------------------------------------------------------    20446
LOC106996293.glutathione.hydrolase5.proenzyme-like-GGT1.rhesus.       aatcctcctgtgtcagcctcctaagtagctgggataacaggcatccgttatcacatccag    136597

Papio.anubis.clone.rp41-133b2.8034-40080.revcompl.Baboon              ctgccttttttttttttcggagaaacaggatctttctgtattgcccaggctgatctcaaa    28996
BCRP3.HUMAN.NCBI.REF                                                  ------------------------------------------------------------    20446
LOC106996293.glutathione.hydrolase5.proenzyme-like-GGT1.rhesus.       ctaattttgtattttcagtacacatggggtttcaccatgttagccgggctgatcttgaa    136657

Papio.anubis.clone.rp41-133b2.8034-40080.revcompl.Baboon              ctcctggcctcaagtgatcctccttcctcagcctcccaaagtgttgggattacaggtgtg    29056
BCRP3.HUMAN.NCBI.REF                                                  ------------------------------------------------------------    20446
LOC106996293.glutathione.hydrolase5.proenzyme-like-GGT1.rhesus.       ctcctgacctcagttgatctatctgtcttgacctcccaaagtgctgggattacaggtgtg    136717

Papio.anubis.clone.rp41-133b2.8034-40080.revcompl.Baboon              agccactgtgtctggcc--tcagtgtctaaatcttgagaag----ccatgtccttcatcg    29110
BCRP3.HUMAN.NCBI.REF                                                  ------------------------------------------------------------    20446
LOC106996293.glutathione.hydrolase5.proenzyme-like-GGT1.rhesus.       agccactgcacccagccaattactaccttcttcttatcaagttgctaatttacttctcag    136777

Papio.anubis.clone.rp41-133b2.8034-40080.revcompl.Baboon              aaggccccctgaactcagatttgtacttcaaggccctggtgtccttggatccagaacaga    29170
BCRP3.HUMAN.NCBI.REF                                                  ------------------------------------------------------------    20446
LOC106996293.glutathione.hydrolase5.proenzyme-like-GGT1.rhesus.       gaatagccaggtgcccaaaatttcccttaaagg--ctaatgtggttcgagtatgtgccct    136835

Papio.anubis.clone.rp41-133b2.8034-40080.revcompl.Baboon              catgaacttgcatctggaggtcttcaggcctgggtggtgtccttcatacatagtccctgg    29230
BCRP3.HUMAN.NCBI.REF                                                  ------------------------------------------------------------    20446
LOC106996293.glutathione.hydrolase5.proenzyme-like-GGT1.rhesus.       ccagatcttatgatgagatgtgatt-----------------------------------    136860

Papio.anubis.clone.rp41-133b2.8034-40080.revcompl.Baboon              aggctgaccttgtcctctatgggcctttagatctcttccatgctcccatcaggtcctcc-    29289
BCRP3.HUMAN.NCBI.REF                                                  ------------------------------------------------------------    20446
LOC106996293.glutathione.hydrolase5.proenzyme-like-GGT1.rhesus.       ------------tccagccagatgcagtggctcacacctgtaatcccaccacctcctcct    136908

Papio.anubis.clone.rp41-133b2.8034-40080.revcompl.Baboon              ---------cctcttcctgtgtcttcaggccctcttcttctcagtcttgccttttgatca    29340
BCRP3.HUMAN.NCBI.REF                                                  ------------------------------------------------------------    20446
LOC106996293.glutathione.hydrolase5.proenzyme-like-GGT1.rhesus.       cctccaattcctcctcctcctccaacacctcctccttctccaactcctcctcctcctcca    136968

Papio.anubis.clone.rp41-133b2.8034-40080.revcompl.Baboon              tttttttcatttcttgcagaggcccctcccttcgggagatgggctgagctgccctcccct    29400
BCRP3.HUMAN.NCBI.REF                                                  ------------------------------------------------------------    20446
LOC106996293.glutathione.hydrolase5.proenzyme-like-GGT1.rhesus.       actcctcctcttcctccacctcctcctcc-------------------------------    136997

Papio.anubis.clone.rp41-133b2.8034-40080.revcompl.Baboon              gtgaacctgctttctctcctctccaggaggtacagaggggactccaagaccgtggccaga    29460
BCRP3.HUMAN.NCBI.REF                                                  ------------------------------------------------------------    20446
LOC106996293.glutathione.hydrolase5.proenzyme-like-GGT1.rhesus.       ------------------------------------------------------------    136997

Papio.anubis.clone.rp41-133b2.8034-40080.revcompl.Baboon              accagacccagaggcccttcttcttgaacgtggtccaggccgtgtcccaggagggggcct    29520
BCRP3.HUMAN.NCBI.REF                                                  ------------------------------------------------------------    20446
LOC106996293.glutathione.hydrolase5.proenzyme-like-GGT1.rhesus.       tccaactcctcctcctctgtttctttatcctcctcctcccctctttca-----------    137046

Papio.anubis.clone.rp41-133b2.8034-40080.revcompl.Baboon              gtgtgtatgccgtctcggacctgaggaagggtggggaggccgcaggctactgagacactg    29580
BCRP3.HUMAN.NCBI.REF                                                  ------------------------------------------------------------    20446
LOC106996293.glutathione.hydrolase5.proenzyme-like-GGT1.rhesus.       ------------------------------------------------------------    137046

Papio.anubis.clone.rp41-133b2.8034-40080.revcompl.Baboon              ctctgcccagagctgaagtccggccccaccatgggtcctatgtccaggccggacatggct    29640
```

```
BCRP3.HUMAN.NCBI.REF                                               ------------------------------------------------------------   20446
LOC106996293.glutathione.hydrolase5.proenzyme-like-GGT1.rhesus.    ------------------------------------------------------------   137046

Papio.anubis.clone.rp41-133b2.8034-40080.revcompl.Baboon           gggggaccaagtactccggcaggatctggacccctggcaggggagtccagctgagagtgg   29700
BCRP3.HUMAN.NCBI.REF                                               ------------------------------------------------------------   20446
LOC106996293.glutathione.hydrolase5.proenzyme-like-GGT1.rhesus.    ------------------------------------------------------------   137046

Papio.anubis.clone.rp41-133b2.8034-40080.revcompl.Baboon           aagaggtggcagggaccagctgggcagatgggaggctgagccccgtccctgacccccttt   29760
BCRP3.HUMAN.NCBI.REF                                               ------------------------------------------------------------   20446
LOC106996293.glutathione.hydrolase5.proenzyme-like-GGT1.rhesus.    ------------------------------------------------------------   137046

Papio.anubis.clone.rp41-133b2.8034-40080.revcompl.Baboon           cccagagcccctggtggccctgaacgggcctctctatccctcctcaggcctcttgcctgg   29820
BCRP3.HUMAN.NCBI.REF                                               ------------------------------------------------------------   20446
LOC106996293.glutathione.hydrolase5.proenzyme-like-GGT1.rhesus.    ----------------------------------------------------tttctttt   137054

Papio.anubis.clone.rp41-133b2.8034-40080.revcompl.Baboon           ggccactctcccacccgctcaacctgtatatcctccagttcaagattaaagaagaggcgg   29880
BCRP3.HUMAN.NCBI.REF                                               ------------------------------------------------------------   20446
LOC106996293.glutathione.hydrolase5.proenzyme-like-GGT1.rhesus.    tcctcctcccctcctacatttcttttttcctcctccttctca------------------   137096

Papio.anubis.clone.rp41-133b2.8034-40080.revcompl.Baboon           acagtggcctgattccatggttctacgtgggggaggggaccatcggacagtgcacagagg   29940
BCRP3.HUMAN.NCBI.REF                                               ------------------------------------------------------------   20446
LOC106996293.glutathione.hydrolase5.proenzyme-like-GGT1.rhesus.    ----------------------------------------------------caggcatg   137104

Papio.anubis.clone.rp41-133b2.8034-40080.revcompl.Baboon           ggtgcctgcaccaggctcctatggttctccacactgcaccaccatggaggtggggaaact   30000
BCRP3.HUMAN.NCBI.REF                                               ------------------------------------------------------------   20446
LOC106996293.glutathione.hydrolase5.proenzyme-like-GGT1.rhesus.    agccactacacaaggcctggctgtttgtttctttctcctcctcctccatttctttttcct   137164

Papio.anubis.clone.rp41-133b2.8034-40080.revcompl.Baboon           gatgctcgcagaccagccgaacctca-----gccaggccacccctggacagagacaagcg   30055
BCRP3.HUMAN.NCBI.REF                                               ------------------------------------------------------------   20446
LOC106996293.glutathione.hydrolase5.proenzyme-like-GGT1.rhesus.    cctcctcttcctccttctcctcctcacaggcaccagccaccacgactcacctggctattt   137224

Papio.anubis.clone.rp41-133b2.8034-40080.revcompl.Baboon           ccacctctgcctccccaacacctcaccactctggtggccaggactcagcagcctgactgg   30115
BCRP3.HUMAN.NCBI.REF                                               ------------------------------------------------------------   20446
LOC106996293.glutathione.hydrolase5.proenzyme-like-GGT1.rhesus.    gcttttcctcctttcctcctcctgctccttctccgcctcccaaattgctgggattacagg   137284

Papio.anubis.clone.rp41-133b2.8034-40080.revcompl.Baboon           ccccagtcctgagagctggttaactgaaaaatcacacaattcacaaatttggaaaagata   30175
BCRP3.HUMAN.NCBI.REF                                               ------------------------------------------------------------   20446
LOC106996293.glutathione.hydrolase5.proenzyme-like-GGT1.rhesus.    cctgagccaccacatctagcta--------------------------------------   137306

Papio.anubis.clone.rp41-133b2.8034-40080.revcompl.Baboon           ctttttctttcttctttcctttcttttttttctttcttttctttctttctttcttttttt   30235
BCRP3.HUMAN.NCBI.REF                                               ------------------------------------------------------------   20446
LOC106996293.glutathione.hydrolase5.proenzyme-like-GGT1.rhesus.    ------------tttcttcttctttttcttcctcttcttcctcctccttcttcacttaat   137354

Papio.anubis.clone.rp41-133b2.8034-40080.revcompl.Baboon           ttttttttttttgagatagggtcttgctctgtcgttcaggctggagtgcagtggtgcaat   30295
BCRP3.HUMAN.NCBI.REF                                               ------------------------------------------------------------   20446
LOC106996293.glutathione.hydrolase5.proenzyme-like-GGT1.rhesus.    tttatttttcttgatatagagtctccctctgttgcccaagctggagtgcagtggtgcgat   137414

Papio.anubis.clone.rp41-133b2.8034-40080.revcompl.Baboon           cacagctcactgcgctcttaacctcctgggctcaagccatccttctgcctcagcctccca   30355
BCRP3.HUMAN.NCBI.REF                                               ------------------------------------------------------------   20446
LOC106996293.glutathione.hydrolase5.proenzyme-like-GGT1.rhesus.    ctcggctcactgcaacctccacctcctgggttca-----------------agcctccca   137457

Papio.anubis.clone.rp41-133b2.8034-40080.revcompl.Baboon           agtaactgggactacagatgt-----aacctggccaatttttacatttttttttttgtaga   30410
BCRP3.HUMAN.NCBI.REF                                               ------------------------------------------------------------   20446
LOC106996293.glutathione.hydrolase5.proenzyme-like-GGT1.rhesus.    agtatctgggattaccagcgtccgccaccatgcccagtcaatttttgtccttttagtaga   137517

Papio.anubis.clone.rp41-133b2.8034-40080.revcompl.Baboon           gacagggtcttgctttgttgcccaggctggtcttgaactcctggtctcaagccatcctcc   30470
BCRP3.HUMAN.NCBI.REF                                               ------------------------------------------------------------   20446
LOC106996293.glutathione.hydrolase5.proenzyme-like-GGT1.rhesus.    gacggggtttcaccatgttggctaggctggtctcaaactcctgacctcatg--atctgtc   137575

Papio.anubis.clone.rp41-133b2.8034-40080.revcompl.Baboon           ctcctcagcctcccaaagtgctgggattataggtgtgagccacta-----tgcccgacaa   30525
BCRP3.HUMAN.NCBI.REF                                               ------------------------------------------------------------   20446
LOC106996293.glutathione.hydrolase5.proenzyme-like-GGT1.rhesus.    cacctccacctcccatagtgctgggattacaggcatgagccactgcacctagccccctgg   137635

Papio.anubis.clone.rp41-133b2.8034-40080.revcompl.Baboon           gattttctttttttttttttccttcaacttgtattttaagttcaggggcccatatgtag-   30584
BCRP3.HUMAN.NCBI.REF                                               ------------------------------------------------------------   20446
LOC106996293.glutathione.hydrolase5.proenzyme-like-GGT1.rhesus.    ttttttgttttttgttttttccttgaaatggagtttcactcttgttgcccaggctggagt   137695

Papio.anubis.clone.rp41-133b2.8034-40080.revcompl.Baboon           gatatgcaggtttgttacatagat------------------------------------   30608
BCRP3.HUMAN.NCBI.REF                                               ------------------------------------------------------------   20446
LOC106996293.glutathione.hydrolase5.proenzyme-like-GGT1.rhesus.    gcaatggcatgatcttccctcactgcaacctccacctcctgggttcaagcgattcacatg   137755

Papio.anubis.clone.rp41-133b2.8034-40080.revcompl.Baboon           ------------------------------------------------------------   30608
BCRP3.HUMAN.NCBI.REF                                               ------------------------------------------------------------   20446
LOC106996293.glutathione.hydrolase5.proenzyme-like-GGT1.rhesus.    ctttagccccccgagtagctgggattacagctgtcagccactgcgctgggcctacagctc   137815

Papio.anubis.clone.rp41-133b2.8034-40080.revcompl.Baboon           -----------------------------------------------------------a   30609
BCRP3.HUMAN.NCBI.REF                                               ------------------------------------------------------------   20446
LOC106996293.glutathione.hydrolase5.proenzyme-like-GGT1.rhesus.    gtttcttaccacataaatctttgcgcctctccacaaaactgccatcagggatgtccccag   137875

Papio.anubis.clone.rp41-133b2.8034-40080.revcompl.Baboon           aacacgtgctttggttgtttgttgcagagatcaac----ccatcacctaggtattaagcc   30665
BCRP3.HUMAN.NCBI.REF                                               ------------------------------------------------------------   20446
LOC106996293.glutathione.hydrolase5.proenzyme-like-GGT1.rhesus.    aaaccattcatcccaggtgccatgcagagaagagcttctcgttctccttttccctttacc   137935

Papio.anubis.clone.rp41-133b2.8034-40080.revcompl.Baboon           cagcatccattagctattcttcctgaagctctcctcccatcccctgactttctttttttt   30725
BCRP3.HUMAN.NCBI.REF                                               ------------------------------------------------------------   20446
LOC106996293.glutathione.hydrolase5.proenzyme-like-GGT1.rhesus.    tcttccctctcacctcatcttgttcattcactcatcccttttccattctcacttttaagc   137995

Papio.anubis.clone.rp41-133b2.8034-40080.revcompl.Baboon           ttttt-------------------------------------------------------   30730
BCRP3.HUMAN.NCBI.REF                                               ------------------------------------------------------------   20446
LOC106996293.glutathione.hydrolase5.proenzyme-like-GGT1.rhesus.    gttaacctttcaaaagcctgtcttcccctacaagtaatgtattgtaactcccgccatcac   138055

Papio.anubis.clone.rp41-133b2.8034-40080.revcompl.Baboon           ------------------------------------------------------------   30730
BCRP3.HUMAN.NCBI.REF                                               ------------------------------------------------------------   20446
LOC106996293.glutathione.hydrolase5.proenzyme-like-GGT1.rhesus.    catatccttctccaacccaccaaactgccaaggatggtaagtccataaactaagaagaaa   138115

Papio.anubis.clone.rp41-133b2.8034-40080.revcompl.Baboon           ------------------------------------------------------------   30730
BCRP3.HUMAN.NCBI.REF                                               ------------------------------------------------------------   20446
LOC106996293.glutathione.hydrolase5.proenzyme-like-GGT1.rhesus.    tgggaaacattcattgcaaactttgcagcccctcagtcacccaatgtgacagcacagatc   138175

Papio.anubis.clone.rp41-133b2.8034-40080.revcompl.Baboon           -------------------ttttttttttcctttgaaacaaagtctcactctgttgccga   30771
BCRP3.HUMAN.NCBI.REF                                               ------------------------------------------------------------   20446
LOC106996293.glutathione.hydrolase5.proenzyme-like-GGT1.rhesus.    ttctggtctttgaagacccttttttttttttttttaagacaggtgtcgctctgtcaccca   138235

Papio.anubis.clone.rp41-133b2.8034-40080.revcompl.Baboon           ggctggagcgcagtggcacaatctcagctcactgcaacttctgcctcccagcttcaagtg   30831
BCRP3.HUMAN.NCBI.REF                                               ------------------------------------------------------------   20446
LOC106996293.glutathione.hydrolase5.proenzyme-like-GGT1.rhesus.    ggttcaagtgcagtggtgcaatcacagctcagtgcagcccccgactcctgtgctcaggtg   138295

Papio.anubis.clone.rp41-133b2.8034-40080.revcompl.Baboon           attctcctgcctccgcctcctgagtagctgggattaca----------------------   30869
BCRP3.HUMAN.NCBI.REF                                               ------------------------------------------------------------   20446
LOC106996293.glutathione.hydrolase5.proenzyme-like-GGT1.rhesus.    atccgcctgcctcagcctcccaagcagctggaactacaggaacacaccaccacacccagc   138355

Papio.anubis.clone.rp41-133b2.8034-40080.revcompl.Baboon           --------------------------tggggtttcaccatgtggccaggctggtcttgaa   30903
BCRP3.HUMAN.NCBI.REF                                               ------------------------------------------------------------   20446
LOC106996293.glutathione.hydrolase5.proenzyme-like-GGT1.rhesus.    taatgtgcttattttttttgtagagatggggtcttgctctgcttaacaggctggtttcaaa   138415

Papio.anubis.clone.rp41-133b2.8034-40080.revcompl.Baboon           atcctgacctcaggtgatccacctgccttggcctcccaaagtgctgggtgattacaggtg   30963
```

```
BCRP3.HUMAN.NCBI.REF                                                ------------------------------------------------------------    20446
LOC106996293.glutathione.hydrolase5.proenzyme-like-GGT1.rhesus.     ctcctggcttcaagggatcctcccaccttggcctcccaaagtgctgg--gattacaggca    138473

Papio.anubis.clone.rp41-133b2.8034-40080.revcompl.Baboon            tgagccacagtggcaactttatttttcataaagagttacaacctgcagggtg--------    31015
BCRP3.HUMAN.NCBI.REF                                                ------------------------------------------------------------    20446
LOC106996293.glutathione.hydrolase5.proenzyme-like-GGT1.rhesus.     tgagtcaccatgcccggtctgaagacttttaaatgctgccatattcaagatacattgaaa    138533

Papio.anubis.clone.rp41-133b2.8034-40080.revcompl.Baboon            ------------------------------------------------------------    31015
BCRP3.HUMAN.NCBI.REF                                                ------------------------------------------------------------    20446
LOC106996293.glutathione.hydrolase5.proenzyme-like-GGT1.rhesus.     ctcacctgtgtttgatgagcctgctttttgcaagtgagtaatataaaacacactgaaata    138593

Papio.anubis.clone.rp41-133b2.8034-40080.revcompl.Baboon            ------------------------------------------------------------    31015
BCRP3.HUMAN.NCBI.REF                                                ------------------------------------------------------------    20446
LOC106996293.glutathione.hydrolase5.proenzyme-like-GGT1.rhesus.     ccttaagcttctcagactttgtaccttcctctggaataatcagtgtatcccaaaagtaaa    138653

Papio.anubis.clone.rp41-133b2.8034-40080.revcompl.Baboon            --------gccatccaccactttcaaaagcatagcctctggcagagaccagaaacaggca    31067
BCRP3.HUMAN.NCBI.REF                                                ------------------------------------------------------------    20446
LOC106996293.glutathione.hydrolase5.proenzyme-like-GGT1.rhesus.     tccataatgaggtccagtttttccttcctccttggctatgaaatagacaagaaaaaggca    138713

Papio.anubis.clone.rp41-133b2.8034-40080.revcompl.Baboon            c-----------------------------------------------------------    31068
BCRP3.HUMAN.NCBI.REF                                                ------------------------------------------------------------    20446
LOC106996293.glutathione.hydrolase5.proenzyme-like-GGT1.rhesus.     agcaagccatgtccacctcactgtaggagactctcctgtttgctttttgactgtatttgg    138773

Papio.anubis.clone.rp41-133b2.8034-40080.revcompl.Baboon            ------------------------------------------------------------    31068
BCRP3.HUMAN.NCBI.REF                                                ------------------------------------------------------------    20446
LOC106996293.glutathione.hydrolase5.proenzyme-like-GGT1.rhesus.     gaagcgggggcctgactgctttcctacttcctaagcacaacttcctttacctaggaaatc    138833

Papio.anubis.clone.rp41-133b2.8034-40080.revcompl.Baboon            ------------------------------------------------------------    31068
BCRP3.HUMAN.NCBI.REF                                                ------------------------------------------------------------    20446
LOC106996293.glutathione.hydrolase5.proenzyme-like-GGT1.rhesus.     ctcagcatgacctacatagattaaaccagtttcttcactttctttccaatattcttacaa    138893

Papio.anubis.clone.rp41-133b2.8034-40080.revcompl.Baboon            ------------------------------------------------------------    31068
BCRP3.HUMAN.NCBI.REF                                                ------------------------------------------------------------    20446
LOC106996293.glutathione.hydrolase5.proenzyme-like-GGT1.rhesus.     ccaaaatatccagaaagggcaaggcaacctgaaaaaatgaggacaggtatattatcccat    138953

Papio.anubis.clone.rp41-133b2.8034-40080.revcompl.Baboon            ------------------------------------------------------------    31068
BCRP3.HUMAN.NCBI.REF                                                ------------------------------------------------------------    20446
LOC106996293.glutathione.hydrolase5.proenzyme-like-GGT1.rhesus.     gagccaaactgccacttacactggttagtcatgaaatcagcaaaattccagatgagctct    139013

Papio.anubis.clone.rp41-133b2.8034-40080.revcompl.Baboon            ------------------------------------------------------------    31068
BCRP3.HUMAN.NCBI.REF                                                ------------------------------------------------------------    20446
LOC106996293.glutathione.hydrolase5.proenzyme-like-GGT1.rhesus.     ccaactacgtattttctatgtttttgatccagacccagatggtactgctctagcagactg    139073

Papio.anubis.clone.rp41-133b2.8034-40080.revcompl.Baboon            ------------------------------------------------------------    31068
BCRP3.HUMAN.NCBI.REF                                                ------------------------------------------------------------    20446
LOC106996293.glutathione.hydrolase5.proenzyme-like-GGT1.rhesus.     ttctggtcatcttcactgaacatcagagatggatcctggaattcaaggcaaagagaattt    139133

Papio.anubis.clone.rp41-133b2.8034-40080.revcompl.Baboon            ------------------------------------------------------------    31068
BCRP3.HUMAN.NCBI.REF                                                ------------------------------------------------------------    20446
LOC106996293.glutathione.hydrolase5.proenzyme-like-GGT1.rhesus.     aagaatcagaactggcagaattgtaaatgtcagataaaaaataaagatccacttgattgt    139193

Papio.anubis.clone.rp41-133b2.8034-40080.revcompl.Baboon            ------------------------------------------------------------    31068
BCRP3.HUMAN.NCBI.REF                                                ------------------------------------------------------------    20446
LOC106996293.glutathione.hydrolase5.proenzyme-like-GGT1.rhesus.     gacgaaaatacctgacctcacagggggctgtagtgacagcaggactgactgatgctagga    139253

Papio.anubis.clone.rp41-133b2.8034-40080.revcompl.Baboon            ------------------------------------------------------------    31068
BCRP3.HUMAN.NCBI.REF                                                ------------------------------------------------------------    20446
LOC106996293.glutathione.hydrolase5.proenzyme-like-GGT1.rhesus.     taaagacagccagggaatgatctaacccagaataaaaaggaggtttaaaaaaaaaccaac    139313

Papio.anubis.clone.rp41-133b2.8034-40080.revcompl.Baboon            ------------------------------------------------------------    31068
BCRP3.HUMAN.NCBI.REF                                                ------------------------------------------------------------    20446
LOC106996293.glutathione.hydrolase5.proenzyme-like-GGT1.rhesus.     atggtcaggcatggtggctcacgcctataatcccagcactttgggaggttgaggccaggc    139373

Papio.anubis.clone.rp41-133b2.8034-40080.revcompl.Baboon            ------------------------------------------------------------    31068
BCRP3.HUMAN.NCBI.REF                                                ------------------------------------------------------------    20446
LOC106996293.glutathione.hydrolase5.proenzyme-like-GGT1.rhesus.     agatcacaaggtcaggagatcgagaccatcttggctaacacggtgaaaccccatttctac    139433

Papio.anubis.clone.rp41-133b2.8034-40080.revcompl.Baboon            ------------------------------------------------------------    31068
BCRP3.HUMAN.NCBI.REF                                                ------------------------------------------------------------    20446
LOC106996293.glutathione.hydrolase5.proenzyme-like-GGT1.rhesus.     aaaaaatacaaaaaatgagctgggcatggtagcgggcacctgtactcccagctactcagg    139493

Papio.anubis.clone.rp41-133b2.8034-40080.revcompl.Baboon            ------------------------------------------------------------    31068
BCRP3.HUMAN.NCBI.REF                                                ------------------------------------------------------------    20446
LOC106996293.glutathione.hydrolase5.proenzyme-like-GGT1.rhesus.     aggctgaggcaggagaatggtgtgaacgtgggaggtggaggctgcagtgagcagagatca    139553

Papio.anubis.clone.rp41-133b2.8034-40080.revcompl.Baboon            ------------------------------------------------------------    31068
BCRP3.HUMAN.NCBI.REF                                                ------------------------------------------------------------    20446
LOC106996293.glutathione.hydrolase5.proenzyme-like-GGT1.rhesus.     cgccactgcactccagcataggcaacagcaagactctgttgaaagaaaagaaagaaagaa    139613

Papio.anubis.clone.rp41-133b2.8034-40080.revcompl.Baboon            ------------------------------------------------------------    31068
BCRP3.HUMAN.NCBI.REF                                                ------------------------------------------------------------    20446
LOC106996293.glutathione.hydrolase5.proenzyme-like-GGT1.rhesus.     agaaagaaagaaagaaagaaagaaagaaagaaagaaagaaagaaagaaagaaagaaagaa    139673

Papio.anubis.clone.rp41-133b2.8034-40080.revcompl.Baboon            ------------------------------------------------------------    31068
BCRP3.HUMAN.NCBI.REF                                                ------------------------------------------------------------    20446
LOC106996293.glutathione.hydrolase5.proenzyme-like-GGT1.rhesus.     agaaagaaagagaaagaaagaaagtcattaattagtaattgctttatttataaatgtaat    139733

Papio.anubis.clone.rp41-133b2.8034-40080.revcompl.Baboon            ------------------------------------------------------------    31068
BCRP3.HUMAN.NCBI.REF                                                ------------------------------------------------------------    20446
LOC106996293.glutathione.hydrolase5.proenzyme-like-GGT1.rhesus.     ttgatattcgattcatcttacttttccatctctatctgctggtacagtcttaaggctgaa    139793

Papio.anubis.clone.rp41-133b2.8034-40080.revcompl.Baboon            ------------------------------------------------------------    31068
BCRP3.HUMAN.NCBI.REF                                                ------------------------------------------------------------    20446
LOC106996293.glutathione.hydrolase5.proenzyme-like-GGT1.rhesus.     ctacactagaagtaaaaatatgccttgggaccaggcatgatggctcatgcctgtcatctc    139853

Papio.anubis.clone.rp41-133b2.8034-40080.revcompl.Baboon            ------------------------------------------------------------    31068
BCRP3.HUMAN.NCBI.REF                                                ------------------------------------------------------------    20446
LOC106996293.glutathione.hydrolase5.proenzyme-like-GGT1.rhesus.     agcagtttgggaggccaaagtgggaggactgcttgagcctagaagttcaagaccagcctg    139913

Papio.anubis.clone.rp41-133b2.8034-40080.revcompl.Baboon            ------------------------------------------------------------    31068
BCRP3.HUMAN.NCBI.REF                                                ------------------------------------------------------------    20446
LOC106996293.glutathione.hydrolase5.proenzyme-like-GGT1.rhesus.     ggaaatatagcaagaccccatctctacaaaaactacacaaagtagccaagcatggtggca    139973

Papio.anubis.clone.rp41-133b2.8034-40080.revcompl.Baboon            ------------------------------------------------------------    31068
BCRP3.HUMAN.NCBI.REF                                                ------------------------------------------------------------    20446
LOC106996293.glutathione.hydrolase5.proenzyme-like-GGT1.rhesus.     cacaccgggggtcccagctacttgggaggctgaggtgggaggactgcttgagccccagag    140033

Papio.anubis.clone.rp41-133b2.8034-40080.revcompl.Baboon            ------------------------------------------------------------    31068
BCRP3.HUMAN.NCBI.REF                                                ------------------------------------------------------------    20446
LOC106996293.glutathione.hydrolase5.proenzyme-like-GGT1.rhesus.     gtcaaagctgcagtgagctgcgtttgcaccactacgctccagcctaggtgacagagcaag    140093

Papio.anubis.clone.rp41-133b2.8034-40080.revcompl.Baboon            ------------------------------------------------------------    31068
BCRP3.HUMAN.NCBI.REF                                                ------------------------------------------------------------    20446
LOC106996293.glutathione.hydrolase5.proenzyme-like-GGT1.rhesus.     accctatcttaaataaatatatgtatgtatgtatgtaaaatgaaattaaaccaggctggg    140153

Papio.anubis.clone.rp41-133b2.8034-40080.revcompl.Baboon            ------------------------------------------------------------    31068
```

```
BCRP3.HUMAN.NCBI.REF                                            ------------------------------------------------------------    20446
LOC106996293.glutathione.hydrolase5.proenzyme-like-GGT1.rhesus.  catggcagctcaggtctggaatcccagcactttgagaggtcaaggcaggaggatgagcac    140213

Papio.anubis.clone.rp41-133b2.8034-40080.revcompl.Baboon        ------------------------------------------------------------    31068
BCRP3.HUMAN.NCBI.REF                                            ------------------------------------------------------------    20446
LOC106996293.glutathione.hydrolase5.proenzyme-like-GGT1.rhesus.  ttgagcccaggagttcaagaccagcctaggcaacacagtgagacccagtctctacaaaaa    140273

Papio.anubis.clone.rp41-133b2.8034-40080.revcompl.Baboon        ------------------------------------------------------------    31068
BCRP3.HUMAN.NCBI.REF                                            ------------------------------------------------------------    20446
LOC106996293.glutathione.hydrolase5.proenzyme-like-GGT1.rhesus.  gtttaaatattagccaggtgtggtgacgcatgcctgtggttccagccacttgggggctg    140333

Papio.anubis.clone.rp41-133b2.8034-40080.revcompl.Baboon        ------------------------------------------------------------    31068
BCRP3.HUMAN.NCBI.REF                                            ------------------------------------------------------------    20446
LOC106996293.glutathione.hydrolase5.proenzyme-like-GGT1.rhesus.  aggagggaggatcatttgagcccaggaggttgagcagtgagctgtgattatgccactgca    140393

Papio.anubis.clone.rp41-133b2.8034-40080.revcompl.Baboon        ------------------------------------------------------------    31068
BCRP3.HUMAN.NCBI.REF                                            ------------------------------------------------------------    20446
LOC106996293.glutathione.hydrolase5.proenzyme-like-GGT1.rhesus.  ctccagcctgggcaacagagtgaggctctctcaaaaaaaaaattatttttaattaaacta    140453

Papio.anubis.clone.rp41-133b2.8034-40080.revcompl.Baboon        ------------------------------------------------------------    31068
BCRP3.HUMAN.NCBI.REF                                            ------------------------------------------------------------    20446
LOC106996293.glutathione.hydrolase5.proenzyme-like-GGT1.rhesus.  aataaattcagctattctagtcatatatcaagacctcaatagccacatgtagttagtggc    140513

Papio.anubis.clone.rp41-133b2.8034-40080.revcompl.Baboon        ------------------------------------------------------------    31068
BCRP3.HUMAN.NCBI.REF                                            ------------------------------------------------------------    20446
LOC106996293.glutathione.hydrolase5.proenzyme-like-GGT1.rhesus.  caccatttcacacagtgcgtataagggacatttctatcattgcaaggcttctttttgaa    140573

Papio.anubis.clone.rp41-133b2.8034-40080.revcompl.Baboon        ------------------------------------------------------------    31068
BCRP3.HUMAN.NCBI.REF                                            ------------------------------------------------------------    20446
LOC106996293.glutathione.hydrolase5.proenzyme-like-GGT1.rhesus.  acaaggtctcactctgtcccccaggtgggagcgcagtggtgcaatcatggcagacggcag    140633

Papio.anubis.clone.rp41-133b2.8034-40080.revcompl.Baboon        ------------------------------------------------------------    31068
BCRP3.HUMAN.NCBI.REF                                            ------------------------------------------------------------    20446
LOC106996293.glutathione.hydrolase5.proenzyme-like-GGT1.rhesus.  ccttgacctactgggctcaaacaatcttcccacctcagcgtcccaagtagctgggattac    140693

Papio.anubis.clone.rp41-133b2.8034-40080.revcompl.Baboon        ------------------------------ttctttttttttttttttttttttgagatg    31098
BCRP3.HUMAN.NCBI.REF                                            ------------------------------------------------------------    20446
LOC106996293.glutathione.hydrolase5.proenzyme-like-GGT1.rhesus.  aggcaagcacaaccataaccaactgatttatttttatttttcttattttcttgagacg    140753

Papio.anubis.clone.rp41-133b2.8034-40080.revcompl.Baboon        gagtctcgctctgttgcccaggttggagtgcagtggcgcgatctcagctcactgcaagct    31158
BCRP3.HUMAN.NCBI.REF                                            ------------------------------------------------------------    20446
LOC106996293.glutathione.hydrolase5.proenzyme-like-GGT1.rhesus.  gagtctcgctctgtcacccaggctagagtgcagaggcacgatgttaactcactataacct    140813

Papio.anubis.clone.rp41-133b2.8034-40080.revcompl.Baboon        ccgcctccc-ggttcacgccattcttctacctcagcctcccgagtagctgggactacagg    31217
BCRP3.HUMAN.NCBI.REF                                            ------------------------------------------------------------    20446
LOC106996293.glutathione.hydrolase5.proenzyme-like-GGT1.rhesus.  ctgcgtccctggttcaagcagttctcctgcttcagcctcctgagtaactgggattacagg    140873

Papio.anubis.clone.rp41-133b2.8034-40080.revcompl.Baboon        cacccgccaccacgcacagctaatttttttttgtatttttagtggagatggggtttcacca    31277
BCRP3.HUMAN.NCBI.REF                                            ------------------------------------------------------------    20446
LOC106996293.glutathione.hydrolase5.proenzyme-like-GGT1.rhesus.  cacacaccaccacacccagctaa-tttttgtatttcttagtagagacgaggtttcacca    140932

Papio.anubis.clone.rp41-133b2.8034-40080.revcompl.Baboon        tgttagccaggatggtctcgatctcctgaccttgtgat----------------------    31315
BCRP3.HUMAN.NCBI.REF                                            ------------------------------------------------------------    20446
LOC106996293.glutathione.hydrolase5.proenzyme-like-GGT1.rhesus.  tgttggtcaagttggactcgaactcctgacctcatgatccacccacctcggcctcccaaa    140992

Papio.anubis.clone.rp41-133b2.8034-40080.revcompl.Baboon        ------------------------------------------------------------    31315
BCRP3.HUMAN.NCBI.REF                                            ------------------------------------------------------------    20446
LOC106996293.glutathione.hydrolase5.proenzyme-like-GGT1.rhesus.  atgctgggattacaggtgtcagccaccatgcccggccgcaactaatcttaaatttttttgt    141052

Papio.anubis.clone.rp41-133b2.8034-40080.revcompl.Baboon        ------------------------------------------------------------    31315
BCRP3.HUMAN.NCBI.REF                                            ------------------------------------------------------------    20446
LOC106996293.glutathione.hydrolase5.proenzyme-like-GGT1.rhesus.  agagatggggtctatgttgtgcagactggtctcaaattcctgggctcaagagagcctctg    141112

Papio.anubis.clone.rp41-133b2.8034-40080.revcompl.Baboon        ------------------------------------------------------------    31315
BCRP3.HUMAN.NCBI.REF                                            ------------------------------------------------------------    20446
LOC106996293.glutathione.hydrolase5.proenzyme-like-GGT1.rhesus.  acctcggtctcccaaagtgctagcattccaggtgtgagccaccacacccagcacctgcag    141172

Papio.anubis.clone.rp41-133b2.8034-40080.revcompl.Baboon        ------------------------------------------------------------    31315
BCRP3.HUMAN.NCBI.REF                                            ------------------------------------------------------------    20446
LOC106996293.glutathione.hydrolase5.proenzyme-like-GGT1.rhesus.  cacccaggtctatcagtgctgacctagaacctctcgggagtttcttaagaattcagaact    141232

Papio.anubis.clone.rp41-133b2.8034-40080.revcompl.Baboon        ------------------------------------------------------------    31315
BCRP3.HUMAN.NCBI.REF                                            ------------------------------------------------------------    20446
LOC106996293.glutathione.hydrolase5.proenzyme-like-GGT1.rhesus.  gggggtattagccaagatggcggcggccgcagtgagttgaggaatcagggcaaagctggg    141292

Papio.anubis.clone.rp41-133b2.8034-40080.revcompl.Baboon        ------------------------------------------------------------    31315
BCRP3.HUMAN.NCBI.REF                                            ------------------------------------------------------------    20446
LOC106996293.glutathione.hydrolase5.proenzyme-like-GGT1.rhesus.  cctgcgtgagattcgcatccacttatgtcagcgcttgcccggcagccagggtgtcaggga    141352

Papio.anubis.clone.rp41-133b2.8034-40080.revcompl.Baboon        ------------------------------------------------------------    31315
BCRP3.HUMAN.NCBI.REF                                            ------------------------------------------------------------    20446
LOC106996293.glutathione.hydrolase5.proenzyme-like-GGT1.rhesus.  cttcattgagaaacgctatgtggagctaaagaaggtgaatcctgacctacccatcctaat    141412

Papio.anubis.clone.rp41-133b2.8034-40080.revcompl.Baboon        ------------------------------------------------------------    31315
BCRP3.HUMAN.NCBI.REF                                            ------------------------------------------------------------    20446
LOC106996293.glutathione.hydrolase5.proenzyme-like-GGT1.rhesus.  ccgcaaatgctccgatgtgcagcccaagctctgggcccgctacgcatttggccaagagaa    141472

Papio.anubis.clone.rp41-133b2.8034-40080.revcompl.Baboon        ------------------------------------------------------------    31315
BCRP3.HUMAN.NCBI.REF                                            ------------------------------------------------------------    20446
LOC106996293.glutathione.hydrolase5.proenzyme-like-GGT1.rhesus.  gaatgtccctttgaacaacttcagtgctgatcaggtaaccagagccctggagaacgtgct    141532

Papio.anubis.clone.rp41-133b2.8034-40080.revcompl.Baboon        ------------------------------------------------------------    31315
BCRP3.HUMAN.NCBI.REF                                            ------------------------------------------------------------    20446
LOC106996293.glutathione.hydrolase5.proenzyme-like-GGT1.rhesus.  aagtggtaaagcctgaagcctccactgaggaataagagcaacagccccagagcctggcct    141592

Papio.anubis.clone.rp41-133b2.8034-40080.revcompl.Baboon        ------------------------------------------------------------    31315
BCRP3.HUMAN.NCBI.REF                                            ------------------------------------------------------------    20446
LOC106996293.glutathione.hydrolase5.proenzyme-like-GGT1.rhesus.  ctgctggacttagtataatgtgaaaaaaatgtgttctcctattcctcataaagcttgtgc    141652

Papio.anubis.clone.rp41-133b2.8034-40080.revcompl.Baboon        ------------------------------------------------------------    31315
BCRP3.HUMAN.NCBI.REF                                            ------------------------------------------------------------    20446
LOC106996293.glutathione.hydrolase5.proenzyme-like-GGT1.rhesus.  tgtaaaatactttctcagggtgttcttgtcctcatctaccctctatcccttactgtgtaa    141712

Papio.anubis.clone.rp41-133b2.8034-40080.revcompl.Baboon        ------------------------------------------------------------    31315
BCRP3.HUMAN.NCBI.REF                                            ------------------------------------------------------------    20446
LOC106996293.glutathione.hydrolase5.proenzyme-like-GGT1.rhesus.  ccactgaggcaaagtagcttaatataaaaataaaactttattctgcctcatcaaaaaaaa    141772

Papio.anubis.clone.rp41-133b2.8034-40080.revcompl.Baboon        ------------------------------------------------------------    31315
BCRP3.HUMAN.NCBI.REF                                            ------------------------------------------------------------    20446
LOC106996293.glutathione.hydrolase5.proenzyme-like-GGT1.rhesus.  aaaaaaaaaagaattcagaactggggccaggcatggtggcccacgcctgtaattccagca    141832

Papio.anubis.clone.rp41-133b2.8034-40080.revcompl.Baboon        ------------------------------------------------------------    31315
BCRP3.HUMAN.NCBI.REF                                            ------------------------------------------------------------    20446
LOC106996293.glutathione.hydrolase5.proenzyme-like-GGT1.rhesus.  ctttgggaggccaaggcaggtggatcacttgaggtcaaaagttcaagaccagcctgacca    141892

Papio.anubis.clone.rp41-133b2.8034-40080.revcompl.Baboon        ------------------------------------------------------------    31315
```

```
BCRP3.HUMAN.NCBI.REF                                            ------------------------------------------------------------    20446
LOC106996293.glutathione.hydrolase5.proenzyme-like-GGT1.rhesus.  acatggtgaaacctcatctctactaaaaaaaaaaaaaaaaaaaaaaagattaggagaaca    141952

Papio.anubis.clone.rp41-133b2.8034-40080.revcompl.Baboon        ------------------------------------------------------------    31315
BCRP3.HUMAN.NCBI.REF                                            ------------------------------------------------------------    20446
LOC106996293.glutathione.hydrolase5.proenzyme-like-GGT1.rhesus.  tggtggtgcatacctgtaatccaagttacttaggaggccaaggcaggagaatcgcttgaa    142012

Papio.anubis.clone.rp41-133b2.8034-40080.revcompl.Baboon        ------------------------------------------------------------    31315
BCRP3.HUMAN.NCBI.REF                                            ------------------------------------------------------------    20446
LOC106996293.glutathione.hydrolase5.proenzyme-like-GGT1.rhesus.  ctgggaggcagaggtagcagtgagtcaagattgtgccacggaactccagcctgggtgaca    142072

Papio.anubis.clone.rp41-133b2.8034-40080.revcompl.Baboon        ------------------------------------------------------------    31315
BCRP3.HUMAN.NCBI.REF                                            ------------------------------------------------------------    20446
LOC106996293.glutathione.hydrolase5.proenzyme-like-GGT1.rhesus.  agaatgaacttctgtctcaaagaaaaaagaaatcagaactggttaccttttcaaaggga    142132

Papio.anubis.clone.rp41-133b2.8034-40080.revcompl.Baboon        ------------------------------------------------------------    31315
BCRP3.HUMAN.NCBI.REF                                            ------------------------------------------------------------    20446
LOC106996293.glutathione.hydrolase5.proenzyme-like-GGT1.rhesus.  tgagcaagggtgcatatccacgagccacttccccctacttgactagtttgcagaagtggc    142192

Papio.anubis.clone.rp41-133b2.8034-40080.revcompl.Baboon        ------------------------------------------------------------    31315
BCRP3.HUMAN.NCBI.REF                                            ------------------------------------------------------------    20446
LOC106996293.glutathione.hydrolase5.proenzyme-like-GGT1.rhesus.  attctgtaagcacgataaatttaagggtgcaaacagaacagcgcagtccactgtgggtgg    142252

Papio.anubis.clone.rp41-133b2.8034-40080.revcompl.Baboon        ---------------------------caggagataggcacttctaaggaggagaggttg    31348
BCRP3.HUMAN.NCBI.REF                                            ------------------------------------------------------------    20446
LOC106996293.glutathione.hydrolase5.proenzyme-like-GGT1.rhesus.  ctgttctttatgtgtcagcgggagtcccaggagctgtgagaaaccagtgtgagctggctg    142312

Papio.anubis.clone.rp41-133b2.8034-40080.revcompl.Baboon        gggcaggagctatatgctgaaggggtt----ggctaaacatacatattcaacaggttaca    31404
BCRP3.HUMAN.NCBI.REF                                            ------------------------------------------------------------    20446
LOC106996293.glutathione.hydrolase5.proenzyme-like-GGT1.rhesus.  gggagggaacgaggggctggatggggttcaggaatccacataaaaaaaaaaaaccgacaa    142372

Papio.anubis.clone.rp41-133b2.8034-40080.revcompl.Baboon        gggagagctatgaatattcatgaaggcagtcctgacacgtgtattgaaagaacatgcatg    31464
BCRP3.HUMAN.NCBI.REF                                            ------------------------------------------------------------    20446
LOC106996293.glutathione.hydrolase5.proenzyme-like-GGT1.rhesus.  gataaagcaacctatctttggataggaagacaaaaaaatgagagggataaaaaaatgagg    142432

Papio.anubis.clone.rp41-133b2.8034-40080.revcompl.Baboon        ------------------------------------------------------------    31464
BCRP3.HUMAN.NCBI.REF                                            ------------------------------------------------------------    20446
LOC106996293.glutathione.hydrolase5.proenzyme-like-GGT1.rhesus.  acaggctgggcgtggtggctcccgtctataatcccaggatttgggtaggctgaagcaggc    142492

Papio.anubis.clone.rp41-133b2.8034-40080.revcompl.Baboon        ------------------------------------------------------------    31464
BCRP3.HUMAN.NCBI.REF                                            ------------------------------------------------------------    20446
LOC106996293.glutathione.hydrolase5.proenzyme-like-GGT1.rhesus.  aaatcacttgaggtcaggagttagaaagaccacttgggcatgggtgataggaggctgtct    142552

Papio.anubis.clone.rp41-133b2.8034-40080.revcompl.Baboon        ------------------------------------------------------------    31464
BCRP3.HUMAN.NCBI.REF                                            ------------------------------------------------------------    20446
LOC106996293.glutathione.hydrolase5.proenzyme-like-GGT1.rhesus.  ggaaacaaggccggtaaggaggccacttttgaggaccaagcgagtggggcagaggcctgg    142612

Papio.anubis.clone.rp41-133b2.8034-40080.revcompl.Baboon        ------------------------------------------------------------    31464
BCRP3.HUMAN.NCBI.REF                                            ------------------------------------------------------------    20446
LOC106996293.glutathione.hydrolase5.proenzyme-like-GGT1.rhesus.  ctgctggcaagaaggcaacgtggacggggtagcaggaggtgagcccaaaggtgaagcaag    142672

Papio.anubis.clone.rp41-133b2.8034-40080.revcompl.Baboon        ------------------------------------------------------------    31464
BCRP3.HUMAN.NCBI.REF                                            ------------------------------------------------------------    20446
LOC106996293.glutathione.hydrolase5.proenzyme-like-GGT1.rhesus.  gggcacactgcagtgggcacagggcaggccaggggaggcgagtgacatctctgcccagag    142732

Papio.anubis.clone.rp41-133b2.8034-40080.revcompl.Baboon        ------------------------------------------------------------    31464
BCRP3.HUMAN.NCBI.REF                                            ------------------------------------------------------------    20446
LOC106996293.glutathione.hydrolase5.proenzyme-like-GGT1.rhesus.  agaacacacaatcacaaagttcaacaccgcttacctggtgaaaccctacaattctgtcgc    142792

Papio.anubis.clone.rp41-133b2.8034-40080.revcompl.Baboon        ------------------------------------------------------------    31464
BCRP3.HUMAN.NCBI.REF                                            ------------------------------------------------------------    20446
LOC106996293.glutathione.hydrolase5.proenzyme-like-GGT1.rhesus.  tctgtactcgctctgaataatgggcttgcgataagtcttacaccaattctcaaactgggg    142852

Papio.anubis.clone.rp41-133b2.8034-40080.revcompl.Baboon        ------------------------------------------------------------    31464
BCRP3.HUMAN.NCBI.REF                                            ------------------------------------------------------------    20446
LOC106996293.glutathione.hydrolase5.proenzyme-like-GGT1.rhesus.  gccagctgcagcagaatcaatgccagttgcccgtagtcgtgataccaagtgtagtagttg    142912

Papio.anubis.clone.rp41-133b2.8034-40080.revcompl.Baboon        ------------------------------------------------------------    31464
BCRP3.HUMAN.NCBI.REF                                            ------------------------------------------------------------    20446
LOC106996293.glutathione.hydrolase5.proenzyme-like-GGT1.rhesus.  ttcacacagatcacgtccacatacggagcccaggaccagagcggcaaagcccgttcagca    142972

Papio.anubis.clone.rp41-133b2.8034-40080.revcompl.Baboon        ------------------------------------------------------------    31464
BCRP3.HUMAN.NCBI.REF                                            ------------------------------------------------------------    20446
LOC106996293.glutathione.hydrolase5.proenzyme-like-GGT1.rhesus.  cccagaagactgcatgactcagcactcacacactacaggggctcttctggcagagaaggt    143032

Papio.anubis.clone.rp41-133b2.8034-40080.revcompl.Baboon        ------------------------------------------------------------    31464
BCRP3.HUMAN.NCBI.REF                                            ------------------------------------------------------------    20446
LOC106996293.glutathione.hydrolase5.proenzyme-like-GGT1.rhesus.  aagaaggggatgtaatcccagcattctggaaggctgaggcaggagagtgacttgcagcca    143092

Papio.anubis.clone.rp41-133b2.8034-40080.revcompl.Baboon        ------------------------------------------------------------    31464
BCRP3.HUMAN.NCBI.REF                                            ------------------------------------------------------------    20446
LOC106996293.glutathione.hydrolase5.proenzyme-like-GGT1.rhesus.  ggagttagagaccagcctgggcaacacagcgagaccctagctctacaaaaactagtagta    143152

Papio.anubis.clone.rp41-133b2.8034-40080.revcompl.Baboon        ------------------------------------------------------------    31464
BCRP3.HUMAN.NCBI.REF                                            ------------------------------------------------------------    20446
LOC106996293.glutathione.hydrolase5.proenzyme-like-GGT1.rhesus.  agaaaattagcatggcacagtggctcgtgcctgtaatcccagcacactgggaggccaagg    143212

Papio.anubis.clone.rp41-133b2.8034-40080.revcompl.Baboon        ------------------------------------------------------------    31464
BCRP3.HUMAN.NCBI.REF                                            ------------------------------------------------------------    20446
LOC106996293.glutathione.hydrolase5.proenzyme-like-GGT1.rhesus.  tgtgaggatcacctgagcccaggagtttgagaccagcctggtcaacattgcaagacccca    143272

Papio.anubis.clone.rp41-133b2.8034-40080.revcompl.Baboon        ------------------------------------------------------------    31464
BCRP3.HUMAN.NCBI.REF                                            ------------------------------------------------------------    20446
LOC106996293.glutathione.hydrolase5.proenzyme-like-GGT1.rhesus.  tttctacaaaacaaaataaaacaaaacaaaacaaaaggctagaatgagaagagctgcctc    143332

Papio.anubis.clone.rp41-133b2.8034-40080.revcompl.Baboon        ------------------------------------------------------------    31464
BCRP3.HUMAN.NCBI.REF                                            ------------------------------------------------------------    20446
LOC106996293.glutathione.hydrolase5.proenzyme-like-GGT1.rhesus.  ctggggctaagaacatccaagtgcattaagttagatcctgaaattaccctgccccacagg    143392

Papio.anubis.clone.rp41-133b2.8034-40080.revcompl.Baboon        ------------------------------------------------------------    31464
BCRP3.HUMAN.NCBI.REF                                            ------------------------------------------------------------    20446
LOC106996293.glutathione.hydrolase5.proenzyme-like-GGT1.rhesus.  caaaaatcatggtcagaaagtggcccaaaggaggcaggcctgtgactgcacactgacact    143452

Papio.anubis.clone.rp41-133b2.8034-40080.revcompl.Baboon        ------------------------------------------------------------    31464
BCRP3.HUMAN.NCBI.REF                                            ------------------------------------------------------------    20446
LOC106996293.glutathione.hydrolase5.proenzyme-like-GGT1.rhesus.  catgatgtgtgcagctgggaagggctatgagaggcagagcagctgccaatgcacagtcct    143512

Papio.anubis.clone.rp41-133b2.8034-40080.revcompl.Baboon        ------------------------------------------------------------    31464
BCRP3.HUMAN.NCBI.REF                                            ------------------------------------------------------------    20446
LOC106996293.glutathione.hydrolase5.proenzyme-like-GGT1.rhesus.  cggccaaagcccagggcccccgccactggaactgactcctgtccgggcagcgctcccatc    143572

Papio.anubis.clone.rp41-133b2.8034-40080.revcompl.Baboon        ------------------------------------------------------------    31464
BCRP3.HUMAN.NCBI.REF                                            ------------------------------------------------------------    20446
LOC106996293.glutathione.hydrolase5.proenzyme-like-GGT1.rhesus.  actgggcttcccctcaccttgccctggagaagcactccctcctgaggggctgatgcagtc    143632

Papio.anubis.clone.rp41-133b2.8034-40080.revcompl.Baboon        ------------------------------------------------------------    31464
```

```
BCRP3.HUMAN.NCBI.REF                                                 ------------------------------------------------------------    20446
LOC106996293.glutathione.hydrolase5.proenzyme-like-GGT1.rhesus.      attgtcacagagaatctctgttttgttttggagacagtctcgctctgttgcccaggctag    143692

Papio.anubis.clone.rp41-133b2.8034-40080.revcompl.Baboon             ------------------------------------------------------------    31464
BCRP3.HUMAN.NCBI.REF                                                 ------------------------------------------------------------    20446
LOC106996293.glutathione.hydrolase5.proenzyme-like-GGT1.rhesus.      aatggagtggtgcaatcttggctcagtgcaacctctgcctcccaggtcaagcactatgag    143752

Papio.anubis.clone.rp41-133b2.8034-40080.revcompl.Baboon             ------------------------------------------------------------    31464
BCRP3.HUMAN.NCBI.REF                                                 ------------------------------------------------------------    20446
LOC106996293.glutathione.hydrolase5.proenzyme-like-GGT1.rhesus.      caatagccggcagttcctcctcaaattaaaaatacaactactatgtcatctagcaatctc    143812

Papio.anubis.clone.rp41-133b2.8034-40080.revcompl.Baboon             ------------------------------------------------------------    31464
BCRP3.HUMAN.NCBI.REF                                                 ------------------------------------------------------------    20446
LOC106996293.glutathione.hydrolase5.proenzyme-like-GGT1.rhesus.      accactggaaaatacaaagtacatggatgtattatgctccctgattacaaatgacataga    143872

Papio.anubis.clone.rp41-133b2.8034-40080.revcompl.Baboon             ------------------------------------------------------------    31464
BCRP3.HUMAN.NCBI.REF                                                 ------------------------------------------------------------    20446
LOC106996293.glutathione.hydrolase5.proenzyme-like-GGT1.rhesus.      agagctctagtgatccagacaatgtggtattggcattcacacaaaaatacacaacaacaa    143932

Papio.anubis.clone.rp41-133b2.8034-40080.revcompl.Baboon             ------------------------------------------------------------    31464
BCRP3.HUMAN.NCBI.REF                                                 ------------------------------------------------------------    20446
LOC106996293.glutathione.hydrolase5.proenzyme-like-GGT1.rhesus.      gacccaacaagggaaccaaatccaaattcatctatggtggacacatttaaaaaagaacac    143992

Papio.anubis.clone.rp41-133b2.8034-40080.revcompl.Baboon             ------------------------------------------------------------    31464
BCRP3.HUMAN.NCBI.REF                                                 ------------------------------------------------------------    20446
LOC106996293.glutathione.hydrolase5.proenzyme-like-GGT1.rhesus.      ccaggggatgggagaccccgggctgccttgggtggggccgcaaaagtgtcctgggatgac    144052

Papio.anubis.clone.rp41-133b2.8034-40080.revcompl.Baboon             ------------------------------------------------------------    31464
BCRP3.HUMAN.NCBI.REF                                                 ------------------------------------------------------------    20446
LOC106996293.glutathione.hydrolase5.proenzyme-like-GGT1.rhesus.      ctgtagctgcctctcacctgggctccggatggtttccactccatgaggatttccatagtt    144112

Papio.anubis.clone.rp41-133b2.8034-40080.revcompl.Baboon             ------------------------------------------------------------    31464
BCRP3.HUMAN.NCBI.REF                                                 ------------------------------------------------------------    20446
LOC106996293.glutathione.hydrolase5.proenzyme-like-GGT1.rhesus.      tctgttcagccactctcagagtcaggagggcttctttggggctgtgaccatctgtcccag    144172

Papio.anubis.clone.rp41-133b2.8034-40080.revcompl.Baboon             --------------------tgacataggactcatgttcactgtaggatggagatg----    31500
BCRP3.HUMAN.NCBI.REF                                                 ------------------------------------------------------------    20446
LOC106996293.glutathione.hydrolase5.proenzyme-like-GGT1.rhesus.      cctttcctgtctccatgacatgccgtgtgacctacgtatactgcaatatgtaggtgcgtg    144232

Papio.anubis.clone.rp41-133b2.8034-40080.revcompl.Baboon             --gtggagacttaatattaaaatgtattaca-----------------------------    31529
BCRP3.HUMAN.NCBI.REF                                                 ------------------------------------------------------------    20446
LOC106996293.glutathione.hydrolase5.proenzyme-like-GGT1.rhesus.      tactgagggcatacagttaagatgacctacaaccgatgctactcgaagtcttccagagat    144292

Papio.anubis.clone.rp41-133b2.8034-40080.revcompl.Baboon             ------------------------------------------------------------    31529
BCRP3.HUMAN.NCBI.REF                                                 ------------------------------------------------------------    20446
LOC106996293.glutathione.hydrolase5.proenzyme-like-GGT1.rhesus.      ttttcagatatccccagaccaaaacaaactagtgacattccatggttttgcaaactggca    144352

Papio.anubis.clone.rp41-133b2.8034-40080.revcompl.Baboon             ------------------------------------------------------------    31529
BCRP3.HUMAN.NCBI.REF                                                 ------------------------------------------------------------    20446
LOC106996293.glutathione.hydrolase5.proenzyme-like-GGT1.rhesus.      ccagcaactgcacagaaacagctccctatgcaccatcagacactctccagacccgcccct    144412

Papio.anubis.clone.rp41-133b2.8034-40080.revcompl.Baboon             ------------------------------------------------------------    31529
BCRP3.HUMAN.NCBI.REF                                                 ------------------------------------------------------------    20446
LOC106996293.glutathione.hydrolase5.proenzyme-like-GGT1.rhesus.      cgctcacaaatacaacacaggagcatgaaccctggcagtcttcactcctaagggccactt    144472

Papio.anubis.clone.rp41-133b2.8034-40080.revcompl.Baboon             ------------------------------------------------------------    31529
BCRP3.HUMAN.NCBI.REF                                                 ------------------------------------------------------------    20446
LOC106996293.glutathione.hydrolase5.proenzyme-like-GGT1.rhesus.      aacagcctctatccatcaggaagcctttgatctgggccaggctgccgacccacaggggtg    144532

Papio.anubis.clone.rp41-133b2.8034-40080.revcompl.Baboon             ------------------------------------------------------------    31529
BCRP3.HUMAN.NCBI.REF                                                 ------------------------------------------------------------    20446
LOC106996293.glutathione.hydrolase5.proenzyme-like-GGT1.rhesus.      aattgtgcctctaatgctgagtgttgcttcccctcactgggctaacccccatccttgcat    144592

Papio.anubis.clone.rp41-133b2.8034-40080.revcompl.Baboon             ------------------------------------------------------------    31529
BCRP3.HUMAN.NCBI.REF                                                 ------------------------------------------------------------    20446
LOC106996293.glutathione.hydrolase5.proenzyme-like-GGT1.rhesus.      atagaagtttcccagggaaactgctgtctgatatccaggagctcatgtgaaatgctccag    144652

Papio.anubis.clone.rp41-133b2.8034-40080.revcompl.Baboon             -aggccaggcacagtggctcacacctataatcctagcacttcatggagccgaggcaggtg    31588
BCRP3.HUMAN.NCBI.REF                                                 ------------------------------------------------------------    20446
LOC106996293.glutathione.hydrolase5.proenzyme-like-GGT1.rhesus.      caggccgggcgcggtggctcaagcctgtaatcccagcactttgggaggccgagacgggtg    144712

Papio.anubis.clone.rp41-133b2.8034-40080.revcompl.Baboon             gatcacatgaggtcaggagttcgagaccagcctggccaacatggtgaaaccccaactcta    31648
BCRP3.HUMAN.NCBI.REF                                                 ------------------------------------------------------------    20446
LOC106996293.glutathione.hydrolase5.proenzyme-like-GGT1.rhesus.      gatcact--aggtcaggagatcgagaccatcctggctaacacggtgaaaccccatctcta    144770

Papio.anubis.clone.rp41-133b2.8034-40080.revcompl.Baboon             ctaaaaa--tataaaaattagccgggcacagtggcgggtgcctgtaatcccatctacttg    31706
BCRP3.HUMAN.NCBI.REF                                                 ------------------------------------------------------------    20446
LOC106996293.glutathione.hydrolase5.proenzyme-like-GGT1.rhesus.      ctaaaaatacaaaaaactagccgggcgaggtggcaggcgcctgtagtcccagctactca    144830

Papio.anubis.clone.rp41-133b2.8034-40080.revcompl.Baboon             ggaggctgaggcaggagaattgcttgaactcaggaggcggaggttgcagtgagccaagat    31766
BCRP3.HUMAN.NCBI.REF                                                 ------------------------------------------------------------    20446
LOC106996293.glutathione.hydrolase5.proenzyme-like-GGT1.rhesus.      ggaggctaagacaggagaatggcccgaacccgggaggcggagcttgcagtgagctgagat    144890

Papio.anubis.clone.rp41-133b2.8034-40080.revcompl.Baboon             ggtgccactggactccagcctaggcaacagagtgagactccatctcaataaaaataataa    31826
BCRP3.HUMAN.NCBI.REF                                                 ------------------------------------------------------------    20446
LOC106996293.glutathione.hydrolase5.proenzyme-like-GGT1.rhesus.      ccggccactacactccagcctgggcgacagagtgagactccgtctcaaaaaaaaaaaaaa    144950

Papio.anubis.clone.rp41-133b2.8034-40080.revcompl.Baboon             aataaaataaaataaaataaaataattacaattaggct--ccatctgtcgaaagatcttt    31884
BCRP3.HUMAN.NCBI.REF                                                 ------------------------------------------------------------    20446
LOC106996293.glutathione.hydrolase5.proenzyme-like-GGT1.rhesus.      aaaaaaaaaaaaaaagctccagcaacctggatggccttggccacctctagtactagcctt    145010

Papio.anubis.clone.rp41-133b2.8034-40080.revcompl.Baboon             t-----------------------------------------------------------    31885
BCRP3.HUMAN.NCBI.REF                                                 ------------------------------------------------------------    20446
LOC106996293.glutathione.hydrolase5.proenzyme-like-GGT1.rhesus.      tccctctgtggggttcactccctgactcttgcttgtgcaggccaacctacagcggctgag    145070

Papio.anubis.clone.rp41-133b2.8034-40080.revcompl.Baboon             ------------------------------------------------------------    31885
BCRP3.HUMAN.NCBI.REF                                                 ------------------------------------------------------------    20446
LOC106996293.glutathione.hydrolase5.proenzyme-like-GGT1.rhesus.      atctcctgctccctcagcctcctgggtcaccctgatggcatcacgctggccagaggctgg    145130

Papio.anubis.clone.rp41-133b2.8034-40080.revcompl.Baboon             -----------------------------------------------------cagggcc    31892
BCRP3.HUMAN.NCBI.REF                                                 ------------------------------------------------------------    20446
LOC106996293.glutathione.hydrolase5.proenzyme-like-GGT1.rhesus.      tgtctgccatcaagaatgaggtacccacaggctgacttccctggtgtgggaagcaatgcc    145190

Papio.anubis.clone.rp41-133b2.8034-40080.revcompl.Baboon             tgaaggcctgcaagtgagcagcctctgaaaaccaaccagagcagttcatggtcaatgatc    31952
BCRP3.HUMAN.NCBI.REF                                                 ------------------------------------------------------------    20446
LOC106996293.glutathione.hydrolase5.proenzyme-like-GGT1.rhesus.      tggggtctccagacagcccacccgaggaaaagaatccagctctgacacagctcaaaga--    145248

Papio.anubis.clone.rp41-133b2.8034-40080.revcompl.Baboon             tcttatcaggcgaaagttactgacattggtctcttgtccagtcagagctgtagttatggc    32012
BCRP3.HUMAN.NCBI.REF                                                 ------------------------------------------------------------    20446
LOC106996293.glutathione.hydrolase5.proenzyme-like-GGT1.rhesus.      -----------ctgtgttatggacatggagagactttccaggcctgtctcctcttctggc    145297

Papio.anubis.clone.rp41-133b2.8034-40080.revcompl.Baboon             tggaagaacagggggtcaataagtcggcatccgtg-------------------------    32047
BCRP3.HUMAN.NCBI.REF                                                 ------------------------------------------------------------    20446
LOC106996293.glutathione.hydrolase5.proenzyme-like-GGT1.rhesus.      tcagtggattcctaggaaaattattgacacactgggcttaaagctctcagcctcgggccc    145357

Papio.anubis.clone.rp41-133b2.8034-40080.revcompl.Baboon             ------------------------------------------------------------    32047
```

```
BCRP3.HUMAN.NCBI.REF                                            ------------------------------------------------------------    20446
LOC106996293.glutathione.hydrolase5.proenzyme-like-GGT1.rhesus.      aaaatgccacagggaatcaaatcagtgtgaccaaggaccaaagacttctccttggctggg    145417

Papio.anubis.clone.rp41-133b2.8034-40080.revcompl.Baboon             ------------------------------------------------------------    32047
BCRP3.HUMAN.NCBI.REF                                            ------------------------------------------------------------    20446
LOC106996293.glutathione.hydrolase5.proenzyme-like-GGT1.rhesus.      aatgggggatcagggttgcagagaccaggggcgcaaagtgatacctgtgccgtaagagaa    145477

Papio.anubis.clone.rp41-133b2.8034-40080.revcompl.Baboon             ------------------------------------------------------------    32047
BCRP3.HUMAN.NCBI.REF                                            ------------------------------------------------------------    20446
LOC106996293.glutathione.hydrolase5.proenzyme-like-GGT1.rhesus.      gaaactggggatctggtccaccccgggctcctcaacctgagtgtgaggccttgctgggcc    145537

Papio.anubis.clone.rp41-133b2.8034-40080.revcompl.Baboon             ------------------------------------------------------------    32047
BCRP3.HUMAN.NCBI.REF                                            ------------------------------------------------------------    20446
LOC106996293.glutathione.hydrolase5.proenzyme-like-GGT1.rhesus.      ctgagctcacctgccccttgggagtccattgtgactgcagtccagaggggtcaatgtcct    145597

Papio.anubis.clone.rp41-133b2.8034-40080.revcompl.Baboon             ------------------------------------------------------------    32047
BCRP3.HUMAN.NCBI.REF                                            ------------------------------------------------------------    20446
LOC106996293.glutathione.hydrolase5.proenzyme-like-GGT1.rhesus.      cactgatgacagaaagaagtcctgggggatgcggagggcatcgtgtagcggcaggcagtc    145657

Papio.anubis.clone.rp41-133b2.8034-40080.revcompl.Baboon             ------------------------------------------------------------    32047
BCRP3.HUMAN.NCBI.REF                                            ------------------------------------------------------------    20446
LOC106996293.glutathione.hydrolase5.proenzyme-like-GGT1.rhesus.      tgggtggtccacaggtgtgcgcttcagcaggtcctgggaagggatgaggtggtgaattcg    145717

Papio.anubis.clone.rp41-133b2.8034-40080.revcompl.Baboon             ------------------------------------------------------------    32047
BCRP3.HUMAN.NCBI.REF                                            ------------------------------------------------------------    20446
LOC106996293.glutathione.hydrolase5.proenzyme-like-GGT1.rhesus.      gagccgtgcttctgggactcaaatgagaagcccctgggcaatgctcagtcctcaccgaaa    145777

Papio.anubis.clone.rp41-133b2.8034-40080.revcompl.Baboon             ------------------------------------------------------------    32047
BCRP3.HUMAN.NCBI.REF                                            ------------------------------------------------------------    20446
LOC106996293.glutathione.hydrolase5.proenzyme-like-GGT1.rhesus.      gaattcaaagggcaggccctcagggacccacactcatgcctgcaaaatctgaactgaaaa    145837

Papio.anubis.clone.rp41-133b2.8034-40080.revcompl.Baboon             ------------------------------------------------------------    32047
BCRP3.HUMAN.NCBI.REF                                            ------------------------------------------------------------    20446
LOC106996293.glutathione.hydrolase5.proenzyme-like-GGT1.rhesus.      ttagaatcggagcagcagggttgactctaaaagggccccagctggcttttctccctgctg    145897

Papio.anubis.clone.rp41-133b2.8034-40080.revcompl.Baboon             ------------------------------------------------------------    32047
BCRP3.HUMAN.NCBI.REF                                            ------------------------------------------------------------    20446
LOC106996293.glutathione.hydrolase5.proenzyme-like-GGT1.rhesus.      cagtctttactcttttcctctggcagaggcagccaggtggacgctcatctcaccacgcag    145957

Papio.anubis.clone.rp41-133b2.8034-40080.revcompl.Baboon             ------------------------------------------------------------    32047
BCRP3.HUMAN.NCBI.REF                                            ------------------------------------------------------------    20446
LOC106996293.glutathione.hydrolase5.proenzyme-like-GGT1.rhesus.      ctgggagagacacccctctacccaccctgcagctgctccaaggatactggagaccgaaag    146017

Papio.anubis.clone.rp41-133b2.8034-40080.revcompl.Baboon             ------------------------------------------------------------    32047
BCRP3.HUMAN.NCBI.REF                                            ------------------------------------------------------------    20446
LOC106996293.glutathione.hydrolase5.proenzyme-like-GGT1.rhesus.      acaggggttggggaagcaaaatattcctgcctccactgagagcaattggccaaatgccaa    146077

Papio.anubis.clone.rp41-133b2.8034-40080.revcompl.Baboon             ------------------------------------------------------------    32047
BCRP3.HUMAN.NCBI.REF                                            ------------------------------------------------------------    20446
LOC106996293.glutathione.hydrolase5.proenzyme-like-GGT1.rhesus.      gctcagtagctaagctctatttaggccctggccctgttggcatggttaccacaggccagg    146137

Papio.anubis.clone.rp41-133b2.8034-40080.revcompl.Baboon             ------------------------------------------------------------    32047
BCRP3.HUMAN.NCBI.REF                                            ------------------------------------------------------------    20446
LOC106996293.glutathione.hydrolase5.proenzyme-like-GGT1.rhesus.      gaggcatactcagggcccaggatccctctgcctgacttttcaaatggtctgttctcagtg    146197

Papio.anubis.clone.rp41-133b2.8034-40080.revcompl.Baboon             ------------------------------------------------------------    32047
BCRP3.HUMAN.NCBI.REF                                            ------------------------------------------------------------    20446
LOC106996293.glutathione.hydrolase5.proenzyme-like-GGT1.rhesus.      actctggtgccctcccacaacttacttggtcaatgggctggtacagcagagctgttgggg    146257

Papio.anubis.clone.rp41-133b2.8034-40080.revcompl.Baboon             ------------------------------------------------------------    32047
BCRP3.HUMAN.NCBI.REF                                            ------------------------------------------------------------    20446
LOC106996293.glutathione.hydrolase5.proenzyme-like-GGT1.rhesus.      acagcagagagaaaaaggacaaagccatgcaaagaatcactcaacacctcagagaccagt    146317

Papio.anubis.clone.rp41-133b2.8034-40080.revcompl.Baboon             ------------------------------------------------------------    32047
BCRP3.HUMAN.NCBI.REF                                            ------------------------------------------------------------    20446
LOC106996293.glutathione.hydrolase5.proenzyme-like-GGT1.rhesus.      ttgtcccccacccaccttgctccccatatgaacacccgcttttgggaagccaaagtggat    146377

Papio.anubis.clone.rp41-133b2.8034-40080.revcompl.Baboon             ------------------------------------------------------------    32047
BCRP3.HUMAN.NCBI.REF                                            ------------------------------------------------------------    20446
LOC106996293.glutathione.hydrolase5.proenzyme-like-GGT1.rhesus.      ccacagcagcctccacctctgttatcactcccagggcagaccaagcagcaagaacaggtg    146437

Papio.anubis.clone.rp41-133b2.8034-40080.revcompl.Baboon             ------------------------------------------------------------    32047
BCRP3.HUMAN.NCBI.REF                                            ------------------------------------------------------------    20446
LOC106996293.glutathione.hydrolase5.proenzyme-like-GGT1.rhesus.      acacacagacctggtgaccctgtcccctccctatccatgcctactctcccatcagaactg    146497

Papio.anubis.clone.rp41-133b2.8034-40080.revcompl.Baboon             ------------------------------------------------------------    32047
BCRP3.HUMAN.NCBI.REF                                            ------------------------------------------------------------    20446
LOC106996293.glutathione.hydrolase5.proenzyme-like-GGT1.rhesus.      gcttcttcgacagctgccccagtcctgtccaggccttacctggctcagagcagatgatcc    146557

Papio.anubis.clone.rp41-133b2.8034-40080.revcompl.Baboon             ------------------------------------------------------------    32047
BCRP3.HUMAN.NCBI.REF                                            ------------------------------------------------------------    20446
LOC106996293.glutathione.hydrolase5.proenzyme-like-GGT1.rhesus.      cctgcacctccaacctgaatcacatggctacacccagcacatgctacagaggccgagcac    146617

Papio.anubis.clone.rp41-133b2.8034-40080.revcompl.Baboon             ------------------------------------------------------------    32047
BCRP3.HUMAN.NCBI.REF                                            ------------------------------------------------------------    20446
LOC106996293.glutathione.hydrolase5.proenzyme-like-GGT1.rhesus.      actctgggtcacatccagaaactccacaataggggaggagacaagctgtttcccaggaag    146677

Papio.anubis.clone.rp41-133b2.8034-40080.revcompl.Baboon             ------------------------------------------------------------    32047
BCRP3.HUMAN.NCBI.REF                                            ------------------------------------------------------------    20446
LOC106996293.glutathione.hydrolase5.proenzyme-like-GGT1.rhesus.      ggcagctccccaggcctggtcattctcagactcctccagtcaaaggccaggcacagccag    146737

Papio.anubis.clone.rp41-133b2.8034-40080.revcompl.Baboon             ------------------------------------------------------------    32047
BCRP3.HUMAN.NCBI.REF                                            ------------------------------------------------------------    20446
LOC106996293.glutathione.hydrolase5.proenzyme-like-GGT1.rhesus.      ctgggatcaccatgcttcactctgacccacacgactgaccttgcctcctctatgggaaga    146797

Papio.anubis.clone.rp41-133b2.8034-40080.revcompl.Baboon             ------------------------------------------------------------    32047
BCRP3.HUMAN.NCBI.REF                                            ------------------------------------------------------------    20446
LOC106996293.glutathione.hydrolase5.proenzyme-like-GGT1.rhesus.      gacccctgcacacagcctcaaacccagacataacaggacaggacacgctgggcgctaggg    146857

Papio.anubis.clone.rp41-133b2.8034-40080.revcompl.Baboon             ------------------------------------------------------------    32047
BCRP3.HUMAN.NCBI.REF                                            ------------------------------------------------------------    20446
LOC106996293.glutathione.hydrolase5.proenzyme-like-GGT1.rhesus.      aggccagccagatctccacacaaaggctctgctcttaagaaggaggtagcctcaggggca    146917

Papio.anubis.clone.rp41-133b2.8034-40080.revcompl.Baboon             ------------------------------------------------------------    32047
BCRP3.HUMAN.NCBI.REF                                            ------------------------------------------------------------    20446
LOC106996293.glutathione.hydrolase5.proenzyme-like-GGT1.rhesus.      aaggtttcctgaagcaactcaggtgaggcctcacatacaagagcccaaaagaggctgcaa    146977

Papio.anubis.clone.rp41-133b2.8034-40080.revcompl.Baboon             ------------------------------------------------------------    32047
BCRP3.HUMAN.NCBI.REF                                            ------------------------------------------------------------    20446
LOC106996293.glutathione.hydrolase5.proenzyme-like-GGT1.rhesus.      aactacaagctcagccagccaagaacccagggccacacccttctgaaacccagactccaa    147037

Papio.anubis.clone.rp41-133b2.8034-40080.revcompl.Baboon             ------------------------------------------------------------    32047
BCRP3.HUMAN.NCBI.REF                                            ------------------------------------------------------------    20446
LOC106996293.glutathione.hydrolase5.proenzyme-like-GGT1.rhesus.      gtgaggatcactaggattcagcagaacagcaggacgtgacagctggaggaccaacactta    147097

Papio.anubis.clone.rp41-133b2.8034-40080.revcompl.Baboon             ------------------------------------------------------------    32047
```

```
BCRP3.HUMAN.NCBI.REF                                                 ------------------------------------------------------------    20446
LOC106996293.glutathione.hydrolase5.proenzyme-like-GGT1.rhesus.      ccaacacccttccagcaagcagctgtgcctcgcccctgcccacatgactcacccaacatc    147157

Papio.anubis.clone.rp41-133b2.8034-40080.revcompl.Baboon             ------------------------------------------------------------    32047
BCRP3.HUMAN.NCBI.REF                                                 ------------------------------------------------------------    20446
LOC106996293.glutathione.hydrolase5.proenzyme-like-GGT1.rhesus.      agtcttgcacctggatgcctctgtccacagggtcatgatctttgacctcctgccatagaa    147217

Papio.anubis.clone.rp41-133b2.8034-40080.revcompl.Baboon             ------------------------------------------------------------    32047
BCRP3.HUMAN.NCBI.REF                                                 ------------------------------------------------------------    20446
LOC106996293.glutathione.hydrolase5.proenzyme-like-GGT1.rhesus.      gtggtgagacacatgatggtggtctgggaagaaggatcctagactgggtgtcccatagga    147277

Papio.anubis.clone.rp41-133b2.8034-40080.revcompl.Baboon             ------------------------------------------------------------    32047
BCRP3.HUMAN.NCBI.REF                                                 ------------------------------------------------------------    20446
LOC106996293.glutathione.hydrolase5.proenzyme-like-GGT1.rhesus.      ccaggttgtgacagatcccacagccaacagtcatgccccagccaacccccaaacgcaaca    147337

Papio.anubis.clone.rp41-133b2.8034-40080.revcompl.Baboon             ------------------------------------------------------------    32047
BCRP3.HUMAN.NCBI.REF                                                 ------------------------------------------------------------    20446
LOC106996293.glutathione.hydrolase5.proenzyme-like-GGT1.rhesus.      gctccccaaagcccttgatgtaaatagcctgtgcactgctaagcagacacctcagggaac    147397

Papio.anubis.clone.rp41-133b2.8034-40080.revcompl.Baboon             ------------------------------------------------------------    32047
BCRP3.HUMAN.NCBI.REF                                                 ------------------------------------------------------------    20446
LOC106996293.glutathione.hydrolase5.proenzyme-like-GGT1.rhesus.      aatctgatggctgtgaagaagatgcagcctcaacagggttccacaagccttctgcaagca    147457

Papio.anubis.clone.rp41-133b2.8034-40080.revcompl.Baboon             ------------------------------------------------------------    32047
BCRP3.HUMAN.NCBI.REF                                                 ------------------------------------------------------------    20446
LOC106996293.glutathione.hydrolase5.proenzyme-like-GGT1.rhesus.      aagccttctttgcttgggtgcctagtcaaagcactagggaagatcttaaaagataaattc    147517

Papio.anubis.clone.rp41-133b2.8034-40080.revcompl.Baboon             ------------------------------------------------------------    32047
BCRP3.HUMAN.NCBI.REF                                                 ------------------------------------------------------------    20446
LOC106996293.glutathione.hydrolase5.proenzyme-like-GGT1.rhesus.      agcattgtgggaggcagaggcaggagtacagcttgaggttaggactttaagaccagcctg    147577

Papio.anubis.clone.rp41-133b2.8034-40080.revcompl.Baboon             ------------------------------------------------------------    32047
BCRP3.HUMAN.NCBI.REF                                                 ------------------------------------------------------------    20446
LOC106996293.glutathione.hydrolase5.proenzyme-like-GGT1.rhesus.      agtgcaatggtgagaccctgtctctacaaaacacaaaaattagcatggcatggtggtaca    147637

Papio.anubis.clone.rp41-133b2.8034-40080.revcompl.Baboon             ------------------------------------------------------------    32047
BCRP3.HUMAN.NCBI.REF                                                 ------------------------------------------------------------    20446
LOC106996293.glutathione.hydrolase5.proenzyme-like-GGT1.rhesus.      tgcccatagtcccagccacttgggaggctgagaagggaggattccttgagcctgggaggt    147697

Papio.anubis.clone.rp41-133b2.8034-40080.revcompl.Baboon             ------------------------------------------------------------    32047
BCRP3.HUMAN.NCBI.REF                                                 ------------------------------------------------------------    20446
LOC106996293.glutathione.hydrolase5.proenzyme-like-GGT1.rhesus.      caaggttgcagtgcccatgatcatgccactgtgttacaacctgggccacagcacaagatc    147757

Papio.anubis.clone.rp41-133b2.8034-40080.revcompl.Baboon             ------------------------------------------------------------    32047
BCRP3.HUMAN.NCBI.REF                                                 ------------------------------------------------------------    20446
LOC106996293.glutathione.hydrolase5.proenzyme-like-GGT1.rhesus.      ctgttacaatcaaacaaacaaacaaaaaaccaaagatgcctgttttctgccccatcaaat    147817

Papio.anubis.clone.rp41-133b2.8034-40080.revcompl.Baboon             ------------------------------------------------------------    32047
BCRP3.HUMAN.NCBI.REF                                                 ------------------------------------------------------------    20446
LOC106996293.glutathione.hydrolase5.proenzyme-like-GGT1.rhesus.      aaacccaaatcgcagggtgacacagggtattaacatttttaaatatctcctttcctgaag    147877

Papio.anubis.clone.rp41-133b2.8034-40080.revcompl.Baboon             ------------------------------------------------------------    32047
BCRP3.HUMAN.NCBI.REF                                                 ------------------------------------------------------------    20446
LOC106996293.glutathione.hydrolase5.proenzyme-like-GGT1.rhesus.      taaatgtatagtaatggctgaaaaccactgagttatgtaaatacacttcatatgacttat    147937

Papio.anubis.clone.rp41-133b2.8034-40080.revcompl.Baboon             ------------------------------------------------------------    32047
BCRP3.HUMAN.NCBI.REF                                                 ------------------------------------------------------------    20446
LOC106996293.glutathione.hydrolase5.proenzyme-like-GGT1.rhesus.      ttgtagccctttcgtgtctagcaatttctacagaccctctccgtgggctttggaagccgg    147997

Papio.anubis.clone.rp41-133b2.8034-40080.revcompl.Baboon             ------------------------------------------------------------    32047
BCRP3.HUMAN.NCBI.REF                                                 ------------------------------------------------------------    20446
LOC106996293.glutathione.hydrolase5.proenzyme-like-GGT1.rhesus.      aatgggcagggccaggtgtgaggtatcctagcacactgaggctagagcacagctcccagc    148057

Papio.anubis.clone.rp41-133b2.8034-40080.revcompl.Baboon             ------------------------------------------------------------    32047
BCRP3.HUMAN.NCBI.REF                                                 ------------------------------------------------------------    20446
LOC106996293.glutathione.hydrolase5.proenzyme-like-GGT1.rhesus.      tcaggggacccatttctgaggctaaggcacccaccagggactcagcctatcttcagagg    148117

Papio.anubis.clone.rp41-133b2.8034-40080.revcompl.Baboon             ------------------------------------------------------------    32047
BCRP3.HUMAN.NCBI.REF                                                 ------------------------------------------------------------    20446
LOC106996293.glutathione.hydrolase5.proenzyme-like-GGT1.rhesus.      gaaaatcacacctcagaaccccagagacccaaaagaaagctcgaatttggcagatacctg    148177

Papio.anubis.clone.rp41-133b2.8034-40080.revcompl.Baboon             ------------------------------------------------------------    32047
BCRP3.HUMAN.NCBI.REF                                                 ------------------------------------------------------------    20446
LOC106996293.glutathione.hydrolase5.proenzyme-like-GGT1.rhesus.      ccagtgggtcctggagcggcagccccagggaaaaccgccattgcactgtcagctccctcc    148237

Papio.anubis.clone.rp41-133b2.8034-40080.revcompl.Baboon             ------------------------------------------------------------    32047
BCRP3.HUMAN.NCBI.REF                                                 ------------------------------------------------------------    20446
LOC106996293.glutathione.hydrolase5.proenzyme-like-GGT1.rhesus.      agccttctctcaggtcccagggggtggaggtctctgactaagctcaaaggtctcaacaaa    148297

Papio.anubis.clone.rp41-133b2.8034-40080.revcompl.Baboon             ------------------------------------------------------------    32047
BCRP3.HUMAN.NCBI.REF                                                 ------------------------------------------------------------    20446
LOC106996293.glutathione.hydrolase5.proenzyme-like-GGT1.rhesus.      cggcgggttgaacctctgaggagttattaacaggcctataattacctcaagactccacct    148357

Papio.anubis.clone.rp41-133b2.8034-40080.revcompl.Baboon             ------------------------------------------------------------    32047
BCRP3.HUMAN.NCBI.REF                                                 ------------------------------------------------------------    20446
LOC106996293.glutathione.hydrolase5.proenzyme-like-GGT1.rhesus.      gaccagggccaaaaatgagtcctgggagggcagccaggaagagagcagagcagagaggaa    148417

Papio.anubis.clone.rp41-133b2.8034-40080.revcompl.Baboon             ------------------------------------------------------------    32047
BCRP3.HUMAN.NCBI.REF                                                 ------------------------------------------------------------    20446
LOC106996293.glutathione.hydrolase5.proenzyme-like-GGT1.rhesus.      acacagggagagggggccctgggaccacatcagatatgggggaaccaggggaacgaggg    148477

Papio.anubis.clone.rp41-133b2.8034-40080.revcompl.Baboon             ------------------------------------------------------------    32047
BCRP3.HUMAN.NCBI.REF                                                 ------------------------------------------------------------    20446
LOC106996293.glutathione.hydrolase5.proenzyme-like-GGT1.rhesus.      cagccctgatgattgtgctccccataggccctgcatgtctgtgtcccctgagaattcagg    148537

Papio.anubis.clone.rp41-133b2.8034-40080.revcompl.Baboon             ------------------------------------------------------------    32047
BCRP3.HUMAN.NCBI.REF                                                 ------------------------------------------------------------    20446
LOC106996293.glutathione.hydrolase5.proenzyme-like-GGT1.rhesus.      gtgaggagctcactcttactcttcacctgttcctgggctctggcaggctggcctgcttcc    148597

Papio.anubis.clone.rp41-133b2.8034-40080.revcompl.Baboon             ------------------------------------------------------------    32047
BCRP3.HUMAN.NCBI.REF                                                 ------------------------------------------------------------    20446
LOC106996293.glutathione.hydrolase5.proenzyme-like-GGT1.rhesus.      tgccccagcctcacccactctccccactcctcaccactgatgtgggcaagttgatctctc    148657

Papio.anubis.clone.rp41-133b2.8034-40080.revcompl.Baboon             ------------------------------------------------------------    32047
BCRP3.HUMAN.NCBI.REF                                                 ------------------------------------------------------------    20446
LOC106996293.glutathione.hydrolase5.proenzyme-like-GGT1.rhesus.      tggtttgctggcctctcactctctgcccaatggcaccgagttaacttctttgcatgcttt    148717

Papio.anubis.clone.rp41-133b2.8034-40080.revcompl.Baboon             ------------------------------------------------------------    32047
BCRP3.HUMAN.NCBI.REF                                                 ------------------------------------------------------------    20446
LOC106996293.glutathione.hydrolase5.proenzyme-like-GGT1.rhesus.      attttctgtgattaaaaagggaagcaattctcactaacgccatttctcagaggagaaaac    148777

Papio.anubis.clone.rp41-133b2.8034-40080.revcompl.Baboon             ------------------------------------------------------------    32047
BCRP3.HUMAN.NCBI.REF                                                 ------------------------------------------------------------    20446
LOC106996293.glutathione.hydrolase5.proenzyme-like-GGT1.rhesus.      aggcacagggatgttctatgacttcctcatggtcaccaagctacaagtgggagctgggct    148837

Papio.anubis.clone.rp41-133b2.8034-40080.revcompl.Baboon             ------------------------------------------------------------    32047
```

```
BCRP3.HUMAN.NCBI.REF                                              ------------------------------------------------------------    20446
LOC106996293.glutathione.hydrolase5.proenzyme-like-GGT1.rhesus.   ttgaatggaggtctacccatgctcctaaatttgagccgtcctccaaagaaaggacagcac    148897

Papio.anubis.clone.rp41-133b2.8034-40080.revcompl.Baboon          ------------------------------------------------------------    32047
BCRP3.HUMAN.NCBI.REF                                              ------------------------------------------------------------    20446
LOC106996293.glutathione.hydrolase5.proenzyme-like-GGT1.rhesus.   tgtaaaagctcagtgaatgtggcaggagacttcactaaggcctgaccaggctggtaccct    148957

Papio.anubis.clone.rp41-133b2.8034-40080.revcompl.Baboon          ------------------------------------------------------------    32047
BCRP3.HUMAN.NCBI.REF                                              ------------------------------------------------------------    20446
LOC106996293.glutathione.hydrolase5.proenzyme-like-GGT1.rhesus.   gatatccaacttctagcctccagaactgggagatacataattctgtttttgacgagctcc    149017

Papio.anubis.clone.rp41-133b2.8034-40080.revcompl.Baboon          ------------------------------------------------------------    32047
BCRP3.HUMAN.NCBI.REF                                              ------------------------------------------------------------    20446
LOC106996293.glutathione.hydrolase5.proenzyme-like-GGT1.rhesus.   ccaggctacggctctttgttgtaacatcctgaactaacccagaagtgcgttccatcatgt    149077

Papio.anubis.clone.rp41-133b2.8034-40080.revcompl.Baboon          ------------------------------------------------------------    32047
BCRP3.HUMAN.NCBI.REF                                              ------------------------------------------------------------    20446
LOC106996293.glutathione.hydrolase5.proenzyme-like-GGT1.rhesus.   gtgtcctattccatcattcagaagggagtcactaacccctcagtgctgtctttgtggatg    149137

Papio.anubis.clone.rp41-133b2.8034-40080.revcompl.Baboon          ------------------------------------------------------------    32047
BCRP3.HUMAN.NCBI.REF                                              ------------------------------------------------------------    20446
LOC106996293.glutathione.hydrolase5.proenzyme-like-GGT1.rhesus.   ctgcccaccctcggctgagatggggcacagggtctgggtctaaataaaattggaaggcac    149197

Papio.anubis.clone.rp41-133b2.8034-40080.revcompl.Baboon          ------------------------------------------------------------    32047
BCRP3.HUMAN.NCBI.REF                                              ------------------------------------------------------------    20446
LOC106996293.glutathione.hydrolase5.proenzyme-like-GGT1.rhesus.   tggaaggtgaaatgtcagaggaggaggatgtctgaatttcgtttaatctcaacctctact    149257

Papio.anubis.clone.rp41-133b2.8034-40080.revcompl.Baboon          ------------------------------------------------------------    32047
BCRP3.HUMAN.NCBI.REF                                              ------------------------------------------------------------    20446
LOC106996293.glutathione.hydrolase5.proenzyme-like-GGT1.rhesus.   ggtgcatcatgaaagaggaggcccagagcaaaagtgtcttctcctgagaccacactgtgg    149317

Papio.anubis.clone.rp41-133b2.8034-40080.revcompl.Baboon          ------------------------------------------------------------    32047
BCRP3.HUMAN.NCBI.REF                                              ------------------------------------------------------------    20446
LOC106996293.glutathione.hydrolase5.proenzyme-like-GGT1.rhesus.   accccaggcccactggagttctcttccatgctacctctcaacgcttctggtatttcttcc    149377

Papio.anubis.clone.rp41-133b2.8034-40080.revcompl.Baboon          ------------------------------------------------------------    32047
BCRP3.HUMAN.NCBI.REF                                              ------------------------------------------------------------    20446
LOC106996293.glutathione.hydrolase5.proenzyme-like-GGT1.rhesus.   atctgtaagtgtgtgcaatatctacagggaacatcacatcatgagttttataagatctat    149437

Papio.anubis.clone.rp41-133b2.8034-40080.revcompl.Baboon          ------------------------------------------------------------    32047
BCRP3.HUMAN.NCBI.REF                                              ------------------------------------------------------------    20446
LOC106996293.glutathione.hydrolase5.proenzyme-like-GGT1.rhesus.   ctcaaacacacctcatgagttccctaggaaggagatgctagcataacccccattgtgcgt    149497

Papio.anubis.clone.rp41-133b2.8034-40080.revcompl.Baboon          ------------------------------------------------------------    32047
BCRP3.HUMAN.NCBI.REF                                              ------------------------------------------------------------    20446
LOC106996293.glutathione.hydrolase5.proenzyme-like-GGT1.rhesus.   gctgggaaggccccaaaacaccaaaggagttctctagggtctcacaactgctaagataaa    149557

Papio.anubis.clone.rp41-133b2.8034-40080.revcompl.Baboon          ------------------------------------------------------------    32047
BCRP3.HUMAN.NCBI.REF                                              ------------------------------------------------------------    20446
LOC106996293.glutathione.hydrolase5.proenzyme-like-GGT1.rhesus.   ggttgtttgacccagaactgtctccttaaaactgggaccctggttacatccaggtcttcc    149617

Papio.anubis.clone.rp41-133b2.8034-40080.revcompl.Baboon          ------------------------------------------------------------    32047
BCRP3.HUMAN.NCBI.REF                                              ------------------------------------------------------------    20446
LOC106996293.glutathione.hydrolase5.proenzyme-like-GGT1.rhesus.   caaggtgctgaaggggcacactttggcataatggtgaaaggtaaggagttgacaggggag    149677

Papio.anubis.clone.rp41-133b2.8034-40080.revcompl.Baboon          ------------------------------------------------------------    32047
BCRP3.HUMAN.NCBI.REF                                              ------------------------------------------------------------    20446
LOC106996293.glutathione.hydrolase5.proenzyme-like-GGT1.rhesus.   ggtgagcggccagaagcccaatgaggccttgagtaggtcttatggaaaggaggagcttac    149737

Papio.anubis.clone.rp41-133b2.8034-40080.revcompl.Baboon          ------------------------------------------------------------    32047
BCRP3.HUMAN.NCBI.REF                                              ------------------------------------------------------------    20446
LOC106996293.glutathione.hydrolase5.proenzyme-like-GGT1.rhesus.   aggatggaacctacaagaagtgacctcacttccttgctgacagaccccaacagaacttag    149797

Papio.anubis.clone.rp41-133b2.8034-40080.revcompl.Baboon          ------------------------------------------------------------    32047
BCRP3.HUMAN.NCBI.REF                                              ------------------------------------------------------------    20446
LOC106996293.glutathione.hydrolase5.proenzyme-like-GGT1.rhesus.   aattctggtaactaggcacccatattatacacacagccccatttccagctcagttggtag    149857

Papio.anubis.clone.rp41-133b2.8034-40080.revcompl.Baboon          ------------------------------------------------------------    32047
BCRP3.HUMAN.NCBI.REF                                              ------------------------------------------------------------    20446
LOC106996293.glutathione.hydrolase5.proenzyme-like-GGT1.rhesus.   ttgacacccaagggaacaacatgttctcttgctgtgtcccgacgtgctgtcgacctcctc    149917

Papio.anubis.clone.rp41-133b2.8034-40080.revcompl.Baboon          ------------------------------------------------------------    32047
BCRP3.HUMAN.NCBI.REF                                              ------------------------------------------------------------    20446
LOC106996293.glutathione.hydrolase5.proenzyme-like-GGT1.rhesus.   gtcgcacaagaggccaaaatgagagtctttctcgagaatatagacattggttcaaccctc    149977

Papio.anubis.clone.rp41-133b2.8034-40080.revcompl.Baboon          ------------------------------------------------------------    32047
BCRP3.HUMAN.NCBI.REF                                              ------------------------------------------------------------    20446
LOC106996293.glutathione.hydrolase5.proenzyme-like-GGT1.rhesus.   accctcgacgcccctggccgtttgccagaaggcacccacaggtaacacaggacccagggc    150037

Papio.anubis.clone.rp41-133b2.8034-40080.revcompl.Baboon          ------------------------------------------------------------    32047
BCRP3.HUMAN.NCBI.REF                                              ------------------------------------------------------------    20446
LOC106996293.glutathione.hydrolase5.proenzyme-like-GGT1.rhesus.   aggcccgttgaggccaggcctcagctgtgtccatcagggcagcgctgagcccctccccat    150097

Papio.anubis.clone.rp41-133b2.8034-40080.revcompl.Baboon          ------------------------------------------------------------    32047
BCRP3.HUMAN.NCBI.REF                                              ------------------------------------------------------------    20446
LOC106996293.glutathione.hydrolase5.proenzyme-like-GGT1.rhesus.   gtgtttgggggaaacaggagaagaggggccctccctggacaggagcaggtaggttgttgg    150157

Papio.anubis.clone.rp41-133b2.8034-40080.revcompl.Baboon          ------------------------------------------------------------    32047
BCRP3.HUMAN.NCBI.REF                                              ------------------------------------------------------------    20446
LOC106996293.glutathione.hydrolase5.proenzyme-like-GGT1.rhesus.   gggtttggagcctgataggtatgtcaggttcacagcccaggccatgccttcaggatggga    150217

Papio.anubis.clone.rp41-133b2.8034-40080.revcompl.Baboon          ------------------------------------------------------------    32047
BCRP3.HUMAN.NCBI.REF                                              ------------------------------------------------------------    20446
LOC106996293.glutathione.hydrolase5.proenzyme-like-GGT1.rhesus.   aggtgactgtcgaggacacagtttgtctgctcagatctgaattccaagacctcaacctgt    150277

Papio.anubis.clone.rp41-133b2.8034-40080.revcompl.Baboon          ------------------------------------------------------------    32047
BCRP3.HUMAN.NCBI.REF                                              ------------------------------------------------------------    20446
LOC106996293.glutathione.hydrolase5.proenzyme-like-GGT1.rhesus.   catctgccggagacctcaagctcctctggctggaggtgtacgcagatcctcctgtgtgcc    150337

Papio.anubis.clone.rp41-133b2.8034-40080.revcompl.Baboon          ------------------------------------------------------------    32047
BCRP3.HUMAN.NCBI.REF                                              ------------------------------------------------------------    20446
LOC106996293.glutathione.hydrolase5.proenzyme-like-GGT1.rhesus.   aaatcctgttggggaagggggtgccagggaggagttctaatggggtatcccactatgggg    150397

Papio.anubis.clone.rp41-133b2.8034-40080.revcompl.Baboon          ------------------------------------------------------------    32047
BCRP3.HUMAN.NCBI.REF                                              ------------------------------------------------------------    20446
LOC106996293.glutathione.hydrolase5.proenzyme-like-GGT1.rhesus.   tcagccaggcaggccactgacacgtcttcgcctggctcactctctttgcagagctccaca    150457

Papio.anubis.clone.rp41-133b2.8034-40080.revcompl.Baboon          ------------------------------------------------------------    32047
BCRP3.HUMAN.NCBI.REF                                              ------------------------------------------------------------    20446
LOC106996293.glutathione.hydrolase5.proenzyme-like-GGT1.rhesus.   cagcagatcaagcaggagctgttggatggattccatttctccaccttcttcaaagaaggg    150517

Papio.anubis.clone.rp41-133b2.8034-40080.revcompl.Baboon          ------------------------------------------------------------    32047
BCRP3.HUMAN.NCBI.REF                                              ------------------------------------------------------------    20446
LOC106996293.glutathione.hydrolase5.proenzyme-like-GGT1.rhesus.   caggggccgcctgctaccaaccacggccagtgctggtctggggtgagactgggcacagaa    150577

Papio.anubis.clone.rp41-133b2.8034-40080.revcompl.Baboon          ------------------------------------------------------------    32047
```

```
BCRP3.HUMAN.NCBI.REF                                               ------------------------------------------------------------    20446
LOC106996293.glutathione.hydrolase5.proenzyme-like-GGT1.rhesus.    gggtctccacagacagggactccagaaaaccagggtgggcccaggactcaaacacgaata    150637

Papio.anubis.clone.rp41-133b2.8034-40080.revcompl.Baboon           ------------------------------------------------------------    32047
BCRP3.HUMAN.NCBI.REF                                               ------------------------------------------------------------    20446
LOC106996293.glutathione.hydrolase5.proenzyme-like-GGT1.rhesus.    tggggactccctaggctctgtcctagggctttcacacttgagtgagccactgtcctctcc    150697

Papio.anubis.clone.rp41-133b2.8034-40080.revcompl.Baboon           ------------------------------------------------------------    32047
BCRP3.HUMAN.NCBI.REF                                               ------------------------------------------------------------    20446
LOC106996293.glutathione.hydrolase5.proenzyme-like-GGT1.rhesus.    caaatgaatctggctgccattagtccccagtggcaacttcgttacaggcaaagtcccttt    150757

Papio.anubis.clone.rp41-133b2.8034-40080.revcompl.Baboon           ------------------------------------------------------------    32047
BCRP3.HUMAN.NCBI.REF                                               ------------------------------------------------------------    20446
LOC106996293.glutathione.hydrolase5.proenzyme-like-GGT1.rhesus.    cacctatcagaggaagatcagagaaaatccttctgttttcactttctgttatgccaggag    150817

Papio.anubis.clone.rp41-133b2.8034-40080.revcompl.Baboon           ------------------------------------------------------------    32047
BCRP3.HUMAN.NCBI.REF                                               ------------------------------------------------------------    20446
LOC106996293.glutathione.hydrolase5.proenzyme-like-GGT1.rhesus.    tatctcagcatgttgctacaagaattgggtacaatggtaacactgtagcaacatactcat    150877

Papio.anubis.clone.rp41-133b2.8034-40080.revcompl.Baboon           ------------------------------------------------------------    32047
BCRP3.HUMAN.NCBI.REF                                               ------------------------------------------------------------    20446
LOC106996293.glutathione.hydrolase5.proenzyme-like-GGT1.rhesus.    actgttaccattttaactccccaccctaattgtacgtctggcaaccggcagctcctctgc    150937

Papio.anubis.clone.rp41-133b2.8034-40080.revcompl.Baboon           ------------------------------------------------------------    32047
BCRP3.HUMAN.NCBI.REF                                               ------------------------------------------------------------    20446
LOC106996293.glutathione.hydrolase5.proenzyme-like-GGT1.rhesus.    cctgccagacctcctccaggatttaaaatccaacacttctacacaaacaagttgggggag    150997

Papio.anubis.clone.rp41-133b2.8034-40080.revcompl.Baboon           ------------------------------------------------------------    32047
BCRP3.HUMAN.NCBI.REF                                               ------------------------------------------------------------    20446
LOC106996293.glutathione.hydrolase5.proenzyme-like-GGT1.rhesus.    tggtgattacaacaaaagaaggtgaagctactctatgcatctgtgtgtaaagtgtcatct    151057

Papio.anubis.clone.rp41-133b2.8034-40080.revcompl.Baboon           ------------------------------------------------------------    32047
BCRP3.HUMAN.NCBI.REF                                               ------------------------------------------------------------    20446
LOC106996293.glutathione.hydrolase5.proenzyme-like-GGT1.rhesus.    cacagaacacatccctgcagacccaccaggcaggagctgtacccacgcatgtcactgtaa    151117

Papio.anubis.clone.rp41-133b2.8034-40080.revcompl.Baboon           ------------------------------------------------------------    32047
BCRP3.HUMAN.NCBI.REF                                               ------------------------------------------------------------    20446
LOC106996293.glutathione.hydrolase5.proenzyme-like-GGT1.rhesus.    tattgcttaagggaggccatgttccacagtgggtcagcctctcagtgctggtggagcctg    151177

Papio.anubis.clone.rp41-133b2.8034-40080.revcompl.Baboon           ------------------------------------------------------------    32047
BCRP3.HUMAN.NCBI.REF                                               ------------------------------------------------------------    20446
LOC106996293.glutathione.hydrolase5.proenzyme-like-GGT1.rhesus.    tcgtgataaaatgtcctttctaggcagcctttctacagggagcataatcccatgtgtgtc    151237

Papio.anubis.clone.rp41-133b2.8034-40080.revcompl.Baboon           ------------------------------------------------------------    32047
BCRP3.HUMAN.NCBI.REF                                               ------------------------------------------------------------    20446
LOC106996293.glutathione.hydrolase5.proenzyme-like-GGT1.rhesus.    ctaataggctggagcagtttcccggggctgccataacatgcacgctgactgaagggctta    151297

Papio.anubis.clone.rp41-133b2.8034-40080.revcompl.Baboon           ------------------------------------------------------------    32047
BCRP3.HUMAN.NCBI.REF                                               ------------------------------------------------------------    20446
LOC106996293.glutathione.hydrolase5.proenzyme-like-GGT1.rhesus.    accacagaaatctggttcctcacagttctggagactggaagtccaacgtcaagctgtcag    151357

Papio.anubis.clone.rp41-133b2.8034-40080.revcompl.Baboon           ------------------------------------------------------------    32047
BCRP3.HUMAN.NCBI.REF                                               ------------------------------------------------------------    20446
LOC106996293.glutathione.hydrolase5.proenzyme-like-GGT1.rhesus.    cctggggtggtttgtctgggcctctctccttgccttggagatggctgacttctatctcgg    151417

Papio.anubis.clone.rp41-133b2.8034-40080.revcompl.Baboon           ------------------------------------------------------------    32047
BCRP3.HUMAN.NCBI.REF                                               ------------------------------------------------------------    20446
LOC106996293.glutathione.hydrolase5.proenzyme-like-GGT1.rhesus.    tcagcacagggtcttctcttcaacttctctgtgcgctgaccacctctccttgtaagaacc    151477

Papio.anubis.clone.rp41-133b2.8034-40080.revcompl.Baboon           ------------------------------------------------------------    32047
BCRP3.HUMAN.NCBI.REF                                               ------------------------------------------------------------    20446
LOC106996293.glutathione.hydrolase5.proenzyme-like-GGT1.rhesus.    ccaggcatattaaattaggggccaccctaatgaactcacttcacctgaattacctcttgg    151537

Papio.anubis.clone.rp41-133b2.8034-40080.revcompl.Baboon           ------------------------------------------------------------    32047
BCRP3.HUMAN.NCBI.REF                                               ------------------------------------------------------------    20446
LOC106996293.glutathione.hydrolase5.proenzyme-like-GGT1.rhesus.    agaactctgtgtccaaacacagtcacattctgagtctctggggttgagaatagaaacagt    151597

Papio.anubis.clone.rp41-133b2.8034-40080.revcompl.Baboon           ------------------------------------------------------------    32047
BCRP3.HUMAN.NCBI.REF                                               ------------------------------------------------------------    20446
LOC106996293.glutathione.hydrolase5.proenzyme-like-GGT1.rhesus.    tacatttggggggaggggacacaattcaacccataaaatacataaatcccctgcggaagac    151657

Papio.anubis.clone.rp41-133b2.8034-40080.revcompl.Baboon           ------------------------------------------------------------    32047
BCRP3.HUMAN.NCBI.REF                                               ------------------------------------------------------------    20446
LOC106996293.glutathione.hydrolase5.proenzyme-like-GGT1.rhesus.    acacacccgggaaagggaaatctgggtgaaaggatgtgcagatagcaagtttcaacagct    151717

Papio.anubis.clone.rp41-133b2.8034-40080.revcompl.Baboon           ------------------------------------------------------------    32047
BCRP3.HUMAN.NCBI.REF                                               ------------------------------------------------------------    20446
LOC106996293.glutathione.hydrolase5.proenzyme-like-GGT1.rhesus.    ctagccagctgactttccaaaagggctccagctgctcacagggtcaccagcagcctctga    151777

Papio.anubis.clone.rp41-133b2.8034-40080.revcompl.Baboon           ------------------------------------------------------------    32047
BCRP3.HUMAN.NCBI.REF                                               ------------------------------------------------------------    20446
LOC106996293.glutathione.hydrolase5.proenzyme-like-GGT1.rhesus.    aggtagctgcttctttgaatgcaacctcccctgaatactaacatgttcttaactttttgc    151837

Papio.anubis.clone.rp41-133b2.8034-40080.revcompl.Baboon           ------------------------------------------------------------    32047
BCRP3.HUMAN.NCBI.REF                                               ------------------------------------------------------------    20446
LOC106996293.glutathione.hydrolase5.proenzyme-like-GGT1.rhesus.    caatctgctgtctgtgtggcagagaaaattaccttgtttcatttccagtttcaaacatac    151897

Papio.anubis.clone.rp41-133b2.8034-40080.revcompl.Baboon           ------------------------------------------------------------    32047
BCRP3.HUMAN.NCBI.REF                                               ------------------------------------------------------------    20446
LOC106996293.glutathione.hydrolase5.proenzyme-like-GGT1.rhesus.    atttgcgtaacctttacagcttcagtacctgcctgtgttttctcttccatgagtcgcct    151957

Papio.anubis.clone.rp41-133b2.8034-40080.revcompl.Baboon           ------------------------------------------------------------    32047
BCRP3.HUMAN.NCBI.REF                                               ------------------------------------------------------------    20446
LOC106996293.glutathione.hydrolase5.proenzyme-like-GGT1.rhesus.    ggcagatcctgggccttgggatgcgcttcttcccaccctagagtcttccggcattttccc    152017

Papio.anubis.clone.rp41-133b2.8034-40080.revcompl.Baboon           ------------------------------------------------------------    32047
BCRP3.HUMAN.NCBI.REF                                               ------------------------------------------------------------    20446
LOC106996293.glutathione.hydrolase5.proenzyme-like-GGT1.rhesus.    cttggccagggctgggatggctgtagctgtttctccagactcacattcatgttaaagaac    152077

Papio.anubis.clone.rp41-133b2.8034-40080.revcompl.Baboon           ------------------------------------------------------------    32047
BCRP3.HUMAN.NCBI.REF                                               ------------------------------------------------------------    20446
LOC106996293.glutathione.hydrolase5.proenzyme-like-GGT1.rhesus.    aaaaacaatcatatccctttaagtcaaagagttcttctagagcatcttgtctggtgtgct    152137

Papio.anubis.clone.rp41-133b2.8034-40080.revcompl.Baboon           ------------------------------------------------------------    32047
BCRP3.HUMAN.NCBI.REF                                               ------------------------------------------------------------    20446
LOC106996293.glutathione.hydrolase5.proenzyme-like-GGT1.rhesus.    gtcactaaaataaggaggggatgagacctccccaggccgttcccttccgtatcagaagtg    152197

Papio.anubis.clone.rp41-133b2.8034-40080.revcompl.Baboon           ------------------------------------------------------------    32047
BCRP3.HUMAN.NCBI.REF                                               ------------------------------------------------------------    20446
LOC106996293.glutathione.hydrolase5.proenzyme-like-GGT1.rhesus.    gagcctcaggtggactagagggaatttcatccagtcctccctgtggaaggtgctctcttc    152257

Papio.anubis.clone.rp41-133b2.8034-40080.revcompl.Baboon           ------------------------------------------------------------    32047
BCRP3.HUMAN.NCBI.REF                                               ------------------------------------------------------------    20446
LOC106996293.glutathione.hydrolase5.proenzyme-like-GGT1.rhesus.    tgacccgggtgctgctgagcaagcatctgacatccaaaagcccgcgtctcctcttctcca    152317

Papio.anubis.clone.rp41-133b2.8034-40080.revcompl.Baboon           ------------------------------------------------------------    32047
```

```
BCRP3.HUMAN.NCBI.REF                                                ------------------------------------------------------------    20446
LOC106996293.glutathione.hydrolase5.proenzyme-like-GGT1.rhesus.     ccatgagggtgatttttgaggattgcagggatgatgctcttcatgcaggctgttgataca    152377

Papio.anubis.clone.rp41-133b2.8034-40080.revcompl.Baboon            ------------------------------------------------------------    32047
BCRP3.HUMAN.NCBI.REF                                                ------------------------------------------------------------    20446
LOC106996293.glutathione.hydrolase5.proenzyme-like-GGT1.rhesus.     gagggtgctattgccccacagctgacattcaccttgaaggcatgaatcattttctccttc    152437

Papio.anubis.clone.rp41-133b2.8034-40080.revcompl.Baboon            ------------------------------------------------------------    32047
BCRP3.HUMAN.NCBI.REF                                                ------------------------------------------------------------    20446
LOC106996293.glutathione.hydrolase5.proenzyme-like-GGT1.rhesus.     tccatcctcacatgcaattttaccttgaaagcctagaacccctttcaccatgggcaccca    152497

Papio.anubis.clone.rp41-133b2.8034-40080.revcompl.Baboon            ------------------------------------------------------------    32047
BCRP3.HUMAN.NCBI.REF                                                ------------------------------------------------------------    20446
LOC106996293.glutathione.hydrolase5.proenzyme-like-GGT1.rhesus.     ggttctccacatgcacaagtccctgaagggttggtgctttttggtgtgagttccagatcc    152557

Papio.anubis.clone.rp41-133b2.8034-40080.revcompl.Baboon            ------------------------------------------------------------    32047
BCRP3.HUMAN.NCBI.REF                                                ------------------------------------------------------------    20446
LOC106996293.glutathione.hydrolase5.proenzyme-like-GGT1.rhesus.     cgatcctctgggggttcatggtgttacactgactcccatgtccctccttgtccccctagt    152617

Papio.anubis.clone.rp41-133b2.8034-40080.revcompl.Baboon            ------------------------------------------------------------    32047
BCRP3.HUMAN.NCBI.REF                                                ------------------------------------------------------------    20446
LOC106996293.glutathione.hydrolase5.proenzyme-like-GGT1.rhesus.     atggaaatgagatctgcaggatgccaactttccagcccagcacaggggagaagctgttgg    152677

Papio.anubis.clone.rp41-133b2.8034-40080.revcompl.Baboon            ------------------------------------------------------------    32047
BCRP3.HUMAN.NCBI.REF                                                ------------------------------------------------------------    20446
LOC106996293.glutathione.hydrolase5.proenzyme-like-GGT1.rhesus.     agtccctggttccagcctttctaactaaacccatctcctcctatgccacctgcctgggtc    152737

Papio.anubis.clone.rp41-133b2.8034-40080.revcompl.Baboon            ------------------------------------------------------------    32047
BCRP3.HUMAN.NCBI.REF                                                ------------------------------------------------------------    20446
LOC106996293.glutathione.hydrolase5.proenzyme-like-GGT1.rhesus.     cctcctgggactttatcaccgtgccacactttttggaactactggttagaaggtgagtgt    152797

Papio.anubis.clone.rp41-133b2.8034-40080.revcompl.Baboon            ------------------------------------------------------------    32047
BCRP3.HUMAN.NCBI.REF                                                ------------------------------------------------------------    20446
LOC106996293.glutathione.hydrolase5.proenzyme-like-GGT1.rhesus.     ccatcccctccaaggcatggaggtgcctctgtctcctactggctgtgtcttgaagatgca    152857

Papio.anubis.clone.rp41-133b2.8034-40080.revcompl.Baboon            ------------------------------------------------------------    32047
BCRP3.HUMAN.NCBI.REF                                                ------------------------------------------------------------    20446
LOC106996293.glutathione.hydrolase5.proenzyme-like-GGT1.rhesus.     cctcttggagactcagttggctcctgtggaacagggagtaatgagaagatgaacctcaca    152917

Papio.anubis.clone.rp41-133b2.8034-40080.revcompl.Baboon            ------------------------------------------------------------    32047
BCRP3.HUMAN.NCBI.REF                                                ------------------------------------------------------------    20446
LOC106996293.glutathione.hydrolase5.proenzyme-like-GGT1.rhesus.     gggttcttgtagggactgaatgatctaagacacagcaaacaaaggggtccgtagagccta    152977

Papio.anubis.clone.rp41-133b2.8034-40080.revcompl.Baboon            ------------------------------------------------------------    32047
BCRP3.HUMAN.NCBI.REF                                                ------------------------------------------------------------    20446
LOC106996293.glutathione.hydrolase5.proenzyme-like-GGT1.rhesus.     aaactctggacaatctgctgtgggtgctgtgattcatgtttattacttttctcttcccac    153037

Papio.anubis.clone.rp41-133b2.8034-40080.revcompl.Baboon            ------------------------------------------------------------    32047
BCRP3.HUMAN.NCBI.REF                                                ------------------------------------------------------------    20446
LOC106996293.glutathione.hydrolase5.proenzyme-like-GGT1.rhesus.     tcactgaagcagctctcagcattctgccgcaatggcccatctctcttgctgtttggaaag    153097

Papio.anubis.clone.rp41-133b2.8034-40080.revcompl.Baboon            ------------------------------------------------------------    32047
BCRP3.HUMAN.NCBI.REF                                                ------------------------------------------------------------    20446
LOC106996293.glutathione.hydrolase5.proenzyme-like-GGT1.rhesus.     gcacatagaaagcaagtctgcaatggcattgctaaaggatgtctgagtttccttctggct    153157

Papio.anubis.clone.rp41-133b2.8034-40080.revcompl.Baboon            ------------------------------------------------------------    32047
BCRP3.HUMAN.NCBI.REF                                                ------------------------------------------------------------    20446
LOC106996293.glutathione.hydrolase5.proenzyme-like-GGT1.rhesus.     ggactttctccttgacacagacagaagaggggtcccttcatgctgaaaaaggagccacag    153217

Papio.anubis.clone.rp41-133b2.8034-40080.revcompl.Baboon            ------------------------------------------------------------    32047
BCRP3.HUMAN.NCBI.REF                                                ------------------------------------------------------------    20446
LOC106996293.glutathione.hydrolase5.proenzyme-like-GGT1.rhesus.     gcccactcagacatctggagaggctcactgggttctccaatggttggggttcactcattc    153277

Papio.anubis.clone.rp41-133b2.8034-40080.revcompl.Baboon            ------------------------------------------------------------    32047
BCRP3.HUMAN.NCBI.REF                                                ------------------------------------------------------------    20446
LOC106996293.glutathione.hydrolase5.proenzyme-like-GGT1.rhesus.     aacacatacatacaaaacacctcatttgtgcatcgttcttcttgtgacttggcctaattt    153337

Papio.anubis.clone.rp41-133b2.8034-40080.revcompl.Baboon            ------------------------------------------------------------    32047
BCRP3.HUMAN.NCBI.REF                                                ------------------------------------------------------------    20446
LOC106996293.glutathione.hydrolase5.proenzyme-like-GGT1.rhesus.     cataaacaaggcagatgacaatccctgggcctcaattctacgcagagtcaggtgctcaca    153397

Papio.anubis.clone.rp41-133b2.8034-40080.revcompl.Baboon            ------------------------------------------------------------    32047
BCRP3.HUMAN.NCBI.REF                                                ------------------------------------------------------------    20446
LOC106996293.glutathione.hydrolase5.proenzyme-like-GGT1.rhesus.     gtagacagaataaacaaatcatatctacagaatgttagaggggaaaccctcatggcctcc    153457

Papio.anubis.clone.rp41-133b2.8034-40080.revcompl.Baboon            ------------------------------------------------------------    32047
BCRP3.HUMAN.NCBI.REF                                                ------------------------------------------------------------    20446
LOC106996293.glutathione.hydrolase5.proenzyme-like-GGT1.rhesus.     tctacatatggtggcatcctcccagattctgactagaatgacggagcccaacaagtataa    153517

Papio.anubis.clone.rp41-133b2.8034-40080.revcompl.Baboon            ------------------------------------------------------------    32047
BCRP3.HUMAN.NCBI.REF                                                ------------------------------------------------------------    20446
LOC106996293.glutathione.hydrolase5.proenzyme-like-GGT1.rhesus.     actgggggacttagggttctgaaaggcctcttcacccacaaaacatgggaggaaatatg    153577

Papio.anubis.clone.rp41-133b2.8034-40080.revcompl.Baboon            ------------------------------------------------------------    32047
BCRP3.HUMAN.NCBI.REF                                                ------------------------------------------------------------    20446
LOC106996293.glutathione.hydrolase5.proenzyme-like-GGT1.rhesus.     tggactctggctggggagagaataaaggagccctggggttcatgtcttataattcccaca    153637

Papio.anubis.clone.rp41-133b2.8034-40080.revcompl.Baboon            ------------------------------------------------------------    32047
BCRP3.HUMAN.NCBI.REF                                                ------------------------------------------------------------    20446
LOC106996293.glutathione.hydrolase5.proenzyme-like-GGT1.rhesus.     acaaggctgacatctggggacttcccctgcaagaggcaaacagtgagttctgtaaatgaa    153697

Papio.anubis.clone.rp41-133b2.8034-40080.revcompl.Baboon            ------------------------------------------------------------    32047
BCRP3.HUMAN.NCBI.REF                                                ------------------------------------------------------------    20446
LOC106996293.glutathione.hydrolase5.proenzyme-like-GGT1.rhesus.     gacttaggtcccttgcaaaggagaggtgaggctggggccacaagtggccactgagagacc    153757

Papio.anubis.clone.rp41-133b2.8034-40080.revcompl.Baboon            ------------------------------------------------------------    32047
BCRP3.HUMAN.NCBI.REF                                                ------------------------------------------------------------    20446
LOC106996293.glutathione.hydrolase5.proenzyme-like-GGT1.rhesus.     catccccagcactgtggcttcccagctctccctgtcctcctccacccaacatctgcccct    153817

Papio.anubis.clone.rp41-133b2.8034-40080.revcompl.Baboon            ------------------------------------------------------------    32047
BCRP3.HUMAN.NCBI.REF                                                ------------------------------------------------------------    20446
LOC106996293.glutathione.hydrolase5.proenzyme-like-GGT1.rhesus.     accctcctaaccccaggaccaggggaaccaaagctggagctttgatgagcaagctgctca    153877

Papio.anubis.clone.rp41-133b2.8034-40080.revcompl.Baboon            ------------------------------------------------------------    32047
BCRP3.HUMAN.NCBI.REF                                                ------------------------------------------------------------    20446
LOC106996293.glutathione.hydrolase5.proenzyme-like-GGT1.rhesus.     caaatctgcctggagctgcagtcttgagtgcccaggtgcacagtgctgtgctccagggcc    153937

Papio.anubis.clone.rp41-133b2.8034-40080.revcompl.Baboon            ------------------------------------------------------------    32047
BCRP3.HUMAN.NCBI.REF                                                ------------------------------------------------------------    20446
LOC106996293.glutathione.hydrolase5.proenzyme-like-GGT1.rhesus.     attggaaagagaatgtcagtgggacgccggggcacacaagggtctgtagagccctgttgc    153997

Papio.anubis.clone.rp41-133b2.8034-40080.revcompl.Baboon            ------------------------------------------------------------    32047
BCRP3.HUMAN.NCBI.REF                                                ------------------------------------------------------------    20446
LOC106996293.glutathione.hydrolase5.proenzyme-like-GGT1.rhesus.     atggccaggtctgccccttccaggacagcactgatggcttggggtagggtggggctgtcc    154057

Papio.anubis.clone.rp41-133b2.8034-40080.revcompl.Baboon            ------------------------------------------------------------    32047
```

```
BCRP3.HUMAN.NCBI.REF                                              ------------------------------------------------------------    20446
LOC106996293.glutathione.hydrolase5.proenzyme-like-GGT1.rhesus.   tctacacaggcagcaagaggccagggacccagaaccacgcaagggtgccctggagggttg    154117

Papio.anubis.clone.rp41-133b2.8034-40080.revcompl.Baboon          ------------------------------------------------------------    32047
BCRP3.HUMAN.NCBI.REF                                              ------------------------------------------------------------    20446
LOC106996293.glutathione.hydrolase5.proenzyme-like-GGT1.rhesus.   tgtgggagaaggccaggcctctgactcagctgtccactccatcaccagcaccaccacctc    154177

Papio.anubis.clone.rp41-133b2.8034-40080.revcompl.Baboon          ------------------------------------------------------------    32047
BCRP3.HUMAN.NCBI.REF                                              ------------------------------------------------------------    20446
LOC106996293.glutathione.hydrolase5.proenzyme-like-GGT1.rhesus.   cattttgttcacctggccccctgaaaacacctcagtttgctgccaggccctgcaacggtc    154237

Papio.anubis.clone.rp41-133b2.8034-40080.revcompl.Baboon          ------------------------------------------------------------    32047
BCRP3.HUMAN.NCBI.REF                                              ------------------------------------------------------------    20446
LOC106996293.glutathione.hydrolase5.proenzyme-like-GGT1.rhesus.   atctttctggataaagctggccttcaggaccttcgcctggcctggactgggcgtggagga    154297

Papio.anubis.clone.rp41-133b2.8034-40080.revcompl.Baboon          ------------------------------------------------------------    32047
BCRP3.HUMAN.NCBI.REF                                              ------------------------------------------------------------    20446
LOC106996293.glutathione.hydrolase5.proenzyme-like-GGT1.rhesus.   ccatcaggaaattgtcctaggccagttggtgcttccggagcccaaggaagccaagccaga    154357

Papio.anubis.clone.rp41-133b2.8034-40080.revcompl.Baboon          ------------------------------------------------------------    32047
BCRP3.HUMAN.NCBI.REF                                              ------------------------------------------------------------    20446
LOC106996293.glutathione.hydrolase5.proenzyme-like-GGT1.rhesus.   tggtgagaagactttccgggggtggtgttttggggctataattcccttaaattccatcca    154417

Papio.anubis.clone.rp41-133b2.8034-40080.revcompl.Baboon          ------------------------------------------------------------    32047
BCRP3.HUMAN.NCBI.REF                                              ------------------------------------------------------------    20446
LOC106996293.glutathione.hydrolase5.proenzyme-like-GGT1.rhesus.   ggcatttctatacttggaaatcccagtgaagagtgactggaatggtgaccttttctcctt    154477

Papio.anubis.clone.rp41-133b2.8034-40080.revcompl.Baboon          ------------------------------------------------------------    32047
BCRP3.HUMAN.NCBI.REF                                              ------------------------------------------------------------    20446
LOC106996293.glutathione.hydrolase5.proenzyme-like-GGT1.rhesus.   ttccagatcctgctccacctcctgggcaacacgcattaacaatgctggccctggagccag    154537

Papio.anubis.clone.rp41-133b2.8034-40080.revcompl.Baboon          ------------------------------------------------------------    32047
BCRP3.HUMAN.NCBI.REF                                              ------------------------------------------------------------    20446
LOC106996293.glutathione.hydrolase5.proenzyme-like-GGT1.rhesus.   caccaccactgctggcggacctgcggcctgctctagagccagagtcacctgtagccctgg    154597

Papio.anubis.clone.rp41-133b2.8034-40080.revcompl.Baboon          ------------------------------------------------------------    32047
BCRP3.HUMAN.NCBI.REF                                              ------------------------------------------------------------    20446
LOC106996293.glutathione.hydrolase5.proenzyme-like-GGT1.rhesus.   atgcacaaggatatctacattcagcatcagcaccagcaccaggggaagggccccctccag    154657

Papio.anubis.clone.rp41-133b2.8034-40080.revcompl.Baboon          ------------------------------------------------------------    32047
BCRP3.HUMAN.NCBI.REF                                              ------------------------------------------------------------    20446
LOC106996293.glutathione.hydrolase5.proenzyme-like-GGT1.rhesus.   gaacagtgctggagccacagtcagccccagagtcaccctgtccctgtcccgggactgtcc    154717

Papio.anubis.clone.rp41-133b2.8034-40080.revcompl.Baboon          ------------------------------------------------------------    32047
BCRP3.HUMAN.NCBI.REF                                              ------------------------------------------------------------    20446
LOC106996293.glutathione.hydrolase5.proenzyme-like-GGT1.rhesus.   agagccaacacactgaggagctgccggacatcacgaccttccctcccaggctgctggctg    154777

Papio.anubis.clone.rp41-133b2.8034-40080.revcompl.Baboon          ------------------------------------------------------------    32047
BCRP3.HUMAN.NCBI.REF                                              ------------------------------------------------------------    20446
LOC106996293.glutathione.hydrolase5.proenzyme-like-GGT1.rhesus.   agcagctgacccttatggatgcggtgagcagctgggcttcgcaggctgtgcctctggcac    154837

Papio.anubis.clone.rp41-133b2.8034-40080.revcompl.Baboon          ------------------------------------------------------------    32047
BCRP3.HUMAN.NCBI.REF                                              ------------------------------------------------------------    20446
LOC106996293.glutathione.hydrolase5.proenzyme-like-GGT1.rhesus.   cagctgtctcaggccagcctgtctctgaggagcggccaatgccctgggtccagtttcagc    154897

Papio.anubis.clone.rp41-133b2.8034-40080.revcompl.Baboon          ------------------------------------------------------------    32047
BCRP3.HUMAN.NCBI.REF                                              ------------------------------------------------------------    20446
LOC106996293.glutathione.hydrolase5.proenzyme-like-GGT1.rhesus.   cccacttcttaccaaccatgggatctggatgagtttcctcacccacaagccttccctgtc    154957

Papio.anubis.clone.rp41-133b2.8034-40080.revcompl.Baboon          ------------------------------------------------------------    32047
BCRP3.HUMAN.NCBI.REF                                              ------------------------------------------------------------    20446
LOC106996293.glutathione.hydrolase5.proenzyme-like-GGT1.rhesus.   tgcatgtggacagcagagatgggacatcgcccgtgccagctgcacagagtgactgtgcag    155017

Papio.anubis.clone.rp41-133b2.8034-40080.revcompl.Baboon          ------------------------------------------------------------    32047
BCRP3.HUMAN.NCBI.REF                                              ------------------------------------------------------------    20446
LOC106996293.glutathione.hydrolase5.proenzyme-like-GGT1.rhesus.   actgaatgacgggggatggacagaaagcaggacagggcaggtgatcactgaggggcaggc    155077

Papio.anubis.clone.rp41-133b2.8034-40080.revcompl.Baboon          ------------------------------------------------------------    32047
BCRP3.HUMAN.NCBI.REF                                              ------------------------------------------------------------    20446
LOC106996293.glutathione.hydrolase5.proenzyme-like-GGT1.rhesus.   agggccatgggtcactcacccagctgctcaggagcctcactaccctcagcacttattagg    155137

Papio.anubis.clone.rp41-133b2.8034-40080.revcompl.Baboon          ------------------------------------------------------------    32047
BCRP3.HUMAN.NCBI.REF                                              ------------------------------------------------------------    20446
LOC106996293.glutathione.hydrolase5.proenzyme-like-GGT1.rhesus.   tacctgatgcatactagattctatggcagacacccaaacagagcccagtgttgcagctgc    155197

Papio.anubis.clone.rp41-133b2.8034-40080.revcompl.Baboon          ------------------------------------------------------------    32047
BCRP3.HUMAN.NCBI.REF                                              ------------------------------------------------------------    20446
LOC106996293.glutathione.hydrolase5.proenzyme-like-GGT1.rhesus.   tgcaaggaaactgcaccagtagggagagaaggagtagagggctgattgggatgggaggaa    155257

Papio.anubis.clone.rp41-133b2.8034-40080.revcompl.Baboon          ------------------------------------------------------------    32047
BCRP3.HUMAN.NCBI.REF                                              ------------------------------------------------------------    20446
LOC106996293.glutathione.hydrolase5.proenzyme-like-GGT1.rhesus.   gatgaggcctcaggatggggaggcctgagccaccttctagttcttggaatgaggatggcc    155317

Papio.anubis.clone.rp41-133b2.8034-40080.revcompl.Baboon          ------------------------------------------------------------    32047
BCRP3.HUMAN.NCBI.REF                                              ------------------------------------------------------------    20446
LOC106996293.glutathione.hydrolase5.proenzyme-like-GGT1.rhesus.   tgggagaaaatgtcactctctcttcccacccttgttggtttctgggacatgatgcattca    155377

Papio.anubis.clone.rp41-133b2.8034-40080.revcompl.Baboon          ------------------------------------------------------------    32047
BCRP3.HUMAN.NCBI.REF                                              ------------------------------------------------------------    20446
LOC106996293.glutathione.hydrolase5.proenzyme-like-GGT1.rhesus.   gggccctcggtgggcagaaaaccaaacccagggactcccacaagtctggagcatatttta    155437

Papio.anubis.clone.rp41-133b2.8034-40080.revcompl.Baboon          ------------------------------------------------------------    32047
BCRP3.HUMAN.NCBI.REF                                              ------------------------------------------------------------    20446
LOC106996293.glutathione.hydrolase5.proenzyme-like-GGT1.rhesus.   aagctttctgagctcaagctcagttcctgccagagactgtggatgactgtgagctcagtc    155497

Papio.anubis.clone.rp41-133b2.8034-40080.revcompl.Baboon          ------------------------------------------------------------    32047
BCRP3.HUMAN.NCBI.REF                                              ------------------------------------------------------------    20446
LOC106996293.glutathione.hydrolase5.proenzyme-like-GGT1.rhesus.   cctgcctgggactgtgggtgactctgagctggggtgtgctgtgtccatgatactctcctc    155557

Papio.anubis.clone.rp41-133b2.8034-40080.revcompl.Baboon          ------------------------------------------------------------    32047
BCRP3.HUMAN.NCBI.REF                                              ------------------------------------------------------------    20446
LOC106996293.glutathione.hydrolase5.proenzyme-like-GGT1.rhesus.   cttccccaaaggatctgttcaagaaggtggagctctacgaatgcttgggctccatctggg    155617

Papio.anubis.clone.rp41-133b2.8034-40080.revcompl.Baboon          ------------------------------------------------------------    32047
BCRP3.HUMAN.NCBI.REF                                              ------------------------------------------------------------    20446
LOC106996293.glutathione.hydrolase5.proenzyme-like-GGT1.rhesus.   gccaacgacatcagaaggggagtgagcacgtggcacccacagtttgtgccaccattgcac    155677

Papio.anubis.clone.rp41-133b2.8034-40080.revcompl.Baboon          ------------------------------------------------------------    32047
BCRP3.HUMAN.NCBI.REF                                              ------------------------------------------------------------    20446
LOC106996293.glutathione.hydrolase5.proenzyme-like-GGT1.rhesus.   acttcaacaggctcgccaactgtgtcaccacctcctgcctcggggaccacagcatgaggg    155737

Papio.anubis.clone.rp41-133b2.8034-40080.revcompl.Baboon          ------------------------------------------------------------    32047
BCRP3.HUMAN.NCBI.REF                                              ------------------------------------------------------------    20446
LOC106996293.glutathione.hydrolase5.proenzyme-like-GGT1.rhesus.   tccaggatagggccagggtggtggagcactggatcaaggtggccagggtaagccatggtt    155797

Papio.anubis.clone.rp41-133b2.8034-40080.revcompl.Baboon          ------------------------------------------------------------    32047
```

```
BCRP3.HUMAN.NCBI.REF                                            ------------------------------------------------------------    20446
LOC106996293.glutathione.hydrolase5.proenzyme-like-GGT1.rhesus.  gggccttgggattccctctctaaaaatggggaactgcctcttctcctccatcggctttca    155857

Papio.anubis.clone.rp41-133b2.8034-40080.revcompl.Baboon         ------------------------------------------------------------    32047
BCRP3.HUMAN.NCBI.REF                                            ------------------------------------------------------------    20446
LOC106996293.glutathione.hydrolase5.proenzyme-like-GGT1.rhesus.  ggattggcatctgtatctctagcctgagccctacacatcccctaggcccttcttcccaca    155917

Papio.anubis.clone.rp41-133b2.8034-40080.revcompl.Baboon         ------------------------------------------------------------    32047
BCRP3.HUMAN.NCBI.REF                                            ------------------------------------------------------------    20446
LOC106996293.glutathione.hydrolase5.proenzyme-like-GGT1.rhesus.  gcttccctgaccttgaccgccatggcccagtggtggctgctcacgtctgacctgggatct    155977

Papio.anubis.clone.rp41-133b2.8034-40080.revcompl.Baboon         ------------------------------------------------------------    32047
BCRP3.HUMAN.NCBI.REF                                            ------------------------------------------------------------    20446
LOC106996293.glutathione.hydrolase5.proenzyme-like-GGT1.rhesus.  tccttgggttaaactgaaatctttctagattagtgacattcactcagccccaggtgtgcc    156037

Papio.anubis.clone.rp41-133b2.8034-40080.revcompl.Baboon         ------------------------------------------------------------    32047
BCRP3.HUMAN.NCBI.REF                                            ------------------------------------------------------------    20446
LOC106996293.glutathione.hydrolase5.proenzyme-like-GGT1.rhesus.  ctcctgaggctccctgggcctctgcttcattcaggaagggagatctcagcagagggggct    156097

Papio.anubis.clone.rp41-133b2.8034-40080.revcompl.Baboon         ------------------------------------------------------------    32047
BCRP3.HUMAN.NCBI.REF                                            ------------------------------------------------------------    20446
LOC106996293.glutathione.hydrolase5.proenzyme-like-GGT1.rhesus.  gaggctgaagtgggtcggactccaactctggacctcacagctcactcttccctctccagg    156157

Papio.anubis.clone.rp41-133b2.8034-40080.revcompl.Baboon         ------------------------------------------------------------    32047
BCRP3.HUMAN.NCBI.REF                                            ------------------------------------------------------------    20446
LOC106996293.glutathione.hydrolase5.proenzyme-like-GGT1.rhesus.  agtgcctacgcctcaacaacttctcctcagtgcacgccatcgtctctgctctgcgcagca    156217

Papio.anubis.clone.rp41-133b2.8034-40080.revcompl.Baboon         ------------------------------------------------------------    32047
BCRP3.HUMAN.NCBI.REF                                            ------------------------------------------------------------    20446
LOC106996293.glutathione.hydrolase5.proenzyme-like-GGT1.rhesus.  acccaatacatcggctacacaagacgtgggcaggagtgtccaggtgaggagggttccctc    156277

Papio.anubis.clone.rp41-133b2.8034-40080.revcompl.Baboon         ------------------------------------------------------------    32047
BCRP3.HUMAN.NCBI.REF                                            ------------------------------------------------------------    20446
LOC106996293.glutathione.hydrolase5.proenzyme-like-GGT1.rhesus.  catgggagcaccagtgttgacttagggacccataggtctccccatgtgccctcaacgact    156337

Papio.anubis.clone.rp41-133b2.8034-40080.revcompl.Baboon         ------------------------------------------------------------    32047
BCRP3.HUMAN.NCBI.REF                                            ------------------------------------------------------------    20446
LOC106996293.glutathione.hydrolase5.proenzyme-like-GGT1.rhesus.  ctgaaaggttcttggagaacagggacgctggaggcagggatgggctggtgggtgtggtca    156397

Papio.anubis.clone.rp41-133b2.8034-40080.revcompl.Baboon         ------------------------------------------------------------    32047
BCRP3.HUMAN.NCBI.REF                                            ------------------------------------------------------------    20446
LOC106996293.glutathione.hydrolase5.proenzyme-like-GGT1.rhesus.  ctaagctgccctggacttctaggcaaggatttccaactcaggactaaggatttttaacca    156457

Papio.anubis.clone.rp41-133b2.8034-40080.revcompl.Baboon         ------------------------------------------------------------    32047
BCRP3.HUMAN.NCBI.REF                                            ------------------------------------------------------------    20446
LOC106996293.glutathione.hydrolase5.proenzyme-like-GGT1.rhesus.  tcaggaacagactggagctaactggagggtgtcaggtgtttgcacccagcagtggaactg    156517

Papio.anubis.clone.rp41-133b2.8034-40080.revcompl.Baboon         ------------------------------------------------------------    32047
BCRP3.HUMAN.NCBI.REF                                            ------------------------------------------------------------    20446
LOC106996293.glutathione.hydrolase5.proenzyme-like-GGT1.rhesus.  tgtccagctggaagctaactgtgaacacgcaggggctcatgtgaagttgagatgggccag    156577

Papio.anubis.clone.rp41-133b2.8034-40080.revcompl.Baboon         ------------------------------------------------------------    32047
BCRP3.HUMAN.NCBI.REF                                            ------------------------------------------------------------    20446
LOC106996293.glutathione.hydrolase5.proenzyme-like-GGT1.rhesus.  gggaggagcatgacagtcccaccctggtcctctggagcccttgtcatcagatgaccccac    156637

Papio.anubis.clone.rp41-133b2.8034-40080.revcompl.Baboon         ------------------------------------------------------------    32047
BCRP3.HUMAN.NCBI.REF                                            ------------------------------------------------------------    20446
LOC106996293.glutathione.hydrolase5.proenzyme-like-GGT1.rhesus.  tggaaactctcacgcaggaagctgagattcactgggttttcaaacaaaagggattggaat    156697

Papio.anubis.clone.rp41-133b2.8034-40080.revcompl.Baboon         ------------------------------------------------------------    32047
BCRP3.HUMAN.NCBI.REF                                            ------------------------------------------------------------    20446
LOC106996293.glutathione.hydrolase5.proenzyme-like-GGT1.rhesus.  tcacaaatctcccccgtgattcccaaatttacccttttttctttcttctatcaatagcaa    156757

Papio.anubis.clone.rp41-133b2.8034-40080.revcompl.Baboon         ------------------------------------------------------------    32047
BCRP3.HUMAN.NCBI.REF                                            ------------------------------------------------------------    20446
LOC106996293.glutathione.hydrolase5.proenzyme-like-GGT1.rhesus.  aagctcaaaatatctaaaagaactctgcaaaaaagacactgcagtgaagagggacctgct    156817

Papio.anubis.clone.rp41-133b2.8034-40080.revcompl.Baboon         ------------------------------------------------------------    32047
BCRP3.HUMAN.NCBI.REF                                            ------------------------------------------------------------    20446
LOC106996293.glutathione.hydrolase5.proenzyme-like-GGT1.rhesus.  gatcaaggtacagtggagtctgggagatgcgggacaagtgtttaagggtcagaggaaaga    156877

Papio.anubis.clone.rp41-133b2.8034-40080.revcompl.Baboon         ------------------------------------------------------------    32047
BCRP3.HUMAN.NCBI.REF                                            ------------------------------------------------------------    20446
LOC106996293.glutathione.hydrolase5.proenzyme-like-GGT1.rhesus.  gtgagtttggaagggcattgatcccagtgtgcagtggttattttgtaatgttttgactta    156937

Papio.anubis.clone.rp41-133b2.8034-40080.revcompl.Baboon         ------------------------------------------------------------    32047
BCRP3.HUMAN.NCBI.REF                                            ------------------------------------------------------------    20446
LOC106996293.glutathione.hydrolase5.proenzyme-like-GGT1.rhesus.  cctactaaaagtggacttgaaaaattccctccatgcctactttgggcaaacaggaggaga    156997

Papio.anubis.clone.rp41-133b2.8034-40080.revcompl.Baboon         ------------------------------------------------------------    32047
BCRP3.HUMAN.NCBI.REF                                            ------------------------------------------------------------    20446
LOC106996293.glutathione.hydrolase5.proenzyme-like-GGT1.rhesus.  ggtgtgtgggtcgatgggcacgtgggggcacgggggcagcaggccctggaaataggatgt    157057

Papio.anubis.clone.rp41-133b2.8034-40080.revcompl.Baboon         ------------------------------------------------------------    32047
BCRP3.HUMAN.NCBI.REF                                            ------------------------------------------------------------    20446
LOC106996293.glutathione.hydrolase5.proenzyme-like-GGT1.rhesus.  ggcaatggctgctgggcttctgagtgagggtgatgagctgcagcattagcaggactctgg    157117

Papio.anubis.clone.rp41-133b2.8034-40080.revcompl.Baboon         ------------------------------------------------------------    32047
BCRP3.HUMAN.NCBI.REF                                            ------------------------------------------------------------    20446
LOC106996293.glutathione.hydrolase5.proenzyme-like-GGT1.rhesus.  ctcccatgctggtccatgctgctggcatggagcttcctccaggctgggggtggtcatgg    157177

Papio.anubis.clone.rp41-133b2.8034-40080.revcompl.Baboon         ------------------------------------------------------------    32047
BCRP3.HUMAN.NCBI.REF                                            ------------------------------------------------------------    20446
LOC106996293.glutathione.hydrolase5.proenzyme-like-GGT1.rhesus.  taggtgggactttccttcttcctcaaactggcagtaattcctcaggaagccaggcctctg    157237

Papio.anubis.clone.rp41-133b2.8034-40080.revcompl.Baboon         ------------------------------------------------------------    32047
BCRP3.HUMAN.NCBI.REF                                            ------------------------------------------------------------    20446
LOC106996293.glutathione.hydrolase5.proenzyme-like-GGT1.rhesus.  ctgctgcttctgtctgcagcgcacctccatgggcagggactgcagtccacactgggggaa    157297

Papio.anubis.clone.rp41-133b2.8034-40080.revcompl.Baboon         ------------------------------------------------------------    32047
BCRP3.HUMAN.NCBI.REF                                            ------------------------------------------------------------    20446
LOC106996293.glutathione.hydrolase5.proenzyme-like-GGT1.rhesus.  agagggaacaacaacagaggaagctcatgtgccagggagtccagtagactgcccagctat    157357

Papio.anubis.clone.rp41-133b2.8034-40080.revcompl.Baboon         ------------------------------------------------------------    32047
BCRP3.HUMAN.NCBI.REF                                            ------------------------------------------------------------    20446
LOC106996293.glutathione.hydrolase5.proenzyme-like-GGT1.rhesus.  gggtcccaatgggcaaactcaggacagatgtatgtgtttgctgggactccccactctgcc    157417

Papio.anubis.clone.rp41-133b2.8034-40080.revcompl.Baboon         ------------------------------------------------------------    32047
BCRP3.HUMAN.NCBI.REF                                            ------------------------------------------------------------    20446
LOC106996293.glutathione.hydrolase5.proenzyme-like-GGT1.rhesus.  ctttgcagacatctgaaaatggtcatgtcacaggattctcaccaattagcagtgacacat    157477

Papio.anubis.clone.rp41-133b2.8034-40080.revcompl.Baboon         -----------------------------------------------------------     32047
BCRP3.HUMAN.NCBI.REF                                            -----------------------------------------------------------     20446
LOC106996293.glutathione.hydrolase5.proenzyme-like-GGT1.rhesus.  gctcatgacaagtatctgggggatccatgcattcctaggggatcctccctgaccagatc     157536
```

```
>Papio.anubis.clone.rp41-133b2.8034-40080.revcompl.Baboon
atcctctccagagggaggcctggttctcagggaacagcaaatgggaagaggtccccagat
cccagggatcagggcttggaccagctggggacacagcccagagggagtgggtctggaagg
gaacagctagacacagcagccttcaccatcggcagcccctcccggcctccctcggggtct
gctccctcctccgggcacagttccaacacctggggcagggttctgggaaaggctggtggg
aggctgttatcacagcccagcacctgagtatcaccagggtcactggggccagggcccagg
tgaagccaggtcggggctctcctttagaagccctgaaaacctggtgataccaaagggccc
acagacaaacagggttttgtgcctgcggagttgagtaccagcgggtctaagccctggagg
gctgtgtccctggggctccccaggggtgagatggaggtgggctcaactggtgtacacgtc
actcctcaatccttattttatttatttaatttaaaaaactatttaaacaaatagagatgg
ggtctcactatgttgaccaggctggtcttaaactcctgacttcaagcaatcctcccatct
cggcctcaaaagtgctaggattacagggatgagccactgcacccagcctcaatccttatt
ttggcctgagaggaaaggccgtggcccagtttgcaggggagaagactgaggctggagggg
caggccttgctctgggtggcacagcagcaagagaagtgggagctggccacgaggcttcct
ggacctgatacgctgctggggtacaccctggttctccaggtcccatggggctcagcccag
gactacttcgggaggtgggagacttaaacccctctttccttctcatagtcccttctccc
atcatttcctgaggaaggacattcagggacctccctggctgtgcctcaggactagaatga
cacccattcctttccctgggcctttgctcaggcggtccctgcaccctggcctctgcctga
ccaggatggtggggagaggagggggggacgtcccctccgctgctgtctccactgttcctgc
tgccctgacctctgggcttccaggactgcagtgggtgggtgggtgggctggcctgagccc
aggaatgcacttcggctcctggttgagcaatgtcactgaggcttgggagtcgggtcgggt
tgggaggaggagtccacaagcccccactgtgaaaggcagccgtgggaacagtctgcctgt
aaacaaccactccagcccaggctgaccaggggctctggctggacataggggcctggcagg
ctgtgtggcctgtaaggacacagtctgtctctgtgcctcagtttctctgctgcccagttg
ggcgtcccagactccaggtgtagacatctggagcaggcagtgctcagctaggaaggaagt
ggggaggactggaggagccacgtgtgaaggattccaacccacatcacctgcacccctgct
gagcctggtcaacagagcccctcagtgggtcctcactcccctggctgcctcccggttagg
caccctgaggcctagggagaacagggccaggccagtgtccccagagaggctgcactgcca
gcacagtaatagcggatttggattcagggaagcagacccgcagccagggtggggaagagc
tgcaggctgggcgtggcacctaggcggcacagcctccctccctggaggcccacgctgcat
ttccaggacagcaagtcccagggatggatggtcccaggtgccaagggctagaggcatggt
ctgtctgcattccccacatggacgtcttgtagtcaccagcgtttgatgctgtcaagtgac
cctgtcctctgtgcagaccgggaagcccttggctaccctggagggttatgggacccaggc
caggctgcagaagcatagggacttgaacccaagttttaagtgacaccactttgtgtcccc
ctccctctgtctctgtttagcgccacctcgatgctgcttgtgctgggccatgcaaagagg
gttaggggatagagatgggagctggggagtgcggctccactctgggagggggcagccttg
ctggatccaggggagatagttgagcagccccagctctgctttcccggagctgccgggaac
cccgggaatggtgtggagattcctgggagccctgcccccacctgcaaccgcagtgcagca
ggcgccaagttctccagcacattgggacagtgtgatcctggcctctggtgagtggcaggc
aggggcctttggacctaccggcagtgagggagttaacacagcagctggctcctctaggca
aggaaaactcccctcagacgctttgctgcctggcctcctgccaggaacaagcaggagctg
aaaactagaagttgaggcgtgagtttggccactccatagtgtgcatttagggagggcagc
agcacgtcacagccgccggccgcaagccatccgtccattcactcatctgtccatctggca
gcccgctgttcagacctatctgtctgtccacccatctgtaagcctgtctctgtcccattg
tctatctgaccatctttctcttactgtcctcttcgtccagctatctggcctgtctgttga
tccatcttcgtgtctgtctgtggccccacctgtttgtccatctgtccaattatctgtgag
tctatctgtgcatcttcttgtccatccatctgcccacccatctgtccctgtgtctgctca
ccggcctccccccctctcctcctgggccacagagccatggcccggggctgtggggccacgg
tcggcctagtcctgctggggctggggctggcgctggctgtcattgtgctggctgtgatcc
tctctcgccaccaagcccccctgcggcccccaggcctttgcccacgctgctgttgctgccg
actccaaggtttgctcggatattggacggtgagtgagaggtggcggggagctgggtggcc
cttggcagccagcccctcctggagaaggtgcgtgtgtgtgagtgtgtgtatgggtgtgtg
gggagtgtgtgtgagtgtgtgattgcgtgcgtgtgtgagagtgtgagtgtgtgtgtgagt
gtgtgggggtgtgggaatgtgtgtgggagtgtgtgtgtgtgagtgtgcatgtgtgtgggt
gtgggggtgaatgtgtgtgattgtgtgggggtatgtgtatgtgtgtgtgtgtgtgtgtgt
gtgcacgtgcactggcccaggaagcaggagccgtgtgtgtgtgggcttcagcacctgcag
ggcttgggcgcaaggaggcagcctcagggcccttgcacagaacaggtggcagggtgtgcc
catggggcagatggtgatttagggacagtcgtgtgtgagtccacacctggctccaggatt
caggaggctcatttgcatatcctaggtgggaaccggtctggccccgcctgaccctgctgg
ccagtgcagccccccttcagtgaggccaattctccaaggctggggtcttctcccagggtca
taggtgaagggcttcagaggctccctgtgtgggtactggcctgctggggtacacacaatg
ctgccacagccagtctgccccaactcccagcccatgaccacatctcgggtctctctgtcc
tggggaggctggtgccccacccctcacatcctctctccctgagtcagggcctgggtctcg
tgagctgagcgactgatacttggtgtcctggatgagggtgtggtggagagaggccacggc
gggtgtttcctgaccctcttccaggaaacccagcccaagggaggccttcgctgctgccac
tgcagagaggacacatacaggaggccccttcctgcccccctgcctcccattgggcccacaa
aagccgggggaagcctcccctccctgcagccacctggtctgcttcccagaagttctgtct
tgcagtccgttgggaggatcccagtgctttgtaaactaaagcaagggaggcgtggccgtt
ctctctctttgttcattcattcaccttttgagtcattccttcctccctccattcccccat
ctgtccatccttccctgccctgattgctcatgccacccccccagcccctcctgacctgat
catttggtttctcttcaggattttttgtctcctcccacagggctgagaatggcagctcag
ggacaagtgggggctggggactgcttggtctccccagtggctcccaggggatttgaggga
ttgatgccagctgccaccccaggctgtgcccctcctctgctcaggaggacatacagagat
gtggcacccacttaaactcaaagttgcaaagatgcaaatgagactggggtctcaggcacc
agagaccaccgtgggcacatggcctttgggagtggggacctgctgccacagatctctgaa
tggagtctggacctgctaggtctccccgagtgactgtctgggggtctccatagtgtgccc
tgctgtgcgcgtgagggtcagtggttggggaggggtctctgctctaatgctccctccgcc
ggcactccctcaaactctcccttggtgaggagagaggatgtggtttgccccagtgtttta
tcgaacaactctctccacttcctgttttcagaagctgggagtggaagagagcctggggct
ggccccagctgctgctgcggaacaggggtcactggacgctgggaccctggccgggctggc
tgggggcctcaggaagaggcctgctgcagcgtcatcctggccaagatccctccctgcagg
ggcccctggccatgctgccgcagggtctgctggggccaccagaagcccatgctcctgcct
ccatctctcccctctgtgctcacctctcaccagcaggccctcccagagtccagtctcctg
ctgtttgtttgtttgtttgtttgagacagtgtcttgctctgtcaccaggctggagtgcag
tggcgcgatttcggctcactgcaacctccacctcctcggttcaaatgattctcctgcctc
agcctcctgagtagctgggactacaggcgccagccaccatgcccagttaatttttgtatt
tttggtagagacagggtttcactatgttggccaggatggtctcaatctcttgacctcatg
atttgcccacctcggcctcccaaactgctgggattacaagagtgagccacggtgcccggc
ccagtctcctactctttcggcaggttttattcttgggattctgctacagccagcaccccg
ggtgtgagttcctaaggcttatgtgagtgtgaacccagcaccatgcctagtagacataca
aaagaagcatggtgacatttccagcataatgactggagatccttgtcaaaaaggtgattt
ttggctgagcatggtggctcacacctgtaatcccagcactttgggaggccgaggcgggtg
gatcacttgacgtcaggagttggagaccagcctgggcaacatggtgaaaccccgtctcta
ctgaaaatacaaaaattagccgggcatggtagcaggtgcctataatcccagctactcggg
aggctgaggcaggagaatcacttgaacctgggaggcaaaggttgcagtaagccaagattg
caccactgcactacagcctgggtgacagagcaagatttggtctcaaaaaacagagagaaa
agtttatatttttgttctaatggttatcttaatatcttcattctataattatatgtttta
cataattataatcactacataagatatactacccctagtactttgtttttttggatattct
gtttgctcctgatggttaatgtatgtgccaacttgccttagttatgatgccccattgttt
ggtcaaatacttgtcagtatcttgctgggaggttatttcatagatgtaattaacattgac
aggcagttgactttaagtgaaacagaatactcaccataatatgggtgggccacctccaat
cagttgaaggccttaagaacaaaaactgaggtttcccagagaagcaggaattctgcttca
agactataacatataaatcctgcctgagtttctggcctgctgactgctctacaggtttta
ggttccagacttcgagatcaactcttaccttaatttatagcctgtgggcatgccctacag
attttaaacttgctagtccccacagtcatgtgagccaattcctaaataaatctctctctg
tctatgtataacctgttggtttagtttctctaaaaaacttttacatccagtttcctggat
gttaagtaataccgaacctagctagtaacttcttcctttttttttttttttttttttttt
gagccggagtttcgctcttgttgcccaggctggagtgcagtggtgagatctcggctcacc
gcaacctccgcctcctgggttcaagcgattctcctgccttagcctcccaagtagctggga
ttacaggcatgtgccaccacacctggctaattttgtatttttggtagaggcagggtttct
ccatgtgggtcagattggtctcaaactcccgaactcatgtgatctgcctgccttggcctc
ccaaagtgctgggattacaggcgtgagccaccgcacccgtctcctagtaatttcttcttt
tccgcgatgtgtgtcttatctctaataatacttttcttcttgaagtctacttcattaaaa
atagtaatgctgggcatggtggctcatgcctgtaatctcagcactttgggaggtcaaggt
gggtggatcgtcaaagcccaggagttcaagaccagcctgggcaacatggtgagactctgc
ctctacaaaaatacaaaaattagctgggtgtggttaatataattctaacttggcacactt
gtagtcccagctacttgggaggttgagggggggagaatcacatgagcctagaagggagaga
ttgctgtgagccaagatcacgtcactgcgctccagcctgggagacagagtgaggctctat
ctcaaaaaaaaagaaaaaaaaaaaaaaagttatacagctttctgggttagtgcatgtatg
atatatttttcattattttccacctttctgtatccttatataaaagccattagttgggtt
ttactttatttccaattactttaatttttattgattgtccttttaaatgtaactaatgat
ttatttgggttgaaagccaccaccagtttgttttccctgcctgttctgtttcttcttatc
tcctctcacatcttgttttgcatttattattttttattattcaatttcctgcttctctata
agtttcataactgtacagtcttggagttattttaaaagatgacagcagattattttagag
```

cttacaacatgcatccttcacttaccaaagtcttaacatgagttagtaccttttttgtt
gatttgtttttcttgagatagagggagtcttgctctgctgtccaggctggagtgcagtg
gagcaatcttggttcactgcaacctccgcctcttaggttcaagcaattctcctgcctcag
tctcctgagtagctgggaccacaggcgtgcaccactatgcccagctaacttttgtttttt
tctagtagagacagggtttcaccatgttggccaggctgaccttgaactcctgaccttaag
agatctgcctgcctcggcgtcctaaagtgttgggattacaggcatgagccaccacgccca
gcctatgagttagtatttctatcctcttcctagttagtacaagaaccttggaacaggaac
gaaatttgcccccaacgacttatatgttaatacttttgtgtattttaaatatgcgtgtgt
gtgtgtatgtgcatagatgtatctgtgtgttttctgtgtttttattcttatttatgttga
gagtatagagctatgtaaaagtaaagagaattgtataatgaagccccgtgtatccattca
atttcaacaacaatcttatggccaagctaatttcatgtatactctttctctttcctgctt
ccttctaccccacattatttcagtgcaaatccagatatataactttacccatacatattt
cagtatattttatttattttaaaccccacaagatatcattttctatactactataatttt
ataccaataacattcatttagatttacccacacatttaccacttctgttaccctttttt
tttttttttgagacagagtgtcgctctgtcacccaggctggcgcaatctgggctcactg
caagctccgcctcccaggttcacgccattctcctgcctcagcctcccgagtagctgggac
tacaggtgcccgccaccatgcccagctaattttttgtatttttagtagagacaggtttc
actgtgttagccaggatggtctcgatctcctaatctcgtgatacacccgcctcggcctcc
caaaatgctgagattacaggcatgagccaccgtgcctggccaccctttgtttttattat
aaaaataactttgggaaaaaatatctttgggcacatggtcaaggatctcctgagggctgt
gacatgggtaaaatatacatatatattccatatatatatatatatatattccactttcac
tcttttgtttttttgagaccaagtctcactctgttacccaggctggagtgcagtggtgtg
atctcagctcactgcaacttctgcctcccaggttcaagtgattctcctgtctcagcctcc
tgagtagctgggattccaggcgtgagccaccaagcctggctaattttttgtacttttttt
ttttttgagatagagtcttgctctgtcgcccaggctggagtgcagtggcacaatctccac
tcactgcaacctccacttccctggttcaagcagtttccctgcctcagcctcccgagtagc
tgggattacaacaggtgcatgacaccatgccctgctaatttttttatatttatggtagag
acagagtttccccatgttggccagactggtcttgaactcctgacctcaggcaattcacct
gccttgacctcccaaagtgcagagattacaggcgtgagtcactgcacctggcccttttt
ttgtttttttaagacagattctcactctgtcccccagactggagtgcagtggcgagatct
cagctccctgcaatctccacctacagggttcaaacgactcttgtgcctcagctttgcaag
tagctgggattacaggtacccaccaccacacctgtctaattttttgtattttttggcagaga
tggggttttaccatgttggccaggctggtctcgaactcctgacctcaggtgatccactca
cctcggccttccaaagtgctgggattacaggcatgagccaccgtggctggccattttcac
ttttgaaggatattgttagtgggcgtagaattctaggttggcagatattttctttcctca
gtttggaaacatggttcccttgtatctgatttatcttgtttttattgggaagccaattct
caatctaattttgctcatttgaaagtaatgtctttttatgttgttttgtttttttgagatg
gagtctcactctgtcgcccaagctggagtgcagctgtgcgatctcagctcactgcaagct
ctgcctcctgggttcaagcaattctctgcctcagcctccaaagtagctgggattacaggt
gtccactaccacgcccagctaattttttgtatttttagtagagatggattttttgccatatt
ggccaggctggtcttgaactcctcagttcaggtgatccacctgcctcagcctcccaaatg
ctgggattacaggcgtgagccgcccctcaccctggccagaaggcagtatctttttttcc
tctggctgctttaaaaagtttgcctttactttgagcagttttacactgatacatttaggt
ggctcttcattccatggcttgattctttttgtccattttagaaaattctcagttttatc
tcttcaagtattacgtcttccccatcctctctctactctccttatgagactccaatttca
catgacctatgccttgttaaagtatcccccatgtctcttaatccatttcctgtatgtttt
atctatttttctccttgtacttcattttgtatattttgtatcaaactgtctcccaattag
ccaggcgtggtggcgggtgcctgtaatcccagctactcagaaggccaaggtgggagaatt
gcttgaacccagggagggaggttgcagtgagcagagatcatgccagatcatgccattgca
ctccagcctgggtgacagagtgagactctgtctcaaaaataaataaataaataaataaaa
ataaaaaccctatctcccagttcacaattttttttttttttttttttttttgcatgtttg
tctctagtctgctgttcaaattcagttcctaattccatttttttttttttttgtgagact
cagtctctatctgctgcccaggctggagtgcagtggtgagatctcaactcactgtagcct
ccacctcccggttcaagcgattctcatgcctcagcctcctgaataactgggattaccacc
atgcctagctcatttttgtatttttagtagaggtggtgcttcgccatgttagccaggcta
gtcttgaactcccagcctcgtgtgattctcctgcctctgcctcccaaagtgctgggatta
taggcctaagccaccactcccagcctccattttaatagttgctagtttttgataaaat
tcttgtttctttatatccttgaatatagatataaagtacttattttaaagtacatggtct
gagagttccataatctggagatcctataggccttttaaaagttgtctgtgctttctctt
gaggtttgttcctgctgtcttatttccttgtttgcttggttgtttttaatttagcactgg
agggtatgtataaaaattgtggccgggcgcggtggctcaagcctgtaatcccagcacttt
gggaggccgagacgggcgaatcacgaggtcaggagatcgagaccatcctggctaacacgg
tgaaaccccgtctctactaaaaaatacaaaaactagccgggcaaggtggcgggcgcctgt
agtcccagctactcgggaggctgaggcaggagaatggcgtaaatccgggaggcagagctt
gcagtgagctgagatccggccactgcactccagcctggacgacagagccagactccgtct
caaaaaaaaaaaaaaaaaaaaaaaaaaaaattgttagaggctgggtgcggtggctcat
gcttgtaatcccagcacttcgggaggccgaggtgggcagatcatgaggtcaggagttcaa
gaccagcctggccaacacagcgaaaccctgtctctactaaaaagacaaaaattagctgag
cgtggtggcgggtggctgtaatcccagctacttgggaggctgagacaggagaattgcttg
aacccaggagatggaggctgcagtgagctgagatcgaggcactgcactccagcctgggca
acagagtgaaatgctgtctcaaataaataaataaataaaaataaaataatttgaggccta
ggagtctgaaattctgggatgtcctttatgcaatttgagtggctgagatgatctgaagct
ggatccagtgctcctgagggctgctctatttctggttgactgtcactcctagagtaagaa
acctgcaccccacgtgtggggcattatggcatcccctcccttagccacgtgagtaggtca
acagcactgctctagaccaggtgtggtggctcacgcctatagtcccagctactcgggaga
ctgaggcaggaagattgcttcaggccaggaatttgagaccagcctgagcaatatgttagg
ttggtacaaaggtaattgcagtttttaccattaaaagtaatggtgaccctgtctcagcaa
aataaaaagcaaaaaaaaaaaaaaaaaaaaaggaagaaagagaaaaaagaaaaaaaaaaa
tcaccaatctatttctcaggattggctgtcgccttgaggggaatgctggccttgtttgtc
tccagccctgtacttctctgcctcctatgcctttaagcatgtttcctatttgctgggctt
ttctagttctctgcgggaattgtggtctgaattacagtctcccattaccaggaagtctga
cttacatttaacatatttctttttttttttttttgagacggagtctcactctgtcgcc
caggctggagtgcagtggcgcaatcttggctcattgcaagctccgcctcccaggtttacg
ccattctcctgcctcagcctcctgagtagctgggactacaggcgcctgccatcatgtccg
gctaatttttttgtatttttagtagagacggggtttcaccgtgttagccaggatggtctca
atctcctgacctcgtgatctgcccgcctcggcctcccaaagtgctaggactacaggtgtg
agccatcgtgcccagccttagcagattttttttttttaatgtaagggagcaaggctgact
ctggggcctgggtaggaggccattgcaataacccaggtgagacgggttggtggcttggac
caatgtggtggagatggaggtagaacctccaacctgagtcgctggtgggttggatgtggg
gaaagaaaaaagtgaccaatccaggacggctcaagggtgtagggcgctgagcaaaagggg
ggagtggttgtgtctatttctaaaacaacgaggactaggagatgagccattttgcgggga
atgttggaaggaaaatcaaaagtacattttaggtcatggtatgtttgaaatgtttcttag
acagccggtgcaggtatcttgtgcatagctgggtataagtgtctggagtgtagggagagg
actggctggagataaaattggggagttaccagcacacaggctgcatctcatgccatgagc
ctagatggagtcacccggggagaacagataggtgcacaaggaccacggaccaagcctggg
gcccccaacatcaggagtcgagggaggtgggaggaaaaccaaagcctggcagggtagggg
aggtgggtgcagggtaccgggccgttccctcttggggctttggtttccccatctgtaacc
attttccccattttgccccggaagccaaaacagttgacaaggctggaagcagacaggttg
gggaagcaagggtgcattccatttatttaggaggtaagacctttgtctgggatgggaatt
taaagtgtttttccctccagtttgtcatttggctttgtttatggtgttttattcttatta
aaattagtgcttatgcctgacttgtttatttcatcttctggctctgtgctctctttccca
ttccaagactacatgtgtccctatggcgcccatgtgtgccacgttgggcccagggagggc
catgtggctcaggagctggggagggtttgtgtccagatggaaccgccaggtccagccaca
gacaccttagccgaaggagggtgctagggccagaggtcccatggggtgggtgcagggacc
caggtccactccctctttgggccttggtttccccatctgcaactgggatttaaatgctgg
cttctgaccgggtgcggtggctcatgtctgtaatcccggcactttgggaggctgaggtgg
gcagatcacctgatgtcgggagttcgagaccagcttgaccaacatggagaaaccctgtct
ctactaaaaatacaaaattagccgggcatggtggcacatgcctgtaatcccagctactcg
ggaggctgaggcaggagaatcacttgaacccgggaggcagaggttgtggtgagctgaaat
cgtgccattgccctatagcctgggcaacaagagtgaaactctgtccaaaaaaaaaagaaaa
agttggcttcttgaggttctggggagcattcctgggtgtaagggaagaggttgtcctcgg
tgagctgtgcaggcctgtgccccagggacagggtcacagtgtaggccaggctgaccagcc
cctcagcactgaagggcccacctcttgctgcccatgatagatggggaggggtaggccaga
gcatgcatttctttcttttctttctttctttctctctctctttctttctttcttttttct
ttctttctttcttttctttctttctttcttttctttcatctttctttttctttctttctt
ttctttcatctttctttttctttctttcttttctttctccttccttccttccttccttcc
ttccttccttccttccctctctctctctctctgtctctctctttctgagatactcttact
ctgttgccaggctggagtgcaatggcgcgattttggctcactgtaacctctgcctgccag
gttcaagcgattctcctgcctcagcctcccgtgtagctgggattacaggtgcccaccatc
acatacagctaagttttgtatttttagtagagacagggtttcaccatgttggccaggctg
gtctcgaactcctgacctcaagtgatccgcctgcctcggcctcccaaagtgctgggatta
caggcgtgagccaccatgcttggcctaggccaaagcatttccataggctcaggccccag
gagaatttgtggggcttgtcagctcaagccccatgccctggtggtgggaaggatcctggg
gccacctgccaaagaccagctttgaagtagttgtggccagagtgtccctagtgagatggg
gagtggagtcccatggtgggctccagggccattgtgagggactaagaggtggcctaggcc
tggttctgcctatggcatatgagctggtgctgctgggagcaatggcaacagaagcacttt

gcaaactgagaagcaccttgtatatggcaaaggtcagtgtaaatggcatcacatttgcag
ctgtagaatgttgtggacctgtgagcagctggatttgatggggaagcctggttgtgttct
gtgacctaatctggggtttggtggcagcagatgggccacctctgtcaccattctccaatg
agtgccagctgagcaagcctagtggggaggcagggggaggggagccccaccgccgactct
gtgccgtgtcccctagagccatcctccagcagcagggctcacccgtggacgccaccatcg
cggctctcgtctgcaccagcgttgtcaatcctcagagcatgggcctgggcggaggggtca
tcttcaccatctacaatgtgacgacaggtgggcccacatgtgacctgccatggggaagat
gggcttgtccacaccacagaggaaccccttctccagccagtcccatccactcaagaccgc
ttctgggtgctcttgggccccctgccctgccctcttcacctcagctgtgcaaagcccttg
tgtcaactcctgtccccctgttagcccagagtccatctcataatgcagagatggaaactg
aggcctggagcaggccaggggctgtctgcagggtccgtgaccgctcacctgccctgccct
actctagggaaggtggaggtcatcaatgcccgggagacagtgccagcccaccacgccccg
agcctgctggaccggtgtgcacaggctctgccgctgggtagaggtgaggccccatggggg
tcccccactcccaccccccaggccactaggactgaccacaacacccccccaaaggatcagt
gcctcatggacaggctgtgactccctggcactgggggacagggagtctatggggaacccc
caaccccaacctgcagagattgagggattgggaaggtccccccttgggaatgggcgagctg
aggtgatcctgggagagggccaggctggacaaaggcctggaaggttgagtggagatgggt
tggtgggacagggtggggctcctggggccagtttgtgcagggccccttgggcctggccat
ggactttgtggggagccacaggtggtctgagcagggagggacatggtcgggtgtgggctc
cagaaagctcactctggctgctgtagctgggggccacacgtaggggtgatggcgaaccc
ctgggaaggcctggaagccacaggtttgagtgctgggatgtgccatgcaggggcccagtg
gatcggggtgcccggggagctccgcggctacgccgaggcccaccgccgccatggccgcct
gccctgggcgcagctgttccagcccaccatcgccctgctccgagggggcatgtggtggc
ccccgtcctcagccagttcctgcacaacggcttcctgcggccttccttgcaggcgtcaac
cctgcggtgagccccacatggtggccctgggctcctgggttcaaggccacatcctgtctg
ggcccagtccacaccccagctctgcctcagtccctcctacactggaggattgacccttgg
ggtgctggggaccttaaggcccagacagagagcactttcctgtttcagtttatggccttc
cggtcccctgtggcacagcctaactcgggtgaggctgcggggagtttgtcgcccaagtgc
taggactccacggtgtggatttgggctctgccaccagccctggccccggcctcctcctcc
ctctctctgtcatggcaggagggcttcaactgccattgtcacccgccccacccagcccac
tggcagcccctcccaggagtgaggagatgacaggccctaggtggacccaggccccactgg
gaaatctgccctccctggaccagggccatgagcggggtgggtggtgtcagtcccaccacc
tcctagggaatctgtcccccggtgtccaccctagcgcctcgggaccctgggtcagggcac
ccccactgtagggtcctgaggactccacccacgagagccccgaaagagtgtggctgcctc
taggacctgcccccaccctgaggctccccccatcccccagccagctcttcttcaacggga
cagaacccctgaggcctcaggacccactcccatggcctgcgctggccaccaccctggaga
ccgtggccacagagggtgcggaggtcttctacacggggaggctgggccagatgctggtgg
aggacattgccaaggaaggtcagcctccttgagatcccatgccctgtccctgctggagga
agagccaagttctgggcactggggggttgagggcaggctccttcccaggccctgagggagt
gctggaggtgcctgggaccccgagaggtcatgcacctagctctgccagcccaaggacccc
tgtggtccaggacctcagagtgcagagcctgcgggttagagccagcagaggggcgttact
gatggtcacaacaggcctgggtagctgagctgaccgttctcacaggctctcaaaggccag
ctccctctcccatcttccccttcacctccccagcctccctgcctctggtctctgtccacc
cctccatgcttgaccccaggggggccggtcctgaggccaccgcctacagaccaaccatgac
cctctgtgtccacaaggctagagcacgagtgggatcccattgtgtgacctgacaccagtg
gctattcctccctgcctcagtttccccatctgttcatggagccctctccgtctcacccca
gggagccagctgacgctgcaggacctggccaaattccagcccgaggtagtggacgctctg
gaggtgcccctgggggactataccctgtactcaccaccgccacctgcaggggggtgccatt
ctcagcttcatcctcaatgtgctaagaggtaaagcccctgcccagagctctggcccccca
cacccgggaactcagactccaagcccagccaaggcccagctcagcctcctcctccataaa
ccgggttcacagatgcacaatgtgaagactgagctgtgtaaaccagtcatgatggtgggt
tttagagctgacctccggctccacccatacgtgaggctttgctgaagggtgtgacatcgt
ttgtccagagtcctcctcccactaggtgaccagtcccagactcagaagcaggtgggctgt
gtgggtggtgggctgcccatcacggacatgcacctgggggagtgaaaccagcccagccca
aggcagcctgcaggggggttctggcagtgccaaggcctggggctgtctgtgctggcttaat
attagtgtcctgtgtctgccattacaaatcaccacaagcttggtggcttaaaataacagc
aatgtgtcctctcacagttctggaagccagaagcccgatatctaggtgtccacagggctg
cattcccttggggggctccaggggagagtctgttctttgccttgttgacctaaataaaaaa
cagaacaaggctgttgtttctttgaacaacattcagagaaaatgatgtttattccagagt
aggcatcgcggtgggaatacccacgctacggtaaactgtgtgtgtgttcagggaggtgaa
ggaagacaaaggttctcaaaaagaaaatgaggaagagtatataattttttttgcttataat
caatgataatttattgtatattttatttttttcattgcattgttattattattattgaga
tggtgtcttgctctgtcacttaggctagagcacagtggcacaatctcggttcactgcaag
ctctacttcctgggttcaagcagttctcctgactcagcctcccaagtagctgggattaca
ggcatgggccaccacatgccgctgattttatctttttttttttttccttttttttttttttg
agacagagtcttgatctgtcgcccagactagagtgcaatggcatgatctcagctcattgc
aacctctgcctctcaggtacaagcgattctcctgcctcagcctctcaagtagctgggatt
acaggtgcctgccatcatacccggttaattttgtatttttggtagagataagtttgcca
tgttgatcaggctggtcttgaactcctgatgtcaggtgatccgcccacctcggcctccca
aagtgctaggattacaggcttgagccaccatgcccagatgattttttttctttttagtaga
gacggggtttcaccatgttggcctggctggtcttgaactcttgaccttaactgacccact
tgccttagcctcccaaagtgctgggatttcagacgtgagccacctctgcctggctatttt
atttttatttcattagttttggggggaacacgtggtgttcggctgcatggaaaagttattt
agtggatgacttctttttttgagatggaatctcactctgtggcccaggctggagagcagt
ggcatgatcttagctcactgcaacctctgcctcccagattcaagcaattctccagtctca
gcctttttttagtaaaaaaggggtttcaccatgttggccaggctggtctcgaactcctgac
ttcaagtgatctgcctgcctcagcctcccaaagtgctgggattacaggcatgagccactg
tgcctgtcctttagtggtgatttctgagaccttggtgcacccattacccaagcagtacac
actgtacctagtgtgtagtcttttatccctcatctgcctcccacccttccccttgagttc
ccacggtccattatagcattatccccctgagacagagtctcgctctgtcaccaaagctgg
ggtgcaatggtgcatctcggttcactacaacctccgccacctggattcaagtgattttcc
tgcctcagcctcccaagtagcgcctgccaccacatctttttttttttttttttttgtatta
ttagtaaagatggggtttcaccatgttggccaggctggtcttgaactcctgacctcaggt
gatccgcctgccttgtcctcccaaagtgctgggattataggtgtgagccaccatgctggg
ctccattatatcattccggtttttttggagatggagtttcactcttgtcacccaggctgga
gtgcaacgcaacggtgagctctcagctcactgcaacctccacctcccggttcaagcgatt
ctcctgcctcagcctccctagtagctaggactacccatgtgtgccaccatgcccagctaa
tttttatattattagtagagacagggtttcatcatgttagccagactggtctcgaactcc
tgaactcaggtgattcacccgccttggcctcccaaagtgctaggattataggcataaact
actgcgcctggcccagtatattgtttttattcctttgcgtcctcatagcttagctttggt
atataaagctaagcttagctttgcgtcctcatagcttagctttggtatataactgttttg
ggcttggcttggtagctcatgcctgtaatcccagcgctttgggaggccaaggcgggcaga
tcacaaggtcaggagattgagaccatcctggatagcacagtgaaaccctgtctctactaa
aaatacaaaaaattagccaggcattgtggtgggcgcctgtagtcccagctgcttgggagg
ctgaggcaggagaatggtgtgaacccaggagatggagcttgcagtgagccgagatcatgc
cactgctctccagcctgggggacagagtgagatccgtctcaaacaaacaaacaaacaaac
aaagaaaaaaaccgttttgaaatgatcatctttggttactgggatgcatggaaaagtgata
agggtggcactagtctgaggctggacaggccactcctgggcagatgtctttgcagaagta
ttttttgtgtaaggttgtgatggcctttgtgtaaggttgtgagttgtttgtttttttgagg
cagggtcttgctctgtcacctgggctggagtgcagtggcactgtcaaggctcactgcagc
ttcaacctcacctcaatcctgtcacctcagccttccaaagtgctgggattacaggtgtga
accaccacatgtggctgcagctctattttgacaattttcaccgcctcttaccacttccag
tggctgccagcgttccttggcttgtggctgcctcactccagtctctgtctccatggtcac
actggtttctcctctgctctgtgtgtcctctcctctgtgtgtctgtcttacaagtacact
gtgatcacattcagggcacatctagataacccaggatcatctcctcctctcaaaatgttt
tacatactttaggctgggtgtggtagctcatgcctgtaatcccagcactttgggaggcta
aggcgggtggatcacttgaggtcaggagttggagaccagcctggctaacacagtgaagcc
ccatctctactaaaaatacaaaaattagccaggcatggtggcgtgcgcctgcagtcctag
ctactcagaaggctgaggcgaaagaataacttgaatccaggaggtggagattgcagtaag
ctgagatcgcgccacacactccagcctgggcgacagaggaagactcctccaaaaaacaca
aaaaaggcggggcatgatggctcatgcctgtaatcccagcactttgggaggccaaggcag
acagatcacttgaggccaggagtttgagaccagcctgaacaacatggcgaaaccctgtct
ctactaaaaatacaaaaaaagaaaaaattagctgggcgtggtggcgaacgcacgtagtat
cagctactcaggagactgaggcacaagaaccccttgaacctgggagatggaggttgcagt
gagctgagactgcaccattgcactccagcctgggcgacagagtgagactctgtctcaaaa
aaaatcgttaacatacttttccacttaaggtattagtcactcttgtgctgtgtaagtagt
atccacaggttttgggaattaggatgtgggtggagctctctggggagggggcaacattc
aaccctttacatagggtgaccccaacctgtgccccgacccctctccagggttcaacttct
cagcagagtctgtggccaggcctgagggagggtgaacatgtaccaccaccttgtggaga
cgctcaagtttgccaggggggcagaggtggaggctggggaccctcgcagccacccgaagc
tccaggtgaggttgctgaggttgctgggatggggggctgtcctctctggctcaggactta
gcatgaaaggagggtcaggcctggcaggggggaggttggagggagatgtatgtggttctag
gccagggcaggactgaaagggatcccggggtggcaggtacgggggtcagatgcaggagtg
gcaccatatctcaaaggacctggagggtgggcagagtctagacttagcctgcgcttgagg
gaggcctggccacaaggtagaggacaggctggaggtggcccccatgggagctgagcttgt
cctgcccttggttctatggatcccgcttggcccctcactgatcctgccacctgcccaccc

accccagaatgcctcccgggacctgctagaggagaccctggcccagctcatccgccaaca
gatcgatggccgggggaccaccagctcagccactacagcttggccgaggcctggggcca
cgggacaggcacatcccatgtgtctgtgctgggggaggatggcagtgccgtggctgccac
cagcaccatcaacacaccgtgcgtggggcctggggaaggctggtggcctcactcctcct
ctcctagacctgcactcccccagccccatgtcccctcacttgtccccatggggcagcacc
ttgcttttgccctttgtctcctcctctgtttcaaaagaggcccccacccctgacacctct
ggctggaagggctgctgctggggtggccccgagctaagacttacctgggaatgggcggcc
tcactcagaagggtgccctgatatgggggcacaggtgggtctttggggacccctcctggg
tgatgccagggagagaacagtggctccagcatgcttcggggcagctgtaaagtgaggggg
tcctgcaaggtgggcagggcaggcggtgtctggtgggagcccaggctctgcctccacggc
tgcagctttggagcgatggtgtattcaccacggacaggcatcatcctcaacaacgagctc
ctggacttatgcgagcgatgcccccggggttccggcaccacccccctcacctggtgagaac
aaggcttcccacctgggatccacaagggcccccccaccaggggagaggagagagggggctg
ggctggggctgcatgctgaccctggatgggtcactgcacttgctaagccgctgtttgct
cagcagtgaatggagacagggtgggtggagctcccggagggtgctggcccccagttccag
gcgagcggtccccatcctccatggtgccctccatcctgatcagcaaagcccaggggtcga
agctagtgattggcggggctggcggggagctcatcatctctgccgtggcccaggtgagtc
tgggggactctgggctccagtgtctcctctctgggcagcatactgtctgactctccctg
gagtggggatgtgagggctgaagtagggtagagggtacccccttctccctgcatccctc
atctctcccccaggccatcatgaacaagttgtggcttggctttgacctgagagcggccat
tgcagcccccatcctgcatgtcaacagtaagggccgtgtggagtacgagcccagcttcag
ccaggtgaggctgaggtccaagctggatgcctagggcagagcccactccccaagtccgtg
ctgctcaaagccacctgagaggaactcagtcactgagattcttaggccaggtacacttca
gctttggggggccataggagttggggacctcgatgggtgaggctgtcagtgacctccagg
ccagttctgtggcctccaagacagacagtagggtgttgtctacactgctcccaggctgag
gatctcagcaacttggtctctctggtctgtcattgatgccatttttcagaagtgagtttt
cctagctggggcctctcagactctccctcatggtgactatttccttgtgtgatttgtaac
ttatatatttgtgactttatttgtaaaagaggttgtttttcctgtggcagtctataaagt
gattttcttctggggcattggtggtaatttttactacaggtgtgtgccaccacacctgga
tagtttttatttttttgtttttcgtagagacagggtctctcgccatgttgcctaggctgg
tcttgaactcctgagctcacgcaatcttcccaccttggcctcccaaaatgctggaattat
aggtgtgagccaccatgtctggcctacaaacatttttttgattccccacaaaagcccagg
gtgcagctccaggggctgaattctcctggttgactcctcctccatccatagacagagact
tgccttccttggagaatctttgggttgatggatgccctttgttatgacctatttaaaaga
ttgataggtccgggcactgtggtcatggctgtaatcccagcactttgggaggcagaggca
ggtggataacctgaggtgaggacttccaggccagcctggccaacatggtgaaaccccgtc
tctagtaaaaatacaaaaattagccaggcatggtggcgcacacctgtaatcccagctact
caggaggctgaggcaggagaatcgcttgaacctgggaggcggaggttgcagtgagctgag
atcgtgccattgcacgatttttgttgcctgggcaacaaacgcaaaattctgtctcaaaaa
aaaaaaaaaaaaagattgatatattttcaagactccaaacttggttcctgaggtcagttc
agttcagctaactgaggttcaaggtcctcaaagctgcctctcctgttatgagatttcttc
ggtctcaagtccaccttccaagcccttagaacctgagaaccatggactgggaccttagtg
tagtaacagagtgtagtccccaacagtcttagtcttcacaccttgagactaaggacctca
gcctgttaggctcaaattcttggagtttgcaggtctccctccccatgtagggtccttcgc
atgacaggaagaccctctcagggctctcaggttccagggatcttagatatggaggatttc
aagatccagggcacatgctttggagccctcctcccttgactctttatttatttatttatt
tatttatttatttatttaggcagtctcactctgtcactcaggctggagtgcagtggcaca
atcttgcctcaccacaacctccgcttcccaggttcaagcaattcttctgcctcagcctct
gagtagctgggactacaggcgtgcaaaccaagcctggctaatttttgtatttttagtaaa
gacggggtttccggccgggcgcggtggctcaagcctgtaatcccagcactttgggaggcc
gagacgggcggatcacgaggtcaggagatcgagaccatcctggctaacacggtgaaaccc
tgtctctactaaaaatacaaaaactagccgggcgaagtggcgggcgcctgtggtccca
gctactcgggaggctgaggcaggagaatggcgtgaacccgggaggcggagcttgcagtga
gctgagatccggccaccgcactccagcctgggcgacagagccagactcagtctcaaaaaa
aaaaaaaaaaaaaaaaaagacggggtttcctcatgttggccaggctggtctggaactccc
aacctcaagtgagctgcccaccttggcctcccaaaatgctgggcttacgagcatgagcca
ctgtgcctggccctcccttgactcttgactgaaggacctttgtcctcgtgaacatcaatt
ctcaggacctttcactctgaggacctgaaatgctgagaatttgggagatgacagtctggg
gactgggattaatggaatccagtggcccacgaacttaaggttctcagttcccttggggag
ttggaatgtcagctattcaggtctagggctttccatggagtaaatcctaaactctgggtt
ggagacttcgagcctccaaggaccttcacagctaaggcccagggactagggtgaggagag
tctttgatcctcagagtcttggagtttggccagtggactctgaggaatggagtctctgag
cactgtaagtccagctttggcttcagtagtgaggatcttggccttcaagtctaaggacag
tgggcaattccacggattgtcaggcacggggaacttatagccaaatgtgcagggctccaa
agacctcatttgccctgtcagcagctcaggccatgtggcatcacctgaagcacctagatg
tctccggaatctcaggccctgcggggtaaggtccccgggccattagtcttttttttttct
ttcttttttttggcttgttattgttgttgagacacagtttcactctgtcacccaggccgga
gtgcagtggcgcgatctcagctcattgcaacctccacctcctgggttcaagtgattctcc
tgcctcagcctcccgagtagctgggattacaggcccacaccaccatgcctggctaatttt
tgtattattagtagagatggggttttgccatgttggccaggctggtctggaactcctgac
ctcaggtatccgcctgcctcggcctcccaaattgctgggattacaggtgtgagtgactgc
acccagccaagttctcaggtcattagtcttaaatggtatctggaaacacggggccctcgt
agcacaggtccctgtactctagaacctctgcccctttacagactggtagttcgtgaggac
catagcccccaggcaaggtacttggcatcttaggaacaagcacaaggctagggctacttt
ggtctctgacttccttgaagtccacaaattcagggtcttgagtctgagcctgtaggcccc
catgccctgagtgtctcacactgtgggactgtgtgtgcctttagcttagggatctaattt
ccttggattcatgggtattcacgtccttatcctagctgaattctccacccacagacacca
gaagggccctaaagttgtgggttcctgggtgtctaaggtcttgacatcgtgtgttgagat
cataggtctttttttgaaaatttttttgaggcaaggtcttgtaaccgcggattcatcaagac
aggggaattgcaatagagaaagagtaattcaggcagagcctgctgggtgggagaccggag
ttttattattactcaaatcagtctccctgaacattcggggagtagagtttttttcgtgttt
gttttgttttgttttgttttgtttttgagacagagtcttgctctgtcgcccaggctggag
tgcagtggcatgatctgtaacctctgcctcctggggtcaagcaattctcatgtctcagcc
tcccaagtagctgggatttcagatgtgtgccaccaagcctggctaatttttgtattttta
gcagaaacagggtttctccatgttggccaggatggtctcgaactcctgacctcaggtgat
ctgcccacctcggcctcccaaagtgctgggattacaggcatgaccaccgcacctggctag
ggtgcagtgtttttaaggacagcttggtgggttgggggaagccaatgagccaggagtgct
gactggtcagggatgaaatcacagggagttggctgggcccggtggctcacgcctgtaatc
ccagcactttgggaggctgaggtgggtggatcacctgaggtcgggagaccagcctggcca
acatggcgaaaacctgtctctactaaaaatacaaaaattagctgggcatagtggcaggca
tctgtaatcccagctactggggaggctgaggcaagagaatcacttgaatctggaaggga
agttgcagtgagccaagatcatgccattgcatgccagcatgggtgacagagcaggactcc
atctcaaaaacagacacacacacacacacaaaccgggaaatcgtagaatcttagggagtt
gaaactgtcttcttgagctgagtcagttcctgggtgggggccacaagatcagatgagcca
gttaatccatctggatggtgccagctgatccatcaagtgcagggtctgcaaaatatctcc
aacactgatcttaggagcagtttagggagggtcagaatcttgtagccttcagctgcatga
ctcctaaaccgtaatttctaatcttgtggctaatgtgagtcctatgaaggcaatctagtg
ctcaggcaagaaggaggtctgctttgggaaagcgctgttactgtctttgttttaaactat
caactaagtttctctcaaagttagttcagcctacacccaggaatgaacaaggacaactgg
gaggttagaagcaagatggagttgattaagttagatctctcactgtctcagtcataattt
tgcaaaggtggtttcagtcttgctcttttgcccaggctggagtgcagtgtcacgatcaag
gctcactgtagcctcaaactcctggcctcaaggatcctcctgcttcagcctcctgagta
gctgggactacaagcacagggccaccacacccgactaatttttttttcttttcttttctt
ttttaataaagacgtgatctcgctatgttgcccaggctggtcttgaactcctgagctcaa
aagatcctcctgcctcagcctccaaaagtgctgggattacaggtgtgagccaccgggccc
agcccaattttttgattttcttgtagagacggtgtcttgctttgttgttgccctggctgct
ctcaaactcctggcctcaagtgatcctcccaccttggctcccaagtggctaacactgtag
gtatgtgccaccatgcctgccttttttttttttcggagaaacaggatctttctgtattgc
ccaggctgatctcaaactcctggcctcaagtgatcctccttcctcagcctcccaaagtgt
tgggattacaggtgtgagccactgtgtctggcctcagtgtctaaatcttgagaagccatg
tccttcatcgaaggccccctgaactcagatttgtacttcaaggccctggtgtccttggat
ccagaacagacatgaacttgcatctggaggtcttcaggcctgggtggtgtccttcataca
tagtccctggaggctgaccttgtcctctatgggcctttagatctcttccatgctcccatc
aggtcctcccctcttcctgtgtcttcaggccctcttcttctcagtcttgccttttgatca
tttttttcatttcttgcagaggcccctcccttcgggagatgggctgagctgccctcccct
gtgaacctgctttctctcctctccaggaggtacagaggggactccaagaccgtggccaga
accagacccagaggcccttcttcttgaacgtggtccaggccgtgtcccaggagggggcct
gtgtgtatgccgtctcggacctgaggaagggtggggaggccgcaggctactgagacactg
ctctgcccagagctgaagtccggccccaccatgggtcctatgtccaggccggacatggct
gggggaccaagtactccggcaggatctggacccctggcaggggagtccagctgagagtgg
aagaggtggcagggaccagctgggcagatggaggctgagccccgtccctgaccccctttt
cccagagcccctggtggccctgaacgggcctctctatccctcctcaggcctcttgcctgg
ggccactctcccacccgctcaacctgtatatcctccagttcaagattaaagaagaggcgg
acagtggcctgattccatggttctacgtgggggaggggaccatcggacagtgcacagagg
ggtgcctgcaccaggctcctatggttctccacactgcaccaccatggaggtggggaaact
gatgctcgcagaccagccgaacctcagccaggccacccctggacagagacaagcgccacc

tctgcctccccaacacctcaccactctggtggccaggactcagcagcctgactggcccca
gtcctgagagctggttaactgaaaaatcacacaattcacaaatttggaaaagatactttt
tctttcttctttcctttcttttttctttcttttctttctttctttctttttttttttt
tttttttgagatagggtcttgctctgtcgttcaggctggagtgcagtggtgcaatcacag
ctcactgcgctcttaacctcctgggctcaagccatccttctgcctcagcctcccaagtaa
ctgggactacagatgtaacctggccaattttacattttttttttgtagagacagggtct
tgctttgttgcccaggctggtcttgaactcctggtctcaagccatcctccctcctcagcc
tcccaaagtgctgggattataggtgtgagccactatgcccgacaagatttcttttttttt
tttttccttcaacttgtattttaagttcaggggcccatatgtaggatatgcaggtttgtt
acatagataaacacgtgctttggttgtttgttgcagagatcaacccatcacctaggtatt
aagcccagcatccattagctattcttcctgaagctctcctcccatcccctgactttcttt
tttttttttttttttttttcctttgaaacaaagtctcactctgttgccgaggctggagc
gcagtggcacaatctcagctcactgcaacttctgcctcccagcttcaagtgattctcctg
cctccgcctcctgagtagctgggattacatggggtttcaccatgtggccaggctggtctt
gaaatcctgacctcaggtgatccacctgccttggcctcccaaagtgctgggtgattacag
gtgtgagccacagtggcaactttatttttcataaagagttacaacctgcagggtggccat
ccaccactttcaaaagcatagcctctggcagagaccagaaacaggcacttcttttttttt
tttttttttttgagatggagtctcgctctgttgcccaggttggagtgcagtggcgcgat
ctcagctcactgcaagctccgcctcccggttcacgccattcttctacctcagcctcccga
gtagctgggactacaggcacccgccaccacgcacagctaattttttttgtatttttagtg
gagatggggtttcaccatgttagccaggatggtctcgatctcctgaccttgtgatcagga
gataggcacttctaaggaggagaggttggggcaggagctatatgctgaaggggttggcta
aacatacatattcaacaggttacagggagagctatgaatattcatgaaggcagtcctgac
acgtgtattgaaagaacatgcatgtgacataggactcatgttcactgtaggatggagatg
gtggagacttaatattaaaatgtattacaaggccaggcacagtggctcacacctataatc
ctagcacttcatggagccgaggcaggtggatcacatgaggtcaggagttcgagaccagcc
tggccaacatggtgaaaccccaactctactaaaaatataaaaattagccgggcacagtgg
cgggtgcctgtaatcccatctacttgggaggctgaggcaggagaattgcttgaactcagg
aggcggaggttgcagtgagccaagatggtgccactggactccagcctaggcaacagagtg
agactccatctcaataaaaataataaaataaaataaaataaaataaaataattacaatta
ggctccatctgtcgaaagatcttttcagggcctgaaggcctgcaagtgagcagcctctga
aaaccaaccagagcagttcatggtcaatgatctcttatcaggcgaaagttactgacattg
gtctcttgtccagtcagagctgtagttatggctggaagaacagggggtcaataagtcggc
atccgtg

>BCRP3.HUMAN.NCBI.REF
actccgtagtgtgcacttggtgagggcagcagctcgccacagctgccagccgtctgtcca
ttcacccatctgtccatctggcagcccgctgttcagacccgtctgtctgtccgcccatct
gtaagcccatctctgtcccattgtctatctgaccatctttctcttactgtcctctttgtc
tagctatctggcctatctgtcgatccatcttcgtgtctgtcttcagcccccacctgtttg
tccatctgtccaattacctgtgagtctatctatgcaccttcttgtccattcatctgccca
cccatctgtccctccgtctgcccaccggcctcccctctccttctgggccgcagagccatg
gcccaggactgcagagccatggttggcctggtcctgctggggctggggcttgtgctggct
gtcattgtgctggctgtggtcctctctcgacaccaggccccatttgacccccggcctttg
cccacgccgctgttgctgctgactccaaggtctgctcggatattggacggtgagtgagac
gtgggaggaagctgggtggcccttggcagccagcccctcctggagaaggcgtgtgtgtga
gagtgtgtgtgtgtgagcatgtgtgtgtgtgagagagtatgtgtcagtgtgtgtgggtat
atgagtgtgagtgtggggtgtgggtgtgtgtgaatgtgtgtgatcgtgtttgggtgtgtg
tatgtgtgagtgtgggtgtgtgtgaatgtgtgtgagtgtgtttgtgtgtatgtgtgagtg
tgggtgggggtatatgagtgtgagtgtgtgggtgggtgtgaacgtgtgtgattgtgtttt
gctgtgtgagggtgtgtgtgactatgagtgtgtgagtgtgggtgtgtgtaaatgtgtgtg
attgtgtgagtgtatgtgtgggtgtgagtgtgtgagtgtgagtatgggggtgtgggtgtg
tgtgaatgtgcgtgattgtgtgtgggtatgtgtgtgtgtgtgagtgtgtgtgtgtgcg
tgtgtgtgcacgtgcactggcccaggcagcaggagccatgtgtgtgggcttcagcacctg
cagggcttgagcgcaaggagacagcctcagggcccttgcacagaacaggcggcagggtgt
gcccgtggggcagatggggacttggggacaatggtggtgtgtgagtccatacctggctcc
aggattcaggaggcccatttgcacatcccaggtgggaacctgtctggccccggctgaccc
tgctggccggtgcaggccccttcagtgaggccaattctccaaggctgcggtcttctccca
gggtcatgggtgaaggggtttggaggctccctgcgtgggtactggcctgctgggttacac
acaatgctgccatagccagtctgcccctacacccagcctggggccacatctcaggtctct
cagtcctgaggagcccggtgccccacccctcacatcctctctccctgagtcagggcctgg
gtctcgtgagctgagtgactgatacttggtgtcctggatgagggcgtgatggagaggggc
cacagcgggtgtttcctgaccctcttccaggaaggtgctgctgccgctgcagggaggaca
catacaggatgccccttcctgccccctgcctcccattgggcccacaaaagccagggcaag
cctcccctccctgccagccacctggtctgcttcccagaaattctgtcttgcaggctgttg
ggaggatcccagtactttgtaaactaaagcaagggaggagtggccgttctctctgttcat
tcattcaccttttcattcattccttcttccctccattcccccatctgtccatccttccct
gccctgattgctcatgccaccgcccccgcagcccctcctgacctggtcctttggtttct
cttcagggatttctgtctcctcccacagggctgagaatggcagctcagggacaagtaggg
gctggggactgcttagtctccccagtggctctcaggggatttgagggtttgacgccagct
gccaccccaggctgtgcccctcctctgctcaggaggacatacaggatgcaacacccactt
aaactcgaagttgcaaagatgcaaatgagactggggtctcaggcaccagagaccacccgt
gggcacgtggcttttgggattggagacctgctgccacagatctctgaagagtctggacct
gctgggtctccccaagtgactctctggggggtctccatagcatgccctgctgtgtgcatga
cggtcactggttgggtaggggtctctactctaaagctccctctgccggcatcccctcgaa
ctctcccttggtgaagagagaggatgtggtttgccccagtgttttatcaaacaactctct
ccacttcctgttttaagaagctgggagtggaagagagcctggggctggccccagctgctg
ctgcgaaacaggggtcactggacgctgggaccctggccgggctggctggaggcctcagga
agaggcctgctacagtgtcatcctggccaagattcctccctgcagaggaccctggccacg
ctgccacagggtctgctggggccaccagaagcccatgctcctgcctccatctctcccctc
tgtgctcacctctcaccaggaggccctcccagagttcagtgtcctgctttttttttttt
ttttagatggtgtctcgttctgtcaccaggctggagtgcagtggcgcgatctcagctcac
tgcaacctctgcttccttggttcaaatgattctcctgcctcagcctcctgagtagctggg
actacaggtgccagccaccacgcccaggtaattttttgtatttttagtagagacggggttt
caccatgttggccaggatggtctctatctcttgattcgcccgccttggcctcccaaagtg
ctggaattacaggagtgagtcatggcacccggcctcatctcctactctttcagcaccagg
ttttactcttgggattctgctacagccgcagcccctgggtgcgagttcctaagctttctg
tgagtgtggacccagcaccgtgcctagtagacatacaaaaggagcatggtgacagtgagg
tctgtcatctccagcataatgactgttttgatccttgtaaaaaaggtgatttttggctgg
gtgtggtggctcacacctgtaatcccagcactttgggaggccgatgggggtggctcactt
gaggtcaggagttggagcccagcctgggcaacatggtgaaaccacgtctctactaaaaat
acaaaaattagctgggcatggtaacggatgcctgtaatcccagctacttgggaggctgag
acaggagaatcacttgaacccaggaggcaaaggttgcggtaagccaagattgtaccactg
cactccagcctgggtgacagagcaagacttggtctcaaaaaaaaaaaaaaaagaaagaaa
gaaaagtttatatttttgttctaatggttatcttaatatcgtcattctataattgtatgt
tttatataattataatagctatataagatataatacccctagtatgttgtttttggata
ttctacttgctcctgatggttaatttatatgtcaacttggctaagctatggtgccccgtt
gtttggtcaaatacttgtcaatatcttgctgggaggttatttcatagatgtgattaacac
tgacagtcagttgactttaagtaaaacagattacccaccataatatgggtgggccacctc
caatcagttgaaggccgtaagaacaaaaactgaggtttcccagagaagcaggaattctgc
ctcaagactgtaacacacaaaccctgcctgagtttctggcctgctgactgctctacagag
tttaggttccagacttcgagatcaactcttacctgaatttatagcctgctggcttgccct
acagattttaaacttgctagtccccacaatcatgtgagccaattcctcaataaatctctc
tctatgtataatctattggtttagtttctctgaaaagctttcacatccagtttcctggat
gttaagaattactgaaactagctagtaacttcttttttttttttttttttttttttttga
gacagagttttgctcttgttgcccaggctggaatgcaatggcacaatctcagctcaccgc
aacctccacttcctgggtccaagcaattctcctccctcagcctcctgagtagctgggatt
acaggcatgtgccaccatgcttggctaattttttgtatttttagtagagacagggcttctc
catgttggtcaggctggtcttgaactcccaacctcaggtgatcagccgccttggcctcac
aaagtgctggaattacaggcatgagccaccgcacctggctcctagtaaattcttcttttc
cgtgatgtgtctcttacctctaataatacttttcttcttttttttttttttgagacggagt
ctcgttctgtcgcccaggcgggagtgctgtggcgcgatctccgctcactgcaagctccgc
cttccgggttcacgccattctcctgcctcaacctcccgagtagctgggactacaggcgcc
cgccactgcgcccggctaattttttgtatttttagtagagacggggtttcaccgtggtct
cgatctcctgacctcgtgatccgcccgcctcggcctcccaaagtgctgggattacaggcg
tgagccaccgcgtccggccatacttttcttcttaaagtctacttcattaaaaatagttat
gctgggcatggtggctcatggctgtaatctcggcactttgttggaggtcgaggtgggtgg
atcactgaagcccaggagttcaagaccaacctgggcaacgtggcgagaccctgcctctac
aaaaaatacaaaaattagctgggtgtggctaatatacacttgtagtcccagctacttggg
atgctgaggtgggagaatcgcttgagcctagaagggagagattgctgtaagccaagatca
catcactgcactccagcctgggagacagagtgaggctctatctccaaaaaaaaaaaaaaa
aaaagttatacagctttcttggttagtgcatgcatgccatatttttcattattttccacc
tctctgtatccttatataaaaggcattagttgggttttactttattttcaattattttaa

tttttattgtccttttaaatgtaactaatgatttatttgggttgaaacccaccaccaatt
tgttttccatgcctattctatttcttcttatctcctctcacatcttgttttggatttatt
atttttattatttaatttcctccttctctattagtttcatagctctgcagtcttagagtt
attttaaaagatgacagtggattattttagagcttacaacatgcatccttcacttatcaa
agtctaacatgagctagtacttttttgttgttgttgagatagagagagtcttcctctgctg
cccaggctggagtgcagtggagcaatcttggttcactgcaacctccacttcttgggttca
agcagttctcctgcctcagtcacctgagtagctgggaccacaggtgtgcaccactatgcc
cggccaattttgtattctttttagtagagacagggtttcaccatgttggccaggctgg
tcttgaactcctgaccttaagagatctgcctacctcggcgtcctaaagtgttgggattac
aggcatgagccaccgcgcccagcctatgagttagtacttctatgctcttcctagtcagta
caagaaccttggaacaggaactaaatttacccccagtgacttatatgctaatatttttgt
gtattttaaatatatatgtgtgcatagatgtatctgtgtgtttttttgtgtttttattctt
atttatgttgagagtgtagagctatgtaagagtaaagagaattgtgtaatgaagccccga
gtatccattcaatttcaacaacaatctcatggccaagctaatttcatgtatactctttcc
tgcttccctctaccccacattatttcagtgcaaatcccagatatataactgtacccatac
atatttcagtatgttttatttattttaaaccccacaatatatcattttctatactactgt
aatttcataccaataacattcatttagatttacccacacgtttacctcttctgttaccct
ttatttttatttataaaaatatctttgggaagaaatatctttcagcacatggtcaaggat
ctcctgagggctatgtcatgggcaaaatatacatatatattccatatatatacacacata
tacacacacacacatatatacacatatacacacacacatatatatattccactttcactt
ttttgtttgtttgtttttttgagaccgagtctcgctctgtggcccaggctggagtgcggtg
gtgggatctcagctcactgcaacttctgcctcctgggttcaggtgattctcctgtctcag
cctcctgagtagctgggattacaggtgttagccatcacgtctggctaattttttgttgttt
tttttttttgagacggagtatcgctctgtcacccaggctggagtgcagtggcaagatct
tgtctcactgcaacctccacctctcaggttcaagcaattcttgtgcctcagcctcctgag
tagctgggattacaggcatccaccaccacatctggctaatttgtgtatttttggtagaga
tggggtttcaccatgttggccaggctggtctcgaacttctgacctcaggtgatccacctg
cctcggccttccaaagtgctgggattacaggcatgagccaccatgcccagccattttcac
ttttgaaggatattgttaatgagcatagaattctaggttggcagatattttctttcctca
gtttgaaaacatgattcccttgtatctgatttctcctgcttttattgggaagccaattct
caatctaattttgctcatttgaaggcaatggctttttctgttgttgtgttttctgaggtg
gagtctcactctgtcacccaggctggactgcagtggtgcaatctcagctcactgcaacct
ctgcctcctgggttcaagtgattcttctgcctcagcctcccaagtagctgggattacagg
tgtccaccatcacacctggctaattgttgtatttttaatagagatgaacttttgccatgt
tggtcaggctgatcccaaactcctcatttcaggtgatccgcccgcctcagcctcccaaat
gctgggattacaggcatgagacagcccacaaccctggcctgcaggcagtatcttttttcc
tctggctgctttgaaaagttttgtctttgttttgagcagtttacactgatgcatttaggt
ggctcctcattccatgacttgattctttttttgtccattttagaaaactgccagctttatc
tcttcaagtattatgtcttccccatcctctctctactctccttatgagactccaatttca
catgacttatgccttgttaaagtatcccccatgtctcttaatccatttcctgtatgttct
atctgtttttctctttgtacttcaatttgtatagtttgtatcaaactatctcccaattag
ccgggcgtggtggtgggtgcctgtaatcccagctacttgggagcctgaggcaggagaatt
gcttgaacccgagaggtggaagttgtagtgagccgagatcatgccactgcactccagcct
gggcaacagagtgagaccctgtctcaataaataaataaataaataaataccagttcacta
ttttttttttatgtttgtgtctagtgtgctgttcaaattgagttcctaattccattttttt
tttagactttttttttttgagtctctatctgttgcccaggctggagttcagtggtgcaatc
tcaactcactgtagcctccacctcccaggttcaagcgattctcatgcctcagcctctcga
gtaactgggattaccaccacgcctaactcattttttgtatttttagtagagatggggttct
gccatgttggccaggctggtcttgaactcttggccttatgtgattggcctacctctgtct
cccaaagtgctgggattataggcctaaaccaccactcccagcctcctttttttttttttt
tttttttgagacggagtctcgctctgtcgcccaggctggagtagagtggtgcgatctcg
gctcactgcaacctccccctcccagttcaagtgattctcctgcctcagcctcccgagtag
ctaggactataggagcatgccaccatgcctggctaattttttgtaattttagtagagatgg
ggattcaccatattggtcaggctggtcttgaacttctgacctcaggtgatctacccacct
cagcctcccaaagtgctgggattacaggcgtgagtcaccacgcctagtgcatccattttt
tgtagttgccagttttctgatgaaattcttaattgtttctttatatccttgatatacatg
taaagaacttattttaaagtacatggtctgatgattttataatctggagatcctatgggc
ctttttaaaagttgtctgtgctttctcttgagctttttttcctgctgtcttatttccttgt
ttgcttagttgtttttaatttggcaatggaagttgtgtataaaaatcgttacaaataatt
ttttttttttttgagatggagtctcgctttgttgcccaagctggagtgcaatgacgtga
tctcggctcactgcaacctctgcatcccagggttcaattctcctacctcagcctcccaag
tagctgggattgcaggcaggtgccagcacgcctggctaattttttgtatttttagtagaga
tgggtttttaccatgttggtcaggctggtctcagactcctgacctcgtgatctgcccacc
tcagcctcccaaagtgctgggattacaggcgtgagccactgcgcccagccagaaataatt
tttaaaaataattttgagccccagcatgatggctcatgcttgtaatcccatcactttggg
aggctgaggcgggcagattgcttgagcctaggagttcaagatcagcctgtacaacatggt
gaaaccccatctctacaaaaaataaaaaaattagctgtgtgtggtggtggtgtgtgcctgt
agtcccagctgtttgggacgctgaggtgggaggctcacttgagcctgggtgatcgaggct
gcagtgagccatgatcctgagactgcactccagcctgggcaacagagtgagatgctgtct
caaataaataaataaataaaaataaaatactttgaggcctaggggtctaaaattctgaga
tctcctttatgcatttgagtgactgagatgatctgaagctggatccagtgctcctgaggg
ctgctttatttctggttgactgtgactcctagagtaagaaacctgcaccccacatgtggg
gcattatggcatcccctccctcagccacatgagtaagtcaacagcactgctctagaccag
gtgtggtggctcacacctatagtcccagctactcgggagactgaggcaggaggattgctt
caggccaggaatttgagaccagccagagcaatatattattaggttggtacaaaagtaatt
gcagtgtttgcctttaaaagtaatggcaaccctgtttcagcaaaataaaaagcaaaaaaa
aaaaaaaaaaaaaaaaagaaaggaagaatcagctgggcgtggtggctcacgcctctaatc
ccagcactttgggaggccaaggcgggcagatcatgagatcaggagatcgagaccatcctg
gctaacacggtgaaaccccatttctactaaaaatacaaaaaattagccgggcatggtggc
aggcgcctgtagtcccagctactcaggaggcggaggcaggagaatggcatgaacccagga
ggtggaggttgcagtgagccgagatcatgccactgcactccagcctgggtgacagagtga
gactccgtctcaaaaaaaaaaaaaaaaagaatcactgctctgtctctcagcctcctcttcc
aagattggccgtcgccttgaggggaatgctggccttgcctgtctccagccctgtacctct
ctgcctcctatgcctttaagcacatgttttctatttgctgggctgtgaaatctgctcttc
atctgatggggtttgctttataggtgactagatccttttctcttggtggttttagaattc
gcattttcacattgaccttaaatagtctgattatagtttgccacggcaaagaccctttgc
attgcattgtttggggatatttgagcctcctctatctggatgtctaatctcttgttagat
gtgagtagttttcattattattttattaatgggctggcatgtgggccgtggttccaggca
ggctcagaggggcagctgcctgatgtctggacagcttctctttctgtcttttcttacctg
gactctgggttgcttgttagctgcttctgccagttctgagttttcaaggggagaggggcc
cagtgatggctgttctttgaaggaaagggaagaatgtctcctgtttaacatgtttctatg
tttccagttacttggttagttagttagagccagggtctctcgctctgttgcgcaggctgg
agtgcaatggcatgatcgtggctcacagcagcctccaccttccaggctcgagcaatgctc
ccacctcagcctctcaagcagctgggactgcaggtatgtgccaccatgcttggctgcctt
tttaaattttttttttttaatacagacaaggtctcactatattgcccaggctggtctta
aactcatgggctcaagtgatactcctgcctcggcctttcaaagtgctgatatcacaggca
gggtttccatttttttaaagctcccagcagtggtataaactcctcctttccagagaaagcg
cactctgtccgcatccctcatgttgtcctctcctgcctctgcttagggttcactccgggg
gaaagtgccacttgagagtttccttttttgtgtgtggttctgactgactgctccctgctca
cagatgctgattctcaggtggggtccctgaggcctggagtgtggcctctgacgaccttc
agggccaggtgtggaatgagagcctgtggccacatggcccccggtgggagacgtcccgcc
gccctttgcttctctgtgccactctggctgcacagttcagagccttgggaaatgttaacc
agtaggacctagacggggaggtgagaaggggtcaccccccaggtgtgcctgtggtgagcc
ttcgtgctgagcaggtgcagggagggaggcccaggtgcacacacctgtgaagtaggggca
gctggctgggctccttgacctgctccagagcttcttattttctggccacttcacctgcag
aaggcccaggtggctgtggcctctagggtcccttgcctgtcctcagctcccaactgggag
gggcagagggaggaggggggtggagaccccaggcagcagggctctgggagcagtggggccc
tgggtcccaggggtgtctggcaggcccctccttactctacgtctcggcctctggatggag
gtgctggctgcagtcgggctctgcctctgactaagggttggggaagtggcgggtgtgggc
tgctgccccgtggggcctctgaacagaccccaggcctctgccaatcatgactccttcct
ttcagctggacccgcaggccctgcaggacagagactggcagcgcgccgtcatcgccatga
atggggtacgtgtccgtgggactctcctggcgcccacttcccccagaaggatagggtggc
ctctgttcatttcaaatcagtcagaggtggctgagcctgaggcagcatctgagagggagc
ctggttggagaagggagggcccccaagagcagaatcaccatgcacgggaatcgtcattca
ttggctggaatgcagttgccagccaggccctgagcatccctcctcaaacaaaggtctcat
ggcaccaccaggacaggtggggcctccactcagggacctggggggctgcccatagaaatgg
agacccctgatttgtctttaggtaccccagaaaggtttagaccttaaaagcaatgacaca
cccaaaaaggcccgggtataaatggtaaaatgttaatatttgagattcttggctttttct
tacattattctgtctttccttcttaattttttaattgttactaagagaaagctggtcacag
tacactataatctcagctactctggaggctgagccaggagaatcactggagcccaagagt
ttgattacagcctgggcaacattgcaagatcccatatctaaaaaaaaaagcaagcaagcaa
gagaagcagcggggattttaggaggtgcttctgcagaaaccagtcgtttatatcatcttc
aacaatcctggctcttgctgaagtagactaggggcttccccgaggggcggctccacctca
tgctgagacctctgcatgccttgggggtggaaatatttgatgagactcccaggggtcctt
gggaccttgggctgtgaggaccagaaggattagaggactgtgccccttctccccactgta
gatcgaagtaaagctctcggtcaagttcaacagcagggagttcagcttgaagaggatgcc

gtcccgaaaacagacaggggtcttcggagtcaagattgctgtggtcaccaagtgagtggg
gaggggcttgggctcacgcactgagggtgcctgtcccttcagctgtttctgcagaaaaga
gcatgtgtgggtctctcctctctgtgcatggccactgcacggtgaggtcaggccccaggg
aacacggcgtcttcagctacctcctgtgtttcctgcaaaccagctcaggaatgtccttgc
caccttgcttggaagcagtaggctggctccaggaactgcccaagtgcagggttttctgcc
cttgcttggaattagtcacggtcccagattcctgttgaatggccataacccctgcccctt
tgtcacgagtcagttgccaagagaagcctgtttggtttgagagcagttcatgcagacata
gaccacttcctctgagaattcatttgcttccccaggatggaatctggctgggcctctgac
cttgctggtcacgtgggccggggcctccatcagtcataccctggactcctatctgtgtct
aaacaccacgccccacccccaactgcacggcagccactcgcatagcactctgggagggct
gtgggcatgagcagcgaggactccatgagcagctcccccagataagccctgctaatgagg
gggcttgcgaagcagctttgatgtgctggtaaatccaggtgcaaaacagaactcaagtta
gggcctccgcacagcactgcgttctaactgtgaaggattcttactctagtgtcctgtgtg
gaggtattggaattgtccattgctaagactcagaggagaaaagcacttagcatcgcagga
cttggagcaccggtgctgaggcaacccttcattcattcgtcggatgtgtgttaaggccca
gggcaggggtcagggattctcctctcacacagcacgtgggtggcaggaccaacaccgggt
ctgacctcccagccgggggcacaggctgctaaccccaggcctggaatctgtcagatgccc
ttcctgtgctgacttgacttagacaggcctcctgaccttcccgcaaaggtcatgtgtgat
tcgcaggggttctggccgcttgaaaggttcctgagaaagcacatgccatgaggacagagc
ttgcagagggaggacaggcatgcagaaggctctgtgtgcagccccagacctgggtacctt
cgtcaccgtcctcaccccacctccgggtgtgcagatagggagcaggcctcctgtgttatg
gcccaagcggggctgttaggacactgagaacattccctcctcccgcaggagagagaggtc
caaggtgccctacatcatgcgccagtgcgtggaggagatcgagcgccgaggcatggagga
ggtgggcatctaccgcgtgtccggtgtggccacggacatccaggcactgaaggcaggctt
caacgtcagtgagtgtcggcctgcgcaggacgggatggaggtgtgggcagtggtgtccgc
gatgagatctcagagtgctccatggcccaggcatgtcacatccttctctgtgtcttttct
tcatttactgttttattattttaaaaaaagagaaaacaagagttgtacaaacagcttcta
tagaagccagtttttacaccatcgtacccactcatgccacttggtggagtggaccagggg
cttctgtggggacttggccttcctgccttgggggtggacaggaggtggaagcccaggact
cagtgcggtctgtccactgccctgtatgaggatgtggtgggcagagggcactgatgaaat
tcagcgcaggccggggctgcagcatctccgcctccatctcaccaaccctcacaggccttg
aaggacccagactggcctcaaatgccaggggagggcactgagaccccagagggtccttcc
cagcatcttcaaagcaacaggattttgtgcctgcagacccttctttgcagcacacaccac
ccaccctgaccaggacccctagaatgcccagcatccctgggagggccctgtggtagtttc
agctccctctgggggcccagaatgaacctggcctgtggtgaggatgtaagcaccaatggc
caattgggtccaaaggaagacaccggttcaaacactgaaaccaatcagattctcccacgg
ccttcctgctatcagacgacactggtgcaggggtggttgctatgtacagggcagagccac
ccaatccccacgcaggcgctgtgtcctgccacgttggcctcctcctggccatcacatcag
gccaagcaggggagaggaatgggaatgcccacgcacccctatcaactctgcagacacaga
accatgcacagctcttgggaggagtcagatgagctgctcaaagcccaggagggacccgca
cagtggtcagtgtggcagggacggtgctttagccaaggcagggatggtgggtgactcact
caggatcttcaaggaggccgctgcatttccgtgctctttccagataacaaggacgtgtcg
gtgatgatgagcgagatggacgtgaacgccatcgcaggcacgctgaagctgtacttccgt
gagctgcccgagcccctcttcactgacgagttctaccccaacttcgcagagggcatcggt
gagcactggaggccttggcctcatgggagacgtctcctccacgtgcactgctgccctcgg
aggctgtgaaaagcgaggtgtgggaacctgagctgtaacccctctgccgtggtcggcatt
ttaacccaacctcaaaaagcaggggaccagaaccgagcctgtcctggaaggccttgccca
tccccagagggctccccatccctactcctcaaggagaccaagaggctgaaatagtcagca
ctgctgtgctatggggtcctaaagtctgctgtcctccttcctgcagaccagggctgaagg
agggtgcctgggtgctcttgccatgggtcctggtccagccaagcatggtttcaaacatga
cctgacccttagtcaacctggaggctgatgtctagagcgggtgctggtgcgtgcagcacc
tgtggcctctgcatcacccttagggcaggtctgcctcccgggcccatgcacagaggacct
ggtctcccagcctgcaggtgcccctgtggtgtccaggacgacgagggggtctctgtgtac
ttggtggggctgggaccctcccacttcccacctccttgtgtccctcactcccctgtttca
ttccatgctgagcctcccctgccttgggctccctggggagggggtggtggcaggagttgc
ccgagggcagctctgcccatgagcagctgctctagcggctcctcctgctgctgttcgccg
ggtgctgctgacccctgcgaggtagagaaaaggcgttcaggtggttcacaccccacacag
gtgcccctcacagggtcctcactggcggccagcgctgtgggtgtgacgatgatgacaagc
ctaaactgcgcaaggactcgtgtcccgggcgctccatgtgaccacctcgggagaggtctc
cggcttgtcgtaacccaggggagtgacccactgcctcctgcagctctttcagacccagtt
gcaaggaagagctgcatgctcaacctgttgttgtccctgccggaggccaacctgctcacc
ttccttttccttctagaccacctggaaaggtagcccagctctcttgtggctgcccaggac
tccaggtctccaggccgttggggtgcccctctgctcccaccagaccccccagcaccaagga
cctttcccccgacccctgtctgcagtaactcactgcttctaaggactagcaccactgcc
acccccacccctgcctctcctctttgccaccctcctccctctgcactgtggccttaacaa
agagctcagagctttggccgtggccagcagtgcacttggaccccccctcttccctcccaag
cacatcatgaagacctccccatcagcccagagctggccccttgtcctgggccactgagac
ccagaagtaccaaggctggagtcagcttgcagcacagccagggtcgaggtcactccctcc
ctgaggactctagcacggcacagcccctctgcctctctcctggtggtggcgttgaaacag
caccctctgcttcggtcctctacagggtggcagagaaggaggcggtcaataaggtgtccc
tgcacaacctcgccactgtctttggccccacgctgctccggccctccgagaaggagagca
agctccctgccaaccccagccagcctgtcaccatgactgacagcaggtccttggaggtca
tgtctcaggtatgggaagacagtctccagcccatgcaaccccagcctgacagaggtggcc
tctgcctgccccacccccagtcctgcccatcttccgacttgcattgtatgtggtggtggc
tgagattcagagagagggacttgcctaggtttgcatggatgggagtgatagggggtgccc
aggccacctcctggtcctgctggtgcaccttgctggggggcttaaaaccaccccaagtgtt
cgggtgtggtggctcatgcctgtaatcccagcactttgggaggccgaggcaggacaactg
aacccaggtgtttgagaccagtctgggcaatgtagcaaaccccatctctagaaaaaatac
aaagaaaaattagtcaggcattgtggcacacatctgtaatcctaggtatctgggaggctg
acacaggaggattgcttgagcccaggagttagaggctgcagtgatccatgatggagccac
tgtactccagcctgggggacagagcaaggccctgtgcatctctaaaataaataatcaccc
cccacccaacaagtcatgccttgtcaggaccccaccccacccccgtctcactgtaagggg
ttcatgacaccagcaggggtttctagcacctgaggtggacttgggggcttgggccccaaa
gacctccccaccagcagctgtgagcccccctctgagccactctcctcttccccactctgc
gagggcaggacgaggtgctgctgtacttcttgcggctggaggccatccctgccctgaaca
gcaagagacagagcatcctgttctccaccgatgtctaaaggtcccagtccatctcctgga
ggcggacagatggcctggaaacctctggctaatcgggccatctgtagagtgggaatcaag
attttctgaggcatccttgggccacccccaggtgtcaggccatctgccaagagacagcgg
cccaaagcagaaggacaggtggcctgggcagatcccgcccaggtctgaaagccccaggct
ggcctcagactgtgggtttttatgtggccacccgagggcgccccaagccagttcatctc
ggagtccaggcctggccctgggagacagggtgaaagcagtggtttttatgaacttaactt
atagagtccaaaagatttctactgaatcacttgtcaagaagcgccctctctggggagaag
ggaacgtgactggattccctcactgttgtatcttgaataaacgctgctgcttcatcctgt
gggggccgtggccctgtccctgtgtgggtggggcctcttccatttccctgacttagaaac
cacactccacttctaacagggtttgagaggcttggtcagcactgggtagcgttttgactc
cattcttggctttcttctttttctttccagaaggatttttgtgcagaaatgggtcttttg
ttgccgtgttagtcctccttggaaggcagctcagaaggcctgtgaaatgtcgggggacag
gacccccagggagggaatcccaggctacgcaccttagggttcgttctccagggagagcga
cctcgtcccccgatcctgaccgcccttccggcccacgctctcctgtttggcttccacagg
cctggacttctctggcttctctgcccacacactccctgcccccagtgtccctgcccctgc
cccagcacaggtgacttcatttctgtcctctcagctcagtggactcgctcatcttttgta
taagtctccacttggtggcagcagcttgctgatgacttgttttaaaactttcatcctaaa
taaccttttgatacttgaatatttttaagttttatacatagtttctaatttttttccgaa
cagatccagatacctaataagatgctggaatgtaatccctggacaatccgtgtcctggca
gcatttggtcttcctctaagcgcctggctccgctgttctcaggagtgggttctgaagtct
ctggagaacaggatacgtggagggttaggaaggggccaggcctagagacgggagactccc
tcccggagcaggtggaggcacaggaccattcgctaccccatctgccggcacctgcggggg
agcccaggcattctttgtaagccctcctgaccacctggctcaaagaaaacagaagcatgg
aggccgccaagtattttcaagaaataatcccatgaacatggcatcactttttttagaaaga
ggggcttggggcaggcagaggagagaagggagatcaaactgagagccaagtttccagacg
gtcctgcaggaggagaggatgcagctgcccagagggaagcaggatcacatttaaggaagt
gtgtggggtccctggatgacaccagcacccagtgcggctctgtctggcaaccgctcccaa
ggtggcaggagtgggtgtcccctgtgtgtcagtgggcagctcctgctgaacccacagctc
actggggagcctgacagtggggccatgtgcctgacactcctctctgcttgtggacctggc
aaggcagggagcagaaaacagagctacttgaaggctttctgtctgcgtctgtgtgcagtg
tggatttagttgtgctttttacttgctgggagagcacagccaccatttacaagcagtgtc
accctcgtgggtggcgaggacagaacaggagcctctgctctctgtacctatctgggcccg
gtgggctcccttgtcctggcttccatctctgtctcagcgaccattcagccctgcacagga
acacatgttgcttagaaaagccaaatccagcccttgtctctgcctcctctggtctcatga
tgtgcatctgttaccttgaaactggaaaccagtctatcaatgtctgtgccaatttttttat
tccctccccaacctccttccccatacgactttttatttatgtaggatgtgtgctgtctaa
tgatgggatgaccacacttttccatgttctaaaagtgctcctctcccacagggtcccagg
gctggtggttgctttgggtctacagctacgtcttacccgcctcctgcctcaacagcctgt
gtggtggcaaagccggtgtggggctggggaacgcagcgttctccaggaggggacccggct
ctccttctgcagtgcaggcgaaggcctagatgccagtgtgacctcccacaaggcgtggct
tccagactccccggccggaagtgatgctttttttgccgcgggccctgggtttgaagcagcc

tggctttctcttggtaagtggctggtgtcttagcagctgcaatctgagctcagccaccta
cacaccaccgtggccgacactttcattaaaagtttcctgagacga

>LOC106996293.glutathione.hydrolase5.proenzyme-like-GGT1.rhesus.
gatcacaaggtcaggagttcaagaccagcctggccaatatggtgaaaccccgtctctacc
aaaaatacaaaaattagccaggcatggtggcgggcttctgtagtcccagctactcaggcg
cctaagacaggagaatgccttgaacctggcggggcggaggttgcagtgagcggagatcat
gccagcctgggcaacagagtaagagtgtcaaaaaaaaaaaattatatatatatgtgtgta
tgtgtatagtgcatagttggaaaagaaaatactggttttcacgggtgattggcagctagc
tgtaagtggaagaagtgcattttcctttctttccaggtcccagagaaaaacctccatgct
gggttgatagcagcagctaggatcagttgcccaatactctggattctcgagatgaagacc
gtctgcagaggctgacctttcctgcaggctcatgtcctaaagcaaattatttgcactcaa
atatcctcccttgtaggacagaaaagaagttcccaagtcttggtcacattgggcctgtgg
agtacagtatcttttctacatatttgcttttgatgttctcagcatccctgcaaggcaggt
tgaggtgtgaggagtctcagtcccagggagctgaaggcagactgcaagagctgggtatgg
atgccgcactcctgggcctgggccgcctgggtttcttcagcctgggaagtacttccaaa
cccactcctcgctagtctcttacaatcctgtgggagggcaaagcctaaatcatcatggct
ccttccaccaactagctgcagatcaggaaagtgaggtccagggaggtcctgactggcttt
gctccagggacaatgaggctagaggtctgttccccacaggaggaccatgatggttctcct
gcttcaaatggcctctttggaaggggcttgctcatgtaggtgaatcctggggctgagcag
cctcagtgacagtccctgaaatggctgcaggtgagggagcagcatgcaggcattaacgtc
acagactgacatcaggccacctagacttacatcctggctcctctggtttctggctctttg
aagttggacaagaaatccccttttttttcttttgagagacagagtcttgctccatcaccc
aggctgtggtgcaatggcgtgatctcagctcactgccacttctaccctccaggttcaagc
aattctcctgcctcagcctcccaagtagctgggattacaggcgctcaccaacatgcctgg
ttaatttttttttccttctatttttagtagagtggggtttcaccatgttggccagcctgg
tcttgaacttctgacctcaggtgatccagcgccttggcctcccaaagtgctggtattaca
ggcgtgagccatgccacccgactgacaagtcccctttcttaacctccttctgcctcagtc
gacttacctggaaaacagagcacctaccttcttcaaatgagaagagaattaaaggagtat
aaggccatcagaacagtacctagcccaagggaggctatggaagtgcttgttgaattaaaa
aatggcttaatctctggcctagtcccaaaggtttgggttcctgccattgccacccagaga
gccctgggtggaggccctgctgacattgaagagtgcctgccctcctcccctcaatgcggt
agacactttccaaaacttacgtcatcttcactccagtgagataatgggaggcttggtgta
cctcgttcctaagatgagggaacaggctgtgagaggaccacatcaccagcaagtgacagg
ctgggaccaaggccaggcctgtctgcctcctagactaggcctgtgtgaggaccggcctta
ctacagcccacttgcttcctcctctgtttagagcttgggctggagctgtcctggccagag
ctctctgcctcaccaggctgcatgagccacaagctgccacttcctggaacttggtggccc
tgccctggacggccttgctggtggtcatgaaggctccgtgaaagctgtgaacacctgcat
ggcagtgaggccagccttccccacttgatccgtttcaggctctggctccgctactacgtc
gtgtgcttctgtccaagaggcagagactgaaaccatctgccgccgcctggattcctgcag
agcttgcctaggacaccagctcaggtccttgtgcaccatcctggtaggaatctctcagtg
acggtggagggtttgtgggctggcttagggccaggggtgggcagaaagcagggcacaagg
gaccggagagcttggcatttctccagcaggagtttcagggagagctcagatgtctgctta
tgtgacaggtttatcagcctgcctggcccagggtgggctctcatagttttgatttaaata
atagcttgctcacagaataagtgcatgttggtttgagctttcattttctcataatcctct
ctgctggcccctcccaccatctaccccatttgcctcactctggggagcctgaacccccct
gttccagagtaggggcatagggaaatgcctctatggcttctgagctgctgggcagggcca
gtccctaaaacaaggagagctgggaggggctggaccccgatggagtgctgagagctgaa
agtcctatttaacaatggggagggagcagaaaccccgaaaagggaaaggcctggccaggt
cccacgcaagtcaggagcagagcagggctgaagcgtgacatctccagtctctccaaagcc
tttgggtgaaaagtccacctaaagcagtgagatcagtggggcctaaggaagatcctttttc
ctcactgcctccatcccagtcctgggacccaacaccgtcctgccctcaccctccttgacc
atctccttctgccctgagaggctgttatgcatccagccagcctgggaatgtgctggaatc
tgtgcaccacggcggatggatacgccgctcacagtcctctgtgggccgtggctgtgagga
aaagtcagagctggtgggttggggtgtgtaggcccaggaggacccctgcctcctccttag
ccttggagcctccctctgctgctgagtttctcctcctctagcaggggcccactgcccccca
gaccttacctcctggctcagcacatgatttccctactctgggcctcagtatcccccgga
catctctaaattcctttcccgcctgccctgcctgtctcctggttgggctgaactgtaact
cctcacacagggctgagggtgactgtggcatcaggcttctggttgggagcccaccctgat
ggtgcttggggaggaatggacatggttcccccctgccagggacagccctaggactaaggcc
ccctgctgctggctatgcccacctccacccccccgcatgctctcccacatggggggaccccc
aaggcctagagggaacacacaggcactttgtggcctgctgtgtctcctaaccccacagag
ctgcccttcgccaagtacaggaggcctgggactgggtactgggggtggcaggcagcactg
taccctgtgggaggcaggcaccaaatcctcccacttagtcactgtggcccctgcctctgg
tctgtgcagccttgtcccccgcccagagggctctggtggcaaggctggcagcccgggctc
gacgtgagcttgctgtggggctccatgtaaagctttgctcctcctggggctcccaacctg
ctccttcgcagctccccttctcaaggagcccaagcgctgcccagctgtgagggtctgctc
cctggaggcctcctttaactccccagcccacagggatggacccaggaccccccatatctta
gactccatggggggatttccccaaggaacacctgggggaaggtcagtgtgacctttgtccc
tccctttttggatagacacgccaccctttctccccaccccagcagcagggctgttgctcag
tgaccctgagatcggtgatgcagaaaagcagaggcgggtgtcactctttattgcggggtc
cacactatgggtgttagggctcctcccactgagggaagcctgagcctctagccggggctg
gcctcctggcctcctatgagtccattctccttctgtccccttaatctcaggctgagcatt
tgcactggatctctgggggcctgtgagtcctcctcgaccttcatggcccactaccatgtg
cttgagggcatcacaagtcagtagcaatgagccaggtgggtggtgggtcacctggtgcag
caggcccggggctcgggtcccagcccagcacctgtggagtcccaggtggaggcaggggtg
gtggttccggctgcaccaccctcaggctcaaggtccaggccctcgtagatggtggggagt
gaggtgcgctggctcagccgccggcgcaggccaaccagcaggggctggggcagggaagcc
acatccacgttgttgcccaggtctacccaggccagtacagggaacttggtggtgtccttg
atggcgtcggtgagcttgcgggcagcagcccgcgtcagccggttgccgttgagcaggagc
tgggtgaggcggggcagcgcccacaggctgggcagcagcaggtgcagcagctcatcactc
agccctgtgaagctcaggtccagcaccgccagcacagcaccgtggctgctcagatagtgt
gtgatgtgctgcacgtcccgtaccgacagtgggatgcctgagaggtccacagtctcctct
gccagtggagtcttctggaggctgcccttgaggctgtgggagggtggcaggcggggatgg
actaggcaaccaggcagtcccccccactgaccagagacagtaacctgtcaggaaaagccca
ggcctatggtctggcaaagccaggttcaaactgtcactttctttcttttacttttttttt
tttttttttgagacagttttactcttgttgcccaggctgaagcgcaatggcacgatctcg
gttcactgcaacctctgcctcctgggttgaaacgattctcctgcctcagcctcctgagta
gctgggattacaggcgtgcaccaccatgcccggctaattttatatttttagtagagacag
ggtttctccatgtttgtcaggctggtctcgaactcctcacctcaggtgatccgaccgcct
cagcctcccaaagtggtgggattacaggcgtgagacaccaagcctggcccaaactgtcac
tttctaagtgggtggccatgggcaatgctcagagcctctgtttcccctttagtgaaaatg
ggacgtggttgttgtgagaattgaatacacacacagtaaggctctatcttcatgaactat
tatttgaaatctcactgtgtgcccagcatctccacacttgttacattgacatctcactta
tttttcagataggtgagataactcttccatggccacacaccactaagcggggaaccagat
ccccaaactgagatgctctgactcttgagcccacatgtttttttccctgtgccacttctg
aggtcgattcttttagagtagggagaaattcttctacaaagggatggctattaggctata
cagggcaccagggctagagagggacaggcccagggtagtgggaggggaggtcctgggtgt
gcaggctggggacctgccctgggggagggcttcctgtgttttctcggaagggaagttcag
agactgagaaaggacccccccctctggccccaccaaccctctaggtctctccccagttac
ctgaacagagccaactaggcagacaggccttttggggggattgacagcagtgagcacaccc
tcaggacccccacctgccatggcagggaacaaccttgtgcggtccaagctcagcgtggcc
tgttaggcccaaggtgggagggcagacaggggaggcctgctaaggtaagctcccggcagc
agttcaggcccctgggcctgggtgcctgtggccagctctgggctgagcctgtgtgtgaag
ctccatggatagagtcactccaggatggagtgtctgagagggacccatggcagagggagg
tactgggcagccacggaggcctcagctttaggcagcctggcagtggcaaagaattcccaa
caaggtacaggaaaatcagggtagagtgtggccttaggtatgccacttctccgaacctca
gtttccttccctctaaaatgagaatcactatccagaatctgcctcccccacaacctgttg
ggaagtcttagctgaaaatgccttctgaggtcacctcagctgttgggtggagggctgggg
tgtccaggtcttgtaagaggccagggattgagggacgtgtgaatggcaggaggcaggacc
gtcccatgggaaatggcaaacgggtcactcagagggctcagcttggcttgggaagcatct
gctgccacaggtcaggctgacttccagggtagggccctcatgtggaccaggtaagggcct
caccaggtgggatttcagcaccagcctcttggtgagagtgcagcctgagaaggaccttg
gaacagcagtgaggaggggcagtggctaggctccccttaccagctctgggtcttcctctg
gcacaggctggacccttgctgctgcttcgagtgaggggtgaggtggtagatgagctgtcg
gcagatcttctccgaggacttccagagctcgtagtcctgtgggagggtagcgtcaccatg
gtgaacctgagcccctcctgcccacctcaggccttggggagaggggcccgggcagccaa
gtgtgtgtgtcagcacagccgtgcaccatgcatcactgagctgcccaccagggcctcccc
ctcccaccccccaacccggctgtagcctggcacagagccaggggaggctgccctctggga
gctggacaggctggtccagtcccgcagtgcccagtgggaggacgcccagacaatgggccc
ttgttccttgtcctcaggcccagggccagcgtcttgggctatcagccggctgcctgccca

ccctcagcacccctcggggacttgccacagggggaggaggccagaccaggcccctcgagg
gagccctgttccattgacgccaatcccagggagtaggaacaagccaggtcagcatgttct
tcttcctttcactctccaagcgagactgccccgtggggtctgggtctgggctgacagggc
tgggggatgtaatgttctacagcaccaccgcctggctctctcttagtcctaactgggctc
ctgcagaggcccagctcagggcagaatccagggtgcgcggcccacaaagctggcactgag
catgctccactgcatcctttctccagtccctctgtgaacagccccaggagcaggcggcac
acacagcagctgcaagccctggccattcctctgatggctgtggggtaggctcagagtcct
aggggttgactatgcagagacctgggcattcagagctggcagggcccatagcggataccc
ttgttttggtgcgggactcactgtgactaggagtcgggggacagtggctggtcacatggc
agcctcatggcggctcgatgctcatggcaggacatctgcaggtgctgtcagagtggggca
gccgtggggtagaggctgcaggtggcaaactccaccccagcaatgaggagggccttgctt
atgaccccagctgttgtgggtggaagcagtgagcacctgtccccaagggtgtctgtacag
gatggagactggtggacagaagggagctctagccaagggcggctctcttctggcccagga
actcagaggctgggggtctggttccgttagtggatttctcagaacagtttgtgtccctca
gggtccccatatgacttctgcccagctagtctcccctgcctgggtccccaggcctggtgc
acagtgctgccacacagaaaacgggtctgcccaggactctgagggtccgctcacacttgc
tgtagctggtggccagcctaatatactgatgctggcagcaggtctcagacagggactgtg
acttgcctaaggccatacagcaagccacagaaggacctagcttcccagacctcagccctg
ggctctgctggcccctgtccaccacagtgcccagcccagcgcccatgcctgccagcctca
ccttcttggggcactgcagttcccgggccaggttcataagcaggtcatgggagatggggt
cgaccaggttgaggaaggccacatctctgtagaggatgtcagtaaggagggacccctcaa
ggcccaggtcctgtgagtgagtggtgaagagaaagttgagtgagccagtcgggtctccca
tctccaggtcccaccccagccagccagatgctggttatgccccttcctgtctccatctcc
tccctaaggctggctgtattctccccacaggcgaccatgggccaactgtccagtggtggg
caaaccctttcatttttccttatttttttgagacagggtcttgctctgtcacccaggctag
agtccagtggtgtgatctcggctcattgtaacctctgcctcctcaagggatcctcccacc
tcagcctcctgaggagctggggctacagataggcactactatgcttagctaattaaaaaa
aattttttgtagagatggggtctcactatgttgcccaggctggtctcaaactcctgggct
caagcgatcctcccaacgtgctgggattacaggtgtaagccacttggccttcgtggaccc
tttggataagtagaggggagggacatgggagccatcagggaagtgataagtgaattccag
aacccttagaccagttccctcgaggtaaagtgttaggctatgacactgccttctctgggc
ttagggcttatcagtgggattctttggggtctctcctcttatggacgggggtttgctctt
cttatgacccctcaaattcccctggccccagcattcccttcagaccctttgccttcccac
ctatcccttggttttcctgagctgctgccagcatctgtttgcggacaggtctggatcctt
cccttgggggcggacctctctctcagatgtctcaaaggcatgctaccctgaagccggcct
caccacccatgccccatgccccagttcagtgtagggcgcctccatgcccatcttcccagg
cactggggccagaagccctggaacccccccaacactctgccctcctcctctctcccccagc
tcacctagtcatcccttctctccccaccactcctgccaggattcaggcctccagcctctt
ctcctacccatccctcaccccaccgcctgcacagacctgccctggccttcctgcctcact
cctccactttctcctcctgtctactcccacaggactggtgtggggctgaacactttcaga
gggtctctctctctctcttttttttttgagaccgagttttgctcctgttgcccaagctgg
agtgcaatggtgtgatgtcagctcactgcaacctctgtctcccaggttcaagcaactctc
ctgcctcagcctcccgagtaggtgggattacaggcatgcgccaccttgcccagctaattt
tgtatttttagtagagatgcggtttctccatgttgatcaggctggtctcgaactcccgac
ttcaggtgatctgcccaccttggcctcccaaagtgttgggattacaggtgtgagccactg
cgtctggcccagagggtctctttagaggagagattgagagctgatatggcagccacagcc
aggaggggcctctagaccccatgtgggccttctccaatccataggtgatatgctgtgtgc
gtgggtcccaagcacaggaagttgggccaggcagggaagtccgtgggtcacagctgcagg
gtccttgcaggttatggagcaggataggcccctggctggacctcaaatcccaacctctga
gacagaaagaggtgacctagctggtctctgaggacccttctttcctccagtttcccaact
aaaatgcccaactggtgtcttgggctccagacggcatcccagactgagatccctgcctct
gggtgcctggctgattcagtgctgctctgctgtttgccacagatgccagctagggaagat
ccagaaggaaggctggctgatgagcctggcagacccaaacagggccccagatcgcaacct
gccagcctgccttccgctggtctagacagctcctcttaaaggggcagctccatttttcca
gcctgctgaacccagagttggaggaggtggagaagggtagagaggaagaagggctctggc
cctggcttgccccagtttttctctgtggaccctgggatgaccacgtctccccatgcctca
gtttccccatttctagtataccttctgcaggccaaacacctgcatggctgactccctgct
tacaggatggggcaggagtggtggcagctgctgcaggggatggggacggggtggtgagga
aagtgacttgcccaggtgatggtgggaccagcatcctgggacgacccagggcttgggatc
tgggtttagtgtacctggtgtgtgtaggagcagggccaccactggaccaagtgttgtgat
gcacagatactaggggtgtgtactctaagggcatgagctgcctgctggagaactggtgat
gcatgcggtggctctgtgtgggctgccatggtgtcttgggatgaggctgcaccggcagac
cagccctgggtcctctctccagggtccagtgcctccccaatcctggaggaaggggaagct
accttcttccattgtctctcaatacaagatgattcgacctcacttgccactcctcccggg
gcccttcccatcttgccagggcctgaatctgagcttttagctccaggctggggccaccct
gaacacacacaacccctctcaaaccgcagagacccggacacactcagcggccccccttctc
cacacacggtccgccggcctcacccagccctcgctccagccggccgccctctagaccact
caagaccccgcgcccagcagcccgtcgcgccctcggaacccgtccgtgcgcgcccaagac
cagctccgcctcgcctcccgacccatacatgcccacaacccataacctcgcgcctcctag
acccccagaccctccctggccgccagccccggggcggccggctccttctctcgcctggcg
caggaggcgcagcagctgccgggcgcgctccggccgccgctcgcggagcgtggactggat
ctcgcgaagccaccgcacccggcgctcgtagggcgcgggcctgcagccggccaccgcccc
ggcctcggagcctgcctcaggcccggcccgccggcccagccgcgcccccatggccaccgc
gcggcgggcgccgaactcacagaaggctggacggtctgcaccccgcctccggggccgccc
atagtcgccccgcccccacccaccccggccccgcccccacccaccccggccccgccccgt
gcgggtatgaaggctgagcgcccgccccaccccgcgccggagcgaggccagtgggttcct
cgatttggcctggtcttactggaggaggccctgcctgtgtcagtcttggcgctagagtcc
aaggacctacagttgccaggggagacgcggagctcggcccagatgaggtggcttttggca
cctctctaccttctggcccaagtgctgactggggaatagtcgctttgacggtgcagagca
ttccaggcgggtgtgggggttccactgcgtgtgccagcattaggtattgcccccacctgc
ttttagaaatctgccctgcatggtgaatctccgtctctactaaaatacaaaaattaggcc
gggcgcgatggctcattcctgtaatcccagcactttgggaggccgaggcagacggaatct
gaggcccggagttcgagaccagcctggctaatatggtgaaaccccatttttactgaaaat
acaaaaaattagcccggcgtggtggtgggcgcctgtaatcccagctacttgggaggctga
ggtaggagaatcaattgaaccggggaggcggaggttgcagtgagccgagatagatggtgc
cactgcactccaccctgggtgacagcaaaactatgtctcagaaaagaaaagaaaagaaga
aaaggaaaaaaaagaaaagagaaaagaaaaggaatctttcctgcagctggcagttgcggt
gtaggacctgctcacactgcatggacactcctgcactggcaaatatttactgcctcctgc
tttttggtttgggcaggcccccacctctgactccagcctggggaataaaccggatgaag
agtgaggttaaccgttaagcccagctctaggcaggctggcagcaggcgggctgttatctg
ccacttcagtcttcctccctgataaggggttgggggggttggggcaggcagggtgagatg
gggccctggctcttctgaggaccagggtgaagtacggacccaccctgctcaaggagtat
cccacttcccagggccccccttcacagaagtctaggtctctgttggaggcctgctgggccc
ctgcagccccaatcccagctctgggaccctcacatctgggtgggggatgtttgaggcact
tatctgcagtccccacttccccaagcaaggagcatgtgtctagcctgtgaggatgggtac
ctgttgtgcaggaaggactgagcaccgtccgagggcatatagtgagggacaacagccaat
cacccgtgtgtgtgtgtgtgtgtgtgtgtgtgtgtgtgtcttttaattaccctatgaaga
ttcaaggttccttcctttgggttaagcactgcctgcccagagatggtagtagggagaggg
cggggtgctaggctctgtcttcctgtggggctgggtccctgagacaagacagaggacagc
aggatgtcttagccccttgaccatccacagacccccaacccctgctggagtggtacttgc
atccccttctcccctggtacctgccagctggacccaacaacctctcctcccgctacagtg
cagccagtattgtcctgaggccaccttggcaccataggtttcctccctggcaaacctgca
ttacggccttgcgtgccttcccccagagccaaggcgctgttgctgaactgaagtggcgac
agaccgggtcagagctttaagcagcagccccagtgccctagggacctctcagacccccctg
cccagagcaggcagcaggtaagccctcctccccttcccctgcagcctcagggctgaagcc
agagctgggagggctcccagatcctgaccatgtcctcccagaggctgagccaggcctgga
cactgtcccctcgaatgtgagaagactgggcagggggagggggagggaggaggggatggag
ggctgagccttgaggcatgaaagaggcaacctaggcagtggtatgggggagtgcccagag
tttagagtcctgtggaaggcagagaactgccccagcccctgcctctcatcctccaccctc
tgtgtccctggctgagaggttcctagaggacgtttttccagcaaaagagggagggtggtg
gctgggcctagatccttgaatgggcttttctctgcctgcttgcagggcgcctagggtaaa
tgggctcttggagacaatttctgacctaacctgacttgaacaggaaccccccagaggcaga
gctatcccctcaggcgtggtggcgtgattccacctcgccagcccatgtgttccacatgga
aggctgctggcccctctccctggggttaccctgagcagcagagctgtgcttgttgagacc
caggggggtggagggattgcgatattgtcctctcagctcccagggctgatgtgggagctca
agggcttaccctggatccttttgggtagccctgccatccctagggagtcagtgaggtcag
aggttgggagctggagaatcaggtagggaggacacagctagacctcaggctctaatggga
acatactaggctccaagtgggacctgccaggagacaggaattgatgacagccacaccttt
ggcgtctcgagccctctgccatccatgcagtggcccagttcaaatgacaggaacacaagg
tccagatggctcagtgggctggtagagggctgtagccagggctggaactggccttgcact
gcctctgggcccttctctacccacctggtggtattcttcaagcctaggaggaaaaaaggc
cagctcagggttaatgtaacctcagcatgtagctgggtggcactgaacctcctggtagcc
actgcaaagaaggatacagaatgatttgggggcattcactaggaggaaggaagcagatgg
atactctgtggagacaggatgtcccctgcgcttgctctacagttacgggttgtcaacctt
ctgcagggccaggaattgtcatgaggctgtagtaggcagaagaacttggcggtggggagg
gtgagatcacaggtataggctcagggcctggattcaaatcccctgggctgccacttgttg

gatgtgtaaccacagacagagtaaggacctgccatagcaaattacacaacccacttccgt
ggcttaaagcaacagaatagttctggaggctagtagtctgaaatccaagtataggacaag
gcacgctccctctgaggactccaggggaagatccttccttgcttcttcagcttctcatgg
ctcctggcaatccttggccttccttggctgcaactgcatcattccagtctctgaaaccat
ctacctgtgtgtctgtgtggccttcacatagccttccttatttctgcaatggggtctcac
tatgttgcccaggctggtcttgaactcctgggtccaagcagtcctcctgcctcagcctcc
caagtagctgggattatgggtgagtgccaccatgccagctcacatggcctccttgtaaga
actctagtggctgggtgctagatcacgcctgtaatccagcacttttggaggctgatgcag
acagatcatgaggtcaggagattgagaccatcctggccaacatggtgaaaccctgtctct
actaaaatacggaaaaaaaaaaaaaaaaattagccaggtgcggtgatgcgtgcttgcagtc
ctagctacttgggaggctgaggcaggggaatcgcttgaacccgggaggcataagttgcag
tgagctgagattgtgcgactctactccagcctggcgacagagcaaggctctatcaaaaaa
acaaaacaaaacaaaacaaaaaaactcaagctactggatttaggactcattcattctttt
attaattttaattttttttttgagacagggtcttactctgttgcccaggctggagtgca
atggcacgatcttgactcactgaagcctccacttcccaggttcaagcaattctcctgcct
cagccttctaagtagctgggcttacaggcgcccggcaccacacctagataatttttgtat
ttttagtagagacaggatttcaccatattggtcaggctggtcttgaactcctgacctcag
gttatccacccccatttacctcccaaagtcctgggattacaggcgtgagccactgtgccc
tgcagggctcattcattctaatcccgtatggtctcatcttaactaattacttatgtagag
accctatttccaaataaggtcatgtgaggaaaaaccaactcaaaccgtgtttttttggctg
aatacggtggctcacgcctatcatcttagcactttgggaggccaaggtgggcggatcact
tgaggctgggagtttgagaccagcctggccaacatagcaaaaccccgtctttactaaaaa
atacaaaaaaatacaaaaattagcctgagtagcaggcgcctgtagtcccagctactcagg
aggctgagggaggagaatcgcttgaacccaggaggtggaggtttcagtgagctgagatag
cacaaccacattccagcctgggtgacagagtgagactccatctcaaaacacacacacaca
cacacacacacacacacacacacacacacacaactactgtgttttccttctgttgtca
caagacaacaatcatcacagaagacttctgtgaccaaatgtgtggattctccccacacac
ccagcaagaatcaattctgcagccgacgccagctggtgtcttccaattcaactcaattct
catgctgtctacctggagatggcctcagaaaccacaggttgagggcttggtcccacaaga
ccacccctcatcccaccagtcacaagtctgggctttgggaacatctgactaatcggcttc
tagttggggttcctacagcaccctctttgggttcaattaatttgctagagtagctcacac
aactcagggagacctgtttgtcggtttatttcaaaggatgttttattttattttctcctc
agacatactttatcatcacttaaagtcagtagctcagaggaaactttaaaagctacaaac
aggcggggcacagtggctcatgcctgtaatcccagcactttgggcggtcgaggcaggtgg
atcacttgaggtcaggagctggagaccagtctggccaacatggtgaaatcccgtctctac
taaaaatacaaaattagctggctgtggtggcaggagcctgtaatcccagctacttaggat
gctgaggcaggataatcatcagctgaacccgagaggcagaagttgcagtgagctgagatc
atgcctctgcgctccagcctgggcaacagagagagagactgtctcaaaaaaaaaaaaaaa
aaagataaaggctacaaatagacagtcagatgaagagatacatagggtaggactggaaga
ctcccaagtacagaagtttctgtcctggtggagttggacacgtggatgagtggggttttt
tattgttgcccaggctggagtacagtggcactaactcggctcactacaacctctgcctcc
tgtgctcaagtgatcctcccctctcagcctcccaagtagctgggaccacaggcatgtgcc
accatgtccagctaatttttttttttttttagttttggtagagatggggtctgtctatat
tgctcaggctgactcctggtctcaagccatccacccacctcgccctcccaaagtgctctg
attataggcgtgagccactgcacctggccgtggatgagttcttattcaccttcctgtcag
cctccacatgaagttccccaaaccccgtcctcttgggcctttttaaaaataaattaatta
aaaaattaaggacagggtctcactatgttgcccaggctggtcttgaactcctggtctcaa
gtgatccacccaccttgtcctcccgtagtgctgggattacaagattagagatgccagctg
tcaggtggctaggtggagtcagtgtaaagtcctagaagatgcattttagtaaaaacagcc
acctgacagctggcatccatccattcagtgaagattgttgggcactgtggatactgtgga
ggaggcagcagtagcagctggccctgcagagtgaggtgcagtgctggagggcttcaggag
gaggtggcattgtgatcggtgtctgaagaatgcctcaggccatgacaatggagccagtgt
aaagtcccaaagatcaggaaggagcaagaggaaaggatagttcatatccaggcacacccc
tccagaaaaaaaaaaaaagaggaaaggatgggcgccagcaaggtaggacaacaggggcctc
tagagtgttttcttcccaggaggggaggggtgtgctcagatttacatgctgagtgcctgg
actgcaatagggaaatagcagaggggctggggcctgtgactgggagtctccaggtgagag
gattggcccgtggggactggcggtcaggtgaggctgggagtgaggtgtagaggtgggagc
agctggacaggactagtgaggatgcaggggcggctgccccgggctagactctggctgctc
tggccgttggaggtctggggaggcagctgcttagctcaggctcgggcacgtccacccggc
acgtctgaaacattagccagtggggatgcagactcgtcagcgagagcattcctgagccca
gcatccaagaggcaaaggaagttgggaaggtgctgctaagaggtgggggaggtccccaaa
agtccaggtgagagcatggcatgtgtggaaagtgctgggcagttttggcatcagtgaaga
cacggggggctgtgtgtgaacaggtggggttgggcgtcaccagggagtctttagacagga
gcgggttggctctcctggagatcaggccatggcttggtgtcgggactggggcatggacag
tcttcctccacccccaacctctccttggagctttgagacactgcagataattagcaggtg
atgtgataaatactacctgtggagggtgccttgggcagggaagacagctctgctctgtcc
tgctctgaggtgctcatcaggaaaggagagaaactactacagacccagtgttggagcctg
gccagaccctgtgaggcctcgaaagtagcctaccaagggaagggaaagcctaccaagata
cccacactttcagtggctggggttgtttgaacccaggactctagggcccatgcctctgc
catcctgcctctgagtttgggagttggacttggtccatcaagggccgggccagccctgct
gctgagtgctcccaggcatgcacaagctgacagttctgaagggcccacgcacttggccat
gctccagcctatgtcctcacatcaaaggtaggtgctggaatagcaaatgctgcagatggc
acaggggtgttggccaggctctgggggcacaggagctctgcccatgcccacagttggcc
tgtgctgacctccctccatcacaactgagtggcacccgccctttcattgagctgaagtct
tccttccaaaatcccttcagtgtggtctttgtggctcagagaggctgtggccaggtacaa
gatctggggtggcccctgcttctctgctctgtggcctttgacagtgggcaggatctgagg
tcaggcccacttgcagcaggtgttacaaagaggcaaattggggcttaatgttggaaagaa
cttgccaaccaactatagtcccgttgtctttggaggtagtgagctccccattggggaagg
tatgtaagccaacctttctgggatgttggaagatcagagaccaaacacttgagattctgt
gatcctagtattaactcaaatgctatgatgcttggatttgaagtcagttctatttagcca
gtttaccaagtgtctccttggtgcaggacaccagggaacacaaaatgtcctggataatac
tgggtttctggctcccagggttccctcagatgttgttccagtggttctagaatataggcc
aggcatggcttgcatctggacccatcagcattgctcgctcaatgtgagcttcatgactgc
agttctcacgcccccacgtgccccctaccccgccccgccatgacccttaccatggccca
ctcagggcacaaggctttctggatgattcctgatactcagtgtggtcagcttcctggacc
tggcagtggggtggggctgcaatgagacgccagtcgcaggtagtggtttgcacagccttc
ctggtgtcgtgggtcactgtcttgccaagtggctttgggcaggtcccttcccctctcagg
ccgcaagttcctatgccaggagcttgttttctcagatacagagctggggcagatcccagc
ccgctgaaacagacagggctttggaggccatggtgggcccatttctcagaggggccgtgt
ggctggatgctgagccaggcactggactggccccttgcgcctcctgtcaggctgaggccc
cggaggcaccgtgggctgctggtgccagcacacaggggtgataaggagatgaaagtcact
agaatggtggggcggcacccgccccgggctgtgttggcagctgtccctggctggaggcca
gcttggccctggaacactcctcaaggaacaaggacacagagagccagctcagccagcctc
cacctagcgccccaatcagcactcccttcttcccatgacccctgccctttgcccggctcc
tggaactggtcaaggagctaatggacacccaccagggtcttaatgagtgagccagggtta
atggttattgggcctggtgacacattcaccagcctcccagggatctcaaagttcagactg
gcctggccttgaaggtcttagctcggccacctccttttccagccttgacacctcggggct
caggaagggcctagggtggtcaagaggtcccctgcagccctcacttcctggaaaaggctc
catttcctggccttcagggttaggtggttccttctgcctggcaggtcctccccaaatcca
atcccagcttgggctggattcccaaggcccctattctggttggtgtctgccccttcccta
gggctcttccccctggggtgaccatactgtctcatgcccagccaggttggggcatgagac
ctggagagaagaatctggaggggccaagatttgatgggagaatgaatgatggaatgaaag
aaggaatttaagcatgtgccttgctaagagtgacaattctggcaagctgcagaccatgtg
tggacctcgatctgagggctgagaggcttgggcctgccagccctgtgggggcacccaccc
catctcatctcattgaatagtgcagagcaggtgagggcatgatggacaaatgggcctcat
gcccctgcttcaactgcctggggtcctccatcagcctgtctgctcagaaccctgaaattg
gggttgaggaaggaccttcctggatcatgtgactcccttcagcccaggtgaccagggtc
cttggagtgacagttcctagagccctgcctgtagccctgtaagttggggagacggcctgt
tgggagaggcatccctgccctggggtcgtagaggtgatctaggctcctcagaccttgtgg
ggcctcagatgcttacatctccagctcctcctggtgtgggcatctggctgcaccccagct
ctgtggactcatctcccagggaacctttggtctaatctgcctcccactgaaacctggcca
cattcagctccaccaaggccttcagagctcaggtctctcattcagggtttgaggccccgc
cagacagacctgcagccgtcccagatcacacagccccagggatgggaccaggagccagca
agtgtcccacctggagcagttcctgtgcctttaaagccttccctccccccaccccgcccc
acccacccccgggccactaggggagggaaggaggagctgggtcacagcagggaatcttac
cttggtttgctggatgaccggaccaggagtcgggtgagccaggaagtgagggtagctggc
tgtgccccagtgctgtgtgacccagaggtgccactcaccctctctgaactggtgaacatc
ataggtggggaggctcaggccagggcactcccaggagtgtctggaggcctgagtttcatt
ctcagctctgccatatgcttgctgcactctagaggagctcctcttcctctccgagcctcg
gtttatgcgcctgtgtggtgggagtgagttgcacttcagggtgaagggggtgagactggt
gtgggcgcaccctgcagaaggatcccacaggtgggcagagccctgggtttttatccgac
tgagtctgggctgggggtggcctccgtttcttgactgatgagtgggtttgagtgaggta
tgcgggtgggtgaaggagggctgggggagtcacctgacttacgtgaagaaacccttgag
acagctgtggcttcttggaattaagagggaagagtgcagcatggagaaactgaggcccgg
gaggatttggcagccctgagtggggtgggggatcagctctggaatagagccagagctg
tgatggggttgggggaccctgtgtccttggccgtgcttgtcccactcctgggctctgtg

tgtggctgtggattggggtgggacagggctggttgtgcgaggggtcattgcccaactcca
gagggcgcctgccacctctcagctctctgtagggatacatatagttccttcaacaggttt
ccagcaggtagaggttattaaatcttactgaaggggtgtttttctaattgtcaactctg
cgccgcaagggtggaaactgtgagagactgattccaactccaggtctgggtggtaagcat
ccagtccaggggtgtagacaggcctggggaagcctcagacatctgtgcgttctcataccc
agggatagtgactccatgctgggggctgtcagcgtaaggggtgggtgggtcctgggctta
cctgcaggtctgagacctcctggggccagctgacctcggtaaatccctttgtctaagct
tcagtttcctgcctgtgaatggggttgcggctgtgctccagtttcacccttgtggctctg
gggttgtggtgaaaaagccatcaagctgggttggagactggctgccgtgtctacctcttc
ctcatactcctctctctgctgcatcctgggaagctgctcagctcagcctagattaggctc
agttgtgtctgtgtgtgcacgtgattgcacctgttggaagtgtgtggtactgacaccaga
gtcagtgtctctgggtgagtgaggcttgcacatttctggggacagggaactcactacctt
atgtgcccagaacaagagctttggggtctggagaagacttctaggccaacccctgcagtc
tttcctcaggtgacatggcttccccagacccacttccctggaggtgccctgtctgcattc
aaggggtagaaggctgactgggacagaacgcgacacactcagcatgtgaggaaaagcctc
cttcattctgtaggccctacctctgttgacatgacctttgataaagtgcctcccttcctg
tctcccctctctggaaccctcagctgcttccacgcttcagctgtgccccatcgcaggcag
ctctctgtctccctactttccccagcccagggttttccttttggggtcagctgcggggt
ctggcccatcctttgcccactcagacattcttcctgcccacctgctgctgagaatcctgc
atgtctcatatgctctcaggcagagaggaaactgctgagcaggccagggcaggggacgga
gccctgtgactctccactttagacccctcccagctgactgctgtggaatgcagccactta
gcagagccaaatcccctgaggttcctgtctgctgtctagccttcagggacaggtcatggg
gccttggttcctgccccgtcattttcccagtcaccctgatcttcaggggaggaacagcc
agggaaagggtttggtcatagcacctccaccctagggctattgaggatctcacagttgtg
tgtctggtgggttctgtccagagcccatttgagaacagtgggtgacaggacaggcctatg
tgacccaggcaggcagcaatattgggtcagccttcgtgtccccttctgtcagctggggca
gcctggaaggatgattgtggggtaggtgttatgggaatagacctcaggtggaggctgcag
gggctctccggcactgtaggcacagcaggcccaggagcaggggaggcagccatgactcgt
ggagcctctggcccatatccttggcagatgagggccacaggggaatgggcagcagtgtcc
aaggtcccctgggctgggcccacggctgttcctggctcagacttcttggtgggctggtca
ggaacatgcagtaacttggggcagttaccaagtggccaaggctgcgctattgggctgtgt
gagcttgggccagctcaggccctctctggaccctgcctttctgggctgttcaggtggttc
cttgggcctggagtgctaatgtttctggatgggcagcagaaccggtctgcactcagggcc
ccaggccatgttcccggaacacacctttagcattgacatcagggtgtggtgaggcccctt
aggctgggttctggtctggtgccaggggcaccactactccactgcctccagagccatctc
tgggacactggctgtgagttcagatgttctgaacagggacagggaaagccagaggaggac
ccagcctggggctagctggaggggctcgtgggcagacagcgccctttggagggaactgag
tctggaaggaaaccctttccccgggtcacagcacctggcacccagggccctcccagggct
gcgtgaactttagtcacatggtgacaggccgagtcaccgtgccaagtcgctgtgcgcctc
cttgctgctgtgacgtcagcctccccttcctcccagccaggctggacctctgtgagaggc
ctgcctgtcctgcaccctgtgcagatgcctcccactgtctgctggggctgatgggcacct
cctggctggtctcttgtactaggtaagctcatgggtcctccggctgctcctgctccttcc
ctgcctctgctcctcctcggaggtggccaccccagatcccagtcccgattcagaggccc
cctgaggagcactgcaggggggccgcaggcgtggctctgagccactctggagagtgggga
gcccagccagttctgtggccgggactttcccaggcagacaagtctgtctcttcctcccca
gcgggtgcagcccagaactgtcttctgaggaagaggtgctctcctgggcccccactgtcc
ccaagcctcaggtaagcccatcagggtcacaaggaaggggatctgggtttgaggccaacc
atggcaactgacttactttctagcctcagtttcccctagggtgtgcagctctcacactgt
ttgggtgacagccagccctctgggcttagacattctttcagtgagtcttgagggtggagg
gatgggaaatggaagcccagggcctcagcagggtgtcttcctccgagctggggacatctg
cctgtgggaggctgggcccaacctcccttgctaacctgccctgggaggaagtgacagggc
ccagcactgcctactcccctccccatgcttcctggcagtctgagcctggctgggtgtccc
ctgctcctccaggagcctacttgggcctttgggtagacagattaacagacaggggaggct
gggtcatggttgggccaccccaggaccctgatggggggctcagctcatgaccctgagcc
tgggagagatgaggccatgccttccagggcactcagcatgacccggcccagtggacggga
ctgggtagcttccttggtgcaggggggttgtcatgctaggatgggggcaccgaccaggcca
ggcccccgccccatgacatgtggtggaaatgtccgtttgttttgttttgtttttttgcttt
tttttttgagatggagtttcactgttgttgcccaagctggagtgcagtggtgcaatcttg
cctcactgcaacttctgcccccccaggttcaagcaattctcctgcctcagctcccgaatag
ctggtattacaggcacctgccaccatacccagctaattttttgtatttttagtggagacg
gggtttcaccatgttggccaggctggtctcaaactcttcacctcaggtgatccacctgc
ttctgcctcccaaagtgctgggattataggcacaagcccctgcatctggccatgtgatag
gaatgttctgtgtccataatggatactgcactttgctggtccagctcctgagtctccttg
gacctccaggaatcggtgtttctatcaggaacccttaaccctgtcccagactccctgctg
ggacccagggtgttggagtggcaagagggctgtcaggcctggtgaagggtgtgagctgtc
ccatggggcagggaggaggcaggacctgttctgtagctggacagacagagccctctagct
gctttctggaagactgaagggcaggtgatgttggagggagggagtgcaggcagggtctct
gagggagttcaggtcagaaacaagtggcacctggattcaggctgtggtggtcacggtggg
gatgaggggctgcttcggattctgctggggatgtggggtggtgcgctgctgcttgactac
tcccagtcccctctgctcttggtgtctgcatccagggctgggaggcggtcaaatgtttca
cactattggccccaggcctgccaagcctggggaggtggccacccttccacgatggcattt
ggatgttccctgtatgtggggagggcacaggggctccattcctagaccacctctgggaca
gtgtgtctggctctgaggtcagatgctctgccctgggacagggtggagtggaggggaacc
cagcttggggctcattggaggggcttgctggcagacactgcactttgtgggaaactgagt
gtgggagggggaacccccctacctctgtcaccacatccctcttcgctgttggggtcatacc
tgtcacctactgccatagccatgagacttcccaagggtcactgctgcacctcactgcaca
gcccagaagggagtccacaggggacatacagtgagcaagagacctgtgcccctcaggcct
cctgggggtgtccccaatgcagccatgataataatcacagccaccattcaccaagccctt
cccacagtctaacctactctattcacaacactctcagcagcaaggcaagtgaggtgctgc
cgtcatccagactggatggttcattgatttgcctgaggccccacggcaggtgagtggcaa
gtccagcctcagagcagggcaggctagctatgctccctgagcccccctttgccatgcttac
cacatgcacatcctgggcttgtgcaggaatgccctgtctcctgcgtgccctgctctgtgc
aaaaccctctttgagctgtgcctgggagatatgctgagagaattcatggaaacaaatgtg
ttactgacagcctctttgcctccagagttcaactggagaccagagaaatcagctagaggc
agagggaggtcacacggagtcccccagaaaggactgggctacgcgcgcttcaggtaacct
tcttgacctttaggagaatgagaaggctgcctgaccagagagtctctgaagaagattctg
tggctacatgctcctgcagagtgtgagggagaccctggttatttcctcagctgtttccac
caaatcctcccatctttcgcggccaacaccccaggcaaggcttggtgcccccgtctgctg
ctggacagtaagtcctggctccgtggcagtgaatctgtggggtactctgattgtgggtac
tatggaagctaaaccccatgctccaggtggggtggagggtcctcaggagactcctggaca
gtgccagactctgggctgaggtggggacacaagagaaaccaggccaggcccgtccctac
cagagcttctccctaagcagtggaggctcagccactgtgagaaggtaggcaaggccctgc
agaaagaggggtgtggaaatatgggggctcccaggaagggcccctgctggagatggggtt
cttaccaggatggactctgaagataagcagggaggatttgggagggcagagatgaggccc
agagcttctgacagagggcatggcctgggcaaaggtctaggggccggacagcctgcacga
gttctgggaagtgggaaggagacactgggcctgtgtttctgaggcctgactttcgactgt
gccctgttggggagggaccagggaatgtatgaggctggacctgaccctgctccttaccct
gtgggtgcagcagagccatgaagaagaggttagtggtgctgggcctgctggccgtggtcc
tggtgctggtcattgttggcctctgtctctggctgccctcggcctccaaggaacctgaca
accacgtgtacactagggctgccgtggctgcagatgccaagcagtgctcggagattggga
ggtgagtggggcagggcatgggggcatgggccatgaaaactgggcaagtggacctgagca
ataccttcacccctctgagacttagtttccccacatgtaagctttgcttggactctctca
gtagcctctgggaaggggacggtgactccgagagcagggtttggatgtcctagagccaaa
cagcgcccctttttctcagttctaagagtctctttctctttggataaactctactgttgta
ttgtttggttgttatttttacttatttcttcatatatcaatttatttagagatggacagt
ctcgctctgtcaccaggctggagtgcagtggtgcaatctcagttaactgcaacctctgcc
tcccaagttcaagtgattctcatgtctaagccttccaagtagctgggattacaggcatgt
gccaccacacctagctaatttgtgtgtgtgtgtgtgtgtgtgtgtgtgtgtgtgtgtgtg
tgtgtttctttttctgagacagagtctcactctgtcacccaggctggagggcagtggtgc
gatcttggcttactgcaacctccacctcccgggttcaagtgattctcctgcctcagcctc
cacagtagctgggactacaggcttatgccaccatgcccggctaattttttgtatttttagt
agagacaggatttcactatgttggccaggctggtcttgaactcctgacctcatgatccac
ctgcctctgcctctcaaagtgctgggattacaggcatgagccaccacacctggcttaatt
atggtgtttttagtagagacagggtttcaccatgttggtcaggctggtctcgaactcctg
acctcaggggatccacccacttgggcctcccaaagtgttgggattacaggtgtgagccac
cacgcctggcttatttttttttttttttaaggtggagtcttactctgtcacccaggctggag
tgtaggggagcgatcattgcccactgcagttcaaactcttgggtttaagtgatccccctg
cctcagattcctaaagtactgggattatagctgtgagccatggtgcctggcctctactgt
tttattctattttagactcctatctcatgttatatacaaagacttaaacagaaaatatct
tatgtttttgatattttgagatagggtcttgttctgtcacccagactggagtgcagcggc
atgatcatagctcactgcagccttgagctcttgggcttaagccatctttccacctcagcc
tcctgagtcactaggactacaggtgtgcatcacacctgactaattaaaaaagactgtttt
gtagagatgaggtctcactatattgcccaggctggtcttgaactcctggcctcaagtgat
cctccaccttggcctcccaaagtgttgagattataggtgtaagccaccatctctagccgg
aaaaaaaaaaaaaaaaaaaaaaagttattaataaaatatagcaatttccctttttgtacc
aattataaaagtcatgtacatttgtttgcttgaaaaagaggaaactatctgggcgaggtg

gttcacacctgtaaccccagcattttgggaggctgaggcgggcagaccacccaaggtcac
gagttcgagaccagcctggccaacatggtgaaactccgtccctattaaaattacaaaata
gttagctgggcgtggtggaatgcacctgtaatcccagctacgcaggaggctgaggcagga
gaattgcttgaacccaggaggcggaggttgcagtgagctaagatcacgccactgcactcc
agcctgggtgactgagtgaaactctatcttagaaaaaaaaagaaaaaagaaaaagaggaa
actgtaatcccagcacttcgggaggttgaggcgataggattgctttagaccatgagttca
agaccagcctgggcaacatagaaagaccctatctctacaaaaagacaaaaaattgccagc
catggtggttcttacctgtagtcccagctactcaggagactgaagtgggaggattacttg
agcccaggaggtcaaggctgcactgagccatgacaatgccactgcacttcatcctggtga
caaggtgagactctgtcttataaaacaaaggccaggcacagtggctcacacctgtaatac
cagcactttgggaggccaaggtgggtggattacttgaacccaggagttcttgaccagcct
gggcaacatggcaaaatccgatctttacaaaacacacaaacaaacaaaattggctgggca
tggtggcatgtgcctatagtcccagctacttgggaggctgaggtgggagggtcaattgag
cccaggagattgaggctgctgtgatctgagatcacaccactgcactccagcctgagcaac
aaagagagacaaaaaaaaaaaaaaaaaaaggcgacaaggtgggtggatcatgagctcaaga
gatggagaccatcctggccaacatagtgaagccccatctctcctaaaaatataaaaatta
gccaggggtggtggcgtgtgcctgtagtcccagctactcgggaggctgaggcaggagaat
ggcgtgaacccaggaggcagaggttgcagtgagctgagatagtgccactgcactcaggcc
tggcaacagagcaagattctgcctcaaaaaaaaaaaagaaaaagaaaaactaaaaacaaaa
accccaaaactcgaatggacttctcttccatcctccgttaggcaggtgggcagcagggtg
tgaatgtggggccaggatggaagcctgcaggttctcacgtctttatgtgccacatggcag
ggacgcactgcaggacggtggctctgcggtggatgcagccattgcagccctgttgtgtat
ggggctcatgaatgcccacagcatgggcatcgggggcggcctgttcctcaccatctacaa
cagcaccacgcgtgagtgccttgggagaggcgagggagaggggcaggggggtgtgggttgg
gccgcggcacagctgggtggtccctgggctcacgcagcataaagggtttgggtgggtctg
cctgcctacctgcttctccttctaggaaaagctgaggtcatcaacgcccgcgaggtggcc
cccaggctggcctttgccagcatgttcaacagctcacagcagtcccagaatggtaagctg
tcctgcaagcttggggtgtgggtgcagagccagctgggccactgggaaggggccttgccc
acaggatcctggccctgtcagggtccaggggcagttctagcacctccatcccttcctggc
cccatagcaccctcccacaatgagtggtcaggaccatcctcaccagggtaaagggctggg
agcttctgttatttctgctaaggcctccggggccaccctgtgcagcacacagagaataat
tattacactagcagaccttatggaccagggctcactggggcccacgctctgctctgtgct
tttcatccatgagtccctcacacccctccctgctcctttggggtaggggatgctatgtgg
gttcccattttacagggtgcagatgctgaggcccagagaggtcatgaaaatcagctgaag
tcatacagctgggaggtggtgaagttaaaattgaacccaagctgtctataccctgccttt
tcaacaggcatcccattcacttatttgttcatttgtggggatggggctctagagtgtgag
gtggagtctctcttttctaatctggtcttcagtggagaggaggcccccaaattccccagg
tacctgaggggaagccactgtccatccaggaagccactgtccatcctcaaaggaggcaca
atagattatgagattaaaaattggaagattgggtgggtctcaacagctcacgcctataat
cctagtacttaaggaggccaagtcaggaggactgcatcagcccaggagtttgagaccagc
ctgggcaacgtagcaagactccgtctctacaaaaaatgaaaaaaaaaaaaaattagccagg
tgtggtggtatgtgtctatggtcccagctcctcgggaggctgaggtgggaggatcacttg
agcccatgaggttgaggctgcagtgagccatgattgtaccactgcactctagcttgggca
acaaagcaagaccctgtcaaaaaaaaaaaaaaaaatgctggatgtggtggctcacgcttgt
aatcccagcactctgggaggctgaggcgggtggatcacctgggtcaggagttcaagacca
tcctggccaacatagtgaaaccctgtctctactaaaagcataataattagccaggcatgt
tggcatgggcctgtaatcccagctactcaggagtctaaggcaggagaatcgcttgaaccc
gggaggtggaggttacagtgagccgagatcatgccactgtactccagcctgggcaacaag
agcgaagctcggtctcaaaaaaaaaaaaaaaagttggaggatggaggggcagggcacactca
ccatagcaagtcttagacctcaggtgaggatcctgggtggtgccctttggagtcttctac
aacatactcaatctttgattttttttttttttttttttttttgagatggagtctcactcctg
ttgcccagactagagtgcagtggtgcaatctctgctcactgcaacctccgcctcccagtt
tcaagtgattctcctgcctcagcctcccatagctgggattacaggttcccggggcccagc
caatttttctatttttagtagagaggggggtttcactatgttgcccaggttggtctcgaa
ctcctgacctcatgatctgcaccccccccgccctcggccttccaaagtgctggaattac
aggtgtgagctactgcaccgaccaatctttcatttttttaatactcattaagaaactcag
catctgtagacatgaagttgctcagggtaagagaatgcaggaatcataggcttggtacct
tgtggatgcttggaatcatttctttttttttttttttttttttgagacggagtctcgctc
tgtcacccaggctggagtgcagtggccggatctcagctcactgcaagctccgcccccgg
gtttacgccattctcctgcctcagcctcccgagtagctgggactacaggcgcccaccatc
tcgcccggctagtttttttgtattttttttagtagagacaaggtttcaccgggttagccatg
atggtctcgatctcctaacctcgtgatccgcccgtctcggcctcctaaagtgctgggatt
acaggcttgagccaccgcgcccggcctgaaatcgtttctttaactagaatgtattgagca
tcatttaaagaatcaggtgttgttcctgaagctggggtgaaaaacaaagatggcagatga
aatctgtgacattccaggtgggaaggagaaactaggcaggtgtgggtgtgttacgggctg
tggaaaaacagacctgggggcctcaaaatctgggattctatggagggtgacctagtcag
ggaggggatatttgagcaaagaccgagaggcaggggtggggtcaggggagctttgtggtg
tctgggggaagaccaagtgatccaggtgtgatgggcagggacagagccctgtggggagca
ggggaggctgcagcaattgacccgaggggaacggcccaggttgtgtgggatctcttaggt
catggtgaagcctctgcctcttgtctcaaagaagtgggggcctgtggagtgtgtgagtgg
agggggatgtatctgacctgtggacccttgggttgctgtgtaagggacagggagcctgt
ggggacctgtccggaggtcactgcagtaattcatggagagggtggtgggaagtggctgac
actggacagacacacttggaagtggagcctgtggatttgaggatgggttgggtgtgatgc
tggtgtagggagtccccgaggaccccagatcctttgtctgtacctggaaggatggggtga
ccctaacggagacaggcagggctctcaggaagcaggtctgcgtggacactcaggagctcg
gttttgggcacgtttaagtttgagatgctttcttcgagcaggcaggtagatacttgcgtc
tggcgatttgaggagcagtccaggccagcaggtcaatttggcagttgtcagtgcttaaat
ggaggctgaagctcagatgtcagacagaccaccaggagagagagcaaagacagagggcag
agcaggagctaggatggcaggcgggggacacacgggtggagccaggtgctgggatgcagg
ggtggctctcagggagagtgatgagcctagtaaagctgagagggggcactgggtctggca
gtgtgggggtcgccggagagcttggcaagtatagtggcatgacagcctgattggagtgag
gtcaggagaagatttttttctgatattgatacatgatattttctatacttatgggtacat
gtgagtgcttgttacatgcatagtgtatggtgatcaaggcagggtatttggggtctccat
caccttgagtatttttcattcctgtttgttagcaccatagtcttctcttccagttactct
gaaatatacaaaatactgttgttaagcattgtcaccccgctctgctgtcaaacattagaa
ctgctgtcttgactgggcctgcggtgcggtggctcatgcctgtcatcccaacaccttgga
aagctgaggtgggtggattacctgaggtcaggagtttgaaaccagccttgccaacatggc
gaaaccccatctctactaaaaatacaaaaattagcccggcgttctggcatgtgcctgtaa
tcccagctacttgggaggctgaggcaggagagttacttgaacccaggaggcggaggttgc
agtgagctgagatcatgccactgaactccagtctgggacacacagcaagactgtctcaaa
aaaaaaaaaaaagaaagaaagaaaaaagaaaaaaaaagaagttctgtctaactgcaaggg
ggaaggaatcatgtgctgggtgtcagaccttccaggatgtatgtgtgcagcttctaagaa
ttgaaaccactggctcttggaaacttgtgccaggcttcagggtgggagaggtagttctag
agccacagccaccaagcctgggagtagccaaatggcccccgtcatctctggcgagagcag
gaaaatacctgggcgcagaggccacggttgtatctttctgggcaaagggtcagtgtctat
agtgtacatatggtcagtgaggctcgggggaagcaggcccaggggtctgtttccagtga
cctcctcgaaagtcagaactggaaggcaaaaccccttataggctgggtgcacctgtagtc
ccagtatgtcgggatgctgaggtgggaggatcacttgaggccaggagtttgagaccagtc
ttggcaacatagcaagaccccgtctctacaaaaaaataaacaataaaaaattaggctcgg
tgtggtggctcacacctgtaatcacagaactttgagaggctgagcggggtggatcacctg
atgttagaaattcaggaccagcctgacgaacctggtgaaacctgtctctactaaaattac
aaaaattagcaaggcgtggtggtgcatgcctgtaatcccagctacttgggaggctgaggc
aggagaattatttgaactcgggaggtggaggttggagtgagccaagattgtgccattgca
ctccagcctgggcaacaagagtgaaactctatctcccccccccaaaaaaagccacatgtgg
tggggtgtgtctatagtctcaggtacttctgaggctgaggtgggaggatcgcttgagcct
ggaaaaccaaggctgcagtgagccgtgattgcaccactccactccagctggggcaacaga
gtgagactctgtctcaaaacaaacaaaaaaccccttatagttggatggggaaactgaggct
gggagaggggacaggacggaggttaaggctcaggtcttgcctctggagcagttgaaaaga
ggaagagagccctctcttggggctggctgattcttgagggtggcctgtgcttgacctggg
ttgggcaggacctgctcttgctttggcacattctggtggagcccatgagtgttacaggat
aagggcttgtggtcagtgagatgggaggggatctggcctggcacaggattttagacatgc
aagcacctgcacagacacctcatcctgggacagcaaaacccagttgcatgctactgcttc
ccctcctgtgccctcctcagacatccctggtccatgtacactcctgcctgccgagcccag
atatgaccttaaacatccccttctgctgggtgtggtggttcactcctggaatctcagctg
cttgggaggctgaggtgggagaattacttgagtccaggagtttgagaccaggctgggaaa
gatggcaagaccccatctcaaaaaagaaaaaaaaaatattttcgtcctaagcctcattg
acccatctgtaaaatgagtcagggaatgtgcctgggatgctgcctgtgagagattctgat
gttccctactcagggccactaactgtagctctctctccccagggggctgtcagtggcgg
tgcccggggagatccgaggctatgagctggcgcaccagcggcatgggcggctgccctggg
ctcgcctcttccggcccagcatccagctggcccgccagggcttccccgtgggcaagggct
tggcagcagccctggaaaacaagcggaccgtcatcgagcagcagcctgtcttgtggtatg
tctgtgggtgcggccccctgacacaggcagtgcaggcacagcccaaggaccctgcaggcc
cctagcagcagtggagcggccctctgccttcaggaccccgcactgataatgggctgagga
gatgcagacccttcccaccacgtgtggggacacattctgaggtggggtcccagtggccac
tgtggctggccgtgtgtcctgagtggcaagggacactaggaggctcccggaaggacatt
gggaggatgagtgctgagtgacagagccaccaacctgtgacaggtgctgccccagctttg

ttccgttctcctgtgtgggtgggtgtggggggtggtctagctgagtccatcccacctgct
tcctcacgtgagccccctctgccccagtgaggtgttctgccgggatggaaaggtgcttcg
ggagggggagagactgaccctgccgcggctggctgacacctacgagacgctggccatcga
gggtgcccaggccttctacaacggcagcctcacggcccagattgtgaaggacatccaggc
agccggtgagtgggtaacctcaggggcctgggtgaggaaccctgcagtggaaaccctgag
ctgtggcccagagccctggggtcttcctgtcctgcctgagcctgcaggaagttcctcgtg
gaggagtgtcagtggccggccatgtgggttcacagctcctgggtatgtcaaagccaagag
aggccttgcggtccaggagagcaagcccctggtggggtaaatgcaggtgtaggcaagagc
cagggctagggaagcactagaatatggcctgaagatccaggaggacttggaggaggtggt
ggctgggctgcagataactttgttaggcagagaaaggaagggattcctagcagaggagca
gctgggctaaggcccagggagagggggctttgattcaccaagagggttacaagggatgag
ggtcaccttgagagaggcgtggggaaggggctttgtggggcaggggcctggagcttggct
gtgactttcttcaggtaatttttgtcactgtttcatggagaaggatgactagtgtgtcga
cctttaccactgaggctggaaattagcatgccaataccctgtctgtctggagctgactcc
aggagaattaagagcctccctccctccctctatccattcatcatgaggagaagaggccaa
gcagcagggacaccggcaggaattctccacttagaaaaggccctctgagctgggcgcggt
ggctcacgtctgtaatcccagcactttgggaggccgaggcaggtggatcacctgacgtca
ggagttcaagaccagtctggccaacatggtgaaaccctgtctctactaaaaatacaaaat
tagtcgggcatggtgggttgtgcctttaatcccagctacttgggaggccacggcaggaga
attgctagaacccgggaggctgaggttgtagtgagccaagattgcaccactgcactccac
agatactccgtctcaaaaaaaaaaaaaaaaaaaaaaaaaaggaaagaaaaggccctctgag
gccaagcttggtgtctcacacctgtaatcccaacactttgggaggctgaggtagaagttg
aggccagaagtccatgacaagcctgggcaacatagtgagactacaaaaaacatattgaca
ctctacataaatatagtgggggtggggctggggaaccagaaaacaaaaaattcaaagcat
actgaaaagaaatacagcaagaccctacaacaaaattaaaatttgctgagtgtggtggtg
ccacctttagcctcagctactggggaggatctgctggaggatcacttgagcccaggagtt
caaggctgtgattaagccactgcactccagcctgggtgacagagcaagatcctatctcta
aaaaagaaaaaggaaaacactgtttggccatagaggagagaaggtagacaagaagccagt
aagccaagcaggcagagggcaggtggccccagcccagctgtggggtggtgagcagtgtca
gggagacaggagttcaagagcgagtgaggttcccaaggggggcaaggttgcctgtggcccc
ctcccagggcacagtcccacgcctccagtagtgcactctgtcctccctggtaggttcagg
cttttccccaccactatggtgcagccatgcttgcccccaacaccactggtgcagctccat
cctccacacaagggtgcagccccttcccagcgcccattcgagctgctgtcccattgcagg
gggcattgtgacagccgaggacctgaacaactaccgcgctgagctgattgagcatccgct
gaacatcagcctgggagatgcggtgctgtacatgcccagcgcgccgctcagcgggcccgt
gctggccctcatcctcaacatccttaaaggtgagtggtcgtgccacagccctgtggtagg
acccatgacacgccccacccctgctgcagctctgctggcccccatgccacgtctttgcat
cactgagctcccagggagtgctcctgtgtcacagctcaccatgtcctgaaggaggcagtg
cagagccacagggctgaagtgggcaatgctcgagggttggaggaggaacaggagtcatca
ggacggagagaggtacaggagctcagggctgcagggcttggtccagagggcatccctggt
ccagtgggtggccctgccacttggtcattgcatgaccagagctgatgcgtgacgtgagat
ccaggttggggagccttcaccttacttcactctcaccacagccttctgaagcagctgctg
ctattggttattaaatgctccttgaggtgggcaaagtggctgaggaaggtgttcaccctc
tgagtccctcagatcctctggggctcagcaacgtgcacctggctctgatcaaccagggta
caacttctcccgggcgagcgtggagaccccggagcagaagggcctaacgtaccaccgcat
cgtagaggcttttcggtttgcctatgccaagaggaccctgcttggggaccccaagtttgt
ggatgtgactgaggtaaggggcagaggctggcccactctaggcgtggggcctgctgtaga
ggcatcaggtgggctccccagggcggctgcagcctcacatatgctttatgaatccattcc
tgccacaaactttgattgcgggcctactgtgtgctcagatggactggtcggtgaccccgg
gtcttggcctctgccccacagaactgacagtgtggggaattagtggccacactcctacct
caagtgctttgcacatgctgtggttctcgagctcagtgctgagatgaggaacgcatgggg
gcattgcagccctcgggcatgaggagaaggacaggtgaaagggagagctccatgctgggt
ccctgtgagaccctgtcatatcccttcccgctgaggatcctcacatccctccttacctac
tcgggtccttggcattcccaggcagacctgcagacccctccacactgaccaggacctcc
ctggaggggcctcagctgcccgggtggtgtcttctctttccccatgagcattctgcacct
ctgactcctgctgtagccagtgacctggtgtcttgtctctttgaggggacagggccacta
cagtgtgtacctctgccctcccttttagctaaggccagctcttccgtccacacgctggct
cagggcactgttcctacaatgctcctctctgcctctgaccatttccttctttataacgga
ttattcccgaaagaaaggcagctcttgtagctctagaaacttccatctgctgcctccttc
tgtcctctgctccccttacagcaaacatggctgggctatgtcctctctcccttcagggtg
tcccctcccctgctctatccccatgccaccagattgccacatccagcctcagtttcccca
tcaggccccactcaacagcatctcacacagctcaccacactctacttgagttttcatttt
gcaaattttcacacctacgaaaatgtaaaaaaaaaacccaaaccgtccaagtgtcaatgtt
ccctttccctagagtccacagtgtggacgtttctgctggatttcctctctctccgtctcc
acccacgtctattgggattcgtgtttttctgagggatttgacagtaggttgctgatgtca
cacctcagggtgtgtctcaagagtgagacttgaacataccacagcaggatgctggggtga
cacaacctactgtcccttctctttacctacagtaggctcccttggctgttttgtttctgt
tgtgtttttgtttggacacagagtctctgtcatccaggttggagtgcagtggtgtgatct
cggctcactgcagcctcaacctccagggctcaagcaatcctcccacttcagcccctacaa
gcagctgggaccacaggcacatgccaccaaacctggctaatttttgtattttttttttaga
gatagggtcttgccacattgcccagactggtctcaaactcctgggctcaaacgatcctcc
cacctcagcctcccaaagcgctgggcttacaggtgtgaaccacagtgctcgacctgtttt
gttgattttaaagcccagggcagtggtctggaagatgtcccacatcctatttgcctttc
tgtttccttaggggcagattcagacagagcaccttgccctgcgattgctaatctgtgggg
gtgttgctgaaactctcctgaggcattttcttcagccttcagggcccatcctctctgtct
cctccttcacctcctcatctcctcctcgccacggccttggggaccttggccaggctcgtg
gcatccagcagcctctcaatgtcaatacctccatgttcatccctcagcccgcccttgccg
gtgcacccatgcttatacatgcgcccatgctcttcaggtctgatgaagcatccaaaggca
agctcctgaccgtcgccaaacctgccccttctgcagggtttacctccctagttggcgcca
tcctcaattcttctctttctcccacccaggccatcatctcttgcctggttgatacccaca
gcctcccctttgggctttatctttatccccctcacaactgccagagggaccctgtgaaaa
cactccccagcctgctcattcctctgcccaaagcctgcgtggcacagagcaaaagccagc
cattatgggacctaggaggtcctgtgggatgggccccagcctgcatcttcatattcttct
cctccctaacccattcactctctgcctatcgcttaccagcctataccacctgcctcaggg
cctttgcactgaccattaaggccgcattccaggctcttttcacatgttgcctcctccgag
aagccctccctgaccactctacccatacctcatgcctctcaattccccttacctggtttg
tggtttcagcactttcctcatgtgcgtttgtttttcttggcgtgagggcagggacctaag
tgtctgttccctgttgattttccagtgccaggcatgcagtgcaaactctagaaatctatt
tttgcatgagtgaatgagtgattgaatgcggcaagggtctggaggctgagggccagacag
acattcagagttgctggaaggcgacagagacagagagtcagactggtcatgcaaggtgct
gggcctgcccttgggccctggggagccatggaaggctgtgggtgccagagggttgtggtc
agagccacagtcgggggccttctgagacctgtgccccctccccaccctccctccccacct
cctcaggccagctctggggtctcggcaggtggtccgcaacatgacctccgagttcttcgc
tgcccagctccgggcccagatctctgatgataccactcacccgatctcctactacaaacc
cgagttctacacaccggatgatgggggcactgcccacctgtctgtcgtcgcagaggacgg
cagtgctgtgtccgccaccagcaccatcaacctctagtaggggctgctgggccgcctggg
tgggacagggccaggggcatgtggtccagggactgcccacttatccagtaaggtggctcc
atcaccccttttcctggtgggaaactgaggcccgaccttggtagcttatcctgggcctct
cagggagtaagtttgagcccaggttgggtcgggcgaggtcaggcgctgtctgacctggct
gggcggtagctttggctccaaggtccgctccccagtcagcgggatcctgttcaatgatga
aatggatgacttcagctctcccagcatcaccaatcagtttggggtgccccccctcacctgc
caatttcatccagccaggtatggggtggaggtctgggggtggagggctggggtggagagg
gggggtgtcctgggcaggcagctaaccagcatccccgccttctcccatcggccacaggga
agcagccgctctcgtccatgtgtccaacgatcatggtgggccaggacggccaggtccgga
tggtggtgggagcttctgggggcacgcagatcaccacggccactgcactggtatgtgtca
ccccttttctccctggcgctgcccaccctgcacagcccccaggccatgctgatcacactc
ccatgccccaggcaatcatctacaacctctggttcggctatgacgtgaagcgggccgtgg
aggagccccggctgcacaaccagcttctacccaacgtcacaacagtggagagaaacattg
accaggtgcgctgggggttggagaaactgagtcacggtgtggggtcccagggcatcctgg
actggaggcctggatcatcatggagtggacaatggttggtgtcctctctctggtgcctgg
gccatctggagcccctgtgccatgagggccaagccacctgctccagtgagacccagcagg
ccccaacctgctcttcctgatgacctggcccaaaatggcaccacctgggctgaggcctgt
gaccacacaggcatggttcaggtggcatctggaaccctgctcaggcttcctgtctcctcc
cacccccaggcagtgactgcagccctggaaacccggcaccatgacactgagatcgcatcc
accttcatcgctgtggtgcaagctgtcgtccacacacctggtggctgggcagctgcctcg
gactccaggaaaggcggggagcctgctggttactgagtgcttggggcggacaaagctgac
cagcaatccagggacaagatactcaccaggaccaggaaggggactttggtgtcccctgtg
agtggtagagcatcacaataaatgaggccactgtgtcaggctccaggcagcctccctggc
ctggctccccactctctgggcctcagtgtattgtgtgtgaaatggaaccatctggctggg
gaggaatggagaggtgggattcggagatcttcacactgcggtcgctggaactagcctcag
tatcttcagcgtggggagagccaggtgcgtggctagggaccaggggaaggtccatgccaa
cccctgccccttcccaccctgatccattggactttggggccaggtgctcccttattgggg
ctgcacagtgacacctaggactagccaccaggggggtgccgcgcccctggtgctttcttag
gcagtgggtggccagctgatgctgggaacctgggcaccttctcagacccatgggcatcca
actcatcctgctaatgacacgggaggtgaagctgagttccaaggaatgggaattgggcat
cacgctagaggaaaacatcttagtcagagccaagcccctgggggtttccaagtataagc

ccagagtgaaacccaagcttgtgaccctctccagagggaggcctggttttcagggaacag
caaatgggaagaggtccccagattccagggatcagggcttggaccagctggggacgcagc
ccagagggagtgggtctggaagggaacagctagacacagcagccttcaccactggcagcc
cctcccggcctccctcggggcctgctccctcctccaagcaccgttccaacacctggggca
gggttctgggaaaggctggtggaggtgggctggtggggggcggtgatcacagcccagcat
ctgggtatcaccaggggcactggggccagggcccaggtgaagccaggtcggggctctcct
ttagaagccccgaaaacctggtgataccaaagggcccacagacaaacagggttttgtgcc
tgcggagttgagtaccaccgggtctaagccctggagggctgtgtccctggggctccccag
gggtgagatggaggtgggctcaactggtgtacccgtcactcctcaatccttattttatgt
atttaatttttaaaaaaatttttatttgaacaaatagagatggggtctcactatgttgac
caggctggtcttaaactcttgacttcaagcagtcctcctagcttggcctccaaagtgcta
ggattactttggggattactttagggatgagtcactgcacgcggcctcaatccttatttt
ggcctgaaaggaaaggctgtggccccgtttgcaggggagaagactgaggctggaggggca
ggccttgctctgggttgcacagcagcaagagaagtgggagctggccatgaggcttcctgg
acccgaagcactggtggggttcaccctggttcttcaggtcccatggggctcagcccagga
ctaccttggtgggggtgggagacttaaatcctctccttcattctcattgtcccttccccc
atcatttcctgaggaagcacattcagggacctccctggctgtgcctcagtccaaaaccag
aatgacacgcattcctttccctgggcctttgctcaggcggtccctgcaccctggcctctg
cctgaccagggtggtggggagaggagggggggacgtcccctccgctgctgtctccactgtt
cctgctgccctggcctctgggcttccaggactgcagtgggtgggtgggtgggctggcctg
agcccaggaatgcacttcggctcctggttgagcaaagtcactgagacttgggagtcgggt
cgggttgggaggaggcgtccacaggcccccactacgaaaggcagctgtgggaacagtctg
cctgtaaacaaccactccagcccaggctgaccaggggctctggctgggacattgggatct
ggcaggctgtgtggcctgtaaggacacagtctgtctctgtgcctcagtttctctgctgcc
cagttgggcgtcccagactccaggtgtagacatctggagcaggcagtgctcagctgggaa
ggaagtggggaggactggaggagccatgtgtgaaggattccaacccacatcacctgcacc
cctgctgagcctggtcaacagagcccctcagtgggtcctcactcccctggctgcctcccg
gttaggcaccctgagggctggggagaacagggccaggccagtgtccccagagaggctgcg
ctgccagcacagtaatagcggatttggattcagggaagcagacccgcagccagggtgggg
aagagctgcaggctgggcgtggcacctaggcggcacagcctccctccctggaggcccacg
ctgcatttccaggacagcaagtcccagggatggatggtcccaggtgccaagggctagagg
catggtctgtctgcattccccacatggacgtcttgtagtcaccagcgtttgatgctgtca
agtcccccctgtcctctctgcggactgagaagcccttggtcatccttaggggggttgtggaa
cccaaaccaggctgcagaagcatagggacttgaacccaagttttaagtgacaccaccttt
tgtcccccctccctcggtctctgttcagttccacttcgatattgcctgtgctgggccatgc
agagagggttaggggatagagatgggaactggggagtggggctccactctcagagagggg
cagccttgctggatccaggggagatagttgagcagccccagctctgctttcccggagctg
ctgggaaccccgggaatggtgtggagattcctgggagctctgcccccacttgacaaccac
agtgcagcaggcaccaagttctcctgcacattgggacagtgtgaccctgggctctggtta
gtggcaggtggggccttgggtcctaccagcagtgagggagttagcacagcagctggctcc
tctagggaaggaaaactcccttcagacactttggtgcctggcctcctgccaggaacaagc
aggagctgaaaactagaagttgaggcataagtttggccactctgtagtgtgtacctgggg
agggcagcagctcgccacagctgccagctgccagccgtctacccattcacctggcagccc
gcttttcagacctgcctgtccacccatctataagcccatctctgtcccgttgtctatctg
accatctttctcttactgtcctctctgtccagcaatctggcctgtctgtcgatccatctt
cttgtctaactgtggccccacctatttgtccatctgtccaattacctttgattctatctg
tgcatcttcttgtccatccatctgcccacccatctgtccctgtgtctgctcactggcctc
ccctctcctcctgggccacagagccatggcccagggctgtgggtccttggtcagcctggt
gctgctggggctggggctggggctggctgtcactgtgctggctgtggtcctctctcgcca
ccagactccctgtggcccccaggcctttgcccacgctactgttgctgctgactccaaggt
ctgctcaaatattggactgtgagtgagacgtgggaggaagctgggtggcctttggcagcc
agcccctcctggagaaggcgtgtgtgtttgagtgtgtgagtgtgtgggcgtgcgtgtgtg
attgcgtgtgtgagtgtgtatgtatgtgtgtgagtggggggtgtgggggtgtgtgaatgt
gtgtgattgtgtttgggggtgtatgtgtgggtgtgtgtgattgtgtttgggtatgtgtgg
gtgtatgtgtgagagtgagtgtagggggtgtgcgtgtgtgaatgtgtgtgattgtgtttg
ggtgtgtgtgtgggagggtgagtgtgactgtgtgggagggggtgtgggtgtgtgtgaatgt
gtgtgattgtgggtatgtgtatgtgtgggtgtgtaagtgcgtgtgtgtgtgggtgtatgt
atgtgtgcgtgtgtgagtgtgtgtgtgcgtgtgtgtacaagtgcactggcccaggaagca
ggagccgtgtgtgtgtgtgggcttcagcacctgcagggcttgggcacaaggaggcagcct
cagggcccttgcacagaacaggtggcagggtgtgcccatggggcagatggtgatttaggg
acagtcatgtgtgagtccacacctggctccaggattcaggagacccatttgcacatccca
ggtggggccagtacaggccccttcagtgaggccaattctccaaggctggggtcttctccc
agggtcataggtgaagggcttcagaggctccctgtgtgggtactggcctgctggggtaca
cacaatgctgccacagccagtctgccccagcttccagctgggggccacatctcgggtttc
tctgtcctggggagcctggtgccccacccctcacatcctctctccctgagtcagggcctg
ggtctcctgagctgagtgactgatacttggtgtcctgaatgagggtgtggtggagagggg
ccacggcgggtgtttcctgaccctcttccaggaaacccagcccaagggaggccttcgctg
ctgccactgcagagaggacacatacaggacgccccttcctgccccctgcctgccattggg
cccacaaaagccggggcaagcctcccctccctgcagccacctggtctgcttcccagaagc
tctgtcttgcaggctgttgggaggatcccagtgctttgtaaactaaagcaagggaggcgt
ggccgttctctctctttgttcattcattcaccttttgagtcatttcttcctccctccatt
accccatctgtccatccttccctgccctgattgctcatgccacacccccagcccctcctg
acctggtcctttggtttctctccatggctttctgtctcctcccacagggctgagaatggc
agctcagggacaagtagagcctggtgactgcttggtctcccgggtggctcctaggggatt
tgagggattgatgcctgctgaggctgtgcccctcctctgctcaggaggacatacagagat
gtggcacccacttaaactcaaagttgcacagatgcaaatgagactggggtctcaggcacc
agagaccaccgtgggcacgtgacctttgggagtggggacctgctgccacagatctctgag
tggagtctggacctactgggtctccccaagtgactgtctgggggtctctgtagcatgccc
tgctgtgtacgtgagggtcagtggttggggaggggtctctgctctaatgcttcctacacc
ggcactccctcaaactctcccttggtgaagagagaggatgtggtttgccccagtgtttta
tcgaacaactctctccacttcctgttttcagaagccgggagtggaagagagcctggggct
ggccccagctgctgctgcggaacaggggtcactggacgctgggaccctggccgggctggc
tgggggcctcaggaagaggcctgctgcagcgtcatcctggccgagatccctccctgcagg
ggcccctggccatgctgccgcagggtctgctggggccaccagaagcccacgctcctgcct
ccatctctgccctgtgtgctcacctctcaccagcaggccctcccagagtccagtctcttc
tgctctttttttgtttgtttgtttttttgagatggtgtctcactctgtcaccaggctggag
tgcagtggcgcaatctcggctcactgaaatttccgtctcctactctttcagcatcaggtt
ttattactgggattctgctacagccagagaccctgggagcagattcctaaggcttatgtg
agtgtggacccagcaccgtgcctagtagacatacaaaaggagcatggtgacagtgaggtc
tgtcatctccagcttaatgactgttttgatccttgtcaaaaaggtgatttttggctgagc
atggtggctcacacctgtaatcccagcactttgggaggccgaggcgggtggatcacttga
ggtcaggagttggagaccagcctgggcaacatggtgaaaccccgtctctactaaaaatac
aaaaattagctgggcatggtagcgggtgcctgtaatcccagctacttgggagtctgagac
aggagaatcacttgaacccaagaggcaaatgttgcagtgagccaagatagcaccactgca
ctacagcctgggtgacagagcaagacttggtctcaaaaaaaaaaaaaaaaagaaaagttta
tatttttgttctaaaacttatcttaatgtcttcattctatattttatataattataagag
ctatataagatatactacccctagtactttgtttttttggatattctattcactcctgatg
gttaatttatgtgtcaactttgctaagctatgatgccctgttgtttggtcaaatacttct
caatatcttgctgggaggttatctcatagatgtgattaacattgacagtcagctgacttt
aggtaaaacaatgtgattaacgctgacagtcagttgactttaagtaaaactgaggtttcc
cagagaagcaggaattctgctttaacactataacatgtaaatcctgcctgagtttctggc
ctgctgactgctctccaggttttaggttccagacttcgagatcaactcttacctgaattt
ataagctgctggttcgccatacagattttaaacttgctagtccccacaaccgtgtgagcc
aattcctaaataaatctctctctatgtataacctattggtttagtttctctaaaaacctt
ttacatctagtttcctggatgttaagtaatactgaaactagctagtaacttcttttcttt
tttttttttgagatggaatttgctcttgttgcccaggctggagtgcagtgccgcgatct
tggctcaccgcaacctccacttcctgggtccaagcgattctcctccctcagcctcccgag
tagctgggattacaggcatgtgccaccatgctcggctaatttttgtatttttagtagaga
tggggcttcttcatgttggtcaggctggtctcgaactcccaacctcaggtgatccacctg
ccttggcctcacaaagtgctgggattacaggcatgagccaccgctcccggctcctcgtaa
cttcttcttttctgtgatatgtctcttatctctaataatactttttcttcttaaagtctac
ttcattaaaaatagtaatgctgggcatggtggctcatgcctgtaatctcggcactttgtt
ggaggttgaggtgggtggatcactgaagcccaggagttcaagaccagcctgggcaacatg
gcaagaccctggctctacagaaaaatacaaaaattagccgggtgtggctaatataattct
aagttagcacacctgtagtcccagctacttgggatgctgaggtgggagaatcgcttgaac
ctagaaggggggagattgctgtgagccaagatcatgtcactgcactccagcctgggagaca
gagtgaggctctatctcaaaaaaaaaaaaaaaaaagaagttatacagctttcttggttaat
gcatgcatatttttcattattttccacctttctgtatccttatataaaaggcattagttg
ggtttttatttccaattagtttaatttttattatccttttaaatgtaactaattatttat
ttgggttgaaagccaccaccaatttgttttccatgcctactctctttcttcttatctcct
cccacatcttgttttgcatttattatttttattatttaatttcctcctctattagttttg
taactgtgcagtcttggagttattttaaaagagacagtagattattttagagcttacaac
atgcatccttcacttaccaaagtctaacatgagctagtactttttttgttgttgtcgtcat
tgagacagagggagtctcgctctgctgtccaggctggagtgcagtggagcaatcttggtt
cactgcaacctctgcctcttgggttcaagcaattctcctgcctcagtctcctgagtagct
gggatcacaggcgtgcaccactatgcccagctaattttgtattctttttttagtagaga

cagggtttcaccatgttggccaggctggtcttgaactcctgaccttaagagatccgcctg
cctcggcgtcccaaaatgttgggattacaggcataaaccactgcgcctagcctatgagtt
agtacttctatccccttcctagtcagtacaagaaccttggaacaggaatgaaatttaccc
ccaatgacttatatgctaatattttgtgtttttaaatatatgtatatgtgcagctggg
tgcggtggctcatgcctgtaatcccagcactttgggaggccgaggcgggcagatcacaag
gtcaggaaatcgagaccatcctggctaacacggtgaaactctgtttctactaaaaataca
aaaaactggctgggcgcaatggctcacacctgtaatcccagcactttaggaggccgaggc
ggaaggacaacctgaggccaggagtttgagaccagcctgaccaacatgcagaaaccccat
ctctactaaaaatacaaaattagtcgggcatgatggcgcatgcctgtaatcccagctact
cgggaggctgaggcaggagaattgcttgaacctgggaggcagaggttgcagtgagctgag
atcgcacctttgcactccagcctgggcaacaagagcaaaactccatctcaaaaaaaaaag
ataaaataaaaaactaactaactaaataatacaaattagtcgggcgtggtggcgcatgcc
tgtaatcccagctactctagaggctgaggcaggagaatggtgtgaacctgggagacggag
attgcagtgagccgagatcgcaccactgcactccagcctgggcgacagagtgagactccg
tctcaaaaaaaaaaaaatatatatatatatatatatatatttatattttgtgtgtgtgcg
tgcatagatgtatctgtgtgtttttttgtgtttttattcttatttatgttgagagtgtag
agctatgtaaaaataaacagaattgtataatgaagccccatgtatccattcaatttcaac
aacaatcttatggccaagctaatttcatgtatactctttctctttcctgcttccttctac
cccacattatttcagtgcaaatcccagatatataactttacccatacatatttcagtatg
ctttatttattttaaaccccacaagatatcatttctatactactataattttataacaa
taacattcatttagatttactcaaacatttacttcttctgttaccctttatttttattta
taaaaatatatttgggaaaaaatatatttgcacacatagtcaaggatctcctgagggcta
tgtcatggccaaaatatacatatatattccatatttatatctatctatatatacacacac
atacacacacacatatatacacacacaaacatatatgtattccactttcactttttatt
tgtttttgagaccgagtctcgctctctcgctctgttgcccaggctggagtgcagtggtg
cgatctcagctcactgcaacttctgcctcctgggttcaagtgattctcctgtctcagcct
ccaaatatctgggattacaggcatgagccaccacgcctggctaattttttttttttttt
tttttttttgagacgaagtctccctctgttgcccaggctggagtgcagtggcacgatctt
ggcgcactgcaacctctactttcctggttcaagcaattcccctgcctcagcctcccgagt
agctgggattacaggtgcacaccaccatgcccagataatttttttgtatttgtagtagag
acagggtttgcctatgatggccagactgttctcaaacttccgacctctggcaatccactc
accttggcctcccaaagtgctggggttataggcatgagccgccaccctgcctggcctgtt
tttttttttttttttttttttttttttttttttgagatagagtctcgttctgtcaccca
ggctggagtgcagtggcaagatcttggctcactgcaacctccgtctctggggttcaagca
attcttgtgcctcaagaattgagtagctgggattacaggcgcccaccaccacatctggct
aatttgtgtatttttggtagagatggagcttcaccatgttggccaggctggtcttgaact
cctgacctcaagtgatccacctatctcggccttccaaagtgctgggattacagacatgag
ccaccatgcccggccattttcacttttgaaggatattgttagtgcacataaaattctagg
ttggcagatattttctttcctcagtttgaaaacatgattcccttgtatttgatttctcct
gtttttattgagaagccaattctcaatctaattttgctcatttgaaggcaatgtcttttt
ttgttgttgtgtttttttgagatggagtctcactctgtcgcccaggctggagtgcagtggc
gtgatctcagttcactgcaagttctgcctcctgggttcacgccattcttctgcctcaggc
tcccgagtagctgggactacaggtgcccacaaccactcccagctaatttttttgtatttttt
agtagagatgggatttcaccatgttagccaggatggtctcgatctcctgaacttgtgatc
cacctgtcctggcctcccaaagtgctgggattacaggtgtgagccaccgcacctggcccc
ttaattgttgtatttttaatagagatgaatttttgccgtgttggtcaggctgatcccgaa
ctcttcgtttcaggtaatccaccctcctcagcctcccaaagtgctgggattacaggtgtg
agcaacccctacaccccggcctgaaggcagtatctttttttcctctggctgctttaaaaag
gtttgtccttggctttgagcagttttacaccgatgcatttaggtggttcttcattctatg
acttcattctttttttgtccattttagaaaattctcagctttatctcttcaagtattacgt
ctttcccatcctctctcttctctccttatgagactccaatttcacatgacttataccttg
ttaaagtatctcccatgtctgttaatccatttcctgtgtgttctgtctatttttctcttt
gtacttcaatttgtacattttgtatcagactatctcccaattagccggacatggtggtgg
ggcacctgtaaccccagctacttgggaggctgaggcaggagaatggcttgaacccgggag
gtgaaggttgcagtgagctgagatcatgccactgtcctccagcctgggcaacagagtaag
gctctgtctcaaataaataaaaaaataaataaataccagttcattatttatttatttattt
tttatgtttgtgtctactgtgctgttcaaattgagttcctaattccatttttttttttttt
tttttgagacacagtctctatctgttgctcaggctggagttcagtggtacgatctcaact
cactgcagcctctgcctcccaggttcaagcgattctcgtgcctcagcctcctgagtaact
gggattaccaccatgcctaactcagttttgtgtttttttttgtttttttttttttttgaga
cggagtctcgctctgtcgcccagcccaggctggagtgcagtggcgcgatctcggctcact
gcaagctccgcctcccgggttcacgccattctcctgcctcagcctcccgagtagctggga
ctacaggcgcccacaaccgcgcccggctaatttttttgtatttttagtagagacggggttt
caccgtggtctcgatctcctgaccttgtgatccgcccgtctcggcctcccaaagtgctgg
gattacaggcgtgagccaccgcgcccggccagttttgtgtttttagtagagacaggtttc
gccatgttggccaggctggtcttgaactcctggcctcatgtgattcaccctgctgtctcc
caacgtgctgggattataggcgtgagccaccactcccagcctccattttttttttttttga
gacggagtctcgctctgttgcccaggctggagtagagtggcacgatctcggctcactgga
acctccacctcccggttcaagagattctcctgtctcaatctcccgagtagctgggactac
aggcacatgccaccatgcctggctaattttttgtaattttagtagagatggggtttcacca
tattggtcaggctggtcttgaactcctgacctcaggtgatccacccacctcagcctccca
aagtgctgggattacaggagtgagtcaccatgcccagtgcgtctattttttttatagtggc
cagttttctgattaaattcttgtttctttatatccttgaatatagatataaagtacttat
tttaaagttcatggtctgacaatttcataatctagagatcctatgggccttttaaaaaat
tgtctctgctttctcttgagctttgttcctgctgtcttatttccttgtttgcttggttgt
ttttaatttggcaatggagggtgtgtataaaaaattgttagaaataattttttttttgaga
tggagtctcgctctgttcccaggctggagtgcaatgacatgatctcggctcaccgcaacc
tccacatcccaggttcaactctcctacctccgcctcccgactagctgggattacaggca
ggtgccagcatgcctggctaattttttgtatttttagtagagatggggtttcaccatgttg
gtcaggctggtctcgaactcctgacctcatgatctgcctgcctcagcctcccaaagtgct
gggattacaggcgtgagccactgtacccagcaagaaataattttaaaaataatttccagc
cccagcacgatggctcatgcttgtaatctcatcactttgggaggctgaggcaggcagatt
gcttgagcctagaagttcaaaatcagcctgcgcaacatggtgaaaccccatctctacaaa
aaataaaaaaactagctaggcgtggtagtgtgtgcctgtagtcccaggtgtttgggatgct
gaggtgggagtctcacttgaaccaaggtgatcgacgctgcagtgagccatgatcctgaga
ctgcactccaacctgggcaacagagtgagatgctgtctcaaataaataaataaaaataaa
ataacatgaggcctagaagtctgaaattctgggatctcccttatgcatttgagcggctga
gatgatctgaagctggatccagtgctcctgagggctgctctatttctggttgactgtcac
tcctagagtaagaaacctgcaccccacgtgtggggcattatggcattgcctccctcagcc
acgtgaataggtcaacagcactgctctagaccaggtgtggtggctcacgcctatagtccc
agctactcaggagactgaagtaggaggattacttcaggccaggaatttgagaccaccctg
agcaatatattaggttggtacaaaagtaagtgtggtttttgccattaaaagtaatagcga
ccctgtctcagcaaaaaaaaaaaaaaaaaaaaaaaaaagggaagaagagaaaaagaatcagc
tgggcatggtggctcacacctgtaatcccagtactttgggaggccgagttgggtggatca
caaggtcaggagattgagaccatcctaacatggtgaaaccccgtctctactgaaaatatg
acaaattagctgggcatggtagtgggcacctgtagtcccagctattcgggaggctgaggc
aggagaatggcgtgaacccaggaggcggaacttgcagtgagccgagatcgcaccactgca
ctccagcctgggtgacagagtgagactccgtctcaaaaaaaaaaaaaagaaagaaagaaag
aaaaaaaccccactgctctgtctctcagcctcctcttccaggattggctgtcatcttgag
gggaatgctggccttgcctgtctccagccttgttcttctctgcctcttatgcctttaagc
acatgttttctatttgctgggctgtgaaatctgcacttcatctgatggggtttgctttat
aggtgactagatccttttctcttggtggttttagaatttgcatcttcacattgactttaa
atagtctgattacagtttgccatggcaaagaccctttgcattgcattgtttggggatatt
tgaccctcttttatctggatgtctaatctcctgttagatgtgagtagttttcattatttc
attaatgggctggcatgtggaccgtggttccaggggggctcagagaggcagccgcctgat
gtctgaccgcttctctttctgtctcttttcttacctggactctgggttgcttgttagctg
cttctgccagttctgagttttcaaggggagagggggcccagtgatggctgttctttgaagg
aaagggaagaatgtctcctgtttaacacatttctatgtttccagttagttagttaggtta
gttagttagttagagccagggtatctcactctgttgcacaggctggagtgcaatggcatg
atcttggctcacagtagcctccacctaccaggctcaagcagtgctcccaccccagcctcc
caagtagctgggactgcaggcatgtgccaccatgcttggctaccttaaaaaaaatttttt
ttttttttttgatacagacgaggtctcactatattgcccaggttggtattaaactcatgg
gctcaagtgatcctcctgccttggcctttctaagtgctgatattacaggcagggcttcaa
tttttttttttttttttttttttttttttgagacggagtctcgctctgtcgcccaagctg
gagtgcagtggccggatctcagctcactgcaagctccgccgcccgagtttacaccattct
cctgcctcagcctcccgagtagctgggactacaggcgcctgccacctcgcccggctagtt
tttttgtatttttttagtagagacggggtttcaccgtgttagccaggatggtcttgatctc
ctgacctcgtgatccgcccgtctcggcctcccaaagtgctgggattacaggcttgagcca
ccgcgcccggcctgggcttcaatttttttaaagctcccagcaggggtattaaagtctccct
ttccagagaaagcgcactctgtccccatccctcatgttatcctctcctgcctctgcttag
ggttcactctgggggaattgccacttgagagattcctttttgtgtgtggcttctgactga
ccggtccctgctcacagctgctgcttctcagggtggggtccctgaggcctggagtgtggc
ctctgaaaaccttcagggccagaagcagaatgagagcctgtggccacatagcccctggtg
ggagacgtcctgccaccctttgcttctctgtgtcactctggctgtacatttcagatccct
gggaaacgttaactggtaggacctagaagggaggtgaggaggggtcacgccccaggtgt
gcctgtggtgagcctttgtgctgagcaggtgcagggagggaggcccaggtgcacacatct

gtgaagtaggggcagctggttgggctccttgacctgctccagaacttcttattttctagc
cacttcacctgcagaaggcccaggtggctgtggtctctagggtcccttgcagtgaacata
ccgcctgtcctcagctcctctctaggcctggtgctgtgctcagcaccgtcgtatgtgtgt
atgtgtgcacgcacgtgaatgtgtgcaggcatcatgaggtgtggtccctgctcttaggca
gcttgccttgtggctgaaatgaaccatcacctgcatcaaggaaacacaaggccagatgca
gtggccttgtctggagtcagatggtggctccgtgacctggcttcactccagaactacctg
gggtttctagttgtgcaaatcccaggtcccacccagattatgagtccagttttctacagg
tggagcccaggaaggtgtatttttaacacgatggtactgctcattcacccagcccagcca
gtggtctctgtgtacccaggaactgctgacacagagctggtgctccccataaaacagaag
acagagccttcacggacgggggacgtgtggcttggtcatgacagggtatttcgtaccagc
acagactgtgttcaggtgacattaaggacaagttcctgcaggctagctgcctggacaggg
ctgggtgggggtagaagaggggtcacagaggggcttcctcccggccgcctcaccagctgc
ttgatttagggcttggctctgggtcatcctgggcctgattctgaaccacgggactggtgt
ggcctgcaggctcctgccacaagctgttcatggtgcaggggggagaacagtgtccacagtt
ccccagacagcagcagtggataccaggcccccaggagttgttactgaagttgctgctgga
caactcgcccttcactgagctccacatagcacccgtggtgatgggagccgggtggagaga
gtctgccagcctgtgcatcagctcccaactgggaggggcagagggaggagggggtgggag
ccccaggcagcagggctctgggagcagtggggccctgggttccaggggtgtctggcaggc
ccctccttactctacctcttttggcctctgggtggagatgctggccacagtcaggctctg
cctctgactaaggactggagaagtggcgggtgtgggctgctgccccgtgcagcctctgaa
cagacgccagggcctctgccaatcatgactccttgctttcagctggacccacaggccctg
caggacagagactgacagtatgctgtcatcaccatgaatggggtatgtgtccgtgggact
tttctggtgcccacatcccccagaagggtagggtggcctctattcatttcaaatcggtca
gaggtggctgagcctgagccagcgtctgacacggagcctggttggaggagggaggttccc
cgaagagcagaatcgccgtgcccgggaatcgtcacactgactgggatgcagttgccagcc
aggccctgagcatccctcctcaaacaaaggtctcatggcaccaccaggacaggtggggcc
tccactcggtgacctggggactgcatgtagaaatggagacctctgatttgtctttaggta
ccccagaaaggtttagaccttaaaagcaatgacacacccaaaaaggcctgggcatatatg
gtaaaatgttaatatttgatgattcttggctttttcttatactattctgtctttcctact
taatttttaattgttattaagaaagagagagtgggcacagtacacctatggtctgagcta
ctctagtggctgagccaggagaatcactggagcccaagagttcgagtacagcctgggcaa
cattgcaagatcccatatttaaaaaaagtaagcaaccaagagaagcagcggggatttta
ggaggtggttctgcagaagccagtcctttacaccatcttcaacaatcctggctcttgctg
aggtagactaggggattccctgaggggcagccctaccccatgctgagacctctgcatgcc
ttcagagtggaaatatttgatgagactccaaggggcccttgagaccttgggctatgaggg
ccagaaagattagtggactatgccctttctcccctctatagattgaagtaaagctcttgg
tcaagttcaacagcagagagttcaacttgaagaggatgccatcatgaaaacaggggtctt
cggagctgagattgctgtggtcaccaagtgagtggggaggggcttgggctcatgcactga
gggtgcatgtcccttcagctgtttctgcagagaagagcatgtgtgggtctctcctctctc
tgtgtggccgctgcgtggtgaggtcaggccccagggaacacttggtgtgttcagctacct
cctgtgtttactcaacaccagctcaggaatgtccttgccaccttgcttggaagcagtagg
gctggctccaggaactgcccgagtgcagttttctgccctcgcttggaatttgtcatggtc
ccaggttcctgttgaatggccataacccctgcccctttgtcacaagtcagttgccaagag
aagcctgttgggtttgagagcagttcttgcagacacagaccacttcctctgagaattcat
ttgcttccccaggttggaatccggctgggcccctgaacttgctggtcatgtgggccgggg
cctccatcaatcataccccggactcctttctgtgtctaaacagcacactcacccccaact
gcacggcagccacttgcgtagcactctgggagggctctgggcatgagcagcgaggactcc
agcagcagcccccccaaataaccactgctaatgagggtggcttgagaagcggctttgatg
tgctcctaaacccagttgcaaaacaaagctaaagttaaggcctcagcacagcgctctgtt
ctaactttgaagtattcttactctagtgtcctgtgtggcggtattggaattgttcagtgc
taggactcagaggagtaaagcacttagcagcgcaggacttagagcgctggtgctgaggca
acccttcattcattcattggatgtgtgttaaggcccaggcccagggcaggggtcagggat
tctcttctcacacagcacgcgggtggcaggaccaacaccgggtctgatctcccagctggg
gacacaggctgctaaccccaggcctggaatctgtcagatgccctttctgtgctgacttta
cttagacaggcctcctgaccttcccacaaagatcatgtgtgacttgcaggggttctggct
gcttgaaaggtcctgagacagtacatgcaatgaggactgagcttgcagagggagcacagg
catgcagaaggttctctgtgcagccccacacctagtcaccttaaccatcatcctcactcc
acctccatagagcaggtctcctgtgtgtggcccacgcagggctgccaggacactgagaac
atttcctcctcccgcaggagacagaggtccgaggtgccctacatcgtacgccagtgcgtg
gaggagctcaagcgccgaggcatggaggaggtgggcatctaccgcgtgtccggagtggcc
acggacatccaggcactgaaggcagccttcgacgtcagtcagtggtggcctggggaggac
aggatggaggtgtgggaggcggtgtccgcgatgagatctcagagtgctccatggtccggg
catgtcacattcttctctgtgtcttttcttcatttacagtgttactatttttaaaaaaga
gaagacaagaattatagaaatagcttctgtagaagccagtttttaaaccatcctagccat
gcatgccacttgctggggtgaaccaggggcttctgtggggccttggccttcctgccttgg
gggtggacaggaggtggaagcccaggactcagtgcagtctgtccactgccctgtgtgacg
atgcggtgggcagaggacactgatgggacccagctcaggctggggctgcagcatctctgc
ctccatttcaccaactctctcaggctatgaaagacatggacctgcctcaagtgccagagg
aggacacagaggccccagagggtcctttccagcatcttcaaagcaacaggattttgtgcc
tgcagacccttctttggggcacacaccactgaccctgaccaggacccctagaatgcctat
cacccctgggtgggccctgtggtaatttccctctgggggcccagaatagacctggcctgc
ggtgaggacgcaagcaccagtggcccattgggtccaaaggaagacattgattcaaacact
gaaaccaatcagattctcccacagccttcctgccatcagaagacactggtgcagggttgg
ttgctatgtacagggcagagtcacccgattcccacgcaggcactgtgtcctgctgtgctg
gcctcctcctggccatcacatcgggccaagcaggggagaggaatgggaatgcccatgcac
ccccatcaactctgcagacacagaaccacacacagctcttgggaggggtcagatgagctg
cttaaagccggggagggacccgcacagtggtcaacatggcagggacggtgctttagccaa
gcctgggatggtgggagactcacttgggatcctgaaggaggccgctgcatttccatgctc
tttccagataacaaggacgtgtcggtgatgatgagcgagatggacgtgaacgccatcgca
gggacgctgaagctgtacttccgtgagctgcccgagcccctcttcaccgacgagttctac
cccaacttcgccgagggcatcggtgagcactggaggccttggcctcatgggagacgtctc
ctccacatgcactgctgcccttggaggctgtgaaaagtgatgtgtgggaacctgagctgt
gccccctctgccatggtcggtgttttaacccaacctcagaaaacaggaccaaaatcaagc
ctgtcctggaagacctcgcccatccccagagggctccccgtccctattcctcaaggagac
caagagggtgaaatggtcagcactgctgtgctgtggggtcctaaagtctgctgtcctcct
tcctgcagaccaggactgaaggagcgcccaggtgctctagccacgggttctggcccagtc
aagcatgggttcaaacctggcctgacccttagtcaacctgcaggctgatggctagagtgg
gtgctggttcatgtggcacctgtagtctccacatcacccttagggcaggtctgcctccca
ggcccatgcacagaggacctgcaggtggccctgtggtgtccaggacaatgagggagtctc
tgcatacttggtggggctggagccctcccacttcccacctctttgtgtccctcactcccg
tttcattccacgccaaacctcccctaccttgggctcccctggggaggggggtggtggcagg
agatgcccaagtgcagctctgtccatgagtgactgctctcgcagctcctcctgctgctgt
tcgccgggtgctgctgacccctgtgaggtggagaaaaggcgttcaggtggctcatacccc
acaccagcgccccttgcagagtcctcactggggggccagagctgtgggactgaggatgatg
acaaccctgggctatgcagggacacgagccccaggcactccacgtaaccacctcaggaga
ggtttctcaggagagcaaaaattacacggggcaggctgggcatggtggctcacgcctata
atcccagcactttgggaggcccaggcgggtggattacctgaggtcaggagttcaagacca
gcctggccaacatggtgaaaccctgtctctactaaaaatacaaaaattagctggacctgg
tggcacatgcctgtaatcccagctactcaggaggctgaggcaggggaattgcttgagccc
ggaggcagaggttgcagtgagctgagatcatgtctctgcactccagcctggctgagagag
caagactctgtcttaaaaaaaaaaaaaaattacacggggcagaagaaagaaggcatggagg
cacgggtgatgggcggagggcccggctgcctgcggcccatcgtgcttgcctgcgcagcct
gggttgggggtgcggagagtgggcgaccatggggtggtgtggcctggccccagctccagc
atcattgtctccacagggacgtgcaggcgctctggaccaatgaccacgcgctggcctggc
acctgagcaatgacttccgagaggaccctgtggcctgggcacgcacttagtgccaggcct
gggaggagctggaggatcagctgcccagtttcctggaggagctgccggactgcccctgca
ccctgacccaggcccgggcggactccggccgcttcttcgtgagcctccaatcagggccca
ggagaggggatgagggtgtcgcctccccactgaggacagcaccaggggaggcagatagag
gtgtcctggagggtggggcgggggtctcagggcacctgcagagttggcctcgggaagggg
atgacagaaccggaggccactgggtgacagccacctgctgctctgcagacggactacggc
tgtgacatggagcagggcagtgtgtgcacctaccaccctggggccgtgcactgtgtgcgc
tctgtgcaggccaggtgagcccccaggctggggccggggtggggactggggacaggggtg
ggctcccaacagtggcctggccgtgacccactggctcccgcagccctcggtacagctcgg
gtcagcagtgctgctacacagcggacgggacgcagctcctgatggctgactccagcagcg
gcagcactcccgaccgcggccatgactggggcgcacccccgttccgcacgccaccccgag
tgcccggcatgtcccactggctctacgatgtcctcagcttctattattgctgcctctggg
cacccgactgcgcccgctacatgcaacggcggccctccaatgactgccgcaactaccagc
ccccgcgactaggtgggtgccatcctgtgccccggaccctgggaaagatcggctgggct
ggggtgcaccccacctgaccctccactctcaccccagcctccgccttcggagacccacac
tttgtgacctttgacggcaccaacttcacattcaatgggcgcggagagtacgtgctgcta
gaggcagtgctgactgatctgagggtgcaggcgcgggcccagccagggaggatgtccaat
ggtgaggccagggctaggggctgctctgggtggcacagggtagatccaaggtgggaggct
ggagccaagtggcgcccgttccgctcccaccaccacaggcacacagacccgtggcacagg
gctgactgcaatggccgtccaggagggcaactcagacgtggtggaggtcaggctggccaa
cgggaccagaggtctggaggtgctgctgaaccaggaggtgctgagcttcgccgagcagag
ctggatggacctgaagggtgagtagtccagccacgtgaggcttcggctgccctcacctc

ctccccattcctgcggggagactgaggggaagccctgggccttcacgcctctcccagccc
tggctagaggcctgggcggtccgacctcaggccttcacacccaccaaggtgcccacatca
tacccacctggtcaaaagccaagaggccaggatgggggacatgtcctccctacagagca
tcccgggagccatctggagggaactcacccggtaacgttcaccgctggcctgcacagagc
cactcttgtgctcctgtcactccctcagtcctcaaaagccactgcaaggtcgccagccct
gcacggttaaggatgtccctggcaagcggtagcgccagcatccgaacccttgcttcagg
gaactgagcgaatgaaaagattcctggttgggatgggtggggacctgggaggggcctgtc
agcactgagggtacaaggaccctcgggacgtgcccaggcaggtgtggctgctgcagccaa
ggccagagggacccacacgtgcatctcagcatgggggttcaccgcaggggctgcacctc
aggcgccgctgggggttccaccacaagcaaggcaggggtggtggagggtcatggtcaaca
gaattggccctggggacgccctcccttcccctgcaggtgccctcacccacagcccctggg
tgcaaccgccgccaccaccgcaaggcctgcaaagatgatgcccaaaaacaccgtgtagct
gcgaactggtggggaagcgagtgtcagcgaggggccctctggggtggtgagtggggacag
gtgcaggcaggtatatgagcagggtgtcctcacctggctggcactccggggtgggtgagg
accaggtgccatcagcctggcaggtgctggtctctgccctggccaggctgtagccgttgt
cacagtggaagtagatggtggaacctgccaggtacctgttgccctccttttgtccgttgg
gaggtggggccagccagccacaggacactactgggatggcggcagtgtgtgctgctgcga
cacaggaagggcaacatgtgagacccccagcccctgtcccgagaccccagggcagccgg
tgggaccgccgagcctacttctaactgcccagcctggaaacaggaacctaaaaagtattg
atgacagagataggagagggctggggccctgctcattttgtctggagcttaactcacttg
aggtaaaagtttgtggctggcagagaggttggtaaatgagggagaaattgcaaaaaggag
agagtcactgattgtaggaatcccaaattctaaatacaactgaggacaaagccctggctg
cccctgcactctgtgtgtgggggaagatgtacggagcccagcttccaactcacttggtca
aatgaggaaactctggggaaaacgcaaagtctctacagtcagcactgcttggggaggaga
gtggctgtgggtgctcaaggaggtggaacttcattccccaggcctgggtcccagaggagg
acggcctgtgtgctgtgctggacggtgatgttcggcttctccccaggtgggagccagcag
ctgctgaggaagggtgatgtggaacaaaggagagagcctgggtctgaggtgtgtggtggt
tttaatggatctgggtttctaatgagacacccccggcgctgatgtgacaaaatcacacca
ttgtgtctagtgttgtatcttccggggcaggagatggtggtttctgtcctgggggctgag
cccctgcaggaggctggaccagcacgggtagatgcagagacgcgtgagcacagggcaggg
tccaccctggggtgggcagagctgggagggtaggcctgcaagggcctcaggggcctgtgc
ttgggtcatgtgcgaggacagtgtggaagaggggacccaggcaggatggggacacccata
accctcctcctgaggacccaaagagaaggcagagctgcctcaaatctttccatatgacag
ttcattttgtgtgtcaaattaggtaggccacagaacccagtcatacactcaaacagaaat
ctagatgtgtccatgacggtatttttaaagatgggattcacatttaaagcagcagacttt
gattagccttcatctgtttttttgcgtttttgttgttgttgttgtttttttgtttttgcttt
gagacagggtcttgctctgttactcaggctggagtgtgggagtgcaaccttggctcactg
cagcctcaacctcctaggctcaaacaatccacccacctccgcctcctgagtagctgggac
cacaggtgtacagccccacatgtggctaattattttattttatttttttgtagagactggg
ttttgccatgttgcccaagctggtctccaattcctgagctcaagtgatccacccacctca
gcctcccaaattgctgggattacaggtgtgagccaccgcacctggccaagttcatcattt
acaatgtgagtcatgtcctttacccaatcagttgacagccttgatagaaataagatgacg
actgagctgttacctctgagatgacaataagaaacacatcacccaggtggagatgacaga
gactttgcttgggtaagagcctatcagatccatgtcaccccaagtccatgttgtaggtga
cagcacagttttagctcagcaacgctcagccccgggccggctgcacccacactctgccca
tcagccccagagccgtgtgtcttgcacgggtctgtgcctcttccctgactgcagctgtgc
ttgctccttggctcaccctttctggttcctctaactttccaccagcatccttcacctccc
cttcctcagatacaagcaggccccatggacccagaacaggattctttcagtacttaagtt
ttacttccatttttttatacttttcaaactaagcttggcttgacccaaatcaacatcttt
cgacctccaccaaagtctccaacgagatatttttatcttaaaccacaaatttctccttga
aaaaacataattagggtgaggcaatatgaacaatggatagagctaaaggttaaactaaat
ataagcatatggccccgtcagatctagttgtttgaggaaaatcaggtgttgtttgaagaa
ctgacaacagaaggcatgcctgagctagacttgggaaaagtcaccagccaagatgactgt
gcaatcccggagaacagaagggcggttttgcttacttctctgtaggtcttgggcactaag
tcttagagttgctatgccgtacattacagtaatagcctcatcctcaggctgcagcccaga
ggtgtgcagaatccctgcattggctgaggcatctttacttccagagaagcagctggggac
tccagagccctggtgtttatttgagtctgaatagtattctcaggagaaactggggaggga
tccctggcttttgctaggaccagagtattgagaagtcatcaacacggaagcacagaagcc
actgctcagggtgcaggtgagtctggcagatgctcccagggatgcagagagggagggcgg
ctgagtcagcatgggctgggagagggaacctgcagacagatagtcctaggtgctaggaca
gtgaccagggttaagctgtttgggggtataagccagaggaggatgacacaggctcaggac
ctgaccctggacatcaaaagactgttcctatctgtgcagtgagagaggaacatgaggagg
aggggatccaggccctggtggacacagccctcctgaaacctgagtgccgtgctgggctgc
acaggcctttttttatcttctccacttgcctggtggggaggggctgttcatgtcaatttgt
ttgatccactggtgatctctgcctcaccctgagaaccagctgaggtctgagcttggttct
ttagacagaggttctgaggggtgagactcagagtttgccccattggagggaccaaggagg
aagcagcttctgggaccttccacagatctgtgagcacgagactctgcccatcttggaagg
acacagctgctgactcctcagcagcaaacacaggctttgctgccctagccctggggagat
aggggggcggcacagctgcgggtgctcctgtgcagaccctgagacagacccacagtgccc
tctgctgctcgctggccaccctgcagtctctcagcagaaactgtgagtggagtcacctga
gagccaggccactgtcaggccacagaatctgtgagtcaagactctggctctgccccgtgt
tactgacaggaataagaatcctttactcatgaggtttttctggatttccaagagaagccc
acgagggaaatctgatgaccatccaatgtgaggaggctgcagggagcggggaatgcagag
ggctctggaagcgaagacctgaacagctccctccacaggggccagggaaggaggcaactg
atagttggatattgtaaatgggaaaatgtctcacatgtctggcctccatcctggatgatt
ttactcttaggccacatacgttgctgcctctcctgccacaggatgcctctcctgagaccc
aataccatggatgggtggctcagcacagcctgggccctggaccctgagagatgagtgtgg
tgcaaaggaacatgctttagtccactccggaaacactagcaaaagaccaggacaaagcag
cagtgagacaggcgtgaggacgtcaaagtgaaaccatgaaagaccactgtggtgaagcag
tggacagagaacaggagtggtggaggcgtccccaggggtaacaccaggttttgtccacgg
aggtacaaatttacttgttcaggtcatctcagtgccctggatcttcttgaagtgaaaaat
cactgaagtctttgtgctttctgacattctgggccctctcttccttcatcttcctttgct
gccccagcttgaagagggtctgcattggcacattcagtgaggggctctgaggcggtggtg
tcctccctgcgtggacattttccaccagaggctgagaggtagatcaagtgctgagttcct
cttctcaaatgcagctcactccccgctctctgaatgagaacctgttatctatgtgtgtt
tctcttatttccacccagggacgcttttgttaaaactgtgtatatctcctctgtttaagg
ggcagcggtggccgcaggtgctcctcatgcttgaaggaatgggaggggagtgtccacagt
ggctcggctccttggctgggcgcaatcactggcagggtcagctccaagtcctcattagac
cagcgcccaggctccttagcccaggaatgtccagaatctgggcacagagcccaggcctca
ggccaccatggcccacctgagcagctcctcctctgcacttcctggggcccggcatggagg
cacagcccccaaggctgctggaggatgaccagggcctatcaacatttgtggagagggtcc
tgaatgggggctcaggcagggtagcattggtcagatttgccccttaggtctcagagaaag
tgctggagagggtgaggctggaggttgggggctgggtggagaacggggttgggaggagag
gaggggggagttgagcccccctcagtgtggcctgcagcgctgggagtggccacatggggg
cagcagagtgtaggaacagcgagtctgggtgccgggaggttgggagggcagtcagccccg
ccctgcagctggatcaacagggttggtcacaactgcagccaaggtccgccacagcccacc
cctctgccgcccctcacctgggccatccccccatctcaggagtgttgaagaggttgcaca
ggttgccccaggtcattcagcaggcactgaccatagggtgggagtccaggcctcccacct
acttggccttgcccttggcgaatgggagagctggggaggcagaggcaggtctcatctgag
gccttcctggaggaggggaatgtgcatgatggggcctgtgtgcgggaacagagctgagag
ggttgcagggcagggtggaaggaggctagcagatgggaatggggtctggttcctgtcttt
ctagacctcactctgttgcttccccattccctgggttttcacctccaggcacggtggggc
tgagggccagccgcttggccagctgggcaggcactttcctccctacagatgaggtaaggc
agggctcccctctcactgtctgaatccccagcgcaccctgtgccatgccaggcctgggcc
accacgtgtcagtgggtgagaggtcagtttcctggtcaaggcctggacatgagggagcgg
gcacaggtttacagggggcccaagaggggcaggggcagcttggcagctcatcaggtccac
tagagggtgtcccagacaagaacagcccccgaactgaagtcaccaaacaggttggattct
cagagctctgattggttggacaaggtgttctgggtagaggaacagtctatgcaaaggctc
agaggaagtgcagtcgtatctgcggaatggcaggggtgggggccagccaaggccacccct
accaggccaaggcagttagacttcatcctaagagcaaagtgtggcctgcctcgggctccc
ccacccccaccaatctccctccaggatgggcccttggtctgcccccgcacctccccctttc
cagcatgagaaagggtgccctgtctgtggccctagtcccagacatcagatgttcccagtg
acaggggcgggccagcctgggaagccccaccacctgccaggggcttttcctggggcctga
aagagacagaggcagggcctgtgcaaaggcggaaggcctggagccggtgcagactctcta
gagacatcaaaggcggtgacctccgcctcaggggggatggtctcccaaacagcagcccagg
gcttgcaggacgggcagccatgggagctttgcgggtggttcacggtggggttgaaatgac
agagtatcagagtcacccagtggcctgtaccgtcgtggtggtaccaatcagagtggggca
gatggtaacccagggctttggaaacacaggtgatgagagagctggggggtgtaacaggag
ctgatgaggtccctgcaggatcccaagacccaccttgggcagaagggcaaagggaggaag
gagtgtgctatgggagccaggtccaggtccccatggaagtgggtgtcacacaggcctgtg
ggccgaggggagtcacagaggtgacagcccagctgcaggtccccgttgactagagcaggg
aagccccacttctctgtgccccctgccctccagcctcccactaggtctcttgttggctga
gcacagtgggtgcggctgacctgggggccaggatctgactcagtacagtacagtacagcc
acagctggggagcgagggacccccctcctctgccacgctgcttccttctgcaagctccagg
gtggccgagtgcctgctgtgggccagagctccactaggcccatcaggaccccccctgtcct
cagggtccagtgctgccaaggaggagggaggggtcggtgcagcgcattgtaggagtggga

agtcagggaggcgtcctggaggagtccggcttcccacaggcccctccaggctttcgcca
gctccctttgttgcaaggatgtaaggatgactgactttggaagccatgccaaccaagtcc
ctgctgacctgatctcccaccactactgttccaggaccaggaaataaacttcttttctga
ttaagccttaatatgtccaattaagccttaatatggaccatttgttcaagcagttggttg
gtcctgagaaaggaaagaaacttctctttggtgagacagggtctcactctgccacccagg
ctggagtgcagtggtgcgatcatagctcatggccgcctcaacctcccaggctcaggtgat
cctcctgcctcagcctcacaagtagctggggccacaggcacgcccaggctggtcttgaac
tcctgggatcaagcaatcctcctgcctcggcctcccaaaatgctgggattacaggcacga
tccactgcacccggccaaaaataaactttttacctgaggaatgagtgcccccttaatta
tcaggcccagagaggcattaaaatgtgacagggtgtcacagcagtcgtgcctcactccca
ccttgatgccacttgctaggtgggttctagactaaccgatgtcaagtagccataaaatgc
catgtgctggacaccatgatgtaggggccacaggaaaacttccccttcaccctctgaagg
ttcaaggaaaaatcagctcacaaaaggcagattaattggaaaacaagccatgcaaattta
ttagcatgcattggggagaatcatagagtcattgccgaatatcccagataaagatgctta
tataccccgcttcttaggggaaagggagatgggaatctctcctttgatactcagctgcag
ccagactcctgcaggaagaccccccaacctcccactcctacaacgcactacactggccact
ccctcctcctcggcagcactggaccctgaggggaggggtctcctgatggcccacagcggg
cacttgatcaccccgggctggcaggagatgggagtgaggatgatttcagaaggacaagta
catgatttgggctggggaggatttttgtttgtttgttgtaagagggagtctacctctgtc
accagcctggaccacccagctacttgggagattgagccactgcccctggcctgggtgggg
agaatttaattggtttaaagaacgtacagggccgggcgcggtggctcatgcctgtaatcc
cagcactttgcgaagctgaggcgggcggatcaggagatcgagatcattttggctaacatg
gtgaaaccccgtctctactaaaactacaaaaaattagccaggtgtggtgggggcatctg
tagtcacagctactcaggaggctgaggcaggagactcgcatgaactcaggaggtggagct
tgcagtgagccgagattgcaccactgcacaccagcctgggcgacagagcgagaccccgtc
tcaaaaaacaaaaacaaaacaaacaaaaaaaaacatacaatggggctgggcacagtggttc
atgcttgtaatcccagcactttgggaggccgaggcaggggggattgcgaggtcaggagttt
cagaccagcctggccaacattgtgaaatcccgcctctactaaaaatataaaaaattagcc
gggtgtggtggtgtgcacctgtaatcccagctacttgggaggctgaggcaggagaatcac
ctgaatctgggaggcacaggttgcagtgagccaagatcatgccactgcacgccagcctgg
gcaacagtgtgagactcggtctcaaaaaaaaaaaaaagaacatacaaacatataatgggcc
tggctcggtggctcatgcctgtaatcccagcactccagagggtgaagcaggcagattatg
agatcaatagatcaagaccatcctggccaacatggtgaaaccccatctttatgaaaaata
caaaaattagccaggcgtggtggtgcacacctgtaatcccagctacttggaaggctgagg
caggagaatggcttgcacctgggaggcggagattgcagtgagccaagatcgcaccactgc
acgccagcctggtgacagagcaagactccatctcaaaaacaaacaaacaaaaacaaacaa
aaccatacagcgccctgggacaaagtctgttgggcctgaagagcaaacaatagattgtga
cgagtctgtccaggtgtgttgacagttttcagtctttcttcctgtgacaggagttcagtt
aaggaagactcacaggaggggccagagctcatggttttcttctttggcagatccagactt
caggcagataaggaacttcagagaacaacttcatcctgagctctggctgacagactgaga
ccgaggcggggagaggtcagagaccttgtggcttcttcttcagttcagtgccatattttg
gggtatcagtttctgagccccagcaataactcatactctatagttcaacagtaaccagcc
aggcaaggagcagtggctcacatctgtaatcctagtgctctgggagatggaggcaagagg
atcgcttgaggccaggagttcaagaccagtatgggcaacatagtggagcctgcctacaaa
cacacacacacacacatttagccatgccagcatgcacctgtaatcgcagctactcaggag
actgaggcaagaggatcatttgagccctggaggatgaggctgcaatgagccatgatgaca
ccactgccaacagagcgagatctcatctcaaaaaataaaataaaataaagccaatcacta
acgcgttattgaaactgcctttgcaaaaaagcataattgaggaaattatgacagtgaaag
aaatcagaggtgaccaactctccaagagtctgaacctcctcaaattgctgctggggataa
catcactattgtaaaacttgagatcagggcttgagatattttgcagaccctgcactccat
ggatcagctgacgccacccagactgctattctggctcaaccagttctgccatcacagcca
ggaacagaagacagcaagaaaaactcatttccacccgctgtgattccatcttcaacctga
ccaatcaacactccccatttcccaagcccctacctgccaaattatctttaaaaactttgg
tcaggcgcctagactcatgtcagtaatcccagtaatccctgcagtttgggagactgaggc
aggcggatcacttgaggtcaggagttcaagatcagcctggccaacacggtgaaacctcgt
ctctactaaaatcgcaataattagctggatgtggtggcacatgcctgtagtcccagctgc
tcaggaggctgaggcatgagaatcacttgaacccagcaggtggaggttgtagtgagcgtc
tcaaaaaaacaaaaagcaaaaaacaaaaaactctgatccccgaatccttggggagactga
tttgagcgataataaaactccgattgactctgcatgaattactctttgtccactgcgact
cccctgtcttgaaaaataggctctgtctaggcagctggcaaggtgaacccattgcagtta
cattacctctctaaaccactgagaattcctgctaaacaattttatatgaaaccactccct
atcctcttttactatttatttatttatttttttgagccagaatctcgctttgtcacccag
gctgcagtgcagtggcgcgatcttggctcattgcagcctccacctcctgggttcaagga
ttcttctgcctcagcctcccaagtagctgggaccaccagtggtgtgcgccaccactccca
gctaatttttgtttgtttgtttgttttgagacgtagttttgctcttgttgcccagctgga
ctgcaatggcacaatcttggctcaccaaaacctctgcctcccaggttcaagcgattctcc
tgcctcagcctcccaagtagctgggattgcaggcatgtgccaccacacccaactaatcta
attttgtatcgttagtagagatggggtttcactctgttggccaggctggtcttaaactcc
caacctcaggtgatctgcccaccttggcctcccaaagtgctgagattacaggtgtgtgcc
accatgcccagtctccttatcctcttttgtctttaaagaaactgcttttgggccgggcgc
ggtggctcaagcctgtaatcccagcactttgggaggccgaggcgggcggatcacaaggtc
acgagatcgagaccacagtgaaaccccgtctctactaaaaatacaaaaaattagccgggt
gcggtggcgggcgcctgtagtcccagctactcaggaggctgaggcaggagaatggtggga
acccgggaggcggagcttgcagtgagccgagatcgcgccactgcactccagcctgggcaa
cagcgtgagactccgtctcaaaaaaaaaaaaaaaaaaaaaaaaaaaaagaaactgcttttg
tggccaggcatggtggctcacgtctgtaatctcaccactttgggaggccaaggcaggtgg
atcatgaggtcaggagttcaagatcagcctggccgagatggtgaaacctcatctctacta
aaaatacaaaacgttagctgggcatggtggtgggcgcctgtaagctactcgtgaggctga
ggcagagaactgctcgaacccggaaggcagaagttgcagtgagccaagatcatgccactg
cactccagcctgggtgacagagtgaaactgtctcaaaaagcaacaacaacaacaacaata
aaaacaaaacaaaactgcttttgggccaggcatggtggttcatgcctgtaatcatagcac
tttgggaggctgagttgggaggatcacctgagttcagaaattggaggccagactgggcaa
catagtgagacaccatctctttaaaaacaaaaacaaacaaacaaacaaaaactgcttgta
acaaaggtcgaatgaagcactccccaaggcaacttggaagtgtgtctggggccactgttc
tcaaccttggcccaaataaactatactaattttgcctcagtttcttcctttaggtcaata
gtccctgtaacaatacaccaaaggcataggtataaaaagatggccttaccttttttcatta
gtgtggaaaataaatactgtgtgtgcaaggtgaaaaaaatctaaacagaacaattcaggg
aactagcccctggctaggggacattgaagctgtgctcagccttttgcttctttaaacact
atggtactgtgtaaataagttcaggttaacctggaggagaaattcacacgagtagatcag
ctggttaaatggcacaagcattttctgtctcaagttatactggtgtaatctcagctcact
gaaacctctgctgagttcaagccattctgcaaatattcacccattgtgagttgtcttttc
actgtttcagtgatgtcttttgttgaacagaatttcttaattttcatatagtccaattta
tcatttttttcttctatactagcttttgtgtcctgtttaagaaaaaattttggtatgcaa
caaaataaaaaaacagaatttaaaaaagagaagcatttacctactctaaggtcacaaaga
tactctctttctttctgtaaatactacttaatattttatttgattatttatttatttatt
tatgtatttttggagatggggtctcactacgttgcccaggctggtttcgaactccttgg
ctcaagtgatcactcggttgctgtatttttagtacagacaggatttcaccatgctgtcct
ggctgatctcgaactcttgacctcgtgatctgcccgcctggggctcctaaagtgctagga
ttacagatgtgagccaccgcgcctggcttgtttgtttttgagatggagtcttgctctgtc
acccaggctggagtgcagtggtgcgatctccactcaatgcaacatcttccttccaggttc
aagcaattctcctgcctcagtctcccaatcagctgagatcacagatgcctgcaaccacaa
ctggctaatttttttgtatttttagtagagacaaggtttcaccatgttggccaggatgct
cttaaactcctgacctcaagtaatccacctacctcagccttccaaagtgctgggattgca
ggcagtgagccacagtggctggcctatttattttgatacttaatttttttctttctttctt
ttcttttccttttttttttttttttttgagatggagtttcactcttgttgcccgacctgg
agtgcaatggtgccatctcggctcaccacaacttctgcctcgcaggttcaagcaattctc
ctgcctcagcctcctgagtagctgggattacaggcacacaccaccacatccagttaattt
tgtatttttagtagagatgggtttctccatgttgatcaggctggtcttgaactcccgac
ctcaggtgatccgcccgcctccacctctcaaactgttgggattacaggcatgagccacca
tgctcggccttgattcttattttctacgtaactgtttaagcgatgaattttcctctgagc
cctgcattagctgtaccctatagggtctgatctgtgctgattatagtgcaacttgaagat
gctgcccttttggtgttgatttcctctgtgtaacaggatgtttcaagagccagctgtaag
agtctctggctgggctgggaatgcacattggctggcactgctggtggtgcagcattgatg
tgagggcagggcctgggcaggttccccagggtgggatgagcagggagggagaggaatggg
cttcctccagggcctcaggaaccaaggaagtctccgaactacctccaggcagggtcagat
gtgcattttccaaagcttattgcattaagagtgaggagtagactgtggtggtgccagcag
cataaagactgaaaagaaataaggaggtggagccagggctgaaccacacagctggacggg
atgggcgggggcagtgggggggggattctacaagtagcacaggagggaggcgaggcgggc
accacagagctttcagctgcctcacggctgacttctatttggcttgttgagtccaaggtg
cctgtgcaccttgtccagatgctctggaggtggttggggaattgctcgtgtatgtgagtc
tgagactgcacacagctcggccaatgccaacatggagatccatcagttcacaagtggtga
acgagccttctctgggtgggtggcagtcccaggttggccaagagtccagggagtgcaccc
tcagtctgcaacagagttagaatccggcaccacggagccagggttgcacccctgctgcgg
ggaacaggagccccttttcctctccctgacatgcagggacaaagttatcatgctctgtgg
gtgccgctgggaggcactgataggaaagactggccaggccttcccaagcaagctccaggc
aacctcccagcaccccggcccagatgtaggtgtggtctgggagcacagagccaggttccc

accgtggggcacctgaggtttgtgtctagcagccttttcttcctttggttccccttttcct
gtttggcccacagggtttgggacaggtggctgctaccaacaggtctcaaggtggccccac
agacctgagccagccacttctccatagccctcccctgcccatgaagactgctcagagggg
ccccattgagagctggatctgggactttggttaaactgcaaggggagaagctctgtcacc
ctgcagggcgtgaagttcagctgaagccattgggaccatggcctgggagcccaactaggg
ggaggctaccacacagagcgcctgagttctggcagcggcccaagagagtcagtgatggcg
gcaggtctgcccgagggtgggccggcttggcccctcatctcaaagtgagccagcttttgc
tccctgaccttcctcctagtctgccagaccaccccttccagccacgcagtcaagcccaca
ttaggaattagccccatccagcccctctcccacatacgacctccaaatatgtccatgtcc
caatccctacaacccaagattagaccttatgtggcaagagtgactttgaagatatgattc
aattaaggattttgagatggggagattatcctggaccatccaggtgggcccactatgccc
ccaaaattcacatgttgaaagctaatcaccaatgtgatagtatcagcaggtggggccttt
gggatgtggttaggtcatgagggtagagccctcatgaatgggattagcgccctataaaag
agaccccagaagccgggcatagtgggctgcacctacagaaccagctactgaggaggctga
ggcaggaagatctcttgggtacagcagagagctctatgatcacgccactgcactccagcc
ttggtgacagagtgagaccctgtctctaaagtaattaaataaaagggaccccagagagct
agctagcttcttccactatgtcagttagaaggcgccatataggccgggcgcggtggctca
agcctgtaatcccagcactttgggaggccgagacaggcggatcacgaggtcaggagatcg
agaccatcctggctaacacggtgaaaccctgtctctactaaaaatacaagaaaattagcc
gggcgaggtggcaggcgcctgtagtcccagctactcgggaggctgaggcaggagaatggc
gtgaacccgggaggcggagcttgcagtgagccgagatcgcgctactgcactccagcctgg
ggcacagagcaagactccgtctcaaaaaaaaaaaaaaaaaaaaagaaggcgccatatatga
ggacgtgggccctcacaggacatcaaatctgccagcaccttgatcttggacttcccagcc
tccagcgctgtgagcaataaatttctgtaaaatgctataagccacccactttccagtatt
tttgttatggcagcccaggcagactaaaacaacctctgtggcacatttggggtgatgtag
gaacagccgatgttcccaggaaccagtgaggggcaggagtcctagatccctctagcgaga
tccccagagagcgccatgtacccgccctgcagctggcaaagctgtttatggaagttaggg
gcctgttggtttagagcggccattctcaaccagggggatgtggcactgtcttgagacagt
tttggttgtcatgagtgggtgtgtgtggtggggagtgctaccgcatcacacaggctgagg
cacgggatgctgctcaacatcctacagtgtggggacggccctcacaacaaagaatcactt
cattcaaaacatggatgagggccaggcgcagtgactcacacctgtaatcccagcatttta
ggaggccgaagcaggaggattgtttgaagccaggagtttgagaccagcctgggcaacatg
gtgaaaccccatctttacaaaaaaagcaaaaaaccagctgggcgtggcgatgcacacctg
tactcccagctacttggaagcctgaggtgggaggatggcttgagcccaggaagtcaaggc
tggagtgagctgtgattgtaacagtacactccaacctgggcaacagagcgagatgctgcc
tcaaaaaaaaaaaaaaaaaattgtacatatagtaaaaattatcaccaatgccctaatgccc
cagaagaaaatcactttacagactgccacaggaaaaacaacctcttttacaaataaagtc
acaaatataaattaaaaattacacaaggaaaaagtcaccatgagggagagtctgagaggc
cccagccaggaaactaaaactcacttctgaaaaatggcatcaaccacagaccagagacca
aggtgctgagattctcagcctgggagcagtgtagacaacaccctgctctctgtcttggag
gccacggaactggcctggaggccacagtggcctcacaaggtccccaactcctatggtccc
caaagctgaagtgtacctggcctaagaatctcagtgactgagttcctctcaggtggcttt
gagcagcatggacctggggagtgggctctgccctaggcatccagcttggacctcagcctc
acctggctgaagctgggctcgtactccacacggcccttactgttgacatgcaggatgggg
gctgcaatggccgctctcaggtcaaagccaagccacagcttgttcatgatggcctggggg
agagatgagggatgcagggagaaaggggtaccctctaccctacttcagccctcacatcc
ccactccagggagagtcagacagtatgctgcccagagaggagacactggagcccagagcc
cccccagactcacctgggccacggcagagatgatgagctccccaccagccccgccaatca
ctagcttcgacccctgggctttgctgatcaggatggagggcaccatggaggacggggacc
gctcgcctggaactgggggccagcaccctccgggagctccatccaccctgtctccattca
ctgctgagcaaacagcagcttagcgagtgcagtgacccatccaggggtcagcatgcagcc
ccagcccagcccccctctctcctctcccctggtgggggcccttgtggaccccgggtggga
agccttgttctcaccaggtgagggggtagtgccggaaccccgggggcatcgctcgcataa
gtccaggagctcgttgttgaggatgatgcctgtccatggtgaatacaccatcgctccaaa
gctgcagccgtggaggcagagcctgggctcccaccagacaccgcctgccctgcccacctt
gcaggacccccctcactttacagctgccccgaagcatgctggagccactgttctctccctg
gcatcacccaggaggggtccccaaagacccacctgtgccccccatatcagggcacccttct
gagtgaggccgcccattcccaggtaagtcttggctcggggccaccccagcagcagccttt
ccagccagagatgtcaggggtgggggcctcttttgaaacagaggaggagacaaaggccaa
aagcaaggtcctgccccatggggacaagtgaggggacatggggctgggggagtgcaggtc
taggagaggaggagtgaggccaccagccttcccccaggccccatgcacggtgtgttgatg
gtgctggtggcagccacggcgctgccatcctcccccagcacagacatgggacatgcctgt
cccatggccccaggcctcggccaagctgtagtgactgagctggtggtccccccggccatc
gaactgttggcggatgagctgggccagggtctcccccagcaggtcctgggaggcattctg
gggtgcgtgggcaagtggcagggtcagtgaggggcaaggcgggatgtgcagaaccaaggg
caggacaagctcagcccccatgggggccacctccagcctgtcctctaccttgtggccagg
cctccctcaagcccaggctaggtctagactctgcccaccctccaggtcctttgagatatg
gtgccactcctgcatctgaccccctgtacctgccaccccgggacccctttcagtcctgccc
tggcctagaaccacatacatctccctccaacctcaccctgctgggcctgacccttctttc
atgctaagtctcaagccagggaggaggacagcccccagcccagcaacctcagtaacctc
acctggagcttcgggtggctgcgagggtcccccagcctccacctctgccccctggcaaac
ttgagcttctccacaaggtggtggtacatgttcaccctcccctcaggcctggccacagac
tctgctgagaagctgaaccctggagagggttcagggcacaggttgcggtcaccctatgta
atggattgaatgttgcccccccgcagaaggattcacccacatcctaattcccaaaacct
gtggatactaccttatacagcacaacagtgaccttaagtggaaaagtatgttaaatatat
atatatataattttttttttttttttttgagacagagtctcactctgttgcccaggctg
gagtgcaatggccctgtctcagctcactgcaacctccgcctcccaggttccagtgattct
tgtgcctcagtctcctgaaactacaggtgttcgccaccatgcccaactaatttttttgta
tgtttagtagggacaaggttttgccatgttgttcaggctggtctcgaactcctggcctca
agtgatctgcctgccttggcctcccaaagtgctaggatcacaggtgtgaaccaccatgcc
cggccttttttgtgtgttttgttttgtttttttttttttgagagggagtcttgctctatc
acccaggctggagtgtgtggcacgatcttgacatgactaaaatagtccctatgaagccaa
tagggccattggtatcatgtagaaggatacaagacacgaaacactgtgtgccagcagact
ggtatcatgtctgtgtctttagcaggagtcctcacagctgtccacacttcagtccagggg
ctattctctggattctgctccccaagtgacacctcaggtgcaaaggaacaaggtcacatg
ggtgggcacagggctacccggcttagaagcctgtgtcccaaaaagaaactgagatttctt
ttaacagtcccacggtcaaagttttcaagagtcccagcaatggacatgcctagttacaaa
caccactgcactcagggaaacccaaagtatggtttacctcttgatttttctccaaagta
aaccaccaaaaagttgaccatagacttcaaaacatttctaacaatagcaaagttcagttg
atggcagacttcaaaaatcgcaattactcttctctaactctagatgcccccattgtttac
aattcattcatagctctcccaattcagatgatgatgaccaactatgggccatttttatat
gtagtctggtaacccaacacagatgacattccgccttttccaggtttccaagggaactga
ctcagaaagttaacccaagttcatatttatttctagagaaagataaaaaaaaatttctaa
ctttattcatgcatacctaatacaagggtacacgaaatttctgagagaccttcaacctca
ctgccaaattttcaagagctgtgacaaaccttaatgtcaaaccaagactgattttatagc
agaactgccatcacatctgtcaggtggatctgccatggactttttgtgtcgtcttcccc
atgtctctgagttgttgaaagtatcacagccccaaacacatttgtttttctttcgctttt
tttttttttttggagatggagtctcactctatctctcaggctggagagcagtggcacaat
ctcggctcactgcaacctccgcctcctgagttcaagcgattctcctgcctcagcctccag
agtagctgggactacaggcatgtgccaccaagcctggctaatttttagagatggggtttc
accatgttggccagactggtctcgaactcctgacctcaggtgatccacccgacttggcct
cccaaagtgctgggattacaggcatgagccactgcgcccggcaggcaaacacatttctag
ttcatgcatacatatgccaggtgggatgaacctgccctggggcccccgatggtgctcatg
ggtagagcagggtgatcccaggctccaagatggcaggcttggatgccggcagcaggtggt
ctggcctgttgttaggcccccctgctggtgcaagcacaaaccagaagtggcaggaagggca
ggtctatccccaggtccctggatggagtacttgggcacttgtggggtgggggttctttgg
gaggggttagcctgtcctcaggccctgtgggagcctacatggacacaggttgtggtgggt
gggctggggtgatcctcaggtccctgaatgtcatccttgggcaccaggggcagacggcct
gggtctgttgtcagtcctccctgtggtatgtgtctgtgcccatggcaacaggtgggcaga
gcaatttccaggctcccgggtggtttgcttgggtggcagtggtgctgactggttgtgagc
ggggtgagtctgcattcaatagccaactcagagaccccagatgccattttccatgacaac
tggcctgtctggacattgtgccatgtggctggccccacactaggccaggcctccatgagt
ataaggaaacatgggaggcaggaggtcggggccctgccctgcggagggagaagaatgggt
cataggcagtggtcattgagcagaagagagaatgagcagaggatgctgcgaggcagtgag
tataaggaatcatatagctgaaggtattcatctgacatataccggaaactaatacagagt
aagaaaatacaaaaaccaaataatccaacaaaacttgggcaaagaatctatagacattgc
tgaaaagaaggcaggaaatgaactaacaggtaaaggaaaagcttgtccacatcactaatc
atcaaaaaaatttaaagcaaagccacactaagagagtgtccccatccacataagatgtat
gttgccaaaaacaaaaacagaattttgaaaaggcaatcaacagtgtagatttgcagagag
ggaaactctttgacactactgatgggaatgtaaattgatgtacacgctatgagcaatagt
cggcagttcctcctcaaatgaaaaatacaactactatgtcatccaggaatctcagcactg
ggaaatacaaagtacatggatgtattatgctccctgattaaaaatgacatagaagagcta
tagtgatccaaacaatatggtattggcattaacacaaaaatatagaacagacacaacgag
ggagccaaataccaaacccaaattcatatatgatagatacattaaaaaaaaaatacaccca
gggaatgggagaccccaggctgccttttgccgggatgctgaagtgtcctggggtctacag
atgccgctcacctgggctctggatggtttctactccgtaaggatttccagagtttcccct

cagccaccctcagagtcagggggctcccatgggccagtgaccatctgtcccagtctttc
gtgtctccatgacacactgtgtgacctatgtatactgcaatatgtaggtgcgtgtattga
aagcgtacagttaagatgacctacaaccgatgctactcgaagtcttccagagatttttca
gatatccccagaccaaaacaaactagtgacattccatggttttgcaaaccagaaccaaca
actgcacagaaacagctccttatgcaccatcagacactctccagacctgccccacattca
caaacctaacgaacgagcacgaacccaggcagtcttcactcctaagggccatttaacaac
ctctatcagtcatgcagcctttgatctgggccaggctgccgacacacaggggtgaattgt
gcctctaatgctgagtgttgcttcccctcactgggctaacccccacccttgcaccaagaa
gggtccctagggaaactgctgttcgacatccaggagcccatgtggaatgttccagcaacc
tggatgacctcggccacctctagtaccagcgtttccctctgtggggctcccttcctggct
cttactcatgaaggctgatctaaagtggctgagatgtcctgctcccttagccacccaggt
caccctgatggcatcacactggccagaggcagatgtctgcattcaagaatgaggtattca
cagtctgacttccctggtgcaggggacaatgtctggcacctccagacagccagaaccagg
taaggaatccagctctgacacagctcaaagactctgtggcagacctggagagattctcca
ggcctgtgtcctctcctggctcactggattcttaggaggattactgagacactgggctta
aagctctcagcctagggcccagaatgccacagggaatcaaatcagggcgatcaaggacta
aaaacgtctccttggctgggaatggggtatcagggttgcagagacaaggggcacagagtg
atgcctgtaccatcagaggatctggtccaccctgggctcctcaacctgagtgtgaggcct
tgctgggacctgagctcaccttccccttgggagtccattgtgactacagtccagcgggat
caatgtcctcactgatgccagacagaagtcctggggggatgcggagggcatcctgtagcag
caggcagtctgggtggtccacaggtgtgtgcttcagcatgtcctgggaagggatgagctg
gtgaattgagggatgtgtttccagggatcagtgagaagcccctggggaatgctcagtcct
caccgagagaattcaaagggcaggccctcagggacccacactcatgcctgcaaaatctga
actgaaaattagaatcggagcagcagggttgactctaaaagggccccagctggcttttct
ccctgctgcagtctttactcttttctcctggcagaggcagccaggtggaggctcatcaaa
ccaagcagctgggagagacacccctctacccaccctgcagctgctccaaggatactggag
accgaaagacaggggttggggaagcaggacgttcctgcctccactgagagcaattggcca
aatgccaagctcagtagctaagctctattgaggccctggccctgttggcatggttaccac
aggccagggaggcatactcagggcccaggatccctctgcctgacttttcaaatggtctgt
tctcagtgactctggtgccctcccacaccgggctcagtcttctgagaaactcctaagccc
tctctggcccttcaggggccttaacactggtgcataggaccaggatgctctgagtgactt
acttggtcaatgggctggtacagcagagctgttggggacagcagagagaaaaaggacaat
gacatacaaaaaaccactcaacacctcacagaccagtttttcccccacccaccttgctc
cccccgtgaacacctgcttgcaggaagccaaagagggtccacagcagcctccacctctgt
tatcactccaatggcagaccaagcagcaagaacaggtgacacacagccctggtgaccgtg
tcccctctctacccatgcctattctcccatcagacttggcttcttcgagggctgccccag
tcctgtccaggccttacctggctcagagcagatgatcccccgcacctcccaccctgaatc
acacggctacacccagcacatgctacagaggcccagcacactctgggtcacatccagaaa
ctctacagtaggggaggggacacgctgtttcccaggaagggcagctccccaggcctggtc
attctcagactccttcagtcaaaggccggccacagccagctgggatcgccatgcttcacg
ctgacaaacatgactggccttgcctcccctatgggaagagacccctgcacacagcctcaa
acccagacataacaggacaggacacgctgggcgctagggaggccagccagatctccacac
aaaggctctgctcttaagcaggaggcagcctcaggggcaaaagtttcctgaagcaactca
ggtcaggcctcacatacaagagccccagaggctgcaaaagcaccagctcagccagccaag
aacccagggccacacccttctgaaacccagacaccaagtgaagatcgctgggattcagca
gaacaggaggacgtgacagctggcggaccaacacttcccaacacccttccagcaagcagc
tgcacccctcctgatgcgagtcaccccacttcggtcctgcacctggatgcctctgtccag
ggtagcatatttggcctcctgtcataggagcagtgagaggcaggatggtggtctgggaag
aaggatcctagactgggtgtcccataggaccaggttgtgacagatcccacagccaacact
catgccccagccaacccccaaacccaatagctccccaaagcccttgatgtaaatagcctg
tgcactgctaagcagacacctcagggaacaatctgatggctgtgaagaagatgcagcctc
aacagggttccacaagccttctgcaagcaaagccttctttgcttgggtgcctactcaaag
cactagggaagatcttaaaagataaattcagcattgtgggaggcagaggcaggagtacag
cttgaggttaggactttaagaccagcctgagtgcaatggtgagaccctgtctctacaaaa
cacaaaaattagcatggcatggtggtacatgcccatagtcccagccacttgggaggctga
gaagggaggattccttgagcccgggaggtcaaggttgcagtgcccatgatcatgccactg
tgttacaacctgggccacagcacaagatcctgttacaatcaaacaaacaaacaaacaacc
aaagatgcctgttttctgccccatcaaataaacccaaatctctgcagggtgacacagggt
atacacatttttaaatatctcctttcctgaagtaaatgtatagtaatggctgaaaaccac
tgagttatgtaaatacacttcctatgacttatttgtagccctttcgtgtctagcaatttc
tacagaccctctccgtgggctttggaagccggaatgggcagggccaggtgtgaggtatcc
tagcacactgaggctagagcacagctcccagctcaggggacccatttctgaggctaagg
cacccaccagggactcagcctatcttcagagggaaaatcacacctcagaaccccagagac
ccaaaagaaagttcgaatttggcacatacctgccagtgggtcctggagtggcagccccag
ggaaaaccgccattgcactgtcagctccctccagccttctctcaggtcccagggggtgga
ggtctctgactaagctcaaaggtctcaacaaacggcgggttgaacctctgaggagttatt
aacaggcctatagttacctcaagactccacctgaccagggccaaaaatgagtcctgggag
ggcagccaggaagacagcagggcagagaggaaacacaggaggagggggccctgggaccac
atcagatatggggaaaccaggggaacgagggcagccctgatgattgtgctccccataggc
cctgcatgtctgtgtcccctgagaattcagggtgaggagctcactcttgcccttcacctg
ttcctgggctctggcaggctggcctgcttcctgccccagcctcacccactctccccactc
ctcaccactgatatgggcaagttgatctctctggtttgctggcctctcactctctgccca
atggcaccgagttaacttctttgcatgctttattttctgtgattaaaaagggaagcaatt
ctcactaacgccatttctcagaggagaaaacaggcacagggatgttctatgacttcctca
cggtcaccaagctacaagtgggagctgggctttgaatggaggtctacccatgctcctaaa
tttgagccgtcctccaaagaaaggacagcattgtaaaagctcagtgaatgtggcaggaga
cttcactaaggcctgaccaggctggtaccctgatatccaacttccagcctccagaactgg
gagaaacataattctgtttctgatgagctccccaggctatggctctttgttgtaacatcc
tgaactagcccagaagtgcgttccatcatgtgtgtcctattccatcattcagaagggagt
cactaacccctcagtgctgtctttgtggatgctgcccaccctcggctgagatggggcaca
gggtctgggtctaaacaaaattggaaggcactggaaggtgcaatgtcagaggaggaggat
gtctgaatttcgtttaatctcaacctctactggtgcatcatgaaagaggaggcccagagc
aaaagtgtcttctcctgagaccacactgtggaccccaggcccactggagttctcttccat
gctacctctcaacgcttcttgtatttcttccatctgtaactgtgtgcaatatctacaggg
aacatcacatcatgagttttgtaagatctatctcaaaacacacctcatgagttccctagg
agggagatgctagcataacccccattgtgcgtgctgggaaggccccaaaacaccaaagga
gttctccagggtctcacaactgctaagataaaggttgtttgacccagaactgtctcctta
aaactgggaccctggttacatccaggtcttcccaaggtgctgaaggggcacactttggca
taatggtgaaaggtaaggagttgatggggaaggtgagcggccagaagcccaatgaggcc
tttgagtaggtcttatggaaaggaggagcttacaggatggaacctacaagatgtgacctc
acttccttgctgacagaccccaacagaacttagaattctggtaactaggcacccatatta
tacacacagccccatttccagctcagttggtagttgacacccaagggaacaacatgttct
cttgctgtgtcccgacgtgctgtcgacctcctcgtcacagaagaggccaaaatgagagtc
tttctcgagaatatagacattggttcaatcctcaccctcgacgcctctggccatttgcca
gaaggcacccacaggtaacacaggacccagggcaggcccatccaggccaggcctcagctg
tgtccatcagggcagcgctgagcccctccccatgtgtttggggaaacaggagaagaggg
gccctccctggacaggagcaggtaggttgttgggggttggagcctggtcggtatgtcag
gttcacagcccaggccatgccttcaggatgggaaggtgagtgtcgaggacatagtttgtc
tgctcagatctgaatcccaagacctcaagcttcatctgccagagacgtcaacatcctctg
gctggaggtctatataaatgctcctatgtgccaaatcctgttgcggaagggggcgccagg
aaggagttctaatggggtatcccactatggggtcagccaggcaggccactgacacctctt
cgcctggctctcgggcaatttctttgcagagctccacacagcagatcaagcaggagctgt
tggatggattccatttctccatcttcttcaaagaagggcaggggccgcctgctaccaacc
atggccagtgctggtctggggtgagattgggcacagaagggtctccacagacagggactc
cagaaaaccagggtggacccaggactcaaatacgaatatggggactcactaggctctgtc
ctagggctttcacacttgagtgagccgctgtcctctcccaaaggaatctggctgccatta
gtccccagtggcaacttggttacaggcaaagtccctttcacctatcagaggaagatcaga
gaaaatccttctgttttcgctttatactatgccaggagtatcccagcatgttgctataag
aattgggtacaatggtaacactgtagaaacatactcatactgttgtcattttaactcccc
accctaagtgtatctccggcaacaggcagctcctctgccctgccgacctcttccaggact
taaaatccaccacttctacacaaacgagttgcgggggtggtgattacaacaaaagaaggt
gaagctcctctatgcatctgtctttaaagtgtcatctcagagaacgcatccctgcagacc
cactgggcaggagctgcaccctcgcatgtcactgtaatattccttaacggaggccctgtt
ccacagtgggtcagcctctcagtgctggtggagcctgtcatgataaaattccctatctag
gcagcctttctataggcagcataatcctaagtgtgtcctaataggctggagcagtttccc
agggctgccataacatgccacgctgactgaagggcttaaccacagaaatctggttcctca
caattctggagactggaagcccaatgtcaagctgtcagtctggggtggtttctctgggcc
tctctccttgccgtggagatggctgacttctatctcggtcagcacagggtcttctcttca
acttctctgtgcgctgcccacctatccttgtaagaaccccaggcatattaacttaggggc
caccctaatgaactcacttcacctgaattacctcttggagaactctgtgtccaaacacag
tcacattctgagtctctggggttaagaatagaaacagttacatttggggaggggacaca
attcaacccataaaatacacaaatcccctgtggaagacacacccgggaaagggaaatctg
ggtgaaaggatgtgcagatagcaagtttcaacagctctagccagctgactttccaaaagg
gctccagctgctcacacggtcaccagcagcctctgaaggtagctgcttctttgaatgcaa
cctcccctgaatactaacatgctcttaactttttgccaatctgctgtctgtgtagcagag
aaaattaccttgtttcatttccagtttcaaacatacatttccgtaacctttcccggtttc

aatacctgcctgcgttttctcttccacaagtcacctggcagatcctaggcgttgggatgc
gcttcctcccaccctggagtcttccggcattttccccttggccagggctgggagggccat
agctgtttctccagactcacattcatgttaaagaacaaaaacaatcatatccttttaagt
caaaggcttcttctagagcatcttgtctggtgcgctgtcactaaaataaggaggggatga
gacctccccaggccgttcccttccatacagaagcggagcctcaggtggcctagagggaat
ttcatccagtcctccctgtggaaggtgctctcctctgacccgggtgctgctgagcaagca
tctgacatccaaaagcccgcatctcctcttctccaccatgagggtgattttgaggattg
cagggatgatgctcttcatgcaggctgttgatacagagggtgctattgccccacagctga
cattcaccttgaaggcatgaatcattttatccttctccatcctcacatgcaattttacct
tgaaagcctagaacccttttcaccatgggcacccagggtctccacatgcacaagtccctg
aagggttgatgcttcttggagtgatttccagagcccggtcctgtggtggttcatggtgtt
acactgactcccatgtccctccttgtccccctagtatgaaaatgagatctgcaggatgcc
aactttccagcccagcacaggggagaagctattggagtccctggttccagcctttctaac
taaacccatctcctcctatgccacctgcctgggtccctcctgggactttatcaccgtgcc
acacttttttggaactactggttagaaggtgagtgtccatcccctccaaggcacggaggtg
cctctgtcccctactggctgtgtcttgaagatgcacctcttggagcctcagttggctcct
gtggaacagggagtaatgagaagatgaagctcacagggctcttgtagggactgaatgatc
taagacacagcaaacaaaggggtccgtagagcctaaaactctggacaatctgctgtgggt
gctgtgattcatgtttattacttttctgttcccactcactgaagcagctctcagcattct
gccgcaatggcccatctctcttgctgtttggaaaggcacatagaaagcaagtctgcaatg
gcattgctaaaggatgtctgagtttccttctggctggactttctccttgacacagacaga
agaggggtcccttcatgctgaaaaaggagccacaggcccactcagacatctggagaggct
cactgggttctccaatggttggggttcactcattcaacacatacatacaaaacacctcat
ttgtgcatcgttcttcttgtgacttggcctaatttcataaacaaggcagatgacaatccc
tgggcctcaattctactcagagtcaggtgctcacagtagacagaataaacaaatcatatc
tacagaatgttagaggggaaaccctcatggcctcctctacatatggtggcatcctcccag
attctgactagaatgacggagcccaacaagtataaactgggggacttagggttctgaaa
ggcctcttcacccacaaaacatgggaggaaatatgtggactctggctggggagagaataa
aggagccctggggttcatgtcttataattcccacaacaaggctgacatttgggaacttcc
cctgcaagaggcaaatagtgagttctgtaaatgaagacttaggtccattgcaaaggagag
gtgagggtggggtcacaactggccactgagagacccatccccaccactgtggcttcccag
ctctccctgtcctcctccacccaacatctgcccctaccctcctaaccccaggaccagggg
aaccaaagctggagctttgatgagcaagctgctcacaaatctgcctggagctgcagtctt
gagtgcccaggtgcacagtgctgtgctccagggccattggaaagagaatgtcagtgggac
gccggggcacacaagggtctgtagagccctgctgcatggccaggtctgccccttccagga
cagcactgatggcttggggtagggtggggctgtcctctacacaggcagcaagaggccagg
gacccagaaccacgcaagggtgccctggagggttgtgtgggagaaggccaggcctctgac
tcagctgtccactccatcaccagcaccaccacctccattttgttcacctggcccccctgaa
aacacctcagtttgctgccaggccctgcaacggtcatctttctggataaagctggccttc
aggaccttcgcctggcctggactgggcgtggaggaccatcaggaaattgtcctaggccag
ttggtgcttccggagcccaaggaggccaagccagatggtgagaagactttccgggggtgg
tgttttggggctataattcccttaaattccatccaggcatttctatacttggaaatccca
gtgaagagtgactggaatggtgaccttttctccttttccagatcctgctccacctcctgg
gcaacacgcattaacaatgctggccctggagccagcaccaccactgctggcggacctgcg
gcctgctctagagccagagtcacctgtagccctggatgcaccaggatatctacattcagc
atcagcaccagcaccaggggaagggcccccctccaggaacagtgctggagccacagtcagc
cccagagtcaccctgtccctgtcccgggactgtccagagccaacacactgaggagctgcc
ggacatcacgaccttccctcccaggctgctggctgagcagctgacccttatggatgcggt
gagcagctgggctttgcaggctgtgcctctggcaccagctgtctcaggccagcctgtctc
tgaggagcggccaatgccctgggtccagtttcagccccacttcttaccaaccatgggatc
tggatgagtttcctcacccacaagccttccctgtctgcatgtggacagcagagatgggac
atcgcccgtgccagctgcacagagtgactgtgcagactgaatgacaggggatggacagaa
agcaggacagggcaggtgatcaccgagggggcaggcagggccatgggtcactcacccagct
gctcaggagcctcactaccctcagcacttattaggtacctgatgcatactagattctatg
gcagacacccaaacagagcccaaggttgcagctgctgcaaggaaactgcaccagtaggga
gagaaggagtagagggctgattgggatgggaggaagatgaggcctcaggatggggaggcc
tgagccaccttctagttcttggaatgaggatggcctgggagaaaatgtcactctctcttc
ccacccttgttggtttctgggacatgatgcattcagggccctcggtgggcagaaaaccaa
acccagggactcccacaagtctggagcatattttaaagctttctgagctcaagctcggtt
cctgccagagactgtggataactgtgagctcagtccctgcctgggactgtgggtgactct
gagctggggtgtgctgtgtccatgacactctcctccttccccaaaggatctgttcaagaa
ggtggagctctacgaatgcttgggctccatctggggccaacgacatcagaaggggagtga
gcacgtggcacccacagtttgtgccaccattgcacacttcaacaggctcgccaactgtgt
caccacctcctgcctcggggaccacagcatgagggtccaggatagggccagggtggtgga
gcactggatcaaggtggccagggtaagccatggttgggccttgggattccctctttaaaa
atggggaactgcctcttctcctccatcggctttcaggattggcatctgtatctctagcct
gagccctacacatcccctaggcccttcttctctgaacttccctgaccttgacccccatgg
cccagtggtggctgctcacgtctgacctgggatcttccttgggttgaactgaaatctttc
tagatgagtgacattcactcagccccaggtgtaccctcctgaggctccctgggcctctgc
ttcattcaagaaggggagatctcagcagagggggctgaggctgaagtgggtctgactccaa
ctctggacctcacagctcactcttccctctccaggagtgcctaagcctcaacaacttctc
ctcagtgcacgccatcgtctctgctctgcgcagcaacccaatacatcggctacacaagac
gtgggcagcagtgtccaggtgaggagctccctccacgggagcaccagtgttgacttaggg
acccataggtctccccatgtgccctcaacgattctgaaaggttcttggagaacagggatg
ctggaggcagggatgggctggtaggtgtggtcactaagctgccctggactcctaggcaag
gatttctaactcaggagtaaggttttttaaccatcaggaacagactggagccaactggag
gctttcaggtgtttgcacccagcagtggaactgtgtccagctggaagctaactgtgaaca
cgcaggggctcatgtgaagtggagatgggccaggggaggagcatgacagtcccaccctgg
tcctctggagcccttgtcatcagacgaccccactggaaactctcacgcaggaagctgaga
ttcactgggttttcaaacaaaagggattggaattcacaaatctcccccgtgattcccaaa
tttaccctttttttctttcttctatcaatagcaaaagctcaaaatatctaaaagaactctg
caaaaaagacactgcagtgaagagggacctgctgatcaaggtacagtggagtctgggaga
tgtgggacaagtgtttaagggtcagaggaaagagtgagtttggaagggcattgatcccgg
tgtgcagtggttattttgtaatgttttgacttacctactaaaagtggacttgaaaaattc
cctccatgcctactttgggcaaacaggaggagaggtgtgtgggtcgatgggcacgtgggg
gcacgggggcagcaggccctggaaataggatgtggcaatggctgctgggcttctgagtga
gggtgatgagctgcagcattagcaggactctggctcccatgctggtccatgctgctggca
tggagcttcctccaggctgggggggtggtcatggtaggtgggactttcttccttcctcaaa
ctggccgcaattccttaggaagccaggcctccgctgctgcttctgtctgcagcgcacctc
catgggcagggactgcagtccacactgggggaaagagggaaccacaacagaggaagctca
tatgccagggagtccagtagactgcccagctatgggtcccaatgggcaaactcaggacag
atgtatgtgtttgctgggactccccactctgccctttgcagacatctgaaaatggtcatg
tcacaggattctcaccaattagcagtgacacatgctcatgacaagtatctgggggatcca
tgcattcctaggggatcctccctgaccagatctcagaaacctccatgcaaatgagaaggc
aacatgtcaccccacccaggattctggaaaaccctgccatgtccgtgagagatgtcgcct
caggaggccacatcctgagtggaggaagagagttctgtgcacggaactcccctgggggat
cactggagaggccaaaccatggatttggcatggcgcaaacccagtttgtgtggcagagac
tccagtggggctcagataggcaggtgccacttaaccaggtctcctaaaatgccctgtccc
tttcccatcaagactctgcaaggctggagacctagacactcagagactccaaagaacaag
accctgatgggtggtggtgctgggatatggggtgagggcagccgagacagagcctccagg
atggggaggaggtgccttctcttttggggccctggggagtcactgcccactcttggtctc
tgtttccttatctggaaaatgaagggatgctgagcctgtagtgcaggcctcacagggtgg
aaatgaggttcaagaaaagaaagcaattggagggtgctcgtgaatggttcttcctcagag
ggatgagggggagaacaatgacaacagctacaggaaacagtactcaggaggtcctgtgag
gtagctgtggttttcatggctctttatagaaaggaaacagtctcagggaggcctggctgc
atgagtgggtgacacacacagggagtgtggagctggccagtggtatgagcactgtgccag
gtgactcacgccagtccctggagatccaagtgtggggtggctggggtgtaactgggggaa
gggaggagagcctcactgtccctgtccctgacacctggcaggcggggagctttaaggtgg
ccacccaggagaggaaccccccagagagcccagatgaggctacagaggcagaagaaggtga
gtgagactgtggcatggagggaccgcaggggatcagaggacagggctcttttccccacca
gctggaggcctccatatcaacacagcgggggcttcctccccagccctgccctcctatggc
cactgggcctggaaaacctccattggagagaccagagcaagggtctgggaaagcagaact
cagagtgtccctagggtaaggctgggaccacaggcccttgtgacttgttaaaatcccacc
atagaggtaactaggagtgattgccagacttggaggtcagctggaatagaggaggcaggg
aattgggaaaggcagctgagggtctttggctgctacaactgggagacctggggacggggg
ttctgggacacaggggctgatgggatttcatgggacagggccttgggcaagcacctgagg
gtcaatactcatcaccactacccctcccacctccccaccccctccatggcacagggcgtgg
tccccttcctgggggattttctgactgagttacacaggttggattcagccatcccggatg
atctggatgtgagtgaccctggggcaggctgcttgggaaccaggatcctgaggcttggga
ggacaggggcctggaaggagcccttagatcttagcccttggaaaacctcctctcctgaga
gcctcatagctgctcctgtgggtgggagtgtcaggcccatctcattacctctgactgaca
gaggctccatggcagtcagcagtccctatccctgagtggtgaagctgcagagctgcctga
ctgcagcgctgctgaggtgtgggctgagctggactcagcctctccctagggtagtcccat
aataggagggtgaaattgagcctttccaagggcaggcaattccaggataactgagcactt
tctttctaagggagacctcagtttctctgtctgtcatctcagagggttggggcagaaggt
ccctgagcctcagctcccatgcccactgccctagagcccggagcctgaggcaggttcacg

ttgtgtcatgtgcacatcccctgaccctggtggccctggcagtagtgcagcatgggaagg
gatggggtggggctgttgtgggccagggacctttttgatgggctctatctgacttccagg
gcaacaccaacaagaggagaaaggtgagcagctgggacattcacgttggatgaggttggg
gatgtggacgtcacagtccaccctggacaggacactccctggctccatcctctacatctt
agttttactgggtttgacacacacaggaggaggagacctatctcagtggagaaagtggga
aaggcactggaatgaaagaccaattcctgcaggaggggctatttacatccaactctgaga
acaggctgggggctgcattgggcccccctcagagaacggccccagggaccagccccttctc
cctgcctgccaaaggtcccctcagcgtcttccacccaggccctgtcagcatcctgtcctc
tgtctctaggaggtccgagttctgcaggaattgcagctactccaagtggctgccatgaat
tacaggcttcagcctctggagaaatttgtcacttgtttctcaagaatggagcagctcagt
gacaaggagaggtgagggcctgggagatgggcagagggtgggagaaagctctccattttt
ttttttttttttgagacggagtctcgctctgccgcccaggctggagtgcagtggccggat
ctcaggtcactgcatgctccaccacccgggttcacgccattctcctgcctcagcctcccg
agtagttgggactacaggcgcccgccaccgcgcccggctagtttttttgtattttttagta
gagacggggtttcaccgtgttagccaggatggtctcgatctcctgacctcgtgatccgcc
cgtctcggcctcccaaagtgctgggattacaggcttgagccaccgcgcccggcctccatt
ttttttaacgtggtctggctctgtcgcccaggctgcactgcagtgtcaccatctctgct
cactgcaacatctgcctcctgggctcaagcccttcctcagcctcccaagcagctgggatt
actgctatccaccaccaaatccggtcgttcccctgttgttgttctggtataggtggggtt
tcacgatgatgtccaggctggtctcaaactcctggcctcaagcaatccacccacctcagc
ctcccaaagtactgacagtacaggtgtcagccaccccacctggcctagaggaggctctcc
tgtggacagcagcagagagcctatggccatgactccactgccagcatcaaaccttgttgc
atggggactgctggggacccaggattccagctggtcaggcaatgagaggggacctgatgt
gtagcttatggtggcctcacagctgcttctctgtcctgcagccacaagctgtcctgtcag
ctggagcccgaatcccagtaggctggaaacatcctgcagtggctggggccgcactgggat
gctggccagaacaccagctctgcatcatccttcacccagaccgtgacaccaggaaaccac
atctaggtggcttgcagctcagctacatcttgaccccactcctcaacaccagctgctcct
cctagccaggatcaggccatgggacttttgcatgtcaggcgggaaaccattttatgttta
ttttctttagtgtataagtaacgggtttttttccttaactttcgttaaaataaaattttaa
aacactactcagaatgttctagtgttggaatgagaatagtaactccagtgcagtaaccgt
ataccagtctttaggattcatagtctagcatgtcacattgaggctatggctgaacaagtc
atggaatagcgtttgcattacgcctgccaactgtttaatgttttccttctttattcaaat
taattactgctaactctgtttaaggaaaacaaaaaataaaacaaacaaactaaaaaacac
ttaaaaaccacagtatgtcacccaatctatgtcacttgttctactgaccagttactctca
aacttaaattctagttaaattcaagttccaccgggttactctactttttttaagttgtaaa
tatttaatgaatcacttaaatatttactgaagggctggagatgggaggtagttatgcaag
tggtagggctggccttctccagaagtcctgtgcagaagggagctggccatgagtctccag
gaaacacagataaacttttcccactatggatcttcccagactttctggcacctctcccct
aagggaacatcaaggaaggagagaattgtttgaacccagtatctaaaggatgtcccacag
cttaatttgaacacaagccccactgtggtatatcagacaaaaacaagtcaaagaaatcaa
catttcagggtctgaaatataatacccccaaatcagcacaaaataaaacacttaaatcca
gtcctggagccacaaatctcctataagaaaagtgggagtttatttcagcaaaacttgtat
ctatgcatgcagtttgcaaaaaagaaagaaaacaaaagcaaaaattatctatggcaacaa
acccttagaccatgcaattgatttggcaatactttttctttcttatttttttgttcttttt
ggatgggacacaaaaatcactgctagcaaatgtgaacacaaacacatgggactatacatt
atcttagagcttctgcatgggaaagagaaacaacgaaccattaaagaaggcatcctgatg
agttcatgtcctttgtagggacatggatgaagctggaaaccatcattctcagcaaactat
tgcaagaacagaaaaccaaacaccgcatgttctcactcataggtgggaattgaacagtga
gaacacttggacacaggaaggggaatatcgcacactggggcttgtggtgggttcgggggt
tgggggagggatagaataggagatatacctaatgtaagtgacgagttaataggtgcagca
catcaacatggcacatgtatacatatgcaacaaacctgcacgttgtgcacatgtaccta
gaacataaagtataataataataataaaagaaggcatcctacagattgaaagaaagttca
gcagaatcgtatctgtgaaaggggttgcaatctaacatgtatgagaaactaacactactc
taactcggaaaatgaacacaacctaatacccaagctaaaactgggcaaagaacctgaaca
ggcacatctgaaaagtaatcagggaattgactgacaggtcacagagaagttgctcagttt
cactaatcctcacacacatgtctaagtgcaaaccacactcagatacctctcactctaatt
agaatgaaacttaccaaaaacaacaaaaaacccatacattcacaggcagtggccaatgta
gaaatgcagaaaaagagattttatactctattggtgagaatgtacattactatacacacg
aagcgtaacagtggaaggtgacttaaatattttacaactacccgttcacccaggaatccc
accactggttacacacagaaagcacatggaatctgttatgttgaagagatatcggccttc
ttatggcaattgaagcactatgcacaatagcgaaggtatccaatccacctacctgttcgt
gcagagataaagggataaagaaactgcagtacacaatggaatcctccttggccatagaaa
tccatgaaaccatgtcatctgcagcaacatacagaaacctggaggacacgaccttcaagg
aaatgagtcaggaagagagagacaaacactgcaagatttcatgcatgtgagaatgagatc
aactttctctctaaacgactttatctcctagaagtagaacattccacagtcgtgaagaga
gcctgggggttggggagttggggcaggaactgggaaatggatacaatattacactcagat
gacaggaataaattcagctgttctattccacagtaggatgtctagagttaactgtatcct
accgtattttccaaaaaaggctgaaaagaaggattctgaatattgcaaccatagagaact
aataaataataactacacaaggtaacagagacaatgccttgctttcattattacacaagg
tacatatgcatggattataatgtcccactctactctttaattgtatacctttactagaga
gcaaattggttttaaggacatagaaataaacaatgctgaagttcatgtgagaccaggaaa
tgccctgcatttccaaagcaattctgagaaatccaaactacattggatgcatcacacccc
ctgatgtgaaatttcactcaaactctagggacctgcttccacatggatgagtggaagaga
acagagaacctgaagtaaacccacacacctcatactacctgatgctggatgaaatacaca
atgataagtaaaagggaaagaactcccttttccatagttttgggataagtggcgagccat
atgcagaagagtaagtacaactaggcctctacttctcactatgtaccaaagttcactcag
atgaatgaaagatgtaaatggaagacctcaaagtacaaaaatcctacaagagaccctagg
aaatacccttcttgacatcagctttgacaaagcatttatatgcctaagatgccacatgac
acggtaacaacagcaataatcaacatgtgggacctaatacactaaagagccaccacacaa
cacaagaaatcacccacagagtagacagacaacatacaggatgagagaaaatgttcccaa
actatgtgcctgaccaaggcctaatatccagaatctaccttaaagaccttacagaaatca
attagccaacccgcctaataattagtaggcaatggatataaacacacacttctcgaaaga
tgatgtgagagcaaccaacaaattgaaaatatgctcaacctaattgtcagagaaatataa
atcaaaagcacaatgagatatatctctcgctggtcagaatgggggttacacagtttaaaa
aaaaaaggacactggcgaggcagcagagaaaggggacactggtccacttttggtgaaaat
gcaaagtacttcagacaccatggaaagcacttcggagattgctcaaagaacttaaaccag
aactaccatctgacccagcaatcacaccactggggtatacacagaggaaaataaatcctt
ctgtgcaaaacacgcatgcacacaaatggtcatggcagcagtatttacaatggcaaacat
gtgaaatcagcctaggtaccggtcaacagtggatcggaaaaggaaaatgtggtacctata
taccacaaaaaactaggcaggaattaaaagaaaaaagaaggaaatcatgtccttggcagc
aacatgaaaggagctggaggccattatctaaagagaaataaggaaaacacagaacaccaa
atgccacatgttctcacttctaaggtggagctaacattgaatacaccctaccgtaaaagt
gaaaacaacagacactggtgactatcagatgaaaaaggaagaagggatgaggtatgggct
gaagacctatctggtgggtccacccactgcctgcatgataaggttgcttggaccccaagt
ctcagtgtcatgcaatataccaaagacagtaactaatctgcctttgtacactttagtctg
taataaaggtggatattatgtaataattacaaacttagaagttaaaagcatgaatgaaaa
acacaaacttctacaattatcaaaacaatcttttatttggcataaactaagaaagtggac
agaatgagtaaaccaaatactcaaccccaatttacatatattgaacacgtgtttcaaaag
aacaccaaaaacacacaatgggcaaaagagagactgttcaatagatgattttgagcaaac
tgaatattcacatagaaaacactgaaataggacccttgtgtcatgcaacacaaaaatcaa
tgcaaaatacattaaagacctaaaactcaattctgaaaccacacaactcctacaagaaaa
catagggtgtgcatgtattttagaaaagaaatccatgcatgtaggctgctaaaggcatta
aaaaaactttaaaacaaggaaacacccatagacaatgtaatggcttggcattttcactca
aaagctggggacgtggttccacagaaccgtggaacacattagaagacccagatataaacc
tgcacatctgaaaccatctgacacttgaaaaaaatcaacaaaaataagtgatggagaaag
gactccctatcagtaagtggtgctgggataagtggctagctagatgtagaataaataaca
ctgggcccctgtgtctcaccatgtacagaaaataactcaaaatgaatcaaagtttgaaat
gcgaaatccagggtgggcgcagtggctcattcctggaatccatgcactttgggagccaaa
gtgggtagatgacttgaagtcaggagttcaagaccagcctggtcgacatagtgaaaccct
gcctctaataaaaaaacaaaagttagctgggcgtggtggcgcgtgcctgtactctcagct
actcaggagattgaggcaagagaatcacttgaacccagaagctggagggtgcagtgagcc
aagatcgctccattgcattccagcctgtacaacagagtgagactcagtctctaaataaat
acataaatacataaaaatgcaagacctgaaatgaaaaagtcctgcacgagaatctagcaa
atacccttctcaacagaggctttggcaaagcatttatatgtcaagtccccaaaagcaatg
gcaacaaaaacaattactgataagtgggacctaatactcaaaagagctgctgcacagaac
aagaaacaaccaacagagtcagcacacagcctatagaatgagggaacatactccccaact
acgcatctgaaaaacgtctaatatccaggatttatcgtaaagaccttaaacaaataaaat
cagaaaaaaaacccaacttataaatgggcatgggacatgaacacacacttaaaagaaggt
gtaccagtaaccaatgagcatgaaaacatgttcgatctcactgatcatcagagaaatgca
aatcagaaacatactgagataccatctcacactggtcagaatggcaattatgacacacag
tccacaacaacagaggctggtggggcagatgagcaaagaaatgcaggtccactgttgggg
gaaatgcaaactagttcagacccccctggagagcagtgtggacatttctcaaagaacctaa
aagagaactaccacccaacgctgcaaccccactcctgaggatctacccaaaggaaaatcc
ttccatccaaaacatgcacgcactcgtatgttcatggcagtactactcacaatggtaaag
acacggaatcagccttggtgcccatcagcagtggatcagagaaaggaaatgtggtacata
cacaccacggaacactacacagccataaaaaacaaatccatgcccttaccagaaacctgg

acagagctgcaggccattacgaacaaaaggcaagaacagaaaaccacaattttcaaaata
gcttcagaggtcagttgtgaatattctctccacagagaaatcataactcaagctcacaga
ggtgccagacactgcaacttcataatgatgctacgtttacacagatcaaattgtcccttg
cactcactggttatacaggcatactctacggcaatgaaactctgtcccattttggtaaca
ttcattctcaccggagtaagatgatatctgagtgtggttttgattgacttctcgtgagga
tcagtaatgttgattcagaatattttacctgtgcatccttctcaggtcttcaggtccttt
gcccagtcttacacatcaaatagaaacaagttcacgataacctggcaaacatctctaacc
acagcaaaatgtaaatcaaaaccacacttagataccatctcattctaattggaatgagtg
ttacaaaaaaagacaaaatcattgaaagaaaggcaaatgctggtgtgtttccagagagag
agagacagagagagacagtcttctgcacagttcgtgagaaggtaaactagtgcacacgct
acagaaaccacttggagattcctccaagccttaaaagactccaattaccatttcaaccag
caattctactactggaaatacactcaaagccattgaaatcaggacaccaaagacagacct
acccacccatgtggatttcagcactgttcccaagagccagtgtaagcatcaacctacctg
cttatccacagacgaaggaagaaagaagctgctccacatgtacacaccgggatactcttc
acacagaaaaccacaatgacatcatatcgtggggaaccacacggttgcacctggagggca
tgatggtaaattaaatgagcaaagggggagagaaacaaacctgagtcatctcacacatgc
agaatctgagaaactctatctcaaagacctggcgagcacaatattggtttccagagattg
ggggaaaacaggggtaatgggcagggattgtaaatgggtacaaagttacacacaaatgag
aggaagaaaattctgttgttctattgcactgcagggtgaccagggttgacagtatcggca
catcactttcaaggcagcttgatagaatactgaaggttctcagcataaaggaataaacaa
tgggaaaaggtaacagaatcactaagtaccctgatttcattgttccacaaggtacgcatg
ggccataatgccacactctaccctctcgttgtattgatcctttactatgcaatacatttg
ttgaaagaaatagaaacaggcgctgctaatattcatatgagaccatgaagtgccctgagt
ttcatcctcagcaatcctgagacatccagactacgtcagattttaaatttcatggaaaag
ccacggacccacttccacacagagcagtggaacacaagagaggaccaagaagttaaacca
cacactgaaaaccatctgatctatcatagaatccacaaaaataagcaaagggaaaaggac
accctgttcaatcactgatgctgaaataagcggctagccatgtgcagaataacactaggc
ccctaactcccaacacacacaaagaggaactcaagatgaatggaagatttcaatgtaaaa
actcaaactattaaaatcttaccagaaaactcgtgaaatacccttctccacataggcttt
ggcaaagcatttagatggctaagtcctgaagagcaacggcaacaaaaacaaaaattggca
agtcagacctaatacctgaaagagctgctgcacagcaagagagactacaacagagtaaac
agacagcgtacagaatgggagaacatgttcccaaactgtgcctctgaccaacgtctgata
tgcagaatctacaaggcccttcaacaagtcaaccagaggaataaacagaaaaagaatatc
ccattaatatacaggccagggatgtgaacacacacttctcaaaagtcgatgtacaagcaa
ctaacaaataggaaaacatgctcaagctcactcattaacagagagctgccaatcaaaagc
acgacgagatgccatcccacgcaggtcacaatggtgaggaccacacagacaaagaacaca
cgctggtgaggcagccaaggaaaggaaacactggtatgccttgggggggaatgaaaactag
tacagacaccattgaaagcagatcagggatttctcaaagagctgaaaacagaactaccat
ctcacccagaaagccctctcctgtgcatccacccaaaggaaaatcaacccttctatccaa
aagacacagacacttgtatgttcatggcagtgttattcacaatgtcaaagacatggaatc
aacctagccatcaacagtggatgagagaccggcacagtgactcacacctgtaattctagc
actttgggaggccgaggcaggtggatcgcttcagttcagaattcaagaccagcctgagca
aaatgatgaaatcccatctctacaataatacaaaaattagctggatgtggtggcgggcac
ctgtagtcctatctaatctggaggctgaggtgggaggatcacctgagcccagaaggtgga
aactgcaggccattgttataagtgaactaaggcccaaatagaaaaccaaacgccacatgt
tctcacttgtaagcgggagctaaactttgaatggactcaaacataaagaagagaacaaca
gacacctggaatgaccaccacaacaataaaaaaacaggagagagggagcgtgtgctgaag
acccaacctgtgggccctctgctcactgcctgggttacggggttaatggaccccaagtc
tcagcgtgatgcaatcaacagatgtaactaacttgcatatgtaccctttaaactataata
ataagagtagaatgcattaaaaaaaatccaaggttagaaccaaagaaaacaataaatgaa
aaaacaatctacttatccaaacaatccatgactgtcacaaacacagaacgaggacctaag
tgacagaaagaggcagccaaatactatattcaacatgatatgtagggaacacatttttca
aaaccccaaaaagacacaatgggaaagggagagtctggttcaattactgattttgagaaa
actgaatctccacaggaaaaacactgaaatagaacccttacaatgcacaatacacaaaat
caacacaaaatgaattaaagacctaaactcaatcttgaatttgtaaaggtcctataagaa
aatatagagtgggtatatattttaggaaacccagtatctatgcaaacaggctgcaaaagg
taaaaaagcatcataacactgaaaaaaaaaaaagtagggaaactacacccagagacaacg
caaggcttggttctgtgtgtgtgtgcatgtgtgcatgcatgtgcacgcacatgcacacat
gtttttgtgtttagtggaagtcgggtgttgaacacaaaaatcactgctcacatacaaaac
tataaccatataagactagggacattttgcaacttctacagggaaaagaaaacaatgaaa
caaatttaaaaaagcatgctaaagactgggagaaactataaaaaatcctaaccctgaaag
aggttgttatctaacacgtacaataaactgatatgactaagtgaaaaacaacaaaaacaa
caaaaaatatccaaggtaaaaggatctaaatagacctgagtgaaaagaaagcaggaaact
gacccacaggtgacagttcctcaatatcactaatcctcacaaaaatgtgaatcaaaacca
tactcggggccgggcgcggtggctcaagcctgtaatcccagcactttgggaggccgagac
gggtggatcacgaggtcaggagatcaagaccatcctggctaacacagtgaaaccccgtct
ctactaaaaaatacaaaaaactagccgggcgaggtagcgggcgcctgtagtcccagctac
acgggaggctgaggcaggagaatggcgtaaacccgggaggcggagcttgcaatgagctga
gatccagccactgcactccagcctgggcgacagagcgagactccgtctcaaaaaaaaaaa
aaaaaaaaaaaaaaaaccatactcggttaccatctaactccacctagaacgagtattac
aaaacacaaaaatcaaaattgttgaaggcaatgctcagtgctgacttgcaagaaaaaaaa
aaaaatcctctacactcttcatggaatgaaaattactatataaactacaaaaaaatttg
gaagttccttaaaaaagtaaaaggtaggccgggtgcagtggctcacacctgtaatctcag
cactttgggaggctgagacaggtggatcacctgaggtcaggagttcgagaccagcctcat
taacatggtgaaatcccatctctactaaaaatacaaaaattagctaggcgtggtggcagg
tgcctgtattctcagccacttgggaggctgaggtgggagaatcacttgaatgcaggaggt
gaagtttgcattgagccgaaattgccccactgcactccagcctgggagacaagagcaaga
ctccgtctttaaaaaaaaaagtacaactgccactccagctgacaatcccaccactgggta
catgtttagagaaaacaaaaaccctgtgttatctgccttttcatgtgtcctgaagcacta
gtcacaatagccgagatgtggaatcaacctacctgtctatccacacatgaagggataaaa
gaaccacagtatatatacacaatgaaacacacatcagctctaatacttcaggaaatcatg
tcatctgcaaccacttggagaaacctggaagacaattaggtgaaatgaaacgagtctgct
agggagagacccacgctgcatgacatcaggcatgtggaatccaaaaaaccttgtctcctt
gaagcacaacttccaataggggttactggaggctggggagagcagggagcctgggcaggg
attggtaacaggtacagagtgatgctcagatacaaggaatccattctggtgttctattgc
acaacagggtgactaggtgattagggtcaacagaatctagtaaatttttttcaaaatcagc
tggaaaatatggttctgaatatcctctccacagagaaataataatgatggaaggtcacac
agatgccagatacgatgatttgatcatgatgctacacaaacatatgtcaaaatgtccctt
gcacccttgggttgggggtgtgtgtatactctatagaaataaaactgccatcaaatgttg
gtagcattcattttaactggagtgagatgaaatccgagtgtggttttctcacacttttgt
gtgaatcagcgatggtgagaatcttctctttgacctgtgcatcaacctcatgtcttcagg
tcctccgcccagggctacataacaaattaaaaacaaattcacaataacctagcaagtatc
acttatcatacacacacaaaaatcaaaaacacactcaagatttcatctcactctacattc
aatggatgccataaaaaaaaaattgaaacatattaaaacacaaaacaatcaaggtgtgga
tttccagaggaggaacttgtctccacatttggtgggaaatggtaaactagtatgcacact
agaagaaaccacttagatgttccttgaaattgtaaaactacaactaccattgtctccagc
aattctattcctagctatatacacagagtgcacgatcaggacatggaagagattatctgt
catcccaggtggaattcagccctatgcccaaaagccaagataagaaatcagccgacctgt
ctatacacagatgaagggatgaagaatctctagtatacagacacaatggaatacttttgc
accatcaaagttcatggaatcctaccatttccagcaatatggctgaacccggagatattc
tgttcaatcaaatcagaaaggcagacaaagaccaagcctgcatgttctcactcatgtggg
agctgaaaaatttaactcactgaaggtgaaaatacagcagtggtcaccacaggctagggg
gaggagaggacatcaaggactggcaataggtacacagagagttacaggagaggaatacat
tctggtattccactccaaagcagggtgatgatacttaacaatatggtcgtgctgttttca
aaatagctactaaggaggattttgaatgttcacacatcgaagatatcaaacctacacgtg
ttcacaagcatgggaaactccctgattttatccatactcaaggtatacaaacaagtatca
caatgtcccaatgcacccctaattctggacatgaagtataaaacaaatgttttatgaaat
gttaacaatacgcggctaaaaattcacatggaaccactaaagacccaaagaacctgaata
tggaaagcaatcccagggaatacaaactcaatgggaggccgcacactccccaatttcaaa
atacatgtaaaacctccacttgggccgggcgcggtggctcaagcctgtaatcccagcact
ttgggaggccgagacgggcggatcacgaggtcaggagatcgagaccatcctggctaacac
agtgaaaccccgtctctactaaaaaatacaaaaaactagccgggcgaggtggtgggcgcc
tgtagtcccagctactcgggaggctgaggcaggagaacggcgtaaacccaggaggcggag
cttgcagtgagctgagatccggccactgcactccagcctgggcggcagagtaagactccg
tctcaaaaaaacaaaaaaacaaaaacaaaaacaaaaacaaaaaacctccactcatccaaa
gactacagtactggcaaaaaaatgaatccatgaacctagggacataagagaacctaaacg
cacacctgtatgcagtcaacacattttttttaaagaatgcctagaagatgcaatgaggct
gggtgctatggcacctgtactcctagtgctttcagagactgagtcaggtgtggtggcgca
caccagcaatcccagccacttgggaagatgaggcaggagaatcgctcgaagcaaggaggc
agatgttgcagagagctgagatctcgccactgcactccagtgtggacgacagcatgagtg
ggactacatcccaaagcggaaaaaagatgagatgaggagtcagtagtctgttcaactgag
cagtctgaaacgactggatagccacatacaaaagaatgaaacaagacataagaccttgat
gttatacaatacacaaatataaactcaaaatacattaaaccctaaacacaagacctaaaa
gcataaaactgctacaggaaaacacagagtgagcttaagtttaggaaaacccgaatctaa
gctcacaggatgtaaaagcaaacaataggaaaaaaaaaaaaaagaaaaacagaaaaaaaac
cacttccaacaacataagtcaaattttctagtttttgttattgactttaccttacaaaaa

caatcactgctagccagtcatgctggctcacatctgtaaatccaggacattgggaggccg
aggcaggtggatcacctgaggtcaggaattcgagaccagactggccaacatggtgaaacc
ctgtctctactaaaaatacaaaaattagccaggagtggtggcgcacacctgtagtcccag
ctacttgggaggctgaggcaagagaatcaatcgaacccgggaagcggaggttgcagtgag
ccaagatcatgacaccacactctagcctgggcaacacagcaagactccgtctcaaaaaaa
aaaaaaaaaggaaaaacaaaaataaaaaaatcacaactaaaataatcaagaaacaaaaac
acaaaaaatcctgaagaagttctgcacattaaagaaagcaatgcaccaaccaacactccg
aaggcattcttcagatagaaaggacattataaggaatcatgcatctgaaactaccacacc
actgaggagcaaaacacacaaaaccaactaacccaaccaaaactgggacacaatcctgaa
cagacatcactaaaaagacaatgcacaattcactaacatgtgaaaagggcgattctcaac
attactaatcatctctaaactgtaaatgaaaaccacactcagatacacactcaccctaat
ttgaacaaataaaagaaaccaattatttcaaacaaattccctggaggaagttgcacacac
agctactctcacactgttgggagaaatcactgttattacagacactatgaaaacctcttg
gagcttcccaaacaatcaaaaaggaaactaccatttaatctggcaattccagcagtgggt
atatacttagggacagtgaaatcactgcatgaaagccatagctgccttcccaggttgacc
gaagcactatacacaatagctaaggtatgcaatcaacctacctatccatcaactgatgaa
gggatgaaggaacttcagtatctggacacaacgaaatacgccccatcaacaaagattgat
gacataatgtcttttgtggcaacacggataaacctggaagacattacactcagtggaatc
agccaggcagggaaacaaaaacactgcatcatctgacccatgaggagactaaaaaagaat
tatctaacggaaagtgaaagtacaatagtggttatcacatgctgggggaggagagggca
tgggctggcattggtaatgggtaccaagttgcacttccaggggaggaattaatgctggtg
atctactccacaggagggtgactacaagtaacaatacggtaggatctctttcaatagagg
tagaaaggagaggaacactgaaggttcacacaacaaacaaataaaaactagacatgggaa
tagatatgcaaaacaccctgacttcatcattcctcaaagtatgcaagtaaacaaaatccc
actgtacccccctaattttggacatgtatgacaaactcaattttaaaactataaatggaca
atggtaaaattcacagagaaccagggaagaccctgaaaatccaaagcacttcccagaaag
agaaaatcacttgattgcctcacgctccgcgatgtcaaattacatcaaaaagctatactt
accgaggcagcaaggaactggcataaaaatagaaacatttatcattggacagtattgggt
agccaacagacaaatctgaatgtacttacagtcaacagatttctcaaaaggccaggcgcg
gtggctcacgtctgtaatcccaacattttgggaggcaacggctggcgaaccacttgatgt
caggagtttgggatcagcctaacatggtgataataaaaatagaaaaattagccagcatgg
tggcatgtgactgtaatcccagctacttgggggctgaggcaggagaatcgcttggatctg
ggaggcagaggttgcagtgagcaaagatcacaccacttcactctagcctgggcaaaaaag
caacactctgtatcaaaacaaacaaacaaacaaacaaaaaacccaaacaaaatgggaaac
agctagtctgttctgtaaatgactttgagaaaactggaaacccacacacaaaacatggaa
acctccttgagcttatccttaatccttatgcaaggtacacaagtgctgtaatgtccccat
tgcaccccctaattctggacatgcagtatgaaccaaacttgtcatgaaatgtaactataca
cagctaaaaattcacatagaactaccatagactctgaatatgcaaagcaatcctaggaaa
tataaacttgtttggaggtctcacactgttcaatttcaaattacatggaaaagatctact
catccaaagactatggcagtggcctaacaatgaaaccatgcagacataacagaacctaaa
cgtacatctgtatggagtcaacacgctgtttttcaagagaatgcctggaagacaaaatg
aggagtgggaggtctgttcaattaatgaatctgaaaatactggatatccacatgaaaaag
aatgaaataggacccttatgttacacaatacacaaataccaacttgaaataaatgaaaac
ctaaacacaagacccaaaagcataaaacttctacaggaaaacacagagtgagtggaagct
cagggcaacttcatctatgctcataggttgcaaaaagaaaaaacaggaaatacatggaaa
atacattcaaaatcaatgaaatgtatcaaaattgtgatttttctttctggcctgacccaa
aatcgctgctaacaaaaggaaaactcaacacgtgaaaaagaaaactcgagaactacggca
tggtaaagggagcaatgaaccaatcaacaatgagaaggcattcttcatatagggaggca
tgatgaggaatcctttatctgaaagaggttgttacatggtatgcacgagaaactaatgct
actaagaagcaaaacatgcaaggccaagtcacctggccaaagttgggcaatgaacctgaa
gagatatttctcaaaagtcccagctactcgggaagctgaggtgggagtgagctgagatcg
cacccctacactccagcaaccatagccagacactgtatcaaaaaaaaaaaaaaaaaggaaa
aaagaaaaaagaaataaactatgcagagaaagataaacatcgcattgtctcactcaggag
gaatctacaaaactttatttcctagaactagtaagcataactttctaaatgggttggggt
tcaaaggtggggcatggtcagagattggaaacaggtataaagttacatattcccatgaga
ggaatgaaacctgtagttcttttccacagcagcagggtgactaggtctaacattatcaga
gtgtacttttcaagaaagctagaaaagaggattctgaatgtcttcacctcactctatgag
gaaagacagatgctaaatacccctgatttcatcattacacaatgtatgcatggaccaaagt
gtctcattctaccctctggtagtacacctttaccacagggtaaatttttgtaaagaaatg
taagggaacaggctgagcatggtggctcatgcctgcaatcccagcactttcagaggctga
ggcaggagtattgcttgaacccaagaagtgcaggctgcagtgagccaagattgcaccact
gcactccagtctgggcaacagagtgagacactgtctcaaaaaataaaaattaaaaaaaaa
aaagatgacgttcaagtgatcaaccaaatgaaaaaatgtgcaacctcagcgcaatgcaac
tcccaaacacaatgagatatcatctcacgttggtcacaatgggcatttttttcaaagtcaa
aacaacatgctggcgaggctgataatctgtcccaaatcaaggcagcaaaattcctgcaaa
gaaaaaaaactaaagcatgtctgccagctgctggtctgtgttagagaaggctctttctag
gggcaccagagggaatggagtgtaattcaaactggaagactttcaaagccagagctagct
cacctattgaggctataccttttaactaaactcaactgcagtagtcaggactcttgttgc
aagtgacagaaacccaactcaaactagcttaagcctaaagacagatttatggactatctc
atggaattcataaacaagatagttctgactctctctcagacctcattggccttaagaaca
atatggagtcagagactcaaacaccactctcttctcttgtcatgtctctgtttctttgag
atttgtcctttttttctcagcaattcccccaatcctctagttcacataagatggagtggag
tctccctctgtcgcccaggcaggagtgcagtggcacagtcttggctcactgcaacctctg
tctcctgggttcaagcgattcttgtgcctcagcttccgagtggctgggatgacaggcatc
tgccaccacgtccagctaacttttgtatttttagtagagacggatttcgccatgttggc
cgggctggtttcgaactcctgacaggtgatccacctgccttggccttgcaaagtgctggg
attacatacgtgagccaccgcgcccggctactgatgcaataatatattcttaactctggt
tcaaagacttttgaagaagcagataatgagcatgacagaattgaaactgatttggggaa
acgattaaaatctccatctaggttgggcgcgatggttcacacctataatcccaacacttt
gggaggccaagacaggcagatcacttgcggtcaggagtttgaaaccagcccagcaaacat
gctgaaaccccatctctactaaaaatacaaaaatttgcttggtgttttggtgtgcacctg
taatcccagctactcaggaagctgagccatgaggatcacttgaacccaggaggtggaggt
tgcagtgagccacgatcacgtcactgcactccagcctgggcaacagagtgagaccctgtc
tcaaaaaaaaaaaaaaaaaaaaaatccccattcatacatgatgataccaaaagatcaaa
agaaagcagaacgatttttttggagcaaaacacactgcttgctgtcaataaaaagattac
agtcctaacttttcccccaagaattctgtcagataatctcaaagttatcaaaatgaaatg
ggattatttaaagatgaaggacttaaagaaaaatacttggccaggcacggtgactaatgc
ttgtaatcccagcactttggaaggccgaggctggtggatcacctgaggtcaggagttcaa
gaccagcctggccaacatcatgaaaccccgtctccactaaacatacaaaaattagccggg
catggtggccggtgcctctaatcccagctactcgggaggatgaggcaagagaatcgctta
aacccaggaggcagagggtgtgcacctgtaatcccagttactggggaggctgaggcacga
gaattgcttgagttcaggagatggaggttgcagtgaactgagatctcaccactgcactcc
agcctgggtgacagaatgagactgcctcaaaaaaaaaaaaaaaaaaaagtgctattttaa
gtgttttatgattaaacttctgtaattgtctattgcactaagttcattatttttattgta
ttaaactttagattcatggggcacatgtgcaggtgtgttccatgagtacactgcaaaatg
ctaaggcttgggcttctattgaactcatcacccaaacactgaccatagtacccaatgact
agtttttcaaccctttcccctccttccctccccacatgtggagttcctagtgtctgttat
ttccatctttatgtccatttgaaccaactgtttagctatttatttatttatttatttttg
agacagagtcttgctccttcaccctggatggagtgcagtggctccatctcagctcactgc
aatattcacctcccgggttcaagcgattctccagcctcagccttctgagtagctgggatt
acaggcaggtgccaccacaccaggcaaacttttgcaattttagtagaaatgggattccac
catgttggccaggctggtatcgaactcctgacctcaagtaatccacctgctttgccctcc
cagagtgctgggattataggcatgagccactgtaccttgcctatttttaaataatactta
attttctttcattttgctaataatttagagtacaattatataatgatattaacttttttt
gggggtgggacagggtctcactctgtcacccaggatggagtgcaatggctcaatcatgc
ttccatgcagcctcaaattcctgggctcatgtgatcctcccacctcagcctcccaaacag
ctgggaccacagccatgcaccaccatgtccagctaatttttctatttttgtggagacagg
gtcttgctatgttgcccgggggtgtcaaattcctgagctcaagcaatctgcccgccttg
gcttcccaaagtgctgggattagaggccgaaaccaccgtccctggcctaataatgtagaa
tcttacttaatgtttcatagtatgattttcaaagtttgtatttgaaatgtatttaaatat
gtagtttgatcttttgaaaaacatttgaattcctaacagattataaaatactattgtgcc
caggaggtggaggttgcaatgaacagagatcaaactgctgcactccagcctgggtgacag
agcaagatgccagctcaaaaaaaaacaaaacaaaacaaaaaaaaactagttgtgttagggt
tcaaaccagagaagcagaaccagtaggaaagatacacacattcagatatacataaacata
tagtgacacacatctgcatagatatagaaacggatggcttctagccaggcacagtggctc
acacctgtaatcccagcactttgggaggccgaggcaggcagatcacctgaggtcaggagt
ttgagaccagcctggccaacatgatgaaactctgtctctaataaaaatacaaaaattagc
caggcatggtggtgcatacctgtagttccagcaaatcgggaggctgaggcaggagaattg
cttgaacccgggaagcagcagttgcagtgagccgagatcacgccactgcactccagcctg
ggtgacaaagctagactccatctctaacaaacaaacaaacaaacaaacaaaaaagctata
tacagacatacatctagatagatagatatattcgtaaaagcttggcttctgcatttgtag
ggttggctaagcaagcccagagctcgcaggactggccgtcaggaacgacaatcaccaccc
aggcagggccaaagcttgcttttcacaggcactcaggaagaaagatccagagggaaggga
gcacaattgtggactcggctgctatttgaagtctgtgttccaggaagtcctaagccctct
cttttctaaaaaaagaaacaaactttttggcttgtggtgccacaagctgtagtctcagct
gctcaggaggttgaggcaggagaattgcttgagcacaggagttagaatctagcctgagca

gcataacaagactttggttctaaaaagcatttaaaaaatagtccggctgcagtggctcac
aaccgtagtcccagcactttgggaggctaaggcaggtgaactgcttgaagtcaggagttc
aagaccagtctggccaacatggtgaaaccccgtctctactaaaaatacaaaaatcagccg
cgcgtggtggcgcatgcctgtaatcccagctacttgggaggctgaggtggaagaattgct
tgaacctgggaggcagaggttgcagtgagccgagatctcgccattgcactccagcctggg
caacaagagcgaaatcccatctaaaaaaaaaatttttttgtaatgaaaaaagttttggag
ataaggggtcttgccctgttgaccaggctggaacacagtggcacagtcacacctcactgc
agcctcaaactcttgggctcaagcaattctccctcctcagccccctagcagctgggact
gtaagtgcataccaccatgctgactaactaaaagaaaaaaaaaatttagagagatgtagt
ctcgctatgttgcccaggctagtctcacactcctgacctcaagatcttcccgccttagcc
tcccaaagtgctaggatgtgagccaccattcccggccccagtaaaccaatactgatacgt
tattattaactaaggttcatcttgattcagatttctttactatttcccctaatgtccttt
tcctgttccaagatctcatccagcagaccatactatgtttagttcttaagtctcctcagg
attgctggagtgcagtgactccatctcagctcactgcaacctccgaccaccaagttcaag
ctattctcctgcctcagcctcccgagttgctgggattacaggcacctgccaccacgccca
gctaattattattatttttttttttgagagggagcctccctctgctccccaggctggagtg
cagtggggcaatcttggctcactgcaacctctgcctcctgggttcaaacaattctcctgc
ctcagcttccctagtagctgggattacatgtgtgtactaccactcactgtcttttttttt
tttttttttggagacagtgtgagatggggtttcaccacattggccaggctggtctagaac
tcctggcctcaggtcatccacccacctcagcctcccaaagtgctgggattacaggtgtca
gccactgtgcccaggctattctgcacttcacttgcaaaatatttttccctttacaataaa
ttgctctatgctgcatcacctttcctgtgtgtttctttttttaaatttttttatttattta
attatttattctttttgaaacagggtctcactatgttgcccaggctggtctctaatcttt
gagctcaagcgatccgctcaccatggcctcccaaagtgctgggatgacaggcattagcca
ccacacccagcctaaattctttgaaactgacaactgcggccccacaacagctgtcaatca
ttgtaccactgccatccagcctggttgacagtgtaaggcctcatctcacaaaaggaaaaa
gatatattaaaaataaataaataaataaatattttttttttccttaaaaacaaggtatccc
agtcatgcacggtagctcacacctgtaatccttgcactttgggaggccaaggcaggtgag
tcatctgaggtcaggagttcaagaccaacccagacctacatggtgaaaccccgtctctac
taaacaaaaaccacaaaaattggctggacatggtggaaggtgcctgtaatccctgctact
agggaggctgagggaggagaatcacttgaacttgggaggtggaggttgcagtgagcccaa
gagtgcaccagtgccctccagtctgggctcctacagagtgagactccagctcaaaaacaa
atacaaacaaaaaagacagggtctccctctgttgcccagaccagagtgcagtgacgcggt
gacagctcactgcacccttgacctcccaggctcaactgattctcctgcctcagtagctgg
gactaccagcacacatcaccatgactggagaattttaaatttttttgtgctgatggtctt
gctatgttgcccagcctggtcttgaactcttggcctgaagtgatcctcctaccaagtcct
ccttacaaagagctgggatgacaggcgtgagccaccgcacccggccccctgaagctgcct
gttccttgagcacgatgtatatcaaggggagctagagtgagagcagcaccgtggggaagg
gacagggctgccaccctgagctatgtcatgttaggccagacccctcttctccaagcgaga
tcaaccacatgagtgatctcaggagcccgtgcccgctcagacagcctggattttagcttc
gtaggtggatgggaacctgtgaaggttcagagatgctggatgaaccttttttcctgggaga
gctttccaaacagggaacaaacagagtcacccggggccccgctgagggaggatcctacca
gaagccgtcatcactgtctcactccacaccggtggtgatggtgatgtcatcgatgtaggt
agttggtgtcgtgtacagaagcacagaccgctgtagtcccgcatagttgaagaagtcaaa
gtctgtgttctgaacaaagtaacccttgggatacctagggcaggaggactgtggttcgct
gggcccttgccatgggtcttttgccctcaggtcacttgcctccccagatcagggcctcct
ctctgtgtaggccaccctgtccctggcaggaagaccacagggtggggcgggaaggtgggt
gcgcgcagtggaggcaggatggtactcacttggagttgtcagttgtgtacggatggtccc
tggtggcagggtggggggcgagcgtgttgttgatggcgacagttgatgacgcagaggcgg
gagggcaggggccccacccggaatagactgctgctgtcagcctcgaaggggaggtagccc
ctttcatgctctagcttgtggacaccaatcacccactgcagacatagggagatatgagaa
gggggtggcaggtcggggcatgaggaggggctctgctatggggcacgccctcacataacc
cgcagcatggggctcaagggcctgccagccagacgagaaaaaggacatcccagtggggta
gatcaggccacctatccacctacacagggcacagcccccagggcagggtaaggcccccca
ctgtcccaccccaggagacaggctgtgggtggaatctgagggtcccatgctgttcagcag
ctgtgcccacacacctgccctgcctgctcctggccacactgaccacaatggcatagaagt
gggcactgccaatcctcagcaccactcttatgtgcaggtcccggatccatcgcttcagca
ggggcacctcccgttcataccacatccagctgacaaaatgctgcagccgccagtcctggc
tgatgtcattgaaaatggaaactggtatgtccaaggtggggcccgactgcgaagagaagg
gccgggctcagctcctaggcccacaaagggatccgggaaaacagtgctgcacagctgtgc
ccccaggcctagcacaagccctgacacatggtgaggactcctggcagaaaagacagataa
cttgctgatgacaggtcaactgccagggcagtttgtgcacctgggccactggggtcagaa
gttcgagaccagcctggccaatatggtgaaaccccatctctactaaaaatacaaaaatta
gctgggggtggtggcggcgcctgtagtcccagctactcaggaggctgaggcaggagaatc
gcttgaacccagaaggcggaggctgcagtgagccaagaccatgccatcgcactccagcct
gggtgacacggcgagactctgtctcaaaaaaaaagaaaaagaaaaagaggatgtagatca
ggcaaacgcggcccagacctcaccggatcccacttctttgctgaccacatccaagtcagg
agggcaacaggggtggcgtctttggcctgatgtcacagtctgtcctctatctgaaccctg
aaaaccaacgtaccttccccctcagcagagccgacatcagaatgtctaaactgtcccct
gctggcgatctctgggcccagctctgtgcagcgcctgctgctccggcctgacgagtgtct
ggtggctcatgcctataatcagtgctttgggaagccaacgtgggaagatggcctgaggcc
agtagttcgagatcagcctggataacatagtgagaccctgtatcttaaaaaacaaaacaa
aacgaaaaagttggtttttgttttgagacagagtttcagtctgtcgcacaggctggagtg
cagtggcttgatctcggctcactgcaacctccgactccctggttcaaacaattctcctgc
ctctgcctcccaagtagctgggattacaggtgctcatcgccacgcccggctaatttttgt
gtttttagtagggacggggtttcaccacgttggccggggtggtcttgaactcctggcctc
cggtaatccgcccgcctcagcctcccaaggtgctgggattacaggtgtgagccaccaagc
ccgaccaagtttcttttctttgtttttttttttttttttttttaattaggcatggcggcc
ccagctaagttgcaaggctggggcgggagaatcgcttgagctcagaagctaggattgtgc
cactccgctgcaacctgggcggcaggaccaaacacagtctcaaaaacatttcaaaaatta
aaaataaatattaggagactgcccgggctcagccttcgcctccggaggcggctggacagg
agcgcttcctcctcctcccgcctcctccgccctggccctcctgggtcccccctagtcaccc
cagtaccccagtcctccaaccccgcgcccccaagcccattgacctttaacctcctccggg
cccccagctcccgggcactcaggtgaaggaggccgcagggcgccggggcttaggccattc
ccaacgccaggcttccgggggcctctcgcaacggtcgcgcctacctctgctcctagaagc
ccgggagtcggttgttggagaagtcggcttggaagcttcagaggccgtccagctccttgc
gctcccgcgatgcgctctgccggatgtacagcatcccgcccagcagcgcctgcgggcagc
cacacaacagccgccggagagccgcccaggacaccgccaccagccaggtcatacttcccg
ctcctcctcttgtcagccagtctgaggggatgagagcggcgcaggggaagccctgcgggc
ttgtcgcctgaggcgcgtgcggcatggcgagcgtgaggccttgggcgccatgtttgttga
gggcgagtgccaggcctggaggggcggggcagtgccaggcgctgctcagtagaacctgtg
gctggaatccagggctaggcctttggggaagcgggacccccaccctcagctccagccaag
aagggcgtcgtctgctgccatcccagcagaggctggatgatccagggccgcaggtcagga
cagaacccccgagattggtggccttactgtttcttcaaggggcacctccacttgctttcc
ccagcaggaattggagcaggccccagcggagcctcttccatgactgctggatgcagcctc
ttgcagggatgcatgtccttgggaggcggctgtagccttgaccttggatctctgggaccc
tgaccctaaccaaggggacagaagttccttcttggctttgaagatacagggagagaggcc
aggtgcagtgggtcaggcctgtaatcccagcactttggggggctgaggtggtcggatca
cctgaggccagtagttcgagaccagcctggccaacacagcgaagccccctctcaaatact
aaaaacagaaaaaaaaaaaaaaaaaaaaaggaggagccaggcatgatagcaggcgcctgt
aatcccagctactcaggaggctgaagctgaagaatcacttgaacccaggaggcgaaggtt
gcagtgagccgaggtcacaccactgcagtccaacctgggtgacagagcaagaatctatct
caaaaaaaaaaaaaaaaaaattgcagggagagggtgccctgctggaccccactttactcc
agtagggaaggcaccgtgtggaagggtagaggaagagatctggagacagtaaaagagaca
taggtttattaaggggactttcacacaggtgcagtggctgtggctggacatgagaaccac
tacatttgtaaaaagcaagcagcttccataacattttcacctagcatcctcgacctgtta
ccggtggaaggtatccgagttcctggtggtgaatccatacacaactgaaacaacctcaat
tcttgcctcctcagaagaaagaatttaaccgagggtcataaagtagaaaccagatagaga
taagttgcagagcaggagtaaaattttattaaaaagcaggccaggaacaggcgtggtggc
tcatgcctgtaatcccagttctttgggaggctgagcccagtggatcacctgagatcagga
gttggagaccagcctggccaacatggtgaaaccatgtctctactaaaaatacaaaaatta
gctggacgtgatggtgagtgcctgtaatcccagctacttgggaggctgaggcaggagaat
ctcttcaacctgggaggtggaggttgtagtgagctgagattgagccactgcactccagcc
tgggtatagagtgagatagtatttaaaaaatggttactaaaaaaactttagaataggaag
taaaggaaagaaggaaagtacaacttggaagagggccaagcggtcgacctgagaaaccaa
gtgcccagcttgacctcttgactcagagtttcacaggttggcatcctttcaggatcttgt
cactcctgattccttagcttggggctccgaacggactttcttctttgaccgtaaattcat
tctgagggtacacatcaattatggctgtcgggtgcctctgccatacagaggggtccctct
ggtcctaggaacctggactggacacagttgtttgtacgattactcctccaccccttttct
catgctcgccaaatccaccttctgagaatggaccttaatactggaaatggagtccccagc
atagtgaaaataaatacattgtagaaactgacaaggcctaaaggctttttcccaaatgtt
tgtgctttttttcttattttatttctttaaaattttacttgtatattcctttagtgaaat
gcttacatgcttaaattaggatatagttcactgtttatgaggcagctttaggccacattt
aacaaaagaaagaaaaagtaggatgcaaaagtttatacagtatgtataaatcagtaattc
aggacgggcttagtggctcacacctgtaatcccagaactttgggagggggaggcaggagg
atgacttgagcccaggagtttgtgaccagcctggacaacataatgagaccactgtctcta

caagaaatacaaaaaaacaaaaaaaaaaattgcccaggcggggtggcacacacctatcatc
ccaggtacatgagaggccgaggcaggagcattgatgaaccccgggaagcagaggttgcag
tgagccaagattgtgccactgcactccagcctaggtgacagagcgagaccccatctcaaa
caaaaaaaaaaaagaaaagaataagactaaagggtgccagaaaagatgccttccttttc
tttttcttttttttttttttttttgagtctcaccctgtcacccagactggagggcaatgg
cccgatctcagctcactgcaacctccacctccctggttcaagtgattctcctgcctcagc
ctcctgagtagctggattacagatgctcaccaccacacccagctagtttttgtattttta
acaacgacagggtttcactatgttaaccaggctggcctcaaactcctgacctcaggtgat
ccacccacctcggcctcccaaagtcctggaattacaggcgtgagccaccacgcctggcca
attcaagtatttcttcagtttacagttggctaagaaagcaaagctttatctaaaaccttg
gggtcagcagaaggggtgttaagtttctgcctgtgagtgtgattccctccaggcccctca
ggaagaaatttggaacaaagaacgccagtcagagttcagtcctcggttcacccttatctg
aggtcgaagggaaagctgtcaccatttttcatccggtggagatctgcgcttccgaaaaca
acttagagacatttgtcaagatgtcatctttactttctataggggatcagatatcttatg
gctgtaaccaacttggatggctattgttctgagctcttattaccttttttctttttctttt
cttttttttttttgagacggagtctcactctgtctcccaggctggagagcagtggtccaat
ctaagcccactgcaccgtccacctcctgggttcaagcaatcctcctgtgtcagcctccta
agtagctgggataacaggcatccgttatcacatccagctaatttttgtattttcagtaca
catggggtttcaccatgttagccgggctgatcttgaactcctgacctcagttgatctatc
tgtcttgacctcccaaagtgctgggattacaggtgtgagccactgcacccagccaattac
taccttcttcttatcaagttgctaatttacttctcaggaatagccaggtgcccaaaattt
cccttaaaggctaatgtggttcgagtatgtgccctccagatcttatgatgagatgtgatt
tccagccagatgcagtggctcacacctgtaatcccaccacctcctcctcctccaattcct
cctcctcctccaacacctcctccttctccaactcctcctcctcctccaactcctcctctt
cctccacctcctcctcctccaactcctcctcctctgtttctttatcctcctcctcccccct
ctttcatttctttttcctcctccccctcctacatttctttttcctcctccttctcacagg
catgagccactacacaaggcctggctgtttgtttctttctcctcctcctccatttctttt
tcctcctcctcttcctccttctcctcctcacaggcaccagccaccacgactcacctggct
atttgcttttcctcctttcctcctcctgctccttctccgcctcccaaattgctgggatta
caggcctgagccaccacatctagctatttcttcttctttttcttcctcttcttcctcctc
cttcttcacttaattttatttttcttgatatagagtctccctctgttgcccaagctggag
tgcagtggtgcgatctcggctcactgcaacctccacctcctgggttcaagcctcccaagt
atctgggattaccagcgtccgccaccatgcccagtcaatttttgtccttttagtagagac
ggggtttcaccatgttggctaggctggtctcaaactcctgacctcatgatctgtccacct
ccacctcccatagtgctgggattacaggcatgagccactgcacctagcccccctggttttt
tgtttttttgtttttttccttgaaatggagtttcactcttgttgcccaggctggagtgcaat
ggcatgatcttccctcactgcaacctccacctcctgggttcaagcgattcacatgcttta
gcccccccgagtagctgggattacagctgtcagccactgcgctgggcctacagctcgtttc
ttaccacataaatctttgcgcctctccacaaaactgccatcagggatgtccccagaaacc
attcatcccaggtgccatgcagagaagagcttctcgttctccttttccctttacctcttc
cctctcacctcatcttgttcattcactcatccctttttccattctcacttttaagcgttaa
cctttcaaaagcctgtcttcccctacaagtaatgtattgtaactcccgccatcaccatat
ccttctccaacccaccaaactgccaaggatggtaagtccataaactaagaagaaatggga
aacattcattgcaaactttgcagcccctcagtcacccaatgtgacagcacagatcttctg
gtctttgaagacccttttttttttttttttaagacaggtgtcgctctgtcacccaggttc
aagtgcagtggtgcaatcacagctcagtgcagcccccgactcctgtgctcaggtgatccg
cctgcctcagcctcccaagcagctggaactacaggaacacaccaccacacccagctaatg
tgcttatttttttgtagagatggggtcttgctctgcttaacaggctggtttcaaactcct
ggcttcaagggatcctcccaccttggcctcccaaagtgctgggattacaggcatgagtca
ccatgcccggtctgaagacttttaaatgctgccatattcaagatacattgaaactcacct
gtgtttgatgagcctgctttttgcaagtgagtaatataaaacacactgaaataccttaag
cttctcagactttgtaccttcctctggaataatcagtgtatcccaaaagtaaatccataa
tgaggtccagtttttccttcctccttggctatgaaatagacaagaaaaaggcaagcaagc
catgtccacctcactgtaggagactctcctgtttgctttttgactgtatttgggaagcgg
gggcctgactgctttcctacttcctaagcacaacttcctttacctaggaaatcctcagca
tgacctacatagattaaaccagtttcttcactttctttccaatattcttacaaccaaaat
atccagaaagggcaaggcaacctgaaaaaatgaggacaggtatattatcccatgagccaa
actgccacttacactggttagtcatgaaatcagcaaaattccagatgagctctccaacta
cgtattttctatgtttttgatccagacccagatggtactgctctagcagactgttctggt
catcttcactgaacatcagagatggatcctggaattcaaggcaaagagaatttaagaatc
agaactggcagaattgtaaatgtcagataaaaaataaagatccacttgattgtgacgaaa
atacctgacctcacaggggggctgtagtgacagcaggactgactgatgctaggataaagac
agccagggaatgatctaacccagaataaaaaggaggtttaaaaaaaaaccaacatggtca
ggcatggtggctcacgcctataatcccagcactttgggaggttgaggccaggcagatcac
aaggtcaggagatcgagaccatcttggctaacacggtgaaaccccatttctacaaaaaat
acaaaaaatgagctgggcatggtagcgggcacctgtactcccagctactcaggaggctga
ggcaggagaatggtgtgaacgtgggaggtggaggctgcagtgagcagagatcacgccact
gcactccagcataggcaacagcaagactctgttgaaagaaaagaaagaaagaaagaaaga
aagaaagaaagaaagaaagaaagaaagaaagaaagaaagaaagaaagaaagaaagaaaga
aagagaaagaaagaaagtcattaattagtaattgctttatttataaatgtaatttgatat
tcgattcatcttacttttccatctctatctgctggtacagtcttaaggctgaactacact
agaagtaaaaatatgccttgggaccaggcatgatggctcatgcctgtcatctcagcagtt
tgggaggccaaagtgggaggactgcttgagcctagaagttcaagaccagcctgggaaata
tagcaagaccccatctctacaaaaactacacaaagtagccaagcatggtggcacacaccg
gggtcccagctacttgggaggctgaggtgggaggactgcttgagccccagaggtcaaag
ctgcagtgagctgcgtttgcaccactacgctccagcctaggtgacagagcaagaccctat
cttaaataaatatatgtatgtatgtatgtaaaatgaaattaaaccaggctgggcatggca
gctcaggtctggaatcccagcactttgagaggtcaaggcaggaggatgagcacttgagcc
caggagttcaagaccagcctaggcaacacagtgagacccagtctctacaaaaagtttaaa
tattagccaggtgtggtgacgcatgcctgtggttccagccacttggggggctgaggaggg
aggatcatttgagcccaggaggttgagcagtgagctgtgattatgccactgcactccagc
ctgggcaacagagtgaggctctctcaaaaaaaaaaattatttttaattaaactaaataaat
tcagctattctagtcatatatcaagacctcaatagccacatgtagttagtggccaccatt
tcacacagtgcgtataagggacatttctatcattgcaaggcttcttttttgaaacaaggt
ctcactctgtcccccaggtgggagcgcagtggtgcaatcatggcagacggcagccttgac
ctactgggctcaaacaatcttcccacctcagcgtcccaagtagctgggattacaggcaag
cacaaccataaccaactgattttatttttttatttttcttatttcttgagacggagtctc
gctctgtcacccaggctagagtgcagaggcacgatgttaactcactataacctctgcgtc
cctggttcaagcagttctcctgcttcagcctcctgagtaactgggattacaggcacacac
caccacacccagctaatttttgtattttcttagtagagacgaggtttcaccatgttggtc
aagttggactcgaactcctgacctcatgatccacccacctcggcctcccaaaatgctggg
attacaggtgtcagccaccatgcccggccgcaactaatcttaaatttttttgtagagatgg
ggtctatgttgtgcagactggtctcaaattcctgggctcaagagagcctctgacctcggt
ctcccaaagtgctagcattccaggtgtgagccaccacacccagcacctgcagcacccagg
tctatcagtgctgacctagaacctctcgggagtttcttaagaattcagaactgggggtat
tagccaagatggcggcggccgcagtgagttgaggaatcagggcaaagctgggcctgcgtg
agattcgcatccacttatgtcagcgcttgcccggcagccagggtgtcagggacttcattg
agaaacgctatgtggagctaaagaaggtgaatcctgacctacccatcctaatccgcaaat
gctccgatgtgcagcccaagctctgggcccgctacgcatttggccaagagaagaatgtcc
ctttgaacaacttcagtgctgatcaggtaaccagagccctggagaacgtgctaagtggta
aagcctgaagcctccactgaggaataagagcaacagccccagagcctggcctctgctgga
cttagtataatgtgaaaaaaatgtgttctcctattcctcataaagcttgtgctgtaaaat
actttctcagggtgttcttgtcctcatctaccctctatcccttactgtgtaaccactgag
gcaaagtagcttaatataaaaataaaactttattctgcctcatcaaaaaaaaaaaaaaaa
aagaattcagaactggggccaggcatggtggcccacgcctgtaattccagcactttggga
ggccaaggcaggtggatcacttgaggtcaaaagttcaagaccagcctgaccaacatggtg
aaacctcatctctactaaaaaaaaaaaaaaaaaaaaaaaaaagattaggagaacatggtggtg
catacctgtaatccaagttacttaggaggccaaggcaggagaatcgcttgaactgggagg
cagaggtagcagtgagtcaagattgtgccacggaactccagcctgggtgacaagaatgaa
cttctgtctcaaagaaaaaagaaatcagaactggttaccttttcaaaggggatgagcaag
ggtgcatatccacgagccacttcccccttacttgactagtttgcagaagtggcattctgta
agcacgataaatttaagggtgcaaacagaacagcgcagtccactgtgggtggctgttctt
tatgtgtcagcgggagtcccaggagctgtgagaaaccagtgtgagctggctggggaggga
acgaggggctggatggggttcaggaatccacataaaaaaaaaaaaaccgacaagataaagc
aacctatctttggataggaagacaaaaaaatgagagggataaaaaaatgaggacaggctg
ggcgtggtggctcccgtctataatcccaggatttgggtaggctgaagcaggcaaatcact
tgaggtcaggagttagaaagaccacttgggcatgggtgataggaggctgtctggaaacaa
ggccggtaaggaggccacttttgaggaccaagcgagtggggcagaggcctggctgctggc
aagaaggcaacgtggacggggtagcaggaggtgagcccaaaggtgaagcaaggggcacac
tgcagtgggcacagggcaggccaggggaggcgagtgacatctctgcccagagagaacaca
caatcacaaagttcaacaccgcttacctggtgaaaccctacaattctgtcgctctgtact
cgctctgaataatgggcttgcgataagtcttacaccaattctcaaactgggggccagctg
cagcagaatcaatgccagttgcccgtagtcgtgataccaagtgtagtagttgttcacaca
gatcacgtccacatacggagcccaggaccagagcggcaaagcccgttcagcacccagaag
actgcatgactcagcactcacacactacaggggctcttctggcagagaaggtaagaaggg
gatgtaatcccagcattctggaaggctgaggcaggagagtgacttgcagccaggagttag

agaccagcctgggcaacacagcgagaccctagctctacaaaaactagtagtaagaaaatt
agcatggcacagtggctcgtgcctgtaatcccagcacactgggaggccaaggtgtgagga
tcacctgagcccaggagtttgagaccagcctggtcaacattgcaagaccccatttctaca
aaacaaaataaaacaaaacaaaacaaaaggctagaatgagaagagctgcctcctggggct
aagaacatccaagtgcattaagttagatcctgaaattaccctgccccacaggcaaaaatc
atggtcagaaagtggcccaaaggaggcaggcctgtgactgcacactgacactcatgatgt
gtgcagctgggaagggctatgagaggcagagcagctgccaatgcacagtcctcggccaaa
gcccagggcccccgccactggaactgactcctgtccgggcagcgctcccatcactgggct
tcccctcaccttgccctggagaagcactccctcctgaggggctgatgcagtcattgtcac
agagaatctctgttttgttttggagacagtctcgctctgttgcccaggctagaatggagt
ggtgcaatcttggctcagtgcaacctctgcctcccaggtcaagcactatgagcaatagcc
ggcagttcctcctcaaattaaaaatacaactactatgtcatctagcaatctcaccactgg
aaaatacaaagtacatggatgtattatgctccctgattacaaatgacatagaagagctct
agtgatccagacaatgtggtattggcattcacacaaaaatacacaacaacaagacccaac
aagggaaccaaatccaaattcatctatggtggacacatttaaaaagaacacccaggga
tgggagaccccgggctgccttgggtggggccgcaaaagtgtcctgggatgacctgtagct
gcctctcacctgggctccggatggtttccactccatgaggatttccatagtttctgttca
gccactctcagagtcaggagggcttctttggggctgtgaccatctgtcccagcctttcct
gtctccatgacatgccgtgtgacctacgtatactgcaatatgtaggtgcgtgtactgagg
gcatacagttaagatgacctacaaccgatgctactcgaagtcttccagagatttttcaga
tatccccagaccaaaacaaactagtgacattccatggttttgcaaactggcaccagcaac
tgcacagaaacagctccctatgcaccatcagacactctccagacccgcccctcgctcaca
aatacaacacaggagcatgaaccctggcagtcttcactcctaagggccacttaacagcct
ctatccatcaggaagcctttgatctgggccaggctgccgacccacaggggtgaattgtgc
ctctaatgctgagtgttgcttcccctcactgggctaaccccatccttgcatatagaagt
ttcccagggaaactgctgtctgatatccaggagctcatgtgaaatgctccagcaggccgg
gcgcggtggctcaagcctgtaatcccagcactttgggaggccgagacgggtggatcacta
ggtcaggagatcgagaccatcctggctaacacggtgaaaccccatctctactaaaaaata
caaaaaactagccgggcgaggtggcaggcgcctgtagtcccagctactcaggaggctaag
acaggagaatggcccgaacccgggaggcggagcttgcagtgagctgagatccggccacta
cactccagcctgggcgacagagtgagactccgtctcaaaaaaaaaaaaaaaaaaaaaaaa
aaaaagctccagcaacctggatggccttggccacctctagtactagcctttccctctgtg
gggttcactccctgactcttgcttgtgcaggccaacctacagcggctgagatctcctgct
ccctcagcctcctgggtcaccctgatggcatcacgctggccagaggctggtgtctgccat
caagaatgaggtacccacaggctgacttccctggtgtgggaagcaatgcctggggtctcc
agacagcccacccgaggaaaagaatccagctctgacacagctcaaagactgtgttatgga
catggagagactttccaggcctgtctcctcttctggctcagtggattcctaggaaaatta
ttgacacactgggcttaaagctctcagcctcgggcccaaaatgccacagggaatcaaatc
agtgtgaccaaggaccaaagacttctccttggctgggaatgggggatcagggttgcagag
accaggggcgcaaagtgatacctgtgccgtaagagaagaaactggggatctggtccaccc
cgggctcctcaacctgagtgtgaggccttgctgggccctgagctcacctgccccttggga
gtccattgtgactgcagtccagaggggtcaatgtcctcactgatgacagaaagaagtcct
gggggatgcggagggcatcgtgtagcggcaggcagtctgggtggtccacaggtgtgcgct
tcagcaggtcctgggaagggatgaggtggtgaattcggagccgtgcttctgggactcaaa
tgagaagcccctgggcaatgctcagtcctcaccgaaagaattcaaagggcaggccctcag
ggacccacactcatgcctgcaaaatctgaactgaaaattagaatcggagcagcagggttg
actctaaaagggccccagctggcttttctccctgctgcagtctttactcttttcctctgg
cagaggcagccaggtggacgctcatctcaccacgcagctgggagagacacccctctaccc
accctgcagctgctccaaggatactggagaccgaaagacaggggttggggaagcaaaata
ttcctgcctccactgagagcaattggccaaatgccaagctcagtagctaagctctattta
ggccctggccctgttggcatggttaccacaggccagggaggcatactcagggcccaggat
ccctctgcctgacttttcaaatggtctgttctcagtgactctggtgccctcccacaactt
acttggtcaatgggctggtacagcagagctgttggggacagcagagagaaaaaggacaaa
gccatgcaaagaatcactcaacacctcagagaccagtttgtcccccacccaccttgctcc
ccatatgaacacccgcttttgggaagccaaagtggatccacagcagcctccacctctgtt
atcactcccagggcagaccaagcagcaagaacaggtgacacacagacctggtgaccctgt
cccctccctatccatgcctactctcccatcagaactggcttcttcgacagctgccccagt
cctgtccaggccttacctggctcagagcagatgatcccctgcacctccaacctgaatcac
atggctacacccagcacatgctacagaggccgagcacactctgggtcacatccagaaact
ccacaataggggaggagacaagctgtttcccaggaagggcagctccccaggcctggtcat
tctcagactcctccagtcaaaggccaggcacagccagctgggatcaccatgcttcactct
gacccacacgactgaccttgcctcctctatgggaagagacccctgcacacagcctcaaac
ccagacataacaggacaggacacgctgggcgctagggaggccagccagatctccacacaa
aggctctgctcttaagaaggaggtagcctcaggggcaaaggtttcctgaagcaactcagg
tgaggcctcacatacaagagcccaaaagaggctgcaaaactacaagctcagccagccaag
aacccagggccacacccttctgaaacccagactccaagtgaggatcactaggattcagca
gaacagcaggacgtgacagctggaggaccaacacttaccaacacccttccagcaagcagc
tgtgcctcgcccctgcccacatgactcacccaacatcagtcttgcacctggatgcctctg
tccacagggtcatgatctttgacctcctgccatagaagtggtgagacacatgatggtggt
ctgggaagaaggatcctagactgggtgtcccataggaccaggttgtgacagatcccacag
ccaacagtcatgccccagccaacccccaaacgcaacagctccccaaagcccttgatgtaa
atagcctgtgcactgctaagcagacacctcagggaacaatctgatggctgtgaagaagat
gcagcctcaacagggttccacaagccttctgcaagcaaagccttctttgcttgggtgcct
agtcaaagcactagggaagatcttaaaagataaattcagcattgtgggaggcagaggcag
gagtacagcttgaggttaggactttaagaccagcctgagtgcaatggtgagaccctgtct
ctacaaaacacaaaaattagcatggcatggtggtacatgcccatagtcccagccacttgg
gaggctgagaagggaggattccttgagcctgggaggtcaaggttgcagtgcccatgatca
tgccactgtgttacaacctgggccacagcacaagatcctgttacaatcaaacaaacaaac
aaaaaaccaaagatgcctgttttctgccccatcaaataaacccaaatcgcagggtgacac
agggtattaacatttttaaatatctcctttcctgaagtaaatgtatagtaatggctgaaa
accactgagttatgtaaatacacttcatatgacttatttgtagccctttcgtgtctagca
atttctacagaccctctccgtgggctttggaagccggaatgggcagggccaggtgtgagg
tatcctagcacactgaggctagagcacagctcccagctcaggggacccatttctgaggc
taaggcacccaccagggactcagcctatcttcagagggaaaatcacacctcagaacccca
gagacccaaaagaaagctcgaatttggcagatacctgccagtgggtcctggagcggcagc
cccagggaaaaccgccattgcactgtcagctccctccagccttctctcaggtcccagggg
gtggaggtctctgactaagctcaaaggtctcaacaaacggcgggttgaacctctgaggag
ttattaacaggcctataattacctcaagactccacctgaccagggccaaaaatgagtcct
gggagggcagccaggaagagagcagagcagagaggaaacacagggagagggggccctggg
accacatcagatatgggggaaccaggggaacgagggcagccctgatgattgtgctcccc
ataggccctgcatgtctgtgtcccctgagaattcagggtgaggagctcactcttactctt
cacctgttcctgggctctggcaggctggcctgcttcctgccccagcctcacccactctcc
ccactcctcaccactgatgtgggcaagttgatctctctggtttgctggcctctcactctc
tgcccaatggcaccgagttaacttctttgcatgctttattttctgtgattaaaaagggaa
gcaattctcactaacgccatttctcagaggagaaaacaggcacagggatgttctatgact
tcctcatggtcaccaagctacaagtgggagctgggctttgaatggaggtctacccatgct
cctaaatttgagccgtcctccaaagaaaggacagcactgtaaaagctcagtgaatgtggc
aggagacttcactaaggcctgaccaggctggtaccctgatatccaacttctagcctccag
aactgggagatacataattctgtttttgacgagctccccaggctacggctctttgttgta
acatcctgaactaacccagaagtgcgttccatcatgtgtgtcctattccatcattcagaa
gggagtcactaacccctcagtgctgtctttgtggatgctgcccaccctcggctgagatgg
ggcacagggtctgggtctaaataaaattggaaggcactggaaggtgaaatgtcagaggag
gaggatgtctgaatttcgtttaatctcaacctctactggtgcatcatgaaagaggaggcc
cagagcaaaagtgtcttctcctgagaccacactgtggaccccaggcccactggagttctc
ttccatgctacctctcaacgcttctggtatttcttccatctgtaagtgtgtgcaatatct
acagggaacatcacatcatgagttttataagatctatctcaaacacacctcatgagttcc
ctaggaaggagatgctagcataaccccattgtgcgtgctgggaaggccccaaaacacca
aaggagttctctagggtctcacaactgctaagataaaggttgtttgacccagaactgtct
ccttaaaactgggaccctggttacatccaggtcttcccaaggtgctgaaggggcacactt
tggcataatggtgaaaggtaaggagttgacaggggagggtgagcggccagaagcccaatg
aggccttgagtaggtcttatggaaaggaggagcttacaggatggaacctacaagaagtga
cctcacttccttgctgacagaccccaacagaacttagaattctggtaactaggcacccat
attatacacacagccccatttccagctcagttggtagttgacacccaaggaacaacatg
ttctcttgctgtgtcccgacgtgctgtcgacctcctcgtcgcacaagaggccaaaatgag
agtctttctcgagaatatagacattggttcaaccctcaccctcgacgcccctggccgttt
gccagaaggcacccacaggtaacacaggacccagggcaggcccgttgaggccaggcctca
gctgtgtccatcagggcagcgctgagcccctccccatgtgtttgggggaaacaggagaag
aggggccctccctggacaggagcaggtaggttgttgggggtttggagcctgataggtatg
tcaggttcacagcccaggccatgccttcaggatgggaaggtgactgtcgaggacacagtt
tgtctgctcagatctgaattccaagacctcaacctgtcatctgccggagacctcaagctc
ctctggctggaggtgtacgcagatcctcctgtgtgccaaatcctgttggggaagggggtg
ccagggaggagttctaatggggtatcccactatggggtcagccaggcaggccactgacac
gtcttcgcctggctcactctctttgcagagctccacacagcagatcaagcaggagctgtt
ggatggattccatttctccaccttcttcaaagaagggcaggggccgcctgctaccaacca
cggccagtgctggtctggggtgagactgggcacagaagggtctccacagacagggactcc
agaaaaccagggtgggcccaggactcaaacacgaatatggggactccctaggctctgtcc

tagggctttcacacttgagtgagccactgtcctctcccaaatgaatctggctgccattag
tccccagtggcaacttcgttacaggcaaagtccctttcacctatcagaggaagatcagag
aaaatccttctgttttcactttctgttatgccaggagtatctcagcatgttgctacaaga
attgggtacaatggtaacactgtagcaacatactcatactgttaccattttaactcccca
ccctaattgtacgtctggcaaccggcagctcctctgccctgccagacctcctccaggatt
taaaatccaacacttctacacaaacaagttgggggagtggtgattacaacaaaagaaggt
gaagctactctatgcatctgtgtgtaaagtgtcatctcacagaacacatccctgcagacc
caccaggcaggagctgtacccacgcatgtcactgtaatattgcttaagggaggccatgtt
ccacagtgggtcagcctctcagtgctggtggagcctgtcgtgataaaatgtcctttctag
gcagcctttctacagggagcataatcccatgtgtgtcctaataggctggagcagtttccc
ggggctgccataacatgcacgctgactgaagggcttaaccacagaaatctggttcctcac
agttctggagactggaagtccaacgtcaagctgtcagcctggggtggtttgtctgggcct
ctctccttgccttggagatggctgacttctatctcggtcagcacagggtcttctcttcaa
cttctctgtgcgctgaccacctctccttgtaagaaccccaggcatattaaattaggggcc
accctaatgaactcacttcacctgaattacctcttggagaactctgtgtccaaacacagt
cacattctgagtctctggggttgagaatagaaacagttacatttgggggaggggacacaa
ttcaacccataaaatacataaatcccctgcggaagacacacacccgggaaagggaaatct
gggtgaaaggatgtgcagatagcaagtttcaacagctctagccagctgactttccaaaag
ggctccagctgctcacagggtcaccagcagcctctgaaggtagctgcttctttgaatgca
acctcccctgaatactaacatgttcttaactttttgccaatctgctgtctgtgtggcaga
gaaaattaccttgtttcatttccagtttcaaacatacatttgcgtaaccttttacagctt
cagtacctgcctgtgttttctcttccatgagtcgcctggcagatcctgggccttgggatg
cgcttcttcccaccctagagtcttccggcattttccccttggccagggctgggatggctg
tagctgtttctccagactcacattcatgttaaagaacaaaaacaatcatatccctttaag
tcaaagagttcttctagagcatcttgtctggtgtgctgtcactaaaataaggaggggatg
agacctccccaggccgttcccttccgtatcagaagtggagcctcaggtggactagaggga
atttcatccagtcctccctgtggaaggtgctctcttctgacccgggtgctgctgagcaag
catctgacatccaaaagcccgcgtctcctcttctccaccatgagggtgattttttgaggat
tgcagggatgatgctcttcatgcaggctgttgatacagagggtgctattgccccacagct
gacattcaccttgaaggcatgaatcattttctccttctccatcctcacatgcaattttac
cttgaaagcctagaacccctttcaccatgggcacccaggttctccacatgcacaagtccc
tgaagggttggtgctttttggtgtgagttccagatcccgatcctctggggggttcatggtg
ttacactgactcccatgtccctccttgtcccccctagtatggaaatgagatctgcaggatg
ccaactttccagcccagcacaggggagaagctgttggagtccctggttccagcctttcta
actaaacccatctcctcctatgccacctgcctgggtccctcctgggactttatcaccgtg
ccacacttttggaactactggttagaaggtgagtgtccatcccctccaaggcatggagg
tgcctctgtctcctactggctgtgtcttgaagatgcacctcttggagactcagttggctc
ctgtggaacagggagtaatgagaagatgaacctcacagggttcttgtagggactgaatga
tctaagacacagcaaacaaaggggtccgtagagcctaaaactctggacaatctgctgtgg
gtgctgtgattcatgtttattacttttctcttcccactcactgaagcagctctcagcatt
ctgccgcaatggcccatctctcttgctgtttggaaaggcacatagaaagcaagtctgcaa
tggcattgctaaaggatgtctgagtttccttctggctggactttctccttgacacagaca
gaagaggggtcccttcatgctgaaaaaggagccacaggcccactcagacatctggagagg
ctcactgggttctccaatggttggggttcactcattcaacacatacatacaaaacacctc
atttgtgcatcgttcttcttgtgacttggcctaatttcataaacaaggcagatgacaatc
cctgggcctcaattctacgcagagtcaggtgctcacagtagacagaataaacaaatcata
tctacagaatgttagaggggaaaccctcatggcctcctctacatatggtggcatcctccc
agattctgactagaatgacggagcccaacaagtataaactgggggggacttagggttctga
aaggcctcttcacccacaaaacatgggaggaaatatgtggactctggctggggagagaat
aaaggagccctggggttcatgtcttataattcccacaacaaggctgacatctggggactt
cccctgcaagaggcaaacagtgagttctgtaaatgaagacttaggtcccttgcaaaggag
aggtgaggctggggccacaagtggccactgagagacccatccccagcactgtggcttccc
agctctccctgtcctcctccacccaacatctgcccctaccctcctaaccccaggaccagg
ggaaccaaagctggagctttgatgagcaagctgctcacaaatctgcctggagctgcagtc
ttgagtgcccaggtgcacagtgctgtgctccagggccattggaaagagaatgtcagtggg
acgccggggcacacaagggtctgtagagccctgttgcatggccaggtctgccccttccag
gacagcactgatggcttggggtagggtggggctgtcctctacacaggcagcaagaggcca
gggacccagaaccacgcaagggtgccctggagggttgtgtgggagaaggccaggcctctg
actcagctgtccactccatcaccagcaccaccacctccattttgttcacctggcccccctg
aaaacacctcagtttgctgccaggccctgcaacggtcatctttctggataaagctggcct
tcaggaccttcgcctggcctggactgggcgtggaggaccatcaggaaattgtcctaggcc
agttggtgcttccggagcccaaggaagccaagccagatggtgagaagactttccgggggt
ggtgttttggggctataattcccttaaattccatccaggcatttctatacttggaaatcc
cagtgaagagtgactggaatggtgaccttttctccttttccagatcctgctccacctcct
gggcaacacgcattaacaatgctggccctggagccagcaccaccactgctggcggacctg
cggcctgctctagagccagagtcacctgtagccctggatgcacaaggatatctacattca
gcatcagcaccagcaccaggggaagggcccccctccaggaacagtgctggagccacagtca
gccccagagtcaccctgtccctgtcccgggactgtccagagccaacacactgaggagctg
ccggacatcacgaccttccctcccaggctgctggctgagcagctgacccttatggatgcg
gtgagcagctgggcttcgcaggctgtgcctctggcaccagctgtctcaggccagcctgtc
tctgaggagcggccaatgccctgggtccagtttcagccccacttcttaccaaccatggga
tctggatgagtttcctcacccacaagccttccctgtctgcatgtggacagcagagatggg
acatcgcccgtgccagctgcacagagtgactgtgcagactgaatgacgggggatggacag
aaagcaggacagggcaggtgatcactgaggggcaggcagggccatgggtcactcacccag
ctgctcaggagcctcactaccctcagcacttattaggtacctgatgcatactagattcta
tggcagacacccaaacagagcccagtgttgcagctgctgcaaggaaactgcaccagtagg
gagagaaggagtagagggctgattgggatgggaggaagatgaggcctcaggatggggagg
cctgagccaccttctagttcttggaatgaggatggcctgggagaaaatgtcactctctct
tcccacccttgttggtttctgggacatgatgcattcagggccctcggtgggcagaaaacc
aaacccagggactcccacaagtctggagcatattttaaagctttctgagctcaagctcag
ttcctgccagagactgtggatgactgtgagctcagtccctgcctgggactgtgggtgact
ctgagctggggtgtgctgtgtccatgatactctcctccttccccaaaggatctgttcaag
aaggtggagctctacgaatgcttgggctccatctggggccaacgacatcagaaggggagt
gagcacgtggcacccacagtttgtgccaccattgcacacttcaacaggctcgccaactgt
gtcaccacctcctgcctcggggaccacagcatgagggtccaggatagggccagggtggtg
gagcactggatcaaggtggccagggtaagccatggttgggccttgggattccctctctaa
aaatggggaactgcctcttctcctccatcggctttcaggattggcatctgtatctctagc
ctgagccctacacatcccctaggcccttcttcccacagcttccctgaccttgaccgccat
ggcccagtggtggctgctcacgtctgacctgggatcttccttgggttaaactgaaatctt
tctagattagtgacattcactcagccccaggtgtgccctcctgaggctccctgggcctct
gcttcattcaggaagggagatctcagcagaggggggctgaggctgaagtgggtcggactcc
aactctggacctcacagctcactcttccctctccaggagtgcctacgcctcaacaacttc
tcctcagtgcacgccatcgtctctgctctgcgcagcaacccaatacatcggctacacaag
acgtgggcaggagtgtccaggtgaggagggttccctccatgggagcaccagtgttgactt
agggacccataggtctccccatgtgccctcaacgactctgaaaggttcttggagaacagg
gacgctggaggcagggatgggctggtgggtgtggtcactaagctgccctggacttctagg
caaggatttccaactcaggactaaggattttaaccatcaggaacagactggagctaact
ggagggtgtcaggtgtttgcacccagcagtggaactgtgtccagctggaagctaactgtg
aacacgcaggggctcatgtgaagttgagatgggccaggggaggagcatgacagtcccacc
ctggtcctctggagcccttgtcatcagatgaccccactggaaactctcacgcaggaagct
gagattcactgggttttcaaacaaaagggattggaattcacaaatctccccccgtgattcc
caaatttacccttttttctttcttctatcaatagcaaaagctcaaaatatctaaaagaac
tctgcaaaaaagacactgcagtgaagagggacctgctgatcaaggtacagtggagtctgg
gagatgcgggacaagtgtttaagggtcagaggaaagagtgagtttggaagggcattgatc
ccagtgtgcagtggttattttgtaatgttttgacttacctactaaaagtggacttgaaaa
attccctccatgcctactttgggcaaacaggaggagaggtgtgtgggtcgatgggcacgt
gggggcacgggggcagcaggccctggaaataggatgtggcaatggctgctgggcttctga
gtgagggtgatgagctgcagcattagcaggactctggctcccatgctggtccatgctgct
ggcatggagcttcctccaggctggggggtggtcatggtaggtgggactttccttcttcct
caaactggcagtaattcctcaggaagccaggcctctgctgctgcttctgtctgcagcgca
cctccatgggcagggactgcagtccacactgggggaaagagggaacaacaacagaggaag
ctcatgtgccagggagtccagtagactgcccagctatgggtcccaatgggcaaactcagg
acagatgtatgtgtttgctgggactccccactctgccctttgcagacatctgaaaatggt
catgtcacaggattctcaccaattagcagtgacacatgctcatgacaagtatctgggggga
tccatgcattcctaggggatcctccctgaccagatc
