# Supplementary material for: An ancestral genomic sequence that serves as a nucleation site for de novo gene birth
Source: PLoS One. 2022 May 12;17(5):e0267864. doi: 10.1371/journal.pone.0267864 (PMC9097989; doi:10.1371/journal.pone.0267864)
Supplement: S7 Fig — (PDF) [file pone.0267864.s007.pdf]

Detection of an ancestral genomic sequence that serves as a nucleation site for de novo gene birth

Nicholas Delihas
Department of Microbiology and Immunology, Renaissance School of Medicine, Stony Brook University, Stony Brook, N.Y., United States of America

S7 Fig. Alignment of the gibbon sequence between *GGT2* and *GGT1* with the human BCRP3 sequence. The gibbon sequence: GGT2.end-endGGT1.48980500-49036766.gibbon is at the end of the file.

```
#
#
#  Percent Identity  Matrix - created by Clustal2.1
#
#

     1: GGT2.end-endGGT1.48980500-49036766.gibbon  100.00   94.55
     2: BCRP3.HUMAN.NCBI.REF                         94.55  100.00
```

```
CLUSTAL O(1.2.4) multiple sequence alignment


GGT2.end-endGGT1.48980500-49036766.gibbon      ctctgggcctcagtgtattgtgtgtgaaatggagccatctggctggggaggaatggagag 60
BCRP3.HUMAN.NCBI.REF                           ------------------------------------------------------------ 0

GGT2.end-endGGT1.48980500-49036766.gibbon      gtgggattcggagatcttcataatgcgggcactggaactagcctcagcatctttagcatg 120
BCRP3.HUMAN.NCBI.REF                           ------------------------------------------------------------ 0

GGT2.end-endGGT1.48980500-49036766.gibbon      gggaaagccaggcacgtggctgggggccaggggaaggttcacaccaagccctgccccttc 180
BCRP3.HUMAN.NCBI.REF                           ------------------------------------------------------------ 0

GGT2.end-endGGT1.48980500-49036766.gibbon      ccaccctgattcctcagactttggggccaggccctcccttactggggctgggcagtgaca 240
BCRP3.HUMAN.NCBI.REF                           ------------------------------------------------------------ 0

GGT2.end-endGGT1.48980500-49036766.gibbon      ctacctaggaccagccaccagggggtgctgcgaccctggcgctttcttaggcagagggtg 300
BCRP3.HUMAN.NCBI.REF                           ------------------------------------------------------------ 0

GGT2.end-endGGT1.48980500-49036766.gibbon      gccagctgatgctgggaacccgggtgccttctcagacccttaggcgtccagctcaccctg 360
BCRP3.HUMAN.NCBI.REF                           ------------------------------------------------------------ 0

GGT2.end-endGGT1.48980500-49036766.gibbon      ccaatgacacgggaggtgaagctgaggtccgaggaatggggactgggcaacaggctggag 420
BCRP3.HUMAN.NCBI.REF                           ------------------------------------------------------------ 0

GGT2.end-endGGT1.48980500-49036766.gibbon      gaaaacatcttggtcagagccacgcccctgggggtttccaaatgcaagcccagagtgaa 480
BCRP3.HUMAN.NCBI.REF                           ------------------------------------------------------------ 0

GGT2.end-endGGT1.48980500-49036766.gibbon      acacaagcttgtgatcctctccagagggaggcctggttctcagggaacagcaaacgggaa 540
BCRP3.HUMAN.NCBI.REF                           ------------------------------------------------------------ 0

GGT2.end-endGGT1.48980500-49036766.gibbon      gatgtccccagatcccagggatcaggggttagaccagccggggacacagcccagagggag 600
BCRP3.HUMAN.NCBI.REF                           ------------------------------------------------------------ 0

GGT2.end-endGGT1.48980500-49036766.gibbon      tgggtccggaaggaaacagctagacacagcagccttcaccatcggcagcccctccaggcc 660
BCRP3.HUMAN.NCBI.REF                           ------------------------------------------------------------ 0

GGT2.end-endGGT1.48980500-49036766.gibbon      tccctcagggtcggctccctcctctgtgcacagttccaacacctggggcagggttctggg 720
BCRP3.HUMAN.NCBI.REF                           ------------------------------------------------------------ 0

GGT2.end-endGGT1.48980500-49036766.gibbon      aagggctggtgggggcggtgatcacagcccagcacctggatatcaccaggggcactggg 780
BCRP3.HUMAN.NCBI.REF                           ------------------------------------------------------------ 0

GGT2.end-endGGT1.48980500-49036766.gibbon      gccagggcccaggtgaggccaggtcggggctatccttcaggagccccgaaaacctggtga 840
BCRP3.HUMAN.NCBI.REF                           ------------------------------------------------------------ 0
```

```
GGT2.end-endGGT1.48980500-49036766.gibbon      ttccaaacggcccacagacaaacagggttttatgcctgcagagtcaagtaccaccgggtc 900
BCRP3.HUMAN.NCBI.REF                           ------------------------------------------------------------ 0

GGT2.end-endGGT1.48980500-49036766.gibbon      tgagccctggagggctgtgtctctggggctctgcaggggtgagatggaggtgggctcaac 960
BCRP3.HUMAN.NCBI.REF                           ------------------------------------------------------------ 0

GGT2.end-endGGT1.48980500-49036766.gibbon      tggtgtacaagtcactcctcaatccttatttatttaatttttaaaaaaaatttaaacca 1020
BCRP3.HUMAN.NCBI.REF                           ------------------------------------------------------------ 0

GGT2.end-endGGT1.48980500-49036766.gibbon      atagagatggggtctcactatgttgaccaggctggtcttaactcctgacttcaagcagtc 1080
BCRP3.HUMAN.NCBI.REF                           ------------------------------------------------------------ 0

GGT2.end-endGGT1.48980500-49036766.gibbon      cccccatctcagtctcccaaagtgctaggattacaggggtgagccactgcacccggcctc 1140
BCRP3.HUMAN.NCBI.REF                           ------------------------------------------------------------ 0

GGT2.end-endGGT1.48980500-49036766.gibbon      aatccttattttggcccgagaggaaaggccgtggccccatttgcaggggagaagaccaag 1200
BCRP3.HUMAN.NCBI.REF                           ------------------------------------------------------------ 0

GGT2.end-endGGT1.48980500-49036766.gibbon      gtcggaagggcaggccttgctctgggtggcacagcagcaagagaagtgggacctggccac 1260
BCRP3.HUMAN.NCBI.REF                           ------------------------------------------------------------ 0

GGT2.end-endGGT1.48980500-49036766.gibbon      gaggcttcctcgacccaacacgctggtggggtacaccctggttctccaggtcccatgggg 1320
BCRP3.HUMAN.NCBI.REF                           ------------------------------------------------------------ 0

GGT2.end-endGGT1.48980500-49036766.gibbon      ctcagcccagggctaccttggggggtggaggacttaaatcctctccttcattctcatcac 1380
BCRP3.HUMAN.NCBI.REF                           ------------------------------------------------------------ 0

GGT2.end-endGGT1.48980500-49036766.gibbon      cccttctcccatcatttcctgaggaaggacattcagggacctgaaggggtggcctgcccc 1440
BCRP3.HUMAN.NCBI.REF                           ------------------------------------------------------------ 0

GGT2.end-endGGT1.48980500-49036766.gibbon      tccacacttgtgggtgtttctcgtcaggtgggacaagagattgagaaaagaaagagacag 1500
BCRP3.HUMAN.NCBI.REF                           ------------------------------------------------------------ 0

GGT2.end-endGGT1.48980500-49036766.gibbon      agacaaaatatagagaaagaaaagtaggcccaggggacctgcactcagcatacagaggac 1560
BCRP3.HUMAN.NCBI.REF                           ------------------------------------------------------------ 0

GGT2.end-endGGT1.48980500-49036766.gibbon      ccaggctggcaccagtctctgagttccctcagtatttatcgatcattatctctaccatct 1620
BCRP3.HUMAN.NCBI.REF                           ------------------------------------------------------------ 0

GGT2.end-endGGT1.48980500-49036766.gibbon      tggagaggggatgtggcagcacaatagggtaatagtggggagtgggtcagcaggaaaac 1680
BCRP3.HUMAN.NCBI.REF                           ------------------------------------------------------------ 0

GGT2.end-endGGT1.48980500-49036766.gibbon      atgtgaacaaatgtctctgtgtcataaacaaggttaagaagaaggtgctggccgggcgtg 1740
BCRP3.HUMAN.NCBI.REF                           ------------------------------------------------------------ 0

GGT2.end-endGGT1.48980500-49036766.gibbon      gtggctcacgcttgtaatcccagcactttaggaggccgaggtgggcggatcacgaggtca 1800
BCRP3.HUMAN.NCBI.REF                           ------------------------------------------------------------ 0

GGT2.end-endGGT1.48980500-49036766.gibbon      ggagatcgagaccacggtgaaaccccgtctctactaaaaatacaaaaaaattagccgggc 1860
BCRP3.HUMAN.NCBI.REF                           ------------------------------------------------------------ 0

GGT2.end-endGGT1.48980500-49036766.gibbon      gtggtggcgggcgcctgtagtcccagctactcggagaggctgaggcatgagaatggcatg 1920
BCRP3.HUMAN.NCBI.REF                           ------------------------------------------------------------ 0

GGT2.end-endGGT1.48980500-49036766.gibbon      aaccggggaggcggagcttgcagtgagccgagatcgcgccactgcactccagcctgggtg 1980
BCRP3.HUMAN.NCBI.REF                           ------------------------------------------------------------ 0

GGT2.end-endGGT1.48980500-49036766.gibbon      acagagcgagactccatctcaaaaaaaaaaaaaaaaaaagaagaaggtgctgtgctttga 2040
BCRP3.HUMAN.NCBI.REF                           ------------------------------------------------------------ 0

GGT2.end-endGGT1.48980500-49036766.gibbon      tatgcacatacataaacatctcaatgcattaaagagcagtattgccagcagcatgtctca 2100
BCRP3.HUMAN.NCBI.REF                           ------------------------------------------------------------ 0

GGT2.end-endGGT1.48980500-49036766.gibbon      cctccagccctaaggcggttttctcctatctcagtagatggaatatacaatcaggtttta 2160
BCRP3.HUMAN.NCBI.REF                           ------------------------------------------------------------ 0

GGT2.end-endGGT1.48980500-49036766.gibbon      cacggagacattccattgcccagggacgagcaggagacagatgccttcctcttatctcaa 2220
BCRP3.HUMAN.NCBI.REF                           ------------------------------------------------------------ 0
```

```
GGT2.end-endGGT1.48980500-49036766.gibbon      ctgcaaagaggccttcgtcttttactaatcctcctcagcacagaccctttatgggtgtca 2280
BCRP3.HUMAN.NCBI.REF                           ------------------------------------------------------------ 0

GGT2.end-endGGT1.48980500-49036766.gibbon      ggctgggggacggtcaggtctttcccttcccacgaggccatatttcaggctatcacatgg 2340
BCRP3.HUMAN.NCBI.REF                           ------------------------------------------------------------ 0

GGT2.end-endGGT1.48980500-49036766.gibbon      ggagaaacctcggacaatacctggttttcctaggcagaggtccctgtggccttctgcagt 2400
BCRP3.HUMAN.NCBI.REF                           ------------------------------------------------------------ 0

GGT2.end-endGGT1.48980500-49036766.gibbon      gttttgtgtccctgcatacttgagattagggagtggtgatgacttttaacaagcattctg 2460
BCRP3.HUMAN.NCBI.REF                           ------------------------------------------------------------ 0

GGT2.end-endGGT1.48980500-49036766.gibbon      ccttcaagcatttgtttaacaaagcacatcctgcatagcccttaatccattaaaccttga 2520
BCRP3.HUMAN.NCBI.REF                           ------------------------------------------------------------ 0

GGT2.end-endGGT1.48980500-49036766.gibbon      gttgacacagcacatgtttctgtgagcacagcgttagggctagggttacagattaacagc 2580
BCRP3.HUMAN.NCBI.REF                           ------------------------------------------------------------ 0

GGT2.end-endGGT1.48980500-49036766.gibbon      atctcaaggcaaaagaatttttcttagtacagaacaaaatggagtctcttatgtctactt 2640
BCRP3.HUMAN.NCBI.REF                           ------------------------------------------------------------ 0

GGT2.end-endGGT1.48980500-49036766.gibbon      tctacatagatagagtaacagtctgatctctctttcttttccccacagggaccttcctgg 2700
BCRP3.HUMAN.NCBI.REF                           ------------------------------------------------------------ 0

GGT2.end-endGGT1.48980500-49036766.gibbon      ctgtgcctcgggtcaggaccagaatgacacacattcctttccctgggcctttgctggggg 2760
BCRP3.HUMAN.NCBI.REF                           ------------------------------------------------------------ 0

GGT2.end-endGGT1.48980500-49036766.gibbon      gtgggggggtccctgcaccctggcctctgcctgaccaggatggtggggagaggaacggg 2820
BCRP3.HUMAN.NCBI.REF                           ------------------------------------------------------------ 0

GGT2.end-endGGT1.48980500-49036766.gibbon      atgtcccccacgctgctgtctccactgttccagctgcccagatctctgggcttccaggac 2880
BCRP3.HUMAN.NCBI.REF                           ------------------------------------------------------------ 0

GGT2.end-endGGT1.48980500-49036766.gibbon      tgcagcgggtgggtggctgggctggcctgagcccaggaatgcacttcagctcctgattga 2940
BCRP3.HUMAN.NCBI.REF                           ------------------------------------------------------------ 0

GGT2.end-endGGT1.48980500-49036766.gibbon      gcaatgtcactgaggcttgggagtcaggtgggggtgggaggaggcgtccaccccccgccc 3000
BCRP3.HUMAN.NCBI.REF                           ------------------------------------------------------------ 0

GGT2.end-endGGT1.48980500-49036766.gibbon      cccaaaagtgagaggcagtcgtgggaacagcctgcctctaaacaaccactccagtccagg 3060
BCRP3.HUMAN.NCBI.REF                           ------------------------------------------------------------ 0

GGT2.end-endGGT1.48980500-49036766.gibbon      ctgaccaggggctctggctggacacaggagcctggcaggctgtgtggcctgtaaggacac 3120
BCRP3.HUMAN.NCBI.REF                           ------------------------------------------------------------ 0

GGT2.end-endGGT1.48980500-49036766.gibbon      agtctgtctctgtgcctcagtttctctgctgcctagatgggggggcctggactccaggtg 3180
BCRP3.HUMAN.NCBI.REF                           ------------------------------------------------------------ 0

GGT2.end-endGGT1.48980500-49036766.gibbon      tagacatctggagcaggcagtgttcagctggggaggaagcgtggaggactgtgggggcca 3240
BCRP3.HUMAN.NCBI.REF                           ------------------------------------------------------------ 0

GGT2.end-endGGT1.48980500-49036766.gibbon      catgggaaggattccagctcacatcacctgcaccgctgctgagcctagtcaacagagccc 3300
BCRP3.HUMAN.NCBI.REF                           ------------------------------------------------------------ 0

GGT2.end-endGGT1.48980500-49036766.gibbon      ctcagtgggtcctcactctcctggctacctcccatttaggcaccctgaggcctggggaga 3360
BCRP3.HUMAN.NCBI.REF                           ------------------------------------------------------------ 0

GGT2.end-endGGT1.48980500-49036766.gibbon      acagagccaggccagtgtccccagagaggctgcgctgccagcacagtaatagtggatttg 3420
BCRP3.HUMAN.NCBI.REF                           ------------------------------------------------------------ 0

GGT2.end-endGGT1.48980500-49036766.gibbon      gattcagggaagcagacctgcagccaaggtgggaaagagctgcaggcagggtgggcccac 3480
BCRP3.HUMAN.NCBI.REF                           ------------------------------------------------------------ 0

GGT2.end-endGGT1.48980500-49036766.gibbon      acatggcacagccccttccctggaggtccatgctgcatttccaggacagcaagtcgcag 3540
BCRP3.HUMAN.NCBI.REF                           ------------------------------------------------------------ 0

GGT2.end-endGGT1.48980500-49036766.gibbon      ggatggatggtgccgggtaccaagggctagaggcatggtctgtctgcattccccacatgg 3600
BCRP3.HUMAN.NCBI.REF                           ------------------------------------------------------------ 0

GGT2.end-endGGT1.48980500-49036766.gibbon      gcgtcttgtagtcaccagcatgtgatgctatcaagtccccctgtcctctgtgcagactgg 3660
```

```
BCRP3.HUMAN.NCBI.REF                              ------------------------------------------------------------ 0

GGT2.end-endGGT1.48980500-49036766.gibbon         gaagcccttggtcaccctgggagggttgggagacccaggccaggctgcagaagcataagg 3720
BCRP3.HUMAN.NCBI.REF                              ------------------------------------------------------------ 0

GGT2.end-endGGT1.48980500-49036766.gibbon         acttgaacccgggtcctgagtggcaccaccttgggtcctccccgccatctgtgttcagct 3780
BCRP3.HUMAN.NCBI.REF                              ------------------------------------------------------------ 0

GGT2.end-endGGT1.48980500-49036766.gibbon         ccaccttgatgctgactaggctgggccatgcagagagggttaggggatagaggtgggagc 3840
BCRP3.HUMAN.NCBI.REF                              ------------------------------------------------------------ 0

GGT2.end-endGGT1.48980500-49036766.gibbon         tggggagcgggactccactctgggagcggggcagccttgccggatccaggggagagagtt 3900
BCRP3.HUMAN.NCBI.REF                              ------------------------------------------------------------ 0

GGT2.end-endGGT1.48980500-49036766.gibbon         gagcggtcccagctctgctttcccagagctgccgggaacccgggtaatggtgtggaggtt 3960
BCRP3.HUMAN.NCBI.REF                              ------------------------------------------------------------ 0

GGT2.end-endGGT1.48980500-49036766.gibbon         cttgggagccctgcccctacctggcaaccgcagtgcagcaggcaccaaattctgcacatt 4020
BCRP3.HUMAN.NCBI.REF                              ------------------------------------------------------------ 0

GGT2.end-endGGT1.48980500-49036766.gibbon         gggacagtgtgaccctgggttctggcgggcggtaggtggggctttgggacctaccggcag 4080
BCRP3.HUMAN.NCBI.REF                              ------------------------------------------------------------ 0

GGT2.end-endGGT1.48980500-49036766.gibbon         tgagggagttaacacagcagctgactcctctaggcaaggaaaactcccctcagaggcttt 4140
BCRP3.HUMAN.NCBI.REF                              ------------------------------------------------------------ 0

GGT2.end-endGGT1.48980500-49036766.gibbon         gctgcctggcctcctgccgggaacaagcaggagctaaaaactagaagttgaggcatgagt 4200
BCRP3.HUMAN.NCBI.REF                              ------------------------------------------------------------ 0

GGT2.end-endGGT1.48980500-49036766.gibbon         ttggacactctgtggtgtgcatctggggagggcagcagcgcgccacagctgccagccacc 4260. 4207 bp
BCRP3.HUMAN.NCBI.REF                              ------actccgtagtgtgcacttggtgagggcagcagctcgccacagctg-------cc 47
                                                        **** ** *******  *** ************ ***********       **

GGT2.end-endGGT1.48980500-49036766.gibbon         agccgtctgtccattcacccatctgtccatctggcagcctgctgttcagacctgtctgtc 4320
BCRP3.HUMAN.NCBI.REF                              agccgtctgtccattcacccatctgtccatctggcagcccgctgttcagacccgtctgtc 107
                                                  *************************************** ************ *******

GGT2.end-endGGT1.48980500-49036766.gibbon         tgtccgcccatctataagcccatctctgtcccattgtctatctgactatctttctcttac 4380
BCRP3.HUMAN.NCBI.REF                              tgtccgcccatctgtaagcccatctctgtcccattgtctatctgaccatctttctcttac 167
                                                  ************* ******************************** *************

GGT2.end-endGGT1.48980500-49036766.gibbon         cgtcctctccgtccagctatctggcctgtctgttgatccatcttcatgtctgtc-tgtgg 4439
BCRP3.HUMAN.NCBI.REF                              tgtcctctttgtctagctatctggcctatctgtcgatccatcttcgtgtctgtcttcagc 227
                                                   *******  *** ************* ***** *********** ******** *  *

GGT2.end-endGGT1.48980500-49036766.gibbon         ccccacctgtttgtccatctgtccaattacttgtgagtctatctatgcttcttcttgtcc 4499
BCRP3.HUMAN.NCBI.REF                              ccccacctgtttgtccatctgtccaattacctgtgagtctatctatgcaccttcttgtcc 287
                                                  ****************************** *****************  **********

GGT2.end-endGGT1.48980500-49036766.gibbon         actcatctgcccacccatctgtccctctgtctgcccaccggcctcccctctcctcctggg 4559
BCRP3.HUMAN.NCBI.REF                              attcatctgcccacccatctgtccctccgtctgcccaccggcctcccctctccttctggg 347
                                                  * ************************* ************************** *****

GGT2.end-endGGT1.48980500-49036766.gibbon         ctgcagagccatggcccgggactgcggagccacggttggcctggtcctgctggggctggg 4619
BCRP3.HUMAN.NCBI.REF                              ccgcagagccatggcccaggactgcagagccatggttggcctggtcctgctggggctggg 407
                                                  * *************** ******* ****** ***************************

GGT2.end-endGGT1.48980500-49036766.gibbon         gctagcgctggctgtcattgtgctggctgtggtcctctctcgacaccaggccccttgtgg 4679
BCRP3.HUMAN.NCBI.REF                              gcttgtgctggctgtcattgtgctggctgtggtcctctctcgacaccaggccccatttga 467
                                                  *** * ************************************************ * **

GGT2.end-endGGT1.48980500-49036766.gibbon         ctcccaggcctttgcccaggctgctgttgccgccgactccaaggtctgctcaaatattgt 4739
BCRP3.HUMAN.NCBI.REF                              c-ccccggcctttgcccacgccgctgttgctgctgactccaaggtctgctcggatattgg 526
                                                  * *** ************ ** ******** ** *****************  ******

GGT2.end-endGGT1.48980500-49036766.gibbon         acggtgagtgagatgtgggaggaagccgggtggcccttggcagccagcccctcctggaga 4799
BCRP3.HUMAN.NCBI.REF                              acggtgagtgagacgtgggaggaagctgggtggcccttggcagccagcccctcctggaga 586
                                                  ************* ************ *********************************

GGT2.end-endGGT1.48980500-49036766.gibbon         aggcgttgtgtgtgagagagtgtgtgtgtgagcgtgtgtgtgtgtgtgtgattatgtg 4859
BCRP3.HUMAN.NCBI.REF                              aggc---gtgtgtgtgagagtgtgtgtgtgagcatgtgtgtgtgtgagagagtatgtg 643
                                                  ****   ******* *************** * *  ************ * ** ******

GGT2.end-endGGT1.48980500-49036766.gibbon         tgagtgtgagtgtgtgggtatatgtgtgagtgtga-gtgtgtgggggtgtgtgtgtgtga 4918
BCRP3.HUMAN.NCBI.REF                              tcagtgtgtgtgggtatatgagtgtgagtgtggggtgtgggtgtgtgtgaatgtgtgtga 703
                                                  * ****** *** **   *   **** * *** *  *** *** * ***  *********

GGT2.end-endGGT1.48980500-49036766.gibbon         ttgtgtttgggtgtgtgtatgtgtgagtttgtggaggtgtgtgagagtatatgtgagtgt 4978
BCRP3.HUMAN.NCBI.REF                              tcgtgtttgggtgtgtgtatgtgtgagtgtg----ggtgtgtgtgaatgtgtgtgagtgt 759
                                                  * ************************** **    ******** ** * * *********

GGT2.end-endGGT1.48980500-49036766.gibbon         gagtgtgtg--------------------------------gggagtgtgggtgggtgt 5005
BCRP3.HUMAN.NCBI.REF                              gtttgtgtgtatgtgtgagtgtgggtgggggtatatgagtgtgagtgtgtgggtgggtgt 819
```

```
                                             *  ******                               * * **************

GGT2.end-endGGT1.48980500-49036766.gibbon      gaatgtgtgtgattgtgtttcggtgtgtgagggtgtgtgtgactgtgaatgtgtgagtgt 5065
BCRP3.HUMAN.NCBI.REF                           gaacgtgtgtgattgtgttttgctgtgtgagggtgtgtgtgactatgagtgtgtga---- 875
                                             *** **************** * ********************* *** *******

GGT2.end-endGGT1.48980500-49036766.gibbon      gagtgtgtgggtgtgtgtaaatgtgtgtgattgtgtgtgagtgtatgtgtgggtgtttgt 5125
BCRP3.HUMAN.NCBI.REF                           ----gtgtgggtgtgtgtaaatgtgtgtgat--tgtgtgagtgtatgtgtgggtgtgagt 929
                                                 ***************************  ***********************  **

GGT2.end-endGGT1.48980500-49036766.gibbon      gggtgagtgtgtcagtatggggggtgtgggtgtgtgtgaatgtgtgtgattgtgtgtggc 5185
BCRP3.HUMAN.NCBI.REF                           gtgtgagtgtgagtat---gggggtgtgggtgtgtgtgaatgtgcgtgattgtgtgtggg 986
                                             * *********    *   ************************* **************

GGT2.end-endGGT1.48980500-49036766.gibbon      tatgtgtatgggtgtgtgtgtgcgcgt------gtgtgtgtgcacgtgcactggcccagg 5239
BCRP3.HUMAN.NCBI.REF                           tatgtgtgtgtgtgtgtgagtgtgtgtgtgtgcgtgtgtgtgcacgtgcactggcccagg 1046
                                             ******* ** ******* *** * **      ***************************

GGT2.end-endGGT1.48980500-49036766.gibbon      aagcaggagccgtgtgtgtgtggtgtgggcttcagcacctgcagggcttgggcacaagga 5299
BCRP3.HUMAN.NCBI.REF                           cagcaggagccatgt-------gtgtgggcttcagcacctgcagggcttgagcgcaagga 1099
                                              ********** ***        **************************** ** ******

GGT2.end-endGGT1.48980500-49036766.gibbon      ggcagcctcagggcccttgcacagaacaggtggcagggtgtgcccgtggggcagatgggg 5359
BCRP3.HUMAN.NCBI.REF                           gacagcctcagggcccttgcacagaacaggcggcagggtgtgcccgtggggcagatgggg 1159
                                             * **************************** *****************************

GGT2.end-endGGT1.48980500-49036766.gibbon      acttggggacaatggtggtgtgtgaatccatacctggctccaggattcaggaggcccatt 5419
BCRP3.HUMAN.NCBI.REF                           acttggggacaatggtggtgtgtgagtccatacctggctccaggattcaggaggcccatt 1219
                                             ************************* **********************************

GGT2.end-endGGT1.48980500-49036766.gibbon      tgcatatcccaggtgggaacctgtctggcccaacctgaccctgctggccggtgcaggccc 5479
BCRP3.HUMAN.NCBI.REF                           tgcacatcccaggtgggaacctgtctggccccggctgaccctgctggccggtgcaggccc 1279
                                             **** ***************************   ***************************

GGT2.end-endGGT1.48980500-49036766.gibbon      cttcagtgaggccaattctccaaggctagggtcttctcccagggtcataggtgaaggggt 5539
BCRP3.HUMAN.NCBI.REF                           cttcagtgaggccaattctccaaggctgcggtcttctcccagggtcatgggtgaaggggt 1339
                                             ***************************  ******************* ***********

GGT2.end-endGGT1.48980500-49036766.gibbon      ttggaggctccctgcgtgggtactggcctgctggggt--acacgatgctgccatagccag 5597
BCRP3.HUMAN.NCBI.REF                           ttggaggctccctgcgtgggtactggcctgctgggttacacacaatgctgccatagccag 1399
                                             ************************************ *   **** ****************

GGT2.end-endGGT1.48980500-49036766.gibbon      tctgccccaacacccagcccggggccacgtctcgggtctctcagtcctggggagccccgt 5657
BCRP3.HUMAN.NCBI.REF                           tctgccctacacccagcctggggccacatctcaggtctctcagtcctgaggagcccggt 1459
                                             ******** ********** ******** **** *************** ******* **

GGT2.end-endGGT1.48980500-49036766.gibbon      gccccacccctcacatcctctctcc--gagtcagggcctgggtctcgtgagctgagtgac 5715
BCRP3.HUMAN.NCBI.REF                           gccccacccctcacatcctctctccctgagtcagggcctgggtctcgtgagctgagtgac 1519
                                             *************************  **********************************

GGT2.end-endGGT1.48980500-49036766.gibbon      tgatacttggtgtcctggatgagggtgtgatggagaggggccacagtgggtgtttcctga 5775
BCRP3.HUMAN.NCBI.REF                           tgatacttggtgtcctggatgagggcgtgatggagaggggccacagcgggtgtttcctga 1579
                                             ************************* ********************* *************

GGT2.end-endGGT1.48980500-49036766.gibbon      ccctcttccaggaaggtgctgctgccgctgcagagaggacacacacaggatgccccttcc 5835
BCRP3.HUMAN.NCBI.REF                           ccctcttccaggaaggtgctgctgccgctgcagggaggacacatacaggatgccccttcc 1639
                                             ********************************* ********* ****************

GGT2.end-endGGT1.48980500-49036766.gibbon      tgcccccctgcctcccattgggcccacgaaatccagggcaagcctcccctccctgccagcc 5895
BCRP3.HUMAN.NCBI.REF                           tgcccccctgcctcccattgggcccacaaaagccagggcaagcctcccctccctgccagcc 1699
                                             *************************** *** ******************************

GGT2.end-endGGT1.48980500-49036766.gibbon      acctggtctgcttcccataaattctgtccttcaggctgttgggaggatcccagtgctttg 5955
BCRP3.HUMAN.NCBI.REF                           acctggtctgcttcccagaaattctgtcttgcaggctgttgggaggatcccagtactttg 1759
                                             ***************** ********** * *********************** *****

GGT2.end-endGGT1.48980500-49036766.gibbon      taaactaaagcaagggaggagtggccattctctctctttgttcattcattcaccttttca 6015
BCRP3.HUMAN.NCBI.REF                           taaactaaagcaagggaggagtggcgttctctc----tgttcattcattcaccttttca 1815
                                             ************************* *******    **********************

GGT2.end-endGGT1.48980500-49036766.gibbon      ttcattccttcctccctccattcccccatctgtccatccttccctgccctgattgctcat 6075
BCRP3.HUMAN.NCBI.REF                           ttcattccttcttccctccattcccccatctgtccatccttccctgccctgattgctcat 1875
                                             *********** ************************************************

GGT2.end-endGGT1.48980500-49036766.gibbon      gcca-----cccgccagcccctcctgacctggtcctttggtttctcttcagggcttcctg 6130
BCRP3.HUMAN.NCBI.REF                           gccaccgcccccccgcagcccctcctgacctggtcctttggtttctcttcagggatttctg 1935
                                             ****     ***  ************************************** ** ***

GGT2.end-endGGT1.48980500-49036766.gibbon      tctcctcccacagggctgagaatggcagctcagggacaagtgggcgctggggactgctta 6190
BCRP3.HUMAN.NCBI.REF                           tctcctcccacagggctgagaatggcagctcagggacaagtaggggctggggactgctta 1995
                                             ***************************************** ** ***************

GGT2.end-endGGT1.48980500-49036766.gibbon      gtctccccagtggctcccaggggattcgagggattgatgccagctgccaccccaggctgt 6250
BCRP3.HUMAN.NCBI.REF                           gtctccccagtggctctcaggggatttgagggtttgacgccagctgccaccccaggctgt 2055
                                             **************** ********* ***** **** **********************

GGT2.end-endGGT1.48980500-49036766.gibbon      gccgctcctatgctcgggaggacatacagagatgcggcacccacttaaactcgaagttgc 6310
BCRP3.HUMAN.NCBI.REF                           gcccctcctctgctcaggaggacatacag-gatgcaacacccacttaaactcgaagttgc 2114
                                             *** ***** ***** ************* *****  ***********************

GGT2.end-endGGT1.48980500-49036766.gibbon      aaagatgcaaatgagactggagtctcaggcaccagagaccacccgtgggcacgtggcttt 6370
BCRP3.HUMAN.NCBI.REF                           aaagatgcaaatgagactggggtctcaggcaccagagaccacccgtgggcacgtggcttt 2174
                                             ******************** ***************************************
```

```
GGT2.end-endGGT1.48980500-49036766.gibbon      tgggagtggggacctgctgccacagatctctgaggagactggacctgctgggtctccccg 6430
BCRP3.HUMAN.NCBI.REF                           tgggattggagacctgctgccacagatctctgaagagtctggacctgctgggtctcccca 2234
                                               ***** *** *********************** *** *********************

GGT2.end-endGGT1.48980500-49036766.gibbon      agggactgtctgggggtctccatagcatgccctgctgtgtgcgtgacagtcagtggttgg 6490
BCRP3.HUMAN.NCBI.REF                           agtgactctctgggggtctccatagcatgccctgctgtgtgcatgacggtcactggttgg 2294
                                               ** **** ********************************** **** **** *******

GGT2.end-endGGT1.48980500-49036766.gibbon      ctagggggtctctactctaaagctccctctgctgacactccctcaaactgtcccttggtg 6550
BCRP3.HUMAN.NCBI.REF                           gta-ggggtctctactctaaagctccctctgccggcatcccctcgaactctcccttggtg 2353
                                                ** **************************** * **  ***** **** **********

GGT2.end-endGGT1.48980500-49036766.gibbon      aagagagaggatgtggtttgccccagtgttttgtcagacaactctctccacttcctgttt 6610
BCRP3.HUMAN.NCBI.REF                           aagagagaggatgtggtttgccccagtgttttatcaaacaactctctccacttcctgttt 2413
                                               ******************************** *** ***********************

GGT2.end-endGGT1.48980500-49036766.gibbon      taagaagctgggagtggaagagagcctggggctggccccagctgctgctgcggaacaggg 6670
BCRP3.HUMAN.NCBI.REF                           taagaagctgggagtggaagagagcctggggctggccccagctgctgctgcgaaacaggg 2473
                                               **************************************************** *******

GGT2.end-endGGT1.48980500-49036766.gibbon      gtcactgggaacaggggtcactggacgctgggaccctggccgggctggctggggggcctc 6730
BCRP3.HUMAN.NCBI.REF                           gtcactgg----------------acgctgggaccctggccgggctggct-ggaggcctc 2516
                                               ********                ************************** ** ******

GGT2.end-endGGT1.48980500-49036766.gibbon      aggaagaggcctgctgcagcatcatcctggccaagattcctccttgcagaggacgctggc 6790
BCRP3.HUMAN.NCBI.REF                           aggaagaggcctgctacagtgtcatcctggccaagattcctccctgcagaggaccctggc 2576
                                               *************** ***  ********************** ********** *****

GGT2.end-endGGT1.48980500-49036766.gibbon      cacgctgccacagggtctgctggggccaccagaagcccatgctcctgcctccatctctcc 6850
BCRP3.HUMAN.NCBI.REF                           cacgctgccacagggtctgctggggccaccagaagcccatgctcctgcctccatctctcc 2636
                                               ************************************************************

GGT2.end-endGGT1.48980500-49036766.gibbon      cctctgtgctcccctctcaccaggaggccctcccagagtccagtctcctgcttttttttt 6910
BCRP3.HUMAN.NCBI.REF                           cctctgtgctcacctctcaccaggaggccctcccagagttcagtgtcctgcttttttttt 2696
                                               *********** *************************** **** ***************

GGT2.end-endGGT1.48980500-49036766.gibbon      ttttttttttttgagacaatgtctcgctctgtcaccaggctggagtgcagtggcacgatc 6970
BCRP3.HUMAN.NCBI.REF                           tttttttt-----agatggtgtctcgttctgtcaccaggctggagtgcagtggcgcgatc 2751
                                               ********     ***   ******* *************************** *****

GGT2.end-endGGT1.48980500-49036766.gibbon      tcggctcactgcaacctctgcctcctcggttcaaatgattctcctgcctcagcctcctga 7030
BCRP3.HUMAN.NCBI.REF                           tcagctcactgcaacctctgcttccttggttcaaatgattctcctgcctcagcctcctga 2811
                                               ** ****************** **** *********************************

GGT2.end-endGGT1.48980500-49036766.gibbon      gtagctgggactacaggtgccagccaccacgcccagctaatttttgtatttttagtagag 7090
BCRP3.HUMAN.NCBI.REF                           gtagctgggactacaggtgccagccaccacgcccaggtaatttttgtatttttagtagag 2871
                                               ************************************ ***********************

GGT2.end-endGGT1.48980500-49036766.gibbon      acggggtttcaccatgttggccagggtggtctctatctcttgacctcgtgatttgcccgc 7150
BCRP3.HUMAN.NCBI.REF                           acggggtttcaccatgttggccaggatggtctctatctct--------tgattcgcccgc 2923
                                               ************************* **************        ***** ******

GGT2.end-endGGT1.48980500-49036766.gibbon      ctcagcctcccaaagtgctgggattacaggagtgagtcatggcgcccggccccatctcct 7210
BCRP3.HUMAN.NCBI.REF                           cttggcctcccaaagtgctggaattacaggagtgagtcatggcacccggcctcatctcct 2983
                                               **  ***************** ********************* ******* ********

GGT2.end-endGGT1.48980500-49036766.gibbon      actctttctttcagcaccaggttttattcttgggattctgctacagccggagcccctggg 7270
BCRP3.HUMAN.NCBI.REF                           ac----tctttcagcaccaggttttactcttgggattctgctacagccgcagcccctggg 3039
                                               **    ******************* *********************** **********

GGT2.end-endGGT1.48980500-49036766.gibbon      tgcaagttcctaaggtttctgtgagtgtggacccagcaccgtgcctagtagacacacaaa 7330
BCRP3.HUMAN.NCBI.REF                           tgcgagttcctaagctttctgtgagtgtggacccagcaccgtgcctagtagacatacaaa 3099
                                               *** ********** *************************************** *****

GGT2.end-endGGT1.48980500-49036766.gibbon      aggagcatggtgacagtga-gtctgtcatctccagcataacgactgttttgatccttgta 7389
BCRP3.HUMAN.NCBI.REF                           aggagcatggtgacagtgaggtctgtcatctccagcataatgactgttttgatccttgta 3159
                                               ******************* ******************** *******************

GGT2.end-endGGT1.48980500-49036766.gibbon      aaaaaggtgatttttggctggatgtggtggctcacacctgtaatcccagcactttgggag 7449
BCRP3.HUMAN.NCBI.REF                           aaaaaggtgatttttggctgggtgtggtggctcacacctgtaatcccagcactttgggag 3219
                                               ********************* **************************************

GGT2.end-endGGT1.48980500-49036766.gibbon      gccaaggcgggtggatcattggaggtcaggagttggagaccagcctgggcaacatggtga 7509
BCRP3.HUMAN.NCBI.REF                           gccgatgggggtggctcacttgaggtcaggagttggagcccagcctgggcaacatggtga 3279
                                               *** * * ****** *** * ***************** *********************

GGT2.end-endGGT1.48980500-49036766.gibbon      aaccccgtctctactaaaaatacaaaaattagccaggcatggtagcgggtgcctgtaatc 7569
BCRP3.HUMAN.NCBI.REF                           aaccacgtctctactaaaaatacaaaaattagctgggcatggtaacggatgcctgtaatc 3339
                                               **** ****************************  ********* *** ***********

GGT2.end-endGGT1.48980500-49036766.gibbon      ccagctacttgggaggctgaggcaggagaattgcttgaacccgggaggcaaaggctgcag 7629
BCRP3.HUMAN.NCBI.REF                           ccagctacttgggaggctgagacaggagaatcacttgaacccaggaggcaaaggttgcgg 3399
                                               ********************* *********  ********* *********** *** *

GGT2.end-endGGT1.48980500-49036766.gibbon      taagccaagatcgtaccactgcactccagcctcggtgacatagcaggacttggtctcaaa 7689
BCRP3.HUMAN.NCBI.REF                           taagccaagattgtaccactgcactccagcctgggtgacagagcaagacttggtctcaaa 3459
                                               *********** ******************** ******* **** **************

GGT2.end-endGGT1.48980500-49036766.gibbon      a-------------aaagacagaaaagtttatatttttgttctaatggttatcttaatat 7736
BCRP3.HUMAN.NCBI.REF                           aaaaaaaaaaaaagaaagaaagaaaagtttatatttttgttctaatggttatcttaatat 3519
                                               *             ***** ****************************************
```

```
GGT2.end-endGGT1.48980500-49036766.gibbon      cttcatcctataat--tatgttttgtataattataatagctatataagatacactacccc 7794
BCRP3.HUMAN.NCBI.REF                           cgtcattctataattgtatgttttatataattataatagctatataagatataatacccc 3579
                                               * **** *******  ******** ************************** * ******

GGT2.end-endGGT1.48980500-49036766.gibbon      tagtacattgttttttggatattctattcgcccctgatggttaatttatgtgtcaacttg 7854
BCRP3.HUMAN.NCBI.REF                           tagtatgttgttttttggatattctacttgctcctgatggttaatttatatgtcaacttg 3639
                                               *****  ******************* * ** ***************** **********

GGT2.end-endGGT1.48980500-49036766.gibbon      gctaagctatggtgccccgttgtttggtcaaatacttgtcaatatcttgctgggaggtta 7914
BCRP3.HUMAN.NCBI.REF                           gctaagctatggtgccccgttgtttggtcaaatacttgtcaatatcttgctgggaggtta 3699
                                               ************************************************************

GGT2.end-endGGT1.48980500-49036766.gibbon      tttcatagatgtgatgaacattgacagtcagttgactttaagtaaaacagattacccacc 7974
BCRP3.HUMAN.NCBI.REF                           tttcatagatgtgattaacactgacagtcagttgactttaagtaaaacagattacccacc 3759
                                               *************** **** ***************************************

GGT2.end-endGGT1.48980500-49036766.gibbon      ataatatgggtgggccacctccaatcagttgaaggccttaagaacaaaaactgaggtttc 8034
BCRP3.HUMAN.NCBI.REF                           ataatatgggtgggccacctccaatcagttgaaggccgtaagaacaaaaactgaggtttc 3819
                                               ************************************* **********************

GGT2.end-endGGT1.48980500-49036766.gibbon      ccagagaagcaggaattccgcttcaagactgtaacacacaaaccctgcctgagtttctgg 8094
BCRP3.HUMAN.NCBI.REF                           ccagagaagcaggaattctgcctcaagactgtaacacacaaaccctgcctgagtttctgg 3879
                                               ****************** ** **************************************

GGT2.end-endGGT1.48980500-49036766.gibbon      cctgctgactgctctacagatgttaggttccagacttcgagatcaactcttacctgaatt 8154
BCRP3.HUMAN.NCBI.REF                           cctgctgactgctctacagagtttaggttccagacttcgagatcaactcttacctgaatt 3939
                                               ********************  **************************************

GGT2.end-endGGT1.48980500-49036766.gibbon      tatagcctgctggcttgccctacagattttaaacttgctagtccccacaatcatgtgagc 8214
BCRP3.HUMAN.NCBI.REF                           tatagcctgctggcttgccctacagattttaaacttgctagtccccacaatcatgtgagc 3999
                                               ************************************************************

GGT2.end-endGGT1.48980500-49036766.gibbon      caattcctaaataaatctctctctgtgtataacctattggtttagtttctctaaaaagct 8274
BCRP3.HUMAN.NCBI.REF                           caattcctcaataaatctctctctatgtataatctattggtttagtttctctgaaaagct 4059
                                               ******** *************** ******* ******************* *******

GGT2.end-endGGT1.48980500-49036766.gibbon      ttcacatccagtttcctggatgttaagtaatactgaaactagctagtaacttcttttttt 8334
BCRP3.HUMAN.NCBI.REF                           ttcacatccagtttcctggatgttaagaattactgaaactagctagtaacttcttttttt 4119
                                               *************************** * ******************************

GGT2.end-endGGT1.48980500-49036766.gibbon      tttttttttttttttgagacagagtttgctcttttttttttttttttttttgggttaatt 8394. 8358 bp
BCRP3.HUMAN.NCBI.REF                           ttttttttttttttttttgaga-------------------------------------- 4142
                                               ***************     ***

GGT2.end-endGGT1.48980500-49036766.gibbon      actatatattttatatgagtgcttgtttaagccaatccaaatagaatttcttaagggatt 8454
BCRP3.HUMAN.NCBI.REF                           ------------------------------------------------------------ 4142

GGT2.end-endGGT1.48980500-49036766.gibbon      tctggctaactatgtcagattttttttttaattaattttttttgcacacaaaacaataaa 8514
BCRP3.HUMAN.NCBI.REF                           ------------------------------------------------------------ 4142

GGT2.end-endGGT1.48980500-49036766.gibbon      cattttctaaaaatacatacaaacaaaaagatgcgtatcaaacatattaggaaggttaca 8574
BCRP3.HUMAN.NCBI.REF                           ------------------------------------------------------------ 4142

GGT2.end-endGGT1.48980500-49036766.gibbon      catgggaagtcggggaatagaaatggggggtgggagttaaaataaatgagagagggactt 8634
BCRP3.HUMAN.NCBI.REF                           ------------------------------------------------------------ 4142

GGT2.end-endGGT1.48980500-49036766.gibbon      tatatggatcagtgataataactcaatcctctatttgacaaagaagagggagtaagaaga 8694
BCRP3.HUMAN.NCBI.REF                           ------------------------------------------------------------ 4142

GGT2.end-endGGT1.48980500-49036766.gibbon      ggaagaaaaagaaagtgggataaaggatcagaaagggaggaaatagaaaaaattagagt 8754
BCRP3.HUMAN.NCBI.REF                           ------------------------------------------------------------ 4142

GGT2.end-endGGT1.48980500-49036766.gibbon      atgactccagggtagacctgttttgttgtcactgagttggttggttggtttgtctgttgt 8814
BCRP3.HUMAN.NCBI.REF                           ------------------------------------------------------------ 4142

GGT2.end-endGGT1.48980500-49036766.gibbon      atttttcatgtttcgccaagttggccagactggtctcgaactcctagcccaaagtgatca 8874
BCRP3.HUMAN.NCBI.REF                           ------------------------------------------------------------ 4142

GGT2.end-endGGT1.48980500-49036766.gibbon      acccgcctcgccccccagagtgccgggaccacaggcgtgagccaccacgtccagccccca 8934
BCRP3.HUMAN.NCBI.REF                           ------------------------------------------------------------ 4142

GGT2.end-endGGT1.48980500-49036766.gibbon      cattgcttctggcctccgtggtagacctcccagacggagcggccaggcagaggagctcct 8994
BCRP3.HUMAN.NCBI.REF                           ------------------------------------------------------------ 4142

GGT2.end-endGGT1.48980500-49036766.gibbon      cacttcttcccagacacggggcggccgggcagaggcgctcctcacttcccagacggggcg 9054
BCRP3.HUMAN.NCBI.REF                           ------------------------------------------------------------ 4142

GGT2.end-endGGT1.48980500-49036766.gibbon      gccaggcagagacgctcctcacttcttcccagacgataagtggtcgggcagaggcgcccc 9114
BCRP3.HUMAN.NCBI.REF                           ------------------------------------------------------------ 4142

GGT2.end-endGGT1.48980500-49036766.gibbon      tcacttcccagacgatgggtggccgggcagaggcgctcctcacttcccagacgatgggtg 9174
```

```
BCRP3.HUMAN.NCBI.REF                             ------------------------------------------------------------ 4142

GGT2.end-endGGT1.48980500-49036766.gibbon        gccgggcagaggcgctcctcacttcccagacggggcggccgggcagaggcgctcctcact 9234
BCRP3.HUMAN.NCBI.REF                             ------------------------------------------------------------ 4142

GGT2.end-endGGT1.48980500-49036766.gibbon        tcccagacggtgggtggccgggcagaggcgctcctcacttcccagacggtgggtggccgg 9294
BCRP3.HUMAN.NCBI.REF                             ------------------------------------------------------------ 4142

GGT2.end-endGGT1.48980500-49036766.gibbon        ggagaggctctcctcacttcccagacggggcggccgggcagaggtgctcctcacttccca 9354
BCRP3.HUMAN.NCBI.REF                             ------------------------------------------------------------ 4142

GGT2.end-endGGT1.48980500-49036766.gibbon        gagggtgggtggccaggcagaggcactcctcacttcccagacaatgggtggccgggcaga 9414
BCRP3.HUMAN.NCBI.REF                             ------------------------------------------------------------ 4142

GGT2.end-endGGT1.48980500-49036766.gibbon        ggctctcctcatttcccagacggggcggccgggcagaggtgctcctcacttcccagacga 9474
BCRP3.HUMAN.NCBI.REF                             ------------------------------------------------------------ 4142

GGT2.end-endGGT1.48980500-49036766.gibbon        tgggtggccgggcagaggcgctcctcacttcccagacggtgggtggccgggcagaggcgc 9534
BCRP3.HUMAN.NCBI.REF                             ------------------------------------------------------------ 4142

GGT2.end-endGGT1.48980500-49036766.gibbon        tcctcacttcccagacggtgggtggccgggcagaggcgctcctcacttcccagacggtgg 9594
BCRP3.HUMAN.NCBI.REF                             ------------------------------------------------------------ 4142

GGT2.end-endGGT1.48980500-49036766.gibbon        gtggccgggcagaggcgctcctcacttcccagacggggcggccgggcagaggcgctcctc 9654
BCRP3.HUMAN.NCBI.REF                             ------------------------------------------------------------ 4142

GGT2.end-endGGT1.48980500-49036766.gibbon        acttcccagacggggcggccgggcagaggtgctcctcacttcccagacgatgggtggccg 9714
BCRP3.HUMAN.NCBI.REF                             ------------------------------------------------------------ 4142

GGT2.end-endGGT1.48980500-49036766.gibbon        ggcagaggcgctcctcacttccaagacagggcggccgggcagaggcgctcctcacttccc 9774
BCRP3.HUMAN.NCBI.REF                             ------------------------------------------------------------ 4142

GGT2.end-endGGT1.48980500-49036766.gibbon        agacggtgggtggccgggcagaggcgctcctcacttcgcagacggtgggtggccgggcag 9834
BCRP3.HUMAN.NCBI.REF                             ------------------------------------------------------------ 4142

GGT2.end-endGGT1.48980500-49036766.gibbon        aggcgctcctcacttcccagacggggcggccgggcagagacgctcctcacttcccagacg 9894
BCRP3.HUMAN.NCBI.REF                             ------------------------------------------------------------ 4142

GGT2.end-endGGT1.48980500-49036766.gibbon        gtgggtggccgggcagaggcgctcctcacttcgcagacggtgggtggccggacagaggcg 9954
BCRP3.HUMAN.NCBI.REF                             ------------------------------------------------------------ 4142

GGT2.end-endGGT1.48980500-49036766.gibbon        ctcttcacttcccagacggggcggccgggcagaggtgctcctcacttcccagacggggcg 10014
BCRP3.HUMAN.NCBI.REF                             ------------------------------------------------------------ 4142

GGT2.end-endGGT1.48980500-49036766.gibbon        gccgggcagaggcgctcctcacttcccagacggtgggtggccgggcagaggcgctcctca 10074
BCRP3.HUMAN.NCBI.REF                             ------------------------------------------------------------ 4142

GGT2.end-endGGT1.48980500-49036766.gibbon        cttcgcagacggtgggtggccgggcagaggcgctcctcacttcccagacggggcggccgg 10134
BCRP3.HUMAN.NCBI.REF                             ------------------------------------------------------------ 4142

GGT2.end-endGGT1.48980500-49036766.gibbon        gcagaggcgctcctcacttcccagacggggcggccgggcagaggcgctcctcacttccca 10194
BCRP3.HUMAN.NCBI.REF                             ------------------------------------------------------------ 4142

GGT2.end-endGGT1.48980500-49036766.gibbon        gacggtgggtggccgggcagaggcgctcctcacttcccagacggggcggccgggcagagg 10254
BCRP3.HUMAN.NCBI.REF                             ------------------------------------------------------------ 4142

GGT2.end-endGGT1.48980500-49036766.gibbon        cgctcctcacttcccagacgatgggtggccgggcagaggcgcccctcacctcttcccaga 10314
BCRP3.HUMAN.NCBI.REF                             ------------------------------------------------------------ 4142

GGT2.end-endGGT1.48980500-49036766.gibbon        tggggcggccgggcagaggcgctcctcacttcccagacgatgggtggccgggcagaggcg 10374
BCRP3.HUMAN.NCBI.REF                             ------------------------------------------------------------ 4142

GGT2.end-endGGT1.48980500-49036766.gibbon        cccctcacctcttcccagacggggcggctgggcagaggcgctcctcacttctacctggac 10434
BCRP3.HUMAN.NCBI.REF                             ------------------------------------------------------------ 4142

GGT2.end-endGGT1.48980500-49036766.gibbon        ggtgcggccgggcagaggcgctcctcacttcttcccggacggggcgcctgggcagaggcg 10494
BCRP3.HUMAN.NCBI.REF                             ------------------------------------------------------------ 4142

GGT2.end-endGGT1.48980500-49036766.gibbon        ctcctcacttcccagacgatgggtgcctgggcagaggcgctcctcacttcccagacgggg 10554
BCRP3.HUMAN.NCBI.REF                             ------------------------------------------------------------ 4142
```

```
GGT2.end-endGGT1.48980500-49036766.gibbon      cgcccgggcagaggcgctcctcacttcccagacgatgggtggccgggcagaggcgctcct 10614
BCRP3.HUMAN.NCBI.REF                           ------------------------------------------------------------ 4142

GGT2.end-endGGT1.48980500-49036766.gibbon      cacttcccagacggggcggccggacagaggggctcctcacttcttcccggacagggcggc 10674
BCRP3.HUMAN.NCBI.REF                           ------------------------------------------------------------ 4142

GGT2.end-endGGT1.48980500-49036766.gibbon      agggcagaggcgctcctcacttcttcccagatggggcggccgggcagaggcgctcttcac 10734
BCRP3.HUMAN.NCBI.REF                           ------------------------------------------------------------ 4142

GGT2.end-endGGT1.48980500-49036766.gibbon      ttcccagacggggcggccaggcagaggcgctcctcacttcttcccagatggggcggccgg 10794
BCRP3.HUMAN.NCBI.REF                           ------------------------------------------------------------ 4142

GGT2.end-endGGT1.48980500-49036766.gibbon      gcagaggcgctcctcacttcccagacggggtggccgggcagaggcgctcttcacttccca 10854
BCRP3.HUMAN.NCBI.REF                           ------------------------------------------------------------ 4142

GGT2.end-endGGT1.48980500-49036766.gibbon      gacggggtggctgggcagaggcgctcttcacttcccagacggggtggccgggcagaggcg 10914
BCRP3.HUMAN.NCBI.REF                           ------------------------------------------------------------ 4142

GGT2.end-endGGT1.48980500-49036766.gibbon      ctcctcacttcttcccagacggggcggccaggcagaggcgctcctcacttcccagacgat 10974
BCRP3.HUMAN.NCBI.REF                           ------------------------------------------------------------ 4142

GGT2.end-endGGT1.48980500-49036766.gibbon      gggtggccaggcagaagcgctcctcacctcccagacgatgggtggccaggcagaggcgct 11034
BCRP3.HUMAN.NCBI.REF                           ------------------------------------------------------------ 4142

GGT2.end-endGGT1.48980500-49036766.gibbon      cctcacctcccagacgatgggtggccgggcagaggcgctcctcacctcccagacgatggg 11094
BCRP3.HUMAN.NCBI.REF                           ------------------------------------------------------------ 4142

GGT2.end-endGGT1.48980500-49036766.gibbon      cggccgggcagagacgctccccacctcccagacggggcggtggccgggcaggggctgcaa 11154
BCRP3.HUMAN.NCBI.REF                           ------------------------------------------------------------ 4142

GGT2.end-endGGT1.48980500-49036766.gibbon      tcccagcaccctggtaggccaaggcaggcggctgggaggcggaggctgccgcgagcccag 11214
BCRP3.HUMAN.NCBI.REF                           ------------------------------------------------------------ 4142

GGT2.end-endGGT1.48980500-49036766.gibbon      accacgccaccgcactccagcccgggcaacaccgagcaccgggtgagcgagactccgtct 11274
BCRP3.HUMAN.NCBI.REF                           ------------------------------------------------------------ 4142

GGT2.end-endGGT1.48980500-49036766.gibbon      gcagtcccagtacctcgggaggctgaggcgggcagagcactcggcgtcaggagctggcga 11334
BCRP3.HUMAN.NCBI.REF                           ------------------------------------------------------------ 4142

GGT2.end-endGGT1.48980500-49036766.gibbon      ccagcgtgggcaacatggcggacgcgcgcctgcaggcaaaggagaaaaagccggcagcgg 11394
BCRP3.HUMAN.NCBI.REF                           ------------------------------------------------------------ 4142

GGT2.end-endGGT1.48980500-49036766.gibbon      tggcgcgcggcggcagtcccaggtagtccgtggcgcgggcagcagtgagccgagtagatt 11454
BCRP3.HUMAN.NCBI.REF                           ------------------------------------------------------------ 4142

GGT2.end-endGGT1.48980500-49036766.gibbon      gcagcctgggccacagaggggaaaaagaagaagaagagaagaagaaagaagaaagaagaa 11514
BCRP3.HUMAN.NCBI.REF                           ------------------------------------------------------------ 4142

GGT2.end-endGGT1.48980500-49036766.gibbon      agaagaagaagaagaagaagaggaagaggaagaggaagaagaagaggaggaagaggagga 11574
BCRP3.HUMAN.NCBI.REF                           ------------------------------------------------------------ 4142

GGT2.end-endGGT1.48980500-49036766.gibbon      agaggaggaggaggaggaggaggaagaggaggaagaagaggaggggggaggggggaggggga 11634
BCRP3.HUMAN.NCBI.REF                           ------------------------------------------------------------ 4142

GGT2.end-endGGT1.48980500-49036766.gibbon      gggggaggaggaggaggaggaggaggaggagagttttgctcttgttgcccaggctggagt 11694. 11664 bp
BCRP3.HUMAN.NCBI.REF                           ----------------------------cagagttttgctcttgttgcccaggctggaat 4174
                                                                            ***************************** *

GGT2.end-endGGT1.48980500-49036766.gibbon      acaatggcacgatctcggctcaccacaacctccacttcctgggtccaagcaattctcctc 11754
BCRP3.HUMAN.NCBI.REF                           gcaatggcacaatctcagctcaccgcaacctccacttcctgggtccaagcaattctcctc 4234
                                                ********* ***** ******* ***********************************

GGT2.end-endGGT1.48980500-49036766.gibbon      cctcagcctcctgagtagctgggattacaggcatgtgccaccatgcttggctaatgttgg 11814
BCRP3.HUMAN.NCBI.REF                           cctcagcctcctgagtagctgggattacaggcatgtgccaccatgcttggctaatttttg 4294
                                               ******************************************************* ** *

GGT2.end-endGGT1.48980500-49036766.gibbon      tatttttagtagagacagggcttctccatgttggtcaggctggtcttgaactcccaacct 11874
BCRP3.HUMAN.NCBI.REF                           tatttttagtagagacagggcttctccatgttggtcaggctggtcttgaactcccaacct 4354
                                               ************************************************************

GGT2.end-endGGT1.48980500-49036766.gibbon      caggtgatccgccgccttggcctcacaaagtgctggaattacaggcatgagccaccgcgc 11934
BCRP3.HUMAN.NCBI.REF                           caggtgatcagccgccttggcctcacaaagtgctggaattacaggcatgagccaccgcac 4414
                                               ********* ************************************************ *
```

```
GGT2.end-endGGT1.48980500-49036766.gibbon       ccggctcctagtaacttcttcttttccgtgatgtgtctcttacctctaata--------- 11985
BCRP3.HUMAN.NCBI.REF                            ctggctcctagtaaattcttcttttccgtgatgtgtctcttacctctaataatactttttc 4474
                                                * ************ ************************************

GGT2.end-endGGT1.48980500-49036766.gibbon       ------------------------------------------------------------ 11985
BCRP3.HUMAN.NCBI.REF                            ttctttttttttttttgagacggagtctcgttctgtcgcccaggcgggagtgctgtggcg 4534

GGT2.end-endGGT1.48980500-49036766.gibbon       ------------------------------------------------------------ 11985
BCRP3.HUMAN.NCBI.REF                            cgatctccgctcactgcaagctccgccttccgggttcacgccattctcctgcctcaacct 4594

GGT2.end-endGGT1.48980500-49036766.gibbon       ------------------------------------------------------------ 11985
BCRP3.HUMAN.NCBI.REF                            cccgagtagctgggactacaggcgcccgccactgcgcccggctaatttttttgtattttta 4654

GGT2.end-endGGT1.48980500-49036766.gibbon       ------------------------------------------------------------ 11985
BCRP3.HUMAN.NCBI.REF                            gtagagacggggtttcaccgtggtctcgatctcctgacctcgtgatccgcccgcctcggc 4714

GGT2.end-endGGT1.48980500-49036766.gibbon       ---------------------------------------------atactttttcttctta 12000
BCRP3.HUMAN.NCBI.REF                            ctcccaaagtgctgggattacaggcgtgagccaccgcgtccggccatactttttcttctta 4774
                                                                                             ***************

GGT2.end-endGGT1.48980500-49036766.gibbon       aaatctacttcattaaaaatagttatgctgggcatggtggctcatgtctgtaatctcggc 12060
BCRP3.HUMAN.NCBI.REF                            aagtctacttcattaaaaatagttatgctgggcatggtggctcatggctgtaatctcggc 4834
                                                ** ******************************************** *************

GGT2.end-endGGT1.48980500-49036766.gibbon       actttgttggaggtcgaggtgggtggatcactgaagcccaggagttcaagaccagcctgg 12120
BCRP3.HUMAN.NCBI.REF                            actttgttggaggtcgaggtgggtggatcactgaagcccaggagttcaagaccaacctgg 4894
                                                ****************************************************** *****

GGT2.end-endGGT1.48980500-49036766.gibbon       gcaacatggcgagaccctgcctctacaaaaaatacaaaaatcagctgggtgtggctaata 12180
BCRP3.HUMAN.NCBI.REF                            gcaacgtggcgagaccctgcctctacaaaaaatacaaaaattagctgggtgtggctaata 4954
                                                ***** *********************************** ******************

GGT2.end-endGGT1.48980500-49036766.gibbon       taattctacgttggtacagttgtagtcccagctacttgggatgctgagatgggagaattg 12240
BCRP3.HUMAN.NCBI.REF                            --------------tacacttgtagtcccagctacttgggatgctgaggtgggagaatcg 5000
                                                              **** ***************************** ********* *

GGT2.end-endGGT1.48980500-49036766.gibbon       cttgagcctagaagggagagattgctgtaagccaagatcacatcactgcactccagcctg 12300
BCRP3.HUMAN.NCBI.REF                            cttgagcctagaagggagagattgctgtaagccaagatcacatcactgcactccagcctg 5060
                                                ************************************************************

GGT2.end-endGGT1.48980500-49036766.gibbon       ggaaacagagtgaggctctatcttcaaaaaaaaaaaaaaaaaaaaaaattatacagctttc 12360
BCRP3.HUMAN.NCBI.REF                            ggagacagagtgaggctctatctcc--aaaaaaaaaaaaaaaaaaagttatacagctttc 5118
                                                *** ******************* *  ****************** *************

GGT2.end-endGGT1.48980500-49036766.gibbon       ttggttagtgcatgaatgatatatttttcattattttccacctctctgtatccttatata 12420
BCRP3.HUMAN.NCBI.REF                            ttggttagtgcatgcatgccatatttttcattattttccacctctctgtatccttatata 5178
                                                ************** ***  ****************************************

GGT2.end-endGGT1.48980500-49036766.gibbon       aaaggcattattagttgggttttactttattttcaattattttaatttttgttgtccttt 12480
BCRP3.HUMAN.NCBI.REF                            aaaggc---attagttgggttttactttattttcaattattttaatttttattgtccttt 5235
                                                ******   ***************************************** *********

GGT2.end-endGGT1.48980500-49036766.gibbon       taaatgtaactaatgatttatttgggttgaaagccaccaccagtttgctttccatgccta 12540
BCRP3.HUMAN.NCBI.REF                            taaatgtaactaatgatttatttgggttgaaacccaccaccaatttgttttccatgccta 5295
                                                ******************************** ********* **** ************

GGT2.end-endGGT1.48980500-49036766.gibbon       ttctgtttcttcttatctcctctcacatcttgttttggatttattatttttattatttaa 12600
BCRP3.HUMAN.NCBI.REF                            ttctatttcttcttatctcctctcacatcttgttttggatttattatttttattatttaa 5355
                                                **** *******************************************************

GGT2.end-endGGT1.48980500-49036766.gibbon       tttcctc---ctctattagtttcataactgtgcagtcttggagttattttaaaagatgac 12657
BCRP3.HUMAN.NCBI.REF                            tttcctccttctctattagtttcatagctctgcagtcttagagttattttaaaagatgac 5415
                                                *******   **************** ** ********* ********************

GGT2.end-endGGT1.48980500-49036766.gibbon       agtggattattttagagcttacaacatgcatccttcacttatcaaagtctcacatgagct 12717
BCRP3.HUMAN.NCBI.REF                            agtggattattttagagcttacaacatgcatccttcacttatcaaagtctaacatgagct 5475
                                                ************************************************** *********

GGT2.end-endGGT1.48980500-49036766.gibbon       agtactttttgttgttgttgttgttgttgagatagagagagtcttcctctgctgcccagg 12777
BCRP3.HUMAN.NCBI.REF                            agtacttt---------ttgttgttgttgagatagagagagtcttcctctgctgcccagg 5526
                                                ********         *******************************************

GGT2.end-endGGT1.48980500-49036766.gibbon       ctggagtgcagtggagcaatcttggttcactgcaacctccacttcttgggttcaagcagt 12837
BCRP3.HUMAN.NCBI.REF                            ctggagtgcagtggagcaatcttggttcactgcaacctccacttcttgggttcaagcagt 5586
                                                ************************************************************

GGT2.end-endGGT1.48980500-49036766.gibbon       tctcctgcctcagtcacctgagtagctgggaccacaggtgtgcaccactatgcccggcta 12897
BCRP3.HUMAN.NCBI.REF                            tctcctgcctcagtcacctgagtagctgggaccacaggtgtgcaccactatgcccggcca 5646
                                                ********************************************************** *

GGT2.end-endGGT1.48980500-49036766.gibbon       atttttgtattcttttttagtagagacagggtttcaccatgttggccaggctggtcttga 12957
BCRP3.HUMAN.NCBI.REF                            atttttgtattcttttttagtagagacagggtttcaccatgttggccaggctggtcttga 5706
                                                ************************************************************

GGT2.end-endGGT1.48980500-49036766.gibbon       actcctgaccttaagaaatctgcctacctcgccatcctaaagtgttgggattacaggcgt 13017
BCRP3.HUMAN.NCBI.REF                            actcctgaccttaagagatctgcctacctcggcgtcctaaagtgttgggattacaggcat 5766
                                                **************** ************** * ************************ *
```

```
GGT2.end-endGGT1.48980500-49036766.gibbon      gagccaccatgcccagcctatgagttagtacttctatcctcttcctagtcagtacaagaa 13077
BCRP3.HUMAN.NCBI.REF                           gagccaccgcgcccagcctatgagttagtacttctatgctcttcctagtcagtacaagaa 5826
                                               ********  *************************** **********************

GGT2.end-endGGT1.48980500-49036766.gibbon      ccttggaacaggaactaaatttacccccagtgacttataggcgaatatttttgtgtattt 13137
BCRP3.HUMAN.NCBI.REF                           ccttggaacaggaactaaatttacccccagtgacttatatgctaatatttttgtgtattt 5886
                                               *************************************** ** *****************

GGT2.end-endGGT1.48980500-49036766.gibbon      taaatatatgtgtgtgcatagatgtatctgtgtgttttttgtgtttctattcttatttat 13197
BCRP3.HUMAN.NCBI.REF                           taaatatatatgtgtgcatagatgtatctgtgtgttttttgtgtttttattcttatttat 5946
                                               ********* ************************************ *************

GGT2.end-endGGT1.48980500-49036766.gibbon      gttgagagtgtggagctatgtaagagtaaagagaattgtgtaatgaagccccatgtatcc 13257
BCRP3.HUMAN.NCBI.REF                           gttgagagtgtagagctatgtaagagtaaagagaattgtgtaatgaagccccgagtatcc 6006
                                               *********** ****************************************  ******

GGT2.end-endGGT1.48980500-49036766.gibbon      actcaatttcaacaacaatcttatggtcaagctaacttcatgtatactctttcctgcttc 13317
BCRP3.HUMAN.NCBI.REF                           attcaatttcaacaacaatctcatggccaagctaatttcatgtatactctttcctgcttc 6066
                                               * ******************* **** ******** ************************

GGT2.end-endGGT1.48980500-49036766.gibbon      cctctaccccacattacttcagtgcaaatcccagatatataactttacccatacatattt 13377
BCRP3.HUMAN.NCBI.REF                           cctctaccccacattatttcagtgcaaatcccagatatataactgtacccatacatattt 6126
                                               **************** ************************** ****************

GGT2.end-endGGT1.48980500-49036766.gibbon      cagtatgttttatttattttaaccccacaagatatcattttctatactactgtaatttt 13437
BCRP3.HUMAN.NCBI.REF                           cagtatgttttatttattttaaccccacaatatatcattttctatactactgtaatttc 6186
                                               ****************************** ****************************

GGT2.end-endGGT1.48980500-49036766.gibbon      ataccaataacattcatttagatttacccacacgtttacctcttctgttaccctttattt 13497
BCRP3.HUMAN.NCBI.REF                           ataccaataacattcatttagatttacccacacgtttacctcttctgttaccctttattt 6246
                                               ************************************************************

GGT2.end-endGGT1.48980500-49036766.gibbon      ttatttatcaaaatatctttgggaaaaaatatctttcagcacatggtcaaggatctcctg 13557
BCRP3.HUMAN.NCBI.REF                           ttatttataaaaatatctttgggaagaaatatctttcagcacatggtcaaggatctcctg 6306
                                               ******** **************** **********************************

GGT2.end-endGGT1.48980500-49036766.gibbon      agggctatgtcataggcaaaatatacatatacattacatatatatacacacatatacaca 13617
BCRP3.HUMAN.NCBI.REF                           agggctatgtcatgggcaaaatatacatatatattccatatatatacacacatatacaca 6366
                                               ************* ***************** *** ************************

GGT2.end-endGGT1.48980500-49036766.gibbon      cacacacatatacacacatacacacacacatatatatattccactttcactttttttgt 13677
BCRP3.HUMAN.NCBI.REF                           cacacacatatatacacatatacacacacatatatatattccactttcactttttttgt 6426
                                               ************ ******* ***************************************

GGT2.end-endGGT1.48980500-49036766.gibbon      ttgtttgttttttgagaccgagtcttgctctgtggcccaggctggagtgcggtggtggga 13737
BCRP3.HUMAN.NCBI.REF                           ttgtttgttttttgagaccgagtctcgctctgtggcccaggctggagtgcggtggtggga 6486
                                               ************************* **********************************

GGT2.end-endGGT1.48980500-49036766.gibbon      tctcagctcactgcaacttctacctcctgggttcaggtgattctccagtctcagcctccc 13797
BCRP3.HUMAN.NCBI.REF                           tctcagctcactgcaacttctgcctcctgggttcaggtgattctcctgtctcagcctcct 6546
                                               ********************* ************************ ************

GGT2.end-endGGT1.48980500-49036766.gibbon      aagtagttgggattacaggtgttagccaccacccctggctaattttttgtatatattttttt 13857
BCRP3.HUMAN.NCBI.REF                           gagtagctgggattacaggtgttagccatcacgtctggctaatttt-------------- 6592
                                                ***** ********************* ***  ************

GGT2.end-endGGT1.48980500-49036766.gibbon      tgagatggagtatcgctctgttgcccaggctggagtgcagtggcgtgatcttggctcact 13917
BCRP3.HUMAN.NCBI.REF                           ------------------------------------------------------------ 6592


GGT2.end-endGGT1.48980500-49036766.gibbon      gcaaccttcatctccctggttcaagcaattcccctgcctcagcctcccaagtagctggga 13977
BCRP3.HUMAN.NCBI.REF                           ------------------------------------------------------------ 6592


GGT2.end-endGGT1.48980500-49036766.gibbon      ttacaggtgcacgccaccatgcccggataatttttttgtatttgtagtagagaccgggtt 14037
BCRP3.HUMAN.NCBI.REF                           ------------------------------------------------------------ 6592


GGT2.end-endGGT1.48980500-49036766.gibbon      ttcctatgatggtcagactggtcttgaacttctgacttcaggcaatccaccccgctcggc 14097
BCRP3.HUMAN.NCBI.REF                           ------------------------------------------------------------ 6592


GGT2.end-endGGT1.48980500-49036766.gibbon      ctcccaaagtgctgggattacaggcatgagccaccatgcctggtctgttttttttttttt 14157
BCRP3.HUMAN.NCBI.REF                           ---------------------------------------------tgttgtttttttttt 6607
                                                                                            **** **********

GGT2.end-endGGT1.48980500-49036766.gibbon      tttgagatggagttttgctctgtcacccaggctggagtgcagtggcaagatcttggctca 14217
BCRP3.HUMAN.NCBI.REF                           tttgagacggagtatcgctctgtcacccaggctggagtgcagtggcaagatcttgtctca 6667
                                               ******* ***** * *************************************** ****

GGT2.end-endGGT1.48980500-49036766.gibbon      ctgcaacctccacctctcgggttcaagcaattcttgtgcctcagcctcctgagtagctgg 14277
BCRP3.HUMAN.NCBI.REF                           ctgcaacctccacctctcaggttcaagcaattcttgtgcctcagcctcctgagtagctgg 6727
                                               ****************** *****************************************

GGT2.end-endGGT1.48980500-49036766.gibbon      gattacaggca-cccccaccaaatctggctaatttgtgtatttttggtagaggtggagtt 14336
BCRP3.HUMAN.NCBI.REF                           gattacaggcatccaccaccacatctggctaatttgtgtatttttggtagagatggggtt 6787
                                               *********** ** ****** ****************************** *** ***

GGT2.end-endGGT1.48980500-49036766.gibbon      tcaccacattggccaggttggtcttgaacttatgacctcaggtgatccacctgcctcagc 14396
BCRP3.HUMAN.NCBI.REF                           tcaccatgttggccaggctggtctcgaacttctgacctcaggtgatccacctgcctcggc 6847
                                               ******  ********* ****** ****** ************************* **

GGT2.end-endGGT1.48980500-49036766.gibbon      cttccaaagtgctgggattacaggcatgagccaccatgcccagccattttcgcttttgaa 14456
```

```
BCRP3.HUMAN.NCBI.REF                           cttccaaagtgctgggattacaggcatgagccaccatgcccagccattttcacttttgaa 6907
                                               *************************************************** ********

GGT2.end-endGGT1.48980500-49036766.gibbon      ggatattgttagtgaacatagaattctaggttggcagatattttctttcctcagtttgaa 14516
BCRP3.HUMAN.NCBI.REF                           ggatattgttaatgagcatagaattctaggttggcagatattttctttcctcagtttgaa 6967
                                               *********** *** ********************************************

GGT2.end-endGGT1.48980500-49036766.gibbon      aacatgattcccttgtatctgatttctcctgcttttattgggaagccaattctcaatcta 14576
BCRP3.HUMAN.NCBI.REF                           aacatgattcccttgtatctgatttctcctgcttttattgggaagccaattctcaatcta 7027
                                               ************************************************************

GGT2.end-endGGT1.48980500-49036766.gibbon      atttgctcatttgaaggcaatggcttttttatgttgttgtgttttttgagatggagtctc 14636
BCRP3.HUMAN.NCBI.REF                           atttgctcatttgaaggcaatggcttttttctgttgttgtgttttctgaggtggagtctc 7087
                                               ****************************** ************** **** *********

GGT2.end-endGGT1.48980500-49036766.gibbon      actctgtcgcccaggctggagtgcagtggtgcaatttcagctcactgcaacctctgcctc 14696
BCRP3.HUMAN.NCBI.REF                           actctgtcacccaggctggactgcagtggtgcaatctcagctcactgcaacctctgcctc 7147
                                               ******** *********** ************** ************************

GGT2.end-endGGT1.48980500-49036766.gibbon      ctgggctcaagtgattcttctgcctcagcctcccaagcagctgggattacaggtgtccac 14756
BCRP3.HUMAN.NCBI.REF                           ctgggttcaagtgattcttctgcctcagcctcccaagtagctgggattacaggtgtccac 7207
                                               ***** ******************************* **********************

GGT2.end-endGGT1.48980500-49036766.gibbon      catcacacccggctaattgttgtatttttaatagagatgaacttttgccatgttggtcag 14816
BCRP3.HUMAN.NCBI.REF                           catcacacctggctaattgttgtatttttaatagagatgaacttttgccatgttggtcag 7267
                                               ********* **************************************************

GGT2.end-endGGT1.48980500-49036766.gibbon      gctgatcccaaattcctcatttcaggtgatccacctgcctcagcctcccaaatgctggga 14876
BCRP3.HUMAN.NCBI.REF                           gctgatcccaaactcctcatttcaggtgatccgcccgcctcagcctcccaaatgctggga 7327
                                               ************ ******************* ** ************************

GGT2.end-endGGT1.48980500-49036766.gibbon      ttacaggcatgagccagcccccaaccccggcctgaaggcagtatctttttccctctggct 14936
BCRP3.HUMAN.NCBI.REF                           ttacaggcatgagacagcccacaaccctggcctgcaggcagtatctttttttcctctggct 7387
                                               ************* ****** ****** ****** *************** *********

GGT2.end-endGGT1.48980500-49036766.gibbon      gctttgaaaagttttgtctttgttttgagcagtttacactgatgcatttaggtggctctt 14996
BCRP3.HUMAN.NCBI.REF                           gctttgaaaagttttgtctttgttttgagcagtttacactgatgcatttaggtggctcct 7447
                                               ********************************************************** *

GGT2.end-endGGT1.48980500-49036766.gibbon      cattctatgacttgattcttttttgtccattttagaaaattctcagctttatctcttcaa 15056
BCRP3.HUMAN.NCBI.REF                           cattccatgacttgattcttttttgtccattttagaaaactgccagctttatctcttcaa 7507
                                               ***** ********************************* *  *****************

GGT2.end-endGGT1.48980500-49036766.gibbon      gtgttacatcttccccatcctctctctactctccttatgaggctcaaatttcacatgact 15116
BCRP3.HUMAN.NCBI.REF                           gtattatgtcttccccatcctctctctactctccttatgagactccaatttcacatgact 7567
                                               ** ***  ********************************* *** **************

GGT2.end-endGGT1.48980500-49036766.gibbon      tatgccttgttaaagtatctcccatgtctcttaatccatttcctggatgttctatctatt 15176
BCRP3.HUMAN.NCBI.REF                           tatgccttgttaaagtatcccccatgtctcttaatccatttcctgtatgttctatctgtt 7627
                                               ******************* ************************* *********** **

GGT2.end-endGGT1.48980500-49036766.gibbon      tttctctttgtacttcaatttgtatattttgtatcaaactatctcccaattagccaggcg 15236
BCRP3.HUMAN.NCBI.REF                           tttctctttgtacttcaatttgtatagtttgtatcaaactatctcccaattagccgggcg 7687
                                               ************************** **************************** ****

GGT2.end-endGGT1.48980500-49036766.gibbon      tggtggtgggtgcctgtaatcccagctacttgggaggctgaggcaggagaattgcttgaa 15296
BCRP3.HUMAN.NCBI.REF                           tggtggtgggtgcctgtaatcccagctacttgggagcctgaggcaggagaattgcttgaa 7747
                                               ************************************ ***********************

GGT2.end-endGGT1.48980500-49036766.gibbon      cccaggagatggaggttgcagtgaaccgagatcatgccactgcactccagtctgggcaac 15356
BCRP3.HUMAN.NCBI.REF                           cccgagaggtggaagttgtagtgagccgagatcatgccactgcactccagcctgggcaac 7807
                                               ***  *** **** **** ***** ************************* *********

GGT2.end-endGGT1.48980500-49036766.gibbon      acagtgagaccctgtttc----aataaataaataagtaaataccagttcactcttttttt 15412
BCRP3.HUMAN.NCBI.REF                           agagtgagaccctgtctcaataaataaataaataaataaataccagttcactattttttt 7867
                                               * ************* **    ************* **************** *******

GGT2.end-endGGT1.48980500-49036766.gibbon      ttatgtttgtgtctagtgtgctgttcaaattgagttcctaattccattttttttttgaga 15472
BCRP3.HUMAN.NCBI.REF                           ttatgtttgtgtctagtgtgctgttcaaattgagttcctaattccattttttttttagac 7927
                                               ********************************************************

GGT2.end-endGGT1.48980500-49036766.gibbon      cttttttttttttgagtctctatctgttgcccaggctggagctcagtggtgcaatctcaac 15532
BCRP3.HUMAN.NCBI.REF                           --ttttttttttgagtctctatctgttgcccaggctggagttcagtggtgcaatctcaac 7985
                                                 ************************************** *******************

GGT2.end-endGGT1.48980500-49036766.gibbon      tcactgtagcctccacctcccaggttcaagcgattcttgtgcctcagcctcctgagtaac 15592
BCRP3.HUMAN.NCBI.REF                           tcactgtagcctccacctcccaggttcaagcgattctcatgcctcagcctctcgagtaac 8045
                                               *************************************  ************  *******

GGT2.end-endGGT1.48980500-49036766.gibbon      tgggattaccaccacgcctaattcatttttgtatttttagtagagatggggttttgccat 15652
BCRP3.HUMAN.NCBI.REF                           tgggattaccaccacgcctaactcatttttgtatttttagtagagatggggttctgccat 8105
                                               ********************* ******************************* ******

GGT2.end-endGGT1.48980500-49036766.gibbon      gttggccaggctggtcttgaactcctggcctcatgtgattggcctaactctgtctcccaa 15712
BCRP3.HUMAN.NCBI.REF                           gttggccaggctggtcttgaactcttggccttatgtgattggcctacctctgtctcccaa 8165
                                               ************************ ****** ************** *************

GGT2.end-endGGT1.48980500-49036766.gibbon      ag-gctgggattataggcctcagccaccactcccagcctccatttttttttttttttttt 15771
BCRP3.HUMAN.NCBI.REF                           agtgctgggattataggcctaaaccaccactcccagcctcc-tttttttttttttttttt 8224
                                               ** ***************** * ****************** ******************

GGT2.end-endGGT1.48980500-49036766.gibbon      ttttgagacagagtcttgctctgtcgcccaggctggagtagagtggtgcgatctcggcac 15831
BCRP3.HUMAN.NCBI.REF                           ttttgagacggagtctcgctctgtcgcccaggctggagtagagtggtgcgatctcggctc 8284
```

```
                                               ********* ****** ***************************************** *

GGT2.end-endGGT1.48980500-49036766.gibbon      actgcaacctccccctcctagttcaagcaattctcctgcctcagcctcccgagtagctgg 15891
BCRP3.HUMAN.NCBI.REF                           actgcaacctccccctcccagttcaagtgattctcctgcctcagcctcccgagtagctag 8344
                                               ****************** ********  ***************************** *

GGT2.end-endGGT1.48980500-49036766.gibbon      gactacagccacatgccaccatgcctggctaatttttgtaattttagtagagatgaggtt 15951
BCRP3.HUMAN.NCBI.REF                           gactataggagcatgccaccatgcctggctaatttttgtaattttagtagagatggggat 8404
                                               ***** **   ******************************************** ** *

GGT2.end-endGGT1.48980500-49036766.gibbon      tcaccatattggtcaggctggtcttgaacttctgacctcaggtgatccacccacctcagc 16011
BCRP3.HUMAN.NCBI.REF                           tcaccatattggtcaggctggtcttgaacttctgacctcaggtgatctacccacctcagc 8464
                                               *********************************************** ************

GGT2.end-endGGT1.48980500-49036766.gibbon      cttccaaagtgctgggattacaggcgtgagtcaccacgcctagtgcgcccattttttata 16071
BCRP3.HUMAN.NCBI.REF                           ctcccaaagtgctgggattacaggcgtgagtcaccacgcctagtgcatccattttttgta 8524
                                               ** *******************************************  ********* **

GGT2.end-endGGT1.48980500-49036766.gibbon      gttgccagttttctgatgaaattcttaattgtttctttatatcctcgatatacatataaa 16131
BCRP3.HUMAN.NCBI.REF                           gttgccagttttctgatgaaattcttaattgtttctttatatccttgatatacatgtaaa 8584
                                               ********************************************* ********* ****

GGT2.end-endGGT1.48980500-49036766.gibbon      gtac-----ttaaagtacatggtctgacgatttcataatctggagatcctatgggccttt 16186
BCRP3.HUMAN.NCBI.REF                           gaacttattttaaagtacatggtctgatgattttataatctggagatcctatgggccttt 8644
                                               * **     ****************** ***** **************************

GGT2.end-endGGT1.48980500-49036766.gibbon      ttaaaagttgtctgtgctttctcttgagctttgttcctggtgtcttatttccttgtttgc 16246
BCRP3.HUMAN.NCBI.REF                           ttaaaagttgtctgtgctttctcttgagcttttttcctgctgtcttatttccttgtttgc 8704
                                               ******************************** ****** ********************

GGT2.end-endGGT1.48980500-49036766.gibbon      ttggttgtttttaatttggcaatggaagttgtgtatacaaattgttacaaataa-----t 16301
BCRP3.HUMAN.NCBI.REF                           ttagttgtttttaatttggcaatggaagttgtgtataaaaatcgttacaaataatttttt 8764
                                               ** ********************************** **** ***********     *

GGT2.end-endGGT1.48980500-49036766.gibbon      tttttttttgagatggagtctcgctttgttccccaagctggagtgcaatgatgtgatctc 16361
BCRP3.HUMAN.NCBI.REF                           tttttttttgagatggagtctcgctttgttgcccaagctggagtgcaatgacgtgatctc 8824
                                               ****************************** ******************** ********

GGT2.end-endGGT1.48980500-49036766.gibbon      ggctcactgcaacctctgcaacccagggttcgattctcctacctctgcctctcaagtagc 16421
BCRP3.HUMAN.NCBI.REF                           ggctcactgcaacctctgcatcccagggttcaattctcctacctcagcctcccaagtagc 8884
                                               ******************** ********** ************* ***** ********

GGT2.end-endGGT1.48980500-49036766.gibbon      tgggattacaggcaggtgccagcatgcctggctaatttttgtatttttagtagagatgag 16481
BCRP3.HUMAN.NCBI.REF                           tgggattgcaggcaggtgccagcacgcctggctaatttttgtatttttagtagagatggg 8944
                                               ******* **************** ********************************* *

GGT2.end-endGGT1.48980500-49036766.gibbon      ttttcaccatgttggtcaggctggtctgaaactcctgacctcatgatctgcccacctcag 16541
BCRP3.HUMAN.NCBI.REF                           tttttaccatgttggtcaggctggtctcagactcctgacctcgtgatctgcccacctcag 9004
                                               **** ********************** * ************ *****************

GGT2.end-endGGT1.48980500-49036766.gibbon      cctcccaaagtgctgggattacaggcgtgagccactgcgcccagctagaaataattttta 16601
BCRP3.HUMAN.NCBI.REF                           cctcccaaagtgctgggattacaggcgtgagccactgcgcccagccagaaataattttta 9064
                                               ******************************************** ***************

GGT2.end-endGGT1.48980500-49036766.gibbon      aaaataatttcaagccccagcatgatggctcatgtttgtaatcccatcgttttgggaggc 16661
BCRP3.HUMAN.NCBI.REF                           aaaataattttgagccccagcatgatggctcatgcttgtaatcccatcactttgggaggc 9124
                                               **********  ********************** *************  **********

GGT2.end-endGGT1.48980500-49036766.gibbon      tgaggcgggcagattgcttgagcctaggagttcaagatcagcctgtgcaacgtggtgaaa 16721
BCRP3.HUMAN.NCBI.REF                           tgaggcgggcagattgcttgagcctaggagttcaagatcagcctgtacaacatggtgaaa 9184
                                               ********************************************** **** ********

GGT2.end-endGGT1.48980500-49036766.gibbon      ccccatctctacaaaaaataaaaaattagctgggt---gtggtggtgtgtgcctgtagtc 16778
BCRP3.HUMAN.NCBI.REF                           ccccatctctacaaaaaataaaaaattagctgtgtgtggtggtggtgtgtgcctgtagtc 9244
                                               ******************************** **   **********************

GGT2.end-endGGT1.48980500-49036766.gibbon      ccagctgtttgggacgctgaggtgggaggctcatttgagcctgggtgattgagtctgcag 16838
BCRP3.HUMAN.NCBI.REF                           ccagctgtttgggacgctgaggtgggaggctcacttgagcctgggtgatcgaggctgcag 9304
                                               ********************************* *************** *** ******

GGT2.end-endGGT1.48980500-49036766.gibbon      tgagccatgatcctgagactgcactccagcttgggcagcagagtgagatgctgtctcaga 16898
BCRP3.HUMAN.NCBI.REF                           tgagccatgatcctgagactgcactccagcctgggcaacagagtgagatgctgtctcaaa 9364
                                               ****************************** ****** ******************** *

GGT2.end-endGGT1.48980500-49036766.gibbon      taaataaataaataaaaataaaataatttgaggcctaggggtctgaaattctgggatctc 16958
BCRP3.HUMAN.NCBI.REF                           taaataaataaataaaaataaaatactttgaggcctaggggtctaaaattctgagatctc 9424
                                               ************************* ****************** ******** ******

GGT2.end-endGGT1.48980500-49036766.gibbon      ctttatgcatttgagtgactgagatggtttgaagctggatccagtgctcctgagggctgc 17018
BCRP3.HUMAN.NCBI.REF                           ctttatgcatttgagtgactgagatgatctgaagctggatccagtgctcctgagggctgc 9484
                                               ************************** * *******************************

GGT2.end-endGGT1.48980500-49036766.gibbon      tctatttctggttgactgtgactcttagagtgagaaacctgcaccccacgtgcggggcat 17078
BCRP3.HUMAN.NCBI.REF                           tttatttctggttgactgtgactcctagagtaagaaacctgcaccccacatgtggggcat 9544
                                               * ********************** ****** ***************** ** *******

GGT2.end-endGGT1.48980500-49036766.gibbon      tatggcatcccctccctcagccacatgagcaggtccacagcactgctctagaccaggtgt 17138
BCRP3.HUMAN.NCBI.REF                           tatggcatcccctccctcagccacatgagtaagtcaacagcactgctctagaccaggtgt 9604
                                               ***************************** * *** ************************

GGT2.end-endGGT1.48980500-49036766.gibbon      ggtggctcacgcctatagtcccagctactcaggagactgaggcaggaggattgcttcagg 17198
BCRP3.HUMAN.NCBI.REF                           ggtggctcacacctatagtcccagctactcgggagactgaggcaggaggattgcttcagg 9664
                                               ********** ******************* *****************************
```

```
GGT2.end-endGGT1.48980500-49036766.gibbon       ccaggaatttgagaccagccagagcaata---tattaggttggtacaaaagtaattgcgg 17255
BCRP3.HUMAN.NCBI.REF                            ccaggaatttgagaccagccagagcaatatattattaggttggtacaaaagtaattgcag 9724
                                                *****************************   ************************** *

GGT2.end-endGGT1.48980500-49036766.gibbon       tttttgcctttaaaagtaatggcgaccctgtgttagcaaaataaaaagcaaaaaaaaaaa 17315
BCRP3.HUMAN.NCBI.REF                            tgtttgcctttaaaagtaatggcaaccctgtttcagcaaaataaaaagcaaaaaaaaaaa 9784
                                                * ********************* ******* * **************************

GGT2.end-endGGT1.48980500-49036766.gibbon       aaaaagaaaggaagaattaaaaaaagaatcagctgggcatggtggctcacacctctaatc 17375
BCRP3.HUMAN.NCBI.REF                            aaaaaaaaaaaaag----aaaggaagaatcagctgggcgtggtggctcacgcctctaatc 9840
                                                ***** ***  ***    ***  *************** *********** *********

GGT2.end-endGGT1.48980500-49036766.gibbon       ccagcacttttgggaggccaaggcgggcagatcatgagatcaggagatccagatcatcct 17435
BCRP3.HUMAN.NCBI.REF                            ccagcac-tttgggaggccaaggcgggcagatcatgagatcaggagatcgagaccatcct 9899
                                                ******* ***************************************** *** ******

GGT2.end-endGGT1.48980500-49036766.gibbon       ggctaacacggtgaaaccctgtttctaccaaaaatacaaaaaattagccgggcgtggtgg 17495
BCRP3.HUMAN.NCBI.REF                            ggctaacacggtgaaaccccatttctactaaaaatacaaaaaattagccgggcatggtgg 9959
                                                *******************  ******* ************************ ******

GGT2.end-endGGT1.48980500-49036766.gibbon       caggcgcctgtagtcccagctactcaggaggctgaggcaggagaatggcatgagcccagg 17555
BCRP3.HUMAN.NCBI.REF                            caggcgcctgtagtcccagctactcaggaggcggaggcaggagaatggcatgaacccagg 10019
                                                ******************************** ******************** ******

GGT2.end-endGGT1.48980500-49036766.gibbon       agatggagcttgcagtgagccgagatcatgccgctgcactccaggctgggtgacagagta 17615
BCRP3.HUMAN.NCBI.REF                            aggtggaggttgcagtgagccgagatcatgccactgcactccagcctgggtgacagagtg 10079
                                                ** ***** *********************** *********** **************

GGT2.end-endGGT1.48980500-49036766.gibbon       agactccgtttcaaaaaaaaaaaaaaaaaaaaaaagaatcactgctctgtctctcagcctcc 17675
BCRP3.HUMAN.NCBI.REF                            agactccgtctc-----aaaaaaaaaaaaaaaaagaatcactgctctgtctctcagcctcc 10134
                                                ********* **     *******************************************

GGT2.end-endGGT1.48980500-49036766.gibbon       tctttcaggattggcggtcgccttgaggggaatgctggccttgcctgtctccagccctgt 17735
BCRP3.HUMAN.NCBI.REF                            tcttccaagattggccgtcgccttgaggggaatgctggccttgcctgtctccagccctgt 10194
                                                **** ** ******* ********************************************

GGT2.end-endGGT1.48980500-49036766.gibbon       acttctctgcctcctatgcctttaagcacatgttttctatttgctgggctgtgaaatctg 17795
BCRP3.HUMAN.NCBI.REF                            acctctctgcctcctatgcctttaagcacatgttttctatttgctgggctgtgaaatctg 10254
                                                ** *********************************************************

GGT2.end-endGGT1.48980500-49036766.gibbon       ctcttcatctcatggggtttgctttataggtcactagatccttttctcttggtggtttta 17855
BCRP3.HUMAN.NCBI.REF                            ctcttcatctgatggggtttgctttataggtgactagatccttttctcttggtggtttta 10314
                                                ********** ******************** ****************************

GGT2.end-endGGT1.48980500-49036766.gibbon       gaatttgcattttcacattgactttaaatagtctgactatagtttgccacagcaaagacc 17915
BCRP3.HUMAN.NCBI.REF                            gaattcgcattttcacattgaccttaaatagtctgattatagtttgccacggcaaagacc 10374
                                                ***** **************** ************* ************* *********

GGT2.end-endGGT1.48980500-49036766.gibbon       ctttgcattgcattgtttggggatatttgagcctcctctatctggatgtctaatctcttg 17975
BCRP3.HUMAN.NCBI.REF                            ctttgcattgcattgtttggggatatttgagcctcctctatctggatgtctaatctcttg 10434
                                                ************************************************************

GGT2.end-endGGT1.48980500-49036766.gibbon       ttagatgtgagtggttatcattattatcttattaatgggctggcatgtgggctgtggttc 18035
BCRP3.HUMAN.NCBI.REF                            ttagatgtgagtagttttcattattattttattaatgggctggcatgtgggccgtggttc 10494
                                                ************ *** ********** ************************ *******

GGT2.end-endGGT1.48980500-49036766.gibbon       caggcgggctcagaggggcagctgccttgatgtctggacagcttctctttcggtttcttt 18095
BCRP3.HUMAN.NCBI.REF                            caggcaggctcagaggggcagctgcc-tgatgtctggacagcttctctttc--tgtcttt 10551
                                                ***** ******************** ************************  * *****

GGT2.end-endGGT1.48980500-49036766.gibbon       tcttacctggactctgggttgcgtgttagctgcatctgccagttctgagttttcaagggg 18155
BCRP3.HUMAN.NCBI.REF                            tcttacctggactctgggttgcttgttagctgcttctgccagttctgagttttcaagggg 10611
                                                ********************** ********** **************************

GGT2.end-endGGT1.48980500-49036766.gibbon       agaggggcccagtgatggctgttctttgaaagaaagggaagaatgtctcctgcttaacat 18215
BCRP3.HUMAN.NCBI.REF                            agaggggcccagtgatggctgttctttgaaggaaagggaagaatgtctcctgtttaacat 10671
                                                ****************************** ********************* *******

GGT2.end-endGGT1.48980500-49036766.gibbon       gtttctgtgtttccagttac----tttgttagttagagccagggtctctcgctctgttgc 18271
BCRP3.HUMAN.NCBI.REF                            gtttctatgtttccagttacttggttagttagttagagccagggtctctcgctctgttgc 10731
                                                ****** *************    ** *********************************

GGT2.end-endGGT1.48980500-49036766.gibbon       gcaggctggagtgcaatggcatgatcttggctcacagcagtctccaccttccaggcttga 18331
BCRP3.HUMAN.NCBI.REF                            gcaggctggagtgcaatggcatgatcgtggctcacagcagcctccaccttccaggctcga 10791
                                                ************************** ************* **************** **

GGT2.end-endGGT1.48980500-49036766.gibbon       gcaatgctcccacctcagcctctcaagcagctgggactgcaggtatgtgccaccatgctt 18391
BCRP3.HUMAN.NCBI.REF                            gcaatgctcccacctcagcctctcaagcagctgggactgcaggtatgtgccaccatgctt 10851
                                                ************************************************************

GGT2.end-endGGT1.48980500-49036766.gibbon       ggctacctttttaatttttttttttttaaatacagacaaggtctcactatattgcccagg 18451
BCRP3.HUMAN.NCBI.REF                            ggctgcctttttaaattttttttttttttaatacagacaaggtctcactatattgcccagg 10911
                                                **** ********* ************ ********************************

GGT2.end-endGGT1.48980500-49036766.gibbon       ctggtcttaaactcatgggctcaagtgatcctcctgcttcggcctttcaaagtgctgata 18511
BCRP3.HUMAN.NCBI.REF                            ctggtcttaaactcatgggctcaagtgatactcctgcctcggcctttcaaagtgctgata 10971
                                                ***************************** ******* **********************

GGT2.end-endGGT1.48980500-49036766.gibbon       ttacaggcagggtttcagttttttaaagctcccagcagtggtattaaactctccctttcc 18571
BCRP3.HUMAN.NCBI.REF                            tcacaggcagggtttccattttttaaagctcccagcagtggta-taaactcctcctttcc 11030
                                                * **************  ************************* *******  *******
```

```
GGT2.end-endGGT1.48980500-49036766.gibbon      agagaaagcgcaccctgtccgcatccctcatgttatcctctcctgcctctgcttagggtt 18631
BCRP3.HUMAN.NCBI.REF                           agagaaagcgcactctgtccgcatccctcatgttgtcctctcctgcctctgcttagggtt 11090
                                               ************* ******************** *************************

GGT2.end-endGGT1.48980500-49036766.gibbon      cactctgggggaaagtgccacttgagagattcctttttgtgtgtggttctgactgaccgc 18691
BCRP3.HUMAN.NCBI.REF                           cactccgggggaaagtgccacttgagagtttcctttttgtgtgtggttctgactgactgc 11150
                                               ***** ********************** **************************** **

GGT2.end-endGGT1.48980500-49036766.gibbon      tccctgctcacagatgctgcttctcagggtggggtccctgaggcctggagcatggcctct 18751
BCRP3.HUMAN.NCBI.REF                           tccctgctcacagatgctgattctcagggtggggtccctgaggcctggagtgtggcctct 11210
                                               ******************* ******************************  ********

GGT2.end-endGGT1.48980500-49036766.gibbon      gacaaccttcagggccagatgcagaatgacagcctgtgaccacacagcccctggtgggag 18811
BCRP3.HUMAN.NCBI.REF                           gacgaccttcagggccaggtgtggaatgagagcctgtggccacatggcccccggtgggag 11270
                                               *** ************** **  ****** ******** *****  ***** ********

GGT2.end-endGGT1.48980500-49036766.gibbon      acgtcctgccaccctttgcttctctgtgccactctggctgcacatttcagatccttggga 18871
BCRP3.HUMAN.NCBI.REF                           acgtcccgccgccctttgcttctctgtgccactctggctgcacagttcagagccttggga 11330
                                               ****** *** ********************************* ****** ********

GGT2.end-endGGT1.48980500-49036766.gibbon      aacgttaaccagtaggacctagaaggggatgtgaggaggggtcacccccccaggtgtgcct 18931
BCRP3.HUMAN.NCBI.REF                           aatgttaaccagtaggacctagacggggaggtgagaaggggtcacccccccaggtgtgcct 11390
                                               ** ******************** ***** ***** ************************

GGT2.end-endGGT1.48980500-49036766.gibbon      gtggtgaatcttcgtgctgagcagatgcagggagggaggcccaggtgcacacacctgtga 18991
BCRP3.HUMAN.NCBI.REF                           gtggtgagccttcgtgctgagcaggtgcagggagggaggcccaggtgcacacacctgtga 11450
                                               *******  *************** ***********************************

GGT2.end-endGGT1.48980500-49036766.gibbon      agtaggggcagctggctgggctccttgacctgctccagagcttctccttattttctggcc 19051
BCRP3.HUMAN.NCBI.REF                           agtaggggcagctggctgggctccttgacctgctccagagctt---cttattttctggcc 11507
                                               *******************************************   **************

GGT2.end-endGGT1.48980500-49036766.gibbon      acttcacctgcagaaggctcaggtggctgtggcctctagggtcccttgcagcggaaatga 19111. 19102 bp
BCRP3.HUMAN.NCBI.REF                           acttcacctgcagaaggcccaggtggctgtggcctctagggtcccttgcc---------- 11557
                                               ****************** ******************************

GGT2.end-endGGT1.48980500-49036766.gibbon      tgcctgtcctcagcccctctctaggcctgacactgtgctcagcactgtcgtgtgtgtgta 19171
BCRP3.HUMAN.NCBI.REF                           ------------------------------------------------------------ 11557

GGT2.end-endGGT1.48980500-49036766.gibbon      tatgtgcacacatgtgaatgtgtgcagacatcatgaggtgtggtccctgctcttaggcag 19231
BCRP3.HUMAN.NCBI.REF                           ------------------------------------------------------------ 11557

GGT2.end-endGGT1.48980500-49036766.gibbon      cttgccttgtggctgaaatgaaccatcacctgaatcaagggatggaaacacaaggtcaga 19291
BCRP3.HUMAN.NCBI.REF                           ------------------------------------------------------------ 11557

GGT2.end-endGGT1.48980500-49036766.gibbon      tgccatgaccttctatggagtcagatggtggctccgtgacctggcttcactccagaacta 19351
BCRP3.HUMAN.NCBI.REF                           ------------------------------------------------------------ 11557

GGT2.end-endGGT1.48980500-49036766.gibbon      cctggggtttctagttgtgcaaatcccaggtcccactcagattatgagtccagttctctg 19411
BCRP3.HUMAN.NCBI.REF                           ------------------------------------------------------------ 11557

GGT2.end-endGGT1.48980500-49036766.gibbon      caggtggaacccagggaggtgtatttttaacaagatggtactgctcattcacccagccca 19471
BCRP3.HUMAN.NCBI.REF                           ------------------------------------------------------------ 11557

GGT2.end-endGGT1.48980500-49036766.gibbon      gccagtggtctcggcatacccaggaaccgctgacatagagcttgtgctcccgataaaatg 19531
BCRP3.HUMAN.NCBI.REF                           ------------------------------------------------------------ 11557

GGT2.end-endGGT1.48980500-49036766.gibbon      ggaaacagcattcatggacgggggacatgtggcgtggtcatgacagtcacggtgttatat 19591
BCRP3.HUMAN.NCBI.REF                           ------------------------------------------------------------ 11557

GGT2.end-endGGT1.48980500-49036766.gibbon      accctgcacataacatagcacagactgtgtttgggtgacattaaggacgagctcctgcag 19651
BCRP3.HUMAN.NCBI.REF                           ------------------------------------------------------------ 11557

GGT2.end-endGGT1.48980500-49036766.gibbon      gctagccgtctggatagtgccgggtgggggtagaagttagaggggtcacagaggggcttc 19711
BCRP3.HUMAN.NCBI.REF                           ------------------------------------------------------------ 11557

GGT2.end-endGGT1.48980500-49036766.gibbon      ctcccagcgccctcatcagctgcttgatttaggccttggttctgggtccttctgggcctg 19771
BCRP3.HUMAN.NCBI.REF                           ------------------------------------------------------------ 11557

GGT2.end-endGGT1.48980500-49036766.gibbon      attctgaaccatgggactggtgtggcctgcaggctcctcccaccacaagctgttcgtggt 19831
BCRP3.HUMAN.NCBI.REF                           ------------------------------------------------------------ 11557

GGT2.end-endGGT1.48980500-49036766.gibbon      gcaggggagaacagtttccacagttccccagacagcagctgtggatgccaggcccccag 19891
BCRP3.HUMAN.NCBI.REF                           ------------------------------------------------------------ 11557

GGT2.end-endGGT1.48980500-49036766.gibbon      gagttgttactgaagttgctgctgaacagctcgcctttcactgagctccacatagcaccc 19951
BCRP3.HUMAN.NCBI.REF                           ------------------------------------------------------------ 11557

GGT2.end-endGGT1.48980500-49036766.gibbon      gtggtgatgggagccggatagacagagcctgccagcctgcacatcagctcccaactggga 20011   19990 bp
```

```
BCRP3.HUMAN.NCBI.REF                          --------------------------------------tgtcctcagctcccaactggga 11579
                                                                                       * *****************

GGT2.end-endGGT1.48980500-49036766.gibbon     ggggcagagggaggagggggtggggaccccaggcagcagggctctgggagcagtggggcc 20071
BCRP3.HUMAN.NCBI.REF                          ggggcagagggaggagggggtggagaccccaggcagcagggctctgggagcagtggggcc 11639
                                              *********************** ************************************

GGT2.end-endGGT1.48980500-49036766.gibbon     ctggattccaggggtgtccggcaggcccttccttactctacctctctcggcctctggatg 20131
BCRP3.HUMAN.NCBI.REF                          ctgggtcccaggggtgtctggcaggcccctccttactctac--gtctcggcctctggatg 11697
                                              **** * *********** ********* ************   ****************

GGT2.end-endGGT1.48980500-49036766.gibbon     gaggtgctggcctcggtcaggctctgcctctgactaagggctggagaagtggcgggcgtg 20191
BCRP3.HUMAN.NCBI.REF                          gaggtgctggctgcagtcgggctctgcctctgactaagggttggggaagtggcgggtgtg 11757
                                              ***********  * *** ********************* *** *********** ***

GGT2.end-endGGT1.48980500-49036766.gibbon     ggctgctgccccgtggggcctctgaacagaccccagggcctctgccaatcatgactcctt 20251
BCRP3.HUMAN.NCBI.REF                          ggctgctgccccgtggggcctctgaacagaccccagggcctctgccaatcatgactcctt 11817
                                              ************************************************************

GGT2.end-endGGT1.48980500-49036766.gibbon     gctttcagctggacccacaggccctgcaggacagagactggcagtgcaccatcatcgcca 20311
BCRP3.HUMAN.NCBI.REF                          cctttcagctggacccgcaggccctgcaggacagagactggcagcgcgccgtcatcgcca 11877
                                               *************** *************************** ** ** *********

GGT2.end-endGGT1.48980500-49036766.gibbon     tgaatggggtacgtgtctgtgggactctcctggtgcccacttccctcagaaggatagggt 20371
BCRP3.HUMAN.NCBI.REF                          tgaatggggtacgtgtccgtgggactctcctggcgcccacttcccccagaaggatagggt 11937
                                              ***************** *************** *********** **************

GGT2.end-endGGT1.48980500-49036766.gibbon     ggcctctgttcatttcaaatcagtcagaggtggctgagcctgaggcggcatctgagaggg 20431
BCRP3.HUMAN.NCBI.REF                          ggcctctgttcatttcaaatcagtcagaggtggctgagcctgaggcagcatctgagaggg 11997
                                              ********************************************** *************

GGT2.end-endGGT1.48980500-49036766.gibbon     agcctggctggaggagggagggcccccgaagagcagaatcaccatgcacgggaatcgcca 20491
BCRP3.HUMAN.NCBI.REF                          agcctggttggagaagggagggccccc-aagagcagaatcaccatgcacgggaatcgtca 12056
                                              ******* ***** ************* ***************************** **

GGT2.end-endGGT1.48980500-49036766.gibbon     ttcactggctgggatgcagttaccagccaggccctgagcatccctcctcaaacaaaggtc 20551
BCRP3.HUMAN.NCBI.REF                          ttcattggctggaatgcagttgccagccaggccctgagcatccctcctcaaacaaaggtc 12116
                                              **** ******* ******** **************************************

GGT2.end-endGGT1.48980500-49036766.gibbon     tcatggcaccaccaggacaggtggggcctccacttggggacctgggggctgcccgtagaa 20611
BCRP3.HUMAN.NCBI.REF                          tcatggcaccaccaggacaggtggggcctccactcagggacctgggggctgcccatagaa 12176
                                              **********************************  ****************** *****

GGT2.end-endGGT1.48980500-49036766.gibbon     atggagacccctgatttgtctttaggtaccctagaaaggcttagaccttaaaagttaatg 20671
BCRP3.HUMAN.NCBI.REF                          atggagacccctgatttgtctttaggtaccccagaaaggtttagaccttaaaag-caatg 12235
                                              ******************************* ******* **************  ****

GGT2.end-endGGT1.48980500-49036766.gibbon     acacacccaaaaaggcccaggtataaatggtaaaatgttaatatttgagattcttggctt 20731
BCRP3.HUMAN.NCBI.REF                          acacacccaaaaaggcccgggtataaatggtaaaatgttaatatttgagattcttggctt 12295
                                              ****************** *****************************************

GGT2.end-endGGT1.48980500-49036766.gibbon     tttcttatattattctgtctttcctacttaatttttaattgttactaagaaagagaaagc 20791
BCRP3.HUMAN.NCBI.REF                          tttcttacattattctgtctttccttcttaatttttaattgttact----aagagaaagc 12351
                                              ******* ***************** ********************    **********

GGT2.end-endGGT1.48980500-49036766.gibbon     tggtcacggtacacctataatctcagctactctggaggctgagccaggagaatcactgga 20851
BCRP3.HUMAN.NCBI.REF                          tggtcacagtaca-ctataatctcagctactctggaggctgagccaggagaatcactgga 12410
                                              ******* ***** **********************************************

GGT2.end-endGGT1.48980500-49036766.gibbon     gcccaagagttcgattacagcctgggcaacattgcaagatcccatatcttaaaaaaaagt 20911
BCRP3.HUMAN.NCBI.REF                          gcccaagagtttgattacagcctgggcaacattgcaagatcccatatctaaaaaaaaagc 12470
                                              *********** ************************************* *********

GGT2.end-endGGT1.48980500-49036766.gibbon     aagcaaccaagagaagcaacagggattttaggagatggttctgcagaagccagtccttta 20971
BCRP3.HUMAN.NCBI.REF                          aagcaagcaagagaagcagcggggattttaggaggtgcttctgcagaaaccagtcgttta 12530
                                              ****** *********** * ************* ** ********** ****** ****

GGT2.end-endGGT1.48980500-49036766.gibbon     caccatcttcaacaatcctggctcttgctgaagtagactaggggcttccccgaggggcga 21031
BCRP3.HUMAN.NCBI.REF                          tatcatcttcaacaatcctggctcttgctgaagtagactaggggcttccccgaggggcgg 12590
                                               * ********************************************************

GGT2.end-endGGT1.48980500-49036766.gibbon     ctccacctcatgctgagacctctgcatgccttggggggtggaaatatttgatgaaact-c 21090
BCRP3.HUMAN.NCBI.REF                          ctccacctcatgctgagacctctgcatgcctt-gggggtggaaatatttgatgagactcc 12649
                                              ******************************** ********************* *** *

GGT2.end-endGGT1.48980500-49036766.gibbon     caggggcccttgggaccttgggctatgaggaccagcaggattagaggactgtaccccttc 21150
BCRP3.HUMAN.NCBI.REF                          caggggtccttgggaccttgggctgtgaggaccagaaggattagaggactgtgccccttc 12709
                                              ****** ***************** ********** **************** *******

GGT2.end-endGGT1.48980500-49036766.gibbon     tccccactatagattgaagtaaagctctcagtcaagttcaacagcagggagttcagcttg 21210
BCRP3.HUMAN.NCBI.REF                          tccccactgtagatcgaagtaaagctctcggtcaagttcaacagcagggagttcagcttg 12769
                                              ******** ***** ************** ******************************

GGT2.end-endGGT1.48980500-49036766.gibbon     aagaggatgccgtcccgaaaacagacaggggtcttcggagtcaagattgcagtggtcacc 21270
BCRP3.HUMAN.NCBI.REF                          aagaggatgccgtcccgaaaacagacaggggtcttcggagtcaagattgctgtggtcacc 12829
                                              ************************************************** *********

GGT2.end-endGGT1.48980500-49036766.gibbon     aagtgagtggggaggggcttgggctcatgcactgagggcgcctgtcccttcagctgtttc 21330
BCRP3.HUMAN.NCBI.REF                          aagtgagtggggaggggcttgggctcacgcactgagggtgcctgtcccttcagctgtttc 12889
                                              *************************** ********** *********************

GGT2.end-endGGT1.48980500-49036766.gibbon     tgcagaaaagagcatgtgtgggtctctcctctctgtgcgtggccgctgcacggtgaggtc 21390
BCRP3.HUMAN.NCBI.REF                          tgcagaaaagagcatgtgtgggtctctcctctctgtgcatggccactgcacggtgaggtc 12949
```

```
                                                  ************************************** ***** ***************

GGT2.end-endGGT1.48980500-49036766.gibbon         aggccccagggaacacttggtgtgttcagctgcctcctgtgtttcctccaaaccagctca 21450
BCRP3.HUMAN.NCBI.REF                              aggccccagggaacac--ggcgtcttcagctacctcctgtgtttcctgcaaaccagctca 13007
                                                  ****************  ** ** ******* *************** ************

GGT2.end-endGGT1.48980500-49036766.gibbon         ggaatgtccttgccgccttgcttggaagcagtaggctggctccaggaactgcccaagtgc 21510
BCRP3.HUMAN.NCBI.REF                              ggaatgtccttgccaccttgcttggaagcagtaggctggctccaggaactgcccaagtgc 13067
                                                  ************** *********************************************

GGT2.end-endGGT1.48980500-49036766.gibbon         agggttttctgcccttgcttggaatttgtcacggtcccagtttcctgctgaatggttgta 21570
BCRP3.HUMAN.NCBI.REF                              agggttttctgcccttgcttggaattagtcacggtcccagattcctgttgaatggccata 13127
                                                  ************************** ************* ****** *******   **

GGT2.end-endGGT1.48980500-49036766.gibbon         acccctgcccctttgtcacgagtcagttgccaagagaagcctgtttgttggtttgagagc 21630
BCRP3.HUMAN.NCBI.REF                              acccctgcccctttgtcacgagtcagttgccaagagaagcctgt---ttggtttgagagc 13184
                                                  ********************************************   *************

GGT2.end-endGGT1.48980500-49036766.gibbon         agttcttgcagacacagatcacctcctctgagaattcatttgcttccccaggatggaatc 21690
BCRP3.HUMAN.NCBI.REF                              agttcatgcagacatagaccacttcctctgagaattcatttgcttccccaggatggaatc 13244
                                                  ***** ******** *** *** *************************************

GGT2.end-endGGT1.48980500-49036766.gibbon         tggctgggcccctgaccttgctggtcacgtgggccagggcctccatcagtcataccctgg 21750
BCRP3.HUMAN.NCBI.REF                              tggctgggcctctgaccttgctggtcacgtgggccggggcctccatcagtcataccctgg 13304
                                                  ********** ************************ ************************

GGT2.end-endGGT1.48980500-49036766.gibbon         agtcctatctgtgtctaaacaccactcctcacccccagctgcacggcagccacttgcata 21810
BCRP3.HUMAN.NCBI.REF                              actcctatctgtgtctaaacaccacgccccacccccaactgcacggcagccactcgcata 13364
                                                  * *********************** ** ******** **************** *****

GGT2.end-endGGT1.48980500-49036766.gibbon         gcactctgggagggctctgggcatgagcagcgaggactccatcagcagttcccccaaata 21870
BCRP3.HUMAN.NCBI.REF                              gcactctgggagggctgtgggcatgagcagcgaggactccatgagcagctcccccagata 13424
                                                  **************** ************************* ***** ******* ***

GGT2.end-endGGT1.48980500-49036766.gibbon         agccctgctaatgagggtgcttgagaagcagctttgatgtgctcctaaatccagttgcaa 21930
BCRP3.HUMAN.NCBI.REF                              agccctgctaatgagggggcttgcgaagcagctttgatgtgctggtaaatccaggtgcaa 13484
                                                  ***************** ***** *******************  ********* *****

GGT2.end-endGGT1.48980500-49036766.gibbon         aacagaactaaagttaaggcctctgcacagcactgtgttctaactttgaagtattcttac 21990
BCRP3.HUMAN.NCBI.REF                              aacagaactcaagttagggcctccgcacagcactgcgttctaactgtgaaggattcttac 13544
                                                  ********* ****** ****** *********** ********* ***** ********

GGT2.end-endGGT1.48980500-49036766.gibbon         tctagtgtcctgtgtggtggtattggaattgtccattgctaagactcagaggagaaaagc 22050
BCRP3.HUMAN.NCBI.REF                              tctagtgtcctgtgtggaggtattggaattgtccattgctaagactcagaggagaaaagc 13604
                                                  ***************** ******************************************

GGT2.end-endGGT1.48980500-49036766.gibbon         acttagcatcgcaggacttggagcactggtgctgaggcaacccttcattcattcgtcgga 22110
BCRP3.HUMAN.NCBI.REF                              acttagcatcgcaggacttggagcaccggtgctgaggcaacccttcattcattcgtcgga 13664
                                                  ************************** *********************************

GGT2.end-endGGT1.48980500-49036766.gibbon         tgtgtgttaaggcccaggcccagggcaggggtcggggattctactctcacacggcacgtg 22170
BCRP3.HUMAN.NCBI.REF                              tgtgtgtta------aggcccagggcaggggtcagggattctcctctcacacagcacgtg 13718
                                                  *********      ****************** ******** ********* *******

GGT2.end-endGGT1.48980500-49036766.gibbon         ggtggcaagaccaacaccgggtctgatctcccagctgggggcacaggctgctaaccccag 22230
BCRP3.HUMAN.NCBI.REF                              ggtggcaggaccaacaccgggtctgacctcccagccgggggcacaggctgctaaccccag 13778
                                                  ******* ****************** ******** ************************

GGT2.end-endGGT1.48980500-49036766.gibbon         gcctggaatctatcagatgcccttcctgtgctgactttacttagacaggcctccagacct 22290
BCRP3.HUMAN.NCBI.REF                              gcctggaatctgtcagatgcccttcctgtgctgacttgacttagacaggcctcctgacct 13838
                                                  *********** ************************* **************** *****

GGT2.end-endGGT1.48980500-49036766.gibbon         tcctgcaaagatcatgtgtgacttccaggggttctggccgcttgaaatg-tcctgagaaa 22349
BCRP3.HUMAN.NCBI.REF                              tcccgcaaaggtcatgtgtgattcgcaggggttctggccgcttgaaaggttcctgagaaa 13898
                                                  *** ****** ********** *  ********************** * **********

GGT2.end-endGGT1.48980500-49036766.gibbon         gtacatgcaatgaggacagagcttgcagagggaggacaggcatgcagaaggctctgtgcg 22409
BCRP3.HUMAN.NCBI.REF                              gcacatgccatgaggacagagcttgcagagggaggacaggcatgcagaaggctctgtgtg 13958
                                                  * ****** ************************************************* *

GGT2.end-endGGT1.48980500-49036766.gibbon         cagccccagacctgggcaccttcgccaccatcctcactccacatcc------------ac 22457
BCRP3.HUMAN.NCBI.REF                              cagccccagacctgggtaccttcgtcaccgtcctcaccccacctccgggtgtgcagatag 14018
                                                  **************** ******* **** ******* **** ***            * 

GGT2.end-endGGT1.48980500-49036766.gibbon         aaagcaggtctcctgtg-tgtggcccaagcagggctgtcaggacactgagaacattccct 22516
BCRP3.HUMAN.NCBI.REF                              ggagcaggcctcctgtgttatggcccaagcggggctgttaggacactgagaacattccct 14078
                                                    ****** ******** * ********** ******* *********************

GGT2.end-endGGT1.48980500-49036766.gibbon         cctcccgcaggagagagaggtccaaggtgccctacatcatgcgccagtgcgtggaggaga 22576
BCRP3.HUMAN.NCBI.REF                              cctcccgcaggagagagaggtccaaggtgccctacatcatgcgccagtgcgtggaggaga 14138
                                                  ************************************************************

GGT2.end-endGGT1.48980500-49036766.gibbon         tcgagcgccgaggcatggaggaggtgggcctctaccgcatgtccggagtggtcgtggaca 22636
BCRP3.HUMAN.NCBI.REF                              tcgagcgccgaggcatggaggaggtgggcatctaccgcgtgtccggtgtggccacggaca 14198
                                                  ***************************** ******** ******* **** *  *****

GGT2.end-endGGT1.48980500-49036766.gibbon         tccaggcactgaaggcagccttcgacgtcagtgagtgttggcctggggaggacagaacgg 22696
BCRP3.HUMAN.NCBI.REF                              tccaggcactgaaggcaggcttcaacgtcagtgagtgtcggcctgcgcaggacgggatgg 14258
                                                  ****************** **** ************** ****** * ***** * * **

GGT2.end-endGGT1.48980500-49036766.gibbon         aggtgtggtcagcggtgtccgtgatgagatctcagagcgctgcatggcccaggcatgtca 22756
BCRP3.HUMAN.NCBI.REF                              aggtgtgggcagtggtgtccgcgatgagatctcagagtgctccatggcccaggcatgtca 14318
                                                  ******** *** ******** *************** *** ******************
```

```
GGT2.end-endGGT1.48980500-49036766.gibbon       catccttctctgtgtcttttcttcatttactgtgttgttatttttaaaaaagagaagaca 22816
BCRP3.HUMAN.NCBI.REF                            catccttctctgtgtcttttcttcatttactgttttattattttaaaaaaagagaaaaca 14378
                                                ********************************* ** ******* *********** ***

GGT2.end-endGGT1.48980500-49036766.gibbon       agagttgtagaaaaagcctctgtagaagccagtttttaaaccatcctagccacgcatgcc 22876
BCRP3.HUMAN.NCBI.REF                            agagttgtacaaacagcttctatagaagccagtttttacaccatcgtacccactcatgcc 14438
                                                ********* *** *** *** **************** ****** ** **** ******

GGT2.end-endGGT1.48980500-49036766.gibbon       acttgctggggtggaccaggggcttctgccgggccttggcctttctgccttgggggtgga 22936
BCRP3.HUMAN.NCBI.REF                            acttggtggagtggaccaggggcttctgtggggacttggccttcctgccttgggggtgga 14498
                                                ***** *** ******************  *** ********* ****************

GGT2.end-endGGT1.48980500-49036766.gibbon       caggaggtggaagcccaggactcagtgcggtctgtccactgccctgtgtgaggatgcggt 22996
BCRP3.HUMAN.NCBI.REF                            caggaggtggaagcccaggactcagtgcggtctgtccactgccctgtatgaggatgtggt 14558
                                                *********************************************** ******** ***

GGT2.end-endGGT1.48980500-49036766.gibbon       gggcagagggcactggtgggacccagctcaggctggggctgcagcatctctgcctccatc 23056
BCRP3.HUMAN.NCBI.REF                            gggcagagggcactgatgaaattcagcgcaggccggggctgcagcatctccgcctccatc 14618
                                                *************** **  *  **** ***** **************** *********

GGT2.end-endGGT1.48980500-49036766.gibbon       tcaacaaccctcacaggctatgaaggacctggacctgcctcaaatgccaggggagggcac 23116
BCRP3.HUMAN.NCBI.REF                            tcaccaaccctcacaggccttgaaggacccagactggcctcaaatgccaggggagggcac 14678
                                                *** **************  *********  ***  ************************

GGT2.end-endGGT1.48980500-49036766.gibbon       tgaggccccagagggtccttcccagcatcttcaaagcaacaggattttgtgcctgcagac 23176
BCRP3.HUMAN.NCBI.REF                            tgagaccccagagggtccttcccagcatcttcaaagcaacaggattttgtgcctgcagac 14738
                                                **** *******************************************************

GGT2.end-endGGT1.48980500-49036766.gibbon       ccttctttggggcacacaccactgaccctgaccaggacacctagaattcccagcacccc- 23235
BCRP3.HUMAN.NCBI.REF                            ccttctttgcagcacacaccacccaccctgaccaggacccctagaatgcccagcatccct 14798
                                                *********  ***********  ************** ******** ******* ***

GGT2.end-endGGT1.48980500-49036766.gibbon       ----------------------------------aggcccagaatggacctggcctgtgg 23261
BCRP3.HUMAN.NCBI.REF                            gggagggccctgtggtagtttcagctccctctgggggcccagaatgaacctggcctgtgg 14858
                                                                                   *********** *************

GGT2.end-endGGT1.48980500-49036766.gibbon       tggggaggtaagtaccagtggacaattggatccaaaggaagacacaggttcaaacactga 23321
BCRP3.HUMAN.NCBI.REF                            tgaggatgtaagcaccaatggccaattgggtccaaaggaagacaccggttcaaacactga 14918
                                                ** *** ***** **** *** ******* *************** **************

GGT2.end-endGGT1.48980500-49036766.gibbon       aaccaatcagattctcacatggccttcctgctatcagaagacactggtgcaggggtggtt 23381
BCRP3.HUMAN.NCBI.REF                            aaccaatcagattctcccacggccttcctgctatcagacgacactggtgcaggggtggtt 14978
                                                **************** ** ****************** *********************

GGT2.end-endGGT1.48980500-49036766.gibbon       gctatgcacagggcagagccacccaatccccacgcaggcactgtgtcctgccacactggc 23441
BCRP3.HUMAN.NCBI.REF                            gctatgtacagggcagagccacccaatccccacgcaggcgctgtgtcctgccacgttggc 15038
                                                ****** ******************************** **************  ****

GGT2.end-endGGT1.48980500-49036766.gibbon       ctcctcccggacatcacatcgggccaaccagaggacaggaataggaatgcccacgcaccc 23501
BCRP3.HUMAN.NCBI.REF                            ctcctcctggccatcacatcaggccaagcaggggagaggaatgggaatgcccacgcaccc 15098
                                                ******* ** ********* ****** *** *** ****** *****************

GGT2.end-endGGT1.48980500-49036766.gibbon       ccatcaactctgcagacacagaaccaggcacagctcttgggaggagtcagatgagctgct 23561
BCRP3.HUMAN.NCBI.REF                            ctatcaactctgcagacacagaaccatgcacagctcttgggaggagtcagatgagctgct 15158
                                                * ************************ *********************************

GGT2.end-endGGT1.48980500-49036766.gibbon       caaagcccgggagggacccgcactgtggtcagtgtggcagggatggtgctttagccaagg 23621
BCRP3.HUMAN.NCBI.REF                            caaagcccaggagggacccgcacagtggtcagtgtggcagggacggtgctttagccaagg 15218
                                                ******** ************** ******************* ****************

GGT2.end-endGGT1.48980500-49036766.gibbon       cagggatggcaggtgactcactcgggatcctcaaggaggctgctgcatttctgtgctctt 23681
BCRP3.HUMAN.NCBI.REF                            cagggatggtgggtgactcactcaggatcttcaaggaggccgctgcatttccgtgctctt 15278
                                                *********  ************ ***** ********** ********** ********

GGT2.end-endGGT1.48980500-49036766.gibbon       tccagataacaaggacgtgttggtgatgatcagcgagatggacgtgaacgccattgcagg 23741
BCRP3.HUMAN.NCBI.REF                            tccagataacaaggacgtgtcggtgatgatgagcgagatggacgtgaacgccatcgcagg 15338
                                                ******************** ********* *********************** *****

GGT2.end-endGGT1.48980500-49036766.gibbon       cacgctgaagctgcacttccgtgagctgcccgagcccctcttcactgacgagttctaccc 23801
BCRP3.HUMAN.NCBI.REF                            cacgctgaagctgtacttccgtgagctgcccgagcccctcttcactgacgagttctaccc 15398
                                                ************* **********************************************

GGT2.end-endGGT1.48980500-49036766.gibbon       caacttcgcagagggcattggtgagcactggaggccttggcctcgtgggagacatctcct 23861
BCRP3.HUMAN.NCBI.REF                            caacttcgcagagggcatcggtgagcactggaggccttggcctcatgggagacgtctcct 15458
                                                ****************** ************************* ******** ******

GGT2.end-endGGT1.48980500-49036766.gibbon       ccacgtgcactgctgccctcggaggctgtgaaaagtgaggtgtgggaacctgagctgtgt 23921
BCRP3.HUMAN.NCBI.REF                            ccacgtgcactgctgccctcggaggctgtgaaaagcgaggtgtgggaacctgagctgtaa 15518
                                                *********************************** **********************

GGT2.end-endGGT1.48980500-49036766.gibbon       cccctctgccacggttggcgttttaacccaacctcaaaaagtgggggaccaaaactgagc 23981
BCRP3.HUMAN.NCBI.REF                            cccctctgccgtggtcggcattttaacccaacctcaaaaagcaggggaccagaaccgagc 15578
                                                **********  *** *** *********************  ******** *** ****

GGT2.end-endGGT1.48980500-49036766.gibbon       ctgtcccagaagccctcgcccatccccagagggctccccgtccctattcctcaaggagac 24041
BCRP3.HUMAN.NCBI.REF                            ctgtcctggaaggccttgcccatccccagagggctccccatccctactcctcaaggagac 15638
                                                ******  **** *** ********************** ****** *************

GGT2.end-endGGT1.48980500-49036766.gibbon       caagagggtgaaatggtcagcactgccctgctgtagggtcctaaagtctgctg---tcct 24098
BCRP3.HUMAN.NCBI.REF                            caagaggctgaaatagtcagcactgctgtgctatggggtcctaaagtctgctgtcctcct 15698
                                                ******* ****** ***********  **** * ******************   ****
```

```
GGT2.end-endGGT1.48980500-49036766.gibbon      tcctgcagaccagggctaaacaagggcattcaggtgctctagccaagggtcctggcccag 24158
BCRP3.HUMAN.NCBI.REF                           tcctgcagaccagggctgaaggagggtgcctgggtgctcttgccatgggtcctggtccag 15758
                                               ***************** **  ****      ******** **** ********* ****

GGT2.end-endGGT1.48980500-49036766.gibbon      tcaagcacaagttcaaacctgggctgaccctcagtcaacctggaggctgatgtctaaagt 24218
BCRP3.HUMAN.NCBI.REF                           ccaagcatggtttcaaacatgacctgacccttagtcaacctggaggctgatgtctagagc 15818
                                                ******    ******* **  ******** ************************ **

GGT2.end-endGGT1.48980500-49036766.gibbon      gggtg-tagtgtgtgcagcgcctgtatcctccacatcacccttacagcaggtctgcctcc 24277
BCRP3.HUMAN.NCBI.REF                           gggtgctggtgcgtgcagcacctgtggcctctgcatcacccttagggcaggtctgcctcc 15878
                                               ***** * *** ******* *****  ****  ***********  **************

GGT2.end-endGGT1.48980500-49036766.gibbon      caggcccatgcacagaggacctgctctcccagcctgcaggtgcccctgtggtgtccagga 24337
BCRP3.HUMAN.NCBI.REF                           cgggcccatgcacagaggacctggtctcccagcctgcaggtgcccctgtggtgtccagga 15938
                                               * ********************* ************************************

GGT2.end-endGGT1.48980500-49036766.gibbon      tgacgagggggtctctgcatacttggtggggctgggg-cctcccacttcccacctccttg 24396
BCRP3.HUMAN.NCBI.REF                           cgacgagggggtctctgtgtacttggtggggctgggaccctcccacttcccacctccttg 15998
                                                ****************  *****************  **********************

GGT2.end-endGGT1.48980500-49036766.gibbon      tgtccctcactcccctgtttcattccatgccgagcctcccctgccttgggctcccctggg 24456
BCRP3.HUMAN.NCBI.REF                           tgtccctcactcccctgtttcattccatgctgagcctcccctgccttgggct-ccctggg 16057
                                               ****************************** ********************* *******

GGT2.end-endGGT1.48980500-49036766.gibbon      gagggggtggtggcaggagttgcccaagggcagctctgcccatgagcagctgctctagcg 24516
BCRP3.HUMAN.NCBI.REF                           gagggggtggtggcaggagttgcccgagggcagctctgcccatgagcagctgctctagcg 16117
                                               ************************* **********************************

GGT2.end-endGGT1.48980500-49036766.gibbon      gctcctgctgctgctgttcgccgtgtgctgctgacccctgtgaggtagagaaaaggcgtt 24576
BCRP3.HUMAN.NCBI.REF                           gctcctcctgctgctgttcgccgggtgctgctgacccctgcgaggtagagaaaaggcgtt 16177
                                               ****** **************** **************** *******************

GGT2.end-endGGT1.48980500-49036766.gibbon      caggtggttcacaccccacacaggtgcccctcacacggtcctcactggtggccagcgctg 24636
BCRP3.HUMAN.NCBI.REF                           caggtggttcacaccccacacaggtgcccctcacagggtcctcactggcggccagcgctg 16237
                                               *********************************** ************ ***********

GGT2.end-endGGT1.48980500-49036766.gibbon      tgggtgtgacgatg-tgacgagcctaaactgcgcaaggactcgtgtcccgggcgctccat 24695
BCRP3.HUMAN.NCBI.REF                           tgggtgtgacgatgatgacaagcctaaactgcgcaaggactcgtgtcccgggcgctccat 16297
                                               ************** **** ****************************************

GGT2.end-endGGT1.48980500-49036766.gibbon      gtgaccacctcgggagaggtctccggcttgttgtaacccagaggagtaacccaccgcctc 24755
BCRP3.HUMAN.NCBI.REF                           gtgaccacctcgggagaggtctccggcttgtcgtaacccaggggagtgacccactgcctc 16357
                                               ******************************* ********* ***** ****** *****

GGT2.end-endGGT1.48980500-49036766.gibbon      ctgcagctctttcagacccggttgcaaagaagagctgcatgctcaacctgctactgtccc 24815
BCRP3.HUMAN.NCBI.REF                           ctgcagctctttcagacccagttgcaaggaagagctgcatgctcaacctgttgttgtccc 16417
                                               ******************* ******* ********************** *  ******

GGT2.end-endGGT1.48980500-49036766.gibbon      tgccgggggccaacctgctcaccttccttttccttctagaccacctggaaaggtagccca 24875
BCRP3.HUMAN.NCBI.REF                           tgccggaggccaacctgctcaccttccttttccttctagaccacctggaaaggtagccca 16477
                                               ****** *****************************************************

GGT2.end-endGGT1.48980500-49036766.gibbon      gctctcttgtggctgcccaggactccaggtctcctggacg-cggggtgcccctctgctcc 24934
BCRP3.HUMAN.NCBI.REF                           gctctcttgtggctgcccaggactccaggtctccaggccgttggggtgcccctctgctcc 16537
                                               ********************************** ** **  ******************

GGT2.end-endGGT1.48980500-49036766.gibbon      caccagaccccccagcaccaaggaccttttcccctgacccctgtctgcagtaactcactgc 24994
BCRP3.HUMAN.NCBI.REF                           caccagaccccccagcaccaaggaccttttcccccgacccctgtctgcagtaactcactgc 16597
                                               ********************************** *************************

GGT2.end-endGGT1.48980500-49036766.gibbon      -tctaaggactagcaccactgccaccccccgcccctgcctctcctctttgccaccctcctc 25053
BCRP3.HUMAN.NCBI.REF                           ttctaaggactagcaccactgccacccccac----------------------------c 16629
                                                **************************** *                             *

GGT2.end-endGGT1.48980500-49036766.gibbon      cctgcctctcctctttgccaccctcctccctctgcactgtggccttaacaaagagctcag 25113
BCRP3.HUMAN.NCBI.REF                           cctgcctctcctctttgccaccctcctccctctgcactgtggccttaacaaagagctcag 16689
                                               ************************************************************

GGT2.end-endGGT1.48980500-49036766.gibbon      agctttggccatggccagcagtgcacttgaacccctctcttccctcccaaccacatcatg 25173
BCRP3.HUMAN.NCBI.REF                           agctttggccgtggccagcagtgcacttggacccccctcttccctcccaagcacatcatg 16749
                                               ********** ****************** ****** ************** *********

GGT2.end-endGGT1.48980500-49036766.gibbon      aagacctccccaccagcccagagctggcccctagtcctgggccactgagacccagaagta 25233
BCRP3.HUMAN.NCBI.REF                           aagacctccccatcagcccagagctggccccttgtcctgggccactgagacccagaagta 16809
                                               ************ ******************* ***************************

GGT2.end-endGGT1.48980500-49036766.gibbon      ccagggctgaagtcagcttgcagcacagccagggtcgaggtcactccctccctgaagact 25293
BCRP3.HUMAN.NCBI.REF                           ccaaggctggagtcagcttgcagcacagccagggtcgaggtcactccctccctgaggact 16869
                                               *** ***** ********************************************** ****

GGT2.end-endGGT1.48980500-49036766.gibbon      caagcatggcacagcccctctgcctctctcctggtggtggcattgtaatgacaccctctg 25353
BCRP3.HUMAN.NCBI.REF                           ctagcacggcacagcccctctgcctctctcctggtggtggcgttgaaacagcaccctctg 16929
                                               * **** ********************************** *** **   *********

GGT2.end-endGGT1.48980500-49036766.gibbon      ctttggtcctctacagggtggcagagaaggaggcagtcaataagatgtccctgcacaacc 25413
BCRP3.HUMAN.NCBI.REF                           cttcggtcctctacagggtggcagagaaggaggcggtcaataaggtgtccctgcacaacc 16989
                                               *** ****************************** ********* ***************

GGT2.end-endGGT1.48980500-49036766.gibbon      tcgccactgtctttggccccaagctgctccagccttccgagaaggagagcaagctccctg 25473
BCRP3.HUMAN.NCBI.REF                           tcgccactgtctttggccccacgctgctccggccctccgagaaggagagcaagctccctg 17049
                                               ********************* ******** *** *************************

GGT2.end-endGGT1.48980500-49036766.gibbon      ccaatccaagccagcctatcacaatgactgacagctggtccttggaggtcacgtcccagg 25533
```

```
BCRP3.HUMAN.NCBI.REF                            ccaaccccagccagcctgtcaccatgactgacagcaggtccttggaggtcatgtctcagg 17109
                                                **** ** ********* **** ************ *************** *** ****

GGT2.end-endGGT1.48980500-49036766.gibbon       tatgggaagacaggctccagcccatgccaccccagcctgacagaggtggcctctgcctgc 25593
BCRP3.HUMAN.NCBI.REF                            tatgggaagacagtctccagcccatgcaaccccagcctgacagaggtggcctctgcctgc 17169
                                                ************* ************* ********************************

GGT2.end-endGGT1.48980500-49036766.gibbon       cccaccccag-cctgcccatcttcctacttgcatcgtatgtggtggtggctgagattca 25652
BCRP3.HUMAN.NCBI.REF                            cccaccccagtcctgcccatcttccgacttgcattgtatgtggtggtggctgagattca 17229
                                                *********** ************** ******** ************************

GGT2.end-endGGT1.48980500-49036766.gibbon       gagagagagacttgcctaggttcgcatggatgggagtgataggggatgcccagtccacct 25712
BCRP3.HUMAN.NCBI.REF                            gagagagggacttgcctaggtttgcatggatgggagtgatagggggtgcccaggccacct 17289
                                                ******* ************** ********************** ******* ******

GGT2.end-endGGT1.48980500-49036766.gibbon       cctggtcctgcggatgcaccttgctgggggcttaaaaccaccccaagtgttcgggtgtgg 25772
BCRP3.HUMAN.NCBI.REF                            cctggtcctgctggtgcaccttgctgggggcttaaaaccaccccaagtgttcgggtgtgg 17349
                                                *********** * **********************************************

GGT2.end-endGGT1.48980500-49036766.gibbon       tggctcatgcctgtaatcccagcactttgtgagaccgaggcaggacaaccgaacccaggt 25832
BCRP3.HUMAN.NCBI.REF                            tggctcatgcctgtaatcccagcactttgggaggccgaggcaggacaactgaacccaggt 17409
                                                ***************************** *** *************** **********

GGT2.end-endGGT1.48980500-49036766.gibbon       gtttgagaccagtctgggcaatgtagcaaaccccgtctctagaaaaaatacaaagaaaaa 25892
BCRP3.HUMAN.NCBI.REF                            gtttgagaccagtctgggcaatgtagcaaaccccatctctagaaaaaatacaaagaaaaa 17469
                                                ********************************** *************************

GGT2.end-endGGT1.48980500-49036766.gibbon       ttagccaggcattgtggcacacacctgtaatcctaggtatctgggaggctgacacaggag 25952
BCRP3.HUMAN.NCBI.REF                            ttagtcaggcattgtggcacacatctgtaatcctaggtatctgggaggctgacacaggag 17529
                                                **** ****************** ************************************

GGT2.end-endGGT1.48980500-49036766.gibbon       gattgcttgagcccaggagttagaggctgcagtgacctatgatggagccactgtactcca 26012
BCRP3.HUMAN.NCBI.REF                            gattgcttgagcccaggagttagaggctgcagtgatccatgatggagccactgtactcca 17589
                                                *********************************** * **********************

GGT2.end-endGGT1.48980500-49036766.gibbon       gcctgggggacagagcaaggccctgtgcatctctaaaataaacaa--ccccccacccaa 26070
BCRP3.HUMAN.NCBI.REF                            gcctgggggacagagcaaggccctgtgcatctctaaaataaataatcacccccacccaa 17649
                                                ****************************************** **   ************

GGT2.end-endGGT1.48980500-49036766.gibbon       caagtcataccttgtcaggaccccaccccacccccgtctcactgtaaggggttcatgaca 26130
BCRP3.HUMAN.NCBI.REF                            caagtcatgccttgtcaggaccccaccccacccccgtctcactgtaaggggttcatgaca 17709
                                                ******** ***************************************************

GGT2.end-endGGT1.48980500-49036766.gibbon       ccagcaggggtttctagcacctgaggtggacttgggggcttgggtcccaaagacctcccc 26190
BCRP3.HUMAN.NCBI.REF                            ccagcaggggtttctagcacctgaggtggacttgggggcttgggccccaaagacctcccc 17769
                                                ********************************************* **************

GGT2.end-endGGT1.48980500-49036766.gibbon       accagcagctgtgagctcccctctgagccactctcctcttccccactctgcccgggcagg 26250
BCRP3.HUMAN.NCBI.REF                            accagcagctgtgagccccccctctgagccactctcctcttccccactctgcgagggcagg 17829
                                                **************** **********************************  *******

GGT2.end-endGGT1.48980500-49036766.gibbon       tccaggtgctgctgtacttcctgtggctggaggccatccctgccctggacagcaagagac 26310
BCRP3.HUMAN.NCBI.REF                            acgaggtgctgctgtacttcttgcggctggaggccatccctgccctgaacagcaagagac 17889
                                                 * ***************** ** *********************** ************

GGT2.end-endGGT1.48980500-49036766.gibbon       agagcatcctgttctccaccaatgtctaaaggtcccagtccatctcctggaggcggacag 26370
BCRP3.HUMAN.NCBI.REF                            agagcatcctgttctccaccgatgtctaaaggtcccagtccatctcctggaggcggacag 17949
                                                ******************** ***************************************

GGT2.end-endGGT1.48980500-49036766.gibbon       acggcctggaaaactctggctaatcaaggccatctgtagagtgagaatcaagattttctg 26430
BCRP3.HUMAN.NCBI.REF                            atggcctggaaacctctggctaatc-gggccatctgtagagtgggaatcaagattttctg 18008
                                                * ********** ************  **************** ****************

GGT2.end-endGGT1.48980500-49036766.gibbon       aggcatccttggcccccccacaagtgtcaggccatctgccaagagacagcggcccaaag 26490
BCRP3.HUMAN.NCBI.REF                            aggcatccttggccacccc-caggtgtcaggccatctgccaagagacagcggcccaaag 18067
                                                ************** **** ** *************************************

GGT2.end-endGGT1.48980500-49036766.gibbon       cagaaggacaggtggcctgggcagatcccgcccaggtctgagagccccaggctggcctca 26550
BCRP3.HUMAN.NCBI.REF                            cagaaggacaggtggcctgggcagatcccgcccaggtctgaaagccccaggctggcctca 18127
                                                ***************************************** ******************

GGT2.end-endGGT1.48980500-49036766.gibbon       gactgtgggtttttttatgtggccacccgagggcgcccccaagccagttcatctcggagtc 26610
BCRP3.HUMAN.NCBI.REF                            gactgtgggtttttttatgtggccacccgagggcg-ccccaagccagttcatctcggagtc 18186
                                                *********************************** ************************

GGT2.end-endGGT1.48980500-49036766.gibbon       caggcctggccctgggagacagggtgaaaggagttgtttttatgaacttaacttacagag 26670
BCRP3.HUMAN.NCBI.REF                            caggcctggccctgggagacagggtgaaagcagtggtttttatgaacttaacttatagag 18246
                                                ****************************** *** ******************** ****

GGT2.end-endGGT1.48980500-49036766.gibbon       tttaaaagatttctacgggatcacgtgtcaagatgcgccctctctggggagaaggaacg 26730
BCRP3.HUMAN.NCBI.REF                            tccaaaagatttctactgaatcacttgtcaagaagcgccctctctggggagaaggaacg 18306
                                                *  ************* * ***** ******** **************************

GGT2.end-endGGT1.48980500-49036766.gibbon       tgaccggattccctcactgttgtatctggaataaacgctgctgcttcatcctgtggggcc 26790
BCRP3.HUMAN.NCBI.REF                            tgactggattccctcactgttgtatcttgaataaacgctgctgcttcatcctgtgggggc 18366
                                                **** ********************** ****************************** *

GGT2.end-endGGT1.48980500-49036766.gibbon       aagg--------cctgtgtgggtggggcctcttccatttccctgacttagaaaccacact 26842
BCRP3.HUMAN.NCBI.REF                            cgtggccctgtccctgtgtgggtggggcctcttccatttccctgacttagaaaccacact 18426
                                                   *        ************************************************

GGT2.end-endGGT1.48980500-49036766.gibbon       ccactcagaacagggttggagaggcttagtcagcactggggagcgtttggactccattct 26902
BCRP3.HUMAN.NCBI.REF                            ccacttctaacagggtttgagaggcttggtcagcactgggtagcgttttgactccattct 18486
```

```
                                               *****   ********* ********* ************ ******* ***********

GGT2.end-endGGT1.48980500-49036766.gibbon      tggctttcttctttttctttccagaaggacttttgtgcagaaatgggtcttttgttgccg 26962
BCRP3.HUMAN.NCBI.REF                           tggctttcttctttttctttccagaaggatttttgtgcagaaatgggtcttttgttgccg 18546
                                               ***************************** ******************************

GGT2.end-endGGT1.48980500-49036766.gibbon      tgtttgtcctccttggaaggcagctccaggaggcccatgaaatgtcgggggacaggaccc 27022
BCRP3.HUMAN.NCBI.REF                           tgttagtcctccttggaaggcagct-cagaaggcctgtgaaatgtcgggggacaggaccc 18605
                                               **** ******************** *** *****  ***********************

GGT2.end-endGGT1.48980500-49036766.gibbon      ccagggagggaatcccaggctacgcactttagggttcattctccagggagagcgacctcg 27082
BCRP3.HUMAN.NCBI.REF                           ccagggagggaatcccaggctacgcaccttagggttcgttctccagggagagcgacctcg 18665
                                               *************************** ********* **********************

GGT2.end-endGGT1.48980500-49036766.gibbon      tctcccgatcctgaccacccttccagcccacgctctcctgtttggcttccacaggcctgg 27142
BCRP3.HUMAN.NCBI.REF                           tcccccgatcctgaccgcccttccggcccacgctctcctgtttggcttccacaggcctgg 18725
                                               ** ************* ******* ***********************************

GGT2.end-endGGT1.48980500-49036766.gibbon      acttctctggcttctctgcccacacactccctgccccaagtgtccctgcccctg-cccag 27201
BCRP3.HUMAN.NCBI.REF                           acttctctggcttctctgcccacacactccctgcccccagtgtccctgcccctgccccag 18785
                                               ************************************* **************** *****

GGT2.end-endGGT1.48980500-49036766.gibbon      cacagctgactccatttctgtcctctcagctcagtggactcgctcaacttttgtaaaagt 27261
BCRP3.HUMAN.NCBI.REF                           cacaggtgacttcatttctgtcctctcagctcagtggactcgctcatcttttgtataagt 18845
                                               ***** ***** ********************************** ******** ****

GGT2.end-endGGT1.48980500-49036766.gibbon      ctccacttggtgatagcagcttgccgatgacttgttttaaaactttcatcctaaataacc 27321
BCRP3.HUMAN.NCBI.REF                           ctccacttggtggcagcagcttgctgatgacttgttttaaaactttcatcctaaataacc 18905
                                               ************  ********** ***********************************

GGT2.end-endGGT1.48980500-49036766.gibbon      tttgatacttgaatatttttaagttttatacgtagtttctaatttttttccccaacagat 27381
BCRP3.HUMAN.NCBI.REF                           tttgatacttgaatatttttaagttttatacatagtttctaattttttttccgaacagat 18965
                                               ******************************* ***************** ** *******

GGT2.end-endGGT1.48980500-49036766.gibbon      ccagatacctaataagatgctggaatgtaatccctggacaatccgtgtcctggcagcatt 27441
BCRP3.HUMAN.NCBI.REF                           ccagatacctaataagatgctggaatgtaatccctggacaatccgtgtcctggcagcatt 19025
                                               ************************************************************

GGT2.end-endGGT1.48980500-49036766.gibbon      tggtcttcccataagtgcctggcttcgctgttctcaggagtgggttctgaagtctctgga 27501
BCRP3.HUMAN.NCBI.REF                           tggtcttcctctaagcgcctggctccgctgttctcaggagtgggttctgaagtctctgga 19085
                                               *********  **** ******** ***********************************

GGT2.end-endGGT1.48980500-49036766.gibbon      gaacaggatacgtggagggttaggaaggggccaggcctagagacaggagactccctccca 27561
BCRP3.HUMAN.NCBI.REF                           gaacaggatacgtggagggttaggaaggggccaggcctagagacgggagactccctcccg 19145
                                               ******************************************** ************** 

GGT2.end-endGGT1.48980500-49036766.gibbon      gagcaggtggaggcacaggaccatttgctaccccatctgccggcacctgcaggggagccc 27621
BCRP3.HUMAN.NCBI.REF                           gagcaggtggaggcacaggaccattcgctaccccatctgccggcacctgcgggggagccc 19205
                                               ************************* ************************ *********

GGT2.end-endGGT1.48980500-49036766.gibbon      aggcattctttgtaaactctcctgaccacctggct-aaagaaaacagaagcatggaggcc 27680
BCRP3.HUMAN.NCBI.REF                           aggcattctttgtaagccctcctgaccacctggctcaaagaaaacagaagcatggaggcc 19265
                                               *************** * ***************** ************************

GGT2.end-endGGT1.48980500-49036766.gibbon      gccaagtattttcaagaaataaccccatgaacattgcaccactgttttagaaaaaggggc 27740
BCRP3.HUMAN.NCBI.REF                           gccaagtattttcaagaaataatcccatgaacatggcatcacttttttagaaagaggggc 19325
                                               ********************** *********** *** **** ********* ******

GGT2.end-endGGT1.48980500-49036766.gibbon      ttggggcaggcagagaaggagagaggggggcagggcagtgtaacagaggagaggggcgaa 27800
BCRP3.HUMAN.NCBI.REF                           ttggggcaggcagaggag------------------------------------------ 19343
                                               *************** **

GGT2.end-endGGT1.48980500-49036766.gibbon      ctgaggggaggagcagccgataacatgagaaattctaaaaaaaaaaagcggcatggtggc 27860
BCRP3.HUMAN.NCBI.REF                           ------------------------------------------------------------ 19343


GGT2.end-endGGT1.48980500-49036766.gibbon      tgttccagctttcggctgagcgggtcctgcggagggaggggaggaggattgacagctgg 27920
BCRP3.HUMAN.NCBI.REF                           ------------------------------------------------------------ 19343


GGT2.end-endGGT1.48980500-49036766.gibbon      caaaaaaaaaaaaaaaaaaaaaaaaaaaaaaaaaaaaaaaaaaaagacgtctacaggagga 27980
BCRP3.HUMAN.NCBI.REF                           ---------agaagggagatcaaactgagagccaagtttccagacggtcctgcaggagga 19394
                                                        * **   * *  ***   * *   **      * *     ** ********

GGT2.end-endGGT1.48980500-49036766.gibbon      gaggatgcagctgcccagagggaagcaggatcacatttaaggaagtgtgtggggtccctg 28040
BCRP3.HUMAN.NCBI.REF                           gaggatgcagctgcccagagggaagcaggatcacatttaaggaagtgtgtggggtccctg 19454
                                               ************************************************************

GGT2.end-endGGT1.48980500-49036766.gibbon      gatgacaccagcacccagtgcaac----tctggcgaccgctcccaaggtgggaggagtgg 28096
BCRP3.HUMAN.NCBI.REF                           gatgacaccagcacccagtgcggctctgtctggcaaccgctcccaaggtggcaggagtgg 19514
                                               *********************  *    ****** **************** ********

GGT2.end-endGGT1.48980500-49036766.gibbon      gtg-cccctgtgtgtcagtgggcagctcctgctgagcccacagctcactggggagcctga 28155
BCRP3.HUMAN.NCBI.REF                           gtgtcccctgtgtgtcagtgggcagctcctgctgaacccacagctcactggggagcctga 19574
                                               *** ******************************* ************************

GGT2.end-endGGT1.48980500-49036766.gibbon      cagcggggccatgcgcctgacactcctctctgcttgtggacctggtgaggcagggagcag 28215
BCRP3.HUMAN.NCBI.REF                           cagtggggccatgtgcctgacactcctctctgcttgtggacctggcaaggcagggagcag 19634
                                               *** ********* *******************************  *************

GGT2.end-endGGT1.48980500-49036766.gibbon      aaaacagagccacttgaaggctttctgtctgcgtctgtgtgcagtgtggatttagttgtg 28275
BCRP3.HUMAN.NCBI.REF                           aaaacagagctacttgaaggctttctgtctgcgtctgtgtgcagtgtggatttagttgtg 19694
                                               ********** *************************************************
```

```
GGT2.end-endGGT1.48980500-49036766.gibbon        cttttttcttgctgggagagcacagccaccatttacaagcagtgtcaccctcgtgggtgg 28335
BCRP3.HUMAN.NCBI.REF                             ctttttacttgctgggagagcacagccaccatttacaagcagtgtcaccctcgtgggtgg 19754
                                                 ****** *****************************************************

GGT2.end-endGGT1.48980500-49036766.gibbon        tgaggacagaacaggatcctctgctctctgtacctatctgggcccagtgggctcccctgt 28395
BCRP3.HUMAN.NCBI.REF                             cgaggacagaacaggagcctctgctctctgtacctatctgggcccggtgggctcccttgt 19814
                                                  *************** **************************** ********** ***

GGT2.end-endGGT1.48980500-49036766.gibbon        cctggcttccatctctgtctcagcgaccatccagccctgcgcaggaacacatgttgcttt 28455
BCRP3.HUMAN.NCBI.REF                             cctggcttccatctctgtctcagcgaccattcagccctgcacaggaacacatgttgctta 19874
                                                 ****************************** ********* ******************

GGT2.end-endGGT1.48980500-49036766.gibbon        gaaaagccaaatccagcccttgtctcggtctcctctggtctcatgatgtgcatctgttac 28515
BCRP3.HUMAN.NCBI.REF                             gaaaagccaaatccagcccttgtctctgcctcctctggtctcatgatgtgcatctgttac 19934
                                                 ************************** * *******************************

GGT2.end-endGGT1.48980500-49036766.gibbon        cttgaaactggaaaccagtctatcaatgtctgtgccaa-ttttgttccctctccaacct 28574
BCRP3.HUMAN.NCBI.REF                             cttgaaactggaaaccagtctatcaatgtctgtgccaattttttattccctccccaacct 19994
                                                 ************************************** ***** ******* *******

GGT2.end-endGGT1.48980500-49036766.gibbon        ccttccccttatgactttttatttacgtaggttgtgtgctgtctaatgatgggatgacca 28634
BCRP3.HUMAN.NCBI.REF                             ccttccccatacgactttttatttatgtaggatgtgtgctgtctaatgatgggatgacca 20054
                                                 ******** ** ************* ***** ****************************

GGT2.end-endGGT1.48980500-49036766.gibbon        cacttttccatgttctaaaagtgctcctctcccgcagggccccagggctgatggttgctt 28694
BCRP3.HUMAN.NCBI.REF                             cacttttccatgttctaaaagtgctcctctcccacagggtcccagggctggtggttgctt 20114
                                                 ********************************* ***** ********** *********

GGT2.end-endGGT1.48980500-49036766.gibbon        tgggtctacagctatgtcttacccgcctcctggctcgaaagcctgtgtggtggcaaagcc 28754
BCRP3.HUMAN.NCBI.REF                             tgggtctacagctacgtcttacccgcctcctgcctcaacagcctgtgtggtggcaaagcc 20174
                                                 ************** ***************** *** * *********************

GGT2.end-endGGT1.48980500-49036766.gibbon        ggtgcggggctggggaacgcggcgttctccaggaggggacccggctctccttctgcagt 28814
BCRP3.HUMAN.NCBI.REF                             ggtgtggggctggggaacgcagcgttctccagga-ggggacccggctctccttctgcagt 20233
                                                 **** *************** ************* *************************

GGT2.end-endGGT1.48980500-49036766.gibbon        gcaggcaaaggcctagatgccagtgtgacctcccacaaggcatggcttccagactcccca 28874
BCRP3.HUMAN.NCBI.REF                             gcaggcgaaggcctagatgccagtgtgacctcccacaaggcgtggcttccagactccccg 20293
                                                 ****** ********************************** *****************

GGT2.end-endGGT1.48980500-49036766.gibbon        accagaagtgatgctttttttgcctcgggccctgggtttgaagcagcctggctttctctcg 28934
BCRP3.HUMAN.NCBI.REF                             gccggaagtgatgctttttttgccgcgggccctgggtttgaagcagcctggctttctcttg 20353
                                                  ** ******************* ********************************** *

GGT2.end-endGGT1.48980500-49036766.gibbon        gtaagtggctggtgtcttagcagctgcaatctgagctcagccacctacacaccatcgtgg     28994
BCRP3.HUMAN.NCBI.REF                             gtaagtggctggtgtcttagcagctgcaatctgagctcagccacctacacaccaccgtg-     20412
                                                 ****************************************************** ****

GGT2.end-endGGT1.48980500-49036766.gibbon        gggaatgaactgccgacactttcattaaaaagtttcctgagacgacttgcgtgcatgttg     29054  29039bp
BCRP3.HUMAN.NCBI.REF                             -----------gccgacactttcattaaaaagtttcctgagacga---------------     20446
                                                            **********************************
```

End of human BCRP3

```
GGT2.end-endGGT1.48980500-49036766.gibbon        atttcatgatgagcgccgctgggaagaagccctgagccggtggggtggtgctggagcggc 29114
BCRP3.HUMAN.NCBI.REF                             ------------------------------------------------------------ 20446

GGT2.end-endGGT1.48980500-49036766.gibbon        aggtgcagtgatggggctgggtgcccaggaggcctccgtgctcaatcaggccacagtggc 29174
BCRP3.HUMAN.NCBI.REF                             ------------------------------------------------------------ 20446

GGT2.end-endGGT1.48980500-49036766.gibbon        caagcccaggctgcagggaaggccggcctgggggtgtgggtgagcacaggtaggcgcca 29234
BCRP3.HUMAN.NCBI.REF                             ------------------------------------------------------------ 20446

GGT2.end-endGGT1.48980500-49036766.gibbon        gctgggcagtgttaggatgctggagcagcatccctaactccactgagtggggtagtctgg 29294
BCRP3.HUMAN.NCBI.REF                             ------------------------------------------------------------ 20446

GGT2.end-endGGT1.48980500-49036766.gibbon        ttggggcagggactgctgttgctttggcagagagagatgatccccactggggagaggctg 29354
BCRP3.HUMAN.NCBI.REF                             ------------------------------------------------------------ 20446

GGT2.end-endGGT1.48980500-49036766.gibbon        ttctgactctgcaggtgggaccgggacagatggccaccagggtgacccggctggtcttcc 29414
BCRP3.HUMAN.NCBI.REF                             ------------------------------------------------------------ 20446

GGT2.end-endGGT1.48980500-49036766.gibbon        tttgctgtgctgagccctgggacatggaggattcccgccacgcacagcctgggcccgggt 29474
BCRP3.HUMAN.NCBI.REF                             ------------------------------------------------------------ 20446

GGT2.end-endGGT1.48980500-49036766.gibbon        tcttacctgtggccaccgctctggcacgagcccctcagtcttgggtggtttctgcctggt 29534
BCRP3.HUMAN.NCBI.REF                             ------------------------------------------------------------ 20446

GGT2.end-endGGT1.48980500-49036766.gibbon        ccgggattcggtgctgctgctgagtccagcctttccaccacctccgcatgggctgtgggt 29594
BCRP3.HUMAN.NCBI.REF                             ------------------------------------------------------------ 20446

GGT2.end-endGGT1.48980500-49036766.gibbon        gttgtcagctgcctcccgccttggcttcagtagctcacccagcttacagggtagctgccc 29654
BCRP3.HUMAN.NCBI.REF                             ------------------------------------------------------------ 20446
```

```
GGT2.end-endGGT1.48980500-49036766.gibbon      tgggctggagatgggcacgcaccctgggtcctacttgaatgaatgcagcttgaggagacc 29714
BCRP3.HUMAN.NCBI.REF                           ------------------------------------------------------------ 20446

GGT2.end-endGGT1.48980500-49036766.gibbon      cggccacatccactgggccacaggttaccctcggcaatgcccacatcagccatcagcctc 29774
BCRP3.HUMAN.NCBI.REF                           ------------------------------------------------------------ 20446

GGT2.end-endGGT1.48980500-49036766.gibbon      agcctccccaggagagcaaggctcacatgacaaaggctgcccgtggccggtgaggtggct 29834
BCRP3.HUMAN.NCBI.REF                           ------------------------------------------------------------ 20446

GGT2.end-endGGT1.48980500-49036766.gibbon      gagcccagccaggacttttgtcggactcccaggatgtggctctgctcgtgagctgcctgg 29894
BCRP3.HUMAN.NCBI.REF                           ------------------------------------------------------------ 20446

GGT2.end-endGGT1.48980500-49036766.gibbon      tcagctctctcggggtgagaggggcctgtcacacgggcccctgcctgcagtgtgaccctt 29954
BCRP3.HUMAN.NCBI.REF                           ------------------------------------------------------------ 20446

GGT2.end-endGGT1.48980500-49036766.gibbon      ttcagctcctctcagcagccctgcctgaggagtgtcaccaccaccatgatcatttccctg 30014
BCRP3.HUMAN.NCBI.REF                           ------------------------------------------------------------ 20446

GGT2.end-endGGT1.48980500-49036766.gibbon      acactgcgagggtgtagggacgtcctgggtagagacagggcctgtggcagcagcaggctc 30074
BCRP3.HUMAN.NCBI.REF                           ------------------------------------------------------------ 20446

GGT2.end-endGGT1.48980500-49036766.gibbon      aggggtgccctgaactggtgggctggggacctggtggagaccacaccaagggctgcacaa 30134
BCRP3.HUMAN.NCBI.REF                           ------------------------------------------------------------ 20446

GGT2.end-endGGT1.48980500-49036766.gibbon      ggggacgagcctccacccttgcctctccgcaggcctcagcagcccctcacacaggcagaa 30194
BCRP3.HUMAN.NCBI.REF                           ------------------------------------------------------------ 20446

GGT2.end-endGGT1.48980500-49036766.gibbon      gggttgacactgggtcctgccctcactgcaagagctacgagtgccacgtgctgttctgcc 30254
BCRP3.HUMAN.NCBI.REF                           ------------------------------------------------------------ 20446

GGT2.end-endGGT1.48980500-49036766.gibbon      caatctggtgtctgcaggggaggaaagggctgctgctggcccatttctgagcgttcagca 30314
BCRP3.HUMAN.NCBI.REF                           ------------------------------------------------------------ 20446

GGT2.end-endGGT1.48980500-49036766.gibbon      cctaagggtgacagcactgtctgtccctaccctccgggtcctgtttgaaaatcaaaccca 30374
BCRP3.HUMAN.NCBI.REF                           ------------------------------------------------------------ 20446

GGT2.end-endGGT1.48980500-49036766.gibbon      tgctcacgggcctatcttttgttcttttagagacagggtctcactttgtcacccaagctg 30434
BCRP3.HUMAN.NCBI.REF                           ------------------------------------------------------------ 20446

GGT2.end-endGGT1.48980500-49036766.gibbon      gagtgcagtggtgcgattatagctcaatgcagcctccaatccccggactcaagggaccct 30494
BCRP3.HUMAN.NCBI.REF                           ------------------------------------------------------------ 20446

GGT2.end-endGGT1.48980500-49036766.gibbon      cctgcctcagcctgccaagtagcttggactatagctgggtgccattgcacctgttttatt 30554
BCRP3.HUMAN.NCBI.REF                           ------------------------------------------------------------ 20446

GGT2.end-endGGT1.48980500-49036766.gibbon      attattttgtagacatggggtctggctatgttgtccaggctattctcaaaattcccggcc 30614
BCRP3.HUMAN.NCBI.REF                           ------------------------------------------------------------ 20446

GGT2.end-endGGT1.48980500-49036766.gibbon      tcaagcaattctcctgcctcggcctctcaaatgttgggattacaggtgtgaggcaaggca 30674
BCRP3.HUMAN.NCBI.REF                           ------------------------------------------------------------ 20446

GGT2.end-endGGT1.48980500-49036766.gibbon      cccagctcagccacagagccctactgcatctctcttactaggagcaagagccgactgccc 30734
BCRP3.HUMAN.NCBI.REF                           ------------------------------------------------------------ 20446

GGT2.end-endGGT1.48980500-49036766.gibbon      cctcctccccattccagagtgttggggctctgttcagccgaggctgggccactggcatgg 30794
BCRP3.HUMAN.NCBI.REF                           ------------------------------------------------------------ 20446

GGT2.end-endGGT1.48980500-49036766.gibbon      cccagggagtgggatcattcactgctgccccaaatctgagatcattccacctcgacaaga 30854
BCRP3.HUMAN.NCBI.REF                           ------------------------------------------------------------ 20446

GGT2.end-endGGT1.48980500-49036766.gibbon      ctccctcatccaatccctttacttgacagctggggaaaccaatgtgcacagagcaccccc 30914
BCRP3.HUMAN.NCBI.REF                           ------------------------------------------------------------ 20446

GGT2.end-endGGT1.48980500-49036766.gibbon      agctcactcggggtctcagagctgatccgtgagcagaggctgagatcctgggatcttgtc 30974
BCRP3.HUMAN.NCBI.REF                           ------------------------------------------------------------ 20446

GGT2.end-endGGT1.48980500-49036766.gibbon      ccccagctgccccgcaagcttgctccctttctgctggaagagatggggccggacctcgac 31034
BCRP3.HUMAN.NCBI.REF                           ------------------------------------------------------------ 20446
```

```
GGT2.end-endGGT1.48980500-49036766.gibbon      cggcagccctggcctggacatgactgtgctcgtgcaggtattcaggcccagatgccccgg 31094
BCRP3.HUMAN.NCBI.REF                           ------------------------------------------------------------ 20446

GGT2.end-endGGT1.48980500-49036766.gibbon      catcatttttttttctttttttcttttttttttttttgagacagtgtctcactctgtcgc 31154
BCRP3.HUMAN.NCBI.REF                           ------------------------------------------------------------ 20446

GGT2.end-endGGT1.48980500-49036766.gibbon      ccaagctggagtgcagtggcatgatattggctcactgcaacctctgcctcctggttaaag 31214
BCRP3.HUMAN.NCBI.REF                           ------------------------------------------------------------ 20446

GGT2.end-endGGT1.48980500-49036766.gibbon      tgattctcctgcctcagccttccaagtagctgggactataggcttgtaccaccacgcctg 31274
BCRP3.HUMAN.NCBI.REF                           ------------------------------------------------------------ 20446

GGT2.end-endGGT1.48980500-49036766.gibbon      actaattattgtatttttactagagacggggtttccccatgttggccaggctcgtgtcaa 31334
BCRP3.HUMAN.NCBI.REF                           ------------------------------------------------------------ 20446

GGT2.end-endGGT1.48980500-49036766.gibbon      actcctgacttcaggtgatccacctgccttggcctcccaaagtgctgggattacaggcgt 31394
BCRP3.HUMAN.NCBI.REF                           ------------------------------------------------------------ 20446

GGT2.end-endGGT1.48980500-49036766.gibbon      gagccacggtgtcatttaaatgtagtgagaggccgggcacggtggctcctgcctgtaatc 31454
BCRP3.HUMAN.NCBI.REF                           ------------------------------------------------------------ 20446

GGT2.end-endGGT1.48980500-49036766.gibbon      ccagtactttgagaggacgaggctgtcagatcacctaagttcaggagttcgagaccagcc 31514
BCRP3.HUMAN.NCBI.REF                           ------------------------------------------------------------ 20446

GGT2.end-endGGT1.48980500-49036766.gibbon      tggccaacatggtgaaaccgtgtctctacaaaaaaatagaaaaaaaatccctgcgtggtg 31574
BCRP3.HUMAN.NCBI.REF                           ------------------------------------------------------------ 20446

GGT2.end-endGGT1.48980500-49036766.gibbon      gtgcgtacctgtagtcccagttactcaggaggctgaggcatgagaattgcttaatcctca 31634
BCRP3.HUMAN.NCBI.REF                           ------------------------------------------------------------ 20446

GGT2.end-endGGT1.48980500-49036766.gibbon      gaggcggaggctgcagtgagctgagatggcgccactgcactccagcctgggtgacagagc 31694
BCRP3.HUMAN.NCBI.REF                           ------------------------------------------------------------ 20446

GGT2.end-endGGT1.48980500-49036766.gibbon      aagactttgtctctaaataattaaataaataaatatggccgagcacggtgccttaggcct 31754
BCRP3.HUMAN.NCBI.REF                           ------------------------------------------------------------ 20446

GGT2.end-endGGT1.48980500-49036766.gibbon      gtaatcccaacactttgggaggctgagggaggtggttcatgaggtcagcagtccgagacc 31814
BCRP3.HUMAN.NCBI.REF                           ------------------------------------------------------------ 20446

GGT2.end-endGGT1.48980500-49036766.gibbon      agcctggccaagacggtgaaacactgtctctactaaaaatacaaaaattagccagctgtg 31874
BCRP3.HUMAN.NCBI.REF                           ------------------------------------------------------------ 20446

GGT2.end-endGGT1.48980500-49036766.gibbon      gtggcaggcacctgtaatcccagctactcgggacactgaggcaggagaatcgcttgaacc 31934
BCRP3.HUMAN.NCBI.REF                           ------------------------------------------------------------ 20446

GGT2.end-endGGT1.48980500-49036766.gibbon      tggaaggcagaggttgcagtgagccgagatgtaccactgcactctagcctgggtgatgga 31994
BCRP3.HUMAN.NCBI.REF                           ------------------------------------------------------------ 20446

GGT2.end-endGGT1.48980500-49036766.gibbon      gcaagactccatctcaaataaataaattaataaatacagagcaagattccatcgcaaata 32054
BCRP3.HUMAN.NCBI.REF                           ------------------------------------------------------------ 20446

GGT2.end-endGGT1.48980500-49036766.gibbon      aataaataaatgtacacctgtaatcctagcactttgggaggctaagacaggtcgatcacc 32114
BCRP3.HUMAN.NCBI.REF                           ------------------------------------------------------------ 20446

GGT2.end-endGGT1.48980500-49036766.gibbon      tgacgtcaggagttcgagaccagcccgaccgatatggcgaaaccccatctctactaaaaa 32174
BCRP3.HUMAN.NCBI.REF                           ------------------------------------------------------------ 20446

GGT2.end-endGGT1.48980500-49036766.gibbon      tacaaaaattagccgagcattttgacgtgtgcctgtcgtcccagatacttgggaggctga 32234
BCRP3.HUMAN.NCBI.REF                           ------------------------------------------------------------ 20446

GGT2.end-endGGT1.48980500-49036766.gibbon      gacaggagaattgcttgaacccaggaggtggaggttgcagtgagccgagatctcggctga 32294
BCRP3.HUMAN.NCBI.REF                           ------------------------------------------------------------ 20446

GGT2.end-endGGT1.48980500-49036766.gibbon      ggcaggagaattgcttgaacccaggaggcggaggtagcagtgagccaagatcacaccatt 32354
BCRP3.HUMAN.NCBI.REF                           ------------------------------------------------------------ 20446

GGT2.end-endGGT1.48980500-49036766.gibbon      gcgctccaccctggcggcaagagtgagactgtctcaaaaaacaaaaacaaacaaacaaac 32414
BCRP3.HUMAN.NCBI.REF                           ------------------------------------------------------------ 20446
```

```
GGT2.end-endGGT1.48980500-49036766.gibbon      aaaaaaaaacatacctgaaaataataaaagctgatacgacaaagccatagctaacctact 32474
BCRP3.HUMAN.NCBI.REF                           ------------------------------------------------------------ 20446

GGT2.end-endGGT1.48980500-49036766.gibbon      atagaatggggaaaagttgaaagcatttcctctgtaaacaggaacaagacaggatgcccg 32534
BCRP3.HUMAN.NCBI.REF                           ------------------------------------------------------------ 20446

GGT2.end-endGGT1.48980500-49036766.gibbon      ttctcaccactcctattcgacatcacacaatcaggcaagagaaaacaataaaaggcatcc 32594
BCRP3.HUMAN.NCBI.REF                           ------------------------------------------------------------ 20446

GGT2.end-endGGT1.48980500-49036766.gibbon      acactggaaaagaggacatcgaattcttcttgtctgatgaagatgtgatcttggatctag 32654
BCRP3.HUMAN.NCBI.REF                           ------------------------------------------------------------ 20446

GGT2.end-endGGT1.48980500-49036766.gibbon      aagcatgtaaaggctccaccagaaaagccctagacttgataaataaattaatacagtcag 32714
BCRP3.HUMAN.NCBI.REF                           ------------------------------------------------------------ 20446

GGT2.end-endGGT1.48980500-49036766.gibbon      ttgcaggatacagaatcaacaacaacaacaacaaaatcagcagcatttctatacaccaat 32774
BCRP3.HUMAN.NCBI.REF                           ------------------------------------------------------------ 20446

GGT2.end-endGGT1.48980500-49036766.gibbon      aatggtctggttgggaaagaaattaagaaggcaatcccatttacaacagcctctgcctcc 32834
BCRP3.HUMAN.NCBI.REF                           ------------------------------------------------------------ 20446

GGT2.end-endGGT1.48980500-49036766.gibbon      caaagtgccaccgcgcctggcctttttttttaagacagagtctcagccgggcgccgtggc 32894
BCRP3.HUMAN.NCBI.REF                           ------------------------------------------------------------ 20446

GGT2.end-endGGT1.48980500-49036766.gibbon      tcacgccattaatcccagcactttggtaggccgaggcgggctgatgacgaggtcaggtga 32954
BCRP3.HUMAN.NCBI.REF                           ------------------------------------------------------------ 20446

GGT2.end-endGGT1.48980500-49036766.gibbon      tcgagaccatcctggctaacacggtgaaaccccgtctgtactaaaaatacaaaaaattag 33014
BCRP3.HUMAN.NCBI.REF                           ------------------------------------------------------------ 20446

GGT2.end-endGGT1.48980500-49036766.gibbon      ccaggtgtggtggcgggcgcctgtagtcccagccactccagagtctgaggcaggagaatg 33074
BCRP3.HUMAN.NCBI.REF                           ------------------------------------------------------------ 20446

GGT2.end-endGGT1.48980500-49036766.gibbon      gcgtgaacccagtaggcggagcttgcggtgagccgagatcaggccactggaaatccagcc 33134
BCRP3.HUMAN.NCBI.REF                           ------------------------------------------------------------ 20446

GGT2.end-endGGT1.48980500-49036766.gibbon      tgggcgacagagggagactccgtctaaaaaaaaaaaaaaaaaaaaaaaaaatcagagtct 33194
BCRP3.HUMAN.NCBI.REF                           ------------------------------------------------------------ 20446

GGT2.end-endGGT1.48980500-49036766.gibbon      ggctgtgttgcccaggctggagtgcagtggcgcgatctcggcgcatcaccatccctgccc 33254
BCRP3.HUMAN.NCBI.REF                           ------------------------------------------------------------ 20446

GGT2.end-endGGT1.48980500-49036766.gibbon      cgcctccgggttcaagtgattctcctgtctcagccgcccgagtagctggtactacaggcg 33314
BCRP3.HUMAN.NCBI.REF                           ------------------------------------------------------------ 20446

GGT2.end-endGGT1.48980500-49036766.gibbon      cgtgccaccatgtctcactaaatttgtatttttactagagacggggtttcactatgttgg 33374
BCRP3.HUMAN.NCBI.REF                           ------------------------------------------------------------ 20446

GGT2.end-endGGT1.48980500-49036766.gibbon      ccaggcttttctccaactcctgatctcctgatccgcccaccccgacctcccaaagtgctg 33434
BCRP3.HUMAN.NCBI.REF                           ------------------------------------------------------------ 20446

GGT2.end-endGGT1.48980500-49036766.gibbon      ggatgcgtgagccccccacacctggccactatttttctttctttcttttgtgtgtgtgtg 33494
BCRP3.HUMAN.NCBI.REF                           ------------------------------------------------------------ 20446

GGT2.end-endGGT1.48980500-49036766.gibbon      tgtgtgcgtttgtgtgtgtgtgtgcgtttgtgtgtgtgtgtgagacgaagtttcgctc 33554
BCRP3.HUMAN.NCBI.REF                           ------------------------------------------------------------ 20446

GGT2.end-endGGT1.48980500-49036766.gibbon      ttgttgcccagattggagtgcaatggtgctatctcagctcactgcaatcccggcctgagc 33614
BCRP3.HUMAN.NCBI.REF                           ------------------------------------------------------------ 20446

GGT2.end-endGGT1.48980500-49036766.gibbon      aggagagcaggaatcttcagcgatccactggcggatctgcagccattgtaagcgcttagt 33674
BCRP3.HUMAN.NCBI.REF                           ------------------------------------------------------------ 20446

GGT2.end-endGGT1.48980500-49036766.gibbon      cttcccatatcttttgcgcgcgtgcctctccttccagtacctatcccgcaagggtccccc 33734
BCRP3.HUMAN.NCBI.REF                           ------------------------------------------------------------ 20446

GGT2.end-endGGT1.48980500-49036766.gibbon      agcctcccccctatcgccagcaggtgctgagagcgcgccattgcactccagcctgggggac 33794
BCRP3.HUMAN.NCBI.REF                           ------------------------------------------------------------ 20446

GGT2.end-endGGT1.48980500-49036766.gibbon      cagagcgaaactctatcttaaaaaaaaaggacgaaaattttggaaaaatatggaagaaac 33854
```

```
BCRP3.HUMAN.NCBI.REF                            ------------------------------------------------------------ 20446

GGT2.end-endGGT1.48980500-49036766.gibbon       caaatggattttagctcaactaactcgtaattattcagtgtctatttttgcaagaaac 33914
BCRP3.HUMAN.NCBI.REF                            ------------------------------------------------------------ 20446

GGT2.end-endGGT1.48980500-49036766.gibbon       catatatttcatgtctacaatcagggccagagtcccagctctcaagtgtgggtgtttcct 33974
BCRP3.HUMAN.NCBI.REF                            ------------------------------------------------------------ 20446

GGT2.end-endGGT1.48980500-49036766.gibbon       aagcaaattgaagaacacaggcataaaagtgcattgaattaataaaattttctccctgt 34034
BCRP3.HUMAN.NCBI.REF                            ------------------------------------------------------------ 20446

GGT2.end-endGGT1.48980500-49036766.gibbon       ctctcgctctcttatttatttatttatttatttatttatttatttattttttttgaga 34094
BCRP3.HUMAN.NCBI.REF                            ------------------------------------------------------------ 20446

GGT2.end-endGGT1.48980500-49036766.gibbon       cggaggttcgcactgtcacccaggctggagtgcagtggcgagatctcaggtcatgccacc 34154
BCRP3.HUMAN.NCBI.REF                            ------------------------------------------------------------ 20446

GGT2.end-endGGT1.48980500-49036766.gibbon       gcgcccggcctttttctcggccgggcgcggtggctcacgcctttaatcccagcactttg 34214
BCRP3.HUMAN.NCBI.REF                            ------------------------------------------------------------ 20446

GGT2.end-endGGT1.48980500-49036766.gibbon       gtaggccgaggcgagctgatcacgaggtcaggagatcgacaccatcctggctaacacggt 34274
BCRP3.HUMAN.NCBI.REF                            ------------------------------------------------------------ 20446

GGT2.end-endGGT1.48980500-49036766.gibbon       gaaacccctctctactaaaaatagaaaaaattagccgggtgtggtcgcgagcgcctgta 34334
BCRP3.HUMAN.NCBI.REF                            ------------------------------------------------------------ 20446

GGT2.end-endGGT1.48980500-49036766.gibbon       gtcccagctactccagaggctgaggcaggagaatggcgtgaaccctctaggcggagcttg 34394
BCRP3.HUMAN.NCBI.REF                            ------------------------------------------------------------ 20446

GGT2.end-endGGT1.48980500-49036766.gibbon       cggtgagccgagatcaggccactggaatccagcctgggcgacagagggagactccgtctc 34454
BCRP3.HUMAN.NCBI.REF                            ------------------------------------------------------------ 20446

GGT2.end-endGGT1.48980500-49036766.gibbon       aaaaaaaaaaaaaaaaaaaaaaaaagacagagtctggctccgttgcccaggctggagtgc 34514
BCRP3.HUMAN.NCBI.REF                            ------------------------------------------------------------ 20446

GGT2.end-endGGT1.48980500-49036766.gibbon       agtgacgcgatctcggcgcatcacaatccctgccccgaccccgggttcaagtcattgtgc 34574
BCRP3.HUMAN.NCBI.REF                            ------------------------------------------------------------ 20446

GGT2.end-endGGT1.48980500-49036766.gibbon       tgtctcagccgcccgagtagctggtactacacgcgcgtgccaccatgtctgactaaattt 34634
BCRP3.HUMAN.NCBI.REF                            ------------------------------------------------------------ 20446

GGT2.end-endGGT1.48980500-49036766.gibbon       gtatttttactagagacggggtttcactatgttggccaggctggtctccaattcctgatc 34694
BCRP3.HUMAN.NCBI.REF                            ------------------------------------------------------------ 20446

GGT2.end-endGGT1.48980500-49036766.gibbon       tcctgatccgcccgccccgacatcccaaagtgctgggatgcatgagcccccacacctggc 34754
BCRP3.HUMAN.NCBI.REF                            ------------------------------------------------------------ 20446

GGT2.end-endGGT1.48980500-49036766.gibbon       cactatttttctttctttcttttgtgtgtgtgtgtgcctgtgacgaagtttcgctct 34814
BCRP3.HUMAN.NCBI.REF                            ------------------------------------------------------------ 20446

GGT2.end-endGGT1.48980500-49036766.gibbon       tgttgcccaggttggagtgcaatggtgccatctcagctcactgcaatccccgcctgagca 34874
BCRP3.HUMAN.NCBI.REF                            ------------------------------------------------------------ 20446

GGT2.end-endGGT1.48980500-49036766.gibbon       gtagagcaggaatcttcagcgatccactgggggatctccagccattgtgcgcgcctggtc 34934
BCRP3.HUMAN.NCBI.REF                            ------------------------------------------------------------ 20446

GGT2.end-endGGT1.48980500-49036766.gibbon       ttcccattgctttgtaagagcgcctctccttccagtacctatcccgcaagcgtcccccag 34994
BCRP3.HUMAN.NCBI.REF                            ------------------------------------------------------------ 20446

GGT2.end-endGGT1.48980500-49036766.gibbon       cctccccccatcgccagcaggtgctgagatcccgccattgcactccaccctgggggacaa 35054
BCRP3.HUMAN.NCBI.REF                            ------------------------------------------------------------ 20446

GGT2.end-endGGT1.48980500-49036766.gibbon       gagcgaaactccatctcaaaaagaaaaaaaaaaggatgaaaattttgggaaaatatggaa 35114
BCRP3.HUMAN.NCBI.REF                            ------------------------------------------------------------ 20446

GGT2.end-endGGT1.48980500-49036766.gibbon       gaaaccaaatggatttctagctcaactaaatcgtaattattcactgtcaattttttgcaag 35174
BCRP3.HUMAN.NCBI.REF                            ------------------------------------------------------------ 20446

GGT2.end-endGGT1.48980500-49036766.gibbon       aaaccatacatttcatgtccacaatcagggccacagtcccagctctcaagtgtgggtgtt 35234
BCRP3.HUMAN.NCBI.REF                            ------------------------------------------------------------ 20446
```

```
GGT2.end-endGGT1.48980500-49036766.gibbon      tcctaaggaaattgaagaacacaggcataaaagtgcattaaattaataaaccttttctc 35294
BCRP3.HUMAN.NCBI.REF                           ------------------------------------------------------------ 20446

GGT2.end-endGGT1.48980500-49036766.gibbon      tctgtctctctctctctcttttttttttttgagacggaggttggcactgtcacccaggc 35354
BCRP3.HUMAN.NCBI.REF                           ------------------------------------------------------------ 20446

GGT2.end-endGGT1.48980500-49036766.gibbon      tggagtgcagtggcgagacatccggtcactgcaggctctgcctcccgagttcacgccact 35414
BCRP3.HUMAN.NCBI.REF                           ------------------------------------------------------------ 20446

GGT2.end-endGGT1.48980500-49036766.gibbon      ctcctgcctcagcctccggagtagctgagactacaggcgcctgccaccacgcccggctaa 35474
BCRP3.HUMAN.NCBI.REF                           ------------------------------------------------------------ 20446

GGT2.end-endGGT1.48980500-49036766.gibbon      tttttgtagttttagaagacggggtttcgctatgttggccaggctggtctccaactcct 35534
BCRP3.HUMAN.NCBI.REF                           ------------------------------------------------------------ 20446

GGT2.end-endGGT1.48980500-49036766.gibbon      gacctcgtgatccgccctcctctgccattcaaactgccaccgcgcccggcctttattttt 35594
BCRP3.HUMAN.NCBI.REF                           ------------------------------------------------------------ 20446

GGT2.end-endGGT1.48980500-49036766.gibbon      aggacacagtctctgccgggcgcggtggctcacgcctttaatctcagcactttggtaggc 35654
BCRP3.HUMAN.NCBI.REF                           ------------------------------------------------------------ 20446

GGT2.end-endGGT1.48980500-49036766.gibbon      cgaggcgggctgatgacgaggtcaggtgatcgagaccatcctggctaacacggtgaaacc 35714
BCRP3.HUMAN.NCBI.REF                           ------------------------------------------------------------ 20446

GGT2.end-endGGT1.48980500-49036766.gibbon      ccgtctgtactaaaaacacaaaaaattagccaggtgtggtggcgggcgcctgtagtccca 35774
BCRP3.HUMAN.NCBI.REF                           ------------------------------------------------------------ 20446

GGT2.end-endGGT1.48980500-49036766.gibbon      gctactccagagtctgaggcaggagaatggcgtgaacccagtaggcggagcttgcggtga 35834
BCRP3.HUMAN.NCBI.REF                           ------------------------------------------------------------ 20446

GGT2.end-endGGT1.48980500-49036766.gibbon      gccgagatcaggccactggaaatccagcctgggcgacagagggagactccgtctaaaaaa 35894
BCRP3.HUMAN.NCBI.REF                           ------------------------------------------------------------ 20446

GGT2.end-endGGT1.48980500-49036766.gibbon      aaaaaaaaaaaagaaaaaaaaaatcagagtctggctgtgttgcccaggctggagtgcagt 35954
BCRP3.HUMAN.NCBI.REF                           ------------------------------------------------------------ 20446

GGT2.end-endGGT1.48980500-49036766.gibbon      ggcgcgatctcggcgcatcaccatccctgccccgcctccgggttcaagtgattctcctgt 36014
BCRP3.HUMAN.NCBI.REF                           ------------------------------------------------------------ 20446

GGT2.end-endGGT1.48980500-49036766.gibbon      ctcagccgcccgagtagctggtactacaggcgcgtgccaccatgtctgactaaatttgta 36074
BCRP3.HUMAN.NCBI.REF                           ------------------------------------------------------------ 20446

GGT2.end-endGGT1.48980500-49036766.gibbon      tttttactagagacggggtttcactatgttggccaggcttttctccaactcctgatctcc 36134
BCRP3.HUMAN.NCBI.REF                           ------------------------------------------------------------ 20446

GGT2.end-endGGT1.48980500-49036766.gibbon      tgatccgcccaccccgacctcccaaagtgctgggatgcgtgagcccccacacctggccac 36194
BCRP3.HUMAN.NCBI.REF                           ------------------------------------------------------------ 20446

GGT2.end-endGGT1.48980500-49036766.gibbon      tatttttctttctttcttttgtgtgtgtgtgtgtgtgcgcgtttgtgtgtgtgtgtg 36254
BCRP3.HUMAN.NCBI.REF                           ------------------------------------------------------------ 20446

GGT2.end-endGGT1.48980500-49036766.gibbon      tttgtgtgtgtgtgtgtgacgaagtttcgctcttgttgcccagattggagtgcaatggtg 36314
BCRP3.HUMAN.NCBI.REF                           ------------------------------------------------------------ 20446

GGT2.end-endGGT1.48980500-49036766.gibbon      ctatctcagctcactgcaatcccggcctgagcaggagagcaggaatcttcagcgatccac 36374
BCRP3.HUMAN.NCBI.REF                           ------------------------------------------------------------ 20446

GGT2.end-endGGT1.48980500-49036766.gibbon      tggcggatctgcagccattgtaagcgcttagtcttcccatatcttttgcgcgcgtgcctc 36434
BCRP3.HUMAN.NCBI.REF                           ------------------------------------------------------------ 20446

GGT2.end-endGGT1.48980500-49036766.gibbon      tccttccagtacctatcccgcaagggtcccccagcctcccccctatcgccagcaggtgctg 36494
BCRP3.HUMAN.NCBI.REF                           ------------------------------------------------------------ 20446

GGT2.end-endGGT1.48980500-49036766.gibbon      agatcgcgccattgcactccagcctgggggaccagagcgaaactctatcttaaaaaaaaa 36554
BCRP3.HUMAN.NCBI.REF                           ------------------------------------------------------------ 20446

GGT2.end-endGGT1.48980500-49036766.gibbon      ggacgaaaattttggaaaaatatggaagaaaccaaatggattttagctcaactaactcg 36614
BCRP3.HUMAN.NCBI.REF                           ------------------------------------------------------------ 20446
```

```
GGT2.end-endGGT1.48980500-49036766.gibbon      taattattcagtgtctattttttgcaagaaaccacatatttcatgtctacaatcagggcc 36674
BCRP3.HUMAN.NCBI.REF                           ------------------------------------------------------------ 20446

GGT2.end-endGGT1.48980500-49036766.gibbon      agagtcccagctctcaagtgtgggtgtttcctaagcaaattgaagaacacaggcataaaa 36734
BCRP3.HUMAN.NCBI.REF                           ------------------------------------------------------------ 20446

GGT2.end-endGGT1.48980500-49036766.gibbon      gtgcattgaattaataaaatttttctccctgtctctcgctctcttatttatttatttatt 36794
BCRP3.HUMAN.NCBI.REF                           ------------------------------------------------------------ 20446

GGT2.end-endGGT1.48980500-49036766.gibbon      tatttatttttgagacggaggttcgcactgtcacccaggctggagtgcagtggcgagat 36854
BCRP3.HUMAN.NCBI.REF                           ------------------------------------------------------------ 20446

GGT2.end-endGGT1.48980500-49036766.gibbon      ctcaggtcatgccaccgcgcccggcctttttttctcggccgggcgcggtggctcacgcctt 36914
BCRP3.HUMAN.NCBI.REF                           ------------------------------------------------------------ 20446

GGT2.end-endGGT1.48980500-49036766.gibbon      taatcccagcactttggtaggccgaggcgagctgatcacgaggtcaggagatcgacacca 36974
BCRP3.HUMAN.NCBI.REF                           ------------------------------------------------------------ 20446

GGT2.end-endGGT1.48980500-49036766.gibbon      tcctggctaacacggtgaaacccctctctactaaaaatagaaaaaattagccgggtgtg 37034
BCRP3.HUMAN.NCBI.REF                           ------------------------------------------------------------ 20446

GGT2.end-endGGT1.48980500-49036766.gibbon      gtcgcgagcgcctgtagtcccagctactccagaggctgaggcaggagaatggcgtgaacc 37094
BCRP3.HUMAN.NCBI.REF                           ------------------------------------------------------------ 20446

GGT2.end-endGGT1.48980500-49036766.gibbon      cgctaggcggagcttgcggtgagccgagatcaggccactggaatccagcctgggcgacag 37154
BCRP3.HUMAN.NCBI.REF                           ------------------------------------------------------------ 20446

GGT2.end-endGGT1.48980500-49036766.gibbon      agggagactccgtctcaaaaaaaaaaagacagagtctggctccgttgcccaggctggagt 37214
BCRP3.HUMAN.NCBI.REF                           ------------------------------------------------------------ 20446

GGT2.end-endGGT1.48980500-49036766.gibbon      gcagtgacgcgatctcggcgcatcgcaatccctgccccgaccccgggttcaagtcattgt 37274
BCRP3.HUMAN.NCBI.REF                           ------------------------------------------------------------ 20446

GGT2.end-endGGT1.48980500-49036766.gibbon      gctgtctcagccgcccgagtagctggtactacacgcgcgtgccaccatgtctgactaaat 37334
BCRP3.HUMAN.NCBI.REF                           ------------------------------------------------------------ 20446

GGT2.end-endGGT1.48980500-49036766.gibbon      gtgtatttttactagagacggggtagacggggtttcactatgttggccaggctggtctcc 37394
BCRP3.HUMAN.NCBI.REF                           ------------------------------------------------------------ 20446

GGT2.end-endGGT1.48980500-49036766.gibbon      aattcctgatctcctgatccgctcgccccgacatcccaaagtgctgggatgcatgagccc 37454
BCRP3.HUMAN.NCBI.REF                           ------------------------------------------------------------ 20446

GGT2.end-endGGT1.48980500-49036766.gibbon      ccacacctggccactatttttttctttctttcttttgtgtgtgtgtgtgtgtgcctgtgac 37514
BCRP3.HUMAN.NCBI.REF                           ------------------------------------------------------------ 20446

GGT2.end-endGGT1.48980500-49036766.gibbon      gaagggtcgctcttgttgcccaggctggagtgcaatggtgcgatctcagctcactgcaat 37574
BCRP3.HUMAN.NCBI.REF                           ------------------------------------------------------------ 20446

GGT2.end-endGGT1.48980500-49036766.gibbon      ccccgcctgagcaggagaacaggaatcttcagcgatccacgggcagatctgcagccattg 37634
BCRP3.HUMAN.NCBI.REF                           ------------------------------------------------------------ 20446

GGT2.end-endGGT1.48980500-49036766.gibbon      ttggtacctgttcttcccgcgtcctttgtgcccgcgtctctctttccagtacctactgca 37694
BCRP3.HUMAN.NCBI.REF                           ------------------------------------------------------------ 20446

GGT2.end-endGGT1.48980500-49036766.gibbon      tgccccccaacgtccgcctcccgccattgccagcaagtgcgttgcgcgggtaccttgctg 37754
BCRP3.HUMAN.NCBI.REF                           ------------------------------------------------------------ 20446

GGT2.end-endGGT1.48980500-49036766.gibbon      cgttaaggtcgctctgtcactggcgccattatgtgcacacgcagccactccctcaggttt 37814
BCRP3.HUMAN.NCBI.REF                           ------------------------------------------------------------ 20446

GGT2.end-endGGT1.48980500-49036766.gibbon      aaaaggcgcgttgcccggccacataaaggtcgctctgtcactggcgccattatgtgcaca 37874
BCRP3.HUMAN.NCBI.REF                           ------------------------------------------------------------ 20446

GGT2.end-endGGT1.48980500-49036766.gibbon      cgcagccactccctcaggtttcctaggcgcgttgcctggccacataaaggtcgctctgtc 37934
BCRP3.HUMAN.NCBI.REF                           ------------------------------------------------------------ 20446

GGT2.end-endGGT1.48980500-49036766.gibbon      actggcgccattatgtgcacacgcactccctcaggtttaaaaggcgcattggccggcaac 37994
BCRP3.HUMAN.NCBI.REF                           ------------------------------------------------------------ 20446
```

```
GGT2.end-endGGT1.48980500-49036766.gibbon      acagctcattgctggcttagcctttggccaagttggtagctccacgaggacgctcagagc 38054
BCRP3.HUMAN.NCBI.REF                           ------------------------------------------------------------ 20446

GGT2.end-endGGT1.48980500-49036766.gibbon      ccagctctggagagctgaagcatccgaccgttccccactgctcccaggagcggttacctg 38114
BCRP3.HUMAN.NCBI.REF                           ------------------------------------------------------------ 20446

GGT2.end-endGGT1.48980500-49036766.gibbon      ggcactctgtgccccttattcctgtccgggcccaggccgaggacctgccagtagggctca 38174
BCRP3.HUMAN.NCBI.REF                           ------------------------------------------------------------ 20446

GGT2.end-endGGT1.48980500-49036766.gibbon      attgcctggagcccgttcagcccatccccaagttcactttgcttgtgggatctccccgtt 38234
BCRP3.HUMAN.NCBI.REF                           ------------------------------------------------------------ 20446

GGT2.end-endGGT1.48980500-49036766.gibbon      gctcctgcccctggtctgagtggcaggccatcttgcaagcaccgggacacttcgcatcag 38294
BCRP3.HUMAN.NCBI.REF                           ------------------------------------------------------------ 20446

GGT2.end-endGGT1.48980500-49036766.gibbon      tggtgtcaagacaatccttccgtgatcctgcaagccctgtcttccttccgggatcagcaa 38354
BCRP3.HUMAN.NCBI.REF                           ------------------------------------------------------------ 20446

GGT2.end-endGGT1.48980500-49036766.gibbon      gccagtgctgtgtgctccgaattccagggcatcctccagctcagccactgcactgagcac 38414
BCRP3.HUMAN.NCBI.REF                           ------------------------------------------------------------ 20446

GGT2.end-endGGT1.48980500-49036766.gibbon      aaggactctctgtggggcccaggagcaggaagtcacccctttggggcccacaacacccgg 38474
BCRP3.HUMAN.NCBI.REF                           ------------------------------------------------------------ 20446

GGT2.end-endGGT1.48980500-49036766.gibbon      ctgtccccagactcgtgaccagggaaggcagtgttgaggagagcaaggcagggatgcctg 38534
BCRP3.HUMAN.NCBI.REF                           ------------------------------------------------------------ 20446

GGT2.end-endGGT1.48980500-49036766.gibbon      agcaggacaaagaccccagagtccaaggatttgatgatcacggaagggtccccaaggtca 38594
BCRP3.HUMAN.NCBI.REF                           ------------------------------------------------------------ 20446

GGT2.end-endGGT1.48980500-49036766.gibbon      ccagggatgcaccgagtgcatttcggcccctgcgggacaatggaggcctctctcccttcg 38654
BCRP3.HUMAN.NCBI.REF                           ------------------------------------------------------------ 20446

GGT2.end-endGGT1.48980500-49036766.gibbon      tgcccaggcctgggcctctgcacacagtcctccatgcccagaggacagaagccagagaca 38714
BCRP3.HUMAN.NCBI.REF                           ------------------------------------------------------------ 20446

GGT2.end-endGGT1.48980500-49036766.gibbon      agcagaggccccagacctcctgcacgagctcaggcacaaaacgaaacgccatctcgagct 38774
BCRP3.HUMAN.NCBI.REF                           ------------------------------------------------------------ 20446

GGT2.end-endGGT1.48980500-49036766.gibbon      cctacagctccacgggaggcttcccgtggctaaagcggaggagggggccagcctcatccc 38834
BCRP3.HUMAN.NCBI.REF                           ------------------------------------------------------------ 20446

GGT2.end-endGGT1.48980500-49036766.gibbon      actgccagccaaccctcagttcctcaaagaaggtcagtgagaacagacctcaggctgtct 38894
BCRP3.HUMAN.NCBI.REF                           ------------------------------------------------------------ 20446

GGT2.end-endGGT1.48980500-49036766.gibbon      cttcgggtcacactcagcgtgcaccaaggcagacgctcgcccccaggaaaggctccccca 38954
BCRP3.HUMAN.NCBI.REF                           ------------------------------------------------------------ 20446

GGT2.end-endGGT1.48980500-49036766.gibbon      gatcccaggcctctaggccctgtggacgcaagtttcccctgctgccacgcaggcgagggg 39014
BCRP3.HUMAN.NCBI.REF                           ------------------------------------------------------------ 20446

GGT2.end-endGGT1.48980500-49036766.gibbon      agcctctgatgatgccacctccgttagagctggggttccgggtcactgctgaagacctgg 39074
BCRP3.HUMAN.NCBI.REF                           ------------------------------------------------------------ 20446

GGT2.end-endGGT1.48980500-49036766.gibbon      accgggagaaggaggctgcgttccggcggatcaacagggcactgcaggttgaggccaagg 39134
BCRP3.HUMAN.NCBI.REF                           ------------------------------------------------------------ 20446

GGT2.end-endGGT1.48980500-49036766.gibbon      ccatctcggactgcagaccctcaaggccttcctacacttcgtgctcacctgcagcagggg 39194
BCRP3.HUMAN.NCBI.REF                           ------------------------------------------------------------ 20446

GGT2.end-endGGT1.48980500-49036766.gibbon      cttctggtctgccttctgtttctaaagcacccagtatggatgcacagcagggaagacaca 39254
BCRP3.HUMAN.NCBI.REF                           ------------------------------------------------------------ 20446

GGT2.end-endGGT1.48980500-49036766.gibbon      acccccaagacggcctgggcctagtggccccccctagcttctgctgcaggacccccctcca 39314
BCRP3.HUMAN.NCBI.REF                           ------------------------------------------------------------ 20446

GGT2.end-endGGT1.48980500-49036766.gibbon      cagttcctgtgtttgggatgcagcacagaccaccaggcccccctcctgttcgtctcctcat 39374
BCRP3.HUMAN.NCBI.REF                           ------------------------------------------------------------ 20446

GGT2.end-endGGT1.48980500-49036766.gibbon      ttccccttcctcccaactttttctacttctgggactcagcccaggtcctctggctgtttg 39434
```

```
BCRP3.HUMAN.NCBI.REF                            ------------------------------------------------------------ 20446

GGT2.end-endGGT1.48980500-49036766.gibbon       ctctacccttcggctcctcatttccccttcctcccaccttttctacttctgggactcag 39494
BCRP3.HUMAN.NCBI.REF                            ------------------------------------------------------------ 20446

GGT2.end-endGGT1.48980500-49036766.gibbon       cccaggtcctctgcctgattgctgcacccttcccagctgtaagcatggacggaagcattt 39554
BCRP3.HUMAN.NCBI.REF                            ------------------------------------------------------------ 20446

GGT2.end-endGGT1.48980500-49036766.gibbon       ctggagccagttccagcccgccacccacgcccatggaaattgacagtagtgaggcggacg 39614
BCRP3.HUMAN.NCBI.REF                            ------------------------------------------------------------ 20446

GGT2.end-endGGT1.48980500-49036766.gibbon       gagctcggcattccgtcagaagaaaccctttaaagagaaaggccgattggtaatagggca 39674
BCRP3.HUMAN.NCBI.REF                            ------------------------------------------------------------ 20446

GGT2.end-endGGT1.48980500-49036766.gibbon       tgagggggcctgaacaccaggggggccccaggcagagcccttccatggtgactgtgggac 39734
BCRP3.HUMAN.NCBI.REF                            ------------------------------------------------------------ 20446

GGT2.end-endGGT1.48980500-49036766.gibbon       ctggcacagggagcaactctgttgggtggcacttttgtttttttttgttgttgttgtttgc 39794
BCRP3.HUMAN.NCBI.REF                            ------------------------------------------------------------ 20446

GGT2.end-endGGT1.48980500-49036766.gibbon       cagatgccaaataaatatttttattaactttctttctgtacttcacttttgtgtcatcaa 39854
BCRP3.HUMAN.NCBI.REF                            ------------------------------------------------------------ 20446

GGT2.end-endGGT1.48980500-49036766.gibbon       catttatggcattaacctaaacagaagccccagtcaattaaagaatagaaaagataaaca 39914
BCRP3.HUMAN.NCBI.REF                            ------------------------------------------------------------ 20446

GGT2.end-endGGT1.48980500-49036766.gibbon       tttttagaactgtaaagcgtgttctaagagtttcttggccattttacttttctttttatt 39974
BCRP3.HUMAN.NCBI.REF                            ------------------------------------------------------------ 20446

GGT2.end-endGGT1.48980500-49036766.gibbon       taattatttttttatttgagttcccttagtatttattgatcattcttgggtgtttctcg 40034
BCRP3.HUMAN.NCBI.REF                            ------------------------------------------------------------ 20446

GGT2.end-endGGT1.48980500-49036766.gibbon       gagaggggacgtggcagggtcatgggataatagtggagagaaggtcagcagataaacacg 40094
BCRP3.HUMAN.NCBI.REF                            ------------------------------------------------------------ 20446

GGT2.end-endGGT1.48980500-49036766.gibbon       tgaacaaaggtgtctggctttcctaggcagaggtccctgcggccttccgcagtgttcgtg 40154
BCRP3.HUMAN.NCBI.REF                            ------------------------------------------------------------ 20446

GGT2.end-endGGT1.48980500-49036766.gibbon       tccctgggtacttgagattagggagtggtgatgactcttaacgagcatgctgccttcaag 40214
BCRP3.HUMAN.NCBI.REF                            ------------------------------------------------------------ 20446

GGT2.end-endGGT1.48980500-49036766.gibbon       catctgtttaacgaagcacatcttgcacagcccttaatccatgtaaccctgagttgacac 40274
BCRP3.HUMAN.NCBI.REF                            ------------------------------------------------------------ 20446

GGT2.end-endGGT1.48980500-49036766.gibbon       agcacatgtttcagagagcacgagtttgggggtaaggttatagattaagagcatcccaag 40334
BCRP3.HUMAN.NCBI.REF                            ------------------------------------------------------------ 20446

GGT2.end-endGGT1.48980500-49036766.gibbon       gcagaatttttcttagtacagaacaaaatggagtatcctatgtctacttctttctacaca 40394
BCRP3.HUMAN.NCBI.REF                            ------------------------------------------------------------ 20446

GGT2.end-endGGT1.48980500-49036766.gibbon       gacacagtaacaatctgatctctctttcttttccccacatttccccttttcttttctttttt 40454
BCRP3.HUMAN.NCBI.REF                            ------------------------------------------------------------ 20446

GGT2.end-endGGT1.48980500-49036766.gibbon       tttttttttgagacggagtctcgctctgtggcccaggctggagctcagtggcgcgatctc 40514
BCRP3.HUMAN.NCBI.REF                            ------------------------------------------------------------ 20446

GGT2.end-endGGT1.48980500-49036766.gibbon       cactcactgcaagctctgcctcccgggttcacgtcattctcctgcctcagtctcccgagt 40574
BCRP3.HUMAN.NCBI.REF                            ------------------------------------------------------------ 20446

GGT2.end-endGGT1.48980500-49036766.gibbon       agctgggactacaggcgcccgccaccacgcccggctaattatttttgtattttttttagt 40634
BCRP3.HUMAN.NCBI.REF                            ------------------------------------------------------------ 20446

GGT2.end-endGGT1.48980500-49036766.gibbon       agagacggggtttcaccgtggtctcgatctcctgacctcgtgatccgcccgcctcggcct 40694
BCRP3.HUMAN.NCBI.REF                            ------------------------------------------------------------ 20446

GGT2.end-endGGT1.48980500-49036766.gibbon       cccgaagtgctgggattacaagcgtgagccatcgcgcccggcctccatttttttttttttt 40754
BCRP3.HUMAN.NCBI.REF                            ------------------------------------------------------------ 20446

GGT2.end-endGGT1.48980500-49036766.gibbon       taagatagagtttcgctcttgtcccccatgctggagtgcaatggcgcgatctcagcacct 40814
BCRP3.HUMAN.NCBI.REF                            ------------------------------------------------------------ 20446
```

```
GGT2.end-endGGT1.48980500-49036766.gibbon      gctggcaatggtgggaggctgagggacgttcgcaggataggtactggaaggagaggcgcc 40874
BCRP3.HUMAN.NCBI.REF                           ------------------------------------------------------------ 20446

GGT2.end-endGGT1.48980500-49036766.gibbon      cgcacaaaagacatgggaaggccaggcgcgcacaagagccgcagatccgccagtggatcg 40934
BCRP3.HUMAN.NCBI.REF                           ------------------------------------------------------------ 20446

GGT2.end-endGGT1.48980500-49036766.gibbon      ctgaagattcctgctctcctgctcagaccaggattgcagtgagctgagatcgcaccattg 40994
BCRP3.HUMAN.NCBI.REF                           ------------------------------------------------------------ 20446

GGT2.end-endGGT1.48980500-49036766.gibbon      cacttcaacctgggcaacaagagcgaaacttcgtcacacacacacacacacacacacaca 41054
BCRP3.HUMAN.NCBI.REF                           ------------------------------------------------------------ 20446

GGT2.end-endGGT1.48980500-49036766.gibbon      cacacaacgcgcgcgcgcgcacacacacacacacacacacacacacaaaagaaagaaa 41114
BCRP3.HUMAN.NCBI.REF                           ------------------------------------------------------------ 20446

GGT2.end-endGGT1.48980500-49036766.gibbon      gaaaaaatagtggccaggtgtgggggctcacggatcccagcactttgggatggcagggcg 41174
BCRP3.HUMAN.NCBI.REF                           ------------------------------------------------------------ 20446

GGT2.end-endGGT1.48980500-49036766.gibbon      ggcggatcaggagattaggagttggagaccagcccggccaacatagtgaaaccccgtctc 41234
BCRP3.HUMAN.NCBI.REF                           ------------------------------------------------------------ 20446

GGT2.end-endGGT1.48980500-49036766.gibbon      tagtaaaaatacaaatttagtcagacatggtggcacgcgcgtgtagtaccagctactcgg 41294
BCRP3.HUMAN.NCBI.REF                           ------------------------------------------------------------ 20446

GGT2.end-endGGT1.48980500-49036766.gibbon      gcggctgagacaggagaatcacttgaacccagaggcggggcagggattgtgatgcgccga 41354
BCRP3.HUMAN.NCBI.REF                           ------------------------------------------------------------ 20446

GGT2.end-endGGT1.48980500-49036766.gibbon      gatcgcgccactgcactccagcctgggcaacagagccagaatcttttttttttttttttt 41414
BCRP3.HUMAN.NCBI.REF                           ------------------------------------------------------------ 20446

GGT2.end-endGGT1.48980500-49036766.gibbon      tgagacggagtcttcctctgtcgcccaggctggattccagtggcctgatctcagctcacc 41474
BCRP3.HUMAN.NCBI.REF                           ------------------------------------------------------------ 20446

GGT2.end-endGGT1.48980500-49036766.gibbon      gcaagctccacctagcgggttcacgccattctcctgcctcagcctctggagtagctggga 41534
BCRP3.HUMAN.NCBI.REF                           ------------------------------------------------------------ 20446

GGT2.end-endGGT1.48980500-49036766.gibbon      ctacaggtgcccgccaccacacccggctaatttttttgtatttttagtagagacggggttt 41594
BCRP3.HUMAN.NCBI.REF                           ------------------------------------------------------------ 20446

GGT2.end-endGGT1.48980500-49036766.gibbon      caccgtgttagccaggatggtctcgatcacctgacctcgtcatccgcccgcctcggccta 41654
BCRP3.HUMAN.NCBI.REF                           ------------------------------------------------------------ 20446

GGT2.end-endGGT1.48980500-49036766.gibbon      ccaaagtgctgggattaaaggcgtgagccacggcgcccggctgagactctgtcttaaaga 41714
BCRP3.HUMAN.NCBI.REF                           ------------------------------------------------------------ 20446

GGT2.end-endGGT1.48980500-49036766.gibbon      aaaaggccgggcgcggtggcactttgggaggcagaggcgggcggatcacgaggtcaggag 41774
BCRP3.HUMAN.NCBI.REF                           ------------------------------------------------------------ 20446

GGT2.end-endGGT1.48980500-49036766.gibbon      ttggagaccagcctggccaacatagcgaaaccccgtctctactaaaactacaaagaatta 41834
BCRP3.HUMAN.NCBI.REF                           ------------------------------------------------------------ 20446

GGT2.end-endGGT1.48980500-49036766.gibbon      gccgggcgtggtggcgggcgcctgtagtcccagctactccggaggctgaggcaggagagt 41894
BCRP3.HUMAN.NCBI.REF                           ------------------------------------------------------------ 20446

GGT2.end-endGGT1.48980500-49036766.gibbon      ggcctgaacttgggaggcggaggttgcagtgacctgagatctcgccactgcactccagcc 41954
BCRP3.HUMAN.NCBI.REF                           ------------------------------------------------------------ 20446

GGT2.end-endGGT1.48980500-49036766.gibbon      tgggtgacagtgtgaacctccgtctcaaaaaaaaaaaaagagagagagagagacggagag 42014
BCRP3.HUMAN.NCBI.REF                           ------------------------------------------------------------ 20446

GGT2.end-endGGT1.48980500-49036766.gibbon      aaaaagtttattaatttaatgcacttttatgcctgtgttcttcaatttgcttaggaaaca 42074
BCRP3.HUMAN.NCBI.REF                           ------------------------------------------------------------ 20446

GGT2.end-endGGT1.48980500-49036766.gibbon      cccacacttgagagctgggactgtggccctgattgtggacatgaaatatatggtttcttg 42134
BCRP3.HUMAN.NCBI.REF                           ------------------------------------------------------------ 20446

GGT2.end-endGGT1.48980500-49036766.gibbon      caaaaattgacagtgaatgattacgatttagttgagctagaaatccacttcgtttcttcc 42194
BCRP3.HUMAN.NCBI.REF                           ------------------------------------------------------------ 20446
```

```
GGT2.end-endGGT1.48980500-49036766.gibbon      atatttttccaaaactttcatcctttttttttttttttttttgagatggagtttcgctct 42254
BCRP3.HUMAN.NCBI.REF                           ------------------------------------------------------------ 20446

GGT2.end-endGGT1.48980500-49036766.gibbon      tgtcccccaggctgaagtggaatggcgccatctcagcacctgctggcaatgggggaggc 42314
BCRP3.HUMAN.NCBI.REF                           ------------------------------------------------------------ 20446

GGT2.end-endGGT1.48980500-49036766.gibbon      tgggggacgctcgcgggataggtactggaaggagaggcgctcttacaaagcaatgggaag 42374
BCRP3.HUMAN.NCBI.REF                           ------------------------------------------------------------ 20446

GGT2.end-endGGT1.48980500-49036766.gibbon      accaggcgcgcacaatggctgcagatccgccagtggatcactgaagatgcctgctctact 42434
BCRP3.HUMAN.NCBI.REF                           ------------------------------------------------------------ 20446

GGT2.end-endGGT1.48980500-49036766.gibbon      gctcaggcggggattgcagtgagctgagatcgcaccattgcactccagcctgggcaacaa 42494
BCRP3.HUMAN.NCBI.REF                           ------------------------------------------------------------ 20446

GGT2.end-endGGT1.48980500-49036766.gibbon      gagcgaaacttcgtaacagacacacacacacacacacacacacaaaagaaagaaagaa 42554
BCRP3.HUMAN.NCBI.REF                           ------------------------------------------------------------ 20446

GGT2.end-endGGT1.48980500-49036766.gibbon      aaaatagtggccaggtgtggcggctcacgcatcccagcactttgggatgtcagggcgggc 42614
BCRP3.HUMAN.NCBI.REF                           ------------------------------------------------------------ 20446

GGT2.end-endGGT1.48980500-49036766.gibbon      ggatcaggatatcaggagttggagaccagcctggccaacatagtgaaaccccgtctctag 42674
BCRP3.HUMAN.NCBI.REF                           ------------------------------------------------------------ 20446

GGT2.end-endGGT1.48980500-49036766.gibbon      taaaaatacaaatttagtcagacatggtggcacgcgcgtgtagtaccagctactcgggcg 42734
BCRP3.HUMAN.NCBI.REF                           ------------------------------------------------------------ 20446

GGT2.end-endGGT1.48980500-49036766.gibbon      gctgagacaggagaatcacttgaacccagaggcggggcagggattgtgatgcgccgagat 42794
BCRP3.HUMAN.NCBI.REF                           ------------------------------------------------------------ 20446

GGT2.end-endGGT1.48980500-49036766.gibbon      cgcgccactgcactccagcctgggcaacagagccagaatctttttttttttttttttgag 42854
BCRP3.HUMAN.NCBI.REF                           ------------------------------------------------------------ 20446

GGT2.end-endGGT1.48980500-49036766.gibbon      acagagtcttcctctgtcgcccaggctggattccagtggcctgatctcagctcaccgcaa 42914
BCRP3.HUMAN.NCBI.REF                           ------------------------------------------------------------ 20446

GGT2.end-endGGT1.48980500-49036766.gibbon      gctccgcctaccgggttcacgccattctcctgcctcagcctctggagtagctgggactac 42974
BCRP3.HUMAN.NCBI.REF                           ------------------------------------------------------------ 20446

GGT2.end-endGGT1.48980500-49036766.gibbon      aggtgcccgccaccacacctggctaatttttttgtatttttagtagagacggggtttcacc 43034
BCRP3.HUMAN.NCBI.REF                           ------------------------------------------------------------ 20446

GGT2.end-endGGT1.48980500-49036766.gibbon      gtgttagccaggatggtctcgatcacctgacctcgtgatgagcccccctcggcctaccaa 43094
BCRP3.HUMAN.NCBI.REF                           ------------------------------------------------------------ 20446

GGT2.end-endGGT1.48980500-49036766.gibbon      agtgctaggattaaaggcgtgagccaccaatgtgctgggattaaaggcgtgagcccacgg 43154
BCRP3.HUMAN.NCBI.REF                           ------------------------------------------------------------ 20446

GGT2.end-endGGT1.48980500-49036766.gibbon      tgcctggcggagactctgtcttaaaaaaaaaggccgggcgcggtggcagtttgaaaggcgg 43214
BCRP3.HUMAN.NCBI.REF                           ------------------------------------------------------------ 20446

GGT2.end-endGGT1.48980500-49036766.gibbon      aggcgggggattgcactccagccttggtgatagagtgagactcaatctcaataaataaat 43274
BCRP3.HUMAN.NCBI.REF                           ------------------------------------------------------------ 20446

GGT2.end-endGGT1.48980500-49036766.gibbon      aaataaataaataaataaataaataaataaatgatactaggacaaaaatcataaggacat 43334
BCRP3.HUMAN.NCBI.REF                           ------------------------------------------------------------ 20446

GGT2.end-endGGT1.48980500-49036766.gibbon      tacataaaccatgttatgctggtgatttttgaaaatatcaaaacagttttatagagaact 43394
BCRP3.HUMAN.NCBI.REF                           ------------------------------------------------------------ 20446

GGT2.end-endGGT1.48980500-49036766.gibbon      ataactcaggccaggtggggtggctaacacctgtaatcccagcactttaggaggccaagg 43454
BCRP3.HUMAN.NCBI.REF                           ------------------------------------------------------------ 20446

GGT2.end-endGGT1.48980500-49036766.gibbon      ttggcggatcacctgacgtcaggagtttgagaccagcctggccaacatagtaaaacccca 43514
BCRP3.HUMAN.NCBI.REF                           ------------------------------------------------------------ 20446

GGT2.end-endGGT1.48980500-49036766.gibbon      actctactaaaaatacaaaaattagctgggcgtggtggcacacgcttgtaatcccagcta 43574
BCRP3.HUMAN.NCBI.REF                           ------------------------------------------------------------ 20446
```

```
GGT2.end-endGGT1.48980500-49036766.gibbon      ttcgggagcctgaggcaggagaatcacttgaacccgggaggcggaggttgcagtgagcca 43634
BCRP3.HUMAN.NCBI.REF                           ------------------------------------------------------------ 20446

GGT2.end-endGGT1.48980500-49036766.gibbon      agatctggtgactgcagtctagcctgggcatcagagaaattccatctcaaaagaaaaata 43694
BCRP3.HUMAN.NCBI.REF                           ------------------------------------------------------------ 20446

GGT2.end-endGGT1.48980500-49036766.gibbon      tgacttcataaaaacgaagtaagaagaaacagaaaattatgtctagatctagaaccatta 43754
BCRP3.HUMAN.NCBI.REF                           ------------------------------------------------------------ 20446

GGT2.end-endGGT1.48980500-49036766.gibbon      aaggaattgaaggtttattttaaaatctgtatcccatcccctcaaaaatctcacaaaaaa 43814
BCRP3.HUMAN.NCBI.REF                           ------------------------------------------------------------ 20446

GGT2.end-endGGT1.48980500-49036766.gibbon      caaaaccaaaggaaagcacctggcccagatgattttgtagataagtctggcaaacattaa 43874
BCRP3.HUMAN.NCBI.REF                           ------------------------------------------------------------ 20446

GGT2.end-endGGT1.48980500-49036766.gibbon      aaaaaaacagaaagtctttatcttctacaaaattttaaaaaaataattaattaatgtatt 43934
BCRP3.HUMAN.NCBI.REF                           ------------------------------------------------------------ 20446

GGT2.end-endGGT1.48980500-49036766.gibbon      tatttctgagaggaagtctcactctgtagcccagactggagtgcagtgctgtgatctcca 43994
BCRP3.HUMAN.NCBI.REF                           ------------------------------------------------------------ 20446

GGT2.end-endGGT1.48980500-49036766.gibbon      ctcaccgcaaactccgcctcccaggttcaagtgattctcctgcttcagcctccacagtag 44054
BCRP3.HUMAN.NCBI.REF                           ------------------------------------------------------------ 20446

GGT2.end-endGGT1.48980500-49036766.gibbon      ctgggattacaggtgtgcctggctaatttttttgtagttttagtagagatggggtttcacc 44114
BCRP3.HUMAN.NCBI.REF                           ------------------------------------------------------------ 20446

GGT2.end-endGGT1.48980500-49036766.gibbon      acgttagccagggtgttcttgaactcccgacctcaggtaatccgccgcctcggcctccca 44174
BCRP3.HUMAN.NCBI.REF                           ------------------------------------------------------------ 20446

GGT2.end-endGGT1.48980500-49036766.gibbon      aagtgaatgagccacagtgcctggcctacataatctcttttttttttttttgagacggag 44234
BCRP3.HUMAN.NCBI.REF                           ------------------------------------------------------------ 20446

GGT2.end-endGGT1.48980500-49036766.gibbon      tctggctctgtcacccaggctggagtgcagtggcgcgatctgggctgactgcaagctccg 44294
BCRP3.HUMAN.NCBI.REF                           ------------------------------------------------------------ 20446

GGT2.end-endGGT1.48980500-49036766.gibbon      cctcccgggttcacgccattctgctgcctcagcctccggagtagctgggactacaagcgc 44354
BCRP3.HUMAN.NCBI.REF                           ------------------------------------------------------------ 20446

GGT2.end-endGGT1.48980500-49036766.gibbon      ctgccacaaaacccggctaatttttttgtatttttagtagagacggggtttcaccgtgtta 44414
BCRP3.HUMAN.NCBI.REF                           ------------------------------------------------------------ 20446

GGT2.end-endGGT1.48980500-49036766.gibbon      gccagcatggtctcgacctcctgtccttgtgatctgcccaccttggcctcccaaagtgct 44474
BCRP3.HUMAN.NCBI.REF                           ------------------------------------------------------------ 20446

GGT2.end-endGGT1.48980500-49036766.gibbon      gggattacaggcgtgagccaccgcccccagccctggcctacataatcattcagaaaaaga 44534
BCRP3.HUMAN.NCBI.REF                           ------------------------------------------------------------ 20446

GGT2.end-endGGT1.48980500-49036766.gibbon      agcaaatggacttctccccaactcattgtgaatttagtataagcttaatacgaaaccaga 44594
BCRP3.HUMAN.NCBI.REF                           ------------------------------------------------------------ 20446

GGT2.end-endGGT1.48980500-49036766.gibbon      cataaatatacaaggaggcaaaagtatagactaacttcttacggatatatatgcaaaaat 44654
BCRP3.HUMAN.NCBI.REF                           ------------------------------------------------------------ 20446

GGT2.end-endGGT1.48980500-49036766.gibbon      gctaaataaaatattagcaaataatccagaaatggattaaacatgtatcacgaccaagtt 44714
BCRP3.HUMAN.NCBI.REF                           ------------------------------------------------------------ 20446

GGT2.end-endGGT1.48980500-49036766.gibbon      gggttttcctaggaaactaagatgatgtaataaaagaaaaaactattaaagtagtacaaa 44774
BCRP3.HUMAN.NCBI.REF                           ------------------------------------------------------------ 20446

GGT2.end-endGGT1.48980500-49036766.gibbon      ctgtcaaaggaaaaaacctgtatgatcattgcaacgggttcatttgcgacataatgtaac 44834
BCRP3.HUMAN.NCBI.REF                           ------------------------------------------------------------ 20446

GGT2.end-endGGT1.48980500-49036766.gibbon      ttttttcttttttttttaagtctttttggctgtaagtttattcaatgcaaaataatcctct 44894
BCRP3.HUMAN.NCBI.REF                           ------------------------------------------------------------ 20446

GGT2.end-endGGT1.48980500-49036766.gibbon      ccaattttactgacgtggctgaccacgtccacgaccaaatctgcctctaaactggaattc 44954
BCRP3.HUMAN.NCBI.REF                           ------------------------------------------------------------ 20446

GGT2.end-endGGT1.48980500-49036766.gibbon      ggttgctgacccagccccagcctcggctttcttgtcggcaccagggggcagagcactccc 45014
```

```
BCRP3.HUMAN.NCBI.REF                                  ------------------------------------------------------------ 20446

GGT2.end-endGGT1.48980500-49036766.gibbon             tctgtaggtagctctgtgggcctcccctcttgtgagtcctgcaggtcgctcaccctccag 45074
BCRP3.HUMAN.NCBI.REF                                  ------------------------------------------------------------ 20446

GGT2.end-endGGT1.48980500-49036766.gibbon             acctttaggccgaggtctgccagtccctggacggctgtggcatggggtggcaggcacaat 45134
BCRP3.HUMAN.NCBI.REF                                  ------------------------------------------------------------ 20446

GGT2.end-endGGT1.48980500-49036766.gibbon             ctccgggggcagatgaaggtaatcatggagatactggataccctcattggtaaggtacga 45194
BCRP3.HUMAN.NCBI.REF                                  ------------------------------------------------------------ 20446

GGT2.end-endGGT1.48980500-49036766.gibbon             gcagacatctctccaggcaaattgttccttcacgtagtagcctcgggactatgtgacatg 45254
BCRP3.HUMAN.NCBI.REF                                  ------------------------------------------------------------ 20446

GGT2.end-endGGT1.48980500-49036766.gibbon             aaggttgggcacatcttcttttttttttctttttgacatgagtctcactctgtcgcccag 45314
BCRP3.HUMAN.NCBI.REF                                  ------------------------------------------------------------ 20446

GGT2.end-endGGT1.48980500-49036766.gibbon             gctggagtgcaggggtgcgatctgggctcactgcaacctccgcccccctgggttcaagcga 45374
BCRP3.HUMAN.NCBI.REF                                  ------------------------------------------------------------ 20446

GGT2.end-endGGT1.48980500-49036766.gibbon             ttctcctgcctcagcctccggattagctgggattacaggcacgcaccaccacatccagct 45434
BCRP3.HUMAN.NCBI.REF                                  ------------------------------------------------------------ 20446

GGT2.end-endGGT1.48980500-49036766.gibbon             aattttttatatttttggtagagacagggtttcatcatgttggctaggctggtctcaaac 45494
BCRP3.HUMAN.NCBI.REF                                  ------------------------------------------------------------ 20446

GGT2.end-endGGT1.48980500-49036766.gibbon             ttctgacctcaagtgatccacctgccttggcctcccaaagtgctgggattagaggcatga 45554
BCRP3.HUMAN.NCBI.REF                                  ------------------------------------------------------------ 20446

GGT2.end-endGGT1.48980500-49036766.gibbon             gccaccatggccggcctgtcctagtctttatatgcccatatatttgagcccttcttgact 45614
BCRP3.HUMAN.NCBI.REF                                  ------------------------------------------------------------ 20446

GGT2.end-endGGT1.48980500-49036766.gibbon             aggttcccagagaatcggtgcttggcttgcctggacacggaataaattgatttattgata 45674
BCRP3.HUMAN.NCBI.REF                                  ------------------------------------------------------------ 20446

GGT2.end-endGGT1.48980500-49036766.gibbon             aataaatgttgtgcccacaatgacttgcctcataacttttttagtagtcaggatcctccca 45734
BCRP3.HUMAN.NCBI.REF                                  ------------------------------------------------------------ 20446

GGT2.end-endGGT1.48980500-49036766.gibbon             cagtagactcttgtaactacaagaatatttttttgttggtgttgcatgtagagtttcctg 45794
BCRP3.HUMAN.NCBI.REF                                  ------------------------------------------------------------ 20446

GGT2.end-endGGT1.48980500-49036766.gibbon             taaaatggcttccaagatatttttcatttctagtaaagaatctgtgagttgatttttaag 45854
BCRP3.HUMAN.NCBI.REF                                  ------------------------------------------------------------ 20446

GGT2.end-endGGT1.48980500-49036766.gibbon             tagtattatgtctaccttatgtggaaggtaagagatagccctttgttttcctcaggaggt 45914
BCRP3.HUMAN.NCBI.REF                                  ------------------------------------------------------------ 20446

GGT2.end-endGGT1.48980500-49036766.gibbon             tttaattatatgatctccttggagaagtaaaggaaactctggagcttcctgtgtggctgt 45974
BCRP3.HUMAN.NCBI.REF                                  ------------------------------------------------------------ 20446

GGT2.end-endGGT1.48980500-49036766.gibbon             gaatgtatcattagctttgggcagggctcccagagctgtgaaccctgtcctgaggagcac 46034
BCRP3.HUMAN.NCBI.REF                                  ------------------------------------------------------------ 20446

GGT2.end-endGGT1.48980500-49036766.gibbon             tgggacattctagtgagcgttcaggccacccagattgcagcctcgtaaatgacgggttta 46094
BCRP3.HUMAN.NCBI.REF                                  ------------------------------------------------------------ 20446

GGT2.end-endGGT1.48980500-49036766.gibbon             tttctctaatgagagtggtttgtgtcttcacccaaataatattttaatattaaattacga 46154
BCRP3.HUMAN.NCBI.REF                                  ------------------------------------------------------------ 20446

GGT2.end-endGGT1.48980500-49036766.gibbon             caaatattttaaattttaaagtaggtatatataaattgtatgattttaaaaataatttca 46214
BCRP3.HUMAN.NCBI.REF                                  ------------------------------------------------------------ 20446

GGT2.end-endGGT1.48980500-49036766.gibbon             ataattgtttaaaaaagtaattttaatacatttcaataatttattttagactcaaggggt 46274
BCRP3.HUMAN.NCBI.REF                                  ------------------------------------------------------------ 20446

GGT2.end-endGGT1.48980500-49036766.gibbon             acacccacaggtttgttgcatgggtatgttgcacgatgctgaggtttggggtacggatcc 46334
BCRP3.HUMAN.NCBI.REF                                  ------------------------------------------------------------ 20446

GGT2.end-endGGT1.48980500-49036766.gibbon             cgtcacccaggtagtgagcatagtttttcaactcatgcccatccctcattcccttcttcc 46394
BCRP3.HUMAN.NCBI.REF                                  ------------------------------------------------------------ 20446
```

```
GGT2.end-endGGT1.48980500-49036766.gibbon      aattagtatgattacaactaagaattttacttttacagctctaatttaatacatattaat 46454
BCRP3.HUMAN.NCBI.REF                           ------------------------------------------------------------ 20446

GGT2.end-endGGT1.48980500-49036766.gibbon      ctagacctggcaataagtattctgctctaagaaaggttttatgttaaataacatgatagt 46514
BCRP3.HUMAN.NCBI.REF                           ------------------------------------------------------------ 20446

GGT2.end-endGGT1.48980500-49036766.gibbon      tagtcccttcaaaataaaaaatttcgatttaagaataccagcacgggcccggcgtggtgg 46574
BCRP3.HUMAN.NCBI.REF                           ------------------------------------------------------------ 20446

GGT2.end-endGGT1.48980500-49036766.gibbon      ctcaagcttgtaatcccagcactttgggaggccgagggcagatcatctgaggtcaggagt 46634
BCRP3.HUMAN.NCBI.REF                           ------------------------------------------------------------ 20446

GGT2.end-endGGT1.48980500-49036766.gibbon      tcgagaccagcctggccaacatggtgaaacctatctctactaaaaatacaaaaattagcc 46694
BCRP3.HUMAN.NCBI.REF                           ------------------------------------------------------------ 20446

GGT2.end-endGGT1.48980500-49036766.gibbon      agacgtggtggcaggtgcccgtaatcccagctactcgggaggctgagggaggagaagcgc 46754
BCRP3.HUMAN.NCBI.REF                           ------------------------------------------------------------ 20446

GGT2.end-endGGT1.48980500-49036766.gibbon      ttgaacccgggagggagaggatgtagtgagccaagatcatgccgtcgcactccagactgg 46814
BCRP3.HUMAN.NCBI.REF                           ------------------------------------------------------------ 20446

GGT2.end-endGGT1.48980500-49036766.gibbon      aggagaagagcaagacttcgtctcaaaagcaaaaagtataccagcactggggacaacatt 46874
BCRP3.HUMAN.NCBI.REF                           ------------------------------------------------------------ 20446

GGT2.end-endGGT1.48980500-49036766.gibbon      ggacaagtagacaaatctagaaggggcaggttgagctgtgtagttttagtgttgttacag 46934
BCRP3.HUMAN.NCBI.REF                           ------------------------------------------------------------ 20446

GGT2.end-endGGT1.48980500-49036766.gibbon      tttgttgatatgttgtgaaatattcattgagatcaaaatctggcattcctaacttgtgtt 46994
BCRP3.HUMAN.NCBI.REF                           ------------------------------------------------------------ 20446

GGT2.end-endGGT1.48980500-49036766.gibbon      atgtggaggccacatggagtggcagaagtgaagtggggtttgaattcagaccgttgttcc 47054
BCRP3.HUMAN.NCBI.REF                           ------------------------------------------------------------ 20446

GGT2.end-endGGT1.48980500-49036766.gibbon      tcaagaatgaattgagctggagagaattacttgcaatctctgatccttagttcctccatc 47114
BCRP3.HUMAN.NCBI.REF                           ------------------------------------------------------------ 20446

GGT2.end-endGGT1.48980500-49036766.gibbon      tagacatacccacctgggatcgtgtgaggattcagtgaactgtaagaggtacctgcctgg 47174
BCRP3.HUMAN.NCBI.REF                           ------------------------------------------------------------ 20446

GGT2.end-endGGT1.48980500-49036766.gibbon      gagaggctcaaggccttcaatgcctttccctgttattttgggagtagtacatctgttttg 47234
BCRP3.HUMAN.NCBI.REF                           ------------------------------------------------------------ 20446

GGT2.end-endGGT1.48980500-49036766.gibbon      gtgtttttaagaaacactgtttgacaaatacatgaggcatacttcatggactattgttgt 47294
BCRP3.HUMAN.NCBI.REF                           ------------------------------------------------------------ 20446

GGT2.end-endGGT1.48980500-49036766.gibbon      ttcagattattcttagaacacagaggcaggatccacagtgtttttatgaggagagctact 47354
BCRP3.HUMAN.NCBI.REF                           ------------------------------------------------------------ 20446

GGT2.end-endGGT1.48980500-49036766.gibbon      ttttattctcccaaaccttggctttcctggattagaggtctcagtgatgtgtgctgatgt 47414
BCRP3.HUMAN.NCBI.REF                           ------------------------------------------------------------ 20446

GGT2.end-endGGT1.48980500-49036766.gibbon      gacacccttgagcaaagttggagaagagatggtggtaatttccaccacttccccttctcc 47474
BCRP3.HUMAN.NCBI.REF                           ------------------------------------------------------------ 20446

GGT2.end-endGGT1.48980500-49036766.gibbon      aagacactggaagtgctgtgaactgtgctccttagctcctctgctctgtaatctctgccc 47534
BCRP3.HUMAN.NCBI.REF                           ------------------------------------------------------------ 20446

GGT2.end-endGGT1.48980500-49036766.gibbon      gggggctctagaggggctctaggcaggagtgtcagccagtgattcctccgtcttgaccag 47594
BCRP3.HUMAN.NCBI.REF                           ------------------------------------------------------------ 20446

GGT2.end-endGGT1.48980500-49036766.gibbon      gtagggacccaggatcttctggtgaattctgatttgttttttttatgagcctgagggtcat 47654
BCRP3.HUMAN.NCBI.REF                           ------------------------------------------------------------ 20446

GGT2.end-endGGT1.48980500-49036766.gibbon      tttaggaatttgtggaagcatctgtgattgtctcaatggttggtgggtgctataggcatt 47714
BCRP3.HUMAN.NCBI.REF                           ------------------------------------------------------------ 20446

GGT2.end-endGGT1.48980500-49036766.gibbon      tatttaatacgtagggcccatggaattcaaggcttagtgcggtgacagttctgtaaaaca 47774
BCRP3.HUMAN.NCBI.REF                           ------------------------------------------------------------ 20446
```

```
GGT2.end-endGGT1.48980500-49036766.gibbon      gaacttttcctatgatctattattagaggtaatagatctattattggaggtaagagacac 47834
BCRP3.HUMAN.NCBI.REF                           ------------------------------------------------------------ 20446

GGT2.end-endGGT1.48980500-49036766.gibbon      atcacggaaaagaagaagttactaggagccgggcacggtggctcatgcctgtaattccag 47894
BCRP3.HUMAN.NCBI.REF                           ------------------------------------------------------------ 20446

GGT2.end-endGGT1.48980500-49036766.gibbon      cactttgtgaggccaaggcagcggatcacctgaggttgggagttcaagaccagcctgacc 47954
BCRP3.HUMAN.NCBI.REF                           ------------------------------------------------------------ 20446

GGT2.end-endGGT1.48980500-49036766.gibbon      aacatggagaagccctgtctctactaaaaatacaaaaattagccaagcatggtggcacat 48014
BCRP3.HUMAN.NCBI.REF                           ------------------------------------------------------------ 20446

GGT2.end-endGGT1.48980500-49036766.gibbon      gcctgtaatcccagctactcaggaggctgagggaggagaattgcttggacccaggaagtg 48074
BCRP3.HUMAN.NCBI.REF                           ------------------------------------------------------------ 20446

GGT2.end-endGGT1.48980500-49036766.gibbon      gaggttgtggtgagccgagatcgtgccattgtactccagcctgggcaacaagagcaaaac 48134
BCRP3.HUMAN.NCBI.REF                           ------------------------------------------------------------ 20446

GGT2.end-endGGT1.48980500-49036766.gibbon      tctgtctcaaaaaaaaaaaaaaaaaaaaaaaaaaaaaaaaaaaagaagttactagctagt 48194
BCRP3.HUMAN.NCBI.REF                           ------------------------------------------------------------ 20446

GGT2.end-endGGT1.48980500-49036766.gibbon      ttcagtattacttaacatccaggaaactggatgtgaaagctttttagagaaactaaacca 48254
BCRP3.HUMAN.NCBI.REF                           ------------------------------------------------------------ 20446

GGT2.end-endGGT1.48980500-49036766.gibbon      ataggttatacacagagagagatttatttaggaattggctcacatgattgtggggactag 48314
BCRP3.HUMAN.NCBI.REF                           ------------------------------------------------------------ 20446

GGT2.end-endGGT1.48980500-49036766.gibbon      caagtttaaaatctgtagggcaagccagcaggctataaattcaggtaagagttgatctca 48374
BCRP3.HUMAN.NCBI.REF                           ------------------------------------------------------------ 20446

GGT2.end-endGGT1.48980500-49036766.gibbon      aagtctggaacctaacatctgtagagcagtctgcaggccagaaactcaggcagggtttgt 48434
BCRP3.HUMAN.NCBI.REF                           ------------------------------------------------------------ 20446

GGT2.end-endGGT1.48980500-49036766.gibbon      gtgttacagtcttgaagcggaattcctgcttctctgggaaacctcagtttttgttcttaa 48494
BCRP3.HUMAN.NCBI.REF                           ------------------------------------------------------------ 20446

GGT2.end-endGGT1.48980500-49036766.gibbon      ggccttcaactgattggaggtggcccacccatattatggtgggtaatctgttttacttaa 48554
BCRP3.HUMAN.NCBI.REF                           ------------------------------------------------------------ 20446

GGT2.end-endGGT1.48980500-49036766.gibbon      agtcaactgactgtcaatgttcatcacatctatgaaataacctcccagcaagatattgac 48614
BCRP3.HUMAN.NCBI.REF                           ------------------------------------------------------------ 20446

GGT2.end-endGGT1.48980500-49036766.gibbon      aagtatttgaccaaacaacggggcaccatagcttagccaagttgacacataaattaacca 48674
BCRP3.HUMAN.NCBI.REF                           ------------------------------------------------------------ 20446

GGT2.end-endGGT1.48980500-49036766.gibbon      tcaggggcgaatagaatatccaaaaaacaacgtactaggggtagtgtatcttatatagct 48734
BCRP3.HUMAN.NCBI.REF                           ------------------------------------------------------------ 20446

GGT2.end-endGGT1.48980500-49036766.gibbon      attataattatacaaaacataattataggatgacgatattaagataaccattagaacaaa 48794
BCRP3.HUMAN.NCBI.REF                           ------------------------------------------------------------ 20446

GGT2.end-endGGT1.48980500-49036766.gibbon      aatatgaacttttctgtctttttttgagaccaagtcctgctatgtcaccgaggctggagt 48854
BCRP3.HUMAN.NCBI.REF                           ------------------------------------------------------------ 20446

GGT2.end-endGGT1.48980500-49036766.gibbon      gcagtggtacaatcttggcttactgcagcctttgcctcccgggttcaagcaattctcctg 48914
BCRP3.HUMAN.NCBI.REF                           ------------------------------------------------------------ 20446

GGT2.end-endGGT1.48980500-49036766.gibbon      cctcagcctcccgagtagctgggattacaggcacccgctaccatgcctggctaatttttg 48974
BCRP3.HUMAN.NCBI.REF                           ------------------------------------------------------------ 20446

GGT2.end-endGGT1.48980500-49036766.gibbon      tatttttagtagagacggggtttcaccatgttgcccaggctggtctccaactcctgacct 49034
BCRP3.HUMAN.NCBI.REF                           ------------------------------------------------------------ 20446

GGT2.end-endGGT1.48980500-49036766.gibbon      ccaatgatccacccgccttggcctcccaaagtgctgggattacaggtgtgagccaccaca 49094
BCRP3.HUMAN.NCBI.REF                           ------------------------------------------------------------ 20446

GGT2.end-endGGT1.48980500-49036766.gibbon      tccagccaaaaatcacctttttttacaaggatcaaaacagtccttatgctgcagatgacag 49154
BCRP3.HUMAN.NCBI.REF                           ------------------------------------------------------------ 20446
```

```
GGT2.end-endGGT1.48980500-49036766.gibbon      actcactgtcaccatgctccttttgtgtgtctactaggcacggtgctgggtccacactca 49214
BCRP3.HUMAN.NCBI.REF                           ------------------------------------------------------------ 20446

GGT2.end-endGGT1.48980500-49036766.gibbon      cagaaaccttaggaactcgcacccaggggctccggctgtagcagaatcccaagaataaaa 49274
BCRP3.HUMAN.NCBI.REF                           ------------------------------------------------------------ 20446

GGT2.end-endGGT1.48980500-49036766.gibbon      cctggtgctgacagaaagagtaggagatggggccgggcgccatgactcactcctgtaatc 49334
BCRP3.HUMAN.NCBI.REF                           ------------------------------------------------------------ 20446

GGT2.end-endGGT1.48980500-49036766.gibbon      ccagcactttgggaggctgaggcgggcaaatcacgaggtcaagagatagagaccaccctg 49394
BCRP3.HUMAN.NCBI.REF                           ------------------------------------------------------------ 20446

GGT2.end-endGGT1.48980500-49036766.gibbon      gccaacatggtgaaaccccgtctctactaaaaatacaaaaattagctgggtgtggtggct 49454
BCRP3.HUMAN.NCBI.REF                           ------------------------------------------------------------ 20446

GGT2.end-endGGT1.48980500-49036766.gibbon      ggcacctgtagtcccagctactcaggaggctgaggcaggagaatcatttgaaccgaggag 49514
BCRP3.HUMAN.NCBI.REF                           ------------------------------------------------------------ 20446

GGT2.end-endGGT1.48980500-49036766.gibbon      gcagaggttgcagtgagccgagatcgtgccactgcactccagcctggtgacagagcgagg 49574
BCRP3.HUMAN.NCBI.REF                           ------------------------------------------------------------ 20446

GGT2.end-endGGT1.48980500-49036766.gibbon      cattgtctcaaaaaaaaaaaaaaaaaagcaggagactggactctgggagggcctcctggt 49634
BCRP3.HUMAN.NCBI.REF                           ------------------------------------------------------------ 20446

GGT2.end-endGGT1.48980500-49036766.gibbon      gagaggggagcacagaggggagagatggaggcaggagcatgggcttctggtggccccagc 49694
BCRP3.HUMAN.NCBI.REF                           ------------------------------------------------------------ 20446

GGT2.end-endGGT1.48980500-49036766.gibbon      agaccctgtggcagcgtggccagcgtcctctgcaaggaggaatcttggccaggatgatgc 49754
BCRP3.HUMAN.NCBI.REF                           ------------------------------------------------------------ 20446

GGT2.end-endGGT1.48980500-49036766.gibbon      tgcagcaagcttcttcctgaggcccccagccagcccggccagggtcccagcgtccagtg 49814
BCRP3.HUMAN.NCBI.REF                           ------------------------------------------------------------ 20446

GGT2.end-endGGT1.48980500-49036766.gibbon      accccctgttccgcagcagcagctggggccagccccaggctctcttccactcccagcttct 49874
BCRP3.HUMAN.NCBI.REF                           ------------------------------------------------------------ 20446

GGT2.end-endGGT1.48980500-49036766.gibbon      taaaacaggaagtggagagagttgtctgacaaagcactggggcaaaccacatcctctctc 49934
BCRP3.HUMAN.NCBI.REF                           ------------------------------------------------------------ 20446

GGT2.end-endGGT1.48980500-49036766.gibbon      ttcaccaagggacagtttgagggagtgtcagcagagggagctttagagtagagacccccct 49994
BCRP3.HUMAN.NCBI.REF                           ------------------------------------------------------------ 20446

GGT2.end-endGGT1.48980500-49036766.gibbon      agccaaccactgactgtcacgcacacagcagggcatgctatggagacccccagacagtcc 50054
BCRP3.HUMAN.NCBI.REF                           ------------------------------------------------------------ 20446

GGT2.end-endGGT1.48980500-49036766.gibbon      ctcggggagacccagcaggtccagtctcctcagagatctgtggcagcaggtccccactcc 50114
BCRP3.HUMAN.NCBI.REF                           ------------------------------------------------------------ 20446

GGT2.end-endGGT1.48980500-49036766.gibbon      caaaagccacgtgcccacgggtggtctctggtgcctgagactccagtctcatttgcatct 50174
BCRP3.HUMAN.NCBI.REF                           ------------------------------------------------------------ 20446

GGT2.end-endGGT1.48980500-49036766.gibbon      ttgcaacttcgagtttaagtgggtgccgcatctctgtatgtcctcccgagcataggagcg 50234
BCRP3.HUMAN.NCBI.REF                           ------------------------------------------------------------ 20446

GGT2.end-endGGT1.48980500-49036766.gibbon      gcacagcctggggtggcagctggcatcaatccctcgaatcccctgggagccactggggag 50294
BCRP3.HUMAN.NCBI.REF                           ------------------------------------------------------------ 20446

GGT2.end-endGGT1.48980500-49036766.gibbon      actaagcagtccccagcgcccacttgtccctgagctgccattctcagccctgtgggagga 50354
BCRP3.HUMAN.NCBI.REF                           ------------------------------------------------------------ 20446

GGT2.end-endGGT1.48980500-49036766.gibbon      gacaggaagccctgaagagaaaccaaaggaccaggtcaggaggggctgggggtggcatg 50414
BCRP3.HUMAN.NCBI.REF                           ------------------------------------------------------------ 20446

GGT2.end-endGGT1.48980500-49036766.gibbon      agcaatcagggcagggaaggatggacagatgggggaatggagggaggaaggaatgaatga 50474
BCRP3.HUMAN.NCBI.REF                           ------------------------------------------------------------ 20446

GGT2.end-endGGT1.48980500-49036766.gibbon      aaaggtgaatgaatgaacaaagagagagaatggccactcctcccttgctttagtttacaa 50534
BCRP3.HUMAN.NCBI.REF                           ------------------------------------------------------------ 20446

GGT2.end-endGGT1.48980500-49036766.gibbon      agcactgggatcctcccaacagcctgaaggacagaatttatgggaagcagaccaggtggc 50594
```

```
BCRP3.HUMAN.NCBI.REF                          ------------------------------------------------------------ 20446

GGT2.end-endGGT1.48980500-49036766.gibbon     tggcagggaggggaggcttgccctggatttcgtgggcccaatgggaggcaggggcagga 50654
BCRP3.HUMAN.NCBI.REF                          ------------------------------------------------------------ 20446

GGT2.end-endGGT1.48980500-49036766.gibbon     aggggcatcctgtgtgtgtcctctctgcagcggcagcaacaccttcctggaagagggtca 50714
BCRP3.HUMAN.NCBI.REF                          ------------------------------------------------------------ 20446

GGT2.end-endGGT1.48980500-49036766.gibbon     ggaaacacccactgtggcccctctccatcacaccctcatccaggacaccaagtatcagtc 50774
BCRP3.HUMAN.NCBI.REF                          ------------------------------------------------------------ 20446

GGT2.end-endGGT1.48980500-49036766.gibbon     actcagctcacaagatccaggccctgactcggagagaggatgtgaggggtggggcacggg 50834
BCRP3.HUMAN.NCBI.REF                          ------------------------------------------------------------ 20446

GGT2.end-endGGT1.48980500-49036766.gibbon     gctccccaggactgagagacccgagacgtggccccgggctgggtgttggggcagactggc 50894
BCRP3.HUMAN.NCBI.REF                          ------------------------------------------------------------ 20446

GGT2.end-endGGT1.48980500-49036766.gibbon     tatggcagcatcgtgtaccccagcaggccagtacccacgcagggagcctccaaaccoctt 50954
BCRP3.HUMAN.NCBI.REF                          ------------------------------------------------------------ 20446

GGT2.end-endGGT1.48980500-49036766.gibbon     cacctatgaccctgggagaagaccctagccttggagaattggcctcactgaaggggcctg 51014
BCRP3.HUMAN.NCBI.REF                          ------------------------------------------------------------ 20446

GGT2.end-endGGT1.48980500-49036766.gibbon     caccggccagcagggtcaggttgggccagacaggttcccacctgggatatgcaaatgggc 51074
BCRP3.HUMAN.NCBI.REF                          ------------------------------------------------------------ 20446

GGT2.end-endGGT1.48980500-49036766.gibbon     ctcctgaatcctggagccaggtatggattcacacaccaccattgtccccaagtccccatc 51134
BCRP3.HUMAN.NCBI.REF                          ------------------------------------------------------------ 20446

GGT2.end-endGGT1.48980500-49036766.gibbon     tgccccacgggcacaccctgccacctgttctgtgcaagggccctgaggctgcctccttgc 51194
BCRP3.HUMAN.NCBI.REF                          ------------------------------------------------------------ 20446

GGT2.end-endGGT1.48980500-49036766.gibbon     gcccaagccctgcaggtgctgaagcccacaccacacacacacggctcctgcttcctgggc 51254
BCRP3.HUMAN.NCBI.REF                          ------------------------------------------------------------ 20446

GGT2.end-endGGT1.48980500-49036766.gibbon     cagtgcacgtgcacacacacacgcgcacacacacactcacacacccacccatacacatac 51314
BCRP3.HUMAN.NCBI.REF                          ------------------------------------------------------------ 20446

GGT2.end-endGGT1.48980500-49036766.gibbon     ccacacacaatcacacacattcacacacacccacacccccatactcacacactcaccca 51374
BCRP3.HUMAN.NCBI.REF                          ------------------------------------------------------------ 20446

GGT2.end-endGGT1.48980500-49036766.gibbon     caaacacccacacatacactcacacacaatcacacacatttacacacacccacacactca 51434
BCRP3.HUMAN.NCBI.REF                          ------------------------------------------------------------ 20446

GGT2.end-endGGT1.48980500-49036766.gibbon     cactcacacattcacacacaccctcacacaccgaaacacaatcacacacattcacaccca 51494
BCRP3.HUMAN.NCBI.REF                          ------------------------------------------------------------ 20446

GGT2.end-endGGT1.48980500-49036766.gibbon     cccacactcccccacacactcacactcacatatactctcacaccccccacacacataca 51554
BCRP3.HUMAN.NCBI.REF                          ------------------------------------------------------------ 20446

GGT2.end-endGGT1.48980500-49036766.gibbon     cacacccaaacacaatcacacacattcacacacacccacacaccccacacactcacact 51614
BCRP3.HUMAN.NCBI.REF                          ------------------------------------------------------------ 20446

GGT2.end-endGGT1.48980500-49036766.gibbon     cacacatacacacactcaaacacaatcacacacattcacacacacaccccacacactcat 51674
BCRP3.HUMAN.NCBI.REF                          ------------------------------------------------------------ 20446

GGT2.end-endGGT1.48980500-49036766.gibbon     actcacacatatatgcacacactcacactcacacataatcacacacacacacacacacgc 51734
BCRP3.HUMAN.NCBI.REF                          ------------------------------------------------------------ 20446

GGT2.end-endGGT1.48980500-49036766.gibbon     tcacacacactctctctcacacacaacgccttctccaggaggggctggctgccaagggcc 51794
BCRP3.HUMAN.NCBI.REF                          ------------------------------------------------------------ 20446

GGT2.end-endGGT1.48980500-49036766.gibbon     acccggcttcctcccacatctcactcaccgtacaatatttgagcagaccttggagtcggt 51854
BCRP3.HUMAN.NCBI.REF                          ------------------------------------------------------------ 20446

GGT2.end-endGGT1.48980500-49036766.gibbon     ggcaacagcagcgtgggcaaaggcctgggagccacagggggcctggtgtcgagagaggac 51914
BCRP3.HUMAN.NCBI.REF                          ------------------------------------------------------------ 20446

GGT2.end-endGGT1.48980500-49036766.gibbon     cacagccagcacaatgacagccagcgctagccccagccccagcaggaccaggccaaccgt 51974
BCRP3.HUMAN.NCBI.REF                          ------------------------------------------------------------ 20446
```

```
GGT2.end-endGGT1.48980500-49036766.gibbon      ggctccgcagtcccgggccatggctctgcagcccaggaggagaggggaggccggtgggca 52034
BCRP3.HUMAN.NCBI.REF                           ------------------------------------------------------------ 20446

GGT2.end-endGGT1.48980500-49036766.gibbon      gacagagggacagatgggtgggcagatgagtggacaagaagaagcatagatagactcaca 52094
BCRP3.HUMAN.NCBI.REF                           ------------------------------------------------------------ 20446

GGT2.end-endGGT1.48980500-49036766.gibbon      agtaattggacagatggacaaacaggtggggccacagacagacatgaagatgaatcgaca 52154
BCRP3.HUMAN.NCBI.REF                           ------------------------------------------------------------ 20446

GGT2.end-endGGT1.48980500-49036766.gibbon      gacaggccagatagctggacggagaggacagtaagagaaagatagtcagatagacaatgg 52214
BCRP3.HUMAN.NCBI.REF                           ------------------------------------------------------------ 20446

GGT2.end-endGGT1.48980500-49036766.gibbon      gacagagatgggcttatagatgggcggacagacagacaggtctgaacagcaggctgccag 52274
BCRP3.HUMAN.NCBI.REF                           ------------------------------------------------------------ 20446

GGT2.end-endGGT1.48980500-49036766.gibbon      atagacagatgggtgaatggacagacggctggcggctggcagctgtggcgtgctgctgcc 52334
BCRP3.HUMAN.NCBI.REF                           ------------------------------------------------------------ 20446

GGT2.end-endGGT1.48980500-49036766.gibbon      ctccccagatgcacaccacagagtgtccaaactcatgcctcaacttctagttttcagctc 52394
BCRP3.HUMAN.NCBI.REF                           ------------------------------------------------------------ 20446

GGT2.end-endGGT1.48980500-49036766.gibbon      ctgcttgttcctggcaggaggccaggcagcaaagcctctgaggggagttttccttgccta 52454
BCRP3.HUMAN.NCBI.REF                           ------------------------------------------------------------ 20446

GGT2.end-endGGT1.48980500-49036766.gibbon      gaggagtcagctgctgtgttaactccctcactgccggtaggtcccaaagccccacctacc 52514
BCRP3.HUMAN.NCBI.REF                           ------------------------------------------------------------ 20446

GGT2.end-endGGT1.48980500-49036766.gibbon      gcccgccagaacccagggtcacactgtcccaatgtgcagaatttggtgcctgctgcactg 52574
BCRP3.HUMAN.NCBI.REF                           ------------------------------------------------------------ 20446

GGT2.end-endGGT1.48980500-49036766.gibbon      cggttgccaggtaggggcagggctcccaagaacctccacaccattacccgggttcccggc 52634
BCRP3.HUMAN.NCBI.REF                           ------------------------------------------------------------ 20446

GGT2.end-endGGT1.48980500-49036766.gibbon      agctctgggaaagcagagctgggaccgctcaactctctcccctggatccggcaaggctgc 52694
BCRP3.HUMAN.NCBI.REF                           ------------------------------------------------------------ 20446

GGT2.end-endGGT1.48980500-49036766.gibbon      cccgctcccagagtggagtcccgctccccagctcccacctctatcccctaaccctctctg 52754
BCRP3.HUMAN.NCBI.REF                           ------------------------------------------------------------ 20446

GGT2.end-endGGT1.48980500-49036766.gibbon      catggcccagcctagtcagcatcaaggtggagctgaacacagatggcggggaggacccaa 52814
BCRP3.HUMAN.NCBI.REF                           ------------------------------------------------------------ 20446

GGT2.end-endGGT1.48980500-49036766.gibbon      ggtggtgccactcaggacccgggttcaagtccttatgcttctgcagcctggcctgggtct 52874
BCRP3.HUMAN.NCBI.REF                           ------------------------------------------------------------ 20446

GGT2.end-endGGT1.48980500-49036766.gibbon      cccaaccctcccagggtgaccaagggcttcccagtctgcacagagaggacaggggggactt 52934
BCRP3.HUMAN.NCBI.REF                           ------------------------------------------------------------ 20446

GGT2.end-endGGT1.48980500-49036766.gibbon      gatagcatcaaatgctggtgactacaagatgcccatgtggggaatgcagacagaccatgc 52994
BCRP3.HUMAN.NCBI.REF                           ------------------------------------------------------------ 20446

GGT2.end-endGGT1.48980500-49036766.gibbon      ctctagcccttggtacctggcaccatccatccctgcgacttgctgtcctggaaatgcagc 53054
BCRP3.HUMAN.NCBI.REF                           ------------------------------------------------------------ 20446

GGT2.end-endGGT1.48980500-49036766.gibbon      atggacctccagggagggggctgtgccatgtgggcgcccaccccgcctgcagctctttc 53114
BCRP3.HUMAN.NCBI.REF                           ------------------------------------------------------------ 20446

GGT2.end-endGGT1.48980500-49036766.gibbon      ccaccctggctgcaggtctgcttccctgaatccaaatccactattactgtgctggcagcg 53174
BCRP3.HUMAN.NCBI.REF                           ------------------------------------------------------------ 20446

GGT2.end-endGGT1.48980500-49036766.gibbon      cagcctctctggggacactggcctggctctgttctccccaggcctcagggtgcctaaatg 53234
BCRP3.HUMAN.NCBI.REF                           ------------------------------------------------------------ 20446

GGT2.end-endGGT1.48980500-49036766.gibbon      ggaggtagccaggagagtgaggacccactgaggggctctgttgactaggctcagcagtgg 53294
BCRP3.HUMAN.NCBI.REF                           ------------------------------------------------------------ 20446

GGT2.end-endGGT1.48980500-49036766.gibbon      tgcaggtgatgtgagctggaatccttcccatgtggcccccacagtcctccacgcttcctc 53354
BCRP3.HUMAN.NCBI.REF                           ------------------------------------------------------------ 20446
```

```
GGT2.end-endGGT1.48980500-49036766.gibbon      cccagctgaacactgcctgctccagatgtctacacctggagtccaggccccccatctag 53414
BCRP3.HUMAN.NCBI.REF                           ------------------------------------------------------------ 20446

GGT2.end-endGGT1.48980500-49036766.gibbon      gcagcagagaaactgaggcacagagacagactgtgtccttacaggccacacagcctgcca 53474
BCRP3.HUMAN.NCBI.REF                           ------------------------------------------------------------ 20446

GGT2.end-endGGT1.48980500-49036766.gibbon      ggctcctgtgtccagccagagctcctggtcagcctggactggagtggttgtttagaggca 53534
BCRP3.HUMAN.NCBI.REF                           ------------------------------------------------------------ 20446

GGT2.end-endGGT1.48980500-49036766.gibbon      ggctgttcccacgactgcctctcatggtgggcagggggtgggggtggacgcctcctccc 53594
BCRP3.HUMAN.NCBI.REF                           ------------------------------------------------------------ 20446

GGT2.end-endGGT1.48980500-49036766.gibbon      acccccacctgactcccaagcctcagtgacattgctcaatcaggagctgaagtgcattcc 53654
BCRP3.HUMAN.NCBI.REF                           ------------------------------------------------------------ 20446

GGT2.end-endGGT1.48980500-49036766.gibbon      tgggctcaggccagcccagccacccacccgctgcagtcctggaagcccagagatctgggc 53714
BCRP3.HUMAN.NCBI.REF                           ------------------------------------------------------------ 20446

GGT2.end-endGGT1.48980500-49036766.gibbon      agctggaacagtggagacagcagcgtgggggacgtcccgctcctctccccaccatcctgg 53774
BCRP3.HUMAN.NCBI.REF                           ------------------------------------------------------------ 20446

GGT2.end-endGGT1.48980500-49036766.gibbon      tcaggcagaggccagggtgcagggaccccccacccccagcaaaggcccagggaaagga 53834
BCRP3.HUMAN.NCBI.REF                           ------------------------------------------------------------ 20446

GGT2.end-endGGT1.48980500-49036766.gibbon      atgtgtgtcattctggtcctgacccgaggcacagccaggaaggtccctgtggggaaaaga 53894
BCRP3.HUMAN.NCBI.REF                           ------------------------------------------------------------ 20446

GGT2.end-endGGT1.48980500-49036766.gibbon      aagagagatcagactgttactctatctatgtagaaagtagacatgagagactccattttg 53954
BCRP3.HUMAN.NCBI.REF                           ------------------------------------------------------------ 20446

GGT2.end-endGGT1.48980500-49036766.gibbon      ttctgtactaagaaaaattcttttgccttgagatgctgttaatctgtaaccctagcccta 54014
BCRP3.HUMAN.NCBI.REF                           ------------------------------------------------------------ 20446

GGT2.end-endGGT1.48980500-49036766.gibbon      accctgtgctcacagaaacatgtgctgtgtcaactcaaggtttcatggattaagagctac 54074
BCRP3.HUMAN.NCBI.REF                           ------------------------------------------------------------ 20446

GGT2.end-endGGT1.48980500-49036766.gibbon      gcaggatgtgctttgttaaacaaatgcttgaaggcagaatgcttgttaaaagtcatcacc 54134
BCRP3.HUMAN.NCBI.REF                           ------------------------------------------------------------ 20446

GGT2.end-endGGT1.48980500-49036766.gibbon      actccctaatctcaagtatgcaaggacacaaaacactgcagaaggccgcagggacctctg 54194
BCRP3.HUMAN.NCBI.REF                           ------------------------------------------------------------ 20446

GGT2.end-endGGT1.48980500-49036766.gibbon      cctaggaaaaccaggtattgtccaaggtttctccccatgtgatagcctgaaatatggcct 54254
BCRP3.HUMAN.NCBI.REF                           ------------------------------------------------------------ 20446

GGT2.end-endGGT1.48980500-49036766.gibbon      cctgggaagggaaagacctgaccgtcccccagcccgacacccataaagggtctgtgctga 54314
BCRP3.HUMAN.NCBI.REF                           ------------------------------------------------------------ 20446

GGT2.end-endGGT1.48980500-49036766.gibbon      ggaggattagtaaaaaacgaaggcctctttgcagttgagataagaggaaggcatttgtct 54374
BCRP3.HUMAN.NCBI.REF                           ------------------------------------------------------------ 20446

GGT2.end-endGGT1.48980500-49036766.gibbon      cctgctcgtccctgggcaatggaatgtctccgtgtaaaacccgattgtatattccatcta 54434
BCRP3.HUMAN.NCBI.REF                           ------------------------------------------------------------ 20446

GGT2.end-endGGT1.48980500-49036766.gibbon      ctgagataggagaaaaccgccttagggctggaggtgagacatgatgctggcaatactgct 54494
BCRP3.HUMAN.NCBI.REF                           ------------------------------------------------------------ 20446

GGT2.end-endGGT1.48980500-49036766.gibbon      ctttaatgcattgagatgtttatgtatgtgcatatcaaagcacagcaccttcttcttaac 54554
BCRP3.HUMAN.NCBI.REF                           ------------------------------------------------------------ 20446

GGT2.end-endGGT1.48980500-49036766.gibbon      cttgtttatgacacagagacatttgttcacatgttttcctgctgaccctctccccactat 54614
BCRP3.HUMAN.NCBI.REF                           ------------------------------------------------------------ 20446

GGT2.end-endGGT1.48980500-49036766.gibbon      taccctattgtgctgccacatccccctctccaagatggtagagataatgatcgataaata 54674
BCRP3.HUMAN.NCBI.REF                           ------------------------------------------------------------ 20446

GGT2.end-endGGT1.48980500-49036766.gibbon      ctgagggaactcagagactggtgccagcctgggtcctctgtatgctgagtgcaggtcccc 54734
BCRP3.HUMAN.NCBI.REF                           ------------------------------------------------------------ 20446
```

```
GGT2.end-endGGT1.48980500-49036766.gibbon      tgggcctacttttctttctctatattttgtctctgtctctttcttttctcagtctcttgt 54794
BCRP3.HUMAN.NCBI.REF                           ------------------------------------------------------------ 20446

GGT2.end-endGGT1.48980500-49036766.gibbon      cccacctgacgagaaacacccacaagtgtggaggggcaggccaccccttcaggtccctga 54854
BCRP3.HUMAN.NCBI.REF                           ------------------------------------------------------------ 20446

GGT2.end-endGGT1.48980500-49036766.gibbon      atgtccttcctcaggaaatgatgggagaaggggtgatgagaatggaggagaggatttaag 54914
BCRP3.HUMAN.NCBI.REF                           ------------------------------------------------------------ 20446

GGT2.end-endGGT1.48980500-49036766.gibbon      tcctccacccccaaggtagtcctgggctgagccccatgggacctggagaaccagggtgt 54974
BCRP3.HUMAN.NCBI.REF                           ------------------------------------------------------------ 20446

GGT2.end-endGGT1.48980500-49036766.gibbon      accccaccagcgtgtcgggtcgaggaagccccgtggccaggtccccttctcttgctgct 55034
BCRP3.HUMAN.NCBI.REF                           ------------------------------------------------------------ 20446

GGT2.end-endGGT1.48980500-49036766.gibbon      gtgccacccagagcaaggcctgcccttccgacctcggtcttctcccctgcaagtggggcc 55094
BCRP3.HUMAN.NCBI.REF                           ------------------------------------------------------------ 20446

GGT2.end-endGGT1.48980500-49036766.gibbon      acagcctttcctctcaggccaaaataaggattgaggatgggtgcagtggctcacccctgt 55154
BCRP3.HUMAN.NCBI.REF                           ------------------------------------------------------------ 20446

GGT2.end-endGGT1.48980500-49036766.gibbon      aatcctagcactttgggagactgagatgggggactgcttgaagtcaggagttaagacca 55214
BCRP3.HUMAN.NCBI.REF                           ------------------------------------------------------------ 20446

GGT2.end-endGGT1.48980500-49036766.gibbon      gcctggtcaacatagtgagaccccatctcaattggtttaaattttaaaaaaaaaattaa 55274
BCRP3.HUMAN.NCBI.REF                           ------------------------------------------------------------ 20446

GGT2.end-endGGT1.48980500-49036766.gibbon      ataaataaggattgaggagtgacttgtacaccagttgagcccacctccatctcacccctg 55334
BCRP3.HUMAN.NCBI.REF                           ------------------------------------------------------------ 20446

GGT2.end-endGGT1.48980500-49036766.gibbon      cagagccccagagacacagccctccagggctcagacccggtggtacttgactctgcaggc 55394
BCRP3.HUMAN.NCBI.REF                           ------------------------------------------------------------ 20446

GGT2.end-endGGT1.48980500-49036766.gibbon      ataaaaccctgtttgtctatgggccgtttggaatcaccaggttttcggggctcctgaagg 55454
BCRP3.HUMAN.NCBI.REF                           ------------------------------------------------------------ 20446

GGT2.end-endGGT1.48980500-49036766.gibbon      atagccccgacctggcctcacctgggccctggccccagtgcccctggtgatatccaggtg 55514
BCRP3.HUMAN.NCBI.REF                           ------------------------------------------------------------ 20446

GGT2.end-endGGT1.48980500-49036766.gibbon      ctgggctgtgatcaccgcccccaccagcccttcccagaaccctgccccaggtgttggaa 55574
BCRP3.HUMAN.NCBI.REF                           ------------------------------------------------------------ 20446

GGT2.end-endGGT1.48980500-49036766.gibbon      ctgtgcacagaggagggagcaggccccgagggaggcctggaggggctgccgatggtgaag 55634
BCRP3.HUMAN.NCBI.REF                           ------------------------------------------------------------ 20446

GGT2.end-endGGT1.48980500-49036766.gibbon      gctgctgtgtctagctgtttccttccggacccactccctctgggctgcatccccggctgg 55694
BCRP3.HUMAN.NCBI.REF                           ------------------------------------------------------------ 20446

GGT2.end-endGGT1.48980500-49036766.gibbon      tctaacccctgatccctgggatctggggacatcttcccgtttgctgttccctgagaacca 55754
BCRP3.HUMAN.NCBI.REF                           ------------------------------------------------------------ 20446

GGT2.end-endGGT1.48980500-49036766.gibbon      ggcctccctctggagaggatcacaagcttgtgtttcactctgggcttgcatttggaaacc 55814
BCRP3.HUMAN.NCBI.REF                           ------------------------------------------------------------ 20446

GGT2.end-endGGT1.48980500-49036766.gibbon      ccccaggggcgtggctctgaccaagatgttttcctccagcctgttgcccagtccccattc 55874
BCRP3.HUMAN.NCBI.REF                           ------------------------------------------------------------ 20446

GGT2.end-endGGT1.48980500-49036766.gibbon      ctcggacctcagcttcacttcccgtgtcattggcaggatcagctggacgcctaaggatct 55934
BCRP3.HUMAN.NCBI.REF                           ------------------------------------------------------------ 20446

GGT2.end-endGGT1.48980500-49036766.gibbon      gagaaggcacccgggttcccagcatcagctggccaccctctgcctaagaaactgccaggg 55994
BCRP3.HUMAN.NCBI.REF                           ------------------------------------------------------------ 20446

GGT2.end-endGGT1.48980500-49036766.gibbon      tcgcagcacccccctggtggctggtcctaggtagtgtcactgcccagccccagtaagggag 56054
BCRP3.HUMAN.NCBI.REF                           ------------------------------------------------------------ 20446

GGT2.end-endGGT1.48980500-49036766.gibbon      ggcctggccccaaattccgagggatcaggttgggaagggacagggcttggtgtgaacctt 56114
BCRP3.HUMAN.NCBI.REF                           ------------------------------------------------------------ 20446

GGT2.end-endGGT1.48980500-49036766.gibbon      cccctggcccccagccacgtgcctggctttccctatgctaaagatgctgaggctagttcc 56174
```

```
BCRP3.HUMAN.NCBI.REF                          ------------------------------------------------------------ 20446


GGT2.end-endGGT1.48980500-49036766.gibbon      agtgcccgcattatgaagatctccgaatcccacctctccattcctccccagccagatggc 56234
BCRP3.HUMAN.NCBI.REF                          ------------------------------------------------------------ 20446


GGT2.end-endGGT1.48980500-49036766.gibbon      tccatttcacacacaatacactgaggcccagag  56267
BCRP3.HUMAN.NCBI.REF                          ---------------------------------  20446
```

==

======

>GGT2.end-endGGT1.48980500-49036766.gibbon.rev.compl
ctctgggcctcagtgtattgtgtgtgaaatggagccatctggctggggaggaatggagag
gtgggattcggagatcttcataatgcgggcactggaactagcctcagcatctttagcatg
gggaaagccaggcacgtggctgggggccaggggaaggttcacaccaagccctgccccttc
ccaccctgattcctcagactttggggccaggccctcccttactggggctgggcagtgaca
ctacctaggaccagccaccaggggggtgctgcgaccctggcgctttcttaggcagagggtg
gccagctgatgctgggaacccgggtgccttctcagacccttaggcgtccagctcaccctg
ccaatgacacgggaggtgaagctgaggtccgaggaatggggactgggcaacaggctggag
gaaaacatcttggtcagagccacgcccctgggggtttccaaatgcaagcccagagtgaa
acacaagcttgtgatcctctccagagggaggcctggttctcagggaacagcaaacgggaa
gatgtccccagatcccagggatcaggggttagaccagccggggacacagcccagagggag
tgggtccggaaggaaacagctagacacagcagccttcaccatcggcagcccctccaggcc
tccctcagggtcggctccctcctctgtgcacagttccaacacctggggcagggttctggg
aagggctggtggggggcggtgatcacagcccagcacctggatatcaccaggggcactggg
gccagggcccaggtgaggccaggtcggggctatccttcaggagccccgaaaacctggtga
ttccaaacggcccacagacaaacagggttttatgcctgcagagtcaagtaccaccgggtc
tgagccctggagggctgtgtctctggggctctgcaggggtgagatggaggtgggctcaac
tggtgtacaagtcactcctcaatccttatttatttaatttttaaaaaaaatttaaacca
atagagatggggtctcactatgttgaccaggctggtcttaactcctgacttcaagcagtc
cccccatctcagtctcccaaagtgctaggattacaggggtgagccactgcacccggcctc
aatccttattttggcccgagaggaaaggccgtggccccatttgcaggggagaagaccaag
gtcggaagggcaggccttgctctgggtggcacagcagcaagagaagtgggacctggccac
gaggcttcctcgacccaacacgctggtggggtacaccctggttctccaggtcccatgggg
ctcagcccagggctaccttggggggtggaggacttaaatcctctccttcattctcatcac
cccttctcccatcatttcctgaggaaggacattcagggacctgaaggggtggcctgcccc
tccacacttgtgggtgtttctcgtcaggtgggacaagagattgagaaaagaaagagacag
agacaaaatatagagaaagaaaagtaggcccaggggacctgcactcagcatacagaggac
ccaggctggcaccagtctctgagttccctcagtatttatcgatcattatctctaccatct
tggagaggggggatgtggcagcacaatagggtaatagtggggagtgggtcagcaggaaaac
atgtgaacaaatgtctctgtgtcataaacaaggttaagaagaaggtgctggccgggcgtg
gtggctcacgcttgtaatcccagcactttaggaggccgaggtgggcggatcacgaggtca
ggagatcgagaccacggtgaaaccccgtctctactaaaaatacaaaaaaattagccgggc
gtggtggcgggcgcctgtagtcccagctactcggagaggctgaggcatgagaatggcatg
aaccggggaggcggagcttgcagtgagccgagatcgcgccactgcactccagcctgggtg
acagagcgagactccatctcaaaaaaaaaaaaaaaaaaagaagaaggtgctgtgctttga
tatgcacatacataaacatctcaatgcattaaagagcagtattgccagcagcatgtctca
cctccagccctaaggcggttttctcctatctcagtagatggaatatacaatcaggtttta
cacggagacattccattgcccagggacgagcaggagacagatgccttcctcttatctcaa
ctgcaaagaggccttcgtcttttactaatcctcctcagcacagaccctttatgggtgtca
ggctgggggacggtcaggtctttcccttcccacgaggccatatttcaggctatcacatgg
ggagaaacctcggacaatacctggttttcctaggcagaggtccctgtggccttctgcagt
gttttgtgtccctgcatacttgagattagggagtggtgatgacttttaacaagcattctg
ccttcaagcatttgtttaacaaagcacatcctgcatagcccttaatccattaaaccttga
gttgacacagcacatgtttctgtgagcacagcgttagggctagggttacagattaacagc
atctcaaggcaaaagaatttttcttagtacagaacaaaatggagtctcttatgtctactt
tctacatagatagagtaacagtctgatctctctttcttttccccacagggaccttcctgg
ctgtgcctcgggtcaggaccagaatgacacacattcctttccctgggcctttgctgggg
gtgggggggtccctgcaccctggcctctgcctgaccaggatggtggggagaggaacggg
atgtcccccacgctgctgtctccactgttccagctgcccagatctctgggcttccaggac
tgcagcgggtgggtggctgggctggcctgagcccaggaatgcacttcagctcctgattga
gcaatgtcactgaggcttgggagtcaggtgggggtgggaggaggcgtccacccccccgccc
cccaaaagtgagaggcagtcgtgggaacagcctgcctctaaacaaccactccagtccagg
ctgaccaggggctctggctggacacaggagcctggcaggctgtgtggcctgtaaggacac
agtctgtctctgtgcctcagtttctctgctgcctagatggggggcctggactccaggtg
tagacatctggagcaggcagtgttcagctggggaggaagcgtggaggactgtgggggcca
catgggaaggattccagctcacatcacctgcaccgctgctgagcctagtcaacagagccc
ctcagtgggtcctcactctcctggctacctcccatttaggcaccctgaggcctggggaga
acagagccaggccagtgtccccagagaggctgcgctgccagcacagtaatagtggatttg
gattcagggaagcagacctgcagccaaggtgggaaagagctgcaggcagggtgggcccac

acatggcacagccccttccctggaggtccatgctgcatttccaggacagcaagtcgcag
ggatggatggtgccgggtaccaagggctagaggcatggtctgtctgcattccccacatgg
gcgtcttgtagtcaccagcatgtgatgctatcaagtcccctgtcctctgtgcagactgg
gaagcccttggtcaccctgggagggttgggagacccaggccaggctgcagaagcataagg
acttgaacccgggtcctgagtggcaccaccttgggtcctccccgccatctgtgttcagct
ccaccttgatgctgactaggctgggccatgcagagagggttaggggatagaggtgggagc
tggggagcgggactccactctgggagcggggcagccttgccggatccaggggagagagtt
gagcggtcccagctctgctttcccagagctgccgggaacccgggtaatggtgtggaggtt
cttgggagccctgcccctacctggcaaccgcagtgcagcaggcaccaaattctgcacatt
gggacagtgtgaccctgggttctggcgggcggtaggtggggctttgggacctaccggcag
tgagggagttaacacagcagctgactcctctaggcaaggaaaactcccctcagaggcttt
gctgcctggcctcctgccgggaacaagcaggagctaaaaactagaagttgaggcatgagt
ttggacactctgtggtgtgcatctggggagggcagcagcgcgccacagctgccagccacc
agccgtctgtccattcacccatctgtccatctggcagcctgctgttcagacctgtctgtc
tgtccgcccatctataagcccatctctgtcccattgtctatctgactatctttctcttac
cgtcctctccgtccagctatctggcctgtctgttgatccatcttcatgtctgtctgtggc
cccacctgtttgtccatctgtccaattacttgtgagtctatctatgcttcttcttgtcca
ctcatctgcccacccatctgtccctctgtctgcccaccggcctcccctctcctcctgggc
tgcagagccatggcccgggactgcggagccacggttggcctggtcctgctggggctgggg
ctagcgctggctgtcattgtgctggctgtggtcctctctcgacaccaggccccttgtggc
tcccaggcctttgcccaggctgctgttgccgccgactccaaggtctgctcaaatattgta
cggtgagtgagatgtgggaggaagccgggtggcccttggcagccagcccctcctggagaa
ggcgttgtgtgtgagagagtgtgtgtgtgagcgtgtgtgtgtgtgtgtgtgattatgtgt
gagtgtgagtgtgtgggtatatgtgtgagtgtgagtgtgtgggggtgtgtgtgtgtgatt
gtgtttgggtgtgtgtatgtgtgagtttgtggaggtgtgtgagagtatatgtgagtgtga
gtgtgtggggagtgtgggtgggtgtgaatgtgtgtgattgtgtttcggtgtgtgagggtg
tgtgtgactgtgaatgtgtgagtgtgagtgtgtgggtgtgtgtaaatgtgtgtgattgtg
tgtgagtgtatgtgtgggtgtttgtgggtgagtgtgtcagtatggggggtgtgggtgtgt
gtgaatgtgtgtgattgtgtgtggctatgtgtatgggtgtgtgtgtgcgcgtgtgtgtgt
gcacgtgcactggcccaggaagcaggagccgtgtgtgtgtggtgtgggcttcagcacctg
cagggcttgggcacaaggaggcagcctcagggcccttgcacagaacaggtggcagggtgt
gcccgtggggcagatggggacttggggacaatggtggtgtgtgaatccatacctggctcc
aggattcaggaggcccatttgcatatcccaggtgggaacctgtctggcccaacctgaccc
tgctggccggtgcaggccccttcagtgaggccaattctccaaggctagggtcttctccca
gggtcataggtgaaggggtttggaggctccctgcgtgggtactggcctgctggggtacac
gatgctgccatagccagtctgccccaacacccagcccggggccacgtctcgggtctctca
gtcctggggagccccgtgccccacccctcacatcctctctccgagtcagggcctgggtct
cgtgagctgagtgactgatacttggtgtcctggatgagggtgtgatggagaggggccaca
gtgggtgtttcctgaccctcttccaggaaggtgctgctgccgctgcagagaggacacaca
caggatgccccttcctgccccctgcctcccattgggcccacgaaatccagggcaagcctc
ccctccctgccagccacctggtctgcttcccataaattctgtccttcaggctgttgggag
gatcccagtgctttgtaaactaaagcaagggaggagtggccattctctctctttgttcat
tcattcaccttttcattcattccttcctccctccattccccccatctgtccatccttccct
gccctgattgctcatgccacccgccagcccctcctgacctggtcctttggtttctcttca
gggcttcctgtctcctcccacagggctgagaatggcagctcagggacaagtgggcgctgg
ggactgcttagtctccccagtggctcccaggggattcgagggattgatgccagctgccac
cccaggctgtgccgctcctatgctcgggaggacatacagagatgcggcacccacttaaac
tcgaagttgcaaagatgcaaatgagactggagtctcaggcaccagagaccacccgtgggc
acgtggcttttgggagtggggacctgctgccacagatctctgaggagactggacctgctg
ggtctccccgagggactgtctgggggtctccatagcatgccctgctgtgtgcgtgacagt
cagtggttggctagggggtctctactctaaagctccctctgctgacactccctcaaactg
tcccttggtgaagagagaggatgtggtttgccccagtgttttgtcagacaactctctcca
cttcctgttttaagaagctgggagtggaagagagcctggggctggccccagctgctgctg
cggaacaggggtcactgggaacaggggtcactggacgctgggaccctggccgggctggct
gggggcctcaggaagaggcctgctgcagcatcatcctggccaagattcctccttgcaga
ggacgctggccacgctgccacagggtctgctggggccaccagaagcccatgctcctgcct
ccatctctcccctctgtgctcccctctcaccaggaggccctcccagagtccagtctcctg
cttttttttttttttttttttgagacaatgtctcgctctgtcaccaggctggagtgcag
tggcacgatctcggctcactgcaacctctgcctcctcggttcaaatgattctcctgcctc
agcctcctgagtagctgggactacaggtgccagccaccacgcccagctaatttttgtatt
tttagtagagacggggtttcaccatgttggccagggtggtctctatctcttgacctcgtg
atttgcccgcctcagcctcccaaagtgctgggattacaggagtgagtcatggcgcccggc
cccatctcctactctttctttcagcaccaggttttattcttgggattctgctacagccgg
agcccctgggtgcaagttcctaaggtttctgtgagtgtggacccagcaccgtgcctagta
gacacacaaaaggagcatggtgacagtgagtctgtcatctccagcataacgactgttttg
atccttgtaaaaaaggtgatttttggctggatgtggtggctcacacctgtaatcccagca
ctttgggaggccaaggcgggtggatcattggaggtcaggagttggagaccagcctgggca
acatggtgaaaccccgtctctactaaaaatacaaaaattagccaggcatggtagcgggtg
cctgtaatcccagctacttgggaggctgaggcaggagaattgcttgaacccgggaggcaa
aggctgcagtaagccaagatcgtaccactgcactccagcctcggtgacatagcaggactt
ggtctcaaaaaaagacagaaaagtttatatttttgttctaatggttatcttaatatcttc
atcctataattatgttttgtataattataatagctatataagatacactacccctagtac

attgtttttggatattctattcgcccctgatggttaatttatgtgtcaacttggctaag
ctatggtgccccgttgtttggtcaaatacttgtcaatatcttgctgggaggttatttcat
agatgtgatgaacattgacagtcagttgactttaagtaaaacagattacccaccataata
tgggtgggccacctccaatcagttgaaggccttaagaacaaaaactgaggtttcccagag
aagcaggaattccgcttcaagactgtaacacacaaaccctgcctgagtttctggcctgct
gactgctctacagatgttaggttccagacttcgagatcaactcttacctgaatttatagc
ctgctggcttgccctacagattttaaacttgctagtccccacaatcatgtgagccaattc
ctaaataaatctctctctgtgtataacctattggtttagtttctctaaaaagctttcaca
tccagtttcctggatgttaagtaatactgaaactagctagtaacttcttttttttttttt
tttttttttgagacagagttttgctctttttttttttttttttttgggttaattactata
tattttatatgagtgcttgtttaagccaatccaaatagaatttcttaagggatttctggc
taactatgtcagattttttttttaattaattttttttgcacacaaaacaataaacatttt
ctaaaaatacatacaaacaaaaagatgcgtatcaaacatattaggaaggttacacatggg
aagtcggggaatagaaatgggggtgggagttaaaataaatgagagagggactttatatg
gatcagtgataataactcaatcctctatttgacaaagaagagggagtaagaagaggaaga
aaaagaaagtgggataaaggatcagaaagggaggaaaatagaaaaaattagagtatgact
ccagggtagacctgttttgttgtcactgagttggttggttggtttgtctgttgtattttt
catgtttcgccaagttggccagactggtctcgaactcctagcccaaagtgatcaacccgc
ctcgcccccccagagtgccgggaccacaggcgtgagccaccacgtccagcccccacattgc
ttctggcctccgtggtagacctcccagacggagcggccaggcagaggagctcctcacttc
ttcccagacacggggcggccgggcagaggcgctcctcacttcccagacggggcggccagg
cagagacgctcctcacttcttcccagacgataagtggtcgggcagaggcgcccctcactt
cccagacgatgggtggccgggcagaggcgctcctcacttcccagacgatgggtggccggg
cagaggcgctcctcacttcccagacggggcggccgggcagaggcgctcctcacttcccag
acggtgggtggccgggcagaggcgctcctcacttcccagacggtgggtggccggggagag
gctctcctcacttcccagacggggcggccgggcagaggtgctcctcacttcccagagggt
gggtggccaggcagaggcactcctcacttcccagacaatgggtggccgggcagaggctct
cctcatttcccagacggggcggccgggcagaggtgctcctcacttcccagacgatgggtg
gccgggcagaggcgctcctcacttcccagacggtgggtggccgggcagaggcgctcctca
cttcccagacggtgggtggccgggcagaggcgctcctcacttcccagacggtgggtggcc
gggcagaggcgctcctcacttcccagacggggcggccgggcagaggcgctcctcacttcc
cagacggggcggccgggcagaggtgctcctcacttcccagacgatgggtggccgggcaga
ggcgctcctcacttccaagacagggcggccgggcagaggcgctcctcacttcccagacgg
tgggtggccgggcagaggcgctcctcacttcgcagacggtgggtggccgggcagaggcgc
tcctcacttcccagacggggcggccgggcagagacgctcctcacttcccagacggtgggt
ggccgggcagaggcgctcctcacttcgcagacggtgggtggccggacagaggcgctcttc
acttcccagacggggcggccgggcagaggtgctcctcacttcccagacggggcggccggg
cagaggcgctcctcacttcccagacggtgggtggccgggcagaggcgctcctcacttcgc
agacggtgggtggccgggcagaggcgctcctcacttcccagacggggcggccgggcagag
gcgctcctcacttcccagacggggcggccgggcagaggcgctcctcacttcccagacggt
gggtggccgggcagaggcgctcctcacttcccagacggggcggccgggcagaggcgctcc
tcacttcccagacgatgggtggccgggcagaggcgcccctcacctcttcccagatggggc
ggccgggcagaggcgctcctcacttcccagacgatgggtggccgggcagaggcgcccctc
acctcttcccagacggggcggctgggcagaggcgctcctcacttctacctggacggtgcg
gccgggcagaggcgctcctcacttcttcccggacggggcgcctgggcagaggcgctcctc
acttcccagacgatgggtgcctgggcagaggcgctcctcacttcccagacggggcgcccg
ggcagaggcgctcctcacttcccagacgatgggtggccgggcagaggcgctcctcacttc
ccagacggggcggccggacagaggggctcctcacttcttcccggacagggcggcagggca
gaggcgctcctcacttcttcccagatggggcggccgggcagaggcgctcttcacttccca
gacggggcggccaggcagaggcgctcctcacttcttcccagatggggcggccgggcagag
gcgctcctcacttcccagacggggtggccgggcagaggcgctcttcacttcccagacggg
gtggctgggcagaggcgctcttcacttcccagacggggtggccgggcagaggcgctcctc
acttcttcccagacggggcggccaggcagaggcgctcctcacttcccagacgatgggtgg
ccaggcagaagcgctcctcacctcccagacgatgggtggccaggcagaggcgctcctcac
ctcccagacgatgggtggccgggcagaggcgctcctcacctcccagacgatgggcggccg
ggcagagacgctccccacctcccagacggggcggtggccgggcaggggctgcaatcccag
caccctggtaggccaaggcaggcggctgggaggcggaggctgccgcgagcccagaccacg
ccaccgcactccagcccgggcaacaccgagcaccgggtgagcgagactccgtctgcagtc
ccagtacctcgggaggctgaggcgggcagagcactcggcgtcaggagctggcgaccagcg
tgggcaacatggcggacgcgcgcctgcaggcaaaggagaaaaagccggcagcggtggcgc
gcggcggcagtcccaggtagtccgtggcgcgggcagcagtgagccgagtagattgcagcc
tgggccacagaggggaaaaagaagaagaagagaagaagaaagaagaaagaagaaagaaga
agaagaagaagaggaagaggaagaggaagaagaagaggaggaagaggaggaagagga
ggaggaggaggaggaagaggaggaagaagaggaggggaggggaggggaggggga
ggaggaggaggaggaggaggagagttttgctcttgttgcccaggctggagtacaatg
gcacgatctcggctcaccacaacctccacttcctgggtccaagcaattctcctccctcag
cctcctgagtagctgggattacaggcatgtgccaccatgcttggctaatgttggtatttt
tagtagagacagggcttctccatgttggtcaggctggtcttgaactcccaacctcaggtg
atccgccgccttggcctcacaaagtgctggaattacaggcatgagccaccgcgcccggct
cctagtaacttcttcttttccgtgatgtgtctcttacctctaataatacttttcttctta
aaatctacttcattaaaaatagttatgctgggcatggtggctcatgtctgtaatctcggc
actttgttggaggtcgaggtgggtggatcactgaagcccaggagttcaagaccagcctgg

gcaacatggcgagaccctgcctctacaaaaaatacaaaaatcagctgggtgtggctaata
taattctacgttggtacagttgtagtcccagctacttgggatgctgagatgggagaattg
cttgagcctagaagggagagattgctgtaagccaagatcacatcactgcactccagcctg
ggaaacagagtgaggctctatcttcaaaaaaaaaaaaaaaaaaaaaattatacagctttc
ttggttagtgcatgaatgatatattttcattattttccacctctctgtatccttatata
aaaggcattattagttgggttttactttattttcaattattttaatttttgttgtccttt
taaatgtaactaatgatttatttgggttgaaagccaccaccagtttgctttccatgccta
ttctgtttcttcttatctcctctcacatcttgttttggatttattatttttattatttaa
tttcctcctctattagtttcataactgtgcagtcttggagttattttaaaagatgacagt
ggattattttagagcttacaacatgcatccttcacttatcaaagtctcacatgagctagt
actttttgttgttgttgttgttgttgagatagagagagtcttcctctgctgcccaggctg
gagtgcagtggagcaatcttggttcactgcaacctccacttcttgggttcaagcagttct
cctgcctcagtcacctgagtagctgggaccacaggtgtgcaccactatgcccggctaatt
tttgtattctttttttagtagagacagggtttcaccatgttggccaggctggtcttgaact
cctgaccttaagaaatctgcctacctcgccatcctaaagtgttgggattacaggcgtgag
ccaccatgcccagcctatgagttagtacttctatcctcttcctagtcagtacaagaacct
tggaacaggaactaaatttacccccagtgacttataggcgaatatttttgtgtattttaa
atatatgtgtgtgcatagatgtatctgtgtgtttttttgtgtttctattcttatttatgtt
gagagtgtggagctatgtaagagtaaagagaattgtgtaatgaagccccatgtatccact
caatttcaacaacaatcttatggtcaagctaacttcatgtatactctttcctgcttccct
ctaccccacattacttcagtgcaaatcccagatatataactttacccatacatatttcag
tatgttttatttattttaaaccccacaagatatcattttctatactactgtaattttata
ccaataacattcatttagatttacccacacgtttacctcttctgttaccctttattttta
tttatcaaaatatctttgggaaaaaatatctttcagcacatggtcaaggatctcctgagg
gctatgtcataggcaaaatatacatatacattacatatatatacacacatatacacacac
acacatatacacacatacacacacacacatatatatattccactttcactttttttgtttg
tttgttttttgagaccgagtcttgctctgtggcccaggctggagtgcggtggtgggatct
cagctcactgcaacttctacctcctgggttcaggtgattctccagtctcagcctcccaag
tagttgggattacaggtgttagccaccacccctggctaattttttgtatatatttttttga
gatggagtatcgctctgttgcccaggctggagtgcagtggcgtgatcttggctcactgca
accttcatctccctggttcaagcaattcccctgcctcagcctcccaagtagctgggatta
caggtgcacgccaccatgcccggataattttttttgtatttgtagtagagaccgggttttc
ctatgatggtcagactggtcttgaacttctgacttcaggcaatccaccccgctcggcctc
ccaaagtgctgggattacaggcatgagccaccatgcctggtctgtttttttttttttttt
gagatggagttttgctctgtcacccaggctggagtgcagtggcaagatcttggctcactg
caacctccacctctcgggttcaagcaattcttgtgcctcagcctcctgagtagctgggat
tacaggcacccccaccaaatctggctaatttgtgtatttttggtagaggtggagtttcac
cacattggccaggttggtcttgaacttatgacctcaggtgatccacctgcctcagccttc
caaagtgctgggattacaggcatgagccaccatgcccagccattttcgcttttgaaggat
attgttagtgaacatagaattctaggttggcagatattttctttcctcagtttgaaaaca
tgattcccttgtatctgatttctcctgcttttattgggaagccaattctcaatctaattt
tgctcatttgaaggcaatggctttttatgttgttgtgttttttgagatggagtctcactc
tgtcgcccaggctggagtgcagtggtgcaatttcagctcactgcaacctctgcctcctgg
gctcaagtgattcttctgcctcagcctcccaagcagctgggattacaggtgtccaccatc
acacccggctaattgttgtatttttaatagagatgaacttttgccatgttggtcaggctg
atcccaaattcctcatttcaggtgatccacctgcctcagcctcccaaatgctgggattac
aggcatgagccagcccccaaccccggcctgaaggcagtatctttttccctctggctgctt
tgaaaagttttgtctttgttttgagcagtttacactgatgcatttaggtggctcttcatt
ctatgacttgattctttttttgtccattttagaaaattctcagctttatctcttcaagtgt
tacatcttccccatcctctctctactctccttatgaggctcaaatttcacatgacttatg
ccttgttaaagtatctcccatgtctcttaatccatttcctggatgttctatctatttttc
tctttgtacttcaatttgtatattttgtatcaaactatctcccaattagccaggcgtggt
ggtgggtgcctgtaatcccagctacttgggaggctgaggcaggagaattgcttgaaccca
ggagatggaggttgcagtgaaccgagatcatgccactgcactccagtctgggcaacacag
tgagaccctgtttcaataaataaataagtaaataccagttcactctttttttttttatgttt
gtgtctagtgtgctgttcaaattgagttcctaattccatttttttttttgagacttttttt
ttttgagtctctatctgttgcccaggctggagctcagtggtgcaatctcaactcactgta
gcctccacctcccaggttcaagcgattcttgtgcctcagcctcctgagtaactgggatta
ccaccacgcctaattcatttttgtatttttagtagagatggggttttgccatgttggcca
ggctggtcttgaactcctggcctcatgtgattggcctaactctgtctcccaaaggctggg
attataggcctcagccaccactcccagcctccattttttttttttttttttttttgagac
agagtcttgctctgtcgcccaggctggagtagagtggtgcgatctcggcacactgcaacc
tccccctcctagttcaagcaattctcctgcctcagcctcccgagtagctgggactacagc
cacatgccaccatgcctggctaatttttgtaattttagtagagatgaggtttcaccatat
tggtcaggctggtcttgaacttctgacctcaggtgatccacccacctcagccttccaaag
tgctgggattacaggcgtgagtcaccacgcctagtgcgcccatttttttatagttgccagt
tttctgatgaaattcttaattgtttctttatatcctcgatatacatataaagtacttaaa
gtacatggtctgacgatttcataatctggagatcctatgggcctttttaaaagttgtctg
tgctttctcttgagctttgttcctggtgtcttatttccttgtttgcttggttgtttttaa
tttggcaatggaagttgtgtatacaaattgttacaaataattttttttttgagatggagt
ctcgctttgttccccaagctggagtgcaatgatgtgatctcggctcactgcaacctctgc
aacccagggttcgattctcctacctctgcctctcaagtagctgggattacaggcaggtgc

cagcatgcctggctaatttttgtatttttagtagagatgagttttcaccatgttggtcag
gctggtctgaaactcctgacctcatgatctgcccacctcagcctcccaaagtgctgggat
tacaggcgtgagccactgcgcccagctagaaataatttttaaaaataatttcaagcccca
gcatgatggctcatgtttgtaatcccatcgttttgggaggctgaggcgggcagattgctt
gagcctaggagttcaagatcagcctgtgcaacgtggtgaaaccccatctctacaaaaaat
aaaaaattagctgggtgtggtggtgtgtgcctgtagtcccagctgtttgggacgctgagg
tgggaggctcatttgagcctgggtgattgagtctgcagtgagccatgatcctgagactgc
actccagcttgggcagcagagtgagatgctgtctcagataaataaataaataaaaataaa
ataatttgaggcctaggggtctgaaattctgggatctcctttatgcatttgagtgactga
gatggtttgaagctggatccagtgctcctgagggctgctctatttctggttgactgtgac
tcttagagtgagaaacctgcaccccacgtgcggggcattatggcatcccctccctcagcc
acatgagcaggtccacagcactgctctagaccaggtgtggtggctcacgcctatagtccc
agctactcaggagactgaggcaggaggattgcttcaggccaggaatttgagaccagccag
agcaatatattaggttggtacaaaagtaattgcggtttttgcctttaaaagtaatggcga
ccctgtgttagcaaaataaaaagcaaaaaaaaaaaaaaaaagaaaggaagaattaaaaaaa
gaatcagctgggcatggtggctcacacctctaatcccagcacttttgggaggccaaggcg
ggcagatcatgagatcaggagatccagatcatcctggctaacacggtgaaaccctgtttc
taccaaaaatacaaaaaattagccgggcgtggtggcaggcgcctgtagtcccagctactc
aggaggctgaggcaggagaatggcatgagcccaggagatggagcttgcagtgagccgaga
tcatgccgctgcactccaggctgggtgacagagtaagactccgtttcaaaaaaaaaaaaa
aaaaaaaagaatcactgctctgtctctcagcctcctctttcaggattggcggtcgccttg
aggggaatgctggccttgcctgtctccagccctgtacttctctgcctcctatgcctttaa
gcacatgttttctatttgctgggctgtgaaatctgctcttcatctcatggggtttgcttt
ataggtcactagatccttttctcttggtggttttagaatttgcattttcacattgacttt
aaatagtctgactatagtttgccacagcaaagaccctttgcattgcattgtttggggata
tttgagcctcctctatctggatgtctaatctcttgttagatgtgagtggttatcattatt
atcttattaatgggctggcatgtgggctgtggttccaggcgggctcagaggggcagctgc
cttgatgtctggacagcttctctttcggtttcttttcttacctggactctgggttgcgtg
ttagctgcatctgccagttctgagttttcaaggggagaggggcccagtgatggctgttct
ttgaaagaaagggaagaatgtctcctgcttaacatgtttctgtgtttccagttactttgt
tagttagagccagggtctctcgctctgttgcgcaggctggagtgcaatggcatgatcttg
gctcacagcagtctccaccttccaggcttgagcaatgctcccacctcagcctctcaagca
gctgggactgcaggtatgtgccaccatgcttggctaccttttaatttttttttttttaa
atacagacaaggtctcactatattgcccaggctggtcttaaactcatgggctcaagtgat
cctcctgcttcggcctttcaaagtgctgatattacaggcagggtttcagtttttaaagc
tcccagcagtggtattaaactctccctttccagagaaagcgcaccctgtccgcatccctc
atgttatcctctcctgcctctgcttagggttcactctgggggaaagtgccacttgagaga
ttcctttttgtgtgtggttctgactgaccgctccctgctcacagatgctgcttctcaggg
tggggtccctgaggcctggagcatggcctctgacaaccttcagggccagatgcagaatga
cagcctgtgaccacacagcccctggtgggagacgtcctgccaccctttgcttctctgtgc
cactctggctgcacatttcagatccttgggaaacgttaaccagtaggacctagaagggga
tgtgaggaggggtcaccccccaggtgtgcctgtggtgaatcttcgtgctgagcagatgca
gggagggaggcccaggtgcacacacctgtgaagtaggggcagctggctgggctccttgac
ctgctccagagcttctccttattttctggccacttcacctgcagaaggctcaggtggctg
tggcctctagggtcccttgcagcggaaatgatgcctgtcctcagcccctctctaggcctg
acactgtgctcagcactgtcgtgtgtgtgtatatgtgcacacatgtgaatgtgtgcagac
atcatgaggtgtggtccctgctcttaggcagcttgccttgtggctgaaatgaaccatcac
ctgaatcaagggatggaaacacaaggtcagatgccatgaccttctatggagtcagatggt
ggctccgtgacctggcttcactccagaactacctggggtttctagttgtgcaaatcccag
gtcccactcagattatgagtccagttctctgcaggtggaacccagggaggtgtattttta
acaagatggtactgctcattcacccagcccagccagtggtctcggcatacccaggaaccg
ctgacatagagcttgtgctcccgataaaatgggaaacagcattcatggacgggggacatg
tggcgtggtcatgacagtcacggtgttatataccctgcacataacatagcacagactgtg
tttgggtgacattaaggacgagctcctgcaggctagccgtctggatagtgccgggtgggg
gtagaagttagaggggtcacagaggggcttcctcccagcgccctcatcagctgcttgatt
taggccttggttctgggtccttctgggcctgattctgaaccatgggactggtgtggcctg
caggctcctcccaccacaagctgttcgtggtgcaggggggagaacagtttccacagttccc
cagacagcagctgtggatgccaggcccccaggagttgttactgaagttgctgctgaacag
ctcgcctttcactgagctccacatagcacccgtggtgatgggagccggatagacagagcc
tgccagcctgcacatcagctcccaactgggaggggcagagggaggaggggggtggggaccc
caggcagcagggctctgggagcagtggggccctggattccaggggtgtccggcaggccct
tccttactctacctctctcggcctctggatggaggtgctggcctcggtcaggctctgcct
ctgactaagggctggagaagtggcgggcgtgggctgctgccccgtggggcctctgaacag
accccagggcctctgccaatcatgactccttgctttcagctggacccacaggccctgcag
gacagagactggcagtgcaccatcatcgccatgaatggggtacgtgtctgtgggactctc
ctggtgcccacttccctcagaaggatagggtggcctctgttcatttcaaatcagtcagag
gtggctgagcctgaggcggcatctgagagggagcctggctggaggagggagggcccccga
agagcagaatcaccatgcacgggaatcgccattcactggctgggatgcagttaccagcca
ggccctgagcatccctcctcaaacaaaggtctcatggcaccaccaggacaggtggggcct
ccacttggggacctgggggctgcccgtagaaatggagacccctgatttgtctttaggtac
cctagaaaggcttagaccttaaaagttaatgacacacccaaaaaggcccaggtataaatg
gtaaaatgttaatatttgagattcttggctttttcttatattattctgtctttcctactt

aatttttaattgttactaagaaagagaaagctggtcacggtacacctataatctcagcta
ctctggaggctgagccaggagaatcactggagcccaagagttcgattacagcctgggcaa
cattgcaagatcccatatcttaaaaaaaagtaagcaaccaagagaagcaacagggatttt
aggagatggttctgcagaagccagtcctttacaccatcttcaacaatcctggctcttgct
gaagtagactaggggcttccccgaggggcgactccacctcatgctgagacctctgcatgc
cttggggggtggaaatatttgatgaaactccaggggcccttgggaccttgggctatgagg
accagcaggattagaggactgtaccccttctccccactatagattgaagtaaagctctca
gtcaagttcaacagcagggagttcagcttgaagaggatgccgtcccgaaaacagacaggg
gtcttcggagtcaagattgcagtggtcaccaagtgagtggggaggggcttgggctcatgc
actgagggcgcctgtcccttcagctgtttctgcagaaaagagcatgtgtgggtctctcct
ctctgtgcgtggccgctgcacggtgaggtcaggccccagggaacacttggtgtgttcagc
tgcctcctgtgtttcctccaaaccagctcaggaatgtccttgccgccttgcttggaagca
gtaggctggctccaggaactgcccaagtgcagggttttctgcccttgcttggaatttgtc
acggtcccagtttcctgctgaatggttgtaacccctgcccctttgtcacgagtcagttgc
caagagaagcctgtttgttggtttgagagcagttcttgcagacacagatcacctcctctg
agaattcatttgcttccccaggatggaatctggctgggcccctgaccttgctggtcacgt
gggccagggcctccatcagtcataccctggagtcctatctgtgtctaaacaccactcctc
acccccagctgcacggcagccacttgcatagcactctgggagggctctgggcatgagcag
cgaggactccatcagcagttcccccaaataagccctgctaatgagggtgcttgagaagca
gctttgatgtgctcctaaatccagttgcaaaacagaactaaagttaaggcctctgcacag
cactgtgttctaactttgaagtattcttactctagtgtcctgtgtggtggtattggaatt
gtccattgctaagactcagaggagaaaagcacttagcatcgcaggacttggagcactggt
gctgaggcaacccttcattcattcgtcggatgtgtgttaaggcccaggcccagggcaggg
gtcggggattctactctcacacggcacgtgggtggcaagaccaacaccgggtctgatctc
ccagctgggggcacaggctgctaaccccaggcctggaatctatcagatgcccttcctgtg
ctgactttacttagacaggcctccagaccttcctgcaaagatcatgtgtgacttccaggg
gttctggccgcttgaaatgtcctgagaaagtacatgcaatgaggacagagcttgcagagg
gaggacaggcatgcagaaggctctgtgcgcagccccagacctgggcaccttcgccaccat
cctcactccacatccacaaagcaggtctcctgtgtgtggcccaagcagggctgtcaggac
actgagaacattccctcctcccgcaggagagagaggtccaaggtgccctacatcatgcgc
cagtgcgtggaggagatcgagcgccgaggcatggaggaggtgggcctctaccgcatgtcc
ggagtggtcgtggacatccaggcactgaaggcagccttcgacgtcagtgagtgttggcct
ggggaggacagaacggaggtgtggtcagcggtgtccgtgatgagatctcagagcgctgca
tggcccaggcatgtcacatccttctctgtgtcttttcttcatttactgtgttgttatttt
taaaaaagagaagacaagagttgtagaaaaagcctctgtagaagccagtttttaaaccat
cctagccacgcatgccacttgctggggtggaccaggggcttctgccgggccttggccttt
ctgccttggggggtggacaggaggtggaagcccaggactcagtgcggtctgtccactgccc
tgtgtgaggatgcggtgggcagagggcactggtgggacccagctcaggctggggctgcag
catctctgcctccatctcaacaaccctcacaggctatgaaggacctggacctgcctcaaa
tgccaggggagggcactgaggccccagagggtccttcccagcatcttcaaagcaacagga
ttttgtgcctgcagacccttctttggggcacacaccactgaccctgaccaggacacctag
aattcccagcaccccaggcccagaatggacctggcctgtggtggggaggtaagtaccagt
ggacaattggatccaaaggaagacacaggttcaaacactgaaaccaatcagattctcaca
tggccttcctgctatcagaagacactggtgcaggggtggttgctatgcacagggcagagc
cacccaatccccacgcaggcactgtgtcctgccacactggcctcctcccggacatcacat
cgggccaaccagaggacaggaataggaatgcccacgcacccccatcaactctgcagacac
agaaccaggcacagctcttgggaggagtcagatgagctgctcaaagcccgggagggaccc
gcactgtggtcagtgtggcagggatggtgctttagccaaggcagggatggcaggtgactc
actcgggatcctcaaggaggctgctgcatttctgtgctctttccagataacaaggacgtg
ttggtgatgatcagcgagatggacgtgaacgccattgcaggcacgctgaagctgcacttc
cgtgagctgcccgagcccctcttcactgacgagttctaccccaacttcgcagagggcatt
ggtgagcactggaggccttggcctcgtgggagacatctcctccacgtgcactgctgccct
cggaggctgtgaaaagtgaggtgtgggaacctgagctgtgtcccctctgccacggttggc
gttttaacccaacctcaaaaagtggggaccaaaactgagcctgtcccagaagccctcgc
ccatccccagagggctccccgtccctattcctcaaggagaccaagagggtgaaatggtca
gcactgccctgctgtagggtcctaaagtctgctgtccttcctgcagaccagggctaaaca
agggcattcaggtgctctagccaagggtcctggcccagtcaagcacaagttcaaacctgg
gctgaccctcagtcaacctggaggctgatgtctaaagtgggtgtagtgtgtgcagcgcct
gtatcctccacatcacccttacagcaggtctgcctcccaggcccatgcacagaggacctg
ctctcccagcctgcaggtgcccctgtggtgtccaggatgacgaggggggtctctgcatact
tggtggggctggggcctcccacttcccacctccttgtgtccctcactcccctgtttcatt
ccatgccgagcctcccctgccttgggctcccctggggaggggggtggtggcaggagttgcc
caagggcagctctgcccatgagcagctgctctagcggctcctgctgctgctgttcgccgt
gtgctgctgacccctgtgaggtagagaaaaggcgttcaggtggttcacaccccacacagg
tgcccctcacacggtcctcactggtggccagcgctgtgggtgtgacgatgtgacgagcct
aaactgcgcaaggactcgtgtcccgggcgctccatgtgaccacctcgggagaggtctccg
gcttgttgtaacccagaggagtaacccaccgcctcctgcagctctttcagacccggttgc
aaagaagagctgcatgctcaacctgctactgtccctgccgggggccaacctgctcacctt
ccttttccttctagaccacctggaaaggtagcccagctctcttgtggctgcccaggactc
caggtctcctggacgcggggtgcccctctgctcccaccagacccccagcaccaaggacct
tttcccctgacccctgtctgcagtaactcactgctctaaggactagcaccactgccaccc
ccgcccctgcctctcctctttgccaccctcctccctgcctctcctctttgccaccctcct

ccctctgcactgtggccttaacaaagagctcagagctttggccatggccagcagtgcact
tgaacccctctcttccctcccaaccacatcatgaagacctccccaccagcccagagctgg
cccctagtcctgggccactgagacccagaagtaccagggctgaagtcagcttgcagcaca
gccagggtcgaggtcactccctccctgaagactcaagcatggcacagcccctctgcctct
ctcctggtggtggcattgtaatgacaccctctgctttggtcctctacagggtggcagaga
aggaggcagtcaataagatgtccctgcacaacctcgccactgtctttggccccaagctgc
tccagccttccgagaaggagagcaagctccctgccaatccaagccagcctatcacaatga
ctgacagctggtccttggaggtcacgtcccaggtatgggaagacaggctccagcccatgc
caccccagcctgacagaggtggcctctgcctgccccacccccagcctgcccatcttccta
cttgcatcgtatgtggtggtggctgagattcagagagagagacttgcctaggttcgcatg
gatgggagtgataggggatgcccagtccacctcctggtcctgcggatgcaccttgctggg
ggcttaaaaccaccccaagtgttcgggtgtggtggctcatgcctgtaatcccagcacttt
gtgagaccgaggcaggacaaccgaacccaggtgtttgagaccagtctgggcaatgtagca
aaccccgtctctagaaaaaatacaaagaaaaattagccaggcattgtggcacacacctgt
aatcctaggtatctgggaggctgacacaggaggattgcttgagcccaggagttagaggct
gcagtgacctatgatggagccactgtactccagcctgggggacagagcaaggccctgtgc
atctctaaaataaacaacccccccacccaacaagtcataccttgtcaggaccccacccca
cccccgtctcactgtaaggggttcatgacaccagcaggggtttctagcacctgaggtgga
cttgggggcttgggtcccaaagacctccccaccagcagctgtgagctcccctctgagcca
ctctcctcttccccactctgcccgggcaggtccaggtgctgctgtacttcctgtggctgg
aggccatccctgccctggacagcaagagacagagcatcctgttctccaccaatgtctaaa
ggtcccagtccatctcctggaggcggacagacggcctggaaaactctggctaatcaaggc
catctgtagagtgagaatcaagattttctgaggcatccttgggcccccccacaagtgtca
ggccatctgccaagagacagcggcccaaagcagaaggacaggtggcctgggcagatcccg
cccaggtctgagagccccaggctggcctcagactgtgggtttttttatgtggccacccgag
ggcgcccccaagccagttcatctcggagtccaggcctggccctgggagacagggtgaaag
gagttgtttttatgaacttaacttacagagtttaaaagatttctacgggatcacgtgtca
agatgcgccctctctggggagaagggaacgtgaccggattccctcactgttgtatctgga
ataaacgctgctgcttcatcctgtggggccaaggcctgtgtgggtggggcctcttccatt
tccctgacttagaaaccacactccactcagaacagggttggagaggcttagtcagcactg
gggagcgtttggactccattcttggctttcttctttttctttccagaaggacttttgtgc
agaaatgggtcttttgttgccgtgtttgtcctccttggaaggcagctccaggaggcccat
gaaatgtcgggggacaggacccccagggagggaatcccaggctacgcactttagggttca
ttctccagggagagcgacctcgtctcccgatcctgaccacccttccagcccacgctctcc
tgtttggcttccacaggcctggacttctctggcttctctgcccacacactccctgcccca
agtgtccctgcccctgcccagcacagctgactccatttctgtcctctcagctcagtggac
tcgctcaacttttgtaaaagtctccacttggtgatagcagcttgccgatgacttgtttta
aaactttcatcctaaataaccttttgatacttgaatatttttaagttttatacgtagttt
ctaatttttccccaacagatccagatacctaataagatgctggaatgtaatccctggac
aatccgtgtcctggcagcatttggtcttcccataagtgcctggcttcgctgttctcagga
gtgggttctgaagtctctggagaacaggatacgtggagggttaggaaggggccaggccta
gagacaggagactccctcccagagcaggtggaggcacaggaccatttgctaccccatctg
ccggcacctgcaggggagcccaggcattctttgtaaactctcctgaccacctggctaaag
aaaacagaagcatggaggccgccaagtattttcaagaaataaccccatgaacattgcacc
actgttttagaaaaaggggcttggggcaggcagagaaggagagagggggggcagggcagtg
taacagaggagaggggcgaactgaggggaggagcagccgataacatgagaaattctaaaa
aaaaaaagcggcatggtggctgttccagctttcggctgagcgggtcctgcggagggaggg
ggaggaggattgacagctggcaaaaaaaaaaaaaaaaaaaaaaaaaaaaaaaaaaaaaaaa
aaaagacgtctacaggaggagaggatgcagctgcccagagggaagcaggatcacatttaa
ggaagtgtgtggggtccctggatgacaccagcacccagtgcaactctggcgaccgctccc
aaggtgggaggagtgggtgcccctgtgtgtcagtgggcagctcctgctgagcccacagct
cactggggagcctgacagcggggccatgcgcctgacactcctctctgcttgtggacctgg
tgaggcagggagcagaaaacagagccacttgaaggctttctgtctgcgtctgtgtgcagt
gtggatttagttgtgctttttttcttgctgggagagcacagccaccatttacaagcagtgt
caccctcgtgggtggtgaggacagaacaggatcctctgctctctgtacctatctgggccc
agtgggctcccctgtcctggcttccatctctgtctcagcgaccatccagccctgcgcagg
aacacatgttgctttgaaaagccaaatccagcccttgtctcggtctcctctggtctcatg
atgtgcatctgttaccttgaaactggaaaccagtctatcaatgtctgtgccaatttttgt
tccctctccaacctccttccccttatgactttttatttacgtaggttgtgtgctgtctaa
tgatgggatgaccacacttttccatgttctaaaagtgctcctctcccgcagggccccagg
gctgatggttgctttgggtctacagctatgtcttacccgcctcctggctcgaaagcctgt
gtggtggcaaagccggtgcggggctggggaacgcggcgttctccaggagggggacccggc
tctccttctgcagtgcaggcaaaggcctagatgccagtgtgacctcccacaaggcatggc
ttccagactccccaaccagaagtgatgctttttttgcctcgggccctgggtttgaagcagc
ctggctttctctcggtaagtggctggtgtcttagcagctgcaatctgagctcagccacct
acacaccatcgtgggggaatgaactgccgacactttcattaaaaagtttcctgagacgac
ttgcgtgcatgttgatttcatgatgagcgccgctgggaagaagccctgagccggtggggt
ggtgctggagcggcaggtgcagtgatggggctgggtgcccaggaggcctccgtgctcaat
caggccacagtggccaagcccaggctgcagggaaggccggcctgggggtgtgggtgagc
acaggtaggcgccagctgggcagtgttaggatgctggagcagcatccctaactccactga
gtggggtagtctggttggggcagggactgctgttgctttggcagagagagatgatcccca
ctggggagaggctgttctgactctgcaggtgggaccgggacagatggccaccagggtgac

ccggctggtcttcctttgctgtgctgagccctgggacatggaggattcccgccacgcaca
gcctgggcccgggttcttacctgtggccaccgctctggcacgagcccctcagtcttgggt
ggtttctgcctggtccgggattcggtgctgctgctgagtccagcctttccaccacctccg
catgggctgtgggtgttgtcagctgcctcccgccttggcttcagtagctcacccagctta
cagggtagctgccctgggctggagatgggcacgcaccctgggtcctacttgaatgaatgc
agcttgaggagacccggccacatccactgggccacaggttaccctcggcaatgcccacat
cagccatcagcctcagcctccccaggagagcaaggctcacatgacaaaggctgcccgtgg
ccggtgaggtggctgagcccagccaggacttttgtcggactcccaggatgtggctctgct
cgtgagctgcctggtcagctctctcggggtgagaggggcctgtcacacgggcccctgcct
gcagtgtgacccttttcagctcctctcagcagccctgcctgaggagtgtcaccaccacca
tgatcatttccctgacactgcgagggtgtagggacgtcctgggtagagacagggcctgtg
gcagcagcaggctcaggggtgccctgaactggtgggctggggacctggtggagaccacac
caagggctgcacaaggggacgagcctccacccttgcctctccgcaggcctcagcagcccc
tcacacaggcagaagggttgacactgggtcctgccctcactgcaagagctacgagtgcca
cgtgctgttctgcccaatctggtgtctgcaggggaggaaagggctgctgctggcccattt
ctgagcgttcagcacctaagggtgacagcactgtctgtccctaccctccgggtcctgttt
gaaaatcaaacccatgctcacgggcctatcttttgttcttttagagacagggtctcactt
tgtcacccaagctggagtgcagtggtgcgattatagctcaatgcagcctccaatccccgg
actcaagggaccctcctgcctcagcctgccaagtagcttggactatagctgggtgccatt
gcacctgttttattattattttgtagacatggggtctggctatgttgtccaggctattct
caaaattcccggcctcaagcaattctcctgcctcggcctctcaaatgttgggattacagg
tgtgaggcaaggcacccagctcagccacagagccctactgcatctctcttactaggagca
agagccgactgccccctcctccccattccagagtgttggggctctgttcagccgaggctg
ggccactggcatggcccagggagtgggatcattcactgctgccccaaatctgagatcatt
ccacctcgacaagactccctcatccaatccctttacttgacagctggggaaaccaatgtg
cacagagcacccccagctcactcggggtctcagagctgatccgtgagcagaggctgagat
cctgggatcttgtcccccagctgccccgcaagcttgctccctttctgctggaagagatgg
ggccggacctcgaccggcagccctggcctggacatgactgtgctcgtgcaggtattcagg
cccagatgccccggcatcatttttttttctttttttctttttttttttttttgagacagtg
tctcactctgtcgcccaagctggagtgcagtggcatgatattggctcactgcaacctctg
cctcctggttaaagtgattctcctgcctcagccttccaagtagctgggactataggcttg
taccaccacgcctgactaattattgtatttttactagagacggggtttccccatgttggc
caggctcgtgtcaaactcctgacttcaggtgatccacctgccttggcctcccaaagtgct
gggattacaggcgtgagccacggtgtcatttaaatgtagtgagaggccgggcacggtggc
tcctgcctgtaatcccagtactttgagaggacgaggctgtcagatcacctaagttcagga
gttcgagaccagcctggccaacatggtgaaaccgtgtctctacaaaaaaatagaaaaaaa
atccctgcgtggtggtgcgtacctgtagtcccagttactcaggaggctgaggcatgagaa
ttgcttaatcctcagaggcggaggctgcagtgagctgagatggcgccactgcactccagc
ctgggtgacagagcaagactttgtctctaaataattaaataaataaatatggccgagcac
ggtgccttaggcctgtaatcccaacactttgggaggctgagggaggtggttcatgaggtc
agcagtccgagaccagcctggccaagacggtgaaacactgtctctactaaaaatacaaaa
attagccagctgtggtggcaggcacctgtaatcccagctactcgggacactgaggcagga
gaatcgcttgaacctggaaggcagaggttgcagtgagccgagatgtaccactgcactcta
gcctgggtgatggagcaagactccatctcaaataaataaattaataaatacagagcaaga
ttccatcgcaaataaataaataaatgtacacctgtaatcctagcactttgggaggctaag
acaggtcgatcacctgacgtcaggagttcgagaccagcccgaccgatatggcgaaacccc
atctctactaaaaatacaaaaattagccgagcattttgacgtgtgcctgtcgtcccagat
acttgggaggctgagacaggagaattgcttgaacccaggaggtggaggttgcagtgagcc
gagatctcggctgaggcaggagaattgcttgaacccaggaggcggaggtagcagtgagcc
aagatcacaccattgcgctccaccctggcggcaagagtgagactgtctcaaaaaacaaaa
acaaacaaacaaacaaaaaaaaacatacctgaaaataataaaagctgatacgacaaagcc
atagctaacctactatagaatggggaaaagttgaaagcatttcctctgtaaacaggaaca
agacaggatgcccgttctcaccactcctattcgacatcacacaatcaggcaagagaaaac
aataaaaggcatccacactggaaaagaggacatcgaattcttcttgtctgatgaagatgt
gatcttggatctagaagcatgtaaaggctccaccagaaaagccctagacttgataaataa
attaatacagtcagttgcaggatacagaatcaacaacaacaacaacaaaatcagcagcat
ttctatacaccaataatggtctggttgggaaagaaattaagaaggcaatcccatttacaa
cagcctctgcctcccaaagtgccaccgcgcctggcctttttttttaagacagagtctcag
ccgggcgccgtggctcacgccattaatcccagcactttggtaggccgaggcgggctgatg
acgaggtcaggtgatcgagaccatcctggctaacacggtgaaaccccgtctgtactaaaa
atacaaaaaattagccaggtgtggtggcgggcgcctgtagtcccagccactccagagtct
gaggcaggagaatggcgtgaacccagtaggcggagcttgcggtgagccgagatcaggcca
ctggaaatccagcctgggcgacagagggagactccgtctaaaaaaaaaaaaaaaaaaaaa
aaaaatcagagtctggctgtgttgcccaggctggagtgcagtggcgcgatctcggcgcat
caccatccctgccccgcctccgggttcaagtgattctcctgtctcagccgcccgagtagc
tggtactacaggcgcgtgccaccatgtctcactaaatttgtatttttactagagacgggg
tttcactatgttggccaggcttttctccaactcctgatctcctgatccgcccaccccgac
ctcccaaagtgctgggatgcgtgagcccccacacctggccactatttttttctttctttct
tttgtgtgtgtgtgtgtgtgcgtttgtgtgtgtgtgtgcgtttgtgtgtgtgtgtgtgag
acgaagtttcgctcttgttgcccagattggagtgcaatggtgctatctcagctcactgca
atcccggcctgagcaggagagcaggaatcttcagcgatccactggcggatctgcagccat
tgtaagcgcttagtcttcccatatcttttgcgcgcgtgcctctccttccagtacctatcc

cgcaagggtcccccagcctccccctatcgccagcaggtgctgagagcgcgccattgcact
ccagcctgggggaccagagcgaaactctatcttaaaaaaaaaggacgaaaattttggaaa
aatatggaagaaaccaaatggattttagctcaactaactcgtaattattcagtgtctat
tttttgcaagaaaccatatatttcatgtctacaatcagggccagagtcccagctctcaag
tgtgggtgtttcctaagcaaattgaagaacacaggcataaaagtgcattgaattaataaa
atttttctccctgtctctcgctctcttatttatttatttatttatttatttattta
tttatttttgagacggaggttcgcactgtcacccaggctggagtgcagtggcgagatct
caggtcatgccaccgcgcccggcctttttctcggccgggcgcggtggctcacgccttta
atcccagcactttggtaggccgaggcgagctgatcacgaggtcaggagatcgacaccatc
ctggctaacacggtgaaacccccctctctactaaaaatagaaaaaattagccgggtgtggt
cgcgagcgcctgtagtcccagctactccagaggctgaggcaggagaatggcgtgaaccct
ctaggcggagcttgcggtgagccgagatcaggccactggaatccagcctgggcgacagag
ggagactccgtctcaaaaaaaaaaaaaaaaaaaaaaaaagacagagtctggctccgttgc
ccaggctggagtgcagtgacgcgatctcggcgcatcacaatccctgccccgaccccgggt
tcaagtcattgtgctgtctcagccgcccgagtagctggtactacacgcgcgtgccaccat
gtctgactaaatttgtatttttactagagacggggtttcactatgttggccaggctggtc
tccaattcctgatctcctgatccgcccgccccgacatcccaaagtgctgggatgcatgag
cccccacacctggccactatttttttctttctttcttttgtgtgtgtgtgtgtgcctgtga
cgaagtttcgctcttgttgcccaggttggagtgcaatggtgccatctcagctcactgcaa
tccccgcctgagcagtagagcaggaatcttcagcgatccactgggggatctccagccatt
gtgcgcgcctggtcttcccattgctttgtaagagcgcctctccttccagtacctatcccg
caagcgtcccccagcctccccccatcgccagcaggtgctgagatcccgccattgcactcc
accctgggggacaagagcgaaactccatctcaaaaagaaaaaaaaaaaggatgaaaatttt
gggaaaatatggaagaaaccaaatggatttctagctcaactaaatcgtaattattcactg
tcaattttttgcaagaaaccatacatttcatgtccacaatcagggccacagtcccagctct
caagtgtgggtgtttcctaaggaaattgaagaacacaggcataaaagtgcattaaattaa
taaacctttttctctctgtctctctctctctctcttttttttttttgagacggaggttggc
actgtcacccaggctggagtgcagtggcgagacatccggtcactgcaggctctgcctccc
gagttcacgccactctcctgcctcagcctccggagtagctgagactacaggcgcctgcca
ccacgcccggctaatttttttgtagttttagaagacggggtttcgctatgttggccaggct
ggtctccaactcctgacctcgtgatccgccctcctctgccattcaaactgccaccgcgcc
cggcctttattttttaggacacagtctctgccgggcgcggtggctcacgcctttaatctca
gcactttggtaggccgaggcgggctgatgacgaggtcaggtgatcgagaccatcctggct
aacacggtgaaaccccgtctgtactaaaaacacaaaaaattagccaggtgtggtggcggg
cgcctgtagtcccagctactccagagtctgaggcaggagaatggcgtgaacccagtaggc
ggagcttgcggtgagccgagatcaggccactggaaatccagcctgggcgacagagggaga
ctccgtctaaaaaaaaaaaaaaaaaagaaaaaaaaaaatcagagtctggctgtgttgccca
ggctggagtgcagtggcgcgatctcggcgcatcaccatccctgccccgcctccgggttca
agtgattctcctgtctcagccgcccgagtagctggtactacaggcgcgtgccaccatgtc
tgactaaatttgtatttttactagagacggggtttcactatgttggccaggcttttctcc
aactcctgatctcctgatccgcccaccccgacctcccaaagtgctgggatgcgtgagccc
ccacacctggccactatttttttctttctttcttttgtgtgtgtgtgtgtgtgtgcgcgtt
tgtgtgtgtgtgtgtttgtgtgtgtgtgtgtgacgaagtttcgctcttgttgcccagatt
ggagtgcaatggtgctatctcagctcactgcaatcccggcctgagcaggagagcaggaat
cttcagcgatccactggcggatctgcagccattgtaagcgcttagtcttcccatatcttt
tgcgcgcgtgcctctccttccagtacctatcccgcaagggtcccccagcctccccctatc
gccagcaggtgctgagatcgcgccattgcactccagcctgggggaccagagcgaaactct
atcttaaaaaaaaaggacgaaaattttggaaaaatatggaagaaaccaaatggattttta
gctcaactaactcgtaattattcagtgtctattttttgcaagaaaccacatatttcatgt
ctacaatcagggccagagtcccagctctcaagtgtgggtgtttcctaagcaaattgaaga
acacaggcataaaagtgcattgaattaataaaatttttctccctgtctctcgctctctta
tttatttatttatttatttatttttgagacggaggttcgcactgtcacccaggctggag
tgcagtggcgagatctcaggtcatgccaccgcgcccggcctttttctcggccgggcgcg
gtggctcacgcctttaatcccagcactttggtaggccgaggcgagctgatcacgaggtca
ggagatcgacaccatcctggctaacacggtgaaacccccctctctactaaaaatagaaaaa
attagccgggtgtggtcgcgagcgcctgtagtcccagctactccagaggctgaggcagga
gaatggcgtgaacccgctaggcggagcttgcggtgagccgagatcaggccactggaatcc
agcctgggcgacagagggagactccgtctcaaaaaaaaaaaaagacagagtctggctccgtt
gcccaggctggagtgcagtgacgcgatctcggcgcatcgcaatccctgccccgaccccgg
gttcaagtcattgtgctgtctcagccgcccgagtagctggtactacacgcgcgtgccacc
atgtctgactaaatgtgtatttttactagagacggggtagacggggtttcactatgttgg
ccaggctggtctccaattcctgatctcctgatccgctcgccccgacatcccaaagtgctg
ggatgcatgagcccccacacctggccactatttttttctttctttcttttgtgtgtgtgtg
tgtgtgcctgtgacgaagggtcgctcttgttgcccaggctggagtgcaatggtgcgatct
cagctcactgcaatccccgcctgagcaggagaacaggaatcttcagcgatccacgggcag
atctgcagccattgttggtacctgttcttcccgcgtcctttgtgcccgcgtctctctttc
cagtacctactgcatgccccccaacgtccgcctcccgccattgccagcaagtgcgttgcg
cgggtaccttgctgcgttaaggtcgctctgtcactggcgccattatgtgcacacgcagcc
actccctcaggtttaaaaggcgcgttgcccggccacataaaggtcgctctgtcactggcg
ccattatgtgcacacgcagccactccctcaggtttcctaggcgcgttgcctggccacata
aaggtcgctctgtcactggcgccattatgtgcacacgcactccctcaggtttaaaaggcg
cattggccggcaacacagctcattgctggcttagcctttggccaagttggtagctccacg

aggacgctcagagcccagctctggagagctgaagcatccgaccgttccccactgctccca
ggagcggttacctgggcactctgtgccccttattcctgtccgggcccaggccgaggacct
gccagtagggctcaattgcctggagcccgttcagcccatccccaagttcactttgcttgt
gggatctccccgttgctcctgcccctggtctgagtggcaggccatcttgcaagcaccggg
acacttcgcatcagtggtgtcaagacaatccttccgtgatcctgcaagccctgtcttcct
tccgggatcagcaagccagtgctgtgtgctccgaattccagggcatcctccagctcagcc
actgcactgagcacaaggactctctgtggggcccaggagcaggaagtcacccctttgggg
cccacaacacccggctgtccccagactcgtgaccagggaaggcagtgttgaggagagcaa
ggcagggatgcctgagcaggacaaagaccccagagtccaaggatttgatgatcacggaag
ggtccccaaggtcaccagggatgcaccgagtgcatttcggcccctgcgggacaatggagg
cctctctcccttcgtgcccaggcctgggcctctgcacacagtcctccatgcccagaggac
agaagccagagacaagcagaggccccagacctcctgcacgagctcaggcacaaaacgaaa
cgccatctcgagctcctacagctccacgggaggcttcccgtggctaaagcggaggagggg
gccagcctcatcccactgccagccaaccctcagttcctcaaagaaggtcagtgagaacag
acctcaggctgtctcttcgggtcacactcagcgtgcaccaaggcagacgctcgcccccag
gaaaggctcccccagatcccaggcctctaggccctgtggacgcaagtttcccctgctgcc
acgcaggcgaggggagcctctgatgatgccacctccgttagagctggggttccgggtcac
tgctgaagacctggaccgggagaaggaggctgcgttccggcggatcaacagggcactgca
ggttgaggccaaggccatctcggactgcagaccctcaaggccttcctacacttcgtgctc
acctgcagcaggggcttctggtctgccttctgtttctaaagcacccagtatggatgcaca
gcagggaagacacaacccccaagacggcctgggcctagtggccccccctagcttctgctgc
agggacccccctccacagttcctgtgtttgggatgcagcacagaccaccaggccccctcct
gttcgtctcctcatttccccttcctcccaactttttctacttctgggactcagcccaggt
cctctggctgtttgctctacccttcggctcctcatttccccttcctcccaccttttttcta
cttctgggactcagcccaggtcctctgcctgattgctgcacccttcccagctgtaagcat
ggacggaagcatttctggagccagttccagcccgccacccacgcccatggaaattgacag
tagtgaggcggacggagctcggcattccgtcagaagaaaccctttaaagagaaaggccga
ttggtaatagggcatgaggggggcctgaacaccagggggcccccaggcagagcccttccat
ggtgactgtgggacctggcacagggagcaactctgttgggtggcacttttgtttttttttg
ttgttgttgtttgccagatgccaaataaatattttattaactttctttctgtacttcac
ttttgtgtcatcaacatttatggcattaacctaaacagaagccccagtcaattaaagaat
agaaaagataaacatttttagaactgtaaagcgtgttctaagagtttcttggccatttta
cttttctttttatttaattatttttttttatttgagttcccttagtatttattgatcattc
ttgggtgtttctcggagaggggacgtggcagggtcatgggataatagtggagagaaggtc
agcagataaacacgtgaacaaaggtgtctggctttcctaggcagaggtccctgcggcctt
ccgcagtgttcgtgtccctgggtacttgagattagggagtggtgatgactcttaacgagc
atgctgccttcaagcatctgtttaacgaagcacatcttgcacagcccttaatccatgtaa
ccctgagttgacacagcacatgtttcagagagcacgagtttgggggtaaggttatagatt
aagagcatcccaaggcagaatttttcttagtacagaacaaaatggagtatcctatgtcta
cttctttctacacagacacagtaacaatctgatctctctttcttttccccacatttcccc
ttttctttcttttttttttttttgagacggagtctcgctctgtggcccaggctggagctc
agtggcgcgatctccactcactgcaagctctgcctcccgggttcacgtcattctcctgcc
tcagtctcccgagtagctgggactacaggcgcccgccaccacgcccggctaattattttt
gtatttttttttagtagagacggggtttcaccgtggtctcgatctcctgacctcgtgatcc
gcccgcctcggcctcccgaagtgctgggattacaagcgtgagccatcgcgcccggcctcc
atttttttttttttttaagatagagtttcgctcttgtcccccatgctggagtgcaatggcg
cgatctcagcacctgctggcaatggtgggaggctgagggacgttcgcaggataggtactg
gaaggagaggcgcccgcacaaaagacatgggaaggccaggcgcgcacaagagccgcagat
ccgccagtggatcgctgaagattcctgctctcctgctcagaccaggattgcagtgagctg
agatcgcaccattgcacttcaacctgggcaacaagagcgaaacttcgtcacacacacaca
cacacacacacacacacacaacgcgcgcgcgcgcgcacacacacacacacacacacacac
acaaaagaaagaaagaaaaaatagtggccaggtgtgggggctcacggatcccagcacttt
gggatggcagggcgggcggatcaggagattaggagttggagaccagcccggccaacatag
tgaaaccccgtctctagtaaaaatacaaatttagtcagacatggtggcacgcgcgtgtag
taccagctactcgggcggctgagacaggagaatcacttgaacccagaggcggggcaggga
ttgtgatgcgccgagatcgcgccactgcactccagcctgggcaacagagccagaatcttt
ttttttttttttttgagacggagtcttcctctgtcgcccaggctggattccagtggcct
gatctcagctcaccgcaagctccacctagcgggttcacgccattctcctgcctcagcctc
tggagtagctgggactacaggtgcccgccaccacacccggctaatttttttgtatttttag
tagagacggggtttcaccgtgttagccaggatggtctcgatcacctgacctcgtcatccg
cccgcctcggcctaccaaagtgctgggattaaaggcgtgagccacggcgcccggctgaga
ctctgtcttaaagaaaaaggccgggcgcggtggcactttgggaggcagaggcgggcggat
cacgaggtcaggagttggagaccagcctggccaacatagcgaaaccccgtctctactaaa
actacaaagaattagccgggcgtggtggcgggcgcctgtagtcccagctactccggaggc
tgaggcaggagagtggcctgaacttgggaggcggaggttgcagtgacctgagatctcgcc
actgcactccagcctgggtgacagtgtgaacctccgtctcaaaaaaaaaaaaaagagagag
agagagacggagagaaaaagtttattaatttaatgcacttttatgcctgtgttcttcaat
ttgcttaggaaacacccacacttgagagctgggactgtggccctgattgtggacatgaaa
tatatggtttcttgcaaaaattgacagtgaatgattacgatttagttgagctagaaatcc
acttcgtttcttccatatttttccaaaactttcatcctttttttttttttttttttgaga
tggagtttcgctcttgtcccccaggctgaagtggaatggcgccatctcagcacctgctgg
caatgggggggaggctgggggacgctcgcgggataggtactggaaggagaggcgctcttac

aaagcaatgggaagaccaggcgcgcacaatggctgcagatccgccagtggatcactgaag
atgcctgctctactgctcaggcggggattgcagtgagctgagatcgcaccattgcactcc
agcctgggcaacaagagcgaaacttcgtaacagacacacacacacacacacacacacaca
aaagaaagaaagaaaaaatagtggccaggtgtggcggctcacgcatcccagcactttggg
atgtcagggcgggcggatcaggatatcaggagttggagaccagcctggccaacatagtga
aaccccgtctctagtaaaaatacaaatttagtcagacatggtggcacgcgcgtgtagtac
cagctactcgggcggctgagacaggagaatcacttgaacccagaggcggggcagggattg
tgatgcgccgagatcgcgccactgcactccagcctgggcaacagagccagaatctttttt
ttttttttttgagacagagtcttcctctgtcgcccaggctggattccagtggcctgatc
tcagctcaccgcaagctccgcctaccgggttcacgccattctcctgcctcagcctctgga
gtagctgggactacaggtgcccgccaccacacctggctaatttttttgtatttttagtaga
gacggggtttcaccgtgttagccaggatggtctcgatcacctgacctcgtgatgagcccc
cctcggcctaccaaagtgctaggattaaaggcgtgagccaccaatgtgctgggattaaag
gcgtgagcccacggtgcctggcggagactctgtcttaaaaaaaaggccgggcgcggtggc
agtttgaaaggcggaggcgggggattgcactccagccttggtgatagagtgagactcaat
ctcaataaataaataaataaataaataaataaataaataaatgatactaggacaaa
aatcataaggacattacataaaccatgttatgctggtgatttttgaaaatatcaaaacag
ttttatagagaactataactcaggccaggtggggtggctaacacctgtaatcccagcact
ttaggaggccaaggttggcggatcacctgacgtcaggagtttgagaccagcctggccaac
atagtaaaaccccaactctactaaaaatacaaaaattagctgggcgtggtggcacacgct
tgtaatcccagctattcgggagcctgaggcaggagaatcacttgaacccgggaggcggag
gttgcagtgagccaagatctggtgactgcagtctagcctgggcatcagagaaattccatc
tcaaaagaaaaatatgacttcataaaaacgaagtaagaagaaacagaaaattatgtctag
atctagaaccattaaaggaattgaaggtttattttaaaatctgtatcccatcccctcaaa
aatctcacaaaaaacaaaaccaaaggaaagcacctggcccagatgattttgtagataagt
ctggcaaacattaaaaaaaaacagaaagtctttatcttctacaaaattttaaaaaaataa
ttaattaatgtatttatttctgagaggaagtctcactctgtagcccagactggagtgcag
tgctgtgatctccactcaccgcaaactccgcctcccaggttcaagtgattctcctgcttc
agcctccacagtagctgggattacaggtgtgcctggctaatttttttgtagttttagtaga
gatggggtttcaccacgttagccagggtgttcttgaactcccgacctcaggtaatccgcc
gcctcggcctcccaaagtgaatgagccacagtgcctggcctacataatctctttttttttt
tttttgagacggagtctggctctgtcacccaggctggagtgcagtggcgcgatctgggct
gactgcaagctccgcctcccgggttcacgccattctgctgcctcagcctccggagtagct
gggactacaagcgcctgccacaaaacccggctaatttttttgtatttttagtagagacggg
gtttcaccgtgttagccagcatggtctcgacctcctgtccttgtgatctgcccaccttgg
cctcccaaagtgctgggattacaggcgtgagccaccgcccccagccctggcctacataat
cattcagaaaaagaagcaaatggacttctccccaactcattgtgaatttagtataagctt
aatacgaaaccagacataaatatacaaggaggcaaaagtatagactaacttcttacggat
atatatgcaaaaatgctaaataaaatattagcaaataatccagaaatggattaaacatgt
atcacgaccaagttgggttttcctaggaaactaagatgatgtaataaaagaaaaaactat
taaagtagtacaaactgtcaaaggaaaaaacctgtatgatcattgcaacgggttcatttg
cgacataatgtaactttttttcttttttttttaagtctttttggctgtaagtttattcaatg
caaaataatcctctccaattttactgacgtggctgaccacgtccacgaccaaatctgcct
ctaaactggaattcggttgctgacccagccccagcctcggctttcttgtcggcaccaggg
ggcagagcactccctctgtaggtagctctgtgggcctcccctcttgtgagtcctgcaggt
cgctcaccctccagacctttaggccgaggtctgccagtccctggacggctgtggcatggg
gtggcaggcacaatctccgggggcagatgaaggtaatcatggagatactggataccctca
ttggtaaggtacgagcagacatctctccaggcaaattgttccttcacgtagtagcctcgg
gactatgtgacatgaaggttgggcacatcttctttttttttttctttttgacatgagtctc
actctgtcgcccaggctggagtgcaggggtgcgatctgggctcactgcaacctccgcccc
ctgggttcaagcgattctcctgcctcagcctccggattagctgggattacaggcacgcac
caccacatccagctaatttttttatatttttggtagagacagggtttcatcatgttggcta
ggctggtctcaaacttctgacctcaagtgatccacctgccttggcctcccaaagtgctgg
gattagaggcatgagccaccatggccggcctgtcctagtctttatatgcccatatatttg
agcccttcttgactaggttcccagagaatcggtgcttggcttgcctggacacggaataaa
ttgatttattgataaataaatgttgtgcccacaatgacttgcctcataactttttagtag
tcaggatcctcccacagtagactcttgtaactacaagaatatttttttgttggtgttgca
tgtagagtttcctgtaaaatggcttccaagatatttttcatttctagtaaagaatctgtg
agttgatttttaagtagtattatgtctaccttatgtggaaggtaagagatagccctttgt
tttcctcaggaggttttaattatatgatctccttggagaagtaaaggaaactctggagct
tcctgtgtggctgtgaatgtatcattagctttgggcagggctcccagagctgtgaaccct
gtcctgaggagcactgggacattctagtgagcgttcaggccacccagattgcagcctcgt
aaatgacgggtttatttctctaatgagagtggtttgtgtcttcacccaaataatatttta
atattaaattacgacaaatattttaaattttaaagtaggtatatataaattgtatgattt
taaaaataatttcaataattgtttaaaaaagtaattttaatacatttcaataatttattt
tagactcaaggggtacacccacaggtttgttgcatgggtatgttgcacgatgctgaggtt
tggggtacggatcccgtcacccaggtagtgagcatagtttttcaactcatgcccatccct
cattcccttcttccaattagtatgattacaactaagaattttacttttacagctctaatt
taatacatattaatctagacctggcaataagtattctgctctaagaaaggttttatgtta
aataacatgatagttagtcccttcaaaataaaaaatttcgatttaagaataccagcacgg
gcccggcgtggtggctcaagcttgtaatcccagcactttgggaggccgagggcagatcat
ctgaggtcaggagttcgagaccagcctggccaacatggtgaaacctatctctactaaaaa

tacaaaaattagccagacgtggtggcaggtgcccgtaatcccagctactcgggaggctga
gggaggagaagcgcttgaacccgggagggagaggatgtagtgagccaagatcatgccgtc
gcactccagactggaggagaagagcaagacttcgtctcaaaagcaaaaagtataccagca
ctggggacaacattggacaagtagacaaatctagaaggggcaggttgagctgtgtagttt
tagtgttgttacagtttgttgatatgttgtgaaatattcattgagatcaaaatctggcat
tcctaacttgtgttatgtggaggccacatggagtggcagaagtgaagtggggtttgaatt
cagaccgttgttcctcaagaatgaattgagctggagagaattacttgcaatctctgatcc
ttagttcctccatctagacatacccacctgggatcgtgtgaggattcagtgaactgtaag
aggtacctgcctgggagaggctcaaggccttcaatgcctttccctgttattttgggagta
gtacatctgttttggtgtttttaagaaacactgtttgacaaatacatgaggcatacttca
tggactattgttgtttcagattattcttagaacacagaggcaggatccacagtgtttta
tgaggagagctacttttattctcccaaaccttggctttcctggattagaggtctcagtg
atgtgtgctgatgtgacacccttgagcaaagttggagaagagatggtggtaatttccacc
acttccccttctccaagacactggaagtgctgtgaactgtgctccttagctcctctgctc
tgtaatctctgcccgggggctctagaggggctctaggcaggagtgtcagccagtgattcc
tccgtcttgaccaggtagggacccaggatcttctggtgaattctgatttgtttttttatg
agcctgagggtcattttaggaatttgtggaagcatctgtgattgtctcaatggttggtgg
gtgctataggcatttatttaatacgtagggcccatggaattcaaggcttagtgcggtgac
agttctgtaaaacagaacttttcctatgatctattattagaggtaatagatctattattg
gaggtaagagacacatcacggaaaagaagaagttactaggagccgggcacggtggctcat
gcctgtaattccagcactttgtgaggccaaggcagcggatcacctgaggttgggagttca
agaccagcctgaccaacatggagaagccctgtctctactaaaaatacaaaaattagccaa
gcatggtggcacatgcctgtaatcccagctactcaggaggctgagggaggagaattgctt
ggacccaggaagtggaggttgtggtgagccgagatcgtgccattgtactccagcctgggc
aacaagagcaaaactctgtctcaaaaaaaaaaaaaaaaaaaaaaaaaaaaaaaaaaaaga
agttactagctagtttcagtattacttaacatccaggaaactggatgtgaaagcttttta
gagaaactaaaccaataggttatacacagagagagatttatttaggaattggctcacatg
attgtggggactagcaagtttaaaatctgtagggcaagccagcaggctataaattcaggt
aagagttgatctcaaagtctggaacctaacatctgtagagcagtctgcaggccagaaact
caggcagggtttgtgtgttacagtcttgaagcggaattcctgcttctctgggaaacctca
gtttttgttcttaaggccttcaactgattggaggtggcccacccatattatggtgggtaa
tctgttttacttaaagtcaactgactgtcaatgttcatcacatctatgaaataacctccc
agcaagatattgacaagtatttgaccaaacaacggggcaccatagcttagccaagttgac
acataaattaaccatcaggggcgaatagaatatccaaaaaacaacgtactaggggtagtg
tatcttatatagctattataattatacaaaacataattataggatgacgatattaagata
accattagaacaaaaatatgaacttttctgtctttttttgagaccaagtcctgctatgtc
accgaggctggagtgcagtggtacaatcttggcttactgcagcctttgcctcccgggttc
aagcaattctcctgcctcagcctcccgagtagctgggattacaggcacccgctaccatgc
ctggctaatttttgtatttttagtagagacggggtttcaccatgttgcccaggctggtct
ccaactcctgacctccaatgatccacccgccttggcctcccaaagtgctgggattacagg
tgtgagccaccacatccagccaaaaatcacctttttttacaaggatcaaaacagtccttat
gctgcagatgacagactcactgtcaccatgctccttttgtgtgtctactaggcacggtgc
tgggtccacactcacagaaaccttaggaactcgcacccaggggctccggctgtagcagaa
tcccaagaataaaacctggtgctgacagaaagagtaggagatggggccgggcgccatgac
tcactcctgtaatcccagcactttgggaggctgaggcgggcaaatcacgaggtcaagaga
tagagaccaccctggccaacatggtgaaaccccgtctctactaaaaatacaaaaattagc
tgggtgtggtggctggcacctgtagtcccagctactcaggaggctgaggcaggagaatca
tttgaaccgaggaggcagaggttgcagtgagccgagatcgtgccactgcactccagcctg
gtgacagagcgaggcattgtctcaaaaaaaaaaaaaaaaaaagcaggagactggactctgg
gagggcctcctggtgagaggggagcacagaggggagagatggaggcaggagcatgggctt
ctggtggccccagcagaccctgtggcagcgtggccagcgtcctctgcaaggaggaatctt
ggccaggatgatgctgcagcaagcttcttcctgaggcccccagccagcccggccagggt
cccagcgtccagtgacccctgttccgcagcagcagctggggccagccccaggctctcttc
cactcccagcttcttaaaacaggaagtggagagagttgtctgacaaagcactggggcaaa
ccacatcctctctcttcaccaagggacagtttgagggagtgtcagcagagggagctttag
agtagagacccccctagccaaccactgactgtcacgcacacagcagggcatgctatggaga
cccccagacagtccctcggggagacccagcaggtccagtctcctcagagatctgtggcag
caggtccccactcccaaaagccacgtgcccacgggtggtctctggtgcctgagactccag
tctcatttgcatctttgcaacttcgagtttaagtgggtgccgcatctctgtatgtcctcc
cgagcataggagcggcacagcctggggtggcagctggcatcaatccctcgaatcccctgg
gagccactggggagactaagcagtccccagcgcccacttgtccctgagctgccattctca
gccctgtgggaggagacaggaagccctgaagagaaaccaaaggaccaggtcaggaggggc
tggggggtggcatgagcaatcagggcagggaaggatggacagatgggggaatggagggag
gaaggaatgaatgaaaaggtgaatgaatgaacaaagagagagaatggccactcctccctt
gctttagtttacaaagcactgggatcctcccaacagcctgaaggacagaatttatgggaa
gcagaccaggtggctggcagggaggggaggcttgccctggatttcgtgggcccaatggga
ggcagggggcaggaaggggcatcctgtgtgtgtcctctctgcagcggcagcaacaccttc
ctggaagagggtcaggaaacacccactgtggcccctctccatcacaccctcatccaggac
accaagtatcagtcactcagctcacaagatccaggccctgactcggagagaggatgtgag
gggtggggcacggggctccccaggactgagagacccgagacgtggccccgggctgggtgt
tggggcagactggctatggcagcatcgtgtaccccagcaggccagtacccacgcagggag
cctccaaacccccttcacctatgaccctgggagaagaccctagccttggagaattggcctc

actgaaggggcctgcaccggccagcagggtcaggttgggccagacaggttcccacctggg
atatgcaaatgggcctcctgaatcctggagccaggtatggattcacacaccaccattgtc
cccaagtccccatctgccccacgggcacaccctgccacctgttctgtgcaagggccctga
ggctgcctccttgcgcccaagccctgcaggtgctgaagcccacaccacacacacacggct
cctgcttcctgggccagtgcacgtgcacacacacacgcgcacacacacactcacacaccc
acccatacacatacccacacacaatcacacacattcacacacacccacacccccccatact
cacacactcacccacaaacacccacacatacactcacacacaatcacacacatttacaca
cacccacacactcacactcacacattcacacacaccctcacacaccgaaacacaatcaca
cacattcacacccacccacactcccccacacactcacactcacatatactctcacacccc
cccacacacatacacacacccaaacacaatcacacacattcacacacacccacacacccc
cacacactcacactcacacatacacacactcaaacacaatcacacacattcacacacaca
ccccacacactcatactcacacatatatgcacacactcacactcacacataatcacacac
acacacacacacgctcacacacactctctctcacacacaacgccttctccaggaggggct
ggctgccaagggccacccggcttcctcccacatctcactcaccgtacaatatttgagcag
accttggagtcggtggcaacagcagcgtgggcaaaggcctgggagccacagggggcctgg
tgtcgagagaggaccacagccagcacaatgacagccagcgctagccccagccccagcagg
accaggccaaccgtggctccgcagtcccgggccatggctctgcagcccaggaggagaggg
gaggccggtgggcagacagagggacagatgggtgggcagatgagtggacaagaagaagca
tagatagactcacaagtaattggacagatggacaaacaggtggggccacagacagacatg
aagatgaatcgacagacaggccagatagctggacggagaggacagtaagagaaagatagt
cagatagacaatgggacagagatgggcttatagatgggcggacagacagacaggtctgaa
cagcaggctgccagatagacagatgggtgaatggacagacggctggcggctggcagctgt
ggcgtgctgctgccctccccagatgcacaccacagagtgtccaaactcatgcctcaactt
ctagttttcagctcctgcttgttcctggcaggaggccaggcagcaaagcctctgaggga
gttttccttgcctagaggagtcagctgctgtgttaactccctcactgccggtaggtccca
aagccccacctaccgcccgccagaacccagggtcacactgtcccaatgtgcagaatttgg
tgcctgctgcactgcggttgccaggtaggggcagggctcccaagaacctccacaccatta
cccgggttcccggcagctctgggaaagcagagctgggaccgctcaactctctcccctgga
tccggcaaggctgccccgctcccagagtggagtcccgctccccagctcccacctctatcc
cctaaccctctctgcatggcccagcctagtcagcatcaaggtggagctgaacacagatgg
cggggaggacccaaggtggtgccactcaggacccgggttcaagtccttatgcttctgcag
cctggcctgggtctcccaaccctcccagggtgaccaagggcttcccagtctgcacagaga
ggacaggggacttgatagcatcaaatgctggtgactacaagatgcccatgtggggaatg
cagacagaccatgcctctagcccttggtacctggcaccatccatccctgcgacttgctgt
cctggaaatgcagcatggacctccagggagggggctgtgccatgtgggcgcccaccccg
cctgcagctctttcccaccctggctgcaggtctgcttccctgaatccaaatccactatta
ctgtgctggcagcgcagcctctctggggacactggcctggctctgttctccccaggcctc
agggtgcctaaatgggaggtagccaggagagtgaggacccactgaggggctctgttgact
aggctcagcagtggtgcaggtgatgtgagctggaatccttcccatgtggcccccacagtc
ctccacgcttcctccccagctgaacactgcctgctccagatgtctacacctggagtccag
gccccccatctaggcagcagagaaactgaggcacagagacagactgtgtccttacaggc
cacacagcctgccaggctcctgtgtccagccagagctcctggtcagcctggactggagtg
gttgtttagaggcaggctgttcccacgactgcctctcatggtgggcagggggggtggggt
ggacgcctcctcccacccccacctgactcccaagcctcagtgacattgctcaatcaggag
ctgaagtgcattcctgggctcaggccagcccagccacccacccgctgcagtcctggaagc
ccagagatctgggcagctggaacagtggagacagcagcgtgggggacgtcccgctcctct
ccccaccatcctggtcaggcagaggccagggtgcagggacccccccacccccccagcaaag
gcccagggaaaggaatgtgtgtcattctggtcctgacccgaggcacagccaggaaggtcc
ctgtggggaaaagaaagagagatcagactgttactctatctatgtagaaagtagacatga
gagactccattttgttctgtactaagaaaaattcttttgccttgagatgctgttaatctg
taaccctagccctaaccctgtgctcacagaaacatgtgctgtgtcaactcaaggtttcat
ggattaagagctacgcaggatgtgctttgttaaacaaatgcttgaaggcagaatgcttgt
taaaagtcatcaccactccctaatctcaagtatgcaaggacacaaaacactgcagaaggc
cgcagggacctctgcctaggaaaaccaggtattgtccaaggtttctccccatgtgatagc
ctgaaatatggcctcctgggaagggaaagacctgaccgtcccccagcccgacacccataa
agggtctgtgctgaggaggattagtaaaaaacgaaggcctctttgcagttgagataagag
gaaggcatttgtctcctgctcgtccctgggcaatggaatgtctccgtgtaaaacccgatt
gtatattccatctactgagataggagaaaaccgccttagggctggaggtgagacatgatg
ctggcaatactgctctttaatgcattgagatgtttatgtatgtgcatatcaaagcacagc
accttcttcttaaccttgtttatgacacagagacatttgttcacatgttttcctgctgac
cctctccccactattaccctattgtgctgccacatccccctctccaagatggtagagata
atgatcgataaatactgagggaactcagagactggtgccagcctgggtcctctgtatgct
gagtgcaggtcccctgggcctacttttctttctctatattttgtctctgtctctttcttt
tctcagtctcttgtcccacctgacgagaaacacccacaagtgtggagggggcaggccaccc
cttcaggtccctgaatgtccttcctcaggaaatgatgggagaaggggtgatgagaatgga
ggagaggatttaagtcctccaccccccaaggtagtcctgggctgagccccatgggacctg
gagaaccagggtgtaccccaccagcgtgtcgggtcgaggaagccccgtggccaggtcccc
cttctcttgctgctgtgccacccagagcaaggcctgcccttccgacctcggtcttctccc
ctgcaagtggggccacagcctttcctctcaggccaaaataaggattgaggatgggtgcag
tggctcacccctgtaatcctagcactttgggagactgagatgggggactgcttgaagtc
aggagttaagaccagcctggtcaacatagtgagaccccatctcaattggtttaaattttt
aaaaaaaaaattaaataaataaggattgaggagtgacttgtacaccagttgagcccacct

ccatctcacccctgcagagccccagagacacagccctccagggctcagacccggtggtac
ttgactctgcaggcataaaaccctgtttgtctatgggccgtttggaatcaccaggttttc
ggggctcctgaaggatagccccgacctggcctcacctgggccctggccccagtgcccctg
gtgatatccaggtgctgggctgtgatcaccgcccccccaccagcccttcccagaaccctgc
cccaggtgttggaactgtgcacagaggagggagcaggccccgagggaggcctggaggggc
tgccgatggtgaaggctgctgtgtctagctgtttccttccggacccactccctctgggct
gcatccccggctggtctaacccctgatccctgggatctggggacatcttcccgtttgctg
ttccctgagaaccaggcctccctctggagaggatcacaagcttgtgtttcactctgggct
tgcatttggaaacccccccaggggcgtggctctgaccaagatgttttcctccagcctgttg
cccagtccccattcctcggacctcagcttcacttcccgtgtcattggcaggatcagctgg
acgcctaaggatctgagaaggcacccgggttcccagcatcagctggccaccctctgccta
agaaactgccagggtcgcagcacccccctggtggctggtcctaggtagtgtcactgcccag
ccccagtaagggagggcctggccccaaattccgagggatcaggttgggaagggacagggc
ttggtgtgaaccttcccctggcccccagccacgtgcctggctttccctatgctaaagatg
ctgaggctagttccagtgcccgcattatgaagatctccgaatcccacctctccattcctc
cccagccagatggctccatttcacacacaatacactgaggcccagag
