# Supplementary material for: An ancestral genomic sequence that serves as a nucleation site for de novo gene birth
Source: PLoS One. 2022 May 12;17(5):e0267864. doi: 10.1371/journal.pone.0267864 (PMC9097989; doi:10.1371/journal.pone.0267864)
Supplement: S8 Fig — (PDF) [file pone.0267864.s008.pdf]

Department of Microbiology and Immunology, Renaissance School of Medicine, Stony Brook University, Stony Brook, N.Y., United States of America

S8 Fig. Alignment of the orangutan BCRP3 sequence with the human *BCRP3* gene sequence.

```
#
#
#  Percent Identity  Matrix - created by Clustal2.1
#
#

     1: GGT1.end.-Cabin1.start.8575573-8602125.orangutan  100.00   76.77
     2: BCRP3.REF.NEW                                      76.77  100.00



CLUSTAL O(1.2.4) multiple sequence alignment


GGT1.end.-Cabin1.start.8575573-8602125.orangutan      ctctgggcctcagtgtattgtgtgtgaaatggagccatctggctggggaggaacagagag 60
BCRP3.REF.NEW                                         ------------------------------------------------------------ 0

GGT1.end.-Cabin1.start.8575573-8602125.orangutan      gtgggattcggagatcttcacaatgcgggcactggagctagcctcagcatcttcagcatg 120
BCRP3.REF.NEW                                         ------------------------------------------------------------ 0

GGT1.end.-Cabin1.start.8575573-8602125.orangutan      gggagagccaggcgcgtagctgggggccagggaaggttcacaccaagccctgccccttcc 180
BCRP3.REF.NEW                                         ------------------------------------------------------------ 0

GGT1.end.-Cabin1.start.8575573-8602125.orangutan      caccctgatccctcggactttggggccaggccctcccttactggggctgggcagtgacac 240
BCRP3.REF.NEW                                         ------------------------------------------------------------ 0

GGT1.end.-Cabin1.start.8575573-8602125.orangutan      tacctaggatcagccaccaggggtgccgcgaccctggcgctttcttaggcagagggtgg 300
BCRP3.REF.NEW                                         ------------------------------------------------------------ 0

GGT1.end.-Cabin1.start.8575573-8602125.orangutan      ccagctgatgctgggaaccccgggcgctttctcagacccttaggcgtccagctcaccctg 360
BCRP3.REF.NEW                                         ------------------------------------------------------------ 0

GGT1.end.-Cabin1.start.8575573-8602125.orangutan      ctgatgacacgggaggtgaagctgaggtctgaggaatggggactgggcaacaggctggag 420
BCRP3.REF.NEW                                         ------------------------------------------------------------ 0

GGT1.end.-Cabin1.start.8575573-8602125.orangutan      gaaaacatctcggtcagagccacgcccctgggggtttccaagtttaagcccggagtgaa 480
BCRP3.REF.NEW                                         ------------------------------------------------------------ 0

GGT1.end.-Cabin1.start.8575573-8602125.orangutan      acccaagcttgtgatcctctccagagggaggcctggttctcaggcaacagcaaacgggaa 540
BCRP3.REF.NEW                                         ------------------------------------------------------------ 0

GGT1.end.-Cabin1.start.8575573-8602125.orangutan      gaggtccccagatcccagggatcagggcttggaccagccggggacgcagcccagagggag 600
BCRP3.REF.NEW                                         ------------------------------------------------------------ 0

GGT1.end.-Cabin1.start.8575573-8602125.orangutan      tgggtccggaaggaaacagctagacacagcagccttcaccatcgggagcccctgcaggcc 660
BCRP3.REF.NEW                                         ------------------------------------------------------------ 0

GGT1.end.-Cabin1.start.8575573-8602125.orangutan      tcccttggggcctgctccctcctctgtgcacagttccaacacctggggcaggattctggg 720
BCRP3.REF.NEW                                         ------------------------------------------------------------ 0

GGT1.end.-Cabin1.start.8575573-8602125.orangutan      aagggctggtggaggtgggctggttgggggcggtgatcactgcccagcacctggatatca 780
BCRP3.REF.NEW                                         ------------------------------------------------------------ 0

GGT1.end.-Cabin1.start.8575573-8602125.orangutan      ccaggggcactggggccagggcccaggtgaggccaggtcgggactatccttcaggagccc 840
BCRP3.REF.NEW                                         ------------------------------------------------------------ 0

GGT1.end.-Cabin1.start.8575573-8602125.orangutan      cgaaaacctggtgattccaaagggcccacagacaaacggggttttatgcctgtggagtca 900
BCRP3.REF.NEW                                         ------------------------------------------------------------ 0

GGT1.end.-Cabin1.start.8575573-8602125.orangutan      agtaccaccgggtctgagctctggaggactgtgtctctggggctccgcaggggtgagatg 960
BCRP3.REF.NEW                                         ------------------------------------------------------------ 0
```

```
GGT1.end.-Cabin1.start.8575573-8602125.orangutan      gaggtgggctcaactggtgtacaagtcactcctcaatccttattttatttatttaatttt 1020
BCRP3.REF.NEW                                         ------------------------------------------------------------ 0

GGT1.end.-Cabin1.start.8575573-8602125.orangutan      tttaaaaaaaatttaaaccaatagagatggggtctcactatgttgaccaggctggtctta 1080
BCRP3.REF.NEW                                         ------------------------------------------------------------ 0

GGT1.end.-Cabin1.start.8575573-8602125.orangutan      actcctgacttcaagcagtccccccatctcagtctcccaaagtgctaggattacaggggt 1140
BCRP3.REF.NEW                                         ------------------------------------------------------------ 0

GGT1.end.-Cabin1.start.8575573-8602125.orangutan      gagccactgcacccagcctcaatccttattttggcctgagaggaaaggccgtggcccatt 1200
BCRP3.REF.NEW                                         ------------------------------------------------------------ 0

GGT1.end.-Cabin1.start.8575573-8602125.orangutan      tgcaggggagaagactgaggctggaggggcaggccttgctctgggttgcacagcagcaag 1260
BCRP3.REF.NEW                                         ------------------------------------------------------------ 0

GGT1.end.-Cabin1.start.8575573-8602125.orangutan      agaagtgggagctggccacgaggcttcctcgacccaacacgctggtggggtacaccctgg 1320
BCRP3.REF.NEW                                         ------------------------------------------------------------ 0

GGT1.end.-Cabin1.start.8575573-8602125.orangutan      ttctccaggtcctatggggctcagcccaggactacctcggggggtgggggacttaaatcc 1380
BCRP3.REF.NEW                                         ------------------------------------------------------------ 0

GGT1.end.-Cabin1.start.8575573-8602125.orangutan      tctccttcattctcatcgccccttccctcatcatttcctgaggaaggacattcagggacc 1440
BCRP3.REF.NEW                                         ------------------------------------------------------------ 0

GGT1.end.-Cabin1.start.8575573-8602125.orangutan      tgaaggggtggcctgcccctccacacctgtggatgtttctcatcaggtgggacaagagac 1500
BCRP3.REF.NEW                                         ------------------------------------------------------------ 0

GGT1.end.-Cabin1.start.8575573-8602125.orangutan      tgagaaaagaaagagacacagagacaaagtatagagaaagaaaagtgggcccaggggacc 1560
BCRP3.REF.NEW                                         ------------------------------------------------------------ 0

GGT1.end.-Cabin1.start.8575573-8602125.orangutan      tgcactcagcatacggaggacccatgctggcaccagtctctgagttccctagtatttatt 1620
BCRP3.REF.NEW                                         ------------------------------------------------------------ 0

GGT1.end.-Cabin1.start.8575573-8602125.orangutan      gatcattatctctaccatctcggagaggggggatgtggcaggacaatagggtaatagtggg 1680
BCRP3.REF.NEW                                         ------------------------------------------------------------ 0

GGT1.end.-Cabin1.start.8575573-8602125.orangutan      gagagggtcagcaggaaaacatgtgaacaaatgtctctgtgtcataaacaaggttaagaa 1740
BCRP3.REF.NEW                                         ------------------------------------------------------------ 0

GGT1.end.-Cabin1.start.8575573-8602125.orangutan      aaaggtgctgtgctttgatgtgcatatacataaacatctcaatgccttaaagacagtatt 1800
BCRP3.REF.NEW                                         ------------------------------------------------------------ 0

GGT1.end.-Cabin1.start.8575573-8602125.orangutan      gccgccagcatgtctcacctccagccctaaggcagttttctcctatctcagtagatggaa 1860
BCRP3.REF.NEW                                         ------------------------------------------------------------ 0

GGT1.end.-Cabin1.start.8575573-8602125.orangutan      tatacaatcgggttttacaccgagacattccattgcccagggatgagcaggagagagatg 1920
BCRP3.REF.NEW                                         ------------------------------------------------------------ 0

GGT1.end.-Cabin1.start.8575573-8602125.orangutan      ccttcctcttatctcaactgcaaagaggtcttccttcctcttttactaatcctcctcagc 1980
BCRP3.REF.NEW                                         ------------------------------------------------------------ 0

GGT1.end.-Cabin1.start.8575573-8602125.orangutan      acagaccctttacgggtgttgggctgggggacggtcaggtctttcccttcccacgaggcc 2040
BCRP3.REF.NEW                                         ------------------------------------------------------------ 0

GGT1.end.-Cabin1.start.8575573-8602125.orangutan      atatttcagactgtcacatggggagaaaccttggacaatacctggctttcctaggcagag 2100
BCRP3.REF.NEW                                         ------------------------------------------------------------ 0

GGT1.end.-Cabin1.start.8575573-8602125.orangutan      gtccctgaggccttccgcagtgttttgtgtccctatttacttgagattagggagtggtga 2160
BCRP3.REF.NEW                                         ------------------------------------------------------------ 0

GGT1.end.-Cabin1.start.8575573-8602125.orangutan      tgacttttaacaagcatgctgccttcaagcatttgtttaacaaagcacatcctgcacagc 2220
BCRP3.REF.NEW                                         ------------------------------------------------------------ 0

GGT1.end.-Cabin1.start.8575573-8602125.orangutan      ccttaatccattaaaccttgagtcgacgcagtacatgtttctgtgagcacagggttgggg 2280
BCRP3.REF.NEW                                         ------------------------------------------------------------ 0

GGT1.end.-Cabin1.start.8575573-8602125.orangutan      ctagggttacagattaacagcatttcaaggcaaaagaatttttcttactacagaacaaaa 2340
BCRP3.REF.NEW                                         ------------------------------------------------------------ 0
```

```
GGT1.end.-Cabin1.start.8575573-8602125.orangutan      tggagcctcttacgtctacttctttctacatagacacagtaacagtctgatatctctttc 2400
BCRP3.REF.NEW                                         ------------------------------------------------------------ 0

GGT1.end.-Cabin1.start.8575573-8602125.orangutan      ttttccccacagggaccttcctggctgtgcctcgggtcaggaccagaatgacacccattc 2460
BCRP3.REF.NEW                                         ------------------------------------------------------------ 0

GGT1.end.-Cabin1.start.8575573-8602125.orangutan      atttccttgggcctttgctcgggcggtccctgcaccctggcctctgcttaccaggatggt 2520
BCRP3.REF.NEW                                         ------------------------------------------------------------ 0

GGT1.end.-Cabin1.start.8575573-8602125.orangutan      ggggagaggagggggggatgtcccccacgctgctgtctccactgttcctgctgcccaggcc 2580
BCRP3.REF.NEW                                         ------------------------------------------------------------ 0

GGT1.end.-Cabin1.start.8575573-8602125.orangutan      tctgggcttccaggactgcagcgggcgggtgggtgggtgggctggcctgagcccaggaat 2640
BCRP3.REF.NEW                                         ------------------------------------------------------------ 0

GGT1.end.-Cabin1.start.8575573-8602125.orangutan      aaacttcagctcctggctgagcaatgtcactgaggcttgggagtcgggtgggggcgggag 2700
BCRP3.REF.NEW                                         ------------------------------------------------------------ 0

GGT1.end.-Cabin1.start.8575573-8602125.orangutan      gaggcgtccacaggcccccccaccgcgagaggcagccgtgggaacagcctgcctctaaaca 2760
BCRP3.REF.NEW                                         ------------------------------------------------------------ 0

GGT1.end.-Cabin1.start.8575573-8602125.orangutan      atcactgcagcccaggctgaccaggggctctggctggacataggggcctggcaggctgtg 2820
BCRP3.REF.NEW                                         ------------------------------------------------------------ 0

GGT1.end.-Cabin1.start.8575573-8602125.orangutan      tgtcctgtaagggcacagtctgtctctgtgcctcagtttctctgctgcccagatggaggg 2880
BCRP3.REF.NEW                                         ------------------------------------------------------------ 0

GGT1.end.-Cabin1.start.8575573-8602125.orangutan      gcccggactccaggtgcagacatctggagcaggcagtgttcagctggagaggaagcggag 2940
BCRP3.REF.NEW                                         ------------------------------------------------------------ 0

GGT1.end.-Cabin1.start.8575573-8602125.orangutan      aggactgtgggggccatgtgggaaggattccagcccacatcacctgcacccctgctgagc 3000
BCRP3.REF.NEW                                         ------------------------------------------------------------ 0

GGT1.end.-Cabin1.start.8575573-8602125.orangutan      ctggtcaacagagcccctcagtgggtcctcactctcctggctgccccccattaggcatcc 3060
BCRP3.REF.NEW                                         ------------------------------------------------------------ 0

GGT1.end.-Cabin1.start.8575573-8602125.orangutan      tgaggcctggggagaacagagccaggccagtgtccccagagaggctgcgctgccagcaca 3120
BCRP3.REF.NEW                                         ------------------------------------------------------------ 0

GGT1.end.-Cabin1.start.8575573-8602125.orangutan      gtagtagcgaatttggattcagggaagcagacctgcagccagggtgggaaagagctgcag 3180
BCRP3.REF.NEW                                         ------------------------------------------------------------ 0

GGT1.end.-Cabin1.start.8575573-8602125.orangutan      gcggggtggggccctcacatggcacagccccccctccctggaggtccatgctgcatttcca 3240
BCRP3.REF.NEW                                         ------------------------------------------------------------ 0

GGT1.end.-Cabin1.start.8575573-8602125.orangutan      ggacagcaagtcccagggatggatggtgcgggtaccaagggctagaggcatggtctgtct 3300
BCRP3.REF.NEW                                         ------------------------------------------------------------ 0

GGT1.end.-Cabin1.start.8575573-8602125.orangutan      gcatttcccacatgggcgtcttgtagtcaccagcatttgatgctatcaagtccccctgtc 3360
BCRP3.REF.NEW                                         ------------------------------------------------------------ 0

GGT1.end.-Cabin1.start.8575573-8602125.orangutan      ctctgtgcagactgggaagcccttggtcaccctgggggggttgggagacccaggccaggc 3420
BCRP3.REF.NEW                                         ------------------------------------------------------------ 0

GGT1.end.-Cabin1.start.8575573-8602125.orangutan      tgcagaagcataaggatttgaacccgggtcctgagtgacaccaccttgtgtccccctccc 3480
BCRP3.REF.NEW                                         ------------------------------------------------------------ 0

GGT1.end.-Cabin1.start.8575573-8602125.orangutan      tctatctatgttcagctccaccttgacgctgactaggctgggccatgcagagagggttag 3540
BCRP3.REF.NEW                                         ------------------------------------------------------------ 0

GGT1.end.-Cabin1.start.8575573-8602125.orangutan      gggacagaggtgggagctggggagcggggctccactctgggagggggggcagccttgccgg 3600
BCRP3.REF.NEW                                         ------------------------------------------------------------ 0

GGT1.end.-Cabin1.start.8575573-8602125.orangutan      atccaggggagttgagcggccccagctctgctttcccagagctgccgggaacccggggaa 3660
BCRP3.REF.NEW                                         ------------------------------------------------------------ 0

GGT1.end.-Cabin1.start.8575573-8602125.orangutan      tggtgtggaggttcttgggagctcttcccctacctggcaaccgcagcgcagcaggcacca 3720
BCRP3.REF.NEW                                         ------------------------------------------------------------ 0
```

```
GGT1.end.-Cabin1.start.8575573-8602125.orangutan       aattctgcacattgcgacagtgtgaccctgggctctggtgggcggtaggtgaggccttgg 3780
BCRP3.REF.NEW                                          ------------------------------------------------------------ 0

GGT1.end.-Cabin1.start.8575573-8602125.orangutan       gacctaccagcagtgagggagttaatatagcagctggctcctctaggcgaggaaaactcc 3840
BCRP3.REF.NEW                                          ------------------------------------------------------------ 0

GGT1.end.-Cabin1.start.8575573-8602125.orangutan       ccccagacgctttgctgcctggcctcctgccaggaacaagcaggagctgaaaactaggag 3900
BCRP3.REF.NEW                                          ------------------------------------------------------------ 0

GGT1.end.-Cabin1.start.8575573-8602125.orangutan       ttgaggcataagtttggccactctgtggtgtgcatctggggagggcagcagcgcgccaca 3960
BCRP3.REF.NEW                                          -------------------actccgtagtgtgcacttggtgagggcagcagctcgccaca 41
                                                                          **** ** *******  *** ************ *******

GGT1.end.-Cabin1.start.8575573-8602125.orangutan       gctgccagccaccagccatctgcctattcacctgtctgcccatctggcagcctgctgttc 4020
BCRP3.REF.NEW                                          gctg-------ccagccgtctgtccattcacccatctgtccatctggcagcccgctgttc 94
                                                       ****       ****** **** * *******  **** ************* *******

GGT1.end.-Cabin1.start.8575573-8602125.orangutan       agacctgtctgtctgtctgcccatccataagcccatctctgtcccattgtctatctgact 4080
BCRP3.REF.NEW                                          agacccgtctgtctgtccgcccatctgtaagcccatctctgtcccattgtctatctgacc 154
                                                       ***** *********** *******  ********************************

GGT1.end.-Cabin1.start.8575573-8602125.orangutan       atctttctcttactgtcctctccatccagctatctggcctgtctgtcgatccatcctcat 4140
BCRP3.REF.NEW                                          atctttctcttactgtcctctttgtctagctatctggcctatctgtcgatccatcttcgt 214
                                                       *********************   ** ************* ************** ** *

GGT1.end.-Cabin1.start.8575573-8602125.orangutan       gtctgtc-tgtggccccacctgttcatccatctgtccaattacctgtgagtctacctctg 4199
BCRP3.REF.NEW                                          gtctgtcttcagcccccacctgtttgtccatctgtccaattacctgtgagtctatctatg 274
                                                       ******* *  * ***********  **************************** ** **

GGT1.end.-Cabin1.start.8575573-8602125.orangutan       catcttcttgtccattcctctgctcacccatctgtccctccgtctgcccacaggcctccc 4259
BCRP3.REF.NEW                                          caccttcttgtccattcatctgcccacccatctgtccctccgtctgcccaccggcctccc 334
                                                       ** ************** ***** *************************** ********

GGT1.end.-Cabin1.start.8575573-8602125.orangutan       ctctccttctggggcgcagagccatggcccaggactgcggagccacggtcggcctggtcc 4319
BCRP3.REF.NEW                                          ctctccttctgggccgcagagccatggcccaggactgcagagccatggttggcctggtcc 394
                                                       ************* ************************ ****** *** **********

GGT1.end.-Cabin1.start.8575573-8602125.orangutan       tgctggggctggggctggggctggggctggggctggggctggctggcattgtgctggctg 4379
BCRP3.REF.NEW                                          t------------------gctggggctggggcttgtgctggctgtcattgtgctggctg 436. 395-396
BCRP3 seq
                                                       *                  *************** * ******** **************
```

Between 345-346 bp of BCRP3 a repeat seq gctggggct probably polymerase stuttering added in orangutan that is not in Rhesus,baboon, chimpLOC112206721.putative.PO.etc.

```
GGT1.end.-Cabin1.start.8575573-8602125.orangutan       tggtcctctcttgccaccaggccccctgtggcccccaggcctttgcccacgctgctgttg 4439
BCRP3.REF.NEW                                          tggtcctctctcgacaccaggccccatttgaccccc-ggcctttgcccacgccgctgttg 495
                                                       *********** * *********** * ** ***** *************** *******

GGT1.end.-Cabin1.start.8575573-8602125.orangutan       ctgctgactccaaggactgctcaaatgttgtacagtaagtgagacgtgggaggaagctgg 4499
BCRP3.REF.NEW                                          ctgctgactccaaggtctgctcggatattggacggtgagtgagacgtgggaggaagctgg 555
                                                       *************** ******  ** *** ** ** ***********************

GGT1.end.-Cabin1.start.8575573-8602125.orangutan       gtggcccttggcagccagcccctcctggagaaggcatgtatgtgtgtgagagagtgtgtg 4559
BCRP3.REF.NEW                                          gtggcccttggcagccagcccctcctggagaaggc----gtgtgtgtgagagtgtgtgtg 611
                                                       ***********************************     ************ *******

GGT1.end.-Cabin1.start.8575573-8602125.orangutan       tgtgagcatatgtgtgtgtgtgattatgtgtgagtgtgagtctgtgtgggtgtgagtgtg 4619
BCRP3.REF.NEW                                          tgtgagcatgtgtgtgtgtgagagagtatgtgtcagtgtgtgtgggtatatgagtgtgag 671
                                                       ********* ********** **   * ****   *** ** ** **   ** * *** *

GGT1.end.-Cabin1.start.8575573-8602125.orangutan       tatggggtgtgagtgtgtgtgaatgtgtgtgattgtgtttgggtgtgtgtgtatgtgtga 4679
BCRP3.REF.NEW                                          tgtggggtgtgggtgtgtgtgaatgtgtgtgatcgtgtttgggtgtgtgtatgtgtgagt 731
                                                       * ********* ********************* **************** * **** *

GGT1.end.-Cabin1.start.8575573-8602125.orangutan       gtgtgtggggggtgtgaatgtgtgtgattgtatttgggtgtgtgtatgtgtgagtgcgtg 4739
BCRP3.REF.NEW                                          gtgggt---gtgtgtgaatgtgtgtgagtgtgtttgtgtgtatgtgtgagtgtgggtggg 788
                                                       *** **   * **************** *** **** **** *** ** *** * * * *

GGT1.end.-Cabin1.start.8575573-8602125.orangutan       ggggtgtgtgggtgtgtgagagtatatgtatgagtgtgtgggggggtgtgggtgggtgtg 4799
BCRP3.REF.NEW                                          ggtatatgagtgtgagt----------------------------gtgtgggtgggtgtg 820
                                                       **  * ** * *** **                            ***************

GGT1.end.-Cabin1.start.8575573-8602125.orangutan       aatgtgtgtgattgtatttcagtgtgtaagggtgtgtgtgactgcgagtgtgtgactgag 4859
BCRP3.REF.NEW                                          aacgtgtgtgattgtgttttgctgtgtgagggtgtgtgtgactatgagtgtgtga----- 875
                                                       ** ************ ***   ***** ***************  **********

GGT1.end.-Cabin1.start.8575573-8602125.orangutan       tgtgtgggtgtgtgggtgtgtgtaaatgtgtatgactgtgtgagtgtatgtatgggtgtt 4919
BCRP3.REF.NEW                                          ---------gtgtgggtgtgtgtaaatgtgtgtgattgtgtgagtgtatgtgtgggtgtg 926
                                                                ********************** *** *************** *******

GGT1.end.-Cabin1.start.8575573-8602125.orangutan       tgtgggtgtgtgagagtgtgtgagtgtgagtatgggggggtgtgggtg--tgtgtgaatg 4977
BCRP3.REF.NEW                                          agtgtgtgagtgtgagtatgggggtgtgggtgtgtgtgaatgtgcgtgattgtgtgtggg 986
                                                        *** *** *** **** ** * ***** ** ** * *  **** ***  ******   *

GGT1.end.-Cabin1.start.8575573-8602125.orangutan       tgtgtgcttgtgtgtgagtgtgcgtgcgtgcgtgtgtgtgtgcacgtgcactggcccagg 5037
BCRP3.REF.NEW                                          tatgtgtgtgtgtgtgtgagtgtgtgtgtgtgcgtgtgtgtgcacgtgcactggcccagg 1046
                                                       * ****  ******** * *** *** *** * ***************************

GGT1.end.-Cabin1.start.8575573-8602125.orangutan       aagcaggagccgtgtgtgtgtgggcttcagcacctgcagggcttgggcgcaaggaagcag 5097
BCRP3.REF.NEW                                          cagcaggagcc--atgtgtgtgggcttcagcacctgcagggcttgagcgcaaggagacag 1104
```

```
                                                       **********   ******************************* *********  ***

GGT1.end.-Cabin1.start.8575573-8602125.orangutan      cctcagggcccttgcacagaacaggtggcagggtgtgcccgtgaggcagatggggacttg 5157
BCRP3.REF.NEW                                         cctcagggcccttgcacagaacaggcggcagggtgtgcccgtggggcagatggggacttg 1164
                                                      ************************* ***************** ****************

GGT1.end.-Cabin1.start.8575573-8602125.orangutan      gggacaatggtggtgtgtgaatccatacctggctccaggattcaggaggcccatttgcat 5217
BCRP3.REF.NEW                                         gggacaatggtggtgtgtgagtccatacctggctccaggattcaggaggcccatttgcac 1224
                                                      ******************** **************************************

GGT1.end.-Cabin1.start.8575573-8602125.orangutan      atcccaggtgggaaca-gtctggccccgcctgaccctgctggccggtgcaggcctcttca 5276
BCRP3.REF.NEW                                         atcccaggtgggaacctgtctggccccggctgaccctgctggccggtgcaggccccttca 1284
                                                      ***************  *********** ************************* *****

GGT1.end.-Cabin1.start.8575573-8602125.orangutan      gtaaggccaattctccaaggctgaggtcttctcccggagtcataggtgaaggagtttgga 5336
BCRP3.REF.NEW                                         gtgaggccaattctccaaggctgcggtcttctcccagggtcatgggtgaaggggtttgga 1344
                                                      ** ******************** *********** * ***** ******** *******

GGT1.end.-Cabin1.start.8575573-8602125.orangutan      ggctccctgcgtgggtactggcctgctgaggtacacacaatgctgccatagccagtctgc 5396
BCRP3.REF.NEW                                         ggctccctgcgtgggtactggcctgctgggttacacacaatgctgccatagccagtctgc 1404
                                                      **************************** * *****************************

GGT1.end.-Cabin1.start.8575573-8602125.orangutan      cctaactcccagcctggggccacatctcgggtctctcagtcctggggagcccggtacccc 5456
BCRP3.REF.NEW                                         ccctacacccagcctggggccacatctcaggtctctcagtcctgaggagcccggtgcccc 1464
                                                      **  ** ********************* *************** ********** ****

GGT1.end.-Cabin1.start.8575573-8602125.orangutan      acccctcacatcctctctccctgagtcagggcctgggtctcgtgagctgagtgactgata 5516
BCRP3.REF.NEW                                         acccctcacatcctctctccctgagtcagggcctgggtctcgtgagctgagtgactgata 1524
                                                      ************************************************************

GGT1.end.-Cabin1.start.8575573-8602125.orangutan      cttggtgtcctggatgagggcgtgatggagaggggccacagcgggtgtttcctgaccctc 5576
BCRP3.REF.NEW                                         cttggtgtcctggatgagggcgtgatggagaggggccacagcgggtgtttcctgaccctc 1584
                                                      ************************************************************

GGT1.end.-Cabin1.start.8575573-8602125.orangutan      ttccaggaagcccagcccaagggaggcctccgctgctgccgctgcagagaggacacatac 5636
BCRP3.REF.NEW                                         ttccaggaag-------------------gtgctgctgccgctgcagggaggacacatac 1625
                                                      **********                     **************** ************

GGT1.end.-Cabin1.start.8575573-8602125.orangutan      aggatgccccttcctgccccctgcctcccactgggcccacaagagccagggcaagcctcc 5696
BCRP3.REF.NEW                                         aggatgccccttcctgccccctgcctcccattgggcccacaaaagccagggcaagcctcc 1685
                                                      ****************************** *********** *****************

GGT1.end.-Cabin1.start.8575573-8602125.orangutan      cctccctgccagccacctgctctgc-tcccagaagttctgtcttgtaggctgttgggagg 5755
BCRP3.REF.NEW                                         cctccctgccagccacctggtctgcttcccagaaattctgtcttgcaggctgttgggagg 1745
                                                      ******************* ***** ******** ********** **************

GGT1.end.-Cabin1.start.8575573-8602125.orangutan      atcccagtgctttgtaaactagagcaagggaggagtggccattctctctctttgttcatt 5815
BCRP3.REF.NEW                                         atcccagtactttgtaaactaaagcaagggaggagtggccgttctctc----tgttcatt 1801
                                                      ******** ************ ****************** *******    ********

GGT1.end.-Cabin1.start.8575573-8602125.orangutan      cattcaccttttcattcattccttcctccctccattcccccatctgtccatccttccccg 5875
BCRP3.REF.NEW                                         cattcaccttttcattcattccttcttccctccattcccccatctgtccatccttccctg 1861
                                                      ************************* ******************************** *

GGT1.end.-Cabin1.start.8575573-8602125.orangutan      ccctgattgctcatgcca------cccccagcccctcctgacctggtcctttggtttctc 5929
BCRP3.REF.NEW                                         ccctgattgctcatgccaccgccccccgcagcccctcctgacctggtcctttggtttctc 1921
                                                      ******************      *** ********************************

GGT1.end.-Cabin1.start.8575573-8602125.orangutan      ctcagggctttctgtctcctcctgcagggctgagaatggcagctcaggcgcaagtggggg 5989
BCRP3.REF.NEW                                         ttcagggatttctgtctcctcccacagggctgagaatggcagctcagggacaagtagggg 1981
                                                       ****** **************  ************************  ***** ****

GGT1.end.-Cabin1.start.8575573-8602125.orangutan      ctggggactgctcggtctcaccactggctcccaggggatttgagggattgacgtcagctg 6049
BCRP3.REF.NEW                                         ctggggactgcttagtctccccagtggctctcaggggatttgagggtttgacgccagctg 2041
                                                      ************  ***** *** ****** *************** ****** ******

GGT1.end.-Cabin1.start.8575573-8602125.orangutan      ccaccccaggctgtgcacctcctctgctcaggaggacatacagagatgcggcacccactt 6109
BCRP3.REF.NEW                                         ccaccccaggctgtgcccctcctctgctcaggaggacatacag-gatgcaacacccactt 2100
                                                      **************** ************************** *****  *********

GGT1.end.-Cabin1.start.8575573-8602125.orangutan      aaactcgaagttgcaaagatgcaaatgagactggggtctcaggcaccagagaccacccgt 6169
BCRP3.REF.NEW                                         aaactcgaagttgcaaagatgcaaatgagactggggtctcaggcaccagagaccacccgt 2160
                                                      ************************************************************

GGT1.end.-Cabin1.start.8575573-8602125.orangutan      gggcacgtggcttttgggagtggggacctgctgccacaaatctctgggtggagtctggat 6229
BCRP3.REF.NEW                                         gggcacgtggcttttgggattggagacctgctgccacagatctctga--agagtctggac 2218
                                                      ******************* *** ************** *******    *********

GGT1.end.-Cabin1.start.8575573-8602125.orangutan      ctgctgggtctccccgagtga----ctgggggtctccatagcgtgccctgctgtgtgcgt 6285
BCRP3.REF.NEW                                         ctgctgggtctccccaagtgactctctgggggtctccatagcatgccctgctgtgtgcat 2278
                                                      *************** *****    ***************** *************** *

GGT1.end.-Cabin1.start.8575573-8602125.orangutan      gacggtcactggttgggtaggggactctactctaaagctccctccgccggcactccctcg 6345
BCRP3.REF.NEW                                         gacggtcactggttgggtaggggtctctactctaaagctccctctgccggcatcccctcg 2338
                                                      *********************** ******************** *******  ******

GGT1.end.-Cabin1.start.8575573-8602125.orangutan      aactctcccttggtgaagagacaggatgtggtttgccccagtgttttgtcaaacaactct 6405
BCRP3.REF.NEW                                         aactctcccttggtgaagagagaggatgtggtttgccccagtgttttatcaaacaactct 2398
                                                      ********************* ************************** ***********

GGT1.end.-Cabin1.start.8575573-8602125.orangutan      ctccacttcctgttttaagaagctgggagtggaagagagcctggggctggtcccagctgc 6465
BCRP3.REF.NEW                                         ctccacttcctgttttaagaagctgggagtggaagagagcctggggctggccccagctgc 2458
                                                      ************************************************** *********
```

```
GGT1.end.-Cabin1.start.8575573-8602125.orangutan      tgctgcgaaacaggggtcactggacactgggaccctggctgggctgactggaggcctcag 6525
BCRP3.REF.NEW                                         tgctgcgaaacaggggtcactggacgctgggaccctggccgggctggctggaggcctcag 2518
                                                      ************************* ************* ****** *************

GGT1.end.-Cabin1.start.8575573-8602125.orangutan      gaagaggcctgctgcagcgtcatcctggccaagattcctccctgccgaggaccctggcca 6585
BCRP3.REF.NEW                                         gaagaggcctgctacagtgtcatcctggccaagattcctccctgcagaggaccctggcca 2578
                                                      ************* *** *************************** **************

GGT1.end.-Cabin1.start.8575573-8602125.orangutan      cgctgccacagggtctgctggggccaccagaaacccatgctcctgcctccatctctcccc 6645
BCRP3.REF.NEW                                         cgctgccacagggtctgctggggccaccagaagcccatgctcctgcctccatctctcccc 2638
                                                      ******************************** ***************************

GGT1.end.-Cabin1.start.8575573-8602125.orangutan      tctgtgctcacctctcaccaggaggccctcccagagttcagtctcctgcttttttttttt 6705
BCRP3.REF.NEW                                         tctgtgctcacctctcaccaggaggccctcccagagttcagtgtcctgcttttttttttt 2698
                                                      ****************************************** *****************

GGT1.end.-Cabin1.start.8575573-8602125.orangutan      tttttttgagatggtgtctcgctctgtcaccaggctgcagtgcagtggcctgatctcag 6765
BCRP3.REF.NEW                                         tttttt---agatggtgtctcgttctgtcaccaggctggagtgcagtggcgcgatctcag 2755
                                                      ******   ************* *************** ***********  ********

GGT1.end.-Cabin1.start.8575573-8602125.orangutan      ctcactgcaacctctgcctcctcggttcaaatgattctcctgcctcagcctcctgagtag 6825
BCRP3.REF.NEW                                         ctcactgcaacctctgcttccttggttcaaatgattctcctgcctcagcctcctgagtag 2815
                                                      ***************** **** *************************************

GGT1.end.-Cabin1.start.8575573-8602125.orangutan      ctgggactacaggtgccaaccaccatgcccagctaatttttgtatttttagtagagatgg 6885
BCRP3.REF.NEW                                         ctgggactacaggtgccagccaccacgcccaggtaatttttgtatttttagtagagacgg 2875
                                                      ****************** ****** ****** ************************ **

GGT1.end.-Cabin1.start.8575573-8602125.orangutan      gatttcaccatgttggccaggatggtctctatctcttgacctcacgattcgcccgcctcg 6945
BCRP3.REF.NEW                                         ggtttcaccatgttggccaggatggtctctatctctt--------gattcgcccgccttg 2927
                                                      * ***********************************        ************* *

GGT1.end.-Cabin1.start.8575573-8602125.orangutan      gcctcccaaagtgctggcattacaggagtgagtcatggtgcccggccccgtctcctactc 7005
BCRP3.REF.NEW                                         gcctcccaaagtgctggaattacaggagtgagtcatggcacccggcctcatctcctactc 2987
                                                      ***************** ********************  ******* * **********

GGT1.end.-Cabin1.start.8575573-8602125.orangutan      tttcagcaccaggttttattcttgggattctgctacagccggagcccctgggtgagagtt 7065
BCRP3.REF.NEW                                         tttcagcaccaggttttactcttgggattctgctacagccgcagcccctgggtgcgagtt 3047
                                                      ****************** ********************** ************ *****

GGT1.end.-Cabin1.start.8575573-8602125.orangutan      cctaaggtttctgtgagtgtggacccagcaccatgcctagtagacatacaaaaggagcat 7125
BCRP3.REF.NEW                                         cctaagctttctgtgagtgtggacccagcaccgtgcctagtagacatacaaaaggagcat 3107
                                                      ****** ************************* ***************************

GGT1.end.-Cabin1.start.8575573-8602125.orangutan      ggtgacagtgaggtctgtcatctccagcataatgactgttttgatccttgtaaaaaaggt 7185
BCRP3.REF.NEW                                         ggtgacagtgaggtctgtcatctccagcataatgactgttttgatccttgtaaaaaaggt 3167
                                                      ************************************************************

GGT1.end.-Cabin1.start.8575573-8602125.orangutan      gatttttggctgggcgtggtggctcatacctgtaatcccagcactttgggaggccaaggg 7245
BCRP3.REF.NEW                                         gatttttggctgggtgtggtggctcacacctgtaatcccagcactttgggaggccgatgg 3227
                                                      ************** *********** **************************** * **

GGT1.end.-Cabin1.start.8575573-8602125.orangutan      gggtggatcacttgaggtcaggagttggaaaccagcctgggcaacatggtgaaaccacgt 7305
BCRP3.REF.NEW                                         gggtggctcacttgaggtcaggagttggagcccagcctgggcaacatggtgaaaccacgt 3287
                                                      ****** **********************  *****************************

GGT1.end.-Cabin1.start.8575573-8602125.orangutan      ctctactaaaagtgcaaaaattcgctgagcttggtagcgggtgcctgtaatcccagctac 7365
BCRP3.REF.NEW                                         ctctactaaaaatacaaaaattagctgggcatggtaacggatgcctgtaatcccagctac 3347
                                                      *********** * ******** **** ** ***** *** *******************

GGT1.end.-Cabin1.start.8575573-8602125.orangutan      ttgggaggctgagacaggagaatcacttgaacccaggaggcaaagtttgcagtaagccaa 7425
BCRP3.REF.NEW                                         ttgggaggctgagacaggagaatcacttgaacccaggaggcaaaggttgcggtaagccaa 3407
                                                      ********************************************* **** *********

GGT1.end.-Cabin1.start.8575573-8602125.orangutan      gattgcaccactgcactccagcctgggtgacagagcaagacttggtctcaaaaaa----- 7480
BCRP3.REF.NEW                                         gattgtaccactgcactccagcctgggtgacagagcaagacttggtctcaaaaaaaaaaa 3467
                                                      ***** *************************************************

GGT1.end.-Cabin1.start.8575573-8602125.orangutan      ------aaagaaagaaaagtttatatttttgttctaatggttatcttaatatcttcatcc 7534
BCRP3.REF.NEW                                         aaaaagaaagaaagaaaagtttatatttttgttctaatggttatcttaatatcgtcattc 3527
                                                            *********************************************** **** *

GGT1.end.-Cabin1.start.8575573-8602125.orangutan      tataattatatgttttatataattataatagctatataagatatactacccctagtatgt 7594
BCRP3.REF.NEW                                         tataattgtatgttttatataattataatagctatataagatataatacccctagtatgt 3587
                                                      ******* ************************************* **************

GGT1.end.-Cabin1.start.8575573-8602125.orangutan      tgttttttggatattctattcgctcctgccggttaatttatgtgtcaacttggctaagct 7654
BCRP3.REF.NEW                                         tgttttttggatattctacttgctcctgatggttaatttatatgtcaacttggctaagct 3647
                                                      ****************** * *******  *********** ******************

GGT1.end.-Cabin1.start.8575573-8602125.orangutan      atggtgccccgttgtttggtcaaatacttgtcaatatcttgctgggaggttatttcatag 7714
BCRP3.REF.NEW                                         atggtgccccgttgtttggtcaaatacttgtcaatatcttgctgggaggttatttcatag 3707
                                                      ************************************************************

GGT1.end.-Cabin1.start.8575573-8602125.orangutan      atgtgattaacactgacagtcacttgactttaagtaaaacagatcacccaccataatatg 7774
BCRP3.REF.NEW                                         atgtgattaacactgacagtcagttgactttaagtaaaacagattacccaccataatatg 3767
                                                      ********************** ********************* ***************

GGT1.end.-Cabin1.start.8575573-8602125.orangutan      ggtgggccacctccaatcagttgaaggccgtaagaacaaaaactgaggtttcccagagaa 7834
BCRP3.REF.NEW                                         ggtgggccacctccaatcagttgaaggccgtaagaacaaaaactgaggtttcccagagaa 3827
                                                      ************************************************************
```

```
GGT1.end.-Cabin1.start.8575573-8602125.orangutan      gcaggaattctgcttcaagactataacacacaaaccctgcctgagtgtctggcctgctga 7894
BCRP3.REF.NEW                                         gcaggaattctgcctcaagactgtaacacacaaaccctgcctgagtttctggcctgctga 3887
                                                      ************* ******** *********************** *************

GGT1.end.-Cabin1.start.8575573-8602125.orangutan      ctgctctacagatttaggttccagacttcgagatcaactcttacctgaatttatagcct 7954
BCRP3.REF.NEW                                         ctgctctacagagtttaggttccagacttcgagatcaactcttacctgaatttatagcct 3947
                                                      ************ ***********************************************

GGT1.end.-Cabin1.start.8575573-8602125.orangutan      gctggcttgccctacagattttaaacttgctagttcccccagtcatgtgagccaattcct 8014
BCRP3.REF.NEW                                         gctggcttgccctacagattttaaacttgctagtccccacaatcatgtgagccaattcct 4007
                                                      ********************************** *** ** ******************

GGT1.end.-Cabin1.start.8575573-8602125.orangutan      aaataaatctctctctatgtataacctattggtttagtttctgtaaaaagctttcacatc 8074
BCRP3.REF.NEW                                         caataaatctctctctatgtataatctattggtttagtttctctgaaaagctttcacatc 4067
                                                       *********************** ***************** * ***************

GGT1.end.-Cabin1.start.8575573-8602125.orangutan      cagtttcctggatgttaagtaatactgaaactagctagtaacttc----------ttttt 8124
BCRP3.REF.NEW                                         cagtttcctggatgttaagaattactgaaactagctagtaacttcttttttttttttttt 4127
                                                      ******************* * ***********************          *****

GGT1.end.-Cabin1.start.8575573-8602125.orangutan      ttttttttttttgagacagagttttgctcttgttgcccaggctggaatgcaatggcacaat 8184
BCRP3.REF.NEW                                         ttttttttttttgagacagagttttgctcttgttgcccaggctggaatgcaatggcacaat 4187
                                                      ************************************************************

GGT1.end.-Cabin1.start.8575573-8602125.orangutan      ctcagctcactgcaacctccacttcctgggtccaagcaattctcctccctcagcctcctg 8244
BCRP3.REF.NEW                                         ctcagctcaccgcaacctccacttcctgggtccaagcaattctcctccctcagcctcctg 4247
                                                      ********** *************************************************

GGT1.end.-Cabin1.start.8575573-8602125.orangutan      agtagctgagattacaggcatgtgccaccatgcttggctaatttttgtatttttagtaga 8304
BCRP3.REF.NEW                                         agtagctgggattacaggcatgtgccaccatgcttggctaatttttgtatttttagtaga 4307
                                                      ******** ***************************************************

GGT1.end.-Cabin1.start.8575573-8602125.orangutan      gacggggcttctccatgttggtcaggctggtcttgaactcccgacctcaggtgatccgcc 8364
BCRP3.REF.NEW                                         gacagggcttctccatgttggtcaggctggtcttgaactcccaacctcaggtgatcagcc 4367
                                                      *** ************************************** ************* ***

GGT1.end.-Cabin1.start.8575573-8602125.orangutan      gccttggcctcacagagtgctgggattacaggcatgagccaccgtgcccggctcctagta 8424
BCRP3.REF.NEW                                         gccttggcctcacaaagtgctggaattacaggcatgagccaccgcacctggctcctagta 4427
                                                      ************** ******** ********************  ** ***********

GGT1.end.-Cabin1.start.8575573-8602125.orangutan      acttcttcttttccgtgatgtgtctcttatctctaata---------------------- 8462
BCRP3.REF.NEW                                         aattcttcttttccgtgatgtgtctcttacctctaataatacttttcttctttttttttt 4487 4466
                                                      * *************************** ********
```

Alu insertion in BCRP3?

```
GGT1.end.-Cabin1.start.8575573-8602125.orangutan      ------------------------------------------------------------ 8462
BCRP3.REF.NEW                                         tttgagacggagtctcgttctgtcgcccaggcgggagtgctgtggcgcgatctccgctca 4547


GGT1.end.-Cabin1.start.8575573-8602125.orangutan      ------------------------------------------------------------ 8462
BCRP3.REF.NEW                                         ctgcaagctccgccttccgggttcacgccattctcctgcctcaacctcccgagtagctgg 4607


GGT1.end.-Cabin1.start.8575573-8602125.orangutan      ------------------------------------------------------------ 8462
BCRP3.REF.NEW                                         gactacaggcgcccgccactgcgcccggctaatttttttgtatttttagtagagacggggt 4667


GGT1.end.-Cabin1.start.8575573-8602125.orangutan      ------------------------------------------------------------ 8462
BCRP3.REF.NEW                                         ttcaccgtggtctcgatctcctgacctcgtgatccgcccgcctcggcctcccaaagtgct 4727


GGT1.end.-Cabin1.start.8575573-8602125.orangutan      --------------------------------atacttttcttcttaaagtctacttcat 8490
BCRP3.REF.NEW                                         gggattacaggcgtgagccaccgcgtccggccatacttttcttcttaaagtctacttcat 4787 4760
                                                                                      ****************************

GGT1.end.-Cabin1.start.8575573-8602125.orangutan      taaaaacagttatgctgggcatggtggctcatgcctgtaatctcggcactttgttggagg 8550
BCRP3.REF.NEW                                         taaaaatagttatgctgggcatggtggctcatggctgtaatctcggcactttgttggagg 4847
                                                      ****** ************************** **************************

GGT1.end.-Cabin1.start.8575573-8602125.orangutan      tcgaggtgggtggttcactgaagcccaggagttcaagaccagcctgggcaatatggcgag 8610
BCRP3.REF.NEW                                         tcgaggtgggtggatcactgaagcccaggagttcaagaccaacctgggcaacgtggcgag 4907
                                                      ************* *************************** *********  *******

GGT1.end.-Cabin1.start.8575573-8602125.orangutan      accctgcctctacaaaaaatacaaaaattagctgggtgtggctaatataattctaagttg 8670
BCRP3.REF.NEW                                         accctgcctctacaaaaaatacaaaaattagctgggtgtggctaatat------------ 4955
                                                      ************************************************

GGT1.end.-Cabin1.start.8575573-8602125.orangutan      gcacacttgtagtcccagctacttgggatgctgaggtgggagaatcacttgagcctagaa 8730
BCRP3.REF.NEW                                         --acacttgtagtcccagctacttgggatgctgaggtgggagaatcgcttgagcctagaa 5013
                                                        ******************************************** *************

GGT1.end.-Cabin1.start.8575573-8602125.orangutan      gggagagattgctgtaagccaagatcacatcactgcactccagcctgggagatagagtga 8790
BCRP3.REF.NEW                                         gggagagattgctgtaagccaagatcacatcactgcactccagcctgggagacagagtga 5073
                                                      **************************************************** *******

GGT1.end.-Cabin1.start.8575573-8602125.orangutan      gggtctatctcaaaaaaaaaaaaaaaaaaaaaagttatacagctttcttggttagtgcat 8850
BCRP3.REF.NEW                                         ggctctatctc--caaaaaaaaaaaaaaaaaaagttatacagctttcttggttagtgcat 5131
                                                      ** ********   **********************************************

GGT1.end.-Cabin1.start.8575573-8602125.orangutan      gcatgatatatttatatttttcattattttccacctctctgtatccttatataaaaggc 8910
BCRP3.REF.NEW                                         gcatgc-------catatttttcattattttccacctctctgtatccttatataaaaggc 5184
                                                      *****         **********************************************

GGT1.end.-Cabin1.start.8575573-8602125.orangutan      attagttgggttttactttattttcaattattttaatttttgttgtccttttaaatgtaa 8970
```

```
BCRP3.REF.NEW                                     attagttgggttttactttattttcaattattttaatttttattgtccttttaaatgtaa 5244
                                                  ***************************************** ******************

GGT1.end.-Cabin1.start.8575573-8602125.orangutan  ctaatgatttatttgggttgaaagccactaccaatttgtttccccatgcctattctgttt 9030
BCRP3.REF.NEW                                     ctaatgatttatttgggttgaaacccaccaccaatttgttt-tccatgcctattctattt 5303
                                                  *********************** **** ************  ************* ***

GGT1.end.-Cabin1.start.8575573-8602125.orangutan  cttcttatctcctctcacatcttgttttggatttattatttttattatttaatttcctcc 9090
BCRP3.REF.NEW                                     cttcttatctcctctcacatcttgttttggatttattatttttattatttaatttcctcc 5363
                                                  ************************************************************

GGT1.end.-Cabin1.start.8575573-8602125.orangutan  tt---tattagtttcataactgtgcagtcttggagttattttaaaagatgacagtgaatt 9147
BCRP3.REF.NEW                                     ttctctattagtttcatagctctgcagtcttagagttattttaaaagatgacagtggatt 5423
                                                  **   ************* ** ********* ************************ ***

GGT1.end.-Cabin1.start.8575573-8602125.orangutan  attttagagcttacaacatgcatccttcacttatcaaagtctaacatgagctagtacttt 9207
BCRP3.REF.NEW                                     attttagagcttacaacatgcatccttcacttatcaaagtctaacatgagctagtacttt 5483
                                                  ************************************************************

GGT1.end.-Cabin1.start.8575573-8602125.orangutan  ttgttgttgttgttgttgagatagagagagtcttcctctgctgcccaggctggagtgtaa 9267
BCRP3.REF.NEW                                     ------ttgttgttgttgagatagagagagtcttcctctgctgcccaggctggagtgcag 5537
                                                        *************************************************** *

GGT1.end.-Cabin1.start.8575573-8602125.orangutan  tggagcaatcttggttcactgcaacctccacttcttgggttcaagcaattctcctgcctc 9327
BCRP3.REF.NEW                                     tggagcaatcttggttcactgcaacctccacttcttgggttcaagcagttctcctgcctc 5597
                                                  *********************************************** ************

GGT1.end.-Cabin1.start.8575573-8602125.orangutan  agtcacctgagtagctgggaccacaggtgtgcaccactatgcccggctaatttttgtatt 9387
BCRP3.REF.NEW                                     agtcacctgagtagctgggaccacaggtgtgcaccactatgcccggccaatttttgtatt 5657
                                                  *********************************************** ************

GGT1.end.-Cabin1.start.8575573-8602125.orangutan  ctgtttttgtagagacagggtttcaccatgttggccaggctggtcttgaattcctgacct 9447
BCRP3.REF.NEW                                     cttttttagtagagacagggtttcaccatgttggccaggctggtcttgaactcctgacct 5717
                                                  ** **** ****************************************** *********

GGT1.end.-Cabin1.start.8575573-8602125.orangutan  taagagatctgcctacctcggcgtcctaaagtgttgggattacaggcatgagccaccgtg 9507
BCRP3.REF.NEW                                     taagagatctgcctacctcggcgtcctaaagtgttgggattacaggcatgagccaccgcg 5777
                                                  ********************************************************** *

GGT1.end.-Cabin1.start.8575573-8602125.orangutan  cccagcctatgagttagtacttctatcctcttcctagtcagtacaggaaccttggaacag 9567
BCRP3.REF.NEW                                     cccagcctatgagttagtacttctatgctcttcctagtcagtacaagaaccttggaacag 5837
                                                  ************************** ****************** **************

GGT1.end.-Cabin1.start.8575573-8602125.orangutan  gaactaaatttaccccccagtgacttatatgctaatatttttgtgtattttaaatatatg 9627
BCRP3.REF.NEW                                     gaactaaattta-cccccagtgacttatatgctaatatttttgtgtattttaaatatata 5896
                                                  ************ **********************************************

GGT1.end.-Cabin1.start.8575573-8602125.orangutan  tgtgtgcatagatgtatctgtgtgtttttt-tgtttttattcttatttatgttgagagtg 9686
BCRP3.REF.NEW                                     tgtgtgcatagatgtatctgtgtgttttttgtgtttttattcttatttatgttgagagtg 5956
                                                  ****************************** *****************************

GGT1.end.-Cabin1.start.8575573-8602125.orangutan  tagagctatgtaagagtaaagagaattgtgtaatgaagccccatgtatccattcaatttc 9746
BCRP3.REF.NEW                                     tagagctatgtaagagtaaagagaattgtgtaatgaagccccgagtatccattcaatttc 6016
                                                  ******************************************  ****************

GGT1.end.-Cabin1.start.8575573-8602125.orangutan  aacaacaatcttatggccaagctaatttcatgtatactctttcctgcttccctctacccc 9806
BCRP3.REF.NEW                                     aacaacaatctcatggccaagctaatttcatgtatactctttcctgcttccctctacccc 6076
                                                  *********** ************************************************

GGT1.end.-Cabin1.start.8575573-8602125.orangutan  acattatttcagtgcaaatcccagatatataactttacccatacatatttcagtatgttt 9866
BCRP3.REF.NEW                                     acattatttcagtgcaaatcccagatatataactgtacccatacatatttcagtatgttt 6136
                                                  ********************************** *************************

GGT1.end.-Cabin1.start.8575573-8602125.orangutan  tatttattttaaaccccacaagatatcattttctatactactgtaattttataccaataa 9926
BCRP3.REF.NEW                                     tatttattttaaaccccacaatatatcattttctatactactgtaatttcataccaataa 6196
                                                  ********************* *************************** **********

GGT1.end.-Cabin1.start.8575573-8602125.orangutan  cattcatttagatttacccacatgtttacctcttctgttacgctttatttttatttataa 9986
BCRP3.REF.NEW                                     cattcatttagatttacccacacgtttacctcttctgttaccctttatttttatttataa 6256
                                                  ********************** ******************* *****************

GGT1.end.-Cabin1.start.8575573-8602125.orangutan  aaatatctttgggaaaaaatatctttcagcacatggtcaaggatctcctgagggctatat 10046
BCRP3.REF.NEW                                     aaatatctttgggaagaaatatctttcagcacatggtcaaggatctcctgagggctatgt 6316
                                                  *************** ****************************************** *

GGT1.end.-Cabin1.start.8575573-8602125.orangutan  catgggcaaaatatacatatatattccatatatatacacacatatacacacacacacaca 10106
BCRP3.REF.NEW                                     catgggcaaaatatacatatatattccatatatatacacacatatacacacacacacata 6376
                                                  ********************************************************** *

GGT1.end.-Cabin1.start.8575573-8602125.orangutan  tatacacatacacacacacacacacacacgtattcgactttcacttttttttttgtttgt 10166
BCRP3.REF.NEW                                     tatacacatatacacacacacatatata--tattccactttcacttttttgtttgtttgt 6434
                                                  ********** *********** * * *  ***** ************** *********

GGT1.end.-Cabin1.start.8575573-8602125.orangutan  tttttgagaccgagtctggcactgtggcccaggctggagtgtggtggtgggatctcagct 10226
BCRP3.REF.NEW                                     tttttgagaccgagtctcgctctgtggcccaggctggagtgcggtggtgggatctcagct 6494
                                                  ***************** ** ******************** ******************

GGT1.end.-Cabin1.start.8575573-8602125.orangutan  cactgcaacttctacctcctgggttcaagtgagtctcctgactcagcctcccgagtagct 10286
BCRP3.REF.NEW                                     cactgcaacttctgcctcctgggttcaggtgattctcctgtctcagcctcctgagtagct 6554
                                                  ************* ************* **** ******* ********** ********

GGT1.end.-Cabin1.start.8575573-8602125.orangutan  gggattgcaggtgttagccaccatgcctggctaatttatatatatatatattttttttttg 10346
BCRP3.REF.NEW                                     gggattacaggtgttagccatcacgtctggctaatttttgt------------------- 6595
```

```
                                                      ****** ************* ** * *********** * *
GGT1.end.-Cabin1.start.8575573-8602125.orangutan      agacggagtatcgctctgttgcccaggctggagtgcagtggcgtgatctcggctcactgc 10406
BCRP3.REF.NEW                                         ------------------------------------------------------------ 6595

GGT1.end.-Cabin1.start.8575573-8602125.orangutan      aacctccacctccctggttcaagcaatttccctgcctcagcctcccaagtagctgggatt 10466
BCRP3.REF.NEW                                         ------------------------------------------------------------ 6595

GGT1.end.-Cabin1.start.8575573-8602125.orangutan      acaggtgcacaccaccatgcccagataatttttttgtatttgtagtagagaccgggtttc 10526
BCRP3.REF.NEW                                         ------------------------------------------------------------ 6595

GGT1.end.-Cabin1.start.8575573-8602125.orangutan      cctatcatggtcagactggtcttgaacttctgacttcaggcaatctgcccacctcggcct 10586
BCRP3.REF.NEW                                         ------------------------------------------------------------ 6595

GGT1.end.-Cabin1.start.8575573-8602125.orangutan      cccaaagtgctgggattataggcatgagccaccgtgctggcctgtttttttttttttgag 10646
BCRP3.REF.NEW                                         ------------------------------------------tgtttttttttttttgag 6613
                                                                                                   ******************
```

Insertion in orangutan

```
GGT1.end.-Cabin1.start.8575573-8602125.orangutan      atggagttttgctctgtcacccaggctggagtgcagtggcaagatcttggctcactgcaa 10706
BCRP3.REF.NEW                                         acggagtatcgctctgtcacccaggctggagtgcagtggcaagatcttgtctcactgcaa 6673
                                                      * ***** * *************************************** **********

GGT1.end.-Cabin1.start.8575573-8602125.orangutan      cctccacctctcgggttcaagcaattcttgtgcctcagcctcctgagtagctgggattat 10766
BCRP3.REF.NEW                                         cctccacctctcaggttcaagcaattcttgtgcctcagcctcctgagtagctgggattac 6733
                                                      ************ **********************************************

GGT1.end.-Cabin1.start.8575573-8602125.orangutan      aggcacccaccaccacatctggctaatttgtgtatttttggtagagatggggtttcacca 10826
BCRP3.REF.NEW                                         aggcatccaccaccacatctggctaatttgtgtatttttggtagagatggggtttcacca 6793
                                                      ***** ******************************************************

GGT1.end.-Cabin1.start.8575573-8602125.orangutan      tgttggccaggctggtctcgaacttctgacctcaggtgatccacctgcctcggccttcca 10886
BCRP3.REF.NEW                                         tgttggccaggctggtctcgaacttctgacctcaggtgatccacctgcctcggccttcca 6853
                                                      ************************************************************

GGT1.end.-Cabin1.start.8575573-8602125.orangutan      aagtgctgggattacaggcatgagccaccatgctgggccattttcgcttttgaaggatat 10946
BCRP3.REF.NEW                                         aagtgctgggattacaggcatgagccaccatgcccagccattttcacttttgaaggatat 6913
                                                      *********************************   ********* **************

GGT1.end.-Cabin1.start.8575573-8602125.orangutan      tgttagtgagcatagaattctaggttggcagatattttctttcctcagtttgaaaacatg 11006
BCRP3.REF.NEW                                         tgttaatgagcatagaattctaggttggcagatattttctttcctcagtttgaaaacatg 6973
                                                      ***** ******************************************************

GGT1.end.-Cabin1.start.8575573-8602125.orangutan      attcccttgtatctgatttctcctgcttttattgggaagccaattctcaatctaattttg 11066
BCRP3.REF.NEW                                         attcccttgtatctgatttctcctgcttttattgggaagccaattctcaatctaattttg 7033
                                                      ************************************************************

GGT1.end.-Cabin1.start.8575573-8602125.orangutan      ctcatttgaaggcaatggcttttta---tgttgtgttttttgagatggagtctcactctg 11123
BCRP3.REF.NEW                                         ctcatttgaaggcaatggctttttctgttgttgtgttttctgaggtggagtctcactctg 7093
                                                      ************************    *********** **** ***************

GGT1.end.-Cabin1.start.8575573-8602125.orangutan      tcgcccaggctggagtgcagtggtgcaatctcagctcactgcaacctctgcctcctgggt 11183
BCRP3.REF.NEW                                         tcacccaggctggactgcagtggtgcaatctcagctcactgcaacctctgcctcctgggt 7153
                                                      ** *********** *********************************************

GGT1.end.-Cabin1.start.8575573-8602125.orangutan      tcaagtgattcttctgcctcagcctcccaagtagctgggattacaggtgtccaccatcac 11243
BCRP3.REF.NEW                                         tcaagtgattcttctgcctcagcctcccaagtagctgggattacaggtgtccaccatcac 7213
                                                      ************************************************************

GGT1.end.-Cabin1.start.8575573-8602125.orangutan      atccagctaattgttgtatttttaatagagatgaacttttgccatgttggtcaggctgat 11303
BCRP3.REF.NEW                                         acctggctaattgttgtatttttaatagagatgaacttttgccatgttggtcaggctgat 7273
                                                      * *  *******************************************************

GGT1.end.-Cabin1.start.8575573-8602125.orangutan      cccaaactcctcatttcaggtgattcgcccacctcagcctcccaaatgctgggattacag 11363
BCRP3.REF.NEW                                         cccaaactcctcatttcaggtgatccgcccgcctcagcctcccaaatgctgggattacag 7333
                                                      ************************ ***** *****************************

GGT1.end.-Cabin1.start.8575573-8602125.orangutan      gcatgagccagcccccaaccccgacctgaaggcagtatcttttttcttctggctgctttg 11423
BCRP3.REF.NEW                                         gcatgagacagcccacaaccctggcctgcaggcagtatcttttttcctctggctgctttg 7393
                                                      ******* ****** ****** * **** ***************** *************

GGT1.end.-Cabin1.start.8575573-8602125.orangutan      aaaagttttgtctttgttttgagtagtttacactgatgcatttaggtggctcttcattct 11483
BCRP3.REF.NEW                                         aaaagttttgtctttgttttgagcagtttacactgatgcatttaggtggctcctcattcc 7453
                                                      *********************** **************************** ******

GGT1.end.-Cabin1.start.8575573-8602125.orangutan      ataacttgattcttttttgtccattttagaaaattctcagctttatctcttcaagtatta 11543
BCRP3.REF.NEW                                         atgacttgattcttttttgtccattttagaaaactgccagctttatctcttcaagtatta 7513
                                                      ** ****************************** *  ***********************

GGT1.end.-Cabin1.start.8575573-8602125.orangutan      tgtcttccccatcctctctctactctcctcatgggactccaatttcacatggcttatacc 11603
BCRP3.REF.NEW                                         tgtcttccccatcctctctctactctccttatgagactccaatttcacatgacttatgcc 7573
                                                      ***************************** *** ***************** ***** **

GGT1.end.-Cabin1.start.8575573-8602125.orangutan      ttgttaaagtatctcccacgtctcttaatccatttcccctatgttctatctatttttctc 11663
BCRP3.REF.NEW                                         ttgttaaagtatcccccatgtctcttaatccatttcctgtatgttctatctgtttttctc 7633
                                                      ************* **** ******************  ************ ********

GGT1.end.-Cabin1.start.8575573-8602125.orangutan      tttgtgcttcaatttgtatattttgtatcaaactatctcccaattagccggggggtgtgg 11723
BCRP3.REF.NEW                                         tttgtacttcaatttgtatagtttgtatcaaactatctcccaattagccgggc---gtgg 7690
```

```
                                                      ***** ************** *******************************    ****

GGT1.end.-Cabin1.start.8575573-8602125.orangutan      tggcgggtgcctgtaatcccagctacttgggaggctgagacaggagaattgcttgaaccc 11783
BCRP3.REF.NEW                                         tggtgggtgcctgtaatcccagctacttgggagcctgaggcaggagaattgcttgaaccc 7750
                                                      *** ***************************** ***** ********************

GGT1.end.-Cabin1.start.8575573-8602125.orangutan      gggaggtggaggttgcagtgagccgagatcatgccaccgcactccagcctgggcaacaga 11843
BCRP3.REF.NEW                                         gagaggtggaagttgtagtgagccgagatcatgccactgcactccagcctgggcaacaga 7810
                                                      * ******** **** ********************* **********************

GGT1.end.-Cabin1.start.8575573-8602125.orangutan      gtgagaccctgtctc----aataaataaataaataaataccagttcac--ttttttttta 11897
BCRP3.REF.NEW                                         gtgagaccctgtctcaataaataaataaataaataaataccagttcactattttttttta 7870
                                                      ***************    *****************************  **********

GGT1.end.-Cabin1.start.8575573-8602125.orangutan      tgtttgtgtctagtgtactgttcaaattgagttcctaattccatttttttttttgagac 11957
BCRP3.REF.NEW                                         tgtttgtgtctagtgtgctgttcaaattgagttcctaattccattttttttt----aga 7926
                                                      **************** ************************************

GGT1.end.-Cabin1.start.8575573-8602125.orangutan      tttttttttttgagtctctatctgttgcccaggctggagttcagtggtgcaatctcaact 12017
BCRP3.REF.NEW                                         cttttttttttgagtctctatctgttgcccaggctggagttcagtggtgcaatctcaact 7986
                                                       ***********************************************************

GGT1.end.-Cabin1.start.8575573-8602125.orangutan      cactgtagtctccacctccctggttcaagcgattctcgtgcttcagcctcccgagtaact 12077
BCRP3.REF.NEW                                         cactgtagcctccacctcccaggttcaagcgattctcatgcctcagcctctcgagtaact 8046
                                                      ******** *********** **************** *** ******** *********

GGT1.end.-Cabin1.start.8575573-8602125.orangutan      gggattaccaccatgcctaactcatttttgtatttttagtagagatggggttttgccatg 12137
BCRP3.REF.NEW                                         gggattaccaccacgcctaactcatttttgtatttttagtagagatggggttctgccatg 8106
                                                      ************* ************************************** *******

GGT1.end.-Cabin1.start.8575573-8602125.orangutan      ttggccaggctggtcttgaactcctggcctcatgtgattggcctacctctgtctcccaaa 12197
BCRP3.REF.NEW                                         ttggccaggctggtcttgaactcttggccttatgtgattggcctacctctgtctcccaaa 8166
                                                      *********************** ****** *****************************

GGT1.end.-Cabin1.start.8575573-8602125.orangutan      gtgctgggattataggcctgagccaccactcccagcctccattattattattatttttttt 12257
BCRP3.REF.NEW                                         gtgctgggattataggcctaaaccaccactcccagcctcc--tttttttttttttttttt 8224
                                                      ******************* * ******************  * ** ** ** *******

GGT1.end.-Cabin1.start.8575573-8602125.orangutan      ttttgagatggagtctcgctctgtcgcccaggctggagtagagtggtgcgatctcggctc 12317
BCRP3.REF.NEW                                         ttttgagacggagtctcgctctgtcgcccaggctggagtagagtggtgcgatctcggctc 8284
                                                      ******** ***************************************************

GGT1.end.-Cabin1.start.8575573-8602125.orangutan      actgcaacctccccctcccagttcaagcgattctcctgcctcagcctcccgagtagctgg 12377
BCRP3.REF.NEW                                         actgcaacctccccctcccagttcaagtgattctcctgcctcagcctcccgagtagctag 8344
                                                      *************************** ******************************* *

GGT1.end.-Cabin1.start.8575573-8602125.orangutan      gactacaggtgcatgccaccatgcctggctaatttttgtaattttagtagagatggggtt 12437
BCRP3.REF.NEW                                         gactataggagcatgccaccatgcctggctaatttttgtaattttagtagagatggggat 8404
                                                      ***** *** ************************************************ *

GGT1.end.-Cabin1.start.8575573-8602125.orangutan      tcaccatattggtcaggctggtcttgaacttctgacctcatgaag--------------- 12482
BCRP3.REF.NEW                                         tcaccatattggtcaggctggtcttgaacttctgacctcaggtgatctacccacctcagc 8464
                                                      **************************************** *

GGT1.end.-Cabin1.start.8575573-8602125.orangutan      ------------------------------------------------------------ 12482
BCRP3.REF.NEW                                         ctcccaaagtgctgggattacaggcgtgagtcaccacgcctagtgcatccatttttgta 8524


GGT1.end.-Cabin1.start.8575573-8602125.orangutan      -------------------------ttcttgtttctttatatccttgatatacatataaa 12517
BCRP3.REF.NEW                                         gttgccagttttctgatgaaattcttaattgtttctttatatccttgatatacatgtaaa 8584
                                                                               *  *************************** ****

GGT1.end.-Cabin1.start.8575573-8602125.orangutan      gtacttattttaaagtacatggtctgacgattttataatctggagatcctatgggccttt 12577
BCRP3.REF.NEW                                         gaacttattttaaagtacatggtctgatgattttataatctggagatcctatgggccttt 8644
                                                      * ************************* ********************************

GGT1.end.-Cabin1.start.8575573-8602125.orangutan      ttaaaagttgtctgtgctttctcttgagctttgttcctgctgtcttatttccttgtttgc 12637
BCRP3.REF.NEW                                         ttaaaagttgtctgtgctttctcttgagcttttttcctgctgtcttatttccttgtttgc 8704
                                                      ******************************** ***************************

GGT1.end.-Cabin1.start.8575573-8602125.orangutan      ttggttgtttttaatttggcaatggaagttgtgtataaaaattgtcctctccctcacccc 12697 12697
BCRP3.REF.NEW                                         ttagttgtttttaatttggcaatggaagttgtgtataaaaatcg---------------- 8748
                                                      ** *************************************** *
```

>GGT1.end.-Cabin1.start.8575573-8602125.orangutan .12697-14242 bp. Found in many genes Consists entirely of retrotransposons and a simple repeat

```
GGT1.end.-Cabin1.start.8575573-8602125.orangutan      ctcccctcccctcccctcccctccctcccctctccctctccgtctccctctccctc 12757
BCRP3.REF.NEW                                         ------------------------------------------------------------ 8748


GGT1.end.-Cabin1.start.8575573-8602125.orangutan      tccctctccccttacttcggtctccctctccttctttcttcgatctccctcttcttcggt 12817
BCRP3.REF.NEW                                         ------------------------------------------------------------ 8748


GGT1.end.-Cabin1.start.8575573-8602125.orangutan      ctccctctccttctttctttgatctccctctgttaccgaagctggactgtactgccgtga 12877
BCRP3.REF.NEW                                         ------------------------------------------------------------ 8748


GGT1.end.-Cabin1.start.8575573-8602125.orangutan      tctcagctcgctgcaacctccctgcctcgggctcctgtgattctcctgcctggcctactg 12937
BCRP3.REF.NEW                                         ------------------------------------------------------------ 8748


GGT1.end.-Cabin1.start.8575573-8602125.orangutan      agtgcctgggattgcaggcgcgtgccgccacacctgattggtttttgtatttttgtggag 12997
BCRP3.REF.NEW                                         ------------------------------------------------------------ 8748
```

```
GGT1.end.-Cabin1.start.8575573-8602125.orangutan      acggggtttcgccgtgttgaccagctggtctccagcttctggcctccagcttctggcctc 13057
BCRP3.REF.NEW                                         ------------------------------------------------------------ 8748

GGT1.end.-Cabin1.start.8575573-8602125.orangutan      cagtgatctgcctgcctcggcctcccgaggtgctgggattgcagacggagtctcgctcac 13117
BCRP3.REF.NEW                                         ------------------------------------------------------------ 8748

GGT1.end.-Cabin1.start.8575573-8602125.orangutan      tcaatgctcaatgttgcccaggctggagtgcagtggcgtgatctcggctcgctacaacct 13177
BCRP3.REF.NEW                                         ------------------------------------------------------------ 8748

GGT1.end.-Cabin1.start.8575573-8602125.orangutan      ccacctcccagccgcctgccttggcctcccaaagtgctaagattacagcctctgccctgc 13237
BCRP3.REF.NEW                                         ------------------------------------------------------------ 8748

GGT1.end.-Cabin1.start.8575573-8602125.orangutan      tgccaccccgtctaggaagtgaggagcgtctctgcctggccgcccatcgtctgggatgtg 13297
BCRP3.REF.NEW                                         ------------------------------------------------------------ 8748

GGT1.end.-Cabin1.start.8575573-8602125.orangutan      aggagcccctctgcccggccgccccgtctgggaagtgaggagcgcctctgcccggccgcc 13357
BCRP3.REF.NEW                                         ------------------------------------------------------------ 8748

GGT1.end.-Cabin1.start.8575573-8602125.orangutan      ccgtctggaaagtgaggagcgcctctgcccggccgccccgtctgggaagtgaggagtgcc 13417
BCRP3.REF.NEW                                         ------------------------------------------------------------ 8748

GGT1.end.-Cabin1.start.8575573-8602125.orangutan      tttgcctggccgccaccccgtcgaggaggaagtgaggagtgcctctgcccggccgccccg 13477
BCRP3.REF.NEW                                         ------------------------------------------------------------ 8748

GGT1.end.-Cabin1.start.8575573-8602125.orangutan      tctgggaggtgaggagcgcctctgcctggccgccccatctgggaggtgaggagcacctct 13537
BCRP3.REF.NEW                                         ------------------------------------------------------------ 8748

GGT1.end.-Cabin1.start.8575573-8602125.orangutan      gcctggccggcggccctgtctggggggtgaggagcgcctctgcctggccgccaccccatc 13597
BCRP3.REF.NEW                                         ------------------------------------------------------------ 8748

GGT1.end.-Cabin1.start.8575573-8602125.orangutan      tgggaggaagtgaggagcgcctctgcccggccgccccgtctgggaggtgaggagcgcctc 13657
BCRP3.REF.NEW                                         ------------------------------------------------------------ 8748

GGT1.end.-Cabin1.start.8575573-8602125.orangutan      tgcctggccgccccgtctgggaggtgaggagcacctctgcccggcggccctgtctgggag 13717
BCRP3.REF.NEW                                         ------------------------------------------------------------ 8748

GGT1.end.-Cabin1.start.8575573-8602125.orangutan      gtgaggagcgcctctgcctggccgccaccccgtctgggaggaagtgaggagcgcctctgc 13777
BCRP3.REF.NEW                                         ------------------------------------------------------------ 8748

GGT1.end.-Cabin1.start.8575573-8602125.orangutan      ccagctgccccatctgggaggtgaggagcatctctgcccggccgccccgtctgggaagtg 13837
BCRP3.REF.NEW                                         ------------------------------------------------------------ 8748

GGT1.end.-Cabin1.start.8575573-8602125.orangutan      atgagcgcctctgcgcggccgccccatctgggaggagaaattattctgccttgggaggct 13897
BCRP3.REF.NEW                                         ------------------------------------------------------------ 8748

GGT1.end.-Cabin1.start.8575573-8602125.orangutan      gttgatatatggccttgcccccagccccgtgctctctgaaacatgtgctgtgtcaactca 13957
BCRP3.REF.NEW                                         ------------------------------------------------------------ 8748

GGT1.end.-Cabin1.start.8575573-8602125.orangutan      gggttaaatggattaagggcggtgcaagatgtgctttgttaaacagatgcttgaaggcag 14017
BCRP3.REF.NEW                                         ------------------------------------------------------------ 8748

GGT1.end.-Cabin1.start.8575573-8602125.orangutan      catgctcattaagagtcatcaccactccctaatctcaagtaccagggacacaaacactgc 14077
BCRP3.REF.NEW                                         ------------------------------------------------------------ 8748

GGT1.end.-Cabin1.start.8575573-8602125.orangutan      ggagggccagagggccgcagggacctctgcctaggaaaaccagagacctttgttcatgtg 14137
BCRP3.REF.NEW                                         ------------------------------------------------------------ 8748

GGT1.end.-Cabin1.start.8575573-8602125.orangutan      tttatctgctgaccttctctccactattatcttatgaccctgccatatcccccctctccga 14197
BCRP3.REF.NEW                                         ------------------------------------------------------------ 8748

GGT1.end.-Cabin1.start.8575573-8602125.orangutan      gaaacacccaagaatgagcaataaatactttaaaaaaaaatttttttacaaataa-----t 14252    14242
bp
BCRP3.REF.NEW                                         ---------------------------------------------ttacaaataattttttt 8764
                                                                                                   **********     *

GGT1.end.-Cabin1.start.8575573-8602125.orangutan      tttttttttgagatggagtctggctttgttgcccaagctggagtgcaatgacatgatctc 14312
BCRP3.REF.NEW                                         tttttttttgagatggagtctcgctttgttgcccaagctggagtgcaatgacgtgatctc 8824
                                                      ******************** ****************************** *******

GGT1.end.-Cabin1.start.8575573-8602125.orangutan      ggctcactacaacctctgcatcccacggttcaattctcctacctcagcctcccaagtagc 14372
BCRP3.REF.NEW                                         ggctcactgcaacctctgcatcccagggttcaattctcctacctcagcctcccaagtagc 8884
```

```
                                                    ******** **************** **********************************

GGT1.end.-Cabin1.start.8575573-8602125.orangutan    tgggattacaggcaggtgccagcacgcctggctaatttttgtatttttagtagagatggg 14432
BCRP3.REF.NEW                                       tgggattgcaggcaggtgccagcacgcctggctaatttttgtatttttagtagagatggg 8944
                                                    ******* ****************************************************

GGT1.end.-Cabin1.start.8575573-8602125.orangutan    ttttcaccatgttggtcaggctggtctcaaactcctgacctcgtgatctgcccacctcag 14492
BCRP3.REF.NEW                                       tttttaccatgttggtcaggctggtctcagactcctgacctcgtgatctgcccacctcag 9004
                                                    **** ************************ ******************************

GGT1.end.-Cabin1.start.8575573-8602125.orangutan    cctcccaaagtgctggaattacaggcgtgagccactgcgcccagccagaaataattttta 14552
BCRP3.REF.NEW                                       cctcccaaagtgctgggattacaggcgtgagccactgcgcccagccagaaataattttta 9064
                                                    **************** *******************************************

GGT1.end.-Cabin1.start.8575573-8602125.orangutan    aaaataatttcgagccccagcatgatggctcatgcttgtaatcccatcactttgggaggc 14612
BCRP3.REF.NEW                                       aaaataattttgagccccagcatgatggctcatgcttgtaatcccatcactttgggaggc 9124
                                                    ********** *************************************************

GGT1.end.-Cabin1.start.8575573-8602125.orangutan    tgaggcaggcagattgcttgagcctaggagttcaagatcagcctgcgcaacatggtgaaa 14672
BCRP3.REF.NEW                                       tgaggcgggcagattgcttgagcctaggagttcaagatcagcctgtacaacatggtgaaa 9184
                                                    ****** ***************************************  *************

GGT1.end.-Cabin1.start.8575573-8602125.orangutan    ccccatctctacaaaaaataaaaaattagctgggt---gtggtggtgtgtacctgtagtc 14729
BCRP3.REF.NEW                                       ccccatctctacaaaaaataaaaaattagctgtgtgtggtggtggtgtgtgcctgtagtc 9244
                                                    ******************************** **   ************ *********

GGT1.end.-Cabin1.start.8575573-8602125.orangutan    ccagctgtttgggacgctgaggtgggaggcttacttgagcctgggtgatcgaggctgcag 14789
BCRP3.REF.NEW                                       ccagctgtttgggacgctgaggtgggaggctcacttgagcctgggtgatcgaggctgcag 9304
                                                    ******************************* ****************************

GGT1.end.-Cabin1.start.8575573-8602125.orangutan    tgagccatgatcctg-------actccagcctgggcaacagagtgagatgctgtctcaaa 14842
BCRP3.REF.NEW                                       tgagccatgatcctgagactgcactccagcctgggcaacagagtgagatgctgtctcaaa 9364
                                                    ***************       **************************************

GGT1.end.-Cabin1.start.8575573-8602125.orangutan    taaataaataaataaaaataaaataatttgaggcctaggagtctgaaattctgagatctc 14902
BCRP3.REF.NEW                                       taaataaataaataaaaataaaatactttgaggcctaggggtctaaaattctgagatctc 9424
                                                    ************************ ************** **** ***************

GGT1.end.-Cabin1.start.8575573-8602125.orangutan    ctttatgcatttgagtgactgagatgatctgaagctggatccagtgctcctgagggctgc 14962
BCRP3.REF.NEW                                       ctttatgcatttgagtgactgagatgatctgaagctggatccagtgctcctgagggctgc 9484
                                                    ************************************************************

GGT1.end.-Cabin1.start.8575573-8602125.orangutan    tttatttctggttgactgtgactcctagagtaagaaacctgcaccccatgtgtggggcat 15022
BCRP3.REF.NEW                                       tttatttctggttgactgtgactcctagagtaagaaacctgcaccccacatgtggggcat 9544
                                                    ************************************************  **********

GGT1.end.-Cabin1.start.8575573-8602125.orangutan    tatggcatcccctccctcagccacatgagtaagtcaacagcactgctctagaccaggtgt 15082
BCRP3.REF.NEW                                       tatggcatcccctccctcagccacatgagtaagtcaacagcactgctctagaccaggtgt 9604
                                                    ************************************************************

GGT1.end.-Cabin1.start.8575573-8602125.orangutan    ggtggctcacgcctatagtcctagctactcgggagactgaggcaggaggattgcttcagg 15142
BCRP3.REF.NEW                                       ggtggctcacacctatagtcccagctactcgggagactgaggcaggaggattgcttcagg 9664
                                                    ********** ********** **************************************

GGT1.end.-Cabin1.start.8575573-8602125.orangutan    ccaggaatttgagaccagccagagcaatatattattagattggtacaaaagtaattgcgg 15202
BCRP3.REF.NEW                                       ccaggaatttgagaccagccagagcaatatattattaggttggtacaaaagtaattgcag 9724
                                                    ************************************** ******************* *

GGT1.end.-Cabin1.start.8575573-8602125.orangutan    tttttgccattaaaagtaatggcaaccctgtttcagcaaaataaaaagcaaaaaaaaaaa 15262
BCRP3.REF.NEW                                       tgtttgcctttaaaagtaatggcaaccctgtttcagcaaaataaaaagcaaaaaaaaaaa 9784
                                                    * ****** ***************************************************

GGT1.end.-Cabin1.start.8575573-8602125.orangutan    aaaagaaaggaagaattaaaaacagaatcagctgggcgtggtggctcacgcctctaatcc 15322
BCRP3.REF.NEW                                       aaaaaaa---aaaaaagaaaggaagaatcagctgggcgtggtggctcacgcctctaatcc 9841
                                                    **** **   ** **  ***   *************************************

GGT1.end.-Cabin1.start.8575573-8602125.orangutan    cagcactttgggaggccaaggcgggcagatcatgagatcaggagatcgagaccatcctgg 15382
BCRP3.REF.NEW                                       cagcactttgggaggccaaggcgggcagatcatgagatcaggagatcgagaccatcctgg 9901
                                                    ************************************************************

GGT1.end.-Cabin1.start.8575573-8602125.orangutan    ctaacacggtgaaaccccatttctactaaaaatacaaaaaattatctgggagtggtggca 15442
BCRP3.REF.NEW                                       ctaacacggtgaaaccccatttctactaaaaatacaaaaaattagccgggcatggtggca 9961
                                                    ******************************************** * ***  ********

GGT1.end.-Cabin1.start.8575573-8602125.orangutan    ggcgcctatagtcccagctactcaggaggctgaggcaggagaatggcatgaacccaggag 15502
BCRP3.REF.NEW                                       ggcgcctgtagtcccagctactcaggaggcggaggcaggagaatggcatgaacccaggag 10021
                                                    ******* ********************** *****************************

GGT1.end.-Cabin1.start.8575573-8602125.orangutan    gtggagcttgcagtgagccgagatcatgccgctgcactccagcctgggtgacagagtgag 15562
BCRP3.REF.NEW                                       gtggaggttgcagtgagccgagatcatgccactgcactccagcctgggtgacagagtgag 10081
                                                    ****** *********************** *****************************

GGT1.end.-Cabin1.start.8575573-8602125.orangutan    actccgtctc----aaaaaaaaaaaagaatcactgctctgtctctcagcctcctcttcca 15618
BCRP3.REF.NEW                                       actccgtctcaaaaaaaaaaaaaaaaagaatcactgctctgtctctcagcctcctcttcca 10141
                                                    **********    **********************************************

GGT1.end.-Cabin1.start.8575573-8602125.orangutan    ggattggccgtcgccttgagggaagtgctggccttgcctgtctccagccctgtacttctc 15678
BCRP3.REF.NEW                                       agattggccgtcgccttgaggggaatgctggccttgcctgtctccagccctgtacctctc 10201
                                                     ********************* * ****************************** ****

GGT1.end.-Cabin1.start.8575573-8602125.orangutan    tgcctcctatgcctttaagcacgtgttttctatttgctgggctgtgaaatctgctcttca 15738
BCRP3.REF.NEW                                       tgcctcctatgcctttaagcacatgttttctatttgctgggctgtgaaatctgctcttca 10261
                                                    ********************** *************************************
```

```
GGT1.end.-Cabin1.start.8575573-8602125.orangutan      tctgatggggtttgctttataggtgactagatccttttctcttggtggttttagaatttg 15798
BCRP3.REF.NEW                                         tctgatggggtttgctttataggtgactagatccttttctcttggtggttttagaattcg 10321
                                                      ********************************************************** *

GGT1.end.-Cabin1.start.8575573-8602125.orangutan      cattttcacattgactttaaatagtctgaatatagtttgccacggcaaagaccctttgca 15858
BCRP3.REF.NEW                                         cattttcacattgaccttaaatagtctgattatagtttgccacggcaaagaccctttgca 10381
                                                      *************** ************* ******************************

GGT1.end.-Cabin1.start.8575573-8602125.orangutan      ttgcattgtttggggatatttgagcctcctctctctggatgtttaatctcttgttagatg 15918
BCRP3.REF.NEW                                         ttgcattgtttggggatatttgagcctcctctatctggatgtctaatctcttgttagatg 10441
                                                      ******************************** ********* *****************

GGT1.end.-Cabin1.start.8575573-8602125.orangutan      tgagtagttttcattattattttactaaataggt-------------------------- 15952
BCRP3.REF.NEW                                         tgagtagttttcattattattttattaatgggctggcatgtgggccgtggttccaggcag 10501
                                                      ************************ ***   * *
```

END OF IDENTITY WITH BCRP3 at 10476 bp at the TE/Alu/LINE.end.but no BCRP3

```
GGT1.end.-Cabin1.start.8575573-8602125.orangutan      ------------------------------------------------------------ 15952
BCRP3.REF.NEW                                         gctcagaggggcagctgcctgatgtctggacagcttctctttctgtcttttcttacctgg 10561

GGT1.end.-Cabin1.start.8575573-8602125.orangutan      ------------------------------------------------------------ 15952
BCRP3.REF.NEW                                         actctgggttgcttgttagctgcttctgccagttctgagttttcaaggggagaggggccc 10621

GGT1.end.-Cabin1.start.8575573-8602125.orangutan      ------------------------------------------------------------ 15952
BCRP3.REF.NEW                                         agtgatggctgttctttgaaggaaagggaagaatgtctcctgtttaacatgtttctatgt 10681

GGT1.end.-Cabin1.start.8575573-8602125.orangutan      ------ttttgtgtttttgtttttttttttgaggagtctcgctctgttgctcaggctgga 16006
BCRP3.REF.NEW                                         ttccagttacttggttagttagttagagccagggtctctcgctctgttgcgcaggctgga 10741
                                                            **   ** **   *  **        **  ************** *********

GGT1.end.-Cabin1.start.8575573-8602125.orangutan      gtgcagtgacttgatcttggctcactgcaacctctgcctcctgagtttaagtgattctcc 16066
BCRP3.REF.NEW                                         gtgcaatggcatgatcgtggctcacagcagcctccaccttccaggctcgagcaatgctcc 10801
                                                      ***** ** * ***** ******** *** ****  *** *   * *  **  ** ****

GGT1.end.-Cabin1.start.8575573-8602125.orangutan      tgcctcagcctcccaagtagctgggactacaggtgcccgccaccacgctcagc------- 16119
BCRP3.REF.NEW                                         cacctcagcctctcaagcagctgggactgcaggtatgtgccaccatgcttggctgccttt 10861
                                                        ********** **** ********** *****    ******* ***  **

GGT1.end.-Cabin1.start.8575573-8602125.orangutan      -taattttttgtgtttttagtagagatggggtttcactgtgttggccaggatggtctcta 16178
BCRP3.REF.NEW                                         ttaaatttttttttttttaatacagacaaggtctcactatattgcccaggctggtcttaa 10921
                                                       *** ***** * ****** ** ***   *** ***** * *** ***** ******  *

GGT1.end.-Cabin1.start.8575573-8602125.orangutan      tctcctgacctca--tgatctgcctgcctcagcctcccaaagtgctgggattacaggcat 16236
BCRP3.REF.NEW                                         actcatgggctcaagtgatactcctgcctcggcctttcaaagtgctgatatcacaggc-- 10979
                                                       *** **  ****  ****   ******** ****  **********  ** ******

GGT1.end.-Cabin1.start.8575573-8602125.orangutan      gagccaccgcgcctggcttattaaacaggttttaaaatcttttgatctctcttcacttt 16296
BCRP3.REF.NEW                                         ------------------------------------------------------------ 10979

GGT1.end.-Cabin1.start.8575573-8602125.orangutan      ccagaacaccaaaactttgaatatttggtcactttttattatcccaaatgtctggaaggc 16356
BCRP3.REF.NEW                                         ------------------------------------------------------------ 10979

GGT1.end.-Cabin1.start.8575573-8602125.orangutan      tttgttcatttttcttcttcatttttctccaactggattgttctaaaaaaactgtatttg 16416
BCRP3.REF.NEW                                         ------------------------------------------------------------ 10979

GGT1.end.-Cabin1.start.8575573-8602125.orangutan      agctctgagatttttctcctactttgcctagcctattgttgaaactttcaaacatattt 16476
BCRP3.REF.NEW                                         ------------------------------------------------------------ 10979

GGT1.end.-Cabin1.start.8575573-8602125.orangutan      tgtatttcttacaatgagttctccagtcccagaatttctgtttggtttttaaaaaaacat 16536
BCRP3.REF.NEW                                         ------------------------------------------------------------ 10979

GGT1.end.-Cabin1.start.8575573-8602125.orangutan      ctctctctccttggtgactttctcactcatatcctcaattgttttcctgaggtttttgta 16596
BCRP3.REF.NEW                                         ------------------------------------------------------------ 10979

GGT1.end.-Cabin1.start.8575573-8602125.orangutan      ttgttttcagatttctcttgcatctcattgtttttataaaatcaatttttttgagctgtt 16656
BCRP3.REF.NEW                                         ------------------------------------------------------------ 10979

GGT1.end.-Cabin1.start.8575573-8602125.orangutan      tatctgggatgtgaagaatttctttgttgaaatcctagtggtggagaattattgtgttct 16716
BCRP3.REF.NEW                                         -------------agggtttccattttttaaagctcccagcagtggtataaactcctcct 11026
                                                                   * *  ** * ** ** *** *     *  *  * ** *     * **

GGT1.end.-Cabin1.start.8575573-8602125.orangutan      tttggggtgtggtatttccttatgttttcatgattccagtgtatgttgatatggcacat 16776
BCRP3.REF.NEW                                         ttccagagaaagcgcactctgtccgcatccctcatgttgtcctctcctgcctctgcttag 11086
                                                      **   * *   *     **  *  *  * * * **       * *  **     **  *

GGT1.end.-Cabin1.start.8575573-8602125.orangutan      ctggtatacagtcacttcttcctattcttgaatttgctttccttttgtgggggagctggg 16836
BCRP3.REF.NEW                                         ggttcactccgggggaaagtgccacttgagagtttcctttttgtgtgtggttctgactga 11146
                                                           *  * *        * * * *   ** *** ****   * *****    *   *

GGT1.end.-Cabin1.start.8575573-8602125.orangutan      cttcttcctggacatgtgtctatggtgttggttgagtagggcactttggctttttttctg 16896
```

```
BCRP3.REF.NEW                                         ctgctccctgctcacagat----------------------------------------- 11165
                                                      ** ** ****  **    *

GGT1.end.-Cabin1.start.8575573-8602125.orangutan      ggtgcacgcagtagtgaattctctgtatgatttcattggctaataaatagcattagtgat 16956
BCRP3.REF.NEW                                         ------------------------------------------------------------ 11165

GGT1.end.-Cabin1.start.8575573-8602125.orangutan      atctgcagtctcctcggtgtgttagggtacagttatcagcagagctggttgtgcggttgt 17016
BCRP3.REF.NEW                                         ------------------------------------------------------------ 11165

GGT1.end.-Cabin1.start.8575573-8602125.orangutan      tctgggaactggattgccacataggcccatctcaggcttcagtggtagagatagtgggct 17076
BCRP3.REF.NEW                                         ----------------------------------------gctgattctcagggtggggt 11185
                                                                                                ** *    *  ***** *

GGT1.end.-Cabin1.start.8575573-8602125.orangutan      aaatgtgtctgtccttgtgccatggggcggcatacagtggcccctgtggt---------- 17126
BCRP3.REF.NEW                                         ccctgaggcctggagtgtggcctctgacgaccttcagggccaggtgtggaatgagagcct 11245
                                                         ** * *      **** * *  * ** * * *** * *   *****

GGT1.end.-Cabin1.start.8575573-8602125.orangutan      ------------------------------------------------------------ 17126
BCRP3.REF.NEW                                         gtggccacatggcccccggtgggagacgtcccgccgccctttgcttctctgtgccactct 11305

GGT1.end.-Cabin1.start.8575573-8602125.orangutan      ------------------------------------------------------------ 17126
BCRP3.REF.NEW                                         ggctgcacagttcagagccttgggaaatgttaaccagtaggacctagacggggaggtgag 11365

GGT1.end.-Cabin1.start.8575573-8602125.orangutan      ---gggtcaacttgttggtctctgagtggctttcgtggctgctggtctggtagcagtgga 17183
BCRP3.REF.NEW                                         aaggggtcaccccccaggtgtgcctgtggtgagccttcgtgctg-------agcaggtgc 11418
                                                         ****** *     *** *    ****    * *   *****       *****  *

GGT1.end.-Cabin1.start.8575573-8602125.orangutan      ctgcatggatgggcaggtacttaagacagtgggcagctggca--tggcatgggtcatgac 17241
BCRP3.REF.NEW                                         agggagggaggcccaggtgcacacacctgtgaagtaggggcagctggctgggctccttga 11478
                                                        * * *** *  ***** *  *   * ***       ****  ****  ** ** *

GGT1.end.-Cabin1.start.8575573-8602125.orangutan      agttgcagtgattaaatgctcttctggatccaatacactgatgttggcagtggttgtgtg 17301
BCRP3.REF.NEW                                         cctgctccagagcttcttattttctgg--ccacttcacctgcagaaggcccaggtggctg 11536
                                                        *      **     *  * ******  *** * ***        *     * **  **

GGT1.end.-Cabin1.start.8575573-8602125.orangutan      ggctgtgcatgccagcctccaggccagcagatggtacttgcacattggagtccgatgagg 17361
BCRP3.REF.NEW                                         tggcctctagggtcccttgcctgtcctcagctcccaactgggaggggcagagggaggagg 11596
                                                       *   *  * *    * * *  * *  *** *   *  **      * **   ** ****

GGT1.end.-Cabin1.start.8575573-8602125.orangutan      tggt-------------------------------------------------------- 17365
BCRP3.REF.NEW                                         gggtggagaccccaggcagcagggctctgggagcagtggggccctgggtcccaggggtgt 11656
                                                       ***

GGT1.end.-Cabin1.start.8575573-8602125.orangutan      -----------------------agtgggagggtattgagggccaacttcagtcttttga 17402
BCRP3.REF.NEW                                         ctggcaggcccctccttactctacgtctcggcctctggatggaggtgctggctgcagtcg 11716
                                                                              **    *  * * ** **      *   *    *

GGT1.end.-Cabin1.start.8575573-8602125.orangutan      gactctgcctctgggtcatgatttgggttgtgtaatcccaagg----------------- 17445
BCRP3.REF.NEW                                         ggctctgcctctgactaagggttggggaagtggcgggtgtgggctgctgccccgtggggc 11776
                                                      * ***********  * * * ** ***  ***         **

GGT1.end.-Cabin1.start.8575573-8602125.orangutan      ----------------------------accctgaatcctgcattctgtctcaagaggca 17477
BCRP3.REF.NEW                                         ctctgaacagaccccagggcctctgccaatcatgactccttcctttcagctggacccgca 11836
                                                                                  * * *** **** * **    **  *   ***

GGT1.end.-Cabin1.start.8575573-8602125.orangutan      gacaaagcaggtgaagccagacggggtgggctttcactcaggcccatcaattgtgactgt 17537
BCRP3.REF.NEW                                         ggccctgcag-----gacagagactggcagcgcgccgtcatcgccatgaatggggtacgt 11891
                                                      * *   ****     * ****    *   **   *  ***   **** *** * *   **

GGT1.end.-Cabin1.start.8575573-8602125.orangutan      gtgaaccagccctgattggcaggcaat--------------------------------- 17564
BCRP3.REF.NEW                                         gtccgtgggactctcctggcgcccacttcccccagaaggatagggtggcctctgttcatt 11951
                                                      **      * *     ****   ** *

GGT1.end.-Cabin1.start.8575573-8602125.orangutan      ------------------------------------------------------------ 17564
BCRP3.REF.NEW                                         tcaaatcagtcagaggtggctgagcctgaggcagcatctgagagggagcctggttggaga 12011

GGT1.end.-Cabin1.start.8575573-8602125.orangutan      -----------------gaacaatagtcaggccccaggctaaatctgtagatgagtgata 17607
BCRP3.REF.NEW                                         agggagggcccccaagagcagaatcaccatgcacgggaatcgtcattcattggctggaat 12071
                                                                       * * ***   ** ** *  *  *     *  *   *   **

GGT1.end.-Cabin1.start.8575573-8602125.orangutan      gcagcttccatgcagagcc-------------------------------actgccccag 17636
BCRP3.REF.NEW                                         gcagttgccagccaggccctgagcatccctcctcaaacaaaggtctcatggcaccaccag 12131
                                                      **** * ***  ***  **                                *  * ****

GGT1.end.-Cabin1.start.8575573-8602125.orangutan      gaggggtggggccacctacagtggccacagccatggcatgccggtggcggatgcacatcc 17696
BCRP3.REF.NEW                                         gacaggtggggcctccactcagggacctgggggctgcccatagaaatggagacccctgat 12191
                                                      **  ********* **      ** *   *     **     *     *    * *

GGT1.end.-Cabin1.start.8575573-8602125.orangutan      ttctcatggcccagtcctggcaggctttctactcagtccca------------------- 17737
BCRP3.REF.NEW                                         ttgtctttaggtaccccagaaaggtttagaccttaaaagcaatgacacacccaaaaaggc 12251
                                                      ** ** *     *  ** *  *** **    ** *    **

GGT1.end.-Cabin1.start.8575573-8602125.orangutan      ------------------------------------------------------------ 17737
BCRP3.REF.NEW                                         ccgggtataaatggtaaaatgttaatatttgagattcttggctttttcttacattattct 12311

GGT1.end.-Cabin1.start.8575573-8602125.orangutan      ------------------------------------------------------------ 17737
BCRP3.REF.NEW                                         gtctttccttcttaatttttaattgttactaagagaaagctggtcacagtacactataat 12371
```

```
GGT1.end.-Cabin1.start.8575573-8602125.orangutan      ------------------------------------------------------------ 17737
BCRP3.REF.NEW                                         ctcagctactctggaggctgagccaggagaatcactggagcccaagagtttgattacagc 12431

GGT1.end.-Cabin1.start.8575573-8602125.orangutan      ------------------------------------------------------------ 17737
BCRP3.REF.NEW                                         ctgggcaacattgcaagatcccatatctaaaaaaaaagcaagcaagcaagagaagcagcg 12491

GGT1.end.-Cabin1.start.8575573-8602125.orangutan      ------------------------------------------------------------ 17737
BCRP3.REF.NEW                                         gggattttaggaggtgcttctgcagaaaccagtcgtttatatcatcttcaacaatcctgg 12551

GGT1.end.-Cabin1.start.8575573-8602125.orangutan      ------------------------------------------------------------ 17737
BCRP3.REF.NEW                                         ctcttgctgaagtagactaggggcttccccgaggggcggctccacctcatgctgagacct 12611

GGT1.end.-Cabin1.start.8575573-8602125.orangutan      ------------------------------------------------------------ 17737
BCRP3.REF.NEW                                         ctgcatgccttgggggtggaaatatttgatgagactcccaggggtccttgggaccttggg 12671

GGT1.end.-Cabin1.start.8575573-8602125.orangutan      -------------------------gctttcacagcagacccaacggtctgtctagaccc 17772
BCRP3.REF.NEW                                         ctgtgaggaccagaaggattagaggactgtgccccttctccccactgtagatcgaagtaa 12731
                                                                                ** *  *      *** ** **   ** *

GGT1.end.-Cabin1.start.8575573-8602125.orangutan      agccttctgtgcatagctcccaactcagctccacgccataggagcccttgcctagctcag 17832
BCRP3.REF.NEW                                         agctctcggtcaa---gttcaacagcagggagttcagcttgaagaggatgccgtcccgaa 12788
                                                      ***  ** **  *    * * *   ***          * * **    ****   *  *

GGT1.end.-Cabin1.start.8575573-8602125.orangutan      gacagagtatcagcagcaacttgtatcttgttcaaaccccagtctggttctggtggtgcc 17892
BCRP3.REF.NEW                                         aacagacaggggtcttcggagtcaagattgctgtggtcaccaagtgagtggggaggggct 12848
                                                       *****       *  *    *  *  *** *     * *    **  *  ** ** **

GGT1.end.-Cabin1.start.8575573-8602125.orangutan      cactttccagcactgatggctgcagcctgtgccacacttgcttc---------------- 17936
BCRP3.REF.NEW                                         tgggctcacgcactgagggtgcctgtcccttcagctgtttctgcagaaaagagcatgtgt 12908
                                                           **  ******* **   * * *  * *  *  ** ** *

GGT1.end.-Cabin1.start.8575573-8602125.orangutan      ------------------------------------------------------------ 17936
BCRP3.REF.NEW                                         gggtctctcctctctgtgcatggccactgcacggtgaggtcaggccccagggaacacggc 12968

GGT1.end.-Cabin1.start.8575573-8602125.orangutan      ------------------------------------------------------------ 17936
BCRP3.REF.NEW                                         gtcttcagctacctcctgtgtttcctgcaaaccagctcaggaatgtccttgccaccttgc 13028

GGT1.end.-Cabin1.start.8575573-8602125.orangutan      ------------------------------------------------------------ 17936
BCRP3.REF.NEW                                         ttggaagcagtaggctggctccaggaactgcccaagtgcagggttttctgcccttgcttg 13088

GGT1.end.-Cabin1.start.8575573-8602125.orangutan      ------------------------------------------------------------ 17936
BCRP3.REF.NEW                                         gaattagtcacggtcccagattcctgttgaatggccataacccctgcccctttgtcacga 13148

GGT1.end.-Cabin1.start.8575573-8602125.orangutan      ------------------------------------------------------------ 17936
BCRP3.REF.NEW                                         gtcagttgccaagagaagcctgtttggtttgagagcagttcatgcagacatagaccactt 13208

GGT1.end.-Cabin1.start.8575573-8602125.orangutan      ------------------------------------------------------------ 17936
BCRP3.REF.NEW                                         cctctgagaattcatttgcttccccaggatggaatctggctgggcctctgaccttgctgg 13268

GGT1.end.-Cabin1.start.8575573-8602125.orangutan      ------------------------------------------------------------ 17936
BCRP3.REF.NEW                                         tcacgtgggccggggcctccatcagtcataccctggactcctatctgtgtctaaacacca 13328

GGT1.end.-Cabin1.start.8575573-8602125.orangutan      ------------------------------------------------------------ 17936
BCRP3.REF.NEW                                         cgccccacccccaactgcacggcagccactcgcatagcactctgggagggctgtgggcat 13388

GGT1.end.-Cabin1.start.8575573-8602125.orangutan      ------------------------------------------------------------ 17936
BCRP3.REF.NEW                                         gagcagcgaggactccatgagcagctcccccagataagccctgctaatgagggggcttgc 13448

GGT1.end.-Cabin1.start.8575573-8602125.orangutan      ---------------------------------------------ctagttccggctgca 17951
BCRP3.REF.NEW                                         gaagcagctttgatgtgctggtaaatccaggtgcaaaacagaactcaagttagggcctcc 13508
                                                                                                   * ****  ***  *

GGT1.end.-Cabin1.start.8575573-8602125.orangutan      agaacccatccctttttttttgagacggggtttcactcttgttgcc-------------- 17997
BCRP3.REF.NEW                                         gcacagcactgcgttctaactgtgaaggattcttactctagtgtcctgtgtggaggtatt 13568
                                                        *   **   * ** *   ** ** **  * * ***** **  **

GGT1.end.-Cabin1.start.8575573-8602125.orangutan      ------------------------------------------------------------ 17997
BCRP3.REF.NEW                                         ggaattgtccattgctaagactcagaggagaaaagcacttagcatcgcaggacttggagc 13628

GGT1.end.-Cabin1.start.8575573-8602125.orangutan      ------------------------------------------------------------ 17997
BCRP3.REF.NEW                                         accggtgctgaggcaacccttcattcattcgtcggatgtgtgttaaggcccagggcaggg 13688

GGT1.end.-Cabin1.start.8575573-8602125.orangutan      -----------------caagctgaagtgcaatggcacaatctccgctcactgcaacctc 18040
BCRP3.REF.NEW                                         gtcagggattctcctctcacacagcacgtgggtggcaggaccaacaccgggtctgacctc 13748
                                                                       **  * * *      *****  * *  * *    *   *****
```

```
GGT1.end.-Cabin1.start.8575573-8602125.orangutan      cacctcccggg------------------------------------------------- 18051
BCRP3.REF.NEW                                         ccagccgggggcacaggctgctaaccccaggcctggaatctgtcagatgcccttcctgtg 13808
                                                      *    *  ***

GGT1.end.-Cabin1.start.8575573-8602125.orangutan      ------------------------------ttcaagcgattctcctgcctcagcctcctga 18082
BCRP3.REF.NEW                                         ctgacttgacttagacaggcctcctgaccttcccgcaaaggtcatgtgtgattcgcaggg 13868
                                                                                ***  ** *   ** **  * *  * *  *

GGT1.end.-Cabin1.start.8575573-8602125.orangutan      gtagctggaattacaggcacccaccaccatgcccagctaatctttgcatttttagtaga- 18141
BCRP3.REF.NEW                                         gttctggccgcttgaaaggttcctgagaaagcacatgccatgaggacagagcttgcagag 13928
                                                      **    *    *  *      *   *  * ** **    **     **    * * ***

GGT1.end.-Cabin1.start.8575573-8602125.orangutan      ----gacagggtttcaccatgttggccaggctggtcttgaactcctgacctcaggcaatc 18197
BCRP3.REF.NEW                                         ggaggacaggcatgcagaaggctctgtgtgcagccccagacctgggtaccttcgtcaccg 13988
                                                          ******  * **  * * *       ** *  *  ** **    ****  * **

GGT1.end.-Cabin1.start.8575573-8602125.orangutan      cacctgcctcagccccccgttttgctatagctacctaagcttcagagaggatttgggatcc 18257
BCRP3.REF.NEW                                         tcctcaccccacctccgggtgtgcagatagggagcaggcctcctgtgttatggcccaagc 14048
                                                        *   ** ** * ** * * ***            * ** **        **     *  *

GGT1.end.-Cabin1.start.8575573-8602125.orangutan      agggcagtctcccttcctggagatgttacctcttgctgtctcccagcaggtccttaactg 18317
BCRP3.REF.NEW                                         ggggctg----------------------------------------------------- 14055
                                                       **** *

GGT1.end.-Cabin1.start.8575573-8602125.orangutan      agattcacagactgagaacatcaaagcgcactcccgttgcctggactgcacggtgccc-- 18375
BCRP3.REF.NEW                                         ---ttaggacactgagaacattccctcctcccgcaggagagaggtccaaggtgcccta 14112
                                                         **   * ***********     *   *  * *  *   *   * ** *******

GGT1.end.-Cabin1.start.8575573-8602125.orangutan      ----------------------cagagggaaagtggagtatagaacgtctctctcaccct 18413
BCRP3.REF.NEW                                         catcatgcgccagtgcgtggaggagatcgagcgccgaggcatggaggaggtgggcatcta 14172
                                                                            ***  **  *  ***    * * *       ** *

GGT1.end.-Cabin1.start.8575573-8602125.orangutan      ctgcctgtaatgaggagtcattcccagctcctggctgatctctac---caagtttggtga 18470
BCRP3.REF.NEW                                         ccgcgtgtccggtgtggccacggacatccaggcactgaaggcaggcttcaacgtcagtga 14232
                                                      * ** ***   * *  * **    ** *      ****   *      ***  *  ****

GGT1.end.-Cabin1.start.8575573-8602125.orangutan      ctcttttccttctcctccctacttctttgtgttttccatctcttc--tgttgagatctca 18528
BCRP3.REF.NEW                                         gtgtcggcctgcgcaggacgggatggaggtgtgggcagtggtgtccgcgatgagatctca 14292
                                                       * *   *** * *    *    *    ****   *  *    **   * **********

GGT1.end.-Cabin1.start.8575573-8602125.orangutan      tgttctctctagg------------ataatattcaaagtgtggttgtctgcacacttttt 18576
BCRP3.REF.NEW                                         gagtgctccatggcccaggcatgtcacatccttctctgtgtcttttcttcatttactgtt 14352
                                                         *    *  **             * *   ***   ****  **   *       * **

GGT1.end.-Cabin1.start.8575573-8602125.orangutan      cgattcttata-------agtaaatcagatgactatgaattgcttctaggcaaccatgtt 18629
BCRP3.REF.NEW                                         ttattattttaaaaaaagagaaaacaagagttgtacaaacagctctatagaagccagtt 14412
                                                        *** ** **       ** ***  ***    **  **  *******   ** *  ***

GGT1.end.-Cabin1.start.8575573-8602125.orangutan      aaaaaaa------aaaaaatgagccccttcagtaagaaccacaaggctcaccttgaact 18683
BCRP3.REF.NEW                                         tttacaccatcgtacccactcatgccacttggtggagtggaccaggggcttctgtgggga 14472
                                                         * *       *   * * *  **  **     **     ** **    *  **

GGT1.end.-Cabin1.start.8575573-8602125.orangutan      ctgcaatcccagagcaaactggaggcccagagaccctgattccagccccacgccttccct 18743
BCRP3.REF.NEW                                         cttggccttcctgccttggggggtggacaggaggtggaagcccaggactcagtgcggtctg 14532
                                                      **       *    *     ** ** *  ***          *  * * **   *   *

GGT1.end.-Cabin1.start.8575573-8602125.orangutan      gcagctttgccctccatgtgggtggtgtgaggctcttcccttgctggatcccagtggtca 18803
BCRP3.REF.NEW                                         tccac--tgccctgtatgaggatgtggtgggcagagggcactgatgaaattcagcgcagg 14590
                                                       *  *  ******  *** ** **  *** *       *  ** ** *   *** *

GGT1.end.-Cabin1.start.8575573-8602125.orangutan      gagtctctggacagtagacagacgcccttggctgagctcccttcagtcctggagctggca 18863
BCRP3.REF.NEW                                         ccggggctg--cagcatctccgcctccatctcaccaaccctcacaggccttgaaggaccc 14648
                                                        *   ***  *** *      *  ** *  *      **   *** *** **     *

GGT1.end.-Cabin1.start.8575573-8602125.orangutan      ggacacccccctacatcctagcgttaactccactctgccctccctgagtcactcctagaac 18923
BCRP3.REF.NEW                                         agactggcctcaaatgccaggg--gagggcactgagacccagagggtccttcccagcat 14706
                                                       ***   **  * ** * ** *   *   ****  * ** *   * ***  *** ** *

GGT1.end.-Cabin1.start.8575573-8602125.orangutan      ccttgtccttccccatacttatcataaagcttcttattaacattattctcatgtgtctg 18983
BCRP3.REF.NEW                                         cttcaaagcaacaggattttgtgcctgcagacccttctttgcagcacacaccacccaccc 14766
                                                      * *        *       *   * *  **   *** **  **  *  * *      *

GGT1.end.-Cabin1.start.8575573-8602125.orangutan      tgtcctgtagacccaaggggagaaagccttggtttgtgtgagttttgatgtgtgaggtct 19043
BCRP3.REF.NEW                                         tgaccagga-cccctagaatgcccagcatccctgggagggccctgtggtagtttcagctc 14825
                                                      ** ** * *  *** **       *** *   *  * * *   * ** *   *   *

GGT1.end.-Cabin1.start.8575573-8602125.orangutan      gaactaactgctgagtctcctgtggcccaatcaatggagtgcaggcagtagcacaacttg 19103
BCRP3.REF.NEW                                         cctctggggggcccagaatgaacctggcctgtggtgaggatgtaagcacca--atggccaa 14883
                                                         **    **  **  *      * **  *     *  ** * ***  *  *   *

GGT1.end.-Cabin1.start.8575573-8602125.orangutan      agggctacaatgatggacaccaatgtaaa------------------------------- 19132
BCRP3.REF.NEW                                         ttgggtccaaaggaagacaccggttcaaacactgaaaccaatcagattctcccacggcct 14943
                                                        ** * *** *   ******  *  ***

GGT1.end.-Cabin1.start.8575573-8602125.orangutan      ------------------------------------------------------------ 19132
BCRP3.REF.NEW                                         tcctgctatcagacgacactggtgcaggggtggttgctatgtacagggcagagccaccca 15003

GGT1.end.-Cabin1.start.8575573-8602125.orangutan      ------------------------------------------------------------ 19132
BCRP3.REF.NEW                                         atccccacgcaggcgctgtgtcctgccacgttggcctcctcctggccatcacatcaggcc 15063
```

```
GGT1.end.-Cabin1.start.8575573-8602125.orangutan      ------------------------------------------------------------ 19132
BCRP3.REF.NEW                                         aagcaggggagaggaatgggaatgcccacgcacccctatcaactctgcagacacagaacc 15123

GGT1.end.-Cabin1.start.8575573-8602125.orangutan      ----------------------------------------------------------ag 19134
BCRP3.REF.NEW                                         atgcacagctcttgggaggagtcagatgagctgctcaaagcccaggagggacccgcacag 15183
                                                                                                                  **

GGT1.end.-Cabin1.start.8575573-8602125.orangutan      tgttctgtgtttaagggatggtggctcaggatcaagcgggccccagggagggatagtctg 19194
BCRP3.REF.NEW                                         tggtcagtgtggcagggacggtgctttag------------ccaaggcagggatgg--tg 15229
                                                      ** ** ****   ***** ****  * **            ** *** ****** *  **

GGT1.end.-Cabin1.start.8575573-8602125.orangutan      ggagactcattctgcctcttaactactgctgctcccccacctacatacacgtatcaccca 19254
BCRP3.REF.NEW                                         ggtgactcactcaggatcttcaaggaggccgct---gcatttccgtgctgctcttccagata 15286
                                                      ** ****** ** *  **** *     ** ***    **  * * * * * *  **   *

GGT1.end.-Cabin1.start.8575573-8602125.orangutan      gagagtatgggcctgggatgaag------------------------------------- 19277
BCRP3.REF.NEW                                         acaaggacgtgtcggtgatgatgagcgagatggacgtgaacgccatcgcaggcacgctga 15346
                                                         ** * * * * * ***** *

GGT1.end.-Cabin1.start.8575573-8602125.orangutan      ------------------------------------------------------------ 19277
BCRP3.REF.NEW                                         agctgtacttccgtgagctgcccgagcccctcttcactgacgagttctaccccaacttcg 15406

GGT1.end.-Cabin1.start.8575573-8602125.orangutan      ------------------------------------------------------------ 19277
BCRP3.REF.NEW                                         cagagggcatcggtgagcactggaggccttggcctcatgggagacgtctcctccacgtgc 15466

GGT1.end.-Cabin1.start.8575573-8602125.orangutan      --------cttctgggccagggagaagaaggaatgaaaaatcgcttctgctctgtcacca 19329
BCRP3.REF.NEW                                         actgctgccctcggaggctgtgaaaagcgaggtgtgggaacctgagctgtaacccctctg 15526
                                                              * ** * * * * ** ***   *       ** *    ***      * *

GGT1.end.-Cabin1.start.8575573-8602125.orangutan      aaggaaatgttaattgtagtatgcctcatatccctgagcagcgtgtggcatcctgctttg 19389
BCRP3.REF.NEW                                         ccgtggtcggcattttaacccaacctcaaaaagcaggggaccagaaccgagcctgtcctg 15586
                                                        *     *  * **  *     ***** *   * * * * *       * ****   **

GGT1.end.-Cabin1.start.8575573-8602125.orangutan      aaaaaa------------------------------------------------------ 19395
BCRP3.REF.NEW                                         gaaggccttgcccatccccagagggctccccatccctactcctcaaggagaccaagaggc 15646
                                                       **

GGT1.end.-Cabin1.start.8575573-8602125.orangutan      ----------------------------------agaggattcattgagcatgtctttac 19421
BCRP3.REF.NEW                                         tgaaatagtcagcactgctgtgctatggggtcctaaagtctgctgtcctccttcctgcag 15706
                                                                                        * **  * *  *   * *  **  *

GGT1.end.-Cabin1.start.8575573-8602125.orangutan      tctttttcagaaatagggatagcaagagactatttgcatgggaacaatctaaagctagct 19481
BCRP3.REF.NEW                                         accagggctgaaggaggg-tgcctgggtgctcttgccatgggtcctggtccagccaagca 15765
                                                       *     * ***  **** *  *  *   ** **  ******  *      *  * ***

GGT1.end.-Cabin1.start.8575573-8602125.orangutan      agattttaattctaagaagggactaaaacacttccagtttgcagctaatatgtacagagc 19541
BCRP3.REF.NEW                                         tggtttcaaacatgacctgacccttagtca-----acctggaggctgatgtctagagcgg 15820
                                                       * *** **   * *   *   ** *  **      *  * *  *** ** * ** ** *

GGT1.end.-Cabin1.start.8575573-8602125.orangutan      tttaaggt---------------------------------------------------- 19549
BCRP3.REF.NEW                                         gtgctggtgcgtgcagcacctgtggcctctgcatcacccttagggcaggtctgcctcccg 15880
                                                       *   ***

GGT1.end.-Cabin1.start.8575573-8602125.orangutan      ------------------------------------------------------------ 19549
BCRP3.REF.NEW                                         ggcccatgcacagaggacctggtctcccagcctgcaggtgcccctgtggtgtccaggacg 15940

GGT1.end.-Cabin1.start.8575573-8602125.orangutan      -----------------------------------------------catcactcctgtc 19562
BCRP3.REF.NEW                                         acgagggggtctctgtgtacttggtggggctgggaccctcccacttcccacctccttgtg 16000
                                                                                                     *  *   * ***

GGT1.end.-Cabin1.start.8575573-8602125.orangutan      ctcacaacaacaaagctgaacaaactggaaatccacaactcttcttagatccatcagaga 19622
BCRP3.REF.NEW                                         tccctcactcccctgtttcattccatgctgagcctcccctgccttgggctccctggggag 16060
                                                        *   **  *   * *  *     **   * ** *  **    *  * *** *  *

GGT1.end.-Cabin1.start.8575573-8602125.orangutan      agtgagatcacagctgaaccactgctctgtgacttgcaaaga------------------ 19664
BCRP3.REF.NEW                                         ggggtggtggcaggagttgcccgagggcagctctgcccatgagcagctgctctagcggct 16120
                                                       * * *  ***  *   * *          **  * * **

GGT1.end.-Cabin1.start.8575573-8602125.orangutan      ---------------cagcagatgcagagaatcacagctcagctgagcagaggcctgtgt 19709
BCRP3.REF.NEW                                         cctcctgctgctgttcgccgggtgctgctgacccctgcgaggtagagaaaaggcgttcag 16180
                                                                     *  * * *** *   * * * **   *  *** * **** *

GGT1.end.-Cabin1.start.8575573-8602125.orangutan      tggagccagtacatgggcaggcaaatttcagaccgtaattggt--------gagttgcgg 19761
BCRP3.REF.NEW                                         gtggttcacaccccacacaggtgcccctcacagggtcctcactggcggccagcgctgtgg 16240
                                                        *   **   *     ****      *** *  **  *   *        * * ** **

GGT1.end.-Cabin1.start.8575573-8602125.orangutan      gaggctcagtgaggaccagcttgagaggtgaacactctggagggaggggctgttttacgg 19821
BCRP3.REF.NEW                                         gtgtgacgatgatgacaagcctaaactgcgcaaggactcgtgtcccgggcgctccatgtg 16300
                                                      * *   *  *** *** *** * *   * * *    ** * *    ****  *      *

GGT1.end.-Cabin1.start.8575573-8602125.orangutan      ggatcct----------------------------------------------------- 19828
BCRP3.REF.NEW                                         accacctcgggagaggtctccggcttgtcgtaacccaggggagtgacccactgcctcctg 16360
                                                          ***

GGT1.end.-Cabin1.start.8575573-8602125.orangutan      ------------------------------------------------------------ 19828
BCRP3.REF.NEW                                         cagctctttcagacccagttgcaaggaagagctgcatgctcaacctgttgttgtccctgc 16420

GGT1.end.-Cabin1.start.8575573-8602125.orangutan      ------------------------------------------------------------ 19828
```

```
BCRP3.REF.NEW                                         cggaggccaacctgctcaccttccttttccttctagaccacctggaaaggtagcccagct 16480

GGT1.end.-Cabin1.start.8575573-8602125.orangutan      ---------------------------------------------cacattttcatacat 19843
BCRP3.REF.NEW                                         ctcttgtggctgcccaggactccaggtctccaggccgttggggtgcccctctgctcccac 16540
                                                                                                    * * * * *   **

GGT1.end.-Cabin1.start.8575573-8602125.orangutan      ttaacctccaggaacaccccc attctcatgttctcagggtgaagataagagaaaactatc 19903
BCRP3.REF.NEW                                         cagacccccagcaccaaggaccttttcccccgacccctgtctgcagtaactcactgcttc 16600
                                                         *** **** * **    * ** **       *   **    *  *    *     **

GGT1.end.-Cabin1.start.8575573-8602125.orangutan      ctcgggcttccagcaggggga-gaaaaagggtaactgttttgaaatatgcccagagtatt 19962
BCRP3.REF.NEW                                         taaggactagcaccactgccacccccacccctgcctctcctctttgccaccctcctccct 16660
                                                         ** **  ** **  *  *     *    *  ** *  *        ***       *

GGT1.end.-Cabin1.start.8575573-8602125.orangutan      ctagtctccttactcaaaaatgagagctaagctatgaggatgcaaaggcgtaagacctgc 20022
BCRP3.REF.NEW                                         ctgcactgtggccttaacaaagagctcagagctttggccgtggccagcagt--------- 16711
                                                      **   **     ** ** ** ***  *  **** **    **   **  **

GGT1.end.-Cabin1.start.8575573-8602125.orangutan      aaattgggttgagtgtctactgctcaggagatgggtgcaccaaaatctcacacatcccca 20082
BCRP3.REF.NEW                                         ---------------------------gcacttggaccccctcttccctcccaagcaca 16744
                                                                                  *   * **  * **    ** * * **  * **

GGT1.end.-Cabin1.start.8575573-8602125.orangutan      tgaaagaacttactcatgtaaccgtgtaccacctgctcccccaaaacccctgaaaataaa 20142
BCRP3.REF.NEW                                         tcatgaagacctccccatcagcccagagctggccccttgtcctgggccactgagacccag 16804
                                                      * *   *     * *    * **  *  *   *  **   **    ** **** *   *

GGT1.end.-Cabin1.start.8575573-8602125.orangutan      aaata------------------------------------------------------- 20147
BCRP3.REF.NEW                                         aagtaccaaggctggagtcagcttgcagcacagccagggtcgaggtcactccctccctga 16864
                                                      ** **

GGT1.end.-Cabin1.start.8575573-8602125.orangutan      ------------------------------------------------------------ 20147
BCRP3.REF.NEW                                         ggactctagcacggcacagcccctctgcctctctcctggtggtggcgttgaaacagcacc 16924

GGT1.end.-Cabin1.start.8575573-8602125.orangutan      -----------tttttaaaaaagtgaaagataaaactgccatcaagaaaaactatttttg 20196
BCRP3.REF.NEW                                         ctctgcttcggtcctctacagggtggcagagaaggaggcggtca--ataaggtgtccctg 16982
                                                                 *  *  * *  ***  *** **    **  ***  * **  * *   **

GGT1.end.-Cabin1.start.8575573-8602125.orangutan      cagaacatcgcctctatcctttcaatctcaccaaaggggaaaactgagaggcactcctgg 20256
BCRP3.REF.NEW                                         cacaacctcgccactgtctttggccccacgctgctccggccctccgagaaggagagcaag 17042
                                                      ** *** ***** ** ** **     * * *      **    * **** * *   *  *

GGT1.end.-Cabin1.start.8575573-8602125.orangutan      ------------------------------aggccacagcacagggccttggactcccta 20286
BCRP3.REF.NEW                                         ctccctgccaaccccagccagcctgtcaccatgactgacagcaggtccttggaggtcatg 17102
                                                                                     * * *  *   **** *******   * *

GGT1.end.-Cabin1.start.8575573-8602125.orangutan      gaagacttatcacaggact-------------------------------agagaacact 20315
BCRP3.REF.NEW                                         tctcaggtatgggaagacagtctccagcccatgcaaccccagcctgacagaggtggcctc 17162
                                                          *  ***   * ***                              **    *

GGT1.end.-Cabin1.start.8575573-8602125.orangutan      ttcctccctccacaccacacctctccgtcctcaggacccctgtatat-tacaggaagcgc 20374
BCRP3.REF.NEW                                         tgcctgccccaccccccagtcctgcccatcttccgacttgcattgtatgtggtggtggctg 17222
                                                      * *** ** *  * ***  ***  ** ** ** *     *  * *** *   **  **

GGT1.end.-Cabin1.start.8575573-8602125.orangutan      aacagaaggaactgctaatctcagaccctgtgatgaagaagtct---------------- 20418
BCRP3.REF.NEW                                         agattcagagagagggacttgcctaggtttgcatggatgggagtgatagggggtgcccag 17282
                                                      *     **  *  *  * *  *  *   *   *** *   *  *

GGT1.end.-Cabin1.start.8575573-8602125.orangutan      -----------------ctagtgaaccccaaaacaatagggaagatgaaaacatgcccg 20461
BCRP3.REF.NEW                                         gccacctcctggtcctgctggtgcaccttgctgggggcttaaaaccaccccaagtgttcg 17342
                                                                       ** *** ***                **       *  **  **

GGT1.end.-Cabin1.start.8575573-8602125.orangutan      ggcgcagtggcccacgcttgtaatcccagcactttgggaggccgaggtgggcagatcacc 20521
BCRP3.REF.NEW                                         ggtgtggtggctcatgcctgtaatcccagcactttgggaggccga----ggcaggacaac 17398
                                                      ** *  ***** ** ** **************************    *****  ** *

GGT1.end.-Cabin1.start.8575573-8602125.orangutan      tgaggtcaggagttcaagaccagcctggccaacatggtgaaaccctgtgcctactaaaaa 20581
BCRP3.REF.NEW                                         tgaacccaggtgtttgagaccagtctgggcaatgtag-caaaccccatctctagaaaaaa 17457
                                                      ***   **** ***  ******* **** ***  * *  ******  *  ***  *****

GGT1.end.-Cabin1.start.8575573-8602125.orangutan      tacaaaaaattggctgggtgtggtgggggtgtgc--ctgtagtcctaagtactagggagg 20639
BCRP3.REF.NEW                                         tacaaagaaaaattagtcaggcattgtggcacacatctgtaatcctaggtatctgggagg 17517
                                                      ****** **      *   *   * * **    *  ***** ***** ***   ******

GGT1.end.-Cabin1.start.8575573-8602125.orangutan      ctgaggcaggagaattgcctgaacccaggtggcagaggttgcagtgagccaagatagtgc 20699
BCRP3.REF.NEW                                         ctgacacaggaggattgcttgagcccaggagttagaggctgcagtgatccatgatggagc 17577
                                                      ****  ****** ***** *** ****** *  ***** ******** *** *** * **

GGT1.end.-Cabin1.start.8575573-8602125.orangutan      caccgcactccagcctgggcaacagagagagactccgtctcaaaaaaaaaaaaaaaaagta 20759
BCRP3.REF.NEW                                         cactgtactccagcctgggggacagagcaaggccctgtgcatctcaaaataaataatca 17637
                                                      *** * *************  ******  *  ** * *  *     **** *** **  *

GGT1.end.-Cabin1.start.8575573-8602125.orangutan      ttgtagtggttccaccaccatgtaaccttggccgtccctttttcttctctctgcctcagtt 20819
BCRP3.REF.NEW                                         ------------------------------------------------------------ 17637

GGT1.end.-Cabin1.start.8575573-8602125.orangutan      tcctgcataaaatggataagatgaagacaatagcgctgcaagatttggaatggtacttgg 20879
BCRP3.REF.NEW                                         ------------------------------------------------------------ 17637

GGT1.end.-Cabin1.start.8575573-8602125.orangutan      tattaagcagagacttggtgtgggtcttttttttttttttgagacagagtctcacgctctg 20939
BCRP3.REF.NEW                                         ------------------------------------------------------------ 17637
```

```
GGT1.end.-Cabin1.start.8575573-8602125.orangutan      gctcaggctggagtgcagtggcacgatctccactcactgcaacttctgcctcctgagttc 20999
BCRP3.REF.NEW                                         ---------------------------ccccccacccaacaagtcatgccttgtcaggac 17670
                                                                                 * ** * * *  *** *  *****  * **  *

GGT1.end.-Cabin1.start.8575573-8602125.orangutan      aagcaactctcctgcctcagcctcccaagtagctgggattacaggcatgtgccaccatgc 21059
BCRP3.REF.NEW                                         ------------------------------------------------------------ 17670

GGT1.end.-Cabin1.start.8575573-8602125.orangutan      ccagttaatttttatacttttagtggagatggggttttcccatgttggccaggctggtct 21119
BCRP3.REF.NEW                                         ------------------------------------------------------------ 17670

GGT1.end.-Cabin1.start.8575573-8602125.orangutan      gaaactcctgacctcaggtgatccacctgcctcggcctccgaaaatgctgggattagagg 21179
BCRP3.REF.NEW                                         ------------------------------------------------------------ 17670

GGT1.end.-Cabin1.start.8575573-8602125.orangutan      aatgagccaccaagcccagcctgtttgttttgagacagggtctcactctgttgcccaggc 21239
BCRP3.REF.NEW                                         ------------------------------------------------------------ 17670

GGT1.end.-Cabin1.start.8575573-8602125.orangutan      tggagtgcagcagtgcgatcatggctcactgcagcctcaacctccctgggctcaggtgat 21299
BCRP3.REF.NEW                                         ------------------------------------------------------------ 17670

GGT1.end.-Cabin1.start.8575573-8602125.orangutan      cctcctacctcagcctcccaagtagctgagactacaagcatgggccactacacctggcta 21359
BCRP3.REF.NEW                                         ------------------------------------------------------------ 17670

GGT1.end.-Cabin1.start.8575573-8602125.orangutan      atttttatatttttttgtagagatggggtttcaccatgttgcccaagctggtcttaaactc 21419
BCRP3.REF.NEW                                         ------------------------------------------------------------ 17670

GGT1.end.-Cabin1.start.8575573-8602125.orangutan      ctgggctcaggagctccactcgcctcagcctcccaaagtgctgggattacaggtgtgaac 21479
BCRP3.REF.NEW                                         ------------------------------------------------------------ 17670

GGT1.end.-Cabin1.start.8575573-8602125.orangutan      caccatgccccgcctggtggtggtgatgtcaaggtgtgggggtgatgggagcccagtgcc 21539
BCRP3.REF.NEW                                         ------------------------------------------------------------ 17670

GGT1.end.-Cabin1.start.8575573-8602125.orangutan      gtaatcagttggtcagtgttggtagctggctcgctcccatgaagacccagctcctgtccc 21599
BCRP3.REF.NEW                                         -----------------------------------------cccaccccacccccgtctc 17689
                                                                                                  ****  * ** *** *

GGT1.end.-Cabin1.start.8575573-8602125.orangutan      tctgcctggggctcttgccttgtaccagtgtcatgctctcgtcttgtagtttttgagggt 21659
BCRP3.REF.NEW                                         actgtaaggggttcatgacaccagcaggggtttctagcacctgaggtggacttgggggct 17749
                                                       ***   **** ** ** *     *  * **      * * *   ** *  ** * ** *

GGT1.end.-Cabin1.start.8575573-8602125.orangutan      caggaatttacaatggcttaacagggtgaagttggctcaggtct--cccacgtggttgca 21717
BCRP3.REF.NEW                                         tgggccccaaagacctccccaccagcagctgtgagccccccctctgagccactctcctctt 17809
                                                        **     *  *   *   **  *  *  **  ** *   ***   ****     *

GGT1.end.-Cabin1.start.8575573-8602125.orangutan      ctcaagctatcatcagtgcatctgagctcccttggggtgttggcagcctcaggtcctcac 21777
BCRP3.REF.NEW                                         ccccactctgcgagggcaggacgaggtgctgctgtacttcttgcggctggaggccatccc 17869
                                                      * * *     *    *     *   *  *   **   *  * ** **   *** * ** *

GGT1.end.-Cabin1.start.8575573-8602125.orangutan      tg--gttattggccagaggcctcggctccttctgtgggcctttctataaacagctgagtg 21835
BCRP3.REF.NEW                                         tgccctgaacagcaagagacagagcatcctgttctccaccgatgtctaaaggtcccagtc 17929
                                                      **   * *   ** **** *   *  ****  * *   **  * * ****   *  ***

GGT1.end.-Cabin1.start.8575573-8602125.orangutan      tccctggatcgtggtgactggttctccccagagccat---tgaccgcaccgggagaacac 21892
BCRP3.REF.NEW                                         catctcctggaggcggacagatggcctggaaacctctggctaatcgggccatctgtagag 17989
                                                         **       *  *** * *   *   * * *  *   * * **  **    * * *

GGT1.end.-Cabin1.start.8575573-8602125.orangutan      agaggaagccacggtgtcttttataacctaatctcagcagcaactgagatctctgtaggc 21952
BCRP3.REF.NEW                                         tg-ggaatcaagatttctgaggcatccttgggccacccccaggtgtcaggccatctgcc 18048
                                                       * **** * *   * ***     * ***     ** *  **  **  *   *    * *

GGT1.end.-Cabin1.start.8575573-8602125.orangutan      cacacacacctcc------ctggggtgtgggaaggcctggtggatgccagggccaggggt 22006
BCRP3.REF.NEW                                         aagagacagcggcccaaagcagaaggacaggtggcctgggcagatcccgcccaggtctga 18108
                                                       * * *** *  *      * *  *    **  * *  **  *** **          *

GGT1.end.-Cabin1.start.8575573-8602125.orangutan      agcggagtagggggctagaggctgcctgctttccctctgcctgcctgtcccaccatcaag 22066
BCRP3.REF.NEW                                         aagccccaggctggcctcagactgtgggtttttttatgtggccacccgagggcgccccaag 18168
                                                      *        *  ***   ** ***   * ***   * ** *  ** *      *  ****

GGT1.end.-Cabin1.start.8575573-8602125.orangutan      caggccaaagctgtaattcacgtccttctctcctctaactgttagatctaagaaagagga 22126
BCRP3.REF.NEW                                         ccagttcatctcggagtccaggcctggccctgggagacagggt--------gaaagcagt 18220
                                                      *  *   *    * * * ** * *   * **     *   * *        *****  *

GGT1.end.-Cabin1.start.8575573-8602125.orangutan      tctgtttcctagcataccttcttcagcctggagagttgatccttttctattcccattccc 22186
BCRP3.REF.NEW                                         ggtttttatgaacttaacttatagagtccaaaagatttct-------------------- 18260
                                                        * ***   * * ** *** *  ** *   *   **  *

GGT1.end.-Cabin1.start.8575573-8602125.orangutan      aaaaccaaattaaatggccttttgacaggatggaactcttgaatgacaccaggggcaggg 22246
BCRP3.REF.NEW                                         --------actgaatcacttgtcaagaagcgccctctctggggagaagggaacgtgactg 18312
                                                              * * ***  * * *  * * *      **** *   **    *  *  *  *

GGT1.end.-Cabin1.start.8575573-8602125.orangutan      ggcggccctcagctgagaatgtgcaggcctggcctcttcctaat-------ggcccctgc 22299
BCRP3.REF.NEW                                         gat-tccctcactgttgtatcttgaataaacgctgctgcttcatcctgtgggggccgtgg 18371
                                                      *    ******     * ** *  *      **  ** * * **       ** ** **
```

```
GGT1.end.-Cabin1.start.8575573-8602125.orangutan      actcggcacttcctgagcagccccgctccgcttccatacctgcctgcctcagggctgccg 22359
BCRP3.REF.NEW                                         ccctgtccctgtgtgggtggggcctcttccatt-------tccctgacttagaaaccaca 18424
                                                       *  * * **   ** *  *  ** ** *  **       * **** ** **      *

GGT1.end.-Cabin1.start.8575573-8602125.orangutan      cagatccgcaagcagagggcgtgtggctggccatgttggagagggcgtcccttgagggtc 22419
BCRP3.REF.NEW                                         ctccacttctaacagggtttgagaggcttggtcagcactgggtagcgttttgactccatt 18484
                                                      *    *  * * *** *   * * **** *    *     *   ****          *

GGT1.end.-Cabin1.start.8575573-8602125.orangutan      ctcagagctttctggggtggtagcacagccaagctgaaacaggcatgtctccccagatcc 22479
BCRP3.REF.NEW                                         cttggctttcttctttttctttccagaaggattttgtgcagaaatgggtcttttgttgc 18544
                                                      **  *   * *      *  *  ** *   *   *    ***  ***  **    * * *

GGT1.end.-Cabin1.start.8575573-8602125.orangutan      catcccacaaactctagaaaggtgtaagcaccaggaacaagctggccccagcagggtcca 22539
BCRP3.REF.NEW                                         cgtgttagtcctccttggaagg-----cagctcagaaggcctgtgaaatgtcgggggaca 18599
                                                      * *   *      ** * ****        *   ***       *      * ***  **

GGT1.end.-Cabin1.start.8575573-8602125.orangutan      ggcatcccaggcacgctgcaactggctgctgcaggggatggcaggttctgcctgcagagg 22599
BCRP3.REF.NEW                                         ggacccccagggagggaatcccaggctac-gcaccttagggttcgttctccagggagagc 18658
                                                      **   ****** * *      * **** * ***    * **   ***** *  * ****

GGT1.end.-Cabin1.start.8575573-8602125.orangutan      cgtttaccgaccggagagtgtccaggcctggagccacagctggtctggggaggaggctgg 22659
BCRP3.REF.NEW                                         gacctcgtcccccgatcctgaccgcccttccggcccacgct------------------- 18699
                                                          *     ** **   ** **   * *   ***   ***

GGT1.end.-Cabin1.start.8575573-8602125.orangutan      gagccagggctgaacaatggctgagccacctgggaggacagatttgaggatggattctct 22719
BCRP3.REF.NEW                                         ------------------------------------------------------------ 18699

GGT1.end.-Cabin1.start.8575573-8602125.orangutan      caggacactgtactcaacgtgtgagacagccaggagggcaggtgcagtggggtccctgga 22779
BCRP3.REF.NEW                                         ------------------------------------------------------------ 18699

GGT1.end.-Cabin1.start.8575573-8602125.orangutan      gggtgctgttcccagggtgggcagccctgcctccagtgggagggtggctgcaggaggcag 22839
BCRP3.REF.NEW                                         ------------------------------------------------------------ 18699

GGT1.end.-Cabin1.start.8575573-8602125.orangutan      gcagggtgctctcaggtgcccctggtccctagagaagagctcacccatctgcccaggctg 22899
BCRP3.REF.NEW                                         ------------------------------------------------------------ 18699

GGT1.end.-Cabin1.start.8575573-8602125.orangutan      tgctgaccaggccgtggccccgtgcagcatttgcacccagagagctcggggtcagccagg 22959
BCRP3.REF.NEW                                         ------------------------------------------------------------ 18699

GGT1.end.-Cabin1.start.8575573-8602125.orangutan      gctctcatgtccccacaggggccctggcactgcctgtccaggcatctctctcctctgggc 23019
BCRP3.REF.NEW                                         ------------------------------------------------------------ 18699

GGT1.end.-Cabin1.start.8575573-8602125.orangutan      atgtgcttatctcctttgccaggtgcctgcaagccacaggccaggacctggttccatgaa 23079
BCRP3.REF.NEW                                         -------------------ctcctgtttggcttccacaggcctggacttctct------- 18733
                                                                         *   **  **    ********* **** *   *

GGT1.end.-Cabin1.start.8575573-8602125.orangutan      cagcagctagaacagtgcctggccttagatgaggaagacccctctctctgaggcaatcag 23139
BCRP3.REF.NEW                                         ------------------------------------------------------------ 18733

GGT1.end.-Cabin1.start.8575573-8602125.orangutan      gccatctagagtgaggggagtggagcaaggagggagggtggaacaggggttcccaagtca 23199
BCRP3.REF.NEW                                         ------------------------------------------------------------ 18733

GGT1.end.-Cabin1.start.8575573-8602125.orangutan      agactcaagtaaggtcaccacccctgagcatggtgaaggggggctggaatgatggccttgt 23259
BCRP3.REF.NEW                                         ------------------------------------------------------------ 18733

GGT1.end.-Cabin1.start.8575573-8602125.orangutan      gcaggtccctagacgccaggggggagacagagccaagcccaggagcttccagggccagccc 23319
BCRP3.REF.NEW                                         ------------------------------------------ggcttctctgcccacaca 18751
                                                                                                 *****   * ***  *

GGT1.end.-Cabin1.start.8575573-8602125.orangutan      agacctggcacctggctcatgggtctgccccgaggagcagggatgtagcaggacgagccg 23379
BCRP3.REF.NEW                                         ctccctgcccccagtgtccctgcccctgccccagcacaggtgacttcatttctgtcctct 18811
                                                         **** * ** *  **   *  *   *** ** *   * **  *            *

GGT1.end.-Cabin1.start.8575573-8602125.orangutan      cagacaaaacctctcagacaccaagttgtaaaaggaagggctttattcagctgggagcat 23439
BCRP3.REF.NEW                                         cagctcagtggactcgctcatctt-ttgtataag---------tctccacttggtggcag 18861
                                                      ***   *     ***   ** *   ***** ***         * * **  ***  ***

GGT1.end.-Cabin1.start.8575573-8602125.orangutan      cggcaagctactgccttaaaatctgagctccctgagtgcacaatttctgtctcttttaag 23499
BCRP3.REF.NEW                                         cagcttgctgatgactt------------------------------------------- 18878
                                                      * **  ***  ** ***

GGT1.end.-Cabin1.start.8575573-8602125.orangutan      ggctcacaacattaaagattttacatgaaagggtcgtgattgatttgagtaagcaagggg 23559
BCRP3.REF.NEW                                         ------------------------------------------------------------ 18878

GGT1.end.-Cabin1.start.8575573-8602125.orangutan      tacgtgacaggggctgcatgcaccggtggccagagagaaacagaacagggcagggagttt 23619
BCRP3.REF.NEW                                         --------------------------------------------------------gttt 18882
                                                                                                              ****

GGT1.end.-Cabin1.start.8575573-8602125.orangutan      cacaacgtttttctatacaatgtctggaatctatgaataacatcggtttctaagttatga 23679
BCRP3.REF.NEW                                         taaaactttcatcctaaataaccttttgatacttgaata-------tttttaagttttat 18935
                                                       * *** **  **   *  *    *   **   ******       *** ****** *
```

```
GGT1.end.-Cabin1.start.8575573-8602125.orangutan      gttgatttttaactactgggtttgggccaggcaggcccaggcctggtttcaggcctggtg 23739
BCRP3.REF.NEW                                         acatagtttctaat---------------------------------------------- 18949
                                                          * ***  * *

GGT1.end.-Cabin1.start.8575573-8602125.orangutan      ccgggctgcctgtctttggttttatttccttgttgtttttcttaaagcaggtactgagt 23799
BCRP3.REF.NEW                                         -----------------------------------ttttttccgaacagatccagatacc 18974
                                                                                         *******  **   *   *   *

GGT1.end.-Cabin1.start.8575573-8602125.orangutan      ataaaacaatatagaataatatgagagggtcttcctcttccttcaggga----------- 23848
BCRP3.REF.NEW                                         taataagatgctggaatgtaatccctggacaatccgtgtcctggcagcatttggtcttcc 19034
                                                        * ** *   * ****   **    **    ***   ****    * *

GGT1.end.-Cabin1.start.8575573-8602125.orangutan      ------------------------------------------------------------ 23848
BCRP3.REF.NEW                                         tctaagcgcctggctccgctgttctcaggagtgggttctgaagtctctggagaacaggat 19094

GGT1.end.-Cabin1.start.8575573-8602125.orangutan      -------------------------------------gagttgccccagtggcacaggat 23871
BCRP3.REF.NEW                                         acgtggagggttaggaaggggccaggcctagagacgggagactccctcccggagcaggtg 19154
                                                                                           ***   ***    **  ****

GGT1.end.-Cabin1.start.8575573-8602125.orangutan      tttggcctggccacttgcttcccacagataactcaagtcgtgggggagctccagacagcc 23931
BCRP3.REF.NEW                                         gaggcacaggaccattcgctaccccatctgccggcacctgcgggggagc-ccaggcattc 19213
                                                         *  * ** *  **   * ** **  *  *   *   * ******** **** **  *

GGT1.end.-Cabin1.start.8575573-8602125.orangutan      cttttctgcaaactggaccccccaggt--------------------aggcccctcccctc 23971
BCRP3.REF.NEW                                         tttgtaagccctcctgaccacctggctcaaagaaaacagaagcatggaggccgccaagta 19273
                                                       ** *  **   *  **** ** **                       * **  *   *

GGT1.end.-Cabin1.start.8575573-8602125.orangutan      aggcccccacatagtgctcctcacagggcggccgggtcctgggcaacaggactgagaaaa 24031
BCRP3.REF.NEW                                         ttttcaagaaataatcccatgaacatggcatcactttttttagaaagaggggcttggggca 19333
                                                          *   * *** * *     *** ***  *    *  *  *  *   ** **  *   *

GGT1.end.-Cabin1.start.8575573-8602125.orangutan      ggcctgctc---agtgggtgtgtgtccagccccggtgagccctgacctgcctgcaggagg 24088
BCRP3.REF.NEW                                         ggcagaggagagaagggagatcaaactgagagccaagtttccagacggtcctgcaggagg 19393
                                                      ***          *  **   *    *      *   *   ** ***   ***********

GGT1.end.-Cabin1.start.8575573-8602125.orangutan      tgggggagcggctgggaggatggcaggagga-ggcttctgaggaactcaaccttgtcaga 24147
BCRP3.REF.NEW                                         agaggatgcagctgcccagagggaagcaggatcacatttaaggaagtgtgtggggtccct 19453
                                                       * **  ** ****    ** ** ** ****   * * * ***** *       ***

GGT1.end.-Cabin1.start.8575573-8602125.orangutan      cattgtttcaggggctggacctgggaatgacagtgagcataagagggagtggcccaaggg 24207
BCRP3.REF.NEW                                         ggatgacaccagcacccagtgcggctctgtctggcaaccgctcccaaggtggcaggagtg 19513
                                                         **   *  *  *       **   ** * *  * *          *****   ** *

GGT1.end.-Cabin1.start.8575573-8602125.orangutan      gatgggggcttgagacaaggcctcagccagcatggggccagcaggtgactgctgggctag 24267
BCRP3.REF.NEW                                         ggtgtcccctgtgtgtcagtgggcagctcctgctgaacccacagctcactggggagcctg 19573
                                                      * **    **       **    ****       *  **  *** * ****  * **  *

GGT1.end.-Cabin1.start.8575573-8602125.orangutan      gcagaggcctcagcagccatggccatgcccattgggctgctctgtctgaggctcctgccc 24327
BCRP3.REF.NEW                                         acagtgg-------------ggccatgtgcctgacactcctctctgcttgtggacctggc 19620
                                                       *** **              *******  * *    ** **** *    *    *    *

GGT1.end.-Cabin1.start.8575573-8602125.orangutan      caggctggctgaggccttgtcactcctgctgaccaaggagaggagctggctcccgctgct 24387
BCRP3.REF.NEW                                         aaggcag----------------------------------------------------- 19627
                                                       **** *

GGT1.end.-Cabin1.start.8575573-8602125.orangutan      ggagaggacggagggcctcctggcagaggagcgggatggggaggcgggggctggaaaggc 24447
BCRP3.REF.NEW                                         ggagcagaaaacagagctacttgaaggctttctgtctgcgtctgtgtgcagtgtggattt 19687
                                                      ****  **     *  ** ** * **     * *  ** *   * * *   **   *

GGT1.end.-Cabin1.start.8575573-8602125.orangutan      agtggttgatgaccagtgtggtgggagagtccaggcctgagtgagaagctggggagcagc 24507
BCRP3.REF.NEW                                         agttgtgcttttttac--ttgctgggagagcacagccaccatttacaagcagtgtcac--- 19742
                                                      *** **   *        ** ********  *** *   * * * **** * *   *

GGT1.end.-Cabin1.start.8575573-8602125.orangutan      aagggaagcgagggccgtgtggagggtggaggcagggcctggaggcctgggaaccgggga 24567
BCRP3.REF.NEW                                         ------------------------------------------------------------ 19742

GGT1.end.-Cabin1.start.8575573-8602125.orangutan      ccgatgctgagtaatcacctggccagtagccacaggcacagcatttgcccctcaggccca 24627
BCRP3.REF.NEW                                         ------------------------------------------------------------ 19742

GGT1.end.-Cabin1.start.8575573-8602125.orangutan      tcccggccttggcccaactgagcccaattcctggcccctgcctcaggacagggaaggtgt 24687
BCRP3.REF.NEW                                         ------------------------------------------------cctcgtgggtgg 19754
                                                                                                      *   *  ****

GGT1.end.-Cabin1.start.8575573-8602125.orangutan      agctggctgggcaggagccacagtgccagcttccactttggtcttagttggattgtttgg 24747
BCRP3.REF.NEW                                         cgaggacagaacaggagcctctgctctctgtacctatctgggcccggtgggctcccttgt 19814
                                                       *  * * *  ******** * *  *    * **  * *** *   ** ** *   ***

GGT1.end.-Cabin1.start.8575573-8602125.orangutan      gttttggtcctgacctcgtttctgaatgctgcttatgtgagctggag-----ggagagtg 24802
BCRP3.REF.NEW                                         cctggcttccatctctgtctcagcgaccattcagccctgcacaggaacacatgttgctta 19874
                                                        *    ***     **   *     *   * *     **  * ***      *  *  *

GGT1.end.-Cabin1.start.8575573-8602125.orangutan      gacaccgccccccccatcccctctcactgccttgtgatgagatctta-------------- 24848
BCRP3.REF.NEW                                         gaaaagccaaatccagcccttgtctctgcctcctctggtctcatgatgtgcatctgttac 19934
                                                      ** *   *    *** *** * ** ******  *   *     * *

GGT1.end.-Cabin1.start.8575573-8602125.orangutan      ------------------------------------------------------------ 24848
BCRP3.REF.NEW                                         cttgaaactggaaaccagtctatcaatgtctgtgccaatttttttattccctccccaacct 19994

GGT1.end.-Cabin1.start.8575573-8602125.orangutan      ------------------------------------------------------------ 24848
```

```
BCRP3.REF.NEW                                         ccttccccatacgactttttatttatgtaggatgtgtgctgtctaatgatgggatgacca 20054

GGT1.end.-Cabin1.start.8575573-8602125.orangutan      ------------------------------------------------------------ 24848
BCRP3.REF.NEW                                         cacttttccatgttctaaaagtgctcctctcccacagggtcccagggctggtggttgctt 20114

GGT1.end.-Cabin1.start.8575573-8602125.orangutan      ------------------------------------------------------------ 24848
BCRP3.REF.NEW                                         tgggtctacagctacgtcttacccgcctcctgcctcaacagcctgtgtggtggcaaagcc 20174

GGT1.end.-Cabin1.start.8575573-8602125.orangutan      ----------------------------------------------------------ga 24850
BCRP3.REF.NEW                                         ggtgtggggctggggaacgcagcgttctccaggaggggacccggctctccttctgcagtg 20234

GGT1.end.-Cabin1.start.8575573-8602125.orangutan      gaggctgggccttgaagactcaggtgtggccccactgggtcaggtgtacagccctctcgg 24910
BCRP3.REF.NEW                                         caggcgaaggcctagatgccagtgtgacctcccacaaggcgtggcttccagactccccgg 20294
                                                       ****   * * *  *  *    ***    *****  **   **  * *** *  * ***

GGT1.end.-Cabin1.start.8575573-8602125.orangutan      gaggcggaggagccctctgcgggtggggagccagcccctaccagcgtc-----ctcttgg 24965
BCRP3.REF.NEW                                         ccggaagtgatgctttttgccgcgggccctgggtttgaagcagcctggctttctcttgg 20354
                                                        **  * *  **  * *    * ***      *     * **** *      *******

GGT1.end.-Cabin1.start.8575573-8602125.orangutan      gtggtgagtggtttcttggcagcaccaacctgggccgccctgtggaggggagagccgcct 25025
BCRP3.REF.NEW                                         taagtggctggtgtcttagcagctgcaatctgagct---------------------cag 20393
                                                         ***  **** **** *****  *** *** **                      *

GGT1.end.-Cabin1.start.8575573-8602125.orangutan      cagccaggaccccagcgttggccacactcccctgcccagtgtcccagggcacaccgtggg 25085
BCRP3.REF.NEW                                         ccacctacacaccaccgtggccgacactttcattaaaagtttcctgagacga------- 20446
                                                      *  **   ** *** *** * * *****  * *    *   * ** * *   *

GGT1.end.-Cabin1.start.8575573-8602125.orangutan      cccataggacctggcacagatgcataggagcctgcggggtggatgagtccattacctgca 25145
BCRP3.REF.NEW                                         ------------------------------------------------------------ 20446

GGT1.end.-Cabin1.start.8575573-8602125.orangutan      gtgagcctgtgtccccacagagggagaagagctggcgagagtggcagagggcaccggctt 25205
BCRP3.REF.NEW                                         ------------------------------------------------------------ 20446

GGT1.end.-Cabin1.start.8575573-8602125.orangutan      cccaccccaggagccacggcacagtccgcaggtgaagacggcccccacaagttccccggc 25265
BCRP3.REF.NEW                                         ------------------------------------------------------------ 20446

GGT1.end.-Cabin1.start.8575573-8602125.orangutan      agagccacactgctggccggcagaggctgccccgggcacaggcgccgagcccacctgcag 25325
BCRP3.REF.NEW                                         ------------------------------------------------------------ 20446

GGT1.end.-Cabin1.start.8575573-8602125.orangutan      ccagggtaaggcgagctggaagcagcccagcaccagcccccaggaggctgcctcacagct 25385
BCRP3.REF.NEW                                         ------------------------------------------------------------ 20446

GGT1.end.-Cabin1.start.8575573-8602125.orangutan      ggcagccagggtgggtttcctggcctggccttgaagggcagggcctgcggatgccccatg 25445
BCRP3.REF.NEW                                         ------------------------------------------------------------ 20446

GGT1.end.-Cabin1.start.8575573-8602125.orangutan      gaatgagatggggaggaaatgggggtagtgcaggccggacaggggagccaagggagaggc 25505
BCRP3.REF.NEW                                         ------------------------------------------------------------ 20446

GGT1.end.-Cabin1.start.8575573-8602125.orangutan      ccaggctgaagagtttaacgcgtggcccattgtgtcaggggctgggtagagagggacccc 25565
BCRP3.REF.NEW                                         ------------------------------------------------------------ 20446

GGT1.end.-Cabin1.start.8575573-8602125.orangutan      agagccttattccgcccctggcatcacggaggggtactagcgagggcaggcggcctgctt 25625
BCRP3.REF.NEW                                         ------------------------------------------------------------ 20446

GGT1.end.-Cabin1.start.8575573-8602125.orangutan      gggcccactgcagaacccaggctgagcccagagtggggaaagaggagcaggcatggacag 25685
BCRP3.REF.NEW                                         ------------------------------------------------------------ 20446

GGT1.end.-Cabin1.start.8575573-8602125.orangutan      ggcggagcatgggcctggtgacagacacttgagggacatcagcagcaggcagaagggtgg 25745
BCRP3.REF.NEW                                         ------------------------------------------------------------ 20446

GGT1.end.-Cabin1.start.8575573-8602125.orangutan      acgggttgggctgggccaagcaggcgtccccgtcgacagccatgtcagattctagccctg 25805
BCRP3.REF.NEW                                         ------------------------------------------------------------ 20446

GGT1.end.-Cabin1.start.8575573-8602125.orangutan      gcctcagtccggcctgttcccaccagctgccccaaccagccaggccccttggggactcct 25865
BCRP3.REF.NEW                                         ------------------------------------------------------------ 20446

GGT1.end.-Cabin1.start.8575573-8602125.orangutan      cctggcctgggcctcccacagaccactcgggatggcaggacctggcgccagcccctgggg 25925
BCRP3.REF.NEW                                         ------------------------------------------------------------ 20446

GGT1.end.-Cabin1.start.8575573-8602125.orangutan      gccaggcatgggctctgtgtaaaccccattccttttaaagccatggggctgcagaggaat 25985
BCRP3.REF.NEW                                         ------------------------------------------------------------ 20446

GGT1.end.-Cabin1.start.8575573-8602125.orangutan      gtggagacgagccaggccagccgggtcctcccagctcacgcagctccctagaggctgcga 26045
BCRP3.REF.NEW                                         ------------------------------------------------------------ 20446
```

```
GGT1.end.-Cabin1.start.8575573-8602125.orangutan      ggcctgcaactggcccagccttcaggctctgggcaaactcagccccttgctccccctgga 26105
BCRP3.REF.NEW                                         ------------------------------------------------------------ 20446

GGT1.end.-Cabin1.start.8575573-8602125.orangutan      ggatgaggcgaaagtggatgccctggggccagctcagtgaccatgcctggctgagcgacc 26165
BCRP3.REF.NEW                                         ------------------------------------------------------------ 20446

GGT1.end.-Cabin1.start.8575573-8602125.orangutan      agctctgggcttcagtgtcctccctggggcatagacaccagtagaacatgcatcctagga 26225
BCRP3.REF.NEW                                         ------------------------------------------------------------ 20446

GGT1.end.-Cabin1.start.8575573-8602125.orangutan      tggttgtggaaatacgacagttcatgcaagcataatgtctggaggtcttgcagaacccag 26285
BCRP3.REF.NEW                                         ------------------------------------------------------------ 20446

GGT1.end.-Cabin1.start.8575573-8602125.orangutan      caaggctcagggagtctcaggtcctgggctttgagttcatggtgttactgggtgaggttc 26345
BCRP3.REF.NEW                                         ------------------------------------------------------------ 20446

GGT1.end.-Cabin1.start.8575573-8602125.orangutan      acagtcccagcaccccacagctcagagccagagttgggtccctgcaggcctgagccaagt 26405
BCRP3.REF.NEW                                         ------------------------------------------------------------ 20446

GGT1.end.-Cabin1.start.8575573-8602125.orangutan      atttgcccagggtagaagccttggagcctgccagccagcacagactacacagcataaagg 26465
BCRP3.REF.NEW                                         ------------------------------------------------------------ 20446

GGT1.end.-Cabin1.start.8575573-8602125.orangutan      cccaggaccgggggggcgagataaagtgctcactgtgtgatgcggcaggcaggaggtgct 26525
BCRP3.REF.NEW                                         ------------------------------------------------------------ 20446

GGT1.end.-Cabin1.start.8575573-8602125.orangutan      cttggtacctttgtcttccagaggggca   26553
BCRP3.REF.NEW                                         ----------------------------   20446
```
