# Supplementary material for: An ancestral genomic sequence that serves as a nucleation site for de novo gene birth
Source: PLoS One. 2022 May 12;17(5):e0267864. doi: 10.1371/journal.pone.0267864 (PMC9097989; doi:10.1371/journal.pone.0267864)
Supplement: S9 Fig — Data kindly provided by Dr. Jessica Storer. (PDF) [file pone.0267864.s009.pdf]

Detection of an ancestral genomic sequence that serves as a nucleation site for de novo gene birth

Nicholas Delihas
Department of Microbiology and Immunology, Renaissance School of Medicine, Stony Brook University, Stony Brook, N.Y., United States of America

S9 Fig. Array of tandem TE repeats in orangutan sequnce. Left, orangutan; right, human *BCRP3* gene sequence tandem TE repeats. The three SVA_A the one LIMEg insertions in the orangutan are highlighted in red. Data kindly provided by Dr. Jessica Storer.

Orangutan

L1MC5	LINE/L1
L1MEg	LINE/L1
(AC)n	Simple_repe
AluSg	SINE/Alu
AluSx	SINE/Alu
AluSx1	SINE/Alu
L1MEg	LINE/L1
AluSx1	SINE/Alu
L1MEg	LINE/L1
AluSx	SINE/Alu
L1MEg	LINE/L1
AluSx	SINE/Alu
AluSx4	SINE/Alu
L1MEg	LINE/L1
(C)n	Simple_repe
SVA_A	Retroposon/
SVA_A	Retroposon/
SVA_A	Retroposon/
AluSx	SINE/Alu
AluJb	SINE/Alu
L1MEg	LINE/L1
FLAM_A	SINE/Alu
MADE1	DNA/TcMar-
A-rich	Low_comple
AluY	SINE/Alu
L1M2	LINE/L1

Human *BCRP3* gene

[illegible]	[illegible]
L1MC5	LINE/L1
L1MEg	LINE/L1
MER4E1	LTR/ERV1
(AT)n	Simple_repe
(ATATACACA	Simple_repe
(AT)n	Simple_repe
AluSg	SINE/Alu
AluSx1	SINE/Alu
AluSg	SINE/Alu
L1MEg	LINE/L1
AluSx1	SINE/Alu
L1MEg	LINE/L1
AluSg4	SINE/Alu
L1MEg	LINE/L1
AluSx	SINE/Alu
AluSx	SINE/Alu
L1MEg	LINE/L1
AluSx	SINE/Alu
L1MEg	LINE/L1
AluJb	SINE/Alu
L1MEg	LINE/L1
FLAM_A	SINE/Alu
MADE1	DNA/TcMar-
(A)n	Simple_repe
AluY	SINE/Alu
L1M2	LINE/L1
[illegible]	[illegible]
