# Supplementary material for: An ancestral genomic sequence that serves as a nucleation site for de novo gene birth
Source: PLoS One. 2022 May 12;17(5):e0267864. doi: 10.1371/journal.pone.0267864 (PMC9097989; doi:10.1371/journal.pone.0267864)
Supplement: S10 Fig — (PDF) [file pone.0267864.s010.pdf]

Detection of an ancestral genomic sequence that serves as a nucleation site for de novo gene birth

Nicholas Delihas
Department of Microbiology and Immunology, Renaissance School of Medicine, Stony Brook University, Stony Brook, N.Y., United States of America

S10 Fig. Alignment of the sequence between genes *LOC112206721-LOC112206738* present in the chimpanzee genomic GGT-spacer duplication locus with the human *BCRP3* and the human sequence that is between *GGT1* and *POM121L10P*. A diagram from the NCBI gene website showing the duplication site that contains the *LOC100610580* glutathione hydrolase light chain 2-like protein is also shown below.

Chimpanzee duplication of GGT locus

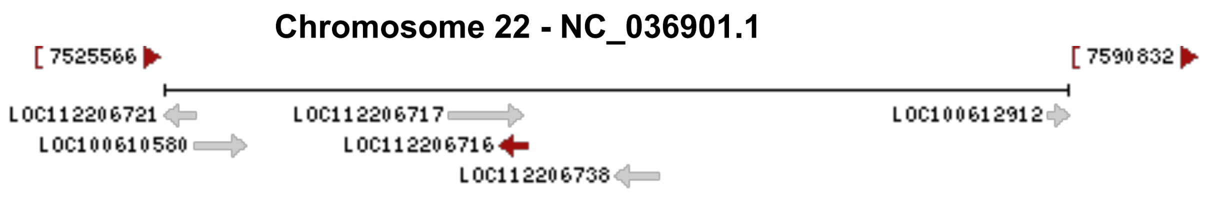


```
#
#
#  Percent Identity  Matrix - created by Clustal2.1
#
#

     1: LOC112206721.putative.POM121-like_protein1.START.LOC112206738  100.00   97.67   93.70
     2: BCRP3.REF.NEW                                                    97.67  100.00  100.00
     3: GGT1.start-POM121L10P.end.24583750-24659147.ref                  93.70  100.00  100.00
```

```
CLUSTAL O(1.2.4) multiple sequence alignment


LOC112206721.putative.POM121-like,protein1.START.LOC112206738      ------------------------------------------------------------   0
BCRP3.REF.NEW                                                      ------------------------------------------------------------   0
GGT1.start-POM121L10P.end.24583750-24659147.ref                    GCCTGGATTCTCCCAGAGATTGCCTGGAACACCAGCTCGGGTCCTCGCACACCATCCTGG   60

LOC112206721.putative.POM121-like,protein1.START.LOC112206738      ------------------------------------------------------------   0
BCRP3.REF.NEW                                                      ------------------------------------------------------------   0
GGT1.start-POM121L10P.end.24583750-24659147.ref                    TAGGAATCTCTCAGTGACAATTGAGAGTTTGTGGGCTGGCTTAGGGCCAGTGGTGGGCAG   120

LOC112206721.putative.POM121-like,protein1.START.LOC112206738      ------------------------------------------------------------   0
BCRP3.REF.NEW                                                      ------------------------------------------------------------   0
GGT1.start-POM121L10P.end.24583750-24659147.ref                    GGAGTCAGCAGGGCGCAAGGGACTGGAGAGCTTGGCATTTCTCCAACAGGAGTTTCAGGG   180

LOC112206721.putative.POM121-like,protein1.START.LOC112206738      ------------------------------------------------------------   0
BCRP3.REF.NEW                                                      ------------------------------------------------------------   0
GGT1.start-POM121L10P.end.24583750-24659147.ref                    AGAGCTCAGAGGCCTGCTTATGTGACAGGTTTATCAGCTTGCCTGGCCCAGGGCCGGCTC   240

LOC112206721.putative.POM121-like,protein1.START.LOC112206738      ------------------------------------------------------------   0
BCRP3.REF.NEW                                                      ------------------------------------------------------------   0
GGT1.start-POM121L10P.end.24583750-24659147.ref                    TCATACCGTTTTGATTTAAATAATAGATTGCTCACAGAATAAATGTATGTTGGTTTAAGC   300

LOC112206721.putative.POM121-like,protein1.START.LOC112206738      ------------------------------------------------------------   0
BCRP3.REF.NEW                                                      ------------------------------------------------------------   0
GGT1.start-POM121L10P.end.24583750-24659147.ref                    TTTCACTTTCTCATAATCCTCTCTGCTGGCCCCTCCCACCATCTACCCCATTTTGCCTCA   360

LOC112206721.putative.POM121-like,protein1.START.LOC112206738      ------------------------------------------------------------   0
BCRP3.REF.NEW                                                      ------------------------------------------------------------   0
GGT1.start-POM121L10P.end.24583750-24659147.ref                    CTCTGGGGAGCCTGAACACCCTGTTCCAGAGTAGGGGCATAGGGAAACACTTCTATGGCT   420

LOC112206721.putative.POM121-like,protein1.START.LOC112206738      ------------------------------------------------------------   0
BCRP3.REF.NEW                                                      ------------------------------------------------------------   0
GGT1.start-POM121L10P.end.24583750-24659147.ref                    TCTGAGCTGCTGGGCAGGGCCAGTCCCTAAAACAAGGAGAGCTGGGAGGGAGCTGGACCC   480

LOC112206721.putative.POM121-like,protein1.START.LOC112206738      ------------------------------------------------------------   0
BCRP3.REF.NEW                                                      ------------------------------------------------------------   0
GGT1.start-POM121L10P.end.24583750-24659147.ref                    CAGGATGGAGTGCTGAGAGCTGAAAGTCCTATTTAACAGAGGGGAGGGAGCAGAGATCCC   540

LOC112206721.putative.POM121-like,protein1.START.LOC112206738      ------------------------------------------------------------   0
BCRP3.REF.NEW                                                      ------------------------------------------------------------   0
GGT1.start-POM121L10P.end.24583750-24659147.ref                    AAAAAGGGAAAGGCCTGGCCAGGTCCCACAGCAAGTCAGGAGCAGAGCGGGCCTGAACTG   600

LOC112206721.putative.POM121-like,protein1.START.LOC112206738      ------------------------------------------------------------   0
BCRP3.REF.NEW                                                      ------------------------------------------------------------   0
GGT1.start-POM121L10P.end.24583750-24659147.ref                    TGAGATCTCCAATCTCTCCAAAGCCTTTGGGTAAGAAGTCCGCCTAAAGCAGTGAGATCA   660

LOC112206721.putative.POM121-like,protein1.START.LOC112206738      ------------------------------------------------------------   0
BCRP3.REF.NEW                                                      ------------------------------------------------------------   0
GGT1.start-POM121L10P.end.24583750-24659147.ref                    GTGGGGTCTGAGGAAAATCCTTTTCCTCACTGCCTCCATCCCAGTCCTGGGACCCAACAC   720

LOC112206721.putative.POM121-like,protein1.START.LOC112206738      ------------------------------------------------------------   0
BCRP3.REF.NEW                                                      ------------------------------------------------------------   0
GGT1.start-POM121L10P.end.24583750-24659147.ref                    CATCCTGTCCTCACCCTCCTTGGCCATCTCCTTTTGCCCTGAGAAGCTGTTATGCATCCA   780

LOC112206721.putative.POM121-like,protein1.START.LOC112206738      ------------------------------------------------------------   0
BCRP3.REF.NEW                                                      ------------------------------------------------------------   0
GGT1.start-POM121L10P.end.24583750-24659147.ref                    GCCAGACTGGGAATGTGCTGGAATCCATGCACCACAGGGGATGGATGCGCCGCTTACAGT   840

LOC112206721.putative.POM121-like,protein1.START.LOC112206738      ------------------------------------------------------------   0
BCRP3.REF.NEW                                                      ------------------------------------------------------------   0
GGT1.start-POM121L10P.end.24583750-24659147.ref                    CCCTGTGGGCCATGGCTGTGAGGGAAAGTCAGAGCTGGTGGCTGGGGTGTGTAGGCCCAG   900

LOC112206721.putative.POM121-like,protein1.START.LOC112206738      ------------------------------------------------------------   0
BCRP3.REF.NEW                                                      ------------------------------------------------------------   0
GGT1.start-POM121L10P.end.24583750-24659147.ref                    GAGGACCCCTGCCTCCTCCTTAGCCTTGGAGCCCCCCTCTGCTGCTGAGTCTTTCCTCAT   960

LOC112206721.putative.POM121-like,protein1.START.LOC112206738      ------------------------------------------------------------   0
BCRP3.REF.NEW                                                      ------------------------------------------------------------   0
GGT1.start-POM121L10P.end.24583750-24659147.ref                    CTAGCAGGGCCCCACTGCCCCCAGACCTTACCTCCTGGCTTAGCACATGACTTCCCCACT   1020

LOC112206721.putative.POM121-like,protein1.START.LOC112206738      ------------------------------------------------------------   0
BCRP3.REF.NEW                                                      ------------------------------------------------------------   0
GGT1.start-POM121L10P.end.24583750-24659147.ref                    CTGGGCCTCAGTATCCCCCCGGACATCTCTAAATTCCTTTCCCGCCTGCCCTGCCTGTCT   1080

LOC112206721.putative.POM121-like,protein1.START.LOC112206738      ------------------------------------------------------------   0
```

```
BCRP3.REF.NEW                                              ------------------------------------------------------------   0
GGT1.start-POM121L10P.end.24583750-24659147.ref            TCTGGTTGGGCTGAACTGTAACTCCCCACACAGGGCTGAGGGTGACTGTGGCATTAGGCT   1140

LOC112206721.putative.POM121-like,protein1.START.LOC112206738   ------------------------------------------------------------   0
BCRP3.REF.NEW                                              ------------------------------------------------------------   0
GGT1.start-POM121L10P.end.24583750-24659147.ref            TCTGGCTGGGAGCCCACCCTGATGGTGCTTGGGGAGGAAGGGAGACACTTCCCCCTGCCA   1200

LOC112206721.putative.POM121-like,protein1.START.LOC112206738   ------------------------------------------------------------   0
BCRP3.REF.NEW                                              ------------------------------------------------------------   0
GGT1.start-POM121L10P.end.24583750-24659147.ref            AGGGCAGCCCTAGGACTACTCTGCCCCCAAGGCCCCCTGCTGCTGGTGATGCGCACCTCC   1260

LOC112206721.putative.POM121-like,protein1.START.LOC112206738   ------------------------------------------------------------   0
BCRP3.REF.NEW                                              ------------------------------------------------------------   0
GGT1.start-POM121L10P.end.24583750-24659147.ref            ACCCGCTGCATGCTCTCCCACACGGGGACTCCCACACGGGGACCCCCAGGGCCTGGAGGG   1320

LOC112206721.putative.POM121-like,protein1.START.LOC112206738   ------------------------------------------------------------   0
BCRP3.REF.NEW                                              ------------------------------------------------------------   0
GGT1.start-POM121L10P.end.24583750-24659147.ref            AACACACAAGCACTTTGTCTCTGGCTTGCTGTCTCCTAACCCCACAGAGCTGCCCCTCGC   1380

LOC112206721.putative.POM121-like,protein1.START.LOC112206738   ------------------------------------------------------------   0
BCRP3.REF.NEW                                              ------------------------------------------------------------   0
GGT1.start-POM121L10P.end.24583750-24659147.ref            CAAGTACAAGAGGCCTGGGGCTGGGTCCTGAGGGTGGCAAGCAGCACTGTACCCTGTGGG   1440

LOC112206721.putative.POM121-like,protein1.START.LOC112206738   ------------------------------------------------------------   0
BCRP3.REF.NEW                                              ------------------------------------------------------------   0
GGT1.start-POM121L10P.end.24583750-24659147.ref            AGGCAGGCACCAAGTCCTCTCACTTAGTCGGCTGTGGCCCCTGCCTCTGGTCTGTGCAGC   1500

LOC112206721.putative.POM121-like,protein1.START.LOC112206738   ------------------------------------------------------------   0
BCRP3.REF.NEW                                              ------------------------------------------------------------   0
GGT1.start-POM121L10P.end.24583750-24659147.ref            CTTGTCCCCTGCCCAGAGGGCTGTGGTGGTGAGGCTGGCAGCCTGGGCTCAGACGTGAGC   1560

LOC112206721.putative.POM121-like,protein1.START.LOC112206738   ------------------------------------------------------------   0
BCRP3.REF.NEW                                              ------------------------------------------------------------   0
GGT1.start-POM121L10P.end.24583750-24659147.ref            TTGCTGTGGGGCTCCATGTAGAGCTTTGCTCCTCCTGGGGCTCCCGACCCGCTCCTTCGC   1620

LOC112206721.putative.POM121-like,protein1.START.LOC112206738   ------------------------------------------------------------   0
BCRP3.REF.NEW                                              ------------------------------------------------------------   0
GGT1.start-POM121L10P.end.24583750-24659147.ref            AGCTCCCCTTCTCAAGGAGCCCAAGCCCTGCCCAGATGTGAGGGCCCGCTCCCTGGAGGC   1680

LOC112206721.putative.POM121-like,protein1.START.LOC112206738   ------------------------------------------------------------   0
BCRP3.REF.NEW                                              ------------------------------------------------------------   0
GGT1.start-POM121L10P.end.24583750-24659147.ref            CTCCTCTAACTCTCCAGCCCACAGGGATGGACCCAGGACCCCCACATCTTAGACTCCATG   1740

LOC112206721.putative.POM121-like,protein1.START.LOC112206738   ------------------------------------------------------------   0
BCRP3.REF.NEW                                              ------------------------------------------------------------   0
GGT1.start-POM121L10P.end.24583750-24659147.ref            GGGGACTTGCCCAAAGAGCACCTGGGGGAAGGTCAGTGTGACCTTTGTCCCACCCTTTTG   1800

LOC112206721.putative.POM121-like,protein1.START.LOC112206738   ------------------------------------------------------------   0
BCRP3.REF.NEW                                              ------------------------------------------------------------   0
GGT1.start-POM121L10P.end.24583750-24659147.ref            GATAGACACAGCCACCCTTTCTCCCCACCCCAGCAGCAGGGCTGTTGCTCAGTGACCCTG   1860

LOC112206721.putative.POM121-like,protein1.START.LOC112206738   ------------------------------------------------------------   0
BCRP3.REF.NEW                                              ------------------------------------------------------------   0
GGT1.start-POM121L10P.end.24583750-24659147.ref            AGGTTGGTGATGCAGAAAAGCAGAGGCGGGTGTCACTCTTTATTGCGGGGTCCACACTGT   1920

LOC112206721.putative.POM121-like,protein1.START.LOC112206738   ------------------------------------------------------------   0
BCRP3.REF.NEW                                              ------------------------------------------------------------   0
GGT1.start-POM121L10P.end.24583750-24659147.ref            GGGTGCTGGGGCCCCTCCCACTGAGGGAAGGCTGAGCCTCTAGCCAGGGCTGGCCACCTG   1980

LOC112206721.putative.POM121-like,protein1.START.LOC112206738   ------------------------------------------------------------   0
BCRP3.REF.NEW                                              ------------------------------------------------------------   0
GGT1.start-POM121L10P.end.24583750-24659147.ref            GCCACCTATGAGTCCATTCTTCTGTCCCCTTAATCTCAGGCTGAGCATTTACACTGGATT   2040

LOC112206721.putative.POM121-like,protein1.START.LOC112206738   ------------------------------------------------------------   0
BCRP3.REF.NEW                                              ------------------------------------------------------------   0
GGT1.start-POM121L10P.end.24583750-24659147.ref            TCTGGGGGCCTGTGAGTCCTCCTTGACCTTCATCGCCCACTATCATGTGCTTGAGAGCAT   2100

LOC112206721.putative.POM121-like,protein1.START.LOC112206738   ------------------------------------------------------------   0
BCRP3.REF.NEW                                              ------------------------------------------------------------   0
GGT1.start-POM121L10P.end.24583750-24659147.ref            CACAAGTCAGTAGCAATGAGCCAGGTGGGTGGTGGGTCACCTGGCACAGCAGGCCTGGGG   2160

LOC112206721.putative.POM121-like,protein1.START.LOC112206738   ------------------------------------------------------------   0
BCRP3.REF.NEW                                              ------------------------------------------------------------   0
GGT1.start-POM121L10P.end.24583750-24659147.ref            CTCGGGTCCCAGCCCAGCAGCTGTGGAGTCCCAGGTGGAGGCAGGGGTGGTGGCCCCGGC   2220

LOC112206721.putative.POM121-like,protein1.START.LOC112206738   ------------------------------------------------------------   0
BCRP3.REF.NEW                                              ------------------------------------------------------------   0
GGT1.start-POM121L10P.end.24583750-24659147.ref            CGCACTGCCCTCAGGCTCAAGGTCCAGGCCCTCGTAGATGGTGGGGAGTGAGGTGCGCTG   2280

LOC112206721.putative.POM121-like,protein1.START.LOC112206738   ------------------------------------------------------------   0
BCRP3.REF.NEW                                              ------------------------------------------------------------   0
GGT1.start-POM121L10P.end.24583750-24659147.ref            GCTCAGCCGCCGGCGCAGGCCGACCAGCAGGGGCTGGGGCAGGGAAGCCACATCCACGTT   2340

LOC112206721.putative.POM121-like,protein1.START.LOC112206738   ------------------------------------------------------------   0
BCRP3.REF.NEW                                              ------------------------------------------------------------   0
GGT1.start-POM121L10P.end.24583750-24659147.ref            GTTGCCCAGGTCCACCCAAGCCAAAGCAGGGAACTTGGTGGTGTCCTTGATGGCATCAGT   2400

LOC112206721.putative.POM121-like,protein1.START.LOC112206738   ------------------------------------------------------------   0
BCRP3.REF.NEW                                              ------------------------------------------------------------   0
GGT1.start-POM121L10P.end.24583750-24659147.ref            GAGCTTGCGGGCAGTGGCCCGCGTCAGTCGGTTGCCGTTGAGCAGGAGCTGGGTGAGGCG   2460

LOC112206721.putative.POM121-like,protein1.START.LOC112206738   ------------------------------------------------------------   0
BCRP3.REF.NEW                                              ------------------------------------------------------------   0
GGT1.start-POM121L10P.end.24583750-24659147.ref            GGGCAGCGCCCACAGGCTGGGCAGCAGGAGGTGCAGCAGCTCATCACTCAGCCCCGTGAA   2520

LOC112206721.putative.POM121-like,protein1.START.LOC112206738   ------------------------------------------------------------   0
BCRP3.REF.NEW                                              ------------------------------------------------------------   0
GGT1.start-POM121L10P.end.24583750-24659147.ref            GCTCAGGTCCAGCACCGCCAGCACAGCACCATGGCTGCTCAGGTAGCGTGTGATGTGTTG   2580

LOC112206721.putative.POM121-like,protein1.START.LOC112206738   ------------------------------------------------------------   0
BCRP3.REF.NEW                                              ------------------------------------------------------------   0
GGT1.start-POM121L10P.end.24583750-24659147.ref            CACGTCCTGTGTCGACAGCGGGATGCCTGAGAGGTCCACAGTCTCCCCTGCCAGCAGAGT   2640

LOC112206721.putative.POM121-like,protein1.START.LOC112206738   ------------------------------------------------------------   0
BCRP3.REF.NEW                                              ------------------------------------------------------------   0
GGT1.start-POM121L10P.end.24583750-24659147.ref            CTTCTGGAGGCTGCTCTTGAGGCTATGGGAGAGTGGCAGGTGGGGATGGACTAGGCAACC   2700

LOC112206721.putative.POM121-like,protein1.START.LOC112206738   ------------------------------------------------------------   0
BCRP3.REF.NEW                                              ------------------------------------------------------------   0
GGT1.start-POM121L10P.end.24583750-24659147.ref            AGGCAGTCCCCCCACTGACCACAGACAGTGACCTGTCGGGAACAGCCCAGGCCTACAGTC   2760

LOC112206721.putative.POM121-like,protein1.START.LOC112206738   ------------------------------------------------------------   0
BCRP3.REF.NEW                                              ------------------------------------------------------------   0
GGT1.start-POM121L10P.end.24583750-24659147.ref            TGGCAAAGCCAGATTCAAACTGTGTCTTTCGTATTTTTTGAGACAAAGTTTCGCTCTTTT   2820

LOC112206721.putative.POM121-like,protein1.START.LOC112206738   ------------------------------------------------------------   0
```

```
BCRP3.REF.NEW                                                  ------------------------------------------------------------   0
GGT1.start-POM121L10P.end.24583750-24659147.ref                TGTCCAGGCTGGAGTGCAGTGGCACAATCTCGGCTTACGGCAACCTCTGCCTCCCGGATT   2880

LOC112206721.putative.POM121-like,protein1.START.LOC112206738  ------------------------------------------------------------   0
BCRP3.REF.NEW                                                  ------------------------------------------------------------   0
GGT1.start-POM121L10P.end.24583750-24659147.ref                CAAGCGATTCTCCTGCTTCAGCCTCCCAAGTAGCTGGGATTAAAGGCATGTGCCACCACG   2940

LOC112206721.putative.POM121-like,protein1.START.LOC112206738  ------------------------------------------------------------   0
BCRP3.REF.NEW                                                  ------------------------------------------------------------   0
GGT1.start-POM121L10P.end.24583750-24659147.ref                CCTGGCTAATTTTGTATTTTTAGTAGAGGCGGGGTTTCTCCATCTTTGTCAGGCTGGTCT   3000

LOC112206721.putative.POM121-like,protein1.START.LOC112206738  ------------------------------------------------------------   0
BCRP3.REF.NEW                                                  ------------------------------------------------------------   0
GGT1.start-POM121L10P.end.24583750-24659147.ref                CGAACTCCTGACCTCAGGTGATCTGACCGCCTCAGCCTCCCAAAGTGCTGGGATTACAGG   3060

LOC112206721.putative.POM121-like,protein1.START.LOC112206738  ------------------------------------------------------------   0
BCRP3.REF.NEW                                                  ------------------------------------------------------------   0
GGT1.start-POM121L10P.end.24583750-24659147.ref                CGTGAGCCACTGCGCCCGGGCCCAAACTGTCACTTTCTAAGTGGGTGGCCATGGGCAATG   3120

LOC112206721.putative.POM121-like,protein1.START.LOC112206738  ------------------------------------------------------------   0
BCRP3.REF.NEW                                                  ------------------------------------------------------------   0
GGT1.start-POM121L10P.end.24583750-24659147.ref                CTCAGAGGCTCAGTTTCCCCTTTGGTGAAAATGGGACGTGGTTGTTGTGAGGATTAAATG   3180

LOC112206721.putative.POM121-like,protein1.START.LOC112206738  ------------------------------------------------------------   0
BCRP3.REF.NEW                                                  ------------------------------------------------------------   0
GGT1.start-POM121L10P.end.24583750-24659147.ref                CACACACAGTAAGGCTCTTTCTTCATGAACTATTATTTGAAATCTCACTGTGTGCCCAGC   3240

LOC112206721.putative.POM121-like,protein1.START.LOC112206738  ------------------------------------------------------------   0
BCRP3.REF.NEW                                                  ------------------------------------------------------------   0
GGT1.start-POM121L10P.end.24583750-24659147.ref                ATCCCACACCTGTTTTATTGACATCTCACTTATTTTTCAGATAGGTGAAGTAACTCTTCC   3300

LOC112206721.putative.POM121-like,protein1.START.LOC112206738  ------------------------------------------------------------   0
BCRP3.REF.NEW                                                  ------------------------------------------------------------   0
GGT1.start-POM121L10P.end.24583750-24659147.ref                ATGGCCACACACCACTAAGCGGGGAACCAGGTCCTCAAACTGAGATGCTCTGACTCTTGA   3360

LOC112206721.putative.POM121-like,protein1.START.LOC112206738  ------------------------------------------------------------   0
BCRP3.REF.NEW                                                  ------------------------------------------------------------   0
GGT1.start-POM121L10P.end.24583750-24659147.ref                GCCCACATGTTTTTTTCCCTGTGCCCCTTCTGATGTCGATTCTTTTAGGGTAGGGAGAAA   3420

LOC112206721.putative.POM121-like,protein1.START.LOC112206738  ------------------------------------------------------------   0
BCRP3.REF.NEW                                                  ------------------------------------------------------------   0
GGT1.start-POM121L10P.end.24583750-24659147.ref                TTCTCCTACAAAGGGACGGCTATTAGGCTGTACAGGGCACCGGGGATAGAGAGGGACAGG   3480

LOC112206721.putative.POM121-like,protein1.START.LOC112206738  ------------------------------------------------------------   0
BCRP3.REF.NEW                                                  ------------------------------------------------------------   0
GGT1.start-POM121L10P.end.24583750-24659147.ref                CCCACAGTAGTGAAAGGGGAGGTCCTGGGTGTGCAGGCTGGGGGCCGGCCATGAGGGAGG   3540

LOC112206721.putative.POM121-like,protein1.START.LOC112206738  ------------------------------------------------------------   0
BCRP3.REF.NEW                                                  ------------------------------------------------------------   0
GGT1.start-POM121L10P.end.24583750-24659147.ref                GCTCCCCGTGTTTTCTCAGTTAGAAGGGAAGTTCAGAGACTGAGAAGGACCCCCGCCCCC   3600

LOC112206721.putative.POM121-like,protein1.START.LOC112206738  ------------------------------------------------------------   0
BCRP3.REF.NEW                                                  ------------------------------------------------------------   0
GGT1.start-POM121L10P.end.24583750-24659147.ref                ACCAACCCTCTAGGTCTCTCCCCAGTTATCTGAACAGAGCCCACTAGGCAGACAGGCCTT   3660

LOC112206721.putative.POM121-like,protein1.START.LOC112206738  ------------------------------------------------------------   0
BCRP3.REF.NEW                                                  ------------------------------------------------------------   0
GGT1.start-POM121L10P.end.24583750-24659147.ref                TTGGGGGACTGACAGCAGTGAGCACACCCTCAGGACCCCCACCAGCCATGGCAGGGAACA   3720

LOC112206721.putative.POM121-like,protein1.START.LOC112206738  ------------------------------------------------------------   0
BCRP3.REF.NEW                                                  ------------------------------------------------------------   0
GGT1.start-POM121L10P.end.24583750-24659147.ref                ACCTTGTGGGGTCCAAGCTCAGCGTGGCCTATTAGGCCCAAGGTGGGACAGGGGTGGAGG   3780

LOC112206721.putative.POM121-like,protein1.START.LOC112206738  ------------------------------------------------------------   0
BCRP3.REF.NEW                                                  ------------------------------------------------------------   0
GGT1.start-POM121L10P.end.24583750-24659147.ref                GCAGACAGGGGAGGCCTGCTAGGGTAAGCTCCCGGCAGCAGTTCAGGCCTCTGGGCCTGG   3840

LOC112206721.putative.POM121-like,protein1.START.LOC112206738  ------------------------------------------------------------   0
BCRP3.REF.NEW                                                  ------------------------------------------------------------   0
GGT1.start-POM121L10P.end.24583750-24659147.ref                GTGCCTGTGGCCAGCTCTGGACTGAGCCTGTGTGTGAGGCTCCATGGATGGAGTCACTCC   3900

LOC112206721.putative.POM121-like,protein1.START.LOC112206738  ------------------------------------------------------------   0
BCRP3.REF.NEW                                                  ------------------------------------------------------------   0
GGT1.start-POM121L10P.end.24583750-24659147.ref                AGGATGGAGTGTCTGAGAGGGACCCATGGCAGAGGGAGCTATTGGGCAGCCACGGAAGCC   3960

LOC112206721.putative.POM121-like,protein1.START.LOC112206738  ------------------------------------------------------------   0
BCRP3.REF.NEW                                                  ------------------------------------------------------------   0
GGT1.start-POM121L10P.end.24583750-24659147.ref                TCAGTTTTAGGCAGCCTGGCAGTGGCAAAGAATTCCCAACAAGGTACAGGAAAATCAGGG   4020

LOC112206721.putative.POM121-like,protein1.START.LOC112206738  ------------------------------------------------------------   0
BCRP3.REF.NEW                                                  ------------------------------------------------------------   0
GGT1.start-POM121L10P.end.24583750-24659147.ref                TAGAGTGTGGCCTTAGGCAAGCCACTTCTCCGAACCTGTTTCCTTCCCTCTAAAATGAGA   4080

LOC112206721.putative.POM121-like,protein1.START.LOC112206738  ------------------------------------------------------------   0
BCRP3.REF.NEW                                                  ------------------------------------------------------------   0
GGT1.start-POM121L10P.end.24583750-24659147.ref                ATCGCGATCCAGAATCTGCCTCCCCCGCAACCTGTTGGGAAGTCTCAGCTGAAGATGCCT   4140

LOC112206721.putative.POM121-like,protein1.START.LOC112206738  ------------------------------------------------------------   0
BCRP3.REF.NEW                                                  ------------------------------------------------------------   0
GGT1.start-POM121L10P.end.24583750-24659147.ref                ACTGAGGTCACCTTGGCTGTTGGGTGGAGGGGTGGGGTGTCCAGGCCGTGTAACAGACCA   4200

LOC112206721.putative.POM121-like,protein1.START.LOC112206738  ------------------------------------------------------------   0
BCRP3.REF.NEW                                                  ------------------------------------------------------------   0
GGT1.start-POM121L10P.end.24583750-24659147.ref                GGGATTGAGGGATGTATGAATGGCAGGAGGCAGGACCGTCCTGTGGGAAATGCCAAACGG   4260

LOC112206721.putative.POM121-like,protein1.START.LOC112206738  ------------------------------------------------------------   0
BCRP3.REF.NEW                                                  ------------------------------------------------------------   0
GGT1.start-POM121L10P.end.24583750-24659147.ref                GTCACTCAGAGGGTGCAGCTTGGCTTGGGGAGCATCTGCTACCACGGGCCAGGCTGACTT   4320

LOC112206721.putative.POM121-like,protein1.START.LOC112206738  ------------------------------------------------------------   0
BCRP3.REF.NEW                                                  ------------------------------------------------------------   0
GGT1.start-POM121L10P.end.24583750-24659147.ref                CCAGGGTAGGGCCCTCATGCGGACCAGGTGAGGGCCTCACCAGGTGGGATTTCAGCACCA   4380

LOC112206721.putative.POM121-like,protein1.START.LOC112206738  ------------------------------------------------------------   0
BCRP3.REF.NEW                                                  ------------------------------------------------------------   0
GGT1.start-POM121L10P.end.24583750-24659147.ref                GCCTCTTGGTGAGAGTGCAGGTGTCAACCTGAGAAGGGACCTTGGAGCAGCAGTGAGGAG   4440

LOC112206721.putative.POM121-like,protein1.START.LOC112206738  ------------------------------------------------------------   0
BCRP3.REF.NEW                                                  ------------------------------------------------------------   0
GGT1.start-POM121L10P.end.24583750-24659147.ref                GGGCAGTGGCTGGGCTACCCTTACCAGCTCTGGGTCTTCCTCTGGCGCAGGCTGGACCCT   4500

LOC112206721.putative.POM121-like,protein1.START.LOC112206738  ------------------------------------------------------------   0
BCRP3.REF.NEW                                                  ------------------------------------------------------------   0
GGT1.start-POM121L10P.end.24583750-24659147.ref                TGCTGCTGCTTCGAGTGAGGGGTGAGGTGGTAGATGAGCTGTCGGCAGATCTTGTCCGAG   4560

LOC112206721.putative.POM121-like,protein1.START.LOC112206738  ------------------------------------------------------------   0
```

```
BCRP3.REF.NEW                                                   ------------------------------------------------------------   0
GGT1.start-POM121L10P.end.24583750-24659147.ref                 GACTTCCAGAGCTCATAGTCCTGTGGGAGGGCAGTGTCACCATGGTGAATCTGAGCCCCT   4620

LOC112206721.putative.POM121-like,protein1.START.LOC112206738   ------------------------------------------------------------   0
BCRP3.REF.NEW                                                   ------------------------------------------------------------   0
GGT1.start-POM121L10P.end.24583750-24659147.ref                 CCTGCCCACCTCAGGGCCTTGGGGAGAGGGACCCAGGCAGCCAAGTGTGTGTGTGAGCAC   4680

LOC112206721.putative.POM121-like,protein1.START.LOC112206738   ------------------------------------------------------------   0
BCRP3.REF.NEW                                                   ------------------------------------------------------------   0
GGT1.start-POM121L10P.end.24583750-24659147.ref                 AGTCATGCACCATGCATCACCGAGTTGCCCACCAGGGCCTCCCCCTCCTACCCCCCAACC   4740

LOC112206721.putative.POM121-like,protein1.START.LOC112206738   ------------------------------------------------------------   0
BCRP3.REF.NEW                                                   ------------------------------------------------------------   0
GGT1.start-POM121L10P.end.24583750-24659147.ref                 CGGCTGTGGCCTGGCACAGAGCCAGGGGAGGCTGCCCTCTGGGAGCTGGACAGGCTGGTC   4800

LOC112206721.putative.POM121-like,protein1.START.LOC112206738   ------------------------------------------------------------   0
BCRP3.REF.NEW                                                   ------------------------------------------------------------   0
GGT1.start-POM121L10P.end.24583750-24659147.ref                 CAGTCCCGCAGTGCCCGGCGGGAGGAAGCCCAGACAATGAGCCCTTGTTCCCTGTCCTCG   4860

LOC112206721.putative.POM121-like,protein1.START.LOC112206738   ------------------------------------------------------------   0
BCRP3.REF.NEW                                                   ------------------------------------------------------------   0
GGT1.start-POM121L10P.end.24583750-24659147.ref                 GGCCCAGGGCCAGCGTCTCGGGCTATCAGCCGGCTGCCTGCCCACCCTCAGCACCCTTCG   4920

LOC112206721.putative.POM121-like,protein1.START.LOC112206738   ------------------------------------------------------------   0
BCRP3.REF.NEW                                                   ------------------------------------------------------------   0
GGT1.start-POM121L10P.end.24583750-24659147.ref                 GGGACTTGCCACAGGGGGAGGAGGCCAGACCAGGCCCCTCGAGGGAGCCGTGCTCCACTG   4980

LOC112206721.putative.POM121-like,protein1.START.LOC112206738   ------------------------------------------------------------   0
BCRP3.REF.NEW                                                   ------------------------------------------------------------   0
GGT1.start-POM121L10P.end.24583750-24659147.ref                 ACGCCAATCCCAGGGAGTAGGAACAAGCCAGGTCAGCGTGTTCTTCTTCCTCTCACTCTC   5040

LOC112206721.putative.POM121-like,protein1.START.LOC112206738   ------------------------------------------------------------   0
BCRP3.REF.NEW                                                   ------------------------------------------------------------   0
GGT1.start-POM121L10P.end.24583750-24659147.ref                 CAAGCGAGACTGCGCCTGCGGGGTCTGGGTCTGGGCTGACAGGGCTGGGGGATGTAATGT   5100

LOC112206721.putative.POM121-like,protein1.START.LOC112206738   ------------------------------------------------------------   0
BCRP3.REF.NEW                                                   ------------------------------------------------------------   0
GGT1.start-POM121L10P.end.24583750-24659147.ref                 TTTACAGCACCACCACCTGGCTCTCTCTCAGTCCTAACTGGGCTCCGGCAGAGGCCCAGC   5160

LOC112206721.putative.POM121-like,protein1.START.LOC112206738   ------------------------------------------------------------   0
BCRP3.REF.NEW                                                   ------------------------------------------------------------   0
GGT1.start-POM121L10P.end.24583750-24659147.ref                 TCAGGGTGGAATCCGAGGTGCGCGGCCCACAAAGCTGGCACTGAGCATGCTCCACTGCAT   5220

LOC112206721.putative.POM121-like,protein1.START.LOC112206738   ------------------------------------------------------------   0
BCRP3.REF.NEW                                                   ------------------------------------------------------------   0
GGT1.start-POM121L10P.end.24583750-24659147.ref                 CCTTTATCCAGTCCCTCTGTGAGCAGCCCCAGGAGCAGGCAGCACACACAGCAGCTGCAA   5280

LOC112206721.putative.POM121-like,protein1.START.LOC112206738   ------------------------------------------------------------   0
BCRP3.REF.NEW                                                   ------------------------------------------------------------   0
GGT1.start-POM121L10P.end.24583750-24659147.ref                 GCCCTGGCTGTTCCTGTGATGGCCGTGGGCTAGGCGCAGAGTCCTAGGGGCCGACTGTGC   5340

LOC112206721.putative.POM121-like,protein1.START.LOC112206738   ------------------------------------------------------------   0
BCRP3.REF.NEW                                                   ------------------------------------------------------------   0
GGT1.start-POM121L10P.end.24583750-24659147.ref                 AGAGACCTGGGCATTCAGAGCTGGCAGGGCCCAGAGCGGATACCCTGGTTTTGGTTGGGG   5400

LOC112206721.putative.POM121-like,protein1.START.LOC112206738   ------------------------------------------------------------   0
BCRP3.REF.NEW                                                   ------------------------------------------------------------   0
GGT1.start-POM121L10P.end.24583750-24659147.ref                 ACTCACTGTGACTAGGAGTCTGGGTCCGGTGGCTGGTCACAGCCACACATCCTGGGGCTG   5460

LOC112206721.putative.POM121-like,protein1.START.LOC112206738   ------------------------------------------------------------   0
BCRP3.REF.NEW                                                   ------------------------------------------------------------   0
GGT1.start-POM121L10P.end.24583750-24659147.ref                 TGTCGATGCTCATGGCAGGACATCTGCAGGTGCTGTCAGCATGGGGTTCTGGGCAGCTGT   5520

LOC112206721.putative.POM121-like,protein1.START.LOC112206738   ------------------------------------------------------------   0
BCRP3.REF.NEW                                                   ------------------------------------------------------------   0
GGT1.start-POM121L10P.end.24583750-24659147.ref                 GGGGTGGAGGCTGCAGGTGGCAAACTCCACCCCAGCAAGGAGGAGGGCCTTGCTTATGAC   5580

LOC112206721.putative.POM121-like,protein1.START.LOC112206738   ------------------------------------------------------------   0
BCRP3.REF.NEW                                                   ------------------------------------------------------------   0
GGT1.start-POM121L10P.end.24583750-24659147.ref                 CCCAGCTGTTGTAGGGGAAAGCAGTGAGCACCCGTCCGCAAGGGTGTCTGTACAGGATGG   5640

LOC112206721.putative.POM121-like,protein1.START.LOC112206738   ------------------------------------------------------------   0
BCRP3.REF.NEW                                                   ------------------------------------------------------------   0
GGT1.start-POM121L10P.end.24583750-24659147.ref                 AGACCTGCGGACAGACGGGGGCTCTAGCCGAGAGCGGCTCTCTTCTGGCCCAGGAATTCA   5700

LOC112206721.putative.POM121-like,protein1.START.LOC112206738   ------------------------------------------------------------   0
BCRP3.REF.NEW                                                   ------------------------------------------------------------   0
GGT1.start-POM121L10P.end.24583750-24659147.ref                 GAGGCTAGGAGTTTGTTTCCGTTGGTGGATTTCTCAGAACAGTCTGTGACCCGCAGGGTC   5760

LOC112206721.putative.POM121-like,protein1.START.LOC112206738   ------------------------------------------------------------   0
BCRP3.REF.NEW                                                   ------------------------------------------------------------   0
GGT1.start-POM121L10P.end.24583750-24659147.ref                 CCCATATGACTTCTGCCCAGCTATAGTCTTCCTGGTCTGGGCCCCCAGGCCTGGTGCACA   5820

LOC112206721.putative.POM121-like,protein1.START.LOC112206738   ------------------------------------------------------------   0
BCRP3.REF.NEW                                                   ------------------------------------------------------------   0
GGT1.start-POM121L10P.end.24583750-24659147.ref                 GTGCTGCCACACAGAAAATGGGTCTGCCCAGGACTCTGAGGGTCCTGCAAAGCACAGCTC   5880

LOC112206721.putative.POM121-like,protein1.START.LOC112206738   ------------------------------------------------------------   0
BCRP3.REF.NEW                                                   ------------------------------------------------------------   0
GGT1.start-POM121L10P.end.24583750-24659147.ref                 ACACTTGCTCTAGCTGGTGGCCAGCCTAATATACTGATGCTGGCAGCAGGTCTCAGACAG   5940

LOC112206721.putative.POM121-like,protein1.START.LOC112206738   ------------------------------------------------------------   0
BCRP3.REF.NEW                                                   ------------------------------------------------------------   0
GGT1.start-POM121L10P.end.24583750-24659147.ref                 GGACAGTGACTTGCCTAAGGCCACACAGCAAGCCGCAGAAGGACCTAGCCTCCCAGTCCC   6000

LOC112206721.putative.POM121-like,protein1.START.LOC112206738   ------------------------------------------------------------   0
BCRP3.REF.NEW                                                   ------------------------------------------------------------   0
GGT1.start-POM121L10P.end.24583750-24659147.ref                 CAGCCCTGGGCTCTGTTGGCCCCCCTGTCCACCACAGTGCCCAGCCCAGGGCCCGTGCCT   6060

LOC112206721.putative.POM121-like,protein1.START.LOC112206738   ------------------------------------------------------------   0
BCRP3.REF.NEW                                                   ------------------------------------------------------------   0
GGT1.start-POM121L10P.end.24583750-24659147.ref                 GCCAGCCTCACCTTCTTGGGGCACTGCAGGTCCCGGGCCAGGTTCACAAGCAGGTCATGG   6120

LOC112206721.putative.POM121-like,protein1.START.LOC112206738   ------------------------------------------------------------   0
BCRP3.REF.NEW                                                   ------------------------------------------------------------   0
GGT1.start-POM121L10P.end.24583750-24659147.ref                 GAGATGGGGTCGACCGGGTTGAGGAAGGCCACATCTCTGTAGAGGATATCAGGAAGGAGG   6180

LOC112206721.putative.POM121-like,protein1.START.LOC112206738   ------------------------------------------------------------   0
BCRP3.REF.NEW                                                   ------------------------------------------------------------   0
GGT1.start-POM121L10P.end.24583750-24659147.ref                 GTCCTCTCAAGGCCCAGGTCCTGTGAGTGGTGAAGAGAGAGTTGAGTGAGCCCGTTGGGT   6240

LOC112206721.putative.POM121-like,protein1.START.LOC112206738   ------------------------------------------------------------   0
BCRP3.REF.NEW                                                   ------------------------------------------------------------   0
GGT1.start-POM121L10P.end.24583750-24659147.ref                 CTCCCATCTCGAGGCACCACCCCAGCCAGCCAGATGCTGCTTATGCCCGTTCCTGTCTCT   6300

LOC112206721.putative.POM121-like,protein1.START.LOC112206738   ------------------------------------------------------------   0
```

```
BCRP3.REF.NEW                                                  ------------------------------------------------------------   0
GGT1.start-POM121L10P.end.24583750-24659147.ref                CCATCTCCTCCCTAAGGCTGGCTGGATTCTCCCCACAGGCGGCCATGGGCCAACAGTCCA   6360

LOC112206721.putative.POM121-like,protein1.START.LOC112206738  ------------------------------------------------------------   0
BCRP3.REF.NEW                                                  ------------------------------------------------------------   0
GGT1.start-POM121L10P.end.24583750-24659147.ref                GTGGCGGGTGGCCCCCTTCTTTTTTTGCTTATTTATTTTTTGAGACAGGGTCTCACCCTG   6420

LOC112206721.putative.POM121-like,protein1.START.LOC112206738  ------------------------------------------------------------   0
BCRP3.REF.NEW                                                  ------------------------------------------------------------   0
GGT1.start-POM121L10P.end.24583750-24659147.ref                TCACCCAGGCTGGAGTGCAGTGGCGTGATCTCAGTTCAGTGTAACATCTGCCTCCTCAAG   6480

LOC112206721.putative.POM121-like,protein1.START.LOC112206738  ------------------------------------------------------------   0
BCRP3.REF.NEW                                                  ------------------------------------------------------------   0
GGT1.start-POM121L10P.end.24583750-24659147.ref                GAATCCTCCCACTTCAGCCTCCTGAGGAGCTGGGGCTATAGACAGGCACCACTATGCTCA   6540

LOC112206721.putative.POM121-like,protein1.START.LOC112206738  ------------------------------------------------------------   0
BCRP3.REF.NEW                                                  ------------------------------------------------------------   0
GGT1.start-POM121L10P.end.24583750-24659147.ref                GCTAATTAAAAAAAAAATGTTTTGTAGAAATGGGGTCTCACTATGTTGCCCAGACTGGTC   6600

LOC112206721.putative.POM121-like,protein1.START.LOC112206738  ------------------------------------------------------------   0
BCRP3.REF.NEW                                                  ------------------------------------------------------------   0
GGT1.start-POM121L10P.end.24583750-24659147.ref                TCAAACTCCTGGGCTCAAGCGATCCTCCCAAAGTGCTGGGATTACAGGTGTGAGCCACTC   6660

LOC112206721.putative.POM121-like,protein1.START.LOC112206738  ------------------------------------------------------------   0
BCRP3.REF.NEW                                                  ------------------------------------------------------------   0
GGT1.start-POM121L10P.end.24583750-24659147.ref                AGCCTTGGAGGACCCTTTGGATAAGTAGAGGGGAGGGACATGGGAGCCCTCAGGGAAGTG   6720

LOC112206721.putative.POM121-like,protein1.START.LOC112206738  ------------------------------------------------------------   0
BCRP3.REF.NEW                                                  ------------------------------------------------------------   0
GGT1.start-POM121L10P.end.24583750-24659147.ref                GTAAGTGAATCCCAGAACCCTTAGACCAGTTCCCTTGAGGTAGTGTTGTGCTGTGACACT   6780

LOC112206721.putative.POM121-like,protein1.START.LOC112206738  ------------------------------------------------------------   0
BCRP3.REF.NEW                                                  ------------------------------------------------------------   0
GGT1.start-POM121L10P.end.24583750-24659147.ref                GCCTTCTCTGGGGTTAGGACTTGTCAGTGGGATTCTTTGGGGTCTTTCGTCTTATGGTGG   6840

LOC112206721.putative.POM121-like,protein1.START.LOC112206738  ------------------------------------------------------------   0
BCRP3.REF.NEW                                                  ------------------------------------------------------------   0
GGT1.start-POM121L10P.end.24583750-24659147.ref                GGGGGTTTGCTCTTCTTATGACCCCTCAAATTCCCCTGTCCCCCAGTGTTCCCTTCAGAT   6900

LOC112206721.putative.POM121-like,protein1.START.LOC112206738  ------------------------------------------------------------   0
BCRP3.REF.NEW                                                  ------------------------------------------------------------   0
GGT1.start-POM121L10P.end.24583750-24659147.ref                CCTCTGCCTTCCCACCTATCCCTTGGTTTTCCTAAGCTGCGGCCAGCATCTGTTTGCGGA   6960

LOC112206721.putative.POM121-like,protein1.START.LOC112206738  ------------------------------------------------------------   0
BCRP3.REF.NEW                                                  ------------------------------------------------------------   0
GGT1.start-POM121L10P.end.24583750-24659147.ref                CAGGTATGGATCCTTCCCTTGGGGGCGGACCTCTCTCTCAGATGTCTCAAAGGCATGCCA   7020

LOC112206721.putative.POM121-like,protein1.START.LOC112206738  ------------------------------------------------------------   0
BCRP3.REF.NEW                                                  ------------------------------------------------------------   0
GGT1.start-POM121L10P.end.24583750-24659147.ref                CCCTGAAGCCAGCCTCACCACCCATGCCCCAGCTCAGTATAGGGCACCTCCATGACCATC   7080

LOC112206721.putative.POM121-like,protein1.START.LOC112206738  ------------------------------------------------------------   0
BCRP3.REF.NEW                                                  ------------------------------------------------------------   0
GGT1.start-POM121L10P.end.24583750-24659147.ref                TTCCCAGGCACTGGGGCCAGAAGCTCTGGAACCCCCCAGCACTCTTCCGTCCTCCTCTCT   7140

LOC112206721.putative.POM121-like,protein1.START.LOC112206738  ------------------------------------------------------------   0
BCRP3.REF.NEW                                                  ------------------------------------------------------------   0
GGT1.start-POM121L10P.end.24583750-24659147.ref                CCCCCAGCTCACCTAGTCATCCCTTCTCTCTCCCCACCACTCCTGCCAAGATTCAGGCCT   7200

LOC112206721.putative.POM121-like,protein1.START.LOC112206738  ------------------------------------------------------------   0
BCRP3.REF.NEW                                                  ------------------------------------------------------------   0
GGT1.start-POM121L10P.end.24583750-24659147.ref                CCAGCCTCCTCTCCTACCCATCCCTCACCCCACCGCCTGCATAGACCTGCCCTGGCCTTC   7260

LOC112206721.putative.POM121-like,protein1.START.LOC112206738  ------------------------------------------------------------   0
BCRP3.REF.NEW                                                  ------------------------------------------------------------   0
GGT1.start-POM121L10P.end.24583750-24659147.ref                CTGCCTCACTCCTCCACTTTCTCCTCCTGTCTACTCCCACAGGACTGGTGTGGGGCTGAA   7320

LOC112206721.putative.POM121-like,protein1.START.LOC112206738  ------------------------------------------------------------   0
BCRP3.REF.NEW                                                  ------------------------------------------------------------   0
GGT1.start-POM121L10P.end.24583750-24659147.ref                TACTCTTTCAGAGGGTCTCTTTGTTTTGAGACGGAGTTTTGCTCTTGTTGCCCGGGCTGG   7380

LOC112206721.putative.POM121-like,protein1.START.LOC112206738  ------------------------------------------------------------   0
BCRP3.REF.NEW                                                  ------------------------------------------------------------   0
GGT1.start-POM121L10P.end.24583750-24659147.ref                GGCGCAGTGGTGCGAGGTCAGCTCACCGCAACCTCCGCCTCCCGGGTTCAAGCAATTCTC   7440

LOC112206721.putative.POM121-like,protein1.START.LOC112206738  ------------------------------------------------------------   0
BCRP3.REF.NEW                                                  ------------------------------------------------------------   0
GGT1.start-POM121L10P.end.24583750-24659147.ref                CTGCCTCAGCCTCCTGAGTAGCTGGGATTGCAGGCATGTGCCACCACGCCCAGCTAATTT   7500

LOC112206721.putative.POM121-like,protein1.START.LOC112206738  ------------------------------------------------------------   0
BCRP3.REF.NEW                                                  ------------------------------------------------------------   0
GGT1.start-POM121L10P.end.24583750-24659147.ref                TGTATTTTTAGCAGGGAAGGGGTTTCTCCATGTTGGTCAGGCTGGTCTCGAATTCCCGAC   7560

LOC112206721.putative.POM121-like,protein1.START.LOC112206738  ------------------------------------------------------------   0
BCRP3.REF.NEW                                                  ------------------------------------------------------------   0
GGT1.start-POM121L10P.end.24583750-24659147.ref                TTCAGGTGATCTGCCTGCCTCAGCCTCCCAAAGTGCTGGGATTATAGGCGTGAGCCACTG   7620

LOC112206721.putative.POM121-like,protein1.START.LOC112206738  ------------------------------------------------------------   0
BCRP3.REF.NEW                                                  ------------------------------------------------------------   0
GGT1.start-POM121L10P.end.24583750-24659147.ref                CATCTGGCCCAGAGGGTCTCTTTAGAGGACATTTTGAGAGCTGATGGGGCAGCCACAGCC   7680

LOC112206721.putative.POM121-like,protein1.START.LOC112206738  ------------------------------------------------------------   0
BCRP3.REF.NEW                                                  ------------------------------------------------------------   0
GGT1.start-POM121L10P.end.24583750-24659147.ref                AGGAGGGGCCTCTAGACCCCATGTGGGCCTTCTCCAATCCATAGGTGATATGCTCTGTGC   7740

LOC112206721.putative.POM121-like,protein1.START.LOC112206738  ------------------------------------------------------------   0
BCRP3.REF.NEW                                                  ------------------------------------------------------------   0
GGT1.start-POM121L10P.end.24583750-24659147.ref                GTGGGTCCCAAGCACAGGAAGTTGGGCCAGGCAGGGGAGGCCGTGGGTCACAGCTGCAGG   7800

LOC112206721.putative.POM121-like,protein1.START.LOC112206738  ------------------------------------------------------------   0
BCRP3.REF.NEW                                                  ------------------------------------------------------------   0
GGT1.start-POM121L10P.end.24583750-24659147.ref                GTCCCTGCAGGTTATGGAGCAGGATAGGCCCCGGCTGGACCTCAAATCCCAACCTCTGAG   7860

LOC112206721.putative.POM121-like,protein1.START.LOC112206738  ------------------------------------------------------------   0
BCRP3.REF.NEW                                                  ------------------------------------------------------------   0
GGT1.start-POM121L10P.end.24583750-24659147.ref                ACACAAAGAGGTGGTCTCTGAGGATCCTTCTTTCCCTCTAGTTTCCCAGCTAAAATGCCC   7920

LOC112206721.putative.POM121-like,protein1.START.LOC112206738  ------------------------------------------------------------   0
BCRP3.REF.NEW                                                  ------------------------------------------------------------   0
GGT1.start-POM121L10P.end.24583750-24659147.ref                AACTGGTGTCTTGGACTCCAGGCAGCATCCCAGACTGAGATCTGTGCCTCTGGTTGTCTG   7980

LOC112206721.putative.POM121-like,protein1.START.LOC112206738  ------------------------------------------------------------   0
BCRP3.REF.NEW                                                  ------------------------------------------------------------   0
GGT1.start-POM121L10P.end.24583750-24659147.ref                GCTGATTCAGTGCTGCTCTGCTGTTTGCCACAGATGCCAGCTAGGGAAGATCCAGGAGGA   8040

LOC112206721.putative.POM121-like,protein1.START.LOC112206738  ------------------------------------------------------------   0
```

```
BCRP3.REF.NEW                                                    ------------------------------------------------------------   0
GGT1.start-POM121L10P.end.24583750-24659147.ref                  AGGCTGGCTGATGAGCCTGGCAGACCCAGACAGGGCCCCAGACCGCAACCTGCCAGCCTG   8100

LOC112206721.putative.POM121-like,protein1.START.LOC112206738    ------------------------------------------------------------   0
BCRP3.REF.NEW                                                    ------------------------------------------------------------   0
GGT1.start-POM121L10P.end.24583750-24659147.ref                  CCTTCCATGGGTCTAGACAGCTCCTCTTAAACGGACAGCTCCATTTTTCCAGCCTGCTGA   8160

LOC112206721.putative.POM121-like,protein1.START.LOC112206738    ------------------------------------------------------------   0
BCRP3.REF.NEW                                                    ------------------------------------------------------------   0
GGT1.start-POM121L10P.end.24583750-24659147.ref                  ATCCAGAGTTGGAGGAGGTGGAGAAGGGAAGGGAGGAAGAAGGGCTCTGGCCCTGGCTTG   8220

LOC112206721.putative.POM121-like,protein1.START.LOC112206738    ------------------------------------------------------------   0
BCRP3.REF.NEW                                                    ------------------------------------------------------------   0
GGT1.start-POM121L10P.end.24583750-24659147.ref                  CCCCACATTTTCTCTGTGGGCCCTGGGGTGACCACTTCGCTCCATGCCTCAGTTTCCCCA   8280

LOC112206721.putative.POM121-like,protein1.START.LOC112206738    ------------------------------------------------------------   0
BCRP3.REF.NEW                                                    ------------------------------------------------------------   0
GGT1.start-POM121L10P.end.24583750-24659147.ref                  TTTCTAATCTACCTTCTGCAGGCCGAACACCTGCATGGCTGACTTCCTGCTCACAGGATG   8340

LOC112206721.putative.POM121-like,protein1.START.LOC112206738    ------------------------------------------------------------   0
BCRP3.REF.NEW                                                    ------------------------------------------------------------   0
GGT1.start-POM121L10P.end.24583750-24659147.ref                  GGGCAGGTAGTGGTGGTAGCTGCTGTAGGGGGTGGGGACGGGGTGGTGAGGGAAGTGGCA   8400

LOC112206721.putative.POM121-like,protein1.START.LOC112206738    ------------------------------------------------------------   0
BCRP3.REF.NEW                                                    ------------------------------------------------------------   0
GGT1.start-POM121L10P.end.24583750-24659147.ref                  TGCCCAGGTGATTGGGGGGACCAGCATCCTGGGACAACCCAGGGGTTGGGATCTGGGTTT   8460

LOC112206721.putative.POM121-like,protein1.START.LOC112206738    ------------------------------------------------------------   0
BCRP3.REF.NEW                                                    ------------------------------------------------------------   0
GGT1.start-POM121L10P.end.24583750-24659147.ref                  AGTGTACCTGGTGTGTGTAGGAGCAGAGCCACCACCGGACCGAGTGTTGTGATGCACAGA   8520

LOC112206721.putative.POM121-like,protein1.START.LOC112206738    ------------------------------------------------------------   0
BCRP3.REF.NEW                                                    ------------------------------------------------------------   0
GGT1.start-POM121L10P.end.24583750-24659147.ref                  TGCTGGGGATGTGTACTCTAAGGGCATGAGCCGCCTGTTGGAGAACTGGTGATGCATGGA   8580

LOC112206721.putative.POM121-like,protein1.START.LOC112206738    ------------------------------------------------------------   0
BCRP3.REF.NEW                                                    ------------------------------------------------------------   0
GGT1.start-POM121L10P.end.24583750-24659147.ref                  TGGCTCTCTGTGGGCTGCCATGGTGTCTTGGGGTGAGGCTGCATCCCTAGACCAGCCCTG   8640

LOC112206721.putative.POM121-like,protein1.START.LOC112206738    ------------------------------------------------------------   0
BCRP3.REF.NEW                                                    ------------------------------------------------------------   0
GGT1.start-POM121L10P.end.24583750-24659147.ref                  GGTCCTCTCTCCAGGGTCCACTGTCTCCCGGATCCTGGAGGAGGGGGAAGTTACCCTCTT   8700

LOC112206721.putative.POM121-like,protein1.START.LOC112206738    ------------------------------------------------------------   0
BCRP3.REF.NEW                                                    ------------------------------------------------------------   0
GGT1.start-POM121L10P.end.24583750-24659147.ref                  CCATTGTCTCTCAATACAAGATGATTTGACCTCACTTGCCACTCCTCCCGGGCCCCTCCC   8760

LOC112206721.putative.POM121-like,protein1.START.LOC112206738    ------------------------------------------------------------   0
BCRP3.REF.NEW                                                    ------------------------------------------------------------   0
GGT1.start-POM121L10P.end.24583750-24659147.ref                  ATCTTGCCAGGGCCTGAATTTGGGCTCTTAGGTCCAGGCTACCGGGCCACCCTCAACACA   8820

LOC112206721.putative.POM121-like,protein1.START.LOC112206738    ------------------------------------------------------------   0
BCRP3.REF.NEW                                                    ------------------------------------------------------------   0
GGT1.start-POM121L10P.end.24583750-24659147.ref                  CACAACCCCTCTCAAACCCCAAAGACCCGGACACACTCAGCAGCCCCCTCCTCCACACGG   8880

LOC112206721.putative.POM121-like,protein1.START.LOC112206738    ------------------------------------------------------------   0
BCRP3.REF.NEW                                                    ------------------------------------------------------------   0
GGT1.start-POM121L10P.end.24583750-24659147.ref                  TCCGCCGACCTCACCCAGCCCTCACTCCAGGCGGTCGCCCTCTCGCCCACGCAAGACCCC   8940

LOC112206721.putative.POM121-like,protein1.START.LOC112206738    ------------------------------------------------------------   0
BCRP3.REF.NEW                                                    ------------------------------------------------------------   0
GGT1.start-POM121L10P.end.24583750-24659147.ref                  GCGTGCTGCAGCCTGTCGCGCCCTCGGAACCCGCCTGCGCGCGCCAGAGACCAGCTCCGC   9000

LOC112206721.putative.POM121-like,protein1.START.LOC112206738    ------------------------------------------------------------   0
BCRP3.REF.NEW                                                    ------------------------------------------------------------   0
GGT1.start-POM121L10P.end.24583750-24659147.ref                  CTCGCCTCCCGGCGGATACAGGCCCACAACCCATAGCCCCGCGCCTCCCAGACCCCCAGA   9060

LOC112206721.putative.POM121-like,protein1.START.LOC112206738    ------------------------------------------------------------   0
BCRP3.REF.NEW                                                    ------------------------------------------------------------   0
GGT1.start-POM121L10P.end.24583750-24659147.ref                  CCCCCAGACCCTCCCTGGCCGCCAGCCCAGGGGCGGACGGCTCCTTCTCTCGCCTGGCGC   9120

LOC112206721.putative.POM121-like,protein1.START.LOC112206738    ------------------------------------------------------------   0
BCRP3.REF.NEW                                                    ------------------------------------------------------------   0
GGT1.start-POM121L10P.end.24583750-24659147.ref                  AGGAGGCGCAGCAGCTGCCGGGCGCGCTCCGGCCGCCGCTCGCGGAGCGTGGACTGGATC   9180

LOC112206721.putative.POM121-like,protein1.START.LOC112206738    ------------------------------------------------------------   0
BCRP3.REF.NEW                                                    ------------------------------------------------------------   0
GGT1.start-POM121L10P.end.24583750-24659147.ref                  TCGCGGAGCCACCGCACCCGGCGCTCGTAGGGCGCGGGCCCGCAGCCGGCCGCCGCCCCG   9240

LOC112206721.putative.POM121-like,protein1.START.LOC112206738    ------------------------------------------------------------   0
BCRP3.REF.NEW                                                    ------------------------------------------------------------   0
GGT1.start-POM121L10P.end.24583750-24659147.ref                  GCCTCAGAGCCAGCCTCGGGCCCGGCCCGCCGGCCCAGCCGCGCCCCCATGGCCGCCGCG   9300

LOC112206721.putative.POM121-like,protein1.START.LOC112206738    ------------------------------------------------------------   0
BCRP3.REF.NEW                                                    ------------------------------------------------------------   0
GGT1.start-POM121L10P.end.24583750-24659147.ref                  CGATGGTCGCCGAACCCACAGGAGGCCGGGCTGTCTGCGCCCCGCTCCCGGGGCCGCCCC   9360

LOC112206721.putative.POM121-like,protein1.START.LOC112206738    ------------------------------------------------------------   0
BCRP3.REF.NEW                                                    ------------------------------------------------------------   0
GGT1.start-POM121L10P.end.24583750-24659147.ref                  TAGTCGCCCCGCCTCCAATCACCGCGGCCCTGCCCCGTCCGGGTCCGAAGGCTGAGCGCC   9420

LOC112206721.putative.POM121-like,protein1.START.LOC112206738    ------------------------------------------------------------   0
BCRP3.REF.NEW                                                    ------------------------------------------------------------   0
GGT1.start-POM121L10P.end.24583750-24659147.ref                  CGCCCCACCCCGCGCCGGAGCGAGGCCAGTGGGTTCCTCGGTTTGGCCTGATCACACTGG   9480

LOC112206721.putative.POM121-like,protein1.START.LOC112206738    ------------------------------------------------------------   0
BCRP3.REF.NEW                                                    ------------------------------------------------------------   0
GGT1.start-POM121L10P.end.24583750-24659147.ref                  AGGAGGCCCTGCTTGCGTCAGTCTTGGCGCTAGAACCCGAGGGCCTGGAGCCGCCAGGGG   9540

LOC112206721.putative.POM121-like,protein1.START.LOC112206738    ------------------------------------------------------------   0
BCRP3.REF.NEW                                                    ------------------------------------------------------------   0
GGT1.start-POM121L10P.end.24583750-24659147.ref                  AGACGCGGAGCTCGGCCGAGACGAGGTGGCTTTTGGCACCTCTTTACCCTCTGGCCCCAG   9600

LOC112206721.putative.POM121-like,protein1.START.LOC112206738    ------------------------------------------------------------   0
BCRP3.REF.NEW                                                    ------------------------------------------------------------   0
GGT1.start-POM121L10P.end.24583750-24659147.ref                  TGCTGACTGGGGAATAGTCGCTTTGACCGTGCAAAGAGCATTCTAGGCGGGGTGGGGGGG   9660

LOC112206721.putative.POM121-like,protein1.START.LOC112206738    ------------------------------------------------------------   0
BCRP3.REF.NEW                                                    ------------------------------------------------------------   0
GGT1.start-POM121L10P.end.24583750-24659147.ref                  TTCTGCTGTGTGTGCCAGTGTTAGGTATTGCCCCCATTGCTTTTAGAAATATGCCCTGCA   9720

LOC112206721.putative.POM121-like,protein1.START.LOC112206738    ------------------------------------------------------------   0
BCRP3.REF.NEW                                                    ------------------------------------------------------------   0
GGT1.start-POM121L10P.end.24583750-24659147.ref                  TGGTGAATCTCCGTCTCTACTAAAATACAAAAATTAGGCCGGGCATGGTGGCTCACGCCT   9780

LOC112206721.putative.POM121-like,protein1.START.LOC112206738    ------------------------------------------------------------   0
```

```
BCRP3.REF.NEW                                                    ------------------------------------------------------------   0
GGT1.start-POM121L10P.end.24583750-24659147.ref                  GTAATCCCAGCACTTTGGGGGGCCGAGGCAGGCGGAATCTAAGGTCAGGAGTTCAAGACT   9840

LOC112206721.putative.POM121-like,protein1.START.LOC112206738    ------------------------------------------------------------   0
BCRP3.REF.NEW                                                    ------------------------------------------------------------   0
GGT1.start-POM121L10P.end.24583750-24659147.ref                  AGCCTGGCTAACATGGTGAAACCACATTTTTATTGAAAATACAAAAAATTAGCCGGGCGT   9900

LOC112206721.putative.POM121-like,protein1.START.LOC112206738    ------------------------------------------------------------   0
BCRP3.REF.NEW                                                    ------------------------------------------------------------   0
GGT1.start-POM121L10P.end.24583750-24659147.ref                  GGTGGCGGGCGCCTGTAATCCCAGCTATTCGGGAGGTTGAGGTAGGAGAATCAATTGAAC   9960

LOC112206721.putative.POM121-like,protein1.START.LOC112206738    ------------------------------------------------------------   0
BCRP3.REF.NEW                                                    ------------------------------------------------------------   0
GGT1.start-POM121L10P.end.24583750-24659147.ref                  CTGGGAGGCGGAGGTTACAGTGAGCCGAGATGGCGCCACCGCACTCTACCCTGGGTGACA   10020

LOC112206721.putative.POM121-like,protein1.START.LOC112206738    ------------------------------------------------------------   0
BCRP3.REF.NEW                                                    ------------------------------------------------------------   0
GGT1.start-POM121L10P.end.24583750-24659147.ref                  GAGCAAAACTCTGTCTCAAAAAAAAAAAAAAGAAAAGAAAAGAAATCTCTGCAGCTGGCA   10080

LOC112206721.putative.POM121-like,protein1.START.LOC112206738    ------------------------------------------------------------   0
BCRP3.REF.NEW                                                    ------------------------------------------------------------   0
GGT1.start-POM121L10P.end.24583750-24659147.ref                  GTTGTGGTGTAGGACCTGCTCACGCTACATGGACACTCCTGCACTGGCAAATATTTACTG   10140

LOC112206721.putative.POM121-like,protein1.START.LOC112206738    ------------------------------------------------------------   0
BCRP3.REF.NEW                                                    ------------------------------------------------------------   0
GGT1.start-POM121L10P.end.24583750-24659147.ref                  CCTCCTGCTTCTTGGTTTGGGGCAGGCCCCCACCTCTGACCCCAGCCTGGGAAATAAACC   10200

LOC112206721.putative.POM121-like,protein1.START.LOC112206738    ------------------------------------------------------------   0
BCRP3.REF.NEW                                                    ------------------------------------------------------------   0
GGT1.start-POM121L10P.end.24583750-24659147.ref                  GGACGAAGAGTCAGGTTAACCGTTAAGCCCAGCTCTGGGCAAGCTGGCAGCAGGCGGGCT   10260

LOC112206721.putative.POM121-like,protein1.START.LOC112206738    ------------------------------------------------------------   0
BCRP3.REF.NEW                                                    ------------------------------------------------------------   0
GGT1.start-POM121L10P.end.24583750-24659147.ref                  GTATCTGCCGCTGGCTTCAGTCTGCCTCCCTGACAAGGGGTGGGGGTGGCGGCAGGCAGA   10320

LOC112206721.putative.POM121-like,protein1.START.LOC112206738    ------------------------------------------------------------   0
BCRP3.REF.NEW                                                    ------------------------------------------------------------   0
GGT1.start-POM121L10P.end.24583750-24659147.ref                  GTGAGATGGGGGCCCTGGCTCCTCTGAGGACCAGGGTGAGGTACGGACCCACCCAGCTCA   10380

LOC112206721.putative.POM121-like,protein1.START.LOC112206738    ------------------------------------------------------------   0
BCRP3.REF.NEW                                                    ------------------------------------------------------------   0
GGT1.start-POM121L10P.end.24583750-24659147.ref                  AGGTGTATCCCACTTCCCAGGGCTCCCTCAGCAGAAGCCTAGGTCTCTGCTGCAGGCCTG   10440

LOC112206721.putative.POM121-like,protein1.START.LOC112206738    ------------------------------------------------------------   0
BCRP3.REF.NEW                                                    ------------------------------------------------------------   0
GGT1.start-POM121L10P.end.24583750-24659147.ref                  CTGGGCCCCTGCAGCCCCAATCCCGGCTCTGGGACGCTCACATCTCCTGCGTGGGGAACG   10500

LOC112206721.putative.POM121-like,protein1.START.LOC112206738    ------------------------------------------------------------   0
BCRP3.REF.NEW                                                    ------------------------------------------------------------   0
GGT1.start-POM121L10P.end.24583750-24659147.ref                  TTTGAGGCACTGATCTGCAGTTCCCACTTCTCTAAGCAAGGAGCACGTGTTTAGCCTGTG   10560

LOC112206721.putative.POM121-like,protein1.START.LOC112206738    ------------------------------------------------------------   0
BCRP3.REF.NEW                                                    ------------------------------------------------------------   0
GGT1.start-POM121L10P.end.24583750-24659147.ref                  AGGACTGGTACCTGTTGTGCAGGAAGGGCTGAGCACTGTCCGAGGGCATATATGAGGGAC   10620

LOC112206721.putative.POM121-like,protein1.START.LOC112206738    ------------------------------------------------------------   0
BCRP3.REF.NEW                                                    ------------------------------------------------------------   0
GGT1.start-POM121L10P.end.24583750-24659147.ref                  GACAGCCAAGCACCCGTGTGTATGTGTGTGTGTGTGTGTGTGTGTGTGTGTGTGTGTCTG   10680

LOC112206721.putative.POM121-like,protein1.START.LOC112206738    ------------------------------------------------------------   0
BCRP3.REF.NEW                                                    ------------------------------------------------------------   0
GGT1.start-POM121L10P.end.24583750-24659147.ref                  CTAATTACCCTATGAAGATTCAAGGTTCCTTCCTTTGGGTTAAGCACTGCCTGCCCAGAG   10740

LOC112206721.putative.POM121-like,protein1.START.LOC112206738    ------------------------------------------------------------   0
BCRP3.REF.NEW                                                    ------------------------------------------------------------   0
GGT1.start-POM121L10P.end.24583750-24659147.ref                  ATGGCAGTGGGGAGAGGGCAGGGTGCTAGGCTCTGTCATCCTGTGGGGCTGGGCCCCTGA   10800

LOC112206721.putative.POM121-like,protein1.START.LOC112206738    ------------------------------------------------------------   0
BCRP3.REF.NEW                                                    ------------------------------------------------------------   0
GGT1.start-POM121L10P.end.24583750-24659147.ref                  GACAAGGCAGAGGACAGCAGGATGTCTTAGCCCCTTGACCATCCACGGACCCCCAACCCC   10860

LOC112206721.putative.POM121-like,protein1.START.LOC112206738    ------------------------------------------------------------   0
BCRP3.REF.NEW                                                    ------------------------------------------------------------   0
GGT1.start-POM121L10P.end.24583750-24659147.ref                  TGCTGGAGCAGTGCTTGCACCCCCTTCTCCCCTGGCACCTGCCAGCTGGACCCAACAGCC   10920

LOC112206721.putative.POM121-like,protein1.START.LOC112206738    ------------------------------------------------------------   0
BCRP3.REF.NEW                                                    ------------------------------------------------------------   0
GGT1.start-POM121L10P.end.24583750-24659147.ref                  TCTCCTCCCACTCCAGTGCAGCCAGCATTGTCCTGGGGCCACCTTGGCACCACAGGTTTC   10980

LOC112206721.putative.POM121-like,protein1.START.LOC112206738    ------------------------------------------------------------   0
BCRP3.REF.NEW                                                    ------------------------------------------------------------   0
GGT1.start-POM121L10P.end.24583750-24659147.ref                  CTCCCTGGCAAACCTGCATGACGGCCTTGCGTGCCCTCCCCCAGGGCCAAGGCGCTGTTG   11040

LOC112206721.putative.POM121-like,protein1.START.LOC112206738    ------------------------------------------------------------   0
BCRP3.REF.NEW                                                    ------------------------------------------------------------   0
GGT1.start-POM121L10P.end.24583750-24659147.ref                  CTGAACTGAAGTGGCAACCTACCCGGTCAGAGCTTTAAGCTGCAGCTCTGATGCCCCAGG   11100

LOC112206721.putative.POM121-like,protein1.START.LOC112206738    ------------------------------------------------------------   0
BCRP3.REF.NEW                                                    ------------------------------------------------------------   0
GGT1.start-POM121L10P.end.24583750-24659147.ref                  GACCTCTCAGACCCCCCTGCCCAGAGCAGGCAGCCCGGTAAGCCCTCCTCCTCTTCCCCT   11160

LOC112206721.putative.POM121-like,protein1.START.LOC112206738    ------------------------------------------------------------   0
BCRP3.REF.NEW                                                    ------------------------------------------------------------   0
GGT1.start-POM121L10P.end.24583750-24659147.ref                  GCAGCCTCAGGGCTGGAGCCACAGCTGGGAGGGCTCCCAGATCCTGACCATATCCTCCCA   11220

LOC112206721.putative.POM121-like,protein1.START.LOC112206738    ------------------------------------------------------------   0
BCRP3.REF.NEW                                                    ------------------------------------------------------------   0
GGT1.start-POM121L10P.end.24583750-24659147.ref                  GAGGCTGAGCCAGGCCTGGGCACTGTCCCCTCAAATGTGAGAATACTGGGCAGGGGGAGG   11280

LOC112206721.putative.POM121-like,protein1.START.LOC112206738    ------------------------------------------------------------   0
BCRP3.REF.NEW                                                    ------------------------------------------------------------   0
GGT1.start-POM121L10P.end.24583750-24659147.ref                  GGAGAAAGGAGGGAATGGAGGGCTGAGCCTGGAGGCATGAAAGGGGCAACCTAGGCAGCG   11340

LOC112206721.putative.POM121-like,protein1.START.LOC112206738    ------------------------------------------------------------   0
BCRP3.REF.NEW                                                    ------------------------------------------------------------   0
GGT1.start-POM121L10P.end.24583750-24659147.ref                  GTATGGGGGAGTGCTCAGAGCTTAGAGTCCTGTGGAAGGCAGAGAACTGCCCCAGCCCCT   11400

LOC112206721.putative.POM121-like,protein1.START.LOC112206738    ------------------------------------------------------------   0
BCRP3.REF.NEW                                                    ------------------------------------------------------------   0
GGT1.start-POM121L10P.end.24583750-24659147.ref                  GCCTCTCATCCCCCACCCTCTGTGTCCCTGGCCGAGAGGTTCCTGGAGGACATTTTTCCA   11460

LOC112206721.putative.POM121-like,protein1.START.LOC112206738    ------------------------------------------------------------   0
BCRP3.REF.NEW                                                    ------------------------------------------------------------   0
GGT1.start-POM121L10P.end.24583750-24659147.ref                  GCAAAAGAGGGAGGGTGGTGTCTGGGCCTAGATCCTTGGATGGGCTTTTCTCTGCCTGCT   11520

LOC112206721.putative.POM121-like,protein1.START.LOC112206738    ------------------------------------------------------------   0
```

```
BCRP3.REF.NEW                                                       ------------------------------------------------------------   0
GGT1.start-POM121L10P.end.24583750-24659147.ref                     TGTGGGGTGCCTAGGGCGAGTGGGCTTTTGGAGACAATTTCTGGCCTAACCTGACTTGGA   11580

LOC112206721.putative.POM121-like,protein1.START.LOC112206738       ------------------------------------------------------------   0
BCRP3.REF.NEW                                                       ------------------------------------------------------------   0
GGT1.start-POM121L10P.end.24583750-24659147.ref                     CAGCAGCCCCCAGAGGCACAGCTCTCCCCTCAGGCATGGGGGCATGATTCCACCTCGCTA   11640

LOC112206721.putative.POM121-like,protein1.START.LOC112206738       ------------------------------------------------------------   0
BCRP3.REF.NEW                                                       ------------------------------------------------------------   0
GGT1.start-POM121L10P.end.24583750-24659147.ref                     GCCCACATGTGTTCTTCACGGAGGGCTGCTGGCCCGTCTCCCTGGGGTTACCCTGAGCAG   11700

LOC112206721.putative.POM121-like,protein1.START.LOC112206738       ------------------------------------------------------------   0
BCRP3.REF.NEW                                                       ------------------------------------------------------------   0
GGT1.start-POM121L10P.end.24583750-24659147.ref                     CAGAGCTGTGTTTGTTGAGACTCCATGGGGTGGAGGGATTGCGATGTTGTCCTCTCAGTT   11760

LOC112206721.putative.POM121-like,protein1.START.LOC112206738       ------------------------------------------------------------   0
BCRP3.REF.NEW                                                       ------------------------------------------------------------   0
GGT1.start-POM121L10P.end.24583750-24659147.ref                     CCCGGGGCTGATGTGGAAGCTCAAGGGCTTGCCCTGGATTCTTCAGGGTAGCCCTGCCAT   11820

LOC112206721.putative.POM121-like,protein1.START.LOC112206738       ------------------------------------------------------------   0
BCRP3.REF.NEW                                                       ------------------------------------------------------------   0
GGT1.start-POM121L10P.end.24583750-24659147.ref                     CCCTAGTGAGTCAGTGAGTTGGGAGGTTGGGGGCTGGAGAATCAGGTAGGGAGGACACAG   11880

LOC112206721.putative.POM121-like,protein1.START.LOC112206738       ------------------------------------------------------------   0
BCRP3.REF.NEW                                                       ------------------------------------------------------------   0
GGT1.start-POM121L10P.end.24583750-24659147.ref                     CTAGACCTCAGGCCCTGATGGGAACGTACTAGGCTCCAAGTGGGACCTGCCAGGAGACAA   11940

LOC112206721.putative.POM121-like,protein1.START.LOC112206738       ------------------------------------------------------------   0
BCRP3.REF.NEW                                                       ------------------------------------------------------------   0
GGT1.start-POM121L10P.end.24583750-24659147.ref                     GAATTGATGACAGCCACACCTTTGGAGTCTCAAGCCCTCTGCCACCCATGCGGTGGCCTA   12000

LOC112206721.putative.POM121-like,protein1.START.LOC112206738       ------------------------------------------------------------   0
BCRP3.REF.NEW                                                       ------------------------------------------------------------   0
GGT1.start-POM121L10P.end.24583750-24659147.ref                     GTTCAAATGACAGGAACACAAGGTCCAGCTGGCCCAATGGGCAGGTAGAGGGCTGCAGCC   12060

LOC112206721.putative.POM121-like,protein1.START.LOC112206738       ------------------------------------------------------------   0
BCRP3.REF.NEW                                                       ------------------------------------------------------------   0
GGT1.start-POM121L10P.end.24583750-24659147.ref                     AGGGCTGGAACTGGCCTTGCACTGCCTCTGGGCCCTTCTCTACCCACCCAGTAGTATTGT   12120

LOC112206721.putative.POM121-like,protein1.START.LOC112206738       ------------------------------------------------------------   0
BCRP3.REF.NEW                                                       ------------------------------------------------------------   0
GGT1.start-POM121L10P.end.24583750-24659147.ref                     TCAAGCCCAGGAGGGAAAAAGGCCAGCTCAGGGTTAATGTTACCTCAGCAAGTAGCTGAG   12180

LOC112206721.putative.POM121-like,protein1.START.LOC112206738       ------------------------------------------------------------   0
BCRP3.REF.NEW                                                       ------------------------------------------------------------   0
GGT1.start-POM121L10P.end.24583750-24659147.ref                     TGGCACTGAGCCTCCTGGTAGCCACTGTAAAGAAGGACACAGAATGGTTTGGGGGCATTC   12240

LOC112206721.putative.POM121-like,protein1.START.LOC112206738       ------------------------------------------------------------   0
BCRP3.REF.NEW                                                       ------------------------------------------------------------   0
GGT1.start-POM121L10P.end.24583750-24659147.ref                     GCTAGGAGGAAGGAAGCAGATGGATACTCTATGGAGACAGAATGTCCTCTGTGTTTGCTC   12300

LOC112206721.putative.POM121-like,protein1.START.LOC112206738       ------------------------------------------------------------   0
BCRP3.REF.NEW                                                       ------------------------------------------------------------   0
GGT1.start-POM121L10P.end.24583750-24659147.ref                     TACAGGAACGGGTTGTCAACCTTCTGCAGGGCCAGGAATTGTCATGAGGCTGTAGTAGGC   12360

LOC112206721.putative.POM121-like,protein1.START.LOC112206738       ------------------------------------------------------------   0
BCRP3.REF.NEW                                                       ------------------------------------------------------------   0
GGT1.start-POM121L10P.end.24583750-24659147.ref                     AGCAGAGCTTGGCGGGGTGAGGCGAGATCACAGGTATAGGCTCAGGGCCTGGATTCAAAT   12420

LOC112206721.putative.POM121-like,protein1.START.LOC112206738       ------------------------------------------------------------   0
BCRP3.REF.NEW                                                       ------------------------------------------------------------   0
GGT1.start-POM121L10P.end.24583750-24659147.ref                     CCCCTGGGCTGCCACTTGTTGGATGTGTAACCATAGGCAGAGTAAGGACCTGCCATAGCA   12480

LOC112206721.putative.POM121-like,protein1.START.LOC112206738       ------------------------------------------------------------   0
BCRP3.REF.NEW                                                       ------------------------------------------------------------   0
GGT1.start-POM121L10P.end.24583750-24659147.ref                     AATTACCACAAACTCAGTGGCTTAAAGCAACAGAATAGTTCTGGAGGCTAAAAGTCTGAA   12540

LOC112206721.putative.POM121-like,protein1.START.LOC112206738       ------------------------------------------------------------   0
BCRP3.REF.NEW                                                       ------------------------------------------------------------   0
GGT1.start-POM121L10P.end.24583750-24659147.ref                     ATCCAAGTATAGGCCAAGCCACACTCCCTTTGAGGACTCCAGGGGAAGATCCTTCCTTGT   12600

LOC112206721.putative.POM121-like,protein1.START.LOC112206738       ------------------------------------------------------------   0
BCRP3.REF.NEW                                                       ------------------------------------------------------------   0
GGT1.start-POM121L10P.end.24583750-24659147.ref                     CTCTTCAGCTTCTGGTGGCTCCTGGCAATCCTTGGCCTTCCTTGGCTGCTGCTGCATCAC   12660

LOC112206721.putative.POM121-like,protein1.START.LOC112206738       ------------------------------------------------------------   0
BCRP3.REF.NEW                                                       ------------------------------------------------------------   0
GGT1.start-POM121L10P.end.24583750-24659147.ref                     TCCAGTCTCTGAAACCATCTACCCTATGTGTCTGTGTGGCCTTCACATGGCATTCCTTAT   12720

LOC112206721.putative.POM121-like,protein1.START.LOC112206738       ------------------------------------------------------------   0
BCRP3.REF.NEW                                                       ------------------------------------------------------------   0
GGT1.start-POM121L10P.end.24583750-24659147.ref                     TTTTGCAGTGGGGTCTCACTATGTTGCCCAGGCTGGTCTTGAACTCCTGGGTCCAAGCAG   12780

LOC112206721.putative.POM121-like,protein1.START.LOC112206738       ------------------------------------------------------------   0
BCRP3.REF.NEW                                                       ------------------------------------------------------------   0
GGT1.start-POM121L10P.end.24583750-24659147.ref                     TCCTCCTGCTTCAGCCTCCCAAGTAGCTGGGATTATGAGTGAGTGCCACCATGCCCGGCT   12840

LOC112206721.putative.POM121-like,protein1.START.LOC112206738       ------------------------------------------------------------   0
BCRP3.REF.NEW                                                       ------------------------------------------------------------   0
GGT1.start-POM121L10P.end.24583750-24659147.ref                     CACATGGCCTCCTTGTAATAACGCTAGTGCCTGGGTGCGGTGGCTCACGCCTGTAATCCC   12900

LOC112206721.putative.POM121-like,protein1.START.LOC112206738       ------------------------------------------------------------   0
BCRP3.REF.NEW                                                       ------------------------------------------------------------   0
GGT1.start-POM121L10P.end.24583750-24659147.ref                     AGCACTTTTGGAGGCTGCGGTGGACGGACCATGAGGTCAGGAAATTGAGGCCATCCTGTC   12960

LOC112206721.putative.POM121-like,protein1.START.LOC112206738       ------------------------------------------------------------   0
BCRP3.REF.NEW                                                       ------------------------------------------------------------   0
GGT1.start-POM121L10P.end.24583750-24659147.ref                     CAACATGGTGAAACCCTGTCTCTACTAAAATACAAAAAAAAAAAAAAAAAAAAAATTAGCC   13020

LOC112206721.putative.POM121-like,protein1.START.LOC112206738       ------------------------------------------------------------   0
BCRP3.REF.NEW                                                       ------------------------------------------------------------   0
GGT1.start-POM121L10P.end.24583750-24659147.ref                     TGGTGTGGTGACACATGCCTGTAGTCCCAGCTACTTGGGAGGCTGAGACAGGGGAATCGC   13080

LOC112206721.putative.POM121-like,protein1.START.LOC112206738       ------------------------------------------------------------   0
BCRP3.REF.NEW                                                       ------------------------------------------------------------   0
GGT1.start-POM121L10P.end.24583750-24659147.ref                     TTGAACCCGGGACGCAGAAGTTGCAGTGAGCTGAGATTGCGTGGTGACTTCACTCCAGCC   13140

LOC112206721.putative.POM121-like,protein1.START.LOC112206738       ------------------------------------------------------------   0
BCRP3.REF.NEW                                                       ------------------------------------------------------------   0
GGT1.start-POM121L10P.end.24583750-24659147.ref                     TGGTGACAGAGCGAGACTCTGTCTCAAAAAAAAAAAAAAAAAAAAAAAGAACTGAAGCTAC   13200

LOC112206721.putative.POM121-like,protein1.START.LOC112206738       ------------------------------------------------------------   0
BCRP3.REF.NEW                                                       ------------------------------------------------------------   0
GGT1.start-POM121L10P.end.24583750-24659147.ref                     TGGATTTAGGGCTCATTCATTCTTTTTTTAATTTTAATTTTTTTTTTTTTTTGAGACAGT   13260

LOC112206721.putative.POM121-like,protein1.START.LOC112206738       ------------------------------------------------------------   0
```

```
BCRP3.REF.NEW                                                      ------------------------------------------------------------   0
GGT1.start-POM121L10P.end.24583750-24659147.ref                    CTCACTCTGTTAGCCCAGGCTGGAGTGCAATGGCATGATCTTGGCTCACTGCAACCTCCA   13320

LOC112206721.putative.POM121-like,protein1.START.LOC112206738      ------------------------------------------------------------   0
BCRP3.REF.NEW                                                      ------------------------------------------------------------   0
GGT1.start-POM121L10P.end.24583750-24659147.ref                    CCTCCCAGGTTCAAGCGATTCTCCTGCCTCAGCCTTCCAAGTAGCTGGGATTACAGGTGC   13380

LOC112206721.putative.POM121-like,protein1.START.LOC112206738      ------------------------------------------------------------   0
BCRP3.REF.NEW                                                      ------------------------------------------------------------   0
GGT1.start-POM121L10P.end.24583750-24659147.ref                    CCGCCACCACGCCCAGCTAATTTTTGTATTTTTAGTAGAGACAGCGTTTCACCATATTGG   13440

LOC112206721.putative.POM121-like,protein1.START.LOC112206738      ------------------------------------------------------------   0
BCRP3.REF.NEW                                                      ------------------------------------------------------------   0
GGT1.start-POM121L10P.end.24583750-24659147.ref                    TCAGGCTGGTCTTGAACTCCTGACCTCAGGTGATCCACCCCTGTCAGCCTCCCAGTGTTG   13500

LOC112206721.putative.POM121-like,protein1.START.LOC112206738      ------------------------------------------------------------   0
BCRP3.REF.NEW                                                      ------------------------------------------------------------   0
GGT1.start-POM121L10P.end.24583750-24659147.ref                    GGATTACAGGCGTGAGCCACTGTGCCCAACAGGGCTCATTCATTCTAATCCCGTATGATC   13560

LOC112206721.putative.POM121-like,protein1.START.LOC112206738      ------------------------------------------------------------   0
BCRP3.REF.NEW                                                      ------------------------------------------------------------   0
GGT1.start-POM121L10P.end.24583750-24659147.ref                    TCATCTTAACTAATTACTTATGTAGAGACTCTATTTCCAAATAAGGTCGTGTGAGGAAAA   13620

LOC112206721.putative.POM121-like,protein1.START.LOC112206738      ------------------------------------------------------------   0
BCRP3.REF.NEW                                                      ------------------------------------------------------------   0
GGT1.start-POM121L10P.end.24583750-24659147.ref                    ACCAACTCAAACTGTGTTTTTTGGCCTGGCGTGGTGGCTCACACCTATAATTCCAGCACT   13680

LOC112206721.putative.POM121-like,protein1.START.LOC112206738      ------------------------------------------------------------   0
BCRP3.REF.NEW                                                      ------------------------------------------------------------   0
GGT1.start-POM121L10P.end.24583750-24659147.ref                    TTGGGGAGGCTAAGGTGGGCGGATCACTTGAGGCTAGGAGTTTGAGACCAGCCTGGCCAA   13740

LOC112206721.putative.POM121-like,protein1.START.LOC112206738      ------------------------------------------------------------   0
BCRP3.REF.NEW                                                      ------------------------------------------------------------   0
GGT1.start-POM121L10P.end.24583750-24659147.ref                    CATAGCAAAACCCCATCTTCACTAAAAAATACAAAACAAAAATTAGCTGGGTATGGTGGC   13800

LOC112206721.putative.POM121-like,protein1.START.LOC112206738      ------------------------------------------------------------   0
BCRP3.REF.NEW                                                      ------------------------------------------------------------   0
GGT1.start-POM121L10P.end.24583750-24659147.ref                    ATGTGCCAGTAGTCCCAGCTACTCAGGAGGCTGAGGCAGGAGAATCGCTTGAACCTAGGA   13860

LOC112206721.putative.POM121-like,protein1.START.LOC112206738      ------------------------------------------------------------   0
BCRP3.REF.NEW                                                      ------------------------------------------------------------   0
GGT1.start-POM121L10P.end.24583750-24659147.ref                    GGCGGAGGTTTCAGTGAGCCGAGATAGCACCACGGCATTCCAGCCTGGGTGACAGAGTGA   13920

LOC112206721.putative.POM121-like,protein1.START.LOC112206738      ------------------------------------------------------------   0
BCRP3.REF.NEW                                                      ------------------------------------------------------------   0
GGT1.start-POM121L10P.end.24583750-24659147.ref                    GACTCCATCTCAAAAACACACACACACACACACACACAAAACAACTGTGTTTCGCCTCTG   13980

LOC112206721.putative.POM121-like,protein1.START.LOC112206738      ------------------------------------------------------------   0
BCRP3.REF.NEW                                                      ------------------------------------------------------------   0
GGT1.start-POM121L10P.end.24583750-24659147.ref                    TTCTCACAAGACAATAATCATCACAGAAGACTTCTTGTAACCAAATTTGTGGATTCTCCC   14040

LOC112206721.putative.POM121-like,protein1.START.LOC112206738      ------------------------------------------------------------   0
BCRP3.REF.NEW                                                      ------------------------------------------------------------   0
GGT1.start-POM121L10P.end.24583750-24659147.ref                    ACACACCCAGCAAGAATCAATTCTGCAGCAGACTCCAGCTGGTGTCTTCCAATTCAACTC   14100

LOC112206721.putative.POM121-like,protein1.START.LOC112206738      ------------------------------------------------------------   0
BCRP3.REF.NEW                                                      ------------------------------------------------------------   0
GGT1.start-POM121L10P.end.24583750-24659147.ref                    AATTCTGGTGCTGTCTACCTGGAGATGGCCTCAGAAACCACAGGTTGAGGGCTTGGTCCC   14160

LOC112206721.putative.POM121-like,protein1.START.LOC112206738      ------------------------------------------------------------   0
BCRP3.REF.NEW                                                      ------------------------------------------------------------   0
GGT1.start-POM121L10P.end.24583750-24659147.ref                    ACAAGACCAACCCCCATCCCACCAGTCACAAGTCTGGGCATTGGGAACATCTGACTAACT   14220

LOC112206721.putative.POM121-like,protein1.START.LOC112206738      ------------------------------------------------------------   0
BCRP3.REF.NEW                                                      ------------------------------------------------------------   0
GGT1.start-POM121L10P.end.24583750-24659147.ref                    GGCTTCTAGTTGGGGTTCCAAGGCCCCCTCTTTGGGTTCAATTAATTTGCTAGAGTAGCT   14280

LOC112206721.putative.POM121-like,protein1.START.LOC112206738      ------------------------------------------------------------   0
BCRP3.REF.NEW                                                      ------------------------------------------------------------   0
GGT1.start-POM121L10P.end.24583750-24659147.ref                    CACACAACTCAGGGAGACCTGTTTATCGGTTTATTTCAAAGGATGTTTTATTTTATTTTC   14340

LOC112206721.putative.POM121-like,protein1.START.LOC112206738      ------------------------------------------------------------   0
BCRP3.REF.NEW                                                      ------------------------------------------------------------   0
GGT1.start-POM121L10P.end.24583750-24659147.ref                    TCCTCAGACATACTTTATCATCCCTTAAAGTCAGTAGCTCAAAGGAAACTTTAAAAGCTA   14400

LOC112206721.putative.POM121-like,protein1.START.LOC112206738      ------------------------------------------------------------   0
BCRP3.REF.NEW                                                      ------------------------------------------------------------   0
GGT1.start-POM121L10P.end.24583750-24659147.ref                    TAAATAGGCCGGGCACGGTGGCTCACGCCTGTAATCCCAGCACTTTGGGAGGTCGAGGCA   14460

LOC112206721.putative.POM121-like,protein1.START.LOC112206738      ------------------------------------------------------------   0
BCRP3.REF.NEW                                                      ------------------------------------------------------------   0
GGT1.start-POM121L10P.end.24583750-24659147.ref                    GGTGGATCACTTGAGGTCAGGAGTTGGAGACCAGCCTGGCCAACATGGTGAAACTCCATC   14520

LOC112206721.putative.POM121-like,protein1.START.LOC112206738      ------------------------------------------------------------   0
BCRP3.REF.NEW                                                      ------------------------------------------------------------   0
GGT1.start-POM121L10P.end.24583750-24659147.ref                    TCTACTAAAAGTACAAAATAAGCTGGGTGTGGTGGCAGGAGCCTGTAATCCCAGCTACTT   14580

LOC112206721.putative.POM121-like,protein1.START.LOC112206738      ------------------------------------------------------------   0
BCRP3.REF.NEW                                                      ------------------------------------------------------------   0
GGT1.start-POM121L10P.end.24583750-24659147.ref                    GGGAGGCTGAGGCAGGAGAATCATCGGTTGAACCCGAGGGGTGCAAGTTGCAGTGAGCCA   14640

LOC112206721.putative.POM121-like,protein1.START.LOC112206738      ------------------------------------------------------------   0
BCRP3.REF.NEW                                                      ------------------------------------------------------------   0
GGT1.start-POM121L10P.end.24583750-24659147.ref                    AGATCACACCTCTGCACTCTAGCATGGGCAACAGAGCGAGACTGAGACTCCGTCTCAAAA   14700

LOC112206721.putative.POM121-like,protein1.START.LOC112206738      ------------------------------------------------------------   0
BCRP3.REF.NEW                                                      ------------------------------------------------------------   0
GGT1.start-POM121L10P.end.24583750-24659147.ref                    AAAAAAAAAAAAAAAAAGATAAAGGCTACAAATAAACAGTCAGATGAAGAGATACATAGG   14760

LOC112206721.putative.POM121-like,protein1.START.LOC112206738      ------------------------------------------------------------   0
BCRP3.REF.NEW                                                      ------------------------------------------------------------   0
GGT1.start-POM121L10P.end.24583750-24659147.ref                    GTAAAGACTGGAAGACTCCTAAGTACAGAAGTTTCTGTCCTGGTAGAGCTGGACACATGG   14820

LOC112206721.putative.POM121-like,protein1.START.LOC112206738      ------------------------------------------------------------   0
BCRP3.REF.NEW                                                      ------------------------------------------------------------   0
GGT1.start-POM121L10P.end.24583750-24659147.ref                    ATGAGTTGGTTTTTTTTGTTGTTGCCCAGGCTGGAGTACAGTGGCACTATCTCAGCTCAC   14880

LOC112206721.putative.POM121-like,protein1.START.LOC112206738      ------------------------------------------------------------   0
BCRP3.REF.NEW                                                      ------------------------------------------------------------   0
GGT1.start-POM121L10P.end.24583750-24659147.ref                    TGCAGCCTCTGCCTCCCGTGCTCAAGTGATCCTCCCCCCTCAGCCTCCCAAGTAGCTGGG   14940

LOC112206721.putative.POM121-like,protein1.START.LOC112206738      ------------------------------------------------------------   0
BCRP3.REF.NEW                                                      ------------------------------------------------------------   0
GGT1.start-POM121L10P.end.24583750-24659147.ref                    ACCACAGGCATGTGCCACTATGCCCAGCTAACTTTTTTATTTTTGTAGAGATGGGGTCTC   15000

LOC112206721.putative.POM121-like,protein1.START.LOC112206738      ------------------------------------------------------------   0
```

```
BCRP3.REF.NEW                                                  ------------------------------------------------------------   0
GGT1.start-POM121L10P.end.24583750-24659147.ref                TCTATTTTGCCCAGGCTGGTCGTGAACTCCTGGGCTCAAGCCATCCACCCACCTCGCCCT   15060

LOC112206721.putative.POM121-like,protein1.START.LOC112206738  ------------------------------------------------------------   0
BCRP3.REF.NEW                                                  ------------------------------------------------------------   0
GGT1.start-POM121L10P.end.24583750-24659147.ref                CCCAAAGCGCTGGGATTATAGGCATGAGCCACTGCACCTGGCCATGGATGAGTTCTTGTT   15120

LOC112206721.putative.POM121-like,protein1.START.LOC112206738  ------------------------------------------------------------   0
BCRP3.REF.NEW                                                  ------------------------------------------------------------   0
GGT1.start-POM121L10P.end.24583750-24659147.ref                CCCCTTCCTGTCAGCCTCCACATGAAACTCCCCAAACCCTGTCCTCTTGGGCCTTTTTTA   15180

LOC112206721.putative.POM121-like,protein1.START.LOC112206738  ------------------------------------------------------------   0
BCRP3.REF.NEW                                                  ------------------------------------------------------------   0
GGT1.start-POM121L10P.end.24583750-24659147.ref                AAAAAATTAATTAAGAAATTAAAGACAGGGTCTCTCTATGTTTCCCAGGCTGCTCTTGAA   15240

LOC112206721.putative.POM121-like,protein1.START.LOC112206738  ------------------------------------------------------------   0
BCRP3.REF.NEW                                                  ------------------------------------------------------------   0
GGT1.start-POM121L10P.end.24583750-24659147.ref                CTCCTAGTCTCAAGTGATCCACCCACCTCGTCCTCCCATAGTGCTGGGATTACAAGATTA   15300

LOC112206721.putative.POM121-like,protein1.START.LOC112206738  ------------------------------------------------------------   0
BCRP3.REF.NEW                                                  ------------------------------------------------------------   0
GGT1.start-POM121L10P.end.24583750-24659147.ref                GAGATGCCAGCTGTCAGGTGGCTAGGTGGATTCAGTGTAAAGTCCTAGAAGATGCATTTT   15360

LOC112206721.putative.POM121-like,protein1.START.LOC112206738  ------------------------------------------------------------   0
BCRP3.REF.NEW                                                  ------------------------------------------------------------   0
GGT1.start-POM121L10P.end.24583750-24659147.ref                AGTAAAAACAGCTAGCCACCTGACAGCTGGCATCCATCCATTCAGTGAAGATTGTTGGGC   15420

LOC112206721.putative.POM121-like,protein1.START.LOC112206738  ------------------------------------------------------------   0
BCRP3.REF.NEW                                                  ------------------------------------------------------------   0
GGT1.start-POM121L10P.end.24583750-24659147.ref                ACTGTGGATACTGTGGAGGAGGCAGCAGTCGCAGCCGGCCTGCAGAGTGAGATGCAGTGC   15480

LOC112206721.putative.POM121-like,protein1.START.LOC112206738  ------------------------------------------------------------   0
BCRP3.REF.NEW                                                  ------------------------------------------------------------   0
GGT1.start-POM121L10P.end.24583750-24659147.ref                CAGAGGGCTTCAGGAGGAGGTGGCATTGTGATTGATGTCTGAAGAGTGTCTCCATGATAA   15540

LOC112206721.putative.POM121-like,protein1.START.LOC112206738  ------------------------------------------------------------   0
BCRP3.REF.NEW                                                  ------------------------------------------------------------   0
GGT1.start-POM121L10P.end.24583750-24659147.ref                TGGAGTCAGTGCAAAGTCCCAAAGACTGGGAAGGAGCAAGAGGAAAGGATAGCTCATATC   15600

LOC112206721.putative.POM121-like,protein1.START.LOC112206738  ------------------------------------------------------------   0
BCRP3.REF.NEW                                                  ------------------------------------------------------------   0
GGT1.start-POM121L10P.end.24583750-24659147.ref                CAGCTGCCCCACTCCAAAAAAAAAAAAAAAAAGAGGAAAGGACGGGCACCAGCAAGGTAG   15660

LOC112206721.putative.POM121-like,protein1.START.LOC112206738  ------------------------------------------------------------   0
BCRP3.REF.NEW                                                  ------------------------------------------------------------   0
GGT1.start-POM121L10P.end.24583750-24659147.ref                GACAAGGGGGGCCTCTGGAGTGTTTTCTTCTCAGTTGGGGAGGGGTGTGCTCAGATTTGC   15720

LOC112206721.putative.POM121-like,protein1.START.LOC112206738  ------------------------------------------------------------   0
BCRP3.REF.NEW                                                  ------------------------------------------------------------   0
GGT1.start-POM121L10P.end.24583750-24659147.ref                ATGCCAAGTGCCTGGGCTGCAGTAGGGAAACAGCAGAGGGGCTGGGACCTGTGATTGGGA   15780

LOC112206721.putative.POM121-like,protein1.START.LOC112206738  ------------------------------------------------------------   0
BCRP3.REF.NEW                                                  ------------------------------------------------------------   0
GGT1.start-POM121L10P.end.24583750-24659147.ref                GTCTCCAGGTGAGAGGGTTGGCCTATGGGGACTGGAGGTCAGGTGAGGCTGGTAGTGGGT   15840

LOC112206721.putative.POM121-like,protein1.START.LOC112206738  ------------------------------------------------------------   0
BCRP3.REF.NEW                                                  ------------------------------------------------------------   0
GGT1.start-POM121L10P.end.24583750-24659147.ref                TATAGAGGTGGGAGCAGCTGGACAGGCCCAGTGAGGATGCAGGGGTAGCTGCCCAGGGCT   15900

LOC112206721.putative.POM121-like,protein1.START.LOC112206738  ------------------------------------------------------------   0
BCRP3.REF.NEW                                                  ------------------------------------------------------------   0
GGT1.start-POM121L10P.end.24583750-24659147.ref                AGACTCTGGCTGCTCTGGTGGCAGTTGCTTAGCTCAGGCTTGGGCACGTCCACCTGGCGC   15960

LOC112206721.putative.POM121-like,protein1.START.LOC112206738  ------------------------------------------------------------   0
BCRP3.REF.NEW                                                  ------------------------------------------------------------   0
GGT1.start-POM121L10P.end.24583750-24659147.ref                ATCTGAAACATTAGCCAGCAGGGCGCAGACTCGTCAGTGAGAGCATTCCTGAGCCCAGCA   16020

LOC112206721.putative.POM121-like,protein1.START.LOC112206738  ------------------------------------------------------------   0
BCRP3.REF.NEW                                                  ------------------------------------------------------------   0
GGT1.start-POM121L10P.end.24583750-24659147.ref                TCCAAGAAGCGAGGGAGGTCGGGAAGGTGCTGCTATGAGGCAGGGGGAGGTCCCCAAAAG   16080

LOC112206721.putative.POM121-like,protein1.START.LOC112206738  ------------------------------------------------------------   0
BCRP3.REF.NEW                                                  ------------------------------------------------------------   0
GGT1.start-POM121L10P.end.24583750-24659147.ref                TCCAGGTGAGAGCATGGCATGCGTGGAAAGTGCTGCGCAGTTTTGGCATCAGTGAAGTCA   16140

LOC112206721.putative.POM121-like,protein1.START.LOC112206738  ------------------------------------------------------------   0
BCRP3.REF.NEW                                                  ------------------------------------------------------------   0
GGT1.start-POM121L10P.end.24583750-24659147.ref                CAGGGGGCTGTGGGTGAACAGGTGTGCTTGGGCATGTCACCAGGGAGGCCTTGAGACAGG   16200

LOC112206721.putative.POM121-like,protein1.START.LOC112206738  ------------------------------------------------------------   0
BCRP3.REF.NEW                                                  ------------------------------------------------------------   0
GGT1.start-POM121L10P.end.24583750-24659147.ref                AGCGGGTTGGCTTTCCTGGAGATCAGGCTGTGGCATGGTGTCGGGACTGGGGCATGGACA   16260

LOC112206721.putative.POM121-like,protein1.START.LOC112206738  ------------------------------------------------------------   0
BCRP3.REF.NEW                                                  ------------------------------------------------------------   0
GGT1.start-POM121L10P.end.24583750-24659147.ref                GTCTTCCTCCACCCCCATCCCCTCCTTGGAGCTTTCAGACACTGCAGATAATTACCAGGT   16320

LOC112206721.putative.POM121-like,protein1.START.LOC112206738  ------------------------------------------------------------   0
BCRP3.REF.NEW                                                  ------------------------------------------------------------   0
GGT1.start-POM121L10P.end.24583750-24659147.ref                GATGTGATAAATACTACCTGTGGAGGGTGCCTTGGGCAGGGAGGACAGGGCGGCCAGGAG   16380

LOC112206721.putative.POM121-like,protein1.START.LOC112206738  ------------------------------------------------------------   0
BCRP3.REF.NEW                                                  ------------------------------------------------------------   0
GGT1.start-POM121L10P.end.24583750-24659147.ref                GGGGGCCCTTTGGGCCCCTGCTCTGAGCTGCTCATCAGGAAAGGGGAGAAGCTCCTACAG   16440

LOC112206721.putative.POM121-like,protein1.START.LOC112206738  ------------------------------------------------------------   0
BCRP3.REF.NEW                                                  ------------------------------------------------------------   0
GGT1.start-POM121L10P.end.24583750-24659147.ref                GCCTGGTGTTGGAGCCTAGCCAGACCCTGTGAGGCCTTGAAAGTAGGAGGTGCATTGTCT   16500

LOC112206721.putative.POM121-like,protein1.START.LOC112206738  ------------------------------------------------------------   0
BCRP3.REF.NEW                                                  ------------------------------------------------------------   0
GGT1.start-POM121L10P.end.24583750-24659147.ref                GTGCATTGTCATCTCAAGGAATCCACCCAGCAACTGTTCTAGTTATCCCTGCTCTCTAGG   16560

LOC112206721.putative.POM121-like,protein1.START.LOC112206738  ------------------------------------------------------------   0
BCRP3.REF.NEW                                                  ------------------------------------------------------------   0
GGT1.start-POM121L10P.end.24583750-24659147.ref                TAGGAGACTGAGGCTAGGAAGGGAAAGGCTGCTGTGATACCCACACTATCAGTGGCTGGG   16620

LOC112206721.putative.POM121-like,protein1.START.LOC112206738  ------------------------------------------------------------   0
BCRP3.REF.NEW                                                  ------------------------------------------------------------   0
GGT1.start-POM121L10P.end.24583750-24659147.ref                GTTGAGTTTAAACCCAGGGACTCTAGGGCCCATGCCTCTGCCATCCTGCCTCTGAGTTTT   16680

LOC112206721.putative.POM121-like,protein1.START.LOC112206738  ------------------------------------------------------------   0
BCRP3.REF.NEW                                                  ------------------------------------------------------------   0
GGT1.start-POM121L10P.end.24583750-24659147.ref                GGAGCTGGACTTGTTCCATCAAGTGCCAGGCCAGCCCTGCTGCTGGGTGCTCCCAGGCAT   16740

LOC112206721.putative.POM121-like,protein1.START.LOC112206738  ------------------------------------------------------------   0
```

```
BCRP3.REF.NEW                                                    ------------------------------------------------------------   0
GGT1.start-POM121L10P.end.24583750-24659147.ref                  TCACAAGCTGACAATTCTGAAGGGCCCACGCACTTGGCCATGCTTCAGTCTGTGTCCTGA   16800

LOC112206721.putative.POM121-like,protein1.START.LOC112206738    ------------------------------------------------------------   0
BCRP3.REF.NEW                                                    ------------------------------------------------------------   0
GGT1.start-POM121L10P.end.24583750-24659147.ref                  CATCACAGGTGGGTGCTGGAATAACAAATGCTACAAATGGCACTGGGGGTGTTGGCCAGG   16860

LOC112206721.putative.POM121-like,protein1.START.LOC112206738    ------------------------------------------------------------   0
BCRP3.REF.NEW                                                    ------------------------------------------------------------   0
GGT1.start-POM121L10P.end.24583750-24659147.ref                  CTCTGGGGGCACAGGAGTTCTGCCCATGCCCACAGCTGGCCTGTGCTGACCTCCCTCCAT   16920

LOC112206721.putative.POM121-like,protein1.START.LOC112206738    ------------------------------------------------------------   0
BCRP3.REF.NEW                                                    ------------------------------------------------------------   0
GGT1.start-POM121L10P.end.24583750-24659147.ref                  CACAGCTGAGTAGCACCAGCACTTCCATTGAGCTGAAGTCTTCCTCCCATTCCTTCAGTG   16980

LOC112206721.putative.POM121-like,protein1.START.LOC112206738    ------------------------------------------------------------   0
BCRP3.REF.NEW                                                    ------------------------------------------------------------   0
GGT1.start-POM121L10P.end.24583750-24659147.ref                  TGGTCTTTGTGGCTCAGAGAGGCTGTGGCCAGGTACAGGATCTGGAGTGGCCCCTGCTTC   17040

LOC112206721.putative.POM121-like,protein1.START.LOC112206738    ------------------------------------------------------------   0
BCRP3.REF.NEW                                                    ------------------------------------------------------------   0
GGT1.start-POM121L10P.end.24583750-24659147.ref                  TCTGCTCTGTGACCTTAGACAGTGGGCAGGATCTGAGGCCAGGCCCACCTGCAGCAGGTG   17100

LOC112206721.putative.POM121-like,protein1.START.LOC112206738    ------------------------------------------------------------   0
BCRP3.REF.NEW                                                    ------------------------------------------------------------   0
GGT1.start-POM121L10P.end.24583750-24659147.ref                  TTACAAAGAGGCAAATTGGCGCTTAATGTTGGTAAGAACTTGCCAGCCAACCAGAGTCCC   17160

LOC112206721.putative.POM121-like,protein1.START.LOC112206738    ------------------------------------------------------------   0
BCRP3.REF.NEW                                                    ------------------------------------------------------------   0
GGT1.start-POM121L10P.end.24583750-24659147.ref                  ACTATCTTTGGAGGTAGTGAGCTCCCCATTGGGGAAGGTATGTAAGCCAACCTTTCTGGG   17220

LOC112206721.putative.POM121-like,protein1.START.LOC112206738    ------------------------------------------------------------   0
BCRP3.REF.NEW                                                    ------------------------------------------------------------   0
GGT1.start-POM121L10P.end.24583750-24659147.ref                  ATGCTGGAAGATCAGAGAGCAAACACTTGAGATTCTGTGATCCTAATATTGAATCCAATG   17280

LOC112206721.putative.POM121-like,protein1.START.LOC112206738    ------------------------------------------------------------   0
BCRP3.REF.NEW                                                    ------------------------------------------------------------   0
GGT1.start-POM121L10P.end.24583750-24659147.ref                  CTATGATGTTTGGATTTGAAGTCAGTTCTATTTAGCCAGTTTACCAAGTGTCTCCTTGGT   17340

LOC112206721.putative.POM121-like,protein1.START.LOC112206738    ------------------------------------------------------------   0
BCRP3.REF.NEW                                                    ------------------------------------------------------------   0
GGT1.start-POM121L10P.end.24583750-24659147.ref                  GCAGGACACCAGGAGACACAAAATGTCCTGGATAATATTGGGTTTCTGGCTCCCAGGGGT   17400

LOC112206721.putative.POM121-like,protein1.START.LOC112206738    ------------------------------------------------------------   0
BCRP3.REF.NEW                                                    ------------------------------------------------------------   0
GGT1.start-POM121L10P.end.24583750-24659147.ref                  CCCTGAGATGTTGTTCCAGCGGTTCTGGAGTATGGGCCAGGCACCGCTTGCATCTGGACC   17460

LOC112206721.putative.POM121-like,protein1.START.LOC112206738    ------------------------------------------------------------   0
BCRP3.REF.NEW                                                    ------------------------------------------------------------   0
GGT1.start-POM121L10P.end.24583750-24659147.ref                  GATCCGCATTGTTCGATCATTGTGAGCCTCATGATGGCAGTTCGGGATGTCATGCCCCCA   17520

LOC112206721.putative.POM121-like,protein1.START.LOC112206738    ------------------------------------------------------------   0
BCRP3.REF.NEW                                                    ------------------------------------------------------------   0
GGT1.start-POM121L10P.end.24583750-24659147.ref                  TGCGCCCCCCAACCCCACCCCGCCATGGCCCTCACCATGGCCCACTCAGGGCATGAGGCT   17580

LOC112206721.putative.POM121-like,protein1.START.LOC112206738    ------------------------------------------------------------   0
BCRP3.REF.NEW                                                    ------------------------------------------------------------   0
GGT1.start-POM121L10P.end.24583750-24659147.ref                  TTCTGGGTGATTGCTGGTATTCAGTGTTGTCAGCTTCTTGGACCTGTCAGTGGGGGTGGG   17640

LOC112206721.putative.POM121-like,protein1.START.LOC112206738    ------------------------------------------------------------   0
BCRP3.REF.NEW                                                    ------------------------------------------------------------   0
GGT1.start-POM121L10P.end.24583750-24659147.ref                  GCTGCAATGAGATGCCAGTTGCAGGAAGTGGTTTGCACAGCCTTCCTGGTGTCATGGGTC   17700

LOC112206721.putative.POM121-like,protein1.START.LOC112206738    ------------------------------------------------------------   0
BCRP3.REF.NEW                                                    ------------------------------------------------------------   0
GGT1.start-POM121L10P.end.24583750-24659147.ref                  ACTCTCTTGCCGAGTGGCTTTGGGCAGGTCCCTTCCCCTCTCAGGCTGCAGGTTCCTATG   17760

LOC112206721.putative.POM121-like,protein1.START.LOC112206738    ------------------------------------------------------------   0
BCRP3.REF.NEW                                                    ------------------------------------------------------------   0
GGT1.start-POM121L10P.end.24583750-24659147.ref                  CCAGGAGCTCCCTTCTTGCCCCACCAGGGCCTTGTTTTCCCAGATACAGAGGTGGGGCAG   17820

LOC112206721.putative.POM121-like,protein1.START.LOC112206738    ------------------------------------------------------------   0
BCRP3.REF.NEW                                                    ------------------------------------------------------------   0
GGT1.start-POM121L10P.end.24583750-24659147.ref                  ATCCCAGCCCGTTGAAACAGACTGGGCTTTGGAGGCCACAGTGGGCCCATTTCTCAGAGG   17880

LOC112206721.putative.POM121-like,protein1.START.LOC112206738    ------------------------------------------------------------   0
BCRP3.REF.NEW                                                    ------------------------------------------------------------   0
GGT1.start-POM121L10P.end.24583750-24659147.ref                  GACTGTGTGGCTGGATGCTGAGCCAGGCACTGGACTGGCCCTTTGAGCCTCCTGTCTGGC   17940

LOC112206721.putative.POM121-like,protein1.START.LOC112206738    ------------------------------------------------------------   0
BCRP3.REF.NEW                                                    ------------------------------------------------------------   0
GGT1.start-POM121L10P.end.24583750-24659147.ref                  CAAGGCCCCTGAGGCACTGTGGGCTGCTGGTGCCAGCACACAAGGGTGATAAGGAGATGA   18000

LOC112206721.putative.POM121-like,protein1.START.LOC112206738    ------------------------------------------------------------   0
BCRP3.REF.NEW                                                    ------------------------------------------------------------   0
GGT1.start-POM121L10P.end.24583750-24659147.ref                  AAGTCACTAGGATGGTGGGGCGGCACCCGCCCCGGGCTGTGTTGGCAGCTGCCCCTGGCT   18060

LOC112206721.putative.POM121-like,protein1.START.LOC112206738    ------------------------------------------------------------   0
BCRP3.REF.NEW                                                    ------------------------------------------------------------   0
GGT1.start-POM121L10P.end.24583750-24659147.ref                  GGAGGCCAGCTTGGCCCTGGAACCCTCCTCAAGGAACAAGGACACAAAAGAGCCAGCTCA   18120

LOC112206721.putative.POM121-like,protein1.START.LOC112206738    ------------------------------------------------------------   0
BCRP3.REF.NEW                                                    ------------------------------------------------------------   0
GGT1.start-POM121L10P.end.24583750-24659147.ref                  GCCAGCCTCTACCTAGCACCCCAATCAGCACTCCCTTCTTCCCATGACCCCTACCCTCTG   18180

LOC112206721.putative.POM121-like,protein1.START.LOC112206738    ------------------------------------------------------------   0
BCRP3.REF.NEW                                                    ------------------------------------------------------------   0
GGT1.start-POM121L10P.end.24583750-24659147.ref                  CCCAGCTCCTGTAACTGGCCAAGGAGCTAATGGACACCCACCAGGGTCTTAATGAGTGAG   18240

LOC112206721.putative.POM121-like,protein1.START.LOC112206738    ------------------------------------------------------------   0
BCRP3.REF.NEW                                                    ------------------------------------------------------------   0
GGT1.start-POM121L10P.end.24583750-24659147.ref                  CCAGGGTTAATGGGTTACTGGGCCCGGTGACACATTCACCAGCCTCCCAGGGCTCTCAAA   18300

LOC112206721.putative.POM121-like,protein1.START.LOC112206738    ------------------------------------------------------------   0
BCRP3.REF.NEW                                                    ------------------------------------------------------------   0
GGT1.start-POM121L10P.end.24583750-24659147.ref                  GTCCACACTGGCCTGGCCTTGAACGTCTTAGCTCGGCCACCTCCTTTTCCAGCTTTGACA   18360

LOC112206721.putative.POM121-like,protein1.START.LOC112206738    ------------------------------------------------------------   0
BCRP3.REF.NEW                                                    ------------------------------------------------------------   0
GGT1.start-POM121L10P.end.24583750-24659147.ref                  CCTCGGGGCTCAGGACGGGCCTAGGGTGGTCAAGAGGTCCCCTGCAGCCCTCACTTCCTG   18420

LOC112206721.putative.POM121-like,protein1.START.LOC112206738    ------------------------------------------------------------   0
BCRP3.REF.NEW                                                    ------------------------------------------------------------   0
GGT1.start-POM121L10P.end.24583750-24659147.ref                  GAAAAGGCTCCATTTCCTGGCCTTCAGGCTCAGGTGGTTCCTTCCGCCTGGCAGGTCCTC   18480

LOC112206721.putative.POM121-like,protein1.START.LOC112206738    ------------------------------------------------------------   0
```

```
BCRP3.REF.NEW                                                  ------------------------------------------------------------   0
GGT1.start-POM121L10P.end.24583750-24659147.ref                CCCAAATCCAATCCCAGCTCGGGCTGGATTCCCAAGGCCCCTATTCTGGTGTCTGCCCCT   18540

LOC112206721.putative.POM121-like,protein1.START.LOC112206738  ------------------------------------------------------------   0
BCRP3.REF.NEW                                                  ------------------------------------------------------------   0
GGT1.start-POM121L10P.end.24583750-24659147.ref                TCCCTAGGGCTCTTCCCCCTGGGGTGACCATACTCTCATGCCCAGCCAGGTCAGGGCTCC   18600

LOC112206721.putative.POM121-like,protein1.START.LOC112206738  ------------------------------------------------------------   0
BCRP3.REF.NEW                                                  ------------------------------------------------------------   0
GGT1.start-POM121L10P.end.24583750-24659147.ref                TTGAGAGAAGAATCTGGAGGGGCCAGGATTTGATGGGAGAATGAATGATGGAATGAAAGA   18660

LOC112206721.putative.POM121-like,protein1.START.LOC112206738  ------------------------------------------------------------   0
BCRP3.REF.NEW                                                  ------------------------------------------------------------   0
GGT1.start-POM121L10P.end.24583750-24659147.ref                AGGAACGTAAGCGTGAGCCTTGCTAAGAGTGACAATACTGGCAGGCTGCAGACCATGTGT   18720

LOC112206721.putative.POM121-like,protein1.START.LOC112206738  ------------------------------------------------------------   0
BCRP3.REF.NEW                                                  ------------------------------------------------------------   0
GGT1.start-POM121L10P.end.24583750-24659147.ref                GGACCTCGATCTGAGGGCTTAGAGGCTTCGGCCTGCCAGCGCTGTGGGGGCACCCACCCC   18780

LOC112206721.putative.POM121-like,protein1.START.LOC112206738  ------------------------------------------------------------   0
BCRP3.REF.NEW                                                  ------------------------------------------------------------   0
GGT1.start-POM121L10P.end.24583750-24659147.ref                ATCTCATTGAATGGGGCAGAGCAGGTGAGGGCATGAGATGGACAGATGGGCCTCATCCCC   18840

LOC112206721.putative.POM121-like,protein1.START.LOC112206738  ------------------------------------------------------------   0
BCRP3.REF.NEW                                                  ------------------------------------------------------------   0
GGT1.start-POM121L10P.end.24583750-24659147.ref                CTGCTTCCGTTGCCTGGGGTCCTCGGTCAGCCTGTTTGCTCAGACCCCTGAAATTGGGGG   18900

LOC112206721.putative.POM121-like,protein1.START.LOC112206738  ------------------------------------------------------------   0
BCRP3.REF.NEW                                                  ------------------------------------------------------------   0
GGT1.start-POM121L10P.end.24583750-24659147.ref                TCAAGGAAGGACCCTCCTGGATTATGTGACTCCCTTCAGTCCAGGTGACCAGGGTCCTTG   18960

LOC112206721.putative.POM121-like,protein1.START.LOC112206738  ------------------------------------------------------------   0
BCRP3.REF.NEW                                                  ------------------------------------------------------------   0
GGT1.start-POM121L10P.end.24583750-24659147.ref                GAGTGACAGCTCCTAGAGCCCCACCTGTAGTCCTGCAAGCTGGGGAGAGGGCCTGTTAGG   19020

LOC112206721.putative.POM121-like,protein1.START.LOC112206738  ------------------------------------------------------------   0
BCRP3.REF.NEW                                                  ------------------------------------------------------------   0
GGT1.start-POM121L10P.end.24583750-24659147.ref                AGAGGCATCCCCTGCCCTGGGGTTGTAGAGGTGAACTAGGCTCCTCAGACCTTGTGGGGC   19080

LOC112206721.putative.POM121-like,protein1.START.LOC112206738  ------------------------------------------------------------   0
BCRP3.REF.NEW                                                  ------------------------------------------------------------   0
GGT1.start-POM121L10P.end.24583750-24659147.ref                CTCAGATGCTTACATCTCCAGCCCCTCCTGGCGTGGGCATCTGGCTGCAGCCCAGCTCTG   19140

LOC112206721.putative.POM121-like,protein1.START.LOC112206738  ------------------------------------------------------------   0
BCRP3.REF.NEW                                                  ------------------------------------------------------------   0
GGT1.start-POM121L10P.end.24583750-24659147.ref                TGGCCCCATCTCCCAGGGAACCTTTGGTCTAATCTGCCTCCCGCTGAAACCCAGCCTCAT   19200

LOC112206721.putative.POM121-like,protein1.START.LOC112206738  ------------------------------------------------------------   0
BCRP3.REF.NEW                                                  ------------------------------------------------------------   0
GGT1.start-POM121L10P.end.24583750-24659147.ref                TCAGCCCCACCGAGGCTTTCAGAGTTCAGGTCTCTCATTCAGGGTTTGAGACCCCACCGG   19260

LOC112206721.putative.POM121-like,protein1.START.LOC112206738  ------------------------------------------------------------   0
BCRP3.REF.NEW                                                  ------------------------------------------------------------   0
GGT1.start-POM121L10P.end.24583750-24659147.ref                GCTCAGAGACACCTGCAGCCTGCAGCGGTCCCAGATCACAGCCTCAGGGATGGGACCAGG   19320

LOC112206721.putative.POM121-like,protein1.START.LOC112206738  ------------------------------------------------------------   0
BCRP3.REF.NEW                                                  ------------------------------------------------------------   0
GGT1.start-POM121L10P.end.24583750-24659147.ref                AGCCAGCCCACATCCCACCTGCAGCAGTTCCTGTGCCTTTAAAGCCTCCCCTCCCCCCGC   19380

LOC112206721.putative.POM121-like,protein1.START.LOC112206738  ------------------------------------------------------------   0
BCRP3.REF.NEW                                                  ------------------------------------------------------------   0
GGT1.start-POM121L10P.end.24583750-24659147.ref                CCCGCCCCCAGGCCACTAGGGGAGGGAAGGAGGAGCTGGGTCACAGCAGGGAATCTTAGC   19440

LOC112206721.putative.POM121-like,protein1.START.LOC112206738  ------------------------------------------------------------   0
BCRP3.REF.NEW                                                  ------------------------------------------------------------   0
GGT1.start-POM121L10P.end.24583750-24659147.ref                TTGGTTTTGGTGTGCTGCTGGATGACCAGACCGGGCGTCGGGTGAGCCCAGAAGTGAGAG   19500

LOC112206721.putative.POM121-like,protein1.START.LOC112206738  ------------------------------------------------------------   0
BCRP3.REF.NEW                                                  ------------------------------------------------------------   0
GGT1.start-POM121L10P.end.24583750-24659147.ref                CAGTTGGCTGTGCCCCAGTGCTGTGTGACCCAGAGGCGCCGCTCACCCTCTCTGAGCTGG   19560

LOC112206721.putative.POM121-like,protein1.START.LOC112206738  ------------------------------------------------------------   0
BCRP3.REF.NEW                                                  ------------------------------------------------------------   0
GGT1.start-POM121L10P.end.24583750-24659147.ref                TGGACATCATAGGTGGGGAAGCTCAGGTCAGGGCACTCCCATGAGTGTCTGGAGGCCTGA   19620

LOC112206721.putative.POM121-like,protein1.START.LOC112206738  ------------------------------------------------------------   0
BCRP3.REF.NEW                                                  ------------------------------------------------------------   0
GGT1.start-POM121L10P.end.24583750-24659147.ref                GTCCCATTCTCAGCTCTGCCATATGCTTGCTGCGCTCTAGAGGAGTTCCTCTTCCTCTCC   19680

LOC112206721.putative.POM121-like,protein1.START.LOC112206738  ------------------------------------------------------------   0
BCRP3.REF.NEW                                                  ------------------------------------------------------------   0
GGT1.start-POM121L10P.end.24583750-24659147.ref                AAGCCTCGGTTTATGTACCCGTGCAGTGGGAGTGAGTTGCACTTCGGGGTGAAGGGGGCA   19740

LOC112206721.putative.POM121-like,protein1.START.LOC112206738  ------------------------------------------------------------   0
BCRP3.REF.NEW                                                  ------------------------------------------------------------   0
GGT1.start-POM121L10P.end.24583750-24659147.ref                AGACTTGTGTGGGCGCATCCTGCAGAAGGATCCCACAGGTGGGCAGAGCCCTGGGTTCTT   19800

LOC112206721.putative.POM121-like,protein1.START.LOC112206738  ------------------------------------------------------------   0
BCRP3.REF.NEW                                                  ------------------------------------------------------------   0
GGT1.start-POM121L10P.end.24583750-24659147.ref                TATCCGACTGGGTCTGGGCTGGGGGCCTCTGTTTCTTGGCTGATGAGTTTATGTGAGTTT   19860

LOC112206721.putative.POM121-like,protein1.START.LOC112206738  ------------------------------------------------------------   0
BCRP3.REF.NEW                                                  ------------------------------------------------------------   0
GGT1.start-POM121L10P.end.24583750-24659147.ref                GAGTGAGGTATGCGAGTGGGTGAAGGAGGGCTGGGGGGAGTCACCTGACTTGTGCGAAGA   19920

LOC112206721.putative.POM121-like,protein1.START.LOC112206738  ------------------------------------------------------------   0
BCRP3.REF.NEW                                                  ------------------------------------------------------------   0
GGT1.start-POM121L10P.end.24583750-24659147.ref                AGCTCTTGAGAGAGCCGTGGCTTCTTGGAATTAAGAGGAAAGAGTGCAGCATGAACAGAC   19980

LOC112206721.putative.POM121-like,protein1.START.LOC112206738  ------------------------------------------------------------   0
BCRP3.REF.NEW                                                  ------------------------------------------------------------   0
GGT1.start-POM121L10P.end.24583750-24659147.ref                AGGCCCTGGAGGATCTGGCAGCCCTGAGTGGGGGCGGGGGGTCAGCTCTGGAGTAGAGCC   20040

LOC112206721.putative.POM121-like,protein1.START.LOC112206738  ------------------------------------------------------------   0
BCRP3.REF.NEW                                                  ------------------------------------------------------------   0
GGT1.start-POM121L10P.end.24583750-24659147.ref                AGAGCTGTGATGGGGTTGGGGGACCCTGTGTCCTTGGGCCATGCTTGCCTCGCTCCTGGG   20100

LOC112206721.putative.POM121-like,protein1.START.LOC112206738  ------------------------------------------------------------   0
BCRP3.REF.NEW                                                  ------------------------------------------------------------   0
GGT1.start-POM121L10P.end.24583750-24659147.ref                TTCTGTTTGTGGCTGTGGATTGGGGTGGGGCAGGGCCGGTTGTGCGAGGGGTCATTGCCC   20160

LOC112206721.putative.POM121-like,protein1.START.LOC112206738  ------------------------------------------------------------   0
BCRP3.REF.NEW                                                  ------------------------------------------------------------   0
GGT1.start-POM121L10P.end.24583750-24659147.ref                AACTCCAGGGGGCGCCTGCCACCTCTCAGCTATATATATAGGGATATATATAGTTCTTTC   20220

LOC112206721.putative.POM121-like,protein1.START.LOC112206738  ------------------------------------------------------------   0
```

```
BCRP3.REF.NEW                                                  ------------------------------------------------------------   0
GGT1.start-POM121L10P.end.24583750-24659147.ref                GACAGGTTTCCAGCAGGTAGTGGTTATTAAATCTTACTGAAGGGGTGTTTTTTCTGATTC   20280

LOC112206721.putative.POM121-like,protein1.START.LOC112206738  ------------------------------------------------------------   0
BCRP3.REF.NEW                                                  ------------------------------------------------------------   0
GGT1.start-POM121L10P.end.24583750-24659147.ref                TCGGCTCTGTGCCGCAAGGGTGGAAACTGTGAGAGACAGATTCCAACTCCACGTCTGGGT   20340

LOC112206721.putative.POM121-like,protein1.START.LOC112206738  ------------------------------------------------------------   0
BCRP3.REF.NEW                                                  ------------------------------------------------------------   0
GGT1.start-POM121L10P.end.24583750-24659147.ref                AGTAAGCATCCAGTCCAGGGCTGTAGGCAGTCCTGGGGAAGACGCCAGAGATCTGTGCAT   20400

LOC112206721.putative.POM121-like,protein1.START.LOC112206738  ------------------------------------------------------------   0
BCRP3.REF.NEW                                                  ------------------------------------------------------------   0
GGT1.start-POM121L10P.end.24583750-24659147.ref                TCTCATATCGAGGGATAGCGACTCCAGGCTGGGGGCTGGCAGGGTAAGGGGTGGGTGGGT   20460

LOC112206721.putative.POM121-like,protein1.START.LOC112206738  ------------------------------------------------------------   0
BCRP3.REF.NEW                                                  ------------------------------------------------------------   0
GGT1.start-POM121L10P.end.24583750-24659147.ref                CCTGGGCTTACCCGCAGGTCTGCAGACTTCCTGGGGCCAGCTGACCTCGTAAAATCCCTT   20520

LOC112206721.putative.POM121-like,protein1.START.LOC112206738  ------------------------------------------------------------   0
BCRP3.REF.NEW                                                  ------------------------------------------------------------   0
GGT1.start-POM121L10P.end.24583750-24659147.ref                TTGTCTAAGCTTCAGTTTCCTGCCTGTGAATGGGGTTGGGGCTGTGCTCTGGTTTCACCC   20580

LOC112206721.putative.POM121-like,protein1.START.LOC112206738  ------------------------------------------------------------   0
BCRP3.REF.NEW                                                  ------------------------------------------------------------   0
GGT1.start-POM121L10P.end.24583750-24659147.ref                TTGTGGCTCTGGGGTTGTGGTGACAAAGCCATCAAGCTGGGTTGAAGGATTAACCAGGAA   20640

LOC112206721.putative.POM121-like,protein1.START.LOC112206738  ------------------------------------------------------------   0
BCRP3.REF.NEW                                                  ------------------------------------------------------------   0
GGT1.start-POM121L10P.end.24583750-24659147.ref                ACTTCAGACTGGCTGCCGTGTCTACCTCTTCCTCATACTCCTCTCTCTGCTGCATCCTGG   20700

LOC112206721.putative.POM121-like,protein1.START.LOC112206738  ------------------------------------------------------------   0
BCRP3.REF.NEW                                                  ------------------------------------------------------------   0
GGT1.start-POM121L10P.end.24583750-24659147.ref                GAAGCTGCTCTGCTCAGCCTAAATGAGGCTCAGTTGTGTGTGTGCGCACGTGCTTGCACG   20760

LOC112206721.putative.POM121-like,protein1.START.LOC112206738  ------------------------------------------------------------   0
BCRP3.REF.NEW                                                  ------------------------------------------------------------   0
GGT1.start-POM121L10P.end.24583750-24659147.ref                TGTGTTGGAAGTGGGTGATACTGACACCAGAGTCTGTGTCTCTGGGTGAGTGAGGCTTGC   20820

LOC112206721.putative.POM121-like,protein1.START.LOC112206738  ------------------------------------------------------------   0
BCRP3.REF.NEW                                                  ------------------------------------------------------------   0
GGT1.start-POM121L10P.end.24583750-24659147.ref                ACATTTCTCGGGACAGGGAACTCACTACCTTATGTGCCCAGGACAAGAGCTGTGGGGTCT   20880

LOC112206721.putative.POM121-like,protein1.START.LOC112206738  ------------------------------------------------------------   0
BCRP3.REF.NEW                                                  ------------------------------------------------------------   0
GGT1.start-POM121L10P.end.24583750-24659147.ref                GGAGAAGACTTCTAGGCCAGCCCCTGCAGTCCTTCCTCAGGTGACATGGCTTCCCCAGAC   20940

LOC112206721.putative.POM121-like,protein1.START.LOC112206738  ------------------------------------------------------------   0
BCRP3.REF.NEW                                                  ------------------------------------------------------------   0
GGT1.start-POM121L10P.end.24583750-24659147.ref                CCACTTCCCCCTAGGTGCCCTCTCTGCATTCAGGGGGTAGAGGGCTGACTGGGACAGAAT   21000

LOC112206721.putative.POM121-like,protein1.START.LOC112206738  ------------------------------------------------------------   0
BCRP3.REF.NEW                                                  ------------------------------------------------------------   0
GGT1.start-POM121L10P.end.24583750-24659147.ref                GTGACACACTCAGCATGTGAGGAAAAGCCTCCTTCATTCTGTAGGCCCTACCTCTATTAA   21060

LOC112206721.putative.POM121-like,protein1.START.LOC112206738  ------------------------------------------------------------   0
BCRP3.REF.NEW                                                  ------------------------------------------------------------   0
GGT1.start-POM121L10P.end.24583750-24659147.ref                CATGTCCTTTGATAAAGTGCCTCCCCTCCTGTCTCCCCTCTCTGGAATCCTCAGCTGCTG   21120

LOC112206721.putative.POM121-like,protein1.START.LOC112206738  ------------------------------------------------------------   0
BCRP3.REF.NEW                                                  ------------------------------------------------------------   0
GGT1.start-POM121L10P.end.24583750-24659147.ref                CCAGGCTTCAGCTGTGCCCCATTGAAGGCAGCTCTGTCTCCCTACTTTCCCCAGCCCAGG   21180

LOC112206721.putative.POM121-like,protein1.START.LOC112206738  ------------------------------------------------------------   0
BCRP3.REF.NEW                                                  ------------------------------------------------------------   0
GGT1.start-POM121L10P.end.24583750-24659147.ref                GTTTTCCTTTTGGGGTCAGCTGCAGGGATCTGGGCCATCCTTTGCCCACTCAGACTTTCT   21240

LOC112206721.putative.POM121-like,protein1.START.LOC112206738  ------------------------------------------------------------   0
BCRP3.REF.NEW                                                  ------------------------------------------------------------   0
GGT1.start-POM121L10P.end.24583750-24659147.ref                TTCTGCCCACCTGCTGCTGTGAATCCTGTATGTCATATATATATATAAAATATGTAATAT   21300

LOC112206721.putative.POM121-like,protein1.START.LOC112206738  ------------------------------------------------------------   0
BCRP3.REF.NEW                                                  ------------------------------------------------------------   0
GGT1.start-POM121L10P.end.24583750-24659147.ref                ATTATATATTATATGTAATATATAATATATAAAGTATATTATATAATATGTATTATATTA   21360

LOC112206721.putative.POM121-like,protein1.START.LOC112206738  ------------------------------------------------------------   0
BCRP3.REF.NEW                                                  ------------------------------------------------------------   0
GGT1.start-POM121L10P.end.24583750-24659147.ref                TATATTATATAATATGTAATATATTATATAATATATAATATGTATTATATTATATATTAT   21420

LOC112206721.putative.POM121-like,protein1.START.LOC112206738  ------------------------------------------------------------   0
BCRP3.REF.NEW                                                  ------------------------------------------------------------   0
GGT1.start-POM121L10P.end.24583750-24659147.ref                ATAATATGTAATATATTATATAATATATAATATGTATTATATTATATATTATATAATATG   21480

LOC112206721.putative.POM121-like,protein1.START.LOC112206738  ------------------------------------------------------------   0
BCRP3.REF.NEW                                                  ------------------------------------------------------------   0
GGT1.start-POM121L10P.end.24583750-24659147.ref                TATTATATTATATATTATATAATATGTATTATATATTATATAATATGTATTATATATTAT   21540

LOC112206721.putative.POM121-like,protein1.START.LOC112206738  ------------------------------------------------------------   0
BCRP3.REF.NEW                                                  ------------------------------------------------------------   0
GGT1.start-POM121L10P.end.24583750-24659147.ref                ATAATATGTATTATATATTATATAATATGTATTATATATTATATAATATGTATTATATAT   21600

LOC112206721.putative.POM121-like,protein1.START.LOC112206738  ------------------------------------------------------------   0
BCRP3.REF.NEW                                                  ------------------------------------------------------------   0
GGT1.start-POM121L10P.end.24583750-24659147.ref                TATATAATATGTATTATATATTATATAATATTATAATATGTATTATATATTATATAATAT   21660

LOC112206721.putative.POM121-like,protein1.START.LOC112206738  ------------------------------------------------------------   0
BCRP3.REF.NEW                                                  ------------------------------------------------------------   0
GGT1.start-POM121L10P.end.24583750-24659147.ref                TATATAATATGTATTATATATTATATAACAATATATAATGTGTATTATATATAATATAAT   21720

LOC112206721.putative.POM121-like,protein1.START.LOC112206738  ------------------------------------------------------------   0
BCRP3.REF.NEW                                                  ------------------------------------------------------------   0
GGT1.start-POM121L10P.end.24583750-24659147.ref                ATTATATAATGTGTATTATATATTATATAATATTATATAATGTGTATTATATATTATATA   21780

LOC112206721.putative.POM121-like,protein1.START.LOC112206738  ------------------------------------------------------------   0
BCRP3.REF.NEW                                                  ------------------------------------------------------------   0
GGT1.start-POM121L10P.end.24583750-24659147.ref                ATATTATATAATGTGTATTATATATTATATAATATTATATAATGTGTATTATATATATAA   21840

LOC112206721.putative.POM121-like,protein1.START.LOC112206738  ------------------------------------------------------------   0
BCRP3.REF.NEW                                                  ------------------------------------------------------------   0
GGT1.start-POM121L10P.end.24583750-24659147.ref                TATTATATAATGTGTATTATATATATAATATTATATAATGTGTATTATATATATAATATT   21900

LOC112206721.putative.POM121-like,protein1.START.LOC112206738  ------------------------------------------------------------   0
BCRP3.REF.NEW                                                  ------------------------------------------------------------   0
GGT1.start-POM121L10P.end.24583750-24659147.ref                ATATAATGTGTATTATATATTTAATATTATATAATGTGTATTATATATTTAATATTATAT   21960

LOC112206721.putative.POM121-like,protein1.START.LOC112206738  ------------------------------------------------------------   0
```

```
BCRP3.REF.NEW                                                     ------------------------------------------------------------   0
GGT1.start-POM121L10P.end.24583750-24659147.ref                   AATGTGTATTATATATTATATAATATTATATAATGTGTATTATATATTATATAATATTAT   22020

LOC112206721.putative.POM121-like,protein1.START.LOC112206738     ------------------------------------------------------------   0
BCRP3.REF.NEW                                                     ------------------------------------------------------------   0
GGT1.start-POM121L10P.end.24583750-24659147.ref                   ATAATGTGTATTATATATTATATAATATATGATGTGTATTATATATTATATATTATATGA   22080

LOC112206721.putative.POM121-like,protein1.START.LOC112206738     ------------------------------------------------------------   0
BCRP3.REF.NEW                                                     ------------------------------------------------------------   0
GGT1.start-POM121L10P.end.24583750-24659147.ref                   TGTGTATTATATATTATATAATATATGATGTGTATTATATATTATATAATATATGATGTG   22140

LOC112206721.putative.POM121-like,protein1.START.LOC112206738     ------------------------------------------------------------   0
BCRP3.REF.NEW                                                     ------------------------------------------------------------   0
GGT1.start-POM121L10P.end.24583750-24659147.ref                   TATTATATATTATATAATATTATATGATGTGTATTATATATTATATATAATATATAATAT   22200

LOC112206721.putative.POM121-like,protein1.START.LOC112206738     ------------------------------------------------------------   0
BCRP3.REF.NEW                                                     ------------------------------------------------------------   0
GGT1.start-POM121L10P.end.24583750-24659147.ref                   TATATATAATATATTACATATAATATATCATATATTATATTTATATAATTTATATAATAT   22260

LOC112206721.putative.POM121-like,protein1.START.LOC112206738     ------------------------------------------------------------   0
BCRP3.REF.NEW                                                     ------------------------------------------------------------   0
GGT1.start-POM121L10P.end.24583750-24659147.ref                   ATCATATATTATATTTATATAATTTATATAATATATCATATATTATATTTATATAATTTA   22320

LOC112206721.putative.POM121-like,protein1.START.LOC112206738     ------------------------------------------------------------   0
BCRP3.REF.NEW                                                     ------------------------------------------------------------   0
GGT1.start-POM121L10P.end.24583750-24659147.ref                   TATAATATATCATATATTATATTTATATAATTTATATAATATATCATATAAATATATTAT   22380

LOC112206721.putative.POM121-like,protein1.START.LOC112206738     ------------------------------------------------------------   0
BCRP3.REF.NEW                                                     ------------------------------------------------------------   0
GGT1.start-POM121L10P.end.24583750-24659147.ref                   ATATAGTATTATATATATAAAATTATACTTTAAGTTCTAGGGTACATGTGCACAACGTAC   22440

LOC112206721.putative.POM121-like,protein1.START.LOC112206738     ------------------------------------------------------------   0
BCRP3.REF.NEW                                                     ------------------------------------------------------------   0
GGT1.start-POM121L10P.end.24583750-24659147.ref                   AGGTTTGTTACATATGTATACATGTGCCATGTTGGTGTGCTGCACCCATTAACTTGTCAT   22500

LOC112206721.putative.POM121-like,protein1.START.LOC112206738     ------------------------------------------------------------   0
BCRP3.REF.NEW                                                     ------------------------------------------------------------   0
GGT1.start-POM121L10P.end.24583750-24659147.ref                   TTACATTAGGTATATCTCCTAATGCTATCCCTCCCCGTTCCCCTCACCCCACAACAGGCC   22560

LOC112206721.putative.POM121-like,protein1.START.LOC112206738     ------------------------------------------------------------   0
BCRP3.REF.NEW                                                     ------------------------------------------------------------   0
GGT1.start-POM121L10P.end.24583750-24659147.ref                   CCAGTGTGTGATGTTCCCCTTCCTGTGTCCAAGTGTTCTCATTGTTTAATTCCAACCTAT   22620

LOC112206721.putative.POM121-like,protein1.START.LOC112206738     ------------------------------------------------------------   0
BCRP3.REF.NEW                                                     ------------------------------------------------------------   0
GGT1.start-POM121L10P.end.24583750-24659147.ref                   GAGTGAGAACATGCATGTCTTATATGCTCTCAGGCAGAGAGGAAACTGCTGAGCAGGCCG   22680

LOC112206721.putative.POM121-like,protein1.START.LOC112206738     ------------------------------------------------------------   0
BCRP3.REF.NEW                                                     ------------------------------------------------------------   0
GGT1.start-POM121L10P.end.24583750-24659147.ref                   GGGCAGGGGACAGAGCCCTGTGGCTCTCCACTTTAGACCCCTCCTGGCTGACTGCCATGG   22740

LOC112206721.putative.POM121-like,protein1.START.LOC112206738     ------------------------------------------------------------   0
BCRP3.REF.NEW                                                     ------------------------------------------------------------   0
GGT1.start-POM121L10P.end.24583750-24659147.ref                   AATGCAGCCACTTAGCAGGGCCAAATCCCCTGATGTTCCTGTTGGCTGTCTAGCCTTCAG   22800

LOC112206721.putative.POM121-like,protein1.START.LOC112206738     ------------------------------------------------------------   0
BCRP3.REF.NEW                                                     ------------------------------------------------------------   0
GGT1.start-POM121L10P.end.24583750-24659147.ref                   GGACAGGTCATGGGACCTTGGTTCCTGCCCTGTCATCTTCCCAGTCACCCTGATCTTCAC   22860

LOC112206721.putative.POM121-like,protein1.START.LOC112206738     ------------------------------------------------------------   0
BCRP3.REF.NEW                                                     ------------------------------------------------------------   0
GGT1.start-POM121L10P.end.24583750-24659147.ref                   GGGGAGGAATAGCCTGGGAAAGGACTTGGTCACAGCACCTCCACCCTAGGGCTATTGAGG   22920

LOC112206721.putative.POM121-like,protein1.START.LOC112206738     ------------------------------------------------------------   0
BCRP3.REF.NEW                                                     ------------------------------------------------------------   0
GGT1.start-POM121L10P.end.24583750-24659147.ref                   ATCTCACAGTTGTGTGTCTGGTGGGTTCTGTCCAGAGCCCATTTGAGAGCAGTGGATGAC   22980

LOC112206721.putative.POM121-like,protein1.START.LOC112206738     ------------------------------------------------------------   0
BCRP3.REF.NEW                                                     ------------------------------------------------------------   0
GGT1.start-POM121L10P.end.24583750-24659147.ref                   AGGACAGGCCTATGTGACCCAGGCAGGCAGCAATATTGGGTCAGCCTTCATGTCCCCTTC   23040

LOC112206721.putative.POM121-like,protein1.START.LOC112206738     ------------------------------------------------------------   0
BCRP3.REF.NEW                                                     ------------------------------------------------------------   0
GGT1.start-POM121L10P.end.24583750-24659147.ref                   TGTCAGCTGGGGCAGCCTGGAAGCATGATTGTGGGGTAGGTGTTATGGGCACGGAATAGA   23100

LOC112206721.putative.POM121-like,protein1.START.LOC112206738     ------------------------------------------------------------   0
BCRP3.REF.NEW                                                     ------------------------------------------------------------   0
GGT1.start-POM121L10P.end.24583750-24659147.ref                   CCTCAGGTGGAGGCTGCAGGGGCTCTCCGGCACCGTAGACACAGCAGGCCCAGGAGCAGG   23160

LOC112206721.putative.POM121-like,protein1.START.LOC112206738     ------------------------------------------------------------   0
BCRP3.REF.NEW                                                     ------------------------------------------------------------   0
GGT1.start-POM121L10P.end.24583750-24659147.ref                   GGAGGGAGCCATGACTCAGGGAGACTCTGGCCCATATCCTTGGCAGATGAGGGCCACAGG   23220

LOC112206721.putative.POM121-like,protein1.START.LOC112206738     ------------------------------------------------------------   0
BCRP3.REF.NEW                                                     ------------------------------------------------------------   0
GGT1.start-POM121L10P.end.24583750-24659147.ref                   GGAATGGGCAGCACTGTCCAAAGTCCCCTGGGCTGGGTCCGCAGCTGTTCCTGGCTCAGA   23280

LOC112206721.putative.POM121-like,protein1.START.LOC112206738     ------------------------------------------------------------   0
BCRP3.REF.NEW                                                     ------------------------------------------------------------   0
GGT1.start-POM121L10P.end.24583750-24659147.ref                   CTTCTTGGTGGGCTGGTCAGAAACATGCAGTAACTTGGGGCAGTTACCAGGTGGCCAAGC   23340

LOC112206721.putative.POM121-like,protein1.START.LOC112206738     ------------------------------------------------------------   0
BCRP3.REF.NEW                                                     ------------------------------------------------------------   0
GGT1.start-POM121L10P.end.24583750-24659147.ref                   CTGCACTGCTGGGCTCTGTGAGCTTGGGCCAGCTCAGGCCCTCTCTGGGCCCTGCCTTTC   23400

LOC112206721.putative.POM121-like,protein1.START.LOC112206738     ------------------------------------------------------------   0
BCRP3.REF.NEW                                                     ------------------------------------------------------------   0
GGT1.start-POM121L10P.end.24583750-24659147.ref                   TGGGCTGTTCGGGTGGTTCCTTGGGCCTGGGGTGCTAATGTTTCTGGATGGGCAGCAGAA   23460

LOC112206721.putative.POM121-like,protein1.START.LOC112206738     ------------------------------------------------------------   0
BCRP3.REF.NEW                                                     ------------------------------------------------------------   0
GGT1.start-POM121L10P.end.24583750-24659147.ref                   CCAGTCTGCACTCAGGGCCCCAGGCCATGTTCCCGGAACACACCTTTAGCATTGACAGCA   23520

LOC112206721.putative.POM121-like,protein1.START.LOC112206738     ------------------------------------------------------------   0
BCRP3.REF.NEW                                                     ------------------------------------------------------------   0
GGT1.start-POM121L10P.end.24583750-24659147.ref                   GCGTGTGGTGAGGCCCCTTAGGCTGGGTTCTGGTCTAGTGCCAGGGGCACCACTACTCCA   23580

LOC112206721.putative.POM121-like,protein1.START.LOC112206738     ------------------------------------------------------------   0
BCRP3.REF.NEW                                                     ------------------------------------------------------------   0
GGT1.start-POM121L10P.end.24583750-24659147.ref                   CCGCCTCCAGAGCCATCTCTGGGACACTGGCTGTGAGTTCAGATGTTCTGAACAGGGACA   23640

LOC112206721.putative.POM121-like,protein1.START.LOC112206738     ------------------------------------------------------------   0
BCRP3.REF.NEW                                                     ------------------------------------------------------------   0
GGT1.start-POM121L10P.end.24583750-24659147.ref                   GGGAGAGCCAGAGGGAACCAGCCTGGGGCTCACTGGAGGGGCTCGTGGGCAGACAGCGCC   23700

LOC112206721.putative.POM121-like,protein1.START.LOC112206738     ------------------------------------------------------------   0
```

```
BCRP3.REF.NEW                                                  ------------------------------------------------------------   0
GGT1.start-POM121L10P.end.24583750-24659147.ref                CTTTGGAGGGAACTGAGTCTGAAAGGAAGGAAACCCTTTCCCCGGCTCATAGCACCTGCC   23760

LOC112206721.putative.POM121-like,protein1.START.LOC112206738  ------------------------------------------------------------   0
BCRP3.REF.NEW                                                  ------------------------------------------------------------   0
GGT1.start-POM121L10P.end.24583750-24659147.ref                ATCCAGGGCCCTCCCAGGGCTGCGTGAACTTTAGTCACGTGGTGACAGGCCGAGTCACCA   23820

LOC112206721.putative.POM121-like,protein1.START.LOC112206738  ------------------------------------------------------------   0
BCRP3.REF.NEW                                                  ------------------------------------------------------------   0
GGT1.start-POM121L10P.end.24583750-24659147.ref                TGCCAAGTCACTGTGCGCCTCCTTGCTGCTGTGACGTCAGCTTCCCCATCCTCCCAGCCA   23880

LOC112206721.putative.POM121-like,protein1.START.LOC112206738  ------------------------------------------------------------   0
BCRP3.REF.NEW                                                  ------------------------------------------------------------   0
GGT1.start-POM121L10P.end.24583750-24659147.ref                GGCTGGACCTCCGTGAGAGGCCTGCCTGTCCTGCACCCTGTGCAGATGCCTCCCACTGTC   23940

LOC112206721.putative.POM121-like,protein1.START.LOC112206738  ------------------------------------------------------------   0
BCRP3.REF.NEW                                                  ------------------------------------------------------------   0
GGT1.start-POM121L10P.end.24583750-24659147.ref                CGCCGGGGCTGCTGGGCACGCCCTGGCTGGTCTCTTGGACTAGGTAAGCTCATGAGTCCT   24000

LOC112206721.putative.POM121-like,protein1.START.LOC112206738  ------------------------------------------------------------   0
BCRP3.REF.NEW                                                  ------------------------------------------------------------   0
GGT1.start-POM121L10P.end.24583750-24659147.ref                CTGGCCGCTCCTGCTCCTTCCCTGCCTCTGCTCCTCCTCGGAGGTGGCCACCCCCAGATC   24060

LOC112206721.putative.POM121-like,protein1.START.LOC112206738  ------------------------------------------------------------   0
BCRP3.REF.NEW                                                  ------------------------------------------------------------   0
GGT1.start-POM121L10P.end.24583750-24659147.ref                CCAGTCCCAATTCGGAGGCCCCCTGAGGAGTGCTGCAGGGGGCCACAGGCGTGGCTCTGA   24120

LOC112206721.putative.POM121-like,protein1.START.LOC112206738  ------------------------------------------------------------   0
BCRP3.REF.NEW                                                  ------------------------------------------------------------   0
GGT1.start-POM121L10P.end.24583750-24659147.ref                GCCACTCTGGAGAGTGGCGGGGGGCCCAGCCAGTTCTGTGGCTGGGACTTTCCCAGGCAG   24180

LOC112206721.putative.POM121-like,protein1.START.LOC112206738  ------------------------------------------------------------   0
BCRP3.REF.NEW                                                  ------------------------------------------------------------   0
GGT1.start-POM121L10P.end.24583750-24659147.ref                ACAAGTCTGTCTCTTCCTCCCCAGCGGGTGCAGCCCAGAACTGTCTTCTGAGGAAGAGGT   24240

LOC112206721.putative.POM121-like,protein1.START.LOC112206738  ------------------------------------------------------------   0
BCRP3.REF.NEW                                                  ------------------------------------------------------------   0
GGT1.start-POM121L10P.end.24583750-24659147.ref                GCTCTCCTGGGCCCCCACTGTCCCCAGGCCTCAGGTAAGCCCATCAGGGTCCCAAGGAAG   24300

LOC112206721.putative.POM121-like,protein1.START.LOC112206738  ------------------------------------------------------------   0
BCRP3.REF.NEW                                                  ------------------------------------------------------------   0
GGT1.start-POM121L10P.end.24583750-24659147.ref                GGGGTCTGGGTTTGAGGCCAACCATGGCAGCTGACCTACTTTCTAGCCTCAGTTTCCCCT   24360

LOC112206721.putative.POM121-like,protein1.START.LOC112206738  ------------------------------------------------------------   0
BCRP3.REF.NEW                                                  ------------------------------------------------------------   0
GGT1.start-POM121L10P.end.24583750-24659147.ref                AGTGTGTGCAGCTCTCACGCTGTTTGGGTGAGAACCAGGCCTCTGGGCTTGGACATTCTT   24420

LOC112206721.putative.POM121-like,protein1.START.LOC112206738  ------------------------------------------------------------   0
BCRP3.REF.NEW                                                  ------------------------------------------------------------   0
GGT1.start-POM121L10P.end.24583750-24659147.ref                TCAGTGAGTTTTTAGGGTGGAGGGATGGGAAATGGAAGCCCAGGACCTCAGCAGGGTGTC   24480

LOC112206721.putative.POM121-like,protein1.START.LOC112206738  ------------------------------------------------------------   0
BCRP3.REF.NEW                                                  ------------------------------------------------------------   0
GGT1.start-POM121L10P.end.24583750-24659147.ref                TTCCTCCGATCCGGGGACATCTGCCTGTGGGAGGCTGGGCCCACCCTCCCTTGCTGACCT   24540

LOC112206721.putative.POM121-like,protein1.START.LOC112206738  ------------------------------------------------------------   0
BCRP3.REF.NEW                                                  ------------------------------------------------------------   0
GGT1.start-POM121L10P.end.24583750-24659147.ref                GCCCTGGGAGGAAGGGACAGGGCCCAGCACTGCCTACTCCCCTCCCTGTTCTTCCCAGCA   24600

LOC112206721.putative.POM121-like,protein1.START.LOC112206738  ------------------------------------------------------------   0
BCRP3.REF.NEW                                                  ------------------------------------------------------------   0
GGT1.start-POM121L10P.end.24583750-24659147.ref                GTCTGAGCCTGGCTGGGTGTCCCCTGCTCCTCCAGCAGCCTACTTGGGCCTTTGGGTAGA   24660

LOC112206721.putative.POM121-like,protein1.START.LOC112206738  ------------------------------------------------------------   0
BCRP3.REF.NEW                                                  ------------------------------------------------------------   0
GGT1.start-POM121L10P.end.24583750-24659147.ref                CAGATTAACAGACAGGCGAGGCTGGGTCATGGTTGGACCACCCCAGGACCCTGACCGGGG   24720

LOC112206721.putative.POM121-like,protein1.START.LOC112206738  ------------------------------------------------------------   0
BCRP3.REF.NEW                                                  ------------------------------------------------------------   0
GGT1.start-POM121L10P.end.24583750-24659147.ref                GCTCAGCTCATGACCCTGAGCCTGGGAGAGATGAGGCCATGCCCTCCAGGGCACTCAGCA   24780

LOC112206721.putative.POM121-like,protein1.START.LOC112206738  ------------------------------------------------------------   0
BCRP3.REF.NEW                                                  ------------------------------------------------------------   0
GGT1.start-POM121L10P.end.24583750-24659147.ref                TGACCCGGCCCAGTGGACGGGACTGGGTAGCTTCCTTGGTGCAGGGGGCTGTCATGCTAG   24840

LOC112206721.putative.POM121-like,protein1.START.LOC112206738  ------------------------------------------------------------   0
BCRP3.REF.NEW                                                  ------------------------------------------------------------   0
GGT1.start-POM121L10P.end.24583750-24659147.ref                GACGGGGTCACTGACCAGGCCAGGCCCCTGCCCCATGACTTGTGGTGGAAATGTCCTTTT   24900

LOC112206721.putative.POM121-like,protein1.START.LOC112206738  ------------------------------------------------------------   0
BCRP3.REF.NEW                                                  ------------------------------------------------------------   0
GGT1.start-POM121L10P.end.24583750-24659147.ref                GTTTTTGTTTTTTTGCATTTTTTTTGAGACGGAGTTTCACTCTTGTTGCCCAGGCTGGAG   24960

LOC112206721.putative.POM121-like,protein1.START.LOC112206738  ------------------------------------------------------------   0
BCRP3.REF.NEW                                                  ------------------------------------------------------------   0
GGT1.start-POM121L10P.end.24583750-24659147.ref                TGCAGTGGTGCAATCTTGCCTCACTGCAACCTCCACCTCCTGGGTTCAAGCAATTCTCCT   25020

LOC112206721.putative.POM121-like,protein1.START.LOC112206738  ------------------------------------------------------------   0
BCRP3.REF.NEW                                                  ------------------------------------------------------------   0
GGT1.start-POM121L10P.end.24583750-24659147.ref                GCCTCAGCTCCTGAGTAGCTGGGATTACAGGCACCTGCCACCATGCCTGGCTAATTTTTT   25080

LOC112206721.putative.POM121-like,protein1.START.LOC112206738  ------------------------------------------------------------   0
BCRP3.REF.NEW                                                  ------------------------------------------------------------   0
GGT1.start-POM121L10P.end.24583750-24659147.ref                GTATTTTTAGTGGAGATGGGGGTTTCACCATGTTGGCCAGGCTGTTCTCAAACTCTTCAC   25140

LOC112206721.putative.POM121-like,protein1.START.LOC112206738  ------------------------------------------------------------   0
BCRP3.REF.NEW                                                  ------------------------------------------------------------   0
GGT1.start-POM121L10P.end.24583750-24659147.ref                CTCAGGTGATCCACCTGCCTCTGCCTCCCAAAGTGCTGGGATTATAGGTGTGAGCCACTG   25200

LOC112206721.putative.POM121-like,protein1.START.LOC112206738  ------------------------------------------------------------   0
BCRP3.REF.NEW                                                  ------------------------------------------------------------   0
GGT1.start-POM121L10P.end.24583750-24659147.ref                CACCCGGCCATGTGATGGGAATGTTCTGTGTCCATAATAGATGCTGCATATTGCTGGCCC   25260

LOC112206721.putative.POM121-like,protein1.START.LOC112206738  ------------------------------------------------------------   0
BCRP3.REF.NEW                                                  ------------------------------------------------------------   0
GGT1.start-POM121L10P.end.24583750-24659147.ref                AGCTCCTGAGGCTCTTTGGACCTCCAGGAATCGGTGTCTCTATCAGGAACCCTTAACCCT   25320

LOC112206721.putative.POM121-like,protein1.START.LOC112206738  ------------------------------------------------------------   0
BCRP3.REF.NEW                                                  ------------------------------------------------------------   0
GGT1.start-POM121L10P.end.24583750-24659147.ref                GACCCGGACTCCCAGCTGGGACCCGGGGTGTCGGAGTGGCAAGAGCGCTGTCAGGCCTGG   25380

LOC112206721.putative.POM121-like,protein1.START.LOC112206738  ------------------------------------------------------------   0
BCRP3.REF.NEW                                                  ------------------------------------------------------------   0
GGT1.start-POM121L10P.end.24583750-24659147.ref                TGAAGGGTGTGAGCTGTCCAACGGGGCAGGGAGGAGGCAGGGCCTGTTCTGCAGTTGGAC   25440

LOC112206721.putative.POM121-like,protein1.START.LOC112206738  ------------------------------------------------------------   0
```

```
BCRP3.REF.NEW                                                   ------------------------------------------------------------   0
GGT1.start-POM121L10P.end.24583750-24659147.ref                 AGACAGAGCCCTCTAGCTGCTTTCTGGAAGACTGAAGGGCAGGTGATGTTGGAGGGAGGG   25500

LOC112206721.putative.POM121-like,protein1.START.LOC112206738   ------------------------------------------------------------   0
BCRP3.REF.NEW                                                   ------------------------------------------------------------   0
GGT1.start-POM121L10P.end.24583750-24659147.ref                 AGTGCAGGCAGGGGCTGTGAGGGAGTTCAGGTCAGAAACAGGTGGCGCCTGGATTCAGGC   25560

LOC112206721.putative.POM121-like,protein1.START.LOC112206738   ------------------------------------------------------------   0
BCRP3.REF.NEW                                                   ------------------------------------------------------------   0
GGT1.start-POM121L10P.end.24583750-24659147.ref                 TGTGGTGGTCACGGTGGGGATGAGGGGCTGCTTTGGATTGTGCTGGGGATGTGGGGTGGT   25620

LOC112206721.putative.POM121-like,protein1.START.LOC112206738   ------------------------------------------------------------   0
BCRP3.REF.NEW                                                   ------------------------------------------------------------   0
GGT1.start-POM121L10P.end.24583750-24659147.ref                 GCGCTGCTGCATGACTACTGCCAGGTCTCTCTGCTCTTAGTGTCTGCATCCAGGGCTGGG   25680

LOC112206721.putative.POM121-like,protein1.START.LOC112206738   ------------------------------------------------------------   0
BCRP3.REF.NEW                                                   ------------------------------------------------------------   0
GGT1.start-POM121L10P.end.24583750-24659147.ref                 AGGGGGTCAAATGAATCACACTATCGGCCCCAGGCCCACCAAGCCTGGGGAGGTGGCCAC   25740

LOC112206721.putative.POM121-like,protein1.START.LOC112206738   ------------------------------------------------------------   0
BCRP3.REF.NEW                                                   ------------------------------------------------------------   0
GGT1.start-POM121L10P.end.24583750-24659147.ref                 CCTTCCATGATGGCATTTGGATGTTCCCTGTGTGTGGGGAGGGCACAGGGACTCCATTCG   25800

LOC112206721.putative.POM121-like,protein1.START.LOC112206738   ------------------------------------------------------------   0
BCRP3.REF.NEW                                                   ------------------------------------------------------------   0
GGT1.start-POM121L10P.end.24583750-24659147.ref                 TAGACCACCTCTGGGACAGTGTGTCTGCCTCTGAGGTCAGACGCTCTGCACTGGGACAGG   25860

LOC112206721.putative.POM121-like,protein1.START.LOC112206738   ------------------------------------------------------------   0
BCRP3.REF.NEW                                                   ------------------------------------------------------------   0
GGT1.start-POM121L10P.end.24583750-24659147.ref                 GTGGAGTGGAGGGAAATCCAGCTTGGGGCTCATTGGAGGGGCTTGCTGGCAGACACCGCC   25920

LOC112206721.putative.POM121-like,protein1.START.LOC112206738   ------------------------------------------------------------   0
BCRP3.REF.NEW                                                   ------------------------------------------------------------   0
GGT1.start-POM121L10P.end.24583750-24659147.ref                 CTTTGTGGGAAACTGACTGTGGGAGAGGGGAACCCCAACCTCTGTCACCACATCCCTCTT   25980

LOC112206721.putative.POM121-like,protein1.START.LOC112206738   ------------------------------------------------------------   0
BCRP3.REF.NEW                                                   ------------------------------------------------------------   0
GGT1.start-POM121L10P.end.24583750-24659147.ref                 CCCTGTTGTCACACCTGTCACCTGCTGCCATAGCCATGAGACTTCCCAAGGGTCACTGCT   26040

LOC112206721.putative.POM121-like,protein1.START.LOC112206738   ------------------------------------------------------------   0
BCRP3.REF.NEW                                                   ------------------------------------------------------------   0
GGT1.start-POM121L10P.end.24583750-24659147.ref                 GCCACTCACTGCACAGCCTGGAAGGGAGTCCACAGGGGACATACAGTGAGCAAGAGACCT   26100

LOC112206721.putative.POM121-like,protein1.START.LOC112206738   ------------------------------------------------------------   0
BCRP3.REF.NEW                                                   ------------------------------------------------------------   0
GGT1.start-POM121L10P.end.24583750-24659147.ref                 GTGCCACTCAGGCCTCCTGGGGGTGTCCCCAGTGCAGCCATGATGATAATCACAGCTACC   26160

LOC112206721.putative.POM121-like,protein1.START.LOC112206738   ------------------------------------------------------------   0
BCRP3.REF.NEW                                                   ------------------------------------------------------------   0
GGT1.start-POM121L10P.end.24583750-24659147.ref                 ATTCACCAAGCCCTGCCCACAGTCTAACCTACTCTATTCACAACACTCCCAGCAGCAAGG   26220

LOC112206721.putative.POM121-like,protein1.START.LOC112206738   ------------------------------------------------------------   0
BCRP3.REF.NEW                                                   ------------------------------------------------------------   0
GGT1.start-POM121L10P.end.24583750-24659147.ref                 CAAGTGAGGTGCTGCCGTCATCCAGGCTGGACAGTTCAGTGATTTGCCTGAGGCCCCACA   26280

LOC112206721.putative.POM121-like,protein1.START.LOC112206738   ------------------------------------------------------------   0
BCRP3.REF.NEW                                                   ------------------------------------------------------------   0
GGT1.start-POM121L10P.end.24583750-24659147.ref                 GCAGGTGAGTGGCAAGTCCAGCATCAGAGCAGGGCAGGCTGGCGGTGCCCCCTGAGCCCC   26340

LOC112206721.putative.POM121-like,protein1.START.LOC112206738   ------------------------------------------------------------   0
BCRP3.REF.NEW                                                   ------------------------------------------------------------   0
GGT1.start-POM121L10P.end.24583750-24659147.ref                 CTTTGCCATGCTGACCACATGCACATCCTGGGCTTCTGCAGGAATGCCCTGTCCCCTACC   26400

LOC112206721.putative.POM121-like,protein1.START.LOC112206738   ------------------------------------------------------------   0
BCRP3.REF.NEW                                                   ------------------------------------------------------------   0
GGT1.start-POM121L10P.end.24583750-24659147.ref                 TGCCCTGCTCCGTGCAAAACCCTCTTTGAGCTGTGCCTGGGAGACATGCTGAGAGAATTC   26460

LOC112206721.putative.POM121-like,protein1.START.LOC112206738   ------------------------------------------------------------   0
BCRP3.REF.NEW                                                   ------------------------------------------------------------   0
GGT1.start-POM121L10P.end.24583750-24659147.ref                 ATGGAAACAAATGTGTTACTGACAGCCTCTTTGCCTCCAGAGTTCAACTGGAGACAGAGA   26520

LOC112206721.putative.POM121-like,protein1.START.LOC112206738   ------------------------------------------------------------   0
BCRP3.REF.NEW                                                   ------------------------------------------------------------   0
GGT1.start-POM121L10P.end.24583750-24659147.ref                 AACCAGCTAGAGGCAGAGGGAGGTAACACGGAGTCCCCCAGAAAGGTCTGGGCTGCGCGT   26580

LOC112206721.putative.POM121-like,protein1.START.LOC112206738   ------------------------------------------------------------   0
BCRP3.REF.NEW                                                   ------------------------------------------------------------   0
GGT1.start-POM121L10P.end.24583750-24659147.ref                 GCTTCAGGTAACCTCCCTTGACCTTCAGGAGAACGAGAAGGCTGCCTGATCAGAGAGTCC   26640

LOC112206721.putative.POM121-like,protein1.START.LOC112206738   ------------------------------------------------------------   0
BCRP3.REF.NEW                                                   ------------------------------------------------------------   0
GGT1.start-POM121L10P.end.24583750-24659147.ref                 CTGAAGAAGATTCTGTGGCTACAGGCTTCAGCAGAGTGTGAGGGAGACCCCGGTTATTTC   26700

LOC112206721.putative.POM121-like,protein1.START.LOC112206738   ------------------------------------------------------------   0
BCRP3.REF.NEW                                                   ------------------------------------------------------------   0
GGT1.start-POM121L10P.end.24583750-24659147.ref                 CTCAGCTATTTCCACCAAATCCTCCTGTCTTTCGTGGCCAACACCCCAGGCAAGGCTTGG   26760

LOC112206721.putative.POM121-like,protein1.START.LOC112206738   ------------------------------------------------------------   0
BCRP3.REF.NEW                                                   ------------------------------------------------------------   0
GGT1.start-POM121L10P.end.24583750-24659147.ref                 GGCCCCCGTCTGCTGCTGGACGGTAAGTCCTGGCCCCGTGGCAGTGAATCTGTGGGGCGC   26820

LOC112206721.putative.POM121-like,protein1.START.LOC112206738   ------------------------------------------------------------   0
BCRP3.REF.NEW                                                   ------------------------------------------------------------   0
GGT1.start-POM121L10P.end.24583750-24659147.ref                 TCTGATTGTGGGCACTATGGAAGCTAAACCCCATGCTCCAGGTGGGGTGGAGGGTCTTCA   26880

LOC112206721.putative.POM121-like,protein1.START.LOC112206738   ------------------------------------------------------------   0
BCRP3.REF.NEW                                                   ------------------------------------------------------------   0
GGT1.start-POM121L10P.end.24583750-24659147.ref                 GAGGACTCCTGGACAGTGCCAGGCTCTAGGCTGGGGTGGGGGACACAGGAGAAACCAGGC   26940

LOC112206721.putative.POM121-like,protein1.START.LOC112206738   ------------------------------------------------------------   0
BCRP3.REF.NEW                                                   ------------------------------------------------------------   0
GGT1.start-POM121L10P.end.24583750-24659147.ref                 CAGGCCCATCCCTACTGGAGCTTCTCCCTAAGCAGTGGAGGCTCAGCCACTGTAAGGAGG   27000

LOC112206721.putative.POM121-like,protein1.START.LOC112206738   ------------------------------------------------------------   0
BCRP3.REF.NEW                                                   ------------------------------------------------------------   0
GGT1.start-POM121L10P.end.24583750-24659147.ref                 TAGGCAAGGCCCTGCAGAAAGAGGGGTGTGGAAATCTGGGGGCTCCCAGGAAGGGCCGCT   27060

LOC112206721.putative.POM121-like,protein1.START.LOC112206738   ------------------------------------------------------------   0
BCRP3.REF.NEW                                                   ------------------------------------------------------------   0
GGT1.start-POM121L10P.end.24583750-24659147.ref                 GCTGGAGATGGGGTTCTTACCAGGATGGGCTCTGAAGATAAGCAGGGAGGATTTGGGAGG   27120

LOC112206721.putative.POM121-like,protein1.START.LOC112206738   ------------------------------------------------------------   0
BCRP3.REF.NEW                                                   ------------------------------------------------------------   0
GGT1.start-POM121L10P.end.24583750-24659147.ref                 GCAGAGATGAGGCCCAGAGCTTCTGGCAGAGGGCATGGCCTGCGCAAAGGTCTGGGGGCC   27180

LOC112206721.putative.POM121-like,protein1.START.LOC112206738   ------------------------------------------------------------   0
```

```
BCRP3.REF.NEW                                                ------------------------------------------------------------   0
GGT1.start-POM121L10P.end.24583750-24659147.ref              GGACAGCCTGCACGTATTCTGGGAAGCGGGAAGGAGACACAGGCCTTGTGTTTCTGAGGC   27240

LOC112206721.putative.POM121-like,protein1.START.LOC112206738 ------------------------------------------------------------   0
BCRP3.REF.NEW                                                ------------------------------------------------------------   0
GGT1.start-POM121L10P.end.24583750-24659147.ref              CCGACTTTAGACTGTGCCCTGTTGGGGAGGGGCCAGGGAATGTCTGAGGCTAGGCCTGAC   27300

LOC112206721.putative.POM121-like,protein1.START.LOC112206738 ------------------------------------------------------------   0
BCRP3.REF.NEW                                                ------------------------------------------------------------   0
GGT1.start-POM121L10P.end.24583750-24659147.ref              CCTGCTTCTTACCCCGTGGGTGCAGCAGAGCCATGAAGAAGAAGTTAGTGGTGCTGGGCC   27360

LOC112206721.putative.POM121-like,protein1.START.LOC112206738 ------------------------------------------------------------   0
BCRP3.REF.NEW                                                ------------------------------------------------------------   0
GGT1.start-POM121L10P.end.24583750-24659147.ref              TGCTGGCCGTGGTCCTGGTGCTGGTCATTGTCGGCCTCTGTCTCTGGCTGCCCTCAGCCT   27420

LOC112206721.putative.POM121-like,protein1.START.LOC112206738 ------------------------------------------------------------   0
BCRP3.REF.NEW                                                ------------------------------------------------------------   0
GGT1.start-POM121L10P.end.24583750-24659147.ref              CCAAGGAACCTGACAACCATGTGTACACCAGGGCTGCCGTGGCCGCGGATGCCAAGCAGT   27480

LOC112206721.putative.POM121-like,protein1.START.LOC112206738 ------------------------------------------------------------   0
BCRP3.REF.NEW                                                ------------------------------------------------------------   0
GGT1.start-POM121L10P.end.24583750-24659147.ref              GCTCGAAGATTGGGAGGTGAGCAGGGCAGGGCATGGGACATGGGCCCTGAAAACTGGGCA   27540

LOC112206721.putative.POM121-like,protein1.START.LOC112206738 ------------------------------------------------------------   0
BCRP3.REF.NEW                                                ------------------------------------------------------------   0
GGT1.start-POM121L10P.end.24583750-24659147.ref              AGTGGACCTGAGCAATACCTTCACCCCCCTGAGACTCAGTTTCCCCATGTGTAAGCTTCG   27600

LOC112206721.putative.POM121-like,protein1.START.LOC112206738 ------------------------------------------------------------   0
BCRP3.REF.NEW                                                ------------------------------------------------------------   0
GGT1.start-POM121L10P.end.24583750-24659147.ref              CTTGGACTCTCTCAGTAGCCTTTGGGAAGGGGACAGTGACTCCGAGAGCAGGGTGTGGGT   27660

LOC112206721.putative.POM121-like,protein1.START.LOC112206738 ------------------------------------------------------------   0
BCRP3.REF.NEW                                                ------------------------------------------------------------   0
GGT1.start-POM121L10P.end.24583750-24659147.ref              CTCTAGAGCCAAACAGGGCCCCTTTTCTCAGTTCTAAGAGTCTCTGTCTCTTTGGAATAA   27720

LOC112206721.putative.POM121-like,protein1.START.LOC112206738 ------------------------------------------------------------   0
BCRP3.REF.NEW                                                ------------------------------------------------------------   0
GGT1.start-POM121L10P.end.24583750-24659147.ref              ACTGCACTGTTTTGTTGTTTGGTTGTTATTTTTACTTATTTCTTCCTATCTATCTATCTA   27780

LOC112206721.putative.POM121-like,protein1.START.LOC112206738 ------------------------------------------------------------   0
BCRP3.REF.NEW                                                ------------------------------------------------------------   0
GGT1.start-POM121L10P.end.24583750-24659147.ref              TCTATCTATCTATCATCTATCTATCTATCTATCTATCTATCTATCTATCTATCTATCTAT   27840

LOC112206721.putative.POM121-like,protein1.START.LOC112206738 ------------------------------------------------------------   0
BCRP3.REF.NEW                                                ------------------------------------------------------------   0
GGT1.start-POM121L10P.end.24583750-24659147.ref              CTATCTATCTACTATCTATCTATCTATTTATTTAGAGATGGAGTTTTGCTCTGTTGCCAG   27900

LOC112206721.putative.POM121-like,protein1.START.LOC112206738 ------------------------------------------------------------   0
BCRP3.REF.NEW                                                ------------------------------------------------------------   0
GGT1.start-POM121L10P.end.24583750-24659147.ref              GCTGGAGTGCAGTGGTGCAATCTCAGTTAACTGCAACCTCCGCCTCCCAAGTTCAAGTGA   27960

LOC112206721.putative.POM121-like,protein1.START.LOC112206738 ------------------------------------------------------------   0
BCRP3.REF.NEW                                                ------------------------------------------------------------   0
GGT1.start-POM121L10P.end.24583750-24659147.ref              TTCTCATGCCTAAGTCTCCCAAGTAGCTGGGATTACAGGCGTGCGCCACCACGCCCAACA   28020

LOC112206721.putative.POM121-like,protein1.START.LOC112206738 ------------------------------------------------------------   0
BCRP3.REF.NEW                                                ------------------------------------------------------------   0
GGT1.start-POM121L10P.end.24583750-24659147.ref              AATTTGTGTGTGTGTGTGTGTGTGTGTGTGTGTGTGTGTGTGTGTGTGTGTGTTTCCAAGAC   28080

LOC112206721.putative.POM121-like,protein1.START.LOC112206738 ------------------------------------------------------------   0
BCRP3.REF.NEW                                                ------------------------------------------------------------   0
GGT1.start-POM121L10P.end.24583750-24659147.ref              AGAGTATCGCTCTGTTACCCAGGCTGGAGGGCAGTGGTGCAATCTTGGCTTACTGCAACC   28140

LOC112206721.putative.POM121-like,protein1.START.LOC112206738 ------------------------------------------------------------   0
BCRP3.REF.NEW                                                ------------------------------------------------------------   0
GGT1.start-POM121L10P.end.24583750-24659147.ref              TCCACCTCCCAGGTTCAAGTGATTCTCCTGCCTCAGCCTCCACAGTAGCTGAGACTACAG   28200

LOC112206721.putative.POM121-like,protein1.START.LOC112206738 ------------------------------------------------------------   0
BCRP3.REF.NEW                                                ------------------------------------------------------------   0
GGT1.start-POM121L10P.end.24583750-24659147.ref              GCATGTGCCACCATGCCCAGCTAATTTTTGTATTTTTAGTAGAGACAGGGTTTTGCTATG   28260

LOC112206721.putative.POM121-like,protein1.START.LOC112206738 ------------------------------------------------------------   0
BCRP3.REF.NEW                                                ------------------------------------------------------------   0
GGT1.start-POM121L10P.end.24583750-24659147.ref              TTGGCCAGGCTGGTCTTGAACTCCTGACCTTGTGATCCTCCCACCTCTGCCTCTCAAAGT   28320

LOC112206721.putative.POM121-like,protein1.START.LOC112206738 --------------------------------------------------------gaca   4
BCRP3.REF.NEW                                                ------------------------------------------------------------   0
GGT1.start-POM121L10P.end.24583750-24659147.ref              GCTGGGATTAAGGTGTGAGCCACTGCTCCTGGCCTAATTATGGTGTTTTTAGTAGAGATG   28380

LOC112206721.putative.POM121-like,protein1.START.LOC112206738 gggtcttgctatgttgtccaggctgatctcaaacacctggcctcaagagatcctcctgct   64
BCRP3.REF.NEW                                                ------------------------------------------------------------   0
GGT1.start-POM121L10P.end.24583750-24659147.ref              GGGTTTCACCATGTTGGTCAGGCTGGTCTTGAACTCCTGACCTCAGGTAATCCACCCACC   28440

LOC112206721.putative.POM121-like,protein1.START.LOC112206738 tgg-cctttcaaagcacttggattattggtgtgagccaccacttcccacatagcctgttc   123
BCRP3.REF.NEW                                                ------------------------------------------------------------   0
GGT1.start-POM121L10P.end.24583750-24659147.ref              TGGGCCTCCCAAAGTGTTGGGATTACAGGTGTGAGCCACCACGCCCGGCTTATTCTTTTT   28500

LOC112206721.putative.POM121-like,protein1.START.LOC112206738 catcacttttacttaatttcacaactttccctgaccattgcctcaaatcagctttttt   183
BCRP3.REF.NEW                                                ------------------------------------------------------------   0
GGT1.start-POM121L10P.end.24583750-24659147.ref              TTTTTTTTTTTTTTTTGGTTAGGAGACAATTTCTTTCTTTCTTTTTTTTTATTTTATTTT   28560

LOC112206721.putative.POM121-like,protein1.START.LOC112206738 ctgacagctgcattgtagtctttgtcagagttatgcacagtctcctgcat---------c   234
BCRP3.REF.NEW                                                ------------------------------------------------------------   0
GGT1.start-POM121L10P.end.24583750-24659147.ref              ATTATTATTATACTTTAAGTTTTAGGGTACATGTGCACAATGTGCAGGTTTGTTACATAT   28620

LOC112206721.putative.POM121-like,protein1.START.LOC112206738 tacggggcctagaagcctgggatctgggggagccacccctgagggcgagtgtctcccctg   294
BCRP3.REF.NEW                                                ------------------------------------------------------------   0
GGT1.start-POM121L10P.end.24583750-24659147.ref              GTACATATGTACACATGTGCCATGTTGGTGTGCTGCACCCACCAACTCATCATTTAGCAT   28680

LOC112206721.putative.POM121-like,protein1.START.LOC112206738 gtgctgtatctgcc----------------------------------------------   308
BCRP3.REF.NEW                                                ------------------------------------------------------------   0
GGT1.start-POM121L10P.end.24583750-24659147.ref              TAGGTATATCTCCCAATGCCATCCCTCCCCCCTCCCCCCAAGGAACACTGTTGCCCAAAC   28740

LOC112206721.putative.POM121-like,protein1.START.LOC112206738 ------------------------------------------------gtcttttcacac   320
BCRP3.REF.NEW                                                ------------------------------------------------------------   0
GGT1.start-POM121L10P.end.24583750-24659147.ref              AGGGACATGAAGGGCTTATTCTTTTTTGTTGTTGTTTTGTTTTGTTTTGTTTTTTGAGAC   28800

LOC112206721.putative.POM121-like,protein1.START.LOC112206738 tgggtgtgacccgaagagacagcctgaggtccgtcctcactcactgtgtttgaggaactg   380
BCRP3.REF.NEW                                                ------------------------------------------------------------   0
GGT1.start-POM121L10P.end.24583750-24659147.ref              GGAGTCTCACGCTGTCTCCCAGGCTGGAGTGCAGTGGCGCAATCTCGGCTCGCTGCAACC   28860

LOC112206721.putative.POM121-like,protein1.START.LOC112206738 agggtcagctggcagtgggatgaggctggcccctcttccgctttagttccgggaggcctc   440
BCRP3.REF.NEW                                                ------------------------------------------------------------   0
GGT1.start-POM121L10P.end.24583750-24659147.ref              TCCGTCTCCTGG-ATTCAGGTGATTCTCCTGCCTCAGCCTCCTGAGTAGCTGATATTACA   28919

LOC112206721.putative.POM121-like,protein1.START.LOC112206738 ccgtagagctgtaggggctcgagatggcctttcatttggggcacgagctggtcccggagg   500
```

```
BCRP3.REF.NEW                                                  ------------------------------------------------------------   0
GGT1.start-POM121L10P.end.24583750-24659147.ref                GGCATCCGCCACCACGCCCGGCTAATTTTTGTATTTTTAGTAGAGACAGGGTTTCACCAT   28979

LOC112206721.putative.POM121-like,protein1.START.LOC112206738  tctgggatctctggttctgacctctgggcacctgctgcagctgtggctgaggcccagaaa   560
BCRP3.REF.NEW                                                  ------------------------------------------------------------   0
GGT1.start-POM121L10P.end.24583750-24659147.ref                AATGGTCTGGCTGGTCTCAAACTCCTGACCTCAGGTGATCCGCCTGCCTTGGCCTCCCAA   29039

LOC112206721.putative.POM121-like,protein1.START.LOC112206738  tgtgaaggccctcca---------------------------------------------   575
BCRP3.REF.NEW                                                  ------------------------------------------------------------   0
GGT1.start-POM121L10P.end.24583750-24659147.ref                AGTGCTGGGATTACAGGCATGAGCCACCATGCCCGGCCGGGCTTATTCTTTTTTTAAGGT   29099

LOC112206721.putative.POM121-like,protein1.START.LOC112206738  ------------------------------------------------------------   575
BCRP3.REF.NEW                                                  ------------------------------------------------------------   0
GGT1.start-POM121L10P.end.24583750-24659147.ref                GGAGTCTTACTCTGTTACCCAGGCTGGAGTATAGGGGAGCGATCATAGCCCACTGCAGCC   29159

LOC112206721.putative.POM121-like,protein1.START.LOC112206738  ------------------------------------------------------------   575
BCRP3.REF.NEW                                                  ------------------------------------------------------------   0
GGT1.start-POM121L10P.end.24583750-24659147.ref                TCAAACTCTTGGGTTTAAGTGATCTTCCCGCCTCAGCTTCCTAAAGTGCTGGGATTACAG   29219

LOC112206721.putative.POM121-like,protein1.START.LOC112206738  ------------------------------------------------------------   575
BCRP3.REF.NEW                                                  ------------------------------------------------------------   0
GGT1.start-POM121L10P.end.24583750-24659147.ref                GTGTGAGCCATGGTGCCTGGCTTCTACTGTTTATTCTATTTAGACTCTTATCTCATGT   29279

LOC112206721.putative.POM121-like,protein1.START.LOC112206738  ------------------------------------------------------------   575
BCRP3.REF.NEW                                                  ------------------------------------------------------------   0
GGT1.start-POM121L10P.end.24583750-24659147.ref                TATATACAAAGATCAGTTCCCTCCTAAAGACTTAAACAGAAAAAATATTATGTTTTTCAT   29339

LOC112206721.putative.POM121-like,protein1.START.LOC112206738  ------------------------------------------------------------   575
BCRP3.REF.NEW                                                  ------------------------------------------------------------   0
GGT1.start-POM121L10P.end.24583750-24659147.ref                ATTTTGAGACAGGGTCTTGTTCTGTCACCCAGGCTGGAGTGCAGTGGCATGATCATAGCT   29399

LOC112206721.putative.POM121-like,protein1.START.LOC112206738  ------------------------------------------------------------   575
BCRP3.REF.NEW                                                  ------------------------------------------------------------   0
GGT1.start-POM121L10P.end.24583750-24659147.ref                CACTGCAGCCTTGAACTCTTGGGCTTAAGCGATCCTCCCACCTCAGCCCCCTGAGTAGCT   29459

LOC112206721.putative.POM121-like,protein1.START.LOC112206738  ------------------------------------------------------------   575
BCRP3.REF.NEW                                                  ------------------------------------------------------------   0
GGT1.start-POM121L10P.end.24583750-24659147.ref                AGGACTACAGGCGTGCATCACACCTGACTAATTAAAAAAGACTGTTTTCTAGAGATGGTC   29519

LOC112206721.putative.POM121-like,protein1.START.LOC112206738  ------------------------------------------------------------   575
BCRP3.REF.NEW                                                  ------------------------------------------------------------   0
GGT1.start-POM121L10P.end.24583750-24659147.ref                TCACTCTATTGCCCAGGCTGGTCTTGAACTCCTGGCCTCAAGTGATCCTCCACCTTGGCC   29579

LOC112206721.putative.POM121-like,protein1.START.LOC112206738  ------------------------------------------------------------   575
BCRP3.REF.NEW                                                  ------------------------------------------------------------   0
GGT1.start-POM121L10P.end.24583750-24659147.ref                TCCCAAAGTGCTGAGATTACAGGTGTAAGCCACCATCTCTAGCCGGGAAAAAAAATGTTA   29639

LOC112206721.putative.POM121-like,protein1.START.LOC112206738  ------------------------------------------------------------   575
BCRP3.REF.NEW                                                  ------------------------------------------------------------   0
GGT1.start-POM121L10P.end.24583750-24659147.ref                TTAATAAAGTATAGCAATTTCCCTTTTTGTCCCAGTTATAAAAGTCATGTACATTTGTTT   29699

LOC112206721.putative.POM121-like,protein1.START.LOC112206738  ----------------------------------tccactccagtagtgaccccaacgtg   601
BCRP3.REF.NEW                                                  ------------------------------------------------------------   0
GGT1.start-POM121L10P.end.24583750-24659147.ref                GCTTGATAAAGAGGAAACTGTCTGGGCAAGGTGGTCCACACCTGTAATCCCAGCACTTTG   29759

LOC112206721.putative.POM121-like,protein1.START.LOC112206738  gggttcagtgtggagggggggaggggctgctgcggcagctgcaggagcagaagtgccacgc   661
BCRP3.REF.NEW                                                  ------------------------------------------------------------   0
GGT1.start-POM121L10P.end.24583750-24659147.ref                GG----AGGTTGAGGCGGGCAGACCACCCGAGGTCAGGAGTTCGAGACCAGCCTGGCCAA   29815

LOC112206721.putative.POM121-like,protein1.START.LOC112206738  cttgttcttctcatgcccgcatccatgctt---------------gcagctgggaagggg   707
BCRP3.REF.NEW                                                  ------------------------------------------------------------   0
GGT1.start-POM121L10P.end.24583750-24659147.ref                CATGGTGCACTCTGTCCCTACTAAAAATATAAAAAGTTAGCCGGGCATGGTGGTGTGCGC   29875

LOC112206721.putative.POM121-like,protein1.START.LOC112206738  caggaatcagcgaggtgacctgggctgagtcctgggaat--------------gggaaga   753
BCRP3.REF.NEW                                                  ------------------------------------------------------------   0
GGT1.start-POM121L10P.end.24583750-24659147.ref                CTGTAATCCCAGCTACTCAGGAGGCTGAGGCAGGGGAATCACTTGAACCCAGGAGGCAGA   29935

LOC112206721.putative.POM121-like,protein1.START.LOC112206738  ggtggcaggaaggggatctgagg-------------------------------------   776
BCRP3.REF.NEW                                                  ------------------------------------------------------------   0
GGT1.start-POM121L10P.end.24583750-24659147.ref                GGTTGCAGTGAGCTAAGATCATGCCACTGCACTCCAGCCTGGGCAACTGAGTGAAACTCC   29995

LOC112206721.putative.POM121-like,protein1.START.LOC112206738  ------------------------------------------------------------   776
BCRP3.REF.NEW                                                  ------------------------------------------------------------   0
GGT1.start-POM121L10P.end.24583750-24659147.ref                CTCTCAGAAAAAAAAAAAGAAAGAAAAGAAAAGAAAAAGAGGAAACTGTAATCCCAGCAC   30055

LOC112206721.putative.POM121-like,protein1.START.LOC112206738  ------------------------------------------------------------   776
BCRP3.REF.NEW                                                  ------------------------------------------------------------   0
GGT1.start-POM121L10P.end.24583750-24659147.ref                TTTGGGAGGTTGAGGCGATAGGATTGCTTTAGACCATGAGTTCGAGACCAGCTTGGGCAA   30115

LOC112206721.putative.POM121-like,protein1.START.LOC112206738  --------------------------aggagaacagggggcctggtggtctgtgcttctt   810
BCRP3.REF.NEW                                                  ------------------------------------------------------------   0
GGT1.start-POM121L10P.end.24583750-24659147.ref                CATAGAAAGACCCTATCTCTACAAAAAAGACAAAAAATTGCCAGGTG--TGGTGGTTCTT   30173

LOC112206721.putative.POM121-like,protein1.START.LOC112206738  cccagacatgggagctgtagaggggacctctgcagcagatgctaagggggccagtaggcc   870
BCRP3.REF.NEW                                                  ------------------------------------------------------------   0
GGT1.start-POM121L10P.end.24583750-24659147.ref                ACCTGTAGTCCCAGCTACTCAGCAGACTGAAGTGGGAGGATTGCTTGAGCCCAGGAGGTC   30233

LOC112206721.putative.POM121-like,protein1.START.LOC112206738  caggcagtcttgggacttgtgtgt-ctcctgctgtgcatccatactgggtgctttaga--   927
BCRP3.REF.NEW                                                  ------------------------------------------------------------   0
GGT1.start-POM121L10P.end.24583750-24659147.ref                AAAGCTGCATTGAGCCAAGACTGTGCCACTGCACTTCATCCTGGGTGACAAAGTGAGACC   30293

LOC112206721.putative.POM121-like,protein1.START.LOC112206738  -------------------------aacggcaggcagaccagaagcccctgttgcaagtg   962
BCRP3.REF.NEW                                                  ------------------------------------------------------------   0
GGT1.start-POM121L10P.end.24583750-24659147.ref                CTGTCTCATAAAACAAAGGCTGGGCACAGTGGCTCATGCCTGTAATACCAGCACTTTGGG   30353

LOC112206721.putative.POM121-like,protein1.START.LOC112206738  aggacaaagtgtgggaaggccgtgagggtctgcagtccgagatggccttgtcctcaacct   1022
BCRP3.REF.NEW                                                  ------------------------------------------------------------   0
GGT1.start-POM121L10P.end.24583750-24659147.ref                AGGCCAAGGTGGGTGGATCACTTGAGCACAGGAGTTCTTGACCAGCCTGGGCAACATGAT   30413

LOC112206721.putative.POM121-like,protein1.START.LOC112206738  ------------------------------------------------------------   1022
BCRP3.REF.NEW                                                  ------------------------------------------------------------   0
GGT1.start-POM121L10P.end.24583750-24659147.ref                GAAACCCCATCTCTACAAAATACACAAACAAACAAAATTGGCTGGGCATGGTGGCATGTG   30473

LOC112206721.putative.POM121-like,protein1.START.LOC112206738  ------------------------------------------------------------   1022
BCRP3.REF.NEW                                                  ------------------------------------------------------------   0
GGT1.start-POM121L10P.end.24583750-24659147.ref                CCCATAGTCCCAGCTACTTGGGAGGCTGAGATGGGAGGGTCAATTGAGCCCAGGAGACTG   30533

LOC112206721.putative.POM121-like,protein1.START.LOC112206738  ------------------------------------------------------------   1022
BCRP3.REF.NEW                                                  ------------------------------------------------------------   0
GGT1.start-POM121L10P.end.24583750-24659147.ref                AGGCTGCAGTGATCTGAGATCACACCACTGCACTCCAGCCTGAGCAACAAAGAGAGACTT   30593

LOC112206721.putative.POM121-like,protein1.START.LOC112206738  ------------------------------------------------------------   1022
BCRP3.REF.NEW                                                  ------------------------------------------------------------   0
GGT1.start-POM121L10P.end.24583750-24659147.ref                TGTCTCAAAAAAAAAAAAAAAAAAAAAAAAAAAAAGAGGCCAAGGCAGGCGGATCATGAG   30653

LOC112206721.putative.POM121-like,protein1.START.LOC112206738  ------------------------------------------------------------   1022
```

```
BCRP3.REF.NEW                                              ------------------------------------------------------------   0
GGT1.start-POM121L10P.end.24583750-24659147.ref            GTCAAGAGATAGAGACCAGCCTGGCCAACATGGTGAAACCCCGTCTCTACTAAAAATACA   30713

LOC112206721.putative.POM121-like,protein1.START.LOC112206738   ------------------------------------------------------------   1022
BCRP3.REF.NEW                                              ------------------------------------------------------------   0
GGT1.start-POM121L10P.end.24583750-24659147.ref            AAAATTAGCTGGGCGTGGTGGCATGCACCTGTAGTCCCAGCTACTCAGGAGGCTGAGGCA   30773

LOC112206721.putative.POM121-like,protein1.START.LOC112206738   ------------------------------------------------------------   1022
BCRP3.REF.NEW                                              ------------------------------------------------------------   0
GGT1.start-POM121L10P.end.24583750-24659147.ref            GGAGAATGGCTTGAACCCGGGAGGCAGAGGTTGCAGTGAGCTGAGATCGTGCCACTGCAC   30833

LOC112206721.putative.POM121-like,protein1.START.LOC112206738   ------------------------------------------------------------   1022
BCRP3.REF.NEW                                              ------------------------------------------------------------   0
GGT1.start-POM121L10P.end.24583750-24659147.ref            TCCAGCCTGGCAATAGAGCAAGACTCCATCTCAAAAAAAAAAAAAAAAAGAAAGAAAGAA   30893

LOC112206721.putative.POM121-like,protein1.START.LOC112206738   ------------------------------------------------------------   1022
BCRP3.REF.NEW                                              ------------------------------------------------------------   0
GGT1.start-POM121L10P.end.24583750-24659147.ref            ACTAAAAACAAAAACCCCAAAACTCGAATGGACTTCTCTTCCATCCTCCTTTGGGCAGGT   30953

LOC112206721.putative.POM121-like,protein1.START.LOC112206738   --gcagtgcactgttgatgcgctggaaggccgcctccttctcccggtccaggtcttcaac   1080
BCRP3.REF.NEW                                              ------------------------------------------------------------   0
GGT1.start-POM121L10P.end.24583750-24659147.ref            GGGCAGCAGGGTGTGTATGCGGGGCCAGGGTGGAAGCCTGCAGGTTCTCATGCCTTTATG   31013

LOC112206721.putative.POM121-like,protein1.START.LOC112206738   agtgacccggtaccccagctctaaggga------------ggtggcagcatcaaaggctc   1128
BCRP3.REF.NEW                                              ------------------------------------------------------------   0
GGT1.start-POM121L10P.end.24583750-24659147.ref            TGCCACATGGCAGGGATGCACTGCGGGACGGTGGCTCTGCGGTGGATGCAGCCATTGCAG   31073

LOC112206721.putative.POM121-like,protein1.START.LOC112206738   ccctcgcctgcgtgg---------------------------------------------   1143
BCRP3.REF.NEW                                              ------------------------------------------------------------   0
GGT1.start-POM121L10P.end.24583750-24659147.ref            CCCTGTTGTGTGTGGGGCTCATGAATGCCCACAGCATGGGCATCGGGGGTGGCCTCTTCC   31133

LOC112206721.putative.POM121-like,protein1.START.LOC112206738   -------------cagcaggggaaacttgtgtgtactgggcctagaggcctcggatgtgg   1190
BCRP3.REF.NEW                                              ------------------------------------------------------------   0
GGT1.start-POM121L10P.end.24583750-24659147.ref            TCACCATCTACAACAGCACCACACGTGAGTGCCTCGGGAGAGGAGAGGGAGAGGGGCAGG   31193

LOC112206721.putative.POM121-like,protein1.START.LOC112206738   aggagtcattcctgagggcgagtgtctgccctggtgctatatctgctgccttttcacact   1250
BCRP3.REF.NEW                                              ------------------------------------------------------------   0
GGT1.start-POM121L10P.end.24583750-24659147.ref            GGGTGTGGGTTGGGCCGAGGCACAGCTG----GGCGGTCCCCAGGCTCACGTGGCATAAA   31249

LOC112206721.putative.POM121-like,protein1.START.LOC112206738   gggtgtgacctgaagagacagcctgaggcctgtcctcactcactgtctttgaggaactga   1310
BCRP3.REF.NEW                                              ------------------------------------------------------------   0
GGT1.start-POM121L10P.end.24583750-24659147.ref            GGGTTTGGGTGGGCGGGCCTGCCTACCTGCTTCTCCTTCTAGGAAAAGCTGAGGTCATCA   31309

LOC112206721.putative.POM121-like,protein1.START.LOC112206738   gggtcagctggcagtgggatgaggctggccccctcctccactttagccccggcaagcctc   1370
BCRP3.REF.NEW                                              ------------------------------------------------------------   0
GGT1.start-POM121L10P.end.24583750-24659147.ref            ACGCCCGCGAGGTG-GCCCCCAGGCTGGCCTTTGCCACCATGTTCAACAGCTCGGAGCAG   31368

LOC112206721.putative.POM121-like,protein1.START.LOC112206738   ccgtggagctgtaggagctggagatggcatttcggttggtgcaggagctcgtccag----   1426
BCRP3.REF.NEW                                              ------------------------------------------------------------   0
GGT1.start-POM121L10P.end.24583750-24659147.ref            TCCCAGAAGGGTAAGCCATGCGGCAGACTTGGGGCGTGGGTGCAGAGCTGGCTGAGCCAC   31428

LOC112206721.putative.POM121-like,protein1.START.LOC112206738   ------------------------------------------------------------   1426
BCRP3.REF.NEW                                              ------------------------------------------------------------   0
GGT1.start-POM121L10P.end.24583750-24659147.ref            CGGGAAGGGGCCTTGCCCACAGGAGCCTGCTCCCGTCAGGGTTCAGGGGCAGTTCTGTCA   31488

LOC112206721.putative.POM121-like,protein1.START.LOC112206738   ------------------------------------------------------------   1426
BCRP3.REF.NEW                                              ------------------------------------------------------------   0
GGT1.start-POM121L10P.end.24583750-24659147.ref            CCCCCCATCCCTTCCTGTCCCCATAGCACCCTCCCACAATGAGTGGTCAGGACCATCATC   31548

LOC112206721.putative.POM121-like,protein1.START.LOC112206738   ---------gaggtctgggatgtctggttatatctgat----------------------   1455
BCRP3.REF.NEW                                              ------------------------------------------------------------   0
GGT1.start-POM121L10P.end.24583750-24659147.ref            ACCATGGTAAAGGGCCGGGAGCTTCTGTTATTTCTGCTAAGGCCTCCGGGGCCACTCTGT   31608

LOC112206721.putative.POM121-like,protein1.START.LOC112206738   ------------------------------------------------------------   1455
BCRP3.REF.NEW                                              ------------------------------------------------------------   0
GGT1.start-POM121L10P.end.24583750-24659147.ref            GCAGCACATGGAGAGAATAATTATTATGCTAGCAGACCTCATGGACCAGGGCTCACTGGG   31668

LOC112206721.putative.POM121-like,protein1.START.LOC112206738   ------------------------------------------------------------   1455
BCRP3.REF.NEW                                              ------------------------------------------------------------   0
GGT1.start-POM121L10P.end.24583750-24659147.ref            GCCCACACTCTGCTCTGTGCTTTTCACCCATGAGCCTCTCACAACCCTCCCTGCTCCTTT   31728

LOC112206721.putative.POM121-like,protein1.START.LOC112206738   ------------------------------------------------------------   1455
BCRP3.REF.NEW                                              ------------------------------------------------------------   0
GGT1.start-POM121L10P.end.24583750-24659147.ref            GGGCTAGGGGATGCTGTGTGGATTCCCATTTTACAGGGTGGGGATGCTGAGGCTCAGACA   31788

LOC112206721.putative.POM121-like,protein1.START.LOC112206738   ------------------------------------------------------------   1455
BCRP3.REF.NEW                                              ------------------------------------------------------------   0
GGT1.start-POM121L10P.end.24583750-24659147.ref            GGTCACGCAAATCAGTTGAGGTCACACAGCTGGGAGGTGGTGAAGCTAAAATTGAACCCA   31848

LOC112206721.putative.POM121-like,protein1.START.LOC112206738   ----------------------------------------------ttctgacctctggg   1469
BCRP3.REF.NEW                                              ------------------------------------------------------------   0
GGT1.start-POM121L10P.end.24583750-24659147.ref            GGCTGTCTATACCGTGCCTTTTCAACAGGCATCCCATTCACTCATTTGTTCATTTGTGGG   31908

LOC112206721.putative.POM121-like,protein1.START.LOC112206738   catggaggtctgtctgcagaggcccaggc-------------ctgggcacaaagggagag   1516
BCRP3.REF.NEW                                              ------------------------------------------------------------   0
GGT1.start-POM121L10P.end.24583750-24659147.ref            GATGGTGCTCTAGAATGTGAGGTGGAGTCTCTCTTTTCTAATCTGGTCTTAAGTGGAGAG   31968

LOC112206721.putative.POM121-like,protein1.START.LOC112206738   aggcctccattgtcccgcaggggccgaaatgcagactgtgcaccctcggtgacctcaggg   1576
BCRP3.REF.NEW                                              ------------------------------------------------------------   0
GGT1.start-POM121L10P.end.24583750-24659147.ref            GAGGCCCCCAAATTCCCCAGGTACCTGAAGGGAAGCCACTGTCCATCCAGGAAGCCACTG   32028

LOC112206721.putative.POM121-like,protein1.START.LOC112206738   acccttctctga------------------------------------------------   1588
BCRP3.REF.NEW                                              ------------------------------------------------------------   0
GGT1.start-POM121L10P.end.24583750-24659147.ref            TCTGTCCTCAAAGGTGGTACAGTAGATTGTGAGATAAAAGTTGGAGGATGGGAGGGTCTC   32088

LOC112206721.putative.POM121-like,protein1.START.LOC112206738   ------------------------------------------------------------   1588
BCRP3.REF.NEW                                              ------------------------------------------------------------   0
GGT1.start-POM121L10P.end.24583750-24659147.ref            AACAACTCACGCCTCTAATCCTAGCACTTTAGGAGGCCAAGTCAGGAGGAGCGCATGAGC   32148

LOC112206721.putative.POM121-like,protein1.START.LOC112206738   ------------------------------------------------------------   1588
BCRP3.REF.NEW                                              ------------------------------------------------------------   0
GGT1.start-POM121L10P.end.24583750-24659147.ref            CCAGGAGTTTGAGACCTGCCTGGGCAACATAGCAAGACTCCATCTCTACAAAAAACGGAA   32208

LOC112206721.putative.POM121-like,protein1.START.LOC112206738   ------------------------------------------------------------   1588
BCRP3.REF.NEW                                              ------------------------------------------------------------   0
GGT1.start-POM121L10P.end.24583750-24659147.ref            AAAAAAATTAGCTAGGTGTGGCGGTGTGTGCTTATGTTCCCAGCTACTTGGGAGGCTAAG   32268

LOC112206721.putative.POM121-like,protein1.START.LOC112206738   ------------------------------------------------------------   1588
BCRP3.REF.NEW                                              ------------------------------------------------------------   0
GGT1.start-POM121L10P.end.24583750-24659147.ref            GTGGGAGGATCACTTGAGCCCAGGAGGTTGAGGCTGCAGTGAGCCATGATTGTACCACTA   32328

LOC112206721.putative.POM121-like,protein1.START.LOC112206738   ------------------------------------------------------------   1588
BCRP3.REF.NEW                                              ------------------------------------------------------------   0
GGT1.start-POM121L10P.end.24583750-24659147.ref            CACTCTAGCTTGGGCAACAAAGTGAGACCCTGCCAAAAAAAAAAAAAAAGGCTGGGCCTTT   32388

LOC112206721.putative.POM121-like,protein1.START.LOC112206738   ------------------------------------------------------------   1588
```

```
BCRP3.REF.NEW                                                  ------------------------------------------------------------   0
GGT1.start-POM121L10P.end.24583750-24659147.ref                TTTTCACAGGCTCACGCCTGTAATCCCAGCACTTTGGGAGGCTGAGGCAGATGGATCACC   32448

LOC112206721.putative.POM121-like,protein1.START.LOC112206738  ------------------------------------------------------------   1588
BCRP3.REF.NEW                                                  ------------------------------------------------------------   0
GGT1.start-POM121L10P.end.24583750-24659147.ref                TAGGTCAGGAGTTCAAGACCAGCCTGGCCAACATAGTGAAACCCTTTCTCTACTAAAAGT   32508

LOC112206721.putative.POM121-like,protein1.START.LOC112206738  ------------------------------------------------------------   1588
BCRP3.REF.NEW                                                  ------------------------------------------------------------   0
GGT1.start-POM121L10P.end.24583750-24659147.ref                ACAATAATTAGTCAGGCGTGGTGGCACAGGCCTGTAATCCCAGCTACTCAGGAGGCTAAG   32568

LOC112206721.putative.POM121-like,protein1.START.LOC112206738  ------------------------------------------------------------   1588
BCRP3.REF.NEW                                                  ------------------------------------------------------------   0
GGT1.start-POM121L10P.end.24583750-24659147.ref                GCAGGAGAATCGCTTGAACCCAGGAGGAGGAGATTGCAGTGAGCCGAGATCATGCCACTG   32628

LOC112206721.putative.POM121-like,protein1.START.LOC112206738  ------------------------------------------------------------   1588
BCRP3.REF.NEW                                                  ------------------------------------------------------------   0
GGT1.start-POM121L10P.end.24583750-24659147.ref                CACTCCAGTCTGGGCAACAAGAATGAAGCTCCGTCTCAAAAAAAAAAAAAGTTGGAGGAT   32688

LOC112206721.putative.POM121-like,protein1.START.LOC112206738  ------------------------------------------------------------   1588
BCRP3.REF.NEW                                                  ------------------------------------------------------------   0
GGT1.start-POM121L10P.end.24583750-24659147.ref                GGAGGGGCAGGACACACTCACCATAGCAGGTCTTAGACTTCAGGTGGGGGTCCTGGGTGG   32748

LOC112206721.putative.POM121-like,protein1.START.LOC112206738  ------------------------------------------------------------   1588
BCRP3.REF.NEW                                                  ------------------------------------------------------------   0
GGT1.start-POM121L10P.end.24583750-24659147.ref                TGCCCTTTGGAGTCTTCTGCAACATACTCAATCTTTGACTTTTTTCTTTTTTTTTTCTTT   32808

LOC112206721.putative.POM121-like,protein1.START.LOC112206738  ------------tcaccaggattctcttggactctagggt-ccttgtcctgctcaggtat   1635
BCRP3.REF.NEW                                                  ------------------------------------------------------------   0
GGT1.start-POM121L10P.end.24583750-24659147.ref                TTTTTTTTTTGTCACCCAGACTGTCGCCCAGGCTGGAGTGCAGTGGCACGATCTCGGCT   32868

LOC112206721.putative.POM121-like,protein1.START.LOC112206738  ccctgccccgctctccttgagggccctcaacactatcttccctggacacaagtctgggga   1695
BCRP3.REF.NEW                                                  ------------------------------------------------------------   0
GGT1.start-POM121L10P.end.24583750-24659147.ref                CACTGCAAGCTCTGCCTCCTGAGTTCACGCCATTCTCCTGCCTCAGCCTC----------   32918

LOC112206721.putative.POM121-like,protein1.START.LOC112206738  cagccgggtgttgtggaccccaaaggggtgactcccggctcctgggccccacagagagtc   1755
BCRP3.REF.NEW                                                  ------------------------------------------------------------   0
GGT1.start-POM121L10P.end.24583750-24659147.ref                -----------------CCGAATAGCTGGGACTACAGGCGCCT-GCCACCACGCCCAGCT   32960

LOC112206721.putative.POM121-like,protein1.START.LOC112206738  catgttct----------------------------------------------------   1763
BCRP3.REF.NEW                                                  ------------------------------------------------------------   0
GGT1.start-POM121L10P.end.24583750-24659147.ref                AATTTTTGTATTTTTAGTAGAGACGGGTTTCACTATGTTGCCCAGGCTGGTCTCGAT   33020

LOC112206721.putative.POM121-like,protein1.START.LOC112206738  ------------------------------------------------------------   1763
BCRP3.REF.NEW                                                  ------------------------------------------------------------   0
GGT1.start-POM121L10P.end.24583750-24659147.ref                CTCCTGACCTCGTGATCCCCCGGCCTCGGCCTCCCAAAGTGCTGGAATTACAGGCGTGAG   33080

LOC112206721.putative.POM121-like,protein1.START.LOC112206738  ------------------------------------------------------------   1763
BCRP3.REF.NEW                                                  ------------------------------------------------------------   0
GGT1.start-POM121L10P.end.24583750-24659147.ref                CCACTGTGCCCGATCAATCTTTCATTTTTTTTAATACTCATTGAGAAACTCAGCATTTG   33140

LOC112206721.putative.POM121-like,protein1.START.LOC112206738  ------------------------------------------------------------   1763
BCRP3.REF.NEW                                                  ------------------------------------------------------------   0
GGT1.start-POM121L10P.end.24583750-24659147.ref                TAGACATGAAGTTGCTCAGGGTAAGAGAATGCGGGAATCATAGGCTTGGCACCTTGTGGA   33200

LOC112206721.putative.POM121-like,protein1.START.LOC112206738  ------------------------------------------------------------   1763
BCRP3.REF.NEW                                                  ------------------------------------------------------------   0
GGT1.start-POM121L10P.end.24583750-24659147.ref                CTCTTAGAATCATTTATTTAACTTGAATGTATTGAGTATTCTCTTAAAGAATCAGCTGTT   33260

LOC112206721.putative.POM121-like,protein1.START.LOC112206738  ------------------------------------------------------------   1763
BCRP3.REF.NEW                                                  ------------------------------------------------------------   0
GGT1.start-POM121L10P.end.24583750-24659147.ref                GTTCCTGAAGCTGGGGTGAAAAACAAAGATGGCAGATGAAATCTGTGACACTCCAGGTGG   33320

LOC112206721.putative.POM121-like,protein1.START.LOC112206738  ------------------------------------------------------------   1763
BCRP3.REF.NEW                                                  ------------------------------------------------------------   0
GGT1.start-POM121L10P.end.24583750-24659147.ref                GAGGAGAAACTAGGCAGGTGCAGGTGTGTTACGGGCTGTAGAAAAACAGGTCTGGAGGGC   33380

LOC112206721.putative.POM121-like,protein1.START.LOC112206738  --------------cagtgcagtggctgagctggaggacgccctggaactcggagcacac   1809
BCRP3.REF.NEW                                                  ------------------------------------------------------------   0
GGT1.start-POM121L10P.end.24583750-24659147.ref                CTCAAAATCTGGGACTGTATAGAGGGTGACCTAGTCAGGGAAGGGGACATCTGAGCAAAG   33440

LOC112206721.putative.POM121-like,protein1.START.LOC112206738  agcactggcttgctgtggtacctgtgcaatcaaattgaaggcaggatcccaggaaggaac   1869
BCRP3.REF.NEW                                                  ------------------------------------------------------------   0
GGT1.start-POM121L10P.end.24583750-24659147.ref                ACCCAGAGGCAGAGATGGGGTCAGGGGAGTTATCTCCTGGTCTGCTATCCAGGTGTGATG   33500

LOC112206721.putative.POM121-like,protein1.START.LOC112206738  gcagggcttgcaggatcacggaa-------------------------------------   1892
BCRP3.REF.NEW                                                  ------------------------------------------------------------   0
GGT1.start-POM121L10P.end.24583750-24659147.ref                GCAGGGACAGAGCCCTGTGGGGAGCTGGGGAGGCTGCAGCAAGTGATCCAAGGGAAATGG   33560

LOC112206721.putative.POM121-like,protein1.START.LOC112206738  ------------------------------------------------------------   1892
BCRP3.REF.NEW                                                  ------------------------------------------------------------   0
GGT1.start-POM121L10P.end.24583750-24659147.ref                CCCAGGTTGTGTGGGATCTCTTAGGTTATGGTGAAGCCTCTGCTTCCTGTCTGAGGGAAG   33620

LOC112206721.putative.POM121-like,protein1.START.LOC112206738  ------------------------------------------------------------   1892
BCRP3.REF.NEW                                                  ------------------------------------------------------------   0
GGT1.start-POM121L10P.end.24583750-24659147.ref                TGGGGGCTCGTGGAGTATGTGAGTGGAAGGGGATGGATCTGGCCTATGGACCCCGTGGGT   33680

LOC112206721.putative.POM121-like,protein1.START.LOC112206738  -------------------aaccttcttagcgttgtcttgacaccactgatgtcaagtgt   1933
BCRP3.REF.NEW                                                  ------------------------------------------------------------   0
GGT1.start-POM121L10P.end.24583750-24659147.ref                TGCTGTGTAAGGGGCAGGGAGCATGTGGGGACCTGTCTGGAGGTCACTGCAGTAATTCGT   33740

LOC112206721.putative.POM121-like,protein1.START.LOC112206738  ccgggtgcttgtaggatgacctgccactcagtccacgggcaggagcaacggggagatccc   1993
BCRP3.REF.NEW                                                  ------------------------------------------------------------   0
GGT1.start-POM121L10P.end.24583750-24659147.ref                GGAGAGGGTGCTGGGAAGTGG-------CTGACGCTGGACAGACACACTTGGAAGTGGAG   33793

LOC112206721.putative.POM121-like,protein1.START.LOC112206738  acaagcaaagtgaactgggggatgggctgaa-----------cgggctccaggcaactga   2042
BCRP3.REF.NEW                                                  ------------------------------------------------------------   0
GGT1.start-POM121L10P.end.24583750-24659147.ref                TCTGTGGATTTGAGGATGGGTTGGGTGTGACGCTTGTGTAGGGAGTCCCCGGGGACCCCT   33853

LOC112206721.putative.POM121-like,protein1.START.LOC112206738  gccctactggcaggtcctcggcctg-ggcccgaacaggaaggaggagcacagagtgccca   2101
BCRP3.REF.NEW                                                  ------------------------------------------------------------   0
GGT1.start-POM121L10P.end.24583750-24659147.ref                GATCTTTTGTCTGTACCTGGAAGGATGGGGTGACCCTAATGGAGACAGGCAGGGTTCTCA   33913

LOC112206721.putative.POM121-like,protein1.START.LOC112206738  ggtaaccgctcctgggagcagtggggaaccgtcggttg----cttgaactctcgagagct   2157
BCRP3.REF.NEW                                                  ------------------------------------------------------------   0
GGT1.start-POM121L10P.end.24583750-24659147.ref                GGAAGCAGGTTGAGCAGACACTCAGGAGCTCGGTTTTGGGCACGTTGAAGTTTGAGATGC   33973

LOC112206721.putative.POM121-like,protein1.START.LOC112206738  gggctctgagcgtcctcgtccagccgccaactcggccaacgg---------ctaagccag   2208
BCRP3.REF.NEW                                                  ------------------------------------------------------------   0
GGT1.start-POM121L10P.end.24583750-24659147.ref                TTTCTTCGAGCAGGCAGGTAGATCCTCAGGTCTGGTGATCGGAGGAGCAGCCCAGGCCAG   34033

LOC112206721.putative.POM121-like,protein1.START.LOC112206738  cagtttcttctgttgccgggcaacgcgccttttaaacctgagggagcgggcgcgtgagca   2268
BCRP3.REF.NEW                                                  ------------------------------------------------------------   0
GGT1.start-POM121L10P.end.24583750-24659147.ref                CAGGTCAATTTGAGAGTTGTCAGTGCATAAATGGAGGCTGAAGCTCAGATGTCAAGCAGA   34093

LOC112206721.putative.POM121-like,protein1.START.LOC112206738  catcatggcgc-----ccgtgacagagcgagcttaacggattaataagcgcagccaggta   2323
```

```
BCRP3.REF.NEW                                                  ------------------------------------------------------------   0
GGT1.start-POM121L10P.end.24583750-24659147.ref                CCACCAGGAGAGAGAGCAAAGACAGAGGGGAGAGTAGGAGCTAGGATGGCAGGCGGGGGA   34153

LOC112206721.putative.POM121-like,protein1.START.LOC112206738  cccgcg---caaggcacttgctggcaatggcgggaggc-------ggacgtgggggtca   2373
BCRP3.REF.NEW                                                  ------------------------------------------------------------   0
GGT1.start-POM121L10P.end.24583750-24659147.ref                GACTCGGGTGGAGCCAGGTGCTGGGATGCAGGGGCAGCTCTCAGGGAAAGTGATGAGCCC   34213

LOC112206721.putative.POM121-like,protein1.START.LOC112206738  tgcaataggtactggaaggagagaggcgggcacaaaggtcgcgggaggaacaggtgccca   2433
BCRP3.REF.NEW                                                  ------------------------------------------------------------   0
GGT1.start-POM121L10P.end.24583750-24659147.ref                AGTAAAGCTGAGAGGGGGCACTGGGTCTGGCAGTGTGGGGGTCACCAGAGAACTTGGCAA   34273

LOC112206721.putative.POM121-like,protein1.START.LOC112206738  caatgc----------ggcagatctgcccgtggatcactgaagattcctgctctcctgct   2483
BCRP3.REF.NEW                                                  ------------------------------------------------------------   0
GGT1.start-POM121L10P.end.24583750-24659147.ref                GTGTGCTGGCATGAGAGTCTGATTGGCCTGAGGTCAGGAGAAGATTTTTTTTCTGATATT   34333

LOC112206721.putative.POM121-like,protein1.START.LOC112206738  ------------------------------------------------------------   2483
BCRP3.REF.NEW                                                  ------------------------------------------------------------   0
GGT1.start-POM121L10P.end.24583750-24659147.ref                GATACATGATATTTTCTATATTTATGGGTACATGTGAGTGCTTGTTACATGCATAGAGTG   34393

LOC112206721.putative.POM121-like,protein1.START.LOC112206738  ------------------------------------------------------------   2483
BCRP3.REF.NEW                                                  ------------------------------------------------------------   0
GGT1.start-POM121L10P.end.24583750-24659147.ref                TATAATGATCAAGGCAGGGTATTTGGAGTCTCCGTCACCTTGAATATTTTTCATTTCTGG   34453

LOC112206721.putative.POM121-like,protein1.START.LOC112206738  ------------------------------------------------------------   2483
BCRP3.REF.NEW                                                  ------------------------------------------------------------   0
GGT1.start-POM121L10P.end.24583750-24659147.ref                GTGTTAGCACCATAGTCCTCTCTTCGTTACTTTGAAATATACAAAATACTGTTGCTAAGC   34513

LOC112206721.putative.POM121-like,protein1.START.LOC112206738  ------------------------------------------------------------   2483
BCRP3.REF.NEW                                                  ------------------------------------------------------------   0
GGT1.start-POM121L10P.end.24583750-24659147.ref                ATCGTCACCCTGGTCTGCTATCAAAGATTAGAACTTCTCCTGTCTTGGCTGGGCACGGTG   34573

LOC112206721.putative.POM121-like,protein1.START.LOC112206738  ------------------------------------------------------------   2483
BCRP3.REF.NEW                                                  ------------------------------------------------------------   0
GGT1.start-POM121L10P.end.24583750-24659147.ref                GCTCACACCTGTCATCCCAGCACCTTGGAAGGCTGAGGTGGGTGGATGACCTGAGGTCAG   34633

LOC112206721.putative.POM121-like,protein1.START.LOC112206738  ------------------------------------------------------------   2483
BCRP3.REF.NEW                                                  ------------------------------------------------------------   0
GGT1.start-POM121L10P.end.24583750-24659147.ref                GAGTTTGAAACCAGCCTGGCCAACATGGCAAAACCCCATCTGTACTAAAAATACAAAAAT   34693

LOC112206721.putative.POM121-like,protein1.START.LOC112206738  ------------------------------------------------------------   2483
BCRP3.REF.NEW                                                  ------------------------------------------------------------   0
GGT1.start-POM121L10P.end.24583750-24659147.ref                TAGCCTGGCGTGCTGGCCTGTGCCTGTAATACCAGCTACTCGGGAGGCTGAGGCAGGAGA   34753

LOC112206721.putative.POM121-like,protein1.START.LOC112206738  ------------------------------------------------------------   2483
BCRP3.REF.NEW                                                  ------------------------------------------------------------   0
GGT1.start-POM121L10P.end.24583750-24659147.ref                ATCGCTTGAACCTGGGAGGCGGAGGTTGTAGTGAGCTGAGATCATGCCACTGCACTGTAG   34813

LOC112206721.putative.POM121-like,protein1.START.LOC112206738  ------------------------------------------------------------   2483
BCRP3.REF.NEW                                                  ------------------------------------------------------------   0
GGT1.start-POM121L10P.end.24583750-24659147.ref                TCTGGGAGACAGAGCAAGACTCCATCTCAAAAACAAACAAAGAAGAAAAACAAAACAAAA   34873

LOC112206721.putative.POM121-like,protein1.START.LOC112206738  ------------------------------------------------------------   2483
BCRP3.REF.NEW                                                  ------------------------------------------------------------   0
GGT1.start-POM121L10P.end.24583750-24659147.ref                CAAAAAATAACTTCTGTCTAACTACAAGGTGGAAGGAATCATGTGCTGGGTGTCAGACCT   34933

LOC112206721.putative.POM121-like,protein1.START.LOC112206738  ------------------------------------------------------------   2483
BCRP3.REF.NEW                                                  ------------------------------------------------------------   0
GGT1.start-POM121L10P.end.24583750-24659147.ref                TCCGGGATGTATGTGCAGCTTCTAGGAATTGAAACCACCAGCTCTTGGAAACTTGTGCCA   34993

LOC112206721.putative.POM121-like,protein1.START.LOC112206738  ------------------------------------------------------------   2483
BCRP3.REF.NEW                                                  ------------------------------------------------------------   0
GGT1.start-POM121L10P.end.24583750-24659147.ref                GGCTTCAGGGTGGGAGAGGCAGTTCTAGAGCCACAGCTGCCAAGCCAAGCCAAATGGCCC   35053

LOC112206721.putative.POM121-like,protein1.START.LOC112206738  ------------------------------------------------------------   2483
BCRP3.REF.NEW                                                  ------------------------------------------------------------   0
GGT1.start-POM121L10P.end.24583750-24659147.ref                CATCATCTCTCGCAAGAGCAGGAGAGTCCCTGGGGGCAGAGGCCATAGTTGTACCTTTCT   35113

LOC112206721.putative.POM121-like,protein1.START.LOC112206738  ------------------------------------------------------------   2483
BCRP3.REF.NEW                                                  ------------------------------------------------------------   0
GGT1.start-POM121L10P.end.24583750-24659147.ref                GGGCAAAGGGTCAGTGTCTGTAGTGTCCTTATGGGCAGCGGGGCTCAGGGGGGAAGCAGG   35173

LOC112206721.putative.POM121-like,protein1.START.LOC112206738  ------------------------------------------------------------   2483
BCRP3.REF.NEW                                                  ------------------------------------------------------------   0
GGT1.start-POM121L10P.end.24583750-24659147.ref                CCCAGGGGTGTGTTTCCAATGACCTCCTCAAAAGTCAGAACTGGAAGGCAAAACCCCTTA   35233

LOC112206721.putative.POM121-like,protein1.START.LOC112206738  ------------------------------------------------------------   2483
BCRP3.REF.NEW                                                  ------------------------------------------------------------   0
GGT1.start-POM121L10P.end.24583750-24659147.ref                TAGGCTGAGTGCACCTGTAATCCCAGCATGTTGGGATGCTGAGGTGGGAGGATTGCTTGA   35293

LOC112206721.putative.POM121-like,protein1.START.LOC112206738  ------------------------------------------------------------   2483
BCRP3.REF.NEW                                                  ------------------------------------------------------------   0
GGT1.start-POM121L10P.end.24583750-24659147.ref                GGCCAGAGTTTGAGACCAGTCTTGGCAACGTAGCAAGACCCCTGTCTCTACAAAAAATAA   35353

LOC112206721.putative.POM121-like,protein1.START.LOC112206738  ------------------------------------------------------------   2483
BCRP3.REF.NEW                                                  ------------------------------------------------------------   0
GGT1.start-POM121L10P.end.24583750-24659147.ref                AAATAAAAAATTAGGCTGGGAATGGTGGCTCACGCCTGTAATCCCAGAACTCTGAGAGGC   35413

LOC112206721.putative.POM121-like,protein1.START.LOC112206738  ------------------------------------------------------------   2483
BCRP3.REF.NEW                                                  ------------------------------------------------------------   0
GGT1.start-POM121L10P.end.24583750-24659147.ref                TGAGGAGGATGGATCACCTGATGTTAGGAGTTCAAGACCAGCCTGACCAACCTGGTGAAA   35473

LOC112206721.putative.POM121-like,protein1.START.LOC112206738  ------------------------------------------------------------   2483
BCRP3.REF.NEW                                                  ------------------------------------------------------------   0
GGT1.start-POM121L10P.end.24583750-24659147.ref                CCCCGTCTCTACTAAAAATACAAAAATTAGCAAGGCATGGTGGTGCATGCCTGTAATCCC   35533

LOC112206721.putative.POM121-like,protein1.START.LOC112206738  ------------------------------------------gaggtggagattgcagtg   2501
BCRP3.REF.NEW                                                  ------------------------------------------------------------   0
GGT1.start-POM121L10P.end.24583750-24659147.ref                AGCTACTTGGGAGGCTGAGGCAGGAGAATTGCTTGAACTCGGGAGGTGGAGGTTGGAGTG   35593

LOC112206721.putative.POM121-like,protein1.START.LOC112206738  agctgagatcgcaccattgcactccagcctgggccacaagtgcaaaactcagtctccaga   2561
BCRP3.REF.NEW                                                  ------------------------------------------------------------   0
GGT1.start-POM121L10P.end.24583750-24659147.ref                AGCCAAGATTGTGCCATTGCACTCCAGCCTGGGCAACAAGAGTGAAACTCCATCTCAGAA   35653

LOC112206721.putative.POM121-like,protein1.START.LOC112206738  ttaaaaaaagaaaaagaaaaaaa-------------------------------------   2584
BCRP3.REF.NEW                                                  ------------------------------------------------------------   0
GGT1.start-POM121L10P.end.24583750-24659147.ref                AAAAAAAAAAAAAAAAAAAAGCCCTGTGTAGTGGGGTATGTCTGTAGTCTTAGGTACTTC   35713

LOC112206721.putative.POM121-like,protein1.START.LOC112206738  ------------------------------------------------------------   2584
BCRP3.REF.NEW                                                  ------------------------------------------------------------   0
GGT1.start-POM121L10P.end.24583750-24659147.ref                AGAGGCTGAGGTAGGAGGATCGCTTGAGCCTGGGAAGCCAAGGCTGCAGTGAGCCATGAT   35773

LOC112206721.putative.POM121-like,protein1.START.LOC112206738  ------------------------------------------------------------   2584
BCRP3.REF.NEW                                                  ------------------------------------------------------------   0
GGT1.start-POM121L10P.end.24583750-24659147.ref                TGCACCACTGCACTCCAGCTGGGACAACAGAATGAGACCCTGTCTCAAAACAGACAAACA   35833

LOC112206721.putative.POM121-like,protein1.START.LOC112206738  ------------------------------------------------------------   2584
```

```
BCRP3.REF.NEW                                                  ------------------------------------------------------------   0
GGT1.start-POM121L10P.end.24583750-24659147.ref                AACAAAAACCCTTATAGTTGGATGGAGAAACTGAGGCTGGGAGAGGGGACAGGACGGAGG   35893

LOC112206721.putative.POM121-like,protein1.START.LOC112206738  ------------------------------------------------------------   2584
BCRP3.REF.NEW                                                  ------------------------------------------------------------   0
GGT1.start-POM121L10P.end.24583750-24659147.ref                TTAAGGCTCAGTCTTGCCTCTCTGGGGCCGTAGAAAAGAGGCAGGGAGCCCTTTCTTGGG   35953

LOC112206721.putative.POM121-like,protein1.START.LOC112206738  ------------------------------------------------------------   2584
BCRP3.REF.NEW                                                  ------------------------------------------------------------   0
GGT1.start-POM121L10P.end.24583750-24659147.ref                GCTGGCTGTGTCTTGAAGGTGGCCTGTGCTTGACCTCGGTCAAGGTGGGATCTGCTCTTG   36013

LOC112206721.putative.POM121-like,protein1.START.LOC112206738  ------------------------------------------------------------   2584
BCRP3.REF.NEW                                                  ------------------------------------------------------------   0
GGT1.start-POM121L10P.end.24583750-24659147.ref                TTTTGGCACATTCTCGTGGAGCCCATGAGTCTTACAGGATAAGGCCTTGTGGTCAGTGAG   36073

LOC112206721.putative.POM121-like,protein1.START.LOC112206738  ------------------------------------------------------------   2584
BCRP3.REF.NEW                                                  ------------------------------------------------------------   0
GGT1.start-POM121L10P.end.24583750-24659147.ref                ATGGGAGGGGGTCTGGCCTGGCACAGGATTTTAGACATGCAGGCACCTGCACAGACAGAC   36133

LOC112206721.putative.POM121-like,protein1.START.LOC112206738  ------------------------------------------------------------   2584
BCRP3.REF.NEW                                                  ------------------------------------------------------------   0
GGT1.start-POM121L10P.end.24583750-24659147.ref                ACCTCATCCTGGGACAGCAAAACCCAGCCGCATGCTACTGCTTCCCCTGCTGTGCCCTCC   36193

LOC112206721.putative.POM121-like,protein1.START.LOC112206738  ------------------------------------------------------------   2584
BCRP3.REF.NEW                                                  ------------------------------------------------------------   0
GGT1.start-POM121L10P.end.24583750-24659147.ref                TCAGACATCCCTGGTCCATGTACACTCCTACCTGCTGAGCCCCTCCTAAAAAAAAAAATT   36253

LOC112206721.putative.POM121-like,protein1.START.LOC112206738  ------------------------------------------------------------   2584
BCRP3.REF.NEW                                                  ------------------------------------------------------------   0
GGT1.start-POM121L10P.end.24583750-24659147.ref                AAACATCCCCTCCTAAAAAAAAACAATTAAAATTAAAAAATAAATTTAAAAATTAAAAAT   36313

LOC112206721.putative.POM121-like,protein1.START.LOC112206738  ------------------------------------------------------------   2584
BCRP3.REF.NEW                                                  ------------------------------------------------------------   0
GGT1.start-POM121L10P.end.24583750-24659147.ref                CCCCTTCTGCTAGGTGTGCTGTTCACGCCTGTAATCTCAGCGACTCAGGAGGCTGAGGTG   36373

LOC112206721.putative.POM121-like,protein1.START.LOC112206738  ------------------------------------------------------------   2584
BCRP3.REF.NEW                                                  ------------------------------------------------------------   0
GGT1.start-POM121L10P.end.24583750-24659147.ref                GGAGGATCGCTTGAATCCAGGAGTTCGAGATCGGGCTGGGCAAGATGGCAAGACCCCAAC   36433

LOC112206721.putative.POM121-like,protein1.START.LOC112206738  ------------------------------------------------------------   2584
BCRP3.REF.NEW                                                  ------------------------------------------------------------   0
GGT1.start-POM121L10P.end.24583750-24659147.ref                TCAAAAAAGAAAAAAAAAAAATCTTTCCTCCTAAGCCTCATTGCCCCATCTGTAAAATGA   36493

LOC112206721.putative.POM121-like,protein1.START.LOC112206738  ------------------------------------------------------------   2584
BCRP3.REF.NEW                                                  ------------------------------------------------------------   0
GGT1.start-POM121L10P.end.24583750-24659147.ref                GTCAGGGACTGTGCCTGGGATGCTGCCTGCGAGAGATCCCGATGTCCCCCACTCAGGGTC   36553

LOC112206721.putative.POM121-like,protein1.START.LOC112206738  ------------------------------------------------------------   2584
BCRP3.REF.NEW                                                  ------------------------------------------------------------   0
GGT1.start-POM121L10P.end.24583750-24659147.ref                ACTAACTATGGCTCTCTCTCCCCAGGGGGGCTGTCGGTGGCGGTGCCTGGGGAGATCCGA   36613

LOC112206721.putative.POM121-like,protein1.START.LOC112206738  ------------------------------------------------------------   2584
BCRP3.REF.NEW                                                  ------------------------------------------------------------   0
GGT1.start-POM121L10P.end.24583750-24659147.ref                GGCTATGAGCTGGCACACCAGCGGCATGGGCGGCTGCCCTGGGCTCGCCTCTTCCAGCCC   36673

LOC112206721.putative.POM121-like,protein1.START.LOC112206738  ------------------------------------------------------------   2584
BCRP3.REF.NEW                                                  ------------------------------------------------------------   0
GGT1.start-POM121L10P.end.24583750-24659147.ref                AGCATCCAGCTGGCCCGCCAGGGCTTCCCCGTGGGCAAGGGCTTGGCGGCAGCCCTGGAA   36733

LOC112206721.putative.POM121-like,protein1.START.LOC112206738  ------------------------------------------------------------   2584
BCRP3.REF.NEW                                                  ------------------------------------------------------------   0
GGT1.start-POM121L10P.end.24583750-24659147.ref                AACAAGCGGACCGTCATCGAGCAGCAGCCTGTCTTGTGGTATGTCTGTGGGTGCGGCCCC   36793

LOC112206721.putative.POM121-like,protein1.START.LOC112206738  ------------------------------------------------------------   2584
BCRP3.REF.NEW                                                  ------------------------------------------------------------   0
GGT1.start-POM121L10P.end.24583750-24659147.ref                CTGACACAGGCAGGGCAGGCACAGCCCAAGGACCTTGCAGGCCGTAGCAGCAGTGGAGCA   36853

LOC112206721.putative.POM121-like,protein1.START.LOC112206738  ------------------------------------------------------------   2584
BCRP3.REF.NEW                                                  ------------------------------------------------------------   0
GGT1.start-POM121L10P.end.24583750-24659147.ref                GCCCTCTGCCTTCAGGACCCTGTGCTGATAATGGGATGAGGAGATACAGACCCTTCCCAC   36913

LOC112206721.putative.POM121-like,protein1.START.LOC112206738  ------------------------------------------------------------   2584
BCRP3.REF.NEW                                                  ------------------------------------------------------------   0
GGT1.start-POM121L10P.end.24583750-24659147.ref                CACGTGTGGGGACACATTCTGAGCGTGGGGTCCCAGTGGCCACTGTGGCTGGCCATGTGT   36973

LOC112206721.putative.POM121-like,protein1.START.LOC112206738  ------------------------------------------------------------   2584
BCRP3.REF.NEW                                                  ------------------------------------------------------------   0
GGT1.start-POM121L10P.end.24583750-24659147.ref                CCTGAGTGGCAAGGGACACTAGGAAGCTCCCGGAAGGGACACTAGGAAGACGAGCGCTGA   37033

LOC112206721.putative.POM121-like,protein1.START.LOC112206738  ------------------------------------------------------------   2584
BCRP3.REF.NEW                                                  ------------------------------------------------------------   0
GGT1.start-POM121L10P.end.24583750-24659147.ref                GTGACAGGGCCACCCACCTGTGACAGGCGCTGCCCCTGTTCTGCTCCGTTCTCCTGTGTG   37093

LOC112206721.putative.POM121-like,protein1.START.LOC112206738  ------------------------------------------------------------   2584
BCRP3.REF.NEW                                                  ------------------------------------------------------------   0
GGT1.start-POM121L10P.end.24583750-24659147.ref                GACGGGTGTGAGGGGTGGTCTAGCTGAGTCCACCCCACCTGCTGCCTCACATGAGCCCCC   37153

LOC112206721.putative.POM121-like,protein1.START.LOC112206738  ------------------------------------------------------------   2584
BCRP3.REF.NEW                                                  ------------------------------------------------------------   0
GGT1.start-POM121L10P.end.24583750-24659147.ref                TCTGCCCCAGTGAGGTGTTCTGCCGGGATAGAAAGGTGCTTCGGGAGGGGGAGAGACTGA   37213

LOC112206721.putative.POM121-like,protein1.START.LOC112206738  ------------------------------------------------------------   2584
BCRP3.REF.NEW                                                  ------------------------------------------------------------   0
GGT1.start-POM121L10P.end.24583750-24659147.ref                CCCTGCCGCAGCTGGCTGACACCTACGAGACGCTGGCCATCGAGGGTGCCCAGGCCTTCT   37273

LOC112206721.putative.POM121-like,protein1.START.LOC112206738  ------------------------------------------------------------   2584
BCRP3.REF.NEW                                                  ------------------------------------------------------------   0
GGT1.start-POM121L10P.end.24583750-24659147.ref                ACAACGGCAGCCTCACGGCCCAGATTGTGAAGGACATCCAGGCGGCCGGTGAGTGGGTAA   37333

LOC112206721.putative.POM121-like,protein1.START.LOC112206738  ------------------------------------------------------------   2584
BCRP3.REF.NEW                                                  ------------------------------------------------------------   0
GGT1.start-POM121L10P.end.24583750-24659147.ref                CCTCAGGGGCCTGGGTGAGGAACTCTGCAGTGGAAACCCTGAGCTGTAGCCCAGAGCTAT   37393

LOC112206721.putative.POM121-like,protein1.START.LOC112206738  ------------------------------------------------------------   2584
BCRP3.REF.NEW                                                  ------------------------------------------------------------   0
GGT1.start-POM121L10P.end.24583750-24659147.ref                GGGGTCCTCCTGTCTTGCCTGAGCCTGCAGGAAGTTCCTGGTGGAGGAGGGTCAGTGACT   37453

LOC112206721.putative.POM121-like,protein1.START.LOC112206738  ------------------------------------------------------------   2584
BCRP3.REF.NEW                                                  ------------------------------------------------------------   0
GGT1.start-POM121L10P.end.24583750-24659147.ref                GGCCATGTGGGTCCACAGCTCCTGCTTATATCAAAACCAAGAGAGGCCACACAGTCCAGG   37513

LOC112206721.putative.POM121-like,protein1.START.LOC112206738  ------------------------------------------------------------   2584
BCRP3.REF.NEW                                                  ------------------------------------------------------------   0
GGT1.start-POM121L10P.end.24583750-24659147.ref                AGAGCAAGTCCCTGTTGGGGTAAATGCAGGTGTAGGCAAGAGCCAGGGCTAGGGAAGCAC   37573

LOC112206721.putative.POM121-like,protein1.START.LOC112206738  ------------------------------------------------------------   2584
```

```
BCRP3.REF.NEW                                                  ------------------------------------------------------------   0
GGT1.start-POM121L10P.end.24583750-24659147.ref                TAGAATACAGCCTGAAGATCCAGGAGGACTTCTTGGAGGAGGTGGCGGCTGGGCTGCAGA   37633

LOC112206721.putative.POM121-like,protein1.START.LOC112206738  ------------------------------------------------------------   2584
BCRP3.REF.NEW                                                  ------------------------------------------------------------   0
GGT1.start-POM121L10P.end.24583750-24659147.ref                TAACTTTGTTAGGCAGAGAGAGGAAGGGATTCCTAGCAGAGGAACAGCTGGGCTAAGGCC   37693

LOC112206721.putative.POM121-like,protein1.START.LOC112206738  ------------------------------------------------------------   2584
BCRP3.REF.NEW                                                  ------------------------------------------------------------   0
GGT1.start-POM121L10P.end.24583750-24659147.ref                CAGTAGAGGGCGCTTTGATTCACCAAGAGGGTTACAAGGGATGAGGGTCTCCTTGAGAGA   37753

LOC112206721.putative.POM121-like,protein1.START.LOC112206738  ------------------------------------------------------------   2584
BCRP3.REF.NEW                                                  ------------------------------------------------------------   0
GGT1.start-POM121L10P.end.24583750-24659147.ref                GGCATGGGGAAGGGGATTTGTGGGGCAGGGGCCTGGAGCTTGGCTGTGGCTTTCTTCAGG   37813

LOC112206721.putative.POM121-like,protein1.START.LOC112206738  ------------------------------------------------------------   2584
BCRP3.REF.NEW                                                  ------------------------------------------------------------   0
GGT1.start-POM121L10P.end.24583750-24659147.ref                TAATTTTTGTCACGTTTCATGGAGGAGGGTGATTAGCGTGTCGACCTTTACCACTGAGGC   37873

LOC112206721.putative.POM121-like,protein1.START.LOC112206738  ------------------------------------------------------------   2584
BCRP3.REF.NEW                                                  ------------------------------------------------------------   0
GGT1.start-POM121L10P.end.24583750-24659147.ref                TGGAAATTGGCATGCCAATACCCTGTCTGTCTGGAGCTGACTCCAGGAGAATTAAGAGCC   37933

LOC112206721.putative.POM121-like,protein1.START.LOC112206738  ------------------------------------------------------------   2584
BCRP3.REF.NEW                                                  ------------------------------------------------------------   0
GGT1.start-POM121L10P.end.24583750-24659147.ref                TCCCTCCTCTATTCATTCATCATGAGGAGAAGAGGCCAAGCGGCAGGGAGACTGGCAGGA   37993

LOC112206721.putative.POM121-like,protein1.START.LOC112206738  -----------------------agaggccgggcgtggcggcttatgcctataatcctag   2621
BCRP3.REF.NEW                                                  ------------------------------------------------------------   0
GGT1.start-POM121L10P.end.24583750-24659147.ref                ATTCTCCAGTTAGAAAAGGCCCTCTGAGCCAGGCGCGGTGGCTCACGTCTGTAATCCCAG   38053

LOC112206721.putative.POM121-like,protein1.START.LOC112206738  cactttgggaggtcggggcagacggatcac--gagatcaggagttggagaccagcctggc   2679
BCRP3.REF.NEW                                                  ------------------------------------------------------------   0
GGT1.start-POM121L10P.end.24583750-24659147.ref                CACTTTGGGAGGCCGAGGCGGGTGGATCACCTGAGGTCAGGAGTTCAAGACCAGCCTGGC   38113

LOC112206721.putative.POM121-like,protein1.START.LOC112206738  caacatagtgaaactccgtctctagtaaaaatacaaatttagtcagacatggtggcacgc   2739
BCRP3.REF.NEW                                                  ------------------------------------------------------------   0
GGT1.start-POM121L10P.end.24583750-24659147.ref                CAACATGGCGAAACCCTGTCTCTACTAAAAATGCAAAATTAGTCAGGCATGGTAGGTTGT   38173

LOC112206721.putative.POM121-like,protein1.START.LOC112206738  gcctgtagtcccagctactccggcggctgagacaggagaatcacttgaacccggggg---   2796
BCRP3.REF.NEW                                                  ------------------------------------------------------------   0
GGT1.start-POM121L10P.end.24583750-24659147.ref                GCCTGTAATCCCAGCTACTTGGGAGGCTGTGCCAGGAGAATCGCTAGAACCTGGGGGGCT   38233

LOC112206721.putative.POM121-like,protein1.START.LOC112206738  ------------------------------------------------------------   2796
BCRP3.REF.NEW                                                  ------------------------------------------------------------   0
GGT1.start-POM121L10P.end.24583750-24659147.ref                GAGGTTGCAGTGAGCCGAGATTGCACCACTGCACTCCACAGAGTGAGATTCCATCTCAAA   38293

LOC112206721.putative.POM121-like,protein1.START.LOC112206738  ------------------------------------------------------------   2796
BCRP3.REF.NEW                                                  ------------------------------------------------------------   0
GGT1.start-POM121L10P.end.24583750-24659147.ref                AAAAAAAAAAGAAAGAAAGAAAAGGCCCTCTGAGGCCAAGCTTGGTGTCTCATGCCTGTA   38353

LOC112206721.putative.POM121-like,protein1.START.LOC112206738  ------------------------------------------------------------   2796
BCRP3.REF.NEW                                                  ------------------------------------------------------------   0
GGT1.start-POM121L10P.end.24583750-24659147.ref                ATCCCAACACTTTGGGAGGCTGAGGTAGAAGTTGAGGCCAGGAGGTCCAAGACAAGCCTG   38413

LOC112206721.putative.POM121-like,protein1.START.LOC112206738  ------------------------------------------------------------   2796
BCRP3.REF.NEW                                                  ------------------------------------------------------------   0
GGT1.start-POM121L10P.end.24583750-24659147.ref                GGCAACATAGTGAGTCTACAAAAAAAATATTGAGAAACTACATAAATATAGTGGGGGTGG   38473

LOC112206721.putative.POM121-like,protein1.START.LOC112206738  ------------------------------------------------------------   2796
BCRP3.REF.NEW                                                  ------------------------------------------------------------   0
GGT1.start-POM121L10P.end.24583750-24659147.ref                GGGTGGGGAACAAGAAAACAAAAAATTCAAAGCATAGTGAAAAGAAATATAGCAAAACCC   38533

LOC112206721.putative.POM121-like,protein1.START.LOC112206738  ------------------------------------------------------------   2796
BCRP3.REF.NEW                                                  ------------------------------------------------------------   0
GGT1.start-POM121L10P.end.24583750-24659147.ref                GGCCGGGCATGGTGGCTCACGCCTGTAATCCCAGCACTTTGAGAGGCCGAGGCGTGTGGA   38593

LOC112206721.putative.POM121-like,protein1.START.LOC112206738  ------------------------------------------------------------   2796
BCRP3.REF.NEW                                                  ------------------------------------------------------------   0
GGT1.start-POM121L10P.end.24583750-24659147.ref                TCACGGGGTCAGGAGATCGAGACCATCCTGGCTAACACAGTGAAACCTGTCTCTACTAAA   38653

LOC112206721.putative.POM121-like,protein1.START.LOC112206738  ------------------------------------------------------------   2796
BCRP3.REF.NEW                                                  ------------------------------------------------------------   0
GGT1.start-POM121L10P.end.24583750-24659147.ref                AATACAAAAAAATTAGCCACGTGTGGTGGCGGGCGCCTGCAGTCCCAGCTACTTGGGAGG   38713

LOC112206721.putative.POM121-like,protein1.START.LOC112206738  -----------------------cgggcggggaagggattgtgatgcgccgagatcgcgc   2833
BCRP3.REF.NEW                                                  ------------------------------------------------------------   0
GGT1.start-POM121L10P.end.24583750-24659147.ref                CTGAGGCAGGAGAATGGCGTGAACCTGGGAGGTGGAGCTTGCAGTGAGCCGATATCGCGC   38773

LOC112206721.putative.POM121-like,protein1.START.LOC112206738  cactgcactccagcctgggcaacagagccagactc-------------------------   2868
BCRP3.REF.NEW                                                  ------------------------------------------------------------   0
GGT1.start-POM121L10P.end.24583750-24659147.ref                CACTGCACTCCAGCCTGGGCGATAGAGCGAGACTCCGTCTCAAAAAACAAAAAAAAAGTA   38833

LOC112206721.putative.POM121-like,protein1.START.LOC112206738  ------------------------------------------------------------   2868
BCRP3.REF.NEW                                                  ------------------------------------------------------------   0
GGT1.start-POM121L10P.end.24583750-24659147.ref                GCAAAACACAACAAAAAAATTAAAATTTGCTGGGTGTAGTTGTGCCTTTAGTCTCAGCTA   38893

LOC112206721.putative.POM121-like,protein1.START.LOC112206738  ------------------------------------------------------------   2868
BCRP3.REF.NEW                                                  ------------------------------------------------------------   0
GGT1.start-POM121L10P.end.24583750-24659147.ref                CTGGGGAGGGTCTGCTGGAGGATCACTTGAGCCCAGGAGTTCAAGGCTGTGATTAAGCCA   38953

LOC112206721.putative.POM121-like,protein1.START.LOC112206738  ------------------------------------------------------------   2868
BCRP3.REF.NEW                                                  ------------------------------------------------------------   0
GGT1.start-POM121L10P.end.24583750-24659147.ref                CTGCACTCCAGCCTGGGTGACAGAGCAAGATCCTATCTCTAAAAAAAAAAAAGAAAAGAA   39013

LOC112206721.putative.POM121-like,protein1.START.LOC112206738  ------------------------------------------------------------   2868
BCRP3.REF.NEW                                                  ------------------------------------------------------------   0
GGT1.start-POM121L10P.end.24583750-24659147.ref                AACACTCTGTGGCCACAGATGAGAGAAGGCAGACAGGAAGCCAGTAAGCCAAGCAGGCAA   39073

LOC112206721.putative.POM121-like,protein1.START.LOC112206738  ------------------------------------------------------------   2868
BCRP3.REF.NEW                                                  ------------------------------------------------------------   0
GGT1.start-POM121L10P.end.24583750-24659147.ref                AGGGCAGGTGGCCCCAGCCCGGCTGTTGGGTGGTGAGCAGTGTCAGGGAGACAGGAGTGC   39133

LOC112206721.putative.POM121-like,protein1.START.LOC112206738  ------------------------------------------------------------   2868
BCRP3.REF.NEW                                                  ------------------------------------------------------------   0
GGT1.start-POM121L10P.end.24583750-24659147.ref                CGGAGCTGATGAGGTTCCCAAGGAGGTGAGGTTGCCTGTGGCCCCCTCCCAGGGCACAGT   39193

LOC112206721.putative.POM121-like,protein1.START.LOC112206738  ------------------------------------------------------------   2868
BCRP3.REF.NEW                                                  ------------------------------------------------------------   0
GGT1.start-POM121L10P.end.24583750-24659147.ref                CCCACCCCTCCAGTAGTGCACTCTGTCCTCCCTGGTAGGTACAGGCTTTTCCCCACCACC   39253

LOC112206721.putative.POM121-like,protein1.START.LOC112206738  ------------------------------------------------------------   2868
BCRP3.REF.NEW                                                  ------------------------------------------------------------   0
GGT1.start-POM121L10P.end.24583750-24659147.ref                ATGGTGCAGCCATGCCTGCCCCCAACACCACTGCTGCAGCTCCATCCTCCACGCAGTGGT   39313

LOC112206721.putative.POM121-like,protein1.START.LOC112206738  ------------------------------------------------------------   2868
```

```
BCRP3.REF.NEW                                                     ------------------------------------------------------------   0
GGT1.start-POM121L10P.end.24583750-24659147.ref                   GCAGCCCCATCCCAGCACCCATTTGAGCTGCTGTCCCATTGCAGGGGGCATTGTGACAGC   39373

LOC112206721.putative.POM121-like,protein1.START.LOC112206738     ------------------------------------------------------------   2868
BCRP3.REF.NEW                                                     ------------------------------------------------------------   0
GGT1.start-POM121L10P.end.24583750-24659147.ref                   TGAGGACCTGAACAACTACCGTGCTGAGCTGATCGAGCACCCGCTGAACATCAGCCTGGG   39433

LOC112206721.putative.POM121-like,protein1.START.LOC112206738     ------------------------------------------------------------   2868
BCRP3.REF.NEW                                                     ------------------------------------------------------------   0
GGT1.start-POM121L10P.end.24583750-24659147.ref                   AGACGTGGTGCTGTACATGCCCAGTGCGCCGCTCAGCGGGCCCGTGCTGGCCCTCATCCT   39493

LOC112206721.putative.POM121-like,protein1.START.LOC112206738     ------------------------------------------------------------   2868
BCRP3.REF.NEW                                                     ------------------------------------------------------------   0
GGT1.start-POM121L10P.end.24583750-24659147.ref                   CAACATCCTCAAAGGTGAGTGGTCGCACCACAGCCGTGTGGTAGGACCCATGACACTGCC   39553

LOC112206721.putative.POM121-like,protein1.START.LOC112206738     ------------------------------------------------------------   2868
BCRP3.REF.NEW                                                     ------------------------------------------------------------   0
GGT1.start-POM121L10P.end.24583750-24659147.ref                   TCTCTCTCCCCACGCCCCACCCCTCCTGCATCTCTGCTCGCCCCCCATGCCACGTCTTTC   39613

LOC112206721.putative.POM121-like,protein1.START.LOC112206738     ------------------------------------------------------------   2868
BCRP3.REF.NEW                                                     ------------------------------------------------------------   0
GGT1.start-POM121L10P.end.24583750-24659147.ref                   CATCACTGAGCTCCCGAGGTGTGTCCCTGCGTCACAGTTCACCATGTCCTGAAGGAGGCA   39673

LOC112206721.putative.POM121-like,protein1.START.LOC112206738     ------------------------------------------------------------   2868
BCRP3.REF.NEW                                                     ------------------------------------------------------------   0
GGT1.start-POM121L10P.end.24583750-24659147.ref                   GTGCAGAGCGACAGGGCTGAAGCGGGCAATGCTCAAGGGTTGGAGGAGGAACAGGAGTCA   39733

LOC112206721.putative.POM121-like,protein1.START.LOC112206738     ------------------------------------------------------------   2868
BCRP3.REF.NEW                                                     ------------------------------------------------------------   0
GGT1.start-POM121L10P.end.24583750-24659147.ref                   TCAGGAGGGAGAGAGGTGCAGGAGCTCAGGGCTGCAGGGCCGGTCCAGAGGGTACCCCGG   39793

LOC112206721.putative.POM121-like,protein1.START.LOC112206738     ------------------------------------------------------------   2868
BCRP3.REF.NEW                                                     ------------------------------------------------------------   0
GGT1.start-POM121L10P.end.24583750-24659147.ref                   TCCAGTGGGTGACCCTGCCACTTGGTCATTGAATGGCCAGAGCTGATGCGTGACGTGAGG   39853

LOC112206721.putative.POM121-like,protein1.START.LOC112206738     ------------------------------------------------------------   2868
BCRP3.REF.NEW                                                     ------------------------------------------------------------   0
GGT1.start-POM121L10P.end.24583750-24659147.ref                   TCCAGGCTCGGGAGCCCTCACCTTACTTCACTCTCACCACAGCCTTCTGAAGCAGCTGCT   39913

LOC112206721.putative.POM121-like,protein1.START.LOC112206738     ------------------------------------------------------------   2868
BCRP3.REF.NEW                                                     ------------------------------------------------------------   0
GGT1.start-POM121L10P.end.24583750-24659147.ref                   GCTATTGGTTATTAAACGCTCCTTGAGGTGGGCAAAGTGGCTCAGGCAGGTGTTAACCCT   39973

LOC112206721.putative.POM121-like,protein1.START.LOC112206738     ------------------------------------------------------------   2868
BCRP3.REF.NEW                                                     ------------------------------------------------------------   0
GGT1.start-POM121L10P.end.24583750-24659147.ref                   CTGAGCCCCTCAGAGCCTCTGGGGCTCAGCAACATGCACCTGGCTCTGATCAACCAGGGT   40033

LOC112206721.putative.POM121-like,protein1.START.LOC112206738     ------------------------------------------------------------   2868
BCRP3.REF.NEW                                                     ------------------------------------------------------------   0
GGT1.start-POM121L10P.end.24583750-24659147.ref                   ACAACTTCTCCCGGGAGAGCGTGGAGAGCCCCGAGCAGAAGGGCCTGACGTACCACCGCA   40093

LOC112206721.putative.POM121-like,protein1.START.LOC112206738     ------------------------------------------------------------   2868
BCRP3.REF.NEW                                                     ------------------------------------------------------------   0
GGT1.start-POM121L10P.end.24583750-24659147.ref                   TCGTAGAGGCTTTCCGGTTTGCCTACGCCAAGAGGACCCTGCTTGGGGACCCCAAGTTTG   40153

LOC112206721.putative.POM121-like,protein1.START.LOC112206738     ------------------------------------------------------------   2868
BCRP3.REF.NEW                                                     ------------------------------------------------------------   0
GGT1.start-POM121L10P.end.24583750-24659147.ref                   TGGATGTGACTGAGGTAAGGGGCAGGGGCTGGCCCACTGTGGGTGTGGGGCCTGCCATAG   40213

LOC112206721.putative.POM121-like,protein1.START.LOC112206738     ------------------------------------------------------------   2868
BCRP3.REF.NEW                                                     ------------------------------------------------------------   0
GGT1.start-POM121L10P.end.24583750-24659147.ref                   AGGCATCAGGTGGGCTCCCCAGGGTGGCTACAGCCTCACATATGCTTTATGAATCCATTC   40273

LOC112206721.putative.POM121-like,protein1.START.LOC112206738     ------------------------------------------------------------   2868
BCRP3.REF.NEW                                                     ------------------------------------------------------------   0
GGT1.start-POM121L10P.end.24583750-24659147.ref                   CTGCCACAGACTTCGATTGCGGGCCTACTGTGTGCTCGGATGGACTCGTGGGTGACCCCC   40333

LOC112206721.putative.POM121-like,protein1.START.LOC112206738     ------------------------------------------------------------   2868
BCRP3.REF.NEW                                                     ------------------------------------------------------------   0
GGT1.start-POM121L10P.end.24583750-24659147.ref                   AGTCTTGGCTTCTGCCGCACAGAACTGACAGTGTGGGGAATTAGTGGCCACCCTCCTACC   40393

LOC112206721.putative.POM121-like,protein1.START.LOC112206738     ------------------------------------------------------------   2868
BCRP3.REF.NEW                                                     ------------------------------------------------------------   0
GGT1.start-POM121L10P.end.24583750-24659147.ref                   TCAGGTCCTTTGCACATGCTGTGGTTCTCGAGTGCTCAGTGCTGAGATGAGGAATGCATG   40453

LOC112206721.putative.POM121-like,protein1.START.LOC112206738     ------------------------------------------------------------   2868
BCRP3.REF.NEW                                                     ------------------------------------------------------------   0
GGT1.start-POM121L10P.end.24583750-24659147.ref                   GGGGCATTGCAGCCCTCGGGCATGGTGAGATGGATGGGTGAAAGGGAGAGGGCCAGGTGA   40513

LOC112206721.putative.POM121-like,protein1.START.LOC112206738     ------------------------------------------------------------   2868
BCRP3.REF.NEW                                                     ------------------------------------------------------------   0
GGT1.start-POM121L10P.end.24583750-24659147.ref                   ACAGAGACCTTGGCCACCCACTCCCTGTCACTCCAAACTAACGCTTCCCAGATGCCACCC   40573

LOC112206721.putative.POM121-like,protein1.START.LOC112206738     ------------------------------------------------------------   2868
BCRP3.REF.NEW                                                     ------------------------------------------------------------   0
GGT1.start-POM121L10P.end.24583750-24659147.ref                   TCAGCCCTGCACCACCTGACCCACTCACTCAGTAGTTGCCCCAGCTCCACACTGGGTCCC   40633

LOC112206721.putative.POM121-like,protein1.START.LOC112206738     ------------------------------------------------------------   2868
BCRP3.REF.NEW                                                     ------------------------------------------------------------   0
GGT1.start-POM121L10P.end.24583750-24659147.ref                   TATAAGACCCTGTCATATCCCTTCCCGCTGAGGATCCTCACATCCCTCCTTACCTACTTG   40693

LOC112206721.putative.POM121-like,protein1.START.LOC112206738     ------------------------------------------------------------   2868
BCRP3.REF.NEW                                                     ------------------------------------------------------------   0
GGT1.start-POM121L10P.end.24583750-24659147.ref                   GGTCCTTGGCATTCCTGGGCGGATCTGCAGACCCCCCCACACTGACCAGTGACCTCCCAG   40753

LOC112206721.putative.POM121-like,protein1.START.LOC112206738     ------------------------------------------------------------   2868
BCRP3.REF.NEW                                                     ------------------------------------------------------------   0
GGT1.start-POM121L10P.end.24583750-24659147.ref                   GAGGGGCGTCAGCTGCCCAGGTGGTGTCTTCTCTTTCCCCGTGAGCATTCTGCACCTCTG   40813

LOC112206721.putative.POM121-like,protein1.START.LOC112206738     ------------------------------------------------------------   2868
BCRP3.REF.NEW                                                     ------------------------------------------------------------   0
GGT1.start-POM121L10P.end.24583750-24659147.ref                   ACTCCCGCTGCAGCCAGTGACCTGGTGTCTTGTCTCTCTGAGGGGACAGAGCCACTGCAG   40873

LOC112206721.putative.POM121-like,protein1.START.LOC112206738     ------------------------------------------------------------   2868
BCRP3.REF.NEW                                                     ------------------------------------------------------------   0
GGT1.start-POM121L10P.end.24583750-24659147.ref                   TGTGTCCCTCTGCCCTCCTTTTTGGCTAAGGCCAGCTCCTTCATCTACTCGCTGGCTCGG   40933

LOC112206721.putative.POM121-like,protein1.START.LOC112206738     ------------------------------------------------------------   2868
BCRP3.REF.NEW                                                     ------------------------------------------------------------   0
GGT1.start-POM121L10P.end.24583750-24659147.ref                   GGTGCTGTTCCTACAATGCTCCTTTCTGCCTCTGATGATTTACTTCTTTATCACGGCTTA   40993

LOC112206721.putative.POM121-like,protein1.START.LOC112206738     ------------------------------------------------------------   2868
BCRP3.REF.NEW                                                     ------------------------------------------------------------   0
GGT1.start-POM121L10P.end.24583750-24659147.ref                   TTCCCGAAAGAAAGGCAGCTGTTGTTGCTCTAGAAACTTCTATCTGCTGCCTCCTTCTGT   41053

LOC112206721.putative.POM121-like,protein1.START.LOC112206738     ------------------------------------------------------------   2868
```

```
BCRP3.REF.NEW                                              ------------------------------------------------------------   0
GGT1.start-POM121L10P.end.24583750-24659147.ref            CCTTTGCTCCTCTTAGAGCAAACATGGCTGGGCTATGTCCTCTCTCCCTTCAGGGTATCC   41113

LOC112206721.putative.POM121-like,protein1.START.LOC112206738      ------------------------------------------------------------   2868
BCRP3.REF.NEW                                              ------------------------------------------------------------   0
GGT1.start-POM121L10P.end.24583750-24659147.ref            CCTCCCCTGCTCTATCCCCATGCCACCAGATCGCCATGTCCAGCCTCAGTTTCCCCATCA   41173

LOC112206721.putative.POM121-like,protein1.START.LOC112206738      ------------------------------------------------------------   2868
BCRP3.REF.NEW                                              ------------------------------------------------------------   0
GGT1.start-POM121L10P.end.24583750-24659147.ref            GGCCCCACTCAGCAGCATCTCACACAGCTCACCACACTCTCCTCGAGTTTTCATTTTGCA   41233

LOC112206721.putative.POM121-like,protein1.START.LOC112206738      ------------------------------------------------------------   2868
BCRP3.REF.NEW                                              ------------------------------------------------------------   0
GGT1.start-POM121L10P.end.24583750-24659147.ref            AATTTTCGCACCTACAAAAATGTAAAAAAACCCCAAAACTGCCCAAGCATCAATGCTCCC   41293

LOC112206721.putative.POM121-like,protein1.START.LOC112206738      ------------------------------------------------------------   2868
BCRP3.REF.NEW                                              ------------------------------------------------------------   0
GGT1.start-POM121L10P.end.24583750-24659147.ref            TTTTCCTGGAGTCTGCAGTGTGGATGTTTCTGTCGGATTTCCTAACTCTCTCCGTCTCCA   41353

LOC112206721.putative.POM121-like,protein1.START.LOC112206738      ------------------------------------------------------------   2868
BCRP3.REF.NEW                                              ------------------------------------------------------------   0
GGT1.start-POM121L10P.end.24583750-24659147.ref            CCCACATCTATTGGGATTCGTGTTTTTCTGAGGGATTCCACAGTAGGTTACTGATGTCGC   41413

LOC112206721.putative.POM121-like,protein1.START.LOC112206738      ------------------------------------------------------------   2868
BCRP3.REF.NEW                                              ------------------------------------------------------------   0
GGT1.start-POM121L10P.end.24583750-24659147.ref            ACCTCAGGGTGTGTCTCAAAAGTGAGACTTGAACATAGCACAGCAGGATGTCGGGGTGAC   41473

LOC112206721.putative.POM121-like,protein1.START.LOC112206738      ------------------------------------------------------------   2868
BCRP3.REF.NEW                                              ------------------------------------------------------------   0
GGT1.start-POM121L10P.end.24583750-24659147.ref            ACAACCTGTTGTCCCTTCTCTTTACCTACAGTAGGCTCCCTTGGCTGTTTTGTTTTTGTT   41533

LOC112206721.putative.POM121-like,protein1.START.LOC112206738      ------------------------------------------------------------   2868
BCRP3.REF.NEW                                              ------------------------------------------------------------   0
GGT1.start-POM121L10P.end.24583750-24659147.ref            GTGTTTTTGTTTGTACACAGAGTCTCTGTCATCCAGGTTGGAGTGCAGTGGTGTGATCTT   41593

LOC112206721.putative.POM121-like,protein1.START.LOC112206738      ------------------------------------------------------------   2868
BCRP3.REF.NEW                                              ------------------------------------------------------------   0
GGT1.start-POM121L10P.end.24583750-24659147.ref            GGCTCACTGCAGCCTCAACCTCCAGGGCTCAAGCAATCCTCCCACTTCAGCCCCCTGAAC   41653

LOC112206721.putative.POM121-like,protein1.START.LOC112206738      ------------------------------------------------------------   2868
BCRP3.REF.NEW                                              ------------------------------------------------------------   0
GGT1.start-POM121L10P.end.24583750-24659147.ref            AGCTGGGACCAGAGGCACGTGCCACCAAACCTGGATAATTTTGTATTTTTTTAGAGAC   41713

LOC112206721.putative.POM121-like,protein1.START.LOC112206738      ------------------------------------------------------------   2868
BCRP3.REF.NEW                                              ------------------------------------------------------------   0
GGT1.start-POM121L10P.end.24583750-24659147.ref            AGGGTCTTGCCCAGACTGGTCTCAAACTCCCGGGATCAAACTATCTTCCCACCTCAGCCT   41773

LOC112206721.putative.POM121-like,protein1.START.LOC112206738      ------------------------------------------------------------   2868
BCRP3.REF.NEW                                              ------------------------------------------------------------   0
GGT1.start-POM121L10P.end.24583750-24659147.ref            CCCAAAGCGCTGGGATTACAGGTGTGTTTTTTTTTTGAGACAGAGTCTCGCTCTGTTGC   41833

LOC112206721.putative.POM121-like,protein1.START.LOC112206738      ------------------------------------------------------------   2868
BCRP3.REF.NEW                                              ------------------------------------------------------------   0
GGT1.start-POM121L10P.end.24583750-24659147.ref            CCAGGCTGGAGTGCAGTGGCGCTATCTCGGCTCACTGTAAGCTCCGCCTCCTGGGTTCAC   41893

LOC112206721.putative.POM121-like,protein1.START.LOC112206738      ------------------------------------------------------------   2868
BCRP3.REF.NEW                                              ------------------------------------------------------------   0
GGT1.start-POM121L10P.end.24583750-24659147.ref            GCCATTCTCCTGCCTCAGCCTCCCGAGTAGCTGGGATTACAGGCGCCCGCCACACCGCCC   41953

LOC112206721.putative.POM121-like,protein1.START.LOC112206738      ------------------------------------------------------------   2868
BCRP3.REF.NEW                                              ------------------------------------------------------------   0
GGT1.start-POM121L10P.end.24583750-24659147.ref            AGCTAATTTTTTGTATTTTTAGTAGAGAGGGGGTTTCACCGTGATCGCGATCTCCTGACC   42013

LOC112206721.putative.POM121-like,protein1.START.LOC112206738      ------------------------------------------------------------   2868
BCRP3.REF.NEW                                              ------------------------------------------------------------   0
GGT1.start-POM121L10P.end.24583750-24659147.ref            TCGTGATCCGCCTGCCTCGGCCTCCCAAAGTGCTGGGATTACAGGCATGAGCCACCGTGC   42073

LOC112206721.putative.POM121-like,protein1.START.LOC112206738      ------------------------------------------------------------   2868
BCRP3.REF.NEW                                              ------------------------------------------------------------   0
GGT1.start-POM121L10P.end.24583750-24659147.ref            CTGGCCTGTTTTGTTGATTTTTAAAGCCCAGGGCAGTAGTCTTGGAAAATGTCCCACATC   42133

LOC112206721.putative.POM121-like,protein1.START.LOC112206738      ------------------------------------------------------------   2868
BCRP3.REF.NEW                                              ------------------------------------------------------------   0
GGT1.start-POM121L10P.end.24583750-24659147.ref            GTGGATTTGCCTTTCTGTTTCCTTGAGGGCAGATTCAGACAGAACACCTTTCCCTGGGAT   42193

LOC112206721.putative.POM121-like,protein1.START.LOC112206738      ---------------------------------tttttttttttttttttttttttgaga   2895
BCRP3.REF.NEW                                              ------------------------------------------------------------   0
GGT1.start-POM121L10P.end.24583750-24659147.ref            TGGTCAACTAATCCGTGGGGTGTTGCTGAAACTTTATTTTATTTTATTTTATTTTTGAGA   42253

LOC112206721.putative.POM121-like,protein1.START.LOC112206738      cgcagtctccct------------ctgtcgcccaggctggattccagtggcctgatctcg   2943
BCRP3.REF.NEW                                              ------------------------------------------------------------   0
GGT1.start-POM121L10P.end.24583750-24659147.ref            CGGAGTCTCGCTCTGTCCCCCAGGCTGTCCCCCAGGCTGGAGTGCAGTGGCGGGATCTCC   42313
```

GGT region with GGT1.new.NCBI.RefSeqGene sequence too

```
LOC112206721.putative.POM121-like,protein1.START.LOC112206738      gctcaccgcaaactccgcctaccgggttcacgccattctcctgcctcagcctctggagta   3003
BCRP3.REF.NEW                                              ------------------------------------------------------------   0
GGT1.start-POM121L10P.end.24583750-24659147.ref            GCTCACTGCAAGCTCCGCCTCCCAGGTTCACGCCATTCTCCTGCCTCAGCCCCCCAAGTA   42373

LOC112206721.putative.POM121-like,protein1.START.LOC112206738      gctcggactacaggcgcccgccaccacacccggctaatttttttggatttttagtagagac   3063
BCRP3.REF.NEW                                              ------------------------------------------------------------   0
GGT1.start-POM121L10P.end.24583750-24659147.ref            GCTGGGACTACATGTGCCCACCACCGCGCCCAGCTAATTTTTTGTATTTTTAGTAGAGAC   42433

LOC112206721.putative.POM121-like,protein1.START.LOC112206738      ggggtttcaccatgttagccaggatggtctcgatctcctgacctcgtgatcagcct----   3119
BCRP3.REF.NEW                                              ------------------------------------------------------------   0
GGT1.start-POM121L10P.end.24583750-24659147.ref            GGGGTTTCACTGTGTTAGCCAGGATGGTCTCGATCTCCTGACGTCGTGATCCGCCCTCCC   42493

LOC112206721.putative.POM121-like,protein1.START.LOC112206738      ------------------gcttcggcctaccaaagtgctgtgattaaaggcgtcagccac   3161
BCRP3.REF.NEW                                              ------------------------------------------------------------   0
GGT1.start-POM121L10P.end.24583750-24659147.ref            AAAGTGCTGGGATTACAGGCCTCGGCCTCCCAAAGTGCTGGGATTACAGGCGTGAGCCAC   42553

LOC112206721.putative.POM121-like,protein1.START.LOC112206738      cgcgcccggtgttgaaactctcttgaggcattttcttcggccttcgggtccatcctctgt   3221
BCRP3.REF.NEW                                              ------------------------------------------------------------   0
GGT1.start-POM121L10P.end.24583750-24659147.ref            CGCGCCCGGTGTTGAAACTCCCTTGAGGCATTTTCTTCGGCCTTCGGGTCCATCCTCTGT   42613

LOC112206721.putative.POM121-like,protein1.START.LOC112206738      ctcctccttgacctcctcatctcctcctcgccactgccttggggaccttggccaggctca   3281
BCRP3.REF.NEW                                              ------------------------------------------------------------   0
GGT1.start-POM121L10P.end.24583750-24659147.ref            CTCCTCCTTGACCTCCTCAT---CTCCTCGCCACTGCCTTGGGGACCTTGGCCAGGCTCA   42670

LOC112206721.putative.POM121-like,protein1.START.LOC112206738      tggcatccagcagccactcaatgtcaatacctccatgttcatctctcagctcgcccttgc   3341
BCRP3.REF.NEW                                              ------------------------------------------------------------   0
GGT1.start-POM121L10P.end.24583750-24659147.ref            TGGCATCCAGCAGCCACTCAATGTCAATACCTCCATGTTCATCTCTCAGCCCGCCCTTGC   42730

LOC112206721.putative.POM121-like,protein1.START.LOC112206738      ccatgaacccatgcttatttatttattttttttt-atatgtgcacccatgctcttcgggtc   3400
BCRP3.REF.NEW                                              ------------------------------------------------------------   0
GGT1.start-POM121L10P.end.24583750-24659147.ref            CCGTGAACCCATGCTTATTTATTTATTTTTTTTAATACGTGCACCCATGCTCTTCAGGTC   42790
```

```
LOC112206721.putative.POM121-like,protein1.START.LOC112206738      tgatgaagtatccaaaatcaagctcctgaccatccccaaacctgccccttctgcagggtt   3460
BCRP3.REF.NEW                                                      ------------------------------------------------------------   0
GGT1.start-POM121L10P.end.24583750-24659147.ref                    TGATGAAGTATCCAAAATCAAGCTCCTGACCATCCCCAAACCTGCCCCTTCTGCAGGGTT   42850

LOC112206721.putative.POM121-like,protein1.START.LOC112206738      tacctcgctagtcagcgccatccttgattcttctctttctcccacccaggccatcatctc   3520
BCRP3.REF.NEW                                                      ------------------------------------------------------------   0
GGT1.start-POM121L10P.end.24583750-24659147.ref                    TACCTCGCTAGTCGGCGCCATCCTTGATTCTTCTCTTTCTCCCACCCAGGCCATCATCTC   42910

LOC112206721.putative.POM121-like,protein1.START.LOC112206738      ttgcctggttgatacccacagcctcccctttgggctttatccttatccctgtcatagctg   3580
BCRP3.REF.NEW                                                      ------------------------------------------------------------   0
GGT1.start-POM121L10P.end.24583750-24659147.ref                    TTGCCTGGTTGATACCCACAGCCTCCCCTTTGGGCTTTATCCTTATCCCCATCATAGCTG   42970

LOC112206721.putative.POM121-like,protein1.START.LOC112206738      ccagagggaccctgtgaaaacactccccagcctgctcattcctctgccctaagcctgcat   3640
BCRP3.REF.NEW                                                      ------------------------------------------------------------   0
GGT1.start-POM121L10P.end.24583750-24659147.ref                    CCAGAGGGATCCTGTGAAAACACTCCCCAGCCTGCTCATTCCTCTGCCCTAAGCCTGCAT   43030

LOC112206721.putative.POM121-like,protein1.START.LOC112206738      ggcacagagcaaaagccagttgttatgggacctaggaggtcctgtgggatgggccccagc   3700
BCRP3.REF.NEW                                                      ------------------------------------------------------------   0
GGT1.start-POM121L10P.end.24583750-24659147.ref                    GGCACAGAGCAAAAGCCAGTTGTTATGGGACCTAGGAAGTCCTGTGGGATGGGCCCCAGC   43090

LOC112206721.putative.POM121-like,protein1.START.LOC112206738      ctgcatcttcatcctcttctccccacccccctccattcactctctgcctatcgctcacca   3760
BCRP3.REF.NEW                                                      ------------------------------------------------------------   0
GGT1.start-POM121L10P.end.24583750-24659147.ref                    CTGCATCTTCATCCTCTTCTCCCCACCCCACTCCATTCACTCTCTGCCTATCACTCACCA   43150

LOC112206721.putative.POM121-like,protein1.START.LOC112206738      gcctataccacctgcctcagggcctttgcactgaccatttaggccacattccaggctctt   3820
BCRP3.REF.NEW                                                      ------------------------------------------------------------   0
GGT1.start-POM121L10P.end.24583750-24659147.ref                    GCCTATACCACCTGCCTCAGGGCCTTTGCACTGACCATTTAGGCCACATTCCAGGCTCTT   43210

LOC112206721.putative.POM121-like,protein1.START.LOC112206738      ttcacatgttgcctcctctgagaagccctccctgaccactctgcccatacctcatgcctc   3880
BCRP3.REF.NEW                                                      ------------------------------------------------------------   0
GGT1.start-POM121L10P.end.24583750-24659147.ref                    TTCACACGTTGCCTCCTCTGAGAAGCCCTCCCTGACCACTCTGCCCATACCTCATGCCTC   43270

LOC112206721.putative.POM121-like,protein1.START.LOC112206738      ttgattccccttacctggcttgtggtttcagcactttccctgcgtgtgtttgtttttctt   3940
BCRP3.REF.NEW                                                      ------------------------------------------------------------   0
GGT1.start-POM121L10P.end.24583750-24659147.ref                    TTGATTCCCCTTACCTGGCTTGTGGTTTCAGCACTTTCCCTGTGTGTGTTTGTTTTTCTT   43330

LOC112206721.putative.POM121-like,protein1.START.LOC112206738      ggcatgagggcaggacctaagtgtctgttccctgttgattccccagtgccaggcatgcag   4000
BCRP3.REF.NEW                                                      ------------------------------------------------------------   0
GGT1.start-POM121L10P.end.24583750-24659147.ref                    GGCATGAGAGCAGGACCTAAGTGTCTGTTCCCTGTTGATTCCCCAGTGCCAGGCATGCAG   43390

LOC112206721.putative.POM121-like,protein1.START.LOC112206738      tgcaaactctagaaatatttttttgcatgaaggaatgagtgattgaatgcagcaagggtct   4060
BCRP3.REF.NEW                                                      ------------------------------------------------------------   0
GGT1.start-POM121L10P.end.24583750-24659147.ref                    TGCAAACTCTAGAAATATTTTTTGCATGAAGGAATGAGTGATTGAATGCAGCAAGGGTCT   43450

LOC112206721.putative.POM121-like,protein1.START.LOC112206738      ggaggctgaggaccaggcagacagacattcagagttgctggaacgcgacagagacaggga   4120
BCRP3.REF.NEW                                                      ------------------------------------------------------------   0
GGT1.start-POM121L10P.end.24583750-24659147.ref                    GGAGGCTGAGGACCAGGCAGACAGACATTCAGAGTTGCTGGAACGCGACAGAGACAGGGA   43510

LOC112206721.putative.POM121-like,protein1.START.LOC112206738      gtcagactggtcatgcaagatcctgggcctgcccttgggtcctggggagccatggaaggt   4180
BCRP3.REF.NEW                                                      ------------------------------------------------------------   0
GGT1.start-POM121L10P.end.24583750-24659147.ref                    GTCAGACTGGTCATGCAAGGTCCTGGGCCTGCCCTTGGGTCCTGGGGAGCCACGGAAGGT   43570

LOC112206721.putative.POM121-like,protein1.START.LOC112206738      tgtgggtgccagagggttgtggtcagagccacagtcaggggccttctgagacctgtgccc   4240
BCRP3.REF.NEW                                                      ------------------------------------------------------------   0
GGT1.start-POM121L10P.end.24583750-24659147.ref                    TGTGGGTGCCAGAGGGTTGCGGTCAGAGTCACAGTCAAGGGCCTTCTGAGACCTGTGCCC   43630

LOC112206721.putative.POM121-like,protein1.START.LOC112206738      cctccccaccctccctccccacctcctcaggccagctctggggtctcggcaggtggtccg   4300
BCRP3.REF.NEW                                                      ------------------------------------------------------------   0
GGT1.start-POM121L10P.end.24583750-24659147.ref                    CCTCCCCACCCTCCCTCCCCACCTCCTCAGGCCAGCTCTGGGGTCTCGGCAGGTGGTCCG   43690

LOC112206721.putative.POM121-like,protein1.START.LOC112206738      cagcatgacctctgagttcttcgctgcccagctccaggcccagatctctgacgacaccac   4360
BCRP3.REF.NEW                                                      ------------------------------------------------------------   0
GGT1.start-POM121L10P.end.24583750-24659147.ref                    CAACATGACCTCCGAGTTCTTCGCTGCCCAGCTCCGGGCCCAGATCTCTGACGACACCAC   43750

LOC112206721.putative.POM121-like,protein1.START.LOC112206738      tcacccgatctcctgctacaagcccgagttctacacgccggttgatgggggcactgctca   4420
BCRP3.REF.NEW                                                      ------------------------------------------------------------   0
GGT1.start-POM121L10P.end.24583750-24659147.ref                    TCACCCGATCTCCTACTACAAGCCCGAGTTCTACACGCCGGATGACGGGGGCACTGCTCA   43810

LOC112206721.putative.POM121-like,protein1.START.LOC112206738      cctgtctgtcgtcgcagaggacggcagtgctgtgtccgccaccagcaccatgaacctcta   4480
BCRP3.REF.NEW                                                      ------------------------------------------------------------   0
GGT1.start-POM121L10P.end.24583750-24659147.ref                    CCTGTCTGTCGTCGCAGAGGACGGCAGTGCTGTGTCCGCCACCAGCACCATCAACCTCTA   43870

LOC112206721.putative.POM121-like,protein1.START.LOC112206738      gtaggggctgctgggccgcctgggtgggaaagggccaggggcgggtggcccagggactgc   4540
BCRP3.REF.NEW                                                      ------------------------------------------------------------   0
GGT1.start-POM121L10P.end.24583750-24659147.ref                    GTAGGGGCTGCTGGGCCGCCTGGGTGGGAAAGGGCCAGGGGCGGGTGGCCCAGGGACTGC   43930

LOC112206721.putative.POM121-like,protein1.START.LOC112206738      ccacttatccagtaaggtggctccgtcacctcttttcctggtgggaaactgaggcccaac   4600
BCRP3.REF.NEW                                                      ------------------------------------------------------------   0
GGT1.start-POM121L10P.end.24583750-24659147.ref                    CCACTTATCCAGTAAGGTGGCTCCGTCACCTCTTTTCCTGGTGGGAAACTGAGGCCCAAC   43990

LOC112206721.putative.POM121-like,protein1.START.LOC112206738      cttggtagcttatcctgggcctctcagtgagtatgtttgagcctcagtgggtggataggg   4660
BCRP3.REF.NEW                                                      ------------------------------------------------------------   0
GGT1.start-POM121L10P.end.24583750-24659147.ref                    CTTGGTAGCTTATCCTGGGCCTCTCAGTGAGTATGTTTGAGCCTCAGTGGGTGGATAGGG   44050

LOC112206721.putative.POM121-like,protein1.START.LOC112206738      accaggctgggccaggcaaggtcgggcactgtctgacctggctgggcggtagctttggct   4720
BCRP3.REF.NEW                                                      ------------------------------------------------------------   0
GGT1.start-POM121L10P.end.24583750-24659147.ref                    ACCAGGCTGGGCCAGGCAAGGTCGGGCACTGTCTGACCTGGCTGGGCGGTAGCTTTGGCT   44110

LOC112206721.putative.POM121-like,protein1.START.LOC112206738      ccaaggtccgctccccggtcagcgggatcctgttcaatgatgaaatggacgacttcagct   4780
BCRP3.REF.NEW                                                      ------------------------------------------------------------   0
GGT1.start-POM121L10P.end.24583750-24659147.ref                    CCAAGGTCCGCTCCCCGGTCAGCGGGATCCTGTTCAATAATGAAATGGACGACTTCAGCT   44170

LOC112206721.putative.POM121-like,protein1.START.LOC112206738      ctcccaacatcaccaacgagtttggggtgccccccctcacctgccaatttcatccagccag   4840
BCRP3.REF.NEW                                                      ------------------------------------------------------------   0
GGT1.start-POM121L10P.end.24583750-24659147.ref                    CTCCCAGCATCACCAACGAGTTTGGGGTACCCCCCTCACCTGCCAATTTCATCCAGCCAG   44230

LOC112206721.putative.POM121-like,protein1.START.LOC112206738      gtatggggtggaggtccgggggtgggggactggggtggagaggggcgggtgtcctgggca   4900
BCRP3.REF.NEW                                                      ------------------------------------------------------------   0
GGT1.start-POM121L10P.end.24583750-24659147.ref                    GTATGGGGTGGAGGTCCGGGGGTGGGGGACTGGGGTGGAGAGGGGCGGGTGTCCTGGGCA   44290

LOC112206721.putative.POM121-like,protein1.START.LOC112206738      ggcagctgacgggcatccctgtcttctcccatcggccgcagggaagcagccgctctcgtc   4960
BCRP3.REF.NEW                                                      ------------------------------------------------------------   0
GGT1.start-POM121L10P.end.24583750-24659147.ref                    GGCAGCTGACGGGCATCCCTGTCTTCTCCCATCGGCCGCAGGGAAGCAGCCGCTCTCGTC   44350

LOC112206721.putative.POM121-like,protein1.START.LOC112206738      catgtgcccgacaatcatggtgggccaggacggccaggtccggatggttgtgggagctgc   5020
BCRP3.REF.NEW                                                      ------------------------------------------------------------   0
GGT1.start-POM121L10P.end.24583750-24659147.ref                    CATGTGCCCGACGATCATGGTGGGCCAGGACGGCCAGGTCCGGATGGTGGTGGGAGCTGC   44410

LOC112206721.putative.POM121-like,protein1.START.LOC112206738      tgggggcacgcagatcaccacggccactgcactggtatgtgtcacaccttttctccctgg   5080
BCRP3.REF.NEW                                                      ------------------------------------------------------------   0
GGT1.start-POM121L10P.end.24583750-24659147.ref                    TGGGGGCACACAGATCACCACGGCCACTGCACTGGTATGTGTCACACCTTTTCTCCCTGG   44470

LOC112206721.putative.POM121-like,protein1.START.LOC112206738      ccgtgcccaccctgcacagcccccaagccatgctgatcacactcccatgccccaggccat   5140
BCRP3.REF.NEW                                                      ------------------------------------------------------------   0
GGT1.start-POM121L10P.end.24583750-24659147.ref                    CCGTGCCCACCCTGCACAGCCCCCAAGCCATGCTGATCACACTCCCATGCCCCAGGCCAT   44530
```

```
LOC112206721.putative.POM121-like,protein1.START.LOC112206738      catctacaacctctggttcggctatgacgtgaagagggccgtggaggagccccggctgca   5200
BCRP3.REF.NEW                                                      ------------------------------------------------------------   0
GGT1.start-POM121L10P.end.24583750-24659147.ref                    CATCTACAACCTCTGGTTCGGCTATGACGTGAAGCGGGCCGTGGAGGAGCCCCGGCTGCA   44590

LOC112206721.putative.POM121-like,protein1.START.LOC112206738      caaccagcttctgcccaacgtcatgacagtggagagaaacattgaccaggtgggccgggg   5260
BCRP3.REF.NEW                                                      ------------------------------------------------------------   0
GGT1.start-POM121L10P.end.24583750-24659147.ref                    CAACCAGCTTCTGCCCAACGTCACGACAGTGGAGAGAAACATTGACCAGGTGGGCCGGGG   44650

LOC112206721.putative.POM121-like,protein1.START.LOC112206738      gttggagaaactgagtcacggtgtggggccccagggcatcctgggctggaggcctggatc   5320
BCRP3.REF.NEW                                                      ------------------------------------------------------------   0
GGT1.start-POM121L10P.end.24583750-24659147.ref                    GTTGGAGAAACTGAGTCAAGGTGTGGGGCCCCAGGGCATCCTGGGCTGGAGGCCTGGATC   44710

LOC112206721.putative.POM121-like,protein1.START.LOC112206738      atcacagagtggacaattgttggtgtcctctctctagtgcctgggccatctggagcccct   5380
BCRP3.REF.NEW                                                      ------------------------------------------------------------   0
GGT1.start-POM121L10P.end.24583750-24659147.ref                    ATCACAGAGTGGACAATGGTTGGTGTCCTCTCTCTAGTGCCTGGGCCATCTGGAGCCCCT   44770

LOC112206721.putative.POM121-like,protein1.START.LOC112206738      gtgacatgagggccaagcccctgctccagtgagacccagtaggccccaacctgcccttc   5440
BCRP3.REF.NEW                                                      ------------------------------------------------------------   0
GGT1.start-POM121L10P.end.24583750-24659147.ref                    GTGCCATGAGGGCCAAGCCCCCTGCTCCAGTGAGACCCAGCAGGCCCCAACCTGCTCTTC   44830

LOC112206721.putative.POM121-like,protein1.START.LOC112206738      ctgatgacctggcccgaaatggcaccacctgggctgaggcctgtgaccacacaggtgtgg   5500
BCRP3.REF.NEW                                                      ------------------------------------------------------------   0
GGT1.start-POM121L10P.end.24583750-24659147.ref                    CTGATGACCTGGCCCGAAATGGCACCACCTGGGCTGAGGCCTGTGACCACACAGATGTGG   44890

LOC112206721.putative.POM121-like,protein1.START.LOC112206738      ttcaggtggcatctggagccttgctcaggcttcccctctcctcccacccccaggcagtga   5560
BCRP3.REF.NEW                                                      ------------------------------------------------------------   0
GGT1.start-POM121L10P.end.24583750-24659147.ref                    TTCAGGTGGCATCTGGAGCCCTGCTCAGGCTTCCCCTCTCCTCCCACCCCCAGGCAGTGA   44950

LOC112206721.putative.POM121-like,protein1.START.LOC112206738      ctgcagccctggagacccggcaccaccgcacccagatcgcgtccaccttcatcgctgtgg   5620
BCRP3.REF.NEW                                                      ------------------------------------------------------------   0
GGT1.start-POM121L10P.end.24583750-24659147.ref                    CTGCAGCCCTGGAGACCCGGCACCATCACACCCAGATCGCGTCCACCTTCATCGCTGTGG   45010

LOC112206721.putative.POM121-like,protein1.START.LOC112206738      tgcaagccatcgtccgcacggctggtggctgggcagctgcctcggactccaggaaaggcg   5680
BCRP3.REF.NEW                                                      ------------------------------------------------------------   0
GGT1.start-POM121L10P.end.24583750-24659147.ref                    TGCAAGCCATCGTCCGCACGGCTGGTGGCTGGGCAGCTGCCTCGGACTCCAGGAAAGGCG   45070

LOC112206721.putative.POM121-like,protein1.START.LOC112206738      gggagcctgccggctactgagtgctccaggcggacaaggctgacaagcaatccagggaca   5740
BCRP3.REF.NEW                                                      ------------------------------------------------------------   0
GGT1.start-POM121L10P.end.24583750-24659147.ref                    GGGAGCCTGCCGGCTACTGAGTGCTCCAGGAGGACAAGGCTGACAAGCAATCCAGGGACA   45130

LOC112206721.putative.POM121-like,protein1.START.LOC112206738      agatactcaccaggaccaggaaggggactctggggga ccggcttcccctgtgagcagcag   5800
BCRP3.REF.NEW                                                      ------------------------------------------------------------   0
GGT1.start-POM121L10P.end.24583750-24659147.ref                    AGATACTCACCAGGACCAGGAAGGGGACTCTGGGGGACCGGCTTCCCCTGTGAGCAGCAG   45190

LOC112206721.putative.POM121-like,protein1.START.LOC112206738      agcagcacaataaatgaggc----------------------------------------   5820
BCRP3.REF.NEW                                                      ------------------------------------------------------------   0
GGT1.start-POM121L10P.end.24583750-24659147.ref                    AGCAGCACAATAAATGAGGCCACTGTGCCAGGCTCCAGGTGGCCTCCCTGGCCTGTCTCC   45250

LOC112206721.putative.POM121-like,protein1.START.LOC112206738      --------------------------------------------------------actg   5824
BCRP3.REF.NEW                                                      ------------------------------------------------------------   0
GGT1.start-POM121L10P.end.24583750-24659147.ref                    CCACTCTCTGGGCCTCAGTGTATTGTGTGTGAAATGGAGCCATCTGGCTGGGGAGGAACA   45310

LOC112206721.putative.POM121-like,protein1.START.LOC112206738      gagaggcgggattcggagatcttcacgctgcggtcgctggaactagcctcagcatcttca   5884
BCRP3.REF.NEW                                                      ------------------------------------------------------------   0
GGT1.start-POM121L10P.end.24583750-24659147.ref                    GAGAGGTGGGATTCGGAGATCTTCACAATGCGGGCACTGGAACTAGCCTCAGCATCTTCA   45370

LOC112206721.putative.POM121-like,protein1.START.LOC112206738      gcatggggaaagccaggcacgtggctgggggccaggggaaggtccatgccaagccctgcc   5944
BCRP3.REF.NEW                                                      ------------------------------------------------------------   0
GGT1.start-POM121L10P.end.24583750-24659147.ref                    GCATGGGGAGAGCCAGGCACATGGCTGGGGGCCAGGGGAAGGTTCACACCAAGCTCTGCC   45430

LOC112206721.putative.POM121-like,protein1.START.LOC112206738      ccttcccaccctgatccctcggactttggggccaggccctcccttccctgggctgggcag   6004
BCRP3.REF.NEW                                                      ------------------------------------------------------------   0
GGT1.start-POM121L10P.end.24583750-24659147.ref                    CCTTCCCACCCTGATCCCTCGGACTTTGGGGCCAGGCCCTCCCTTACTGGGGCTGGGCAG   45490

LOC112206721.putative.POM121-like,protein1.START.LOC112206738      caacaccacctaggaccagccaccagagggtgcagcgaccccggccctttcttaggcaga   6064
BCRP3.REF.NEW                                                      ------------------------------------------------------------   0
GGT1.start-POM121L10P.end.24583750-24659147.ref                    TGACACTACCTAGGATCAGCCACCAGGGGGTACCACGACCCTGGCACTTTCTTAGGCAGA   45550

LOC112206721.putative.POM121-like,protein1.START.LOC112206738      gagtggccagctgatgccgggaacccgggtgccttctcagacccgtaggcgtccagctca   6124
BCRP3.REF.NEW                                                      ------------------------------------------------------------   0
GGT1.start-POM121L10P.end.24583750-24659147.ref                    GGGTGGCCAGCTGATGCTGGGAACCCGGGTGCCTTCTTAGACCCGTAGGCGTCCAGCTCA   45610

LOC112206721.putative.POM121-like,protein1.START.LOC112206738      ccctgccgaggacacgggaggtgaagctgaggtctgaggagtggggattgggcaagaggc   6184
BCRP3.REF.NEW                                                      ------------------------------------------------------------   0
GGT1.start-POM121L10P.end.24583750-24659147.ref                    CCCTGCCGATGACACTGGAGGTGAAGCTGAGGTCCGAGGAATGGGGACTGGGCAACAGGC   45670

LOC112206721.putative.POM121-like,protein1.START.LOC112206738      tggaggaaaacatctcggtcagagccaagcccctgggggtttccaagtttaagcccaga   6244
BCRP3.REF.NEW                                                      ------------------------------------------------------------   0
GGT1.start-POM121L10P.end.24583750-24659147.ref                    TGGAGGAAAACATCTCGGTCAGAGCCACGCCCCTGGGGGGTTTCCAAGTTTAAGCCCAGA   45730

LOC112206721.putative.POM121-like,protein1.START.LOC112206738      gtgaaacccaagcttgtgatcctctccagagggaggcctggttctcagggaacagcaaac   6304
BCRP3.REF.NEW                                                      ------------------------------------------------------------   0
GGT1.start-POM121L10P.end.24583750-24659147.ref                    GTGAAACCCAAGCTTGTGATCCTCTCCAGAGGGAGGCCTGGTTCTCAGGGAACAGCAAAC   45790

LOC112206721.putative.POM121-like,protein1.START.LOC112206738      tggaagaggtccccagatcccagggatcagggcttggaccagccggggacgcagcccaga   6364
BCRP3.REF.NEW                                                      ------------------------------------------------------------   0
GGT1.start-POM121L10P.end.24583750-24659147.ref                    GGGAAGATGTCCCCAGATCCCAGGGATCAGGGCTTGGACCAGCCGGGGACGCAGCCCAGA   45850

LOC112206721.putative.POM121-like,protein1.START.LOC112206738      gggagtgggtctggaagggaacaactagacacagcagtcttcaccatcggcagcccctcc   6424
BCRP3.REF.NEW                                                      ------------------------------------------------------------   0
GGT1.start-POM121L10P.end.24583750-24659147.ref                    GGGAGTGGGTCCAGAAGGAAACAGCTAGACACAGCAGCCTTCACCATCGGCAGCCCCTCC   45910

LOC112206721.putative.POM121-like,protein1.START.LOC112206738      aggcctccctcggggcctgctccctcctccaggcacagttccaacacctggggcaggatt   6484
BCRP3.REF.NEW                                                      ------------------------------------------------------------   0
GGT1.start-POM121L10P.end.24583750-24659147.ref                    AGGCCTCCCTCAGGGCCTGCTCCCTCCTCTGTGCACAGTTCCAACACCTGGGGCAGGGTT   45970

LOC112206721.putative.POM121-like,protein1.START.LOC112206738      ctgggaaaggctggtggaggtaggctggtgaggggtggtggtcacagcccagcagcaggg   6544
BCRP3.REF.NEW                                                      ------------------------------------------------------------   0
GGT1.start-POM121L10P.end.24583750-24659147.ref                    CTGGGAAGGGCTGGTGGAGGTGGGCTGGTGGGAGGCGGTGATCACAGCCCAGCAC-----   46025

LOC112206721.putative.POM121-like,protein1.START.LOC112206738      gcactctggatatcaccaggggcactctggccaggtcccaggtgaggccaggtcggggct   6604
BCRP3.REF.NEW                                                      ------------------------------------------------------------   0
GGT1.start-POM121L10P.end.24583750-24659147.ref                    -----CTGGATATCACCAGGGGCACTGGGGCCAGGGGCCAGGTGAGGCCAGGTCGGGGCT   46080

LOC112206721.putative.POM121-like,protein1.START.LOC112206738      atccttcaggagtcccgaaaacctggtgataccaaagggcccacggacaaacagggtttt   6664
BCRP3.REF.NEW                                                      ------------------------------------------------------------   0
GGT1.start-POM121L10P.end.24583750-24659147.ref                    ATCCTTCAGGATCCCCGAAGACCTGGTGATTCCAAAGGGCCCATAGACAAACAGGGTTTT   46140

LOC112206721.putative.POM121-like,protein1.START.LOC112206738      gtgcctgcagagtagagtaccaccgggtctgagccctggagggctgtgtctctggggctc   6724
BCRP3.REF.NEW                                                      ------------------------------------------------------------   0
GGT1.start-POM121L10P.end.24583750-24659147.ref                    CTGCCTGTGGAGTCAAGTCCCACTGGGTCTGAGCTCTGGAGGGCTGTGTCTCTGGGGCTC   46200

LOC112206721.putative.POM121-like,protein1.START.LOC112206738      ccag--------------------ttgagtgatgtacgagtcactcctcaatccttattt   6764
BCRP3.REF.NEW                                                      ------------------------------------------------------------   0
GGT1.start-POM121L10P.end.24583750-24659147.ref                    TGCAGGGGTGAGATGGAGGTGGGCTCAACTGGTGTACAAGTCACTCTTCAATCCTTATTT   46260
```

```
LOC112206721.putative.POM121-like,protein1.START.LOC112206738      tatttatttaa---tttaaaaaattatttaaacaaatagagatggggtctcactatgttg   6821
BCRP3.REF.NEW                                                      ------------------------------------------------------------   0
GGT1.start-POM121L10P.end.24583750-24659147.ref                    TATTTATTTAATTTTTTTAAAAAAAATTTAAACCAATAGAGATGGGGTCTCACTATGTTG   46320

LOC112206721.putative.POM121-like,protein1.START.LOC112206738      accacgctggacttaaactcctgacttcaagcagtccccccatctcagtctcccaaagtg   6881
BCRP3.REF.NEW                                                      ------------------------------------------------------------   0
GGT1.start-POM121L10P.end.24583750-24659147.ref                    ATCAGGCTGGTCTT-AACTCCTGACTTCAAGCAGTCCCCCCATCTCAGTCTCCCAAAGTG   46379

LOC112206721.putative.POM121-like,protein1.START.LOC112206738      ctaggattacagaggtgagccactgcagccagcctcaatccttattttggcctgagagga   6941
BCRP3.REF.NEW                                                      ------------------------------------------------------------   0
GGT1.start-POM121L10P.end.24583750-24659147.ref                    CTAGGATTACAGGGGTGAGACACTGCACCCGGCCTCAATCCTTATTTTGGCCTGAGAGGA   46439

LOC112206721.putative.POM121-like,protein1.START.LOC112206738      caggccgtggcctcattttcaggggagaagactgaggctggaggggcaggccttgctctg   7001
BCRP3.REF.NEW                                                      ------------------------------------------------------------   0
GGT1.start-POM121L10P.end.24583750-24659147.ref                    AAGGCCGTGGCCCCATTTGCAGGGGAGAAGACTGAAGCTGGAGGGGCAGGCCTTGCTCTG   46499

LOC112206721.putative.POM121-like,protein1.START.LOC112206738      ggttgcacagcagcaagagaagtgagagctggccacgaggcttcctggacccgacacgct   7061
BCRP3.REF.NEW                                                      ------------------------------------------------------------   0
GGT1.start-POM121L10P.end.24583750-24659147.ref                    GGTTGCACAGCAGCAACAGAAGTGGGAGCTGGCCACGAGGCTTCCTCGACTCGACACACT   46559

LOC112206721.putative.POM121-like,protein1.START.LOC112206738      ggtggggtacaccctggttctccaggtcccatggggctcagcccaggactacctcggggg   7121
BCRP3.REF.NEW                                                      ------------------------------------------------------------   0
GGT1.start-POM121L10P.end.24583750-24659147.ref                    GGTGGGGTACACCCTGGTTCTCCAGGTCCCATGGGGCTCAGCCCAGGACTACCTCGGGGG   46619

LOC112206721.putative.POM121-like,protein1.START.LOC112206738      gtgagggacttaaatcctctccttcattctcatcgccccttcccccaccatttcctgagg   7181
BCRP3.REF.NEW                                                      ------------------------------------------------------------   0
GGT1.start-POM121L10P.end.24583750-24659147.ref                    GTGAGGGACTTAAATCCTCTCCTTCATTCTCATCGCCCCTTCCCCCATCATTTCCTGAGG   46679
```

THIS,46690 0N GGT1.start-POM121L10P.end.24583750-24659147.ref IS POSITION 51899BP ON GGT1.new.NCBI.RefSeqGene THAT ENDS AT 52255BP

```
LOC112206721.putative.POM121-like,protein1.START.LOC112206738      aaggacatt---------------------------------------------------   7190
BCRP3.REF.NEW                                                      ------------------------------------------------------------   0
GGT1.start-POM121L10P.end.24583750-24659147.ref                    AAGGACATTCAGGGACCTGAAGGAGCGGCCTGCCCCTCCACATCTGTGGGTGTTTCTCAT   46739

LOC112206721.putative.POM121-like,protein1.START.LOC112206738      ------------------------------------------------------------   7190
BCRP3.REF.NEW                                                      ------------------------------------------------------------   0
GGT1.start-POM121L10P.end.24583750-24659147.ref                    CAGGTGGGACAAGAGACTGAGAAAAGAAAGAGACACAGAGACAAAGTATAGAGAAAGAAA   46799

LOC112206721.putative.POM121-like,protein1.START.LOC112206738      ------------------------------------------------------------   7190
BCRP3.REF.NEW                                                      ------------------------------------------------------------   0
GGT1.start-POM121L10P.end.24583750-24659147.ref                    AGTGGGCCCAGGGGACCTGCGCTCAGCATACAGAGGCCCCACGCTGGCATCAGTCTCTGA   46859

LOC112206721.putative.POM121-like,protein1.START.LOC112206738      ------------------------------------------------------------   7190
BCRP3.REF.NEW                                                      ------------------------------------------------------------   0
GGT1.start-POM121L10P.end.24583750-24659147.ref                    GTTCCCTAGTATTTATTGATCATTATCTCTACCATCTCAGAGAGGGGGATGTAGCAGGAC   46919

LOC112206721.putative.POM121-like,protein1.START.LOC112206738      ------------------------------------------------------------   7190
BCRP3.REF.NEW                                                      ------------------------------------------------------------   0
GGT1.start-POM121L10P.end.24583750-24659147.ref                    AATATGGTAATAGTGGGGAGAGGGTCAGCAGGAAAACACGTGAACAAATGTCTCTGTGTC   46979

LOC112206721.putative.POM121-like,protein1.START.LOC112206738      ------------------------------------------------------------   7190
BCRP3.REF.NEW                                                      ------------------------------------------------------------   0
GGT1.start-POM121L10P.end.24583750-24659147.ref                    ATAAACAAGGTTAAGAAAAAGGTGCTGTGCTTTGATGTGCATATACATAAACATCTCAAT   47039

LOC112206721.putative.POM121-like,protein1.START.LOC112206738      ------------------------------------------------------------   7190
BCRP3.REF.NEW                                                      ------------------------------------------------------------   0
GGT1.start-POM121L10P.end.24583750-24659147.ref                    GCATTAAAGAGCAGTATTGCCACCAGCATGTCCCACCTCCAGCCCTAAGGCAGTTTTCTC   47099

LOC112206721.putative.POM121-like,protein1.START.LOC112206738      ------------------------------------------------------------   7190
BCRP3.REF.NEW                                                      ------------------------------------------------------------   0
GGT1.start-POM121L10P.end.24583750-24659147.ref                    CTATCTCAGTAGATGGAATATACAATTGGGTTTTACACATTCCTTTGCCCAGGGACGATC   47159

LOC112206721.putative.POM121-like,protein1.START.LOC112206738      ------------------------------------------------------------   7190
BCRP3.REF.NEW                                                      ------------------------------------------------------------   0
GGT1.start-POM121L10P.end.24583750-24659147.ref                    AGGAGACAGATGCCTTCCTCTTATCTCAACTGCAAAGAGGCCTTCCTTCCTCTTATACTA   47219

LOC112206721.putative.POM121-like,protein1.START.LOC112206738      ------------------------------------------------------------   7190
BCRP3.REF.NEW                                                      ------------------------------------------------------------   0
GGT1.start-POM121L10P.end.24583750-24659147.ref                    ATCCTCCTCAGCACAGACCCTTTACGGGTGTCGGGCTGGGGAACGGTCAGGTCTTTTCCT   47279

LOC112206721.putative.POM121-like,protein1.START.LOC112206738      ------------------------------------------------------------   7190
BCRP3.REF.NEW                                                      ------------------------------------------------------------   0
GGT1.start-POM121L10P.end.24583750-24659147.ref                    TCCCACAAGGCCATATTTCAGACTGTCACATGGGGAGAAACCTTGGACAATACCTGGCTT   47339

LOC112206721.putative.POM121-like,protein1.START.LOC112206738      ------------------------------------------------------------   7190
BCRP3.REF.NEW                                                      ------------------------------------------------------------   0
GGT1.start-POM121L10P.end.24583750-24659147.ref                    TCCTAGGCAGAGGTCCCTGCGGCCTTCTGCAGTGTTTTGTGCCCCTGCTTACTTGAGATT   47399

LOC112206721.putative.POM121-like,protein1.START.LOC112206738      ------------------------------------------------------------   7190
BCRP3.REF.NEW                                                      ------------------------------------------------------------   0
GGT1.start-POM121L10P.end.24583750-24659147.ref                    AGGGAGTGGTGATGACTTTTAACAAGCATGCTGCCTTCAAGCATTTGTTTAACAAAGCAC   47459

LOC112206721.putative.POM121-like,protein1.START.LOC112206738      ------------------------------------------------------------   7190
BCRP3.REF.NEW                                                      ------------------------------------------------------------   0
GGT1.start-POM121L10P.end.24583750-24659147.ref                    ATCCTGCACAGCCCTGAATCCATTAAACCTTGAGTCGACACAGTACTTGTTTCTGTGAGC   47519

LOC112206721.putative.POM121-like,protein1.START.LOC112206738      ------------------------------------------------------------   7190
BCRP3.REF.NEW                                                      ------------------------------------------------------------   0
GGT1.start-POM121L10P.end.24583750-24659147.ref                    ACAGGGTTGGGGATAGGGTTACAGATTAACAGCATCTCAAGGCAAAAGAATTTTTCTTAC   47579

LOC112206721.putative.POM121-like,protein1.START.LOC112206738      ------------------------------------------------------------   7190
BCRP3.REF.NEW                                                      ------------------------------------------------------------   0
GGT1.start-POM121L10P.end.24583750-24659147.ref                    TACAGAACAAAATGGAGCCTCTTACGTCTACTTCTTTCTACATAGACACAGTAACAGTCT   47639

LOC112206721.putative.POM121-like,protein1.START.LOC112206738      ---------------------cagggaccttcctggctgtgcctcggatcaagaccagaa   7229
BCRP3.REF.NEW                                                      ------------------------------------------------------------   0
GGT1.start-POM121L10P.end.24583750-24659147.ref                    GATATCTCTTTCTTTTCCCCACAGGGACCTTCCTGGCTGTGCCTCGGATCAGGACCAGAA   47699

LOC112206721.putative.POM121-like,protein1.START.LOC112206738      tgacacccattcctttccctgggcctttgctcaggtggtccctgcaccctggcctctgcc   7289
BCRP3.REF.NEW                                                      ------------------------------------------------------------   0
GGT1.start-POM121L10P.end.24583750-24659147.ref                    TGACACCCATTCATTTCCCTGGGCCTTTGCTCCGGTGGTCCCTGCACCCTGGCCTCTGCC   47759

LOC112206721.putative.POM121-like,protein1.START.LOC112206738      tgaccaggatggtggggagaggagggggacgtcccccacgctgctgtctccactgttcc   7349
BCRP3.REF.NEW                                                      ------------------------------------------------------------   0
GGT1.start-POM121L10P.end.24583750-24659147.ref                    TGACGAGGATGGTGGGGAGAGGAGGGGGACATCCCCCACGCTGCTGTCTCCACTGT---   47816

LOC112206721.putative.POM121-like,protein1.START.LOC112206738      tgctgcccaggcctctgagcttccaggactgcagcgggtgggtgggtgggctggcctaag   7409
BCRP3.REF.NEW                                                      ------------------------------------------------------------   0
GGT1.start-POM121L10P.end.24583750-24659147.ref                    ---------GGCCTCTGAGCTTCCAGGACTGCAGCGGGTGGGTGGGTGGCCTGGCCTAAG   47867

LOC112206721.putative.POM121-like,protein1.START.LOC112206738      cccaggaatgcacttcagctcctggttgagcaatgtcactgaggcttgggagtcgggtgg   7469
BCRP3.REF.NEW                                                      ------------------------------------------------------------   0
GGT1.start-POM121L10P.end.24583750-24659147.ref                    CCCAGGAATGCACTTCAGCTCCTGGTTGAGCAATGTCACTGAGGCTTGGGAGTCGGGTGG   47927

LOC112206721.putative.POM121-like,protein1.START.LOC112206738      gggcgggaggaggcgtccacaggcccccc--accgcgagaggcagccgtgggaacagcct   7527
```

```
BCRP3.REF.NEW                                             ------------------------------------------------------------   0
GGT1.start-POM121L10P.end.24583750-24659147.ref           GGACGGGAGGAGGCGTCCGCAGGCCCCCCCTACCGTGAGAGGCAGCCGTGGGAACAGCCT   47987

LOC112206721.putative.POM121-like,protein1.START.LOC112206738  gcctctaaacaatcactccagtccaggctgaccaggggctctggctggacataggggcct   7587
BCRP3.REF.NEW                                             ------------------------------------------------------------   0
GGT1.start-POM121L10P.end.24583750-24659147.ref           ACCTCTAAACAATCGCTGCAGCCCAGGCTGACCAGGGGCTCTGGCCGGACATAGGGGCCT   48047

LOC112206721.putative.POM121-like,protein1.START.LOC112206738  ggcaggctgtgtggcctgtaaggacacagtctgtctctgtgcctcagtttctctgctgcc   7647
BCRP3.REF.NEW                                             ------------------------------------------------------------   0
GGT1.start-POM121L10P.end.24583750-24659147.ref           GGCAGGCTGTGTGGCCTGTAAGGACACAGTCTGTCTCTGTGCCTCAGTTTCTCTGCTGCC   48107

LOC112206721.putative.POM121-like,protein1.START.LOC112206738  cagatgggggggcccggattccaggtgtagacatctggagcaggcagtgttcagctgggg   7707
BCRP3.REF.NEW                                             ------------------------------------------------------------   0
GGT1.start-POM121L10P.end.24583750-24659147.ref           CAGATGGAGAGGCCCAGACTCCAGGTGTAGACATCTGGAGCAGGCAGTGTTCAGCTGGGG   48167

LOC112206721.putative.POM121-like,protein1.START.LOC112206738  aggaagcggggaggactgtgggggccacatgggaaggattccagcccacatcacctgcac   7767
BCRP3.REF.NEW                                             ------------------------------------------------------------   0
GGT1.start-POM121L10P.end.24583750-24659147.ref           AGGGAGCGGGGAGGACTATGGGGGCCACGTGGGAAGAAGTCCAGCCCACATCACCTGCAC   48227

LOC112206721.putative.POM121-like,protein1.START.LOC112206738  ccctgctgagcctggtcaacggagcccctcagtgggtcctcactcccctggctgcctccc   7827
BCRP3.REF.NEW                                             ------------------------------------------------------------   0
GGT1.start-POM121L10P.end.24583750-24659147.ref           CCCTGCTGAGCCTGGTCAACAGAGGCCCTCAGTGGGTCCTCACTCTCCTGGCTGCCTCCC   48287

LOC112206721.putative.POM121-like,protein1.START.LOC112206738  atttaggcaccctgaggcctggggagaacagagccaggccagtgtccctagagaggctgc   7887
BCRP3.REF.NEW                                             ------------------------------------------------------------   0
GGT1.start-POM121L10P.end.24583750-24659147.ref           ATTTAGGCACCCTGAGGCCTGGGGAGAACAGAGCCAGGCCAGTGTCCCCAGAGAGGCTGC   48347

LOC112206721.putative.POM121-like,protein1.START.LOC112206738  gctgccggcacagtaatagcggatttggattcagggaagcagacccgcatccagggtggg   7947
BCRP3.REF.NEW                                             ------------------------------------------------------------   0
GGT1.start-POM121L10P.end.24583750-24659147.ref           GCTGCCAGCACAGTAGTAGCAGATTTGGATTCAGGGAAGTAGACCTGCAGCCAGGGTGGG   48407

LOC112206721.putative.POM121-like,protein1.START.LOC112206738  aaagagctgcaggcagggcgtggcctcca-----cacagcctgcctccctggaggtccat   8002
BCRP3.REF.NEW                                             ------------------------------------------------------------   0
GGT1.start-POM121L10P.end.24583750-24659147.ref           AAAGAGCTGCAGGCGGGGTGGAGCCCCCACATGGCACAGCCCCCCTCCTTGGAGGTCTAT   48467

LOC112206721.putative.POM121-like,protein1.START.LOC112206738  gctgcatttccaggacagcaagtcct--ggacaaatggtcccggatgccaaaggctagag   8060
BCRP3.REF.NEW                                             ------------------------------------------------------------   0
GGT1.start-POM121L10P.end.24583750-24659147.ref           GCTGCATTTCCAGGACAGCAAGTCCCAGGGATGGATGGTGCCTGGTGCCAAGGGCTAGAG   48527

LOC112206721.putative.POM121-like,protein1.START.LOC112206738  gcatggtctgtctgcattccccacatgggcgtcttgtagtcaccagcatttgatgctgtc   8120
BCRP3.REF.NEW                                             ------------------------------------------------------------   0
GGT1.start-POM121L10P.end.24583750-24659147.ref           GCATGGTCTGTCTGCATTCCCTACAGGGGCATCTTGTAGTCACCAGCATTTGATGCTGTC   48587

LOC112206721.putative.POM121-like,protein1.START.LOC112206738  aagtcccccctgtcctctgtgcagactgggaagcccttggccaccct--ggggagttgtgg   8178
BCRP3.REF.NEW                                             ------------------------------------------------------------   0
GGT1.start-POM121L10P.end.24583750-24659147.ref           AAGTCCCCCTGTCCTCTGTGCAGACTGGGAAGCCCTTGGTCACCCTGGGGGGGTTGGGG   48647

LOC112206721.putative.POM121-like,protein1.START.LOC112206738  gacccaggccaggctgcagaagcataaggacttgaacccaagttttaagtgacaccacct   8238
BCRP3.REF.NEW                                             ------------------------------------------------------------   0
GGT1.start-POM121L10P.end.24583750-24659147.ref           GACCCAGGCCAGGCTGCAGAAACATAAGGACTTGAACCCGGGTCCTGAGTGACACCACCT   48707

LOC112206721.putative.POM121-like,protein1.START.LOC112206738  tgtgtcccccctccctctgtctctgtttagaaccaccttgatgctgactaggctgggccat   8298
BCRP3.REF.NEW                                             ------------------------------------------------------------   0
GGT1.start-POM121L10P.end.24583750-24659147.ref           TGGGTCCTCCTCCCTCTGCCTCTGTTCAGCTCCACCTTGATGGTGACTAGGCTGGGCCAT   48767

LOC112206721.putative.POM121-like,protein1.START.LOC112206738  gcggagagggttaggggatagagatgagagctggggagcagggctccactctgggagggg   8358
BCRP3.REF.NEW                                             ------------------------------------------------------------   0
GGT1.start-POM121L10P.end.24583750-24659147.ref           GCGGAGAGGGTTAGGGGATAGAGATGGGAGCTGGGGAGCAGGGCTCCACTCTGGGAGGGG   48827

LOC112206721.putative.POM121-like,protein1.START.LOC112206738  ggcagcctcgccggatccaggggagagagttgagcggccccagctctgctttcccagagc   8418
BCRP3.REF.NEW                                             ------------------------------------------------------------   0
GGT1.start-POM121L10P.end.24583750-24659147.ref           GGCAGCCTTGCCGGATCCAGGG--CAGAGTTAAGCGGCCCCAGCTCTGCTTTCCTAGAGC   48885

LOC112206721.putative.POM121-like,protein1.START.LOC112206738  tgccgggaacccggggaatggtgtggaggttccagggagccctgcccctacctggcaacc   8478
BCRP3.REF.NEW                                             ------------------------------------------------------------   0
GGT1.start-POM121L10P.end.24583750-24659147.ref           TGCTGAGAACCCGGGAAATGGTGTGGAGGTTCCGGGGAGCCCTGCCCCTACCTGGCAACC   48945

LOC112206721.putative.POM121-like,protein1.START.LOC112206738  acagtgcagcaggcaccaagttctcctgcacattgcgacagtgtgaccctgggctctggc   8538
BCRP3.REF.NEW                                             ------------------------------------------------------------   0
GGT1.start-POM121L10P.end.24583750-24659147.ref           GCAGTGCAGCAGGCACCAAGTTCTCCTGCACATTGCGACAGTGTGACCCTGGGCTCTGGC   49005

LOC112206721.putative.POM121-like,protein1.START.LOC112206738  gggcagtaggtggggcctttggacctaccagcagtgagggagttaacacagcagctgact   8598
BCRP3.REF.NEW                                             ------------------------------------------------------------   0
GGT1.start-POM121L10P.end.24583750-24659147.ref           GGGCAGTAGGTGGGGCCTTTGGACCTACCAGCAGTGAGGGAGTTAACACAGCAGCTGACT   49065

LOC112206721.putative.POM121-like,protein1.START.LOC112206738  cctctaggcaaggaaaactcccctcagatgctttgctgcctggcctcctgccagcaacaa   8658
BCRP3.REF.NEW                                             ------------------------------------------------------------   0
GGT1.start-POM121L10P.end.24583750-24659147.ref           CCTCTAGGCAAGGAAAACTCCCCTCAGACGCTTTGCTGCCTGGCCTCCTGCCAGCAACAA   49125

LOC112206721.putative.POM121-like,protein1.START.LOC112206738  gcaggagctgaaaactagaagttgaggcgtgagtttggccactccgtagtgtgcacttgg   8718
BCRP3.REF.NEW                                             ----------------------------------------actccgtagtgtgcacttgg   20
GGT1.start-POM121L10P.end.24583750-24659147.ref           GCAGGAGCTGAAAACCAGAAGTTGAGGCGTGAGTTTGGTCACTCCGTAGTGTGCACTTGG   49185
                                                                                                  ********************
```

START of BCRP3 SEQ

```
LOC112206721.putative.POM121-like,protein1.START.LOC112206738  tgagggcag----------cagctgccagccgtctgtccattcacccatctgtccatctg   8768
BCRP3.REF.NEW                                             tgagggcagcagctcgccacagctgccagccgtctgtccattcacccatctgtccatctg   80
GGT1.start-POM121L10P.end.24583750-24659147.ref           TGAGGGCAGCAGCTCGCCACAGCTGCCAGCCGTCTGTCCATTCACCCATCTGTCCATCTG   49245
                                                          *********          *****************************************

LOC112206721.putative.POM121-like,protein1.START.LOC112206738  gcagcccgctgttcagac----ctgtctgtctgcccatctgtaagcccatctctgtccca   8824
BCRP3.REF.NEW                                             gcagcccgctgttcagacccgtctgtctgtccgcccatctgtaagcccatctctgtccca   140
GGT1.start-POM121L10P.end.24583750-24659147.ref           GCAGCCCGCTGTTCAGACCCGTCTGTCTGTCCGCCCATCTGTAAGCCCATCTCTGTCCCA   49305
                                                          ******************    ********* ****************************

LOC112206721.putative.POM121-like,protein1.START.LOC112206738  ttgtctatctgaccatctttctcttactgtcctctttgtctagctatctggc----ctgt   8880
BCRP3.REF.NEW                                             ttgtctatctgaccatctttctcttactgtcctctttgtctagctatctggcctatctgt   200
GGT1.start-POM121L10P.end.24583750-24659147.ref           TTGTCTATCTGACCATCTTTCTCTTACTGTCCTCTTTGTCTAGCTATCTGGCCTATCTGT   49365
                                                          ****************************************************    ****

LOC112206721.putative.POM121-like,protein1.START.LOC112206738  cgatccatctttgtgtctgtcttcagcccccacctgtttgtccatctgtccaattacctg   8940
BCRP3.REF.NEW                                             cgatccatcttcgtgtctgtcttcagcccccacctgtttgtccatctgtccaattacctg   260
GGT1.start-POM121L10P.end.24583750-24659147.ref           CGATCCATCTTCGTGTCTGTCTTCAGCCCCCACCTGTTTGTCCATCTGTCCAATTACCTG   49425
                                                          *********** ************************************************

LOC112206721.putative.POM121-like,protein1.START.LOC112206738  tgagtctatctatgcatcttcttgtccattcatctgcccacccgtctgtccctccgtctg   9000
BCRP3.REF.NEW                                             tgagtctatctatgcaccttcttgtccattcatctgcccacccatctgtccctccgtctg   320
GGT1.start-POM121L10P.end.24583750-24659147.ref           TGAGTCTATCTATGCACCTTCTTGTCCATTCATCTGCCCACCCATCTGTCCCTCCGTCTG   49485
                                                          **************** ************************** ****************

LOC112206721.putative.POM121-like,protein1.START.LOC112206738  cccaccggcctcccctctccttctgggccgcagagccatggcccaggactgcagagccat   9060
BCRP3.REF.NEW                                             cccaccggcctcccctctccttctgggccgcagagccatggcccaggactgcagagccat   380
GGT1.start-POM121L10P.end.24583750-24659147.ref           CCCACCGGCCTCCCCTCTCCTTCTGGGCCGCAGAGCCATGGCCCAGGACTGCAGAGCCAT   49545
                                                          ************************************************************

LOC112206721.putative.POM121-like,protein1.START.LOC112206738  ggttggcctggtcctgctggggctggggctggcgctggctgtcattgtgctggctgtggt   9120
BCRP3.REF.NEW                                             ggttggcctggtcctgctggggctggggcttgtgctggctgtcattgtgctggctgtggt   440
GGT1.start-POM121L10P.end.24583750-24659147.ref           GGTTGGCCTGGTCCTGCTGGGGCTGGGGCTTGTGCTGGCTGTCATTGTGCTGGCTGTGGT   49605
                                                          ****************************** * **************************

LOC112206721.putative.POM121-like,protein1.START.LOC112206738  cctctctcgacaccaggccccatgtggctcccaggcctttgctcacgctgctgctgctgc   9180
BCRP3.REF.NEW                                             cctctctcgacaccaggccccatttgac-ccccggcctttgcccacgccgctgttgctgc   499
GGT1.start-POM121L10P.end.24583750-24659147.ref           CCTCTCTCGACACCAGGCCCCATTTGAC-CCCCGGCCTTTGCCCACGCCGCTGTTGCTGC   49664
                                                          *********************** ** * *** ********* ***** **** ******
```

```
LOC112206721.putative.POM121-like,protein1.START.LOC112206738      tgactccaaggtctgctcaaatattgtatggtgagtgagacgtgggaggaagctgggtgg   9240
BCRP3.REF.NEW                                                      tgactccaaggtctgctcggatattggacggtgagtgagacgtgggaggaagctgggtgg   559
GGT1.start-POM121L10P.end.24583750-24659147.ref                    TGACTCCAAGGTCTGCTCGGATATTGGACGGTGAGTGAGACGTGGGAGGAAGCTGGGTGG   49724
                                                                   *****************  ****** * ********************************

LOC112206721.putative.POM121-like,protein1.START.LOC112206738      cccttggcagccagcccctcctggagaaggcgtgtgtgtgtgagcatgtgtgtgtgtgtg   9300
BCRP3.REF.NEW                                                      cccttggcagccagcccctcctggagaaggcgtgtgtgtgagagtgtgtgtgtgtgagca   619
GGT1.start-POM121L10P.end.24583750-24659147.ref                    CCCTTGGCAGCCAGCCCCTCCTGGAGAAGGCGTGTGTGTGAGAGTGTGTGTGTGTGAGCA   49784
                                                                   *************************************** ***   ********** *

LOC112206721.putative.POM121-like,protein1.START.LOC112206738      tgagattatgtgtgagtgtgagtgt--gtgtgggtatatgtgtgagtgtgtttgtggagt   9358
BCRP3.REF.NEW                                                      tgtgtgtgtgtgagagagtatgtgtcagtgtgtgtgggtatatgagtgtgagtgtggggt   679
GGT1.start-POM121L10P.end.24583750-24659147.ref                    TGTGTGTGTGTGAGAGAGTATGTGTCAGTGTGTGTGGGTATATGAGTGTGAGTGTGGGGT   49844
                                                                   ** *  * **** *** **  ****  ***** **   * * ********  ***** **

LOC112206721.putative.POM121-like,protein1.START.LOC112206738      gtgggggtgtgtgaatgtgtgtgatcgtgtttgggtgtgtgtatgtgtgagtgtgg--gt   9416
BCRP3.REF.NEW                                                      gtgggtgtgtgtgaatgtgtgtgatcgtgtttgggtgtgtgtatgtgtgagtgtgggtgt   739
GGT1.start-POM121L10P.end.24583750-24659147.ref                    GTGGGTGTGTGTGAATGTGTGTGATCGTGTTTGGGTGTGTGTATGTGTGAGTGTGGGTGT   49904
                                                                   ***** **************************************************  **

LOC112206721.putative.POM121-like,protein1.START.LOC112206738      gtgtgaatgtgtgtgattgtgtttgtgtatgtgtgagtgtgtgggtgtgtgtgggtgtgt   9476
BCRP3.REF.NEW                                                      gtgtgaatgtgtgtgagtgtgtttgtgtatgtgtgagtgtggg-----------------   784
GGT1.start-POM121L10P.end.24583750-24659147.ref                    GTGTGAATGTGTGTGAGTGTGTTTGTGTATGTGTGAGTGTGGG-----------------   49949
                                                                   *************** *********** * **** * *******

LOC112206721.putative.POM121-like,protein1.START.LOC112206738      gagcgtatatgtgagtgtgagtgtgtgggggtgtgggtgggtgtgaatgtgtgtgattgt   9536
BCRP3.REF.NEW                                                      ---------tgggggtatatgagtgtgagtgtgtgggtgggtgtgaacgtgtgtgattgt   835
GGT1.start-POM121L10P.end.24583750-24659147.ref                    ---------TGGGGGTATATGAGTGTGAGTGTGTGGGTGGGTGTGAACGTGTGTGATTGT   50000
                                                                            ** * ** *  * ***** * **************** ***********

LOC112206721.putative.POM121-like,protein1.START.LOC112206738      gttacgctgtgtgaggttgtgtgtgactgagtgtgtgagtgtgagtgtgtgggtgtgtgt   9596
BCRP3.REF.NEW                                                      gttttgctgtgtgagggtgtgtgtgac------tatgagtgtgtgagtgtgggtgtgtgt   889
GGT1.start-POM121L10P.end.24583750-24659147.ref                    GTTTTGCTGTGTGAGGGTGTGTGTGAC------TATGAGTGTGTGAGTGTGGGTGTGTGT   50054
                                                                   ***  *********** **********      * ******** * *************

LOC112206721.putative.POM121-like,protein1.START.LOC112206738      aaatgtgtgtgattgtgtgagtgtatgtgggtgtgggtgtgtgtgagtgtgagtgtgtgg   9656
BCRP3.REF.NEW                                                      aaatgtgtgtgattgtgtgagtgtatgtgtgggtgtgagtgtgtgagtgtgagtat----   945
GGT1.start-POM121L10P.end.24583750-24659147.ref                    AAATGTGTGTGATTGTGTGAGTGTATGTGTGGGTGTGAGTGTGTGAGTGTGAGTAT----   50110
                                                                   ***************************** * *** * *************** *

LOC112206721.putative.POM121-like,protein1.START.LOC112206738      gtgtgtgtaaatgtgtgtgattgtgtgagtgtatgtgggtgtgggtgtgtgtgagtgtgt   9716
BCRP3.REF.NEW                                                      ------------------------------------------------------------   945
GGT1.start-POM121L10P.end.24583750-24659147.ref                    ------------------------------------------------------------   50110


LOC112206721.putative.POM121-like,protein1.START.LOC112206738      gagtgtgagtagggggggtgtgggtgtgtgtgaatgtgtgtgattgtgtgtgggtatgtg   9776
BCRP3.REF.NEW                                                      -------------gggggtgtgggtgtgtgtgaatgtgcgtgattgtgtgtgggtatgtg   992
GGT1.start-POM121L10P.end.24583750-24659147.ref                    -------------GGGGGTGTGGGTGTGTGTGAATGTGCGTGATTGTGTGTGGGTATGTG   50157
                                                                                ************************* ********************

LOC112206721.putative.POM121-like,protein1.START.LOC112206738      tatgtgtgggtgtgtgagcgtgtgtgtgcgcgtgtgtgtgtgcacacgtgcactggccca   9836
BCRP3.REF.NEW                                                      tgtgt----gtgtgtgagtgtgtgtgtgtgcgtgtgtgtg----cacgtgcactggccca   1044
GGT1.start-POM121L10P.end.24583750-24659147.ref                    TGTGT----GTGTGTGAGTGTGTGTGTGTGCGTGTGTGTG----CACGTGCACTGGCCCA   50209
                                                                   * ***    ********* ********* ***********    ***************

LOC112206721.putative.POM121-like,protein1.START.LOC112206738      ggaagcaggagccgtgtgtgtgggcttcagcacctgcagggcttgagcgcaaggagacag   9896
BCRP3.REF.NEW                                                      ggcagcaggagccatgtgtgtgggcttcagcacctgcagggcttgagcgcaaggagacag   1104
GGT1.start-POM121L10P.end.24583750-24659147.ref                    GGCAGCAGGAGCCATGTGTGTGGGCTTCAGCACCTGCAGGGCTTGAGCGCAAGGAGACAG   50269
                                                                   ** ********** **********************************************

LOC112206721.putative.POM121-like,protein1.START.LOC112206738      cctcagggcccttgcacagaacaggtggcagggtgtgcccgtggggcagatggggacttg   9956
BCRP3.REF.NEW                                                      cctcagggcccttgcacagaacaggcggcagggtgtgcccgtggggcagatggggacttg   1164
GGT1.start-POM121L10P.end.24583750-24659147.ref                    CCTCAGGGCCCTTGCACAGAACAGGCGGCAGGGTGTGCCCGTGGGGCAGATGGGGACTTG   50329
                                                                   ************************* **********************************

LOC112206721.putative.POM121-like,protein1.START.LOC112206738      gggacaatggtggtgtgtgagtccatacctggctccaggattcaggaggcccatttgcat   10016
BCRP3.REF.NEW                                                      gggacaatggtggtgtgtgagtccatacctggctccaggattcaggaggcccatttgcac   1224
GGT1.start-POM121L10P.end.24583750-24659147.ref                    GGGACAATGGTGGTGTGTGAGTCCATACCTGGCTCCAGGATTCAGGAGGCCCATTTGCAC   50389
                                                                   ***********************************************************

LOC112206721.putative.POM121-like,protein1.START.LOC112206738      atcccaggtgggaacctgtctggccccagctgaccctgctggccggtgcaggtcccttca   10076
BCRP3.REF.NEW                                                      atcccaggtgggaacctgtctggccccggctgaccctgctggccggtgcaggccccttca   1284
GGT1.start-POM121L10P.end.24583750-24659147.ref                    ATCCCAGGTGGGAACCTGTCTGGCCCCGGCTGACCCTGCTGGCCGGTGCAGGCCCCTTCA   50449
                                                                   *************************** ************************ *******

LOC112206721.putative.POM121-like,protein1.START.LOC112206738      gtgaggccaattctccaaggctggggtcttctcccagggtcatgggcgaaggggtttgga   10136
BCRP3.REF.NEW                                                      gtgaggccaattctccaaggctgcggtcttctcccagggtcatgggtgaaggggtttgga   1344
GGT1.start-POM121L10P.end.24583750-24659147.ref                    GTGAGGCCAATTCTCCAAGGCTGCGGTCTTCTCCCAGGGTCATGGGTGAAGGGGTTTGGA   50509
                                                                   *********************** ********************** *************

LOC112206721.putative.POM121-like,protein1.START.LOC112206738      ggctccctgcgtgggtactggcctgctggggtacacacaatgctgccatagccagtctgc   10196
BCRP3.REF.NEW                                                      ggctccctgcgtgggtactggcctgctgggttacacacaatgctgccatagccagtctgc   1404
GGT1.start-POM121L10P.end.24583750-24659147.ref                    GGCTCCCTGCGTGGGTACTGGCCTGCTGGGTTACACACAATGCTGCCATAGCCAGTCTGC   50569
                                                                   ****************************** *****************************

LOC112206721.putative.POM121-like,protein1.START.LOC112206738      cccaacacccagcccggggccacatctcaggtctctcagtcctgaggagcccggtgcccc   10256
BCRP3.REF.NEW                                                      ccctacacccagcctggggccacatctcaggtctctcagtcctgaggagcccggtgcccc   1464
GGT1.start-POM121L10P.end.24583750-24659147.ref                    CCCTACACCCAGCCTGGGGCCACATCTCAGGTCTCTCAGTCCTGAGGAGCCCGGTGCCCC   50629
                                                                   *** ********** *********************************************

LOC112206721.putative.POM121-like,protein1.START.LOC112206738      acccctcacatcttctctccctgagtcagggcctggatctcgtgagctgagtgacttgat   10316
BCRP3.REF.NEW                                                      acccctcacatcctctctccctgagtcagggcctgggtctcgtgagctgagtgac-tgat   1523
GGT1.start-POM121L10P.end.24583750-24659147.ref                    ACCCCTCACATCCTCTCTCCCTGAGTCAGGGCCTGGGTCTCGTGAGCTGAGTGAC-TGAT   50688
                                                                   ************ *********************** ****************** ****

LOC112206721.putative.POM121-like,protein1.START.LOC112206738      acttggtgtcctggatgagggcgtgatggagaggggccacagtgggtgtttcctgaccct   10376
BCRP3.REF.NEW                                                      acttggtgtcctggatgagggcgtgatggagaggggccacagcgggtgtttcctgaccct   1583
GGT1.start-POM121L10P.end.24583750-24659147.ref                    ACTTGGTGTCCTGGATGAGGGCGTGATGGAGAGGGGCCACAGCGGGTGTTTCCTGACCCT   50748
                                                                   ****************************************** *****************

LOC112206721.putative.POM121-like,protein1.START.LOC112206738      cttccaggaaggtgctgctgccgctgcagggaggacacatacaggatgccccttcctgcc   10436
BCRP3.REF.NEW                                                      cttccaggaaggtgctgctgccgctgcagggaggacacatacaggatgccccttcctgcc   1643
GGT1.start-POM121L10P.end.24583750-24659147.ref                    CTTCCAGGAAGGTGCTGCTGCCGCTGCAGGGAGGACACATACAGGATGCCCCTTCCTGCC   50808
                                                                   ************************************************************

LOC112206721.putative.POM121-like,protein1.START.LOC112206738      ccctgcctcccattgggcccacaaaagccagggcaagcctcccctccttgccagccacct   10496
BCRP3.REF.NEW                                                      ccctgcctcccattgggcccacaaaagccagggcaagcctcccctccctgccagccacct   1703
GGT1.start-POM121L10P.end.24583750-24659147.ref                    CCCTGCCTCCCATTGGGCCCACAAAAGCCAGGGCAAGCCTCCCCTCCCTGCCAGCCACCT   50868
                                                                   *********************************************** ************

LOC112206721.putative.POM121-like,protein1.START.LOC112206738      ggtctgcttcccagaaattctgtcttgcaggctgttgggaggatcccagtactttgtaaa   10556
BCRP3.REF.NEW                                                      ggtctgcttcccagaaattctgtcttgcaggctgttgggaggatcccagtactttgtaaa   1763
GGT1.start-POM121L10P.end.24583750-24659147.ref                    GGTCTGCTTCCCAGAAATTCTGTCTTGCAGGCTGTTGGGAGGATCCCAGTACTTTGTAAA   50928
                                                                   ************************************************************

LOC112206721.putative.POM121-like,protein1.START.LOC112206738      ctaaagcaagggaggagtggccattctctctctttgttcattcattcaccttttcattca   10616
BCRP3.REF.NEW                                                      ctaaagcaagggaggagtggccgttctctc----tgttcattcattcaccttttcattca   1819
GGT1.start-POM121L10P.end.24583750-24659147.ref                    CTAAAGCAAGGGAGGAGTGGCCGTTCTCTC----TGTTCATTCATTCACCTTTTCATTCA   50984
                                                                   ********************** *******    **************************

LOC112206721.putative.POM121-like,protein1.START.LOC112206738      ttccttcttccctccattcccccatctgtccatccttccctgccctgattgctcatgcca   10676
BCRP3.REF.NEW                                                      ttccttcttccctccattcccccatctgtccatccttccctgccctgattgctcatgcca   1879
GGT1.start-POM121L10P.end.24583750-24659147.ref                    TTCCTTCTTCCCTCCATTCCCCCATCTGTCCATCCTTCCCTGCCCTGATTGCTCATGCCA   51044
                                                                   ************************************************************

LOC112206721.putative.POM121-like,protein1.START.LOC112206738      ----cccccccagcccctcctgacctggtcctttggtttctcttcagggctttctgtctc   10732
BCRP3.REF.NEW                                                      ccgcccccgcagcccctcctgacctggtcctttggtttctcttcagggatttctgtctc   1939
GGT1.start-POM121L10P.end.24583750-24659147.ref                    CCGCCCCCCGCAGCCCCTCCTGACCTGGTCCTTTGGTTTCTCTTCAGGGATTTCTGTCTC   51104
                                                                       ***** **************************************** **********

LOC112206721.putative.POM121-like,protein1.START.LOC112206738      ctcccacagggctgagaatggcagctcagggacaagtgggggctggggactgcttagtct   10792
BCRP3.REF.NEW                                                      ctcccacagggctgagaatggcagctcagggacaagtaggggctggggactgcttagtct   1999
GGT1.start-POM121L10P.end.24583750-24659147.ref                    CTCCCACAGGGCTGAGAATGGCAGCTCAGGGACAAGTAGGGGCTGGGGACTGCTTAGTCT   51164
                                                                   ************************************* **********************

LOC112206721.putative.POM121-like,protein1.START.LOC112206738      ccccagtggctctcaggggatttgagggtttgacgccagctgccaccccaggctgtgccc   10852
BCRP3.REF.NEW                                                      ccccagtggctctcaggggatttgagggtttgacgccagctgccaccccaggctgtgccc   2059
GGT1.start-POM121L10P.end.24583750-24659147.ref                    CCCCAGTGGCTCTCAGGGGATTTGAGGGTTTGACGCCAGCTGCCACCCCAGGCTGTGCCC   51224
                                                                   ************************************************************

LOC112206721.putative.POM121-like,protein1.START.LOC112206738      ctcctctgctcaggaggacatacagagatgcgacacccacttaaactcaaagttgcaaag   10912
BCRP3.REF.NEW                                                      ctcctctgctcaggaggacatacag-gatgcaacacccacttaaactcgaagttgcaaag   2118
GGT1.start-POM121L10P.end.24583750-24659147.ref                    CTCCTCTGCTCAGGAGGACATACAG-GATGCAACACCCACTTAAACTCGAAGTTGCAAAG   51283
                                                                   ************************* ***** **************** ***********
```

```
LOC112206721.putative.POM121-like,protein1.START.LOC112206738      atgcaaatgagactggggtctcaggcaccagagaccacccttggtcacgtggcttttggg   10972
BCRP3.REF.NEW                                                       atgcaaatgagactggggtctcaggcaccagagaccacccgtgggcacgtggcttttggg   2178
GGT1.start-POM121L10P.end.24583750-24659147.ref                     ATGCAAATGAGACTGGGGTCTCAGGCACCAGAGACCACCCGTGGGCACGTGGCTTTTGGG   51343
                                                                    **************************************** *** ***************

LOC112206721.putative.POM121-like,protein1.START.LOC112206738      agtggggatctgctgccacagatctctgaagagtctagacctgctgggtctccccgagtg   11032
BCRP3.REF.NEW                                                       attggagacctgctgccacagatctctgaagagtctggacctgctgggtctccccaagtg   2238
GGT1.start-POM121L10P.end.24583750-24659147.ref                     ATTGGAGACCTGCTGCCACAGATCTCTGAAGAGTCTGGACCTGCTGGGTCTCCCCAAGTG   51403
                                                                    * *** ** *************************** ****************** ****

LOC112206721.putative.POM121-like,protein1.START.LOC112206738      actgtctggggggtctccatagcatgccctgctgtgtgcgtgacggtcactggttgggtag   11092
BCRP3.REF.NEW                                                       actctctggggggtctccatagcatgccctgctgtgtgcatgacggtcactggttgggtag   2298
GGT1.start-POM121L10P.end.24583750-24659147.ref                     ACTCTCTGGGGGTCTCCATAGCATGCCCTGCTGTGTGCATGACGGTCACTGGTTGGGTAG   51463
                                                                    *** ********************************** *********************

LOC112206721.putative.POM121-like,protein1.START.LOC112206738      gggtctctactctaaagctccttctgccggcatcccctcgaactctcccttggtgaagag   11152
BCRP3.REF.NEW                                                       gggtctctactctaaagctccctctgccggcatcccctcgaactctcccttggtgaagag   2358
GGT1.start-POM121L10P.end.24583750-24659147.ref                     GGGTCTCTACTCTAAAGCTCCCTCTGCCGGCATCCCCTCGAACTCTCCCTTGGTGAAGAG   51523
                                                                    ********************* **************************************

LOC112206721.putative.POM121-like,protein1.START.LOC112206738      agaggatgtggtttgccccagtgttttagcaaacaactctctccacttcctgttttaaga   11212
BCRP3.REF.NEW                                                       agaggatgtggtttgccccagtgttttatcaaacaactctctccacttcctgttttaaga   2418
GGT1.start-POM121L10P.end.24583750-24659147.ref                     AGAGGATGTGGTTTGCCCCAGTGTTTTATCAAACAACTCTCTCCACTTCCTGTTTTAAGA   51583
                                                                    **************************** *******************************

LOC112206721.putative.POM121-like,protein1.START.LOC112206738      agctgggagtggaagagagactggggctggccccagctgctgctgtgaaacaggggtcac   11272
BCRP3.REF.NEW                                                       agctgggagtggaagagagcctggggctggccccagctgctgctgcgaaacaggggtcac   2478
GGT1.start-POM121L10P.end.24583750-24659147.ref                     AGCTGGGAGTGGAAGAGAGCCTGGGGCTGGCCCCAGCTGCTGCTGCGAAACAGGGGTCAC   51643
                                                                    ******************* ************************* **************

LOC112206721.putative.POM121-like,protein1.START.LOC112206738      tggacgctgggaccctggccgggctggctggaggcctcaggaagaggcctgctacagcgt   11332
BCRP3.REF.NEW                                                       tggacgctgggaccctggccgggctggctggaggcctcaggaagaggcctgctacagtgt   2538
GGT1.start-POM121L10P.end.24583750-24659147.ref                     TGGACGCTGGGACCCTGGCCGGGCTGGCTGGAGGCCTCAGGAAGAGGCCTGCTACAGTGT   51703
                                                                    ********************************************************* **

LOC112206721.putative.POM121-like,protein1.START.LOC112206738      catcctggccaagattcctccctgcagaggaccctggccacactgccacagggtctgctg   11392
BCRP3.REF.NEW                                                       catcctggccaagattcctccctgcagaggaccctggccacgctgccacagggtctgctg   2598
GGT1.start-POM121L10P.end.24583750-24659147.ref                     CATCCTGGCCAAGATTCCTCCCTGCAGAGGACCCTGGCCACGCTGCCACAGGGTCTGCTG   51763
                                                                    ***************************************** ******************

LOC112206721.putative.POM121-like,protein1.START.LOC112206738      gggccaccagaagcccatgctcctgcctccatctctcccctctgtgctcacctctcacca   11452
BCRP3.REF.NEW                                                       gggccaccagaagcccatgctcctgcctccatctctcccctctgtgctcacctctcacca   2658
GGT1.start-POM121L10P.end.24583750-24659147.ref                     GGGCCACCAGAAGCCCATGCTCCTGCCTCCATCTCTCCCCTCTGTGCTCACCTCTCACCA   51823
                                                                    ************************************************************

LOC112206721.putative.POM121-like,protein1.START.LOC112206738      ggaggccctcccagagttcagtgtcctgcttttttttttttttttttttttttgagacag   11512
BCRP3.REF.NEW                                                       ggaggccctcccagagttcagtgtcctgcttttttttttttttttt--------agatgg   2710
GGT1.start-POM121L10P.end.24583750-24659147.ref                     GGAGGCCCTCCCAGAGTTCAGTGTCCTGCTTTTTTTTTTTTTTTTT--------AGATGG   51875
                                                                    **********************************************        ***  *

LOC112206721.putative.POM121-like,protein1.START.LOC112206738      tgtctcactctgtcaccaggctggagtacagtggcgcgatctcagctcactgcaacctct   11572
BCRP3.REF.NEW                                                       tgtctcgttctgtcaccaggctggagtgcagtggcgcgatctcagctcactgcaacctct   2770
GGT1.start-POM121L10P.end.24583750-24659147.ref                     TGTCTCGTTCTGTCACCAGGCTGGAGTGCAGTGGCGCGATCTCAGCTCACTGCAACCTCT   51935
                                                                    ******  ******************* ********************************

LOC112206721.putative.POM121-like,protein1.START.LOC112206738      gcttccttggttcaaatgattctcctgcctcagcctcctgagtagctgggactacaggtg   11632
BCRP3.REF.NEW                                                       gcttccttggttcaaatgattctcctgcctcagcctcctgagtagctgggactacaggtg   2830
GGT1.start-POM121L10P.end.24583750-24659147.ref                     GCTTCCTTGGTTCAAATGATTCTCCTGCCTCAGCCTCCTGAGTAGCTGGGACTACAGGTG   51995
                                                                    ************************************************************

LOC112206721.putative.POM121-like,protein1.START.LOC112206738      ccagccaccacacccagctaatttttgtatttttagtagagacggggtttcaccatgttg   11692
BCRP3.REF.NEW                                                       ccagccaccacgcccaggtaatttttgtatttttagtagagacggggtttcaccatgttg   2890
GGT1.start-POM121L10P.end.24583750-24659147.ref                     CCAGCCACCACGCCCAGGTAATTTTTGTATTTTTAGTAGAGACGGGGTTTCACCATGTTG   52055
                                                                    *********** ***** ******************************************

LOC112206721.putative.POM121-like,protein1.START.LOC112206738      gccaggatggtctctatctcttgattcgcccgccttggcctcccaaagtgctggcattac   11752
BCRP3.REF.NEW                                                       gccaggatggtctctatctcttgattcgcccgccttggcctcccaaagtgctggaattac   2950
GGT1.start-POM121L10P.end.24583750-24659147.ref                     GCCAGGATGGTCTCTATCTCTTGATTCGCCCGCCTTGGCCTCCCAAAGTGCTGGAATTAC   52115
                                                                    ****************************************************** *****

LOC112206721.putative.POM121-like,protein1.START.LOC112206738      aggagtgagtcatggcgcccggccccatctcctactctttcagccccaggttttattctt   11812
BCRP3.REF.NEW                                                       aggagtgagtcatggcacccggcctcatctcctactctttcagcaccaggttttactctt   3010
GGT1.start-POM121L10P.end.24583750-24659147.ref                     AGGAGTGAGTCATGGCACCCGGCCTCATCTCCTACTCTTTCAGCACCAGGTTTTACTCTT   52175
                                                                    **************** ******* ******************* ********** ****

LOC112206721.putative.POM121-like,protein1.START.LOC112206738      gggattctgctacagccggagcccctgggtgcgagctcctaagctttctgtgagtgtgga   11872
BCRP3.REF.NEW                                                       gggattctgctacagccgcagcccctgggtgcgagttcctaagctttctgtgagtgtgga   3070
GGT1.start-POM121L10P.end.24583750-24659147.ref                     GGGATTCTGCTACAGCCGCAGCCCCTGGGTGCGAGTTCCTAAGCTTTCTGTGAGTGTGGA   52235
                                                                    ****************** **************** ************************

LOC112206721.putative.POM121-like,protein1.START.LOC112206738      cccagcactgtgcctagtagacatacaaaaggagcatggtgacagtgaggtctgtcatct   11932
BCRP3.REF.NEW                                                       cccagcaccgtgcctagtagacatacaaaaggagcatggtgacagtgaggtctgtcatct   3130
GGT1.start-POM121L10P.end.24583750-24659147.ref                     CCCAGCACCGTGCCTAGTAGACATACAAAAGGAGCATGGTGACAGTGAGGTCTGTCATCT   52295
                                                                    ******** ***************************************************

LOC112206721.putative.POM121-like,protein1.START.LOC112206738      ccagcataatgactgttttgatgcttgtgaaaaaggtgatttttggctgggtgtggtggc   11992
BCRP3.REF.NEW                                                       ccagcataatgactgttttgatccttgtaaaaaaggtgatttttggctgggtgtggtggc   3190
GGT1.start-POM121L10P.end.24583750-24659147.ref                     CCAGCATAATGACTGTTTTGATCCTTGTAAAAAAGGTGATTTTTGGCTGGGTGTGGTGGC   52355
                                                                    ********************** ***** *******************************

LOC112206721.putative.POM121-like,protein1.START.LOC112206738      tcacacctgtaatcccagcactttgggaggccgagggggtggctcacttgaggtcagga   12052
BCRP3.REF.NEW                                                       tcacacctgtaatcccagcactttgggaggccgatgggggtggctcacttgaggtcagga   3250
GGT1.start-POM121L10P.end.24583750-24659147.ref                     TCACACCTGTAATCCCAGCACTTTGGGAGGCCGATGGGGGTGGCTCACTTGAGGTCAGGA   52415
                                                                    ********************************** *************************

LOC112206721.putative.POM121-like,protein1.START.LOC112206738      gttggagaccagcctgggcaacatggtgaaaccacgtctctactaaaaatacaaaaatta   12112
BCRP3.REF.NEW                                                       gttggagcccagcctgggcaacatggtgaaaccacgtctctactaaaaatacaaaaatta   3310
GGT1.start-POM121L10P.end.24583750-24659147.ref                     GTTGGAGCCCAGCCTGGGCAACATGGTGAAACCACGTCTCTACTAAAAATACAAAAATTA   52475
                                                                    ******* ****************************************************

LOC112206721.putative.POM121-like,protein1.START.LOC112206738      gctgggcatggtagcgggtgcctataatcccagctacttgggaggctgagacaggagaat   12172
BCRP3.REF.NEW                                                       gctgggcatggtaacggatgcctgtaatcccagctacttgggaggctgagacaggagaat   3370
GGT1.start-POM121L10P.end.24583750-24659147.ref                     GCTGGGCATGGTAACGGATGCCTGTAATCCCAGCTACTTGGGAGGCTGAGACAGGAGAAT   52535
                                                                    ************* *** ***** ************************************

LOC112206721.putative.POM121-like,protein1.START.LOC112206738      cacttgaacccaggaggcaaaggttgcagtaagccaagattgcgccactgcactccagcc   12232
BCRP3.REF.NEW                                                       cacttgaacccaggaggcaaaggttgcggtaagccaagattgtaccactgcactccagcc   3430
GGT1.start-POM121L10P.end.24583750-24659147.ref                     CACTTGAACCCAGGAGGCAAAGGTTGCGGTAAGCCAAGATTGTACCACTGCACTCCAGCC   52595
                                                                    *************************** **************  ****************

LOC112206721.putative.POM121-like,protein1.START.LOC112206738      tgggtgacagagcaagacttggtctcaaaaaaaaaaaaaaaaaagaaagaaagaaaagttt   12292
BCRP3.REF.NEW                                                       tgggtgacagagcaagacttggtctc-aaaaaaaaaaaaaaaagaaagaaagaaaagttt   3489
GGT1.start-POM121L10P.end.24583750-24659147.ref                     TGGGTGACAGAGCAAGACTTGGTCTC-AAAAAAAAAAAAAAAAGAAAGAAAGAAAAGTTT   52654
                                                                    ************************** *********************************

LOC112206721.putative.POM121-like,protein1.START.LOC112206738      atatttttgttctaatggttatcttaatatcttcattctataattatatgttttatataa   12352
BCRP3.REF.NEW                                                       atatttttgttctaatggttatcttaatatcgtcattctataattgtatgttttatataa   3549
GGT1.start-POM121L10P.end.24583750-24659147.ref                     ATATTTTTGTTCTAATGGTTATCTTAATATCGTCATTCTATAATTGTATGTTTTATATAA   52714
                                                                    ******************************* ************* **************

LOC112206721.putative.POM121-like,protein1.START.LOC112206738      ttataatagctatataagatataatacccctagtatgttgtttttggatattctactcg   12412
BCRP3.REF.NEW                                                       ttataatagctatataagatataatacccctagtatgttgtttttggatattctacttg   3609
GGT1.start-POM121L10P.end.24583750-24659147.ref                     TTATAATAGCTATATAAGATATAATACCCCTAGTATGTTGTTTTTTGGATATTCTACTTG   52774
                                                                    ********************************************************** *

LOC112206721.putative.POM121-like,protein1.START.LOC112206738      ctcctgatggttaatttatgtgtcaacttggctaagctatggtgccccgttgtttggtca   12472
BCRP3.REF.NEW                                                       ctcctgatggttaatttatatgtcaacttggctaagctatggtgccccgttgtttggtca   3669
GGT1.start-POM121L10P.end.24583750-24659147.ref                     CTCCTGATGGTTAATTTATATGTCAACTTGGCTAAGCTATGGTGCCCCGTTGTTTGGTCA   52834
                                                                    ******************* ****************************************

LOC112206721.putative.POM121-like,protein1.START.LOC112206738      aatacttgtcaatatcttgctgggaggttatttcatagatgtgattaacactgacagtca   12532
BCRP3.REF.NEW                                                       aatacttgtcaatatcttgctgggaggttatttcatagatgtgattaacactgacagtca   3729
GGT1.start-POM121L10P.end.24583750-24659147.ref                     AATACTTGTCAATATCTTGCTGGGAGGTTATTTCATAGATGTGATTAACACTGACAGTCA   52894
                                                                    ************************************************************

LOC112206721.putative.POM121-like,protein1.START.LOC112206738      gttgactttaagtaaaacagattacccaccataatatgggtgggccacctccaatca-tt   12591
BCRP3.REF.NEW                                                       gttgactttaagtaaaacagattacccaccataatatgggtgggccacctccaatcagtt   3789
GGT1.start-POM121L10P.end.24583750-24659147.ref                     GTTGACTTTAAGTAAAACAGATTACCCACCATAATATGGGTGGGCCACCTCCAATCAGTT   52954
                                                                    ********************************************************* **

LOC112206721.putative.POM121-like,protein1.START.LOC112206738      gaaggccttaagaacaaaaactgaggtttcccagagaagcaggaattctgcttcaagact   12651
BCRP3.REF.NEW                                                       gaaggccgtaagaacaaaaactgaggtttcccagagaagcaggaattctgcctcaagact   3849
GGT1.start-POM121L10P.end.24583750-24659147.ref                     GAAGGCCGTAAGAACAAAAACTGAGGTTTCCCAGAGAAGCAGGAATTCTGCCTCAAGACT   53014
                                                                    ******* ******************************************* ********
```

```
LOC112206721.putative.POM121-like,protein1.START.LOC112206738    gtaacacacaaaccctgcctgagtttctggcctgctgactgctctacagattttaggttc   12711
BCRP3.REF.NEW                                                    gtaacacacaaaccctgcctgagtttctggcctgctgactgctctacagagtttaggttc   3909
GGT1.start-POM121L10P.end.24583750-24659147.ref                  GTAACACACAAACCCTGCCTGAGTTTCTGGCCTGCTGACTGCTCTACAGAGTTTAGGTTC   53074
                                                                 ************************************************** *********

LOC112206721.putative.POM121-like,protein1.START.LOC112206738    cagacttcgagatcaactcttacctgaatttatagcctgctggcttgccctacagatttt   12771
BCRP3.REF.NEW                                                    cagacttcgagatcaactcttacctgaatttatagcctgctggcttgccctacagatttt   3969
GGT1.start-POM121L10P.end.24583750-24659147.ref                  CAGACTTCGAGATCAACTCTTACCTGAATTTATAGCCTGCTGGCTTGCCCTACAGATTTT   53134
                                                                 ************************************************************

LOC112206721.putative.POM121-like,protein1.START.LOC112206738    aaacttgctagtccccacaatcatgtaagccaattcctaaataaatctctctctatgtat   12831
BCRP3.REF.NEW                                                    aaacttgctagtccccacaatcatgtgagccaattcctcaataaatctctctctatgtat   4029
GGT1.start-POM121L10P.end.24583750-24659147.ref                  AAACTTGCTAGTCCCCACAATCATGTGAGCCAATTCCTCAATAAATCTCTCTCTATGTAT   53194
                                                                 ************************** *********** *********************

LOC112206721.putative.POM121-like,protein1.START.LOC112206738    aacctattggtttagtttctctgaaaagctttcacatccagtttcctggatgttaagaat   12891
BCRP3.REF.NEW                                                    aatctattggtttagtttctctgaaaagctttcacatccagtttcctggatgttaagaat   4089
GGT1.start-POM121L10P.end.24583750-24659147.ref                  AATCTATTGGTTTAGTTTCTCTGAAAAGCTTTCACATCCAGTTTCCTGGATGTTAAGAAT   53254
                                                                 ** *********************************************************

LOC112206721.putative.POM121-like,protein1.START.LOC112206738    tattggaactagctagtaacttcttctttttttttttttttttttttttttgagacagag   12951
BCRP3.REF.NEW                                                    tactgaaactagctagtaacttct--ttttttttttttttttttttttttttgagacagag   4147
GGT1.start-POM121L10P.end.24583750-24659147.ref                  TACTGAAACTAGCTAGTAACTTCT--TTTTTTTTTTTTTTTTTTTTTTTTTTGAGACAGAG   53312
                                                                 ** ** ******************  **********************************

LOC112206721.putative.POM121-like,protein1.START.LOC112206738    ttttgctcttgttgcccaggctggaatgcaatggcacaatcgcagctcaccgcaacctcc   13011
BCRP3.REF.NEW                                                    ttttgctcttgttgcccaggctggaatgcaatggcacaatctcagctcaccgcaacctcc   4207
GGT1.start-POM121L10P.end.24583750-24659147.ref                  TTTTGCTCTTGTTGCCCAGGCTGGAATGCAATGGCACAATCTCAGCTCACCGCAACCTCC   53372
                                                                 ***************************************** ******************

LOC112206721.putative.POM121-like,protein1.START.LOC112206738    acttcctgggtccaaacaattctcctccctcagcctcctgagtagctgggattacaggca   13071
BCRP3.REF.NEW                                                    acttcctgggtccaagcaattctcctccctcagcctcctgagtagctgggattacaggca   4267
GGT1.start-POM121L10P.end.24583750-24659147.ref                  ACTTCCTGGGTCCAAGCAATTCTCCTCCCTCAGCCTCCTGAGTAGCTGGGATTACAGGCA   53432
                                                                 *************** ********************************************

LOC112206721.putative.POM121-like,protein1.START.LOC112206738    tgtgccaccatgcttggctaatttttgtatttttagtagagacagggcttctccatgttg   13131
BCRP3.REF.NEW                                                    tgtgccaccatgcttggctaatttttgtatttttagtagagacagggcttctccatgttg   4327
GGT1.start-POM121L10P.end.24583750-24659147.ref                  TGTGCCACCATGCTTGGCTAATTTTTGTATTTTTAGTAGAGACAGGGCTTCTCCATGTTG   53492
                                                                 ************************************************************

LOC112206721.putative.POM121-like,protein1.START.LOC112206738    gtcaggctggtcttgaactcccaacctcaggtgatcagccgccttggcctcacaaagtgc   13191
BCRP3.REF.NEW                                                    gtcaggctggtcttgaactcccaacctcaggtgatcagccgccttggcctcacaaagtgc   4387
GGT1.start-POM121L10P.end.24583750-24659147.ref                  GTCAGGCTGGTCTTGAACTCCCAACCTCAGGTGATCAGCCGCCTTGGCCTCACAAAGTGC   53552
                                                                 ************************************************************

LOC112206721.putative.POM121-like,protein1.START.LOC112206738    tggaattacaggcatgagccactgcgcctggctcctagtaaattcttcttttccgtgatg   13251
BCRP3.REF.NEW                                                    tggaattacaggcatgagccaccgcacctggctcctagtaaattcttcttttccgtgatg   4447
GGT1.start-POM121L10P.end.24583750-24659147.ref                  TGGAATTACAGGCATGAGCCACCGCACCTGGCTCCTAGTAAATTCTTCTTTTCCGTGATG   53612
                                                                 ********************** ** **********************************

LOC112206721.putative.POM121-like,protein1.START.LOC112206738    tgtctcttacctctaata------------------------------------------   13269
BCRP3.REF.NEW                                                    tgtctcttacctctaataatactttcttctttttttttttttgagacggagtctcgttc   4507.   4467-4759bp
GGT1.start-POM121L10P.end.24583750-24659147.ref                  TGTCTCTTACCTCTAATAATACTTTTCTTCTTTTTTTTTTTTTGAGACGGAGTCTCGTTC   53672
                                                                 ******************
```

Alu seq. missing in chimp

```
LOC112206721.putative.POM121-like,protein1.START.LOC112206738    ------------------------------------------------------------   13269
BCRP3.REF.NEW                                                    tgtcgcccaggcgggagtgctgtggcgcgatctccgctcactgcaagctccgccttccgg   4567
GGT1.start-POM121L10P.end.24583750-24659147.ref                  TGTCGCCCAGGCGGGAGTGCTGTGGCGCGATCTCCGCTCACTGCAAGCTCCGCCTTCCGG   53732

LOC112206721.putative.POM121-like,protein1.START.LOC112206738    ------------------------------------------------------------   13269
BCRP3.REF.NEW                                                    gttcacgccattctcctgcctcaacctcccgagtagctgggactacaggcgcccgccact   4627
GGT1.start-POM121L10P.end.24583750-24659147.ref                  GTTCACGCCATTCTCCTGCCTCAACCTCCCGAGTAGCTGGGACTACAGGCGCCCGCCACT   53792

LOC112206721.putative.POM121-like,protein1.START.LOC112206738    ------------------------------------------------------------   13269
BCRP3.REF.NEW                                                    gcgcccggctaatttttgtatttttagtagagacggggtttcaccgtggtctcgatctc   4687
GGT1.start-POM121L10P.end.24583750-24659147.ref                  GCGCCCGGCTAATTTTTGTATTTTTAGTAGAGACGGGGTTTCACCGTGGTCTCGATCTC   53852

LOC112206721.putative.POM121-like,protein1.START.LOC112206738    ------------------------------------------------------------   13269
BCRP3.REF.NEW                                                    ctgacctcgtgatccgcccgcctcggcctcccaaagtgctgggattacaggcgtgagcca   4747
GGT1.start-POM121L10P.end.24583750-24659147.ref                  CTGACCTCGTGATCCGCCCGCCTCGGCCTCCCAAAGTGCTGGGATTACAGGCGTGAGCCA   53912

LOC112206721.putative.POM121-like,protein1.START.LOC112206738    ------------atactttcttcttaaagtctacttcattaaaaatagttatgctgggc   13317
BCRP3.REF.NEW                                                    ccgcgtccggccatactttcttcttaaagtctacttcattaaaaatagttatgctgggc   4807.  4759bp
GGT1.start-POM121L10P.end.24583750-24659147.ref                  CCGCGTCCGGCCATACTTTTCTTCTTAAAGTCTACTTCATTAAAAATAGTTATGCTGGGC   53972
                                                                             ************************************************

LOC112206721.putative.POM121-like,protein1.START.LOC112206738    atggtggctcatgcctgtaatctcggcactttgttggaggttgaggtgggtggatcactg   13377
BCRP3.REF.NEW                                                    atggtggctcatggctgtaatctcggcactttgttggaggtcgaggtgggtggatcactg   4867
GGT1.start-POM121L10P.end.24583750-24659147.ref                  ATGGTGGCTCATGGCTGTAATCTCGGCACTTTGTTGGAGGTCGAGGTGGGTGGATCACTG   54032
                                                                 ************* *************************** ******************

LOC112206721.putative.POM121-like,protein1.START.LOC112206738    aagcccaggagttcaagagcagcctgggcaacatggcgagaccctgcctctacaaaaaat   13437
BCRP3.REF.NEW                                                    aagcccaggagttcaagaccaacctgggcaacgtggcgagaccctgcctctacaaaaaat   4927
GGT1.start-POM121L10P.end.24583750-24659147.ref                  AAGCCCAGGAGTTCAAGACCAACCTGGGCAACGTGGCGAGACCCTGCCTCTACAAAAAAT   54092
                                                                 ****************** ** ********** ***************************

LOC112206721.putative.POM121-like,protein1.START.LOC112206738    acaaaaattagctgggtgtggctaatataattctaagttggcacacttgtagtcccagct   13497
BCRP3.REF.NEW                                                    acaaaaattagctgggtgtggctaatat--------------acacttgtagtcccagct   4973
GGT1.start-POM121L10P.end.24583750-24659147.ref                  ACAAAAATTAGCTGGGTGTGGCTAATAT--------------ACACTTGTAGTCCCAGCT   54138
                                                                 ****************************              ******************

LOC112206721.putative.POM121-like,protein1.START.LOC112206738    acttgggatgctgaggtgggagaatcgcttgagcctagaagggagagattgctgtaaccc   13557
BCRP3.REF.NEW                                                    acttgggatgctgaggtgggagaatcgcttgagcctagaagggagagattgctgtaagcc   5033
GGT1.start-POM121L10P.end.24583750-24659147.ref                  ACTTGGGATGCTGAGGTGGGAGAATCGCTTGAGCCTAGAAGGGAGAGATTGCTGTAAGCC   54198
                                                                 ********************************************************* **

LOC112206721.putative.POM121-like,protein1.START.LOC112206738    aagatcacatcactgcactccagcctgggagacagagtgaggctctatctccaaaaaaaa   13617
BCRP3.REF.NEW                                                    aagatcacatcactgcactccagcctgggagacagagtgaggctctatctcc--------   5085
GGT1.start-POM121L10P.end.24583750-24659147.ref                  AAGATCACATCACTGCACTCCAGCCTGGGAGACAGAGTGAGGCTCTATCTCC--------   54250
                                                                 ****************************************************

LOC112206721.putative.POM121-like,protein1.START.LOC112206738    aaaaaaaaaaaaaaaaaaaagcgatacagctttcttggttagtgcatgcatgacatattt   13677
BCRP3.REF.NEW                                                    -aaaaaaaaaaaaaaaaaaagttatacagctttcttggttagtgcatgcatgccatattt   5144
GGT1.start-POM121L10P.end.24583750-24659147.ref                  -AAAAAAAAAAAAAAAAAAAGTTATACAGCTTTCTTGGTTAGTGCATGCATGCCATATTT   54309
                                                                  ********************  ***************************** *******

LOC112206721.putative.POM121-like,protein1.START.LOC112206738    ttcattattttccacctctctgtatccttatataaaaggcattagttgggttttacttta   13737
BCRP3.REF.NEW                                                    ttcattattttccacctctctgtatccttatataaaaggcattagttgggttttacttta   5204
GGT1.start-POM121L10P.end.24583750-24659147.ref                  TTCATTATTTTCCACCTCTCTGTATCCTTATATAAAAGGCATTAGTTGGGTTTTACTTTA   54369
                                                                 ************************************************************

LOC112206721.putative.POM121-like,protein1.START.LOC112206738    ttttcagttattttaacttttattgtccttttaaatgtaactaatgatttatttgggttg   13797
BCRP3.REF.NEW                                                    ttttcaattattttaattttttattgtccttttaaatgtaactaatgatttatttgggttg   5264
GGT1.start-POM121L10P.end.24583750-24659147.ref                  TTTTCAATTATTTTAATTTTTATTGTCCTTTTAAATGTAACTAATGATTTATTTGGGTTG   54429
                                                                 ****** ********* *******************************************

LOC112206721.putative.POM121-like,protein1.START.LOC112206738    aaacccaccaccaatttgttttccatgcctattctatttcttcttatctcctctcacatc   13857
BCRP3.REF.NEW                                                    aaacccaccaccaatttgttttccatgcctattctatttcttcttatctcctctcacatc   5324
GGT1.start-POM121L10P.end.24583750-24659147.ref                  AAACCCACCACCAATTTGTTTTCCATGCCTATTCTATTTCTTCTTATCTCCTCTCACATC   54489
                                                                 ************************************************************

LOC112206721.putative.POM121-like,protein1.START.LOC112206738    ttgttttggatttattatttttattatttttttatttaatttcctccttctctattagtt   13917
BCRP3.REF.NEW                                                    ttgttttggatttattattttta--------ttatttaatttcctccttctctattagtt   5376
GGT1.start-POM121L10P.end.24583750-24659147.ref                  TTGTTTTGGATTTATTATTTTTA--------TTATTTAATTTCCTCCTTCTCTATTAGTT   54541
                                                                 ***********************        *****************************

LOC112206721.putative.POM121-like,protein1.START.LOC112206738    tcataactgtgcagtcttagagttattttaaaagatgac--tggattattttagagctta   13975
BCRP3.REF.NEW                                                    tcatagctctgcagtcttagagttattttaaaagatgacagtggattattttagagctta   5436
GGT1.start-POM121L10P.end.24583750-24659147.ref                  TCATAGCTCTGCAGTCTTAGAGTTATTTTAAAAGATGACAGTGGATTATTTTAGAGCTTA   54601
                                                                 ***** ** ******************************  *******************

LOC112206721.putative.POM121-like,protein1.START.LOC112206738    caacatgcatccttcacttatcaaagtctaacatgagctagtacttt---ttgttgttga   14032
BCRP3.REF.NEW                                                    caacatgcatccttcacttatcaaagtctaacatgagctagtactttttgttgttgttga   5496
GGT1.start-POM121L10P.end.24583750-24659147.ref                  CAACATGCATCCTTCACTTATCAAAGTCTAACATGAGCTAGTACTTTTTGTTGTTGTTGA   54661
                                                                 ***********************************************   **********

LOC112206721.putative.POM121-like,protein1.START.LOC112206738    gatagagagagtcttcctctgctgcccaggctggagtgcagtggagcaatcttggttcac   14092
BCRP3.REF.NEW                                                    gatagagagagtcttcctctgctgcccaggctggagtgcagtggagcaatcttggttcac   5556
GGT1.start-POM121L10P.end.24583750-24659147.ref                  GATAGAGAGAGTCTTCCTCTGCTGCCCAGGCTGGAGTGCAGTGGAGCAATCTTGGTTCAC   54721
```

```
                                                                   ************************************************************

LOC112206721.putative.POM121-like,protein1.START.LOC112206738      tgcaacctccacttcttgggttcaagcaattctcctgcctcagtcacctgagtagctggg   14152
BCRP3.REF.NEW                                                      tgcaacctccacttcttgggttcaagcagttctcctgcctcagtcacctgagtagctggg   5616
GGT1.start-POM121L10P.end.24583750-24659147.ref                    TGCAACCTCCACTTCTTGGGTTCAAGCAGTTCTCCTGCCTCAGTCACCTGAGTAGCTGGG   54781
                                                                   **************************** *******************************

LOC112206721.putative.POM121-like,protein1.START.LOC112206738      accacaggtgtgcaccactatgcccggctaattttgtattctttttagtagagacagg   14212
BCRP3.REF.NEW                                                      accacaggtgtgcaccactatgcccggccaattttgtattctttttagtagagacagg   5676
GGT1.start-POM121L10P.end.24583750-24659147.ref                    ACCACAGGTGTGCACCACTATGCCCGGCCAATTTTTGTATTCTTTTTAGTAGAGACAGG   54841
                                                                   **************************** *******************************

LOC112206721.putative.POM121-like,protein1.START.LOC112206738      ggttcaccatgttggccaggctggtcttgaactcctgaccttaagagatctgcttacctc   14272
BCRP3.REF.NEW                                                      gtttcaccatgttggccaggctggtcttgaactcctgaccttaagagatctgcctacctc   5736
GGT1.start-POM121L10P.end.24583750-24659147.ref                    GTTTCACCATGTTGGCCAGGCTGGTCTTGAACTCCTGACCTTAAGAGATCTGCCTACCTC   54901
                                                                   * *************************************************** ******

LOC112206721.putative.POM121-like,protein1.START.LOC112206738      ggcgtcctaaagtgttgggattacaggcgtgagccaccacgcccagcctatgagttagta   14332
BCRP3.REF.NEW                                                      ggcgtcctaaagtgttgggattacaggcatgagccaccgcgcccagcctatgagttagta   5796
GGT1.start-POM121L10P.end.24583750-24659147.ref                    GGCGTCCTAAAGTGTTGGGATTACAGGCATGAGCCACCGCGCCCAGCCTATGAGTTAGTA   54961
                                                                   **************************** ********* *********************

LOC112206721.putative.POM121-like,protein1.START.LOC112206738      cttctatcctcttcctagtcagtacaagaaccttggaacaggaactaaatttacccccag   14392
BCRP3.REF.NEW                                                      cttctatgctcttcctagtcagtacaagaaccttggaacaggaactaaatttacccccag   5856
GGT1.start-POM121L10P.end.24583750-24659147.ref                    CTTCTATGCTCTTCCTAGTCAGTACAAGAACCTTGGAACAGGAACTAAATTTACCCCCAG   55021
                                                                   ******* ****************************************************

LOC112206721.putative.POM121-like,protein1.START.LOC112206738      tgacttatatgctaatatttttgtgtattttaaatacaca-------------aaatctg   14439
BCRP3.REF.NEW                                                      tgacttatatgctaatatttttgtgtattttaaatatatatgtgtgcatagatgtatctg   5916
GGT1.start-POM121L10P.end.24583750-24659147.ref                    TGACTTATATGCTAATATTTTTGTGTATTTTAAATATATATGTGTGCATAGATGTATCTG   55081
                                                                   ************************************ * *               *****

LOC112206721.putative.POM121-like,protein1.START.LOC112206738      tatgtttttgtgtttttattcttatttatgttgagagtgtagagctatgtaagagtaaa   14499
BCRP3.REF.NEW                                                      tgtgtttttgtgtttttattcttatttatgttgagagtgtagagctatgtaagagtaaa   5976
GGT1.start-POM121L10P.end.24583750-24659147.ref                    TGTGTTTTTGTGTTTTTATTCTTATTTATGTTGAGAGTGTAGAGCTATGTAAGAGTAAA   55141
                                                                   * **********************************************************

LOC112206721.putative.POM121-like,protein1.START.LOC112206738      gagaattgtgtaatgaagccccgagtatccattcaatttcaacaacaatcttatggccaa   14559
BCRP3.REF.NEW                                                      gagaattgtgtaatgaagccccgagtatccattcaatttcaacaacaatctcatggccaa   6036
GGT1.start-POM121L10P.end.24583750-24659147.ref                    GAGAATTGTGTAATGAAGCCCCGAGTATCCATTCAATTTCAACAACAATCTCATGGCCAA   55201
                                                                   *************************************************** ********

LOC112206721.putative.POM121-like,protein1.START.LOC112206738      gctaatttcatgtatactctttcctgcttccctctaccccacattatttcagtgcaaatc   14619
BCRP3.REF.NEW                                                      gctaatttcatgtatactctttcctgcttccctctaccccacattatttcagtgcaaatc   6096
GGT1.start-POM121L10P.end.24583750-24659147.ref                    GCTAATTTCATGTATACTCTTTCCTGCTTCCCTCTACCCCACATTATTTCAGTGCAAATC   55261
                                                                   ************************************************************

LOC112206721.putative.POM121-like,protein1.START.LOC112206738      ccagatatataactgtacccatacatatttcagtatgttttatttattttaaaccccaca   14679
BCRP3.REF.NEW                                                      ccagatatataactgtacccatacatatttcagtatgttttatttattttaaaccccaca   6156
GGT1.start-POM121L10P.end.24583750-24659147.ref                    CCAGATATATAACTGTACCCATACATATTTCAGTATGTTTTATTTATTTTAAACCCCACA   55321
                                                                   ************************************************************

LOC112206721.putative.POM121-like,protein1.START.LOC112206738      agatatcattttctatactactgtaatttcataccaataacattcatttagatttaccca   14739. 14739 bp
BCRP3.REF.NEW                                                      atatatcattttctatactactgtaatttcataccaataacattcatttagatttaccca   6216
GGT1.start-POM121L10P.end.24583750-24659147.ref                    ATATATCATTTTCTATACTACTGTAATTTCATACCAATAACATTCATTTAGATTTACCCA   55381
                                                                   * **********************************************************

LOC112206721.putative.POM121-like,protein1.START.LOC112206738      cacatctacctcttctgttaccctttatttttatttataaaaatatctttgggaaaaaat   14799
BCRP3.REF.NEW                                                      cacgtttacctcttctgttaccctttatttttatttataaaaatatctttgggaagaaat   6276
GGT1.start-POM121L10P.end.24583750-24659147.ref                    CACGTTTACCTCTTCTGTTACCCTTTATTTTTATTTATAAAAATATCTTTGGGAAGAAAT   55441
                                                                   *** * ************************************************* ****

LOC112206721.putative.POM121-like,protein1.START.LOC112206738      atctttcagcacatggtcaaggatctcctgagggctatgtcatgggcaaaatatacatat   14859
BCRP3.REF.NEW                                                      atctttcagcacatggtcaaggatctcctgagggctatgtcatgggcaaaatatacatat   6336
GGT1.start-POM121L10P.end.24583750-24659147.ref                    ATCTTTCAGCACATGGTCAAGGATCTCCTGAGGGCTATGTCATGGGCAAAATATACATAT   55501
                                                                   ************************************************************

LOC112206721.putative.POM121-like,protein1.START.LOC112206738      atattccatatatatacacacatatacacacacacatatatacacatatacacacacaca   14919
BCRP3.REF.NEW                                                      atattccatatatatacacacatatacacacacacacatatatacacatatacacacaca   6396
GGT1.start-POM121L10P.end.24583750-24659147.ref                    ATATTCCATATATATACACACATATACACACACACACATATATACACATATACACACACA   55561
                                                                   ************************************ ***** *** *** *********

LOC112206721.putative.POM121-like,protein1.START.LOC112206738      catatatatattccactttcactttttgtttgtttgtttttgagaccgagtcttgctc   14979
BCRP3.REF.NEW                                                      catatatatattccactttcactttttgtttgtttgtttttgagaccgagtctcgctc   6456
GGT1.start-POM121L10P.end.24583750-24659147.ref                    CATATATATATTCCACTTTCACTTTTTGTTTGTTTGTTTTTGAGACCGAGTCTCGCTC   55621
                                                                   ******************************************************* ****

LOC112206721.putative.POM121-like,protein1.START.LOC112206738      tgtggcccaggctggagtgcggtggtgggatctcagctcactgcaacttctacctcctgg   15039
BCRP3.REF.NEW                                                      tgtggcccaggctggagtgcggtggtgggatctcagctcactgcaacttctgcctcctgg   6516
GGT1.start-POM121L10P.end.24583750-24659147.ref                    TGTGGCCCAGGCTGGAGTGCGGTGGTGGGATCTCAGCTCACTGCAACTTCTGCCTCCTGG   55681
                                                                   *************************************************** ********

LOC112206721.putative.POM121-like,protein1.START.LOC112206738      gttcaggtgattctcctgtctcagcctcctgagtagctgggattacaggtgttagccatc   15099
BCRP3.REF.NEW                                                      gttcaggtgattctcctgtctcagcctcctgagtagctgggattacaggtgttagccatc   6576
GGT1.start-POM121L10P.end.24583750-24659147.ref                    GTTCAGGTGATTCTCCTGTCTCAGCCTCCTGAGTAGCTGGGATTACAGGTGTTAGCCATC   55741
                                                                   ************************************************************
```

Alu seq. missing in human BCRP3

```
LOC112206721.putative.POM121-like,protein1.START.LOC112206738      acgtctggctaattttttttgttttttttttttgagacggagtatcgctctgttgcccag   15159 15121 bp
BCRP3.REF.NEW                                                      acgtctggctaattttgttg----------------------------------------   6597
GGT1.start-POM121L10P.end.24583750-24659147.ref                    ACGTCTGGCTAATTTTGTTG----------------------------------------   55762
                                                                   **************** ***

LOC112206721.putative.POM121-like,protein1.START.LOC112206738      gctggagtgcagtggcgtgatctcggctcactgcaacctccatctccctggttcaagcaa   15219
BCRP3.REF.NEW                                                      ------------------------------------------------------------   6597
GGT1.start-POM121L10P.end.24583750-24659147.ref                    ------------------------------------------------------------   55762


LOC112206721.putative.POM121-like,protein1.START.LOC112206738      ttcccctgctcagcctcccaagtagctgggattacaggtgcacgccaccatgcccggata   15279
BCRP3.REF.NEW                                                      ------------------------------------------------------------   6597
GGT1.start-POM121L10P.end.24583750-24659147.ref                    ------------------------------------------------------------   55762


LOC112206721.putative.POM121-like,protein1.START.LOC112206738      atttttttgtatttgtagtagaaaccgggtttccctatgatggtcagactggtcttgaac   15339
BCRP3.REF.NEW                                                      ------------------------------------------------------------   6597
GGT1.start-POM121L10P.end.24583750-24659147.ref                    ------------------------------------------------------------   55762


LOC112206721.putative.POM121-like,protein1.START.LOC112206738      ttctgacttcaggcaatccgcccacctcggcctctcaaagtgctgggattataggcatga   15399
BCRP3.REF.NEW                                                      ------------------------------------------------------------   6597
GGT1.start-POM121L10P.end.24583750-24659147.ref                    ------------------------------------------------------------   55762


LOC112206721.putative.POM121-like,protein1.START.LOC112206738      gccaccatgcctggcctgtttttttttttttttgagatggagttttgctctgtcacccag   15459 15419 bp
BCRP3.REF.NEW                                                      --------------------ttttttttttttttgagacggagtatcgctctgtcacccag   6637
GGT1.start-POM121L10P.end.24583750-24659147.ref                    --------------------TTTTTTTTTTTTTGAGACGGAGTATCGCTCTGTCACCCAG   55802
                                                                                       ***************** ***** * **************

LOC112206721.putative.POM121-like,protein1.START.LOC112206738      gctggagtgcagtggcaagatcttgtctcactgcaacctccacctctcgggttaaagcaa   15519
BCRP3.REF.NEW                                                      gctggagtgcagtggcaagatcttgtctcactgcaacctccacctctcaggttcaagcaa   6697
GGT1.start-POM121L10P.end.24583750-24659147.ref                    GCTGGAGTGCAGTGGCAAGATCTTGTCTCACTGCAACCTCCACCTCTCAGGTTCAAGCAA   55862
                                                                   ************************************************ **** ******

LOC112206721.putative.POM121-like,protein1.START.LOC112206738      ttcttgtgcctcagcctcctgagtagctgggattacaggcatccaccaccacatctggct   15579
BCRP3.REF.NEW                                                      ttcttgtgcctcagcctcctgagtagctgggattacaggcatccaccaccacatctggct   6757
GGT1.start-POM121L10P.end.24583750-24659147.ref                    TTCTTGTGCCTCAGCCTCCTGAGTAGCTGGGATTACAGGCATCCACCACCACATCTGGCT   55922
                                                                   ************************************************************

LOC112206721.putative.POM121-like,protein1.START.LOC112206738      aatttgtgtatttttggtagagatggggtttcaccatgttggccaggcaggtctcgaact   15639
BCRP3.REF.NEW                                                      aatttgtgtatttttggtagagatggggtttcaccatgttggccaggctggtctcgaact   6817
GGT1.start-POM121L10P.end.24583750-24659147.ref                    AATTTGTGTATTTTTGGTAGAGATGGGGTTTCACCATGTTGGCCAGGCTGGTCTCGAACT   55982
                                                                   ************************************************ ***********

LOC112206721.putative.POM121-like,protein1.START.LOC112206738      tctgacctcaggtgatccacctgcatcggccttccaaagtgctgggattacaggcatgag   15699
BCRP3.REF.NEW                                                      tctgacctcaggtgatccacctgcctcggccttccaaagtgctgggattacaggcatgag   6877
GGT1.start-POM121L10P.end.24583750-24659147.ref                    TCTGACCTCAGGTGATCCACCTGCCTCGGCCTTCCAAAGTGCTGGGATTACAGGCATGAG   56042
                                                                   ************************ ***********************************

LOC112206721.putative.POM121-like,protein1.START.LOC112206738      ccaccatgcccagccattttcacttttgaaggatattgttagtgagcatagaattctagg   15759
BCRP3.REF.NEW                                                      ccaccatgcccagccattttcacttttgaaggatattgttaatgagcatagaattctagg   6937
GGT1.start-POM121L10P.end.24583750-24659147.ref                    CCACCATGCCCAGCCATTTTCACTTTTGAAGGATATTGTTAATGAGCATAGAATTCTAGG   56102
                                                                   ***************************************** ******************

LOC112206721.putative.POM121-like,protein1.START.LOC112206738      ttggcagatatttctttcctcagtttgaaaacatgattcccttgtatctgatttctcct   15819
BCRP3.REF.NEW                                                      ttggcagatatttctttcctcagtttgaaaacatgattcccttgtatctgatttctcct   6997
```

```
GGT1.start-POM121L10P.end.24583750-24659147.ref                 TTGGCAGATATTTTCTTTCCTCAGTTTGAAAACATGATTCCCTTGTATCTGATTTCTCCT   56162
                                                                ************************************************************

LOC112206721.putative.POM121-like,protein1.START.LOC112206738   gcttttattgggaagccaattctcaatctaattttgctcatttgaaggcaatggcttttt   15879
BCRP3.REF.NEW                                                   gcttttattgggaagccaattctcaatctaattttgctcatttgaaggcaatggcttttt   7057
GGT1.start-POM121L10P.end.24583750-24659147.ref                 GCTTTTATTGGGAAGCCAATTCTCAATCTAATTTTGCTCATTTGAAGGCAATGGCTTTTT   56222
                                                                ************************************************************

LOC112206721.putative.POM121-like,protein1.START.LOC112206738   atgttgttgtgttttctgagatggagtctcactctgtcacccaggctggactgcagtggt   15939
BCRP3.REF.NEW                                                   ctgttgttgtgttttctgaggtggagtctcactctgtcacccaggctggactgcagtggt   7117
GGT1.start-POM121L10P.end.24583750-24659147.ref                 CTGTTGTTGTGTTTTCTGAGGTGGAGTCTCACTCTGTCACCCAGGCTGGACTGCAGTGGT   56282
                                                                 ******************* ***************************************

LOC112206721.putative.POM121-like,protein1.START.LOC112206738   gcaatctcagctcactgcaacctctgcctcctgggttcaagtgattcttctgcctcagcc   15999
BCRP3.REF.NEW                                                   gcaatctcagctcactgcaacctctgcctcctgggttcaagtgattcttctgcctcagcc   7177
GGT1.start-POM121L10P.end.24583750-24659147.ref                 GCAATCTCAGCTCACTGCAACCTCTGCCTCCTGGGTTCAAGTGATTCTTCTGCCTCAGCC   56342
                                                                ************************************************************

LOC112206721.putative.POM121-like,protein1.START.LOC112206738   tcccaagtagctgggattacaggtgtccaccatcacaactggctaattgttgtattttta   16059
BCRP3.REF.NEW                                                   tcccaagtagctgggattacaggtgtccaccatcacacctggctaattgttgtattttta   7237
GGT1.start-POM121L10P.end.24583750-24659147.ref                 TCCCAAGTAGCTGGGATTACAGGTGTCCACCATCACACCTGGCTAATTGTTGTATTTTTA   56402
                                                                ************************************* **********************

LOC112206721.putative.POM121-like,protein1.START.LOC112206738   atagagatgaacttttgccatgttggtcaggctgatcccaaactcctcatttcaggtgat   16119
BCRP3.REF.NEW                                                   atagagatgaacttttgccatgttggtcaggctgatcccaaactcctcatttcaggtgat   7297
GGT1.start-POM121L10P.end.24583750-24659147.ref                 ATAGAGATGAACTTTTGCCATGTTGGTCAGGCTGATCCCAAACTCCTCATTTCAGGTGAT   56462
                                                                ************************************************************

LOC112206721.putative.POM121-like,protein1.START.LOC112206738   ccgcccgcctcagcctcccaaatgctgggattacaggcatgagccagcccccaaccctgg   16179
BCRP3.REF.NEW                                                   ccgcccgcctcagcctcccaaatgctgggattacaggcatgagacagcccacaaccctgg   7357
GGT1.start-POM121L10P.end.24583750-24659147.ref                 CCGCCCGCCTCAGCCTCCCAAATGCTGGGATTACAGGCATGAGACAGCCCACAACCCTGG   56522
                                                                ******************************************* ****** *********

LOC112206721.putative.POM121-like,protein1.START.LOC112206738   cctgcaggcagtatctttttttcctctggctgctttgaaaagttttgtctttgttttgagc   16239
BCRP3.REF.NEW                                                   cctgcaggcagtatctttttttcctctggctgctttgaaaagttttgtctttgttttgagc   7417
GGT1.start-POM121L10P.end.24583750-24659147.ref                 CCTGCAGGCAGTATCTTTTTTTCCTCTGGCTGCTTTGAAAAGTTTTGTCTTTGTTTTGAGC   56582
                                                                ************************************************************

LOC112206721.putative.POM121-like,protein1.START.LOC112206738   agtttacactgatgcatttaggtggctcttcattccatgacttgactcttttttgtccat   16299
BCRP3.REF.NEW                                                   agtttacactgatgcatttaggtggctcctcattccatgacttgattcttttttgtccat   7477
GGT1.start-POM121L10P.end.24583750-24659147.ref                 AGTTTACACTGATGCATTTAGGTGGCTCCTCATTCCATGACTTGATTCTTTTTTGTCCAT   56642
                                                                **************************** **************** **************

LOC112206721.putative.POM121-like,protein1.START.LOC112206738   tttagaaaactgccagatttatctcttcaagtattacgtcttccccatcctctctctact   16359
BCRP3.REF.NEW                                                   tttagaaaactgccagctttatctcttcaagtattatgtcttccccatcctctctctact   7537
GGT1.start-POM121L10P.end.24583750-24659147.ref                 TTTAGAAAACTGCCAGCTTTATCTCTTCAAGTATTATGTCTTCCCCATCCTCTCTCTACT   56702
                                                                **************** ******************* ***********************

LOC112206721.putative.POM121-like,protein1.START.LOC112206738   ctccttatgagactccaatttcacatgacttatgccttgttaaagtatcccccatgtctc   16419
BCRP3.REF.NEW                                                   ctccttatgagactccaatttcacatgacttatgccttgttaaagtatcccccatgtctc   7597
GGT1.start-POM121L10P.end.24583750-24659147.ref                 CTCCTTATGAGACTCCAATTTCACATGACTTATGCCTTGTTAAAGTATCCCCCATGTCTC   56762
                                                                ************************************************************

LOC112206721.putative.POM121-like,protein1.START.LOC112206738   ttagtccatttcctgtatgttctatctatttttctctttgtacttcaatttgtatagttt   16479
BCRP3.REF.NEW                                                   ttaatccatttcctgtatgttctatctgtttttctctttgtacttcaatttgtatagttt   7657
GGT1.start-POM121L10P.end.24583750-24659147.ref                 TTAATCCATTTCCTGTATGTTCTATCTGTTTTTCTCTTTGTACTTCAATTTGTATAGTTT   56822
                                                                *** *********************** ********************************

LOC112206721.putative.POM121-like,protein1.START.LOC112206738   gtatcaaactatctcccaattagctgggcgtggtggtaggtgcctgtaatcccagctact   16539
BCRP3.REF.NEW                                                   gtatcaaactatctcccaattagccgggcgtggtggtgggtgcctgtaatcccagctact   7717
GGT1.start-POM121L10P.end.24583750-24659147.ref                 GTATCAAACTATCTCCCAATTAGCCGGGCGTGGTGGTGGGTGCCTGTAATCCCAGCTACT   56882
                                                                ************************ ************ **********************

LOC112206721.putative.POM121-like,protein1.START.LOC112206738   tgggaggctgaggcaggagaattgcttgaacccgagaggtggaagttgtagtgagctgag   16599
BCRP3.REF.NEW                                                   tgggagcctgaggcaggagaattgcttgaacccgagaggtggaagttgtagtgagccgag   7777
GGT1.start-POM121L10P.end.24583750-24659147.ref                 TGGGAGCCTGAGGCAGGAGAATTGCTTGAACCCGAGAGGTGGAAGTTGTAGTGAGCCGAG   56942
                                                                ****** ************************************************* ***

LOC112206721.putative.POM121-like,protein1.START.LOC112206738   atcatgccactgcactccagcctgggcaacagagtgagaccctgtctcaataaataaata   16659
BCRP3.REF.NEW                                                   atcatgccactgcactccagcctgggcaacagagtgagaccctgtctcaataaataaata   7837
GGT1.start-POM121L10P.end.24583750-24659147.ref                 ATCATGCCACTGCACTCCAGCCTGGGCAACAGAGTGAGACCCTGTCTCAATAAATAAATA   57002
                                                                ************************************************************

LOC112206721.putative.POM121-like,protein1.START.LOC112206738   aataaatacataaataccagttcactattttttttttatgtttgtgtctagtgtgctgttc   16719
BCRP3.REF.NEW                                                   aataa----ataaataccagttcactattttttttttatgtttgtgtctagtgtgctgttc   7893
GGT1.start-POM121L10P.end.24583750-24659147.ref                 AATAA----ATAAATACCAGTTCACTATTTTTTTTTTATGTTTGTGTCTAGTGTGCTGTTC   57058
                                                                *****    ***************************************************

LOC112206721.putative.POM121-like,protein1.START.LOC112206738   aaattgagttcctaattccatttttttttttgagactttttttttttgagtctctatctg   16779
BCRP3.REF.NEW                                                   aaattgagttcctaattccattttttttttt---agactttttttttttgagtctctatctg   7950
GGT1.start-POM121L10P.end.24583750-24659147.ref                 AAATTGAGTTCCTAATTCCATTTTTTTTTTT---AGACTTTTTTTTTTTGAGTCTCTATCTG   57115
                                                                ******************************   ***************************

LOC112206721.putative.POM121-like,protein1.START.LOC112206738   ttgcccaggctggagttcagtggtgcaatctcagctcactgtagcctccacctcccaggt   16839
BCRP3.REF.NEW                                                   ttgcccaggctggagttcagtggtgcaatctcaactcactgtagcctccacctcccaggt   8010
GGT1.start-POM121L10P.end.24583750-24659147.ref                 TTGCCCAGGCTGGAGTTCAGTGGTGCAATCTCAACTCACTGTAGCCTCCACCTCCCAGGT   57175
                                                                ********************************* **************************

LOC112206721.putative.POM121-like,protein1.START.LOC112206738   tcaagcgattctcatgcctcagcctctcgagtaactgggattaccaccacgcctaactca   16899
BCRP3.REF.NEW                                                   tcaagcgattctcatgcctcagcctctcgagtaactgggattaccaccacgcctaactca   8070
GGT1.start-POM121L10P.end.24583750-24659147.ref                 TCAAGCGATTCTCATGCCTCAGCCTCTCGAGTAACTGGGATTACCACCACGCCTAACTCA   57235
                                                                ************************************************************

LOC112206721.putative.POM121-like,protein1.START.LOC112206738   tttttgtatttt-gtagagatggggttctgccatgttagccaggctggtcttgaactcc   16958
BCRP3.REF.NEW                                                   tttttgtattttagtagagatggggttctgccatgttggccaggctggtcttgaactct   8130
GGT1.start-POM121L10P.end.24583750-24659147.ref                 TTTTTGTATTTTAGTAGAGATGGGGTTCTGCCATGTTGGCCAGGCTGGTCTTGAACTCT   57295
                                                                ************ ************************ *********************

LOC112206721.putative.POM121-like,protein1.START.LOC112206738   tggccttatgtaattggcctacctctgtctcccaaagtgctgggattataggcctaagcc   17018
BCRP3.REF.NEW                                                   tggccttatgtgattggcctacctctgtctcccaaagtgctgggattataggcctaaacc   8190
GGT1.start-POM121L10P.end.24583750-24659147.ref                 TGGCCTTATGTGATTGGCCTACCTCTGTCTCCCAAAGTGCTGGGATTATAGGCCTAAACC   57355
                                                                *********** ********************************************* **

LOC112206721.putative.POM121-like,protein1.START.LOC112206738   actactcccagcctcc-ttttttttttttttttttttgagacggagtctctctctgttg   17077
BCRP3.REF.NEW                                                   accactcccagcctcctttttttttttttttttttttgagacggagtctcgctctgtcg   8250
GGT1.start-POM121L10P.end.24583750-24659147.ref                 ACCACTCCCAGCCTCCTTTTTTTTTTTTTTTTTTTTTGAGACGGAGTCTCGCTCTGTCG   57415
                                                                ** ************* ********************************** ****** *

LOC112206721.putative.POM121-like,protein1.START.LOC112206738   cccaggctggagtagagtggtgcgatctcggctcactgcaacctcccccctcccagttcaa   17137
BCRP3.REF.NEW                                                   cccaggctggagtagagtggtgcgatctcggctcactgcaacctcccccctcccagttcaa   8310
GGT1.start-POM121L10P.end.24583750-24659147.ref                 CCCAGGCTGGAGTAGAGTGGTGCGATCTCGGCTCACTGCAACCTCCCCCCTCCCAGTTCAA   57475
                                                                ************************************************************

LOC112206721.putative.POM121-like,protein1.START.LOC112206738   gtgattctcctgcctcagcctcccgagtagctgggactataggagcatgccaccatgcct   17197
BCRP3.REF.NEW                                                   gtgattctcctgcctcagcctcccgagtagctaggactataggagcatgccaccatgcct   8370
GGT1.start-POM121L10P.end.24583750-24659147.ref                 GTGATTCTCCTGCCTCAGCCTCCCGAGTAGCTAGGACTATAGGAGCATGCCACCATGCCT   57535
                                                                ******************************** ***************************

LOC112206721.putative.POM121-like,protein1.START.LOC112206738   ggctaattttgtaattttagtagagatggggtttcaccatattggtcaggctggtcttg   17257
BCRP3.REF.NEW                                                   ggctaattttgtaattttagtagagatggggattcaccatattggtcaggctggtcttg   8430
GGT1.start-POM121L10P.end.24583750-24659147.ref                 GGCTAATTTTGTAATTTTAGTAGAGATGGGGATTCACCATATTGGTCAGGCTGGTCTTG   57595
                                                                ******************************* ****************************

LOC112206721.putative.POM121-like,protein1.START.LOC112206738   aacttctgacctcaggtgatccacccacctcagcctcccaaagtgctgggattacaggcg   17317
BCRP3.REF.NEW                                                   aacttctgacctcaggtgatctacccacctcagcctcccaaagtgctgggattacaggcg   8490
GGT1.start-POM121L10P.end.24583750-24659147.ref                 AACTTCTGACCTCAGGTGATCTACCCACCTCAGCCTCCCAAAGTGCTGGGATTACAGGCG   57655
                                                                ********************* **************************************

LOC112206721.putative.POM121-like,protein1.START.LOC112206738   taagtcaccacgcctagtgcatccatttttgtagttgccagttttctgatgaaattctt   17377
BCRP3.REF.NEW                                                   tgagtcaccacgcctagtgcatccatttttgtagttgccagttttctgatgaaattctt   8550
GGT1.start-POM121L10P.end.24583750-24659147.ref                 TGAGTCACCACGCCTAGTGCATCCATTTTTGTAGTTGCCAGTTTTCTGATGAAATTCTT   57715
                                                                * **********************************************************

LOC112206721.putative.POM121-like,protein1.START.LOC112206738   aattatttctttatatccttgatacacatgtaaagaatttattttaaagtacatggtctg   17437
BCRP3.REF.NEW                                                   aattgtttctttatatccttgatatacatgtaaagaacttattttaaagtacatggtctg   8610
GGT1.start-POM121L10P.end.24583750-24659147.ref                 AATTGTTTCTTTATATCCTTGATATACATGTAAAGAACTTATTTTAAAGTACATGGTCTG   57775
                                                                **** ******************* ************ **********************

LOC112206721.putative.POM121-like,protein1.START.LOC112206738   atgattttatattctggagatcctataggcctttttcaaagttgtctgtgctttctcttg   17497
BCRP3.REF.NEW                                                   atgattttataatctggagatcctatgggcctttttaaaagttgtctgtgctttctcttg   8670
GGT1.start-POM121L10P.end.24583750-24659147.ref                 ATGATTTTATAATCTGGAGATCCTATGGGCCTTTTTAAAAGTTGTCTGTGCTTTCTCTTG   57835
                                                                *********** ************** ********* ***********************

LOC112206721.putative.POM121-like,protein1.START.LOC112206738   agttttgttcctgctgtcttatttccttgtttgcttggttgtttttaatttggcaatgaa   17557
BCRP3.REF.NEW                                                   agctttttttcctgctgtcttatttccttgtttgcttagttgtttttaatttggcaatgga   8730
```

```
GGT1.start-POM121L10P.end.24583750-24659147.ref                    AGCTTTTTTCCTGCTGTCTTATTTCCTTGTTTGCTTAGTTGTTTTTAATTTGGCAATGGA   57895
                                                                   ** *** ****************************** ********************* *

LOC112206721.putative.POM121-like,protein1.START.LOC112206738      agttgtgtataaaaattgttacaaataa-tttttttttttgagatgtggattctcgctt   17616
BCRP3.REF.NEW                                                      agttgtgtataaaaatcgttacaaataatttttttttttttttgagatggagtctcgctt   8790
GGT1.start-POM121L10P.end.24583750-24659147.ref                    AGTTGTGTATAAAAATCGTTACAAATAATTTTTTTTTTTTTTTGAGATGGAGTCTCGCTT   57955
                                                                   **************** *********** ************  **  **** ********

LOC112206721.putative.POM121-like,protein1.START.LOC112206738      tgttgcccaagctggagtgcaatgatgtgatctcggctcactgcaacctctgcatcccag   17676
BCRP3.REF.NEW                                                      tgttgcccaagctggagtgcaatgacgtgatctcggctcactgcaacctctgcatcccag   8850
GGT1.start-POM121L10P.end.24583750-24659147.ref                    TGTTGCCCAAGCTGGAGTGCAATGACGTGATCTCGGCTCACTGCAACCTCTGCATCCCAG   58015
                                                                   ************************* **********************************

LOC112206721.putative.POM121-like,protein1.START.LOC112206738      ggttcacttctcctacctcagcctcccaagtagctgggattgcaggcaggtgccagcacg   17736
BCRP3.REF.NEW                                                      ggttcaattctcctacctcagcctcccaagtagctgggattgcaggcaggtgccagcacg   8910
GGT1.start-POM121L10P.end.24583750-24659147.ref                    GGTTCAATTCTCCTACCTCAGCCTCCCAAGTAGCTGGGATTGCAGGCAGGTGCCAGCACG   58075
                                                                   ****** *****************************************************

LOC112206721.putative.POM121-like,protein1.START.LOC112206738      cctggctaattttgtatttttagtagagatgggttttcaccatgttggtcaggctggtc   17796
BCRP3.REF.NEW                                                      cctggctaattttgtatttttagtagagatgggtttttaccatgttggtcaggctggtc   8970
GGT1.start-POM121L10P.end.24583750-24659147.ref                    CCTGGCTAATTTTGTATTTTTAGTAGAGATGGGTTTTTACCATGTTGGTCAGGCTGGTC   58135
                                                                   ************************************** *********************

LOC112206721.putative.POM121-like,protein1.START.LOC112206738      tcagactcctgacctcgtgatctgcccacctcagcctcccaaagtgctgggattacaggc   17856
BCRP3.REF.NEW                                                      tcagactcctgacctcgtgatctgcccacctcagcctcccaaagtgctgggattacaggc   9030
GGT1.start-POM121L10P.end.24583750-24659147.ref                    TCAGACTCCTGACCTCGTGATCTGCCCACCTCAGCCTCCCAAAGTGCTGGGATTACAGGC   58195
                                                                   ************************************************************

LOC112206721.putative.POM121-like,protein1.START.LOC112206738      gtgagccactgcacccagccagaaataatttttaaaaataatttcaagcccaagcatgat   17916
BCRP3.REF.NEW                                                      gtgagccactgcgcccagccagaaataatttttaaaaataattttgagccccagcatgat   9090
GGT1.start-POM121L10P.end.24583750-24659147.ref                    GTGAGCCACTGCGCCCAGCCAGAAATAATTTTTAAAAATAATTTTGAGCCCCAGCATGAT   58255
                                                                   ************ *******************************  ***** ********

LOC112206721.putative.POM121-like,protein1.START.LOC112206738      ggctcatgcttgtaatcccatcactttgggaggctgaggcaggcagattgcttgagccta   17976
BCRP3.REF.NEW                                                      ggctcatgcttgtaatcccatcactttgggaggctgaggcgggcagattgcttgagccta   9150
GGT1.start-POM121L10P.end.24583750-24659147.ref                    GGCTCATGCTTGTAATCCCATCACTTTGGGAGGCTGAGGCGGGCAGATTGCTTGAGCCTA   58315
                                                                   **************************************** *******************

LOC112206721.putative.POM121-like,protein1.START.LOC112206738      ggagttcaagatcagcctgcgcaacatggtgaaaccccatctctacaaaaaataaaaaat   18036
BCRP3.REF.NEW                                                      ggagttcaagatcagcctgtacaacatggtgaaaccccatctctacaaaaaataaaaaat   9210
GGT1.start-POM121L10P.end.24583750-24659147.ref                    GGAGTTCAAGATCAGCCTGTACAACATGGTGAAACCCCATCTCTACAAAAAATAAAAAAT   58375
                                                                   *******************  ***************************************

LOC112206721.putative.POM121-like,protein1.START.LOC112206738      tagctgggtgtggttgtggtgtgtgcctgtagtcccagctgtttgggacgctgaagtggg   18096
BCRP3.REF.NEW                                                      tagctgtgtgtggtggtggtgtgtgcctgtagtcccagctgtttgggacgctgaggtggg   9270
GGT1.start-POM121L10P.end.24583750-24659147.ref                    TAGCTGTGTGTGGTGGTGGTGTGTGCCTGTAGTCCCAGCTGTTTGGGACGCTGAGGTGGG   58435
                                                                   ****** ******* *************************************** *****

LOC112206721.putative.POM121-like,protein1.START.LOC112206738      aggctcacttgagcctgggtgatcgaggctgcagtgagccatgatcctgagactgtactc   18156
BCRP3.REF.NEW                                                      aggctcacttgagcctgggtgatcgaggctgcagtgagccatgatcctgagactgcactc   9330
GGT1.start-POM121L10P.end.24583750-24659147.ref                    AGGCTCACTTGAGCCTGGGTGATCGAGGCTGCAGTGAGCCATGATCCTGAGACTGCACTC   58495
                                                                   ******************************************************* ****

LOC112206721.putative.POM121-like,protein1.START.LOC112206738      cagcctgggcaacagagtgagatgctgtctcaaataaataaataaataaaaataaaataa   18216
BCRP3.REF.NEW                                                      cagcctgggcaacagagtgagatgctgtctcaaataaataaataaataaaaataaaatac   9390
GGT1.start-POM121L10P.end.24583750-24659147.ref                    CAGCCTGGGCAACAGAGTGAGATGCTGTCTCAAATAAATAAATAAATAAAAATAAAATAC   58555
                                                                   ***********************************************************

LOC112206721.putative.POM121-like,protein1.START.LOC112206738      tttgaggcctaggggtctgaaattctgagatctcctttatgcatttgagtgactgagatg   18276
BCRP3.REF.NEW                                                      tttgaggcctaggggtctaaaattctgagatctcctttatgcatttgagtgactgagatg   9450
GGT1.start-POM121L10P.end.24583750-24659147.ref                    TTTGAGGCCTAGGGGTCTAAAATTCTGAGATCTCCTTTATGCATTTGAGTGACTGAGATG   58615
                                                                   ****************** *****************************************

LOC112206721.putative.POM121-like,protein1.START.LOC112206738      atctgaagctggatccagtgctcctgagggctgctttatttctggttgactgtgactcct   18336
BCRP3.REF.NEW                                                      atctgaagctggatccagtgctcctgagggctgctttatttctggttgactgtgactcct   9510
GGT1.start-POM121L10P.end.24583750-24659147.ref                    ATCTGAAGCTGGATCCAGTGCTCCTGAGGGCTGCTTTATTTCTGGTTGACTGTGACTCCT   58675
                                                                   ************************************************************

LOC112206721.putative.POM121-like,protein1.START.LOC112206738      agagtaagaaacctgcaccccacgtgtggggcattatggcatcccctccctcagccacat   18396
BCRP3.REF.NEW                                                      agagtaagaaacctgcaccccacatgtggggcattatggcatcccctccctcagccacat   9570
GGT1.start-POM121L10P.end.24583750-24659147.ref                    AGAGTAAGAAACCTGCACCCCACATGTGGGGCATTATGGCATCCCCTCCCTCAGCCACAT   58735
                                                                   *********************** ************************************

LOC112206721.putative.POM121-like,protein1.START.LOC112206738      gagtaagtcaacagcactgctctagaccaggtgtggtggctcacgcctatagtcccagct   18456
BCRP3.REF.NEW                                                      gagtaagtcaacagcactgctctagaccaggtgtggtggctcacacctatagtcccagct   9630
GGT1.start-POM121L10P.end.24583750-24659147.ref                    GAGTAAGTCAACAGCACTGCTCTAGACCAGGTGTGGTGGCTCACACCTATAGTCCCAGCT   58795
                                                                   ******************************************** ***************

LOC112206721.putative.POM121-like,protein1.START.LOC112206738      actcgggagactgaggcaggaggattgcttcaggccaggaatttgagaccagccagagca   18516
BCRP3.REF.NEW                                                      actcgggagactgaggcaggaggattgcttcaggccaggaatttgagaccagccagagca   9690
GGT1.start-POM121L10P.end.24583750-24659147.ref                    ACTCGGGAGACTGAGGCAGGAGGATTGCTTCAGGCCAGGAATTTGAGACCAGCCAGAGCA   58855
                                                                   ************************************************************

LOC112206721.putative.POM121-like,protein1.START.LOC112206738      atatattattaggttggtacaaaagtaattgcagtttttgccattaaaagtaatggcaac   18576
BCRP3.REF.NEW                                                      atatattattaggttggtacaaaagtaattgcagtgtttgcctttaaaagtaatggcaac   9750
GGT1.start-POM121L10P.end.24583750-24659147.ref                    ATATATTATTAGGTTGGTACAAAAGTAATTGCAGTGTTTGCCTTTAAAAGTAATGGCAAC   58915
                                                                   *********************************** ****** *****************

LOC112206721.putative.POM121-like,protein1.START.LOC112206738      cctgtttcagcaaaataaaaagcaaaaaaaaaaaaaaaaaggaagaatgaaaaaaagaat   18636
BCRP3.REF.NEW                                                      cctgtttcagcaaaataaaaagcaaaaaaaaaaaaaaaaaaaaaaa-gaaaggaagaat   9809
GGT1.start-POM121L10P.end.24583750-24659147.ref                    CCTGTTTCAGCAAAATAAAAAGCAAAAAAAAAAAAAAAAAAAAAAA-GAAAGGAAGAAT   58974
                                                                   ***************************************  ** ** ****  ******

LOC112206721.putative.POM121-like,protein1.START.LOC112206738      caaccgggcatggtggctcacgcctctaatcccagcactttaggaggccaaggcgggcag   18696
BCRP3.REF.NEW                                                      cagctgggcgtggtggctcacgcctctaatcccagcactttgggaggccaaggcgggcag   9869
GGT1.start-POM121L10P.end.24583750-24659147.ref                    CAGCTGGGCGTGGTGGCTCACGCCTCTAATCCCAGCACTTTGGGAGGCCAAGGCGGGCAG   59034
                                                                   ** * **** ******************************* ******************

LOC112206721.putative.POM121-like,protein1.START.LOC112206738      atcatgagatcaggagatcgagaccatcctggctaacacggtgaaaccccgtttctacta   18756
BCRP3.REF.NEW                                                      atcatgagatcaggagatcgagaccatcctggctaacacggtgaaaccccatttctacta   9929
GGT1.start-POM121L10P.end.24583750-24659147.ref                    ATCATGAGATCAGGAGATCGAGACCATCCTGGCTAACACGGTGAAACCCCATTTCTACTA   59094
                                                                   ************************************************** *********

LOC112206721.putative.POM121-like,protein1.START.LOC112206738      aaaatacaaaaaattagccgggcgtggtggcaggcgcctgtagtcccagctactcaggag   18816
BCRP3.REF.NEW                                                      aaaatacaaaaaattagccgggcatggtggcaggcgcctgtagtcccagctactcaggag   9989
GGT1.start-POM121L10P.end.24583750-24659147.ref                    AAAATACAAAAAATTAGCCGGGCATGGTGGCAGGCGCCTGTAGTCCCAGCTACTCAGGAG   59154
                                                                   *********************** ************************************

LOC112206721.putative.POM121-like,protein1.START.LOC112206738      gcggaggcaggagaatggcatgaacccaggaggtagagcttgcagtgagccgagatcatg   18876
BCRP3.REF.NEW                                                      gcggaggcaggagaatggcatgaacccaggaggtggaggttgcagtgagccgagatcatg   10049
GGT1.start-POM121L10P.end.24583750-24659147.ref                    GCGGAGGCAGGAGAATGGCATGAACCCAGGAGGTGGAGGTTGCAGTGAGCCGAGATCATG   59214
                                                                   ********************************** *** ********************

LOC112206721.putative.POM121-like,protein1.START.LOC112206738      ccactgcactccagcctgggtgacagagtgagactccgtctc--aaaaaaaaaaaaaaaga   18934
BCRP3.REF.NEW                                                      ccactgcactccagcctgggtgacagagtgagactccgtctcaaaaaaaaaaaaaaaaaga   10109
GGT1.start-POM121L10P.end.24583750-24659147.ref                    CCACTGCACTCCAGCCTGGGTGACAGAGTGAGACTCCGTCTCAAAAAAAAAAAAAAAAAGA   59274
                                                                   ******************************************  *****************

LOC112206721.putative.POM121-like,protein1.START.LOC112206738      atcactgctctgtctctcagcctcctcttccaggattggccgtcgccttgaggggaatgc   18994
BCRP3.REF.NEW                                                      atcactgctctgtctctcagcctcctcttccaagattggccgtcgccttgaggggaatgc   10169
GGT1.start-POM121L10P.end.24583750-24659147.ref                    ATCACTGCTCTGTCTCTCAGCCTCCTCTTCCAAGATTGGCCGTCGCCTTGAGGGGAATGC   59334
                                                                   ******************************** ***************************

LOC112206721.putative.POM121-like,protein1.START.LOC112206738      tggccttgcctgtctccagccctgtacctctctgcctcctatgcctttaagcacatgttt   19054
BCRP3.REF.NEW                                                      tggccttgcctgtctccagccctgtacctctctgcctcctatgcctttaagcacatgttt   10229
GGT1.start-POM121L10P.end.24583750-24659147.ref                    TGGCCTTGCCTGTCTCCAGCCCTGTACCTCTCTGCCTCCTATGCCTTTAAGCACATGTTT   59394
                                                                   ************************************************************

LOC112206721.putative.POM121-like,protein1.START.LOC112206738      tctatttgctgggctgtgaaatctgctcttcatctgatggggtttgctttataggtgact   19114
BCRP3.REF.NEW                                                      tctatttgctgggctgtgaaatctgctcttcatctgatggggtttgctttataggtgact   10289
GGT1.start-POM121L10P.end.24583750-24659147.ref                    TCTATTTGCTGGGCTGTGAAATCTGCTCTTCATCTGATGGGGTTTGCTTTATAGGTGACT   59454
                                                                   ************************************************************

LOC112206721.putative.POM121-like,protein1.START.LOC112206738      agattcttttctcttggcggttttagaatttgcattttcacattgaccttaaatagtctg   19174
BCRP3.REF.NEW                                                      agatcctttttctcttggtggttttagaattcgcattttcacattgaccttaaatagtctg   10349
GGT1.start-POM121L10P.end.24583750-24659147.ref                    AGATCCTTTTCTCTTGGTGGTTTTAGAATTCGCATTTTCACATTGACCTTAAATAGTCTG   59514
                                                                   **** ************ *********** ******************************

LOC112206721.putative.POM121-like,protein1.START.LOC112206738      attatagtttgccacggcaaagaccctttgcattgcattgtttggggatatttgagcctc   19234
BCRP3.REF.NEW                                                      attatagtttgccacggcaaagaccctttgcattgcattgtttggggatatttgagcctc   10409
GGT1.start-POM121L10P.end.24583750-24659147.ref                    ATTATAGTTTGCCACGGCAAAGACCCTTTGCATTGCATTGTTTGGGGATATTTGAGCCTC   59574
                                                                   ************************************************************

LOC112206721.putative.POM121-like,protein1.START.LOC112206738      ctctatctggatgtctaatctcttgttagatgtgagtagttttcattattattttattaa   19294. 19292 bp
BCRP3.REF.NEW                                                      ctctatctggatgtctaatctcttgttagatgtgagtagttttcattattattttattaa   10469
```

```
GGT1.start-POM121L10P.end.24583750-24659147.ref                   CTCTATCTGGATGTCTAATCTCTTGTTAGATGTGAGTAGTTTTCATTATTATTTTATTAA   59634
                                                                  ************************************************************

LOC112206721.putative.POM121-like,protein1.START.LOC112206738     tgggctggcatgtgggccgtggttccaggcgggctcagaggggcagctgcctgatgtctg   19354
BCRP3.REF.NEW                                                     tgggctggcatgtgggccgtggttccaggcaggctcagaggggcagctgcctgatgtctg   10529
GGT1.start-POM121L10P.end.24583750-24659147.ref                   TGGGCTGGCATGTGGGCCGTGGTTCCAGGCAGGCTCAGAGGGGCAGCTGCCTGATGTCTG   59694
                                                                  ****************************** *****************************

LOC112206721.putative.POM121-like,protein1.START.LOC112206738     gacagcttctctttctgtcttttcttacctggactctgggttgcttgttagctgcttctg   19414
BCRP3.REF.NEW                                                     gacagcttctctttctgtcttttcttacctggactctgggttgcttgttagctgcttctg   10589
GGT1.start-POM121L10P.end.24583750-24659147.ref                   GACAGCTTCTCTTTCTGTCTTTTCTTACCTGGACTCTGGGTTGCTTGTTAGCTGCTTCTG   59754
                                                                  ************************************************************

LOC112206721.putative.POM121-like,protein1.START.LOC112206738     ccagttctgag-tttcaaggggagaggggcccagtgatggctgttctttgaaggaaaggg   19473
BCRP3.REF.NEW                                                     ccagttctgagttttcaaggggagaggggcccagtgatggctgttctttgaaggaaaggg   10649
GGT1.start-POM121L10P.end.24583750-24659147.ref                   CCAGTTCTGAGTTTTCAAGGGGAGAGGGGCCCAGTGATGGCTGTTCTTTGAAGGAAAGGG   59814
                                                                  *********** ************************************************

LOC112206721.putative.POM121-like,protein1.START.LOC112206738     aagaatgtctcctgtttaacatgtttctatgtttccagttacttggttagttagttagag   19533
BCRP3.REF.NEW                                                     aagaatgtctcctgtttaacatgtttctatgtttccagttacttggttagttagttagag   10709
GGT1.start-POM121L10P.end.24583750-24659147.ref                   AAGAATGTCTCCTGTTTAACATGTTTCTATGTTTCCAGTTACTTGGTTAGTTAGTTAGAG   59874
                                                                  ************************************************************

LOC112206721.putative.POM121-like,protein1.START.LOC112206738     ccagggtctctcgctctgttgcgtaggctggagtgcaatggcatgatcgtggctcacagc   19593
BCRP3.REF.NEW                                                     ccagggtctctcgctctgttgcgcaggctggagtgcaatggcatgatcgtggctcacagc   10769
GGT1.start-POM121L10P.end.24583750-24659147.ref                   CCAGGGTCTCTCGCTCTGTTGCGCAGGCTGGAGTGCAATGGCATGATCGTGGCTCACAGC   59934
                                                                  *********************** ************************************

LOC112206721.putative.POM121-like,protein1.START.LOC112206738     agcctccaccttccaggctcgagcaatgctcccacctcagcctctcaagcagctgggact   19653
BCRP3.REF.NEW                                                     agcctccaccttccaggctcgagcaatgctcccacctcagcctctcaagcagctgggact   10829
GGT1.start-POM121L10P.end.24583750-24659147.ref                   AGCCTCCACCTTCCAGGCTCGAGCAATGCTCCCACCTCAGCCTCTCAAGCAGCTGGGACT   59994
                                                                  ************************************************************

LOC112206721.putative.POM121-like,protein1.START.LOC112206738     gcaggtatgtgccaccatgcttggctgcctttttaatttttttttttttttaatacagac   19713
BCRP3.REF.NEW                                                     gcaggtatgtgccaccatgcttggctgcctttttaa-atttttttttttttaatacagac   10888
GGT1.start-POM121L10P.end.24583750-24659147.ref                   GCAGGTATGTGCCACCATGCTTGGCTGCCTTTTTAA-ATTTTTTTTTTTTTAATACAGAC   60053
                                                                  ************************************  **********************

LOC112206721.putative.POM121-like,protein1.START.LOC112206738     agggtctcactatattgcccaggctggtcttaaactcatgggctcaagtgatcctcctgc   19773
BCRP3.REF.NEW                                                     aaggtctcactatattgcccaggctggtcttaaactcatgggctcaagtgatactcctgc   10948
GGT1.start-POM121L10P.end.24583750-24659147.ref                   AAGGTCTCACTATATTGCCCAGGCTGGTCTTAAACTCATGGGCTCAAGTGATACTCCTGC   60113
                                                                  * ************************************************** *******

LOC112206721.putative.POM121-like,protein1.START.LOC112206738     cttggcctttcaaagtgctgatatcacaggcagggtttccgtttttaaagctcccagca   19833
BCRP3.REF.NEW                                                     ctcggcctttcaaagtgctgatatcacaggcagggtttccatttttaaagctcccagca   11008
GGT1.start-POM121L10P.end.24583750-24659147.ref                   CTCGGCCTTTCAAAGTGCTGATATCACAGGCAGGGTTTCCATTTTTAAAGCTCCCAGCA   60173
                                                                  ** ************************************* *******************

LOC112206721.putative.POM121-like,protein1.START.LOC112206738     gtggtatgaaactcctcctttccagagaaagcgcactctgtccgcatccctcatgttgtc   19893
BCRP3.REF.NEW                                                     gtggtat-aaactcctcctttccagagaaagcgcactctgtccgcatccctcatgttgtc   11067
GGT1.start-POM121L10P.end.24583750-24659147.ref                   GTGGTAT-AAACTCCTCCTTTCCAGAGAAAGCGCACTCTGTCCGCATCCCTCATGTTGTC   60232
                                                                  ******* ****************************************************

LOC112206721.putative.POM121-like,protein1.START.LOC112206738     ctctcctgcctctgcttagggttcactccgggggaaagtgccacttgagagtttcctttt   19953
BCRP3.REF.NEW                                                     ctctcctgcctctgcttagggttcactccgggggaaagtgccacttgagagtttcctttt   11127
GGT1.start-POM121L10P.end.24583750-24659147.ref                   CTCTCCTGCCTCTGCTTAGGGTTCACTCCGGGGGAAAGTGCCACTTGAGAGTTTCCTTTT   60292
                                                                  ************************************************************

LOC112206721.putative.POM121-like,protein1.START.LOC112206738     tgtgtgtggttctgactgaccgctccctgctcacagatgctgcttctcagggtggggtcc   20013
BCRP3.REF.NEW                                                     tgtgtgtggttctgactgactgctccctgctcacagatgctgattctcagggtggggtcc   11187
GGT1.start-POM121L10P.end.24583750-24659147.ref                   TGTGTGTGGTTCTGACTGACTGCTCCCTGCTCACAGATGCTGATTCTCAGGGTGGGGTCC   60352
                                                                  ******************** ********************* *****************

LOC112206721.putative.POM121-like,protein1.START.LOC112206738     ctgaggcctggagtgtggcctctgacgaccttcagggccaggtgcggaatgagagcctgt   20073
BCRP3.REF.NEW                                                     ctgaggcctggagtgtggcctctgacgaccttcagggccaggtgtggaatgagagcctgt   11247
GGT1.start-POM121L10P.end.24583750-24659147.ref                   CTGAGGCCTGGAGTGTGGCCTCTGACGACCTTCAGGGCCAGGTGTGGAATGAGAGCCTGT   60412
                                                                  ******************************************** ***************

LOC112206721.putative.POM121-like,protein1.START.LOC112206738     ggccacatggcccctggtgggagacgtcccgccgccctttgcttctctgtgccactctgg   20133
BCRP3.REF.NEW                                                     ggccacatggcccccggtgggagacgtcccgccgccctttgcttctctgtgccactctgg   11307
GGT1.start-POM121L10P.end.24583750-24659147.ref                   GGCCACATGGCCCCCGGTGGGAGACGTCCCGCCGCCCTTTGCTTCTCTGTGCCACTCTGG   60472
                                                                  ************** *********************************************

LOC112206721.putative.POM121-like,protein1.START.LOC112206738     ctgcacagttcagagccttgggaaatgttaaccagtaggacctagaaggggaggtgagaa   20193
BCRP3.REF.NEW                                                     ctgcacagttcagagccttgggaaatgttaaccagtaggacctagacggggaggtgagaa   11367
GGT1.start-POM121L10P.end.24583750-24659147.ref                   CTGCACAGTTCAGAGCCTTGGGAAATGTTAACCAGTAGGACCTAGACGGGGAGGTGAGAA   60532
                                                                  ********************************************** *************

LOC112206721.putative.POM121-like,protein1.START.LOC112206738     ggggtca-cccccaggtgtgcctgtggtgagccttcgtgctgagcaggtgcagggaggga   20252
BCRP3.REF.NEW                                                     ggggtcacccccccaggtgtgcctgtggtgagccttcgtgctgagcaggtgcagggaggga   11427
GGT1.start-POM121L10P.end.24583750-24659147.ref                   GGGGTCACCCCCCAGGTGTGCCTGTGGTGAGCCTTCGTGCTGAGCAGGTGCAGGGAGGGA   60592
                                                                  ******* ****************************************************

LOC112206721.putative.POM121-like,protein1.START.LOC112206738     ggcccaggtgcacacacctgtgaagtaggggcagctggctgggctccttgacctgctcca   20312
BCRP3.REF.NEW                                                     ggcccaggtgcacacacctgtgaagtaggggcagctggctgggctccttgacctgctcca   11487
GGT1.start-POM121L10P.end.24583750-24659147.ref                   GGCCCAGGTGCACACACCTGTGAAGTAGGGGCAGCTGGCTGGGCTCCTTGACCTGCTCCA   60652
                                                                  ************************************************************

LOC112206721.putative.POM121-like,protein1.START.LOC112206738     gagcttcttatttctggccacttcacctgcagaaggcccaggtggctgtggcctctagg   20372
BCRP3.REF.NEW                                                     gagcttcttatttctggccacttcacctgcagaaggcccaggtggctgtggcctctagg   11547
GGT1.start-POM121L10P.end.24583750-24659147.ref                   GAGCTTCTTATTTCTGGCCACTTCACCTGCAGAAGGCCCAGGTGGCTGTGGCCTCTAGG   60712
                                                                  ************************************************************

LOC112206721.putative.POM121-like,protein1.START.LOC112206738     gtcccttgcctgtcctcagctcccaactgggaggggcagagggaggagggggtggagacc   20432
BCRP3.REF.NEW                                                     gtcccttgcctgtcctcagctcccaactgggaggggcagagggaggagggggtggagacc   11607
GGT1.start-POM121L10P.end.24583750-24659147.ref                   GTCCCTTGCCTGTCCTCAGCTCCCAACTGGGAGGGGCAGAGGGAGGAGGGGGTGGAGACC   60772
                                                                  ************************************************************

LOC112206721.putative.POM121-like,protein1.START.LOC112206738     ccaggcagcagggctctgggagcagtggggccctgggttccaggggtgtctggcaggccc   20492
BCRP3.REF.NEW                                                     ccaggcagcagggctctgggagcagtggggccctgggtcccaggggtgtctggcaggccc   11667
GGT1.start-POM121L10P.end.24583750-24659147.ref                   CCAGGCAGCAGGGCTCTGGGAGCAGTGGGGCCCTGGGTCCCAGGGGTGTCTGGCAGGCCC   60832
                                                                  ************************************** *********************

LOC112206721.putative.POM121-like,protein1.START.LOC112206738     ctccttactctacgtctctcggcctctggatggaggtgctggccgcagtcaggctctgcc   20552
BCRP3.REF.NEW                                                     ctccttactctacg--tctcggcctctggatggaggtgctggctgcagtcgggctctgcc   11725
GGT1.start-POM121L10P.end.24583750-24659147.ref                   CTCCTTACTCTACG--TCTCGGCCTCTGGATGGAGGTGCTGGCTGCAGTCGGGCTCTGCC   60890
                                                                  **************  *************************** ****** *********

LOC112206721.putative.POM121-like,protein1.START.LOC112206738     tctgactaagggttggagaagtgtcggatgtgggctgctgccccgtggggcctctgaaca   20612
BCRP3.REF.NEW                                                     tctgactaagggttggggaagtggcgggtgtgggctgctgccccgtggggcctctgaaca   11785
GGT1.start-POM121L10P.end.24583750-24659147.ref                   TCTGACTAAGGGTTGGGGAAGTGGCGGGTGTGGGCTGCTGCCCCGTGGGGCCTCTGAACA   60950
                                                                  **************** ****** *** ********************************

LOC112206721.putative.POM121-like,protein1.START.LOC112206738     gaccccagggcctctgccaatcatgactccttgctttcagctggacccgcaggccctgca   20672
BCRP3.REF.NEW                                                     gaccccagggcctctgccaatcatgactccttcctttcagctggacccgcaggccctgca   11845
GGT1.start-POM121L10P.end.24583750-24659147.ref                   GACCCCAGGGCCTCTGCCAATCATGACTCCTTCCTTTCAGCTGGACCCGCAGGCCCTGCA   61010
                                                                  ******************************* ****************************

LOC112206721.putative.POM121-like,protein1.START.LOC112206738     ggacagagactggcagcgcaccatcatctccatgaatgtggtacatgtccgtgggactct   20732
BCRP3.REF.NEW                                                     ggacagagactggcagcgcgccgtcatcgccatgaatggggtacgtgtccgtgggactct   11905
GGT1.start-POM121L10P.end.24583750-24659147.ref                   GGACAGAGACTGGCAGCGCGCCGTCATCGCCATGAATGGGGTACGTGTCCGTGGGACTCT   61070
                                                                  ******************* ** ***** ********* ***** ***************

LOC112206721.putative.POM121-like,protein1.START.LOC112206738     cctggtgcccacttcccccagaaggatagggtggcctctgttcatttcaaatcagtcaga   20792
BCRP3.REF.NEW                                                     cctggcgcccacttcccccagaaggatagggtggcctctgttcatttcaaatcagtcaga   11965
GGT1.start-POM121L10P.end.24583750-24659147.ref                   CCTGGCGCCCACTTCCCCCAGAAGGATAGGGTGGCCTCTGTTCATTTCAAATCAGTCAGA   61130
                                                                  ***** ******************************************************

LOC112206721.putative.POM121-like,protein1.START.LOC112206738     ggtggctgagcctgaggcggcatctgagagggagcctggttggagaagggagggccccca   20852
BCRP3.REF.NEW                                                     ggtggctgagcctgaggcagcatctgagagggagcctggttggagaagggagggccccca   12025
GGT1.start-POM121L10P.end.24583750-24659147.ref                   GGTGGCTGAGCCTGAGGCAGCATCTGAGAGGGAGCCTGGTTGGAGAAGGGAGGGCCCCCA   61190
                                                                  ****************** *****************************************

LOC112206721.putative.POM121-like,protein1.START.LOC112206738     agagcagaatcaccatgcacaggaatcgtcattcattggctggaatgcagttgccagcca   20912
BCRP3.REF.NEW                                                     agagcagaatcaccatgcacgggaatcgtcattcattggctggaatgcagttgccagcca   12085
GGT1.start-POM121L10P.end.24583750-24659147.ref                   AGAGCAGAATCACCATGCACGGGAATCGTCATTCATTGGCTGGAATGCAGTTGCCAGCCA   61250
                                                                  ******************** ***************************************

LOC112206721.putative.POM121-like,protein1.START.LOC112206738     ggccctgagcttccctcctgaaacaaaggtctcatggcaccaccaggacaggtggggcct   20972
BCRP3.REF.NEW                                                     ggccctgagcatccctcctcaaacaaaggtctcatggcaccaccaggacaggtggggcct   12145
GGT1.start-POM121L10P.end.24583750-24659147.ref                   GGCCCTGAGCATCCCTCCTCAAACAAAGGTCTCATGGCACCACCAGGACAGGTGGGGCCT   61310
                                                                  ********** ******** ****************************************

LOC112206721.putative.POM121-like,protein1.START.LOC112206738     ccactcagggacctgggggctgcccatagaaatggagacccctgatttgtctttaggtac   21032
BCRP3.REF.NEW                                                     ccactcagggacctgggggctgcccatagaaatggagacccctgatttgtctttaggtac   12205
```

```
GGT1.start-POM121L10P.end.24583750-24659147.ref                   CCACTCAGGGACCTGGGGGCTGCCCATAGAAATGGAGACCCCTGATTTGTCTTTAGGTAC   61370
                                                                  ************************************************************

LOC112206721.putative.POM121-like,protein1.START.LOC112206738     cccagaaaggtttagaccttaaaagcaatgacacacccaaaaaggcccgggtataaatgg   21092
BCRP3.REF.NEW                                                     cccagaaaggtttagaccttaaaagcaatgacacacccaaaaaggcccgggtataaatgg   12265
GGT1.start-POM121L10P.end.24583750-24659147.ref                   CCCAGAAAGGTTTAGACCTTAAAAGCAATGACACACCCAAAAAGGCCCGGGTATAAATGG   61430
                                                                  ************************************************************

LOC112206721.putative.POM121-like,protein1.START.LOC112206738     taaaatgttaatatttgagattcttggcttttcttacattattctgtctttcctactta   21152
BCRP3.REF.NEW                                                     taaaatgttaatatttgagattcttggcttttcttacattattctgtctttccttctta   12325
GGT1.start-POM121L10P.end.24583750-24659147.ref                   TAAAATGTTAATATTTGAGATTCTTGGCTTTTCTTACATTATTCTGTCTTTCCTTCTTA   61490
                                                                  ******************************************************* ****

LOC112206721.putative.POM121-like,protein1.START.LOC112206738     atttttaattgttactaagagaaagctggtcacagtacactataatctcagctactctgg   21212
BCRP3.REF.NEW                                                     atttttaattgttactaagagaaagctggtcacagtacactataatctcagctactctgg   12385
GGT1.start-POM121L10P.end.24583750-24659147.ref                   ATTTTTAATTGTTACTAAGAGAAAGCTGGTCACAGTACACTATAATCTCAGCTACTCTGG   61550
                                                                  ************************************************************

LOC112206721.putative.POM121-like,protein1.START.LOC112206738     aggctgagccaggagaatcactggagcccaagagtttgattacagcctgggcaacattgc   21272
BCRP3.REF.NEW                                                     aggctgagccaggagaatcactggagcccaagagtttgattacagcctgggcaacattgc   12445
GGT1.start-POM121L10P.end.24583750-24659147.ref                   AGGCTGAGCCAGGAGAATCACTGGAGCCCAAGAGTTTGATTACAGCCTGGGCAACATTGC   61610
                                                                  ************************************************************

LOC112206721.putative.POM121-like,protein1.START.LOC112206738     aagatcccatatcttaaaaaaaagtaagcaagcaagagaagcagtggggatttaggaga   21332
BCRP3.REF.NEW                                                     aagatcccatatctaaaaaaaaagcaagcaagcaagagaagcagcggggatttaggagg   12505
GGT1.start-POM121L10P.end.24583750-24659147.ref                   AAGATCCCATATCTAAAAAAAAAGCAAGCAAGCAAGAGAAGCAGCGGGGATTTAGGAGG   61670
                                                                  ************** ********* ******************* ************** 

LOC112206721.putative.POM121-like,protein1.START.LOC112206738     tggttctgcagaaaccagtcgtttacatcatcttcaacaatcctggctcttgctgaagta   21392
BCRP3.REF.NEW                                                     tgcttctgcagaaaccagtcgtttatatcatcttcaacaatcctggctcttgctgaagta   12565
GGT1.start-POM121L10P.end.24583750-24659147.ref                   TGCTTCTGCAGAAACCAGTCGTTTATATCATCTTCAACAATCCTGGCTCTTGCTGAAGTA   61730
                                                                  ** ********************** **********************************

LOC112206721.putative.POM121-like,protein1.START.LOC112206738     gactaggggcttccccgaggggcggctccacctcatgctgagacctctgcatgccttggg   21452
BCRP3.REF.NEW                                                     gactaggggcttccccgaggggcggctccacctcatgctgagacctctgcatgccttggg   12625
GGT1.start-POM121L10P.end.24583750-24659147.ref                   GACTAGGGGCTTCCCCGAGGGGCGGCTCCACCTCATGCTGAGACCTCTGCATGCCTTGGG   61790
                                                                  ************************************************************

LOC112206721.putative.POM121-like,protein1.START.LOC112206738     ggtggaaatatttgatgagactcccaggggtccttgggaccttgggctatgaggaccaga   21512
BCRP3.REF.NEW                                                     ggtggaaatatttgatgagactcccaggggtccttgggaccttgggctgtgaggaccaga   12685
GGT1.start-POM121L10P.end.24583750-24659147.ref                   GGTGGAAATATTTGATGAGACTCCCAGGGGTCCTTGGGACCTTGGGCTGTGAGGACCAGA   61850
                                                                  ************************************************ ***********

LOC112206721.putative.POM121-like,protein1.START.LOC112206738     aggattagaggactgtgccccttctccccactgtagatcgaagtaaagctctcggtcaag   21572
BCRP3.REF.NEW                                                     aggattagaggactgtgccccttctccccactgtagatcgaagtaaagctctcggtcaag   12745
GGT1.start-POM121L10P.end.24583750-24659147.ref                   AGGATTAGAGGACTGTGCCCCTTCTCCCCACTGTAGATCGAAGTAAAGCTCTCGGTCAAG   61910
                                                                  ************************************************************

LOC112206721.putative.POM121-like,protein1.START.LOC112206738     ttcaacagcagggagttcagcttgaagaggatgccatcccagaaacagacaggggtcttc   21632
BCRP3.REF.NEW                                                     ttcaacagcagggagttcagcttgaagaggatgccgtcccgaaaacagacaggggtcttc   12805
GGT1.start-POM121L10P.end.24583750-24659147.ref                   TTCAACAGCAGGGAGTTCAGCTTGAAGAGGATGCCGTCCCGAAAACAGACAGGGGTCTTC   61970
                                                                  *********************************** ****  ******************

LOC112206721.putative.POM121-like,protein1.START.LOC112206738     -gagtcaagattgctgtggtcaccaagtgagtggggaggggcttgggctcacgcactgag   21691
BCRP3.REF.NEW                                                     ggagtcaagattgctgtggtcaccaagtgagtggggaggggcttgggctcacgcactgag   12865
GGT1.start-POM121L10P.end.24583750-24659147.ref                   GGAGTCAAGATTGCTGTGGTCACCAAGTGAGTGGGGAGGGGCTTGGGCTCACGCACTGAG   62030
                                                                   ***********************************************************

LOC112206721.putative.POM121-like,protein1.START.LOC112206738     ggtgcctatcccttcagctgtttctgcagaaaagagcatgtgtgggtctctcctctctgt   21751
BCRP3.REF.NEW                                                     ggtgcctgtcccttcagctgtttctgcagaaaagagcatgtgtgggtctctcctctctgt   12925
GGT1.start-POM121L10P.end.24583750-24659147.ref                   GGTGCCTGTCCCTTCAGCTGTTTCTGCAGAAAAGAGCATGTGTGGGTCTCTCCTCTCTGT   62090
                                                                  ******* ****************************************************

LOC112206721.putative.POM121-like,protein1.START.LOC112206738     gcgtggccactgcacggtgaggtcaggccccagggaacactgcgtgttcagctacctcct   21811
BCRP3.REF.NEW                                                     gcatggccactgcacggtgaggtcaggccccagggaacacggcgtcttcagctacctcct   12985
GGT1.start-POM121L10P.end.24583750-24659147.ref                   GCATGGCCACTGCACGGTGAGGTCAGGCCCCAGGGAACACGGCGTCTTCAGCTACCTCCT   62150
                                                                  ** ************************************* **** **************

LOC112206721.putative.POM121-like,protein1.START.LOC112206738     gtgtttcctgcaaaccagctcaggaatgtccttgccaccttgcttggaagcggtaggctg   21871
BCRP3.REF.NEW                                                     gtgtttcctgcaaaccagctcaggaatgtccttgccaccttgcttggaagcagtaggctg   13045
GGT1.start-POM121L10P.end.24583750-24659147.ref                   GTGTTTCCTGCAAACCAGCTCAGGAATGTCCTTGCCACCTTGCTTGGAAGCAGTAGGCTG   62210
                                                                  *************************************************** ********

LOC112206721.putative.POM121-like,protein1.START.LOC112206738     gctccaggaactgcccaagtgcagggttttctgcccttgcttggaattagtcatggtccc   21931
BCRP3.REF.NEW                                                     gctccaggaactgcccaagtgcagggttttctgcccttgcttggaattagtcacggtccc   13105
GGT1.start-POM121L10P.end.24583750-24659147.ref                   GCTCCAGGAACTGCCCAAGTGCAGGGTTTTCTGCCCTTGCTTGGAATTAGTCACGGTCCC   62270
                                                                  ***************************************************** ******

LOC112206721.putative.POM121-like,protein1.START.LOC112206738     agattcctgttgaatgtttgtaacccctgcccctttgtcatgagtcagttgccaagagaa   21991
BCRP3.REF.NEW                                                     agattcctgttgaatggccataacccctgcccctttgtcacgagtcagttgccaagagaa   13165
GGT1.start-POM121L10P.end.24583750-24659147.ref                   AGATTCCTGTTGAATGGCCATAACCCCTGCCCCTTTGTCACGAGTCAGTTGCCAAGAGAA   62330
                                                                  ****************    ******************** *******************

LOC112206721.putative.POM121-like,protein1.START.LOC112206738     gcctgtttcttggtttgagagcagttcatgcagacacagaccacttcctctgagaattta   22051
BCRP3.REF.NEW                                                     gcctgt---ttggtttgagagcagttcatgcagacatagaccacttcctctgagaattca   13222
GGT1.start-POM121L10P.end.24583750-24659147.ref                   GCCTGT---TTGGTTTGAGAGCAGTTCATGCAGACATAGACCACTTCCTCTGAGAATTCA   62387
                                                                  ******   *************************** ********************* *

LOC112206721.putative.POM121-like,protein1.START.LOC112206738     tttgcttccctaggatggaatctggctgggcctctgaccttgctggtcacgtgggccggg   22111
BCRP3.REF.NEW                                                     tttgcttccccaggatggaatctggctgggcctctgaccttgctggtcacgtgggccggg   13282
GGT1.start-POM121L10P.end.24583750-24659147.ref                   TTTGCTTCCCCAGGATGGAATCTGGCTGGGCCTCTGACCTTGCTGGTCACGTGGGCCGGG   62447
                                                                  ********** *************************************************

LOC112206721.putative.POM121-like,protein1.START.LOC112206738     gcctccattagtcatacctggactcctatctgtgtctaaacaccacgccccacccccaa   22171
BCRP3.REF.NEW                                                     gcctccatcagtcatacctggactcctatctgtgtctaaacaccacgccccacccccaa   13342
GGT1.start-POM121L10P.end.24583750-24659147.ref                   GCCTCCATCAGTCATACCCTGGACTCCTATCTGTGTCTAAACACCACGCCCCACCCCCAA   62507
                                                                  ******** ***************************************************

LOC112206721.putative.POM121-like,protein1.START.LOC112206738     ctgcatggcagccactcgcatagcactctgggagggctgtgggcatgagcagcgaggact   22231
BCRP3.REF.NEW                                                     ctgcacggcagccactcgcatagcactctgggagggctgtgggcatgagcagcgaggact   13402
GGT1.start-POM121L10P.end.24583750-24659147.ref                   CTGCACGGCAGCCACTCGCATAGCACTCTGGGAGGGCTGTGGGCATGAGCAGCGAGGACT   62567
                                                                  ***** ******************************************************

LOC112206721.putative.POM121-like,protein1.START.LOC112206738     ccatcagcagctcccccagataagccctgctaatgagggggcttgcgaagcagctttgat   22291
BCRP3.REF.NEW                                                     ccatgagcagctcccccagataagccctgctaatgagggggcttgcgaagcagctttgat   13462
GGT1.start-POM121L10P.end.24583750-24659147.ref                   CCATGAGCAGCTCCCCCAGATAAGCCCTGCTAATGAGGGGGCTTGCGAAGCAGCTTTGAT   62627
                                                                  **** *******************************************************

LOC112206721.putative.POM121-like,protein1.START.LOC112206738     gtgctggtaaatccaggtgcaaaacagaactcaagttagggcctctgcacagcactgcgt   22351
BCRP3.REF.NEW                                                     gtgctggtaaatccaggtgcaaaacagaactcaagttagggcctccgcacagcactgcgt   13522
GGT1.start-POM121L10P.end.24583750-24659147.ref                   GTGCTGGTAAATCCAGGTGCAAAACAGAACTCAAGTTAGGGCCTCCGCACAGCACTGCGT   62687
                                                                  ********************************************* **************

LOC112206721.putative.POM121-like,protein1.START.LOC112206738     tctaactgtgaaggattcttactctagtgtcctgtgtggaggtattggaattgtccattg   22411
BCRP3.REF.NEW                                                     tctaactgtgaaggattcttactctagtgtcctgtgtggaggtattggaattgtccattg   13582
GGT1.start-POM121L10P.end.24583750-24659147.ref                   TCTAACTGTGAAGGATTCTTACTCTAGTGTCCTGTGTGGAGGTATTGGAATTGTCCATTG   62747
                                                                  ************************************************************

LOC112206721.putative.POM121-like,protein1.START.LOC112206738     ctaagactcagaggagaaaagcacttagcatcgcaggacttggagcaccggtgctgaggc   22471
BCRP3.REF.NEW                                                     ctaagactcagaggagaaaagcacttagcatcgcaggacttggagcaccggtgctgaggc   13642
GGT1.start-POM121L10P.end.24583750-24659147.ref                   CTAAGACTCAGAGGAGAAAAGCACTTAGCATCGCAGGACTTGGAGCACCGGTGCTGAGGC   62807
                                                                  ************************************************************

LOC112206721.putative.POM121-like,protein1.START.LOC112206738     aacccttcattcattcgtcggatgtgtgttaaggcccagggcaggggtcagggattctcc   22531
BCRP3.REF.NEW                                                     aacccttcattcattcgtcggatgtgtgttaaggcccagggcaggggtcagggattctcc   13702
GGT1.start-POM121L10P.end.24583750-24659147.ref                   AACCCTTCATTCATTCGTCGGATGTGTGTTAAGGCCCAGGGCAGGGGTCAGGGATTCTCC   62867
                                                                  ************************************************************

LOC112206721.putative.POM121-like,protein1.START.LOC112206738     tctcacacagcacgtgggtggcaggaccaacaccgggtctgatctcccagccgggggcac   22591
BCRP3.REF.NEW                                                     tctcacacagcacgtgggtggcaggaccaacaccgggtctgacctcccagccgggggcac   13762
GGT1.start-POM121L10P.end.24583750-24659147.ref                   TCTCACACAGCACGTGGGTGGCAGGACCAACACCGGGTCTGACCTCCCAGCCGGGGGCAC   62927
                                                                  ****************************************** *****************

LOC112206721.putative.POM121-like,protein1.START.LOC112206738     aggctgctaaccccaggcctggaatctgtcagatgcccttcctgtgctgacttgacttag   22651
BCRP3.REF.NEW                                                     aggctgctaaccccaggcctggaatctgtcagatgcccttcctgtgctgacttgacttag   13822
GGT1.start-POM121L10P.end.24583750-24659147.ref                   AGGCTGCTAACCCCAGGCCTGGAATCTGTCAGATGCCCTTCCTGTGCTGACTTGACTTAG   62987
                                                                  ************************************************************

LOC112206721.putative.POM121-like,protein1.START.LOC112206738     acaggcctcctgaccttcccgcaaaggtcatgtgtgattcgcaggggttctggccgcttg   22711
BCRP3.REF.NEW                                                     acaggcctcctgaccttcccgcaaaggtcatgtgtgattcgcaggggttctggccgcttg   13882
GGT1.start-POM121L10P.end.24583750-24659147.ref                   ACAGGCCTCCTGACCTTCCCGCAAAGGTCATGTGTGATTCGCAGGGGTTCTGGCCGCTTG   63047
                                                                  ************************************************************

LOC112206721.putative.POM121-like,protein1.START.LOC112206738     aaaggttcctgagaaagtacatgcaatgaggacagagcttgcagagggaggacaggcatg   22771
BCRP3.REF.NEW                                                     aaaggttcctgagaaagcacatgccatgaggacagagcttgcagagggaggacaggcatg   13942
```

```
GGT1.start-POM121L10P.end.24583750-24659147.ref                 AAAGGTTCCTGAGAAAGCACATGCCATGAGGACAGAGCTTGCAGAGGGAGGACAGGCATG   63107
                                                                ***************** ****** ***********************************

LOC112206721.putative.POM121-like,protein1.START.LOC112206738   cagaaggctctgtgtgcagcgccagacctgggtaccttcgtcaccgtcctcaccccacct   22831
BCRP3.REF.NEW                                                   cagaaggctctgtgtgcagccccagacctgggtaccttcgtcaccgtcctcaccccacct   14002
GGT1.start-POM121L10P.end.24583750-24659147.ref                 CAGAAGGCTCTGTGTGCAGCCCCAGACCTGGGTACCTTCGTCACCGTCCTCACCCCACCT   63167
                                                                ******************** ***************************************

LOC112206721.putative.POM121-like,protein1.START.LOC112206738   ccgggtgtgcagatagggagcaggtctcctgtgttatggcccaagcagggctgttaggac   22891
BCRP3.REF.NEW                                                   ccgggtgtgcagatagggagcaggcctcctgtgttatggcccaagcggggctgttaggac   14062
GGT1.start-POM121L10P.end.24583750-24659147.ref                 CCGGGTGTGCAGATAGGGAGCAGGCCTCCTGTGTTATGGCCCAAGCGGGGCTGTTAGGAC   63227
                                                                ************************ ********************* *************

LOC112206721.putative.POM121-like,protein1.START.LOC112206738   actgagagcattccctcctcccgcaggagagagaggtccaaggtgccctacatcgtgcac   22951
BCRP3.REF.NEW                                                   actgagaacattccctcctcccgcaggagagagaggtccaaggtgccctacatcatgcgc   14122
GGT1.start-POM121L10P.end.24583750-24659147.ref                 ACTGAGAACATTCCCTCCTCCCGCAGGAGAGAGAGGTCCAAGGTGCCCTACATCATGCGC   63287
                                                                ******* ********************************************** *** *

LOC112206721.putative.POM121-like,protein1.START.LOC112206738   cagtgcgtggaggagatcgagcgccgaggcatggaggaggtgggcatctaccgcgtgtcc   23011
BCRP3.REF.NEW                                                   cagtgcgtggaggagatcgagcgccgaggcatggaggaggtgggcatctaccgcgtgtcc   14182
GGT1.start-POM121L10P.end.24583750-24659147.ref                 CAGTGCGTGGAGGAGATCGAGCGCCGAGGCATGGAGGAGGTGGGCATCTACCGCGTGTCC   63347
                                                                ************************************************************

LOC112206721.putative.POM121-like,protein1.START.LOC112206738   ggccagtgtggccgcagacatccaggcactgaaggcagccttcgacgccagtgagtgtcg   23071
BCRP3.REF.NEW                                                   g----gtgtggccacggacatccaggcactgaaggcaggcttcaacgtcagtgagtgtcg   14238
GGT1.start-POM121L10P.end.24583750-24659147.ref                 G----GTGTGGCCACGGACATCCAGGCACTGAAGGCAGGCTTCAACGTCAGTGAGTGTCG   63403
                                                                *    ******** * ********************** **** *** ************

LOC112206721.putative.POM121-like,protein1.START.LOC112206738   gcctgcgcaggacgggatggaggtgtgggcagtggtgtccgcaatgagatctcagagtgc   23131
BCRP3.REF.NEW                                                   gcctgcgcaggacgggatggaggtgtgggcagtggtgtccgcgatgagatctcagagtgc   14298
GGT1.start-POM121L10P.end.24583750-24659147.ref                 GCCTGCGCAGGACGGGATGGAGGTGTGGGCAGTGGTGTCCGCGATGAGATCTCAGAGTGC   63463
                                                                ****************************************** *****************

LOC112206721.putative.POM121-like,protein1.START.LOC112206738   tccatggcccaggcatgtcacatccttctctgtgtcttttcttcatttactgttttatta   23191
BCRP3.REF.NEW                                                   tccatggcccaggcatgtcacatccttctctgtgtcttttcttcatttactgttttatta   14358
GGT1.start-POM121L10P.end.24583750-24659147.ref                 TCCATGGCCCAGGCATGTCACATCCTTCTCTGTGTCTTTTCTTCATTTACTGTTTTATTA   63523
                                                                ************************************************************

LOC112206721.putative.POM121-like,protein1.START.LOC112206738   ttttaaaaaaagagaaaacaagagttgtacaaacagcttccatagaagccagtttttaca   23251
BCRP3.REF.NEW                                                   ttttaaaaaaagagaaaacaagagttgtacaaacagcttctatagaagccagtttttaca   14418
GGT1.start-POM121L10P.end.24583750-24659147.ref                 TTTTAAAAAAAGAGAAAACAAGAGTTGTACAAACAGCTTCTATAGAAGCCAGTTTTTACA   63583
                                                                *************************************** ********************

LOC112206721.putative.POM121-like,protein1.START.LOC112206738   ccatcgtacccactcatgccacttggtggagtggaccaggggcttctgtggggacttggc   23311
BCRP3.REF.NEW                                                   ccatcgtacccactcatgccacttggtggagtggaccaggggcttctgtggggacttggc   14478
GGT1.start-POM121L10P.end.24583750-24659147.ref                 CCATCGTACCCACTCATGCCACTTGGTGGAGTGGACCAGGGGCTTCTGTGGGGACTTGGC   63643
                                                                ************************************************************

LOC112206721.putative.POM121-like,protein1.START.LOC112206738   cttcctgccttggggggtggacaggaggtggaagcccaggactcagtgcggtctgtccact   23371
BCRP3.REF.NEW                                                   cttcctgccttggggggtggacaggaggtggaagcccaggactcagtgcggtctgtccact   14538
GGT1.start-POM121L10P.end.24583750-24659147.ref                 CTTCCTGCCTTGGGGGGTGGACAGGAGGTGGAAGCCCAGGACTCAGTGCGGTCTGTCCACT   63703
                                                                ************************************************************

LOC112206721.putative.POM121-like,protein1.START.LOC112206738   gccctgtatgaggatgtggtgggcagagggcactgatgaaattcagcgcaggctggggct   23431
BCRP3.REF.NEW                                                   gccctgtatgaggatgtggtgggcagagggcactgatgaaattcagcgcaggccggggct   14598
GGT1.start-POM121L10P.end.24583750-24659147.ref                 GCCCTGTATGAGGATGTGGTGGGCAGAGGGCACTGATGAAATTCAGCGCAGGCCGGGGCT   63763
                                                                ***************************************************** ******

LOC112206721.putative.POM121-like,protein1.START.LOC112206738   gcagcatctccgcctccatctcaccaaccctcacaggctttgaaggacccagacctgcct   23491
BCRP3.REF.NEW                                                   gcagcatctccgcctccatctcaccaaccctcacaggccttgaaggacccagactggcct   14658
GGT1.start-POM121L10P.end.24583750-24659147.ref                 GCAGCATCTCCGCCTCCATCTCACCAACCCTCACAGGCCTTGAAGGACCCAGACTGGCCT   63823
                                                                ************************************** ***************  ****

LOC112206721.putative.POM121-like,protein1.START.LOC112206738   caaatgccaggggagggcactgagaccccagagggtccttcccagcatcttcaaagcaac   23551
BCRP3.REF.NEW                                                   caaatgccaggggagggcactgagaccccagagggtccttcccagcatcttcaaagcaac   14718
GGT1.start-POM121L10P.end.24583750-24659147.ref                 CAAATGCCAGGGGAGGGCACTGAGACCCCAGAGGGTCCTTCCCAGCATCTTCAAAGCAAC   63883
                                                                ************************************************************

LOC112206721.putative.POM121-like,protein1.START.LOC112206738   aggattttgtgcctgcagacccttctttggggcacacaccacccaccctgaccaggaccc   23611
BCRP3.REF.NEW                                                   aggattttgtgcctgcagacccttctttgcagcacacaccacccaccctgaccaggaccc   14778
GGT1.start-POM121L10P.end.24583750-24659147.ref                 AGGATTTTGTGCCTGCAGACCCTTCTTTGCAGCACACACCACCCACCCTGACCAGGACCC   63943
                                                                *****************************  *****************************

LOC112206721.putative.POM121-like,protein1.START.LOC112206738   ctagaatgcccagcatccctgggagggccctgtggtagtttcagctccctctggggtcc   23671
BCRP3.REF.NEW                                                   ctagaatgcccagcatccctgggagggccctgtggtagtttcagctccctctgggggccc   14838
GGT1.start-POM121L10P.end.24583750-24659147.ref                 CTAGAATGCCCAGCATCCCTGGGAGGGCCCTGTGGTAGTTTCAGCTCCCTCTGGGGGCCC   64003
                                                                ********************************************************* **

LOC112206721.putative.POM121-like,protein1.START.LOC112206738   agaatgaacctcgcctgtggtgaggatgtaagcaccaatggccaattgggtccaaaggaa   23731
BCRP3.REF.NEW                                                   agaatgaacctggcctgtggtgaggatgtaagcaccaatggccaattgggtccaaaggaa   14898
GGT1.start-POM121L10P.end.24583750-24659147.ref                 AGAATGAACCTGGCCTGTGGTGAGGATGTAAGCACCAATGGCCAATTGGGTCCAAAGGAA   64063
                                                                *********** ************************************************

LOC112206721.putative.POM121-like,protein1.START.LOC112206738   gacaccggttcaaacactgaaaccaatcagattctcccacggccttcctgctatcagaag   23791
BCRP3.REF.NEW                                                   gacaccggttcaaacactgaaaccaatcagattctcccacggccttcctgctatcagacg   14958
GGT1.start-POM121L10P.end.24583750-24659147.ref                 GACACCGGTTCAAACACTGAAACCAATCAGATTCTCCCACGGCCTTCCTGCTATCAGACG   64123
                                                                ********************************************************** *

LOC112206721.putative.POM121-like,protein1.START.LOC112206738   acactggtgcaggggtggttgctatgtacagggcagagccacccaatccccatgcaggcg   23851
BCRP3.REF.NEW                                                   acactggtgcaggggtggttgctatgtacagggcagagccacccaatccccacgcaggcg   15018
GGT1.start-POM121L10P.end.24583750-24659147.ref                 ACACTGGTGCAGGGGTGGTTGCTATGTACAGGGCAGAGCCACCCAATCCCCACGCAGGCG   64183
                                                                **************************************************** *******

LOC112206721.putative.POM121-like,protein1.START.LOC112206738   ctgtgtcctgccatgctggcctcctcctggccatcacatcaggccaagcaggggagagga   23911
BCRP3.REF.NEW                                                   ctgtgtcctgccacgttggcctcctcctggccatcacatcaggccaagcaggggagagga   15078
GGT1.start-POM121L10P.end.24583750-24659147.ref                 CTGTGTCCTGCCACGTTGGCCTCCTCCTGGCCATCACATCAGGCCAAGCAGGGGAGAGGA   64243
                                                                ************* * ********************************************

LOC112206721.putative.POM121-like,protein1.START.LOC112206738   atgggaatgcccacgcacccctatcaactctgcagacacagaaccatgcacagctcttgg   23971
BCRP3.REF.NEW                                                   atgggaatgcccacgcacccctatcaactctgcagacacagaaccatgcacagctcttgg   15138
GGT1.start-POM121L10P.end.24583750-24659147.ref                 ATGGGAATGCCCACGCACCCCTATCAACTCTGCAGACACAGAACCATGCACAGCTCTTGG   64303
                                                                ************************************************************

LOC112206721.putative.POM121-like,protein1.START.LOC112206738   gaggagtcagatgagctgctcaaagcccaggagggacccacacagtggtcagcatggcag   24031
BCRP3.REF.NEW                                                   gaggagtcagatgagctgctcaaagcccaggagggacccgcacagtggtcagtgtggcag   15198
GGT1.start-POM121L10P.end.24583750-24659147.ref                 GAGGAGTCAGATGAGCTGCTCAAAGCCCAGGAGGGACCCGCACAGTGGTCAGTGTGGCAG   64363
                                                                *************************************** ************  ******

LOC112206721.putative.POM121-like,protein1.START.LOC112206738   ggacggtgctttagccaaggcagggatggcgggtgactcactcaggatcttcaaggaggc   24091
BCRP3.REF.NEW                                                   ggacggtgctttagccaaggcagggatggtgggtgactcactcaggatcttcaaggaggc   15258
GGT1.start-POM121L10P.end.24583750-24659147.ref                 GGACGGTGCTTTAGCCAAGGCAGGGATGGTGGGTGACTCACTCAGGATCTTCAAGGAGGC   64423
                                                                **************************** *******************************

LOC112206721.putative.POM121-like,protein1.START.LOC112206738   cgctgcatttccctgctctttccagataacaaggacgtgtcggtgatgatgagcgagacg   24151
BCRP3.REF.NEW                                                   cgctgcatttccgtgctctttccagataacaaggacgtgtcggtgatgatgagcgagatg   15318
GGT1.start-POM121L10P.end.24583750-24659147.ref                 CGCTGCATTTCCGTGCTCTTTCCAGATAACAAGGACGTGTCGGTGATGATGAGCGAGATG   64483
                                                                ************ ********************************************* *

LOC112206721.putative.POM121-like,protein1.START.LOC112206738   gacatgaacgccatcgcaggcacgctgaagctgtacttccgtgagctgcccgagcccctc   24211
BCRP3.REF.NEW                                                   gacgtgaacgccatcgcaggcacgctgaagctgtacttccgtgagctgcccgagcccctc   15378
GGT1.start-POM121L10P.end.24583750-24659147.ref                 GACGTGAACGCCATCGCAGGCACGCTGAAGCTGTACTTCCGTGAGCTGCCCGAGCCCCTC   64543
                                                                *** ********************************************************

LOC112206721.putative.POM121-like,protein1.START.LOC112206738   ttcactgacaagttctaccccaacttcgcagagggcatcggtgagcactggaggccttgg   24271
BCRP3.REF.NEW                                                   ttcactgacgagttctaccccaacttcgcagagggcatcggtgagcactggaggccttgg   15438
GGT1.start-POM121L10P.end.24583750-24659147.ref                 TTCACTGACGAGTTCTACCCCAACTTCGCAGAGGGCATCGGTGAGCACTGGAGGCCTTGG   64603
                                                                ********* **************************************************

LOC112206721.putative.POM121-like,protein1.START.LOC112206738   cctcatgggagacgtctccgccacgtgcactgctgccctcagaggctgtgaaaagcgagg   24331
BCRP3.REF.NEW                                                   cctcatgggagacgtctcctccacgtgcactgctgccctcggaggctgtgaaaagcgagg   15498
GGT1.start-POM121L10P.end.24583750-24659147.ref                 CCTCATGGGAGACGTCTCCTCCACGTGCACTGCTGCCCTCGGAGGCTGTGAAAAGCGAGG   64663
                                                                ******************* ******************** *******************

LOC112206721.putative.POM121-like,protein1.START.LOC112206738   tgtgggaacctgagctgtaacccctctgccatggtcagaattttaacccaacctcaaaaa   24391
BCRP3.REF.NEW                                                   tgtgggaacctgagctgtaacccctctgccgtggtcggcattttaacccaacctcaaaaa   15558
GGT1.start-POM121L10P.end.24583750-24659147.ref                 TGTGGGAACCTGAGCTGTAACCCCTCTGCCGTGGTCGGCATTTTAACCCAACCTCAAAAA   64723
                                                                ****************************** ***** * *********************

LOC112206721.putative.POM121-like,protein1.START.LOC112206738   gcaggggaccagaaccgagcctgtcctggaaggccttgcccatccccagagggctcccca   24451
BCRP3.REF.NEW                                                   gcaggggaccagaaccgagcctgtcctggaaggccttgcccatccccagagggctcccca   15618
GGT1.start-POM121L10P.end.24583750-24659147.ref                 GCAGGGGACCAGAACCGAGCCTGTCCTGGAAGGCCTTGCCCATCCCCAGAGGGCTCCCCA   64783
                                                                ************************************************************

LOC112206721.putative.POM121-like,protein1.START.LOC112206738   tccctattcctcaaggaggccaagtgggtgaaatggtcagcactgccgtgctgtggggtc   24511
BCRP3.REF.NEW                                                   tccctactcctcaaggagaccaagaggctgaaatagtcagcactgctgtgctatggggtc   15678
```

```
GGT1.start-POM121L10P.end.24583750-24659147.ref                      TCCCTACTCCTCAAGGAGACCAAGAGGCTGAAATAGTCAGCACTGCTGTGCTATGGGGTC   64843
                                                                     ****** *********** ***** ** ****** *********** ***** *******

LOC112206721.putative.POM121-like,protein1.START.LOC112206738        ctaaagtctgctgtcctccttcctgcagaccagggctaaacactggtgcccaggtgctct   24571
BCRP3.REF.NEW                                                        ctaaagtctgctgtcctccttcctgcagaccagggctgaaggagggtgcctgggtgctct   15738
GGT1.start-POM121L10P.end.24583750-24659147.ref                      CTAAAGTCTGCTGTCCTCCTTCCTGCAGACCAGGGCTGAAGGAGGGTGCCTGGGTGCTCT   64903
                                                                     ************************************* **    ******  ********

LOC112206721.putative.POM121-like,protein1.START.LOC112206738        tgccatgggtcctggtccagccaagcatggtttcaaacatgacctgacccttagtcaacc   24631
BCRP3.REF.NEW                                                        tgccatgggtcctggtccagccaagcatggtttcaaacatgacctgacccttagtcaacc   15798
GGT1.start-POM121L10P.end.24583750-24659147.ref                      TGCCATGGGTCCTGGTCCAGCCAAGCATGGTTTCAAACATGACCTGACCCTTAGTCAACC   64963
                                                                     ************************************************************

LOC112206721.putative.POM121-like,protein1.START.LOC112206738        tggaggctgatgtctagagtgggtgctggtgcgtgcagcacctgtggcctctgcatcacc   24691
BCRP3.REF.NEW                                                        tggaggctgatgtctagagcgggtgctggtgcgtgcagcacctgtggcctctgcatcacc   15858
GGT1.start-POM121L10P.end.24583750-24659147.ref                      TGGAGGCTGATGTCTAGAGCGGGTGCTGGTGCGTGCAGCACCTGTGGCCTCTGCATCACC   65023
                                                                     ******************* ****************************************

LOC112206721.putative.POM121-like,protein1.START.LOC112206738        cttagggcaggtctgcctcccgggcccatgcacagaggacctggtctcccagcctgcagg   24751
BCRP3.REF.NEW                                                        cttagggcaggtctgcctcccgggcccatgcacagaggacctggtctcccagcctgcagg   15918
GGT1.start-POM121L10P.end.24583750-24659147.ref                      CTTAGGGCAGGTCTGCCTCCCGGGCCCATGCACAGAGGACCTGGTCTCCCAGCCTGCAGG   65083
                                                                     ************************************************************

LOC112206721.putative.POM121-like,protein1.START.LOC112206738        tgtccctgtgatgtccaggacgacgaggggatctctgcgtacttggtggggctgggactc   24811
BCRP3.REF.NEW                                                        tgcccctgtggtgtccaggacgacgagggggtctctgtgtacttggtggggctgggaccc   15978
GGT1.start-POM121L10P.end.24583750-24659147.ref                      TGCCCCTGTGGTGTCCAGGACGACGAGGGGGTCTCTGTGTACTTGGTGGGGCTGGGACCC   65143
                                                                     ** ******* ******************* ****** ******************** *

LOC112206721.putative.POM121-like,protein1.START.LOC112206738        tcccacttcccacctccttgtgtccctcactcccctgtttcattccatgctgagcctccc   24871
BCRP3.REF.NEW                                                        tcccacttcccacctccttgtgtccctcactcccctgtttcattccatgctgagcctccc   16038
GGT1.start-POM121L10P.end.24583750-24659147.ref                      TCCCACTTCCCACCTCCTTGTGTCCCTCACTCCCCTGTTTCATTCCATGCTGAGCCTCCC   65203
                                                                     ************************************************************

LOC112206721.putative.POM121-like,protein1.START.LOC112206738        ctgccttgggctcctctggggagggggtggtggcaggagttgtccaagggcagctctgcc   24931
BCRP3.REF.NEW                                                        ctgccttgggctcc-ctggggagggggtggtggcaggagttgcccgagggcagctctgcc   16097
GGT1.start-POM121L10P.end.24583750-24659147.ref                      CTGCCTTGGGCTCC-CTGGGGAGGGGGTGGTGGCAGGAGTTGCCCGAGGGCAGCTCTGCC   65262
                                                                     ************** *************************** ** **************

LOC112206721.putative.POM121-like,protein1.START.LOC112206738        cacgagcagctgctctagcggctcctcctgctgctgttcaccgggtgctgctgacccctg   24991
BCRP3.REF.NEW                                                        catgagcagctgctctagcggctcctcctgctgctgttcgccgggtgctgctgacccctg   16157
GGT1.start-POM121L10P.end.24583750-24659147.ref                      CATGAGCAGCTGCTCTAGCGGCTCCTCCTGCTGCTGTTCGCCGGGTGCTGCTGACCCCTG   65322
                                                                     ** ************************************ ********************

LOC112206721.putative.POM121-like,protein1.START.LOC112206738        cgaggtagagaaaaggcgttcaggtggttcacaccccacacaggtgcccctcacagggtc   25051
BCRP3.REF.NEW                                                        cgaggtagagaaaaggcgttcaggtggttcacaccccacacaggtgcccctcacagggtc   16217
GGT1.start-POM121L10P.end.24583750-24659147.ref                      CGAGGTAGAGAAAAGGCGTTCAGGTGGTTCACACCCCACACAGGTGCCCCTCACAGGGTC   65382
                                                                     ************************************************************

LOC112206721.putative.POM121-like,protein1.START.LOC112206738        ctcactggtggccagcgctgtgggtgtgacgatgatgacaagcctaaactgcacaaggac   25111
BCRP3.REF.NEW                                                        ctcactggcggccagcgctgtgggtgtgacgatgatgacaagcctaaactgcgcaaggac   16277
GGT1.start-POM121L10P.end.24583750-24659147.ref                      CTCACTGGCGGCCAGCGCTGTGGGTGTGACGATGATGACAAGCCTAAACTGCGCAAGGAC   65442
                                                                     ******** ******************************************* *******

LOC112206721.putative.POM121-like,protein1.START.LOC112206738        tcgtgtcccgggtgctccatgtgaccacctcgggagaggtctccggcttgtcgtaaccca   25171
BCRP3.REF.NEW                                                        tcgtgtcccgggcgctccatgtgaccacctcgggagaggtctccggcttgtcgtaaccca   16337
GGT1.start-POM121L10P.end.24583750-24659147.ref                      TCGTGTCCCGGGCGCTCCATGTGACCACCTCGGGAGAGGTCTCCGGCTTGTCGTAACCCA   65502
                                                                     ************ ***********************************************

LOC112206721.putative.POM121-like,protein1.START.LOC112206738        ggggagtaacacactgcctcctgcagctctttcagacccagttgcaaagaagagctgcat   25231
BCRP3.REF.NEW                                                        ggggagtgacccactgcctcctgcagctctttcagacccagttgcaaggaagagctgcat   16397
GGT1.start-POM121L10P.end.24583750-24659147.ref                      GGGGAGTGACCCACTGCCTCCTGCAGCTCTTTCAGACCCAGTTGCAAGGAAGAGCTGCAT   65562
                                                                     ******* ** ************************************* ***********

LOC112206721.putative.POM121-like,protein1.START.LOC112206738        gttcaacc---tgttgtccctgccggaggccaacctgctcaccttccttttccttctaga   25288
BCRP3.REF.NEW                                                        gctcaacctgttgttgtccctgccggaggccaacctgctcaccttccttttccttctaga   16457
GGT1.start-POM121L10P.end.24583750-24659147.ref                      GCTCAACCTGTTGTTGTCCCTGCCGGAGGCCAACCTGCTCACCTTCCTTTTCCTTCTAGA   65622
                                                                     * ******   *************************************************

LOC112206721.putative.POM121-like,protein1.START.LOC112206738        ccacctggaaaggtagcccagctctcttgtggctgcccaggactccaggtctccaggccg   25348
BCRP3.REF.NEW                                                        ccacctggaaaggtagcccagctctcttgtggctgcccaggactccaggtctccaggccg   16517
GGT1.start-POM121L10P.end.24583750-24659147.ref                      CCACCTGGAAAGGTAGCCCAGCTCTCTTGTGGCTGCCCAGGACTCCAGGTCTCCAGGCCG   65682
                                                                     ************************************************************

LOC112206721.putative.POM121-like,protein1.START.LOC112206738        ctggggtgcccctctgctcccaccagacccccagcaccaaggaccttttcccccgacccc   25408
BCRP3.REF.NEW                                                        ttggggtgcccctctgctcccaccagacccccagcaccaaggaccttttcccccgacccc   16577
GGT1.start-POM121L10P.end.24583750-24659147.ref                      TTGGGGTGCCCCTCTGCTCCCACCAGACCCCCAGCACCAAGGACCTTTTCCCCCGACCCC   65742
                                                                      ***********************************************************

LOC112206721.putative.POM121-like,protein1.START.LOC112206738        tgtctgcagtaactcactgcttctaaggactagcaccactgccacccccaccactgcctc   25468
BCRP3.REF.NEW                                                        tgtctgcagtaactcactgcttctaaggactagcaccactgccacccccacccctgcctc   16637
GGT1.start-POM121L10P.end.24583750-24659147.ref                      TGTCTGCAGTAACTCACTGCTTCTAAGGACTAGCACCACTGCCACCCCCACCCCTGCCTC   65802
                                                                     **************************************************** *******

LOC112206721.putative.POM121-like,protein1.START.LOC112206738        tcctctttgccaccctcctccctctgcactgtggccttaacaaagagctcagagctttgg   25528
BCRP3.REF.NEW                                                        tcctctttgccaccctcctccctctgcactgtggccttaacaaagagctcagagctttgg   16697
GGT1.start-POM121L10P.end.24583750-24659147.ref                      TCCTCTTTGCCACCCTCCTCCCTCTGCACTGTGGCCTTAACAAAGAGCTCAGAGCTTTGG   65862
                                                                     ************************************************************

LOC112206721.putative.POM121-like,protein1.START.LOC112206738        ccgtggccagcagtgcacttggacccgcctcttccctcccaagcacatcatgaagacctc   25588
BCRP3.REF.NEW                                                        ccgtggccagcagtgcacttggaccccccctcttccctcccaagcacatcatgaagacctc   16757
GGT1.start-POM121L10P.end.24583750-24659147.ref                      CCGTGGCCAGCAGTGCACTTGGACCCCCCTCTTCCCTCCCAAGCACATCATGAAGACCTC   65922
                                                                     ************************** *********************************

LOC112206721.putative.POM121-like,protein1.START.LOC112206738        cccatcagcccagagctggccccttgtcccggccactgagacccagaagtaccaaggct   25648
BCRP3.REF.NEW                                                        cccatcagcccagagctggccccttgtcctggccactgagacccagaagtaccaaggct   16817
GGT1.start-POM121L10P.end.24583750-24659147.ref                      CCCATCAGCCCAGAGCTGGCCCCTTGTCCTGGCCACTGAGACCCAGAAGTACCAAGGCT   65982
                                                                     ***************************** ******************************

LOC112206721.putative.POM121-like,protein1.START.LOC112206738        ggagtcagcttgcagcacagccagggtcgaggtcactccctccctgaggactctagcacg   25708
BCRP3.REF.NEW                                                        ggagtcagcttgcagcacagccagggtcgaggtcactccctccctgaggactctagcacg   16877
GGT1.start-POM121L10P.end.24583750-24659147.ref                      GGAGTCAGCTTGCAGCACAGCCAGGGTCGAGGTCACTCCCTCCCTGAGGACTCTAGCACG   66042
                                                                     ************************************************************

LOC112206721.putative.POM121-like,protein1.START.LOC112206738        gcacagcccctctgcctctctcctggtggtggcgttgaaacagcaccctctgctttggtc   25768
BCRP3.REF.NEW                                                        gcacagcccctctgcctctctcctggtggtggcgttgaaacagcaccctctgcttcggtc   16937
GGT1.start-POM121L10P.end.24583750-24659147.ref                      GCACAGCCCCTCTGCCTCTCTCCTGGTGGTGGCGTTGAAACAGCACCCTCTGCTTCGGTC   66102
                                                                     ******************************************************* ****

LOC112206721.putative.POM121-like,protein1.START.LOC112206738        ctctacagggtggcagagaaggaggtggtcaataagatgtccctgcacaacctcgccact   25828
BCRP3.REF.NEW                                                        ctctacagggtggcagagaaggaggcggtcaataaggtgtccctgcacaacctcgccact   16997
GGT1.start-POM121L10P.end.24583750-24659147.ref                      CTCTACAGGGTGGCAGAGAAGGAGGCGGTCAATAAGGTGTCCCTGCACAACCTCGCCACT   66162
                                                                     ************************* ********** ***********************

LOC112206721.putative.POM121-like,protein1.START.LOC112206738        gtctttggccccacgctgctccggccctccgagaaggagagcaagctccctgccaacccc   25888
BCRP3.REF.NEW                                                        gtctttggccccacgctgctccggccctccgagaaggagagcaagctccctgccaacccc   17057
GGT1.start-POM121L10P.end.24583750-24659147.ref                      GTCTTTGGCCCCACGCTGCTCCGGCCCTCCGAGAAGGAGAGCAAGCTCCCTGCCAACCCC   66222
                                                                     ************************************************************

LOC112206721.putative.POM121-like,protein1.START.LOC112206738        agccagcccatcaccatgactgacagcaggtccttggaggtcatgtcccaggtatgggaa   25948
BCRP3.REF.NEW                                                        agccagcctgtcaccatgactgacagcaggtccttggaggtcatgtctcaggtatgggaa   17117
GGT1.start-POM121L10P.end.24583750-24659147.ref                      AGCCAGCCTGTCACCATGACTGACAGCAGGTCCTTGGAGGTCATGTCTCAGGTATGGGAA   66282
                                                                     ********  ************************************* ************

LOC112206721.putative.POM121-like,protein1.START.LOC112206738        gacagtctccagcccatgcaaccccagcctgacagaggtggcctctgcctgccccacccc   26008
BCRP3.REF.NEW                                                        gacagtctccagcccatgcaaccccagcctgacagaggtggcctctgcctgccccacccc   17177
GGT1.start-POM121L10P.end.24583750-24659147.ref                      GACAGTCTCCAGCCCATGCAACCCCAGCCTGACAGAGGTGGCCTCTGCCTGCCCCACCCC   66342
                                                                     ************************************************************

LOC112206721.putative.POM121-like,protein1.START.LOC112206738        cagtcctgcccatcttccaacttgcattgtatgtggtggtggctgagattcagagacagg   26068
BCRP3.REF.NEW                                                        cagtcctgcccatcttccgacttgcattgtatgtggtggtggctgagattcagagagagg   17237
GGT1.start-POM121L10P.end.24583750-24659147.ref                      CAGTCCTGCCCATCTTCCGACTTGCATTGTATGTGGTGGTGGCTGAGATTCAGAGAGAGG   66402
                                                                     ****************** ************************************* ***

LOC112206721.putative.POM121-like,protein1.START.LOC112206738        gacttgcctaggtttgcatggatgggagtgataggggtgcccaggccacctcctggtcc   26128
BCRP3.REF.NEW                                                        gacttgcctaggtttgcatggatgggagtgataggggtgcccaggccacctcctggtcc   17297
GGT1.start-POM121L10P.end.24583750-24659147.ref                      GACTTGCCTAGGTTTGCATGGATGGGAGTGATAGGGGTGCCCAGGCCACCTCCTGGTCC   66462
                                                                     ************************************************************

LOC112206721.putative.POM121-like,protein1.START.LOC112206738        tgct-gtgcaccttgctgggggcttaaaaccaccccaagtgttcgggtgtggtggctcat   26187
BCRP3.REF.NEW                                                        tgctggtgcaccttgctgggggcttaaaaccaccccaagtgttcgggtgtggtggctcat   17357
GGT1.start-POM121L10P.end.24583750-24659147.ref                      TGCTGGTGCACCTTGCTGGGGGCTTAAAACCACCCCAAGTGTTCGGGTGTGGTGGCTCAT   66522
                                                                     **** *******************************************************

LOC112206721.putative.POM121-like,protein1.START.LOC112206738        gcctgtaatcccagcactttgggaggccgaggcaggacaactgaacccaggtgtttgaga   26247
BCRP3.REF.NEW                                                        gcctgtaatcccagcactttgggaggccgaggcaggacaactgaacccaggtgtttgaga   17417
```

```
GGT1.start-POM121L10P.end.24583750-24659147.ref                  GCCTGTAATCCCAGCACTTTGGGAGGCCGAGGCAGGACAACTGAACCCAGGTGTTTGAGA   66582
                                                                 ************************************************************

LOC112206721.putative.POM121-like,protein1.START.LOC112206738    ccagtctgggcaatgtagcaaaccccatctccagaaaaaatacaaagaaaaattaggcag   26307
BCRP3.REF.NEW                                                    ccagtctgggcaatgtagcaaaccccatctctagaaaaaatacaaagaaaaattagtcag   17477
GGT1.start-POM121L10P.end.24583750-24659147.ref                  CCAGTCTGGGCAATGTAGCAAACCCCATCTCTAGAAAAAATACAAAGAAAAATTAGTCAG   66642
                                                                 ******************************* ************************ ***

LOC112206721.putative.POM121-like,protein1.START.LOC112206738    gcattgtggcacacatctgtaatcctaggtatctgggaggctgacacaggaggattgctt   26367
BCRP3.REF.NEW                                                    gcattgtggcacacatctgtaatcctaggtatctgggaggctgacacaggaggattgctt   17537
GGT1.start-POM121L10P.end.24583750-24659147.ref                  GCATTGTGGCACACATCTGTAATCCTAGGTATCTGGGAGGCTGACACAGGAGGATTGCTT   66702
                                                                 ************************************************************

LOC112206721.putative.POM121-like,protein1.START.LOC112206738    gagcccaggagttagaggctgcagtgatccatgatggagccactgtactccagcctgggg   26427
BCRP3.REF.NEW                                                    gagcccaggagttagaggctgcagtgatccatgatggagccactgtactccagcctgggg   17597
GGT1.start-POM121L10P.end.24583750-24659147.ref                  GAGCCCAGGAGTTAGAGGCTGCAGTGATCCATGATGGAGCCACTGTACTCCAGCCTGGGG   66762
                                                                 ************************************************************

LOC112206721.putative.POM121-like,protein1.START.LOC112206738    gacagagcaaggccctgtgcatctctaaaataaataaccacccccacccaacaagtcat   26487
BCRP3.REF.NEW                                                    gacagagcaaggccctgtgcatctctaaaataaataatcacccccacccaacaagtcat   17657
GGT1.start-POM121L10P.end.24583750-24659147.ref                  GACAGAGCAAGGCCCTGTGCATCTCTAAAATAAATAATCACCCCCACCCAACAAGTCAT   66822
                                                                 ************************************* **********************

LOC112206721.putative.POM121-like,protein1.START.LOC112206738    gccttgtcaggaccccaccccaccccgtctcactgtaaggggttgatgacaccagcagg   26547
BCRP3.REF.NEW                                                    gccttgtcaggaccccaccccaccccgtctcactgtaaggggttcatgacaccagcagg   17717
GGT1.start-POM121L10P.end.24583750-24659147.ref                  GCCTTGTCAGGACCCCACCCCACCCCGTCTCACTGTAAGGGGTTCATGACACCAGCAGG   66882
                                                                 ******************************************** ***************

LOC112206721.putative.POM121-like,protein1.START.LOC112206738    ggtttctagcacctgaggtggacttggggtcttgggccccaaagacctccccaccagcag   26607
BCRP3.REF.NEW                                                    ggtttctagcacctgaggtggacttgggggcttgggccccaaagacctccccaccagcag   17777
GGT1.start-POM121L10P.end.24583750-24659147.ref                  GGTTTCTAGCACCTGAGGTGGACTTGGGGGCTTGGGCCCCAAAGACCTCCCCACCAGCAG   66942
                                                                 ***************************** ******************************

LOC112206721.putative.POM121-like,protein1.START.LOC112206738    ctgtgagccccccctccgagccactctcctcttccccactctgcgagggcaggtcgagttg   26667
BCRP3.REF.NEW                                                    ctgtgagccccccctctgagccactctcctcttccccactctgcgagggcaggacgaggtg   17837
GGT1.start-POM121L10P.end.24583750-24659147.ref                  CTGTGAGCCCCCCTCTGAGCCACTCTCCTCTTCCCCACTCTGCGAGGGCAGGACGAGGTG   67002
                                                                 *************** ************************************ **** **

LOC112206721.putative.POM121-like,protein1.START.LOC112206738    ctgctgtacttcttgtggctggaggccatccctgccctggacagcaagagacagagcatc   26727
BCRP3.REF.NEW                                                    ctgctgtacttcttgcggctggaggccatccctgccctgaacagcaagagacagagcatc   17897
GGT1.start-POM121L10P.end.24583750-24659147.ref                  CTGCTGTACTTCTTGCGGCTGGAGGCCATCCCTGCCCTGAACAGCAAGAGACAGAGCATC   67062
                                                                 *************** *********************** ********************

LOC112206721.putative.POM121-like,protein1.START.LOC112206738    ctgttctccactgatgtctaaaggtcccagtccatctcctggaggcggacagatggcctg   26787
BCRP3.REF.NEW                                                    ctgttctccaccgatgtctaaaggtcccagtccatctcctggaggcggacagatggcctg   17957
GGT1.start-POM121L10P.end.24583750-24659147.ref                  CTGTTCTCCACCGATGTCTAAAGGTCCCAGTCCATCTCCTGGAGGCGGACAGATGGCCTG   67122
                                                                 *********** ************************************************

LOC112206721.putative.POM121-like,protein1.START.LOC112206738    gaaacctctggctaatcaggccatctgtagagtgggaatcaagattttctgcggcatcct   26847
BCRP3.REF.NEW                                                    gaaacctctggctaatcgggccatctgtagagtgggaatcaagattttctgaggcatcct   18017
GGT1.start-POM121L10P.end.24583750-24659147.ref                  GAAACCTCTGGCTAATCGGGCCATCTGTAGAGTGGGAATCAAGATTTTCTGAGGCATCCT   67182
                                                                 ***************** ********************************* ********

LOC112206721.putative.POM121-like,protein1.START.LOC112206738    tgggccacccccaggtgtcaggccatctgccaagagacagcggcccaaagcagaaggaca   26907
BCRP3.REF.NEW                                                    tgggccacccccaggtgtcaggccatctgccaagagacagcggcccaaagcagaaggaca   18077
GGT1.start-POM121L10P.end.24583750-24659147.ref                  TGGGCCACCCCCAGGTGTCAGGCCATCTGCCAAGAGACAGCGGCCCAAAGCAGAAGGACA   67242
                                                                 ************************************************************

LOC112206721.putative.POM121-like,protein1.START.LOC112206738    ggtggcctgggcagatcccacccaggtctgaaagccccaggctggcctcagactgtgggt   26967
BCRP3.REF.NEW                                                    ggtggcctgggcagatcccgcccaggtctgaaagccccaggctggcctcagactgtgggt   18137
GGT1.start-POM121L10P.end.24583750-24659147.ref                  GGTGGCCTGGGCAGATCCCGCCCAGGTCTGAAAGCCCCAGGCTGGCCTCAGACTGTGGGT   67302
                                                                 ******************* ****************************************

LOC112206721.putative.POM121-like,protein1.START.LOC112206738    tttttatgtggccacccgagggcgccccaagccagttcatctcggagtccaggcctggcc   27027
BCRP3.REF.NEW                                                    tttttatgtggccacccgagggcgccccaagccagttcatctcggagtccaggcctggcc   18197
GGT1.start-POM121L10P.end.24583750-24659147.ref                  TTTTTATGTGGCCACCCGAGGGCGCCCCAAGCCAGTTCATCTCGGAGTCCAGGCCTGGCC   67362
                                                                 ************************************************************

LOC112206721.putative.POM121-like,protein1.START.LOC112206738    ctgggagacagggtgaaagcagtggtttttatgaacttaacttgtagagtctaaaagatt   27087
BCRP3.REF.NEW                                                    ctgggagacagggtgaaagcagtggtttttatgaacttaacttatagagtccaaaagatt   18257
GGT1.start-POM121L10P.end.24583750-24659147.ref                  CTGGGAGACAGGGTGAAAGCAGTGGTTTTTATGAACTTAACTTATAGAGTCCAAAAGATT   67422
                                                                 ******************************************* ******* ********

LOC112206721.putative.POM121-like,protein1.START.LOC112206738    tctactgaatcacttgtcaagatgctccctctctggggagaagggaacgtgaccggattc   27147
BCRP3.REF.NEW                                                    tctactgaatcacttgtcaagaagcgccctctctggggagaagggaacgtgactggattc   18317
GGT1.start-POM121L10P.end.24583750-24659147.ref                  TCTACTGAATCACTTGTCAAGAAGCGCCCTCTCTGGGGAGAAGGGAACGTGACTGGATTC   67482
                                                                 ********************** ** *************************** ******

LOC112206721.putative.POM121-like,protein1.START.LOC112206738    cctcactgttgtatcttgaataaacgctgctgcttcatcctgtggggccatggccctgt   27207
BCRP3.REF.NEW                                                    cctcactgttgtatcttgaataaacgctgctgcttcatcctgtggggccgtggccctgt   18377
GGT1.start-POM121L10P.end.24583750-24659147.ref                  CCTCACTGTTGTATCTTGAATAAACGCTGCTGCTTCATCCTGTGGGGGCCGTGGCCCTGT   67542
                                                                 ************************************************** *********

LOC112206721.putative.POM121-like,protein1.START.LOC112206738    ccctgtgtgggtggggcctcttccatttccctgacttagaaaccacactccacttagaac   27267
BCRP3.REF.NEW                                                    ccctgtgtgggtggggcctcttccatttccctgacttagaaaccacactccacttctaac   18437
GGT1.start-POM121L10P.end.24583750-24659147.ref                  CCCTGTGTGGGTGGGGCCTCTTCCATTTCCCTGACTTAGAAACCACACTCCACTTCTAAC   67602
                                                                 *******************************************************  ***

LOC112206721.putative.POM121-like,protein1.START.LOC112206738    agggtttgagaggcttagtcagcactgggtagcgttttgactccattcttggctttcttc   27327
BCRP3.REF.NEW                                                    agggtttgagaggcttggtcagcactgggtagcgttttgactccattcttggctttcttc   18497
GGT1.start-POM121L10P.end.24583750-24659147.ref                  AGGGTTTGAGAGGCTTGGTCAGCACTGGGTAGCGTTTTGACTCCATTCTTGGCTTTCTTC   67662
                                                                 **************** *******************************************

LOC112206721.putative.POM121-like,protein1.START.LOC112206738    tttttctttccagaaggattttgtgcagaaatgggtcttttgttgccgtgttagtcctc   27387
BCRP3.REF.NEW                                                    tttttctttccagaaggattttgtgcagaaatgggtcttttgttgccgtgttagtcctc   18557
GGT1.start-POM121L10P.end.24583750-24659147.ref                  TTTTTCTTTCCAGAAGGATTTTGTGCAGAAATGGGTCTTTTGTTGCCGTGTTAGTCCTC   67722
                                                                 ************************************************************

LOC112206721.putative.POM121-like,protein1.START.LOC112206738    cttggaaggcagctcagaaggcccgtgaaacgtcggggacaggacccccagggagggaa   27447
BCRP3.REF.NEW                                                    cttggaaggcagctcagaaggcctgtgaaatgtcggggacaggacccccagggagggaa   18617
GGT1.start-POM121L10P.end.24583750-24659147.ref                  CTTGGAAGGCAGCTCAGAAGGCCTGTGAAATGTCGGGGACAGGACCCCCAGGGAGGGAA   67782
                                                                 *********************** ****** *****************************

LOC112206721.putative.POM121-like,protein1.START.LOC112206738    tcccaggccacgcactttagggttcgttctccagggagagcgacctcgtcccccaatcct   27507
BCRP3.REF.NEW                                                    tcccaggctacgcaccttagggttcgttctccagggagagcgacctcgtcccccgatcct   18677
GGT1.start-POM121L10P.end.24583750-24659147.ref                  TCCCAGGCTACGCACCTTAGGGTTCGTTCTCCAGGGAGAGCGACCTCGTCCCCCGATCCT   67842
                                                                 ******** ****** ************************************** *****

LOC112206721.putative.POM121-like,protein1.START.LOC112206738    gaccgcccttccggcccacgctctcctgtttggcttccacaggcctggacttctctggct   27567
BCRP3.REF.NEW                                                    gaccgcccttccggcccacgctctcctgtttggcttccacaggcctggacttctctggct   18737
GGT1.start-POM121L10P.end.24583750-24659147.ref                  GACCGCCCTTCCGGCCCACGCTCTCCTGTTTGGCTTCCACAGGCCTGGACTTCTCTGGCT   67902
                                                                 ************************************************************

LOC112206721.putative.POM121-like,protein1.START.LOC112206738    tctctgcccacacactccctg-ccccagtgtccctgcccctgccccagcacaggtgactt   27626
BCRP3.REF.NEW                                                    tctctgcccacacactccctgccccccagtgtccctgcccctgccccagcacaggtgactt   18797
GGT1.start-POM121L10P.end.24583750-24659147.ref                  TCTCTGCCCACACACTCCCTGCCCCCAGTGTCCCTGCCCCTGCCCCAGCACAGGTGACTT   67962
                                                                 ********************* **************************************

LOC112206721.putative.POM121-like,protein1.START.LOC112206738    catttctgtcctctcagctcagtggactcgctcaactttgtataagtctccacttggtg   27686
BCRP3.REF.NEW                                                    catttctgtcctctcagctcagtggactcgctcatctttgtataagtctccacttggtg   18857
GGT1.start-POM121L10P.end.24583750-24659147.ref                  CATTTCTGTCCTCTCAGCTCAGTGGACTCGCTCATCTTTTGTATAAGTCTCCACTTGGTG   68022
                                                                 ********************************** *************************

LOC112206721.putative.POM121-like,protein1.START.LOC112206738    gcagcagcttgctgatgacttgttttaaaactttcatcctaaataaccttttgatgcttg   27746
BCRP3.REF.NEW                                                    gcagcagcttgctgatgacttgttttaaaactttcatcctaaataaccttttgatacttg   18917
GGT1.start-POM121L10P.end.24583750-24659147.ref                  GCAGCAGCTTGCTGATGACTTGTTTTAAAACTTTCATCCTAAATAACCTTTTGATACTTG   68082
                                                                 ******************************************************* ****

LOC112206721.putative.POM121-like,protein1.START.LOC112206738    aatattttaagttttatacgtagtttctaa-ttttttcctcaacagatccagatacctaa   27805
BCRP3.REF.NEW                                                    aatattttaagttttatacatagtttctaattttttttccgaacagatccagatacctaa   18977
GGT1.start-POM121L10P.end.24583750-24659147.ref                  AATATTTTAAGTTTTATACATAGTTTCTAATTTTTTTTCCGAACAGATCCAGATACCTAA   68142
                                                                 ******************* ********** ****** *  ********************

LOC112206721.putative.POM121-like,protein1.START.LOC112206738    taagatgctggaatgtaatccctggacagtccgtgtcctggcagcattt-gtcttcctct   27864
BCRP3.REF.NEW                                                    taagatgctggaatgtaatccctggacaatccgtgtcctggcagcatttggtcttcctct   19037
GGT1.start-POM121L10P.end.24583750-24659147.ref                  TAAGATGCTGGAATGTAATCCCTGGACAATCCGTGTCCTGGCAGCATTTGGTCTTCCTCT   68202
                                                                 **************************** ******************** **********

LOC112206721.putative.POM121-like,protein1.START.LOC112206738    aagcgcctggctccgctgttctcaggagtgggttctgaagtctctggagaacaggatacg   27924
BCRP3.REF.NEW                                                    aagcgcctggctccgctgttctcaggagtgggttctgaagtctctggagaacaggatacg   19097
GGT1.start-POM121L10P.end.24583750-24659147.ref                  AAGCGCCTGGCTCCGCTGTTCTCAGGAGTGGGTTCTGAAGTCTCTGGAGAACAGGATACG   68262
                                                                 ************************************************************

LOC112206721.putative.POM121-like,protein1.START.LOC112206738    tggagggttaggaaggggccaggcctagagatgggagactccctcccggagcaggtggag   27984
BCRP3.REF.NEW                                                    tggagggttaggaaggggccaggcctagagacgggagactccctcccggagcaggtggag   19157
```

```
GGT1.start-POM121L10P.end.24583750-24659147.ref                 TGGAGGGTTAGGAAGGGGCCAGGCCTAGAGACGGAGACTCCCTCCCGGAGCAGGTGGAG   68322
                                                                ******************************* ****************************

LOC112206721.putative.POM121-like,protein1.START.LOC112206738   gcacaggaccattcgctaccccatctgccgacacctgcgggggagcccaggcattctttg   28044
BCRP3.REF.NEW                                                   gcacaggaccattcgctaccccatctgccggcacctgcgggggagcccaggcattctttg   19217
GGT1.start-POM121L10P.end.24583750-24659147.ref                 GCACAGGACCATTCGCTACCCCATCTGCCGGCACCTGCGGGGGAGCCCAGGCATTCTTTG   68382
                                                                ****************************** *****************************

LOC112206721.putative.POM121-like,protein1.START.LOC112206738   taagccctcctgaccacctggctcaaagaaaacagaagcatggaggccg-caagtatttt   28103
BCRP3.REF.NEW                                                   taagccctcctgaccacctggctcaaagaaaacagaagcatggaggccgccaagtatttt   19277
GGT1.start-POM121L10P.end.24583750-24659147.ref                 TAAGCCCTCCTGACCACCTGGCTCAAAGAAAACAGAAGCATGGAGGCCGCCAAGTATTTT   68442
                                                                ************************************************* **********

LOC112206721.putative.POM121-like,protein1.START.LOC112206738   caagaaataat-ccatgaacatggcatcacttttttagaaagaggggctt-gggcaggca   28161
BCRP3.REF.NEW                                                   caagaaataatcccatgaacatggcatcacttttttagaaagaggggcttggggcaggca   19337
GGT1.start-POM121L10P.end.24583750-24659147.ref                 CAAGAAATAATCCCATGAACATGGCATCACTTTTTTAGAAAGAGGGGCTTGGGGCAGGCA   68502
                                                                *********** ************************************** *********

LOC112206721.putative.POM121-like,protein1.START.LOC112206738   gaggagagaagagagagcaaactgagagccaagtttccagacggtcctgcaggaggagag   28221
BCRP3.REF.NEW                                                   gaggagagaagggagatcaaactgagagccaagtttccagacggtcctgcaggaggagag   19397
GGT1.start-POM121L10P.end.24583750-24659147.ref                 GAGGAGAGAAGGGAGATCAAACTGAGAGCCAAGTTTCCAGACGGTCCTGCAGGAGGAGAG   68562
                                                                *********** **** *******************************************

LOC112206721.putative.POM121-like,protein1.START.LOC112206738   gatgcagctgcccagagggaagcaggatcacatttaaggaagtgtgtggggtccctggat   28281
BCRP3.REF.NEW                                                   gatgcagctgcccagagggaagcaggatcacatttaaggaagtgtgtggggtccctggat   19457
GGT1.start-POM121L10P.end.24583750-24659147.ref                 GATGCAGCTGCCCAGAGGGAAGCAGGATCACATTTAAGGAAGTGTGTGGGGTCCCTGGAT   68622
                                                                ************************************************************

LOC112206721.putative.POM121-like,protein1.START.LOC112206738   gacaccagcacccagtgcggctctgtctggcaaccgctcccaaggtgggaggagtgggtg   28341
BCRP3.REF.NEW                                                   gacaccagcacccagtgcggctctgtctggcaaccgctcccaaggtggcaggagtgggtg   19517
GGT1.start-POM121L10P.end.24583750-24659147.ref                 GACACCAGCACCCAGTGCGGCTCTGTCTGGCAACCGCTCCCAAGGTGGCAGGAGTGGGTG   68682
                                                                ************************************************* **********

LOC112206721.putative.POM121-like,protein1.START.LOC112206738   tcccctgtgtgtcagtgggcagctcctgctgaacccgcagctcactggggagcctgacag   28401
BCRP3.REF.NEW                                                   tcccctgtgtgtcagtgggcagctcctgctgaacccacagctcactggggagcctgacag   19577
GGT1.start-POM121L10P.end.24583750-24659147.ref                 TCCCCTGTGTGTCAGTGGGCAGCTCCTGCTGAACCCACAGCTCACTGGGGAGCCTGACAG   68742
                                                                ************************************ ***********************

LOC112206721.putative.POM121-like,protein1.START.LOC112206738   tggggccatgtgcctgacactcctctctgcttgtggacctggcaaggcagggagcagaaa   28461
BCRP3.REF.NEW                                                   tggggccatgtgcctgacactcctctctgcttgtggacctggcaaggcagggagcagaaa   19637
GGT1.start-POM121L10P.end.24583750-24659147.ref                 TGGGGCCATGTGCCTGACACTCCTCTCTGCTTGTGGACCTGGCAAGGCAGGGAGCAGAAA   68802
                                                                ************************************************************

LOC112206721.putative.POM121-like,protein1.START.LOC112206738   acagagccacttgaaggctttctgtctgcgtctgtgtgcagtgtggatttagttgtgctt   28521
BCRP3.REF.NEW                                                   acagagctacttgaaggctttctgtctgcgtctgtgtgcagtgtggatttagttgtgctt   19697
GGT1.start-POM121L10P.end.24583750-24659147.ref                 ACAGAGCTACTTGAAGGCTTTCTGTCTGCGTCTGTGTGCAGTGTGGATTTAGTTGTGCTT   68862
                                                                ******* ****************************************************

LOC112206721.putative.POM121-like,protein1.START.LOC112206738   ttttcttgcggggagagcacagccaccatttacaagcagtgtcaccctcgtgggtggcga   28581
BCRP3.REF.NEW                                                   tttacttgctgggagagcacagccaccatttacaagcagtgtcaccctcgtgggtggcga   19757
GGT1.start-POM121L10P.end.24583750-24659147.ref                 TTTACTTGCTGGGAGAGCACAGCCACCATTTACAAGCAGTGTCACCCTCGTGGGTGGCGA   68922
                                                                *** ***** **************************************************

LOC112206721.putative.POM121-like,protein1.START.LOC112206738   ggacagaacaggagcctctgctctctgtacctatctgggcccggtgggctcccctgtcct   28641
BCRP3.REF.NEW                                                   ggacagaacaggagcctctgctctctgtacctatctgggcccggtgggctcccttgtcct   19817
GGT1.start-POM121L10P.end.24583750-24659147.ref                 GGACAGAACAGGAGCCTCTGCTCTCTGTACCTATCTGGGCCCGGTGGGCTCCCTTGTCCT   68982
                                                                ***************************************************** ******

LOC112206721.putative.POM121-like,protein1.START.LOC112206738   ggcttccatctctgtctcagcgaccattcagccctgcacaggaacacatgttgcttagaa   28701
BCRP3.REF.NEW                                                   ggcttccatctctgtctcagcgaccattcagccctgcacaggaacacatgttgcttagaa   19877
GGT1.start-POM121L10P.end.24583750-24659147.ref                 GGCTTCCATCTCTGTCTCAGCGACCATTCAGCCCTGCACAGGAACACATGTTGCTTAGAA   69042
                                                                ************************************************************

LOC112206721.putative.POM121-like,protein1.START.LOC112206738   aagccaaatccagcccttgtctctgcctcctctggtctcatgatgtgcatctattacctt   28761
BCRP3.REF.NEW                                                   aagccaaatccagcccttgtctctgcctcctctggtctcatgatgtgcatctgttacctt   19937
GGT1.start-POM121L10P.end.24583750-24659147.ref                 AAGCCAAATCCAGCCCTTGTCTCTGCCTCCTCTGGTCTCATGATGTGCATCTGTTACCTT   69102
                                                                **************************************************** *******

LOC112206721.putative.POM121-like,protein1.START.LOC112206738   gaaactggaaaccagtctatcaatgtctgtgccaatttttattccctccccaacctcct   28821
BCRP3.REF.NEW                                                   gaaactggaaaccagtctatcaatgtctgtgccaatttttattccctccccaacctcct   19997
GGT1.start-POM121L10P.end.24583750-24659147.ref                 GAAACTGGAAACCAGTCTATCAATGTCTGTGCCAATTTTTATTCCCTCCCCAACCTCCT   69162
                                                                ************************************************************

LOC112206721.putative.POM121-like,protein1.START.LOC112206738   tccccatacgactttttatttatgtaggatgtgtgctgtctaatgatgggatgaccacac   28881
BCRP3.REF.NEW                                                   tccccatacgactttttatttatgtaggatgtgtgctgtctaatgatgggatgaccacac   20057
GGT1.start-POM121L10P.end.24583750-24659147.ref                 TCCCCATACGACTTTTTATTTATGTAGGATGTGTGCTGTCTAATGATGGGATGACCACAC   69222
                                                                ************************************************************

LOC112206721.putative.POM121-like,protein1.START.LOC112206738   ttttccatgttctaaaagtgctcctctcccgcagggtcccaggctggtggttgctttgg   28941
BCRP3.REF.NEW                                                   ttttccatgttctaaaagtgctcctctcccacagggtcccaggctggtggttgctttgg   20117
GGT1.start-POM121L10P.end.24583750-24659147.ref                 TTTTCCATGTTCTAAAAGTGCTCCTCTCCCACAGGGTCCCAGGCTGGTGGTTGCTTTGG   69282
                                                                ****************************** *****************************

LOC112206721.putative.POM121-like,protein1.START.LOC112206738   gtctacagctacgtcttacccgcctcctgcctcaaaagcctgtgtggtggcaaagccg--   28999
BCRP3.REF.NEW                                                   gtctacagctacgtcttacccgcctcctgcctcaacagcctgtgtggtggcaaagccggt   20177
GGT1.start-POM121L10P.end.24583750-24659147.ref                 GTCTACAGCTACGTCTTACCCGCCTCCTGCCTCAACAGCCTGTGTGGTGGCAAAGCCGGT   69342
                                                                *********************************** **********************

LOC112206721.putative.POM121-like,protein1.START.LOC112206738   tgggggctggggaacgcagcgttctccaggagggggacccggctctccttctgcaatgca   29059
BCRP3.REF.NEW                                                   gtggggctggggaacgcagcgttctccagga-ggggacccggctctccttctgcagtgca   20236
GGT1.start-POM121L10P.end.24583750-24659147.ref                 GTGGGGCTGGGGAACGCAGCGTTCTCCAGGA-GGGGACCCGGCTCTCCTTCTGCAGTGCA   69401
                                                                  ***************************** *********************** ****

LOC112206721.putative.POM121-like,protein1.START.LOC112206738   ggcaaaggcctagatgccagtgtgacctcccacaaggcatggcttccagactccccgacc   29119
BCRP3.REF.NEW                                                   ggcgaaggcctagatgccagtgtgacctcccacaaggcgtggcttccagactccccggcc   20296
GGT1.start-POM121L10P.end.24583750-24659147.ref                 GGCGAAGGCCTAGATGCCAGTGTGACCTCCCACAAGGCGTGGCTTCCAGACTCCCCGGCC   69461
                                                                *** ********************************** ****************** **

LOC112206721.putative.POM121-like,protein1.START.LOC112206738   agaagtgatgcttttttgcctcgggccctgggtttgaagcagcctggctttctcttggta   29179
BCRP3.REF.NEW                                                   ggaagtgatgcttttttgccgcgggccctgggtttgaagcagcctggctttctcttggta   20356
GGT1.start-POM121L10P.end.24583750-24659147.ref                 GGAAGTGATGCTTTTTTGCCGCGGGCCCTGGGTTTGAAGCAGCCTGGCTTTCTCTTGGTA   69521
                                                                 ******************* ***************************************

LOC112206721.putative.POM121-like,protein1.START.LOC112206738   agtggctggtgtcttagcagctgcaatctgagctcagccacctacacaccaccgtggccg   29239
BCRP3.REF.NEW                                                   agtggctggtgtcttagcagctgcaatctgagctcagccacctacacaccaccgtggccg   20416
GGT1.start-POM121L10P.end.24583750-24659147.ref                 AGTGGCTGGTGTCTTAGCAGCTGCAATCTGAGCTCAGCCACCTACACACCACCGTGGCCG   69581
                                                                ************************************************************

LOC112206721.putative.POM121-like,protein1.START.LOC112206738   acactttcattaaaaagtttcctgagacgacttgtgtgcatgttgatttcatgatcagcg   29299
BCRP3.REF.NEW                                                   acactttcattaaaaagtttcctgagacga------------------------------   20446
GGT1.start-POM121L10P.end.24583750-24659147.ref                 ACACTTTCATTAAAAAGTTTCCTGAGACGACTTGCGTGCATGTTGACTTCATGATCAGCG   69641
                                                                ******************************
```

End of BCRP3 sequence

```
LOC112206721.putative.POM121-like,protein1.START.LOC112206738   ccgctgggaagaaccccagagccggtggggtggggctggaagcagcaggtgcagtgatgg   29359
BCRP3.REF.NEW                                                   ------------------------------------------------------------   20446
GGT1.start-POM121L10P.end.24583750-24659147.ref                 CCGCTGGGAAGAACCCCAGAGCCGGTGGGGTGGGGCTGGAAGCAGCAGGTGCAGTGATAG   69701

LOC112206721.putative.POM121-like,protein1.START.LOC112206738   ggctgggtgcccaggagtcctcagtgctcaatcaggccaaggtggccaagcccaggctgc   29419
BCRP3.REF.NEW                                                   ------------------------------------------------------------   20446
GGT1.start-POM121L10P.end.24583750-24659147.ref                 GGCTGGGTGCCCAGGAGGCCTCAGTGCTCAATCAGGCCAAGGTGGCCAAGCCCAGGCTGC   69761

LOC112206721.putative.POM121-like,protein1.START.LOC112206738   agggaaggctggcctgggggtgtgggtgagcacaggcaggcaccagctgggcagtatta   29479
BCRP3.REF.NEW                                                   ------------------------------------------------------------   20446
GGT1.start-POM121L10P.end.24583750-24659147.ref                 AGGGAAGGCCGGCCTGGGGGTGTGGGTGAGCACAGGCAGGCACCAGCTGGGCAGTGTTA   69821

LOC112206721.putative.POM121-like,protein1.START.LOC112206738   ggatgctggagcagcatccgtaaccccactgactggggtagtctggttggggcagggacc   29539
BCRP3.REF.NEW                                                   ------------------------------------------------------------   20446
GGT1.start-POM121L10P.end.24583750-24659147.ref                 GGATGCTGGAGCAGCATCCGTAACTCCACTGAGTGGGGTAGTCTGGTTGGGGCAGGGACC   69881

LOC112206721.putative.POM121-like,protein1.START.LOC112206738   gctattgctttggcagagagagatgatccccactggggagaggctgttctgactctgcag   29599
BCRP3.REF.NEW                                                   ------------------------------------------------------------   20446
GGT1.start-POM121L10P.end.24583750-24659147.ref                 GCTGTTGCTTTGGCAGAGAGAGAGGATCCCCACTGGGGAGAGGCTGTTCTGACTCTGCAG   69941

LOC112206721.putative.POM121-like,protein1.START.LOC112206738   gtgggacagggacagatggccaccagggtgacccggctggtcttcctttgctacgctaag   29659
BCRP3.REF.NEW                                                   ------------------------------------------------------------   20446
GGT1.start-POM121L10P.end.24583750-24659147.ref                 GTGGGACAGGGACAGATGGCCACCAGGGTGACCCGGCTGGTCTTCCTTTGCTATGCTAAG   70001
```

```
LOC112206721.putative.POM121-like,protein1.START.LOC112206738      ccctgggacatggaggattcccgccacacacagcccgggcccgggttcttacctgtggcc   29719
BCRP3.REF.NEW                                                      ------------------------------------------------------------   20446
GGT1.start-POM121L10P.end.24583750-24659147.ref                    CCCTGGGACATGGAGGATTCCTGC--CACACAGCCTGGGCCCGGGTTCTTACCTGTGGCC   70059

LOC112206721.putative.POM121-like,protein1.START.LOC112206738      accgctctggcacgagcccctcagtcttgggtggtttctgcctggtccgggatttggtgt   29779
BCRP3.REF.NEW                                                      ------------------------------------------------------------   20446
GGT1.start-POM121L10P.end.24583750-24659147.ref                    ACCGCTCTGGCATGAGCCCCTCAGTCTTGGGTGGTTTCTGCCTGGTCCGGGATTTGGTGT   70119

LOC112206721.putative.POM121-like,protein1.START.LOC112206738      tcctgctgagtccagcctttctaccacctcctcatgggccgtgggtggtgttgtcagctg   29839
BCRP3.REF.NEW                                                      ------------------------------------------------------------   20446
GGT1.start-POM121L10P.end.24583750-24659147.ref                    TGCTGCTGAGTCCAGCCTTTCTGCCACCTCCGCATGGGCCGTGGGTGGTGATGTCAGCTG   70179

LOC112206721.putative.POM121-like,protein1.START.LOC112206738      cctcccaccttggcttcagtagctcacccagcttacaggggagctgccctgggctggaga   29899
BCRP3.REF.NEW                                                      ------------------------------------------------------------   20446
GGT1.start-POM121L10P.end.24583750-24659147.ref                    CCTCCCACCTTGGCTTCAGTAGCTCACCCAGCTTACAGGGGAGCTGCCCTGGGCTGGAGA   70239

LOC112206721.putative.POM121-like,protein1.START.LOC112206738      tgggcatgcaccctgggtcctacttgaatgaatgcagcttgaggagagccggccatatac   29959
BCRP3.REF.NEW                                                      ------------------------------------------------------------   20446
GGT1.start-POM121L10P.end.24583750-24659147.ref                    TGGGCATGCACCCTGGGTCCTACTTGAATGAATGCAGCTTGAGGAGACCCGGCCATATAC   70299

LOC112206721.putative.POM121-like,protein1.START.LOC112206738      actgggccacaggttaccctcggcaatgcccacatcagccgtcagcctgagcct-cccag   30018
BCRP3.REF.NEW                                                      ------------------------------------------------------------   20446
GGT1.start-POM121L10P.end.24583750-24659147.ref                    ACTGGGCCACAGGTTACCCTCGGCAATGCCCACATCAGCCGTCAGCCTGAGCCTCCCCAG   70359

LOC112206721.putative.POM121-like,protein1.START.LOC112206738      gagagcaaggctcacacgacaaaggctgcccgtggccaatgaggtggctgagcccagcca   30078
BCRP3.REF.NEW                                                      ------------------------------------------------------------   20446
GGT1.start-POM121L10P.end.24583750-24659147.ref                    GAGAGCAAGGCTCACACGACAAAGGCTGCCCGTGGCCAGTGAGGTGGCTGAGCCCAGCCA   70419

LOC112206721.putative.POM121-like,protein1.START.LOC112206738      gaacctttctcggactcccgggatgtggctctgctcgtgagctgcctggtcagctctctc   30138
BCRP3.REF.NEW                                                      ------------------------------------------------------------   20446
GGT1.start-POM121L10P.end.24583750-24659147.ref                    GGACCTTTCTCGGACTCCCGGGATGTGGCTCTGCTCGTGAGCTGCCTGGTCAGCTCTCTC   70479

LOC112206721.putative.POM121-like,protein1.START.LOC112206738      ggggtgagaggggcttgtcacacgggcccctgcctgcagtgtgacccttctcagcttctc   30198
BCRP3.REF.NEW                                                      ------------------------------------------------------------   20446
GGT1.start-POM121L10P.end.24583750-24659147.ref                    GGGGTGAGAGGGGCTTGTCACACGGGCCCCTGCCTGCAGTGTGACCCTTCTCAGCTTCTC   70539

LOC112206721.putative.POM121-like,protein1.START.LOC112206738      tcagcagccctgcctgcagagtgtcaccaccaccatgatcatttccctgacactgcgagg   30258
BCRP3.REF.NEW                                                      ------------------------------------------------------------   20446
GGT1.start-POM121L10P.end.24583750-24659147.ref                    TCAGCAGCCCTGCCTGCAGAGTGTCACCACCACCATGATCATTTCCCTGACACTGCGAGG   70599

LOC112206721.putative.POM121-like,protein1.START.LOC112206738      gtgggggatgtcctgggtagagacagggcccgt-gcagcagcaggctcaggggcgccct   30317
BCRP3.REF.NEW                                                      ------------------------------------------------------------   20446
GGT1.start-POM121L10P.end.24583750-24659147.ref                    GTCGGGGACGTCCTGGGTAGAGACAGGGCCCGTGGCAGCAGCAGGCTCAGGGGCGCCCT   70659

LOC112206721.putative.POM121-like,protein1.START.LOC112206738      acactggtgggctggggacctggtggagaccacaccaagggctggacaaggggacgagcc   30377
BCRP3.REF.NEW                                                      ------------------------------------------------------------   20446
GGT1.start-POM121L10P.end.24583750-24659147.ref                    ACACTGGTGGGCTGGGGACCTGGTGGAGACCACGCCAAGGGCTGGACAAGGGGACGAGCC   70719

LOC112206721.putative.POM121-like,protein1.START.LOC112206738      tccaccctggcctctccgcaggcctcagcagcccctcccacaggcagaagggttgacact   30437
BCRP3.REF.NEW                                                      ------------------------------------------------------------   20446
GGT1.start-POM121L10P.end.24583750-24659147.ref                    TCCACCCTGGCCTCTCCGCAGGCCTCAGCAGCCCCTCCCACAGGCAGAAGGGTTGACACT   70779

LOC112206721.putative.POM121-like,protein1.START.LOC112206738      gggttctgccctcactgcaagagctgcaagtgccacgtgctgttctgcccaatctggtgt   30497
BCRP3.REF.NEW                                                      ------------------------------------------------------------   20446
GGT1.start-POM121L10P.end.24583750-24659147.ref                    GGGTTCTGCCCTCACTGCAAGAGCTGCAAGTGCCACGTGCTGTTCTGCCCAATCTGGTGT   70839

LOC112206721.putative.POM121-like,protein1.START.LOC112206738      ctgcaggtgagggaagggctgccgctggcccgtttctgagtgttcagcacctaagggtga   30557
BCRP3.REF.NEW                                                      ------------------------------------------------------------   20446
GGT1.start-POM121L10P.end.24583750-24659147.ref                    CTGCAGGTGAGGAAAGGACTGCCGCTGGCCCGTTTCTGAGTGTTCAGCACCTAAGGGTGA   70899

LOC112206721.putative.POM121-like,protein1.START.LOC112206738      cagcactgtctgtccctaccctccgggtcctgtttgaaaatcaaacccatgctcacaggc   30617
BCRP3.REF.NEW                                                      ------------------------------------------------------------   20446
GGT1.start-POM121L10P.end.24583750-24659147.ref                    CAGCACTGTCTGTCCCTACCCTCCGGGTCCTGTTTGAAGATCAAACCCATGCTCACAGGA   70959

LOC112206721.putative.POM121-like,protein1.START.LOC112206738      caattttttttttcttttagagacagggtctcactttgtcacccaagctggagtgcagtgg   30677
BCRP3.REF.NEW                                                      ------------------------------------------------------------   20446
GGT1.start-POM121L10P.end.24583750-24659147.ref                    CAATTTTTTTTTCTTTTAGAGACAGGGTCTCACTTTGTCACCCAAGCTGGAGTGCAGTGG   71019

LOC112206721.putative.POM121-like,protein1.START.LOC112206738      tgcgattatagctcaatgcagcctccaattcccggactcaagggaccttcctgcctcagc   30737
BCRP3.REF.NEW                                                      ------------------------------------------------------------   20446
GGT1.start-POM121L10P.end.24583750-24659147.ref                    TGCGATTATAGCTCAATGCAGCCTCCAATTCCCGGACTCAAGGGACCTTCCTGCCTCAGC   71079

LOC112206721.putative.POM121-like,protein1.START.LOC112206738      ctgccaagtagcttggactatagctgtgtgttttattattattttgtagacatggggtct   30797
BCRP3.REF.NEW                                                      ------------------------------------------------------------   20446
GGT1.start-POM121L10P.end.24583750-24659147.ref                    CTGCCAAGTAGCTTGGACTATAGCTGTGTGTTTTATTATTATTTTGTAGACATGGGGTCT   71139

LOC112206721.putative.POM121-like,protein1.START.LOC112206738      ggctatgttgtccaggctattctcaaaattcccggcctcaagcaatcctcccacctcggc   30857
BCRP3.REF.NEW                                                      ------------------------------------------------------------   20446
GGT1.start-POM121L10P.end.24583750-24659147.ref                    GGCTATGTTGTCCAGGCTATTCTCAAAATTCCCGGCCTCAAGCAATCCTCCCGCCTCAGC   71199

LOC112206721.putative.POM121-like,protein1.START.LOC112206738      ctctcaaaggttgggattacaggtgtgaggcaaggcacccagctcagccacagagcccta   30917
BCRP3.REF.NEW                                                      ------------------------------------------------------------   20446
GGT1.start-POM121L10P.end.24583750-24659147.ref                    CTCTCAAAGGTTGGGATTACAGGTGTGAGGCAAGGCACCCAGCTCAGCCACAGAGCCCTA   71259

LOC112206721.putative.POM121-like,protein1.START.LOC112206738      ttgcatctctcttactaggagcaagagctgactgccccctcatccccattccagagtgtt   30977
BCRP3.REF.NEW                                                      ------------------------------------------------------------   20446
GGT1.start-POM121L10P.end.24583750-24659147.ref                    TTGCATCTCTCTTACTAGGAGCAAGAGCTGACTGCCCCCTCATCCCCATTCCAGAGTGTT   71319

LOC112206721.putative.POM121-like,protein1.START.LOC112206738      ggggctgtgttcagccaaggccgggccactggcatggcccagggagcgggatcattcact   31037
BCRP3.REF.NEW                                                      ------------------------------------------------------------   20446
GGT1.start-POM121L10P.end.24583750-24659147.ref                    GGGGCTGTGTTGAGCCGAGGCCGGGCCACTGGCATGGCTCAGGGAGCGGGATCATTCACT   71379

LOC112206721.putative.POM121-like,protein1.START.LOC112206738      gctgccccaaatctgagatcattccactttgacaagacttcctcatccaatccctttact   31097
BCRP3.REF.NEW                                                      ------------------------------------------------------------   20446
GGT1.start-POM121L10P.end.24583750-24659147.ref                    GCTGCCCCAAATCTGAGATCATTCCACCTTGACAAGACTTCCTCATCCAATCCCTTTACT   71439

LOC112206721.putative.POM121-like,protein1.START.LOC112206738      tgacagctggggaaaccaatgcgcacagagcacccccagctcactcggggtctcagagct   31157
BCRP3.REF.NEW                                                      ------------------------------------------------------------   20446
GGT1.start-POM121L10P.end.24583750-24659147.ref                    TGACAGCTGGGGAAACCAATGCGCACAGAGCACCCCCAGCTCACTCGGGGTCTCGGAGCT   71499

LOC112206721.putative.POM121-like,protein1.START.LOC112206738      gatccataagcggaggctgagatcctgggatcttgtcccccagcctccctgcaagcttac   31217
BCRP3.REF.NEW                                                      ------------------------------------------------------------   20446
GGT1.start-POM121L10P.end.24583750-24659147.ref                    GATCCATGAGCGGAGGCTGAGATCCTGGGATCTTGTCCCCCAGCCTCCCGGCAAGCTTAC   71559

LOC112206721.putative.POM121-like,protein1.START.LOC112206738      tacctttctgctgaaagagatggggccggacctcgaccagcagccctggcctggacatga   31277
BCRP3.REF.NEW                                                      ------------------------------------------------------------   20446
GGT1.start-POM121L10P.end.24583750-24659147.ref                    TCCCTTTCTGCTGAAAGAGATGGGGCCGGACCTCGACCAGCAGCCCTGGCCTGGACATGA   71619

LOC112206721.putative.POM121-like,protein1.START.LOC112206738      ctgtgctcatgcaggtattgaggccgagatgccccggcatcatatgtttttctattttct   31337
BCRP3.REF.NEW                                                      ------------------------------------------------------------   20446
GGT1.start-POM121L10P.end.24583750-24659147.ref                    CTGTGCTCATGCAGGTATTGAGGCCGAGACGCCCTGGCATCATATGTTTTTCTGTTTTC-   71678

LOC112206721.putative.POM121-like,protein1.START.LOC112206738      tttttttttttttgagacggtgtctcactctgtcacccaagctggagtgcagtggcatg   31397
BCRP3.REF.NEW                                                      ------------------------------------------------------------   20446
GGT1.start-POM121L10P.end.24583750-24659147.ref                    -TTTTTTTTTTTTGAGACGGTGTCTCACTCTGTCACCCAAGCTGGAGTGCAGTGACATG   71737
```

```
LOC112206721.putative.POM121-like,protein1.START.LOC112206738      atattggctcactgcaacctctgcctcccagttaaagtgattctcctgcctcagccttcc   31457
BCRP3.REF.NEW                                                      ------------------------------------------------------------   20446
GGT1.start-POM121L10P.end.24583750-24659147.ref                    ATATTGGCTCACTGCAACCTCTGCCTCCCGGTTAAAGTGATTCTCCTGCCTCAGCCTTCC   71797

LOC112206721.putative.POM121-like,protein1.START.LOC112206738      aagtatctaggcctacaggcttgtaccaccacgcctgactaatttgtgcatttttactag   31517
BCRP3.REF.NEW                                                      ------------------------------------------------------------   20446
GGT1.start-POM121L10P.end.24583750-24659147.ref                    AAGTAGCTGGGCCTACAGGCTTGTACCACCATGCCTGACTAATTTGTGCATTTTTACTAG   71857

LOC112206721.putative.POM121-like,protein1.START.LOC112206738      agacggggtttccccatgttggccaggctcgtgtcgaactcctgacctcaagtgatccac   31577
BCRP3.REF.NEW                                                      ------------------------------------------------------------   20446
GGT1.start-POM121L10P.end.24583750-24659147.ref                    AGACGGGGTTTCCCCATGTTGGCCAGGCTCATGTCGAACTCCTGACCTCAAGTGATCCAC   71917

LOC112206721.putative.POM121-like,protein1.START.LOC112206738      ctgccttggcctcccaaagtgccaggattacaggcatgagccatggcgtcacttaaatgt   31637
BCRP3.REF.NEW                                                      ------------------------------------------------------------   20446
GGT1.start-POM121L10P.end.24583750-24659147.ref                    CTGCCTTGGCCTCCCAAAGTGCCGGGATTACAGGCATGAGCCATGGCGTCACTTAAATGT   71977

LOC112206721.putative.POM121-like,protein1.START.LOC112206738      agtgagaggccgggcacagtggctcatgcctgtaatcccagtactttgagaggacgaggc   31697
BCRP3.REF.NEW                                                      ------------------------------------------------------------   20446
GGT1.start-POM121L10P.end.24583750-24659147.ref                    AGTGAGAGGCCGGGCACAGTGGCTCATGCCTGTAATCCCAGTACTTTGAGAGGACGAGGC   72037

LOC112206721.putative.POM121-like,protein1.START.LOC112206738      tgtcagatcacctaaggtcaggagttcgagaccagcctggccaacatggtgaaaccgtgt   31757
BCRP3.REF.NEW                                                      ------------------------------------------------------------   20446
GGT1.start-POM121L10P.end.24583750-24659147.ref                    TGTCAGATCACCTAAGGTCAGGAGTTTGAGACCAGCCTGGCCAACATGGTGAAACCGTGT   72097

LOC112206721.putative.POM121-like,protein1.START.LOC112206738      ctctaaaaaaaatagaaaaaaatatccctgcatggtggtgagtacctgtagtcccagtta   31817
BCRP3.REF.NEW                                                      ------------------------------------------------------------   20446
GGT1.start-POM121L10P.end.24583750-24659147.ref                    CTCTAAAAAAAATAGAAAAAAATATCCCTGCATGGTGGTGAGTACCCGTAGTCCCAGTTA   72157

LOC112206721.putative.POM121-like,protein1.START.LOC112206738      ctcaggaggctgaggcatgagaatcgcttaaacctcggaggcggaggctccagtgagctg   31877
BCRP3.REF.NEW                                                      ------------------------------------------------------------   20446
GGT1.start-POM121L10P.end.24583750-24659147.ref                    CTCAGGAGGCTGAGGCATGAGAATCGCTTAAACCTCGGAGGCGGAGGCTGCAGTGAGCTG   72217

LOC112206721.putative.POM121-like,protein1.START.LOC112206738      agatggcgccactgcactccagcctgggtgacagagcaagactttgtctctaaataatta   31937
BCRP3.REF.NEW                                                      ------------------------------------------------------------   20446
GGT1.start-POM121L10P.end.24583750-24659147.ref                    AGATGGCGCCACTGCACTCCAGCCTGGGTGACAGAGCAAGACTTTGTCTCTAAATATTTA   72277

LOC112206721.putative.POM121-like,protein1.START.LOC112206738      aataaataaatatggccgagcatggtgccttaggcctgtagtcccaacactttgggaggc   31997
BCRP3.REF.NEW                                                      ------------------------------------------------------------   20446
GGT1.start-POM121L10P.end.24583750-24659147.ref                    AATAAATAAATATGGCCGAGCATGGTGCCTTAGGCCTGTAGTCCCAACACTTTGGGAGGC   72337

LOC112206721.putative.POM121-like,protein1.START.LOC112206738      tgaggca-gtggttcatgaggtcaggaacccgagaccagcctggccaagatggtgaaaca   32056
BCRP3.REF.NEW                                                      ------------------------------------------------------------   20446
GGT1.start-POM121L10P.end.24583750-24659147.ref                    TGAGGCAGGTGGTTCATGAGGTCAGGAGCCCGAGACCAGCCTGGCCAAGATGGTGAAACA   72397

LOC112206721.putative.POM121-like,protein1.START.LOC112206738      ctgtctctactaaaaatacaaaaattagccagctgtggtggcaggcacctgtaatcccag   32116
BCRP3.REF.NEW                                                      ------------------------------------------------------------   20446
GGT1.start-POM121L10P.end.24583750-24659147.ref                    CTGTCTCTACTAAAAATACAAAAATTAGCCAGCTGTGGTGGCAGGCACCTGTAATCCCAG   72457

LOC112206721.putative.POM121-like,protein1.START.LOC112206738      ctacttgggacactgaggcaggagaatcacttgaaactggaagtcagaggttgcagggag   32176
BCRP3.REF.NEW                                                      ------------------------------------------------------------   20446
GGT1.start-POM121L10P.end.24583750-24659147.ref                    CTACTTGGGACACTGAGGCAGGAGAATCACTTGAAACTGGAAGTCAGAGGTTGCAGGGAG   72517

LOC112206721.putative.POM121-like,protein1.START.LOC112206738      ccgagattgcaccactgcactctagcctgggcgatggagcaagactccatctcaaataaa   32236
BCRP3.REF.NEW                                                      ------------------------------------------------------------   20446
GGT1.start-POM121L10P.end.24583750-24659147.ref                    CCGAGATTGCACCACTGCACTCTAGCCTGGGCGATGGAGCAAGACTCCATCTCAAATAAA   72577

LOC112206721.putative.POM121-like,protein1.START.LOC112206738      taaattaataaatacagagcaagattccatct----taaataaataaataaacatacacc   32292
BCRP3.REF.NEW                                                      ------------------------------------------------------------   20446
GGT1.start-POM121L10P.end.24583750-24659147.ref                    TAAATTAATAAATACAGAGCAAGATTCCATCTTAAATAAATAAATAAATAAACATACACC   72637

LOC112206721.putative.POM121-like,protein1.START.LOC112206738      tgtaatcctagcagttcgggaggctaagacaggtcgatcacctgaggtcaggagttcgag   32352
BCRP3.REF.NEW                                                      ------------------------------------------------------------   20446
GGT1.start-POM121L10P.end.24583750-24659147.ref                    TGTAATCCTAGCAGTTCGGGAGGCTAAGACAGGTCGATCACCTGAGGTCAGGAGTTCGAG   72697

LOC112206721.putative.POM121-like,protein1.START.LOC112206738      accagcctgaccaatatggcaaaactccatctctactaaaaatacaaaaattagccaggc   32412
BCRP3.REF.NEW                                                      ------------------------------------------------------------   20446
GGT1.start-POM121L10P.end.24583750-24659147.ref                    ACCAGCCTGACCAATATGGCAAAACTCCATCTCTACTAAAAATACAAAAATTAGCCAGGC   72757

LOC112206721.putative.POM121-like,protein1.START.LOC112206738      gttttgacgtgtgcctgtagtcccagctacttgggaggctgagacaggagaattgcttga   32472
BCRP3.REF.NEW                                                      ------------------------------------------------------------   20446
GGT1.start-POM121L10P.end.24583750-24659147.ref                    GTTTTGACGTGTGCCTGTAGTCCCAGCTACTTGGGAGGCTGAGACAGGAGAATTGCTTGA   72817

LOC112206721.putative.POM121-like,protein1.START.LOC112206738      acccaagaggtggaggttgcagtgagccgagatctcggctgcacttcagcctgggtgaca   32532
BCRP3.REF.NEW                                                      ------------------------------------------------------------   20446
GGT1.start-POM121L10P.end.24583750-24659147.ref                    ACCCAAGAGGTGGAGGTTGCAGTGAGCCAAGATCTCGGCTGCACTTCAGCCTGGGTGACA   72877

LOC112206721.putative.POM121-like,protein1.START.LOC112206738      gagtgagactgtctcaaaaggaataaaaaaaatacaaaataaaaaaaatgtagtaagatt   32592
BCRP3.REF.NEW                                                      ------------------------------------------------------------   20446
GGT1.start-POM121L10P.end.24583750-24659147.ref                    GAGTGAGACTGTCTCAAAAGGAATAAAAAAAATACAAAATAAAAAAAATGTAGTAAGATT   72937

LOC112206721.putative.POM121-like,protein1.START.LOC112206738      gcagagtcgtgccgcggaagcgtgctggtcctatccatgtagtgaaggctgatttcatac   32652
BCRP3.REF.NEW                                                      ------------------------------------------------------------   20446
GGT1.start-POM121L10P.end.24583750-24659147.ref                    GCAGAGTCGTGCCGCGGAAGCGTGCTGGTCCTATCCATGTAGTGAAGGCTGATTTCATAC   72997

LOC112206721.putative.POM121-like,protein1.START.LOC112206738      acaaatgtcacaagaacttttttttctttttc-------tttgagacggagtatggccctg   32705
BCRP3.REF.NEW                                                      ------------------------------------------------------------   20446
GGT1.start-POM121L10P.end.24583750-24659147.ref                    ACAAATGTCACAAGAACTTTTTTTCTTTTTCTTTTTTTTTTGAGACGGAGTATGGCTCTG   73057

LOC112206721.putative.POM121-like,protein1.START.LOC112206738      tcacccaggctggagtgcagtggcgcgatctcagctcactgcaagctctgcctcccgggt   32765
BCRP3.REF.NEW                                                      ------------------------------------------------------------   20446
GGT1.start-POM121L10P.end.24583750-24659147.ref                    TCACCCAGGCTGGATTGCAGTGGCGCGATCTCAGCTCACTACAAGCTCTGCCTCCCGTGT   73117

LOC112206721.putative.POM121-like,protein1.START.LOC112206738      ttatgccgttctcctgcctcagcctcccaagtagctgggactacaggcctgtgcagcaga   32825
BCRP3.REF.NEW                                                      ------------------------------------------------------------   20446
GGT1.start-POM121L10P.end.24583750-24659147.ref                    TTATGCCGTTCTCCTGCCTCAGCCTCCCAAGTAGCTGGGACTACAGGCCTGTGCAGCAGA   73177

LOC112206721.putative.POM121-like,protein1.START.LOC112206738      tgcagggggccactaggcccaggcagtcttgggacttgtgtgtctcctgctgtgcatcc   32885
BCRP3.REF.NEW                                                      ------------------------------------------------------------   20446
GGT1.start-POM121L10P.end.24583750-24659147.ref                    TGCAGGGGGGCCACTAGGCCCAGGCAGTCTTGGGACTTGTGTGTCTCCTGCTGTGCATCC   73237

LOC112206721.putative.POM121-like,protein1.START.LOC112206738      atactgggtgctttagaaacggcaggcagaccagaagcccctgttgcaagtgaggacaaa   32945
BCRP3.REF.NEW                                                      ------------------------------------------------------------   20446
GGT1.start-POM121L10P.end.24583750-24659147.ref                    ATACTGGGTGCTTTAGAAACGGCAGGCAGACCAGAAGCCCCTGTTGCAAGTGAGGACAAA   73297

LOC112206721.putative.POM121-like,protein1.START.LOC112206738      gtgtgggaaggccgtgagggtctgcagtccgagatggccttgtcctcaacctgcagtgca   33005
BCRP3.REF.NEW                                                      ------------------------------------------------------------   20446
GGT1.start-POM121L10P.end.24583750-24659147.ref                    GTGTGGGAAGGCCGTGAGGGTCTGCAGTCCGAGATGGCCTGGTCCTCAACCTGCAGTGCA   73357

LOC112206721.putative.POM121-like,protein1.START.LOC112206738      ctgttgatgcgctggaaggccgcctccttctcccggtccaggtcttcagcagtgacccgg   33065
BCRP3.REF.NEW                                                      ------------------------------------------------------------   20446
GGT1.start-POM121L10P.end.24583750-24659147.ref                    CTGTTGATGCGCTGGAAGGCCGCCTCCTTCTCCCGGTCCAGGTCTTCAGCAGTGACCCGG   73417

LOC112206721.putative.POM121-like,protein1.START.LOC112206738      taccccatctctaagggaggtggcagcatcaaaggctcccctcgcctgcgtggcagcaga   33125
BCRP3.REF.NEW                                                      ------------------------------------------------------------   20446
GGT1.start-POM121L10P.end.24583750-24659147.ref                    TACCCCATCTCTAAGGGAGGTGGCAGCATCAAAGGCTCCCCTCGCCTGCGTGGCAGCAGG   73477
```

```
LOC112206721.putative.POM121-like,protein1.START.LOC112206738      ggaaacttgcatctacggggcctagaggcctgggatctgcgggagccacccctgggggcg   33185
BCRP3.REF.NEW                                                      ------------------------------------------------------------   20446
GGT1.start-POM121L10P.end.24583750-24659147.ref                    GGAAACTTGCATCTACGGGGCCTAGAGGCCTGGGATCTGGGGGAGCCACCCCTGGGGGCG   73537

LOC112206721.putative.POM121-like,protein1.START.LOC112206738      agtgtctgccctggtgctgtatctgccgtcttttcacactgggtgtgacccgaagagaca   33245
BCRP3.REF.NEW                                                      ------------------------------------------------------------   20446
GGT1.start-POM121L10P.end.24583750-24659147.ref                    AGTGTCTGCCCTGGTGCTGTATCTGCCGTCTTTTCACACTGGATGTGACCCGAAGAGACA   73597

LOC112206721.putative.POM121-like,protein1.START.LOC112206738      gcctgaggtccgccgtcactcagtgtgtttcaggaactgagggtcagctggcagtgggat   33305
BCRP3.REF.NEW                                                      ------------------------------------------------------------   20446
GGT1.start-POM121L10P.end.24583750-24659147.ref                    GCCTGAGGTCCGTCCTCACTCAGTGTGTTTCAGGAACTGAGGGTCAGCTGGCAGTGGGAT   73657

LOC112206721.putative.POM121-like,protein1.START.LOC112206738      gaggctggcccctcttccgctttagttccgggaggcctcccgtagagctgtaggggctcg   33365
BCRP3.REF.NEW                                                      ------------------------------------------------------------   20446
GGT1.start-POM121L10P.end.24583750-24659147.ref                    GAGGCTGGCCCCTCTTCCGCTTTCGTTCCGGGAGGCCTCCCGTAGAGCTGTAGGGGCTCG   73717

LOC112206721.putative.POM121-like,protein1.START.LOC112206738      agatggcctttcgtttggggcacgagctggtccaggaggtctgggatctctggttctgac   33425
BCRP3.REF.NEW                                                      ------------------------------------------------------------   20446
GGT1.start-POM121L10P.end.24583750-24659147.ref                    AGATGGCATTTCATTTGGGGCACGAGCTGGTCCGGGAGGTCTGGGATCTCTGGTTCTGAC   73777

LOC112206721.putative.POM121-like,protein1.START.LOC112206738      ctctgggcacctgctgcagctgtggctgaggcccagaaatgtgaaggccctccatccact   33485
BCRP3.REF.NEW                                                      ------------------------------------------------------------   20446
GGT1.start-POM121L10P.end.24583750-24659147.ref                    CTCTGGGCACCTGCTGCAGCTGTGGCTGAGGCCCAGAAATGTGAAGGGCCTCCATCCACT   73837

LOC112206721.putative.POM121-like,protein1.START.LOC112206738      ccagtagtgaccccaacgtggggttcagtgtggagggggggaggggctgctgcggcagctg   33545
BCRP3.REF.NEW                                                      ------------------------------------------------------------   20446
GGT1.start-POM121L10P.end.24583750-24659147.ref                    CCAGTAGTGACCCCAACGTGGGGTTCAGTGTGGAGGGGGGAGGGGCTGCTGTGGCAGCTG   73897

LOC112206721.putative.POM121-like,protein1.START.LOC112206738      caggagcagaagtgccacgccttgttcttctcatgcccgcatccatgcttgcagctggga   33605
BCRP3.REF.NEW                                                      ------------------------------------------------------------   20446
GGT1.start-POM121L10P.end.24583750-24659147.ref                    CAGGAGCAGAAGTGCCACGCCTTGTTCTTCTCATGCCTGCATCCATGCTTGCAGCTGGGA   73957

LOC112206721.putative.POM121-like,protein1.START.LOC112206738      agggggcaggaatcagcgaggtgacctgggctgagtcctgggaatgggaagaggtggcag   33665
BCRP3.REF.NEW                                                      ------------------------------------------------------------   20446
GGT1.start-POM121L10P.end.24583750-24659147.ref                    AGGGGGCAGGAATCAGCGAGGTGACCTGGGCTGAGTCCTGGGAATCAGAAGAGGTGGCAG   74017

LOC112206721.putative.POM121-like,protein1.START.LOC112206738      gaaggggatctgaggaggagaacagggggcctggtggtctgtgcttcttcccagacatgg   33725
BCRP3.REF.NEW                                                      ------------------------------------------------------------   20446
GGT1.start-POM121L10P.end.24583750-24659147.ref                    GAAGGGGATCTGAGGAGGAGAACAGGGGGCCTGGTGGTCTGTGCTTCTTCCCAGACACGG   74077

LOC112206721.putative.POM121-like,protein1.START.LOC112206738      gagctgtagaggggacctctgcagcagatgctaa-ggggccagtaggcccaggcagtctt   33784
BCRP3.REF.NEW                                                      ------------------------------------------------------------   20446
GGT1.start-POM121L10P.end.24583750-24659147.ref                    GAGCTGTAGAGGGGACCTCTGCAGCAGATGCTAAGGGGGCCAGTAGGCCCAGGCAGTCTT   74137

LOC112206721.putative.POM121-like,protein1.START.LOC112206738      gggacttgtgtgtctcctgctgtgcatccatactgggtgctttagaaacggcaggcagac   33844
BCRP3.REF.NEW                                                      ------------------------------------------------------------   20446
GGT1.start-POM121L10P.end.24583750-24659147.ref                    GGGACTTGTGTGTCTCCTGCTGTGCATCCATACTGGATGCTTTAGAAACAGCAGGCAGAC   74197

LOC112206721.putative.POM121-like,protein1.START.LOC112206738      cagaagcccctgttgcaagtgaggacaaagtgtgggaaggccgtgagggtctgcagtccg   33904
BCRP3.REF.NEW                                                      ------------------------------------------------------------   20446
GGT1.start-POM121L10P.end.24583750-24659147.ref                    CAGAAGCCCCTGTTGCAAGTGAGGACAAAGTGTGGGAAGGCCATGAGGGTCTGCAGTCCG   74257

LOC112206721.putative.POM121-like,protein1.START.LOC112206738      agatggccttgtcctcaacctgcagtgcactgttgatgcgctggaaggccgcctccttct   33964
BCRP3.REF.NEW                                                      ------------------------------------------------------------   20446
GGT1.start-POM121L10P.end.24583750-24659147.ref                    AGATGGCCTTGTCCTCAACCTGCAGTGCACTGTTGATGCGCTGGAATGCCGCCTCCTTCT   74317

LOC112206721.putative.POM121-like,protein1.START.LOC112206738      cccggtccaggtcttcagcagtgacccggtaccccagctctaagggaggtggcagcatca   34024
BCRP3.REF.NEW                                                      ------------------------------------------------------------   20446
GGT1.start-POM121L10P.end.24583750-24659147.ref                    CCCGGTCCAGGTCTTCAACAGTGACCCGGTACCCCAGCTCTAAGGGAGGTGGCAGCATCA   74377

LOC112206721.putative.POM121-like,protein1.START.LOC112206738      aaggctcccctcgcctgcgtggcagcaggggaaacttgtgtgtactgggcctagaggcct   34084
BCRP3.REF.NEW                                                      ------------------------------------------------------------   20446
GGT1.start-POM121L10P.end.24583750-24659147.ref                    AAGGCTCCCCTCGCCTGCGTGGCAGCAGGGGAAACTTGTGTGTACTGGGCCTAGAGGCCT   74437

LOC112206721.putative.POM121-like,protein1.START.LOC112206738      cagatgtggaggagtcattcctgagggcgagtgtctgccctggtgctatatctgctgcct   34144
BCRP3.REF.NEW                                                      ------------------------------------------------------------   20446
GGT1.start-POM121L10P.end.24583750-24659147.ref                    CGGATGTGGAGGAGTCATTCCTGAGGGTGAGTGTCTGCCCTGGTGCTATATCTGCTGCCT   74497

LOC112206721.putative.POM121-like,protein1.START.LOC112206738      tttcacactgggtgtgacctgaagagacagcctgaggcctgtcctcactcactgtctttg   34204
BCRP3.REF.NEW                                                      ------------------------------------------------------------   20446
GGT1.start-POM121L10P.end.24583750-24659147.ref                    TTTCACACTGGGTGTGACCTGAAGAGACAGCCTGAGGCCTGTCCTCACTCACTGTCTTTG   74557

LOC112206721.putative.POM121-like,protein1.START.LOC112206738      aggaactgagggtcagctggcagtgggatgaggctggcccctcctccgctttagccccg   34264
BCRP3.REF.NEW                                                      ------------------------------------------------------------   20446
GGT1.start-POM121L10P.end.24583750-24659147.ref                    AGGAACTGAGGGTCAGCTGGCAGTGGGATGAGGCTGGCCCCCTCCTCCGCTTTAGCCCCG   74617

LOC112206721.putative.POM121-like,protein1.START.LOC112206738      gcaagcctcccgtggagctgtaggagctggagatggcatttcggttggtgcaggagctcg   34324
BCRP3.REF.NEW                                                      ------------------------------------------------------------   20446
GGT1.start-POM121L10P.end.24583750-24659147.ref                    GCAAGCCTCCCGTGGAGCTGTAGGAGCTGGAGATGGCATTTCGGTTGGTGCAGGAGCTCG   74677

LOC112206721.putative.POM121-like,protein1.START.LOC112206738      tccaggaggtctgggatgtctgcttatatctgatttctgacctctgggcatggaggtctg   34384
BCRP3.REF.NEW                                                      ------------------------------------------------------------   20446
GGT1.start-POM121L10P.end.24583750-24659147.ref                    TCCAGGAGGTCTGGGATGTCTGGTTATATCTGATTTCTGACCTCTGGGCATGGAGGTCTG   74737

LOC112206721.putative.POM121-like,protein1.START.LOC112206738      tctgcagaggcccgggcctgggcacaaagggagagaggcctccattgtcccgcaggggct   34444
BCRP3.REF.NEW                                                      ------------------------------------------------------------   20446
GGT1.start-POM121L10P.end.24583750-24659147.ref                    TCTGCAGAGGCCCGGGCCTGGGCACAAAGGGAGAGAGGCCTCCATTGTCCCGCAGGGGCC   74797

LOC112206721.putative.POM121-like,protein1.START.LOC112206738      gaaatgtagaccgtgcatccccggtgacctcggggacccttctctgatcaccaggattct   34504
BCRP3.REF.NEW                                                      ------------------------------------------------------------   20446
GGT1.start-POM121L10P.end.24583750-24659147.ref                    GAAATGCAGACGGTGCATCCCCGGTGACCTCGGGGACCCTTCTCTGATCACCAGGATTCT   74857

LOC112206721.putative.POM121-like,protein1.START.LOC112206738      cttggactctagggtccttgtcctgctcaggcatccctgccccactctccttgagggccc   34564
BCRP3.REF.NEW                                                      ------------------------------------------------------------   20446
GGT1.start-POM121L10P.end.24583750-24659147.ref                    CTTGGACTCTAGGGTCCTTGTCCTGCTCAGGCATCCCTGCCCCGCTCTCCTTGAGGGCCC   74917

LOC112206721.putative.POM121-like,protein1.START.LOC112206738      tcaacactatcttccctggacacaagtctggggacagccgggtgttgtggaccccaaagg   34624
BCRP3.REF.NEW                                                      ------------------------------------------------------------   20446
GGT1.start-POM121L10P.end.24583750-24659147.ref                    TCAACACTATCTTCCCTGGACACAAGTCTGGGGACAGCCGGGTGTTGTGGACCCCAAAGG   74977

LOC112206721.putative.POM121-like,protein1.START.LOC112206738      ggtgactcccggctcctgggccccacagagagtccatgttctcagtgcagtggctgagct   34684
BCRP3.REF.NEW                                                      ------------------------------------------------------------   20446
GGT1.start-POM121L10P.end.24583750-24659147.ref                    GGTGACTCCCGGCTCCTGGGCCCCACAGAGAGTCCATGTTCTCAGTGCAGTGGCTGAGCT   75037

LOC112206721.putative.POM121-like,protein1.START.LOC112206738      ggaggacgccctggaactcggagcacacagcactggcttgctgtggtacctgtgcaatca   34744
BCRP3.REF.NEW                                                      ------------------------------------------------------------   20446
GGT1.start-POM121L10P.end.24583750-24659147.ref                    GGAGGACGCCCTGGAACTCGGAGCACACAGCACTGGCTTGCTGTGGTACCTGTGCAATCA   75097

LOC112206721.putative.POM121-like,protein1.START.LOC112206738      aattgaaggcaggatcccaggaaggaacgcagggcttgcaggatcacggaaaaccttctt   34804
BCRP3.REF.NEW                                                      ------------------------------------------------------------   20446
GGT1.start-POM121L10P.end.24583750-24659147.ref                    AATTGAAGGCAGGATCCCAGGAAGGAACGCAGGGCTTGCAGGATCACCGAAAACCTTCTT   75157

LOC112206721.putative.POM121-like,protein1.START.LOC112206738      agcgttgtcttgacaccactgatgtcaagtgtccgggtgcttgtaggatggcctgccact   34864
BCRP3.REF.NEW                                                      ------------------------------------------------------------   20446
GGT1.start-POM121L10P.end.24583750-24659147.ref                    AGAGTTGTCTTGACACCACTGATGTCAAGTGTCCGGGTGCTTGTAGGATGGCCTGCCACT   75217
```

```
LOC112206721.putative.POM121-like,protein1.START.LOC112206738      cagtccacgggcaggagcaacggggagatcccacaagcaaagtgaactggggg atgggct   34924
BCRP3.REF.NEW                                                      ------------------------------------------------------------   20446
GGT1.start-POM121L10P.end.24583750-24659147.ref                    CAGTCCACGGGCAGGAGCAACGGGGAGATCCCACAAGCAAAGTGAACTGGGGGATGGGCT   75277


LOC112206721.putative.POM121-like,protein1.START.LOC112206738      gaacgggctccaggcaactgagccctactggcaggtcctcggcctgggcccgaacaggaa   34984
BCRP3.REF.NEW                                                      ------------------------------------------------------------   20446
GGT1.start-POM121L10P.end.24583750-24659147.ref                    GAACGGGCTCCAGGCAACTGAGCCCTACTGGCAGGTCCTCGGCCTGGGCCCGAACAGGAA   75337


LOC112206721.putative.POM121-like,protein1.START.LOC112206738      ggaggggcacagagtgcccaggtagccgctcctgggagcagtggggaaccgtcggttgct   35044
BCRP3.REF.NEW                                                      ------------------------------------------------------------   20446
GGT1.start-POM121L10P.end.24583750-24659147.ref                    GGAGGGGCACAGAGTGCCCAGGTAACCGCTCCTGGGAGCAGTGGGGAACCGTCGGTTGCT   75397

MOST BUT NOT ALL POM121L10P IN CHIMP
LOC112206721.putative.POM121-like,protein1.START.LOC112206738      tgaactctcgagagctgggctctgagcgtcctcgtccagccgccaactcggccaaaggct   35104
BCRP3.REF.NEW                                                      ------------------------------------------------------------   20446
GGT1.start-POM121L10P.end.24583750-24659147.ref                    T-----------------------------------------------------------   75398


LOC112206721.putative.POM121-like,protein1.START.LOC112206738      aagccagcagtttcttctgttgccgggcaacgcgccttttaaacctgagggagcgggcgc   35164
BCRP3.REF.NEW                                                      ------------------------------------------------------------   20446
GGT1.start-POM121L10P.end.24583750-24659147.ref                    ------------------------------------------------------------   75398


LOC112206721.putative.POM121-like,protein1.START.LOC112206738      gtgagcacatcatggcgcccgtgacagagcgagcttaacggattaataagcgcagccagg   35224
BCRP3.REF.NEW                                                      ------------------------------------------------------------   20446
GGT1.start-POM121L10P.end.24583750-24659147.ref                    ------------------------------------------------------------   75398


LOC112206721.putative.POM121-like,protein1.START.LOC112206738      tacccgcgcgaggcacttgctggcaatggcgggaggcggacgtgggggtcatgcaatag   35284
BCRP3.REF.NEW                                                      ------------------------------------------------------------   20446
GGT1.start-POM121L10P.end.24583750-24659147.ref                    ------------------------------------------------------------   75398


LOC112206721.putative.POM121-like,protein1.START.LOC112206738      gtactggaaggagagaggcgggcacaaaggtcgcgggaggaacaggcgcccacagtggcg   35344
BCRP3.REF.NEW                                                      ------------------------------------------------------------   20446
GGT1.start-POM121L10P.end.24583750-24659147.ref                    ------------------------------------------------------------   75398


LOC112206721.putative.POM121-like,protein1.START.LOC112206738      gcagatctgcccgtggatcactgaagattcctgctctcctgctgaggtggagattgcagt   35404
BCRP3.REF.NEW                                                      ------------------------------------------------------------   20446
GGT1.start-POM121L10P.end.24583750-24659147.ref                    ------------------------------------------------------------   75398


LOC112206721.putative.POM121-like,protein1.START.LOC112206738      gagctgagatcgcaccattgcactccagcctgggccacaagtgcaaaactcag     35457
BCRP3.REF.NEW                                                      -----------------------------------------------------     20446
GGT1.start-POM121L10P.end.24583750-24659147.ref                    -----------------------------------------------------     75398

+++++++++++++++++++++++++++++++++++++++++++++++++++++++
```

```
>LOC112206721.putative.POM121-like,protein1.START.LOC112206738.putative.POM121-like protein1.END.7525566-7561122.chimp
        1 gacagggtct tgctatgttg tccaggctga tctcaaacac ctggcctcaa gagatcctcc
       61 tgcttggcct ttcaaagcac ttggattatt ggtgtgagcc accacttccc acatagcctg
      121 ttccatcact ttttacttaa tttcacaact ttccctgacc attgcctcaa atcagctttt
      181 tttctgacag ctgcattgta gtctttgtca gagttatgca cagtctcctg catctacggg
      241 gcctagaagc ctgggatctg ggggagccac ccctgagggc gagtgtctcc cctggtgctg
      301 tatctgccgt cttttcacac tgggtgtgac ccgaagagac agcctgaggt ccgtcctcac
      361 tcactgtgtt tgaggaactg agggtcagct ggcagtggga tgaggctggc ccctcttccg
      421 ctttagttcc gggaggcctc ccgtagagct gtaggggctc gagatggcct ttcatttggg
      481 gcacgagctg gtcccggagg tctgggatct ctggttctga cctctgggca cctgctgcag
      541 ctgtggctga ggcccagaaa tgtgaaggcc ctccatccac tccagtagtg accccaacgt
      601 ggggttcagt gtggaggggg gaggggctgc tgcggcagct gcaggagcag aagtgccacg
      661 ccttgttctt ctcatgcccg catccatgct tgcagctggg aagggggcag gaatcagcga
      721 ggtgacctgg gctgagtcct gggaatggga agaggtggca ggaaggggat ctgaggagga
      781 gaacaggggg cctggtggtc tgtgcttctt cccagacatg ggagctgtag aggggacctc
      841 tgcagcagat gctaaggggg ccagtaggcc caggcagtct tgggacttgt gtgtctcctg
      901 ctgtgcatcc atactgggtg ctttagaaac ggcaggcaga ccagaagccc ctgttgcaag
      961 tgaggacaaa gtgtgggaag gccgtgaggg tctgcagtcc gagatggcct tgtcctcaac
     1021 ctgcagtgca ctgttgatgc gctggaaggc cgcctccttc tcccggtcca ggtcttcaac
     1081 agtgacccgg taccccagct ctaagggagg tggcagcatc aaaggctccc ctcgcctgcg
     1141 tggcagcagg ggaaacttgt gtgtactggg cctagaggcc tcggatgtgg aggagtcatt
     1201 cctgagggcg agtgtctgcc ctggtgctat atctgctgcc ttttcacact gggtgtgacc
     1261 tgaagagaca gcctgaggcc tgtcctcact cactgtcttt gaggaactga gggtcagctg
     1321 gcagtgggat gaggctggcc ccctcctcca ctttagcccc ggcaagcctc ccgtggagct
     1381 gtaggagctg gagatggcat ttcggttggt gcaggagctc gtccaggagg tctgggatgt
     1441 ctggttatat ctgatttctg acctctgggc atggaggtct gtctgcagag gcccaggcct
     1501 gggcacaaag ggagagaggc ctccattgtc ccgcaggggc cgaaatgcag actgtgcacc
     1561 ctcggtgacc tcagggaccc ttctctgatc accaggattc tcttggactc tagggtcctt
     1621 gtcctgctca ggtatccctg ccccgctctc cttgagggcc ctcaacacta tcttccctgg
     1681 acacaagtct ggggacagcc gggtgttgtg gaccccaaag ggtgactccc cggctcctgg
     1741 gccccacaga gagtccatgt tctcagtgca gtggctgagc tggaggacgc cctggaactc
     1801 ggagcacaca gcactggctt gctgtggtac ctgtgcaatc aaattgaagg caggatccca
     1861 ggaaggaacg cagggcttgc aggatcacgg aaaaccttct tagcgttgtc ttgacaccac
     1921 tgatgtcaag tgtccgggtg cttgtaggat gacctgccac tcagtccacg ggcaggagca
     1981 acggggagat cccacaagca aagtgaactg ggggatgggc tgaacgggct ccaggcaact
     2041 gagccctact ggcaggtcct cggcctgggc ccgaacagga aggaggagca cagagtgccc
     2101 aggtaaccgc tcctgggagc agtggggaac cgtcggttgc ttgaactctc gagagctggg
     2161 ctctgagcgt cctcgtccag ccgccaactc ggccaacggc taagccagca gtttcttctg
     2221 ttgccgggca acgcgccttt taaacctgag ggagcgggcg cgtgagcaca tcatggcgcc
     2281 cgtgacagag cgagcttaac ggattaataa gcgcagccag gtacccgcgc aaggcacttg
     2341 ctggcaatgg cgggaggcgg acgtgggggg tcatgcaata ggtactggaa ggagagaggc
     2401 gggcacaaag gtcgcgggag gaacaggtgc ccacaatgcg gcagatctgc ccgtggatca
     2461 ctgaagattc ctgctctcct gctgaggtgg agattgcagt gagctgagat cgcaccattg
     2521 cactccagcc tgggccacaa gtgcaaaact cagtctccag attaaaaaaa gaaaaagaaa
     2581 aaaaagaggc cgggcgtggc ggcttatgcc tataatccta gcactttggg aggtcggggc
     2641 agacggatca cgagatcagg agttggagac cagcctggcc aacatagtga aactccgtct
     2701 ctagtaaaaa tacaaattta gtcagacatg gtggcacgcg cctgtagtcc cagctactcc
     2761 ggcggctgag acaggagaat cacttgaacc cgggggcggg cggggaaggg attgtgatgc
     2821 gccgagatcg cgccactgca ctccagcctg ggcaacagag ccagactctt tttttttttt
     2881 tttttttttt tgagacgcag tctccctctg tcgcccaggc tggattccag tggcctgatc
     2941 tcggctcacc gcaaactccg cctaccgggt tcacgccatt ctcctgcctc agcctctgga
     3001 gtagctcgga ctacaggcgc ccgccaccac acccggctaa ttttttggat ttttagtaga
     3061 gacggggttt caccatgtta gccaggatgg tctcgatctc ctgacctcgt gatcagcctg
     3121 cttcggccta ccaaagtgct gtgattaaag gcgtcagcca ccgcgcccgg tgttgaaact
     3181 ctcttgaggc attttcttcg gccttcgggt ccatcctctg tctcctcctt gacctcctca
     3241 tctcctcctc gccactgcct tggggacctt ggccaggctc atggcatcca gcagccactc
     3301 aatgtcaata cctccatgtt catctctcag ctcgcccttg cccatgaacc catgcttatt
     3361 tatttatttt ttttatatgt gcacccatgc tcttcgggtc tgatgaagta tccaaaatca
     3421 agctcctgac catccccaaa cctgcccctt ctgcagggtt tacctcgcta gtcagcgcca
     3481 tccttgattc ttctctttct cccacccagg ccatcatctc ttgcctggtt gatacccaca
     3541 gcctcccctt tgggctttat ccttatccct gtcatagctg ccagagggac cctgtgaaaa
     3601 cactccccag cctgctcatt cctctgccct aagcctgcat ggcacagagc aaaagccagt
     3661 tgttatggga cctaggaggt cctgtgggat gggccccagc ctgcatcttc atcctcttct
     3721 ccccaccccc ctccattcac tctctgccta tcgctcacca gcctatacca cctgcctcag
     3781 ggcctttgca ctgaccattt aggccacatt ccaggctctt ttcacatgtt gcctcctctg
     3841 agaagccctc cctgaccact ctgcccatac ctcatgcctc ttgattcccc ttacctggct
     3901 tgtggtttca gcactttccc tgcgtgtgtt tgtttttctt ggcatgaggg caggacctaa
     3961 gtgtctgttc cctgttgatt ccccagtgcc aggcatgcag tgcaaactct agaaatattt
     4021 tttgcatgaa ggaatgagtg attgaatgca gcaagggtct ggaggctgag gaccaggcag
     4081 acagacattc agagttgctg gaacgcgaca gagacaggga gtcagactgg tcatgcaaga
     4141 tcctgggcct gcccttgggt cctggggagc catggaaggt tgtgggtgcc agagggttgt
     4201 ggtcagagcc acagtcaggg gccttctgag acctgtgccc cctccccacc ctccctcccc
     4261 acctcctcag gccagctctg ggtctcggc aggtggtccg cagcatgacc tctgagttct
     4321 tcgctgccca gctccaggcc cagatctctg acgacaccac tcacccgatc tcctgctaca
     4381 agcccgagtt ctacacgccg gttgatgggg gcactgctca cctgtctgtc gtcgcagagg
     4441 acggcagtgc tgtgtccgcc accagcacca tgaacctcta gtaggggctg ctgggccgcc
     4501 tgggtgggaa agggccaggg gcgggtggcc cagggactgc ccacttatcc agtaaggtgg
     4561 ctccgtcacc tcttttcctg gtgggaaact gaggcccaac cttggtagct tatcctgggc
     4621 ctctcagtga gtatgtttga gcctcagtgg gtggataggg accaggctgg gccaggcaag
     4681 gtcgggcact gtctgacctg gctgggcggt agctttggct ccaaggtccg ctccccggtc
     4741 agcgggatcc tgttcaatga tgaaatggac gacttcagct ctcccaacat caccaacgag
     4801 tttggggtgc ccccctcacc tgccaatttc atccagccag gtatggggtg gaggtccggg
     4861 ggtgggggac tggggtggag aggggcgggt gtcctgggca ggcagctgac gggcatccct
     4921 gtcttctccc atcggccgca gggaagcagc cgctctcgtc catgtgcccg acaatcatgg
     4981 tgggccagga cggccaggtc cggatggttg tgggagctgc tgggggcacg cagatcacca
     5041 cggccactgc actggtatgt gtcacacctt ttctccctgg ccgtgcccac cctgcacagc
     5101 ccccaagcca tgctgatcac actcccatgc cccaggccat catctacaac ctctggttcg
     5161 gctatgacgt gaagagggcc gtggaggagc cccggctgca caaccagctt ctgcccaacg
     5221 tcatgacagt ggagagaaac attgaccagg tgggccgggg gttggagaaa ctgagtcacg
     5281 gtgtggggcc ccagggcatc ctgggctgga ggcctggatc atcacagagt ggacaattgt
     5341 tggtgtcctc tctctagtgc ctgggccatc tggagcccct gtgacatgag ggccaagccc
     5401 cctgctccag tgagacccag taggccccaa cctgcccttc ctgatgacct ggcccgaaat
```

5461 ggcaccacct gggctgaggc ctgtgaccac acaggtgtgg ttcaggtggc atctggagcc
5521 ttgctcaggc ttcccctctc ctcccacccc caggcagtga ctgcagccct ggagacccgg
5581 caccaccgca cccagatcgc gtccaccttc atcgctgtgg tgcaagccat cgtccgcacg
5641 gctggtggct gggcagctgc ctcggactcc aggaaaggcg gggagcctgc cggctactga
5701 gtgctccagg cggacaaggc tgacaagcaa tccagggaca agatactcac caggaccagg
5761 aaggggactc tgggggaccg gcttcccctg tgagcagcag agcagcacaa taaatgaggc
5821 actggagagg cgggattcgg agatcttcac gctgcggtcg ctggaactag cctcagcatc
5881 ttcagcatgg ggaaagccag gcacgtggct gggggccagg ggaaggtcca tgccaagccc
5941 tgccccttcc caccctgatc cctcggactt tggggccagg ccctcccttc cctgggctgg
6001 gcagcaacac cacctaggac cagccaccag agggtgcagc gaccccggcc ctttcttagg
6061 cagagagtgg ccagctgatg ccgggaaccc gggtgccttc tcagacccgt aggcgtccag
6121 ctcaccctgc cgaggacacg ggaggtgaag ctgaggtctg aggagtgggg attgggcaag
6181 aggctggagg aaaacatctc ggtcagagcc aagcccctgg ggggtttcca agtttaagcc
6241 cagagtgaaa cccaagcttg tgatcctctc cagagggagg cctggttctc agggaacagc
6301 aaactggaag aggtccccag atcccaggga tcagggcttg gaccagccgg ggacgcagcc
6361 cagagggagt gggtctggaa gggaacaact agacacagca gtcttcacca tcggcagccc
6421 ctccaggcct ccctcggggc ctgctccctc ctccaggcac agttccaaca cctggggcag
6481 gattctggga aaggctggtg gaggtaggct ggtgaggggt ggtggtcaca gcccagcagc
6541 aggggcactc tggatatcac caggggcact ctggccaggt cccaggtgag gccaggtcgg
6601 ggctatcctt caggagtccc gaaaacctgg tgataccaaa gggcccacgg acaaacaggg
6661 ttttgtgcct gcagagtaga gtaccaccgg gtctgagccc tggagggctg tgtctctggg
6721 gctcccagtt gagtgatgta cgagtcactc ctcaatcctt attttattta tttaatttaa
6781 aaaattattt aaacaaatag agatggggtc tcactatgtt gaccacgctg gacttaaact
6841 cctgacttca agcagtcccc ccatctcagt ctcccaaagt gctaggatta cagaggtgag
6901 ccactgcagc cagcctcaat ccttattttg gcctgagagg acaggccgtg gcctcatttt
6961 caggggagaa gactgaggct ggagggcag gccttgctct gggttgcaca gcagcaagag
7021 aagtgagagc tggccacgag gcttcctgga cccgacacgc tggtggggta caccctggtt
7081 ctccaggtcc catggggctc agcccaggac tacctcgggg ggtgagggac ttaaatcctc
7141 tccttcattc tcatcgcccc ttcccccacc atttcctgag gaaggacatt cagggacctt
7201 cctggctgtg cctcggatca agaccagaat gacacccatt cctttccctg ggcctttgct
7261 caggtggtcc ctgcaccctg gcctctgcct gaccaggatg gtggggagag gaggggggac
7321 gtcccccacg ctgctgtctc cactgttcct gctgcccagg cctctgagct tccaggactg
7381 cagcgggtgg gtgggtgggc tggcctaagc ccaggaatgc acttcagctc ctggttgagc
7441 aatgtcactg aggcttggga gtcgggtggg ggcgggagga ggcgtccaca ggccccccac
7501 cgcgagaggc agccgtggga acagcctgcc tctaaacaat cactccagtc caggctgacc
7561 aggggctctg gctggacata ggggcctggc aggctgtgtg gcctgtaagg acacagtctg
7621 tctctgtgcc tcagtttctc tgctgcccag atgggggggc ccggattcca ggtgtagaca
7681 tctggagcag gcagtgttca gctggggagg aagcggggag gactgtgggg gccacatggg
7741 aaggattcca gcccacatca cctgcacccc tgctgagcct ggtcaacgga gcccctcagt
7801 gggtcctcac tcccctggct gcctcccatt taggcaccct gaggcctggg gagaacagag
7861 ccaggccagt gtccctagag aggctgcgct gccggcacag taatagcgga tttggattca
7921 gggaagcaga cccgcatcca gggtgggaaa gagctgcagg cagggcgtgg cctccacaca
7981 gcctgcctcc ctggaggtcc atgctgcatt tccaggacag caagtcctgg acaaatggtc
8041 ccggatgcca aaggctagag gcatggtctg tctgcattcc ccacatgggc gtcttgtagt
8101 caccagcatt tgatgctgtc aagtcccccct gtcctctgtg cagactggga agcccttggc
8161 caccctgggg agttgtggga cccaggccag gctgcagaag cataaggact tgaacccaag
8221 ttttaagtga caccaccttg tgtcccccctc cctctgtctc tgtttagaac caccttgatg
8281 ctgactaggc tgggccatgc ggagagggtt aggggataga gatgagagct ggggagcagg
8341 gctccactct gggagggggg cagcctcgcc ggatccaggg gagagagttg agcggcccca
8401 gctctgcttt cccagagctg ccgggaaccc ggggaatggt gtggaggttc cagggagccc
8461 tgcccctacc tggcaaccac agtgcagcag gcaccaagtt ctcctgcaca ttgcgacagt
8521 gtgaccctgg gctctggcgg gcagtaggtg gggcctttgg acctaccagc agtgagggag
8581 ttaacacagc agctgactcc tctaggcaag gaaaactccc ctcagatgct ttgctgcctg
8641 gcctcctgcc agcaacaagc aggagctgaa aactagaagt tgaggcgtga gtttggccac
8701 tccgtagtgt gcacttggtg agggcagcag ctgccagccg tctgtccatt cacccatctg
8761 tccatctggc agcccgctgt tcagacctgt ctgtctgccc atctgtaagc ccatctctgt
8821 cccattgtct atctgaccat ctttctctta ctgtcctctt tgtctagcta tctggcctgt
8881 cgatccatct ttgtgtctgt cttcagcccc cacctgtttg tccatctgtc caattacctg
8941 tgagtctatc tatgcatctt cttgtccatt catctgccca cccgtctgtc cctccgtctg
9001 cccaccggcc tcccctctcc ttctgggccg cagagccatg gcccaggact gcagagccat
9061 ggttggcctg gtcctgctgg ggctggggct ggcgctggct gtcattgtgc tggctgtggt
9121 cctctctcga caccaggccc catgtggctc ccaggccttt gctcacgctg ctgctgctgc
9181 tgactccaag gtctgctcaa atattgtatg gtgagtgaga cgtgggagga agctgggtgg
9241 cccttggcag ccagcccctc ctggagaagg cgtgtgtgtg tgagcatgtg tgtgtgtgtg
9301 tgagattatg tgtgagtgtg agtgtgtgtg ggtatatgtg tgagtgtgtt tgtggagtgt
9361 gggggtgtgt gaatgtgtgt gatcgtgttt gggtgtgtgt atgtgtgagt gtgggtgtgt
9421 gaatgtgtgt gattgtgttt gtgtatgtgt gagtgtgtgg gtgtgtgtgg gtgtgtgagc
9481 gtatatgtga gtgtgagtgt gtgggggtgt gggtgggtgt gaatgtgtgt gattgtgtta
9541 cgctgtgtga ggttgtgtgt gactgagtgt gtgagtgtga gtgtgtgggt gtgtgtaaat
9601 gtgtgtgatt gtgtgagtgt atgtgggtgt gggtgtgtgt gagtgtgagt gtgtgggtgt
9661 gtgtaaatgt gtgtgattgt gtgagtgtat gtgggtgtgg gtgtgtgtga gtgtgtgagt
9721 gtgagtaggg ggggtgtggg tgtgtgtgaa tgtgtgtgat tgtgtgtggg tatgtgtatg
9781 tgtgggtgtg tgagcgtgtg tgtgcgcgtg tgtgtgtgca cacgtgcact ggcccaggaa
9841 gcaggagccg tgtgtgtggg cttcagcacc tgcagggctt gagcgcaagg agacagcctc
9901 agggcccttg cacagaacag gtggcagggt gtgcccgtgg ggcagatggg gacttgggga
9961 caatggtggt gtgtgagtcc atacctggct ccaggattca ggaggcccat ttgcatatcc
10021 caggtgggaa cctgtctggc cccagctgac cctgctggcc ggtgcaggtc ccttcagtga
10081 ggccaattct ccaaggctgg ggtcttctcc cagggtcatg ggcgaagggg tttggaggct
10141 ccctgcgtgg gtactggcct gctggggtac acacaatgct gccatagcca gtctgcccca
10201 acacccagcc cggggccaca tctcaggtct ctcagtcctg aggagcccgg tgccccaccc
10261 ctcacatctt ctctccctga gtcagggcct ggatctcgtg agctgagtga cttgatactt
10321 ggtgtcctgg atgagggcgt gatggagagg ggccacagtg ggtgtttcct gaccctcttc
10381 caggaaggtg ctgctgccgc tgcagggagg acacatacag gatgcccctt cctgcccccct
10441 gcctcccatt gggcccacaa aagccagggc aagcctcccc tccttgccag ccacctggtc
10501 tgcttcccag aaattctgtc ttgcaggctg ttgggaggat cccagtactt tgtaaactaa
10561 agcaagggag gagtggccat tctctctctt tgttcattca ttcacctttt cattcattcc
10621 ttcttccctc cattccccca tctgtccatc cttccctgcc ctgattgctc atgccacccc
10681 cccagcccct cctgacctgg tcctttggtt tctcttcagg gctttctgtc tcctcccaca
10741 gggctgagaa tggcagctca gggacaagtg ggggctgggg actgcttagt ctccccagtg
10801 gctctcaggg gatttgaggg tttgacgcca gctgccaccc caggctgtgc ccctcctctg
10861 ctcaggagga catacagaga tgcgacaccc acttaaactc aaagttgcaa agatgcaaat
10921 gagactgggg tctcaggcac cagagaccac ccttggtcac gtggcttttg ggagtgggga
10981 tctgctgcca cagatctctg aagagtctag acctgctggg tctccccgag tgactgtctg
11041 ggggtctcca tagcatgccc tgctgtgtgc gtgacggtca ctggttgggt aggggtctct
11101 actctaaagc tccttctgcc ggcatcccct cgaactctcc cttggtgaag agagaggatg
11161 tggtttgccc cagtgtttta gcaaacaact ctctccactt cctgttttaa gaagctggga
11221 gtggaagaga gactggggct ggccccagct gctgctgtga aacaggggtc actggacgct
11281 gggaccctgg ccgggctggc tggaggcctc aggaagaggc ctgctacagc gtcatcctgg
11341 ccaagattcc tccctgcaga ggaccctggc cacactgcca cagggtctgc tggggccacc
11401 agaagcccat gctcctgcct ccatctctcc cctctgtgct cacctctcac caggaggccc
11461 tcccagagtt cagtgtcctg cttttttttt tttttttttt tttttgagac agtgtctcac
11521 tctgtcacca ggctggagta cagtggcgcg atctcagctc actgcaacct ctgcttcctt
11581 ggttcaaatg attctcctgc ctcagcctcc tgagtagctg ggactacagg tgccagccac
11641 cacacccagc taatttttgt atttttagta gagacggggt ttcaccatgt tggccaggat
11701 ggtctctatc tcttgattcg cccgccttgg cctcccaaag tgctggcatt acaggagtga
11761 gtcatggcgc ccggccccat ctcctactct ttcagcccca ggttttattc ttgggattct
11821 gctacagccg gagcccctgg gtgcgagctc ctaagctttc tgtgagtgtg gacccagcac
11881 tgtgcctagt agacatacaa aaggagcatg gtgacagtga ggtctgtcat ctccagcata
11941 atgactgttt tgatgcttgt gaaaaaggtg atttttggct gggtgtggtg gctcacacct
12001 gtaatcccag cactttggga ggccgagggg ggtggctcac ttgaggtcag gagttggaga
12061 ccagcctggg caacatggtg aaaccacgtc tctactaaaa atacaaaaat tagctgggca
12121 tggtagcggg tgcctataat cccagctact tgggaggctg agacaggaga atcacttgaa
12181 cccaggaggc aaaggttgca gtaagccaag attgcgccac tgcactccag cctgggtgac
12241 agagcaagac ttggtctcaa aaaaaaaaaa aaaaagaaag aaagaaaagt ttatattttt
12301 gttctaatgg ttatcttaat atcttcattc tataattata tgttttatat aattataata
12361 gctatataag atataatacc cctagtatgt tgttttttgg atattctact cgctcctgat
12421 ggttaattta tgtgtcaact tggctaagct atggtgcccc gttgtttggt caaatacttg
12481 tcaatatctt gctgggaggt tatttcatag atgtgattaa cactgacagt cagttgactt
12541 taagtaaaac agattaccca ccataatatg ggtgggccac ctccaatcat tgaaggcctt
12601 aagaacaaaa actgaggttt cccagagaag caggaattct gcttcaagac tgtaacacac
12661 aaaccctgcc tgagtttctg gcctgctgac tgctctacag attttaggtt ccagacttcg
12721 agatcaactc ttacctgaat ttatagcctg ctggcttgcc ctacagattt taaacttgct
12781 agtccccaca atcatgtaag ccaattccta aataaatctc tctctatgta taacctattg
12841 gtttagtttc tctgaaaagc tttcacatcc agtttcctgg atgttaagaa ttattggaac
12901 tagctagtaa cttcttcttt tttttttttt tttttttttt ttgagacaga gttttgctct
12961 tgttgcccag gctggaatgc aatggcacaa tcgcagctca ccgcaacctc cacttcctgg
13021 gtccaaacaa ttctcctccc tcagcctcct gagtagctgg gattacaggc atgtgccacc
13081 atgcttggct aatttttgta tttttagtag agacagggct tctccatgtt ggtcaggctg
13141 gtcttgaact cccaacctca ggtgatcagc cgccttggcc tcacaaagtg ctggaattac
13201 aggcatgagc cactgcgcct ggctcctagt aaattcttct tttccgtgat gtgtctctta
13261 cctctaataa tacttttctt cttaaagtct acttcattaa aaatagttat gctgggcatg
13321 gtggctcatg cctgtaatct cggcactttg ttggaggttg aggtgggtgg atcactgaag
13381 cccaggagtt caagagcagc ctgggcaaca tggcgagacc ctgcctctac aaaaaataca
13441 aaaattagct gggtgtggct aatataattc taagttggca cacttgtagt cccagctact
13501 tgggatgctg aggtgggaga atcgcttgag cctagaaggg agagattgct gtaacccaag
13561 atcacatcac tgcactccag cctgggagac agagtgaggc tctatctcca aaaaaaaaaa
13621 aaaaaaaaaa aaaaaaagcg atacagcttt cttggttagt gcatgcatga catatttttc
13681 attattttcc acctctctgt atccttatat aaaaggcatt agttgggttt tactttattt
13741 tcagttattt taacttttat tgtcctttta aatgtaacta atgatttatt tgggttgaaa
13801 cccaccacca atttgttttc catgcctatt ctatttcttc ttatctcctc tcacatcttg
13861 ttttggattt attattttta ttattttttt atttaatttc ctccttctct attagtttca
13921 taactgtgca gtcttagagt tattttaaaa gatgactgga ttattttaga gcttacaaca
13981 tgcatccttc acttatcaaa gtctaacatg agctagtact ttttgttgtt gagatagaga
14041 gagtcttcct ctgctgccca ggctggagtg cagtggagca atcttggttc actgcaacct
14101 ccacttcttg ggttcaagca attctcctgc ctcagtcacc tgagtagctg ggaccacagg

14161 tgtgcaccac tatgcccggc taatttttgt attctttttt agtagagaca ggggttcacc
14221 atgttggcca ggctggtctt gaactcctga ccttaagaga tctgcttacc tcggcgtcct
14281 aaagtgttgg gattacaggc gtgagccacc acgcccagcc tatgagttag tacttctatc
14341 ctcttcctag tcagtacaag aaccttggaa caggaactaa atttaccccc agtgacttat
14401 atgctaatat ttttgtgtat tttaaataca caaaatctgt atgtttttttg tgtttttatt
14461 cttatttatg ttgagagtgt agagctatgt aagagtaaag agaattgtgt aatgaagccc
14521 cgagtatcca ttcaatttca acaacaatct tatggccaag ctaatttcat gtatactctt
14581 tcctgcttcc ctctacccca cattatttca gtgcaaatcc cagatatata actgtaccca
14641 tacatatttc agtatgtttt atttatttta aaccccacaa gatatcattt tctatactac
14701 tgtaatttca taccaataac attcatttag atttacccac acatctacct cttctgttac
14761 cctttatttt tatttataaa aatatctttg ggaaaaaata tctttcagca catggtcaag
14821 gatctcctga gggctatgtc atgggcaaaa tatacatata tattccatat atatacacac
14881 atatacacac acacatatat acacatatac acacacacac atatatatat tccactttca
14941 ctttttttgtt tgtttgtttt ttgagaccga gtcttgctct gtggcccagg ctggagtgcg
15001 gtggtgggat ctcagctcac tgcaacttct acctcctggg ttcaggtgat tctcctgtct
15061 cagcctcctg agtagctggg attacaggtg ttagccatca cgtctggcta atttttttttg
15121 tttttttttt ttgagacgga gtatcgctct gttgcccagg ctggagtgca gtggcgtgat
15181 ctcggctcac tgcaacctcc atctccctgg ttcaagcaat tcccctgctc agcctcccaa
15241 gtagctggga ttacaggtgc acgccaccat gcccggataa tttttttgta tttgtagtag
15301 aaaccgggtt tccctatgat ggtcagactg gtcttgaact tctgacttca ggcaatccgc
15361 ccacctcggc ctctcaaagt gctgggatta taggcatgag ccaccatgcc tggcctgttt
15421 tttttttttt ttgagatgga gttttgctct gtcacccagg ctggagtgca gtggcaagat
15481 cttgtctcac tgcaacctcc acctctcggg ttaaagcaat tcttgtgcct cagcctcctg
15541 agtagctggg attacaggca tccaccacca catctggcta atttgtgtat ttttggtaga
15601 gatggggttt caccatgttg gccaggcagg tctcgaactt ctgacctcag gtgatccacc
15661 tgcatcggcc ttccaaagtg ctgggattac aggcatgagc caccatgccc agccattttc
15721 acttttgaag gatattgtta gtgagcatag aattctaggt tggcagatat tttctttcct
15781 cagtttgaaa acatgattcc cttgtatctg atttctcctg cttttattgg gaagccaatt
15841 ctcaatctaa ttttgctcat ttgaaggcaa tggcttttta tgttgttgtg ttttctgaga
15901 tggagtctca ctctgtcacc caggctggac tgcagtggtg caatctcagc tcactgcaac
15961 ctctgcctcc tgggttcaag tgattcttct gcctcagcct cccaagtagc tgggattaca
16021 ggtgtccacc atcacaactg gctaattgtt gtatttttaa tagagatgaa cttttgccat
16081 gttggtcagg ctgatcccaa actcctcatt tcaggtgatc cgcccgcctc agcctcccaa
16141 atgctgggat tacaggcatg agccagcccc caaccctggc ctgcaggcag tatctttttt
16201 cctctggctg ctttgaaaag ttttgtcttt gttttgagca gtttacactg atgcatttag
16261 gtggctcttc attccatgac ttgactcttt tttgtccatt ttagaaaact gccagattta
16321 tctcttcaag tattacgtct tccccatcct ctctctactc tccttatgag actccaattt
16381 cacatgactt atgccttgtt aaagtatccc ccatgtctct tagtccattt cctgtatgtt
16441 ctatctattt ttctctttgt acttcaattt gtatagtttg tatcaaacta tctcccaatt
16501 agctgggcgt ggtggtaggt gcctgtaatc ccagctactt gggaggctga ggcaggagaa
16561 ttgcttgaac ccgagaggtg gaagttgtag tgagctgaga tcatgccact gcactccagc
16621 ctgggcaaca gagtgagacc ctgtctcaat aaataaataa ataaatacat aaataccagt
16681 tcactatttt tttttatgtt tgtgtctagt gtgctgttca aattgagttc ctaattccat
16741 tttttttttt tgagactttt ttttttgagt ctctatctgt tgcccaggct ggagttcagt
16801 ggtgcaatct cagctcactg tagcctccac ctcccaggtt caagcgattc tcatgcctca
16861 gcctctcgag taactgggat taccaccacg cctaactcat ttttgtattt ttgtagagat
16921 ggggttctgc catgttagcc aggctggtct tgaactcctg gccttatgta attggcctac
16981 ctctgtctcc caaagtgctg ggattatagg cctaagccac tactcccagc ctcctttttt
17041 tttttttttt tttttgagac ggagtctctc tctgttgccc aggctggagt agagtggtgc
17101 gatctcggct cactgcaacc tcccctccc agttcaagtg attctcctgc ctcagcctcc
17161 cgagtagctg ggactatagg agcatgccac catgcctggc taatttttgt aattttagta
17221 gagatggggt ttcaccatat tggtcaggct ggtcttgaac ttctgacctc aggtgatcca
17281 cccacctcag cctcccaaag tgctgggatt acaggcgtaa gtcaccacgc ctagtgcatc
17341 catttttttgt agttgccagt tttctgatga aattcttaat tatttcttta tatccttgat
17401 acacatgtaa agaatttatt ttaaagtaca tggtctgatg attttatatt ctggagatcc
17461 tataggcctt tttcaaagtt gtctgtgctt tctcttgagt tttgttcctg ctgtcttatt
17521 tccttgtttg cttggttgtt tttaatttgg caatgaaagt tgtgtataaa aattgttaca
17581 aataattttt tttttttgag atgtggattc tcgctttgtt gcccaagctg gagtgcaatg
17641 atgtgatctc ggctcactgc aacctctgca tcccagggtt cacttctcct acctcagcct
17701 cccaagtagc tgggattgca ggcaggtgcc agcacgcctg gctaattttt gtatttttag
17761 tagagatggg ttttcaccat gttggtcagg ctggtctcag actcctgacc tcgtgatctg
17821 cccacctcag cctcccaaag tgctgggatt acaggcgtga gccactgcac ccagccagaa
17881 ataattttta aaaataattt caagcccaag catgatggct catgcttgta atcccatcac
17941 tttgggaggc tgaggcaggc agattgcttg agcctaggag ttcaagatca gcctgcgcaa
18001 catggtgaaa ccccatctct acaaaaaata aaaaattagc tgggtgtggt tgtggtgtgt
18061 gcctgtagtc ccagctgttt gggacgctga agtgggaggc tcacttgagc ctgggtgatc
18121 gaggctgcag tgagccatga tcctgagact gtactccagc ctgggcaaca gagtgagatg
18181 ctgtctcaaa taaataaata aataaaaata aaataatttg aggcctaggg gtctgaaatt
18241 ctgagatctc ctttatgcat ttgagtgact gagatgatct gaagctggat ccagtgctcc
18301 tgagggctgc tttatttctg gttgactgtg actcctagag taagaaacct gcaccccacg
18361 tgtggggcat tatggcatcc cctccctcag ccacatgagt aagtcaacag cactgctcta
18421 gaccaggtgt ggtggctcac gcctatagtc ccagctactc gggagactga ggcaggagga
18481 ttgcttcagg ccaggaattt gagaccagcc agagcaatat attattaggt tggtacaaaa
18541 gtaattgcag tttttgccat taaaagtaat ggcaaccctg tttcagcaaa ataaaaagca
18601 aaaaaaaaaa aaaaaaggaa gaatgaaaaa aagaatcaac cgggcatggt ggctcacgcc
18661 tctaatccca gcactttagg aggccaaggc gggcagatca tgagatcagg agatcgagac
18721 catcctggct aacacggtga aaccccgttt ctactaaaaa tacaaaaaat tagccgggcg
18781 tggtggcagg cgcctgtagt cccagctact caggaggcgg aggcaggaga atggcatgaa
18841 cccaggaggt agagcttgca gtgagccgag atcatgccac tgcactccag cctgggtgac
18901 agagtgagac tccgtctcaa aaaaaaaaaa aagaatcact gctctgtctc tcagcctcct
18961 cttccaggat tggccgtcgc cttgagggga atgctggcct tgcctgtctc cagccctgta
19021 cctctctgcc tcctatgcct ttaagcacat gttttctatt tgctgggctg tgaaatctgc
19081 tcttcatctg atggggtttg ctttataggt gactagattc ttttctcttg gcggttttag
19141 aatttgcatt ttcacattga ccttaaatag tctgattata gtttgccacg gcaaagaccc
19201 tttgcattgc attgtttggg gatatttgag cctcctctat ctggatgtct aatctcttgt
19261 tagatgtgag tagttttcat tattatttta ttaatgggct ggcatgtggg ccgtggttcc
19321 aggcgggctc agaggggcag ctgcctgatg tctggacagc ttctctttct gtcttttctt
19381 acctggactc tgggttgctt gttagctgct tctgccagtt ctgagtttca aggggagagg
19441 ggcccagtga tggctgttct ttgaaggaaa gggaagaatg tctcctgttt aacatgtttc
19501 tatgtttcca gttacttggt tagttagtta gagccagggt ctctcgctct gttgcgtagg
19561 ctggagtgca atggcatgat cgtggctcac agcagcctcc accttccagg ctcgagcaat
19621 gctcccacct cagcctctca agcagctggg actgcaggta tgtgccacca tgcttggctg
19681 ccttttttaat tttttttttt ttttaataca gacagggtct cactatattg cccaggctgg
19741 tcttaaactc atgggctcaa gtgatcctcc tgccttggcc tttcaaagtg ctgatatcac
19801 aggcagggtt tccgtttttt aaagctccca gcagtggtat gaaactcctc ctttccagag
19861 aaagcgcact ctgtccgcat ccctcatgtt gtcctctcct gcctctgctt agggttcact
19921 ccgggggaaa gtgccacttg agagtttcct ttttgtgtgt ggttctgact gaccgctccc
19981 tgctcacaga tgctgcttct cagggtgggg tccctgaggc ctggagtgtg gcctctgacg
20041 accttcaggg ccaggtgcgg aatgagagcc tgtggccaca tggcccctgg tgggagacgt
20101 cccgccgccc tttgcttctc tgtgccactc tggctgcaca gttcagagcc ttgggaaatg
20161 ttaaccagta ggacctagaa ggggaggtga gaaggggtca cccccaggtg tgcctgtggt
20221 gagccttcgt gctgagcagg tgcagggagg gaggcccagg tgcacacacc tgtgaagtag
20281 gggcagctgg ctgggctcct tgacctgctc cagagcttct tattttctgg ccacttcacc
20341 tgcagaaggc ccaggtggct gtggcctcta gggtcccttg cctgtcctca gctcccaact
20401 gggaggggca gagggaggag ggggtggaga ccccaggcag cagggctctg ggagcagtgg
20461 ggccctgggt tccaggggtg tctggcaggc ccctccttac tctacgtctc tcggcctctg
20521 gatggaggtg ctggccgcag tcaggctctg cctctgacta agggttggag aagtgtcgga
20581 tgtgggctgc tgccccgtgg ggcctctgaa cagaccccag ggcctctgcc aatcatgact
20641 ccttgctttc agctggaccc gcaggccctg caggacagag actggcagcg caccatcatc
20701 tccatgaatg tggtacatgt ccgtgggact ctcctggtgc ccacttcccc cagaaggata
20761 gggtggcctc tgttcatttc aaatcagtca gaggtggctg agcctgaggc ggcatctgag
20821 agggagcctg gttggagaag ggagggcccc caagagcaga atcaccatgc acaggaatcg
20881 tcattcattg gctggaatgc agttgccagc caggccctga gcttccctcc tgaaacaaag
20941 gtctcatggc accaccagga caggtggggc ctccactcag ggacctgggg gctgcccata
21001 gaaatggaga cccctgattt gtctttaggt accccagaaa ggtttagacc ttaaaagcaa
21061 tgacacaccc aaaaaggccc gggtataaat ggtaaaatgt taatatttga gattcttggc
21121 tttttcttac attattctgt ctttcctact taatttttaa ttgttactaa gagaaagctg
21181 gtcacagtac actataatct cagctactct ggaggctgag ccaggagaat cactggagcc
21241 caagagtttg attacagcct gggcaacatt gcaagatccc atatcttaaa aaaaagtaag
21301 caagcaagag aagcagtggg gattttagga gatggttctg cagaaaccag tcgtttacat
21361 catcttcaac aatcctggct cttgctgaag tagactaggg gcttccccga ggggcggctc
21421 cacctcatgc tgagacctct gcatgccttg ggggtggaaa tatttgatga gactcccagg
21481 ggtccttggg accttgggct atgaggacca gaaggattag aggactgtgc cccttctccc
21541 cactgtagat cgaagtaaag ctctcggtca agttcaacag cagggagttc agcttgaaga
21601 ggatgccatc ccagaaacag acaggggtct tcgagtcaag attgctgtgg tcaccaagtg
21661 agtggggagg ggcttgggct cacgcactga gggtgcctat cccttcagct gtttctgcag
21721 aaaagagcat gtgtgggtct ctcctctctg tgcgtggcca ctgcacggtg aggtcaggcc
21781 ccagggaaca ctgcgtgttc agctacctcc tgtgtttcct gcaaaccagc tcaggaatgt
21841 ccttgccacc ttgcttggaa gcggtaggct ggctccagga actgcccaag tgcagggttt
21901 tctgcccttg cttggaatta gtcatggtcc cagattcctg ttgaatgttt gtaacccctg
21961 cccctttgtc atgagtcagt tgccaagaga agcctgtttc ttggtttgag agcagttcat
22021 gcagacacag accacttcct ctgagaattt atttgcttcc ctaggatgga atctggctgg
22081 gcctctgacc ttgctggtca cgtgggccgg ggcctccatt agtcataccc tggactccta
22141 tctgtgtcta aacaccacgc cccaccccca actgcatggc agccactcgc atagcactct
22201 gggagggctg tgggcatgag cagcgaggac tccatcagca gctcccccag ataagccctg
22261 ctaatgaggg ggcttgcgaa gcagctttga tgtgctggta aatccaggtg caaaacagaa
22321 ctcaagttag ggcctctgca cagcactgcg ttctaactgt gaaggattct tactctagtg
22381 tcctgtgtgg aggtattgga attgtccatt gctaagactc agaggagaaa agcacttagc
22441 atcgcaggac ttggagcacc ggtgctgagg caacccttca ttcattcgtc ggatgtgtgt
22501 taaggcccag ggcaggggtc agggattctc ctctcacaca gcacgtgggt ggcaggacca
22561 acaccgggtc tgatctccca gccgggggca caggctgcta accccaggcc tggaatctgt
22621 cagatgccct tcctgtgctg acttgactta gacaggcctc ctgaccttcc cgcaaaggtc
22681 atgtgtgatt cgcaggggtt ctggccgctt gaaaggttcc tgagaaagta catgcaatga
22741 ggacagagct tgcagaggga ggacaggcat gcagaaggct ctgtgtgcag cgccagacct
22801 gggtaccttc gtcaccgtcc tcaccccacc tccgggtgtg cagatagga gcaggtctcc

22861 tgtgttatgg cccaagcagg gctgttagga cactgagagc attccctcct cccgcaggag
22921 agagaggtcc aaggtgccct acatcgtgca ccagtgcgtg gaggagatcg agcgccgagg
22981 catggaggag gtgggcatct accgcgtgtc cggccagtgt ggccgcagac atccaggcac
23041 tgaaggcagc cttcgacgcc agtgagtgtc ggcctgcgca ggacgggatg gaggtgtggg
23101 cagtggtgtc cgcaatgaga tctcagagtg ctccatggcc caggcatgtc acatccttct
23161 ctgtgtcttt tcttcattta ctgttttatt attttaaaaa aagagaaaac aagagttgta
23221 caaacagctt ccatagaagc cagtttttac accatcgtac ccactcatgc cacttggtgg
23281 agtggaccag gggcttctgt ggggacttgg ccttcctgcc ttgggggtgg acaggaggtg
23341 gaagcccagg actcagtgcg gtctgtccac tgccctgtat gaggatgtgg tgggcagagg
23401 gcactgatga aattcagcgc aggctggggc tgcagcatct ccgcctccat ctcaccaacc
23461 ctcacaggct ttgaaggacc cagacctgcc tcaaatgcca ggggagggca ctgagacccc
23521 agagggtcct tcccagcatc ttcaaagcaa caggattttg tgcctgcaga cccttctttg
23581 gggcacacac cacccaccct gaccaggacc cctagaatgc ccagcatccc tgggagggcc
23641 ctgtggtagt ttcagctccc tctgggggtc cagaatgaac ctcgcctgtg gtgaggatgt
23701 aagcaccaat ggccaattgg gtccaaagga agacaccggt tcaaacactg aaaccaatca
23761 gattctccca cggccttcct gctatcagaa gacactggtg caggggtggt tgctatgtac
23821 agggcagagc cacccaatcc ccatgcaggc gctgtgtcct gccatgctgg cctcctcctg
23881 gccatcacat caggccaagc aggggagagg aatgggaatg cccacgcacc cctatcaact
23941 ctgcagacac agaaccatgc acagctcttg ggaggagtca gatgagctgc tcaaagccca
24001 ggagggaccc acacagtggt cagcatggca gggacggtgc tttagccaag gcagggatgg
24061 cgggtgactc actcaggatc ttcaaggagg ccgctgcatt tccctgctct ttccagataa
24121 caaggacgtg tcggtgatga tgagcgagac ggacatgaac gccatcgcag gcacgctgaa
24181 gctgtacttc cgtgagctgc ccgagcccct cttcactgac aagttctacc ccaacttcgc
24241 agagggcatc ggtgagcact ggaggccttg gcctcatggg agacgtctcc gccacgtgca
24301 ctgctgccct cagaggctgt gaaaagcgag gtgtgggaac ctgagctgta acccctctgc
24361 catggtcaga attttaaccc aacctcaaaa agcaggggac cagaaccgag cctgtcctgg
24421 aaggccttgc ccatccccag agggctcccc atccctattc ctcaaggagg ccaagtgggt
24481 gaaatggtca gcactgccgt gctgtggggt cctaaagtct gctgtcctcc ttcctgcaga
24541 ccagggctaa acactggtgc ccaggtgctc ttgccatggg tcctggtcca gccaagcatg
24601 gtttcaaaca tgacctgacc cttagtcaac ctggaggctg atgtctagag tgggtgctgg
24661 tgcgtgcagc acctgtggcc tctgcatcac ccttagggca ggtctgcctc ccgggcccat
24721 gcacagagga cctggtctcc cagcctgcag gtgtccctgt gatgtccagg acgacgaggg
24781 gatctctgcg tacttggtgg ggctgggact ctcccacttc ccacctcctt gtgtccctca
24841 ctcccctgtt tcattccatg ctgagcctcc cctgccttgg gctcctctgg ggagggggtg
24901 gtggcaggag ttgtccaagg gcagctctgc ccacgagcag ctgctctagc ggctcctcct
24961 gctgctgttc accgggtgct gctgacccct gcgaggtaga gaaaaggcgt tcaggtggtt
25021 cacaccccac acaggtgccc ctcacagggt cctcactggt ggccagcgct gtgggtgtga
25081 cgatgatgac aagcctaaac tgcacaagga ctcgtgtccc gggtgctcca tgtgaccacc
25141 tcgggagagg tctccggctt gtcgtaaccc aggggagtaa cacactgcct cctgcagctc
25201 tttcagaccc agttgcaaag aagagctgca tgttcaacct gttgtccctg ccggaggcca
25261 acctgctcac cttccttttc cttctagacc acctggaaag gtagcccagc tctcttgtgg
25321 ctgcccagga ctccaggtct ccaggccgct ggggtgcccc tctgctccca ccagaccccc
25381 agcaccaagg accttttccc ccgacccctg tctgcagtaa ctcactgctt ctaaggacta
25441 gcaccactgc cacccccacc actgcctctc ctctttgcca ccctcctccc tctgcactgt
25501 ggccttaaca aagagctcag agctttggcc gtggccagca gtgcacttgg acccgcctct
25561 tccctcccaa gcacatcatg aagacctccc catcagccca gagctggccc cttgtcccgg
25621 gccactgaga cccagaagta ccaaggctgg agtcagcttg cagcacagcc agggtcgagg
25681 tcactccctc cctgaggact ctagcacggc acagcccctc tgcctctctc ctggtggtgg
25741 cgttgaaaca gcaccctctg ctttggtcct ctacagggtg gcagagaagg aggtggtcaa
25801 taagatgtcc ctgcacaacc tcgccactgt ctttggcccc acgctgctcc ggccctccga
25861 gaaggagagc aagctccctg ccaaccccag ccagcccatc accatgactg acagcaggtc
25921 cttggaggtc atgtcccagg tatgggaaga cagtctccag cccatgcaac cccagcctga
25981 cagaggtggc ctctgcctgc cccaccccca gtcctgccca tcttccaact tgcattgtat
26041 gtggtggtgg ctgagattca gagacaggga cttgcctagg tttgcatgga tgggagtgat
26101 aggggggtgcc caggccacct cctggtcctg ctgtgcacct tgctgggggc ttaaaaccac
26161 cccaagtgtt cgggtgtggt ggctcatgcc tgtaatccca gcactttggg aggccgaggc
26221 aggacaactg aacccaggtg tttgagacca gtctgggcaa tgtagcaaac cccatctcca
26281 gaaaaaatac aaagaaaaat taggcaggca ttgtggcaca catctgtaat cctaggtatc
26341 tgggaggctg acacaggagg attgcttgag cccaggagtt agaggctgca gtgatccatg
26401 atggagccac tgtactccag cctgggggac agagcaaggc cctgtgcatc tctaaaataa
26461 ataaccaccc cccacccaac aagtcatgcc ttgtcaggac cccaccccac ccccgtctca
26521 ctgtaagggg ttgatgacac cagcaggggt ttctagcacc tgaggtggac ttggggtctt
26581 gggccccaaa gacctcccca ccagcagctg tgagcccccc tccgagccac tctcctcttc
26641 cccactctgc gagggcaggt cgagttgctg ctgtacttct tgtggctgga ggccatccct
26701 gccctggaca gcaagagaca gagcatcctg ttctccactg atgtctaaag gtcccagtcc
26761 atctcctgga ggcggacaga tggcctggaa acctctggct aatcaggcca tctgtagagt
26821 gggaatcaag attttctgcg gcatccttgg gccaccccca ggtgtcaggc catctgccaa
26881 gagacagcgg cccaaagcag aaggacaggt ggcctgggca gatcccaccc aggtctgaaa
26941 gccccaggct ggcctcagac tgtgggtttt ttatgtggcc acccgagggc gccccaagcc
27001 agttcatctc ggagtccagg cctggccctg ggagacaggg tgaaagcagt ggtttttatg
27061 aacttaactt gtagagtcta aaagatttct actgaatcac ttgtcaagat gctccctctc
27121 tggggagaag ggaacgtgac cggattccct cactgttgta tcttgaataa acgctgctgc
27181 ttcatcctgt gggggccatg gccctgtccc tgtgtgggtg gggcctcttc catttccctg
27241 acttagaaac cacactccac ttagaacagg gtttgagagg cttagtcagc actgggtagc
27301 gttttgactc cattcttggc tttcttcttt ttctttccag aaggattttt gtgcagaaat
27361 gggtcttttg ttgccgtgtt agtcctcctt ggaaggcagc tcagaaggcc cgtgaaacgt
27421 cgggggacag gacccccagg gagggaatcc caggccacgc actttagggt tcgttctcca
27481 gggagagcga cctcgtcccc caatcctgac cgcccttccg gcccacgctc tcctgtttgg
27541 cttccacagg cctggacttc tctggcttct ctgcccacac actccctgcc ccagtgtccc
27601 tgcccctgcc ccagcacagg tgacttcatt tctgtcctct cagctcagtg gactcgctca
27661 acttttgtat aagtctccac ttggtggcag cagcttgctg atgacttgtt ttaaaacttt
27721 catcctaaat aaccttttga tgcttgaata tttttaagtt ttatacgtag tttctaattt
27781 ttcctcaaca gatccagata cctaataaga tgctggaatg taatccctgg acagtccgtg
27841 tcctggcagc atttgtcttc ctctaagcgc ctggctccgc tgttctcagg agtgggttct
27901 gaagtctctg gagaacagga tacgtggagg gttaggaagg ggccaggcct agagatggga
27961 gactccctcc cggagcaggt ggaggcacag gaccattcgc taccccatct gccgacacct
28021 gcgggggagc ccaggcattc tttgtaagcc ctcctgacca cctggctcaa agaaaacaga
28081 agcatggagg ccgcaagtat tttcaagaaa taatccatga acatggcatc actttttag
28141 aaagaggggc ttgggcaggc agaggagaga agagagagca aactgagagc caagtttcca
28201 gacggtcctg caggaggaga ggatgcagct gcccagaggg aagcaggatc acatttaagg
28261 aagtgtgtgg ggtccctgga tgacaccagc acccagtgcg gctctgtctg gcaaccgctc
28321 ccaaggtggg aggagtgggt gtcccctgtg tgtcagtggg cagctcctgc tgaacccgca
28381 gctcactggg gagcctgaca gtggggccat gtgcctgaca ctcctctctg cttgtggacc
28441 tggcaaggca gggagcagaa aacagagcca cttgaaggct ttctgtctgc gtctgtgtgc
28501 agtgtggatt tagttgtgct tttttcttgc ggggagagca cagccaccat ttacaagcag
28561 tgtcaccctc gtgggtggcg aggacagaac aggagcctct gctctctgta cctatctggg
28621 cccggtgggc tcccctgtcc tggcttccat ctctgtctca gcgaccattc agccctgcac
28681 aggaacacat gttgcttaga aaagccaaat ccagcccttg tctctgcctc ctctggtctc
28741 atgatgtgca tctattacct tgaaactgga aaccagtcta tcaatgtctg tgccaatttt
28801 ttattccctc cccaacctcc ttccccatac gactttttat ttatgtagga tgtgtgctgt
28861 ctaatgatgg gatgaccaca cttttccatg ttctaaaagt gctcctctcc cgcagggtcc
28921 cagggctggt ggttgctttg ggtctacagc tacgtcttac ccgcctcctg cctcaaaagc
28981 ctgtgtggtg gcaaagccgt g

29102 ggggctggg gaacgcagcg ttctccagga gggggacccg gctctccttc tgcaatgcag
29161 gcaaaggcct agatgccagt gtgacctccc acaaggcatg gcttccagac tccccgacca
29221 gaagtgatgc ttttttgcct cgggccctgg gtttgaagca gcctggcttt ctcttggtaa
29281 gtggctggtg tcttagcagc tgcaatctga gctcagccac ctacacacca ccgtggccga
29341 cactttcatt aaaaagtttc ctgagacgac ttgtgtgcat gttgatttca tgatcagcgc
29401 cgctgggaag aaccccagag ccggtggggt ggggctggaa gcagcaggtg cagtgatggg
29461 gctgggtgcc caggagtcct cagtgctcaa tcaggccaag gtggccaagc ccaggctgca
29521 gggaaggctg gcctgggggg tgtgggtgag cacaggcagg caccagctgg gcagtattag
29581 gatgctggag cagcatccgt aaccccactg actggggtag tctggttggg gcagggaccg
29641 ctattgcttt ggcagagaga gatgatcccc actggggaga ggctgttctg actctgcagg
29701 tgggacaggg acagatggcc accagggtga cccggctggt cttcctttgc tacgctaagc
29761 cctgggacat ggaggattcc cgccacacac agcccgggcc cgggttctta cctgtggcca
29821 ccgctctggc acgagcccct cagtcttggg tggtttctgc ctggtccggg atttggtgtt
29881 cctgctgagt ccagcctttc taccacctcc tcatgggccg tgggtggtgt tgtcagctgc
29941 ctcccacctt ggcttcagta gctcacccag cttacagggg agctgccctg ggctggagat
30001 gggcatgcac cctgggtcct acttgaatga atgcagcttg aggagagccg gccatataca
30061 ctgggccaca ggttaccctc ggcaatgccc acatcagccg tcagcctgag cctcccagga
30121 gagcaaggct cacacgacaa aggctgcccg tggccaatga ggtggctgag cccagccaga
30181 acctttctcg gactcccggg atgtggctct gctcgtgagc tgcctggtca gctctctcgg
30241 ggtgagaggg gcttgtcaca cgggcccctg cctgcagtgt gacccttctc agcttctctc
30301 agcagccctg cctgcagagt gtcaccacca ccatgatcat ttccctgaca ctgcgagggt
30361 gggggatgt cctgggtaga gacagggccc gtgcagcagc aggctcaggg gcgccctaca
30421 ctggtgggct ggggacctgg tggagaccac accaagggct ggacaagggg acgagcctcc
30481 accctggcct ctccgcaggc ctcagcagcc cctcccacag gcagaagggt tgacactggg
30541 ttctgccctc actgcaagag ctgcaagtgc cacgtgctgt tctgcccaat ctggtgtctg
30601 caggtgaggg aagggctgcc gctggcccgt ttctgagtgt tcagcaccta agggtgacag
30661 cactgtctgt ccctaccctc cgggtcctgt ttgaaaatca aacccatgct cacaggccaa
30721 tttttttttc ttttagagac agggtctcac tttgtcaccc aagctggagt gcagtggtgc
30781 gattatagct caatgcagcc tccaattccc ggactcaagg gaccttcctg cctcagcctg
30841 ccaagtagct tggactatag ctgtgtgttt tattattatt ttgtagacat ggggtctggc
30901 tatgttgtcc aggctattct caaaattccc ggcctcaagc aatcctccca cctcggcctc
30961 tcaaaggttg ggattacagg tgtgaggcaa ggcacccagc tcagccacag agccctattg
31021 catctctctt actaggagca agagctgact gccccctcat ccccattcca gagtgttggg
31081 gctgtgttca gccaaggccg ggccactggc atggcccagg gagcgggatc attcactgct
31141 gccccaaatc tgagatcatt ccactttgac aagacttcct catccaatcc ctttacttga
31201 cagctgggga aaccaatgcg cacagagcac ccccagctca ctcggggtct cagagctgat
31261 ccataagcgg aggctgagat cctgggatct tgtcccccag cctccctgca agcttactac
31321 ctttctgctg aaagagatgg ggccggacct cgaccagcag ccctggcctg gacatgactg
31381 tgctcatgca ggtattgagg ccgagatgcc ccggcatcat atgtttttct attttctttt

```
    31441 tttttttttt tgagacggtg tctcactctg tcacccaagc tggagtgcag tggcatgata
    31501 ttggctcact gcaacctctg cctcccagtt aaagtgattc tcctgcctca gccttccaag
    31561 tatctaggcc tacaggcttg taccaccacg cctgactaat ttgtgcattt ttactagaga
    31621 cggggtttcc ccatgttggc caggctcgtg tcgaactcct gacctcaagt gatccacctg
    31681 ccttggcctc ccaaagtgcc aggattacag gcatgagcca tggcgtcact taaatgtagt
    31741 gagaggccgg gcacagtggc tcatgcctgt aatcccagta ctttgagagg acgaggctgt
    31801 cagatcacct aaggtcagga gttcgagacc agcctggcca acatggtgaa accgtgtctc
    31861 taaaaaaaat agaaaaaaat atccctgcat ggtggtgagt acctgtagtc ccagttactc
    31921 aggaggctga ggcatgagaa tcgcttaaac ctcggaggcg gaggctccag tgagctgaga
    31981 tggcgccact gcactccagc ctgggtgaca gagcaagact ttgtctctaa ataattaaat
    32041 aaataaatat ggccgagcat ggtgccttag gcctgtagtc ccaacacttt gggaggctga
    32101 ggcagtggtt catgaggtca ggaacccgag accagcctgg ccaagatggt gaaacactgt
    32161 ctctactaaa aatacaaaaa ttagccagct gtggtggcag gcacctgtaa tcccagctac
    32221 ttgggacact gaggcaggag aatcacttga aactggaagt cagaggttgc agggagccga
    32281 gattgcacca ctgcactcta gcctgggcga tggagcaaga ctccatctca aataaataaa
    32341 ttaataaata cagagcaaga ttccatctta aataaataaa taaacataca cctgtaatcc
    32401 tagcagttcg ggaggctaag acaggtcgat cacctgaggt caggagttcg agaccagcct
    32461 gaccaatatg gcaaaactcc atctctacta aaaatacaaa aattagccag gcgttttgac
    32521 gtgtgcctgt agtcccagct acttgggagg ctgagacagg agaattgctt gaacccaaga
    32581 ggtggaggtt gcagtgagcc gagatctcgg ctgcacttca gcctgggtga cagagtgaga
    32641 ctgtctcaaa aggaataaaa aaaatacaaa ataaaaaaaa tgtagtaaga ttgcagagtc
    32701 gtgccgcgga agcgtgctgg tcctatccat gtagtgaagg ctgatttcat acacaaatgt
    32761 cacaagaact ttttttcttt ttctttgaga cggagtatgg ccctgtcacc caggctggag
    32821 tgcagtggcg cgatctcagc tcactgcaag ctctgcctcc cgggtttatg ccgttctcct
    32881 gcctcagcct cccaagtagc tgggactaca ggcctgtgca gcagatgcag gggggccact
    32941 aggcccaggc agtcttggga cttgtgtgtc tcctgctgtg catccatact gggtgcttta
    33001 gaaacggcag gcagaccaga agcccctgtt gcaagtgagg acaaagtgtg ggaaggccgt
    33061 gagggtctgc agtccgagat ggccttgtcc tcaacctgca gtgcactgtt gatgcgctgg
    33121 aaggccgcct ccttctcccg gtccaggtct tcagcagtga cccggtaccc catctctaag
    33181 ggaggtggca gcatcaaagg ctcccctcgc ctgcgtggca gcagaggaaa cttgcatcta
    33241 cggggcctag aggcctggga tctgcgggag ccacccctgg gggcgagtgt ctgccctggt
    33301 gctgtatctg ccgtcttttc acactgggtg tgacccgaag agacagcctg aggtccgccg
    33361 tcactcagtg tgtttcagga actgagggtc agctggcagt gggatgaggc tggcccctct
    33421 tccgctttag ttccgggagg cctcccgtag agctgtaggg gctcgagatg gcctttcgtt
    33481 tggggcacga gctggtccag gaggtctggg atctctggtt ctgacctctg ggcacctgct
    33541 gcagctgtgg ctgaggccca gaaatgtgaa ggccctccat ccactccagt agtgacccca
    33601 acgtggggtt cagtgtggag gggggagggg ctgctgcggc agctgcagga gcagaagtgc
    33661 cacgccttgt tcttctcatg cccgcatcca tgcttgcagc tgggaagggg gcaggaatca
    33721 gcgaggtgac ctgggctgag tcctgggaat gggaagaggt ggcaggaagg ggatctgagg
    33781 aggagaacag gggggcctggt ggtctgtgct tcttcccaga catgggagct gtagagggga
    33841 cctctgcagc agatgctaag gggccagtag gcccaggcag tcttgggact tgtgtgtctc
    33901 ctgctgtgca tccatactgg gtgctttaga aacggcaggc agaccagaag cccctgttgc
    33961 aagtgaggac aaagtgtggg aaggccgtga gggtctgcag tccgagatgg ccttgtcctc
    34021 aacctgcagt gcactgttga tgcgctggaa ggccgcctcc ttctcccggt ccaggtcttc
    34081 agcagtgacc cggtacccca gctctaaggg aggtggcagc atcaaaggct cccctcgcct
    34141 gcgtggcagc aggggaaact tgtgtgtact gggcctagag gcctcagatg tggaggagtc
    34201 attcctgagg gcgagtgtct gccctggtgc tatatctgct gccttttcac actgggtgtg
    34261 acctgaagag acagcctgag gcctgtcctc actcactgtc tttgaggaac tgagggtcag
    34321 ctggcagtgg gatgaggctg gcccctcct ccgctttagc cccggcaagc ctcccgtgga
    34381 gctgtaggag ctggagatgg catttcggtt ggtgcaggag ctcgtccagg aggtctggga
    34441 tgtctgctta tatctgattt ctgacctctg ggcatggagg tctgtctgca gaggcccggg
    34501 cctgggcaca aagggagaga ggcctccatt gtcccgcagg ggctgaaatg tagaccgtgc
    34561 atccccggtg acctcgggga cccttctctg atcaccagga ttctcttgga ctctagggtc
    34621 cttgtcctgc tcaggcatcc ctgccccact ctccttgagg gccctcaaca ctatcttccc
    34681 tggacacaag tctggggaca gccgggtgtt gtggacccca aaggggtgac tcccggctcc
    34741 tgggccccac agagagtcca tgttctcagt gcagtggctg agctggagga cgccctggaa
    34801 ctcggagcac acagcactgg cttgctgtgg tacctgtgca atcaaattga aggcaggatc
    34861 ccaggaagga acgcagggct tgcaggatca cggaaaacct tcttagcgtt gtcttgacac
    34921 cactgatgtc aagtgtccgg gtgcttgtag gatggcctgc cactcagtcc acgggcagga
    34981 gcaacgggga gatcccacaa gcaaagtgaa ctgggggatg ggctgaacgg gctccaggca
    35041 actgagccct actggcaggt cctcggcctg ggcccgaaca ggaaggaggg gcacagagtg
    35101 cccaggtagc cgctcctggg agcagtgggg aaccgtcggt tgcttgaact ctcgagagct
    35161 gggctctgag cgtcctcgtc cagccgccaa ctcggccaaa ggctaagcca gcagtttctt
    35221 ctgttgccgg gcaacgcgcc ttttaaacct gagggagcgg gcgcgtgagc acatcatggc
    35281 gcccgtgaca gagcgagctt aacggattaa taagcgcagc caggtacccg cgcgaggcac
    35341 ttgctggcaa tggcgggagg cggacgtggg gggtcatgca ataggtactg gaaggagaga
    35401 ggcgggcaca aaggtcgcgg gaggaacagg cgcccacagt ggcggcagat ctgcccgtgg
    35461 atcactgaag attcctgctc tcctgctgag gtggagattg cagtgagctg agatcgcacc
    35521 attgcactcc agcctgggcc acaagtgcaa aactcag
//


>BCRP3.REF.NEW
        1 actccgtagt gtgcacttgg tgagggcagc agctcgccac agctgccagc cgtctgtcca
       61 ttcacccatc tgtccatctg gcagcccgct gttcagaccc gtctgtctgt ccgcccatct
      121 gtaagcccat ctctgtccca ttgtctatct gaccatcttt ctcttactgt cctctttgtc
      181 tagctatctg gcctatctgt cgatccatct tcgtgtctgt cttcagcccc cacctgtttg
      241 tccatctgtc caattacctg tgagtctatc tatgcacctt cttgtccatt catctgccca
      301 cccatctgtc cctccgtctg cccaccggcc tcccctctcc ttctgggccg cagagccatg
      361 gcccaggact gcagagccat ggttggcctg gtcctgctgg ggctggggct tgtgctggct
      421 gtcattgtgc tggctgtggt cctctctcga caccaggccc catttgaccc ccggcctttg
      481 cccacgccgc tgttgctgct gactccaagg tctgctcgga tattggacgg tgagtgagac
      541 gtgggaggaa gctgggtggc ccttggcagc cagcccctcc tggagaaggc gtgtgtgtga
      601 gagtgtgtgt gtgtgagcat gtgtgtgtgt gagagagtat gtgtcagtgt gtgtgggtat
      661 atgagtgtga gtgtggggtg tgggtgtgtg tgaatgtgtg tgatcgtgtt tgggtgtgtg
      721 tatgtgtgag tgtgggtgtg tgtgaatgtg tgtgagtgtg tttgtgtgta tgtgtgagtg
      781 tgggtggggg tatatgagtg tgagtgtgtg ggtgggtgtg aacgtgtgtg attgtgtttt
      841 gctgtgtgag ggtgtgtgtg actatgagtg tgtgagtgtg ggtgtgtgta aatgtgtgtg
      901 attgtgtgag tgtatgtgtg ggtgtgagtg tgtgagtgtg agtatggggg tgtgggtgtg
      961 tgtgaatgtg cgtgattgtg tgtgggtatg tgtgtgtgtg tgtgagtgtg tgtgtgtgcg
     1021 tgtgtgtgca cgtgcactgg cccaggcagc aggagccatg tgtgtgggct tcagcacctg
     1081 cagggcttga gcgcaaggag acagcctcag ggcccttgca cagaacaggc ggcagggtgt
     1141 gcccgtgggg cagatgggga cttggggaca atggtggtgt gtgagtccat acctggctcc
     1201 aggattcagg aggcccattt gcacatccca ggtgggaacc tgtctggccc cggctgaccc
     1261 tgctggccgg tgcaggcccc ttcagtgagg ccaattctcc aaggctgcgg tcttctccca
     1321 gggtcatggg tgaaggggtt tggaggctcc ctgcgtgggt actggcctgc tgggttacac
     1381 acaatgctgc catagccagt ctgcccctac acccagcctg gggccacatc tcaggtctct
     1441 cagtcctgag gagcccggtg ccccacccct cacatcctct ctccctgagt cagggcctgg
     1501 gtctcgtgag ctgagtgact gatacttggt gtcctggatg agggcgtgat ggagaggggc
     1561 cacagcgggt gtttcctgac cctcttccag gaaggtgctg ctgccgctgc agggaggaca
     1621 catacaggat gccccttcct gccccctgcc tcccattggg cccacaaaag ccagggcaag
     1681 cctcccctcc ctgccagcca cctggtctgc ttcccagaaa ttctgtcttg caggctgttg
     1741 ggaggatccc agtactttgt aaactaaagc aagggaggag tggccgttct ctctgttcat
     1801 tcattcacct tttcattcat tccttcttcc ctccattccc ccatctgtcc atccttccct
     1861 gccctgattg ctcatgccac cgccccccgc agcccctcct gacctggtcc tttggtttct
     1921 cttcagggat ttctgtctcc tcccacaggg ctgagaatgg cagctcaggg acaagtaggg
     1981 gctggggact gcttagtctc cccagtggct ctcaggggat ttgagggttt gacgccagct
     2041 gccaccccag gctgtgcccc tcctctgctc aggaggacat acaggatgca acacccactt
     2101 aaactcgaag ttgcaaagat gcaaatgaga ctggggtctc aggcaccaga gaccacccgt
     2161 gggcacgtgg cttttgggat tggagacctg ctgccacaga tctctgaaga gtctggacct
     2221 gctgggtctc cccaagtgac tctctggggg tctccatagc atgccctgct gtgtgcatga
     2281 cggtcactgg ttgggtaggg gtctctactc taaagctccc tctgccggca tcccctcgaa
     2341 ctctcccttg gtgaagagag aggatgtggt ttgccccagt gttttatcaa acaactctct
     2401 ccacttcctg ttttaagaag ctgggagtgg aagagagcct ggggctggcc ccagctgctg
     2461 ctgcgaaaca ggggtcactg gacgctggga ccctggccgg gctggctgga ggcctcagga
     2521 agaggcctgc tacagtgtca tcctggccaa gattcctccc tgcagaggac cctggccacg
     2581 ctgccacagg gtctgctggg gccaccagaa gcccatgctc ctgcctccat ctctcccctc
     2641 tgtgctcacc tctcaccagg aggccctccc agagttcagt gtcctgcttt tttttttttt
     2701 ttttagatgg tgtctcgttc tgtcaccagg ctggagtgca gtggcgcgat ctcagctcac
     2761 tgcaacctct gcttccttgg ttcaaatgat tctcctgcct cagcctcctg agtagctggg
     2821 actacaggtg ccagccacca cgcccaggta atttttgtat ttttagtaga gacggggttt
     2881 caccatgttg gccaggatgg tctctatctc ttgattcgcc cgccttggcc tcccaaagtg
     2941 ctggaattac aggagtgagt catggcaccc ggcctcatct cctactcttt cagcaccagg
     3001 ttttactctt gggattctgc tacagccgca gcccctgggt gcgagttcct aagctttctg
     3061 tgagtgtgga cccagcaccg tgcctagtag acatacaaaa ggagcatggt gacagtgagg
     3121 tctgtcatct ccagcataat gactgttttg atccttgtaa aaaaggtgat ttttggctgg
     3181 gtgtggtggc tcacacctgt aatcccagca ctttgggagg ccgatggggg tggctcactt
     3241 gaggtcagga gttggagccc agcctgggca acatggtgaa accacgtctc tactaaaaat
     3301 acaaaaatta gctgggcatg gtaacggatg cctgtaatcc cagctacttg ggaggctgag
     3361 acaggagaat cacttgaacc caggaggcaa aggttgcggt aagccaagat tgtaccactg
     3421 cactccagcc tgggtgacag agcaagactt ggtctcaaaa aaaaaaaaaa aagaaagaaa
     3481 gaaaagttta tatttttgtt ctaatggtta tcttaatatc gtcattctat aattgtatgt
     3541 tttatataat tataatagct atataagata taatacccct agtatgttgt tttttggata
     3601 ttctacttgc tcctgatggt taatttatat gtcaacttgg ctaagctatg gtgccccgtt
     3661 gtttggtcaa atacttgtca atatcttgct gggaggttat ttcatagatg tgattaacac
     3721 tgacagtcag ttgactttaa gtaaaacaga ttacccacca taatatgggt gggccacctc
     3781 caatcagttg aaggccgtaa gaacaaaaac tgaggtttcc cagagaagca ggaattctgc
     3841 ctcaagactg taacacacaa accctgcctg agtttctggc ctgctgactg ctctacagag
     3901 tttaggttcc agacttcgag atcaactctt acctgaattt atagcctgct ggcttgccct
     3961 acagatttta aacttgctag tccccacaat catgtgagcc aattcctcaa taaatctctc
     4021 tctatgtata atctattggt ttagtttctc tgaaaagctt tcacatccag tttcctggat
     4081 gttaagaatt actgaaacta gctagtaact tctttttttt tttttttttt tttttttga
     4141 gacagagttt tgctcttgtt gcccaggctg gaatgcaatg gcacaatctc agctcaccgc
     4201 aacctccact tcctgggtcc aagcaattct cctccctcag cctcctgagt agctgggatt
     4261 acaggcatgt gccaccatgc ttggctaatt tttgtatttt tagtagagac agggcttctc
```

```
 4321 catgttggtc aggctggtct tgaactccca acctcaggtg atcagccgcc ttggcctcac
 4381 aaagtgctgg aattacaggc atgagccacc gcacctggct cctagtaaat tcttcttttc
 4441 cgtgatgtgt ctcttacctc taataatact tttcttcttt tttttttttt gagacggagt
 4501 ctcgttctgt cgcccaggcg ggagtgctgt ggcgcgatct ccgctcactg caagctccgc
 4561 cttccgggtt cacgccattc tcctgcctca acctcccgag tagctgggac tacaggcgcc
 4621 cgccactgcg cccggctaat tttttgtatt tttagtagag acggggtttc accgtggtct
 4681 cgatctcctg acctcgtgat ccgcccgcct cggcctccca aagtgctggg attacaggcg
 4741 tgagccaccg cgtccggcca tacttttctt cttaaagtct acttcattaa aaatagttat
 4801 gctgggcatg gtggctcatg gctgtaatct cggcactttg ttggaggtcg aggtgggtgg
 4861 atcactgaag cccaggagtt caagaccaac ctgggcaacg tggcgagacc ctgcctctac
 4921 aaaaaataca aaaattagct gggtgtggct aatatacact tgtagtccca gctacttggg
 4981 atgctgaggt gggagaatcg cttgagccta gaagggagag attgctgtaa gccaagatca
 5041 catcactgca ctccagcctg ggagacagag tgaggctcta tctccaaaaa aaaaaaaaaa
 5101 aaaagttata cagctttctt ggttagtgca tgcatgccat atttttcatt attttccacc
 5161 tctctgtatc cttatataaa aggcattagt tgggttttac tttattttca attattttaa
 5221 tttttattgt ccttttaaat gtaactaatg atttatttgg gttgaaaccc accaccaatt
 5281 tgttttccat gcctattcta tttcttctta tctcctctca catcttgttt tggatttatt
 5341 atttttatta tttaatttcc tccttctcta ttagtttcat agctctgcag tcttagagtt
 5401 attttaaaag atgacagtgg attattttag agcttacaac atgcatcctt cacttatcaa
 5461 agtctaacat gagctagtac tttttgttgt tgttgagata gagagagtct tcctctgctg
 5521 cccaggctgg agtgcagtgg agcaatcttg gttcactgca acctccactt cttgggttca
 5581 agcagttctc ctgcctcagt cacctgagta gctgggacca caggtgtgca ccactatgcc
 5641 cggccaattt ttgtattctt ttttagtaga gacagggttt caccatgttg gccaggctgg
 5701 tcttgaactc ctgaccttaa gagatctgcc tacctcggcg tcctaaagtg ttgggattac
 5761 aggcatgagc caccgcgccc agcctatgag ttagtacttc tatgctcttc ctagtcagta
 5821 caagaacctt ggaacaggaa ctaaatttac ccccagtgac ttatatgcta atatttttgt
 5881 gtattttaaa tatatatgtg tgcatagatg tatctgtgtg ttttttgtgt ttttattctt
 5941 atttatgttg agagtgtaga gctatgtaag agtaaagaga attgtgtaat gaagccccga
 6001 gtatccattc aatttcaaca acaatctcat ggccaagcta atttcatgta tactctttcc
 6061 tgcttccctc taccccacat tatttcagtg caaatcccag atatataact gtacccatac
 6121 atatttcagt atgttttatt tattttaaac cccacaatat atcattttct atactactgt
 6181 aatttcatac caataacatt catttagatt tacccacacg tttacctctt ctgttaccct
 6241 ttatttttat ttataaaaat atctttggga agaaatatct ttcagcacat ggtcaaggat
 6301 ctcctgaggg ctatgtcatg ggcaaaatat acatatatat tccatatata tacacacata
 6361 tacacacaca cacatatata cacatataca cacacacata tatatattcc actttcactt
 6421 ttttgtttgt ttgtttttttg agaccgagtc tcgctctgtg gcccaggctg gagtgcggtg
 6481 gtgggatctc agctcactgc aacttctgcc tcctgggttc aggtgattct cctgtctcag
 6541 cctcctgagt agctgggatt acaggtgtta gccatcacgt ctggctaatt tttgttgttt
 6601 tttttttttt gagacggagt atcgctctgt cacccaggct ggagtgcagt ggcaagatct
 6661 tgtctcactg caacctccac ctctcaggtt caagcaattc ttgtgcctca gcctcctgag
 6721 tagctgggat tacaggcatc caccaccaca tctggctaat ttgtgtattt ttggtagaga
 6781 tggggtttca ccatgttggc caggctggtc tcgaacttct gacctcaggt gatccacctg
 6841 cctcggcctt ccaaagtgct gggattacag gcatgagcca ccatgcccag ccattttcac
 6901 ttttgaagga tattgttaat gagcatagaa ttctaggttg gcagatattt tctttcctca
 6961 gtttgaaaac atgattccct tgtatctgat ttctcctgct tttattggga agccaattct
 7021 caatctaatt ttgctcattt gaaggcaatg gctttttctg ttgttgtgtt ttctgaggtg
 7081 gagtctcact ctgtcaccca ggctggactg cagtggtgca atctcagctc actgcaacct
 7141 ctgcctcctg ggttcaagtg attcttctgc ctcagcctcc caagtagctg ggattacagg
 7201 tgtccaccat cacacctggc taattgttgt atttttaata gagatgaact tttgccatgt
 7261 tggtcaggct gatcccaaac tcctcatttc aggtgatccg cccgcctcag cctcccaaat
 7321 gctgggatta caggcatgag acagcccaca accctggcct gcaggcagta tctttttttcc
 7381 tctggctgct ttgaaaagtt ttgtctttgt tttgagcagt ttacactgat gcatttaggt
 7441 ggctcctcat tccatgactt gattcttttt tgtccatttt agaaaactgc cagctttatc
 7501 tcttcaagta ttatgtcttc cccatcctct ctctactctc cttatgagac tccaatttca
 7561 catgacttat gccttgttaa agtatccccc atgtctctta atccatttcc tgtatgttct
 7621 atctgttttt ctctttgtac ttcaatttgt atagtttgta tcaaactatc tcccaattag
 7681 ccgggcgtgg tggtgggtgc ctgtaatccc agctacttgg gagcctgagg caggagaatt
 7741 gcttgaaccc gagaggtgga agttgtagtg agccgagatc atgccactgc actccagcct
 7801 gggcaacaga gtgagaccct gtctcaataa ataaataaat aaataaatac cagttcacta
 7861 ttttttttta tgtttgtgtc tagtgtgctg ttcaaattga gttcctaatt ccattttttt
 7921 tttagacttt tttttttgag tctctatctg ttgcccaggc tggagttcag tggtgcaatc
 7981 tcaactcact gtagcctcca cctcccaggt tcaagcgatt ctcatgcctc agcctctcga
 8041 gtaactggga ttaccaccac gcctaactca tttttgtatt tttagtagag atggggttct
 8101 gccatgttgg ccaggctggt cttgaactct tggccttatg tgattggcct acctctgtct
 8161 cccaaagtgc tgggattata ggcctaaacc accactccca gcctcctttt tttttttttt
 8221 ttttttttga gacggagtct cgctctgtcg cccaggctgg agtagagtgg tgcgatctcg
 8281 gctcactgca acctccccct cccagttcaa gtgattctcc tgcctcagcc tcccgagtag
 8341 ctaggactat aggagcatgc caccatgcct ggctaatttt tgtaatttta gtagagatgg
 8401 ggattcacca tattggtcag gctggtcttg aacttctgac ctcaggtgat ctacccacct
 8461 cagcctccca aagtgctggg attacaggcg tgagtcacca cgcctagtgc atccattttt
 8521 tgtagttgcc agttttctga tgaaattctt aattgtttct ttatatcctt gatatacatg
 8581 taaagaactt attttaaagt acatggtctg atgattttat aatctggaga tcctatgggc
 8641 ctttttaaaa gttgtctgtg ctttctcttg agcttttttc ctgctgtctt atttccttgt
 8701 ttgcttagtt gtttttaatt tggcaatgga agttgtgtat aaaaatcgtt acaaataatt
 8761 tttttttttt tttgagatgg agtctcgctt tgttgcccaa gctggagtgc aatgacgtga
 8821 tctcggctca ctgcaacctc tgcatcccag ggttcaattc tcctacctca gcctcccaag
 8881 tagctgggat tgcaggcagg tgccagcacg cctggctaat ttttgtattt ttagtagaga
 8941 tgggttttta ccatgttggt caggctggtc tcagactcct gacctcgtga tctgcccacc
 9001 tcagcctccc aaagtgctgg gattacaggc gtgagccact gcgcccagcc agaaataatt
 9061 tttaaaaata attttgagcc ccagcatgat ggctcatgct tgtaatccca tcactttggg
 9121 aggctgaggc gggcagattg cttgagccta ggagttcaag atcagcctgt acaacatggt
 9181 gaaaccccat ctctacaaaa aataaaaaat tagctgtgtg tggtggtggt gtgtgcctgt
 9241 agtcccagct gtttgggacg ctgaggtggg aggctcactt gagcctgggt gatcgaggct
 9301 gcagtgagcc atgatcctga gactgcactc cagcctgggc aacagagtga gatgctgtct
 9361 caaataaata aataaataaa aataaaatac tttgaggcct aggggtctaa aattctgaga
 9421 tctcctttat gcatttgagt gactgagatg atctgaagct ggatccagtg ctcctgaggg
 9481 ctgctttatt tctggttgac tgtgactcct agagtaagaa acctgcaccc cacatgtggg
 9541 gcattatggc atcccctccc tcagccacat gagtaagtca acagcactgc tctagaccag
 9601 gtgtggtggc tcacacctat agtcccagct actcgggaga ctgaggcagg aggattgctt
 9661 caggccagga atttgagacc agccagagca atatattatt aggttggtac aaaagtaatt
 9721 gcagtgtttg cctttaaaag taatggcaac cctgtttcag caaaataaaa agcaaaaaaa
 9781 aaaaaaaaaa aaaaaaagaa aggaagaatc agctgggcgt ggtggctcac gcctctaatc
 9841 ccagcacttt gggaggccaa ggcgggcaga tcatgagatc aggagatcga gaccatcctg
 9901 gctaacacgg tgaaacccca tttctactaa aaatacaaaa aattagccgg gcatggtggc
 9961 aggcgcctgt agtcccagct actcaggagg cggaggcagg agaatggcat gaacccagga
10021 ggtggaggtt gcagtgagcc gagatcatgc cactgcactc cagcctgggt gacagagtga
10081 gactccgtct caaaaaaaaa aaaaaaagaa tcactgctct gtctctcagc ctcctcttcc
10141 aagattggcc gtcgccttga ggggaatgct ggccttgcct gtctccagcc ctgtacctct
10201 ctgcctccta tgcctttaag cacatgtttt ctatttgctg ggctgtgaaa tctgctcttc
10261 atctgatggg gtttgcttta taggtgacta gatccttttc tcttggtggt tttagaattc
10321 gcattttcac attgacctta aatagtctga ttatagtttg ccacggcaaa gaccctttgc
10381 attgcattgt ttggggatat ttgagcctcc tctatctgga tgtctaatct cttgttagat
10441 gtgagtagtt ttcattatta ttttattaat gggctggcat gtgggccgtg gttccaggca
10501 ggctcagagg ggcagctgcc tgatgtctgg acagcttctc tttctgtctt ttcttacctg
10561 gactctgggt tgcttgttag ctgcttctgc cagttctgag ttttcaaggg gagaggggcc
10621 cagtgatggc tgttctttga aggaaaggga agaatgtctc ctgtttaaca tgtttctatg
10681 tttccagtta cttggttagt tagttagagc cagggtctct cgctctgttg cgcaggctgg
10741 agtgcaatgg catgatcgtg gctcacagca gcctccacct tccaggctcg agcaatgctc
10801 ccacctcagc ctctcaagca gctgggactg caggtatgtg ccaccatgct tggctgcctt
10861 tttaaatttt tttttttttta atacagacaa ggtctcacta tattgcccag gctggtctta
10921 aactcatggg ctcaagtgat actcctgcct cggcctttca aagtgctgat atcacaggca
10981 gggtttccat tttttaaagc tcccagcagt ggtataaact cctcctttcc agagaaagcg
11041 cactctgtcc gcatccctca tgttgtcctc tcctgcctct gcttagggtt cactccgggg
11101 gaaagtgcca cttgagagtt tcctttttgt gtgtggttct gactgactgc tccctgctca
11161 cagatgctga ttctcagggt ggggtccctg aggcctggag tgtggcctct gacgaccttc
11221 agggccaggt gtggaatgag agcctgtggc cacatggccc ccggtgggag acgtcccgcc
11281 gccctttgct tctctgtgcc actctggctg cacagttcag agccttggga aatgttaacc
11341 agtaggacct agacggggag gtgagaaggg gtcacccccc aggtgtgcct gtggtgagcc
11401 ttcgtgctga gcaggtgcag ggagggaggc ccaggtgcac acacctgtga agtaggggca
11461 gctggctggg ctccttgacc tgctccagag cttcttattt tctggccact tcacctgcag
11521 aaggcccagg tggctgtggc ctctagggtc ccttgcctgt cctcagctcc caactgggag
11581 gggcagaggg aggaggggt ggagacccca ggcagcaggg ctctgggagc agtggggccc
11641 tgggtcccag gggtgtctgg caggcccctc cttactctac gtctcggcct ctggatggag
11701 gtgctggctg cagtcgggct ctgcctctga ctaagggttg gggaagtggc gggtgtgggc
11761 tgctgccccg tggggcctct gaacagaccc cagggcctct gccaatcatg actccttcct
11821 ttcagctgga cccgcaggcc ctgcaggaca gagactggca gcgcgccgtc atcgccatga
11881 atggggtacg tgtccgtggg actctcctgg cgcccacttc ccccagaagg atagggtggc
11941 ctctgttcat ttcaaatcag tcagaggtgg ctgagcctga ggcagcatct gagagggagc
12001 ctggttggag aagggagggc ccccaagagc agaatcacca tgcacgggaa tcgtcattca
12061 ttggctggaa tgcagttgcc agccaggccc tgagcatccc tcctcaaaca aaggtctcat
12121 ggcaccacca ggacaggtgg ggcctccact cagggacctg gggctgccc atagaaatgg
12181 agacccctga tttgtcttta ggtaccccag aaaggtttag accttaaaag caatgacaca
12241 cccaaaaagg cccgggtata aatggtaaaa tgttaatatt tgagattctt ggctttttct
12301 tacattattc tgtctttcct tcttaatttt taattgttac taagagaaag ctggtcacag
12361 tacactataa tctcagctac tctggaggct gagccaggag aatcactgga gcccaagagt
12421 ttgattacag cctgggcaac attgcaagat cccatatcta aaaaaaaagc aagcaagcaa
12481 gagaagcagc ggggatttta ggaggtgctt ctgcagaaac cagtcgttta tatcatcttc
12541 aacaatcctg gctcttgctg aagtagacta ggggcttccc cgaggggcgg ctccacctca
12601 tgctgagacc tctgcatgcc ttgggggtgg aaatatttga tgagactccc aggggtcctt
12661 gggaccttgg gctgtgagga ccagaaggat tagaggactg tgccccttct ccccactgta
12721 gatcgaagta aagctctcgg tcaagttcaa cagcagggag ttcagcttga agaggatgcc
12781 gtcccgaaaa cagacagggg tcttcggagt caagattgct gtggtcacca agtgagtggg
12841 gaggggcttg ggctcacgca ctgagggtgc ctgtcccttc agctgtttct gcagaaaaga
12901 gcatgtgtgg gtctctcctc tctgtgcatg gccactgcac ggtgaggtca ggccccaggg
12961 aacacggcgt cttcagctac ctcctgtgtt tcctgcaaac cagctcagga atgtccttgc
```

```
13021 caccttgctt ggaagcagta ggctggctcc aggaactgcc caagtgcagg gttttctgcc
13081 cttgcttgga attagtcacg gtcccagatt cctgttgaat ggccataacc cctgcccctt
13141 tgtcacgagt cagttgccaa gagaagcctg tttggtttga gagcagttca tgcagacata
13201 gaccacttcc tctgagaatt catttgcttc cccaggatgg aatctggctg ggcctctgac
13261 cttgctggtc acgtgggccg gggcctccat cagtcatacc ctggactcct atctgtgtct
13321 aaacaccacg ccccaccccc aactgcacgg cagccactcg catagcactc tgggagggct
13381 gtgggcatga gcagcgagga ctccatgagc agctccccca gataagccct gctaatgagg
13441 gggcttgcga agcagctttg atgtgctggt aaatccaggt gcaaaacaga actcaagtta
13501 gggcctccgc acagcactgc gttctaactg tgaaggattc ttactctagt gtcctgtgtg
13561 gaggtattgg aattgtccat tgctaagact cagaggagaa aagcacttag catcgcagga
13621 cttggagcac cggtgctgag gcaacccttc attcattcgt cggatgtgtg ttaaggccca
13681 gggcaggggt cagggattct cctctcacac agcacgtggg tggcaggacc aacaccgggt
13741 ctgacctccc agccgggggc acaggctgct aaccccaggc ctggaatctg tcagatgccc
13801 ttcctgtgct gacttgactt agacaggcct cctgaccttc ccgcaaaggt catgtgtgat
13861 tcgcaggggt tctggccgct tgaaaggttc ctgagaaagc acatgccatg aggacagagc
13921 ttgcagaggg aggacaggca tgcagaaggc tctgtgtgca gccccagacc tgggtacctt
13981 cgtcaccgtc ctcaccccac ctccgggtgt gcagataggg agcaggcctc ctgtgttatg
14041 gcccaagcgg ggctgttagg acactgagaa cattccctcc tcccgcagga gagagaggtc
14101 caaggtgccc tacatcatgc gccagtgcgt ggaggagatc gagcgccgag gcatggagga
14161 ggtgggcatc taccgcgtgt ccggtgtggc cacggacatc caggcactga aggcaggctt
14221 caacgtcagt gagtgtcggc ctgcgcagga cgggatggag gtgtgggcag tggtgtccgc
14281 gatgagatct cagagtgctc catggcccag gcatgtcaca tccttctctg tgtcttttct
14341 tcatttactg ttttattatt ttaaaaaaag agaaaacaag agttgtacaa acagcttcta
14401 tagaagccag tttttacacc atcgtaccca ctcatgccac ttggtggagt ggaccagggg
14461 cttctgtggg gacttggcct tcctgccttg gggtggaca ggaggtggaa gcccaggact
14521 cagtgcggtc tgtccactgc cctgtatgag gatgtggtgg gcagagggca ctgatgaaat
14581 tcagcgcagg ccggggctgc agcatctccg cctccatctc accaaccctc acaggccttg
14641 aaggacccag actggcctca aatgccaggg gagggcactg agaccccaga gggtccttcc
14701 cagcatcttc aaagcaacag gattttgtgc ctgcagaccc ttctttgcag cacacaccac
14761 ccaccctgac caggacccct agaatgccca gcatccctgg gagggccctg tggtagtttc
14821 agctccctct gggggcccag aatgaacctg gcctgtggtg aggatgtaag caccaatggc
14881 caattgggtc caaaggaaga caccggttca aacactgaaa ccaatcagat tctcccacgg
14941 ccttcctgct atcagacgac actggtgcag gggtggttgc tatgtacagg gcagagccac
15001 ccaatcccca cgcaggcgct gtgtcctgcc acgttggcct cctcctggcc atcacatcag
15061 gccaagcagg ggagaggaat gggaatgccc acgcacccct atcaactctg cagacacaga
15121 accatgcaca gctcttggga ggagtcagat gagctgctca aagcccagga gggacccgca
15181 cagtggtcag tgtggcaggg acggtgcttt agccaaggca gggatggtgg gtgactcact
15241 caggatcttc aaggaggccg ctgcatttcc gtgctctttc cagataacaa ggacgtgtcg
15301 gtgatgatga gcgagatgga cgtgaacgcc atcgcaggca cgctgaagct gtacttccgt
15361 gagctgcccg agcccctctt cactgacgag ttctacccca acttcgcaga gggcatcggt
15421 gagcactgga ggccttggcc tcatgggaga cgtctcctcc acgtgcactg ctgccctcgg
15481 aggctgtgaa aagcgaggtg tgggaacctg agctgtaacc cctctgccgt ggtcggcatt
15541 ttaacccaac ctcaaaaagc aggggaccag aaccgagcct gtcctggaag gccttgccca
15601 tccccagagg gctccccatc cctactcctc aaggagacca agaggctgaa atagtcagca
15661 ctgctgtgct atggggtcct aaagtctgct gtcctccttc ctgcagacca gggctgaagg
15721 agggtgcctg ggtgctcttg ccatgggtcc tggtccagcc aagcatggtt tcaaacatga
15781 cctgaccctt agtcaacctg gaggctgatg tctagagcgg gtgctggtgc gtgcagcacc
15841 tgtggcctct gcatcaccct tagggcaggt ctgcctcccg ggcccatgca cagaggacct
15901 ggtctcccag cctgcaggtg cccctgtggt gtccaggacg acgagggggt ctctgtgtac
15961 ttggtggggc tgggaccctc ccacttccca cctccttgtg tccctcactc ccctgtttca
16021 ttccatgctg agcctcccct gccttgggct ccctggggag ggggtggtgg caggagttgc
16081 ccgagggcag ctctgcccat gagcagctgc tctagcggct cctcctgctg ctgttcgccg
16141 ggtgctgctg accccctgcga ggtagagaaa aggcgttcag gtggttcaca ccccacacag
16201 gtgcccctca cagggtcctc actggcggcc agcgctgtgg gtgtgacgat gatgacaagc
16261 ctaaactgcg caaggactcg tgtcccgggc gctccatgtg accacctcgg gagaggtctc
16321 cggcttgtcg taacccaggg gagtgaccca ctgcctcctg cagctctttc agacccagtt
16381 gcaaggaaga gctgcatgct caacctgttg ttgtccctgc cggaggccaa cctgctcacc
16441 ttccttttcc ttctagacca cctggaaagg tagcccagct ctcttgtggc tgcccaggac
16501 tccaggtctc caggccgttg gggtgcccct ctgctcccac cagaccccca gcaccaagga
16561 ccttttcccc cgacccctgt ctgcagtaac tcactgcttc taaggactag caccactgcc
16621 acccccaccc ctgcctctcc tctttgccac cctcctccct ctgcactgtg gccttaacaa
16681 agagctcaga gctttggccg tggccagcag tgcacttgga ccccccctctt ccctcccaag
16741 cacatcatga agacctcccc atcagcccag agctggcccc ttgtcctggg ccactgagac
16801 ccagaagtac caaggctgga gtcagcttgc agcacagcca gggtcgaggt cactccctcc
16861 ctgaggactc tagcacggca cagcccctct gcctctctcc tggtggtggc gttgaaacag
16921 caccctctgc ttcggtcctc tacagggtgg cagagaagga ggcggtcaat aaggtgtccc
16981 tgcacaacct cgccactgtc tttggcccca cgctgctccg gccctccgag aaggagagca
17041 agctccctgc caaccccagc cagcctgtca ccatgactga cagcaggtcc ttggaggtca
17101 tgtctcaggt atgggaagac agtctccagc ccatgcaacc ccagcctgac agaggtggcc
17161 tctgcctgcc ccacccccag tcctgcccat cttccgactt gcattgtatg tggtggtggc
17221 tgagattcag agagagggac ttgcctaggt ttgcatggat gggagtgata gggggtgccc
17281 aggccacctc ctggtcctgc tggtgcacct tgctgggggc ttaaaaccac cccaagtgtt
17341 cgggtgtggt ggctcatgcc tgtaatccca gcactttggg aggccgaggc aggacaactg
17401 aacccaggtg tttgagacca gtctgggcaa tgtagcaaac cccatctcta gaaaaaatac
17461 aaagaaaaat tagtcaggca ttgtggcaca catctgtaat cctaggtatc tgggaggctg
17521 acacaggagg attgcttgag cccaggagtt agaggctgca gtgatccatg atggagccac
17581 tgtactccag cctgggggac agagcaaggc cctgtgcatc tctaaaataa ataatcaccc
17641 cccacccaac aagtcatgcc ttgtcaggac cccaccccac ccccgtctca ctgtaagggg
17701 ttcatgacac cagcaggggt ttctagcacc tgaggtggac ttgggggctt gggccccaaa
17761 gacctcccca ccagcagctg tgagcccccc tctgagccac tctcctcttc cccactctgc
17821 gagggcagga cgaggtgctg ctgtacttct tgcggctgga ggccatccct gccctgaaca
17881 gcaagagaca gagcatcctg ttctccaccg atgtctaaag gtcccagtcc atctcctgga
17941 ggcggacaga tggcctggaa acctctggct aatcgggcca tctgtagagt gggaatcaag
18001 attttctgag gcatccttgg gccaccccca ggtgtcaggc catctgccaa gagacagcgg
18061 cccaaagcag aaggacaggt ggcctgggca gatcccgccc aggtctgaaa gccccaggct
18121 ggcctcagac tgtgggtttt ttatgtggcc acccgagggc gccccaagcc agttcatctc
18181 ggagtccagg cctggccctg ggagacaggg tgaaagcagt ggtttttatg aacttaactt
18241 atagagtcca aaagatttct actgaatcac ttgtcaagaa gcgccctctc tggggagaag
18301 ggaacgtgac tggattccct cactgttgta tcttgaataa acgctgctgc ttcatcctgt
18361 gggggccgtg gccctgtccc tgtgtgggtg gggcctcttc catttccctg acttagaaac
18421 cacactccac ttctaacagg gtttgagagg cttggtcagc actgggtagc gttttgactc
18481 cattcttggc tttcttcttt ttctttccag aaggattttt gtgcagaaat gggtcttttg
18541 ttgccgtgtt agtcctcctt ggaaggcagc tcagaaggcc tgtgaaatgt cgggggacag
18601 gacccccagg gagggaatcc caggctacgc accttagggt tcgttctcca gggagagcga
18661 cctcgtcccc cgatcctgac cgcccttccg gcccacgctc tcctgtttgg cttccacagg
18721 cctggacttc tctggcttct ctgcccacac actccctgcc cccagtgtcc ctgcccctgc
18781 cccagcacag gtgacttcat ttctgtcctc tcagctcagt ggactcgctc atcttttgta
18841 taagtctcca cttggtggca gcagcttgct gatgacttgt tttaaaactt tcatcctaaa
18901 taaccttttg atacttgaat atttttaagt tttatacata gtttctaatt tttttccgaa
18961 cagatccaga tacctaataa gatgctggaa tgtaatccct ggacaatccg tgtcctggca
19021 gcatttggtc ttcctctaag cgcctggctc cgctgttctc aggagtgggt tctgaagtct
19081 ctggagaaca ggatacgtgg agggttagga aggggccagg cctagagacg ggagactccc
19141 tcccggagca ggtggaggca caggaccatt cgctacccca tctgccggca cctgcggggg
19201 agcccaggca ttctttgtaa gccctcctga ccacctggct caaagaaaac agaagcatgg
19261 aggccgccaa gtattttcaa gaaataatcc catgaacatg gcatcacttt tttagaaaga
19321 ggggcttggg gcaggcagag gagagaaggg agatcaaact gagagccaag tttccagacg
19381 gtcctgcagg aggagaggat gcagctgccc agagggaagc aggatcacat ttaaggaagt
19441 gtgtggggtc cctggatgac accagcaccc agtgcggctc tgtctggcaa ccgctcccaa
19501 ggtggcagga gtgggtgtcc cctgtgtgtc agtgggcagc tcctgctgaa cccacagctc
19561 actggggagc ctgacagtgg ggccatgtgc ctgacactcc tctctgcttg tggacctggc
19621 aaggcaggga gcagaaaaca gagctacttg aaggctttct gtctgcgtct gtgtgcagtg
19681 tggatttagt tgtgcttttt acttgctggg agagcacagc caccatttac aagcagtgtc
19741 accctcgtgg gtggcgagga cagaacagga gcctctgctc tctgtaccta tctgggcccg
19801 gtgggctccc ttgtcctggc ttccatctct gtctcagcga ccattcagcc ctgcacagga
19861 acacatgttg cttagaaaag ccaaatccag cccttgtctc tgcctcctct ggtctcatga
19921 tgtgcatctg ttaccttgaa actggaaacc agtctatcaa tgtctgtgcc aatttttat
19981 tccctcccca acctccttcc ccatacgact ttttatttat gtaggatgtg tgctgtctaa
20041 tgatgggatg accacacttt tccatgttct aaaagtgctc ctctcccaca gggtcccagg
20101 gctggtggtt gctttgggtc tacagctacg tcttacccgc ctcctgcctc aacagcctgt
20161 gtggtggcaa agccggtgtg gggctgggga acgcagcgtt ctccaggagg ggacccggct
20221 ctccttctgc agtgcaggcg aaggcctaga tgccagtgtg acctcccaca aggcgtggct
20281 tccagactcc ccggccggaa gtgatgcttt tttgccgcgg gccctgggtt tgaagcagcc
20341 tggctttctc ttggtaagtg gctggtgtct tagcagctgc aatctgagct cagccaccta
20401 cacaccaccg tggccgacac tttcattaaa aagtttcctg agacga
//
```
