# Supplementary material for: An ancestral genomic sequence that serves as a nucleation site for de novo gene birth
Source: PLoS One. 2022 May 12;17(5):e0267864. doi: 10.1371/journal.pone.0267864 (PMC9097989; doi:10.1371/journal.pone.0267864)
Supplement: S11 Fig — (PDF) [file pone.0267864.s011.pdf]

**Detection of an ancestral genomic sequence that serves as a nucleation site for de novo gene birth**

Nicholas Delihas
Department of Microbiology and Immunology, Renaissance School of Medicine, Stony Brook University, Stony Brook, N.Y., United States of America

**S11 Fig.** Alignment of the chimpanzee sequence between *GGT1* and *LOC749026* with the *FAM247A sequ*ence in humans. The FAM247 sequence starts at the 3′ end of the chimpanzee spacer that is linked to *GGT1*.

**Chromosome 22 - NC_036901.1**

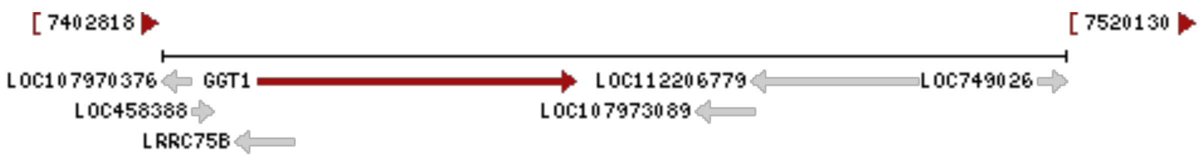


```
=====================================================================================================================

#
#
#  Percent Identity  Matrix - created by Clustal2.1
#
#

     1: GGT1end-LOC749026.end.7456450-7520130.chimp  100.00   97.76
     2: FAM247.LOC105372935.ref.human                 97.76  100.00


CLUSTAL O(1.2.4) multiple sequence alignment


GGT1end-LOC749026.end.7456450-7520130.chimp      ctctgggcctcagtgtattctgtgtgaaatggagccatctggctggggaggaacagagag 60
FAM247.LOC105372935.ref.human                    ------------------------------------------------------------ 0

GGT1end-LOC749026.end.7456450-7520130.chimp      gtgggattcggagatcttcacaatgtgggcactggaactagcctcaacatcttcagcatg 120
FAM247.LOC105372935.ref.human                    ------------------------------------------------------------ 0

GGT1end-LOC749026.end.7456450-7520130.chimp      gggagagccaggcacatggctgggggccaggggaaggttcacaccaagctctgccccttc 180
FAM247.LOC105372935.ref.human                    ------------------------------------------------------------ 0

GGT1end-LOC749026.end.7456450-7520130.chimp      ccaccctgatccctcggactttggggccaggccctcccttactggggctgggcagtgaca 240
FAM247.LOC105372935.ref.human                    ------------------------------------------------------------ 0

GGT1end-LOC749026.end.7456450-7520130.chimp      ctacctaggatcagccaccgggggtgccacgaccctggcactttcttaggcagagggtg 300
FAM247.LOC105372935.ref.human                    ------------------------------------------------------------ 0

GGT1end-LOC749026.end.7456450-7520130.chimp      gccagctgatgctgggaacccgggcgccttctcagacccgtaggcgtccagctcaccctg 360
FAM247.LOC105372935.ref.human                    ------------------------------------------------------------ 0

GGT1end-LOC749026.end.7456450-7520130.chimp      ccgatgacactggaggtgaagctgaggtccgaggaatggggactgggcaacaggctggag 420
FAM247.LOC105372935.ref.human                    ------------------------------------------------------------ 0

GGT1end-LOC749026.end.7456450-7520130.chimp      gaaaacatctcggtcagagccacgcccctgggggtttccaagtttaagcccagagtgaa 480
FAM247.LOC105372935.ref.human                    ------------------------------------------------------------ 0

GGT1end-LOC749026.end.7456450-7520130.chimp      acccaagcttgtgatcctctccagagggaggcctggttctcagggaacagcaaacgggaa 540
FAM247.LOC105372935.ref.human                    ------------------------------------------------------------ 0

GGT1end-LOC749026.end.7456450-7520130.chimp      gatgtccccagatcccagggatcagggcttggaccagccggggacgcagcccagagggag 600
FAM247.LOC105372935.ref.human                    ------------------------------------------------------------ 0

GGT1end-LOC749026.end.7456450-7520130.chimp      tgggtccggaaggaaacagctcgacacagcagccttcaccatcggcagcccctccaggcc 660
FAM247.LOC105372935.ref.human                    ------------------------------------------------------------ 0

GGT1end-LOC749026.end.7456450-7520130.chimp      tccctcggggcctgctccctcctctgtgcacagttccaacacctggggcagggttctggg 720
FAM247.LOC105372935.ref.human                    ------------------------------------------------------------ 0

GGT1end-LOC749026.end.7456450-7520130.chimp      aagggctggtggaggtgggctggtgggaggcggtgatcacagcccagcacctggatatca 780
FAM247.LOC105372935.ref.human                    ------------------------------------------------------------ 0

GGT1end-LOC749026.end.7456450-7520130.chimp      ccaggggcactggggccaggggccaggtgaggccaggtcggggctatccttcaggagccc 840
FAM247.LOC105372935.ref.human                    ------------------------------------------------------------ 0

GGT1end-LOC749026.end.7456450-7520130.chimp      cgaaaacctggtgattccaaagggcccatagacaaacagggttttctgcctgtggagtca 900
FAM247.LOC105372935.ref.human                    ------------------------------------------------------------ 0

GGT1end-LOC749026.end.7456450-7520130.chimp      agtcccactgggtctgagctctggagggctgtgtctctggggctctgcaagggtgagatg 960
FAM247.LOC105372935.ref.human                    ------------------------------------------------------------ 0

GGT1end-LOC749026.end.7456450-7520130.chimp      gaggtgggctcaaatggtgtacaagtcactcctcaatccttattttatttatttaatttt 1020
FAM247.LOC105372935.ref.human                    ------------------------------------------------------------ 0

GGT1end-LOC749026.end.7456450-7520130.chimp      tttaaaaaaatttaaaccaatagagatggggtctcactatgttgatcaggctggtcttaa 1080
FAM247.LOC105372935.ref.human                    ------------------------------------------------------------ 0

GGT1end-LOC749026.end.7456450-7520130.chimp      ctcctgacttcaagcagtccccccatgtcagtctcccaaagtgctaggattacaggggtg 1140
FAM247.LOC105372935.ref.human                    ------------------------------------------------------------ 0

GGT1end-LOC749026.end.7456450-7520130.chimp      agccactgcacccggcctcaatccttattttggcctgagaggaaaggccgtggccccatt 1200
FAM247.LOC105372935.ref.human                    ------------------------------------------------------------ 0

GGT1end-LOC749026.end.7456450-7520130.chimp      tgcaggggagaagactgaagctggaggggcaggccttgctctgggttgcacagcagcaag 1260
FAM247.LOC105372935.ref.human                    ------------------------------------------------------------ 0

GGT1end-LOC749026.end.7456450-7520130.chimp      agaagtgggagctggccacgaggcttcctcgactcgacacactggtggggtacaccctgg 1320
FAM247.LOC105372935.ref.human                    ------------------------------------------------------------ 0

GGT1end-LOC749026.end.7456450-7520130.chimp      ttctccaggtcccatggggctcagcccaggactacctcgggggtgagggacttaaatcc 1380
FAM247.LOC105372935.ref.human                    ------------------------------------------------------------ 0

GGT1end-LOC749026.end.7456450-7520130.chimp      tctccttcattctcatcaccccttcccccatcatttcctgaggaaggacattcagggacc 1440
FAM247.LOC105372935.ref.human                    ------------------------------------------------------------ 0

GGT1end-LOC749026.end.7456450-7520130.chimp      tgaaggagcggcctgcccctccacatctgtgggtgtttctcatcaggtgggacaagagac 1500
```

```
FAM247.LOC105372935.ref.human                 ------------------------------------------------------------ 0

GGT1end-LOC749026.end.7456450-7520130.chimp   tgagaaaagaaagagacacagagacaaagtatagagaaagaaaagtgggcccaggggacc 1560
FAM247.LOC105372935.ref.human                 ------------------------------------------------------------ 0

GGT1end-LOC749026.end.7456450-7520130.chimp   tgcgctcagcatatggaggacccacgctggcaccagtctctgagttccctagtatttatt 1620
FAM247.LOC105372935.ref.human                 ------------------------------------------------------------ 0

GGT1end-LOC749026.end.7456450-7520130.chimp   gatcattatctctaccatctcagagaggggatgtggcaggacaacatggtaatagtggg 1680
FAM247.LOC105372935.ref.human                 ------------------------------------------------------------ 0

GGT1end-LOC749026.end.7456450-7520130.chimp   gcgagggtcagcaggaaaacacgtgaacaaatgtctctgtgtcataaacaaggttaagaa 1740
FAM247.LOC105372935.ref.human                 ------------------------------------------------------------ 0

GGT1end-LOC749026.end.7456450-7520130.chimp   aaaggtgctgtgctttgatgtgcatatacataaacatctcaatgcattaaagagcagtat 1800
FAM247.LOC105372935.ref.human                 ------------------------------------------------------------ 0

GGT1end-LOC749026.end.7456450-7520130.chimp   tgccaccagcatgtcccacctccagccctaaggcagttttctcctatctcagtagatgga 1860
FAM247.LOC105372935.ref.human                 ------------------------------------------------------------ 0

GGT1end-LOC749026.end.7456450-7520130.chimp   atatacaattgggttttacaccgagacattcctttgcccagggacgatcaggagacagat 1920
FAM247.LOC105372935.ref.human                 ------------------------------------------------------------ 0

GGT1end-LOC749026.end.7456450-7520130.chimp   gccttcctcttgtctcaactgcaaagaggccttccttcctcttatactaatcctcctcag 1980
FAM247.LOC105372935.ref.human                 ------------------------------------------------------------ 0

GGT1end-LOC749026.end.7456450-7520130.chimp   cacagaccctttacgggtgtcgggctgggggacggtcaggtctttctcttcccacgaggc 2040
FAM247.LOC105372935.ref.human                 ------------------------------------------------------------ 0

GGT1end-LOC749026.end.7456450-7520130.chimp   catatttcagactgtcacatggggagaaaccttggacaatacctggctttcctaggcaga 2100
FAM247.LOC105372935.ref.human                 ------------------------------------------------------------ 0

GGT1end-LOC749026.end.7456450-7520130.chimp   ggtccctgcggccttctgcagtgttttgtgtccctgcttacttgagattagggagtggtg 2160
FAM247.LOC105372935.ref.human                 ------------------------------------------------------------ 0

GGT1end-LOC749026.end.7456450-7520130.chimp   atgactttaacaagcatgttgccttaaagcatttgtttaacaaagcacatcctgcatag 2220
FAM247.LOC105372935.ref.human                 ------------------------------------------------------------ 0

GGT1end-LOC749026.end.7456450-7520130.chimp   ccctaaatccattaaaccttgagtcgacacagtacatgtttctgtgagcacagggttggg 2280
FAM247.LOC105372935.ref.human                 ------------------------------------------------------------ 0

GGT1end-LOC749026.end.7456450-7520130.chimp   gatagggttacagattaacagcatctcaaggcaaaagaatttttcttactacagaacaaa 2340
FAM247.LOC105372935.ref.human                 ------------------------------------------------------------ 0

GGT1end-LOC749026.end.7456450-7520130.chimp   atggagcctcttacgtctacttctttctacatagacacagtaacagtctgatatctcttt 2400
FAM247.LOC105372935.ref.human                 ------------------------------------------------------------ 0

GGT1end-LOC749026.end.7456450-7520130.chimp   cttttccccacagggaccttcctggctgtgcctaggatcaggaccagaatgacacccatt 2460
FAM247.LOC105372935.ref.human                 ------------------------------------------------------------ 0

GGT1end-LOC749026.end.7456450-7520130.chimp   catttccctgggcctttgctcgggcggtccctgcaccctggcctctgcctgaccaggatg 2520
FAM247.LOC105372935.ref.human                 ------------------------------------------------------------ 0

GGT1end-LOC749026.end.7456450-7520130.chimp   gtggggagaggagggggatgtcccccacgctgctgtctccactgttcctgctgcccagg 2580
FAM247.LOC105372935.ref.human                 ------------------------------------------------------------ 0

GGT1end-LOC749026.end.7456450-7520130.chimp   cctctgagcttccaggactgcagcgggtgggtgggtggcctggcctaagcccaggaatgc 2640
FAM247.LOC105372935.ref.human                 ------------------------------------------------------------ 0

GGT1end-LOC749026.end.7456450-7520130.chimp   acttcagctcctggtagagcaatgtcactgaggcttgggagtcgggtggggacgggagga 2700
FAM247.LOC105372935.ref.human                 ------------------------------------------------------------ 0

GGT1end-LOC749026.end.7456450-7520130.chimp   ggcgtccgcaggccccccctaccgtgagaggcagccgtgggaacagcctacctctaaacaa 2760
FAM247.LOC105372935.ref.human                 ------------------------------------------------------------ 0

GGT1end-LOC749026.end.7456450-7520130.chimp   tcactgtagcccaggctgaccaggggctctggccggacagaggggcctggcaggctgtgt 2820
FAM247.LOC105372935.ref.human                 ------------------------------------------------------------ 0

GGT1end-LOC749026.end.7456450-7520130.chimp   ggcctgtaaggacacagtctgtctctgtgcctcagtttctctgctgcccagatggagagg 2880
FAM247.LOC105372935.ref.human                 ------------------------------------------------------------ 0

GGT1end-LOC749026.end.7456450-7520130.chimp   cccagactccaggtgtagacatctggagcaggcagtgttcagctggggagggagcggtga 2940
FAM247.LOC105372935.ref.human                 ------------------------------------------------------------ 0

GGT1end-LOC749026.end.7456450-7520130.chimp   ggactatgggggccacgtgggaagaagtccagcccacatcacctgcacccctgctgagcc 3000
FAM247.LOC105372935.ref.human                 ------------------------------------------------------------ 0

GGT1end-LOC749026.end.7456450-7520130.chimp   tggtcaacagaggccctcagtgggtcctcactctcctggctgcctcccatttaggcaccc 3060
FAM247.LOC105372935.ref.human                 ------------------------------------------------------------ 0

GGT1end-LOC749026.end.7456450-7520130.chimp   tgaggcctggggagaacagagccaggccagtgtccccagagaggctgcgctgccagcaca 3120
FAM247.LOC105372935.ref.human                 ------------------------------------------------------------ 0

GGT1end-LOC749026.end.7456450-7520130.chimp   gtagtagcagatttggattcagggaagtagacctgcagccagggtgggaaagagctgcag 3180
FAM247.LOC105372935.ref.human                 ------------------------------------------------------------ 0

GGT1end-LOC749026.end.7456450-7520130.chimp   gcggggtggagcccccacatggcacagccccccctccctggaggtctgtgctgcatttcca 3240
FAM247.LOC105372935.ref.human                 ------------------------------------------------------------ 0

GGT1end-LOC749026.end.7456450-7520130.chimp   ggacagcaagtcccagggatggatggtgccaggtgccaagggctagaggcatggtctgtc 3300
FAM247.LOC105372935.ref.human                 ------------------------------------------------------------ 0

GGT1end-LOC749026.end.7456450-7520130.chimp   tgcattccccacaggggcgtcttatagtcaccagcatttgatgctgtcaagtccccctgt 3360
FAM247.LOC105372935.ref.human                 ------------------------------------------------------------ 0

GGT1end-LOC749026.end.7456450-7520130.chimp   cctctgtgcagactgggaagcccttggtcaccctgggggggttgggggacccaggccagg 3420
FAM247.LOC105372935.ref.human                 ------------------------------------------------------------ 0

GGT1end-LOC749026.end.7456450-7520130.chimp   ctgcagaaacataaggacttgaacccgggtcctgagtgacaccaccttgggtcctcctcc 3480
FAM247.LOC105372935.ref.human                 ------------------------------------------------------------ 0

GGT1end-LOC749026.end.7456450-7520130.chimp   ctctgcctctgttcagctccaccttgatgctgactaggctgggccatgcggagagggtta 3540
FAM247.LOC105372935.ref.human                 ------------------------------------------------------------ 0

GGT1end-LOC749026.end.7456450-7520130.chimp   ggggatagagatgggagctggggagcagggctccactctgggagggggggcagccttgccg 3600
FAM247.LOC105372935.ref.human                 ------------------------------------------------------------ 0

GGT1end-LOC749026.end.7456450-7520130.chimp   gatccagggcagagttaagcggccccagctctgctttcctagagctgctgagaacccagg 3660
FAM247.LOC105372935.ref.human                 ------------------------------------------------------------ 0
```

```
GGT1end-LOC749026.end.7456450-7520130.chimp      aaatggtgtggagtttccggggaaccctgcccctacctggcaaccgcagtgcagcaggca 3720
FAM247.LOC105372935.ref.human                    ------------------------------------------------------------ 0

GGT1end-LOC749026.end.7456450-7520130.chimp      ccaagttctcctgcacattgcgacagtgtgaccctgggctctggcgggcggtaggtgggg 3780
FAM247.LOC105372935.ref.human                    ------------------------------------------------------------ 0

GGT1end-LOC749026.end.7456450-7520130.chimp      cctttggacctaccagcagtgagggagttaacacagcagctgactcctctaggcaaggaa 3840
FAM247.LOC105372935.ref.human                    ------------------------------------------------------------ 0

GGT1end-LOC749026.end.7456450-7520130.chimp      aactcccctcagatgctttgctgcctggcctcctgccagcaacaagcaggagctgaaaac 3900. 3893bp
FAM247.LOC105372935.ref.human                    -----------------------------------------------------tgaaaac 7
                                                                                                      *******
```

# Start of FAM247A sequence

```
GGT1end-LOC749026.end.7456450-7520130.chimp      tagaagttgaggcgtgagtttggccactccgtagtgtgcacttggtgagggcagcagctc 3960
FAM247.LOC105372935.ref.human                    tagaagttgaggcatgagtttggccactccgtagtgtgcacttggtgagggcagcagctc 67
                                                 ************* **********************************************

GGT1end-LOC749026.end.7456450-7520130.chimp      gccacagctgccagccgtctgtccattcacccatctgtccatctggcagcccgctgttca 4020
FAM247.LOC105372935.ref.human                    gccacagctgccagccatctgtccattcacccatctgtccatctggcagcccgctgttca 127
                                                 **************** *******************************************

GGT1end-LOC749026.end.7456450-7520130.chimp      gacccgtctgtctgtccgcccatctgtaagcccatctctgtcccattgtctatctgacca 4080
FAM247.LOC105372935.ref.human                    gacctgtctgtctgtccgcccatctgtaagcccatctctgtcccattgtctatctgacca 187
                                                 **** *******************************************************

GGT1end-LOC749026.end.7456450-7520130.chimp      tctttctcttactgtcctctttatctagctatctggcctatccgtcgatccatcttcgtg 4140
FAM247.LOC105372935.ref.human                    tctttctcttactgtcctctttgtctagctatctggcctgtctgtcgatccatcttcgtg 247
                                                 ********************** **************** ** *****************

GGT1end-LOC749026.end.7456450-7520130.chimp      tctgtcttcagcccccacctgtttgtccatctgtccaattacctgtgagtctatctatgc 4200
FAM247.LOC105372935.ref.human                    tctgtcttcagcccccacctgtttgtccatctgtccaattacctgtgagtctatctatgc 307
                                                 ************************************************************

GGT1end-LOC749026.end.7456450-7520130.chimp      atcttcttgtccattcatctgcccacccatctgtccctccgtctgcccaccggcctcccc 4260
FAM247.LOC105372935.ref.human                    atcttcttgtccattcatctgcccacccatctgtccctccatctgcccaccggcctcccc 367
                                                 **************************************** *******************

GGT1end-LOC749026.end.7456450-7520130.chimp      tctccttctgggccgcagagccatggcccaggactgcagagccatggttggcctggtcct 4320
FAM247.LOC105372935.ref.human                    tctccttctgggccgcagagccatggcccaggactacggagccatgggtgacctggtcct 427
                                                 *********************************** * ********* ** *********

GGT1end-LOC749026.end.7456450-7520130.chimp      ------gctggggctggggctggcgctggctgtcattgtgctggctgtggtcctctctcg 4374
FAM247.LOC105372935.ref.human                    gctggggctggggctggggctggcgctggctgtcattgtgctggctgtggtcctctctcg 487
                                                       ******************************************************

GGT1end-LOC749026.end.7456450-7520130.chimp      acaccaggccccatttgacccccggcctttgcccacgccgctgttgctgctgactccaag 4434
FAM247.LOC105372935.ref.human                    acaccaggccccatttgacccccggcctttgcccacgccgctgttgctgctgactccaag 547
                                                 ************************************************************

GGT1end-LOC749026.end.7456450-7520130.chimp      gtctgctcaaatattgtacggtgagtgagacgtgggaggaagctgggtggcccttggcag 4494
FAM247.LOC105372935.ref.human                    gtcttctcaaatattgtacggtgagtgagacgtgggaggaagctgggtggcctttggcag 607
                                                 **** *********************************************** *******

GGT1end-LOC749026.end.7456450-7520130.chimp      ccagcccctcctggagaaggcgtgtatgtgagagtgtgtgtgtgagcatgtgtgtgtgtg 4554
FAM247.LOC105372935.ref.human                    ccagcccctcctggagaaggc------------gtgtgtgtgtgagcatgtgtgtgtgtg 655
                                                 *********************            ***************************

GGT1end-LOC749026.end.7456450-7520130.chimp      agagattatgtgtcagtgtgtgtgggtatatgtgtgagtgtgagtgtttgtggggtgtgg 4614
FAM247.LOC105372935.ref.human                    agagattatgtgtgagtgtgtgtgggtatatgtgtgagt----gtgtttgtggggtgtgg 711
                                                 ************* *************************    *****************

GGT1end-LOC749026.end.7456450-7520130.chimp      gtgtgtgtgaatgtgtgtgatcgtgtttgggtgtgtgtatgtgtgagtgtgg--gtgtgt 4672
FAM247.LOC105372935.ref.human                    gtgtgtgtgaatgtgtgtgatcgtgtttgggtgtgtgtatgtgtgagtgtgggtgtgtgt 771
                                                 ****************************************************  ******

GGT1end-LOC749026.end.7456450-7520130.chimp      gaatgtgtgtgattgtgtttgtgtgtatgtgt----gagtgtgtgggtgtgtgtgggtat 4728
FAM247.LOC105372935.ref.human                    gaatgtgtgtgattgtgtttgtgtatgtgtgtgtgtgggtgtgtgtgagtatatgtgagtgt 831
                                                 ************************ * *****    * ******* ** * **** ** *

GGT1end-LOC749026.end.7456450-7520130.chimp      gtgagtgtgagtgtgtgggtgggtgtgaacgtgtgtgattgtgttttgctgtgtgagggt 4788
FAM247.LOC105372935.ref.human                    gagtgtgtgggggtgtgggtgggtgtgaatgtgtgtgattgtgtttcgctgtgtgagggt 891
                                                 * * ***** * ***************** **************** *************

GGT1end-LOC749026.end.7456450-7520130.chimp      gtgtgtgactatgagtgtgtgagtgtgggtgtgtgtaaatgtgtgtgattgtgtgagtgt 4848
FAM247.LOC105372935.ref.human                    gtgtgtgactgtgagtgtgtgagtgtgggtgtgtg--ggtgtgtgtaaatgtgtgagtgt 949
                                                 ********** ************************    ******* * ***********

GGT1end-LOC749026.end.7456450-7520130.chimp      atgtgtgggtgtgagtgtgtgagtgtgagtatgggggtgggtgggtgtgaatgtgtgtga 4908
FAM247.LOC105372935.ref.human                    gagtatgggggggtgggtatgtgtgaatgtgtgtgattgtgtgtgggtatatttgtgggg 1009
                                                   ** **** * * * ** ** ***   ** ** *  ** *** * **  ** **** *

GGT1end-LOC749026.end.7456450-7520130.chimp      ttgtgtgtgtgtgtgtgagtgtgtgtgtgcgcgtgtgtgtgcacgtgcactggcccagga 4968
FAM247.LOC105372935.ref.human                    gtgtgtgtgtgtgtgcacgtgt----------gtgtgtgtgcacgtgcactggcccagga 1059
                                                  **************   ****          ****************************

GGT1end-LOC749026.end.7456450-7520130.chimp      agcaggagccatgtgtgtgggcttcagcacctgcagggcttgagagcaaggagacagcct 5028
FAM247.LOC105372935.ref.human                    agcaggagccgtgtgtgtgggcttcagcacctgcagggcttgagcgcaaggagacagcct 1119
                                                 ********** ******************************** ****************

GGT1end-LOC749026.end.7456450-7520130.chimp      cagggcccttgcacagaacaggtggcaggtgtgcccgtggggcagatggggacttgagg 5088
FAM247.LOC105372935.ref.human                    cagggcccttgcacagaacaggcggcaggtgtgcccgtggggcagatggggacttgggg 1179
                                                 ********************** ********************************** **

GGT1end-LOC749026.end.7456450-7520130.chimp      acaatggtggtgtgtgagtccatacctggctccaggattcaggaggcccatttgcacatc 5148
FAM247.LOC105372935.ref.human                    acaatggtggtgtgtgagtccatacctggctccaggattcaggaggcccatttgcatatc 1239
                                                 ******************************************************** ***

GGT1end-LOC749026.end.7456450-7520130.chimp      ccaggtgggaacctgtctggccccgcctgaccctgctggcc-gtgcaggccccttcagtg 5207
FAM247.LOC105372935.ref.human                    ccaggtgggaacctgtctggccccgcctgaccctgctggccggcgcaggccccttcagtg 1299
                                                 ***************************************** * ****************

GGT1end-LOC749026.end.7456450-7520130.chimp      aggccaattctccaaggctggggtcttctcccagggtcatgggcgaaggggtttggaggc 5267
FAM247.LOC105372935.ref.human                    aggccaattctccaaggctgcggtcttctcccagggtcatgggtgaaggggtttggaggc 1359
                                                 ******************** ********************** ****************

GGT1end-LOC749026.end.7456450-7520130.chimp      tccctgcgtgggtactggcctgctggggtacacacaatgctgccatagccagtctgcccc 5327
FAM247.LOC105372935.ref.human                    tccctgcgtgggtactggcctgctggggtacacacaatgctgccatagccagtctgcccc 1419
                                                 ************************************************************

GGT1end-LOC749026.end.7456450-7520130.chimp      aacacccagcccggggccacatctcaggtctctcagtcctgaggagcccggtgccccacc 5387
FAM247.LOC105372935.ref.human                    tacacccagcccggggccacatctcaggtctctcagtcctgaggagcccggtgccccacc 1479
                                                  ***********************************************************

GGT1end-LOC749026.end.7456450-7520130.chimp      cctcacatcctctctccctgagtcagggcctgggtctcgtgagccgagtgactgatactt 5447
FAM247.LOC105372935.ref.human                    cctcacatcctctctccctgagtcagggcctgggtctcgtgagctgagtgactgatactt 1539
                                                 ******************************************** ***************

GGT1end-LOC749026.end.7456450-7520130.chimp      ggtgtcctggatgagggcgtgatggagaggggccacagtgggtgtttcctgaccctcttc 5507
FAM247.LOC105372935.ref.human                    ggtgtcctggatgagggcgtggtggagaggggccacagcgggtgtttcctgaccctcttc 1599
                                                 ********************* **************** *********************

GGT1end-LOC749026.end.7456450-7520130.chimp      caggaaggtgctgctgccgctgcagggaggacacatacaggatgccccttcctgcgccct 5567
FAM247.LOC105372935.ref.human                    caggaaggtgctgctgccgctgcagggaggacacacacaggatgccccttcttgcccccct 1659
                                                 *********************************** *************** *** ****

GGT1end-LOC749026.end.7456450-7520130.chimp      gcctcccattgggcccacaaaagccagggcaagcctcccctccttgccagccacctggtc 5627
FAM247.LOC105372935.ref.human                    gcctcccattgggcccacaaaagccagggcaagcctcccctccctgccagccacctggtc 1719
                                                 ******************************************* ****************

GGT1end-LOC749026.end.7456450-7520130.chimp      tgcttcccagaaattctgtcttgcaggctgttgggaggatcccagtactttgtaaactaa 5687
FAM247.LOC105372935.ref.human                    tgcttcccagaaattctgtcttgcaggctgttgggaggatcccagtactttgtaaactaa 1779
                                                 ************************************************************

GGT1end-LOC749026.end.7456450-7520130.chimp      agcaagggaggagtggccattctctctctttgttcattcattcaacttttcattcattcc 5747
FAM247.LOC105372935.ref.human                    agcaagggaggagtggccgttctctctctttgttcattcattcaccttttcattcattcc 1839
                                                 ****************** ************************* ***************

GGT1end-LOC749026.end.7456450-7520130.chimp      ttcttccctccattcccccatctgtccatccttccctgccctgattgctcttgccaccgc 5807
FAM247.LOC105372935.ref.human                    ttcttccctccattcccccatctgtgcatccttccctgccctgattgctcatgccac--c 1897
```

```
                                                 ************************* ************************ ******  *

GGT1end-LOC749026.end.7456450-7520130.chimp      ccccccccagcccctcctgacctggtcctttggtttctcttcagggctttctgtctcctcc 5867
FAM247.LOC105372935.ref.human                    ccccccccagcccctcctgacctggtcctttggtttctcttcagggctttctgtctcctcc 1957
                                                 ************************************************************

GGT1end-LOC749026.end.7456450-7520130.chimp      cacagggctgagaatggcagctcagggacaagtaggggctggggactgcttagtctcccc 5927
FAM247.LOC105372935.ref.human                    cacagggctgagaatggcagctcagggacaagtaggggctggggactgcttagtctcccc 2017
                                                 ************************************************************

GGT1end-LOC749026.end.7456450-7520130.chimp      agtggctctcaggggatttgagggtttgacgccagctgccaccccaggttgtgcccctcc 5987
FAM247.LOC105372935.ref.human                    agtggctctcaggggatttgagggtttgacgccagctgccaccccaggctgtgcccctcc 2077
                                                 ************************************************ ***********

GGT1end-LOC749026.end.7456450-7520130.chimp      tctgctcaggaggacatacag-gatgcgacactcacttaaacacaaagttgcaaagatgc 6046
FAM247.LOC105372935.ref.human                    tctgctcgggaggacatacagagatgcgacacccacttaaactcgaagttgcaaagatgc 2137
                                                 ******* ************* ********** ********* * ***************

GGT1end-LOC749026.end.7456450-7520130.chimp      aaatgagactggggtctcaggcaccagagaccacccgtgggcacgtggcttttgggagtg 6106
FAM247.LOC105372935.ref.human                    aaatgagactggggtctcaggcaccagagaccacccgtgggcacgtggcttttgggagtg 2197
                                                 ************************************************************

GGT1end-LOC749026.end.7456450-7520130.chimp      gagacctgctgccacagatctctgaagagtctggacctgctgggtctccccgagtgactg 6166
FAM247.LOC105372935.ref.human                    gggacctgctgccacagatctctgaagagtctggacctgctgggtctccccgagtgactg 2257
                                                 * **********************************************************

GGT1end-LOC749026.end.7456450-7520130.chimp      tctgggggtctccatagcatgccctgctgtgtgcgtgacggtcactggttgggtaggggc 6226
FAM247.LOC105372935.ref.human                    tctgggggtctccatagcatgccctgctgtgtgcgtgacggtcactggttgggtaggggt 2317
                                                 ***********************************************************

GGT1end-LOC749026.end.7456450-7520130.chimp      ctctactctaaagctccctctgccggcatcccctcgaactctcccttggtgaagagagag 6286
FAM247.LOC105372935.ref.human                    ctctactctaaagctccctctgccggcatcccctccaactctcccttggtga--agagag 2375
                                                 *********************************** ****************  ******

GGT1end-LOC749026.end.7456450-7520130.chimp      gatgtggtttgccccagtgttttatcaaacaactctctccacttcctgttttaagaagct 6346
FAM247.LOC105372935.ref.human                    gatgtggtttgccccagtgttttatcaaacaactctctccacttccagttttaagaagct 2435
                                                 ********************************************** *************

GGT1end-LOC749026.end.7456450-7520130.chimp      gggagtggaagagagcctggggctggccccagctgctgctgcaaaacaggggtcactgga 6406
FAM247.LOC105372935.ref.human                    gggagtggaagagagcctggggctggccccagctgctgctgtgaaacaggggtcactgga 2495
                                                 *****************************************  *****************

GGT1end-LOC749026.end.7456450-7520130.chimp      tgctgggtccctggccgggctggctggaggcctcaggaagaggcctgctgcagcgtcatc 6466
FAM247.LOC105372935.ref.human                    cgctgggaccctggccgggctggctggaggcctcaggaagaggcctgctacagcgtcatc 2555
                                                  ****** ***************************************** **********

GGT1end-LOC749026.end.7456450-7520130.chimp      ctggccaagattcctccctgcagaggaccctggccacactgccacagggtctgctggggc 6526
FAM247.LOC105372935.ref.human                    ctggccaagattcctccctgcagaggaccctggccacgctgccacagggtctgctggggc 2615
                                                 ************************************* **********************

GGT1end-LOC749026.end.7456450-7520130.chimp      caccagaagcccatgctcctgcctccatctctcccctctgtgctcacctctcaccaggag 6586
FAM247.LOC105372935.ref.human                    caccagaagcccatgctcctgcctccatctctcccctctgtgctcacctctcaccaggag 2675
                                                 ************************************************************

GGT1end-LOC749026.end.7456450-7520130.chimp      accctcccagagttcagtgtcctgctttttttttttttttttttttagatggtgtctcg 6646
FAM247.LOC105372935.ref.human                    gccctcccagagttcagtgtcctgcttttttttttttttttgt----gacggtgtctca 2731
                                                  ***************************************** *    ** ********

GGT1end-LOC749026.end.7456450-7520130.chimp      ctctgtcaccaggctggagtgcagtggcgcgatctcagctcactgcaacctctgcttcct 6706
FAM247.LOC105372935.ref.human                    ctctgtcaccaggctggagtgcagtggcgcgatctcagcttactgcaacctctgcttcct 2791
                                                 **************************************** *******************

GGT1end-LOC749026.end.7456450-7520130.chimp      tggttcaaatgattctcctgcctcagcctcctgagtagctgggactacaggtgccagcca 6766
FAM247.LOC105372935.ref.human                    cggttcaaatgattctcctgcctcagcctcctgagtagctgggactacaggtgccagcca 2851
                                                  ***********************************************************

GGT1end-LOC749026.end.7456450-7520130.chimp      ccacacccagctaatttttgtattttcagtagagacagggtttcaccatgttggccagga 6826
FAM247.LOC105372935.ref.human                    ccacgccccgctaatttctgtatttttagtagagacggggtttcaccgtgttggccagga 2911
                                                 **** *** ******** ******** ********* ********** ************

GGT1end-LOC749026.end.7456450-7520130.chimp      tggtctctatctcttgattcacccgccttggccacccaaagtgctggcattacaggagtg 6886
FAM247.LOC105372935.ref.human                    tggtctctatctcttgattcacccgccttggccacccaaagtgctggcattacaggagtg 2971
                                                 ************************************************************

GGT1end-LOC749026.end.7456450-7520130.chimp      agtcatggcacccggcctcatctcctactctttcagcaccaggttttattcttgggattc 6946
FAM247.LOC105372935.ref.human                    agtcatggcacctggcctcatctcctactctttcagcaccaggttttattcttgggattc 3031
                                                 ************ ***********************************************

GGT1end-LOC749026.end.7456450-7520130.chimp      tgctacagccggagcccctgggtgcgagctcctaagctttctgtgagtgtggacccagca 7006
FAM247.LOC105372935.ref.human                    tgctacagccggagcccctgggtgcgagttcctaaggtttctgtgagtgtggacccagca 3091
                                                 **************************** ******* ***********************

GGT1end-LOC749026.end.7456450-7520130.chimp      ctgtgcctagtagacatacaaaaggagcatggtgacagtgaggtctgtcatctccagcgt 7066
FAM247.LOC105372935.ref.human                    ccgtgcctagtagacatacaaaaggagcatggtgacagtgaggtctgtcatctccagcat 3151
                                                 * ******************************************************** *

GGT1end-LOC749026.end.7456450-7520130.chimp      aatgactgttttgatccttgtgaaaaaggtgattttggctgggtgtggtggctcacacc 7126
FAM247.LOC105372935.ref.human                    aatgactgttttgatccttgtaaaaaaggtgattttggctgggtgtggtggctcacacc 3211
                                                 ********************* **************************************

GGT1end-LOC749026.end.7456450-7520130.chimp      tgtaatcccagcactttgggaggccgaggagggtggctcacttgaggtcaggagttggag 7186
FAM247.LOC105372935.ref.human                    tgtaatcccagcactttgggaggccgagggggtggctcacttgaggtcaggagttggag 3271
                                                 ***************************** ******************************

GGT1end-LOC749026.end.7456450-7520130.chimp      accagcctgggcaacatggtgaatccatgtctctactaaaaatacaaaaattagctgggc 7246
FAM247.LOC105372935.ref.human                    accagcctgggcaacatggtgaaaccatgtctctactaaaaatacaaaaattagctgggc 3331
                                                 *********************** ************************************

GGT1end-LOC749026.end.7456450-7520130.chimp      atggtagcaggtgcctgtaatcccagctacttgggagtctgagacaggagaatcacttga 7306
FAM247.LOC105372935.ref.human                    atggtagcaggtgcctgtaatcccagatacttgggaggctgagacaggagaatcacttga 3391
                                                 ************************** ********** **********************

GGT1end-LOC749026.end.7456450-7520130.chimp      acccaggaggcaaaggttgcagtaagccaagattgcgccactgcactccagcctgggtga 7366
FAM247.LOC105372935.ref.human                    acccaggaggcaaaggtttcagtaagccaagattgcaccactgcactccagcctgggtga 3451
                                                 ****************** ***************** ***********************

GGT1end-LOC749026.end.7456450-7520130.chimp      cagagcaagacttggtctcaaaaaaaaaaa----aaaaaaagaaaagtttatatttttgt 7422
FAM247.LOC105372935.ref.human                    cagagcaagacttggtctcaaaaaaaaaaaaaagaaagaaagaaaagtttatatttttgt 3511
                                                 ******************************    *** **********************

GGT1end-LOC749026.end.7456450-7520130.chimp      tctaatggttatcttaatatcttcattctataattatatgttttatataat-----tagc 7477
FAM247.LOC105372935.ref.human                    tctaatggttatcttaatatcgtcattctataattatatgttttatataattataatagc 3571
                                                 ********************* *****************************     ****

GGT1end-LOC749026.end.7456450-7520130.chimp      tatataagatataatacccctagtatgttgttttttggatattctactcgctcctgatgg 7537
FAM247.LOC105372935.ref.human                    tatataagatataatacccctagtatgttgttttttggatattctacttgctcctgatgg 3631
                                                 ************************************************ ***********

GGT1end-LOC749026.end.7456450-7520130.chimp      ttaatttatgtgtcaacttggctaagctatggtgccccgttgtttggtcaaatacttgtc 7597
FAM247.LOC105372935.ref.human                    ttaatttatgtgtcaacttggctaagctatggtgtcctgttgtttggtcaaatacttgtc 3691
                                                 ********************************** ** **********************

GGT1end-LOC749026.end.7456450-7520130.chimp      aatatcttgctgggaggttatttcatagatgtgattaacactgacagtcagttgacttta 7657
FAM247.LOC105372935.ref.human                    aatatcttgctgggaggttatttcatagatgtgattaacactgacagtcaattgacttta 3751
                                                 ************************************************** *********

GGT1end-LOC749026.end.7456450-7520130.chimp      agtaaaacagattacccaccataatatgggtgggccacctccaatcacttgaaggcctta 7717
FAM247.LOC105372935.ref.human                    agtaaaacagattacccaccataatatgggtgggccacctccaatcagttgaaggcctta 3811
                                                 *********************************************** ************

GGT1end-LOC749026.end.7456450-7520130.chimp      agaacaaaaactgaggtttcccagagaagcaggaattctgcttcaagactgtaacacaca 7777
FAM247.LOC105372935.ref.human                    agaacaaaaactgaggtttcccagagaagcaggaattctgcttcaagactgtaacacaca 3871
                                                 ************************************************************

GGT1end-LOC749026.end.7456450-7520130.chimp      aaccctgcctgagtttctggcttgctgactgctctacagattttaggttccagacttcga 7837
FAM247.LOC105372935.ref.human                    aaccctgcctgagtttctggcctgctgactgctctacagattttaggttccagacttcga 3931
                                                 ********************* **************************************

GGT1end-LOC749026.end.7456450-7520130.chimp      gatcaactcttacctgaatttatagcctgctggcttgccctacagattttt-aaacttgct 7896
FAM247.LOC105372935.ref.human                    gatcaactcttacctgaatttatagcctgctggcttgccctacagatttttaaaacttgct 3991
                                                 ************************************************** *********

GGT1end-LOC749026.end.7456450-7520130.chimp      agtccccacaatcatgtgagccaattcctcaataaatctctctctatgtataatctattg 7956
FAM247.LOC105372935.ref.human                    agtccccacaatcatgtgagccaattcctaaataaatctctctctatgtataatctattg 4051
                                                 ***************************** ******************************
```

```
GGT1end-LOC749026.end.7456450-7520130.chimp      gtttagtttctctgaaaaactttcacatccagtttcctggatgttaagaattaccgaaac 8016
FAM247.LOC105372935.ref.human                    gtttagtttctctgaaaaactttcacatccagtttcctggttgttaagaattaccgaaac 4111
                                                 **************************************** *******************

GGT1end-LOC749026.end.7456450-7520130.chimp      tagctagtaacttc-----------ttttttttgagacagagttttgctcttgttgccca 8065
FAM247.LOC105372935.ref.human                    tagctagtaacttctttttttttttttttttttgagacagagttttgctcttgttgccca 4171
                                                 **************           ***********************************

GGT1end-LOC749026.end.7456450-7520130.chimp      ggctggaatgcaatggcacaatctcagctcaccgcaacctccacttcctgggtccaagca 8125
FAM247.LOC105372935.ref.human                    ggctggaatgcaatggcacaatctcagctcaccgcaacctccacttcctgggtccaagca 4231
                                                 ************************************************************

GGT1end-LOC749026.end.7456450-7520130.chimp      attctcctccctcagcctcctgagtagctgggattacaggcatgtgccaccatgcttggc 8185
FAM247.LOC105372935.ref.human                    attctcctccctcagcctcctgagtagctgggattacaggcatgtgccaccatgcttggc 4291
                                                 ************************************************************

GGT1end-LOC749026.end.7456450-7520130.chimp      taatttttgtatttttagtagagacagggcttctccatgttggtcaggctggtcttgaac 8245
FAM247.LOC105372935.ref.human                    taatttttgtatttttagtagagacagggcttctccatgttggtcaggctggtcttgaac 4351
                                                 ************************************************************

GGT1end-LOC749026.end.7456450-7520130.chimp      tcccaacctcaggtgatccgccgccttggcctcacaaagtgctggaattacaggcacgag 8305
FAM247.LOC105372935.ref.human                    tcccaacctcaggtgatccgccgccttggcctcacaaagtgctggaattacaggcacgag 4411
                                                 ************************************************************

GGT1end-LOC749026.end.7456450-7520130.chimp      ccactgtgcctggctcctagtaaattcttcttttccgtgatgtgtctcttacctctaata 8365
FAM247.LOC105372935.ref.human                    ccattgcgcctggctcctagtaaattcttcttttctgtgatgtgtctcttacctctaata 4471
                                                 *** ** **************************** ************************

GGT1end-LOC749026.end.7456450-7520130.chimp      atacttttcttcttaaagtctacttcattaaaaatagttatgctgggcatggtggctcat 8425
FAM247.LOC105372935.ref.human                    atacttttcttcttaaagtctacttcattaaaaatagttatgctgggcatggtggctcat 4531
                                                 ************************************************************

GGT1end-LOC749026.end.7456450-7520130.chimp      ggctgtaatcttggcactttgttggaggtcgaggtgggtggatcactgaagcccaggagt 8485
FAM247.LOC105372935.ref.human                    ggctgtaatcttggcactttgctggaggtcgaggtgggtggatcactgaagcccaggagt 4591
                                                 ********************* **************************************

GGT1end-LOC749026.end.7456450-7520130.chimp      tcaagaccaacctgggcgacatggtgagaccctgcctctacaaaaaatacaaaaattagc 8545
FAM247.LOC105372935.ref.human                    tcaagaccaacctgggcaacatggcgagaccctgcctctacaaaaaatacaaaaattagc 4651
                                                 ***************** ****** ***********************************

GGT1end-LOC749026.end.7456450-7520130.chimp      tgggtgtggctaatataattctaagttggcacacttgtagtcccagctacttgggatgct 8605
FAM247.LOC105372935.ref.human                    tgggtgtggctaatataattctaagttggcacacttgtagtcccagctacttgggatgct 4711
                                                 ************************************************************

GGT1end-LOC749026.end.7456450-7520130.chimp      gaggtgggagaatcgcttgagcctagaagggagagattgctgtaacccaagatcacatca 8665
FAM247.LOC105372935.ref.human                    gaggtgggagaatcgcttgagcctagaagggagagattgctgtaagccaagatcacatca 4771
                                                 ********************************************* **************

GGT1end-LOC749026.end.7456450-7520130.chimp      ttgcactccagcctgggagac--agtgaggctctatctcc-----aaaaaaaaaaaaaaa 8718
FAM247.LOC105372935.ref.human                    ctgcactccagcctgggagacagagtgaggctctatctccaaaaaaaaaaaaaaaaaaaa 4831
                                                  ********************  *****************     ***************

GGT1end-LOC749026.end.7456450-7520130.chimp      aaaaaaagttatacagctttcttggttagtgcatgcatgacatatttttcattatcttcc 8778
FAM247.LOC105372935.ref.human                    aaaaaaagttatacagctttcttggttagtacatgcatgacatatttttcatgatcttcc 4891
                                                 ****************************** ********************* *******

GGT1end-LOC749026.end.7456450-7520130.chimp      acctctctgtattcttatataaaaggcattagttgggttttactttattttcaattattt 8838
FAM247.LOC105372935.ref.human                    acctctctgtacccttatataaaaggcattagttgggttttactttattttcaattattt 4951
                                                 ***********  ***********************************************

GGT1end-LOC749026.end.7456450-7520130.chimp      taacttttattgtccttttaaatgtaactaatgatttatttgggttgaaacccaccacca 8898
FAM247.LOC105372935.ref.human                    taatttttattgtccttttaaatgtaactaatgatttatttgggttgaaacccaccacca 5011
                                                 *** ********************************************************

GGT1end-LOC749026.end.7456450-7520130.chimp      atttgttttccatgcctattctatttcttcttatctcctctcacatcttgttttggattt 8958
FAM247.LOC105372935.ref.human                    atttgttttccatgcctattctatttcttcttatctcctctcacatcttgttttgga--- 5068
                                                 *********************************************************

GGT1end-LOC749026.end.7456450-7520130.chimp      attatttttattatttttattatttaatttcctccttctctattagtttcataactgtgc 9018
FAM247.LOC105372935.ref.human                    ------tttattatttttattatttaatttcctccttctctattagtttcataactgtgc 5122
                                                       ******************************************************

GGT1end-LOC749026.end.7456450-7520130.chimp      agtcttagagttattttaaaagatgactggattattttagagcttacaacatgcatcctt 9078
FAM247.LOC105372935.ref.human                    agtcttagagttattttaaaagatgactggattattttagagcttacaacatgcatcctt 5182
                                                 ************************************************************

GGT1end-LOC749026.end.7456450-7520130.chimp      cacttatcaaagtctaacatgagctagtactttttgttgttgttgagatagagagagtct 9138
FAM247.LOC105372935.ref.human                    cccttatcaaagtctaacatgagctagtactttttgttgtggttgagatagagagagtct 5242
                                                 * *************************************** ******************

GGT1end-LOC749026.end.7456450-7520130.chimp      tcctctgctgcccaggctggagtgcagtggagcaatcttggttcactgcaaccttcactt 9198
FAM247.LOC105372935.ref.human                    tcctctgctgcccaggctggagtgcagtggagcaatcttggttcactgcaacctccactt 5302
                                                 ****************************************************** *****

GGT1end-LOC749026.end.7456450-7520130.chimp      cttgggttcaagcaattctcctgcctcagtcacctgagtagctgcgaccacaggtgtgca 9258
FAM247.LOC105372935.ref.human                    cttgggttcaagcaattctcctgcctcagtcacctgagtagctgggaccacaggtgtgca 5362
                                                 ******************************************** ***************

GGT1end-LOC749026.end.7456450-7520130.chimp      ccactatgcccggccaatttttgtattcttttttagtagagacagggggttcaccatgtt 9318
FAM247.LOC105372935.ref.human                    ccactatgcccggccaatttttgtattctttttcagtagagaca-gggtttcaccatgtt 5421
                                                 ********************************* ********** *** ***********

GGT1end-LOC749026.end.7456450-7520130.chimp      ggccaggctggtcttgaactcctgaccttaagagatctgcttacctcggcgtcctaaagt 9378
FAM247.LOC105372935.ref.human                    ggccaggctggtcttgaactccggaccttaagagatctgcctacctcggcatcctaaagt 5481
                                                 ********************** ***************** ********* *********

GGT1end-LOC749026.end.7456450-7520130.chimp      gttgggattacaggcgtgagccaccacgcccagcctatgagttagtacttctatcctctt 9438
FAM247.LOC105372935.ref.human                    gttgggattacaggcgtgagccaccgcgcccagcctatgagttagtacttctatcctctt 5541
                                                 ************************* **********************************

GGT1end-LOC749026.end.7456450-7520130.chimp      cctagtcagtacaagaaccttggaacaggaactaaatttacccccagtgacttatatgct 9498
FAM247.LOC105372935.ref.human                    cctagtcagtacaagaaccttggaacaggaactaaatttacccccagtgacttatatgct 5601
                                                 ************************************************************

GGT1end-LOC749026.end.7456450-7520130.chimp      aatatttttgtgtatttttaaatatatgtgtgtgcatagatgtatctgtgtgttttttgtg 9558
FAM247.LOC105372935.ref.human                    aatatttttgtgtattttaaatatgtgtgtgtgcatagatgtatctgtgtgttttttgtg 5661
                                                 ************************ ***********************************

GGT1end-LOC749026.end.7456450-7520130.chimp      tttttattcttatttatgttgagagtgtagagctatgtaagagtaaaaagaattgtgtaa 9618
FAM247.LOC105372935.ref.human                    tttttattcttatttatgttgagagtgtagagctatgtaagagtaaagagaattgtgtaa 5721
                                                 *********************************************** ************

GGT1end-LOC749026.end.7456450-7520130.chimp      tgaagccccgagtatccattcaatttcaacaacaatcttatggccaagctaatttcatgt 9678
FAM247.LOC105372935.ref.human                    tgaagccccgagtatccattcaatttcaacaacaatcttatggccaagctcatttcatgt 5781
                                                 ************************************************** *********

GGT1end-LOC749026.end.7456450-7520130.chimp      atactctttcctgcttccctctaccccacattatttcagtgcaaatcccagatatataac 9738
FAM247.LOC105372935.ref.human                    atactctttcctgcttccctctaccccacattatttcagtgcaaatcccagatatataac 5841
                                                 ************************************************************

GGT1end-LOC749026.end.7456450-7520130.chimp      tgtacccatacatatttcagtatgttttatttattttaaaccccacaagatatcattttc 9798
FAM247.LOC105372935.ref.human                    tgtaccaatacatatttcagtatgttttatttattttaaaccccacaagatatcattttc 5901
                                                 ****** *****************************************************

GGT1end-LOC749026.end.7456450-7520130.chimp      tatactactgtaattttataccaataacattcatttagatttacccacacgtttacccta 9858
FAM247.LOC105372935.ref.human                    tatactactgtaattttataccaataacattcatttagatttacccacacgtttacccta 5961
                                                 ************************************************************

GGT1end-LOC749026.end.7456450-7520130.chimp      ccctccgggtcctgtttgaaaatcaaacccatgctcacaggccaatttttttttctttta 9918
FAM247.LOC105372935.ref.human                    ccctccgggtcctgtttgaaaatcaagcccatgctcacaggccaa-ttttttttctttta 6020
                                                 ************************** ****************** **************

GGT1end-LOC749026.end.7456450-7520130.chimp      gagacagggtctcactttgtcacccaagctggagtgcagtggtgcgattatagctcaatg 9978
FAM247.LOC105372935.ref.human                    gagacagggtctcactttgtcacccaagctggagtgcagtggtgcgattatagctcaatg 6080
                                                 ************************************************************

GGT1end-LOC749026.end.7456450-7520130.chimp      cagcctccaattcctggactgaagggaccctcctgcctcagcctgccaagtagcttggac 10038
FAM247.LOC105372935.ref.human                    cagcctccaattcctggactcaagggaccctcctgcctcagcctgccaagtagcttggac 6140
                                                 ******************** ***************************************

GGT1end-LOC749026.end.7456450-7520130.chimp      tatagctgtatgttttattattattttgtagacatggggtctggctatgttgtccaggct 10098
FAM247.LOC105372935.ref.human                    tatagctgtgtgttttcttattattttgtagacatggggtctggctatgttgtccaggct 6200
                                                 ********* ****** ********************************************
```

```
GGT1end-LOC749026.end.7456450-7520130.chimp      attctcaaaattcctggcctcgagcaatcctcctgcctcggcctctcaaaggttgggatt 10158
FAM247.LOC105372935.ref.human                    attctcaaaattcccggcctcgagcaatcctcctgcctcggcctctcaaaggttgggatt 6260
                                                 ************** *********************************************

GGT1end-LOC749026.end.7456450-7520130.chimp      acaggtgtgaggcaaggcacccagctcagccacagagccctgttgcatctctcttactag 10218
FAM247.LOC105372935.ref.human                    acaggtgtgaggcaaggcacccagctcagccacagagccctgttgcatctctcttactag 6320
                                                 ************************************************************

GGT1end-LOC749026.end.7456450-7520130.chimp      gagcaagagctgactgccccctcatccccattccagagtgttggagctgtgttcagccaa 10278
FAM247.LOC105372935.ref.human                    gagcaagagctgactgccccctcatccccattccagagtgttgggctgtgttcagccga 6380
                                                 ******************************************** ************* *

GGT1end-LOC749026.end.7456450-7520130.chimp      ggccgggccactggcatggcccagggagtgggatcattcactgctgccccaaagctgaga 10338
FAM247.LOC105372935.ref.human                    ggccgggccactggcatggcccagggagcgggatcattcactgctgccccaaatctgaga 6440
                                                 **************************** ************************ ******

GGT1end-LOC749026.end.7456450-7520130.chimp      tcattccaccttgacaagacttcctcatccaatccctttacttgacagctggggaaacca 10398
FAM247.LOC105372935.ref.human                    tcattccaccttgacaagacttcctcatccaatccctttacttgacagctggggaaacca 6500
                                                 ************************************************************

GGT1end-LOC749026.end.7456450-7520130.chimp      atgcgcacagagcacccccagctcactcagggtctcagagctgatccatgagcagaggcc 10458
FAM247.LOC105372935.ref.human                    atgcgcacagagcacccccagctcactcggggtctcagagctgatccatgagcagaggct 6560
                                                 **************************** *******************************

GGT1end-LOC749026.end.7456450-7520130.chimp      gagatcctgggatcttgtcccccagccgccccgcaagcttactccctttctgctggaaga 10518
FAM247.LOC105372935.ref.human                    gagatcctgggatcttgtcccccagccgccctgcaagcttactccctttctgctggaaga 6620
                                                 ******************************* ****************************

GGT1end-LOC749026.end.7456450-7520130.chimp      gatggggccggacctcgaccagcagccctggcctggacatgactgtgctcacgcaggtat 10578
FAM247.LOC105372935.ref.human                    gatggggccggacctcgaccagcagccctggcctggacatgactgtgctcacccaggtat 6680
                                                 **************************************************** *******

GGT1end-LOC749026.end.7456450-7520130.chimp      tgaggccgtgatgccccggcatcatatttttttctcttttcttttctttttttttttttg 10638
FAM247.LOC105372935.ref.human                    tgaggccgagatgccccggcatcatatgtttttctc----tctttttttctttttttttg 6736
                                                 ******** ****************** ********    ******* * **********

GGT1end-LOC749026.end.7456450-7520130.chimp      agacggtgtctcactctgtcacccaagctggagtgcagtggaatgatattggctcactgc 10698
FAM247.LOC105372935.ref.human                    agacagtatctcactctgtcacccaagctggagtgcagtggcatgatattggctcactgc 6796
                                                 **** ** ********************************* ******************

GGT1end-LOC749026.end.7456450-7520130.chimp      aacctctgcctcccggttaaagtgattctcctgcctcagtcttccaagtagctgggccta 10758
FAM247.LOC105372935.ref.human                    aacctctgcctcccgcttaaagtgattctcctgcctcagtcttccaagtagctgggccta 6856
                                                 *************** ********************************************

GGT1end-LOC749026.end.7456450-7520130.chimp      caggcttgtaccaccacgcctgactaatttttgtatttttactagagacggggtttcccc 10818
FAM247.LOC105372935.ref.human                    caggcttgtaccaccacacctgactaatttttgtatttttactagagacggggtttcccc 6916
                                                 ***************** ******************************************

GGT1end-LOC749026.end.7456450-7520130.chimp      atgttggccaggctcgtgtcaaacttctgacctcagctgatccacctgccttggcctccc 10878
FAM247.LOC105372935.ref.human                    atgttggccaggctcgtgtcgaactcctgacctcaggtgatccacctgccttggcctccc 6976
                                                 ******************** **** ********** ***********************

GGT1end-LOC749026.end.7456450-7520130.chimp      aaagtgctaggattacaggcatgagccatggcgtcacttaaatgtagtgagaggccgggc 10938
FAM247.LOC105372935.ref.human                    aaagtgctaggattacaggcatgagccatggcgtcacttaaatgtagtgagaggccgggc 7036
                                                 ************************************************************

GGT1end-LOC749026.end.7456450-7520130.chimp      aaggggctcatgcctgtaatcccagtacttagagaggacgaggctgtcagatcacctaag 10998
FAM247.LOC105372935.ref.human                    aaggggctcatgcctgtaatcccagtgctttgagaggacgaggctgtcagatcacctaag 7096
                                                 ************************** *** *****************************

GGT1end-LOC749026.end.7456450-7520130.chimp      gtcaggagttcgagaccagcctggccaacatggtgaaactgtgtctctacaaaaaaaaga 11058
FAM247.LOC105372935.ref.human                    gtcaggagttcgagaccagcctggccaacatggtgaaactgtgtctctacaaaaaaatag 7156
                                                 *********************************************************

GGT1end-LOC749026.end.7456450-7520130.chimp      aaaaaaaaatccctgcgtggtggcgagtatctgtagtcctagttactcaggaggctgagg 11118
FAM247.LOC105372935.ref.human                    aaaaaaaaatccctgcgtggtggcaagtatctgtagtcccagttactcaggaggctgagg 7216
                                                 ************************ ************** ********************

GGT1end-LOC749026.end.7456450-7520130.chimp      catgagaatcgcttaaacctcggaggcggaggctgcagtgagctgagatggcaccactgc 11178
FAM247.LOC105372935.ref.human                    catgagaattgcttaaacctcggaggcggaggctgcagtgagctgagatggcgccactgc 7276
                                                 ********* ****************************************** *******

GGT1end-LOC749026.end.7456450-7520130.chimp      actccagcctgggtgacagagcaagactttgtctctaaataattaaataaataaatatgg 11238
FAM247.LOC105372935.ref.human                    actccagcctgggtgacagagcaagactttgtctctaaataattaaataaataaatatgg 7336
                                                 ************************************************************

GGT1end-LOC749026.end.7456450-7520130.chimp      ccgagcatggtgccttaggcctgtaatcccaacacttcgggaggctgaggcaggtggttc 11298
FAM247.LOC105372935.ref.human                    ccgagcatggtgccttaggcctgtaatcccaacactttgggaggctgaggcaggtggttc 7396
                                                 ************************************* **********************

GGT1end-LOC749026.end.7456450-7520130.chimp      atgaggtcaggagcccgagaccagcctggccaagatggtgaaacactgtctctactaaaa 11358
FAM247.LOC105372935.ref.human                    atgaggtcaggagcccgagaccagcctggccaagatggtgaaacactgtctctactaaaa 7456
                                                 ************************************************************

GGT1end-LOC749026.end.7456450-7520130.chimp      atacaaaaattaaccagctgtggtggcaggcacctgtaatcccagctacttgggacactg 11418
FAM247.LOC105372935.ref.human                    atacaaaaattagccagctgtggtggcaggcacctgtaatcccagctacttgggacactg 7516
                                                 ************ ***********************************************

GGT1end-LOC749026.end.7456450-7520130.chimp      aggcaggagaatcgcttgaacctggaaggcagaggttgcaatgagctgagattgcaccac 11478
FAM247.LOC105372935.ref.human                    aggcaggagaatcgcttgaacctggaaggcagaggttgcaatgagccgagattgcaccgc 7576
                                                 ********************************************* ************ *

GGT1end-LOC749026.end.7456450-7520130.chimp      tgcactctagcctgggcaatggagcaagactccgtctcaaataaataaattaataaatac 11538
FAM247.LOC105372935.ref.human                    tgcactctagcctgggcgatggagcaagactccatctcaaataaataaattaataaatac 7636
                                                 ***************** *************** **************************

GGT1end-LOC749026.end.7456450-7520130.chimp      agagcaatattccatctcaaataaataaataaatgtacacctgtaatcctagcactttgg 11598
FAM247.LOC105372935.ref.human                    agagcaagattccatctcaaataaataaataaatgtacacctgtaatcctagcactttgg 7696
                                                 ******* ****************************************************

GGT1end-LOC749026.end.7456450-7520130.chimp      gaggctaagacaggtcgatcacctgaggtcaggagttcgagaccagcctgaccaatatgg 11658
FAM247.LOC105372935.ref.human                    gaggctaagacaggtcgatcacctgaggtcaggagttcgagaccagcctgaccaatatgg 7756
                                                 ************************************************************

GGT1end-LOC749026.end.7456450-7520130.chimp      caaaactccatctctactaaaaatacaaaaattagccgggcgttttgacgtgtgcctgta 11718
FAM247.LOC105372935.ref.human                    caaaactccatctctactaaaaatacaaaaattagccgggcgttttgacgtgtgcctgta 7816
                                                 ************************************************************

GGT1end-LOC749026.end.7456450-7520130.chimp      gtcccagctacttgggaggctgagacaagagaattgcttgaacccaggaggtggaggttg 11778
FAM247.LOC105372935.ref.human                    gtcccagctacttgggaggctgagacaagagaattgcttgaacccaagaggtggaggttg 7876
                                                 ********************************************** *************

GGT1end-LOC749026.end.7456450-7520130.chimp      cagtgagccgagatctcggctgcacttcagcctgggtgacagagtgagactctgtctcaa 11838
FAM247.LOC105372935.ref.human                    cagtgagccgagatctcggctgcacttcagcctgggtgacagagtgagactctgtctcaa 7936
                                                 ************************************************************

GGT1end-LOC749026.end.7456450-7520130.chimp      aaggaataaataaaatacaaagtaaaaaaaaatgtagtaagattgcagagtcgtgccgcg 11898
FAM247.LOC105372935.ref.human                    aaggaataaataaaatacaaagtaaaaaaaaatgtagtaagattgcagagtcgtgccgca 7996
                                                 ***********************************************************

GGT1end-LOC749026.end.7456450-7520130.chimp      gaagcgtgctggtcctatccatgtagtgaaggctgatttcatacacaaatgtcacaagaa 11958
FAM247.LOC105372935.ref.human                    gaagcgtgctggtcctatccatgtagtgaaggctgatttcatacacaaatgtcagaagaa 8056
                                                 ****************************************************** *****

GGT1end-LOC749026.end.7456450-7520130.chimp      cttttcttttcttttcttt---ttttttttttttttgagacggagtctcgttgtgtcacc 12015
FAM247.LOC105372935.ref.human                    cttttcttttcttttcttttcttttttttttttttttgagacggagtctcgctctgtcacc 8116
                                                 *******************   **************************** * *******

GGT1end-LOC749026.end.7456450-7520130.chimp      caggctggagtgcagtggtgcgatctcggctcactgcaagctctgcctcccgggtttatg 12075
FAM247.LOC105372935.ref.human                    caggctggagtgcagtggtgtgatctaggctcactgcaagctctgcctccctggtttatg 8176
                                                 ******************** ***** ************************ ********

GGT1end-LOC749026.end.7456450-7520130.chimp      ccattctcctgcctcagcctcctgagtagctgggactacaggcgcctgccacctagccca 12135
FAM247.LOC105372935.ref.human                    ccattctcctgcctcagcctcctcagtagctgggactacaggcgcctgccacctagccca 8236
                                                 *********************** ************************************

GGT1end-LOC749026.end.7456450-7520130.chimp      actaatttttttgtacttttagaggagatggggtttcaccgcgttagccaggatagtctc 12195
FAM247.LOC105372935.ref.human                    gctaatttttttgtacttttagaggagatggggtttcaccgcgttagccaggatagtctc 8296
                                                  ***********************************************************

GGT1end-LOC749026.end.7456450-7520130.chimp      gatctcctgacctcgtgatccgcccgtctcggcctcccaaagtgctgagattacaggcat 12255
FAM247.LOC105372935.ref.human                    aatctcctgacctcgtgatccgcccgtctcggcctcccaaagtgctgggattacaggcat 8356
                                                  ********************************************** ************

GGT1end-LOC749026.end.7456450-7520130.chimp      gagccaccacacctggccttcttatatgcttttattgcatttgagcgtacctcttttaca 12315
```

```
FAM247.LOC105372935.ref.human                     gagccaccacacccggccttcttatatgcttttattgcatttgagcgtacctcttttaca 8416
                                                  ************* **********************************************

GGT1end-LOC749026.end.7456450-7520130.chimp       gcgaagatc-ttttttgaaattttattttatttttagagatggagtcttgctttgttgcc 12374
FAM247.LOC105372935.ref.human                     gcgaagatcttttttttaaattttattttatttttagagatggagtcttgctttgttgcc 8476
                                                  ********* ****** *******************************************

GGT1end-LOC749026.end.7456450-7520130.chimp       caggctggagcactgtggtgtgatcatagctcactgcagccttgaactcccaggcacagg 12434
FAM247.LOC105372935.ref.human                     caggctggagcactgtggtgtgatcatagctcactgcagccttgaactcccgggcacagg 8536
                                                  *************************************************** ********

GGT1end-LOC749026.end.7456450-7520130.chimp       tgatcctcccacctcagcctcctgaatagctgggactacaggcatgcaccaccatgcctg 12494
FAM247.LOC105372935.ref.human                     tgatcctcccacctcagcctcctgaatagctgggactacaggcatgcaccaccatgcctg 8596
                                                  ************************************************************

GGT1end-LOC749026.end.7456450-7520130.chimp       gcatattttaaaaatgtttgtagagatgaggtctcgctatgttgccaggcttgtctcaaa 12554
FAM247.LOC105372935.ref.human                     gcatattttaaagatgtttgtagagatgaggtctcgctatgttgccaggcttgtctcaaa 8656
                                                  ************ ***********************************************

GGT1end-LOC749026.end.7456450-7520130.chimp       ctactgggctcaagccatcgatccatttcagcctcccaaagtgcttggattataggcatg 12614
FAM247.LOC105372935.ref.human                     ctactgggctcaagccatccatccatttcagcctcccaaagtgcttggattataggcatg 8716
                                                  ******************* ****************************************

GGT1end-LOC749026.end.7456450-7520130.chimp       agcaccgcgcctggccatcacactgtttttttgtttgtttgtttgtttgttttgagatgg 12674
FAM247.LOC105372935.ref.human                     agcactgcgcctggccatcacactgtttttttgtttgtttgtttgtttgttttgagatgg 8776
                                                  ***** ******************************************************

GGT1end-LOC749026.end.7456450-7520130.chimp       agtcttgctctgtcgcccaggctggagtgcagtagtgggatctcacctcattgcaagctc 12734
FAM247.LOC105372935.ref.human                     agtcttgctctgtcgcccaggctggagtgcagtagtgggatctcacctcattgcaagctc 8836
                                                  ************************************************************

GGT1end-LOC749026.end.7456450-7520130.chimp       cgccttgtgggttcacgccattctcctgcctcagcctcccgagtagctgggattacaggc 12794
FAM247.LOC105372935.ref.human                     cgccttgtgggttcacgccattctcctgcctcagcctcctgagtagctgggattacaggc 8896
                                                  *************************************** ********************

GGT1end-LOC749026.end.7456450-7520130.chimp       gcccgccaccacgcctggctaattttttgtatttttagtagagacgggatttcaccgtgt 12854
FAM247.LOC105372935.ref.human                     gcccgccaccacgcctggctaattttttgtatttttagtagagacgggatttcaccgtgt 8956
                                                  ************************************************************

GGT1end-LOC749026.end.7456450-7520130.chimp       tagccaggatggtctcgatctcctgacctcgtgatctgcccgccttggcctctcaaagtg 12914
FAM247.LOC105372935.ref.human                     tagccaggatggtctcgatctcctgac--cgtgatctgcccgcctcggcctctcaaagtg 9014
                                                  ***************************  **************** **************

GGT1end-LOC749026.end.7456450-7520130.chimp       ctgggattacaggcatgagccaataactatttttataatcattaactaccgaggagaagc 12974
FAM247.LOC105372935.ref.human                     ctgggattacaggcatgagccaataaatatttttataatcattaactaccgaggagaagc 9074
                                                  ************************** *********************************

GGT1end-LOC749026.end.7456450-7520130.chimp       tggagagaaaaaaggtggatgaagttaaggcagagagactttgtaagtttcttagctcag 13034
FAM247.LOC105372935.ref.human                     tggagagaaaaaaggtggatgaagttaaggcagagagactttgtaagtttcttagctgag 9134
                                                  ********************************************************* **

GGT1end-LOC749026.end.7456450-7520130.chimp       cttttggagactgtatatcagagatcctatttgaggtgattttcagctacagagataggc 13094
FAM247.LOC105372935.ref.human                     cttttggagactgtatatcagagatcctatttgaggtgattttcggctacagagataggc 9194
                                                  ******************************************** ***************

GGT1end-LOC749026.end.7456450-7520130.chimp       ctggtcatttgaaaaataacggattaggtgaagcttgcgtttgaaatcctccttctactt 13154
FAM247.LOC105372935.ref.human                     ctggtcatttgaaaaataacggattaggtgaagcttgcgtttgaaatcctccttctactt 9254
                                                  ************************************************************

GGT1end-LOC749026.end.7456450-7520130.chimp       ttcatctttctctcttgtttattctgagcatccatcttagacacccaatctttgtcactt 13214
FAM247.LOC105372935.ref.human                     ctcatctttctctcttgtttattctgaacatccatcttagacacccaatctttgtcactt 9314
                                                   ************************** ********************************

GGT1end-LOC749026.end.7456450-7520130.chimp       tgtggttgtcattgatttccctgttattgagattagaggttggcagactttctctgtaaa 13274
FAM247.LOC105372935.ref.human                     tgtggttgtcattgatttccctgttattgagattagaggttggcagactttctctgtaaa 9374
                                                  ************************************************************

GGT1end-LOC749026.end.7456450-7520130.chimp       gggctggagagaaagtacttcagactttgtggtctgtgcagtgtctgttgcaaccactta 13334
FAM247.LOC105372935.ref.human                     gggctggagagaaagtacttcagactttgtggtctgtgcagtgtctgttgcaaccactta 9434
                                                  ************************************************************

GGT1end-LOC749026.end.7456450-7520130.chimp       actctgccctatagagcaaaagcagccgtagacagtacatggacagatgagcatggctgg 13394
FAM247.LOC105372935.ref.human                     actctgccctgtagagcaaaagcagccgtagacagtacatgggcagatgagcatggctgg 9494
                                                  ********** ******************************* *****************

GGT1end-LOC749026.end.7456450-7520130.chimp       ggtccaattacatttacttgcaaaaagagggttggagactgggtgtgatctcacaccttt 13454
FAM247.LOC105372935.ref.human                     ggtccagttacatttacttgcaaaaagagggttggagactgggtgcgatctcccaccttt 9554
                                                  ****** ************************************** ****** *******

GGT1end-LOC749026.end.7456450-7520130.chimp       aatcccagcactttgggaggccgaggcaggaggatcactttaagtcaggagttcaagacc 13514
FAM247.LOC105372935.ref.human                     aatcccggcactttgggaggccgaggcaggaggatcacttcaagccaggagttcaagacc 9614
                                                  ****** ********************************* *** ***************

GGT1end-LOC749026.end.7456450-7520130.chimp       agcctgggcaacaaagcaagactccatctctacaaaaaaaataaaaattattatagctag 13574
FAM247.LOC105372935.ref.human                     agcctgggcaacaaagcaagactccatctctac-aaaaaaataaaaattattatagctag 9673
                                                  ********************************* **************************

GGT1end-LOC749026.end.7456450-7520130.chimp       gcatggtggtacacacccgtagtcctagctactcaggaagctaaaactgaggcaggaggg 13634
FAM247.LOC105372935.ref.human                     gcatggtggtacacacccgtagtcctagctactcaggaagctaaaactgaggcaggaggg 9733
                                                  ************************************************************

GGT1end-LOC749026.end.7456450-7520130.chimp       tcagttgagcccaggagcacgaggctgtggtgagctattactgtgccagtgcactccagc 13694
FAM247.LOC105372935.ref.human                     tcagttgagcccaggagcacgaggctgtggtgagctattattgtgccagtgcactccagc 9793
                                                  **************************************** *******************

GGT1end-LOC749026.end.7456450-7520130.chimp       ctggtgacagagcaagaacggtctcgtcaaaaaataaacaaaaggctgggcacggtggct 13754
FAM247.LOC105372935.ref.human                     ctggtgacagagcaagaaccgtctcatcaaaaaataaacaaaaggctgggcacggtggct 9853
                                                  ******************* ***** **********************************

GGT1end-LOC749026.end.7456450-7520130.chimp       cacgcctgtaatccctgcactttgggagactgaggtgggcagatcatgaggtcaggagat 13814
FAM247.LOC105372935.ref.human                     cacgcctgtaatccctgcactttgggagactaaggtgggcagatcatgaggtcaggagat 9913
                                                  ******************************* ****************************

GGT1end-LOC749026.end.7456450-7520130.chimp       tgagaccatcctggctaacacggtgaaaccctgtctctactaaaaatacaaaaagttagc 13874
FAM247.LOC105372935.ref.human                     tgagaccatcctggctaacacggtgaaaccctgtctctactaaaaatacaaaaagttagc 9973
                                                  ************************************************************

GGT1end-LOC749026.end.7456450-7520130.chimp       tgggcgtggtggcgggcacctgtagtcccagctactcgggaggctgaggagggagaattg 13934
FAM247.LOC105372935.ref.human                     cgggcgtggtggtgggcgcctgtagtcccagccactcgggaggctgaggagggagaatcg 10033
                                                   *********** **** ************** ************************* *

GGT1end-LOC749026.end.7456450-7520130.chimp       tttgaacttgggaggcggaggttgcagtgagccaagattgtgccactgcactctagcctg 13994
FAM247.LOC105372935.ref.human                     tttgaacctgggaggcggaggttgcagtgagccaaggttgtgccactgcactctagcctg 10093
                                                  ******* **************************** ***********************

GGT1end-LOC749026.end.7456450-7520130.chimp       ggctacagggcaagactccatt---aaaaaaaaaaaaaaccagcaaaaaccaaacaaaac 14051
FAM247.LOC105372935.ref.human                     ggctacagggcaagactccattaaaaaaaaaaaaaaaaaccagcaaaaaccaaacaaaac 10153
                                                  **********************   ***********************************

GGT1end-LOC749026.end.7456450-7520130.chimp       ataatgcatgttctctcttataaatgggagctaaacatggggactcattgacttaaagat 14111
FAM247.LOC105372935.ref.human                     ataatgcatgttctctcttataaatgggagctaaacatggggactcattgacttaaagat 10213
                                                  ************************************************************

GGT1end-LOC749026.end.7456450-7520130.chimp       ggcaacaactgggaactgctggatggggagggaggggaggggtgaaaggccaactgttgg 14171
FAM247.LOC105372935.ref.human                     ggcaacaactgggaactgctggatggggagggaggggaggggtgaaaggccaactgttgg 10273
                                                  ************************************************************

GGT1end-LOC749026.end.7456450-7520130.chimp       ggagtatgctcatatccatgtgacaaacctgcacatgtgcccgctgaatctaaaataaaa 14231
FAM247.LOC105372935.ref.human                     ggagtatgctcatatccatgtgacaaacctgcacatgtgcccgctgaatctaaaataaaa 10333
                                                  ************************************************************

GGT1end-LOC749026.end.7456450-7520130.chimp       gttgaaagtagattttaaaaaccccaagagggctgggtttggcttgtgtgtccatagctt 14291
FAM247.LOC105372935.ref.human                     gttgaaagtagatttaaaaaaccccaagagggctgggtttggcttgtgtgtccatagctt 10393
                                                  *************** ********************************************

GGT1end-LOC749026.end.7456450-7520130.chimp       gttaacctccactttagatattaattaatagaaacatagtgcttatcttcccaggccacc 14351
FAM247.LOC105372935.ref.human                     gttaacctccgctttagatattaactaatagaaacatagtgcttatcttcccaggccacc 10453
                                                  ********** ************* ***********************************

GGT1end-LOC749026.end.7456450-7520130.chimp       tattttgttcctctccaaggtgatggatagatgaaggcttaatccagcggcctggaagtt 14411
FAM247.LOC105372935.ref.human                     tattttgttcctctccaaggtgatggatagatgaaggcctaatccagccgcctggaagtt 10513
                                                  ************************************** ********* ***********

GGT1end-LOC749026.end.7456450-7520130.chimp       tgctgacgcttgtcctgtcagggattaatgaagcattgttttctgatgaaggtttcatgc 14471
FAM247.LOC105372935.ref.human                     tgctgacgcttgtcctgtcacggattaatgaagcattgttttctgatgaaggtttcatgc 10573
```

```
                                                   ******************** ***************************************

GGT1end-LOC749026.end.7456450-7520130.chimp        cgctgtgctgatgtgtcttctcttctctctaggcaggaaactgcatatcttctggtttac 14531
FAM247.LOC105372935.ref.human                      cgctgtgctgatgtgtcttctcttctctctaggcaggaaactgcatatcttctggtttac 10633
                                                   ************************************************************

GGT1end-LOC749026.end.7456450-7520130.chimp        atgaagatggagtgctaatggaaatgcccaaaaccttcagagattgacacgctgtcattt 14591
FAM247.LOC105372935.ref.human                      atgaagatggagtgctaatggaaatgcccaaaaccttcagagattgacacgctgtcattt 10693
                                                   ************************************************************

GGT1end-LOC749026.end.7456450-7520130.chimp        tccatttccgttcctggatctacggagtcttctaagagattttgcaatgaggagaagcat 14651
FAM247.LOC105372935.ref.human                      tccatttccgttcctggatctacggagtcttctaagagattttgcaatgaggagaagcac 10753
                                                   ***********************************************************

GGT1end-LOC749026.end.7456450-7520130.chimp        tgttttcaaactatataactgagccttatttataattagggatattatcaaaatatgtaa 14711
FAM247.LOC105372935.ref.human                      tgttttcaaactatataactgagccttatttataattagggatattatcaaaatatgtaa 10813
                                                   ************************************************************

GGT1end-LOC749026.end.7456450-7520130.chimp        ccatgaggcccctcaggtcctgatcagtcagaatggatgctttcaccagcagacccggcc 14771
FAM247.LOC105372935.ref.human                      ccatgaggcccctcaggtcctgatcagtcagaatggatgctttcaccagcagacccggcc 10873
                                                   ************************************************************

GGT1end-LOC749026.end.7456450-7520130.chimp        atgtggctgctcggtcctgggtgctcgctgctgtgcaagacattagccctttagttatga 14831
FAM247.LOC105372935.ref.human                      atgtggctgctcggtcctgggtgctcgctgctgtgcaagacattagccctttagttatga 10933
                                                   ************************************************************

GGT1end-LOC749026.end.7456450-7520130.chimp        gcctgtgggaacttcaggggttcccagtggggagagcagtggcagtgggaggcatctggg 14891
FAM247.LOC105372935.ref.human                      gcctgtgggaacttcaggggttcccagtggggagagcagtggcagtgggaggcatctggg 10993
                                                   ************************************************************

GGT1end-LOC749026.end.7456450-7520130.chimp        ggccaaaggtcagtggcagggggtatttcagtattatacaactgctgtgaccagacttgt 14951
FAM247.LOC105372935.ref.human                      ggccaaaggtcagtggcagggggtatttcagtattatacaactgctgtgaccagacttgt 11053
                                                   ************************************************************

GGT1end-LOC749026.end.7456450-7520130.chimp        atactggctgaatatcagtgctgtttgtaatttttcactttgagaaccaacattaattcc 15011
FAM247.LOC105372935.ref.human                      atactggctgaatatcagtgctgtttgtaatttttcactttgagaaccaacattaattcc 11113
                                                   ************************************************************

GGT1end-LOC749026.end.7456450-7520130.chimp        atatgaatcaagtgttttgtaactgctgttcatttattcagcaaatatttattgatcatc 15071
FAM247.LOC105372935.ref.human                      atatgaatcaagtgttttgtaactgctattcatttattcagcaaatatttattgatcatc 11173
                                                   *************************** ********************************

GGT1end-LOC749026.end.7456450-7520130.chimp        tcttctccataagatagtgtgataaacacagtcatgaataaagttattttccacaaaagg 15131
FAM247.LOC105372935.ref.human                      tcttctccataagatagtgtgataaacacagtcatgaataaagttattttccacaaaa-- 11231
                                                   **********************************************************
```

**End of FAM247A sequence**

```
GGT1end-LOC749026.end.7456450-7520130.chimp        actttgcagttttaacgggggggcagtagggattgtgctatagaaattcaaaggcaaggga 15191
FAM247.LOC105372935.ref.human                      ------------------------------------------------------------ 11231

GGT1end-LOC749026.end.7456450-7520130.chimp        agtcacttctgttgtgaggccctgggaggagcctccaggctggaaagggttaaggtggag 15251
FAM247.LOC105372935.ref.human                      ------------------------------------------------------------ 11231

GGT1end-LOC749026.end.7456450-7520130.chimp        gtctccgataggggcagcgtccacagtggactggctgcaaaaggccgtgctcagcattca 15311
FAM247.LOC105372935.ref.human                      ------------------------------------------------------------ 11231

GGT1end-LOC749026.end.7456450-7520130.chimp        gacagcatcacacactctgcttttctctaccagggaggcaggtggggaaggatagcgatg 15371
FAM247.LOC105372935.ref.human                      ------------------------------------------------------------ 11231

GGT1end-LOC749026.end.7456450-7520130.chimp        ggaaggcaggcggagctcagaatgtggaaggggatccagtaaggcttggaagtttgcacc 15431
FAM247.LOC105372935.ref.human                      ------------------------------------------------------------ 11231

GGT1end-LOC749026.end.7456450-7520130.chimp        tgatctggtgggtggtgaggagcccttgaagggacaaggaggcgaggagcactcagctgt 15491
FAM247.LOC105372935.ref.human                      ------------------------------------------------------------ 11231

GGT1end-LOC749026.end.7456450-7520130.chimp        gttcttacactgatctgccactggggttagagacaaacgtggtgggaatggaaagccacg 15551
FAM247.LOC105372935.ref.human                      ------------------------------------------------------------ 11231

GGT1end-LOC749026.end.7456450-7520130.chimp        cacagtcactagcgcctctgggggggagaatggatgtggctggtgagagaacagggggggcc 15611
FAM247.LOC105372935.ref.human                      ------------------------------------------------------------ 11231

GGT1end-LOC749026.end.7456450-7520130.chimp        cagggagagtccggcaccaacctgggggggggagcccagtgggtgtgagcacccccacttt 15671
FAM247.LOC105372935.ref.human                      ------------------------------------------------------------ 11231

GGT1end-LOC749026.end.7456450-7520130.chimp        agagatgaagtgatggagacattcagatgtttaaccccttgttcaagattccatacttga 15731
FAM247.LOC105372935.ref.human                      ------------------------------------------------------------ 11231

GGT1end-LOC749026.end.7456450-7520130.chimp        taaatggcagatcaaactcccaacataaaatgtgggtcatttctttattattttatttgt 15791
FAM247.LOC105372935.ref.human                      ------------------------------------------------------------ 11231

GGT1end-LOC749026.end.7456450-7520130.chimp        attggttaacaatgatcagccatgcaagaataaatgattattgtaaaatctgcaaacaat 15851
FAM247.LOC105372935.ref.human                      ------------------------------------------------------------ 11231

GGT1end-LOC749026.end.7456450-7520130.chimp        gtagatatgtagagaatcccttccttggagcttgaccttgtcagacaggtatagatgagt 15911
FAM247.LOC105372935.ref.human                      ------------------------------------------------------------ 11231

GGT1end-LOC749026.end.7456450-7520130.chimp        gttcgggggcagccgtaaaaactgccagagactgggctgcttataacaaacgcgtggtct 15971
FAM247.LOC105372935.ref.human                      ------------------------------------------------------------ 11231

GGT1end-LOC749026.end.7456450-7520130.chimp        cccagaagcccacattcaaggtgtccaaaggcctggctcctggaggctctggaggagagc 16031
FAM247.LOC105372935.ref.human                      ------------------------------------------------------------ 11231

GGT1end-LOC749026.end.7456450-7520130.chimp        ctgttccctgtctctcagcttctgccggttgccagcaagtgttgccgttcattcactcca 16091
FAM247.LOC105372935.ref.human                      ------------------------------------------------------------ 11231

GGT1end-LOC749026.end.7456450-7520130.chimp        gccactgcctccatctgcacacagcaaaacagcatatcctgaagtgctcaaccttatagc 16151
FAM247.LOC105372935.ref.human                      ------------------------------------------------------------ 11231

GGT1end-LOC749026.end.7456450-7520130.chimp        cattattttaaaatatccggaagacacaggaccgtgggagtggctgttggagaaattttc 16211
FAM247.LOC105372935.ref.human                      ------------------------------------------------------------ 11231

GGT1end-LOC749026.end.7456450-7520130.chimp        atgaaggaagaaagattacaactaagttttaaaatgctagttttgtttgttttgtcttgg 16271
FAM247.LOC105372935.ref.human                      ------------------------------------------------------------ 11231

GGT1end-LOC749026.end.7456450-7520130.chimp        agaggaggtaaaagtgggagtaaaaatagggagtttggtgtaaggtgggaaaagcaaagg 16331
FAM247.LOC105372935.ref.human                      ------------------------------------------------------------ 11231

GGT1end-LOC749026.end.7456450-7520130.chimp        atccccgcatggatgggctgaagggtgtgatgggagaacagtgagaagtacgtttgggga 16391
FAM247.LOC105372935.ref.human                      ------------------------------------------------------------ 11231

GGT1end-LOC749026.end.7456450-7520130.chimp        agcaattggaaatagcagctaagcttaatcacaatctatcaaaagggacttgttgaagaa 16451
FAM247.LOC105372935.ref.human                      ------------------------------------------------------------ 11231

GGT1end-LOC749026.end.7456450-7520130.chimp        ttaatgtgtgactagaaacagggaggttatgggcttgtcagctcgacagtgggcactcag 16511
FAM247.LOC105372935.ref.human                      ------------------------------------------------------------ 11231

GGT1end-LOC749026.end.7456450-7520130.chimp        ttccactaacaaatgatgcccgtgtgggcagacagaatgatggacagacagaatgatggg 16571
FAM247.LOC105372935.ref.human                      ------------------------------------------------------------ 11231

GGT1end-LOC749026.end.7456450-7520130.chimp        caggcagatgaatgcatgggctttacgtgaaacaggtccacttggttgctgacaagatac 16631
FAM247.LOC105372935.ref.human                      ------------------------------------------------------------ 11231
```

```
GGT1end-LOC749026.end.7456450-7520130.chimp      tgttttaaagttccattttgccatacttcgaacagcttgtcattagctcaatttagccac 16691
FAM247.LOC105372935.ref.human                    ------------------------------------------------------------ 11231

GGT1end-LOC749026.end.7456450-7520130.chimp      atgtaaaatcactaaggcagacttccagagttcccacatgaaaatcaaatgtaaaccagc 16751
FAM247.LOC105372935.ref.human                    ------------------------------------------------------------ 11231

GGT1end-LOC749026.end.7456450-7520130.chimp      agtgacctgcttcaacaccatcatcggaagtcagaagttgaactctttttgatgtttaa 16811
FAM247.LOC105372935.ref.human                    ------------------------------------------------------------ 11231

GGT1end-LOC749026.end.7456450-7520130.chimp      agcctgcataatattcgctgtattattattcagcatattactatttccttcgtgatggaa 16871
FAM247.LOC105372935.ref.human                    ------------------------------------------------------------ 11231

GGT1end-LOC749026.end.7456450-7520130.chimp      atttggtttatcccaattttctattatatcaaaacaccgctacatagaaaatccccatgc 16931
FAM247.LOC105372935.ref.human                    ------------------------------------------------------------ 11231

GGT1end-LOC749026.end.7456450-7520130.chimp      acatatttctcctaattgtggaaatattttacatagaagactctagacatgggatgaaat 16991
FAM247.LOC105372935.ref.human                    ------------------------------------------------------------ 11231

GGT1end-LOC749026.end.7456450-7520130.chimp      tcccaggttattggaattttaaaatagataggtacttccaaattgacctcttacaaatta 17051
FAM247.LOC105372935.ref.human                    ------------------------------------------------------------ 11231

GGT1end-LOC749026.end.7456450-7520130.chimp      tatgaattcgtaagcttccaactgttatggagttacccattttgagaaatctgtgctaaa 17111
FAM247.LOC105372935.ref.human                    ------------------------------------------------------------ 11231

GGT1end-LOC749026.end.7456450-7520130.chimp      aggacccaaacaatgctgatgacaatgatcaggataataagtacgctgggaagacaacaa 17171
FAM247.LOC105372935.ref.human                    ------------------------------------------------------------ 11231

GGT1end-LOC749026.end.7456450-7520130.chimp      aatgatttagatcttagacaagtcattctaggtgtctccactgtttcagttcttgcgttc 17231
FAM247.LOC105372935.ref.human                    ------------------------------------------------------------ 11231

GGT1end-LOC749026.end.7456450-7520130.chimp      gttcattcttgtgctttttcgttttaccaaataaaatagctccttgatgtcatacgaatc 17291
FAM247.LOC105372935.ref.human                    ------------------------------------------------------------ 11231

GGT1end-LOC749026.end.7456450-7520130.chimp      catgctatgcttaatgagtattggttagtaaaatgcctataactagtaatcttcatctat 17351
FAM247.LOC105372935.ref.human                    ------------------------------------------------------------ 11231

GGT1end-LOC749026.end.7456450-7520130.chimp      gcaattaaatattaattcataaaacacttcaaatgtaaacaataattagtaaatgaaaag 17411
FAM247.LOC105372935.ref.human                    ------------------------------------------------------------ 11231

GGT1end-LOC749026.end.7456450-7520130.chimp      tacataatacctcaattagaaaaaaatcactccattaaaaagacattatttgtgtgataa 17471
FAM247.LOC105372935.ref.human                    ------------------------------------------------------------ 11231

GGT1end-LOC749026.end.7456450-7520130.chimp      aagagattgccattttttgtatttttctacaaggttaaagaaaactaagtcaacttataca 17531
FAM247.LOC105372935.ref.human                    ------------------------------------------------------------ 11231

GGT1end-LOC749026.end.7456450-7520130.chimp      agtgaattttaaaagactttagggcgggcatggtggctcacgcctgtaatcccagcactt 17591
FAM247.LOC105372935.ref.human                    ------------------------------------------------------------ 11231

GGT1end-LOC749026.end.7456450-7520130.chimp      taagaggccgaggagggcagatcacctgaggtcaggagttcgaaaccagcctgaccaaca 17651
FAM247.LOC105372935.ref.human                    ------------------------------------------------------------ 11231

GGT1end-LOC749026.end.7456450-7520130.chimp      tggtgaaatctcatttctactaaaaatacaaaaaaattagcccagtgtggtggcacgtgc 17711
FAM247.LOC105372935.ref.human                    ------------------------------------------------------------ 11231

GGT1end-LOC749026.end.7456450-7520130.chimp      ctataatctcagctacttgggaggctgagacaggagaatagtttgaacctgggaggcgga 17771
FAM247.LOC105372935.ref.human                    ------------------------------------------------------------ 11231

GGT1end-LOC749026.end.7456450-7520130.chimp      ggttgcagtgaaccaggatcgcaccattgcactccagcttgggcaacaagagcgaaactc 17831
FAM247.LOC105372935.ref.human                    ------------------------------------------------------------ 11231

GGT1end-LOC749026.end.7456450-7520130.chimp      catctcaaaaataaataaataaataaataaataaataaataaaagcctttaaccca 17891
FAM247.LOC105372935.ref.human                    ------------------------------------------------------------ 11231

GGT1end-LOC749026.end.7456450-7520130.chimp      gaatgctgagtaaattggccaaaaatgctaacctatgcatttcaatactataggagttgc 17951
FAM247.LOC105372935.ref.human                    ------------------------------------------------------------ 11231

GGT1end-LOC749026.end.7456450-7520130.chimp      atgggtagaaataactagatgaaatacttctggtatttcaccttcccaacccacacgagc 18011
FAM247.LOC105372935.ref.human                    ------------------------------------------------------------ 11231

GGT1end-LOC749026.end.7456450-7520130.chimp      cagtgtttttctgtgaataacaaaaacagcagaatttacttgcctctccataagaggtta 18071
FAM247.LOC105372935.ref.human                    ------------------------------------------------------------ 11231

GGT1end-LOC749026.end.7456450-7520130.chimp      ccacttctgtgtgttcccctgaaacaggtggtggctgggtgagaaggtggacggcactag 18131
FAM247.LOC105372935.ref.human                    ------------------------------------------------------------ 11231

GGT1end-LOC749026.end.7456450-7520130.chimp      ggcaggagatgggggctccagtatcgtgggtgagcttcctaaacctctgcaactttcagc 18191
FAM247.LOC105372935.ref.human                    ------------------------------------------------------------ 11231

GGT1end-LOC749026.end.7456450-7520130.chimp      ccctaaatgggatgagccatcagaattttttagcacaatgcccagaacaaagtaaggattt 18251
FAM247.LOC105372935.ref.human                    ------------------------------------------------------------ 11231

GGT1end-LOC749026.end.7456450-7520130.chimp      gacaaatgacgcctctctccacattgttctgtcatcagccaccgcatcctgtacctccaa 18311
FAM247.LOC105372935.ref.human                    ------------------------------------------------------------ 11231

GGT1end-LOC749026.end.7456450-7520130.chimp      gcccactgggctccggctgtttccatcacatggagaatgactcagagcctggcctccagc 18371
FAM247.LOC105372935.ref.human                    ------------------------------------------------------------ 11231

GGT1end-LOC749026.end.7456450-7520130.chimp      caccctcctggcctttctgcttctcactctgccactggctcctcatggaccaccagcctg 18431
FAM247.LOC105372935.ref.human                    ------------------------------------------------------------ 11231

GGT1end-LOC749026.end.7456450-7520130.chimp      ggtgtcctcagacataccacacacttcactgtgggagtcacgcagccctcactgctcctt 18491
FAM247.LOC105372935.ref.human                    ------------------------------------------------------------ 11231

GGT1end-LOC749026.end.7456450-7520130.chimp      cgccagggagccacagggctttcctcctcaggaggactctgcaagcagctggatgaaggg 18551
FAM247.LOC105372935.ref.human                    ------------------------------------------------------------ 11231

GGT1end-LOC749026.end.7456450-7520130.chimp      ccctcccgtcttcatcttccttaattttttgtcacagttctccttccttccactcagtgca 18611
FAM247.LOC105372935.ref.human                    ------------------------------------------------------------ 11231

GGT1end-LOC749026.end.7456450-7520130.chimp      gcgcacactgattgatcctccatcttccccaaaagacaggaacagcatgagcagtagaga 18671
FAM247.LOC105372935.ref.human                    ------------------------------------------------------------ 11231

GGT1end-LOC749026.end.7456450-7520130.chimp      gtagattccaatgatagaaaaaatagtcagtgatttctcatttccattgatcatcaatga 18731
FAM247.LOC105372935.ref.human                    ------------------------------------------------------------ 11231

GGT1end-LOC749026.end.7456450-7520130.chimp      agaaaatgtatcctgaaggtcacgtacctcctatgggactgctgcatcctcagcctcctg 18791
FAM247.LOC105372935.ref.human                    ------------------------------------------------------------ 11231
```

```
GGT1end-LOC749026.end.7456450-7520130.chimp      aatttcagcccagcaccttcctccccagcacagcaacaggtcagcccttaccagcatccc 18851
FAM247.LOC105372935.ref.human                    ------------------------------------------------------------ 11231

GGT1end-LOC749026.end.7456450-7520130.chimp      tctcttattgcctttgtgcagagccagcaccagggccaggggaggccttgggattgtccc 18911
FAM247.LOC105372935.ref.human                    ------------------------------------------------------------ 11231

GGT1end-LOC749026.end.7456450-7520130.chimp      tccccaacaatctgtgaaacaatcctttatgtcaccaacaaagcacagccttatgcactg 18971
FAM247.LOC105372935.ref.human                    ------------------------------------------------------------ 11231

GGT1end-LOC749026.end.7456450-7520130.chimp      gtggtcagtccctcccaacacctctgtcactgtaaagctggcaggcaaccctccaaggtt 19031
FAM247.LOC105372935.ref.human                    ------------------------------------------------------------ 11231

GGT1end-LOC749026.end.7456450-7520130.chimp      ggccttcccaagcactgcacctctaggtgacagagcacgtccttaccttgaagcctgggc 19091
FAM247.LOC105372935.ref.human                    ------------------------------------------------------------ 11231

GGT1end-LOC749026.end.7456450-7520130.chimp      gcccagtctatcctgtccaatgagcgagctgtggagaagggggggattccgggttaagggg 19151
FAM247.LOC105372935.ref.human                    ------------------------------------------------------------ 11231

GGT1end-LOC749026.end.7456450-7520130.chimp      agactagcagggctcctgcttttatgttgccctgttgggaaggctattaaagaaacacaa 19211
FAM247.LOC105372935.ref.human                    ------------------------------------------------------------ 11231

GGT1end-LOC749026.end.7456450-7520130.chimp      agtgctaagcagtgaggatagaacatgttttcattatttaaaccaatacattccacagat 19271
FAM247.LOC105372935.ref.human                    ------------------------------------------------------------ 11231

GGT1end-LOC749026.end.7456450-7520130.chimp      ggaataataagaaatgctacaaccaagctaactgaatccaacagcatataaaaaagataa 19331
FAM247.LOC105372935.ref.human                    ------------------------------------------------------------ 11231

GGT1end-LOC749026.end.7456450-7520130.chimp      tccacaatgattcaagtgggtttcatactagggatgcagggacggtttaacatacgcaag 19391
FAM247.LOC105372935.ref.human                    ------------------------------------------------------------ 11231

GGT1end-LOC749026.end.7456450-7520130.chimp      tcaataaatgtgatacatcacataaataaaactaaaaacaaaaatcacatgataatctga 19451
FAM247.LOC105372935.ref.human                    ------------------------------------------------------------ 11231

GGT1end-LOC749026.end.7456450-7520130.chimp      atagatgcagaaaaagcctttgacaaaatccagcatttctttatgattaaaaccgttcat 19511
FAM247.LOC105372935.ref.human                    ------------------------------------------------------------ 11231

GGT1end-LOC749026.end.7456450-7520130.chimp      caaaatcagcatagaacggacataccttaaggtaataaaagctatctatgacaaacccac 19571
FAM247.LOC105372935.ref.human                    ------------------------------------------------------------ 11231

GGT1end-LOC749026.end.7456450-7520130.chimp      agccaacattttcctgaatgggggagagttgaaagcattcctcctgaggaagggaacaag 19631
FAM247.LOC105372935.ref.human                    ------------------------------------------------------------ 11231

GGT1end-LOC749026.end.7456450-7520130.chimp      acaaagatgcccacgttcaccacttctcaacacagtgctgttcactacagcattggttat 19691
FAM247.LOC105372935.ref.human                    ------------------------------------------------------------ 11231

GGT1end-LOC749026.end.7456450-7520130.chimp      aagagcaagactggaaacagaacaaatggatacccatagcggggtgcttaagtaattttg 19751
FAM247.LOC105372935.ref.human                    ------------------------------------------------------------ 11231

GGT1end-LOC749026.end.7456450-7520130.chimp      ggaatagtcatggggtgcagtactttatagctctgaaacaatacaatggatttacatttg 19811
FAM247.LOC105372935.ref.human                    ------------------------------------------------------------ 11231

GGT1end-LOC749026.end.7456450-7520130.chimp      aaatgtggaatgataactaaggtgcattgcccagtgatatatgcagaggtgcagaggact 19871
FAM247.LOC105372935.ref.human                    ------------------------------------------------------------ 11231

GGT1end-LOC749026.end.7456450-7520130.chimp      ttgtgtaaacacgatcacacatcagcctgcattccaggtgcatgcttctatttgcacatg 19931
FAM247.LOC105372935.ref.human                    ------------------------------------------------------------ 11231

GGT1end-LOC749026.end.7456450-7520130.chimp      gattgcagggatgatatgcaaacaaaaatgttgacttggtgtttggaagttcagagtgga 19991
FAM247.LOC105372935.ref.human                    ------------------------------------------------------------ 11231

GGT1end-LOC749026.end.7456450-7520130.chimp      agggaaacttccttgctaaccttttatgatatttagagtttctaaatgtgaatacataat 20051
FAM247.LOC105372935.ref.human                    ------------------------------------------------------------ 11231

GGT1end-LOC749026.end.7456450-7520130.chimp      acatttagaaatcttagttaataagaaaagcctctgttcctggcctcttgctggcacatg 20111
FAM247.LOC105372935.ref.human                    ------------------------------------------------------------ 11231

GGT1end-LOC749026.end.7456450-7520130.chimp      tcaggtggaaatggggctgtcatgctaatgtgtgcaaactgagaaaaatccaagaatggg 20171
FAM247.LOC105372935.ref.human                    ------------------------------------------------------------ 11231

GGT1end-LOC749026.end.7456450-7520130.chimp      agtctgctttttcatcatacaaataattgttaatagaaacagtatgataattgctcatt 20231
FAM247.LOC105372935.ref.human                    ------------------------------------------------------------ 11231

GGT1end-LOC749026.end.7456450-7520130.chimp      gatataccatgcatattctattagataataataatttctgaaatttgaactatacttaca 20291
FAM247.LOC105372935.ref.human                    ------------------------------------------------------------ 11231

GGT1end-LOC749026.end.7456450-7520130.chimp      catggaaattgaaatatatggatgaaacattgtggcttctataggcaattgttttattgg 20351
FAM247.LOC105372935.ref.human                    ------------------------------------------------------------ 11231

GGT1end-LOC749026.end.7456450-7520130.chimp      cattttacaaactgatcatcattcctcatggcacgggtctatgtgatattaagtagcttg 20411
FAM247.LOC105372935.ref.human                    ------------------------------------------------------------ 11231

GGT1end-LOC749026.end.7456450-7520130.chimp      ttatgcttgggaaaggcagtgatgaccacaagaatgacttcaactactaaagtacaatgg 20471
FAM247.LOC105372935.ref.human                    ------------------------------------------------------------ 11231

GGT1end-LOC749026.end.7456450-7520130.chimp      agatttcaacaatgttttgtttaatatttaaatatttcatgtgctcccaggctttttctc 20531
FAM247.LOC105372935.ref.human                    ------------------------------------------------------------ 11231

GGT1end-LOC749026.end.7456450-7520130.chimp      accctaatagctctcatccatatcatgtgggtcccattaatacagatacctccgaatgca 20591
FAM247.LOC105372935.ref.human                    ------------------------------------------------------------ 11231

GGT1end-LOC749026.end.7456450-7520130.chimp      ccactcttccattatatccagtcaattgctggttaccttgggcctacaactgggggaggg 20651
FAM247.LOC105372935.ref.human                    ------------------------------------------------------------ 11231

GGT1end-LOC749026.end.7456450-7520130.chimp      caggggctgctggccacctcctcatctacagtaagagtcaatgagcagttaaatggatac 20711
FAM247.LOC105372935.ref.human                    ------------------------------------------------------------ 11231

GGT1end-LOC749026.end.7456450-7520130.chimp      tgaaaaccatttatcctgctggagtgagaaataaatggtttctttcaatagcgtagtaaa 20771
FAM247.LOC105372935.ref.human                    ------------------------------------------------------------ 11231

GGT1end-LOC749026.end.7456450-7520130.chimp      atgcatcttttccaaactatttatatgactcaaggcccatctcaatttcagatgtggtta 20831
FAM247.LOC105372935.ref.human                    ------------------------------------------------------------ 11231

GGT1end-LOC749026.end.7456450-7520130.chimp      gcctcaattcctgattctcaccaaggtgtgtaatgtcatccacagcccagtgcagaggaa 20891
FAM247.LOC105372935.ref.human                    ------------------------------------------------------------ 11231

GGT1end-LOC749026.end.7456450-7520130.chimp      cacgtgctgccgtcagactgccagggtccgatcccgcctcctcactcaccctgggagatc 20951
FAM247.LOC105372935.ref.human                    ------------------------------------------------------------ 11231
```

```
GGT1end-LOC749026.end.7456450-7520130.chimp      cctttaagccaggagtcaacagtgaggatggaaacatgagtgctttttaaagtcctgaaa 21011
FAM247.LOC105372935.ref.human                    ------------------------------------------------------------ 11231

GGT1end-LOC749026.end.7456450-7520130.chimp      gttcagaggcagactgtcaatttctcctgcacccctgggcacacaccaggagaactttgt 21071
FAM247.LOC105372935.ref.human                    ------------------------------------------------------------ 11231

GGT1end-LOC749026.end.7456450-7520130.chimp      ctccaggatcaagtaagtgcctgtgagagagttgtgtccctcagattctgttcaccacag 21131
FAM247.LOC105372935.ref.human                    ------------------------------------------------------------ 11231

GGT1end-LOC749026.end.7456450-7520130.chimp      gtgacactcgatgcaaccccaaacctcttctgcacaatcccaaggggtgctgactaatcc 21191
FAM247.LOC105372935.ref.human                    ------------------------------------------------------------ 11231

GGT1end-LOC749026.end.7456450-7520130.chimp      aacccaaaggctgtgatgtttggcagaggcagaaaagaaaaggccaggtgttccgggaaa 21251
FAM247.LOC105372935.ref.human                    ------------------------------------------------------------ 11231

GGT1end-LOC749026.end.7456450-7520130.chimp      gatcaccttcaaataacacagcactctcatagcccagagagacagttctaactattatgc 21311
FAM247.LOC105372935.ref.human                    ------------------------------------------------------------ 11231

GGT1end-LOC749026.end.7456450-7520130.chimp      caataaacccggaaaagaccaaatccaatttgacacatatttccttttttcgttttgattt 21371
FAM247.LOC105372935.ref.human                    ------------------------------------------------------------ 11231

GGT1end-LOC749026.end.7456450-7520130.chimp      catgcccccctccctcaacctcccaagcagcatggataatccccgaaggcccctgggaact 21431
FAM247.LOC105372935.ref.human                    ------------------------------------------------------------ 11231

GGT1end-LOC749026.end.7456450-7520130.chimp      ctctccaattggatcttacgtggaaagtagttacccacctacaaatccccatcatcagat 21491
FAM247.LOC105372935.ref.human                    ------------------------------------------------------------ 11231

GGT1end-LOC749026.end.7456450-7520130.chimp      atgctctccacaatcaaatctttagaaacacaaacaccaggataagtcattagagagagg 21551
FAM247.LOC105372935.ref.human                    ------------------------------------------------------------ 11231

GGT1end-LOC749026.end.7456450-7520130.chimp      cccacccactcctcccaccctagctgaagccacggtgcttcgcacaggatcccctggtgt 21611
FAM247.LOC105372935.ref.human                    ------------------------------------------------------------ 11231

GGT1end-LOC749026.end.7456450-7520130.chimp      ttcctctgggctcacagatatccctacagcctctctggacattgttttatacttgcaaaa 21671
FAM247.LOC105372935.ref.human                    ------------------------------------------------------------ 11231

GGT1end-LOC749026.end.7456450-7520130.chimp      tcatttgctctcaccagaccccaaatcctccttcccaaaaggagcccagaatcaggtttc 21731
FAM247.LOC105372935.ref.human                    ------------------------------------------------------------ 11231

GGT1end-LOC749026.end.7456450-7520130.chimp      tgtaccctggagatggcgcttttcctcaggaagtgagttatttcagggtacgtatcatt 21791
FAM247.LOC105372935.ref.human                    ------------------------------------------------------------ 11231

GGT1end-LOC749026.end.7456450-7520130.chimp      ctccagtgtcaatggctcctgcaattatagaaaagaaaacattaggagggtgaaatgatg 21851
FAM247.LOC105372935.ref.human                    ------------------------------------------------------------ 11231

GGT1end-LOC749026.end.7456450-7520130.chimp      ccatacacgtcacacagatctgatagtctctcgacaacttgagagagaaaataagaaggg 21911
FAM247.LOC105372935.ref.human                    ------------------------------------------------------------ 11231

GGT1end-LOC749026.end.7456450-7520130.chimp      gtatagtgattgagtcaaaggtcgaagtcccccaaaactggcacggaagacacctgtgga 21971
FAM247.LOC105372935.ref.human                    ------------------------------------------------------------ 11231

GGT1end-LOC749026.end.7456450-7520130.chimp      aaagacaagaccttttcccacagaatttatctttaaagtgtatctagattggcagtttca 22031
FAM247.LOC105372935.ref.human                    ------------------------------------------------------------ 11231

GGT1end-LOC749026.end.7456450-7520130.chimp      caactcttaatccatgggggaaaactgctgtggagggaaacacctctgcattgcagtgga 22091
FAM247.LOC105372935.ref.human                    ------------------------------------------------------------ 11231

GGT1end-LOC749026.end.7456450-7520130.chimp      tcgtggatgctgccatctaccacaccccagtgtgcctggcatgggttggtgagaggctgc 22151
FAM247.LOC105372935.ref.human                    ------------------------------------------------------------ 11231

GGT1end-LOC749026.end.7456450-7520130.chimp      caatcaatagcaccacaccaagggaattgcagatgtcataaatagtccacattggcagat 22211
FAM247.LOC105372935.ref.human                    ------------------------------------------------------------ 11231

GGT1end-LOC749026.end.7456450-7520130.chimp      gttcatgtctacatttgattaaactgcagatgacattgataatgcacactggcagatgtt 22271
FAM247.LOC105372935.ref.human                    ------------------------------------------------------------ 11231

GGT1end-LOC749026.end.7456450-7520130.chimp      catgtctacatctgattggaaagaagccaggaaagtaacatttctgttcaagacaaagaa 22331
FAM247.LOC105372935.ref.human                    ------------------------------------------------------------ 11231

GGT1end-LOC749026.end.7456450-7520130.chimp      aagtgtcttacattggcagcgtcttctttttacagatgtcttgtacagtgtcctcatta 22391
FAM247.LOC105372935.ref.human                    ------------------------------------------------------------ 11231

GGT1end-LOC749026.end.7456450-7520130.chimp      gcaatgtcatatacagcgtccttattagcgaattcgtatacagcatcctcattagcgatg 22451
FAM247.LOC105372935.ref.human                    ------------------------------------------------------------ 11231

GGT1end-LOC749026.end.7456450-7520130.chimp      ccatatacagcgtcctcgttagtgatgtcgtatagagcgtcctcattagcgatgtcatat 22511
FAM247.LOC105372935.ref.human                    ------------------------------------------------------------ 11231

GGT1end-LOC749026.end.7456450-7520130.chimp      acaatgtcctcattagcgatgtcatatacagcaacctcattagctatgtcttgtaaagca 22571
FAM247.LOC105372935.ref.human                    ------------------------------------------------------------ 11231

GGT1end-LOC749026.end.7456450-7520130.chimp      tccttattagcgatgtcatatacaacgtcctcattagcgatgtcgtatacagcgtcctcg 22631
FAM247.LOC105372935.ref.human                    ------------------------------------------------------------ 11231

GGT1end-LOC749026.end.7456450-7520130.chimp      ttagcgatgtcttgtacggtgtccttataagcaatgtcgtctacagcgtcctcgttagca 22691
FAM247.LOC105372935.ref.human                    ------------------------------------------------------------ 11231

GGT1end-LOC749026.end.7456450-7520130.chimp      tgccttgtgggtgtcattagcgatgtcatatacagcatcctcattggtgatgtcttatat 22751
FAM247.LOC105372935.ref.human                    ------------------------------------------------------------ 11231

GGT1end-LOC749026.end.7456450-7520130.chimp      ggtgtcctcattagcgatgttgtgtacagcgtcctcgttagcaatgccttgtacagtgtc 22811
FAM247.LOC105372935.ref.human                    ------------------------------------------------------------ 11231

GGT1end-LOC749026.end.7456450-7520130.chimp      ctcgttagcgatgtcatatacagtgtcctcattagcgatggcttgtacactgtcctcatt 22871
FAM247.LOC105372935.ref.human                    ------------------------------------------------------------ 11231

GGT1end-LOC749026.end.7456450-7520130.chimp      agtgatgtcgtgtacagcatcctcgttagcgtgccttgtacggtgtcattagcgatgtcg 22931
FAM247.LOC105372935.ref.human                    ------------------------------------------------------------ 11231

GGT1end-LOC749026.end.7456450-7520130.chimp      tatacagcgtcataattagcgatgtcttatacggtgtcatcattagcgatgttgtgtaca 22991
FAM247.LOC105372935.ref.human                    ------------------------------------------------------------ 11231

GGT1end-LOC749026.end.7456450-7520130.chimp      gcgtcatcgttagcgatgccttgtatggtgtcctcattagtgatgtcgtatacaatgtcc 23051
FAM247.LOC105372935.ref.human                    ------------------------------------------------------------ 11231

GGT1end-LOC749026.end.7456450-7520130.chimp      tcatcagcgatgtcatatatagcgtcctcattagtgatgtcgtatacagcgtgctcacta 23111
FAM247.LOC105372935.ref.human                    ------------------------------------------------------------ 11231

GGT1end-LOC749026.end.7456450-7520130.chimp      gcgatgtctttttttttatatatactttaagttttagggtacatgtgcacattgtgcag 23171
```

```
FAM247.LOC105372935.ref.human                     ------------------------------------------------------------ 11231

GGT1end-LOC749026.end.7456450-7520130.chimp       gttagttacatatgtatacatgtgccatgctggtgcgctgcacccactaactcatcatct 23231
FAM247.LOC105372935.ref.human                     ------------------------------------------------------------ 11231

GGT1end-LOC749026.end.7456450-7520130.chimp       agcattaggtatatctcccgatgctatccctccacccccccaccacaacagtccccagag 23291
FAM247.LOC105372935.ref.human                     ------------------------------------------------------------ 11231

GGT1end-LOC749026.end.7456450-7520130.chimp       tgtgatattccccttcctgtgtccatgtgatcttattgttcaattcccacctatgagtga 23351
FAM247.LOC105372935.ref.human                     ------------------------------------------------------------ 11231

GGT1end-LOC749026.end.7456450-7520130.chimp       gaatatgcggtgtttggtttttttgttcttgcgatagttactagcgatgtcttatatggtg 23411
FAM247.LOC105372935.ref.human                     ------------------------------------------------------------ 11231

GGT1end-LOC749026.end.7456450-7520130.chimp       tcctcattagcaatgtcgtgtacagcgtccacgttagcatgccttgtcggtgccattagc 23471
FAM247.LOC105372935.ref.human                     ------------------------------------------------------------ 11231

GGT1end-LOC749026.end.7456450-7520130.chimp       aatgtcgtataaagcgtcctcattggtgatgtcttgtacagtgccctcattagcgatgtt 23531
FAM247.LOC105372935.ref.human                     ------------------------------------------------------------ 11231

GGT1end-LOC749026.end.7456450-7520130.chimp       gtgtacagtgtacttgttagcgatggcttgtacgatgtcctcgttaacatgccttgtacg 23591
FAM247.LOC105372935.ref.human                     ------------------------------------------------------------ 11231

GGT1end-LOC749026.end.7456450-7520130.chimp       gtgtcattagcgatgtcatatatggcatcctcattagcgatggcgatgccctgggcggcg 23651
FAM247.LOC105372935.ref.human                     ------------------------------------------------------------ 11231

GGT1end-LOC749026.end.7456450-7520130.chimp       tcctcgttggcgatgccctgggcggcgtcctcgttggtgatgccgtggacggcgtcctcc 23711
FAM247.LOC105372935.ref.human                     ------------------------------------------------------------ 11231

GGT1end-LOC749026.end.7456450-7520130.chimp       ttggcgataccctgggcggcatcctcgttggcgatgccctgggcggcgtcctccttggcg 23771
FAM247.LOC105372935.ref.human                     ------------------------------------------------------------ 11231

GGT1end-LOC749026.end.7456450-7520130.chimp       atgccatggacggcgtccacgttggcgatgccctgggcggcgtcctccttggcgatgcca 23831
FAM247.LOC105372935.ref.human                     ------------------------------------------------------------ 11231

GGT1end-LOC749026.end.7456450-7520130.chimp       tggacggcgtccacgttggcgatgccctggacggcgaccacgttggcgatgccctgggcg 23891
FAM247.LOC105372935.ref.human                     ------------------------------------------------------------ 11231

GGT1end-LOC749026.end.7456450-7520130.chimp       gcgtcctcgttggcgatgccctggacggcgaccacgttggcgatgccctgggcggcatcc 23951
FAM247.LOC105372935.ref.human                     ------------------------------------------------------------ 11231

GGT1end-LOC749026.end.7456450-7520130.chimp       tcgttggcgatgccctggatggcgaccacgttggcgatgccctgggcggcgtcctcgttg 24011
FAM247.LOC105372935.ref.human                     ------------------------------------------------------------ 11231

GGT1end-LOC749026.end.7456450-7520130.chimp       gcgatgccctggacggcgaccacgttggcgatgccctgggcggcgtcctccttggcgatg 24071
FAM247.LOC105372935.ref.human                     ------------------------------------------------------------ 11231

GGT1end-LOC749026.end.7456450-7520130.chimp       ccctgggcggcatcctcgttggcgattccgtgggcggcgtcctcgctggtgatgccgtgg 24131
FAM247.LOC105372935.ref.human                     ------------------------------------------------------------ 11231

GGT1end-LOC749026.end.7456450-7520130.chimp       gcggcgtcctcgctggcgatgccgtgggcggcgtcctcgttggcgatgccctgggcggcg 24191
FAM247.LOC105372935.ref.human                     ------------------------------------------------------------ 11231

GGT1end-LOC749026.end.7456450-7520130.chimp       tcctcgttgtcgatgcccttgtcggcggcctcgttagcgatgtcgtgtacagtgtccttg 24251
FAM247.LOC105372935.ref.human                     ------------------------------------------------------------ 11231

GGT1end-LOC749026.end.7456450-7520130.chimp       ttagcgatgtcgtgtgtggcgtcctcgttagtgatgtcgtgtacggtgtcctcatgggga 24311
FAM247.LOC105372935.ref.human                     ------------------------------------------------------------ 11231

GGT1end-LOC749026.end.7456450-7520130.chimp       gctagaagaacacagagttaaggtcagtgccctggtggtggagactgaatcacccagggg 24371
FAM247.LOC105372935.ref.human                     ------------------------------------------------------------ 11231

GGT1end-LOC749026.end.7456450-7520130.chimp       cttgcttggtgtggtgcatggaggtggctgatcacagcatgggccaagctgatgctggga 24431
FAM247.LOC105372935.ref.human                     ------------------------------------------------------------ 11231

GGT1end-LOC749026.end.7456450-7520130.chimp       catcctcccaggtggacctgcactagtgaagctaagggatgtggctcagaacactttctg 24491
FAM247.LOC105372935.ref.human                     ------------------------------------------------------------ 11231

GGT1end-LOC749026.end.7456450-7520130.chimp       cagtgggaatcagtttccaggttcaggtatgcattatccggtgaagtggggaaatataaa 24551
FAM247.LOC105372935.ref.human                     ------------------------------------------------------------ 11231

GGT1end-LOC749026.end.7456450-7520130.chimp       aaataaaaattgacaaattcatgaaaagccttccatgagtgcaagtgtgagtttttttatc 24611
FAM247.LOC105372935.ref.human                     ------------------------------------------------------------ 11231

GGT1end-LOC749026.end.7456450-7520130.chimp       cactttacattcagtatgcattcacacatacaaaatatttttacaagaaatcagaaattt 24671
FAM247.LOC105372935.ref.human                     ------------------------------------------------------------ 11231

GGT1end-LOC749026.end.7456450-7520130.chimp       taatttttgtcagttatgttaaatctaacttagctgccaacataaagattctatctcatt 24731
FAM247.LOC105372935.ref.human                     ------------------------------------------------------------ 11231

GGT1end-LOC749026.end.7456450-7520130.chimp       tacttggtctcgagaaaatctagcacatagtaagtagaccaaaatgtttattaaatgaaa 24791
FAM247.LOC105372935.ref.human                     ------------------------------------------------------------ 11231

GGT1end-LOC749026.end.7456450-7520130.chimp       acacagagcagagatagggggggctgctaggcagactgggttgcacttgattacctggatg 24851
FAM247.LOC105372935.ref.human                     ------------------------------------------------------------ 11231

GGT1end-LOC749026.end.7456450-7520130.chimp       ataataaactgcacaaaacctcggtcaaattaatattgaaactgccttttgcttgggctc 24911
FAM247.LOC105372935.ref.human                     ------------------------------------------------------------ 11231

GGT1end-LOC749026.end.7456450-7520130.chimp       gtttcccttgcggaagaaggatgaccaagaagatgaacaggaaagaaatgagaaacagag 24971
FAM247.LOC105372935.ref.human                     ------------------------------------------------------------ 11231

GGT1end-LOC749026.end.7456450-7520130.chimp       gcctttgcttagtagctaaaggccaccttctgtaacatgaaatagtctgcaagtggcctt 25031
FAM247.LOC105372935.ref.human                     ------------------------------------------------------------ 11231

GGT1end-LOC749026.end.7456450-7520130.chimp       gaactctgccgtgatttagtgacagagttccctcatgtcttctacccaggttgaagtcca 25091
FAM247.LOC105372935.ref.human                     ------------------------------------------------------------ 11231

GGT1end-LOC749026.end.7456450-7520130.chimp       gcaaaattgcgactgtcctctttacaacttgcgagaccacactgcttctgcatttgcctg 25151
FAM247.LOC105372935.ref.human                     ------------------------------------------------------------ 11231

GGT1end-LOC749026.end.7456450-7520130.chimp       ttgtatgtatgagatttacacttgttttaaagcaacattttgtttcagttgggctggtgg 25211
FAM247.LOC105372935.ref.human                     ------------------------------------------------------------ 11231

GGT1end-LOC749026.end.7456450-7520130.chimp       ccatacccggcactagccagtcaatagtgagatggctcctcatggaggaggcttggcttg 25271
FAM247.LOC105372935.ref.human                     ------------------------------------------------------------ 11231

GGT1end-LOC749026.end.7456450-7520130.chimp       aggctgaaggtctttaacccacatatacaagagagttgccactaagggatggaagccagg 25331
FAM247.LOC105372935.ref.human                     ------------------------------------------------------------ 11231
```

```
GGT1end-LOC749026.end.7456450-7520130.chimp      ctaataaccaagtgccacacagagttcctatctgtccctcctcaccattttggctggca 25391
FAM247.LOC105372935.ref.human                    ------------------------------------------------------------ 11231

GGT1end-LOC749026.end.7456450-7520130.chimp      ggatttgagcattttagggcttgggaagatagtattactaaatctactaaaatacatcac 25451
FAM247.LOC105372935.ref.human                    ------------------------------------------------------------ 11231

GGT1end-LOC749026.end.7456450-7520130.chimp      ccatccttatagactttggccagttgctgagcaaattaacttcacaactgaagtgggcca 25511
FAM247.LOC105372935.ref.human                    ------------------------------------------------------------ 11231

GGT1end-LOC749026.end.7456450-7520130.chimp      cactggcctttgtggtcccccactcctcttagaatttgtgagcatggggcctactggagg 25571
FAM247.LOC105372935.ref.human                    ------------------------------------------------------------ 11231

GGT1end-LOC749026.end.7456450-7520130.chimp      agggtggaggtggaggaggggggatcaggaaaataactgatattagcctcaatatatgg 25631
FAM247.LOC105372935.ref.human                    ------------------------------------------------------------ 11231

GGT1end-LOC749026.end.7456450-7520130.chimp      gtgatgcaagaatctgtacaacaaactctcataacacacatttatatatgtagcaaacct 25691
FAM247.LOC105372935.ref.human                    ------------------------------------------------------------ 11231

GGT1end-LOC749026.end.7456450-7520130.chimp      gcacatcctgcacatgtacccctgaacttaaaagttaaaaaaaaaaaaagggatctgtga 25751
FAM247.LOC105372935.ref.human                    ------------------------------------------------------------ 11231

GGT1end-LOC749026.end.7456450-7520130.chimp      gctgagccaaacacctggggatctttgtgcttttgacacactgatgactatgccggtccg 25811
FAM247.LOC105372935.ref.human                    ------------------------------------------------------------ 11231

GGT1end-LOC749026.end.7456450-7520130.chimp      tggggagatgagcctataactgccctgggttgtgtgaccatggaggccactttatgatga 25871
FAM247.LOC105372935.ref.human                    ------------------------------------------------------------ 11231

GGT1end-LOC749026.end.7456450-7520130.chimp      tgggcagtgtctggggccttttgggctcgttgctttagggcttatacatgaatgctggac 25931
FAM247.LOC105372935.ref.human                    ------------------------------------------------------------ 11231

GGT1end-LOC749026.end.7456450-7520130.chimp      tccctgtgtggtggtgaacaccccatgactaagtgcatgtcagcgtcagcactggcccac 25991
FAM247.LOC105372935.ref.human                    ------------------------------------------------------------ 11231

GGT1end-LOC749026.end.7456450-7520130.chimp      actcctgggttcgtgttttcacttttcattcaggaactcgggagctggggccactccct 26051
FAM247.LOC105372935.ref.human                    ------------------------------------------------------------ 11231

GGT1end-LOC749026.end.7456450-7520130.chimp      tggcccttcaggttctccacctgagcagtggggataataaggcagacccggggatggctc 26111
FAM247.LOC105372935.ref.human                    ------------------------------------------------------------ 11231

GGT1end-LOC749026.end.7456450-7520130.chimp      tggtgagggtggaggagtcactgtacagagagagtagagcgggggtggattttattgtta 26171
FAM247.LOC105372935.ref.human                    ------------------------------------------------------------ 11231

GGT1end-LOC749026.end.7456450-7520130.chimp      gaagtggacactggttattgggttgtataagtgggaaatctctcctgagaaaacacacag 26231
FAM247.LOC105372935.ref.human                    ------------------------------------------------------------ 11231

GGT1end-LOC749026.end.7456450-7520130.chimp      cctcacctgtacagaaacacacacattcacaccacacgatgcagcctcacacaagacacc 26291
FAM247.LOC105372935.ref.human                    ------------------------------------------------------------ 11231

GGT1end-LOC749026.end.7456450-7520130.chimp      accaatcctcaagcacccaactcagcaccacccaaaagggagcacaattgcttcctcaaa 26351
FAM247.LOC105372935.ref.human                    ------------------------------------------------------------ 11231

GGT1end-LOC749026.end.7456450-7520130.chimp      atttggccataattttccctggggaattcaggttttaaaaaaacacttcccctatactt 26411
FAM247.LOC105372935.ref.human                    ------------------------------------------------------------ 11231

GGT1end-LOC749026.end.7456450-7520130.chimp      attcctatcacaatcccaggatcagggaggctcttcacattgaaaccaggcaaggatgcc 26471
FAM247.LOC105372935.ref.human                    ------------------------------------------------------------ 11231

GGT1end-LOC749026.end.7456450-7520130.chimp      acacctttcttggcatccagattgttttcttggcaagtgattccagaatacttactagat 26531
FAM247.LOC105372935.ref.human                    ------------------------------------------------------------ 11231

GGT1end-LOC749026.end.7456450-7520130.chimp      tcacacctcagaggggccacctgcaccacctgcaatacagaaacaaagctttttgagggg 26591
FAM247.LOC105372935.ref.human                    ------------------------------------------------------------ 11231

GGT1end-LOC749026.end.7456450-7520130.chimp      tatgtcatgttgtggattgtttgcacaaggctctgtttctctcaatgaatactgaaaact 26651
FAM247.LOC105372935.ref.human                    ------------------------------------------------------------ 11231

GGT1end-LOC749026.end.7456450-7520130.chimp      tgatcagaaagtgtagtcaacttcaaggcctccaaaacaagggtaggatacacactggaa 26711
FAM247.LOC105372935.ref.human                    ------------------------------------------------------------ 11231

GGT1end-LOC749026.end.7456450-7520130.chimp      aagacatcagcttctggatggtggatctctcaggtccacgtaggttggcaagtgcaaaat 26771
FAM247.LOC105372935.ref.human                    ------------------------------------------------------------ 11231

GGT1end-LOC749026.end.7456450-7520130.chimp      actgaatccaaggagaagacattgcttccaaggacaaggaccccaaggatacagtctaca 26831
FAM247.LOC105372935.ref.human                    ------------------------------------------------------------ 11231

GGT1end-LOC749026.end.7456450-7520130.chimp      acctgaagccatcacagctaaatgccattttggattacatatcagttgctaagagtcact 26891
FAM247.LOC105372935.ref.human                    ------------------------------------------------------------ 11231

GGT1end-LOC749026.end.7456450-7520130.chimp      tcttcctccccctcagaaaactgcatttaatacctgtcatggacattgtcatttttttcac 26951
FAM247.LOC105372935.ref.human                    ------------------------------------------------------------ 11231

GGT1end-LOC749026.end.7456450-7520130.chimp      atgtaaagtcagttgaaaaagaaagacaccaagaaaggaacatttctatttcagagaaag 27011
FAM247.LOC105372935.ref.human                    ------------------------------------------------------------ 11231

GGT1end-LOC749026.end.7456450-7520130.chimp      caaggcaaccttaccctcgcgttgactggcctctctccatctcctctgtccttgtgaact 27071
FAM247.LOC105372935.ref.human                    ------------------------------------------------------------ 11231

GGT1end-LOC749026.end.7456450-7520130.chimp      agagactcctcagaggctaggaggacacagagcaacagttagtcatagatgcttttgttc 27131
FAM247.LOC105372935.ref.human                    ------------------------------------------------------------ 11231

GGT1end-LOC749026.end.7456450-7520130.chimp      atgagttattcagggagctctgcttaatgtggacaacaggacagtgtgtgtggatgtgtt 27191
FAM247.LOC105372935.ref.human                    ------------------------------------------------------------ 11231

GGT1end-LOC749026.end.7456450-7520130.chimp      tcattaaaagcatagcttgagctcctgctagaaaatcttccctcgtggaaagacaggcaa 27251
FAM247.LOC105372935.ref.human                    ------------------------------------------------------------ 11231

GGT1end-LOC749026.end.7456450-7520130.chimp      gaacgaggagctaaggagcaagaaatagagtccctggcattttgccgatggcaacttaag 27311
FAM247.LOC105372935.ref.human                    ------------------------------------------------------------ 11231

GGT1end-LOC749026.end.7456450-7520130.chimp      aaaatgggaatgagtcagtctacaaatggtactgaagcacatgctatagtttgatgagag 27371
FAM247.LOC105372935.ref.human                    ------------------------------------------------------------ 11231

GGT1end-LOC749026.end.7456450-7520130.chimp      tcccactgctcacactgtgaggtttgaaacccagctaaatggttttctaaacctgtaaaa 27431
FAM247.LOC105372935.ref.human                    ------------------------------------------------------------ 11231

GGT1end-LOC749026.end.7456450-7520130.chimp      acaatattagcttgcaggatttatgtcccaagactacttttacctctgaggatccacagt 27491
FAM247.LOC105372935.ref.human                    ------------------------------------------------------------ 11231
```

```
GGT1end-LOC749026.end.7456450-7520130.chimp      ggctgtcactgcagttatgtgttttagcattttgcacttgaataaaagcaaagtttaata 27551
FAM247.LOC105372935.ref.human                    ------------------------------------------------------------ 11231

GGT1end-LOC749026.end.7456450-7520130.chimp      gatagattggattcaattctaggcaaaacagtctatggtatttattcactaatcctttgt 27611
FAM247.LOC105372935.ref.human                    ------------------------------------------------------------ 11231

GGT1end-LOC749026.end.7456450-7520130.chimp      tataagtgctgatgggagaattagaaatactgaaattatatcctttaaaattaattaaag 27671
FAM247.LOC105372935.ref.human                    ------------------------------------------------------------ 11231

GGT1end-LOC749026.end.7456450-7520130.chimp      cataattattaatcacacaatatttttcatccaggcctcctttctttgtcatgcatgc 27731
FAM247.LOC105372935.ref.human                    ------------------------------------------------------------ 11231

GGT1end-LOC749026.end.7456450-7520130.chimp      atattaattgaggatggagaatatctacgcttgttcaggccagccaacatacgacagttt 27791
FAM247.LOC105372935.ref.human                    ------------------------------------------------------------ 11231

GGT1end-LOC749026.end.7456450-7520130.chimp      acttcaagagaggagacatgggttgaatgctggtgtgttttaactctgcagcgcaaacag 27851
FAM247.LOC105372935.ref.human                    ------------------------------------------------------------ 11231

GGT1end-LOC749026.end.7456450-7520130.chimp      ttgcaacaagtgtggtgaactaatcaccagatggccctttgctgccttatttgtcattgt 27911
FAM247.LOC105372935.ref.human                    ------------------------------------------------------------ 11231

GGT1end-LOC749026.end.7456450-7520130.chimp      gccttacatgtagcttgcaggatttgattacgcttatgttttgtggtgatcatactttca 27971
FAM247.LOC105372935.ref.human                    ------------------------------------------------------------ 11231

GGT1end-LOC749026.end.7456450-7520130.chimp      actattcctaaaatactgcttcagtcttatctgtttggggtcaactgctgaggatttcat 28031
FAM247.LOC105372935.ref.human                    ------------------------------------------------------------ 11231

GGT1end-LOC749026.end.7456450-7520130.chimp      acaaattaatgaagtttgtgaatctaaagttctacacaaaggtggaaatatttgcaaatc 28091
FAM247.LOC105372935.ref.human                    ------------------------------------------------------------ 11231

GGT1end-LOC749026.end.7456450-7520130.chimp      atttatcttgtaagagactaaaatttagaatctattttaaatatatttaagatatattca 28151
FAM247.LOC105372935.ref.human                    ------------------------------------------------------------ 11231

GGT1end-LOC749026.end.7456450-7520130.chimp      aaaatctacaacaaactgactaaataaaaatcagacaactctttaaaaatgggcaaaaga 28211
FAM247.LOC105372935.ref.human                    ------------------------------------------------------------ 11231

GGT1end-LOC749026.end.7456450-7520130.chimp      cttcaacatatatttccctaaagaagatacagccacagatagtagcacaggaaaagctgc 28271
FAM247.LOC105372935.ref.human                    ------------------------------------------------------------ 11231

GGT1end-LOC749026.end.7456450-7520130.chimp      tcaggatcattagtcattagggaaatgcaaatgaaaaacacaagcagccaccaatataca 28331
FAM247.LOC105372935.ref.human                    ------------------------------------------------------------ 11231

GGT1end-LOC749026.end.7456450-7520130.chimp      cctactaggatgatttaaaggaaaataagtgtgaagaaggacataaagaaattgtaaccc 28391
FAM247.LOC105372935.ref.human                    ------------------------------------------------------------ 11231

GGT1end-LOC749026.end.7456450-7520130.chimp      tgatacattgatggtagaaatggataaagttgcagccactgtgaaaaacagtctgcagtg 28451
FAM247.LOC105372935.ref.human                    ------------------------------------------------------------ 11231

GGT1end-LOC749026.end.7456450-7520130.chimp      gctcagaaggttaaatatagaacccctgttggacccaggaactctactcttaggcacccc 28511
FAM247.LOC105372935.ref.human                    ------------------------------------------------------------ 11231

GGT1end-LOC749026.end.7456450-7520130.chimp      aaagaatagagaacagaaatcaaacagatgtttgtatactaatgtttatagcatcacttt 28571
FAM247.LOC105372935.ref.human                    ------------------------------------------------------------ 11231

GGT1end-LOC749026.end.7456450-7520130.chimp      tcacaggagccaaaaggtggaaataatccaaccatcagtgaacaaatgaatgtaataaaa 28631
FAM247.LOC105372935.ref.human                    ------------------------------------------------------------ 11231

GGT1end-LOC749026.end.7456450-7520130.chimp      gcaaggtggtctgcatgcaatgctacatcatccatctgtaaaaaatgaacataattttga 28691
FAM247.LOC105372935.ref.human                    ------------------------------------------------------------ 11231

GGT1end-LOC749026.end.7456450-7520130.chimp      tagatgatacaacatgggtggacattgagaacactatgcttagtgaaataagccagacac 28751
FAM247.LOC105372935.ref.human                    ------------------------------------------------------------ 11231

GGT1end-LOC749026.end.7456450-7520130.chimp      aaaaggaatatattgtataactgtaattacatgaagtgcctagaatagtcaaattcatac 28811
FAM247.LOC105372935.ref.human                    ------------------------------------------------------------ 11231

GGT1end-LOC749026.end.7456450-7520130.chimp      aagagaaagtgggataggaatcaccatgggctggaaataggggggaaggtgctatattgct 28871
FAM247.LOC105372935.ref.human                    ------------------------------------------------------------ 11231

GGT1end-LOC749026.end.7456450-7520130.chimp      tattgtggacaaggttttgtaagaaatcatcaaaattgtgggtgtagatagtggtgttgg 28931
FAM247.LOC105372935.ref.human                    ------------------------------------------------------------ 11231

GGT1end-LOC749026.end.7456450-7520130.chimp      ttatgcaaccctgtgaatatattgaatgccatggagtgcacactttggttaaaaggttca 28991
FAM247.LOC105372935.ref.human                    ------------------------------------------------------------ 11231

GGT1end-LOC749026.end.7456450-7520130.chimp      aatgataaatattgtgttatatatatttccccacgatagaaaacacgcacagccaagccc 29051
FAM247.LOC105372935.ref.human                    ------------------------------------------------------------ 11231

GGT1end-LOC749026.end.7456450-7520130.chimp      agatgccagtcttgttagctgccttcctttaccttcaagagtgggctgaagcttgtccaa 29111
FAM247.LOC105372935.ref.human                    ------------------------------------------------------------ 11231

GGT1end-LOC749026.end.7456450-7520130.chimp      tctttcaaggttgctgaagactgtatgatggaagtcatctgcattgggaaagaaattaat 29171
FAM247.LOC105372935.ref.human                    ------------------------------------------------------------ 11231

GGT1end-LOC749026.end.7456450-7520130.chimp      ggagagaggagaaaacttgagaatccacactactcaccctgcagggccaagaactctgtc 29231
FAM247.LOC105372935.ref.human                    ------------------------------------------------------------ 11231

GGT1end-LOC749026.end.7456450-7520130.chimp      tcccatgcattgctgtcctgtctcagtatttcctgtgaccacctcctttttcaactgaag 29291
FAM247.LOC105372935.ref.human                    ------------------------------------------------------------ 11231

GGT1end-LOC749026.end.7456450-7520130.chimp      actttgtacctgaaggggttcccaggtttttcacctcggcccttgtcaggactgatcctc 29351
FAM247.LOC105372935.ref.human                    ------------------------------------------------------------ 11231

GGT1end-LOC749026.end.7456450-7520130.chimp      tcaactactgaccatttcacctccattcatgtccatgccacatcaggctgtgttgtctag 29411
FAM247.LOC105372935.ref.human                    ------------------------------------------------------------ 11231

GGT1end-LOC749026.end.7456450-7520130.chimp      atggaatgaatccaccccaaatgtccctttctggaggaagccaccattatgctgtacctc 29471
FAM247.LOC105372935.ref.human                    ------------------------------------------------------------ 11231

GGT1end-LOC749026.end.7456450-7520130.chimp      caagcataatggtacgtccacacacaccaaggcacctcgctcatgcaaggtgtgtgtcct 29531
FAM247.LOC105372935.ref.human                    ------------------------------------------------------------ 11231

GGT1end-LOC749026.end.7456450-7520130.chimp      ctaacaaagtttcacgctctaaacccagataacttttcaaacccaagttctgttgattcc 29591
FAM247.LOC105372935.ref.human                    ------------------------------------------------------------ 11231

GGT1end-LOC749026.end.7456450-7520130.chimp      cctactttgggtgctccatagatgctcatttgtctactaaacactgccccaggcaattaa 29651
FAM247.LOC105372935.ref.human                    ------------------------------------------------------------ 11231
```

```
GGT1end-LOC749026.end.7456450-7520130.chimp      atattccaaagtgaccagcagaatttttatgttaattctgacattgcgttgttagtacaa 29711
FAM247.LOC105372935.ref.human                    ------------------------------------------------------------ 11231

GGT1end-LOC749026.end.7456450-7520130.chimp      gtgtttttccccttcaaatttatgtctttgttactgatagatgtaactgataatgcgtt 29771
FAM247.LOC105372935.ref.human                    ------------------------------------------------------------ 11231

GGT1end-LOC749026.end.7456450-7520130.chimp      tttcagctatgttgccaagcatatttatataaaaatatactcagattgttttcagaattt 29831
FAM247.LOC105372935.ref.human                    ------------------------------------------------------------ 11231

GGT1end-LOC749026.end.7456450-7520130.chimp      gacaaagatgatagcaacaatgataatcttatttgttttatactaatctttatgtgttac 29891
FAM247.LOC105372935.ref.human                    ------------------------------------------------------------ 11231

GGT1end-LOC749026.end.7456450-7520130.chimp      tttcatcatttcttacatattggggcctaccatacattgtacggtgaaattagtgctatg 29951
FAM247.LOC105372935.ref.human                    ------------------------------------------------------------ 11231

GGT1end-LOC749026.end.7456450-7520130.chimp      caccatggtagaaatatcaattggcaaaagtaatttagaaaatagttcccttgtttctta 30011
FAM247.LOC105372935.ref.human                    ------------------------------------------------------------ 11231

GGT1end-LOC749026.end.7456450-7520130.chimp      aattaggctgggtgtggtggctcatgcctataatcccagcactttgggaggcagagatgg 30071
FAM247.LOC105372935.ref.human                    ------------------------------------------------------------ 11231

GGT1end-LOC749026.end.7456450-7520130.chimp      gtggatcacctgaggctaggagtttgagactagcctgaccaacacagcaaaatcctgtct 30131
FAM247.LOC105372935.ref.human                    ------------------------------------------------------------ 11231

GGT1end-LOC749026.end.7456450-7520130.chimp      ctactgaaaatacaaaaattatccaggcatggtggcgtgtgccagttgtcccagctactc 30191
FAM247.LOC105372935.ref.human                    ------------------------------------------------------------ 11231

GGT1end-LOC749026.end.7456450-7520130.chimp      gggaggttgaggcacgagaattgcttaaacctgggaggtggaggttccagtgagctgaga 30251
FAM247.LOC105372935.ref.human                    ------------------------------------------------------------ 11231

GGT1end-LOC749026.end.7456450-7520130.chimp      ttgtgccactgcactccagcctgggtctcagaaaaaaaaaatttttttgaccaaaatgt 30311
FAM247.LOC105372935.ref.human                    ------------------------------------------------------------ 11231

GGT1end-LOC749026.end.7456450-7520130.chimp      cattatgcattatgtgactgtatatgaatgctaaaagctacattactcatcaaagaaaat 30371
FAM247.LOC105372935.ref.human                    ------------------------------------------------------------ 11231

GGT1end-LOC749026.end.7456450-7520130.chimp      aacaaagcaattaaatgtccattaactgataaatgaataaacactatctgtatgagtaaa 30431
FAM247.LOC105372935.ref.human                    ------------------------------------------------------------ 11231

GGT1end-LOC749026.end.7456450-7520130.chimp      cacagcagactatgaaggaaaacacatgaccagcacatgctaacacgtcaattaacttca 30491
FAM247.LOC105372935.ref.human                    ------------------------------------------------------------ 11231

GGT1end-LOC749026.end.7456450-7520130.chimp      aacatagtatgctaaatgaaggaagtcagattccaaatacatatatatgtccatttctat 30551
FAM247.LOC105372935.ref.human                    ------------------------------------------------------------ 11231

GGT1end-LOC749026.end.7456450-7520130.chimp      taagcaagtgggaaatttatggagatggaatgtcacagcagtattgcttagggctggaga 30611
FAM247.LOC105372935.ref.human                    ------------------------------------------------------------ 11231

GGT1end-LOC749026.end.7456450-7520130.chimp      tgggggtggggattaactgccagtgtgcaagagagaatttgggtgagggaaacatattta 30671
FAM247.LOC105372935.ref.human                    ------------------------------------------------------------ 11231

GGT1end-LOC749026.end.7456450-7520130.chimp      aattagatcgtggtgatgggtgcacacagtatcaatttaataaagcatcaaattgtagac 30731
FAM247.LOC105372935.ref.human                    ------------------------------------------------------------ 11231

GGT1end-LOC749026.end.7456450-7520130.chimp      cttttcagtgggcaaactttatggtaggttcacacccaatataggtgttaaaaataaatt 30791
FAM247.LOC105372935.ref.human                    ------------------------------------------------------------ 11231

GGT1end-LOC749026.end.7456450-7520130.chimp      aatgttacggaaattcttgtcgggtttttaacaagccaagagatatgctgtgaaagcagc 30851
FAM247.LOC105372935.ref.human                    ------------------------------------------------------------ 11231

GGT1end-LOC749026.end.7456450-7520130.chimp      attaattcaaatggttgtcacagttcacttaaagttaatcggatagttgtcctacaaata 30911
FAM247.LOC105372935.ref.human                    ------------------------------------------------------------ 11231

GGT1end-LOC749026.end.7456450-7520130.chimp      tagggtgaatgttattcatgaatttcctgaatctattgcaataatcacatttttttccat 30971
FAM247.LOC105372935.ref.human                    ------------------------------------------------------------ 11231

GGT1end-LOC749026.end.7456450-7520130.chimp      taaactcttgaggtagctaattttatttattgcattttcaatgttaatctactatttcat 31031
FAM247.LOC105372935.ref.human                    ------------------------------------------------------------ 11231

GGT1end-LOC749026.end.7456450-7520130.chimp      atattgagattaactcacattagtcagaatttacagtattttaaaatatcacagaattta 31091
FAM247.LOC105372935.ref.human                    ------------------------------------------------------------ 11231

GGT1end-LOC749026.end.7456450-7520130.chimp      atttaccttatctggttttggtttcaagactatactagccatttcatttaattgtacatg 31151
FAM247.LOC105372935.ref.human                    ------------------------------------------------------------ 11231

GGT1end-LOC749026.end.7456450-7520130.chimp      tagggtattctaatttatggaaaactattacattttccttgatttttttttttttagaaa 31211
FAM247.LOC105372935.ref.human                    ------------------------------------------------------------ 11231

GGT1end-LOC749026.end.7456450-7520130.chimp      ttacttctagagatctatatggtagaatccatggagaattgttttaattcttcattcatg 31271
FAM247.LOC105372935.ref.human                    ------------------------------------------------------------ 11231

GGT1end-LOC749026.end.7456450-7520130.chimp      tcttcagtgggtataggattggtcatattggtcatagttttctgctcggatttcaataag 31331
FAM247.LOC105372935.ref.human                    ------------------------------------------------------------ 11231

GGT1end-LOC749026.end.7456450-7520130.chimp      aaacttgtggaagaacctgaagggtgggatctttgagggagcctaagacagagcaagaca 31391
FAM247.LOC105372935.ref.human                    ------------------------------------------------------------ 11231

GGT1end-LOC749026.end.7456450-7520130.chimp      agctaagaaggagggcagtgccacagcagaactgccattgatgccccctcgcctagattg 31451
FAM247.LOC105372935.ref.human                    ------------------------------------------------------------ 11231

GGT1end-LOC749026.end.7456450-7520130.chimp      cggaagagacatccagctgtagacactgaggtgcaggaaaacaatggagcaccatcagag 31511
FAM247.LOC105372935.ref.human                    ------------------------------------------------------------ 11231

GGT1end-LOC749026.end.7456450-7520130.chimp      aaagcagtgcccaggaacaaggaggcactgatggtggcaaggggcaaagacagctgccac 31571
FAM247.LOC105372935.ref.human                    ------------------------------------------------------------ 11231

GGT1end-LOC749026.end.7456450-7520130.chimp      gaggctgttcacatgagggtctcaggctgcatagacacccacaccagctgaggggtcctg 31631
FAM247.LOC105372935.ref.human                    ------------------------------------------------------------ 11231

GGT1end-LOC749026.end.7456450-7520130.chimp      gttttcataaagtgtgtggctcagccaggccaccaacaagcagttcacaaacagtagtaa 31691
FAM247.LOC105372935.ref.human                    ------------------------------------------------------------ 11231

GGT1end-LOC749026.end.7456450-7520130.chimp      tacgacactttccaaagaccttacttgagtaacacggtgatcctcacaaatttccaatca 31751
FAM247.LOC105372935.ref.human                    ------------------------------------------------------------ 11231

GGT1end-LOC749026.end.7456450-7520130.chimp      ggatggtcgcacagttcctcctgctttaggactcagagcctgcccgtggtcacagtgggt 31811
FAM247.LOC105372935.ref.human                    ------------------------------------------------------------ 11231

GGT1end-LOC749026.end.7456450-7520130.chimp      aggtgcagactctgaagatgcactttggtcagagaccccgctgaactctgtctaatgagg 31871
```

```
FAM247.LOC105372935.ref.human                    ------------------------------------------------------------ 11231

GGT1end-LOC749026.end.7456450-7520130.chimp      acctctgtcctgtctgctgaccaccggtcagaggtgcaggctgcagtggggagtaagaat 31931
FAM247.LOC105372935.ref.human                    ------------------------------------------------------------ 11231

GGT1end-LOC749026.end.7456450-7520130.chimp      gccaccttctcaatgtttggaaaactccctgccagaactgagaatggccctttctaagca 31991
FAM247.LOC105372935.ref.human                    ------------------------------------------------------------ 11231

GGT1end-LOC749026.end.7456450-7520130.chimp      gaaggcaagctcagactaaagaaggaggccgaacacatcaggttggcagattgccaaaga 32051
FAM247.LOC105372935.ref.human                    ------------------------------------------------------------ 11231

GGT1end-LOC749026.end.7456450-7520130.chimp      ttcactcagggagagcccacatcctgggccatcttgggtggtggcaagatgaggtagacg 32111
FAM247.LOC105372935.ref.human                    ------------------------------------------------------------ 11231

GGT1end-LOC749026.end.7456450-7520130.chimp      actgcttttgcaacacatacctgacaacaaaaaatcaacaactgtaaaagagccacaaaa 32171
FAM247.LOC105372935.ref.human                    ------------------------------------------------------------ 11231

GGT1end-LOC749026.end.7456450-7520130.chimp      tccccaaatatttgcaaattagcaatgcacttttaaataactcatgggttaaagaagaag 32231
FAM247.LOC105372935.ref.human                    ------------------------------------------------------------ 11231

GGT1end-LOC749026.end.7456450-7520130.chimp      tctcaatagaaaattaaaaatacttttaactacattaaaagaaaatgtgacttggcaaga 32291
FAM247.LOC105372935.ref.human                    ------------------------------------------------------------ 11231

GGT1end-LOC749026.end.7456450-7520130.chimp      tttctggatgtagcaaaagcagtccttagagggaaatctatagcattggatgcaatatac 32351
FAM247.LOC105372935.ref.human                    ------------------------------------------------------------ 11231

GGT1end-LOC749026.end.7456450-7520130.chimp      taaaaatcacaagacctaaaatcagtaatatcatgtttcaattagggaactatagaaaat 32411
FAM247.LOC105372935.ref.human                    ------------------------------------------------------------ 11231

GGT1end-LOC749026.end.7456450-7520130.chimp      agaggaatgcaatggaaagcaagtaaaagtaataaacaacatcacagaaatcaataaaat 32471
FAM247.LOC105372935.ref.human                    ------------------------------------------------------------ 11231

GGT1end-LOC749026.end.7456450-7520130.chimp      taaaacactgaaatcatcagaaaatcaataaaaccaaaagctggttctttgatatgctca 32531
FAM247.LOC105372935.ref.human                    ------------------------------------------------------------ 11231

GGT1end-LOC749026.end.7456450-7520130.chimp      ttacaatgaatgaattgatatgcaggctaaccaagaaaaagaagataacacaaatgacca 32591
FAM247.LOC105372935.ref.human                    ------------------------------------------------------------ 11231

GGT1end-LOC749026.end.7456450-7520130.chimp      atttcagaaataaaagaggagccatctctactgaactgttaggcattaaaaggaatatca 32651
FAM247.LOC105372935.ref.human                    ------------------------------------------------------------ 11231

GGT1end-LOC749026.end.7456450-7520130.chimp      tgaacagttctatgaccgcagtttgataacctcagtgaaatgtatcaattccttgaaagg 32711
FAM247.LOC105372935.ref.human                    ------------------------------------------------------------ 11231

GGT1end-LOC749026.end.7456450-7520130.chimp      caatcttcccaaggtcatgctaggatcctaatttgaataaacttatgtctattaaataag 32771
FAM247.LOC105372935.ref.human                    ------------------------------------------------------------ 11231

GGT1end-LOC749026.end.7456450-7520130.chimp      ttgaattcacattaagagcattccgaaaaagaaagcaccaggcccagatggtttctctca 32831
FAM247.LOC105372935.ref.human                    ------------------------------------------------------------ 11231

GGT1end-LOC749026.end.7456450-7520130.chimp      tgaaatctaccgagttcttcaacaggtgaataaaaagacaaaaattcatttaatgcaata 32891
FAM247.LOC105372935.ref.human                    ------------------------------------------------------------ 11231

GGT1end-LOC749026.end.7456450-7520130.chimp      ttatttggtgatttaatgtgccatttttgccattaaggcataaaaaagacatgaaagca 32951
FAM247.LOC105372935.ref.human                    ------------------------------------------------------------ 11231

GGT1end-LOC749026.end.7456450-7520130.chimp      gctaaagcgtacatcaatttagtgcaataaattcatctgaaaaaactacataatatatga 33011
FAM247.LOC105372935.ref.human                    ------------------------------------------------------------ 11231

GGT1end-LOC749026.end.7456450-7520130.chimp      ttccaactatatgacattctggaaaaggcaaagctgaagcgatagtaaaaatattaatag 33071
FAM247.LOC105372935.ref.human                    ------------------------------------------------------------ 11231

GGT1end-LOC749026.end.7456450-7520130.chimp      ttgccaaggtttctggagaaagaggacagagattaatgagaagagaggatttttagggaa 33131
FAM247.LOC105372935.ref.human                    ------------------------------------------------------------ 11231

GGT1end-LOC749026.end.7456450-7520130.chimp      gtgaacattttctttatgagaccataagggtgaacataatgttttaaatatttcaaaatt 33191
FAM247.LOC105372935.ref.human                    ------------------------------------------------------------ 11231

GGT1end-LOC749026.end.7456450-7520130.chimp      catatatatgtataacagaaagaatgaacattatgcaaatgtagacttcagataataatg 33251
FAM247.LOC105372935.ref.human                    ------------------------------------------------------------ 11231

GGT1end-LOC749026.end.7456450-7520130.chimp      tgtcaatattttctcattattctagcaaatgtaccacagtaatgtaagatgttactaata 33311
FAM247.LOC105372935.ref.human                    ------------------------------------------------------------ 11231

GGT1end-LOC749026.end.7456450-7520130.chimp      ggtgaaattaggaagtgagggtgaggagacagaataatatgggaacttcgtgtattatat 33371
FAM247.LOC105372935.ref.human                    ------------------------------------------------------------ 11231

GGT1end-LOC749026.end.7456450-7520130.chimp      actcaattttatttatttatttatttatttatttatttatttatttatttatttatttt 33431
FAM247.LOC105372935.ref.human                    ------------------------------------------------------------ 11231

GGT1end-LOC749026.end.7456450-7520130.chimp      gagatggagtttcactcttgtcacccaggctggagtgcaatggcatgatcttggctcact 33491
FAM247.LOC105372935.ref.human                    ------------------------------------------------------------ 11231

GGT1end-LOC749026.end.7456450-7520130.chimp      gcaacctctgcctcctgggttcaagcgattctcctgcctcagcctcctgagtagctggga 33551
FAM247.LOC105372935.ref.human                    ------------------------------------------------------------ 11231

GGT1end-LOC749026.end.7456450-7520130.chimp      ttacaggcacctgccaccacacccggctaatttttttgtatttttagtagagatggggtt 33611
FAM247.LOC105372935.ref.human                    ------------------------------------------------------------ 11231

GGT1end-LOC749026.end.7456450-7520130.chimp      tcaccatgttggccaggctggtctccaactcctgacctcagatgatccgcctgccttggc 33671
FAM247.LOC105372935.ref.human                    ------------------------------------------------------------ 11231

GGT1end-LOC749026.end.7456450-7520130.chimp      ctcctaaagtgctgggattataagtgtgagccaccacgcccggccatatgctcagttttt 33731
FAM247.LOC105372935.ref.human                    ------------------------------------------------------------ 11231

GGT1end-LOC749026.end.7456450-7520130.chimp      atgtcaatttaaaactctctaaagaaatatattaattgaaaaataaaaatatagcaccac 33791
FAM247.LOC105372935.ref.human                    ------------------------------------------------------------ 11231

GGT1end-LOC749026.end.7456450-7520130.chimp      tctttcagggagatctatgcttatgtttaacaaccaggtaagttctagacattagcttga 33851
FAM247.LOC105372935.ref.human                    ------------------------------------------------------------ 11231

GGT1end-LOC749026.end.7456450-7520130.chimp      aacattgtctatcattcaacatgaaccaaaattgacttttaagtagatatttacttttgt 33911
FAM247.LOC105372935.ref.human                    ------------------------------------------------------------ 11231

GGT1end-LOC749026.end.7456450-7520130.chimp      ggtggtagcaatatttactgaccaggcaaattagaatcctgacacattaaaaatatggc 33971
FAM247.LOC105372935.ref.human                    ------------------------------------------------------------ 11231

GGT1end-LOC749026.end.7456450-7520130.chimp      ttagtctcttcgtagtttcctcttacatatgggacactgaatactccccgcaattgcaat 34031
FAM247.LOC105372935.ref.human                    ------------------------------------------------------------ 11231
```

```
GGT1end-LOC749026.end.7456450-7520130.chimp      tcttgaatcaacttaattaatgaacttccacagtaccttcttgtgggtacctcttcttct 34091
FAM247.LOC105372935.ref.human                    ------------------------------------------------------------ 11231

GGT1end-LOC749026.end.7456450-7520130.chimp      ttacccgggagccatgaggtctccgacactggttggtgtgcacagcatatcttcttgtat 34151
FAM247.LOC105372935.ref.human                    ------------------------------------------------------------ 11231

GGT1end-LOC749026.end.7456450-7520130.chimp      tctctatcagagaagatgctggttaatgcatttacaatagatagggctgttgacatcttg 34211
FAM247.LOC105372935.ref.human                    ------------------------------------------------------------ 11231

GGT1end-LOC749026.end.7456450-7520130.chimp      ctgacagaagaccagagggaaaatagtgataatctgttctaagtttaaacttatgatcct 34271
FAM247.LOC105372935.ref.human                    ------------------------------------------------------------ 11231

GGT1end-LOC749026.end.7456450-7520130.chimp      tttctttacaggcttccaagcagagcccactgaatcaaagttgggtttcaggaagatcac 34331
FAM247.LOC105372935.ref.human                    ------------------------------------------------------------ 11231

GGT1end-LOC749026.end.7456450-7520130.chimp      ggagttcagtgagcactcaacacctctatcagacagactgcgtgggcagtgccttcctgg 34391
FAM247.LOC105372935.ref.human                    ------------------------------------------------------------ 11231

GGT1end-LOC749026.end.7456450-7520130.chimp      agaggagaatacagcaggatgactgtgagtgcagggctggtgcagagtgggggcccggat 34451
FAM247.LOC105372935.ref.human                    ------------------------------------------------------------ 11231

GGT1end-LOC749026.end.7456450-7520130.chimp      tcaaattccactaagccatgtggacctggcaagctcatgtcctccctctgccctcagttc 34511
FAM247.LOC105372935.ref.human                    ------------------------------------------------------------ 11231

GGT1end-LOC749026.end.7456450-7520130.chimp      tctgcactgtcataatgtaattttagcaatactttttaggccctatttaggccctacttc 34571
FAM247.LOC105372935.ref.human                    ------------------------------------------------------------ 11231

GGT1end-LOC749026.end.7456450-7520130.chimp      ttagtatcacagtacagggctaaaaaatcactaaatacaggaaaaccttagagaggactg 34631
FAM247.LOC105372935.ref.human                    ------------------------------------------------------------ 11231

GGT1end-LOC749026.end.7456450-7520130.chimp      gtacttcagtaatgttctctaagtgtttactacatgccaggaggaataagctggacactt 34691
FAM247.LOC105372935.ref.human                    ------------------------------------------------------------ 11231

GGT1end-LOC749026.end.7456450-7520130.chimp      agcagtggcggaatatggagggggaatttggatggctccggggcagtggagcatgctttc 34751
FAM247.LOC105372935.ref.human                    ------------------------------------------------------------ 11231

GGT1end-LOC749026.end.7456450-7520130.chimp      ctgttcggcttttccgtgggcatgatgccttatggtttatggaggacatcagccctgcag 34811
FAM247.LOC105372935.ref.human                    ------------------------------------------------------------ 11231

GGT1end-LOC749026.end.7456450-7520130.chimp      ggggtgcagaggaggggctgtggctgagattttacacttgagggtgctgacattcagaga 34871
FAM247.LOC105372935.ref.human                    ------------------------------------------------------------ 11231

GGT1end-LOC749026.end.7456450-7520130.chimp      tgataagtgacgagcagaacctcaaccccgctgagtgaaggacctgagatgggaaatgta 34931
FAM247.LOC105372935.ref.human                    ------------------------------------------------------------ 11231

GGT1end-LOC749026.end.7456450-7520130.chimp      tttggttccctagagagagagattcctgaaaaactgccacctcttcatcacgccctgtgc 34991
FAM247.LOC105372935.ref.human                    ------------------------------------------------------------ 11231

GGT1end-LOC749026.end.7456450-7520130.chimp      cagagacccaagagacccctcactgtctttctccagtcctcctagcccaaggtgtgtggg 35051
FAM247.LOC105372935.ref.human                    ------------------------------------------------------------ 11231

GGT1end-LOC749026.end.7456450-7520130.chimp      tggacaaaggtgatgctctggaggaaatgcctgagataaggacaggtccttaatgataaa 35111
FAM247.LOC105372935.ref.human                    ------------------------------------------------------------ 11231

GGT1end-LOC749026.end.7456450-7520130.chimp      gaattctcctttctctttcagatccttgacctcccagtatgacagcttaaaggctgtcat 35171
FAM247.LOC105372935.ref.human                    ------------------------------------------------------------ 11231

GGT1end-LOC749026.end.7456450-7520130.chimp      ctctgtggcctgcctcccctttcccttcaccctgccagctgcctctcagtgactgtctcc 35231
FAM247.LOC105372935.ref.human                    ------------------------------------------------------------ 11231

GGT1end-LOC749026.end.7456450-7520130.chimp      tccagtgactacactgagggaccagggactgcttgcctcccgaggctgctcagaccttcc 35291
FAM247.LOC105372935.ref.human                    ------------------------------------------------------------ 11231

GGT1end-LOC749026.end.7456450-7520130.chimp      gacactgcaaaatgattgtcaaaagatgggtctgcaaagagtaacttcccttccattgat 35351
FAM247.LOC105372935.ref.human                    ------------------------------------------------------------ 11231

GGT1end-LOC749026.end.7456450-7520130.chimp      caaacctgaatatgcaagctactgtgaattaactggaaaagtggccatgtgggctggtgc 35411
FAM247.LOC105372935.ref.human                    ------------------------------------------------------------ 11231

GGT1end-LOC749026.end.7456450-7520130.chimp      tttggtgatttaatgaattaagtctgcaacccccactgcctccttgactattgatcagag 35471
FAM247.LOC105372935.ref.human                    ------------------------------------------------------------ 11231

GGT1end-LOC749026.end.7456450-7520130.chimp      ctgcctgcaataaggtctggctaagaatgggcagtggctgcatcagctctgggtaaaatt 35531
FAM247.LOC105372935.ref.human                    ------------------------------------------------------------ 11231

GGT1end-LOC749026.end.7456450-7520130.chimp      tgacctaaaatgaccaatctcattcactaacctcaccatagtcttatgggttcaatggac 35591
FAM247.LOC105372935.ref.human                    ------------------------------------------------------------ 11231

GGT1end-LOC749026.end.7456450-7520130.chimp      ccgtccaatcctttgctctgttctctccatcaccttcctgtgtaattttcctccaccatg 35651
FAM247.LOC105372935.ref.human                    ------------------------------------------------------------ 11231

GGT1end-LOC749026.end.7456450-7520130.chimp      cacataatagaaacatggcacaggggagctaatcacctcttttatcccccacttcaggct 35711
FAM247.LOC105372935.ref.human                    ------------------------------------------------------------ 11231

GGT1end-LOC749026.end.7456450-7520130.chimp      cacacataagtttatagtaaaagccttttcaaatgactgctttaactgctgctatagcat 35771
FAM247.LOC105372935.ref.human                    ------------------------------------------------------------ 11231

GGT1end-LOC749026.end.7456450-7520130.chimp      gtgtcatcagttgaatggaatctgtcatgtgactttaagcaaccctttgttgagagacaa 35831
FAM247.LOC105372935.ref.human                    ------------------------------------------------------------ 11231

GGT1end-LOC749026.end.7456450-7520130.chimp      gattcaatactagggacagtattctagtgtactacatcattgattttatgttatgaagat 35891
FAM247.LOC105372935.ref.human                    ------------------------------------------------------------ 11231

GGT1end-LOC749026.end.7456450-7520130.chimp      catcatttattgaaaatgtataaataatgaagcccagccttactcttcaatgctgtgtgt 35951
FAM247.LOC105372935.ref.human                    ------------------------------------------------------------ 11231

GGT1end-LOC749026.end.7456450-7520130.chimp      gtaaatccactgagtgtgctgacccccatgcttgtacccacctgctaacacagaaagagt 36011
FAM247.LOC105372935.ref.human                    ------------------------------------------------------------ 11231

GGT1end-LOC749026.end.7456450-7520130.chimp      ccattcagaagggaggcacagctccagcactgaggctgtccataccagcttcacaagaga 36071
FAM247.LOC105372935.ref.human                    ------------------------------------------------------------ 11231

GGT1end-LOC749026.end.7456450-7520130.chimp      gttgccacaaggacgacggatacccagataacaaccaaatggtaatttgagtacttaatg 36131
FAM247.LOC105372935.ref.human                    ------------------------------------------------------------ 11231

GGT1end-LOC749026.end.7456450-7520130.chimp      gtcatgatccctaaagtgtgtagctcagagggcttgtggtgataactccatcaagactct 36191
FAM247.LOC105372935.ref.human                    ------------------------------------------------------------ 11231
```

```
GGT1end-LOC749026.end.7456450-7520130.chimp      aaagcatctctccaattcttattggacttgatccatgtcttgaggagacccagctatgac 36251
FAM247.LOC105372935.ref.human                    ------------------------------------------------------------ 11231

GGT1end-LOC749026.end.7456450-7520130.chimp      acgcaggcaccacattgtcctacttagtgcctcccttagtgtttcagaacctgtgatttg 36311
FAM247.LOC105372935.ref.human                    ------------------------------------------------------------ 11231

GGT1end-LOC749026.end.7456450-7520130.chimp      atcagaaacatgggctttctatgttggtttcacactaaggactatgtgacacctgcagga 36371
FAM247.LOC105372935.ref.human                    ------------------------------------------------------------ 11231

GGT1end-LOC749026.end.7456450-7520130.chimp      agatgtctacatagctacctggattatgagatcatgaggctctcttatgtgagggatggc 36431
FAM247.LOC105372935.ref.human                    ------------------------------------------------------------ 11231

GGT1end-LOC749026.end.7456450-7520130.chimp      gtttgggatctctgcaggcgtgggtaattccaggcatagagggtgctggaactcccttgc 36491
FAM247.LOC105372935.ref.human                    ------------------------------------------------------------ 11231

GGT1end-LOC749026.end.7456450-7520130.chimp      atggtgaatagtgatctcttcactggctgataaatagaggttgtagttcaggccttcaac 36551
FAM247.LOC105372935.ref.human                    ------------------------------------------------------------ 11231

GGT1end-LOC749026.end.7456450-7520130.chimp      attagcaccgtatgagtaaacattttgactcttcactatgcagcaagtgaaccagggcac 36611
FAM247.LOC105372935.ref.human                    ------------------------------------------------------------ 11231

GGT1end-LOC749026.end.7456450-7520130.chimp      atttatttatgtggcttagtttctccatctggcatgtgggctcaataaacaagctcacaa 36671
FAM247.LOC105372935.ref.human                    ------------------------------------------------------------ 11231

GGT1end-LOC749026.end.7456450-7520130.chimp      catatgggcatgatgatgatgaggtgtgaactaatgtaagtaaagtatgtggtctgattt 36731
FAM247.LOC105372935.ref.human                    ------------------------------------------------------------ 11231

GGT1end-LOC749026.end.7456450-7520130.chimp      gttaaattaagaaaaatggcactgagagttgtgctgggtaaacacaacatttttttcctg 36791
FAM247.LOC105372935.ref.human                    ------------------------------------------------------------ 11231

GGT1end-LOC749026.end.7456450-7520130.chimp      ggggaaacacacatagacacacattcacaagcaaatcatgcagacgtgcacacagaccac 36851
FAM247.LOC105372935.ref.human                    ------------------------------------------------------------ 11231

GGT1end-LOC749026.end.7456450-7520130.chimp      ctcaccccacccccgccctaatacacacatacccacacacaacctaatgtgaacacgttc 36911
FAM247.LOC105372935.ref.human                    ------------------------------------------------------------ 11231

GGT1end-LOC749026.end.7456450-7520130.chimp      ccagaaactatacatagataaaaagagtatgtcacctggaaaaccagtttcttttactat 36971
FAM247.LOC105372935.ref.human                    ------------------------------------------------------------ 11231

GGT1end-LOC749026.end.7456450-7520130.chimp      accctacatcctcattcccaccagatgtcttggatcatggaggctctccagacaaaagcc 37031
FAM247.LOC105372935.ref.human                    ------------------------------------------------------------ 11231

GGT1end-LOC749026.end.7456450-7520130.chimp      agcagttaagctccagatttcctatagaatccttttctaacaaccagtgagtgattccag 37091
FAM247.LOC105372935.ref.human                    ------------------------------------------------------------ 11231

GGT1end-LOC749026.end.7456450-7520130.chimp      aatacgtaccattgaatgtgctccctgaagtcacctgtaattagagaaggaaaacactct 37151
FAM247.LOC105372935.ref.human                    ------------------------------------------------------------ 11231

GGT1end-LOC749026.end.7456450-7520130.chimp      gagaatcaggctatgctatggatggctcacacaggtcttttgttcacttggaaactctgg 37211
FAM247.LOC105372935.ref.human                    ------------------------------------------------------------ 11231

GGT1end-LOC749026.end.7456450-7520130.chimp      gtaaccaagattggaaataaggttcaagtcaaaagccccaactctagagtagagttccct 37271
FAM247.LOC105372935.ref.human                    ------------------------------------------------------------ 11231

GGT1end-LOC749026.end.7456450-7520130.chimp      taggaaagcacaggagcttttcttgaagaatgtttctgtctaggtaattttggagtagca 37331
FAM247.LOC105372935.ref.human                    ------------------------------------------------------------ 11231

GGT1end-LOC749026.end.7456450-7520130.chimp      attgcagaattcttatctaaagtggaaagcttgttcctgaagaaaacatcccttaacacg 37391
FAM247.LOC105372935.ref.human                    ------------------------------------------------------------ 11231

GGT1end-LOC749026.end.7456450-7520130.chimp      cagtgtactatctgacactgccaattttgcacgtcctctggaatcaggtgtcagttggta 37451
FAM247.LOC105372935.ref.human                    ------------------------------------------------------------ 11231

GGT1end-LOC749026.end.7456450-7520130.chimp      aaatacacctcctccatccccaaggaaatattatctaacatctataatgtagtggataat 37511
FAM247.LOC105372935.ref.human                    ------------------------------------------------------------ 11231

GGT1end-LOC749026.end.7456450-7520130.chimp      tttcccatagctgatatcaactgaaaaataaaggatccaagaaaacaacatttacatctt 37571
FAM247.LOC105372935.ref.human                    ------------------------------------------------------------ 11231

GGT1end-LOC749026.end.7456450-7520130.chimp      aggcaaagacaggctactttaccttggtagtagaggagggctcccttttcacacgccttt 37631
FAM247.LOC105372935.ref.human                    ------------------------------------------------------------ 11231

GGT1end-LOC749026.end.7456450-7520130.chimp      tggaaggcttcttcgggtcacctaggggatgtggagggacacagcatggctgtcagttca 37691
FAM247.LOC105372935.ref.human                    ------------------------------------------------------------ 11231

GGT1end-LOC749026.end.7456450-7520130.chimp      ttggcagtgctactcatgaatgactcagggactggaacttaggggtgtgcctggttaaca 37751
FAM247.LOC105372935.ref.human                    ------------------------------------------------------------ 11231

GGT1end-LOC749026.end.7456450-7520130.chimp      agcatggaatgagcttctcctggaccatcttcttcatggaccaaggaaggcaaagaaaga 37811
FAM247.LOC105372935.ref.human                    ------------------------------------------------------------ 11231

GGT1end-LOC749026.end.7456450-7520130.chimp      gcagcaaggaaatgagagtagagcccttggctttccaggtaatggcaaatgaaagcaacg 37871
FAM247.LOC105372935.ref.human                    ------------------------------------------------------------ 11231

GGT1end-LOC749026.end.7456450-7520130.chimp      tgaaataatcaaactccaaatgaacaaatgctaaaatacatgctaggattcaaccacagc 37931
FAM247.LOC105372935.ref.human                    ------------------------------------------------------------ 11231

GGT1end-LOC749026.end.7456450-7520130.chimp      atcctgtcacttcttcagaccctttaaaagcccagcaggactgccactaccttcttgaca 37991
FAM247.LOC105372935.ref.human                    ------------------------------------------------------------ 11231

GGT1end-LOC749026.end.7456450-7520130.chimp      tctaccaagtccctttcaacctccacagacccacatacactgctactgcatttatcatgg 38051
FAM247.LOC105372935.ref.human                    ------------------------------------------------------------ 11231

GGT1end-LOC749026.end.7456450-7520130.chimp      agggtatagggttctgcctggtttatgtgtgaatttttaaaaactagatttaataccat 38111
FAM247.LOC105372935.ref.human                    ------------------------------------------------------------ 11231

GGT1end-LOC749026.end.7456450-7520130.chimp      gcaccagcattaattatatttatttcttttcttggttatgaaaataatcagccaggcata 38171
FAM247.LOC105372935.ref.human                    ------------------------------------------------------------ 11231

GGT1end-LOC749026.end.7456450-7520130.chimp      gtggctcacacctgtaatcccagcagtttggaaggtggaggtgggcggatcatttgaggt 38231
FAM247.LOC105372935.ref.human                    ------------------------------------------------------------ 11231

GGT1end-LOC749026.end.7456450-7520130.chimp      caggagttcgagaccagcctgaccaacatggtaaaaccccatctcaactaaaaaaaaaaa 38291
FAM247.LOC105372935.ref.human                    ------------------------------------------------------------ 11231

GGT1end-LOC749026.end.7456450-7520130.chimp      aaaaaaaaaaaaaacccagctactcaggaggctgaggcaggacaatcccttgaacctgga 38351
FAM247.LOC105372935.ref.human                    ------------------------------------------------------------ 11231
```

```
GGT1end-LOC749026.end.7456450-7520130.chimp      ggcagagattgcagtgagctgagattgcaccactgcactccagcctgagtgacagagact 38411
FAM247.LOC105372935.ref.human                    ------------------------------------------------------------ 11231

GGT1end-LOC749026.end.7456450-7520130.chimp      tcatctcaaaaaataaaaaagtaaaaaatcaggccaggtgtggtggctcacgcctgtaat 38471
FAM247.LOC105372935.ref.human                    ------------------------------------------------------------ 11231

GGT1end-LOC749026.end.7456450-7520130.chimp      cccagcactttgggaggccgaggcgggtggatcatgaggtgagaagattgagaccatcct 38531
FAM247.LOC105372935.ref.human                    ------------------------------------------------------------ 11231

GGT1end-LOC749026.end.7456450-7520130.chimp      ggctaacagagtgaaaccccgtctctactaaaaacacaaaaactttgccaggcgtggtgg 38591
FAM247.LOC105372935.ref.human                    ------------------------------------------------------------ 11231

GGT1end-LOC749026.end.7456450-7520130.chimp      cacgtgcctgtagtcccagctactcgggaggctgaggcaggagaatcgcttgaacacagg 38651
FAM247.LOC105372935.ref.human                    ------------------------------------------------------------ 11231

GGT1end-LOC749026.end.7456450-7520130.chimp      aggcagaggttgcagtgagctgagatcatgccactgcactccagcctgggcaacagcgca 38711
FAM247.LOC105372935.ref.human                    ------------------------------------------------------------ 11231

GGT1end-LOC749026.end.7456450-7520130.chimp      agactgtctcaaaaaaaaaaaaaaaaaaaaaacctcccctaaaaaataatcaattattaa 38771
FAM247.LOC105372935.ref.human                    ------------------------------------------------------------ 11231

GGT1end-LOC749026.end.7456450-7520130.chimp      agattagaaaatactgaaatacttatatttaaaataaccactataaccatacattttcc 38831
FAM247.LOC105372935.ref.human                    ------------------------------------------------------------ 11231

GGT1end-LOC749026.end.7456450-7520130.chimp      gttcagtcttcctttgccctgtgtttgcagtcatcatgtgaggggctgcctatgttcagc 38891
FAM247.LOC105372935.ref.human                    ------------------------------------------------------------ 11231

GGT1end-LOC749026.end.7456450-7520130.chimp      ccaggcaacccacagcgagagagggcagagcggggagatggcccctggtgagcactgagg 38951
FAM247.LOC105372935.ref.human                    ------------------------------------------------------------ 11231

GGT1end-LOC749026.end.7456450-7520130.chimp      ctcttcaaaccagttgccgagggagttgcaagcagggtgacacatgaggctcattattta 39011
FAM247.LOC105372935.ref.human                    ------------------------------------------------------------ 11231

GGT1end-LOC749026.end.7456450-7520130.chimp      gtggtattttgtgttcttacttgtcttgtccccaggctgtgaagctcacagacttttttt 39071
FAM247.LOC105372935.ref.human                    ------------------------------------------------------------ 11231

GGT1end-LOC749026.end.7456450-7520130.chimp      tttttaacatttctataggggtcatttgaagttcataacaccatcaaacatcagtacaag 39131
FAM247.LOC105372935.ref.human                    ------------------------------------------------------------ 11231

GGT1end-LOC749026.end.7456450-7520130.chimp      gtcttcccattatttaccggctttgggatatgtgctgaggagtggccacgcactgaagga 39191
FAM247.LOC105372935.ref.human                    ------------------------------------------------------------ 11231

GGT1end-LOC749026.end.7456450-7520130.chimp      atgtgaccagttgtcccaagtgttgtccttcaactcaaaatccgcaaccagttcaaaagc 39251
FAM247.LOC105372935.ref.human                    ------------------------------------------------------------ 11231

GGT1end-LOC749026.end.7456450-7520130.chimp      atttgggcttttatgtgcctttcacataaagactatgacacagctaagctattcatgtac 39311
FAM247.LOC105372935.ref.human                    ------------------------------------------------------------ 11231

GGT1end-LOC749026.end.7456450-7520130.chimp      taatacctgccctgagctgtgtgaccacagacaccaccctataatgtggccagtgtgtag 39371
FAM247.LOC105372935.ref.human                    ------------------------------------------------------------ 11231

GGT1end-LOC749026.end.7456450-7520130.chimp      gaactctacaggctcagatcattccagacatacatgcagatgttggaactcccttgcaca 39431
FAM247.LOC105372935.ref.human                    ------------------------------------------------------------ 11231

GGT1end-LOC749026.end.7456450-7520130.chimp      gtgactcatgatcccaaggctgagtgctgagggcaggtcagtagttcagagcctcagcat 39491
FAM247.LOC105372935.ref.human                    ------------------------------------------------------------ 11231

GGT1end-LOC749026.end.7456450-7520130.chimp      tggggctgtatgcttgggttcccatcttggctcttttgctgaggggctcataagcttggg 39551
FAM247.LOC105372935.ref.human                    ------------------------------------------------------------ 11231

GGT1end-LOC749026.end.7456450-7520130.chimp      cacattcatcactgtctttttctacccagcgaaaggtgatagtaaatcaatcatgccatt 39611
FAM247.LOC105372935.ref.human                    ------------------------------------------------------------ 11231

GGT1end-LOC749026.end.7456450-7520130.chimp      tgtttgtggtgatggccgaatgatttgatgtaggtaaagtgtacagtataagtgctcatg 39671
FAM247.LOC105372935.ref.human                    ------------------------------------------------------------ 11231

GGT1end-LOC749026.end.7456450-7520130.chimp      aggcttaattatcagtgaaatgtcttgctttagatttcacagcttaataaggctcagagg 39731
FAM247.LOC105372935.ref.human                    ------------------------------------------------------------ 11231

GGT1end-LOC749026.end.7456450-7520130.chimp      cttattcacgcccaggattctgaactcccaagtcattgttctatgacatacacctgcttg 39791
FAM247.LOC105372935.ref.human                    ------------------------------------------------------------ 11231

GGT1end-LOC749026.end.7456450-7520130.chimp      gcctaaaaccgaccactttatcataatgcagacccagttttgaaagaagcagataaaatt 39851
FAM247.LOC105372935.ref.human                    ------------------------------------------------------------ 11231

GGT1end-LOC749026.end.7456450-7520130.chimp      gtcaaaagtactgtgtttgcctcagcgcacactcagtgcgctccacacaaagggcatcac 39911
FAM247.LOC105372935.ref.human                    ------------------------------------------------------------ 11231

GGT1end-LOC749026.end.7456450-7520130.chimp      catctgccacaatggcccagtggcctggttggcacagacagtggcagagcaaagggcaag 39971
FAM247.LOC105372935.ref.human                    ------------------------------------------------------------ 11231

GGT1end-LOC749026.end.7456450-7520130.chimp      agatccctaatcccatcaccatggtcgatagggcatcatagacattccagggtgaaggca 40031
FAM247.LOC105372935.ref.human                    ------------------------------------------------------------ 11231

GGT1end-LOC749026.end.7456450-7520130.chimp      caatccacattgtgaggtccaactgctgccatgtagacaggtgtgtttttacatgtacag 40091
FAM247.LOC105372935.ref.human                    ------------------------------------------------------------ 11231

GGT1end-LOC749026.end.7456450-7520130.chimp      gaagatcattgaagataagtgttttatatccatggttaacgatgagatgaccatgaaatg 40151
FAM247.LOC105372935.ref.human                    ------------------------------------------------------------ 11231

GGT1end-LOC749026.end.7456450-7520130.chimp      aacaccagtgtactgggtggagcagcttctctattcagtcttctgcactaaagcctgtga 40211
FAM247.LOC105372935.ref.human                    ------------------------------------------------------------ 11231

GGT1end-LOC749026.end.7456450-7520130.chimp      aacaatatcatcttgccttatttactaacaaatacaagtgcctctaaacttagacagttt 40271
FAM247.LOC105372935.ref.human                    ------------------------------------------------------------ 11231

GGT1end-LOC749026.end.7456450-7520130.chimp      ccaagtcatggaactgatgagcacttagctcctgcagagagctctggatgctgggtcggg 40331
FAM247.LOC105372935.ref.human                    ------------------------------------------------------------ 11231

GGT1end-LOC749026.end.7456450-7520130.chimp      agaacaaagacacacaatacatcaaaacagcattaacaagtaaacaggttttcaaagctc 40391
FAM247.LOC105372935.ref.human                    ------------------------------------------------------------ 11231

GGT1end-LOC749026.end.7456450-7520130.chimp      tccacatgcaaatttacacaattatccttttaatttttatcttcatatatatatacataa 40451
FAM247.LOC105372935.ref.human                    ------------------------------------------------------------ 11231

GGT1end-LOC749026.end.7456450-7520130.chimp      tctacttgcttctgagtataaataaaactgtatgttcttagttaatagtctctaccaatt 40511
FAM247.LOC105372935.ref.human                    ------------------------------------------------------------ 11231

GGT1end-LOC749026.end.7456450-7520130.chimp      cattccatttatctctctgagttgaaatactgcatctcattggataacaaaaaaaaaatt 40571
```

```
FAM247.LOC105372935.ref.human                    ------------------------------------------------------------ 11231

GGT1end-LOC749026.end.7456450-7520130.chimp      tgactaagattacaatggaaaggtgagtaggttgggtgattgactgtgattgacaattcc 40631
FAM247.LOC105372935.ref.human                    ------------------------------------------------------------ 11231

GGT1end-LOC749026.end.7456450-7520130.chimp      atgattctggataacttccaaagcataaaaataaatgtgagttttatttcacacgtagac 40691
FAM247.LOC105372935.ref.human                    ------------------------------------------------------------ 11231

GGT1end-LOC749026.end.7456450-7520130.chimp      aatacacatacttattactttaaaaaattaatatgtgaatggaactgacttactacaaat 40751
FAM247.LOC105372935.ref.human                    ------------------------------------------------------------ 11231

GGT1end-LOC749026.end.7456450-7520130.chimp      ttattaaagtaaatacatatttcacaaaagaaagaagagaggaagggaaaaacatgttaa 40811
FAM247.LOC105372935.ref.human                    ------------------------------------------------------------ 11231

GGT1end-LOC749026.end.7456450-7520130.chimp      aaacaaagggaggtacattttattgtgtgaaaagcctccaacccatccctactactgtgg 40871
FAM247.LOC105372935.ref.human                    ------------------------------------------------------------ 11231

GGT1end-LOC749026.end.7456450-7520130.chimp      ctttgttccaaatatttggaaagtaatgatttcataagttattaattaggttaaaaactg 40931
FAM247.LOC105372935.ref.human                    ------------------------------------------------------------ 11231

GGT1end-LOC749026.end.7456450-7520130.chimp      cattacaatagactttgccatattctcccctggagaataacttaatctgtggggtggggg 40991
FAM247.LOC105372935.ref.human                    ------------------------------------------------------------ 11231

GGT1end-LOC749026.end.7456450-7520130.chimp      atggaacgttgaaggatgcaggatgtaaaaggaaattatatatatatattatatatatta 41051
FAM247.LOC105372935.ref.human                    ------------------------------------------------------------ 11231

GGT1end-LOC749026.end.7456450-7520130.chimp      catattatatataatatattacatattatatacattatatataatatattatatattata 41111
FAM247.LOC105372935.ref.human                    ------------------------------------------------------------ 11231

GGT1end-LOC749026.end.7456450-7520130.chimp      tataatgtatgtaatatacacattatatattaaatatataaataatatatattataaatt 41171
FAM247.LOC105372935.ref.human                    ------------------------------------------------------------ 11231

GGT1end-LOC749026.end.7456450-7520130.chimp      ataatatataatatatatgatatataatatatgcagtatatatgatatatattatattgt 41231
FAM247.LOC105372935.ref.human                    ------------------------------------------------------------ 11231

GGT1end-LOC749026.end.7456450-7520130.chimp      atatattattatatacaatataatatataatatataatgtataatatataatatatatta 41291
FAM247.LOC105372935.ref.human                    ------------------------------------------------------------ 11231

GGT1end-LOC749026.end.7456450-7520130.chimp      tatgtatagtatatataatatataatatatacagtatatataatatataatatatataca 41351
FAM247.LOC105372935.ref.human                    ------------------------------------------------------------ 11231

GGT1end-LOC749026.end.7456450-7520130.chimp      gtatatataatatatattatatataatatataatccataatatatatgttatatattaca 41411
FAM247.LOC105372935.ref.human                    ------------------------------------------------------------ 11231

GGT1end-LOC749026.end.7456450-7520130.chimp      tatacagtatatataatatatattacatatacagtatatataatatatattatatataca 41471
FAM247.LOC105372935.ref.human                    ------------------------------------------------------------ 11231

GGT1end-LOC749026.end.7456450-7520130.chimp      gtatatataatatatattatatatacagtatatataatatatattatagatataatatct 41531
FAM247.LOC105372935.ref.human                    ------------------------------------------------------------ 11231

GGT1end-LOC749026.end.7456450-7520130.chimp      ataatttgggaatttgggaataaactgaatcccaattcacactgggactataccagctgc 41591
FAM247.LOC105372935.ref.human                    ------------------------------------------------------------ 11231

GGT1end-LOC749026.end.7456450-7520130.chimp      caccatgcctggctaatttttttgtatttttcatagagacagagtttcactgtattggcca 41651
FAM247.LOC105372935.ref.human                    ------------------------------------------------------------ 11231

GGT1end-LOC749026.end.7456450-7520130.chimp      ggatggtcttgatctcctcaccttgtgattctcttgcattggcctcccaaagtgctggga 41711
FAM247.LOC105372935.ref.human                    ------------------------------------------------------------ 11231

GGT1end-LOC749026.end.7456450-7520130.chimp      ttacaggcctgagccaagatacatatttttttaaatgaagaaaaatttcaaaggtactctg 41771
FAM247.LOC105372935.ref.human                    ------------------------------------------------------------ 11231

GGT1end-LOC749026.end.7456450-7520130.chimp      cttggtacaataatcaaatatataaattgaggaatgaaacataaccatgaaacatattta 41831
FAM247.LOC105372935.ref.human                    ------------------------------------------------------------ 11231

GGT1end-LOC749026.end.7456450-7520130.chimp      taactgcatatggaaaatacagatgataatttttttaaataacatattttgaaagcattaa 41891
FAM247.LOC105372935.ref.human                    ------------------------------------------------------------ 11231

GGT1end-LOC749026.end.7456450-7520130.chimp      ctagtaatttgaaaagatcacatttgacagggcagtatgaacataccttgaatgcagcca 41951
FAM247.LOC105372935.ref.human                    ------------------------------------------------------------ 11231

GGT1end-LOC749026.end.7456450-7520130.chimp      cacaggttcctcataagaaaaatcaaaatcagggaaaatgaaaccacagaggttcaatct 42011
FAM247.LOC105372935.ref.human                    ------------------------------------------------------------ 11231

GGT1end-LOC749026.end.7456450-7520130.chimp      gctctgatctttgaaaaactcagcacagatagtggcacttaggactaagggcaggagatc 42071
FAM247.LOC105372935.ref.human                    ------------------------------------------------------------ 11231

GGT1end-LOC749026.end.7456450-7520130.chimp      cctaatcccatcaccatggcgatagggcataaacattccagggtgaaggcacaatccaca 42131
FAM247.LOC105372935.ref.human                    ------------------------------------------------------------ 11231

GGT1end-LOC749026.end.7456450-7520130.chimp      ctgtgaggtccaattgctgccatgcaggcaggtgtgcttttacatgtacagggaggtcat 42191
FAM247.LOC105372935.ref.human                    ------------------------------------------------------------ 11231

GGT1end-LOC749026.end.7456450-7520130.chimp      tgaaggcttagtgttttgtttcaaaaactgaatcccaagcccacacattattatgctgtg 42251
FAM247.LOC105372935.ref.human                    ------------------------------------------------------------ 11231

GGT1end-LOC749026.end.7456450-7520130.chimp      cttcttaaaacaagttatgagatgggaaataaggcacccccaaatatatatatatataat 42311
FAM247.LOC105372935.ref.human                    ------------------------------------------------------------ 11231

GGT1end-LOC749026.end.7456450-7520130.chimp      tatacataatataatatatataattatatataatataatatatataatttctttttacat 42371
FAM247.LOC105372935.ref.human                    ------------------------------------------------------------ 11231

GGT1end-LOC749026.end.7456450-7520130.chimp      cctgcatccttatatcacatataatatatcatatataatataataatatataattattat 42431
FAM247.LOC105372935.ref.human                    ------------------------------------------------------------ 11231

GGT1end-LOC749026.end.7456450-7520130.chimp      atataatataatcattcttaattattatatataatacaataatatataattattatatat 42491
FAM247.LOC105372935.ref.human                    ------------------------------------------------------------ 11231

GGT1end-LOC749026.end.7456450-7520130.chimp      aatataataatatataattattatatataatacaataatatataattattatatataata 42551
FAM247.LOC105372935.ref.human                    ------------------------------------------------------------ 11231

GGT1end-LOC749026.end.7456450-7520130.chimp      taagaatatataattattatatataatatataattattatatataatataagaatatata 42611
FAM247.LOC105372935.ref.human                    ------------------------------------------------------------ 11231

GGT1end-LOC749026.end.7456450-7520130.chimp      atatatataattattatatataatatataattatatgtataatattatatatcatataat 42671
FAM247.LOC105372935.ref.human                    ------------------------------------------------------------ 11231

GGT1end-LOC749026.end.7456450-7520130.chimp      gatatataatgtatattatatataatatattatataatacataatatatatattacatct 42731
FAM247.LOC105372935.ref.human                    ------------------------------------------------------------ 11231
```

```
GGT1end-LOC749026.end.7456450-7520130.chimp      aatattatgtataatatatcatgtatattatgtataatatatcataacataatatatatt 42791
FAM247.LOC105372935.ref.human                    ------------------------------------------------------------ 11231

GGT1end-LOC749026.end.7456450-7520130.chimp      ataatataatataatatatatatattatatttatataatatcatatataatatataatat 42851
FAM247.LOC105372935.ref.human                    ------------------------------------------------------------ 11231

GGT1end-LOC749026.end.7456450-7520130.chimp      attatatttgtataatatcatatataatatataatatattatatttatataatataatat 42911
FAM247.LOC105372935.ref.human                    ------------------------------------------------------------ 11231

GGT1end-LOC749026.end.7456450-7520130.chimp      ataatatataatatattatatttatataatatcatgtataatatattatatttatataat 42971
FAM247.LOC105372935.ref.human                    ------------------------------------------------------------ 11231

GGT1end-LOC749026.end.7456450-7520130.chimp      atcatatataatgtattatatttatataatatcatatataatatattatatttatataat 43031
FAM247.LOC105372935.ref.human                    ------------------------------------------------------------ 11231

GGT1end-LOC749026.end.7456450-7520130.chimp      atcatatataatatatttatatatcatatataaatatcatatataatatttatataatat 43091
FAM247.LOC105372935.ref.human                    ------------------------------------------------------------ 11231

GGT1end-LOC749026.end.7456450-7520130.chimp      ataatattatattacataataatatcatataatatataatatattatgatatatataata 43151
FAM247.LOC105372935.ref.human                    ------------------------------------------------------------ 11231

GGT1end-LOC749026.end.7456450-7520130.chimp      taatataatataattatatataatataatgtataatatatatcttatatgatatataata 43211
FAM247.LOC105372935.ref.human                    ------------------------------------------------------------ 11231

GGT1end-LOC749026.end.7456450-7520130.chimp      tatatcttatatgatatataatatatatcatatatgatatataatatataatatattata 43271
FAM247.LOC105372935.ref.human                    ------------------------------------------------------------ 11231

GGT1end-LOC749026.end.7456450-7520130.chimp      tattatgtatcataaatatattatacatcatagattatatataaaatatatcatagatta 43331
FAM247.LOC105372935.ref.human                    ------------------------------------------------------------ 11231

GGT1end-LOC749026.end.7456450-7520130.chimp      catataatatatcatagattacatataatatatatcatagattatatataatatatatca 43391
FAM247.LOC105372935.ref.human                    ------------------------------------------------------------ 11231

GGT1end-LOC749026.end.7456450-7520130.chimp      tagattatatatattatatatcatagattgtatatattataaatgatttatattatatat 43451
FAM247.LOC105372935.ref.human                    ------------------------------------------------------------ 11231

GGT1end-LOC749026.end.7456450-7520130.chimp      aatctatgatatataatatataatctatgaaatatattagatcatatataatataatatt 43511
FAM247.LOC105372935.ref.human                    ------------------------------------------------------------ 11231

GGT1end-LOC749026.end.7456450-7520130.chimp      ttatatattatatcatatgtattatattatatattatatattatgtattattatataata 43571
FAM247.LOC105372935.ref.human                    ------------------------------------------------------------ 11231

GGT1end-LOC749026.end.7456450-7520130.chimp      cataatatataattgtataatatatattatatattatatactacattatatgtcatataa 43631
FAM247.LOC105372935.ref.human                    ------------------------------------------------------------ 11231

GGT1end-LOC749026.end.7456450-7520130.chimp      tataatatataataagtcatattatatattatatataatatatattataagtcatattat 43691
FAM247.LOC105372935.ref.human                    ------------------------------------------------------------ 11231

GGT1end-LOC749026.end.7456450-7520130.chimp      atattgtatataatatatattataagtcatattatatattatatacataatataaatata 43751
FAM247.LOC105372935.ref.human                    ------------------------------------------------------------ 11231

GGT1end-LOC749026.end.7456450-7520130.chimp      tataatttatttttagacagagtcttgttctgttgcacaggctggggtgcaatggcacca 43811
FAM247.LOC105372935.ref.human                    ------------------------------------------------------------ 11231

GGT1end-LOC749026.end.7456450-7520130.chimp      tcttggctcactgcaacctctgcctcacgggttcaagcaattgtcctgcctcagcctccc 43871
FAM247.LOC105372935.ref.human                    ------------------------------------------------------------ 11231

GGT1end-LOC749026.end.7456450-7520130.chimp      aggtagctgggactacagcacactgggactacaccagctgccaccatgcctggctaattt 43931
FAM247.LOC105372935.ref.human                    ------------------------------------------------------------ 11231

GGT1end-LOC749026.end.7456450-7520130.chimp      tttgtatttttggtagagacagggtttcactgtattgggcaggatgttcttgatctcctc 43991
FAM247.LOC105372935.ref.human                    ------------------------------------------------------------ 11231

GGT1end-LOC749026.end.7456450-7520130.chimp      accttgtgatcctcttgccttggcctcccaaagtgctgggattacaggcctgagccaaga 44051
FAM247.LOC105372935.ref.human                    ------------------------------------------------------------ 11231

GGT1end-LOC749026.end.7456450-7520130.chimp      tacatatttttttaaatgaagaaaaatttcaaaggtactctgcttggtacagtaatcaaat 44111
FAM247.LOC105372935.ref.human                    ------------------------------------------------------------ 11231

GGT1end-LOC749026.end.7456450-7520130.chimp      atataaattgaggaataaaacataaccatgaaacatatttataactacatatggaaaata 44171
FAM247.LOC105372935.ref.human                    ------------------------------------------------------------ 11231

GGT1end-LOC749026.end.7456450-7520130.chimp      cggaggataatttttttaaataacatattttgaaagcattaactagtaatttgaaaagatc 44231
FAM247.LOC105372935.ref.human                    ------------------------------------------------------------ 11231

GGT1end-LOC749026.end.7456450-7520130.chimp      acatttgacagggcagtatgaacataccttgaatgcagccacacaggttccccatgagaa 44291
FAM247.LOC105372935.ref.human                    ------------------------------------------------------------ 11231

GGT1end-LOC749026.end.7456450-7520130.chimp      taatcaaaatcagggaaaatgaaaccacaaaggttcaatctgctctgacctttgaaaaac 44351
FAM247.LOC105372935.ref.human                    ------------------------------------------------------------ 11231

GGT1end-LOC749026.end.7456450-7520130.chimp      tcagcacagatagtggcacttaggaccaaaggcaggagatccctaatcccatcaccatgg 44411
FAM247.LOC105372935.ref.human                    ------------------------------------------------------------ 11231

GGT1end-LOC749026.end.7456450-7520130.chimp      cgatagggcataaacattccagggtgaaggcacaatccacactgtgaggtccaactgctg 44471
FAM247.LOC105372935.ref.human                    ------------------------------------------------------------ 11231

GGT1end-LOC749026.end.7456450-7520130.chimp      ccatgcagacaggtgtgcttttacatgtacaggaaggtcattgaaggctcagtgttttgt 44531
FAM247.LOC105372935.ref.human                    ------------------------------------------------------------ 11231

GGT1end-LOC749026.end.7456450-7520130.chimp      ttcaaaaactgaatcctaagcccacacattattatgctgtgcttcttaaaataagttatg 44591
FAM247.LOC105372935.ref.human                    ------------------------------------------------------------ 11231

GGT1end-LOC749026.end.7456450-7520130.chimp      agatgggaaatagggcacccccaaatatatatatgtaaaaatatatacaacatataatat 44651
FAM247.LOC105372935.ref.human                    ------------------------------------------------------------ 11231

GGT1end-LOC749026.end.7456450-7520130.chimp      atataacatatatataatttccttttacatcctgcatccttatattatatataattatta 44711
FAM247.LOC105372935.ref.human                    ------------------------------------------------------------ 11231

GGT1end-LOC749026.end.7456450-7520130.chimp      tatataattatgtataataattatatataatacatatgatatatattatataatatatgg 44771
FAM247.LOC105372935.ref.human                    ------------------------------------------------------------ 11231

GGT1end-LOC749026.end.7456450-7520130.chimp      tatatattatataatatatatgatatatatgatatatattatatatgatatatattatat 44831
FAM247.LOC105372935.ref.human                    ------------------------------------------------------------ 11231

GGT1end-LOC749026.end.7456450-7520130.chimp      tatataatatatattatataatatataatataatatatattctatatgatgcatattata 44891
FAM247.LOC105372935.ref.human                    ------------------------------------------------------------ 11231
```

```
GGT1end-LOC749026.end.7456450-7520130.chimp      taatatatgatatatgttatatgtaatatatgatatatattatatgtaatatatgatata 44951
FAM247.LOC105372935.ref.human                    ------------------------------------------------------------ 11231

GGT1end-LOC749026.end.7456450-7520130.chimp      tattatatgtaatatatgatatatattatataatgtatatatgatgtataacatatatta 45011
FAM247.LOC105372935.ref.human                    ------------------------------------------------------------ 11231

GGT1end-LOC749026.end.7456450-7520130.chimp      tatataatatcctataatatataatatgtaagatatattacatatgtcatataatatata 45071
FAM247.LOC105372935.ref.human                    ------------------------------------------------------------ 11231

GGT1end-LOC749026.end.7456450-7520130.chimp      ttttatgtcatataagatatattatatacctaatatatttattatatattatatatgttt 45131
FAM247.LOC105372935.ref.human                    ------------------------------------------------------------ 11231

GGT1end-LOC749026.end.7456450-7520130.chimp      tatattatgtatataatatataatatatataatgtgacatataatatatatgatatataa 45191
FAM247.LOC105372935.ref.human                    ------------------------------------------------------------ 11231

GGT1end-LOC749026.end.7456450-7520130.chimp      ttgtatatcatatattatatattatatattgtataatatattatataatatataatatac 45251
FAM247.LOC105372935.ref.human                    ------------------------------------------------------------ 11231

GGT1end-LOC749026.end.7456450-7520130.chimp      attatatatatataattttttttttttgacagagtcttgttctgttgcacaggctggagt 45311
FAM247.LOC105372935.ref.human                    ------------------------------------------------------------ 11231

GGT1end-LOC749026.end.7456450-7520130.chimp      gcaatggcgccatcttggctcactgcaacctctgtctcacgggttcaagcgattgtcctg 45371
FAM247.LOC105372935.ref.human                    ------------------------------------------------------------ 11231

GGT1end-LOC749026.end.7456450-7520130.chimp      cctcagcctcccaggtacctaggactacaccacactgggactacaccagctgccaccatg 45431
FAM247.LOC105372935.ref.human                    ------------------------------------------------------------ 11231

GGT1end-LOC749026.end.7456450-7520130.chimp      ccaggctaattttctgtatttttggtagagacaggattttactgtattgggcaggatggt 45491
FAM247.LOC105372935.ref.human                    ------------------------------------------------------------ 11231

GGT1end-LOC749026.end.7456450-7520130.chimp      cttgatctcctcaccttgtgatcctcttgccttggcctcccaaagtgctgggattacagg 45551
FAM247.LOC105372935.ref.human                    ------------------------------------------------------------ 11231

GGT1end-LOC749026.end.7456450-7520130.chimp      cctgagccaagatacatgtttttaaatgaagaaaatttcaaaggtactctgcttggta 45611
FAM247.LOC105372935.ref.human                    ------------------------------------------------------------ 11231

GGT1end-LOC749026.end.7456450-7520130.chimp      caataatcaaatatataaattgaggaataaaacataaccatgaaacatatttataactgc 45671
FAM247.LOC105372935.ref.human                    ------------------------------------------------------------ 11231

GGT1end-LOC749026.end.7456450-7520130.chimp      atatggaaaatacggaggataatttttaaataacatattttgaaagcattaactagtaa 45731
FAM247.LOC105372935.ref.human                    ------------------------------------------------------------ 11231

GGT1end-LOC749026.end.7456450-7520130.chimp      tttgaaaagatcgcatttgacaggccagtatgaacataccttgaatgcagccacacaggt 45791
FAM247.LOC105372935.ref.human                    ------------------------------------------------------------ 11231

GGT1end-LOC749026.end.7456450-7520130.chimp      tccccataagaaaaatcaaaatcagggaaaatgaaaccacagaggttcaatctgctctga 45851
FAM247.LOC105372935.ref.human                    ------------------------------------------------------------ 11231

GGT1end-LOC749026.end.7456450-7520130.chimp      cctttgaaaaactcagcacagacagtggcacttaggaccaaaggcaggagatccctaatc 45911
FAM247.LOC105372935.ref.human                    ------------------------------------------------------------ 11231

GGT1end-LOC749026.end.7456450-7520130.chimp      ccatcaccatggcgatagggcataaacattccagggtgaaggcacaatccacactgtgag 45971
FAM247.LOC105372935.ref.human                    ------------------------------------------------------------ 11231

GGT1end-LOC749026.end.7456450-7520130.chimp      gtccaactgctgccatgcagacaggtgtgcttttacatgtacaggaaggtcattgaaggc 46031
FAM247.LOC105372935.ref.human                    ------------------------------------------------------------ 11231

GGT1end-LOC749026.end.7456450-7520130.chimp      tcagtgttttgtttcaaaaactgaatcccaagcccacatattattatgctttgcttctta 46091
FAM247.LOC105372935.ref.human                    ------------------------------------------------------------ 11231

GGT1end-LOC749026.end.7456450-7520130.chimp      aaataagttatgagatgggaaattagggcacccccaaatatatatatataatacataata 46151
FAM247.LOC105372935.ref.human                    ------------------------------------------------------------ 11231

GGT1end-LOC749026.end.7456450-7520130.chimp      taatatataatatatataacatatataatttccttttacatcctgcatccttatattata 46211
FAM247.LOC105372935.ref.human                    ------------------------------------------------------------ 11231

GGT1end-LOC749026.end.7456450-7520130.chimp      tataatattatatataattatatataatattatatataattatatataatattatatata 46271
FAM247.LOC105372935.ref.human                    ------------------------------------------------------------ 11231

GGT1end-LOC749026.end.7456450-7520130.chimp      atataatatataattatatataatgattatataatataatatatataattatatataata 46331
FAM247.LOC105372935.ref.human                    ------------------------------------------------------------ 11231

GGT1end-LOC749026.end.7456450-7520130.chimp      taatatataattatatatattatataattatgtatattattatatgtgtaatatagtaat 46391
FAM247.LOC105372935.ref.human                    ------------------------------------------------------------ 11231

GGT1end-LOC749026.end.7456450-7520130.chimp      atataattatatatgtaatatataatatataatatatttatattattatatataatgtat 46451
FAM247.LOC105372935.ref.human                    ------------------------------------------------------------ 11231

GGT1end-LOC749026.end.7456450-7520130.chimp      tatataatatatatgatataatatattatatataatatattatatcatatatattatata 46511
FAM247.LOC105372935.ref.human                    ------------------------------------------------------------ 11231

GGT1end-LOC749026.end.7456450-7520130.chimp      taatataatattatatatgtatattatataatatatattatacataatatatcataatat 46571
FAM247.LOC105372935.ref.human                    ------------------------------------------------------------ 11231

GGT1end-LOC749026.end.7456450-7520130.chimp      attacatatcataatataatatataagatatattatatataatatataaaattatataat 46631
FAM247.LOC105372935.ref.human                    ------------------------------------------------------------ 11231

GGT1end-LOC749026.end.7456450-7520130.chimp      atatgatatattatatataatataatataattatatataatataatataattatatataa 46691
FAM247.LOC105372935.ref.human                    ------------------------------------------------------------ 11231

GGT1end-LOC749026.end.7456450-7520130.chimp      tataatatacataatatatatcatatattatatataatatatatcatttattatatataa 46751
FAM247.LOC105372935.ref.human                    ------------------------------------------------------------ 11231

GGT1end-LOC749026.end.7456450-7520130.chimp      tatatatcatttattatattatatatcatatataatatatatcatttattatattatata 46811
FAM247.LOC105372935.ref.human                    ------------------------------------------------------------ 11231

GGT1end-LOC749026.end.7456450-7520130.chimp      tcatatgtaatatatatcatatatagtatatatcatatatatcatatatataatcgtata 46871
FAM247.LOC105372935.ref.human                    ------------------------------------------------------------ 11231

GGT1end-LOC749026.end.7456450-7520130.chimp      tcatatatattatatatactacatgtcatatattaagtttggggatagcccagaattata 46931
FAM247.LOC105372935.ref.human                    ------------------------------------------------------------ 11231

GGT1end-LOC749026.end.7456450-7520130.chimp      tatggattttcaactgcacaggagctggtcccctaacccttgcactgttcaaggatcaac 46991
FAM247.LOC105372935.ref.human                    ------------------------------------------------------------ 11231

GGT1end-LOC749026.end.7456450-7520130.chimp      agtatttaaaatactatacattttattcatattttagagtgtaatacttcttctcattaa 47051
FAM247.LOC105372935.ref.human                    ------------------------------------------------------------ 11231
```

```
GGT1end-LOC749026.end.7456450-7520130.chimp      aaaaaattaactataaaacagcctcaggaaggtctttcaggagttcttccagaagaaagc 47111
FAM247.LOC105372935.ref.human                    ------------------------------------------------------------ 11231

GGT1end-LOC749026.end.7456450-7520130.chimp      cttgttatcatagaaaatgacagctgcatgtatgttattgcctctgaagaccctgaagac 47171
FAM247.LOC105372935.ref.human                    ------------------------------------------------------------ 11231

GGT1end-LOC749026.end.7456450-7520130.chimp      ctttcactgggacaagatttggagatggaagacagtgatattgattatcctgacccttac 47231
FAM247.LOC105372935.ref.human                    ------------------------------------------------------------ 11231

GGT1end-LOC749026.end.7456450-7520130.chimp      gaggctaagctagtgtgtgtgtttctatcttagttttaacaaaattttaaaacataaa 47291
FAM247.LOC105372935.ref.human                    ------------------------------------------------------------ 11231

GGT1end-LOC749026.end.7456450-7520130.chimp      aaataaaaataaataaagcttatcaaataaggatataaagtatttctgtacagctgttca 47351
FAM247.LOC105372935.ref.human                    ------------------------------------------------------------ 11231

GGT1end-LOC749026.end.7456450-7520130.chimp      atgtgtttttgttttaagatgtgttattatgaaagaatcaaaaaattaaaaaaattaaaa 47411
FAM247.LOC105372935.ref.human                    ------------------------------------------------------------ 11231

GGT1end-LOC749026.end.7456450-7520130.chimp      gtttataaagttatgataagctaagatgaacttgttaaagaaagaaaaaaattaaataca 47471
FAM247.LOC105372935.ref.human                    ------------------------------------------------------------ 11231

GGT1end-LOC749026.end.7456450-7520130.chimp      tttagtgtagcctaagtgtacagtgcatataaagtctatagttatgtacagtaatatcct 47531
FAM247.LOC105372935.ref.human                    ------------------------------------------------------------ 11231

GGT1end-LOC749026.end.7456450-7520130.chimp      aggccttttcattcactcaccaagtactcactgactcatcagagcaactgccagtcctgc 47591
FAM247.LOC105372935.ref.human                    ------------------------------------------------------------ 11231

GGT1end-LOC749026.end.7456450-7520130.chimp      aacctctgttcatgctaagtgtcctatactggtgtaccactctcttaacttttgtaatat 47651
FAM247.LOC105372935.ref.human                    ------------------------------------------------------------ 11231

GGT1end-LOC749026.end.7456450-7520130.chimp      atatatatatttttttctttcttttttggagaaggagttttattttgttgcccaggctg 47711
FAM247.LOC105372935.ref.human                    ------------------------------------------------------------ 11231

GGT1end-LOC749026.end.7456450-7520130.chimp      gagtgcaatggcacgatcttggctcactgcaacctctgcctccggggttcaagtgattct 47771
FAM247.LOC105372935.ref.human                    ------------------------------------------------------------ 11231

GGT1end-LOC749026.end.7456450-7520130.chimp      cctgcctcagcctcacgagtagctgggattacaggcccacgccagcactcccagctaatt 47831
FAM247.LOC105372935.ref.human                    ------------------------------------------------------------ 11231

GGT1end-LOC749026.end.7456450-7520130.chimp      ttgtatttttagtacagacggagtttctccatgttggtcaggctggtctcgaacttctga 47891
FAM247.LOC105372935.ref.human                    ------------------------------------------------------------ 11231

GGT1end-LOC749026.end.7456450-7520130.chimp      cctcaggtgatccgcccgcctcggtctcccaatgtactatgtttttactgtatcttttcc 47951
FAM247.LOC105372935.ref.human                    ------------------------------------------------------------ 11231

GGT1end-LOC749026.end.7456450-7520130.chimp      atgtttagatattactattgtgatataactgcctacagcagcggtccccaaagttttgac 48011
FAM247.LOC105372935.ref.human                    ------------------------------------------------------------ 11231

GGT1end-LOC749026.end.7456450-7520130.chimp      accagggactggttttgtgaaagataatttttccacagattggggatggctttgggctga 48071
FAM247.LOC105372935.ref.human                    ------------------------------------------------------------ 11231

GGT1end-LOC749026.end.7456450-7520130.chimp      aactgttccacctcagatcatcaggcattagttagattctttttttctttttttttaga 48131
FAM247.LOC105372935.ref.human                    ------------------------------------------------------------ 11231

GGT1end-LOC749026.end.7456450-7520130.chimp      ctgagtctcccactgttgccaggctggagtgcagtggcacaatctcggctcactgcaacc 48191
FAM247.LOC105372935.ref.human                    ------------------------------------------------------------ 11231

GGT1end-LOC749026.end.7456450-7520130.chimp      tctgcctcccgggttcaagtgattctcctgtctcagtcccccgagtagctgggactacag 48251
FAM247.LOC105372935.ref.human                    ------------------------------------------------------------ 11231

GGT1end-LOC749026.end.7456450-7520130.chimp      gcatgcgccaccacacccagctaattttgtatttttagtagagacggggtttccatgtt 48311
FAM247.LOC105372935.ref.human                    ------------------------------------------------------------ 11231

GGT1end-LOC749026.end.7456450-7520130.chimp      ggccaggattgtcttgaccttgtgatccacctgcctcggcctcccagagtgttgggatta 48371
FAM247.LOC105372935.ref.human                    ------------------------------------------------------------ 11231

GGT1end-LOC749026.end.7456450-7520130.chimp      caggcgtgagccacagcacccagcctagttagattctcataaggagcacataacctagat 48431
FAM247.LOC105372935.ref.human                    ------------------------------------------------------------ 11231

GGT1end-LOC749026.end.7456450-7520130.chimp      cccttgtatgtgcagttcacaataggatttgcactcctgtgaagatctaatgctgctgct 48491
FAM247.LOC105372935.ref.human                    ------------------------------------------------------------ 11231

GGT1end-LOC749026.end.7456450-7520130.chimp      gatctgacaggaggcggagctcaggcagtaatgctcggctggccagccactcatctcctg 48551
FAM247.LOC105372935.ref.human                    ------------------------------------------------------------ 11231

GGT1end-LOC749026.end.7456450-7520130.chimp      ctttgtgcccagttcctaaaaggacacgaaccggtgctggtccatggcttgggtgttggg 48611
FAM247.LOC105372935.ref.human                    ------------------------------------------------------------ 11231

GGT1end-LOC749026.end.7456450-7520130.chimp      gaccactggcctactgtattcagcacagtgacatgctgcacaggtgtgtagtccaggagc 48671
FAM247.LOC105372935.ref.human                    ------------------------------------------------------------ 11231

GGT1end-LOC749026.end.7456450-7520130.chimp      aataggctgtacgatatagcctaggtatgtagtaggctacactgtctaggtttgtataaa 48731
FAM247.LOC105372935.ref.human                    ------------------------------------------------------------ 11231

GGT1end-LOC749026.end.7456450-7520130.chimp      gtacactctttggtgtctgcacagccacaaaatcacctaatgatgcatttcttggaatat 48791
FAM247.LOC105372935.ref.human                    ------------------------------------------------------------ 11231

GGT1end-LOC749026.end.7456450-7520130.chimp      attaccattaaatgagatttacctgtattagtgtcatctcaggtttattgtctagtaatt 48851
FAM247.LOC105372935.ref.human                    ------------------------------------------------------------ 11231

GGT1end-LOC749026.end.7456450-7520130.chimp      ttaaaagtatttgtatattctttcccagacacatcatcatactctggtgcatatctctat 48911
FAM247.LOC105372935.ref.human                    ------------------------------------------------------------ 11231

GGT1end-LOC749026.end.7456450-7520130.chimp      tttattccctagacccactcttaatttttctgcattctgctttgttccttaggaggctca 48971
FAM247.LOC105372935.ref.human                    ------------------------------------------------------------ 11231

GGT1end-LOC749026.end.7456450-7520130.chimp      cctgaattggatacttcagagatctcccttaccctcatgcttttattggggttcagctaa 49031
FAM247.LOC105372935.ref.human                    ------------------------------------------------------------ 11231

GGT1end-LOC749026.end.7456450-7520130.chimp      tggagggggtggcagaagataggtgagagaagagtgtgtttatccctcacctctgcccct 49091
FAM247.LOC105372935.ref.human                    ------------------------------------------------------------ 11231

GGT1end-LOC749026.end.7456450-7520130.chimp      gcagggtcagcacagtcagactgtgtccctttgctgaagatcacagcttctgtctggtgc 49151
FAM247.LOC105372935.ref.human                    ------------------------------------------------------------ 11231

GGT1end-LOC749026.end.7456450-7520130.chimp      cttcttggcaagctggcactgtctctagtcaggtgactgcttggccccttactacttaca 49211
FAM247.LOC105372935.ref.human                    ------------------------------------------------------------ 11231

GGT1end-LOC749026.end.7456450-7520130.chimp      ctcttctgtctggtttctttgtgtctttcctgctgtgctgtaagtagtccctttattaga 49271
```

```
FAM247.LOC105372935.ref.human                   ------------------------------------------------------------ 11231

GGT1end-LOC749026.end.7456450-7520130.chimp     atccatccacattacccagttatataaagacaaatagttggtcactgatctagttcactc 49331
FAM247.LOC105372935.ref.human                   ------------------------------------------------------------ 11231

GGT1end-LOC749026.end.7456450-7520130.chimp     cctgagaatcagccttcatcaaagatccctaacagtcagatattctgttggccgctccat 49391
FAM247.LOC105372935.ref.human                   ------------------------------------------------------------ 11231

GGT1end-LOC749026.end.7456450-7520130.chimp     tcctgctccccattattctagttgtgtcttccttgtctctagtgcagtcacaaagcataa 49451
FAM247.LOC105372935.ref.human                   ------------------------------------------------------------ 11231

GGT1end-LOC749026.end.7456450-7520130.chimp     gcgcctccactgtcacttgttctttgcaactctaggatcacatcattccccttgaaatca 49511
FAM247.LOC105372935.ref.human                   ------------------------------------------------------------ 11231

GGT1end-LOC749026.end.7456450-7520130.chimp     gagaacctgcaatctccccaagcccacaaaattcagccgtcacagtacttttcaagcatg 49571
FAM247.LOC105372935.ref.human                   ------------------------------------------------------------ 11231

GGT1end-LOC749026.end.7456450-7520130.chimp     ccagcagccctccccaacaaacgaatccttaatttcttggagaaacatcttctggggctt 49631
FAM247.LOC105372935.ref.human                   ------------------------------------------------------------ 11231

GGT1end-LOC749026.end.7456450-7520130.chimp     cctgtgggacacagtgggggtcggcttggcatgtggatcacacctgatattttacccccc 49691
FAM247.LOC105372935.ref.human                   ------------------------------------------------------------ 11231

GGT1end-LOC749026.end.7456450-7520130.chimp     acctcctactattcctattcctttgtatgcacctctctttattatgctaggaaagctctg 49751
FAM247.LOC105372935.ref.human                   ------------------------------------------------------------ 11231

GGT1end-LOC749026.end.7456450-7520130.chimp     gcaacacaacctcaaaataagcaggccagtgtggattcacagtttctgtcagacaaacaa 49811
FAM247.LOC105372935.ref.human                   ------------------------------------------------------------ 11231

GGT1end-LOC749026.end.7456450-7520130.chimp     acttgagcaccccttctaacttcttgggctaagactttaacttcagaatctctatgtatc 49871
FAM247.LOC105372935.ref.human                   ------------------------------------------------------------ 11231

GGT1end-LOC749026.end.7456450-7520130.chimp     tatgttgataaacttagcctatgtcaagattatattctgcatgccttagtcaaacaccat 49931
FAM247.LOC105372935.ref.human                   ------------------------------------------------------------ 11231

GGT1end-LOC749026.end.7456450-7520130.chimp     gtaaatctatgcctcatatattctctaggctttagctgatagggattagcaaagtagtgt 49991
FAM247.LOC105372935.ref.human                   ------------------------------------------------------------ 11231

GGT1end-LOC749026.end.7456450-7520130.chimp     ggttgttttggtctttaaacaatgtccatttctaccagctttccattgcactctttttt 50051
FAM247.LOC105372935.ref.human                   ------------------------------------------------------------ 11231

GGT1end-LOC749026.end.7456450-7520130.chimp     tttttttttttttgagatggagtcttgctctgtcgcccaggctggagtgcagtggcccg 50111
FAM247.LOC105372935.ref.human                   ------------------------------------------------------------ 11231

GGT1end-LOC749026.end.7456450-7520130.chimp     atctcggctcactgcaagctctgcctcccgggttcacgccattctcctgcctcagcctcc 50171
FAM247.LOC105372935.ref.human                   ------------------------------------------------------------ 11231

GGT1end-LOC749026.end.7456450-7520130.chimp     cgagtagctgggactacaggcgtgtgccaccacacacggctaatttttgtatttttagt 50231
FAM247.LOC105372935.ref.human                   ------------------------------------------------------------ 11231

GGT1end-LOC749026.end.7456450-7520130.chimp     agagatggggtctcactgtgttagccaggatggtctcgatctcctgacctcgtgatcggc 50291
FAM247.LOC105372935.ref.human                   ------------------------------------------------------------ 11231

GGT1end-LOC749026.end.7456450-7520130.chimp     ccaccttggcctcccaaagtattgggattacaggcgtgagccaccgcaccgggccgactc 50351
FAM247.LOC105372935.ref.human                   ------------------------------------------------------------ 11231

GGT1end-LOC749026.end.7456450-7520130.chimp     cattgcactcttacagaccatgttgacaaaattataactgcaacagtcaactacaatagc 50411
FAM247.LOC105372935.ref.human                   ------------------------------------------------------------ 11231

GGT1end-LOC749026.end.7456450-7520130.chimp     aattttacctctcatgtaccactttaaaaaacacatcattcccaataaccacatttgatg 50471
FAM247.LOC105372935.ref.human                   ------------------------------------------------------------ 11231

GGT1end-LOC749026.end.7456450-7520130.chimp     atttaagtaatcatgataccaccgtacactacagatgatcaggtttctgtctcctcccga 50531
FAM247.LOC105372935.ref.human                   ------------------------------------------------------------ 11231

GGT1end-LOC749026.end.7456450-7520130.chimp     caagaaggtcaggaattcattcagactgaaacaggtcagcaaaccacttccagattctca 50591
FAM247.LOC105372935.ref.human                   ------------------------------------------------------------ 11231

GGT1end-LOC749026.end.7456450-7520130.chimp     tctataaagatctatttctagaaccatttctagtacctatacaatattagaatcaagtca 50651
FAM247.LOC105372935.ref.human                   ------------------------------------------------------------ 11231

GGT1end-LOC749026.end.7456450-7520130.chimp     ggaagcagaaaacattctagatattttaaacagaaaacaattcatggagagatcaattac 50711
FAM247.LOC105372935.ref.human                   ------------------------------------------------------------ 11231

GGT1end-LOC749026.end.7456450-7520130.chimp     aagagtatgtgtagcaaaagatgaaagggtcaggttgcttaagtaacttgataaatctct 50771
FAM247.LOC105372935.ref.human                   ------------------------------------------------------------ 11231

GGT1end-LOC749026.end.7456450-7520130.chimp     gcttcttttgtgttgggtgataaaaatgttgagcagagatcagtggcagatgatccaagg 50831
FAM247.LOC105372935.ref.human                   ------------------------------------------------------------ 11231

GGT1end-LOC749026.end.7456450-7520130.chimp     cgtcagctgtccaagctcagcatctggagccggtgctgaggaaatgtgcatttctaggtc 50891
FAM247.LOC105372935.ref.human                   ------------------------------------------------------------ 11231

GGT1end-LOC749026.end.7456450-7520130.chimp     tgggcgccattggaccatcactactgtccttgaagctgccaccgtgagaactcacatctc 50951
FAM247.LOC105372935.ref.human                   ------------------------------------------------------------ 11231

GGT1end-LOC749026.end.7456450-7520130.chimp     agctgctatagccacagctgatattgtcattgcttccagaattatttcccttgctccatc 51011
FAM247.LOC105372935.ref.human                   ------------------------------------------------------------ 11231

GGT1end-LOC749026.end.7456450-7520130.chimp     gtggggatcccgcaatactgctgctgctgaaatcactgttgttgctccttccagaactgt 51071
FAM247.LOC105372935.ref.human                   ------------------------------------------------------------ 11231

GGT1end-LOC749026.end.7456450-7520130.chimp     cggcactgcagcattattatcactgcagctaccactggagatggctacagacaccagaaa 51131
FAM247.LOC105372935.ref.human                   ------------------------------------------------------------ 11231

GGT1end-LOC749026.end.7456450-7520130.chimp     cagaagagcacctctcccctcctccggcagtgtgactttcagtcggctcctctcgtagcc 51191
FAM247.LOC105372935.ref.human                   ------------------------------------------------------------ 11231

GGT1end-LOC749026.end.7456450-7520130.chimp     agatggtatagaaatgacacaccaatgtccttcagaacatacatgcaaaatattttaaat 51251
FAM247.LOC105372935.ref.human                   ------------------------------------------------------------ 11231

GGT1end-LOC749026.end.7456450-7520130.chimp     atattagaaattctattccccttagaaaactaagttcacctacatatttttcaaaaatcc 51311
FAM247.LOC105372935.ref.human                   ------------------------------------------------------------ 11231

GGT1end-LOC749026.end.7456450-7520130.chimp     atcagcaatacagaataaaggcattggttctatacagcgttgcaagaatattttaacaat 51371
FAM247.LOC105372935.ref.human                   ------------------------------------------------------------ 11231

GGT1end-LOC749026.end.7456450-7520130.chimp     agaaggtattttaatgtcattcaccacattaggagattgaaaaagggaaaacacaggatt 51431
FAM247.LOC105372935.ref.human                   ------------------------------------------------------------ 11231
```

```
GGT1end-LOC749026.end.7456450-7520130.chimp      accttagcagatgtaggaaaaaaataagatggtaaaatacaacattcatttatattaaaa 51491
FAM247.LOC105372935.ref.human                    ------------------------------------------------------------ 11231

GGT1end-LOC749026.end.7456450-7520130.chimp      tctttataccaaactaggaaaagcagacaaatttcttaacctgataagcttatctataac 51551
FAM247.LOC105372935.ref.human                    ------------------------------------------------------------ 11231

GGT1end-LOC749026.end.7456450-7520130.chimp      aaaaccatcgacaccaccatatgtaatgttgaagcataatcgttatttttataaaagct 51611
FAM247.LOC105372935.ref.human                    ------------------------------------------------------------ 11231

GGT1end-LOC749026.end.7456450-7520130.chimp      aggaaagggaatgagatggctgttgacactgctcgtatttatcattgtcctggggtttct 51671
FAM247.LOC105372935.ref.human                    ------------------------------------------------------------ 11231

GGT1end-LOC749026.end.7456450-7520130.chimp      ggccagttcagttaagataggacaaagaaatattatagaaagaggaagaaaaaaagaaaa 51731
FAM247.LOC105372935.ref.human                    ------------------------------------------------------------ 11231

GGT1end-LOC749026.end.7456450-7520130.chimp      cacatacgttgtcattgtgtggagtttctgtttgtttgtttatgtgtttttgtgagatgg 51791
FAM247.LOC105372935.ref.human                    ------------------------------------------------------------ 11231

GGT1end-LOC749026.end.7456450-7520130.chimp      tgtctcactctgtcgcccaggctggagtacagtggtgtgatcttggctcactgcaacctc 51851
FAM247.LOC105372935.ref.human                    ------------------------------------------------------------ 11231

GGT1end-LOC749026.end.7456450-7520130.chimp      attgtgtggagatttgtaattacccatagaaaatccaagaaaatttgtggatgcactaat 51911
FAM247.LOC105372935.ref.human                    ------------------------------------------------------------ 11231

GGT1end-LOC749026.end.7456450-7520130.chimp      caaatcagcaaggttttcagatatgaaggcaaagtaaacagaaaatgaaattcttataaa 51971
FAM247.LOC105372935.ref.human                    ------------------------------------------------------------ 11231

GGT1end-LOC749026.end.7456450-7520130.chimp      cagtagcaaccaattaaaaatattatattataatagcacattcatggtaacataaaacat 52031
FAM247.LOC105372935.ref.human                    ------------------------------------------------------------ 11231

GGT1end-LOC749026.end.7456450-7520130.chimp      taatgtatctaagaattaattcagaaaacaaatacatgatctttcatttaattttattaa 52091
FAM247.LOC105372935.ref.human                    ------------------------------------------------------------ 11231

GGT1end-LOC749026.end.7456450-7520130.chimp      agttatttcaataaatataccaccagtatttttttagaacttgattggaaaagtcatatt 52151
FAM247.LOC105372935.ref.human                    ------------------------------------------------------------ 11231

GGT1end-LOC749026.end.7456450-7520130.chimp      aaagtaatcaatggaaaggaagaaccaaagtgattttgaacaataatagcctatcaatat 52211
FAM247.LOC105372935.ref.human                    ------------------------------------------------------------ 11231

GGT1end-LOC749026.end.7456450-7520130.chimp      ttgaatgtgttttaattataataattaaaatggtttgctttggctcagtgatacaccgtt 52271
FAM247.LOC105372935.ref.human                    ------------------------------------------------------------ 11231

GGT1end-LOC749026.end.7456450-7520130.chimp      aaacaatgggaaaaaatggaagcaccaagaaatagatccataggtatgctgaatacattg 52331
FAM247.LOC105372935.ref.human                    ------------------------------------------------------------ 11231

GGT1end-LOC749026.end.7456450-7520130.chimp      gacttgtaataaaagatatgatactaaaagtcagtagaaaaattatactatgcaataaat 52391
FAM247.LOC105372935.ref.human                    ------------------------------------------------------------ 11231

GGT1end-LOC749026.end.7456450-7520130.chimp      gatgtcgaaaaaacatataaatttttatccatatataaataaaaatctgtatattacagc 52451
FAM247.LOC105372935.ref.human                    ------------------------------------------------------------ 11231

GGT1end-LOC749026.end.7456450-7520130.chimp      atatcacaaaatcctgtatctatttaggttaaagactcaaatcttaaactacttgtgaaa 52511
FAM247.LOC105372935.ref.human                    ------------------------------------------------------------ 11231

GGT1end-LOC749026.end.7456450-7520130.chimp      ttacttatgaatattacatgtttaagatatttaaaagtatatacagagaactaatgacgt 52571
FAM247.LOC105372935.ref.human                    ------------------------------------------------------------ 11231

GGT1end-LOC749026.end.7456450-7520130.chimp      tgggcaacaaatggcttattaatcaaatgccagaatgcatgaagaagaaaagaaaagaaa 52631
FAM247.LOC105372935.ref.human                    ------------------------------------------------------------ 11231

GGT1end-LOC749026.end.7456450-7520130.chimp      aataaattagactgtattaaaatgaaaaatcctaggcatcctaaataaaggttaaatgca 52691
FAM247.LOC105372935.ref.human                    ------------------------------------------------------------ 11231

GGT1end-LOC749026.end.7456450-7520130.chimp      agccagaaaattgggtaaaatatttacaaaatatataattgaaaagtataatatccatta 52751
FAM247.LOC105372935.ref.human                    ------------------------------------------------------------ 11231

GGT1end-LOC749026.end.7456450-7520130.chimp      tataaagaggatttctacaaattaatgatataaaataaatagtttagtagaaaaaatgat 52811
FAM247.LOC105372935.ref.human                    ------------------------------------------------------------ 11231

GGT1end-LOC749026.end.7456450-7520130.chimp      taagaactactccacaatattcacaacagatgataaatagtgaattattgtgaaaagaca 52871
FAM247.LOC105372935.ref.human                    ------------------------------------------------------------ 11231

GGT1end-LOC749026.end.7456450-7520130.chimp      attgaaaagagcaaggtacaattcaacagcaataagactttatgcccttgtctataaaaa 52931
FAM247.LOC105372935.ref.human                    ------------------------------------------------------------ 11231

GGT1end-LOC749026.end.7456450-7520130.chimp      cacaaaactatgccaagggctttaggaaccgtgaatttatatacggcgaaggggagttta 52991
FAM247.LOC105372935.ref.human                    ------------------------------------------------------------ 11231

GGT1end-LOC749026.end.7456450-7520130.chimp      aatctgccactttgaagagtttttggaaatatatagtaaagttgaaaatatatacatat 53051
FAM247.LOC105372935.ref.human                    ------------------------------------------------------------ 11231

GGT1end-LOC749026.end.7456450-7520130.chimp      gtgtatatatatatacccacacacatacacatacacactagaaattccacaaggtaaacc 53111
FAM247.LOC105372935.ref.human                    ------------------------------------------------------------ 11231

GGT1end-LOC749026.end.7456450-7520130.chimp      ttctccaagagaatacacacacatttgcagaagagatgtccaaaggtgtttgttgaaaca 53171
FAM247.LOC105372935.ref.human                    ------------------------------------------------------------ 11231

GGT1end-LOC749026.end.7456450-7520130.chimp      ttattggtaatagctaaaaattggaaataatcttaaagctcattactaggaaaatggaaa 53231
FAM247.LOC105372935.ref.human                    ------------------------------------------------------------ 11231

GGT1end-LOC749026.end.7456450-7520130.chimp      agtgaatcgtggtatgtgcatataatgtaaaactatacatcagttaaaattaattgtctg 53291
FAM247.LOC105372935.ref.human                    ------------------------------------------------------------ 11231

GGT1end-LOC749026.end.7456450-7520130.chimp      aatttatatggatcaatttttatatattgaaacatcgttgcattccaggaataaatccca 53351
FAM247.LOC105372935.ref.human                    ------------------------------------------------------------ 11231

GGT1end-LOC749026.end.7456450-7520130.chimp      cttggtcatggtgtataatccttttaatatgatgctgaattcagtatggtagttttttt 53411
FAM247.LOC105372935.ref.human                    ------------------------------------------------------------ 11231

GGT1end-LOC749026.end.7456450-7520130.chimp      tttagacaaagttttgctcttgttgcctaggctgaagtgcaatggtatgatcttggctca 53471
FAM247.LOC105372935.ref.human                    ------------------------------------------------------------ 11231

GGT1end-LOC749026.end.7456450-7520130.chimp      ccacaacctccacctcccgggttcaagcgattctcttgtctcagcctcccgagtagctga 53531
FAM247.LOC105372935.ref.human                    ------------------------------------------------------------ 11231

GGT1end-LOC749026.end.7456450-7520130.chimp      ggttacaggcatgcaccaccacacatggctaatttttgtatttttagtagagacagagttt 53591
FAM247.LOC105372935.ref.human                    ------------------------------------------------------------ 11231
```

```
GGT1end-LOC749026.end.7456450-7520130.chimp      ctccatgttggtcaggctggtcttgaactcctgacctcaggtgatccacccacctcagcc 53651
FAM247.LOC105372935.ref.human                    ------------------------------------------------------------ 11231

GGT1end-LOC749026.end.7456450-7520130.chimp      tcccaaagtgctggaattacaggcatgagccaccatgccccacccagtatgctagtattt 53711
FAM247.LOC105372935.ref.human                    ------------------------------------------------------------ 11231

GGT1end-LOC749026.end.7456450-7520130.chimp      ttagagtttttgcatcaatgatcagaggcatattgatctgttgttttattttcttatagt 53771
FAM247.LOC105372935.ref.human                    ------------------------------------------------------------ 11231

GGT1end-LOC749026.end.7456450-7520130.chimp      gtttttggctttggtatcagggtaatgctggcctcatagaaagagttaagaggtattcac 53831
FAM247.LOC105372935.ref.human                    ------------------------------------------------------------ 11231

GGT1end-LOC749026.end.7456450-7520130.chimp      tcctcttccactctttggaaatgtttgaaaagaatcagtgttagttcttctttaaatgtt 53891
FAM247.LOC105372935.ref.human                    ------------------------------------------------------------ 11231

GGT1end-LOC749026.end.7456450-7520130.chimp      tggcagaactcaccagtaaagctatcaggtccaggacttttctttgtcatgagattgtct 53951
FAM247.LOC105372935.ref.human                    ------------------------------------------------------------ 11231

GGT1end-LOC749026.end.7456450-7520130.chimp      tattactgactcaattcccttactaggttaggtctattctgactttctgtttctttataa 54011
FAM247.LOC105372935.ref.human                    ------------------------------------------------------------ 11231

GGT1end-LOC749026.end.7456450-7520130.chimp      ttgagtcttggtaggttttgtgtgtctaggaattcatctatttcatctgggttatccaat 54071
FAM247.LOC105372935.ref.human                    ------------------------------------------------------------ 11231

GGT1end-LOC749026.end.7456450-7520130.chimp      ttatgagcacacaattatttatagtaatcttttataatcctttctaggctgggaatggtg 54131
FAM247.LOC105372935.ref.human                    ------------------------------------------------------------ 11231

GGT1end-LOC749026.end.7456450-7520130.chimp      ggtcatgcctgtaattccaataatttgggaggcggaggtgggaaaattccttgaggccag 54191
FAM247.LOC105372935.ref.human                    ------------------------------------------------------------ 11231

GGT1end-LOC749026.end.7456450-7520130.chimp      gagttcaagatcagcctggataacatggcaagatcctgtctcttaaaaaaaaaaaaaaag 54251
FAM247.LOC105372935.ref.human                    ------------------------------------------------------------ 11231

GGT1end-LOC749026.end.7456450-7520130.chimp      agctggcatagtggcgagcccaggaattcaaggctgcagtgagccgattgtgccactgat 54311
FAM247.LOC105372935.ref.human                    ------------------------------------------------------------ 11231

GGT1end-LOC749026.end.7456450-7520130.chimp      tgtggttgcatctgattgttgccagggcaatggagctagaccttgtctctaaagtataga 54371
FAM247.LOC105372935.ref.human                    ------------------------------------------------------------ 11231

GGT1end-LOC749026.end.7456450-7520130.chimp      cagacacttgtctctaaaacatagacaggcagatagacagacagacagacagacagaaat 54431
FAM247.LOC105372935.ref.human                    ------------------------------------------------------------ 11231

GGT1end-LOC749026.end.7456450-7520130.chimp      atataaagatattctcaaatacatttctgttttaagtaatttagattgtttccctttttcc 54491
FAM247.LOC105372935.ref.human                    ------------------------------------------------------------ 11231

GGT1end-LOC749026.end.7456450-7520130.chimp      ttagtcttgtcagttttattaatctttttgaagaaccaacttttggttttgtttttttatt 54551
FAM247.LOC105372935.ref.human                    ------------------------------------------------------------ 11231

GGT1end-LOC749026.end.7456450-7520130.chimp      gtttttctattctcttttccatttatctctgccctgatctctattgtttccttccttctg 54611
FAM247.LOC105372935.ref.human                    ------------------------------------------------------------ 11231

GGT1end-LOC749026.end.7456450-7520130.chimp      ctaactttgggtttagttttattcttctttttctagtttcttaatgtgtaaacttatgtt 54671
FAM247.LOC105372935.ref.human                    ------------------------------------------------------------ 11231

GGT1end-LOC749026.end.7456450-7520130.chimp      gttgaattgaggtgtttctttttttaatgtgtttatagctataaatttcctccttagcac 54731
FAM247.LOC105372935.ref.human                    ------------------------------------------------------------ 11231

GGT1end-LOC749026.end.7456450-7520130.chimp      tcctttcaccacaactcataagttgtgtttctttgttgtttctttgtttttttgagacaga 54791
FAM247.LOC105372935.ref.human                    ------------------------------------------------------------ 11231

GGT1end-LOC749026.end.7456450-7520130.chimp      gtttcattctgtcacccaggctggagtgcagttgtgttagctcagtggtgcaacctctgc 54851
FAM247.LOC105372935.ref.human                    ------------------------------------------------------------ 11231

GGT1end-LOC749026.end.7456450-7520130.chimp      ctcctgggttcaagaaattctcatgcctcagcctcctgggtagctgggactacaggcacg 54911
FAM247.LOC105372935.ref.human                    ------------------------------------------------------------ 11231

GGT1end-LOC749026.end.7456450-7520130.chimp      taccaccactcccagctaatttttgtgtttttagtagagacggggtttcaccatgttggc 54971
FAM247.LOC105372935.ref.human                    ------------------------------------------------------------ 11231

GGT1end-LOC749026.end.7456450-7520130.chimp      ctggctgatcttgaactcctgacctctagctatccatctccctcagtctcccaaagtgct 55031
FAM247.LOC105372935.ref.human                    ------------------------------------------------------------ 11231

GGT1end-LOC749026.end.7456450-7520130.chimp      gggattacaggtgtgagccaccacaactggccacaacccataagtttggatatagtgtgc 55091
FAM247.LOC105372935.ref.human                    ------------------------------------------------------------ 11231

GGT1end-LOC749026.end.7456450-7520130.chimp      tttcattttcatttgtctttaagaattttataatttctttgtgatttctttgatccattg 55151
FAM247.LOC105372935.ref.human                    ------------------------------------------------------------ 11231

GGT1end-LOC749026.end.7456450-7520130.chimp      gttgtttgagagtgtgttgtttaaattctactaatttgtgaactttttaaaatcttctgt 55211
FAM247.LOC105372935.ref.human                    ------------------------------------------------------------ 11231

GGT1end-LOC749026.end.7456450-7520130.chimp      tattgatttctaactttatcctgttgtggtcagagaagacactttgtatgatgtctatcg 55271
FAM247.LOC105372935.ref.human                    ------------------------------------------------------------ 11231

GGT1end-LOC749026.end.7456450-7520130.chimp      ttttgaatctactgagctttttttcctttttgggacagggtctcgttctgtcagtggtac 55331
FAM247.LOC105372935.ref.human                    ------------------------------------------------------------ 11231

GGT1end-LOC749026.end.7456450-7520130.chimp      aatggaacattttctttgtcatgaagtacaggctggagtacagtggtacaatctctgctc 55391
FAM247.LOC105372935.ref.human                    ------------------------------------------------------------ 11231

GGT1end-LOC749026.end.7456450-7520130.chimp      actgcagcttcaacctcctgggctcctaagcaattctcacgcttcagcctcccaagaagc 55451
FAM247.LOC105372935.ref.human                    ------------------------------------------------------------ 11231

GGT1end-LOC749026.end.7456450-7520130.chimp      tgggatcacaggcacgcaccaccacgcccagctaatttttgtatttttagtagagatggg 55511
FAM247.LOC105372935.ref.human                    ------------------------------------------------------------ 11231

GGT1end-LOC749026.end.7456450-7520130.chimp      gtttcaccatgttggccaggctgatgtcaagctcctggcttcaagcaatccacccgcctc 55571
FAM247.LOC105372935.ref.human                    ------------------------------------------------------------ 11231

GGT1end-LOC749026.end.7456450-7520130.chimp      agcctcccaaattactctgggattacagatgtgagccactgcacccagcccctattgaga 55631
FAM247.LOC105372935.ref.human                    ------------------------------------------------------------ 11231

GGT1end-LOC749026.end.7456450-7520130.chimp      attaatttgtgatgtgttatatggtctatcctgaaaatgtcccatgtatacttgagaagt 55691
FAM247.LOC105372935.ref.human                    ------------------------------------------------------------ 11231

GGT1end-LOC749026.end.7456450-7520130.chimp      atgtgtattatgttgttgggtagaatatcctgtgtatgtctgttaggtctagttgttttt 55751
FAM247.LOC105372935.ref.human                    ------------------------------------------------------------ 11231
```

```
GGT1end-LOC749026.end.7456450-7520130.chimp      tcaggtgttgttcaagtgctgtatttccttacttatgtctggtctggttgttttatttat 55811
FAM247.LOC105372935.ref.human                    ------------------------------------------------------------ 11231

GGT1end-LOC749026.end.7456450-7520130.chimp      ttgtgaaatgaagacaagggacaaaatcctaagacatccccccttaaaagggaaggaccac 55871
FAM247.LOC105372935.ref.human                    ------------------------------------------------------------ 11231

GGT1end-LOC749026.end.7456450-7520130.chimp      ccacagaaaaggactgagagacccccagaatctatagcttagctaattgatggtctttct 55931
FAM247.LOC105372935.ref.human                    ------------------------------------------------------------ 11231

GGT1end-LOC749026.end.7456450-7520130.chimp      ctcctgaagttagtaaagagtggaaaagatgactccttcttcaaacatgaagaaagcaat 55991
FAM247.LOC105372935.ref.human                    ------------------------------------------------------------ 11231

GGT1end-LOC749026.end.7456450-7520130.chimp      ctaagtcttcaaagaacaggaagcatgagaaaatatgacaccaccaaagaaacaaaatga 56051
FAM247.LOC105372935.ref.human                    ------------------------------------------------------------ 11231

GGT1end-LOC749026.end.7456450-7520130.chimp      aactccagtggctgactccaaagacatgaagatctacaaattgcctgacaaagaattcaa 56111
FAM247.LOC105372935.ref.human                    ------------------------------------------------------------ 11231

GGT1end-LOC749026.end.7456450-7520130.chimp      ataatcatcttagggtgacatcagcaagatggaagaacaggaggccctccactcacctct 56171
FAM247.LOC105372935.ref.human                    ------------------------------------------------------------ 11231

GGT1end-LOC749026.end.7456450-7520130.chimp      ccccacacaaacaatgatctggcagccattcatggacaaaactgcctttgcaagagtttt 56231
FAM247.LOC105372935.ref.human                    ------------------------------------------------------------ 11231

GGT1end-LOC749026.end.7456450-7520130.chimp      aatatccaggcaggaggtgacaaaactctagcaaagcccaaaaccaaggagagctgcttt 56291
FAM247.LOC105372935.ref.human                    ------------------------------------------------------------ 11231

GGT1end-LOC749026.end.7456450-7520130.chimp      gagaaggcaggtccacacaccagtgacaggttcccagttgcagactgaaagcagctttgt 56351
FAM247.LOC105372935.ref.human                    ------------------------------------------------------------ 11231

GGT1end-LOC749026.end.7456450-7520130.chimp      cctcctatggacttgggtccagctccactagatcatggtcttgcaactagccccatccac 56411
FAM247.LOC105372935.ref.human                    ------------------------------------------------------------ 11231

GGT1end-LOC749026.end.7456450-7520130.chimp      taagcacctaggaggaaccatgaccatttatgcccccagtaacaggccttctaaccatgg 56471
FAM247.LOC105372935.ref.human                    ------------------------------------------------------------ 11231

GGT1end-LOC749026.end.7456450-7520130.chimp      tcctgactgcagaaactaaagcagccctgacccggcttcagccccactctactacagtca 56531
FAM247.LOC105372935.ref.human                    ------------------------------------------------------------ 11231

GGT1end-LOC749026.end.7456450-7520130.chimp      gtcttgcctgcccaggaattcagtgatgcctttacatgcccttggtaacaggcctgctga 56591
FAM247.LOC105372935.ref.human                    ------------------------------------------------------------ 11231

GGT1end-LOC749026.end.7456450-7520130.chimp      tctcagtctcagctatggacactaaagtagccctgtgactccattccagtcttgctttgc 56651
FAM247.LOC105372935.ref.human                    ------------------------------------------------------------ 11231

GGT1end-LOC749026.end.7456450-7520130.chimp      cacagtctgggcacagtccagcccatcctgggacctggtgaaagacataccatccattct 56711
FAM247.LOC105372935.ref.human                    ------------------------------------------------------------ 11231

GGT1end-LOC749026.end.7456450-7520130.chimp      cctaataagaggaccaccaatcttgaacccaaatgtggtcaccaaagctgtcctgtgact 56771
FAM247.LOC105372935.ref.human                    ------------------------------------------------------------ 11231

GGT1end-LOC749026.end.7456450-7520130.chimp      tggctctagccctgctctactgtggtctggaggcagtcctgtcttcccatggacctacct 56831
FAM247.LOC105372935.ref.human                    ------------------------------------------------------------ 11231

GGT1end-LOC749026.end.7456450-7520130.chimp      agtaaaccagcaggagctcaatctgggacccacagggagctataccagtccatgcccttg 56891
FAM247.LOC105372935.ref.human                    ------------------------------------------------------------ 11231

GGT1end-LOC749026.end.7456450-7520130.chimp      gtaataggcctgatatctgaagacttgactgtagaactagaagtagccccatgacctggc 56951
FAM247.LOC105372935.ref.human                    ------------------------------------------------------------ 11231

GGT1end-LOC749026.end.7456450-7520130.chimp      tccagtcctgttcgatcagggtatcagaggcagtccagttctcctgaggatccagcagaa 57011
FAM247.LOC105372935.ref.human                    ------------------------------------------------------------ 11231

GGT1end-LOC749026.end.7456450-7520130.chimp      actgcatcaccaacctatgccttgatagcaggcctgccaatcagagattcagctgcagac 57071
FAM247.LOC105372935.ref.human                    ------------------------------------------------------------ 11231

GGT1end-LOC749026.end.7456450-7520130.chimp      caggcagcagccatgtgatatggctccaggcccacttgactgtgatccagtcaagggatc 57131
FAM247.LOC105372935.ref.human                    ------------------------------------------------------------ 11231

GGT1end-LOC749026.end.7456450-7520130.chimp      cagcaggaaaaggtttgcacctgctgaaaccaatctataaagacagaaagagattgggca 57191
FAM247.LOC105372935.ref.human                    ------------------------------------------------------------ 11231

GGT1end-LOC749026.end.7456450-7520130.chimp      tggtggctcacacctgtaatcccagcactttgggaggccaaggcgggcagaccacttgag 57251
FAM247.LOC105372935.ref.human                    ------------------------------------------------------------ 11231

GGT1end-LOC749026.end.7456450-7520130.chimp      gccaggagtttgagaccagcctggacaacacggtgaaactctgtctctacaaaaaataca 57311
FAM247.LOC105372935.ref.human                    ------------------------------------------------------------ 11231

GGT1end-LOC749026.end.7456450-7520130.chimp      aaaaaattagccaggcatggtggcacatgcctgtgatcccagctgcttgggaggctgagg 57371
FAM247.LOC105372935.ref.human                    ------------------------------------------------------------ 11231

GGT1end-LOC749026.end.7456450-7520130.chimp      caggagaatcatttgaacctgggaggtggaggttacagtgagctgagatcacgccactgc 57431
FAM247.LOC105372935.ref.human                    ------------------------------------------------------------ 11231

GGT1end-LOC749026.end.7456450-7520130.chimp      actccagctgggtgacagagcaagactctgtcccaaaaaaaaaaaaaaaaaaaaaaagaa 57491
FAM247.LOC105372935.ref.human                    ------------------------------------------------------------ 11231

GGT1end-LOC749026.end.7456450-7520130.chimp      gaggtatttgcttcttcatagacattaatgaaagtctgtagatcatgaagaatcagcaaa 57551
FAM247.LOC105372935.ref.human                    ------------------------------------------------------------ 11231

GGT1end-LOC749026.end.7456450-7520130.chimp      tatgacactaccaaaataaactcataaatctccagtaatcaaccccccaaaaatagagat 57611
FAM247.LOC105372935.ref.human                    ------------------------------------------------------------ 11231

GGT1end-LOC749026.end.7456450-7520130.chimp      ctacaaattgcctgacaattcaaaataattaatttaagatagctcagtgaaatttactag 57671
FAM247.LOC105372935.ref.human                    ------------------------------------------------------------ 11231

GGT1end-LOC749026.end.7456450-7520130.chimp      aatgtatatatcaactcaataatatcaagaaaatagtagatgaacaaaactaaaagttca 57731
FAM247.LOC105372935.ref.human                    ------------------------------------------------------------ 11231

GGT1end-LOC749026.end.7456450-7520130.chimp      ataaagatacagaaataataagaaccaaacagaaattctagaactgaagaatacaatgaa 57791
FAM247.LOC105372935.ref.human                    ------------------------------------------------------------ 11231

GGT1end-LOC749026.end.7456450-7520130.chimp      taaaatgaaaatgcaagctgggtgccgtggcggacgcgtgtaatcccagcactttgggag 57851
FAM247.LOC105372935.ref.human                    ------------------------------------------------------------ 11231

GGT1end-LOC749026.end.7456450-7520130.chimp      gccgaggcaggcggatcacttgaggtcaagagttcaagaccagcctggccaacatgccaa 57911
FAM247.LOC105372935.ref.human                    ------------------------------------------------------------ 11231

GGT1end-LOC749026.end.7456450-7520130.chimp      aaccccatctgtattaaaaatacaaaaattagccaggcatggtggcatatgcctgtaatc 57971
```

```
FAM247.LOC105372935.ref.human                    ------------------------------------------------------------ 11231

GGT1end-LOC749026.end.7456450-7520130.chimp      ctagctactagggaggctgaggcaggagaatcgcttgaaccgggaggtggaagttggagt 58031
FAM247.LOC105372935.ref.human                    ------------------------------------------------------------ 11231

GGT1end-LOC749026.end.7456450-7520130.chimp      gagccaagatggcaccactgcattcaagcctgggtgacacagactctgtctcaaaaatat 58091
FAM247.LOC105372935.ref.human                    ------------------------------------------------------------ 11231

GGT1end-LOC749026.end.7456450-7520130.chimp      ataaaaataaataaatgaatgaaatgaaaatgcaatagagaatttcaattgcagacttga 58151
FAM247.LOC105372935.ref.human                    ------------------------------------------------------------ 11231

GGT1end-LOC749026.end.7456450-7520130.chimp      tcaaacagaagaaagaatctgtgaaattaaagacaggacatttgaaatatcctgtcagaa 58211
FAM247.LOC105372935.ref.human                    ------------------------------------------------------------ 11231

GGT1end-LOC749026.end.7456450-7520130.chimp      gaccagaaagaaaaaaaagtttataaagtaaaaaccttggggatttataggacaccacca 58271
FAM247.LOC105372935.ref.human                    ------------------------------------------------------------ 11231

GGT1end-LOC749026.end.7456450-7520130.chimp      tgaagtgggtcaaacatattatggatttcctagaagtaaaaaagatagaaaaggcctgaa 58331
FAM247.LOC105372935.ref.human                    ------------------------------------------------------------ 11231

GGT1end-LOC749026.end.7456450-7520130.chimp      agtatatttgaagaaataatgggtaaaaactttccaaattcttagagccaagtgggcttc 58391
FAM247.LOC105372935.ref.human                    ------------------------------------------------------------ 11231

GGT1end-LOC749026.end.7456450-7520130.chimp      cagacacatgaagttcagaggccccaaaaaaggccagcccaaagaccataataccaacat 58451
FAM247.LOC105372935.ref.human                    ------------------------------------------------------------ 11231

GGT1end-LOC749026.end.7456450-7520130.chimp      gtattataattaaaatatcaaaagtcaaagacagagactttcaaaagcagcaagaagaaa 58511
FAM247.LOC105372935.ref.human                    ------------------------------------------------------------ 11231

GGT1end-LOC749026.end.7456450-7520130.chimp      ggaattcatcacatacaaggaaacttcaataaggctatcagtggatttctcagcagaaac 58571
FAM247.LOC105372935.ref.human                    ------------------------------------------------------------ 11231

GGT1end-LOC749026.end.7456450-7520130.chimp      cttataagccaggagagaataggatgctatattcaatgtattggaagaaaaaaactgcca 58631
FAM247.LOC105372935.ref.human                    ------------------------------------------------------------ 11231

GGT1end-LOC749026.end.7456450-7520130.chimp      atcaggaagaccacaacttgtaaagctgtccttcagaaatgaaaaagagatgataaggtc 58691
FAM247.LOC105372935.ref.human                    ------------------------------------------------------------ 11231

GGT1end-LOC749026.end.7456450-7520130.chimp      tttcccagactcccaccaaaaaaattctaagggagttcatcaccactgcacctgccttat 58751
FAM247.LOC105372935.ref.human                    ------------------------------------------------------------ 11231

GGT1end-LOC749026.end.7456450-7520130.chimp      aaggcagtttttcaagttgaaataaaaggattctgattagcaacattaaaacattaagat 58811
FAM247.LOC105372935.ref.human                    ------------------------------------------------------------ 11231

GGT1end-LOC749026.end.7456450-7520130.chimp      attaagttcacaggtaggccgggcgcggtggctcacgcctgtaatcccagcactttggga 58871
FAM247.LOC105372935.ref.human                    ------------------------------------------------------------ 11231

GGT1end-LOC749026.end.7456450-7520130.chimp      ggccaaggcgggtggatcacaaggtcaggagagggagatcatgctggctaacacggtgaa 58931
FAM247.LOC105372935.ref.human                    ------------------------------------------------------------ 11231

GGT1end-LOC749026.end.7456450-7520130.chimp      accccgtctctactaaaaatacaaaaaattagccaggcgtggtggcgggcgcctgtagtc 58991
FAM247.LOC105372935.ref.human                    ------------------------------------------------------------ 11231

GGT1end-LOC749026.end.7456450-7520130.chimp      ccagttactcgggaggccgaggcaggagaatgacttgaacccgggaggcagagcttgcag 59051
FAM247.LOC105372935.ref.human                    ------------------------------------------------------------ 11231

GGT1end-LOC749026.end.7456450-7520130.chimp      tgagccgagatcatggacactgccctccagcctgggcgacagagcgagactctgtctcaa 59111
FAM247.LOC105372935.ref.human                    ------------------------------------------------------------ 11231

GGT1end-LOC749026.end.7456450-7520130.chimp      aaaaaaaaagttcacagggaaaggtaagtatatagtcacattcataacactgtaatatt 59171
FAM247.LOC105372935.ref.human                    ------------------------------------------------------------ 11231

GGT1end-LOC749026.end.7456450-7520130.chimp      gcagtggtggtgtataaatcacttttcactgtagtataaaagttaaaatgtaaaagtatt 59231
FAM247.LOC105372935.ref.human                    ------------------------------------------------------------ 11231

GGT1end-LOC749026.end.7456450-7520130.chimp      aggcggcccgggcgcggggcagcggctgtggggaggcggggaggcggggaggcgggggc 59291
FAM247.LOC105372935.ref.human                    ------------------------------------------------------------ 11231

GGT1end-LOC749026.end.7456450-7520130.chimp      ctggccggacacccctgcgcccccctccccgcacccgggcggagggcggcctcttcccccct 59351
FAM247.LOC105372935.ref.human                    ------------------------------------------------------------ 11231

GGT1end-LOC749026.end.7456450-7520130.chimp      ccccctcccccgccacccccggcagccgcctcccccaggacacaggaggcgggcggaggc 59411
FAM247.LOC105372935.ref.human                    ------------------------------------------------------------ 11231

GGT1end-LOC749026.end.7456450-7520130.chimp      cgggtccgcgcagcgggcgacttgcagcgtgggccgggtcgaggtcgggggggcggttt 59471
FAM247.LOC105372935.ref.human                    ------------------------------------------------------------ 11231

GGT1end-LOC749026.end.7456450-7520130.chimp      cgggggctgggaagctgggggccggggacaggagggtgggggggccggggaaaggaggg 59531
FAM247.LOC105372935.ref.human                    ------------------------------------------------------------ 11231

GGT1end-LOC749026.end.7456450-7520130.chimp      ctggggggccggggacaggagggccatgcaggcagtggcaagtgggcggcgagggctccg 59591
FAM247.LOC105372935.ref.human                    ------------------------------------------------------------ 11231

GGT1end-LOC749026.end.7456450-7520130.chimp      tggggcaggtggacggggatcagcgcctggggctgctccgttccccaggcgggtgtcgtg 59651
FAM247.LOC105372935.ref.human                    ------------------------------------------------------------ 11231

GGT1end-LOC749026.end.7456450-7520130.chimp      gctctggcctccatgcaactccaggtctgcgcgccccgcgctgctggagccccagtcaaa 59711
FAM247.LOC105372935.ref.human                    ------------------------------------------------------------ 11231

GGT1end-LOC749026.end.7456450-7520130.chimp      agtctatttaaaaagcagagagaggatgcttccgtctgagtggagtgatgaagacctgat 59771
FAM247.LOC105372935.ref.human                    ------------------------------------------------------------ 11231

GGT1end-LOC749026.end.7456450-7520130.chimp      ccctgggccatttgggaacactagctgcctttcatcacagtcaacctggactcagagaat 59831
FAM247.LOC105372935.ref.human                    ------------------------------------------------------------ 11231

GGT1end-LOC749026.end.7456450-7520130.chimp      gtcaagagcttgttggttgggtcaagaatgaatctaggcatgacgtcatagtttatagtc 59891
FAM247.LOC105372935.ref.human                    ------------------------------------------------------------ 11231

GGT1end-LOC749026.end.7456450-7520130.chimp      atccttttaaacctgcaaagaagcatgtgcaggtttaaagttatttcacgggtactgctt 59951
FAM247.LOC105372935.ref.human                    ------------------------------------------------------------ 11231

GGT1end-LOC749026.end.7456450-7520130.chimp      gccaatctttggaggatgtgaagcctgcagagaaataaagtgtcgcccctctgcgcgtcg 60011
FAM247.LOC105372935.ref.human                    ------------------------------------------------------------ 11231

GGT1end-LOC749026.end.7456450-7520130.chimp      ctccccatctgctagaatgtttctcatgaatgctcctccagtggttgctctccagcccaa 60071
FAM247.LOC105372935.ref.human                    ------------------------------------------------------------ 11231

GGT1end-LOC749026.end.7456450-7520130.chimp      atgggaggcctctgtcccgccagggagctttagattcccccggtgcttctcggaggctga 60131
FAM247.LOC105372935.ref.human                    ------------------------------------------------------------ 11231
```

```
GGT1end-LOC749026.end.7456450-7520130.chimp      caagggcgtggagagcatgtcggtgagcacccgggtgcagatgctcatcagcacgctgca 60191
FAM247.LOC105372935.ref.human                    ------------------------------------------------------------ 11231

GGT1end-LOC749026.end.7456450-7520130.chimp      gagcgacagggctgctaggggcaccagcgatgagcgcgctgcgcagagggggcagaggga 60251
FAM247.LOC105372935.ref.human                    ------------------------------------------------------------ 11231

GGT1end-LOC749026.end.7456450-7520130.chimp      tgccacgacgccaggcctgctgccaagtccaccgtgcacaaggagctgcctgcgttggct 60311
FAM247.LOC105372935.ref.human                    ------------------------------------------------------------ 11231

GGT1end-LOC749026.end.7456450-7520130.chimp      gcctgtggtcttgttgctgactttgaccccatggggaggaggaaactgcagactttggc 60371
FAM247.LOC105372935.ref.human                    ------------------------------------------------------------ 11231

GGT1end-LOC749026.end.7456450-7520130.chimp      ccattggtgctagattcagacagtgacgattccgtggaccgggacattgaggaggccatc 60431
FAM247.LOC105372935.ref.human                    ------------------------------------------------------------ 11231

GGT1end-LOC749026.end.7456450-7520130.chimp      caggagtacctgaaggtaaagagtggagccgcacagcccggggccagcggggcccagcca 60491
FAM247.LOC105372935.ref.human                    ------------------------------------------------------------ 11231

GGT1end-LOC749026.end.7456450-7520130.chimp      tgcgcagccttccagggctgcaggcggaggcagtagatgtaagccggaactggctcacag 60551
FAM247.LOC105372935.ref.human                    ------------------------------------------------------------ 11231

GGT1end-LOC749026.end.7456450-7520130.chimp      cagtgcccaactgccctgtgttccccaaaacttgtacctggctcagatggtgtggccctg 60611
FAM247.LOC105372935.ref.human                    ------------------------------------------------------------ 11231

GGT1end-LOC749026.end.7456450-7520130.chimp      gcagccaggtgggatccagcaaggaccagggctctgcctccccagtcagcatgagcagag 60671
FAM247.LOC105372935.ref.human                    ------------------------------------------------------------ 11231

GGT1end-LOC749026.end.7456450-7520130.chimp      atgactcctttgagcagagcatcagggcagaaatagaacagtttctgaatgagaaaagac 60731
FAM247.LOC105372935.ref.human                    ------------------------------------------------------------ 11231

GGT1end-LOC749026.end.7456450-7520130.chimp      agcatgagacccaaaaatgtgatgggtcagtggagaagaaaccagacacacatgaaaatt 60791
FAM247.LOC105372935.ref.human                    ------------------------------------------------------------ 11231

GGT1end-LOC749026.end.7456450-7520130.chimp      cggccaagtcactctcgaaatcccaccaagagccggctacaaaggtggtgcaccggcagg 60851
FAM247.LOC105372935.ref.human                    ------------------------------------------------------------ 11231

GGT1end-LOC749026.end.7456450-7520130.chimp      gcctgatgggcgtccaggagttcgccttctgcagacctcccccggttagcaaagacaaac 60911
FAM247.LOC105372935.ref.human                    ------------------------------------------------------------ 11231

GGT1end-LOC749026.end.7456450-7520130.chimp      gtgcagcccagaaccctcaggtccaaggtcatgaccacgcaggagaactagggcagcaca 60971
FAM247.LOC105372935.ref.human                    ------------------------------------------------------------ 11231

GGT1end-LOC749026.end.7456450-7520130.chimp      aagccagcaacccccgccgcccttcagaagcagtacagaataaaagtgggattaaaagga 61031
FAM247.LOC105372935.ref.human                    ------------------------------------------------------------ 11231

GGT1end-LOC749026.end.7456450-7520130.chimp      gcgccagcaccgcaaggaggggaaagcgagtcacgagcgccatacaggcgcccgaggcgt 61091
FAM247.LOC105372935.ref.human                    ------------------------------------------------------------ 11231

GGT1end-LOC749026.end.7456450-7520130.chimp      ccgactccagcagcgacgacggcattgaggaggccatccagctgtaccaggtgcagaaaa 61151
FAM247.LOC105372935.ref.human                    ------------------------------------------------------------ 11231

GGT1end-LOC749026.end.7456450-7520130.chimp      cacgcaaggaggccgacggggacccgccccagagggcccagctccaagaggaaagagcac 61211
FAM247.LOC105372935.ref.human                    ------------------------------------------------------------ 11231

GGT1end-LOC749026.end.7456450-7520130.chimp      ctgcccctcccgcacacagcacaagtagcgccacaaaaagtgccttgccagagacccaca 61271
FAM247.LOC105372935.ref.human                    ------------------------------------------------------------ 11231

GGT1end-LOC749026.end.7456450-7520130.chimp      ggaaaacacccagcaagaagaagccagtgcccaccaagaccacggaccctggtccagggg 61331
FAM247.LOC105372935.ref.human                    ------------------------------------------------------------ 11231

GGT1end-LOC749026.end.7456450-7520130.chimp      atctggacgctgaccattcccccaagatcccaaaggaaaccaaagctccacctccaacga 61391
FAM247.LOC105372935.ref.human                    ------------------------------------------------------------ 11231

GGT1end-LOC749026.end.7456450-7520130.chimp      gcccggcttccaggagcaagtttgtggaatggtcctcttgccaggcagacacctccgctg 61451
FAM247.LOC105372935.ref.human                    ------------------------------------------------------------ 11231

GGT1end-LOC749026.end.7456450-7520130.chimp      agctgatgtgtgtagaagcagtcctggacattttcaagacggtcctgccggccctatgga 61511
FAM247.LOC105372935.ref.human                    ------------------------------------------------------------ 11231

GGT1end-LOC749026.end.7456450-7520130.chimp      gggcagcgatgggtccctgtccgcaagcccactcttctactcccccaacgtgccttcccg 61571
FAM247.LOC105372935.ref.human                    ------------------------------------------------------------ 11231

GGT1end-LOC749026.end.7456450-7520130.chimp      ctctgacggtgacagtagctccgtggacagcgacgacagcattgagcagaaaatctggac 61631
FAM247.LOC105372935.ref.human                    ------------------------------------------------------------ 11231

GGT1end-LOC749026.end.7456450-7520130.chimp      gtttttggccttcaaggtgcagtctggaagtctgctggccagaggtgagagctgcccgca 61691
FAM247.LOC105372935.ref.human                    ------------------------------------------------------------ 11231

GGT1end-LOC749026.end.7456450-7520130.chimp      ggctgcccagggcccattttcaccacctggcctcagcagccagaccggcagccccaaggc 61751
FAM247.LOC105372935.ref.human                    ------------------------------------------------------------ 11231

GGT1end-LOC749026.end.7456450-7520130.chimp      ccctctctctaaaacactggacccactcctggctgcaaaaggaagcatagaggcggcggc 61811
FAM247.LOC105372935.ref.human                    ------------------------------------------------------------ 11231

GGT1end-LOC749026.end.7456450-7520130.chimp      caagtgaggccatccactcccaagaacattcgggaggtggggaaagagggtggccaggat 61871
FAM247.LOC105372935.ref.human                    ------------------------------------------------------------ 11231

GGT1end-LOC749026.end.7456450-7520130.chimp      gcggaccacagccaggggagagccgggcccggccatgaggggcgggaccttcccatccag 61931
FAM247.LOC105372935.ref.human                    ------------------------------------------------------------ 11231

GGT1end-LOC749026.end.7456450-7520130.chimp      ggcacagccagcgaggcctcgggatgggagggcgccgctagggtgggacctttccatcca 61991
FAM247.LOC105372935.ref.human                    ------------------------------------------------------------ 11231

GGT1end-LOC749026.end.7456450-7520130.chimp      gggcacagccagcgaggccccgggaggagagggcgccgctagggtgcctggtgacactcg 62051
FAM247.LOC105372935.ref.human                    ------------------------------------------------------------ 11231

GGT1end-LOC749026.end.7456450-7520130.chimp      catgtcacagggccagggtaagacagacgaggcaaggcacctagacaagaagaagagctc 62111
FAM247.LOC105372935.ref.human                    ------------------------------------------------------------ 11231

GGT1end-LOC749026.end.7456450-7520130.chimp      cgaagacaaaagcagttccctggacagtgacaaggacctggacacagccatcaaggactt 62171
FAM247.LOC105372935.ref.human                    ------------------------------------------------------------ 11231

GGT1end-LOC749026.end.7456450-7520130.chimp      gttaaggtccaagtgaaagctcaagaagaggtccaaggagcccagggctgtgtgcaggaa 62231
FAM247.LOC105372935.ref.human                    ------------------------------------------------------------ 11231

GGT1end-LOC749026.end.7456450-7520130.chimp      gaaggtcaggttcagcacctcccagacgcacttccaggagcagatgggcaggctcctgag 62291
FAM247.LOC105372935.ref.human                    ------------------------------------------------------------ 11231
```

```
GGT1end-LOC749026.end.7456450-7520130.chimp      agagtggaaaaacaggcacctgcaggtgctgaagagctgcctaagtccaagagagacagc 62351
FAM247.LOC105372935.ref.human                    ------------------------------------------------------------ 11231


GGT1end-LOC749026.end.7456450-7520130.chimp      tgcgagggctccaggaagaaaccccccagtgtctttggcagcagggcagagaggacgagc 62411
FAM247.LOC105372935.ref.human                    ------------------------------------------------------------ 11231


GGT1end-LOC749026.end.7456450-7520130.chimp      cccagccttcctggtgaggagacccgcttctgcctccgcctccgaagagaatctattccc 62471
FAM247.LOC105372935.ref.human                    ------------------------------------------------------------ 11231


GGT1end-LOC749026.end.7456450-7520130.chimp      cagagagtcccagggcccagctcccagccctggctccttgtctgacaacagcagttcagt 62531
FAM247.LOC105372935.ref.human                    ------------------------------------------------------------ 11231


GGT1end-LOC749026.end.7456450-7520130.chimp      ggacagcgacgatagcatcgaactggagattaggaagtttttggtggaaaaggccaagga 62591
FAM247.LOC105372935.ref.human                    ------------------------------------------------------------ 11231


GGT1end-LOC749026.end.7456450-7520130.chimp      gtcggtgagcagttcagaagttcaggcagagggccccaccgctctcgggacagggggccc 62651
FAM247.LOC105372935.ref.human                    ------------------------------------------------------------ 11231


GGT1end-LOC749026.end.7456450-7520130.chimp      agccaggccagaggtaccttgcaggaaggagccggccccactgcctggcatgtgcacacg 62711
FAM247.LOC105372935.ref.human                    ------------------------------------------------------------ 11231


GGT1end-LOC749026.end.7456450-7520130.chimp      gagccagagggccgggggggtcccacatttggctgaagggcatcgaggcgcagggagcg 62771
FAM247.LOC105372935.ref.human                    ------------------------------------------------------------ 11231


GGT1end-LOC749026.end.7456450-7520130.chimp      caggagcacagggcgcagcgtgcctgctcagccagggtgggaaggggctccccactgatc 62831
FAM247.LOC105372935.ref.human                    ------------------------------------------------------------ 11231


GGT1end-LOC749026.end.7456450-7520130.chimp      cggcggaggggatcatgcgccacccaggaggaccagcggcagtgtctccgccaaggtctc 62891
FAM247.LOC105372935.ref.human                    ------------------------------------------------------------ 11231


GGT1end-LOC749026.end.7456450-7520130.chimp      tcagtgagcaggagaaatgtttacgtttacaaagaccagagcccacgaggggctgagcct 62951
FAM247.LOC105372935.ref.human                    ------------------------------------------------------------ 11231


GGT1end-LOC749026.end.7456450-7520130.chimp      gctgccaaaagtgcttttggtcagcggcccagctgtgccacagcgggcaccgaggcagga 63011
FAM247.LOC105372935.ref.human                    ------------------------------------------------------------ 11231


GGT1end-LOC749026.end.7456450-7520130.chimp      ggcgccaggaggacctttcacgtgggctgcaggaaccggagcttcctgacccccagcccg 63071
FAM247.LOC105372935.ref.human                    ------------------------------------------------------------ 11231


GGT1end-LOC749026.end.7456450-7520130.chimp      ggagctgagagggacgctggagcccaggccaaccgcgccccgccctggagtgactttgcc 63131
FAM247.LOC105372935.ref.human                    ------------------------------------------------------------ 11231


GGT1end-LOC749026.end.7456450-7520130.chimp      caccacagtcggctgcccagcccatgggcacttcgctccaaaggtagagatgcggcatgg 63191
FAM247.LOC105372935.ref.human                    ------------------------------------------------------------ 11231


GGT1end-LOC749026.end.7456450-7520130.chimp      agggggcggcattgggagagagagacaaggggtccgagggccccgcccggggcctgccca 63251
FAM247.LOC105372935.ref.human                    ------------------------------------------------------------ 11231


GGT1end-LOC749026.end.7456450-7520130.chimp      gcctgccccttgcgggcttctccccgctgctgtccacccagctcttccactttggaaagg 63311
FAM247.LOC105372935.ref.human                    ------------------------------------------------------------ 11231


GGT1end-LOC749026.end.7456450-7520130.chimp      gtgtctcctgggggggcagggagaccggcctcttcagcccctacccaggactgcctctgc 63371
FAM247.LOC105372935.ref.human                    ------------------------------------------------------------ 11231


GGT1end-LOC749026.end.7456450-7520130.chimp      agggcctgtccttctcggccttcagggagtcccaggccgggcccagccctgtctttggaa 63431
FAM247.LOC105372935.ref.human                    ------------------------------------------------------------ 11231


GGT1end-LOC749026.end.7456450-7520130.chimp      gcccacacttgctggcgaagaaggactgcggtcaccggccaagcaggaaggtacagacag 63491
FAM247.LOC105372935.ref.human                    ------------------------------------------------------------ 11231


GGT1end-LOC749026.end.7456450-7520130.chimp      ggctgagtttgcacaacaggaagagctctggctcggaggaaaggattttagacctgaggt 63551
FAM247.LOC105372935.ref.human                    ------------------------------------------------------------ 11231


GGT1end-LOC749026.end.7456450-7520130.chimp      atccacgaagggtcaatcagtagagatgaccaggaccaggacaccttgggcagtgacgcc 63611
FAM247.LOC105372935.ref.human                    ------------------------------------------------------------ 11231


GGT1end-LOC749026.end.7456450-7520130.chimp      agtgacttcagcaacacctccgcagaggtgttgctggtgcagtggtggcagctcagtagt 63671
FAM247.LOC105372935.ref.human                    ------------------------------------------------------------ 11231


GGT1end-LOC749026.end.7456450-7520130.chimp      gaaggtataa 63681
FAM247.LOC105372935.ref.human                    ---------- 11231
===========================================================
```

```
>GGT1end-LOC749026.end.7456450-7520130.chimp
        1 ctctgggcct cagtgtattc tgtgtgaaat ggagccatct ggctggggag gaacagagag
       61 gtgggattcg gagatcttca caatgtgggc actggaacta gcctcaacat cttcagcatg
      121 gggagagcca ggcacatggc tgggggccag gggaaggttc acaccaagct ctgccccttc
      181 ccaccctgat ccctcggact ttggggccag gccctccctt actggggctg ggcagtgaca
      241 ctacctagga tcagccaccg gggggtgcca cgaccctggc actttcttag gcagagggtg
      301 gccagctgat gctgggaacc cgggcgcctt ctcagacccg taggcgtcca gctcaccctg
      361 ccgatgacac tggaggtgaa gctgaggtcc gaggaatggg gactgggcaa caggctggag
      421 gaaaacatct cggtcagagc cacgcccctg gggggtttcc aagtttaagc ccagagtgaa
      481 acccaagctt gtgatcctct ccagagggag gcctggttct cagggaacag caaacgggaa
      541 gatgtcccca gatcccaggg atcagggctt ggaccagccg gggacgcagc ccagagggag
      601 tgggtccgga aggaaacagc tcgacacagc agccttcacc atcggcagcc cctccaggcc
      661 tccctcgggg cctgctccct cctctgtgca cagttccaac acctggggca gggttctggg
      721 aagggctggt ggaggtgggc tggtgggagg cggtgatcac agcccagcac ctggatatca
      781 ccaggggcac tggggccagg ggccaggtga ggccaggtcg gggctatcct tcaggagccc
      841 cgaaaacctg gtgattccaa agggcccata gacaaacagg gttttctgcc tgtggagtca
      901 agtcccactg ggtctgagct ctggagggct gtgtctctgg ggctctgcaa gggtgagatg
      961 gaggtgggct caaatggtgt acaagtcact cctcaatcct tattttattt atttaatttt
     1021 tttaaaaaaa tttaaaccaa tagagatggg gtctcactat gttgatcagg ctggtcttaa
     1081 ctcctgactt caagcagtcc ccccatgtca gtctcccaaa gtgctaggat tacaggggtg
     1141 agccactgca cccggcctca atccttattt tggcctgaga ggaaaggccg tggccccatt
     1201 tgcaggggag aagactgaag ctggaggggc aggccttgct ctgggttgca cagcagcaag
     1261 agaagtggga gctggccacg aggcttcctc gactcgacac actggtgggg tacaccctgg
     1321 ttctccaggt cccatggggc tcagcccagg actacctcgg ggggtgaggg acttaaatcc
     1381 tctccttcat tctcatcacc ccttccccca tcatttcctg aggaaggaca ttcagggacc
     1441 tgaaggagcg gcctgcccct ccacatctgt gggtgtttct catcaggtgg gacaagagac
     1501 tgagaaaaga aagagacaca gagacaaagt atagagaaag aaaagtgggc ccaggggacc
     1561 tgcgctcagc atatggagga cccacgctgg caccagtctc tgagttccct agtatttatt
     1621 gatcattatc tctaccatct cagagagggg gatgtggcag gacaacatgg taatagtggg
     1681 gcgagggtca gcaggaaaac acgtgaacaa atgtctctgt gtcataaaca aggttaagaa
     1741 aaaggtgctg tgctttgatg tgcatataca taaacatctc aatgcattaa agagcagtat
     1801 tgccaccagc atgtcccacc tccagcccta aggcagtttt ctcctatctc agtagatgga
     1861 atatacaatt gggttttaca ccgagacatt cctttgccca gggacgatca ggagacagat
     1921 gccttcctct tgtctcaact gcaaagaggc cttccttcct cttatactaa tcctcctcag
     1981 cacagaccct ttacgggtgt cgggctgggg gacggtcagg tctttctctt cccacgaggc
     2041 catatttcag actgtcacat ggggagaaac cttggacaat acctggcttt cctaggcaga
     2101 ggtccctgcg gccttctgca gtgttttgtg tccctgctta cttgagatta gggagtggtg
     2161 atgactttta acaagcatgt tgccttaaag catttgttta acaaagcaca tcctgcatag
     2221 ccctaaatcc attaaacctt gagtcgacac agtacatgtt tctgtgagca cagggttggg
     2281 gataggttta cagattaaca gcatctcaag gcaaaagaat ttttcttact acagaacaaa
     2341 atggagcctc ttacgtctac ttctttctac atagacacag taacagtctg atatctcttt
     2401 cttttcccca cagggacctt cctggctgtg cctaggatca ggaccagaat gacacccatt
     2461 catttccctg ggcctttgct cgggcggtcc ctgcaccctg gcctctgcct gaccaggatg
     2521 gtggggagag gagggggat gtcccccacg ctgctgtctc cactgttcct gctgcccagg
     2581 cctctgagct tccaggactg cagcgggtgg gtgggtggcc tggcctaagc ccaggaatgc
```

2641 acttcagctc ctggtagagc aatgtcactg aggcttggga gtcgggtggg gacgggagga
2701 ggcgtccgca ggccccccta ccgtgagagg cagccgtggg aacagcctac ctctaaacaa
2761 tcactgtagc ccaggctgac caggggctct ggccggacag aggggcctgg caggctgtgt
2821 ggcctgtaag gacacagtct gtctctgtgc ctcagtttct ctgctgccca gatggagagg
2881 cccagactcc aggtgtagac atctggagca ggcagtgttc agctggggag ggagcggtga
2941 ggactatggg ggccacgtgg gaagaagtcc agcccacatc acctgcaccc ctgctgagcc
3001 tggtcaacag aggccctcag tgggtcctca ctctcctggc tgcctcccat ttaggcaccc
3061 tgaggcctgg ggagaacaga gccaggccag tgtccccaga gaggctgcgc tgccagcaca
3121 gtagtagcag atttggattc agggaagtag acctgcagcc agggtgggaa agagctgcag
3181 gcggggtgga gcccccacat ggcacagccc ccctccctgg aggtctgtgc tgcatttcca
3241 ggacagcaag tcccagggat ggatggtgcc aggtgccaag ggctagaggc atggtctgtc
3301 tgcattcccc acaggggcgt cttatagtca ccagcatttg atgctgtcaa gtccccctgt
3361 cctctgtgca gactgggaag cccttggtca ccctgggggg gttgggggac ccaggccagg
3421 ctgcagaaac ataaggactt gaacccgggt cctgagtgac accaccttgg gtcctcctcc
3481 ctctgcctct gttcagctcc accttgatgc tgactaggct gggccatgcg gagagggtta
3541 ggggatagag atgggagctg gggagcaggg ctccactctg ggaggggggc agccttgccg
3601 gatccagggc agagttaagc ggccccagct ctgctttcct agagctgctg agaacccagg
3661 aaatggtgtg gagtttccgg ggaaccctgc ccctacctgg caaccgcagt gcagcaggca
3721 ccaagttctc ctgcacattg cgacagtgtg accctgggct ctggcgggcg gtaggtgggg
3781 cctttggacc taccagcagt gagggagtta acacagcagc tgactcctct aggcaaggaa
3841 aactcccctc agatgctttg ctgcctggcc tcctgccagc aacaagcagg agctgaaaac
3901 tagaagttga ggcgtgagtt tggccactcc gtagtgtgca cttggtgagg gcagcagctc
3961 gccacagctg ccagccgtct gtccattcac ccatctgtcc atctggcagc ccgctgttca
4021 gacccgtctg tctgtccgcc catctgtaag cccatctctg tcccattgtc tatctgacca
4081 tctttctctt actgtcctct ttatctagct atctggccta tccgtcgatc catcttcgtg
4141 tctgtcttca gcccccacct gtttgtccat ctgtccaatt acctgtgagt ctatctatgc
4201 atcttcttgt ccattcatct gcccacccat ctgtccctcc gtctgcccac cggcctcccc
4261 tctccttctg ggccgcagag ccatggccca ggactgcaga gccatggttg gcctggtcct
4321 gctggggctg gggctggcgc tggctgtcat tgtgctggct gtggtcctct ctcgacacca
4381 ggccccattt gacccccggc ctttgcccac gccgctgttg ctgctgactc caaggtctgc
4441 tcaaatattg tacggtgagt gagacgtggg aggaagctgg gtggcccttg gcagccagcc
4501 cctcctggag aaggcgtgta tgtgagagtg tgtgtgtgag catgtgtgtg tgtgagagat
4561 tatgtgtcag tgtgtgtggg tatatgtgtg agtgtgagtg tttgtggggt gtgggtgtgt
4621 gtgaatgtgt gtgatcgtgt ttgggtgtgt gtatgtgtga gtgtgggtgt gtgaatgtgt
4681 gtgattgtgt ttgtgtgtat gtgtgagtgt gtgggtgtgt gtgggtatgt gagtgtgagt
4741 gtgtgggtgg gtgtgaacgt gtgtgattgt gttttgctgt gtgagggtgt gtgtgactat
4801 gagtgtgtga gtgtgggtgt gtgtaaatgt gtgtgattgt gtgagtgtat gtgtgggtgt
4861 gagtgtgtga gtgtgagtat gggggtgggt gggtgtgaat gtgtgtgatt gtgtgtgtgt
4921 gtgtgagtgt gtgtgtgcgc gtgtgtgtgc acgtgcactg gcccaggaag caggagccat
4981 gtgtgtgggc ttcagcacct gcagggcttg agagcaagga gacagcctca gggcccttgc
5041 acagaacagg tggcagggtg tgcccgtggg gcagatgggg acttgaggac aatggtggtg
5101 tgtgagtcca tacctggctc caggattcag gaggcccatt tgcacatccc aggtgggaac
5161 ctgtctggcc ccgcctgacc ctgctggccg tgcaggcccc ttcagtgagg ccaattctcc
5221 aaggctgggg tcttctccca gggtcatggg cgaaggggtt tggaggctcc ctgcgtgggt
5281 actggcctgc tggggtacac acaatgctgc catagccagt ctgccccaac acccagcccg
5341 gggccacatc tcaggtctct cagtcctgag gagcccggtg ccccacccct cacatcctct
5401 ctccctgagt caggggcctgg gtctcgtgag ccgagtgact gatacttggt gtcctggatg
5461 agggcgtgat ggagaggggc cacagtgggt gtttcctgac cctcttccag gaaggtgctg
5521 ctgccgctgc agggaggaca catacaggat gccccttcct gcgccctgcc tcccattggg
5581 cccacaaaag ccagggcaag cctcccctcc ttgccagcca cctggtctgc ttcccagaaa
5641 ttctgtcttg caggctgttg ggaggatccc agtactttgt aaactaaagc aagggaggag
5701 tggccattct ctctctttgt tcattcattc aacttttcat tcattccttc ttccctccat
5761 tcccccatct gtccatcctt ccctgccctg attgctcttg ccaccgcccc ccccagcccc
5821 tcctgacctg gtcctttggt ttctcttcag ggctttctgt ctcctcccac agggctgaga
5881 atggcagctc agggacaagt aggggctggg gactgcttag tctccccagt ggctctcagg
5941 ggatttgagg gtttgacgcc agctgccacc ccaggttgtg cccctcctct gctcaggagg
6001 acatacagga tgcgacactc acttaaacac aaagttgcaa agatgcaaat gagactgggg
6061 tctcaggcac cagagaccac ccgtgggcac gtggcttttg ggagtggaga cctgctgcca
6121 cagatctctg aagagtctgg acctgctggg tctccccgag tgactgtctg gggtctcca
6181 tagcatgccc tgctgtgtgc gtgacggtca ctggttgggt aggggcctct actctaaagc
6241 tccctctgcc ggcatcccct cgaactctcc cttggtgaag agagaggatg tggtttgccc
6301 cagtgtttta tcaaacaact ctctccactt cctgttttaa gaagctggga gtggaagaga
6361 gcctggggct ggccccagct gctgctgcaa aacaggggtc actggatgct gggtccctgg
6421 ccgggctggc tggaggcctc aggaagaggc ctgctgcagc gtcatcctgg ccaagattcc
6481 tccctgcaga ggaccctggc cacactgcca cagggtctgc tggggccacc agaagcccat
6541 gctcctgcct ccatctctcc cctctgtgct cacctctcac caggagaccc tcccagagtt
6601 cagtgtcctg cttttttttt tttttttttt tttagatggt gtctcgctct gtcaccaggc
6661 tggagtgcag tggcgcgatc tcagctcact gcaacctctg cttccttggt tcaaatgatt
6721 ctcctgcctc agcctcctga gtagctggga ctacaggtgc cagccaccac acccagctaa
6781 tttttgtatt ttcagtagag acagggtttc accatgttgg ccaggatggt ctctatctct
6841 tgattcaccc gccttggcca cccaaagtgc tggcattaca ggagtgagtc atggcacccg
6901 gcctcatctc ctactctttc agcaccaggt tttattcttg ggattctgct acagccggag
6961 cccctgggtg cgagctccta agctttctgt gagtgtggac ccagcactgt gcctagtaga
7021 catacaaaag gagcatggtg acagtgaggt ctgtcatctc cagcgtaatg actgttttga
7081 tccttgtgaa aaaggtgatt tttggctggg tgtggtggct cacacctgta atcccagcac
7141 tttgggaggc cgaggagggt ggctcacttg aggtcaggag ttggagacca gcctgggcaa
7201 catggtgaat ccatgtctct actaaaaata caaaaattag ctgggcatgg tagcaggtgc
7261 ctgtaatccc agctacttgg gagtctgaga caggagaatc acttgaaccc aggaggcaaa
7321 ggttgcagta agccaagatt gcgccactgc actccagcct gggtgacaga gcaagacttg
7381 gtctcaaaaa aaaaaaaaaa aaagaaaagt ttatattttt gttctaatgg ttatcttaat
7441 atcttcattc tataattata tgttttatat aattagctat ataagatata ataccccta g
7501 tatgttgttt tttggatatt ctactcgctc ctgatggtta atttatgtgt caacttggct
7561 aagctatggt gccccgttgt ttggtcaaat acttgtcaat atcttgctgg gaggttattt
7621 catagatgtg attaacactg acagtcagtt gactttaagt aaaacagatt acccaccata
7681 atatgggtgg gccacctcca atcacttgaa ggccttaaga acaaaaactg aggtttccca
7741 gagaagcagg aattctgctt caagactgta acacacaaac cctgcctgag tttctggctt
7801 gctgactgct ctacagattt taggttccag acttcgagat caactcttac ctgaatttat
7861 agcctgctgg cttgccctac agattttaaa cttgctagtc cccacaatca tgtgagccaa
7921 ttcctcaata aatctctctc tatgtataat ctattggttt agtttctctg aaaaactttc
7981 acatccagtt tcctggatgt taagaattac cgaaactagc tagtaacttc ttttttttga
8041 gacagagttt tgctcttgtt gcccaggctg gaatgcaatg gcacaatctc agctcaccgc
8101 aacctccact tcctgggtcc aagcaattct cctccctcag cctcctgagt agctgggatt
8161 acaggcatgt gccaccatgc ttggctaatt tttgtatttt tagtagagac agggcttctc
8221 catgttggtc aggctggtct tgaactccca acctcaggtg atccgccgcc ttggcctcac
8281 aaagtgctgg aattacaggc acgagccact gtgcctggct cctagtaaat tcttcttttc
8341 cgtgatgtgt ctcttacctc taataatact tttcttctta aagtctactt cattaaaaat
8401 agttatgctg ggcatggtgg ctcatggctg taatcttggc actttgttgg aggtcgaggt
8461 gggtggatca ctgaagccca ggagttcaag accaacctgg gcgacatggt gagaccctgc
8521 ctctacaaaa aatacaaaaa ttagctgggt gtggctaata taattctaag ttggcacact
8581 tgtagtccca gctacttggg atgctgaggt gggagaatcg cttgagccta gaagggagag
8641 attgctgtaa cccaagatca catcattgca ctccagcctg ggagacagtg aggctctatc
8701 tccaaaaaaa aaaaaaaaaa aaaaagttat acagctttct tggttagtgc atgcatgaca
8761 tatttttcat tatcttccac ctctctgtat tcttatataa aaggcattag ttgggtttta
8821 ctttattttc aattatttta acttttattg tccttttaaa tgtaactaat gatttatttg
8881 ggttgaaacc caccaccaat ttgttttcca tgcctattct atttcttctt atctcctctc
8941 acatcttgtt ttggatttat tatttttatt atttttatta tttaatttcc tccttctcta
9001 ttagtttcat aactgtgcag tcttagagtt attttaaaag atgactggat tattttagag
9061 cttacaacat gcatccttca cttatcaaag tctaacatga gctagtactt tttgttgttg
9121 ttgagataga gagagtcttc ctctgctgcc caggctggag tgcagtggag caatcttggt
9181 tcactgcaac cttcacttct tgggttcaag caattctcct gcctcagtca cctgagtagc
9241 tgcgaccaca ggtgtgcacc actatgcccg gccaattttt gtattctttt ttagtagaga
9301 caggggggttc accatgttgg ccaggctggt cttgaactcc tgaccttaag agatctgctt
9361 acctcggcgt cctaaagtgt tgggattaca ggcgtgagcc accacgccca gcctatgagt
9421 tagtacttct atcctcttcc tagtcagtac aagaaccttg gaacaggaac taaatttacc
9481 cccagtgact tatatgctaa tatttttgtg tattttaaat atatgtgtgt gcatagatgt
9541 atctgtgtgt tttttgtgtt tttattctta tttatgttga gagtgtagag ctatgtaaga
9601 gtaaaaagaa ttgtgtaatg aagccccgag tatccattca atttcaacaa caatcttatg
9661 gccaagctaa tttcatgtat actctttcct gcttccctct accccacatt atttcagtgc
9721 aaatcccaga tatataactg tacccataca tatttcagta tgttttattt attttaaacc
9781 ccacaagata tcattttcta tactactgta attttatacc aataacattc atttagattt
9841 acccacacgt ttaccctacc ctccgggtcc tgtttgaaaa tcaaacccat gctcacaggc
9901 caattttttt ttcttttaga gacagggtct cactttgtca cccaagctgg agtgcagtgg
9961 tgcgattata gctcaatgca gcctccaatt cctggactga agggaccctc ctgcctcagc
10021 ctgccaagta gcttggacta tagctgtatg ttttattatt attttgtaga catggggtct
10081 ggctatgttg tccaggctat tctcaaaatt cctggcctcg agcaatcctc ctgcctcggc
10141 ctctcaaagg ttgggattac aggtgtgagg caaggcaccc agctcagcca cagagccctg
10201 ttgcatctct cttactagga gcaagagctg actgcccccct catccccatt ccagagtgtt
10261 ggagctgtgt tcagccaagg ccgggccact ggcatggccc agggagtggg atcattcact
10321 gctgccccaa agctgagatc attccacctt gacaagactt cctcatccaa tccctttact
10381 tgacagctgg ggaaaccaat gcgcacagag cacccccagc tcactcaggg tctcagagct
10441 gatccatgag cagaggccga gatcctggga tcttgtcccc cagccgcccc gcaagcttac
10501 tccctttctg ctggaagaga tggggccgga cctcgaccag cagccctggc ctggacatga
10561 ctgtgctcac gcaggtattg aggccgtgat gccccggcat catatttttt tctctttttc
10621 tttttctttt tttttttgag acggtgtctc actctgtcac ccaagctgga gtgcagtgga
10681 atgatattgg ctcactgcaa cctctgcctc ccggttaaag tgattctcct gcctcagtct
10741 tccaagtagc tgggcctaca ggcttgtacc accacgcctg actaattttt gtatttttac
10801 tagagacggg gtttccccat gttggccagg ctcgtgtcaa acttctgacc tcagctgatc
10861 cacctgcctt ggcctcccaa agtgctagga ttacaggcat gagccatggc gtcacttaaa
10921 tgtagtgaga ggccgggcaa ggggctcatg cctgtaatcc cagtacttag agaggacgag
10981 gctgtcagat cacctaaggt caggagttcg agaccagcct ggccaacatg gtgaaactgt
11041 gtctctacaa aaaaaagaaa aaaaaaatcc ctgcgtggtg gcgagtatct gtagtcctag
11101 ttactcagga ggctgaggca tgagaatcgc ttaaacctcg gaggcggagg ctgcagtgag
11161 ctgagatggc accactgcac tccagcctgg gtgacagagc aagactttgt ctctaaataa
11221 ttaaataaat aaatatggcc gagcatggtg ccttaggcct gtaatcccaa cacttcggga
11281 ggctgaggca ggtggttcat gaggtcagga gcccgagacc agcctggcca agatggtgaa

11341 acactgtctc tactaaaat acaaaaatta accagctgtg gtggcaggca cctgtaatcc
11401 cagctacttg ggacactgag gcaggagaat cgcttgaacc tggaaggcag aggttgcaat
11461 gagctgagat tgcaccactg cactctagcc tgggcaatgg agcaagactc cgtctcaaat
11521 aaataaatta ataaatacag agcaatattc catctcaaat aaataaataa atgtacacct
11581 gtaatcctag cactttggga ggctaagaca ggtcgatcac ctgaggtcag gagttcgaga
11641 ccagcctgac caatatggca aaactccatc tctactaaaa atacaaaaat tagccgggcg
11701 ttttgacgtg tgcctgtagt cccagctact tgggaggctg agacaagaga attgcttgaa
11761 cccaggaggt ggaggttgca gtgagccgag atctcggctg cacttcagcc tgggtgacag
11821 agtgagactc tgtctcaaaa ggaataaata aaatacaaag taaaaaaaaa tgtagtaaga
11881 ttgcagagtc gtgccgcgga agcgtgctgg tcctatccat gtagtgaagg ctgatttcat
11941 acacaaatgt cacaagaact tttcttttct ttttcttttt tttttttttt tgagacggag
12001 tctcgttgtg tcacccaggc tggagtgcag tggtgcgatc tcggctcact gcaagctctg
12061 cctcccgggt ttatgccatt ctcctgcctc agcctcctga gtagctggga ctacaggcgc
12121 ctgccaccta gcccaactaa tttttttgta cttttagagg agatggggtt tcaccgcgtt
12181 agccaggata gtctcgatct cctgacctcg tgatccgccc gtctcggcct cccaaagtgc
12241 tgagattaca ggcatgagcc accacacctg gccttcttat atgcttttat tgcatttgag
12301 cgtacctctt ttacagcgaa gatctttttt gaaattttat tttattttta gagatggagt
12361 cttgctttgt tgcccaggct ggagcactgt ggtgtgatca tagctcactg cagccttgaa
12421 ctcccaggca caggtgatcc tcccacctca gcctcctgaa tagctgggac tacaggcatg
12481 caccaccatg cctggcatat tttaaaaatg tttgtagaga tgaggtctcg ctatgttgcc
12541 aggcttgtct caaactactg ggctcaagcc atcgatccat ttcagcctcc caaagtgctt
12601 ggattatagg catgagcacc gcgcctggcc atcacactgt tttttgtttt gtttgtttgt
12661 ttgttttgag atggagtctt gctctgtcgc ccaggctgga gtgcagtagt gggatctcac
12721 ctcattgcaa gctccgcctt gtgggttcac gccattctcc tgcctcagcc tcccgagtag
12781 ctgggattac aggcgcccgc caccacgcct ggctaatttt ttgtattttt agtagagacg
12841 ggatttcacc gtgttagcca ggatggtctc gatctcctga cctcgtgatc tgcccgcctt
12901 ggcctctcaa agtgctggga ttacaggcat gagccaataa ctatttttat aatcattaac
12961 taccgaggag aagctggaga gaaaaaaggt ggatgaagtt aaggcagaga gactttgtaa
13021 gtttcttagc tcagcttttg gagactgtat atcagagatc ctatttgagg tgattttcag
13081 ctacagagat aggcctggtc atttgaaaaa taacggatta ggtgaagctt gcgtttgaaa
13141 tcctccttct acttttcatc tttctctctt gtttattctg agcatccatc ttagacaccc
13201 aatctttgtc actttgtggt tgtcattgat ttccctgtta ttgagattag aggttggcag
13261 actttctctg taaagggctg gagagaaagt acttcagact ttgtggtctg tgcagtgtct
13321 gttgcaacca cttaactctg ccctatagag caaaagcagc cgtagacagt acatggacag
13381 atgagcatgg ctggggtcca attacattta cttgcaaaaa gagggttgga gactgggtgt
13441 gatctcacac ctttaatccc agcactttgg gaggccgagg caggaggatc actttaagtc
13501 aggagttcaa gaccagcctg ggcaacaaag caagactcca tctctacaaa aaaaataaaa
13561 attattatag ctaggcatgg tggtacacac ccgtagtcct agctactcag gaagctaaaa
13621 ctgaggcagg agggtcagtt gagcccagga gcacgaggct gtggtgagct attactgtgc
13681 cagtgcactc cagcctggtg acagagcaag aacggtctcg tcaaaaaata aacaaaaggc
13741 tgggcacggt ggctcacgcc tgtaatccct gcactttggg agactgaggt gggcagatca
13801 tgaggtcagg agattgagac catcctggct aacacggtga aaccctgtct ctactaaaaa
13861 tacaaaaagt tagctgggcg tggtggcggg cacctgtagt cccagctact cgggaggctg
13921 aggagggaga attgtttgaa cttgggaggc ggaggttgca gtgagccaag attgtgccac
13981 tgcactctag cctgggctac agggcaagac tccattaaaa aaaaaaaaaa ccagcaaaaa
14041 ccaaacaaaa cataatgcat gttctctctt ataaatggga gctaaacatg gggactcatt
14101 gacttaaaga tggcaacaac tgggaactgc tggatgggga gggaggggag gggtgaaagg
14161 ccaactgttg gggagtatgc tcatatccat gtgacaaacc tgcacatgtg cccgctgaat
14221 ctaaaataaa agttgaaagt agattttaaa aaccccaaga gggctgggtt tggcttgtgt
14281 gtccatagct tgttaacctc cactttagat attaattaat agaaacatag tgcttatctt
14341 cccaggccac ctattttgtt cctctccaag gtgatggata gatgaaggct taatccagcg
14401 gcctggaagt ttgctgacgc ttgtcctgtc agggattaat gaagcattgt tttctgatga
14461 aggtttcatg ccgctgtgct gatgtgtctt ctcttctctc taggcaggaa actgcatatc
14521 ttctggttta catgaagatg gagtgctaat ggaaatgccc aaaaccttca gagattgaca
14581 cgctgtcatt ttccatttcc gttcctggat ctacggagtc ttctaagaga ttttgcaatg
14641 aggagaagca ttgttttcaa actatataac tgagccttat ttataattag ggatattatc
14701 aaaatatgta accatgaggc ccctcaggtc ctgatcagtc agaatggatg ctttcaccag
14761 cagacccggc catgtggctg ctcggtcctg ggtgctcgct gctgtgcaag acattagccc
14821 tttagttatg agcctgtggg aacttcaggg gttcccagtg gggagagcag tggcagtggg
14881 aggcatctgg gggccaaagg tcagtggcag gggtatttc agtattatac aactgctgtg
14941 accagacttg tatactggct gaatatcagt gctgtttgta atttttcact ttgagaacca
15001 acattaattc catatgaatc aagtgttttg taactgctgt tcatttattc agcaaatatt
15061 tattgatcat ctcttctcca taagatagtg tgataaacac agtcatgaat aaagttattt
15121 tccacaaaag gactttgcag ttttaacggg gggcagtagg gattgtgcta tagaaattca
15181 aaggcaaggg aagtcacttc tgttgtgagg ccctgggagg agcctccagg ctggaaaggg
15241 ttaaggtgga ggtctccgat aggggcagcg tccacagtgg actggctgca aaaggccgtg
15301 ctcagcattc agacagcatc acacactctg cttttctcta ccagggaggc aggtggggaa
15361 ggatagcgat gggaaggcag gcggagctca gaatgtggaa ggggatccag taaggcttgg
15421 aagtttgcac ctgatctggt gggtggtgag gagcccttga agggacaagg aggcgaggag
15481 cactcagctg tgttcttaca ctgatctgcc actggggtta gagacaaacg tggtgggaat
15541 ggaaagccac gcacagtcac tagcgcctct gggggagaa tggatgtggc tggtgagaga
15601 acagggggc ccagggagag tccggcacca acctgggggg ggagcccagt gggtgtgagc
15661 acccccactt tagagatgaa gtgatggaga cattcagatg tttaacccct tgttcaagat
15721 tccatacttg ataaatggca gatcaaactc ccaacataaa atgtgggtca tttctttatt
15781 attttatttg tattggttaa caatgatcag ccatgcaaga ataaatgatt attgtaaaat
15841 ctgcaaacaa tgtagatatg tagagaatcc cttccttgga gcttgacctt gtcagacagg
15901 tatagatgag tgttcggggg cagccgtaaa aactgccaga gactgggctg cttataacaa
15961 acgcgtggtc tcccagaagc ccacattcaa ggtgtccaaa ggcctggctc ctggaggctc
16021 tggaggagag cctgttccct gtctctcagc ttctgccggt tgccagcaag tgttgccgtt
16081 cattcactcc agccactgcc tccatctgca cacagcaaaa cagcatatcc tgaagtgctc
16141 aaccttatag ccattatttt aaaatatccg gaagacacag gaccgtggga gtggctgttg
16201 gagaaatttt catgaaggaa gaaagattac aactaagttt taaaatgcta gttttgtttg
16261 ttttgtcttg gagaggaggt aaaagtggga gtaaaaatag ggagtttggt gtaaggtggg
16321 aaaagcaaag gatccccgca tggatgggct gaagggtgtg atgggagaac agtgagaagt
16381 acgtttgggg aagcaattgg aaatagcagc taagcttaat cacaatctat caaaagggac
16441 ttgttgaaga attaatgtgt gactagaaac agggaggtta tgggcttgtc agctcgacag
16501 tgggcactca gttccactaa caaatgatgc ccgtgtgggc agacagaatg atggacagac
16561 agaatgatgg gcaggcagat gaatgcatgg gctttacgtg aaacaggtcc acttggttgc
16621 tgacaagata ctgttttaaa gttccatttt gccatacttc gaacagcttg tcattagctc
16681 aatttagcca catgtaaaat cactaaggca gacttccaga gttcccacat gaaaatcaaa
16741 tgtaaaccag cagtgacctg cttcaacacc atcatcggaa gtcagaagtt gaactctttt
16801 ttgatgttta aagcctgcat aatattcgct gtattattat tcagcatatt actatttcct
16861 tcgtgatgga aatttggttt atcccaattt tctattatat caaaacaccg ctacatagaa
16921 aatccccatg cacatatttc tcctaattgt ggaaatattt tacatagaag actctagaca
16981 tgggatgaaa ttcccaggtt attggaattt taaaatagat aggtacttcc aaattgacct
17041 cttacaaatt atatgaattc gtaagcttcc aactgttatg gagttaccca ttttgagaaa
17101 tctgtgctaa aaggacccaa acaatgctga tgacaatgat caggataata agtacgctgg
17161 gaagacaaca aaatgattta gatcttagac aagtcattct aggtgtctcc actgtttcag
17221 ttcttgcgtt cgttcattct tgtgcttttt cgttttacca aataaaatag ctccttgatg
17281 tcatacgaat ccatgctatg cttaatgagt attggttagt aaaatgccta taactagtaa
17341 tcttcatcta tgcaattaaa tattaattca taaaacactt caaatgtaaa caataattag
17401 taaatgaaaa gtacataata cctcaattag aaaaaaatca ctccattaaa aagacattat
17461 ttgtgtgata aaagagattg ccatttttgt atttttctac aaggttaaag aaaactaagt
17521 caacttatac aagtgaattt taaaagactt tagggcgggc atggtggctc acgcctgtaa
17581 tcccagcact ttaagaggcc gaggagggca gatcacctga ggtcaggagt tcgaaaccag
17641 cctgaccaac atggtgaaat ctcatttcta ctaaaaatac aaaaaaatta gcccagtgtg
17701 gtggcacgtg cctataatct cagctacttg ggaggctgag acaggagaat agtttgaacc
17761 tgggaggcgg aggttgcagt gaaccaggat cgcaccattg cactccagct tgggcaacaa
17821 gagcgaaact ccatctcaaa aataaataaa taaataaata aataaataaa taaataaaag
17881 cctttaaccc agaatgctga gtaaattggc caaaaatgct aacctatgca tttcaatact
17941 ataggagttg catgggtaga aataactaga tgaaatactt ctggtatttc accttcccaa
18001 cccacacgag ccagtgtttt tctgtgaata acaaaaacag cagaatttac ttgcctctcc
18061 ataagaggtt accacttctg tgtgttcccc tgaaacaggt ggtggctggg tgagaaggtg
18121 gacggcacta gggcaggaga tgggggctcc agtatcgtgg gtgagcttcc taaacctctg
18181 caactttcag cccctaaatg ggatgagcca tcagaatttt tagcacaatg cccagaacaa
18241 agtaaggatt tgacaaatga cgcctctctc cacattgttc tgtcatcagc caccgcatcc
18301 tgtacctcca agcccactgg gctccggctg tttccatcac atggagaatg actcagagcc
18361 tggcctccag ccaccctcct ggcctttctg cttctcactc tgccactggc tcctcatgga
18421 ccaccagcct gggtgtcctc agacatacca cacacttcac tgtgggagtc acgcagccct
18481 cactgctcct tcgccaggga gccacagggc tttcctcctc aggaggactc tgcaagcagc
18541 tggatgaagg gccctcccgt cttcatcttc cttaattttt gtcacagttc tccttccttc
18601 cactcagtgc agcgcacact gattgatcct ccatcttccc caaaagacag gaacagcatg
18661 agcagtagag agtagattcc aatgatagaa aaaatagtca gtgatttctc atttccattg
18721 atcatcaatg aagaaaatgt atcctgaagg tcacgtacct cctatgggac tgctgcatcc
18781 tcagcctcct gaatttcagc ccagcacctt cctccccagc acagcaacag gtcagccctt
18841 accagcatcc ctctcttatt gcctttgtgc agagccagca ccagggccag gggaggcctt
18901 gggattgtcc ctccccaaca atctgtgaaa caatccttta tgtcaccaac aaagcacagc
18961 cttatgcact ggtggtcagt ccctcccaac acctctgtca ctgtaaagct ggcaggcaac
19021 cctccaaggt tggccttccc aagcactgca cctctaggtg acagagcacg tccttacctt
19081 gaagcctggg cgcccagtct atcctgtcca atgagcgagc tgtggagaag gggggattcc
19141 gggttaaggg gagactagca gggctcctgc ttttatgttg ccctgttggg aaggctatta
19201 aagaaacaca aagtgctaag cagtgaggat agaacatgtt ttcattattt aaaccaatac
19261 attccacaga tggaataata agaaatgcta caaccaagct aactgaatcc aacagcatat
19321 aaaaaagata atccacaatg attcaagtgg gtttcatact agggatgcag ggacggttta
19381 acatacgcaa gtcaataaat gtgatacatc acataaataa aactaaaaac aaaaatcaca
19441 tgataatctg aatagatgca gaaaaagcct ttgacaaaat ccagcatttc tttatgatta
19501 aaaccgttca tcaaaatcag catagaacgg acatacctta aggtaataaa agctatctat
19561 gacaaaccca cagccaacat tttcctgaat gggggagagt tgaaagcatt cctcctgagg
19621 aagggaacaa gacaaagatg cccacgttca ccacttctca acacagtgct gttcactaca
19681 gcattggtta taagagcaag actggaaaca gaacaaatgg atacccatag cggggtgctt
19741 aagtaatttt gggaatagtc atggggtgca gtactttata gctctgaaac aatacaatgg
19801 atttacattt gaaatgtgga atgataacta aggtgcattg cccagtgata tatgcagagg
19861 tgcagaggac tttgtgtaaa cacgatcaca catcagcctg cattccaggt gcatgcttct
19921 atttgcacat ggattgcagg gatgatatgc aaacaaaaat gttgacttgg tgtttggaag
19981 ttcagagtgg aagggaaact tccttgctaa ccttttatga tatttagagt ttctaaatgt

20041 gaatacataa tacatttaga aatcttagtt aataagaaaa gcctctgttc ctggcctctt
20101 gctggcacat gtcaggtgga aatggggctg tcatgctaat gtgtgcaaac tgagaaaaat
20161 ccaagaatgg gagtctgctt ttttcatcat acaaataatt gttaatagaa acagtatgat
20221 aattgctcat tgatatacca tgcatattct attagataat aataatttct gaaatttgaa
20281 ctatacttac acatggaaat tgaaatatat ggatgaaaca ttgtggcttc tataggcaat
20341 tgttttattg gcattttaca aactgatcat cattcctcat ggcacgggtc tatgtgatat
20401 taagtagctt gttatgcttg ggaaaggcag tgatgaccac aagaatgact tcaactacta
20461 aagtacaatg gagatttcaa caatgttttg tttaatattt aaatatttca tgtgctccca
20521 ggctttttct caccctaata gctctcatcc atatcatgtg ggtcccatta atacagatac
20581 ctccgaatgc accactcttc cattatatcc agtcaattgc tggttacctt gggcctacaa
20641 ctgggggagg gcaggggctg ctggccacct cctcatctac agtaagagtc aatgagcagt
20701 taaatggata ctgaaaacca tttatcctgc tggagtgaga aataaatggt ttctttcaat
20761 agcgtagtaa aatgcatctt ttccaaacta tttatatgac tcaaggccca tctcaatttc
20821 agatgtggtt agcctcaatt cctgattctc accaaggtgt gtaatgtcat ccacagccca
20881 gtgcagagga acacgtgctg ccgtcagact gccagggtcc gatcccgcct cctcactcac
20941 cctgggagat ccctttaagc caggagtcaa cagtgaggat ggaaacatga gtgcttttta
21001 aagtcctgaa agttcagagg cagactgtca atttctcctg cacccctggg cacacaccag
21061 gagaactttg tctccaggat caagtaagtg cctgtgagag agttgtgtcc ctcagattct
21121 gttcaccaca ggtgacactc gatgcaaccc caaacctctt ctgcacaatc ccaaggggtg
21181 ctgactaatc caacccaaag gctgtgatgt ttggcagagg cagaaaagaa aaggccaggt
21241 gttccgggaa agatcacctt caaataacac agcactctca tagcccagag agacagttct
21301 aactattatg ccaataaacc cggaaaagac caaatccaat ttgacacata tttccttttt
21361 cgttttgatt tcatgccccc tccctcaacc tcccaagcag catggataat ccccgaaggc
21421 ccctgggaac tctctccaat tggatcttac gtggaaagta gttacccacc tacaaatccc
21481 catcatcaga tatgctctcc acaatcaaat ctttagaaac acaaacacca ggataagtca
21541 ttagagagag gcccacccac tcctcccacc ctagctgaag ccacggtgct tcgcacagga
21601 tcccctggtg tttcctctgg gctcacagat atccctacag cctctctgga cattgtttta
21661 tacttgcaaa atcatttgct ctcaccagac cccaaatcct ccttcccaaa aggagcccag
21721 aatcaggttt ctgtaccctg gagatggcgc tttttcctca ggaagtgagt tatttcaggg
21781 tacgtatcat tctccagtgt caatggctcc tgcaattata gaaaagaaaa cattaggagg
21841 gtgaaatgat gccatacacg tcacacagat ctgatagtct ctcgacaact tgagagagaa
21901 aataagaagg ggtatagtga ttgagtcaaa ggtcgaagtc ccccaaaact ggcacggaag
21961 acacctgtgg aaaagacaag accttttccc acagaattta tctttaaagt gtatctagat
22021 tggcagtttc acaactctta atccatgggg gaaaactgct gtggagggaa acacctctgc
22081 attgcagtgg atcgtggatg ctgccatcta ccacacccca gtgtgcctgg catgggttgg
22141 tgagaggctg ccaatcaata gcaccacacc aagggaattg cagatgtcat aaatagtcca
22201 cattggcaga tgttcatgtc tacatttgat taaactgcag atgacattga taatgcacac
22261 tggcagatgt tcatgtctac atctgattgg aaagaagcca ggaaagtaac atttctgttc
22321 aagacaaaga aaagtgtctt acattggcag cgtcttcttt tttacagatg tcttgtacag
22381 tgtcctcatt agcaatgtca tatacagcgt ccttattagc gaattcgtat acagcatcct
22441 cattagcgat gccatataca gcgtcctcgt tagtgatgtc gtatagagcg tcctcattag
22501 cgatgtcata tacaatgtcc tcattagcga tgtcatatac agcaacctca ttagctatgt
22561 cttgtaaagc atccttatta gcgatgtcat atacaacgtc ctcattagcg atgtcgtata
22621 cagcgtcctc gttagcgatg tcttgtacgg tgtccttata agcaatgtcg tctacagcgt
22681 cctcgttagc atgccttgtg ggtgtcatta gcgatgtcat atacagcatc ctcattggtg
22741 atgtcttata tggtgtcctc attagcgatg ttgtgtacag cgtcctcgtt agcaatgcct
22801 tgtacagtgt cctcgttagc gatgtcatat acagtgtcct cattagcgat ggcttgtaca
22861 ctgtcctcat tagtgatgtc gtgtacagca tcctcgttag cgtgccttgt acggtgtcat
22921 tagcgatgtc gtatacagcg tcataattag cgatgtctta tacggtgtca tcattagcga
22981 tgttgtgtac agcgtcatcg ttagcgatgc cttgtatggt gtcctcatta gtgatgtcgt
23041 atacaatgtc ctcatcagcg atgtcatata tagcgtcctc attagtgatg tcgtatacag
23101 cgtgctcact agcgatgtct ttttttttta tatatacttt aagttttagg gtacatgtgc
23161 acattgtgca ggttagttac atatgtatac atgtgccatg ctggtgcgct gcacccacta
23221 actcatcatc tagcattagg tatatctccc gatgctatcc ctccaccccc ccaccacaac
23281 agtccccaga gtgtgatatt ccccttcctg tgtccatgtg atcttattgt tcaattccca
23341 cctatgagtg agaatatgcg gtgtttggtt ttttgttctt gcgatagtta ctagcgatgt
23401 cttatatggt gtcctcatta gcaatgtcgt gtacagcgtc cacgttagca tgccttgtcg
23461 gtgccattag caatgtcgta taaagcgtcc tcattggtga tgtcttgtac agtgccctca
23521 ttagcgatgt tgtgtacagt gtacttgtta gcgatggctt gtacgatgtc ctcgttaaca
23581 tgccttgtac ggtgtcatta gcgatgtcat atatggcatc ctcattagcg atggcgatgc
23641 cctgggcggc gtcctcgttg gcgatgccct gggcggcgtc ctcgttggtg atgccgtgga
23701 cggcgtcctc cttggcgata ccctgggcgg catcctcgtt ggcgatgccc tgggcggcgt
23761 cctccttggc gatgccatgg acggcgtcca cgttggcgat gccctgggcg gcgtcctcct
23821 tggcgatgcc atggacggcg tccacgttgg cgatgccctg gacggcgacc acgttggcga
23881 tgccctgggc ggcgtcctcg ttggcgatgc cctggacggc gaccacgttg gcgatgccct
23941 gggcggcatc ctcgttggcg atgccctgga tggcgaccac gttggcgatg ccctgggcgg
24001 cgtcctcgtt ggcgatgccc tggacggcga ccacgttggc gatgccctgg gcggcgtcct
24061 ccttggcgat gccctgggcg gcatcctcgt tggcgattcc gtgggcggcg tcctcgctgg
24121 tgatgccgtg ggcggcgtcc tcgctggcga tgccgtgggc ggcgtcctcg ttggcgatgc
24181 cctgggcggc gtcctcgttg tcgatgccct tgtcggcggc ctcgttagcg atgtcgtgta
24241 cagtgtcctt gttagcgatg tcgtgtgtgg cgtcctcgtt agtgatgtcg tgtacggtgt
24301 cctcatgggg agctagaaga acacagagtt aaggtcagtg ccctggtggt ggagactgaa
24361 tcacccaggg gcttgcttgg tgtggtgcat ggaggtggct gatcacagca tgggccaagc
24421 tgatgctggg acatcctccc aggtggacct gcactagtga agctaaggga tgtggctcag
24481 aacactttct gcagtgggaa tcagtttcca ggttcaggta tgcattatcc ggtgaagtgg
24541 ggaaatataa aaaataaaaa ttgacaaatt catgaaaagc cttccatgag tgcaagtgtg
24601 agttttttat ccactttaca ttcagtatgc attcacacat acaaaatatt tttacaagaa
24661 atcagaaatt ttaattttg tcagttatgt taaatctaac ttagctgcca acataaagat
24721 tctatctcat ttacttggtc tcgagaaaat ctagcacata gtaagtagac caaaatgttt
24781 attaaatgaa aacacagagc agagataggg gggctgctag gcagactggg ttgcacttga
24841 ttacctggat gataataaac tgcacaaaac ctcggtcaaa ttaatattga aactgccttt
24901 tgcttgggct cgtttccctt gcggaagaag gatgaccaag aagatgaaca ggaaagaaat
24961 gagaaacaga ggcctttgct tagtagctaa aggccacctt ctgtaacatg aaatagtctg
25021 caagtggcct tgaactctgc cgtgatttag tgacagagtt ccctcatgtc ttctacccag
25081 gttgaagtcc agcaaaattg cgactgtcct ctttacaact tgcgagacca cactgcttct
25141 gcatttgcct gttgtatgta tgagatttac acttgtttta aagcaacatt ttgtttcagt
25201 tgggctggtg gccatacccg gcactagcca gtcaatagtg agatggctcc tcatggagga
25261 ggcttggctt gaggctgaag gtctttaacc cacatataca agagagttgc cactaaggga
25321 tggaagccag gctaataacc aagtgccaca cagagttcct atctgtccct cctcaccatt
25381 tttggctggc aggatttgag cattttaggg cttgggaaga tagtattact aaatctacta
25441 aaatacatca cccatcctta tagactttgg ccagttgctg agcaaattaa cttcacaact
25501 gaagtgggcc acactggcct ttgtggtccc ccactcctct tagaatttgt gagcatgggg
25561 cctactggag gagggtggag gtggaggagg gggggatcag gaaaataact gatattagcc
25621 tcaatatatg ggtgatgcaa gaatctgtac aacaaactct cataacacac atttatatat
25681 gtagcaaacc tgcacatcct gcacatgtac ccctgaactt aaaagttaaa aaaaaaaaaa
25741 gggatctgtg agctgagcca aacacctggg gatctttgtg cttttgacac actgatgact
25801 atgccggtcc gtggggagat gagcctataa ctgccctggg ttgtgtgacc atggaggcca
25861 ctttatgatg atgggcagtg tctggggcct tttgggctcg ttgctttagg gcttatacat
25921 gaatgctgga ctccctgtgt ggtggtgaac accccatgac taagtgcatg tcagcgtcag
25981 cactggccca cactcctggg ttcgtgtttt cactttttca ttcaggaact cgggagctgg
26041 ggccactccc ttggcccttc aggttctcca cctgagcagt ggggataata aggcagaccc
26101 ggggatggct ctggtgaggg tggaggagtc actgtacaga gagagtagag cgggggtgga
26161 ttttattgtt agaagtggac actggttatt gggttgtata agtgggaaat ctctcctgag
26221 aaaacacaca gcctcacctg tacagaaaca cacacattca caccacacga tgcagcctca
26281 cacaagacac caccaatcct caagcaccca actcagcacc acccaaaagg gagcacaatt
26341 gcttcctcaa aatttggcca taatttttcc ctggggaatt caggttttaa aaaaacactt
26401 cccctatact tattcctatc acaatcccag gatcagggag gctcttcaca ttgaaaccag
26461 gcaaggatgc cacacctttc ttggcatcca gattgttttc ttggcaagtg attccagaat
26521 acttactaga ttcacacctc agaggggcca cctgcaccac ctgcaataca gaaacaaagc
26581 tttttgaggg gtatgtcatg ttgtggattg tttgcacaag gctctgtttc tctcaatgaa
26641 tactgaaaac ttgatcagaa agtgtagtca acttcaaggc ctccaaaaca agggtaggat
26701 acacactgga aaagacatca gcttctggat ggtggatctc tcaggtccac gtaggttggc
26761 aagtgcaaaa tactgaatcc aaggagaaga cattgcttcc aaggacaagg accccaagga
26821 tacagtctac aacctgaagc catcacagct aaatgccatt ttggattaca tatcagttgc
26881 taagagtcac ttcttcctcc ccctcagaaa actgcattta atacctgtca tggacattgt
26941 catttttca catgtaaagt cagttgaaaa agaaagacac caagaaagga acatttctat
27001 ttcagagaaa gcaaggcaac cttaccctcg cgttgactgg cctctctcca tctcctctgt
27061 ccttgtgaac tagagactcc tcagaggcta ggaggacaca gagcaacagt tagtcataga
27121 tgcttttgtt catgagttat tcagggagct ctgcttaatg tggacaacag gacagtgtgt
27181 gtggatgtgt ttcattaaaa gcatagcttg agctcctgct agaaaatctt ccctcgtgga
27241 aagacaggca agaacgagga gctaaggagc aagaaataga gtccctggca ttttgccgat
27301 ggcaacttaa gaaaatggga atgagtcagt ctacaaatgg tactgaagca catgctatag
27361 tttgatgaga gtcccactgc tcacactgtg aggtttgaaa cccagctaaa tggttttcta
27421 aacctgtaaa aacaatatta gcttgcagga tttatgtccc aagactactt ttacctctga
27481 ggatccacag tggctgtcac tgcagttatg tgttttagca ttttgcactt gaataaaagc
27541 aaagtttaat agatagattg gattcaattc taggcaaaac agtctatggt atttattcac
27601 taatcctttg ttataagtgc tgatgggaga attagaaata ctgaaattat atcctttaaa
27661 attaattaaa gcataattat taatcacaca atattttttc atccaggcct ccttttcttt
27721 gtcatgcatg catattaatt gaggatggag aatatctacg cttgttcagg ccagccaaca
27781 tacgacagtt tacttcaaga gaggagacat gggttgaatg ctggtgtgtt ttaactctgc
27841 agcgcaaaca gttgcaacaa gtgtggtgaa ctaatcacca gatggccctt tgctgcctta
27901 tttgtcattg tgccttacat gtagcttgca ggatttgatt acgcttatgt tttgtggtga
27961 tcatactttc aactattcct aaaatactgc ttcagtctta tctgtttggg gtcaactgct
28021 gaggatttca tacaaattaa tgaagtttgt gaatctaaag ttctacacaa aggtggaaat
28081 atttgcaaat catttatctt gtaagagact aaaatttaga atctatttta aatatattta
28141 agatatattc aaaaatctac aacaaactga ctaaataaaa atcagacaac tctttaaaaa
28201 tgggcaaaag acttcaacat atatttccct aaagaagata cagccacaga tagtagcaca
28261 ggaaaagctg ctcaggatca ttagtcatta gggaaatgca aatgaaaaac acaagcagcc
28321 accaatatac acctactagg atgatttaaa ggaaaataag tgtgaagaag gacataaaga
28381 aattgtaacc ctgatacatt gatggtagaa atggataaag ttgcagccac tgtgaaaaac
28441 agtctgcagt ggctcagaag gttaaatata gaacccctgt tggacccagg aactctactc
28501 ttaggcaccc caaagaatag agaacagaaa tcaaacagat gtttgtatac taatgtttat
28561 agcatcactt ttcacaggag ccaaaaggtg gaaataatcc aaccatcagt gaacaaatga
28621 atgtaataaa agcaaggtgg tctgcatgca atgctacatc atccatctgt aaaaaatgaa
28681 cataattttg atagatgata caacatgggt ggacattgag aacactatgc ttagtgaaat

28741 aagccagaca caaaaggaat atattgtata actgtaatta catgaagtgc ctagaatagt
28801 caaattcata caagagaaag tgggatagga atcaccatgg gctggaaata gggggaaggt
28861 gctatattgc ttattgtgga caaggttttg taagaaatca tcaaaattgt gggtgtagat
28921 agtggtgttg gttatgcaac cctgtgaata tattgaatgc catggagtgc acactttggt
28981 taaaaggttc aaatgataaa tattgtgtta tatatatttc cccacgatag aaaacacgca
29041 cagccaagcc cagatgccag tcttgttagc tgccttcctt taccttcaag agtgggctga
29101 agcttgtcca atctttcaag gttgctgaag actgtatgat ggaagtcatc tgcattggga
29161 aagaaattaa tggagagagg agaaaacttg agaatccaca ctactcaccc tgcagggcca
29221 agaactctgt ctcccatgca ttgctgtcct gtctcagtat ttcctgtgac cacctccttt
29281 ttcaactgaa gactttgtac ctgaaggggt tcccaggttt ttcacctcgg cccttgtcag
29341 gactgatcct ctcaactact gaccatttca cctccattca tgtccatgcc acatcaggct
29401 gtgttgtcta gatggaatga atccacccca aatgtccctt tctggaggaa gccaccatta
29461 tgctgtacct ccaagcataa tggtacgtcc acacacacca aggcacctcg ctcatgcaag
29521 gtgtgtgtcc tctaacaaag tttcacgctc taaacccaga taacttttca aacccaagtt
29581 ctgttgattc ccctactttg ggtgctccat agatgctcat ttgtctacta aacactgccc
29641 caggcaatta aatattccaa agtgaccagc agaattttta tgttaattct gacattgcgt
29701 tgttagtaca agtgttttc ccccttcaaa tttatgtctt tgttactgat agatgtaact
29761 gataatgcgt ttttcagcta tgttgccaag catatttata taaaaatata ctcagattgt
29821 tttcagaatt tgacaaagat gatagcaaca atgataatct tatttgtttt atactaatct
29881 ttatgtgtta ctttcatcat ttcttacata ttggggccta ccatacattg tacggtgaaa
29941 ttagtgctat gcaccatggt agaaatatca attggcaaaa gtaatttaga aaatagttcc
30001 cttgtttctt aaattaggct gggtgtggtg gctcatgcct ataatcccag cactttggga
30061 ggcagagatg ggtggatcac ctgaggctag gagtttgaga ctagcctgac caacacagca
30121 aaatcctgtc tctactgaaa atacaaaaat tatccaggca tggtggcgtg tgccagttgt
30181 cccagctact cgggaggttg aggcacgaga attgcttaaa cctgggaggt ggaggttcca
30241 gtgagctgag attgtgccac tgcactccag cctgggtctc agaaaaaaaa aatttttttt
30301 gaccaaaatg tcattatgca ttatgtgact gtatatgaat gctaaaagct acattactca
30361 tcaaagaaaa taacaaagca attaaatgtc cattaactga taaatgaata aacactatct
30421 gtatgagtaa acacagcaga ctatgaagga aaacacatga ccagcacatg ctaacacgtc
30481 aattaacttc aaacatagta tgctaaatga aggaagtcag attccaaata catatatatg
30541 tccatttcta ttaagcaagt gggaaattta tggagatgga atgtcacagc agtattgctt
30601 agggctggag atgggggtgg ggattaactg ccagtgtgca agagagaatt tgggtgaggg
30661 aaacatattt aaattagatc gtggtgatgg gtgcacacag tatcaattta ataaagcatc
30721 aaattgtaga ccttttcagt gggcaaactt tatggtaggt tcacacccaa tataggtgtt
30781 aaaaataaat taatgttacg gaaattcttg tcgggttttt aacaagccaa gagatatgct
30841 gtgaaagcag cattaattca aatggttgtc acagttcact taaagttaat cggatagttg
30901 tcctacaaat atagggtgaa tgttattcat gaatttcctg aatctattgc aataatcaca
30961 ttttttcca ttaaactctt gaggtagcta attttattta ttgcattttc aatgttaatc
31021 tactatttca tatattgaga ttaactcaca ttagtcagaa tttacagtat tttaaaatat
31081 cacagaattt aatttacctt atctggtttt ggtttcaaga ctatactagc catttcattt
31141 aattgtacat gtagggtatt ctaatttatg gaaaactatt acattttcct tgattttttt
31201 tttttagaa attacttcta gagatctata tggtagaatc catggagaat tgttttaatt
31261 cttcattcat gtcttcagtg ggtataggat tggtcatatt ggtcatagtt ttctgctcgg
31321 atttcaataa gaaacttgtg gaagaacctg aagggtggga tctttgaggg agcctaagac
31381 agagcaagac aagctaagaa ggagggcagt gccacagcag aactgccatt gatgcccccct
31441 cgcctagatt gcggaagaga catccagctg tagacactga ggtgcaggaa aacaatggag
31501 caccatcaga gaaagcagtg cccaggaaca aggaggcact gatggtggca aggggcaaag
31561 acagctgcca cgaggctgtt cacatgaggg tctcaggctg catagacacc cacaccagct
31621 gaggggtcct ggttttcata aagtgtgtgg ctcagccagg ccaccaacaa gcagttcaca
31681 aacagtagta atacgacact ttccaaagac cttacttgag taacacggtg atcctcacaa
31741 atttccaatc aggatggtcg cacagttcct cctgctttag gactcagagc ctgcccgtgg
31801 tcacagtggg taggtgcaga ctctgaagat gcactttggt cagagacccc gctgaactct
31861 gtctaatgag gacctctgtc ctgtctgctg accaccggtc agaggtgcag gctgcagtgg
31921 ggagtaagaa tgccaccttc tcaatgtttg gaaaactccc tgccagaact gagaatggcc
31981 ctttctaagc agaaggcaag ctcagactaa agaaggaggc cgaacacatc aggttggcag
32041 attgccaaag attcactcag ggagagccca catcctgggc catcttgggt ggtggcaaga
32101 tgaggtagac gactgctttt gcaacacata cctgacaaca aaaaatcaac aactgtaaaa
32161 gagccacaaa atccccaaat atttgcaaat tagcaatgca cttttaaata actcatgggt
32221 taaagaagaa gtctcaatag aaaattaaaa atacttttaa ctacattaaa agaaaatgtg
32281 acttggcaag atttctggat gtagcaaaag cagtccttag agggaaatct atagcattgg
32341 atgcaatata ctaaaaatca caagacctaa aatcagtaat atcatgtttc aattagggaa
32401 ctatagaaaa tagaggaatg caatggaaag caagtaaaag taataaacaa catcacagaa
32461 atcaataaaa ttaaaacact gaaatcatca gaaaatcaat aaaaccaaaa gctggttctt
32521 tgatatgctc attacaatga atgaattgat atgcaggcta accaagaaaa agaagataac
32581 acaaatgacc aatttcagaa ataaaagagg agccatctct actgaactgt taggcattaa
32641 aaggaatatc atgaacagtt ctatgaccgc agtttgataa cctcagtgaa atgtatcaat
32701 tccttgaaag gcaatcttcc caaggtcatg ctaggatcct aatttgaata aacttatgtc
32761 tattaaataa gttgaattca cattaagagc attccgaaaa agaaagcacc aggcccagat
32821 ggtttctctc atgaaatcta ccgagttctt caacaggtga ataaaaagac aaaaattcat
32881 ttaatgcaat attatttggt gatttaatgt gccatttttt gccattaagg cataaaaaag
32941 acatgaaagc agctaaagcg tacatcaatt tagtgcaata aattcatctg aaaaaactac
33001 ataatatatg attccaacta tatgacattc tggaaaaggc aaagctgaag cgatagtaaa
33061 aatattaata gttgccaagg tttctggaga aagaggacag agattaatga gaagagagga
33121 tttttaggga agtgaacatt ttctttatga gaccataagg gtgaacataa tgttttaaat
33181 atttcaaaat tcatatatat gtataacaga aagaatgaac attatgcaaa tgtagacttc
33241 agataataat gtgtcaatat tttctcatta ttctagcaaa tgtaccacag taatgtaaga
33301 tgttactaat aggtgaaatt aggaagtgag ggtgaggaga cagaataata tgggaacttc
33361 gtgtattata tactcaattt ttatttattt atttatttat ttatttattt atttatttat
33421 ttatttattt tgagatggag tttcactctt gtcacccagg ctggagtgca atggcatgat
33481 cttggctcac tgcaacctct gcctcctggg ttcaagcgat tctcctgcct cagcctcctg
33541 agtagctggg attacaggca cctgccacca cacccggcta atttttttgt atttttagta
33601 gagatggggt ttcaccatgt tggccaggct ggtctccaac tcctgacctc agatgatccg
33661 cctgccttgg cctcctaaag tgctgggatt ataagtgtga gccaccacgc ccggccatat
33721 gctcagtttt tatgtcaatt taaaactctc taaagaaata tattaattga aaaataaaaa
33781 tatagcacca ctctttcagg gagatctatg cttatgttta acaaccaggt aagttctaga
33841 cattagcttg aaacattgtc tatcattcaa catgaaccaa aattgacttt taagtagata
33901 tttacttttg tggtggtagc aatatttact gaccaggcaa attagaatcc tgacacatta
33961 aaaaatatgg cttagtctct tcgtagtttc ctcttacata tgggacactg aatactcccc
34021 gcaattgcaa ttcttgaatc aacttaatta atgaacttcc acagtacctt cttgtgggta
34081 cctcttcttc tttacccggg agccatgagg tctccgacac tggttggtgt gcacagcata
34141 tcttcttgta ttctctatca gagaagatgc tggttaatgc atttacaata gatagggctg
34201 ttgacatctt gctgacagaa gaccagaggg aaaatagtga taatctgttc taagtttaaa
34261 cttatgatcc ttttctttac aggcttccaa gcagagccca ctgaatcaaa gttgggtttc
34321 aggaagatca cggagttcag tgagcactca acacctctat cagacagact gcgtgggcag
34381 tgccttcctg gagaggagaa tacagcagga tgactgtgag tgcagggctg gtgcagagtg
34441 ggggcccgga ttcaaattcc actaagccat gtggacctgg caagctcatg tcctccctct
34501 gccctcagtt ctctgcactg tcataatgta attttagcaa tactttttag gccctattta
34561 ggccctactt cttagtatca cagtacaggg ctaaaaaatc actaaataca ggaaaacctt
34621 agagaggact ggtacttcag taatgttctc taagtgttta ctacatgcca ggaggaataa
34681 gctggacact tagcagtggc ggaatatgga gggggaattt ggatggctcc ggggcagtgg
34741 agcatgcttt cctgttcggc ttttccgtgg gcatgatgcc ttatggttta tggaggacat
34801 cagccctgca gggggtgcag aggaggggct gtggctgaga ttttacactt gagggtgctg
34861 acattcagag atgataagtg acgagcagaa cctcaacccc gctgagtgaa ggacctgaga
34921 tgggaaatgt atttggttcc ctagagagag agattcctga aaaactgcca cctcttcatc
34981 acgccctgtg ccagagaccc aagagacccc tcactgtctt tctccagtcc tcctagccca
35041 aggtgtgtgg gtggacaaag gtgatgctct ggaggaaatg cctgagataa ggacaggtcc
35101 ttaatgataa agaattctcc tttctctttc agatccttga cctcccagta tgacagctta
35161 aaggctgtca tctctgtggc ctgcctcccc tttcccttca ccctgccagc tgcctctcag
35221 tgactgtctc ctccagtgac tacactgagg gaccagggac tgcttgcctc ccgaggctgc
35281 tcagaccttc cgacactgca aaatgattgt caaaagatgg gtctgcaaag agtaacttcc
35341 cttccattga tcaaacctga atatgcaagc tactgtgaat taactggaaa agtggccatg
35401 tgggctggtg ctttggtgat ttaatgaatt aagtctgcaa cccccactgc ctccttgact
35461 attgatcaga gctgcctgca ataaggtctg gctaagaatg ggcagtggct gcatcagctc
35521 tgggtaaaat ttgacctaaa atgaccaatc tcattcacta acctcaccat agtcttatgg
35581 gttcaatgga cccgtccaat cctttgctct gttctctcca tcaccttcct gtgtaatttt
35641 cctccaccat gcacataata gaaacatggc acaggggagc taatcacctc ttttatcccc
35701 cacttcaggc tcacacataa gtttatagta aaagcctttt caaatgactg ctttaactgc
35761 tgctatagca tgtgtcatca gttgaatgga atctgtcatg tgactttaag caaccctttg
35821 ttgagagaca agattcaata ctagggacag tattctagtg tactacatca ttgattttat
35881 gttatgaaga tcatcattta ttgaaaatgt ataaataatg aagcccagcc ttactcttca
35941 atgctgtgtg tgtaaatcca ctgagtgtgc tgacccccat gcttgtaccc acctgctaac
36001 acagaaagag tccattcaga agggaggcac agctccagca ctgaggctgt ccataccagc
36061 ttcacaagag agttgccaca aggacgacgg atacccagat aacaaccaaa tggtaatttg
36121 agtacttaat ggtcatgatc cctaaagtgt gtagctcaga gggcttgtgg tgataactcc
36181 atcaagactc taaagcatct ctccaattct tattggactt gatccatgtc ttgaggagac
36241 ccagctatga cacgcaggca ccacattgtc ctacttagtg cctcccttag tgtttcagaa
36301 cctgtgattt gatcagaaac atgggctttc tatgttggtt tcacactaag gactatgtga
36361 cacctgcagg aagatgtcta catagctacc tggattatga gatcatgagg ctctcttatg
36421 tgagggatgg cgtttgggat ctctgcaggc gtgggtaatt ccaggcatag agggtgctgg
36481 aactcccttg catggtgaat agtgatctct tcactggctg ataaatagag gttgtagttc
36541 aggccttcaa cattagcacc gtatgagtaa acattttgac tcttcactat gcagcaagtg
36601 aaccagggca catttattta tgtggcttag tttctccatc tggcatgtgg gctcaataaa
36661 caagctcaca acatatgggc atgatgatga tgaggtgtga actaatgtaa gtaaagtatg
36721 tggtctgatt tgttaaatta agaaaaatgg cactgagagt tgtgctgggt aaacacaaca
36781 ttttttttcct gggggaaaca cacatagaca cacattcaca agcaaatcat gcagacgtgc
36841 acacagacca cctcacccca cccccgccct aatacacaca tacccacaca caacctaatg
36901 tgaacacgtt cccagaaact atacatagat aaaaagagta tgtcacctgg aaaaccagtt
36961 tcttttacta taccctacat cctcattccc accagatgtc ttggatcatg gaggctctcc
37021 agacaaaagc cagcagttaa gctccagatt tcctatagaa tccttttcta acaaccagtg
37081 agtgattcca gaatacgtac cattgaatgt gctccctgaa gtcacctgta attagagaag
37141 gaaaacactc tgagaatcag gctatgctat ggatggctca cacaggtctt ttgttcactt
37201 ggaaactctg ggtaaccaag attggaaata aggttcaagt caaaagcccc aactctagag
37261 tagagttccc ttaggaaagc acaggagctt ttcttgaaga atgtttctgt ctaggtaatt
37321 ttggagtagc aattgcagaa ttcttatcta aagtggaaag cttgttcctg aagaaaacat
37381 cccttaacac gcagtgtact atctgacact gccaattttg cacgtcctct ggaatcaggt

37441 gtcagttggt aaaatacacc tcctccatcc ccaaggaaat attatctaac atctataatg
37501 tagtggataa ttttcccata gctgatatca actgaaaaat aaaggatcca agaaaacaac
37561 atttacatct taggcaaaga caggctactt taccttggta gtagaggagg gctccctttt
37621 cacacgcctt ttggaaggct tcttcgggtc acctagggga tgtggaggga cacagcatgg
37681 ctgtcagttc attggcagtg ctactcatga atgactcagg gactggaact taggggtgtg
37741 cctggttaac aagcatggaa tgagcttctc ctggaccatc ttcttcatgg accaaggaag
37801 gcaaagaaag agcagcaagg aaatgagagt agagcccttg gctttccagg taatggcaaa
37861 tgaaagcaac gtgaaataat caaactccaa atgaacaaat gctaaaatac atgctaggat
37921 tcaaccacag catcctgtca cttcttcaga ccctttaaaa gcccagcagg actgccacta
37981 ccttcttgac atctaccaag tccctttcaa cctccacaga cccacataca ctgctactgc
38041 atttatcatg gagggtatag ggttctgcct ggtttatgtg tgaatttttt aaaaactaga
38101 tttaatacca tgcaccagca ttaattatat ttatttcttt tcttggttat gaaaataatc
38161 agccaggcat agtggctcac acctgtaatc ccagcagttt ggaaggtgga ggtgggcgga
38221 tcatttgagg tcaggagttc gagaccagcc tgaccaacat ggtaaaaccc catctcaact
38281 aaaaaaaaaa aaaaaaaaaa aaaaacccag ctactcagga ggctgaggca ggacaatccc
38341 ttgaacctgg aggcagagat tgcagtgagc tgagattgca ccactgcact ccagcctgag
38401 tgacagagac ttcatctcaa aaaataaaaa agtaaaaaat caggccaggt gtggtggctc
38461 acgcctgtaa tcccagcact ttgggaggcc gaggcgggtg gatcatgagg tgagaagatt
38521 gagaccatcc tggctaacag agtgaaaccc cgtctctact aaaaacacaa aaactttgcc
38581 aggcgtggtg gcacgtgcct gtagtcccag ctactcggga ggctgaggca ggagaatcgc
38641 ttgaacacag gaggcagagg ttgcagtgag ctgagatcat gccactgcac tccagcctgg
38701 gcaacagcgc aagactgtct caaaaaaaaa aaaaaaaaaa aaacctcccc taaaaaataa
38761 tcaattatta aagattagaa aatactgaaa tacttatatt ttaaaataac cactataacc
38821 atacattttc cgttcagtct tcctttgccc tgtgtttgca gtcatcatgt gaggggctgc
38881 ctatgttcag cccaggcaac ccacagcgag agagggcaga gcggggagat ggcccctggt
38941 gagcactgag gctcttcaaa ccagttgccg agggagttgc aagcagggtg acacatgagg
39001 ctcattattt agtggtattt tgtgttctta cttgtcttgt ccccaggctg tgaagctcac
39061 agactttttt tttttaaca tttctatagg ggtcatttga agttcataac accatcaaac
39121 atcagtacaa ggtcttccca ttatttaccg gctttgggat atgtgctgag gagtggccac
39181 gcactgaagg aatgtgacca gttgtcccaa gtgttgtcct tcaactcaaa atccgcaacc
39241 agttcaaaag catttgggct tttatgtgcc tttcacataa agactatgac acagctaagc
39301 tattcatgta ctaatacctg ccctgagctg tgtgaccaca gacaccaccc tataatgtgg
39361 ccagtgtgta ggaactctac aggctcagat cattccagac atacatgcag atgttggaac
39421 tcccttgcac agtgactcat gatcccaagg ctgagtgctg agggcaggtc agtagttcag
39481 agcctcagca ttggggctgt atgcttgggt tcccatcttg gctcttttgc tgaggggctc
39541 ataagcttgg gcacattcat cactgtcttt ttctacccag cgaaaggtga tagtaaatca
39601 atcatgccat ttgtttgtgg tgatggccga atgatttgat gtaggtaaag tgtacagtat
39661 aagtgctcat gaggcttaat tatcagtgaa atgtcttgct ttagatttca cagcttaata
39721 aggctcagag gcttattcac gcccaggatt ctgaactccc aagtcattgt tctatgacat
39781 acacctgctt ggcctaaaac cgaccacttt atcataatgc agacccagtt ttgaaagaag
39841 cagataaaat tgtcaaaagt actgtgtttg cctcagcgca cactcagtgc gctccacaca
39901 aagggcatca ccatctgcca caatggccca gtggcctggt tggcacagac agtggcagag
39961 caaagggcaa gagatcccta atcccatcac catggtcgat agggcatcat agacattcca
40021 gggtgaaggc acaatccaca ttgtgaggtc caactgctgc catgtagaca ggtgtgtttt
40081 tacatgtaca ggaagatcat tgaagataag tgttttatat ccatggttaa cgatgagatg
40141 accatgaaat gaacaccagt gtactgggtg gagcagcttc tctattcagt cttctgcact
40201 aaagcctgtg aaacaatatc atcttgcctt atttactaac aaatacaagt gcctctaaac
40261 ttagacagtt tccaagtcat ggaactgatg agcacttagc tcctgcagag agctctggat
40321 gctgggtcgg gagaacaaag acacacaata catcaaaaca gcattaacaa gtaaacaggt
40381 tttcaaagct ctccacatgc aaatttacac aattatcctt ttaattttta tcttcatata
40441 tatatacata atctacttgc ttctgagtat aaataaaact gtatgttctt agttaatagt
40501 ctctaccaat tcattccatt tatctctctg agttgaaata ctgcatctca ttggataaca
40561 aaaaaaaaat ttgactaaga ttacaatgga aaggtgagta ggttgggtga ttgactgtga
40621 ttgacaattc catgattctg gataacttcc aaagcataaa aataaatgtg agttttattt
40681 cacacgtaga caatacacat acttattact ttaaaaaatt aatatgtgaa tggaactgac
40741 ttactacaaa tttattaaag taaatacata tttcacaaaa gaaagaagag aggaagggaa
40801 aaacatgtta aaaacaaagg gaggtacatt ttattgtgtg aaaagcctcc aacccatccc
40861 tactactgtg gctttgttcc aaatatttgg aaagtaatga tttcataagt tattaattag
40921 gttaaaaact gcattacaat agactttgcc atattctccc ctggagaata acttaatctg
40981 tggggtgggg gatggaacgt tgaaggatgc aggatgtaaa aggaaattat atatatatat
41041 tatatatatt acatattata tataatatat tacatattat atacattata tataatatat
41101 tatatattat atataatgta tgtaatatac acattatata ttaaatatat aaataatata
41161 tattataaat tataatatat aatatatatg atatataata tatgcagtat atatgatata
41221 tattatattg tatatattat tatatacaat ataatatata atatataatg tataatatat
41281 aatatatatt atatgtatag tatatataat atataatata tacagtatat ataatatata
41341 atatatatac agtatatata atatatatta tatataatat ataatccata atatatatgt
41401 tatatattac atatacagta tatataatat atattacata tacagtatat ataatatata
41461 ttatatatac agtatatata atatatatta tatatacagt atatataata tatattatag
41521 atataatatc tataatttgg gaatttggga ataaactgaa tcccaattca cactgggact
41581 ataccagctg ccaccatgcc tggctaattt tttgtatttt tcatagagac agagtttcac
41641 tgtattggcc aggatggtct tgatctcctc accttgtgat tctcttgcat tggcctccca
41701 aagtgctggg attacaggcc tgagccaaga tacatatttt ttaaatgaag aaaaatttca
41761 aaggtactct gcttggtaca ataatcaaat atataaattg aggaatgaaa cataaccatg
41821 aaacatattt ataactgcat atggaaaata cagatgataa ttttttaaat aacatatttt
41881 gaaagcatta actagtaatt tgaaaagatc acatttgaca gggcagtatg aacatacctt
41941 gaatgcagcc acacaggttc ctcataagaa aaatcaaaat cagggaaaat gaaaccacag
42001 aggttcaatc tgctctgatc tttgaaaaac tcagcacaga tagtggcact taggactaag
42061 ggcaggagat ccctaatccc atcaccatgg cgatagggca taaacattcc agggtgaagg
42121 cacaatccac actgtgaggt ccaattgctg ccatgcaggc aggtgtgctt ttacatgtac
42181 agggaggtca ttgaaggctt agtgttttgt ttcaaaaact gaatcccaag cccacacatt
42241 attatgctgt gcttcttaaa acaagttatg agatgggaaa taaggcaccc ccaaatatat
42301 atatatataa ttatacataa tataatatat ataattatat ataatataat atatataatt
42361 tctttttaca tcctgcatcc ttatatcaca tataatatat catatataat ataataatat
42421 ataattatta tatataatat aatcattctt aattattata tataatacaa taatatataa
42481 ttattatata taatataata atatataatt attatatata atacaataat atataattat
42541 tatatataat ataagaatat ataattatta tatataatat ataattatta tatataatat
42601 aagaatatat aatatatata attattatat ataatatata attatatgta taatattata
42661 tatcatataa tgatatataa tgtatattat atataatata ttatataata cataatatat
42721 atattacatc taatattatg tataatatat catgtatatt atgtataata tatcataaca
42781 taatatatat tataatataa tataatatat atatattata tttatataat atcatatata
42841 atatataata tattatattt gtataatatc atatataata tataatatat tatatttata
42901 taatataata tataatatat aatatattat atttatataa tatcatgtat aatatattat
42961 atttatataa tatcatatat aatgtattat atttatataa tatcatatat aatatattat
43021 atttatataa tatcatatat aatatattta tatatcatat ataaatatca tatataatat
43081 ttatataata tataatatta tattacataa taatatcata taatatataa tatattatga
43141 tatatataat ataatataat ataattatat ataatataat gtataatata tatcttatat
43201 gatatataat atatatctta tatgatatat aatatatatc atatatgata tataatatat
43261 aatatattat atattatgta tcataaatat attatacatc atagattata tataaaatat
43321 atcatagatt acatataata tatcatagat tacatataat atatatcata gattatatat
43381 aatatatatc atagattata tatattatat atcatagatt gtatatatta taaatgattt
43441 atattatata taatctatga tatataatat ataatctatg aaatatatta gatcatatat
43501 aatataatat tttatatatt atatcatatg tattatatta tatattatat attatgtatt
43561 attatataat acataatata taattgtata atatatatta tatattatat actacattat
43621 atgtcatata atataatata taataagtca tattatatat tatatataat atatattata
43681 agtcatatta tatattgtat ataatatata ttataagtca tattatatat tatatacata
43741 atataaatat atataattta tttttagaca gagtcttgtt ctgttgcaca ggctggggtg
43801 caatggcacc atcttggctc actgcaacct ctgcctcacg ggttcaagca attgtcctgc
43861 ctcagcctcc caggtagctg ggactacagc acactgggac tacaccagct gccaccatgc
43921 ctggctaatt ttttgtattt ttggtagaga cagggtttca ctgtattggg caggatgttc
43981 ttgatctcct caccttgtga tcctcttgcc ttggcctccc aaagtgctgg gattacaggc
44041 ctgagccaag atacatattt tttaaatgaa gaaaaatttc aaaggtactc tgcttggtac
44101 agtaatcaaa tatataaatt gaggaataaa acataaccat gaaacatatt tataactaca
44161 tatggaaaat acggaggata attttttaaa taacatattt tgaaagcatt aactagtaat
44221 ttgaaaagat cacatttgac agggcagtat gaacatacct tgaatgcagc cacacaggtt
44281 ccccatgaga ataatcaaaa tcagggaaaa tgaaaccaca aaggttcaat ctgctctgac
44341 ctttgaaaaa ctcagcacag atagtggcac ttaggaccaa aggcaggaga tccctaatcc
44401 catcaccatg gcgatagggc ataaacattc cagggtgaag gcacaatcca cactgtgagg
44461 tccaactgct gccatgcaga caggtgtgct tttacatgta caggaaggtc attgaaggct
44521 cagtgttttg tttcaaaaac tgaatcctaa gcccacacat tattatgctg tgcttcttaa
44581 aataagttat gagatgggaa atagggcacc cccaaatata tatatgtaaa aatatataca
44641 acatataata tatataacat atatataatt tcctttttaca tcctgcatcc ttatattata
44701 tataattatt atatataatt atgtataata attatatata atacatatga tatatattat
44761 ataatatatg gtatatatta tataatatat atgatatata tgatatatat tatatatgat
44821 atatattata ttatataata tatattatat aatatataat ataatatata ttctatatga
44881 tgcatattat ataatatatg atatatgtta tatgtaatat atgatatata ttatatgtaa
44941 tatatgatat atattatatg taatatatga tatatattat ataatgtata tatgatgtat
45001 aacatatatt atatataata tcctataata tataatatgt aagatatatt acatatgtca
45061 tataatatat attttatgtc atataagata tattatatac ctaatatatt tattatatat
45121 tatatatgtt ttatattatg tatataatat ataatatata taatgtgaca tataatatat
45181 atgatatata attgtatatc atatattata tattatatat tgtataatat attatataat
45241 atataatata cattatatat atataatttt ttttttttga cagagtcttg ttctgttgca
45301 caggctggag tgcaatggcg ccatcttggc tcactgcaac ctctgtctca cgggttcaag
45361 cgattgtcct gcctcagcct cccaggtacc taggactaca ccacactggg actacaccag
45421 ctgccaccat gccaggctaa ttttctgtat ttttggtaga gacaggattt tactgtattg
45481 ggcaggatgg tcttgatctc ctcaccttgt gatcctcttg ccttggcctc ccaaagtgct
45541 gggattacag gcctgagcca agatacatgt tttttaaatg aagaaaaatt tcaaaggtac
45601 tctgcttggt acaataatca aatatataaa ttgaggaata aaacataacc atgaaacata
45661 tttataactg catatggaaa atacggagga taattttttta aataacatat tttgaaagca
45721 ttaactagta atttgaaaag atcgcatttg acaggccagt atgaacatac cttgaatgca
45781 gccacacagg ttccccataa gaaaaatcaa aatcagggaa aatgaaacca cagaggttca
45841 atctgctctg acctttgaaa aactcagcac agacagtggc acttaggacc aaaggcagga
45901 gatccctaat cccatcacca tggcgatagg gcataaacat tccagggtga aggcacaatc
45961 cacactgtga ggtccaactg ctgccatgca gacaggtgtg cttttacatg tacaggaagg
46021 tcattgaagg ctcagtgttt tgtttcaaaa actgaatccc aagcccacat attattatgc
46081 tttgcttctt aaaataagtt atgagatggg aaattagggc acccccaaat atatatatat

46141 aatacataat ataatatata atatatataa catatataat ttccttttac atcctgcatc
46201 cttatattat atataatatt atatataatt atatataata ttatatataa ttatatataa
46261 tattatatat aatataatat ataattatat ataatgatta tataatataa tatatataat
46321 tatatataat ataatatata attatatata ttatataatt atgtatatta ttatatgtgt
46381 aatatagtaa tatataatta tatatgtaat atataatata taatatattt atattattat
46441 atataatgta ttatataata tatatgatat aatatattat atataatata ttatatcata
46501 tatattatat ataatataat attatatatg tatattatat aatatatatt atacataata
46561 tatcataata tattacatat cataatataa tatataagat atattatata taatatataa
46621 aattatataa tatatgatat attatatata atataatata attatatata atataatata
46681 attatatata atataatata cataatatat atcatatatt atatataata tatatcattt
46741 attatatata atatatatca tttattatat tatatatcat atataatata tatcatttat
46801 tatattatat atcatatgta atatatatca tatatagtat atatcatata tatcatatat
46861 ataatcgtat atcatatata ttatatatac tacatgtcat atattaagtt tggggatagc
46921 ccagaattat atatggattt tcaactgcac aggagctggt cccctaaccc ttgcactgtt
46981 caaggatcaa cagtatttaa aatactatac attttattca tattttagag tgtaatactt
47041 cttctcatta aaaaaaatta actataaaac agcctcagga aggtctttca ggagttcttc
47101 cagaagaaag ccttgttatc atagaaaatg acagctgcat gtatgttatt gcctctgaag
47161 accctgaaga cctttcactg ggacaagatt tggagatgga agacagtgat attgattatc
47221 ctgaccctta cgaggctaag ctagtgtgtg tgtttctatc ttagttttta acaaaaattt
47281 taaaacataa aaaataaaaa taaataaagc ttatcaaata aggatataaa gtatttctgt
47341 acagctgttc aatgtgtttt tgttttaaga tgtgttatta tgaaagaatc aaaaaattaa
47401 aaaaattaaa agtttataaa gttatgataa gctaagatga acttgttaaa gaaagaaaaa
47461 aattaaatac atttagtgta gcctaagtgt acagtgcata taaagtctat agttatgtac
47521 agtaatatcc taggcctttt cattcactca ccaagtactc actgactcat cagagcaact
47581 gccagtcctg caacctctgt tcatgctaag tgtcctatac tggtgtacca ctctcttaac
47641 ttttgtaata tatatatata tttttttctt tctttttttg gagaaggagt tttattttgt
47701 tgcccaggct ggagtgcaat ggcacgatct tggctcactg caacctctgc ctccggggtt
47761 caagtgattc tcctgcctca gcctcacgag tagctgggat tacaggccca cgccagcact
47821 cccagctaat tttgtatttt tagtacagac ggagtttctc catgttggtc aggctggtct
47881 cgaacttctg acctcaggtg atccgcccgc ctcggtctcc caatgtacta tgtttttact
47941 gtatcttttc catgtttaga tattactatt gtgatataac tgcctacagc agcggtcccc
48001 aaagttttga caccagggac tggttttgtg aaagataatt tttccacaga ttggggatgg
48061 ctttgggctg aaactgttcc acctcagatc atcaggcatt agttagattc ttttttttct
48121 tttttttttag actgagtctc ccactgttgc caggctggag tgcagtggca caatctcggc
48181 tcactgcaac ctctgcctcc cgggttcaag tgattctcct gtctcagtcc cccgagtagc
48241 tgggactaca ggcatgcgcc accacaccca gctaattttt gtatttttag tagagacggg
48301 gtttccatgt tggccaggat tgtcttgacc ttgtgatcca cctgcctcgg cctcccagag
48361 tgttgggatt acaggcgtga gccacagcac ccagcctagt tagattctca taaggagcac
48421 ataacctaga tcccttgtat gtgcagttca caataggatt tgcactcctg tgaagatcta
48481 atgctgctgc tgatctgaca ggaggcggag ctcaggcagt aatgctcggc tggccagcca
48541 ctcatctcct gctttgtgcc cagttcctaa aaggacacga accggtgctg gtccatggct
48601 tgggtgttgg ggaccactgg cctactgtat tcagcacagt gacatgctgc acaggtgtgt
48661 agtccaggag caataggctg tacgatatag cctaggtatg tagtaggcta cactgtctag
48721 gtttgtataa agtacactct ttggtgtctg cacagccaca aaatcaccta atgatgcatt
48781 tcttggaata tattaccatt aaatgagatt tacctgtatt agtgtcatct caggtttatt
48841 gtctagtaat tttaaaagta tttgtatatt ctttcccaga cacatcatca tactctggtg
48901 catatctcta ttttattccc tagacccact cttaattttt ctgcattctg ctttgttcct
48961 taggaggctc acctgaattg gatacttcag agatctccct taccctcatg cttttattgg
49021 ggttcagcta atggaggggg tggcagaaga taggtgagag aagagtgtgt ttatccctca
49081 cctctgcccc tgcagggtca gcacagtcag actgtgtccc tttgctgaag atcacagctt
49141 ctgtctggtg ccttcttggc aagctggcac tgtctctagt caggtgactg cttggcccct
49201 tactacttac actcttctgt ctggtttctt tgtgtctttc ctgctgtgct gtaagtagtc
49261 cctttattag aatccatcca cattacccag ttatataaag acaaatagtt ggtcactgat
49321 ctagttcact ccctgagaat cagccttcat caaagatccc taacagtcag atattctgtt
49381 ggccgctcca ttcctgctcc ccattattct agttgtgtct tccttgtctc tagtgcagtc
49441 acaaagcata agcgcctcca ctgtcacttg ttctttgcaa ctctaggatc acatcattcc
49501 ccttgaaatc agagaacctg caatctcccc aagcccacaa aattcagccg tcacagtact
49561 tttcaagcat gccagcagcc ctccccaaca aacgaatcct taatttcttg gagaaacatc
49621 ttctggggct tcctgtggga cacagtgggg gtcggcttgg catgtggatc acacctgata
49681 tttttacccc cacctcctac tattcctatt cctttgtatg cacctctctt tattatgcta
49741 ggaaagctct ggcaacacaa cctcaaaata agcaggccag tgtggattca cagtttctgt
49801 cagacaaaca aacttgagca ccccttctaa cttcttgggc taagacttta acttcagaat
49861 ctctatgtat ctatgttgat aaacttagcc tatgtcaaga ttatattctg catgccttag
49921 tcaaacacca tgtaaatcta tgcctcatat attctctagg ctttagctga tagggattag
49981 caaagtagtg tggttgtttt ggtctttaaa caatgtccat tttctaccag ctttccattg
50041 cactcttttt tttttttttt tttttgagat ggagtcttgc tctgtcgccc aggctggagt
50101 gcagtggccc gatctcggct cactgcaagc tctgcctccc gggttcacgc cattctcctg
50161 cctcagcctc ccgagtagct gggactacag gcgtgtgcca ccacacacgg ctaatttttt
50221 gtatttttag tagagatggg gtctcactgt gttagccagg atggtctcga tctcctgacc
50281 tcgtgatcgg cccaccttgg cctcccaaag tattgggatt acaggcgtga gccaccgcac
50341 cgggccgact ccattgcact cttacagacc atgttgacaa aattataact gcaacagtca
50401 actacaatag caattttacc tctcatgtac cactttaaaa aacacatcat tcccaataac
50461 cacatttgat gatttaagta atcatgatac caccgtacac tacagatgat caggtttctg
50521 tctcctcccg acaagaaggt caggaattca ttcagactga aacaggtcag caaaccactt
50581 ccagattctc atctataaag atctatttct agaaccattt ctagtaccta tacaatatta
50641 gaatcaagtc aggaagcaga aaacattcta gatattttaa acagaaaaca attcatggag
50701 agatcaatta caagagtatg tgtagcaaaa gatgaaaggg tcaggttgct taagtaactt
50761 gataaatctc tgcttctttt gtgttgggtg ataaaaatgt tgagcagaga tcagtggcag
50821 atgatccaag gcgtcagctg tccaagctca gcatctggag ccggtgctga ggaaatgtgc
50881 atttctaggt ctgggcgcca ttggaccatc actactgtcc ttgaagctgc caccgtgaga
50941 actcacatct cagctgctat agccacagct gatattgtca ttgcttccag aattatttcc
51001 cttgctccat cgtggggatc ccgcaatact gctgctgctg aaatcactgt tgttgctcct
51061 tccagaactg tcggcactgc agcattatta tcactgcagc taccactgga gatggctaca
51121 gacaccagaa acagaagagc acctctcccc tcctccggca gtgtgacttt cagtcggctc
51181 ctctcgtagc cagatggtat agaaatgaca caccaatgtc cttcagaaca tacatgcaaa
51241 atattttaaa tatattagaa attctattcc ccttagaaaa ctaagttcac ctacatattt
51301 ttcaaaaatc catcagcaat acagaataaa ggcattggtt ctatacagcg ttgcaagaat
51361 attttaacaa tagaaggtat tttaatgtca ttcaccacat taggagattg aaaaagggaa
51421 aacacaggat taccttagca gatgtaggaa aaaaataaga tggtaaaata caacattcat
51481 ttatattaaa atctttatac caaactagga aaagcagaca aatttcttaa cctgataagc
51541 ttatctataa caaaaccatc gacaccacca tatgtaatgt tgaagcataa tcgttatttt
51601 ttataaaagc taggaaaggg aatgagatgg ctgttgacac tgctcgtatt tatcattgtc
51661 ctggggtttc tggccagttc agttaagata ggacaaagaa atattataga aagaggaaga
51721 aaaaaagaaa acacatacgt tgtcattgtg tggagtttct gtttgtttgt ttatgtgttt
51781 ttgtgagatg gtgtctcact ctgtcgccca ggctggagta cagtggtgtg atcttggctc
51841 actgcaacct cattgtgtgg agatttgtaa ttacccatag aaaatccaag aaaatttgtg
51901 gatgcactaa tcaaatcagc aaggttttca gatatgaagg caaagtaaac agaaaatgaa
51961 attcttataa acagtagcaa ccaattaaaa atattatatt ataatagcac attcatggta
52021 acataaaaca ttaatgtatc taagaattaa ttcagaaaac aaatacatga tctttcattt
52081 aattttatta aagttatttc aataaatata ccaccagtat ttttttagaa cttgattgga
52141 aaagtcatat taaagtaatc aatggaaagg aagaaccaaa gtgattttga acaataatag
52201 cctatcaata tttgaatgtg ttttaattat aataattaaa atggtttgct ttggctcagt
52261 gatacaccgt taaacaatgg gaaaaaatgg aagcaccaag aaatagatcc ataggtatgc
52321 tgaatacatt ggacttgtaa taaaagatat gatactaaaa gtcagtagaa aaattatact
52381 atgcaataaa tgatgtcgaa aaaacatata aatttttatc catatataaa taaaaatctg
52441 tatattacag catatcacaa aatcctgtat ctatttaggt taaagactca aatcttaaac
52501 tacttgtgaa attacttatg aatattacat gtttaagata tttaaaagta tatacagaga
52561 actaatgacg ttgggcaaca aatggcttat taatcaaatg ccagaatgca tgaagaagaa
52621 aagaaaagaa aaataaatta gactgtatta aaatgaaaaa tcctaggcat cctaaataaa
52681 ggttaaatgc aagccagaaa attgggtaaa atatttacaa aatatataat tgaaaagtat
52741 aatatccatt atataaagag gatttctaca aattaatgat ataaaataaa tagtttagta
52801 gaaaaaatga ttaagaacta ctccacaata ttcacaacag atgataaata gtgaattatt
52861 gtgaaaagac aattgaaaag agcaaggtac aattcaacag caataagact ttatgccctt
52921 gtctataaaa acacaaaact atgccaaggg ctttaggaac cgtgaattta tatacggcga
52981 aggggagttt aaatctgcca ctttgaagag ttttttggaa atatatagta aagttgaaaa
53041 tatatacata tgtgtatata tatataccca cacacataca catacacact agaaattcca
53101 caaggtaaac cttctccaag agaatacaca cacatttgca gaagagatgt ccaaaggtgt
53161 ttgttgaaac attattggta atagctaaaa attggaaata atcttaaagc tcattactag
53221 gaaaatggaa aagtgaatcg tggtatgtgc atataatgta aaactataca tcagttaaaa
53281 ttaattgtct gaatttatat ggatcaattt ttatatattg aaacatcgtt gcattccagg
53341 aataaatccc acttggtcat ggtgtataat ccttttaata tgatgctgaa ttcagtatgg
53401 tagttttttt ttttagacaa agttttgctc ttgttgccta ggctgaagtg caatggtatg
53461 atcttggctc accacaacct ccacctcccg ggttcaagcg attctcttgt ctcagcctcc
53521 cgagtagctg aggttacagg catgcaccac cacacatggc taattttgta tttttagtag
53581 agacagagtt tctccatgtt ggtcaggctg gtcttgaact cctgacctca ggtgatccac
53641 ccacctcagc ctcccaaagt gctggaatta caggcatgag ccaccatgcc ccacccagta
53701 tgctagtatt tttagagttt ttgcatcaat gatcagaggc atattgatct gttgttttat
53761 tttcttatag tgtttttggc tttggtatca gggtaatgct ggcctcatag aaagagttaa
53821 gaggtattca ctcctcttcc actctttgga aatgtttgaa aagaatcagt gttagttctt
53881 ctttaaatgt ttggcagaac tcaccagtaa agctatcagg tccaggactt ttctttgtca
53941 tgagattgtc ttattactga ctcaattccc ttactaggtt aggtctattc tgactttctg
54001 tttctttata attgagtctt ggtaggtttt gtgtgtctag gaattcatct atttcatctg
54061 ggttatccaa tttatgagca cacaattatt tatagtaatc ttttataatc ctttctaggc
54121 tgggaatggt gggtcatgcc tgtaattcca ataatttggg aggcggaggt gggaaaattc
54181 cttgaggcca ggagttcaag atcagcctgg ataacatggc aagatcctgt ctcttaaaaa
54241 aaaaaaaaaa gagctggcat agtggcgagc ccaggaattc aaggctgcag tgagccgatt
54301 gtgccactga ttgtggttgc atctgattgt tgccagggca atggagctag accttgtctc
54361 taaagtatag acagacactt gtctctaaaa catagacagg cagatagaca gacagacaga
54421 cagacagaaa tatataaaga tattctcaaa tacatttctg ttttaagtaa tttagattgt
54481 ttccctttc cttagtcttg tcagttttat taatcttttt gaagaaccaa cttttggttt
54541 tgttttttat tgtttttcta ttctcttttc catttatctc tgccctgatc tctattgttt
54601 ccttccttct gctaactttg ggtttagttt tattcttctt tttctagttt cttaatgtgt
54661 aaacttatgt tgttgaattg aggtgtttct ttttttaatg tgtttatagc tataaatttc
54721 ctccttagca ctcctttcac cacaactcat aagttgtgtt tctttgttgt ttctttgttt
54781 tttgagacag agtttcattc tgtcacccag gctggagtgc agttgtgtta gctcagtggt

54841 gcaacctctg cctcctgggt tcaagaaatt ctcatgcctc agcctcctgg gtagctggga
54901 ctacaggcac gtaccaccac tcccagctaa tttttgtgtt tttagtagag acggggtttc
54961 accatgttgg cctggctgat cttgaactcc tgacctctag ctatccatct ccctcagtct
55021 cccaaagtgc tgggattaca ggtgtgagcc accacaactg gccacaaccc ataagtttgg
55081 atatagtgtg ctttcatttt catttgtctt taagaatttt ataatttctt tgtgatttct
55141 ttgatccatt ggttgtttga gagtgtgttg tttaaattct actaatttgt gaacttttta
55201 aaatcttctg ttattgattt ctaactttat cctgttgtgg tcagagaaga cactttgtat
55261 gatgtctatc gttttgaatc tactgagctt ttttcctttt ttgggacagg gtctcgttct
55321 gtcagtggta caatggaaca ttttctttgt catgaagtac aggctggagt acagtggtac
55381 aatctctgct cactgcagct tcaacctcct gggctcctaa gcaattctca cgcttcagcc
55441 tcccaagaag ctgggatcac aggcacgcac caccacgccc agctaatttt tgtattttta
55501 gtagagatgg ggtttcacca tgttggccag gctgatgtca agctcctggc ttcaagcaat
55561 ccacccgcct cagcctccca aattactctg ggattacaga tgtgagccac tgcacccagc
55621 ccctattgag aattaatttg tgatgtgtta tatggtctat cctgaaaatg tcccatgtat
55681 acttgagaag tatgtgtatt atgttgttgg gtagaatatc ctgtgtatgt ctgttaggtc
55741 tagttgtttt ttcaggtgtt gttcaagtgc tgtatttcct tacttatgtc tggtctggtt
55801 gttttattta tttgtgaaat gaagacaagg gacaaaatcc taagacatcc cccttaaaag
55861 ggaaggacca cccacagaaa aggactgaga gacccccaga atctatagct tagctaattg
55921 atggtctttc tctcctgaag ttagtaaaga gtggaaaaga tgactccttc ttcaaacatg
55981 aagaaagcaa tctaagtctt caaagaacag gaagcatgag aaaatatgac accaccaaag
56041 aaacaaaatg aaactccagt ggctgactcc aaagacatga agatctacaa attgcctgac
56101 aaagaattca aataatcatc ttagggtgac atcagcaaga tggaagaaca ggaggccctc
56161 cactcacctc tccccacaca aacaatgatc tggcagccat tcatggacaa aactgccttt
56221 gcaagagttt taatatccag gcaggaggtg acaaaactct agcaaagccc aaaaccaagg
56281 agagctgctt tgagaaggca ggtccacaca ccagtgacag gttcccagtt gcagactgaa
56341 agcagctttg tcctcctatg gacttgggtc cagctccact agatcatggt cttgcaacta
56401 gccccatcca ctaagcacct aggaggaacc atgaccattt atgcccccag taacaggcct
56461 tctaaccatg gtcctgactg cagaaactaa agcagccctg acccggcttc agccccactc
56521 tactacagtc agtcttgcct gcccaggaat tcagtgatgc ctttacatgc ccttggtaac
56581 aggcctgctg atctcagtct cagctatgga cactaaagta gccctgtgac tccattccag
56641 tcttgctttg ccacagtctg ggcacagtcc agcccatcct gggacctggt gaaagacata
56701 ccatccattc tcctaataag aggaccacca atcttgaacc caaatgtggt caccaaagct
56761 gtcctgtgac ttggctctag ccctgctcta ctgtggtctg gaggcagtcc tgtcttccca
56821 tggacctacc tagtaaacca gcaggagctc aatctgggac ccacagggag ctataccagt
56881 ccatgccctt ggtaataggc ctgatatctg aagacttgac tgtagaacta gaagtagccc
56941 catgacctgg ctccagtcct gttcgatcag ggtatcagag gcagtccagt tctcctgagg
57001 atccagcaga aactgcatca ccaacctatg ccttgatagc aggcctgcca atcagagatt
57061 cagctgcaga ccaggcagca gccatgtgat atggctccag gcccacttga ctgtgatcca
57121 gtcaagggat ccagcaggaa aaggtttgca cctgctgaaa ccaatctata aagacagaaa
57181 gagattgggc atggtggctc acacctgtaa tcccagcact ttgggaggcc aaggcgggca
57241 gaccacttga ggccaggagt ttgagaccag cctggacaac acggtgaaac tctgtctcta
57301 caaaaaatac aaaaaaatta gccaggcatg gtggcacatg cctgtgatcc cagctgcttg
57361 ggaggctgag gcaggagaat catttgaacc tgggaggtgg aggttacagt gagctgagat
57421 cacgccactg cactccagct gggtgacaga gcaagactct gtcccaaaaa aaaaaaaaaa
57481 aaaaaaaaga agaggtattt gcttcttcat agacattaat gaaagtctgt agatcatgaa
57541 gaatcagcaa atatgacact accaaaataa actcataaat ctccagtaat caacccccca
57601 aaaatagaga tctacaaatt gcctgacaat tcaaaataat taatttaaga tagctcagtg
57661 aaatttacta gaatgtatat atcaactcaa taatatcaag aaaatagtag atgaacaaaa
57721 ctaaaagttc aataaagata cagaaataat aagaaccaaa cagaaattct agaactgaag
57781 aatacaatga ataaaatgaa aatgcaagct gggtgccgtg gcggacgcgt gtaatcccag
57841 cactttggga ggccgaggca ggcggatcac ttgaggtcaa gagttcaaga ccagcctggc
57901 caacatgcca aaccccatc tgtattaaaa atacaaaaat tagccaggca tggtggcata
57961 tgcctgtaat cctagctact agggaggctg aggcaggaga atcgcttgaa ccgggaggtg
58021 gaagttggag tgagccaaga tggcaccact gcattcaagc ctgggtgaca cagactctgt
58081 ctcaaaaata tataaaaata aataaatgaa tgaaatgaaa atgcaataga gaatttcaat
58141 tgcagacttg atcaaacaga agaaagaatc tgtgaaatta aagacaggac atttgaaata
58201 tcctgtcaga agaccagaaa gaaaaaaaag tttataaagt aaaaaccttg gggatttata
58261 ggacaccacc atgaagtggg tcaaacatat tatggatttc ctagaagtaa aaaagataga
58321 aaaggcctga aagtatattt gaagaaataa tgggtaaaaa ctttccaaat tcttagagcc
58381 aagtgggctt ccagacacat gaagttcaga ggccccaaaa aaggccagcc caaagaccat
58441 aataccaaca tgtattataa ttaaaatatc aaaagtcaaa gacagagact ttcaaaagca
58501 gcaagaagaa aggaattcat cacatacaag gaaacttcaa taaggctatc agtggatttc
58561 tcagcagaaa ccttataagc caggagagaa taggatgcta tattcaatgt attggaagaa
58621 aaaaactgcc aatcaggaag accacaactt gtaaagctgt ccttcagaaa tgaaaaagag
58681 atgataaggt ctttcccaga ctcccaccaa aaaaattcta agggagttca tcaccactgc
58741 acctgcctta taaggcagtt tttcaagttg aaataaaagg attctgatta gcaacattaa
58801 aacattaaga tattaagttc acaggtaggc cgggcgcggt ggctcacgcc tgtaatccca
58861 gcactttggg aggccaaggc gggtggatca caaggtcagg agagggagat catgctggct
58921 aacacggtga aaccccgtct ctactaaaaa tacaaaaaat tagccaggcg tggtggcggg
58981 cgcctgtagt cccagttact cgggaggccg aggcaggaga atgacttgaa cccgggaggc
59041 agagcttgca gtgagccgag atcatggaca ctgccctcca gcctgggcga cagagcgaga
59101 ctctgtctca aaaaaaaaaa agttcacagg gaaaggtaag tatatagtca cattcataac
59161 actgtaatat tgcagtggtg gtgtataaat cacttttcac tgtagtataa aagttaaaat
59221 gtaaaagtat taggcggccc gggcgcgggg cagcggctgt ggggaggcgg ggaggcgggg
59281 aggcgggggg cctggccgga cacccctgcg ccccctcccc gcacccgggc ggagggcggc
59341 ctcttccccc tccccctccc ccgccacccc cggcagccgc ctcccccagg acacaggagg
59401 cgggcggagg ccgggtccgc gcagcgggcg acttgcagcg tgggccgggt cgaggtcggg
59461 gggggcggtt tcgggggctg ggaagctggg gggccgggga caggagggtg ggggggccgg
59521 ggaaaggagg gctgggggc cggggacagg agggccatgc aggcagtggc aagtgggcgg
59581 cgagggctcc gtggggcagg tggacgggga tcagcgcctg gggctgctcc gttccccagg
59641 cgggtgtcgt ggctctggcc tccatgcaac tccaggtctg cgcgccccgc gctgctggag
59701 ccccagtcaa aagtctattt aaaaagcaga gagaggatgc ttccgtctga gtggagtgat
59761 gaagacctga tccctgggcc atttgggaac actagctgcc tttcatcaca gtcaacctgg
59821 actcagagaa tgtcaagagc ttgttggttg ggtcaagaat gaatctaggc atgacgtcat
59881 agtttatagt catcctttta aacctgcaaa gaagcatgtg caggtttaaa gttatttcac
59941 gggtactgct tgccaatctt tggaggatgt gaagcctgca gagaaataaa gtgtcgcccc
60001 tctgcgcgtc gctccccatc tgctagaatg tttctcatga atgctcctcc agtggttgct
60061 ctccagccca aatgggaggc ctctgtcccg ccagggagct ttagattccc ccggtgcttc
60121 tcggaggctg acaagggcgt ggagagcatg tcggtgagca cccgggtgca gatgctcatc
60181 agcacgctgc agagcgacag ggctgctagg ggcaccagcg atgagcgcgc tgcgcagagg
60241 gggcagaggg atgccacgac gccaggcctg ctgccaagtc caccgtgcac aaggagctgc
60301 ctgcgttggc tgcctgtggt cttgttgctg actttgaccc catgggggag gaggaaactg
60361 cagactttgg cccattggtg ctagattcag acagtgacga ttccgtggac cgggacattg
60421 aggaggccat ccaggagtac ctgaaggtaa agagtggagc cgcacagccc ggggccagcg
60481 gggcccagcc atgcgcagcc ttccagggct gcaggcggag gcagtagatg taagccggaa
60541 ctggctcaca gcagtgccca actgccctgt gttccccaaa acttgtacct ggctcagatg
60601 gtgtggccct ggcagccagg tgggatccag caaggaccag ggctctgcct ccccagtcag
60661 catgagcaga gatgactcct ttgagcagag catcagggca gaaatagaac agtttctgaa
60721 tgagaaaaga cagcatgaga cccaaaaatg tgatgggtca gtggagaaga aaccagacac
60781 acatgaaaat tcggccaagt cactctcgaa atcccaccaa gagccggcta caaaggtggt
60841 gcaccggcag ggcctgatgg gcgtccagga gttcgccttc tgcagacctc ccccggttag
60901 caaagacaaa cgtgcagccc agaaccctca ggtccaaggt catgaccacg caggagaact
60961 agggcagcac aaagccagca acccccgccg cccttcagaa gcagtacaga ataaaagtgg
61021 gattaaaagg agcgccagca ccgcaaggag gggaaagcga gtcacgagcg ccatacaggc
61081 gcccgaggcg tccgactcca gcagcgacga cggcattgag gaggccatcc agctgtacca
61141 ggtgcagaaa acacgcaagg aggccgacgg ggacccgccc cagagggccc agctccaaga
61201 ggaaagagca cctgcccctc ccgcacacag cacaagtagc gccacaaaaa gtgccttgcc
61261 agagacccac aggaaaacac ccagcaagaa gaagccagtg cccaccaaga ccacggaccc
61321 tggtccaggg gatctggacg ctgaccattc ccccaagatc ccaaaggaaa ccaaagctcc
61381 acctccaacg agcccggctt ccaggagcaa gtttgtggaa tggtcctctt gccaggcaga
61441 cacctccgct gagctgatgt gtgtagaagc agtcctggac attttcaaga cggtcctgcc
61501 ggccctatgg agggcagcga tgggtccctg tccgcaagcc cactcttcta ctcccccaac
61561 gtgccttccc gctctgacgg tgacagtagc tccgtggaca gcgacgacag cattgagcag
61621 aaaatctgga cgtttttggc cttcaaggtg cagtctggaa gtctgctggc cagaggtgag
61681 agctgcccgc aggctgccca gggcccattt tcaccacctg gcctcagcag ccagaccggc
61741 agccccaagg cccctctctc taaaacactg gacccactcc tggctgcaaa aggaagcata
61801 gaggcggcgg ccaagtgagg ccatccactc ccaagaacat tcgggaggtg gggaaagagg
61861 gtggccagga tgcggaccac agccagggga gagccgggcc cggccatgag gggcggacc
61921 ttcccatcca gggcacagcc agcgaggcct cgggatggga gggcgccgct agggtgggac
61981 ctttccatcc agggcacagc cagcgaggcc ccgggaggag agggcgccgc tagggtgcct
62041 ggtgacactc gcatgtcaca gggccagggt aagacagacg aggcaaggca cctagacaag
62101 aagaagagct ccgaagacaa aagcagttcc ctggacagtg acaaggacct ggacacagcc
62161 atcaaggact tgttaaggtc caagtgaaag ctcaagaaga ggtccaagga gcccaggct
62221 gtgtgcagga agaaggtcag gttcagcacc tcccagacgc acttccagga gcagatgggc
62281 aggctcctga gagagtggaa aaacaggcac ctgcaggtgc tgaagagctg cctaagtcca
62341 agagagacag ctgcgagggc tccaggaaga aaccccccag tgtctttggc agcagggcag
62401 agaggacgag ccccagcctt cctggtgagg agacccgctt ctgcctccgc ctccgaagag
62461 aatctattcc ccagagagtc ccaggcccca gctcccagcc ctggctcctt gtctgacaac
62521 agcagttcag tggacagcga cgatagcatc gaactggaga ttaggaagtt tttggtggaa
62581 aaggccaagg agtcggtgag cagttcagaa gttcaggcag agggccccac cgctctcggg
62641 acaggggggcc cagccaggcc agaggtacct tgcaggaagg agccggcccc actgcctggc
62701 atgtgcacac ggagccagag ggccgggggg ggtcccacat ttggctgaag ggcatcgagg
62761 cgcagggagc gcaggagcac agggcgcagc gtgcctgctc agccagggtg ggaaggggct
62821 ccccactgat ccggcggagg ggatcatgcg ccacccagga ggaccagcgg cagtgtctcc
62881 gccaaggtct ctcagtgagc aggagaaatg tttacgttta caaagaccag agcccacgag
62941 gggctgagcc tgctgccaaa agtgcttttg gtcagcggcc cagctgtgcc acagcgggca
63001 ccgaggcagg aggcgccagg aggacctttc acgtgggctg caggaaccgg agcttcctga
63061 cccccagccc gggagctgag agggacgctg gagcccaggc caaccgcgcc ccgccctgga
63121 gtgactttgc ccaccacagt cggctgccca gcccatgggc acttcgctcc aaaggtagag
63181 atgcggcatg gagggggcgg cattgggaga gagagacaag gggtccgagg gccccgcccg
63241 gggcctgccc agcctgcccc ttgcgggctt ctccccgctg ctgtccaccc agctcttcca
63301 ctttggaaag ggtgtctcct ggggggcag ggagaccggc ctcttcagcc cctacccagg
63361 actgcctctg cagggcctgt ccttctcggc cttcagggag tcccaggccg ggcccagccc
63421 tgtctttgga agcccacact tgctggcgaa gaaggactgc ggtcaccggc caagcaggaa
63481 ggtacagaca gggctgagtt tgcacaacag gaagagctct ggctcggagg aaaggatttt

```
    63541 agacctgagg tatccacgaa gggtcaatca gtagagatga ccaggaccag gacaccttgg
    63601 gcagtgacgc cagtgacttc agcaacacct ccgcagaggt gttgctggtg cagtggtggc
    63661 agctcagtag tgaaggtata a
//

>FAM247.LOC105372935.ref.human
        1 tgaaaactag aagttgaggc atgagtttgg ccactccgta gtgtgcactt ggtgagggca
       61 gcagctcgcc acagctgcca gccatctgtc cattcaccca tctgtccatc tggcagcccg
      121 ctgttcagac ctgtctgtct gtccgcccat ctgtaagccc atctctgtcc cattgtctat
      181 ctgaccatct ttctcttact gtcctctttg tctagctatc tggcctgtct gtcgatccat
      241 cttcgtgtct gtcttcagcc cccacctgtt tgtccatctg tccaattacc tgtgagtcta
      301 tctatgcatc ttcttgtcca ttcatctgcc cacccatctg tccctccatc tgcccaccgg
      361 cctcccctct ccttctgggc cgcagagcca tggcccagga ctacggagcc atgggtgacc
      421 tggtcctgct ggggctgggg ctggggctgg cgctggctgt cattgtgctg gctgtggtcc
      481 tctctcgaca ccaggcccca tttgaccccc ggcctttgcc cacgccgctg ttgctgctga
      541 ctccaaggtc ttctcaaata ttgtacggtg agtgagacgt gggaggaagc tgggtggcct
      601 ttggcagcca gcccctcctg gagaaggcgt gtgtgtgtga gcatgtgtgt gtgtgagaga
      661 ttatgtgtga gtgtgtgtgg gtatatgtgt gagtgtgttt gtggggtgtg ggtgtgtgtg
      721 aatgtgtgtg atcgtgtttg ggtgtgtgta tgtgtgagtg tgggtgtgtg tgaatgtgtg
      781 tgattgtgtt tgtgtatgtg tgtgtgggtg tgtgtgagta tatgtgagtg tgagtgtgtg
      841 ggggtgtggg tgggtgtgaa tgtgtgtgat tgtgtttcgc tgtgtgaggg tgtgtgtgac
      901 tgtgagtgtg tgagtgtggg tgtgtgggtg tgtgtaaatg tgtgagtgtg agtatggggg
      961 ggtgggtatg tgtgaatgtg tgtgattgtg tgtgggtata tttgtggggg tgtgtgtgtg
     1021 tgtgcacgtg tgtgtgtgtg cacgtgcact ggcccaggaa gcaggagccg tgtgtgtggg
     1081 cttcagcacc tgcagggctt gagcgcaagg agacagcctc agggcccttg cacagaacag
     1141 gcggcagggt gtgcccgtgg ggcagatggg gacttgggga caatggtggt gtgtgagtcc
     1201 atacctggct ccaggattca ggaggcccat ttgcatatcc caggtgggaa cctgtctggc
     1261 cccgcctgac cctgctggcc ggcgcaggcc ccttcagtga ggccaattct ccaaggctgc
     1321 ggtcttctcc cagggtcatg ggtgaagggg tttggaggct ccctgcgtgg gtactggcct
     1381 gctggggtac acacaatgct gccatagcca gtctgcccct acacccagcc cggggccaca
     1441 tctcaggtct ctcagtcctg aggagcccgg tgccccaccc ctcacatcct ctctccctga
     1501 gtcagggcct gggtctcgtg agctgagtga ctgatacttg gtgtcctgga tgagggcgtg
     1561 gtggagaggg gccacagcgg gtgtttcctg accctcttcc aggaaggtgc tgctgccgct
     1621 gcagggagga cacacacagg atgccccttc ttgccccctg cctcccattg ggcccacaaa
     1681 agccagggca agcctcccct ccctgccagc cacctggtct gcttcccaga aattctgtct
     1741 tgcaggctgt tgggaggatc ccagtacttt gtaaactaaa gcaagggagg agtggccgtt
     1801 ctctctcttt gttcattcat tcacctttc attcattcct tcttccctcc attcccccat
     1861 ctgtgcatcc ttccctgccc tgattgctca tgccaccccc ccccagcccc tcctgacctg
     1921 gtcctttggt ttctcttcag ggctttctgt ctcctcccac agggctgaga atggcagctc
     1981 agggacaagt aggggctggg gactgcttag tctccccagt ggctctcagg ggatttgagg
     2041 gtttgacgcc agctgccacc ccaggctgtg cccctcctct gctcgggagg acatacagag
     2101 atgcgacacc cacttaaact cgaagttgca aagatgcaaa tgagactggg gtctcaggca
     2161 ccagagacca cccgtgggca cgtggctttt gggagtgggg acctgctgcc acagatctct
     2221 gaagagtctg gacctgctgg gtctccccga gtgactgtct gggggtctcc atagcatgcc
     2281 ctgctgtgtg cgtgacggtc actggttggg taggggtctc tactctaaag ctccctctgc
     2341 cggcatcccc tccaactctc ccttggtgaa gagaggatgt ggtttgcccc agtgttttat
     2401 caaacaactc tctccacttc cagttttaag aagctgggag tggaagagag cctggggctg
     2461 gccccagctg ctgctgtgaa acaggggtca ctggacgctg ggaccctggc cgggctggct
     2521 ggaggcctca ggaagaggcc tgctacagcg tcatcctggc caagattcct ccctgcagag
     2581 gaccctggcc acgctgccac agggtctgct ggggccacca gaagcccatg ctcctgcctc
     2641 catctctccc ctctgtgctc acctctcacc aggaggccct cccagagttc agtgtcctgc
     2701 tttttttttt tttttttgtg acggtgtctc actctgtcac caggctggag tgcagtggcg
     2761 cgatctcagc ttactgcaac ctctgcttcc tcggttcaaa tgattctcct gcctcagcct
     2821 cctgagtagc tgggactaca ggtgccagcc accacgcccc gctaatttct gtatttttag
     2881 tagagacggg gtttcaccgt gttggccagg atggtctcta tctcttgatt cacccgcctt
     2941 ggccacccaa agtgctggca ttacaggagt gagtcatggc acctggcctc atctcctact
     3001 ctttcagcac caggttttat tcttgggatt ctgctacagc cggagcccct gggtgcgagt
     3061 tcctaaggtt tctgtgagtg tggacccagc accgtgccta gtagacatac aaaaggagca
     3121 tggtgacagt gaggtctgtc atctccagca taatgactgt tttgatcctt gtaaaaaagg
     3181 tgatttttgg ctgggtgtgg tggctcacac ctgtaatccc agcactttgg gaggccgagg
     3241 ggggtggctc acttgaggtc aggagttgga gaccagcctg ggcaacatgg tgaaaccatg
     3301 tctctactaa aaatacaaaa attagctggg catggtagca ggtgcctgta atcccagata
     3361 cttgggaggc tgagacagga gaatcacttg aacccaggag gcaaaggttt cagtaagcca
     3421 agattgcacc actgcactcc agcctgggtg acagagcaag acttggtctc aaaaaaaaaa
     3481 aaaagaaaga aagaaaagtt tatatttttg ttctaatggt tatcttaata tcgtcattct
     3541 ataattatat gttttatata attataatag ctatataaga tataataccc ctagtatgtt
     3601 gtttttttgga tattctactt gctcctgatg gttaatttat gtgtcaactt ggctaagcta
     3661 tggtgtcctg ttgtttggtc aaatacttgt caatatcttg ctgggaggtt atttcataga
     3721 tgtgattaac actgacagtc aattgacttt aagtaaaaca gattacccac cataatatgg
     3781 gtgggccacc tccaatcagt tgaaggcctt aagaacaaaa actgaggttt cccagagaag
     3841 caggaattct gcttcaagac tgtaacacac aaaccctgcc tgagtttctg gcctgctgac
     3901 tgctctacag attttaggtt ccagacttcg agatcaactc ttacctgaat ttatagcctg
     3961 ctggcttgcc ctacagattt taaaacttgc tagtccccac aatcatgtga gccaattcct
     4021 aaataaatct ctctctatgt ataatctatt ggtttagttt ctctgaaaaa ctttcacatc
     4081 cagtttcctg gttgttaaga attaccgaaa ctagctagta acttcttttt tttttttttt
     4141 ttttgagaca gagttttgct cttgttgccc aggctggaat gcaatggcac aatctcagct
     4201 caccgcaacc tccacttcct gggtccaagc aattctcctc cctcagcctc ctgagtagct
     4261 gggattacag gcatgtgcca ccatgcttgg ctaatttttg tatttttagt agagacaggg
     4321 cttctccatg ttggtcaggc tggtcttgaa ctcccaacct caggtgatcc gccgccttgg
     4381 cctcacaaag tgctggaatt acaggcacga gccattgcgc ctggctccta gtaaattctt
     4441 cttttctgtg atgtgtctct tacctctaat aatacttttc ttcttaaagt ctacttcatt
     4501 aaaaatagtt atgctgggca tggtggctca tggctgtaat cttggcactt tgctggaggt
     4561 cgaggtgggt ggatcactga agcccaggag ttcaagacca acctgggcaa catggcgaga
     4621 ccctgcctct acaaaaaata caaaaattag ctgggtgtgg ctaatataat tctaagttgg
     4681 cacacttgta gtcccagcta cttgggatgc tgaggtggga gaatcgcttg agcctagaag
     4741 ggagagattg ctgtaagcca agatcacatc actgcactcc agcctgggag acagagtgag
     4801 gctctatctc caaaaaaaaa aaaaaaaaaa aaaaaaaagt tatacagctt tcttggttag
     4861 tacatgcatg acatattttt catgatcttc cacctctctg taccсttata taaaaggcat
     4921 tagttgggtt ttactttatt ttcaattatt ttaattttta ttgtcctttt aaatgtaact
     4981 aatgatttat ttgggttgaa acccaccacc aatttgtttt ccatgcctat tctatttctt
     5041 cttatctcct ctcacatctt gttttggatt tattattttt attatttaat ttcctccttc
     5101 tctattagtt tcataactgt gcagtcttag agttatttta aaagatgact ggattatttt
     5161 agagcttaca acatgcatcc ttcccttatc aaagtctaac atgagctagt actttttgtt
     5221 gtggttgaga tagagagagt cttcctctgc tgcccaggct ggagtgcagt ggagcaatct
     5281 tggttcactg caacctccac ttcttgggtt caagcaattc tcctgcctca gtcacctgag
     5341 tagctgggac cacaggtgtg caccactatg cccggccaat ttttgtattc tttttcagta
     5401 gagacagggt ttcaccatgt tggccaggct ggtcttgaac tccggacctt aagagatctg
     5461 cctacctcgg catcctaaag tgttgggatt acaggcgtga gccaccgcgc ccagcctatg
     5521 agttagtact tctatcctct tcctagtcag tacaagaacc ttggaacagg aactaaattt
     5581 acccccagtg acttatatgc taatattttt gtgtatttta aatatgtgtg tgtgcataga
     5641 tgtatctgtg tgttttttgt gtttttattc ttatttatgt tgagagtgta gagctatgta
     5701 agagtaaaga gaattgtgta atgaagcccc gagtatccat tcaatttcaa caacaatctt
     5761 atggccaagc tcatttcatg tatactcttt cctgcttccc tctaccccac attatttcag
     5821 tgcaaatccc agatatataa ctgtaccaat acatatttca gtatgtttta tttattttaa
     5881 accccacaag atatcatttt ctatactact gtaattttat accaataaca ttcatttaga
     5941 tttacccaca cgtttaccct accctccggg tcctgtttga aaatcaagcc catgctcaca
     6001 ggccaatttt ttttctttta gagacagggt ctcactttgt cacccaagct ggagtgcagt
     6061 ggtgcgatta tagctcaatg cagcctccaa ttcctggact caagggaccc tcctgcctca
     6121 gcctgccaag tagcttggac tatagctgtg tgttttctta ttattttgta gacatggggt
     6181 ctggctatgt tgtccaggct attctcaaaa ttcccggcct cgagcaatcc tcctgcctcg
     6241 gcctctcaaa ggttgggatt acaggtgtga ggcaaggcac ccagctcagc cacagagccc
     6301 tgttgcatct ctcttactag gagcaagagc tgactgcccc ctcatcccca ttccagagtg
     6361 ttggggctgt gttcagccga ggccgggcca ctggcatggc ccagggagcg ggatcattca
     6421 ctgctgcccc aaatctgaga tcattccacc ttgacaagac ttcctcatcc aatcccttta
     6481 cttgacagct ggggaaacca atgcgcacag agcaccccca gctcactcgg ggtctcagag
     6541 ctgatccatg agcagaggct gagatcctgg gatcttgtcc cccagccgcc ctgcaagctt
     6601 actccctttc tgctggaaga gatggggccg gacctcgacc agcagccctg gcctggacat
     6661 gactgtgctc acccaggtat tgaggccgag atgccccggc atcatatgtt tttctctctt
     6721 tttttctttt tttttgagac agtatctcac tctgtcaccc aagctggagt gcagtggcat
     6781 gatattggct cactgcaacc tctgcctccc gcttaaagtg attctcctgc ctcagtcttc
     6841 caagtagctg ggcctacagg cttgtaccac cacacctgac taatttttgt atttttacta
     6901 gagacggggt ttccccatgt tggccaggct cgtgtcgaac tcctgacctc aggtgatcca
     6961 cctgccttgg cctcccaaag tgctaggatt acaggcatga gccatggcgt cacttaaatg
     7021 tagtgagagg ccgggcaagg ggctcatgcc tgtaatccca gtgctttgag aggacgaggc
     7081 tgtcagatca cctaaggtca ggagttcgag accagcctgg ccaacatggt gaaactgtgt
     7141 ctctacaaaa aaatagaaaa aaaaatccct gcgtggtggc aagtatctgt agtcccagtt
     7201 actcaggagg ctgaggcatg agaattgctt aaacctcgga ggcggaggct gcagtgagct
     7261 gagatggcgc cactgcactc cagcctgggt gacagagcaa gactttgtct ctaaataatt
     7321 aaataaataa atatggccga gcatggtgcc ttaggcctgt aatcccaaca ctttgggagg
     7381 ctgaggcagg tggttcatga ggtcaggagc ccgagaccag cctggccaag atggtgaaac
     7441 actgtctcta ctaaaaatac aaaaattagc cagctgtggt ggcaggcacc tgtaatccca
     7501 gctacttggg acactgaggc aggagaatcg cttgaacctg gaaggcagag gttgcaatga
     7561 gccgagattg caccgctgca ctctagcctg ggcgatggag caagactcca tctcaaataa
     7621 ataaattaat aaatacagag caagattcca tctcaaataa ataaataaat gtacacctgt
     7681 aatcctagca ctttgggagg ctaagacagg tcgatcacct gaggtcagga gttcgagacc
     7741 agcctgacca atatggcaaa actccatctc tactaaaaat acaaaaatta gccgggcgtt
     7801 ttgacgtgtg cctgtagtcc cagctacttg ggaggctgag acaagagaat tgcttgaacc
     7861 caagaggtgg aggttgcagt gagccgagat ctcggctgca cttcagcctg ggtgacagag
     7921 tgagactctg tctcaaaagg aataaataaa atacaaagta aaaaaaaatg tagtaagatt
     7981 gcagagtcgt gccgcagaag cgtgctggtc ctatccatgt agtgaaggct gatttcatac
     8041 acaaatgtca gaagaacttt tcttttcttt ttcttttctt tttttttttt ttgagacgga
     8101 gtctcgctct gtcacccagg ctggagtgca gtggtgtgat ctaggctcac tgcaagctct
     8161 gcctccctgg tttatgccat tctcctgcct cagcctcctc agtagctggg actacaggcg
     8221 cctgccacct agcccagcta attttttttgt acttttagag gagatggggt ttcaccgcgt
     8281 tagccaggat agtctcaatc tcctgacctc gtgatccgcc cgtctcggcc tcccaaagtg
```

```
 8341 ctgggattac aggcatgagc caccacaccc ggccttctta tatgctttta ttgcatttga
 8401 gcgtacctct tttacagcga agatcttttt tttaaatttt attttatttt tagagatgga
 8461 gtcttgcttt gttgcccagg ctggagcact gtggtgtgat catagctcac tgcagccttg
 8521 aactcccggg cacaggtgat cctcccacct cagcctcctg aatagctggg actacaggca
 8581 tgcaccacca tgcctggcat attttaaaga tgtttgtaga gatgaggtct cgctatgttg
 8641 ccaggcttgt ctcaaactac tgggctcaag ccatccatcc atttcagcct cccaaagtgc
 8701 ttggattata ggcatgagca ctgcgcctgg ccatcacact gtttttttgt ttgtttgttt
 8761 gtttgttttg agatggagtc ttgctctgtc gcccaggctg gagtgcagta gtgggatctc
 8821 acctcattgc aagctccgcc ttgtgggttc acgccattct cctgcctcag cctcctgagt
 8881 agctgggatt acaggcgccc gccaccacgc ctggctaatt ttttgtattt ttagtagaga
 8941 cgggatttca ccgtgttagc caggatggtc tcgatctcct gaccgtgatc tgcccgcctc
 9001 ggcctctcaa agtgctggga ttacaggcat gagccaataa atatttttat aatcattaac
 9061 taccgaggag aagctggaga gaaaaaaggt ggatgaagtt aaggcagaga gactttgtaa
 9121 gtttcttagc tgagcttttg gagactgtat atcagagatc ctatttgagg tgattttcgg
 9181 ctacagagat aggcctggtc atttgaaaaa taacggatta ggtgaagctt gcgtttgaaa
 9241 tcctccttct acttctcatc tttctctctt gtttattctg aacatccatc ttagacaccc
 9301 aatctttgtc actttgtggt tgtcattgat ttccctgtta ttgagattag aggttggcag
 9361 actttctctg taaagggctg gagagaaagt acttcagact ttgtggtctg tgcagtgtct
 9421 gttgcaacca cttaactctg ccctgtagag caaaagcagc cgtagacagt acatgggcag
 9481 atgagcatgg ctggggtcca gttacattta cttgcaaaaa gagggttgga gactgggtgc
 9541 gatctcccac ctttaatccc ggcactttgg gaggccgagg caggaggatc acttcaagcc
 9601 aggagttcaa gaccagcctg ggcaacaaag caagactcca tctctacaaa aaaataaaaa
 9661 ttattatagc taggcatggt ggtacacacc cgtagtccta gctactcagg aagctaaaac
 9721 tgaggcagga gggtcagttg agcccaggag cacgaggctg tggtgagcta ttattgtgcc
 9781 agtgcactcc agcctggtga cagagcaaga accgtctcat caaaaaataa acaaaaggct
 9841 gggcacggtg gctcacgcct gtaatccctg cactttggga gactaaggtg ggcagatcat
 9901 gaggtcagga gattgagacc atcctggcta acacggtgaa accctgtctc tactaaaaat
 9961 acaaaaagtt agccgggcgt ggtggtgggc gcctgtagtc ccagccactc gggaggctga
10021 ggagggagaa tcgtttgaac ctgggaggcg gaggttgcag tgagccaagg ttgtgccact
10081 gcactctagc ctgggctaca gggcaagact ccattaaaaa aaaaaaaaaa aaccagcaaa
10141 aaccaaacaa aacataatgc atgttctctc ttataaatgg gagctaaaca tggggactca
10201 ttgacttaaa gatggcaaca actgggaact gctggatggg gagggagggg aggggtgaaa
10261 ggccaactgt tggggagtat gctcatatcc atgtgacaaa cctgcacatg tgcccgctga
10321 atctaaaata aaagttgaaa gtagatttaa aaaaccccaa gagggctggg tttggcttgt
10381 gtgtccatag cttgttaacc tccgctttag atattaacta atagaaacat agtgcttatc
10441 ttcccaggcc acctattttg ttcctctcca aggtgatgga tagatgaagg cctaatccag
10501 ccgcctggaa gtttgctgac gcttgtcctg tcacggatta atgaagcatt gttttctgat
10561 gaaggtttca tgccgctgtg ctgatgtgtc ttctcttctc tctaggcagg aaactgcata
10621 tcttctggtt tacatgaaga tggagtgcta atggaaatgc ccaaaacctt cagagattga
10681 cacgctgtca ttttccattt ccgttcctgg atctacggag tcttctaaga gattttgcaa
10741 tgaggagaag cactgttttc aaactatata actgagcctt atttataatt agggatatta
10801 tcaaaatatg taaccatgag gcccctcagg tcctgatcag tcagaatgga tgctttcacc
10861 agcagacccg gccatgtggc tgctcggtcc tgggtgctcg ctgctgtgca agacattagc
10921 cctttagtta tgagcctgtg ggaacttcag gggttcccag tggggagagc agtggcagtg
10981 ggaggcatct gggggccaaa ggtcagtggc agggggtatt tcagtattat acaactgctg
11041 tgaccagact tgtatactgg ctgaatatca gtgctgtttg taatttttca ctttgagaac
11101 caacattaat tccatatgaa tcaagtgttt tgtaactgct attcatttat tcagcaaata
11161 tttattgatc atctcttctc cataagatag tgtgataaac acagtcatga ataaagttat
11221 tttccacaaa a
```
