# Supplementary material for: An ancestral genomic sequence that serves as a nucleation site for de novo gene birth
Source: PLoS One. 2022 May 12;17(5):e0267864. doi: 10.1371/journal.pone.0267864 (PMC9097989; doi:10.1371/journal.pone.0267864)
Supplement: S12 Fig — (PDF) [file pone.0267864.s012.pdf]

An ancestral genomic sequence that serves as a nucleation site for *de novo* gene birth

Nicholas Delihas

Department of Microbiology and Immunology, Renaissance School of Medicine, Stony Brook University, Stony Brook, N.Y., United States of America

S12 Fig. a. RepeatMasker analysis of the FAM247 sequence. b. Secondary structure predictions of FAM247 imperfect palindrome. The program used was with the one provided by the Mathews Lab- University of Rochester (http://rna.urmc.rochester.edu/RNAstructureWeb/Servers/Predict1/Predict1.html).

a.

+	score	% div.	% del.	% ins.	query sequence	position in query- begin	end	(left)	C +	matching repeat	repeat class/family	-position in repeat- (left) begin	end end	begin (left)	linkage id/graphic	
+	22	32.9	1.3	6.1	FAM247	101	254	(10977)	+	(TCTG)n	Simple_repeat	1	147	(0)	1	
+	12	18.0	10.0	0.0	FAM247	428	477	(10754)	+	(GCTGTG)n	Simple_repeat	1	55	(0)	2	
+	189	21.6	0.2	1.0	FAM247	629	1040	(10191)	+	(GT)n	Simple_repeat	1	409	(0)	3	
+	16	24.8	0.0	0.0	FAM247	1812	1854	(9377)	+	(TTCA)n	Simple_repeat	1	43	(0)	4	
+	1947	11.8	3.5	0.0	FAM247	2701	2988	(8243)	C	AluY	SINE/Alu	(13)	298	1	5	
+	2312	12.0	0.0	0.0	FAM247	3189	3497	(7734)	+	AluSx	SINE/Alu	1	309	(3)	6	
+	2702	15.5	0.9	0.2	FAM247	3626	4077	(7154)	+	MLT2C2	LTR/ERVL	3	457	(7)	7	
+	2217	12.9	0.3	0.3	FAM247	4123	4425	(6806)	C	AluSx	SINE/Alu	(8)	304	2	8	
+	271	22.0	24.0	0.7	FAM247	4451	4512	(6719)	C	L1MEg	LINE/L1	(3749)	2397	2335	9	
+	1900	15.4	0.6	6.2	FAM247	4513	4838	(6393)	+	AluJb	SINE/Alu	2	310	(2)	10	
+	511	20.9	16.7	3.2	FAM247	4839	5215	(6016)	C	L1MEg	LINE/L1	(3812)	2334	1895	9	
+	1976	14.2	0.0	1.4	FAM247	5232	5516	(5715)	C	AluSx	SINE/Alu	(31)	281	1	11	
+	301	25.2	2.9	1.9	FAM247	5517	5621	(5610)	C	L1MEg	LINE/L1	(4332)	1910	1805	9	
+	573	25.4	5.8	1.9	FAM247	5662	5866	(5365)	C	L1MC5	LINE/L1	(4)	7943	7731	12	
+	333	16.9	0.0	11.5	FAM247	5854	5960	(5271)	C	L1MEg	LINE/L1	(4430)	1812	1717	9	*
+	1423	18.8	6.5	0.7	FAM247	6007	6285	(4946)	C	AluJb	SINE/Alu	(16)	296	2	13	
+	216	39.9	2.9	1.4	FAM247	6448	6587	(4644)	C	MIR3	SINE/MIR	(36)	172	31	14	
+	2110	13.9	0.3	0.0	FAM247	6709	7011	(4220)	C	AluSx	SINE/Alu	(3)	309	6	15	
+	1847	15.6	0.3	0.7	FAM247	7029	7331	(3900)	+	AluSx	SINE/Alu	1	302	(10)	16	
+	2065	13.4	0.7	0.0	FAM247	7335	7625	(3606)	+	AluSx	SINE/Alu	1	293	(19)	17	
+	2062	13.4	0.7	0.7	FAM247	7673	7966	(3265)	+	AluSx	SINE/Alu	19	312	(0)	18	
+	2439	9.0	0.0	0.3	FAM247	8063	8374	(2857)	C	AluY	SINE/Alu	(0)	311	1	19	
+	1780	18.1	0.7	1.0	FAM247	8426	8732	(2499)	C	AluJb	SINE/Alu	(6)	306	1	20	
+	2366	7.8	0.7	0.0	FAM247	8742	9036	(2195)	C	AluY	SINE/Alu	(2)	309	13	21	
+	786	24.4	1.1	0.0	FAM247	9349	9524	(1707)	C	MER58A	DNA/hAT-Charlie	(1)	223	46	22	
+	1548	18.3	1.6	3.7	FAM247	9531	9836	(1395)	+	AluJb	SINE/Alu	1	300	(12)	23	
+	2270	10.5	0.7	0.0	FAM247	9837	10132	(1099)	+	AluY	SINE/Alu	1	298	(13)	24	
+	569	13.4	7.7	3.0	FAM247	10139	10340	(891)	+	L1PA16	LINE/L1	5896	6157	(9)	25	
+	451	27.6	9.9	8.0	FAM247	10794	11076	(155)	+	MamRep38	DNA/hAT-Tip100	8	295	(0)	26	
+	185	33.9	0.0	0.0	FAM247	11141	11202	(29)	C	L2b	LINE/L2	(0)	3375	3314	27	

Fig. b.

FAM247 imperfect palindrome: *tgaaaactag aagttgaggc atgagtttgg*

DNA secondary structure

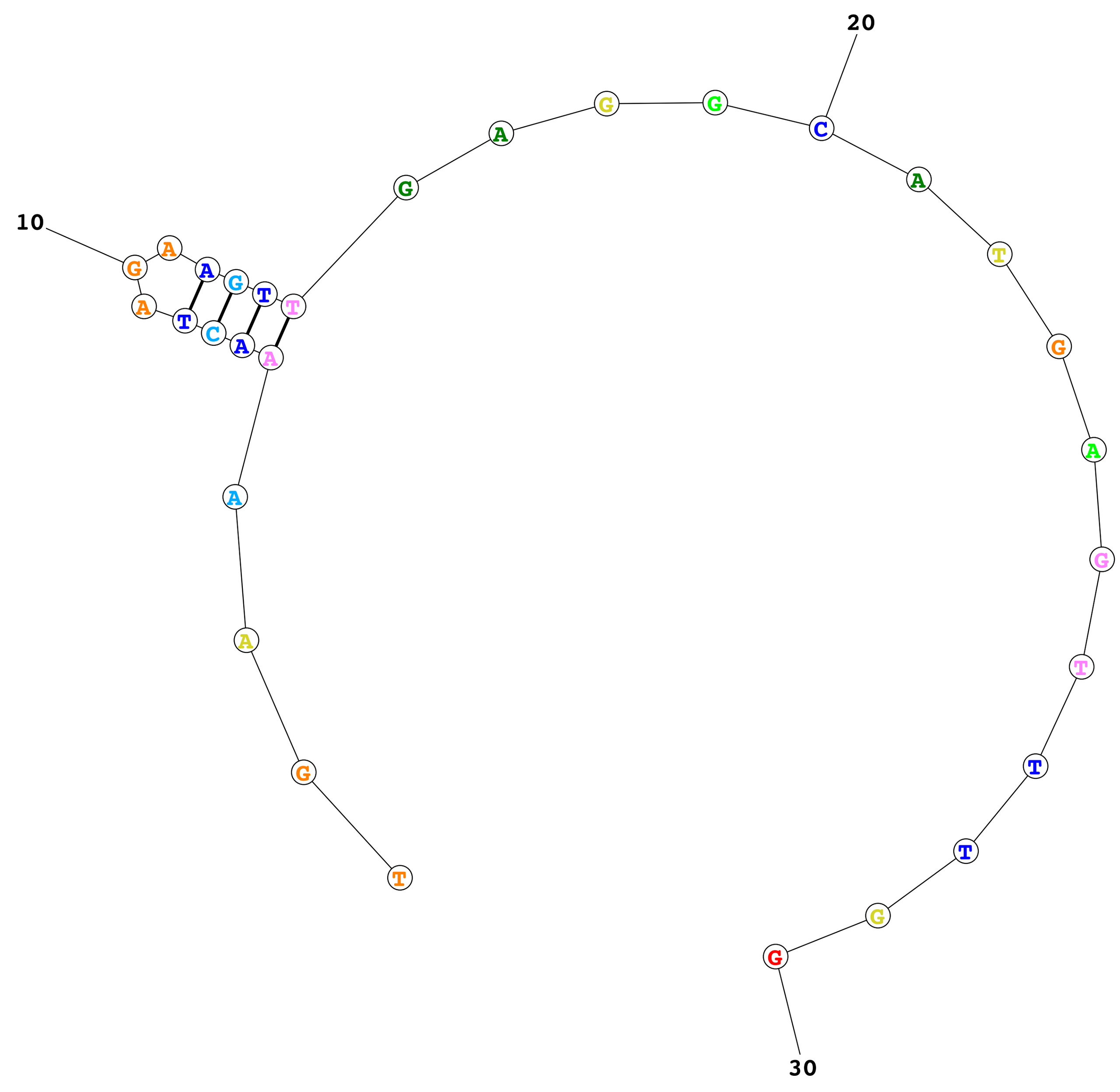

Probability >= 99%
99% > Probability >= 95%
95% > Probability >= 90%
90% > Probability >= 80%
80% > Probability >= 70%
70% > Probability >= 60%
60% > Probability >= 50%
50% > Probability
ENERGY = -0.9 01/09/22 08:37:18

RNA secondary structure

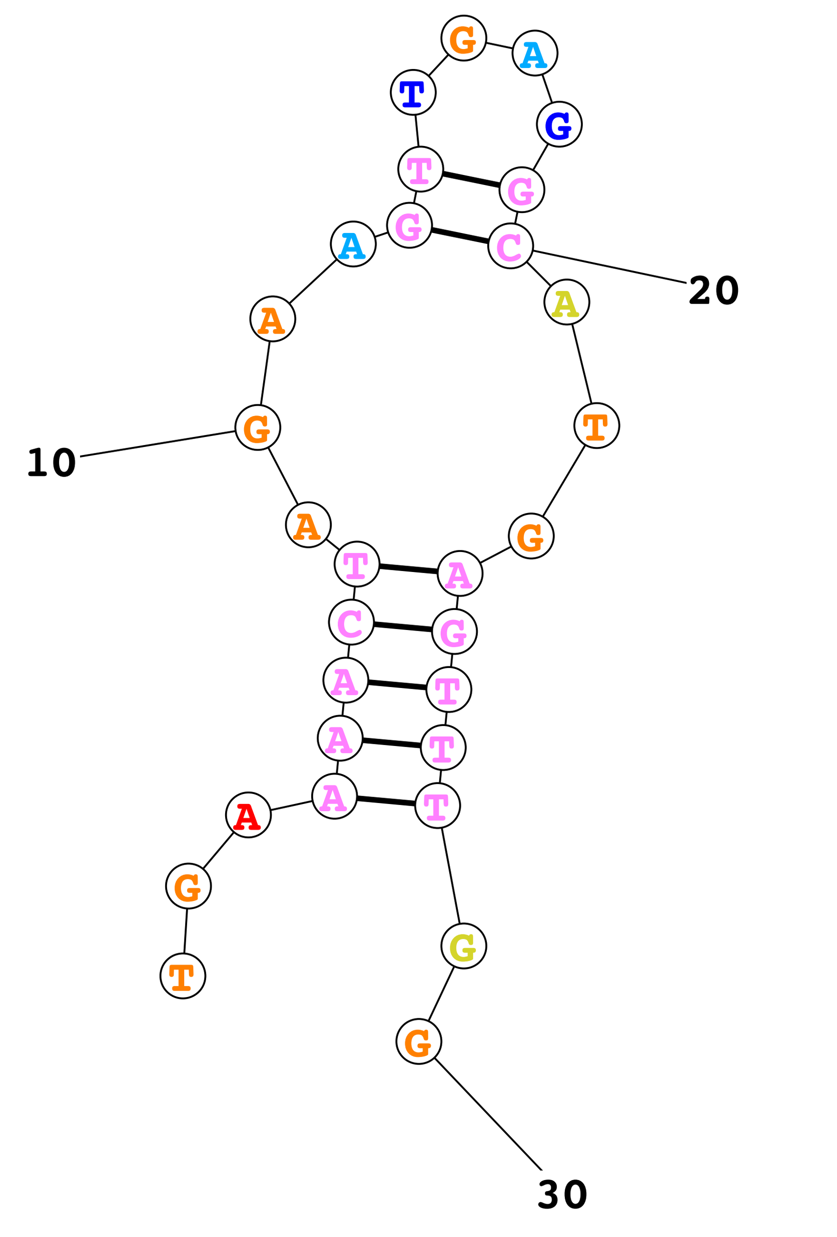

Probability >= 99%
99% > Probability >= 95%
95% > Probability >= 90%
90% > Probability >= 80%
80% > Probability >= 70%
70% > Probability >= 60%
60% > Probability >= 50%
50% > Probability
ENERGY = -1.2 01/09/22 08:33:46
